# Phylogenetic and Molecular Characteristics of Wild Bird-Origin Avian Influenza Viruses Circulating in Poland in 2018−2022: Reassortment, Multiple Introductions, and Wild Bird–Poultry Epidemiological Links

**DOI:** 10.1155/2024/6661672

**Published:** 2024-04-12

**Authors:** Kamila Dziadek, Edyta Świętoń, Edyta Kozak, Krzysztof Wyrostek, Karolina Tarasiuk, Natalia Styś-Fijoł, Krzysztof Śmietanka

**Affiliations:** ^1^Department of Poultry Diseases, National Veterinary Research Institute, Pulawy 24-100, Poland; ^2^Department of Omic Analyses, National Veterinary Research Institute, Pulawy 24-100, Poland

## Abstract

Since 2020, a significant increase in the severity of H5N*x* highly pathogenic avian influenza (HPAI) epidemics in poultry and wild birds has been observed in Poland. To further investigate the genetic diversity of HPAI H5N*x* viruses of clade 2.3.4.4b, HPAIV-positive samples collected from dead wild birds in 2020–2022 were phylogenetically characterized. In addition, zoonotic potential and possible reassortment between HPAIVs and LPAIVs circulating in the wild avifauna in Poland have been examined. The genome-wide phylogenetic analysis revealed the presence of three different avian influenza virus (AIV) subtypes (H5N8, H5N5, and H5N1) during the HPAI 2020/2021 season, while in the next HPAI 2021/2022 epidemic only one H5N1 subtype encompassing seven various genotypes (G1–G7) was confirmed. No reassortment events between LPAIVs (detected in the framework of active surveillance) and HPAIVs circulating in Poland have been captured, but instead, epidemiological links between wild birds and poultry due to bidirectional, i.e., wild bird-to-poultry and poultry-to-wild bird HPAIV transmission were evident. Furthermore, at least five independent H5N8 HPAIV introductions into the Baltic Sea region related to unprecedented mass mortality among swans in February–March 2021 in Poland, as well as a general tendency of current H5N*x* viruses to accumulate specific mutations associated with the ability to break the interspecies barrier were identified. These results highlight the importance of continuous active and passive surveillance for AI to allow a rapid response to emerging viruses.

## 1. Introduction

Avian influenza (AI) is a highly contagious viral disease of wild and domestic birds caused by viruses belonging to the Alphainfluenzavirus (Influenza A virus) genus from the Orthomyxoviridae family [1]. The avian influenza virus (AIV) genome is organized into eight negative sense, single-stranded RNA gene segments encoding at least 11 structural and nonstructural proteins. Based on the surface glycoproteins, i.e., haemagglutinin (HA) and neuraminidase (NA), the AIVs are classified into different subtypes (H1–H16 and N1–N9). The AIV genome structure, i.e., the presence of RNA as virus genetic material associated with error-prone replication (genetic drift) as well as genome segmentation enabling reassortment (genetic shift) determines the high genetic variability of AIVs [2]. The further ability of HA to spontaneously mutate allows the distinction between low pathogenic (LP) and highly pathogenic (HP) virus strains, which cause diseases associated with contrasting clinical manifestations: low pathogenic avian influenza (LPAI) related to mild or asymptomatic infection and highly pathogenic avian influenza (HPAI) leading to fatal systemic illness [3]. Hence, the disease's devastating consequences for the poultry industry as well as bird species diversity around the world are obvious. Moreover, AI is still considered a public health concern due to the possible virus transmission from birds to mammals [4]. Given the far-reaching social and economic consequences of AIV infections, continuous surveillance of AIVs harbored in wild birds seems to be crucial for identifying virus introductions and reassortment events, wild bird–poultry epidemiological links, and the increase in zoonotic potential, as shown in the present study.

Since the first detection of the HPAI H5N1 A/Goose/Guangdong/1/1996 (Gs/Gd) virus lineage in China's Guangdong Province in 1996 [5], continuous diversification and spatiotemporal expansion of the HPAIV H5N1 Gs/Gd lineage, supported by long-distance bird migrations, has been observed. In Poland, the first introduction of the HPAI H5N1 Gs/Gd lineage (clade 2.2) was confirmed in spring 2006 in wild birds, followed by the subsequent lineage's incursion into wild and domestic bird populations in late 2007 [6]. Globally, the HPAIV H5N1 Gs/Gd lineage of clade 2 was identified as the main cause of the majority of HPAI outbreaks until 2008. Subsequently, the evolution of the H5N1 Gs/Gd lineage and further reassortment of subclade 2.3.4.4 with LPAIVs resulted in the emergence of virus strains comprising different NA subtypes, i.e., N2, N5, N6, or N8, which are now collectively referred to as H5N*x* [7]. These viruses, specifically H5N8 and H5N5 strains belonging to the 2.3.4.4b clade, led to the H5N*x* HPAI 2016/2017 epidemic in Poland that was unprecedented in the number of outbreaks in poultry and wild birds at that time [8]. Since the following HPAI H5N8 epidemic in 2019/2020, Poland has annually faced consecutive HPAI outbreaks of increasing intensity and range caused by H5N*x* viruses of the 2.3.4.4.b clade [9, 10]. Bearing in mind the fact that Poland is one of the largest producers and exporters of poultry in Europe and in the world, the consequences of recurring HPAI epidemics involve enormous economic losses. Apart from disease outbreaks in poultry, the huge impact of HPAIV on the natural environment is worth noting as evidenced by the mass mortality of seabirds, i.e., gulls and terns, in Poland and other European countries in 2023 [11]. Recently, HPAIV (H5N1) clade 2.3.4.4b infections of domestic cats were confirmed in Poland [12] which indicates that more attention should be paid to the zoonotic potential of HPAIV due to its potential threat to public health.

To fill in the knowledge gap regarding the genetic and epidemiological characteristics of HPAI H5N*x* viruses circulating in wild birds in Poland between 2020 and 2022, a comprehensive phylogenetic analysis with particular emphasis on genotype analysis, investigation of wild bird-poultry connections, and assessment of zoonotic potential was carried out. The period covered by the study includes seasons of HPAI, during which the scope of the epidemic and its impact on bird health were the greatest in history. Furthermore, to investigate the possible reassortment between LPAIVs and HPAIVs viral sequences originating from active surveillance of live birds in Poland were also included in the study. The results obtained enable a deeper understanding of the intricate interactions among the host, the virus, and the environment in the context of a highly dynamic epidemiological situation of HPAI at the interface of the health of wild and domestic birds as well as public health in recent years.

## 2. Materials and Methods

### 2.1. Samples

A total of 52 AIV-positive wild bird samples collected between 2018 and 2022 in Poland in the frame of active and passive surveillance for avian influenza were selected for the study. Active surveillance-positive samples (*n* = 9) originating from anseriform and charadriiform bird species included six different AIV subtypes, among them one coinfection was present. The remaining samples (*n* = 43) were submitted to the National Reference Laboratory in Poland as passive surveillance samples collected mainly from wild birds found dead during two consecutive HPAI seasons 2020/2021 (*n* = 20) and 2021/2022 (*n* = 23).  Table 1 summarizes information about samples used in this study.

### 2.2. Sample Preparation

Depending on the surveillance type, various sample matrices were available for further diagnostics, e.g., cloacal and/or oropharyngeal swabs (active surveillance) or internal organs including brain tissue (passive surveillance) (Table 1). According to WOAH [13] (previously OIE) diagnostic manual, pooled organ samples were prepared as 20% homogenates in phosphate-buffered saline (PBS), while 10% PBS solution was used for intestines. Taking into account the neurotropism of HPAIV strains, 20% of brain tissue homogenates were prepared separately for each individual increasing the chance of subsequent detection of viral RNA. Cloacal and oropharyngeal swabs were immersed in 1 mL PBS each and pooled in a 1 : 1 ratio for further examination if collected from the same individual.

**Figure 1:**
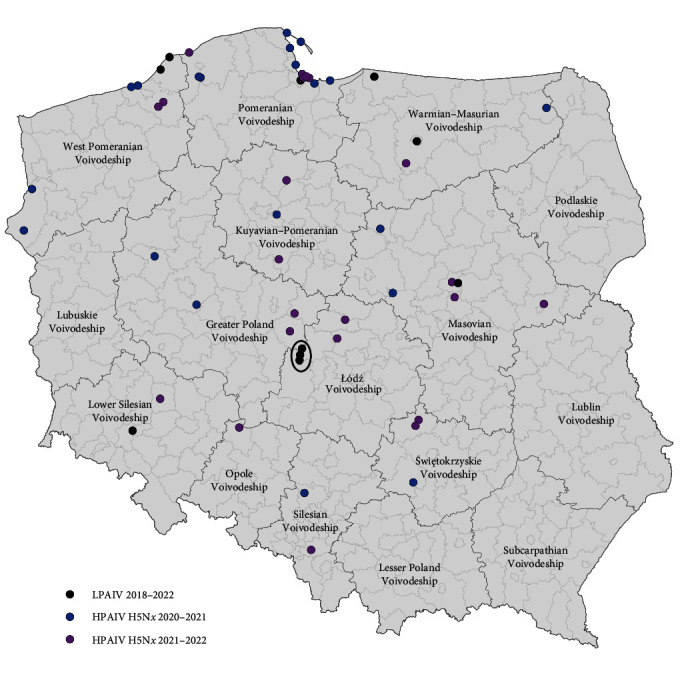
Geographical distribution of AIVs involved in the study. The location of the Jeziorsko artificial reservoir was marked with an ellipse.

Detailed information about all clinical samples including the geographical distribution of discussed AI cases is summarized in Figure 1 and *Supplementary 1*.

### 2.3. RNA Extraction

From prepared homogenates or PBS-immersed swabs, RNA was extracted manually using two commercial kits: (a) Syngen Viral Minikit PLUS (Syngen) and (b) RNAeasy Mini Kit (Qiagen) or an automated RNA isolation in IndiMag 48s extraction machine using IndiMag Pathogen Kit (Indical Biosciences) was performed according to the manufacturer's instructions. In the case of one active surveillance sample virus propagation in specific pathogen-free (SPF) embryonated chicken eggs preceded the final nucleic acid isolation step, where allantoic–amniotic fluid was used as RNA extraction matrix. The use of different extraction methods resulted from the implementation of new diagnostic protocols and laboratory equipment over the years covered by the study.

### 2.4. Detection of AIV and AIV Subtyping

The presence of AIV RNA was confirmed in a real-time RT-PCR targeting viral matrix gene following the procedure described by Spackman et al. [14]. The RT-qPCR was run in an ABI 7500 real-time PCR system using QuantiTect Probe RT-PCR Kit (Qiagen) according to the manufacturer's protocol. For RT-qPCR-positive samples, hemagglutinin subtyping was performed starting from H5 and H7 confirmation [15, 16], possibly followed by subsequent detection of the remaining HA and NA subtypes [17].

### 2.5. Whole Genome Sequencing

#### 2.5.1. Preamplification of All Eight AIV Segment Genes

To obtain full-length AIV sequences, RNA samples with a *C*_*q*_ value < 30 were amplified using SuperScript III One-Step RT-PCR System with Platinum Taq High Fidelity DNA Polymerase (ThermoFisher Scientific). For this purpose, primers targeting conserved 3′ and 5′UTRs of all eight viral genes [18] with slight modifications improving amplification of the longest AIV segments as PB2, PB1, and PA [19] were used.

#### 2.5.2. Purification and Quality/Quantity Assessment of RT-PCR Products

The RT-PCR products were purified using magnetic beads Agentcourt AMPure XP Clean-Up (Beckman Coulter) or KAPA Pure Beads (Roche). The quantity and quality assessment of DNA samples was performed using NanoDrop One (ThermoFisher Scientific) and/or Qubit 3.0 fluorometer (ThermoFisher Scientific) taking into account commonly recommended DNA purity parameters such as A260/A280 ratio (1.8–2.0), A260/A230 ratio (2.0–2.2) and the minimal DNA input for library preparation specified in the manufacturer's instructions.

#### 2.5.3. Library Preparation

Depending on the sequencing facility library preparation was performed using three different commercial kits, i.e., KAPA HyperPlus (Roche), Illumina DNA Prep (Illumina), or Nextera XT DNA Library Preparation Kit (Illumina) following the manufacturer's manual. The quality of libraries was evaluated with Fragment Analyzer (Advanced Analytical) or Bioanalyzer 2100 (Agilent) and sporadically based on a 1.5% agarose gel electrophoresis image.

#### 2.5.4. High-Throughput Sequencing

Paired-end sequencing (2 × 150 bp or 2 × 300 bp) was performed on Illumina NGS platforms (MiSeq, NextSeq 550, or iSeq 100) using corresponding Illumina reagent kits (MiSeq Reagent Kit v3, NextSeq 500/550 High Output Kit v2.5, iSeq 100 i1 Reagent v2). Run quality monitoring was guaranteed by adding PhiX Control Library v3 (Illumina) as 1%–5% of the total sequencing volume.

### 2.6. Next-Generation Sequencing Data Analysis

#### 2.6.1. Quality Assessment and Filtering of Raw Data

The widely used FastQC software [20] was used for the basic quality evaluation of raw sequencing data generated on Illumina platforms. Filtering and trimming of poor-quality reads and adapters were performed with Trimmomatic [21] applying minimum thresholds such as quality score ≥ Q20 and read length ≥ 50 bp, which ensure the minimum quality of raw data suitable for intended research purposes, i.e., generation of consensus sequences and phylogeny while preventing excessive loss of data.

#### 2.6.2. Read Mapping to Reference Genomes

Filtered reads were mapped to a reference sequence using BWA [22] and preprocessed according to the GATK best practices (https://gatk.broadinstitute.org/) to produce analysis-ready BAM files, i.e., BAM files generated after implementation of specific tools enabling additional technical error correction (MarkDuplicates, BaseRecalibrator). Reference genomes used for Illumina read mapping were selected from consensus sequences of different AIV subtypes available in a public database, i.e., GISAID EpiFlu [23]. Additionally, read coverage and read distribution were inspected in the analysis-ready BAM files using tablet [24].

#### 2.6.3. Generation of Consensus Sequences

Variant call format (VCF) files were generated from analysis-ready BAM files using two different variant callers, i.e., BCFtools [25] or LoFreq [26] with applied thresholds such as minimum mapping quality ≥30 and minimum read coverage ≥450. Finally, an in-house protocol was used for the generation of consensus sequences from the VCF files. According to the IUPAC nucleotide code, each base contributing 25%–75% to a single position in the genome was incorporated into the consensus sequence through the use of degenerate base symbols.

LPAIV and HPAIV sequences from Poland were deposited in the GISAID database (https://gisaid.org/) under accession numbers listed in *Supplementary 1*.

### 2.7. Phylogenetic Analysis

BLAST [27] was used to identify and include publicly available sequences from the GISAID EpiFlu database [23] which showed the highest similarity to AIV sequences from Poland (*Supplementary 2*). Initially, all selected protein-coding sequences were aligned in MAFFT v7 [28]. Maximum likelihood trees were generated for all eight AIV segments on the IQ-TREE web server [29] using automatic substitution model selection and ultrafast bootstrap approximation approach (1,000 replications). The phylogenetic trees were visualized in FigTree v1.4.4 (http://tree.bio.ed.ac.uk/software/figtree/). Identification of phylogenetic relationships between virus strains was assessed based on tree topology (branching pattern) and bootstrap value (BS). Only bootstrap-supported phylogenetic relationships (BS > 95), e.g., in the case of wild bird-poultry connections were considered significant.

### 2.8. Analysis of Molecular Markers

The molecular analysis aimed to identify markers affecting AIV biological properties [30–32] was carried out manually in MEGA7 Software [33] after prior visualization of the protein alignment including all Polish viruses and the reference strains mentioned in the literature [30–32]. Mutations in HA genes were identified based on the protein alignment of various HA subtypes and numbered according to the recommended numbering scheme published by Burke and Smith [32] allowing direct comparison of amino acid (aa) changes between virus strains of different HA subtypes.

## 3. Results

### 3.1. Classification of Samples

The phylogenetic analysis revealed that all HPAI H5N*x* viruses belonged to the H5 Goose/Guangdong lineage 2.3.4.4b and were predominantly related to HPAI viruses circulating in Europe at that time. Both the H2N3 LPAIV case and the active surveillance samples clustered with subtype-specific sequences available in GISAID EpiFlu [23]. However, the scarcity of sequences of currently circulating LPAIVs disallows any further conclusion regarding their phylogenetic relationships.

### 3.2. Genotypes

#### 3.2.1. Passive Surveillance—HPAIV H5Nx Samples

**Figure 2:**
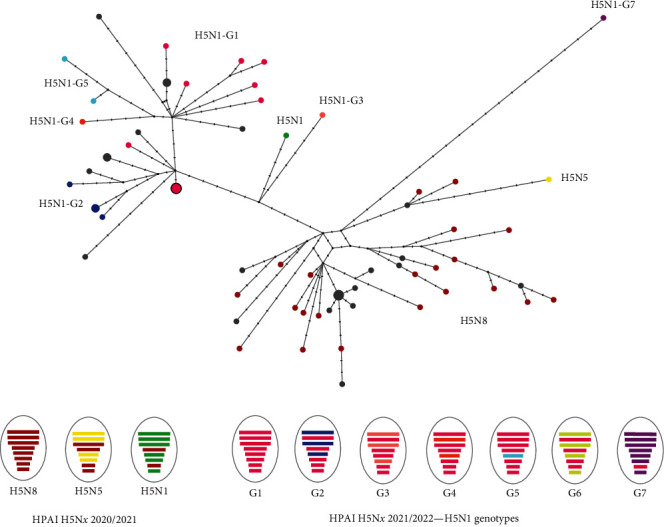
Graphical representations of HPAIV subtypes and genotypes detected during two consecutive H5N*x* HPAI seasons in 2020–2022 in Poland along with their evolutionary relationships. (a) Genetic network of HA (H5) sequences collected from wild birds (colorful circles) and poultry (gray circles). Small-diameter circles represent unique sequences, while large-diameter circles encompass groups of phylogenetically similar sequences. (b) Identified HPAIV subtypes/genotypes along with undergoing reassortment events. For reassortants, AIV genes clustering phylogenetically with seasonally dominant genotypes such as H5N8 and H5N1-G1 were colored in red and pink, respectively. Exchanged gene segments are indicated corresponding to the color scheme used in the HA genetic network (H5N1-G6 was not included in the network analysis).

During the HPAI 2020/2021 season, three AIV subtypes were identified (H5N8, H5N5, and H5N1). Apart from single H5N5 and H5N1 detection, all wild bird samples collected between December 2020 and May 2021 belonged to the H5N8 subtype and showed similar gene segment constellation forming one genotype (Figure 2). All sequences were found to be similar to H5N8 viruses discovered during this period in other European countries (*Supplementary 3*). In February 2021, a tufted duck infected by H5N5 HPAIV was found dead in a seaside town in the West Pomeranian Voivodeship in Poland. The phylogenetic analysis of this sample (MB061/21) disclosed reassortment events of PB2, PB1, NP, and NA segments with Eurasian LPAIV strains in comparison to the remaining viral genes which showed a high sequence similarity to HPAI 2020/2021 H5N8 viruses. In the case of HPAIV H5N1 detected in April 2021 in white stork, all gene segments except for HA and MP clustered with AIV sequences of the H5N1 subtype circulating in Europe at a time, whereas the HA and MP genes were similar to H5N8 sequences from 2020/2021 HPAI outbreaks (Figure 2 and *Supplementary 3*).

In the following AI 2021/2022 epidemic only one HPAIV H5N1 subtype encompassing at least seven different genotypes was observed (Figure 2). The most dominating genotype (G1) was regularly detected from November 2021 to February 2022 in six distinct Voivodeships in Poland. Its reassortment with other HPAI viruses circulating in Europe was confirmed by the simultaneous identification of four additional H5N1 genotypes (G3, G4, G5, and G6) with varying contributions of G1 in their gene constellations (Figure 2). For instance, genotype G6 discovered in Silesian Voivodeship as a single detection (MB078/22) shared only half of the genes with the G1 genotype, whereas in the case of G5 genotype (MB028/22, MB058/22) almost all gene segments except for NP grouped in a G1-like cluster (*Supplementary 3*). The latter could also be distinguished from the G4 genotype (MB042/22) by the lack of reassortment of the PB1 gene segment which represented the only structural difference between the two H5N1 genotypes (G4 and G5). In the case of the G3 genotype, discovered in November 2021 in the Greater Poland Voivodeship (MB490/21), reassortment of three gene segments (PB2, PA, and NA) was confirmed while maintaining the G1-like scaffold. In January 2022, a new G1 reassortant (herein referred to as G2) was first detected in a mute swan found dead in the Opole Voivodeship (MB034/22). A few months later, the presence of this genotype was also confirmed in samples from seabirds originating from the Baltic Sea regions suggesting that G2 has been in circulation since at least early 2022 without redetection until May–July of the same year (*Supplementary 1*). As a common feature of all aforementioned genotypes (G1–G6), the clustering of their MP and HA genes into one phylogenetically related group could be determined (Figure 2 and *Supplementary 3*). The most phylogenetically distant H5N1 genotype (G7) was represented by one sample (MB122/22) derived from a mute swan found dead near Lake Jemiołowo in the Warmian–Masurian Voivodeship in April 2022. The phylogenetic analysis confirmed the closest relationship of almost all of its viral segments to HPAIV sequences from China, South Korea, and Japan dated to late autumn 2021 (Figure 2 and *Supplementary 3*). However, the virus' MP gene segment clustered together with H5N*x* sequences from the two previous HPAI seasons in Poland indicating its reassortment.

#### 3.2.2. Passive and Active Surveillance—LPAIVs of Different Subtypes

For two active surveillance samples collected at the Jeziorsko artificial reservoir (P079w24/18 and P079w25/18), the presence of AIV coinfection including H3–H12-N5–N8 subtypes was confirmed (Table 1 and Figure 1). As a consequence, the genome-wide phylogenetic analysis encompassed the remaining seven swab samples collected in the frame of active surveillance between 2018 and 2021 and one low-pathogenic wild bird case from July 2022 (MB152/22) (*Supplementary 1*). No clear phylogenetic relationships between LPAIV samples collected in the same year at different locations in Poland were found. For the most part, the samples were located on divergent branches in the phylogenetic tree clustering with various LPAIVs of different subtypes (*Supplementary 3*). Given the geographical distribution of these samples, any reassortment and further evolutionary relationships could be ruled out.

### 3.3. Reassortment between LPAIVs and HPAIVs

No evidence of reassortment between active surveillance LPAIVs and HPAIVs was detected in Poland during the 2020/2021 and 2021/2022 avian influenza epidemics. Based on the branching pattern of the phylogenetic trees, the role of these LPAIVs as potential donors for internal gene segments of HPAIVs could be excluded (*Supplementary 3*).

### 3.4. Poultry–Wild Bird Connections

**Figure 3:**
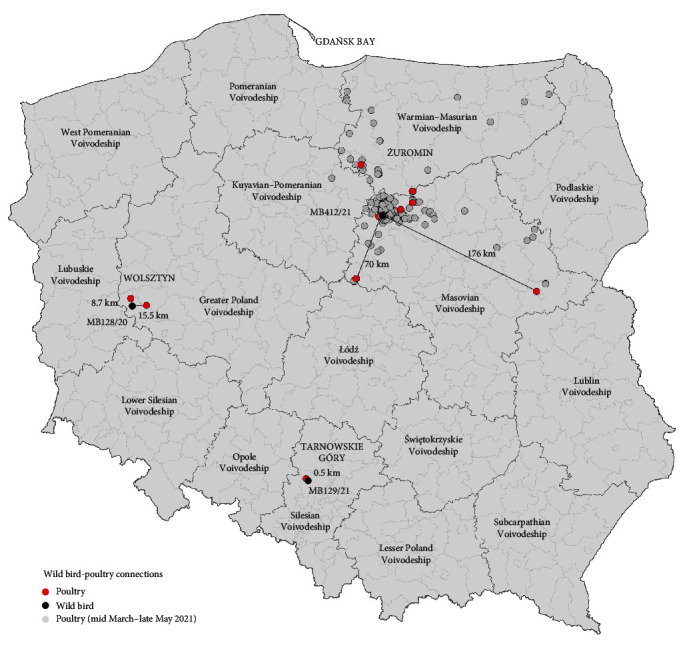
Geographical representation of phylogenetically confirmed epidemiological links between wild birds (black dots) and poultry farms (red dots) during the H5N*x* HPAI 2020/2021 epidemic in Poland. The high HPAIV contamination indicated by the enormous number of HPAIV outbreaks in poultry in three neighboring voivodeships in Poland (Masovian Voivodeship, Warmian–Masurian Voivodeship, Kuyavian–Pomeranian Voivodeship) between mid-March and May 2021 is presented (gray dots).

Despite the ongoing HPAI 2020/2021 epidemic in poultry since the 24 November 2020 in Poland, the first case of H5N8 HPAIV infection in a wild bird (MB128/20) was confirmed only 2 weeks later in Wolsztyn (Greater Poland Voivodeship). Given its bootstrap-supported 100% identity to HA sequences from H5N8-infected poultry (i.e., A/turkey/Poland/475/2020, A/chicken/Poland/476/2020) including further spatiotemporal relationships, an epidemiological link is very likely (Table 2 and Figure 3). Similarly, there may be a possible epidemiological connection between the buzzard (MB129/21) found dead near the poultry farm where H5N8 HPAIV infection was detected in February 2021 (only data for PB2 is available) (Table 2 and Figure 3). Both H5N8-positive wild birds, i.e., tundra bean goose (MB128/20) and buzzard (MB129/21) were found as a result of active searches for wild bird carcasses during epizootic investigations conducted in areas, where HPAIV outbreaks in poultry were confirmed at that time. Furthermore, the phylogenetic analysis revealed a bootstrap-supported wild bird-poultry connection between a white stork sample (MB412/21) and multiple H5N8 HPAIVs detected in late March–May 2021 on poultry farms (i.e., chicken and turkey flocks) located mostly in the area with the highest poultry density in Poland, such as Żuromin (Masovian Voivodeship). The phylogenetic clustering of H5N8 HPAIV sequences originating from Żuromin-distant poultry farms (i.e., H1168, H1184, and H1289) indicates further virus spread beyond the Żuromin district with the highest HPAIV contamination, probably caused by human activity (Table 2, Figure 3, and *Supplementary 3*).

### 3.5. Different Virus Introductions during the HPAI 2020/2021 Season

**Figure 4:**
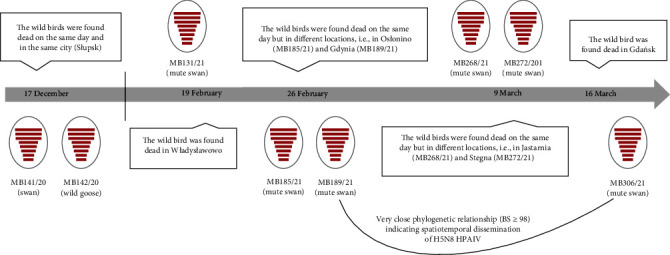
Timeline representing different H5N8 HPAIV introductions observed in Poland during the 2020/2021 H5N*x* HPAI season. Vertical bar demarcates between HPAIVs detected in December 2020 in north-western Poland (Słupsk, West Pomeranian Voivodeship) and HPAIVs identified over 3.5 weeks in February–March 2021 in north-central Poland (along Gdańsk Bay, Pomeranian Voivodeship).

At the beginning of 2021, mass mortality of mute swans along Puck Bay (part of Gdańsk Bay) in Poland was reported. The sequencing results confirmed the occurrence of at least five independent H5N8 HPAIV introductions over a period of 3.5 weeks (February–March) into this region (Figure 4, *Supplementary 3*, and *Supplementary 4*). The significant sequence diversity of investigated samples generally precludes, with the exception of two samples, any common epidemiological and evolutionary links. BLAST results for individual AIV gene segments showed a great variety regarding the geographical distribution of the most related sequences which strongly supports the hypothesis about separate virus incursions in the Pomeranian Voivodeship in Poland (Table 3). In contrast, a very close bootstrap-supported phylogenetic relationship (BS ≥ 98) was found between two samples (MB189/21 and MB306/21) submitted within 2.5 weeks from different seaside towns located 40 km apart (Figure 4 and *Supplementary 3*). This finding confirmed the ongoing circulation of H5N8 HPAIV in the swan population and its further spread in the southeastern bay of the Baltic Sea.

The evidence of more than one virus introduction in the Pomeranian Voivodeship existed already at the beginning of the HPAI 2020/2021 season, when the phylogenetic analysis of two viruses from anseriform bird samples collected on the 17 December 2020 in Słupsk suggested their incursions from different geographical origins (Figure 4 and *Supplementary 3*). BLAST top hits showed the closest relationship with sequences from Germany (MB142/20) or other countries located in the Baltic and North Sea region (MB141/20).

### 3.6. Molecular Analysis including AIV Genetic Markers

The high pathogenicity of HPAIV H5 samples collected over two HPAI seasons in Poland was confirmed by the presence of an identical polybasic cleavage site (PLREKRRKR/GLF). However, sequencing of two mute swan samples (MB490-L1/21 and MB122/22) revealed their unique amino acid sequences, i.e., SLREKRRKR/GLF and PLRERRRKR/GLF, respectively. LPAIVs of different subtypes displayed a variety of hemagglutinin cleavage site (HACS) motifs, including single amino acid (aa) differences within the subtype as detected in H9 samples. Interestingly, in H3 viruses the HACS did not change despite the spatiotemporal differences between all samples included in the analysis (*Supplementary 5*).

Molecular characterization of all sequences allowed the identification of genetic markers affecting AIV biological properties [30–32], especially those associated with virulence, polymerase activity and virus replication, antiviral response, or virus receptor binding (Table 4). If specific mutations were present in the LPAIVs, their simultaneous detection could often be confirmed in most H5 HPAIV sequences (Table 4). However, seasonal patterns in the maintenance of mutations within internal gene segments were noticeable among HPAIVs of the H5 subtype. For instance, the PB2-I292V [34, 35] mutation or PB1-F2 truncation could be found only in H5N8 sequences from the HPAI 2020/2021 season, while other nonsynonymous substitutions, e.g., N66S in PB1-F2 [43, 44] or Q400P in PA genes [46] were detected exclusively during the following HPAI H5N1 2021/2022 epidemic. Furthermore, no genetic markers within M2 and NS2/NEP proteins were identified as well as very little evidence of the possible antiviral resistance [73] was determined (*Supplementary 6*).

## 4. Discussion

The 2020/2021 HPAI H5N*x* epidemic in Europe was the most severe in terms of the number and duration of outbreaks in poultry, captive, and wild birds [74]. Poland was one of the most affected countries within the European Union, with an estimated loss of 14 million domestic birds culled as a consequence of numerous HPAI H5N8 outbreaks in poultry [10]. Since then, the previously significant seasonality of HPAI has become less prominent with regard to the continuous H5N*x* outbreaks occurring throughout the summer months in 2021, followed by the emergence of a new H5N1 HPAIV of 2.3.4.4b clade and its continued spatiotemporal spread in Europe. It raises concerns about the possibility of HPAI becoming endemic [75].

Similarly to other European countries [76–78], the HPAI H5N*x* 2020/2021 season in Poland was dominated by the H5N8 subtype detected in nearly all investigated samples from wild and domestic birds. The H5N8 sequences were phylogenetically related to viruses circulating at this time on the European continent and formed one genotype identified in Poland repeatedly until August 2021. In contrast to the reports from Germany [76], the Netherlands [77], and the United Kingdom [78], demonstrating the presence of a variety of different HPAIV subtypes, e.g., H5N1, H5N3, H5N4, and H5N5 including their genotypes or even LPAIV detections, only two non-H5N8 HPAIV wild bird cases were detected in Poland in spring 2021. This includes the H5N1 white stork case and one H5N5 detection in a tufted duck, both showing high relatedness of their HA and MP gene segments to the dominant H5N8 genotype present in Poland (Figure 2). A similar observation regarding the preservation of these genes with simultaneous frequent interchangeability of the remaining segments of the AIV genome resulting in novel reassortants has already been discussed for the HPAI H5N*x* 2020/2021 season [76] and applies also to the Polish HPAIV H5N*x* strains. Furthermore, the tendency has been retained in the following HPAI H5N1 2021/2022 epidemic as multiple reassortment events in at least six Polish H5N1 genotypes (G1–G6) involved particularly gene segments other than HA and MP (Figure 2). A closer insight into the genome configuration of these viruses disclosed a significant contribution of the G1 in the scaffold of the remaining genotypes which might be associated with its comparable largest geographical distribution and ongoing circulation in Poland until February 2021. The presumed frequent occurrence of the G1 genotype could have promoted its progressive segment gene exchange with other viruses, resulting in new low-frequency reassortants, i.e., sporadically detected at different locations and time points (G3–G6). The genome-wide phylogenetic analysis of the six Polish H5N1 genotypes showed high similarity to viruses circulating to a large extent in other countries of the European continent. Conversely, the last genotype (G7) identified in spring 2022 in a mute swan was in a close phylogenetic relationship with HPAIVs of Asian origin, especially those identified in late autumn 2021 in South Korea [79], China [80], and Japan (*Supplementary 3*). Following the logic of bird migrations, it seems very likely that the virus was introduced from Asia to Europe as early as late summer 2021, circulated in the environment as a minority variant, and initially remained undiscovered. The clustering of the virus' MP gene with the Polish H5N*x* HPAI sequences from 2020 to 2021 supports the hypothesis about its silent circulation until the virus increased its prevalence in the current virus population becoming detectable while simultaneously undergoing reassortment with other AIV strains. However, its prolonged circulation did not result in further G7 observations either in poultry or wild birds.

Apart from the genotype analysis, one possible way of the emergence of new HPAIVs in Poland has been looked further into. In general, two different pathways are relevant, such as a rapid transition of LPAIV to HPAIV via the acquisition of a polybasic HACS or reassortment between HPAIVs and LPAIVs [7]. Since LPAIVs of H5 and H7 subtypes have not been detected in poultry in Poland since 1995 [81] and no emergence of HPAIV from a low-pathogenic progenitor has been recorded so far, the main emphasis has been placed on the investigation of the possible reassortment events between LPAI viruses from active surveillance and HPAIVs H5N*x* from 2020 to 2022. Based on the sampling time, only five LPAIVs of various subtypes circulated in different Voivodeships in Poland (*n* = 5) in the period of August 2020 and February 2021 might have served as potential internal gene donors during the HPAI H5N*x* 2020/2021 epidemic (*Supplementary 1*). Despite the strong spatiotemporal correspondence of one H9N7 LPAIV (P075/21 collected from a black-headed gull in Gdańsk, 9 February 2021) to H5N8 HPAIVs from the Pomeranian Voivodeship, no exchanges of internal genes between the strains were discovered, but they cannot be ruled out.

Nevertheless, the phylogenetic analysis enabled the identification of at least five independent H5N8 HPAIV introductions into Gdańsk Bay associated with unprecedented mass mortality among swans observed from February 2021 (Figure 4). Along with Germany and Denmark, Poland was at this time one of the countries with the highest number of HPAIV-related wild bird deaths with a noticeable peak in detections between February and March 2021 [82]. As already discussed [10], the significant decrease in outdoor temperatures can be seen as the most likely reason for the massive influx of wild birds from different parts of Europe to urban areas around Gdańsk as these human-adjacent habitats might facilitate the birds' survival through better access to food and water. As a consequence, diverse H5N8 viruses of different origins had been circulating within a few weeks in a small geographical area favoring further virus spread or even reassortment with locally disseminated LPAIVs (Table 3). As an example, the minimal nucleotide difference between the viruses detected in swans within 2.5 weeks at distanced locations (MB189/21, MB306/21) excludes separate virus introductions, but, on the contrary, indicates within-host evolution of the same H5N8 HPAIV and thus strongly supports the hypothesis about local virus transmission between birds (Figure 4, Table 3, and *Supplementary 3*). Interestingly, despite the very small number of wild birds reported dead in the first phase of the H5N*x* 2020/2021 epidemic in Poland, two separate H5N8 HPAIV introductions were captured on the same day in the same town in northern Poland (MB141/20, MB142/20) (Figure 4). These findings suggest that the observed low HPAIV detection rate in wild birds does not necessarily coincide with the degree of genetic diversity of circulating viruses.

Further sequence analysis of the Polish HPAIVs showed at least three likely epidemiological links between wild and domestic birds during the H5N*x* HPAI 2020/2021 epidemic (Table 2 and Figure 3). Although for tundra bean goose (MB128/20) and buzzard (MB129/21), the phylogenetic analysis was based on single genes only, the results of epizootic investigations and the spatial proximity of the wild birds to HPAI outbreaks in poultry support the suspicion of an epidemiological connection (Table 2 and Figure 3). It seems particularly significant that the wild bird carcasses, MB128/20 and MB129/21, were not accidental findings, but results of active searches initiated immediately after confirmation of HPAIV outbreaks in Wolsztyn (Greater Poland Voivodeship) and Tarnowskie Góry (Silesian Voivodeship), respectively. In general, the main cause of HPAI outbreaks in poultry at the beginning of the epidemic in Poland was identified as primary virus introductions via wild birds and to some extent secondary virus spread [10]. Hence, the confirmation of two H5N8 HPAIV outbreaks in poultry in Wolsztyn (A/turkey/Poland/475/2020, A/chicken/Poland/476/2020) within a short period of time and their close phylogenetic relationship to the tundra bean goose (MB128/20), even if the wild bird carcass was found several days later, corresponds to this route of virus transmission. However, the phylogenetic relationship of the HPAI outbreak in Tarnowskie Góry (A/turkey/Poland/116/2021) with the buzzard found dead more than one week later within the HPAI 3 km protection zone (MB129/21) does not fit into the aforementioned epidemiological scenario. In this case, the buzzard's predatory behavior in the HPAIV-contaminated area could have led to its viral infection rather than any direct contact between the wild and domestic birds. This can be corroborated by the fact that tracheal and cloacal swabs taken from the buzzard's carcass tested positive for H5N8 HPAIV indicating early-stage virus infection which occurred with high probability later than the HPAI outbreak in the nearby turkey farm. Over time, at the end of March 2021, the H5N8 HPAIV was introduced into high poultry density areas such as Żuromin and Mława in the Masovian Voivodeship causing the most drastic economic losses to the poultry industry to date in Poland [10]. The H5N8 sequences from the Żuromin district obtained from late-March to mid-May 2021 clustered together with the white stork case (MB412/21, collected in May 2021) suggesting one initial source of H5N8 HPAIV introduction resulting in multiple HPAI outbreaks due to secondary virus spread via human activity. The occurrence of HPAIV outbreaks in locations distant from the Żuromin district, but showing high phylogenetic relatedness to the H5N8 HPAIV present in this region, i.e., H1168, H1184, and H1289 (Table 2 and Figure 3), can be considered even greater evidence of human contribution to the scale of this epidemic. However, secondary infections of wild birds living in the highly HPAIV-contaminated zone around Żuromin and their involvement in the chain of further virus spread, i.e., bidirectional virus transmission (wild bird-to-poultry and poultry-to-wild bird transmission) over this period are also very likely. Given the collection date of the white stork sample (MB412/21), it seems very plausible that the second mentioned route of transmission, i.e., HPAIV spillover from poultry to wild birds, took place.

Taking into account the reports of HPAIV infections in various mammal species in other European countries [83–85], a comprehensive molecular analysis of the Polish virus strains was carried out regarding pathogenicity and possible zoonotic potential. Apart from the polybasic HACS being considered one of the most important virulence determinants of AIVs, multiple other genetic markers impacting virus-host interactions and infection dynamics were identified [30–32] (Table 4 and *Supplementary 6*). These results are in line with the findings published by Byrne et al. [78] and highlight the general tendency of current H5N*x* viruses to accumulate specific mutations, especially those associated with the ability to break interspecies barrier, e.g., increasing virus binding to *α*2,6 receptors, or polymerase activity in mammalian cells (Table 4). Interestingly, most of these mutations have been already present in LPAIVs circulating in Poland since 2018 which proves that the process of their fixation was not directly related to the large scale of the H5N*x* HPAI 2020/2021 epidemic but started much earlier.

Nonetheless, the discussed results must be seen in light of some limitations. Since the main criterion for a sample to be included in the study was its sequencing suitability considered as a proper *C*_*q*_ value (*C*_*q*_ < 30, RT-qPCR against the M gene), the obtained data may not entirely reflect the epidemiological situation in Poland, e.g., in terms of the number of genotypes and subtypes detected. Due to regional differences in passive surveillance efforts regarding the intensity and regularity of wild bird carcass searches, there is a very likely underestimation of the number of AIV-positive wild animals and geographical sampling bias. In addition, the lack of clear wild bird-poultry connections during the HPAI H5N*x* 2021/2022 epidemic is due to the ongoing whole genome sequencing of HPAI outbreaks in poultry at the time of writing this manuscript which disallowed the analysis but does not preclude such epidemiological links. Future studies should consider increasing both the effectiveness of passive and active surveillance strategies and the sequencing capacity which may translate into a deeper understanding of the HPAIV spread dynamics in Poland.

## 5. Conclusions

The study results fill in the knowledge gap regarding the phylogenetic and molecular characteristics of wild bird-origin AIVs circulating in Poland between 2018 and 2022. In addition to an increasing number of mutations associated with zoonotic potential, AIVs showed both various genetic compositions as multiple reassortants and a high genetic diversity within a subtype. Recognizing wild birds as an important source of virus introductions into poultry farms, more effort needs to be put into active and passive AIV surveillance in Poland in order to allow a rapid response to emerging viruses. Currently, with the increasing number of AIV cases in mammals, the continuous monitoring of AIVs in avifauna becomes at the same time an essential part of human safety protection in the context of the One Health concept.

## Figures and Tables

**Table 1:** A summary of AIV-positive wild bird samples included in the present study.

Sample source	Collection period	AIV subtype	Host species	Sample matrix	Total
Active surveillance	2018–2021	LPAIV H3N8	Mallard duck	Cloacal and/or oropharyngeal swab	2
		LPAIV H3-H12-N5-N8^*∗*^	Common teal		2
		LPAIV H5N2	Mallard duck		1
		LPAIV H9N2	Mute swan, mallard duck		2
		LPAIV H9N7	Black-headed gull		1
		LPAIV H16N3	Herring gull		1

Passive surveillance	2020–2021	HPAIV H5N1	White stork	Internal organs including brain tissue	1
		HPAIV H5N5	Tufted duck		1
		HPAIV H5N8	Tundra bean goose (Mute) swan Wild goose buzzard White stork		18

Passive surveillance	2021–2022	LPAIV H2N3	Swan	Internal organs including brain tissue	1
		HPAIV H5N1	(Mute) swan Greylag goose Crane Mallard duck Hawk White-fronted goose Herring gull Black-headed gull Sandwich tern Common tern Common murre		22

An asterisk ( ^*∗*^) indicates coinfection of two different AIV subtypes.

**Table 2:** Epidemiological links between H5N8 HPAIV outbreaks in wild birds and poultry during the H5N*x* HPAI 2020/2021 season in Poland.

Wild bird (collection date, species)	Poultry (collection date)	Distance (km)	Suspected virus transmission route
MB128/20 (8 December 2020, tundra bean goose)	A/turkey/Poland/475/2020 (3 December 2020)	15.5	Wild bird-to-poultry-spillover of HPAIV as the primary virus introduction, followed by a secondary virus spread within the area
	A/chicken/Poland/476/2020 (4 December 2020)	8.7	

MB129/21 (19 February 2021, buzzard)	A/turkey/Poland/116/2021 (11 February 2021)	0.5	Poultry-to-wild bird spillover of HPAIV due to the buzzard's predatory behavior in the HPAIV-contaminated area

MB412/21 (2 May 2021, white stork)	A/chicken/Poland/H293/2021 (23 March 2021)	7.3	Poultry-to-wild bird spillover of HPAIV due to the high infection pressure resulting from the massive environment contamination with HPAIV
	A/chicken/Poland/H712/2021 (16 April 2021)	19.3	
	A/chicken/Poland/H928/202 (25 April 2021)	39.7	
	A/chicken/Poland/H984/2021 (28 April 2021)	9.5	
	A/chicken/Poland/H1084/2021 (07 May 2021)	33.7	
	A/chicken/Poland/H1161/2021 (13 May 2021)	4.5	
	A/turkey/Poland/H1168/2021 (13 May 2021)	56	
	A/turkey/Poland/H1184/2021 (14 May 2021)	70	
	A/turkey/Poland/H1289/2021 (24 May 2021)	176	

Straight line distance between HPAIV-positive poultry farms and collection spots of dead wild birds is given. The sequence names of H5N8 HPAIV obtained from poultry include the host species.

**Table 3:** Country or countries of origin of the most related sequences (BLAST top hits) to the H5N8 HPAIVs identified along Gdańsk Bay (Pomeranian Voivodeship) in February–March 2021.

Segment	MB131/21	MB185/21	MB189/21	MB268/21	MB272/21	MB306/21
PB2	CZ/RU	SE	NL	ES/CZ	DE	NL
PB1	RU	FI/SE/CZ/NL	NL	CZ	DE	NL
PA	RU	SE	ES/NL/CZ/SE	CZ	DE	ES/NL/CZ/SE
HA	RU/NL	SE/NL/CZ	NL	ES/CZ	DE	NL
NP	DE	FI/EE/SE	NL	CZ	DE	NL
NA	DE/CZ/RU	FI/SE/LV/NL	NL	CZ	DE	NL
MP	DE/CZ	SE/NL	NL	CZ/DE	DE	NL
NS	RU/DE/CZ	SE/FI/EE	DE/NL/CZ	DE/NL/CZ	DE	DE/NL/CZ

BLAST top hits originating from more than one country were listed in the order of decreasing similarity to the Polish sequences (GISAID database status from November 2022). Two-letter country codes were used: CZ, Czechia; RU, Russian Federation; NL, Netherlands; DE, Germany; SE, Sweden; FI, Finland; EE, Estonia; LV, Latvia; and ES, Spain.

**Table 4:** Molecular markers changing AIV biological properties [30–32] detected in HPAIV sequences of the H5 subtype in Poland from 2020 to 2022.

Segment	Mutation	*N*	Phenotype
PB2	I292V ^*∗*^	18	Polymerase activity in mammalian cells [34, 35] ↑, virulence in mice ↑ [34]
PB2	K389R ^*∗*^	40	Polymerase activity and replication in mammalian cells ↑ [36]
PB2	K526R	1	Polymerase activity in mammalian cells ↑ [37]
PB2	V598T ^*∗*^	41	Polymerase activity and replication in mammalian cells ↑, virulence in mice ↑ [36]
PB2	S715N ^*∗*^	42	Virulence in mice ↓ [38]
PB2	L89V + G309D ^*∗*^	38	Polymerase activity in mammalian cells ↑, virulence in mice ↑ [39]
PB2	L89V + G309D + T339K + R477G + I495V + K627E + A676T ^*∗*^	35	Polymerase activity in mammalian cells ↑, virulence in mice ↑ [39]
PB1	D3*V* ^*∗*^	42	Polymerase activity and viral replication in avian and mammalian cells [40]
PB1	D622G ^*∗*^	42	Polymerase activity and virulence in mice ↑ [41]
PB1	S678N	1	Replication in avian and mammalian cells ↑ [42]
PB1-F2	N66*S* ^*∗*^	21	Viral replication, antiviral response, and virulence in mice ↑ [43, 44]
PA	S37*A* ^*∗*^	42	Polymerase activity in mammalian cells ↑ [45]
PA	K158R	2	Polymerase activity in mammalian cells ↑ [40]
PA	P190S ^*∗*^	41	Virulence in mice ↓ [46]
PA	N383D ^*∗*^	41	Polymerase activity in avian and mammalian cells ↑ [47, 48]
PA	Q400*P*	12	Virulence in mice ↓ [46]
PA	N409S ^*∗*^	42	Polymerase activity in mammalian cells ↑ [45]
HA	S123*P*	41	Virus binding to *α*2,6 receptors ↑ [49]
HA	S133A ^*∗*^	41	Pseudovirus binding to *α*2,6 receptors ↑ [50]
HA	S154N	42	Virus binding to *α*2,6 receptors ↑ [51]
HA	T156A ^*∗*^	41	Virus binding to *α*2,6 receptors ↑, transmission to guinea pig ↑ [51, 52]
HA	V182N ^*∗*^	41	Virus binding to *α*2,6 receptors ↑, Virus binding to *α*2,3 receptors ↓ [53]
HA	V210I ^*∗*^	1	Virus binding to *α*2,6 receptors ↑ [54]
HA	K394E ^*∗*^	42	Fusion pH ↑, HA stability ↓, virulence in mice ↓ [55]
HA	S107R + T108I	41	Fusion pH ↑, Virulence in chickens and mice ↑ [56]
HA	K218Q + S223R	42	Virus binding to *α*2,3 and *α*2,6 receptors ↑ [57]
NP	I41V	1	Polymerase activity in mammalian cells ↑ [58]
NP	M105V ^*∗*^	30	Virulence in chickens ↑ [59]
NP	A184K ^*∗*^	42	Replication in avian cells ↑, virulence in chickens ↑, host immune response ↑ [60]
NP	N319K	2	Polymerase activity and replication in mammalian cells [61, 62] ↑
M1	N30*D* ^*∗*^	42	Virulence in mice ↑ [63]
M1	I43*M* ^*∗*^	42	Virulence in mice, chickens, and ducks ↑ [64]
M1	T215A ^*∗*^	42	Virulence in mice ↑ [63]
NS1	P42*S* ^*∗*^	41	Virulence in mice ↑, antiviral immune response in mice ↓ [65]
NS1	I106M ^*∗*^	42	Virus replication and virulence in mice ↑ [66]
NS1	C138F ^*∗*^	42	Virus replication in mammalian cells ↑, interferon response ↓ [67]
NS1	V149A ^*∗*^	42	Virulence in chickens ↑, interferon response in avian cells ↓ [68]
NS1	L103F + I106M ^*∗*^	41	Virulence in mice ↓ [69]
NS1	K55E + K66E + C138F ^*∗*^	22	Virus replication in mammalian cells ↑, interferon response ↓ [67]
NS1	^227^ESEV^230^ (PDZ domain) ^*∗*^	22	Virus replication in mice ↑ [70], virulence in mice ↑ [70, 71], and virus replication in mammalian and avian cells ↓ [72]

Asterisk ( ^*∗*^) indicates mutations present also in LPAIVs from Poland. *N* = number of HPAIV sequences having the mutation, ↑ = increased, ↓ = decreased. For mutations identified in HA gene, H5 numbering was used.

## Data Availability

The data and material used to support the findings of this study are included in the supplementary information files. LPAIV and HPAIV sequences from Poland were deposited in the GISAID database (https://gisaid.org/) under accession numbers listed in *Supplementary 1*.

## Supplementary Materials

Supplementary 1 Detailed sample list.

Supplementary 2 Sequence alignments.

Supplementary 3 Phylogenetic trees.

Supplementary 4 Different H5N8 introductions.

Supplementary 5 HACS motifs.

Supplementary 6 Genetic markers.
