# Supplementary material for: Phylogenetic and Molecular Characteristics of Wild Bird-Origin Avian Influenza Viruses Circulating in Poland in 2018−2022: Reassortment, Multiple Introductions, and Wild Bird–Poultry Epidemiological Links
Source: Transbound Emerg Dis. 2024 Apr 12;2024:6661672. doi: 10.1155/2024/6661672 (PMC12017110; doi:10.1155/2024/6661672)
Supplement: Supplementary 1 — Detailed sample list. [file 6661672.f1.pdf]

Group	No.	Surveillance type	Sample ID	Sequence name	Isolate ID	Collection/Submission date	City (incl. further details)	County	Voivodeship	Species	Sample type	Subtype/genotype
ACTIVE SURVEILLANCE 2018-2021 (LPAIVs)	1	active	P074/18 w5	A/mallard/Poland/P074w5/2018	EPI_ISL_17978924	05.09.2018	Darłowo	sławieński	West Pomeranian Voivodeship	mallard duck	cloacal swab	H3N8
	2	active	P077/18 w1	A/mallard/Poland/P077w1/2018	EPI_ISL_17978925	12.09.2018	Braniewo	braniewski	Warmian-Masurian Voivodeship	mallard duck	cloacal swab	H5N2
	3	active	P079/18 w24	A/common_teal/Poland/P079w24_mixed_H3N8/2018<br>A/common_teal/Poland/P079w24_mixed_H12N5/2018	EPI_ISL_17982396<br>EPI_ISL_17982398	14.09.2018	Jeziorsko (artificial reservoir)	sieradzki	Łódź Voivodeship	common teal	cloacal swab	H3-H12-N8-N5 (co-infection)
	4	active	P079/18 w25	A/common_teal/Poland/P079w25_mixed_H3N8/2018<br>A/common_teal/Poland/P079w25_mixed_H12N5/2018	EPI_ISL_17982400<br>EPI_ISL_17982402	14.09.2018	Jeziorsko (artificial reservoir)	sieradzki	Łódź Voivodeship	common teal	cloacal swab	H3-H12-N8-N5 (co-infection)
	5	active	P060/20 w2	A/mallard/Poland/P060w2/2020	EPI_ISL_17978926	25.08.2020	Jawor	jaworski	Lower Silesian Voivodeship	mallard duck	cloacal swab	H3N8
	6	active	P071/20 w1	A/mute_swan/Poland/P071/2020	EPI_ISL_17978927	18.09.2020	Jeziorsko (artificial reservoir)	sieradzki	Łódź Voivodeship	mute swan	cloacal swab	H9N2
	7	active	P092/20	A/herring_gull/Poland/P092/2020	EPI_ISL_17978928	28.10.2020	Jarosławiec	sławieński	West Pomeranian Voivodeship	herring gull	cloacal swab	H16N3
	8	active	P096/20 w2	A/mallard/Poland/P096/2020	EPI_ISL_17978951	02.11.2020	Olsztyn	olsztyński	Warmian-Masurian Voivodeship	mallard duck	cloacal swab	H9N2
	9	active	P075/21	A/black-headed_gull/Poland/P075/2021	EPI_ISL_17978956	09.02.2021	Gdańsk	gdański	Pomeranian Voivodeship	black-headed gull	cloacal swab	H9N7
HPAI 2020-2021 (H5N8, H5N5, H5N1)	10	passive	MB132/20	A/tundra_bean_goose/Poland/MB132/2020	EPI_ISL_1220094	11.12.2020	Gryfino	gryfiński	West Pomeranian Voivodeship	tundra bean goose	internal organs (liver, duodenum, pancreas, lungs), tracheal + cloacal swab*	H5N8
	11	passive	MB141/20	A/swan/Poland/MB141/2020	EPI_ISL_846623	17.12.2020	Słupsk	słupski	Pomeranian Voivodeship	swan	internal organs (spleen, kidneys, lungs, liver, duodenum, pancreas, trachea + oropharynx)	H5N8
	12	passive	MB142/20	A/wild_goose/Poland/MB142/2020	EPI_ISL_846624	17.12.2020	Słupsk	słupski	Pomeranian Voivodeship	wild goose	cloacal + tracheal swab*, internal organ (spleen, kidneys, liver, pancreas), intestines	H5N8
	13	passive	MB021/21	A/mute_swan/Poland/MB021-21-22VIR5675-11/2021	EPI_ISL_18244181	19.01.2021	Zławieś Wielka	toruński	Kuyavian-Pomeranian Voivodeship	mute swan	internal organs (lungs, spleen, kidneys, liver), intestines	H5N8
	14	passive	MB061/21	A/tufted_duck/Poland/MB061/2021	EPI_ISL_1255051	03.02.2021	Mielno	koszaliński	West Pomeranian Voivodeship	tufted duck	internal organs (lungs, trachea), tracheal + cloacal swab*	H5N5
	15	passive	MB122/21	A/swan/Poland/MB122-21_22VIR5675-9/2021	EPI_ISL_18244493	18.02.2021	Kórnickie Lake (Kórnik)	poznański	Greater Poland Voivodeship	swan	parenchymal organs, intestines	H5N8
	16	passive	MB129/21	A/buzzard/Poland/MB129-21_22VIR5675-7/2021	EPI_ISL_18244810	19.02.2021	Brynek (Tworóg Commune)	tarnogórski	Silesian Voivodeship	buzzard	cloacal + tracheal swab*	H5N8
	17	passive	MB131/21	A/mute_swan/Poland/MB131/2021	EPI_ISL_2111625	19.02.2021	Władysławowo (Puck Bay)	pucki	Pomeranian Voivodeship	mute swan	interal organs (trachea, liver, kidney, lung, heart, spleen)	H5N8
	18	passive	MB185/21	A/mute_swan/Poland/MB185/2021	EPI_ISL_2199986	26.02.2021	Beka-Osłonino	pucki	Pomeranian Voivodeship	mute swan	internal organs (spleen, lung, kidney, intestines, heart, liver, trachea)	H5N8
	19	passive	MB189/21	A/mute_swan/Poland/MB189/2021	EPI_ISL_2113167	26.02.2021	Gdynia (Beach Redłowo)	pucki	Pomeranian Voivodeship	mute swan	internal organs	H5N8
	20	passive	MB268/21	A/mute_swan/Poland/MB268/2021	EPI_ISL_2113493	09.03.2021	Jastarnia (Puck Bay, beach)	pucki	Pomeranian Voivodeship	mute swan	internal organs (heart, spleen, brain, trachea, kidney, liver, lungs), intestines	H5N8
	21	passive	MB272/21	A/mute_swan/Poland/MB272/2021	EPI_ISL_2114013	09.03.2021	Stegna (Stegna Commune)	nowodworski	Pomeranian Voivodeship	mute swan	oropharyngeal + cloacal swab*, internal organs, intestines	H5N8
	22	passive	MB277/21	A/buzzard/Poland/MB277-21_22VIR5675-18/2021	EPI_ISL_18244823	09.03.2021	Wola Ładowska (Iłów Commune)	sochaczewski	Masovian Voivodeship	buzzard	tracheal + cloacal swab*, brain tissue	H5N8
	23	passive	MB292/21	A/mute_swan/Poland/MB292-21_22VIR5675-14/2021	EPI_ISL_18245197	11.03.2021	Sarbinowo (Mielno Commune)	koszaliński	West Pomeranian Voivodeship	mute swan	internal organs (trachea, lungs, liver, kidney, spleen), brain tissue	H5N8
	24	passive	MB306/21	A/mute_swan/Poland/MB306-21_22VIR5675-20/2021	EPI_ISL_18245531	16.03.2021	Gdańsk (Przegalińska Street 115-131, Martwa Wisła river)	gdański	Pomeranian Voivodeship	mute swan	internal organs (lung, trachea), intestines, brain tissue (mute swan no.1)	H5N8
	25	passive	MB363/21	A/white_stork/Poland/MB363-21_22VIR5675-15/2021	EPI_ISL_18245534	01.04.2021	Możne 2G (Olecko Commune)	olecki	Warmian-Masurian Voivodeship	white stork	brain, internal organs, intestines	H5N8
	26	passive	MB372/21	A/mute_swan/Poland/MB372-21_22VIR5675-19/2021	EPI_ISL_18245535	07.04.2021	Jezioro Samołęskie (Wronki Commune)	szamotulski	Greater Poland Voivodeship	mute swan	internal organs (liver, spleen, kidney, lung, trachea), intestines, brain, cloacal + oropharyngeal swab*	H5N8
	27	passive	MB391/21	A/white_stork/Poland/MB391/2021	EPI_ISL_2681045	20.04.2021	Łysaków Drugi	jędrzejowski	Świętokrzyskie (Holy Cross) Voivodeship	white stork	cloacal + tracheal swab*, brain tissue	H5N1
	28	passive	MB396/21	A/mute_swan/Poland/MB396_21RS1385-19/2021	EPI_ISL_3102078	21.04.2021	Morzycko Lake	gryfiński	West Pomeranian Voivodeship	mute swan	internal organs, oropharyngeal + cloacal swab*	H5N8
	29	passive	MB412/21	A/white_stork/Poland/MB412_21RS1385-11/2021	EPI_ISL_3102070	02.05.2021	Bieżuń (at the end of the Sierpecka Street on the right)	żuromiński	Masovian Voivodeship	white stork	oroparyngeal + cloacal swab*, brain	H5N8
HPAI 2021-2022 (H5N1) + LPAIV H2N3	30	passive	MB490/21	A/mute_swan/Poland/MB490-L1/2021	EPI_ISL_6937114	09.11.2021	Koło	kolski	Greater Poland Voivodship	mute swan	oropharyngeal + cloacal swab*, internal organs (heart, liver, lungs), intestines	H5N1 (G3)
	31	passive	MB503/21	A/greylag_goose/Poland/MB503_21RS3290-18/2021	EPI_ISL_18245810	22.11.2021	Rybakówka	siedlecki	Masovian Voivodeship	greylag goose	internal organs, intestines	H5N1 (G1)
	32	passive	MB528/21	A/crane/Poland/MB528/2021	EPI_ISL_18245811	10.12.2021	Gledzianówek 25	łęczycki	Łódź Voivodeship	crane	tracheal + cloacal swab*, brain	H5N1 (G1)
	33	active (virological)	MW542/21	A/wild_bird/Poland/MW542/2021	EPI_ISL_18245812	20.12.2021	Turek (Turek Commune)	turecki	Greater Poland Voivodeship	mallard duck	cloacal swab -> virus isolate	H5N1 (G1)
	34	passive	MB544/21	A/hawk/Poland/MB544/2021	EPI_ISL_18245813	22.12.2021	Parzęczew (Ozorkowska Street 10, 95-045)	zgierski	Łódź Voivodeship	hawk	tracheal + cloacal swab*, brain	H5N1 (G1)
	35	passive	MB550/21	A/mute_swan/Poland/MB550/2021	EPI_ISL_18245814	23.12.2021	Niecemino Lake (Polanów Commune)	koszaliński	West Pomeranian Voivodeship	mute swan	tracheal + cloacal swab*, internal organs (lung, liver, spleen, kidney), intestines	H5N1 (G1)
	36	passive	MB551/21	A/mute_swan/Poland/MB551/2021	EPI_ISL_18245815	23.12.2021	Świecie (86-105)	świecki	Kuyavian-Pomeranian Voivodship	mute swan	brain, interal organs (trachea, lungs, liver, pancreas, kidney, spleen), intestines	H5N1 (G1)
	37	passive	MB008/22	A/mute_swan/Poland/MB008-22_22VIR5675-16/2022	EPI_ISL_18245597	05.01.2022	Końskie (pond in the Browarna Street)	konecki	Świętokrzyskie (Holy Cross) Voivodship	mute swan	internal organs (heart, liver, lungs), intestines, cloacal + tracheal swab*	H5N1 (G1)
	38	passive	MB020/22	A/mute_swan/Poland/MB020-22_22VIR5675-3/2022	EPI_ISL_18245613	11.01.2022	Mostowo (Rosnowskie Lake, Manowo Commune)	koszaliński	West Pomeranian Voivodship	mute swan	internal organs (lungs, spleen, kidney, liver), intestines, tracheal + cloacal swab*, brain	H5N1 (G1)
	39	passive	MB028/22	A/white-fronted_goose/Poland/MB028-22_22VIR5675-1/2022	EPI_ISL_18245742	14.01.2022	Kruszwica (88-150, Gopło Lake)	inowrocławski	Kuyavian-Pomeranian Voivodship	white-fronted goose	internal organs (pancreas, lungs, trachea, spleen, liver, kidney), intestines, brain	H5N1 (G5)
	40	passive	MB034/22	A/mute_swan/Poland/MB034-22_22VIR5675-12/2022	EPI_ISL_18245743	18.01.2022	Michalice (pond, Namysłów Commune)	namysłowski	Opole Voivodship	mute swan	internal organs (liver, spleen, trachea, kidney), intestines, brain	H5N1 (G2)
	41	passive	MB040/22	A/mute_swan/Poland/MB040-22_22VIR5675-8/2022	EPI_ISL_18245744	20.01.2022	Końskie (pond in the Browarna Street)	konecki	Świętokrzyskie (Holy Cross) Voivodship	mute swan	internal organs (heart, liver, lung)	H5N1 (G1)
	42	passive	MB042/22	A/mute_swan/Poland/MB042-22_22VIR5675-6/2022	EPI_ISL_18245789	24.01.2022	Wrzosy (56-100 Wołów Commune, ponds)	wołowski	Lower Silesian Voivodship	mute swan	liver, stomach, heart	H5N1 (G4)
	43	passive	MB058/22	A/swan/Poland/MB058-22_22VIR5675-2/2022	EPI_ISL_18245790	03.02.2022	sample collection in Wieliszew	legionowski	Masovian Voivodship	swan	brain, tracheal + cloacal swab*	H5N1 (G5)
	44	passive	MB078/22	A/swan/Poland/MB078_22VIR2515-7/2022	EPI_ISL_11922813	15.02.2022	Brzeźce (Pokój Street, near to the lake)	pszczyński	Silesian Voivodeship	swan	internal organs (lung, trachea, liver, spleen, kidney), brain, intestines	H5N1 (G6)
	45	passive	MB083/22	A/swan/Poland/MB083_22VIR2515-8/2022	EPI_ISL_11922814	18.02.2022	Warszawa (Żerański Canal)	Warszawa	Masovian Voivodship	swan	oropahryngeal + cloacal swab*	H5N1 (G1)
	46	passive	MB122/22	A/mute_swan/Poland/MB122/2022	EPI_ISL_18245816	07.04.2022	Jemiołowo Lake	olsztyński	Warmian-Masurian Voivodship	mute swan	internal organs (trachea, pancreas), brain, intestines	H5N1 (G7)
	47	passive	MB138/22	A/herring_gull/Poland/MB138/2022	EPI_ISL_14917979	30.05.2022	Pruszcz Gdański	gdański	Pomeranian Voivodship	herring gull	internal organs (trachea, pancreas)	H5N1 (G2)
	48	passive	MB139/22	A/black-headed_gull/Poland/MB139/2022	EPI_ISL_14917999	30.05.2022	Pruszcz Gdański	gdański	Pomeranian Voivodship	black-headed gull	internal organs (trachea, pancreas, lung)	H5N1 (G2)
	49	passive	MB142/22	A/sandwich_tern/Poland/MB142/2022	EPI_ISL_18245817	15.06.2022	Pruszcz Gdański	gdański	Pomeranian Voivodship	sandwich tern	trachea, intestines	H5N1 (G2)
	50	passive	MB143/22	A/common_tern/Poland/MB143/2022	EPI_ISL_18245818	15.06.2022	Pruszcz Gdański	gdański	Pomeranian Voivodship	common tern	trachea, intestines	H5N1 (G2)
	51	passive	MB151/22	A/common_murre/Poland/MB151/2022	EPI_ISL_14917968	13.07.2022	Ustka	słupski	Pomeranian Voivodship	common murre	cloacal + tracheal swab*, brain	H5N1 (G2)
	52	passive	MB152/22	A/swan/Poland/MB152/2022	EPI_ISL_18245819	15.07.2022	Białobrzegi	legionowski	Masovian Voivodship	swan	brain + tracheal + cloacal swab*	H2N3

* oropharyngeal/tracheal and cloacal swabs taken additionally from wild bird carcasses
