# Supplementary material for: Phylogenetic and Molecular Characteristics of Wild Bird-Origin Avian Influenza Viruses Circulating in Poland in 2018−2022: Reassortment, Multiple Introductions, and Wild Bird–Poultry Epidemiological Links
Source: Transbound Emerg Dis. 2024 Apr 12;2024:6661672. doi: 10.1155/2024/6661672 (PMC12017110; doi:10.1155/2024/6661672)
Supplement: Supplementary 2 — Sequence alignments. [file 6661672.f2.pdf]

PB2

Isolate ID	Sequence
EPI_ISL_11259284	>A/goose/Ireland/3869_22VIR2064-2/2022_H5N1_2022-02-14<br>ATGGAGAGAATAAAAGAGCTAAGAGATTTGATGTCGCAGTCTCGCACTCGCGAGATACTGACAAAAACCACCGTGGACCATATGGCCATAATCAAGAAATATACATCAGGAAGACAGGAGAAGAACCCTGCACTTAGGATGAAGTGGATGATGGCAATGAAATATCCGATTACAGCAGACAAAAGGATAATGGAGATGATCCCTGAAAGAAACGAGCAAGGTCAGACT<br>CTTTGGAGCAAAACAAATGATGCTGGATCGGATAGAGTAATGGTGTCACCTCTGGCTGTGACGTGGTGGAATAGAAATGGACCAACAACAAGTACCGTCCATTACCCAAAGGTCTATAAAACCTACTTTGAAAAGGTTGAAAGGTTAAAGCATGGAACCTTCGGCCCTGTCCATTTCCGGAATCAGGTTAAGATACGCCGCAGAGTTGACATAAACCCGGGCCATGCAGA<br>CCTCAGTGCTAAAGAAGCACAAGACGTCATCATGGAGGTCGTTTTCCCAAATGAAGTCGGAGCCAGAATATTGACATCAGAGTCACAGTTGACAATTACAAAAGAAAAGAAGGAGGAACTCCAGGACTGTAAGATTGCCCCTCTAATGGTGGCATACATGTTGGAGAGAGAACTGGTTCGAAAAACCAGATTCCTGCCAGTAGCTGGCGGAACAAGTAGCGTATATATCG<br>AAGTGTTGCACTTGACTCAAGGAACCTGCTGGGAACAAATGTATACGCCAGGAGGAGAAGTGAGAAATGATGACGTTGACCAAAGTTTAATTATTGCTGCCAGAAATATCGTTAGGAGGGCAACAGTATCAGCAGACCCATTGGCTTCACTACTGGAGATGTGCCATAGTACACAGATTGGCGGGATAAGAATGGTAGACATTCTTAGACAGAACCCAACAGAAGAGCA<br>AGCCGTGGATATATGCAAAGCAGCAATGGGTTTAAGAATCAGTTCATCCTTCAGTTTTGGAGGTTTCACTTTCAAAAGGACAAGCGGATCATCTGTCAAAAGAGAAGAGGAAGTGCTCACCGGCAACCTCCAAACATTGAAAATAAGAGTACATGAAGGGTATGAGGAATTCACAATGGTTGGGCGGAGAGCAACAGCCATTCTAAGGAAAGCAACCAGAAGGCTGATC<br>CAATTGATAGTAAGTGGAAGAGACGAGCAGTCAATCGCCGAAGCGATCATAGTGGCAATGGTGTTCTCTCAAGAGGATTGCATGATAAAGGCTGTACGAGGTGATTTAAATTTTGTCAATAGAGCGAATCAGCGGCTCAATCCTATGCATCAGCTCCTGAGGCATTTCCAAAAGGATGCAAAGGTACTATTCCAAAACTGGGGAATTGAACCCATTGACAATGTCATGGGA<br>ATGATAGGAATATTGCCTGATATGACCCCCAGCACAGAGATGTCACTAAGAGGAGTGAGGGTCAGTAAAATGGGAGTGGATGAATATTCCAGTACTGAGAGGGTGGTCGTGAGTATTGATCGCTTCTTGAGGGTACGAGACCAGAGAGGAAATGTACTCTTGTCTCCCGAAGAGGTCAGTGAAACACAGGGAACAGAGAAGCTAACGATAACATATTCATCATCCATGA<br>TGTGGGAAATTAATGGCCCTGAGTCAGTGCTAGTTAACACATATCAATGGATCATCAGAAACTGGGAAACTGTGAAGATTCAGTGGTCCCAAGACCCTACAATGCTATACAACAAGATGGAGTTTGAGCCTTTTCAGTCCTTGGTGCCTAAGGCAGCCAGAGGCCAGTACAGTGGATTTGTAAGGACCCTATTCCAGCAGATGCGTGATGTGCTGGGAACCTTTGACACTGT<br>CCAGATAATAAAGCTACTTCCATTTGCAGCAGCACCACCGGAACAGAGTAGGATGCAGTTCTCTTCTCTAACTGTAAACGTAAGGGGTTCAGGAATGAGAATACTTGTGAGAGGCAACTCCCCTGTGTTCAACTATAATAAAGCAACCAAGAGGCTCATAGTCCTTGGAAAGGATGCTGGTGCATTGACAGAAGACCCAGGTGAGGGGACAGCAGGAGTGGAGTCTGCG<br>GTATTGAGAGGGTTCCTAATTCTGGGCAAAGAGGACAAAAGATATGGACCAGCGCTGAGCATCAATGAATTGAGCAATCTTGCGAAAGGGGAGAAGGCTAATGTGTTGATAGGGCAAGGAGACGTGGTGTTGGTGATGAAACGGAAACGGGACTCTAGCATACTTACTGACAGCCAGACAGCGACCAAAAGAATTCGGATGGCCATCAATTA
EPI_ISL_11259280	>A/greylag_goose/Ireland/033062_22VIR1325-7/2021_H5N1_2021-11-15<br>ATGGAGAGAATAAAAGAGCTAAGAGATTTGATGTCGCAGTCTCGCACTCGCGAGATACTGACAAAAACCACCGTGGACCATATGGCCATAATCAAGAAATATACATCAGGAAGACAGGAGAAGAACCCTGCACTTAGGATGAAGTGGATGATGGCAATGAAATATCCGATTACAGCAGACAAAAGGATAATGGAGATGATCCCTGAAAGAAACGAGCAAGGTCAGACT<br>CTTTGGAGCAAAACAAATGATGCTGGATCGGATAGAGTAATGGTGTCACCTCTGGCTGTGACGTGGTGGAATAGAAATGGACCAACAACAAGTACCGTCCATTACCCAAAGGTCTATAAAACCTACTTTGAAAAGGTTGAAAGGTTAAAGCATGGAACCTTCGGCCCTGTCCATTTCCGGAATCAGGTTAAGATACGCCGCAGAGTTGACATAAACCCGGGCCATGCAGA<br>CCTCAGTGCTAAAGAAGCACAAGACGTCATCATGGAGGTCGTTTTCCCAAATGAAGTCGGAGCCAGAATATTGACATCAGAGTCACAGTTGACAATTACAAAAGAAAAGAAGGAGGAACTCCAGGACTGTAAGATTGCCCCTCTAATGGTGGCATACATGTTGGAGAGAGAACTGGTTCGAAAAACCAGATTCCTGCCAGTAGCTGGCGGAACAAGTAGCGTATATATCG<br>AAGTGTTGCACTTGACTCAAGGAACCTGCTGGGAACAAATGTATACGCCAGGAGGAGAAGTGAGAAATGATGACGTTGACCAAAGTTTAATTATTGCTGCCAGAAATATCGTTAGGAGGGCAACAGTATCAGCAGACCCATTGGCTTCACTACTGGAGATGTGCCATAGTACACAGATTGGCGGGATAAGAATGGTAGACATTCTTAGACAGAACCCAACAGAAGAGCA<br>AGCCGTGGATATATGCAAAGCAGCAATGGGTTTAAGAATCAGTTCATCCTTCAGTTTTGGAGGTTTCACTTTCAAAAGGACAAGCGGATCATCTGTCAAAAGAGAAGAGGAAGTGCTCACCGGCAACCTCCAAACATTGAAAATAAGAGTACATGAAGGGTATGAGGAATTCACAATGGTTGGGCGGAGAGCAACAGCCATTCTAAGGAAAGCAACCAGAAGGCTGATC<br>CAATTGATAGTAAGTGGAAGAGACGAGCAGTCAATCGCCGAAGCGATCATAGTGGCAATGGTGTTCTCTCAAGAGGATTGCATGATAAAGGCTGTACGAGGTGATTTAAATTTTGTCAATAGAGCGAATCAGCGGCTCAATCCTATGCATCAGCTCCTGAGGCATTTCCAAAAGGATGCAAAGGTACTATTCCAAAACTGGGGAATTGAACCCATTGACAATGTCATGGGA<br>ATGATAGGAATATTGCCTGATATGACCCCCAGCACAGAGATGTCACTAAGAGGAGTGAGGGTCAGTAAAATGGGAGTGGATGAATATTCCAGTACTGAGAGGGTGGTCGTGAGTATTGATCGCTTCTTGAGGGTACGAGACCAGAGAGGAAATGTACTCTTGTCTCCCGAAGAGGTCAGTGAAACACAGGGAACAGAGAAGCTAACGATAACATATTCATCATCCATGA<br>TGTGGGAAATTAATGGCCCTGAGTCAGTGCTAGTTAACACATATCAATGGATCATCAGAAACTGGGAAACTGTGAAGATTCAGTGGTCCCAAGACCCTACAATGCTATACAACAAGATGGAGTTTGAGCCTTTTCAGTCCTTGGTGCCTAAGGCAGCCAGAGGCCAGTACAGTGGATTTGTAAGGACCCTATTCCAGCAGATGCGTGATGTGCTGGGAACCTTTGACACTGT<br>CCAGATAATAAAGCTACTTCCATTTGCAGCAGCACCACCGGAACAGAGTAGGATGCAGTTCTCTTCTCTAACTGTAAACGTAAGGGGTTCAGGAATGAGAATACTTGTGAGAGGCAACTCCCCTGTGTTCAACTATAATAAAGCAACCAAGAGGCTCATAGTCCTTGGAAAGGATGCTGGTGCATTGACAGAAGACCCAGGTGAGGGGACAGCAGGAGTGGAGTCTGCG<br>GTATTGAGAGGGTTCCTAATTCTGGGCAAAGAGGACAAAAGATATGGACCAGCGCTGAGCATCAATGAATTGAGCAATCTTGCGAAAGGGGAGAAGGCTAATGTGTTGATAGGGCAAGGAGACGTGGTGTTGGTGATGAAACGGAAACGGGACTCTAGCATACTTACTGACAGCCAGACAGCGACCAAAAGAATTCGGATGGCCATCAATTA
EPI_ISL_7733633	>A/turkey/Italy/21VIR9143-2/2021_H5N1_2021-11-03<br>ATGGAGAGAATAAAAGAGCTAAGAGATTTGATGTCGCAGTCTCGCACTCGCGAGATACTGACAAAAACCACCGTGGACCATATGGCCATAATCAAGAAATATACATCAGGAAGACAGGAGAAGAACCCTGCACTTAGGATGAAGTGGATGATGGCAATGAAATATCCGATTACAGCAGACAAAAGGATAATGGAGATGATCCCTGAAAGAAACGAGCAAGGTCAGACT<br>CTTTGGAGCAAAACAAATGATGCTGGATCGGATAGAGTAATGGTGTCACCTCTGGCTGTGACGTGGTGGAATAGAAATGGACCAACAACAAGTACAGTCCATTACCCAAAGGTCTATAAAACCTACTTTGAAAAGGTTGAAAGGTTAAAGCATGGAACCTTCGGCCCTGTCCATTTCCGGAATCAGGTTAAGATACGCCGCAGAGTTGACATAAACCCGGGCCATGCAGA<br>CCTCAGTGCTAAAGAAGCACAAGACGTCATCATGGAGGTCGTTTTCCCAAATGAAGTCGGAGCCAGAATATTGACATCAGAGTCACAGTTAACAATTACAAAAGAAAAGAAGGAGGAACTCCAGGACTGTAAGATTGCCCCTCTAATGGTGGCATACATGTTGGAGAGAGAACTGGTTCGAAAAACCAGATTCCTGCCAGTAGCTGGCGGAACAAGTAGCGTATATATCG<br>AAGTGTTGCACTTGACTCAAGGAACCTGCTGGGAACAAATGTATACGCCAGGAGGAGAAGTGAGAAATGATGACGTTGACCAAAGTTTAATTATTGCTGCCAGAAATATCGTTAGGAGAGCAACAGTATCAGCAGACCCATTGGCTTCACTACTGGAGATGTGCCATAGTACACAGATTGGCGGGATAAGAATGGTAGACATTCTTAGACAGAACCCAACAGAAGAGCA<br>AGCCGTGGATATATGCAAAGCAGCAATGGGTTTAAGAATCAGTTCATCCTTCAGTTTTGGAGGTTTCACTTTCAAAAGGACAAGCGGATCATCTGTCAAAAGAGAAGAGGAAGTGCTCACCGGCAACCTCCAAACATTGAAAATAAGAGTACATGAAGGGTATGAGGAATTCACAATGGTTGGGCGGAGAGCAACAGCCATTCTAAGGAAAGCAACCAGAAGGCTGATC<br>CAATTGATAGTAAGTGGAAAAGACGAGCAGTCAATCGCCGAAGCGATCATAGTGGCAATGGTGTTCTCTCAAGAGGATTGCATGATAAAGGCTGTACGAGGTGATTTAAATTTTGTCAATAGAGCGAATCAGCGGCTCAATCCTATGCATCAGCTCCTGAGGCATTTCCAAAAGGATGCAAAGGTACTATTCCAAAACTGGGGAATTGAACCCATTGACAATGTCATGGGA<br>ATGATAGGAATATTGCCTGATATGACTCCCAGCACAGAGATGTCACTAAGAGGAGTGAGGGTCAGTAAAATGGGAGTGGATGAATATTCCAGTACTGAGAGGGTGGTCGTGAGTATTGATCGCTTCTTGAGGGTACGAGACCAGAGAGGAAATGTACTCTTGTCTCCCGAAGAGGTCAGTGAAACACAGGGAACAGAGAAGCTAACGATAACATATTCATCATCCATGA<br>TGTGGGAAATTAATGGCCCTGAGTCAGTGCTAGTTAACACATATCAATGGGTCATCAGAAACTGGGAAACTGTGAAGATTCAGTGGTCCCAAGACCCTACAATGCTATACAACAAGATGGAGTTTGAGCCTTTTCAGTCCTTGGTGCCCAAGGCAGCCAGAGGCCAGTACAGTGGATTTGTAAGGACCTTATTCCAGCAGATGCGTGATGTGCTGGGAACCTTTGACACTG<br>TCCAGATAATAAAGCTACTTCCATTTGCAGCAGCACCACCGGAACAGAGTAGGATGCAGTTCTCTTCTCTAACTGTAAACGTAAGGGGTTCAGGAATGAGAATACTTGTGAGAGGCAACTCCCCTGTGTTCAACTATAATAAGGCAACCAAGAGGCTCATAGTCCTTGGAAAGGATGCTGGTGCATTGACAGAAGACCCAGGTGAGGGGACAGCAGGAGTGGAGTCTGCG<br>GTATTGAGAGGGTTCCTAATTCTGGGCAAAGAGGACAAAAGATATGGACCAGCGCTGAGCATCAATGAATTGAGCAATCTTGCGAAAGGGGAGAAGGCTAATGTGTTGATAGGGCAAGGAGACGTGGTGTTGGTGATGAAACGGAAACGGGACTCTAGCATACTTACTGACAGCCAGACAGCGACCAAAAGAATTCGGATGGCCATCAATTA
EPI_ISL_7733630	>A/chicken/Italy/21VIR9691-2/2021_H5N1_2021-11-15<br>ATGGAGAGAATAAAAGAGCTAAGAGATTTGATGTCGCAGTCTCGCACTCGCGAGATACTGACAAAAACCACCGTGGACCATATGGCCATAATCAAGAAATATACATCAGGAAGACAGGAGAAGAACCCTGCACTTAGGATGAAGTGGATGATGGCAATGAAATATCCGATTACAGCAGACAAAAGGATAATGGAGATGATCCCTGAAAGAAACGAGCAAGGTCAGACT<br>CTTTGGAGCAAAACAAATGATGCTGGATCGGATAGAGTAATGGTGTCACCTCTGGCTGTGACGTGGTGGAATAGAAATGGACCAACAACAAGTACAGTCCATTACCCAAAGGTCTATAAAACCTACTTTGAAAAGGTTGAAAGGTTAAAGCATGGAACCTTCGGCCCTGTCCATTTCCGGAATCAGGTTAAGATACGCCGCAGAGTTGACATAAACCCGGGCCATGCAGA<br>CCTCAGTGCTAAAGAAGCACAAGACGTCATCATGGAGGTCGTTTTCCCAAATGAAGTCGGAGCCAGAATATTGACATCAGAGTCACAGTTAACAATTACAAAAGAAAAGAAGGAGGAACTCCAGGACTGTAAGATTGCCCCTCTAATGGTGGCATACATGTTGGAGAGAGAACTGGTTCGAAAAACCAGATTCCTGCCAGTAGCTGGCGGAACAAGTAGCGTATATATCG<br>AAGTGTTGCACTTGACTCAAGGAACCTGCTGGGAACAAATGTATACGCCAGGAGGAGAAGTGAGAAATGATGACGTTGACCAAAGTTTAATTATTGCTGCCAGAAATATCGTTAGGAGAGCAACAGTATCAGCAGACCCATTGGCTTCACTACTGGAGATGTGCCATAGTACACAGATTGGCGGGATAAGAATGGTAGACATTCTTAGACAGAACCCAACAGAAGAGCA<br>AGCCGTGGATATATGCAAAGCAGCAATGGGTTTAAGAATCAGTTCATCCTTCAGTTTTGGAGGTTTCACTTTCAAAAGGACAAGCGGATCATCTGTCAAAAGAGAAGAGGAAGTGCTCACCGGCAACCTCCAAACATTGAAAATAAGAGTACATGAAGGGTATGAGGAATTCACAATGGTTGGGCGGAGAGCAACAGCCATTCTAAGGAAAGCAACCAGAAGGCTGATC<br>CAATTGATAGTAAGTGGAAAAGACGAGCAGTCAATCGCCGAAGCGATCATAGTGGCAATGGTGTTCTCTCAAGAGGATTGCATGATAAAGGCTGTACGAGGTGATTTAAATTTTGTCAATAGAGCGAATCAGCGGCTCAATCCTATGCATCAGCTCCTGAGGCATTTCCAAAAGGATGCAAAGGTACTATTCCAAAACTGGGGAATTGAACCCATTGACAATGTCATGGGA<br>ATGATAGGAATATTGCCTGATATGACTCCCAGCACAGAGATGTCACTAAGAGGAGTGAGGGTCAGTAAAATGGGAGTGGATGAATATTCCAGTACTGAGAGGGTGGTCGTGAGTATTGATCGCTTCTTGAGGGTACGAGACCAGAGAGGAAATGTACTCTTGTCTCCCGAAGAGGTCAGTGAAACACAGGGAACAGAGAAGCTAACGATAACATATTCATCATCCATGA<br>TGTGGGAAATTAATGGCCCTGAGTCAGTGCTAGTTAACACATATCAATGGGTCATCAGAAACTGGGAAACTGTGAAGATTCAGTGGTCCCAAGACCCTACAATGCTATACAACAAGATGGAGTTTGAGCCTTTTCAGTCCTTGGTGCCCAAGGCAGCCAGAGGCCAGTACAGTGGATTTGTAAGGACCTTATTCCAGCAGATGCGTGATGTGCTGGGAACCTTTGACACTG<br>TCCAGATAATAAAGCTACTTCCATTTGCAGCAGCACCACCGGAACAGAGTAGGATGCAGTTCTCTTCTCTAACTGTAAACGTAAGGGGTTCAGGAATGAGAATACTTGTGAGAGGCAACTCCCCTGTGTTCAACTATAATAAGGCAACCAAGAGGCTCATAGTCCTTGGAAAGGATGCTGGTGCATTGACAGAAGACCCAGGTGAGGGGACAGCAGGAGTGGAGTCTGCG<br>GTATTGAGAGGGTTCCTAATTCTGGGCAAAGAGGACAAAAGATATGGACCAGCGCTGAGCATCAATGAATTGAGCAATCTTGCGAAAGGGGAGAAGGCTAATGTGTTGATAGGGCAAGGAGACGTGGTGTTGGTGATGAAACGGAAACGGGACTCTAGCATACTTACTGACAGCCAGACAGCGACCAAAAGAATTCGGATGGCCATCAATTA
EPI_ISL_9603819	>A/goose/Czech_Republic/22608-2T/2021_H5N1_2021-11-18<br>ATGGAGAGAATAAAAGAGCTAAGAGATTTGATGTCGCAGTCTCGCACTCGCGAGATACTGACAAAAACCACCGTGGACCATATGGCCATAATCAAGAAATATACATCAGGAAGACAGGAGAAGAACCCTGCACTTAGGATGAAGTGGATGATGGCAATGAAATATCCGATTACAGCAGACAAAAGGATAATGGAGATGATCCCTGAAAGAAACGAGCAAGGTCAGACT<br>CTTTGGAGCAAAACAAATGATGCTGGATCGGATAGAGTAATGGTGTCACCTCTGGCTGTGACGTGGTGGAATAGAAATGGACCAACAACAAGTACAGTCCATTACCCAAAGGTCTATAAAACCTACTTTGAAAAGGTTGAAAGGTTAAAGCATGGAACCTTCGGCCCTGTCCATTTCCGGAATCAGGTTAAGATACGCCGCAGAGTTGACATAAACCCGGGCCATGCAGA<br>CCTCAGTGCTAAAGAAGCACAAGACGTCATCATGGAGGTCGTTTTCCCAAATGAAGTCGGAGCCAGAATATTGACATCAGAGTCACAGTTAACAATTACAAAAGAAAAGAAGGAGGAACTCCAGGACTGTAAGATTGCCCCTCTAATGGTGGCATACATGTTGGAGAGAGAACTGGTTCGAAAAACCAGATTCCTGCCAGTAGCTGGCGGAACAAGTAGCGTATATATCG<br>AAGTGTTGCACTTGACTCAAGGAACCTGCTGGGAACAAATGTATACGCCAGGAGGAGAAGTGAGAAATGATGACGTTGACCAAAGTTTAATTATTGCTGCCAGAAATATCGTTAGGAGAGCAACAGTATCAGCAGACCCATTGGCTTCACTACTGGAGATGTGCCATAGTACACAGATTGGCGGGATAAGAATGGTAGACATTCTTAGACAGAACCCAACAGAAGAGCA<br>AGCCGTGGATATATGCAAAGCAGCAATGGGTTTAAGAATCAGTTCATCCTTCAGTTTTGGAGGTTTCACTTTCAAAAGGACAAGCGGATCATCTGTCAAAAGAGAAGAGGAAGTGCTCACCGGCAACCTCCAAACATTGAAAATAAGAGTACATGAAGGGTATGAGGAATTCACAATGGTTGGGCGGAGAGCAACAGCCATTCTAAGGAAAGCAACCAGAAGGCTGATC<br>CAATTGATAGTAAGTGGAAAAGACGAGCAGTCAATCGCCGAAGCGATCATAGTGGCAATGGTGTTCTCTCAAGAGGATTGCATGATAAAGGCTGTACGAGGTGATTTAAATTTTGTCAATAGAGCGAATCAGCGGCTCAATCCTATGCATCAGCTCCTGAGGCATTTCCAAAAGGATGCAAAGGTACTATTCCAAAACTGGGGAATTGAACCCATTGACAATGTCATGGGA<br>ATGATAGGAATATTGCCTGATATGACTCCCAGCACAGAGATGTCACTAAGAGGAGTGAGGGTCAGTAAAATGGGAGTGGATGAATATTCCAGTACTGAGAGGGTGGTCGTGAGTATTGATCGCTTCTTGAGGGTACGAGACCAGAGAGGAAATGTACTCTTGTCTCCCGAAGAGGTCAGTGAAACACAGGGAACAGAGAAGCTAACGATAACATATTCATCATCCATGA<br>TGTGGGAAATTAATGGCCCTGAGTCAGTGCTAGTTAACACATATCAATGGGTCATCAGAAACTGGGAAACTGTGAAGATTCAGTGGTCCCAAGACCCTACAATGCTATACAACAAGATGGAGTTTGAGCCTTTTCAGTCCTTGGTGCCCAAGGCAGCCAGAGGCCAGTACAGTGGATTTGTAAGGACCTTATTCCAGCAGATGCGTGATGTGCTGGGAACCTTTGACACTG<br>TCCAGATAATAAAGCTACTTCCATTTGCAGCAGCACCACCGGAACAGAGTAGGATGCAGTTCTCTTCTCTAACTGTAAACGTAAGGGGTTCAGGAATGAGAATACTTGTGAGAGGCAACTCCCCTGTGTTCAACTATAATAAGGCAACCAAGAGGCTCATAGTCCTTGGAAAGGATGCTGGTGCATTGACAGAAGACCCAGGTGAGGGGACAGCAGGAGTGGAGTCTGCG<br>GTATTGAGAGGGTTCCTAATTCTGGGCAAAGAGGACAAAAGATATGGACCAGCGCTGAGCATCAATGAATTGAGCAATCTTGCGAAAGGGGAGAAGGCTAATGTGTTGATAGGGCAAGGAGACGTGGTGTTGGTGATGAAACGGAAACGGGACTCTAGCATACTTACTGACAGCCAGACAGCGACCAAAAGAATTCGGATGGCCATCAATTA
EPI_ISL_11327090	>A/chicken/Czech_Republic/63/2022_H5N1_2022-01-03<br>ATGGAGAGAATAAAAGAGCTAAGAGATTTGATGTCGCAGTCTCGCACTCGCGAGATACTGACAAAAACCACCGTGGACCATATGGCCATAATCAAGAAATATACATCAGGAAGACAGGAGAAGAACCCTGCACTTAGGATGAAGTGGATGATGGCAATGAAATATCCGATTACAGCAGACAAAAGGATAATGGAGATGATCCCTGAAAGAAACGAGCAAGGTCAGACT<br>CTTTGGAGCAAAACAAATGATGCTGGATCGGATAGAATAATGGTGTCACCTCTGGCTGTGACGTGGTGGAATAGAAATGGACCAACAACAAGTACCGTCCATTACCCAAAGGTCTATAAAACCTACTTTGAAAAGGTTGAAAGGTTAAAGCATGGAACCTTCGGCCCTGTCCATTTCCGGAATCAGGTTAAGATACGCCGCAGAGTTGACATAAACCCGGGCCATGCAGAC<br>CTCAGTGCTAAAGAAGCACAAGACGTCATCATGGAGGTCGTTTTCCCAAATGAAGTCGGAGCCAGAATATTGACATCAGAGTCACAGTTAACAATTACAAAAGAAAAGAAGGAGGAACTCCAGGACTGTAAGATTGCCCCTCTAATGGTGGCATACATGTTGGAGAGAGAACTGGTTCGAAAAACCAGATTCCTGCCAGTAGCTGGCGGAACAAGTAGCGTATATATCGA<br>AGTGTTACACTTGACTCAAGGAACCTGCTGGGAACAAATGTATACGCCAGGAGGAGAAGTGAGAAATGATGACGTTGACCAAAGTTTAATTATTGCTGCCAGAAATATCGTTAGGAGGGCAACAGTATCAGCAGACCCATTGGCTTCACTACTGGAGATGTGCCATAGTACACAGATTGGCGGGATAAGAATGGTAGACATTCTTAGACAGAACCCAACAGAAGAGCAA<br>GCCGTGGATATATGCAAAGCAGCAATGGGTTTAAGAATCAGTTCATCCTTCAGTTTTGGAGGTTTCACTTTCAAAAGGACAAGCGGATCATCTGTCAAAAGAGAAGAGGAAGTGCTCACAGGCAACCTCCAAACATTGAAAATAAGAGTACATGAAGGGTATGAGGAATTCACAATGGTTGGACGGAGAGCAACAGCCATTCTAAGGAAAGCAACCAGAAGGCTAATCC<br>AATTGATAGTAAGTGGAAGAGACGAGCAGTCAATCGCCGAAGCGATCATAGTGGCAATGGTGTTCTCTCAAGAGGATTGCATGATAAAGGCTGTACGAGGTGATTTAAATTTTGTCAATAGAGCGAATCAGCGGCTCAATCCTATGCATCAGCTCCTGAGGCATTTCCAAAAGGATGCAAAGGTACTATTCCAAAACTGGGGAATTGAACCCATTGACAATGTCATGGGAA<br>TGATAGGAATATTGCCTGATATGACCCCCAGCACAGAGATGTCACTAAGAGGAGTGAGGGTCAGTAAAATGGGAGTGGATGAATATTCCAGTACTGAGAGGGTGGTCGTGAGTATTGATCGCTTCTTGAGGGTACGAGACCAGAGAGGAAATGTACTCTTGTCTCCCGAAGAGGTCAGTGAAACACAGGGAACAGAGAAGCTAACGATAACATATTCATCATCCATGAT<br>GTGGGAAATTAATGGCCCTGAGTCAGTGCTAGTTAACACATATCAATGGATCATCAGAAACTGGGAAACTGTGAAGATTCAGTGGTCCCAAGACCCTACAATGCTATACAACAAGATGGAGTTTGAGCCTTTTCAGTCCTTGGTGCCTAAGGCAGCCAGAGGCCAGTACAGTGGATTTGTAAGGACCCTATTCCAGCAGATGCGTGATGTGCTGGGAACCTTTGACACTGT<br>CCAGATAATAAAGCTACTTCCATTTGCAGCAGCACCACCGGAACAGAGTAGGATGCAGTTCTCTTCTCTAACTGTAAACGTAAGGGGTTCAGGAATGAGAATACTTGTGAGAGGCAACTCCCCTGTGTTCAACTATAATAAAGCAACCAAGAGGCTCATAGTCCTTGGAAAGGATGCTGGTGCATTGACAGAAGACCCAGGTGAGGGGACAGCAGGAGTGGAGTCTGCG<br>GTATTGAGAGGGTTCCTAATTCTGGGCAAAGAGGACAAAAGATATGGACCAGCGCTGAGCATCAATGAATTGAGCAATCTTGCGAAAGGGGAGAAGGCTAATGTGTTGATAGGGCAAGGAGACGTGGTGTTGGTGATGAAACGGAAACGGGACTCTAGCATACTTACTGACAGCCAGACAGCGACCAAAAGAATTCGGATGGCCATCAATTA
EPI_ISL_11007535	>A/swan/Slovenia/13_22VIR777-8/2022_H5N1_2022-01-03<br>ATGGAGAGAATAAAAGAGCTAAGAGATTTGATGTCGCAGTCTCGCACTCGCGAGATACTGACAAAAACCACCGTGGACCATATGGCCATAATCAAGAAATATACATCAGGAAGACAGGAGAAGAACCCTGCACTTAGGATGAAGTGGATGATGGCAATGAAATATCCGATTACAGCAGACAAAAGGATAATGGAAATGATCCCTGAAAGAAACGAGCAAGGTCAGACT<br>CTTTGGAGCAAAACAAATGATGCTGGATCGGATAGAATAATGGTGTCACCTCTGGCTGTGACGTGGTGGAATAGAAATGGACCAACAACAAGTACCGTCCATTACCCAAAGGTCTATAAAACCTACTTTGAAAAGGTTGAAAGGTTAAAGCATGGAACCTTCGGCCCTGTCCATTTCCGGAATCAGGTTAAGATACGCCGCAGAGTTGACATAAACCCGGGCCATGCAGAC<br>CTCAGTGCTAAAGAAGCACAAGACGTCATCATGGAGGTCGTTTTCCCAAATGAAGTCGGAGCCAGAATATTGACATCAGAGTCACAGTTAACAATTACAAAAGAAAAGAAGGAGGAACTCCAGGACTGTAAGATTGCCCCTCTAATGGTGGCATACATGTTGGAGAGAGAACTGGTTCGAAAAACCAGATTCCTGCCAGTAGCTGGCGGAACAAGTAGCGTATATATCGA<br>AGTGTTGCACTTGACTCAAGGAACCTGCTGGGAACAAATGTATACGCCAGGAGGAGAAGTGAGAAATGATGACGTTGACCAAAGTTTAATTATTGCTGCCAGAAATATCGTTAGGAGGGCAACAGTATCAGCAGACCCATTGGCTTCACTACTGGAGATGTGCCATAGTACACAGATTGGCGGGATAAGAATGGTAGACATTCTTAGACAGAACCCAACAGAAGAGCAA<br>GCCGTGGATATATGCAAAGCAGCAATGGGTTTAAGAATCAGTTCATCCTTCAGTTTTGGAGGTTTCACTTTCAAAAGGACAAGCGGATCATCTGTCAAAAGAGAAGAGGAAGTGCTCACAGGCAACCTCCAAACATTGAAAATAAGAGTACATGAAGGGTATGAGGAATTCACAATGGTTGGACGGAGAGCAACAGCCATTCTAAGGAAAGCAACCAGAAGGCTAATCC<br>AATTGATAGTAAGTGGAAGAGACGAGCAGTCAATCGCCGAAGCGATCATAGTGGCAATGGTGTTCTCTCAAGAGGATTGCATGATAAAGGCTGTACGAGGTGATTTAAATTTTGTCAATAGAGCGAATCAGCGGCTCAATCCTATGCATCAGCTCCTGAGGCATTTCCAAAAGGATGCAAAGGTACTATTCCAAAACTGGGGAATTGAACCCATTGACAATGTCATGGGAA<br>TGATAGGAATATTGCCTGATATGACCCCCAGCACAGAGATGTCACTAAGAGGAGTGAGGGTCAGTAAAATGGGAGTGGATGAATATTCCAGTACTGAGAGGGTGGTCGTGAGTATTGATCGCTTCTTGAGGGTACGAGACCAGAGAGGAAATGTACTCTTGTCTCCCGAAGAGGTCAGTGAAACACAGGGAACAGAGAAGCTAACGATAACATATTCATCATCCATGAT<br>GTGGGAAATTAATGGCCCTGAGTCAGTGCTAGTTAACACATATCAATGGATCATCAGAAACTGGGAAACTGTGAAGATTCAGTGGTCCCAAGACCCTACAATGCTATACAACAAGATGGAGTTTGAGCCTTTTCAGTCCTTGGTGCCTAAGGCAGCCAGAGGCCAGTACAGTGGATTTGTAAGGACCCTATTCCAGCAGATGCGTGATGTGCTGGGAACCTTTGACACTGT<br>CCAGATAATAAAGCTACTTCCATTTGCAGCAGCACCACCGGAACAGAGTAGGATGCAGTTCTCTTCTCTAACTGTAAACGTAAGGGGTTCAGGAATGAGAATACTTGTGAGAGGCAACTCCCCTGTGTTCAACTATAATAAAGCAACCAAGAGGCTCATAGTCCTTGGAAAGGATGCTGGTGCATTGACAGAAGACCCAGGTGAGGGGACAGCAGGAGTGGAGTCTGCG<br>GTATTGAGAGGGTTCCTAATTCTGGGCAAAGAGGACAAAAGATATGGACCAGCGCTGAGCATCAATGAATTGAGCAATCTTGCGAAAGGGGAGAAGGCTAATGTGTTGATAGGGCAAGGAGACGTGGTGTTGGTGATGAAACGGAAACGGGACTCTAGCATACTTACTGACAGCCAGACAGCGACCAAAAGAATTCGGATGGCCATCAATTA
EPI_ISL_10347326	>A/long-eared_owl/Germany-NI/AI09037/2021_H5N1_2021-12-28<br>ATGGAGAGAATAAAAGAGCTAAGAGATTTGATGTCGCAGTCTCGCACTCGCGAGATACTGACAAAAACCACCGTGGACCATATGGCCATAATCAAGAAATATACATCAGGAAGACAGGAGAAGAACCCTGCACTTAGGATGAAGTGGATGATGGCAATGAAATATCCGATTACAGCAGACAAAAGGATAATGGAGATGATCCCTGAAAGAAACGAGCAAGGTCAGACT<br>CTTTGGAGCAAAACAAATGATGCTGGATCGGATAGAGTAATGGTGTCACCTCTGGCTGTGACGTGGTGGAATAGAAATGGACCAACAACAAGTACCGTCCATTACCCAAAGGTCTATAAAACCTACTTTGAAAAGGTTGAAAGGTTAAAGCATGGAACCTTCGGCCCTGTCCATTTCCGGAATCAGGTTAAGATACGCCGCAGAGTTGACATAAACCCGGGCCATGCAGA<br>CCTCAGTGCTAAAGAAGCACAAGACGTCATCATGGAGGTCGTTTTCCCAAATGAAGTCGGAGCCAGAATATTGACATCAGAGTCACAGTTGACAATTACAAAAGAAAAGAAGGAGGAACTCCAGGACTGTAAGATTGCCCCTCTAATGGTGGCATACATGTTGGAGAGAGAACTGGTTCGAAAAACCAGATTCCTGCCAGTAGCTGGCGGAACAAGTAGCGTATATATCG<br>AAGTGTTGCACTTGACTCAAGGAACCTGCTGGGAACAAATGTATACGCCAGGAGGAGAAGTGAGAAATGATGACGTTGACCAAAGTTTAATTATTGCTGCCAGAAATATCGTTAGGAGGGCAACAGTATCAGCAGACCCATTGGCTTCACTACTGGAGATGTGCCATAGTACACAGATTGGCGGGATAAGAATGGTAGACATTCTTAGACAGAACCCAACAGAAGAGCA<br>AGCCGTGGATATATGCAAAGCAGCAATGGGTTTAAGAATCAGTTCATCCTTCAGTTTTGGAGGTTTCACTTTCAAAAGGACAAGCGGATCATCTGTCAAAAGAGAAGAGGAAGTGCTCACCGGCAACCTCCAAACATTGAAAATAAGAGTACATGAAGGGTATGAGGAATTCACAATGGTTGGGCGGAGAGCAACAGCCATTCTAAGGAAAGCAACCAGAAGGCTGATC<br>CAATTGATAGTAAGTGGAAGAGACGAGCAGTCAATCGCCGAAGCGATCATAGTGGCAATGGTGTTCTCTCAAGAGGATTGCATGATAAAGGCTGTACGAGGTGATTTAAATTTTGTCAATAGAGCGAATCAGCGGCTCAATCCTATGCATCAGCTCCTGAGGCATTTCCAAAAGGATGCAAAGGTACTATTCCAAAACTGGGGAATTGAACCCATTGACAATGTCATGGGA<br>ATGATAGGAATATTGCCTGATATGACCCCCAGCACAGAGATGTCACTAAGAGGAGTGAGGGTCAGTAAAATGGGAGTGGATGAATATTCCAGTACTGAGAGGGTGGTCGTGAGTATTGATCGCTTCTTGAGGGTACGAGACCAGAGAGGAAATGTACTCTTGTCTCCCGAAGAGGTCAGTGAAACACAGGGAACAGAGAAGCTAACGATAACATATTCATCATCCATGA<br>TGTGGGAAATTAATGGCCCTGAGTCAGTGCTAGTTAACACATATCAATGGATCATCAGAAACTGGGAAACTGTGAAGATTCAGTGGTCCCAAGACCCTACAATGCTATACAACAAGATGGAGTTTGAGCCTTTTCAGTCCTTGGTGCCTAAGGCAGCCAGAGGCCAGTACAGTGGATTTGTAAGGACCCTATTCCAGCAGATGCGTGATGTGCTGGGAACCTTTGACACTGT<br>CCAGATAATAAAGCTACTTCCATTTGCAGCAGCACCACCGGAACAGAGTAGGATGCAGTTCTCTTCTCTAACTGTAAACGTAAGGGGTTCAGGAATGAGAATACTTGTGAGAGGCAACTCCCCTGTGTTCAACTATAATAAAGCAACCAAGAGGCTCATAGTCCTTGGAAAGGATGCTGGTGCATTGACAGAAGACCCAGGTGAGGGGACAGCAGGAGTGGAGTCTGCG<br>GTATTGAGAGGGTTCCTAATTCTGGGCAAAGAGGACAAAAGATATGGACCAGCGCTGAGCATCAATGAATTGAGCAATCTTGCGAAAGGGGAGAAGGCTAATGTGTTGATAGGGCAAGGAGACGTGGTGTTGGTGATGAAACGGAAACGGGACTCTAGCATACTTACTGACAGCCAGACAGCGACCAAAAGAATTCGGATGGCCATCAATTA
EPI_ISL_9261749	>A/Caspian_gull/Netherlands/1/2022_H5N1_2022-01-14<br>ATGGAGAGAATAAAAGAGCTAAGAGATTTGATGTCGCAGTCTCGCACTCGCGAGATACTGACAAAAACCACCGTGGACCATATGGCCATAATCAAGAAATATACATCAGGAAGACAGGAGAAGAACCCTGCACTTAGGATGAAGTGGATGATGGCAATGAAATATCCGATTACAGCAGACAAAAGGATAATGGAGATGATCCCTGAAAGAAACGAGCAAGGTCAGACT<br>CTTTGGAGCAAAACAAATGATGCTGGATCGGATAGAGTAATGGTGTCACCTCTGGCTGTGACGTGGTGGAATAGAAATGGACCAACAACAAGTACCGTCCATTACCCAAAGGTCTATAAAACCTACTTTGAAAAGGTTGAAAGGTTAAAGCATGGAACCTTCGGCCCTGTCCATTTCCGGAATCAGGTTAAGATACGCCGCAGAGTTGACATAAACCCGGGCCATGCAGA<br>CCTCAGTGCTAAAGAAGCACAAGACGTCATCATGGAGGTCGTTTTCCCAAATGAAGTCGGAGCCAGAATATTGACATCAGAGTCACAGTTGACAATTACAAAAGAAAAGAAGGAGGAACTCCAGGACTGTAAGATTGCCCCTCTAATGGTGGCATACATGTTGGAGAGAGAACTGGTTCGAAAAACCAGATTCCTGCCAGTAGCTGGCGGAACAAGTAGCGTATATATCG<br>AAGTGTTGCACTTGACTCAAGGAACCTGCTGGGAACAAATGTATACGCCAGGAGGAGAAGTGAGAAATGATGACGTTGACCAAAGTTTAATTATTGCTGCCAGAAATATCGTTAGGAGGGCAACAGTATCAGCAGACCCATTGGCTTCACTACTGGAGATGTGCCATAGTACACAGATTGGCGGGATAAGAATGGTAGACATTCTTAGACAGAACCCAACAGAAGAGCA<br>AGCCGTGGATATATGCAAAGCAGCAATGGGTTTAAGAATCAGTTCATCCTTCAGTTTTGGAGGTTTCACTTTCAAAAGGACAAGCGGATCATCTGTCAAAAGAGAAGAGGAAGTGCTCACCGGCAACCTCCAAACATTGAAAATAAGAGTACATGAAGGGTATGAGGAATTCACAATGGTTGGGCGGAGAGCAACAGCCATTCTAAGGAAAGCAACCAGAAGGCTGATC<br>CAATTGATAGTAAGTGGAAGAGACGAGCAGTCAATCGCCGAAGCGATCATAGTGGCAATGGTGTTCTCTCAAGAGGATTGCATGATAAAGGCTGTACGAGGTGATTTAAATTTTGTCAATAGAGCGAATCAGCGGCTCAATCCTATGCATCAGCTCCTGAGGCATTTCCAAAAGGATGCAAAGGTACTATTCCAAAACTGGGGAATTGAACCCATTGACAATGTCATGGGA<br>ATGATAGGAATATTGCCTGATATGACCCCCAGCACAGAGATGTCACTAAGAGGAGTGAGGGTCAGTAAAATGGGAGTGGATGAATATTCCAGTACTGAGAGGGTGGTCGTGAGTATTGATCGCTTCTTGAGGGTACGAGACCAGAGAGGAAATGTACTCTTGTCTCCCGAAGAGGTCAGTGAAACACAGGGAACAGAGAAGCTAACGATAACATATTCATCATCCATGA<br>TGTGGGAAATTAATGGCCCTGAGTCAGTGCTAGTTAACACATATCAATGGATCATCAGAAACTGGGAAACTGTGAAGATTCAGTGGTCCCAAGACCCTACAATGCTATACAACAAGATGGAGTTTGAGCCTTTTCAGTCCTTGGTGCCTAAGGCAGCCAGAGGCCAGTACAGTGGATTTGTAAGGACCCTATTCCAGCAGATGCGTGATGTGCTGGGAACCTTTGACACTGT<br>CCAGATAATAAAGCTACTTCCATTTGCAGCAGCACCACCGGAACAGAGTAGGATGCAGTTCTCTTCTCTAACTGTAAACGTAAGGGGTTCAGGAATGAGAATACTTGTGAGAGGCAACTCCCCTGTGTTCAACTATAATAAAGCAACCAAGAGGCTCATAGTCCTTGGAAAGGATGCTGGTGCATTGACAGAAGACCCAGGTGAGGGGACAGCAGGAGTGGAGTCTGCG<br>GTATTGAGAGGGTTCCTAATTCTGGGCAAAGAGGACAAAAGATATGGACCAGCGCTGAGCATCAATGAATTGAGCAATCTTGCGAAAGGGGAGAAGGCTAATGTGTTGATAGGGCAAGGAGACGTGGTGTTGGTGATGAAACGGAAACGGGACTCTAGCATACTTACTGACAGCCAGACAGCGACCAAAAGAATTCGGATGGCCATCAATTA
EPI_ISL_9029965	>A/Greylag_goose/England/054503/2021_H5N1_2021-10-30<br>ATGGAGAGAATAAAAGAGCTAAGAGATTTGATGTCGCAGTCTCGCACTCGCGAGATACTGACAAAAACCACCGTGGACCATATGGCCATAATCAAGAAATATACATCAGGAAGACAGGAGAAGAACCCTGCACTTAGGATGAAGTGGATGATGGCAATGAAATATCCGATTACAGCAGACAAAAGGATAATGGAGATGATCCCTGAAAGAAACGAGCAAGGTCAGACT<br>CTTTGGAGCAAAACAAATGATGCTGGATCGGATAGAGTAATGGTGTCACCTCTGGCTGTGACGTGGTGGAATAGAAATGGACCAACAACAAGTACCGTCCATTACCCAAAGGTCTATAAAACCTACTTTGAAAAGGTTGAAAGGTTAAAGCATGGAACCTTCGGCCCTGTCCATTTCCGGAATCAGGTTAAGATACGCCGCAGAGTTGACATAAACCCGGGCCATGCAGA<br>CCTCAGTGCTAAAGAAGCACAAGACGTCATCATGGAGGTCGTTTTCCCAAATGAAGTCGGAGCCAGAATATTGACATCAGAGTCACAGTTGACAATTACAAAAGAAAAGAAGGAGGAACTCCAGGACTGTAAGATTGCCCCTCTAATGGTGGCATACATGTTGGAGAGAGAACTGGTTCGAAAAACCAGATTCCTGCCAGTAGCTGGCGGAACAAGTAGCGTATATATCG<br>AAGTGTTGCACTTGACTCAAGGAACCTGCTGGGAACAAATGTATACGCCAGGAGGAGAAGTGAGAAATGATGACGTTGACCAAAGTTTAATTATTGCTGCCAGAAATATCGTTAGGAGGGCAACAGTATCAGCAGACCCATTGGCTTCACTACTGGAGATGTGCCATAGTACACAGATTGGCGGGATAAGAATGGTAGACATTCTTAGACAGAACCCAACAGAAGAGCA<br>AGCCGTGGATATATGCAAAGCAGCAATGGGTTTAAGAATCAGTTCATCCTTCAGTTTTGGAGGTTTCACTTTCAAAAGGACAAGCGGATCATCTGTCAAAAGAGAAGAGGAAGTGCTCACCGGCAACCTCCAAACATTGAAAATAAGAGTACATGAAGGGTATGAGGAATTCACAATGGTTGGGCGGAGAGCAACAGCCATTCTAAGGAAAGCAACCAGAAGGCTGATC<br>CAATTGATAGTAAGTGGAAGAGACGAGCAGTCAATCGCCGAAGCGATCATAGTGGCAATGGTGTTCTCTCAAGAGGATTGCATGATAAAGGCTGTACGAGGTGATTTAAATTTTGTCAATAGAGCGAATCAGCGGCTCAATCCTATGCATCAGCTCCTGAGGCATTTCCAAAAGGATGCAAAGGTACTATTCCAAAACTGGGGAATTGAACCCATTGACAATGTCATGGGA<br>ATGATAGGAATATTGCCTGATATGACCCCCAGCACAGAGATGTCACTAAGAGGAGTGAGGGTCAGTAAAATGGGAGTGGATGAATATTCCAGTACTGAGAGGGTGGTCGTGAGTATTGATCGCTTCTTGAGGGTACGAGACCAGAGAGGAAATGTACTCTTGTCTCCCGAAGAGGTCAGTGAAACACAGGGAACAGAGAAGCTAACGATAACATATTCATCATCCATGA<br>TGTGGGAAATTAATGGCCCTGAGTCAGTGCTAGTTAACACATATCAATGGATCATCAGAAACTGGGAAACTGTGAAGATTCAGTGGTCCCAAGACCCTACAATGCTATACAACAAGATGGAGTTTGAGCCTTTTCAGTCCTTGGTGCCTAAGGCAGCCAGAGGCCAGTACAGTGGATTTGTAAGGACCCTATTCCAGCAGATGCGTGATGTGCTGGGAACCTTTGACACTGT<br>CCAGATAATAAAGCTACTTCCATTTGCAGCAGCACCACCGGAACAGAGTAGGATGCAGTTCTCTTCTCTAACTGTAAACGTAAGGGGTTCAGGAATGAGAATACTTGTGAGAGGCAACTCCCCTGTGTTCAACTATAATAAAGCAACCAAGAGGCTCATAGTCCTTGGAAAGGATGCTGGTGCATTGACAGAAGACCCAGGTGAGGGGACAGCAGGAGTGGAGTCTGCG<br>GTATTGAGAGGGTTCCTAATTCTGGGCAAAGAGGACAAAAGATATGGACCAGCGCTGAGCATCAATGAATTGAGCAATCTTGCGAAAGGGGAGAAGGCTAATGTGTTGATAGGGCAAGGAGACGTGGTGTTGGTGATGAAACGGAAACGGGACTCTAGCATACTTACTGACAGCCAGACAGCGACCAAAAGAATTCGGATGGCCATCAATTA
EPI_ISL_14388434	>A/Buteo_buteo/belgium/334_0013/2021_H5N1_2021-12-24<br>ATGGAGAGAATAAAAGAGCTAAGAGATTTGATGTCGCAGTCTCGCACTCGCGAGATACTGACAAAAACCACCGTGGACCATATGGCCATAATCAAGAAATATACATCAGGAAGACAGGAGAAGAACCCTGCACTTAGGATGAAGTGGATGATGGCAATGAAATATCCGATTACAGCAGACAAAAGGATAATGGAGATGATCCCTGAAAGAAACGAGCAAGGTCAGACT<br>CTTTGGAGCAAAACAAATGATGCTGGATCGGATAGAATAATGGTGTCACCTCTGGCTGTGACGTGGTGGAATAGAAATGGACCAACAACAAGTACCGTCCATTACCCAAAGGTCTATAAAACCTACTTTGAAAAGGTTGAAAGGTTAAAGCATGGAACCTTCGGCCCTGTCCATTTCCGGAATCAGGTTAAGATACGCCGCAGAGTTGACATAAACCCGGGCCATGCAGAC<br>CTCAGTGCTAAAGAAGCACAAGACGTCATCATGGAGGTCGTTTTCCCAAATGAAGTCGGAGCCAGAATATTGACATCAGAGTCACAGTTAACAATTACAAAAGAAAAGAAGGAGGAACTCCAGGACTGTAAGATTGCCCCTCTAATGGTGGCATACATGTTGGAGAGAGAACTGGTTCGAAAAACCAGATTCCTGCCAGTAGCTGGAGGAACAAGTAGCGTATATATCG<br>AAGTGTTACACTTGACTCAAGGAACCTGCTGGGAACAAATGTATACGCCAGGAGGAGAAGTGAGAAATGATGACGTTGACCAAAGTTTAATTATTGCTGCCAGAAATATCGTTAGGAGGGCAACAGTATCAGCAGACCCATTGGCTTCACTACTGGAGATGTGCCATAGTACACAGATTGGCGGGATAAGAATGGTAGACATTCTTAGACAGAACCCAACAGAAGAGCA<br>AGCCGTGGATATATGCAAAGCAGCAATGGGTTTAAGAATCAGTTCATCCTTCAGTTTTGGAGGTTTCACTTTCAAAAGGACAAGCGGATCATCTGTCAAAAGAGAAGAGGAAGTGCTCACAGGCAACCTCCAAACATTGAAAATAAGAGTACATGAAGGGTATGAGGAATTCACAATGGTTGGACGGAGAGCAACAGCCATTCTAAGGAAAGCAACCAGAAGGCTAATC<br>CAATTGATAGTAAGTGGAAGAGACGAGCAGTCAATCGCCGAAGCGATCATAGTGGCAATGGTGTTCTCTCAAGAGGATTGCATGATAAAGGCTGTACGAGGTGATTTAAATTTTGTCAATAGAGCGAATCAGCGGCTCAATCCTATGCATCAGCTCCTGAGGCATTTCCAAAAGGATGCAAAGGTACTATTCCAAAACTGGGGAATTGAACCCATTGACAATGTCATGGGA<br>ATGATAGGAATATTGCCTGATATGACCCCCAGCACAGAGATGTCACTAAGAGGAGTGAGGGTCAGTAAAATGGGAGTGGATGAATATTCCAGTACTGAGAGGGTGGTCGTGAGTATTGATCGCTTCTTGAGGGTACGAGACCAGAGAGGAAATGTACTCTTGTCTCCCGAAGAGGTCAGTGAAACACAGGGAACAGAGAAGCTAACGATAACATATTCATCATCCATGA<br>TGTGGGAAATTAATGGCCCTGAGTCAGTGCTAGTTAACACATATCAATGGATCATCAGAAACTGGGAAACTGTGAAGATTCAGTGGTCCCAAGACCCTACAATGCTATACAACAAGATGGAGTTTGAGCCTTTTCAGTCCTTGGTGCCTAAGGCAGCCAGAGGCCAGTACAGTGGATTTGTAAGGACCCTATTCCAGCAGATGCGTGATGTGCTGGGAACCTTTGACACTGT<br>CCAGATAATAAAGCTACTTCCATTTGCAGCAGCACCACCGGAACAGAGTAGGATGCAGTTCTCTTCTCTAACTGTAAACGTAAGGGGTTCAGGAATGAGAATACTTGTGAGAGGCAACTCCCCTGTGTTCAACTATAATAAAGCAACCAAGAGGCTCATAGTCCTTGGAAAGGATGCTGGTGCATTGACAGAAGACCCAGGTGAGGGGACAGCAGGAGTGGAGTCTGCG<br>GTATTGAGAGGGTTCCTAATTCTGGGCAAAGAGGACAAAAGATATGGACCAGCGCTGAGCATCAATGAATTGAGCAATCTTGCGAAAGGGGAGAAGGCTAATGTGTTGATAGGGCAAGGAGACGTGGTGTTGGTGATGAAACGGAAACGGGACTCTAGCATACTTACTGACAGCCAGACAGCGACCAAAAGAATTCGGATGGCCATCAATTA
EPI_ISL_14388346	>A/Tyto_alba/Belgium/334_0012/2021_H5N1_2021-12-23<br>ATGGAGAGAATAAAAGAGCTAAGAGATTTGATGTCGCAGTCTCGCACTCGCGAGATACTGACAAAAACCACCGTGGACCATATGGCCATAATCAAGAAATATACATCAGGAAGACAGGAGAAGAACCCTGCACTTAGGATGAAGTGGATGATGGCAATGAAATATCCGATTACAGCAGACAAAAGGATAATGGAGATGATCCCTGAAAGAAACGAGCAAGGTCAGACT<br>CTTTGGAGCAAAACAAATGATGCTGGATCGGATAGAATAATGGTGTCACCTCTGGCTGTGACGTGGTGGAATAGAAATGGACCAACAACAAGTACCGTCCATTACCCAAAGGTCTATAAAACCTACTTTGAAAAGGTTGAAAGGTTAAAGCATGGAACCTTCGGCCCTGTCCATTTCCGGAATCAGGTTAAGATACGCCGCAGAGTTGACATAAACCCGGGCCATGCAGAC<br>CTCAGTGCTAAAGAAGCACAAGACGTCATCATGGAGGTCGTTTTCCCAAATGAAGTCGGAGCCAGAATATTGACATCAGAGTCACAGTTAACAATTACAAAAGAAAAGAAGGAGGAACTCCAGGACTGTAAGATTGCCCCTCTAATGGTGGCATACATGTTGGAGAGAGAACTGGTTCGAAAAACCAGATTCCTGCCAGTAGCTGGAGGAACAAGTAGCGTATATATCG<br>AAGTGTTACACTTGACTCAAGGAACCTGCTGGGAACAAATGTATACGCCAGGAGGAGAAGTGAGAAATGATGACGTTGACCAAAGTTTAATTATTGCTGCCAGAAATATCGTTAGGAGGGCAACAGTATCAGCAGACCCATTGGCTTCACTACTGGAGATGTGCCATAGTACACAGATTGGCGGGATAAGAATGGTAGACATTCTTAGACAGAACCCAACAGAAGAGCA<br>AGCCGTGGATATATGCAAAGCAGCAATGGGTTTAAGAATCAGTTCATCCTTCAGTTTTGGAGGTTTCACTTTCAAAAGGACAAGCGGATCATCTGTCAAAAGAGAAGAGGAAGTGCTCACAGGCAACCTCCAAACATTGAAAATAAGAGTACATGAAGGGTATGAGGAATTCACAATGGTTGGACGGAGAGCAACAGCCATTCTAAGGAAAGCAACCAGAAGGCTAATC<br>CAATTGATAGTAAGTGGAAGAGACGAGCAGTCAATCGCCGAAGCGATCATAGTGGCAATGGTGTTCTCTCAAGAGGATTGCATGATAAAGGCTGTACGAGGTGATTTAAATTTTGTCAATAGAGCGAATCAGCGGCTCAATCCTATGCATCAGCTCCTGAGGCATTTCCAAAAGGATGCAAAGGTACTATTCCAAAACTGGGGAATTGAACCCATTGACAATGTCATGGGA<br>ATGATAGGAATATTGCCTGATATGACCCCCAGCACAGAGATGTCACTAAGAGGAGTGAGGGTCAGTAAAATGGGAGTGGATGAATATTCCAGTACTGAGAGGGTGGTCGTGAGTATTGATCGCTTCTTGAGGGTACGAGACCAGAGAGGAAATGTACTCTTGTCTCCCGAAGAGGTCAGTGAAACACAGGGAACAGAGAAGCTAACGATAACATATTCATCATCCATGA<br>TGTGGGAAATTAATGGCCCTGAGTCAGTGCTAGTTAACACATATCAATGGATCATCAGAAACTGGGAAACTGTGAAGATTCAGTGGTCCCAAGACCCTACAATGCTATACAACAAGATGGAGTTTGAGCCTTTTCAGTCCTTGGTGCCTAAGGCAGCCAGAGGCCAGTACAGTGGATTTGTAAGGACCCTATTCCAGCAGATGCGTGATGTGCTGGGAACCTTTGACACTGT<br>CCAGATAATAAAGCTACTTCCATTTGCAGCAGCACCACCGGAACAGAGTAGGATGCAGTTCTCTTCTCTAACTGTAAACGTAAGGGGTTCAGGAATGAGAATACTTGTGAGAGGCAACTCCCCTGTGTTCAACTATAATAAAGCAACCAAGAGGCTCATAGTCCTTGGAAAGGATGCTGGTGCATTGACAGAAGACCCAGGTGAGGGGACAGCAGGAGTGGAGTCTGCG<br>GTATTGAGAGGGTTCCTAATTCTGGGCAAAGAGGACAAAAGATATGGACCAGCGCTGAGCATCAATGAATTGAGCAATCTTGCGAAAGGGGAGAAGGCTAATGTGTTGATAGGGCAAGGAGACGTGGTGTTGGTGATGAAACGGAAACGGGACTCTAGCATACTTACTGACAGCCAGACAGCGACCAAAAGAATTCGGATGGCCATCAATTA

EPI_ISL_7778754	>A/Withe-tiled_eagle/Estonia/TA2124126-1_21VIR10433-11/2021_H5N1_2021-10-12<br>ATGGAGAGAATAAAAGAGCTAAGAGATTTGATGTCGCAGTCTCGCACTCGCGAGATACTGACAAAAACCACCGTGGACCATATGGCCATAATCAAGAAATATACATCAGGAAGACAGGAGAAGAACCCTGCACTTAGGATGAAGTGGATGATGGCAATGAAATATCCGATTACAGCAGACAAAAGGATAATGGAGATGATCCCTGAAAGAAACGAGCAAGGTCAGACT<br>CTTTGGAGCAAAACAAATGATGCTGGATCGGATAGAATAATGGTGTCACCTCTGGCTGTGACGTGGTGGAATAGAAATGGACCAACAACAAGTACCGTCCATTACCCAAAGGTCTATAAAACCTACTTTGAAAAGGTTGAAAGGTTAAAGCATGGAACCTTCGGCCCTGTCCATTTCCGGAATCAGGTTAAGATACGCCGCAGAGTTGACATAAACCCGGGCCATGCAGAC<br>CTCAGTGCTAAAGAAGCACAAGACGTCATCATGGAGGTCGTTTTCCCAAATGAAGTCGGAGCCAGAATATTGACATCAGAGTCACAGTTAACAATTACAAAAGAAAAGAAGGAGGAACTCCAGGACTGTAAGATTGCCCCTCTAATGGTGGCATACATGTTGGAGAGAGAACTGGTTCGAAAAACCAGATTCCTGCCAGTAGCTGGCGGAACAAGTAGCGTATATATCGA<br>AGTGTTGCACTTGACTCAAGGAACCTGCTGGGAACAAATGTATACGCCAGGAGGAGAAGTGAGAAATGATGACGTTGACCAAAGTTTAATTATTGCTGCCAGAAATATCGTTAGGAGGGCAACAGTATCAGCAGACCCATTGGCTTCACTACTGGAGATGTGCCATAGTACACAGATTGGCGGGATAAGAATGGTAGACATTCTTAGACAGAACCCAACAGAAGAGCAA<br>GCCGTGGATATATGCAAAGCAGCAATGGGTTTAAGAATCAGTTCATCCTTCAGTTTTGGAGGTTTCACTTTCAAAAGGACAAGCGGATCATCTGTCAAAAGAGAAGAGGAAGTGCTCACCGGCAACCTCCAAACATTGAAAATAAGAGTACATGAAGGGTATGAGGAATTCRCAATGGTTGGGCGGAGAGCAACAGCCATTCTAAGGAAAGCAACCAGAAGGCTGATCC<br>AATTGATAGTAAGTGGAAGAGACGAGCAGTCAATCGCCGAAGCGATCATAGTGGCAATGGTGTTCTCTCAAGAGGATTGCATGATAAAGGCTGTACGAGGTGATTTAAATTTTGTCAATAGAGCGAATCAGCGGCTCAATCCTATGCATCAGCTCCTGAGGCATTTCCAAAAGGATGCAAAGGTACTATTCCAAAACTGGGGAATTGAACCCATTGACAATGTCATGGGAA<br>TGATAGGAATATTGCCTGATATGACCCCCAGCACAGAGATGTCACTAAGAGGAGTGAGGGTCAGTAAAATGGGAGTGGATGAATATTCCAGTACTGAGAGGGTGGTCGTGAGTATTGATCGCTTCTTGAGGGTACGAGACCAGAGAGGAAATGTACTCTTGTCTCCCGAAGAGGTCAGTGAAACACAGGGAACAGAGAAGCTAACGATAACATATTCATCATCCATGAT<br>GTGGGAAATTAATGGCCCTGAGTCAGTGCTAGTTAACACATATCAATGGATCATCAGAAACTGGGAAACTGTGAAGATTCAGTGGTCCCAAGACCCTACAATGCTATACAACAAGATGGAGTTTGAGCCTTTTCAGTCCTTGGTGCCTAAGGCAGCCAGAGGCCAGTACAGTGGATTTGTAAGGACCCTATTCCAGCAGATGCGTGATGTGCTGGGAACCTTTGACACTGT<br>CCAGATAATAAAGCTACTTCCATTTGCAGCAGCACCACCGGAACAGAGTAGGATGCAGTTCTCTTCTCTGACTGTAAACGTAAGGGGTTCAGGAATGAGAATACTTGTGAGAGGCAACTCCCCTGTGTTCAACTATAATAAAGCAACCAAGAGGCTCATAGTCCTTGGAAAGGATGCTGGTGCATTGACAGAAGACCCAGGTGAGGGGACAGCAGGAGTGGAGTCTGCG<br>GTATTGAGAGGGTTCCTAATTCTGGGCAAAGAGGACAAAAGATATGGACCAGCGCTGAGCATCAATGAATTGAGCAATCTTGCGAAAGGGGAGAAGGCTAATGTGTTGATAGGGCAAGGAGACGTGGTGTTGGTGATGAAACGGAAACGGGACTCTAGCATACTTACTGACAGCCAGACAGCGACCAAAAGAATTCGGATGGCCATCAATTA
EPI_ISL_1184524	>A/mallard/Novosibirsk_region/3445k/2020_H1N1_2020-08-29<br>ATGGAGAGAATAAAAGAACTAAGAGATTTGATGTCGCAGTCTCGCACTCGCGAGATACTGACAAAAACCACGGTGGACCATATGGCCATAATTAAGAAATACACATCAGGAAGACAGGAGAAGAACCCTGCCCTTAGGATGAAGTGGATGATGGCAATGAAATACCCGATTACAGCAGACAAAAGAATAATGGAAATGATCCCTGAAAGAAATGAACAAGGCCAGACT<br>CTCTGGAGCAAAACAAGTGATGCTGGGTCAGACAGAGTAATGGTGTCACCTCTGGCTGTGACATGGTGGAATAGAAATGGACCGACGACAAGTACAGTCCACTATCCAAAGGTCTACAAAACCTACTTTGAAAAAGTCGAAAGGTTGAAACATGGAACCTTCGGTCCTGTTCACTTTCGGAATCAGGTTAAAATACGCCGCAGGGTTGACATAAACCCGGGCCATGCAGA<br>TCTCAGTGCCAAAGAAGCACAAGATGTCATCATGGAGGTCGTTTTCCCAAACGAAGTTGGAGCAAGGATATTGACATCAGAGTCACAATTAACAATAACAAAGGAAAAGAAGGAGGAGCTTCAGGACTGCAAGATCGCCCCTTTAATGGTGGCATACATGTTGGAGAGAGAACTGGTTCGCAAAACCAGATTCCTACCAGTAGCTGGCGGAACAAGCAGCGTATATATC<br>GAGGTATTGCATTTGACTCAAGGGACCTGCTGGGAACAAATGTACACACCGGGAGGGGAGGTGAGAAATGATGATGTCGATCAGAGTTTGATTATTGCTGCTAGAAATATTGTTAGGAGAGCAACAGTGTCGGCAGACCCGTTGGCTTCGCTCTTGGAGATGTGCCATAGTACACAAATTGGCGGGATAAGAATGGTGGACATCCTTAGACAGAACCCAACAGAAGAGC<br>AAGCTGTGGATATATGCAAAGCAGCAATGGGTTTGAGAATCAGTTCATCCTTCAGCTTTGGAGGTTTCACTTTTAAAAGGACAAGTGGGTCATCTGTCAAAAGAGAAGAAGAAGTGCTCACGGGCAACCTCCAAACACTGAAAATAAGAGTACATGAAGGATATGAAGAATTCACAATGGTTGGGCGAAGAGCTACGGCCATTCTAAGGAAAGCAACCAGAAGGCTGAT<br>CCAATTAATAGTGAGTGGGAGAGACGAGCAGTCAATCGCTGAAGCGATTATAGTGGCAATGGTTTTCTCACAAGAGGACTGCATGATAAAAGCAGTGCGAGGTGATTTGAACTTTGTTAACAGGGCAAATCAGCGGCTAAATCCTATGCACCAACTTCTGAGACATTTCCAAAAGGATGCAAAGGTGCTGTTTCAAAATTGGGGAATTGAACCCATTGACAATGTCATGGG<br>AATGATCGGGATACTGCCTGACATGACCCCCAGCACAGAGATGTCACTGAGAGGAGTGAGAGTCAGTAAAATGGGAGTGGATGAATATTCCAGTACTGAGAGAGTGGTCGTAAGCATTGATCGTTTCTTAAGAGTCCGAGACCAGAGGGGAAACGTACTCTTGTCTCCTGAAGAAGTTAGTGAAACACAGGGAACAGAGAAACTGACGATAACATATTCATCATCTATG<br>ATGTGGGAAATCAATGGTCCGGAGTCAGTACTGGTCAACACATATCAATGGATCATTAGAAATTGGGAAACTGTGAAGATTCAGTGGTCCCAAGACCCCACAATGTTGTACAATAAGATGGAGTTTGAGCCTTTCCAATCCTTGGTGCCCAAGGCTGCCAGAGGCCAGTACAGTGGATTTGTGAGGACGTTATTCCAGCAGATGCGTGATGTGCTGGGGACATTTGACACT<br>GTCCAAATAATAAAGCTCCTACCATTTGCAGCAGCCCCACCGGAACAGAGTAGGATGCAGTTCTCTTCTCTGACTGTGAATGTAAGAGGTTCAGGAATGAGAATACTTGTGAGAGGCAACTCCCCTGTGTTCAACTATAACAAAGCAACCAAGAGACTCACAGTTCTTGGAAAGGATGCAGGTGCATTGACAGAAGATCCAGACGAGGGAACAGCAGGAGTGGAGTCTGC<br>GGTGTTAAGAGGATTTCTAATTCTGGGCAAAGAAGACAAAAGATATGGACCAGCATTGAGCATTAACGAATTGAGCAATCTTGCGAAAGGGGAGAAGGCTAATGTGTTGATAGGGCAAGGAGACGTGGTGTTGGTAATGAAACGGAAACGGGACTCTAGCATACTTACTGACAGCCAGACAGCGACCAAAAGGATTCGGATGGCCATCAATTA
EPI_ISL_11633614	>A/Cygnus_columbianus/Hubei/121/2021_H5N1_2021-12-20<br>ATGGAGAGAATAAAAGAACTAAGAGATTTGATGTCGCAGTCTCGCACTCGCGAGATACTGACAAAAACCACGGTGGACCATATGGCCATAATTAAGAAATACACATCAGGAAGACAGGAGAAGAACCCTGCCCTTAGGATGAAGTGGATGATGGCAATGAAATACCCGATTACAGCAGACAAAAGAATAATGGAAATGATCCCTGAAAGAAATGAACAAGGCCAGACT<br>CTCTGGAGCAAAACAAGTGATGCTGGATCAGACAGAGTAATGGTGTCACCCCTGGCTGTGACATGGTGGAATAGAAATGGACCGACGACAAGTACAGTCCACTATCCAAAGGTCTACAAAACCTACTTTGAAAAAGTCGAAAGGTTGAAACATGGAACCTTCGGTCCTGTTCACTTTCGGAATCAGGTTAAAATACGCCGCAGGGTTGACATAAACCCGGGCCATGCAGA<br>TCTCAGCGCCAAAGAAGCACAAGATGTCATCATGGAGGTCGTTTTCCCAAACGAAGTTGGAGCAAGGATATTGACATCAGAGTCACAATTAACAATAACAAAGGAAAAGAAGGAGGAGCTTCAGGACTGCAAGATCGCCCCTTTAATGGTGGCATACATGTTGGAGAGAGAACTGGTTCGCAAAACCAGATTCCTACCAGTAGCTGGCGGAACAAGCAGCGTATATATC<br>GAGGTATTGCATTTGACTCAAGGGACCTGCTGGGAACAAATGTACACACCGGGAGGGGAGGTGAGAAATGATGATGTCGATCAGAGTTTGATTATTGCTGCTAGAAATATTGTTAGGAGAGCAACAGTGTCGGCAGACCCGTTGGCTTCGCTCTTGGAGATGTGCCATAGTACACAAATTGGCGGGATGAGAATGGTGGACATCCTTAGACAGAACCCAACAGAAGAGC<br>AAGCTGTGGATATATGCAAAGCAGCAATGGGTTTGAGAATCAGTTCATCCTTCAGCTTTGGAGGTTTCACTTTTAAAAGGACAAGTGGGTCATCTGTCAAAAGAGAAGAAGAAGTGCTCACGGGCAACCTCCAAACACTGAAAATAAGAGTACATGAAGGATATGAAGAATTCACAATGGTTGGGCGAAGAGCTACGGCCATTCTAAGGAAAGCAACCAGAAGGCTGAT<br>CCAATTAATAGTGAGTGGGAGAGACGAGCAGTCAATCGCTGAAGCTATTATAGTGGCAATGGTTTTCTCACAAGAGGACTGCATGATAAAAGCAGTGCGAGGTGATTTGAACTTTGTTAACAGAGCAAATCAGCGGCTAAATCCTATGCACCAACTTCTGAGACATTTCCAAAAGGATGCAAAGGTGCTGTTTCAAAATTGGGGAATTGAACCCATTGACAATGTCATGGG<br>AATGATCGGGATACTGCCTGACATGACCCCCAGCACAGAGATGTCACTGAGAGGAGTGAGAGTCAGTAAAATGGGAGTGGATGAATATTCCAGCACTGAGAGAGTGGTCGTAAGCATTGATCGTTTCTTAAGAGTCCGAGACCAGAGGGGAAACGTACTCTTGTCTCCTGAAGAAGTTAGTGAAACACAGGGAACAGAGAAACTGACGATAACATATTCATCGTCTATG<br>ATGTGGGAAATCAATGGTCCGGAGTCAGTACTGGTCAACACATATCAATGGATCATTAGAAATTGGGAAACTGTGAAGATTCAGTGGTCCCAAGACCCCACAATGTTGTACAATAAGATGGAGTTTGAACCTTTCCAATCCTTGGTGCCCAAGGCTGCCAGAGGCCAGTACAGTGGATTTGTGAGGACGTTATTCCAGCAGATGCGTGATGTGCTGGGGACATTTGACACT<br>GTCCAAATAATAAAGCTCCTACCATTTGCAGCAGCCCCACCGGAACAGAGTAGGATGCAGTTCTCTTCTCTGACTGTGAATGTAAGAGGTTCAGGAATGAGAATACTTGTGAGAGGCAACTCCCCTGTGTTCAACTATAACAAAGCAACCAAGAGACTCACAGTTCTTGGAAAGGATGCAGGTGCGTTGACAGAAGATCCAGACGAGGGAACAGCAGGAGTGGAGTCTGC<br>GGTGTTAAGAGGATTTCTAATTCTGGGCAAAGAAGACAAAAGATATGGACCAGCATTGAGCATTAACGAATTGAGCAATCTTGCGAAAGGGGAGAAGGCTAATGTGTTGATAGGGCAAGGAGACGTGGTGTTGGTAATGAAACGGAAACGGGACTCTAGCATACTTACTGACAGCCAGACAGCGACCAAAAGGATTCGGATGGCCATCAATTA
EPI_ISL_7987343	>A/teal/Italy/21VIR8923-73/2021_H5N3_2021-10-29<br>ATGGAGAGAATAAAAGAACTAAGAGATTTGATGTCGCAGTCTCGCACTCGCGAGATACTGACAAAAACTACGGTGGACCATATGGCCATAATTAAGAAATACACATCAGGAAGACAGGAGAAGAACCCTGCCCTTAGGATGAAGTGGATGATGGCAATGAAATACCCGATTACAGCAGACAAAAGAATAATGGAAATGATCCCTGAAAGGAATGAACAAGGCCAGACT<br>CTCTGGAGCAAAACAAGTGATGCTGGATCAGACAGAGTAATGGTGTCACCTCTGGCTGTGACATGGTGGAATAGAAATGGACCGACGACAAGTACAGTCCACTATCCAAAGGTCTACAAAACCTACTTTGAAAAAGTCGAAAGGTTGAAACATGGAACCTTCGGTCCTGTTCACTTTCGGAATCAGGTTAAGATACGCCGCAGGGTTGACATAAACCCGGGCCATGCAGA<br>TCTCAGTGCCAAAGAAGCACAAGATGTCATCATGGAGGTCGTTTTTCCAAACGAAGTTGGAGCAAGGATATTGACATCAGAGTCACAATTAACAATAACAAAGGAAAAGAAGGAGGAGCTTCAGGACTGCAAGATCGCCCCTTTAATGGTGGCATACATGTTGGAGAGAGAACTGGTTCGCAAAACCAGATTCCTACCAGTAGCTGGCGGAACAAGCAGCGTATATATC<br>GAGGTATTGCATTTGACCCAAGGGACCTGCTGGGAACAAATGTACACACCGGGAGGGGAGGTGAGAAATGATGATGTCGATCAGAGTTTGATTATTGCTGCTAGAAATATTGTTAGGAGAGCAACAGTGTCGGCAGACCCGTTGGCTTCGCTCTTGGAGATGTGCCATAGTACACAAATTGGCGGGATAAGAATGGTGGACATCCTTAGACAGAACCCAACAGAAGAGC<br>AAGCTGTGGATATATGCAAAGCAGCAATGGGTTTGAGAATCAGTTCATCCTTCAGCTTTGGAGGTTTCACTTTTAAAAGGACAAGTGGGTCATCTGTCAAAAGAGAAGAAGAAGTGCTCACGGGCAACCTCCAAACACTGAAAATAAGAGTACATGAAGGATATGAGGAATTCACAATGGTTGGGCGAAGAGCTACAGCCATTCTAAGGAAAGCAACCAGAAGGCTGAT<br>CCAATTAATAGTGAGTGGGAGAGACGAGCAGTCAATCGCTGAAGCGATTATAGTGGCAATGGTTTTCTCACAAGAGGACTGCATGATAAAAGCAGTGCGAGGTGATTTGAACTTTGTTAACAGAGCAAATCAGCGGCTAAATCCTATGCACCAACTTCTGAGACATTTCCAAAAGGATGCAAAGGTGCTGTTTCAAAATTGGGGAATTGAACCCATTGACAATGTCATGGG<br>AATGATCGGGATACTGCCTGACATGACCCCCAGCACAGAGATGTCACTGAGAGGAGTAAGAGTTAGTAAAATGGGAGTGGATGAATATTCCAGTACTGAGAGAGTGGTCGTAAGCATTGATCGTTTCTTAAGAGTCCGAGACCAGAGGGGAAACGTACTCTTGTCTCCTGAAGAAGTTAGTGAAACACAGGGAACAGAGAAACTGACGATAACATATTCATCGTCTATGA<br>TGTGGGAAATCAATGGTCCGGAGTCAGTACTGGTCAACACATATCAATGGATCATTAGAAATTGGGAAACTGTGAAGATTCAGTGGTCCCAAGACCCCACAATGTTGTACAATAAGATGGAGTTTGAGCCTTTCCAATCCTTGGTGCCCAAGGCTGCCAGAGGCCAGTACAGTGGATTTGTGAGGACGTTATTCCAGCAGATGCGTGATGTGCTGGGGACATTTGACACTG<br>TCCAAATAATAAAGCTCCTACCATTTGCAGCAGCCCCACCGGAACAGAGTAGGATGCAGTTCTCTTCTCTGACTGTGAATGTAAGAGGTTCAGGAATGAGAATACTTGTGAGAGGCAACTCCCCTGTGTTCAACTATAACAAAGCAACCAAGAGACTCACAGTTCTTGGAAAGGATGCAGGTGCATTGACAGAAGATCCAGACGAGGGAACAGCAGGAGTGGAGTCTGCG<br>GTGTTAAGAGGATTTCTAATTCTGGGCAAAGAAGACAAAAGATATGGACCAGCATTGAGCATTAACGAATTGAGCAATCTTGCGAAAGGGGAGAAGGCTAATGTGTTGATAGGGCAAGGAGACGTGGTGTTGGTAATGAAACGGAAACGGGACTCTAGCATACTTACTGACAGCCAGACAGCGACCAAAAGGATTCGGATGGCCATCAATTA
EPI_ISL_11007534	>A/seagull/Slovenia/2075_22VIR777-7/2021_H5N1_2021-12-30<br>ATGGAGAGAATAAAAGAACTAAGAGATTTGATGTCGCAGTCTCGCACTCGCGAGATACTGACAAAAACCACGGTGGACCATATGGCCATAATTAAGAAATACACATCAGGAAGACAGGAGAAGAACCCTGCCCTTAGGATGAAGTGGATGATGGCAATGAAATACCCGATTACAGCAGACAAAAGAATAATGGAAATGATCCCTGAAAGAAATGAACAAGGCCAGACT<br>CTCTGGAGCAAAACAAGTGATGCTGGATCAGACAGAGTAATGGTGTCACCTCTGGCTGTGACATGGTGGAACAGAAATGGACCGACGACAAGTACAGTCCACTATCCAAAGGTCTACAAAACCTACTTTGAAAAAGTTGAAAGGTTGAAACATGGAACCTTCGGTCCTGTTCACTTTCGGAATCAGGTTAAAATACGCCGCAGGGTTGACATAAACCCGGGCCATGCAGA<br>TCTCAGTGCCAAAGAAGCACAAGATGTCATCATGGAGGTCGTTTTCCCAAACGAAGTTGGAGCAAGGATATTGACATCAGAGTCACAATTAACAATAACAAAGGAAAAGAAGGAGGAGCTTCAGGACTGCAAGATCGCCCCTTTAATGGTGGCATACATGTTGGAGAGAGAACTGGTTCGCAAAACCAGATTCCTACCAGTAGCTGGCGGAACAAGCAGCGTATATATC<br>GAGGTGTTGCATTTGACTCAAGGGACCTGCTGGGAACAAATGTACACACCGGGAGGGGAGGTGAGAAATGATGATGTCGATCAGAGTTTGATTATTGCTGCTAGAAATATTGTTAGGAGAGCAACAGTGTCGGCAGACCCGTTGGCTTCGCTCTTGGAGATGTGCCATAGTACACAAATTGGCGGGATAAGAATGGTGGACATCCTTAGACAGAACCCAACAGAAGAGC<br>AAGCTGTGGATATATGCAAAGCAGCAATGGGTTTAAGAATCAGTTCATCCTTCAGCTTTGGAGGTTTCACTTTTAAAAGAACAAGTGGGTCATCTGTCAAAAGAGAAGAAGAAGTGCTCACGGGCAACCTCCAAACACTGAAAATAAGAGTACATGAAGGATATGAAGAATTCACAATGGTTGGGCGAAGAGCTACGGCCATTCTAAGGAAAGCAACCAGAAGGCTGATC<br>CAATTAATAGTGAGTGGGAGAGACGAGCAGTCAATCGCTGAAGCGATTATAGTGGCAATGGTTTTCTCACAAGAGGACTGCATGATAAAAGCAGTGCGAGGTGATTTGAACTTTGTTAACAGAGCAAATCAGCGGCTAAATCCTATGCACCAACTTCTGAGACATTTCCAAAAGGATGCAAAGGTGCTGTTTCAAAATTGGGGGACTGAACCCATTGACAATGTCATGGG<br>AATGATCGGGATACTGCCTGACATGACCCCTAGCACAGAGATGTCACTGAGAGGAGTGAGAGTCAGTAAAATGGGAGTGGATGAATATTCCAGTACTGAGAGAGTGGTCGTAAGCATTGATCGTTTCTTAAGAGTCCGAGACCAGAGGGGAAACGTACTCTTGTCTCCTGAAGAAGTTAGTGAAACACAGGGAACAGAGAAACTGACGATAACATATTCATCGTCTATG<br>ATGTGGGAAATCAATGGTCCGGAGTCAGTACTGGTCAACACATATCAATGGATCATTAGAAATTGGGAAACTGTGAAGATTCAGTGGTCCCAAGACCCCACAATGTTGTACAATAAAATGGAGTTTGAGCCTTTCCAATCCTTGGTGCCCAAGGCTGCCAGAGGCCAGTACAGTGGATTTGTGAGGACGTTATTCCAGCAGATGCGTGATGTGCTGGGGACATTTGACACT<br>GTCCAAATAATAAAGCTCCTACCATTTGCAGCAGCCCCACCGGAACAGAGTAGGATGCAGTTCTCTTCTCTGACTGTGAATGTAAGAGGTTCAGGAATGAGAATACTTGTGAGAGGTAACTCCCCTGTGTTCAACTATAACAAAGCAACCAAGAGACTCACAGTTCTTGGAAAGGATGCAGGTGCATTGACAGAAGATCCAGACGAGGGAACAGCAGGAGTGGAGTCTGC<br>GGTGTTAAGAGGATTTCTAATTCTGGGCAAAGAAGACAAAAGGTATGGACCAGCATTGAGCATTAACGAGTTGAGCAATCTTGCGAAAGGGGAGAAGGCTAATGTGTTGATAGGGCAAGGAGACGTGGTGTTGGTAATGAAACGGAAACGGGACTCTAGCATACTTACTGACAGCCAGACAGCGACCAAAAGGATTCGGATGGCCATCAATTA
EPI_ISL_11007530	>A/swan/Slovenia/2049_22VIR777-3/2021_H5N1_2021-12-27<br>ATGGAGAGAATAAAAGAACTAAGAGATTTGATGTCGCAGTCTCGCACTCGCGAGATACTGACAAAAACCACGGTGGACCATATGGCCATAATTAAGAAATACACATCAGGAAGACAGGAGAAGAACCCTGCCCTTAGGATGAAGTGGATGATGGCAATGAAATACCCGATTACAGCAGACAAAAGAATAATGGAAATGATCCCTGAAAGAAATGAACAAGGCCAGACT<br>CTCTGGAGCAAAACAAGTGATGCTGGATCAGACAGAGTAATGGTGTCACCTCTGGCTGTGACATGGTGGAACAGAAATGGACCGACGACAAGTACAGTCCACTATCCAAAGGTCTACAAAACCTACTTTGAAAAAGTTGAAAGGTTGAAACATGGAACCTTCGGTCCTGTTCACTTTCGGAATCAGGTTAAAATACGCCGCAGGGTTGACATAAACCCGGGCCATGCAGA<br>TCTCAGTGCCAAAGAAGCACAAGATGTCATCATGGAGGTCGTTTTCCCAAACGAAGTTGGAGCAAGGATATTGACATCAGAGTCACAATTAACAATAACAAAGGAAAAGAAGGAGGAGCTTCAGGACTGCAAGATCGCCCCTTTAATGGTGGCATACATGTTGGAGAGAGAACTGGTTCGCAAAACCAGATTCCTACCAGTAGCTGGCGGAACAAGCAGCGTATATATC<br>GAGGTGTTGCATTTGACTCAAGGGACCTGCTGGGAACAAATGTACACACCGGGAGGGGAGGTGAGAAATGATGATGTCGATCAGAGTTTGATTATTGCTGCTAGAAATATTGTTAGGAGAGCAACAGTGTCGGCAGACCCGTTGGCTTCGCTCTTGGAGATGTGCCATAGTACACAAATTGGCGGGATAAGAATGGTGGACATCCTTAGACAGAACCCAACAGAAGAGC<br>AAGCTGTGGATATATGCAAAGCAGCAATGGGTTTAAGAATCAGTTCATCCTTCAGCTTTGGAGGTTTCACTTTTAAAAGAACAAGTGGGTCATCTGTCAAAAGAGAAGAAGAAGTGCTCACGGGCAACCTCCAAACACTGAAAATAAGAGTACATGAAGGATATGAAGAATTCACAATGGTTGGGCGAAGAGCTACGGCCATTCTAAGGAAAGCAACCAGAAGGCTGATC<br>CAATTAATAGTGAGTGGGAGAGACGAGCAGTCAATCGCTGAAGCGATTATAGTGGCAATGGTTTTCTCACAAGAGGACTGCATGATAAAAGCAGTGCGAGGTGATTTGAACTTTGTTAACAGAGCAAATCAGCGGCTAAATCCTATGCACCAACTTCTGAGACATTTCCAAAAGGATGCAAAGGTGCTGTTTCAAAATTGGGGGACTGAACCCATTGACAATGTCATGGG<br>AATGATCGGGATACTGCCTGACATGACCCCTAGCACAGAGATGTCACTGAGAGGAGTGAGAGTCAGTAAAATGGGAGTGGATGAATATTCCAGTACTGAGAGAGTGGTCGTAAGCATTGATCGTTTCTTAAGAGTCCGAGACCAGAGGGGAAACGTACTCTTGTCTCCTGAAGAAGTTAGTGAAACACAGGGAACAGAGAAACTGACGATAACATATTCATCGTCTATG<br>ATGTGGGAAATCAATGGTCCGGAGTCAGTACTGGTCAACACATATCAATGGATCATTAGAAATTGGGAAACTGTGAAGATTCAGTGGTCCCAAGACCCCACAATGTTGTACAATAAAATGGAGTTTGAGCCTTTCCAATCCTTGGTGCCCAAGGCTGCCAGAGGCCAGTACAGTGGATTTGTGAGGACGTTATTCCAGCAGATGCGTGATGTGCTGGGGACATTTGACACT<br>GTCCAAATAATAAAGCTCCTACCATTTGCAGCAGCCCCACCGGAACAGAGTAGGATGCAGTTCTCTTCTCTGACTGTGAATGTAAGAGGTTCAGGAATGAGAATACTTGTGAGAGGTAACTCCCCTGTGTTCAACTATAACAAAGCAACCAAGAGACTCACAGTTCTTGGAAAGGATGCAGGTGCATTGACAGAAGATCCAGACGAGGGAACAGCAGGAGTGGAGTCTGC<br>GGTGTTAAGAGGATTTCTAATTCTGGGCAAAGAAGACAAAAGGTATGGACCAGCATTGAGCATTAACGAGTTGAGCAATCTTGCGAAAGGGGAGAAGGCTAATGTGTTGATAGGGCAAGGAGACGTGGTGTTGGTAATGAAACGGAAACGGGACTCTAGCATACTTACTGACAGCCAGACAGCGACCAAAAGGATTCGGATGGCCATCAATTA
EPI_ISL_338357	>A/Tufted_Duck/Novosibirsk_region/989k/2018_H3N8_2018-10-01<br>ATGGAGAGAATAAAAGAACTAAGAGATTTGATGTCGCAGTCTCGCACTCGCGAGATACTGACAAAAACCACTGTGGACCATATGGCCATAATAAAGAAATACACGTCAGGAAGACAGGAGAAGAACCCTGCCCTCAGAATGAAATGGATGATGGCAATGAAATATCCGATTACAGCAGACAAAAGAATAATGGAGATGATCCCTGAAAGAAATGAGCAAGGTCAGACT<br>CTCTGGAGCAAAGCAAATGATGCTGGATCAGACAGAGTGATGGTGTCACCTCTGGCTGTGACGTGGTGGAATAGAAATGGGCCAACGACAAGTACAGTCCACTATCCAAAGGTCTACAAAACCTACTTTGAAAAGGTTGAAAGGTTGAAACATGGAACCTTCGGTCCTGTTCACTTCCGAAATCAGGTTAAAATACGCCGCAGGGTTGACATAAACCCGGGCCATGCAGA<br>TCTCAGTGCCAAAGAAGCACAGGATGTCATCATGGAGGTCGTCTTCCCAAATGAAGTTGGAGCCAGGATATTGACATCAGAGTCACAATTAACAATAACAAAGGAAAAGAAGGAGGAACTTCAGGACTGTAAGATTGCTCCTTTGATGGTGGCATACATGTTGGAGAGAGAACTGGTTCGCAAAACCAGATTTTTACCAGTAGCTGGCGGAACAAGCAGCGTATATATCG<br>AGGTGTTGCATTTGACTCAAGGGACCTGCTGGGAACAAATGTACACACCGGGAGGGGAGGTGAGAAATGATGATGTTGATCAGAGTTTGATCATCGCTGCTAGGAATATCGTTAGGAGAGCAACAGTATCAGCAGACCCATTGGCTTCGCTCTTGGAGATGTGCCACAGTACACAGATTGGCGGGGGAAGGATGGTGGACATTCTTAGACAGAACCCAACAGAAGAGC<br>AAGCTGTGGATATATGCAAAGCAGCAATGGGTTTAAGAATCAGTTCATCTTTCAGCTTTGGAGGTTTCACTTTCAAAAGGACAAGTGGGTCGTCTGTCAAAAGAGAGGAAGAAGTGCTCACGGGCAACCTTCAAACACTGAAAATAAGAGTACATGAAGGATATGAGGAATTCACAATGGTTGGGCGAAGAGCTACAGCCATTTTGAGGAAAGCAACCAGAAGGCTGATT<br>CAATTAATAGTGAGTGGGAGAGACGAGCAGTCAATCGCTGAAGCAATCATAGTGGCAATGGTTTTCTCACAAGAGGACTGCATGATAAAAGCAGTACGAGGTGATCTGAATTTTGTTAACAGAGCGAATCAGCGGCTAAATCCTATGCATCAACTCCTGAGGCATTTCCAAAAGGATGCAAAGGTATTGTTTCAAAACTGGGGGATTGAACCAATTGACAATGTAATGGG<br>GATGATCGGAATACTGCCTGACATGACCCCCAGCACAGAGATGTCACTGAGAGGAGTGAGAGTCAGCAAAATGGGAGTGGATGAATATTCCAGTACTGAGAGAGTGGTCGTGAGCATTGATCGTTTCTTGAGGGTCCGAGATCAGAGGGGAAACGTGCTCTTGTCTCCTGAGGAAGTTAGTGAAACACAGGGAACAGAGAAACTGACGATAACATATTCATCGTCCATG<br>ATGTGGGAAATCAATGGTCCGGAATCAGTGCTAGTCAACACATACCAATGGATCATTAGAAATTGGGAAGCTGTGAAGATTCAGTGGTCCCAAGACCCTACAATGTTGTACAATAAGATGGAATTTGAGCCCTTCCAATCCTTGGTGCCCAAGGCTGCCAGAGGCCAGTATAGTGGGTTTGTGAGGACATTGTTCCAGCAGATGCGTGATGTGCTGGGGACATTTGACACT<br>GTCCAAATAATAAAGCTCCTACCATTTGCAGCAGCCCCACCGGAACAGAGTAGGATGCAGTTCTCCTCTCTGACTGTGAACGTAAGAGGTTCAGGAATGAGAATACTTGTGAGAGGCAATTCCCCTGTGTTCAACTATAACAAGGCAACCAAGAGACTCACAGTTCTTGGAAAGGATGCAGGTGCCTTGACAGAAGATCCAGATGAGGGAACAGCAGGAGTGGAATCTGC<br>GGTATTAAGAGGATTTCTAATTCTGGGCAAAGAGGACAAAAGATATGGACCAGCATTGAGCATCAACGAATTGAGCAATCTTGCGAAAGGGGAGAAGGCTAATGTGTTGATAGGGCAAGGAGACGTGGTGTTGGTAATGAAACGGAAACGGGACTCTAGCATACTTACTGACAGCCAGACAGCGACCAAAAGAATTCGGATGGCCATCAATTA
EPI_ISL_1240999	>A/mallard/Novosibirsk_region/3513k/2020_H4N6_2020-08-29<br>ATGGAGAGAATAAAAGAACTAAGAGATTTGATGTCGCAGTCTCGCACTCGCGAGATACTGACAAAAACCACTGTAGACCATATGGCCATAATAAAGAAATATACGTCAGGAAGACAGGAGAAGAACCCTGCCCTCAGAATGAAATGGATGATGGCAATGAAATATCCGATTACAGCAGACAAAAGAATAATGGAGATGATCCCTGAAAGAAATGAGCAAGGCCAGACT<br>CTCTGGAGCAAAACAAATGATGCTGGATCAGACAGAGTGATGGTGTCACCTCTGGCTGTGACGTGGTGGAATAGAAATGGGCCAACGACAAGTACAGTCCACTATCCAAAGGTCTACAAAACCTACTTTGAAAAGGTTGAAAGGTTGAAACATGGAACCTTCGGTCCTGTTCGCTTCCGAAATCAGGTCAAAATACGCCGCAGGGTTGACATAAACCCGGGTCATGCAGA<br>TCTCAGTGCCAAAGAAGCACAGGATGTCATCATGGAGGTCGTCTTCCCAAATGAAGTTGGAGCCAGGATACTGACATCAGAGTCACAATTAACAATAACAAAGGAAAAGAAGGAGGAACTTCAGGACTGTAAGATTGCTCCTTTGATGGTGGCATACATGTTGGAGAGAGAACTGGTTCGCAAAACCAGATTTTTACCAGTAGCTGGCGGAACAAGCAGCGTATATATCG<br>AGGTGTTGCATTTGACTCAAGGGACCTGCTGGGAACAAATGTACACACCGGGAGGGGAGGTGAGAAATGATGATGTTGATCAGAGTTTGATCATCGCTGCTAGGAATATCGTTAGGAGAGCAACAGTATCAGCAGATCCATTGGCTTCGCTCTTGGAGATGTGCCACAGTACACAGATTGGCGGGGTAAGGATGGTGGACATTCTTAGACAGAACCCAACAGAAGAGCA<br>AGCTGTGGATATATGCAAAGCAGCAATGGGTTTAAGAATCAGTTCATCCTTCAGCTTTGGAGGTTTCACTTTCAAAAGGACAAGTGGGTCGTCTGTCAAAAGAGAGGAAGAAGTGCTCACAGGCAACCTTCAAACACTGAAAATAAGGGTACATGAAGGGTATGAGGAATTCACAATGGTTGGGCGAAGAGCTACAGCCATTTTGAGGAAAGCAACCAGAAGGCTGATC<br>CAATTAATAGTGAGTGGGAGAGATGAGCAGTCAATCGCTGAAGCAATCATAGTGGCAATGGTTTTCTCACAAGAGGACTGCATGATAAAAGCAGTACGAGGTGATCTGAATTTTGTTAACAGAGCGAATCAGCGGCTAAATCCTATGCATCAGCTCCTAAGGCATTTCCAAAAGGATGCAAAGGTGTTGTTTCAAAACTGGGGGATTGAACCAATTGACAATGTAATGGG<br>GATGATCGGGATACTGCCTGACATGACCCCCAGCACAGAAATGTCACTGAGAGGAGTGAGAGTCAGCAAAATGGGAGTGGATGAATATTCCAGCACTGAGAGAGTGGTCGTGAGCATTGATCGTTTCTTAAGGGTCCGAGATCAGAGGGGAAACGTGCTCTTGTCCCCTGAGGAAGTTAGTGAAACACAGGGAACAGAGAAACTGACGATAACATATTCATCGTCCATG<br>ATGTGGGAAATCAATGGTCCGGAATCAGTGCTAGTCAACACATATCAATGGATCATTAGAAATTGGGAAACTGTGAAGATTCAGTGGTCCCAAGACCCTACAATGTTGTACAATAAGATGGAATTTGAGCCCTTCCAGTCCTTGGTGCCCAAGGCTGCCAGAGGCCAGTATAGTGGGTTTGTGAGGACATTGTTCCAGCAGATGCGTGATGTGCTGGGAACATTTGACACT<br>GTCCAAATAATAAAGCTCCTACCATTTGCAGCAGCCCCACCGGAACAGAGTAGGATGCAGTTCTCCTCTCTGACTGTGAACGTAAGAGGTTCAGGAATGAGAATACTTGTGAGAGGCAATTCCCCTGTGTTCAACTATAACAAGGCAACCAAGAGACTCACAGTTCTTGGAAAGGATGCAGGTGCCTTGACAGAAGATCCAGATGAGGGAACAGCAGGAGTGGAATCTGC<br>GGTATTAAGAGGATTTCTAATTCTGGGCAAAGAGGATAAAAGATATGGACCAGCATTGAGCATCAACGAATTGAGCAATCTTGCGAAAGGGGAGAAGGCTAATGTATTGATAGGGCAAGGAGACGTGGTGTTGGTAATGAAACGGAAACGGGACTCTAGCATACTTACTGACAGCCAGACAGCGACCAAAAGAATTCGGATGGCCATCAATTA
EPI_ISL_1005678	>A/common_teal/Chany_Lake/4/2020_H10N7_2020-08-29<br>ATGGAGAGAATAAAAGAACTAAGAGATTTGATGTCGCAGTCTCGCACTCGCGAGATACTGACAAAAACCACTGTAGACCATATGGCCATAATAAAGAAATATACGTCAGGAAGACAGGAGAAGAACCCTGCCCTCAGGATGAAATGGATGATGGCAATGAAATATCCGATTACAGCAGACAAAAGAATAATGGAGATGATCCCTGAAAGAAATGAGCAAGGCCAGACT<br>CTCTGGAGCAAAACAAATGATGCTGGATCAGACAGAGTGATGGTATCACCTCTGGCTGTGACGTGGTGGAATAGAAATGGGCCAACGACAAGTACAGTCCACTATCCAAAGGTCTACAAAACCTACTTTGAAAAGGTTGAAAGGTTGAAACATGGAACCTTCGGACCTGTTCACTTCCGAAATCAGGTCAAAATACGCCGCAGGGTTGACATAAACCCGGGTCATGCAGA<br>TCTCAGTGCCAAAGAAGCACAGGATGTCATCATGGAGGTCGTCTTCCCAAATGAAGTTGGAGCCAGGATACTGACATCAGAATCACAATTAACAATAACAAAGGAAAAGAAGGAGGAACTTCAGGACTGTAAGATTGCTCCTTTGATGGTGGCATACATGTTGGAGAGAGAACTGGTTCGCAAAACCAGATTTTTACCAGTAGCTGGCGGAACAAGCAGCGTATATATCG<br>AGGTGTTGCATTTGACTCAAGGGACCTGCTGGGAACAAATGTACACACCGGGAGGGGAGGTGAGAAATGATGATGTTGATCAGAGTTTGATCATCGCTGCTAGGAATATCGTTAGGAGAGCAACAGTATCAGCAGACCCATTGGCTTCGCTCTTGGAGATGTGCCACAGTACACAGATTGGCGGAGTAAGGATGGTGGACATTCTTAGACAGAACCCAACAGAAGAGCA<br>AGCTGTGGATATATGCAAAGCAGCAATGGGTTTAAGAATCAGTTCATCCTTCAGCTTTGGAGGTTTCACTTTCAAAAGGACAAGTGGGTCGTCTGTCAAAAAAGAGGAAGAAGTGCTCACGGGCAACCTTCAAACACTGAAAATAAGAGTACATGAAGGATATGAGGAATTCACAATGGTTGGGCGAAGAGCTACAGCCATTTTGAGGAAAGCAACCAGAAGGCTGATC<br>CAATTAATAGTGAGTGGGAGAGACGAGCAGTCAATCGCTGAAGCAATCATAGTGGCAATGGTTTTCTCACAAGAGGACTGCATGATAAAAGCAGTACGAGGTGATCTGAATTTTGTTAACAGAGCGAATCAGCGGCTAAATCCTATGCATCAGCTCTTGAGGCATTTCCAAAAGGATGCAAAGGTGTTATTTCAGAACTGGGGGATTGAACCAATTGATAATGTAATGGG<br>GATGATCGGGATACTGCCTGACATGACCCCCAGCACAGAAATGTCACTGAGAGGAGTGAGAGTCAGCAAAATGGGAGTGGATGAATATTCCAGCACTGAGAGAGTGGTCGTGAGCATTGATCGTTTCTTAAGGGTCCGAGATCAGAGGGGAAACGTGCTCTTGTCTCCAGAGGAAGTTAGTGAAACACAGGGAACAGAGAAACTGACGATAACATATTCATCGTCCATG<br>ATGTGGGAAATCAATGGTCCGGAATCAGTGCTAGTCAACACATATCAATGGATCATTAGAAATTGGGAAACTGTGAAGATTCAGTGGTCCCAAGACCCTACAATGTTGTACAATAAGATGGAATTTGAGCCCTTCCAATCCTTGGTGCCCAAGGCTGCCAGAGGCCAGTATAGTGGGTTTGTGAGGACATTGTTCCAGCAGATGCGTGATGTGCTGGGAACATTTGACACT<br>GTCCAAATAATAAAGCTCCTACCATTTGCAGCAGCCCCACCGGAACAGAGTAGGATGCAGTTCTCCTCTCTGACTGTGAACGTAAGAGGTTCAGGAATGAGAATACTTGTGAGAGGCAATTCCCCTGTGTTCAACTATAACAAGGCAACCAAGAGACTCACAGTTCTTGGAAAGGATGCAGGTGCCTTGACAGAAGATCCAGATGAGGGAACAGCAGGAGTGGAATCTGC<br>GGTATTAAGAGGATTTCTAATTTTGGGCAAAGAGGATAAAAGATATGGACCAGCATTGAGCATCAACGAATTGAGCAATCTTGCGAAAGGGGAGAAGGCTAATGTGTTGATAGGGCAAGGAGACGTGGTGTTGGTAATGAAACGGAAACGGGACTCTAGCATACTTACTGACAGCCAGACAGCGACCAAAAGAATTCGGATGGCCATCAATTA
EPI_ISL_11211731	>A/duck/Bangladesh/19D1818/2021_H5N1_2021-12-22<br>ATGGAGAGAATAAAAGAACTAAGAGATTTGATGTCGCAGTCTCGCACTCGCGAGATACTGACAAAAACCACTGTAGACCACATGGCCATAATAAAGAAATATACGTCAGGAAGACAGGAGAAGAATCCTGCCCTCAGAATGAAATGGATGATGGCAATGAAATATCCGATTACAGCAGATAAAAGAATAATGGAGATGATCCCTGAAAGAAATGAGCAAGGCCAGACT<br>CTCTGGAGCAAAACAAATGATGCTGGATCAGACAGAGTGATGGTGTCACCTCTGGCTGTGACGTGGTGGAATAGAAATGGGCCAACGACAAGTACAGTCCACTATCCAAAGGTCTACAAAACCTACTTTGAAAAGGTTGAAAGGTTGAAACATGGAACCTTCGGTCCTGTTCACTTCCGAAATCAGGTCAAAATACGCCGCAGGGTTGACATAAACCCGGGTCATGCAGA<br>TCTCAGTGCCAAAGAAGCACAGGATGTCATCATGGAGGTTGTCTTCCCAAATGAAGTTGGAGCCAGGATACTGACATCAGAGTCACAATTAACAATAACAAAGGAAAAGAAGGAGGAACTTCAGGACTGTAAGATTGCTCCTTTGATGGTGGCATACATGTTGGAGAGAGAACTGGTTCGCAAAACCAGATTTTTACCAGTAGCTGGCGGAACAAGCAGCGTGTATATCG<br>AGGTGTTGCATTTGACTCAAGGGACCTGCTGGGAACAAATGTACACACCGGGAGGGGAGGTGAGAAATGATGATGTTGATCAGAGTTTGATCATCGCTGCTAGGAATATTGTCAGGAGAGCAACAGTATCAGCAGACCCATTGGCTTCGCTCTTGGAGATGTGCCACAGTACACAGATTGGCGGGATAAGGATGGTGGACATTCTTAGACAGAACCCAACAGAAGAGCA<br>AGCTGTGGATATATGCAAAGCAGCAATGGGTTTAAGGATCAGTTCATCCTTCAGCTTTGGAGGTTTCACTTTCAAAAGGACAAGTGGGTCGTCTGTCAAAAGAGAGGAAGAAGTGCTCACGGGCAACCTTCAAACACTGARAATAAGGATACATGAAGGATATGAGGAATTCACAATGGTTGGGCGAAGAGCTACAGCCATTTTGAGGAAAGCAACCAGAAGGCTGATC<br>CAGTTAATAGTGAGTGGGAGAGATGAGCAGTCAATCGCTGAAGCAATCATAGTGGCAATGGTTTTTTCACAAGAGGACTGCATGATAAAAGCAGTACGAGGTGATCTGAATTTTGTTAACAGAGCGAATCAGCGGCTAAATCCTATGCATCAGCTCCTAAGGCATTTCCAGAAAGATGCAAAGGTGTTGTTTCAAAACTGGGGGATTGAACCAATTGACAATGTAATGGG<br>GATGATCGGGATACTGCCTGACATGACCCCCAGCACAGAAATGTCACTGAGAGGAGTGAGAGTCAGCAAAATGGGAGTGGATGAATATTCCAGCACTGAGAGAGTGGTCGTGAGCATTGATCGTTTCTTAAGGGTCCGAGATCAGAGGGGAAACGTGCTCTTGTCTCCTGAGGAAGTTAGTGAAACACAGGGAACAGAGAAACTGACGATAACATATTCATCGTCCATG<br>ATGTGGGAAATCAATGGTCCGGAATCAGTGCTTGTCAACACATATCAATGGATCATTAGAAATTGGGAAACTGTGAAGATTCAGTGGTCCCAAGACCCTACAATGTTGTACAATAAGATGGAATTTGAGCCCTTCCAATCCTTGGTGCCCAAGGCTGCCAGAGGCCAGTATAGTGGGTTTGTGAGGACATTGTTCCAGCAGATGCGTGATGTGCTGGGAACATTTGACACT<br>GTCCAAATAATAAAGCTCCTACCATTTGCAGCAGCCCCACCAGAACAGAGTAGGATGCAGTTCTCCTCTCTGACTGTGAACGTAAGAGGTTCAGGAATGAGAATACTTGTGAGAGGCAATTCCCCTGTGTTCAACTATAACAAGGCAACCAAGAGACTCACAGTTCTTGGAAAGGATGCAGGTGCCTTGACAGAAGATCCAGATGAGGGAACAGCAGGAGTGGAGTCTGC<br>GGTATTGAGAGGATTTCTAATTCTGGGCAAAGAGGATAAAAGATATGGACCAGCATTGAGCATCAACGAATTGAGCAATCTTGCGAAAGGGGAGAAGGCTAATGTGCTGATAGGGCAAGGAGACGTGGTGTTGGTAATGAAACGGAAACGGGACTCTAGCATACTTACTGACAGCCAGACAGCGACCAAAAGAATTCGGATGGCCATCAATTA
EPI_ISL_5865695	>A/green-winged-teal/Georgia/DT-22246/2020_H5N8_2020-09-29<br>ATGGAGAGAATAAAAGAACTAAGAGATTTGATGTCGCAGTCTCGCACTCGCGAGATACTAACAAAAACCACTGTGGACCATATGGCCATAATAAAGAAATACACATCAGGAAGACAGGAGAAGAACCCTGCCCTCAGGATGAAATGGATGATGGCAATGAAATATCCTATTACAGCTGACAAAAGAATAATGGAGATGATCCCTGAAAGGAATGAGCAAGGTCAGACT<br>CTCTGGAGCAAAACAAATGATGCTGGATCAGACAGAGTGATGGTCTCACCTCTGGCTGTGACATGGTGGAATAGAAATGGCCAACAACAAGTACAGTACACTACCCAAAAGTCTACAAAACCTACTTTGAAAAGGTAGAAAGGTTGAAACATGGAACCTTTGGTCCTGTTCACTTTCGAAATCAGGTTAAGATACGCCGCAGGGTTGACATAAACCCGGGCCATGCAGA<br>TCTCAGTGCCAAAGAAGCACAAGATGTCATCATGGAGGTTGTTTTCCCAAATGAAGTTGGAGCCAGGATCTTGACATCAGAATCACAATTAACAATAACAAGGGAAAAGAAGGAGGAACTTCAGGATTGCAAGATTGCTCCTTTGATGGTGGCATACATGTTGGAAAGAGAACTGGTTCGCAAGACCAGATTTTTACCAGTAGCTGGCGGAACAAGCAGCGTATACATCG<br>AGGTATTGCATTTGACTCAAGGGACCTGCTGGGAACAAATGTACACACCAGGAGGGGAGGTGAGAAATGATGATGTTGATCAGAGTTTGATCATTGCTGCTAGAAATATAGTTAGGAGGGCAACAGTATCAGCAGACCCTTTGGCTTCGCTCTTGGAAATGTGCCACAGTACACAAATTGGTGGAGTAAGGATGGTGGACATTCTTAGGCAGAACCCAACAGAGGAGCA<br>AGCTGTGGATATATGCAAAGCAGCAATGGGTTTAAGAATCAGTTCATCCTTCAGCTTTGGAGGTTTCACTTTCAAAAGGACAAGTGGGTCGTCTGTCAAAAGAGAAGAAGAAATACTCACTGGCAACCTCCAAACACTGAAAGTAAGAATACATGAAGGATATGAGGAATTCACAATGGTTGGGCGAAGAGCTACAGCCATTTTGAGGAAAGCAACCAGGAGACTGATCC<br>AATTAATAGTGAGTGGAAGAGACGAGCAGTCAATCGCTGAAGCAATCATAGTGGCAATGGTTTTCTCACAGGAGGATTGCATGATAAAAGCAGTACGAGGTGATTTGAATTTTGTCAACAGAGCGAATCAGCGGCTAAATCCTATGCATCAACTTCTGAGGCATTTCCAAAAGGATGCAAAAGTGCTGTTTCAAAACTGGGGGATTGAACCAATTGACAATGTAATGGGG<br>ATGATCGGGATACTGCCTGACATGACCCCCAGCACAGAGATGTCACTGAGAGGAGTGAGAGTCAGCAAAATGGGAGTGGATGAATATTCCAGTACTGAGAGAGTGGTCGTGAGCATTGATCGCTTCTTGAGAGTCCGAGATCAGAGGGGAAATGTGCTTTTGTCTCCTGAGGAAGTTAGTGAAACACATGGAACAGAGAAACTGACGATAACGTATTCATCGTCTATGAT<br>GTGGGAAATCAATGGTCCGGAATCCGTGCTAGTCAACACATATCAATGGATCATTAGAAGTTGGGAAACTGTGAAGATTCAATGGTCCCAGGACCCTACGATGTTGTACAATAAGATGGAATTTGAGCCCTTCCAATCCTTGGTGCCCAAGGCTGCTAGAGGCCAGTATAGTGGGTTTGTGAGGACATTATTCCAACAGATGCGTGATGTGTTGGGGACATTTGACACTGTC<br>CAAATAATAAAGCTCCTACCATTTGCAGCAGCCCCACCGGAACAGAGTAGGATGCAATTTTCCTCTCTGACTGTGAACGTAAGAGGTTCAGGAATGAGAATACTTGTGAGGGGCAACTCCCCTGTGTTCAACTATAATAAGGCCACCAAGAGACTCACAGTTCTTGGAAAGGATGCAGGCGCCTTGACAGAATATCCAGATGAGGGAACAGCAGGAGTGGAGTCTGCAGT<br>ATTAAGAGGATTTCTAATTCTGGGCAAAGAGGACAAAAGATATGGACCAGCATTGAGCATCAACGAATTGAGCAATCTTGCGAAAGGGGAAAAGGCTAATGTGTTGATAGGACAAGGAGACGTGGTGTTGGTAATGAAACGGAAACGGGACTCTAGCATACTTACTGACAGCCAGACAGCGACCAAAAGAATTCGGATGGCCATCAATTA
EPI_ISL_5863641	>A/mallard/Georgia/DT-22495/2020_H5N8_2020-10-09<br>ATGGAGAGAATAAAAGAACTAAGAGATTTGATGTCGCAGTCTCGCACTCGCGAGATACTAACAAAAACCACTGTGGACCATATGGCCATAATAAAGAAATACACATCAGGAAGACAGGAGAAGAACCCTGCCCTCAGGATGAAATGGATGATGGCAATGAAATATCCTATTACAGCTGACAAAAGAATAATGGAGATGATCCCTGAAAGGAATGAGCAAGGTCAGACT<br>CTCTGGAGCAAAACAAATGATGCTGGATCAGACAGAGTGATGGTCTCACCTCTGGCTGTGACATGGTGGAATAGAAATGGCCAACAACAAGTACAGTACACTACCCAAAAGTCTACAAAACCTACTTTGAAAAGGTAGAAAGGTTGAAACATGGAACCTTTGGTCCTGTTCACTTTCGAAATCAGGTTAAGATACGCCGCAGGGTTGACATAAACCCGGGCCATGCAGA<br>TCTCAGTGCCAAAGAAGCACAAGATGTCATCATGGAGGTTGTTTTCCCAAATGAAGTTGGAGCCAGGATCTTGACATCAGAATCACAATTAACAATAACAAGGGAAAAGAAGGAGGAACTTCAGGATTGCAAGATTGCTCCTTTGATGGTGGCATACATGTTGGAAAGAGAACTGGTTCGCAAGACCAGATTTTTACCAGTAGCTGGCGGAACAAGCAGCGTATACATCG<br>AGGTATTGCATTTGACTCAAGGGACCTGCTGGGAACAAATGTACACACCAGGAGGGGAGGTGAGAAATGATGATGTTGATCAGAGTTTGATCATTGCTGCTAGAAATATAGTTAGGAGGGCAACAGTATCAGCAGACCCTTTGGCTTCGCTCTTGGAAATGTGCCACAGTACACAAATTGGTGGAGTAAGGATGGTGGACATTCTTAGGCAGAACCCAACAGAGGAGCA<br>AGCTGTGGATATATGCAAAGCAGCAATGGGTTTAAGAATCAGTTCATCCTTCAGCTTTGGAGGTTTCACTTTCAAAAGGACAAGTGGGTCGTCTGTCAAAAGAGAAGAAGAAATACTCACTGGCAACCTCCAAACACTGAAAGTAAGAATACATGAAGGATATGAGGAATTCACAATGGTTGGGCGAAGAGCTACAGCCATTTTGAGGAAAGCAACCAGGAGACTGATCC<br>AATTAATAGTGAGTGGAAGAGACGAGCAGTCAATCGCTGAAGCAATCATAGTGGCAATGGTTTTCTCACAGGAGGATTGCATGATAAAAGCAGTACGAGGTGATTTGAATTTTGTCAACAGAGCGAATCAGCGGCTAAATCCTATGCATCAACTTCTGAGGCATTTCCAAAAGGATGCAAAAGTGCTGTTTCAAAACTGGGGGATTGAACCAATTGACAATGTAATGGGG<br>ATGATCGGGATACTGCCTGACATGACCCCCAGCACAGAGATGTCACTGAGAGGAGTGAGAGTCAGCAAAATGGGAGTGGATGAATATTCCAGTACTGAGAGAGTGGTCGTGAGCATTGATCGCTTCTTGAGAGTCCGAGATCAGAGGGGAAATGTGCTTTTGTCTCCTGAGGAAGTTAGTGAAACACATGGAACAGAGAAACTGACGATAACGTATTCATCGTCTATGAT<br>GTGGGAAATCAATGGTCCGGAATCCGTGCTAGTCAACACATATCAATGGATCATTAGAAGTTGGGAAACTGTGAAGATTCAATGGTCCCAGGACCCTACGATGTTGTACAATAAGATGGAATTTGAGCCCTTCCAATCCTTGGTGCCCAAGGCTGCTAGAGGCCAGTATAGTGGGTTTGTGAGGACATTATTCCAACAGATGCGTGATGTGTTGGGGACATTTGACACTGTC<br>CAAATAATAAAGCTCCTACCATTTGCAGCAGCCCCACCGGAACAGAGTAGGATGCAATTTTCCTCTCTGACTGTGAACGTAAGAGGTTCAGGAATGAGAATACTTGTGAGGGGCAACTCCCCTGTGTTCAACTATAATAAGGCCACCAAGAGACTCACAGTTCTTGGAAAGGATGCAGGCGCCTTGACAGAATATCCAGATGAGGGAACAGCAGGAGTGGAGTCTGCAGT<br>ATTAAGAGGATTTCTAATTCTGGGCAAAGAGGACAAAAGATATGGACCAGCATTGAGCATCAACGAATTGAGCAATCTTGCGAAAGGGGAAAAGGCTAATGTGTTGATAGGACAAGGAGACGTGGTGTTGGTAATGAAACGGAAACGGGACTCTAGCATACTTACTGACAGCCAGACAGCGACCAAAAGAATTCGGATGGCCATCAATTA

EPI_ISL_683752	>A/Eurasian_wigeon/Italy/20VIR7301-34/2020_H5N8_2020-11-21<br>ATGGAGAGAATAAAAGAACTAAGAGATTTGATGTCGCAGTCTCGCACTCGCGAGATACTAACAAAAACCACTGTGGACCATATGGCCATAATAAAGAAATACACATCAGGAAGACAGGAGAAGAACCCTGCCCTCAGGATGAAATGGATGATGGCAATGAAATATCCTATTACAGCTGACAAAAGAATAATGGAGATGATCCCTGAAAGGAATGAGCAAGGTCAGACT<br>CTCTGGAGCAAAACAAATGATGCTGGATCAGACAGAGTGATGGTCTCACCTCTGGCTGTGACATGGTGGAATAGAAATGGGCCAACAACAAGTACAGTACACTACCCAAAAGTCTACAAAACCTACTTTGAAAAGGTAGAAAGGTTGAAACATGGAACCTTTGGTCCTGTTCACTTCGAAATCAGGTTAAGATACGCCGCAGGGTTGACATAAACCCGGGCCATGCAGA<br>TCTCAGTGCCAAAGAAGCACAGGATGTCATCATGGAGGTTGTTTTCCCAAATGAAGTTGGAGCCAGGATCTTGACATCAGAATCACAATTAACAATAACAAGGGAAAAGAAGGAGGAACTTCAGGATTGCAAGATTGCTCCTTTGATGGTGGCATACATGTTGGAAAGAGAACTGGTTCGCAAGACCAGATTTTTACCAGTAGCTGGCGGAACAAGCAGCGTATACATCG<br>AGGTATTGCATTTGACTCAAGGGACCTGCTGGGAACAAATGTACACACCAGGAGGGGAGGTGAGAAATGATGATGTTGATCAGAGTTTGATCATTGCTGCTAGAAATATAGTTAGGAGGGCAACAGTATCAGCAGACCCTTTGGCTTCGCTCTTGGAAATGTGCCACAGTACACAAATTGGTGGAGTAAGGATGGTGGACATTCTTAGGCAGAACCCAACAGAGGAGCA<br>AGCTGTGGATATATGCAAAGCAGCAATGGGTTTAAGAATCAGTTCATCCTTCAGCTTTGGAGGTTTCACTTTCAAAAGGACAAGTGGGTCGTCTGTCAAAAGAGAAGAAGAAATACTCACTGGCAACCTCCAAACACTGAAAGTAAGAATACATGAAGGATATGAGGAATTCACAATGGTTGGGCGAAGAGCTACAGCCATTTTGAGGAAAGCAACCAGGAGACTGATCC<br>AATTAATAGTGAGTGGAAGAGACGAGCAGTCAATCGCTGAAGCAATCATAGTGGCAATGGTTTTCTCACAGGAGGATTGCATGATAAAAGCAGTACGAGGTGATTTGAATTTTGTCAACAGAGCGAATCAGCGGCTAAATCCTATGCATCAACTTCTGAGGCATTTCCAAAAGGATGCAAAAGTGCTGTTTCAAAACTGGGGGATTGAACCAATTGACAATGTAATGGGG<br>ATGATCGGGATACTGCCTGACATGACCCCCAGCACAGAGATGTCACTGAGAGGAGTGAGAGTCAGCAAAATGGGAGTGGATGAATATTCCAGTACTGAGAGAGTGGTCGTGAGCATTGATCGCTTCTTGAGAGTCCGAGATCAGAGGGGAAATGTGCTTTTGTCTCCTGAGGAAGTTAGTGAAACACATGGAACAGAGAAACTGACGATAACGTATTCATCGTCTATGAT<br>GTGGGAAATCAATGGTCCGGAATCCGTGCTAGTCAACACATATCAATGGATCATTAGAAGTTGGGAAACTGTGAAGATTCAATGGTCCCAGGACCCTACGATGTTGTACAATAAGATGGAATTTGAGCCCTTCCAATCCTTGGTGCCCAAGGCTGCTAGAGGCCAGTATAGTGGGTTTGTGAGGACATTATTCCAACAGATGCGTGATGTGTTGGGGACATTTGACACTGTC<br>CAAATAATAAAGCTCCTACCATTTGCAGCAGCCCCACCGGAACAGAGTAGGATGCAATTTTCCTCTCTGACTGTGAACGTAAGAGGTTCAGGAATGAGAATACTTGTGAGGGGCAACTCCCCTGTGTTCAACTATAATAAGGCCACCAAGAGACTCACAGTTCTTGGAAAGGATGCAGGCGCCTTGACAGAATATCCAGATGAGGGAACAGCAGGAGTGGAGTCTGCAGT<br>ATTAAGAGGATTTCTAATTCTGGGCAAAGAGGACAAAAGATATGGACCAGCATTGAGCATCAACGAATTGAGCAATCTTGCGAAAGGGGAAAAGGCTAATGTGTTGATAGGACAAGGAGACGTGGTGTTGGTAATGAAACGGAAACGGGACTCTAGCATACTTACTGACAGCCAGACAGCGACCAAAAGAATTCGGATGGCCATCAATTA
EPI_ISL_1191587	>A/chicken/Czech_Republic/1566-2/2021_H5N8_2021-01-22<br>ATGGAGAGAATAAAAGAACTAAGAGATTTGATGTCGCAGTCTCGCACTCGCGAGATACTAACAAAAACCACTGTGGACCATATGGCCATAATAAAGAAATACACATCAGGAAGACAGGAGAAGAACCCTGCCCTCAGGATGAAATGGATGATGGCAATGAAATATCCTATTACAGCTGACAAAAGAATAATGGAGATGATCCCTGAAAGGAATGAGCAAGGTCAGACT<br>CTCTGGAGCAAAACAAATGATGCTGGATCAGACAGAGTGATGGTCTCACCCCTGGCTGTGACATGGTGGAATAGAAATGGGCCAACAACAAGTACAGTACACTACCCAAAAGTCTACAAAACCTACTTTGAAAAGGTAGAAAGGTTGAAACATGGAACCTTTGGTCCTGTTCACTTTCGAAATCAGGTTAAGATACGCCGCAGGGTTGACATAAACCCGGGCCATGCAGA<br>TCTCAGTGCCAAAGAAGCACAGGATGTCATCATGGAGGTTGTTTTCCCAAATGAAGTTGGAGCCAGGATCTTGACATCAGAATCACAATTAACAATAACAAGAGAAAAGAAGGAGGAACTTCAGGATTGCAAGATTGCTCCTTTGATGGTGGCATACATGTTGGAAAGAGAACTGGTTCGCAAGACCAGATTTTTACCAGTAGCTGGCGGAACAAGCAGCGTATACATCG<br>AGGTATTGCATTTGACTCAAGGGACCTGCTGGGAACAAATGTACACACCAGGAGGGGAGGTGAGAAATGATGATGTTGATCAGAGTTTGATCATTGCTGCTAGAAATATAGTTAGGAGGGCAACAGTATCAGCAGACCCTTTGGCTTCGCTCTTGGAAATGTGCCACAGTACACAAATTGGTGGAATAAGGATGGTGGACATTCTTAGGCAGAACCCAACAGAGGAGCA<br>AGCTGTGGATATATGCAAAGCAGCAATGGGTTTAAGAATCAGTTCATCCTTCAGCTTTGGAGGTTTCACTTTCAAAAGGACAAGTGGGTCGTCTGTCAAAAGAGAAGAAGAAATACTCACTGGCAACCTCCAAACACTGAAAGTAAGAATACATGAAGGATATGAGGAATTCACAATGGTTGGGCGAAGAGCTACAGCCATTTTGAGGAAAGCAACCAGGAGACTGATCC<br>AATTAATAGTGAGTGGAAGAGACGAGCAGTCAATCGCTGAAGCAATCATAGTGGCAATGGTTTTCTCACAGGAGGATTGCATGATAAAAGCAGTACGAGGTGATTTGAATTTTGTCAACAGAGCGAATCAGCGGCTAAATCCTATGCATCAACTTCTGAGGCATTTCCAAAAGGATGCAAAAGTGCTGTTTCAAAACTGGGGGATTGAACCAATTGACAATGTAATGGGG<br>ATGATCGGGATACTGCCTGACATGACCCCCAGCACAGAGATGTCACTGAGAGGAGTGAGAGTCAGCAAAATGGGAGTGGATGAATATTCCAGTACTGAGAGAGTGGTCGTGAGCATTGATCGCTTCTTGAGAGTCCGAGATCAGAGGGGAAATGTGCTTTTGTCTCCTGAGGAAGTTAGTGAAACACATGGAACAGAGAAACTGACGATAACGTATTCATCGTCTATGAT<br>GTGGGAAATCAATGGTCCGGAATCCGTGCTAGTCAACACATATCAATGGATCATTAGAAGTTGGGAAACTGTGAAGATTCAATGGTCCCAGGACCCTACGATGTTGTACAATAAGATGGAATTTGAGCCCTTCCAATCCTTGGTGCCCAAGGCTGCTAGAGGCCAGTATAGTGGGTTTGTGAGGACATTATTCCAACAGATGCGTGATGTGTTGGGGACATTTGACACTGTC<br>CAAATAATAAAGCTCCTACCATTTGCAGCAGCCCCACCGGAACAGAGTAGGATGCAATTTTCCTCTCTGACTGTGAACGTAAGAGGTTCAGGAATGAGAATACTTGTGAGGGGCAACTCCCCTGTGTTCAACTATAATAAGGCCACCAAGAGACTCACAGTTCTTGGAAAGGATGCAGGCGCCTTGACAGAATATCCAGATGAGGGAACAGCAGGAGTTGAGTCTGCAGT<br>ATTAAGAGGATTTCTAATTCTGGGCAAAGAGGACAAAAGATATGGACCAGCATTGAGCATCAACGAATTGAGCAATCTTGCGAAAGGGGAAAAGGCTAATGTGTTGATAGGACAAGGAGACGTGGTGTTGGTAATGAAACGGAAACGGGACTCTAGCATACTTACTGACAGCCAGACAGCGACCAAAAGAATTCGGATGGCCATCAATTA
EPI_ISL_5098160	>A/domestic_duck/Germany-BB/AI01437/2021_H5N8_2021-02-17<br>ATGGAGAGAATAAAAGAACTAAGAGATTTGATGTCGCAGTCTCGCACTCGCGAGATACTAACAAAAACCACTGTGGACCATATGGCCATAATAAAGAAATACACATCAGGAAGACAGGAGAAGAACCCTGCCCTCAGGATGAAATGGATGATGGCAATGAAATATCCTATTACAGCTGACAAAAGAATAATGGAGATGATCCCTGAAAGGAATGAGCAAGGTCAGACT<br>CTCTGGAGCAAAACAAATGATGCTGGATCAGACAGAGTGATGGTCTCACCTCTGGCTGTGACATGGTGGAATAGAAATGGGCCAACAACAAGTACAGTACACTACCCAAAAGTCTACAAAACCTACTTTGAAAAGGTAGAAAGGTTGAAACATGGAACCTTTGGTCCTGTTCACTTTCGAAATCAGGTTAAGATACGCCGCAGGGTTGACATAAACCCGGGCCATGCAGA<br>TCTCAGTGCCAAAGAAGCACAGGATGTCATCATGGAGGTTGTTTTCCCAAATGAAGTTGGAGCCAGGATCTTGACATCAGAATCACAATTAACAATAACAAGGGAAAAGAAGGAGGAACTTCAGGATTGCAAGATTGCTCCTTTGATGGTGGCATACATGTTGGAAAGAGAACTGGTTCGCAAGACCAGATTTTTACCAGTAGCTGGCGGAACAAGCAGCGTATACATCG<br>AGGTATTGCATTTGACTCAAGGGACCTGCTGGGAACAAATGTACACACCAGGAGGGGAGGTGAGAAATGATGATGTTGATCAGAGTTTGATCATTGCTGCTAGAAATATAGTTAGGAGGGCAACAGTATCAGCAGACCCATTGGCTTCGCTTTTGGAAATGTGCCACAGTACACAAATTGGTGGAGTAAGGATGGTGGACATTCTTAGGCAGAACCCAACAGAGGAGCA<br>AGCTGTGGATATATGCAAAGCAGCAATGGGTTTAAGAATCAGTTCATCCTTCAGCTTTGGAGGTTTCACTTTCAAAAGGACAAGTGGGTCGTCTGTCAAAAGAGAAGAAGAAATACTCACTGGCAACCTCCAAACACTGAAAGTAAGAATACATGAAGGATATGAGGAATTCACAATGGTTGGGCGAAGAGCTACAGCCATTTTGAGGAAAGCAACCAGGAGACTGATCC<br>AATTAATAGTGAGTGGAAGAGACGAGCAGTCAATCGCTGAAGCAATCATAGTGGCAATGGTTTTCTCACAGGAGGATTGCATGATAAAAGCAGTACGAGGTGATTTGAATTTTGTCAACAGAGCGAATCAGCGGCTAAATCCTATGCATCAACTTCTGAGGCATTTCCAAAAGGATGCAAAAGTGCTGTTTCAAAACTGGGGGATTGAACCAATTGACAATGTAATGGGG<br>ATGATCGGGATACTGCCTGACATGACCCCCAGCACAGAGATGTCACTGAGAGGAGTGAGAGTCAGCAAAATGGGAGTGGATGAATATTCCAGTACTGAGAGAGTGGTCGTGAGCATTGATCGCTTCTTGAGAGTCCGAGATCAGAGGGGAAATGTGCTTCTGTCTCCTGAGGAAGTTAGTGAAACACATGGAACAGAGAAACTGACGATAACGTATTCATCGTCTATGA<br>TGTGGGAAATCAATGGACCGGAATCCGTGCTAGTCAACACATATCAATGGATCATTAGAAATTGGGAAACTGTGAAGATTCAGTGGTCCCAGGACCCTACGATGTTATACAATAAGATGGAATTTGAGCCCTTCCAATCCTTGGTGCCCAAGGCTGCTAGAGGCCAGTATAGTGGGTTTGTGAGGACATTATTCCAACAGATGCGTGATGTGTTGGGAACATTTGACACTGT<br>CCAAATAATAAAGCTCCTACCATTTGCAGCAGCCCCACCGGAACAGAGTAGGATGCAATTTTCCTCTCTGACTGTGAACGTAAGAGGTTCAGGAATGAGAATACTTGTGAGGGGCAACTCCCCTGTGTTCAACTATAATAAGGCCACCAAGAGACTCACAGTTCTTGGAAAGGATGCAGGCGCCTTGACAGAATATCCAGATGAGGGAACAGCAGGAGTGGAGTCTGCAG<br>TATTAAGAGGATTTCTAATTCTGGGCAAAGAGGACAAAAGATATGGACCAGCATTGAGCATCAACGAATTGAGCAATCTTGCGAAAGGGGAAAAGGCTAATGTGTTGATAGGACAAGGAGACGTGGTGTTGGTAATGAAACGGAAACGGGACTCTAGCATACTTACTGACAGCCAGACAGCGACCAAAAGAATTCGGATGGCCATCAATTA
EPI_ISL_5102132	>A/chicken/Germany-BY/AI02064/2021_H5N8_2021-03-01<br>ATGGAGAGAATAAAAGAACTAAGAGATTTGATGTCGCAGTCTCGCACTCGCGAGATACTAACAAAAACCACTGTGGACCATATGGCCATAATAAAGAAATACACATCAGGAAGACAGGAGAAGAACCCTGCCCTCAGGATGAAATGGATGATGGCAATGAAATATCCTATTACAGCTGACAAAAGAATAATGGAGATGATCCCTGAAAGGAATGAGCAAGGTCAGACT<br>CTCTGGAGCAAAACAAATGATGCTGGATCAGACAGAGTGATGGTCTCACCTCTGGCTGTGACATGGTGGAATAGAAATGGGCCAACAACAAGTACAGTACACTACCCAAAAGTCTACAAAACCTACTTTGAAAAGGTAGAAAGGTTGAAACATGGAACCTTTGGTCCTGTTCACTTTCGAAATCAGGTTAAGATACGCCGCAGGGTTGACATAAACCCGGGCCATGCAGA<br>TCTCAGTGCCAAAGAAGCACAGGATGTCATCATGGAGGTTGTTTTCCCAAATGAAGTTGGAGCCAGGATCTTGACATCAGAATCACAATTAACAATAACAAGGGAAAAGAAGGAGGAACTTCAGGATTGCAAGATTGCTCCTTTGATGGTGGCATACATGTTGGAAAGAGAACTGGTTCGCAAGACCAGATTTTTACCAGTAGCTGGCGGAACAAGCAGCGTATACATCG<br>AGGTATTGCATTTGACTCAAGGGACCTGCTGGGAACAAATGTACACACCAGGAGGGGAGGTGAGAAATGATGATGTTGATCAGAGTTTGATCATTGCTGCTAGAAATATAGTTAGGAGGGCAACAGTATCAGCAGACCCATTGGCTTCGCTTTTGGAAATGTGCCACAGTACACAAATTGGTGGAGTAAGGATGGTGGACATTCTTAGGCAGAACCCAACAGAGGAGCA<br>AGCTGTGGATATATGCAAAGCAGCAATGGGTTTAAGAATCAGTTCATCCTTCAGCTTTGGAGGTTTCACTTTCAAAAGGACAAGTGGGTCGTCTGTCAAAAGAGAAGAAGAAATACTCACTGGCAACCTCCAAACACTGAAAGTAAGAATACATGAAGGATATGAGGAATTCACAATGGTTGGGCGAAGAGCTACAGCCATTTTGAGGAAAGCAACCAGGAGACTGATCC<br>AATTAATAGTGAGTGGAAGAGACGAGCAGTCAATCGCTGAAGCAATCATAGTGGCAATGGTTTTCTCACAGGAGGATTGCATGATAAAAGCAGTACGAGGTGATTTGAATTTTGTCAACAGAGCGAATCAGCGGCTAAATCCTATGCATCAACTTCTGAGGCATTTCCAAAAGGATGCAAAAGTGCTGTTTCAAAACTGGGGGATTGAACCAATTGACAATGTAATGGGG<br>ATGATCGGGATACTGCCTGACATGACCCCCAGCACAGAGATGTCACTGAGAGGAGTGAGAGTCAGCAAAATGGGAGTGGATGAATATTCCAGTACTGAGAGAGTGGTCGTGAGCATTGATCGCTTCTTGAGAGTCCGAGATCAGAGGGGAAATGTGCTTCTGTCTCCTGAGGAAGTTAGTGAAACACATGGAACAGAGAAACTGACGATAACGTATTCATCGTCTATGA<br>TGTGGGAAATCAATGGACCGGAATCCGTGCTAGTCAACACATATCAATGGATCATTAGAAATTGGGAAACTGTGAAGATTCAGTGGTCCCAGGACCCTACGATGTTATACAATAAGATGGAATTTGAGCCCTTCCAATCCTTGGTGCCCAAGGCTGCTAGAGGCCAGTATAGTGGGTTTGTGAGGACATTATTCCAACAGATGCGTGATGTGTTGGGAACATTTGACACTGT<br>CCAAATAATAAAGCTCCTACCATTTGCAGCAGCCCCACCGGAACAGAGTAGGATGCAATTTTCCTCTCTGACTGTGAACGTAAGAGGTTCAGGAATGAGAATACTTGTGAGGGGCAACTCCCCTGTGTTCAACTATAATAAGGCCACCAAGAGACTCACAGTTCTTGGAAAGGATGCAGGCGCCTTGACAGAATATCCAGATGAGGGAACAGCAGGAGTGGAGTCTGCAG<br>TATTAAGAGGATTTCTAATTCTGGGCAAAGAGGACAAAAGATATGGACCAGCATTGAGCATCAACGAATTGAGCAATCTTGCGAARGGGGAAAAGGCTAATGTGTTGATAGGACAAGGAGACGTGGTGTTGGTAATGAAACGGAAACGGGACTCTAGCATACTTACTGACAGCCAGACAGCGACCAAAAGAATTCGGATGGCCATCAATTA
EPI_ISL_7778759	>A/mute_swan/Estonia/TA2106419-1_21VIR7512-3/2021_H5N8_2021-03-04<br>ATGGAGAGAATAAAAGAACTAAGAGATTTGATGTCACAGTCTCGCACTCGCGAGATACTAACAAAAACCACTGTGGACCATATGGCCATAATAAAGAAATACACATCAGGGAGACAGGAGAAGAACCCTGCCCTCAGGATGAAATGGATGATGGCAATGAAATATCCTATTACAGCTGACAAAAGAATAATGGAGATGATCCCTGAAAGGAATGAGCAAGGTCAGACT<br>CTCTGGAGCAAAACAAATGATGCTGGATCAGACAGAGTGATGGTCTCACCTCTGGCTGTGACATGGTGGAATAGAAACGGGCCAACAACAAGTACAGTACACTACCCAAAAGTCTACAAAACCTACTTTGAAAAGGTGGAAAGGTTGAAACATGGAACCTTTGGTCCTGTTCACTTTCGAAATCAGGTTAAGATACGCCGCAGGGTTGACATAAACCCGGGCCATGCAGA<br>TCTCAGTGCCAAAGAAGCACAGGATGTCATCATGGAGGTTGTTTTCCCAAATGAAGTTGGAGCCAGGATCTTGACATCAGAATCACAATTAACAATAACAAGGGAAAAGAAGGAGGAACTTCAGGATTGCAAGATTGCTCCTTTGATGGTGGCATACATGTTGGAAAGAGAACTGGTTCGCAAGACCAGATTTTTACCAGTAGCTGGCGGAACAAGCAGCGTGTACATCG<br>AGGTATTGCATTTGACTCAAGGGACCTGCTGGGAACAAATGTACACACCAGGAGGGGAGGTGAGAAATGATGATGTTGATCAGAGTTTGATCATTGCTGCTAGAAATATAGTTAGGAGGGCAACAGTATCAGCAGACCCATTGGCTTCGCTCTTGGAAATGTGCCACAGTACACAAATTGGTGGAGTGAGGATGGTGGACATTCTTAGGCAGAACCCAACAGAGGAGCA<br>AGCTGTGGATATATGCAAAGCAGCAATGGGTTTAAGAATCAGTTCATCCTTCAGCTTTGGAGGTTTCACTTTCAAAAGGACAAGTGGGTCGTCTGTCAAAAGAGAAGAAGAAATACTCACTGGCAACCTCCAAACACTGAAAGTAAGAATACATGAAGGATATGAGGAATTCACAATGGTTGGGCGAAGAGCTACAGCCATTTTGAGGAAAGCAACCAGGAGACTGATCC<br>AATTAATAGTGAGCGGACGAGACGAGCAGTCAATCGCTGAAGCAATCATAGTGGCAATGGTTTTCTCACAGGAGGATTGCATGATAAAAGCAGTACGAGGTGATTTGAATTTTGTCAACAGAGCGAATCAGCGGCTAAATCCTATGCATCAACTTCTGAGGCATTTCCAAAAGGATGCAAAAGTGCTGTTTCAAAACTGGGGGATTGAACCAATTGACAATGTAATGGGG<br>ATGATCGGGATACTGCCTGACATGACCCCCAGCACAGAGATGTCACTGAGAGGAGTGAGAGTCAGCAAAATGGGAGTGGATGAATATTCCAGTACTGAGAGAGTGGTCGTGAGCATTGATCGCTTCTTGAGAGTCCGAGATCAGAGGGGAAATGTGCTTCTGTCTCCTGAGGAAGTTAGTGAAACACATGGAACAGAGAAACTGACGATAACGTATTCATCGTCTATGA<br>TGTGGGAAATCAATGGTCCGGAATCCGTGCTAGTCAACACATATCAATGGATCATTAGAAATTGGGAAACTGTGAAGATTCAGTGGTCCCAGGACCCTACGATGTTGTACAATAAGATGGAATTTGAGCCCTTTCAATCCTTGGTGCCCAAGGCTGCTAGAGGCCAGTATAGTGGGTTTGTGAGGACATTATTCCAACAGATGCGTGATGTGTTGGGGACATTTGACACTGT<br>CCAAATAATAAAGCTCCTACCATTTGCAGCAGCCCCACCGGAACAGAGTAGGATGCAATTTTCCTCTCTGACTGTGAACGTAAGAGGTTCAGGAATGAGAATACTTGTGAGGGGCAACTCCCCTGTGTTCAACTATAATAAGGCCACCAAGAGACTCACAGTTCTTGGAAAGGATGCAGGCGCCTTGACAGAATATCCAGATGAGGGAACAGCAGGAGTGGAGTCTGCAG<br>TATTAAGAGGATTTCTAATTCTGGGCAAAGAGGACAAAAGATATGGACCAGCATTGAGCATCAACGAATTGAGCAATCTTGCGAAAGGGGAAAAGGCTAATGTGTTGATAGGACAAGGAGACGTGGTGTTGGTAATGAAACGGAAACGGGACTCTAGCATACTTACTGACAGCCAGACAGCGACCAAAAGAATTCGGATGGCCATCAATTA
EPI_ISL_7778769	>A/pheasant/Finland/499_21VIR7689-1/2021_H5N8_2021-01-01<br>ATGGAGAGAATAAAAGAACTAAGAGATTTGATGTCACAGTCTCGCACTCGCGAGATACTAACAAAAACCACTGTGGACCATATGGCCATAATAAAGAAATACACATCAGGGAGACAGGAGAAGAACCCTGCCCTCAGGATGAAATGGATGATGGCAATGAAATATCCTATTACAGCTGACAAAAGAATAATGGAGATGATCCCTGAAAGGAATGAGCAAGGTCAGACT<br>CTCTGGAGCAAAACAAATGATGCTGGATCAGACAGAGTGATGGTCTCACCTCTGGCTGTGACATGGTGGAATAGAAACGGGCCAACAACAAGTACAGTACACTACCCAAAAGTCTACAAAACCTACTTTGAAAAGGTGGAAAGGTTGAAACATGGAACCTTTGGTCCTGTTCACTTTCGAAATCAGGTTAAGATACGCCGCAGGGTTGACATAAACCCGGGCCATGCAGA<br>TCTCAGTGCCAAAGAAGCACAGGATGTCATCATGGAGGTTGTTTTCCCAAATGAAGTTGGAGCCAGGATCTTGACATCAGAATCACAATTAACAATAACAAGGGAAAAGAAGGAGGAACTTCAGGATTGCAAGATTGCTCCTTTGATGGTGGCATACATGTTGGAAAGAGAACTGGTTCGCAAGACCAGATTTTTACCAGTAGCTGGCGGAACAAGCAGCGTGTACATCG<br>AGGTATTGCATTTGACTCAAGGGACCTGCTGGGAACAAATGTACACACCAGGAGGGGAGGTGAGAAATGATGATGTTGATCAGAGTTTGATCATTGCTGCTAGAAATATAGTTAGGAGGGCAACAGTATCAGCAGACCCATTGGCTTCGCTCTTGGAAATGTGCCACAGTACACAAATTGGTGGAGTGAGGATGGTGGACATTCTTAGGCAGAACCCAACAGAGGAGCA<br>AGCTGTGGATATATGCAAAGCAGCAATGGGTTTAAGAATCAGTTCATCCTTCAGCTTTGGAGGTTTCACTTTCAAAAGGACAAGTGGGTCGTCTGTCAAAAGAGAAGAAGAAATACTCACTGGCAACCTCCAAACACTGAAAGTAAGAATACATGAAGGATATGAGGAATTCACAATGGTTGGGCGAAGAGCTACAGCCATTTTGAGGAAAGCAACCAGGAGACTAATCC<br>AATTAATAGTGAGTGGACGAGACGAGCAGTCAATCGCTGAAGCAATCATAGTGGCAATGGTTTTCTCACAGGAGGATTGCATGATAAAAGCAGTACGAGGTGATTTGAATTTTGTCAACAGAGCGAATCAGCGGCTAAATCCTATGCATCAACTTCTGAGGCATTTCCAAAAGGATGCAAAAGTGCTGTTTCAAAACTGGGGGATTGAACCAATTGACAATGTAATGGGG<br>ATGATCGGGATACTGCCTGACATGACCCCCAGCACAGAGATGTCACTGAGAGGAGTGAGAGTCAGCAAAATGGGAGTGGATGAATATTCCAGTACTGAGAGAGTGGTCGTGAGCATTGATCGCTTCTTGAGAGTCCGAGATCAGAGGGGAAATGTGCTTCTGTCTCCTGAGGAAGTTAGTGAAACACATGGAACAGAGAAACTGACGATAACGTATTCATCGTCTATGA<br>TGTGGGAAATCAATGGTCCGGAATCCGTGCTAGTCAACACATATCAATGGATCATTAGAAATTGGGAAACTGTGAAGATTCAGTGGTCCCAGGACCCTACGATGTTGTACAATAAGATGGAATTTGAGCCCTTTCAATCCTTGGTGCCCAAGGCTGCTAGAGGCCAGTATAGTGGGTTTGTGAGGACATTATTCCAACAGATGCGTGATGTGTTGGGGACATTTGACACTGT<br>CCAAATAATAAAGCTCCTACCATTTGCAGCAGCCCCACCGGAACAGAGTAGGATGCAATTTTCCTCTCTGACTGTGAACGTAAGAGGTTCAGGAATGAGAATACTTGTGAGGGGCAACTCCCCTGTGTTCAACTATAATAAGGCCACCAAGAGACTCACAGTTCTTGGAAAGGATGCAGGCGCCTTGACAGAATATCCAGATGAGGGAACAGCAGGAGTGGAGTCTGCAG<br>TATTAAGAGGATTTCTAATTCTGGGCAAAGAGGACAAAAGATATGGACCAGCATTGAGCATCAACGAATTGAGCAATCTTGCGAAAGGGGAAAAGGCTAATGTGTTGATAGGACAAGGAGACGTGGTGTTGGTAATGAAACGGAAACGGGACTCTAGCATACTTACTGACAGCCAGACAGCGACCAAAAGAATTCGGATGGCCATCAATTA
EPI_ISL_7778760	>A/mute_swan/Estonia/TA2106615_21VIR7512-4/2021_H5N8_2021-03-04<br>ATGGAGAGAATAAAAGAACTAAGAGATTTGATGTCACAGTCTCGCACTCGCGAGATACTAACAAAAACCACTGTGGACCATATGGCCATAATAAAGAAATACACATCAGGGAGACAGGAGAAGAACCCTGCCCTCAGGATGAAATGGATGATGGCAATGAAATATCCTATTACAGCTGACAAAAGAATAATGGAGATGATCCCTGAAAGGAATGAGCAAGGTCAGACT<br>CTCTGGAGCAAAACAAATGATGCTGGATCAGACAGAGTGATGGTCTCACCTCTGGCTGTGACATGGTGGAATAGAAACGGGCCAACAACAAGTACAGTACACTACCCAAAAGTCTACAAAACCTACTTTGAAAAGGTGGAAAGGTTGAAACATGGAACCTTTGGTCCTGTTCACTTTCGAAATCAGGTTAAGATACGCCGCAGGGTTGACATAAACCCGGGCCATGCAGA<br>TCTCAGTGCCAAAGAAGCACAGGATGTCATCATGGAGGTTGTTTTCCCAAATGAAGTTGGAGCCAGGATCTTGACATCAGAATCACAATTAACAATAACAAGGGAAAAGAAGGAGGAACTTCAGGATTGCAAGATTGCTCCTTTGATGGTGGCATACATGTTGGAAAGAGAACTGGTTCGCAAGACCAGATTTTTACCAGTAGCTGGCGGAACAAGCAGCGTGTACATCG<br>AGGTATTGCATTTGACTCAAGGGACCTGCTGGGAACAAATGTACACACCAGGAGGGGAGGTGAGAAATGATGATGTTGATCAGAGTTTGATCATTGCTGCTAGAAATATAGTTAGGAGGGCAACAGTATCAGCAGACCCATTGGCTTCGCTCTTGGAAATGTGCCACAGTACACAAATTGGTGGAGTGAGGATGGTGGACATTCTTAGGCAGAACCCAACAGAGGAGCA<br>AGCTGTGGATATATGCAAAGCAGCAATGGGTTTAAGAATCAGTTCATCCTTCAGCTTTGGAGGTTTCACTTTCAAAAGGACAAGTGGGTCGTCTGTCAAAAGAGAAGAAGAAATACTCACTGGCAACCTCCAAACACTGAAAGTAAGAATACATGAAGGATATGAGGAATTCACAATGGTTGGGCGAAGAGCTACAGCCATTTTGAGGAAAGCAACCAGGAGACTGATCC<br>AATTAATAGTGAGCGGACGAGACGAGCAGTCAATCGCTGAAGCAATCATAGTGGCAATGGTTTTCTCACAGGAGGATTGCATGATAAAAGCAGTACGAGGTGATTTGAATTTTGTCAACAGAGCGAATCAGCGGCTAAATCCTATGCATCAACTTCTGAGGCATTTCCAAAAGGATGCAAAAGTGCTGTTTCAAAACTGGGGGATTGAACCAATTGACAATGTAATGGGG<br>ATGATCGGGATACTGCCTGACATGACCCCCAGCACAGAGATGTCACTGAGAGGAGTGAGAGTCAGCAAAATGGGAGTGGATGAATATTCCAGTACTGAGAGAGTGGTCGTGAGCATTGATCGCTTCTTGAGAGTCCGAGATCAGAGGGGAAATGTGCTTCTGTCTCCTGAGGAAGTTAGTGAAACACATGGAACAGAGAAACTGACGATAACGTATTCATCGTCTATGA<br>TGTGGGAAATCAATGGTCCGGAATCCGTGCTAGTCAACACATATCAATGGATCATTAGAAATTGGGAAACTGTGAAGATTCAGTGGTCCCAGGACCCTACGATGTTGTACAATAAGATGGAATTTGAGCCCTTTCAATCCTTGGTGCCCAAGGCTGCTAGAGGCCAGTATAGTGGGTTTGTGAGGACATTATTCCAACAGATGCGTGATGTGTTGGGGACATTTGACACTGT<br>CCAAATAATAAAGCTCCTACCATTTGCAGCAGCCCCACCGGAACAGAGTAGGATGCAATTTTCCTCTCTGACTGTGAACGTAAGAGGTTCAGGAATGAGAATACTTGTGAGGGGCAACTCCCCTGTGTTCAACTATAATAAGGCCACCAAGAGACTCACAGTTCTTGGAAAGGATGCAGGCGCCTTGACAGAATATCCAGATGAGGGAACAGCAGGAGTGGAGTCTGCAG<br>TATTAAGAGGATTTCTAATTCTGGGCAAAGAGGACAAAAGATATGGACCAGCATTGAGCATCAACGAATTGAGCAATCTTGCGAAAGGGGAAAAGGCTAATGTGTTGATAGGACAAGGAGACGTGGTGTTGGTAATGAAACGGAAACGGGACTCTAGCATACTTACTGACAGCCAGACAGCGACCAAAAGAATTCGGATGGCCATCAATTA
EPI_ISL_1139169	>A/peregrine_falcon/Netherlands/20018821-002/2020_H5N8_2020-11-25<br>ATGGAGAGAATAAAAGAACTAAGAGATTTGATGTCACAGTCTCGCACTCGCGAGATACTAACAAAAACCACTGTGGACCATATGGCCATAATAAAGAAATACACATCAGGGAGACAGGAGAAGAACCCTGCCCTCAGGATGAAATGGATGATGGCAATGAAATATCCTATTACAGCTGACAAAAGAATAATGGAGATGATCCCTGAAAGGAATGAGCAAGGTCAGACT<br>CTCTGGAGCAAAACAAATGATGCTGGATCAGACAGAGTGATGGTCTCACCTCTGGCTGTGACATGGTGGAATAGAAACGGGCCAACAACAAGTACAGTACACTACCCAAAAGTCTACAAAACCTACTTTGAAAAGGTGGAAAGGTTGAAACATGGAACCTTTGGTCCTGTTCACTTTCGAAATCAGGTTAAGATACGCCGCAGGGTTGACATAAACCCGGGCCATGCAGA<br>TCTCAGTGCCAAAGAAGCACAGGATGTCATCATGGAGGTTGTTTTCCCAAATGAAGTTGGAGCCAGGATCTTGACATCAGAATCACAATTAACAATAACAAGGGAAAAGAAGGAGGAACTTCAGGATTGCAAGATTGCTCCTTTGATGGTGGCATACATGTTGGAAAGAGAACTGGTTCGCAAGACCAGATTTTTACCAGTAGCTGGCGGAACAAGCAGCGTGTACATCG<br>AGGTATTGCATTTGACTCAAGGGACCTGCTGGGAACAAATGTACACACCAGGAGGGGAGGTGAGAAATGATGATGTTGATCAGAGTTTGATCATTGCTGCTAGAAATATAGTTAGGAGGGCAACAGTATCAGCAGACCCATTGGCTTCGCTCTTGGAAATGTGCCACAGTACACAAATTGGTGGAGTGAGGATGGTGGACATTCTTAGGCAGAACCCAACAGAGGAGCA<br>AGCTGTGGATATATGCAAAGCAGCAATGGGTTTAAGAATCAGTTCATCCTTCAGCTTTGGAGGTTTCACTTTCAAAAGGACAAGTGGGTCGTCTGTCAAAAGAGAAGAAGAAATACTCACTGGCAACCTCCAAACACTGAAAGTAAGAATACATGAAGGATATGAGGAATTCACAATGGTTGGGCGAAGAGCTACAGCCATTTTGAGGAAAGCAACCAGGAGACTGATCC<br>AATTAATAGTGAGTGGACGAGACGAGCAGTCAATCGCTGAAGCAATCATAGTGGCAATGGTTTTCTCACAGGAGGATTGCATGATAAAAGCAGTACGAGGTGATTTGAATTTTGTCAACAGAGCGAATCAGCGGCTAAATCCTATGCATCAACTTCTGAGGCATTTCCAAAAGGATGCAAAAGTGCTGTTTCAAAACTGGGGGATTGAACCAATTGACAATGTAATGGGG<br>ATGATCGGGATACTGCCTGACATGACCCCCAGCACAGAGATGTCACTGAGAGGAGTGAGAGTCAGCAAAATGGGAGTGGATGAATATTCCAGTACTGAGAGAGTGGTCGTGAGCATTGATCGCTTCTTGAGAGTCCGAGATCAGAGGGGAAATGTGCTTCTGTCTCCTGAGGAAGTTAGTGAAACACATGGAACAGAGAAACTGACGATAACGTATTCATCGTCTATGA<br>TGTGGGAAATCAATGGTCCGGAATCCGTGCTAGTCAACACATATCAATGGATCATTAGAAATTGGGAAACTGTGAAGATTCAGTGGTCCCAGGACCCTACGATGTTGTACAATAAGATGGAATTTGAGCCCTTTCAATCCTTGGTGCCCAAGGCTGCTAGAGGCCAGTATAGTGGGTTTGTGAGGACATTATTCCAACAGATGCGTGATGTGTTGGGGACATTTGACACTGT<br>CCAAATAATAAAGCTCCTACCATTTGCAGCAGCCCCACCGGAACAGAGTAGGATGCAATTTTCCTCTCTGACTGTGAACGTAAGAGGTTCAGGAATGAGAATACTTGTGAGGGGCAACTCCCCTGTGTTCAACTATAATAAGGCCACCAAGAGACTCACAGTTCTTGGAAAGGATGCAGGCGCCTTGACAGAATATCCAGATGAGGGAACAGCAGGAGTGGAGTCTGCAG<br>TATTAAGAGGATTTCTAATTCTGGGCAAAGAGGACAAAAGATATGGACCAGCATTGAGCATCAACGAATTGAGCAATCTTGCGAAAGGGGAAAAGGCTAATGTGTTGATAGGACAAGGAGACGTGGTGTTGGTAATGAAACGGAAACGGGACTCTAGCATACTTACTGACAGCCAGACAGCGACCAAAAGAATTCGGATGGCCATCAATTA
EPI_ISL_826296	>A/barnacle_goose/Sweden/SVA201221SZ0154/KN003828/2020_H5N8_2020-12-15<br>ATGGAGAGAATAAAAGAACTAAGAGATTTGATGTCACAGTCTCGCACTCGCGAGATACTAACAAAAACCACTGTGGACCATATGGCCATAATAAAGAAATACACATCAGGGAGACAGGAGAAGAACCCTGCTCTCAGGATGAAATGGATGATGGCAATGAAATATCCTATTACAGCTGACAAAAGAATAATGGAGATGATCCCTGAAAGGAATGAGCAAGGTCAGACTC<br>TCTGGAGCAAAACAAATGATGCTGGATCAGACAGAGTGATGGTCTCACCTCTGGCTGTGACATGGTGGAATAGAAACGGGCCAACAACAAGTACAGTACACTACCCAAAAGTCTACAAAACCTACTTTGAAAAGGTGGAAAGGTTGAAACATGGAACCTTTGGTCCTGTTCACTTTCGAAATCAGGTTAAGATACGCCGCAGGGTTGACATAAACCCGGGCCATGCAGAT<br>CTCAGTGCCAAAGAAGCACAGGATGTCATCATGGAGGTTGTTTTCCCAAATGAAGTTGGAGCCAGGATCTTGACATCAGAATCACAATTAACAATAACAAGGGAAAAGAAGGAGGAACTTCAGGATTGCAAGATTGCTCCTTTGATGGTGGCATACATGTTGGAAAGAGAACTGGTTCGCAAGACCAGATTTTTACCAGTAGCTGGCGGAACAAGCAGCGTGTACATCGA<br>GGTATTGCATTTGACTCAAGGGACCTGCTGGGAACAAATGTACACACCAGGAGGGGAGGTGAGAAATGATGATGTTGATCAGAGTTTGATCATTGCTGCTAGAAATATAGTTAGGAGGGCAACAGTATCAGCAGACCCATTGGCTTCGCTCTTGGAAATGTGCCACAGTACACAAATTGGTGGAGTGAGGATGGTGGACATTCTTAGGCAGAACCCAACAGAGGAGCAA<br>GCTGTGGATATATGCAAAGCAGCAATGGGTTTAAGAATCAGTTCATCCTTCAGCTTTGGAGGTTTCACTTTCAAAAGGACAAGTGGGTCGTCTGTCAAAAGAGAAGAAGAAATACTCACTGGCAACCTCCAAACACTGAAAGTAAGAATACATGAAGGATATGAGGAATTCACAATGGTTGGGCGAAGAGCTACAGCCATTTTGAGGAAAGCAACCAGGAGACTGATCCA<br>ATTAATAGTGAGTGGACGAGACGAGCAGTCAATCGCTGAAGCAATCATAGTGGCAATGGTTTTCTCACAGGAGGATTGCATGATAAAAGCAGTACGAGGTGATTTGAATTTTGTCAACAGAGCGAATCAGCGGCTAAATCCTATGCATCAACTTCTGAGGCATTTCCAAAAGGATGCAAAAGTGCTGTTTCAAAACTGGGGGATTGAACCAATTGACAATGTAATGGGGAT<br>GATCGGGATACTGCCTGACATGACCCCCAGCACAGAGATGTCACTGAGAGGAGTGAGAGTCAGCAAAATGGGAGTGGATGAATATTCCAGTACTGAGAGAGTGGTCGTGAGCATTGATCGCTTCTTGAGAGTCCGAGATCAGAGGGGAAATGTGCTTCTGTCTCCTGAGGAAGTTAGTGAAACACATGGAACAGAGAAACTGACGATAACGTATTCATCGTCTATGATG<br>TGGGAAATCAATGGTCCGGAATCCGTGCTAGTCAACACATATCAATGGATCATTAGAAATTGGGAAACTGTGAAGATTCAGTGGTCCCAGGACCCTACGATGTTGTACAATAAGATGGAATTTGAGCCCTTTCAATCCTTGGTGCCCAAGGCTGCTAGAGGCCAGTATAGTGGGTTTGTGAGGACATTATTCCAACAGATGCGTGATGTGTTGGGGACATTTGACACTGTCC<br>AAATAATAAAGCTCCTACCATTTGCAGCAGCCCCACCGGAACAGAGTAGGATGCAATTTTCCTCTCTGACTGTGAACGTAAGAGGTTCAGGAATGAGAATACTTGTGAGGGGCAACTCCCCTGTGTTCAACTATAATAAGGCCACCAAGAGACTCACAGTTCTTGGAAAGGATGCAGGCGCCTTGACAGAATATCCAGATGAGGGAACAGCAGGAGTGGAGTCTGCAGTA<br>TTAAGAGGATTTCTAATTCTGGGCAAAGAGGACAAAAGATATGGACCAGCATTGAGCATCAACGAATTGAGCAATCTTGCGAAAGGGGAAAAGGCTAATGTGTTGATAGGACAAGGAGACGTGGTGTTGGTAATGAAACGGAAACGGGACTCTAGCATACTTACTGACAGCCAGACAGCGACCAAAAGAATTCGGATGGCCATCAATTA
EPI_ISL_13175667	>A/Common_pheasant/Sweden/SVA210224SZ0005/KN000542-IP8/2021_H5N8_2021-02-24<br>ATGGAGAGAATAAAAGAACTAAGAGATTTGATGTCACAGTCTCGCACTCGCGAGATACTAACAAAAACCACTGTGGACCATATGGCCATAATAAAGAAATACACATCAGGGAGACAGGAGAAGAACCCTGCTCTCAGGATGAAATGGATGATGGCAATGAAATATCCTATTACAGCTGACAAAAGAATAATGGAGATGATCCCTGAAAGGAATGAGCAAGGTCAGACTC<br>TCTGGAGCAAAACAAATGATGCTGGATCAGACAGAGTGATGGTCTCACCTCTGGCTGTGACATGGTGGAATAGAAACGGGCCAACAACAAGTACAGTACACTACCCAAAAGTCTACAAAACCTACTTTGAAAAGGTGGAAAGGTTGAAACATGGAACCTTTGGTCCTGTTCACTTTCGAAATCAGGTTAAGATACGCCGCAGGGTTGACATAAACCCGGGCCATGCAGAT<br>CTCAGTGCCAAAGAAGCACAGGATGTCATCATGGAGGTTGTTTTCCCAAATGAAGTTGGAGCCAGGATCTTGACATCAGAATCACAATTAACAATAACAAGGGAAAAGAAGGAGGAACTTCAGGATTGCAAGATTGCTCCTTTGATGGTGGCATACATGTTGGAAAGAGAACTGGTTCGCAAGACCAGATTTTTACCAGTAGCTGGCGGAACAAGCAGCGTGTACATCGA<br>GGTATTGCATTTGACTCAAGGGACCTGCTGGGAACAAATGTACACACCAGGAGGGGAGGTGAGAAATGATGATGTTGATCAGAGTTTGATCATTGCTGCTAGAAATATAGTTAGGAGGGCAACAGTATCAGCGGACCCATTGGCTTCGCTCTTGGAAATGTGCCACAGTACACAAATTGGTGGAGTGAGGATGGTGGACATTCTTAGGCAGAACCCAACAGAGGAGCAA<br>GCTGTGGATATATGCAAAGCAGCAATGGGTTTAAGAATCAGTTCATCCTTCAGCTTTGGAGGTTTCACTTTCAAAAGGACAAGTGGGTCGTCTGTCAAAAGAGAAGAAGAAATACTCACTGGCAACCTCCAAACACTGAAAGTAAGAATACATGAAGGATATGAGGAATTCACAATGGTTGGGCGAAGAGCTACAGCCATTTTGAGGAAAGCAACCAGGAGACTGATCCA<br>ATTAATAGTGAGTGGACGAGACGAGCAGTCAATCGCTGAAGCAATCATAGTGGCAATGGTTTTCTCACAGGAGGATTGCATGATAAAAGCAGTACGAGGTGATTTGAATTTTGTCAACAGAGCGAATCAGCGGCTAAATCCTATGCATCAACTTCTGAGGCATTTCCAAAAGGATGCAAAAGTGCTGTTTCAAAACTGGGGGATTGAACCAATTGACAATGTAATGGGGAT<br>GATCGGGATACTGCCTGACATGACCCCCAGCACAGAGATGTCACTGAGAGGAGTGAGAGTCAGCAAAATGGGAGTGGATGAATATTCCAGTACTGAGAGAGTGGTCGTGAGCATTGATCGCTTCTTGAGAGTCCGAGATCAGAGGGGAAATGTGCTTCTGTCTCCTGAGGAAGTTAGTGAAACACATGGAACAGAGAAACTGACGATAACGTATTCATCGTCTATGATG<br>TGGGAAATCAATGGTCCGGAATCCGTGCTAGTCAACACATATCAATGGATCATTAGAAATTGGGAAACTGTGAAGATTCAGTGGTCCCAGGACCCTACGATGTTGTACAATAAGATGGAATTTGAGCCCTTTCAATCCTTGGTGCCCAAGGCTGCTAGAGGCCAGTATAGTGGGTTTGTGAGGACATTATTCCAACAGATGCGTGATGTGTTGGGGACATTTGACACTGTCC<br>AAATAATAAAGCTCCTACCATTTGCAGCAGCCCCACCGGAACAGAGTAGGATGCAATTTTCCTCTCTGACTGTGAACGTAAGAGGTTCAGGAATGAGAATACTTGTGAGGGGCAACTCCCCTGTGTTCAACTATAATAAGGCCACCAAGAGACTCACAGTTCTTGGAAAGGATGCAGGCGCCTTGACAGAATATCCAGATGAGGGAACAGCAGGAGTGGAGTCTGCAGTA<br>TTAAGAGGATTTCTAATTCTGGGCAAAGAGGACAAAAGATATGGACCAGCATTGAGCATCAACGAATTGAGCAATCTTGCGAAAGGGGAAAAGGCTAATGTGTTGATAGGACAAGGAGACGTGGTGTTGGTAATGAAACGGAAACGGGACTCTAGCATACTTACTGACAGCCAGACAGCGACCAAAAGAATTCGGATGGCCATCAATTA
EPI_ISL_647969	>A/Turkey/Sweden/SVA201114SZ0001/20KN303106/2020_H5N8_2020-11-13<br>ATGGAGAGAATAAAAGAACTAAGAGATTTGATGTCGCAGTCTCGCACTCGCGAGATACTAACAAAAACCACTGTGGACCATATGGCCATAATAAAGAAATACACATCAGGGAGACAGGAGAAGAACCCTGCCCTCAGGATGAAATGGATGATGGCAATGAAATATCCTATTACAGCTGACAAAAGAATAATGGAGATGATCCCTGAAAGGAATGAGCAAGGTCAGACT<br>CTCTGGAGCAAAACAAATGATGCTGGATCAGACAGAGTGATGGTCTCACCTCTGGCTGTGACATGGTGGAATAGAAATGGGCCAACAACAAGTACAGTACACTACCCAAAAGTCTACAAAACCTACTTTGAAAAGGTAGAAAGGTTGAAACATGGAACCTTTGGTCCTGTTCACTTTCGAAATCAGGTTAAGATACGCCGCAGGGTTGACATAAACCCGGGCCATGCAGA<br>TCTCAGTGCCAAAGAAGCACAGGATGTCATCATGGAGGTTGTTTTCCCAAATGAAGTTGGAGCCAGGATCTTGACATCAGAATCACAATTAACAATAACAATGGAAAAGAAGGAGGAACTTCAGGATTGCAAGATTGCTCCTTTGATGGTGGCATACATGTTGGAAAGAGAACTGGTTCGCAAGACCAGATTTTTACCAGTAGCTGGCGGAACAAGCAGCGTATACATCG<br>AGGTATTGCATTTGACTCAAGGGACCTGCTGGGAACAAATGTACACACCAGGAGGGGAGGTGAGAAATGATGATGTTGATCAGAGTTTGATCATTGCTGCTAGAAATATAGTTAGGAGGGCAACAGTATCAGCAGACCCATTGGCTTCGCTCTTGGAAATGTGCCACAGTACACAAATTGGTGGAGTGAGGATGGTGGACATTCTTAGGCAGAACCCAACAGAGGAGCA<br>AGCTGTGGATATATGCAAAGCAGCAATGGGTTTAAGAATCAGTTCATCCTTCAGCTTTGGAGGTTTCACTTTCAAAAGGACAAGTGGGTCGTCTGTCAAAAGAGAAGAAGAAATACTCACTGGCAACCTCCAAACACTGAAAGTAAGAATACATGAAGGATATGAGGAATTCACAATGGTTGGGCGAAGAGCTACAGCCATTTTGAGGAAAGCAACCAGGAGACTGATCC<br>AATTAATAGTGAGTGGACGAGACGAACAGTCAATCGCTGAAGCAATCATAGTGGCAATGGTTTTCTCACAGGAGGATTGCATGATAAAAGCAGTACGAGGTGATTTGAATTTTGTCAACAGAGCGAATCAGCGGCTAAATCCTATGCATCAACTTCTGAGGCATTTCCAAAAGGATGCAAAAGTGCTGTTTCAAAACTGGGGGATTGAACCAATTGACAATGTAATGGGG<br>ATGATCGGGATACTGCCTGACATGACCCCCAGCACAGAGATGTCACTGAGAGGAGTGAGAGTCAGCAAAATGGGAGTGGATGAATATTCCAGTACTGAGAGAGTGGTCGTGAGCATTGATCGCTTCTTGAGAGTCCGAGATCAGAGGGGAAATGTGCTTCTGTCTCCTGAGGAAGTTAGTGAAACACATGGAACAGAGAAACTGACGATAACGTATTCATCGTCTATGA<br>TGTGGGAAATCAATGGTCCGGAATCCGTGCTAGTCAACACATATCAATGGATCATTAGAAATTGGGAAACTGTGAAGATTCAGTGGTCCCAGGACCCTACGATGCTGTACAATAAGATGGAATTTGAGCCCTTCCAATCCTTGGTGCCCAAGGCTGCTAGAGGCCAGTATAGTGGGTTTGTGAGGACATTATTCCAACAGATGCGTGATGTGTTGGGGACATTTGACACTGT<br>CCAAATAATAAAGCTCCTACCATTTGCAGCAGCCCCACCGGAACAGAGTAGGATGCAATTTTCCTCTCTGACTGTGAACGTAAGAGGTTCAGGAATGAGAATACTTGTGAGGGGCAACTCCCCTGTGTTCAACTATAATAAGACCACCAAGAGACTCACAGTTCTTGGAAAGGATGCAGGCGCCTTGACAGAATATCCAGATGAGGGAACAGCAGGAGTGGAGTCTGCAG<br>TATTAAGAGGATTTCTAATTCTGGGCAAAGAGGACAAAAGATATGGACCAGCATTGAGCATCAACGAATTGAGCAATCTTGCGAAAGGGGAAAAGGCTAATGTGTTGATAGGACAAGGAGACGTGGTGTTGGTAATGAAACGGAAACGGGACTCTAGCATACTTACTGACAGCCAGACAGCGACCAAAAGAATTCGGATGGCCATCAATTA
EPI_ISL_5095555	>A/chicken/Germany-NI/AI00547/2021_H5N8_2021-01-12<br>ATGGAGAGAATAAAAGAACTAAGAGATTTGATGTCGCAGTCTCGCACTCGCGAGATACTAACAAAAACCACTGTGGACCATATGGCCATAATAAAGAAATACACATCAGGGAGACAGGAGAAGAACCCTGCCCTCAGGATGAAATGGATGATGGCAATGAAATATCCTATTACAGCTGACAAAAGAATAATGGAGATGATCCCTGAAAGGAATGAGCAAGGTCAGACT<br>CTCTGGAGCAAAACAAATGATGCTGGATCAGACAGAGTGATGGTCTCACCTCTGGCTGTGACATGGTGGAATAGAAATGGGCCAACAACAAGTACAGTACACTATCCAAAAGTCTACAAAACCTACTTTGAAAAGGTAGAAAGGTTGAAACATGGAACCTTTGGTCCTGTTCACTTTCGAAATCAGGTTAAGATACGCCGCAGGGTTGACATAAACCCGGGCCATGCAGA<br>TCTCAGTGCCAAAGAAGCACAGGATGTCATCATGGAGGTTGTTTTCCCAAATGAAGTTGGAGCCAGGATCTTGACATCAGAATCACAATTAACAATAACAATGGAAAAGAAGGAGGAACTTCAGGATTGCAAGATTGCTCCTTTGATGGTGGCATACATGTTGGAAAGAGAACTGGTTCGCAAGACCAGATTTTTACCAGTAGCTGGCGGAACAAGCAGCGTATACATCG<br>AGGTATTGCATTTGACTCAAGGGACCTGCTGGGAACAAATGTACACACCAGGAGGGGAGGTGAGAAATGATGATGTTGATCAGAGTTTGATCATTGCTGCTAGAAATATAGTTAGGAGGGCAACAGTATCAGCAGACCCATTGGCTTCGCTCTTGGAAATGTGCCACAGTACACAAATTGGTGGAGTGAGGATGGTGGACATTCTTAGGCAGAACCCAACAGAGGAGCA<br>AGCTGTGGATATATGCAAAGCAGCAATGGGTTTAAGAATCAGTTCATCCTTCAGCTTTGGAGGTTTCACTTTCAAAAGGACAAGTGGGTCGTCTGTCAAAAGAGAAGAAGAAATACTCACTGGCAACCTCCAAACACTGAAAGTAAGAATACATGAAGGATATGAGGAATTCACAATGGTTGGGCGAAGAGCTACAGCCATTTTGAGGAAAGCAACCAGGAGACTGATCC<br>AATTAATAGTGAGTGGACGAGACGAACAGTCAATCGCTGAAGCAATCATAGTGGCAATGGTTTTCTCACAGGAGGATTGCATGATAAAAGCAGTACGAGGTGATTTGAATTTTGTCAACAGAGCGAATCAGCGGCTAAATCCTATGCATCAACTTCTGAGGCATTTCCAAAAGGATGCAAAAGTGCTGTTTCAAAACTGGGGGATTGAACCAATTGACAATGTAATGGGG<br>ATGATCGGGATACTGCCTGACATGACCCCCAGCACAGAGATGTCACTGAGAGGAGTGAGAGTCAGCAAAATGGGAGTGGATGAATATTCCAGTACTGAGAGAGTGGTCGTGAGCATTGATCGCTTCTTGAGAGTCCGAGATCAGAGGGGAAATGTGCTTCTGTCTCCTGAGGAAGTTAGTGAAACACATGGAACAGAGAAACTGACGATAACGTATTCATCGTCTATGA<br>TGTGGGAAATCAATGGTCCGGAATCCGTGCTAGTCAACACATATCAATGGATCATTAGAAATTGGGAAACTGTGAAGATTCAGTGGTCCCAGGACCCTACGATGTTGTACAATAAGATGGAATTTGAGCCCTTCCAATCCTTGGTGCCCAAGGCTGCTAGAGGCCAGTATAGTGGGTTTGTGAGGACATTATTCCAACAGATGCGTGATGTGTTGGGGACATTTGACACTGT<br>CCAAATAATAAAGCTCCTACCATTTGCAGCAGCCCCACCGGAACAGAGTAGGATGCAATTTTCCTCTCTGACTGTGAACGTAAGAGGTTCAGGAATGAGAATACTTGTGAGGGGCAACTCCCCTGTGTTCAACTATAATAAGACCACCAAGAGACTCACAGTTCTTGGAAAGGATGCAGGCGCCTTGACAGAATATCCAGATGAGGGAACAGCAGGAGTGGAGTCTGCAG<br>TATTAAGAGGATTTCTAATTCTGGGCAAAGAGGACAAAAGATATGGACCAGCATTGAGCATCAACGAATTGAGCAATCTTGCGAAAGGGGAAAAGGCTAATGTGTTGATAGGACAAGGAGACGTGGTGTTGGTAATGAAACGGAAACGGGACTCTAGCATACTTACTGACAGCCAGACAGCGACCAAAAGAATTCGGATGGCCATCAATTA

EPI_ISL_666890	>A/turkey/Croatia/104/2020_H5N8_2020-11-17<br>ATGGAGAGAATAAAAGAACTAAGAGATTTGATGTCGCAGTCTCGCACTCGCGAGATACTAACAAAAACCACTGTGGACCATATGGCCATAATAAAGAAATACACATCAGGGAGACAGGAGAAGAACCCTGCCCTCAGGATGAAATGGATGATGGCAATGAAATATCCTATTACAGCTGACAAAAGAATAATGGAGATGATCCCTGAAAGGAATGAGCAAGGTCAGACT<br>CTCTGGAGCAAAACAAATGATGCTGGATCAGACAGAGTGATGGTCTCACCTCTGGCTGTGACATGGTGGAATAGAAATGGGCCAACAACAAGTACAGTACACTACCCAAAAGTCTACAAAACCTACTTTGAAAAGGTAGAAAGGTTGAAACATGGAACCTTTGGTCCTGTTCACTTTCGAAATCAGGTTAAGATACGCCGCAGGGTTGACATAAACCCGGGCCATGCAGA<br>TCTCAGTGCCAAAGAAGCACAGGATGTCATCATGGAGGTTGTTTTCCCAAATGAAGTTGGAGCCAGGATCTTGACATCAGAATCACAATTAACAATAACAATGGAAAAGAAGGAGGAACTTCAGGATTGCAAGATTGCTCCTTTGATGGTGGCATACATGTTGGAAAGAGAACTGGTTCGCAAGACCAGATTTTTACCAGTAGCTGGCGGAACAAGCAGCGTATACATCG<br>AGGTATTGCATTTGACTCAAGGGACCTGCTGGGAACAAATGTACACACCAGGAGGGGAGGTGAGAAATGATGATGTTGATCAGAGTTTGATCATTGCTGCTAGAAATATAGTTAGGAGGGCAACAGTATCAGCAGACCCATTGGCTTCGCTCTTGGAAATGTGCCACAGTACACAAATTGGTGGAGTGAGGATGGTGGACATTCTTAGGCAGAACCCAACAGAGGAGCA<br>AGCTGTGGATATATGCAAAGCAGCAATGGGTTTAAGAATCAGTTCATCCTTCAGCTTTGGAGGTTTCACTTTCAAAAGGACAAGTGGGTCGTCTGTCAAAAGAGAAGAAGAAATACTCACTGGCAACCTCCAAACACTGAAAGTAAGAATACATGAAGGATATGAGGAATTCACAATGGTTGGGCGAAGAGCTACAGCCATTTTGAGGAAAGCAACCAGGAGACTGATCC<br>AATTAATAGTGAGTGGACGAGACGAACAGTCAATCGCTGAAGCAATCATAGTGGCAATGGTTTTCTCACAGGAGGATTGCATGATAAAAGCAGTACGAGGTGATTTGAATTTTGTCAACAGAGCGAATCAGCGGCTAAATCCTATGCATCAACTTCTGAGGCATTTCCAAAAGGATGCAAAAGTGCTGTTTCAAAACTGGGGGATTGAACCAATTGACAATGTAATGGGG<br>ATGATCGGGATACTGCCTGACATGACCCCCAGCACAGAGATGTCACTGAGAGGAGTGAGAGTCAGCAAAATGGGAGTGGATGAATATTCCAGTACTGAGAGAGTGGTCGTGAGCATTGATCGCTTCTTGAGAGTCCGAGATCAGAGGGGAAATGTGCTTCTGTCTCCTGAGGAAGTTAGTGAAACACATGGAACAGAGAAACTGACGATAACGTATTCATCGTCTATGA<br>TGTGGGAAATCAATGGTCCGGAATCCGTGCTAGTCAACACATATCAATGGATCATTAGAAATTGGGAAACTGTGAAGATTCAGTGGTCCCAGGACCCTACGATGTTGTACAATAAGATGGAATTTGAGCCCTTCCAATCCTTGGTGCCCAAGGCTGCTAGAGGCCAGTATAGTGGGTTTGTGAGGACATTATTCCAACAGATGCGTGATGTGTTGGGGACATTTGACACTGT<br>CCAAATAATAAAGCTCCTACCATTTGCAGCAGCCCCACCGGAACAGAGTAGGATGCAATTTTCCTCTCTGACTGTGAACGTAAGAGGTTCAGGAATGAGAATACTTGTGAGGGGCAACTCCCCTGTGTTCAACTATAATAAGACCACCAAGAGACTCACAGTTCTTGGAAAGGATGCAGGCGCCTTGACAGAATATCCAGATGAGGGAACAGCAGGAGTGGAGTCTGCAG<br>TATTAAGAGGATTTCTAATTCTGGGCAAAGAGGACAAAAGATATGGACCAGCATTGAGCATCAACGAATTGAGCAATCTTGCGAAAGGGGAAAAGGCTAATGTGTTGATAGGACAAGGAGACGTGGTGTTGGTAATGAAACGGAAACGGGACTCTAGCATACTTACTGACAGCCAGACAGCGACCAAAAGAATTCGGATGGCCATCAATTA
EPI_ISL_5066996	>A/turkey/Germany-NI/AI03432/2020_H5N8_2020-12-20<br>ATGGAGAGAATAAAAGAACTAAGAGATTTGATGTCGCAGTCTCGCACTCGCGAGATACTAACAAAAACCACTGTGGACCATATGGCCATAATAAAGAAATACACATCAGGGAGACAGGAGAAGAACCCTGCCCTCAGGATGAAATGGATGATGGCAATGAAATATCCTATTACAGCTGACAAAAGAATAATGGAGATGATCCCTGAAAGGAATGAGCAAGGTCAGACT<br>CTCTGGAGCAAAACAAATGATGCTGGATCAGACAGAGTGATGGTCTCACCTCTGGCTGTGACATGGTGGAATAGAAATGGGCCAACAACAAGTACAGTACACTACCCAAAAGTCTACAAAACCTACTTTGAAAAGGTAGAAAGGTTGAAACATGGAACCTTTGGTCCTGTTCACTTTCGAAATCAGGTTAAGATACGCCGCAGGGTTGACATAAACCCGGGCCATGCAGA<br>TCTCAGTGCCAAAGAAGCACAGGATGTCATCATGGAGGTTGTTTTCCCAAATGAAGTTGGAGCCAGGATCTTGACATCAGAATCACAATTAACAATAACAATGGAAAAGAAGGAGGAACTTCAGGATTGCAAGATTGCTCCTTTGATGGTGGCATACATGTTGGAAAGAGAACTGGTTCGCAAGACCAGATTTTTACCAGTAGCTGGCGGAACAAGCAGCGTATACATCG<br>AGGTATTGCATTTGACTCAAGGGACCTGCTGGGAACAAATGTACACACCAGGAGGGGAGGTGAGAAATGATGATGTTGATCAGAGTTTGATCATTGCTGCTAGAAATATAGTTAGGAGGGCAACAGTATCAGCAGACCCATTGGCTTCGCTCTTGGAAATGTGCCACAGTACACAAATTGGTGGAGTGAGGATGGTGGACATTCTTAGGCAGAACCCAACAGAGGAGCA<br>AGCTGTGGATATATGCAAAGCAGCAATGGGTTTAAGAATCAGTTCATCCTTCAGCTTTGGAGGTTTCACTTTCAAAAGGACAAGTGGGTCGTCTGTCAAAAGAGAAGAAGAAATACTCACTGGCAACCTCCAAACACTGAAAGTAAGAATACATGAAGGATATGAGGAATTCACAATGGTTGGGCGAAGAGCTACAGCCATTTTGAGGAAAGCAACCAGGAGACTGATCC<br>AATTAATAGTGAGTGGACGAGACGAACAGTCAATCGCTGAAGCAATCATAGTGGCAATGGTTTTCTCACAGGAGGATTGCATGATAAAAGCAGTACGAGGTGATTTGAATTTTGTCAACAGAGCGAATCAGCGGCTAAATCCTATGCATCAACTTCTGAGGCATTTCCAAAAGGATGCAAAAGTGCTGTTTCAAAACTGGGGGATTGAACCAATTGACAATGTAATGGGG<br>ATGATCGGGATACTGCCTGACATGACCCCCAGCACAGAGATGTCACTGAGAGGAGTGAGAGTCAGCAAAATGGGAGTGGATGAATATTCCAGTACTGAGAGAGTGGTCGTGAGCATTGATCGCTTCTTGAGAGTCCGAGATCAGAGGGGAAATGTGCTTCTGTCTCCTGAGGAAGTTAGTGAAACACATGGAACAGAGAAACTGACGATAACGTATTCATCGTCTATGA<br>TGTGGGAAATCAATGGTCCGGAATCCGTGCTAGTCAACACATATCAATGGATCATTAGAAATTGGGAAACTGTGAAGATTCAGTGGTCCCAGGACCCTACGATGTTGTACAATAAGATGGAATTTGAGCCCTTCCAATCCTTGGTGCCCAAGGCTGCTAGAGGCCAGTATAGTGGGTTTGTGAGGACATTATTCCAACAGATGCGTGATGTGTTGGGGACATTTGACACTGT<br>CCAAATAATAAAGCTCCTACCATTTGCAGCAGCCCCACCGGAACAGAGTAGGATGCAATTTTCCTCTCTGACTGTGAACGTAAGAGGTTCAGGAATGAGAATACTTGTGAGGGGCAACTCCCCTGTGTTCAACTATAATAAGACCACCAAGAGACTCACAGTTCTTGGAAAGGATGCAGGCGCCTTGACAGAATATCCAGATGAGGGAACAGCAGGAGTGGAGTCTGCAG<br>TATTAAGAGGATTTCTAATTCTGGGCAAAGAGGACAAAAGATATGGACCAGCATTGAGCATCAACGAATTGAGCAATCTTGCGAAAGGGGAAAAGGCTAATGTGTTGATAGGACAAGGAGACGTGGTGTTGGTAATGAAACGGAAACGGGACTCTAGCATACTTACTGACAGCCAGACAGCGACCAAAAGAATTCGGATGGCCATCAATTA
EPI_ISL_5143586	>A/chicken/Germany-SN/AI03496/2021_H5N8_2021-03-24<br>ATGGAGAGAATAAAAGAACTAAGAGATTTGATGTCGCAGTCTCGCACTCGCGAGATACTAACAAAAACCACTGTGGACCATATGGCCATAATAAAGAAATACACATCAGGGAGACAGGAGAAGAACCCTGCCCTCAGGATGAAATGGATGATGGCAATGAAATATCCTATTACAGCTGACAAAAGAATAATGGAGATGATCCCTGAAAGGAATGAGCAAGGTCAGACT<br>CTCTGGAGCAAAACAAATGATGCTGGATCAGACAGAGTGATGGTCTCACCTCTGGCTGTGACGTGGTGGAATAGAAATGGGCCAACAACAAGTACAGTACACTACCCAAAAGTCTACAAAACCTACTTTGAAAAGGTAGAAAGGTTGAAACATGGAACCTTTGGTCCTGTTCACTTTCGAAATCAGGTTAAGATACGCCGCAGGGTTGACACAAACCCGGGCCATGCAGA<br>TCTCAGTGCCAAAGAAGCACAGGATGTCATCATGGAGGTTGTTTTCCCAAATGAAGTTGGAGCCAGGATCTTGACATCAGAATCACAATTAACAATAACAATGGAAAAGAAGGAGGAACTTCAGGATTGCAAGATTGCTCCTTTGATGGTGGCATACATGTTGGAAAGAGAACTGGTTCGCAAGACCAGGTTTTTACCAGTAGCTGGCGGAACAAGCAGCGTATACATCG<br>AGGTATTGCATTTGACTCAAGGGACCTGCTGGGAACAAATGTACACACCAGGAGGGGAGGTGAGAAATGATGATGTTGATCAGAGTTTGATCATTGCTGCTAGAAATATAGTTAGGAGGGCAACAGTATCAGCAGACCCATTGGCTTCGCTCTTGGAAATGTGCCACAGTACACAAATTGGTGGAGTGAGGATGGTGGACATTCTTAGGCAGAACCCAACAGAGGAGCA<br>AGCTGTGGATATATGCAAAGCAGCAATGGGTTTAAGAATCAGTTCATCCTTCAGCTTTGGAGGTTTCACTTTCAAAAGGACAAGTGGGTCGTCTGTCAAAAGAGAAGAAGAAATACTCACTGGCAACCTCCAAACACTGAAAGTAAGAATACATGAAGGATATGAGGAATTCACAATGGTTGGGCGAAGAGCTACAGCCATTTTGAGGAAGGCAACCAGGAGACTGATCC<br>AATTAATAGTGAGTGGACGAGACGAACAGTCAATCGCTGAGGCAATCATAGTGGCAATGGTTTTCTCACAGGAGGATTGCATGATAAAAGCAGTACGAGGTGATTTGAATTTTGTCAACAGAGCGAATCAGCGGCTAAATCCTATGCATCAACTTCTGAGGCATTTCCAAAAAGATGCAAAAGTGCTGTTTCAAAACTGGGGGATTGAACCAATTGACAATGTAATGGGG<br>ATGATCGGGATACTGCCTGACATGACCCCCAGCACAGAGATGTCACTGAGAGGAGTGAGAGTCAGCAAAATGGGAGTGGATGAATATTCCAGTACTGAGAGAGTGGTCGTGAGCATTGATCGCTTCTTGAGAGTCCGAGATCAGAGGGGAAATGTGCTTCTGTCTCCTGAGGAAGTTAGTGAAACACATGGAACAGAGAAACTGACGATAACGTATTCATCGTCCATGA<br>TGTGGGAAATCAATGGTCCGGAATCCGTGCTAGTCAACACATATCAATGGATCATTAGAAATTGGGAAACTGTGAAGATTCAGTGGTCCCAGGACCCTACGATGTTGTACAATAAGATGGAATTTGAGCCCTTCCAATCCTTGGTGCCTAAGGCTGCTAGAGGCCAGTATAGTGGGTTTGTGAGGACATTATTCCAACAGATGCGTGATGTGTTGGGGACATTTGACACTGT<br>CCAAATAATAAAGCTCCTACCATTTGCAGCAGCCCCACCGGAACAGAGTAGGATGCAATTTTCCTCTCTGACTGTGAACGTAAGAGGTTCAGGAATGAGAATACTTGTGAGGGGCAACTCCCCTGTGTTCAACTATAATAAGACCACCAAGAGACTCACAGTTCTTGGAAAGGATGCAGGCGCCTTGACAGAATATCCAGATGAGGGAACAGCAGGAGTGGAGTCTGCAG<br>TATTAAGAGGATTTCTAATTCTGGGCAAAGAGGACAAAAGATATGGACCAGCATTGAGCATCAACGAATTGAGCAATCTTGCGAAAGGGGAAAAGGCCAATGTGTTGATAGGACAAGGAGACGTGGTGTTGGTAATGAAACGGAAACRGGACTCTAGCATACTTACTGACAGCCAGACAGCGACCAAAAGAATTCGGATGGCCATCAATTA
EPI_ISL_5095319	>A/turkey/Germany-NI/AI00402/2021_H5N8_2021-01-06<br>ATGGAGAGAATAAAAGAACTAAGAGATTTGATGTCGCAGTCTCGCACTCGCGAGATACTAACAAAAACCACTGTGGACCATATGGCCATAATAAAGAAATACACATCAGGGAGACAGGAGAAGAACCCTGCCCTCAGGATGAAATGGATGATGGCAATGAAATATCCTATTACAGCTGACAAAAGAATAATGGAGATGATCCCTGAAAGGAATGAGCAAGGTCAGACT<br>CTCTGGAGCAAAACAAATGATGCTGGATCAGACAGAGTGATGGTCTCACCTCTGGCTGTGACATGGTGGAATAGAAATGGGCCAACAACAAGTACAGTACACTACCCAAAAGTCTACAAAACCTACTTTGAAAAGGTAGAAAGGTTGAAACATGGAACCTTTGGTCCTGTTCACTTTCGAAATCAGGTTAAGATACGCCGCAGGGTTGACATAAACCCGGGCCATGCAGA<br>TCTCAGTGCCAAAGAAGCACAGGATGTCATCATGGAGGTTGTTTTCCCAAATGAAGTTGGAGCCAGGATCTTGACATCAGAATCACAATTAACAATAACAATGGAAAAGAAGGAGGAACTTCAGGATTGCAAGATTGCTCCTTTGATGGTGGCATACATGTTGGAAAGAGAACTGGTTCGCAAGACCAGATTTTTACCAGTAGCTGGCGGAACAAGCAGCGTATACATCG<br>AGGTATTGCATTTGACTCAAGGGACCTGCTGGGAACAAATGTACACACCAGGAGGGGAGGTGAGAAATGATGATGTTGATCAGAGTTTGATCATTGCTGCTAGAAATATAGTTAGGAGGGCAACAGTATCAGCAGACCCATTGGCTTCGCTCTTGGAAATGTGCCACAGTACACAAATTGGTGGAGTGAGGATGGTGGACATTCTTAGGCAGAACCCAACAGAGGAGCA<br>AGCTGTGGATATATGCAAAGCAGCAATGGGTTTAAGAATCAGTTCATCCTTCAGCTTTGGAGGTTTCACTTTCAAAAGGACAAGTGGGTCGTCTGTCAAAAGAGAAGAAGAAATACTCACTGGCAACCTCCAAACACTGAAAGTAAGAATACATGAAGGATATGAGGAATTCACAATGGTTGGGCGAAGAGCTACAGCCATTTTGAGGAAAGCAACCAGGAGACTGATCC<br>AATTAATAGTGAGTGGACGAGACGAACAGTCAATCGCTGAAGCAATCATAGTGGCAATGGTTTTCTCACAGGAGGATTGCATGATAAAAGCAGTACGAGGTGATTTGAATTTTGTCAACAGAGCGAATCAGCGGCTAAATCCTATGCATCAACTTCTGAGGCATTTCCAAAAGGATGCAAAAGTGCTGTTTCAAAACTGGGGGATTGAACCAATTGACAATGTAATGGGG<br>ATGATCGGGATACTGCCTGACATGACCCCCAGCACAGAGATGTCACTGAGAGGAGTGAGAGTCAGCAAAATGGGAGTGGATGAATATTCCAGTACTGAGAGAGTGGTCGTGAGCATTGATCGCTTCTTGAGAGTCCGAGATCAGAGGGGAAATGTGCTTCTGTCTCCTGAGGAAGTTAGTGAAACACATGGAACAGAGAAACTGACGATAACGTATTCATCGTCTATGA<br>TGTGGGAAATCAATGGTCCGGAATCCGTGCTAGTCAACACATATCAATGGATCATTAGAAATTGGGAAACTGTGAAGATTCAGTGGTCCCAGGACCCTACGATGTTGTACAATAAGATGGAATTTGAGCCCTTCCAATCCTTGGTGCCCAAGGCTGCTAGAGGCCAGTATAGTGGGTTTGTGAGGACATTATTCCAACAGATGCGTGATGTGTTGGGGACATTTGACACTGT<br>CCAAATAATAAAGCTCCTACCATTTGCAGCAGCCCCACCGGAACAGAGTAGGATGCAATTTTCCTCTCTGACTGTGAACGTAAGAGGTTCAGGAATGAGAATACTTGTGAGGGGCAACTCCCCTGTGTTCAACTATAATAAGACCACCAAGAGACTCACAGTTCTTGGAAAGGATGCAGGCGCCTTGACAGAATATCCAGATGAGGGAACAGCAGGAGTGGAGTCTGCAG<br>TATTAAGAGGATTTCTAATTCTGGGCAAAGAGGACAAAAGATATGGACCAGCATTGAGCATCAACGAATTGAGCAATCTTGCGAAAGGGGAAAAGGCTAATGTGTTGATAGGACAAGGAGACGTGGTGTTGGTAATGAAACGGAAACGGGACTCTAGCATACTTACTGACAGCCAGACAGCGACCAAAAGAATTCGGATGGCCATCAATTA
EPI_ISL_2681044	>A/chicken/Romania/12448_21VIR3734-3/2021_H5N8_2021-05-05<br>ATGGAGAGAATAAAAGAACTAAGAGATTTGATGTCGCAGTCTCGCACTCGCGAGATACTAACAAAAACCACTGTGGACCATATGGCCATAATAAAGAAATACACATCAGGGAGACAGGAGAAGAACCCTGCCCTCAGGATGAAATGGATGATGGCAATGAAATATCCTATTACAGCTGACAAAAGAATAATGGAGATGATCCCTGAAAGGAATGAGCAAGGTCAGACT<br>CTCTGGAGCAAAACAAATGATGCTGGATCAGACAGAGTGATGGTCTCACCTCTGGCTGTGACGTGGTGGAATAGAAATGGGCCAACAACAAGTACAGTACACTACCCAAAAGTCTACAAAACCTACTTTGAAAAGGTAGAAAGGTTGAAACATGGAACCTTTGGTCCTGTTCACTTTCGAAATCAGGTTAAGATACGCCGCAGGGTTGACATAAACCCGGGCCATGCAGA<br>TCTCAGTGCCAAAGAAGCACAGGATGTCATCATGGAGGTTGTTTTCCCAAATGAAGTTGGAGCCAGGATCTTGACATCAGAATCACAATTAACAATAACAATGGAAAAGAAGGAGGAACTTCAGGATTGCAAGATTGCTCCTTTGATGGTGGCATACATGTTGGAAAGAGAACTGGTTCGCAAGACCAGATTTTTACCAGTAGCTGGCGGAACAAGCAGCGTATACATCG<br>AGGTATTGCATTTGACTCAAGGGACCTGCTGGGAACAAATGTACACACCAGGAGGGGAGGTGAGAAATGATGATGTTGATCAGAGTTTGATCATTGCTGCTAGAAATATAGTTAGGAGGGCAACAGTATCAGCAGACCCATTGGCTTCGCTCTTGGAAATGTGCCACAGTACACAAATTGGTGGAGTGAGGATGGTGGACATTCTTAGGCAGAACCCAACAGAGGAGCA<br>AGCTGTGGATATATGCAAAGCAGCAATGGGTTTAAGAATCAGTTCATCCTTCAGCTTTGGAGGTTTCACTTTCAAAAGGACAAGTGGGTCGTCTGTCAAAAGAGAAGAAGAAATACTCACTGGCAACCTCCAAACACTGAAAGTAAGAATACATGAAGGATATGAGGAATTCACAATGGTTGGGCGAAGAGCTACAGCCATTTTGAGGAAAGCAACCAGGAGACTGATCC<br>AATTAATAGTGAGTGGACGAGACGAACAGTCAATCGCTGAGGCAATCATAGTGGCAATGGTTTTCTCACAGGAGGATTGCATGATAAAAGCAGTACGAGGTGATTTGAATTTTGTCAACAGAGCGAATCAGCGGCTAAATCCTATGCATCAACTTCTGAGGCATTTCCAAAAAGATGCAAAAGTGCTGTTTCAAAACTGGGGGATTGAACCAATTGACAATGTAATGGGG<br>ATGATCGGGATACTGCCTGACATGACCCCCAGCACAGAGATGTCACTGAGAGGAGTGAGAGTCAGCAAAATGGGAGTGGATGAATATTCCAGTACTGAGAGAGTGGTCGTGAGCATTGATCGCTTCTTGAGAGTCCGAGATCAGAGGGGAAATGTGCTTCTGTCTCCTGAGGAAGTTAGTGAAACACATGGAACAGAGAAACTGACGATAACGTATTCATCGTCTATGA<br>TGTGGGAAATCAATGGTCCGGAATCCGTGCTAGTCAACACATATCAATGGATCATTAGAAATTGGGAAACTGTGAAGATTCAGTGGTCCCAGGACCCTACGATGTTGTACAATAAGATGGAATTTGAGCCCTTCCAATCCTTGGTGCCCAAGGCTGCTAGAGGCCAGTATAGTGGGTTTGTGAGGACATTATTCCAACAGATGCGTGATGTGTTGGGGACATTTGACACTGT<br>CCAAATAATAAAGCTCCTACCATTTGCAGCAGCCCCACCGGAACAGAGTAGGATGCAATTTTCCTCTCTGACTGTGAACGTAAGAGGTTCAGGAATGAGAATACTTGTGAGGGGCAACTCCCCTGTGTTCAACTATAATAAGACCACCAAGAGACTCACAGTTCTTGGAAAGGATGCAGGCGCMTTGACAGAATATCCAGATGAGGGAACAGCAGGAGTGGAGTCTGCA<br>GTATTAAGAGGATTTCTAATTCTGGGCAAAGAGGACAAAAGATATGGACCAGCATTGAGCATCAACGAATTGAGCAATCTTGCGAAAGGGGAAAAGGCTAATGTGTTGATAGGACAAGGAGACGTGGTGTTGGTAATGAAACGGAAACGGGACTCTAGCATACTTACTGACAGCCAGACAGCGACCAAAAGAATTCGGATGGCCATCAATTA
EPI_ISL_12437700	>A/duck/Czech_Republic/6017/2021_H5N8_2021-03-26<br>ATGGAGAGAATAAAAGAACTAAGAGATTTGATGTCGCAGTCTCGCACTCGCGAGATACTAACAAAAACCACTGTGGACCATATGGCCATAATAAAGAAATACACATCAGGGAGACAGGAGAAGAACCCTGCCCTCAGGATGAAATGGATGATGGCAATGAAATATCCTATTACAGCTGACAAAAGAATAATGGAGATGATCCCTGAAAGGAATGAGCAAGGTCAGACT<br>CTCTGGAGCAAAACAAATGATGCTGGATCAGACAGAGTGATGGTCTCACCTCTGGCTGTGACGTGGTGGAATAGAAATGGGCCAACAACAAGTACAGTACACTACCCAAAAGTCTACAAAACCTACTTTGAAAAGGTAGAAAGGTTGAAACATGGAACCTTTGGTCCTGTTCACTTTCGAAATCAGGTTAAGATACGCCGCAGGGTTGACATAAACCCGGGCCATGCAGA<br>TCTCAGTGCCAAAGAAGCACAGGATGTCATCATGGAGGTTGTTTTCCCAAATGAAGTTGGAGCCAGGATCTTGACATCAGAATCACAATTAACAATAACAATGGAAAAGAAGGAGGAACTTCAGGATTGCAAGATTGCTCCTTTGATGGTGGCATACATGTTGGAAAGAGAACTGGTTCGCAAGACCAGATTTTTACCAGTAGCTGGCGGAACAAGCAGCGTATACATCG<br>AGGTATTGCATTTGACTCAAGGGACCTGCTGGGAACAAATGTACACACCAGGAGGGGAGGTGAGAAATGATGATGTTGATCAGAGTTTGATCATTGCTGCTAGAAATATAGTTAGGAGGGCAACAGTATCAGCAGACCCATTGGCTTCGCTCTTGGAAATGTGCCACAGTACACAAATTGGTGGAGTGAGGATGGTGGACATTCTTAGGCAGAACCCAACAGAGGAGCA<br>AGCTGTGGATATATGCAAAGCAGCAATGGGTTTAAGAATCAGTTCATCCTTCAGCTTTGGAGGTTTCACTTTCAAAAGGACAAGTGGGTCGTCTGTCAAAAGAGAAGAAGAAATACTCACTGGCAACCTCCAAACACTGAAAGTAAGAATACATGAAGGATATGAGGAATTCACAATGGTTGGGCGAAGAGCTACAGCCATTTTGAGGAAAGCAACCAGGAGACTGATCC<br>AATTAATAGTGAGTGGACGAGACGAACAGTCAATCGCTGAGGCAATCATAGTGGCAATGGTTTTCTCACAGGAGGATTGCATGATAAAAGCAGTACGAGGTGATTTGAATTTTGTCAACAGAGCGAATCAGCGGCTAAATCCTATGCATCAACTTCTGAGGCATTTCCAAAAAGATGCAAAAGTGCTGTTTCAAAACTGGGGGATTGAACCAATTGACAATGTAATGGGG<br>ATGATCGGGATACTGCCTGACATGACCCCCAGCACAGAGATGTCACTGAGAGGAGTGAGAGTCAGCAAAATGGGAGTGGATGAATATTCCAGTACTGAGAGAGTGGTCGTGAGCATTGATCGCTTCTTGAGAGTCCGAGATCAGAGGGGAAATGTGCTTCTGTCTCCTGAGGAAGTTAGTGAAACACATGGAACAGAGAAACTGACGATAACGTATTCATCGTCTATGA<br>TGTGGGAAATCAATGGTCCGGAATCCGTGCTAGTCAACACATATCAATGGATCATTAGAAATTGGGAAACTGTGAAGATTCAGTGGTCCCAGGACCCTACGATGTTGTACAATAAGATGGAATTTGAGCCCTTCCAATCCTTGGTGCCCAAGGCTGCTAGAGGCCAGTATAGTGGGTTTGTGAGGACATTATTCCAACAGATGCGTGATGTGTTGGGGACATTTGACACTGT<br>CCAAATAATAAAGCTCCTACCATTTGCAGCAGCCCCACCGGAACAGAGTAGGATGCAATTTTCCTCTCTGACTGTGAACGTAAGAGGTTCAGGAATGAGAATACTTGTGAGGGGCAACTCCCCTGTGTTCAACTATAATAAGACCACCAAGAGACTCACAGTTCTTGGAAAGGATGCAGGCGCCTTGACAGAATATCCAGATGAGGGAACAGCAGGAGTGGAGTCTGCAG<br>TATTAAGAGGATTTCTAATTCTGGGCAAAGAGGACAAAAGATATGGACCAGCATTGAGCATCAACGAATTGAGCAATCTTGCGAAAGGGGAAAAGGCTAATGTGTTGATAGGACAAGGAGACGTGGTGTTGGTAATGAAACGGAAACGGGACTCTAGCATACTTACTGACAGCCAGACAGCGACCAAAAGAATTCGGATGGCCATCAATTA
EPI_ISL_1697189	>A/chicken/Czech_Republic/6532-2/2021_H5N8_2021-04-01<br>ATGGAGAGAATAAAAGAACTAAGAGATTTGATGTCGCAGTCTCGCACTCGCGAGATACTAACAAAAACCACTGTGGACCATATGGCCATAATAAAGAAATACACATCAGGGAGACAGGAGAAGAACCCTGCCCTCAGGATGAAATGGATGATGGCAATGAAATATCCTATTACAGCTGACAAAAGAATAATGGAGATGATCCCTGAAAGGAATGAGCAAGGTCAGACT<br>CTCTGGAGCAAAACAAATGATGCTGGATCAGACAGAGTGATGGTCTCACCTCTGGCTGTGACGTGGTGGAATAGAAATGGGCCAACAACAAGTACAGTACACTACCCAAAAGTCTACAAAACCTACTTTGAAAAGGTAGAAAGGTTGAAACATGGAACCTTTGGTCCTGTTCACTTTCGAAATCAGGTTAAGATACGCCGCAGGGTTGACACAAACCCGGGCCATGCAGA<br>TCTCAGTGCCAAAGAAGCACAGGATGTCATCATGGAGGTTGTTTTCCCAAATGAAGTTGGAGCCAGGATCTTGACATCAGAATCACAATTAACAATAACAATGGAAAAGAAGGAGGAACTTCAGGATTGCAAGATTGCTCCTTTGATGGTGGCATACATGTTGGAAAGAGAACTGGTTCGCAAGACCAGATTTTTACCAGTAGCTGGCGGAACAAGCAGCGTATACATCG<br>AGGTATTGCATTTGACTCAAGGGACCTGCTGGGAACAAATGTACACACCAGGAGGGGAGGTGAGAAATGATGATGTTGATCAGAGTTTGATCATTGCTGCTAGAAATATAGTTAGGAGGGCAACAGTATCAGCAGACCCATTGGCTTCGCTCTTGGAAATGTGCCACAGTACACAAATTGGTGGAGTGAGGATGGTGGACATTCTTAGGCAGAACCCAACAGAGGAGCA<br>AGCTGTGGATATATGCAAAGCAGCAATGGGTTTAAGAATCAGTTCATCCTTCAGCTTTGGAGGTTTCACTTTCAAAAGGACAAGTGGGTCGTCTGTCAAAAGAGAAGAAGAAATACTCACTGGCAACCTCCAAACACTGAAAGTAAGAATACATGAAGGATATGAGGAATTCACAATGGTTGGGCGAAGAGCTACAGCCATTTTGAGGAAAGCAACCAGGAGACTGATCC<br>AATTAATAGTGAGTGGACGAGACGAACAGTCAATCGCTGAGGCAATCATAGTGGCAATGGTTTTCTCACAGGAGGATTGCATGATAAAAGCAGTACGAGGTGATTTGAATTTTGTCAACAGAGCGAATCAGCGGCTAAATCCTATGCATCAACTTCTGAGGCATTTCCAAAAAGATGCAAAAGTGCTGTTTCAAAACTGGGGGATTGAACCAATTGACAATGTAATGGGG<br>ATGATCGGGATACTGCCTGACATGACCCCCAGCACAGAGATGTCACTGAGAGGAGTGAGAGTCAGCAAAATGGGAGTGGATGAATATTCCAGTACTGAGAGAGTGGTCGTGAGCATTGATCGCTTCTTGAGAGTCCGAGATCAGAGGGGAAATGTGCTTCTGTCTCCTGAGGAAGTTAGTGAAACACATGGAACAGAGAAACTGACGATAACGTATTCATCGTCCATGA<br>TGTGGGAAATCAATGGTCCGGAATCCGTGCTAGTCAACACATATCAATGGATCATTAGAAATTGGGAAACTGTGAAGATTCAGTGGTCCCAGGACCCTACGATATTGTACAATAAGATGGAATTTGAGCCCTTCCAATCCTTGGTGCCCAAGGCTGCTAGAGGCCAGTATAGTGGGTTTGTGAGGACATTATTCCAACAGATGCGTGATGTGTTGGGGACATTTGACACTGT<br>CCAAATAATAAAGCTCCTACCATTTGCAGCAGCCCCACCGGAACAGAGTAGGATGCAATTTTCCTCTCTGACTGTGAACGTAAGAGGTTCAGGAATGAGAATACTTGTGAGGGGCAACTCCCCTGTGTTCAACTATAATAAGACCACCAAGAGACTCACAGTTCTTGGAAAGGATGCAGGCGCCTTGACAGAATATCCAGATGAGGGAACAGCAGGAGTGGAGTCTGCAG<br>TATTAAGAGGATTTCTAATTCTGGGCAAAGAGGACAAAAGATATGGACCAGCATTGAGCATCAACGAATTGAGCAATCTTGCGAAAGGGGAAAAGGCTAATGTGTTGATAGGACAAGGAGACGTGGTGTTGGTAATGAAACGGAAACGGGACTCTAGCATACTTACTGACAGCCAGACAGCGACCAAAAGAATTCGGATGGCCATCAATTA
EPI_ISL_1941582	>A/chicken/Czech_Republic/7100/2021_H5N8_2021-04-08<br>ATGGAGAGAATAAAAGAACTAAGAGATTTGATGTCGCAGTCTCGCACTCGCGAGATACTAACAAAAACCACTGTGGACCATATGGCCATAATAAAGAAATACACATCAGGGAGACAGGAGAAGAACCCTGCCCTCAGGATGAAATGGATGATGGCAATGAAATATCCTATTACAGCTGACAAAAGAATAATGGAGATGATCCCTGAAAGGAATGAGCAAGGTCAGACT<br>CTCTGGAGCAAAACAAATGATGCTGGATCAGACAGAGTGATGGTCTCACCTCTGGCTGTGACGTGGTGGAATAGAAATGGGCCAACAACAAGTACAGTACACTACCCAAAAGTCTACAAAACCTACTTTGAAAAGGTAGAAAGGTTGAAACATGGAACCTTTGGTCCTGTTCACTTTCGAAATCAGGTTAAGATACGCCGCAGGGTTGACACAAACCCGGGCCATGCAGA<br>TCTCAGTGCCAAAGAAGCACAGGATGTCATCATGGAGGTTGTTTTCCCAAATGAAGTTGGAGCCAGGATCTTGACATCAGAATCACAATTAACAATAACAATGGAAAAGAAGGAGGAACTTCAGGATTGCAAGATTGCTCCTTTGATGGTGGCATACATGTTGGAAAGAGAACTGGTTCGCAAGACCAGATTTTTACCAGTAGCTGGCGGAACAAGCAGCGTATACATCG<br>AGGTATTGCATTTGACTCAAGGGACCTGCTGGGAACAAATGTACACACCAGGAGGGGAGGTGAGAAATGATGATGTTGATCAGAGTTTGATCATTGCTGCTAGAAATATAGTTAGGAGGGCAACAGTATCAGCAGACCCATTGGCTTCGCTCTTGGAAATGTGCCACAGTACACAAATTGGTGGAGTGAGGATGGTGGACATTCTTAGGCAGAACCCAACAGAGGAGCA<br>AGCTGTGGATATATGCAAAGCAGCAATGGGTTTAAGAATCAGTTCATCCTTCAGCTTTGGAGGTTTCACTTTCAAAAGGACAAGTGGGTCGTCTGTCAAAAGAGAAGAAGAAATACTCACTGGCAACCTCCAAACACTGAAAGTAAGAATACATGAAGGATATGAGGAATTCACAATGGTTGGGCGAAGAGCTACAGCCATTTTGAGGAAAGCAACCAGGAGACTGATCC<br>AATTAATAGTGAGTGGACGAGACGAACAGTCAATCGCTGAGGCAATCATAGTGGCAATGGTTTTCTCACAGGAGGATTGCATGATAAAAGCAGTACGAGGTGATTTGAATTTTGTCAACAGAGCGAATCAGCGGCTAAATCCTATGCATCAACTTCTGAGGCATTTCCAAAAAGATGCAAAAGTGCTGTTTCAAAACTGGGGGATTGAACCAATTGACAATGTAATGGGG<br>ATGATCGGGATACTGCCTGACATGACCCCCAGCACAGAGATGTCACTGAGAGGAGTGAGAGTCAGCAAAATGGGAGTGGATGAATATTCCAGTACTGAGAGAGTGGTCGTGAGCATTGATCGCTTCTTGAGAGTCCGAGATCAGAGGGGAAATGTGCTTCTGTCTCCTGAGGAAGTTAGTGAAACACATGGAACAGAGAAACTGACGATAACGTATTCATCGTCCATGA<br>TGTGGGAAATCAATGGTCCGGAATCCGTGCTAGTCAACACATATCAATGGATCATTAGAAATTGGGAAACTGTGAAGATTCAGTGGTCCCAGGACCCTACGATGTTGTACAATAAGATGGAATTTGAGCCCTTCCAATCCTTGGTGCCCAAGGCTGCTAGAGGCCAGTATAGTGGGTTTGTGAGGACATTATTCCAACAGATGCGTGATGTGTTGGGGACATTTGACACTGT<br>CCAAATAATAAAGCTCCTACCATTTGCAGCAGCCCCACCGGAACAGAGTAGGATGCAATTTTCCTCTCTGACTGTGAACGTAAGAGGTTCAGGAATGAGAATACTTGTGAGGGGCAACTCCCCTGTGTTCAACTATAATAAGACCACCAAGAGACTCACAGTTCTTGGAAAGGATGCAGGCGCCTTGACAGAATATCCAGATGAGGGAACAGCAGGAGTGGAGTCTGCAG<br>TATTAAGAGGATTTCTAATTCTGGGCAAAGAGGACAAAAGATATGGACCAGCATTGAGCATCAACGAATTGAGCAATCTTGCGAAAGGGGAAAAGGCTAATGTGTTGATAGGACAAGGAGACGTGGTGTTGGTAATGAAACGGAAACGGGACTCTAGCATACTTACTGACAGCCAGACAGCGACCAAAAGAATTCGGATGGCCATCAATTA
EPI_ISL_12210335	>A/Falco_peregrinus/Belgium/4055_0002/2022_H5N1_2022-03-19<br>ATGGAGAGAATAAAAGAACTAAGAGATTTAATGTCGCAGTCTCGCACTCGCGAGATACTGACAAAAACCACTGTGGACCATATGGCCATAATAAAGAAATACACATCAGGAAGACAGGAGAAGAACCCTGCCCTCAGGATGAAGTGGATGATGGCAATGAAATATCCTATTACAGCAGACAAAAGAATAATGGAGACGATCCCTGAAAGGAATGAGCAAGGTCAGACT<br>CTCTGGAGCAAAACAAATGACGCTGGATCAGACAGAGTGATGGTGTCACCTCTGGCTGTGACATGGTGGAATAGAAATGGGCCAACGACAAGTACAGTCCATTACCCAAAGGTTTACAAAACCTACTTTGAAAAAGTGGAAAGATTGAAACATGGAACCTTTGGCCCTGTTCACTTTCGAAATCAGGTTAAGATACGCCGCAGGGTTGACATAAACCCGGGTCATGCAGA<br>TCTCAGTGCCAAAGAAGCACAGGATGTCATCATGGAAGTCGTTTTCCCAAATGAAGTTGGAGCCAGGATATTGACATCAGAATCACAATTAACAATAACAAAGGGAAAGAAGGAGGAACTTCAGGACTGTAAGATTGCTCCTTTGATGGTGGCATACATGTTGGAGAGAGAACTGGTTCGAAAAACCAGATTTTTACCAGTAGCTGGCGGAACAAGCAGCGTATATATCG<br>AAGTATTGCATTTGACTCAAGGGACCTGCTGGGAACAAATGTACACACCGGGAGGGGAAGTGAGAAATGATGATGTTGATCAGAGTTTGATCATTGCTGCTAGAAATATCGTTAGGAGAGCAACAGTATCAGCAGACCCATTGGCTTCGCTCTTGGAAATGTGCCACAGCACACAAATTGGCGGGATAAGGATGGTAGACATTCTTAGACAGAACCCAACAGAAGAGCA<br>AGCCGTGGATATATGCAAAGCAGCAATGGGTTTAAGAATCAGTTCATCCTTCAGCTTTGGAGGTTTCACTTTCAAAAGAACAAGTGGATTGTCTGTCAAAAGAGAAGAAGAAGTGCTCACAGGCAACCTCCAAACACTGAAAATAAGAGTACATGAAGGATATGAGGAATTCACAATGGTTGGACGGAGAGCTACAGCCATTTTAAGGAAAGCAACCAGAAGGCTGATCC<br>AATTAATAGTGAGTGGAAGAGACGAGCAGTCAATCGCTGAAGCAATCATAGTGGCAATGGTTTTCTCACAAGAGGACTGCATGATAAAAGCAGTACGAGGTGATCTGAACTTTGTTAATAGAGCAAATCAGCGACTAAATCCTATGCATCAACTTCTGAGGCATTTCCAAAAGGATGCAAAGGTGCTGTTTCAAAACTGGGGGGTTGAACCAATTGACAATGTAATGGGG<br>ATGATTGGAATACTGCCTGACATGACCCCCAGCACGGAGATGTCACTGAGAGGAGTGAGAGTCAGCAAAATGGGAGTGGATGAATATTCCAGCACTGAGAGAGTGGTCGTGAGCATTGATCGTTTCTTGAGAGTCCGAGATCAAAGAGGAAACGTGCTCCTGTCTCCGGAGGAAGTTAGTGAAACACAGGGAACAGAGAAACTGACGATAACATATTCATCGTCCATGA<br>TGTGGGAAATCAATGGTCCGGAATCCGTGCTAGTCAACACATATCAATGGATCATCAGAAATTGGGAGACTGTGAAGATTCAATGGTCCCAAGACCCTACGATGTTGTACAACAAGATGGAATTTGAGCCCTTCCAATCCTTGGTGCCCAAGGCTGCCAGAGGCCAGTATAGTGGATTTGTGAGGACATTGTTCCAGCAGATGCGTGATGTGCTGGGAACATTTGATACTG<br>TCCAAATAATAAAGCTCCTGCCTTTTGCTGCAGCCCCACCGGAACAGAGTAGGATGCAGTTTTCCTCTCTGACTGTGAACGTAAGAGGTTCAGGAATGAGAATACTTGTGAGAGGCAACTCCCCTGTGTTCAACTATAACAAGGCCACCAAGAGACTCACAGTTCTTGGAAAGGATGCAGGTGCCTTGACAGAAGATCCGGATGAGGGAACAGCAGGAGTAGAGTCTGCA<br>GTATTAAGAGGATTTCTAATTCTGGGCAAAGAGGACAAAAGATATGGACCAGCATTGAGCATTAACGAACTGAGCAATCTTGCGAAAGGGGAAAAGGCTAATGTGTTGATAGGGCAAGGAGACGTGGTGTTGGTAATGAAACGGAAACGGGACTCTAGCATACTTACTGACAGCCAGACAGCGACCAAAAGAATTCGGATGGCCATCAATTA
EPI_ISL_5463801	>A/chicken/Saratov/29-07V/2021_H5N1_2021-09-30<br>ATGGAGAGAATAAAAGAACTAAGAGATTTAATGTCGCAGTCTCGCACTCGCGAGATACTGACAAAAACCACTGTGGACCATATGGCCATAATAAAGAAATACACATCAGGAAGACAGGAGAAGAACCCTGCCCTCAGGATGAAGTGGATGATGGCAATGAAATATCCTATTACAGCAGACAAAAGAATAATGGAGATGATCCCTGAAAGGAATGAGCAAGGTCAGACT<br>CTCTGGAGCAAAACAAATGACGCTGGATCAGACAGAGTGATGGTGTCACCTCTGGCTGTGACATGGTGGAATAGAAATGGGCCAACGACAAGTACAGTCCATTACCCAAAGGTTTACAAAACCTACTTTGAAAAAGTGGAAAGATTGAAACATGGAACCTTTGGCCCTGTTCACTTTCGAAATCAGGTTAAGATACGCCGCAGGGTTGACATAAACCCGGGTCATGCAGA<br>TCTCAGTGCCAAAGAAGCACAGGATGTCATCATGGAAGTCGTTTTCCCAAATGAAGTTGGAGCCAGGATATTGACATCAGAATCACAATTAACAATAACAAAGGAAAAGAAGGAGGAACTTCAGGACTGTAAGATTGCTCCTTTGATGGTGGCATACATGTTGGAGAGAGAACTGGTTCGAAAAACCAGATTTTTACCAGTAGCTGGCGGAACAAGCAGCGTATATATCG<br>AAGTATTGCATTTGACTCAAGGGACCTGCTGGGAACAAATGTACACACCGGGAGGGGAAGTGAGAAATGATGATGTTGATCAGAGTTTGATCATTGCTGCTAGAAATATCGTTAGGAGAGCAACAGTATCAGCAGACCCATTGGCTTCGCTCTTGGAAATGTGCCACAGTACACAAATTGGCGGGATAAGGATGGTAGACATTCTTAGACAGAACCCAACAGAAGAGCA<br>AGCCGTGGATATATGCAAAGCAGCAATGGGTTTAAGAATCAGTTCATCCTTCAGCTTTGGAGGTTTCACTTTCAAAAGAACAAGTGGATCGTCTGTCAAAAGAGAAGAAGAAGTGCTCACAGGCAACCTCCAAACACTGAAAATAAGAGTACATGAAGGATATGAGGAATTCACAATGGTTGGACGGAGAGCTACAGCCATTTTAAGGAAAGCAACCAGAAGGCTGATCC<br>AATTAATAGTGAGTGGAAGAGACGAGCAGTCAATCGCTGAAGCAATCATAGTGGCAATGGTTTTCTCACAAGAGGACTGCATGATAAAAGCAGTACGAGGTGATCTGAACTTTGTTAATAGAGCAAATCAGCGACTAAATCCTATGCATCAACTTCTGAGGCATTTCCAAAAGGATGCAAAGGTGCTGTTTCAAAACTGGGGGGTTGAACCAATTGACAATGTAATGGGG<br>ATGATTGGAATACTGCCTGACATGACCCCCAGCACGGAGATGTCACTGAGAGGAGTGAGAGTCAGCAAAATGGGAGTGGATGAATATTCCAGTACTGAGAGAGTGGTCGTGAGCATTGATCGTTTCTTGAGAGTCCGAGATCAAAGAGGAAACGTGCTCCTGTCTCCTGAGGAAGTTAGTGAAACACAGGGAACAGAGAAACTGACGATAACATATTCATCGTCCATGA<br>TGTGGGAAATCAATGGTCCGGAATCCGTGCTAGTCAACACATATCAATGGATCATCAGAAATTGGGAGACTGTGAAGATTCAATGGTCCCAAGACCCTACGATGTTGTACAACAAGATGGAATTTGAGCCCTTCCAATCCTTGGTGCCCAAGGCTGCCAGAGGCCAGTATAGTGGATTTGTGAGGACATTGTTCCAGCAGATGCGTGATGTGCTGGGAACATTTGATACTG<br>TCCAAATAATAAAGCTCCTGCCTTTTGCTGCAGCCCCACCGGAACAGAGTAGGATGCAGTTTTCCTCTCTGACTGTGAACGTAAGAGGTTCAGGAATGAGAATACTTGTGAGAGGCAACTCCCCTGTGTTCAACTATAACAAGGCCACCAAGAGACTCACAGTTCTTGGAAAGGATGCAGGTGCCTTGACAGAAGATCCAGATGAGGGAACAGCAGGAGTAGAGTCTGCA<br>GTATTAAGAGGATTTCTAATTCTGGGCAAAGAGGACAAAAGATATGGACCAGCATTGAGCATCAACGAACTGAGCAATCTTGCGAAAGGGGAAAAGGCTAATGTATTGATAGGGCAAGGAGACGTGGTGTTGGTAATGAAACGGAAACGGGACTCTAGCATACTTACTGACAGCCAGACAGCGACCAAAAGAATTCGGATGGCCATCAATTA
EPI_ISL_5463802	>A/duck/Saratov/29-08V/2021_H5N1_2021-09-30<br>ATGGAGAGAATAAAAGAACTAAGAGATTTAATGTCGCAGTCTCGCACTCGCGAGATACTGACAAAAACCACTGTGGACCATATGGCCATAATAAAGAAATACACATCAGGAAGACAGGAGAAGAACCCTGCCCTCAGGATGAAGTGGATGATGGCAATGAAATATCCTATTACAGCAGACAAAAGAATAATGGAGATGATCCCTGAAAGGAATGAGCAAGGTCAGACT<br>CTCTGGAGCAAAACAAATGACGCTGGATCAGACAGAGTGATGGTGTCACCTCTGGCTGTGACATGGTGGAATAGAAATGGGCCAACGACAAGTACAGTCCATTACCCAAAGGTTTACAAAACCTACTTTGAAAAAGTGGAAAGATTGAAACATGGAACCTTTGGCCCTGTTCACTTTCGAAATCAGGTTAAGATACGCCGCAGGGTTGACATAAACCCGGGTCATGCAGA<br>TCTCAGTGCCAAAGAAGCACAGGATGTCATCATGGAAGTCGTTTTCCCAAATGAAGTTGGAGCCAGGATATTGACATCAGAATCACAATTAACAATAACAAAGGAAAAGAAGGAGGAACTTCAGGACTGTAAGATTGCTCCTTTGATGGTGGCATACATGTTGGAGAGAGAACTGGTTCGAAAAACCAGATTTTTACCAGTAGCTGGCGGAACAAGCAGCGTATATATCG<br>AAGTATTGCATTTGACTCAAGGGACCTGCTGGGAACAAATGTACACACCGGGAGGGGAAGTGAGAAATGATGATGTTGATCAGAGTTTGATCATTGCTGCTAGAAATATCGTTAGGAGAGCAACAGTATCAGCAGACCCATTGGCTTCGCTCTTGGAAATGTGCCACAGTACACAAATTGGCGGGATAAGGATGGTAGACATTCTTAGACAGAACCCAACAGAAGAGCA<br>AGCCGTGGATATATGCAAAGCAGCAATGGGTTTAAGAATCAGTTCATCCTTCAGCTTTGGAGGTTTCACTTTCAAAAGAACAAGTGGATCGTCTGTCAAAAGAGAAGAAGAAGTGCTCACAGGCAACCTCCAAACACTGAAAATAAGAGTACATGAAGGATATGAGGAATTCACAATGGTTGGACGGAGAGCTACAGCCATTTTAAGGAAAGCAACCAGAAGGCTGATCC<br>AATTAATAGTGAGTGGAAGAGACGAGCAGTCAATCGCTGAAGCAATCATAGTGGCAATGGTTTTCTCACAAGAGGACTGCATGATAAAAGCAGTACGAGGTGATCTGAACTTTGTTAATAGAGCAAATCAGCGACTAAATCCTATGCATCAACTTCTGAGGCATTTCCAAAAGGATGCAAAGGTGCTGTTTCAAAACTGGGGGGTTGAACCAATTGACAATGTAATGGGG<br>ATGATTGGAATACTGCCTGACATGACCCCCAGCACGGAGATGTCACTGAGAGGAGTGAGAGTCAGCAAAATGGGAGTGGATGAATATTCCAGTACTGAGAGAGTGGTCGTGAGCATTGATCGTTTCTTGAGAGTCCGAGATCAAAGAGGAAACGTGCTCCTGTCTCCTGAGGAAGTTAGTGAAACACAGGGAACAGAGAAACTGACGATAACATATTCATCGTCCATGA<br>TGTGGGAAATCAATGGTCCGGAATCCGTGCTAGTCAACACATATCAATGGATCATCAGAAATTGGGAGACTGTGAAGATTCAATGGTCCCAAGACCCTACGATGTTGTACAACAAGATGGAATTTGAGCCCTTCCAATCCTTGGTGCCCAAGGCTGCCAGAGGCCAGTATAGTGGATTTGTGAGGACATTGTTCCAGCAGATGCGTGATGTGCTGGGAACATTTGATACTG<br>TCCAAATAATAAAGCTCCTGCCTTTTGCTGCAGCCCCACCGGAACAGAGTAGGATGCAGTTTTCCTCTCTGACTGTGAACGTAAGAGGTTCAGGAATGAGAATACTTGTGAGAGGCAACTCCCCTGTGTTCAACTATAACAAGGCCACCAAGAGACTCACAGTTCTTGGAAAGGATGCAGGTGCCTTGACAGAAGATCCAGATGAGGGAACAGCAGGAGTAGAGTCTGCA<br>GTATTAAGAGGATTTCTAATTCTGGGCAAAGAGGACAAAAGATATGGACCAGCATTGAGCATCAACGAACTGAGCAATCTTGCGAAAGGGGAAAAGGCTAATGTGTTGATAGGGCAAGGAGACGTGGTGTTGGTAATGAAACGGAAACGGGACTCTAGCATACTTACTGACAGCCAGACAGCGACCAAAAGAATTCGGATGGCCATCAATTA
EPI_ISL_15364796	>A/Mallard/Netherlands/9/2022_H5N1_2022-09-11<br>ATGGAGAGAATAAAAGAACTAAGAGATTTAATGTCGCAGTCTCGCACTCGCGAGATACTGACAAAAACCACTGTGGACCATATGGCCATAATAAAGAAATACACATCAGGAAGACAGGAGAAGAACCCTGCCCTCAGGATGAAGTGGATGATGGCAATGAAATATCCTATTACAGCAGACAAAAGAATAATGGAGATGATCCCTGAAAGGAATGAGCAAGGTCAGACT<br>CTCTGGAGCAAAACAAATGACGCTGGATCAGACAGAGTGATGGTGTCACCTCTGGCTGTGACATGGTGGAATAGAAATGGGCCAACGACAAGTACAGTCCATTACCCAAAGGTTTACAAAACCTACTTTGAAAAAGTGGAAAGATTGAAACATGGAACCTTTGGCCCTGTTCACTTTCGAAATCAGGTTAAGATACGCCGCAGGGTTGACGTGAACCCGGGTCATGCAGA<br>TCTCAGTGCCAAAGAAGCACAGGATGTCATCATGGAAGTCGTTTTCCCAAATGAAGTTGGAGCCAGGATATTGACATCAGAATCACAATTAACAATAACAAAGGAAAAGAAGGAGGAACTTCAGGACTGTAAGATTGCTCCTTTGATGGTGGCATACATGTTGGAGAGAGAACTGGTTCGAAAAACCAGATTTTTACCAGTAGCTGGCGGAACAAGCAGCGTATATATCG<br>AAGTATTGCATTTGACTCAAGGGACCTGCTGGGAACAAATGTACACACCGGGAGGGnAAGTGAGAAATGATGATGTTGATCAGAGTTTGATCATTGCTGCTAGAAATATCGTTAGGAGAGCAACAGTATCAGCAGACCCATTGGCTTCGCTCTTGGAAATGTGCCACAGCACACAAATTGGCGGGATAAGGATGGTAGACATTCTTAGACAGAACCCAACAGAAGAGCA<br>AGCCGTGGATATATGCAAAGCAGCAATGGGTTTAAGAATCAGTTCATCCTTCAGCTTTGGAGGTTTCACTTTCAAAAGAACAAGTGGATCGTCTGTCAAAATAGAAGAAGAAGTGCTCACAGGCAACCTCCAAACACTGAAAATAAGAGTACATGAAGGATATGAGGAATTCACAATGGTTGGACGGAGAGCTACAGCCATTTTGAGGAAAGCAACCAGAAGGCTGATCC<br>AATTAATAGTGAGTGGAAGAGACGAGCAGTCAATCGCTGAAGCAATCATAGTGGCAATGGTTTTCTCACAAGAGGACTGCATGATAAAAGCAGTACGAGGTGATCTGAACTTTGTTAATAGAGCAAATCAGCGACTAAATCCTATGCATCAACTTCTGAGGCATTTCCAAAAGGATGCAAAGGTGCTGTTTCAAAACTGGGGGnTTGAACCAATTGACAATGTAATGGGG<br>ATGATTGGAATACTGCCTGACATGACCCCCAGCACGGAGATGTCACTGAGAGGAGTGAGAGTCAGCAAAATGGGAGTGGATGAATATTCCAGTACTGAGAGAGTGGTCGCGAGCATTGATCGTTTCTTGAGAGTCCGAGATCAAAGAGGAAACGTGCTCCTGTCTCCGGAGGAAGTTAGTGAAACACAGGGAACTGAGAAACTGACGATAACATATTCATCGTCCATGA<br>TGTGGGAAATAAATGGTCCGGAGTCCGTGCTAGTCAACACATATCAATGGATCATCAGAAATTGGGAGACTGTGAAGATTCAATGGTCCCAAGACCCTACGATGTTGTACAACAAGATGGAATTTGAGCCCTTCCAATCCTTGGTGCCCAAGGCTGCCAGAGGCCAGTATAGTGGATTTGTGAGGACATTGTTCCAGCAGATGCGTGATGTGCTGGGAACATTTGATACTG<br>TCCAAATAATAAAGCTCCTGCCTTTTGCTGCAGCCCCTCCGGAACAGAGTAGGATGCAGTTTTCCTCTCTGACTGTGAACGTAAGAGGTTCAGGAATGAGAATACTTGTGAGAGGCAACTCCCCTGTGTTCAACTATAACAAGGCCACCAAGAGACTCACAGTTCTTGGAAAGGATGCAGGTGCCTTGACAGAAGATCCAGATGAGGGAACAGAAGGAGTAGAGTCTGCA<br>GTATTAAGAGGATTTCTAATTCTGGGCAAAGAGGACAAAAGATATGGACCAGCATTGAGCATTAACGAACTGAGCAATCTTGCGAAAGGGGAAAAGGCTAATGTGTTGATAGGGCAAGGAGACGTGGTGTTGGTAATGAAACGGAAACGGGACTCTAGCATACTTACTGACAGCCAGACAGCGACCAAAAGAATTCGGATGGCCATCAATTA

EPI_ISL_13613225	>A/Black-headed_gull/Netherlands/7/2022_H5N1_2022-06-10<br>ATGGAGAGAATAAAAGAACTAAGAGATTTAATGTCGCAGTCTCGCACTCGCGAGATACTGACAAAAACCACTGTGGACCATATGGCCATAATAAAGAAATACACATCAGGAAGACAGGAGAAGAACCCTGCCCTCAGGATGAAGTGGATGATGGCAATGAAATATCCTATTACAGCAGACAAAAGAATAATGGAGATGATCCCTGAAAGGAATGAGCAAGGTCAGACT<br>CTCTGGAGCAAAACAAATGACGCTGGATCAGACAGAGTGATGGTGTCACCTCTGGCTGTGACATGGTGGAATAGAAATGGCCAACGACAAGTACAGTCCATTACCCAAAGGTTTACAAAACCTACTTTGAAAAAGTGGAAAGATTGAAACATGGAACCTTTGGCCCTGTTCACTTTCGAAATCAGGTTAAGATACGCCGCAGGGTTGACGTGAACCCGGGTCATGCAGA<br>TCTCAGTGCCAAAGAAGCACAGGATGTCATCATGGAAGTCGTTTTCCCAAATGAAGTTGGAGCCAGGATATTGACATCAGAATCACAATTAACAATAACAAAGGAAAAGAAGGAGGAACTTCAGGACTGTAAGATTGCTCCTTTGATGGTGGCATACATGTTGGAGAGAGAACTGGTTCGAAAAACCAGATTTTTACCAGTAGCTGGCGGAACAAGCAGCGTATATATCG<br>AAGTATTGCATTTGACTCAAGGGACCTGCTGGGAACAAATGTACACACCGGGAGGGnAAGTGAGAAATGATGATGTTGATCAGAGTTTGATCATTGCTGCTAGAAATATCGTTAGGAGAGCAACAGTATCAGCAGACCCATTGGCTTCGCTCTTGGAAATGTGCCACAGCACACAAATTGGCGGGATAAGGATGGTAGACATTCTTAGACAGAACCCAACAGAAGAGCA<br>AGCCGTGGATATATGCAAAGCAGCAATGGGTTTAAGAATCAGTTCATCCTTCAGCTTTGGAGGTTTCACTTTCAAAAGAACAAGTGGATCGTCTGTCAAAATAGAAGAAGAAGTGCTCACAGGCAACCTCCAAACACTGAAAATAAGAGTACATGAAGGATATGAGGAATTCACAATGGTTGGACGGAGAGCTACAGCCATTTTGAGGAAAGCAACCAGAAGGCTGATCC<br>AATTAATAGTGAGTGGAAGAGACGAGCAGTCAATCGCTGAAGCAATCATAGTGGCAATGGTTTTCTCACAAGAGGACTGCATGATAAAAGCAGTACGAGGTGATCTGAACTTTGTTAATAGAGCAAATCAGCGACTAAATCCTATGCATCAACTTCTGAGGCATTTCCAAAAGGATGCAAAGGTGCTGTTTCAAAACTGGGGGnTTGAACCAATTGACAATGTAATGGGG<br>ATGATTGGAATACTGCCTGACATGACCCCCAGCACGGAGATGTCACTGAGAGGAGTGAGAGTCAGCAAAATGGGAGTGGATGAATATTCCAGTACTGAGAGAGTGGTCGTGAGCATTGATCGTTTCTTGAGAGTCCGAGATCAAAGAGGAAACGTGCTCCTGTCTCCGGAGGAAGTTAGTGAAACACAGGGAACTGAGAAACTGACGATAACATATTCATCGTCCATGA<br>TGTGGGAAATCAATGGTCCGGAGTCCGTGCTAGTCAACACATATCAATGGATCATCAGAAATTGGGAGACTGTGAAGATTCAATGGTCCCAAGACCCTACGATGTTGTACAACAAGATGGAATTTGAGCCCTTCCAATCCTTGGTGCCCAAGGCTGCCAGAGGCCAGTATAGTGGATTTGTGAGGACATTGTTCCAGCAGATGCGTGATGTGCTGGGAACATTTGATACTG<br>TCCAAATAATAAAGCTCCTGCCTTTTGCTGCAGCCCCTCCGGAACAGAGTAGGATGCAGTTTTCCTCTCTGACTGTGAACGTAAGAGGTTCAGGAATGAGAATACTTGTGAGAGGCAACTCCCCTGTGTTCAACTATAACAAGGCCACCAAGAGACTCACAGTTCTTGGAAAGGATGCAGGTGCCTTGACAGAAGATCCAGATGAGGGAACAGAAGGAGTAGAGTCTGCA<br>GTATTAAGAGGATTTCTAATTCTGGGCAAAGAGGACAAAAGATATGGACCAGCATTGAGCATTAACGAACTGAGCAATCTTGCGAAAGGGGAAAAGGCTAATGTGTTGATAGGGCAAGGAGACGTGGTGTTGGTAATGAAACGGAAACGGGACTCTAGCATACTTACTGACAGCCAGACAGCGACCAAAAGAATTCGGATGGCCATCAATTA
EPI_ISL_14233919	>A/Eurasian_Spoonbill/Netherlands/3/2022_H5N1_2022-07-24<br>ATGGAGAGAATAAAAGAACTAAGAGATTTAATGTCGCAGTCTCGCACTCGCGAGATACTGACAAAAACCACTGTGGACCATATGGCCATAATAAAGAAATACACATCAGGAAGACAGGAGAAGAACCCTGCCCTCAGGATGAAGTGGATGATGGCAATGAAATATCCTATTACAGCAGACAAAAGAATAATGGAGATGATCCCTGAAAGGAATGAGCAAGGTCAGACT<br>CTCTGGAGCAAAACAAATGACGCTGGATCAGACAGAGTGATGGTGTCACCTCTGGCTGTGACATGGTGGAATAGAAATGGCCAACGACAAGTACAGTCCATTACCCAAAGGTTTACAAAACCTACTTTGAAAAAGTGGAAAGATTGAAACATGGAACCTTTGGCCCTGTTCACTTTCGAAATCAGGTTAAGATACGCCGCAGGGTTGACGTAAACCCGGGTCATGCAGA<br>TCTCAGTGCCAAAGAAGCACAGGATGTCATCATGGAAGTCGTTTTCCCAAATGAAGTTGGAGCCAGGATATTGACATCAGAATCACAATTAACAATAACAAAGGAAAAGAAGGAGGAACTTCAGGACTGTAAGATTGCTCCTTTGATGGTGGCATACATGTTGGAGAGAGAACTGGTTCGAAAAACCAGATTTTTACCAGTAGCTGGCGGAACAAGCAGCGTATATATCG<br>AAGTATTGCATTTGACTCAAGGGACCTGCTGGGAACAAATGTACACACCGGGAGGnnAAGTGAGAAATGATGATGTTGATCAGAGTTTGATCATTGCTGCTAGAAATATCGTTAGGAGAGCAACAGTATCAGCAGACCCATTGGCTTCGCTCTTGGAAATGTGCCACAGCACACAAATTGGCGGGATAAGGATGGTAGACATTCTTAGACAGAACCCAACAGAAGAGCAA<br>GCCGTGGATATATGCAAAGCAGCAATGGGTTTAAGAATCAGTTCATCCTTCAGCTTTGGAGGTTTCACTTTCAAAAGAACAAGTGGATCGTCTGTCAAAATAGAAGAAGAAGTGCTCACAGGCAACCTCCAAACACTGAAAATAAGAGTACATGAAGGATATGAGGAATTCACAATGGTTGGACGGAGAGCTACAGCCATTTTAAGGAAAGCAACCAGAAGGCTGATCCA<br>ATTAATAGTGAGTGGAAGAGACGAGCAGTCAATCGCTGAAGCAATCATAGTAGCAATGGTTTTCTCACAAGAGGACTGCATGATAAAAGCAGTACGAGGTGATCTGAACTTTGTTAATAGAGCAAATCAGCGACTAAATCCTATGCATCAACTTCTGAGGCATTTCCAAAAGGATGCAAAGGTGCTGTTTCAAAACTGGGGGnTTGAACCAATTGACAATGTAATGGGGAT<br>GATTGGAATACTGCCTGACATGACCCCCAGCACGGAGATGTCACTGAGAGGAGTGAGAGTCAGCAAAATGGGAGTGGATGAATATTCCAGTACTGAGAGAGTGGTCGTGAGCATTGATCGTTTCTTGAGAGTCCGAGATCAAAGAGGAAACGTGCTCCTGTCTCCGGAAGAAGTTAGTGAAACACAGGGAACTGAGAAACTGACGATAACATATTCATCGTCCATGATG<br>TGGGAAATCAATGGTCCGGAATCCGTGCTAGTCAACACATATCAATGGATCATCAGAAATTGGGAGACTGTGAAGATTCAATGGTCCCAAGACCCTACGATGTTGTACAACAAGATGGAATTTGAGCCCTTCCAATCCTTGGTGCCCAAGGCTGCCAGAGGCCAGTATAGTGGATTTGTGAGGACATTGTTCCAGCAGATGCGTGATGTGCTGGGAACATTTGATACTGTC<br>CAAATAATAAAGCTCCTGCCTTTTGCTGCAGCCCCTCCGGAACAGAGTAGGATGCAGTTTTCCTCTCTGACTGTGAACGTAAGAGGTTCAGGAATGAGAATACTTGTGAGAGGCAACTCCCCTGTGTTCAACTATAACAAGGCCACCAAGAGACTCACAGTTCTTGGAAAGGATGCAGGTGCCTTGACAGAAGATCCAGATGAGGGAACAGAAGGAGTAGAGTCTGCAGT<br>ATTAAGAGGATTTCTAATTCTGGGCAAAGAGGACAAAAGATATGGACCAGCATTGAGCATTAACGAACTGAGCAATCTTGCGAAAGGGGAAAAGGCTAATGTGTTGATAGGGCAAGGAGACGTGGTGTTGGTAATGAAACGGAAACGGGACTCTAGCATACTTACTGACAGCCAGACAGCGACCAAAAGAATTCGGATGGCCATCAATTA
EPI_ISL_13429298	>A/Common_Tern/Netherlands/1/2022_H5N1_2022-06-03<br>ATGGAGAGAATAAAAGAACTAAGAGATTTAATGTCGCAGTCTCGCACTCGCGAGATACTGACAAAAACCACTGTGGACCATATGGCCATAATAAAGAAATACACATCAGGAAGACAGGAGAAGAACCCTGCCCTCAGGATGAAGTGGATGATGGCAATGAAATATCCTATTACAGCAGACAAAAGAATAATGGAGATGATCCCTGAAAGGAATGAGCAAGGTCAGACT<br>CTCTGGAGCAAAACAAATGACGCTGGATCAGACAGAGTGATGGTGTCACCTCTGGCTGTGACATGGTGGAATAGAAATGGCCAACGACAAGTACAGTCCATTACCCAAAGGTTTACAAAACCTACTTTGAAAAAGTGGAAAGATTGAAACATGGAACCTTTGGCCCTGTTCACTTTCGAAATCAGGTTAAGATACGCCGCAGGGTTGACGTAAACCCGGGTCATGCAGA<br>TCTCAGTGCCAAAGAAGCACAGGATGTCATCATGGAAGTCGTTTTCCCAAATGAAGTTGGAGCCAGGATATTGACATCAGAATCACAATTAACAATAACAAAGGAAAAGAAGGAGGAACTTCAGGACTGTAAGATTGCTCCTTTGATGGTGGCATACATGTTGGAGAGAGAACTGGTTCGAAAAACCAGATTTTTACCAGTAGCTGGCGGAACAAGCAGCGTATATATCG<br>AAGTATTGCATTTGACTCAAGGGACCTGCTGGGAACAAATGTACACACCGGGAGGGnAAGTGAGAAATGATGATGTTGATCAGAGTTTGATCATTGCTGCTAGAAATATCGTTAGGAGAGCAACAGTATCAGCAGACCCATTGGCTTCGCTCTTGGAAATGTGCCACAGCACACAAATTGGCGGGATAAGGATGGTAGACATTCTTAGACAGAACCCAACAGAAGAGCA<br>AGCCGTGGATATATGCAAAGCAGCAATGGGTTTAAGAATCAGTTCATCCTTCAGCTTTGGAGGTTTCACTTTCAAAAGAACAAGTGGATCGTCTGTCAAAATAGAAGAAGAAGTGCTCACAGGCAACCTCCAAACACTGAAAATAAGAGTACATGAAGGATATGAGGAATTCACAATGGTTGGACGGAGAGCTACAGCCATTTTAAGGAAAGCAACCAGAAGGCTGATCC<br>AATTAATAGTGAGTGGAAGAGACGAGCAGTCAATCGCTGAAGCAATCATAGTGGCAATGGTTTTCTCACAAGAGGACTGCATGATAAAAGCAGTACGAGGTGATCTGAACTTTGTTAATAGAGCAAATCAGCGACTAAATCCTATGCATCAACTTCTGAGGCATTTCCAAAAGGATGCAAAGGTGCTGTTTCAAAACTGGGGGnTTGAACCAATTGACAATGTAATGGGG<br>ATGATTGGAATACTGCCTGACATGACCCCCAGCACGGAGATGTCACTGAGAGGAGTGAGAGTCAGCAAAATGGGAGTGGATGAATATTCCAGTACTGAGAGAGTGGTCGTGAGCATTGATCGTTTCTTGAGAGTCCGAGATCAAAGAGGAAACGTGCTCCTGTCTCCGGAGGAAGTTAGTGAAACACAGGGAACTGAGAAACTGACGATAACATATTCATCGTCCATGA<br>TGTGGGAAATCAATGGTCCGGAATCCGTGCTAGTCAACACATATCAATGGATCATCAGAAATTGGGAGACTGTGAAGATTCAATGGTCCCAAGACCCTACGATGTTGTACAACAAGATGGAATTTGAGCCCTTCCAATCCTTGGTGCCCAAGGCTGCCAGAGGCCAGTATAGTGGATTTGTGAGGACATTGTTCCAGCAGATGCGTGATGTGCTGGGAACATTTGATACTG<br>TCCAAATAATAAAGCTCCTGCCTTTTGCTGCAGCCCCTCCGGAACAGAGTAGGATGCAGTTTTCCTCTCTGACTGTGAACGTAAGAGGTTCAGGAATGAGAATACTTGTGAGAGGCAACTCCCCTGTGTTCAACTATAACAAGGCCACCAAGAGACTCACAGTTCTTGGAAAGGATGCAGGTGCCTTGACAGAAGATCCAGATGAGGGAACAGAAGGAGTAGAGTCTGCA<br>GTATTAAGAGGATTTCTAATTCTGGGCAAAGAGGACAAAAGATATGGACCAGCATTGAGCATTAACGAACTGAGCAATCTTGCGAAAGGGGAAAAGGCTAATGTGTTGATAGGGCAAGGAGACGTGGTGTTGGTAATGAAACGGAAACGGGACTCTAGCATACTTACTGACAGCCAGACAGCGACCAAAAGAATTCGGATGGCCATCAATTA
EPI_ISL_13778475	>A/Sandwich_Tern/Netherlands/12/2022_H5N1_2022-06-07<br>ATGGAGAGAATAAAAGAACTAAGAGATTTAATGTCGCAGTCTCGCACTCGCGAGATACTGACAAAAACCACTGTGGACCATATGGCCATAATAAAGAAATACACATCAGGAAGACAGGAGAAGAACCCTGCCCTCAGGATGAAGTGGATGATGGCAATGAAATATCCTATTACAGCAGACAAAAGAATAATGGAGATGATCCCTGAAAGGAATGAGCAAGGTCAGACT<br>CTCTGGAGCAAAACAAATGACGCTGGATCAGACAGAGTGATGGTGTCACCTCTGGCTGTGACATGGTGGAATAGAAATGGCCAACGACAAGTACAGTCCATTACCCAAAGGTTTACAAAACCTACTTTGAAAAAGTGGAAAGATTGAAACATGGAACCTTTGGCCCTGTTCACTTTCGAAATCAGGTTAAGATACGCCGCAGGGTTGACGTAAACCCGGGTCATGCAGA<br>TCTCAGTGCCAAAGAAGCACAGGATGTCATCATGGAAGTCGTTTTCCCAAATGAAGTTGGAGCCAGGATATTGACATCAGAATCACAATTAACAATAACAAAGGAAAAGAAGGAGGAACTTCAGGACTGTAAGATTGCTCCTTTGATGGTGGCATACATGTTGGAGAGAGAACTGGTTCGAAAAACCAGATTTTTACCAGTAGCTGGCGGAACAAGCAGCGTATATATCG<br>AAGTATTGCATTTGACTCAAGGGACCTGCTGGGAACAAATGTACACACCGGGAGGGnAAGTGAGAAATGATGATGTTGATCAGAGTTTGATCATTGCTGCTAGAAATATCGTTAGGAGAGCAACAGTATCAGCAGACCCATTGGCTTCGCTCTTGGAAATGTGCCACAGCACACAAATTGGCGGGATAAGGATGGTAGACATTCTTAGACAGAACCCAACAGAAGAGCA<br>AGCCGTGGATATATGCAAAGCAGCAATGGGTTTAAGAATCAGTTCATCCTTCAGCTTTGGAGGTTTCACTTTCAAAAGAACAAGTGGATCGTCTGTCAAAATAGAAGAAGAAGTGCTCACAGGCAACCTCCAAACACTGAAAATAAGAGTACATGAAGGATATGAGGAATTCACAATGGTTGGACGGAGAGCTACAGCCATTTTAAGGAAAGCAACCAGAAGGCTGATCC<br>AATTAATAGTGAGTGGAAGAGACGAGCAGTCAATCGCTGAAGCAATCATAGTGGCAATGGTTTTCTCACAAGAGGACTGCATGATAAAAGCAGTACGAGGTGATCTGAACTTTGTTAATAGAGCAAATCAGCGACTAAATCCTATGCATCAACTTCTGAGGCATTTCCAAAAGGATGCAAAGGTGCTGTTTCAAAACTGGGGGnTTGAACCAATTGACAATGTAATGGGG<br>ATGATTGGAATACTGCCTGACATGACCCCCAGCACGGAGATGTCACTGAGAGGAGTGAGAGTCAGCAAAATGGGAGTGGATGAATATTCCAGTACTGAGAGAGTGGTCGTGAGCATTGATCGTTTCTTGAGAGTCCGAGATCAAAGAGGAAACGTGCTCCTGTCTCCGGAGGAAGTTAGTGAAACACAGGGAACTGAGAAACTGACGATAACATATTCATCGTCCATGA<br>TGTGGGAAATCAATGGTCCGGAATCCGTGCTAGTCAACACATATCAATGGATCATCAGAAATTGGGAGACTGTGAAGATTCAATGGTCCCAAGACCCTACGATGTTGTACAACAAGATGGAATTTGAGCCCTTCCAATCCTTGGTGCCCAAGGCTGCCAGAGGCCAGTATAGTGGATTTGTGAGGACATTGTTCCAGCAGATGCGTGATGTGCTGGGAACATTTGATACTG<br>TCCAAATAATAAAGCTCCTGCCTTTTGCTGCAGCCCCTCCGGAACAGAGTAGGATGCAGTTTTCCTCTCTGACTGTGAACGTAAGAGGTTCAGGAATGAGAATACTTGTGAGAGGCAACTCCCCTGTGTTCAACTATAACAAGGCCACCAAGAGACTCACAGTTCTTGGAAAGGATGCAGGTGCCTTGACAGAAGATCCAGATGAGGGAACAGAAGGAGTAGAGTCTGCA<br>GTATTAAGAGGATTTCTAATTCTGGGCAAAGAGGACAAAAGATATGGACCAGCATTGAGCATTAACGAACTGAGCAATCTTGCGAAAGGGGAAAAGGCTAATGTGTTGATAGGGCAAGGAGACGTGGTGTTGGTAATGAAACGGAAACGGGACTCTAGCATACTTACTGACAGCCAGACAGCGACCAAAAGAATTCGGATGGCCATCAATTA
EPI_ISL_234408	>A/mallard/Poland/141/2015_H5N1_2015-11-26<br>ATGGAGAGAATAAAAGAACTAAGAGATTTGATGTCGCAGTCTCGCACTCGCGAGATACTGACAAAAACCACTGTGGACCATATGGCCATAATAAAGAAGTACACGTCAGGAAGACAGGAGAAGAATCCTGCCCTTAGAATGAAATGGATGATGGCAATGAAATATCCGATTACAGCAGACAAAAGGATAATGGAGATGATCCCTGAAAGAAATGAGCAGGGTCAGACT<br>CTCTGGAGCAAAACAAATGATGCTGGATCAGACAGAGTGATGGTGTCACCTCTGGCAGTGACATGGTGGAATAGAAATGGGCCAACGACAAGTACAGTCCATTATCCAAAGGTCTACAAAACCTACTTTGAAAAGGTTGAAAGGTTGAAACATGGAACCTTCGGTCCTGTTCACTTTCGAAATCAAGTTAAAATACGCCGCAGGGTTGACATAAACCCGGGCCATGCAGA<br>TCTCAGTGCTAAAGAAGCACAAGATGTCATCATGGAGGTCGTTTTCCCAAACGAAGTTGGAGCCAGGATATTGACATCAGAGTCACAATTAACAATAACAAAAGAAAAGAAGGAGGAGCTTCAGGACTGTAAGATTGCTCCTTTGATGGTGGCATACATGTTGGAGAGAGAACTGGTTCGCAAAACCAGATTCCTGCCCGTAGCTGGCGGGACAAGCAGCGTGTATATCG<br>AGGTGTTGCATTTGACTCAAGGGACCTGCTGGGAACAAATGTACACACCGGGAGGGGAGGTGAGAAATGATGATGTTGATCAAAGTTTGATCATTGCTGCTAGAAATATTGTTAGAAGAGCAACAGTATCAGCAGACCCGTTGGCTTCGCTTTTGGAGATGTGCCATAGTACACAAATTGGCGGGATAAGGATGGTGGACATTCTTAGACAGAACCCAACAGAAGAGCA<br>AGCTGTGGATATATGCAAAGCAGCAATGGGTCTAAGAATCAGTTCATCCTTCAGCTTTGGAGGGTTCACCTTCAAACGGACAAGTGGGTCATCTGTCAAAAGAGAAGAAGAAGTGCTCACAGGCAACCTCCAAACACTGAAAATAAGAGTACATGAAGGATATGAGGAATTCACAATGGTTGGGCGAAGAGCGACAGCCATTCTAAGGAAAGCAACCAGAAGGCTAATC<br>CAATTAATAGTGAGTGGGAGAGACGAGCAGTCAATCGCTGAAGCGATCATAGTGGCAATGGTTTTCTCACAAGAGGATTGCATGATAAAAGCAGTACGAGGTGATTTGAATTTTGTTAACAGAGCAAATCAGCGGCTGAATCCTATGCACCAACTTCTGAGGCATTTCCAAAAGGACGCAAAGGTACTGTTTCAAAATTGGGGAATTGAACCAATTGACAATGTCATGGG<br>GATGATTGGAATACTGCCTGACATGACCCCCAGCACAGAGATGTCACTGAGAGGAGTGAGAGTCAGTAAAATGGGAGTGGATGAATATTCCAGTACTGAGAGAGTGGTCGTGAGCATTGATCGTTTCTTGAGAGTCCGAGATCAGAGGGGAAACGTGCTCTTGTCTCCCGAAGAAGTTAGTGAAACACAGGGAACAGAGAAACTGACGATAACATATTCATCGTCCATG<br>ATGTGGGAAATCAATGGTCCAGAATCGGTGCTAGTCAACACATATCAATGGATAATTAGAAATTGGGAAACTGTGAAGATTCAATGGTCCCAAGACCCTACAATGTTGTACAACAAGATGGAGTTTGAGCCCTTCCAATCCTTGGTGCCTAAGGCTGCTAGAGGCCAGTATAGTGGGTTTGTGAGGACGTTATTTCAGCAGATGCGTGATGTGCTAGGGACATTTGACACT<br>GTCCAAATAATAAAGCTCCTACCATTTGCAGCAGCCCCACCAGAACAGAGTAGGATGCAGTTCTCTTCTTTGACTGTAAATGTAAGAGGTTCAGGAATGAGAATACTTGTGAGAGGCAACTCCCCTGTGTTCAACTATAACAAGGCAACCAAGAGACTCACAGTTCTTGGAAAAGATGCAGGTGCTTTAACAGAAGATCCAGATGAGGGAACAGCAGGAGTGGAATCTGC<br>GGTATTAAGAGGATTTCTAATTCTGGGCAAAGAAGACAAAAGATATGGACCAGCATTGAGCATCAATGAGTTGAGTAATCTTGCGAAAGGGGAAAAGGCTAATGTGTTGATAGGGCAAGGAGACGTGGTGTTGGTAATGAAACGGAAACGGGACTCTAGCATACTTACTGACAGCCAGACAGCGACCAAAAGAATTCGGATGGCCATCAATTA
EPI_ISL_822837	>A/green_sandpiper/Netherlands/19011490-013/2019_H5N1_2019-08-13<br>ATGGAGAGAATAAAAGAACTAAGAGATTTGATGTCGCAGTCTCGCACTCGCGAGATACTGACAAAAACCACTGTGGACCATATGGCCATAATAAAGAAATACACGTCAGGAAGACAGGAGAAGAATCCTGCCCTTAGAATGAAATGGATGATGGCAATGAAATATCCGATTACAGCAGACAAAAGAATAATGGAGATGATCCCTGAAAGAAATGAGCAGGGTCAGACT<br>CTCTGGAGCAAAACAAATGACGCCGGATCAGACAGAGTGATGGTGTCACCTCTGGCAGTGACATGGTGGAATAGAAATGGGCCAACGACAAGTACAGTCCACTATCCAAAGGTCTACAAAACCTACTTTGAAAAGGTTGAAAGGTTGAAACATGGAACTTTCGGTCCTGTCCACTTTCGAAATCAGGTTAAAATACGCCGCAGGGTTGACATAAACCCGGGCCATGCAGA<br>TCTCAGTGCTAAAGAAGCACAAGATGTCATCATGGAGGTCGTTTTCCCAAACGAAGTTGGAGCCAGGATATTGACATCAGAATCACAATTAACAATAACAAAAGAAAAGAAGGAGGAGCTTCAGGACTGTAAGATTGCTCCTTTGATGGTGGCATACATGTTGGAGAGAGAACTGGTTCGCAAAACCAGATTCTTGCCCGTAGCTGGCGGGACAAGCAGCGTGTACATCG<br>AGGTGTTGCATTTGACTCAGGGGACCTGCTGGGAACAAATGTACACACCGGGAGGGGAGGTGAGAAATGATGATGTTGATCAAAGTTTGATCATTGCCGCTAGAAATATTGTTAGAAGAGCAACAGTATCAGCAGACCCGTTAGCTTCGCTTTTGGAGATGTGCCATAGTACACAAATTGGCGGGATAAGGATGGTGGACATTCTTAGACAGAACCCTACAGAAGAGCAA<br>GCTGTGGATATATGCAAAGCAGCAATGGGTCTAAGAATCAGTTCATCCTTCAGCTTTGGAGGGTTCACCTTCAAACGGACAAGTGGGTCATCTGTCAAAAGAGAAGAAGAAGTGCTCACAGGCAACCTCCAAACACTGAAAATAAGAGTACATGAAGGATATGAGGAATTTACAATGGTTGGGCGAAGAGCGACAGCCATTCTAAGGAAAGCAACCAGACGGCTAATCC<br>AATTAATAGTGAGTGGGAGAGACGAGCAGTCAATCGCTGAAGCGATCATAGTGGCAATGGTTTTCTCACAAGAGGATTGCATGATAAAAGCAGTACGAGGTGATTTGAATTTTGTTAACAGAGCAAATCAGCGGCTGAATCCTATGCATCAACTTCTGAGGCATTTCCAAAAGGACGCAAAGGTGCTGTTTCAAAATTGGGGAATTGAACCAATTGACAATGTCATGGGG<br>ATGATTGGAATACTGCCTGACATGACCCCCAGCACAGAGATGTCACTGAGAGGAGTGAGGGTCAGTAAAATGGGAGTGGATGAATATTCCAGTACTGAGAGAGTGGTCGTGAGCATTGATCGTTTCTTGAGAGTCCGAGATCAGAGGGGAAACGTGCTCTTGTCCCCCGAAGAAGTTAGTGAAACACAGGGAACAGAGAAACTGACGATAACATATTCATCGTCCATGA<br>TGTGGGAAATCAATGGTCCAGAATCGGTGCTAGTCAACACATATCAATGGATCATTAGAAATTGGGAAACTGTGAAGATTCAATGGTCCCAAGACCCTACAATGCTGTACAACAAGATGGAGTTTGAGCCCTTCCAATCCTTGGTGCCTAAGGCTGCTAGAGGCCAGTATAGCGGGTTTGTGAGGACGTTGTTTCAGCAGATGCGTGATGTGCTGGGGACATTTGACACTG<br>TTCAAATAATAAAGCTCCTACCGTTTGCAGCAGCCCCACCAGAACAGAGTAGGATGCAGTTCTCTTCTCTGACTGTAAATGTGAGAGGTTCAGGAATGAGAATACTTGTGAGAGGCAACTCCCCTGTGTTCAACTATAACAAGGCAACTAAGAGACTCACAGTTCTTGGAAAGGATGCAGGTGCTTTAACAGAAGATCCAGATGAGGGAACAGCAGGAGTGGAATCTGCG<br>GTATTAAGAGGGTTTCTAATTCTGGGCAAAGAAGACAAAAGATATGGACCAGCATTGAGCATCAATGAGTTGAGTAATCTTGCGAAAGGGGAAAAGGCTAATGTGTTGATAGGGCAAGGAGACGTGGTGTTGGTAATGAAACGGAAACGGGACTCTAGCATACTTACTGACAGCCAGACAGCGACCAAAAGAATTCGGATGGCCATCAATTA
EPI_ISL_819124	>A/mallard/Netherlands/19001282-001/2019_H5N1_2019-01-23<br>ATGGAGAGAATAAAAGAACTAAGAGATTTGATGTCGCAGTCTCGCACTCGCGAGATACTGACAAAAACCACTGTGGACCATATGGCCATAATAAAGAAATACACGTCAGGAAGACAGGAGAAGAATCCTGCTCTTAGAATGAAATGGATGATGGCAATGAAATATCCGATTACAGCAGACAAAAGAATAATGGAGATGATCCCTGAAAGAAATGAGCAGGGTCAGACT<br>CTCTGGAGCAAAACAAATGATGCCGGATCAGACAGAGTGATGGTGTCACCTCTGGCAGTGACATGGTGGAATAGAAATGGGCCAACGACAAGTACCGTCCACTATCCAAAGGTCTACAAAACCTACTTTGAAAAGGTTGAAAGGTTGAAACATGGAACCTTCGGTCCTGTCCACTTTCGAAATCAGGTTAAAATACGCCGCAGGGTTGACATAAACCCGGGCCATGCAGA<br>TCTCAGTGCTAAAGAAGCACAAGATGTCATCATGGAGGTCGTTTTCCCAAACGAAGTTGGAGCCAGGATATTGACATCAGAGTCACAATTAACAATAACAAAAGAAAAGAAGGAGGAGCTTCAGGACTGTAAGATTGCTCCTTTGATGGTGGCATACATGTTGGAGAGAGAACTGGTTCGCAAAACCAGATTCCTGCCCGTAGCTGGCGGGACAAGCAGCGTGTACATCG<br>AGGTGTTGCATTTGACTCAGGGGACCTGCTGGGAACAAATGTACACACCGGGAGGGGAGGTGAGAAATGATGATGTTGATCAAAGTTTGATCATTGCCGCTAGAAATATTGTTAGAAGAGCAACAGTATCAGCAGACCCGTTAGCTTCGCTTTTGGAGATGTGCCATAGTACACAAATTGGCGGGATAAGGATGGTGGACATTCTTAGACAGAACCCTACAGAAGAGCAA<br>GCTGTGGATATATGCAAAGCAGCAATGGGTCTAAGAATCAGTTCATCCTTCAGCTTTGGAGGGTTCACCTTCAAACGGACAAGTGGGTCATCTGTCAAAAGAGAAGAAGAAGTGCTCACAGGCAACCTCCAAACACTGAAAATAAGAGTACATGAAGGATATGAGGAATTTACAATGGTTGGGCGAAGAGCGACAGCCATTCTAAGGAAAGCAACCAGACGGCTAATCC<br>AATTAATAGTGAGTGGGAGAGACGAGCAGTCAATCGCTGAAGCGATCATAGTGGCAATGGTTTTCTCACAAGAGGATTGCATGATAAAAGCAGTACGAGGTGATTTAAATTTTGTTAACAGAGCTAATCAGCGGCTGAATCCTATGCATCAACTTCTGAGGCATTTCCAAAAGGACGCAAAGGTGCTGTTTCAAAATTGGGGAATTGAACCAATTGACAATGTCATGGGGA<br>TGATTGGAATACTGCCTGACATGACCCCCAGCACAGAGATGTCACTGAGAGGAGTGAGAGTCAGTAAAATGGGAGTGGATGAGTATTCCAGTACTGAGAGAGTGGTCGTGAGCATTGATCGTTTCTTGAGAGTCCGAGATCAGAGGGGAAACGTGCTCTTGTCCCCCGAAGAAGTTAGTGAAACACAGGGAACAGAGAAACTGACGATAACATATTCATCGTCCATGAT<br>GTGGGAAATCAATGGTCCAGAATCGGTGCTAGTCAACACATATCAATGGATCATTAGAAATTGGGAAACTGTAAAGATTCAATGGTCCCAAGACCCTACAATGCTGTACAACAAGATGGAGTTTGAGCCCTTCCAATCCTTGGTGCCTAAGGCTGCTAGAGGCCAGTATAGCGGGTTTGTGAGGACGTTGTTTCAGCAGATGCGTGATGTGCTGGGGACATTTGACACTGT<br>TCAAATAATAAAGCTCCTACCGTTTGCAGCAGCCCCACCAGAACAGAGTAGGATGCAGTTCTCTTCTCTGACTGTAAATGTAAGAGGTTCGGGAATGAGAATACTTGTGAGAGGCAACTCCCCTGTGTTCAACTATAACAAGGCAACCAAGAGACTCACAGTTCTTGGAAAGGATGCAGGTGCTTTAACAGAAGATCCAGATGAGGGAACAGCAGGAGTGGAATCTGCGG<br>TATTAAGAGGGTTTCTAATTCTGGGCAAAGAAGACAAAAGATATGGACCAGCATTGAGCATCAATGAGTTGAGTAATCTTGCGAAAGGGGAAAAGGCTAATGTGTTGATAGGGCAAGGAGACGTGGTGTTGGTAATGAAACGGAAACGGGACTCTAGCATACTTACTGACAGCCAGACAGCGACCAAAAGAATTCGGATGGCCATCAATTA
EPI_ISL_399472	>A/Anas_platyrhynchos/Belgium/2322/2015_H11N7_2015-09-29<br>ATGGAGAGAATAAAAGAACTAAGAGATTTGATGTCGCAGTCTCGCACTCGCGAGATACTGACAAAAACCACTGTGGACCATATGGCCATAATAAAGAAATACACGTCAGGAAGACAGGAGAAGAATCCTGCCCTTAGAATGAAATGGATGATGGCAATGAAATATCCGATTACAGCAGACAAAAGGATAATGGAGATGATCCCTGAAAGAAATGAGCAGGGTCAGACT<br>CTCTGGAGCAAAACAAATGATGCTGGATCAGACAGAGTGATGGTGTCACCTCTGGCAGTGACATGGTGGAATAGAAATGGGCCAACGACAAGTACAGTCCATTATCCAAAGGTCTACAAAACCTACTTTGAAAAGGTTGAAAGATTGAAACATGGAACCTTCGGTCCTGTTCACTTTCGAAATCAGGTTAAAATACGCCGCAGGGTTGACATAAACCCGGGCCATGCAGA<br>TCTCAGTGCTAAAGAAGCACAAGATGTCATCATGGAGGTCGTTTTCCCAAACGAAGTTGGAGCCAGGATATTGACATCAGAGTCACAATTAACAATAACAAAAGAAAAGAAGGAGGAGCTTCAGGACTGTAAGATTGCTCCTTTGATGGTGGCATACATGTTGGAGAGAGAACTGGTTCGCAAAACCAGATTCCTGCCCGTAGCTGGCGGGACAAGCAGCGTGTATATCG<br>AGGTGTTGCATTTGACTCAAGGGACCTGCTGGGAACAAATGTACACACCGGGAGGGGAGGTGAGAAATGATGATGTTGATCAAAGTTTGATCATTGCTGCTAGAAATATTGTTAGAAGAGCAACAGTATCAGCAGACCCGTTAGCTTCGCTTTTGGAGATGTGCCATAGTACACAAATTGGCGGGATAAGGATGGTGGACATTCTTAGACAGAACCCAACAGAAGAGCAA<br>GCTGTGGATATATGCAAAGCAGCAATGGGTCTAAGAATCAGTTCATCCTTCAGCTTTGGAGGGTTCACCTTCAAACGGACAAGTGGGTCATCTGTCAAAAGAGAAGAAGAAGTGCTCACAGGCAACCTCCAAACACTGAAAATAAGAGTACATGAAGGATATGAGGAATTCACAATGGTTGGGCGAAGAGCGACAGCCATTCTAAGGAAAGCAACCAGAAGGCTAATCC<br>AATTAATAGTGAGTGGGAGAGACGAGCAGTCAATTGCTGAAGCGATCATAGTGGCAATGGTTTTCTCACAAGAGGATTGCATGATAAAAGCAGTACGAGGTGATTTGAATTTTGTTAACAGAGCAAATCAGCGGCTGAATCCTATGCATCAACTTCTGAGGCATTTCCAAAAGGACGCAAAGGTGCTGTTTCAAAATTGGGGAATTGAACCAATTGACAATGTCATGGGGA<br>TGATTGGAATACTGCCTGACATGACCCCCAGCACAGAGATGTCACTGAGAGGAGTGAGAGTCAGTAAAATGGGAGTGGATGAATATTCCAGTACTGAGAGAGTGGTCGTGAGCATTGATCGTTTCTTGAGAGTCCGAGATCAGAGGGGAAACGTGCTCTTGTCCCCCGAAGAAGTTAGTGAAACACAGGGAACAGAGAAACTGACGATAACATATTCATCGTCCATGAT<br>GTGGGAAATCAATGGTCCAGAATCGGTGCTAGTCAACACATATCAATGGATCATTAGAAATTGGGAAACTGTGAAGATTCAATGGTCCCAAGACCCTACAATGTTGTACAACAAGATGGAGTTTGAGCCCTTCCAATCCTTGGTGCCTAAGGCTGCTAGAGGCCAGTATAGTGGGTTTGTGAGGACGTTATTTCAGCAGATGCGTGATGTGCTGGGGACATTTGACACTGTT<br>CAAATAATAAAGCTCCTACCGTTTGCAGCAGCCCCACCAGAACAGAGTAGGATGCAGTTCTCTTCTTTGACTGTAAATGTAAGAGGTTCAGGAATGAGAATACTTGTGAGAGGCAACTCCCCTGTGTTCAACTATAACAAGGCAACCAAGAGACTCACAGTTCTTGGAAAGGATGCAGGTGCTTTAACAGAAGATCCAGATGAGGGAACAGCAGGAGTGGAATCTGCGGT<br>ATTAAGAGGGTTTCTAATTCTGGGCAAAGAAGACAAAAGATATGGACCAGCATTGAGCATCAATGAGTTGAGTAATCTTGCGAAAGGGGAAAAGGCTAATGTGTTGATAGGGCAAGGAGACGTGGTGTTGGTAATGAAACGGAAACGGGACTCTAGCATACTTACTGACAGCCAGACAGCGACCAAAAGAATTCGGATGGCCATCAATTA
EPI_ISL_14388281	>A/Cygnus_olor/Belgium/11638_0002/2021_H10N4_2021-09-06<br>ATGGAGAGAATAAAAGAACTAAGAGATTTGATGTCGCAGTCTCGCACTCGCGAGATACTGACAAAAACCACTGTGGACCATATGGCCATAATAAAGAAATACACGTCAGGAAGACAGGAGAAGAATCCTGCCCTTAGAATGAAATGGATGATGGCAATGAAATATCCGATTACAGCAGACAAAAGGATAATGGAGATGATCCCTGAAAGAAATGAGCAGGGTCAGACT<br>CTCTGGAGCAAGACAAATGATGCCGGATCAGACAGAGTGATGGTGTCACCTCTGGCAGTGACATGGTGGAATAGAAATGGGCCAACGACAAGTACAGTCCACTATCCAAAGGTCTACAAAACCTACTTTGAAAAGGTTGAAAGGTTGAAACATGGAACCTTCGGTCCTGTCCACTTTCGAAATCAGGTTAAAATACGCCGCAGGGTTGACATAAACCCGGGCCATGCAGA<br>TCTCAGTGCTAAAGAAGCACAAGATGTCATCATGGAGGTCGTTTTCCCAAACGAAGTTGGAGCCAGGATATTGACATCAGAGTCACAATTAACAATAACAAAAGAAAAGAAGGAGGAGCTTCAGGACTGTAAGATTGCCCCTTTGATGGTAGCATACATGTTGGAGAGAGAACTGGTTCGCAAAACCAGATTCCTGCCCGTAGCTGGCGGGACAAGCAGCGTGTACATCG<br>AGGTGTTGCATTTGACTCAGGGGACCTGCTGGGAACAAATGTACACACCGGGAGGGGAGGTGAGAAATGATGATGTTGATCAAAGTTTGATCATTGCTGCTAGAAATATTGTTAGAAGAGCAACAGTATCAGCAGACCCGTTAGCTTCGCTTTTGGAGATGTGCCATAGTACACAAATTGGCGGGATAAGGATGGTGGACATTCTTAGACAGAACCCTACAGAAGAGCAA<br>GCTGTGGATATATGCAAAGCAGCAATGGGTCTAAGAATCAGTTCATCCTTCAGCTTTGGAGGGTTCACCTTCAAACGGACAAGTGGGTCATCTGTCAAAAGAGAAGAAGAAGTGCTCACAGGCAACCTCCAAACACTGAAAATAAGAGTACATGAAGGATATGAGGAATTCACAATGGTTGGGCGAAGAGCGACAGCCATTCTAAGGAAAGCAACCAGAAGGCTAATCC<br>AATTAATAGTGAGTGGGAGAGACGAGCAGTCAATCGCTGAAGCGATAATAGTGGCAATGGTTTTTTCACAAGAGGATTGCATGATAAAAGCAGTACGAGGTGATTTGAATTTCGTTAACAGAGCAAATCAGCGGCTGAATCCTATGCATCAACTTCTGAGGCATTTCCAAAAGGACGCAAAGGTGCTGTTTCAAAATTGGGGAATTGAACCAATTGACAATGTCATGGGG<br>ATGATTGGAATACTGCCTGACATGACCCCCAGCACAGAAATGTCACTGAGAGGAGTGAGAGTCAGTAAAATGGGAGTGGATGAATATTCCAGTACTGAGAGAGTGGTCGTGAGCATTGATCGTTTCTTGAGAGTCCGAGATCAGAGGGGAAACGTGCTCTTGTCCCCCGAAGAAGTTAGTGAAACACAGGGAACAGAGAAACTGACGATAACATATTCATCGTCCATGA<br>TGTGGGAAATCAATGGTCCAGAATCGGTGCTGGTCAACACATATCAATGGATCATTAGAAATTGGGAAACTGTGAAGATTCAATGGTCCCAAGACCCTACAATGTTGTACAACAAGATGGAGTTTGAGCCCTTCCAATCCTTGGTGCCTAAGGCTGCTAGAGGCCAGTATAGTGGGTTTGTGAGAACGTTGTTTCAGCAGATGCGTGATGTGCTGGGGACATTTGACACTGT<br>TCAAATAATAAAGCTCCTACCGTTTGCAGCAGCCCCACCAGAACAGAGTAGGATGCAGTTCTCTTCTTTGACTGTAAATGTAAGAGGTTCAGGAATGAGAATACTTGTGAGAGGCAACTCCCCTGTGTTCAACTATAACAAGGCAACCAAGAGACTCACAGTTCTTGGAAAGGATGCAGGTGCTTTAACAGAAGATCCAGATGAGGGGACAGCAGGAGTGGAATCTGCGG<br>TATTAAGAGGGTTTCTAATTCTGGGCAAAGAAGATAAAAGATATGGACCAGCATTGAGCATCAATGAGTTGAGTAATCTTGCGAAAGGGGAAAAGGCTAATGTGTTGATAGGGCAAGGAGACGTGGTGTTGGTAATGAAACGGAAACGGGACTCTAGCATACTTACTGACAGTCAGACAGCGACCAAAAGAATTCGGATGGCCATCAATTA
EPI_ISL_399580	>A/Anas_crecca/Belgium/9685/2016_H6N2_2016-10-13<br>ATGGAGAGAATAAAAGAACTAAGAGATTTGATGTCGCAGTCTCGCACTCGCGAGATACTGACAAAAACCACTGTGGACCATATGGCCATAATAAAGAAATACACGTCAGGAAGACAGGAGAAGAATCCTGCCCTTAGAATGAAATGGATGATGGCAATGAAATATCCGATTACAGCAGACAAAAGAATAATGGAGATGATCCCTGAAAGAAATGAGCAGGGTCAGACT<br>CTCTGGAGCAAAACAAATGATGCCGGATCAGACAGAGTGATGGTGTCACCTCTGGCAGTGACATGGTGGAATAGAAATGGGCCAACGACAAGTACAGTCCACTATCCAAAGGTCTACAAAACCTACTTTGAAAAGGTTGAAAGGTTGAAACATGGAACCTTCGGTCCTGTCCACTTTCGAAATCAGGTTAAAATACGCCGCAGGGTTGACATAAACCCGGGCCATGCAGA<br>TCTCAGTGCTAAAGAAGCACAAGATGTCATCATGGAGGTCGTTTTCCCAAACGAAGTTGGAGCCAGGATATTGACATCAGAGTCACAATTAACAATAACAAAAGAAAAGAAGGAGGAGCTTCAGGACTGTAAGATTGCTCCTTTGATGGTGGCATACATGTTGGAGAGAGAACTGGTTCGCAAAACCAGATTCCTGCCCGTAGCTGGCGGGACAAGCAGCGTGTACATCG<br>AAGTGTTGCATTTGACTCAGGGGACCTGCTGGGAACAAATGTACACACCGGGAGGGGAGGTGAGAAATGATGATGTTGATCAAAGTTTGATCATTGCCGCTAGAAATATTGTTAGAAGAGCAACAGTATCAGCAGACCCGTTAGCTTCGCTTTTGGAGATGTGCCATAGTACACAAATTGGCGGGATAAGGATGGTGGACATTCTTAGACAGAACCCTACAGAAGAGCAA<br>GCTGTGGATATATGCAAAGCAGCAATGGGTCTAAGAATCAGTTCATCCTTCAGCTTTGGAGGGTTCACCTTCAAACGGACAAGTGGGTCATCTGTCAAAAGAGAAGAAGAAGTGCTCACAGGCAACCTCCAAACACTGAAAATAAGAGTACATGAAGGATATGAGGAATTTACAATGGTTGGGCGAAGAGCGACAGCCATTCTAAGGAAAGCAACCAGAAGGCTAATCC<br>AATTAATAGTGAGTGGGAGAGACGAGCAGTCAATCGCTGAAGCGATCATAGTGGCAATGGTTTTCTCACAAGAGGATTGCATGATAAAAGCAGTACGAGGTGATTTGAATTTTGTTAACAGAGCAAATCAGCGGCTGAATCCTATGCATCAACTTCTGAGGCATTTCCAAAAGGACGCAAAGGTGCTGTTTCAAAATTGGGGAATGGAACCAATTGACAATGTCATGGGG<br>ATGATTGGAATACTGCCTGACATGACCCCCAGCACAGAGATGTCACTGAGAGGAGTGAGAGTCAGTAAAATGGGAGTGGATGAATATTCCAGTACTGAGAGAGTGGTCGTGAGCATTGATCGTTTCTTGAGAGTCCGAGATCAGAGGGGAAACGTGCTCTTGTCCCCCGAAGAAGTTAGTGAAACACAGGGAACAGAGAAACTGACGATAACATATTCATCGTCCATGA<br>TGTGGGAAATCAATGGTCCAGAATCGGTGCTAGTCAACACATATCAATGGATCATTAGAAATTGGGAAACTGTGAAGATTCAATGGTCCCAAGACCCTACAATGTTGTACAACAAGATGGAGTTTGAGCCCTTCCAATCCTTGGTGCCTAAGGCTGCTAGAGGCCAGTATAGCGGGTTTGTGAGGACGTTGTTTCAGCAGATGCGTGATGTGCTGGGGACATTTGACACTG<br>TTCAAATAATAAAGCTACTACCGTTTGCAGCAGCCCCACCAGAACAGAGTAGGATGCAGTTCTCTTCTCTGACTGTAAATGTAAGAGGTTCAGGAATGAGAATACTTGTGAGAGGCAACTCCCCTGTGTTCAACTATAACAAGGCAACCAAGAGACTCACAGTTCTTGGAAAGGATGCAGGTGCTTTAACAGAAGATCCAGATGAGGGAACAGCAGGAGTGGAATCTGCG<br>GTATTAAGAGGGTTTCTAATTCTGGGCAAAGAAGACAAAAGATATGGACCAGCATTGAGCATCAATGAGTTGAGTAATCTTGCGAAAGGGGAAAAGGCTAATGTGTTGATAGGGCAAGGAGACGTGGTGTTGGTAATGAAACGGAAACGGGACTCTAGCATACTTACTGACAGCCAGACAGCGACCAAAAGAATTCGGATGGCCATCAATTA
EPI_ISL_502612	>A/Anas_platyrhynchos/Belgium/7827/2018_H3N8_2018-01-09<br>ATGGAGAGAATAAAAGAACTAAGAGATTTGATGTCGCAGTCTCGCACTCGCGAGATACTGACAAAAACCACTGTGGACCATATGGCCATAATAAAGAAATACACGTCAGGAAGACAGGAGAAGAATCCTGCCCTTAGAATGAAATGGATGATGGCAATGAAATATCCGATTACAGCAGACAAAAGAATAATGGAGATGATCCCTGAAAGAAATGAGCAGGGTCAGACT<br>CTCTGGAGCAAAACAAATGATGCAGGATCAGACAGAGTGATGGTGTCACCTCTGGCAGTGACATGGTGGAATAGAAATGGGCCAACGACAAGTACAGTCCACTATCCAAAGGTCTACAAAACCTACTTTGAAAAGGTTGAAAGGTTGAAACATGGAACCTTCGGTCCTGTCCACTTTCGAAATCAGGTTAAAATACGCCGCAGGGTTGACATAAACCCGGGCCATGCGGA<br>TCTCAGTGCTAAAGAAGCACAAGATGTCATCATGGAGGTCGTTTTCCCAAACGAAGTTGGAGCCAGGATATTGACATCAGAGTCACAATTAACAATAACAAAAGAAAAGAAGGAGGAGCTTCAGGACTGTAAGATTGCTCCTTTGATGGTGGCATACATGTTGGAGAGAGAACTGGTTCGCAAAACCAGATTCCTGCCCGTAGCTGGCGGGACAAGCAGCGTGTACATCG<br>AGGTGTTGCATTTGACTCAGGGGACCTGCTGGGAGCAAATGTACACACCGGGAGGGGAGGTGAGAAATGATGATGTTGATCAAAGTTTGATCATTGCCGCTAGAAATATTGTTAGAAGGGCAACAGTATCAGCAGACCCGTTAGCTTCGCTTTTGGAGATGTGCCATAGTACGCAAATTGGCGGGATAAGGATGGTGGACATTCTTAGACAGAACCCTACAGAAGAGCA<br>AGCTGTGGATATATGCAAAGCAGCAATGGGTCTAAGAATCAGTTCATCCTTCAGCTTTGGAGGGTTCACCTTCAAACGGACAAGTGGGTCATCTGTCAAAAGAGAAGAAGAAGTGCTCACAGGCAACCTCCAAACACTGAAAATAAGAGTACATGAAGGATATGAGGAATTTACAATGGTTGGGCGAAGAGCGACAGCCATTCTAAGGAAAGCAACCAGAAGGCTAATC<br>CAATTAATAGTGAGTGGGAGAGACGAGCAGTCAATCGCTGAAGCGATCATAGTGGCAATGGTTTTCTCACAAGAGGATTGCATGATAAAAGCAGTACGAGGTGATTTGAATTTTGTTAACAGAGCAAATCAGCGGCTGAATCCTATGCATCAACTTCTGAGGCATTTCCAAAAGGACGCAAAGGTGCTGTTTCAAAATTGGGGAATTGAACCAATTGACAATGTTATGGGG<br>ATGATTGGAATACTGCCTGACATGACCCCCAGCACAGAGATGTCACTGAGAGGAGTGAGAGTCAGTAAAATGGGAGTGGATGAATATTCCAGTACTGAGAGAGTGGTCGTGAGCATTGATCGTTTCTTGAGAGTCCGAGATCAGAGGGGAAACGTGCTCTTGTCCCCCGAAGAAGTTAGTGAAACACAGGGAACAGAGAAACTGACGATAACATATTCATCGTCCATGA<br>TGTGGGAAATCAATGGTCCAGAATCGGTGCTAGTCAACACATATCAATGGATCATTAGAAATTGGGAAACTGTGAAGATTCAATGGTCCCAAGACCCTACAATGTTGTACAACAAGATGGAGTTTGAGCCCTTCCAATCCTTGGTGCCTAAGGCTGCTAGAGGCCAGTATAGCGGGTTTGTGAGGACGTTGTTTCAGCAGATGCGTGATGTGCTGGGGACATTTGACACTG<br>TTCAAATAATAAAGCTCCTACCGTTTGCAGCAGCCCCACCAGAACAGAGTAGGATGCAGTTCTCTTCTCTGACTGTAAATGTAAGAGGTTCAGGAATGAGAATACTTGTGAGAGGCAACTCCCCTGTGTTCAACTATAACAAGGCAACCAAGAGACTCACAGTCCTTGGAAAGGATGCAGGTGCTTTAACAGAAGATCCAGATGAGGGAACAGCAGGAGTGGAATCTGCG<br>GTATTAAGAGGGTTTCTAATTCTGGGCAAAGAAGACAAAAGATATGGACCAGCATTGAGCATCAATGAGTTGAGTAATCTTGCGAAAGGGGAAAAGGCTAATGTGTTGATAGGGCAAGGAGACGTGGTGTTGGTAATGAAACGGAAACGGGACTCTAGCATACTTACTGACAGCCAGACAGCGACCAAAAGAATTCGGATGGCCATCAATTA
EPI_ISL_15535272	>A/domestic_goose/Poland/H397-N/2022_H5N1_2022-09-20<br>ATGGAGAGAATAAAAGAACTAAGAGATTTAATGTCGCAGTCTCGCACTCGCGAGATACTGACAAAAACCACTGTGGACCATATGGCCATAATAAAGAAATACACATCAGGAAGACAGGAGAAGAACCCTGCCCTCAGGATGAAGTGGATGATGGCAATGAAATATCCTATTACAGCAGACAAAAGAATAATGGAGATGATCCCTGAAAGGAATGAGCAAGGTCAGACT<br>CTCTGGAGCAAAACAAATGACGCTGGATCAGACAGAGTGATGGTGTCACCTCTGGCTGTGACATGGTGGAATAGAAATGGCCAACGACAAGTACAGTCCATTACCCAAAGGTTTACAAAACCTACTTTGAAAAAGTGGAAAGATTGAAACATGGAACCTTTGGCCCTGTTCACTTTCGAAATCAGGTTAAGATACGCCGCAGGGTTGACGTAAACCCGGGTCATGCAGA<br>TCTCAGTGCCAAAGAAGCACAGGATGTCATCATGGAAGTCGTTTTCCCAAATGAAGTTGGAGCCAGGATATTGACATCAGAATCACAATTAACAATAACAAAGGAAAAGAAGGAGGAACTTCAGGACTGCAAGATTGCTCCTTTGATGGTGGCATACATGTTGGAGAGAGAACTGGTTCGAAAAACCAGATTTTTACCAGTAGCTGGCGGAACAAGCAGCGTATATATCG<br>AAGTATTGCATTTGACTCAAGGGACCTGCTGGGAACAAATGTACACACCGGGAGGGGAAGTGAGAAATGATGATGTTGATCAGAGTTTGATCATTGCTGCTAGAAATATAGTTAGGAGAGCAACAGTATCAGCAGACCCATTGGCTTCGCTCTTGGAAATGTGCCACAGCACACAAATTGGCGGGATAAGGATGGTAGACATTCTTAGACAGAACCCAACAGAAGAGCA<br>AGCCGTGGATATATGCAAAGCAGCAATGGGTTTAAGAATCAGTTCATCCTTCAGCTTTGGAGGTTTCACTTTCAAAAGAACAAGTGGATCGTCTGTCAAAATAGAAGAAGAAGTGCTCACAGGCAACCTCCAAACACTGAAAATAAGAGTACATGAAGGATATGAGGAATTCACAATGGTTGGACGGAGAGCTACAGCCATTTTAAGGAAAGCAACCAGAAGGCTGATCC<br>AATTAATAGTGAGTGGAAGAGACGAGCAGTCAATCGCTGAAGCAATCATAGTGGCAATGGTTTTCTCACAAGAGGACTGCATGATAAAAGCAGTACGAGGTGATCTGAACTTTGTTAATAGAGCAAATCAGCGACTAAATCCTATGCATCAACTTCTGAGGCATTTCCAAAAGGATGCAAAGGTGCTGTTTCAAAACTGGGGGGTTGAACCAATTGACAATGTAATGGGG<br>ATGATTGGAATACTGCCTGACATGACCCCCAGCACGGAGATGTCACTGAGAGGAGTAAGAGTCAGCAAAATGGGAGTGGATGAATATTCCAGTACTGAGAGAGTGGTCGTGAGCATTGATCGTTTCTTGAGAGTCCGAGATCAAAGAGGAAACGTGCTCCTGTCTCCGGAGGAAGTTAGTGAAACACAGGGAACTGAGAAACTGACGATAACATATTCATCGTCCATGA<br>TGTGGGAAATCAATGGTCCGGAATCCGTGCTAGTCAACACATATCAATGGATCATCAGAAATTGGGAGACTGTGAAGATTCAATGGTCCCAAGACCCTACGATGTTGTACAACAAGATGGAATTTGAGCCCTTCCAATCCTTGGTGCCCAAGGCTGCCAGAGGCCAGTATAGTGGATTTGTGAGGACATTGTTCCAGCAGATGCGTGATGTGCTGGGAACATTTGATACTG<br>TCCAAATAATAAAGCTCCTGCCTTTTGCTGCAGCCCCTCCGGAACAGAGTAGGATGCAGTTTTCCTCTCTGACTGTGAACGTAAGAGGTTCAGGAATGAGAATACTTGTGAGAGGCAACTCCCCTGTGTTCAACTATAACAAGGCCACCAAGAGACTCACAGTTCTTGGAAAGGATGCAGGTGCCTTGACAGAAGATCCAGATGAGGGAACAGAAGGAGTAGAGTCTGCA<br>GTATTAAGAGGATTTCTAATTCTGGGCAAAGAGGACAAAAGATATGGACCTGCATTGAGCATTAACGAACTGAGCAATCTTGCGAAAGGGGAAAAGGCTAATGTGTTGATAGGGCAAGGAGACGTGGTGTTGGTAATGAAACGGAAACGGGACTCTAGCATACTTACTGACAGCCAGACAGCGACCAAAAGAATTCGGATGGCCATCAATTA

EPI_ISL_18245819	>A/swan/Poland/MB152/2022_H2N3_2022-07-15<br>ATGGAGAGAATAAAAGAACTAAGAGATTTGATGTCGCAGTCTCGCACTCGCGAGATACTGACAAAAACCACTGTGGACCATATGGCCATAATAAAGAAATACACATCAGGAAGACAAGAGAAGAATCCTGCCCTTAGAATGAAATGGATGATGGCAATGAAATATCCGATTACAGCAGACAAAAGAATAATGGAGATGATCCCTGAAAGAAATGAGCAGGGTCAGACT<br>CTCTGGAGCAAAACAAATGATGCCGGATCAGACAGAGTGATGGTGTCACCTCTGGCAGTGACATGGTGGAATAGAAATGGGCCAACGACAAGTACAGTCCACTATCCAAAGGTATACAAAACCTACTTTGAAAAGGTTGAAAGGTTGAAACATGGAACCTTCGGTCCTGTCCATTTTCGAAATCAGGTTAAAATACGCCGCAGGGTTGACATAAACCCGGGCCATGCAGA<br>TCTCAGTGCTAAAGAAGCGCAAGATGTCATCATGGAGGTCGTTTTCCCAAACGAAGTTGGAGCCAGGATATTGACATCAGAGTCACAATTAACAATAACAAAAGAAAAGAAGGAGGAGCTTCAGGACTGTAAGATTGCTCCCTTGATGGTGGCATACATGTTGGAGAGAGAGCTGGTTCGCAAAACCAGATTCCTGCCCGTAGCTGGCGGGACAAGCAGCGTGTACATC<br>GAGGTGTTGCATTTGACTCAGGGGACCTGCTGGGAACAAATGTACACGCCGGGAGGGGAGGTGAGAAATGATGATGTTGATCAAAGTTTGATCATTGCCGCTAGAAATATTGTTAGAAGAGCAACAGTATCAGCAGACCCGTTAGCTTCGCTTTTGGAGATGTGCCATAGTACACAAATTGGCGGGATAAGGATGGTGGACATTCTTAGACAGAACCCTACAGAAGAGC<br>AAGCTGTGGATATATGCAAAGCAGCAATGGGTCTAAGAATCAGTTCATCCTTCAGTTTTGGAGGGTTCACCTTCAAACGGACAAGTGGGTCATCTGTCAAAAGAGAAGAAGAGGTGCTCACAGGCAACCTCCAAACACTGAAAATAAGAGTACATGAAGGATATGAGGAATTTACAATGGTTGGGCGAAGAGCGACAGCCATTCTAAGGAAAGCAACCAGAAGGCTAAT<br>CCAATTAATAGTGAGTGGGAGAGACGAGCAGTCAATCGCTGAAGCGATCATAGTGGCAATGGTTTTCTCACAAGAGGATTGCATGATAAAAGCAGTACGAGGTGATTTGAATTTTGTTAACAGAGCAAATCAGCGGCTGAATCCTATGCATCAACTCCTGAGGCATTTCCAAAAGGACGCAAAGGTGCTGTTTCAAAATTGGGGAGTTGAACCAATTGACAATGTCATGG<br>GGATGATTGGAATACTGCCTGACATGACCCCCAGCACAGAGATGTCACTGAGAGGGGTGAGAGTCAGTAAAATGGGAGTGGATGAATATTCCAGTACTGAGAGAGTGGTCGTGAGCATTGATCGTTTCTTGAGAGTCCGAGATCAGAGGGGAAATGTGCTCTTGTCCCCCGAAGAAGTTAGTGAAACACAGGGAACAGAGAAACTGACAATAACATATTCATCGTCCAT<br>GATGTGGGAAATCAATGGTCCAGAATCGGTGCTAGTTAACACATATCAATGGATCATTAGAAATTGGGAAACTGTGAAAATTCAATGGTCCCAAGACCCTACAATGTTGTACAACAAGATGGAGTTTGAGCCCTTCCAATCCTTGGTGCCTAAGGCTGCTAGAGGCCAGTACAGCGGGTTTGTGAGGACGTTGTTTCAGCAGATGCGTGATGTGCTGGGGACATTTGACAC<br>TGTTCAAATAATAAAGCTCCTACCGTTTGCAGCAGCCCCACCAGAACAGAGTAGGATGCAGTTCTCTTCTCTGACTGTAAATGTAAGAGGTTCAGGAATGAGAATACTTGTGAGAGGCAACTCCCCTGTGTTCAACTATAACAAGGCAACCAAGAGACTCACAGTTCTTGGAAAGGATGCAGGTGCTTTAACAGAAGATCCAGATGAGGGAACAGCGGGAGTGGAATCTG<br>CGGTATTAAGAGGGTTTCTAATTCTAGGCAAAGAAGACAAAAGATATGGACCAGCATTGAGCATCAATGAGTTGAGTAATCTTGCGAAAGGGGAGAAGGCTAATGTGTTGATAGGGCAAGGAGACGTGGTGTTGGTAATGAAACGGAAACGGGACTCTAGCATACTTACTGACAGCCAGACAGCGACCAAAAGAATTCGGATGGCCATCAATTA
internal NVRI repository	>A/turkey/Poland/H02-T1/2022_H5N1_2022-01-01<br>ATGGAGAGAATAAAAGAGCTAAGAGATTTGATGTCGCAGTCTCGCACTCGCGAGATACTGACAAAAACCACCGTGGACCATATGGCCATAATCAAGAAATATACATCAGGAAGACAGGAGAAGAACCCTGCACTTAGGATGAAGTGGATGATGGCAATGAAATATCCGATTACAGCAGACAAAAGGATAATGGAGATGATCCCTGAAAGAAACGAGCAAGGTCAGACT<br>CTTTGGAGCAAAACAAATGATGCTGGATCGGATAGAGTAATGGTGTCACCTCTGGCTGTGACGTGGTGGAATAGAAATGGACCAACAACAAGTACCGTCCATTACCCAAAGGTCTATAAGACCTACTTTGAAAAGGTTGAAAGGTTAAAGCATGGAACCTTCGGCCCTGTCCATTTCCGGAATCAGGTTAAGATACGCCGCAGAGTTGACATAAACCCGGGCCATGCAGA<br>CCTCAGTGCTAAAGAAGCACAGGACGTCATCATGGAGGTCGTTTTCCCAAATGAAGTCGGAGCCAGAATATTGACATCAGAGTCACAGTTGACAATTACAAAAGAAAAGAAGGAGGAACTCCAGGACTGTAAGATTGCCCCTCTAATGGTGGCATACATGTTGGAGAGAGAACTGGTTCGAAAAACCAGATTCCTGCCAGTAGCTGGCGGAACAAGTAGCGTATATATC<br>GAAGTGTTGCACTTGACTCAAGGAACCTGCTGGGAACAAATGTATACGCCAGGAGGAGAAGTGAGAAATGATGACGTTGACCAAAGTTTAATTATTGCTGCCAGAAATATCGTTAGGAGGGCAACAGTATCAGCAGACCCATTGGCTTCACTACTGGAGATGTGCCATAGTACACAGATTGGCGGGATAAGAATGGTAGACATTCTTAGACAGAACCCAACAGAAGAGC<br>AAGCCGTGGATATATGCAAAGCAGCAATGGGTTTAAGAATCAGTTCATCCTTCAGTTTTGGAGGTTTCACTTTCAAAAGGACAAGCGGATCATCTGTCAAAAGAGAAGAGGAAGTGCTCACCGGCAACCTCCAAACATTGAAAATAAGAGTACATGAAGGGTATGAGGAATTCACAATGGTTGGGCGGAGAGCAACAGCCATTCTAAGGAAAGCAACCAGAAGGCTGAT<br>CCAATTGATAGTAAGTGGAAGAGACGAGCAGTCAATCGCCGAAGCGATCATAGTGGCAATGGTGTTCTCTCAAGAGGATTGCATGATAAAGGCTGTACGAGGTGATTTAAATTTTGTCAATAGAGCGAATCAGCGGCTCAATCCTATGCATCAGCTCCTGAGGCATTTCCAAAAGGATGCAAAGGTACTATTCCAAAACTGGGGAATTGAACCCATTGACAATGTCATGGG<br>AATGATAGGAATATTGCCTGATATGACCCCCAGCACAGAGATGTCACTAAGAGGAGTGAGGGTCAGTAAAATGGGAGTGGATGAATATTCCAGTACTGAGAGGGTGGTCGTGAGTATTGATCGCTTCTTGAGGGTACGAGACCAGAGAGGAAATGTACTCTTGTCTCCCGAAGAGGTCAGTGAAACACAGGGAACAGAGAAGCTAACGATAACATATTCATCATCCATG<br>ATGTGGGAAATCAATGGCCCTGAGTCAGTGCTAGTTAACACATATCAATGGATCATCAGAAACTGGGAAACTGTGAAGATTCAGTGGTCCCAAGACCCTACAATGCTATACAACAAGATGGAGTTTGAGCCTTTTCAGTCCTTGGTGCCTAAGGCAGCCAGAGGCCAGTACAGTGGATTTGTAAGGACCCTATTCCAGCAGATGCGTGATGTGCTGGGAACCTTTGACACT<br>GTCCAGATAATAAAGCTACTTCCATTTGCAGCAGCACCACCGGAACAGAGTAGGATGCAGTTCTCTTCTCTAACTGTAAACGTAAGGGGTTCAGGAATGAGAATACTTGTGAGAGGCAACTCCCCTGTGTTCAACTATAATAAAGCAACCAAGAGGCTCATAGTCCTTGGCAAGGATGCTGGTGCATTGACARAAGACCCAGGTGAGGGGACAGCAGGAGTGGAGTCTGC<br>GGTATTGAGAGGGTTCCTAATTCTGGGCAAAGAGGACAAAAGATATGGACCAGCGCTGAGCATCAATGAATTGAGCAATCTTGCGAAAGGAGAGAAGGCTAATGTGTTGATAGGGCAAGGAGACGTGGTGTTGGTGATGAAACGGAAACGGGACTCTAGCATACTTACTGACAGCCAGACAGCGACCAAAAGAATTCGGATGGCCATCAATTA
internal NVRI repository	>A/turkey/Poland/H1910-T3/2021_H5N1_2021-11-01<br>ATGGAGAGAATAAAAGAGCTAAGAGATTTGATGTCGCAGTCTCGCACTCGCGAGATACTGACAAAAACCACCGTGGACCATATGGCCATAATCAAGAAATATACATCAGGAAGACAGGAGAAGAACCCTGCACTTAGGATGAAGTGGATGATGGCAATGAAATATCCGATTACAGCAGACAAAAGGATAATGGAGATGATCCCTGAAAGAAACGAGCAAGGTCAGACT<br>CTTTGGAGCAAAACAAATGATGCTGGATCGGATAGAGTAATGGTGTCACCTCTGGCTGTGACGTGGTGGAATAGAAATGGACCAACAACAAGTACAGTCCATTACCCAAAGGTCTATAAAACCTACTTTGAAAAGGTTGAAAGGTTAAAGCATGGAACCTTCGGCCCTGTCCATTTCCGGAATCAGGTTAAGATACGCCGCAGAGTTGACATAAACCCGGGCCATGCAGA<br>CCTCAGTGCTAAAGAAGCACAAGACGTCATCATGGAGGTCGTTTTCCCAAATGAAGTCGGAGCCAGAATATTGACATCAGAGTCACAGTTAACAATTACAAAAGAAAAGAAGGAGGAACTCCAGGACTGTAAGATTGCCCCTCTAATGGTGGCATACATGTTGGAGAGAGAACTGGTTCGAAAAACCAGATTCCTGCCAGTAGCTGGCGGAACAAGTAGCGTATATATCG<br>AAGTGTTGCACTTGACTCAAGGAACCTGCTGGGAACAAATGTATACGCCAGGAGGAGAAGTGAGAAATGATGACGTTGACCAAAGTTTAATTATTGCTGCCAGAAATATCGTTAGGAGAGCAACAGTATCAGCAGACCCATTGGCTTCACTACTGGAGATGTGCCATAGTACACAGATTGGCGGGATAAGAATGGTAGACATTCTTAGACAGAACCCAACAGAAGAGCA<br>AGCTGTGGATATATGCAAAGCAGCAATGGGTTTAAGAATCAGTTCATCCTTCAGTTTTGGAGGTTTCACTTTCAAAAGGACAAGCGGATCATCTGTCAAAAGAGAAGAGGAAGTGCTCACCGGCAACCTCCAAACATTGAAAATAAGAGTACATGAAGGGTATGAGGAATTCACAATGGTTGGGCGGAGAGCAACAGCCATTCTAAGGAAAGCAACCAGAAGGCTGATC<br>CAATTGATAGTAAGTGGAAAAGACGAGCAGTCAATCGCCGAAGCGATCATAGTGGCAATGGTGTTCTCTCAAGAGGATTGCATGATAAAGGCTGTACGAGGTGATTTAAATTTTGTCAATAGAGCGAATCAGCGGCTCAATCCTATGCATCAGCTCCTGAGGCATTTCCAAAAGGATGCAAAGGTACTATTCCAAAACTGGGGAATTGAACCCATTGACAATGTCATGGGA<br>ATGATAGGAATATTGCCTGATATGACTCCCAGCACAGAGATGTCACTAAGAGGAGTGAGGGTCAGTAAAATGGGAGTGGATGAATATTCCAGTACTGAGAGGGTGGTCGTGAGTATTGATCGCTTCTTGAGGGTACGAGACCAGAGAGGAAATGTACTCTTGTCTCCCGAAGAGGTCAGTGAAACACAGGGAACAGAGAAGCTAACGATAACATATTCATCATCCATGA<br>TGTGGGAAATTAATGGCCCTGAGTCAGTGCTAGTTAACACATATCAATGGGTCATCAGAAACTGGGAAACTGTGAAGATTCAGTGGTCCCAAGACCCTACAATGCTATACAACAGGATGGAGTTTGAGCCTTTTCAGTCCTTGGTGCCCAAGGCAGCCAGAGGCCAGTACAGTGGATTTGTAAGGACCTTATTCCAGCAGATGCGTGATGTGCTGGGAACCTTTGACACTG<br>TCCAGATAATAAAGCTACTTCCATTTGCAGCAGCACCACCGGAACAGAGTAGGATGCAGTTCTCTTCTCTAACTGTAAACGTAAGGGGTTCAGGAATGAGAATACTTGTGAGAGGCAACTCCCCTGTGTTCAACTATAATAAGGCAACCAAGAGGCTCATAGTCCTTGGAAAGGATGCTGGTGCATTGACAGAAGACCCAGGTGAGGGGACAGCAGGAGTGGAGTCTGCG<br>GTATTGAGAGGGTTCCTAATTCTGGGCAAAGAGGACAAAAGATATGGACCAGCGCTGAGCATCAATGAATTGAGCAATCTTGCGAAAGGGGAGAAGGCTAATGTGTTGATAGGGCAAGGAGACGTGGTATTGGTGATGAAACGGAAACGGGACTCTAGCATACTTACTGACAGCCAGACAGCGACCAAAAGAATTCGGATGGCCATCAATTA
EPI_ISL_6929970	>A/turkey/Poland/H1911-N/2021_H5N1_2021-11-01<br>ATGGAGAGAATAAAAGAGCTAAGAGATTTGATGTCGCAGTCTCGCACTCGCGAGATACTGACAAAAACCACCGTGGACCATATGGCCATAATCAAGAAATATACATCAGGAAGACAGGAGAAGAACCCTGCACTTAGGATGAAGTGGATGATGGCAATGAAATATCCGATTACAGCAGACAAAAGGATAATGGAGATGATCCCTGAAAGAAACGAGCAAGGTCAGACT<br>CTTTGGAGCAAAACAAATGATGCTGGATCGGATAGAGTAATGGTGTCACCTCTGGCTGTGACGTGGTGGAATAGAAATGGACCAACAACAAGTACAGTCCATTACCCAAAGGTCTATAAAACCTACTTTGAAAAGGTTGAAAGGTTAAAGCATGGAACCTTCGGCCCTGTCCATTTCCGGAATCAGGTTAAGATACGCCGCAGAGTTGACATAAACCCGGGCCATGCAGA<br>CCTCAGTGCTAAAGAAGCACAAGACGTCATCATGGAGGTCGTTTTCCCAAATGAAGTCGGAGCCAGAATATTGACATCAGAGTCACAGTTAACAATTACAAAAGAAAAGAAGGAGGAACTCCAGGACTGTAAGATTGCCCCTCTAATGGTGGCATACATGTTGGAGAGAGAACTGGTTCGAAAAACCAGATTCCTGCCAGTAGCTGGCGGAACAAGTAGCGTATATATCG<br>AAGTGTTGCACTTGACTCAAGGAACCTGCTGGGAACAAATGTATACGCCAGGAGGAGAAGTGAGAAATGATGACGTTGACCAAAGTTTAATTATTGCTGCCAGAAATATCGTTAGGAGAGCAACAGTATCAGCAGACCCATTGGCTTCACTACTGGAGATGTGCCATAGTACACAGATTGGCGGGATAAGAATGGTAGACATTCTTAGACAGAACCCAACAGAAGAGCA<br>AGCTGTGGATATATGCAAAGCAGCAATGGGTTTAAGAATCAGTTCATCCTTCAGTTTTGGAGGTTTCACTTTCAAAAGGACAAGCGGATCATCTGTCAAAAGAGAAGAGGAAGTGCTCACCGGCAACCTCCAAACATTGAAAATAAGAGTACATGAAGGGTATGAGGAATTCACAATGGTTGGGCGGAGAGCAACAGCCATTCTAAGGAAAGCAACCAGAAGGCTGATC<br>CAATTGATAGTAAGTGGAAAAGACGAGCAGTCAATCGCCGAAGCGATCATAGTGGCAATGGTGTTCTCTCAAGAGGATTGCATGATAAAGGCTGTACGAGGTGATTTAAATTTTGTCAATAGAGCGAATCAGCGGCTCAATCCTATGCATCAGCTCCTGAGGCATTTCCAAAAGGATGCAAAGGTACTATTCCAAAACTGGGGAATTGAACCCATTGACAATGTCATGGGA<br>ATGATAGGAATATTGCCTGATATGACTCCCAGCACAGAGATGTCACTAAGAGGAGTGAGGGTCAGTAAAATGGGAGTGGATGAATATTCCAGTACTGAGAGGGTGGTCGTGAGTATTGATCGCTTCTTGAGGGTACGAGACCAGAGAGGAAATGTACTCTTGTCTCCCGAAGAGGTCAGTGAAACACAGGGAACAGAGAAGCTAACGATAACATATTCATCATCCATGA<br>TGTGGGAAATTAATGGCCCTGAGTCAGTGCTAGTTAACACATATCAATGGGTCATCAGAAACTGGGAAACTGTGAAGATTCAGTGGTCCCAAGACCCTACAATGCTATACAACAGGATGGAGTTTGAGCCTTTTCAGTCCTTGGTGCCCAAGGCAGCCAGAGGCCAGTACAGTGGATTTGTAAGGACCTTATTCCAGCAGATGCGTGATGTGCTGGGAACCTTTGACACTG<br>TCCAGATAATAAAGCTACTTCCATTTGCAGCAGCACCACCGGAACAGAGTAGGATGCAGTTCTCTTCTCTAACTGTAAACGTAAGGGGTTCAGGAATGAGAATACTTGTGAGAGGCAACTCCCCTGTGTTCAACTATAATAAGGCAACCAAGAGGCTCATAGTCCTTGGAAAGGATGCTGGTGCATTGACAGAAGACCCAGGTGAGGGGACAGCAGGAGTGGAGTCTGCG<br>GTATTGAGAGGGTTCCTAATTCTGGGCAAAGAGGACAAAAGATATGGACCAGCGCTGAGCATCAATGAATTGAGCAATCTTGCGAAAGGGGAGAAGGCTAATGTGTTGATAGGGCAAGGAGACGTGGTATTGGTGATGAAACGGAAACGGGACTCTAGCATACTTACTGACAGCCAGACAGCGACCAAAAGAATTCGGATGGCCATCAATTA
EPI_ISL_6930238	>A/turkey/Poland/H1913-T1/2021_H5N1_2021-11-02<br>ATGGAGAGAATAAAAGAGCTAAGAGATTTGATGTCGCAGTCTCGCACTCGCGAGATACTGACAAAAACCACCGTGGACCATATGGCCATAATCAAGAAATATACATCAGGAAGACAGGAGAAGAACCCTGCACTTAGGATGAAGTGGATGATGGCAATGAAATATCCGATTACAGCAGACAAAAGGATAATGGAGATGATCCCTGAAAGAAACGAGCAAGGTCAGACT<br>CTTTGGAGCAAAACAAATGATGCTGGATCGGATAGAGTAATGGTGTCACCTCTGGCTGTGACGTGGTGGAATAGAAATGGACCAACAACAAGTACAGTCCATTACCCAAAGGTCTATAAAACCTACTTTGAAAAGGTTGAAAGGTTAAAGCATGGAACCTTCGGCCCTGTCCATTTCCGGAATCAGGTTAAGATACGCCGCAGAGTTGACATAAACCCGGGCCATGCAGA<br>CCTCAGTGCTAAAGAAGCACAAGACGTCATCATGGAGGTCGTTTTCCCAAATGAAGTCGGAGCCAGAATATTGACATCAGAGTCACAGTTAACAATTACAAAAGAAAAGAAGGAGGAACTCCAGGACTGTAAGATTGCCCCTCTAATGGTGGCATACATGTTGGAGAGAGAACTGGTTCGAAAAACCAGATTCCTGCCAGTAGCTGGAGGAACAAGTAGCGTATATATCG<br>AAGTGTTGCACTTGACTCAAGGAACCTGCTGGGAACAAATGTATACGCCAGGAGGAGAAGTGAGAAATGATGACGTTGACCAAAGTTTAATTATTGCTGCCAGAAATATCGTTAGGAGAGCAACAGTATCAGCAGACCCATTGGCTTCACTACTGGAGATGTGCCATAGTACACAGATTGGCGGGATAAGAATGGTAGACATTCTTAGACAGAACCCAACAGAAGAGCA<br>AGCTGTGGATATATGCAAAGCAGCAATGGGTTTAAGAATCAGTTCATCCTTCAGTTTTGGAGGTTTCACTTTCAAAAGGACAAGCGGATCATCTGTCAAAAGAGAAGAGGAAGTGCTCACCGGCAACCTCCAAACATTGAAAATAAGAGTACATGAAGGGTATGAGGAATTCACAATGGTTGGGCGGAGAGCAACAGCCATTCTAAGGAAAGCAACCAGAAGGCTGATC<br>CAATTGATAGTAAGTGGAAAAGACGAGCAGTCAATCGCCGAAGCGATCATAGTGGCAATGGTGTTCTCTCAAGAGGATTGCATGATAAAGGCTGTACGAGGTGATTTAAATTTTGTCAATAGAGCGAATCAGCGGCTCAATCCTATGCATCAGCTCCTGAGGCATTTCCAAAAGGATGCAAAGGTACTATTCCAAAACTGGGGAATTGAACCCATTGACAATGTCATGGGA<br>ATGATAGGAATATTGCCTGATATGACTCCCAGCACAGAGATGTCACTAAGAGGAGTGAGGGTCAGTAAAATGGGAGTGGATGAATATTCCAGTACTGAGAGGGTGGTCGTGAGTATTGATCGCTTCTTGAGGGTACGAGACCAGAGAGGAAATGTACTCTTGTCTCCCGAAGAGGTCAGTGAAACACAGGGAACAGAGAAGCTAACGATAACATATTCATCATCCATGA<br>TGTGGGAAATTAATGGCCCTGAGTCAGTGCTAGTTAACACATATCAATGGGTCATCAGAAACTGGGAAACTGTGAAGATTCAGTGGTCCCAAGACCCTACAATGCTATACAACAGGATGGAGTTTGAGCCTTTTCAGTCCTTGGTGCCCAAGGCAGCCAGAGGCCAGTACAGTGGATTTGTAAGGACCTTATTCCAGCAGATGCGTGATGTGCTGGGAACCTTTGACACTG<br>TCCAGATAATAAAGCTACTTCCATTTGCAGCAGCACCACCGGAACAGAGTAGGATGCAGTTCTCTTCTCTAACTGTAAACGTAAGGGGTTCAGGAATGAGAATACTTGTGAGAGGCAACTCCCCTGTGTTCAACTATAATAAGGCAACCAAGAGGCTCATAGTCCTTGGAAAGGATGCTGGTGCATTGACAGAAGACCCAGGTGAGGGGACAGCAGGAGTGGAGTCTGCG<br>GTATTGAGAGGGTTCCTAATTCTGGGCAAAGAGGACAAAAGATATGGACCAGCGCTGAGCATCAATGAATTGAGCAATCTTGCGAAAGGGGAGAAGGCTAATGTGTTGATAGGGCAAGGAGACGTGGTATTGGTGATGAAACGGAAACGGGACTCTAGCATACTTACTGACAGCCAGACAGCGACCAAAAGAATTCGGATGGCCATCAATTA
internal NVRI repository	>A/turkey/Poland/H1923_21RS3290-1/2021_H5N1_2021-11-03<br>ATGGAGAGAATAAAAGAGCTAAGAGATTTGATGTCGCAGTCTCGCACTCGCGAGATACTGACAAAAACCACCGTGGACCATATGGCCATAATCAAGAAATATACATCAGGAAGACAGGAGAAGAACCCTGCACTTAGGATGAAGTGGATGATGGCAATGAAATATCCGATTACAGCAGACAAAAGGATAATGGAGATGATCCCTGAAAGAAACGAGCAAGGTCAGACT<br>CTTTGGAGCAAAACAAATGATGCTGGATCGGATAGAGTAATGGTGTCACCTCTGGCTGTGACGTGGTGGAATAGAAATGGACCAACAACAAGTACAGTCCATTACCCAAAGGTCTATAAAACCTACTTTGAAAAGGTTGAAAGGTTAAAGCATGGAACCTTCGGCCCTGTCCATTTCCGGAATCAGGTTAAGATACGCCGCAGAGTTGACATAAACCCGGGCCATGCAGA<br>CCTCAGTGCTAAAGAAGCACAAGACGTCATCATGGAGGTCGTTTTCCCAAATGAAGTCGGAGCCAGAATATTGACATCAGAGTCACAGTTAACAATTACAAAAGAAAAGAAGGAGGAACTCCAGGACTGTAAGATTGCCCCTCTAATGGTGGCATACATGTTGGAGAGAGAACTGGTTCGAAAAACCAGATTCCTGCCAGTAGCTGGCGGAACAAGTAGCGTATATATCG<br>AAGTGTTGCACTTGACTCAAGGAACCTGCTGGGAACAAATGTATACGCCAGGAGGAGAAGTGAGAAATGATGACGTTGACCAAAGTTTAATTATTGCTGCCAGAAATATCGTTAGGAGAGCAACAGTATCAGCAGACCCATTGGCTTCACTACTGGAGATGTGCCATAGTACACAGATTGGCGGGATAAGAATGGTAGACATTCTTAGACAGAACCCAACAGAAGAGCA<br>AGCTGTGGATATATGCAAAGCAGCAATGGGTTTAAGAATCAGTTCATCCTTCAGTTTTGGAGGTTTCACTTTCAAAAGGACAAGCGGATCATCTGTCAAAAGAGAAGAGGAAGTGCTCACCGGCAACCTCCAAACATTGAAAATAAGAGTACATGAAGGGTATGAGGAATTCACAATGGTTGGGCGGAGAGCAACAGCCATTCTAMGGAAAGCAACCAGAAGGCTGATC<br>CAATTGATAGTAAGTGGAAAAGACGAGCAGTCAATCGCCGAAGCGATCATAGTGGCAATGGTGTTCTCTCAAGAGGATTGCATGATAAAGGCTGTACGAGGTGATTTAAATTTTGTCAATAGAGCGAATCAGCGGCTCAATCCTATGCATCAGCTCCTGAGGCATTTCCAAAAGGATGCAAAGGTACTATTCCAAAACTGGGGAATTGAACCCATTGACAATGTCATGGGA<br>ATGATAGGAATATTGCCTGATATGACTCCCAGCACAGAGATGTCACTAAGAGGAGTGAGGGTCAGTAAAATGGGAGTGGATGAATATTCCAGTACTGAGAGGGTGGTCGTGAGTATTGATCGCTTCTTGAGGGTACGAGACCAGAGAGGAAATGTACTCTTGTCTCCCGAAGAGGTCAGTGAAACACAGGGAACAGAGAAGCTAACGATAACATATTCATCATCCATGA<br>TGTGGGAAATTAATGGCCCTGAGTCAGTGCTAGTTAACACATATCAATGGGTCATCAGAAACTGGGAAACTGTGAAGATTCAGTGGTCCCAAGACCCTACAATGCTATACAACAGGATGGAGTTTGAGCCTTTTCAGTCCTTGGTGCCCAAGGCAGCCAGAGGCCAGTACAGTGGATTTGTAAGGACCTTATTCCAGCAGATGCGTGATGTGCTGGGAACCTTTGACACTG<br>TCCAGATAATAAAGCTACTTCCATTTGCAGCAGCACCACCGGAACAGAGTAGGATGCAGTTCTCTTCTCTAACTGTAAACGTAAGGGGTTCAGGAATGAGAATACTTGTGAGAGGCAACTCCCCTGTGTTCAACTATAATAAGGCAACCAAGAGGCTCATAGTCCTTGGAAAGGATGCTGGTGCATTGACAGAAGACCCAGGTGAGGGGACAGCAGGAGTGGAGTCTGCG<br>GTATTGAGAGGGTTCCTAATTCTGGGCAAAGAGGACAAAAGATATGGACCAGCGCTGAGCATCAATGAATTGAGCAATCTTGCGAAAGGGGAGAAGGCTAATGTGTTGATAGGGCAAGGAGACGTGGTATTGGTGATGAAACGGAAACGGGACTCTAGCATACTTACTGACAGCCAGACAGCGACCAAAAGAATTCGGATGGCCATCAATTA
EPI_ISL_6930564	>A/turkey/Poland/H1924-T1/2021_H5N1_2021-11-03<br>ATGGAGAGAATAAAAGAGCTAAGAGATTTGATGTCGCAGTCTCGCACTCGCGAGATACTGACAAAAACCACCGTGGACCATATGGCCATAATCAAGAAATATACATCAGGAAGACAGGAGAAGAACCCTGCACTTAGGATGAAGTGGATGATGGCAATGAAATATCCGATTACAGCAGACAAAAGGATAATGGAGATGATCCCTGAAAGAAACGAGCAAGGTCAGACT<br>CTTTGGAGCAAAACAAATGATGCTGGATCGGATAGAGTAATGGTGTCACCTCTGGCTGTGACGTGGTGGAATAGAAATGGACCAACAACAAGTACAGTCCATTACCCAAAGGTCTATAAAACCTACTTTGAAAAGGTTGAAAGGTTAAAGCATGGAACCTTCGGCCCTGTCCATTTCCGGAATCAGGTTAAGATACGCCGCAGAGTTGACATAAACCCGGGCCATGCAGA<br>CCTCAGTGCTAAAGAAGCACAAGACGTCATCATGGAGGTCGTTTTCCCAAATGAAGTCGGAGCCAGAATATTGACATCAGAGTCACAGTTAACAATTACAAAAGAAAAGAAGGAGGAACTCCAGGACTGTAAGATTGCCCCTCTAATGGTGGCATACATGTTGGAGAGAGAACTGGTTCGAAAAACCAGATTCCTGCCAGTAGCTGGCGGAACAAGTAGCGTATATATCG<br>AAGTGTTGCACTTGACTCAAGGAACCTGCTGGGAACAAATGTATACGCCAGGAGGAGAAGTGAGAAATGATGACGTTGACCAAAGTTTAATTATTGCTGCCAGAAATATCGTTAGGAGAGCAACAGTATCAGCAGACCCATTGGCTTCACTACTGGAGATGTGCCATAGTACACAGATTGGCGGGATAAGAATGGTAGACATTCTTAGACAGAACCCAACAGAAGAGCA<br>AGCTGTGGATATATGCAAAGCAGCAATGGGTTTAAGAATCAGTTCATCCTTCAGTTTTGGAGGTTTCACTTTCAAAAGGACAAGCGGATCATCTGTCAAAAGAGAAGAGGAAGTGCTCACCGGCAACCTCCAAACATTGAAAATAAGAGTACATGAAGGGTATGAGGAATTCACAATGGTTGGGCGGAGAGCAACAGCCATTCTAAGGAAAGCAACCAGAAGGCTGATC<br>CAATTGATAGTAAGTGGAAAAGACGAGCAGTCAATCGCCGAAGCGATCATAGTGGCAATGGTGTTCTCTCAAGAGGATTGCATGATAAAGGCTGTACGAGGTGATTTAAATTTTGTCAATAGAGCGAATCAGCGGCTCAATCCTATGCATCAGCTCCTGAGGCATTTCCAAAAGGATGCAAAGGTACTATTCCAAAACTGGGGAATTGAACCCATTGACAATGTCATGGGA<br>ATGATAGGAATATTGCCTGATATGACTCCCAGCACAGAGATGTCACTAAGAGGAGTGAGGGTCAGTAAAATGGGAGTGGATGAATATTCCAGTACTGAGAGGGTGGTCGTGAGTATTGATCGCTTCTTGAGGGTACGAGACCAGAGAGGAAATGTACTCTTGTCTCCCGAAGAGGTCAGTGAAACACAGGGAACAGAGAAGCTAACGATAACATATTCATCATCCATGA<br>TGTGGGAAATTAATGGCCCTGAGTCAGTGCTAGTTAACACATATCAATGGGTCATCAGAAACTGGGAAACTGTGAAGATTCAGTGGTCCCAAGACCCTACAATGCTATACAACAGGATGGAGTTTGAGCCTTTTCAGTCCTTGGTGCCCAAGGCAGCCAGAGGCCAGTACAGTGGATTTGTAAGGACCTTATTCCAGCAGATGCGTGATGTGCTGGGAACCTTTGACACTG<br>TCCAGATAATAAAGCTACTTCCATTTGCAGCAGCACCACCGGAACAGAGTAGGATGCAGTTCTCTTCTCTAACTGTAAACGTAAGGGGTTCAGGAATGAGAATACTTGTGAGAGGCAACTCCCCTGTGTTCAACTATAATAAGGCAACCAAGAGGCTCATAGTCCTTGGAAAGGATGCTGGTGCATTGACAGAAGACCCAGGTGAGGGGACAGCAGGAGTGGAGTCTGCG<br>GTATTGAGAGGGTTCCTAATTCTGGGCAAAGAGGACAAAAGATATGGACCAGCGCTGAGCATCAATGAATTGAGCAATCTTGCGAAAGGGGAGAAGGCTAATGTGTTGATAGGGCAAGGAGACGTGGTATTGGTGATGAAACGGAAACGGGACTCTAGCATACTTACTGACAGCCAGACAGCGACCAAAAGAATTCGGATGGCCATCAATTA
internal NVRI repository	>A/turkey/Poland/H1941_21RS3290-3/2021_H5N1_2021-11-06<br>ATGGAGAGAATAAAAGAGCTAAGAGATTTGATGTCGCAGTCTCGCACTCGCGAGATACTGACAAAAACCACCGTGGACCATATGGCCATAATCAAGAAATATACATCAGGAAGACAGGAGAAGAACCCTGCACTTAGGATGAAGTGGATGATGGCAATGAAATATCCGATTACAGCAGACAAAAGGATAATGGAGATGATCCCTGAAAGAAACGAGCAAGGTCAGACT<br>CTTTGGAGCAAAACAAATGATGCTGGATCGGGTAGAGTAATGGTGTCACCTCTGGCTGTGACGTGGTGGAATAGAAATGGACCAACAACAAGTACAGTCCATTACCCAAAGGTCTATAAAACCTACTTTGAAAAGGTTGAAAGGTTAAAGCATGGAACCTTCGGCCCTGTCCATTTCCGGAATCAGGTTAAGATACGCCGCAGAGTTGACATAAACCCGGGCCATGCAGA<br>CCTCAGTGCTAAAGAAGCACAAGACGTCATCATGGAGGTCGTTTTCCCAAATGAAGTCGGAGCCAGAATATTGACATCAGAGTCACAGTTAACAATTACAAAAGAAAAGAAGGAGGAACTCCAGGACTGTAAGATTGCCCCTCTAATGGTGGCATACATGTTGGAGAGAGAACTGGTTCGAAAAACCAGATTCCTGCCAGTAGCTGGCGGAACAAGTAGCGTATATATCG<br>AAGTGTTGCACTTGACTCAAGGAACCTGCTGGGAACAAATGTATACGCCAGGAGGAGAAGTGAGAAATGATGACGTTGACCAAAGTTTAATTATTGCTGCCAGAAATATCGTTAGGAGAGCAACAGTATCAGCAGACCCATTGGCTTCACTACTGGAGATGTGCCATAGTACACAGATTGGCGGGATAAGAATGGTAGACATTCTTAGACAGAACCCAACAGAAGAGCA<br>AGCCGTGGATATATGCAAAGCAGCAATGGGTTTAAGAATCAGTTCATCCTTCAGTTTTGGAGGTTTCACTTTCAAAAGGACAAGCGGGTCATCTGTCAAAAGAGAAGAGGAAGTGCTCACCGGCAACCTCCAAACATTGAAAATAAGAGTACATGAAGGGTATGAGGAATTCACAATGGTTGGGCGGAGAGCAACAGCCATTCTAAGGAAAGCAACCAGAAGGCTGATC<br>CAATTGATAGTAAGTGGAAGAGACGAGCAGTCAATCGCCGAAGCGATCATAGTGGCAATGGTGTTCTCTCAAGAGGATTGCATGATAAAGGCTGTACGAGGTGATTTAAATTTTGTCAATAGAGCGAATCAGCGGCTCAATCCTATGCATCAGCTCCTGAGGCATTTCCAAAAGGATGCAAAGGTACTATTCCAAAACTGGGGAATAGAACCCATTGACAATGTCATGGG<br>AATGATAGGAATATTGCCTGATATGACCCCCAGCACAGAGATGTCACTAAGAGGAGTGAGGGTCAGTAAAATGGGAGTGGATGAATATTCCAGTACTGAGAGGGTGGTCGTGAGTATTGATCGCTTCTTGAGGGTACGAGACCAGAGAGGAAATGTACTCTTGTCTCCCGAAGAGGTCAGTGAAACACAGGGAACAGAGAAGCTAACGATAACATATTCATCATCCATG<br>ATGTGGGAAATTAATGGCCCTGAGTCAGTGCTAGTTAACACATATCAATGGATCATCAGAAACTGGGAAACTGTGAAGATTCAGTGGTCCCAAGACCCTACAATGCTATACAACAAGATGGAGTTTGAGCCTTTTCAGTCCTTGGTGCCTAAGGCAGCCAGAGGCCAGTACAGTGGATTTGTAAGGACCCTATTCCAGCAGATGCGTGATGTGCTGGGAACCTTTGACACT<br>GTCCAGATAATAAAGCTACTTCCATTTGCAGCAGCACCACCGGAACAGAGTAGGATGCAGTTCTCTTCTCTAACTGTAAACGTAAGGGGTTCAGGAATGAGAATACTTGTGAGAGGCAACTCCCCTGTGTTCAACTATAATAAGGCAACCAAGAGGCTCATAGTCCTTGGAAAGGATGCTGGTGCATTGACAGAAGACCCAGGTGAGGGGACAGCAGGAGTGGAGTCTGC<br>GGTATTGAGAGGGTTCCTAATTCTGGGCAAAGAGGACAAAAGATATGGACCAGCGCTGAGCATCAATGAATTGAGCAATCTTGCGAAAGGGGAGAAGGCTAATGTGTTGATAGGGCAAGGAGACGTGGTGTTGGTGATGAAACGGAAACGGGACTCTAGCATACTTACTGACAGCCAGACAGCGACCAAAAGAATTCGGATGGCCATCAATTA
EPI_ISL_6935584	>A/turkey/Poland/H1944-N/2021_H5N1_2021-11-08<br>ATGGAGAGAATAAAAGAGCTAAGAGATTTGATGTCGCAGTCTCGCACTCGCGAGATACTGACAAAAACCACCGTGGACCATATGGCCATAATCAAGAAATATACATCAGGAAGACAGGAGAAGAACCCTGCACTTAGGATGAAGTGGATGATGGCAATGAAATATCCGATTACAGCAGACAAAAGGATAATGGAGATGATCCCTGAAAGAAACGAGCAAGGTCAGACT<br>CTTTGGAGCAAAACAAATGATGCTGGATCGGATAGAGTAATGGTGTCACCTCTGGCTGTGACGTGGTGGAATAGAAATGGACCAACAACAAGTACAGTCCATTACCCAAAGGTCTATAAAACCTACTTTGAAAAGGTTGAAAGGTTAAAGCATGGAACCTTCGGCCCTGTCCATTTCCGGAATCAGGTTAAGATACGCCGCAGAGTTGACATAAACCCGGGCCATGCAGA<br>CCTCAGTGCTAAAGAAGCACAAGACGTCATCATGGAGGTCGTTTTCCCAAATGAAGTCGGAGCCAGAATATTGACATCAGAGTCACAGTTAACAATTACAAAAGAAAAGAAGGAGGAACTCCAGGACTGTAAGATTGCCCCTCTAATGGTGGCATACATGTTGGAGAGAGAACTGGTTCGAAAAACCAGATTCCTGCCAGTAGCTGGCGGAACAAGTAGCGTATATATCG<br>AAGTGTTGCACTTGACTCAAGGAACCTGCTGGGAACAAATGTATACGCCAGGAGGAGAAGTGAGAAATGATGACGTTGACCAAAGTTTAATTATTGCTGCCAGAAATATCGTTAGGAGAGCAACAGTATCAGCAGACCCATTGGCTTCACTACTGGAGATGTGCCATAGTACACAGATTGGCGGGATAAGAATGGTAGACATTCTTAGACAGAACCCAACAGAAGAGCA<br>AGCTGTGGATATATGCAAAGCAGCAATGGGTTTAAGAATCAGTTCATCCTTCAGTTTTGGAGGTTTCACTTTCAAAAGGACAAGCGGATCATCTGTCAAAAGAGAAGAGGAAGTGCTCACCGGCAACCTCCAAACATTGAAAATAAGAGTACATGAAGGGTATGAGGAATTCACAATGGTTGGGCGGAGAGCAACAGCCATTCTAAGGAAAGCAACCAGAAGGCTGATC<br>CAATTGATAGTAAGTGGAAAAGACGAGCAGTCAATCGCCGAAGCGATCATAGTGGCAATGGTGTTCTCTCAAGAGGATTGCATGATAAAGGCTGTACGAGGTGATTTAAATTTTGTCAATAGAGCGAATCAGCGGCTCAATCCTATGCATCAGCTCCTGAGGCATTTCCAAAAGGATGCAAAGGTACTATTCCAAAACTGGGGAATTGAACCCATTGACAATGTCATGGGA<br>ATGATAGGAATATTGCCTGATATGACTCCCAGCACAGAGATGTCACTAAGAGGAGTGAGGGTCAGTAAAATGGGAGTGGATGAATATTCCAGTACTGAGAGGGTGGTCGTGAGTATTGATCGCTTCTTGAGGGTACGAGACCAGAGAGGAAATGTACTCTTGTCTCCCGAAGAGGTCAGTGAAACACAGGGAACAGAGAAGCTAACGATAACATATTCATCATCCATGA<br>TGTGGGAAATTAATGGCCCTGAGTCAGTGCTAGTTAACACATATCAATGGGTCATCAGAAACTGGGAAACTGTGAAGATTCAGTGGTCCCAAGACCCTACAATGCTATACAACAGGATGGAGTTTGAGCCTTTTCAGTCCTTGGTGCCCAAGGCAGCCAGAGGCCAGTACAGTGGATTTGTAAGGACCTTATTCCAGCAGATGCGTGATGTGCTGGGAACCTTTGACACTG<br>TCCAGATAATAAAGCTACTTCCATTTGCAGCAGCACCACCGGAACAGAGTAGGATGCAGTTCTCTTCTCTAACTGTAAACGTAAGGGGTTCAGGAATGAGAATACTTGTGAGAGGCAACTCCCCTGTGTTCAACTATAATAAGGCAACCAAGAGGCTCATAGTCCTTGGAAAGGATGCTGGTGCATTGACAGAAGACCCAGGTGAGGGGACAGCAGGAGTGGAGTCTGCG<br>GTATTGAGAGGGTTCCTAATTCTGGGCAAAGAGGACAAAAGATATGGACCAGCGCTGAGCATCAATGAATTGAGCAATCTTGCGAAAGGGGAGAAGGCTAATGTGTTGATAGGGCAAGGAGACGTGGTATTGGTGATGAAACGGAAACGGGACTCTAGCATACTTACTGACAGCCAGACAGCGACCAAAAGAATTCGGATGGCCATCAATTA
internal NVRI repository	>A/turkey/Poland/H1977_21RS3290-10/2021_H5N1_2021-11-16<br>ATGGAGAGAATAAAAGAGCTAAGAGATTTGATGTCGCAGTCTCGCACTCGCGAGATACTGACAAAAACCACCGTGGACCATATGGCCATAATCAAGAAATATACATCAGGAAGACAGGAGAAGAACCCTGCACTTAGGATGAAGTGGATGATGGCAATGAAATATCCGATTACAGCAGACAAAAGGATAATGGAGATGATCCCTGAAAGAAACGAGCAAGGTCAGACT<br>CTTTGGAGCAAAACAAATGATGCTGGATCGGATAGAGTAATGGTGTCACCTCTGGCTGTGACGTGGTGGAATAGAAATGGACCAACAACAAGTACAGTCCATTACCCAAAGGTCTATAAAACCTACTTTGAAAAGGTTGAAAGGTTAAAGCATGGAACCTTCGGCCCTGTCCATTTCCGGAATCAGGTTAAGATACGCCGCAGAGTTGACATAAACCCGGGCCATGCAGA<br>CCTCAGTGCTAAAGAAGCACAAGACGTCATCATGGAGGTCGTTTTCCCAAATGAAGTCGGAGCCAGAATATTGACATCAGAGTCACAGTTAACAATTACAAAAGAAAAGAAGGAGGAACTCCAGGACTGTAAGATTGCCCCTCTAATGGTGGCATACATGTTGGAGAGAGAACTGGTTCGAAAAACCAGATTCCTGCCAGTAGCTGGCGGAACAAGTAGCGTATATATCG<br>AAGTGTTGCACTTGACTCAAGGAACCTGCTGGGAACAAATGTATACGCCAGGAGGAGAAGTGAGAAATGATGACGTTGACCAAAGTTTAATTATTGCTGCCAGAAATATCGTTAGGAGAGCAACAGTATCAGCAGACCCATTGGCTTCACTACTGGAGATGTGCCATAGTACACAGATTGGCGGGATARGAATGGTAGACATTCTTAGACAGAACCCAACAGAAGAGCA<br>AGCTGTGGATATATGCAAAGCAGCAATGGGTTTAAGAATCAGTTCATCCTTCAGTTTTGGAGGTTTCACTTTCAAAAGGACAAGCGGATCATCTGTCAAAAGAGAAGAGGAAGTGCTCACCGGCAACCTCCAAACATTGAAAATAAGAGTACATGAAGGGTATGAGGAATTCACAATGGTTGGGCGGAGAGCAACAGCCATTCTAAGGAAAGCAACCAGAAGGCTGATC<br>CAATTGATAGTAAGTGGAAAAGACGAGCAGTCAATCGCCGAAGCGATCATAGTGGCAATGGTGTTCTCTCAAGAGGATTGCATGATAAAGGCTGTACGAGGTGATTTAAATTTTGTCAATAGAGCGAATCAGCGGCTCAATCCTATGCATCAGCTCCTGAGGCATTTCCAAAAGGATGCAAAGGTACTATTCCAAAACTGGGGAATTGAACCCATTGACAATGTCATGGGA<br>ATGATAGGAATATTGCCTGATATGACTCCCAGCACAGAGATGTCACTAAGAGGAGTGAGGGTCAGTAAAATGGGAGTGGATGAATATTCCAGTACTGAGAGGGTGGTCGTGAGTATTGATCGCTTCTTGAGGGTACGAGACCAGAGAGGAAATGTACTCTTGTCTCCCGAAGAGGTCAGTGAAACACAGGGAACAGAGAAGCTAACGATAACATATTCATCATCCATGA<br>TGTGGGAAATTAATGGCCCTGAGTCAGTGCTAGTTAACACATATCAATGGGTCATCAGAAACTGGGAAACTGTGAAGATTCAGTGGTCCCAAGACCCTACAATGCTATACAACAGGATGGAGTTTGAGCCTTTTCAGTCCTTGGTGCCCAAGGCAGCCAGAGGCCAGTACAGTGGATTTGTAAGGACCTTATTCCAGCAGATGCGTGATGTGCTGGGAACCTTTGACACTG<br>TCCAGATAATAAAGCTACTTCCATTTGCAGCAGCACCACCGGAACAGAGTAGGATGCAGTTCTCTTCTCTAACTGTAAACGTAAGGGGTTCAGGAATGAGAATACTTGTGAGAGGCAACTCCCCTGTGTTCAACTATAATAAGGCAACCAAGAGGCTCATAGTCCTTGGAAAGGATGCTGGTGCATTGACAGAAGACCCAGGTGAGGGGACAGCAGGAGTGGAGTCTGCG<br>GTATTGAGAGGGTTCCTAATTCTGGGCAAAGAGGACAAAAGATATGGACCAGCGCTGAGCATCAATGAATTGAGCAATCTTGCGAAAGGGGAGAAGGCTAATGTGTTGATAGGGCAAGGAGACGTGGTATTGGTGATGAAACGGAAACGGGACTCTAGCATACTTACTGACAGCCAGACAGCGACCAAAAGAATTCGGATGGCCATCAATTA
internal NVRI repository	>A/turkey/Poland/H2043-T2/2021_H5N1_2021-11-27<br>ATGGAGAGAATAAAAGAGCTAAGAGATTTGATGTCGCAGTCTCGCACTCGCGAGATACTGACAAAAACCACCGTGGACCATATGGCCATAATCAAGAAATATACATCAGGAAGACAGGAGAAGAACCCTGCACTTAGGATGAAGTGGATGATGGCAATGAAATATCCGATTACAGCAGACAAAAGGATAATGGAGATGATCCCTGAAAGAAACGAGCAAGGTCAGACT<br>CTTTGGAGCAAAACAAATGATGCTGGATCGGATAGAGTAATGGTGTCACCTCTGGCTGTGACGTGGTGGAATAGAAATGGACCAACAACAAGTACAGTCCATTACCCAAAGGTCTATAAAACCTACTTTGAAAAGGTTGAAAGGTTAAAGCATGGAACCTTCGGCCCTGTCCATTTCCGGAATCAGGTTAAGATACGCCGCAGAGTTGACATAAACCCGGGCCATGCAGA<br>CCTCAGTGCTAAAGAAGCACAAGACGTCATCATGGAGGTCGTTTTCCCAAATGAAGTCGGAGCCAGAATATTGACATCAGAGTCACAGTTAACAATTACAAAAGAAAAGAAGGAGGAACTCCAGGACTGTAAGATTGCCCCTCTAATGGTGGCATACATGTTGGAGAGAGAACTGGTTCGAAAAACCAGATTCCTGCCAGTAGCTGGCGGAACAAGTAGCGTATATATCG<br>AAGTGTTGCACTTGACTCAAGGAACCTGCTGGGAACAAATGTATACGCCAGGAGGAGAAGTGAGAAATGATGACGTTGACCAGAGTTTAATTATTGCTGCCAGAAATATAGTTAGGAGAGCAACAGTATCAGCAGACCCATTGGCTTCACTACTGGAGATGTGCCATGGTACACAGATTGGCGGGATAAGAATGGTAGACATTCTTAGACAGAACCCAACAGAAGAGCA<br>AGCCGTGGATATATGCAAAGCAGCAATGGGTTTAAGRATCAGTTCATCCTTCAGTTTTGGAGGTTTCACTTTCAAAAGGACAAGCGGATCATCTGTCAAAAGAGAAGAGGAAGTGCTCACCGGCAACCTCCAAACATTGAAAATAAGAGTACATGAAGGGTATGAGGAATTCACAATGGTTGGGCGGAGAGCAACAGCCATTCTAAGGAAAGCAACCAGAAGGCTGATC<br>CAATTGATAGTAAGTGGAAGAGACGAGCAGTCAATCGCCGAAGCGATCATAGTGGCAATGGTGTTCTCTCAAGAGGATTGCATGATAAAGGCTGTACGAGGTGATTTAAATTTTGTCAATAGAGCGAATCAGCGGCTCAATCCTATGCATCAGCTCCTGAGGCATTTCCAAAAGGATGCAAAGGTACTATTCCAAAACTGGGGAATTGAACCCATTGACAATGTCATGGGA<br>ATGATAGGAATATTGCCCGATATGACCCCCAGCACAGAGATGTCACTAAGAGGAGTGAGGGTCAGCAAAATGGGAGTGGATGAATATTCCAGTACTGAGAGGGTGGTCGTGAGTATTGATCGCTTCTTGAGGGTACGAGACCAGAGAGGAAATGTACTCTTGTCTCCCGAAGAGGTCAGTGAAACACAGGGAACAGAGAAGCTAACGATAACATATTCATCATCAATGA<br>TGTGGGAAATTAATGGCCCTGAGTCAGTGCTAGTTAACACATATCAATGGATCATCAGAAACTGGGAAACTGTGAAGATTCAGTGGTCCCAAGACCCTACAATGCTATACAACAAGATGGAGTTTGAGCCTTTTCAGTCCTTGGTGCCTAAGGCAGCCAGAGGCCAGTACAGTGGATTTGTAAGGACCCTATTCCAGCAGATGCGTGATGTGCTGGGAACCTTTGACACTGT<br>CCAGATAATAAAGCTACTTCCATTTGCAGCAGCACCACCGGAACAGAGTAGGATGCAGTTCTCTTCTCTAACTGTAAACGTAAGGGGTTCAGGAATGAGAATACTTGTGAGAGGCAACTCCCCTGTGTTCAACTATAATAAGGCAACCAAGAGGCTCATAGTCCTTGGAAAGGATGCTGGTGCATTGACAGAAGACCCAGGTGAGGGGACAGCAGGAGTGGAGTCTGCG<br>GTATTGAGAGGGTTCCTAATTCTGGGCAAAGAGGACAAAAGATATGGACCAGCGCTGAGCATCAATGAATTGAGCAATCTTGCGAAAGGGGAGAAGGCTAATGTGTTGATAGGGCAAGGAGACGTGGTGTTGGTGATGAAACGGAAACGGGACTCTAGCATACTTACTGACAGCCAGACAGCGACCAAAAGAATTCGGATGGCCATCAATTA
internal NVRI repository	>A/turkey/Poland/H2113-T1/2021_H5N1_2021-12-15<br>ATGGAGAGAATAAAAGAGCTAAGAGATTTGATGTCGCAGTCTCGCACTCGCGAGATACTGACAAAAACCACCGTGGACCATATGGCCATAATCAAGAAATATACATCAGGAAGACAGGAGAAGAACCCTGCACTTAGGATGAAGTGGATGATGGCAATGAAATATCCGATTACAGCAGACAAAAGGATAATGGAGATGATCCCTGAAAGAAACGAGCAAGGTCAGACT<br>CTTTGGAGCAAAACAAATGATGCTGGATCGGATAGAGTAATGGTGTCACCTCTGGCTGTGACGTGGTGGAATAGAAATGGACCAACAACAAGTACCGTCCATTACCCAAAGGTCTATAAAACCTACTTTGAAAAGGTTGAAAGGTTAAAGCATGGAACCTTCGGCCCTGTCCATTTCCGGAATCAGGTTAAGATACGCCGCAGAGTTGACATAAACCCGGGCCATGCAGA<br>CCTCAGTGCTAAAGAAGCACAAGACGTCATCATGGAGGTCGTTTTCCCAAATGAAGTCGGAGCCAGAATATTGACATCAGAGTCACAGTTGACAATTACAAAAGAAAAGAAGGAGGAACTCCAGGACTGTAAGATTGCCCCTCTAATGGTGGCATACATGTTGGAGAGAGAACTGGTTCGAAAAACCAGATTCCTGCCAGTAGCTGGCGGAACAAGTAGCGTATATATCG<br>AAGTGTTGCACTTGACTCAAGGAACCTGCTGGGAACAAATGTATACGCCAGGAGGAGAAGTGAGAAATGATGACGTTGACCAAAGTTTAATTATTGCTGCCAGAAATATCGTTAGGAGGGCAACAGTATCAGCAGACCCATTGGCTTCACTACTGGAGATGTGCCATAGTACACAGATTGGCGGGATAAGAATGGTAGACATTCTTAGACAGAACCCAACAGAAGAGCA<br>AGCCGTGGATATATGCAAAGCAGCAATGGGTTTAAGAATCAGTTCATCCTTCAGTTTTGGAGGTTTCACTTTCAAAAGGACAAGCGGATCATCTGTCAAAAGAGAAGAGGAAGTGCTCACCGGCAACCTCCAAACATTGAAAATAAGAGTACATGAAGGGTATGAGGAATTCACAATGGTTGGGCGGAGAGCAACAGCCATTCTAAGGAAAGCAACCAGAAGGCTGATC<br>CAATTGATAGTAAGTGGAAGAGACGAGCAGTCAATCGCCGAAGCGATCATAGTGGCAATGGTGTTCTCTCAAGAGGATTGCATGATAAAGGCTGTACGAGGTGATTTAAATTTTGTCAATAGAGCGAATCAGCGGCTCAATCCTATGCATCAGCTCCTGAGGCATTTCCAAAAGGATGCAAAGGTACTATTCCAAAACTGGGGAATTGAACCCATTGACAATGTCATGGGA<br>ATGATAGGAATATTGCCTGATATGACCCCCAGCACAGAGATGTCACTAAGAGGAGTGAGGGTCAGTAAAATGGGAGTGGATGAATATTCCAGTACTGAGAGGGTGGTCGTAAGTATTGATCGCTTCTTGAGGGTACGAGACCAGAGAGGAAATGTACTCTTGTCTCCCGAAGAGGTCAGTGAAACACAGGGAACAGAGAAGCTAACGATAACATATTCATCATCCATGA<br>TGTGGGAAATTAATGGCCCTGAGTCAGTGCTAGTTAACACATATCAATGGATCATCAGAAACTGGGAAACTGTGAAGATTCAGTGGTCCCAAGACCCTACAATGCTATACAACAAGATGGAGTTTGAGCCTTTTCAGTCCTTGGTGCCTAAGGCAGCCAGAGGCCAGTACAGTGGATTTGTAAGGACCCTATTCCAGCAGATGCGTGATGTGCTGGGAACCTTTGACACTGT<br>CCAGATAATAAAGCTACTTCCATTTGCAGCAGCACCACCGGAACAGAGTAGGATGCAGTTCTCTTCTCTAACTGTAAACGTAAGGGGTTCAGGAATGAGAATACTTGTGAGAGGCAACTCCCCTGTGTTCAACTATAATAAAGCAACCAAGAGGCTCATAGTCCTTGGAAAGGATGCTGGTGCATTGACAGAAGACCCAGGTGAGGGGACAGCAGGAGTGGAGTCTGCG<br>GTATTGAGAGGGTTCCTAATTCTGGGCAAAGAGGACAAAAGATATGGACCAGCGCTGAGCATCAATGAATTGAGCAATCTTGCGAAAGGGGAGAAGGCTAATGTGTTGATAGGGCAAGGAGACGTGGTGTTGGTGATGAAACGGAAACGGGACTCTAGCATACTTACTGACAGCCAGACAGCGACCAAAAGAATTCGGATGGCCATCAATTA

internal NVRI repository	>A/turkey/Poland/H2118-N/2021_H5N1_2021-12-16<br>ATGGAGAGAATAAAAGAGCTAAGAGATTTGATGTCGCAATCTCGCACTCGCGAGATACTGACAAAAACCACCGTGGACCATATGGCCATAATCAAGAAATATACATCAGGAAGACAGGAGAAGAACCCTGCACTTAGGATGAAGTGGATGATGGCAATGAAATATCCGATTACAGCAGACAAAAGGATAATGGAGATGATCCCTGAAAGAAACGAGCAAGGTCAGACT<br>CTTTGGAGCAAAACAAATGATGCTGGATCGGATAGAGTAATGGTGTCACCTCTGGCTGTGACGTGGTGGAATAGAAATGGACCAACAACAAGTACCGTCCATTACCCAAAGGTCTATAAAACCTACTTTGAAAAGGTTGAAAGGTTAAAGCATGGAACCTTCGGCCCTGTCCATTTCCGGAATCAGGTTAAGATACGCCGCAGAGTTGACATAAACCCGGGCCATGCAGA<br>CCTCAGTGCTAAAGAAGCACAGGACGTCATCATGGAGGTCGTTTTCCCAAATGAAGTCGGAGCCAGAATATTGACATCAGAGTCACAGTTGACAATTACAAAAGAAAAGAAGGAGGAACTCCAGGACTGTAAGATTGCCCCTCTAATGGTGGCATACATGTTGGAGAGAGAACTGGTTCGAAAAACCAGATTCCTGCCAGTAGCTGGCGGAACAAGTAGCGTATATATC<br>GAAGTGTTGCACTTGACTCAAGGAACCTGCTGGGAACAAATGTATACGCCAGGAGGAGAAGTGAGAAATGATGACGTTGACCAAAGTTTAATTATTGCTGCCAGAAATATCGTTAGGAGGGCAACAGTATCAGCAGACCCATTGGCTTCACTACTGGAGATGTGCCATAGTACACAGATTGGCGGGATAAGAATGGTAGACATTCTTAGACAGAACCCAACAGAAGAGC<br>AAGCCGTGGATATATGCAAAGCAGCAATGGGTTTAAGAATCAGTTCATCCTTCAGTTTTGGAGGTTTCACTTTCAAAAGGACAAGCGGATCATCTGTCAAAAGAGAAGAGGAAGTGCTCACCGGCAACCTCCAAACATTGAAAATAAGAGTACATGAAGGGTATGAGGAATTCACAATGGTTGGGCGGAGAGCAACAGCCATTCTAAGGAAAGCAACCAGAAGGCTGAT<br>CCAATTGATAGTAAGTGGAAGAGACGAGCAGTCAATCGCCGAAGCGATCATAGTGGCAATGGTGTTCTCTCAAGAGGATTGCATGATAAAGGCTGTACGAGGTGATTTAAATTTTGTCAATAGAGCGAATCAGCGGCTCAATCCTATGCATCAGCTCCTGAGGCATTTCCAAAAGGATGCAAAGGTACTATTCCAAAACTGGGGAATTGAACCCATTGACAATGTCATGGG<br>AATGATAGGAATATTGCCTGATATGACCCCCAGCACAGAGATGTCACTAAGAGGAGTGAGGGTCAGTAAAATGGGAGTGGATGAATATTCCAGTACTGAGAGGGTGGTCGTGAGTATTGATCGCTTCTTGAGGGTACGAGACCAGAGAGGAAATGTACTCTTGTCTCCCGAAGAGGTCAGTGAAACACAGGGAACAGAGAAGCTAACGATAACATATTCATCATCCATG<br>ATGTGGGAAATCAATGGCCCTGAGTCAGTGCTAGTTAACACATATCAATGGATCATCAGAAACTGGGAAACTGTGAAGATTCAGTGGTCCCAAGACCCTACAATGCTATACAACAAGATGGAGTTTGAGCCTTTTCAGTCCTTGGTGCCTAAGGCAGCCAGAGGCCAGTACAGTGGATTTGTAAGGACCCTATTCCAGCAGATGCGTGATGTGCTGGGAACCTTTGACACT<br>GTCCAGATAATAAAGCTACTTCCATTTGCAGCAGCACCACCGGAACAGAGTAGGATGCAGTTCTCTTCTCTAACTGTAAACGTAAGGGGTTCAGGAATGAGAATACTTGTGAGAGGCAACTCCCCTGTGTTCAACTATAATAAAGCAACCAAGAGGCTCATAGTCCTTGGAAAGGATGCTGGTGCATTGACAGAAGACCCAGGTGAGGGGACAGCAGGAGTGGAGTCTGC<br>GGTATTGAGAGGGTTCCTAATTCTGGGCAAAGAGGACAAAAGATATGGACCAGCGCTGAGCATCAATGAATTGAGCAATCTTGCGAAAGGGGAGAAGGCTAATGTGTTGATAGGGCAAGGAGACGTGGTGTTGGTGATGAAACGGAAACGGGACTCTAGCATACTTACTGACAGCCAGACAGCGACCAAAAGAATTCGGATGGCCATCAATTA
internal NVRI repository	>A/turkey/Poland/H2179-T4/2021_H5N1_2021-12-29<br>ATGGAGAGAATAAAAGAACTAAGAGATTTGATGTCGCAGTCTCGCACTCGCGAGATACTGACAAAAACCACGGTGGACCATATGGCCATAATTAAGAAATACACATCAGGAAGACAGGAGAAGAACCCTGCCCTTAGGATGAAGTGGATGATGGCAATGAAATACCCGATTACAGCAGACAAAAGAATAATGGAAATGATCCCTGAAAGAAATGAACAAGGCCAGACT<br>CTCTGGAGCAAAACAAGTGATGCTGGATCAGACAGAGTAATGGTGTCACCTCTGGCTGTGACATGGTGGAACAGAAATGGACCGACGACAAGTACAGTCCACTATCCAAAGGTCTACAAAACCTACTTTGAAAAAGTTGAAAGGTTGAAACATGGAACCTTCGGTCCTGTTCACTTTCGGAATCAGGTTAAAATACGCCGCAGGGTTGACATAAACCCGGGCCATGCAGA<br>TCTCAGTGCCAAAGAAGCACAAGATGTCATCATGGAGGTTGTTTTCCCAAACGAAGTTGGAGCAAGGATATTGACATCAGAGTCACAATTAACAATAACAAAGGAAAAGAAGGAGGAGCTTCAGGACTGCAAGATCGCCCCTTTAATGGTGGCATACATGTTGGAGAGAGAACTGGTTCGCAAAACCAGATTCCTACCAGTAGCTGGCGGAACAAGCAGCGTATATATC<br>GAGGTGTTGCATTTGACTCAAGGGACCTGCTGGGAACAAATGTACACACCGGGAGGGGAGGTGAGAAATGATGATGTCGATCAGAGTTTGATTATTGCTGCTAGAAATATTGTTAGGAGAGCAACAGTGTCGGCAGACCCGTTGGCTTCGCTCTTGGAGATGTGCCATAGTACACAAATTGGCGGGATAAGAATGGTGGACATCCTTAGACAGAACCCAACAGAAGAGC<br>AAGCTGTGGATATATGCAAAGCAGCAATGGGTTTGAGAATCAGTTCATCCTTCAGCTTTGGAGGTTTCACTTTTAAAAGAACAAGTGGGTCATCTGTCAAAAGAGAAGAAGAAGTGCTCACGGGCAACCTCCAAACACTGAAAATAAGAGTACATGAAGGATATGAAGAATTCACAATGGTTGGGCGAAGAGCTACGGCCATTCTAAGGAAAGCAACCAGAAGGCTGAT<br>CCAATTAATAGTGAGTGGGAGAGACGAGCAGTCAATCGCTGAAGCGATTATAGTGGCAATGGTTTTCTCACAAGAGGACTGCATGATAAAAGCAGTGCGAGGTGATTTGAACTTTGTTAACAGAGCAAATCAGCGGCTAAATCCTATGCACCAACTTCTGAGACATTTCCAAAAGGATGCAAAGGTGCTGTTTCAAAATTGGGGGACTGAACCCATTGACAATGTCATGG<br>GAATGATCGGGATACTGCCTGACATGACCCCTAGCACAGAGATGTCACTGAGAGGAGTGAGAGTCAGTAAAATGGGAGTGGATGAATATTCCAGTACTGAGAGAGTGGTCGTAAGCATTGATCGTTTCTTAAGAGTCCGWGACCAGAGGGGAAACGTACTCTTGTCTCCTGAAGAAGTTAGTGAAACACAGGGAACAGAGAAACTGACGATAACATATTCATCGTCTAT<br>GATGTGGGAAATCAATGGTCCGGAGTCAGTACTGGTCAACACATATCAATGGATCATTAGAAATTGGGAAACTGTGAAGATTCAGTGGTCCCAAGACCCCACAATGTTGTACAATAAAATGGAGTTTGAGCCTTTCCAATCCTTGGTGCCCAAGGCTGCCAGAGGCCAGTACAGTGGATTTGTGAGGACGTTATTCCAGCAGATGCGTGATGTGCTGGGGACATTTGACAC<br>TGTCCAAATAATAAAGCTCCTACCATTTGCAGCAGCCCCACCGGAACAGAGTAGGATGCAGTTCTCTTCTCTGACTGTGAATGTAAGAGGTTCAGGAATGAGAATACTTGTGAGAGGTAACTCCCCTGTGTTCAACTATAACAAAGCAACCAAGAGACTCACAGTTCTTGGAAAGGATGCAGGTGCATTGACAGAAGATCCAGACGAGGGAACAGCAGGAGTGGAGTCTG<br>CGGTGTTAAGAGGATTTCTAATTCTGGGCAAAGAAGACAAAAGGTATGGACCAGCATTGAGCATTAACGAATTGAGCAATCTTGCGAAAGGGGAGAAGGCTAATGTGTTGATAGGGCAAGGAGACGTGGTGTTGGTAATGAAACGGAAACGGGACTCTAGCATACTTACTGACAGCCAGACAGCGACCAAAAGGATTCGGATGGCCATCAATTA
internal NVRI repository	>A/turkey/Poland/H2188-Ni/2021_H5N1_2021-12-30<br>ATGGAGAGAATAAAAGAACTAAGAGATTTGATGTCGCAGTCTCGCACTCGCGAGATACTGACAAAAACCACTGTGGACCATATGGCCATAATAAAGAAATACACATCAGGAAGACAGGAGAAGAACCCTGCCCTCAGGATGAAGTGGATGATGGCAATGAAATATCCTATTACAGCAGACAAAAGAATAATGGAGATGATCCCTGAAAGGAATGAGCAAGGTCAGACT<br>CTCTGGAGCAAAACAAATGACGCTGGATCAGACAGAGTGATGGTGTCACCTCTGGCTGTGACATGGTGGAATAGAAATGGGCCAACGACAAGTACAGTCCATTACCCAAAGGTTTACAAAACCTACTTTGAAAAAGTGGAAAGATTGAAACATGGAACCTTTGGCCCTGTTCACTTTCGAAATCAGGTTAAGATACGCCGCAGGGTTGACATAAACCCGGGTCATGCAGA<br>TCTCAGTGCCAAAGAAGCACAGGATGTCATCATGGAAGTCGTTTTCCCAAATGAAGTTGGAGCCAGGATATTGACATCAGAATCACAATTAACAATAACAAAGGAAAAGAAGGAGGAACTTCAGGACTGTAAGATTGCTCCTTTGATGGTGGCATACATGTTGGAGAGAGAACTGGTTCGAAAAACCAGATTTTTACCAGTAGCTGGCGGAACAAGCAGCGTATATATCG<br>AAGTATTGCATTTGACTCAAGGGACCTGCTGGGAACAAATGTACACACCGGGAGGGGAAGTGAGAAATGATGATGTTGATCAGAGTTTGATCATTGCTGCTAGAAACATCGTTAGGAGAGCAACAGTATCAGCAGACCCATTGGCTTCGCTCTTGGAAATGTGCCACAGTACACAAATTGGCGGGATAAGGATGGTAGACATTCTTAGACAGAACCCAACAGAAGAGCA<br>AGCCGTGGATATATGCAAAGCAGCAATGGGTTTAAGAATCAGTTCATCCTTCAGCTTTGGAGGTTTCACTTTCAAAAGAACAAGTGGATCGTCTGTCAAAAGAGAAGAAGAAGTGCTCACAGGCAACCTCCAAACACTGAAAATAAGAGTACATGAAGGATATGAGGAATTCACAATGGTTGGACGGAGAGCTACAGCCATTTTAAGGAAAGCAACCAGAAGGCTGATCC<br>AATTAATAGTGAGTGGAAGAGACGAGCAGTCAATCGCTGAAGCAATCATAGTGGCAATGGTTTTCTCACAAGAGGACTGCATGATAAAAGCAGTACGAGGTGATCTGAACTTTGTTAATAGAGCAAATCAGCGACTAAATCCTATGCATCAACTTCTGAGGCATTTCCAAAAGGATGCAAAGGTGCTGTTTCAAAACTGGGGGGTTGAACCAATTGACAATGTAATGGGG<br>ATGATTGGAATACTGCCTGACATGACCCCCAGCACGGAGATGTCACTGAGAGGAGTGAGAGTCAGCAAAATGGGAGTGGATGAATATTCCAGTACTGAGAGAGTGGTCGTGAGCATTGATCGTTTCTTGAGAGTCCGAGATCAAAGAGGAAACGTGCTCCTGTCTCCTGAGGAAGTTAGTGAAACACAGGGAACAGAGAAACTGACGATAACATATTCATCGTCCATGA<br>TGTGGGAAATCAATGGTCCGGAATCCGTGCTAGTCAACACATATCAATGGATCATCAGAAATTGGGAGACTGTGAAGATTCAATGGTCCCAAGACCCTACGATGTTGTACAACAAGATGGAATTTGAGCCCTTCCAATCCTTGGTGCCCAAGGCTGCCAGAGGCCAGTATAGTGGATTTGTGAGAACATTGTTCCAGCAGATGCGTGATGTGCTGGGAACATTTGATACTGT<br>CCAAATAATAAAGCTCCTGCCTTTTGCTGCAGCCCCACCGGAACAGAATAGGATGCAGTTTTCCTCTCTGACTGTGAACGTAAGAGGTTCAGGAATGAGAATACTTGTGAGAGGCAACTCCCCTGTGTTCAACTATAACAAGGCCACCAAGAGACTCACAGTTCTTGGAAAGGATGCAGGTGCCTTGACAGAAGATCCAGATGAGGGARCAGCAGGAGTAGAGTCTGCAG<br>TATTAAGAGGATTTCTAATTCTGGGCAAAGAGGACAAAAGATATGGACCAGCATTGAGCATCAACGAACTGAGCAATCTTGCGAAAGGGGAAAAGGCTAATGTGTTGATAGGGCAAGGAGACGTGGTGTTGGTAATGAAACGGAAACGGGACTCTAGCATACTTACTGACAGCCAGACAGCGACCAAAAGAATTCGGATGGCCATCAATTA
internal NVRI repository	>A/turkey/Poland/H296/2022_H5N1_2022-04-21<br>ATGGAGAGAATAAAAGAACTAAGAGATTTGATGTCGCAGTCTCGCACTCGCGAGATACTGACAAAAACCACGGTGGACCATATGGCCATAATTAAGAAMTACACATCAGGAAGACAGGAGAAGAACCCTGCCCTTAGGATGAAGTGGATGATGGCAATGAAATACCCGATTACAGCAGACAAAAGAATAATGGAAATGATCCCTGAAAGAAATGAACAAGGCCAGAC<br>TCTCTGGAGCAAAACAAATGATGCTGGATCAGACAGAGTAATGGTGTCACCTCTGGCTGTGACATGGTGGAACAGAAATGGACCGACGACAAGTACAGTCCACTATCCAAAGGTCTACAAAACCTACTTTGGAAAAGTTGAAAGGTTGAAACATGGAACCTTCGGTCCTGTTCACTTTCGGAATCAGGTTAAAATACGCCGCAGGGTTGACATAAACCCGGGCCATGCAG<br>ATCTCAGTGCCAAAGAAGCACAAGATGTCATCATGGAGGTCGTTTTCCCAAACGAAGTTGGAGCAAGGACATTGACATCAGAGTCACAATTAACAGTAACAAAGGAAAAGAAGGAGGAGCTTCAGGACTGCAAGATCGCCCCTTTAATGGTGGCATACATGTTGGAGAGAGAACTGGTTCGCAAAACCAGATTCCTACCAGTAGCTGGYGGAACAAGCAGCGTATATAT<br>CGAGGTGTTGCATTTGACTCAAGGGACCTGCTGGGAACAAATGTACACACCGGGAGGGGAGGTGAGAAATGATGATGTCGATCAGAGTTTGATTATTGCTGCTAGAAATATTGTTAGGAGAGCAACAGTGTCGGCAGACCCGTTGGCTTCGCTCTTGGAGATGTGCCATAGTACACAAATTGGCGGGATAAGAATGGTGGACATCCTTAGACAGAACCCAACAGAAGAG<br>CAAGCTGTGGATATATGCAAAGCAGCAATGGGTTTAAGAATCAGTTCATCCTTCAGCTTTGGAGGTTTCACTTTTAAAAGAACAAGTGGGTCATCTGTCAAAAGAGAAGAAGAAGTGCTCACGGGCAACCTCCAAACACTGAAAATAAGAGTACATGAAGGATATGAAGAATTCACAATGGTTGGGCGAAGAGCTACGGCCATTCTAAGGAAAGCAACCAGAAGGCTGA<br>CCCAATTAATAGTGAGTGGGAGAGACGAGCAGTCAATCGCTGAAGCGATTATAGTGGCAATGGTTTTCTCACAAGAGGACTGCATGATAAAAGCAGTGCGAGGTGATTTGAACTTTGTTAACAGAGCAAATCAGCGGCTAAATCCTATGCACCAACTTCTGAGACATTTCCAAAAGGATGCAAAGGTGCTGTTTCAAAATTGGGGGACTGAACCCATTGACAATGTCATGG<br>GAATGATCGGGATACTGCCTGACATGACCCCTAGCACAGAGATGTCACTGAGAGGAGTGAGAGTCAGTAAAATGGGAGTGGATGAATATTCCAGTACTGAGAGAGTGGTCGTAAGCATTGATCGTTTCTTAAGAGTCCGAGACCAGAGGGGAAACGTACTCTTGTCTCCTGAAGAAGTTAGTGAAACACAGGGAACAGAGAAACTGACGATAACATATTCATCGTCTAT<br>GATGTGGGAAATCAATGGTCCGGAGTCAGTACTGGTCAACACATATCAATGGATCATTAGAAATTGGGAAACTGTGAAGATTCAGTGGTCCCAAGACCCCACAATGTTGTACAATAAAATGGAGTTTGAGCCTTTCCAATCCTTGGTGCCCAAGGCTGCCAGAGGCCAGTACAGTGGATTTGTAAGGACGTTATTCCAGCAGATGCGTGATGTGCTGGGGACATTTGACAC<br>TGTCCAAATAATAAAGCTCCTACCATTTGCATCAGCCCCACCGGAACAGAGTAGGATGCAGTTCTCTTCTCTGACTGTGAATGTAAGAGGTTCAGGAATGAGAATACTTGTGAGAGGTAACTCCCCTGTGTTCAACTATAACAAAGCAACCAAGAGACTCACAGTTCTTGGAAAGGATGCAGGTGCATTGACAGAAGATCCAGACGAGGGAACAGCAGGAGTGGAGTCTG<br>CGGTGTTAAGAGGATTTCTAATTCTGGGCAAAGAAGACAAAAGGTATGGACCAACATTGAGCATTAACGAATTGAGCAATCTTGCGAAAGGGGAGAAGGCTAATGTGTTGATAGGGCAAGGAGACGTGGTGTTGGTAATGAAACGGAAACGGGACTCTAGCATACTTACTGACAGCCAGACAGCGACCAAAAGGATTCGGATGGCCATCAATTA
internal NVRI repository	>A/turkey/Poland/H297/2022_H5N1_2022-04-21<br>ATGGAGAGAATAAAAGAACTAAGAGATTTGATGTCGCAGTCTCGCACTCGCGAGATACTGACAAAAACCACGGTGGACCATATGGCCATAATTAAGAAATACACATCAGGAAGACAGGAGAAGAACCCTGCCCTTAGGATGAAGTGGATGATGGCAATGAAATACCCGATTACAGCAGACAAAAGAATAATGGAAATGATCCCTGAAAGAAATGAACAAGGCCAGACT<br>CTCTGGAGCAAAACAAATGATGCTGGATCAGACAGAGTAATGGTGTCACCTCTGGCTGTGACATGGTGGAACAGAAATGGACCGACGACAAGTACAGTCCACTATCCAAAGGTCTACAAAACCTACTTTGGAAAAGTTGAAAGGTTGAAACATGGAACCTTCGGTCCTGTTCACTTTCGGAATCAGGTTAAAATACGCCGCAGGGTTGACATAAACCCGGGCCATGCAGA<br>TCTCAGTGCCAAAGAAGCACAAGATGTCATCATGGAGGTCGTTTTCCCAAACGAAGTTGGAGCAAGGACATTGACATCAGAGTCACAATTAACAGTAACAAAGGAAAAGAAGGAGGAGCTTCAGGACTGCAAGATCGCCCCTTTAATGGTGGCATACATGTTGGAGAGAGAACTGGTTCGCAAAACCAGATTCCTACCAGTAGCTGGCGGAACAAGCAGCGTATATATC<br>GAGGTGTTGCATTTGACTCAAGGGACCTGCTGGGAACAAATGTACACACCGGGAGGGGAGGTGAGAAATGATGATGTCGATCAGAGTTTGATTATTGCTGCTAGAAATATTGTTAGGAGAGCAACAGTGTCGGCAGACCCGTTGGCTTCGCTCTTGGAGATGTGCCATAGTACACAAATTGGCGGGATAAGAATGGTGGACATCCTTAGACAGAACCCAACAGAAGAGC<br>AAGCTGTGGATATATGCAAAGCAGCAATGGGTTTAAGAATCAGTTCATCCTTCAGCTTTGGAGGTTTCACTTTTAAAAGAACAAGTGGGTCATCTGTCAAAAGAGAAGAAGAAGTGCTCACGGGCAACCTCCAAACACTGAAAATAAGAGTACATGAAGGATATGAAGAATTCACAATGGTTGGGCGAAGAGCTACGGCCATTCTAAGGAAAGCAACCAGAAGGCTGAC<br>CCAATTAATAGTGAGTGGGAGAGACGAGCAGTCAATCGCTGAAGCGATTATAGTGGCAATGGTTTTCTCACAAGAGGACTGCATGATAAAAGCAGTGCGAGGTGATTTGAACTTTGTTAACAGAGCAAATCAGCGGCTAAATCCTATGCACCAACTTCTGAGACATTTCCAAAAGGATGCAAAGGTGCTGTTTCAAAATTGGGGGACTGAACCCATTGACAATGTCATGG<br>GAATGATCGGGATACTGCCTGACATGACCCCTAGCACAGAGATGTCACTGAGAGGAGTGAGAGTCAGTAAAATGGGAGTGGATGAATATTCCAGTACTGAGAGAGTGGTCGTAAGCATTGATCGTTTCTTAAGAGTCCGAGACCAGAGGGGAAACGTACTCTTGTCTCCTGAAGAAGTTAGTGAAACACAGGGAACAGAGAAACTGACGATAACATATTCATCGTCTAT<br>GATGTGGGAAATCAATGGTCCGGAGTCAGTACTGGTCAACACATATCAATGGATCATTAGAAATTGGGAAACTGTGAAGATTCAGTGGTCCCAAGACCCCACAATGTTGTACAATAAAATGGAGTTTGAGCCTTTCCAATCCTTGGTGCCCAAGGCTGCCAGAGGCCAGTACAGTGGATTTGTAAGGACGTTATTCCAGCAGATGCGTGATGTGCTGGGGACATTTGACAC<br>TGTCCAAATAATAAAGCTCCTACCATTTGCATCAGCCCCACCGGAACAGAGTAGGATGCAGTTCTCTTCTCTGACTGTGAATGTAAGAGGTTCAGGAATGAGAATACTTGTGAGAGGTAACTCCCCTGTGTTCAACTATAACAAAGCAACCAAGAGACTCACAGTTCTTGGAAAGGATGCAGGTGCATTGACAGAAGATCCAGACGAGGGAACAGCAGGAGTGGAGTCTG<br>CGGTGTTAAGAGGATTTCTAATTCTGGGCAAAGAAGACAAAAGGTATGGACCAACATTGAGCATTAACGAATTGAGCAATCTTGCGAAAGGGGAGAAGGCTAATGTGTTGATAGGGCAAGGAGACGTGGTGTTGGTAATGAAACGGAAACGGGACTCTAGCATACTTACTGACAGCCAGACAGCGACCAAAAGGATTCGGATGGCCATCAATTA
internal NVRI repository	>A/turkey/Poland/H303/2022_H5N1_2022-04-26<br>ATGGAGAGAATAAAAGAACTAAGAGATTTGATGTCGCAGTCTCGCACTCGCGAGATACTGACAAAAACCACGGTGGACCATATGGCCATAATTAAGAAATACACATCAGGAAGACAGGAGAAGAACCCTGCCCTTAGGATGAAGTGGATGATGGCAATGAAATACCCGATTACAGCAGACAAAAGAATAATGGAAATGATCCCTGAAAGAAATGAACAAGGCCAGACT<br>CTCTGGAGCAAAACAAATGATGCTGGATCAGACAGAGTAATGGTGTCACCTCTGGCTGTGACATGGTGGAACAGAAATGGACCGACGACAAGTACAGTCCACTATCCAAAGGTCTACAAAACCTACTTTGGAAAAGTTGAAAGGTTGAAACATGGAACCTTCGGTCCTGTTCACTTTCGGAATCAGGTTAAAATACGCCGCAGGGTTGACATAAACCCGGGCCATGCAGA<br>TCTCAGTGCCAAAGAAGCACAAGATGTCATCATGGAGGTCGTTTTCCCAAACGAAGTTGGAGCAAGGACGTTGACATCAGAGTCACAATTAACAGTAACAAAGGAAAAGAAGGAGGAGCTTCAGGACTGCAAGATCGCCCCTTTAATGGTGGCATACATGTTGGAGAGAGAACTGGTTCGCAAAACCAGATTCCTACCAGTAGCTGGCGGAACAAGCAGCGTATATATC<br>GAGGTGTTGCATTTGACTCAAGGGACCTGCTGGGAACAAATGTACACACCGGGAGGGGAGGTGAGAAATGATGATGTCGATCAGAGTTTGATTATTGCTGCTAGAAATATTGTTAGGAGAGCAACAGTGTCGGCAGACCCGTTGGCTTCGCTCTTGGAGATGTGCCATAGTACACAAATTGGCGGGATAAGAATGGTGGACATCCTTAGACAGAACCCAACAGAAGAGC<br>AAGCTGTGGATATATGCAAAGCAGCAATGGGTTTAAGAATCAGTTCATCCTTCAGCTTTGGAGGTTTCACTTTTAAAAGAACAAGTGGGTCATCTGTCAAAAGAGAAGAAGAAGTGCTCACGGGCAACCTCCAAACACTGAAAATAAGAGTACATGAAGGATATGAAGAATTCACAATGGTTGGGCGAAGAGCTACGGCCATTCTAAGGAAAGCAACCAGAAGGCTGAC<br>CCAATTAATAGTGAGTGGGAGAGACGAGCAGTCAATCGCTGAAGCGATTATAGTGGCAATGGTTTTCTCACAAGAGGACTGCATGATAAAAGCAGTGCGAGGTGATTTGAACTTTGTTAACAGAGCAAATCAGCGGCTAAATCCTATGCACCAACTTCTGAGACATTTCCAAAAGGATGCAAAGGTGCTGTTTCAAAATTGGGGGACTGAACCCATTGACAATGTCATGG<br>GAATGATCGGGATACTGCCTGACATGACCCCTAGCACAGAGATGTCACTGAGAGGAGTGAGAGTCAGTAAAATGGGAGTGGATGAATATTCCAGTACTGAGAGAGTGGTCGTAAGCATTGATCGTTTCTTAAGAGTCCGAGACCAGAGGGGAAACGTACTCTTGTCTCCTGAAGAAGTTAGTGAAACACAGGGAACAGAGAAACTGACGATAACATATTCATCGTCTAT<br>GATGTGGGAAATCAATGGTCCGGAGTCAGTACTGGTCAACACATATCAATGGATCATTAGAAATTGGGAAACTGTGAAGATTCAGTGGTCCCAAGACCCCACAATGTTGTACAATAAAATGGAGTTTGAGCCTTTCCAATCCTTGGTGCCCAAGGCTGCCAGAGGCCAGTACAGTGGATTTGTAAGGACGTTATTCCAGCAGATGCGTGATGTGCTGGGGACATTTGACAC<br>TGTCCAAATAATAAAGCTCCTACCATTTGCATCAGCCCCACCGGAACAGAGTAGGATGCAGTTCTCTTCTCTGACTGTGAATGTAAGAGGTTCAGGAATGAGAATACTTGTGAGAGGTAACTCCCCTGTGTTCAACTATAACAAAGCAACCAAGAGACTCACAGTTCTTGGAAAGGATGCAGGTGCATTGACAGAAGATCCAGACGAGGGAACAGCAGGAGTGGAGTCTG<br>CGGTGTTAAGAGGATTTCTAATTCTGGGCAAAGAAGACAAAAGGTATGGACCAACATTGAGCATTAACGAATTGAGCAATCTTGCGAAAGGGGAGAAGGCTAATGTGTTGATAGGGCAAGGAGACGTGGTGTTGGTAATGAAACGGAAACGGGACTCTAGCATACTTACTGACAGCCAGACAGCGACCAAAAGGATTCGGATGGCCATCAATTA
internal NVRI repository	>A/turkey/Poland/H32-T/2022_H5N1_2022-01-09<br>ATGGAGAGAATAAAAGAGCTAAGAGATTTGATGTCGCAGTCTCGCACTCGCGAGATACTGACAAAAACCACCGTGGACCATATGGCCATAATCAAGAAATATACATCAGGAAGACAGGAGAAGAACCCTGCACTTAGGATGAAGTGGATGATGGCAATGAAATATCCGATTACAGCAGACAAAAGGATAATGGAGATGATCCCTGAAAGAAACGAGCAAGGTCAGACT<br>CTTTGGAGCAAAACAAATGATGCTGGATCGGATAGAGTAATGGTGTCACCTCTGGCTGTGACGTGGTGGAATAGAAATGGACCAACAACAAGTACCGTCCATTACCCAAAGGTCTATAAAACCTACTTTGAAAAGGTTGAAAGGTTAAAGCATGGAACCTTCGGCCCTGTCCATTTCCGGAATCAGGTTAAGATACGCCGCAGAGTTGACATAAACCCGGGCCATGCAGA<br>CCTCAGTGCTAAAGAAGCACAAGACGTCATCATGGAGGTCGTTTTCCCAAATGAAGTCGGAGCCAGAATATTGACATCAGAGTCACAGTTAACAATTACAAAAGAAAAGAAGGAGGAACTCCAGGACTGTAAGATTGCCCCTCTAATGGTGGCATACATGTTGGAGAGAGAACTGGTTCGAAAAACCAGATTCCTGCCAGTAGCTGGCGGAACAAGTAGCGTATATATCG<br>AAGTGTTGCACTTGACTCAAGGAACCTGCTGGGAACAAATGTATACGCCAGGAGGAGAAGTGAGAAATGATGACGTTGACCAAAGTTTAATTATTGCTGCCAGAAATATCGTTAGGAGGGCAACAGTATCAGCAGACCCATTGGCTTCACTACTGGAGATGTGCCATAGTACACAGATTGGCGGGATAAGAATGGTAGACATTCTTAGACAGAACCCAACAGAAGAGCA<br>AGCCGTGGATATATGCAAAGCAGCAATGGGTTTAAGAATCAGTTCATCCTTCAGTTTTGGAGGTTTCACTTTCAAAAGGACAAGCGGATCATCTGTCAAAAGAGAAGAGGAAGTGCTCACCGGCAACCTCCAAACATTGAAAATAAGAGTACATGAAGGGTATGAGGAATTCACAATGGTTGGGCGGAGAGCAACAGCCATTCTAAGGAAAGCAACCAGAAGGCTGATC<br>CAATTGATAGTAAGTGGAAGAGACGAGCAGTCAATCGCCGAAGCGATCATAGTGGCAATGGTGTTCTCTCAAGAGGATTGCATGATAAAGGCTGTACGAGGTGATTTAAATTTTGTCAATAGAGCGAATCAGCGGCTCAATCCTATGCATCAGCTCCTGAGGCATTTCCAAAAGGATGCAAAGGTACTATTCCAAAACTGGGGAATTGAACCCATTGACAATGTCATGGGA<br>ATGATAGGAATATTGCCTGATATGACCCCCAGCACAGAGATGTCACTAAGAGGAGTGAGGGTCAGTAAAATGGGAGTGGATGAATATTCCAGTACTGAGAGGGTGGTCGTGAGTATTGATCGCTTCTTGAGGGTACGAGACCAGAGAGGAAATGTACTCTTGTCTCCCGAAGAGGTCAGTGAAACACAGGGAACAGAGAAGCTAACGATAACATATTCATCATCCATGA<br>TGTGGGAAATTAATGGCCCTGAGTCAGTGCTAGTTAACACATATCAATGGATCATCAGAAACTGGGAAACTGTGAAGATTCAGTGGTCCCAAGACCCTACAATGCTATACAACAAGATGGAGTTTGAGCCTTTTCAGTCCTTGGTGCCTAAGGCAGCCAGAGGCCAGTACAGTGGATTTGTAAGGACCCTATTCCAGCAGATGCGTGATGTGCTGGGAACCTTTGACACTGT<br>CCAGATAATAAAGCTACTTCCATTTGCAGCAGCACCACCGGAACAGAGTAGGATGCAGTTCTCTTCTCTAACTGTAAACGTAAGGGGTTCAGGAATGAGAATACTTGTGAGAGGCAACTCCCCTGTGTTCAACTATAATAAAGCAACCAAGAGGCTCATAGTCCTTGGAAAGGATGCTGGTGCATTGACAGAAGACCCAGGTGAGGGGACAGCAGGAGTGGAGTCTGCG<br>GTATTGAGAGGGTTCCTAATTCTGGGCAAAGAGGACAAAAGATATGGACCAGCGCTGAGCATCAATGAATTGAGCAATCTTGCGAAAGGGGAGAAGGCTAATGTGTTGATAGGGCAAGGAGACGTGGTGTTGGTGATGAAACGGAAACGGGACTCTAGCATACTTACTGACAGCCAGACAGCGACCAAAAGAATTCGGATGGCCATCAATTA
internal NVRI repository	>A/turkey/Poland/H35-T/2022_H5N1_2022-01-09<br>ATGGAGAGAATAAAAGAGCTAAGAGATTTGATGTCGCAGTCTCGCACTCGCGAGATACTGACAAAAACCACCGTGGACCATATGGCCATAATCAAGAAATATACATCAGGAAGACAGGAGAAGAACCCTGCACTTAGGATGAAGTGGATGATGGCAATGAAATATCCGATTRCAGCAGACAAAAGGATAATGGAGATGATCCCTGAAAGAAACGAGCAAGGTCAGACT<br>CTTTGGAGCAAAACAAATGATGCTGGATCGGATAGAGTAATGGTGTCACCTCTGGCTGTGACGTGGTGGAATAGAAATGGACCAACAACAAGTACCGTCCATTACCCAAAGGTCTATAAGACCTACTTTGAAAAGGTTGAAAGGTTAAAGCATGGAACCTTCGGCCCTGTCCATTTCCGGAATCAGGTTAAGATACGCCGCAGAGTTGACATAAACCCGGGCCATGCAGA<br>CCTCAGTGCTAAAGAAGCACAGGACGTCATCATGGAGGTCGTTTTCCCAAATGAAGTCGGAGCCAGAATATTGACATCAGAGTCACAGTTGACAATTACAAAAGAAAAGAAGGAGGAACTCCAGGACTGTAAGATTGCCCCTCTAATGGTGGCATACATGTTGGAGAGAGAACTGGTTCGAAAAACCAGATTCCTGCCAGTAGCTGGCGGAACAAGTAGCGTATATATC<br>GAAGTGTTGCACTTGACTCAAGGAACCTGCTGGGAACAAATGTATACGCCAGGAGGAGAAGTGAGAAATGATGACGTTGACCAAAGTTTAATTATTGCTGCCAGAAATATCGTTAGGAGGGCAACAGTATCAGCAGACCCATTGGCTTCACTACTGGAGATGTGCCATAGTACACAGATTGGCGGGATAAGAATGGTAGACATTCTTAGACAGAACCCAACAGAAGAGC<br>AAGCCGTGGATATATGCAAAGCAGCAATGGGTTTAAGAATCAGTTCATCCTTCAGTTTTGGAGGTTTCACTTTCAAAAGGACAAGCGGATCATCTGTCAAAAGAGAAGAGGAAGTGCTCACCGGCAACCTCCAAACATTGAAAATAAGAGTACATGAAGGGTATGAGGAATTCACAATGGTTGGGCGGAGAGCAACAGCCATTCTAAGGAAAGCAACCAGAAGGCTGAT<br>CCAATTGATAGTAAGTGGAAGAGACGAGCAGTCAATCGCCGAAGCGATCATAGTGGCAATGGTGTTCTCTCAAGAGGATTGCATGATAAAGGCTGTACGAGGTGATTTAAATTTTGTCAATAGAGCGAATCAGCGGCTCAATCCTATGCATCAGCTCCTGAGGCATTTCCAAAAGGATGCAAAGGTACTATTCCAAAACTGGGGAATTGAACCCATTGACAATGTCATGGG<br>AATGATAGGAATATTGCCTGATATGACCCCCAGCACAGAGATGTCACTAAGAGGAGTGAGGGTCAGTAAAATGGGAGTGGATGAATATTCCAGTACTGAGAGGGTGGTCGTGAGTATTGATCGCTTCTTGAGGGTACGAGACCAGAGAGGAAATGTACTCTTGTCTCCCGAAGAGGTCAGTGAAACACAGGGAACAGAGAAGCTAACGATAACATATTCATCATCCATG<br>ATGTGGGAAATCAATGGCCCTGAGTCAGTGCTAGTTAACACATATCAATGGATCATCAGAAACTGGGAAACTGTGAAGATTCAGTGGTCCCAAGACCCTACAATGCTATACAACAAGATGGAGTTTGAGCCTTTTCAGTCCTTGGTGCCTAAGGCAGCCAGAGGCCAGTACAGTGGATTTGTAAGGACCCTATTCCAGCAGATGCGTGATGTGCTGGGAACCTTTGACACT<br>GTCCAGATAATAAAGCTACTTCCATTTGCAGCAGCACCACCGGAACAGAGTAGGATGCAGTTCTCTTCTCTAACTGTAAACGTAAGGGGTTCAGGAATGAGAATACTTGTGAGAGGCAACTCCCCTGTGTTCAACTATAATAAAGCAACCAAGAGGCTCATAGTCCTTGGCAAGGATGCTGGTGCATTGACAGAAGACCCAGGTGAGGGGACAGCAGGAGTGGAGTCTGC<br>GGTATTGAGAGGGTTCCTAATTCTGGGCAAAGAGGACAAAAGATATGGACCAGCGCTGAGCATCAATGAATTGAGCAATCTTGCGAAAGGAGAGAAGGCTAATGTGTTGATAGGGCAAGGAGACGTGGTGTTGGTGATGAAACGGAAACGGGACTCTAGCATACTTACTGACAGCCAGACAGCGACCAAAAGAATTCGGATGGCCATCAATTA
EPI_ISL_18245817	>A/sandwich_stern/Poland/MB142/2022_H5N1_2022-06-15<br>ATGGAGAGAATAAAAGAACTAAGAGATTTAATGTCACAGTCTCGCACTCGCGAGATACTGACAAAAACCACTGTGGACCATATGGCCATAATAAAGAAATACACATCAGGAAGACAGGAGAAGAACCCTGCCCTCAGGATGAAGTGGATGATGGCAATGAAATATCCTATTACAGCAGACAAAAGAATAATGGAGATGATCCCTGAAAGGAATGAGCAAGGTCAGACT<br>CTCTGGAGCAAAACAAATGACGCTGGATCAGACAGAGTGATGGTGTCACCTCTGGCTGTGACATGGTGGAATAGAAATGGGCCAACGACAAGTACAGTCCATTACCCAAAGGTTTACAAAACCTACTTTGAAAAAGTGGAAAGATTGAAACATGGAACCTTTGGCCCTGTTCACTTTCGAAATCAGGTTAAGATACGCCGCAGGGTTGACATAAACCCGGGTCATGCAGA<br>TCTCAGTGCCAAAGAAGCACAGGATGTCATCATGGAAGTCGTTTTCCCAAATGAAGTTGGAGCCAGGATATTGACATCAGAATCACAATTAACAATAACAAAGGAAAAGAAGGAGGAACTTCAGGACTGTAAGATTGCTCCTTTGATGGTGGCATACATGTTGGAGAGAGAACTGGTTCGAAAAACCAGATTTTTACCAGTAGCTGGCGGAACAAGCAGCGTATATATCG<br>AAGTATTGCATTTGACTCAAGGGACCTGCTGGGAACAAATGTACACACCGGGAGGGGAAGTGAGAAATGATGATGTTGATCAGAGTTTGATCATTGCTGCTAGAAATATCGTTAGGAGAGCAACAGTATCAGCAGACCCATTGGCTTCGCTCTTGGAAATGTGCCACAGCACACAAATTGGCGGGATAAGGATGGTAGACATTCTTAGACAGAACCCAACAGAAGAGCA<br>AGCCGTGGATATATGCAAAGCAGCAATGGGTTTAAGAATCAGTTCATCCTTCAGCTTTGGAGGTTTCACTTTCAAAAGAACAAGTGGATCGTCTGTCAAAAGAGAAGAAGAAGTGCTCACAGGCAACCTCCAAACACTGAAAATAAGAGTACATGAAGGATATGAGGAATTCACAATGGTTGGACGGAGAGCTACAGCCATTTTAAGGAAAGCAACCAGAAGGCTGATCC<br>AATTAATAGTGAGTGGAAGAGACGAGCAGTCAATCGCTGAAGCAATCATAGTGGCAATGGTTTTCTCACAAGAGGACTGCATGATAAAAGCAGTACGAGGTGATCTGAACTTTGTTAATAGAGCAAATCAGCGACTAAATCCTATGCATCAACTTCTGAGGCATTTCCAAAAGGATGCAAAGGTGCTGTTTCAAAACTGGGGGGTTGAACCAATTGACAATGTAATGGGG<br>ATGATTGGAATACTGCCTGACATGACCCCCAGCACGGAGATGTCACTGAGAGGAGTGAGAGTTAGCAAAATGGGAGTGGATGAATATTCCAGTACTGAGAGAGTGGTCGTGAGCATTGATCGTTTCTTGAGAGTCCGAGATCAAAGAGGAAACGTGCTCCTGTCTCCGGAGGAAGTTAGTGAAACACAGGGAACAGAGAAACTGACGATAACATATTCATCGTCCATGA<br>TGTGGGAAATCAATGGTCCGGAATCCGTGCTAGTCAACACATATCAATGGATCATCAGAAATTGGGAGACTGTGAAGATTCAATGGTCCCAAGACCCTACGATGTTGTACAATAAGATGGAATTTGAGCCCTTCCAATCCTTGGTGCCCAAGGCTGCCAGAGGCCAGTATAGTGGATTTGTGAGGACATTGTTCCAGCAGATGCGTGATGTGCTGGGAACATTTGATACTGT<br>CCAAATAATAAAGCTCCTGCCTTTTGCTGCAGCCCCACCGGAACAGAGTAGGATGCAGTTTTCCTCTCTGACTGTGAACGTAAGAGGTTCAGGAATGAGAATACTTGTGAGAGGCAACTCCCCTGTGTTCAACTATAACAAGGCCACCAAGAGACTCACAGTTCTTGGAAAGGATGCAGGTGCCTTGACAGAAGATCCAGATGAGGGAACAGCAGGAGTAGAGTCTGCAG<br>TATTAAGAGGATTTCTAATTCTGGGCAAAGAGGACAAAAGATATGGACCAGCATTGAGCATCAACGAACTGAGCAATCTTGCGAAAGGGGAAAAGGCTAATGTGTTGATAGGGCAAGGAGACGTGGTGTTGGTAATGAAACGGAAACGGGACTCTAGCATACTTACTGACAGCCAGACAGCGACCAAAAGAATTCGGATGGCCATCAATTA
internal NVRI repository	>A/laying_hen/Poland/H205/2022_H5N1_2022-03-08<br>ATGGAGAGAATAAAAGAACTAAGAGATTTGATGTCGCAGTCTCGCACTCGCGAGATACTGACAAAAACCACGGTGGACCATATGGCCATAATTAAGAAATACACATCAGGAAGACAGGAGAAGAACCCTGCCCTTAGGATGAAGTGGATGATGGCAATGAAATACCCGATTACAGCAGACAAAAGAATAATGGAAATGATCCCTGAAAGAAATGAACAAGGCCAGACT<br>CTCTGGAGCAAAACAAATGATGCTGGATCAGACAGAGTAATGGTGTCACCTCTGGCTGTGACATGGTGGAACAGAAATGGACCGACGACAAGTACAGTCCACTATCCAAAGGTCTACAAAACCTACTTTGGAAAAGTTGAAAGGTTGAAACATGGAACCTTCGGTCCTGTTCACTTTCGGAATCAGGTTAAAATACGCCGCAGGGTTGACATAAACCCGGGCCATGCAGA<br>TCTCAGTGCCAAAGAAGCACAAGATGTCATCATGGAGGTCGTTTTCCCAAACGAAGTTGGAGCAAGGACATTGACATCAGAGTCACAATTAACAGTAACAAAGGAAAAGAAGGAGGAGCTTCAGGACTGCAAGATCGCCCCTTTAATGGTGGCATACATGTTGGAGAGAGAACTGGTTCGCAAAACCAGATTCCTACCAGTAGCTGGCGGAACAAGCAGCGTATATATC<br>GAGGTGTTGCATTTGACTCAAGGRACCTGCTGGGAACAAATGTACACACCGGGAGGGGAGGTGAGAAATGATGATGTCGATCAGAGTTTGATTATTGCTGCTAGAAATATTGTTAGGAGAGCAACAGTGTCGGCAGACCCGTTGGCTTCGCTCTTGGAGATGTGCCATAGTACACAAATTGGCGGGATAAGAATGGTGGACATCCTTAGACAGAACCCAACAGAAGAGC<br>AAGCTGTGGATATATGCAAAGCAGCAATGGGTTTAAGAATCAGTTCATCCTTCAGCTTTGGAGGTTTCACTTTTAAAAGAACAAGTGGGTCATCTGTCAAAAGAGAAGAAGAAGTGCTCACGGGCAACCTCCAAACACTGAAAATAAGAGTACATGAAGGATATGAAGAATTCACAATGGTTGGGCGAAGAGCTACGGCCATTCTAAGGAAAGCAACCAGAAGGCTGAC<br>CCAATTAATAGTGAGTGGGAGAGACGAGCAGTCAATCGCTGAAGCGATTATAGTGGCAATGGTTTTCTCACAAGAGGACTGCATGATAAAAGCAGTGCGAGGTGATTTGAACTTTGTTAACAGAGCAAATCAGCGGCTAAATCCTATGCACCAACTTCTGAGACATTTCCAAAAGGATGCAAAGGTGCTGTTTCAAAATTGGGGGACTGAACCCATTGACAATGTCATGG<br>GAATGATCGGGATACTGCCTGACATGACCCCTAGCACAGAGATGTCACTGAGAGGAGTGAGAGTCAGTAAAATGGGAGTGGATGAATATTCCAGTACTGAGAGAGTGGTCGTAAGCATTGATCGTTTCTTAAGAGTCCGAGACCAGAGGGGAAACGTACTCTTGTCTCCTGAAGAAGTTAGTGAAACACAGGGAACAGAGAAACTGACGATAACATATTCATCGTCTAT<br>GATGTGGGAAATCAATGGTCCGGAGTCAGTACTGGTCAACACATATCAATGGATCATTAGAAATTGGGAAACTGTGAAGATTCAGTGGTCCCAAGACCCCACAATGTTGTACAATAAAATGGAGTTTGAGCCTTTCCAATCCTTGGTGCCCAAGGCTGCCAGAGGCCAGTACAGTGGATTTGTAAGGACGTTATTCCAGCAGATGCGTGATGTGCTGGGGACATTTGACAC<br>TGTCCAAATAATAAAGCTCCTACCATTTGCAGCAGCCCCACCGGAACAGAGTAGGATGCAGTTCTCTTCTCTGACTGTGAATGTAAGAGGTTCAGGAATGAGAATACTTGTGAGAGGTAACTCCCCTGTGTTCAACTATAACAAAGCAACCAAGAGACTCACAGTTCTTGGAAAGGATGCAGGTGCATTGACAGAAGATCCAGACGAGGGAACAGCAGGAGTGGAGTCTG<br>CGGTGTTAAGAGGATTTCTAATTCTGGGCAAAGAAGACAAAAGGTATGGACCAGCATTGAGCATTAACGAATTGAGCAATCTTGCGAAAGGGGAGAAGGCTAATGTGTTGATAGGGCAAGGAGACGTGGTGTTGGTAATGAAACGGAAACGGGACTCTAGCATACTTACTGACAGCCAGACAGCGACCAAAAGGATTCGGATGGCCATCAATTA
internal NVRI repository	>A/laying_hen/Poland/H2157-N/2021_H5N1_2021-12-27<br>ATGGAGAGAATAAAAGAGCTAAGAGATTTGATGTCGCAGTCTCGCACTCGCGAGATACTGACAAAAACCACCGTGGACCATATGGCCATAATCAAGAAATATACATCAGGAAGACAGGAGAAGAACCCTGCACTTAGGATGAAGTGGATGATGGCAATGAAATATCCGATTACAGCAGACAAAAGGATAATGGAGATGATCCCTGAAAGAAACGAGCAAGGTCAGACT<br>CTTTGGAGCAAAACAAATGATGCTGGATCGGATAGAGTAATGGTGTCACCTCTGGCTGTGACGTGGTGGAATAGAAATGGACCAACAACAAGTACCGTCCATTACCCAAAGGTCTATAAAACCTACTTTGAAAAGGTTGAAAGGTTAAAGCATGGAACCTTCGGCCCTGTCCATTTCCGGAATCAGGTTAAGATACGCCGCAGAGTTGACATAAACCCGGGCAATGCAGA<br>CCTCAGTGCTAAAGAAGCACAAGACGTCATCATGGAGGTCGTTTTCCCAAATGAAGTCGGAGCCAGAATATTGACATCAGAGTCACAGTTGACAATTACAAAAGAAAAGAAAGAGGAACTCCAGGACTGTAAGATTGCTCCTCTAATGGTGGCATACATGTTGGAGAGAGAACTGGTTCGAAAAACCAGATTCCTGCCAGTAGCTGGCGGAACAAGTAGCGTATATATCG<br>AAGTGTTGCACTTGACTCAAGGAACCTGCTGGGAACAAATGTATACGCCAGGAGGAGAAGTGAGAAATGATGACGTTGACCAAAGTTTAATTATTGCTGCCAGAAATATCGTTAGGAGGGCAACAGTATCAGCAGACCCATTGGCTTCACTACTGGAGATGTGCCATAGTACACAGATTGGCGGGATAAGAATGGTAGACATTCTTAGACAGAACCCAACAGAAGAGCA<br>AGCCGTGGATATATGCAAAGCAGCAATGGGTTTAAGAATCAGTTCATCCTTCAGTTTCGGAGGTTTCACTTTCAAAAGGACAAGCGGATCATCTGTCAAAAGAGAAGAGGAAGTGCTCACCGGCAACCTCCAAACATTGAAAATAAGAGTACATGAAGGGTATGAGGAATTCACAATGGTTGGGCGGAGAGCAACAGCCATTCTAAGGAAAGCAACCAGAAGGCTGATC<br>CAATTGATAGTAAGTGGAAGAGACGAGCAGTCAATCGCCGAAGCGATCATAGTGGCAATGGTGTTCTCTCAAGAGGATTGCATGATAAAGGCTGTACGAGGTGATTTAAATTTTGTCAATAGAGCGAATCAGCGGCTCAATCCTATGCATCAGCTCCTGAGGCATTTCCAAAAGGATGCAAAGGTACTATTCCAAAACTGGGGAATTGAACCCATTGACAATGTCATGGGA<br>ATGATAGGAATATTGCCTGATATGACCCCCAGCACAGAGATGTCACTAAGAGGAGTGAGGGTCAGTAAAATGGGAGTGGATGAATATTCCAGTACTGAGAGGGTGGTCGTGAGTATTGATCGCTTCTTGAGGGTACGAGACCAGAGAGGAAATGTACTCTTGTCTCCCGAAGAGGTCAGTGAAACACAGGGAACAGAGAAGCTAACGATAACATATTCATCATCCATGA<br>TGTGGGAAATTAATGGCCCTGAGTCAGTGCTAGTTAACACATATCAATGGATCATCAGAAACTGGGAAACTGTGAAGATTCAGTGGTCCCAAGACCCTACAATGCTATACAACAAGATGGAGTTTGAGCCTTTTCAGTCCTTGGTGCCTAAGGCAGCCAGAGGCCAGTACAGTGGATTTGTAAGGACCCTATTCCAGCAGATGCGTGATGTGCTGGGAACCTTTGACACTGT<br>CCAGATAATAAAGCTACTTCCATTTGCAGCAGCACCACCGGAACAGAGTAGGATGCAGTTCTCTTCTCTAACTGTAAACGTAAGGGGTTCAGGAATGAGAATACTTGTGAGAGGCAACTCCCCTGTGTTCAACTATAATAAAGCAACCAAGAGGCTCATAGTCCTTGGAAAGGATGCTGGTGCATTGACAGAAGACCCAGGTGAGGGGGCAGCAGGAGTGGAGTCTGCG<br>GTATTGAGAGGGTTCCTAATTCTGGGCAAAGAGGACAAAAGATATGGACCAGCGCTGAGCATCAATGAATTGAGCAATCTTGCGAAAGGGGAGAAGGCTAATGTGTTGATAGGGCAAGGAGACGTGGTGTTGGTGATGAAACGGAAACGGGACTCTAGCATACTTACTGACAGCCAGACAGCGACCAAAAGAATTCGGATGGCCATCAATTA
internal NVRI repository	>A/laying_hen/Poland/H2159-T3/2021_H5N1_2021-12-27<br>ATGGAGAGAATAAAAGAACTAAGAGATTTGATGTCGCAGTCTCGCACTCGCGAGATACTGACAAAAACCACGGTGGACCATATGGCCATAATTAAGAAATACACATCAGGAAGACAGGAGAAGAACCCTGCCCTTAGGATGAAGTGGATGATGGCAATGAAATACCCGATTACAGCAGACAAAAGAATAATGGAAATGATCCCTGAAAGAAATGAACAAGGCCAGACT<br>CTCTGGAGCAAAACAAATGATGCTGGATCAGACAGAGTAATGGTGTCACCTCTGGCTGTGACATGGTGGAACAGAAATGGACCGACGACAAGTACAGTCCACTATCCAAAGGTCTACAAAACCTACTTTGGAAAAGTTGAAAGGTTGAAACATGGAACCTTCGGTCCTGTTCACTTTCGGAATCAGGTTAAAATACGCCGCAGGGTTGACATAAACCCGGGCCATGCAGA<br>TCTCAGTGCCAAAGAAGCACAAGATGTCATCATGGAGGTCGTTTTCCCAAACGAAGTTGGAGCAAGGACATTGACATCAGAGTCACAATTAACAGTAACAAAGGAAAAGAAGGAGGAGCTTCAGGACTGCAAGATCGCCCCTTTAATGGTGGCATACATGTTGGAGAGAGAACTGGTTCGCAAAACCAGATTCCTACCAGTAGCTGGCGGAACAAGCAGCGTATATATC<br>GAGGTGTTGCATTTGACTCAAGGGACCTGCTGGGAACAAATGTACACACCGGGAGGGGAGGTGAGAAATGATGATGTCGATCAGAGTTTGATTATTGCTGCTAGAAATATTGTTAGGAGAGCAACAGTGTCGGCAGACCCGTTGGCTTCGCTCTTGGAGATGTGCCATAGTACACAAATTGGCGGGATAAGAATGGTGGACATCCTTAGACAGAACCCAACAGAAGAGC<br>AAGCTGTGGATATATGCAAAGCAGCAATGGGTTTAAGAATCAGTTCATCCTTCAGCTTTGGAGGTTTCACTTTTAAAAGAACAAGTGGGTCATCTGTCAAAAGAGAAGAAGAAGTGCTCACGGGCAACCTCCAAACACTGAAAATAAGAGTACATGAAGGATATGAAGAATTCACAATGGTTGGGCGAAGAGCTACGGCCATTCTAAGGAAAGCAACCAGAAGGCTGAC<br>CCAATTAATAGTGAGTGGGAGAGACGAGCAGTCAATCGCTGAAGCGATTATAGTGGCAATGGTTTTCTCACAAGAGGACTGCATGATAAAAGCAGTGCGAGGTGATTTGAACTTTGTTAACAGAGCAAATCAGCGGCTAAATCCTATGCACCAACTTCTGAGACATTTCCAAAAGGATGCAAAGGTGCTGTTTCAAAATTGGGGGACTGAACCCATTGACAATGTCATGG<br>GAATGATCGGGATACTGCCTGACATGACCCCTAGCACAGAGATGTCACTGAGAGGAGTGAGAGTCAGTAAAATGGGAGTGGATGAATATTCCAGTACTGAGAGAGTGGTCGTAAGCATTGATCGTTTCTTAAGAGTCCGAGACCAGAGGGGAAACGTACTCTTGTCTCCTGAAGAAGTTAGTGAAACACAGGGAACAGAGAAACTGACGATAACATATTCATCGTCTAT<br>GATGTGGGAAATCAATGGTCCGGAGTCAGTACTGGTCAACACATATCAATGGATCATTAGAAATTGGGAAACTGTGAAGATTCAGTGGTCCCAAGACCCCACAATGTTGTACAATAAAATGGAGTTTGAGCCTTTCCAATCCTTGGTGCCCAAGGCTGCCAGAGGCCAGTACAGTGGATTTGTAAGGACGTTATTCCAGCAGATGCGTGATGTGCTGGGGACATTTGACAC<br>TGTCCAAATAATAAAGCTCCTACCATTTGCAGCAGCCCCACCGGAACAGAGTAGGATGCAGTTCTCTTCTCTGACTGTGAATGTAAGAGGTTCAGGAATGAGAATACTTGTGAGAGGTAACTCCCCTGTGTTCAACTATAACAAAGCAACCAAGAGACTCACAGTTCTTGGAAAGGATGCAGGTGCATTGACAGAAGATCCAGACGAGGGAACAGCAGGAGTGGAGTCTG<br>CGGTGTTAAGAGGATTTCTAATTCTGGGCAAAGAAGACAAAAGGTATGGACCAGCATTGAGCATTAACGAATTGAGCAATCTTGCGAAAGGGGAGAAGGCTAATGTGTTGATAGGGCAAGGAGACGTGGTGTTGGTAATGAAACGGAAACGGGACTCTAGCATACTTACTGACAGCCAGACAGCGACCAAAAGGATTCGGATGGCCATCAATTA

internal NVRI repository	>A/laying_hen/Poland/H283/2022_H5N1_2022-04-12<br>ATGGAGAGAATAAAAGAACTAAGAGATTTGATGTCGCAGTCTCGCACTCGCGAGATACTGACAAAAACCACGGTGGACCATATGGCCATAATTAAGAAATACACATCAGGAAGACAGGAGAAGAACCCTGCCCTTAGGATGAAGTGGATGATGGCAATGAAATACCCGATTACAGCAGACAAAAGAATAATGGAAATGATCCCTGAAAGAAATGAACAAGGCCAGACT<br>CTCTGGAGCAAAACAAATGATGCTGGATCAGACAGAGTAATGGTGTCACCTCTGGCTGTGACATGGTGGAACAGAAATGGACCGACGACAAGTACAGTCCACTATCCAAAGGTCTACAAAACCTACTTTGGAAAAGTTGAAAGGTTGAAACATGGAACCTTCGGTCCTGTTCACTTTCGGAATCAGGTTAAAATACGCCGCAGGGTTGACATAAACCCGGGCCATGCAGA<br>TCTCAGTGCCAAAGAAGCACAAGATGTCATCATGGAGGTCGTTTTCCCAAACGAAGTTGGAGCAAGGACATTGACATCAGAGTCACAATTAACAGTAACAAAGGAAAAGAAGGAGGAGCTTCAGGACTGCAAGATCGCCCCTTTAATGGTGGCATACATGTTGGAGAGAGAACTGGTTCGCAAAACCAGATTCCTACCAGTAGCTGGCGGAACAAGCAGCGTATATATC<br>GAGGTGTTGCATTTGACTCAAGGGACCTGCTGGGAACAAATGTACACACCGGGAGGGGAGGTGAGAAATGATGATGTCGATCAGAGTTTGATTATTGCTGCTAGAAATATTGTTAGRAGAGCAACAGTGTCGGCAGACCCGTTGGCTTCGCTCTTGGAGATGTGCCATAGTACACAAATTGGCGGGATAAGAATGGTGGACATCCTTAGACAGAACCCAACAGAAGAGC<br>AAGCTGTGGATATATGCAAAGCAGCAATGGGTTTAAGAATCAGTTCATCCTTCAGCTTTGGAGGTTTCACTTTTAAAAGAACAAGTGGGTCATCTGTCAAAAGAGAAGAAGAAGTGCTCACGGGCAACCTCCAAACACTGAAAATAAGAGTACATGAAGGATATGAAGAATTCACAATGGTTGGGCGAAGAGCTACGGCCATTCTAAGGAAAGCAACCAGAAGGCTGAC<br>CCAATTAATAGTGAGTGGGAGAGACGAGCAGTCAATCGCTGAAGCGATTATAGTGGCAATGGTTTTCTCACAAGAGGACTGCATGATAAAAGCAGTGCGAGGTGATTTGAACTTTGTTAACAGAGCAAATCAGCGGCTAAATCCTATGCACCAACTTCTGAGACATTTCCAAAAGGATGCAAAGGTGCTGTTTCAAAATTGGGGGACTGAACCCATTGACAATGTCATGG<br>GAATGATCGGGATACTGCCTGACATGACCCCTAGCACAGAGATGTCACTGAGAGGAGTGAGAGTCAGTAAAATGGGAGTGGATGAATATTCCAGTACTGAGAGAGTGGTCGTAAGCATTGATCGTTTCTTAAGAGTCCGAGACCAGAGGGGAAACGTACTCTTGTCTCCTGAAGAAGTTAGTGAAACACAGGGAACAGAGAAACTGACGATAACATATTCATCGTCTAT<br>GATGTGGGAAATCAATGGTCCGGAGTCAGTACTGGTCAACACATATCAATGGATCATTAGAAATTGGGAAACTGTGAAGATTCAGTGGTCCCAAGACCCCACAATGTTGTACAATAAAATGGAGTTTGAGCCTTTCCAATCCTTGGTGCCCAAGGCTGCCAGAGGCCAGTACAGTGGATTTGTAAGGACGTTATTCCAGCAGATGCGTGATGTGCTGGGGACATTTGACAC<br>TGTCCAAATAATAAAGCTCCTACCATTTGCATCAGCCCCACCGGAACAGAGTAGGATGCAGTTCTCTTCTCTGACTGTGAATGTAAGAGGTTCAGGAATGAGAATACTTGTRAGAGGTAACTCCCCTGTGTTCAACTATAACAAAGCAACCAAGAGACTCACAGTTCTTGGAAAGGATGCAGGTGCATTGACAGAAGATCCAGACGAGGGAACAGCAGGAGTGGAGTCTG<br>CGGTGTTAAGAGGATTTCTAATTCTGGGCAAAGAAGACAAAAGGTATGGACCAACATTGAGCATTAACGAATTGAGCAATCTTGCGAAAGGGGAGAAGGCTAATGTGTTGATAGGGCAAGGAGACGTGGTGTTGGTAATGAAACGGAAACGGGACTCTAGCATACTTACTGACAGCCAGACAGCGACCAAAAGGATTCGGATGGCCATCAATTA
internal NVRI repository	>A/laying_hen/Poland/H290-T/2022_H5N1_2022-04-20<br>ATGGAGAGAATAAAAGAACTAAGAGATTTGATGTCGCAGTCTCGCACTCGCGAGATACTGACAAAAACCACGGTGGACCATATGGCCATAATTAAGAAATACACATCAGGAAGACAGGAGAAGAACCCTGCCCTTAGGATGAAGTGGATGATGGCAATGAAATACCCGATTACAGCAGACAAAAGAATAATGGAAATGATCCCTGAAAGAAATGAACAAGGCCAGACT<br>CTCTGGAGCAAAACAAATGATGCTGGATCAGACAGAGTAATGGTGTCACCTCTGGCTGTGACATGGTGGAACAGAAATGGACCGACGACAAGTACAGTCCACTATCCAAAGGTCTACAAAACCTACTTTGGAAAAGTTGAAAGGTTGAAACATGGAACCTTCGGTCCTGTTCACTTTCGGAATCAGGTTAAAATACGCCGCAGGGTTGACATAAACCCGGGCCATGCAGA<br>TCTCAGTGCCAAAGAAGCACAAGATGTCATCATGGAGGTCGTTTTCCCAAACGAAGTTGGAGCAAGGACATTGACATCAGAGTCACAATTAACAGTAACAAAGGAAAAGAAGGAGGAGCTTCAGGACTGCAAGATCGCCCCTTTAATGGTGGCATACATGTTGGAGAGAGAACTGGTTCGCAAAACCAGATTCCTACCAGTAGCTGGCGGAACAAGCAGCGTATATATC<br>GAGGTGTTGCATTTGACTCAAGGGACCTGCTGGGAACAAATGTACACACCGGGAGGGGAGGTGAGAAATGATGATGTCGATCAGAGTTTGATTATTGCTGCTAGAAATATTGTTAGGAGAGCAACAGTGTCGGCAGACCCGTTGGCTTCGCTCTTGGAGATGTGCCATAGTACACAAATTGGCGGGATAAGAATGGTGGACATCCTTAGACAGAACCCAACAGAAGAGC<br>AAGCTGTGGATATATGCAAAGCAGCAATGGGTTTAAGAATCAGTTCATCCTTCAGCTTTGGAGGTTTCACTTTTAAAAGAACAAGTGGGTCATCTGTCAAAAGAGAAGAAGAAGTGCTCACGGGCAACCTCCAAACACTGAAAATAAGAGTACATGAAGGATATGAAGAATTCACAATGGTTGGGCGAAGAGCTACGGCCATTCTAAGGAAAGCAACCAGAAGGCTGAC<br>CCAATTAATAGTGAGTGGGAGAGACGAGCAGTCAATCGCTGAAGCGATTATAGTGGCAATGGTTTTCTCACAAGAGGACTGCATGATAAAAGCAGTGCGAGGTGATTTGAACTTTGTTAACAGAGCAAATCAGCGGCTAAATCCTATGCACCAACTTCTGAGACATTTCCAAAAGGATGCAAAGGTGCTGTTTCAAAATTGGGGGACTGAACCCATTGACAATGTCATGG<br>GAATGATCGGGATACTGCCTGACATGACCCCTAGCACAGAGATGTCACTGAGAGGAGTGAGAGTCAGTAAAATGGGAGTGGATGAATATTCCAGTACTGAGAGAGTGGTCGTAAGCATTGATCGTTTCTTAAGAGTCCGAGACCAGAGGGGAAACGTACTCTTGTCTCCTGAAGAAGTTAGTGAAACACAGGGAACAGAGAAACTGACGATAACATATTCATCGTCTAT<br>GATGTGGGAAATCAATGGTCCGGAGTCAGTACTGGTCAACACATATCAATGGATCATTAGAAATTGGGAAACTGTGAAGATTCAGTGGTCCCAAGACCCCACAATGTTGTACAATAAAATGGAGTTTGAGCCTTTCCAATCCTTGGTGCCCAAGGCTGCCAGAGGCCAGTACAGTGGATTTGTAAGGACGTTATTCCAGCAGATGCGTGATGTGCTGGGGACATTTGACAC<br>TGTCCAAATAATAAAGCTCCTACCATTTGCATCAGCCCCACCGGAACAGAGTAGGATGCAGTTCTCTTCTCTGACTGTGAATGTAAGAGGTTCAGGAATGAGAATACTTGTGAGAGGTAACTCCCCTGTGTTCAACTATAACAAAGCAACCAAGAGACTCACAGTTCTTGGAAAGGATGCAGGTGCATTGACAGAAGATCCAGACGAGGGAACAGCAGGAGTGGAGTCTG<br>CGGTGTTAAGAGGATTTCTAATTCTGGGCAAAGAAGACAAAAGGTATGGACCAACATTGAGCATTAACGAATTGAGCAATCTTGCGAAAGGGGAGAAGGCTAATGTGTTGATAGGGCAAGGAGACGTGGTGTTGGTAATGAAACGGAAACGGGACTCTAGCATACTTACTGACAGCCAGACAGCGACCAAAAGGATTCGGATGGCCATCAATTA
internal NVRI repository	>A/laying_hen/Poland/H363/2022_H5N1_2022-07-20<br>ATGGAGAGAATAAAAGAACTAAGAGATTTAATGTCACAGTCTCGCACTCGCGAGATACTAACAAAAACCACTGTGGACCATATGGCCATAATAAAGAAATACACATCAGGAAGACAGGAGAAGAACCCTGCTCTTAGGATGAAATGGATGATGGCAATGAAGTACCCGATTACAGCAGACAAAAGAATAATGGAGATGATCCCTGAAAGAAATGAGCAAGGCCAAACT<br>CTCTGGAGCAAAACAAATGATGCTGGATCAGACAGAGTGATGGTGTCACCTCTGGCTGTGACATGGTGGAATAGAAATGGGCCAACGACAAGCACAGTCCATTATCCAAAGGTTTACAAAACCTACTTTGAAAAAGTCGAAAGGTTGAAACATGGGACCTTTGGTCCTGTTCACTTTCGAAATCAGGTCAAAATACGCCGCAGGGTCGACATAAACCCGGGCCATGCAGA<br>TCTCAGTGCTAAGGAAGCACAAGACGTCATCATGGAGGTCGTTTTCCCAAACGAAGTTGGAGCCAGGATATTGACATCAGAGTCACAATTAACAATAACAAAGGAAAAGAAGGAGGAGCTTCAAGACTGTAAGATTGCTCCTTTGATGGTGGCATACATGTTGGAGAGGGAACTGGTCCGCAAAACCAGATTCCTACCGGTAGCTGGCGGGACAAGCAGTGTGTATATC<br>GAGGTGCTGCATTTGACTCAAGGGACCTGCTGGGAACAAATGTACACACCGGGAGGAGAGGTGAGAAATGATGATGTCGATCAAAGTTTGATTATTGCTGCTAGAAATATTGTCAGGAGAGCAACAGTATCAGCAGACCCGTTGGCTTCACTCTTGGAGATGTGCCATAGTACACAAATTGGAGGAATAAGGATGGTTGACATTCTTAGACAAAACCCAACAGAAGAACA<br>AGCTGTAGATATATGCAAAGCAGCAATGGGTCTAAGAATCAGTTCATCCTTCAGCTTTGGAGGTTTCACTTTCAAAAGGACAAGTGGGTCATCTGTCAAGAAAGAAGAAGAAGTGCTCACAGGCAACCTCCAAACATTGAAAATAAGGGTGCACGAAGGATATGAAGAATTCACAATGGTTGGGCGAAGAGCGACCGCCATTTTAAGGAAAGCAACCAGAAGGTTGATA<br>CAATTAATAGTGAGTGGGAGGGACGAGCAGTCAATTGCTGAAGCAATCATAGTGGCAATGGTTTTCTCACAAGAAGACTGCATGATAAAGGCAGTACGAGGTGATTTGAATTTTGTCAACAGAGCAAACCAGCGGCTGAATCCTATGCATCAACTTCTAAGACATTTCCAGAAGGATGCAAAAGTGCTGTTTCAGAACTGGGGAATTGAACCCATTGACAATGTCATGGG<br>GATGATCGGAATACTGCCTGACATGACCCAAGCACAGATATGTCTCTGAGAGGAGTGAGGGTCAGTAAAATGGGAGTGGATGAATATTCCAGTACTGAGAGGGTTGTCGTGAGCATTGATCGTTTCTTAAGGGTCCGAGATCAAAGGGGAAATGTACTCCTGTCTCCCGAGGAGGTCAGTGAAACACAAGGAACAGAGAAGCTAACGATAACATATTCGTCGTCAATG<br>ATGTGGGAAATCAATGGTCCGGAATCAGTGCTGGTCAACACATATCAATGGATTATTAGAAATTGGGAAACTGTGAAAATTCAGTGGTCACAAGACCCTACAATGTTATACAATAAGATGGAGTTTGAGCCCTTCCAATCCTTGGTGCCTAAGGCTGCCAGAGGCCAGTATAGTGGATTCGTGAGGACGTTGTTCCAGCAGATGCGTGATGTATTGGGGACATTTGACACT<br>GTTCAAATAATAAAGCTCCTACCATTTGCAGCAGCCCCACCAGAACAGAGTAGGATGCAGTTCTCTTCTCTAACTGTGAATATAAGGGGTTCAGGGATGAGAATACTTATGAGAGGCAATTCCCCTGTGTTCAACTATAACAAGGCAACCAAGAGGCTCACAGTCCTTGGAAAAGATGCAGGTGCCTTAACAGAAGATCCAGACGAGGGAACAGCAGGAGTGGAATCTGC<br>GGTATTAAGAGGATTTCTAATTCTGGGCAAAGAAGATAAGAGATATGGCCCAGCGTTGAGCATCAACGAATTGAGCAATCTTGCGAAAGGGGAGAAAGCTAATGTGTTGATAGGGCAAGGAGACGTGGTGTTGGTAATGAAACGGAAACGGGACTCTAGCATACTTACTGACAGCCAGACAGCGACCAAAAGGATTCGGATGGCCATCAATTA
internal NVRI repository	>A/domestic_duck/Poland/01/2022_H5N1_2021-12-31<br>ATGGAGAGAATAAAAGAACTAAGAGATTTGATGTCGCAGTCTCGCACTCGCGAGATACTGACAAAAACCACTGTGGACCATATGGCCATAATAAAGAAATACACATCAGGAAGACAGGAGAAGAACCCTGCCCTCAGGATGAAGTGGATGATGGCAATGAAATATCCTATTACAGCAGACAAAAGAATAATGGAGATGATCCCTGAAAGGAATGAGCAAGGTCAGACT<br>CTCTGGAGCAAAACAAATGACGCTGGATCAGACAGAGTGATGGTGTCACCTCTGGCTGTGACATGGTGGAATAGAAATGGGCCAACGACAAGTACAGTCCATTACCCAAAGGTTTACAAAACCTACTTTGAAAAAGTGGAAAGATTGAAACATGGAACCTTTGGCCCTGTTCACTTTCGAAATCAGGTTAAGATACGCCGCAGGGTTGACATAAACCCGGGTCATGCAGA<br>TCTCAGTGCCAAAGAAGCACAGGATGTCATCATGGAAGTCGTTTTCCCAAATGAAGTTGGAGCCAGGATATTGACATCAGAATCACAATTAACAATAACAAAGGAAAAGAAGGAGGAACTTCAGGACTGTAAGATTGCTCCTTTGATGGTGGCATACATGTTGGAGAGAGAACTGGTTCGAAAAACCAGATTTTTACCAGTAGCTGGCGGAACAAGCAGCGTATATATCG<br>AAGTATTGCATTTGACTCAAGGGACCTGCTGGGAACAAATGTACACACCGGGAGGGGAAGTGAGAAATGATGATGTTGATCAGAGTTTGATCATTGCTGCTAGAAACATCGTTAGGAGAGCAACAGTATCAGCAGACCCATTGGCTTCGCTCTTGGAAATGTGCCACAGTACACAAATTGGCGGGATAAGGATGGTAGACATTCTTAGACAGAACCCAACAGAAGAGCA<br>AGCCGTGGATATATGCAAAGCAGCAATGGGTTTAAGAATCAGTTCATCCTTCAGCTTTGGAGGTTTCACTTTCAAAAGAACAAGTGGATCGTCTGTCAAAAGAGAAGAAGAAGTGCTCACAGGCAACCTCCAAACACTGAAAATAAGAGTACATGAAGGATATGAGGAATTCACAATGGTTGGACGGAGAGCTACAGCCATTTTAAGGAAAGCAACCAGAAGGCTGATCC<br>AATTAATAGTGAGTGGAAGAGACGAGCAGTCAATCGCTGAAGCAATCATAGTGGCAATGGTTTTCTCACAAGAGGACTGCATGATAAAAGCAGTACGAGGTGATCTGAACTTTGTTAATAGAGCAAATCAGCGACTAAATCCTATGCATCAACTTCTGAGGCATTTCCAAAAGGATGCAAAGGTGCTGTTTCAAAACTGGGGGGTTGAACCAATTGACAATGTAATGGGG<br>ATGATTGGAATACTGCCTGACATGACCCCCAGCACGGAGATGTCACTGAGAGGAGTGAGAGTCAGCAAAATGGGAGTGGATGAGTATTCCAGTACTGAGAGAGTGGTCGTGAGCATTGATCGTTTCTTGAGAGTCCGAGATCAAAGAGGAAACGTGCTCCTGTCTCCTGAGGAAGTTAGTGAAACACAGGGAACAGAGAAACTGACGATAACATATTCATCGTCCATGA<br>TGTGGGAAATCAATGGTCCGGAATCCGTGCTAGTCAACACATATCAATGGATCATCAGAAATTGGGAGACTGTGAAGATTCAATGGTCCCAAGACCCTACGATGTTGTACAACAAGATGGAATTTGAGCCCTTCCAATCCTTGGTGCCCAAGGCTGCCAGAGGCCAGTATAGTGGATTTGTGAGAACATTGTTCCAGCAGATGCGTGATGTGCTGGGAACATTTGATACTGT<br>CCAAATAATAAAGCTCCTGCCTTTTGCTGCAGCCCCACCGGAACAGAATAGGATGCAGTTTTCCTCTCTGACTGTGAACGTAAGAGGTTCAGGAATGAGAATACTTGTGAGAGGCAACTCCCCTGTGTTCAACTATAACAAGGCCACCAAGAGACTCACAGTTCTTGGAAAGGATGCAGGTGCCTTGACAGAAGATCCAGATGAGGGAACAGCAGGAGTAGAGTCTGCAG<br>TATTAAGAGGATTTCTAATTCTGGGCAAAGAGGACAAAAGATATGGACCAGCATTGAGCATCAACGAACTGAGCAATCTTGCGAAAGGGGAAAAGGCTAATGTGTTGATAGGGCAAGGAGACGTGGTGTTGGTAATGAAACGGAAACGGGACTCTAGCATACTTACTGACAGCCAGACAGCGACCAAAAGAATTCGGATGGCCATCAATTA
internal NVRI repository	>A/domestic_duck/Poland/2160/2021_H5N1_2021-12-27<br>ATGGAGAGAATAAAAGAACTAAGAGATTTGATGTCGCAGTCTCGCACTCGCGAGATACTGACAAAAACCACTGTGGACCATATGGCCATAATAAAGAAATACACATCAGGAAGACAGGAGAAGAACCCTGCCCTCAGGATGAAGTGGATGATGGCAATGAAATATCCTATTACAGCAGACAAAAGAATAATGGAGATGATCCCTGAAAGGAATGAGCAAGGTCAGACT<br>CTCTGGAGCAAAACAAATGACGCTGGATCAGACAGAGTGATGGTGTCACCTCTGGCTGTGACATGGTGGAATAGAAATGGGCCAACGACAAGTACAGTCCATTACCCAAAGGTTTACAAAACCTACTTTGAAAAAGTGGAAAGATTGAAACATGGAACCTTTGGCCCTGTTCACTTTCGAAATCAGGTTAAGATACGCCGCAGGGTTGACATAAACCCGGGTCATGCAGA<br>TCTCAGTGCCAAAGAAGCACAGGATGTCATCATGGAAGTCGTTTTCCCAAATGAAGTTGGAGCCAGGATATTGACATCAGAATCACAATTAACAATAACAAAGGAAAAGAAGGAGGAACTTCAGGACTGTAAGATTGCTCCTTTGATGGTGGCATACATGTTGGAGAGAGAACTGGTTCGAAAAACCAGATTTTTACCAGTAGCTGGCGGAACAAGCAGCGTATATATCG<br>AAGTATTGCATTTGACTCAAGGGACCTGCTGGGAACAAATGTACACACCGGGAGGGGAAGTGAGAAATGATGATGTTGATCAGAGTTTGATCATTGCTGCTAGAAACATCGTTAGGAGAGCAACAGTATCAGCAGACCCATTGGCTTCGCTCTTGGAAATGTGCCACAGTACACAAATTGGCGGGATAAGGATGGTAGACATTCTTAGACAGAACCCAACAGAAGAGCA<br>AGCCGTGGATATATGCAAAGCAGCAATGGGTTTAAGAATCAGTTCATCCTTCAGCTTTGGAGGTTTCACTTTCAAAAGAACAAGTGGATCGTCTGTCAAAAGAGAAGAAGAAGTGCTCACAGGCAACCTCCAAACACTGAAAATAAGAGTACATGAAGGATATGAGGAATTCACAATGGTTGGACGGAGAGCTACAGCCATTTTAAGGAAAGCAACCAGAAGGCTGATCC<br>AATTAATAGTGAGTGGAAGAGACGAGCAGTCAATCGCTGAAGCAATCATAGTGGCAATGGTTTTCTCACAAGAGGACTGCATGATAAAAGCAGTACGAGGTGATCTGAACTTTGTTAATAGAGCAAATCAGCGACTAAATCCTATGCATCAACTTCTGAGGCATTTCCAAAAGGATGCAAAGGTGCTGTTTCAAAACTGGGGGGTTGAACCAATTGACAATGTAATGGGG<br>ATGATTGGAATACTGCCTGACATGACCCCCAGCACGGAGATGTCACTGAGAGGAGTGAGAGTCAGCAAAATGGGAGTGGATGAATATTCCAGTACTGAGAGAGTGGTCGTGAGCATTGATCGTTTCTTGAGAGTCCGAGATCAAAGAGGAAACGTGCTCCTGTCTCCTGAGGAAGTTAGTGAAACACAGGGAACAGAGAAACTGACGATAACATATTCATCGTCCATGA<br>TGTGGGAAATCAATGGTCCGGAATCCGTGCTAGTCAACACATATCAATGGATCATCAGAAATTGGGAGACTGTGAAGATTCAATGGTCCCAAGACCCTACGATGTTGTACAACAAGATGGAATTTGAGCCCTTCCAATCCTTGGTGCCCAAGGCTGCCAGAGGCCAGTATAGTGGATTTGTGAGAACATTGTTCCAGCAGATGCGTGATGTGCTGGGAACATTTGATACTGT<br>CCAAATAATAAAGCTCCTGCCTTTTGCTGCAGCCCCACCGGAACAGAATAGGATGCAGTTTTCCTCTCTGACTGTGAACGTAAGAGGTTCAGGAATGAGAATACTTGTGAGAGGCAACTCCCCTGTGTTCAACTATAACAAGGCCACCAAGAGACTCACAGTTCTTGGAAAGGATGCAGGTGCCTTGACAGAAGATCCAGATGAGGGAACAGCAGGAGTAGAGTCTGCAG<br>TATTAAGAGGATTTCTAATTCTGGGCAAAGAGGACAAAAGATATGGACCAGCATTGAGCATCAACGAACTGAGCAATCTTGCGAAAGGGGAAAAGGCTAATGTGTTGATAGGGCAAGGAGACGTGGTGTTGGTAATGAAACGGAAACGGGACTCTAGCATACTTACTGACAGCCAGACAGCGACCAAAAGAATTCGGATGGCCATCAATTA
internal NVRI repository	>A/domestic_duck/Poland/2177/2021_H5N1_2021-12-29<br>ATGGAGAGAATAAAAGAACTAAGAGATTTGATGTCGCAGTCTCGCACTCGCGAGATACTGACAAAAACCACTGTGGACCATATGGCCATAATAAAGAAATACACATCAGGAAGACAGGAGAAGAACCCTGCCCTCAGGATGAAGTGGATGATGGCAATGAAATATCCTATTACAGCAGACAAAAGAATAATGGAGATGATCCCTGAAAGGAATGAGCAAGGTCAGACT<br>CTCTGGAGCAAAACAAATGACGCTGGATCAGACAGAGTGATGGTGTCACCTCTGGCTGTGACATGGTGGAATAGAAATGGGCCAACGACAAGTACAGTCCATTACCCAAAGGTTTACAAAACCTACTTTGAAAAAGTGGAAAGATTGAAACATGGAACCTTTGGCCCTGTTCACTTTCAAAATCAGGTTAAGATACGCCGCAGGGTTGACATAAACCCGGGTCATGCAGA<br>TCTCAGTGCCAAAGAAGCACAGGATGTCATCATGGAAGTCGTTTTCCCAAATGAAGTTGGAGCCAGGATATTGACATCAGAATCACAATTAACAATAACAAAGGAAAAGAAGGAGGAACTTCAGGACTGTAAGATTGCTCCTTTGATGGTGGCATACATGTTGGAGAGAGAACTGGTTCGAAAAACCAGATTTTTACCAGTAGCTGGCGGAACAAGCAGCGTATATATCG<br>AAGTATTGCATTTGACTCAAGGGACCTGCTGGGAACAAATGTACACACCGGGAGGGGAAGTGAGAAATGATGATGTTGATCAGAGTTTGATCATTGCTGCTAGAAACATCGTTAGGAGAGCAACAGTATCAGCAGACCCATTGGCTTCGCTCTTGGAAATGTGCCACAGTACACAAATTGGCGGGATAAGGATGGTAGACATTCTTAGACAGAACCCAACAGAAGAGCA<br>AGCCGTGGATATATGCAAAGCAGCAATGGGTTTAAGAATCAGTTCATCCTTCAGCTTTGGAGGTTTCACTTTCAAAAGAACAAGTGGATCGTCTGTCAAAAGAGAAGAAGAAGTGCTCACAGGCAACCTCCAAACACTGAAAATAAGAGTACATGAAGGATATGAGGAATTCACAATGGTTGGACGGAGAGCTACAGCCATTTTAAGGAAAGCAACCAGAAGGCTGATCC<br>AATTAATAGTGAGTGGAAGAGACGAGCAGTCAATCGCTGAAGCAATCATAGTGGCAATGGTTTTCTCACAAGAGGACTGCATGATAAAAGCAGTACGAGGTGATCTGAACTTTGTTAATAGAGCAAATCAGCGACTAAATCCTATGCATCAACTTCTGAGGCATTTCCAAAAGGATGCAAAGGTGCTGTTTCAAAACTGGGGGGTTGAACCAATTGACAATGTAATGGGG<br>ATGATTGGAATACTGCCTGACATGACCCCCAGCACGGAGATGTCACTGAGAGGAGTGAGAGTCAGCAAAATGGGAGTGGATGAATATTCCAGTACTGAGAGAGTGGTCGTGAGCATTGATCGTTTCTTGAGAGTCCGAGATCAAAGAGGAAACGTGCTCCTGTCTCCTGAGGAAGTTAGTGAAACACAGGGAACAGAAAAACTGACGATAACATATTCATCGTCCATGA<br>TGTGGGAAATCAATGGTCCGGAATCCGTGCTAGTCAACACATATCAATGGATCATCAGAAATTGGGAGACTGTGAAGATTCAATGGTCCCAAGACCCTACGATGTTGTACAACAAGATGGAATTTGAGCCCTTCCAATCCTTGGTGCCCAAGGCTGCCAGAGGCCAGTATAGTGGATTTGTGAGAACATTGTTCCAGCAGATGCGTGATGTGCTGGGAACATTTGATACTGT<br>CCAAATAATAAAGCTCCTGCCTTTTGCTGCAGCCCCACCGGAACAGAATAGGATGCAGTTTTCCTCTCTGACTGTGAACGTAAGAGGTTCAGGAATGAGAATACTTGTGAGAGGCAACTCCCCTGTGTTCAACTATAACAAGGCCACCAAGAGACTCACAGTTCTTGGAAAGGATGCAGGTGCCTTGACAGAAGATCCAGATGAGGGAACAGCAGGAGTAGAGTCTGCAG<br>TATTAAGAGGATTTCTAATTCTGGGCAAAGAGGACAAAAGATATGGACCAGCATTGAGCATCAACGAACTGAGCAATCTTGCGAAAGGGGAAAAGGCTAATGTGTTGATAGGGCAAGGAGACGTGGTGTTGGTAATGAAACGGAAACGGGACTCTAGCATACTTACTGACAGCCAGACAGCGACCAAAAGAATTCGGATGGCCATCAATTA
internal NVRI repository	>A/domestic_duck/Poland/H144/2022_H5N1_2022-02-13<br>ATGGAGAGAATAAAAGAGCTAAGAGATTTGATGTCGCAGTCTCGCACTCGCGAGATACTGACAAAAACCACCGTGGACCATATGGCCATAATCAAGAAATATACATCAGGAAGACAGGAGAAGAACCCTGCACTTAGGATGAAGTGGATGATGGCAATGAAATATCCGATTACAGCAGACAAAAGGATAATGGAGATGATCCCTGAAAGAAACGAGCAAGGTCAGACT<br>CTTTGGAGCAAAACAAATGATGCTGGATCGGATAGAATAATGGTGTCACCTCTGGCTGTGACGTGGTGGAATAGAAATGGACCAACAACAAGTACCGTCCATTACCCAAAGGTCTATAAAACCTACTTTGAAAAGGTTGAAAGGTTAAAGCATGGAACCTTCGGCCCTGTCCATTTCCGGAATCAGGTTAAGATACGCCGCAGAGTTGACATAAACCCGGGCCATGCAGAC<br>CTCAGTGCTAAAGAAGCACAAGACGTCATCATGGAGGTCGTTTTCCCAAATGAAGTCGGAGCCAGAATATTGACATCAGAGTCACAGTTAACAATTACAAAAGAAAAGAAGGAGGAACTCCAGGACTGTAAGATTGCCCCTCTAATGGTGGCATACATGTTGGAGAGAGAACTGGTTCGAAAAACCAGATTCCTGCCAGTAGCTGGCGGAACAAGTAGCGTATATATCGA<br>AGTGTTGCACTTGACTCAAGGAACCTGCTGGGAACAAATGTATACGCCAGGAGGAGAAGTGAGAAATGATGACGTTGACCAAAGTTTAATTATTGCTGCCAGAAATATCGTTAGGAGGGCAACAGTATCAGCAGACCCATTGGCTTCACTACTGGAGATGTGCCATAGTACACAGATTGGCGGGATAAGAATGGTAGACATTCTTAGACAGAACCCAACAGAAGAGCAA<br>GCCGTGGATATATGCAAAGCAGCAATGGGTTTAAGAATCAGTTCATCCTTCAGTTTTGGAGGTTTCACTTTCAAAAGGACAAGTGGATCATCTGTCAAAAGAGAAGAGGAAGTGCTCACAGGCAACCTCCAAACATTGAAAATAAGAGTACATGAAGGGTATGAGGAATTCACAATGGTTGGACGGAGAGCAACAGCCATTCTAAGGAAAGCAACCAGAAGGCTAATCC<br>AATTGATAGTAAGTGGAAGAGACGAGCAGTCAATCGCCGAAGCGATCATAGTGGCAATGGTGTTCTCTCAAGAGGATTGCATGATAAAGGCTGTACGAGGTGATTTAAATTTTGTCAATAGAGCGAATCAGCGGCTCAATCCTATGCATCAGCTCCTGAGGCATTTCCAAAAGGATGCAAAGGTACTATTCCAAAACTGGGGAATTGAACCCATTGACAATGTCATGGGAA<br>TGATAGGAATATTGCCTGATATGACCCCCAGCACAGAGATGTCACTAAGAGGAGTGAGGGTCAGTAAAATGGGAGTGGATGAATATTCCAGTACTGAGAGGGTGGTCGTGAGTATTGATCGCTTCTTGAGGGTACGAGACCAGAGAGGAAATGTACTCTTGTCTCCCGAAGAGGTCAGTGAAACACAGGGAACAGAGAAGCTAACGATAACATATTCATCATCCATGAT<br>GTGGGAAATTAATGGCCCTGAGTCAGTGCTAGTTAACACATATCAATGGATCATCAGAAACTGGGAAACTGTGAAGATTCAGTGGTCCCAAGACCCTACAATGCTATACAACAAGATGGAGTTTGAGCCTTTTCAGTCCTTGGTGCCTAAGGCAGCCAGAGGCCAGTACAGTGGATTTGTAAGGACCCTATTCCAGCAGATGCGTGATGTGCTGGGAACCTTTGACACTGT<br>CCAGATAATAAAGCTACTTCCATTTGCAGCAGCACCACCGGAACAGAGTAGGATGCAGTTCTCTTCTCTAACTGTAAACGTAAGGGGTTCAGGAATGAGAATACTTGTGAGAGGCAACTCCCCTGTGTTCAACTATAATAAAGCAACCAAGAGGCTCATAGTCCTTGGAAAGGATGCTGGTGCATTGACAGAAGACCCAGGTGAGGGGACAGCAGGAGTGGAGTCTGCG<br>GTATTGAGAGGGTTCCTAATTCTGGGCAAAGAGGACAAAAGATATGGACCAGCGCTGAGCATCAATGAATTGAGCAATCTTGCGAAAGGGGAGAAGGCTAATGTGTTGATAGGGCAAGGAGACGTGGTGTTGGTGATGAAACGGAAACGGGACTCTAGCATACTTACTGACAGCCAGACAGCGACCAAAAGAATTCGGATGGCCATCAATTA
internal NVRI repository	>A/domestic_duck/Poland/H146/2022_H5N1_2022-02-14<br>ATGGAGAGAATAAAAGAGCTAAGAGATTTGATGTCGCAGTCTCGCACYCGCGAGATACTGACAAAAACCACCGTGGACCATATGGCCATAATCAAGAAATATACATCAGGAAGACAGGAGAAGAACCCTGCACTTAGGATGAAGTGGATGATGGCAATGAAATATCCGATTACAGCAGACAAAAGGATAATGGAGATGATCCCTGAAAGAAACGAGCAAGGTCAGACT<br>CTTTGGAGCAAAACAAATGATGCTGGATCGGATAGAATAATGGTGTCACCTCTGGCTGTGACGTGGTGGAATAGAAATGGACCAACAACAAGTACCGTCCATTACCCAAAGGTCTATAAAACCTACTTTGAAAAGGTTGAAAGGTTAAAGCATGGAACCTTCGGCCCTGTYCATTTCCGGAATCAGGTTAAGATACGCCGCAGAGTTGACATAAACCCGGGCCATGCAGAC<br>CTCAGTGCTAAAGAAGCACAAGACATCATCATGGAGGTCGTTTTCCCAAATGAAGTCGGAGCCAGAATATTGACATCAGAGTCACAGTTAACAATTACAAAAGAAAAGAAGGAGGAACTCCAGGACTGTAAGATTGCCCCTCTAATGGTGGCATACATGTTGGAGAGAGAACTGGTTCGAAAAACCAGATTCCTGCCAGTAGCTGGCGGAACAAGTAGCGTATATATCGA<br>AGTGTTGCACTTGACTCAAGGAACCTGCTGGGAACAAATGTATACGCCAGGAGGAGAAGTGAGAAATGATGACGTTGACCAAAGTTTAATTATTGCTGCCAGAAATATCGTTAGGAGGGCAACAGTATCAGCAGACCCATTGGCTTCACTACTGGAGATGTGCCATAGTACACAGATTGGCGGGATAAGAATGGTAGACATTCTTAGACAGAACCCAACAGAAGAGCAA<br>GCCGTGGATATATGCAAAGCAGCAATGGGTTTAAGAATCAGTTCATCCTTCAGTTTTGGAGGTTTCACTTTCAAAAGGACAAGTGGATCATCTGTCAAAAGAGAAGAGGAAGTGCTCACAGGCAACCTCCAAACATTGAAAATAAGAGTACATGAAGGGTATGAGGAATTCACAATGGTTGGACGGAGAGCAACAGCCATTCTAAGGAAAGCAACCAGAAGGCTAATCC<br>AATTGATAGTAAGTGGAAGAGACGAGCAGTCAATCGCCGAAGCGATCATAGTGGCAATGGTGTTCTCTCAAGAGGATTGCATGATAAAGGCTGTACGAGGTGATTTAAATTTTGTCAATAGAGCGAATCAGCGGCTCAATCCTATGCATCAGCTCCTGAGGCATTTCCAAAAGGATGCAAAGGTACTATTCCAAAACTGGGGAATTGAACCCATTGACAATGTCATGGGAA<br>TGATAGGAATATTGCCTGATATGACCCCCAGCACAGAGATGTCACTAAGAGGAGTGAGGGTCAGTAAAATGGGAGTGGATGAATATTCCAGTACTGAGAGGGTGGTCGTGAGTATTGATCGCTTCTTGAGGGTACGAGACCAGAGAGGAAATGTACTCTTGTCTCCCGAAGAGGTCAGTGAAACACAGGGAACAGAGAAGCTAACGATAACATATTCATCATCCATGAT<br>GTGGGAAATTAATGGCCCTGAGTCAGTGCTAGTTAACACATATCAATGGATCATCAGAAACTGGGAAACTGTGAAGATTCAGTGGTCCCAAGACCCTACAATGCTATACAACAAGATGGAGTTTGAGCCTTTTCAGTCCTTGGTGCCTAAGGCAGCCAGAGGCCAGTACAGTGGATTTGTAAGGACCCTATTCCAGCAGATGCGTGATGTGCTGGGAACCTTTGACACTGT<br>CCAGATAATAAAGCTACTTCCATTTGCAGCAGCACCACCGGAACAGAGTAGGATGCAGTTCTCTTCTCTAACTGTAAACGTAAGGGGTTCAGGAATGAGAATACTTGTGAGAGGCAACTCCCCTGTGTTCAACTATAATAAAGCAACCAAGAGGCTCATAGTCCTTGGAAAGGATGCTGGTGCATTGACAGAAGACCCAGGTGAGGGGACAGCAGGAGTGGAGTCTGCG<br>GTATTGAGAGGGTTCCTAATTCTGGGCAAAGAGGACAAAAGATATGGACCAGCGCTGAGCATCAATGAATTGAGCAATCTTGCGAAAGGGGAGAAGGCTAATGTGTTGATAGGGCAAGGAGACGTGGTGTTGGTGATGAAACGGAAACGGGACTCTAGCATACTTACTGACAGCCAGACAGCGACCAAAAGAATTCGGATGGCCATCAATTA
internal NVRI repository	>A/domestic_duck/Poland/H158/2022_H5N1_2022-02-19<br>ATGGAGAGAATAAAAGAACTAAGAGATTTGATGTCGCAGTCTCGCACTCGCGAGATACTGACAAAAACCACTGTGGACCATATGGCCATAATAAAGAAATACACATCAGGAAGACAGGAGAAGAACCCTGCCCTCAGGATGAAGTGGATGATGGCAATGAAATATCCTATTACAGCAGACAAAAGAATAATGGAGATGATCCCTGAAAGGAATGAGCAAGGTCAGACT<br>CTCTGGAGCAAAACAAATGACGCTGGATCAGACAGAGTGATGGTGTCACCTCTGGCTGTGACATGGTGGAATAGAAATGGGCCAACGACAAGTACAGTCCATTACCCAAAGGTTTACAAAACCTACTTTGAAAAAGTGGAAAGATTGAAACATGGAACCTTTGGCCCTGTTCACTTTCAAAATCAGGTTAAGATACGCCGCAGGGTTGACATAAACCCGGGTCATGCAGA<br>TCTCAGTGCCAAAGAAGCACAGGATGTCATCATGGAAGTCGTTTTCCCAAATGAAGTTGGAGCCAGGATATTGACATCAGAATCACAATTAACAATAACAAAGGAAAAGAAGGAGGAACTTCAGGACTGTAAGATTGCTCCTTTGATGGTGGCATACATGTTGGAGAGAGAACTGGTTCGAAAAACCAGATTTTTACCAGTAGCTGGCGGAACAAGCAGCGTATATATCG<br>AAGTATTGCATTTGACTCAAGGGACCTGCTGGGAACAAATGTACACACCGGGAGGGGAAGTGAGAAATGATGATGTTGATCAGAGTTTGATCATTGCTGCTAGAAACATCGTTAGGAGAGCAACAGTATCAGCAGACCCATTGGCTTCGCTCTTGGAAATGTGCCACAGTACACAAATTGGCGGGATAAGGATGGTAGACATTCTTAGACAGAACCCAACAGAAGAGCA<br>AGCCGTGGATATATGCAAAGCAGCAATGGGTTTAAGAATCAGTTCATCCTTCAGCTTTGGAGGTTTCACTTTCAAAAGAACAAGTGGATCGTCTGTCAAAAGAGAAGAAGAAGTGCTCACAGGCAACCTCCAAACACTGAAAATAAGAGTACATGAAGGATATGAGGAATTCACAATGGTTGGACGGAGAGCTACAGCCATTTTAAGGAAAGCAACCAGAAGGCTGATCC<br>AATTAATAGTGAGTGGAAGAGACGAGCAGTCAATCGCTGAAGCAATCATAGTGGCAATGGTTTTCTCACAAGAGGACTGCATGATAAAAGCAGTACGAGGTGATCTGAACTTTGTTAATAGAGCAAATCAGCGACTAAATCCTATGCATCAACTTCTGAGGCATTTCCAAAAGGATGCAAAGGTGCTGTTTCAAAACTGGGGGGTTGAACCAATTGACAATGTAATGGGG<br>ATGATTGGAATACTGCCTGACATGACCCCCAGCACGGAGATGTCACTGAGAGGAGTGAGAGTCAGCAAAATGGGAGTGGATGAATATTCCAGTACTGAGAGAGTGGTCGTGAGCATTGATCGTTTCTTGAGAGTCCGAGATCAAAGAGGAAACGTGCTCCTGTCTCCTGAGGAAGTTAGTGAAACACAGGGAACAGAAAAACTGACGATAACATATTCATCGTCCATGA<br>TGTGGGAAATCAATGGTCCGGAATCCGTGCTAGTCAACACATATCAATGGATCATCAGAAATTGGGAGACTGTGAAGATTCAATGGTCCCAAGACCCTACGATGTTGTACAACAAGATGGAATTTGAGCCCTTCCAATCCTTGGTGCCCAAGGCTGCCAGAGGCCAGTATAGTGGATTTGTGAGAACATTGTTCCAGCAGATGCGTGATGTGCTGGGAACATTTGATACTGT<br>CCAAATAATAAAGCTCCTGCCTTTTGCTGCAGCCCCACCGGAACAGAATAGGATGCAGTTTTCCTCTCTGACTGTGAACGTAAGAGGTTCAGGAATGAGAATACTTGTGAGAGGCAACTCCCCTGTGTTCAACTATAACAAGGCCACCAAGAGACTCACAGTTCTTGGAAAGGATGCAGGTGCCTTGACAGAAGATCCAGATGAGGGAACAGCAGGAGTAGAGTCTGCAG<br>TATTAAGAGGATTTCTAATTCTGGGCAAAGAGGACAAAAGATATGGACCAGCATTGAGCATCAACGAACTGAGCAATCTTGCGAAAGGGGAAAAGGCTAATGTGTTGATAGGGCAAGGAGACGTGGTGTTGGTAATGAAACGGAAACGGGACTCTAGCATACTTACTGACAGCCAGACAGCGACCAAAAGAATTCGGATGGCCATCAATTA
internal NVRI repository	>A/domestic_duck/Poland/H189/2022_H5N1_2022-03-04<br>ATGGAGAGAATAAAAGAACTAAGAGATTTAATGTCACAGTCTCGCACTCGCGAGATACTAACAAAAACCACTGTGGACCATATGGCCATAATAAAGAAATACACATCAGGAAGACAGGAGAAGAACCCTGCTCTTAGGATGAAATGGATGATGGCAATGAAGTACCCGATTACAGCAGACAAAAGAATAATGGAGATGATCCCTGAAAGAAATGAGCAAGGCCAAACT<br>CTCTGGAGCAAAACAAATGATGCTGGATCAGACAGAGTGATGGTGTCACCTCTGGCTGTGACATGGTGGAATAGAAATGGGCCAACGACAAGCACAGTCCATTATCCAAAGGTTTACAAAACCTACTTTGAAAAAGTCGAAAGGTTGAAACATGGGACCTTTGGTCCTGTTCACTTTCGAAATCAGGTCAAAATACGCCGCAGGGTCGACATAAACCCGGGCCATGCAGA<br>TCTCAGTGCTAAGGAAGCACAAGACGTCATCATGGAGGTCGTTTTCCCAAACGAAGTTGGAGCCAGGATATTGACATCAGAGTCACAATTAACAATAACAAAGGAAAAGAAGGAGGAGCTTCAAGACTGTAAGATTGCTCCTTTGATGGTGGCATACATGTTGGAGAGGGAACTGGTCCGCAAAACCAGATTCCTACCGGTAGCTGGCGGGACAAGCAGTGTGTATATC<br>GAGGTGCTGCATTTGACTCAAGGGACCTGCTGGGAACAAATGTACACACCGGGAGGAGAGGTGAGAAATGATGATGTCGATCAAAGTTTGATTATTGCTGCTAGAAATATTGTCAGGAGAGCAACAGTATCAGCAGACCCGTTGGCTTCACTCTTGGAGATGTGCCATAGTACACAAATTGGAGGAATAAGGATGGTTGACATTCTTAGACAAAACCCAACAGAAGAACA<br>AGCTGTAGATATATGCAAAGCAGCAATGGGTCTAAGAATCAGTTCATCCTTCAGCTTTGGAGGTTTCACTTTCAAAAGGACAAGTGGGTCATCTGTCAAGAAAGAAGAAGAAGTGCTCACAGGCAACCTCCAAACATTGAAAATAAGGGTACACGAAGGATATGAAGAATTCACAATGGTTGGGCGAAGAGCGACCGCCATTTTAAGGAAAGCAACCAGAAGGTTGATA<br>CAATTAATAGTGAGTGGGAGGGACGAGCAGTCAATTGCTGAAGCAATCATAGTGGCAATGGTTTTCTCACAAGAAGACTGCATGATAAAGGCAGTACGAGGTGATTTGAATTTTGTCAACAGAGCAAACCAGCGGCTGAATCCTATGCATCAACTTCTAAGACATTTCCAGAAGGATGCAAAAGTGCTGTTTCAGAACTGGGGAATTGAACCCATTGACAATGTCATGGG<br>GATGATCGGAATACTGCCTGACATGACCCAAGCACAGATATGTCTCTGAGAGGAGTGAGGGTCAGTAAAATGGGAGTGGATGAATATTCCAGTACTGAGAGGGTTGTCGTGAGCATTGATCGTTTCTTAAGGGTCCGAGATCAACGGGGAAATGTACTCCTGTCTCCCGAGGAGGTCAGTGAAACACAAGGAACAGAGAAGCTAACGATAACATATTCGTCGTCAATG<br>ATGTGGGAAATCAATGGTCCGGAATCAGTGCTGGTCAACACATATCAATGGATTATTAGAAATTGGGAAACTGTGAAAATTCAGTGGTCACAAGACCCTACAATGTTATACAATAAGATGGAGTTTGAGCCCTTCCAATCCTTGGTGCCTAAGGCTGCCAGAGGCCAGTATAGTGGATTCGTGAGGACGTTGTTCCAGCAGATGCGTGATGTATTGGGGACATTTGACACT<br>GTTCAAATAATAAAGCTCCTACCATTTGCAGCAGCCCCACCAGAACAGAGTAGGATGCAGTTCTCTTCTCTAACTGTGAATATAAGGGGTTCAGGGATGAGAATACTTATGAGAGGCAATTCCCCTGTGTTCAACTATAACAAGGCAACCAAGAGGCTCACAGTCCTTGGAAAAGATGCAGGTGCCTTAACAGAAGATCCAGACGAGGGAACAGCAGGAGTGGAATCTGC<br>GGTATTAAGAGGATTTCTAATTCTGGGCAAAGAAGATAAGAGATATGGCCCAGCGTTGAGCATCAACGAATTGAGCAATCTTGCGAAAGGGGAGAAAGCTAATGTGTTGATAGGGCAAGGAGACGTGGTGTTGGTAATGAAACGGAAACGGGACTCTAGCATACTTACTGACAGCCAGACAGCGACCAAAAGGATTCGGATGGCCATCAATTA
EPI_ISL_6934175	>A/domestic_duck/Poland/H1942-N/2021_H5N1_2021-11-07<br>ATGGAGAGAATAAAAGAGCTAAGAGATTTGATGTCGCAGTCTCGCACTCGCGAGATACTGACAAAAACCACCGTGGACCATATGGCCATAATCAAGAAATATACATCAGGAAGACAGGAGAAGAACCCTGCACTTAGGATGAAGTGGATGATGGCAATGAAATATCCGATTACAGCAGACAAAAGGATAATGGAGATGATCCCTGAAAGAAACGAGCAAGGTCAGACT<br>CTTTGGAGCAAAACAAATGATGCTGGATCGGATAGAATAATGGTGTCACCTCTGGCTGTGACGTGGTGGAATAGAAATGGACCAACAACAAGTACCGTCCATTACCCAAAGGTCTATAAAACCTACTTTGAAAAGGTTGAAAGGTTAAAGCATGGAACCTTCGGCCCTGTCCATTTCCGGAATCAGGTTAAGATACGCCGCAGAGTTGACATAAACCCGGGCCATGCAGAC<br>CTCAGTGCTAAAGAAGCACAAGACGTCATCATGGAGGTCGTTTTCCCAAATGAAGTCGGAGCCAGAATATTGACATCAGAGTCACAGTTAACAATTACAAAAGAAAAGAAGGAGGAACTCCAGGACTGTAAGATTGCCCCTCTAATGGTGGCATACATGTTGGAGAGAGAACTGGTTCGAAAAACCAGATTCCTGCCAGTAGCTGGCGGAACAAGTAGCGTATATATCGA<br>AGTGTTGCACTTGACTCAAGGAACCTGCTGGGAACAAATGTATACGCCAGGAGGAGAAGTGAGAAATGATGACGTTGACCAAAGTTTAATTATTGCTGCCAGAAATATCGTTAGGAGGGCAACAGTATCAGCAGACCCATTGGCTTCACTACTGGAGATGTGCCATAGTACACAGATTGGCGGGATAAGAATGGTAGACATTCTTAGACAGAACCCAACAGAAGAGCAA<br>GCCGTGGATATATGCAAAGCAGCAATGGGTTTAAGAATCAGTTCATCCTTCAGTTTTGGAGGTTTCACTTTCAAAAGGACAAGTGGATCATCTGTCAAAAGAGAAGAGGAAGTGCTCACAGGCAACCTCCAAACATTGAAAATAAGAGTACATGAAGGGTATGAGGAATTCACAATGGTTGGACGGAGAGCAACAGCCATTCTAAGGAAAGCAACCAGAAGGCTAATCC<br>AATTGATAGTAAGTGGAAGAGACGAGCAGTCAATCGCCGAAGCGATCATAGTGGCAATGGTGTTCTCTCAAGAGGATTGCATGATAAAGGCTGTACGAGGTGATTTAAATTTTGTCAATAGAGCGAATCAGCGGCTCAATCCTATGCATCAGCTCCTGAGGCATTTCCAAAAGGATGCAAAGGTACTATTCCAAAACTGGGGAATTGAACCCATTGACAATGTCATGGGAA<br>TGATAGGAATATTGCCTGATATGACCCCCAGCACAGAGATGTCACTAAGAGGAGTGAGGGTCAGTAAAATGGGAGTGGATGAATATTCCAGTACTGAGAGGGTGGTCGTGAGTATTGATCGCTTCTTGAGGGTACGAGACCAGAGAGGAAATGTACTCTTGTCTCCCGAAGAGGTCAGTGAAACACAGGGAACAGAGAAGCTAACGATAACATATTCATCATCCATGAT<br>GTGGGAAATTAATGGCCCTGAGTCAGTGCTAGTTAACACATATCAATGGATCATCAGAAACTGGGAAACTGTGAAGATTCAGTGGTCCCAAGACCCTACAATGCTATACAACAAGATGGAGTTTGAGCCTTTTCAGTCCTTGGTGCCTAAGGCAGCCAGAGGCCAGTACAGTGGATTTGTAAGGACCCTATTCCAGCAGATGCGTGATGTGCTGGGAACCTTTGACACTGT<br>CCAGATAATAAAGCTACTTCCATTTGCAGCAGCACCACCGGAACAGAGTAGGATGCAGTTCTCTTCTCTAACTGTAAACGTAAGGGGTTCAGGAATGAGAATACTTGTGAGAGGCAACTCCCCTGTGTTCAACTATAATAAAGCAACCAAGAGGCTCATAGTCCTTGGAAAGGATGCTGGTGCATTGACAGAAGACCCAGGTGAGGGGACAGCAGGAGTGGAGTCTGCG<br>GTATTGAGAGGGTTCCTAATTCTGGGCAAAGAGGACAAAAGATATGGACCAGCGCTGAGCATCAATGAATTGAGCAATCTTGCGAAAGGGGAGAAGGCTAATGTGTTGATAGGGCAAGGAGACGTGGTGTTGGTGATGAAACGGAAACGGGACTCTAGCATACTTACTGACAGCCAGACAGCGACCAAAAGAATTCGGATGGCCATCAATTA
internal NVRI repository	>A/domestic_duck/Poland/H2020-N/2021_H5N1_2021-11-23<br>ATGGAGAGAATAAAAGAGCTAAGAGATTTGATGTCGCAGTCTCGCACTCGCGAGATACTGACAAAAACCACCGTGGACCATATGGCCATAATCAAGAAATATACATCAGGAAGACAGGAGAAGAACCCTGCACTTAGGATGAAGTGGATGATGGCAATGAAATATCCGATTACAGCAGACAAAAGGATAATGGAGATGATCCCTGAAAGAAACGAGCAAGGTCAGACT<br>CTTTGGAGCAAAACAAATGATGCTGGATCGGATAGAATAATGGTGTCACCTCTGGCTGTGACGTGGTGGAATAGAAATGGACCAACAACAAGTACCGTCCATTACCCAAAGGTCTATAAAACCTACTTTGAAAAGGTTGAAAGGTTAAAGCATGGAACCTTCGGCCCTGTCCATTTCCGGAATCAGGTTAAGATACGCCGCAGAGTTGACATAAACCCGGGCCATGCAGAC<br>CTCAGTGCTAAAGAAGCACAAGACGTCATCATGGAGGTCGTTTTCCCAAATGAAGTCGGAGCCAGAATATTGACATCAGAGTCACAGTTAACAATTACAAAAGAAAAGAAGGAGGAACTCCAGGACTGTAAGATTGCCCCTCTAATGGTGGCATACATGTTGGAGAGAGAACTGGTTCGAAAAACCAGATTCCTGCCAGTAGCTGGCGGAACAAGTAGCGTATATATCGA<br>AGTGTTGCACTTGACTCAAGGAACCTGCTGGGAACAAATGTATACGCCAGGAGGAGAAGTGAGAAATGATGACGTTGACCAAAGTTTAATTATTGCTGCCAGAAATATCGTTAGGAGGGCAACAGTATCAGCAGACCCATTGGCTTCACTACTGGAGATGTGCCATAGTACACAGATTGGCGGGATAAGAATGGTAGACATTCTTAGACAGAACCCAACAGAAGAGCAA<br>GCCGTGGATATATGCAAAGCAGCAATGGGTTTAAGAATCAGTTCATCCTTCAGTTTTGGAGGTTTCACTTTCAAAAGGACAAGTGGATCATCTGTCAAAAGAGAAGAGGAAGTGCTCACAGGCAACCTCCAAACATTGAAAATAAGAGTACATGAAGGGTATGAGGAATTCACAATGGTTGGACGGAGAGCAACAGCCATTCTAAGGAAAGCAACCAGAAGGCTAATCC<br>AATTGATAGTAAGTGGAAGAGACGAGCAGTCAATCGCCGAAGCGATCATAGTGGCAATGGTGTTCTCTCAAGAGGATTGCATGATAAAGGCTGTACGAGGTGATTTAAATTTTGTCAATAGAGCGAATCAGCGGCTCAATCCTATGCATCAGCTCCTGAGGCATTTCCAAAAGGATGCAAAGGTACTATTCCAAAACTGGGGAATTGAACCCATTGACAATGTCATGGGAA<br>TGATAGGAATATTGCCTGATATGACCCCCAGCACAGAGATGTCACTAAGAGGAGTGAGGGTCAGTAAAATGGGAGTGGATGAATATTCCAGTACTGAGAGGGTGGTCGTGAGTATTGATCGCTTCTTGAGGGTACGAGACCAGAGAGGAAATGTACTCTTGTCTCCCGAAGAGGTCAGTGAAACACAGGGAACAGAGAAGCTAACGATAACATATTCATCATCCATGAT<br>GTGGGAAATTAATGGCCCTGAGTCAGTGCTAGTTAACACATATCAATGGATCATCAGAAACTGGGAAACTGTGAAGATTCAGTGGTCCCAAGACCCTACAATGCTATACAACAAGATGGAGTTTGAGCCTTTTCAGTCCTTGGTGCCTAAGGCAGCCAGAGGCCAGTACAGTGGATTTGTAAGGACCCTATTCCAGCAGATGCGTGATGTGCTGGGAACCTTTGACACTGT<br>CCAGATAATAAAGCTACTTCCATTTGCAGCAGCACCACCGGAACAGAGTAGGATGCAGTTCTCTTCTCTAACTGTAAACGTAAGGGGTTCAGGAATGAGAATACTTGTGAGAGGCAACTCCCCTGTGTTCAACTATAATAAAGCAACCAAGAGGCTCATAGTCCTTGGAAAGGATGCTGGTGCATTGACAGAAGACCCAGGTGAGGGGACAGCAGGAGTGGAGTCTGCG<br>GTATTGAGAGGGTTCCTAATTCTGGGCAAAGAGGACAAAAGATATGGACCAGCGCTGAGCATCAATGAATTGAGCAATCTTGCGAAAGGGGAGAAGGCTAATGTGTTGATAGGGCAAGGAGACGTGGTGTTGGTGATGAAACGGAAACGGGACTCTAGCATACTTACTGACAGCCAGACAGCGACCAAAAGAATTCGGATGGCCATCAATTA

internal NVRI repository	>A/domestic_duck/Poland/H2045-T1/2021_H5N1_2021-11-26<br>[illegible]
internal NVRI repository	>A/domestic_duck/Poland/H2077-J/2021_H5N1_2021-12-04<br>[illegible]
internal NVRI repository	>A/domestic_duck/Poland/H2078-N/2021_H5N1_2022-12-05<br>[illegible]
internal NVRI repository	>A/domestic_duck/Poland/H2085-T2/2021_H5N1_2021-12-06<br>[illegible]
internal NVRI repository	>A/domestic_duck/Poland/H2099-N/2021_H5N1_2021-12-11<br>[illegible]
internal NVRI repository	>A/domestic_duck/Poland/H2102-N/2021_H5N1_2021-12-13<br>[illegible]
internal NVRI repository	>A/domestic_duck/Poland/H2117-T1/2021_H5N1_2021-12-16<br>[illegible]
internal NVRI repository	>A/domestic_duck/Poland/H2126-N/2021_H5N1_2021-12-20<br>[illegible]
internal NVRI repository	>A/domestic_duck/Poland/H2153_T3/2021_H5N1_2021-12-25<br>[illegible]
internal NVRI repository	>A/domestic_duck/Poland/H2154_N/2021_H5N1_2021-12-24<br>[illegible]
internal NVRI repository	>A/domestic_goose/Poland/33/2022_H5N1_2022-01-09<br>[illegible]
internal NVRI repository	>A/domestic_goose/Poland/53/2022_H5N1_2022-01-12<br>[illegible]

EPI_ISL_6931008	>A/domestic_goose/Poland/H1931-T1/2021_H5N1_2021-11-03<br>ATGGAGAGAATAAAAGAGCTAAGAGATTTGATGTCGCAGTCTCGCACTCGCGAGATACTGACAAAAACCACCGTGGACCATATGGCCATAATCAAGAAATATACATCAGGAAGACAGGAGAAGAACCCTGCACTTAGGATGAAGTGGATGATGGCAATGAAATATCCGATTACAGCAGACAAAAGGATAATGGAGATGATCCCTGAAAGAAACGAGCAAGGTCAGACT<br>CTTTGGAGCAAAACAAATGATGCTGGATCGGGTAGAGTAATGGTGTCACCTCTGGCTGTGACGTGGTGGAATAGAAATGGACCAACAACAAGTACAGTCCATTACCCAAAGGTCTATAAAACCTACTTTGAAAAGGTTGAAAGGTTAAAGCATGGAACCTTCGGCCCTGTCCATTTCCGGAATCAGGTTAAGATACGCCGCAGAGTTGACATAAACCCGGGCCATGCAGA<br>CCTCAGTGCTAAAGAAGCACAAGACGTCATCATGGAGGTCGTTTTCCCAAATGAAGTCGGAGCCAGAATATTGACATCAGAGTCACAGTTAACAATTACAAAAGAAAAGAAGGAGGAACTCCAGGACTGTAAGATTGCCCCTCTAATGGTGGCATACATGTTGGAGAGAGAACTGGTTCGAAAAACCAGATTCCTGCCAGTAGCTGGCGGAACAAGTAGCGTATATATCG<br>AAGTGTTGCACTTGACTCAAGGAACCTGCTGGGAACAAATGTATACGCCAGGAGGAGAAGTGAGAAATGATGACGTTGACCAAAGTTTAATTATTGCTGCCAGAAATATCGTTAGGAGAGCAACAGTATCAGCAGACCCATTGGCTTCACTACTGGAGATGTGCCATAGTACACAGATTGGCGGGATAAGAATGGTAGACATTCTTAGACAGAACCCAACAGAAGAGCA<br>AGCCGTGGATATATGCAAAGCAGCAATGGGTTTAAGAATCAGTTCATCCTTCAGTTTTGGAGGTTTCACTTTCAAAAGGACAAGCGGATCATCTGTCAAAAGAGAAGAGGAAGTGCTCACCGGCAACCTCCAAACATTGAAAATAAGAGTACATGAAGGGTATGAGGAATTCACAATGGTTGGGCGGAGAGCAACAGCCATTCTAAGGAAAGCAACCAGAAGGCTGATC<br>CAATTGATAGTAAGTGGAAGAGACGAGCAGTCAATCGCCGAAGCGATCATAGTGGCAATGGTGTTCTCTCAAGAGGATTGCATGATAAAGGCTGTACGAGGTGATTTAAATTTTGTCAATAGAGCGAATCAGCGGCTCAATCCTATGCATCAGCTCCTGAGGCATTTCCAAAAGGATGCAAAGGTACTATTCCAAAACTGGGGAATAGAACCCATTGACAATGTCATGGG<br>AATGATAGGAATATTGCCTGATATGACCCCCAGCACAGAGATGTCACTAAGAGGAGTGAGGGTCAGTAAAATGGGAGTGGATGAATATTCCAGTACTGAGAGGGTGGTCGTGAGTATTGATCGCTTCTTGAGGGTACGAGACCAGAGAGGAAATGTACTCTTGTCTCCCGAAGAGGTCAGTGAAACACAGGGAACAGAGAAGCTAACGATAACATATTCATCATCCATG<br>ATGTGGGAAATTAATGGCCCTGAGTCAGTGCTAGTTAACACATATCAATGGATCATCAGAAACTGGGAAACTGTGAAGATTCAGTGGTCCCAAGACCCTACAATGCTATACAACAAGATGGAGTTTGAGCCTTTTCAGTCCTTGGTGCCTAAGGCAGCCAGAGGCCAGTACAGTGGATTTGTAAGGACCCTATTCCAGCAGATGCGTGATGTGCTGGGAACCTTTGACACT<br>GTCCAGATAATAAAGCTACTTCCATTTGCAGCAGCACCACCGGAACAGAGTAGGATGCAGTTCTCTTCTCTAACTGTAAACGTAAGGGGTTCAGGAATGAGAATACTTGTGAGAGGCAACTCCCCTGTGTTCAACTATAATAAGGCAACCAAGAGGCTCATAGTCCTTGGAAAGGATGCTGGTGCATTGACAGAAGACCCAGGTGAGGGGACAGCAGGAGTGGAGTCTGC<br>GGTATTGAGAGGGTTCCTAATTCTGGGCAAAGAGGACAAAAGATATGGACCAGCGCTGAGCATCAATGAATTGAGCAATCTTGCGAAAGGGGAGAAGGCTAATGTGTTGATAGGGCAAGGAGACGTGGTGTTGGTGATGAAACGGAAACGGGACTCTAGCATACTTACTGACAGCCAGACAGCGACCAAAAGAATTCGGATGGCCATCAATTA
internal NVRI repository	>A/domestic_goose/Poland/H28-T/2022_H5N1_2022-01-05<br>ATGGAGAGAATAAAAGAACTAAGAGATTTAATGTCGCAGTCTCGCACTCGCGAGATACTGACAAAAACCACTGTGGACCATATGGCCATAATAAAGAAATACACATCAGGAAGACAGGAGAAGAACCCGGCCCTCAGGATGAAGTGGATGATGGCAATGAAATATCCTATTACAGCAGACAAAAGAATAATGGAGATGATCCCTGAAAGGAATGAGCAAGGTCAGACT<br>CTCTGGAGCAAAACAAATGACGCTGGATCAGACAGAGTGATGGTGTCACCTCTGGCTGTGACATGGTGGAATAGAAATGGGCCAACAACAAGTACAGTCCATTACCCAAAGGTTTACAAAACCTACTTTGAAAAAGTGGAAAGATTGAAACATGGAACCTTTGGCCCTGTTCACTTTCGAAATCAGGTTAAGATACGCCGCAGGGTTGACATAAACCCGGGTCATGCAGA<br>TCTCAGTGCCAAAGAAGCACAGGATGTCATCATGGAAGTCGTTTTCCCAAATGAAATTGGAGCCAGGACATTGACATCAGAATCACAATTAACAATAACAAAGGAAAAGAAGGAGGAACTTCAGGACTGTAAGATTGCTCCTTTGATGGTGGCATACATGTTGGAGAGAGAACTGGTTCGAAAAACCAGATTTTTACCAGTAGCTGGCGGAACAAGCAGCGTATATATCG<br>AAGTATTGCATTTGACTCAAGGGACCTGCTGGGAACAAATGTACACACCGGGAGGGGAAGTGAGAAATGATGATGTTGATCAGAGTTTGATCATTGCTGCTAGAAATATCGTTAGGAGAGCAACAGTATCAGCAGACCCATTGGCTTCGCTCTTGGAAATGTGCCACAGTACACAAATTGGCGGGATAAGGATGGTAGACATTCTTAGACAGAACCCAACAGAAGAGCA<br>AGCCGTGGATATATGCAAAGCAGCAATGGGTTTAAGAATCAGTTCATCCTTCAGCTTTGGAGGTTTCACTTTCAAAAGAACAAGTGGATCGTCTGTCAAAAGAGAAGAAGAAGTGCTCACAGGCAACCTCCAAACACTGAAAATAAGAGTACATGAAGGATATGAGGAATTCACAATGGTTGGACGGAGAGCTACAGCCATTTTAAGGAAAGCAACCAGAAGGCTGATCC<br>AATTAATAGTGAGTGGAAGAGACGAGCAGTCAATCGCTGAAGCAATCATAGTGGCAATGGTTTTCTCACAAGAGGACTGCATGATAAAAGCAGTACGAGGTGATCTGAACTTTGTTAATAGAGCAAATCAGCGACTAAATCCTATGCATCAACTTCTGAGGCATTTCCAAAAGGATGCAAAGGTGCTGTTTCAAAACTGGGGGGTTGAACCAATTGACAATGTAATGGGG<br>ATGATTGGAATACTGCCTGACATGACCCCCAGCACGGAGATGTCACTGAGAGGAGTGAGAGTCAGCAAAATGGGAGTGGATGAATATTCCAGTACTGAGAGAGTGGTCGTGAGCATTGATCGTTTCTTGAGAGTCCGAGATCAAAGAGGAAACGTGCTCCTGTCTCCTGAGGAAGTTAGTGAAACACAGGGAACAGAGAAACTGACGATAACATATTCATCGTCCATGA<br>TGTGGGAAATCAATGGTCCGGAATCCGTGCTAGTCAACACATATCAATGGATCATCAGAAATTGGGAGACTGTGAAGATTCAATGGTCCCAAGACCCTACGATGTTGTACAACAAGATGGAATTTGAGCCCTTCCAATCCTTGGTGCCCAAGGCTGCCAGAGGCCAGTATAGTGGATTTGTGAGGACATTGTTCCAGCAGATGCGTGATGTGCTGGGAACATTTGATACTG<br>TCCAAATAATAAAGCTCCTGCCTTTTGCAGCAGCCCCACCGGAACAGAGTAGGATGCAGTTTTCCTCTCTGACTGTGAACGTAAGAGGTTCAGGAATGAGAATACTTGTGAGAGGCAACTCCCCTGTGTTCAACTATAACAAGGCCACCAAGAGACTCACAGTTCTTGGAAAGGATGCAGGTGCCTTGACAGAAGATCCAGATGAGGGAACAGCAGGAGTAGAGTCTGCA<br>GTATTAAGAGGATTTCTAATTCTGGGCAAAGAGGACAAAAGATATGGACCAGCATTGAGTATCAACGAACTGAGCAATCTTGCGAAAGGGGAAAAGGCTAATGTGTTGATAGGGCAAGGAGACGTGGTGTTGGTAATGAAACGGAAACGGGACTCTAGCATACTTACTGACAGCCAGACAGCGACCAAAAGAATTCGGATGGCCATCAATTA
EPI_ISL_14917951	>A/domestic_goose/Poland/H371/2022_H5N1_2022-07-21<br>ATGGAGAGAATAAAAGAACTAAGAGATTTAATGTCACAGTCTCGCACTCGCGAGATACTAACAAAAACCACTGTGGACCATATGGCCATAATAAAGAAATACACATCAGGAAGACAGGAGAAGAACCCTGCTCTTAGGATGAAATGGATGATGGCAATGAAGTACCCGATTACAGCAGACAAAAGAATAATGGAGATGATCCCTGAAAGAAATGAGCAAGGCCAAACT<br>CTCTGGAGCAAAACAAATGATGCTGGATCAGACAGAGTGATGGTGTCACCTCTGGCTGTGACATGGTGGAATAGAAATGGGCCAACGACAAGCACAGTCCATTATCCAAAGGTTTACAAAACCTACTTTGAAAAAGTCGAAAGGTTGAAACATGGGACCTTTGGTCCTGTTCACTTTCGAAATCAGGTCAAAATACGCCGCAGGGTCGACATAAACCCGGGCCATGCAGA<br>TCTCAGTGCTAAGGAAGCACAAGACGTCATCATGGAGGTCGTTTTCCCAAACGAAGTTGGAGCCAGGATATTGACATCAGAGTCACAATTAACAATAACAAAGGAAAAGAAGGAGGAGCTTCAAGACTGTAAGATTGCTCCTTTGATGGTGGCATACATGTTGGAGAGGGAACTGGTCCGCAAAACCAGATTCCTACCGGTAGCTGGCGGGACAAGCAGTGTGTATATC<br>GAGGTGCTGCATTTGACTCAAGGGACCTGCTGGGAACAAATGTACACACCGGGAGGAGAGGTGAGAAATGATGATGTCGATCAAAGTTTGATTATTGCTGCTAGAAATATTGTCAGGAGAGCAACAGTATCAGCAGACCCGTTGGCTTCACTCTTGGAGATGTGCCATAGTACACAAATTGGAGGAATAAGGATGGTTGACATTCTTAGACAAAACCCAACAGAAGAACA<br>AGCTGTAGATATATGCAAAGCAGCAATGGGTCTAAGAATCAGTTCATCCTTCAGCTTTGGAGGTTTCACTTTCAAAAGGACAAGTGGGTCATCTGTCAAGAAAGAAGAAGAAGTGCTCACAGGCAACCTCCAAACATTGAAAATAAGGGTACACGAAGGATATGAAGAATTCACAATGGTTGGGCGAAGAGCGACCGCCATTTTAAGGAAAGCAACCAGAAGGTTGATA<br>CAATTAATAGTGAGTGGGAGGGACGAGCAGTCAATTGCTGAAGCAATCATAGTGGCAATGGTTTTCTCACAAGAAGACTGCATGATAAAGGCAGTACGAGGTGATTTGAATTTTGTCAACAGAGCAAACCAGCGGCTGAATCCYATGCATCAACTTCTAAGACATTTCCAGAAGGATGCAAAAGTGCTGTTTCAGAACTGGGGAATTGAACCCATTGACAATGTCATGGG<br>GATGATCGGAATACTGCCTGACATGACCCCAAGCACAGATATGTCTCTGAGAGGAGTGAGGGTCAGTAAAATGGGAGTGGATGAATATTCCAGTACTGAGAGGGTTGTCGTGAGCATTGATCGTTTCTTAAGGGTCCGAGATCAAAGGGGAAATGTACTCCTGTCTCCCGAGGAGGTCAGTGAAACACAAGGAACAGAGAAGCTAACGATAACATATTCGTCGTCAATG<br>ATGTGGGAAATCAATGGTCCGGAATCAGTGCTGGTCAACACATATCAATGGATTATTAGAAATTGGGAAACTGTGAAAATTCAGTGGTCACAAGACCCTACAATGTTATACAATAAGATGGAGTTTGAGCCCTTCCAATCCTTGGTGCCTAAGGCTGCCAGAGGCCAGTATAGTGGATTCGTGAGGACGTTGTTCCAGCAGATGCGTGATGTATTGGGGACATTTGACACT<br>GTTCAAATAATAAAGCTCCTACCATTTGCAGCAGCCCCACCAGAACAGAGTAGGATGCAGTTCTCTTCTCTAACTGTGAATATAAGGGGTTCAGGGATGAGAATACTTATGAGAGGCAATTCCCCTGTGTTCAACTATAACAAGGCAACCAAGAGGCTCACAGTCCTTGGAAAAGATGCAGGTGCCTTAACAGAAGATCCAGACGAGGGAACAGCAGGAGTGGAATCTGC<br>GGTATTAAGAGGATTTCTAATTCTGGGCAAAGAAGATAAGAGATATGGCCCAGCGTTGAGCATCAACGAATTGAGCAATCTTGCGAAAGGGGAGAAAGCTAATGTGTTGATAGGGCAAGGAGACGTGGTGTTGGTAATGAAACGGAAACGGGACTCTAGCATACTTACTGACAGCCAGACAGCGACCAAAAGGATTCGGATGGCCATCAATTA
EPI_ISL_11922810	>A/duck/Poland/H126_22VIR2515-4/2022_H5N1_2022-02-08<br>ATGGAGAGAATAAAAGAACTAAGAGATTTAATGTCGCAGTCTCGCACTCGCGAGATACTGACAAAAACCACTGTGGACCATATGGCCATAATAAAGAAATACACATCAGGAAGACAGGAGAAGAACCCGGCCCTCAGGATGAAGTGGATGATGGCAATGAAATATCCTATTACAGCAGACAAAAGAATAATGGAGATGATCCCTGAAAGGAATGAGCAAGGTCAGACT<br>CTCTGGAGCAAAACAAATGACGCTGGATCAGACAGAGTGATGGTGTCACCTCTGGCTGTGACATGGTGGAATAGAAATGGGCCAACGACAAGTACAGTCCATTACCCAAAGGTTTACAAAACCTACTTTGAAAAAGTGGAAAGATTGAAACATGGAACCTTTGGCCCTGTTCACTTTCGAAATCAGGTTAAGATACGCCGCAGGGTTGACATAAACCCGGGTCATGCAGA<br>TCTCAGTGCCAAAGAAGCACAGGATGTCATCATGGAAGTCGTTTTCCCAAATGAAGTTGGAGCCAGGACATTGACATCAGAATCACAATTAACAATAACAAAGGAAAAGAAGGAGGAACTTCAGGACTGTAAGATTGCTCCTTTGATGGTGGCATACATGTTGGAGAGAGAACTGGTTCGAAAAACCAGATTTTTACCAGTAGCTGGCGGAACAAGCAGCGTATATATCG<br>AAGTATTGCATTTGACTCAAGGGACCTGCTGGGAACAAATGTACACACCGGGAGGGGAAGTGAGAAATGATGATGTTGATCAGAGTTTGATCATTGCTGCTAGAAATATCGTTAGGAGAGCAACAGTATCAGCAGACCCATTGGCTTCGCTCTTGGAAATGTGCCACAGTACACAAATTGGCGGGATAAGGATGGTAGACATTCTTAGACAGAACCCAACAGAAGAGCA<br>AGCCGTGGATATATGCAAAGCWGCAATGGGTTTAAGAATCAGTTCATCCTTCAGCTTTGGAGGTTTCACTTTCAAAAGAACAAGTGGATCGTCTGTCAAAAGAGAAGAAGAAGTGCTCACAGGCAACCTCCAAACACTGAAAATAAGAGTACATGAAGGATATGAGGAATTCACAATGGTTGGACGGAGAGCTACAGCCATTTTAAGGAAAGCAACCAGAAGGCTGATC<br>CAATTAATAGTGAGTGGAAGAGACGAGCAGTCAATCGCTGAAGCAATCATAGTGGCAATGGTTTTCTCACAAGAGGACTGCATGATAAAAGCAGTACGAGGTGATCTGAACTTTGTTAATAGAGCAAATCAGCGACTAAATCCGATGCATCAACTTCTGAGGCATTTCCAAAAGGATGCAAAGGTGCTGTTTCAAAACTGGGGGGTTGAACCAATTGACAATGTAATGGG<br>GATGATTGGAATACTGCCTGACATGACCCCCAGCACGGAGATGTCACTGAGAGGAGTGAGAGTCAGCAAAATGGGAGTGGATGAATATTCCAGTACTGAGAGAGTGGTCGTGAGCATTGATCGTTTCTTGAGAGTCCGAGATCAAAGAGGAAACGTGCTCCTGTCTCCTGAGGAAGTTAGTGAAACACAGGGAACAGAGAAACTGACGATAACATATTCATCGTCCATG<br>ATGTGGGAAATCAATGGTCCGGAATCCGTGCTAGTCAACACATATCAATGGATCATCAGAAATTGGGAGACTGTGAAGATTCAATGGTCCCAAGACCCTACGATGTTGTACAACAAGATGGAATTTGAGCCCTTCCAATCCTTGGTGCCCAAGGCTGCCAGAGGCCAGTATAGTGGATTTGTGAGGACATTGTTCCAGCAGATGCGTGATGTGCTGGGAACATTTGATACT<br>GTCCAAATAATAAAGCTCCTGCCTTTTGCAGCAGCCCCACCGGAACAGAGTAGGATGCAGTTTTCCTCTCTGACTGTGAACGTAAGAGGTTCAGGAATGAGAATACTTGTGAGAGGCAACTCCCCTGTGTTCAACTATAACAAGGCCATCAAGAGACTCACAGTTCTTGGAAAGGATGCAGGTGCCTTGACAGAAGATCCAGATGAGGGAACAGCAGGAGTAGAGTCTGC<br>AGTATTAAGAGGATTTCTAATTCTGGGCAAAGAGGACAAAAGATATGGACCAGCATTGAGTATCAACGAACTGAGCAATCTTGCGAAAGGGGAAAAGGCTAATGTGTTGATAGGGCAAGGAGACGTGGTGTTGGTAATGAAACGGAAACGGGACTCTAGCATACTTACTGACAGCCAGACAGCGACCAAAAGAATTCGGATGGCCATCAATTA
EPI_ISL_11922808	>A/duck/Poland/H188_22VIR2515-2/2022_H5N1_2022-03-02<br>ATGGAGAGAATAAAAGAACTAAGAGATTTAATGTCACAGTCTCGCACTCGCGAGATACTAACAAAAACCACTGTGGACCATATGGCCATAATAAAGAAATACACATCAGGAAGACAGGAGAAGAACCCTGCTCTTAGGATGAAATGGATGATGGCAATGAAGTACCCGATTACAGCAGACAAAAGAATAATGGAGATGATCCCTGAAAGAAATGAGCAAGGCCAAACT<br>CTCTGGAGCAAAACAAATGATGCTGGATCAGACAGAGTGATGGTGTCACCTCTGGCTGTGACATGGTGGAATAGAAATGGGCCAACGACAAGCACAGTCCATTATCCAAAGGTTTACAAAACCTACTTTGAAAAAGTCGAAAGGTTGAAACATGGGACCTTTGGTCCTGTTCACTTTCGAAATCAGGTCAAAATACGCCGCAGGGTCGACATAAACCCGGGCCATGCAGA<br>TCTCAGTGCTAAGGAAGCACAAGACGTCATCATGGAGGTCGTTTTCCCAAACGAAGTTGGAGCCAGGATATTGACATCAGAGTCACAATTAACAATAACAAAGGAAAAGAAGGAGGAGCTTCAAGACTGTAAGATTGCTCCTTTGATGGTGGCATACATGTTGGAGAGGGAACTGGTCCGCAAAACCAGATTCCTACCGGTAGCTGGCGGGACAAGCAGTGTGTATATC<br>GAGGTGCTGCATTTGACTCAAGGGACCTGCTGGGAACAAATGTACACACCGGGAGGAGAGGTGAGAAATGATGATGTCGATCAAAGTTTGATTATTGCTGCTAGAAATATTGTCAGGAGAGCAACAGTATCAGCAGACCCGTTGGCTTCACTCTTGGAGATGTGCCATAGTACACAAATTGGAGGAATAAGGATGGTTGACATTCTTAGACAAAACCCAACAGAAGAACA<br>AGCTGTAGATATATGCAAAGCAGCAATGGGTCTAAGAATCAGTTCATCCTTCAGCTTTGGAGGTTTCACTTTCAAAAGGACAAGTGGGTCATCTGTCAAGAAAGAAGAAGAAGTGCTCACAGGCAACCTCCAAACATTGAAAATAAGGGTACACGAAGGATATGAAGAATTCACAATGGTTGGGCGAAGAGCGACCGCCATTTTAAGGAAAGCAACCAGAAGGTTGATA<br>CAATTAATAGTGAGTGGGAGGGACGAGCAGTCAATTGCTGAAGCAATCATAGTGGCAATGGTTTTCTCACAAGAAGACTGCATGATAAAGGCAGTACGAGGTGATTTGAATTTTGTCAACAGAGCAAACCAGCGGCTGAATCCTATGCATCAACTTCTAAGACATTTCCAGAAGGATGCAAAAGTGCTGTTTCAGAACTGGGGAATTGAACCCATTGACAATGTCATGGG<br>GATGATCGGAATACTGCCTGACATGACCCCAAGCACAGATTTGTCTCTGAGAGGAGTGAGGGTCAGTAAAATGGGAGTGGATGAATATTCCAGTACTGAGAGGGTTGTCGTGAGCATTGATCGTTTCTTAAGGGTCCGAGATCAACGGGGAAATGTACTCCTGTCTCCCGAGGAGGTCAGTGAAACACAAGGAACAGAGAAGCTAACGATAACATATTCGTCGTCAATGA<br>TGTGGGAAATCAATGGTCCGGAATCAGTGCTGGTCAACACATATCAATGGATTATTAGAAATTGGGAAACTGTGAAAATTCAGTGGTCACAAGACCCTACAATGTTATACAATAAGATGGAGTTTGAGCCCTTCCAATCCTTGGTGCCTAAGGCTGCCAGAGGCCAGTATAGTGGATTCGTGAGGACGTTGTTCCAGCAGATGCGTGATGTATTGGGGACATTTGACACTGT<br>TCAAATAATAAAGCTCCTACCATTTGCAGCAGCCCCACCAGAACAGAGTAGGATGCAGTTCTCTTCTCTAACTGTGAATATAAGGGGTTCAGGGATGAGAATACTTATGAGAGGCAATTCCCCTGTGTTCAACTATAACAAGGCAACCAAGAGGCTCACAGTCCTTGGAAAAGATGCAGGTGCCTTAACAGAAGATCCAGACGAGGGAACAGCAGGAGTGGAATCTGCGG<br>TATTAAGAGGATTTCTAATTCTGGGCAAAGAAGATAAGAGATATGGCCCAGCGTTGAGCATCAACGAATTGAGCAATCTTGCGAAAGGGGAGAAAGCTAATGTGTTGATAGGGCAAGGAGACGTGGTGTTGGTAATGAAACGGAAACGGGACTCTAGCATACTTACTGACAGCCAGACAGCGACCAAAAGGATTCGGATGGCCATCAATTA
internal NVRI repository	>A/duck/Poland/H1968_21RS3290-5/2021_H5N1_2021-11-12<br>ATGGAGAGAATAAAAGAGCTAAGAGATTTGATGTCGCAGTCTCGCACTCGCGAGATACTGACAAAAACCACCGTGGACCATATGGCCATAATCAAGAAATATACATCAGGAAGACAGGAGAAGAACCCTGCACTTAGGATGAAGTGGATGATGGCAATGAAATATCCGATTACAGCAGACAAAAGGATAATGGAGATGATCCCTGAAAGAAACGAGCAAGGTCAGACT<br>CTTTGGAGCAAAACAAATGATGCTGGATCGGATAGAATAATGGTGTCACCTCTGGCTGTGACGTGGTGGAATAGAAATGGACCAACAACAAGTACCGTCCATTACCCAAAGGTCTATAAAACCTACTTTGAAAAGGTTGAAAGGTTAAAGCATGGAACCTTCGGCCCTGTCCATTTCCGGAATCAGGTTAAGATACGCCGCAGAGTTGACATAAACCCGGGCCATGCAGAC<br>CTCAGTGCTAAAGAAGCACAAGACGTCATCATGGAGGTCGTTTTCCCAAATGAAGTCGGAGCCAGAATATTGACATCAGAGTCACAGTTAACAATTACAAAAGAAAAGAAGGAGGAACTCCAGGACTGTAAGATTGCCCCTCTAATGGTGGCATACATGTTGGAGAGAGAACTGGTTCGAAAAACCAGATTCCTGCCAGTAGCTGGCGGAACAAGTAGCGTATATATCGA<br>AGTGTTGCACTTGACTCAAGGAACCTGCTGGGAACAAATGTATACGCCAGGAGGAGAAGTGAGAAATGATGACGTTGACCAAAGTTTAATTATTGCTGCCAGAAATATCGTTAGGAGGGCAACAGTATCAGCAGACCCATTGGCTTCACTACTGGAGATGTGCCATAGTACACAGATTGGCGGGATAAGAATGGTAGACATTCTTAGACAGAACCCAACAGAAGAGCAA<br>GCCGTGGATATATGCAAAGCAGCAATGGGTTTAAGAATCAGTTCATCCTTCAGTTTTGGAGGTTTCACTTTCAAAAGGACAAGTGGATCATCTGTCAAAAGAGAAGAGGAAGTGCTCACAGGCAACCTCCAAACATTGAAAATAAGAGTACATGAAGGGTATGAGGAATTCACAATGGTTGGACGGAGAGCAACAGCCATTCTAAGGAAAGCAACCAGAAGGCTAATCC<br>AATTGATAGTAAGTGGAAGAGACGAGCAGTCAATCGCCGAAGCGATCATAGTGGCAATGGTGTTCTCTCAAGAGGATTGCATGATAAAGGCTGTACGAGGTGATTTAAATTTTGTCAATAGAGCGAATCAGCGGCTCAATCCTATGCATCAGCTCCTGAGGCATTTCCAAAAGGATGCAAAGGTACTATTCCAAAACTGGGGAATTGAACCCATTGACAATGTCATGGGAA<br>TGATAGGAATATTGCCTGATATGACCCCCAGCACAGAGATGTCACTAAGAGGAGTGAGGGTCAGTAAAATGGGAGTGGATGAATATTCCAGTACTGAGAGGGTGGTCGTGAGTATTGATCGCTTCTTGAGGGTACGAGACCAGAGAGGAAATGTACTCTTGTCTCCCGAAGAGGTCAGTGAAACACAGGGAACAGAGAAGCTAACGATAACATATTCATCATCCATGAT<br>GTGGGAAATTAATGGCCCTGAGTCAGTGCTAGTTAACACATATCAATGGATCATCAGAAACTGGGAAACTGTGAAGATTCAGTGGTCCCAAGACCCTACAATGCTATACAACAAGATGGAGTTTGAGCCTTTTCAGTCCTTGGTGCCTAAGGCAGCCAGAGGCCAGTACAGTGGATTTGTAAGGACCCTATTCCAGCAGATGCGTGATGTGCTGGGAACCTTTGACACTGT<br>CCAGATAATAAAGCTACTTCCATTTGCAGCAGCACCACCGGAACAGAGTAGGATGCAGTTCTCTTCTCTAACTGTAAACGTAAGGGGTTCAGGAATGAGAATACTTGTGAGAGGCAACTCCCCTGTGTTCAACTATAATAAAGCAACCAAGAGGCTCATAGTCCTTGGAAAGGATGCTGGTGCATTGACAGAAGACCCAGGTGAGGGGACAGCAGGAGTGGAGTCTGCG<br>GTATTGAGAGGGTTCCTAATTCTGGGCAAAGAGGACAAAAGATATGGACCAGCGCTGAGCATCAATGAATTGAGCAATCTTGCGAAAGGGGAGAAGGCTAATGTGTTGATAGGGCAAGGAGACGTGGTGTTGGTGATGAAACGGAAACGGGACTCTAGCATACTTACTGACAGCCAGACAGCGACCAAAAGAATTCGGATGGCCATCAATTA
internal NVRI repository	>A/duck/Poland/H1969_21RS3290-6/2021_H5N1_2021-11-12<br>ATGGAGAGAATAAAAGAGCTAAGAGATTTGATGTCGCAGTCTCGCACTCGCGAGATACTGACAAAAACCACCGTGGACCATATGGCCATAATCAAGAAATATACATCAGGAAGACAGGAGAAGAACCCTGCACTTAGGATGAAGTGGATGATGGCAATGAAATATCCGATTACAGCAGACAAAAGGATAATGGAGATGATCCCTGAAAGAAACGAGCAAGGTCAGACT<br>CTTTGGAGCAAAACAAATGATGCTGGATCGGATAGAATAATGGTGTCACCTCTGGCTGTGACGTGGTGGAATAGAAATGGACCAACAACAAGTACCGTCCATTACCCAAAGGTCTATAAAACCTACTTTGAAAAGGTTGAAAGGTTAAAGCATGGAACCTTCGGCCCTGTCCATTTCCGGAATCAGGTTAAGATACGCCGCAGAGTTGACATAAACCCGGGCCATGCAGAC<br>CTCAGTGCTAAAGAAGCACAAGACGTCATCATGGAGGTCGTTTTCCCAAATGAAGTCGGAGCCAGAATATTGACATCAGAGTCACAGTTAACAATTACAAAAGAAAAGAAGGAGGAACTCCAGGACTGTAAGATTGCCCCTCTAATGGTGGCATACATGTTGGAGAGAGAACTGGTTCGAAAAACCAGATTCCTGCCAGTAGCTGGCGGAACAAGTAGCGTATATATCGA<br>AGTGTTGCACTTGACTCAAGGAACCTGCTGGGAACAAATGTATACGCCAGGAGGAGAAGTGAGAAATGATGACGTTGACCAAAGTTTAATTATTGCTGCCAGAAATATCGTTAGGAGGGCAACAGTATCAGCAGACCCATTGGCTTCACTACTGGAGATGTGCCATAGTACACAGATTGGCGGGATAAGAATGGTAGACATTCTTAGACAGAACCCAACAGAAGAGCAA<br>GCCGTGGATATATGCAAAGCAGCAATGGGTTTAAGAATCAGTTCATCCTTCAGTTTTGGAGGTTTCACTTTCAAAAGGACAAGTGGATCATCTGTCAAAAGAGAAGAGGAAGTGCTCACAGGCAACCTCCAAACATTGAAAATAAGAGTACATGAAGGGTATGAGGAATTCACAATGGTTGGACGGAGAGCAACAGCCATTCTAAGGAAAGCAACCAGAAGGCTAATCC<br>AATTGATAGTAAGTGGAAGAGACGAGCAGTCAATCGCCGAAGCGATCATAGTGGCAATGGTGTTCTCTCAAGAGGATTGCATGATAAAGGCTGTACGAGGTGATTTAAATTTTGTCAATAGAGCGAATCAGCGGCTCAATCCTATGCATCAGCTCCTGAGGCATTTCCAAAAGGATGCAAAGGTACTATTCCAAAACTGGGGAATTGAACCCATTGACAATGTCATGGGAA<br>TGATAGGAATATTGCCTGATATGACCCCCAGCACAGAGATGTCACTAAGAGGAGTGAGGGTCAGTAAAATGGGAGTGGATGAATATTCCAGTACTGAGAGGGTGGTCGTGAGTATTGATCGCTTCTTGAGGGTACGAGACCAGAGAGGAAATGTACTCTTGTCTCCCGAAGAGGTCAGTGAAACACAGGGAACAGAGAAGCTAACGATAACATATTCATCATCCATGAT<br>GTGGGAAATTAATGGCCCTGAGTCAGTGCTAGTTAACACATATCAATGGATCATCAGAAACTGGGAAACTGTGAAGATTCAGTGGTCCCAAGACCCTACAATGCTATACAACAAGATGGAGTTTGAGCCTTTTCAGTCCTTGGTGCCTAAGGCAGCCAGAGGCCAGTACAGTGGATTTGTAAGGACCCTATTCCAGCAGATGCGTGATGTGCTGGGAACCTTTGACACTGT<br>CCAGATAATAAAGCTACTTCCATTTGCAGCAGCACCACCGGAACAGAGTAGGATGCAGTTCTCTTCTCTAACTGTAAACGTAAGGGGTTCAGGAATGAGAATACTTGTGAGAGGCAACTCCCCTGTGTTCAACTATAATAAAGCAACCAAGAGGCTCATAGTCCTTGGAAAGGATGCTGGTGCATTGACAGAAGACCCAGGTGAGGGGACAGCAGGAGTGGAGTCTGCG<br>GTATTGAGAGGGTTCCTAATTCTGGGCAAAGAGGACAAAAGATATGGACCAGCGCTGAGCATCAATGAATTGAGCAATCTTGCGAAAGGGGAGAAGGCTAATGTGTTGATAGGGCAAGGAGACGTGGTGTTGGTGATGAAACGGAAACGGGACTCTAGCATACTTACTGACAGCCAGACAGCGACCAAAAGAATTCGGATGGCCATCAATTA
internal NVRI repository	>A/duck/Poland/H1971_21RS3290-8/2021_H5N1_2021-11-13<br>ATGGAGAGAATAAAAGAGCTAAGAGATTTGATGTCGCAGTCTCGCACTCGCGAGATACTGACAAAAACCACCGTGGACCATATGGCCATAATCAAGAAATATACATCAGGAAGACAGGAGAAGAACCCTGCACTTAGGATGAAGTGGATGATGGCAATGAAATATCCGATTACAGCAGACAAAAGGATAATGGAGATGATCCCTGAAAGAAACGAGCAAGGTCAGACT<br>CTTTGGAGCAAAACAAATGATGCTGGATCGGATAGAATAATGGTGTCACCTCTGGCTGTGACGTGGTGGAATAGAAATGGACCAACAACAAGTACCGTCCATTACCCAAAGGTCTATAAAACCTACTTTGAAAAGGTTGAAAGGTTAAAGCATGGAACCTTCGGCCCTGTCCATTTCCGGAATCAGGTTAAGATACGCCGCAGAGTTGACATAAACCCGGGCCATGCAGAC<br>CTCAGTGCTAAAGAAGCACAAGACGTCATCATGGAGGTCGTTTTCCCAAATGAAGTCGGAGCCAGAATATTGACATCAGAGTCACAGTTAACAATTACAAAAGAAAAGAAGGAGGAACTCCAGGACTGTAAGATTGCCCCTCTAATGGTGGCATACATGTTGGAGAGAGAACTGGTTCGAAAAACCAGATTCCTGCCAGTAGCTGGCGGAACAAGTAGCGTATATATCGA<br>AGTGTTGCACTTGACTCAAGGAACCTGCTGGGAACAAATGTATACGCCAGGAGGAGAAGTGAGAAATGATGACGTTGACCAAAGTTTAATTATTGCTGCCAGAAATATCGTTAGGAGGGCAACAGTATCAGCAGACCCATTGGCTTCACTACTGGAGATGTGCCATAGTACACAGATTGGCGGGATAAGAATGGTAGACATTCTTAGACAGAACCCAACAGAAGAGCAA<br>GCCGTGGATATATGCAAAGCAGCAATGGGTTTAAGAATCAGTTCATCCTTCAGTTTTGGAGGTTTCACTTTCAAAAGGACAAGTGGATCATCTGTCAAAAGAGAAGAGGAAGTGCTCACAGGCAACCTCCAAACATTGAAAATAAGAGTACATGAAGGGTATGAGGAATTCACAATGGTTGGACGGAGAGCAACAGCCATTCTAAGGAAAGCAACCAGAAGGCTAATCC<br>AATTGATAGTAAGTGGAAGAGACGAGCAGTCAATCGCCGAAGCGATCATAGTGGCAATGGTGTTCTCTCAAGAGGATTGCATGATAAAGGCTGTACGAGGTGATTTAAATTTTGTCAATAGAGCGAATCAGCGGCTCAATCCTATGCATCAGCTCCTGAGGCATTTCCAAAAGGATGCAAAGGTACTATTCCAAAACTGGGGAATTGAACCCATTGACAATGTCATGGGAA<br>TGATAGGAATATTGCCTGATATGACCCCCAGCACAGAGATGTCACTAAGAGGAGTGAGGGTCAGTAAAATGGGAGTGGATGAATATTCCAGTACTGAGAGGGTGGTCGTGAGTATTGATCGCTTCTTGAGGGTACGAGACCAGAGAGGAAATGTACTCTTGTCTCCCGAAGAGGTCAGTGAAACACAGGGAACAGAGAAGCTAACGATAACATATTCATCATCCATGAT<br>GTGGGAAATTAATGGCCCTGAGTCAGTGCTAGTTAACACATATCAATGGATCATCAGAAACTGGGAAACTGTGAAGATTCAGTGGTCCCAAGACCCTACAATGCTATACAACAAGATGGAGTTTGAGCCTTTTCAGTCCTTGGTGCCTAAGGCAGCCAGAGGCCAGTACAGTGGATTTGTAAGGACCCTATTCCAGCAGATGCGTGATGTGCTGGGAACCTTTGACACTGT<br>CCAGATAATAAAGCTACTTCCATTTGCAGCAGCACCACCGGAACAGAGTAGGATGCAGTTCTCTTCTCTAACTGTAAACGTAAGGGGTTCAGGAATGAGAATACTTGTGAGAGGCAACTCCCCTGTGTTCAACTATAATAAAGCAACCAAGAGGCTCATAGTCCTTGGAAAGGATGCTGGTGCATTGACAGAAGACCCAGGTGAGGGGACAGCAGGAGTGGAGTCTGCG<br>GTATTGAGAGGGTTCCTAATTCTGGGCAAAGAGGACAAAAGATATGGACCAGCGCTGAGCATCAATGAATTGAGCAATCTTGCGAAAGGGGAGAAGGCTAATGTGTTGATAGGGCAAGGAGACGTGGTGTTGGTGATGAAACGGAAACGGGACTCTAGCATACTTACTGACAGCCAGACAGCGACCAAAAGAATTCGGATGGCCATCAATTA
internal NVRI repository	>A/duck/Poland/H1980_21RS3290-11/2021_H5N1_2021-11-16<br>ATGGAGAGAATAAAAGAGCTAAGAGATTTGATGTCGCAGTCTCGCACTCGCGAGATACTGACAAAAACCACCGTGGACCATATGGCCATAATCAAGAAATATACATCAGGAAGACAGGAGAAGAACCCTGCACTTAGGATGAAGTGGATGATGGCAATGAAATATCCGATTACAGCAGACAAAAGGATAATGGAGATGATCCCTGAAAGAAACGAGCAAGGTCAGACT<br>CTTTGGAGCAAAACAAATGATGCTGGATCGGATAGAATAATGGTGTCACCTCTGGCTGTGACGTGGTGGAATAGAAATGGACCAACAACAAGTACCGTCCATTACCCAAAGGTCTATAAAACCTACTTTGAAAAGGTTGAAAGGTTAAAGCATGGAACCTTCGGCCCTGTCCATTTCCGGAATCAGGTTAAGATACGCCGCAGAGTTGACATAAACCCGGGCCATGCAGAC<br>CTCAGTGCTAAAGAAGCACAAGACGTCATCATGGAGGTCGTTTTCCCAAATGAAGTCGGAGCCAGAATATTGACATCAGAGTCACAGTTAACAATTACAAAAGAAAAGAAGGAGGAACTCCAGGACTGTAAGATTGCCCCTCTAATGGTGGCATACATGTTGGAGAGAGAACTGGTTCGAAAAACCAGATTCCTGCCAGTAGCTGGCGGAACAAGTAGCGTATATATCGA<br>AGTGTTGCACTTGACTCAAGGAACCTGCTGGGAACAAATGTATACGCCAGGAGGAGAAGTGAGAAATGATGACGTTGACCAAAGTTTAATTATTGCTGCCAGAAATATCGTTAGGAGGGCAACAGTATCAGCAGACCCATTGGCTTCACTACTGGAGATGTGCCATAGTACACAGATTGGCGGGATAAGAATGGTAGACATTCTTAGACAGAACCCAACAGAAGAGCAA<br>GCCGTGGATATATGCAAAGCAGCAATGGGTTTAAGAATCAGTTCATCCTTCAGTTTTGGAGGTTTCACTTTCAAAAGGACAAGTGGATCATCTGTCAAAAGAGAAGAGGAAGTGCTCACAGGCAACCTCCAAACATTGAAAATAAGAGTACATGAAGGGTATGAGGAATTCACAATGGTTGGACGGAGAGCAACAGCCATTCTAAGGAAAGCAACCAGAAGGCTAATCC<br>AATTGATAGTAAGTGGAAGAGACGAGCAGTCAATCGCCGAAGCGATCATAGTGGCAATGGTGTTCTCTCAAGAGGATTGCATGATAAAGGCTGTACGAGGTGATTTAAATTTTGTCAATAGAGCGAATCAGCGGCTCAATCCTATGCATCAGCTCCTGAGGCATTTCCAAAAGGATGCAAAGGTACTATTCCAAAACTGGGGAATTGAACCCATTGACAATGTCATGGGAA<br>TGATAGGAATATTGCCTGATATGACCCCCAGCACAGAGATGTCACTAAGAGGAGTGAGGGTCAGCAAAATGGGAGTGGATGAATATTCCAGTACTGAGAGGGTGGTCGTGAGTATTGATCGCTTCTTGAGGGTACGAGACCAGAGAGGAAATGTACTCTTGTCTCCCGAAGAGGTCAGTGAAACACAGGGAACAGAGAAGCTAACGATAACATATTCATCATCCATGAT<br>GTGGGAAATTAATGGCCCTGAGTCAGTGCTAGTTAACACATATCAATGGATCATCAGAAACTGGGAAACTGTGAAGATTCAGTGGTCCCAAGACCCTACAATGCTATACAACAAGATGGAGTTTGAGCCTTTTCAGTCCTTGGTGCCTAAGGCAGCCAGAGGCCAGTACAGTGGATTTGTAAGGACCCTATTCCAGCAGATGCGTGATGTGCTGGGAACCTTTGACACTGT<br>CCAGATAATAAAGCTACTTCCATTTGCAGCAGCACCACCGGAACAGAGTAGGATGCAGTTCTCTTCTCTAACTGTAAACGTAAGGGGTTCAGGAATGAGAATACTTGTGAGAGGCAACTCCCCTGTGTTCAACTATAATAAAGCAACCAAGAGGCTCATAGTCCTTGGAAAGGATGCTGGTGCATTGACAGAAGACCCAGGTGAGGGGACAGCAGGAGTGGAGTCTGCG<br>GTATTGAGAGGGTTCCTAATTCTGGGCAAAGAGGACAAAAGATATGGACCAGCGCTGAGCATCAATGAATTGAGCAATCTTGCGAAAGGGGAGAAGGCTAATGTGTTGATAGGGCAAGGAGACGTGGTGTTGGTGATGAAACGGAAACGGGACTCTAGCATACTTACTGACAGCCAGACAGCGACCAAAAGAATTCGGATGGCCATCAATTA
internal NVRI repository	>A/duck/Poland/H1982_21RS3290-12/2021_H5N1_2021-11-16<br>ATGGAGAGAATAAAAGAGCTAAGAGATTTGATGTCGCAGTCTCGCACTCGCGAGATACTGACAAAAACCACCGTGGACCATATGGCCATAATCAAGAAATATACATCAGGAAGACAGGAGAAGAACCCTGCACTTAGGATGAAGTGGATGATGGCAATGAAATATCCGATTACAGCAGACAAAAGGATAATGGAGATGATCCCTGAAAGAAACGAGCAAGGTCAGACT<br>CTTTGGAGCAAAACAAATGATGCTGGATCGGATAGAATAATGGTGTCACCTCTGGCTGTGACGTGGTGGAATAGAAATGGACCAACAACAAGTACCGTCCATTACCCAAAGGTCTATAAAACCTACTTTGAAAAGGTTGAAAGGTTAAAGCATGGAACCTTCGGCCCTGTCCATTTCCGGAATCAGGTTAAGATACGCCGCAGAGTTGACATAAACCCGGGCCATGCAGAC<br>CTCAGTGCTAAAGAAGCACAAGACGTCATCATGGAGGTCGTTTTCCCAAATGAAGTCGGAGCCAGAATATTGACATCRGAGTCACAGTTAACAATTACAAAAGAAAAGAAGGAGGAACTCCAGGACTGTAAGATTGCCCCTCTAATGGTGGCATACATGTTGGAGAGAGAACTGGTTCGAAAAACCAGATTCCTGCCAGTAGCTGGCGGAACAAGTAGCGTATATATCGA<br>AGTGTTGCACTTGACTCAAGGAACCTGCTGGGAACAAATGTATACGCCAGGAGGAGAAGTGAGAAATGATGACGTTGACCAAAGTTTAATTATTGCTGCCAGAAATATCGTTAGGAGGGCAACAGTATCAGCAGACCCATTGGCTTCACTACTGGAGATGTGCCATAGTACACAGATTGGCGGGATAAGAATGGTAGACATTCTTAGACAGAACCCAACAGAAGAGCAA<br>GCCGTGGATATATGCAAAGCAGCAATGGGTTTAAGAATCAGTTCATCCTTCAGTTTTGGAGGTTTCACTTTCAAAAGGACAAGCGGATCATCTGTCAAAAGAGAAGAGGAAGTGCTCACAGGCAACCTCCAAACATTGAAAATAAGAGTACATGAAGGGTATGAGGAATTCACAATGGTTGGACGGAGAGCAACAGCCATTCTAAGGAAAGCAACCAGAAGGCTAATCC<br>AATTGATAGTAAGTGGAAGAGACGAGCAGTCAATCGCCGAAGCGATCATAGTGGCAATGGTGTTCTCTCAAGAGGATTGCATGATAAAGGCTGTACGAGGTGATTTAAATTTTGTCAATAGAGCGAATCAGCGGCTCAATCCTATGCATCAGCTCCTGAGGCATTTCCAAAAGGATGCAAAGGTACTATTCCAAAACTGGGGAATTGAACCCATTGACAATGTCATGGGAA<br>TGATAGGAATATTGCCTGATATGACCCCCAGCACAGAGATGTCACTAAGAGGAGTGAGGGTCAGTAAAATGGGAGTGGATGAATATTCCAGTACTGAGAGGGTGGTCGTGAGTATTGATCGCTTCTTGAGGGTACGAGACCAGAGAGGAAATGTACTCTTGTCTCCCGAAGAGGTCAGTGAAACACAGGGAACAGAGAAGCTAACGATAACATATTCATCATCCATGAT<br>GTGGGAAATTAATGGCCCTGAGTCAGTGCTAGTTAACACATATCAATGGATCATCAGAAACTGGGAAACTGTGAAGATTCAGTGGTCCCAAGACCCTACAATGCTATACAACAAGATGGAGTTTGAGCCTTTTCAGTCCTTGGTGCCTAAGGCAGCCAGAGGCCAGTACAGTGGATTTGTAAGGACCCTATTCCAGCAGATGCGTGATGTGCTGGGAACCTTTGACACTGT<br>CCAGATAATAAAGCTACTTCCATTTGCAGCAGCACCACCGGAACAGAGTAGGATGCAGTTCTCTTCTCTAACTGTAAACGTAAGGGGTTCAGGAATGAGAATACTTGTGAGAGGCAACTCCCCTGTGTTCAACTATAATAAAGCAACCAAGAGGCTCATAGTCCTTGGAAAGGATGCTGGTGCATTGACAGAAGACCCAGGTGAGGGGACAGCAGGAGTGGAGTCTGCG<br>GTATTGAGAGGGTTCCTAATTCTGGGCAAAGAGGACAAAAGATATGGACCAGCGCTGAGCATCAATGAATTGAGCAATCTTGCGAAAGGGGAGAAGGCTAATGTGTTGATAGGGCAAGGAGACGTGGTGTTGGTGATGAAACGGAAACGGGACTCTAGCATACTTACTGACAGCCAGACAGCGACCAAAAGAATTCGGATGGCCATCAATTA
internal NVRI repository	>A/duck/Poland/H1993_21RS3290-14/2021_H5N1_2021-11-17<br>ATGGAGAGAATAAAAGAGCTAAGAGATTTGATGTCGCAGTCTCGCACTCGCGAGATACTGACAAAAACCACCGTGGACCATATGGCCATAATCAAGAAATATACATCAGGAAGACAGGAGAAGAACCCTGCACTTAGGATGAAGTGGATGATGGCAATGAAATATCCGATTACAGCAGACAAAAGGATAATGGAGATGATCCCTGAAAGAAACGAGCAAGGTCAGACT<br>CTTTGGAGCAAAACAAATGATGCTGGATCGGATAGAATAATGGTGTCACCTCTGGCTGTGACGTGGTGGAATAGAAATGGACCAACAACAAGTACCGTCCATTACCCAAAGGTCTATAAAACCTACTTTGAAAAGGTTGAAAGGTTAAAGCATGGAACCTTCGGCCCTGTCCATTTCCGGAATCAGGTTAAGATACGCCGCAGAGTTGACATAAACCCGGGCCATGCAGAC<br>CTCAGTGCTAAAGAAGCACAAGACGTCATCATGGAGGTCGTTTTCCCAAATGAAGTCGGAGCCAGAATATTGACATCAGAGTCACAGTTAACAATTACAAAAGAAAAGAAGGAGGAACTCCAGGACTGTAAGATTGCCCCTCTAATGGTGGCATACATGTTGGAGAGAGAACTGGTTCGAAAAACCAGATTCCTGCCAGTAGCTGGCGGAACAAGTAGCGTATATATCGA<br>AGTGTTGCACTTGACTCAAGGAACCTGCTGGGAACAAATGTATACGCCAGGAGGAGAAGTGAGAAATGATGACGTTGACCAAAGTTTAATTATTGCTGCCAGAAATATCGTTAGGAGGGCAACAGTATCAGCAGACCCATTGGCTTCACTACTGGAGATGTGCCATAGTACACAGATTGGCGGGATAAGAATGGTAGACATTCTTAGACAGAACCCAACAGAAGAGCAA<br>GCCGTGGATATATGCAAAGCAGCAATGGGTTTAAGAATCAGTTCATCCTTCAGTTTTGGAGGTTTCACTTTCAAAAGGACAAGTGGATCATCTGTCAAAAGAGAAGAGGAAGTGCTCACAGGCAACCTCCAAACATTGAAAATAAGAGTACATGAAGGGTATGAGGAATTCACAATGGTTGGACGGAGAGCAACAGCCATTCTAAGGAAAGCAACCAGAAGGCTAATCC<br>AATTGATAGTAAGTGGAAGAGACGAGCAGTCAATCGCCGAAGCGATCATAGTGGCAATGGTGTTCTCTCAAGAGGATTGCATGATAAAGGCTGTACGAGGTGATTTAAATTTTGTCAATAGAGCGAATCAGCGGCTCAATCCTATGCATCAGCTCCTGAGGCATTTCCAAAAGGATGCAAAGGTACTATTCCAAAACTGGGGAATTGAACCCATTGACAATGTCATGGGAA<br>TGATAGGAATATTGCCTGATATGACCCCCAGCACAGAGATGTCACTAAGAGGAGTGAGGGTCAGTAAAATGGGAGTGGATGAATATTCCAGTACTGAGAGGGTGGTCGTGAGTATTGATCGCTTCTTGAGGGTACGAGACCAGAGAGGAAATGTACTCTTGTCTCCCGAAGAGGTCAGTGAAACACAGGGAACAGAGAAGCTAACGATAACATATTCATCATCCATGAT<br>GTGGGAAATTAATGGCCCTGAGTCAGTGCTAGTTAACACATATCAATGGATCATCAGAAACTGGGAAACTGTGAAGATTCAGTGGTCCCAAGACCCTACAATGCTATACAACAAGATGGAGTTTGAGCCTTTTCAGTCCTTGGTGCCTAAGGCAGCCAGAGGCCAGTACAGTGGATTTGTAAGGACCCTATTCCAGCAGATGCGTGATGTGCTGGGAACCTTTGACACTGT<br>CCAGATAATAAAGCTACTTCCATTTGCAGCAGCACCACCGGAACAGAGTAGGATGCAGTTCTCTTCTCTAACTGTAAACGTAAGGGGTTCAGGAATGAGAATACTTGTGAGAGGCAACTCCCCTGTGTTCAACTATAATAAAGCAACCAAGAGGCTCATAGTCCTTGGAAAGGATGCTGGTGCATTGACAGAAGACCCAGGTGAGGGGACAGCAGGAGTGGAGTCTGCG<br>GTATTGAGAGGGTTCCTAATTCTGGGCAAAGAGGACAAAAGATATGGACCAGCGCTGAGCATCAATGAATTGAGCAATCTTGCGAAAGGGGAGAAGGCTAATGTGTTGATAGGGCAAGGAGACGTGGTGTTGGTGATGAAACGGAAACGGGACTCTAGCATACTTACTGACAGCCAGACAGCGACCAAAAGAATTCGGATGGCCATCAATTA
internal NVRI repository	>A/duck/Poland/H2008_21RS3290-16/2021_H5N1_2021-11-18<br>ATGGAGAGAATAAAAGAGCTAAGAGATTTGATGTCGCAGTCTCGCACTCGCGAGATACTGACAAAAACCACCGTGGACCATATGGCCATAATCAAGAAATATACATCAGGAAGACAGGAGAAGAACCCTGCACTTAGGATGAAGTGGATGATGGCAATGAAATATCCGATTACAGCAGACAAAAGGATAATGGAGATGATCCCTGAAAGAAACGAGCAAGGTCAGACT<br>CTTTGGAGCAAAACAAATGATGCTGGATCGGATAGAATAATGGTGTCACCTCTGGCTGTGACGTGGTGGAATAGAAATGGACCAACAACAAGTACCGTCCATTACCCAAAGGTCTATAAAACCTACTTTGAAAAGGTTGAAAGGTTAAAGCATGGAACCTTCGGCCCTGTCCATTTCCGGAATCAGGTTAAGATACGCCGCAGAGTTGACATAAACCCGGGCCATGCAGAC<br>CTCAGTGCTAAAGAAGCACAAGACGTCATCATGGAGGTCGTTTTCCCAAATGAAGTCGGAGCCAGAATATTGACATCAGAGTCACAGTTAACAATTACAAAAGAAAAGAAGGAGGAACTCCAGGACTGTAAGATTGCCCCTCTAATGGTGGCATACATGTTGGAGAGAGAACTGGTTCGAAAAACCAGATTCCTGCCAGTAGCTGGCGGAACAAGTAGCGTATATATCGA<br>AGTGTTGCACTTGACTCAAGGAACCTGCTGGGAACAAATGTATACGCCAGGAGGAGAAGTGAGAAATGATGACGTTGACCAAAGTTTAATTATTGCTGCCAGAAATATCGTTAGGAGGGCAACAGTATCAGCAGACCCATTGGCTTCACTACTGGAGATGTGCCATAGTACACAGATTGGCGGGATAAGAATGGTAGACATTCTTAGACAGAACCCAACAGAAGAGCAA<br>GCCGTGGATATATGCAAAGCAGCAATGGGTTTAAGAATCAGTTCATCCTTCAGTTTTGGAGGTTTCACTTTCAAAAGGACAAGCGGATCATCTGTCAAAAGAGAAGAGGAAGTGCTCACAGGCAACCTCCAAACATTGAAAATAAGAGTACATGAAGGGTATGAGGAATTCACAATGGTTGGACGGAGAGCAACAGCCATTCTAAGGAAAGCAACCAGAAGGCTAATCC<br>AATTGATAGTAAGTGGAAGAGACGAGCAGTCAATCGCCGAAGCGATCATAGTGGCAATGGTGTTCTCTCAAGAGGATTGCATGATAAAGGCTGTACGAGGTGATTTAAATTTTGTCAATAGAGCGAATCAGCGGCTCAATCCTATGCATCAGCTCCTGAGGCATTTCCAAAAGGATGCAAAGGTACTATTCCAAAACTGGGGAATTGAACCCATTGACAATGTCATGGGAA<br>TGATAGGAATATTGCCTGATATGACCCCCAGCACAGAGATGTCACTAAGAGGAGTGAGGGTCAGTAAAATGGGAGTGGATGAATATTCCAGTACTGAGAGGGTGGTCGTGAGTATTGATCGCTTCTTGAGGGTACGAGACCAGAGAGGAAATGTACTCTTGTCTCCCGAAGAGGTCAGTGAAACACAGGGAACAGAGAAGCTAACGATAACATATTCATCATCCATGAT<br>GTGGGAAATTAATGGCCCTGAGTCAGTGCTAGTTAACACATATCAATGGATCATCAGAAACTGGGAAACTGTGAAGATTCAGTGGTCCCAAGACCCTACAATGCTATACAACAAGATGGAGTTTGAGCCTTTTCAGTCCTTGGTGCCTAAGGCAGCCAGAGGCCAGTACAGTGGATTTGTAAGGACCCTATTCCAGCAGATGCGTGATGTGCTGGGAACCTTTGACACTGT<br>CCAGATAATAAAGCTACTTCCATTTGCAGCAGCACCACCGGAACAGAGTAGGATGCAGTTCTCTTCTCTAACTGTAAACGTAAGGGGTTCAGGAATGAGAATACTTGTGAGAGGCAACTCCCCTGTGTTCAACTATAATAAAGCAACCAAGAGGCTCATAGTCCTTGGAAAGGATGCTGGTGCATTGACAGAAGACCCAGGTGAGGGGACAGCAGGAGTGGAGTCTGCG<br>GTATTGAGAGGGTTCCTAATTCTGGGCAAAGAGGACAAAAGATATGGACCAGCGCTGAGCATCAATGAATTGAGCAATCTTGCGAAAGGGGAGAAGGCTAATGTGTTGATAGGGCAAGGAGACGTGGTGTTGGTGATGAAACGGAAACGGGACTCTAGCATACTTACTGACAGCCAGACAGCGACCAAAAGAATTCGGATGGCCATCAATTA

internal NVRI repository	>A/duck/Poland/H2014_21RS3290-17/2021_H5N1_2021-11-20<br>[illegible]
EPI_ISL_11922811	>A/goose/Poland/H124_22VIR2515-5/2022_H5N1_2022-02-05<br>[illegible]
EPI_ISL_18245818	>A/common_tern/Poland/MB143/2022_H5N1_2022-06-15<br>[illegible]
EPI_ISL_14917915	>A/breeding_hen/Poland/H361/2022_H5N1_2022-07-18<br>[illegible]
EPI_ISL_14917942	>A/breeding_hen/Poland/H364/2022_H5N1_2022-07-21<br>[illegible]
EPI_ISL_11922812	>A/chicken/Poland/H071_22VIR2515-6/2022_H5N1_2022-01-23<br>[illegible]
EPI_ISL_11922809	>A/chicken/Poland/H157_22VIR2515-3/2022_H5N1_2022-02-18<br>[illegible]
EPI_ISL_11922807	>A/chicken/Poland/H182_22VIR2515-1/2022_H5N2_2022-02-28<br>[illegible]
internal NVRI repository	>A/chicken/Poland/H1928_21RS3290-2/2021_H5N1_2021-11-03<br>[illegible]
EPI_ISL_6931288	>A/chicken/Poland/H1940-N/2021_H5N1_2021-11-05<br>[illegible]
internal NVRI repository	>A/chicken/Poland/H1962_21RS3290-4/2021_H5N1_2021-11-11<br>[illegible]
internal NVRI repository	>A/chicken/Poland/H1976_21RS3290-9/2021_H5N1_2021-11-15<br>[illegible]

internal NVRI repository	>A/chicken/Poland/H1983_21RS3290-13/2021_H5N1_2021-11-16<br>[illegible]
internal NVRI repository	>A/chicken/Poland/H2130-T1c/2021_H5N1_2021-12-20<br>[illegible]
internal NVRI repository	>A/chicken/Poland/H2175-N/2021_H5N1_2021-12-18<br>[illegible]
internal NVRI repository	>A/chicken/Poland/H36/2022_H5N1_2022-01-08<br>[illegible]
internal NVRI repository	>A/chicken/Poland/H62-N/2022_H5N1_2022-01-17<br>[illegible]
EPI_ISL_502603	>A/Anas_platyrhynchos/Belgium/11442-39/2016_H5N3_2016-11-04<br>[illegible]
EPI_ISL_7591585	>A/Anas_platyrhynchos/Belgium/827/2020_H3N8_2020-01-19<br>[illegible]
EPI_ISL_7593059	>A/Anas_platyrhynchos/Belgium/8751-0002/2020_H4N6_2020-08-27<br>[illegible]
EPI_ISL_2234811	>A/anser_anser/Spain/102-8-21VIR1230-4/2021_H5N8_2021-01-07<br>[illegible]
EPI_ISL_18245197	>A/mute_swan/Poland/MB292-21_22VIR5675-14/2021_H5N8_2021-03-11<br>[illegible]
EPI_ISL_267243	>A/barnacle_goose/Netherlands/2/2014_H3N6_2014-12-15<br>[illegible]
EPI_ISL_327625	>A/black_skimmer/Chile/C20100/2016_H13N9_2016-12-13<br>[illegible]

EPI_ISL_4031461	>A/black-headed_gull/Denmark/4663-1/2018_H5N6_2018-03-26<br>ATGGAGAGAATAAAAGAACTAAGAGATTTGATGTCGCAGTCTCGCACTCGCGAGATACTGACAAAAACCACTGTGGACCATATGGCCATAATCAAGAAATATACGTCAGGAAGACAGGAGAAGAATCCTGCTCTTAGGATGAAATGGATGATGGCAATGAAATATCCGATTACAGCGGACAAAAGGATAATGGAGATGATCCCTGAAAGAATGAGCAAGGTCAGACT<br>CTTTGGAGCAAAACAAATGATGCTGGATCGGACAGAGTTATGGTGTCACCTCTGGCTGTGACGTGGTGGAATAGAAATGGGCCAACGACAAGTACAGTCCATTATCCAAAGGTCTATAAAACCTACTTTGAAAAGGTCGAAAGGTTAAAGCATGGAACCTTCGGTCCCGTCCACTTTCGAAATCAGGTCAAAATACGCCGCAGGGTTGACATAAACCCAGGCCATGCAGA<br>TCTCAGTGCCAAAGAAGCACAGGATGTCATCATGGAGGTCGTTTTCCCAAATGAAGTCGGAGCCAGGATATTGACATCAGAGTCACAGTTAACAATAACAAAGGAAAAGAAGGAGGAGCTTCAGGACTGTAAGATTGCTCCTTTAATGGTGGCATACATGTTGGAGAGAGAATTGGTTCGCAAAACCAGATTCCTACCAGTAGCTGGTGGGACAAGCAGCGTGTACATTG<br>AAGTGTTGCACTTGACTCAAGGGACCTGCTGGGAACAAATGTACACGCCGGGAGGGGAGGTGAGAAATGATGATGTTGATCAGAGTTTAATTATTGCTGCTCGAAATATTGTTAGGAGAGCAACAGTATCAGCAGACCCATTGGCTTCGCTCTTGGAGATGTGCCATAGTACGCAAATTGGCGGGATAAGGATGGTAGACATCCTTAGACAAAACCCAACGGAAGAGCA<br>AGCTGTGGACATATGCAAAGCTGCAATGGGTCTAAGGATCAGTTCATCCTTCAGCTTTGGAGGTTTCACTTTCAAGAGGACGAGTGGGTCATCTGTCAAAAGAGAAGAAGAAGTGCTCACAGGCAACCTCCAGACATTGAAAATAAGAGTGCATGAAGGATATGAGGAATTCACAATGGTTGGGCGAAGAGCAACAGCTATTCTAAGGAAAGCAACCAGAAGGCTGATC<br>CAATTGATAGTGAGTGGGAGAGACGAGCAGTCAATCGCCGAGGCGATCATAGTGGCAATGGTGTTCTCACAAGAGGACTGTATGATAAAAGCAGTACGAGGTGATCTGAATTTTGTCAACAGAGCAAACCAGCGGTTAAATCCTATGCATCAACTATTGAGGCATTTCCAGAAGGATGCAAAGGTGCTGTTTCAAAACTGGGGAATTGAGCCCATCGACAATGTCATGGG<br>GATGATCGGAATATTACCTGACATGACCCCCAGCACAGAGATGTCACTAAGAGGAGTGAGAGTCAGTAAAATGGGAGTGGATGAATACTCCAGTACTGAGAGAGTGGTTGTGAGCATTGATCGCTTCTTGAGGGTCCGAGACCAGAGGGGAAACGTGCTCTTGTCTCCTGAAGAAGTTAGTGAAACACAGGGAACAGAAAAGCTGACGATAACATATTCATCATCCATG<br>ATGTGGGAAATCAACGGCCCGGAATCAGTGTTAGTGAACACATATCAATGGATCATTAGAAATTGGGAAACTGTGAAAATTCAGTGGTCCCAAGACCCTACAATGCTATACAATAAGATGGAGTTTGAGCCCTTTCAATCCTTGGTGCCTAAGGCTGCCAGAGGTCAGTACAGTGGATTTGTGAGAACGCTATTCCAGCAGATGCGTGATGTGCTGGGGACATTTGACACT<br>GTCCAGATAATAAAGCTGCTACCATTTGCAGCAGCCCCACCAGAACAGAGTAGAATGCAGTTCTCTTCTCTAACCGTGAACGTGAGGGGTTCAGGAATGAGAATACTTGTGAGAGGCAACTCCCCTGTGTTCAACTATAACAAGGCGACCAAGAGGCTTACAGTCCTTGGAAAGGATGCAGGTGCATTGACAGAAGATCCAGATGAGGGAACAGCAGGAGTGGAATCTG<br>CGGTACTAAGAGGATTTCTAATTCTAGGCAAAGAAGACAAAAGATATGGACCAGCATTGAGCATCAACGAATTGAGCAATCTTGCGAAAGGGGAGAAGGCTAATGTGTTGATAGGGCAAGGAGACGTGGTGTTGGTAATGAAACGGAAACGGGACTCTAGCATACTTACTGACAGCCAGACAGCGACCAAAAGAATTCGGATGGCCATCAATTA
EPI_ISL_14917999	>A/black-headed_gull/Poland/MB139/2022_H5N1_2022-05-30<br>ATGGAGAGAATAAAAGAACTAAGAGATTTAATGTCACAGTCTCGCACTCGCGAGATACTGACAAAAACCACTGTGGACCATATGGCCATAATAAAGAAATACACATCAGGAAGACAGGAGAAAAACCCTGCCCTCAGGATGAAGTGGATGATGGCAATGAAATATCCTATTACAGCAGACAAAAGAATAATGGAGATGATCCCTGAAAGGAATGAGCAAGGTCAGACT<br>CTCTGGAGCAAAACAAATGACGCTGGATCAGACAGAGTGATGGTGTCACCTCTGGCTGTGACATGGTGGAATAGAAATGGGCCAACGACAAGTACAGTCCATTACCCAAAGGTTTACAAAACCTACTTTGAAAAAGTGGAAAGATTGAAACATGGAACCTTTGGCCCTGTTCACTTTCGAAATCAGGTTAAGATACGCCGCAGGGTTGACATAAACCCGGGTCATGCAGA<br>TCTCAGTGCCAAAGAAGCACAGGATGTCATCATGGAAGTCGTTTTCCCAAATGAAGTTGGAGCCAGGATATTGACATCAGAATCACAATTAACAATAACAAAGGAAAAGAAGGAGGAACTTCAGGACTGTAAGATTGCTCCTTTGATGGTGGCATACATGTTGGAGAGAGAACTGGTTCGAAAAACCAGATTTTTACCAGTAGCTGGCGGAACAAGCAGCGTATATATCG<br>AAGTATTGCATTTGACTCAAGGGACCTGCTGGGAACAAATGTACACACCGGGAGGGGAAGTGAGAAATGATGATGTTGATCAGAGTTTGATCATTGCTGCTAGAAATATCGTTAGGAGAGCAACAGTATCAGCAGACCCATTGGCTTCGCTCTTGGAAATGTGCCACAGCACACAAATTGGCGGGATAAGGATGGTAGACATTCTTAGACAGAACCCAACAGAAGAGCA<br>AGCCGTGGATATATGCAAAGCAGCAATGGGTTTAAGAATCAGTTCATCCTTCAGCTTTGGAGGTTTCACTTTCAAAAGAACAAGTGGATCGTCTGTCAAAAGAGAAGAAGAAGTGCTCACAGGCAACCTCCAAACACTGAAAATAAGAGTACATGAAGGATATGAGGAATTCACAATGGTTGGACGGAGAGCTACAGCCATTTTAAGGAAAGCAACCAGAAGGCTGATCC<br>AATTAATAGTGAGTGGAAGAGACGAGCAGTCAATCGCTGAAGCAATCATAGTGGCAATGGTTTTCTCACAAGAGGACTGCATGATAAAAGCAGTACGAGGTGATCTGAACTTTGTTAATAGAGCAAATCAGCGACTAAATCCTATGCATCAACTTCTGAGGCATTTCCAAAAGGATGCAAAGGTGCTGTTTCAAAACTGGGGGGTTGAACCAATTGACAATGTAATGGGG<br>ATGATTGGAATACTGCCTGACATGACCCCCAGCACGGAGATGTCACTGAGAGGAGTGAGAGTCAGCAAAATGGGAGTGGATGAATATTCCAGTACTGAGAGAGTGGTCGTGAGCATTGATCGTTTCTTGAGAGTCCGAGATCAAAGAGGAAACGTGCTCCTGTCTCCGGAGGAAGTTAGTGAAACACAGGGAACAGAGAAACTGACGATAACATATTCATCGTCCATGA<br>TGTGGGAAATCAATGGTCCGGAATCCGTGCTAGTCAACACATATCAATGGATCATCAGAAATTGGGAGACTGTGAAGATTCAATGGTCCCAAGACCCTACGATGTTGTACAATAAGATGGAATTTGAGCCCTTCCAATCCTTGGTGCCCAAGGCTGCCAGAGGCCAGTATAGTGGATTTGTGAGGACATTGTTCCAGCAGATGCGTGATGTGCTGGGAACATTTGATACTGT<br>CCAAATAATAAAGCTCCTGCCTTTTGCTGCAGCCCCACCGGAACAGAGTAGGATGCAGTTTTCCTCTCTGACTGTGAACGTAAGAGGTTCAGGAATGAGAATACTTGTGAGAGGCAACTCCCCTGTGTTCAACTATAACAAGGCCACCAAGAGACTCACAGTTCTTGGAAAGGATGCAGGTGCCTTGACAGAAGATCCAGATGAGGGAACAGCAGGAGTAGAGTCTGCAG<br>TATTAAGAGGATTTCTAATTCTGGGCAAAGAGGACAAAAGATATGGACCAGCATTGAGCATCAACGAACTGAGCAATCTTGCGAAAGGGGAAAAGGCTAATGTGTTGATAGGGCAAGGAGACGTGGTGTTGGTAATGAAACGGAAACGGGACTCTAGCATACTTACTGACAGCCAGACAGCGACCAAAAGAATTCGGATGGCCATCAATTA
EPI_ISL_17978956	>A/black-headed_gull/Poland/P075/2021_H9N7_2021-02-09<br>ATGGAGAGAATAAAAGAACTAAGAGATTTGATGTCACAGTCTCGCACTCGCGAGATACTGACAAAAACCACTGTGGACCATATGGCCATAATAAAGAAATACACATCAGGAAGACAAGAGAAGAACCCTGCTCTTAGGATGAAATGGATGATGGCAATGAAATATCCGATTACAGCAGACAAAAGAATAATGGAGATGATCCCTGAAAGAATGAGCAAGGCCAAACT<br>CTCTGGAGCAAAACAAATGATGCTGGATCAGACAGAGTGATGGTATCACCTCTAGCTGTGACATGGTGGAATAGAAATGGGCCAACGACAAGTACAGTCCATTATCCAAAGGTTTACAAAACCTACTTCGAAAAAGTCGAAAGGTTAAAACATGGGACCTTTGGTCCTGTTCACTTTCGAAATCAGGTCAAAATACGCCGCAGGGTCGACATAAACCCGGGCCATGCAGA<br>TCTCAGTGCTAAAGAAGCACAAGATGTCATCATGGAGGTCGTTTTCCCAAACGAAGTTGGAGCCAGGATATTGACATCAGAGTCACAATTAACAATAACAAAGGAAAAGAAGGAGGAGCTTCAAGACTGTAAGATTGCTCCTTTGATGGTGGCATACATGTTGGAGAGGGAACTGGTTCGCAAAACCAGATTCCTACCAGTAGCTGGCGGAACAAGCAGTGTGTATATAG<br>AGGTGTTGCATTTGACTCAAGGGACTTGCTGGGAACAAATGTACACACCGGGAGGAGAGGTGAGAAATGATGATGTCGATCAAAGTTTGATTATTGCTGCTAGAAATATTGTCAGGAGAGCAACAGTATCAGCAGACCCTTTGGCTTCACTCTTGGAGATGTGCCATAGTACACAAATTGGAGGGATAAGGATGGTTGACATTCTTAGACAAAACCCAACAGAAGAACAA<br>GCTGTGGATATATGCAAAGCAGCAATGGGTCTAAGAATCAGTTCATCCTTCAGCTTTGGAGGTTTCACTTTCAAAAGGACAAGTGGGTCATCTGTCAAGAAAGAAGAAGAAGTGCTCACAGGCAATCTCCAAACATTGAAAATAAGGGTACACGAAGGATATGAGGAATTCACAATGGTTGGGCGAAGAGCAACCGCCATTCTAAGGAAAGCAACCAGAAGGTTGATAC<br>AATTAATAGTGAGTGGGAGGGACGAGCAGTCAATTGCTGAAGCGATCATAGTGGCAATGGTTTTTTCACAAGAAGACTGCATGATAAAGGCAGTACGAGGTGATTTGAATTTTGTCAACAGAGCAAACCAGCGGCTAAATCCTATGCATCAACTTCTGAGACATTTCCAGAAGGATGCAAAAGTACTGTTTCAGAACTGGGGAGTTGAATCCATTGACAATGTCATGGGG<br>ATGATCGGAATACTGCCTGACATGACCCCAAGCACAGAGATGTCTCTGAGAGGAGTGAGAGTCAGTAAAATGGGAGTGGATGAATATTCCAGTACTGAGAGGGTTGTTGTGAGCATTGACCGTTTCTTAAGGGTCCGAGATCAAAGGGGAAATGTACTCTTATCTCCCGAGGAGGTCAGTGAAACGCAAGGAACAGAGAAACTAACGATAACATATTCATCGTCCATGAT<br>GTGGGAAATCAATGGTCCGGAATCAGTGCTGGTCAACACATATCAATGGATTATTAGAAATTGGGAAACTGTGAAGATTCAGTGGTCACAAGACCCTACAATGTTATACAATAAGATGGAATTTGAGCCTTTCCAATCCTTGGTGCCCAAGGCTGCCAGAGGCCAGTATAGTGGGTTCGTGAGGACGTTATTCCAGCAGATGCGTGATGTGCTGGGGACATTTGACACTGT<br>CCAAATAATAAAGCTCCTACCATTTGCAGCAGCCCCGCCAGAACAGAGTAGGATGCAGTTCTCTTCTCTGACTGTGAATGTAAGGGGTTCAGGGATGAGAATACTTGTGAGAGGCAATTCCCCTGTGTTCAACTATAACAAGGCAACCAAACGGCTCACAGTCCTTGGAAAGGATGCAGGTGCCTTGACAGAAGATCCAGACGAGGGAACAGCAGGAGTGGAATCTGCG<br>GTGTTAAGAGGATTTCTAATTCTAGGCAAAGAAGATAAGAGGTATGGCCCAGCGTTGAGCATCAACGAATTGAGCAATCTTGCGAAAGGGGAGAAAGCTAATGTGTTGATAGGGCAAGGAGACGTGGTGTTGGTAATGAAACGGAAACGGGACTCTAGCATACTTACTGACAGCCAGACAGCGACCAAAAGGATTCGGATGGCCATCAATTA
EPI_ISL_308785	>A/black-tailed_gull/Weihai/17/2016_H13N8_2016-12-28<br>ATGGAGAGAATAAAAGAACTAAGAGATCTAATGTCGCAGTCTCGCACTCGCGAGATACTCACCAAAACCACTGTGGACCACATGGCAATAATCAAAAAATACACATCGGGAAGGCAAGAGAAAAACCCCGCACTAAGGATGAAATGGATGATGGCAATGAAATACCCAATCACTGCAGAGAAGAGAATAATGGAAATGATTCCTGAAAGGAATGAGCAAGGACAAACC<br>CTTTGGAGTAAAACAAACGATGCCGGATCGGACCGAGTAATGGTATCGCCTCTCGCTGTGACGTGGTGGAACAGGAATGGACCAACAGCAAGTACAGTCCACTACCCAAAGGTATACAAAACTTATTTTGAAAAAGTTGAAAGGTTGAAACACGGGACCTTTGGCCCTGTCCACTTCAGAAATCAAGTTAAGATAAGACGGAGAGTCGACGTAAACCCAGGCCATGCAGA<br>CCTAAGTGCCAAAGAGGCACAGGATGTAATCATGGAAGTTGTCTTCCCAAATGAAGTGGGAGCGAGAATACTGACTTCAGAGTCACAACTGACGGTAACAAAGGAGAAAAAAGAAGAACTCCAGGACTGCAAAATTGCCCCTTTGATGGTTGCATACATGCTAGAGAGGGAATTGGTCCGCAAGACAAGGTTCCTCCCAGTGGCTGGTGGAACAAGCAGTGTCTATATT<br>GAGGTGCTGCACTTAACCCAGGGGACATGCTGGGAGCAGATGTACACTCCAGGAGGAGAGGTGAGGAATGATGATGTAGACCAGAGCTTGATTATTGCTGCCAGAAACATAGTGAGAAGAGCAACTGTATCAGCAGACCCACTAGCATCCCTACTGGAAATGTGCCACAGCACACAGATTGGAGGGGTAAGGATGGTCGACATCCTTCGACAAAATCCAACAGAAGAA<br>CAAGCCGTGGACATATGCAAGGCAGCAATGGGATTGAGGATTAGCTCATCTTTCAGCTTTGGTGGATTCACTTTTAAAAGAACAAGTGGATCTTCAGTAAAAAGAGAAGAAGAAGTGCTTACGGGCAACCTTCAAACATTGAAAATAAGAGTACATGAGGGGTATGAAGAGTTCACAATGGTAGGAAGACGAGCAACTGCTATTATCAGGAAAGCAACCAGAAGGCTGA<br>TCCAGCTAATAGTAAGTGGGAGAGACGAGCAGTCAATTGCTGAAGCAATAATTGTGGCCATGGTGTTCTCACAAGAGGACTGCATGATCAAGGCAGTTCGGGGTGACCTGAATTTTGTCAATAGGGCGAACCAGAGGCTGAATCCAATGCATCAACTCCTGAGGCACTTTCAAAAGGATGCAAAGGTACTTTTTCAAAACTGGGGAATCGAACCCATTGACAGTGTGATG<br>GGGATGATTGGGATATTGCCCGACATGACCCCAAGTACTGAGATGTCGCTGAGGGGGATAAGAGTCAGCAAGACGGGAGTAGATGAATACTCCAGCACAGAAAGGGTCGTAGTGAGCATCGACCGATTTTTAAGAGTTCGAGACCAAAGAGGGAACATACTATTGTCACCTGAAGAAGTTAGCGAGACACAGGGAACAGAGAAATTGACAATCACTTATTCGTCGTCA<br>ATGATGTGGGAGATCAATGGTCCTGAGTCGGTGTTGGTCAATACTTATCAATGGATCATCAGAAACTGGGAAACTGTGAAAATTCAATGGTCACAGGATCCCACAATGTTATATAACAAGATGGAGTTCGAACCATTCCAATCTCTTGTCCCTAAAGCAGCTAGGGGTCAATACAGTGGATTCGTGAGGACATTATTCCAGCAGATGCGGGATGTGCTTGGAACATTTGACA<br>CTGTTCAGATAATAAAACTCCTCCCATTTGCTGCTGCCCCACCAGAACAAAGTAAGATGCAGTTCTCCTCCATGACTGTAAATGTAAGAGGATCAGGGATGAGAATACTGGTAAGAGGCAACTCCCCAGTGTTCAATTACAATAAGGCCACCAAGAGGCTCACTATTCTTGGGAAAGATGCAGGTGCATTGACCGAAGATCCAGATGAAGGCACAGCTGGAATAGAGTCTG<br>CCGTTCTAAGAGGATTCCTCATTTTGGGCAAAGAAGACAAGAGATATGGCCCGGCATTGAGCATCAATGAGCTGAGCAATCTTGCAAAAGGAGAGAAGGCTAATGTGCTAATTGGGCAAGGAGACGTAGTGTTGGTAATGAAACGGAAACGGGACTCTAGCATACTTACTGACAGTCAGACAGCGACCAAAAGAATTCGGATGGCCATCAATTA
EPI_ISL_18244810	>A/buzzard/Poland/MB129-21_22VIR5675-7/2021_2021-02-19<br>ATGGAGAGAATAAAAGAACTAAGAGATTTGATGTCGCAGTCTCGCACTCGCGAGATACTAACAAAAACCACTGTGGACCATATGGCCATAATAAAGAAATACACATCAGGGAGACAGGAGAAGAACCCTGCCCTCAGGATGAAATGGATGATGGCAATGAAATATCCTATTACAGCTGACAAAAGAATAATGGAGATGATCCCTGAAAGGAATGAGCAAGGTCAGACT<br>CTCTGGAGCAAAACAAATGATGCTGGATCAGACAGAGTGATGGTCTCACCTCTGGCTGTGACGTGGTGGAATAGAAATGGGCCAACAACAAGTACAGTACACTACCCAAAAGTCTACAAAACCTACTTTGAAAAGGTAGAAAGGTTGAAACATGGAACCTTTGGTCCTGTTCACTTTCGAAATCAGGTTAAGATACGCCGCAGGGTTGACATAAACCCGGGCCATGCAGA<br>TCTCAGTGCCAAAGAAGCACAGGATGTCATCATGGAGGTTGTTTTCCCAAATGAAGTTGGAGCCAGGATCTTGACATCAGAATCACAATTAACAATAACAATGGAAAAGAAGGAGGAACTTCAGGATTGCAAGATTGCTCCTTTGATGGTGGCATACATGTTGGAAAGAGAACTGGTTCGCAAGACCAGATTTTTACCAGTAGCTGGCGGAACAAGCAGCGTATACATCG<br>AGGTATTGCATTTGACTCAAGGGACCTGCTGGGAACAAATGTACACACCAGGAGGGGAGGTGAGAAATGATGATGTTGATCAGAGTTTGATCATTGCTGCTAGAAATATAGTTAGGAGGGCAACAGTATCAGCAGACCCATTGGCTTCGCTCTTGGAAATGTGCCACAGTACACAAATTGGTGGAGTGAGGATGGTGGACATTCTTAGGCAGAACCCAACAGAGGAGCA<br>AGCTGTGGATATATGCAAAGCAGCAATGGGTTTAAGAATCAGTTCATCCTTCAGCTTTGGAGGTTTCACTTTCAAAAGGACAAGTGGGTCGTCTGTCAAAAGGGAAGAAGAAATACTCACTGGCAACCTCCAAACACTGAAAGTAAGAATACATGAAGGATATGAGGAATTCACAATGGTTGGGCGAAGAGCTACAGCCATTTTGAGGAAAGCAACCAGGAGACTGATCC<br>AATTAATAGTGAGTGGACGAGACGAACAGTCAATCGCTGAGGCAATCATAGTGGCAATGGTTTTCTCACAGGAGGATTGCATGATAAAAGCAGTACGAGGTGATTTGAATTTTGTCAACAGAGCGAATCAGCGGCTAAATCCTATGCATCAACTTCTGAGGCATTTCCAAAAAGATGCAAAAGTGCTGTTTCAAAACTGGGGGATTGAACCAATTGACAATGTAATGGGG<br>ATGATCGGGATACTGCCTGACATGACCCCCAGCACAGAGATGTCACTGAGAGGAGTGAGAGTCAGCAAAATGGGAGTGGATGAATATTCCAGTACTGAGAGAGTGGTCGTGAGCATTGATCGCTTCTTGAGAGTCCGAGATCAGAGGGGAAATGTGCTTCTGTCTCCTGAGGAAGTTAGTGAAACACATGGAACAGAGAAACTGACGATAACGTATTCATCGTCTATGA<br>TGTGGGAAATCAATGGTCCGGAATCCGTGCTAGTCAACACATATCAATGGATCATTAGAAATTGGGAAACTGTGAAGATTCAGTGGTCCCAGGACCCTACGATGTTGTACAATAAGATGGAATTTGAGCCCTTCCAATCCTTGGTGCCCAAGGCTGCTAGAGGCCAGTATAGTGGGTTTGTGAGGACATTATTCCAACAGATGCGTGATGTGTTGGGGACATTTGACACTGT<br>CCAAATAATAAAGCTCCTACCATTTGCAGCAGCCCCACCGGAACAGAGTAGGATGCAATTTTCCTCTATGACTGTGAACGTAAGAGGTTCAGGAATGAGAATACTTGTGAGGGGCAACTCCCCTGTGTTCAACTATAATAAGACCACCAAGAGACTCACAGTTCTTGGAAAGGATGCAGGCGCCTTGACAGAATATCCAGATGAGGGAACAGCAGGAGTGGAGTCTGCAG<br>TATTAAGAGGATTTCTAATTCTGGGCAAAGAGGACAAAAGATATGGACCGGCATTGAGCATCAACGAATTGAGCAATCTTGCGAAAGGGGAAAAGGCTAATGTGTTGATAGGACAAGGAGACGTGGTGTTGGTAATGAAACGGAAACGGGACTCTAGCATACTTACTGACAGCCAGACAGCGACCAAAAGAATTCGGATGGCCATCAATTA
EPI_ISL_18244823	>A/buzzard/Poland/MB277-21_22VIR5675-18/2021_H5N8_2021-03-09<br>ATGGAGAGAATAAAAGAACTAAGAGATTTGATGTCGCAGTCTCGCACTCGCGAGATACTAACAAAAACCACTGTGGACCATATGGCCATAATAAAGAAATACACATCAGGGAGACAGGAGAAGAACCCTGCCCTCAGGATGAAATGGATGATGGCAATGAAATATCCTATTACAGCTGACAAAAGAATAATGGAGATGATCCCTGAAAGGAATGAGCAAGGTCAGACT<br>CTCTGGAGCAAAACAAAAGATGCTGGATCAGACAGAGTGATGGTCTCACCTCTGGCTGTGACATGGTGGAATAGAAATGGGCCAACAACAAGTACAGTACACTACCCAAAAGTCTACAAAACCTACTTTGAAAAGGTAGAAAGGTTGAAACATGGAACCTTTGGTCCTGTTCACTTTCGAAATCAGGTTAAGATACGCCGCAGGGTTGACATAAACCCGGGCCATGCAGA<br>TCTCAGTGCCAAAGAAGCACAGGATGTCATCATGGAGGTTGTTTTCCCAAATGAAGTTGGAGCCAGGATCTTGACATCAGAATCACAATTAACAATAACAATGGAAAAGAAGGAGGAACTTCAGGATTGCAAGATTGCTCCTTTGATGGTGGCATACATGTTGGAAAGAGAACTGGTTCGCAAGACCAGATTTTTACCAGTAGCTGGCGGAACAAGCAGCGTATACATCG<br>AGGTATTGCATTTGACTCAAGGGACCTGCTGGGAACAAATGTACACACCAGGAGGGGAGGTGAGAAATGATGATGTTGATCAGAGTTTGATCATTGCTGCTAGAAATATAGTTAGGAGGGCAACAGTATCAGCAGACCCATTGGCTTCGCTCTTGGAAATGTGCCACAGTACACAAATTGGTGGAGTGAGGATGGTGGATATTCTTAGGCAGAACCCAACAGAGGAGCA<br>AGCTGTGGATATATGCAAAGCAGCAATGGGTTTAAGAATTAGTTCATCCTTCAGCTTTGGAGGTTTCACTTTCAAAAGGACAAGTGGGTCGTCTATCAAAAGAGAAGAAGAAATACTCACTGGCAACCTCCAAACACTGAAAGTAAGAATACATGAAGGATATGAGGAATTCACAATGGTTGGGCGAAGAGCTACAGCCATTTTGAGGAAAGCAACCAGGAGACTGATCC<br>AATTAATAGTGAGTGGACGAGACGAACAGTCAATCGCTGAAGCAATCATAGTGGCAATGGTTTTCTCACAGGAGGATTGCATGATAAAAGCAGTACGAGGTGATTTGAATTTTGTCAACAGAGCGAATCAGCGGCTAAATCCTATGCATCAACTTCTGAGGCATTTCCAAAAGGATGCAAAAGTGCTGTTTCAAAACTGGGGGATTGAACCAATTGACAATGTAATGGGG<br>ATGATCGGGATACTGCCTGACATGACCCCCAGCACAGAGATGTCACTGAGAGGAGTGAGAGTCAGCAAAATGGGAGTGGATGAATATTCCAGTACTGAGAGAGTGGTCGTGAGCATTGATCGCTTCTTGAGAGTCAGAGATCAGAGGGGAAATGTGCTTCTGTCTCCTGAGGAAGTTAGTGAAACACATGGAACAGAGAAACTGACGATAACGTATTCATCGTCTATGA<br>TGTGGGAAATCAATGGTCCGGAATCCGTGCTAGTCAACACATATCAATGGATCATTAGAAATTGGGAAACTGTGAAGATTCAGTGGTCCCAGGACCCTACGATGTTGTACAATAAGATGGAATTTGAGCCCTTCCAATCCTTGGTGCCCAAGGCTGCTAGAGGCCAGTATAGTGGGTTTGTGAGGACATTATTCCAACAGATGCGTGATGTGTTGGGGACATTTGACACTGT<br>CCAAATAATAAAGCTCCTACCATTTGCAGCAGCCCCACCGGAACAGAGTAGGATGCAATTTTCCTCTCTGACTGTGAACGTAAGAGGTTCAGGAATGAGAATACTTGTGAGGGGCAACTCCCCTGTGTTCAACTATAATAAGACCACCAAGAGACTCACAGTTCTTGGAAAGGATGCAGGCGCCTTGACAGAACATCCAGATGAGGGAACAGCAGGAGTGGAGTCTGCAG<br>TATTAAGAGGATTTCTAATTCTGGGCAAAGAGGACAAAAGATATGGACCAGCATTGAGCATCAACGAACTGAGCAATCTTGCGAAAGGGGAAAAGGCTAATGTGTTGATAGGACAAGGAGACGTGGTGTTGGTAATGAAACGGAAACGGGACTCTAGCATACTTACTGACAGCCAGACAGCGACCAAAAGAATTCGGATGGCCATCAATTA
EPI_ISL_9603929	>A/chicken/Czech_Republic/25690/2021_H5N1_2021-12-22<br>ATGGAGAGAATAAAAGAACTAAGAGATTTGATGTCGCAGTCTCGCACTCGCGAGATACTGACAAAAACCACGGTGGACCATATGGCCATAATTAAGAAATACACATCAGGAAGACAGGAGAAGAACCCTGCCCTTAGGATGAAGTGGATGATGGCAATGAAATACCCGATTACAGCAGACAAAAGAATAATGGAAATGATCCCTGAAAGAATGAACAAGGCCAGACT<br>CTCTGGAGCAAAACAAGTGATGCTGGATCAGACAGAGTAATGGTGTCACCTCTGGCTGTGACATGGTGGAATAGAAATGGACCGACGACAAGTACAGTCCACTATCCAAAGGTCTACAAAACCTACTTTGAAAAAGTCGAAAGGTTGAAACATGGAACCTTCGGTCCTGTTCACTTTCGGAATCAGGTTAAAATACGCCGCAGGGTTGACATAAACCCGGGCCATGCAGA<br>TCTCAGTGCCAAAGAAGCACAAGATGTCATCATGGAGGTCGTTTTTCCAAACGAAGTTGGAGCAAGGATATTGACATCAGAGTCACAATTAACAATAACAAAGGAAAAGAAGGAGGAGCTTCAGGGCTGCAAAATCGCCCCTTTAATGGTGGCATACATGTTGGAGAGAGAACTGGTTCGCAAAACCAGATTCCTACCAGTAGCTGGCGGAACAAGCAGCGTATATATC<br>GAGGTATTGCATTTGACTCAAGGGACCTGCTGGGAACAAATGTACACACCGGGAGGRGAGGTGAGAAATGATGATGTCGATCAGAGCTTAATTATTGCTGCTAGAAATATTGTTAGGAGAGCAACAGTGTCGGCAGACCCGTTGGCTTCGCTCTTGGAGATGTGCCATAGTACACAAATTGGCGGGATAAGAATGGTGGACATCCTTAGACAGAACCCAACAGAAGAGC<br>AAGCTGTGGATATATGCAGAGCAGCAATGGGTTTGAGAATCAGTTCATCCTTCAGCTTTGGAGGTTTCACTTTTAAAAGGACAAGTGGGTCATCTGTCAAAAGAGAAGAAGAAGTGCTCACGGGCAACCTCCAAACACTGAAAATAAGAGTACATGAAGGATATGAAGAATTCACAATGGTTGGGCGAAGAGCTACAGCCATTCTAAGGAAAGCAACCAGAAGGCTGAT<br>CCAATTAATAGTGAGTGGGAGAGACGAGCAGTCAATCGCTGAAGCGATTATAGTGGCAATGGTTTTCTCACAAGAGGACTGCATGATAAAAGCAGTGCGAGGTGATTTGAACTTTGTTAATAGAGCAAACCAACGGCTAAATCCTATGCACCAACTTCTGAGACATTTCCAAAAGGATGCAAAGGTGCTGTTTCAAAATTGGGGAATTGAACCCATTGACAATGTCATGGG<br>AATGATCGGGATACTGCCTGACATGACCCCCAGCACAGAGATGTCACTGAGAGGAGTGAGAGTCAGTAAAATGGGAGTGGATGAATATTCCAGTACTGAGAGAGTGGTCGTAAGCATTGATCGTTTCTTAAGAGTCCGAGACCAGAGGGGAAACGTACTCTTGTCTCCTGAAGAAGTTAGTGAAACACAGGGAACAGAGAAACTGACGATAACATACTCATCGTCTATG<br>ATGTGGGAAATCAATGGTCCGGAGTCAGTACTGGTCAATACATATCAATGGATCATTAGAAATTGGGAAGCTGTGAAGATTCAGTGGTCCCAAGACCCCACAATGTTGTACAATAAGATGGAGTTTGAGCCTTTCCAATCCTTGGTGCCCAAGGCTGCCAGAGGCCAGTACAGTGGATTTGTGAGAACGTTATTCCAGCAGATGCGTGATGTGCTGGGGACATTTGAAACT<br>GTCCAAATAATAAAGCTCCTACCATTTGCAGCAGCCCCACCGGAACAGAGTAGGATGCAGTTCTCTTCTCTGACTGTGAATGTAAGAGGTTCAGGGATGAGAATACTTGTGAGAGGCAACTCCCCTGTGTTCAACTATAACAAAGCAACCAAGAGACTCACAGTTCTTGGAAAAGATGCAGGTGCATTGACAGAAGATCCAGACGAGGGAACAGCAGGAGTGGAGTCTGC<br>GGTGTTAAGAGGATTTCTAATTCTGGGCAAAGAAGACAAAAGATATGGACCAGCATTGAGCATTAACGAATTGAGCAATCTTGCGAAAGGGGAGAAGGCTAATGTGTTGATAGGGCAAGGAGACGTGGTGTTGGTAATGAAACGGAAACGGGACTCTAGCATACTTACTGACAGCCAGACAGCGACCAAAAGGATTCGGATGGCCATCAATTA
EPI_ISL_2234825	>A/chicken/Ireland/005930-20VIR5487-1/2020_H6N1_2021-03-01<br>ATGGAGAGAATAAAAGAACTAAGAGATTTGATGTCGCAGTCTCGCACTCGCGAGATACTGACAAAAACCACTGTGGACCATATGGCCATAATCAAGAAATATACGTCAGGAAGACAGGAGAAGAATCCTGCTCTCAGGATGAAATGGATGATGGCAATGAAATATCCGATTACAGCAGACAAAAGGATAATGGAGATGATCCCTGAAAGAAATGAGCAAGGTCAGACT<br>CTTTGGAGCAAAACAAATGATGCTGGATCGGACAGAGTTATGGTGTCACCTTTGGCTGTGACGTGGTGGAATAGAAATGGGCCAACGACAAGTACAGTCCATTATCCAAAGGTCTATAAAACCTACTTTGAAAAGGTCGAAAGGTTGAAGCATGGAACCTTCGGTCCCGTCCACTTTCGGAATCAGGTCAAAATACGCCGCAGGGTTGACATAAATCCAGGCCATGCAGAT<br>CTCAGTGCCAAAGAAGCACAGGATGTCATCATGGAGGTCGTTTTCCCAAATGAAGTCGGAGCCAGGATACTGACATCAGAGTCACAGTTGACAATAACAAAGGAAAAGAAGGAGGAGCTTCAGGATTGTAAGATTGCTCCTTTAATGGTGGCATACATGTTGGAGAGAGAATTGGTTCGCAAAACCAGATTCCTACCAGTAGCTGGTGGGACAAGCAGCGTGTACATTG<br>AGGTGTTGCACTTGACTCAAGGGACCTGTTGGGAACAAATGTACACGCCGGGAGGGGAGGTGAGAAATGATGATGTTGATAAGAGTTTAATTATTGCTGCTCGGAATATTGTTAGGAGAGCAACAGTATCAGCAGACCCATTGGCTTCGCTTTTGGAGATGTGCCATAGTACGCAAATTGGCGGGATAAGGATGGTAGACATCCTTAGACAAAACCCAACGGAAGAGCA<br>AGCTGTGGACATATGCAAAGCTGCAATGGGTCTAAGGATCAGTTCATCCTTCAGCTTTGGAGGTTTCACTTTCAAGAGAACGAGTGGGTCATCTGTCAAAAGAGAAGAAGAAGTGCTCACAGGCAACCTCCAAACATTGAAAATAAGAGTGCATGAAGGATATGAGGAATTCACAATGGTTGGGCGAAGAGCAACAGCTATTCTAAGGAAAGCAACCAGAAGGCTGATC<br>CAATTGATAGTGAGTGGGAGAGACGAGCAGTCAATCGCCGAGGCGATCATAGTGGCAATGGTGTTCTCACAAGAGGACTGTATGATAAAAGCAGTACGAGGTGATCTGAATTTTGTCAACAGAGCAAACCAGCGGTTAAACCCTATGCATCAACTATTGAGGCATTTCCAAAAGGATGCAAAGGTGCTGTTTCAAAACTGGGGAATTGAGCCCATCGACAATGTCATGGG<br>GATGATCGGAATATTACCTGACATGACCCCTAGCACAGAGATGTCACTAAGAGGAGTGAGAGTCAGTAAAATGGGAGTGGATGAGTACTCCAGTACTGAGAGAGTGGTTGTGAGCATTGATCGCTTCCTGAGGGTCCGAGACCAGAGGGGAAACGTGCTCTTGTCTCCTGAAGAAGTTAGTGAAACACAGGGAATAGAAAAGCTGACGATAACATATTCATCATCCATG<br>ATGTGGGAAATCAACGGCCCAGAATCAGTGTTAGTGAACACATATCAATGGATCATTAGAAATTGGGAAACTGTGAAAATTCAATGGTCCCAAGACCCTACAATGCTATACAATAAGATGGAGTTTGAGCCTTTTCAATCTTTGGTGCCTAAGGCTGCCAGGGGTCAGTACAGTGGATTTGTGAGAACGCTATTCCAGCAGATGCGTGATGTGCTGGGGACATTTGACACC<br>GTCCAGATAATAAAGCTGCTACCATTTGCAGCAGCCCCACCAGAACAGAGTAGAATGCAGTTCTCTTCTCTAACCGTGAACGTGAGGGGTTCAGGAATGAGAATACTTGTGAGAGGCAACTCCCCTGTGTTCAACTATAACAAGGCGACCAAGAGGCTTACAGTCCTTGGAAAGGATGCAGGTGCATTGACAGAAGATCCAGATGAGGGAACAGCAGGAGTGGAATCTG<br>CGGTATTAAGAGGATTTCTAATTCTAGGCAAAGAGGACAAAAGATATGGACCAGCATTAAGCATCAACGAATTGAGCAATCTTGCGAAAGGGGAGAAGGCTAATGTGTTGATAGGGCAAGGAGACGTGGTGTTGGTAATGAAACGGAAACGGGACTCTAGCATACTTACTGACAGCCAGACAGCGACCAAAAGAATTCGGATGGCCATCAATTA
EPI_ISL_6829533	>A/chicken/Kagoshima/21A6T/2021_H5N1_2021-11-12<br>ATGGAGAGAATAAAAGAACTAAGAGATTTGATGTCGCAGTCTCGCACTCGCGAGATACTGACAAAAACCACGGTGGACCATATGGCCATAATTAAGAAATACACATCAGGAAGACAGGAGAAGAACCCTGCCCTTAGGATGAAGTGGATGATGGCAATGAAATACCCGATTACAGCAGACAAAAGAATAATGGAAATGATCCCTGAAAGAATGAACAAGGCCAGACT<br>CTCTGGAGCAAAACAAGTGATGCTGGATCAGACAGAGTAATGGTGTCACCTCTGGCTGTGACATGGTGGAATAGAAATGGACCGACGACAAGTACAGTTCACTATCCAAAGGTCTACAAAACCTACTTTGAAAAAGTCGAAAGGTTGAAACATGGAACCTTCGGTCCTGTTCACTTTCGGAATCAGGTTAAAATACGCCGCAGGGTTGACATAAACCCGGGCCATGCAGA<br>CCTCAGTGCCAAAGAAGCACAAGATGTCATCATGGAGGTCGTTTTTCCAAACGAAGTTGGAGCAAGGATATTGGCATCAGAGTCACAATTAACAATAACAAAGGAAAAGAAGGGGGGAGCTTCAGGACTGCAAGATCGCCCCTTTAATGGTGGCATACATGTTGGAGAGAGAACTGGTTCGCAAAACCAGATTCCTACCAGTTGCTGGCGGAACAAGCAGCGTATATATC<br>GAGGTATTGCATCTGACTCAAGGGACCTGCTGGGAACAAATGTACACACCGGGAGGGGAGGTGAGAAATGATGATGTCGATCAGAGTTTGATTATTGCTGCTAGAAACATTGTTAGGAGAGCAACAGTGTCGGCAGACCCGTTGGCTTCGCTCTTGGAGATGTGCCATAGTACACAAATTGGCGGGATAAGAATGGTGGACATCCTTAGACAGAACCCAACAGAAGAGC<br>AAGCTGTGGATATATGCAAAGCAGCAATGGGTTTGAGAATCAGTTCATCCTTCAGCTTTGGAGGTTTCACTTTTAAAAGGACAAGTGGGTCATCTGTCAAAAGGGAAGAAGAAGTGCTCACGGGCAACCTCCAAACACTGAAAATAAGAGTACACGAAGGATATGAAGAATTCACAATGGTTGGGCGAAGAGCTACAGCCATTCTAAGGAAAGCAACCAGAAGGCTGAT<br>CCAATTAATAGTGAGTGGGAGAGACGAGCAGTCAATCGCTGAAGCGATTATAGTGGCAATGGTTTTCTCACAAGAGGACTGCATGATAAAAGCAGTGCGAGGTGATTTGAACTTTGTTAACAGAGCAAATCAGCGGCTAAATCCTATGCACCAACTTCTGAGACATTTCCAAAAGGATGCAAAGGTGCTGTTTCAAAATTGGGGAATTGAACCCATTGACAATGTCATGGG<br>AATGATCGGGATACTGCCTGACATGACCCCCAGCACAGAGATGTCACTGAGAGGAGTGAGAGTCAGTAAAATGGGAGTGGATGAATATTCCAGTACTGAGAGAGTGGTCGTAAGCATTGATCGTTTCTTAAGAGTCCGAGACCAGAGGGGAAACGTACTCTTGTCTCCTGAAGAAGTTAGTGAAACACAGGGAACAGAGAAACTGACGATAACATATTCATCGTCTATG<br>ATGTGGGAAATCAATGGTCCGGAGTCAGTACTGGTCAACACATATCAATGGATCATTAGAAATTGGGAAACTGTGAAGATTCAGTGGTCCCAAGACCCCACAATGTTGTACAATAAGATGGAGTTTGAGCCCTTCCAATCCTTGGTGCCCAAGGCTGCCAGAGGCCAGTACAGTGGATTTGTGAGGACGTTATTCCAGCAGATGCGTGATGTGCTGGGGACATTTGACACT<br>GTCCAAATAATAAAGCTCCTACCATTTGCAGCAGCCCCACCGGAACAGAGTAGGATGCAATTCTCTTCTCTGACTGTGAATGTAAGAGGTTCAGGAATGAGAATACTTGTGAGAGGCAACTCCCCTGTGTTCAACTATAACAAAGCAACCAAGAGACTCACAGTTCTTGGAAAGGATGCAGGTGCATTGACAGAAGATCCAGACGAGGGAACAGCAGGAGTGGAGTCTGC<br>GGTGTTAAGAGGATTCCTAATTCTTGGCAAAGAAGACAAAAGATATGGACCAGCATTGAGCATTAACGAATTGAGCAATCTTGCGAAAGGGGAGAAGGCTAATGTGTTGATAGGGCAAGGAGACGTGGTGTTGGTAATGAAACGGAAACGGGACTCTAGCATACTTACTGACAGCCAGACAGCGACCAAAAGGATTCGGATGGCCATCAATTA
EPI_ISL_2932608	>A/chicken/Kazakhstan/220-B-2-H5N8-4/2020_H5N8_2020-09-01<br>ATGGAGAGAATAAAAGAACTAAGAGATTTGATGTCGCAGTCTCGCACTCGCGAGATACTAACAAAAACCACTGTGGACCATATGGCCATAATAAAGAAATACACATCAGGGAGACAGGAGAAGAACCCTGCCCTCAGGATGAAATGGATGATGGCAATGAAATATCCTATTACAGCTGACAAAAGAATAATGGAGATGATCCCTGAAAGGAATGAGCAAGGTCAGACT<br>CTCTGGAGCAAAACAAATGATGCTGGATCAGACAGAGTGATGGTCTCACCTCTGGCTGTGACATGGTGGAATAGAAATGGGCCAACAACAAGTACAGTACACTACCCAAAAGTCTACAAAACCTACTTTGAAAAGGTAGAAAGGTTGAAACATGGAACCTTTGGTCCTGTTCACTTTCGAAATCAGGTTAAGATACGCCGCAGGGTTGACATAAACCCGGGCCATGCAGA<br>TCTCAGTGCCAAAGAAGCACAGGATGTCATCATGGAGGTTGTTTTCCCAAATGAAGTTGGAGCCAGGATCTTGACATCAGAATCACAATTAACAATAACAATGGAAAAGAAGGAGGAACTTCAGGATTGCAAGATTGCTCCTTTGATGGTGGCATACATGTTGGAAAGAGAACTGGTTCGCAAGACCAGATTTTTACCAGTAGCTGGCGGAACAAGCAGCGTATACATCG<br>AGGTATTGCATTTGACTCAAGGGACCTGCTGGGAACAAATGTACACACCAGGAGGGGAGGTGAGAAATGATGATGTTGATCAGAGTTTGATCATTGCTGCTAGAAATATAGTTAGGAGGGCAACAGTATCAGCAGACCCATTGGCTTCGCTCTTGGAAATGTGCCACAGTACACAAATTGGTGGAGTGAGGATGGTGGACATTCTTAGGCAGAACCCAACAGAGGAGCA<br>AGCTGTGGATATATGCAAAGCAGCAATGGGTTTAAGAATCAGTTCATCCTTCAGCTTTGGAGGTTTCACTTTCAAAAGGACAAGTGGGTCGTCTGTCAAAAGAGAAGAAGAAATACTCACTGGCAACCTCCAAACACTGAAAGTAAGAATACATGAAGGATATGAGGAATTCACAATGGTTGGGCGAAGAGCTACAGCCATTTTGAGGAAAGCAACCAGGAGACTGATCC<br>AATTAATAGTGAGTGGACGAGACGAGCAGTCAATCGCTGAAGCAATCATAGTGGCAATGGTTTTCTCACAGGAGGATTGCATGATAAAAGCAGTACGAGGTGATTTGAATTTTGTCAACAGAGCGAATCAGCGGCTAAATCCTATGCATCAACTTCTGAGGCATTTCCAAAAGGATGCAAAAGTGCTGTTTCAAAACTGGGGGATTGAACCAATTGACAATGTAATGGGG<br>ATGATCGGGATACTGCCTGACATGACCCCCAGCACAGAGGTGTCACTGAGAGGAGTGAGAGTCAGCAAAATGGGAGTGGATGAATATTCCAGTACTGAGAGAGTGGTCGTGAGCATTGATCGCTTCTTGAGAGTCCGAGATCAGAGGGGAAATGTGCTTCTGTCTCCTGAGGAAGTTAGTGAAACACATGGAACAGAGAAACTGACGATAACGTATTCATCGTCTATGA<br>TGTGGGAAATCAATGGTCCGGAATCCGTGCTAGTCAACACATATCAATGGATCATTAGAAATTGGGAAACTGTGAAGATTCAGTGGTCCCAGGACCCTACGATGTTGTACAATAAGATGGAATTTGAGCCCTTCCAATCCTTGGTGCCCAAGGCTGCTAGAGGCCAGTATAGTGGGTTTGTGAGGACATTATTCCAACAGATGCGTGATGTGTTGGGGACATTTGACACTGT<br>CCAAATAATAAAGCTCCTACCATTTGCAGCAGCCCCACCGGAACAGAGTAGGATGCAATTTTCCTCTCTGACTGTGAACGTAAGAGGTTCAGGAATGAGAATACTTGTGAGGGGCAACTCCCCTGTGTTCAACTATAATAAGGCCACCAAGAGACTCACAGTTCTTGGAAAGGATGCAGGCGCCTTGACAGAATATCCAGATGAGGGAACAGCAGGAGTGGAGTCTGCAG<br>TATTAAGAGGATTTCTAATTCTGGGCAAAGAGGACAAAAGATATGGACCAGCATTGAGCATCAACGAATTGAGCAATCTTGCGAAAGGGGAAAAGGCTAATGTGTTGATAGGACAAGGAGACGTGGTGTTGGTAATGAAACGGAAACGGGACTCTAGCATACTTACTGACAGCCAGACAGCGACCAAAAGAATTCGGATGGCCATCAATTA
EPI_ISL_1114763	>A/chicken/Krasnodar/334-03/2021_H5N8_2021-01-05<br>ATGGAGAGAATAAAAGAACTAAGAGATTTGATGTCGCAGTCTCGCACTCGCGAGATACTAACAAAAACCACTGTGGACCATATGGCCATAATAAAGAAATACACATCAGGAAGACAGGAGAAGAACCCTGCCCTCAGGATGAAATGGATGATGGCAATGAAATATCCTATTACAGCTGACAAAAGAATAATGGAGATGATCCCTGAAAGGAATGAGCAAGGTCAGACT<br>CTCTGGAGCAAAACAAATGATGCTGGATCAGACAGAGTGATGGTCTCACCTCTGGCTGTGACATGGTGGAATAGAAATGGGCCAACAACAAGTACAGTACACTACCCAAAAGTCTACAAAACCTACTTTGAAAAGGTAGAAAGGTTGAAACATGGAACCTTTGGTCCTGTTCACTTTCGAAATCAGGTTAAGATACGCCGCAGGGTTGACATAAACCCGGGCCATGCAGA<br>TCTCAGTGCCAAAGAAGCACAGGATGTCATCATGGAGGTTGTTTTCCCAAATGAAGTTGGAGCCAGGATCTTGACATCAGAATCACAATTAACAATAACAAGGGAAAAGAAGGAGGAACTTCAGGATTGCAAGATTGCTCCTTTGATGGTGGCATACATGTTGGAAAGAGAACTGGTTCGCAAGACCAGATTTTTACCAGTAGCTGGCGGAACAAGCAGCGTATACATCG<br>AGGTATTGCATTTGACTCAAGGGACCTGCTGGGAACAAATGTACACACCAGGAGGGGAGGTGAGAAATGATGATGTTGATCAGAGTTTGATCATTGCTGCTAGAAATATAGTTAGGAGGGCAACAGTATCAGCAGACCCATTGGCTTCGCTTTTGGAAATGTGCCACAGTACACAAATTGGTGGAGTAAGGATGGTGGACATTCTTAGGCAGAACCCAACAGAGGAGCA<br>AGCTGTGGATATATGCAAAGCAGCAATGGGTTTAAGAATCAGTTCATCCTTCAGCTTTGGAGGTTTCACTTTCAAAAGGACAAGTGGGTCGTCTGTCAAAAGAGAAGAAGAAATACTCACTGGCAACCTCCAAACACTGAAAGTAAGAATACATGAAGGATATGAGGAATTCACAATGGTTGGGCGAAGAGCTACAGCCATTTTGAGGAAAGCAACCAGGAGACTGATCC<br>AATTAATAGTGAGTGGAAGAGACGAGCAGTCAATCGCTGAAGCAATCATAGTGGCAATGGTTTTCTCACAGGAGGATTGCATGATAAAAGCAGTACGAGGTGATTTGAATTTTGTCAACAGAGCGAATCAGCGGCTAAATCCTATGCATCAACTTCTGAGGCATTTCCAAAAGGATGCAAAAGTGCTGTTTCAAAACTGGGGGATTGAACCAATTGACAATGTAATGGGG<br>ATGATCGGGATACTGCCTGACATGACCCCCAGCACAGAGATGTCACTGAGAGGAGTGAGAGTCAGCAAAATGGGAGTGGATGAATATTCCAGTACTGAGAGAGTGGTCGTGAGCATTGATCGCTTCTTGAGAGTCCGAGATCAGAGGGGAAATGTGCTTCTGTCTCCTGAGGAAGTTAGTGAAACACATGGAACAGAGAAACTGACGATAACGTATTCATCGTCTATGA<br>TGTGGGAAATCAATGGTCCGGAATCCGTGCTAGTCAACACATATCAATGGATCATTAGAAATTGGGAAACTGTGAAGATTCAGTGGTCCCAGGACCCTACGATGTTATACAATAAGATGGAATTTGAGCCCTTCCAATCCTTGGTGCCCAAGGCTGCTAGAGGTCAGTATAGTGGGTTTGTAAGGACATTATTCCAACAGATGCGTGATGTGTTGGGGACATTTGACACTGT<br>CCAAATAATAAAGCTCCTACCATTTGCAGCAGCCCCACCGGAACAGAGTAGGATGCAATTTTCCTCTCTGACTGTGAACGTAAGAGGTTCAGGAATGAGAATACTTGTGAGGGGCAACTCCCCTGTGTTCAACTATAATAAGGCCACCAAGAGACTCACAGTTCTTGGAAAGGATGCAGGCGCCTTGACAGAATATCCAGATGAGGGAACAGCAGGAGTGGAGTCTGCAG<br>TATTAAGAGGATTTCTAATTCTGGGCAAAGAGGACAAAAGATATGGACCAGCATTGAGCATCAACGAATTGAGCAATCTTGCGAAAGGGGAAAAGGCTAATGTGTTGATAGGACAAGGAGACGTGGTGTTGGTAATGAAACGGAAACGGGACTCTAGCATACTTACTGACAGCCAGACAGCGACCAAAAGAATTCGGATGGCCATCAATTA
EPI_ISL_394155	>A/chicken/Netherlands/17013178-006010/2017_H10N7_2017-09-27<br>ATGGAGAGAATAAAAGAGCTAAGAGATTTGATGTCGCAGTCTCGCACTCGCGAGATACTGACAAAAACCACTGTGGACCATATGGCCATAATCAAGAAATATACATCAGGAAGACAGGAGAAGAACCCTGCACTTAGGATGAAATGGATGATGGCAATGAAATATCCGATTACAGCAGACAAGAGGATAATGGAGATGATCCCTGAGAGAAATGAGCAAGGTCAGACT<br>CTTTGGAGCAAAACAAATGATGCTGGATCAGACAGAGTGATGGTGTCACCTCTGGCTGTGACGTGGTGGAATAGAAATGGACCAACAACAAGTACAGTCCATTACCCAAAGGTCTATAAAACCTACTTTGAAAAGGTTGAAAGGTTAAAGCATGGAACCTTCGGCCCTGTTCATTTTCGAAATCAGGTTAAGATACGCCGCAGAGTTGACACAAACCCGGGCCATGCAGA<br>CCTCAGTGCTAAAGAAGCACAAGACGTCATCATGGAGGTCGTTTTCCCAAATGAAGTCGGGGCCAGAATATTGACATCAGAGTCACAGTTAACTATAACAAAGGAAAAAAAGGAGGAACTCCAGGACTGTAAGATTGCCCCTTAATGGTGGCATACATGTTGGAGAGAGAACTGGTTCGAAAAACCAGATTCCTGCCAGTAGCTGGCGGAACAAGCAGCGTATATATC<br>GAGGTGTTGCACTTGACTCAAGGAACCTGCTGGGAACAAATGTATACGCCAGGAGGAGAAGTGAGAAATGATGACGTTGATCAGAGTTTAATTATTGCTGCCAGAAATATCGTTAGGAGAGCAACAGTATCAGCAGACCCATTGGCTTCGCTACTGGAGATGTGCCATAGTACACAAATTGGCGGGATAAGGATGGTAGACATCCTTAGACAGAACCCAACAGAAGAGC<br>AAGCCGTGGATATATGCAAAGCAGCAATGGGTCTAAGAATCAGTTCATCCTTCAGCTTTGGAGGTTTCACTTTCAAAAGGACAAGCGGGTCATCTGTCAAAAGAGAAGAGGAAGTGCTCACCGGCAACCTCCAAACATTGAAAATAAGAGTGCATGAAGGGTATGAGGAATTCACAATGGTTGGGCGGAGAGCAACAGCTATTCTAAGGAAAGCAACCAGAAGGCTGAT<br>CCAATTGATAGTAAGTGGAAGAGACGAGCAGTCAATCGCCGAAGCGATCATAGTGGCAATGGTGTTCTCCCAAGAGGATTGCATGATAAAGGCAGTACGAGGTGATTTAAATTTTGTCAATAGAGCAAATCAGCGGCTCAATCCTATGCATCAACTCCTGAGGCATTTCCAAAAGGATGCAAAGGTACTGTTCCAAAACTGGGGAATTGAACCCATTGACAATGTCATGG<br>GGATGATAGGAATATTGCCTGATATGACACCCAGCACAGAGATGTCACTAAGAGGAGTGAGGGTCAGTAAAATGGGAGTGGATGAATATTCCAGTACTGAGAGGGTGGTCGTGAGTATTGATCGTTTCTTGAGGGTCCGAGACCAGAGAGGAAACGTACTCTTGTCTCCTGAAGAGGTTAGTGAAACACAGGGAACAGAGAAGCTGACGATAACATATTCATCATCCAT<br>GATGTGGGAAATTAATGGTCCGGAATCAGTGTTAGTTAACACATATCAATGGATCATCAGAAACTGGGAAACTGTGAAGATTCAGTGGTCCCAAGACCCTACAATGCTATACAACAAGATGGAGTTTGAGCCCTTTCAGTCCTTGGTGCCTAAGGCGGCCAGAGGCCAGTACAGTGGATTTGTAAGGACTCTATTCCAGCAGATGCGTGATGTGCTGGGGACCTTTGACAC<br>TGTCCAGATAATAAAGCTACTCCCATTTGCAGCAGCCCCACCGGAACAGAGTAGGATGCAGTTCTCTTCTCTAACTGTAAACGTAAGGGGTTCAGGAATGAGAATACTTGTGAGAGGCAACTCCCCTGTGTTCAACTATAACAAGGCAACCAAGAGGCTCACAGTCCTTGGAAAGGATGCTGGTGCATTGACAGAAGACCCAGATGAGGGGACGGCAGGAGTGGAGTCTG<br>CGGTATTGAGAGGGTTCCTAATTCTGGGCAAAGAAGACAAAAGATATGGACCAGCGCTGAGCATCAATGAATTGAGCAATCTTGCGAAAGGGGAGAAGGCTAATGTGTTGATAGGGCAAGGAGACGTGGTGTTGGTGATGAAACGGAAACGGGACTCTAGCATACTTACTGACAGCCAGACAGCGACCAAAAGAATTCGGATGGCCATCAATTA

internal NVRI repository	>A/chicken/Poland/104/2021_H5N8_2021-02-09<br>ATGGAGAGAATAAAAGAACTAAGAGATTTGATGTCGCAGTCTCGCACTCGCGAGATACTAACAAAAACCACTGTGGACCATATGGCCATAATAAAGAAATACACATCAGGAAGACAGGAGAAGAACCCTGCCCTCAGGATGAAATGGATGATGGCAATGAAATATCCTATTACAGCTGACAAAAGAATAATGGAGATGATCCCTGAAAGGAATGAGCAAGGTCAGACT<br>CTCTGGAGCAAAACAAATGATGCTGGATCAGACAGAGTGATGGTCTCACCTCTGGCTGTGACATGGTGGAATAGAAATGGGCCAACAACAAGTACAGTACACTACCCAAAAGTCTACAAAACCTACTTTGAAAAGGTAGAAAGGTTGAAACATGGAACCTTTGGTCCTGTTCACTTTCGAAGTCAGGTTAAGATACGCCGTAGGGTTGACATAAACCCGGGCCATGCAGA<br>TCTCAGTGCCAAAGAAGCACAGGATGTCATCATGGAGGTTGTTTTCCCAAATGAAGTTGGAGCCAGGATCTTGACATCAGAATCACAATTAACAATAACAAGGGAAAAGAAGGAGGAACTTCAGGATTGCAAGATTGCTCCTTTGATGGTGGCATACATGTTGGAAAGAGAACTGGTTCGCAAGACCAGATTTTTACCAGTAGCTGGCGGAACAAGCAGCGTATACATCG<br>AGGTATTGCATTTGACTCAAGGGACCTGCTGGGAACAAATGTACACACCAGGAGGGGAGGTGAGAAATGATGATGTTGATCAGAGTTTGATCGTTGCTGCTAGAAATATAGTTAGGAGGGCAACAGTATCAGCAGACCCATTGGCTTCGCTTTTGGAAATGTGCCACAGTACACAAATTGGTGGAGTAAGGATGGTGGACATTCTTAAGCAGAACCCAACAGAGGAGCA<br>AGCTGTGGATATATGCAAAGCAGCAATGGGTTTAAGAATCAGTTCATCCTTCAGCTTTGGAGGTTTCACTTTCAAAAGGACAAGTGGGTCGTCTGTCAAAAGAGAAGAAGAAATACTCACTGGCAACCTCCAAACACTGAAAGTAAGAATACATGAAGGGTATGAGGAATTCACAATGGTTGGGCGAAGAGCTACAGCCATTTTGAGGAAAGCAACCAGGAGACTGATCC<br>AATTAATAGTGAGTGGAAGAGACGAGCAGTCAATCGCTGAAGCAATCATAGTGGCAATGGTTTTCTCACAGGAGGATTGCATGATAAAAGCAGTACGAGGTGATTTGAATTTTGTCAACAGAGCGAATCAGCGGCTAAATCCTATGCATCAACTTCTGAGGCATTTCCAAAAGGATGCAAAAGTGCTGTTTCAAAACTGGGGGATTGAACCAATTGACAATGTAATGGGG<br>ATGATCGGGATACTGCCTGACATGACCCCCAGCACAGAGATGTCACTGAGAGGAGTGAGAGTCAGCAAAATGGGAGTGGATGAATATTCCAGTACTGAGAGAGTGGTCGTGAGCATTGATCGCTTCTTGAGAGTCCGAGATCAGAGGGGAAATGTGCTTCTGTCTCCTGAGGAAGTTAGTGAAACACATGGAACAGAGAAACTGACGATAACGTATTCATCGTCTATGA<br>TGTGGGAAATCAATGGTCCGGAATCCGTGCTAGTCAACACATATCAATGGATCATTAGAAATTGGGAAACTGTGAAGATTCAGTGGTCCCAGGACCCTACGATGTTATACAATAAGATGGAATTTGAGCCCTTCCAATCCTTGGTGCCCAAGGCTGCTAGAGGCCAGTATAGTGGGTTTGTGAGGACATTATTCCAACAGATGCGTGATGTGTTGGGAACATTTGACACTAT<br>CCAAATAATAAAGCTCCTACCATTTGCAGCAGCCCCACCGGAACAGAGTAGGATGCAATTTTCCTCTCTGACTGTGAACGTAAGAGGTTCAGGAATGAGAATACTTGTGAGGGGCAACTCCCCTGTGTTCAACTATAATAAGGCCACCAAGAGACTCACAGTTCTTGGAAAGGATGCAGGCGCCTTGACAGAATATCCAGATGAGGGAACAGCAGGAGTGGAGTCTGCAG<br>TATTAAGAGGATTTCTAATTCTGGGCAAAGAGGACAAAAGATATGGACCAGCATTGAGCATCAACGAATTGAGCAATCTTGCGAAAGGGGAAAAGGCTAATGTGTTGATAGGACAAGGAGACGTGGTGTTGGTAATGAAACGGAAACGGGACTCTAGCATACTTACTGACAGCCAGACAGCGACCAAAAGAATTCGGATGGCCATCAATTA
EPI_ISL_661177	>A/chicken/Poland/448/2020_H5N8_2020-11-24<br>ATGGAGAGAATAAAAGAACTAAGAGATTTGATGTCGCAGTCTCGCACTCGCGAGATACTAACAAAAACCACTGTGGACCATATGGCCATAATAAAGAAATACACATCAGGAAGACAGGAGAAGAACCCTGCCCTCAGGATGAAATGGATGATGGCAATGAAATATCCTATTACAGCTGACAAAAGAATAATGGAGATGATCCCTGAAAGGAATGAGCAAGGTCAGACT<br>CTCTGGAGCAAAACAAATGATGCTGGATCAGACAGAGTGATGGTCTCACCTCTGGCTGTGACATGGTGGAATAGAAATGGGCCAACAACAAGTACAGTACACTACCCAAAAGTCTACAAAACCTACTTTGAAAAGGTAGAAAGGTTGAAACATGGAACCTTTGGTCCTGTTCACTTTCGAAGTCAGGTTAAGATACGCCGTAGGGTTGACATAAACCCGGGCCATGCAGA<br>TCTCAGTGCCAAAGAAGCACAGGATGTCATCATGGAGGTTGTTTTCCCAAATGAAGTTGGAGCCAGGATCTTGACATCAGAATCACAATTAACAATAACAAGGGAAAAGAAGGAGGAACTTCAGGATTGCAAGATTGCTCCTTTGATGGTGGCATACATGTTGGAAAGAGAACTGGTTCGCAAGACCAGATTTTTACCAGTAGCTGGCGGAACAAGCAGCGTATACATCG<br>AGGTATTGCATTTGACTCAAGGGACCTGCTGGGAACAAATGTACACACCAGGAGGGGAGGTGAGAAATGATGATGTTGATCAGAGTTTGATCGTTGCTGCTAGAAATATAGTTAGGAGGGCAACAGTATCAGCAGACCCATTGGCTTCGCTTTTGGAAATGTGCCACAGTACACAAATTGGTGGAGTAAGGATGGTGGACATTCTTAGGCAGAACCCAACAGAGGAGCA<br>AGCTGTGGATATATGCAAAGCAGCAATGGGTTTAAGAATCAGTTCATCCTTCAGCTTTGGAGGTTTCACTTTCAAAAGGACAAGTGGGTCGTCTGTCAAAAGAGAAGAAGAAATACTCACTGGCAACCTCCAAACACTGAAAGTAAGAATACATGAAGGGTATGAGGAATTCACAATGGTTGGGCGAAGAGCTACAGCCATTTTGAGGAAAGCAACCAGGAGACTGATCC<br>AATTAATAGTGAGTGGAAGAGACGAGCAGTCAATCGCTGAAGCAATCATAGTGGCAATGGTTTTCTCACAGGAGGATTGCATGATAAAAGCAGTACGAGGTGATTTGAATTTTGTCAACAGAGCGAATCAGCGGCTAAATCCTATGCATCAACTTCTGAGGCATTTCCAAAAGGATGCAAAAGTGCTGTTTCAAAACTGGGGGATTGAACCAATTGACAATGTAATGGGG<br>ATGATCGGGATACTGCCTGACATGACCCCCAGCACAGAGATGTCACTGAGAGGAGTGAGAGTCAGCAAAATGGGAGTGGATGAATATTCCAGTACTGAGAGAGTGGTCGTGAGCATTGATCGCTTCTTGAGAGTCCGAGATCAGAGGGGAAATGTGCTTCTGTCTCCTGAGGAAGTTAGTGAAACACATGGAACAGAGAAACTGACGATAACGTATTCATCGTCTATGA<br>TGTGGGAAATCAATGGTCCGGAATCCGTGCTAGTCAACACATATCAATGGATCATTAGAAATTGGGAAACTGTGAAGATTCAGTGGTCCCAGGACCCTACGATGTTATACAATAAGATGGAATTTGAGCCCTTCCAATCCTTGGTGCCCAAGGCTGCTAGAGGCCAGTATAGTGGGTTTGTGAGGACATTATTCCAACAGATGCGTGATGTGTTGGGAACATTTGACACTAT<br>CCAAATAATAAAGCTCCTACCATTTGCAGCAGCCCCACCGGAACAGAGTAGGATGCAATTTTCCTCTCTGACTGTGAACGTAAGAGGTTCAGGAATGAGAATACTTGTGAGGGGCAACTCCCCTGTGTTCAACTATAATAAGGCCACCAAGAGACTCACAGTTCTTGGAAAGGATGCAGGCGCCTTGACAGAATATCCAGATGAGGGAACAGCAGGAGTGGAGTCTGCAG<br>TATTAAGAGGATTTCTAATTCTGGGCAAAGAGGACAAAAGATATGGACCAGCATTGAGCATCAACGAATTGAGCAATCTTGCGAAAGGGGAAAAGGCTAATGTGTTGATAGGACAAGGAGACGTGGTGTTGGTAATGAAACGGAAACGGGACTCTAGCATACTTACTGACAGCCAGACAGCGACCAAAAGAATTCGGATGGCCATCAATTA
EPI_ISL_846601	>A/chicken/Poland/474/2020_H5N8_2020-12-03<br>ATGGAGAGAATAAAAGAACTAAGAGATTTGATGTCACAGTCTCGCACTCGCGAGATACTAACAAAAACCACTGTGGACCATATGGCCATAATAAAGAAATACACATCAGGGAGACAGGAGAAGAACCCTGCTCTCAGGATGAAATGGATGATGGCAATGAAATATCCTATTACAGCTGACAAAAGAATAATGGAGATGATCCCTGAAAGGAATGAGCAAGGTCAGACTC<br>TCTGGAGCAAAACAAATGATGCTGGATCAGACAGAGTGATGGTCTCACCTCTGGCTGTGACATGGTGGAATAGAAACGGGCCAACAACAAGTACAGTACACTACCCAAAAGTCTACAAAACCTACTTTGAAAAGGTGGAAAGGTTGAAACATGGAACCTTTGGTCCTGTTCACTTTCGAAATCAGGTTAAGATACGCCGCAGGGTTGACATAAACCCGGGCCATGCAGAT<br>CTCAGTGCCAAAGAAGCACAGGATGTCATCATGGAGGTTGTTTTCCCAAATGAAGTTGGAGCCAGGATCTTGACATCAGAATCACAATTAACAATAACAAGGGAAAAGAAGGAGGAACTTCAGGATTGCAAGATTGCTCCTTTGATGGTGGCATACATGTTGGAAAGAGAACTGGTTCGCAAGACCAGATTTTTACCAGTAGCTGGCGGAACAAGCAGCGTGTACATCGA<br>GGTATTGCATTTGACTCAAGGGACCTGCTGGGAACAAATGTACACACCAGGAGGGGAGGTGAGAAATGATGATGTTGATCAGAGTTTGATCATTGCTGCTAGAAATATAGTTAGGAGGGCAACAGTATCAGCAGACCCATTGGCTTCGCTCTTGGAAATGTGCCACAGTACACAAATTGGTGGAGTGAGGATGGTGGACATTCTTAGGCAGAACCCAACAGAGGAGCAA<br>GCTGTGGATATATGCAAAGCAGCAATGGGTTTAAGAATCAGTTCATCCTTCAGCTTTGGAGGTTTCACTTTCAAAAGGACAAGTGGGTCGTCTGTCAAAAGAGAAGAAGAAATACTCACTGGCAACCTCCAAACACTGAAAGTAAGAATACATGAAGGATATGAGGAATTCACAATGGTTGGGCGAAGAGCTACAGCCATTTTGAGGAAAGCAACCAGGAGACTGATCCA<br>ATTAATAGTGAGTGGACGAGACGAGCAGTCAATCGCTGAAGCAATCATAGTGGCAATGGTTTTCTCACAGGAGGATTGCATGATAAAAGCAGTACGAGGTGATTTGAATTTTGTCAACAGAGCGAATCAGCGGCTAAATCCTATGCATCAACTTCTGAGGCATTTCCAAAAGGATGCAAAAGTGCTGTTTCAAAACTGGGGGATTGAACCAATTGACAATGTAATGGGGAT<br>GATCGGGATACTGCCTGACATGACCCCCAGCACAGAGATGTCACTGAGAGGAGTGAGAGTCAGCAAAATGGGAGTGGATGAATATTCCAGTACTGAGAGAGTGGTCGTGAGCATTGATCGCTTCTTGAGAGTCCGAGATCAGAGGGGAAATGTGCTTCTGTCTCCTGAGGAAGTTAGTGAAACACATGGAACAGAGAAACTGACGATAACGTATTCATCGTCTATGATG<br>TGGGAAATCAATGGTCCGGAATCCGTGCTAGTCAACACATATCAATGGATCATTAGAAATTGGGAAACTGTGAAGATTCAGTGGTCCCAGGACCCTACGATGTTGTACAATAAGATGGAATTTGAGCCCTTTCAATCCTTGGTGCCCAAGGCTGCTAGAGGCCAGTATAGTGGGTTTGTGAGGACATTATTCCAACAGATGCGTGATGTGTTGGGGACATTTGACACTGTCC<br>AAATAATAAAGCTCCTACCATTTGCAGCAGCCCCACCGGAACAGAGTAGGATGCAATTTTCCTCTCTGACTGTGAACGTAAGAGGTTCAGGAATGAGAATACTTGTGAGGGGCAACTCCCCTGYGTTCAACTATAATAAGGCCACCAAGAGACTCACAGTTCTTGGAAAGGATGCAGGCGCCTTGACAGAATATCCAGATGAGGGAACAGCAGGAGTGGAGTCTGCAGTA<br>TTAAGAGGATTTCTAATTCTGGGCAAAGAGGACAAAAGATATGGACCAGCATTGAGCATCAACGAGTTGAGCAATCTTGCGAAAGGGGAAAAGGCTAATGTGTTGATAGGACAAGGAGACGTGGTGTTGGTRATGAAACGGAAACGGGACTCTAGCATACTTACTGACAGCCAGACAGCGACCAAAAGAATTCGGATGGCCATCAATTA
EPI_ISL_846603	>A/chicken/Poland/476/2020_H5N8_2020-12-04<br>ATGGAGAGAATAAAAGAACTAAGAGATTTGATGTCGCAGTCTCGCACTCGCGAGATACTAACAAAAACCACTGTGGACCATATGGCCATAATAAAGAAATACACATCAGGAAGACAGGAGAAGAACCCTGCCCTCAGGATGAAATGGATGATGGCAATGAAATATCCTATTACAGCTGACAAAAGAATAATGGAGATGATCCCTGAAAGGAATGAGCAAGGTCAGACT<br>CTCTGGAGCAAAACAAATGATGCTGGATCAGACAGAGTGATGGTCTCACCTCTGGCTGTGACATGGTGGAATAGAAATGGGCCAACAACAAGTACAGTACACTACCCAAAAGTCTACAAAACCTACTTTGAAAAGGTAGAAAGGTTGAAACATGGAACCTTTGGTCCTGTTCACTTTCGAAGTCAGGTTAAGATACGCCGTAGGGTTGACATAAACCCGGGCCATGCAGA<br>TCTCAGTGCCAAAGAAGCACAGGATGTCATCATGGAGGTTGTTTTCCCAAATGAAGTTGGAGCCAGGATCTTGACATCAGAATCACAATTAACAATAACAAGGGAAAAGAAGGAGGAACTTCAGGATTGCAAGATTGCTCCTTTGATGGTGGCATACATGTTGGAAAGAGAACTGGTTCGCAAGACCAGATTTTTACCAGTAGCTGGCGGAACAAGCAGCGTATACATCG<br>AGGTATTGCATTTGACTCAAGGGACCTGCTGGGAACAAATGTACACACCAGGAGGGGAGGTGAGAAATGATGATGTTGATCAGAGTTTGATCGTTGCTGCTAGAAATATAGTTAGGAGGGCAACAGTATCAGCAGACCCATTGGCTTCGCTTTTGGAAATGTGCCACAGTACACAAATTGGTGGAGTAAGGATGGTGGACATTCTTAGGCAGAACCCAACAGAGGAGCA<br>AGCTGTGGATATATGCAAAGCAGCAATGGGTTTAAGAATCAGTTCATCCTTCAGCTTTGGAGGTTTCACTTTCAAAAGGACAAGTGGGTCGTCTGTCAAAAGAGAAGAAGAAATACTCACTGGCAACCTCCAAACACTGAAAGTAAGAATACATGAAGGGTATGAGGAATTCACAATGGTTGGGCGAAGAGCTACAGCCATTTTGAGGAAAGCAACCAGGAGACTGATCC<br>AATTAATAGTGAGTGGAAGAGACGAGCAGTCAATCGCTGAAGCAATCATAGTGGCAATGGTTTTCTCACAGGAGGATTGCATGATAAAAGCAGTACGAGGTGATTTGAATTTTGTCAACAGAGCGAATCAGCGGCTAAATCCTATGCATCAACTTCTGAGGCATTTCCAAAAGGATGCAAAAGTGCTGTTTCAAAACTGGGGGATTGAACCAATTGACAATGTAATGGGG<br>ATGATCGGGATACTGCCTGACATGACCCCCAGCACAGAGATGTCACTGAGAGGAGTGAGAGTCAGCAAAATGGGAGTGGATGAATATTCCAGTACTGAGAGAGTGGTCGTGAGCATTGATCGCTTCTTGAGAGTCCGAGATCAGAGGGGAAATGTGCTTCTGTCTCCTGAGGAAGTTAGTGAAACACATGGAACAGAGAAACTGACGATAACGTATTCATCGTCTATGA<br>TGTGGGAAATCAATGGTCCGGAATCCGTGCTAGTCAACACATATCAATGGATCATTAGAAATTGGGAAACTGTGAAGATTCAGTGGTCCCAGGACCCTACGATGTTATACAATAAGATGGAATTTGAGCCCTTCCAATCCTTGGTGCCCAAGGCTGCTAGAGGCCAGTATAGTGGGTTTGTGAGGACATTATTCCAACAGATGCGTGATGTGTTGGGAACATTTGACACTAT<br>CCAAATAATAAAGCTCCTACCATTTGCAGCAGCCCCACCGGAACAGAGTAGGATGCAATTTTCCTCTCTGACTGTGAACGTAAGAGGTTCAGGAATGAGAATACTTGTGAGGGGCAACTCCCCTGTGTTCAACTATAATAAGGCCACCAAGAGACTCACAGTTCTTGGAAAGGATGCAGGCGCCTTGACAGAATATCCAGATGAGGGAACAGCAGGAGTGGAGTCTGCAG<br>TATTAAGAGGATTTCTAATTCTGGGCAAAGAGGACAAAAGATATGGACCAGCATTGAGCATCAACGAATTGAGCAATCTTGCGAAAGGGGAAAAGGCTAATGTGTTGATAGGACAAGGAGACGTGGTGTTGGTAATGAAACGGAAACGGGACTCTAGCATACTTACTGACAGCCAGACAGCGACCAAAAGAATTCGGATGGCCATCAATTA
EPI_ISL_3102072	>A/chicken/Poland/H1084_21RS1385-13/2021_H5N8_2021-05-07<br>ATGGAGAGAATAAAAGAACTAAGAGATTTGATGTCGCAGTCTCGCACTCGCGAGATACTAACAAAAACCACTGTGGACCATATGGCCATAATAAAGAAATACACATCAGGAAGACAGGAGAAGAACCCTGCCCTCAGGATGAAATGGATGATGGCAATGAAATATCCTATTACAGCTGACAAAAGAATAATGGAGATGATCCCTGAAAGGAATGAGCAAGGTCAGACT<br>CTCTGGAGCAAAACAAATGATGCTGGATCAGACAGAGTGATGGTCTCACCTCTGGCTGTGACATGGTGGAATAGAAATGGGCCAACAACAAGTACAGTACACTACCCAAAAGTCTACAAAACCTACTTTGAAAAGGTAGAAAGGTTGAAACATGGAACCTTTGGTCCTGTTCACTTTCGAAATCAGGTTAAGATACGCCGCAGGGTTGACATAAACCCGGGCCATTCAGAT<br>CTCAGTGCCAAAGAAGCACAGGATGTCATCATGGAGGTTGTTTTCCCAAATGAAGTTGGAGCCAGGATCTTGACATCAGAATCACAATTAACAATAACAAGGGAAAAGAAGGAGGAACTTCAGGATTGCAAGATTGCTCCTTTGATGGTGGCATACATGTTGGAAAGAGAACTGGTTCGCAAGACCAGATTTTTACCAGTAGCTGGCGGAACAAGCAGCGTATACATCGA<br>GGTATTGCATTTGACTCAAGGGACCTGCTGGGAACAAATGTACACACCAGGAGGGGAGGTGAGAAATGATGATGTTGATCAGAGTTTGATCATTGCTGCTAGAAATATAGTTAGGAGGGCAACAGTATCAGCAGACCCATTGGCTTCGCTTTTGGAAATGTGCCACAGTACACAAATTGGTGGAGTAAGGATGGTGGACATTCTTAGGCAGAACCCAACAGAGGAGCAA<br>GCTGTGGATATATGCAAAGCAGCAATGGGTTTAAGAATCAGTTCATCCTTCAGCTTTGGAGGTTTCACTTTCAAAAGGACAAGTGGGTCGTCTGTCAAAAGAGAAGAAGAAATACTCACTGGCAACCTCCAAACACTGAAAGTAAGAATACATGAAGGATATGAGGAATTCACAATGGTTGGGCGAAGAGCTACAGCCATTTTGAGGAAAGCAACCAGGAGACTGATCCA<br>ATTAATAGTGAGTGGAAGAGACGAGCAGTCAATCGCTGAAGCAATCATAGTGGCAATGGTTTTCTCACAGGAGGATTGCATGATAAAAGCAGTACGAGGTGATTTGAATTTCGTCAACAGAGCGAATCAGCGGCTAAATCCTATGCATCAACTTCTGAGGCATTTCCAAAAGGATGCAAAAGTGCTGTTTCAAAACTGGGGGATTGAACCAATTGACAATGTAATGGGGA<br>TGATCGGGATACTGCCTGACATGACCCCCAGCACAGAGATGTCACTGAGAGGAGTGAGAGTCAGCAAAATGGGAGTGGATGAATATTCCAGTACTGAGAGAGTGGTCGTGAGCATTGATCGCTTCTTGAGAGTCCGAGATCAGAGGGGAAATGTGCTTCTGTCTCCTGAGGAAGTTAGTGAAACACATGGAACAGAGAAACTGACGRTAACGTATTCATCGTCTATGAT<br>GTGGGAAATCAATGGTCCGGAATCCGTGCTAGTCAACACATATCAATGGATCATTAGAAATTGGGAAACTGTGAAGATTCAGTGGTCCCAGGACCCTACGATGTTATACAACAAGATGGAATTTGAGCCCTTCCAATCCTTGGTGCCCAAGGCTGCTAGAGGCCAGTATAGTGGGTTTGTGAGGACATTATTCCAACAGATGCGTGATGTGTTGGGAACATTTGACACTGTC<br>CAAATAATAAAGCTCCTACCATTTGCAGCAGCCCCACCGGAACAGAGTAGGATGCAATTTTCCTCTCTGACTGTAAACGTAAGAGGTTCAGGAATGAGAATACTTGTGAGGGGCAACTCCCCTGTGTTCAACTATAATAAGGCCACCAAGAGACTCACAGTTCTTGGAAAGGATGCAGGCGCCTTGACAGAATATCCAGATGAGGGAACAGCAGGAGTGGAGTCTGCAGT<br>ATTAAGAGGATTTCTAATTCTGGGCAAAGAGGACAAAAGATATGGACCAGCATTGAGCATCAACGAATTGAGCAATCTTGCGAAAGGGGAAAAGGCTAATGTGTTGATAGGACAAGGAGACGTGGTGTTGGTAATGAAACGGAAACGGGACTCTAGCATACTTACTGACAGCCAGACAGCGACCAAAAGAATTCGGATGGCCATCAATTA
EPI_ISL_3102062	>A/chicken/Poland/H1124_21RS1385-3/2021_H5N8_2021-05-11<br>ATGGAGAGAATAAAAGAACTAAGAGATTTGATGTCGCAGTCTCGCACTCGCGAGATACTAACAAAAACCACTGTGGACCATATGGCCATAATAAAGAAATACACATCAGGGAGACAGGAGAAGAACCCTGCCCTCAGGATGAAATGGATGATGGCAATGAAATATCCTATTACAGCTGACAAAAGAATAATGGAGATGATCCCTGAAAGGAATGAGCAAGGTCAGACT<br>CTCTGGAGCAAAACAAATGATGCTGGATCAGACAGAGTGATGGTCTCACCTCTGGCTGTGACGTGGTGGAATAGAAATGGGCCAACAACAAGTACAGTACACTACCCAAAAGTCTACAAAACCTACTTTGAAAAGGTAGAAAGGTTGAAACATGGAACCTTTGGTCCTGTTCACTTTCGAAATCAGGTTAAGATACGCCGCAGGGTTGACACAAACCCGGGCCATGCAGA<br>TCTCAGTGCCAAAGAAGCACAGGATGTCATCATGGAGGTTGTTTTCCCAAATGAAGTTGGAGCCAGGATCTTGACATCAGAATCACAATTAACAATAACAATGGAAAAGAAGGAGGAACTTCAGGATTGCAAGATTGCTCCTTTGATGGTGGCATACATGTTGGAAAGAGAACTGGTTCGCAAGACCAGATTTTTACCAGTAGCTGGCGGAACAAGCAGCGTATACATCG<br>AGGTATTGCATTTGACTCAAGGGACCTGCTGGGAACAAATGTACACACCAGGAGGGGAGGTGAGAAATGATGATGTTGATCAGAGTTTGATCATTGCTGCTAGAAATATAGTTAGGAGGGCAACAGTATCAGCAGACCCATTGGCTTCGCTCTTGGAAATGTGCCACAGTACACAAATTGGTGGAGTGAGGATGGTGGACATTCTTAGGCAGAACCCAACAGAGGAGCA<br>AGCTGTGGATATATGCAAAGCAGCAATGGGTTTAAGAATCAGTTCATCCTTCAGCTTTGGAGGTTTCACTTTCAAAAGGACAAGTGGGTCGTCTGTCAAAAGAGAAGAAGAAATACTCACTGGCAACCTCCAAACACTGAAAGTAAGAATACATGAAGGATATGAGGAATTCACAATGGTTGGGCGAAGAGCTACAGCCATTTTGAGGAAAGCAACCAGGAGACTGATCC<br>AATTAATAGTGAGTGGACGAGACGAACAGTCAATCGCTGAGGCAATCATAGTGGCAATGGTTTTCTCACAGGAGGATTGCATGATAAAAGCAGTACGAGGTGATTTGAATTTTGTCAACAGAGCGAATCAGCGGCTAAATCCTATGCATCAACTTCTGAGGCATTTCCAAAAAGATGCAAAAGTGCTGTTTCAAAACTGGGGGGTTGAACCAATTGACAATGTAATGGGG<br>ATGATCGGGATACTGCCTGACATGACCCCCAGCACAGAGATGTCACTGAGAGGAGTGAGAGTCAGCAAAATGGGAGTGGATGAATATTCCAGTACTGAGAGAGTGGTCGTGAGCATTGATCGCTTCTTGAGAGTCCGAGATCAGAGGGGAAATGTGCTTCTGTCTCCTGAGGAAGTTAGTGAAACACATGGAACAGAGAAACTGACGATAACGTATTCATCGTCCATGA<br>TGTGGGAAATCAATGGTCCGGAATCCGTGCTAGTCAACACATATCAATGGATCATTAGAAATTGGGAAACTGTGAAGATTCAGTGGTCCCAGGACCCTACGATGTTGTACAATAAGATGGAATTTGAGCCCTTCCAATCCTTGGTGCCCAAGGCTGCTAGAGGCCAGTATAGTGGGTTTGTGAGGACATTATTCCAACAGATGCGTGATGTGTTGGGGACATTTGACACTGT<br>CCAAATAATAAAGCTCCTACCATTTGCAGCAGCCCCACCGGAACAGAGTAGGATGCAATTTTCCTCTCTGACTGTGAACGTAAGAGGTTCAGGAATGAGAATACTTGTGAGGGGCAACTCCCCTGTGTTCAACTATAATAAGACCACCAAGAGACTCACAGTTCTTGGAAAGGATGCAGGCGCCTTGACAGAATATCCAGATGAGGGAACAGCAGGAGTGGAGTCTGCAG<br>TATTAAGAGGATTTCTAATTCTGGGCAAAGAGGACAAAAGATATGGACCAGCATTGAGCATCAACGAATTGAGCAATCTTGCGAAAGGGGAAAAGGCTAATGTGTTGATAGGACAAGGAGACGTGGTGTTGGTAATGAAACGGAAACGAGACTCTAGCATACTTACTGACAGCCAGACAGCGACCAAAAGAATTCGGATGGCCATCAATTA
EPI_ISL_3102069	>A/chicken/Poland/H1161_21RS1385-10/2021_H5N8_2021-05-13<br>ATGGAGAGAATAAAAGAACTAAGAGATTTGATGTCGCAGTCTCGCACTCGCGAGATACTAACAAAAACCACTGTGGACCATATGGCCATAATAAAGAAATACACATCAGGAAGACAGGAGAAGAACCCTGCCCTCAGGATGAAATGGATGATGGCAATGAAATATCCTATTACAGCTGACAAAAGAATAATGGAGATGATCCCTGAAAGGAATGAGCAAGGTCAGACT<br>CTCTGGAGCAAAACAAATGATGCTGGATCAGACAGAGTGATGGTCTCACCTCTGGCTGTGACATGGTGGAATAGAAATGGGCCAACAACAAGTACAGTACACTACCCAAAAGTCTACAAAACCTACTTTGAAAAGGTAGAAAGGTTGAAACATGGAACCTTTGGTCCTGTTCACTTTCGAAATCAGGTTAAGATACGCCGCAGGGTTGACATAAACCCGGGCCATTCAGAT<br>CTCAGTGCCAAAGAAGCACAGGATGTCATCATGGAGGTTGTTTTCCCAAATGAAGTTGGAGCCAGGATCTTGACATCAGAATCACAATTAACAATAACAAGGGAAAAGAAGGAGGAACTTCAGGATTGCAAGATTGCTCCTTTGATGGTGGCATACATGTTGGAAAGAGAACTGGTTCGCAAGACCAGATTTTTACCAGTAGCTGGCGGAACAAGCAGCGTATACATCGA<br>GGTATTGCATTTGACTCAAGGGACCTGCTGGGAACAAATGTACACACCAGGAGGGGAGGTGAGAAATGATGATGTTGATCAGAGTTTGATCATTGCTGCTAGAAATATAGTTAGGAGGGCAACAGTATCAGCAGACCCATTGGCTTCGCTTTTGGAAATGTGCCACAGTACACAAATTGGTGGAGTAAGGATGGTGGACATTCTTAGGCAGAACCCAACAGAGGAGCAG<br>GCTGTGGATATATGCAAAGCAGCAATGGGTTTAAGAATCAGTTCATCCTTCAGCTTTGGAGGTTTCACTTTCAAAAGGACAAGTGGGTCGTCTGTCAAAAGAGAAGAAGAAATACTCACTGGCAACCTCCAAACACTGAAAGTAAGAATACATGAAGGATATGAGGAATTCACAATGGTTGGGCGAAGGGCTACAGCCATTTTGAGGAAAGCAACCAGGAGACTGATCCA<br>ATTAATAGTGAGTGGAAGAGACGAGCAGTCAATCGCTGAAGCAATCATAGTGGCAATGGTTTTCTCACAGGAGGATTGCATGATAAAAGCAGTACGAGGTGATTTGAATTTCGTCAACAGAGCAAATCAGCGGCTAAATCCTATGCATCAACTTCTGAGGCATTTCCAAAAGGATGCAAAAGTGCTGTTTCAAAACTGGGGGATTGAACCAATTGACAATGTAATGGGGA<br>TGATCGGGATACTGCCTGACATGACCCCCAGCACAGAGATGTCACTGAGAGGAGTGAGAGTCAGCAAAATGGGAGTGGATGAATATTCCAGTACTGAGAGAGTGGTCGTGAGCATTGATCGCTTCTTGAGAGTCCGAGATCAGAGGGGAAATGTGCTTCTGTCTCCTGAGGAAGTTAGTGAAACACATGGAACAGAGAAACTGACGATAACGTATTCATCGTCTATGAT<br>GTGGGAAATCAATGGTCCGGAATCCGTGCTAGTCAACACATATCAATGGATCATTAGAAATTGGGAAACTGTGAAGATTCAGTGGTCCCAGGACCCTACGATGTTATACAACAAGATGGARTTTGAGCCCTTCCAATCCTTGGTGCCCAAGGCTGCTAGAGGCCAGTATAGTGGGTTTGTGAGGACATTATTCCAACAGATGCGTGATGTGTTGGGAACATTTGACACTGTC<br>CAAATAATAAAGCTCCTACCATTTGCAGCAGCCCCACCGGAACAGAGTAGGATGCAATTTTCCTCTCTGACTGTAAACGTAAGAGGTTCAGGAATGAGAATACTTGTGAGGGGCAACTCCCCTGTGTTCAACTATAATAAGGCCACCAAGAGACTCACAGTTCTTGGAAAGGATGCAGGCGCCTTGACAGAATATCCAGATGAGGGAACAGCAGGAGTGGAGTCTGCAGT<br>ATTAAGAGGATTTCTAATTCTGGGCAAAGAGGACAAAAGATATGGACCAGCATTGAGCATCAACGAATTGAGCAATCTTGCGAAAGGGGAAAAGGCTAATGTGTTGATAGGACAAGGAGACGTGGTGTTGGTAATGAAACGGAAACGGGACTCTAGCATACTTACTGACAGCCAGACAGCGACCAAAAGAATTCGGATGGCCATCAATTA
EPI_ISL_3102067	>A/chicken/Poland/H293_21RS1385-8/2021_H5N8_2021-03-23<br>ATGGAGAGAATAAAAGAACTAAGAGATTTGATGTCGCAGTCTCGCACTCGCGAGATACTAACAAAAACCACTGTGGACCATATGGCCATAATAAAGAAATACACATCAGGAAGACAGGAGAAGAACCCTGCCCTCAGGATGAAATGGATGATGGCAATGAAATATCCTATTACAGCTGACAAAAGAATAATGGAGATGATCCCTGAAAGGAATGAGCAAGGTCAGACT<br>CTCTGGAGCAAAACAAATGATGCTGGATCAGACAGAGTGATGGTCTCACCTCTGGCTGTGACATGGTGGAATAGAAATGGGCCAACAACAAGTACAGTACACTACCCAAAAGTCTACAAAACCTACTTTGAAAAGGTAGAAAGGTTGAAACATGGAACCTTTGGTCCTGTTCACTTTCGAAATCAGGTTAAGATACGCCGCAGGGTTGACATAAACCCGGGCCATTCAGAT<br>CTCAGTGCCAAAGAAGCACAGGATGTCATCATGGAGGTTGTTTTCCCAAATGAAGTTGGAGCCAGGATCTTGACATCAGAATCACAATTAACAATAACAAGGGAAAAGAAGGAGGAACTTCAGGATTGCAAGATTGCTCCTTTGATGGTGGCATACATGTTGGAAAGAGAACTGGTTCGCAAGACCAGATTTTTACCAGTAGCTGGCGGAACAAGCAGCGTATACATCGA<br>GGTATTGCATTTGACTCAAGGGACCTGCTGGGAACAAATGTACACACCAGGAGGGGAGGTGAGAAATGATGATGTTGATCAGAGTTTGATCATTGCTGCTAGAAATATAGTTAGGAGGGCAACAGTATCAGCAGACCCATTGGCTTCGCTTTTGGAAATGTGCCACAGTACACAAATTGGTGGAGTAAGGATGGTGGACATTCTTAGGCAGAACCCAACAGAGGAGCAA<br>GCTGTGGATATATGCAAAGCAGCAATGGGTTTAAGAATCAGTTCATCCTTCAGCTTTGGAGGTTTCACTTTCAAAAGGACAAGTGGGTCGTCTGTCAAAAGAGAAGAAGAAATACTCACTGGCAACCTCCAAACACTGAAAGTAAGAATACATGAAGGATATGAGGAATTCACAATGGTTGGGCGAAGAGCTACAGCCATTTTGAGGAAAGCAACCAGGAGACTGATCCA<br>ATTAATAGTGAGTGGAAGAGACGAGCAGTCAATCGCTGAAGCAATCATAGTGGCAATGGTTTTCTCACAGGAGGATTGCATGATAAAAGCAGTACGAGGTGATTTGAATTTCGTCAACAGAGCRAATCAGCGGCTAAATCCTATGCATCAACTTCTGAGGCATTTCCAAAAGGATGCAAAAGTGCTGTTTCAAAACTGGGGGATTGAACCAATTGACAATGTAATGGGGAT<br>GATCGGGATACTGCCTGACATGACCCCCAGCACAGAGATGTCACTGAGAGGAGTGAGAGTCAGCAAAATGGGAGTGGATGAATATTCCAGTACTGAGAGAGTGGTCGTGAGCATTGATCGCTTCTTGAGAGTCCGAGATCAGAGGGGAAATGTGCTTCTGTCTCCTGAGGAAGTTAGTGAAACACATGGAACAGAGAAACTGACGATAACGTATTCATCGTCTATGATG<br>TGGGAAATCAATGGTCCGGAATCCGTGCTAGTCAACACATATCAATGGATCATTAGAAATTGGGAAACTGTGAAGATTCAGTGGTCCCAGGACCCTACGATGTTATACAACAAGATGGAATTTGAGCCCTTCCAATCCTTGGTGCCCAAGGCTGCTAGAGGCCAGTATAGTGGGTTTGTGAGGACATTATTCCAACAGATGCGTGATGTGTTGGGAACATTTGACACTGTCC<br>AAATAATAAAGCTCCTACCATTTGCAGCAGCCCCACCGGAACAGAGTAGGATGCAATTTTCCTCTCTGACTGTAAACGTAAGAGGTTCAGGAATGAGAATACTTGTGAGGGGCAACTCCCCTGTGTTCAACTATAATAAGGCCACCAAGAGACTCACAGTTCTTGGAAAGGATGCAGGCGCCTTGACAGAATATCCAGATGAGGGAACAGCAGGAGTGGAGTCTGCAGTA<br>TTAAGAGGATTTCTAATTCTGGGCAAAGAGGACAAAAGATATGGACCAGCATTGAGCATCAACGAATTGAGCAATCTTGCGAAAGGGGAAAAGGCTAATGTGTTGATAGGACAAGGAGACGTGGTGTTGGTAATGAAACGGAAACGGGACTCTAGCATACTTACTGACAGCCAGACAGCGACCAAAAGAATTCGGATGGCCATCAATTA
EPI_ISL_3102071	>A/chicken/Poland/H712_21RS1385-12/2021_H5N8_2021-04-16<br>ATGGAGAGAATAAAAGAACTAAGAGATTTGATGTCGCAGTCTCGCACTCGCGAGATACTAACAAAAACCACTGTGGACCATATGGCCATAATAAAGAAATACACATCAGGAAGACAGGAGAAGAACCCTGCCCTCAGGATGAAATGGATGATGGCAATGAAATATCCTATTACAGCTGACAAAAGAATAATGGAGATGATCCCTGAAAGGAATGAGCAAGGTCAGACT<br>CTCTGGAGCAAAACAAATGATGCTGGATCAGACAGAGTGATGGTCTCACCTCTGGCTGTGACATGGTGGAATAGAAATGGGCCAACAACAAGTACAGTACACTACCCAAAAGTCTACAAAACCTACTTTGAAAAGGTAGAAAGGTTGAAACATGGAACCTTTGGTCCTGTTCACTTTCGAAATCAGGTTAAGATACGCCGCAGGGTTGACATAAACCCGGGCCATTCAGAT<br>CTCAGTGCCAAAGAAGCACAGGATGTCATCATGGAGGTTGTTTTCCCAAATGAAGTTGGAGCCAGGATCTTGACATCAGAATCACAATTAACAATAACAAGGGAAAAGAAGGAGGAACTTCAGGATTGCAAGATTGCTCCTTTGATGGTGGCATACATGTTGGAAAGAGAACTGGTTCGCAAGACCAGATTTTTACCAGTAGCTGGCGGAACAAGCAGCGTATACATCGA<br>GGTATTGCATTTGACTCAAGGGACCTGCTGGGAACAAATGTACACACCAGGAGGGGAGGTGAGAAATGATGATGTTGATCAGAGTTTGATCATTGCTGCTAGAAATATAGTTAGGAGGGCAACAGTATCAGCAGACCCATTGGCTTCGCTTTTGGAAATGTGCCACAGTACACAAATTGGTGGAGTAAGGATGGTGGACATTCTTAGGCAGAACCCAACAGAGGAGCAA<br>GCTGTGGATATATGCAAAGCAGCAATGGGTTTAAGAATCAGTTCATCCTTCAGCTTTGGAGGTTTCACTTTCAAAAGGACAAGTGGGTCGTCTGTCAAAAGAGAAGAAGAAATACTCACTGGCAACCTCCAAACACTGAAAGTAAGAATACATGAAGGATATGAGGAATTCACAATGGTTGGGCGAAGAGCTACAGCCATTTTGAGGAAAGCAACCAGGAGACTGATCCA<br>ATTAATAGTGAGTGGAAGAGACGAGCAGTCAATCGCTGAAGCAATCATAGTGGCAATGGTTTTCTCACAGGAGGATTGCATGATAAAAGCAGTACGAGGTGATTTGAATTTCGTCAACAGAGCGAATCAGCGGCTAAATCCTATGCATCAACTTCTGAGGCATTTCCAAAAGGATGCAAAAGTGCTGTTTCAAAACTGGGGGATTGAACCAATTGACAATGTAATGGGGA<br>TGATCGGGATACTGCCTGACATGACCCCCAGCACAGAGATGTCACTGAGAGGAGTGAGAGTCAGCAAAATGGGAGTGGATGAATATTCCAGTACTGAGAGAGTGGTCGTGAGCATTGATCGCTTCTTGAGAGTCCGAGATCAGAGGGGAAATGTGCTTCTGTCTCCTGAGGAAGTTAGTGAAACACATGGAACAGAGAAACTGACGATAACGTATTCATCGTCTATGAT<br>GTGGGAAATCAATGGTCCGGAATCCGTGCTAGTCAACACATATCAATGGATCATTAGAAATTGGGAAACTGTGAAGATTCAGTGGTCCCAGGACCCTACGATGTTATACAACAAGATGGAATTTGAGCCCTTCCAATCCTTGGTGCCCAAGGCTGCTAGAGGCCAGTATAGTGGGTTTGTGAGGACATTATTCCAACAGATGCGTGATGTGTTGGGAACATTTGACACTGTC<br>CAAATAATAAAGCTCCTACCATTTGCAGCAGCCCCACCGGAACAGAGTAGGATGCAATTTTCCTCTCTGACTGTAAACGTAAGAGGTTCAGGAATGAGAATACTTGTGAGGGGCAACTCCCCTGTGTTCAACTATAATAAGGCCACCAAGAGACTCACAGTTCTTGGAAAGGATGCAGGCGCCTTGACAGAATATCCAGATGAGGGAACAGCAGGAGTGGAGTCTGCAGT<br>ATTAAGAGGATTTCTAATTCTGGGCAAAGAGGACAAAAGATATGGACCAGCATTGAGCATCAACGAATTGAGCAATCTTGCGAAAGGGGAAAAGGCTAATGTGTTGATAGGACAAGGAGACGTGGTGTTGGTAATGAAACGGAAACGGGACTCTAGCATACTTACTGACAGCCAGACAGCGACCAAAAGAATTCGGATGGCCATCAATTA
EPI_ISL_3102066	>A/chicken/Poland/H812_21RS1385-7/2021_H5N8_2021-04-20<br>ATGGAGAGAATAAAAGAACTAAGAGATTTGATGTCGCAGTCTCGCACTCGCGAGATACTAACAAAAACCACTGTGGACCATATGGCCATAATAAAGAAATACACATCAGGAAGACAGGAGAAGAACCCTGCCCTCAGGATGAAATGGATGATGGCAATGAAATATCCTATTACAGCTGACAAAAGAATAATGGAGATGATCCCTGAAAGGAATGAGCAAGGTCAGACT<br>CTCTGGAGCAAAACAAATGATGCTGGATCAGACAGAGTGATGGTCTCACCTCTGGCTGTGACATGGTGGAATAGAAATGGGCCAACAACAAGTACAGTACACTACCCAAAAGTCTACAAAACCTACTTTGAAAAGGTAGAAAGGTTGAAACATGGAACCTTTGGTCCTGTTCACTTTCGAAGTCAGGTTAAGATACGCCGTAGGGTTGACATAAACCCGGGCCATGCAGA<br>TCTCAGTGCCAAAGAAGCACAGGATGTCATCATGGAGGTTGTTTTCCCAAATGAAGTTGGAGCCAGGATCTTGACATCAGAATCACAATTAACAATAACAAGGGAAAAGAAGGAGGAACTTCAGGATTGCAAGATTGCTCCTTTGATGGTGGCATACATGTTGGAAAGAGAACTGGTTCGCAAGACCAGATTTTTACCAGTAGCTGGCGGAACAAGCAGCGTATACATCG<br>AGGTATTGCATTTGACTCAAGGGACCTGCTGGGAACAAATGTACACACCAGGAGGGGAGGTGAGAAATGATGATGTTGATCAGAGTTTGATCGTTGCTGCTAGAAATATAGTTAGGAGGGCAACAGTATCAGCAGACCCATTGGCTTCGCTTTTGGAAATGTGCCACAGTACACAAATTGGTGGAGTAAGGATGGTGGACATTCTTAGGCAGAACCCAACAGAGGAGCA<br>AGCTGTGGATATATGCAAAGCAGCAATGGGTTTAAGAATCAGTTCATCCTTCAGCTTTGGAGGTTTCACTTTCAAAAGGACAAGTGGGTCGTCTGTCAAAAGAGAAGAAGAAATACTCACTGGCAACCTCCAAACACTGAAAGTAAGAATACATGAAGGGTATGAGGAATTCACAATGGTTGGGCGAAGAGCTACAGCCATTTTGAGGAAAGCAACCAGGAGACTGATCC<br>AATTAATAGTGAGTGGAAGAGACGAGCAGTCAATCGCTGAAGCAATCATAGTGGCAATGGTTTTCTCACAGGAGGATTGCATGATAAAAGCAGTACGAGGTGATTTGAATTTTGTCAACAGAGCGAATCAGCGGCTAAATCCTATGCATCAACTTCTGAGGCATTTCCAAAAGGATGCAAAAGTGCTGTTTCAAAACTGGGGGATTGAACCAATTGACAATGTAATGGGG<br>ATGATCGGGATACTGCCTGACATGACCCCCAGCACAGAGATGTCACTGAGAGGAGTGAGAGTCAGCAAAATGGGAGTGGATGAATATTCCAGTACTGAGAGAGTGGTCGTGAGCATTGATCGCTTCTTGAGAGTCCGAGATCAGAGGGGAAATGTGCTTCTGTCTCCTGAGGAAGTTAGTGAAACACATGGAACAGAGAAACTGACGATAACGTATTCATCGTCTATGA<br>TGTGGGAAATCAATGGTCCGGAATCCGTGCTAGTCAACACATATCAATGGATCATTAGAAATTGGGAAACTGTGAAGATTCAGTGGTCCCAGGACCCTACGATGTTATACAATAAGATGGAATTTGAGCCCTTCCAATCCTTGGTGCCCAAGGCTGCTAGAGGCCAGTATAGTGGGTTTGTGAGGACATTATTCCAACAGATGCGTGATGTGTTGGGAACATTTGACACTAT<br>CCAAATAATAAAGCTCCTACCATTTGCAGCAGCCCCACCGGAACAGAGTAGGATGCAATTTTCCTCTCTGACTGTGAACGTAAGAGGTTCAGGAATGAGAATACTTGTGAGGGGCAACTCCCCTGTGTTCAACTATAATAAGGCCACCAAGAGACTCACAGTTCTTGGAAAGGATGCAGGCGCCTTGACAGAATATCCAGATGAGGGAACAGCAGGAGTGGAGTCTGCAG<br>TATTAAGAGGATTTCTAATTCTGGGCAAAGAGGACAAAAGATATGGACCAGCATTGAGCATCAACGAATTGAGCAATCTTGCGAAAGGGGAAAAGGCTAATGTGTTGATAGGACAAGGAGACGTGGTGTTGGTAATGAAACGGAAACGGGACTCTAGCATACTTACTGACAGCCAGACAGCGACCAAAAGAATTCGGATGGCCATCAATTA
EPI_ISL_3102075	>A/chicken/Poland/H928_21RS1385-16/2021_H5N8_2021-04-25<br>ATGGAGAGAATAAAAGAACTAAGAGATTTGATGTCGCAGTCTCGCACTCGCGAGATACTAACAAAAACCACTGTGGACCATATGGCCATAATAAAGAAATACACATCAGGAAGACAGGAGAAGAACCCTGCCCTCAGGATGAAATGGATGATGGCAATGAAATATCCTATTACAGCTGACAAAAGAATAATGGAGATGATCCCTGAAAGGAATGAGCAAGGTCAGACT<br>CTCTGGAGCAAAACAAATGATGCTGGATCAGACAGAGTGATGGTCTCACCTCTGGCTGTGACATGGTGGAATAGAAATGGGCCAACAACAAGTACAGTACACTACCCAAAAGTCTACAAAACCTACTTTGAAAAGGTAGAAAGGTTGAAACATGGAACCTTTGGTCCTGTTCACTTTCGAAATCAGGTTAAGATACGCCGCAGGGTTGACATAAACCCGGGCCATTCAGAT<br>CTCAGTGCCAAAGAAGCACAGGATGTCATCATGGAGGTTGTTTTCCCAAATGAAGTTGGAGCCAGGATCTTGACATCAGAATCACAATTAACAATAACAAGGGAAAAGAAGGAGGAACTTCAGGATTGCAAGATTGCTCCTTTGATGGTGGCATACATGTTGGAAAGAGAACTGGTTCGCAAGACCAGATTTTTACCAGTAGCTGGCGGAACAAGCAGCGTATACATCGA<br>GGTATTGCATTTGACTCAAGGGACCTGCTGGGAACAAATGTACACACCAGGAGGGGAGGTGAGAAATGATGATGTTGATCAGAGTTTGATCATTGCTGCTAGAAATATAGTTAGGAGGGCAACAGTATCAGCAGACCCATTGGCTTCGCTTTTGGAAATGTGCCACAGTACACAAATTGGTGGAGTAAGGATGGTGGACATTCTTAGGCAGAACCCAACAGAGGAGCAA<br>GCTGTGGATATATGCAAAGCAGCAATGGGTTTAAGAATCAGTTCATCCTTCAGCTTTGGAGGTTTCACTTTCAAAAGGACAAGTGGGTCGTCTGTCAAAAGAGAAGAAGAAATACTCACTGGCAACCTCCAAACACTGAAAGTAAGAATACATGAAGGATATGAGGAATTCACAATGGTTGGGCGAAGAGCTACAGCCATTTTGAGGAAAGCAACCAGGAGACTGATCCA<br>ATTAATAGTGAGTGGAAGAGACGAGCAGTCAATCGCTGAAGCAATCATAGTGGCAATGGTTTTCTCACAGGAGGATTGCATGATAAAAGCAGTACGAGGTGATTTGAATTTCGTCAACAGAGCGAATCAGCGGCTAAATCCTATGCATCAACTTCTGAGGCATTTCCAAAAGGATGCAAAAGTGCTGTTTCAAAACTGGGGGATTGAACCAATTGACAATGTAATGGGGA<br>TGATCGGGATACTGCCTGACATGACCCCCAGCACAGAGATGTCACTGAGAGGAGTGAGAGTCAGCAAAATGGGAGTGGATGAATATTCCAGTACTGAGAGAGTGGTCGTGAGCATTGATCGCTTCTTGAGAGTCCGAGATCAGAGGGGAAATGTGCTTCTGTCTCCTGAGGAAGTTAGTGAAACACATGGAACAGAGAAACTGACGATAACGTATTCATCGTCTATGAT<br>GTGGGAAATCAATGGTCCGGAATCCGTGCTAGTCAACACATATCAATGGATCATTAGAAATTGGGAAACTGTGAAGATTCAGTGGTCCCAGGACCCTACGATGTTATACAACAAGATGGAATTTGAGCCCTTCCAATCCTTGGTGCCCAAGGCTGCTAGAGGCCAGTATAGTGGGTTTGTGAGGACATTATTCCAACAGATGCGTGATGTGTTGGGAACATTTGACACTGTC<br>CAAATAATAAAGCTCCTACCATTTGCAGCAGCCCCACCGGAACAGAGTAGGATGCAATTTTCCTCTCTGACTGTAAACGTAAGAGGTTCAGGAATGAGAATACTTGTGAGGGGCAACTCCCCTGTGTTCAACTATAATAAGGCCACCAAGAGACTCACAGTTCTTGGAAAGGATGCAGGCGCCTTGACAGAATATCCAGATGAGGGAACAGCAGGAGTGGAGTCTGCAGT<br>ATTAAGAGGATTTCTAATTCTGGGCAAAGAGGACAAAAGATATGGACCAGCATTGAGCATCAACGAATTGAGCAATCTTGCGAAAGGGGAAAAGGCTAATGTGTTGATAGGACAAGGAGACGTGGTGTTGGTAATGAAACGGAAACGGGACTCTAGCATACTTACTGACAGCCAGACAGCGACCAAAAGAATTCGGATGGCCATCAATTA
EPI_ISL_3102068	>A/chicken/Poland/H984_21RS1385-9/2021_H5N8_2021-04-28<br>ATGGAGAGAATAAAAGAACTAAGAGATTTGATGTCGCAGTCTCGCACTCGCGAGATACTAACAAAAACCACTGTGGACCATATGGCCATAATAAAGAAATACACATCAGGAAGACAGGAGAAGAACCCTGCCCTCAGGATGAAATGGATGATGGCAATGAAATATCCTATTACAGCTGACAAAAGAATAATGGAGATGATCCCTGAAAGGAATGAGCAAGGTCAGACT<br>CTCTGGAGCAAAACAAATGATGCTGGATCAGACAGAGTGATGGTCTCACCTCTGGCTGTGACATGGTGGAATAGAAATGGGCCAACAACAAGTACAGTACACTACCCAAAAGTCTACAAAACCTACTTTGAAAAGGTAGAAAGGTTGAAACATGGAACCTTTGGTCCTGTTCACTTTCGAAATCAGGTTAAGATACGCCGCAGGGTTGACATAAACCCGGGCCATTCAGAT<br>CTCAGTGCCAAAGAAGCACAGGATGTCATCATGGAGGTTGTTTTCCCAAATGAAGTTGGAGCCAGGATCTTGACATCAGAATCACAATTAACAATAACAAGGGAAAAGAAGGAGGAACTTCAGGATTGCAAGATTGCTCCTTTGATGGTGGCATACATGTTGGAAAGAGAACTGGTTCGCAAGACCAGATTTTTACCAGTAGCTGGCGGAACAAGCAGCGTATACATCGA<br>GGTATTGCATTTGACTCAAGGGACCTGCTGGGAACAAATGTACACACCAGGAGGGGAGGTGAGAAATGATGATGTTGATCAGAGTTTGATCATTGCTGCTAGAAATATAGTTAGGAGGGCAACAGTATCAGCAGACCCATTGGCTTCGCTTTTGGAAATGTGCCACAGTACACAAATTGGTGGAGTAAGGATGGTGGACATTCTTAGGCAGAACCCAACAGAGGAGCAA<br>GCTGTGGATATATGCAAAGCAGCAATGGGTTTAAGAATCAGTTCATCCTTCAGCTTTGGAGGTTTCACTTTCAAAAGGACAAGTGGGTCGTCTGTCAAAAGAGAAGAAGAAATACTCACTGGCAACCTCCAAACACTGAAAGTAAGAATACATGAAGGATATGAGGAATTCACAATGGTTGGGCGAAGAGCTACAGCCATTTTGAGGAAAGCAACCAGGAGACTGATCCA<br>ATTAATAGTGAGTGGAAGAGACGAGCAGTCAATCGCTGAAGCAATCATAGTGGCAATGGTTTTCTCACAGGAGGATTGCATGATAAAAGCAGTACGAGGTGATTTGAATTTCGTCAACAGAGCGAATCAGCGGCTAAATCCTATGCATCAACTTCTGAGGCATTTCCAAAAGGATGCAAAAGTGCTGTTTCAAAACTGGGGGATTGAACCAATTGACAATGTAATGGGGA<br>TGATCGGGATACTGCCTGACATGACCCCCAGCACAGAGATGTCACTGAGAGGAGTGAGAGTCAGCAAAATGGGAGTGGATGAATATTCCAGTACTGAGAGAGTGGTCGTGAGCATTGATCGCTTCTTGAGAGTCCGAGATCAGAGGGGAAATGTGCTTCTGTCTCCTGAGGAAGTTAGTGAAACACATGGAACAGAGAAACTGACGATAACGTATTCATCGTCTATGAT<br>GTGGGAAATCAATGGTCCGGAATCCGTGCTAGTCAACACATATCAATGGATCATTAGAAATTGGGAAACTGTGAAGATTCAGTGGTCCCAGGACCCTACGATGTTATACAACAAGATGGAATTTGAGCCCTTCCAATCCTTGGTGCCCAAGGCTGCTAGAGGCCAGTATAGTGGGTTTGTGAGGACATTATTCCAACAGATGCGTGATGTGTTGGGAACATTTGACACTGTC<br>CAAATAATAAAGCTCCTACCATTTGCAGCAGCCCCACCGGAACAGAGTAGGATGCAATTTTCCTCTCTGACTGTAAACGTAAGAGGTTCAGGAATGAGAATACTTGTGAGGGGCAACTCCCCTGTGTTCAACTATAATAAGGCCACCAAGAGACTCACAGTTCTTGGAAAGGATGCAGGCGCCTTGACAGAATATCCAGATGAGGGAACAGCAGGAGTGGAGTCTGCAGT<br>ATTAAGAGGATTTCTAATTCTGGGCAAAGAGGACAAAAGATATGGACCAGCATTGAGCATCAACGAATTGAGCAATCTTGCGAAAGGGGAAAAGGCTAATGTGTTGATAGGACAAGGAGACGTGGTGTTGGTAATGAAACGGAAACGGGACTCTAGCATACTTACTGACAGCCAGACAGCGACCAAAAGAATTCGGATGGCCATCAATTA

EPI_ISL_7622539	>A/Chroicocephalus_ridibundus/Belgium/13464/2020_H13N8_2021-12-09<br>ATGGAGAGAATAAAAGAACTAAGAGATCTAATGTCGCAGTCTCGCACTCGCGAGATACTCACCAAAACCACTGTGGACCACATGGCCATAATCAAAAAATACACATCGGGAAGGCAAGAGAAGAACCCCGCACTAAGAATGAAATGGATGATGGCAATGAAATATCCAATCACCGCAGAGAAGAGAATAGTGGAAATGATTCCTGAAAGGAATGAGCAGGGACAAAC<br>CCTTTGGAGTAAAACAAACGATGCCGGATCAGACCGAGTAATGGTATCGCCTCTCGCTGTGACATGGTGGAACAGGAATGGACCAACAGCAAGTACAGTCCATTACCCAAAGGTATACAAAACTTATTTGAAAAAGTTGAGAGATTGAAACACGGGACCTTTGGCCCTGTCCACTTCAGAAATCAAGTTAAGATAAGACGGAGGGTCGATGTAAACCCGGGCCATGCAG<br>ACCTAAGTGCCAAAGAGGCACAGGATGTAATCATGGAAGTTGTCTTCCCAAATGAGGTTGGAGCAAGAATACTGACGTCAGAGTCACAACTGACGGTAACAAAGGAGAAAAAAGAAGAACTCCAGGACTGCAAAATTGCCCCTTTGATGGTTGCATATATGCTAGAGAGGGAATTGGTCCGGAAGACAAGGTTTCTCCCAGTGGCTGGTGGAACAAGCAGTGTCTATAT<br>TGAGGTGCTGCACTTAACCCAGGGGACATGCTGGGAGCAAATGTACACTCCAGGAGGAGAGGTGAGGAATGATGATGTAGACCAGAGCTTGATTATTGCTGCCAGGAACATAGTGAGGAGAGCAACAGTATCGGCAGACCCACTAGCATCCCTACTGGAAATGTGCCACAGCACACAGATTGGAGGGGTAAGGATGGTAGACATCCTTCGACAAAATCCAACAGAAGA<br>ACAAGCCGTGGACATATGCAAGGCAGCAATGGGATTAAGGATCAGCTCATCTTTCAGCTTTGGTGGATTCACTTTTAAAAGAACAAGTGGATCTTCAGTAAAAAGAGAAGAAGAAGTGCTTACGGGCAACCTTCAAACATTGAAAATAAGAGTGCATGAGGGGTATGAAGAGTTCACAATGGTAGGAAGACGAGCAACTGCTATCATCAGGAAAGCAACCAGGAGGCTG<br>ATTCAGCTGATAGTAAGCGGGAGAGACGAGCAGTCAATTGCTGAAGCAATAATTGTGGCCATGGTGTTCTCACAGGAGGATTGCATGATCAAGGCAGTTCGAGGTGATCTGAATTTTGTCAATAGGGCGAACCAGAGGCTGAACCCAATGCATCAACTCCTAAGACACTTTCAAAAGGATGCAAAAGTGCTTTTTCAAAACTGGGGAATCGAACCCATTGACAGTGTGAT<br>GGGGATGATTGGGATATTGCCAGACATGACCCCAAGTACTGAGATGTCGCTGAGGGGGATAAGAGTCAGCAAGACGGGAGTAGATGAATACTCCAGCACAGAAAGAGTCGTAGTGAGCATCGACCGATTTTTAAGAGTTCGAGACCAAAGAGGGAACATACTATTGTCACCTGAAGAAGTCAGTGAGACACAGGGAACAGAGAAATTGACAATCACCTATTCGTCGTC<br>AATGATGTGGGAGATCAATGGTCCTGAGTCGGTGTTGGTCAATACCTATCAGTGGATCATCAGAAATTGGGAAACTGTGAAAATCCAATGGTCACAGGATCCCACAATGTTATATAACAAGATGGAATTCGAACCATTCCAATCTCTTGTCCCTAAGGCAGCTAGGGGTCAATACAGTGGATTCGTGAGGACATTATTCCAGCAGATGCGGGACGTGCTTGGAACATTTGAC<br>ACTGTTCAAATAATAAAACTCCTCCCATTTGCTGCTGCCCCACCAGAACAAAGTAAGATGCAGTTCTCCTCCATGACTGTAAATGTAAGAGGATCGGGAATGAGAATACTGGTAAGAGGCAACTCCCCAGTGTTCAATTACAATAAGGCCACCAAGAGGCTCACTATTCTCGGGAAAGATGCAGGTGCATTGACCGAAGATCCAGATGAAGGCACAGCTGGAATAGAGTCT<br>GCTGTTTTAAGAGGATTCCTCATTTTGGGCAAAGAAGACAAGAGATATGGCCCGGCATTGAGCATCAATGAGCTGAGCAATCTTGCAAAGGGAGAGAAGGCTAATGTGCTAATTGGGCAAGGAGACGTAGTGTTGGTAATGAAACGGAAACGGGACTCTAGCATACTTACTGACAGTCAGACAGCGACCAAAAGAATTCGGATGGCCATCAATTA
EPI_ISL_2175628	>A/common_buzzard/Netherlands/21021187-001/2021_H5N8_2021-01-04<br>ATGGAGAGAATAAAAGAACTAAGAGATTTGATGTCACAGTCTCGCACTCGCGAGATACTAACAAAAACCACTGTGGACCATATGGCCATAATAAAGAAATACACATCAGGGAGACAGGAGAAGAACCCTGCCCTCAGGATGAAATGGATGATGGCAATGAAATATCCTATTACAGCTGACAAAAGAATAATGGAGATGATCCCTGAAAGGAATGAGCAAGGTCAGACT<br>CTCTGGAGCAAAACAAATGATGCTGGATCAGACAGAGTGATGGTCTCACCTCTGGCTGTGACATGGTGGAATAGAAACGGGCCAACAACAAGTACAGTACACTACCCAAAAGTCTACAAAACCTACTTTGAAAAGGTGGAAAGGTTGAAACATGGAACCTTTGGTCCTGTTCACTTTCGAAATCAGGTTAAGATACGCCGCAGGGTTGACATAAACCCGGGCCATGCAGA<br>TCTCAGTGCCAAAGAAGCACAGGATGTCATCATGGAGGTTGTTTTCCCAAATGAAGTTGGAGCCAGGATCTTGACATCAGAATCACAATTAACAATAACAAGGGAAAAGAAGGAGGAACTTCAGGATTGCAAGATTGCTCCTTTGATGGTGGCATACATGTTGGAAAGAGAACTGGTTCGCAAGACCAGATTTTTACCAGTAGCTGGCGGAACAAGCAGCGTGTACATCG<br>AGGTATTGCATTTGACTCAAGGGACCTGCTGGGAACAAATGTACACACCAGGAGGGGAGGTGAGAAATGATGATGTTGATCAGAGTTTGATCATTGCTGCTAGAAATATAGTTAGGAGGGCAACAGTATCAGCAGACCCATTGGCTTCGCTCTTGGAAATGTGCCACAGTACACAAATTGGTGGAGTGAGGATGGTGGACATTCTTAGGCAGAACCCAACAGAGGAGCA<br>AGCTGTGGATATATGCAAAGCAGCAATGGGTTTAAGAATCAGTTCATCCTTCAGCTTTGGAGGTTTCACTTTCAAAAGGACAAGTGGGTCGTCTGTCAAAAGAGAAGAAGAAATACTCACTGGCAACCTCCAAACACTGAAAGTAAGAATACATGAAGGATATGAGGAATTCACAATGGTTGGGCGAAGAGCTACAGCCATTTTGAGGAAAGCAACCAGGAGACTGATCC<br>AATTAATAGTGAGTGGACGAGACGAGCAGTCAATCGCTGAAGCAATCATAGTGGCAATGGTTTTCTCACAGGAGGATTGCATGATAAAAGCAGTACGAGGTGATTTGAATTTTGTCAACAGAGCGAATCAGCGGCTAAATCCTATGCATCAACTTCTGAGGCATTTCCAAAAGGATGCAAAAGTGCTGTTTCAAAACTGGGGATTGAACCAATTGACAATGTAATGGGG<br>ATGATCGGGATACTGCCTGACATGACCCCCAGCACAGAGATGTCACTGAGAGGAGTGAGAGTCAGCAAAATGGGAGTGGATGAATATTCCAGTACTGAGAGAGTGGTCGTGAGCATTGATCGCTTCTTGAGAGTCCGAGATCAGAGGGGAAATGTGCTTCTGTCTCCTGAGGAAGTTAGTGAAACACATGGAACAGAGAAACTGACGATAACGTATTCATCGTCTATGA<br>TGTGGGAAATCAATGGTCCGGAATCCGTGCTAGTCAACACATATCAATGGATCATTAGAAATTGGGAAACTGTGAAGATTCAGTGGTCCCAGGACCCTACGATGTTGTACAATAAGATGGAATTTGAGCCCTTTCAATCCTTGGTGCCCAAGGCTGCTAGAGGCCAGTATAGTGGGTTTGTGAGGACATTATTCCAACAGATGCGTGATGTGTTGGGGACATTTGACACTGT<br>CCAAATAATAAAGCTCCTACCATTTGCAGCAGCCCCACCGGAACAGAGTAGGATGCAATTTTCCTCTCTGACTGTGAATGTAAGAGGTTCAGGAATGAGAATACTTGTGAGGGGCAACTCCCCTGTGTTCAACTATAATAAGGCCACCAAGAGACTCACAGTTCTTGGAAAGGATGCAGGCGCCTTGACAGAATATCCAGATGAGGGAACAGCAGGAGTGGAGTCTGCAG<br>TATTAAGAGGATTTCTAATTCTGGGCAAAGAGGACAAAAGATATGGACCAGCATTGAGCATCAACGAATTGAGCAATCTTGCGAAAGGGGAAAAGGCTAATGTGTTGATAGGACAAGGAGACGTGGTGTTGGTAATGAAACGGAAACGGGACTCTAGCATACTTACTGACAGCCAGACAGCGACCAAAAGAATTCGGATGGCCATCAATTA
EPI_ISL_14917968	>A/common_murre/Poland/MB151/2022_H5N1_2022-07-13<br>ATGGAGAGAATAAAAGAACTAAGAGATTTAATGTCGCAGTCTCGCACTCGCGAGATACTGACAAAAACCACTGTGGACCATATGGCCATAATAAAGAAATACACATCAGGAAGACAGGAGAAGAACCCTGCCCTCAGGATGAAGTGGATGATGGCAATGAAATATCCTATTACAGCAGACAAAAGAATAATGGAGATGATCCCTGAAAGGAATGAGCAAGGTCAGACT<br>CTCTGGAGCAAAACAAATGACGCTGGATCAGACAGAGTGATGGTGTCACCTCTGGCTGTGACATGGTGGAATAGAAATGGGCCAACGACAAGTACAGTCCATTACCCAAAGGTTTACAAAACCTACTTTGAAAAAGTGGAAAGATTGAAACATGGAACCTTTGGCCCTGTTCACTTTCGAAATCAGGTTAAGATACGCCGCAGGGTTGACATAAACCCGGGTCATGCAGA<br>TCTCAGTGCCAAAGAAGCACAGGATGTCATCATGGAAGTCGTTTTCCCAAATGAAGTTGGAGCCAGGATATTGACATCAGAATCACAATTAACAATAACAAAGGAAAAGAAGRAGGAACTTCAGGACTGTAAGATTGCTCCTTTGATGGTGGCATACATGTTGGAGAGAGAACTGGTTCGAAAAACCAGATTTTTACCAGTAGCTGGCGGAACAAGCAGCGTATATATCG<br>AAGTATTGCATTTGACTCAAGGGACCTGCTGGGAACAAATGTACACACCGGGAGGGGAAGTGAGAAATGATGATGTTGATCAGAGTTTGATCATTGCTGCTAGAAATATCGTTAGGAGAGCAACAGTATCAGCAGACCCATTGGCTTCGCTCTTGGAAATGTGCCACAGCACACAAATTGGCGGGATAAGGATGGTAGACATTCTTAGACAGAACCCAACAGAAGAGCA<br>AGCCGTGGATATATGCAAAGCAGCAATGGGTTTAAGAATCAGTTCATCCTTCAGCTTTGGAGGTTTCACTTTCAAAAGAACAAGTGGGTCGTCTGTCAAAAGAGAAGAAGAAGTGCTCACAGGCAACCTCCAAACACTGAAAATAAGAGTACATGAAGGATATGAGGAATTCACAATGGTTGGACGGAGAGCTACAGCCATTTTAAGGAAAGCAACCAGAAGGCTGATC<br>CAATTAATAGTGAGTGGAAGAGACGAGCAGTCAATCGCTGAAGCAATCATAGTGGCAATGGTTTTCTCACAAGAGGACTGCATGATAAAAGCAGTACGAGGTGATCTGAACTTTGTTAATAGAGCAAATCAGCGACTAAATCCTATGCATCAACTTCTGAGGCATTTCCAAAAGGATGCAAAGGTGCTGTTTCAAAACTGGGGGGTTGAACCAATTGACAATGTAATGGG<br>GATGATTGGAATACTGCCTGACATGACCCCCAGCACGGAGATGTCACTGAGAGGAGTGAGAGTCAGCAAAATGGGAGTGGATGAATAYTCCAGTACTGAGAGAGTGGTCGTGAGCATTGATCGTTTCTTGAGAGTCCGAGATCAAAGAGGAAACGTGCTCCTGTCTCCGGAGGAAGTTAGTGAAACACAGGGAACAGAGAAACTGACGATAACATATTCATCGTCCATG<br>ATGTGGGAAATCAATGGTCCGGAATCCGTGCTAGTCAACACATATCAATGGATCATCAGAAATTGGGAGACTGTGAAGATTCAATGGTCCCAAGACCCTACGATGTTGTACAATAAGATGGAATTTGAGCCCTTCCAATCCTTGGTGCCCAAGGCTGCCAGAGGCCAGTATAGTGGATTTGTGAGGACATTGTTCCAGCAGATGCGTGATGTGCTGGGAACATTTGATACT<br>GTCCAAATAATAAAGCTCCTGCCTTTTGCTGCAGCCCCACCGGAACAGAGTAGGATGCAGTTTTCCTCTCTGACTGTGAACGTAAGAGGTTCAGGAATGAGAATACTTGTGAGAGGCAACTCCCCTGTGTTCAACTATAACAAGGCCACCAAGAGACTCACAGTTCTTGGGAAGGATGCAGGTGCCTTGACAGAAGATCCAGATGAGGGAACAGCAGGAGTAGAGTCTGC<br>AGTATTAAGAGGATTTCTAATTCTGGGCAAAGAGGACAAAAGATATGGACCAGCATTGAGCATCAACGAACTGAGCAATCTTGCGAAAGGGGAAAAGGCTAATGTGTTGATAGGGCAAGGAGACGTGGTGTTGGTAATGAAACGGAAACGGGACTCTAGCATACTTACTGACAGCCAGACAGCGACCAAAAGAATTCGGATGGCCATCAATTA
EPI_ISL_328977	>A/common_teal/Netherlands/1/2015_H11N1_2015-09-29<br>ATGGAGAGAATAAAAGAGCTAAGAGATTTGATGTCGCAGTCTCGCACTCGCGAGATACTGACAAAAACCACTGTGGACCATATGGCCATAATCAAGAAATATACATCAGGAAGACAGGAGAAGAACCCTGCACTTAGGATGAAATGGATGATGGCAATGAAATATCCGATTACAGCAGACAAAAGGATAATGGAGATGATCCCTGAAAGAAATGAGCAGGGTCAGACT<br>CTTTGGAGCAAAACAAATGATGCTGGATCAGACAGAGTGATGGTGTCACCTCTGGCTGTGACGTGGTGGAATAGAAATGGACCAACAACAAGTACAGTCCATTACCCAAAGGTCTATAAAACCTACTTTGAAAAGGTTGAAAGGTTAAAGCATGGAACCTTCGGCCCTGTTCATTTTCGAAATCAGGTTAAGATACGCCGCAGAGTTGACATAAACCCGGGCCATGCAGAC<br>CTCAGTGCTAAAGAAGCACAAGACGTCATCATGGAGGTCGTTTTCCCAAATGAGGTCGGAGCCAGAATATTGACATCAGAGTCACAGTTAACAATAACAAAGGAAAAGAAGGAGGAACTCCAGGACTGTAAGATTGCCCCTTTAATGGTGGCATACATGTTGGAGAGAGAACTGGTTCGAAAAACCAGATTCCTGCCAGTAGCTGGCGGGACAAGCAGCGTATATATCG<br>AGGTGTTGCACTTGACTCAAGGAACCTGCTGGGAACAAATGTATACGCCAGGAGGAGAAGTGAGAAATGATGACGTTGATCAGAGTTTAATTATTGCTGCCAGAAATATCGTTAGGAGAGCAACAGTATCAGCAGACCCATTGGCTTCGCTACTGGAGATGTGCCATAGTACACAAATTGGCGGGATAAGGATGGTAGACATCCTTAGACAGAACCCAACAGAAGAGCA<br>AGCCGTGGATATATGCAAAGCAGCAATGGGTCTAAGAATCAGTTCATCCTTCAGCTTTGGAGGTTTCACTTTCAAAAGGACAAGCGGGTCATCTGTCAAAAGAGAAGAGGAAGTGCTCACCGGCAACCTCCAAACATTGAAGATAAGAGTGCATGAAGGGTATGAGGAATTCACAATGGTTGGGCGGAGAGCAACAGCCATTCTAAGGAAAGCAACCAGAAGGCTGATC<br>CAATTGATAGTAAGTGGGAGAGACGAGCAGTCAATCGCCGAAGCGATCATAGTGGCAATGGTGTTCTCCCAAGAGGATTGCATGATAAAGGCAGTACGAGGTGATTTAAATTTTGTCAATAGAGCGAATCAGCGGCTCAATCCTATGCATCAGCTCCTGAGGCATTTCCAAAAGGATGCAAAGGTACTGTTCCAAAACTGGGGAATTGAATCCATTGACAATGTCATGGG<br>GATGATAGGAATATTGCCTGATATGACACCCAGCACAGAGATGTCACTAAGAGGAGTGAGAGTCAGTAAAATGGGAGTGGATGAATATTCCAGTACTGAGAGGGTGGTCGTTAGTATTGATCGTTTCTTGAGGGTCCGAGACCAGAGAGGAAACGTACTCTTGTCTCCTGAAGAGGTTAGTGAAACACAGGGAACAGAGAAGCTGACGATAACATATTCATCATCCATG<br>ATGTGGGAAATTAATGGTCCGGAATCAGTGTTAGTTAACACATATCAATGGATCATTAGAAACTGGGAAACTGTGAAGATTCAGTGGTCCCAAGACCCTACAATGTTATACAACAAGATGGAGTTTGAGCCCTTTCAGTCCTTGGTGCCTAAGGCGGCCAGAGGCCAGTATAGTGGATTTGTAAGGACTCTATTCCAGCAGATGCGTGATGTGCTGGGGACCTTTGACACTG<br>TCCAGATAATAAAGCTACTCCCATTTGCAGCAGCCCCACCGGAACAGAGTAGGATGCAGTTCTCTTCTCTAACTGTAAACGTAAGGGGTTCAGGAATGAGAATACTTGTGAGAGGCAACTCCCCTGTGTTCAACTATAACAAGGCAACCAAGAGGCTCACAGTCCTTGGAAAGGATGCTGGTGCATTGACAGAAGACCCAGATGAGGGGACGGCAGGAGTGGAGTCTGC<br>GGTATTAAGAGGGTTCCTAATTCTGGGCAAAGAAGACAAAAGATATGGACCAGCGCTGAGCATCAATGAATTGAGCAATCTTGCGAAAGGGGAGAAGGCTAATGTGCTGATAGGGCAAGGAGACGTGGTGTTGGTGATGAAACGGAAACGGGACTCTAGCATACTTACTGACAGCCAGACAGCGACCAAAAGAATTCGGATGGCCATCAATTA
EPI_ISL_18245811	>A/crane/Poland/MB528/2021_H5N1_2021-12-10<br>ATGGAGAGAATAAAAGAGCTAAGAGATTTGATGTCGCAGTCTCGCACTCGCGAGATACTGACAAAAACCACCGTGGACCATATGGCCATAATCAAGAAATATACATCAGGAAGACAGGAGAAGAACCCTGCACTTAGGATGAAGTGGATGATGGCAATGAAATATCCGATTACAGCAGACAAAAGGATAATGGAGATGATCCCTGAAAGAAACGAGCAAGGTCAGACT<br>CTTTGGAGCAAAACAAATGATGCTGGATCGGATAGAGTAATGGTGTCACCTCTGGCTGTGACGTGGTGGAATAGAAATGGACCAACAACAAGTACCGTCCATTACCCAAAGGTCTATAAAACCTACTTTGAAAAGATTGAAAGGTTAAAGCATGGAACCTTCGGCCCTGTCCATTTCCGGAATCAGGTTAAGATACGCCGCAGAGTTGACATAAACCCGGGCCATGCAGAC<br>CTCAGTGCTAAAGAAGCACAAGACGTCATCATGGAGGTCGTTTTCCCAAATGAAGTCGGAGCCAGAATATTGACATCAGAGTCACAGTTGACAATTACAAAAGAAAAGAAGGAGGAACTCCAGGACTGTAAGATTGCCCCTCTAATGGTGGCATACATGTTGGAGAGAGAATTGGTTCGAAAAACCAGATTCCTGCCAGTAGCTGGCGGAACAAGTAGCGTATATATCGA<br>AGTGTTGCACTTGACTCAAGGAACCTGCTGGGAACAAATGTATACGCCAGGAGGAGAAGTGAGAAATGATGACGTTGACCAAAGTTTAATTATTGCTGCCAGAAATATCGTTAGGAGGGCAACAGTATCAGCAGACCCATTGGCTTCACTACTGGAGATGTGCCATAGTACACAGATTGGCGGGATAAGAATGGTAGACATTCTTAGACAGAACCCAACAGAAGAGCAA<br>GCCGTGGATATATGCAAAGCAGCAATGGGTTTAAGAATCAGTTCATCCTTCAGTTTTGGAGGTTTCACTTTCAAAAGGACAAGCGGATCATCTGTCAAAAGAGAAGAGGAAGTGCTCACCGGCAACCTCCAAACATTGAAAATAAGAGTACATGAAGGGTATGAGGAATTCACAATGGTTGGGCGGAGAGCAACAGCCATTCTAAGGAAAGCAACCAGAAGGCTGATCC<br>AATTGATAGTAAGTGGAAGAGACGAGCAGTCAATCGCCGAAGCGATCATAGTGGCAATGGTGTTCTCTCAAGAGGATTGCATGATAAAGGCTGTACGAGGTGATTTAAATTTTGTCAATAGAGCGAATCAGCGGCTCAATCCTATGCATCAGCTCCTGAGGCATTTCCAAAAGGATGCAAAAGTACTATTCCAAAACTGGGGAATTGAACCCATTGACAATGTCATGGGAA<br>TGATAGGAATATTGCCTGATATGACCCCCAGCACAGAGATGTCACTAAGAGGAGTGAGGGTCAGTAAAATGGGAGTGGATGAATATTCCAGTACTGAGAGGGTGGTCGTGAGTATTGATCGCTTCTTGAGGGTACGAGACCAGAGAGGAAATGTACTCTTGTCTCCCGAAGAGGTCAGTGAAACACAGGGAACAGAGAAGCTAACGATAACATATTCATCATCCATGAT<br>GTGGGAAATTAATGGCCCTGAGTCAGTGCTAGTTAACACATATCAATGGATCATCAGAAACTGGGAAAMTGTGAAGATTCAGTGGTCCCAAGACCCTACAATGCTATACAACAAGATGGAGTTTGAGCCTTTTCAGTCCTTGGTGCCTAAGGCAGCCAGAGGCCAGTACAGTGGATTTGTAAGGACCCTATTCCAGCAGATGCGTGATGTGCTGGGAACCTTTGACACTGT<br>CCAGATAATAAAGCTACTTCCATTTGCAGCAGCACCACCGGAACAGAGTAGGATGCAGTTCTCTTCTCTAACTGTAAACGTAAGGGGTTCAGGAATGAGAATACTTGTGAGAGGCAACTCCCCTGTGTTCAACTATAATAAAGCAACCAAGAGGCTCATAGTCCTTGGAAAGGATGCTGGTGCATTGACAGAAGACCCAGGTGAGGGGACAGCAGGAGTAGAGTCTGCGG<br>TATTGAGAGGGTTCCTAATTCTGGGCAAAGAGGACAAAAGATATGGACCAGCGCTGAGCATCAATGAATTGAGCAATCTTGCGAAAGGGGAGAAGGCTAATGTGTTGATAGGGCAAGGAGACGTGGTGTTGGTGATGAAACGGAAACGGGACTCTAGCATACTTACTGACAGCCAGACAGCGACCAAAAGAATTCGGATGGCCATCAATTA
internal NVRI repository	>A/domestic_duck/Poland/H1942-Nd/2021_H5N1_2021-11-07<br>ATGGAGAGAATAAAAGAGCTAAGAGATTTGATGTCGCAGTCTCGCACTCGCGAGATACTGACAAAAACCACCGTGGACCATATGGCCATAATCAAGAAATATACATCAGGAAGACAGGAGAAGAACCCTGCACTTAGGATGAAGTGGATGATGGCAATGAAATATCCGATTACAGCAGACAAAAGGATAATGGAGATGATCCCTGAAAGAAACGAGCAAGGTCAGACT<br>CTTTGGAGCAAAACAAATGATGCTGGATCGGATAGAATAATGGTGTCACCTCTGGCTGTGACGTGGTGGAATAGAAATGGACCAACAACAAGTACCGTCCATTACCCAAAGGTCTATAAAACCTACTTTGAAAAGGTTGAAAGGTTAAAGCATGGAACCTTCGGCCCTGTCCATTTCCGGAATCAGGTTAAGATACGCCGCAGAGTTGACATAAACCCGGGCCATGCAGAC<br>CTCAGTGCTAAAGAAGCACAAGACGTCATCATGGAGGTCGTTTTCCCAAATGAAGTCGGAGCCAGAATATTGACATCAGAGTCACAGTTAACAATTACAAAAGAAAAGAAGGAGGAACTCCAGGACTGTAAGATTGCCCCTCTAATGGTGGCATACATGTTGGAGAGAGAACTGGTTCGAAAAACCAGATTCCTGCCAGTAGCTGGCGGAACAAGTAGCGTATATATCGA<br>AGTGTTGCACTTGACTCAAGGAACCTGCTGGGAACAAATGTATACGCCAGGAGGAGAAGTGAGAAATGATGACGTTGACCAAAGTTTAATTATTGCTGCCAGAAATATCGTTAGGAGGGCAACAGTATCAGCAGACCCATTGGCTTCACTACTGGAGATGTGCCATAGTACACAGATTGGCGGGATAAGAATGGTAGACATTCTTAGACAGAACCCAACAGAAGAGCAA<br>GCCGTGGATATATGCAAAGCAGCAATGGGTTTAAGAATCAGTTCATCCTTCAGTTTTGGAGGTTTCACTTTCAAAAGGACAAGTGGATCATCTGTCAAAAGAGAAGAGGAAGTGCTCACAGGCAACCTCCAAACATTGAAAATAAGAGTACATGAAGGGTATGAGGAATTCACAATGGTTGGACGGAGAGCAACAGCCATTCTAAGGAAAGCAACCAGAAGGCTAATCC<br>AATTGATAGTAAGTGGAAGAGACGAGCAGTCAATCGCCGAAGCGATCATAGTGGCAATGGTGTTCTCTCAAGAGGATTGCATGATAAAGGCTGTACGAGGTGATTTAAATTTTGTCAATAGAGCGAATCAGCGGCTCAATCCTATGCATCAGCTCCTGAGGCATTTCCAAAAGGATGCAAAGGTACTATTCCAAAACTGGGGAATTGAACCCATTGACAATGTCATGGGAA<br>TGATAGGAATATTGCCTGATATGACCCCCAGCACAGAGATGTCACTAAGAGGAGTGAGGGTCAGTAAAATGGGAGTGGATGAATATTCCAGTACTGAGAGGGTGGTCGTGAGTATTGATCGCTTCTTGAGGGTACGAGACCAGAGAGGAAATGTACTCTTGTCTCCCGAAGAGGTCAGTGAAACACAGGGAACAGAGAAGCTAACGATAACATATTCATCATCCATGAT<br>GTGGGAAATTAATGGCCCTGAGTCAGTGCTAGTTAACACATATCAATGGATCATCAGAAACTGGGAAACTGTGAAGATTCAGTGGTCCCAAGACCCTACAATGCTATACAACAAGATGGAGTTTGAGCCTTTTCAGTCCTTGGTGCCTAAGGCAGCCAGAGGCCAGTACAGTGGATTTGTAAGGACCCTATTCCAGCAGATGCGTGATGTGCTGGGAACCTTTGACACTGT<br>CCAGATAATAAAGCTACTTCCATTTGCAGCAGCACCACCGGAACAGAGTAGGATGCAGTTCTCTTCTCTAACTGTAAACGTAAGGGGTTCAGGAATGAGAATACTTGTGAGAGGCAACTCCCCTGTGTTCAACTATAATAAAGCAACCAAGAGGCTCATAGTCCTTGGAAAGGATGCTGGTGCATTGACAGAAGACCCAGGTGAGGGGACAGCAGGAGTGGAGTCTGCG<br>GTATTGAGAGGGTTCCTAATTCTGGGCAAAGAGGACAAAAGATATGGACCAGCGCTGAGCATCAATGAATTGAGCAATCTTGCGAAAGGGGAGAAGGCTAATGTGTTGATAGGGCAAGGAGACGTGGTGTTGGTGATGAAACGGAAACGGGACTCTAGCATACTTACTGACAGCCAGACAGCGACCAAAAGAATTCGGATGGCCATCAATTA
EPI_ISL_5143300	>A/domestic_goose/Germany-MV/AI02558/2021_H5N8_2021-03-10<br>ATGGAGAGAATAAAAGAACTAAGAGATTTGATGTCACAGTCTCGCACTCGCGAGATACTAACAAAAACCACTGTGGACCATATGGCCATAATAAAGAAATACACATCAGGGAGACAGGAGAAGAACCCTGCCCTCAGGATGAAATGGATGATGGCAATGAAATATCCTATTACAGCTGACAAAAGAATAATGGAGATGATCCCTGAAAGGAATGAGCAAGGTCAGACT<br>CTCTGGAGCAAAACAAATGATGCTGGATCAGACAGAGTGATGGTCTCACCTCTGGCTGTGACATGGTGGAATAGAAACGGGCCAACAACAAGTACAGTACACTACCCAAAAGTCTACAAAACCTACTTTGAAAAGGTGGAAAGGTTGAAACATGGAACCTTTGGTCCTGTTCACTTTCGAAATCAGGTTAAGATACGCCGCAGGGTTGACATAAACCCGGGCCATGCAGA<br>TCTCAGTGCCAAAGAAGCACAGGATGTCATCATGGAGGTTGTTTTCCCAAATGAAGTTGGAGCCAGGATCTTGACATCAGAATCACAATTAACAATAACAAGGGAAAAGAAGGAGGAACTTCAGGATTGCAAGATTGCTCCTTTGATGGTGGCATACATGTTGGAAAGAGAACTGGTTCGCAAGACCAGATTTTTACCAGTAGCTGGCGGAACAAGCAGCGTGTACATCG<br>AGGTATTGCATTTGACTCAAGGGACCTGCTGGGAACAAATGTACACACCAGGAGGGGAGGTGAGAAATGATGATGTTGATCAGAGTTTGATCATTGCTGCTAGAAATATAGTTAGGAGGGCAACAGTATCAGCAGACCCATTGGCTTCGCTCTTGGAAATGTGCCACAGTACACAAATTGGTGGAGTGAGGATGGTGGACATTCTTAGGCAGAACCCAACAGAGGAGCA<br>AGCTGTGGATATATGCAAAGCAGCAATGGGTTTAAGAATCAGTTCATCCTTCAGCTTTGGAGGTTTCACTTTCAAAAGGACAAGTGGGTCGTCTGTCAAAAGAGAAGAAGAAATACTCACTGGCAACCTCCAAACACTGAAAGTAAGAATACATGAAGGATATGAGGAATTCACAATGGTTGGGCGAAGAGCTACAGCCATTTTGAGGAAAGCAACCAGGAGACTGATCC<br>AATTAATAGTGAGTGGACGAGACGAGCAGTCAATCGCTGAAGCAATCATAGTGGCAATGGTTTTCTCACAGGAGGATTGCATGATAAAAGCAGTACGAGGTGATTTGAATTTTGTCAACAGAGCGAATCAGCGGCTAAATCCTATGCATCAACTTCTGAGGCATTTCCAAAAGGATGCAAAAGTGCTGTTTCAAAACTGGGGATTGAACCAATTGACAATGTAATGGGG<br>ATGATCGGGATACTGCCTGACATGACCCCCAGCACAGAGATGTCACTGAGAGGAGTGAGAGTCAGCAAAATGGGAGTGGATGAATATTCTAGTACTGAGAGAGTGGTCGTGAGCATTGATCGCTTCTTGAGAGTCCGAGATCAGAGGGGAAATGTGCTTCTGTCTCCTGAGGAAGTTAGTGAAACACATGGAACAGAGAAACTGACGATAACGTATTCATCGTCTATGAT<br>GTGGGAAATCAATGGTCCGGAATCCGTGCTAGTCAACACATATCAATGGATCATTAGAAATTGGGAAACTGTGAAGATTCAGTGGTCCCAGGACCCTACGATGTTGTACAATAAGATGGAATTTGAGCCCTTTCAATCCTTGGTTCCCAAGGCTGCTAGAGGCCAGTATAGTGGGTTTGTGAGGACATTATTCCAACAGATGCGTGATGTGTTGGGGACATTTGACACTGTC<br>CAAATAATAAAGCTCCTACCATTTGCAGCAGCCCCACCGGAACAGAGTAGGATGCAATTTTCCTCTCTGACTGTGAACGTAAGAGGTTCAGGAATGAGAATACTTGTGAGGGGCAACTCCCCTGTGTTCAACTATAATAAGGCCACCAAGAGACTCACAGTTCTTGGAAAGGATGCAGGCGCCTTGACAGAATATCCAGATGAGGGAACAGAAGGAGTGGAGTCTGCAGT<br>ATTAAGAGGATTTCTAATTCTGGGCAAAGAGGACAAAAGATATGGACCAGCATTGAGCATCAACGAATTGAGCAATCTTGCGAAAGGGGAAAAGGCTAATGTGTTGATAGGACAAGGAGACGTGGTGTTGGTAATGAAACGGAAACGGGACTCTAGCATACTTACTGACAGCCAGACAGCGACCAAAAGAATTCGGATGGCCATCAATTA
EPI_ISL_4071639	>A/duck/Bangladesh/42981/2020_H8N4_2020-01-27<br>ATGGAGAGAATAAAAGAGCTAAGAGATTTGATGTCGCAGTCTCGCACTCGCGAGATACTGACAAAAACCACTGTGGACCATATGGCCATAATCAAGAAATATACATCAGGAAGACAGGAGAAGAATCCTGCACTTAGGATGAAATGGATGATGGCAATGAAATATCCGATTACAGCAGACAAAAGGATAATGGAGATGATCCCTGAAAGGAATGAGCAAGGTCAGACT<br>CTTTGGAGCAAAACAAATGATGCTGGATCAGACAGAGTGATGGTGTCACCTCTGGCTGTGACGTGGTGGAATAGAAATGGACCAACAACAAGTACAGTCCATTACCCAAAGGTCTATAAAACCTACTTTGAAAAGGTCGAAAGGTTAAAGCATGGAACCTTCGGCCCTGTTCATTTTCGAAACCAAGTTAAGATACGCCGCAGAGTTGACATAAACCCGGGCCATGCAGA<br>CCTCAGTGCTAAAGAGGCACAAGACGTCATTATGGAGGTCGTTTTCCCAAATGAAGTCGGAGCCAGAATATTGACATCAGAGTCACAGTTAACAATAACAAAGGAAAAGAAGGAGGAACTCCAGGACTGTAAGATTGCCCCCTTAATGGTGGCATACATGTTAGAGAGAGAACTGGTTCGAAAAACCAGATTCCTGCCAGTAGCTGGCGGAACAAGCAGCGTATATATC<br>GAGGTGTTGCACTTGACTCAAGGAACCTGCTGGGAACAAATGTATACGCCAGGAGGAGAAGTGAGAAATGATGACATTGATCAGAGCTTAATTATTGCTGCCAGAAATATCGTTAGGAGAGCAACAGTATCAGCAGACCCATTGGCTTCGCTACTGGAGATGTGCCATAGTACACAAATTGGAGGGATACGGATGGTAGACATCCTCAGACAGAACCCAACAGAAGAGC<br>AAGCTGTGGATATATGCAAAGCAGCAATGGGTCTAAGAATCAGTTCATCCTTCAGCTTTGGAGGTTTCACTTTCAAAAGGACAAGCGGGTCATCTGTCAAAAGAGAAGAGGAAGTGCTCACCGGCAACCTCCAAACATTGAAAATAAGAGTGCATGAAGGGTATGAGGAATTCACAATGGTTGGACGGAGAGCAACAGCCATTCTAAGGAAGGCAACCAGAAGGCTGAT<br>CCAATTGATAGTAAGTGGAAGAGATGAGCAGTCAATCGCCGAAGCGATCATAGTGGCAATGGTGTTCTCCCAAGAGGATTGCATGATAAAAGCAGTACGAGGTGATTTAAATTTTGTCAATAGAGCAAATCAGCGGCTCAATCCTATGCATCAGCTCCTGAGGCATTTCCAAAAGGATGCAAAGGTACTGTTCCAAAACTGGGGAATTGAGCCCATTGATAATGTTATGGG<br>GATGATAGGAATACTGCCTGATATGACACCCAGCACAGAGATGTCACTAAGAGGAGTGAGGGTCAGTAAAATGGGAGTGGATGAATATTCCAGTACTGAGAGGGTGGTCGTGAGTATTGATCGCTTCTTGAGGGTCCGAGACCAGAGAGGAAATGTACTCTTGTCTCCTGAAGAGGTTAGTGAAACACAGGGAACAGAGAAGCTGACGATAACATATTCATCATCCATG<br>ATGTGGGAAATTAATGGTCCGGAATCAGTGTTAGTTAACACATATCAATGGATCATCAGAAACTGGGAAACTGTGAAAATTCAGTGGTCCCAAGACCCTACAATGCTATACAACAAGATGGAGTTTGAGCCTTTTCAGTCCTTGGTGCCTAAGGCGGCCAGAGGCCAGTACAGTGGATTTGTAAGAACTCTATTCCAGCAGATGCGTGATGTGCTGGGGACCTTTGACACTG<br>TCCAGATAATAAAGCTACTCCCATTTGCAGCAGCCCCACCGGAACAGAGTAGGATGCAGTTCTCTTCTCTAACTGTAAATGTAAGGGGTTCAGGAATGAGAATACTTGTGAGAGGCAACTCCCCTGTGTTCAATTATAACAAGGCAACCAAGAGGCTCACAGTCCTTGGAAAGGATGCTGGTGCATTGACAGAAGACCCAGATGAGGGGACGGCAGGAGTGGAGTCTGCG<br>GTATTGAGAGGGTTCCTAATTCTGGGCAAAGAAGACAAAAGGTATGGACCAGCGCTGAGCATCAATGAATTGAGCAATCTTGCGAAAGGGGAGAAGGCTAATGTGTTGATAGGGCAAGGAGACGTGGTGTTGGTGATGAAACGGAAACGGGACTCTAGCATACTTACTGACAGCCAGACAGCGACCAAAAGAATTCGGATGGCCATCAATTA
EPI_ISL_637098	>A/duck/Chelyabinsk/1207-1/2020_H5N8_2020-07-31<br>ATGGAGAGAATAAAAGAACTAAGAGATTTGATGTCGCAGTCTCGCACTCGCGAGATACTAACAAAAACCACTGTGGACCATATGGCCATAATAAAGAAATACACATCAGGAAGACAGGAGAAGAACCCTGCCCTCAGGATGAAATGGATGATGGCAATGAAATATCCTATTACAGCTGACAAAAGAATAATGGAGATGATCCCTGAAAGGAATGAGCAAGGTCAGACT<br>CTCTGGAGCAAAACAAATGATGCTGGATCAGACAGAGTGATGGTCTCACCTCTGGCTGTGACATGGTGGAATAGAAATGGGCCAACAACAAGTACAGTACACTACCCAAAAGTCTACAAAACCTACTTTGAAAAGGTAGAAAGGTTGAAACATGGAACCTTTGGTCCTGTTCACTTTCGAAATCAGGTTAAGATACGCCGCAGGGTTGACATAAACCCGGGCCATGCAGA<br>TCTCAGTGCCAAAGAAGCACAGGATGTCATCATGGAGGTTGTTTTCCCAAATGAAGTTGGAGCCAGGATCTTGACATCAGAATCACAATTAACAATAACAAGGGAAAAGAAGGAGGAACTTCAGGATTGCAAGATTGCTCCTTTGATGGTGGCATACATGTTGGAAAGAGAACTGGTTCGCAAGACCAGATTTTTACCAGTAGCTGGCGGAACAAGCAGCGTATACATCG<br>AGGTATTGCATTTGACTCAAGGGACCTGCTGGGAACAAATGTACACACCAGGAGGGGAGGTGAGAAATGATGATGTTGATCAGAGTTTGATCATTGCTGCTAGAAATATAGTTAGGAGGGCAACAGTATCAGCAGACCCATTGGCTTCGCTCTTGGAAATGTGCCACAGTACACAAATTGGTGGAGTAAGGATGGTGGACATTCTTAGGCAGAACCCAACAGAGGAGCA<br>AGCTGTGGATATATGCAAAGCAGCAATGGGTTTAAGAATCAGTTCATCCTTCAGCTTTGGAGGTTTCACTTTCAAAAGGACAAGTGGGTCGTCTGTCAAAAGAGAAGAAGAAATACTCACTGGCAACCTCCAAACACTGAAAGTAAGAATACATGAAGGATATGAGGAATTCACAATGGTTGGGCGAAGAGCTACAGCCATTTTGAGGAAAGCAACCAGGAGACTGATCC<br>AATTAATAGTGAGTGGAAGAGACGAACAGTCAATCGCTGAAGCAATCATAGTGGCAATGGTTTTCTCACAGGAGGATTGCATGATAAAAGCAGTACGAGGTGATTTGAATTTTGTCAACAGAGCGAATCAGCGGCTAAATCCTATGCATCAACTTCTGAGGCATTTCCAAAAGGATGCAAAAGTGCTGTTTCAAAACTGGGGGATTGAACCAATTGACAATGTAATGGGG<br>ATGATCGGGATACTGCCTGACATGACCCCCAGCACAGAGATGTCACTGAGAGGAGTGAGAGTCAGCAAAATGGGAGTGGATGAATATTCCAGTACTGAGAGAGTGGTCGTGAGCATTGATCGCTTCTTGAGAGTCCGAGATCAGAGGGGAAATGTGCTTCTGTCTCCTGAGGAAGTTAGTGAAACACATGGAACAGAGAAACTGACGATAACGTATTCATCGTCTATGA<br>TGTGGGAAATCAATGGTCCGGAATCCGTGCTAGTCAACACATATCAATGGATCATTAGAAATTGGGAAACTGTGAAGATTCAGTGGTCCCAGGACCCTACGATGTTGTACAATAAGATGGAATTTGAACCCTTCCAATCCTTGGTGCCCAAGGCTGCTAGAGGCCAGTATAGTGGGTTTGTGAGGACATTATTCCAACAGATGCGTGATGTGTTGGGGACATTTGACACTGT<br>CCAAATAATAAAGCTCCTACCATTTGCAGCAGCCCCACCGGAACAGAGTAGGATGCAATTTTCCTCTCTGACTGTGAACGTAAGAGGTTCAGGAATGAGAATACTTGTGAGGGGCAACTCCCCTGTGTTCAACTATAATAAGGCCACCAAGAGACTCACAGTTCTTGGAAAGGATGCAGGCGCCTTGACAGAATATCCAGACGAGGGAACAGCAGGAGTGGAGTCTGCAG<br>TATTAAGAGGATTTCTAATTCTGGGCAAAGAGGACAAAAGATATGGACCAGCATTGAGCATCAACGAATTGAGCAATCTTGCGAAAGGGGAAAAGGCTAATGTGTTGATAGGACAAGGAGACGTGGTGTTGGTAATGAAACGGAAACGGGACTCTAGCATACTTACTGACAGCCAGACAGCGACCAAAAGAATTCGGATGGCCATCAATTA
EPI_ISL_381339	>A/Duck/China/Weihai/2017_H13N8_2017-05<br>ATGGAGAGAATAAAAGAACTAAGAGATCTAATGTCGCAGTCTCGCACTCGCGAGATACTCACCAAAACCACTGTGGACCACATGGCAATAATCAAAAAATACACATCGGGAAGGCAAGAGAAAAACCCCGCACTAAGGATGAAATGGATGATGGCAATGAAATACCCAATCACTGCAGAGAAGAGAATAATGGAAATGATTCCTGAAAGGAATGAGCAAGGACAAACC<br>CTTTGGAGTAAAACAAACGATGCCGGATCGGACCGAGTAATGGTATCGCCTCTCGCTGTGACGTGGTGGAACAGGAATGGACCAACAGCAAGTACAGTCCACTACCCAAAGGTATACAAAACTTATTTTGAAAAAGTTGAAAGGTTGAAACACGGGACCTTTGGCCCTGTCCACTTCAGAAATCAAGTTAAGATAAGACGGAGAGTCGACGTAAACCCAGGCCATGCAGA<br>CCTAAGTGCCAAAGAGGCACAGGATGTAATCATGGAAGTTGTCTTCCCAAATGAAGTGGGAGCGAGAATACTGACTTCAGAGTCACAACTGACGGTAACAAAGGAGAAAAAAGAAGAACTCCAGGACTGCAAAATTGCCCCTTTGATGGTTGCATACATGCTAGAGAGGGAATTGGTCCGCAAGACAAGGTTCCTCCCAGTGGCTGGTGGAACAAGCAGTGTCTATATT<br>GAGGTGCTGCACTTAACCCAGGGGACATGCTGGGAGCAGATGTACACTCCAGGAGGAGAGGTGAGGAATGATGATGTAGACCAGAGCTTGATTATTGCTGCCAGAAACATAGTGAGAAGAGCAACTGTATCAGCAGACCCACTAGCATCCCTACTGGAAATGTGCCACAGCACACAGATTGGAGGGGTAAGGATGGTCGACATCCTTCGACAAAATCCAACAGAAGAA<br>CAAGCCGTGGACATATGCAAGGCAGCAATGGGATTGAGGATTAGCTCATCTTTCAGCTTTGGTGGATTCACTTTTAAAAGAACAAGTGGATCTTCAGTAAAAAGAGAAGAAGAAGTGCTTACGGGCAACCTTCAAACATTGAAAATAAGAGTACATGAGGGGTATGAAGAGTTCACAATGGTAGGAAGACGAGCAACTGCTATTATCAGGAAAGCAACCAGAAGGCTGA<br>TCCAGCTAATAGTAAGTGGGAGAGACGAGCAGTCAATTGCTGAAGCAATAATTGTGGCCATGGTGTTCTCACAAGAGGACTGCATGATCAAGGCAGTTCGGGGTGACCTGAATTTTGTCAATAGGGCGAACCAGAGGCTGAATCCAATGCATCAACTCCTGAGGCACTTTCAAAAGGATGCAAAGGTACTTTTTCAAAACTGGGGAATCGAACCCATTGACAGTGTGATG<br>GGGATGATTGGGATATTGCCCGACATGACCCCAAGTACTGAGATGTCGCTGAGGGGGATAAGAGTCAGCAAGACGGGAGTAGATGAATACTCCAGCACAGAAAGGGTCGTAGTGAGCATCGACCGATTTTTAAGAGTTCGAGACCAAAGAGGGAACATACTATTGTCACCTGAAGAAGTTAGCGAGACACAGGGAACAGAGAAATTGACAATCACTTATTCGTCGTCA<br>ATGATGTGGGAGATCAATGGTCCTGAGTCGGTGTTGGTCAATACCTATCAATGGATCATCAGAAACTGGGAAACTGTGAAAATTCAATGGTCACAGGATCCCACAATGTTATATAACAAGATGGAGTTCGAACCATTCCAATCTCTTGTCCCTAAAGCAGCTAGGGGTCAATACAGTGGATTCGTGAGGACATTATTCCAGCAGATGCGGGATGTGCTTGGAACATTTGACA<br>CTGTTCAGATAATAAAACTCCTCCCATTTGCTGCTGCCCCACCAGAACAAAGTAAGATGCAGTTCTCCTCCATGACTGTAAATGTAAGAGGATCAGGGATGAGAATACTGGTAAGAGGCAACTCCCCAGTGTTCAATTACAATAAGGCCACCAAGAGGCTCACTATTCTTGGGAAAGATGCAGGTGCATTGACCGAAGATCCAGATGAAGGCACAGCTGGAATAGAGTCTG<br>CCGTTCTAAGAGGATTCCTCATTTTGGGCAAAGAAGACAAGAGATATGGCCCGGCATTGAGCATCAATGAGCTGAGCAATCTTGCAAAAGGAGAGAAGGCTAATGTGCTAATTGGGCAAGGAGACGTAGTGTTGGTAATGAAACGGAAACGGGACTCTAGCATACTTACTGACAGTCAGACAGCGACCAAAAGAATTCGGATGGCCATCAATTA
EPI_ISL_12572654	>A/duck/Guangdong/S4518/2021_H5N1_2021-12-08<br>ATGGAGAGAATAAAAGAACTAAGAGATTTGATGTCGCAGTCTCGCATTCGCGAGATACTGACAAAAACCACGGTGGACCATATGGCCATAATTAAGAAATACACATCAGGAAGACAGGAGAAGAACCCTGCCCTTAGGATGAAGTGGATGATGGCAATGAAATACCCGATTACAGCAGACAAAAGAATAATGGAAATGATCCCTGAAAGAAATGAACAAGGCCAGACT<br>CTCTGGAGCAAAACAAGTGATGCTGGATCAGACAGAGTAATGGTGTCACCTCTGGCTGTGACATGGTGGAATAGAAATGGACCGACGACAAGTACAGTCCACTATCCAAAGGTCTACAAAACCTACTTTGAAAAAGTCGAAAGGTTGAAACATGGAACCTTCGGCCCTGTTCACTTTCGGAATCAGGTTAAAATACGCCGCAGGGTTGACATAAACCCGGGCCATGCAGA<br>CCTCAGTGCCAAAGAAGCACAAGATGTCATCATGGAGGTCGTTTTTCCAAACGAAGTTGGAGCAAGGATATTGACATCAGAGTCACAATTAACAATAACAAAGGAAAAGAAGGGGGAGCTTCAGGACTGCAAGATCGCCCCTTTAATGGTGGCATACATGTTGGAGAGAGAACTGGTTCGCAAACCAGATTCCTACCAGTTGCTGGCGGAACAAGCAGCGTATATATC<br>GAGGTATTGCATTTGACTCAAGGGACCTGCTGGGAACAAATGTACACACCGGGAGGGGAGGTGAGAAATGATGATGTCGATCAGAGTTTGATTATTGCTGCTAGAAACATTGTTAGGAGAGCAACAGTGTCGGCAGACCCGTTGGCTTCGCTCTTGGAGATGTGCCATAGTACACAAATTGGCGGGATAAGAATGGTGGACATCCTTAGACAGAACCCAACAGAAGAGC<br>AAGCTGTGGATATATGCAAAGCAGCAATGGGTTTGAGAATCAGTTCATCCTTCAGCTTTGGAGGTTTCACTTTTAAAAGGACAAGTGGGTCATCTGTCAAAAGAGAAGAAGAAGTGCTCACGGGCAACCTCCAAACACTGAAAATAAGAGTACACGAAGGATATGAAGAATTCACAATGGTTGGGCGAAGAGCTACAGCCATTCTAAGGAAAGCAACCAGAAGGCTGAT<br>CCAATTAATAGTGAGTGGGAGAGACGAGCAGTCAATCGCTGAAGCGATTATAGTGGCAATGGTTTTCTCACAAGAGGACTGCATGATAAAAGCAGTGCGAGGTGATTTGAACTTTGTTAACAGAGCAAATCAGCGGCTAAATCCTATGCACCAACTTCTGAGACATTTCCAAAAGGATGCAAAGATGCTGTTTCAAAATTGGGGAATTGAACCCATTGACAATGTCATGGG<br>AATGATCGGGATACTGCCTGACATGACCCCCAGCACAGAGATGTCACTGAGAGGAGTGAGAGTCAGTAAAATGGGAGTGGATGAATATTCCAGTACTGAGAGAGTGGTCGTAAGCATTGATCGTTTCTTAAGAGTCCGAGACCAGAGGGGAAACGTACTCTTGTCTCCTGAAGAAGTTAGTGAAACACAGGGAACAGAGAAACTGACGATAACATATTCATCGTCTATG<br>ATGTGGGAAATCAATGGTCCGGAGTCAGTACTGGTCAACACATATCAATGGATCATTAGAAATTGGGAAACTGTGAAGATTCAGTGGTCCCAAGACCCCACAATGTTGTACAATAAGATGGAGTTTGAACCCTTCCAATCCTTGGTGCCCAAGGCTGCCAGAGGCCAGTACAGTGGATTTGTGAGGACGTTATTCCAGCAGATGCGTGATGTGCTGGGGACATTTGACACT<br>GTCCAAATAATAAAGCTCCTACCATTTGCAGCAGCCCCACCGGAACAGAGTAGGATGCAATTCTCTTCTCTGACTGTGAATGTAAGAGGTTCAGGAATGAGAATACTTGTGAGAGGCAACTCCCCTGTGTTCAACTATAACAAAGCAACCAAGAGACTCACAGTTCTTGGAAAGGATGCAGGTGCATTGACAGAAGATCCAGACGAGGGAACAGCAGGAGTGGAGTCTGC<br>GGTGTTAAGAGGATTCCTAATTCTTGGCAAAGAAGACAAAAGATATGGACCAGCATTGAGCATTAACGAATTGAGCAATCTTGCGAAAGGGGAGAAGGCTAATGTGTTGATAGGGCAAGGAGACGTGGTGTTGGTAATGAAACGGAAACGGGACTCTAGCATACTTACTGACAGCCAGACAGCGACCAAAAGGATTCGGATGGCCATCAATTA
EPI_ISL_12572655	>A/duck/Guangdong/S4525/2021_H5N1_2021-12-08<br>ATGGAGAGAATAAAAGAACTAAGAGATTTGATGTCGCAGTCTCGCATTCGCGAGATACTGACAAAAACCACGGTGGACCATATGGCCATAATTAAGAAATACACATCAGGAAGACAGGAGAAGAACCCTGCCCTTAGGATGAAGTGGATGATGGCAATGAAATACCCGATTACAGCAGACAAAAGAATAATGGAAATGATCCCTGAAAGAAATGAACAAGGCCAGACT<br>CTCTGGAGCAAAACAAGTGATGCTGGATCAGACAGAGTAATGGTGTCACCTCTGGCTGTGACATGGTGGAATAGAAATGGACCGACGACAAGTACAGTCCACTATCCAAAGGTCTACAAAACCTACTTTGAAAAAGTCGAAAGGTTGAAACATGGAACCTTCGGTCCTGTTCACTTTCGGAATCAGGTTAAAATACGCCGCAGGGTTGACATAAACCCGGGCCATGCAGA<br>CCTCAGTGCCAAAGAAGCACAAGATGTCATCATGGAGGTCGTTTTTCCAAACGAAGTTGGAGCAAGGATATTGACATCAGAGTCACAATTAACAATAACAAAGGAAAAGAAGGGGGAGCTTCAGGACTGCAAGATCGCCCCTTTAATGGTGGCATACATGTTGGAGAGAGAACTGGTTCGCAAACCAGATTCCTACCAGTTGCTGGCGGAACAAGCAGCGTATATATC<br>GAGGTATTGCATTTGACTCAAGGGACCTGCTGGGAACAAATGTACACACCGGGAGGGGAGGTGAGAAATGATGATGTCGATCAGAGTTTGATTATTGCTGCTAGAAACATTGTTAGGAGAGCAACAGTGTCGGCAGACCCGTTGGCTTCGCTCTTGGAGATGTGCCATAGTACACAAATTGGCGGGATAAGAATGGTGGACATCCTTAGACAGAACCCAACAGAAGAGC<br>AAGCTGTGGATATATGCAAAGCAGCAATGGGTTTGAGAATCAGTTCATCCTTCAGCTTTGGAGGTTTCACTTTTAAAAGGACAAGTGGGTCATCTGTCAAAAGAGAAGAAGAAGTGCTCACGGGCAACCTCCAAACACTGAAAATAAGAGTACACGAAGGATATGAAGAATTCACAATGGTTGGGCGAAGAGCTACAGCCATTCTAAGGAAAGCAACCAGAAGGCTGAT<br>CCAATTAATAGTGAGTGGGAGAGACGAGCAGTCAATCGCTGAAGCGATTATAGTGGCAATGGTTTTCTCACAAGAGGACTGCATGATAAAAGCAGTGCGAGGTGATTTGAACTTTGTTAACAGAGCAAATCAGCGGCTAAATCCTATGCACCAACTTCTGAGACATTTCCAAAAGGATGCAAAGATGCTGTTTCAAAATTGGGGAATTGAACCCATTGACAATGTCATGGG<br>AATGATCGGGATACTGCCTGACATGACCCCCAGCACAGAGATGTCACTGAGAGGAGTGAGAGTCAGTAAAATGGGAGTGGATGAATATTCCAGTACTGAGAGAGTGGTCGTAAGCATTGATCGTTTCTTAAGAGTCCGAGACCAGAGGGGAAACGTACTCTTGTCTCCTGAAGAAGTTAGTGAAACACAGGGAACAGAGAAACTGACGATAACATATTCATCGTCTATG<br>ATGTGGGAAATCAATGGTCCGGAGTCAGTACTGGTCAACACATATCAATGGATCATTAGAAATTGGGAAACTGTGAAGATTCAGTGGTCCCAAGACCCCACAATGTTGTACAATAAGATGGAGTTTGAACCCTTCCAATCCTTGGTGCCCAAGGCTGCCAGAGGCCAGTACAGTGGATTTGTGAGGACGTTATTCCAGCAGATGCGTGATGTGCTGGGGACATTTGACACT<br>GTCCAAATAATAAAGCTCCTACCATTTGCAGCAGCCCCACCGGAACAGAGTAGGATGCAATTCTCTTCTCTGACTGTGAATGTAAGAGGTTCAGGAATGAGAATACTTGTGAGAGGCAACTCCCCTGTGTTCAACTATAACAAAGCAACCAAGAGACTCACAGTTCTTGGAAAGGATGCAGGTGCATTGACAGAAGATCCAGACGAGGGAACAGCAGGAGTGGAGTCTGC<br>GGTGTTAAGAGGATTCCTAATTCTTGGCAAAGAAGACAAAAGATATGGACCAGCATTGAGCATTAACGAATTGAGCAATCTTGCGAAAGGGGAGAAGGCTAATGTGTTGATAGGGCAAGGAGACGTGGTGTTGGTAATGAAACGGAAACGGGACTCTAGCATACTTACTGACAGCCAGACAGCGACCAAAAGGATTCGGATGGCCATCAATTA

EPI_ISL_12572656	>A/duck/Guizhou/S1321/2022_H5N1_2022-02-22<br>ATGGAGAGAATAAAAGAACTAAGAGATTTGATGTCGCAGTCTCGCACTCGCGAGATACTGACAAAAACCACGGTGGACCATATGGCCATAATTAAGAAATACACATCAGGAAGACAGGAGAAGAACCCTGCCCTTAGGATGAAGTGGATGATGGCAATGAAATACCCGATTACAGCAGACAAAAGAATAATGGAAATGATCCCTGAAAGAAATGAACAAGGCCAGACT<br>CTCTGGAGCAAAACAAGTGATGCTGGATCAGACAGAGTAATGGTGTCACCTCTGGCTGTGACATGGTGGAATAGAAATGGACCGACGACAAGTACAGTCCACTATCCAAAGGTCTACAAAACCTACTTTGAAAAAGTCGAAAGGTTGAAACATGGAACCTTCGGTCCTGTTCACTTTCGGAATCAGGTTAAAATACGCCGCAGGGTTGACATAAACCCGGGCCATGCAGA<br>CCTCAGTGCCAAAGAAGCACAAGATGTCATCATGGAGGTCGTTTTTCCAAACGAAGTTGGAGCAAGGATATTGACATCAGAGTCACAATTAACAATAACAAAGGAAAAGAAGGGGGAGCTTCAGGACTGCAAGATCGCCCCTTTAATGGTGGCATACATGTTGGAGAGAGAACTGGTTCGCAAAACCAGATTCCTACCAGTTGCTGGCGGAACAAGCAGCGTATATATC<br>GAGGTATTGCATTTGACTCAAGGGACCTGCTGGGAACAAATGTACACACCGGGAGGGGAGGTGAGAAATGATGATGTCGATCAGAGTTTGATTATTGCTGCTAGAAACATTGTTAGGAGAGCAACAGTGTCGGCAGACCCGTTGGCTTCGCTCTTGGAGATGTGCCATAGTACACAAATTGGCGGGATAAGAATGGTAGACATCCTTAGACAGAACCCAACAGAAGAGC<br>AAGCTGTGGATATATGCAAAGCAGCAATGGGTTTGAGAATCAGTTCATCTTTCAGCTTTGGAGGTTTCACTTTTAAAAGGACAAGTGGGTCATCTGTCAAAAGAGAAGAAGAAGTGCTCACGGGCAACCTCCAAACACTGAAAATAAGAGTACACGAAGGATATGAAGAATTCACAATGGTTGGGCGAAGAGCTACAGCCATTCTAAGGAAAGCAACCAGAAGGCTGAT<br>CCAATTAATAGTGAGTGGGAGAGACGAGCAGTCAATCGCTGAAGCGATTATAGTGGCAATGGTTTTCTCACAAGAGGACTGCATGATAAAAGCAGTGCGAGGTGATTTGAACTTTGTTAACAGAGCAAATCAGCGGCTAAATCCTATGCACCAACTTCTGAGACATTTCCAAAAGGATGCAAAGGTGCTGTTTCAAAATTGGGGAATTGAACCCATTGACAATGTCATGGG<br>AATGATCGGGATACTGCCTGACATGACCCCCAGCACAGAGATGTCACTGAGAGGAGTGAGAGTCAGTAAAATGGGAGTGGATGAATATTCCAGTACTGAGAGAGTGGTCGTAAGCATTGATCGTTTCTTAAGAGTCCGAGACCAGAGGGGAAACGTACTCTTGTCTCCTGAAGAAGTTAGTGAAACACAGGGAACAGAGAAACTGACGATAACATATTCATCGTCTATG<br>ATGTGGGAAATCAATGGTCCGGAGTCAGTACTGGTCAACACATATCAATGGATCATTAGAAATTGGGAAACTGTGAAGATTCAGTGGTCCCAAGACCCCACAATGTTGTACAATAAGATGGAGTTTGAGCCCTTCCAATCCTTGGTGCCCAAGGCTGCCAGAGGCCAGTACAGTGGATTTGTGAGGACGTTATTCCAGCAGATGCGTGATGTGCTGGGGACATTTGACACT<br>GTCCAAATAATAAAGCTCCTACCATTTGCAGCAGCCCCACCGGAACAGAGTAGGATGCAATTCTCTTCTCTGACTGTAAATGTAAGAGGTTCAGGAATGAGAATACTTGTGAGAGGCAACTCCCCTGTGTTCAACTATAACAAAGCAACCCAAGAGACTCACAGTTCTTGGAAAGGATGCAGGTGCATTGACAGAAGATCCAGACGAGGGAACAGCAGGAGTGGAGTCTGC<br>GGTGTTAAGAGGATTCCTAATTCTTGGCAAAGAAGACAAAAGATATGGACCAGCATTGAGCATTAACGAATTGAGCAATCTTGCGAAAGGGGAGAAGGCTAATGTGTTGATAGGGCAAGGAGACGTGGTGTTGGTAATGAAACGGAAACGGGACTCTAGCATACTTACTGACAGCCAGACAGCGACCAAAAGGATTCGGATGGCCATCAATTA
EPI_ISL_12572659	>A/duck/Hubei/SE220/2022_H5N1_2022-01-10<br>ATGGAGAGAATAAAAGAACTAAGAGATTTGATGTCGCAGTCTCGCACTCGCGAGATACTGACAAAAACCACGGTGGACCATATGGCCATAATTAAGAAATACACATCAGGAAGACAGGAGAAGAACCCTGCCCTTAGGATGAAGTGGATGATGGCAATGAAATACCCGATTACAGCAGACAAAAGAATAATGGAAATGATCCCTGAAAGAAATGAACAAGGCCAGACT<br>CTCTGGAGCAAAACAAGTGATGCTGGATCAGACAGAGTAATGGTGTCACCTCTGGCTGTGACATGGTGGAATAGAAATGGACCGACGACAAGTACAGTCCACTATCCAAAGGTCTACAAAACCTACTTTGAAAAAGTCGAAAGGTTGAAACATGGAACCTTCGGTCCTGTTCACTTTCGGAATCAGGTTAAAATACGCCGCAGGGTTGACATAAACCCGGGCCATGCAGA<br>CCTCAGTGCCAAAGAAGCACAAGATGTCATCATGGAGGTCGTTTTTCCAAACGAAGTTGGAGCAAGGATATTGACATCAGAGTCACAATTAACAATAACAAAGGAAAAGAAGGGGGATCTTCAGGACTGCAAGATCGCCCCTTTAATGGTGGCATACATGTTGGAGAGAGAACTGGTTCGCAAAACCAGATTCCTACCTGTTGCTGGCGGAACAAGCAGCGTATATATCG<br>AGGTATTGCATTTGACTCAAGGGACCTGCTGGGAACAAATGTACACACCGGGAGGGGAGGTGAGAAATGATGATGTCGATCAGAGTTTGATTATTGCTGCTAGAAACATTGTTAGGAGAGCAACAGTGTCGGCAGACCCGTTGGCTTCGCTCTTGGAGATGTGCCATAGTACACAAATTGGCGGGATAAGAATGGTGGACATCCTTAGACAGAACCCAACAGAAGAGCA<br>AGCTGTGGATATATGCAAAGCAGCAATGGGTTTGAGAATCAGTTCATCCTTCAGCTTTGGAGGTTTCACTTTTAAAAGGACAAGTGGGTCATCTGTCAAAAGAGAAGAAGAAGTGCTCACGGGCAACCTCCAAACACTGAAAATAAGAGTACACGAAGGATATGAAGAATTCACAATGGTTGGGCGAAGAGCTACAGCCATTCTAAGGAAAGCAACCAGAAGGCTGATC<br>CAATTAATAGTGAGTGGGAGAGACGAGCAGTCAATCGCTGAAGCGATTATAGTGGCAATGGTTTTCTCACAAGAGGACAGCATGATAAAAGCAGTGCGAGGTGATTTGAACTTTGTTAACAGAGCAAATCAGCGGCTAAATCCTATGCACCAACTTCTGAGACATTTCCAAAAGGATGCAAAGGTGCTGTTTCAAAATTGGGGAATTGAACCCATTGACAATGTCATGGG<br>AATGATCGGGATACTGCCTGACATGACCCCCAGCACAGAGATGTCACTGAGAGGAGTGAGAGTCAGTAAAATGGGAGTGGATGAATATTCCAGTACTGAGAGAGTGGTCGTAAGCATTGATCGTTTCTTAAGAGTCCGAGACCAGAGGGGAAACGTACTCTTGTCTCCTGAAGAAGTTAGTGAAACACAGGGAACAGAGAAACTGACGATAACATATTCATCGTCTATG<br>ATGTGGGAAATCAATGGTCCGGAGTCAGTACTGGTCAACACATATCAATGGATCATTAGAAATTGGGAAACTGTGAAGATTCAGTGGTCCCAAGACCCCACAATGTTGTACAATAAGATGGAGTTTGAGCCCTTCCAATCCTTGGTGCCCAAGGCTGCCAGAGGCCAGTACAGTGGATTTGTGAGGACGTTATTCCAGCAGATGCGTGATGTGCTGGGGACATTTGACACT<br>GTCCAAATAATAAAGCTCCTACCATTTGCAGCAGCCCCACCGGAACAGAGTAGGATGCAATTCTCTTCTCTGACTGTGAATGTAAGAGGTTCAGGAATGAGAATACTTGTGAGAGGCAACTCCCCTGTGTTCAACTATAACAAAGCAACCCAGAGACTCACAGTTCTTGGAAAGGATGCAGGTGCATTGACAGAAGATCCAGACGAGGGAACAGCAGGAGTGGAGTCTGC<br>GGTGTTAAGAGGATTCCTAATTCTTGGCAAAGAAGACAAAAGATATGGACCAGCATTGAGCATTAACGAATTGAGCAATCTTGCGAAAGGGGAGAAGGCTAATGTGTTGATAGGGCAAGGAGACGTGGTGTTGGTAATGAAACGGAAACGGGACTCTAGCATACTTACTGACAGCCAGACAGCGACCAAAAGGATTCGGATGGCCATCAATTA
EPI_ISL_3128527	>A/duck/Kosovo/107-21VIR5162-17/2021_H5N8_2021-06-02<br>ATGGAGAGAATAAAAGAACTAAGAGATTTGATGTCGCAGTCTCGCACTCGCGAGATACTAACAAAAACCACTGTGGACCATATGGCCATAATAAAGAAATACACATCAGGAAGACAGGAGAAGAACCCTGCCCTCAGGATGAAATGGATGATGGCAATGAAATATCCTATTACAGCTGACAAAAGAATAATGGAGATGATCCCTGAAAGGAATGAGCAAGGTCAGACT<br>CTCTGGAGCAAAACAAATGATGCTGGATCAGACAGAGTGATGGTCTCACCTCTGGCTGTGACATGGTGGAATAGAAATGGGCCAACAACAAGTACAGTACACTACCCAAAAGTCTACAAAACCTACTTTGAAAAGGTAGAAAGGTTGAAACATGGAACCTTTGGTCCTGTTCACTTTCGAAATCAGGTTAAGATACGCCGCAGGGTTGACATAAACCCGGGCCATGCAGA<br>TCTCAGTGCCAAAGAAGCACAGGATGTCATCATGGAGGTTGTTTTCCCAAATGAAGTTGGAGCCAGGATCTTGACATCAGAATCACAATTAACAATAACAAGGGAAAAGAAGGAGGAACTTCAGGATTGCAAGATTGCTCCTTTGATGGTGGCATACATGTTGGAAAGAGAACTGGTTCGCAAGACCAGATTTTTACCAGTAGCTGGCGGAACAAGCAGCGTATACATCG<br>AGGTATTGCATTTGACTCAAGGGACCTGCTGGGAACAAATGTACACACCAGGAGGGGAGGTGAGAAATGATGATGTTGATCAGAGTTTGATCATTGCTGCTAGAAATATAGTTAGGAGGGCAACAGTATCAGCAGACCCATTGGCTTCGCTTTTGGAAATGTGCCACAGTACACAAATTGGTGGAGTAAGGATGGTGGACATTCTTAGGCAGAACCCAACAGAGGAGCA<br>AGCTGTGGATATATGCAAAGCAGCAATGGGTTTAAGAATCAGTTCATCCTTCAGCTTTGGAGGTTTCACTTTCAAAAGGACAAGTGGGTCGTCTGTCAAAAGAGAAGAAGAAATACTCACTGGCAACCTCCAAACACTGAAAGTAAGAATACATGAAGGATATGAGGAATTCACAATGGTTGGGCGAAGAGCTACAGCCATTTTGAGGAAAGCAACCAGGAGACTGATCC<br>AATTAATAGTGAGTGGAAGAGACGAGCAGTCAATCGCTGAAGCAATCATAGTGGCAATGGTTTTCTCACAGGAGGATTGCATGATAAAAGCAGTACGAGGTGATTTGAATTTTGTCAACAGAGCGAATCAGCGGCTAAATCCTATGCATCAACTTCTGAGGCATTTCCAAAAGGATGCAAAAGTGCTGTTTCAAAACTGGGGGATTGAACCAATTGACAATGTAATGGGG<br>ATGATCGGGATACTGCCTGACATGACCCCCAGCACAGAGATGTCACTGAGAGGAGTGAGAGTCAGCAAAATGGGAGTGGATGAATATTCCAGTACTGAGAGAGTAGTCGTGAGCATTGATCGCTTCTTGAGAGTCCGAGATCAGAGGGGAAATGTGCTTTTGTCTCCTGAGGAAGTTAGTGAAACACATGGAACAGAGAAACTGACGATAACGTATTCATCGTCTATGAT<br>GTGGGAAATCAATGGTCCGGAATCCGTGCTAGTCAACACATATCAATGGATCATTAGAAATTGGGAAACTGTGAAGATTCAGTGGTCCCAGGACCCTACGATGTTATACAATAAGATGGAATTTGAGCCCTTCCAATCCTTGGTGCCCAAGGCTGCTAGAGGCCAGTATAGTGGGTTTGTGAGGACATTATTCCAACAGATGCGTGATGTGTTGGGGACATTTGACACTGTC<br>CAAATAATAAAGCTCCTACCATTTGCAGCAGCCCCACCGGAACAGAGTAGGATGCAATTTTCCTCTCTGACTGTGAACGTAAGAGGTTCAGGAATGAGAATACTTGTGAGGGGCAACTCCCCTGTGTTCAACTATAATAAGGCCACCAAGAGACTCACAGTTCTTGGAAAGGATGCAGGCGCCTTGACAGAATATCCAGATGAGGGAACAGCAGGAGTGGAGTCTGCAGT<br>ATTAAGAGGATTTCTAATTCTGGGCAAAGAGGACAAAAGATATGGACCAGCATTGAGCATCAACGAATTGAGCAATCTTGCGAAAGGGGAAAAGGCTAATGTGTTGATAGGACAAGGAGACGTGGTGTTGGTAATGAAACGGAAACGGGACTCTAGCATACTTACTGACAGCCAGACAGCGACCAAAAGAATTCGGATGGCCATCAATTA
EPI_ISL_697690	>A/duck/Mongolia/314/2018_H3N6_2018-09-02<br>ATGGAGAGAATAAAAGAACTAAGAGATTTGATGTCACAGTCTCGCACTCGCGAGATACTGACAAAAACCACTGTGGACCATATGGCCATAATAAAGAAATACACATCAGGAAGACAGGAGAAGAACCCTGCTCTTAGGATGAAATGGATGATGGCAATGAAATACCCGATTACAGCAGACAAAAGAATAATGGAGATGATCCCTGAAAGAAATGAGCAAGGCCAAACT<br>CTCTGGAGCAAAACAAATGATGCTGGATCAGACAGAGTGATGGTGTCACCTCTGGCTGTGACATGGTGGAATAGAAATGGGCCAACGACAAGCACAGTCCATTATCCAAAGGTTTACAAGACCTATTTTGAAAAAGTCGAAAGGTTGAAACATGGAACCTTTGGTCCTGTTCACTTTCGGAATCAGGTCAAAATACGCCGCAGGGTCGACATAAACCCGGGCCATGCAGA<br>TCTCAGTGCTAAAGAAGCACAAGATGTCATCATGGAGGTCGTTTTCCCAAACGAAGTTGGAGCCAGGATATTGACATCAGAGTCACAATTAACAATAACAAAGGAAAAGAAGGAGGAGCTTCAAGACTGTAAGATTGCTCCTTTGATGGTGGCATACATGTTGGAGAGGGAACTGGTTCGCAAAACCAGATTCCTACCAGTAGCTGGCGGAACAAGCAGTGTGTATATCG<br>AGGTGTTGCATTTGACTCAAGGGACCTGCTGGGAACAAATGTACACACCGGGAGGAGAGGTGAGAAATGATGATGTCGATCAAAGTTTGATTATTGCTGCTAGAAATATTGTCAGGAGAGCAACAGTATCAGCAGACCCGTTAGCTTCGCTCTTGGAGATGTGCCATAGTACACAAATTGGCGGGATAAGGATGGTTGACATTCTTAGACAAAACCCAACAGAAGAGCAA<br>GCTGTGGATATATGCAAAGCAGCAATGGGTCTAAGAATCAGTTCATCCTTCAGCTTTGGAGGTTTCACTTTCAAAAGGACAAGTGGGTCATCTGTCAAAAAGGAAGAAGAAGTGCTCACAGGCAACCTCCAAACATTGAAAATAAGAGTACACGAAGGATATGAGGAATTCACAATGGTTGGGCGAAGAGCAACAGCCATTCTAAGGAAAGCAACCAGAAGGTTGATAC<br>AATTAATAGTGAGTGGGAGGGACGAGCAGTCAATCGCTGAAGCGATCATAGTGGCAATGGTTTTCTCACAAGAAGACTGCATGATAAAAGCAGTACGAGGTGATTTGAATTTTGTCAACAGAGCAAACCAACGGCTAAATCCTATGCATCAACTTCTGAGACATTTCCAGAAGGATGCAAAAGTGCTGTTTCAGAACTGGGGAGTTGAACCCATTGACAATGTCATGGGG<br>ATGATCGGAATACTGCCTGATATGACCCCAAGCACAGAGATGTCACTGAGAGGAGTGAGAGTCAGTAAAATGGGAGTGGATGAATATTCCAGTACTGAGAGGGTTGTCGTGAGCATTGATCGTTTCTTAAGGGTCCGAGATCAAAGGGGGAATGTACTCTTGTCTCCCGAGGAGGTCAGTGAAGCACAAGGAACAGAGAAGCTGACGATAACATATTCATCGTCCATGA<br>TGTGGGAAATCAATGGTCCGGAATCAGTGCTGGTCAACACATATCAATGGATCATTAGAAATTGGGAAACTGTGAAGATTCAGTGGTCACAAGACCCTACAATGTTATACAATAGGATGGAGTTTGAGCCCTTCCAATCCTTGGTGCCTAAGGCTGCCAGAGGCCAGTATAGTGGATTCGTGAGGACATTATTCCAGCAGATGCGTGATGTGCTGGGGACATTTGACACTG<br>TTCAAATAATAAAGCTCCTACCATTTGCAGCAGCCCCACCAGAACAGAGTAGGATGCAGTTCTCTTCTCTGACTGTGAATGTAAGGGGTTCAGGGATGAGAATACTTGTGAGAGGCAATTCCCCTGTGTTCAACTATAACAAGGCAACCAAGAGGCTCACAGTCCTTGGAAAGGATGCAGGTGCCTTGACAGAAGATCCAGACGAGGGAACAGCAGGAGTGGAATCTGCG<br>GTATTAAGAGGATTTCTAATTCTGGGCAAAGAAGATAAGAGATATGGCCCAGCATTGAGCATCAACGAATTGAGCAATCTTGCGAAAGGGGAGAAAGCTAATGTGTTGATAGGGCAAGGAGACGTGGTGTTGGTAATGAAACGGAAACGGGACTCTAGCATACTTACTGACAGCCAGACAGCGACCAAAAGGATTCGGATGGCCATCAATTA
EPI_ISL_697695	>A/duck/Mongolia/447/2018_H4N6_2018-09-02<br>ATGGAGAGAATAAAAGAGCTAAGAGATTTGATGTCGCAGTCTCGCACTCGCGAGATACTGACAAAAACCACTGTGGACCATATGGCCATAATCAAGAAATATACATCAGGAAGACAGGAGAAGAACCCTGCACTTAGGATGAAATGGATGATGGCAATGAAATATCCGATTACAGCAGACAAAAGGATAATGGAGATGATCCCTGAAAGAAATGAGCAAGGTCAGACT<br>CTTTGGAGCAAAACAAATGATGCTGGATCAGACAGAGTGATGGTGTCACCTCTGGCTGTGACGTGGTGGAATAGAAATGGACCAACAACAAGTACAGTCCATTACCCAAAGGTCTATAAAACCTACTTTGAAAAGGTTGAAAGATTAAAGCACGGAACCTTCGGCCCTGTCCATTTTCGAAATCAGGTTAAGATACGCCGCAGAGTTGACATAAACCCGGGCCATGCAGA<br>CCTCAGTGCTAAAGAAGCACAAGACGTCATCATGGAGGTCGTTTTCCCAAATGAAGTCGGAGCCAGAATATTGACATCAGAGTCACAGTTAACAATAACAAAGGAAAAGAAGGAAGAACTCCAGGACTGTAAGATTGCCCCTTTAATGGTGGCATACATGTTGGAGAGAGAACTGGTTCGAAAAACCAGATTCCTGCCAGTAGCTGGCGGAACAAGCAGCGTATATATTG<br>AGGTGTTGCACTTGACTCAAGGAACCTGCTGGGAACAAATGTATACGCCAGGAGGAGAAGTGAGAAATGATGACGTTGATCAGAGTTTAATTATTGCTGCCAGAAATATCGTTAGGAGAGCAACAGTATCAGCAGACCCATTGGCTTCGCTACTGGAGATGTGCCATAGTACACAAATTGGCGGGATAAGGATGGTAGACATCCTTAGACAGAACCCAACAGAAGAGCA<br>AGCTGTGGATATATGCAAAGCAGCAATGGGTCTAAGAATCAGTTCATCCTTCAGCTTTGGAGGTTTCACTTTCAAAAGGACAAGCGGGTCATCTGTCAAAAGAGAAGAGGAAGTGCTCACCGGTAACCTCCAAACATTGAAAATAAGAGTGCATGAAGGGTATGAGGAATTCACAATGGTTGGACGGAGAGCAACAGCCATTCTAAGGAAAGCAACCAGAAGGCTGATC<br>CAATTGATAGTAAGTGGAAGAGACGAGCAGTCAATCGCCGAAGCGATCATAGTGGCAATGGTGTTCTCCCAAGAGGATTGCATGATAAAGGCAGTACGAGGTGATTTAAATTTTGTCAATAGAGCGAATCAGCGGCTCAATCCTATGCATCAGCTCCTGAGGCATTTCCAAAAGGATGCAAAGGTACTGTTCCAAAACTGGGGAATTGAACCCATTGACAATGTCATGGG<br>AATGATAGGAATATTGCCTGATATGACACCCAGCACAGAGATGTCACTAAGAGGGGTGAGGGTCAGTAAGATGGGAGTGGATGAATATTCCAGTACTGAGAGGGTGGTCGTGAGTATTGACCGCTTCTTGAGGGTCCGAGACCAGAGAGGAAACGTACTCTTATCCCCTGAAGAGGTTAGTGAAACACAGGGAACAGAGAAGCTGACGATAACATATTCATCATCCATG<br>ATGTGGGAAATTAATGGTCCGGAATCAGTGTTAGTTAACACATATCAATGGATCATCAGAAACTGGGAAACTGTGAAGATCCAGTGGTCCCAAGACCCTACAATGCTATACAACAAGATGGAGTTTGAGCCTTTTCAGTCCTTGGTGCCTAAGGCGGCCAGAGGCCAGTACAGTGGATTTGTAAGGACTCTATTCCAGCAGATGCGTGATGTGCTGGGGACCTTTGACACT<br>GTCCAGATAATAAAGCTACTCCCATTTGCAGCAGCCCCACCGGAACAGAGTAGGATGCAGTTCTCTTCTCTAACTGTAAACGTAAGGGGTTCAGGAATGAGAATACTTGTGAGAGGCAACTCCCCTGTGTTCAACTATAACAAGGCAACCAAGAGGCTCACAGTTCTTGGAAAGGATGCTGGTGCATTGACAGAAGACCCAGATGAGGGGACGGCAGGAGTGGAGTCTGC<br>GGTATTGAGAGGGTTCCTAATCCTGGGCAAAGAAGACAAAAGATATGGACCAGCGCTGAGCATCAATGAATTGAGCAATCTTGCGAAAGGGGAGAAGGCTAATGTGTTGATAGGGCAAGGAGACGTGGTGTTGGTGATGAAACGGAAACGGGACTCTAGCATACTTACTGACAGCCAGACAGCGACCAAAAGAATTCGGATGGCCATCAATTA
EPI_ISL_209131	>A/duck/Mongolia/543/2015_H4N6_2015-08-31<br>ATGGAGAGAATAAAAGAACTAAGAGATTTGATGTCACAGTCTCGCACTCGCGAGATACTGACAAAAACCACTGTGGACCATATGGCCATAATAAAGAAATACACATCAGGAAGACAGGAGAAGAACCCTGCTCTTAGGATGAAATGGATGATGGCAATGAAATACCCGATTACAGCAGACAAAAGAATAATGGAGATGATCCCTGAAAGAAATGAGCAAGGCCAAACT<br>CTCTGGAGCAAAACAAATGATGCTGGATCAGACAGAGTGATGGTGTCACCTCTGGCTGTGACATGGTGGAATAGAAATGGGCCAACGACAAGCACAGTCCATTATCCAAAGGTTTACAAAACCTACTTTGAAAAAGTCGAAAGGTTGAAACATGGAACCTTTGGTCCTGTTCACTTTCGAATCAGGTCAAAATACGCCGCAGGGTCGACATAAACCCGGGCCATGCAGA<br>TCTCAGTGCTAAAGAAGCACAAGATGTCATCATGGAGGTCGTTTTCCCAAACGAAGTTGGAGCCAGGATATTGACATCAGAGTCACAATTAACAATAACAAAGGAAAAGAAGGAGGAGCTTCAAGACTGTAAGATTGCTCCTTTGATGGTGGCATACATGTTGGAGAGGGAACTGGTTCGCAAAACCAGATTCCTACCAGTAGCTGGCGGAACAAGCAGTGTGTATATCG<br>AGGTGTTGCATTTGACTCAAGGGACCTGCTGGGAACAAATGTACACACCGGGAGGAGAGGTGAGAAATGATGATGTCGATCAAAGTTTGATTATTGCTGCTAGAAATATTGTCAGGAGAGCAACAGTATCAGCAGACCCGTTGGCTTCGCTCTTGGAGATGTGCCATAGTACACAAATTGGCGGGATAAGGATGGTTGACATTCTTAGACAAAACCCAACAGAAGAGCAA<br>GCTGTGGATATATGCAAAGCAGCAATGGGTCTAAGAATCAGTTCATCCTTCAGCTTTGGAGGTTTCACTTTCAAAAGGACAAGTGGGTCATCTGTCAAGAAGGAAGAAGAAGTGCTCACAGGCAACCTCCAAACATTGAAAATAAGAGTACACGAAGGATATGAGGAATTCACAATGGTTGGGCGAAGAGCAACAGCCATTCTAAGGAAAGCAACCAGAAGGTTGATAC<br>AATTAATAGTGAGTGGGAGGGACGAGCAGTCAATCGCTGAAGCGATCATAGTGGCAATGGTTTTCTCACAAGAAGACTGCATGATAAAAGCAGTACGAGGTGATTTGAATTTTGTCAACAGAGCAAACCAGCGGCTAAATCCTATGCATCAACTTCTGAGGCATTTCCAGAAGGATGCAAAAGTGCTGTTTCAGAACTGGGGAGTTGAACCCATTGACAATGTCATGGGG<br>ATGATCGGAATACTGCCTGACATGACCCCAAGCACAGAGATGTCACTGAGAGGAGTGAGAGTCAGTAAAATGGGAGTGGATGAATATTCCAGTACTGAGAGGGTTGTCGTGAGCATTGATCGTTTCTTAAGGGTCCGAGATCAAAGGGGAAATGTACTCTTATCTCCCGAGGAGGTCAGTGAAACACAAGGAACAGAGAAGCTGACGATAACATATTCATCGTCCATGA<br>TGTGGGAAATCAATGGTCCGGAATCAGTGCTGGTCAACACATATCAATGGATCATTAGAAATTGGGAAACTGTGAAGATTCAGTGGTCACAAGACCCTACAATGTTATACAATAGGATGGAGTTTGAGCCCTTCCAATCCTTGGTGCCTAAGGCTGCCAGAGGCCAGTATAGTGGATTCGTGAGGACATTATTCCAGCAGATGCGTGATGTGCTGGGGACATTTGACACTG<br>TTCAAATAATAAAGCTCCTACCATTTGCAGCAGCCCCACCAGAACAGAGTAGGATGCAGTTCTCTTCTCTGACTGTGAATGTAAGGGGTTCAGGGATGAGAATACTTGTGAGAGGCAATTCCCCTGTGTTCAACTATAACAAGGCAACCAAGAGGCTCACAGTCCTTGGAAAGGATGCAGGTGCCTTGACAGAAGATCCAGACGAGGGAACAGCAGGAGTGGAATCTGCA<br>GTATTAAGAGGATTTCTAATTCTGGGCAAAGAAGATAAGAGATATGGCCCAGCATTGAGCATCAACGAATTGAGCAATCTTGCGAAAGGGGAGAAAGCTAATGTGTTGATAGGGCAAGGAGACGTGGTGTTGGTAATGAAACGGAAACGGGACTCTAGCATACTTACTGACAGCCAGACAGCGACCAAAAGGATTCGGATGGCCATCAATTA
EPI_ISL_503350	>A/duck/Mongolia/619/2019_H3N6_2019-09-13<br>ATGGAGAGAATAAAAGAACTAAGAGATTTGATGTCACAGTCTCGCACTCGCGAGATACTGACAAAAACCACTGTGGACCATATGGCCATAATAAAGAAATACACATCAGGAAGACAGGAGAAGAACCCTGCTCTTAGGATGAAATGGATGATGGCAATGAAATACCCGATTACAGCAGACAAAAGAATAATGGAGATGATCCCTGAAAGAAATGAGCAAGGCCAAACT<br>CTCTGGAGCAAAACAAATGATGCTGGATCAGACAGAGTGATGGTGTCACCTCTGGCTGTGACATGGTGGAATAGAAATGGGCCAACGACAAGCACAGTCCATTATCCAAAGGTTTACAAAACCTACTTTGAAAAAGTCGAAAGGTTGAAACATGGAACCTTTGGTCCTGTTCACTTTCGAATCAGGTCAAAATACGCCGCAGGGTCGACATAAACCCGGGTCATGCAGA<br>TCTCAGTGCTAAAGAAGCACAAGATGTCATCATGGAGGTCGTTTTCCCAAACGAAGTTGGAGCCAGGATATTGACATCAGAGTCACAATTAACAATAACAAAGGAAAAGAAGGAGGAGCTTCAAGACTGTAAGATTGCTCCTTTGATGGTGGCATACATGTTGGAGAGGGAACTGGTTCGAAAAACCAGATTCCTACCAGTAGCTGGCGGAACAAGCAGTGTGTATATCG<br>AGGTGTTGCATTTGACTCAAGGGACCTGCTGGGAACAAATGTACACACCGGGAGGAGAGGTGAGAAATGATGATGTCGATCAAAGTTTGATTATTGCTGCTAGAAATATTGTCAGGAGAGCAACAGTATCAGCAGACCCGTTGGCTTCGCTCTTGGAGATGTGCCATAGTACACAAATTGGCGGGATAAGGATGGTTGACATTCTTAGACAAAACCCAACAGAAGAGCAA<br>GCTGTGGATATATGCAAAGCAGCAATGGGTCTAAGAATCAGTTCATCCTTCAGCTTTGGAGGTTTCACTTTCAAAAGGACAAGTGGGTCATCTGTCAAGAAGGAAGAAGAAGTGCTCACAGGCAACCTCCAAACATTGAAAATAAGAGTACACGAAGGATATGAGGAATTCACAATGGTTGGGCGAAGAGCAACAGCCATTCTAAGGAAAGCAACCAGAAGGTTGATAC<br>AATTAATAGTGAGTGGGAGGGACGAGCAGTCAATCGCTGAAGCGATCATAGTGGCAATGGTTTTCTCACAAGAAGACTGCATGATAAAAGCAGTACGAGGTGATTTGAATTTTGTCAACAGAGCAAACCAGCGGCTAAATCCTATGCATCAACTTCTGAGGCATTTCCAGAAGGATGCAAAAGTACTGTTTCAGAACTGGGGAGTTGAACCCATTGACAATGTCATGGGG<br>ATGATTGGAATACTGCCTGACATGACCCCAAGCACAGAGATGTCACTGAGAGGAGTGAGAGTCAGTAAAATGGGAGTGGATGAATATTCCAGTACTGAGAGGGTTGTCGTGAGCATTGATCGTTTCTTAAGGGTCCGAGATCAAAGGGGAAATGTACTCTTATCTCCCGAGGAGGTCAGTGAAACACAAGGAACAGAGAAGTTGACGATAACATATTCATCGTCCATGAT<br>GTGGGAAATCAATGGTCCGGAATCAGTGCTGGTCAACACATATCAATGGATCATTAGAAATTGGGAAACTGTGAAGATTCAGTGGTCACAAGACCCTACAATGTTATACAATAGGATGGAGTTTGAGCCCTTCCAATCCTTGGTGCCTAAGGCTGCCAGAGGCCAGTATAGTGGATTCGTGAGAACATTATTCCAGCAGATGCGTGATGTGCTGGGGACATTTGACACTGT<br>TCAAATAATAAAGCTCCTACCATTTGCAGCAGCCCCACCAGAACAGAGTAGGATGCAGTTCTCTTCTCTGACTGTGAATGTAAGGGGTTCAGGGATGAGAATACTTGTGAGAGGCAATTCCCCTGTGTTCAACTATAACAAGGCAACCAAGAGGCTCACAGTCCTTGGAAAGGATGCAGGTGCCTTGACAGAAGATCCAGACGAGGGAACAGCAGGAGTGGAATCTGCGG<br>TATTAAGAGGATTTCTAATTCTGGGCAGAGAAGATAAGAGATATGGCCCAGCATTGAGCATCAACGAATTGAGCAATCTTGCGAAAGGGGAGAAAGCTAATGTGTTGATAGGGCAAGGAGACGTGGTGTTGGTAATGAAACGGAAACGGGACTCTAGCATACTTACTGACAGCCAGACAGCGACCAAAAGGATTCGGATGGCCATCAATTA
EPI_ISL_503355	>A/duck/Mongolia/729/2019_H4N6_2019-09-17<br>ATGGAGAGAATAAAAGAACTAAGAGATTTGATGTCACAGTCTCGCACTCGCGAGATACTGACAAAAACCACTGTGGACCATATGGCCATAATAAAGAAATACACATCAGGAAGACAGGAGAAGAACCCTGCTCTTAGGATGAAATGGATGATGGCAATGAAATACCCGATTACAGCAGACAAAAGAATAATGGAGATGATCCCTGAAAGAAATGAGCAAGGCCAAACT<br>CTCTGGAGCAAAACAAATGATGCTGGATCAGACAGAGTGATGGTGTCACCTCTGGCTGTGACATGGTGGAATAGAAATGGGCCAACGACAAGCACAGTCCATTATCCAAAGGTTTACAAAACCTACTTTGAAAAAGTCGAAAGGTTGAAACATGGAACCTTTGGTCCTGTTCACTTTCGAATCAGGTCAAAATACGCCGCAGGGTCGACATAAACCCGGGTCATGCAGA<br>TCTCAGTGCTAAAGAAGCACAAGATGTCATCATGGAGGTCGTTTTCCCAAACGAAGTTGGAGCCAGGATATTGACATCAGAGTCACAATTAACAATAACAAAGGAAAAGAAGGAGGAGCTTCAAGACTGTAAGATTGCTCCTTTGATGGTGGCATACATGTTGGAGAGGGAACTGGTTCGAAAAACCAGATTCCTACCAGTAGCTGGCGGAACAAGCAGTGTGTATATCG<br>AGGTGTTGCATTTGACTCAAGGGACCTGCTGGGAACAAATGTACACACCGGGAGGAGAGGTGAGAAATGATGATGTCGATCAAAGTTTGATTATTGCTGCTAGAAATATTGTCAGGAGAGCAACAGTATCAGCAGACCCGTTGGCTTCGCTCTTGGAGATGTGCCATAGTACACAAATTGGCGGGATAAGGATGGTTGACATTCTTAGACAAAACCCAACAGAAGAGCAA<br>GCTGTGGATATATGCAAAGCAGCAATGGGTCTAAGAATCAGTTCATCCTTCAGCTTTGGAGGTTTCACTTTCAAAAGGACAAGTGGGTCATCTGTCAAGAAGGAAGAAGAAGTGCTCACAGGCAACCTCCAAACATTGAAAATAAGAGTACACGAAGGATATGAGGAATTCACAATGGTTGGGCGAAGAGCAACAGCCATTCTAAGGAAAGCAACCAGAAGGTTGATAC<br>AATTAATAGTGAGTGGGAGGGACGAGCAGTCAATCGCTGAAGCGATCATAGTGGCAATGGTTTTCTCACAAGAAGACTGCATGATAAAAGCAGTACGAGGTGATTTGAATTTTGTCAACAGAGCAAACCAGCGGCTAAATCCTATGCATCAACTTCTGAGGCATTTCCAGAAGGATGCAAAAGTACTGTTTCAGAACTGGGGAGTTGAACCCATTGACAATGTCATGGGG<br>ATGATTGGAATACTGCCTGACATGACCCCAAGCACAGAGATGTCACTGAGAGGAGTGAGAGTCAGTAAAATGGGAGTGGATGAATATTCCAGTACTGAGAGGGTTGTCGTGAGCATTGATCGTTTCTTAAGGGTCCGAGATCAAAGGGGAAATGTACTCTTATCTCCCGAGGAGGTCAGTGAAACACAAGGAACAGAGAAGCTGACGATAACATATTCATCGTCCATGAT<br>GTGGGAAATCAATGGTCCGGAATCAGTGCTGGTCAACACATATCAATGGATCATTAGAAATTGGGAAACTGTGAAGATTCAGTGGTCACAAGACCCTACAATGTTATACAATAGGATGGAGTTTGAGCCCTTCCAATCCTTGGTGCCTAAGGCTGCCAGAGGCCAGTATAGTGGATTCGTGAGAACATTATTCCAGCAGATGCGTGATGTGCTGGGGACATTTGACACTGT<br>TCAAATAATAAAGCTCCTACCATTTGCAGCAGCCCCACCAGAACAGAGTAGGATGCAGTTCTCTTCTCTGACTGTGAATGTAAGGGGTTCAGGGATGAGAATACTTGTGAGAGGCAATTCCCCTGTGTTCAACTATAACAAGGCAACCAAGAGGCTCACAGTCCTTGGAAAGGATGCAGGTGCCTTGACAGAAGATCCAGACGAGGGAACAGCAGGAGTGGAATCTGCGG<br>TATTAAGAGGATTTCTAATTCTGGGCAGAGAAGATAAGAGATATGGCCCAGCATTGAGCATCAACGAATTGAGCAATCTTGCGAAAGGGGAGAAAGCTAATGTGTTGATAGGGCAAGGAGACGTGGTGTTGGTAATGAAACGGAAACGGGACTCTAGCATACTTACTGACAGCCAGACAGCGACCAAAAGGATTCGGATGGCCATCAATTA
EPI_ISL_697687	>A/duck/Moscow/5744/2019_H1N1_2019-10-15<br>ATGGAGAGAATAAAAGAGCTAAGAGATTTGATGTCGCAGTCTCGCACTCGCGAGATACTGACAAAAACCACTGTGGACCATATGGCCATAATCAAGAAATATACATCAGGAAGACAGGAGAAGAACCCTGCACTTAGGATGAAGTGGATGATGGCAATGAAATATCCGATTACAGCAGACAAAAGGATAATGGAGATGATCCCTGAAAGAAATGAGCAAGGTCAGACT<br>CTTTGGAGCAAAACAAATGATGCTGGATCAGACAGAGTGATGGTGTCACCTCTGGCTGTGACGTGGTGGAATAGAAATGGACCAACAACAAGTACAGTCCATTACCCAAAGGTTTATAAAACCTACTTTGAAAAGGTTGAAAGGTTAAAGCATGGAACCTTCGGCCCTGTTCATTTTCGAAATCAGGTTAAGATACGCCGCAGAGTTGACATAAACCCGGGCCATGCAGAC<br>CTCAGTGCTAAAGAAGCACAAGACGTCATCATGGAGGTCGTTTTCCCAAATGAAGTCGGAGCCAGAATATTGACATCAGAATCGCAGTTAACAATAACAAAGGAAAAGAAGGAGGAACTCCAGGACTGTAAGATTGCCCCTTTAATGGTGGCATACATGTTGGAGAGAGAGCTGGTTCGAAAAACCAGATTCCTGCCAGTAGCTGGCGGAACAAGCAGCGTATATATCG<br>AGGTGCTGCACTTGACTCAAGGAACCTGCTGGGAACAAATGTATACGCCAGGAGGAGAAGTGAGAAATGATGACGTTGATCAGAGTTTAATTATTGCTGCCAGAAATATCGTTAGGAGGGCAACAGTATCAGCAGACCCATTGGCTTCGCTACTGGAGATGTGCCATAGTACACAAATTGGCGGGATAAGGATGGTAGACATCCTCAGACAGAACCCAACAGAAGAGCA<br>AGCCGTGGATATATGCAAAGCAGCAATGGGTCTAAGAATCAGTTCATCCTTCAGCTTTGGAGGTTTCACTTTCAAAAGAACAAGTGGGTCATCTGTCAAAAGAGAAGAGGAAGTGCTCACCGGCAACCTCCAAACATTGAAGATAAGAGTGCATGAAGGGTATGAGGGATTCACAATGGTTGGGCGGAGAGCAACAGCCATTCTAAGGAAAGCAACCAGAAGGCTGATC<br>CAATTGATAGTAAGTGGAAGAGACGAGCAGTCAATCGCCGAAGCGATCATAGTGGCAATGGTGTTCTCCCAAGAGGATTGCATGATAAAGGCAGTAAGAGGTGATTTAAATTTTGTCAATAGAGCGAATCAGCGGCTCAATCCTATGCATCAGCTCCTGAGGCATTTCCAAAAGGATGCAAAGGTACTGTTCCAAAACTGGGGAATTGAACCCATTGACAATGTCATGGG<br>GATGATAGGAATATTGCCTGATATGACACCCAGCACAGAGATGTCACTAAGAGGAGTGAGGGTCAGTAAAATGGGAGTGGATGAATATTCCAGTACTGAGAGGGTGGTCGTGAGTATTGATCGTTTCTTAAGGGTCCGAGACCAGAGAGGGAACGTACTCTTATCCCCTGAAGAGGTTAGTGAAACACAGGGAACAGAGAAGCTGACGATAACATATTCATCATCCATG<br>ATGTGGGAAATTAATGGTCCGGAATCAGTGTTAGTTAACACATATCAATGGATCATCAGAAACTGGGAGACTGTGAAGATTCAGTGGTCCCAAGACCCTACAATGCTCTACAACAAGATGGAGTTTGAGCCTTTTCAGTCCTTGGTGCCTAAAGCGGCCAGAGGCCAGTACAGTGGATTTGTAAGGACTCTATTCCAGCAAATGCGTGATGTGCTGGGGACCTTTGACACT<br>GTCCAGATAATAAAGCTACTCCCATTTGCAGCAGCCCCACCGGAACAGAGTAGGATGCAGTTCTCTTCTCTAACTGTAAACGTAAGGGGTTCAGGAATGAGAATACTTGTGAGAGGCAACTCCCCTGTGTTCAACTATAACAAGGCAACCAAGAGGCTCACAGTCCTTGGAAAGGATGCTGGTGCATTGACAGAAGACCCAGATGAGGGGACGGCAGGAGTGGAGTCTG<br>CGGTATTGAGAGGGTTCCTAATTCTGGGCAAAGAAGACAAAAGATATGGACCAGCGCTGAGCATCAATGAATTGAGCAATCTTGCGAAAGGGGAGAAGGCTAATGTGTTGATAGGGCAAGGAGACGTGGTGTTGGTGATGAAACGGAAGCGGGACTCTAGCATACTTACTGACAGCCAGACAGCGACCAAAAGAATTCGGATGGCCATCAATTA
EPI_ISL_825173	>A/duck/Netherlands/18018989-011015/2018_H5N3_2018-12-24<br>ATGGAGAGAATAAAAGAACTAAGAGATTTGATGTCGCAGTCTCGCACTCGCGAGATACTGGCAAAAACCACTGTGGACCATATGGCCATAATAAAGAAATACACGTCAGGAAGACAGGAGAAGAATCCTGCCCTTAGAATGAAATGGATGATGGCAATGAAATATCCGATTACAGCAGACAAAAGAATAATGGAGATGATCCCTGAAAGAAATGAGCAGGGTCAGACT<br>CTCTGGAGCAAAACAAATGATGCCGGATCAGACAGAGTGATGGTGTCACCTCTGGCAGTGACATGGTGGAATAGAAATGGGCCAACAACAAGTACAGTCCACTATCCAAAGGTCTACAAAACCTACTTTGAAAAGGTTGAAAGGTTAAAAAATGGAACCTTCGGTCCTGTCCACTTTCGAAATCAGGTTAAAATACGCCGCAGGGTTGACATAAACCCGGGCCATGCAGA<br>CCTCAGCGCTAAAGAAGCACAAGATGTCATCATGGAGGTCGTTTTCCCAAACGAAGTTGGGGCCAGGATATTGACATCAGAGTCACAATTAACAATAACAAAGGAAAAGAAGGAGGAGCTTCAGGACTGTAAGATTGCTCCTCTGATGGTGGCATACATGTTGGAGAGAGAATTGGTTCGCAAAACCAGATTCCTGCCCGTAGCTGGCGGGACAAGCAGCGTGTACATT<br>GAGGTGTTGCATTTGACTCAGGGGACCTGCTGGGAACAAATGTACACACCGGGAGGGGAGGTGAGAAATGATGATGTTGATCAAAGTTTGATCATTGCCGCTAGAAATATTGTTAGAAGAGCAACAGTATCAGCAGACCCGTTAGCTTCGCTTTTGGAGATGTGCCATAGTACACAAATTGGCGGGATAAGGATGGTGGACATTCTTAGACAGAACCCTACAGAAGAGC<br>AAGCTGTGGATATATGCAAAGCAGCAATGGGTCTAAGAATCAGTTCATCCTTCAGCTTTGGAGGGTTCACCTTCAAACGGACAAGTGGGTCATCTGTCAAAAGAGAAGAAGAAGTGCTCACAGGCAACCTCCAAACACTGAAAATAAGAGTACATGAAGGATATGAGGAATTTACAATGGTTGGGCGAAGAGCGACAGCCATTCTAAGGAAAGCAACCAGAAGGCTAAT<br>CCAATTAATAGTGAGTGGGAGGGACGAGCAGTCAATCGCTGAAGCGATCATAGTGGCAATGGTTTTCTCACAAGAGGATTGCATGATAAAAGCAGTACGAGGTGATTTGAATTTTGTTAACAGAGCAAATCAGCGGCTGAATCCTATGCATCAACTTCTGAGGCATTTCCAAAAGGACGCAAAGGTGCTGTTTCAAAATTGGGGAATTGAGCCAATTGACAATGTCATGG<br>GGATGATTGGAATACTGCCTGACATGACCCCCAGCACAGAGATGTCACTGAGAGGAGTGAGAGTCAGTAAAATGGGAGTGGATGAATATTCCAGTACTGAGAGAGTGGTCGTGAGCATTGATCGTTTCTTGAGAGTCCGGGATCAGAGGGGAAACGTGCTCTTGTCCCCCGAAGAAGTTAGTGAAACACAGGGAACAGAGAAACTGACGATAACATATTCATCGTCCAT<br>GATGTGGGAAATCAATGGTCCAGAATCGGTGCTAGTCAACACATACCAATGGATTATTAGAAATTGGGAAACTGTGAAGATTCAATGGTCCCAAGACCCTACAATGCTGTACAACAAGATGGAATTTGAGCCCTTCCAATCCTTGGTGCCTAAGGCTGCTAGAGGCCAGTATAGCGGGTTTGTGAGGACGTTGTTTCAGCAGATGCGTGATGTGCTGGGGACATTTGACAC<br>TGTTCAAATAATAAAGCTCCTACCGTTTGCAGCAGCCCCACCAGAACAGAGTAGGATGCAGTTCTCTTCTCTGACTGTAAATGTAAGAGGTTCAGGGATGAGAATACTTGTGAGAGGCAACTCCCCTGTGTTCAACTATAACAAGGCAACCAAGAGACTCACAGTTCTTGGAAAGGATGCAGGTGCTTTAACAGAAGATCCAGATGAGGGAACAGCAGGAGTGGAATCTG<br>CGGTATTAAGAGGGTTTCTAATTCTGGGCAAAGAAGACAAAAGATATGGACCAGCATTAAGCATCAATGAGTTGAGTAATCTTGTGAAGGGGGAAAAGGCTAATGTGTTGATAGGGCAAGGAGACGTGGTGTTGGTAATGAAACGGAAACGGGACTCTAGCATACTTACTGACAGCCAGACAGCGACCAAAAGGATTCGGATGGCCATCAATTA
EPI_ISL_3102064	>A/duck/Poland/H1029_21RS1385-5/2021_H5N8_2021-05-01<br>ATGGAGAGAATAAAAGAACTAAGAGATTTGATGTCGCAGTCTCGCACTCGCGAGATACTAACAAAAACCACTGTGGACCATATGGCCATAATAAAGAAATACACATCAGGGAGACAGGAGAAGAACCCTGCCCTCAGGATGAAATGGATGATGGCAATGAAATATCCTATTACAGCTGACAAAAGAATAATGGAGATGATCCCTGAAAGGAATGAGCAAGGTCAGACT<br>CTCTGGAGCAAAACAAATGATGCTGGATCAGACAGAGTGATGGTCTCACCTCTGGCTGTGACGTGGTGGAATAGAAATGGGCCAACAACAAGTACAGTACACTACCCAAAAGTCTACAAAACCTACTTTGAAAAGGTAGAAAGGTTGAAACATGGAACCTTTGGTCCTGTTCACTTTCGAAATCAGGTTAAGATACGCCGCAGGGTTGACACAAACCCGGGCCATGCAGA<br>TCTCAGTGCCAAAGAAGCACAGGATGTCATCATGGAGGTTGTTTTCCCAAATGAAGTTGGAGCCAGGATCTTGACATCAGAATCACAATTAACAATAACAATGGAAAAGAAGGAGGAACTTCAGGATTGCAAGATTGCTCCTTTGATGGTGGCATACATGTTGGAAAGAGAACTGGTTCGCAAGACCAGATTTTTACCAGTAGCTGGCGGAACAAGCAGCGTATACATCG<br>AGGTATTGCATTTGACTCAAGGGACCTGCTGGGAACAAATGTACACACCAGGAGGGGAGGTGAGAAATGATGATGTTGATCAGAGTTTGATCATTGCTGCTAGAAATATAGTTAGGAGGGCAACAGTATCAGCAGACCCATTGGCTTCGCTCTTGGAAATGTGCCACAGTACACAAATTGGTGGAGTGAGGATGGTGGACATTCTTAGGCAGAACCCAACAGAGGAGCA<br>AGCTGTGGATATATGCAAAGCAGCAATGGGTTTAAGAATCAGTTCATCCTTCAGCTTTGGAGGTTTCACTTTCAAAAGGACAAGTGGGTCGTCTGTCAAAAGAGAAGAAGAAATACTCACTGGCAACCTCCAAACACTGAAAGTAAGAATACATGAAGGATACGAGGAATTCACAATGGTTGGGCGAAGAGCTACAGCCATTTTGAGGAAAGCAACCAGGAGACTGATCC<br>AATTAATAGTGAGTGGACGAGACGAACAGTCAATCGCTGAGGCAATCATAGTGGCAATGGTTTTCTCACAGGAGGATTGCATGATAAAAGCAGTACGAGGTGATTTGAATTTTGTCAACAGAGCGAATCAGCGGCTAAATCCTATGCATCAACTTCTGAGGCATTTCCAAAAAGATGCAAAAGTGCTGTTTCAAAACTGGGGGATTGAACCAATTGACAATGTAATGGGG<br>ATGATCGGGATACTGCCTGACATGACCCCCAGCACAGAGATGTCACTGAGAGGAGTGAGAGTCAGCAAAATGGGAGTGGATGAATATTCCAGTACTGAGAGAGTGGTCGTGAGCATTGATCGCTTCTTGAGAGTCCGAGATCAGAGGGGAAATGTGCTTCTGTCTCCTGAGGAAGTTAGTGAAACACATGGAACAGAGAAACTGACGATAACGTATTCATCGTCCATGA<br>TGTGGGAAATCAATGGTCCGGAATCCGTGCTAGTCAACACATATCAATGGATCATTAGAAATTGGGAAACTGTGAAGATTCAGTGGTCCCAGGACCCTACGATGTTGTACAATAAGATGGAATTTGAGCCCTTCCAATCCTTGGTGCCCAAGGCTGCTAGAGGCCAGTATAGTGGGTTTGTGAGGACATTATTCCAACAGATGCGTGATGTGTTGGGGACATTTGACACTGT<br>CCAAATAATAAAGCTCCTACCATTTGCAGCAGCCCCACCGGAACAGAGTAGGATGCAATTTTCCTCTCTGACTGTGAACGTAAGAGGTTCAGGAATGAGAATACTTGTGAGGGGCAACTCCCCTGTGTTCAACTATAATAAGACCACCAAGAGACTCACAGTTCTTGGAAAGGATGCAGGCGCCTTGACAGAATATCCAGATGAGGGAACAGCAGGAGTGGAGTCTGCAG<br>TATTAAGAGGATTTCTAATTCTGGGCAAAGAGAACAAAAGATATGGACCAGCATTGAGCATCAACGAATTGAGCAATCTTGCGAAAGGGGAAAAGGCTAATGTGTTGATAGGACAAGGAGACGTGGTGTTGGTAATGAAACGGAAACGGGACTCTAGCATACTTACTGACAGCCAGACAGCGACCAAAAGAATTCGGATGGCCATCAATTA
EPI_ISL_3102060	>A/duck/Poland/H160_21RS1385-1/2021_H5N8_2021-03-05<br>ATGGAGAGAATAAAAGAACTAAGAGATTTGATGTCGCAGTCTCGCACTCGCGAGATACTAACAAAAACCACTGTGGACCATATGGCCATAATAAAGAAATACACATCAGGGAGACAGGAGAAGAACCCTGCCCTCAGGATGAAATGGATGATGGCAATGAAATATCCTATTACAGCTGACAAAAGAATAATGGAGATGATCCCTGAAAGGAATGAGCAAGGTCAGACT<br>CTCTGGAGCAAAACAAATGATGCTGGATCAGACAGAGTGATGGTCTCACCTCTGGCTGTGACGTGGTGGAATAGAAATGGGCCAACAACAAGTACAGTACACTACCCAAAAGTCTACAAAACCTACTTTGAAAAGGTAGAAAGGTTGAAACATGGAACCTTTGGTCCTGTTCACTTTCGAAATCAGGTTAAGATACGCCGCAGGGTTGACACAAACCCGGGCCATGCAGA<br>TCTCAGTGCCAAAGAAGCACAGGATGTCATCATGGAGGTTGTTTTCCCAAATGAAGTTGGAGCCAGGATCTTGACATCAGAATCACAATTAACAATAACAATGGAAAAGAAGGAGGAACTTCAGGATTGCAAGATTGCTCCTTTGATGGTGGCATACATGTTGGAAAGAGAACTGGTTCGCAAGACCAGATTTTTACCAGTAGCTGGCGGAACAAGCAGCGTATACATCG<br>AGGTATTGCATTTGACTCAAGGGACCTGCTGGGAACAAATGTACACACCAGGAGGGGAGGTGAGAAATGATGATGTTGATCAGAGTTTGATCATTGCTGCTAGAAATATAGTTAGGAGGGCAACAGTATCAGCAGACCCATTGGCTTCGCTCTTGGAAATGTGCCACAGTACACAAATTGGTGGAGTGAGGATGGTGGACATTCTTAGGCAGAACCCAACAGAGGAGCA<br>AGCTGTGGATATATGCAAAGCAGCAATGGGTTTAAGAATCAGTTCATCCTTCAGCTTTGGAGGTTTCACTTTCAAAAGGACAAGTGGGTCGTCTGTCAAAAGAGAAGAAGAAATACTCACTGGCAACCTCCAAACACTGAAAGTAAGAATACATGAAGGATATGAGGAATTCACAATGGTTGGGCGAAGAGCTACAGCCATTTTGAGGAAAGCAACCAGGAGACTGATCC<br>AATTAATAGTGAGTGGACGAGACGAACAGTCAATCGCTGAGGCAATCATAGTGGCAATGGTTTTCTCACAGGAGGATTGCATGATAAAAGCAGTACGAGGTGATTTGAATTTTGTCAACAGAGCGAATCAGCGGCTAAATCCTATGCATCAACTTCTGAGGCATTTCCAAAAAGATGCAAAAGTGCTGTTTCAAAACTGGGGGATTGAACCAATTGACAATGTAATGGGG<br>ATGATCGGGATACTGCCTGACATGACCCCCAGCACAGAGATGTCACTGAGAGGAGTGAGAGTCAGCAAAATGGGAGTGGATGAATATTCCAGTACTGAGAGAGTGGTCGTGAGCATTGATCGCTTCTTGAGAGTCCGAGATCAGAGGGGAAATGTGCTTCTGTCTCCTGAGGAAGTTAGTGAAACACATGGAACAGAGAAACTGACGATAACGTATTCATCGTCCATGA<br>TGTGGGAAATCAATGGTCCGGAATCCGTGCTAGTCAACACATATCAATGGATCATTAGAAATTGGGAAACTGTGAAGATTCAGTGGTCCCAGGACCCTACGATGTTGTACAATAAGATGGAATTTGAGCCCTTCCAATCCTTGGTGCCCAAGGCTGCTAGAGGCCAGTATAGTGGGTTTGTGAGGACATTATTCCAACAGATGCGTGATGTGTTGGGGACATTTGACACTGT<br>CCAAATAATAAAGCTCCTACCATTTGCAGCAGCCCCACCGGAACAGAGTAGGATGCAATTTTCCTCTCTGACTGTGAACGTAAGAGGTTCAGGAATGAGAATACTTGTGAGGGGCAACTCCCCTGTGTTCAACTATAATAAGACCACCAAGAGACTCACAGTTCTTGGAAAGGATGCAGGCGCCTTGACAGAATATCCAGATGAGGGAACAGCAGGAGTGGAGTCTGCAG<br>TATTAAGAGGATTTCTAATTCTGGGCAAAGAGGACAAAAGATATGGACCAGCATTGAGCATCAACGAATTGAGCAATCTTGCGAAAGGGGAAAAGGCTAATGTGTTGATAGGACAAGGAGACGTGGTGTTGGTAATGAAACGGAAACGGGACTCTAGCATACTTACTGACAGCCAGACAGCGACCAAAAGAATTCGGATGGCCATCAATTA

internal NVRI repository	>A/duck/Poland/H1970_21RS3290-7/2021_H5N1_2021-11-13<br>[illegible]
internal NVRI repository	>A/duck/Poland/H2007-21RS3290-15/2021_H5N1_2021-11-17<br>[illegible]
EPI_ISL_3102077	>A/duck/Poland/H514_21RS1385-18/2021_H5N8_2021-04-07<br>[illegible]
EPI_ISL_3102076	>A/duck/Poland/H515_21RS1385-17/2021_H5N8_2021-04-07<br>[illegible]
EPI_ISL_3102061	>A/duck/Poland/H542_21RS1385-2/2021_H5N8_2021-04-09<br>[illegible]
EPI_ISL_5463797	>A/duck/Saratov/29-02V/2021_H5N1_2021-09-30<br>[illegible]
EPI_ISL_333974	>A/duck/Vietnam/NCVD-ND4V3P/2016_H3N8_2016-07-21<br>[illegible]
EPI_ISL_603133	>A/Eurasian_Wigeon/Netherlands/1/2020_H5N1_2020-10-16<br>[illegible]
EPI_ISL_341563	>A/Eurasian-teal/Egypt/P2-29/2017_H6N2_2017-11-19<br>[illegible]
EPI_ISL_9250718	>A/Gallus_gallus/Belgium/11372-0001/2021_H5N8_2021-08-31<br>[illegible]
EPI_ISL_12572661	>A/goose/Guizhou/S1541/2022_H5N1_2022-02-22<br>[illegible]
EPI_ISL_654828	>A/goose/Omsk/30004/2020_H5N8_2020-09-03<br>[illegible]

EPI_ISL_3102065	>A/goose/Poland/H1036_21RS1385-6/2021_H5N8_2021-05-03<br>[illegible]
EPI_ISL_3102063	>A/goose/Poland/H1044_21RS1385-4/2021_H5N8_2021-05-05<br>[illegible]
EPI_ISL_626651	>A/goose/Russian_Federation/Kurgan/1345-25/2020_H5N8_2020-08-20<br>[illegible]
EPI_ISL_4055121	>A/goose/Yunlin/18030020-1/2018_H5N2_2018-03-30<br>[illegible]
EPI_ISL_393962	>A/great_black-headed_gull/Ningxia/2/2018_H16N3_2018-10-23<br>[illegible]
EPI_ISL_8515483	>A/great_egret/Czech_Republic/23609/2021_H5N1_2021-11-28<br>[illegible]
EPI_ISL_8515481	>A/grey_heron/Czech_Republic/23608/2021_H5N1_2021-11-28<br>[illegible]
EPI_ISL_18245810	>A/greylag_goose/Poland/MB503_21RS3290-18/2021_H5N1_2021-11-22<br>[illegible]
EPI_ISL_661312	>A/guinea_fowl/Germany-NW/AI01184/2020_H5N8_2020-09-11<br>[illegible]
EPI_ISL_18245813	>A/hawk/Poland/MB544/2021_H5N1_2021-12-22<br>[illegible]
EPI_ISL_14917979	>A/herring_gull/Poland/MB138/2022_H5N1_2022-05-30<br>[illegible]
EPI_ISL_17978928	>A/herring_gull/Poland/P092/2020_H16N3_2020-10-28<br>[illegible]

EPI_ISL_373079	>A/laughing_gull/Delaware_Bay/458/2018_H13N6_2018-05-22<br>ATGGAGAGAATAAAAGAACTAAGAGATCTAATGTCACAGTCTCGCACTCGCGAGATACTCACCAAAACCACTGTGGACCACATGGCCATAATCAAAAAATACACATCGGGAAGGCAGGAGA[illegible]<br>CTTTGGAGTAAAACAAATGATGCCGGATCGGACCGAGTAATGGTATCGCCTCTCGCTGTGACGTGGTGGAACAGGAATGGACCAACAGCAAGTGCAGTCCACTACCCAAAGGTATACAAAA[illegible]<br>CCTAAGTGCCAAAGAGGCACAGGATGTAATCATGGAAGTTGTCTTCCCAAATGAGGTGGGAGCAAGAATACTGACGTCAGAGTCACAACTGGCGATAACAAAGAGAAAAAAGAAGAACT[illegible]<br>GAGGTGCTGCACTTAACCCAGGGGACATGCTGGGAACAGATGTACACTCCAGGAGGAGAGGTGAGGAATGATGATGTAGACCAGAGCTTGATTATTGCTGCCAGAAACATAGTGAGAAGA[illegible]<br>CAAGCCGTGGACATATGCAAGGCAGCAATGGGGTTGAGGATTAGCTCATCTTTCAGCTTTGGTGGATTCACTTTTAAAAGAACAAGTGGATCTTCAGTAAAAAGAGAAGAAGAAGTGCTTAC[illegible]<br>TCCAGCTAATAGTAAGTGGGAGAGACGAGCAGTCAATTGCTGAAGCAATAATTGTGGCCATGGTGTTCTCACAAGAGGACTGCATGATCAAGGCAGTTCGAGGTGATCTGAATTTTGTCAAT[illegible]<br>GGATGATTGGAATATTGCCCGACATGACCCCAAGTACTGAGATGTCGCTGAGGGGGGTAAGGGTCAGCAAGACGGGAGTAGATGAGTACTCCAGCACAGAAAGGGTCGTAGTGAGTATCG[illegible]<br>TGATGTGGGAGATCAATGGTCCTGAGTCGGTGTTGGTTAATACTTACCAATGGATCATCAGAAACTGGGAAACTGTGAAAATTCAATGGTCACAGGATCCCACAATGTTATATAACAAGATGG[illegible]<br>TGTTCAAATAATAAAACTCCTCCCATTTGCTGCTGCCCCACCAGAACAAAGTAAGATGCAATTCTCCTCCATGACTGTAAATGTTAGAGGATCAGGGATGAGAATACTGGTAAGAGGCAACTC[illegible]<br>CGTTCTAAGAGGATTCCTCATTTTGGGCAAAGAAGACAAGAGATATGGCCCGGCATTGAGCATCAATGAGCTGAGCAATCTTGCAAAGGGAGAGAAGGCTAATGTGCTAATTGGGCAAGGA[illegible]
EPI_ISL_654958	>A/mallard/Italy/20VIR7139-73/2020_H5N8_2020-11-14<br>ATGGAGAGAATAAAAGAACTACGAGATTTGATGTCGCAGTCTCGCACTCGCGAGATACTAACAAAAACCACTGTGGACCATATGGCCATAATAAAGAAATACACATCAGGGAGACAGGAGA[illegible]<br>CTCTGGAGCAAAACAAATGATGCTGGATCAGACAGAGTGATGGTCTCACCTCTGGCTGTGACATGGTGGAATAGAAATGGCCAACAACAAGTACAGTACACTACCCAAAAGTCTACAAAA[illegible]<br>TCTCAGTGCCAAAGAAGCACAGGATGTCATCATGGAGGTTGTTTTCCCAAATGAAGTTGGAGCCAGGATCTTGACATCAGAATCACAATTAACAATAACAATGGAAAAGAAGGAGGAACTTC[illegible]<br>AGGTATTGCATTTGACTCAAGGGACCTGCTGGGAACAAATGTACACACCAGGAGGGGAGGTGAGAAATGATGATGTTGATCAGAGTTTGATCATTGCTGCTAGAAATATAGTTAGGAGGGC[illegible]<br>AGCTGTGGATATATGCAAAGCAGCAATGGGTTTAAGAATCAGTTCATCCTTCAGCTTTGGAGGTTTCACTTTCAAAAGGACAAGTGGGTCGTCTGTCAAAAGAGAAGAAGAAATACTCACTG[illegible]<br>AATTAATAGTGAGTGGACGAGACGAGCAGTCAATCGCTGAAGCAATCATAGTGGCAATGGTTTTCTCACAGGAGGATTGCATGATAAAAGCAGTACGAGGTGATTTGAATTTTGTCAACAGA[illegible]<br>[illegible]
EPI_ISL_373089	>A/Mallard/Netherlands/37/2015_H3N8_2015-08-29<br>[illegible]
EPI_ISL_1241001	>A/mallard/Novosibirsk_region/3541k/2020_H12N5_2020-08-29<br>[illegible]
EPI_ISL_17978926	>A/mallard/Poland/P060w2/2020_H3N8_2020-08-25<br>[illegible]
EPI_ISL_17978924	>A/mallard/Poland/P074w5/2018_H3N8_2018-09-05<br>[illegible]
EPI_ISL_17978925	>A/mallard/Poland/P077w1/2018_H5N2_2018-09-12<br>[illegible]
EPI_ISL_17978951	>A/mallard/Poland/P096/2020_H9N2_2020-11-02<br>[illegible]
EPI_ISL_328964	>A/mallard_duck/Netherlands/11/2015_mixed_2015-06-05<br>[illegible]
EPI_ISL_243608	>A/mallard_duck/Netherlands/15/2011_H6N8_2011-09-14<br>[illegible]
EPI_ISL_6780544	>A/Muscovy_duck/Quang-Ninh/353/2017_H5N6_2017-11-14<br>[illegible]
EPI_ISL_9603920	>A/mute_swan/Czech_Republic/22380/2021_H5N1_2021-11-15<br>[illegible]

EPI_ISL_1399237	>A/mute_swan/Czech_Republic/3549/2021_H5N8_2021-02-19<br>ATGGAGAGAATAAAAGAACTAAGAGATTTGATGTCACAGTCTCGCACTCGCGAGATACTAACAAAAACCACTGTGGACCATATGGCCATAATAAAGAAATACACATCAGGGAGACAGGAGAAGAACCCTGCCCTCAGGATGAAATGGATGATGGCAATGAAATATCCTATTACAGCTGACAAAAGAATAATGGAGATGATCCCTGAAAGGAATGAGCAAGGTCAGACT<br>CTCTGGAGCAGAACAAATGATGCTGGATCAGACAGAGTGATGGTCTCACCTCTGGCTGTGACATGGTGGAATAGAAATGGGCCAACAACAAGTACAGTACACTACCCAAAAGTCTACAAAACCTACTTTGAAAAGGTAGAAAGGTTGAAACATGGAACCTTTGGTCCTGTTCACTTCGAAATCAGGTTAAGATACGCCGCAGGGTTGACATAAACCCGGGCCATGCAGA<br>TCTCAGTGCCAAAGAAGCACAGGATGTCATCATGGAGGTTGTTTTCCCAAATGAAGTTGGAGCCAGGATCTTGACATCAGAATCACAATTAACAATAACAATGGAAAAGAAGGAGGAACTTCAGGATTGCAAGATTGCTCCTTTGATGGTGGCATACATGTTGGAAAGAGAACTGGTTCGCAAGACCAGATTTTTACCAGTAGCTGGCGGAACAAGCAGCGTATACATCG<br>AGGTATTGCATTTGACTCAAGGGACCTGCTGGGAACAAATGTACACACCAGGAGGGGAGGTGAGAAATGATGATGTTGATCAGAGTTTGATCATTGCTGCTAGAAATATAGTTAGGAGGGCAACAGTATCAGCAGACCCATTGGCTTCGCTCTTGGAAATGTGCCACAGTACACAAATTGGTGGAGTGAGGATGGTGGACATTCTTAGGCAGAACCCAACAGAGGAGCA<br>AGCTGTGGATATATGCAAAGCAGCAATGGGTTTAAGAATCAGTTCATCCTTCAGCTTTGGAGGTTTCACTTTCAAAAGGACAAGTGGGTCGTCTGTCAAAAGAGAAGAAGAAATACTCACTGGCAACCTCCAAACACTGAAAGTAAGAATACATGAAGGATATGAGGAATTCACAATGGTTGGGCGAAGAGCTACAGCCATTTTGAGGAAAGCAACCAGGAGACTGATCC<br>AATTAATAGTGAGTGGACGAGACGAACAGTCAATCGCTGAAGCAATCATAGTGGCAATGGTTTTCTCACAGGAGGATTGCATGATAAAAGCAGTACGAGGTGATTTGAATTTTGTCAACAGAGCGAATCAGCGGCTAAATCCTATGCATCAACTTCTGAGGCATTTCCAAAAAGATGCAAAAGTGCTGTTTCAAACTGGGGGATTGAACCAATTGACAATGTAATGGGGA<br>TGATCGGGATACTGCCTGACATGACCCCCAGCACAGAGATGTCACTGAGAGGAGTGAGAGTCAGCAAAATGGGAGTGGATGAATATTCCAGTACTGAGAGAGTGGTCGTGAGCATTGATCGCTTCTTGAGAGTCCGAGATCAGAGGGGAAATGTGCTTCTGTCTCCTGAGGAAGTTAGTGAAACACATGGAACAGAGAAACTGACGATAACGTATTCATCGTCTATGAT<br>GTGGGAAATCAATGGTCCGGAATCCGTGCTAGTCAACACATATCAATGGATCATTAGAAATTGGGAAACTGTGAAGATTCAGTGGTCCCAGGACCCTACGATGTTGTACAATAAGATGGAATTTGAGCCCTTCCAATCCTTGGTGCCCAAGGCTGCTAGAGGCCAGTATAGTGGGTTTGTGAGGACATTATTCCAACAGATGCGTGATGTGTTGGGGACATTTGACACTGTC<br>CAAATAATAAAGCTCCTACCATTTGCAGCAGCCCCACCGGAACAGAGTAGGATGCAATTTTCCTCTCTGACTGTGAACGTAAGAGGTTCAGGAATGAGAATACTTGTGAGGGGCAACTCCCCTGTGTTCAACTATAATAAGACCACCAAGAGACTCACAGTTCTTGGAAAGGATGCAGGCGCCTTGACAGAATATCCAGATGAAGGAACAGCAGGAGTGGAGTCTGCAGT<br>ATTAAGAGGATTTCTAATTCTGGGCAAAGAGGACAAAAGATATGGACCAGCATTGAGCATCAACGAATTGAGCAATCTTGCGAAAGGGGAAAAGGCTAATGTGTTGATAGGACAAGGAGACGTGGTGTTGGTAATGAAACGGAAACGGGACTCTAGCATACTTACTGACAGCCAGACAGCGACCAAAAGAATTCGGATGGCCATCAATTA
EPI_ISL_18245597	>A/mute_swan/Poland/MB008-22_22VIR5675-16/2022_2022-01-05<br>ATGGAGAGAATAAAAGAGCTAAGAGATTTGATGTCGCAGTCTCGCACTCGCGAGATACTGACAAAAACCACCGTGGACCATATGGCCATAATCAAGAAATATACATCAGGAAGACAGGAGAAGAACCCTGCACTTAGGATGAAGTGGATGATGGCAATGAAATATCCGATTACAGCAGACAAAAGGATAATGGAGATGATCCCTGAAAGAAACGAGCAAGGTCAGACT<br>CTTTGGAGCAAAACAAATGATGCTGGATCGGATAGAGTAATGGTGTCACCTCTGGCTGTGACGTGGTGGAATAGAAATGGACCAACAACAAGTACAGTCCATTAnnnnnnnnnnnnnnnnnnnnnnnnnnnnnnnnnnnnnnnnnnnnnnnnnnnnnnnnnnnnnnnnnnnnnnnnnnnnnnnnnnnnnnnnnnnnnnnnnnnnnnnnnnnnnnnAGTTGACATAAACCCGGGCCATGCAGACCTCAGT<br>GCTAAAnnnnnnnnnnnnnnCATCATGGAGGTCGTTTTCCCAAATGAAGTCGGAGCCAGAATATTGACATCAGAGTCACAGTTAACAATTACAAAAGAAAAAAAGGAnnnnCTCCAGGACTGTAAGATTGCCCCTCTAATGGTGGCATACATGTTGGAGAGAGAACTGGTTCGAAAAACCAGATTCCTGCCAGTGGCTGGCGGAACAAGTAGCGTATATATCGAAGTGTTGC<br>ACTTGACTCAAGGAACCTGCTGGGAACAAATGTATACGCCAGGAGGAGAAGTGAGAAATGATGACGTTGACCAAAGTTTAATTATTGCTGCCAGAAATATCGTTAGGAGAGCAACAGTATCAGCAGACCCnnnnnnnnnnnnnnnnnnnnnnnnnnnnnnnnnnnnnnnnnnnnnnnnnnnnnnnnnnnnnnnnnnnnnnnnnnnnnnnnnnnnnnnnnnnnnnnnnnnnnnnnnnnnnnnnnnnnnnnnnn<br>nnnnnnnnnnnnnnnnnnnnnnnnnnnnnnnnnnnnnnnnnnnnnnnnnnnnnnnnnnnnnnnnnnnnnnnnnnnnnnnnnnnnnnnnnnnnnnnnnnnnnnnnnnnnnnnnnnnnnnnnnnnnnnnnnnnnnnnnnnnnnnnnnnnnnnnnnnnnnTGAGGAATTCACAATGGTTGGGCGGAGAGCAACAGCCATTCTAAGGAAAGCAACCAGAAGGCTGATCCAATTGATAGTAAGTGGAAAAGACGAGCA<br>GTCAATCGCCGAAGCGATCATAGTGGCAATGGTGTTCTCTCAAGAGGATTGCATGATAAAGGCTGTACGAGGTGATTTAAATTTTGTCAATAGAGCGAATCAGCGGCTCAATCCTATGCATCAGCTCCTGAGGCATTTCCAAAAGGATGCAAAGGTACTATTCCAAAACTGGGGAATTGAACCCATTGACAATGTCATGGGAATGATAGGAATATTGCCTGATATGACTCCC<br>AGCACAGAGATGTCACTAAGAGGAGTGAGGGTCAGTAAAATGGGAGTGGATGAATATTCCAGTACTGAGAGGGTGGTCGTGAGTATTGATCGCTTCTTGAGGGTACGAGACCAGAGAGGAAATGTACTCTTGTCTCCCGAAGAGGTCAGTGAAACACAGGGAACAGAGAAGCTAACGATAACATATTCATCATCCATGATGTGGGAAATTAATGGCCCTGAGTCAGTGC<br>TAGTTAACACATATCAATGGATCATCAGAAACTGGGAAACTGTGAAGATTCAGTGGTCnnnnnnnnnnnnnnnnnnnnnnnnnnnnnnnnnnnnnnnnnnnnnnnnnnnnnnnnnnTTTCAGnnnnnnnnGCCTAAGGCAGCCAGAGGCCAGTACAGTGGATTTGTAAGGACCTTATTCCAGCAGATGCGTGATGTGCTGGGAACCTTTGACACTGTCCAGATAATAAAGCTACTTCCATTTGCAGCAGC<br>ACCACCGGAACAGAGTAGGATGCAGTTCTCTTCTCTAACTGTAAACGTAAGGGGTTCAGGAATGAGAATACTTGTGAGAGGCAACTCCCCTGTGTTCAACTATAATAAGGCAACCAAGAGGCTCATAGTCCTTGGAAAGGATGCTGGTGCATTGACAGAAGACCCAGGTGAGGGGACAGCAGGAGTGGAGTCTGCGGTATTGAGAGGGTTCCTAATTCTGGGCAAAGAG<br>GACAAAAGATATGGACCAGCGCTGAGCATCAATGAATTGAGCAATCTTGCGAAAGGGGAGAAGGCTAATGTGTTGATAGGGCAAGGAGACGTGGTGTTGGTGATGAAACGGAAACGGGACTCTAGCATACTTACTGACAGCCAGACAGCGACCAAAAGAATTCGGATGGCCATCAATTA
EPI_ISL_18245613	>A/mute_swan/Poland/MB020-22_22VIR5675-3/2022_H5N1_2022-01-11<br>ATGGAGAGAATAAAAGAGCTAAGAGATTTGATGTCGCAGTCTCGCACTCGCGAGATACTGACAAAAACCACCGTGGACCATATGGCCATAATCAAGAAATATACATCAGGAAGACAGGAGAAGAACCCTGCACTTAGGATGAAGTGGATGATGGCAATGAAATATCCGATTACAGCAGACAAAAGGATAATGGAGATGATCCCTGAAAGAAACGAGCAAGGTCAGACT<br>CTTTGGAGCAAAACAAATGATGCTGGATCGGATAGAGTAATGGTGTCACCTCTGGCTGTGACGTGGTGGAATAGAAATGGACCAACAACAAGTRCCGTCCATTACCCAAAGGTCTATAAAACCTACTTTGAAAAGGTTGAAAGGTTAAAGCATGGAACCTTCGGCCCTGTCCATTTCCGGAATCAGGTTAAGATACGCCGCAGAGTTGACATAAACCCGGGCCATGCAGAC<br>CTCAGTGCTAAAGAAGCACAAGACGTCATCATGGAGGTCGTTTTCCCAAATGAAGTCGGAGCCAGAATATTGACATCAGAGTCACAGTTGACAATTACAAAAGAAAAGAAGGAGGAACTCCAGGACTGTAAGATTGCCCCTCTAATGGTGGCATACATGTTGGAGAGAGAACTGGTTCGAAAAACCAGATTCCTGCCAGTAGCTGGCGGAACAAGTAGCGTATATATCG<br>AAGTGTTGCACTTGACCCAAGGAACCTGCTGGGAACAAATGTATACGCCAGGAGGAGAAGTGAGAAATGATGACGTTGACCAAAGTTTAATTATTGCTGCCAGAAATATCGTTAGGAGGGCAACAGTATCAGCAGACCCATTGGCTTCACTACTGGAGATGTGCCATAGTACACAGATTGGCGGGATAAGAAnnnnnnnnnnnnnnnnnnnnGAACCCAACAGAAGAGCAA<br>GCCGTGGATATATGCAAAGCAGCAATGGGTTTAAGGATCAGTTCATCCTTCAGTTTTGGAGGTTTCACTTTCAAAAGGACAAGCGGATCATCTGTCAAAAGAGAAGAGGAAGTGCTCACCGGCAACCTCCAAACATTGAAAATAAGAGTACATGAAGGGTATGAGGAATTCACAATGGTTGGGCGGAGAGCAACAGCCATTCTAAGGAAAGCAACCAGAAGGCTGATCC<br>AATTGATAGTAAGTGGAAGAGACGAGCAGTCAATCGCCGAAGCGATCATAGTGGCAATGGTGTTCTCTCAAGAGGATTGCATGATAAAGGCTGTACGAGGTGATTTAAATTTTGTCAATAGAGCGAATCAGCGGCTCAATCCTATGCATCAGCTCCTGAGGCATTTCCAAAAGGATGCAAAGGTACTATTCCAAAACTGGGGAATTGAACCCATTGACAATGTCATGGGAA<br>TGATAGGAATATTGCCTGATATGACCCCCAGCACAGARATGTCACTAAGAGGAGTGAGGGTCAGTAAAATGGGAGTGGATGAATATTCCAGTACTGAGAGGGTGGTCGTGAGTATTGATCGCTTCTTGAGGGTACGAGACCAGAGAGGAAATGTACTCTTGTCTCCCGAAGAGGTCAGTGAAACACAGGGAACAGAGAAGCTAACGATAACATATTCATCATCCATGAT<br>GTGGGAAATTAATGGCCCTGAGTCAGTGCTAGTTAACACATATCAATGGATCATCAGAAACTGGGAAACTGTGAAGATTCAGTGGTCCCAAGACCCTACAATGCTATACAACAAGATGGAGTTTGAGCCTTTTCAGTCCTTGGTGCCTAAGGCAGCCAGAGGCCAGTACAGTGGATTTGTAAGGACCCTATTCCAGCAGATGCGTGATGTGCTGGGAACCTTTGACACTGT<br>CCAGATAATAAAGCTACTTCCATTTGCAGCAGCACCACCGGAACAGAGTAGGATGCAGTTCTCTTCTCTAACTGTAAACGTAAGGGGTTCAGGAATGAGAATACTTGTGAGAGGCAACTCCCCTGTGTTCAACTATAATAAAGCAACCAAGAGGCTCATAGTCCTTGGAAAGGATGCTGGTGCATTGACAGAAGACCCAGGTGAGGGGACAGCAGGAGTGGAGTCTGCG<br>GTATTGAGAGGGTTCCTAATTCTGGGCAAAGAGGACAAAAGATATGGACCAGCGCTGAGCATCAATGAATTGAGCAATCTTGCGAAAGGGGAGAAGGCTAATGTGTTGATAGGGCAAGGAGACGTGGTGTTGGTGATGAAACGGAAACGGGACTCTAGCATACTTACTGACAGCCAGACAGCGACCAAAAGAATTCGGATGGCCATCAATTA
EPI_ISL_18244181	>A/mute_swan/Poland/MB021-21_22VIR5675-11/2021_H5N8_2021-01-19<br>ATGGAGAGAATAAAAGAACTAAGAGATTTGATGTCGCAGTCTCGCACTCGCGAGATACTAACAAAAACCACTGTGGACCATATGGCCATAATAAAGAAATACACATCAGGGAGACAGGAGAAGAACCCTGCCCTCAGGATGAAATGGATGATGGCAATGAAATATCCTATTACAGCTGACAAAAGAATAATGGAGATGATCCCTGAAAGGAATGAGCAAGGTCAGACT<br>CTCTGGAGCAAAACAAATGATGCTGGATCAGACAGAGTGATGGTCTCACCTCTGGCTGTGACGTGGTGGAATAGAAATGGGCCAACAACAAGTACAGTACACTACCCAAAAGTCTACAAAACCTACTTTGAAAAGGTAGAAAGGTTGAAACATGGAACCTTTGGTCCTGTTCACTTTCGAAATCAGGTTAAGATACGCCGCAGGGTTGACATAAACCCGGGCCATGCAGA<br>TCTCAGTGCCAAAGAAGCACAGGATGTCATCATGGAGGTTGTTTTCCCAAATGAAGTTGGAGCCAGGATCTTGACATCAGAATCACAATTAACAATAACAATGGAAAAGAAGGAGGAACTTCAGGATTGCAAGATTGCTCCTTTGATGGTGGCATACATGTTGGAAAGAGAACTGGTTCGCAAGACCAGATTTTTACCAGTAGCTGGCGGAACAAGCAGCGTATACATCG<br>AGGTATTGCATTTGACTCAAGGGACCTGCTGGGAACAAATGTACACACCAGGAGGGGAGGTGAGAAATGATGATGTTGATCAGAGTTTGATCATTGCTGCTAGAAATATAGTTAGGAGGGCAACAGTATCAGCAGACCCATTGGCTTCGCTCTTGGAAATGTGCCACAGTACACAAATTGGTGGAGTGAGGATGGTGGACATTCTTAGGCAGAACCCAACAGAGGAGCA<br>AGCTGTGGATATATGCAAAGCAGCAATGGGTTTAAGAATCAGTTCATCCTTCAGCTTTGGAGGTTTCACTTTCAAAAGGACAAGTGGGTCGTCTGTCAAAAGAGAAGAAGAAATACTCACTGGCAACCTCCAAACACTGAAAGTAAGAATACATGAAGGATATGAGGAATTCACAATGGTTGGGCGAAGAGCTACAGCCATTTTGAGGAAAGCAACCAGGAGACTGATCC<br>AATTAATAGTGAGTGGACGAGACGAACAGTCAATCGCTGAGGCAATCATAGTGGCAATGGTTTTCTCACAGGAGGATTGCATGATAAAAGCAGTACGAGGTGATTTGAATTTTGTCAACAGAGCGAATCAGCGGCTAAATCCTATGCATCAACTTCTGAGGCATTTCCAAAAAGATGCAAAAGTGCTGTTTCAAACTGGGGGATTGAACCAATTGACAATGTAATGGGG<br>ATGATCGGGATACTGCCTGACATGACCCCCAGCACAGAGATGTCACTGAGAGGAGTGAGAGTCAGCAAAATGGGAGTGGATGAATATTCCAGTACTGAGAGAGTGGTCGTGAGCATTGATCGCTTCTTGAGAGTCCGAGATCAGAGGGGAAATGTGCTTCTGTCTCCTGAGGAAGTTAGTGAAACACATGGAACAGAGAAACTGACGATAACGTATTCATCGTCTATGA<br>TGTGGGAAATCAATGGTCCGGAATCCGTGCTAGTCAACACATATCAATGGATCATTAGAAATTGGGAAACTGTGAAGATTCAGTGGTCCCAGGACCCTACGATGTTGTACAATAAGATGGAATTTGAGCCCTTCCAATCCTTGGTGCCCAAGGCTGCTAGAGGCCAGTATAGTGGGTTTGTGAGGACATTATTCCAACAGATGCGTGATGTGTTGGGGACATTTGACACTGT<br>CCAAATAATAAAGCTCCTACCATTTGCAGCAGCCCCACCGGAACAGAGTAGGATGCAATTTTCCTCTCTGACTGTGAACGTAAGAGGTTCAGGAATGAGAATACTTGTGAGGGGCAACTCCCCTGTGTTCAACTATAATAAGACCACCAAGAGACTCACAGTTCTTGGAAAGGATGCAGGCGCCTTGACAGAATATCCAGATGAGGGAACAGCAGGAGTGGAGTCTGCAG<br>TATTAAGAGGATTTCTAATTCTGGGCAAAGAGGACAAAAGATATGGACCAGCATTGAGCATCAACGAATTGAGCAATCTTGCGAAAGGGGAAAAGGCTAATGTGTTGATAGGACAAGGAGACGTGGTGTTGGTAATGAAACGGAAACGGGACTCTAGCATACTTACTGACAGCCAGACAGCGACCAAAAGAATTCGGATGGCCATCAATTA
EPI_ISL_18245743	>A/mute_swan/Poland/MB034-22_22VIR5675-12/2022_H5N1_2022-01-18<br>ATGGAGAGAATAAAAGAACTAAGAGATTTAATGTCGCAGTCCCGCACTCGCGAGATACTGACAAAAACCACTGTGGACCATATGGCAATAATAAAGAAATACACATCAGGAAGACAGGAGAAGAACCCTGCCCTCAGGATGAAGTGGATGATGGCAATGAAATATCCTATTACAGCAGACAAAAGAATAATGGAGATGATCCCTGAAAGGAATGAGCAAGGTCAGACT<br>CTCTGGAGCAAAACAAATGACGCTGGATCAGACAGAGTGATGGTCTCACCTCTGGCTGTGACATGGTGGAATAGAAATGGGCCAATGACAAGTACAGTCCATTACCCAAAGGTTTACAAAACCTACTTTGAAAAAGTGGAAAGATTGAAACATGGAACCTTTGGCCCTGTTCACTTTCAAAATCAGGTTAAGATACGCCGCAGGGTTGACATAAACCCGGGTCATGCAGA<br>TCTCAGTGCCAAAGAAGCACAGGATGTCATCATGGAAGTCGTTTTCCCAAATGAAGTTGGAGCCAGGATATTGACATCAGAATCACAATTAACAATAACAAAGGAAAAGAAGGAGGAACTTCAGGACTGTAAGATTGCTCCTTTGATGGTGGCATACATGTTGGAGAGAGAACTGGTTCGAAAAACCAGATTTTTACCAGTAGCTGGCGGAACAAGCAGCGTATATATCG<br>AAGTATTGCATTTGACTCAAGGGACCTGCTGGGAACAAATGTACACACCGGGAGGGGAAGTGAGAAATGATGATGTTGATCAGAGTTTGATCATTGCTGCTAGAAATATCGTTAGGAGAGCAACAGTATCAGCAGACCCATTGGCTTCGCTCTTGGAAATGTGCCACAGTACACAAATTGGCGGGATAAGGATGGTAGACATTCTTAGACAGAACCCAACAGAAGAGCA<br>AGCCGTGGATATATGCAAAGCAGCAATGGGTTTAAGAATCAGTTCATCCTTCAGCTTTGGAGGTTTCACTTTCAAAAGAACAAGTGGATCGTCTGTCAAAAGAGAAGAAGAAGTGCTCACAGGCAACCTCCAAACACTGAAAATAAGAGTACATGAAGGATATGAGGAATTCACAATGGTTGGACGGAGAGCTACAGCCATTTTAAGGAAAGCAACCAGAAGGCTGATCC<br>AATTAATAGTGAGTGGAAGAGACGAGCAGTCAATTGCTGAAGCAATCATAGTGGCAATGGTTTTCTCACAAGAGGACTGCATGATAAAAGCAGTACGAGGTGATCTGAACTTTGTTAATAGAGCAAATCAGCGACTAAATCCTATGCATCAACTTCTGAGGCATTTCCAAAAGGATGCAAAGGTGCTGTTTCAAACTGGGGGGTTGAACCAATTGACAGTGTAATGGGG<br>ATGATTGGAATACTGCCTGACATGACCCCCAGCACGGAGATGTCACTGAGAGGAGTGAGAGTCAGCAAAATGGGAGTGGATGAATATTCCAGTACTGAGAGAGTGGTCGTGAGCATTGATCGTTTCTTGAGAGTCCGAGATCAAAGAGGAAACGTGCTCCTGTCCCCTGAGGAAGTTAGTGAAACACAGGGAATAGAGAGACTGACGATAACATATTCATCGTCCATGA<br>TGTGGGAAATCAATGGTCCGGAATCCGTGCTAGTCAACACATATCAATGGATCATCAGAAATTGGGAGACTGTGAAGATTCAATGGTCCCAAGACCCTACGATGTTGTACAACAAGATGGAATTTGAGCCCTTCCAATCCTTGGTGCCCAAGGCTGCCAGAGGCCAGTATAGTGGATTTGTGAGGACATTGTTCCAGCAGATGCGTGATGTGCTGGGAACATTTGATACTG<br>TCCAAATAATAAAGCTCCTGCCTTTTGCAGCAGCCCCACCGGAACAGAGTAGGATGCAGTTTTCCTCTCTGACTGTGAACGTAAGAGGTTCAGGAATGAGAATACTTGTGAGAGGCAACTCCCCTGTGTTCAACTATAACAAGGCCACCAAGAGACTCACAGTTCTTGGAAAGGATGCAGGTGCCTTGACAGAAGATCCAGATGAGGGAACAGCAGGAGTAGAGTCTGCA<br>GTATTAAGAGGATTTCTAATTCTGGGCAAAGAGGACAAAAGATATGGACCAGCATTGAGCATCAACGAACTGAGCAATCTTGCGAAAGGGGAAAAGGCTAATGTGTTGATAGGGCAAGGAGACGTGGTGTTGGTAATGAAACGGAAACGGGACTCTAGCATACTTACTGACAGCCAGACAGCGACCAAAAGAATTCGGATGGCCATCAATTA
EPI_ISL_18245744	>A/mute_swan/Poland/MB040-22_22VIR5675-8/2022_H5N1_2022-01-20<br>ATGGAGAGAATAAAAGAGCTAAGAGATTTGATGTCGCAGTCTCGCACTCGCGAGATACTGACAAAAACCACCGTGGACCATATGGCCATAATCAAGAAATATACATCAGGAAGACAGGAGAAGAACCCTGCACTTAGGATGAAGTGGATGATGGCAATGAAATATCCGATTACAGCAGACAAAAGGATAATGGAGATGATCCCTGAAAGAAACGAGCAAGGTCAGACT<br>CTTTGGAGCAAAACAAATGATGCTGGATCGGATAGAGTAATGGTGTCACCTCTGGCTGTGACGTGGTGGAATAGAAATGGACCAACAACAAGTACAGTCCATTACCCAAAGGTCTATAAAACCTACTTTGnnnnnnnnnnnnnnnnnnnnnnnnnnnnnnnnnnnnnnnnnnnnnnnnnnnnnnnnnnnnnnnnnnnnnnnnnnnnnnnnnnnnnnnnnnnnnnnnnnnnnnnnnnnnnnnnnnnnn<br>nn<br>nnn<br>nn<br>nnn<br>nn<br>nnn<br>nnnnnnnnnnnnnnnnnnnnnnnnnnnnnnnnnnnnnnnnnnnnnnnnnnnnnnnnnnnnnnnnnnnnnnnnnnnnnnnnnnnnnnnnnnnnnnnnnnnnnnnnnnnnnnnnnnnnnnnnnnnnnnnnnnnnnnnnnnnnnnnnnnnnnnnnnnnnnnnnnnnnnnnnnnnnnnnnnnnnTGCAGTTTTCTTCTCTAACTGTAAACGTAAGGGGTTCAGGAATGAGAATACTTGTGAGAGGCAACT<br>CCCCTGTGTTCAACTATAATAAGGCAACCAAGAGGCTCATAGTCCTTGGAAAGGATGCTGGTGCATTGACAGAAGACCCAGGTGAGGGGACAGCAGGAGTGGAGTCTGCGGTATTGAGAGGGTTCCTAATTCTGGGCAAAGAGGACAAAAGATATGGACCAGCGCTGAGCATCAATGAATTGAGCAATCTTGCGAAAGGGGAGAAGGCTAATGTGTTGATAGGGCAAG<br>GAGACGTGGTGTTGGTGATGAAACGGAAACGGGACTCTAGCATACTTACTGACAGCCAGACAGCGACCAAAAGAATTCGGATGGCCATCAATTA
EPI_ISL_18245789	>A/mute_swan/Poland/MB042-22_22VIR5675-6/2022_H5N1_2022-01-24<br>ATGGAGAGAATAAAAGAGCTAAGAGATTTGATGTCGCAGTCTCGCACTCGCGAGATACTGACAAAAACCACCGTGGACCATATGGCCATAATCAAGAAATATACATCAGGAAGACAGGAGAAGAACCCTGCACTTAGGATGAAGTGGATGATGGCAATGAAATATCCGATTACAGCAGACAAAAGGATAATGGAGATGATCCCTGAAAGAAACGAGCAAGGTCAGACT<br>CTTTGGAGCAAAACAAATGATGCTGGATCGGATAGAATAATGGTGTCACCTCTGGCTGTGACGTGGTGGAATAGAAATGGACCAACAACAAGTACCGTCCATTACCCAAAAGTCTATAAAACCTACTTTGAAAAGGTTGAAAGGTTAAAGCATGGAACCTTCGGCCCTGTCCATTTCCGGAATCAGGTTAAGATACGCCGCAGAGTTGACATAAACCCGGGCCATGCAGAC<br>CTCAGTGCTAAAGAAGCACAAGACGTCATCATGGAGGTCGTTTTCCCAAATGAAGTCGGAGCCAGAATATTGACATCAGAGTCACAGTTGACAATTACAAAAGAAAAGAAGGAGGAACTCCAGGACTGTAAGATTGCCCCTCTAATGGTGGCATACATGTTGGAGAGAGAACTGGTTCGAAAAACCAGATTCCTGCCAGTAGCTGGCGGAACAAGTAGCGTATATATCG<br>AAGTGTTGCACTTGACTCAAGGAACCTGCTGGGAACAAATGTATACGCCAGGAGGAGAAGTGAGAAATGATGACGTTGACCAAAGTTTAATTATTGCTGCCAGAAATATCGTTAGGAGGGCAACAGTATCAGCAGACCCATTGGCTTCACTACTGGAGATGTGCCATAGTACACAGATTGGCGGGATAAGAATGGTAGACATTCTTAGACAGAACCCAACAGAAGAGCA<br>AGCCGTGGATATATGCAAAGCAGCAATGGGTTTAAGAATCAGTTCATCCTTCAGTTTTGGAGGTTTCACTTTCAAAAGGACAAGCGGATCATCTGTCAAAAGAGAAGAGGAAGTGCTTACCGGCAACCTCCAAACATTGAAAATAAGAGTACATGAAGGGTATGAGGAATTCACAATGGTTGGGCGGAGAGCAACAGCCATTCTAAGGAAAGCAACCAGAAGGCTGATC<br>CAATTGATAGTAAGTGGAAGAGACGAGCAGTCAATCGCCGAAGCGATCATAGTGGCAATGGTGTTCTCTCAAGAGGATTGCATGATAAAGGCTGTACGAGGTGATTTAAATTTTGTCAATAGAGCGAATCAGCGGCTCAATCCTATGCATCAGCTCCTGAGGCATTTCCAAAAGGATGCAAAGGTACTATTCCAAAACTGGGGAATTGAACCCATTGACAATGTCATGGGA<br>ATGATAGGAATATTGCCTGATATGACCCCCAGCACAGAGATGTCACTAAGAGGAGTGAGGGTCAGTAAAATGGGAGTGGATGAATATTCCAGTACTGAGAGGGTGGTCGTAAGTATTGATCGCTTCTTGAGGGTACGAGACCAGAGAGGAAATGTACTCTTGTCTCCCGAAGAGGTCAGTGAAACACAGGGAACAGAGAAGCTAACGATAACATATTCATCATCCATGA<br>TGTGGGAAATTAATGGCCCTGAGTCAGTGCTAGTTAACACATATCAATGGATCATCAGAAACTGGGAAACTGTGAAGATTCAGTGGTCCCAAGACCCTACAATGCTATACAACAAGATGGAGTTTGAGCCTTTTCAGTCCTTGGTGCCTAAGGCAGCCAAAGGCCAGTACAGTGGATTTGTAAGGACCCTATTCCAGCAGATGCGTGATGTGCTGGGAACCTTTGACACTGT<br>CCAGATAATAAAGCTACTTCCATTTGCAGCAGCACCACCGGAACAGAGTAGGATGCAGTTCTCTTCTCTAACTGTAAACGTAAGGGGTTCAGGAATGAGAATACTTGTGAGAGGCAACTCCCCTGTGTTCAACTATAATAAAGCAACCAAGAGGCTCATAGTCCTTGGAAAGGATGCTGGTGCATTGACAGAAGACCCAGGTGAGGGGACAGCAGGAGTGGAGTCTGCG<br>GTATTGAGAGGGTTCCTAATTCTGGGCAAAGAGGACAAAAGATATGGACCAGCGCTGAGCATCAATGAATTGAGCAATCTTGCGAAAGGGGAGAAGGCTAATGTGTTGATAGGGCAAGGAGACGTGGTGTTGGTGATGAAACGGAAACGGGACTCTAGCATACTTACTGACAGCCAGACAGCGACCAAAAGAATTCGGATGGCCATCAATTA
EPI_ISL_18245816	>A/mute_swan/Poland/MB122/2022_H5N1_2022-04-07<br>ATGGAGAGAATAAAAGAACTAAGAGATTTGATGTCGCAGTCTCGCACTCGCGAGATACTGACAAAAACCACGGTGGACCATATGGCCATAATTAAGAAATACACATCAGGAAGACAGGAGAAGAACCCTGCCCTTAGGATGAAGTGGATGATGGCAATGAAATACCCGATTACAGCAGACAAAAGAATAATGGAAATGATCCCTGAAAGAAATGAACAGGGCCAGACT<br>CTCTGGAGCAAAACAAGTGATGCTGGATCAGACAGAGTAATGGTGTCACCTCTGGCTGTGACATGGTGGAATAGAAATGGACCGACGACAAGTACAGTCCACTATCCAAAGGTCTACAAAACCTACTTTGAAAAAGTCGAAAGGTTGAAACATGGAACCTTCGGTCCTGTTCACTTTCGGAATCAGGTTAAAATACGCCGCAGGGTTGACATAAACCCGGGCCATGCAGA<br>CCTCAGTGCCAAAGAAGCACAAGATGTCATCATGGAGGTCGTTTTTCCAAACGAAGTTGGAGCAAGGATATTGACATCAGAGTCACAATTAACAATAACAAAGGAAAAGAAGGAGGAGCTTCAGGACTGCAAGATCGCCCCTTTAATGGTGGCATACATGTTGGAGAGAGAACTGGTTCGCAAAACCAGATTCCTACCAGTTGCTGGCGGAACAAGCAGCGTATATATCG<br>AGGTATTGCATTTGACTCAAGGGACCTGCTGGGAACAAATGTACACACCGGGAGGGGAGGTGAGAAATGATGATGTCGATCAGAGTTTGATTATTGCTGCTAGAAACATTGTTAGGAGAGCAACAGTGTCGGCAGACCCGTTGGCTTCGCTCTTGGAGATGTGCCATAGTACACAAATTGGCGGGATAAGAATGGTGGACATCCTTAGACAGAACCCAACAGAAGAGCA<br>AGCTGTGGACATATGCAAAGCAGCAATGGGTTTGAGAATCAGTTCATCCTTCAGCTTTGGAGGTTTCACTTTTAAAAGGACAAGTGGGTCATCTGTCAAAAGAGAAGAAGAAGTGCTCACGGGCAACCTCCAAACACTGAAAATAAGAGTACACGAAGGATATGAAGAATTCACAATGGTTGGGAGAAGAGCTACAGCCATTCTAAGGAAAGCAACCAGAAGGCTGATC<br>CAATTAATAGTGAGTGGGAGAGACGAGCAGTCAATCGCTGAAGCGATTATAGTGGCAATGGTTTTCTCACAAGAGGACTGCATGATAAAAGCAGTGCGAGGTGATTTGAACTTTGTTAACAGAGCAAATCAGCGGCTAAATCCTATGCACCAACTTCTGAGACATTTCCAAAAGGATGCAAAGGTGCTGTTTCAAAATTGGGGAATTGAACCCATTGACAATGTCATGGGA<br>ATGATCGGGATACTGCCTGACATGACCCCCAGCACAGAGATGTCACTGAGAGGAGTGAGGGTCAGTAAAATGGGAGTGGATGAATATTCCAGTACTGAGAGAGTGATCGTAAGCATTGATCGTTTCTTAAGAGTCCGAGACCAGAGGGGAAACGTACTCTTGTCTCCTGAAGAAGTTAGTGAAACACAGGGAACAGAGAAACTGACGATAACATATTCATCGTCTATGAT<br>GTGGGAAATCAATGGTCCGGAGTCAGTACTGGTCAACACATATCAATGGATCATTAGAAATTGGGAAACTGTGAAGATTCAGTGGTCCCAAGACCCCACAATGTTGTACAATAAGATGGAGTTTGAGCCCTTCCAATCCTTGGTGCCCAAGGCTGCCAGAGGCCAGTACAGTGGATTTGTGAGGACGTTATTCCAGCAGATGCGTGATGTGCTGGGGACATTTGACACCGT<br>CCAAATAATAAAGCTCCTACCATTTGCAGCAGCCCCACCGGAACAGAGTAGGATGCAATTCTCTTCTCTGACTGTGAATGTAAGAGGTTCAGGAATGAGAATACTTGTGAGAGGCAACTCCCCTGTGTTCAACTATAACAAAGCAACCAAGAGACTCACAGTTCTTGGAAAGGATGCAGGTGCATTGACAGAAGATCCAGACGAGGGAACAGCAGGAGTGGAGTCTGCGG<br>TGTTGAGAGGATTCCTAATTCTGGGCAAAGAAGACAAAAGATATGGACCAGCATTGAGCATTAACGAATTGAGCAATCTTGCGAAAGGGGAGAAGGCTAATGTGTTGATAGGGCAAGGAGACGTGGTGTTGGTAATGAAACGGAAACGGGACTCTAGCATACTTACTGACAGCCAGACAGCGACCAAAAGGATTCGGATGGCCATCAATTA
EPI_ISL_2111625	>A/mute_swan/Poland/MB131/2021_H5N8_2021-02-19<br>ATGGAGAGAATAAAAGAACTAAGAGATTTGATGTCGCAGTCTCGCACTCGCGAGATACTAACAAAAACCACTGTGGACCATATGGCCATAATAAAGAAATACACATCAGGAAGACAGGAGAAGAACCCTGCCCTCAGGATGAAATGGATGATGGCAATGAAATATCCTATTACAGCTGACAAAAGAATAATGGAGATGATCCCTGAAAGGAATGAGCAAGGTCAGACT<br>CTCTGGAGCAAAACAAATGATGCTGGATCAGACAGAGTGATGGTCTCACCCCTGGCTGTGACATGGTGGAATAGAAATGGCCCAACAACAAGTACAGTACACTACCCAAAAGTCTACAAAACCTACTTTGAAAAGGTAGAAAGGTTGAAACATGGAACCTTTGGTCCTGTTCACTTTCGAAATCAGGTTAAGATACGCCGCAGGGTTGACATAAACCCGGGCCATGCAGA<br>TCTCAGTGCCAAAGAAGCACAGGATGTCATCATGGAGGTTGTTTTCCCAAATGAAGTTGGAGCCAGGATCTTGACATCAGAATCACAATTAACAATAACAAGGGAAAAGAAGGAGGAACTTCAGGATTGCAAGATTGCTCCTTTGATGGTGGCATACATGTTGGAAAGAGAACTGGTTCGCAAGACCAGATTTTTACCAGTAGCTGGCGGAACAAGCAGCGTATACATCG<br>AGGTATTGCATTTGACTCAAGGGACCTGCTGGGAACAAATGTACACACCAGGAGGGGAGGTGAGAAATGATGATGTTGATCAGAGTTTGATCATTGCTGCTAGAAATATAGTTAGGAGGGCAACAGTATCAGCAGACCCATTGGCTTCGCTCTTGGAAATGTGCCACAGTACACAAATTGGTGGAGTAAGGATGGTGGACATTCTTAGGCAGAACCCAACAGAGGAGCA<br>AGCTGTGGATATATGCAAAGCAGCAATGGGTTTAAGAATCAGTTCATCCTTCAGCTTTGGAGGTTTCACTTTCAAAAGGACAAGTGGGTCGTCTGTCAAAAGAGAAGAAGAAATACTCACTGGCAACCTCCAAACACTGAAAGTAAGAATACATGAAGGATATGAGGAATTCACAATGGTTGGGCGAAGAGCTACAGCCATTTTGAGGAAAGCAACCAGGAGACTGATCC<br>AATTAATAGTGAGTGGAAGAGACGAGCAGTCAATCGCTGAAGCAATCATAGTGGCAATGGTTTTCTCACAGGAGGATTGCATGATAAAAGCAGTACGAGGTGATTTGAATTTTGTCAACAGAGCGAATCAGCGGCTAAATCCTATGCATCAACTTCTGAGGCATTTCCAAAAGGATGCAAAGTGCTGTTTCAAACTGGGGGATTGAACCAATTGACAATGTAATGGGG<br>ATGATCGGGATACTGCCTGACATGACCCCCAGCACAGAGATGTCACTGAGAGGAGTGAGAGTCAGCAAAATGGGAGTGGATGAATATTCCAGTACTGAGAGAGTGGTCGTGAGCATTGATCGCTTCTTGAGAGTCCGAGATCAGAGGGGAAATGTGCTTCTGTCTCCTGAGGAAGTTAGTGAAACACATGGAACAGAGAAACTGACGATAACGTATTCATCGTCTATGA<br>TGTGGGAAATCAATGGTCCGGAATCCGTGCTAGTCAACACATATCAATGGATCATTAGAAGTTGGGAAACTGTGAAGATTCAATGGTCCCAGGACCCTACGATGTTGTACAATAAGATGGAATTTGAGCCCTTCCAATCCTTGGTGCCCAAGGCTGCTAGAGGCCAGTATAGTGGGTTTGTGAGGACATTATTCCAACAGATGCGTGATGTGTTGGGGACATTTGACACTGT<br>CCAAATAATAAAGCTCCTACCATTTGCAGCAGCCCCACCGGAACAGAGTAGGATGCAATTTTCCTCTCTGACTGTGAACGTAAGAGGTTCAGGAATGAGAATACTTGTGAGGGGCAACTCCCCTGTGTTCAACTATAATAAGGCCACCAAGAGACTCACAGTTCTTGGAAAGGATGCAGGCGCCTTGACAGAATATCCAGATGAGGGAACAGCAGGAGTGGAGTCTGCAG<br>TATTAAGAGGATTTCTAATTCTGGGCAAAGAGGACAAAAGATATGGACCAGCATTGAGCATCAACGAATTGAGCAATCTTGCGAAAGGGGAAAAGGCTAATGTGTTGATAGGACAAGGAGACGTGGTGTTGGTAATGAAACGGAAACGGGACTCTAGCATACTTACTGACAGCCAGACAGCGACCAAAAGAATTCGGATGGCCATCAATTA
EPI_ISL_2113167	>A/mute_swan/Poland/MB189/2021_H5N8_2021-02-26<br>ATGGAGAGAATAAAAGAACTAAGAGATTTGATGTCGCAGTCTCGCACTCGCGAGATACTAACAAAAACCACTGTGGACCATATGGCCATAATAAAGAAATACACATCAGGGAGACAGGAGAAGAACCCTGCCCTCAGGATGAAATGGATGATGGCAATGAAATATCCTATTACAGCTGACAAAAGAATAATGGAGATGATCCCTGAGAGGAATGAGCAAGGTCAGACT<br>CTCTGGAGCAAAACAAATGATGCTGGATCAGACAGAGTGATGGTCTCACCTCTGGCTGTGACATGGTGGAATAGAAATGGGCCAACAACAAGTACAGTACACTACCCAAAAGTCTACAAAACCTACTTTGAAAAGGTAGAAAGGTTGAAACATGGAACCTTTGGTCCTGTTCACTTTCGAAATCAGGTTAAGATACGCCGCAGGGTTGACATAAACCCGGGCCATGCAGA<br>TCTCAGTGTCAAAGAAGCACAGGATGTCATCATGGAGGTTGTTTTCCCAAATGAAGTTGGAGCCAGGATCTTGACATCAGAATCACAATTAACAATAACAATGGAAAAGAAGGAGGAACTTCAGGATTGCAAGATTGCTCCTTTGATGGTGGCATACATGTTGGAAAGAGAACTGGTTCGCAAGACCAGATTTTTACCAGTAGCTGGCGGGACAAGCAGCGTATACATCG<br>AGGTATTGCATTTGACTCAAGGGACCTGCTGGGAACAAATGTACACACCAGGAGGGGAGGTGAGAAATGATGATGTTGATCAGAGTTTGATCATTGCTGCTAGAAATATAGTTAGGAGGGCAACAGTATCAGCAGACCCACTGGCTTCGCTCTTGGAAATGTGCCACAGTACACAAATTGGTGGAGTGAGGATGGTGGACATTCTTAGGCAGAACCCAACAGAGGAGCA<br>AGCTGTGGATATATGCAAAGCAGCAATGGGTTTAAGAATCAGTTCATCCTTCAGCTTTGGAGGTTTCACTTTCAAAAGGACAAGTGGGTCGTCTGTCAAAAGAGAAGAAGAAATACTCACTGGCAACCTCCAAACACTGAAAGTAAGAATACATGAAGGATATGAGGAATTCACAATGGTTGGGCGAAGAGCTACAGCCATTTTGAGGAAAGCAACCAGGAGACTGATCC<br>AATTAATAGTGAGTGGACGAGACGAACAGTCAATCGCTGAAGCAATCATAGTGGCAATGGTTTTCTCACAGGAGGATTGCATGATAAAAGCAGTACGAGGTGATTTGAATTTTGTCAACAGAGCGAATCAGCGGCTAAATCCTATGCATCAACTTCTGAGGCATTTCCAAAAGGATGCAAAAGTGCTGTTTCAAACTGGGGGATTGAACCAATTGACAATGTAATGGGG<br>ATGATCGGGATACTGCCTGACATGACCCCCAGCACAGAGATGTCACTGAGAGGAGTGAGAGTCAGCAAAATGGGAGTGGATGAATATTCCAGTACTGAGAGAGTGGTCGTGAGCATTGATCGCTTCTTGAGAGTCCGAGATCAGAGGGGAAATGTGCTTCTGTCTCCTGAGGAAGTTAGTGAAACACATGGAACAGAGAAACTGACGATAACGTATTCATCGTCTATGA<br>TGTGGGAAATCAATGGTCCGGAATCCGTGCTAGTCAACACATATCAATGGATCATTAGAAATTGGGAAACTGTGAAGATTCAGTGGTCCCAGGACCCTACGATGTTGTACAATAAGATGGAATTTGAGCCCTTCCAATCCTTGGTGCCCAAGGCTGCTAGAGGCCAGTATAGTGGGTTTGTGAGGACATTATTCCAACAGATGCGTGATGTGTTGGGGACATTTGACACTGT<br>CCAAATAATAAAGCTCCTACCATTTGCAGCAGCCCCACCGGAACTGAGTAGGATGCAATTTTCCTCTCTGACTGTGAACGTAAGAGGTTCAGGAATGAGAATACTTGTGAGGGGCAATTCCCCTGTGTTCAACTATAATAAGACCACCAAGAGACTCACAGTTCTTGGAAAGGATGCAGGCGCCTTGACAGAATATCCAGATGAGGGAACAGCAGGAGTGGAGTCTGCAG<br>TATTAAGAGGATTTCTAATTCTGGGCAAAGAGGACAAAAGATATGGACCAGCATTGAGCATCAACGAATTGAGCAATCTTGCGAAAGGGGAAAAGGCTAATGTGTTGATAGGACAAGGAGACGTGGTGTTGGTAATGAAACGGAAACGGGACTCTAGCATACTTACTGACAGCCAGACAGCGACCAAAAGAATTCGGATGGCCATCAATTA
EPI_ISL_2113493	>A/mute_swan/Poland/MB268/2021_H5N8_2021-03-09<br>ATGGAGAGAATAAAAGAACTAAGAGATTTGATGTCACAGTCTCGCACTCGCGAGATACTAACAAAAACCACTGTGGACCATATGGCCATAATAAAGAAATACACATCAGGGAGACAGGAGAAGAACCCTGCCCTCAGGATGAAATGGATGATGGCAATGAAATATCCTATTACAGCTGACAAAAGAATAATGGAGATGATCCCTGAAAGGAATGAGCAAGGTCAGACT<br>CTCTGGAGCAGAACAAATGATGCTGGATCAGACAGAGTGATGGTCTCACCTCTGGCTGTGACATGGTGGAATAGAAATGGGCCAACAACAAGTACAGTACACTACCCAAAAGTCTACAAAACCTACTTTGAAAAGGTAGAAAGGTTGAAACATGGAACCTTTGGTCCTGTTCACTTTCGAAATCAGGTTAAGATACGCCGCAGGGTTGACATAAACCCGGGCCATGCAGA<br>TCTCAGTGCCAAAGAAGCACAGGATGTCATCATGGAGGTTGTTTTCCCAAATGAAGTTGGAGCCAGGATCTTGACATCAGAATCACAATTAACAATAACAATGGAAAAGAAGGAGGAACTTCAGGATTGCAAGATTGCTCCTTTGATGGTGGCATACATGTTGGAAAGAGAACTGGTTCGCAAGACCAGATTTTTACCAGTAGCTGGCGGAACAAGCAGCGTATACATCG<br>AGGTATTGCATTTGACTCAAGGGACCTGCTGGGAACAAATGTACACACCAGGAGGGGAGGTGAGAAATGATGATGTTGATCAGAGTTTGATCATTGCTGCTAGAAATATAGTTAGGAGGGCAACAGTATCAGCAGACCCATTGGCTTCGCTCTTGGAAATGTGCCACAGTACACAAATTGGTGGAGTGAGGATGGTGGACATTCTTAGGCAGAACCCAACAGAGGAGCA<br>AGCTGTGGATATATGCAAAGCAGCAATGGGTTTAAGAATCAGTTCATCCTTCAGCTTTGGAGGTTTCACTTTCAAAAGGACAAGTGGGTCGTCTGTCAAAAGAGAAGAAGAAATACTCACTGGCAACCTCCAAACACTGAAAGTAAGAATACATGAAGGATATGAGGAATTCACAATGGTTGGGCGAAGAGCTACAGCCATTTTGAGGAAAGCAACCAGGAGACTGATCC<br>AATTAATAGTGAGTGGACGAGACGAACAGTCAATCGCTGAAGCAATCATAGTGGCAATGGTTTTCTCACAGGAGGATTGCATGATAAAAGCAGTACGAGGTGATTTGAATTTTGTCAACAGAGCGAATCAGCGGCTAAATCCTATGCATCAACTTCTGAGGCATTTCCAAAAAGATGCAAAAGTGCTGTTTCAAACTGGGGGATTGAACCAATTGACAATGTAATGGGGA<br>TGATCGGGATACTGCCTGACATGACCCCCAGCACAGAGATGTCACTGAGAGGAGTGAGAGTCAGCAAAATGGGAGTGGATGAATATTCCAGTACTGAGAGAGTGGTCGTGAGCATTGATCGCTTCTTGAGAGTCCGAGATCAGAGGGGAAATGTGCTTCTGTCTCCTGAGGAAGTTAGTGAAACACATGGAACAGAGAAACTGACGATAACGTATTCATCGTCTATGAT<br>GTGGGAAATCAATGGTCCGGAATCCGTGCTAGTCAACACATATCAATGGATCATTAGAAATTGGGAAACTGTGAAGATTCAGTGGTCCCAGGACCCTACGATGTTGTACAATAAGATGGAATTTGAGCCCTTCCAATCCTTGGTGCCCAAGGCTGCTAGAGGCCAGTATAGTGGGTTTGTGAGGACATTATTCCAACAGATGCGTGATGTGTTGGGGACATTTGACACTGTC<br>CAAATAATAAAGCTCCTACCATTTGCAGCAGCCCCACCGGAACAGAGTAGGATGCAATTTTCCTCTCTGACTGTGAACGTAAGAGGTTCAGGAATGAGAATACTTGTGAGGGGCAACTCCCCTGTGTTCAACTATAATAAGACCACCAAGAGGCTCACAGTTCTTGGAAAGGATGCAGGCGCCTTGACAGAATATCCAGATGAGGGAACAGCAGGAGTGGAGTCTGCAGT<br>ATTAAGAGGATTTCTAATTCTGGGCAAAGAGGACAAAAGATATGGACCAGCATTGAGCATCAACGAATTGAGCAATCTTGCGAAAGGGGAAAAGGCTAATGTGTTGATAGGACAAGGAGACGTGGTGTTGGTAATGAAACGGAAACGGGACTCTAGCATACTTACTGACAGCCAGACAGCGACCAAAAGAATTCGGATGGCCATCAATTA
EPI_ISL_2114013	>A/mute_swan/Poland/MB272/2021_H5N8_2021-03-09<br>ATGGAGAGAATAAAAGAACTAAGAGATTTGATGTCGCAGTCTCGCACTCGCGAGATACTAACAAAAACCACTGTGGACCATATGGCCATAATAAAGAAATACACATCAGGAAGACAGGAGAAGAACCCTGCCCTCAGGATGAAATGGATGATGGCAATGAAATATCCTATTACAGCTGACAAAAGAATAATGGAGATGATCCCTGAAAGGAATGAGCAAGGTCAGACT<br>CTCTGGAGCAAAACAAATGATGCTGGATCAGACAGAGTGATGGTCTCACCTCTGGCTGTGACATGGTGGAATAGAAATGGGCCAACAACAAGTACAGTACACTACCCAAAAGTCTACAAAACCTACTTTGAAAAGGTAGAAAGGTTGAAACATGGAACCTTTGGTCCTGTTCACTTTCGAAATCAGGTTAAGATACGCCGCAGGGTTGACATAAACCCGGGCCATGCAGA<br>TCTCAGTGCCAAAGAAGCACAGGATGTCATCATGGAGGTTGTTTTCCCAAATGAAGTTGGAGCCAGGATCTTGACATCAGAATCACAATTAACAATAACAAGGGAAAAGAAGGAGGAACTTCAGGATTGCAAGATTGCTCCTTTGATGGTGGCATACATGTTGGAAAGAGAACTGGTTCGCAAGACCAGATTTTTACCAGTAGCTGGCGGAACAAGCAGCGTATACATCG<br>AGGTATTGCATTTGACTCAAGGGACCTGCTGGGAACAAATGTACACACCAGGAGGGGAGGTGAGAAATGATGATGTTGATCAGAGTTTGATCATTGCTGCTAGAAATATAGTTAGGAGGGCAACAGTATCAGCAGACCCATTGGCTTCGCTTTTGGAAATGTGCCACAGTACACAAATTGGTGGAGTAAGGATGGTGGACATTCTTAGGCAGAACCCAACAGAGGAGCA<br>AGCTGTGGATATATGCAAAGCAGCAATGGGTTTAAGAATCAGTTCATCCTTCAGCTTTGGAGGTTTCACTTTCAAAAGGACAAGTGGGTCGTCTGTCAAAAGAGAAGAAGAAATACTCACTGGCAACCTCCAAACACTGAAAGTAAGAATACATGAAGGATATGAGGAATTCACAATGGTTGGGCGAAGAGCTACAGCCATTTTGAGGAAAGCAACCAGGAGACTGATCC<br>AATTAATAGTGAGTGGAAGAGACGAGCAGTCAATCGCTGAAGCAATCATAGTGGCAATGGTTTTCTCACAGGAGGATTGCATGATAAAAGCAGTACGAGGTGATTTGAATTTTGTCAACAGAGCGAATCAGCGGCTAAATCCTATGCATCAACTTCTGAGACATTTCCAAAAGGATGCAAAGTGCTGTTTCAAACTGGGGGATTGAACCAATTGACAATGTAATGGGG<br>ATGATCGGGATACTGCCTGACATGACCCCCAGCACAGAGATGTCACTGAGAGGAGTGAGAGTCAGCAAAATGGGAGTGGATGAATATTCCAGTACTGAGAGAGTGGTCGTGAGCATTGATCGCTTCTTGAGAGTCCGAGATCAGAGGGGAAATGTGCTTCTGTCTCCTGAGGAAGTTAGTGAAACACATGGAACAGAGAAACTGACGATAACGTATTCATCGTCTATGA<br>TGTGGGAAATCAATGGACCGGAATCCGTGCTAGTCAACACATATCAATGGATCATTAGAAATTGGGAAACTGTGAAGATTCAGTGGTCCCAGGACCCTACGATGTTATACAATAAGATGGAATTTGAGCCCTTCCAATCCTTGGTGCCCAAGGCTGCTAGAGGCCAGTATAGTGGGTTTGTGAGGACATTATTCCAACAGATGCGTGATGTGTTGGGAACATTTGACACTGT<br>CCAAATAATAAAGCTCCTACCATTTGCAGCAGCCCCACCGGAACAGAGTAGGATGCAATTTTCCTCTCTGACTGTGAACGTAAGAGGTTCAGGAATGAGAATACTTGTGAGGGGCAACTCCCCTGTGTTCAACTATAATAAGGCCACCAAGAGACTCACAGTTCTTGGAAAGGATGCAGGCGCCTTGACAGAATATCCAGATGAGGGAACAGCAGGAGTGGAGTCTGCAG<br>TATTAAGAGGATTTCTAATTCTGGGCAAAGAGGACAAAAGATATGGACCAGCATTGAGCATCAACGAATTGAGCAATCTTGCGAAAGGGGAAAAGGCTAATGTGTTGATAGGACAAGGAGACGTGGTGTTGGTAATGAAACGGAAACGGGACTCTAGCATACTTACTGACAGCCAGACAGCGACCAAAAGAATTCGGATGGCCATCAATTA

EPI_ISL_18245531	>A/mute_swan/Poland/MB306-21_22VIR5675-20/2021_H5N8_2021-03-16<br>ATGGAGAGAATAAAAGAACTAAGAGATTTGATGTCGCAGTCTCGCACTCGCGAGATACTAACAAAAACCACTGTGGACCATATGGCCAT [illegible]<br>CTCTGGAGCAAAACAAATGATGCTGGATCAGACAGAGTGATGGTCTCACCTCTGGCTGTGACATGGTGGAATAGAAATGGGCCAACAA [illegible]<br>TCTCAGTGTCAAAGAAGCACAGGATGTCATCATGGAGGTTGTTTTCCCAAATGAAGTTGGAGCCAGGATCTTGACATCAGAATCACAAT [illegible]<br>AGGTATTGCATTTGACTCAAGGGACCTGCTGGGAACAAATGTACACACCAGGAGGGGAGGTGAGAAATGATGATGTTGATCAGAGTTT [illegible]<br>AGCTGTGGATATATGCAAAGCAGCAATGGGTTTAAGAATCAGTTCATCCTTCAGCTTTGGAGGTTTCACTTTCAAAAGGACAAGTGGGT [illegible]<br>AATTAATAGTGAGTGGACGAGACGAACAGTCAATCGCTGAAGCAATCATAGTGGCAATGGTTTTCTCACAGGAGGATTGCATGATAAAA [illegible]<br>ATGATCGGGATACTGCCTGACATGACCCCCAGCACAGAGATGTCACTGAGAGGAGTGAGAGTCAGCAAAATGGGAGTGGATGAATATT [illegible]<br>TGTGGGAAATCAATGGTCCGGAATCCGTGCTAGTCAACACATATCAATGGATCATTAGAAATTGGGAAACTGTGAAGATTCAGTGGTCC [illegible]<br>CCAAATAATAAAGCTCCTACCATTTGCAGCAGCCCCACCGGAACAGAGTAGGATGCAATTTTCCTCTCTGACTGTGAACGTAAGAGGTT [illegible]<br>TATTAAGAGGATTTCTAATTCTGGGCAAAGAGGACAAAAGATATGGACCAGCATTGAGCATCAACGAATTGAGCAATCTTGCGAAAGG [illegible]
EPI_ISL_18245535	>A/mute_swan/Poland/MB372-21_22VIR5675-19/2021_H5N8_2021-04-07<br>ATGGAGAGAATAAAAGAACTAAGAGATTTGATGTCGCAGTCCCGCACTCGCGAGATACTAACAAAAACCACTGTGGACCATATGGCCAT [illegible]
EPI_ISL_3102078	>A/mute_swan/Poland/MB396_21RS1385-19/2021_H5N8_2021-04-21<br>[illegible]
EPI_ISL_6937114	>A/mute_swan/Poland/MB490-L1/2021_H5N1_2021-11-08<br>[illegible]
EPI_ISL_18245814	>A/mute_swan/Poland/MB550/2021_H5N1_2021-12-23<br>[illegible]
EPI_ISL_18245815	>A/mute_swan/Poland/MB551/2021_H5N1_2021-12-23<br>[illegible]
EPI_ISL_17978927	>A/mute_swan/Poland/P071/2020_H9N2_2020-09-18<br>[illegible]
EPI_ISL_1139164	>A/northern_goshawk/Netherlands/20018560-002/2020_H5N8_2020-11-22<br>[illegible]
EPI_ISL_1139047	>A/northern_lapwing/Netherlands/20017480-001/2020_H5N8_2020-11-06<br>[illegible]
EPI_ISL_3128524	>A/partridge/Kosovo/96-21VIR5162-14/2021_H5N8_2021-05-31<br>[illegible]
EPI_ISL_668456	>A/Peregrine_falcon/Sweden/SVA201117SZ0467/20KN003345/2020_H5N8_2020-11-10<br>[illegible]
EPI_ISL_4031467	>A/pheasant/Denmark/12106-03/2018_H5N6_2018-08-27<br>[illegible]

EPI_ISL_400288	>A/Shoveler/Omsk_Region/111/2019_H1N2_2019-09-01<br>ATGGAGAGAATAAAAGAACTAAGAGATTTGATGTCGCAGTCTCGCACTCGCGAGATACTGACAAAAACCACGGTGGACCATATGGCCATAATTAAGAAATACACATCAGGAAGACAGGAGAAGAACCCTGCCCTTAGGATGAAGTGGATGATGGCAATGAAATACCCGATTACAGCAGACAAAAGAATAATGGAAATGATCCCTGAAAGAAATGAACAAGGCCAGACT<br>CTCTGGAGCAAAACAAGTGATGCTGGATCAGACAGAGTAATGGTGTCACCTCTGGCTGTGACATGGTGGAATAGAAATGGACCGACGACAAGTACAGTCCACTATCCAAAGGTCTACAAAACCTACTTTGAAAAAGTCGAGAGGTTGAAACATGGAACCTTCGGTCCTGTTCACTTTCGGAATCAGGTTAAAATACGCCGCAGGGTTGACATAAACCCGGGCCATGCAGA<br>TCTCAGTGCCAAAGAAGCACAAGATGTCATCATGGAGGTCGTTTTCCCAAATGAAGTTGGAGCAAGGATATTGACATCAGAGTCACAATTAACAATAACAAAGGAAAAGAAGGAGGAGCTTCAGGACTGCAAGATCGCCCCTTTAATGGTGGCATACATGTTGGAGAGAGAACTGGTTCGCAAAACCAGATTCCTACCAGTAGCTGGCGGAACAAGCAGCGTATATATC<br>GAGGTATTGCATTTGACTCAAGGGACCTGCTGGGAACAAATGTACACACCGGGAGGGGAGGTGAGAAATGATGATGTCGATCAGAGTTTGATCATTGCTGCTAGAAATATTGTTAGGAGAGCAACAGTATCGGCAGACCCGTTGGCTTCGCTCTTGGAGATGTGCCATAGTACACAAATTGGCGGGATAAGAATGGTGGACATCCTTAGACAGAACCCAACAGAAGAGC<br>AAGCTGTGGATATATGCAAAGCAGCAATGGGTTTGAGAATCAGTTCATCCTTCAGCTTTGGAGGTTTCACTTTTAAAAGGACAAGTGGGTCATCTGTCAAAAGAGAAGAAGAAGTGCTCACGGGCAACCTCCAAACACTGAAAATAAGAGTACATGAAGGATATGAAGAATTCACAATGGTTGGGCGAAGAGCTACAGCCATTCTAAGGAAAGCAACCAGAAGGCTGAT<br>CCAATTAATAGTGAGTGGGAGAGACGAGCAGTCAATCGCTGAAGCGATCATAGTGGCAATGGTTTTCTCACAAGAGGATTGCATGATAAAAGCAGTGCGAGGTGATTTGAACTTTGTTAACAGAGCAAATCAGCGGCTAAATCCTATGCACCAACTTCTGAGACATTTCCAAAAGGATGCAAAAGTGCTGTTTCAAAATTGGGGAATTGAACCCATTGACAATGTCATGGG<br>AATGATCGGAATACTGCCTGACATGACCCCCAGCACAGAGATGTCACTGAGAGGAGTGAGGGTCAGTAAAATGGGAGTGGATGAATATTCCAGTACTGAGAGAGTGGTCGTAAGCATTGATCGTTTCTTAAGAGTCCGAGACCAGAGGGGAAACGTACTCTTGTCTCCTGAAGAAGTTAGTGAAACACAGGGAACAGAGAAACTGACGATAACATATTCATCGTCTATG<br>ATGTGGGAAATCAATGGTCCGGAGTCAGTACTGGTCAACACATATCAATGGATCATTAGAAATTGGGAAACTGTGAAGATTCAGTGGTCCCAAGACCCTACAATGTTGTACAATAAGATGGAGTTTGAGCCTTTCCAATCCTTGGTGCCCAAGGCTGCCAGAGGCCAGTACAGTGGATTTGTGAGGACGTTATTCCAGCAGATGCGTGATGTGCTGGGGACATTTGACACT<br>GTCCAAATAATAAAGCTCCTACCATTTGCAGCAGCCCCACCGGAACAGAGTAGGATGCAGTTCTCTTCTCTGACTGTGAATGTAAGAGGTTCAGGAATGAGAATACTTGTGAGAGGCAACTCCCCTGTGTTCAACTATAACAAAGCAACCAAGAGACTCACAGTTCTTGGAAAGGATGCAGGTGCATTGACAGAAGATCCAGACGAGGGAACAGCAGGAGTGGAGTCTGC<br>GGTATTAAGAGGATTTCTAATTCTGGGCAAAGAAGACAAAAGATATGGACCAGCATTGAGCATTAACGAATTGAGCAATCTTGCGAAAGGGGAGAAGGCTAATGTGTTGATAGGGCAAGGAGACGTGGTGTTGGTAATGAAACGGAAACGGGACTCTAGCATACTTACTGACAGCCAGACAGCGACCAAAAGGATTCGGATGGCCATCAATTA
EPI_ISL_277382	>A/swan/East_China/SD588/2015_H13N8_2015-12-26<br>ATGGAGAGAATAAAAGAACTAAGAGATCTAATGTCACAGTCTCGCACTCGCGAGATACTCACCAAAACCACTGTGGACCACATGGCCATAATCAAAAAATACACATCGGGAAGGCAAGAGAAGAACCCCGCACTAAGGATGAAATGGATGATGGCAATGAAATATCCAATCACCGCGGATAAGAGAGTAATGGAAATGATTCCTGAAAGGAATGAGCAGGGACAAACC<br>CTTTGGAGCAAAACAAACGATGCCGGATCGGACCGAGTAATGGTATCGCCTCTCGCTGTGACGTGGTGGAACAGGAATGGTCCAACAGCAAGTACAGTCCACTACCCAAAGGTATACAAGACTTATTTTGAAAAAGTTGAGAGGTTGAAACACGGGACCTTTGGCCCTGTCCACTTCAGAAATCAAGTTAAGATAAGACGGAGGGTCGATGTAAACCCAGGCCATGCAG<br>ACCTAAGTGCCAAAGAGGCACAGGATGTAATCATGGAAGTTGTCTTCCCAAATGAGGTGGGAGCAAGAATACTGACGTCAGAGTCACAATTGACGGTAACAAAGGAGAAAAAAGAAGAACTCCAGGACTGCAAAATTGCCCCTTTGATGGTTGCATACATGCTAGAGAGAGAATTGGTCCGCAAAACGAGGTTCCTCCCAGTGGCTGGTGGAACAAGCAGTGTCTATAT<br>TGAGGTGCTGCACTTAACCCAGGGGACATGCTGGGAGCAGATGTACACTCCAGGAGGGGAGGTGAGGAATGATGATGTAGACCAGAGCTTGATTATTGCTGCCAGGAACATAGTGAGAAGAGCAACAGTATCAGCAGACCCACTAGCATCCCTACTGGAAATGTGCCACAGTACACAGATTGGAGGGATAAGGATGGTTGACATCCTTCGACAAAATCCAACAGAAGA<br>ACAAGCCGTGGACATATGCAAGGCAGCAATGGGATTGAGGATTAGCTCATCTTTCAGCTTTGGTGGATTCACTTTTAAAAGAACAAGTGGATCCTCAGTGAAAAGAGAAGAAGAAGTGCTTACAGGCAACCTTCAAACATTGAAAATAAGAGTGCATGAGGGGTATGAAGAATTCACAATGGTAGGAAGACGAGCAACTGCTATTATCAGGAAAGCAACCAGAAGGCTG<br>ATCCAGCTAATAGTAAGTGGGAGAGACGAGCAGTCAATTGCTGAAGCAATAATTGTGGCCATGGTGTTCTCACAAGAGGACTGCATGATCAAGGCAGTTCGAGGTGATCTGAATTTTGTTAATAGGGCGAACCAGAGGCTGAACCCAATGCATCAACTCCTGAGACACTTTCAAAAAGATGCAAAAGTGCTCTTCCAAAACTGGGGAATTGAACCCATTGACAGTGTGAT<br>GGGGATGGTTGGGATATTGCCCGACATGACCCCAAGTACTGAGATGTCGCTGAGGGGGATAAGAGTCAGCAAGACGGGAGTAGATGAATACTCCAGCACAGAAAGGGTCGTAGTGAGCATCGACCGATTTTTAAGAGTTCGAGACCAACGAGGGAACATACTATTGTCACCTGAAGAAGTCAGCGAGACACAGGGAACAGAGAAATTGACAATCACTTATTCGTCATC<br>AATGATGTGGGAGATCAATGGTCCTGAGTCAGTATTGGTCAATACTTATCAATGGATCATCAGAAACTGGGAGACTGTGAAAATTCAATGGTCACAGGATCCCACAATGTTATATAATAAGATGGAATTCGAACCATTCCAGTCCCTTGTCCCTAAAGCAGCTAGGGGTCAATACAGTGGATTCGTGAGGACATTATTCCAGCAAATGCGGGATGTGCTTGGAACATTTGAC<br>ACTGTTCAGATAATAAAACTCCTCCCATTTGCTGCTGCCCCACCAGAACAAAGTAAGATGCAGTTCTCCTCCATGACTGTAAATGTAAGAGGATCAGGGATGAGAATACTGGTAAGAGGCAACTCCCCAGTGTTCAATTACAATAAGTCCACCAAGAGGCTCACTATTCTCGGGAAAGATGCAGGTGCATTGACCGAAGATCCAGATGAAGGCACAGCTGGAATAGAGTCT<br>GCTGTTTTAAGAGGATTCCTCATTTTGGGCAAAGAAGACAAGAGATATGGACCGGCATTGAGCATCAATGAGCTGAGCAATCTTGCAAAGGGAGAGAAGGCTAATGTGCTAATTGGGCAAGGAGACGTAGTGTTGGTAATGAAACGGAAACGGGACTCTAGCATACTTACTGACAGTCAGACAGCGACCAAAAGAATTCGGATGGCCATCAATTA
EPI_ISL_10434072	>A/swan/Germany-BW/AI00996/2022_H5N2_2022-01-29<br>ATGGAGAGAATAAAAGAACTAAGAGATCTGATGTCGCAGTCTCGCACTCGCGAGATACTGACAAAAACCACTGTGGACCATATGGCCATAATAAAAAAGTACACGTCAGGAAGACAGGAGAAGAATCCTGCTCTTAGGATGAAATGGATGATGGCAATGAAATATCCGATTACAGCAGACAAAAGGATAATGGAGATGATCCCTGAAAGAAATGAGCAGGGTCAGACT<br>CTCTGGAGCAAAACAAATGATGCTGGATCAGACAGAGTGATGGTGTCACCTCTGGCAGTGACATGGTGGAATAGAAATGGGCCAACGACAAGTACAGTCCATTATCCAAAGGTCTACAAAACCTACTTTGAAAAGGTTGAAAGGTTGAAACATGGAACCTTCGGTCCTGTTCACTTTCGAAATCAGGTTAAAATACGCCGCAGGGTTGACATAAACCCCGGCCATGCAGA<br>TCTCAGTGCTAAAGAAGCACAAGATGTCATCATGGAGGTCGTTTTCCCAAACGAAGTTGGAGCCAGGATACTGACATCAGAGTCACAATTAACAATAACAAAAGAAAAGAAGGAGGAACTTCAGAACTGTAAGATTGCTCCTTTGATGGTGGCATACATGTTGGAGAGAGAACTGGTTCGCAAAACCAGATTCCTGCCCGTAGCTGGCGGGACAAGCAGCGTGTACATCG<br>AGGTGTTGCATTTGACTCAAGGGACCTGCTGGGAACAAATGTACACACCGGGAGGGGAGGTGAGAAATGATGATGTTGATCAAAGTTTGATCATTGCTGCTAGAAATATTGTTAGAAGAGCAACAGTATCAGCAGACCCGTTGGCTTCGCTTTTGGAAATGTGCCATAGTACACAAATTGGCGGGATAAGGATGGTGGACATTCTTAGACAGAACCCAACAGAAGAGCAA<br>GCTGTGGATATATGCAAAGCAGCAATGGGTCTAAGAATCAGTTCATCCTTCAGCTTTGGAGGGTTCACCTTCAAACGGACAAGTGGGTCATCTGTCAAAAGAGAAGAAGAAGTGCTCACAGGCAACCTCCAAACACTGAAAATAAGAGTACATGAAGGGTACGAGGAATTCACAATGGTTGGGCGAAGAGCGACAGCTATTCTAAGGAAAGCAACCAGAAGGCTAATCC<br>AATTAATAGTGAGTGGGAGAGACGAGCAGTCAATCGCTGAAGCAATCATAGTAGCAATGGTTTTCTCACAAGAGGATTGCATGATAAAAGCAGTACGAGGTGATTTGAATTTTGTTAACAGAGCAAATCAGCGACTGAATCCTATGCATCAACTTCTGAGGCATTTCCAAAAGGACGCAAAGGTGCTGTTTCAAAATTGGGGAATTGAACCAATTGACAATGTCATGGGGA<br>TGATTGGAATACTGCCTGACATGACCCCCAGCACAGAAATGTCACTGAGAGGAGTAAGAGTCAGCAAAATGGGAGTGGATGAATATTCTAGTACTGAGAGAGTGGTTGTGAGCATTGATCGTTTCTTGAGAGTCCGAGACCAGAGGGGAAATGTGCTCTTGTCTCCCGAAGAAGTTAGTGAAACACAGGGAACAGAGAAACTGACGATAACATATTCATCGTCCATGATG<br>TGGGAAATCAATGGTCCAGAATCAGTGCTAGTCAACACATATCAATGGATCATTAGAAATTGGGAAACTGTGAAGATTCAATGGTCCCAAGACCCTACAATGTTGTACAACAAGATGGAGTTTGAGCCCTTCCAATCCTTGGTGCCTAAGGCTGCTAGAGGCCAGTATAGTGGGTTTGTGAGGACATTATTTCAGCAGATGCGTGATGTGCTGGGGACATTTGACACTGTCC<br>AAATAATAAAACTCCTACCATTTGCAGCGGCCCCACCGGAACAGAGTAGGATGCAGTTCTCTTCTTTGACTGTAAATGTAAGAGGTTCAGGAATGAGAATCCTTGTGAGAGGCAACTCCCCTGTGTTCAACTATAACAAGGCAACCAAGAGACTCACAGTTCTTGGAAAGGATGCAGGTGCTTTAACAGAAGATCCAGATGAGGGAACAGCAGGAGTGGAGTCCGCGGTA<br>TTAAGAGGATTTCTAATTCTGGGCAAGGAAGACAAAAGATATGGGCCAGCATTGAGCATCAATGAATTGAGTAATCTTGCGAAAGGGGAAAAGGCTAATGTGTTGATAGGGCAAGGAGACGTGGTGTTGGTAATGAAACGGAAACGGGACTCTAGCATACTTACTGACAGCCAGACAGCGACCAAAAGAATTCGGATGGCCATCAATTA
EPI_ISL_2932614	>A/swan/Kazakhstan/9-20-B-Talg-39/2020_H5N8_2020-09-21<br>ATGGAGAGAATAAAAGAACTAAGAGATTTGATGTCGCAGTCTCGCACTCGCGAGATACTAACAAAAACCACTGTGGACCATATGGCCATAATAAAGAAATACACATCAGGGAGACAGGAGAAGAACCCTGCCCTCAGGATGAAATGGATGATGGCAATGAAATATCCTATTACAGCTGACAAAAGAATAATGGAGATGATCCCTGAAAGGAATGAGCAAGGTCAGACT<br>CTCTGGAGCAAAACAAATGATGCTGGATCAGACAGAGTGATGGTCTCACCTCTGGCTGTGACATGGTGGAATAGAAATGGGCCAACAACAAGTACAGTACACTACCCAAAAGTCTACAAAACCTACTTTGAAAAGGTAGAAAGGTTGAAACATGGAACCTTTGGTCCTGTTCACTTTCGAAATCAGGTTAAGATACGCCGCAGGGTTGACATAAACCCGGGCCATGCAGA<br>TCTCAGTGCCAAAGAAGCACAGGATGTCATCATGGAGGTTGTTTTCCCAAATGAAGTTGGAGCCAGGATCTTGACATCAGAATCACAATTAACAATAACAAGGGAAAAGAAGGAGGAACTTCAGGATTGCAAGATTGCTCCTTTGATGGTGGCATACATGTTGGAAAGAGAACTGGTTCGCAAGACCAGATTTTTACCAGTAGCTGGCGGAACAAGCAGCGTATACATCG<br>AGGTATTGCATTTGACTCAAGGGACCTGCTGGGAACAAATGTACACACCAGGAGGGGAGGTGAGAAATGATGATGTTGATCAGAGTTTGATCATTGCTGCTAGAAATATAGTTAGGAGGGCAACAGTATCAGCAGACCCATTGGCTTCGCTCTTGGAAATGTGCCACAGTACACAAATTGGTGGAGTGAGGATGGTGGACATTCTTAGGCAGAACCCAACAGAGGAGCA<br>AGCTGTGGATATATGCAAAGCAGCAATGGGTTTAAGAATCAGTTCATCCTTCAGCTTTGGAGGTTTCACTTTCAAAAGGACAAGTGGGTCGTCTGTCAAAAGAGAAGAAGAAATACTCACTGGCAACCTCCAAACACTGAAAGTAAGAATACATGAAGGATATGAGGAATTCACAATGGTTGGGCGAAGAGCTACAGCCATTTTGAGGAAAGCAACCAGGAGACTGATCC<br>AATTAATAGTGAGTGGACGAGACGAGCAGTCAATCGCTGAAGCAATCATAGTGGCAATGGTTTTCTCACAGGAGGATTGCATGATAAAAGCAGTACGAGGTGATTTGAATTTTGTCAACAGAGCGAATCAGCGGCTAAATCCTATGCATCAACTTCTGAGGCATTTCCAAAAGGATGCAAAAGTGCTGTTTCAAAACTGGGGGATTGAACCAATTGACAATGTAATGGGG<br>ATGATCGGGATACTGCCTGACATGACCCCCAGCACAGAGATGTCACTGAGAGGGGTGAGAGTCAGCAAAATGGGAGTGGATGAATATTCCAGTACTGAGAGAGTGGTCGTGAGCATTGATCGCTTCTTGAGAGTCCGAGATCAGAGGGGAAATGTGCTTCTGTCTCCTGAGGAAGTTAGTGAAACACATGGAACAGAGAAACTGACGATAACGTATTCATCGTCTATGA<br>TGTGGGAAATCAATGGTCCGGAATCCGTGCTAGTCAACACATATCAATGGATCATTAGAAATTGGGAAACTGTGAAGATTCAGTGGTCCCAGGACCCTACGATGTTGTACAATAAGATGGAATTTGAGCCCTTTCAATCCTTGGTGCCCAAGGCTGCTAGAGGCCAGTATAGTGGGTTTGTGAGGACATTATTCCAACAGATGCGTGATGTGTTGGGGACATTTGACACTGT<br>CCAAATAATAAAGCTCCTACCATTTGCAGCAGCCCCACCGGAACAGAGTAGGATGCAATTTTCCTCTCTGACTGTGAACGTAAGAGGTTCAGGAATGAGAATACTTGTGAGGGGCAACTCCCCTGTGTTCAACTATAATAAGGCCACCAAGAGACTCACAGTTCTTGGAAAGGATGCAGGCGCCTTGACAGAATATCCAGATGAGGGAACAGCAGGAGTGGAGTCTGCAG<br>TATTAAGAGGATTTCTAATTCTGGGCAAAGAGGACAAAAGATATGGACCAGCATTGAGCATCAACGAATTGAGCAATCTTGCGAAAGGGGAAAAGGCTAATGTGTTGATAGGACAAGGAGACGTGGTGTTGGTAATGAAACGGAAACGGGACTCTAGCATACTTACTGACAGCCAGACAGCGACCAAAAGAATTCGGATGGCCATCAATTA
EPI_ISL_1719909	>A/swan/Lithuania/1258PG1-21VIR2606-2/2021_H5N8_2021-02-26<br>ATGGAGAGAATAAAAGAACTAAGAGATTTGATGTCGCAGTCTCGCACTCGCGAGATACTAACAAAAACCACTGTGGACCATATGGCCATAATAAAGAAATACACATCAGGAAGACAGGAGAAGAACCCTGCCCTCAGGATGAAATGGATGATGGCAATGAAATATCCTATTACAGCTGACAAAAGAATAATGGAGATGATCCCTGAAAGGAATGAGCAAGGTCAGACT<br>CTCTGGAGCAAAACAAATGATGCTGGATCAGACAGAGTGATGGTCTCACCTCTGGCTGTGACATGGTGGAATAGAAATGGGCCAACAACAAGTACAGTACACTACCCAAAAGTCTACAAAACCTACTTTGAAAAGGTAGAAAGGTTGAAACATGGAACCTTTGGTCCTGTTCACTTTCGAAATCAGGTTAAGATACGCCGCAGGGTTGACATAAACCCGGGCCATGCAGA<br>TCTCAGTGCCAAAGAAGCACAGGATGTCATCATGGAGGTTGTTTTCCCAAATGAAGTTGGAGCCAGGATCTTGACATCAGAATCACAATTAACAATAACAAGGGAAAAGAAGGAGGAACTTCAGGATTGCAAGATTGCTCCTTTGATGGTGGCATACATGTTGGAAAGAGAACTGGTTCGCAAGACCAGATTTTTACCAGTAGCTGGCGGAACAAGCAGCGTATACATCG<br>AGGTATTGCATTTGACTCAAGGGACCTGCTGGGAACAAATGTACACACCAGGAGGGGAGGTGAGAAATGATGATGTTGATCAGAGTTTGATCATTGCTGCTAGAAATATAGTTAGGAGGGCAACAGTATCAGCAGACCCATTGGCTTCGCTTTTGGAAATGTGCCACAGTACACAAATTGGTGGAGTAAGAATGGTGGACATTCTTAGGCAGAACCCAACAGAGGAGCA<br>AGCTGTGGATATATGCAAAGCAGCAATGGGTTTAAGAATCAGTTCATCCTTCAGCTTTGGAGGTTTCACTTTCAAAAGGACAAGTGGGTCGTCTGTCAAAAGAGAAGAAGAAATACTCACTGGCAACCTCCAAACACTGAAAGTAAGAATACATGAAGGATATGAGGAATTCACAATGGTTGGGCGAAGAGCTACAGCCATTTTGAGGAAAGCAACCAGGAGACTGATCC<br>AATTAATAGTGAGTGGAAGAGACGAGCAGTCAATCGCTGAAGCAATCATAGTGGCAATGGTTTTCTCACAGGAGGATTGCATGATAAAAGCAGTACGAGGTGATTTGAATTTTGTCAACAGAGCGAATCAGCGGCTAAATCCTATGCATCAACTTCTGAGGCATTTCCAAAAGGATGCAAAAGTGCTGTTTCAAAACTGGGGGATTGAACCAATTGACAATGTAATGGGG<br>ATGATCGGGATACTGCCTGACATGACCCCCAGCACAGAGATGTCACTGAGAGGAGTGAGAGTCAGCAAAATGGGAGTGGATGAATATTCCAGTACTGAGAGAGTGGTCGTGAGCATTGATCGCTTCTTGAGAGTCCGAGATCAGAGGGGAAATGTGCTTCTGTCTCCTGAGGAAGTTAGTGAAACACATGGAACAGAGAAACTGACGATAACGTATTCATCGTCTATGA<br>TGTGGGAAATCAATGGTCCGGAATCCGTGCTAGTCAACACATATCAATGGATCATTAGAAATTGGGAAACTGTGAAGATTCAGTGGTCCCAGGACCCTACGATGTTATACAATAAGATGGAATTTGAGCCCTTCCAATCCTTGGTGCCCAAGGCTGCTAGAGGCCAGTATAGTGGGTTTGTGAGGACATTATTCCAACAGATGCGTGATGTGTTGGGAACATTTGACACTGT<br>CCAAATAATAAAGCTCCTACCATTTGCAGCAGCCCCACCGGAACAGAGTAGGATGCAATTTTCCTCTCTGACTGTGAACGTAAGAGGTTCAGGAATGAGAATACTTGTGAGGGGCAACTCCCCTGTGTTCAACTATAATAAGGCCACCAAGAGACTCACAGTTCTTGGAAAGGATGCAGGCGCCTTGACAGAATATCCAGATGAGGGAACAGCAGGAGTGGAGTCTGCAG<br>TATTAAGAGGATTTCTAATTCTGGGCAAAGAGGACAAAAGATATGGACCAGCATTGAGCATCAACGAATTGAGCAATCTTGCGAAAGGGGAAAAGGCTAATGTGTTGATAGGACAAGGAGACGTGGTGTTGGTAATGAAACGGAAACGGGACTCTAGCATACTTACTGACAGCCAGACAGCGACCAAAAGAATTCGGATGGCCATCAATTA
EPI_ISL_18245790	>A/swan/Poland/MB058-22_22VIR5675-2/2022_H5N1_2022-02-03<br>ATGGAGAGAATAAAAAAGCTAAGAGATTTGATGTCGCAGTCTCGCACTCGCGAGATACTGACAAAAACCACCGTGGACCATATGGCCATAATCAAGAAATATACATCAGGAAGACAGGAGAAGAACCCTGCACTTAGGATGAAGTGGATGATGGCAATGAAATATCCGATTACAGCAGACAAAAGGATAATGGAGATGATCCCTGAAAGAAACGAGCAAGGTCAGACT<br>CTTTGGAGCAAAACAAATGATGCTGGATCGGACAGAGTAATGGTGTCACCTCTGGCTGTGACGTGGTGGAATAGAAATGGACCAACAACAAGTACCGTCCATTACCCAAAGGTCTATAAAACCTACTTTGAAAAGGTTGAAAGGTTAAAGCATGGAACCTTCGGCCCTGTCCATTTCCGGAATCAGGTTAAGATACGCCGCAGAGTTGACATAAACCCGGGCCATGCAGA<br>CCTCAGTGCTAAAGAAGCACAAGACGTCATCATGGAGGTCGTTTTCCCAAATGAAGTCGGAGCCAGAATATTGACATCAGAGTCACAGTTGACAATTACAAAAGAAAAGAAGGAGGAACTCCAGGACTGTAAGATTGCCCCTCTAATGGTGGCATACATGTTGGAGAGAGAACTGGTTCGAAAAACCAGATTCCTGCCAGTAGCTGGCGGAACAAGTAGCGTATATATCG<br>AAGTGTTGCACTTGACTCAAGGAACCTGCTGGGAACAAATGTATACGCCAGGAGGAGAAGTGAGAAATGATGACGTTGACCAAAGTTTAATTATTGCTGCCAGAAATATCGTTAGGAGGGCAACAGTATCAGCAGACCCATTGGCTTCACTACTGGAGATGTGCCATAGTACACAGATTGGCGGGATAAGAATGGTAGACATTCTTAGACAGAACCCAACAGAAGAGCA<br>AGCCGTGGATATATGCAAAGCAGCAATGGGTTTAAGAATCAGTTCATCCTTCAGTTTTGGAGGTTTCACTTTCAAAAGGACAAGCGGATCATCTGTCAAAAGAGAAGAGGAAGTGCTCACCGGCAACCTCCAAACATTGAAAATAAGAGTACATGAAGGGTATGAGGAATTCACAATGGTTGGGCGGAGAGCAACAGCCATTCTAAGGAAAGCAACCAGAAGGCTGATC<br>CAATTGATAGTAAGTGGAAGAGACGAGCAGTCAATCGCCGAAGCGATCATAGTGGCAATGGTGTTCTCTCAAGAGGATTGCATGATAAAGGCTGTACGAGGTGATTTAAATTTTGTCAATAGAGCGAATCAGCGGCTCAATCCTATGCATCAGCTCCTGAGGCATTTCCAAAAGGATGCAAAGGTACTATTCCAAAACTGGGGAATTGAACCCATTGACAATGTCATGGGA<br>ATGATAGGAATATTGCCTGATATGACCCCCAGCACAGAGATGTCACTAAGAGGAGTGAGGGTCAGTAAAATGGGAGTGGATGAATATTCCAGTACTGAGAGGGTGGTCGTAAGTATTGATCGCTTCTTGAGGGTACGAGACCAGAGAGGAAATGTACTCTTGTCTCCCGAAGAGGTCAGTGAAACACAGGGAACAGAGAAGCTAACGATAACATATTCATCATCCATGA<br>TGTGGGAAATTAATGGCCCTGAGTCTGTGCTAGTTAACACATATCAATGGATCATCAGAAACTGGGAAACTGTGAAGATTCAGTGGTCCCAAGACCCTACAATGCTATACAACAAGATGGAGTTTGAGCCTTTTCAGTCCTTGGTGCCTAAGGCAGCCAGAGGCCAGTACAGTGGATTTGTAAGGACCCTATTCCAGCAGATGCGTGATGTGCTGGGAACCTTTGACACTGT<br>CCAGATAATAAAGCTACTTCCATTTGCAGCAGCACCACCGGAACAGAGTAGGATGCAGTTCTCTTCTCTAACTGTAAACGTAAGGGGTTCAGGAATGAGAATACTTGTGAGAGGCAACTCCCCTGTGTTCAACTATAATAAAGCAACCAAGAGGCTCATAGTCCTTGGAAAGGATGCTGGTGCATTGACAGAAGACCCAGGTGAGGGGACAGCAGGAGTGGAGTCTGCG<br>GTATTGAGAGGGTTCCTAATTCTGGGCAAAGAGGACAAAAGATATGGACCAGCACTGAGCATCAATGAATTGAGCAATCTTGCGAAAGGGGAGAAGGCTAATGTGTTGATAGGGCAAGGAGACGTGGTGTTGGTGATGAAACGGAAACGGGACTCTAGCATACTTACTGACAGCCAGACAGCGACCAAAAGAATTCGGATGGCCATCAATTA
EPI_ISL_11922813	>A/swan/Poland/MB078_22VIR2515-7/2022_H5N1_2022-02-15<br>ATGGAGAGAATAAAAGAACTAAGAGATTTAATGTCACAGTCTCGCACTCGCGAGATACTAACAAAAACCACTGTGGACCATATGGCCATAATAAAGAAATACACATCAGGAAGACAGGAGAAGAACCCTGCTCTTAGGATGAAATGGATGATGGCAATGAAGTACCCGATTACAGCAGACAAAAGAATAATGGAGATGATCCCTGAAAGAAATGAGCAAGGCCAAACT<br>CTCTGGAGCAAAACAAATGATGCTGGATCAGAYAGAGTGATGGTGTCACCTCTGGCTGTGACATGGTGGAATAGAAATGGGCCAACGACAAGCACAGTCCATTATCCAAAGGTTTACAAAACCTACTTTGAAAAAGTCGAAAGGTTGAAACATGGGACCTTTGGTCCTGTTCACTTTCGAAATCAGGTCAAAATACGCCGCAGGGTCGACATAAACCCGGGCCATGCAGA<br>TCTCAGTGCTAAGGAAGCACAAGACGTCATCATGGAGGTCGTTTTCCCAAACGAAGTTGGAGCCAGGATATTGACATCAGAATCACAATTAACAATAACAAAGGAAAAGAAGGAGGAGCTTCAAGACTGTAAGATTGCTCCTYTRATGGTGGCATACATGTTGGAGAGGGAACTGGTCCGCAAAACCAGATTCCTACCGGTAGCTGGCGGGACAARCAGTGTGTATATCG<br>AGGTGCTGCATTTGACTCAAGGGACTTGCTGGGAACAAATGTACACACCGGGAGGAGAGGTGAGAAATGATGATGTCGATCAAAGTTTGATTATTGCTGCTAGAAATATTGTCAGGAGAGCAACAGTATCAGCAGACCCGTTGGCTTCACTCTTGGAGATGTGCCATAGTACACAAATTGGAGGAATAAGGATGGTTGACATTCTTAGACAAAACCCAACAGAAGAACAA<br>GCTGTAGATATATGCAAAGCAGCAATGGGTCTAAGAATCAGTTCATCCTTCAGCTTTGGAGGTTTCACTTTCAAAAGGACAAGTGGGTCATCTGTCAAGAAAGAAGAAGAAGTGCTCACAGGCAACCTCCAAACATTGAAAATAAGGGTACACGAAGGATATGAAGAATTCACAATGGTTGGGCGAAGAGCGACCGCCATTTTAAGGAAAGCAACYAGAAGGTTGATACA<br>ATTAATAGTGAGTGGGAGGGACGAGCAGTCAATTGCTGAAGCAATCATAGTGGCAATGGTTTTCTCACAAGAAGACTGCATGATAAAGGCAGTACGAGGTGATTTGAATTTTGTCAACAGAGCAAACCAGCGGCTGAATCCTATGCATCAACTTCTAAGACATTTCCAGAAGGATGCAAAAGTGCTGTTTCAGAACTGGGGAATTGAACCCATTGACAATGTCATGGGGAT<br>GATCGGAATACTGCCTGACATGACCCCAAGCACAGATATGTCTCTGAGAGGAGTGAGGGTCAGTAAAATGGGAGTGGATGAATATTCCAGTACTGAGAGGGTTGTCGTGAGCATTGATCGTTTCTTAAGGGTCCGAGATCAAAGGGGAAATGTACTCCTGTCTCCCGAGGAGGTCAGTGAAACACAAGGAACAGAGAAGCTAACGATAACATATTCGTCGTCAATGATG<br>TGGGAAATCAATGGTCCGGAATCAGTGCTGGTCAACACATATCAATGGATTATTAGAAATTGGGAAACTGTGAAAATTCAGTGGTCACAAGACCCTACAATGTTATACAATAAGATGGAGTTTGAGCCCTTCCAATCCTTGGTGCCTAAGGCTGCCAGAGGCCAGTATAGTGGATTCGTGAGGACGTTGTTCCAGCAGATGCGTGATGTATTGGGGACATTTGACACTGTTC<br>AAATAATAAAGCTCCTACCATTTGCAGCAGCCCCACCAGAACAGAGTAGGATGCAGTTCTCTTCTCTAACTGTGAATGTAAGGGGTTCAGGGATGAGAATACTTATGAGAGGCAATTCCCCTGTGTTCAACTATAACAAGGCAACCAAGAGGCTCACAGTCCTTGGAAAAGATGCAGGTGCCTTAACAGAAGATCCAGACGAGGGAACAGCAGGAGTGGAATCTGCGGTA<br>TTAAGAGGATTTCTAATTCTGGGCAAAGAAGATAAGAGATATGGCCCAGCGTTGAGCATCAACGAATTGAGCAATCTTGCGAAAGGGGAGAAAGCTAATGTGTTGATAGGGCAAGGAGACGTGGTGTTGGTAATGAAACGGAAACGGGACTCTAGCATACTTACTGACAGCCAGACAGCGACCAAAAGGATTCGGATGGCCATCAATTA
EPI_ISL_11922814	>A/swan/Poland/MB083_22VIR2515-8/2022_H5N1_2022-02-18<br>ATGGAGAGAATAAAAGAGCTAAGAGATTTGATGTCGCAGTCTCGCACTCGCGAGATACTGACAAAAACCACCGTGGACCATATGGCCATAATCAAGAAATATACATCAGGAAGACAGGAGAAGAACCCTGCACTTAGGATGAAGTGGATGATGGCAATGAAATATCCGATTACAGCAGACAAAAGGATAATGGAGATGATCCCTGAAAGAAACGAGCAAGGTCAGACT<br>CTTTGGAGCAAAACAAATGATGCTGGATCGGATAGAGTAATGGTGTCACCTCTGGCTGTGACGTGGTGGAATAGAAATGGACCAACAACAAGTACCGTCCATTACCCAAAGGTCTATAAAACCTACTTTGAAAAGGTTGAAAGGTTAAAGCATGGAACCTTCGGCCCTGTCCATTTCCGGAATCAGGTTAAGATACGCCGCAGAGTTGACATAAACCCGGGCCATGCAGA<br>CCTCAGTGCTAAAGAAGCACAAGACGTCATCATGGAGGTCGTTTTCCCAAATGAAGTCGGAGCCAGAATATTGACATCAGAGTCACAGTTGACAATTACAAAAGAAAAGAAGGAGGAACTCCAGGACTGTAAGATTGCTCCTCTAATGGTGGCATACATGTTGGAGAGAGAACTGGTTCGAAAAACCAGATTCCTGCCAGTAGCTGGCGGAACAAGTAGCGTATATATCG<br>AAGTGTTGCACTTGACTCAAGGAACCTGCTGGGAACAAATGTATACGCCAGGAGGAGAAGTGAGAAATGATGACGTTGACCAAAGTTTAATTATTGCTGCCAGAAATATCGTTAGGAGGGCAACAGTATCAGCAGACCCATTGGCTTCACTACTGGAGATGTGCCATAGTACACAGATTGGCGGGATAAGAATGGTAGACATTCTTAGACAGAACCCAACAGAAGAGCA<br>AGCCGTGGATATATGCAAAGCAGCAATGGGTTTAAGAATCAGTTCATCCTTCAGTTTTGGAGGTTTCACTTTCAAAAGGACAAGCGGATCATCTGTCAAAAGAGAAGAGGAAGTGCTCACYGGCAACCTCCAAACATTGAAAATAAGAGTACATGAAGGGTATGAGGAATTCACAATGGTTGGGCGGAGAGCAACAGCCATTCTAAGGAAAGCAACCAGAAGGCTGATC<br>CAATTGATAGTAAGTGGAAGAGACGAGCAGTCAATCGCCGAAGCGATCATAGTGGCAATGGTGTTCTCTCAAGAGGATTGCATGATAAAGGCTGTACGAGGTGATTTAAATTTTGTCAATAGAGCGAATCAGCGGCTCAATCCTATGCATCAGCTCCTGAGGCATTTCCAAAAGGATGCAAAGGTACTATTCCAAAACTGGGGAATTGAACCCATTGACAATGTCATGGGA<br>ATGATAGGAATATTGCCTGATATGACCCCCAGCACAGAGATGTCACTAAGAGGAGTGAGGGTCAGTAAAATGGGAGTGGATGAATATTCCAGTACTGAGAGGGTGGTCGTGAGTATTGATCGCTTCTTGAGGGTACGAGACCAGAGAGGAAATGTACTCTTGTCTCCCGAAGAGGTCAGTGAAACACAGGGAACAGAGAAGCTAACGATAACATATTCATCATCCATGA<br>TGTGGGAAATTAATGGCCCTGAGTCAGTGCTAGTTAACACATATCAATGGATCATCAGAAACTGGGAAACTGTGAAGATTCAGTGGTCCCAAGACCCTACAATGCTATACAACAAGATGGAGTTTGAGCCTTTTCAGTCCTTGGTGCCTAAGGCAGCCAGAGGCCAGTACAGTGGATTTGTACGGACCCTATTCCAGCAGATGCGTGATGTGCTGGGAACATTTGACACTGT<br>CCAGATAATAAAGCTACTTCCATTTGCAGCAGCACCACCGGAACAGAGTAGGATGCAGTTCTCTTCTCTAACTGTAAACGTAAGGGGTTCAGGAATGAGAATACTTGTGAGAGGCAACTCCCCTGTGTTCAACTATAATAAAGCAACCAAGAGGCTCATAGTCCTTGGAAAGGATGCTGGTGCATTGACAGAAGACCCAGGTGAGGGGACAGCAGGAGTGGAGTCTGCG<br>GTATTGAGAGGGTTCCTAATTCTGGGCAAAGAGGACAAAAGATATGGACCAGCGCTGAGCATCAATGAATTGAGCAATCTTGCGAAAGGGGAGAAGGCTAATGTGTTGATAGGGCAAGGAGACGTGGTGTTGGTGATGAAACGGAAACGGGACTCTAGCATACTTACTGACAGCCAGACAGCGACCAAAAGAATTCGGATGGCCATCAATTA
internal NVRI repository	>A/swan/Poland/MB097/2021_H5N8_2021-02-12<br>ATGGAGAGAATAAAAGAACTAAGAGATTTGATGTCGCAGTCTCGCACTCGCGAGATACTAACAAAAACCACTGTGGACCATATGGCCATAATAAAGAAATACACATCAGGAAGACAGGAGAAGAACCCTGCCCTCAGGATGAAATGGATGATGGCAATGAAATATCCTATTACAGCTGACAAAAGAATAATGGAGATGATCCCTGAAAGGAATGAGCAAGGTCAGACT<br>CTCTGGAGCAAAACAAATGATGCTGGATCAGACAGAGTGATGGTCTCACCCTTGGCTGTGACATGGTGGAATAGAAATGGGCCAACAACAAGTACAGTACACTACCCAAAAGTCTACAAAACCTACTTTGAAAAGGTAGAAAGGTTGAAACATGGAACCTTTGGTCCTGTTCACTTTCGAAATCAGGTTAAGATACGCCGCAGGGTTGACATAAACCCGGGCCATGCAGA<br>TCTCAGTGCCAAAGAAGCACAGGATGTCATCATGGAGGTTGTTTTCCCAAATGAAGTTGGAGCCAGGATCTTGACATCAGAATCACAATTAACAATAACAAGGGAAAAGAAGGAGGAACTTCAGGATTGCAAGATTGCTCCTTTGATGGTGGCATACATGTTGGAAAGAGAACTGGTTCGCAAGACCAGATTTTTACCAGTAGCTGGCGGAACAAGCAGCGTATACATCG<br>AGGTATTGCATTTGACTCAAGGGACCTGCTGGGAACAAATGTACACACCAGGAGGGGAGGTGAGAAATGATGATGTTGATCAGAGTTTGATCATTGCTGCTAGAAATATAGTTAGGAGGGCAACAGTATCAGCAGACCCATTGGCTTCGCTTTTGGAAATGTGCCACAGTACACAAATTGGTGGAGTAAGGATGGTGGACATTCTTAGGCAGAACCCAACAGAGGAGCA<br>AGCTGTGGATATATGCAAAGCAGCAATGGGTTTAAGAATCAGTTCATCCTTCAGCTTTGGAGGTTTCACCTTCAAAAGGACAAGTGGGTCGTCTGTCAAAAGAGAAGAAGAAATACTCACTGGCAACCTCCAAACACTGAAAGTAAGAATACATGAAGGATATGAGGAATTCACAATGGTTGGGCGAAGAGCTACAGCCATTTTGAGGAAAGCAACCAGGAGACTGATCC<br>AATTAATAGTGAGTGGAAGAGACGAGCAGTCAATCGCTGAAGCAATCATAGTGGCAATGGTTTTCTCACAGGAGGATTGCATGATAAAAGCAGTACGAGGTGATTTGAATTTTGTCAACAGAGCGAATCAGCGGCTAAATCCTATGCATCAACTTCTGAGGCATTTCCAAAAGGATGCAAAAGTGCTGTTTCAAAACTGGGGGATTGAACCAATTGACAATGTAATGGGG<br>ATGATCGGGATACTGCCTGACATGACCCCCAGCACAGAGATGTCACTGAGAGGAGTGAGAGTCAGCAAAATGGGAGTGGATGAATATTCCAGTACTGAGAGAGTGGTCGTGAGCATTGATCGCTTCTTGAGAGTCCGAGATCAGAGGGGAAATGTGCTTCTGTCTCCTGAGGAAGTTAGTGAAACACATGGAACAGAGAAACTGACGATAACGTATTCATCGTCTATGA<br>TGTGGGAAATCAATGGTCCGGAATCCGTGCTAGTCAACACATATCAATGGATCATTAGAAATTGGGAAACTGTGAAGATTCAGTGGTCCCAGGACCCTACGATGTTATACAATAAGATGGAATTTGAGCCCTTCCAATCCTTGGTGCCCAAGGCTGCTAGAGGCCAGTATAGTGGGTTTGTGAGGACATTATTCCAACAGATGCGTGATGTGTTGGGAACATTTGACACTGT<br>CCAAATAATAAAGCTCCTACCATTTGCAGCGGCCCCACCGGAACAGAGTAGGATGCAATTTTCCTCTCTGACTGTGAACGTAAGAGGTTCAGGAATGAGAATACTTGTGAGGGGCAACTCCCCTGTGTTCAACTATAATAAGGCCACCAAGAGACTCACAGTTCTTGGAAAGGATGCAGGCGCCTTGACAGAATATCCAGATGAGGGAACAGCAGGAGTGGAGTCTGCAG<br>TATTAAGAGGATTTCTAATTCTGGGCAAAGAGGACAAAAGATATGGACCAGCATTGAGCATCAACGAATTGAGCAATCTTGTGAAAGGGGAAAAGGCTAATGTGCTGATAGGACAAGGAGACGTGGTGTTGGTAATGAAACGGAAACGGGACTCTAGCATACTTACTGACAGCCAGACAGCGACCAAAAGAATTCGGATGGCCATCAATTA
EPI_ISL_18244493	>A/swan/Poland/MB122-21_22VIR5675-9/2021_H5N8_2021-02-18<br>ATGGAGAGAATAAAAGAACTAAGAGATTTGATGTCGCAGTCTCGCACTCGCGAGATACTAACAAAAACCACTGTGGACCATATGGCCATAATAAAGAAATACACATCAGGGAGACAGGAGAAGAACCCTGCCCTCAGGATGAAATGGATGATGGCAATGAAATATCCTATTACAGCTGACAAAAGAATAATGGAGATGATCCCTGAAAGGAATGAGCAAGGTCAGACT<br>CTCTGGAGCAAAACAAATGATGCTGGATCAGACAGAGTGATGGTCTCACCTCTGGCTGTGACGTGGTGGAATAGAAATGGGCCAACAACAAGTACAGTACACTACCCAAAAGTCTACAAAACCTACTTTGAAAAGGTAGAAAGGTTGAAACATGGAACCTTTGGTCCTGTTCACTTTCGAAATCAGGTTAAGATACGCCGCAGGGTTGACACAAACCCGGGCCATGCAGA<br>TCTCAGTGCCAAAGAAGCACAGGATGTCATCATGGAGGTTGTTTTCCCAAATGAAGTTGGAGCCAGGATCTTGACATCAGAATCACAATTAACAATAACAATGGAAAAGAAGGAGGAACTTCAGGATTGCAAGATTGCTCCTTTGATGGTGGCATACATGTTGGAAAGAGAACTGGTTCGCAAGACCAGATTTTTACCAGTAGCTGGCGGAACAAGCAGCGTATACATCG<br>AGGTATTGCATTTGACTCAAGGGACCTGCTGGGAACAAATGTACACACCAGGAGGGGAGGTGAGAAATGATGATGTTGATCAGAGTTTGATCATTGCTGCTAGAAATATAGTTAGGAGGGCAACAGTATCAGCAGACCCATTGGCTTCGCTCTTGGAAATGTGCCACAGTACACAAATTGGTGGAGTGAGGATGGTGGACATTCTTAGGCAGAACCCAACAGAGGAGCA<br>AGCTGTGGATATATGCAAAGCAGCAATGGGTTTAAGAATCAGTTCATCCTTCAGCTTTGGAGGTTTCACTTTCAAAAGGACAAGTGGGTCGTCTGTCAAAAGAGAAGAAGAAATACTCACTGGCAACCTCCAAACACTGAAAGTAAGAATACATGAAGGATATGAGGAATTCACAATGGTTGGGCGAAGAGCTACAGCCATTTTGAGGAAAKCAACCAGGAGACTGATCC<br>AATTAATAGTGAGTGGACGAGACGAACAGTCAATCGCTGAGGCAATCATAGTGGCAATGGTTTTCTCACAGGAGGATTGCATGATAAAAGCAGTACGAGGTGATTTGAATTTTGTCAACAGAGCGAATCAGCGGCTAAATCCTATGCATCAACTTCTGAGGCATTTCCAAAAAGATGCAAAAGTGCTGTTTCAAAACTGGGGGATTGAACCAATTGACAATGTAATGGGG<br>ATGATCGGGATACTGCCTGACATGACCCCCAGCACAGAGATGTCACTGAGAGGAGTGAGAGTCAGCAAAATGGGAGTGGATGAATATTCCAGTACTGAGAGAGTGGTCGTGAGCATTGATCGCTTCTTGAGAGTCCGAGATCAGAGGGGAAATGTGCTTCTGTCTCCTGAGGAAGTTAGTGAAACACATGGAACAGAGAAACTGACGATAACGTATTCATCGTCCATGA<br>TGTGGGAAATCAATGGTCCGGAATCCGTGCTAGTCAACACATATCAATGGATCATTAGAAATTGGGAAACTGTGAAGATTCAGTGGTCCCAGGACCCTACGATGTTGTACAATAAGATGGAATTTGAGCCCTTCCAATCCTTGGTGCCCAAGGCTGCTAGAGGCCAGTATAGTGGGTTTGTGAGGACATTATTCCAACAGATGCGTGATGTGTTGGGGACATTTGACACTGT<br>CCAAATAATAAAGCTCCTACCATTTGCAGCAGCCCCACCGGAACAGAGTAGGATGCAATTTTCCTCTCTGACTGTGAACGTAAGAGGTTCAGGAATGAGAATACTTGTGAGGGGCAACTCCCCTGTGTTCAACTATAATAAGACCACCAAGAGACTCACAGTTCTTGGAAAGGATGCAGGCGCCTTGACAGAATATCCAGATGAGGGAACAGCAGGAGTGGAGTCTGCAG<br>TATTAAGAGGATTTCTAATTCTGGGCAAAGAGGACAAAAGATATGGACCAGCATTGAGCATCAACGAATTGAGCAATCTTGCGAAAGGGGAAAAGGCTAATGTGTTGATAGGACAAGGAGACGTGGTGTTGGTAATGAAACGGAAACGGGACTCTAGCATACTTACTGACAGCCAGACAGCGACCAAAAGAATTCGGATGGCCATCAATTA
EPI_ISL_846623	>A/swan/Poland/MB141/2020_H5N8_2020-12-17<br>ATGGAGAGAATAAAAGAACTAAGAGATTTGATGTCACAGTCTCGCACTCGCGAGATACTAACAAAAACCACTGTGGACCATATGGCCATAATAAAGAAATACACATCAGGGAGACAGGAGAAGAACCCTGCCCTCAGGATGAAATGGATGATGGCAATGAAATATCCTATTACAGCTGACAAAAGAATAATGGAGATGATCCCTGAAAGGAATGAGCAAGGTCAGACT<br>CTCTGGAGCAAAACAAATGATGCTGGATCAGACAGAGTGATGGTCTCACCTCTGGCTGTGACATGGTGGAATAGAAACGGGCCAACAACAAGTACAGTACACTACCCAAAAGTCTACAAAACCTACTTTGAAAAGGTGGAAAGGTTGAAACATGGAACCTTTGGTCCTGTTCACTTTCGAAATCAGGTTAAGATACGCCGCAGGGTTGACATAAACCCGGGCCATGCAGA<br>TCTCAGTGCCAAAGAAGCACAGGATGTCATCATGGAGGTTGTTTTCCCAAATGAAGTTGGAGCCAGGATCTTGACATCAGAATCACAATTAACAATAACAAGGGAAAAGAAGGAGGAACTTCAGGATTGCAAGATTGCTCCTTTGATGGTGGCATACATGTTGGAAAGAGAACTGGTTCGCAAGACCAGATTTTTACCAGTAGCTGGCGGAACAAGCAGCGTGTACATCG<br>AGGTATTGCATTTGACTCAAGGGACCTGCTGGGAACAAATGTACACACCAGGAGGGGAGGTGAGAAATGATGATGTTGATCARAGTTTGATCATTGCTGCTAGAAATATAGTTAGGAGGGCAACAGTATCAGCAGACCCATTGGCTTCGCTCTTGGAAATGTGCCACAGTACACAAATTGGTGGAGTGAGGATGGTGGACATTCTTAGGCAGAACCCAACAGAGGAGCA<br>AGCTGTGGATATATGCAAAGCAGCAATGGGTTTAAGAATCAGTTCATCCTTCAGCTTTGGAGGTTTCACTTTCAAAAGGACAAGTGGGTCGTCTGTCAAAAGAGAAGAAGAAATACTCACTGGCAACCTCCAAACACTGAAAGTAAGAATACAYGAAGGATATGAGGAATTCACAATGGTTGGGCGAAGAGCTACAGCCATTTTGAGGAAAGCAACCAGGAGACTGATCC<br>AATTAATAGTGAGTGGACGAGACGAGCAGTCAATCGCTGAAGCAATCATAGTGGCAATGGTTTTCTCACAGGAGGATTGCATGATAAAAGCAGTACGAGGTGATTTGAATTTTGTCAACAGAGCGAATCAGCGGCTAAATCCTATGCATCAACTTCTGAGGCATTTCCAAAAGGATGCAAAAGTGCTGTTTCAAAACTGGGGGATTGAACCAATTGACAATGTAATGGGG<br>ATGATCGGGATACTGCCTGACATGACCCCCAGCACAGAGATGTCACTGAGAGGAGTGAGAGTCAGCAAAATGGGAGTGGATGAATATTCCAGTACTGAGAGAGTGGTCGTGAGCATTGATCGCTTCTTGAGAGTCCGAGATCAGAGGGGAAATGTGCTTCTGTCTCCTGAGGAAGTTAGTGAAACACATGGAACAGAGAAACTGACGATAACGTATTCATCGTCTATGA<br>TGTGGGAAATCAATGGTCCGGAATCCGTGCTAGTCAACACATATCAATGGATCATTAGAAATTGGGAAACTGTGAAGATTCAGTGGTCCCAGGACCCTACGATGTTGTACAATAAGATGGAATTTGAGCCCTTTCAATCCTTGGTGCCCAAGGCTGCTAGAGGCCAGTATAGTGGGTTTGTGAGGACATTATTCCAACAGATGCGTGATGTGTTGGGGACATTTGACACTGT<br>CCAAATAATAAAGCTCCTACCATTTGCAGCAGCCCCACCGGAACAGAGTAGGATGCAATTTTCCTCTCTGACTGTGAACGTAAGAGGTTCAGGAATGAGAATACTTGTGAGGGGCAACTCCCCTGTGTTCAACTATAATAAGGCCACCAAGAGACTCACAGTTCTTGGAAAGGATGCAGGYGCCTTGACAGAATATCCAGATGAGGGAACAGCAGGAGTGGAGTCTGCAG<br>TATTAAGAGGATTTCTAATTCTGGGCAAAGAGGACAAAAGATATGGACCAGCATTGAGCATCAACGAATTGAGCAATCTTGYGAAAGGGGAAAAGGCTAATGTGTTGATAGGACAAGGAGACGTGGTGTTGGTAATGAAACGGAAACGGGACTCTAGCATACTTACTGACAGCCAGACAGCGACCAAAAGAATTCGGATGGCCATCAATTA
EPI_ISL_2199986	>A/mute_swan/Poland/MB185/2021_H5N8_2021-02-26<br>ATGGAGAGAATAAAAGAACTAAGAGATTTGATGTCACAGTCTCGCACTCGCGAGATACTAACAAAAACCACTGTGGACCATATGGCCATAATAAAGAAATACACATCAGGGAGACAGGAGAAGAACCCTGCTCTCAGGATGAAATGGATGATGGCAATGAAATATCCTATTACAGCTGACAAAAGAATAATGGAGATGATCCCTGAAAGGAATGAGCAAGGTCAGACTC<br>TCTGGAGCAAAACAAATGATGCTGGATCAGACAGAGTGATGGTCTCACCTCTGGCTGTGACATGGTGGAATAGAAACGGGCCAACAACAAGTACAGTACACTACCCAAAAGTCTACAAAACCTACTTTGAAAAGGTGGAAAGGTTGAAACATGGAACCTTTGGTCCTGTTCACTTTCGAAATCAGGTTAAGATACGCCGCAGGGTTGACATAAACCCGGGCCATGCAGAT<br>CTCAGTGCCAAAGAAGCACAGGATGTCATCATGGAGGTTGTTTTCCCAAATGAAGTTGGAGCCAGGATCTTGACATCAGAATCACAATTAACAATAACAAGGGAAAAGAAGGAGGAACTTCAGGATTGCAAGATTGCTCCTTTGATGGTGGCATACATGTTGGAAAGAGAACTGGTTCGCAAGACCAGATTTTTACCAGTAGCTGGCGGAACAAGCAGCGTGTACATCGA<br>GGTATTGCATTTGACTCAAGGGACCTGCTGGGAACAAATGTACACACCAGGAGGGGAGGTGAGAAATGATGATGTTGATCAGAGTTTGATCATTGCTGCTAGAAATATAGTTAGGAGGGCAACAGTATCAGCAGACCCATTGGCTTCGCTCTTGGAAATGTGCCACAGTACACAAATTGGTGGAGTGAGGATGGTGGACATTCTTAGGCAGAACCCAACAGAGGAGCAA<br>GCTGTGGATATATGCAAAGCAGCAATGGGTTTAAGAATCAGTTCATCCTTCAGCTTTGGAGGTTTCACTTTCAAAAGGACAAGTGGGTCGTCTGTCAAAAGAGAAGAAGAAATACTAACTGGCAACCTCCAAACACTGAAAGTAAGAATACATGAAGGATATGAGGAATTCACAATGGTTGGGCGAAGAGCTACAGCCATTTTGAGGAAAGCAACCAGGAGACTGATCCA<br>ATTAATAGTGAGTGGACGAGACGAGCAGTCAATCGCTGAAGCAATCATAGTGGCAATGGTTTTCTCACAGGAGGATTGCATGATAAAAGCAGTACGAGGTGATTTGAATTTTGTCAACAGAGCGAATCAGCGGCTAAATCCTATGCATCAACTTCTGAGGCATTTCCAAAAGGATGCAAAAGTGCTGTTTCAAAACTGGGGGATTGAACCAATTGACAATGTAATGGGGAT<br>GATCGGGATACTGCCTGACATGACCCCCAGCACAGAGATGTCACTGAGAGGAGTGAGAGTCAGCAAAATGGGAGTGGATGAATATTCCAGTACTGAGAGAGTGGTCGTGAGCATTGATCGCTTCTTGAGAGTCCGAGATCAGAGGGGAAATGTGCTTCTGTCTCCTGAGGAAGTTAGTGAAACACATGGAACAGAGAAACTTACGATAACGTATTCATCGTCTATGATGT<br>GGGAAATCAATGGTCCGGAATCCGTGCTAGTCAACACATATCAATGGATCATTAGAAATTGGGAAACTGTGAAGATTCAGTGGTCCCAGGACCCTACGATGTTGTACAATAAGATGGAATTTGAGCCCTTTCAATCCTTGGTGCCCAAGGCTGCTAGAGGCCAGTATAGTGGGTTTGTGAGGACATTATTCCAACAGATGCGTGATGTGTTGGGGACATTTGACACTGTCCA<br>AATAATAAAGCTCCTACCATTTGCAGCAGCCCCACCGGAACAGAGTAGGATGCAATTTTCCTCTCTGACTGTGAACGTAAGAGGTTCTGGAATGAGAATACTTGTGAGGGGCAACTCCCCTGTGTTCAACTATAATAAGGCCACCAAGAGACTCACAGTTCTTGGAAAGGATGCAGGCGCCTTGACAGAATATCCAGATGAGGGAACAGCAGGAGTGGAGTCTGCAGTAT<br>TAAGAGGATTTCTAATTCTGGGCAAAGAGGACAAAAGATATGGACCAGCATTGAGCATCAACGAATTGAGCAATCTTGCGAAAGGGGAAAAGGCTAATGTGTTGATAGGACAAGGAGACGTGGTGTTGGTAATGAAACGGAAACGGGACTCTAGCATACTTACTGACAGCCAGACAGCGACCAAAAGAATTCGGATGGCCATCAATTA

EPI_ISL_11922818	>A/swan/Romania/10455_22VIR2749-4/2022_H5N1_2022-02-09<br>ATGGAGAGAATAAAAGAACTAAGAGATTTGATGTCACAGTCTCGCACTCGCGAGATACTGACAAAAACCACGGTGGACCATATGGCCATAATTAAGAAATACACATCAGGAAGACAGGAAAAGAACCCTGCCCTTAGGATGAAGTGGATGATGGCAATGAAATACCCGATTACAGCAGACAAAAGAATAATGGAAATGATCCCTGAAAGAAATGAACAAGGCCAGACT<br>CTCTGGAGCAAAACAAGTGATGCTGGATCAGACAGAGTAATGGTGTCACCTCTGGCTGTGACATGGTGGAATAGAAATGGACCGACGACAAGTACAGTCCATTATCCAAAGGTCTACAAAACCTACTTTGAAAAGGTCGAAAGGTTGAAACATGGAACCTTCGGTCCTGTTCACTTCGGAATCAGGTTAAAATACGCCGCAGGGTTGACATAAACCCGGGCCATGCAGA<br>TCTCAGTGCCAAAGAAGCACAAGATGTCATCATGGAGGTCGTTTTCCCAAACGAGGTTGGAGCAAAGATATTGACATCAGAGTCACAATTAACAATAACAAAGGAAAAGAAGGAGGAGCTTCAGGACTGCAAGATCGCCCCTTTAATGGTGGCATACATGTTGGAGAGGGAACTGGTTCGCAAAACCAGATTCCTACCAGTAGCTGGCGGAACAAGCAGCGTATATATC<br>GAGGTATTGCATTTGACTCAAGGGACCTGCTGGGAACAAATGTACACACCGGGAGGGGAGGTGAGAAATGATGATGTCGATCAGAGTTTGATTATTGCTGCTAGAAATATTGTTAGGAGAGCAACAGTGTCGGCAGACCCGTTGGCTTCGCTCTTGGAGATGTGCCATAGTACACAAATTGGCGGGATAAGGATGGTGGACATCCTTAGACAGAACCCAACAGAAGAGC<br>AAGCTGTGGATATATGCAAAGCAGCAATGGGTTTGAGAATCAGTTCATCCTTCAGCTTTGGAGGTTTCACTTTTAAAAGGACAAGTGGGTCATCTGTCAAAAGAGAAGAAGAAGTGCTCACGGGCAACCTCCAAACACTGAAAATAAGGGTACATGAAGGATACGAAGAATTCACAATGGTTGGGCGAAGAGCTACGGCCATTCTAAGGAAAGCAACCAGAAGGCTGAT<br>CCAATTAATAGTGAGTGGGAGAGACGAGCAGTCAATCGCTGAAGCGATTATAGTGGCAATGGTTTTCTCGCAAGAGGACTGCATGATAAAAGCAGTGCGAGGTGATTTGAACTTTGTTAACAGAGCAAATCAGCGGCTAAATCCTATGCATCAACTTCTGAGACATTTCCAAAAGGATGCAAAGGTGCTGTTTCAAAATTGGGGAATTGAACCCATTGACAATGTCATGGG<br>GATGATCGGGATACTGCCTGACATGACCCCCAGCACAGAGATGTCACTAAGAGGAGTGAGAGTCAGTAAAATGGGAGTGGATGAATATTCCAGTACTGAGAGAGTGGTCGTAAGCATTGATCGTTTCTTAAGAGTCCGAGACCAGAGGGGAAACGTACTCTTGTCTCCTGAAGAAGTTAGTGAAACACAGGGAACAGAGAAACTGACAATAACATATTCATCATCTATG<br>ATGTGGGAAATCAATGGTCCGGAGTCAGTACTGGTCAACACATATCAATGGATCATTAGAAATTGGGAAACTGTGAAGATTCAGTGGTCCCAAGACCCCACAATGTTGTACAATAAGATGGAGTTTGAGCCTTTCCAATCCTTGGTGCCCAAGGCTGCCAGAGGCCAGTACAGTGGATTTGTGAGGACGTTATTCCAGCAGATGCGTGATGTGCTGGGGACATTTGACACT<br>GTCCAAATAATAAAGCTCCTACCATTTGCAGCAGCCCCACCGGAACAGAGTAGGATGCAGTTCTCTTCTCTGACTGTGAATGTAAGAGGTTCAGGAATGAGAATACTTGTGAGAGGCAACTCCCCTGTGTTCAACTATAACAAAGCAACCAAGAGACTCACAGTTCTTGGAAAGGATGCAGGTGCATTGACAGAAGATCCAGACGAGGGAACAGCAGGAGTGGAGTCTGC<br>TGTGTTAAGAGGATTTCTAATTCTGGGCAAAGAAGACAAAAGATATGGACCAGCATTGAGCATTAACGAATTGAGCAATCTTGCGAAAGGGGAGAAGGCTAATGTGTTGATAGGGCAAGGGGACGTGGTGTTGGTAATGAAACGGAAACGGGACTCTAGCATACTTACTGACAGCCAGACAGCGACCAAAAGGATTCGGATGGCCATCAATTA
EPI_ISL_661178	>A/swan/Tumen/1479-2/2020_H5N8_2020-09-10<br>ATGGAGAGAATAAAAGAACTAAGAGATTTGATGTCGCAGTCTCGCACTCGCGAGATACTAACAAAAACCACTGTGGACCATATGGCCATAATAAAGAAATACACATCAGGGAGACAGGAGAAGAACCCTGCCCTCAGGATGAAATGGATGATGGCAATGAAATATCCTATTACAGCTGACAAAAGAATAATGGAGATGATCCCTGAAAGGAATGAGCAAGGTCAGACT<br>CTCTGGAGCAAAACAAATGATGCTGGATCAGACAGAGTGATGGTCTCACCTCTGGCTGTGACATGGTGGAATAGAAATGGGCCAACAACAAGTACAGTACACTACCCAAAAGTCTACAAAACCTACTTTGAAAAGGTAGAAAGGTTGAAACATGGAACCTTTGGTCCTGTTCACTTTCGAAATCAGGTTAAGATACGCCGCAGGGTTGACATAAACCCGGGCCATGCAGA<br>TCTCAGTGCCAAAGAAGCACAGGATGTCATCATGGAGGTTGTTTTCCCAAATGAAGTTGGAGCCAGGATCTTGACATCAGAATCACAATTAACAATAACAAGGGAAAAGAAGGAGGAACTTCAGGATTGCAAGATTGCTCCTTTGATGGTGGCATACATGTTGGAAAGAGAACTGGTTCGCAAGACCAGATTTTTACCAGTAGCTGGCGGAACAAGCAGCGTATACATCG<br>AGGTATTGCATTTGACTCAAGGGACCTGCTGGGAACAAATGTACACACCAGGAGGGGAGGTGAGAAATGATGATGTTGATCAGAGTTTGATCATTGCTGCTAGAAATATAGTTAGGAGGGCAACAGTATCAGCAGACCCATTGGCTTCGCTCTTGGAAATGTGCCACAGTACACAAATTGGTGGAGTGAGGATGGTGGACATTCTTAGGCAGAACCCAACAGAGGAGCA<br>AGCTGTGGATATATGCAAAGCAGCAATGGGTTTAAGAATCAGTTCATCCTTCAGCTTTGGAGGTTTCACTTTCAAAAGGACAAGTGGGTCGTCTGTCAAAAGAGAAGAAGAAATACTCACTGGCAACCTCCAAACACTGAAAGTAAGAATACATGAAGGATATGAGGAATTCACAATGGTTGGGCGAAGAGCTACAGCCATTTTGAGGAAAGCAACCAGGAGACTGATCC<br>AATTAATAGTGAGTGGACGAGACGAGCAGTCAATCGCTGAAGCAATCATAGTGGCAATGGTTTTCTCACAGGAGGATTGCATGATAAAAGCAGTACGAGGTGATTTGAATTTTGTCAACAGAGCGAATCAGCGGCTAAATCCTATGCATCAACTTCTGAGGCATTTCCAAAAGGATGCAAAAGTGCTGTTTCAAAACTGGGGGATTGAACCAATTGACAATGTAATGGGG<br>ATGATCGGGATACTGCCTGACATGACCCCCAGCACAGAGATGTCACTGAGAGGAGTGAGAATCAGCAAAATGGGAGTGGATGAATATTCCAGTACTGAGAGAGTGGTCGTGAGCATTGATCGCTTCTTGAGAGTCCGAGATCAGAGGGGAAATGTGCTTCTGTCTCCTGAGGAAGTTAGTGAAACACATGGAACAGAGAAACTGACGATAACGTACTCATCGTCTATGA<br>TGTGGGAATCAATGGTCCGGAATCCGTGCTAGTCAACACATATCAATGGATCATTAGAAATTGGGAAACTGTGAAGATTCAGTGGTCCCAGGACCCTACGATGTTGTACAATAAGATGGAATTTGAGCCCTTCCAATCCTTGGTGCCCAAGGCTGCTAGAGGCCAGTATAGTGGGTTTGTGAGGACATTATTCCAACAGATGCGTGATGTGTTGGGGACATTTGACACTGT<br>CCAAATAATAAAGCTCCTACCATTTGCAGCAGCCCCACCGGAACAGAGTAGGATGCAATTTTCTCCCTGACTGTGAACGTAAGAGGTTCAGGAATGAGAATACTTGTGAGGGGCAACTCCCCTGTGTTCAACTATAATAAGGCCACCAAGAGACTCACAGTTCTTGGAAAGGATGCAGGCGCCTTGACAGAATATCCAGATGAGGGAACAGCAGGAGTGGAGTCTGCAG<br>TATTAAGAGGATTTCTAATTCTGGGCAAAGAGGACAAAAGATATGGACCAGCATTGAGCATCAACGAATTGAGCAATCTTGCGAAAGGGGAAAAGGCTAATGTGTTGATAGGACAAGGAGACGTGGTGTTGGTAATGAAACGGAAACGGGACTCTAGCATACTTACTGACAGCCAGACAGCGACCAAAAGAATTCGGATGGCCATCAATTA
EPI_ISL_331295	>A/teal/Chany/324/2017_H12N5_2017-09-03<br>ATGGAGAGAATAAAAGAGCTAAGAGATTTGATGTCGCAGTCTCGCACTCGCGAGATACTGACAAAAACCACTGTGGACCATATGGCCATAATCAAGAAATATACATCAGGAAGACAGGAGAAGAACCCTGCACTTAGGATGAAATGGATGATGGCAATGAAATATCCGATTACAGCAGACAAAAGGATAATGGAGATGATCCCTGAAAGAAATGAGCAAGGTCAGACT<br>CTTTGGAGCAAAACAAATGATGCTGGATCAGACAGAGTGATGGTGTCACCTCTGGCTGTGACGTGGTGGAATAGAAATGGACCGACAACAAGTACAGTCCATTACCCAAAGGTCTATAAAACCTACTTTGAAAAGGTTGAAAGGTTAAAGCATGGAACCTTCGGCCCTGTTCATTTTCGAAATCAGGTTAAGATACGCCGCAGAGTTGACATAAACCCGGGCCATGCAGA<br>CCTCAGTGCTAAAGAAGCACAAGACGTCATCATGGAGGTCGTTTTCCCAAATGAAGTCGGAGCCAGAATATTGACATCAGAGTCACAGTTAACAATAACAAAGGAAAAGAAGGAGGAACTCCAGGACTGTAAGATTGCCCCTTTAATGGTGGCATACATGTTGGAGAGAGAACTGGTTCGAAAAACCAGATTCCTGCCAGTAGCTGGCGGAACAAGCAGTGTATATATC<br>GAGGTGTTGCACTTGACTCAAGGAACCTGCTGGGAACAAATGTATACGCCAGGAGGAGAAGTGAGAAATGATGACGTTGATCAGAGTTTAATTATTGCTGCCAGAAATATCGTTAGGAGAGCAACAGTATCAGCAGACCCATTGGCTTCGCTACTGGAGATGTGCCATAGTACACAAATTGGCGGGATAAGGATGGTAGACATCCTTAGACAGAACCCAACAGAAGAGC<br>AAGCTGTGGATATATGCAAAGCAGCAATGGGTCTAAGAATCAGTTCATCCTTCAGCTTTGGAGGTTTCACTTTCAAAAGGACAAGCGGGTCATCTGTCAAAAGAGAAGAGGAAGTGCTCACCGGCAACCTCCAAACATTGAAAATAAGAGTGCATGAAGGGTATGAGGAATTCACAATGGTTGGACGGAGAGCAACAGCCATTCTAAGGAAAGCAACCAGAAGGCTGAT<br>CCAATTGATAGTAAGTGGAAGAGACGAGCAGTCAATCGCCGAAGCGATCATAGTGGCAATGGTGTTCTCCCAAGAGGATTGCATGATAAAGGCAGTACGAGGTGATTTAAATTTTGTCAATAGAGCGAATCAGCGGCTCAATCCTATGCATCAGCTCCTGAGGCATTTCCAAAAGGATGCAAAGGTACTGTTCCAAAACTGGGGAATTGAACCCATTGACAATGTCATGG<br>GGATGATAGGAATATTGCCTGATATGACACCCAGCACAGAGATGTCACTAAGAGGAGTGAGGGTCAGTAAAATGGGAGTGGATGAATATTCCAGTACTGAGAGGGTGGTCGTGAGTATTGATCGCTTCTTGAGGGTCCGAGACCAGAGAGGAAACGTAATCTTGTCTCCTGAAGAGGTTAGTGAAACACAGGGAACAGAGAAGCTGACGATAACATATTCATCATCCAT<br>GATGTGGGAAATTAATGGTCCGGAATCAGTGTTAGTTAACACATATCAATGGGTCATCAGAAACTGGGAAACTGTGAAGATTCAGTGGTCCCAAGACCCTACAATGCTATACAACAAGATGGAGTTTGAGCCTTTTCAGTCCTTGGTGCCTAAGGCGGCCAGAGGCCAGTACAGTGGATTTGTAAGGACTCTATTCCAGCAGATGCGTGATGTGCTGGGGACCTTTGACAC<br>TGTCCAGATAATAAAGCTACTCCCATTTGCAGCAGCCCCACCGGAACAGAGTAGGATGCAGTTCTCTTCTCTAACTGTAAACGTAAGGGGTTCAGGAATGAGAATACTTGTGAGAGGCAACTCCCCTGTGTTCAACTATAACAAGGCAACCAAGAGGCTCACAGTTCTTGGAAAGGATGCTGGTGCATTGACAGAAGACCCAGATGAGGGGACGGCAGGAGTGGAGTCTG<br>CGGTATTGAGAGGGTTCCTAATTCTGGGCAAAGAAGACAAAAGATATGGACCAGCGCTGAGCATCAATGAATTGAGCAATCTTGCGAAAGGGGAGAAGGCTAATGTGTTGATAGGGCAAGGAGACGTGGTGTTGGTGATGAAACGGAAACGGGACTCTAGCATACTTACTGACAGCCAGACAGCGACCAAAAGAATTCGGATGGCCATCAATTA
EPI_ISL_331307	>A/teal/Dagestan/1017/2018_H12N5_2018-01-23<br>ATGGAGAGAATAAAAGAGCTAAGAGATTTGATGTCACAGTCTCGCACTCGCGAGATACTGACAAAAACCACTGTGGACCATATGGCCATAATCAAGAAATATACATCAGGAAGACAGGAGAAGAATCCTGCACTTAGGATGAAATGGATGATGGCAATGAAATATCCGATTACAGCGGACAAAAGGATAACGGAGATGATCCCTGAAAGAAATGAGCAAGGTCAGACT<br>CTTTGGAGCAAAACAAATGATGCTGGATCAGACAGAGTGATGGTGTCACCTCTGGCTGTGACGTGGTGGAATAGAAATGGACCAACAACAAGTACAGTCCATTACCCAAAGGTCTATAAAACCTACTTTGAAAAGGTTGAAAGGTTAAAGCATGGAACCTTCGGCCCTGTTCATTTTCGAAATCAGGTTAAGATACGCCGCAGAGTTGACATAAACCCGGGCCATGCAGAC<br>CTCAGTGCTAAAGAAGCACAAGACGTCATCATGGAGGTCGTTTTCCCAAATGAAGTCGGAGCCAGAATATTGACATCAGAGTCACAGTTAACAATAACAAAGGAAAAGAAGGAGGAACTCCAGGACTGTAAGATTGCCCCTTTAATGGTGGCATACATGTTAGAGAGAGAACTGGTTCGAAAAACCAGATTCCTGCCAGTAGCTGGCGGAACAAGCAGCGTATATATCG<br>AGGTGTTGCACTTGACTCAAGGAACCTGCTGGGAACAAATGTATACGCCAGGAGGAGAAGTGAGAAATGATGACGTTGATCAGAGTTTAATTATTGCTGCCAGAAATATCGTTAGGAGAGCAACAGTATCAGCAGACCCATTGGCTTCGCTACTGGAGATGTGCCATAGTACACAAATTGGCGGGATAAGGATGGTAGACATCCTTAGACAGAACCCAACAGAAGAGCA<br>AGCTGTGGATATATGCAAAGCAGCAATGGGTCTAAGAATCAGTTCATCCTTCAGCTTTGGAGGTTTCACTTTCAAAAGGACAAGCGGGTCATCTGTCAAAAGAGAAGAGGAAGTGCTCACCGGCAACCTCCAAACATTGAAGATAAGAGTGCATGAAGGGTATGAGGAGTTCACAATGGTTGGACGGAGAGCAACAGCCATTCTAAGGAAAGCAACCAGAAGGCTGATC<br>CAATTGATAGTAAGTGGAAGAGACGAGCAGTCAATCGCCGAAGCGATCATAGTGGCAATGGTGTTCTCCCAAGAGGATTGCATGATAAAGGCAGTACGAGGTGACTTAAATTTTGTCAATAGAGCGAATCAGCGGCTCAATCCTATGCATCAGCTCCTGAGGCATTTCCAAAAGGATGCAAAGGTATTGTTCCAAAACTGGGGAATTGAACCCATTGACAATGTCATGGG<br>GATGATAGGAATATTGCCTGATATGACACCCAGCACAGAGATGTCACTAAGAGGAGTGAGGGTCAGTAAAATGGGAGTGGATGAATATTCCAGTACTGAGAGGGTGGTCGTGAGTATTGATCGCTTCTTGAGGGTCCGAGACCAGAGAGGAAACGTACTCTTGTCTCCTGAAGAGGTTAGTGAAACACAGGGAACAGAGAAGCTGACGATAACATATTCATCATCCATG<br>ATGTGGGAAATTAATGGTCCGGAATCAGTGTTAGTTAACACATATCAATGGATCATCAGAAACTGGGAAACTGTGAAGATTCAGTGGTCCCAAGACCCTACAATGCTATATAACAAGATGGAGTTTGAGCCTTTTCAGTCCTTGGTGCCTAAGGCGACCAGAGGCCAGTACAGTGGGTTTGTAAGGACTCTATTCCAGCAGATGCGTGATGTGCTGGGGACCTTTGACACT<br>GTCCAGATAATAAAGCTACTCCCATTTGCAGCAGCCCCACCGGAACAGAGTAGGATGCAGTTCTCTTCTCTAACTGTAAACGTAAGGGGTTCAGGAATGAGAATACTTGTGAGAGGCAACTCCCCTGTGTTCAACTATAACAAGGCAACCAAGAGGCTCACAGTCCTTGGAAAGGATGCTGGTGCATTGACAGAAGACCCAGATGAGGGGACGGCAGGAGTGGAGTCTG<br>CGGTATTGAGAGGGTTCCTAATTCTGGGCAAAGAAGACAAAAGATATGGACCAGCGCTGAGCATCAATGAATTGAGCAATCTTGCGAAAGGGGAGAAGGCTAATGTGTTGATAGGGCAAGGAGACGTGGTGTTGGTGATGAAACGGAAACGGGACTCTAGCATACTTACTGACAGCCAGACAGCGACCAAAAGAATTCGGATGGCCATCAATTA
EPI_ISL_1255051	>A/tufted_duck/Poland/MB061/2021_H5N5_2021-02-01<br>ATGGAGAGAATAAAAGAACTAAGAGATTTGATGTCGCAGTCTCGCACCCGCGAGATACTGACAAAAACTACTGTGGACCATATGGCCATAATCAAGAAATATACGTCAGGAAGACAGGAGAAGAATCCTGCTCTCAGGATGAAATGGATGATGGCAATGAAATATCCGATTACAGCAGACAAAAGGATAATGGAGATGATCCCTGAAAGAAATGAGCAAGGTCAGACT<br>CTTTGGAGCAAAACAAATGATGCTGGATCGGACAGAGTTATGGTGTCACCTTTGGCTGTGACGTGGTGGAATAGAAATGGGCCAACGACAAGTACAGTCCATTATCCAAAGGTCTATAAAACCTACTTTGAAAAGGTCGAAAGGTTAAAGCATGGAACCTTCGGTCCTGTCCACTTTCGAAATCAGGTCAAAATACGCCGCAGGGTTGACATAAATCCAGGCCATGCAGAT<br>CTCAGTGCCAAAGAAGCACAGGATGTCATCATGGAGGTCGTTTTCCCAAATGAAGTCGGAGCCAGGATATTGACATCAGAGTCACAGTTAACAATAACAAAGGAAAAGAAGGAGGAGCTTCAGGACTGTAAGATTGCTCCTTTAATGGTGGCATACATGTTGGAGAGAGAATTGGTTCGCAAAACCAGATTCCTACCAGTAGCTGGTGGGACAAGCAGCGTGTACATTG<br>AGGTGTTGCACTTGACTCAAGGGACCTGTTGGGAACAAATGTACACGCCGGGAGGGGAGGTGAGAAATGATGATGTTGATCAGAGTTTAATTATTGCTGCCCGAAATATTGTTAGGAGAGCAACAGTATCAGCAGACCCATTGGCTTCGCTTTTGGAGATGTGCCATAGTACGCAAATTGGCGGGATAAGGATGGTAGACATCCTTAGACAAAACCCAACGGAAGAGCA<br>AGCTGTGGACATATGCAAAGCTGCAATGGGTCTAAGGATCAGTTCATCCTTCAGCTTTGGAGGTTTCACTTTCAAAAGAACGAGTGGGTCATCTGTCAAAAGAGAAGAAGAAGTGCTCACAGGCAACCTCCAAACATTGAAAATAAGAGTGCATGAAGGATATGAGGAATTCACAATGGCTGGGCGAAGAGCAACAGCTATCCTAAGGAAAGCAACCAGAAGGCTGATC<br>CAATTGATAGTGAGTGGGAGAGACGAGCAGTCAATCGCCGAGGCGATCATAGTGGCAATGGTGTTCTCACAAGAGGACTGTATGATAAAAGCAGTACGAGGTGATCTGAATTTTGTCAACAGAGCAAACCAGCGGTTAAACCCTATGCATCAACTATTGAGGCATTTCCAGAAGGATGCAAAGGTGCTGTTTCAAAACTGGGGAATTGAGCCCATCGACAATGTCATGGG<br>GATGATCGGAATATTACCTGACATGACCCCCAGTACAGAGATGTCACTAAGAGGAGTGAGAGTCAGTAAAATGGGAGTGGATGAGTACTCCAGTACTGAGAGAGTGGTTGTGAGCATTGATCGCTTCTTGAGGGTCCGAGACCAGAGGGGAAACGTGCTCTTGTCTCCTGAAGAAGTTAGTGAAACACAGGGAATAGAGAAGATGACGATAACATATTCATCATCCATG<br>ATGTGGGAAATCAACGGCCCAGAATCAGTGTTAGTGAACACATATCAATGGATCATTAGAAATTGGGAAACTGTGAAAATTCAATGGTCCCAAGACCCTACAATGCTATACAATAAGATGGAGTTTGAGCCCTTTCAATCTTTGGTGCCTAAGGCTGCCAGGGGTCAGTACAGTGGATTTGTGAGAACGCTATTCCAGCAGATGCGTGATGTGCTGGGGACATTTGACACT<br>GTCCAGATAATAAAGCTGCTGCCATTTGCAGCAGCCCCACCAGAACAGAGTAGAATGCAATTCTCTTCTCTAACCGTGAACGTGAGGGGTTCAGGAATGAGAATACTCGTGAGAGGCAACTCCCCTGTGTTCAACTATAACAAGGCGACCAAGAGGCTTACAGTCCTTGGAAAGGATGCAGGTGCATTGACAGAAGATCCAGATGAGGGAACAGCAGGAGTGGAATCTG<br>CGGTATTAAGAGGATTTCTAATTCTAGGCAAAGAAGACAAAAGATATGGGCCAGCATTGAGCATCAACGAATTGAGCAATCTTGCGAAAGGGGAGAAGGCTAATGTTTTGATAGGGCAAGGAGACGTGGTGTTGGTAATGAAACGGAAACGGGACTCTAGCATACTTACTGACAGCCAGACAGCGACCAAAAGAATTCGGATGGCCATCAATTA
EPI_ISL_1220094	>A/tundra-bean-goose/Poland/MB132/2020_H5N8_2020-12-11<br>ATGGAGAGAATAAAAGAACTAAGAGATTTGATGTCGCAGTCTCGCACTCGCGAGATACTAACAAAAACCACTGTGGACCATATGGCCATAATAAAGAAATACACATCAGGAAGACAGGAGAAGAACCCTGCCCTCAGGATGAAATGGATGATGGCAATGAAATATCCTATTACAGCTGACAAAAGAATAATGGAGATGATCCCTGAAAGGAATGAGCAAGGTCAGACT<br>CTCTGGAGCAAAACAAATGATGCTGGATCAGACAGAGTGATGGTCTCACCTCTGGCTGTGACATGGTGGAATAGAAATGGGCCAACAACAAGTACAGTACACTACCCAAAAGTCTACAAAACCTACTTTGAAAAGGTAGAAAGGTTGAAACATGGAACCTTTGGTCCTGTTCACTTTCGAAATCAGGTTAAGATACGCCGCAGGGTTGACATAAACCCGGGCCATGCAGA<br>TCTCAGTGCCAAAGAAGCACAGGATGTCATCATGGAGGTTGTTTTCCCAAATGAAGTTGGAGCCAGGATCTTGACATCAGAATCACAATTAACAATAACAAGGGAAAAGAAGGAGGAACTTCAGGATTGCAAGATTGCTCCTTTGATGGTGGCATACATGTTGGAAAGAGAACTGGTTCGCAAGACCAGATTTTTACCAGTAGCTGGCGGAACAAGCAGCGTATACATCG<br>AGGTATTGCATTTGACTCAAGGGACCTGCTGGGAACAAATGTACACACCAGGAGGGGAGGTGAGAAATGATGATGTTGATCAGAGTTTGATCATTGCTGCTAGAAATATAGTTAGGAGGGCAACAGTATCAGCAGACCCATTGGCTTCGCTTTTGGAAATGTGCCACAGTACACAAATTGGTGGAGTAAGGATGGTGGACATTCTTAGGCAGAACCCAACAGAGGAGCA<br>AGCTGTGGATATATGCAAAGCAGCAATGGGTTTAAGAATCAGTTCATCCTTCAGCTTTGGAGGTTTCACTTTCAAAAGGACAAGTGGGTCGTCTGTCAAAAGAGAAGAAGAAATACTCACTGGCAACCTCCAAACACTGAAAGTAAGAATACATGAAGGATATGAGGAATTCACAATGGTTGGGCGAAGAGCTACAGCCATTTTGAGGAAAGCAACCAGGAGACTGATCC<br>AATTAATAGTGAGTGGAAGAGACGAGCAGTCAATCGCTGAAGCAATCATAGTGGCAATGGTTTTCTCACAGGAGGATTGCATGGTAAAAGCAGTACGAGGTGATTTGAATTTTGTCAACAGAGCGAATCAGCGGCTAAATCCTATGCATCAACTTCTGAGGCATTTCCAAAAGGATGCAAAAGTGCTGTTTCAAAACTGGGGGATTGAACCAATTGACAATGTAATGGGG<br>ATGATCGGGATACTGCCTGACATGACCCCCAGCACAGAGATGTCACTGAGAGGAGTGAGAGTCAGCAAAATGGGAGTGGATGAATATTCCAGTACTGAGAGAGTGGTCGTGAGCATTGATCGCTTCTTGAGAGTCCGAGATCAGAGGGGAAATGTGCTTCTGTCTCCTGAGGAAGTTAGTGAAACACATGGAACAGAGAAACTGACGATAACGTATTCATCGTCTATGA<br>TGTGGGAAATCAATGGTCCGGAATCCGTGCTAGTCAACACATATCAATGGATCATTAGAAATTGGGAAACTGTGAAGATTCAGTGGTCCCAGGACCCTACGATGTTGTACAATAAGATGGAATTTGAGCCCTTCCAATCCTTGGTGCCCAAGGCTGCTAGAGGCCAGTATAGTGGGTTTGTGAGGACATTATTCCAACAGATGCGTGATGTGTTGGGGACATTTGACACTGT<br>CCAAATAATAAAGCTCCTACCATTTGCAGCAGCCCCACCGGAACAGAGTAGGATGCAATTTTCTTCTCTGACTGTGAACGTAAGAGGTTCAGGAATGAGAATACTTGTGAGGGGCAACTCCCCTGTGTTCAACTATAATAAGGCCACCAAGAGACTCACAGTTCTTGGAAAGGATGCAGGCGCCTTGACAGAATATCCAGATGAGGGAACAGCAGGAGTGGAGTCTGCAG<br>TATTAAGAGGATTTCTAATTCTGGGCAAAGAGGACAAAAGATATGGACCAGCATTGAGCATCAACGAATTGAGCAATCTTGCGAAAGGGGAAAAGGCTAATGTGTTGATAGGACAAGGAGACGTGGTGTTGGTAATGAAACGGAAACGGGACTCTAGCATACTTACTGACAGCCAGACAGCGACCAAAAGAATTCGGATGGCCATCAATTA
EPI_ISL_766053	>A/turkey/England/039352/2020_H5N8_2020-12-04<br>ATGGAGAGAATAAAAGAACTAAGAGATTTGATGTCGCAGTCTCGCACTCGCGAGATACTAACAAAAACCACTGTGGACCATATGGCCATAATAAAGAAATACACATCAGGGAGACAGGAGAAGAACCCTGCCCTCAGGATGAAATGGATGATGGCAATGAAATATCCTATTACAGCTGACAAAAGAATAATGGAGATGATCCCTGAAAGGAATGAGCAAGGTCAGACT<br>CTCTGGAGCAAAACAAATGATGCTGGATCAGACAGAGTGATGGTCTCACCTCTGGCTGTGACATGGTGGAATAGAAATGGGCCAACAACAAGTACAGTACACTACCCAAAAGTCTACAAAACCTACTTTGAAAAGGTAGAAAGGTTGAAACATGGAACCTTTGGTCCTGTTCACTTTCGAAATCAGGTTAAGATACGTCGCAGGGTTGACATAAACCCGGGCCATGCAGA<br>TCTCAGTGCCAAAGAAGCACAGGATGTCATCATGGAGGTTGTTTTCCCAAATGAAGTTGGAGCCAGGATCTTGACATCAGAATCACAATTAACAATAACAAGGGAAAAGAAGGAGGAACTTCAGGATTGCAAGATTGCTCCTTTGATGGTGGCATACATGTTGGAAAGAGAACTGGTTCGCAAGACCAGATTTTTACCAGTAGCTGGCGGAACAAGCAGCGTATACATCG<br>AGGTATTGCATTTGACTCAAGGGACCTGCTGGGAACAAATGTACACACCAGGAGGGGAGGTGAGAAATGATGATGTTGATCAGAGTTTGATCATTGCTGCTAGAAATATAGTTAGGAGGGCAACAGTATCAGCAGACCCATTGGCTTCGCTCTTGGAAATGTGCCACAGTACACAAATTGGTGGAGTGAGGATGGTGGACATTCTTAGGCAGAACCCAACAGAGGAGCA<br>AGCTGTGGATATATGCAAAGCAGCAATGGGTTTAAGAATCAGTTCATCCTTCAGCTTTGGAGGTTTCACTTTCAAAAGGACAAGTGGGTCGTCTGTCAAAGGGAAGAAGAAATACTCACTGGCAACCTCCAAACACTGAAAGTAAGAATACATGAAGGATATGAGGAATTCACAATGGTTGGGCGAAGAGCTACAGCCATTTTGAGGAAAGCAACCAGGAGACTGATCC<br>AATTAATAGTGAGTGGACGAGACGAGCAGTCAATCGCTGAAGCAATCATAGTGGCGATGGTTTTCTCACAGGAGGATTGCATGATAAAAGCAGTACGAGGTGATTTGAATTTTGTCAACAGAGCGAATCAGCGGCTAAATCCTATGCATCAACTTCTGAGGCATTTCCAAAAGGATGCAAAAGTGCTGTTTCAAAACTGGGGGATTGAACCAATTGACAATGTAATGGGG<br>ATGATCGGGATACTGCCTGACATGACCCCCAGCACAGAGATGTCACTGAGAGGAGTGAGAGTCAGCAAAATGGGAGTGGATGAATATTCCAGTACTGAGAGAGTGGTCGTGAGCATTGATCGCTTCTTGAGAGTCCGAGATCAGAGGGGAAATGTGCTTCTGTCTCCTGAGGAAGTTAGTGAAACACATGGAACAGAGAAACTGACGATAACGTATTCATCGTCTATGA<br>TGTGGGAAATCAATGGTCCGGAATCCGTGCTAGTCAACACATATCAATGGATCATTAGAAATTGGGAAACTGTGAAGATTCAGTGGTCCCAGGACCCTACGATGTTGTACAATAAGATGGAATTTGAGCCCTTCCAATCCTTGGTGCCCAAGGCTGCTAGAGGCCAGTATAGTGGGTTTGTGAGGACATTGTTCCAACAGATGCGTGATGTGTTGGGGACATTTGACACTGT<br>CCAAATAATAAAGCTCCTACCATTTGCAGCAGCCCCACCGGAACAGAGTAGGATGCAATTTTCCTCTCTGACTGTGAACGTAAGAGGTTCAGGAATGAGAATACTTGTGAGGGGCAACTCCCCTGTGTTCAACTATAATAAGGCCACCAAGAGACTCACAGTTCTTGGAAAGGATGCAGGCGCCTTGACAGAATATCCAGATGAGGGAACAGCAGGAGTGGAGTCTGCAG<br>TATTAAGAGGATTTCTAATTCTGGGCAAAGAGGACAAAAGATATGGACCAGCATTGAGCATCAACGAATTGAGCAATCTTGCGAAAGGGGAAAAGGCTAATGTGTTGATAGGACAAGGAGACGTGGTGTTGGTAATGAAACGGAAACGGGACTCTAGCATACTTACTGACAGCCAGACAGCGACCAAAAGAATTCGGATGGCCATCAATTA
internal NVRI repository	>A/turkey/Poland/116/2021_H5N8_2021-02-11<br>ATGGAGAGAATAAAAGAACTAAGAGATTTGATGTCGCAGTCTCGCACTCGCGAGATACTAACAAAAACCACTGTGGACCATATGGCCATAATAAAGAAATACACATCAGGGAGACAGGAGAAGAACCCTGCCCTCAGGATGAAATGGATGATGGCAATGAAATATCCTATTACAGCTGACAAAAGAATAATGGAGATGATCCCTGAAAGGAATGAGCAAGGTCAGACT<br>CTCTGGAGCAAAACAAATGATGCTGGATCAGACAGAGTGATGGTCTCACCTCTGGCTGTGACGTGGTGGAATAGAAATGGGCCAACAACAAGTACAGTACACTACCCAAAAGTCTACAAAACCTACTTTGAAAAGGTAGAAAGGTTGAAACATGGAACCTTTGGTCCTGTTCACTTTCGAAATCAGGTTAAGATACGCCGCAGGGTTGACATAAACCCGGGCCATGCAGA<br>TCTCAGTGCCAAAGAAGCACAGGATGTCATCATGGAGGTTGTTTTCCCAAATGAAGTTGGAGCCAGGATCTTGACATCAGAATCACAATTAACAATAACAATGGAAAAGAAGGAGGAACTTCAGGATTGCAAGATTGCTCCTTTGATGGTGGCATACATGTTGGAAAGAGAACTGGTTCGCAAGACCAGATTTTTACCAGTAGCTGGCGGAACAAGCAGCGTATACATCG<br>AGGTATTGCATTTGACTCAAGGGACCTGCTGGGAACAAATGTACACACCAGGAGGGGAGGTGAGAAATGATGATGTTGATCAGAGTTTGATCATTGCTGCTAGAAATATAGTTAGGAGGGCAACAGTATCAGCAGACCCATTGGCTTCGCTCTTGGAAATGTGCCACAGTACACAAATTGGTGGAGTGAGGATGGTGGACATTCTTAGGCAGAACCCAACAGAGGAGCA<br>AGCTGTGGATATATGCAAAGCAGCAATGGGTTTAAGAATCAGTTCATCCTTCAGCTTTGGAGGTTTCACTTTCAAAAGGACAAGTGGGTCGTCTGTCAAAAGGGAAGAAGAAATACTCACTGGCAACCTCCAAACACTGAAAGTAAGAATACATGAAGGATATGAGGAATTCACAATGGTTGGGCGAAGAGCTACAGCCATTTTGAGGAAAGCAACCAGGAGACTGATCC<br>AATTAATAGTGAGTGGACGAGACGAACAGTCAATCGCTGAGGCAATCATAGTGGCAATGGTTTTCTCACAGGAGGATTGCATGATAAAAGCAGTACGAGGTGATTTGAATTTTGTCAACAGAGCGAATCAGCGGCTAAATCCTATGCATCAACTTCTGAGGCATTTCCAAAAAGATGCAAAAGTGCTGTTTCAAAACTGGGGATTGAACCAATTGACAATGTAATGGGG<br>ATGATCGGGATACTGCCTGACATGACCCCCAGCACAGAGATGTCACTGAGAGGAGTGAGAGTCAGCAAAATGGGAGTGGATGAATATTCCAGTACTGAGAGAGTGGTCGTGAGCATTGATCGCTTCTTGAGAGTCCGAGATCAGAGGGGAAATGTGCTTCTGTCTCCTGAGGAAGTTAGTGAAACACATGGAACAGAGAAACTGACGATAACGTATTCATCGTCTATGA<br>TGTGGGAAATCAATGGTCCGGAATCCGTGCTAGTCAACACATATCAATGGATCATTAGAAATTGGGAAACTGTGAAGATTCAGTGGTCCCAGGACCCTACGATGTTGTACAATAAGATGGAATTTGAGCCCTTCCAATCCTTGGTGCCCAAGGCTGCTAGAGGCCAGTATAGTGGGTTTGTGAGGACATTATTCCAACAGATGCGTGATGTGTTGGGGACATTTGACACTGT<br>CCAAATAATAAAGCTCCTACCATTTGCAGCAGCCCCACCGGAACAGAGTAGGATGCAATTTTCCTCTATGACTGTGAACGTAAGAGGTTCAGGAATGAGAATACTTGTGAGGGGCAACTCCCCTGTGTTCAACTATAATAAGACCACCAAGAGACTCACAGTTCTTGGAAAGGATGCAGGCGCCTTGACAGAATATCCAGATGAGGGAACAGCAGGAGTGGAGTCTGCAG<br>TATTAAGAGGATTTCTAATTCTGGGCAAAGAGGACAAAAGATATGGACCGGCATTGAGCATCAACGAATTGAGCAATCTTGCGAAAGGGGAAAAGGCTAATGTGTTGATAGGACAAGGAGACGTGGTGTTGGTAATGAAACGGAAACGGGACTCTAGCATACTTACTGACAGCCAGACAGCGACCAAAAGAATTCGGATGGCCATCAATTA
EPI_ISL_779129	>A/turkey/Poland/464/2020_H5N8_2020-12-01<br>ATGGAGAGAATAAAAGAACTAAGAGATTTGATGTCGCAGTCTCGCACTCGCGAGATACTAACAAAAACCACTGTGGACCATATGGCCATAATAAAGAAATACACATCAGGAAGRCAGGAGAAGAACCCTGCCCTCAGGATGAAATGGATGATGGCAATGAAATATCCTATTACAGCTGACAAAAGAATAATGGAGATGATCCCTGAAAGGAATGAGCAAGGTCAGACTC<br>TCTGGAGCAAAACAAATGATGCTGGATCAGACAGAGTGATGGTCTCACCCCTGGCTGTGACATGGTGGAATAGAAATGGGCCAACAACAAGTACAGTACACTACCCAAAAGTCTACAAAACCTACTTTGAAAAGGTAGAAAGGTTGAAACATGGAACCTTTGGTCCTGTTCACTTTCGAAATCAGGTTAAGATACGCCGCAGGGTTGACATAAACCCGGGCCATGCAGAT<br>CTCAGTGCCAAAGAAGCACAGGATGTCATCATGGAGGTTGTTTTCCCAAATGAAGTTGGAGCCAGGATCTTGACATCAGAATCACAATTAACAATAACAAGAGAAAAGAAGGAGGAACTTCAGGATTGCAAGATTGCTCCTTTGATGGTGGCATACATGTTGGAAAGAGAACTGGTTCGCAAGACCAGATTTTTACCAGTAGCTGGCGGAACAAGCAGCGTATACATCGA<br>GGTATTGCATTTGACTCAAGGGACCTGCTGGGAACAAATGTACACACCAGGAGGGGAGGTGAGAAATGATGATGTTGATCAGAGTTTGATCATTGCTGCTAGAAATATAGTTAGGAGGGCAACAGTATCAGCAGACCCTTTGGCTTCGCTCTTGGAAATGTGCCACAGTACACAAATTGGTGGAGTAAGGATGGTGGACATTCTTAGGCAGAACCCAACAGAGGAGCAA<br>GCTGTGGATATATGCAAAGCAGCAATGGGTTTAAGAATCAGTTCATCCTTCAGCTTTGGAGGTTTCACTTTCAAAAGGACAAGTGGGTCGTCTGTCAAAAGAGAAGAAGAAATACTCACTGGCAACCTCCAAACACTGAAAGTAAGAATACATGAAGGATATGAGGAATTCACAATGGTTGGGCGAAGAGCTACAGCCATTTTGAGGAAAGCAACCAGGAGACTGATCCA<br>ATTAATAGTGAGTGGAAGAGACGAGCAGTCAATCGCTGAAGCAATCATAGTGGCAATGGTTTTCTCACAGGAGGATTGCATGATAAAAGCAGTACGAGGTGATTTGAATTTTGTCAACAGAGCGAATCAGCGGCTAAATCCTATGCATCAACTTCTGAGGCATTTCCAAAAGGATGCAAAAGTGCTGTTTCAAAACTGGGGGATTGAACCAATTGACAATGTAATGGGGA<br>TGATCGGGATACTGCCTGACATGACCCCCAGCACAGAGATGTCACTGAGAGGAGTGAGAGTCAGCAAAATGGGAGTGGATGAATATTCCAGTACTGAGAGAGTGGTCGTGAGCATTGATCGCTTCTTGAGAGTCCGAGATCAGAGGGGAAATGTGCTTTTGTCTCCTGAGGAAGTTAGTGAAACACATGGAACAGAGAAACTGACGATAACGTATTCATCGTCTATGAT<br>GTGGGAAATCAATGGTCCGGAATCCGTGCTAGTCAACACATATCAATGGATCATTAGAAGTTGGGAAACTGTGAAGATTCAATGGTCCCAGGACCCTACGATGCTGTACAATAAGATGGAATTTGAGCCCTTCCAATCCTTGGTGCCCAAGGCTGCTAGAGGCCAGTATAGTGGGTTTGTGAGGACATTATTCCAACAGATGCGTGATGTGTTGGGGACATTTGACACTGT<br>CCAAATAATAAAGCTCCTACCATTTGCAGCAGCCCCACCGGAACAGAGTAGGATGCAATTTTCCTCTCTGACTGTGAACGTAAGAGGTTCAGGAATGAGAATACTTGTGAGGGGCAACTCCCCTGTGTTCAACTATAATAAGGCCACCAAGAGACTCACAGTTCTTGGAAAGGATGCAGGCGCCTTGACAGAATATCCAGATGAGGGAACAGCAGGAGTTGAGTCTGCAG<br>TATTAAGAGGATTTCTAATTCTGGGCAAAGAGGACAAAAGATATGGACCAGCATTGAGCATCAACGAATTGAGCAATCTTGCGAAAGGGGAAAAGGCTAATGTGTTGATAGGACAAGGAGACGTGGTGTTGGTAATGAAACGGAAACGGGACTCTAGCATACTTACTGACAGCCAGACAGCGACCAAAAGAATTCGGATGGCCATCAATTA
EPI_ISL_846602	>A/turkey/Poland/475/2020_H5N8_2020-12-03<br>ATGGAGAGAATAAAAGAACTAAGAGATTTGATGTCGCAGTCTCGCACTCGCGAGATACTAACAAAAACCACTGTGGACCATATGGCCATAATAAAGAAATACACATCAGGAAGACAGGAGAAGAACCCTGCCCTCAGGATGAAATGGATGATGGCAATGAAATATCCTATTACAGCTGACAAAAGAATAATGGAGATGATCCCTGAAAGGAATGAGCAAGGTCAGACT<br>CTCTGGAGCAAAACAAATGATGCTGGATCAGACAGAGTGATGGTCTCACCTCTGGCTGTGACATGGTGGAATAGAAATGGGCCAACAACAAGTACAGTACACTACCCAAAAGTCTACAAAACCTACTTTGAAAAGGTAGAAAGGTTGAAACATGGAACCTTTGGTCCTGTTCACTTTCGAAGTCAGGTTAAGATACGCCGTAGGGTTGACATAAACCCGGGCCATGCAGA<br>TCTCAGTGCCAAAGAAGCACAGGATGTCATCATGGAGGTTGTTTTCCCAAATGAAGTTGGAGCCAGGATCTTGACATCAGAATCACAATTAACAATAACAAGGGAAAAGAAGGAGGAACTTCAGGATTGCAAGATTGCTCCTTTGATGGTGGCATACATGTTGGAAAGAGAACTGGTTCGCAAGACCAGATTTTTACCAGTAGCTGGCGGAACAAGCAGCGTATACATCG<br>AGGTATTGCATTTGACTCAAGGGACCTGCTGGGAACAAATGTACACACCAGGAGGGGAGGTGAGAAATGATGATGTTGATCAGAGTTTGATCGTTGCTGCTAGAAATATAGTTAGGAGGGCAACAGTATCAGCAGACCCATTGGCTTCGCTTTTGGAAATGTGCCACAGTACACAAATTGGTGGAGTAAGGATGGTGGACATTCTTAGGCAGAACCCAACAGAGGAGCA<br>AGCTGTGGATATATGCAAAGCAGCAATGGGTTTAAGAATCAGTTCATCCTTCAGCTTTGGAGGTTTCACTTTCAAAAGGACAAGTGGGTCGTCTGTCAAAAGAGAAGAAGAAATACTCACTGGCAACCTCCAAACACTGAAAGTAAGAATACATGAAGGGTATGAGGAATTCACAATGGTTGGGCGAAGAGCTACAGCCATTTTGAGGAAAGCAACCAGGAGACTGATCC<br>AATTAATAGTGAGTGGAAGAGACGAGCAGTCAATCGCTGAAGCAATCATAGTGGCAATGGTTTTCTCACAGGAGGATTGCATGATAAAAGCAGTACGAGGTGATTTGAATTTTGTCAACAGAGCGAATCAGCGGCTAAATCCTATGCATCAACTTCTGAGGCATTTCCAAAAGGATGCAAAAGTGCTGTTTCAAAACTGGGGGATTGAACCAATTGACAATGTAATGGGG<br>ATGATCGGGATACTGCCTGACATGACCCCCAGCACAGAGATGTCACTGAGAGGAGTGAGAGTCAGCAAAATGGGAGTGGATGAATATTCCAGTACTGAGAGAGTGGTCGTGAGCATTGATCGCTTCTTGAGAGTCCGAGATCAGAGGGGAAATGTGCTTCTGTCTCCTGAGGAAGTTAGTGAAACACATGGAACAGAGAAACTGACGATAACGTATTCATCGTCTATGA<br>TGTGGGAAATCAATGGTCCGGAATCCGTGCTAGTCAACACATATCAATGGATCATTAGAAATTGGGAAACTGTGAAGATTCAGTGGTCCCAGGACCCTACGATGTTATACAATAAGATGGAATTTGAGCCCTTCCAATCCTTGGTGCCCAAGGCTGCTAGAGGCCAGTATAGTGGGTTTGTGAGGACATTATTCCAACAGATGCGTGATGTGTTGGGAACATTTGACACTAT<br>CCAAATAATAAAGCTCCTACCATTTGCAGCAGCCCCACCGGAACAGAGTAGGATGCAATTTTCCTCTCTGACTGTGAACGTAAGAGGTTCAGGAATGAGAATACTTGTGAGGGGCAAYTCCCCTGTGTTCAACTATAATAAGGCCACCAAGAGACTCACAGTTCTTGGAAAGGATGCAGGCGCCTTGACAGAATATCCAGATGAGGGAACAGCAGGAGTGGAGTCTGCAG<br>TATTAAGAGGATTTCTAATTCTGGGCAAAGAGGACAAAAGATATGGACCAGCATTGAGCATCAACGAATTGAGCAATCTTGCGAAAGGGGAAAAGGCTAATGTGTTGATAGGACAAGGAGACGTGGTGTTGGTAATGAAACGGAAACGGGACTCTAGCATACTTACTGACAGCCAGACAGCGACCAAAAGAATTCGGATGGCCATCAATTA
EPI_ISL_846604	>A/turkey/Poland/477/2020_H5N8_2020-12-07<br>ATGGAGAGAATAAAAGAACTAAGAGATTTGATGTCGCAGTCTCGCACTCGCGAGATACTAACAAAAACCACTGTGGACCATATGGCCATAATAAAGAAATACACATCAGGGAGACAGGAGAAGAACCCTGCCCTCAGGATGAAATGGATGATGGCAATGAAATATCCTATTRCAGCTGACAAAAGAATAATGGAGATGATCCCTGAAAGGAATGAGCAAGGTCAGACT<br>CTCTGGAGCAAAACAAATGATGCTGGATCAGACAGAGTGATGGTCTCACCTCTCGCTGTGACATGGTGGAATAGAAATGGGCCAACAACAAGTACAGTACACTACCCAAAAGTCTACAAAACCTACTTTGAAAAGGTAGAAAGGTTGAAACATGGAACCTTTGGTCCTGTTCACTTTCGAAATCAGGTTAAGATACGCCGCAGGGTTGACATAAACCCGGGCCATGCAGA<br>TCTCAGTGCCAAAGAAGCACAGGATGTCATCATGGAGGTTGTTTTCCCAAATGAAGTTGGAGCCAGGATCTTGACATCAGAATCACAATTAACAATAACAATGGAAAAGAAGGAGGAACTTCAGGATTGCAAGATTGCTCCTTTGATGGTGGCATACATGTTGGAAAGAGAACTGGTTCGCAAGACCAGATTTTTACCAGTAGCTGGCGGAACAAGCAGCGTATACATCG<br>AGGTATTGCATTTGACTCAAGGGACCTGCTGGGAACAAATGTACACACCAGGAGGGGAGGTGAGAAATGATGATGTTGATCAGAGTTTGATCATTGCTGCTAGAAATATAGTTAGGAGGGCAACAGTATCAGCAGACCCATTGGCTTCGCTCTTGGAAATGTGCCACAGTACACAAATTGGTGGAGTGAGGATGGTGGATATTCTTAGGCAGAACCCAACAGAGGAGCA<br>AGCTGTGGATATATGCAAAGCAGCAATGGGTTTAAGAATCAGTTCATCCTTCAGCTTTGGAGGTTTCACTTTCAAAAGGACAAGTGGGTCGTCTGTCAAAAGAGAAGAAGAAATACTCACTGGCAACCTCCAAACACTGAAAGTAAGAATACATGAAGGATATGAGGAATTCACAATGGTTGGGCGAAGAGCTACAGCCATTTTGAGGAAAGCAACCAGGAGACTGATCC<br>AATTAATAGTGAGTGGACGAGACGAACAGTCAATCGCTGAAGCAATCATAGTGGCAATGGTTTTCTCACAGGAGGATTGCATGATAAAAGCAGTACGAGGTGATTTGAATTTTGTCAACAGAGCGAATCAGCGGCTAAATCCTATGCATCAACTTCTGAGGCATTTCCAAAAGGATGCAAAAGTGCTGTTTCAAAACTGGGGGATTGAACCAATTGACAATGTAATGGGG<br>ATGATCGGGATACTGCCTGACATGACCCCCAGCACAGAGATGTCACTGAGAGGAGTGAGAGTCAGCAAAATGGGAGTGGATGAATATTCCAGTACTGAGAGAGTGGTCGTGAGCATTGATCGCTTCTTGAGAGTCCGAGATCAGAGGGGAAATGTGCTTCTGTCTCCTGAGGAAGTTAGTGAAACACATGGAACAGAGAGACTGACGATAACGTATTCATCGTCTATGA<br>TGTGGGAAATCAATGGTCCGGAATCCGTGCTAGTCAACACATATCAATGGATCATTAGAAATTGGGAAACTGTGAAGATTCAGTGGTCCCAGGACCCTACGATGTTGTACAATAAGATGGAATTTGAGCCCTTCCAATCCTTGGTGCCCAAGGCTGCTAGAGGCCAGTATAGTGGGTTTGTGAGGACATTATTCCAACAGATGCGTGATGTGTTGGGGACATTTGACACTGT<br>CCAAATAATAAAGCTCCTACCATTTGCAGCAGCCCCACCGGAACAGAGTAGGATGCAATTTTCCTCTCTGACTGTGAACGTAAGAGGTTCAGGAATGAGAATACTTGTGAGGGGCAACTCCCCTGTGTTCAACTATAATAAGACCACCAAGAGACTCACAGTTCTTGGAAAGGATGCAGGCGCCTTGACAGAATATCCAGATGAGGGAACAGCAGGAGTGGAGTCTGCAG<br>TATTAAGAGGATTTCTAATTCTGGGCAAAGAGGACAAAAGATATGGACCAGCATTGAGCATCAACGAATTGAGCAATCTTGCGAAAGGGGAAAAGGCTAATGTGTTGATAGGACAAGGAGACGTGGTGTTGGTAATGAAACGGAAACGGGACTCTAGCATACTTACTGACAGCCAGACAGCGACCAAAAGAATTCGGATGGCCATCAATTA
EPI_ISL_3102074	>A/turkey/Poland/H1168_21RS1385-15/2021_H5N8_2021-05-13<br>ATGGAGAGAATAAAAGAACTAAGAGATTTGATGTCGCAGTCTCGCACTCGCGAGATACTAACAAAAACCACTGTGGACCATATGGCCATAATAAAGAAATACACATCAGGAAGACAGGAGAAGAACCCTGCCCTCAGGATGAAATGGATGATGGCAATGAAATATCCTATTACAGCTGACAAAAGAATAATGGAGATGATCCCTGAAAGGAATGAGCAAGGTCAGACT<br>CTCTGGAGCAAAACAAATGATGCTGGATCAGACAGAGTGATGGTCTCACCTCTGGCTGTGACATGGTGGAATAGAAATGGGCCAACAACAAGTACAGTACACTACCCAAAAGTCTACAAAACCTACTTTGAAAAGGTAGAAAGGTTGAAACATGGAACCTTTGGTCCTGTTCACTTTCGAAATCAGGTTAAGATACGCCGCAGGGTTGACATAAACCCGGGCCATTCAGAT<br>CTCAGTGCCAAAGAAGCACAGGATGTCATCATGGAGGTTGTTTTCCCAAATGAAGTTGGAGCCAGGATCTTGACATCAGAATCACAATTAACAATAACAAGGGAAAAGAAGGAGGAACTTCAGGATTGCAAGATTGCTCCTTTGATGGTGGCATACATGTTGGAAAGAGAACTGGTTCGCAAGACCAGATTTTTACCAGTAGCTGGCGGAACAAGCAGCGTATACATCGA<br>GGTATTGCATTTGACTCAAGGGACCTGCTGGGAACAAATGTACACACCAGGAGGGGAGGTGAGAAATGATGATGTTGATCAGAGTTTGATCATTGCTGCTAGAAATATAGTTAGGAGGGCAACAGTATCAGCAGACCCATTGGCTTCGCTTTTGGAAATGTGCCACAGTACACAAATTGGTGGAGTAAGGATGGTGGACATTCTTAGGCAGAACCCAACAGAGGAGCAA<br>GCTGTGGATATATGCAAAGCAGCAATGGGTTTAAGAATCAGTTCATCCTTCAGCTTTGGAGGTTTCACTTTCAAAAGGACAAGTGGGTCGTCTGTCAAAAGAGAAGAAGAAATACTCACTGGCAACCTCCAAACACTGAAAGTAAGAATACATGAAGGATATGAGGAATTCACAATGGTTGGGCGAAGAGCTACAGCCATTTTGAGGAAAGCAACCAGGAGACTGATCCA<br>ATTAATAGTGAGTGGAAGAGACGAGCAGTCAATCGCTGAAGCAATCATAGTGGCAATGGTTTTCTCACAGGAGGATTGCATGATAAAAGCAGTACGAGGTGATTTGAATTTCGTCAACAGAGCGAATCAGCGGCTAAATCCTATGCATCAACTTCTGAGGCATTTCCAAAAGGATGCAAAAGTGCTGTTTCAAAACTGGGGGATTGAACCAATTGACAATGTAATGGGGA<br>TGATCGGGATACTGCCTGACATGACCCCCAGCACAGAGATGTCACTGAGAGGAGTGAGAGTCAGCAAAATGGGAGTGGATGAATATTCCAGTACTGAGAGAGTGGTCGTGAGCATTGATCGCTTCTTGAGAGTCCGAGATCAGAGGGGAAATGTGCTTCTGTCTCCTGAGGAAGTTAGTGAAACACATGGAACAGAGAAACTGACGATAACGTATTCATCGTCTATGAT<br>GTGGGAAATCAATGGTCCGGAATCCGTGCTAGTCAACACATATCAATGGATCATTAGAAATTGGGAAACTGTGAAGATTCAGTGGTCCCAGGACCCTACGATGTTATACAACAAGATGGAATTTGAGCCCTTCCAATCCTTGGTGCCCAAGGCTGCTAGAGGCCAGTATAGTGGGTTTGTGAGGACATTATTCCAACAGATGCGTGATGTGTTGGGAACATTTGACACTGTC<br>CAAATAATAAAGCTCCTACCATTTGCAGCAGCCCCACCGGAACAGAGTAGGATGCAATTTTCCTCTCTGACTGTAAACGTAAGAGGTTCAGGAATGAGAATACTTGTGAGGGGCAACTCCCCTGTGTTCAACTATAATAAGGCCACCAAGAGACTCACAGTTCTTGGAAAGGATGCAGGCGCCTTGACAGAATATCCAGATGAGGGAACAGCAGGAGTGGAGTCTGCAGT<br>ATTAAGAGGATTTCTAATTCTGGGCAAAGAGGACAAAAGATATGGACCAGCATTGAGCATCAACGAATTGAGCAATCTTGCGAAAGGGGAAAAGGCTAATGTGTTGATAGGACAAGGAGACGTGGTGTTGGTAATGAAACGGAAACGGGACTCTAGCATACTTACTGACAGCCAGACAGCGACCAAAAGAATTCGGATGGCCATCAATTA

EPI_ISL_3102073	>A/turkey/Poland/H1184_21RS1385-14/2021_H5N8_2021-05-14<br>ATGGAGAGAATAAAAGAACTAAGAGATTTGATGTCGCAGTCTCGCACTCGCGAGATACTAACAAAAACCACTGTGGACCATATGGCCATAATAAAGAAATACACATCAGGAAGACAGGAGAAGAACCCTGCCCTCAGGATGAAATGGATGATGGCAATGAAATATCCTATTACAGCTGACAAAAGAATAATGGAGATGATCCCTGAAAGGAATGAGCAAGGTCAGACT<br>CTCTGGAGCAAAACAAATGATGCTGGATCAGACAGAGTGATGGTCTCACCTCTGGCTGTGACATGGTGGAATAGAAATGGGCCAACAACAAGTACAGTACACTACCCAAAAGTCTACAAAACCTACTTTGAAAAGGTAGAAAGGTTGAAACATGGAACCTTTGGTCCTGTTCACTTTCGGAATCAGGTTAAGATACGCCGCAGGGTTGACATAAACCCGGGCCATTCAGAT<br>CTCAGTGCCAAAGAAGCACAGGATGTCATCATGGAGGTTGTTTTCCCAAATGAAGTTGGAGCCAGGATCTTGACATCAGAATCACAATTAACAATAACAAGGGAAAAGAAGGAGGAACTTCAGGATTGCAAGATTGCTCCTTTGATGGTGGCATACATGTTGGAAAGAGAACTGGTTCGCAAGACCAGATTTTTACCAGTAGCTGGCGGAACAAGCAGCGTATACATCGA<br>GGTATTGCATTTGACTCAAGGGACCTGCTGGGAACAAATGTACACACCAGGAGGGGAGGTGAGAAATGATGATGTTGATCAGAGTTTGATCATTGCTGCTAGAAATATAGTTAGGAGGGCAACAGTATCAGCAGACCCATTGGCTTCGCTTTTGGAAATGTGCCACAGTACACAAATTGGTGGAGTAAGGATGGTGGACATTCTTAGGCAGAACCCAACAGAGGAGCAA<br>GCTGTGGATATATGCAAAGCAGCAATGGGTTTAAGAATCAGTTCATCCTTCAGCTTTGGAGGTTTCACTTTCAAAAGGACAAGTGGGTCGTCTGTCAAAAGAGAAGAAGAAATACTCACTGGCAACCTCCAAACACTGAAAGTAAGAATACATGAAGGATATGAGGAATTCACAATGGTTGGGCGAAGAGCTACAGCCATTTTGAGGAAAGCAACCAGGAGACTGATCCA<br>ATTAATAGTGAGTGGAAGAGACGAGCAGTCAATCGCTGAAGCAATCATAGTGGCAATGGTTTTCTCACAGGAGGATTGCATGATAAAAGCAGTACGAGGTGATTTGAATTTCGTCAACAGAGCAAATCAGCGGCTAAATCCTATGCATCAACTTCTGAGGCATTTCCAAAAGGATGCAAAAGTGCTGTTTCAAAACTGGGGAATTGAACCAATTGACAATGTAATGGGA<br>TGATCGGGATACTGCCTGACATGACCCCCAGCACAGAGATGTCACTGAGAGGAGTGAGAGTCAGCAAAATGGGAGTGGATGAATATTCCAGTACTGAGAGAGTGGTCGTGAGCATTGATCGCTTCTTGAGAGTCCGAGATCAGAGGGGAAATGTGCTTCTGTCTCCTGAGGAAGTTAGTGAAACACATGGAACAGAGAAACTGACGATAACGTATTCATCGTCTATGAT<br>GTGGGAAATCAATGGTCCGGAATCCGTGCTAGTCAACACATATCAATGGATCATTAGAAATTGGGAAACTGTGAAGATTCAGTGGTCCCAGGACCCTACGATGTTATACAACAAGATGGAATTTGAGCCCTTCCAATCCTTGGTGCCCAAGGCTGCTAGAGGCCAGTATAGTGGGTTTGTGAGGACATTATTCCAACAGATGCGTGATGTGTTGGGAACATTTGACACTGTC<br>CAAATAATAAAGCTCCTACCATTTGCAGCAGCCCCACCGGAACAGAGTAGGATGCAATTTTCCTCTCTGACTGTAAACGTAAGAGGTTCAGGAATGAGAATACTTGTGAGGGGCAACTCCCCTGTGTTCAACTATAATAAGGCCACCAAGAGACTCACAGTTCTTGGAAAGGATGCAGGCGCCTTGACAGAATATCCAGATGAGGGAACAGCAGGAGTGGAGTCTGCAGT<br>ATTAAGAGGATTTCTAATTCTGGGCAAAGAGGACAAAAGATATGGACCAGCATTGAGCATCAACGAATTGAGCAATCTTGCGAAAGGGGAAAAGGCTAATGTGTTGATAGGACAAGGAGACGTGGTGTTGGTAATGAAACGGAAACGGGACTCTAGCATACTTACTGACAGCCAGACAGCGACCAAAAGAATTCGGATGGCCATCAATTA
EPI_ISL_3102079	>A/turkey/Poland/H1289_21RS1385-20/2021_H5N8_2021-05-24<br>ATGGAGAGAATAAAAGAACTAAGAGATTTGATGTCGCAGTCTCGCACTCGCGAGATACTAACAAAAACCACTGTGGACCATATGGCCATAATAAAGAAATACACATCAGGAAGACAGGAGAAGAACCCTGCCCTCAGGATGAAATGGATGATGGCAATGAAATATCCTATTACAGCTGACAAAAGAATAATGGAGATGATCCCTGAAAGGAATGAGCAAGGTCAGACT<br>CTCTGGAGCAAAACAAATGATGCTGGATCAGACAGAGTGATGGTCTCACCTCTGGCTGTGACATGGTGGAATAGAAATGGGCCAACAACAAGTACAGTACACTACCCAAAAGTCTACAAAACCTACTTTGAAAAGGTAGAAAGGTTGAAACATGGAACCTTTGGTCCTGTTCACTTTCGAAATCAGGTTAAGATACGCCGCAGGGTTGACATAAACCCGGGCCATTCAGAT<br>CTCAGTGCCAAAGAAGCACAGGATGTCATCATGGAGGTTGTTTTCCCAAATGAAGTTGGAGCCAGGATCTTGACATCAGAATCACAATTAACAATAACAAGGGAAAAGAAGGAGGAACTTCAGGATTGCAAGATTGCTCCTTTGATGGTGGCATACATGTTGGAAAGAGAACTGGTTCGCAAGACCAGATTTTTACCAGTAGCTGGCGGAACAAGCAGCGTATACATCGA<br>GGTATTGCATTTGACTCAAGGGACCTGCTGGGAACAAATGTACACACCAGGAGGGGAGGTGAGAAATGATGATGTTGATCAGAGTTTGATCATTGCTGCTAGAAATATAGTTAGGAGGGCAACAGTATCAGCAGACCCATTGGCTTCGCTTTTGGAAATGTGCCACAGTACACAAATTGGTGGAGTAAGGATGGTGGACATTCTTAGGCAGAACCCAACAGAGGAGCAA<br>GCTGTGGATATATGCAAAGCAGCAATGGGTTTAAGAATCAGTTCATCCTTCAGCTTTGGAGGTTTCACTTTCAAAAGGACAAGTGGGTCGTCTGTCAAAAGAGAAGAAGAAATACTCACTGGCAACCTCCAAACACTGAAAGTAAGAATACATGAAGGATATGAGGAATTCACAATGGTTGGGCGAAGAGCTACAGCCATTTTAAGGAAAGCAACCAGGAGACTGATCCA<br>ATTAATAGTGAGTGGAAGAGACGAGCAGTCAATCGCTGAAGCAATCATAGTGGCAATGGTTTTCTCACAGGAGGATTGCATGATAAAAGCAGTACGAGGTGATTTGAATTTCGTCAACAGAGCGAATCAGCGGCTAAATCCTATGCATCAACTTCTGAGGCATTTCCAAAAGGATGCAAAAGTGCTGTTTCAAAACTGGGGAATTGAACCAATTGACAATGTAATGGGA<br>TGATCGGGATACTGCCTGACATGACCCCCAGCACAGAGATGTCACTGAGAGGAGTGAGAGTCAGCAAAATGGGAGTGGATGAATATTCCAGTACTGAGAGAGTGGTCGTGAGCATTGATCGCTTCTTGAGAGTCCGAGATCAGAGGGGAAATGTGCTTCTGTCTCCTGAGGAAGTTAGTGAAACACATGGAACAGAGAAACTGACGATAACGTATTCATCGTCTATGAT<br>GTGGGAAATCAATGGTCCGGAATCCGTGCTAGTCAACACATATCAATGGATCATTAGAAATTGGGAAACTGTGAAGATTCAGTGGTCCCAGGACCCTACGATGTTATACAACAAGATGGAATTTGAGCCCTTCCAATCCTTGGTGCCCAAGGCAGCTAGAGGCCAGTATAGTGGGTTTGTGAGGACATTATTCCAACAGATGCGTGATGTGTTGGGAACATTTGACACTGTC<br>CAAATAATAAAGCTCCTACCATTTGCAGCAGCCCCACCGGAACAGAGTAGGATGCAATTTTCCTCTCTGACTGTAAACGTAAGAGGTTCAGGAATGAGAATACTTGTGAGGGGCAACTCCCCTGTGTTCAACTATAATAAGGCCACCAAGAGACTCACAGTTCTTGGAAAGGATGCAGGCGCCTTGACAGAATATCCAGATGAGGGAACAGCAGGAGTGGAGTCTGCAGT<br>ATTAAGAGGATTTCTAATTCTGGGCAAAGAGGACAAAAGATATGGACCAGCATTGAGCATCAACGAATTGAGCAATCTTGCGAAAGGGGAAAAGGCTAATGTGTTGATAGGACAAGGAGACGTGGTGTTGGTAATGAAACGGAAACGGGACTCTAGCATACTTACTGACAGCCAGACAGCGACCAAAAGAATTCGGATGGCCATCAATTA
EPI_ISL_6929958	>A/turkey/Poland/H1910N/2021_H5N1_2021-11-02<br>ATGGAGAGAATAAAAGAGCTAAGAGATTTGATGTCGCAGTCTCGCACTCGCGAGATACTGACAAAAACCACCGTGGACCATATGGCCATAATCAAGAAATATACATCAGGAAGACAGGAGAAGAACCCTGCACTTAGGATGAAGTGGATGATGGCAATGAAATATCCGATTACAGCAGACAAAAGGATAATGGAGATGATCCCTGAAAGAAATGAGCAAGGTCAGACT<br>CTTTGGAGCAAAACAAATGATGCTGGATCGGATAGAGTAATGGTGTCACCTCTGGCTGTGACGTGGTGGAATAGAAATGGACCAACAACAAGTACAGTCCATTACCCAAAGGTCTATAAAACCTACTTTGAAAAGGTTGAAAGGTTAAAGCATGGAACCTTCGGCCCTGTCCATTTCCGGAATCAGGTTAAGATACGCCGCAGAGTTGACATAAACCCGGGCCATGCAGA<br>CCTCAGTGCTAAAGAAGCACAAGACGTCATCATGGAGGTCGTTTTCCCAAATGAAGTCGGAGCCAGAATATTGACATCAGAGTCACAGTTAACAATTACAAAAGAAAAGAAGGAGGAACTCCAGGACTGTAAGATTGCCCCTCTAATGGTGGCATACATGTTGGAGAGAGAACTGGTTCGAAAAACCAGATTCCTGCCAGTAGCTGGCGGAACAAGTAGCGTGTATATATCG<br>AAGTGTTGCACTTGACTCAAGGGACCTGCTGGGAACAAATGTATACGCCAGGAGGAGAAGTGAGAAATGATGACGTTGACCAAAGTTTAATTATTGCTGCCAGAAATATCGTTAGGAGAGCAACAGTATCAGCAGACCCATTGGCTTCACTACTGGAGATGTGCCATAGTACACAGATTGGCGGGATAAGAATGGTAGACATTCTTAGACAGAACCCAACAGAGAGGCA<br>AGCTGTGGATATATGCAAAGCAGCAATGGGTTTAAGAATCAGTTCATCCTTCAGTTTTGGAGGTTTCACTTTCAAAAGGACAAGCGGATCATCTGTCAAAAGAGAAGAGGAAGTGCTCACCGGCAACCTCCAAACATTGAAAATAAGAGTACATGAAGGGTATGAGGAATTCACAATGGTTGGGCGGAGAGCAACAGCCATTCTAAGGAAAGCAACCAGAAGGCTGATC<br>CAATTGATAGTAAGTGGAAAAGACGAGCAGTCAATCGCCGAAGCGATCATAGTGGCAATGGTGTTCTCTCAAGAGGATTGCATGATAAAAGCTGTACGAGGTGATTTAAATTTTGTCAATAGAGCGAATCAGCGGCTCAATCCTATGCATCAGCTCCTGAGGCATTTCCAAAAGGATGCAAAGGTACTATTCCAAAACTGGGGAATTGAACCCATTGACAATGTCATGGGA<br>ATGATAGGAATATTGCCTGATATGACTCCCAGCACAGAGATGTCACTAAGAGGAGTGAGGGTCAGTAAAATGGGAGTGGATGAATATTCCAGTACTGAGAGGGTGGTCGTGAGTATTGATCGCTTCTTGAGGGTACGAGACCAGAGAGGAAATGTACTCTTGTCTCCCGAAGAGGTCAGTGAAACACAGGGAACAGAGAAGCTAACGATAACATATTCATCATCCATGA<br>TGTGGGAAATTAATGGCCCTGAGTCAGTGCTAGTTAACACATATCAATGGATCATCAGAAACTGGGAAACTGTGAAGATTCAGTGGTCCCAAGACCCCACAATGCTATACAACAAGATGGAGTTTGAGCCTTTCCAGTCTCTGGTGCCTAAGGCAGCCAGAGGCCAGTACAGTGGATTTGTAAGGACCTTATTCCAGCAGATGCGTGATGTGCTGGGAACCTTTGACACTG<br>TCCAGATAATAAAGCTACTTCCATTTGCAGCAGCACCACCGGAACAGAGTAGGATGCAGTTCTCTTCTCTAACTGTAAACGTAAGGGGTTCAGGAATGAGAATACTTGTGAGAGGCAACTCCCCTGTGTTCAACTATAATAAGGCAACCAAGAGGCTCATAGTCCTTGGAAAGGATGCTGGTGCATTGACAGAAGACCCAGGTGAGGGGACAGCAGGAGTGGAGTCTGCG<br>GTATTGAGAGGGTTCCTAATTCTGGGCAAAGAGGACAAAAGATATGGACCAGCGCTGAGCATCAATGAATTGAGCAATCTTGCGAAAGGGGAGAAGGCTAATGTGTTGATAGGGCAAGGAGACGTGGTATTGGTGATGAAACGGAAACGGGACTCTAGCATACTTACTGACAGCCAGACAGCGACCAAAAGAATTCGGATGGCCATCAATTA
EPI_ISL_18245534	>A/white_stork/Poland/MB363-21_22VIR5675-15/2021_H5N8_2021-04-01<br>ATGGAGAGAATAAAAGAACTAAGAGATTTGATGTCGCAGTCTCGCACTCGCGAGATACTAACAAAAACCACTGTGGATCATATGGCCATAATAAAGAAATACACATCAGGAAGACAGGAGAAGAACCCTGCCCTCAGGATGAAATGGATGATGGCAATGAAATATCCTATTACAGCTGACAAAAGAATAATGGAGATGATCCCTGAAAGGAATGAGCAAGGTCAGACTC<br>TCTGGAGCAAAACAAATGATGCTGGATCAGACAGAGTGATGGTCTCACCTCTGGCTGTGACATGGTGGAATAGAAATGGGCCAACAACAAGTACAGTACACTACCCAAAAGTCTACAAAACCTACTTTGAAAAGGTAGAAAGGTTGAAACATGGAACCTTTGGTCCTGTTCACTTTCGAAATCAGGTTAAGATACGCCGCAGGGTTGACATAAACCCAGGCCATGCAGA<br>CTCAGTGCCAAAGAAGCACAGGATGTCATCATGGAGGTTGTTTTCCCAAATGAAGTTGGAGCCAGGATCTTGACATCAGAATCACAATTAACAATAACAAGGGAAAAGAAGGAGGAACTTCAGGATTGCAAGATTGCTCCTTTGATGGTGGCATACATGTTGGAAAGAGAACTGGTTCGCAAGACCAGATTTTTACCAGTAGCTGGCGGAACAAGCAGCGTATACATCGA<br>GGTATTGCATTTGACTCAAGGGACCTGCTGGGAACAAATGTACACACCAGGAGGGGAGGTGAGAAATGATGATGTTGATCAGAGTTTGATCATTGCTGCTAGAAATATAGTTAGGAGGGCAACAGTATCAGCAGACCCATTGGCTTCGCTTTTGGAAATGTGCCACAGTACACAAATTGGTGGAGTAAGGATGGTGGACATTCTTAGGCAGAACCCAACAGAGGAGCAA<br>GCTGTGGATATATGCAAAGCAGCAATGGGTTTAAGAATCAGTTCATCCTTCAGCTTTGGAGGTTTCACTTTCAAAAGGACAAGTGGGTCGTCTGTCAAAAGAGAAGAAGAAATACTCACTGGCAACCTCCAAACACTGAAAGTAAGAATACATGAAGGATATGAGGAATTCACAATGGTTGGGCGAAGAGCTACAGCCATTTTGAGGAAAGCAACCAGGAGACTGATCCA<br>ATTAATAGTGAGTGGAAGAGACGAGCAGTCAATCGCTGAAGCAATCATAGTGGCAATGGTTTTCTCACAGGAGGATTGCATGATAAAAGCAGTACGAGGTGATTTGAATTTCGTCAACAGAGCAAATCAGCGGCTAAATCCTATGCATCAACTTCTGAGGCATTTCCAAAAGGATGCAAAAGTGCTGTTTCAAAACTGGGGAATTGAACCAATTGACAATGTAATGGGA<br>TGATCGGGATACTGCCTGACATGACCCCCAGCACAGAGATGTCACTGAGAGGAGTGAGAGTCAGCAAAATGGGAGTGGATGAATATTCCAGTACTGAGAGAGTGGTCGTGAGCATTGATCGCTTCTTGAGAGTCCGAGATCAGAGGGGAAATGTGCTTCTGTCTCCTGAGGAAGTTAGTGAAACACATGGAACAGAGAAACTGACGATAACGTATTCATCGTCTATGAT<br>GTGGGAAATCAATGGTCCGGAATCCGTGCTAGTCAACACATATCAATGGATCATTAGAAATTGGGAAACTGTGAAGATTCAGTGGTCCCAGGACCCTACGATGTTATACAACAAGATGGAATTTGAGCCCTTCCAATCCTTGGTGCCCAAGGCTGCTAGAGGCCAGTATAGTGGGTTTGTGAGGACATTATTCCAACAGATGCGTGATGTGTTGGGAACATTTGACACTGTC<br>CAAATAATAAAGCTCCTACCATTTGCAGCAGCCCCACCGGAACAGAGTAGGATGCAATTTTCCTCTCTGACTGTGAACGTAAGAGGTTCAGGAATGAGAATACTTGTGAGGGGCAACTCCCCTGTGTTCAACTATAATAAGGCCACCAAGAGACTCACAGTTCTTGGAAAGGATGCAGGCGCCTTGACAGAATATCCAGATGAGGGAACAGCAGGAGTGGAGTCTGCAGT<br>ATTAAGAGGATTTCTAATTCTGGGCAAAGAGGACAAAAGATATGGACCAGCATTGAGCATCAACGAATTGAGCAATCTTGCGAAAGGGGAAAAGGCTAATGTGTTGATAGGACAAGGAGACGTGGTGTTGGTAATGAAACGGAAACGGGACTCTAGCATACTTACTGACAGCCAGACAGCGACCAAAAGAATTCGGATGGCCATCAATTA
EPI_ISL_2681045	>A/white_stork/Poland/MB391/2021_H5N1_2021-04-20<br>ATGGAGAGAATAAAAGAGCTAAGAGATTTGATGTCGCAGTCTCGCACTCGCGAGATACTGACAAAAACCACCGTGGACCATATGGCCATAATCAAGAAATATACATCAGGAAGACAGGAGAAGAACCCTGCACTTAGGATGAAGTGGATGATGGCAATGAAATATCCGATTACAGCAGACAAAAGGATAATGGAGATGATCCCTGAAAGAAATGAGCAAGGTCAGACT<br>CTTTGGAGCAAAACAAATGATGCTGGATCAGATAGAGTAATGGTGTCACCTCTGGCTGTGACGTGGTGGAATAGAAATGGACCAACAACAAGTACAGTCCATTACCCAAAGGTCTATAAAACCTACTTTGAAAAGGTTGAAAGGTTAAAGCATGGAACCTTCGGCCCTGTCCATTTCCGGAATCAGGTTAAGATACGCCGCAGAGTTGACATAAACCCGGGCCATGCAGA<br>CCTCAGTGCTAAAGAAGCACAAGACGTCATCATGGAGGTCGTTTTCCCAAATGAAGTCGGAGCCAGAATATTGACATCAGAGTCACAGTTAACAATTACAAAAGAAAAGAAGGAGGAACTCCAGGACTGTAAGATTGCCCCTCTAATGGTGGCATACATGTTGGAGAGAGAACTGGTTCGAAAAACCAGATTCCTGCCAGTAGCTGGCGGAACAAGTAGCGTGTATATATCG<br>AAGTGTTGCACTTGACTCAAGGGACCTGCTGGGAACAAATGTATACGCCAGGAGGAGAAGTGAGAAATGATGACGTTGACCAAAGTTTAATTATTGCTGCCAGAAATATCGTTAGGAGAGCAACAGTATCAGCAGACCCATTGGCTTCACTACTGGAGATGTGCCATAGTACACAGATTGGCGGGATAAGAATGGTAGACATTCTTAGACAGAACCCAACAGAGAGGCA<br>AGCCGTGGATATTTGCAAAGCAGCAATGGGTTTAAGAATCAGTTCATCCTTCAGTTTTGGAGGTTTCACTTTCAAAAGGACAAGCGGATCATCTGTCAAAAGAGAAGAGGAAGTGCTCACCGGCAACCTCCAAACATTGAAAATAAGAGTACATGAGGGGTATGAGGAATTCACAATGGTTGGGCGGAGAGCAACAGCCATTCTAAGGAAAGCAACCAGAAGGCTGATC<br>CAATTGATAGTAAGTGGAAAAGACGAGCAGTCAATCGCCGAAGCGATCATAGTGGCAATGGTGTTCTCTCAAGAGGATTGCATGATAAAAGCTGTACGAGGTGATTTAAATTTTGTCAATAGAGCGAATCAGCGGCTCAATCCTATGCATCAGCTCCTGAGGCATTTCCAAAAGGATGCAAAGGTACTATTCCAAAACTGGGGAATTGAACCCATTGACAATGTCATGGGA<br>ATGATAGGAATATTGCCTGATATAACACCCAGCACAGAGATGTCACTAAGAGGAGTGAGGGTCAGTAAAATGGGAGTGGATGAATATTCTAGTACTGAGAGGGTGGTCGTGAGTATTGATCGCTTCTTGAGGGTACGAGACCAGAGAGGAAATGTACTCTTGTCTCCCGAAGAGGTCAGTGAAACACAGGGAACAGAGAAGCTAACGATAACATATTCATCATCCATGA<br>TGTGGGAAATTAATGGCCCTGAGTCAGTGCTAGTTAACACATATCAATGGATCATCAGAAACTGGGAAACTGTGAAGATTCAGTGGTCCCAAGACCCCACAATGCTATACAACAAGATGGAGTTTGAGCCTTTCCAGTCTCTGGTGCCTAAGGCAGCCAGAGGCCAGTACAGTGGATTTGTAAGGACCTTATTCCAGCAGATGCGTGATGTGCTGGGAACCTTTGACACTG<br>TCAGATAATAAAGCTACTTCCATTTGCAGCAGCACCACCGGAACAGAGTAGGATGCAGTTCTCTTCTCTAACTGTAAACGTAAGGGGTTCAGGAATGAGAATACTTGTGAGAGGCAACTCCCCTGTGTTCAACTATAACAAGGCAACCAAAAGGCTCACAGTCCTTGGAAAGGATGCTGGTGCATTGACAGAAGACCCAGGTGAGGGGACAGCAGGAGTGGAGTCTGCG<br>GTATTGAGAGGGTTCCTAATTCTGGGCAAAGAGGACAAAAGATATGGACCAGCGCTGAGCATCAATGAATTGAGCAATCTTGCGAAAGGGGAGAAGGCTAATGTGTTGATAGGGCAAGGAGACGTGGTGTTGGTGATGAAACGGAAACGGGACTCTAGCATACTTACTGACAGCCAGACAGCGACCAAAAGAATTCGGATGGCCATCAATTA
EPI_ISL_3102070	>A/white_stork/Poland/MB412_21RS1385-11/2021_H5N8_2021-05-02<br>ATGGAGAGAATAAAAGAACTAAGAGATTTGATGTCGCAGTCTCGCACTCGCGAGATACTAACAAAAACCACTGTGGACCATATGGCCATAATAAAGAAATACACATCAGGAAGACAGGAGAAGAACCCTGCCCTCAGGATGAAATGGATGATGGCAATGAAATATCCTATTACAGCTGACAAAAGAATAATGGAGATGATCCCTGAAAGGAATGAGCAAGGTCAGACT<br>CTCTGGAGCAAAACAAATGATGCTGGATCAGACAGAGTGATGGTCTCACCTCTGGCTGTGACATGGTGGAATAGAAATGGGCCAACAACAAGTACAGTACACTACCCAAAAGTCTACAAAACCTACTTTGAAAAGGTAGAAAGGTTGAAACATGGAACCTTTGGTCCTGTTCACTTTCGGAATCAGGTTAAGATACGCCGCAGGGTTGACATAAACCCGGGCCATTCAGAT<br>CTCAGTGCCAAAGAAGCACAGGATGTCATCATGGAGGTTGTTTTCCCAAATGAAGTTGGAGCCAGGATCTTGACATCAGAATCACAATTAACAATAACAAGGGAAAAGAAGGAGGAACTTCAGGATTGCAAGATTGCTCCTTTGATGGTGGCATACATGTTGGAAAGAGAACTGGTTCGCAAGACCAGATTTTTACCAGTAGCTGGCGGAACAAGCAGCGTATACATCGA<br>GGTATTGCATTTGACTCAAGGGACCTGCTGGGAACAAATGTACACACCAGGAGGGGAGGTGAGAAATGATGATGTTGATCAGAGTTTGATCATTGCTGCTAGAAATATAGTTAGGAGGGCAACAGTATCAGCAGACCCATTGGCTTCGCTTTTGGAAATGTGCCACAGTACACAAATTGGTGGAGTAAGGATGGTGGACATTCTTAGGCAGAACCCAACAGAGGAGCAA<br>GCTGTGGATATATGCAAAGCAGCAATGGGTTTAAGAATCAGTTCATCCTTCAGCTTTGGAGGTTTCACTTTCAAAAGGACAAGTGGGTCGTCTGTCAAAAGAGAAGAAGAAATACTCACTGGCAACCTCCAAACACTGAAAGTAAGAATACATGAAGGATATGAGGAATTCACAATGGTTGGGCGAAGAGCTACAGCCATTTTGAGGAAAGCAACCAGGAGACTGATCCA<br>ATTAATAGTGAGTGGAAGAGACGAGCAGTCAATCGCTGAAGCAATCATAGTGGCAATGGTTTTCTCACAGGAGGATTGCATGATAAAAGCAGTACGAGGTGATTTGAATTTCGTCAACAGAGCAAATCAGCGGCTAAATCCTATGCATCAACTTCTGAGGCATTTCCAAAAGGATGCAAAAGTGCTGTTTCAAAACTGGGGAATTGAACCAATTGACAATGTAATGGGA<br>TGATCGGGATACTGCCTGACATGACCCCCAGCACAGAGATGTCACTGAGAGGAGTGAGAGTCAGCAAAATGGGAGTGGATGAATATTCCAGTACTGAGAGAGTGGTCGTGAGCATTGATCGCTTCTTGAGAGTCCGAGATCAGAGGGGAAATGTGCTTCTGTCTCCTGAGGAAGTTAGTGAAACACATGGAACAGAGAAACTGACGATAACGTATTCATCGTCTATGAT<br>GTGGGAAATCAATGGTCCGGAATCCGTGCTAGTCAACACATATCAATGGATCATTAGAAATTGGGAAACTGTGAAGATTCAGTGGTCCCAGGACCCTACGATGTTATACAACAAGATGGAATTTGAGCCCTTCCAATCCTTGGTGCCCAAGGCTGCTAGAGGCCAGTATAGTGGGTTTGTGAGGACATTATTCCAACAGATGCGTGATGTGTTGGGAACATTTGACACTGTC<br>CAAATAATAAAGCTCCTACCATTTGCAGCAGCCCCACCGGAACAGAGTAGGATGCAATTTTCCTCTCTGACTGTAAACGTAAGAGGTTCAGGAATGAGAATACTTGTGAGGGGCAACTCCCCTGTGTTCAACTATAATAAGGCCACCAAGAGACTCACAGTTCTTGGAAAGGATGCAGGCGCCTTGACAGAATATCCAGATGAGGGAACAGCAGGAGTGGAGTCTGCAGT<br>ATTAAGAGGATTTCTAATTCTGGGCAAAGAGGACAAAAGATATGGACCAGCATTGAGCATCAACGAATTGAGCAATCTTGCGAAAGGGGAAAAGGCTAATGTGTTGATAGGACAAGGAGACGTGGTGTTGGTAATGAAACGGAAACGGGACTCTAGCATACTTACTGACAGCCAGACAGCGACCAAAAGAATTCGGATGGCCATCAATTA
EPI_ISL_18245742	>A/white-fronted_goose/Poland/MB028-22_22VIR5675-1/2022_2022-01-14<br>ATGGAGAGAATAAAAGAGCTAAGAGATTTGATGTCGCAGTCTCGCACTCGCGAGATACTGACAAAAACCACCGTGGACCATATGGCCATAATCAAGAAATATACATCAGGAAGACAGGAGAAGAACCCTGCACTTAGGATGAAGTGGATGATGGCAATGAAATATCCGATTACAGCAGACAAAAGGATAATGGAGATGATCCCTGAAAGAAATGAGCAAGGTCAGACT<br>CTTTGGAGCAAAACAAATGATGCTGGATCGGATAGAGTAATGGTGTCACCTCTGGCTGTGACGTGGTGGAATAGAAATGGACCAACAACAAGTACAGTCCATTACCCAAAGGTCTATAAAACCTACTTTGAAAAGGTTGAAAGGTTAAAGCATGGAACCTTCGGCCCTGTCCATTTCCGGAATCAGGTTAAGATACGCCGCAGAGTTGACATAAACCCGGGCCATGCAGA<br>CCTCAGTGCTAAAGAAGCACAAGACGTCATCATGGAGGTCGTTTTCCCAAATGAAGTCGGAGCCAGAATATTGACATCAGAGTCACAGTTAACAATTACAAAAGAAAAGAAGGAGGAACTCCAGGACTGTAAGATTGCCCCTCTAATGGTGGCATACATGTTGGAGAGAGAACTGGTTCGAAAAACCAGATTCCTGCCAGTAGCTGGCGGAACAAGTAGCGTGTATATATCG<br>AAGTGTTGCACTTGACTCAAGGGACCTGCTGGGAACAAATGTATACGCCAGGAGGAGAAGTGAGAAATGATGACGTTGACCAAAGTTTAATTATTGCTGCCAGAAATATCGTTAGGAGAGCAACAGTATCAGCAGACCCATTGGCTTCACTACTGGAGATGTGCCATAGTACACAGATTGGCGGGATAAGAATGGTAGACATTCTTAGACAGAACCCAACAGAGAGGCA<br>AGCCGTGGATATATGCAAAGCAGCAATGGGTTTAAGAATCAGTTCATCCTTCAGTTTTGGAGGTTTCACTTTCAAAAGGACAAGCGGATCATCTGTCAAAAGAGAAGAGGAAGTGCTCACCGGCAACCTCCAAACATTGAAAATAAGAGTACATGAAGGGTATGAGGAATTCACAATGGTTGGGCGGAGAGCAACAGCCATTCTAAGGAAAGCAACCAGAAGGCTGATC<br>CAATTGATAGTAAGTGGAAAAGACGAGCAGTCAATCGCCGAAGCGATCATAGTGGCAATGGTGTTCTCTCAAGAGGATTGCATGATAAAAGCTGTACGAGGTGATTTAAATTTTGTCAATAGAGCGAATCAGCGGCTCAATCCTATGCATCAGCTCCTGAGGCATTTCCAAAAGGATGCAAAGGTACTATTCCAAAACTGGGGAATTGAACCCATTGACAATGTCATGGGA<br>ATGATAGGAATATTGCCTGATATGACCCCCAGCACAGAGATGTCACTAAGAGGAGTGAGGGTCAGTAAAATGGGAGTGGATGAATATTCCAGTACTGAGAGGGTGGTCGTAAGTATTGATCGCTTCTTGAGGGTACGAGACCAGAGAGGAAATGTACTCTTGTCTCCCGAAGAGGTCAGTGAAACACAGGGAACAGAGAAGCTAACGATAACATATTCATCATCCATGA<br>TGTGGGAAATTAATGGCCCTGAGTCAGTGCTAGTTAACACATATCAATGGATCATCAGAAACTGGGAAACTGTGAAGATTCAGTGGTCCCAAGACCCCACAATGCTATACAACAAGATGGAGTTTGAGCCTTTCCAGTCTCTGGTGCCTAAGGCAGCCAGAGGCCAGTACAGTGGATTTGTAAGGACCTTATTCCAGCAGATGCGTGATGTGCTGGGAACCTTTGACACTG<br>CCAGATAATAAAGCTACTTCCATTTGCAGCAGCACCACCGGAACAGAGTAGGATGCAGTTCTCTTCTCTAACTGTAAACGTAAGGGGTTCAGGAATGAGAATACTTGTGAGAGGCAACTCCCCTGTGTTCAACTATAATAAGGCAACCAAGAGGCTCATAGTCCTTGGAAAGGATGCTGGTGCATTGACAGAAGACCCAGGTGAGGGGACAGCAGGAGTGGAGTCTGCG<br>GTATTGAGAGGGTTCCTAATTCTGGGCAAAGAGGACAAAAGATATGGACCAGCGCTGAGCATCAATGAATTGAGCAATCTTGCGAAAGGGGAGAAGGCTAATGTGTTGATAGGGCAAGGAGACGTGGTGTTGGTGATGAAACGGAAACGGGACTCTAGCATACTTACTGACAGCCAGACAGCGACCAAAAGAATTCGGATGGCCATCAATTA
EPI_ISL_2234822	>A/whooper_swan/Romania/10171_21VIR2593-15/2021_H5N5_2021-01-12<br>ATGGAGAGAATAAAAGAACTAAGAGATTTGATGTCGCAGTCTCGCACTCGCGAGATACTAACAAAAACCACGGTGGACCATATGGCCATAATTAAGAAATACACATCAGGAAGACAGGAGAAGAACCCTGCCCTTAGGATGAAGTGGATGATGGCAATGAAATACCCGATTACAGCAGACAAAAGAATAATGGAAATGATCCCTGAAAGAAATGAACAAGGCCAGACT<br>CTCTGGAGCAAAACAAGTGATGCCGGATCAGACAGAGTAATGGTGTCACCTCTGGCTGTGACATGGTGGAATAGAAATGGACCGACGACAAGTACAGTCCACTATCCAAAGGTCTATAAAACCTACTTTGAAAAGGTCGAAAGGTTGAAACATGGAACCTTCGGTCCTGTTCATTTTCGGAATCAGGTTAAAATACGCCGCAGAGTTGATATAAACCCGGGCCATGCAGA<br>TCTCAGTGCCAAAGAAGCACAAGATGTCATCATGGAGGTCGTTTTTCCAAACGAAGTTGGAGCAAGGATATTGACATCAGAGTCACAATTAACAATAACGAAAGAAAAGAAGGAGGAGCTTCAGGACTGCAAGATCGCCCCTTTAATGGTGGCGTACATGTTGGAGAGAGAACTGGTTCGCAAAACCAGATTCCTACCAGTAGCTGGCGGAACAAGCAGCGTATATATC<br>GAGGTATTGCATTTGACTCAAGGGACCTGCTGGGAACAAATGTACACACCGGGAGGGGAGGTGAGAAATGATGATGTCGATCAGAGCTTGATCATTGCTGCTAGAAATATTGTTAGGAGAGCAACAGTATCAGCAGACCCATTGGCTTCTTTAGAGATGTGCCATAGTACACAAATTGGCGGGATAAGAATGGTGGACATCCTTAGACAGAACCCAACAGAGGAGC<br>AAGCTGTGGATATATGCAAAGCAGCAATGGGTTTGAGAATCAGTTCATCCTTCAGCTTTGGAGGTTTCACTTTTAAAAGGACAAGTGGGTCATCTGTCAAAAGAGAAGAGGAAGTTCTCACGGGCAACCTCCAAACACTGAAAATAAGAGTACATGAAGGATATGAAGAATTCACAATGGTTGGGCGAAGAGCAACAGCCATTCTTAGAAAAGCAACCAGAAGGCTGAT<br>CCAATTAATAGTGAGTGGGAGAGACGAGCAGTCAATCGCTGAAGCGATTATAGTGGCAATGGTTTTCTCACAAGAGGATTGCATGATAAAAGCAGTTAGAGGCGATTTGAACTTTGTTAACAGAGCAAATCAGCGGCTAAACCCTATGCATCAACTCCTGAGACATTTCCAAAAGGATGCAAAGGTGCTGTTTCAAAATTGGGGAATTGAACCCATTGACAATGTGATGGG<br>AATGATCGGAATACTGCCTGACATGACCCCCAGCACAGAGATGTCACTGAGAGGAGTGAGAGTCAGTAAAATGGGAGTGGATGAATATTCCAGTACTGAGAGAGTGGTCGTAAGCATTGATCGTTTCTTAAGAGTCCGAGACCAGAGGGGAAATGTGCTTCTGTCTCCTGAAGAAGTTAGTGAAACACAGGGAACAGAGAAACTGACGATAACATATTCATCGTCTATG<br>ATGTGGGAAATCAATGGTCCGGAGTCAGTGCTAGTCAACACATATCAATGGATCATTAGAAATTGGGAAACTGTGAAGATTCAGTGGTCCCAAGACCCCACAATGTTGTACAACAAGATGGAGTTTGAGCCCTTTCAGTCTCTGGTGCCCAAGGCAGCCAGAGGCCAGTACAGTGGATTTGTGAGGACATTATTCCAGCAGATGCGTGATGTGCTGGGGACATTTGACACT<br>GTCCAGATAATAAAGCTCCTACCATTTGCAGCAGCCCCACCGGAACAGAGTAGGATGCAGTTCTCTTCTCTGACTGTGAATGTAAGAGGTTCAGGAATGAGGATACTTGTGAGGGGCAATTCCCCTGTGTTCAACTATAACAAAGCAACCAAGAGACTCACAGTTCTTGGAAAGGATGCAGGTGCATTGACAGAGGATCCAGACGAGGGAACAGCAGGAGTGGAGTCTGC<br>GGTATTAAGAGGATTTCTAATTCTGGGCAAAGAGGACAAAAGATATGGACCAGCATTGAGCATTAACGAATTGAGCAATCTTACGAAAGGGGAGAAGGCCAATGTGTTGATAGGGCAAGGAGACGTGGTGTTGGTAATGAAACGGAAACGGGACTCTAGCATACTTACTGACAGCCAGACAGCGACCAAAAGGATTCGGATGGCCATCAATTA
EPI_ISL_1420637	>A/wigeon/Latvia/23903/2021_H5N8_2021-02-04<br>ATGGAGAGAATAAAAGAACTAAGAGATTTGATGTCACAGTCTCGCACTCGCGAGATACTAACAAAAACCACTGTGGACCATATGGCCATAATAAAGAAATACACATCAGGGAGACAGGAGAAGAACCCTGCCCTCAGGATGAAATGGATGATGGCAATGAAATATCCTATTACAGCTGACAAAAGAATAATGGAGATGATCCCTGAAAGGAATGAGCAAGGTCAGACT<br>CTCTGGAGCAAAACAAATGATGCTGGATCAGACAGAGTGATGGTCTCACCTCTGGCTGTGACATGGTGGAATAGAAACGGGCCAACAACAAGTACAGTACACTACCCAAAAGTCTACAAAACCTACTTTGAAAAGGTGGAAAGGTTGAAACATGGAACCTTTGGTCCTGTTCACTTTCGAAATCAGGTTAAGATACGCCGCAGGGTTGACATAAACCCGGGCCATGCAGA<br>TCTCAGTGCCAAAGAAGCACAGGATGTCATCATGGAGGTTGTTTTCCCAAATGAAGTTGGAGCCAGGATCTTGACATCAGAATCACAATTAACAATAACAAGGGAAAAGAAGGAGGAACTTCAGGATTGCAAGATTGCTCCTTTGATGGTGGCATACATGTTGGAAAGAGAACTGGTTCGCAAGACCAGATTTTTACCAGTAGCTGGCGGAACAAGCAGCGTGTACATCG<br>AGGTATTGCATTTGACTCAAGGGACCTGCTGGGAACAAATGTACACACCAGGAGGGGAGGTGAGAAATGATGATGTTGATCAGAGTTTGATCATTGCTGCTAGAAATATAGTTAGGAGGGCAACAGTATCAGCAGACCCATTGGCTTCGCTTCTGGAAATGTGCCACAGTACACAAATTGGTGGAGTGAGGATGGTGGACATTCTTAGGCAGAACCCAACAGAGGAGCA<br>AGCTGTGGATATATGCAAAGCAGCAATGGGTTTAAGAATCAGTTCATCCTTCAGCTTTGGAGGTTTCACTTTCAAAAGGACAAGCAAGTCGTCTGTCAAAAGAGAAGAAGAAATACTCACTGGCAACCTCCAAACACTGAAAGTAAGAATACATGAAGGATATGAGGAATTCACAATGGTTGGGCGAAGAGCTACAGCCATTTTGAGGAAAGCAACCAGGAGACTGATCC<br>AATTAATAGTGAGTGGAAGAGACGAGCAGTCAATCGCTGAAGCAATCATAGTGGCAATGGTTTTCTCACAGGAGGATTGCATGATAAAAGCAGTACGAGGTGATTTGAATTTCGTCAACAGAGCGAATCAGCGGCTAAATCCTATGCATCAACTTCTGAGGCATTTCCAAAAGGATGCAAAGGTGCTGTTTCAAAACTGGGGAATTGAACCAATTGACAATGTAATGGG<br>ATGATCGGGATACTGCCTGACATGACCCCCAGCACAGAGATGTCACTGAGAGGAGTGAGAGTCAGCAAAATGGGAGTGGATGAATATTCCAGTACTGAGAGAGTGGTCGTGAGCATTGATCGCTTCTTGAGAGTCCGAGATCAGAGGGGAAATGTGCTTCTGTCTCCTGAGGAAGTTAGTGAAACACAGGGAACAGAGAAACTGACGATAACGTATTCATCGTCTATGA<br>TGTGGGAAATCAATGGTCCGGAATCCGTGCTAGTCAACACATATCAATGGATCATTAGAAATTGGGAAACTGTGAAGATTCAGTGGTCCCAGGACCCTACGATGTTATACAACAAGATGGAATTTGAGCCCTTCCAATCCTTGGTGCCCAAGGCTGCTAGAGGCCAGTATAGTGGGTTTGTGAGGACATTATTCCAACAGATGCGTGATGTGTTGGGAACATTTGACACTGT<br>CCAAATAATAAAGCTCCTACCATTTGCAGCAGCCCCACCGGAACAGAGTAGGATGCAATTTTCCTCTCTGACTGTGAACGTAAGAGGTTCAGGAATGAGAATACTTGTGAGGGGCAACTCCCCTGTGTTCAACTATAATAAGGCCACCAAGAGACTCACAGTTCTTGGAAAGGATGCAGGCGCCTTGACAGAATATCCAGATGAGGGAACAGCAGGAGTGGAGTCTGCAG<br>TATTAAGAGGATTTCTAATTCTGGGCAAAGAGGACAAAAGATATGGACCAGCATTGAGCATCAACGAATTGAGCAATCTTGCGAAAGGGGAAAAGGCTAATGTGTTGATAGGACAAGGAGACGTGGTATTGGTAATGAAACGGAAACGGGACTCTAGCATACTTACTGACAGCCAGACAGCGACCAAAAGAATTCGGATGGCCATCAATTA
EPI_ISL_18245812	>A/wild_bird/Poland/MW542/2021_H5N1_2021-12-20<br>ATGGAGAGAATAAAAGAGCTAAGAAATTTGATGTCGCAGTCTCGCACTCGCGAGATACTGACAAAAACCACCGTGGACCATATGGCCATAATCAAGAAATATACATCAGGAAGACAGGAGAAGAACCCTGCACTTAGGATGAAGTGGATGATGGCAATGAAATATCCGATTACAGCAGACAAAAGGATAATGGAGATGATCCCTGAAAGAAATGAGCAAGGTCAGACT<br>CTTTGGAGCAAAACAAATGATGCTGGATCGGATAGAGTAATGGTGTCACCTCTGGCTGTGACGTGGTGGAATAGAAATGGACCAACAACAAGTACAGTCCATTACCCAAAGGTCTATAAAACCTACTTTGAAAAGGTTGAAAGGTTAAAGCATGGAACCTTCGGCCCTGTCCATTTCCGGAATCAGGTTAAGATACGCCGCAGAGTTGACATAAACCCGGGCCATGCAGAC<br>CTCAGTGCTAAAGAAGCACAAGACGTCATCATGGAGGTCGTTTTCCCAAATGAAGTCGGAGCCAGAATATTGACATCAGAGTCACAGTTAACAATTACAAAAGAAAAGAAGGAGGAACTCCAGGACTGTAAGATTGCCCCTCTAATGGTGGCATACATGTTGGAGAGAGAACTGGTTCGAAAAACCAGATTCCTGCCAGTAGCTGGCGGAACAAGTAGCGTGTATATATCGA<br>AGTGTTGCACTTGACTCAAGGGACCTGCTGGGAACAAATGTATACGCCAGGAGGAGAAGTGAGAAATGATGACGTTGACCAAAGTTTAATTATTGCTGCCAGAAATATCGTTAGGAGAGCAACAGTATCAGCAGACCCATTGGCTTCACTACTGGAGATGTGCCATAGTACACAGATTGGCGGGATAAGAATGGTAGACATTCTTAGACAGAACCCAACAGAGAGGCAA<br>GCCGTGGATATATGCAAAGCAGCAATGGGTTTAAGAATCAGTTCATCCTTCAGTTTTGGAGGTTTCACTTTCAAAAGGACAAGTGGATCATCTGTTAAAAGAGAAGAGGAAGTGCTCACAGGCAACCTCCAAACATTGAAAATAAGAGTACATGAAGGGTATGAGGAATTCACAATGGTTGGACGGAGAGCAACAGCCATTCTAAGGAAAGCAACCAGAAGGCTGATCCA<br>ATTGATAGTAAGTGGAAGAGACGAGCAGTCAATCGCCGAAGCGATCATAGTGGCAATGGTGTTCTCTCAAGAGGATTGCATGATAAAAGCTGTACGAGGTGATTTAAATTTTGTCAATAGAGCGAATCAGCGGCTCAATCCTATGCATCAGCTCCTGAGGCATTTCCAAAAGGATGCAAAGGTACTATTCCAAAACTGGGGAATTGAACCCATTGACAATGTCATGGGAAT<br>GATAGGAATATTGCCTGATATGACCCCCAGCACAGAGATGTCACTAAGAGGAGTGAGGGTCAGCAAAATGGGAGTGGATGAATATTCCAGTACTGAGAGGGTGGTCGTGAGTATTGATCGCTTCTTGAGGGTACGAGACCAGAGAGGAAATGTACTCTTGTCTCCCGAAGAGGTCAGTGAAACACAGGGAACAGAGAAGCTAACGATAACATATTCATCATCCATGATG<br>TGGGAAATTAATGGCCCTGAGTCAGTGCTAGTTAACACATATCAATGGATCATCAGAAACTGGGAAACTGTGAAGATTCAGTGGTCCCAAGACCCCACAATGCTATACAACAAGATGGAGTTTGAGCCTTTCCAGTCTCTGGTGCCTAAGGCAGCCAGAGGCCAGTACAGTGGATTTGTAAGGACCTTATTCCAGCAGATGCGTGATGTGCTGGGAACCTTTGACACTGTCC<br>AGATAATAAAGCTACTTCCATTTGCAGCAGCACCACCGGAACAGAGTAGGATGCAGTTCTCTTCTCTAACTGTAAACGTAAGGGGTTCAGGAATGAGAATACTTGTGAGAGGCAACTCCCCTGTGTTCAACTATAATAAAGCAACCAAGAGGCTCATAGTCCTTGGAAAGGATGCTGGTGCATTGACAGAAGACCCAGGTGAGGGGACAGCAGGAGTGGAGTCTGCGGTA<br>TTGAGAGGGTTCCTAATTCTGGGCAAAGAGGACAAAAGATATGGACCAGCGCTGAGCATCAATGAATTGAGCAATCTTGCGAAAGGGGAGAAGGCTAATGTGTTGATAGGGCAAGGAGACGTGGTGTTGGTGATGAAACGGAAACGGGACTCTAGCATACTTACTGACAGCCAGACAGCGACCAAAAGAATTCGGATGGCCATCAATTA
EPI_ISL_846624	>A/wild_goose/Poland/MB142/2020_H5N8_2020-12-17<br>ATGGAGAGAATAAAAGAACTAAGAGATTTGATGTCGCAGTCTCGCACTCGCGAGATACTAACAAAAACCACTGTGGACCATATGGCCATAATAAAGAAATACACATCAGGAAGACAGGAGAAGAACCCTGCCCTCAGGATGAAATGGATGATGGCAATGAAATATCCTATTACAGCTGACAAAAGAATAATGGAGATGATCCCTGAAAGGAATGAGCAAGGTCAGACT<br>CTCTGGAGCAAAACAAATGATGCTGGATCAGACAGAGTGATGGTCTCACCTCTGGCTGTGACATGGTGGAATAGAAATGGGCCAACAACAAGTACAGTACACTACCCAAAAGTCTACAAAACCTACTTTGAAAAGGTAGAAAGGTTGAAACATGGAACCTTTGGTCCTGTTCACTTTCGAAATCAGGTTAAGATACGCCGCAGGGTTGACATAAACCCGGGCCATGCAGA<br>TCTCAGTGCCAAAGAAGCACAGGATGTCATCATGGAGGTTGTTTTCCCAAATGAAGTTGGAGCCAGGATCTTGACATCAGAATCACAATTAACAATAACAAGGGAAAAGAAGGAGGAACTTCAGGATTGCAAGATTGCTCCTTTGATGGTGGCATACATGTTGGAAAGAGAACTGGTTCGCAAGACCAGATTTTTACCAGTAGCTGGCGGAACAAGCAGCGTATACATCG<br>AGGTATTGCATTTGACTCAAGGGACCTGCTGGGAACAAATGTACACACCAGGAGGGGAGGTGAGAAATGATGATGTTGATCAGAGTTTGATCATTGCTGCTAGAAATATAGTTAGGAGGGCAACAGTATCAGCAGACCCATTGGCTTCGCTTTTGGAAATGTGCCACAGTACACAAATTGGTGGAGTAAGGATGGTGGACATTCTTAGGCAGAACCCAACAGAGGAGCA<br>AGCTGTGGATATATGCAAAGCAGCAATGGGTTTAAGAATCAGTTCATCCTTCAGCTTTGGAGGTTTCACTTTCAAAAGGACAAGTGGGTCGTCTGTCAAAAGAGAAGAAGAAATACTCACTGGCAACCTCCAAACACTGAAAGTAAGAATACATGAAGGATATGAGGAATTCACAATGGTTGGGCGAAGAGCTACAGCCATTTTGAGGAAAGCAACCAGGAGACTGATCC<br>AATTAATAGTGAGTGGAAGAGACGAGCAGTCAATCGCTGAAGCAATCATAGTGGCAATGGTTTTCTCACAGGAGGATTGCATGATAAAAGCAGTACGAGGTGATTTGAATTTCGTCAACAGAGCGAATCAGCGGCTAAATCCTATGCATCAACTTCTGAGGCATTTCCAAAAGGATGCAAAGGTGCTGTTTCAAAACTGGGGAATTGAACCAATTGACAATGTAATGGG<br>ATGATCGGGATACTGCCTGACATGACCCCCAGCACAGAGATGTCACTGAGAGGAGTGAGAGTCAGCAAAATGGGAGTGGATGAATATTCCAGTACTGAGAGAGTGGTCGTGAGCATTGATCGCTTCTTGAGAGTCCGAGATCAGAGGGGAAATGTGCTTCTGTCTCCTGAGGAAGTTAGTGAAACACAGGGAACAGAGAAACTGACGATAACGTATTCATCGTCTATGA<br>TGTGGGAAATCAATGGTCCGGAATCCGTGCTAGTCAACACATATCAATGGATCATTAGAAATTGGGAAACTGTGAAGATTCAGTGGTCCCAGGACCCTACGATGTTATACAACAAGATGGAATTTGAGCCCTTCCAATCCTTGGTGCCCAAGGCTGCTAGAGGCCAGTATAGTGGGTTTGTGAGGACATTATTCCAACAGATGCGTGATGTGTTGGGAACATTTGACACTGT<br>CCAAATAATAAAGCTCCTACCATTTGCAGCAGCCCCACCGGAACAGAGTAGGATGCAATTTTCCTCTCTGACTGTGAACGTAAGAGGTTCAGGAATGAGAATACTTGTGAGGGGCAACTCCCCTGTGTTCAACTATAATAAGGCCACCAAGAGACTCACAGTTCTTGGAAAGGATGCAGGCGCCTTGACAGAATATCCAGATGAGGGAACAGCAGGAGTGGAGTCTGCAG<br>TATTAAGAGGATTTCTAATTCTGGGCAAAGAGGACAAAAGATATGGACCAGCATTGAGCATCAACGAATTGAGCAATCTTGCGAAAGGGGAAAAGGCTAATGTGTTGATAGGACAAGGAGACGTGGTGTTGGTAATGAAACGGAAACGGGACTCTAGCATACTTACTGACAGCCAGACAGCGACCAAAAGAATTCGGATGGCCATCAATTA

PB1

Isolate ID	Sequence
EPI_ISL_7778754	>A/Withe-tiled_eagle/Estonia/TA2124126-1_21VIR10433-11/2021_H5N1_2021-10-12<br>ATGGATGTCAATCCGACTTTACTTTTCTTAAAAGTGCCAGCGCAAGATGCCATAAGTACCACATTCCCTTACACTGGAGATCCTCCATACAGCCATGGAACAGGGACAGGATACACAATGGACACAGTCAACAGAACACATCAATACTCAGAGAAGGGAAAATGGACAACAAACACAGAAACCAGAGCACCTCAACTCAACCCAATTGATGGGCCACTACCTGAGGACAACGA<br>ACCGAGCGGATATGCACAAACAGATTGCGTGTTGGAAGCAATGGCTTTCCTTGAAGAGTCCCACCCAGGGATCTTTGAAAACTCTTGTCTTGAAACGATGGAAGTCGTTCAGCAAACAAGAGTGGACAAACTAACTCAAGGTCGCCAGACATATGACTGGACACTGAATAGAAACCAACCAGCTGCAACTGCCCTGGCCAACACTATAGAGGTCTTCAGATCAAACGGTCTAA<br>CAGCCAATGAATCGGGGAGACTAATAGATTTCCTCAAGGATGTGATGGACTCAATGGATAAAGAAGAAATGGAAATAACAACACATTTCCAGAGAAAGAGAAGAGTAAGGGACAACATGACCAAGAAAATGGTCACACAAAGAACAATAGGAAAGAAGAAACAAAGGCTAAACAAGAGGAGCTACTTAATAAGAGCACTGACACTGAATACAATGACAAAAGATGCAGA<br>AAGAGGCAAATTGAAAAGACGGGCGATTGCAACACCAGGGATGCAGATTAGAGGATTTGTGTACTTTGTCGAAACACTGGCAAGGAGCATCTGTGAAAAACTTGAGCAATCTGGACTCCCGGTTGGAGGGAATGAGAAGAAGGCTAAATTGGCAAATGTCGTGAGAAAAATGATGACTAACTCACAAGATACAGAGCTCTCCTTCACAATTACTGGAGATAACACCAAATG<br>GAATGAGAATCAAAATCCTCGGATGTTTCTGGCAATGATAACATACATTACAAGAAACCAACCTGAATGGTTTAGAAATGTCTTGAGTATTGCCCCTATAATGTTCTCGAACAAAATGGCGAGATTGGGAAAAGGGTACATGTTTGAAAGTAAGAGCATGAAGTTACGGACACAAATACCTGCAGAAATGCTTGCAAACATTGACTTAAAATACTTCAATGAATCAACAAGAAA<br>GAAAATCGAAAAAATAAGGCCTCTACTAATAGATGGCACTGCCTCATTGAGTCCTGGAATGATGATGGGCATGTTCAATATGCTGAGTACAGTATTAGGAGTTTCAATCCTAAATCTTGGGCAAAAGAAGTACACCAAAACCACATACTGGTGGGATGGACTCCAATCCTCTGATGATTTCGCCCTCATAGTAAATGCACCGAATCATGAGGGAATACAAGCAGGAGTGGATA<br>GGTTCTATAGGACCTGCAAACTGGTCGGGATCAATATGAGCAAAAAGAAGTCTTACATAAACCGGACTGGAACATTTGAGTTCACAAGCTTTTTCTATCGCTATGGATTTGTGGCTAACTTCAGTATGGAGCTGCCCAGCTTTGGAGTTTCTGGGATCAATGAATCAGCTGACATGAGCATTGGCGTCACAGTGATAAAGAACAACATGATAAACAATGACCTTGGACCAGCAA<br>CAGCTCAAATGGCCCTTCAACTATTCATCAAAGATTACAGGTACACGTACCGATGCCACAGAGGTGACACACAAATTCAAACGAGGAGATCATTCGAGCTGAAGAAGCTGTGGGAACAGACCCGTTCAAAGGCAGGACTGTTGGTGTCAGATGGAGGACCAAATCTATACAACATTCGGAATCTCCATATCCCAGAGGTCTGCCTGAAGTGGGAGCTGATGGACGAAGATTA<br>CCAGGGCAGGTTGTGTAATCCTCTGAACCCATTTGTCAGCCATAGAGAAATTGAGTCCGTAAACAATGCTGTGGTGATGCCAGCCCACGGTCCAGCCAAAAGCATGGAATATGATGCCGTTGCGACTACACACTCATGGATTCCTAAAAGGAATCGTTCCATTCTCAATACCAGTCAAAGGGGAATTCTTGAGGATGAACAGATGTACCAGAAATGCTGCAGTCTATTCGAGAA<br>ATTCTTCCCCAGTAGTTCATACAGGAGACCAGTTGGAATTTCCAGCATGGTGGAGGCCATGGTGTCTAGGGCCCGAATCGATGCACGAATTGATTTCGAATCTGGAAGGATAAAGAAGGAAGAGTTTGCTGAGATCATGAAGATCTGTTCCACCATTGAAGAGCTCAGACGGCAAAAATAG
EPI_ISL_846624	>A/wild_goose/Poland/MB142/2020_H5N8_2020-12-17<br>ATGGATGTCAATCCGACTTTACTTTTCTTAAAAGTGCCAGCGCAAAATGCTATAAGTACTACATTCCCTTACACTGGAGATCCTCCATACAGCCATGGAACAGGAACAGGGTATACCATGGACACAGTAAACAGAACACATCAATACTCAGAAAAGGGAAAGTGGACAACAAACACAGAAACCGGAGCACCCCAACTCAACCCAATTGATGGACCATTACCAGAGGACAATGA<br>GCCAAGCGGATATGCACAAACTGATTGCGTGTTGGAAGCAATGGCTTTCCTTGAAGAATCCCACCCAGGGATATTTGAAAACTCTTGTCTTGAAGCGATGGAAATCGTTCAGCAAACAAGAGTGGACAAACTAACCCAAGGTCGCCAGACTTATGACTGGACACTGAACAGAAACCAACCAGCTGCAACCTCTTTGGCCAACACTATAGAGGTGTTCAGATCGAATGGTCTGA<br>CAGCCAATGAATCAGGGAGACTAATAGATTTCCTCAGGGATGTGATGGAATCAATGGACAAAGAAGAGATGGAAATAACAACACATTTCCAGAGAAAAAGAAGAGTGAGGGACAACATGACTAAGAAGATGGTCACACAAAGAACAATAGGGAAGAAGAAGCAGAGGCTGAACAAGAGAGGCTACTTAATAAGAGCACTGACATTGAATACAATGACCAAAGATGCAGA<br>AAGAGGCAAGTTGAAGAGACGGGCAATTGCAACACCCGGGATGCAGATTAGAGGATTTGTGTACTTCGTTGAAACACTAGCGAGGAGCATCTGTGAGAAACTAGAGCAATCTGGGCTCCCTGTTGGAGGGAATGAGAAGAAGGCTAAATTGGCAAATGTTGTGAGAAAAATGATGACTAACTCACAAGATACAGAGCTCTCCTTTACAATTACTGGAGACAACACCAAATG<br>GAATGAGAATCAAAACCCTCGGATGTTTTTGGCAATGATAACATATATCACAAGAAACCAACCTGAATGGTTTAGAAATGTCTTAAGCATTGCCCCTATAATGTTCTCAAACAAAATGGCGAGATTAGGGAAAGGATACATGTTTGAAAGTAAGAGCATGAAGCTAAGAACACAAATACCAGCAGAGATGCTTACAAATATCGATCTGAAGTATTTCAATGAATCAACGAGAAA<br>AGAAAATCGAGAAAATAAGACCTCTGCTGATTGATGGCACGGCCTCATTGAGTCCTGGGATGATGATGGGCATGTTCAATATGCTGAGCACAGTATTAGGGGTCTCAATCCTGAATCTCGGGCAAAAAAGGTACACCAAAACCACATACTGGTGGGATGGACTTCAATCCTCTGATGATTTCGCTCTCATAGTGAATGCACCGAATCATGAGGGGATACAAGCAGGAGTGGAT<br>AGATTCTATAGGACCTGCAAACTGGTTGGGATCAACATGAGCAAAAAGAAGTCTTACATAAACCGAACAGGAACATTTGAGTTCACAAGTTTTTTCTACCGCTATGGATTTGTAGCCAACTTCAGTATGGAATTACCCAGCTTTGGAGTGTCTGGAATCAATGAATCAGCTGACATGAGCATTGGAGTTACAGTGATAAAAAACAATATGATAAACAATGATCTTGGACCAGCA<br>ACAGCTCAAATGGCTCTTCAGTTATTCATCAAAGACTATAGGTACACATACCGATGCCACAGGGGTGATACACAAATTCAAACGAGGAGATCATTCGAGCTGAAGAAGCTGTGGGAGCAGACCCGTTCAAAGGCAGGGCTGTTGATATCAGACGGGGGGCCAAACCTATACAACATTCGGAATCTCCACATCCCAGAGGTCTGCTTGAAGTGGGAGCTGATGGATGAAGACT<br>ACCAAGGCAGGCTGTGCAATCCTCTGAATCCATTTGTCAGTCATAAAGAGATTGAGTCCGTAAACAATGCTGTAGTAATGCCTGCCCACGGCCCAGCCAAGAGCATGGAATATGATGCTGTTGCGACCACACACTCCTGGATTCCTAAGAGGAACCGTTCCATTCTCAATACCAGCCAAAGGGGAATTCTTGAGGATGAGCAGATGTACCAAAAGTGCTGTAGTCTATTCGAGA<br>AATTCTTCCCCAGCAGTTCATACAGGAGGCAGTTGGAATTTCCAGCATGGTGGAGGCCATGGTGTCTAGGGCCCGAATTGATGCACGCATCGACTTCGAATCTGGAAGGATTAAGAAAGAAGAGTTTGCTGAGATCATGAAGATCTGTTCCACCATTGAAGAGCTCAGACGGCAAAAATAG
EPI_ISL_7733611	>A/Wild_goose/Italy/21VIR10193/2021_H5N1_2021-11-23<br>ATGGATGTCAATCCGACTTTACTTTTCTTAAAAGTGCCAGCGCAAGATGCCATAAGTACCACATTCCCTTACACTGGAGATCCTCCATACAGCCATGGAACAGGGACAGGATACACAATGGACACAGTCAACAGAACACATCAATACTCAGAGAAGGGAAAATGGACAACAAACACAGAAACCAGAGCACCTCAACTCAACCCAATTGATGGGCCACTACCTGAGGACAACGA<br>ACCGAGCGGATATGCACAAACAGATTGCGTGTTGGAAGCAATGGCTTTCCTTGAAGAGTCCCACCCAGGGATCTTTGAAAACTCTTGTCTTGAAACGATGGAAGTCGTTCAGCAAACAAGAGTGGACAAACTAACTCAAGGTCGCCAGACATATGACTGGACACTGAATAGAAACCAACCAGCTGCAACTGCCCTGGCCAACACTATAGAGGTCTTCAGATCAAACGGTCTAA<br>CAGCCAATGAATCGGGGAGACTAATAGATTTCCTCAAGGATGTGATGGACTCAATGGATAAAGAAGAAATGGAAATAACAACACATTTCCAGAGAAAGAGAAGAGTAAGGGACAACATGACCAAGAAAATGGTCACACAAAGAACAATAGGAAAGAAGAAACAAAGGCTAAACAAGAGGAGCTACTTAATAAGAGCACTGACACTGAATACAATGACAAAAGATGCAGA<br>AAGAGGCAAATTGAAAAGACGGGCGATTGCAACACCAGGGATGCAGATTAGAGGATTTGTGTACTTTGTCGAAACACTGGCAAGGAGCATCTGTGAAAAACTTGAGCAATCTGGACTCCCGGTTGGAGGGAATGAGAAGAAGGCTAAATTGGCAAATGTCGTGAGAAAAATGATGACTAACTCACAAGATACAGAGCTCTCCTTCACAATTACTGGAGATAACACCAAATG<br>GAATGAGAATCAAAATCCTCGGATGTTTCTGGCAATGATAACATACATTACAAGAAACCAACCTGAATGGTTTAGAAATGTCTTGAGTATTGCCCCTATAATGTTCTCGAACAAAATGGCGAGATTGGGAAAAGGGTACATGTTTGAAAGTAAGAGCATGAAGTTACGGACACAAATACCTGCAGAAATGCTTGCAAACATTGACTTAAAATACTTCAATGAATCAACAAGAAA<br>GAAAATCGAAAAAATAAGGCCTCTACTAATAGATGGCACTGCCTCATTGAGTCCTGGAATGATGATGGGCATGTTCAATATGCTGAGTACAGTATTAGGAGTTTCAATCCTAAATCTTGGGCAAAAGAAGTACACCAAAACCACATACTGGTGGGATGGACTCCAATCCTCTGATGATTTCGCCCTCATAGTAAATGCACCGAATCATGAGGGAATACAAGCAGGAGTGGATA<br>GGTTCTATAGGACCTGCAAACTGGTCGGGATCAATATGAGCAAAAAGAAGTCTTACATAAACCGGACTGGAACATTTGAGTTCACAAGCTTTTTCTATCGCTATGGATTTGTGGCTAACTTCAGTATGGAGCTGCCCAGCTTTGGAGTTTCTGGGATCAATGAATCAGCTGACATGAGCATTGGCGTCACAGTGATAAAGAACAACATGATAAACAATGACCTTGGACCAGCAA<br>CAGCTCAAATGGCCCTTCAACTATTCATCAAAGATTACAGGTACACGTACCGATGCCACAGAGGTGACACACAAATTCAAACGAGGAGATCATTCGAGCTGAAGAAGCTGTGGGAACAGACCCGTTCAAAGGCAGGACTGTTGGTGTCAGATGGAGGACCAAATCTATACAACATTCGGAATCTCCATATCCCAGAGGTCTGCCTGAAGTGGGAGCTGATGGACGAAGATTA<br>CCAGGGCAGGTTGTGTAATCCTCTGAACCCATTTGTCAGCCATAGAGAAATTGAGTCCGTAAACAATGCTGTGGTGATGCCAGCCCACGGTCCAGCCAAAAGCATGGAATATGATGCCGTTGCGACTACACACTCATGGATTCCTAAAAGGAATCGTTCCATTCTCAATACCAGTCAAAGGGGAATTCTTGAGGATGAACAGATGTACCAGAAATGCTGCAGTCTATTCGAGAA<br>ATTCTTCCCCAGTAGTTCATACAGGAGACCAGTTGGAATTTCCAGCATGGTGGAGGCCATGGTGTCTAGGGCCCGAATCGATGCACGCATTGATTTCGAATCTGGAAGGATCAAGAAGGAAGAGTTTGCTGAGATCATGAAGATCTGTTCCACCATTGAAGAGCTCAGACGGCAAAAATAG
EPI_ISL_5098139	>A/wild_goose/Germany-NI/AI00854/2021_H5N8_2021-01-18<br>ATGGATGTCAATCCGACTTTACTTTTCTTAAAAGTGCCAGCGCAAAATGCTATAAGTACTACATTCCCTTACACTGGAGATCCCCCATACAGCCATGGAACAGGAACAGGGTATACCATGGACACAGTAAACAGAACACATCAATACTCAGAAAAGGGAAAGTGGACAACAAACACAGAAACCGGAGCACCCCAACTCAACCCAATTGATGGACCATTACCAGAGGACAATGA<br>GCCAAGCGGATATGCACAAACTGATTGCGTGTTGGAAGCAATGGCTTTCCTTGAAGAATCCCACCCAGGGATATTTGAAAACTCTTGTCTTGAAGCGATGGAAATCGTTCAGCAAACAAGAGTGGACAAACTAACCCAAGGTCGCCAGACTTATGACTGGACACTGAACAGAAACCAACCAGCTGCAACCTCTTTGGCCAACACTATAGAGGTGTTCAGATCGAATGGTCTGA<br>CAGCCAATGAATCAGGGAGACTGATAGATTTCCTCAGGGATGTGATGGAATCAATGGAAAAAGAAGAGATGGAAGTAACAACACATTTCCAGAGAAAAAGAAGAGTGAGGGACAACATGACTAAGAAGATGGTCACACAAAGAACAATAGGGAAGAAGAAGCAGAGGCTGAACAAGAGGAGTTACTTAATAAGAGCACTGACATTGAATACAATGACCAAAGATGCAGA<br>AAGAGGCAAGTTGAAGAGACGGGCAATTGCAACACCCGGGATGCAGATTAGAGGATTCGTGTACTTCGTTGAAACACTAGCGAGGAGCATCTGTGAGAAACTAGAGCAATCTGGGCTCCCTGTTGGAGGGAATGAGAAGAAGGCTAAATTGGCAAATGTTGTGAGAAAAATGATGACTAACTCACAAGATACAGAGCTCTCCTTTACAATTACTGGAGACAACACCAAATG<br>GAATGAGAATCAAAACCCTCGGATGTTTTTGGCAATGATAACATATATCACAAGAAACCAACCTGAATGGTTTAGAAATGTCTTAAGCATTGCCCCTATAATGTTCTCAAACAAAATGGCGAGATTAGGGAAAGGATACATGTTTGAAAGTAAGAGCATGAAGCTAAGAACACAAATACCAGCAGAGATGCTTACAAATATTGATCTGAAGTATTTCAACGAACCAACGAGAAA<br>AGAAAATCGAGAAAATAAGACCTCTGCTGATTGATGGCACGGCCTCATTGAGTCCTGGGATGATGATGGGCATGTTCAATATGCTGAGCACAGTATTAGGGGTCTCAATCCTGAATCTCGGGCAAAAGAGGTACACCAAAACCACATACTGGTGGGATGGACTCCAATCCTCTGATGATTTCGCTCTCATAGTGAATGCACCGAATCATGAGGGGATACAAGCAGGAGTGGAT<br>AGATTCTATAGGACCTGCAAACTGGTTGGGATCAACATGAGCAAAAAGAAGTCTTACATAAACCGAACAGGAACATTTGAGTTCACAAGTTTTTTCTACCGCTATGGATTTGTAGCCAACTTCAGTATGGAATTACCCAGCTTTGGAGTGTCTGGAATCAATGAATCAGCTGACATGAGCATTGGAGTTACAGTGATAAAAAACAATATGATAAACAATGATCTTGGACCAGCA<br>ACAGCTCAAATGGCTCTTCAGTTATTCATCAAAGACTATAGGTACACATACCGATGCCACAGGGGTGATACACAAATTCAAACGAGGAGATCATTCGAGCTGAAGAAGCTGTGGGAGCAGACCCGTTCAAAGGCAGGGCTGTTGATATCAGACGGGGGGCCAAACCTATACAACATTCGGAATCTCCACATCCCAGAGGTCTGCTTGAAGTGGGAGCTGATGGATGAAGACT<br>ACCAAGGCAGGCTGTGCAATCCTCTGAATCCATTTGTCAGTCATAAAGAGATTGAGTCCGTAAACAATGCTGTAGTAATGCCTGCCCACGGCCCAGCCAAGAGCATGGAATATGATGCTGTTGCGACCACACACTCCTGGATTCCTAAGAGGAACCGTTCCATTCTCAATACCAGCCAAAGGGGAATTCTTGAGGATGAGCAGATGTACCAAAAGTGCTGTAGTCTATTCGAGA<br>AATTCTTCCCCAGCAGTTCATACAGGAGGCAGTTGGAATTTCCAGCATGGTGGAGGCCATGGTGTCTAGGGCCCGAATTGATGCACGCATCGACTTCGAATCTGGAAGGATTAAGAAAGAAGAGTTTGCTGAGATCATGAAGATCTGTTCCACCATTGAAGAGCTCAGACGGCAAAAATAG
EPI_ISL_5098138	>A/wild_goose/Germany-NI/AI00626/2021_H5N8_2021-01-11<br>ATGGATGTCAATCCGACTTTACTTTTCTTAAAAGTGCCAGCGCAAAATGCTATAAGTACTACATTCCCTTACACTGGAGATCCCCCATACAGCCATGGAACAGGAACAGGGTATACCATGGACACAGTAAACAGAACACATCAATACTCAGAAAAGGGAAAGTGGACAACAAACACAGAAACCGGAGCACCCCAACTCAACCCAATTGATGGACCATTACCAGAGGACAATGA<br>GCCAAGCGGATATGCACAAACTGATTGCGTGTTGGAAGCAATGGCTTTCCTTGAAGAATCCCACCCAGGGATATTTGAAAACTCTTGTCTTGAAGCGATGGAAATCGTTCAGCAAACAAGAGTGGACAAACTAACCCAAGGTCGCCAGACTTATGACTGGACACTGAACAGAAACCAACCAGCTGCAACCTCTTTGGCCAACACTATAGAGGTGTTCAGATCGAATGGTCTGA<br>CAGCCAATGAATCAGGGAGACTGATAGATTTCCTCAGGGATGTGATGGAATCAATGGAAAAAGAAGAGATGGAAGTAACAACACATTTCCAGAGAAAAAGAAGAGTGAGGGACAACATGACTAAGAAGATGGTCACACAAAGAACAATAGGGAAGAAGAAGCAGAGGCTGAACAAGAGGAGTTACTTAATAAGAGCACTGACATTGAATACAATGACCAAAGATGCAGA<br>AAGAGGCAAGTTGAAGAGACGGGCAATTGCAACACCCGGGATGCAGATTAGAGGATTCGTGTACTTCGTTGAAACACTAGCGAGGAGCATCTGTGAGAAACTAGAGCAATCTGGGCTCCCTGTTGGAGGGAATGAGAAGAAGGCTAAATTGGCAAATGTTGTGAGAAAAATGATGACTAACTCACAAGATACAGAGCTCTCCTTTACAATTACTGGAGACAACACCAAATG<br>GAATGAGAATCAAAACCCTCGGATGTTTTTGGCAATGATAACATATATCACAAGAAACCAACCTGAATGGTTTAGAAATGTCTTAAGCATTGCCCCTATAATGTTCTCAAACAAAATGGCGAGATTAGGGAAAGGATACATGTTTGAAAGTAAGAGCATGAAGCTAAGAACACAAATACCAGCAGAGATGCTTACAAATATTGATCTGAAGTATTTCAACGAACCAACGAGAAA<br>AGAAAATCGAGAAAATAAGACCTCTGCTGATTGATGGCACGGCCTCATTGAGTCCTGGGATGATGATGGGCATGTTCAATATGCTGAGCACAGTATTAGGGGTCTCAATCCTGAATCTCGGGCAAAAGAGGTACACCAAAACCACATACTGGTGGGATGGACTCCAATCCTCTGATGATTTCGCTCTCATAGTGAATGCACCGAATCATGAGGGGATACAAGCAGGAGTGGAT<br>AGATTCTATAGGACCTGCAAACTGGTTGGGATCAACATGAGCAAAAAGAAGTCTTACATAAACCGAACAGGAACATTTGAGTTCACAAGTTTTTTCTACCGCTATGGATTTGTAGCCAACTTCAGTATGGAATTACCCAGCTTTGGAGTGTCTGGAATCAATGAATCAGCTGACATGAGCATTGGAGTTACAGTGATAAAAAACAATATGATAAACAATGATCTTGGACCAGCA<br>ACAGCTCAAATGGCTCTTCAGTTATTCATCAAAGACTATAGGTACACATACCGATGCCACAGGGGTGATACACAAATTCAAACGAGGAGATCATTCGAGCTGAAGAAGCTGTGGGAGCAGACCCGTTCAAAGGCAGGGCTGTTGATATCAGACGGGGGGCCAAACCTATACAACATTCGGAATCTCCACATCCCAGAGGTCTGCTTGAAGTGGGAGCTGATGGATGAAGACT<br>ACCAAGGCAGGCTGTGCAATCCTCTGAATCCATTTGTCAGTCATAAAGAGATTGAGTCCGTAAACAATGCTGTAGTAATGCCTGCCCACGGCCCAGCCAAGAGCATGGAATATGATGCTGTTGCGACCACACACTCCTGGATTCCTAAGAGGAACCGTTCCATTCTCAATACCAGCCAAAGGGGAATTCTTGAGGATGAGCAGATGTACCAAAAGTGCTGTAGTCTATTCGAGA<br>AATTCTTCCCCAGCAGTTCATACAGGAGGCAGTTGGAATTTCCAGCATGGTGGAGGCCATGGTGTCTAGGGCCCGAATTGATGCACGCATCGACTTCGAATCTGGAAGGATTAAGAAAGAAGAGTTTGCTGAGATCATGAAGATCTGTTCCACCATTGAAGAGCTCAGACGGCAAAAATAG
EPI_ISL_18245812	>A/wild_bird/Poland/MW542/2021_H5N1_2021-12-20<br>ATGGATGTCAATCCGACTTTACTTTTCTTAAAAGTGCCAGCGCAAGATGCCATAAGTACCACATTCCCTTACACTGGAGATCCTCCATACAGCCATGGAACAGGGACAGGATACACAATGGACACAGTCAACAGAACACATCAATACTCAGAGAAGGGAAAATGGACAACAAACACAGAAACCAGAGCACCTCAACTCAACCCAATTGATGGGCCACTACCTGAGGACAACGA<br>ACCGAGCGGATATGCACAAACAGATTGCGTGTTGGAAGCAATGGCTTTCCTTGAAGAGTCCCACCCAGGGATCTTTGAAAACTCTTGTCTTGAAACGATGGAAGTCGTTCAGCAAACAAGAGTGGACAAACTAACTCAAGGTCGCCAGACATATGACTGGACACTGAATAGAAACCAACCAGCTGCAACTGCCCTGGCCAACACTATAGAGGTCTTCAGATCAAACGGTCTAA<br>CAGCCAATGAATCGGGGAGACTAATAGATTTCCTCAAGGATGTGATGGACTCAATGGATAAAGAAGAAATGGAAATAACAACACATTTCCAGAGAAAGAGAAGAGTAAGGGACAACATGACCAAGAAAATGGTCACACAAAGAACAATAGGAAAGAAGAAACAAAGGCTAAACAAGAGGAGCTACTTAATAAGAGCACTGACACTGAATACAATGACAAAAGATGCAGA<br>AAGAGGCAAATTGAAAAGACGGGCGATTGCAACACCAGGGATGCAGATTAGAGGATTTGTGTACTTTGTCGAAACACTGGCAAGGAGCATCTGTGAAAAACTTGAGCAATCTGGACTCCCGGTTGGAGGGAATGAGAAGAAGGCTAAATTGGCAAATGTCGTGAGAAAAATGATGACTAACTCACAAGATACAGAGCTCTCCTTCACAATTACTGGAGATAACACCAAATG<br>GAATGAGAATCAAAATCCTCGGATGTTTCTGGCAATGATAACATACATTACAAGAAACCAACCTGAATGGTTTAGAAATGTCTTGAGTGTTGCCCCTATAATGTTCTCGAACAAAATGGCGAGATTGGGAAAAGGGTACATGTTTGAAAGTAAGAGCATGAAGTTACGGACACAAATACCTGCAGAAATGCTTGCAAACATTGACTTAAAATACTTCAATGAATCAACAAGAAA<br>GAAAATCGAAAAAATAAGGCCTCTACTAATAGATGGCACTGCCTCATTGAGTCCTGGAATGATGATGGGCATGTTCAATATGCTGAGTACAGTATTAGGAGTTTCAATCCTAAATCTTGGGCAAAAGAAGTACACCAAAACCACATACTGGTGGGATGGACTCCAATCCTCTGATGATTTCGCCCTCATAGTAAATGCACCGAATCATGAGGGAATACAAGCAGGAGTGGATA<br>GGTTCTATAGGACCTGCAAACTGGTCGGGATCAATATGAGCAAAAAGAAGTCTTACATAAACCGGACTGGAACATTTGAGTTCACAAGCTTTTTCTATCGCTATGGATTTGTGGCTAACTTCAGTATGGAGCTGCCCAGCTTTGGAGTTTCTGGGATCAATGAATCAGCTGACATGAGCATTGGCGTCACAGTGATAAAGAACAACATGATAAACAATGACCTTGGACCAGCAA<br>CAGCTCAAATGGCCCTTCAACTATTCATCAAAGATTACAGGTACACGTACCGATGCCACAGAGGTGACACACAAATTCAAACGAGGAGATCATTCGAGCTGAAGAAGCTGTGGGAACAGACCCGTTCAAAGGCAGGACTGTTGGTGTCAGATGGAGGACCAAATCTATACAACATTCGGAATCTCCATATCCCAGAGGTCTGCCTGAAGTGGGAGCTGATGGACGAAGATTA<br>CCAGGGCAGGTTGTGTAATCCTCTGAACCCATTTGTCAGCCATAGAGAAATTGAGTCCGTAAACAATGCTGTGGTGATGCCAGCCCACGGTCCAGCCAAAAGCATGGAATATGATGCCGTTGCGACTACACACTCATGGATTCCTAAAAGGAATCGTTCCATTCTCAATACCAGTCAAAGGGGAATTCTTGAGGATGAACAGATGTACCAGAAATGCTGCAGTCTATTCGAGAA<br>ATTCTTCCCCAGTAGTTCATACAGGAGACCAGTTGGAATTTCCAGCATGGTGGAGGCCATGGTGTCTAGGGCCCGAATCGATGCACGAATTGATTTCGAATCTGGAAGGATAAAGAAGGAAGAGTTTGCTGAGATCATGAAGATCTGTTCCACCATTGAAGAGCTCAGACGGCAAAAATAG
EPI_ISL_14065167	>A/white-tailed_eagle/Hokkaido/20220210001/2022_H5N1_2022-02-10<br>ATGGATGTCAATCCGACTTTACTTTTCTTAAAAGTGCCAGCGCAAGATGCCATAAGTACCACATTCCCTTACACTGGAGATCCTCCATACAGCCATGGAACAGGGACAGGATACACAATGGACACAGTCAACAGAACACATCAATACTCAGAGAAGGGAAAATGGACAACAAACACAGAAACCGGAGCACCTCAACTCAACCCAATTGATGGGCCACTACCTGAGGACAACGA<br>ACCGAGCGGATATGCACAAACAGATTGCGTGTTGGAAGCAATGGCTTTCCTTGAGGAGTCCCACCCAGGGATCTTTGAAAACTCTTGTCTTGAAACGATGGAAGTCGTTCAGCAAACAAGAGTGGACAAACTAACTCAAGGTCGCCAGACTTATGACTGGACACTGAATAGAAACCAACCAGCTGCAACTGCCCTGGCCAACACTATAGAGGTCTTCAGATCAAACGGTCTAA<br>CAGCCAATGAATCGGGGAGACTAATAGATTTCCTCAAGGATGTGATGGACTCAATGGATAAAGAAGAAATGGAAATAACAACACATTTCCAGAGAAAGAGAAGAGTAAGGGACAACATGACCAAGAAAATGGTCACACAAAGAACAATAGGAAAGAAGAAACAAAGGCTAAACAAGAGGAGCTACTTAATAAGAGCACTGACACTGAATACAATGACAAAAGATGCAGA<br>AAGAGGCAAATTGAAGAGACGGGCGATTGCAACACCAGGGATGCAGATTAGAGGATTTGTGTACTTTGTCGAAACACTGGCAAGGAGCATCTGTGAAAAACTTGAGCAATCTGGACTCCCGGTTGGAGGGAATGAGAAGAAGGCTAAATTGGCAAATGTCGTGAGAAAAATGATGACTAACTCACAAGATACAGAGCTCTCCTTCACAATTACTGGAGACAACACCAAATG<br>GAATGAGAATCAAAATCCTCGGATGTTTCTGGCAATGATAACATACATTACAAGAAACCAACCTGAATGGTTTAGAAATGTCTTGAGTATTGCCCCTATAATGTTCTCGAACAAAATGGCGAGATTGGGAAAAGGGTACATGTTTGAAAGTAAGAGCATGAAGTTACGGACACAAATACCTGCAGAAATGCTTGCAAACATTGACTTAAAATACTTCAATGAATCAACAAGAAA<br>GAAAATCGAAAAAATAAGGCCTCTACTAATAGATGGCACTGCCTCATTGAGTCCTGGAATGATGATGGGCATGTTCAATATGCTGAGTACAGTATTAGGAGTTTCAATCCTAAATCTTGGGCAAAAGAAGTACACCAAAACCACATACTGGTGGGATGGACTCCAATCCTCTGATGATTTCGCCCTCATAGTAAATGCACCGAATCATGAGGGAATACAAGCAGGAGTGGATA<br>GGTTCTATAGGACCTGCAAACTGGTCGGGATCAATATGAGCAAAAAGAAGTCTTACATAAACCGGACTGGAACATTTGAGTTCACAAGCTTTTTCTATCGCTATGGATTTGTGGCTAACTTCAGTATGGAGCTGCCCAGCTTTGGAGTTTCTGGGATCAATGAATCAGCTGACATGAGCATTGGCGTCACAGTGATAAAGAACAACATGATAAACAATGACCTTGGACCAGCAA<br>CAGCTCAAATGGCCCTTCAACTATTCATCAAAGATTACAGGTACACGTACCGATGCCACAGAGGTGACACACAAATTCAAACGAGGAGATCATTCGAGCTGAAGAAGCTGTGGGAACAGACCCGTTCAAAGGCAGGACTGTTGGTGTCAGATGGAGGACCAAATCTATACAACATTCGGAATCTCCATATCCCAGAGGTCTGCCTGAAGTGGGAGCTGATGGACGAAGATTA<br>CCAGGGCAGGTTGTGTAATCCTCTGAACCCATTTGTCAGCCATAGAGAAATTGAGTCCGTAAACAATGCTGTGGTGATGCCAGCCCACGGTCCAGCCAAAAGCATGGAATATGATGCCGTTGCGACTACACACTCATGGATTCCTAAAAGGAATCGTTCCATTCTCAATACCAGTCAAAGGGGAATTCTTGAGGATGAACAGATGTACCAGAAATGCTGCAGTCTATTCGAGAA<br>ATTCTTCCCCAGTAGTTCATACAGGAGACCAGTTGGAATTTCCAGCATGGTGGAGGCCATGGTGTCTAGGGCCCGAATCGATGCACGCATTGATTTCGAATCTGGAAGGATCAAGAAGGAAGAGTTTGCTGAGATCATGAAGATCTGTTCCACCATTGAAGAGCTCAGACGGCAAAAATAG
EPI_ISL_18245742	>A/white-fronted_goose/Poland/MB028-22_22VIR5675-1/2022_2022-01-14<br>ATGGATGTCAATCCGACTTTACTTTTCTTAAAAGTGCCAGCGCAAGATGCCATAAGTACCACATTCCCTTACACTGGAGATCCTCCATACAGCCATGGAACAGGGACAGGATACACAATGGACACAGTCAACAGAACACATCAATACTCAGAGAAGGGAAAATGGACAACAAACACAGAAACCAGAGCACCTCAACTCAACCCAATTGATGGGCCACTACCTGAGGACAACGA<br>ACCGAGCGGATATGCACAAACAGATTGCGTGTTGGAAGCAATGGCTTTCCTTGAAGAGTCCCACCCAGGGATCTTTGAAAACTCTTGTCTTGAAACGATGGAAGTCGTTCAGCAAACAAGAGTGGACAAACTAACTCAAGGTCGCCAGACATATGACTGGACACTGAATAGAAACCAACCAGCTGCAACTGCCCTGGCCAACACTATAGAGGTCTTCAGATCAAATGGTCTAA<br>CAGCCAATGAATCGGGGAGACTAATAGATTTCCTCAAGGATGTGATGGACTCAATGGATAAAGAAGAAATGGAAATAACAACACATTTCCAGAGAAAGAGAAGAGTAAGGGACAACATGACCAAGAAAATGGTCACACAAAGAACAATAGGAAAGAAGAAACAAAGGCTAAACAAGAGGAGCTACTTAATAAGAGCACTGACACTGAATACAATGACAAAAGATGCAGA<br>AAGAGGCAAATTGAAAAGACGGGCGATTGCAACACCAGGGATGCAGATTAGAGGATTTGTATACTTTGTCGAAACACTGGCAAGGAGCATCTGTGAAAAACTTGAGCAATCTGGACTCCCGGTTGGAGGGAATGAGAAGAAGGCTAAATTGGCAAATGTCGTGAGAAAAATGATGACTAACTCACAAGATACAGAGCTCTCCTTCACAATTACTGGAGATAACACCAAATG<br>GAATGAGAATCAAAATCCTCGGATGTTTCTGGCAATGATAACATACATTACAAGAAACCAACCTGAATGGTTTAGAAATGTCTTGAGTATTGCCCCTATAATGTTCTCGAACAAAATGGCGAGATTGGGAAAAGGGTACATGTTTGAAAGTAAGAGCATGAAGTTACGGACACAAATACCTGCAGAAATGCTTGCAACATTGACTTAAAATACTTCAATGAATCAACAAGAAA<br>GAAAATCGAAAAAATAAGGCCTCTACTAATAGATGGCACTGCCTCATTGAGTCCTGGAATGATGATGGGCATGTTCAATATGCTGAGTACAGTATTAGGAGTTTCAATCCTAAATCTTGGGCAAAAGAAGTACACCAAAACCACATACTGGTGGGATGGACTCCAATCCTCTGATGATTTCGCCCTCATAGTAAATGCACCGAATCATGAGGGAATACAAGCAGGAGTGGATA<br>GGTTCTATAGGACCTGCAAACTGGTCGGGATCAATATGAGCAAAAAGAAGTCTTACATAAACCGGACTGGAACATTTGAGTTCACAAGCTTTTTCTATCGCTATGGATTTGTGGCTAACTTCAGTATGGAGCTGCCCAGCTTTGGGGTTTCTGGGATCAATGAATCAGCTGACATGAGCATTGGCGTCACAGTGATAAAGAACAACATGATAAACAATGACCTTGGACCAGCAA<br>CAGCTCAGATGGCCCTTCAACTATTCATCAAAGATTACAGGTACACGTACCGATGCCACAGAGGTGACACACAAATTCAAACGAGGAGATCATTCGAGCTGAAGAAGCTGTGGGAACAGACCCGTTCAAAGGCAGGACTGTTGGTGTCAGATGGAGGACCAAATCTATACAACATTCGGAATCTCCATATCCCAGAGGTCTGCCTGAAGTGGGAGCTGATGGACGAAGATTA<br>CCAGGGCAGGTTGTGTAATCCTCTGAACCCATTTGTCAGCCATAGAGAAATTGAGTCCGTAAACAATGCTGTGGTGATGCCAGCCCACGGTCCAGCCAAAAGCATGGAATATGATGCCGTTGCGACTACACACTCATGGATTCCTAAAAGGAATCGTTCCATTCTCAATACCAGTCAAAGGGGAATTCTTGAGGATGAACAGATGTACCAGAAATGCTGCAGTCTATTCGAGAA<br>ATTCTTCCCCAGTAGTTCATACAGGAGACCAGTTGGAATTTCCAGCATGGTGGAGGCCATGGTGTCTAGGGCCCGAATCGATGCACGCATTGATTTCGAATCTGGAAGGATCAAGAAGGAAGAGTTTGCTGAGATCATGAAGATCTGTTCCACCATTGAAGAGCTCAGACGGCAAAAATAG
EPI_ISL_267292	>A/white-fronted_goose/Netherlands/5/2011_H6N8_2011-12-14<br>ATGGATGTCAATCCGACTTTACTTTTCTTAAAAGTGCCAGCGCAAAATGCTATAAGTACTACGTTCCCTTACACTGGAGATCCTCCATACAGCCATGGAACAGGAACAGGATACACCATGGACACAGTCAACAGAACACATCAATACTCAGAGAAGGGAAAGTGGACAACGAACACAGAAACTGGGGCGCCCCAACTCAACCCAATTGATGGACCATTACCAGAGGACAATGA<br>GCCAAGCGGATATGCACAAACAGATTGCGTGTTGGAAGCAATGGCTTTCCTTGAAGAATCCCACCCAGGGATATTTGAAAACTCTTGTCTTGAAACGATGGAGGTCGTTCAGCAAACAAGAGTGGACAAACTAACCCAAGGTCGCCAGACTTATGATTGGACACTAAATCGAAACCAACCAGCTGCAACTGCTTTGGCCAACACTATAGAGGTCTTCAGATCGAACGGTCTAAC<br>AGCCAATGAGTCGGGGAGACTAATAGATTTCCTCAAGGATGTGATGGAATCAATGGATAAAGAAGAAATGGAAATAACAACACATTTCCAGAGAAAGAGAAGAGTGAGGGACAACATGACCAAGAAAATGGTCACACAAAGAACGATAGGAAAGAAGAAGCAGAGGCTGAACAAGAGGAGTTACTTAATAAGGGCACTGACATTGAATACAATGACCAAAGATGCAGA<br>AGAGGCAAATTGAAGAGACGGGCAATTGCAACCCCGGGGATGCAGATTAGAGGATTCGTGTACTTTGTCGAAACACTAGCAAGGAGCATCTGTGAAAACTCGAGCAGTCTGGGCTCCCTGTTGGAGGGAATGAGAAGAAGGCCAAATTGGCAAATGTCGTGAGAAAAATGATGACTAACTCGCAAGATACAGAGCTCTCCTTTACAATTACTGGAGACAACACCAAATGG<br>AATGAGAATCAAAATCCTCGGATGTTTTTGGCAATGATAACATACATCACAAGAAACCAACCTGAATGGTTTAGAAATGTCTTGAGCATTGCCCCTATAATGTTCTCAAACAAAATGGCGAGATTAGGGAAAGGATACATGTTTGAGAGTAAGAGCATGAAGCTAAGGACACAAATACCAGCAGAAATGCTTGCAAATATTGACTTGAAATACTTCAATGAATCAACGAGAAA<br>GAAAATCGAGAAAATAAGGCCTCTGCTAATTGATGGCACAGCCTCATTGAGTCCTGGAATGATGATGGGCATGTTCAATATGCTGAGCACAGTATTAGGGGTCTCAATCCTGAATCTTGGACAAAAGAGGTACACCAAAACCACATACTGGTGGGATGGACTCCAATCCTCTGATGATTTCGCTCTCATAGTGAATGCACCGAATCATGAGGGGATACAAGCAGGGGTGGATA<br>GGTTCTATAGGACCTGCAAACTGGTTGGGATCAACATGAGCAAAAAGAAGTCTTACATAAACCGGACAGGAACATTCGAGTTCACAAGCTTTTTCTACCGCTATGGATTTGTAGCCAACTTCAGTATGGAGCTTCCCAGCTTTGGAGTGTCTGGAATCAATGAATCAGCTGACATGAGCATTGGAGTTACAGTGATAAAGAACAATATGATAAACAATGACCTTGGACCAGCAA<br>CAGCTCAAATGGCTCTTCAGCTATTCATCAAGGACTATAGGTACACATACCGATGCCACAGAGGTGACACACAAATTCAAACGAGGAGATCATTCGAGCTGAAGAAGCTGTGGGAGCAGACCCGTTCAAAGGCAGGGCTGTTGATATCAGATGGAGGACCAAATCTATACAACATTCGGAATCTCCACATCCCAGAGGTCTGCTTGAAGTGGGGACTAATGGATGAAGACTA<br>CCAGGGCAGGCTGTGTAATCCTCTGAATCCGTTTGTCAGTCATAAGGAAATTGAGTCCGTAAACAATGCTGTGGTAATGCCTGCCCACGGCCCAGCCAAGAGCATGGAATATGATGCTGTTGCGACTACACACTCATGGATTCCTAAGAGGAACCGTTCCATTCTCAATACCAGCCAGAGGGGAATTCTCGAGGATGAGCAGATGTACCAGAAGTGCTGCAGTCTATTCGAGA<br>AATTCTTCCCCAGTAGTTCATACAGGAGGCAGTTGGAATTTCCAGCATGGTGGAGGCCATGGTGTCCAGGGCCCGAATTGATGCACGCATTGATTTCGAATCTGGAAGGATTAAGAAAGAAGAGTTTGCTGAGATCATGAAGATCTGTTCCACCATTGAAGAGCTCAGACGGCAAAAATAG
EPI_ISL_11259281	>A/white-fronted_goose/Ireland/033181_22VIR1325-8/2021_H5N1_2021-11-15<br>ATGGATGTCAATCCGACTTTACTTTTCTTAAAAGTGCCAGCGCAAGATGCCATAAGTACCACATTCCCTTACACTGGAGATCCTCCATACAGCCATGGAACAGGGACAGGATACACAATGGACACAGTCAACAGAACACATCAATACTCAGAGAAGGGAAAATGGACAACAAACACAGAAACCAGAGCACCTCAACTCAACCCAATTGATGGGCCACTACCTGAGGACAACGA<br>ACCGAGCGGATATGCACAAACAGATTGCGTGTTGGAAGCAATGGCTTTCCTTGAAGAGTCCCATCCAGGGATCTTTGAAAACTCTTGTCTTGAAACGATGGAAGTCGTTCAGCAAACAAGAGTGGACAAACTAACTCAAGGTCGCCAGACATATGACTGGACACTGAATAGAAACCAACCGGCTGCAACTGCCCTGGCCAACACTATAGAGGTCTTCAGATCAAACGGTCTAA<br>CAGCCAATGAATCGGGGAGACTAATAGATTTCCTCAAGGATGTGATGGACTCAATGGATAAAGAAGAAATGGAAATAACAACACATTTCCAGAGAAAGAGAAGAGTAAGGGACAACATGACCAAGAAAATGGTCACACAAAGAACAATAGGAAAGAAGAAACAAAGGCTAAACAAGAGGAGCTACTTAATAAGAGCACTGACACTGAATACAATGACAAAAGATGCAGA<br>AAGAGGCAAATTGAAGAGACGGGCGATTGCAACACCAGGGATGCAGATTAGAGGATTTGTGTACTTTGTCGAAACACTGGCAAGGAGCATCTGTGAAAAACTTGAGCAATCTGGACTCCCGGTTGGAGGGAATGAGAAGAAGGCTAAATTGGCAAATGTCGTGAGAAAAATGATGACTAACTCACAAGATACAGAGCTCTCCTTCACAATTACTGGAGATAACACCAAATG<br>GAATGAGAATCAAAATCCTCGGATGTTTCTGGCAATGATAACATACATTACAAGAAACCAACCTGAATGGTTTAGAAATGTCTTGAGTATTGCCCCTATAATGTTCTCGAACAAAATGGCGAGATTGGGAAAAGGGTACATGTTTGAAAGTAAGAGCATGAAGTTACGGACACAAATACCTGCAGAAATGCTTGCAAACATTGACTTAAAATACTTCAATGAATCAACAAGAAA<br>GAAAATCGAAAAAATAAGGCCTCTACTAATAGATGGCACTGCCTCATTGAGTCCTGGAATGATGATGGGCATGTTCAATATGCTGAGTACAGTATTAGGAGTTTCAATCCTAAATCTTGGGCAAAAGAAGTACACCAAAACCACATACTGGTGGGATGGACTCCAATCCTCTGATGATTTCGCCCTCATAGTAAATGCACCGAATCATGAGGGAATACAAGCAGGAGTGGATA<br>GGTTCTATAGGACCTGCAAACTGGTCGGGATCAATATGAGCAAAAAGAAGTCTTACATAAACCGGACTGGAACATTTGAGTTCACAAGCTTTTTCTATCGCTATGGATTTGTGGCTAACTTCAGTATGGAGCTGCCCAGCTTTGGAGTTTCTGGGATCAATGAATCAGCTGACATGAGCATTGGCATCACAGTGATAAAGAACAACATGATAAACAATGACCTTGGACCAGCAA<br>CAGCTCAAATGGCCCTTCAACTATTCATCAAAGATTACAGGTACACGTACCGATGCCATAGAGGTGACACACAAATTCAAACGAGGAGATCATTCGAGCTGAAGAAGCTGTGGGAACAGACCCGTTCAAAGGCAGGACTGTTGGTGTCAGATGGAGGACCAAATCTATACAACATTCGGAATCTCCATATCCCAGAGGTCTGCCTGAAGTGGGAGCTGATGGACGAAGATTA<br>CCAGGGCAGGTTGTGTAATCCTCTGAACCCATTTGTCAGCCATAGAGAAATTGAGTCCGTAAACAATGCTGTGGTGATGCCAGCCCACGGTCCAGCCAAAAGCATGGAATATGATGCCGTTGCGACTACACACTCATGGATTCCTAAAAGGAATCGTTCCATTCTCAATACCAGTCAAAGGGGAATTCTTGAGGATGAACAGATGTACCAGAAATGCTGCGGTCTATTCGAGAA<br>ATTCTTCCCCAGTAGTTCATACAGGAGACCAGTTGGAATTTCCAGCATGGTGGAGGCCATGGTGTCTAGGGCCCGAATCGATGCACGCATTGATTTCGAATCTGGAAGGATCAAGAAGGAAGAGTTTGCTGAGATCATGAAGATCTGTTCCACCATTGAAGAGCTCAGACGGCAAAAATAG
EPI_ISL_3102070	>A/white_stork/Poland/MB412_21RS1385-11/2021_H5N8_2021-05-02<br>ATGGATGTCAATCCGACTTTACTTTTCTTAAAAGTGCCAGCGCAAAATGCTATAAGTACTACATTCCCTTACACTGGAGATCCTCCATACAGCCATGGAACAGGAACAGGGTATACCATGGACACAGTAAACAGAACACATCAATACTCAGAAAAGGGAAAGTGGACAACAAACACAGAAACCGGAGCACCCCAACTCAACCCAATTGATGGACCATTACCAGAGGACAATGA<br>GCCAAGCGGATATGCACAAACGGATTGCGTGTTGGAAGCAATGGCTTTCCTTGAAAAATCCCACCCAGGGATATTTGAAAACTCTTGTCTTGAAGCGATGGAAATCGTTCAGCAAACAAGAGTGGACAAACTAACCCAAGGTCGCCAGACTTATGACTGGACACTGAACAGAAACCAACCAGCTGCAACCTCTTTGGCCAACACTATAGAGGTGTTCAGATCGAATGGTCTGA<br>CAGCCAATGAATCAGGGAGACTGATAGATTTCCTCAGGGATGTGATGGAATCAATGGACAAAGAAGAGATGGAAGTAACAACACATTTCCAGAGAAAAAGAAGAGTGAGGGACAACATGACTAAGAAGATGGTCACACAAAGAACAATAGGGAAGAAGAAGCAGAGGCTGAACAAGAGGAGTTACTTAATAAGAGCACTGACATTGAATACAATGACCAAAGATGCAGA<br>AAGAGGCAAGTTGAAGAGACGGGCAATTGCAACACCCGGGATGCAGATTAGAGGATTCGTGTACTTCGTTGAAACACTAGCGAGGAGCATCTGTGAGAAACTAGAGCAATCTGGGCTCCCTGTTGGAGGGAATGAGAAGAAGGCTAAATTGGCAAATGTTGTGAGAAAAATGATGACTAACTCACAAGATACAGAGCTCTCCTTTACAATTACTGGAGACAACACCAAATG<br>GAATGAGAATCAAAACCCTCGGATGTTTTTGGCAATGATAACATATATCACAAGAAACCAACCTGAATGGTTTAGAAATGTCTTAAGCATTGCCCCTATAATGTTCTCAAACAAAATGGCGAGATTAGGGAAAGGATACATGTTTGAAAGTAAGAGCATGAAGCTAAGAACACAAATACCAGCAGAGATGCTTACAAATATCGATCTGAAGTATTTCAACGAATCAACGAGAAA<br>AGAAAATCGAGAAAATAAGACCTCTGCTGATTGATGGCACGGCCTCATTGAGTCCTGGGATGATGATGGGCATGTTCAATATGCTGAGCACAGTATTAGGGGTCTCAATCCTGAATCTCGGGCAAAAAAGGTACACCAAAACCACATACTGGTGGGATGGACTTCAATCCTCTGATGATTTCGCTCTCATAGTGAATGCACCGAATCATGAGGGGATACAAGCAGGAGTGGAT<br>AGATTCTATAGGACCTGCAAACTGGTTGGGATCAACATGAGCAAAAAGAAGTCTTACATAAACCGAACAGGAACATTTGAGTTCACAAGTTTTTTCTACCGCTATGGATTTGTAGCCAACTTCAGTATGGAATTACCCAGCTTTGGAGTGTCTGGAATCAATGAATCAGCTGACATGAGCATTGGAGTTACAGTGATAAAAAACAATATGATAAACAATGATCTTGGACCAGCA<br>ACAGCTCAAATGGCTCTTCAGTTATTCATCAAAGACTATAGGTACACATACCGATGCCACAGGGGTGATACACAAATTCAAACGAGGAGATCATTCGAGCTGAAGAAGCTGTGGGAGCAGACCCGTTCAAAGGCAGGGCTGTTGATATCAGACGGGGGGCCAAACCTATACAACATTCGGAATCTCCACATCCCAGAGGTCTGCTTGAAGTGGGAGCTGATGGATGAAGACT<br>ACCAAGGCAGGCTGTGCAATCCTCTGAATCCATTTGTCAGTCATAAAGAGATTGAGTCCGTAAACAATGCTGTAGTAATGCCTGCCCACGGCCCAGCCAAGAGCATGGAATATGATGCTGTTGCGACCACACACTCGTGGATTCCTAAGAGGAACCGTTCCATTCTCAATACCAGCCAAAGGGGAATTCTTGAGGATGAGCAGATGTACCAAAAGTGCTGTAGTCTATTCGAGA<br>AATTCTTCCCCAGCAGTTCATACAGGAGGCAGTTGGAATTTCCAGCATGGTGGAGGCCATGGTGTCTAGGGCCCGAATTGATGCACGCATCGACTTCGAATCTGGAAGGATTAAGAAAGAAGAGTTTGCTGAGATCATGAAGATCTGTTCCACCATTGAAGAGCTCAGACGGCAAAAATAG
EPI_ISL_2681045	>A/white_stork/Poland/MB391/2021_H5N1_2021-04-20<br>ATGGATGTCAATCCGACTTTACTTTTCTTAAAAGTGCCAGCGCAAAATGCCATAAGTACCACATTCCCTTATACTGGAGATCCTCCATACAGCCATGGAACAGGGACAGGATACACAATGGACACAGTCAACAGAACACATCAATACTCAGAGAAGGGAAAATGGACAACAAACACAGAAACCGGAGCACCTCAACTCAACCCAATTGATGGACCACTGCCTGAGGACAACGA<br>ACCGAGCGGATATGCACAAACAGATTGCGTGTTGGAAGCAATGGCTTTCCTTGAGGAGTCCCACCCAGGGATCTTTGAAAACTCTTGTCTTGAAACGATGGAAGTCGTTCAGCAAACAAGAGTGGACAAACTAACTCAAGGTCGCCAGACTTATGACTGGACACTGAATAGAAACCAACCAGCTGCAACTGCCCTGGCCAACACTATAGAGGTCTTCAGATCAAACGGTCTAA<br>CAGCCAATGAATCGGGGAGACTAATAGATTTCCTCAAGGATGTGATGGACTCAATGGAYAAAGAAGAAATGGAAATAACAACACATTTCCAGAGAAAGAGAAGAGTAAGGGACAACATGACCAAGAAAATGGTCACACAAAGAACAATAGGAAAGAAGAAACAAAGGCTAAACAAGAGGAGYTACTTAATAAGAGCACTGACACTGAATACAATGACAAAAGATGCAGA<br>AAGAGGCAAATTGAAGAGACGGGCGATTGCAACACCAGGGATGCAGATTAGAGGATTTGTGTACTTTGTCGAAACACTGGCAAGGAGCATCTGTGAAAAACTTGAGCAATCTGGACTCCCGGTTGGAGGGAATGAGAAGAAGGCTAAATTGGCAAATGTCGTGAGAAAAATGATGACTAACTCACAAGATACAGAGCTCTCCTTCACAATTACTGGAGACAACACCAAATG<br>GAATGAGAATCAAAATCCTCGGATGTTTCTGGCAATGATAACATACATTACAAGAAACCAACCTGAATGGTTTAGAAATGTCTTGAGTATTGCCCCTATAATGTTCTCGAACAAAATGGCGAGATTGGGAAAAGGGTACATGTTTGAAAGTAAGAGCATGAAGTTACGGACACAAATACCTGCAGAAATGCTTGCGAACATTGACTTAAAATACTTCAATGAATCAACAAGAAA<br>GAAAATCGAAAAAATAAGGCCTCTACTAATAGATGGCACTGCCTCATTGAGTCCTGGAATGATGATGGGCATGTTCAATATGCTGAGTACAGTATTAGGAGTTTCAATCCTAAATCTTGGGCAAAAGAAGTACACCAAAACCACATACTGGTGGGATGGACTCCAATCCTCTGATGATTTCGCCCTCATAGTAAATGCACCGAATCATGAGGGAATACAAGCAGGAGTGGATA<br>GGTTCTATAGGACCTGCAAACTGGTCGGGATCAATATGAGCAAAAAGAAGTCTTACATAAACCGGACTGGAACATTTGAGTTCACAAGCTTTTTCTATCGCTATGGATTTGTGGCTAACTTCAGTATGGAGCTGCCCAGCTTTGGAGTTTCTGGGATCAATGAATCAGCTGACATGAGCATTGGCGTAACAGTGATAAAGAACAACATGATAAACAATGACCTTGGACCAGCAA<br>CAGCTCAAATGGCCCTTCAACTATTCATCAAAGATTACAGGTACACGTACCGATGCCACAGAGGTGACACACAAATTCAAACGAGGAGATCATTCGAGCTGAAGAAGCTGTGGGAACAGACCCGTTCAAAGGCAGGACTGTTGGTGTCAGATGGAGGACCAAATCTATACAACATTCGGAATCTCCATATCCCAGAGGTCTGCCTGAAGTGGGAGCTGATGGACGAAGATTA<br>CCAGGGCAGGTTGTGTAATCCTCTGAACCCATTTGTCAGCCATAAAGAAATTGAGTCCGTAAACAATGCTGTGGTGATGCCAGCCCACGGTCCAGCCAAAAGCATGGAATATGATGCCGTTGCGACTACACACTCATGGATTCCTAAAAGGAATCGTTCCATTCTCAATACCAGTCAAAGGGGAATTCTTGAGGATGAACAGATGTACCAGAAATGCTGCAGTCTATTCGAAAA<br>ATTCTTCCCCAGTAGTTCATACAGGAGACCAGTTGGAATTTCCAGCATGGTGGAGGCCATGGTGTCTAGGGCCCGAATCGATGCACGCATTGATTTCGAATCTGGAAGGATCAAGAAGGAAGAGTTTGCTGAGATCATGAAGATCTGTTCCACCATTGAAGAGCTCAGACGGCAAAAATAG

EPI_ISL_18245534	>A/white_stork/Poland/MB363-21_22VIR5675-15/2021_H5N8_2021-04-01<br>ATGGATGTCAATCCGACTTTACTTTTCTTAAAAGTGCCAGCGCAAAATGCTATAAGTACTACATTCCCTTACACTGGAGATCCTCCATACAGCCATGGAACAGGAACAGGGTATACCATGGACACAGTAAACAGAACACATCAATACTCAGAAAAGGGAAAGTGGACAACAAACACAGAAACCGGAGCACCCCAACTCAACCCAATTGATGGACCATTACCAGAGGACAATGA<br>GCCAAGCGGATATGCACAAACTGATTGCGTGTTGGAAGCAATGGCTTTCCTTGAAGAATCCCACCCAGGGATATTTGAAAACTCTTGTCTTGAAGCGATGGAAATCGTTCAGCAAACAAGAGTGGACAAACTAACCCAAGGTCGCCAGACTTATGACTGGACACTGAACAGAAACCAACCAGCTGCAACCTCTTTGGCCAACACTATAGAGGTGTTCAGATCGAATGGTCTGA<br>CAGCCAATGAATCAGGGAGACTGATAGATTTTCTCAGGGATGTGATGGAATCAATGGATAAAGAAGAGATGGAAGTAACAACACATTTCCAGAGAAAAAGAAGAGTGAGGGACAACATGACTAAGAAGATGGTCACACAAAGAACAATAGGGAAGAAGAAGCAGAGGCTGAACAAGAGGAGTTACTTAATAAGAGCACTGACATTGAATACAATGACCAAAGATGCAGA<br>AAGAGGCAAGTTGAAGAGACGGGCAATTGCAACACCCGGGATGCAGATTAGAGGATTCGTGTACTTCGTTGAAACACTAGCGAGGAGCATCTGTGAGAAACTAGAGCAATCTGGGCTCCCTGTTGGAGGGAATGAGAAGAAGGCTAAATTGGCAAATGTTGTGAGAAAAATGATGACTAACTCACAAGATACAGAGCTCTCCTTTACAATTACTGGAGACAACACCAAATG<br>GAATGAGAATCAAAACCCTCGGATGTTTTTGGCAATGATAACATATATCACAAGAAACCAACCTGAATGGTTTAGAAATGTCTTAAGCATTGCCCCTATAATGTTCTCAAACAAAATGGCGAGATTAGGGAAAGGATACATGTTTGAAAGTAAGAGCATGAAGCTAAGAACACAAATACCAGCAGAGATGCTTACAAATATCGATCTGAAGTATTTCAACGAACCAACGAGAA<br>AGAAAATCGAGAAAATAAGACCTCTGCTGATTGATGGCACGGCCTCATTGAGTCCTGGGATGATGATGGGCATGTTCAATATGCTGAGCACAGTATTAGGGGTCTCAATCCTGAATCTCGGGCAAAAAGGTACACCAAAACCACATACTGGTGGGATGGACTTCAATCCTCTGATGATTTCGCTCTCATAGTGAATGCACCGAATCATGAGGGGATACAAGCAGGAGTGGAT<br>AGATTCTATAGGACCTGCAAACTGGTTGGGATCAACATGAGCAAAAAGAAGTCTTACATAAACCGAACAGGAACATTTGAGTTCACAAGTTTTTTCTACCGCTATGGATTTGTAGCTAACTTCAGTATGGAATTACCCAGCTTTGGAGTGTCTGGAATCAATGAATCAGCTGACATGAGCATTGGAGTTACAGTGATAAAAAACAATATGATAAACAATGATCTTGGACCAGCA<br>ACAGCTCAAATGGCTCTTCAGTTATTCATCAAAGATTATAGGTACACATACCGATGCCACAGGGGTGATACACAAATTCAAACGAGGAGATCATTCGAGCTGAAGAAGCTGTGGGAGCAGACCCGTTCAAAGGCAGGGCTGTTGATATCAGACGGGGGGCCAAACCTATACAACATTCGGAATCTCCACATCCCAGAGGTCTGCTTGAAGTGGGAGCTGATGGATGAAGACT<br>ACCAAGGCAGGCTGTGCAATCCTCTGAATCCATTTGTCAGTCATAAAGAGATTGAGTCCGTAAACAATGCTGTAGTAATGCCCGCCCATGGCCCGGCCAAGAGCATGGAATATGATGCTGTTGCGACCACACACTCGTGGATTCCTAAGAGGAATCGTTCCATTCTCAATACCAGCCAAAGGGGAATTCTTGAGGATGAGCAGATGTACCAAAAGTGCTGTAGTCTATTCGAGA<br>AATTCTTCCCCAGCAGTTCATACAGGAGGCCAGTTGGAATTTCCAGCATGGTGGAGGCCATGGTGTCTAGGGCCCGAATTGATGCACGCATCGACTTCGAATCTGGAAGGATTAAGAAAGAAGAGTTTGCTGAGATCATGAAGATCTGTTCCACCATTGAAGAGCTCAGACGGCAAAAATAG
EPI_ISL_7753329	>A/white_stork/Germany-MV/AI05979/2021_H5N1_2021-10-20<br>ATGGATGTCAATCCGACTTTACTTTTCTTAAAAGTGCCAGCGCAAGATGCCATAAGTACCACATTCCCTTACACTGGAGATCCTCCATACAGCCATGGAACAGGGACAGGATACACAATGGACACAGTCAACAGAACACATCAATACTCAGAGAAGGGAAAATGGACAACAAACACAGAAACCGGAGCACCTCAACTCAACCCAATTGATGGGCCACTACCTGAGGACAACGA<br>ACCGAGCGGATATGCACAAACAGATTGCGTGTTGGAAGCAATGGCTTTCCTTGAAGAGTCCCACCCAGGGATCTTTGAAAACTCTTGTCTTGAAACGATGGAAGTCGTTCAGCAAACAAGAGTGGACAAACTAACTCAAGGTCGCCAGACATATGACTGGACACTGAATAGAAACCAACCAGCTGCAACTGCCCTGGCCAACACTATAGAGGTCTTCAGATCAAACGGTCTAA<br>CAACCAATGAATCGGGGAGACTAATAGATTTCCTCAAGGATGTGATGGACTCAATGGATAAAGAAGAAATGGAAATAACAACACATTTCCAGAGAAAGAGAAGAGTAAGGGACAACATGACCAAGAAAATGGTCACACAAAGAACAATAGGAAAGAAGAAACAAAGGCTAACCAAGAGGAGCTACTTAATAAGAGCACTGACACTGAATACAATGACAAAAGATGCAGAA<br>AGAGGCAAATTGAAAAGACGGGCGATTGCAACACCAGGGATGCAGATTAGAGGATTTGTGTACTTTGTCGAAACACTGGCAAGGAGCATCTGTGAAAAACTTGAGCAATCTGGACTCCCCGTTGGAGGGAATGAGAAGAAGGCTAAATTGGCAAATGTCGTGAGAAAAATGATGACTAACTCACAAGATACAGAGCTCTCCTTCACAATTACTGGAGATAACACCAAATGG<br>AATGAGAATCAAAATCCTCGGATGTTTCTGGCAATGATAACATACATTACAAGAAACCAACCTGAATGGTTTAGAAATGTCTTGAGTATTGCCCCTATAATGTTCTCGAACAAAATGGCGAGATTGGGAAAAGGGTACATGTTTGAAAGTAAGAGCATGAAGTTACGGACACAAATACCTGCAGAAATGCTTGCAAACATTGACTTAAAATACTTCAATGAATCAACAAGAAAG<br>AAAATCGAAAAAATAAGGCCTCTACTAATAGATGGCACTGCCTCATTGAGTCCTGGAATGATGATGGGCATGTTCAATATGCTGAGTACAGTATTAGGAGTTTCAATCCTAAATCTTGGGCAAAAGAAGTACACCAAAACCACATACTGGTGGGATGGACTCCAATCCTCTGATGATTTCGCCCTCATAGTAAATGCACCGAATCATGAGGGAATACAAGCAGGAGTGGATAG<br>GTTCTATAGGACCTGCAAACTGGTCGGGATCAATATGAGCAAAAAGAAGTCTTACATAAACCGGACTGGAACATTTGAGTTCACAAGCTTTTTCTATCGCTATGGATTTGTGGCTAACTTCAGTATGGAGCTGCCCAGCTTTGGAGTTTCTGGGATCAATGAATCAGCTGACATGAGCATTGGCATCACAGTGATAAAGAACAACATGATAAACAATGACCTTGGACCAGCAAC<br>AGCTCAAATGGCCCTTCAACTATTCATCAAAGATTACAGGTACACGTACCGATGCCACAGAGGTGACACACAAATTCAAACGAGGAGATCATTCGAGCTGAAGAAGCTGTGGGAACAGACCCGTTCAAAGGCAGGACTGTTGGTGTCAGATGGAGGACCAAATCTATACAACATTCGGAATCTCCATATCCCAGAGGTCTGCCTGAAGTGGGAGCTGATGGACGAAGATTAC<br>CAGGGCAGGTTGTGTAATCCTCTGAACCCATTTGTCAGCCATAGAGAAATTGAGTCCGTAAACAATGCTGTGGTGATGCCAGCCCACGGTCCAGCCAAAAGCATGGAATATGATGCCGTTGCGACTACACACTCATGGATTCCTAAAAGGAATCGTTCCATTCTCAATACCAGTCAAAGGGGAATTCTTGAGGATGAACAGATGTACCAGAAATGCTGCAGTCTATTCGAGAAA<br>TTCTTCCCCAGTAGTTCATACAGGAGACCAGTTGGAATTTCCAGCATGGTGGAGGCCATGGTGTCTAGGGCCCGAATCGATGCACGCATTGATTTCGAATCTGGAAGGATCAAGAAGGAAGAGTTTGCTGAGATCATGAAGATCTGTTCCACCATTGAAGAGCTCAGACGGCAAAAATAG
EPI_ISL_7952129	>A/western_jackdaw/Netherlands/21039297-002/2021_H5N1_2021-11-20<br>ATGGATGTCAATCCGACTTTACTTTTCTTAAAAGTGCCAGCGCAAGATGCCATAAGTACCACATTCCCTTACACTGGAGATCCTCCATACAGCCATGGAACAGGGACAGGATACACAATGGACACAGTCAACAGAACACATCAATACTCAGAGAAGGGAAAATGGACAACAAACACAGAAACCGGAGCACCTCAACTCAACCCAATTGATGGGCCACTACCTGAGGACAACGA<br>ACCGAGCGGATATGCACAAACAGATTGCGTGTTGGAAGCAATGGCTTTCCTTGAAGAGTCCCATCCAGGGATCTTTGAAAACTCTTGTCTTGAAACGATGGAAGTCGTTCAGCAAACAAGAGTGGACAAACTAACTCAAGGTCGCCAGACATATGACTGGACACTGAATAGAAACCAACCAGCTGCAACTGCCCTGGCCAACACTATAGAGGTCTTCAGATCAAACGGTCTAA<br>CAGCCAATGAATCGGGGAGACTAATAGATTTCCTCAAGGATGTGATGGACTCAATGGATAAAGAAGAAATGGAAATAACAACACATTTCCAGAGAAAGAGAAGAGTAAGGGACAACATGACCAAGAAAATGGTCACACAAAGAACAATAGGAAAGAAGAAACAAAGGCTAAACAAGAGGAGCTACTTAATAAGAGCACTGACACTGAATACAATGACAAAAGATGCAGA<br>AAGAGGCAAATTGAAGAGACGGGCGATTGCAACACCAGGGATGCAGATTAGAGGATTTGTGTACTTTGTCGAAACACTGGCAAGGAGCATCTGTGAAAAACTTGAGCAATCTGGACTCCCCGTTGGAGGGAATGAGAAGAAGGCTAAATTGGCAAATGTTGTGAGAAAAATGATGACTAACTCACAAGATACAGAGCTCTCCTTCACAATTACTGGAGATAACACCAAATG<br>GAATGAGAATCAAAATCCTCGAATGTTTCTGGCAATGATAACATACATTACAAGAAACCAACCTGAATGGTTTAGGAATGTCTTGAGTATTGCCCCTATAATGTTCTCGAACAAAATGGCGAGATTGGGAAAAGGGTACATGTTTGAAAGTAAGAGCATGAAGTTACGGACACAAATACCTGCAGAAATGCTTGCAAACATTGACTTAAAATATTTCAATGAATCAACAAGAAA<br>GAAAATCGAAAAAATAAGGCCTCTACTAATAGATGGCACTGCCTCATTGAGTCCTGGAATGATGATGGGCATGTTCAATATGCTGAGTACAGTATTAGGAGTTTCAATCCTAAATCTTGGGCAAAAGAAGTACACCAAAACCACATACTGGTGGGATGGACTCCAATCCTCTGATGATTTCGCCCTCATAGTAAATGCACCGAATCATGAGGGAATACAAGCAGGAGTGGATA<br>GGTTCTATAGGACCTGCAAACTGGTCGGGATCAATATGAGCAAAAAGAAGTCTTACATAAACCGGACTGGAACATTTGAGTTCACAAGCTTTTTCTATCGCTATGGATTTGTGGCTAACTTCAGTATGGAGCTGCCCAGCTTTGGAGTTTCTGGGATCAATGAATCAGCTGACATGAGCATTGGCATCACAGTGATAAAGAACAACATGATAAACAATGACCTTGGACCAGCAA<br>CAGCTCAAATGGCCCTTCAACTATTCATCAAAGATTACAGGTACACGTACCGATGCCATAGAGGTGACACACAAATTCAAACGAGGAGATCATTCGAGCTGAAGAAGCTGTGGGAACAGACCCGTTCAAAGGCAGGACTGTTGGTGTCAGATGGAGGACCAAATCTATACAACATTCGGAATCTCCATATCCCAGAGGTCTGCCTGAAGTGGGAGCTGATGGACGAAGATTA<br>CCAGGGCAGGTTGTGTAATCCTCTGAACCCATTTGTCAGCCATAAAGAAATTGAGTCCGTAAACAATGCTGTGGTGATGCCAGCCCACGGTCCAGCCAAAAGCATGGAATATGATGCCGTTGCGACTACACACTCATGGATTCCTAAAAGGAATCGTTCCATTCTCAATACCAGTCAAAGGGGAATTCTTGAGGATGAACAGATGTACCAGAAATGCTGCGGTCTATTCGAGAA<br>ATTCTTCCCCAGTAGTTCATACAGGAGACCAGTTGGAATTTCCAGCATGGTGGAGGCCATGGTGTCTAGGGCCCGAATCGATGCACGCATTGATTTCGAATCTGGAAGGATCAAGAAGGAAGAGTTTGCTGAGATCATGAAGATCTGTTCCACCATTGAAGAGCTCAGACGGCAAAAATAG
EPI_ISL_14388346	>A/Tyto_alba/Belgium/334_0012/2021_H5N1_2021-12-23<br>ATGGATGTCAATCCGACTTTACTTTTCTTAAAAGTGCCAGCGCAAGATGCCATAAGTACCACATTCCCTTACACTGGAGATCCTCCATACAGCCATGGAACAGGGACAGGATACACAATGGACACAGTCAACAGAACACATCAATACTCAGAGAAGGGAAAATGGACAACAAACACAGAAACCAGAGCACCTCAACTCAACCCAATTGATGGGCCACTACCTGAGGACAACGA<br>ACCGAGCGGATATGCACAAACAGATTGCGTGTTGGAAGCAATGGCTTTCCTTGAAGAGTCCCACCCAGGGATCTTTGAAAACTCTTGTCTTGAAACGATGGAAGTCGTTCAGCAAACAAGAGTGGACAAACTAACTCAAGGTCGCCAGACATATGACTGGACACTGAATAGAAACCAACCAGCTGCAACTGCCCTGGCCAACACTATAGAGGTCTTCAGATCAAACGGTCTAA<br>CAGCCAATGAATCGGGGAGACTAATAGATTTCCTCAAGGATGTGATGGACTCAATGGATAAAGAAGAAATGGAAATAACAACACATTTCCAGAGAAAGAGAAGAGTAAGGGACAACATGACCAAGAAAATGGTCACACAAAGAACAATAGGAAAGAAGAAACAAAGGCTAAACAAGAGGAGCTACTTAATAAGAGCACTGACACTGAATACAATGACAAAAGATGCAGA<br>AAGAGGCAAATTGAAAAGACGGGCGATTGCAACACCAGGGATGCAGATTAGAGGATTTGTGTACTTTGTCGAAACACTGGCAAGGAGCATCTGTGAAAAACTTGAGCAATCTGGACTCCCCGTTGGAGGGAATGAGAAGAAGGCTAAATTGGCAAATGTCGTGAGAAAAATGATGACTAACTCACAAGATACAGAGCTCTCCTTCACAATTACTGGAGATAACACCAAATG<br>GAATGAGAATCAAAATCCTCGGATGTTTCTGGCAATGATAACATACATTACAAGAAACCAACCTGAATGGTTTAGAAATGTCTTGAGTGTTGCCCCTATAATGTTCTCGAACAAAATGGCGAGATTGGGAAAAGGGTACATGTTTGAAAGTAAGAGCATGAAGTTACGGACACAAATACCTGCAGAAATGCTTGCAAACATTGACTTAAAATACTTCAATGAATCAACAAGAAA<br>GAAAATCGAAAAAATAAGGCCTCTACTAATAGATGGCACTGCCTCATTGAGTCCTGGAATGATGATGGGCATGTTCAATATGCTGAGTACAGTATTAGGAGTTTCAATCCTAAATCTTGGGCAAAAGAAGTACACCAAAACCACATACTGGTGGGATGGACTCCAATCCTCTGATGATTTCGCCCTCATAGTAAATGCACCGAATCATGAGGGAATACAAGCAGGAGTGGATA<br>GGTTCTATAGGACCTGCAAACTGGTCGGGATCAATATGAGCAAAAAGAAGTCTTACATAAACCGGACTGGAACATTTGAGTTCACAAGCTTTTTCTATCGCTATGGATTTGTGGCTAACTTCAGTATGGAGCTGCCCAGCTTTGGAGTTTCTGGGATCAATGAATCAGCTGACATGAGCATTGGCGTCACAGTGATAAAGAACAACATGATAAACAATGACCTTGGACCAGCAA<br>CAGCTCAAATGGCCCTTCAACTATTCATCAAAGATTACAGGTACACGTACCGATGCCATAGAGGTGACACACAAATTCAAACGAGGAGATCATTCGAGCTGAAGAAGCTGTGGGAACAGACCCGTTCAAAGGCAGGACTGTTGGTGTCAGATGGAGGACCAAATCTATACAACATTCGGAATCTCCATATCCCAGAGGTCTGCCTGAAGTGGGAGCTGATGGACGAAGATTA<br>CCAGGGCAGGTTGTGTAATCCTCTGAACCCATTTGTCAGCCATAGAGAAATTGAGTCCGTAAACAATGCTGTGGTGATGCCAGCCCACGGTCCAGCCAAAAGCATGGAATATGATGCCGTTGCGACTACACACTCATGGATTCCTAAAAGGAATCGTTCCATTCTCAATACCAGTCAAAGGGGAATTCTTGAGGATGAACAGATGTACCAGAAATGCTGCAGTCTATTCGAGAA<br>ATTCTTCCCCAGTAGTTCATACAGGAGACCAGTTGGAATTTCCAGCATGGTGGAGGCCATGGTGTCTAGGGCCCGAATCGATGCACGAATTGATTTCGAATCTGGAAGGATAAAGAAGGAAGAGTTTGCTGAGATCATGAAGATCTGTTCCACCATTGAAGAGCTCAGACGGCAAAAATAG
EPI_ISL_647969	>A/Turkey/Sweden/SVA201114SZ0001/20KN303106/2020_H5N8_2020-11-13<br>ATGGATGTCAATCCGACTTTACTTTTCTTAAAAGTGCCAGCGCAAAATGCTATAAGTACTACATTCCCTTACACTGGAGATCCCCCATACAGCCATGGAACAGGAACAGGGTATACCATGGACACAGTAAACAGAACACATCAATACTCAGAAAAGGGAAAGTGGGCAACAAACACAGAAACCGGAGCACCCCAACTCAACCCAATTGATGGACCATTACCAGAGGACAATGA<br>GCCAAGCGGATATGCACAAACTGATTGCGTGTTGGAAGCAATGGCTTTCCTTGAAGAATCCCACCCAGGGATATTTGAAAACTCTTGTCTTGAAGCGATGGAAATCGTTCAGCAAACAAGAGTGGACAAACTAACCCAAGGTCGCCAGACTTATGACTGGACACTGAACAGAAACCAACCAGCTGCAACCTCTTTGGCCAACACTATAGAGGTGTTCAGATCGAATGGTCTGA<br>CAGCCAATGAATCAGGGAGACTGATAGATTTTCTCAGGGATGTGATGGAATCAATGGATAAAGAAGAGATGGAAGTAACAACACATTTCCAGAGAAAAAGAAGAGTGAGGGACAACATGACTAAGAAGATGGTCACACAAAGAACAATAGGGAAGAAGAAGCAGAGGCTGAACAAGAGGAGTTACTTAATAAGAGCACTGACATTGAATACAATGACCAAAGATGCAGA<br>AAGAGGCAAGTTGAAGAGACGGGCAATTGCAACACCCGGGATGCAGATTAGAGGATTCGTGTACTTCGTTGAAACACTAGCGAGGAGCATCTGTGAGAAACTAGAGCAATCTGGGCTCCCTGTTGGAGGGAATGAGAAAAAGGCTAAATTGGCAAATGTTGTGAGAAAAATGATGACTAACTCACAAGATACAGAGCTCTCCTTTACAATTACTGGAGACAACACCAAATG<br>GAATGAGAATCAAAACCCTCGGATGTTTTTGGCAATGATAACATATATCACAAGAAACCAACCTGAATGGTTTAGAAATGTCTTAAGCATTGCCCCTATAATGTTCTCAAACAAAATGGCGAGATTAGGGAAAGGATACATGTTTGAAAGTAAGAGCATGAAGCTAAGAACACAAATACCAGCAGAGATGCTTACAAATATTGATCTGAAGTATTTCAACGAACCAACGAGAA<br>AGAAAATCGAGAAAATAAGACCTCTGCTGATTGATGGCACGGCCTCATTGAGTCCTGGGATGATGATGGGCATGTTCAATATGCTGAGCACAGTATTAGGGGTCTCAATCCTGAATCTCGGGCAAAAGAGGTACACCAAAACCACATACTGGTGGGATGGACTTCAATCCTCTGATGATTTCGCTCTCATAGTGAATGCACCGAATCATGAGGGGATACAAGCAGGAGTGGAT<br>AGATTCTATAGGACCTGCAAACTGGTTGGGATCAACATGAGCAAAAAGAAGTCTTACATAAACCGAACAGGAACATTTGAGTTCACAAGTTTTTTCTACCGCTATGGATTTGTAGCCAACTTCAGTATGGAATTACCCAGCTTTGGAGTGTCTGGAATCAATGAATCAGCTGACATGAGCATTGGAGTTACAGTGATAAAAAACAATATGATAAACAATGATCTTGGACCAGCA<br>ACAGCTCAAATGGCTCTTCAGTTATTCATCAAAGACTATAGGTACACATACCGATGCCACAGGGGTGATACACAAATTCAAACGAGGAGATCATTCGAGCTGAAGAAGCTGTGGGAGCAGACCCGTTCAAGGGCAGGGCTGTTGATATCAGACGGGGGGCCAAACCTATACAACATTCGGAATCTCCACATCCCAGAGGTCTGCTTGAAGTGGGAGCTGATGGATGAAGACT<br>ACCAAGGCAGGCTGTGCAATCCTCTGAATCCATTTGTCAGTCATAAAGAGATTGAGTCCGTAAACAATGCTGTAGTAATGCCCGCCCATGGCCCGGCCAAGAGCATGGAATATGATGCTGTTGCGACCACACACTCGTGGATTCCTAAGAGGAACCGTTCCATCCTCAATACCAGCCAAAGGGGAATTCTTGAGGATGAGCAGATGTACCAAAAGTGCTGTAGTCTATTCGAGA<br>AATTCTTCCCCAGCAGTTCATACAGGAGGCCAGTTGGAATTTCCAGCATGGTGGAGGCCATGGTGTCTAGGGCCCGAATTGATGCACGCATCGACTTCGAATCTGGAAGGATTAAGAAAGAAGAGTTTGCTGAGATCATGAAGATCTGTTCCACCATTGAAGAGCTCAGACGGCAAAAATAG
EPI_ISL_1114759	>A/turkey/Rostov-on-Don/332-12/2021_H5N8_2021-01-29<br>ATGGATGTCAATCCGACTTTACTTTTCTTAAAAGTGCCAGCGCAAAATGCTATAAGTACTACATTCCCTTACACTGGAGATCCTCCATACAGCCATGGGACAGGAACAGGGTATACCATGGACACAGTAAACAGAACACATCAATACTCAGAAAAGGGAAAGTGGACAACAAACACAGAAACCGGAGCACCCCAACTCAACCCAATTGATGGACCATTACCAGAGGACAATGA<br>GCCAAGCGGATATGCACAAACTGATTGCGTGTTGGAAGCAATGGCTTTCCTTGAAGAATCCCACCCAGGGATATTTGAAAACTCTTGTCTTGAAGCGATGGAAATCGTTCAGCAAACAAGAGTGGACAAACTAACCCAAGGTCGCCAGACTTATGACTGGACACTGAACAGAAACCAACCAGCTGCAACCTCTTTGGCCAACACTATAGAGGTGTTCAGATCGAATGGTCTGA<br>CAGCCAATGAATCAGGGAGACTGATAGATTTTCTCAGGGATGTGATGGAATCAATGGATAAAGAAGAGATGGAAGTAACAACACATTTCCAGAGAAAAAGAAGAGTGAGGGACAACATGACTAAGAAGATGGTCACACAAAGAACAATAGGGAAGAAGAAGCAGAGGATGAACAAGAGGAGTTACTTGATAAGAGCACTGACATTGAATACAATGACCAAAGATGCAGA<br>AAGAGGCAAGTTGAAGAGACGGGCAATTGCAACACCCGGGATGCAGATTAGAGGATTCGTGTACTTCGTTGAAACACTAGCGAGGAGCATCTGTGAGAAACTAGAGCAATCTGGGCTCCCTGTTGGAGGGAATGAGAAGAAGGCTAAATTGGCAAATGTTGTGAGAAAAATGATGACTAACTCACAAGATACAGAGCTCTCCTTTACAATTACTGGAGACAACACCAAATG<br>GAATGAGAATCAAAACCCTCGGATGTTTTTGGCAATGATAACATATATCACAAGAAACCAACCTGAATGGTTTAGAAATGTCTTAAGCATTGCCCCTATAATGTTCTCAAACAAAATGGCGAGATTAGGGAAAGGATACATGTTTGAAAGTAAGAGCATGAAGCTAAGAACACAAATACCAGCAGAGATGCTTACAAATATTGATCTGAAGTATTTCAACGAACCAACGAGAA<br>AGAAAATCGAGAAAATAAGACCTCTGCTGATTGATGGCACGGCCTCATTGAGTCCTGGGATGATGATGGGCATGTTCAATATGCTGAGCACAGTATTGGGGGTCTCAATCCTGAATCTCGGGCAAAAGAGGTACACCAAAACCACATACTGGTGGGATGGACTTCAATCCTCTGATGATTTCGCTCTCATAGTGAATGCACCGAATCATGAGGGGATACAAGCAGGAGTGGAT<br>AGATTCTATAGGACCTGCAAACTGGTTGGGATCAACATGAGCAAAAAGAAGTCTTACATAAACCGAACAGGAACATTTGAGTTCACAAGTTTTTTCTACCGCTATGGATTTGTAGCTAACTTCAGTATGGAATTACCCAGCTTTGGAGTGTCTGGAATCAATGAATCAGCTGACATGAGCATTGGAGTTACAGTGATAAAAAACAATATGATAAACAATGATCTTGGACCAGCA<br>ACAGCTCAAATGGCTCTTCAGTTATTCATCAAAGACTATAGGTACACATACCGATGCCACAGGGGTGATACACAAATTCAAACGAGGAGATCATTCGAGCTGAAGAAGCTGTGGGAGCAGACCCGTTCAAAGGCAGGGCTGTTGATATCAGACGGGGGGCCAAACCTATACAACATTCGGAACCTCCACATCCCAGAGGTCTGCTTGAAGTGGGAGCTGATGGATGAAGACT<br>ACCAAGGCAGGCTGTGCAATCCTCTGAATCCATTTGTCAGTCATAAAGAGATTGAGTCCGTAAACAATGCTGTAGTAATGCCCGCCCATGGCCCGGCCAAGAGCATGGAATATGATGCTGTTGCGACCACACATTCGTGGATTCCTAAGAGGAACCGCTCCATTCTCAATACCAACCAAAGGGGAATTCTTGAGGATGAGCAGATGTACCAAAAGTGCTGTAGTCTATTCGAGA<br>AATTCTTCCCCAGCAGTTCATACAGGAGGCCAGTTGGAATTTCCAGCATGGTGGAGGCCATGGTGTCTAGGGCCCGAATTGATGCACGCATCGACTTCGAATCTGGAAGGATTAAGAAGGAAGAGTTTGCTGAGATCATGAAGATCTGTTCCACCATTGGAGAGCTCAGACGGCAAAAATAG
internal NVRI repository	>A/turkey/Poland/H35-T/2022_H5N1_2022-01-09<br>ATGGATGTCAATCCGACTTTACTTTTCTTAAAAGTGCCAGCGCAAGATGCCATAAGTACCACATTCCCTTACACTGGAGATCCTCCATACAGCCATGGAACAGGGACAGGATACACAATGGACACAGTCAACAGAACACATCAATACTCAGAGAAGGGAAAATGGACAACAAACACAGAAACCGGAGCACCTCAACTCAACCCAATTGATGGGCCACTACCTGAGGACAACGA<br>ACCGAGCGGATATGCACAAACAGATTGCGTGTTGGAAGCAATGGCTTTCCTTGAAGAGTCCCACCCAGGGATCTTTGAAAACTCTTGTCTTGAAACGATGGAAGTCGTTCAGCAAACAAGAGTGGACAAACTAACTCAAGGTCGCCAGACATATGACTGGACACTGAATAGAAACCAACCAGCTGCAACTGCCCTGGCCAACACTATAGAGGTCTTCAGATCAAACGGTCTAA<br>CAACCAATGAATCGGGGAGACTAATAGATTTCCTCAAGGATGTGATGGACTCAATGGATAAAGAAGAAATGGAAATAACAACACATTTCCAGAGAAAGAGAAGAGTAAGGGACAACATGACCAAGAAAATGGTCACACAAAGAACAATAGGAAAGAAGAAACAAAGGCTAAACAAGAGGAGCTACTTAATAAGAGCACTGACACTGAATACAATGACAAAGGATGCAGA<br>AAGAGGCAAATTGAAAAGACGGGCGATTGCAACACCAGGGATGCAGATTAGAGGATTTGTGTACTTTGTCGAAACACTGGCAAGGAGCATCTGTGAAAAACTTGAGCAATCTGGACTCCCCGTTGGAGGGAATGAGAAGAAGGCTAAATTGGCAAATGTCGTGAGAAAAATGATGACTAACTCACAAGATACAGAGCTCTCCTTCACAATTACTGGAGATAACACCAAATG<br>GAATGAGAATCAAAATCCTCGGATGTTTCTGGCAATGATAACATACATTACAAGAAACCAACCTGAATGGTTTAGAAATGTCTTGAGTATTGCCCCTATAATGTTCTCGAACAAAATGGCGAGATTGGGAAAAGGGTACATGTTTGAAAGTAAGAGCATGAAGTTACGGACACAAATACCTGCAGAAATGCTTGCAAACATTGACTTAAAATACTTCAATGAATCAACAAGAAA<br>GAAAATCGAAAAAATAAGGCCTCTACTAATAGATGGCACTGCCTCATTGAGTCCTGGAATGATGATGGGCATGTTCAATATGCTGAGTACAGTATTAGGAGTTTCAATCCTAAATCTTGGGCAAAAGAAGTACACCAAAACCACATACTGGTGGGATGGACTCCAATCCTCTGATGATTTCGCCCTCATAGTAAATGCACCGAATCATGAGGGAATACAAGCAGGAGTGGATA<br>GGTTCTATAGGACCTGCAAACTGGTCGGGATCAATATGAGCAAAAAGAAGTCTTACATAAACCGGACTGGAACATTTGAGTTCACAAGCTTTTTCTATCGCTATGGATTTGTGGCTAACTTCAGTATGGAGCTACCCAGCTTTGGAGTTTCTGGGATCAATGAATCAGCTGACATGAGCATTGGCATCACAGTGATAAAGAACAACATGATAAACAATGACCTTGGACCAGCAA<br>CTGCTCAAATGGCCCTTCAACTATTCATCAAAGATTACAGGTACACGTACCGATGCCACAGAGGTGACACACAAATTCAAACGAGGAGATCATTCGAGCTGAAGAAGCTGTGGGAACAGACCCGTTCAAAGGCAGGACTGTTGGTGTCAGATGGAGGACCAAATCTATACAACATTCGGAATCTCCATATCCCAGAGGTCTGCCTGAAGTGGGAGCTGATGGACGAAGATTA<br>CCAGGGCAGGTTGTGTAATCCTCTGAACCCATTTGTCAGCCATAGAGAAATTGAGTCCGTAAACAATGCTGTGGTGATGCCAGCCCACGGTCCAGCCAAAAGCATGGAATATGATGCCGTTGCGACTACACACTCATGGATTCCTAAAAGGAATCGTTCCATTCTCAATACCAGTCAAAGGGGAATTCTTGAGGATGAACAGATGTACCAGAAATGCTGCAGTCTATTCGAGAA<br>ATTCTTCCCCAGTAGTTCATACAGGAGACCAGTTGGAATCTCCAGCATGGTGGAGGCCATGGTGTCTAGGGCCCGAATCGATGCACGCATTGATTTCGAGTCTGGAAGGATCAAGAAGGAAGAGTTTGCTGAGATCATGAAGATCTGTTCCACCATTGAAGAGCTCAGACGGCAAAAATAG
internal NVRI repository	>A/turkey/Poland/H32-T/2022_H5N1_2022-01-09<br>ATGGATGTCAATCCGACTTTACTTTTCTTAAAAGTGCCAGCGCAAGATGCCATAAGTACCACATTCCCTTACACTGGAGATCCTCCATACAGCCATGGAACAGGGACAGGATACACAATGGACACAGTCAACAGAACACATCAATACTCAGAGAAGGGAAAATGGAYAACAAACACAGAAACCAGAGCACCTCAACTCAACCCAATTGATGGGCCACTACCTGAGGACAACGA<br>ACCGAGCGGATATGCACAAACAGATTGCGTGTTGGAAGCAATGGCTTTCCTTGAAGAGTCCCACCCAGGGATCTTTGAAAACTCTTGTCTTGAAACGATGGAAGTCGTTCAGCAAACAAGAGTGGACAAACTAACTCAAGGTCGCCAGACATATGACTGGACACTGAATAGAAACCAACCAGCTGCAACTGCCCTGGCCAACACTATAGAGGTCTTCAGATCAAACGGTCTAA<br>CAGCCAATGAATCGGGGAGACTAATAGATTTCCTCAAGGATGTGATGGACTCAATGGATAAAGAAGAAATGGAAATAACAACACATTTCCAGAGAAAGAGAAGAGTAAGGGACAACATGACCAAGAAAATGGTCACACAAAGAACAATAGGAAAGAAGAAACAAAGGCTAAACAAGAGGAGCTACTTAATAAGAGCACTGACACTGAATACAATGACAAAAGATGCAGA<br>AAGAGGCAAATTGAAAAGACGGGCGATTGCAACACCAGGGATGCAGATTAGAGGATTTGTGTACTTTGTCGAAACACTGGCAAGGAGCATCTGTGAAAAACTTGAGCAATCTGGACTCCCCGTTGGAGGGAATGAGAAGAAGGCTAAATTGGCAAATGTCGTGAGAAAAATGATGACTAACTCACAAGATACAGAGCTCTCCTTCACAATTACTGGAGATAACACCAAATG<br>GAATGAGAATCAAAATCCTCGGATGTTTCTGGCAATGATAACATACATTACAAGAAACCAACCTGAATGGTTTAGAAATGTCTTGAGTATTGCCCCTATAATGTTCTCGAACAAAATGGCGAGATTGGGAAAAGGGTACATGTTTGAAAGTAAGAGCATGAAGTTACGGACACAAATACCTGCAGAAATGCTTGCAAACATTGACTTAAAATACTTCAATGAATCAACAAGAAA<br>GAAAATCGAAAAAATAAGGCCTCTACTAATAGATGGCACTGCCTCATTGAGTCCTGGAATGATGATGGGCATGTTCAATATGCTGAGTACAGTATTAGGAGTTTCAATCCTAAATCTTGGGCAAAAGAAGTACACCAAAACCACATACTGGTGGGATGGACTCCAATCCTCTGATGATTTCGCCCTCATAGTAAATGCACCGAATCATGAGGGAATACAAGCAGGAGTGGATA<br>GGTTCTATAGGACCTGCAAACTGGTCGGGATCAATATGAGCAAAAAGAAGTCTTACATAAACCGGACTGGAACATTTGAGTTCACAAGCTTTTTCTATCGCTATGGATTTGTGGCTAACTTCAGTATGGAGCTGCCCAGCTTTGGAGTTTCTGGGATCAATGAATCAGCTGACATGAGCATTGGCGTCACAGTGATAAAGAACAACATGATAAACAATGACCTTGGACCAGCAA<br>CAGCTCAAATGGCCCTTCAACTATTCATCAAAGATTACAGGTACACGTACCGATGCCACAGAGGTGACACACAAATTCAAACGAGGAGATCATTCGAGCTGAAGAAGCTGTGGGAACAGACCCGTTCAAAGGCAGGACTGTTGGTGTCAGATGGAGGACCAAATCTGTACAACATTCGGAATCTCCATATCCCAGAGGTCTGCCTGAAGTGGGAGCTGATGGACGAAGATTA<br>CCAGGGCAGGTTGTGTAATCCTCTGAACCCATTTGTCAGCCATAGAGAAATTGAGTCCGTAAACAATGCTGTGGTGATGCCAGCCCACGGTCCAGCCAAAAGCATGGAATATGATGCCGTTGCGACTACACACTCATGGATTCCTAAAAGGAATCGTTCCATTCTCAATACCAGTCAAAGGGGAATTCTTGAGGATGAACAGATGTACCAGAAATGCTGCAGTCTATTCGAGAA<br>ATTCTTCCCCAGTAGTTCATACAGGAGACCAGTTGGAATTTCCAGCATGGTGGAGGCCATGGTGTCTAGGGCCCGAATCGATGCACGAATTGATTTCGAATCTGGAAGGATCAAGAAGGAAGAGTTTGCTGAGATCATGAAGATCTGTTCCACCATTGAAGAGCTCAGACGGCAAAAATAG
internal NVRI repository	>A/turkey/Poland/H303/2022_H5N1_2022-04-26<br>ATGGATGTCAATCCGACTTTACTTTTCTTAAAAGTGCCAGCGCAAGATGCCATAAGTACCACATTCCCTTACACTGGAGATCCTCCATACAGCCATGGAACAGGGACAGGATACACAATGGACACAGTCAACAGAACACATCAATACTCAGAGAAGGGAAAATGGACAACAAACACAGAAACCGGAGCACCTCAACTCAACCCAATTGATGGGCCACTACCTGAGGACAACGA<br>ACCGAGCGGATATGCACAAACAGATTGCGTGTTGGAAGCAATGGCTTTCCTTGAAGAGTCCCACCCAGGGATCTTTGAAAACTCTTGTCTTGAAACGATGGAAGTCGTTCAGCAAACAAGAGTGGACAAACTAACTCAAGGTCGCCAGACATATGACTGGACACTGAATAGAAACCAACCAGCTGCAACTGCCCTGGCCAACACTATAGAGGTCTTCAGATCAAACGGTCTAA<br>CAGCCAATGAATCGGGGAGACTAATAGATTTCCTCAAGGATGTGATGGACTCAATGGATAAAGAAGAAATGGAAATAACAACACATTTCCAGAGAAAGAGAAGAGTAAGGGACAACATGACCAAGAAAATGGTCACACAAAGAACAATAGGAAAGAAGAAACAAAGGCTAAACAAGAGGAGCTACTTAATAAGAGCACTAACACTGAATACAATGACAAAAGATGCAGA<br>AAGAGGCAAATTGAAGAGACGGGCGATTGCAACACCAGGGATGCAGATTAGAGGATTTGTGTACTTTGTCGAAACACTGGCAAGGAGCATCTGTGAAAAACTTGAGCAATCTGGACTCCCCGTTGGAGGGAATGAGAAGAAGGCTAAATTGGCAAATGTCGTGAGAAAAATGATGACTAACTCACAAGATACAGAGCTCTCCTTCACAATTACTGGAGATAACACCAAATG<br>GAATGAGAATCAAAATCCTCGGATGTTTCTGGCAATGATAACATACATTACAAGAAACCAACCTGAATGGTTTAGAAATGTCTTGAGTATTGCCCCTATAATGTTCTCGAACAAAATGGCGAGATTGGGAAAAGGGTACATGTTTGAAAGTAAGAGCATGAAGTTACGGACACAAATACCTGCAGAAATGCTTGCAAACATTGACTTAAAATACTTCAATGAATCAACAAGAAA<br>GAAAATCGAAAAAATAAGGCCTCTACTAATAGATGGCACTGCCTCATTGAGTCCTGGAATGATGATGGGCATGTTCAATATGCTGAGTACAGTATTAGGAGTTTCAATCCTAAATCTTGGGCAAAAGAAGTACACCAAAACCACATACTGGTGGGATGGACTCCAATCCTCTGATGATTTCGCCCTCATAGTAAATGCACCGAATCATGAGGGAATACAAGCAGGAGTGGATA<br>GGTTCTATAGGACCTGCAAACTGGTCGGGATCAATATGAGCAAAAAGAAGTCTTACATGAACCGGACTGGAACATTTGAGTTCACAAGCTTTTTCTATCGCTATGGATTTGTGGCTAACTTCAGTATGGAGCTGCCCAGCTTTGGAGTTTCTGGGATCAATGAATCAGCTGACATGAGCATTGGCGTCACAGTGATAAAGAACAACATGATAAACAATGACCTTGGACCGGCAA<br>CAGCTCAAATGGCCCTTCAACTATTCATCAAAGATTACAGGTACACGTACCGATGCCACAGAGGTGACACACAAATTCAAACGAGGAGATCATTCGAGCTGAAGAAGCTGTGGGAACAGACCCGTTCAAAGGCAGGACTGTTGGTGTCAGATGGAGGACCAAATCTATACAACATTCGGAATCTCCATATCCCAGAGGTCTGCCTGAAGTGGGAGCTGATGGACGAAGATTA<br>CCAGGGCAGGTTGTGTAATCCTCTGAACCCATTTGTCAGCCATAAAGAACTTGAGTCCGTAAACAATGCTGTGGTGATGCCAGCCCACGGTCCAGCCAAAAGCATGGAATATGATGCCGTTGCGACTACACACTCATGGATTCCTAAAAGGAATCGTTCCATTCTCAATACCAGTCAAAGGGGAATTCTTGAGGATGAACAGATGTACCAGAAATGCTGCAGTCTATTCGAGAA<br>ATTCTTCCCCAGTAGTTCATACAGGAGACCAGTTGGAATTTCCAGCATGGTGGAGGCCATGGTGTCTAGGGCCCGAATCGATGCACGCATTGATTTCGAATCTGGAAGGATTAAGAAGGAAGAGTTTGCTGAGATCATGAAGATCTGTTCCACCATTGAAGAGCTCAGACGGCAAAAATAG
internal NVRI repository	>A/turkey/Poland/H297/2022_H5N1_2022-04-21<br>ATGGATGTCAATCCGACTTTACTTTTCTTAAAAGTGCCAGCGCAAGATGCCATAAGTACCACATTCCCTTACACTGGAGATCCTCCATACAGCCATGGAACAGGGACAGGATACACAATGGACACAGTCAACAGAACACATCAATACTCAGAGAAGGGAAAATGGACAACAAACACAGAAACCGGAGCACCTCAACTCAACCCAATTGATGGGCCACTACCTGAGGACAACGA<br>ACCGAGCGGATATGCACAAACAGATTGCGTGTTGGAAGCAATGGCTTTCCTTGAAGAGTCCCACCCAGGGATCTTTGAAAACTCTTGTCTTGAAACGATGGAAGTCGTTCAGCAAACAAGAGTGGACAAACTAACTCAAGGTCGCCAGACATATGACTGGACACTGAATAGAAACCAACCAGCTGCAACTGCCCTGGCCAACACTATAGAGGTCTTCAGATCAAACGGTCTAA<br>CAGCCAATGAATCGGGGAGACTAATAGATTTCCTCAAGGATGTGATGGACTCAATGGATAAAGAAGAAATGGAAATAACAACACATTTCCAGAGAAAGAGAAGAGTAAGGGACAACATGACCAAGAAAATGGTCACACAAAGAACAATAGGAAAGAAGAAACAAAGGCTAAACAAGAGGAGCTACTTAATAAGAGCACTGACACTGAATACAATGACAAAAGATGCAGA<br>AAGAGGCAAATTGAAGAGACGGGCGATTGCAACACCAGGGATGCAGATTAGAGGATTTGTGTACTTTGTCGAAACACTGGCAAGGAGCATCTGTGAAAAACTTGAGCAATCTGGACTCCCCGTTGGAGGGAATGAGAAGAAGGCTAAATTGGCAAATGTCGTGAGAAAAATGATGACTAACTCACAAGATACAGAGCTCTCCTTCACAATTACTGGAGATAACACCAAATG<br>GAATGAGAATCAAAATCCTCGGATGTTTCTGGCAATGATAACATACATTACAAGAAACCAACCTGAATGGTTTAGAAATGTCTTGAGTATTGCCCCTATAATGTTCTCGAACAAAATGGCGAGATTGGGAAAAGGGTACATGTTTGAAAGTAAGAGCATGAAGTTACGGACACAAATACCTGCAGAAATGCTTGCAAATATTGACTTAAAATACTTCAATGAATCAACAAGAAA<br>GAAAATCGAAAAAATAAGGCCTCTACTAATAGATGGCACTGCCTCATTGAGTCCTGGAATGATGATGGGCATGTTCAATATGCTGAGTACAGTATTAGGAGTTTCAATCCTAAATCTTGGGCAAAAGAAGTACACCAAAACCACATACTGGTGGGATGGACTCCAATCCTCTGATGATTTCGCCCTCATAGTAAATGCACCGAATCATGAGGGAATACAAGCAGGAGTGGATA<br>GGTTCTATAGGACCTGCAAACTGGTCGGGATCAATATGAGCAAAAAGAAGTCTTACATGAACCGGACTGGAACATTTGAGTTCACAAGCTTTTTCTATCGCTATGGATTTGTGGCTAACTTCAGTATGGAGCTGCCCAGCTTTGGAGTTTCTGGGATCAATGAATCAGCTGACATGAGCATTGGCGTCACAGTGATAAAGAACAACATGATAAACAATGACCTTGGACCGGCAA<br>CAGCTCAAATGGCCCTTCAACTATTCATCAAAGATTACAGGTACACGTACCGATGCCACAGAGGTGACACACAAATTCAAACGAGGAGATCATTCGAGCTGAAGAAGCTGTGGGAACAGACCCGTTCAAAGGCAGGACTGTTGGTGTCAGATGGAGGACCAAATCTATACAACATTCGGAATCTCCATATCCCAGAGGTCTGCCTGAAGTGGGAGCTGATGGACGAAGATTA<br>CCAGGGCAGGTTGTGTAATCCTCTGAACCCATTTGTCAGCCATAAAGAACTTGAGTCCGTAAACAATGCTGTGGTGATGCCAGCCCACGGTCCAGCCAAAAGCATGGAATATGATGCCGTTGCGACTACACACTCATGGATTCCTAAAAGGAATCGTTCCATTCTCAATACCAGTCAAAGGGGAATTCTTGAGGATGAACAGATGTACCAGAAATGCTGCAGTCTATTCGAGAA<br>ATTCTTCCCCAGTAGTTCATACAGGAGACCAGTTGGAATTTCCAGCATGGTGGAGGCCATGGTGTCTAGGGCCCGAATCGATGCACGCATTGATTTCGAATCTGGAAGGATTAAGAAGGAAGAGTTTGCTGAGATCATGAAGATCTGTTCCACCATTGAAGAGCTCAGACGGCAAAAATAG
internal NVRI repository	>A/turkey/Poland/H296/2022_H5N1_2022-04-21<br>ATGGATGTCAATCCGACTTTACTTTTCTTAAAAGTGCCAGCGCAAGATGCCATAAGTACCACATTCCCTTACACTGGAGATCCTCCATACAGCCATGGAACAGGGACAGGATACACAATGGACACAGTCAACAGAACACATCAATACTCAGAGAAGGGAAAATGGACAACAAACACAGAAACCGGAGCACCTCAACTCAACCCAATTGATGGGCCACTACCTGAGGACAACGA<br>ACCGAGCGGATATGCACAAACAGATTGCGTGTTGGAAGCAATGGCTTTCCTTGAAGAGTCCCACCCAGGGATCTTTGAAAACTCTTGTCTTGAAACGATGGAAGTCGTTCAGCAAACAAGAGTGGACAAACTAACTCAAGGTCGCCAGACATATGACTGGACACTGAATAGAAACCAACCAGCTGCAACTGCCCTGGCCAACACTATAGAGGTCTTCAGATCAAACGGTCTAA<br>CAGCCAATGAATCGGGGAGACTAATAGATTTCCTCAAGGATGTGATGGACTCAATGGATAAAGAAGAAATGGAAATAACAACACATTTCCAGAGAAAGAGAAGAGTAAGGGACAACATGACCAAGAAAATGGTCACACAAAGAACAATAGGAAAGAAGAAACAAAGGCTAAACAAGAGGAGCTACTTAATAAGAGCACTGACACTGAATACAATGACAAAAGATGCAGA<br>AAGAGGCAAATTGAAGAGACGGGCGATTGCAACACCAGGGATGCAGATTAGAGGATTTGTGTACTTTGTCGAAACACTGGCAAGGAGCATCTGTGAAAAACTTGAGCAATCTGGACTCCCCGTTGGAGGGAATGAGAAGAAGGCTAAATTGGCAAATGTCGTGAGAAAAATGATGACTAACTCACAAGATACAGAGCTCTCCTTCACAATTACTGGAGATAACACCAAATG<br>GAATGAGAATCAAAATCCTCGGATGTTTCTGGCAATGATAACATACATTACAAGAAACCAACCTGAATGGTTTAGAAATGTCTTGAGTATTGCCCCTATAATGTTCTCGAACAAAATGGCGAGATTGGGAAAAGGGTACATGTTTGAAAGTAAGAGCATGAAGTTACGGACACAAATACCTGCAGAAATGCTTGCAAACATTGACTTAAAATACTTCAATGAATCAACAAGAAA<br>GAAAATCGAAAAAATAAGGCCTCTACTAATAGATGGCACTGCCTCATTGAGTCCTGGAATGATGATGGGCATGTTCAATATGCTGAGTACAGTATTAGGAGTTTCAATCCTAAATCTTGGGCAAAAGAAGTACACCAAAACCACATACTGGTGGGATGGACTCCAATCCTCTGATGATTTCGCCCTCATAGTAAATGCACCGAATCATGAGGGAATACAAGCAGGAGTGGATA<br>GGTTCTATAGGACCTGCAAACTGGTCGGGATCAATATGAGCAAAAAGAAGTCTTACATGAACCGGACTGGAACATTTGAGTTCACAAGCTTTTTCTATCGCTATGGATTTGTGGCTAACTTCAGTATGGAGCTGCCCAGCTTTGGAGTTTCTGGGATCAATGAATCAGCTGACATGAGCATTGGCGTCACAGTGATAAAGAACAACATGATAAACAATGACCTTGGACCGGCAA<br>CAGCTCAAATGGCCCTTCAACTATTCATCAAAGATTACAGGTACACGTACCGATGCCACAGAGGTGACACACAAATTCAAACGAGGAGATCATTCGAGCTGAAGAAGCTGTGGGAACAGACCCGTTCAAAGGCAGGACTGTTGGTGTCAGATGGAGGACCAAATCTATACAACATTCGGAATCTCCATATCCCAGAGGTCTGCCTGAAGTGGGAGCTGATGGACGAAGATTA<br>CCAGGGCAGGTTGTGTAATCCTCTGAACCCATTTGTCAGCCATAAAGAACTTGAGTCCGTAAACAATGCTGTGGTGATGCCAGCCCACGGTCCAGCCAAAAGCATGGAATATGATGCCGTTGCGACTACACACTCATGGATTCCTAAAAGGAATCGTTCCATTCTCAATACCAGTCAAAGGGGAATTCTTGAGGATGAACAGATGTACCAGAAATGCTGCAGTCTATTCGAGAA<br>ATTCTTCCCCAGTAGTTCATACAGGAGACCAGTTGGAATTTCCAGCATGGTGGAGGCCATGGTGTCTAGGGCCCGAATCGATGCACGCATTGATTTCGAATCTGGAAGGATTAAGAAGGAAGAGTTTGCTGAGATCATGAAGATCTGTTCCACCATTGAAGAGCTCAGACGGCAAAAATAG
internal NVRI repository	>A/turkey/Poland/H2188-Ni/2021_H5N1_2021-12-30<br>ATGGATGTCAATCCGACTTTACTTTTCTTAAAAGTGCCAGCGCAAGATGCCATAAGTACCACATTCCCTTACACTGGAGATCCTCCATACAGCCATGGAACAGGGACAGGATACACAATGGACACAGTCAACAGAACACATCAATACTCAGAGAAGGGAAAATGGGCAACAAACACAGAAACCGGAGCACCTCAACTCAACCCAATTGATGGGCCACTACCTGAGGACAACGA<br>ACCGAGCGGATATGCACAAACAGATTGCGTGTTGGAAGCAATGGCTTTCCTTGAAGAGTCCCATCCAGGGATCTTTGAAAACTCTTGTCTTGAAACGATGGAAGTCGTTCAGCAAACAAGAGTGGACAAACTAACTCAAGGTCGCCAGACATATGACTGGACACTGAATAGAAACCAACCAGCTGCAACTGCCCTGGCCAACACTATAGAGGTCTTCAGATCAAACGGTCTAA<br>CAGCCAATGAATCGGGGAGACTAATAGATTTCCTCAAGGATGTGATGGACTCAATGGATAAAGAAGAAATGGAAATAACAACACATTTCCAGAGAAAGAGAAGAGTAAGGGACAACATGACCAAGAAAATGGTCACACAAAGAACAATAGGAAAGAAGAAACAAAGGCTAAACAAGAGGAGCTACTTAATAAGAGCACTGACACTGAATACAATGACAAAAGATGCAGA<br>AAGAGGCAAATTGAAGAGACGGGCGATTGCAACACCAGGGATGCAGATTAGAGGATTTGTGTACTTTGTCGAAACACTGGCAAGGAGCATCTGTGAAAAACTTGAGCAATCTGGACTCCCTGTTGGAGGGAATGAGAAGAAGGCTAAATTGGCAAATGTTGTGAGAAAAATGATGACTAACTCACAAGATACAGAGCTCTCCTTCACAATTACTGGAGATAACACCAAATG<br>GAATGAGAATCAAAATCCTCGGATGTTTCTGGCAATGATAACATACATTACAAGAAACCAACCTGAATGGTTTAGGAATGTCTTGAGTATTGCCCCTATAATGTTCTCGAACAAAATGGCGAGATTGGGAAAAGGGTACATGTTTGAAAGTAAGAGCATGAAGTTACGGACACAAATACCTGCAGAAATGCTTGCAAACATTGACTTAAAATACTTCAATGAATCAACAAGAAA<br>GAAAATCGAAAAAATAAGGCCTCTACTAATAGATGGCACTGCCTCATTGAGTCCTGGAATGATGATGGGCATGTTCAATATGCTGAGTACAGTATTGGGAGTTTCAATCCTAAATCTTGGGCAAAAGAAGTACACCAAAACCACATACTGGTGGGATGGACTCCAATCCTCTGATGATTTCGCCCTCATAGTAAATGCACCGAATCATGAGGGAATACAAGCAGGAGTGGATA<br>GGTTCTATAGGACCTGCAAACTGGTCGGGATCAATATGAGCAAAAAGAAGTCTTACATAAACCGGACTGGAACATTTGAGTTCACAAGCTTTTTCTATCGCTATGGATTTGTGGCTAACTTCAGTATGGAGCTGCCCAGCTTTGGGGTTTCTGGGATCAATGAATCAGCTGACATGAGCATTGGCATCACAGTGATAAAGAACAACATGATAAACAATGACCTTGGACCAGCAA<br>CAGCTCAAATGGCCCTTCAACTATTCATCAAAGATTACAGGTACACGTACCGATGCCATAGAGGTGACACACAAATTCAAACGAGGAGATCATTCGAGCTGAAGAAGCTGTGGGAACAGACCCGTTCAAAGGCAGGACTGTTGGTGTCAGATGGAGGACCAAATCTATACAACATTCGGAATCTCCATATCCCAGAGGTCTGCCTGAAGTGGGAGCTGATGGACGAAGATTA<br>CCAGGGCAGGTTGTGTAATCCTCTGAACCCATTTGTCAGCCATAAAGAAATTGAGTCCGTAAACAATGCTGTGGTGATGCCAGCCCACGGTCCAGCCAAAAGCATGGAATATGATGCCGTTGCGACTACACACTCATGGATTCCTAAAAGGAATCGTTCCATTCTCAATACCAGTCAAAGGGGAATTCTTGAGGATGAACAGATGTACCAGAAATGCTGCGGTCTATTCGAGAA<br>ATTCTTCCCCAGTAGTTCATACAGGAGACCAGTTGGAATTTCCAGCATGGTGGAGGCCATGGTGTCTAGGGCCCGAATCGATGCACGCATTGATTTCGAATCTGGAAGGATCAAGAAGGAAGAGTTTGCTGAGATCATGAAGATCTGTTCCACCATTGAAGAGCTCAGACGGCAAAAATAG

internal NVRI repository	>A/turkey/Poland/H2179-T4/2021_H5N1_2021-12-29<br>ATGGATGTCAATCCGACTCTACTTTTCTTAAAAGTGCCAGCGCAAGATGCCATAAGTACAACATTCCCTTACACTGGAGATCCTCCATACAGCCATGGAACAGGGACAGGATACACAATGGACACAGTCAACAGAACACATCAATACTCAGAGAAGGGAAAATGGACAACAAACACAGAAACCGGAGCACCTCAACTCAACCCAATTGATGGGCCACTACCTGAGGACAACGA<br>ACCGAGCGGATATGCACAAACAGATTGCGTGTTGGAAGCAATGGCTTTCCTTGAAGAGTCCCACCCAGGGATCTTTGAAAACTCTTGTCTTGAAACGATGGAAGTCGTTCAGCAAACAAGAGTGGACAAACTAACTCAAGGTCGCCAGACATATGACTGGACACTGAATAGAAACCAACCAGCTGCAACTGCCCTGGCCAACACTATAGAGGTCTTCAGATCAAACGGTCTAA<br>CAGCCAATGAATCGGGGAGACTAATAGATTTCCTCAAGGATGTGATGGACTCAATGGATAAAGAAGAAATGGAAATAACAACACATTTCCAGAGAAAGAGAAGAGTAAGGGACAACATGACCAAGAAAATGGTCACACAAAGAACAATAGGAAAGAAGAAACAAAGGCTAAACAAGAGGAGCTACTTAATAAGAGCACTGACACTGAATACAATGACAAAAGATGCAGA<br>AAGAGGCAAATTGAAGAGACGGGCGATTGCAACACCAGGGATGCAGATTAGAGGATTTGTGTACTTTGTCGAAACACTGGCAAGGAGCATCTGTGAAAAACTTGAGCAATCTGGACTCCCCGTTGGAGGGAATGAGAAGAAGGCTAAATTGGCAAATGTCGTGAGAAAAATGATGACTAACTCACAAGATACAGAGCTCTCCTTCACAATTACTGGAGATAACACCAAATG<br>GAATGAGAATCAAAATCCTCGGATGTTTCTGGCAATGATAACATACATTACAAGAAACCAACCTGAATGGTTTAGAAATGTCTTGAGTATTGCCCCTATAATGTTCTCGAACAAAATGGCGAGATTGGGAAAAGGGTACATGTTTGAAAGTAAGAGCATGAAGTTACGGACACAAATACCTGCAGAAATGCTTGCAAACATTGACTTAAAATACTTCAATGAATCAACAAGAAA<br>GAAAATCGAAAAAATAAGGCCTCTACTAATAGATGGCACTGCCTCATTGAGTCCTGGAATGATGATGGGCATGTTCAATATGCTGAGTACAGTATTAGGAGTTTCAATCCTAAATCTTGGGCAAAAGAAGTACACCAAAACCACATACTGGTGGGATGGACTCCAATCCTCTGATGATTTCGCCCTCATAGTAAATGCACCGAATCATGAGGGAATACAAGCAGGAGTGGATA<br>GGTTCTATAGGACCTGCAAACTGGTCGGGATCAATATGAGCAAAAAGAAGTCTTACATAAACCGGACTGGAACATTTGAGTTCACAAGCTTTTTCTATCGCTATGGATTTGTGGCTAACTTCAGTATGGAGCTGCCCAGCTTTGGAGTTTCTGGGATCAATGAATCAGCTGACATGAGCATTGGCGTCACAGTGATAAAGAACAACATGATAAACAATGACCTTGGACCGGCAA<br>CAGCTCAAATGGCCCTTCAACTATTCATCAAAGATTACAGGTACACGTACCGGTGCCACAGAGGTGACACACAAATTCAAACGAGGAGATCATTCGAGCTGAAGAAGCTGTGGGAACAGACCCGTTCAAAGGCAGGACTGTTGGTGTCAGATGGAGGACCAAATCTATACAACATTCGGAATCTCCATATCCCAGAGGTCTGCCTGAAGTGGGAGCTGATGGACGAAGATTA<br>CCAGGGCAGGTTGTGTAATCCTCTGAACCCATTTGTCAGCCATAAAGAAATTGAGTCCGTAAACAATGCTGTGGTGATGCCAGCCCACGGTCCAGCCAAAAGCATGGAATATGATGCCGTTGCGACTACACACTCATGGATTCCTAAAAGGAATCGTTCCATTCTCAATACCAGTCAAAGGGGAATTCTTGAGGATGAACAGATGTACCAGAAATGCTGCAGTCTATTCGAGAA<br>ATTCTTCCCCAGTAGTTCATACAGGAGACCAGTTGGAATTTCCAGCATGGTGGAGGCCATGGTGTCTAGGGCCCGAATCGATGCACGCATTGATTTCGAATCTGGAAGGATCAAGAAGGAAGAGTTTGCTGAGATCATGAAGATCTGTTCCACCATTGAAGAGCTCAGACGGCAAAAATAG
internal NVRI repository	>A/turkey/Poland/H2118-N/2021_H5N1_2021-12-16<br>ATGGATGTCAATCCGACTTTACTTTTCTTAAAAGTGCCAGCGCAAGATGCCATAAGTACCACATTCCCTTACACTGGAGATCCTCCATACAGCCATGGAACAGGGACAGGATACACAATGGACACAGTCAACAGAACACATCAATACTCAGAGAAGGGAAAATGGACAACAAACACAGAAACCGGAGCACCTCAACTCAACCCAATTGATGGGCCACTACCTGAGGACAACGA<br>ACCGAGCGGATATGCACAAACAGATTGCGTGTTGGAAGCAATGGCTTTCCTTGAAGAGTCCCACCCAGGGATCTTTGAAAACTCTTGTCTTGAAACGATGGAAGTCGTTCAGCAAACAAGAGTGGACAAACTAACTCAAGGTCGCCAGACATATGACTGGACACTGAATAGAAACCAACCAGCTGCAACTGCCCTGGCCAACACTATAGAGGTCTTCAGATCAAACGGTCTAA<br>CAACCAATGAATCGGGGAGACTAATAGATTTCCTCAAGGATGTGATGGACTCAATGGATAAAGAAGAAATGGAAATAACAACACATTTCCAGAGAAAGAGAAGAGTAAGGGACAACATGACCAAGAAAATGGTCACACAAAGAACAATAGGAAAGAAGAAACAAAGGCTAAACAAGAGGAGCTACTTAATAAGAGCACTGACACTGAATACAATGACAAAAGATGCAGA<br>AAGAGGCAAATTGAAAAGACGGGCGATTGCAACACCAGGGATGCAGATTAGAGGATTTGTGTACTTTGTCGAAACACTGGCAAGGAGCATCTGTGAAAAACTTGAGCAATCTGGACTCCCCGTTGGAGGGAATGAGAAGAAGGCTAAATTGGCAAATGTCGTGAGAAAAATGATGACTAACTCACAAGATACAGAGCTCTCCTTCACAATTACTGGAGATAACACCAAATG<br>GAATGAGAATCAAAATCCTCGGATGTTTCTGGCAATGATAACATACATTACAAGAAACCAACCTGAATGGTTTAGAAATGTCTTGAGTATTGCCCCTATAATGTTCTCGAACAAAATGGCGAGATTGGGAAAAGGGTACATGTTTGAAAGTAAGAGCATGAAGTTACGGACACAAATACCTGCAGAAATGCTTGCAAACATTGACTTAAAATACTTCAATGAATCAACAAGAAA<br>GAAAATCGAAAAAATAAGGCCTCTACTAATAGATGGCACTGCCTCATTGAGTCCTGGAATGATGATGGGCATGTTCAATATGCTGAGTACAGTATTAGGAGTTTCAATCCTAAATCTTGGGCAAAAGAAGTACACCAAAACCACATACTGGTGGGATGGACTCCAATCCTCTGATGATTTCGCCCTCATAGTAAATGCACCGAATCATGAGGGAATACAAGCAGGAGTGGATA<br>GGTTCTATAGGACCTGCAAACTGGTCGGGATCAATATGAGCAAAAAGAAGTCTTACATAAACCGGACTGGAACATTTGAGTTCACAAGCTTTTTCTATCGCTATGGATTTGTGGCTAACTTCAGTATGGAGCTGCCCAGCTTTGGAGTTTCTGGGATCAATGAATCAGCTGACATGAGCATTGGCATCACAGTGATAAAGAACAACATGATAAACAATGACCTTGGACCAGCAA<br>CTGCTCAAATGGCCCTTCAACTATTCATCAAAGATTACAGGTACACGTACCGATGCCACAGAGGTGACACACAAATTCAAACGAGGAGATCATTCGAGCTGAAGAAGCTGTGGGAACAGACCCGTTCAAAGGCAGGACTGTTGGTGTCAGATGGAGGACCAAATCTATACAACATTCGGAATCTCCATATCCCAGAGGTCTGCCTGAAGTGGGAGCTGATGGACGAAGATTA<br>CCAGGGCAGGTTGTGTAATCCTCTGAACCCATTTGTCAGCCATAGAGAAATTGAGTCCGTAAACAATGCTGTAGTGATGCCAGCCCACGGTCCAGCCAAAAGCATGGAATATGATGCCGTTGCGACTACACACTCATGGATTCCTAAAAGGAATCGTTCCATTCTCAATACCAGTCAAAGGGGAATTCTTGAGGATGAACAGATGTACCAGAAATGCTGCAGTCTATTCGAGAA<br>ATTCTTCCCCAGTAGTTCATACAGGAGACCAGTTGGAATTTCCAGCATGGTGGAGGCCATGGTGTCTAGGGCCCGAATCGATGCACGCATTGATTTCGAATCTGGAAGGATCAAGAAGGAAGAGTTTGCTGGGATCATGAAGATCTGTTCCACCATTGAAGAGCTCAGACGGCAAAAATAG
internal NVRI repository	>A/turkey/Poland/H2113-T1/2021_H5N1_2021-12-15<br>ATGGATGTCAATCCGACTTTACTTTTCTTAAAAGTGCCAGCGCAAGATGCCATAAGTACCACATTCCCTTACACTGGAGATCCTCCATACAGCCATGGAACAGGGACAGGATACACAATGGACACAGTCAACAGAACACATCAATACTCAGAGAAGGGAAAATGGACAACAAACACAGAAACCGGAGCACCTCAACTCAACCCAATTGATGGGCCACTACCTGAGGACAACGA<br>ACCGAGCGGATATGCACAAACAGATTGCGTGTTGGAAGCAATGGCTTTCCTTGAAGAGTCCCACCCAGGGATCTTTGAAAACTCTTGTCTTGAAACGATGGARGTCGTTCAGCAAACAAGAGTGGACAAACTAACTCAAGGTCGCCAGACATATGACTGGACACTGAATAGAAACCAACCAGCTGCAACTGCCCTGGCCAACACTATAGAGGTCTTCAGATCAAATGGTCTAA<br>CAGCCAATGAATCGGGGAGACTAATAGATTTCCTCAAGGATGTGATGGACTCAATGGATAAAGAAGAAATGGAAATAACAACACATTTCCAGAGAAAGAGAAGAGTAAGGGACAACATGACCAAGAAAATGGTCACACAAAGAACAATAGGAAAGAAGAAACAAAGGCTAAACAAGAGGAGCTACTTAATAAGAGCACTGACACTGAATACAATGACAAAAGATGCAGA<br>AAGAGGCAAATTGAAAAGACGGGCGATTGCAACACCAGGGATGCAGATTAGAGGATTTGTATACTTTGTCGAAACACTGGCAAGGAGCATCTGTGAAAAACTTGAGCAATCTGGACTCCCCGTTGGAGGGAATGAGAAGAAGGCTAAATTGGCAAATGTCGTGAGAAAAATGATGACTAACTCACAAGATACAGAGCTCTCCTTCACAATTACTGGAGATAACACCAAATG<br>GAATGAGAATCAAAATCCTCGGATGTTTCTGGCAATGATAACATACATTACAAGAAACCAACCTGAATGGTTTAGAAATGTCTTGAGTATTGCCCCTATAATGTTCTCGAACAAAATGGCGAGATTGGGAAAAGGGTACATGTTTGAAAGTAAGAGCATGAAGTTACGGACACAAATACCTGCAGAAATGCTTGCAAACATTGACTTAAAATACTTCAATGAATCAACAAGAAA<br>GAAAATCGAAAAAATAAGGCCTCTACTAATAGATGGCACCGCCTCATTGAGTCCTGGAATGATGATGGGCATGTTCAATATGCTGAGTACAGTATTAGGAGTTTCAATCCTAAATCTTGGGCAAAAGAAGTACACCAAAACCACATACTGGTGGGATGGACTCCAATCCTCTGATGATTTCGCCCTCATAGTAAATGCACCGAATCATGAGGGAATACAAGCAGGAGTGGATA<br>GGTTCTATAGGACCTGCAAACTGGTCGGGATCAATATGAGCAAAAAGAAGTCTTACATAAACCGGACTGGAACATTTGAGTTCACAAGCTTTTTCTATCGCTATGGATTTGTGGCTAACTTCAGTATGGAGCTGCCCAGCTTTGGGGTTTCTGGGATCAATGAATCAGCTGACATGAGCATTGGCGTCACAGTGATAAAGAACAACATGATAAACAATGACCTTGGACCAGCAA<br>CAGCTCAGATGGCCCTTCAACTATTCATCAAAGATTACAGGTACACGTACCGATGCCACAGAGGTGACACACAAATTCAAACGAGGAGATCATTCGAGCTGAAGAAGCTGTGGGAACAGACCCGTTCAAAGGCAGGACTGTTGGTGTCAGATGGAGGACCAAATCTATACAACATTCGGAATCTCCATATCCCAGAGGTCTGCCTGAAGTGGGAGCTGATGGACGAAGATTA<br>CCAGGGCAGGTTGTGTAATCCTCTGAACCCATTTGTCAGCCATAGAGAAATTGAGTCCGTAAACAATGCTGTGGTGATGCCAGCCCACGGTCCAGCCAAAAGCATGGAATATGATGCCGTTGCGACTACACACTCATGGATTCCTAAAAGGAATCGTTCCATTCTCAATACCAGTCAAAGGGGAATTCTTGAGGATGAACAGATGTACCAGAAATGCTGCAGTCTATTCGAGAA<br>ATTCTTCCCCAGTAGTTCATACAGGAGACCAGTTGGAATTTCCAGCATGGTGGAGGCCATGGTGTCTAGGGCCCGAATCGATGCACGCATTGATTTCGAATCTGGAAGGATCAAGAAGGAAGAGTTTGCTGAGATCATGAAGATCTGTTCCACCATTGAAGAGCTCAGACGGCAAAAATAG
internal NVRI repository	>A/turkey/Poland/H2043-T2/2021_H5N1_2021-11-27<br>ATGGATGTCAATCCGACTTTACTTTTCTTAAAAGTGCCAGCTCAAGATGCCATAAGTACCACATTCCCTTACACTGGAGATCCTCCATACAGCCATGGAACAGGGACAGGATACACAATGGACACAGTCAACAGAACACATCAATACTCAGAGAAGGGAAAATGGACAACAAACACAGAAACCGGAGCACCTCAACTCAACCCAATTGATGGGCCACTACCTGAGGACAACGA<br>ACCGAGCGGATATGCACAAACAGATTGCGTGTTGGAAGCAATGGCTTTCCTTGAAGAGTCCCACCCAGGGATCTTTGAAAACTCTTGTCTTGAAACGATGGAAGTCGTTCAGCAAACAAGAGTGGACAAACTAACTCAAGGTCGCCAGACATATGACTGGACACTGAATAGAAACCAACCAGCTGCAACTGCCCTGGCCAACACTATAGAGGTCTTCAGATCAAACGGTCTAA<br>CAGCCAATGAATCGGGGAGACTAATAGATTTCCTCAAGGATGTGATGGACTCAATGGATAAAGAAGAAATGGAAATGACAACACATTTCCAGAGAAAGAGAAGAGTAAGGGACAACATGACCAAGAAAATGGTCACACAAAGAACAATAGGAAAGAAGAAACAAAGGCTAAACAAGAGGAGCTACTTAATAAGAGCATTGACACTGAATACAATGACAAAAGATGCAGA<br>AAGAGGCAAATTGAAGAGACGGGCGATTGCAACACCAGGGATGCAGATTAGAGGATTTGTGTACTTTGTCGAAACACTGGCAAGGAGCATCTGTGAAAAACTTGAGCAATCTGGACTCCCCGTTGGAGGGAATGAGAAGAAGGCTAAATTGGCAAATGTCGTGAGAAAAATGATGACTAACTCACAAGATACAGAGCTCTCCTTCACAATTACTGGAGATAACACCAAATG<br>GAATGAGAATCAAAATCCTCGGATGTTTCTGGCAATGATAACATACATTACAAGAAACCAACCTGAATGGTTTAGAAATGTCTTGAGTATTGCCCCTATAATGTTCTCGAACAAAATGGCGAGATTGGGAAAAGGGTACATGTTTGAAAGTAAGAGCATGAAGTTACGGACACAAATACCTGCAGAAATGCTTGCAAACATTGACTTAAAATATTTCAATGAATCAACAAGAAA<br>GAAAATCGAAAAAATAAGGCCTCTACTAATAGATGGCACTGCCTCATTGAGTCCTGGAATGATGATGGGCATGTTCAATATGCTGAGTACMGTATTAGGAGTTTCAATCCTAAATCTTGGGCAAAAGAAGTACACCAAAACCACATACTGGTGGGATGGACTCCAATCCTCTGATGATTTCGCCCTCATAGTAAATGCACCGAATCATGAGGGAATACAAGCAGGAGTGGATA<br>GGTTCTATAGGACCTGCAAACTGGTCGGGATCAATATGAGCAAAAAGAAGTCTTACATAAACCGGACTGGAACATTTGAGTTCACAAGCTTTTTCTATCGCTATGGATTTGTGGCTAACTTCAGTATGGAGCTGCCCAGCTTTGGAGTTTCTGGGATCAATGAATCAGCTGACATGAGCATTGGCGTCACAGTGATAAAGAACAACATGGTAAACAATGACCTTGGACCAGCAA<br>CAGCTCAAATGGCCCTTCAACTATTCATCAAAGATTACAGGTACACGTACCGATGCCACAGAGGTGACACACAAATTCAAACGAGGAGATCATTCGAGCTGAAGAAGCTGTGGGAACAGACCCGTTCAAAGGCAGGACTGTTGGTGTCAGATGGAGGACCAAATCTATACAACATTCGGAATCTCCATATCCCAGAGGTCTGCCTGAAGTGGGAGCTGATGGACGAAGATTA<br>CCAGGGCAGGTTGTGTAATCCTCTGAACCCATTTGTCAGCCATAAAGAAATTGAGTCCGTAAACAATGCTGTGGTGATGCCAGCCCACGGTCCAGCCAAAAGCATGGAATATGATGCCGTTGCGACTACACACTCATGGATTCCTAAAAGGAATCGTTCCATTCTCAATACCAGTCAAAGGGGAATTCTTGAGGATGAACAGATGTACCAGAAATGCTGCAGTCTATTCGAGAA<br>ATTCTTCCCCAGTAGTTCATACAGGAGACCAGTTGGAATTTCCAGCATGGTGGAGGCCATGGTGTCTAGGGCCCGAATCGATGCACGCATTGATTTCGAATCTGGAAGGATCAAGAAGGAGGAGTTTGCTGAGATCATGAAGATCTGTTCCACCATTGAAGAGCTCAGACGGCAAAAATAG
internal NVRI repository	>A/turkey/Poland/H1977_21RS3290-10/2021_H5N1_2021-11-16<br>ATGGATGTCAATCCGACTTTACTTTTCTTAAAAGTGCCAGCGCAAGATGCCATAAGTACCACATTCCCTTACACTGGAGATCCTCCATACAGCCATGGAACAGGGACAGGATACACGATGGACACAGTCAACAGAACACATCAATACTCAGAGAAGGGAAAATGGACAACAAACACAGAAACCGGAGCACCTCAACTCAACCCAATTGATGGGCCACTACCTGAGGACAACGA<br>ACCGAGCGGATATGCACAAACAGATTGCGTGTTGGAAGCAATGGCTTTCCTTGAAGAGTCCCACCCAGGGATCTTTGAAAACTCTTGTCTTGAAACGATGGAAGTCGTTCAGCAAACAAGAGTGGACAAACTAACTCAAGGTCGCCAGACATATGACTGGACACTGAATAGAAACCAACCAGCTGCAACTGCCCTGGCCAACACTATAGAGGTCTTCAGATCAAACGGTCTAA<br>CAGCCAATGAATCGGGGAGACTAATRGATTTCCTCAAGGATGTGATGGACTCAATGGATAAAGAAGAAATGGAAATAACAACACATTTCCAGAGAAAGAGAAGAGTAAGGGACAACATGACCAAGAAAATGGTCACACAAAGAACAATAGGAAAGAAGAAACAAAGGCTAAACAAGAGGAGCTACTTAATAAGAGCACTGACACTGAATACAATGACAAAAGATGCAGA<br>AAGAGGCAAATTGAAGAGACGGGCGATTGCAACACCAGGGATGCAGATTAGAGGATTTGTGTACTTTGTCGAAACACTGGCAAGGAGCATCTGTGAAAAACTTGAGCAATCTGGACTCCCCGTTGGAGGGAATGAGAAGAAGGCTAAATTGGCAAATGTCGTGAGAAAAATGATGACTAACTCACAAGATACAGAGCTCTCCTTCACAATTACTGGAGACAACACCAAATG<br>GAATGAGAATCAAAATCCTCGGATGTTTCTGGCAATGATAACATACATTACAAGAAACCAACCTGAATGGTTTAGAAATGTCTTAAGTATTGCCCCTATAATGTTCTCGAACAAAATGGCGAGATTGGGAAAAGGGTACATGTTTGAAAGTAAGAGCATGAAGTTACGGACACAAATACCTGCAGAAATGCTTGCAAACATTGACTTAAAATACTTCAATGAATCAACAAGAAA<br>GAAAATCGAAAAAATAAGGCCTCTACTAATAGATGGCACTGCCTCATTGAGTCCTGGAATGATGATGGGCATGTTCAATATGCTGAGTACAGTATTAGGAGTTTCAATCCTAAATCTTGGGCAAAAGAAGTACACCAAAACCACATACTGGTGGGATGGACTCCAATCCTCTGATGATTTCGCCCTCATAGTAAATGCACCGAATCATGAGGGAATACAAGCAGGAGTGGATA<br>GGTTCTATAGGACCTGCAAACTGGTCGGGATCAATATGAGCAAAAAGAAGTCTTACATAAACCGGACTGGAACATTTGAGTTCACAAGCTTTTTCTATCGCTATGGATTTGTGGCTAACTTCAGTATGGAGCTGCCCAGCTTTGGGGTTTCTGGGATCAATGAATCAGCTGACATGAGCATTGGCGTCACAGTGATAAAGAACAACATGATAAACAATGACCTTGGACCAGCAA<br>CAGCTCAAATGGCCCTTCAACTATTCATCAAAGATTACAGGTACACGTACCGATGCCACAGAGGTGACACACAAATTCAAACGAGGAGATCATTCGAGCTGAAGAAGCTGTGGGAACAGACCCGTTCAAAGGCAGGACTGTTGGTGTCAGATGGAGGACCAAATCTATACAACATTCGGAATCTCCATATCCCAGAGGTCTGCCTGAAGTGGGAGCTGATGGACGAAGATTA<br>CCAGGGCAGGTTGTGTAATCCTCTGAACCCATTTGTCAGCCATAAAGAAATTGAGTCCGTAAACAATGCTGTGGTGATGCCAGCTCACGGTCCAGCCAAAAGCATGGAATATGATGCCGTTGCGACTACACACTCATGGACTCCTAAAAGGAATCGTTCCATTCTCAATACCAGTCAAAGGGGAATTCTTGAGGATGAACAGATGTACCAGAAATGCTGCAGTCTATTCGAGAA<br>ATTCTTCCCCAGTAGTTCATACAGGAGACCAGTTGGAATTTCCAGCATGGTGGAGGCCATGGTGTCTAGGGCCCGAATCGATGCACGCATTGATTTCGAATCTGGAAGGATCAAGAAGGAAGAGTTTGCTGAGATCATGAAGATCTGTTCCACCATTGAAGAGCTCAGACGGCAAAAATAG
EPI_ISL_6935584	>A/turkey/Poland/H1944-N/2021_H5N1_2021-11-08<br>ATGGATGTCAATCCGACTTTACTTTTCTTAAAAGTGCCAGCGCAAGATGCCATAAGTACCACATTCCCTTACACTGGAGATCCTCCATACAGCCATGGAACAGGGACAGGATACACGATGGACACAGTCAACAGAACACATCAATACTCAGAGAAGGGAAAATGGACAACAAACACAGAAACCGGAGCACCTCAACTCAACCCAATTGATGGGCCACTACCTGAGGACAACGA<br>ACCGAGCGGATATGCACAAACAGATTGCGTGTTGGAAGCAATGGCTTTCCTTGAAGAGTCCCACCCAGGGATCTTTGAAAACTCTTGTCTTGAAACGATGGAAGTCGTTCAGCAAACAAGAGTGGACAAACTAACTCAAGGTCGCCAGACATATGACTGGACACTGAATAGAAACCAACCAGCTGCAACTGCCCTGGCCAACACTATAGAGGTCTTCAGATCAAACGGTCTAA<br>CAGCCAATGAATCGGGGAGACTAATAGATTTCCTCAAGGATGTGATGGACTCAATGGATAAAGAAGAAATGGAAATAACAACACATTTCCAGAGAAAGAGAAGAGTAAGGGACAACATGACCAAGAAAATGGTCACACAAAGAACAATAGGAAAGAAGAAACAAAGGCTAAACAAGAGGAGCTACTTAATAAGAGCACTGACACTGAATACAATGACAAAAGATGCAGA<br>AAGAGGCAAATTGAAGAGACGGGCGATTGCAACACCAGGGATGCAGATTAGAGGATTTGTGTACTTTGTCGAAACACTGGCAAGGAGCATCTGTGAAAAACTTGAGCAATCTGGACTCCCCGTTGGAGGGAATGAGAAGAAGGCTAAATTGGCAAATGTCGTGAGAAAAATGATGACTAACTCACAAGATACAGAGCTCTCCTTCACAATTACTGGAGACAACACCAAATG<br>GAATGAGAATCAAAATCCTCGGATGTTTCTGGCAATGATAACATACATTACAAGAAACCAACCTGAATGGTTTAGAAAYGTCTTAAGTATTGCCCCCATAATGTTCTCGAACAAAATGGCGAGATTGGGAAAAGGGTACATGTTTGAAAGTAAGAGCATGAAGTTACGGACACAAATACCTGCAGAAATGCTTGCAAACATTGACTTAAAATACTTCAATGAATCAACAAGAAA<br>GAAAATCGAAAAAATAAGGCCTCTACTAATAGATGGCACTGCCTCATTGAGTCCTGGAATGATGATGGGCATGTTCAATATGCTGAGTACAGTATTAGGAGTTTCAATCCTAAATCTTGGGCAAAAGAAGTACACCAAAACCACATACTGGTGGGATGGACTCCAATCCTCTGATGATTTCGCCCTCATAGTAAATGCACCGAATCATGAGGGAATACAAGCAGGAGTGGATA<br>GGTTCTATAGGACCTGCAAACTGGTCGGGATCAATATGAGCAAAAAGAAGTCTTACATAAACCGGACTGGAACATTTGAGTTCACAAGCTTTTTCTATCGCTATGGATTTGTGGCTAACTTCAGTATGGAGCTGCCCAGCTTTGGGGTTTCTGGGATCAATGAATCAGCTGACATGAGCATTGGCGTCACAGTGATAAAGAACAACATGATAAACAATGACCTTGGACCAGCAA<br>CAGCTCAAATGGCCCTTCAACTATTCATCAAAGATTACAGGTACACGTACCGATGCCACAGAGGTGACACACAAATTCAAACGAGGAGATCATTCGAGCTGAAGAAGCTGTGGGAACAGACCCGTTCAAAGGCAGGACTGTTGGTGTCAGATGGAGGACCAAATCTATACAACATTCGGAATCTCCATATCCCAGAGGTCTGCCTGAAGTGGGAGCTGATGGACGAAGATTA<br>CCAGGGCAGGTTGTGTAATCCTCTGAACCCATTTGTCAGCCATAAAGAAATTGAGTCCGTAAACAATGCTGTGGTGATGCCAGCTCACGGTCCAGCCAAAAGCATGGAATATGATGCCGTTGCGACTACACACTCATGGACTCCTAAAAGGAATCGTTCCATTCTCAATACCAGTCAAAGGGGAATTCTTGAGGATGAACAGATGTACCAGAAATGCTGCAGTCTATTCGAGAA<br>ATTCTTCCCCAGTAGTTCATACAGGAGACCAGTTGGAATTTCCAGCATGGTGGAGGCCATGGTGTCTAGGGCCCGAATCGATGCACGCATTGATTTCGAATCTGGAAGGATCAAGAAGGAAGAGTTTGCTGAGATCATGAAGATCTGTTCCACCATTGAAGAGCTCAGACGGCAAAAATAG
internal NVRI repository	>A/turkey/Poland/H1941_21RS3290-3/2021_H5N1_2021-11-06<br>ATGGATGTCAATCCGACTTTACTTTTCTTAAAAGTGCCAGCGCAAGATGCCATAAGTACCACATTCCCTTACACTGGAGATCCTCCATACAGCCATGGAACAGGGACAGGATACACAATGGACACGGTCAACAGAACACATCAATACTCAGAGAAGGGAAAGTGGACAACAAACACAGAAACCGGAGCACCTCAACTCAACCCAATTGATGGGCCACTACCTGAGGACAACGA<br>ACCGAGCGGATATGCACAAACAGATTGCGTGTTGGAAGCAATGGCTTTCCTTGAAGAGTCCCACCCAGGGATCTTTGAAAACTCTTGTCTTGAAACGATGGAAGTCGTTCAGCAAACAAGAGTGGACAAACTAACTCAAGGTCGCCAGACATATGACTGGACACTGAATAGAAACCAACCAGCTGCAACTGCCCTGGCCAACACTATAGAGGTCTTCAGATCAAACGGTCTAA<br>CAGCCAATGAATCGGGGAGACTAATAGATTTCCTCAAGGATGTGATGGACTCAATGGATAAAGAANNNNNNNNNNNNNNNNNNNNNNTTTCCAGAGAAAGAGAAGAGTAAGGGACAACATGACCAAGAAAATGGTCACACAAAGAACAATAGGAAAGAAGAAACAAAGGCTAAACAAGAGGAGCTACTTAATAAGAGCACTGACACTGAATACAATGACAAAAGATGCA<br>GAAAGAGGCAAATTGAAGAGACGGGCGATTGCAACACCAGGGATGCAGATTAGAGGATTTGTGTACTTTGTCGAAACACTGGCAAGGAGCATCTGTGAAAAACTTGAGCAATCTGGACTCCCCGTTGGAGGGAATGAGAAGAAGGCTAAATTGGCAAATGTCGTGAGAAAAATGATGACTAACTCACAAGATACAGAGCTCTCCTTCACAATTACTGGAGATAACACCAAA<br>TGGAATGAGAATCAAAATCCTCGGATGTTTCTGGCAATGATAACATACATTACAAGAAACCAACCTGAATGGTTTAGAAATGTCTTGAGTATTGCCCCTATAATGTTCTCGAACAAAATGGCGAGATTGGGAAAAGGGTACATGTTTGAAAGTAAGAGCATGAAGTTACGGACACAAATACCTGCAGAAATGCTTGCAAACATTGACCTAAAATACTTCAATGAATCAACAAGA<br>AAGAAAATCGAAAAAATAAGGCCTCTACTAATAGATGGCACTGCCTCATTGAGTCCTGGAATGATGATGGGCATGTTCAATATGCTGAGTACAGTATTAGGGGTTTCAATCCTAAATCTTGGACAAAAGAAGTACACCAAAACCACATACTGGTGGGATGGACTCCAATCCTCTGATGATTTCGCCCTCATAGTAAATGCACCGAATCATGAGGGAATACAAGCAGGAGTGGA<br>TAGGTTCTATAGGACCTGCAAACTGGTCGGGATCAATATGAGCAAAAAGAAGTCTTACATAAACCGGACTGGAACATTTGAGTTCACAAGCTTTTTCTATCGCTATGGATTTGTGGCTAACTTCAGTATGGAGCTGCCCAGCTTTGGAGTTTCTGGGATCAATGAATCAGCTGATATGAGCATTGGCGTCACAGTGATAAAGAACAACATGATAAACAATGACCTTGGACCAGC<br>AACAGCTCAAATGGCCCTTCAACTATTCATCAAAGATTACAGGTACACGTACCGATGCCACAGAGGTGACACACAAATTCAAACGAGGAGATCATTCGAGCTGAAGAAGCTGTGGGAACAGACCCGTTCAAAGGCAGGACTGTTGGTGTCAGATGGAGGACCAAATCTATACAACATTCGGAATCTCCATATCCCAGAGGTCTGCCTGAAGTGGGAGCTGATGGACGAAGAT<br>TACCAGGGCAGGTTGTGTAATCCTCTGAACCCATTTGTCAGCCATAAAGAAATTGAGTCCGTAAACAATGCTGTGGTGATGCCAGCCCACGGTCCAGCCAAAAGCATGGAATATGATGCCGTTGCGACTACACACTCATGGATTCCTAAAAGGAATCGTTCCATTCTCAATACCAGTCAAAGGGGAATTCTTGAGGATGAACAGATGTACCAGAAATGTTGCAGTCTATTCGAG<br>AAATTCTTCCCCAGTAGTTCATACAGGAGACCAGTTGGAATTTCCAGCATGGTGGAGGCCATGGTGTCTAGGGCCCGAATCGATGCACGCATTGATTTCGAATCTGGAAGGATCAAGAAGGAAGAGTTTGCTGAGATCATGAAGATCTGTTCCACCATTGAAGAGCTCAGACGGCAAAAATAG
EPI_ISL_6930564	>A/turkey/Poland/H1924-T1/2021_H5N1_2021-11-03<br>ATGGATGTCAATCCGACTTTACTTTTCTTAAAAGTGCCAGCGCAAGATGCCATAAGTACCACATTCCCTTACACTGGAGATCCTCCATACAGCCATGGAACAGGGACAGGATACACGATGGACACAGTCAACAGAACACATCAATACTCAGAGAAGGGAAAATGGACAACAAACACAGAAACCGGAGCACCTCAACTCAACCCAATTGATGGGCCACTACCTGAGGACAACGA<br>ACCGAGCGGATATGCACAAACAGATTGCGTGTTGGAAGCAATGGCTTTCCTTGAAGAGTCCCACCCAGGGATCTTTGAAAACTCTTGTCTTGAAACGATGGAAGTCGTTCAGCAAACAAGAGTGGACAAACTAACTCAAGGTCGCCAGACATATGACTGGACACTGAATAGAAACCAACCAGCTGCAACTGCCCTGGCCAACACTATAGAGGTCTTCAGATCAAACGGTCTAA<br>CAGCCAATGAATCGGGGAGACTAATAGATTTCCTCAAGGATGTGATGGACTCAATGGATAAAGAAGAAATGGAAATAACAACACATTTCCAGAGAAAGAGAAGAGTAAGGGACAACATGACCAAGAAAATGGTCACACAAAGAACAATAGGAAAGAAGAAACAAAGGCTAAACAAGAGGAGCTACTTAATAAGAGCACTGACACTGAATACAATGACAAAAGATGCAGA<br>AAGAGGCAAATTGAAGAGACGGGCGATTGCAACACCAGGGATGCAGATTAGAGGATTTGTGTACTTTGTCGAAACACTGGCAAGGAGCATCTGTGAAAAACTTGAGCAATCTGGACTCCCCGTTGGAGGGAATGAGAAGAAGGCTAAATTGGCAAATGTCGTGAGAAAAATGATGACTAACTCACAAGATACAGAGCTCTCCTTCACAATTACTGGAGACAACACCAAATG<br>GAATGAGAATCAAAATCCTCGGATGTTTCTGGCAATGATAACATACATTACAAGAAACCAACCTGAATGGTTTAGAAATGTCTTAAGTATTGCCCCTATAATGTTCTCGAACAAAATGGCGAGATTGGGAAAAGGGTACATGTTTGAAAGTAAGAGCATGAAGTTACGGACACAAATACCTGCAGAAATGCTTGCAAACATTGACTTAAAATACTTCAATGAATCAACAAGAAA<br>GAAAATCGAAAAAATAAGGCCTCTACTAATAGATGGCACTGCCTCATTGAGTCCTGGAATGATGATGGGCATGTTCAATATGCTGAGTACAGTATTAGGAGTTTCAATCCTAAATCTTGGGCAAAAGAAGTACACCAAAACCACATACTGGTGGGATGGACTCCAATCCTCTGATGATTTCGCCCTCATAGTAAATGCACCGAATCATGAGGGAATACAAGCAGGAGTGGATA<br>GGTTCTATAGGACCTGCAAACTGGTCGGGATCAATATGAGCAAAAAGAAGTCTTACATAAACCGGACTGGAACATTTGAGTTCACAAGCTTTTTCTATCGCTATGGATTTGTGGCTAACTTCAGTATGGAGCTGCCCAGCTTTGGGGTTTCTGGGATCAATGAATCAGCTGACATGAGCATTGGCGTCACAGTGATAAAGAACAACATGATAAACAATGACCTTGGACCAGCAA<br>CAGCTCAAATGGCCCTTCAACTATTCATCAAAGATTACAGGTACACGTACCGATGCCACAGAGGTGACACACAAATTCAAACGAGGAGATCATTCGAGCTGAAGAAGCTGTGGGAACAGACCCGTTCAAAGGCAGGACTGTTGGTGTCAGATGGAGGACCAAATCTATACAACATTCGGAATCTCCATATCCCAGAGGTCTGCCTGAAGTGGGAGCTGATGGACGAAGATTA<br>CCAGGGCAGGTTGTGTAATCCTCTGAACCCATTTGTCAGCCATAAAGAAATTGAGTCCGTAAACAATGCTGTGGTGATGCCAGCTCACGGTCCAGCCAAAAGCATGGAATATGATGCCGTTGCGACTACACACTCATGGACTCCTAAAAGGAATCGTTCCATTCTCAATACCAGTCAAAGGGGAATTCTTGAGGATGAACAGATGTACCAGAAATGCTGCAGTCTATTCGAGAA<br>ATTCTTCCCCAGTAGTTCATACAGGAGACCAGTTGGAATTTCCAGCATGGTGGAGGCCATGGTGTCTAGGGCCCGAATCGACGCACGCATTGATTTCGAATCTGGAAGGATCAAGAAGGAAGAGTTTGCTGAGATCATGAAGATCTGTTCCACCATTGAAGAGCTCAGACGGCAAAAATAG
internal NVRI repository	>A/turkey/Poland/H1923_21RS3290-1/2021_H5N1_2021-11-03<br>ATGGATGTCAATCCGACTTTACTTTTCTTAAAAGTGCCAGCGCAAGATGCCATAAGTACCACATTCCCTTACACTGGAGATCCTCCATACAGCCATGGAACAGGGACAGGATACACGATGGACACAGTCAACAGAACACATCAATACTCAGAGAAGGGAAAATGGACAACAAACACAGAAACCGGAGCACCTCAACTCAACCCAATTGATGGGCCACTACCTGAGGACAACGA<br>ACCGAGCGGATATGCACAAACAGATTGCGTGTTGGAAGCAATGGCTTTCCTTGAAGAGTCCCACCCAGGGATCTTTGAAAACTCTTGTCTTGAAACGATGGAAGTCGTTCAGCAAACAAGAGTGGACAAACTAACTCAAGGTCGCCAGACATATGACTGGACACTGAATAGAAACCAACCAGCTGCAACTGCCCTGGCCAACACTATAGAGGTCTTCAGATCAAACGGTCTAA<br>CAGCCAATGAATCGGGGAGACTAATAGATTTCCTCAAGGATGTGATGGACTCAATGGATAAAGAAGAAATGGAAATAACAACACATTTCCAGAGAAAGAGAAGAGTAAGGGACAACATGACCAAGAAAATGGTCACACAAAGAACAATAGGAAAGAAGAAACAAAGGCTAAACAAGAGGAGCTACTTAATAAGAGCACTGACACTGAATACAATGACAAAAGATGCAGA<br>AAGAGGCAAATTGAAGAGACGGGCGATTGCAACACCAGGGATGCAGATTAGAGGATTTGTGTACTTTGTCGAAACACTGGCAAGGAGCATCTGTGAAAAACTTGAGCAATCTGGACNNNNNNNNNNNNNNNNNGAGAAGAAGGCTAAATTGGCAAATGTCGTGAGAAAAATGATGACTAACTCACAAGATACAGAGCTCTCCTTCANNNNNNNNNNNNNNNNNNNNNNN<br>NNNNNNNNNNNNNNNNNNNNNNNNNNNNNNNNNNNNNNNNNNNNNNNNNNNNNNNNNNNNNNNNNNNNNNNNNNNNNNNNGTCTTAAGTATTGCCCCTATAATGTTCNNNNNACAAAATGGCGAGATTGGGAAAAGGGTACATGTTTGAAAGTAAGAGCATGAAGTTACGGACACANNNNNNNNNAGAAATGCTTGCAANNNNNNNCTTAAAAT<br>ANNNNNNNNNNNCAACAAGAAAGAAAATCGAAAAAATAAGGCCTCTACTAATAGATGGCACTGCCTCATTGAGTCCTGGAATGATGATGGGCATGTTCAATATGCTGAGTACAGTATTAGGAGTTTCAATCCTAAATCTTGGGCAAAAGAAGTACACCAAAACCACATACTGGTGGGATGGACTCCAATCCTCTGATGATTTCGCCCTCATAGTAAATGCACCGAATCATGA<br>GGGAATACAAGCAGGAGTGGATAGGTTCTATAGGACCTGCAAACTGGTCGGGATCAATATGAGCAAAAAGAAGTCTTACATAAACCGGACTGGAACATTTGAGTTCACAAGCTTTTTCTATCGCTATGGATTTGTGGCTAACTTCAGTATGGAGCTGCCCAGCTTTGGGGTTTCTGGGATCAATGAATCAGCTGACATGAGCATTGGCGTCACAGTGATAAAGAACAACATGA<br>TAAACAATGACCTTGGACCAGCAACAGCTCAAATGGCCCTTCAACTATTCATCAAAGATTACAGGTACACGTACCGATGCCACAGAGGTGACACACAAATTCAAACGAGGAGATCATTCGAGCTGAAGAAGCTGTGGGAACAGACCCGTTCAAAGGCAGGACTGTTGGTGTCAGATGGAGGACCAAATCTATACAACATTCGGAATCTCCATATCCCAGAGGTCTGCCTGAAG<br>TGGGAGCTGATGGACGAAGATTACCAGGGCAGGTTGTGTAATCCTCTGAACCCATTTGTCAGCCATAAAGAAATTGAGTCCGTAAACAATGCTGTGGTGATGCCAGCTCACGGTCCAGCCAAAAGCATGGAATATGATGCCGTTGCGACTACACACTCATGGACTCCTAAAAGGAATCGTTCCATTCTCAATACCAGTCAAAGGGGAATTCTTGAGGATGAACAGATGTACCA<br>GAAATGCTGCAGTCTATTCGAGAAATTCTTCCCCAGTAGTTCATACAGGAGACCAGTTGGAATTTCCAGCATGGTGGAGGCCATGGTGTCTAGGGCCCGAATCGATGCACGCATTGATTTCGAATCTGGAAGGATCAAGAAGGAAGAGTTTGCTGAGATCATGAAGATCTGTTCCACCATTGAAGAGCTCAGACGGCAAAAATAG
EPI_ISL_6930238	>A/turkey/Poland/H1913-T1/2021_H5N1_2021-11-02<br>ATGGATGTCAATCCGACTTTACTTTTCTTAAAAGTGCCAGCGCAAGATGCCATAAGTACCACATTCCCTTACACTGGAGATCCTCCATACAGCCATGGAACAGGGACAGGATACACGATGGACACAGTCAACAGAACACATCAATACTCAGAGAAGGGAAAATGGACAACAAACACAGAAACCGGAGCACCTCAACTCAACCCAATTGATGGGCCACTACCTGAGGACAACGA<br>ACCGAGCGGATATGCACAAACAGATTGCGTGTTGGAAGCAATGGCTTTCCTTGAAGAGTCCCACCCAGGGATCTTTGAAAACTCTTGTCTTGAAACGATGGAAGTCGTTCAGCAAACAAGAGTGGACAAACTAACTCAAGGTCGCCAGACATATGACTGGACACTGAATAGAAACCAACCAGCTGCAACTGCCCTGGCCAACACTATAGAGGTCTTCAGATCAAACGGTCTAA<br>CAGCCAATGAATCGGGGAGACTAATAGATTTCCTCAAGGATGTGATGGACTCAATGGATAAAGAAGAAATGGAAATAACAACACATTTCCAGAGAAAGAGAAGAGTAAGGGACAACATGACCAAGAAAATGGTCACACAAAGAACAATAGGAAAGAAGAAACAAAGGCTAAACAAGAGGAGCTACTTAATAAGAGCACTGACACTGAATACAATGACAAAAGATGCAGA<br>AAGAGGCAAATTGAAGAGACGGGCGATTGCAACACCAGGGATGCAGATTAGAGGATTTGTGTACTTTGTCGAAACACTGGCAAGGAGCATCTGTGAAAAACTTGAGCAATCTGGACTCCCCGTTGGAGGGAATGAGAAGAAGGCTAAATTGGCAAATGTCGTGAGAAAAATGATGACTAACTCACAAGATACAGAGCTCTCCTTCACAATTACTGGAGACAACACCAAATG<br>GAATGAGAATCAAAATCCTCGGATGTTTCTGGCAATGATAACATACATTACAAGAAACCAACCTGAATGGTTTAGAAATGTCTTAAGTATTGCCCCTATAATGTTCTCGAACAAAATGGCGAGATTGGGAAAAGGGTACATGTTTGAAAGTAAGAGCATGAAGTTACGGACACAAATACCTGCAGAAATGCTTGCAAACATTGACTTAAAATACTTCAATGAATCAACAAGAAA<br>GAAAATCGAAAAAATAAGGCCTCTACTAATAGATGGCACTGCCTCATTGAGTCCTGGAATGATGATGGGCATGTTCAATATGCTGAGTACAGTATTAGGAGTTTCAATCCTAAATCTTGGGCAAAAGAAGTACACCAAAACCACATACTGGTGGGATGGACTCCAATCCTCTGATGATTTCGCCCTCATAGTAAATGCACCGAATCATGAGGGAATACAAGCAGGAGTGGATA<br>GGTTCTATAGGACCTGCAAACTGGTCGGGATCAATATGAGCAAAAAGAAGTCTTACATAAACCGGACTGGAACATTTGAGTTCACAAGCTTTTTCTATCGCTATGGATTTGTGGCTAACTTCAGTATGGAGCTGCCCAGCTTTGGGGTTTCTGGGATCAATGAATCAGCTGACATGAGCATTGGCGTCACAGTGATAAAGAACAACATGATAAACAATGACCTTGGACCAGCAA<br>CAGCTCAAATGGCCCTTCAACTATTCATCAAAGATTACAGGTACACGTACCGATGCCACAGAGGTGACACACAAATTCAAACGAGGAGATCATTCGAGCTGAAGAAGCTGTGGGAACAGACCCGTTCAAAGGCAGGACTGTTGGTGTCAGATGGAGGACCAAATCTATACAACATTCGGAATCTCCATATCCCAGAGGTCTGCCTGAAGTGGGAGCTGATGGACGAAGATTA<br>CCAGGGCAGGTTGTGTAATCCTCTGAACCCATTTGTCAGCCATAAAGAAATTGAGTCCGTAAACAATGCTGTGGTGATGCCAGCTCACGGTCCAGCCAAAAGCATGGAATATGATGCCGTTGCGACTACACACTCATGGACTCCTAAAAGGAATCGTTCCATTCTCAATACCAGTCAAAGGGGAATTCTTGAGGATGAACAGATGTACCAGAAATGCTGCAGTCTATTCGAGAA<br>ATTCTTCCCCAGTAGTTCATACAGGAGACCAGTTGGAATTTCCAGCATGGTGGAGGCCATGGTGTCTAGGGCCCGAATCGATGCACGCATTGATTTCGAATCTGGAAGGATCAAGAAGGAAGAGTTTGCTGAGATCATGAAGATCTGTTCCACCATTGAAGAGCTCAGACGGCAAAAATAG
EPI_ISL_6929970	>A/turkey/Poland/H1911-N/2021_H5N1_2021-11-01<br>ATGGATGTCAATCCGACTTTACTTTTCTTAAAAGTGCCAGCGCAAGATGCCATAAGTACCACATTCCCTTACACTGGAGATCCTCCATACAGCCATGGAACAGGGACAGGATACACGATGGACACAGTCAACAGAACACATCAATACTCAGAGAAGGGAAAATGGACAACAAACACAGAAACCGGAGCACCTCAACTCAACCCAATTGATGGGCCACTACCTGAGGACAACGA<br>ACCGAGCGGATATGCACAAACAGATTGCGTGTTGGAAGCAATGGCTTTCCTTGAAGAGTCCCACCCAGGGATCTTTGAAAACTCTTGTCTTGAAACGATGGAAGTCGTTCAGCAAACAAGAGTGGACAAACTAACTCAAGGTCGCCAGACATATGACTGGACACTGAATAGAAACCAACCAGCTGCAACTGCMCTGGCCAACACTATAGAGGTCTTCAGATCAAACGGTCTAA<br>CAGCCAATGAATCGGGGAGACTAATAGATTTCCTCAAGGATGTGATGGACTCAATGGATAAAGAAGAAATGGAAATAACAACACATTTCCAGAGAAAGAGAAGAGTAAGGGACAACATGACCAAGAAAATGGTCACACAAAGAACAATAGGAAAGAAGAAACAAAGGCTAAACAAGAGGAGCTACTTAATAAGAGCACTGACACTGAATACAATGACAAAAGATGCAGA<br>AAGAGGCAAATTGAAGAGACGGGCGATTGCAACACCAGGGATGCAGATTAGAGGATTTGTGTACTTTGTCGAAACACTGGCAAGGAGCATCTGTGAAAAACTTGAGCAATCTGGACTCCCCGTTGGAGGGAATGAGAAGAAGGCTAAATTGGCAAATGTCGTGAGAAAAATGATGACTAACTCACAAGATACAGAGCTCTCCTTCACAATTACTGGAGACAACACCAAATG<br>GAATGAGAATCAAAATCCTCGGATGTTTCTGGCAATGATAACATACATTACAAGAAACCAACCTGAATGGTTTAGAAATGTCTTAAGTATTGCCCCTATAATGTTCTCGAACAAAATGGCGAGATTGGGAAAAGGGTACATGTTTGAAAGTAAGAGCATGAAGTTACGGACACAAATACCTGCAGAAATGCTTGCAAACATTGACTTAAAATACTTCAATGAATCAACAAGAAA<br>GAAAATCGAAAAAATAAGGCCTCTACTAATAGATGGCACTGCCTCATTGAGTCCTGGAATGATGATGGGCATGTTCAATATGCTGAGTACAGTATTAGGAGTTTCAATCCTAAATCTTGGGCAAAAGAAGTACACCAAAACCACATACTGGTGGGATGGACTCCAATCCTCTGATGATTTCGCCCTCATAGTAAATGCACCGAATCATGAGGGAATACAAGCAGGAGTGGATA<br>GGTTCTATAGGACCTGCAAACTGGTCGGGATCAATATGAGCAAAAAGAAGTCTTACATAAACCGGACTGGAACATTTGAGTTCACAAGCTTTTTCTATCGCTATGGATTTGTGGCTAACTTCAGTATGGAGCTGCCCAGCTTTGGGGTTTCTGGGATCAATGAATCAGCTGACATGAGCATTGGCGTCACAGTGATAAAGAACAACATGATAAACAATGACCTTGGACCAGCAA<br>CAGCTCAAATGGCCCTTCAACTATTCATCAAAGATTACAGGTACACGTACCGATGCCACAGAGGTGACACACAAATTCAAACGAGGAGATCATTCGAGCTGAAGAAGCTGTGGGAACAGACCCGTTCAAAGGCAGGACTGTTGGTGTCAGATGGAGGACCAAATCTATACAACATTCGGAATCTCCATATCCCAGAGGTCTGCCTGAAGTGGGAGCTGATGGACGAAGATTA<br>CCAGGGCAGGTTGTGTAATCCTCTGAACCCATTTGTCAGCCATAAAGAAATTGAGTCCGTAAACAATGCTGTGGTGATGCCAGCTCACGGTCCAGCCAAAAGCATGGAATATGATGCCGTTGCGACTACACACTCATGGACTCCTAAAAGGAATCGTTCCATTCTCAATACCAGTCAAAGGGGAATTCTTGAGGATGAACAGATGTACCAGAAATGCTGCAGTCTATTCGAGAA<br>ATTCTTCCCCAGTAGTTCATACAGGAGACCAGTTGGAATTTCCAGCATGGTGGAGGCCATGGTGTCTAGGGCCCGAATCGATGCACGCATTGATTTCGAATCTGGAAGGATCAAGAAGGAAGAGTTTGCTGAGATCATGAAGATCTGTTCCACCATTGAAGAGCTCAGACGGCAAAAATAG
EPI_ISL_6929958	>A/turkey/Poland/H1910-T3/2021_H5N1_2021-11-01<br>ATGGATGTCAATCCGACTTTACTTTTCTTAAAAGTGCCAGCGCAAGATGCCATAAGTACCACATTCCCTTACACTGGAGATCCTCCATACAGCCATGGAACAGGGACAGGATACACGATGGACACAGTCAACAGAACACATCAATACTCAGAGAAGGGAAAATGGACAACAAACACAGAAACCGGAGCACCTCAACTCAACCCAATTGATGGGCCACTACCTGAGGACAACGA<br>ACCGAGCGGATATGCACAAACAGATTGCGTGTTGGAAGCAATGGCTTTCCTTGAAGAGTCCCACCCAGGGATCTTTGAAAACTCTTGTCTTGAAACGATGGAAGTCGTTCAGCAAACAAGAGTGGACAAACTAACTCAAGGTCGCCAGACATATGACTGGACACTGAATAGAAACCAACCAGCTGCAACTGCCCTGGCCAACACTATAGAGGTCTTCAGATCAAACGGTCTAA<br>CAGCCAATGAATCGGGGAGACTAATAGATTTCCTCAAGGATGTGATGGACTCAATGGATAAAGAAGAAATGGAAATAACAACACATTTCCAGAGAAAGAGAAGAGTAAGGGACAACATGACCAAGAAAATGGTCACACAAAGAACAATAGGAAAGAAGAAACAAAGGCTAAACAAGAGGAGCTACTTAATAAGAGCACTGACACTGAATACAATGACAAAAGATGCAGA<br>AAGAGGCAAATTGAAGAGACGGGCGATTGCAACACCAGGGATGCAGATTAGAGGATTTGTGTACTTTGTCGAAACACTGGCAAGGAGCATCTGTGAAAAACTTGAGCAATCTGGACTCCCCGTTGGAGGGAATGAGAAGAAGGCTAAATTGGCAAATGTCGTGAGAAAAATGATGACTAACTCACAAGATACAGAGCTCTCCTTCACAATTACTGGAGACAACACCAAATG<br>GAATGAGAATCAAAATCCTCGGATGTTTCTGGCAATGATAACATACATTACAAGAAACCAACCTGAATGGTTTAGAAATGTCTTAAGTATTGCCCCTATAATGTTCTCGAACAAAATGGCGAGATTGGGAAAAGGGTACATGTTTGAAAGTAAGAGCATGAAGTTACGGACACAAATACCTGCAGAAATGCTTGCAAACATTGACTTAAAATACTTCAATGAATCAACAAGAAA<br>GAAAATCGAAAAAATAAGGCCTCTACTAATAGATGGCACTGCCTCATTGAGTCCTGGAATGATGATGGGCATGTTCAATATGCTGAGTACAGTATTAGGAGTTTCAATCCTAAATCTTGGGCAAAAGAAGTACACCAAAACCACATACTGGTGGGATGGACTCCAATCCTCTGATGATTTCGCCCTCATAGTAAATGCACCGAATCATGAGGGAATACAAGCAGGAGTGGATA<br>GGTTCTATAGGACCTGCAAACTGGTCGGGATCAATATGAGCAAAAAGAAGTCTTACATAAACCGGACTGGAACATTTGAGTTCACAAGCTTTTTCTATCGCTATGGATTTGTGGCTAACTTCAGTATGGAGCTGCCCAGCTTTGGGGTTTCTGGGATCAATGAATCAGCTGACATGAGCATTGGCGTCACAGTGATAAAGAACAACATGATAAACAATGACCTTGGACCAGCAA<br>CAGCTCAAATGGCCCTTCAACTATTCATCAAAGATTACAGGTACACGTACCGATGCCACAGAGGTGACACACAAATTCAAACGAGGAGATCATTCGAGCTGAAGAAGCTGTGGGAACAGACCCGTTCAAAGGCAGGACTGTTGGTGTCAGATGGAGGACCAAATCTATACAACATTCGGAATCTCCATATCCCAGAGGTCTGCCTGAAGTGGGAGCTGATGGACGAAGATTA<br>CCAGGGCAGGTTGTGTAATCCTCTGAACCCATTTGTCAGCCATAAAGAAATTGAGTCCGTAAACAATGCTGTGGTGATGCCAGCTCACGGTCCAGCCAAAAGCATGGAATATGATGCCGTTGCGACTACACACTCATGGACTCCTAAAAGGAATCGTTCCATTCTCAATACCAGTCAAAGGGGAATTCTTGAGGATGAACAGATGTACCAGAAATGCTGCAGTCTATTCGAGAA<br>ATTCTTCCCCAGTAGTTCATACAGGAGACCAGTTGGAATTTCCAGCATGGTGGAGGCCATGGTGTCTAGGGCCCGAATCGATGCACGCATTGATTTCGAATCTGGAAGGATCAAGAAGGAAGAGTTTGCTGAGATCATGAAGATCTGTTCCACCATTGAAGAGCTCAGACGGCAAAAATAG

EPI_ISL_3102079	>A/turkey/Poland/H1289_21RS1385-20/2021_H5N8_2021-05-24<br>ATGGATGTCAATCCGACTTTACTTTTCTTAAAAGTGCCAGCGCAAAATGCTATAAGTACTACATTCCCTTACACTGGAGATCCTCCATACAGCCATGGAACAGGAACAGGGTATACCATGGACACAGTAAACAGAACACATCAATACTCAGAAAAGGGAAAGTGGACAACAAACACAGAAACCGGAGCACCCCAACTCAACCCAATTGATGGACCATTACCAGAGGACAATGA<br>GCCAAGCGGATATGCACAAACGGATTGCGTGTTGGAAGCAATGGCTTTCCTTGAAGAATCCCACCCAGGGATATTTGAAAACTCTTGTCTTGAAGCGATGGAAATCGTTCAGCAAACAAGAGTGGACAAACTAACCCAAGGTCGCCAGACTTATGACTGGACACTGAACAGAAACCAACCAGCTGCAACCTCTTTGGCCAACACTATAGAGGTGTTCAGATCGAATGGTCTGA<br>CAGCCAATGAATCAGGGAGACTGATAGATTTTCTCAGGGATGTGATGGAATCAATGGATAAAGAAGAGATGGAAGTAACAACACATTTCCAGAGAAAAAGAAGAGTGAGGGACAACATGACTAAGAAGATGGTCACACAAAGAACAATAGGGAAGAAGAAGCAGAGGCTGATCAAGAGGAGTTACTTAATAAGAGCACTGACATTGAATACAATGACCAAAGATGCAGA<br>AAGAGGCAAGTTGAAGAGACGGGCAATTGCAACACCCGGGATGCAGATTAGAGGATTCGTGTACTTCGTTGAAACACTAGCGAGGAGCATCTGTGAGAAACTAGAGCAATCTGGGCTCCCTGTTGGAGGGAATGAGAAGAAGGCTAAATTGGCAAATGTTGTGAGAAAAATGATGACTAACTCACAAGATACAGAGCTCTCCTTTACAATTACTGGAGACAACACCAAATG<br>GAATGAGAATCAAAACCCTCGGATGTTTTTGGCAATGATAACATATATCACAAGAAACCAACCTGAATGGTTTAGAAATGTCTTAAGCATTGCCCCTATAATGTTCTCAAACAAAATGGCGAGATTAGGGAAAGGATACATGTTTGAAAGTAAGAGCATGAAGCTAAGAACACAAATATCAGCAGAGATGCTTACAAATATCGATCTGAAGTATTTCAACGAACCAACGAGAA<br>AGAAAATCGAGAAAATAAGACCTCTGCTGATTGATGGCACGGCTTCATTGAGTCCTGGGATGATGATGGGCATGTTCAATATGCTGAGCACAGTATTAGGGGTCTCAATCCTGAATCTCGGGCAAAAAAGGTACACCAAAACCACATACTGGTGGGATGGACTTCAATCCTCTGATGATTTCGCTCTCATAGTGAATGCACCGAATCATGAGGGGATACAAGCAGGAGTGGAT<br>AGATTCTATAGGACCTGCAAACTGGTTGGGATCAACATGAGCAAAAAGAAGTCGTACATAAACCGAACAGGAACATTTGAGTTCACAAGTTTTTTCTACCGCTATGGATTTGTAGCTAACTTCAGTATGGAATTACCCAGCTTTGGAGTGTCTGGAATCAATGAATCAGCTGACATGAGCATTGGAGTTACAGTGATAAAAAACAATATGATAAACAATGATCTTGGACCAGCA<br>ACAGCTCAAATGGCTCTTCAGTTATTTATCAAAGACTATAGGTACACATACCGATGCCACAGGGGTGATACACAAATTCAAACGAGGAGATCATTCGAGCTGAAGAAGCTGTGGGAGCAGACCCGTTCAAAGGCAGGGCTGTTGATATCAGATGGGGGGCCAAACCTATACAACATTCGGAATCTCCACATCCCAGAGGTCTGCTTGAAGTGGGAGCTGATGGATGAAGACT<br>ACCAAGGCAGGCTGTGCAATCCTCTGAATCCATTTGTCAGTCATAAAGAGATTGAGTCCGTAAACAATGCTGTAGTAATGCCCGCCCATGGCCCGGCCAAGAGCATGGAATATGATGCTGTTGCGACCACACACTCGTGGATTCCTAAGAGGAACCGTTCCATTCTCAATACCAGCCAAAGGGGAATTCTTGAGGATGAGCAGATGTACCAAAAGTGCTGTAGTCTATTCGAGA<br>AATTCTTCCCCAGCAGTTCATACAGGAGGCCAGTTGGAATTTCCAGCATGGTGGAGGCCATGGTGTCTAGGGCCCGAATTGATGCACGCATCGACTTCGAATCTGGAAGGATTAAGAAAGAAGAGTTTGCTGAGATCATGAAGATCTGTTCCACCATTGAAGAGCTCAGACGGCAAAAATAG
EPI_ISL_3102073	>A/turkey/Poland/H1184_21RS1385-14/2021_H5N8_2021-05-14<br>ATGGATGTCAATCCGACTTTACTTTTCTTAAAAGTGCCAGCGCAAAATGCTATAAGTACTACATTCCCTTACACTGGAGATCCTCCATACAGCCATGGAACAGGAACAGGGTATACCATGGACACAGTAAACAGAACACATCAATACTCAGAAAAGGGAAAGTGGACAACAAACACAGAAACCGGAGCACCCCAACTCAACCCAATTGATGGACCATTACCAGAGGACAATGA<br>GCCAAGCGGATATGCACAAACGGATTGCGTGTTGGAAGCAATGGCTTTCCTTGAAAAATCCCACCCAGGGATATTTGAAAACTCTTGTCTTGAAGCGATGGAAATCGTTCAGCAAACAAGAGTGGACAAACTAACCCAAGGTCGCCAGACTTATGACTGGACACTGAACAGAAACCAACCAGCTGCAACCTCTTTGGCCAACACTATAGAGGTGTTCAGATCGAATGGTCTGA<br>CAGCCAATGAATCAGGGAGACTGATAGATTTTCTCAGGGATGTGATGGAATCAATGGATAAAGAAGAGATGGAAGTAACAACACATTTCCAGAGAAAAAGAAGAGTGAGGGACAACATGACTAAGAAGATGGTCACACAAAGAATAATAGGGAAGAAGAAGCAGAGGCTGATCAAGAGGAGTTACTTAATAAGAGCACTGACATTGAATACAATGACCAAAGATGCAGA<br>AAGAGGCAAGTTGAAGAGACGGGCAATTGCAACACCCGGGATGCAGATTAGAGGATTCGTGTACTTCGTTGAAACACTAGCGAGGAGCATCTGTGAGAAACTAGAGCAATCTGGGCTCCCTGTTGGAGGGAATGAGAAGAAGGCTAAATTGGCAAATGTTGTGAGAAAAATGATGACTAACTCACAAGATACAGAGCTCTCCTTTACAATTACTGGAGACAACACCAAATG<br>GAATGAGAATCAAAACCCTCGGATGTTTTTGGCAATGATAACATATATCACAAGAAACCAACCTGAATGGTTTAGAAATGTCTTAAGCATTGCCCCTATAATGTTCTCAAACAAAATGGCGAGATTAGGGAAAGGATACATGTTTGAAAGTAAGAGCATGAAGCTAAGAACACAAATATCAGCAGAGATGCTTACAAATATCGATCTGAAGTATTTCAACGAACCAACGAGAA<br>AGAAAATCGAGAAAATAAGACCTCTGCTGATTGATGGCACGGCTTCATTGAGTCCTGGGATGATGATGGGCATGTTCAATATGCTGAGCACAGTATTAGGGGTCTCAATCCTGAATCTCGGGCAAAAAAGGTACACCAAAACCACATACTGGTGGGATGGACTTCAATCCTCTGATGATTTCGCTCTCATAGTGAATGCACCGAATCATGAGGGGATACAAGCAGGAGTGGAT<br>AGATTCTATAGGACCTGCAAACTGGTTGGGATCAACATGAGCAAAAAGAAGTCTTACATAAACCGAACAGGAACATTTGAGTTCACAAGTTTTTTCTACCGCTATGGATTTGTAGCTAACTTCAGTATGGAATTACCCAGCTTTGGAGTGTCTGGAATCAATGAATCAGCTGACATGAGCATTGGAGTTACAGTGATAAAAAACAATATGATAAACAATGATCTTGGACCAGCA<br>ACAGCTCAAATGGCTCTTCAGTTATTTATCAAAGACTATAGGTACACATACCGATGCCACAGGGGTGATACACAAATTCAAACGAGGAGATCATTCGAGCTGAAGAAGCTGTGGGAGCAGACCCGTTCAAAGGCAGGGCTGTTGATATCAGATGGGGGGCCAAACCTATACAACATTCGGAATCTCCACATCCCAGAGGTCTGCTTGAAGTGGGAGCTGATGGATGAAGACT<br>ACCAAGGCAGGCTGTGCAATCCTCTGAATCCATTTGTCAGTCATAAAGAGATTGAGTCCGTAAACAATGCTGTAGTAATGCCCGCCCATGGCCCGGCCAAGAGCATGGAATATGATGCTGTTGCGACCACACACTCGTGGATTCCTAAGAGGAACCGTTCCATTCTCAATACCAGCCAAAGGGGAATTCTTGAGGATGAGCAGATGTACCAAAAGTGCTGTAGTCTATTCGAGA<br>AATTCTTCCCCAGCAGTTCATACAGGAGGCCAGTTGGAATTTCCAGCATGGTGGAGGCCATGGTGTCTAGGGCCCGAATTGATGCACGCATCGACTTCGAATCTGGAAGGATTAAGAAAGAAGAGTTTGCTGAGATCATGAAGATCTGTTCCACCATTGAAGAGCTCAGACGGCAAAAATAG
EPI_ISL_3102074	>A/turkey/Poland/H1168_21RS1385-15/2021_H5N8_2021-05-13<br>ATGGATGTCAATCCGACTTTACTTTTCTTAAAAGTGCCAGCGCAAAATGCTATAAGTACTACATTCCCTTACACTGGAGATCCTCCATACAGCCATGGAACAGGAACAGGGTATACCATGGACACAGTAAACAGAACACATCAATACTCAGAAAAGGGAAAGTGGACAACAAACACAGAAACCGGAGCACTCCAACTCAACCCAATTGATGGACCATTACCAGAGGACAATGA<br>GCCAAGCGGATATGCACAAACGGATTGCGTGTTGGAAGCAATGGCTTTCCTTGAAGAATCCCACCCAGGGATATTTGAAAACTCTTGTCTTGAAGCGATGGAAATCGTTCAGCAAACAAGAGTGGACAAACTAACCCAAGGTCGCCAGACTTATGACTGGACACTGAACAGAAACCAACCAGCTGCAACCTCTTTGGCCAACACTATAGAGGTGTTCAGATCGAATGGTCTGA<br>CAGCCAATGAATCAGGGAGACTGATAGATTTTCTCAGGGATGTGATGGAATCAATGGATAAAGAAGAGATGGAAGTAACAACACATTTCCAGAGAAAAAGAAGAGTGAGGGACAACATGACTAAGAAGATGGTCACACAAAGAACAATAGGGAAGAAGAAGCAGAGGCTGATCAAGAGGAGTTACTTAATAAGAGCACTGACATTGAATACAATGACCAAAGATGCAGA<br>AAGAGGCAAGTTGAAGAGACGGGCAATTGCAACACCCGGGATGCAGATTAGAGGATTCGTGTACTTCGTTGAAACACTAGCGAGGAGCATCTGTGAGAAACTAGAGCAATCTGGGCTCCCTGTTGGAGGGAATGAGAAGAAGGCTAAATTGGCAAATGTTGTGAGAAAAATGATGACTAACTCACAAGATACAGAGCTCTCCTTTACAATTACTGGAGACAACACCAAATG<br>GAATGAGAATCAAAACCCTCGGATGTTTTTGGCAATGATAACATATATCACAAGAAACCAACCTGAATGGTTTAGAAATGTCTTAAGCATTGCCCCTATAATGTTCTCAAACAAAATGGCGAGATTAGGGAAAGGATACATGTTTGAAAGTAAGAGCATGAAGCTAAGAACACAAATATCAGCGGAGATGCTTACAAATATCGATCTGAAGTATTTCAACGAACCAACGAGAA<br>AGAAAATCGAGAAAATAAGACCTCTGCTGATTGATGGCACGGCTTCATTGAGTCCTGGGATGATGATGGGCATGTTCAATATGCTGAGCACAGTATTAGGGGTCTCAATCCTGAATCTCGGGCAAAAAAGGTACACCAAAACCACATACTGGTGGGATGGACTTCAATCCTCTGATGATTTCGCTCTCATAGTGAATGCACCGAATCATGAGGGGATACAAGCAGGAGTGGAT<br>AGATTCTATAGGACCTGCAAACTGGTTGGGATCAACATGAGCAAAAAGAAGTCTTACATAAACCGAACAGGAACATTTGAGTTCACAAGTTTTTTCTACCGCTATGGATTTGTAGCTAACTTCAGTATGGAATTACCCAGCTTTGGAGTGTCTGGAATCAATGAATCAGCTGACATGAGCATTGGAGTTACAGTGATAAAAAACAATATGATAAACAATGATCTTGGACCAGCA<br>ACAGCTCAAATGGCTCTTCAGTTATTTATCAAAGACTATAGGTACACATACCGATGCCACAGGGGTGATACACAAATTCAAACGAGGAGATCATTCGAGCTGAAGAAGCTGTGGGAGCAGACCCGTTCAAAGGCAGGGCTGTTGATATCAGATGGGGGGCCAAACCTATACAACATTCGGAATCTCCACATCCCAGAGGTCTGCTTGAAGTGGGAGCTGATGGATGAAGACT<br>ACCAAGGCAGGCTGTGCAATCCTCTGAATCCATTTGTCAGTCATAAAGAGATTGAGTCCGTAAACAATGCTGTAGTAATGCCCGCCCATGGCCCGGCCAAGAGCATGGAATATGATGCTGTTGCGACCACACACTCGTGGATTCCTAAGAGGAACCGTTCCATTCTCAATACCAGCCAAAGGGGAATTCTTGAGGATGAGCAGATGTACCAAAAGTGCTGTAGTCTATTCGAGA<br>AATTCTTCCCCAGCAGTTCATACAGGAGGCCAGTTGGAATTTCCAGCATGGTGGAGGCCATGGTGTCTAGGGCCCGAATTGATGCACGCATCGACTTCGAATCTGGAAGGATTAAGAAAGAAGAGTTTGCTGAGATCATGAAGATCTGTTCCACCATTGAAGAGCTCAGACGGCAAAAATAG
internal NVRI repository	>A/turkey/Poland/H02-T1/2022_H5N1_2022-01-01<br>ATGGATGTCAATCCGACTTTACTTTTCTTAAAAGTGCCAGCGCAAGATGCCATAAGTACCACATTCCCTTACACTGGAGATCCTCCATACAGCCATGGAACAGGGACAGGATACACAATGGACACAGTCAACAGAACACATCAATACTCAGAGAAGGGAAAATGGACAACAAACACAGAAACCGGAGCACCTCAACTCAACCCAATTGATGGGCCACTACCTGAGGACAACGA<br>ACCGAGCGGATATGCACAAACAGATTGCGTGTTGGAAGCAATGGCTTTCCTTGAAGAGTCCCACCCAGGGATCTTTGAAAACTCTTGTCTTGAAACGATGGAAGTCGTTCAGCAAACAAGAGTGGACAAACTAACTCAAGGTCGCCAGACATATGACTGGACACTGAATAGAAACCAACCAGCTGCAACTGCCCTGGCCAACACTATAGAGGTCTTCAGATCAAACGGTCTAA<br>CAACCAATGAATCGGGGAGACTAATAGATTTCCTCAAGGATGTGATGGACTCAATGGATAAAGAAGAAATGGAAATAACAACACATTTCCAGAGAAAGAGAAGAGTAAGGGACAACATGACCAAGAAAATGGTCACACAAAGAACAATAGGAAAGAAGAAACAAAGGCTAAACAAGAGGAGCTACTTAATAAGAGCACTGACACTGAATACAATGACAAAGGATGCAGA<br>AAGAGGCAAATTGAAAAGACGGGCGATTGCAACACCAGGGATGCAGATTAGAGGATTTGTGTACTTTGTCGAAACACTGGCAAGGAGCATCTGTGAAAAACTTGAGCAATCTGGACTCCCCGTTGGAGGGAATGAGAAGAAGGCTAAATTGGCAAATGTCGTGAGAAAAATGATGACTAACTCACAAGATACAGAGCTCTCCTTCACAATTACTGGAGATAACACCAAATG<br>GAATGAGAATCAAAATCCTCGGATGTTTCTGGCAATGATAACATACATTACAAGAAACCAACCTGAATGGTTTAGAAATGTCTTGAGTATTGCCCCTATAATGTTCTCGAACAAAATGGCGAGATTGGGAAAAGGGTACATGTTTGAAAGTAAGAGCATGAAGTTACGGACACAAATACCTGCAGAAATGCTTGCAAACATTGACTTAAAATACTTCAATGAATCAACAAGAAA<br>GAAAATCGAAAAAATAAGGCCTCTACTAATAGATGGCACTGCCTCATTGAGTCCTGGAATGATGATGGGCATGTTCAATATGCTGAGTACAGTATTAGGAGTTTCAATCCTAAATCTTGGGCAAAAGAAGTACACCAAAACCACATACTGGTGGGATGGACTCCAATCCTCTGATGATTTCGCCCTCATAGTAAATGCACCGAATCATGAGGGAATACAAGCAGGAGTGGATA<br>GGTTCTATAGGACCTGCAAACTGGTCGGGATCAATATGAGCAAAAAGAAGTCTTACATAAACCGGACTGGAACATTTGAGTTCACAAGCTTTTTCTATCGCTATGGATTTGTGGCTAACTTCAGTATGGAGCTGCCCAGCTTTGGAGTTTCTGGGATCAATGAATCAGCTGACATGAGCATTGGCATCACAGTGATAAAGAACAACATGATAAACAATGACCTTGGACCAGCAA<br>CTGCTCAAATGGCCCTTCAACTATTCATCAAAGATTACAGGTACACGTACCGATGCCACAGAGGTGACACACAAATTCAAACGAGGAGATCATTCGAGCTGAAGAAGCTGTGGGAACAGACCCGTTCAAAGGCAGGRCTGTTGGTGTCAGATGGAGGACCAAATCTATACAACATTCGGAATCTCCATATCCCAGAGGTCTGCCTGAAGTGGGAGCTGATGGACGAAGATTAC<br>CAGGGCAGGTTGTGTAATCCTCTGAACCCATTTGTCAGCCATAGAGAAATTGAGTCCGTAAACAATGCTGTGGTGATGCCAGCCCACGGTCCAGCCAAAAGCATGGAATATGATGCCGTTGCGACTACACACTCATGGATTCCTAAAAGGAATCGTTCCATTCTCAATACCAGTCAAAGGGGAATTCTTGAGGATGAACAGATGTACCAGAAATGCTGCAGTCTATTCGAGAAA<br>TTCTTCCCCAGTAGTTCATACAGGAGACCAGTTGGAATCTCCAGCATGGTGGAGGCCATGGTGTCTAGGGCCCGAATCGATGCACGCATTGATTTCGAGTCTGGAAGGATCAAGAAGGAAGAGTTTGCTGAGATCATGAAGATCTGTTCCACCATTGAAGAGCTCAGACGGCAAAAATAG
EPI_ISL_846604	>A/turkey/Poland/477/2020_H5N8_2020-12-07<br>ATGGATGTCAATCCGACTTTACTTTTCTTAAAAGTGCCAGCGCAAAATGCTATAAGTACTACATTCCCTTACACTGGAGATCCCCCATACAGCCATGGAACAGGAACAGGGTATACCATGGACACAGTAAACAGAACACATCAATACTCAGAAAAGGGAAAGTGGACAACAAACACAGAAACCGGAGCACCCnAACTCAACCCAATTGATGGACCATTACCAGAGGACAATGA<br>GCCAAGCGGATATGCACAAACTGATTGCGTGTTGGAAGCCATGGCTTTCCTTGAAGAATCCCACCCAGGGATATTTGAAAACTCTTGTCTTGAAGCGATGGAAATCGTTCAGCAAACAAGAGTGGACAAACTAACCCAAGGTCGCCAGACTTATGACTGGACACTGAACAGAAACCAACCAGCTGCAACCTCTTTGGCCAACACTATAGAGGTGTTCAGATCGAATGGTCTGA<br>CAGCCAATGAATCAGGGAGACTGATAGATTTTCTCAGGGATGTGATGGAATCAATGGATAAAGAAGAGATGGAAGTAACAACACATTTCCAGAGAAAAAGAAGAGTGAGGGACAACATGACTAAGAAGATGGTCACACAAAGAACAATAGGGAAGAAGAAGCAGAGGCTGAACAAGAGGAGTTACTTAATAAGAGCACTGACATTGAATACAATGACCAAAGATGCAGA<br>AAGAGGCAAGTTGAAGAGACGGGCAATTGCAACACCCGGGATGCAGATTAGAGGATTCGTGTACTTCGTTGAAACACTAGCGAGGAGCATCTGTGAGAAACTAGAGCAATCTGGGCTCCCTGTTGGAGGGAATGAGAAAAAGGCTAAATTGGCAAATGTTGTGAGAAAAATGATGACTAACTCACAAGATACAGAGCTCTCCTTTACAATTACTGGAGACAACACCAAATG<br>GAACGAGAATCAAAACCCTCGGATGTTTTTGGCAATGATAACATATATCACAAGAAACCAACCTGAATGGTTTAGAAATGTCTTAAGCATTGCCCCTATAATGTTCTCAAACAAAATGGCGAGATTAGGGAAAGGATACATGTTTGAAAGTAAGAGCATGAAGCTAAGAACACAAATACCAGCAGAGATGCTTACAAATATTGATCTGAAGTATTTCAACGAACCAACGAGAA<br>AGAAAATCGAGAAAATAAGACCTCTGCTGATTGATGGCACGGCCTCATTGAGTCCTGGGATGATGATGGGCATGTTCAATATGCTGAGCACAGTATTAGGGGTYTCAATCCTGAATCTCGGGCAAAAGAGGTACACCAAAACCACATACTGGTGGGATGGACTTCAATCCTCTGATGATTTCGCTCTCATAGTGAATGCACCGAATCATGAGGGGATACAAGCAGGAGTGGAT<br>AGATTCTATAGGACCTGCAAATTGGTTGGGATCAACATGAGCAAAAAGAAGTCTTACATAAACCGAACAGGAACATTTGAGTTCACAAGTTTTTTCTACCGCTATGGATTTGTAGCCAACTTCAGTATGGAATTGCCCAGCTTTGGAGTGTCTGGAATCAATGAATCAGCTGACATGAGCATTGGAGTTACAGTGATAAAAAACAATATGATAAACAATGATCTTGGACCAGCA<br>ACAGCTCAAATGGCTCTTCAGTTATTCATCAAAGACTATAGGTACACATACCGATGCCACAGGGGTGATACACAAATTCAAACGAGGAGATCATTCGAGCTGAAGAAGCTGTGGGAGCAGACCCGTTCAAAGGCAGGGCTGTTGATATCAGACGGGGGGCCAAACCTATACAACATTCGGAATCTCCACATCCCAGAGGTCTGCTTGAAGTGGGAGCTGATGGATGAAGACT<br>ACCAAGGCAGGCTGTGCAATCCTCTGAATCCATTTGTCAGTCATAAAGAGATTGAGTCCGTAAACAATGCTGTAGTAATGCCCGCCCATGGCCCGGCCAAGAGCATGGAATATGATGCTGTTGCGACCACACACTCGTGGATTCCTAAGAGGAACCGTTCCATCCTCAATACCAGCCAAAGGGGAATTCTTGAGGATGAGCAGATGTACCAAAAGTGCTGTAGTCTATTCGAGA<br>AATTCTTCCCCAGCAGTTCATACAGGAGGCCAGTTGGAATTTCCAGCATGGTGGAGGCCATGGTGTCTAGGGCCCGAATTGATGCACGCATCGACTTCGAATCTGGAAGGATTAAGAAAGAAGAGTTTGCTGAGATCATGAAGATCTGTTCCACCATTGAAGAGCTCAGACGGCAAAAATAG
EPI_ISL_846602	>A/turkey/Poland/475/2020_H5N8_2020-12-03<br>ATGGATGTCAATCCGACTTTACTTTTCTTAAAAGTGCCAGCGCAAAATGCTATAAGTACTACATTCCCTTACACTGGAGATCCTCCATACAGCCATGGAACAGGAACAGGGTATACCATGGACACAGTAAACAGAACACATCAATACTCAGAAAAGGGAAAGTGGACAACAAACACAGAAACCGGAGCACCCCAACTCAACCCAATTGATGGACCATTACCAGAGGACAATGA<br>GCCAAGCGGATATGCACAAACTGATTGCGTGTTGGAAGCAATGGCTTTCCTTGAAGAATCCCACCCAGGGATATTTGAAAACTCTTGTCTTGAAGCAATGGAAATCGTTCAGCAAACAAGAGTGGACAAACTAACCCAAGGTCGCCAGACTTATGACTGGACACTGAACAGAAACCAACCAGCTGCAACCTCTTTGGCCAACACTATAGAGGTGTTCAGATCGAATGGTCTGA<br>CAGCCAATGAATCAGGGAGACTGATAGATTTTCTCAGGGATGTGATGGAATCAATGGACAAAGAAGAGATGGAAGTAACAACACATTTCCAGAGAAAAAGAAGAGTGAGGGACAACATGACTAAGAAGATGGTCACACAAAGAACAATAGGGAAGAAGAAGCAGAGGCTGAACAAGAGGAGTTACTTAATAAGAGCACTGACATTGAATACAATGACCAAAGATGCAGA<br>AAGAGGCAAGTTGAAGAGACGGGCAATTGCAACACCCGGGATGCAGATTAGAGGATTCGTGTACTTCGTTGAAACACTAGCGAGGAGCATCTGTGAGAAACTAGAGCAATCTGGGCTCCCTGTTGGAGGGAATGAGAAGAAGGCTAAATTGGCAAATGTTGTGAGAAAAATGATGACTAACTCACAAGATACAGAGCTCTCCTTTACAATTACTGGAGACAACACCAAATG<br>GAATGAGAATCAAAACCCTCGGATGTTTTTGGCAATGATAACATATATCACAAGAAACCAACCTGAATGGTTTAGAAATGTCTTAAGCATTGCCCCTATAATGTTCTCAAACAAAATGGCGAGATTAGGGAAAGGATACATGTTTGAAAGTAAGAGCATGAAGCTAAGAACACAAATACCAGCAGAGATGCTTACAAATATCGATCTGAAGTATTTCAACGAACCAACGAGAA<br>AGAAAATCGAGAAAATAAGACCTCTGCTGATTGATGGCACGGCCTCATTGAGTCCTGGGATGATGATGGGCATGTTCAATATGCTGAGCACAGTATTAGGGGTCTCAATCCTGAATCTCGGGCAAAAAAGGTACACCAAAACCACATACTGGTGGGATGGACTTCAATCCTCTGATGATTTCGCTCTCATAGTGAATGCACCGAATCATGAGGGGATACAAGCAGGAGTGGAT<br>AGATTCTATAGGACCTGCAAACTGGTTGGGATCAACATGAGCAAAAAGAAGTCTTACATAAACCGAACAGGAACATTTGAGTTCACAAGTTTTTTCTACCGCTATGGATTTGTAGCTAACTTCAGTATGGAATTACCCAGCTTTGGAGTGTCTGGAATCAATGAATCAGCTGACATGAGCATTGGAGTTACAGTGATAAAAAACAATATGATAAACAATGATCTTGGACCAGCA<br>ACAGCTCAAATGGCTCTTCAGTTATTCATCAAAGACTATAGGTACACATACCGATGCCACAGGGGTGATACACAAATTCAAACGAGGAGATCATTCGAGCTGAAGAAGCTGTGGGAGCAGACCCGTTCAAAGGCAGGGCTGTTGATATCAGACGGGGGGCCAAACCTATACAACATTCGGAATCTCCACATCCCAGAGGTCTGCTTGAAGTGGGAGCTGATGGATGAAGACT<br>ACCAAGGCAGGCTGTGCAATCCTCTGAATCCATTTGTCAGTCATAAAGAGATTGAGTCCGTAAACAATGCTGTAGTAATGCCCGCCCATGGCCCGGCCAAGAGCATGGAATATGATGCTGTTGCGACCACACACTCGTGGATTCCTAAGAGGAACCGTTCCATTCTCAATACCAGCCAAAGGGGAATTCTTGAGGATGAGCAGATGTACCAAAAGTGCTGTAGTCTATTCGAGA<br>AATTCTTCCCCAGCAGTTCATACAGGAGGCCAGTTGGAATTTCCAGCATGGTGGAGGCCATGGTGTCTAGGGCCCGAATTGATGCACGCATCGACTTCGAATCTGGAAGGATTAAGAAAGAAGAGTTTGCTGAGATCATGAAGATCTGTTCCACCATTGAAGAGCTCAGACGGCAAAAATAG
EPI_ISL_779129	>A/turkey/Poland/464/2020_H5N8_2020-12-01<br>ATGGATGTCAATCCGACTTTACTTTTCTTAAAAGTGCCAGCGCAAAATGCTATAAGTACTACATTCCCTTACACTGGAGATCCTCCATACAGCCATGGAACAGGAACAGGGTATACCATGGACACAGTAAACAGAACACATCAATACTCAGAAAAGGGAAAGTGGACAACAAACACAGAAACCGGAGCACCCCAACTCAACCCAATTGATGGACCATTACCAGAGGACAATGA<br>GCCAAGCGGATATGCACAAACTGATTGCGTGTTGGAAGCAATGGCTTTCCTTGAAGAATCCCACCCAGGGATATTTGAAAACTCTTGTCTTGAAGCAATGGAAATCGTTCAGCAAACAAGAGTGGACAAACTAACCCAAGGTCGCCAGACTTATGACTGGACACTGAACAGAAACCAACCAGCTGCAACCTCTTTGGCCAACACTATAGAGGTGTTCAGATCGAATGGTCTGA<br>CAGCCAATGAATCAGGGAGACTGATAGATTTTCTCAGGGATGTGATGGAATCAATGGATAAAGAAGAGATGGAAGTAACAACACATTTCCAGAGAAAAAGAAGAGTGAGGGACAACATGACTAAGAAGATGGTCACACAAAGAACAATAGGGAAGAAGAAGCAGAGGCTGAACAAGAGGAGTTACTTAATAAGAGCACTGACATTGAATACAATGACCAAAGATGCAGA<br>AAGAGGCAAGTTGAAGAGACGGGCAATTGCAACACCCGGGATGCAGATTAGAGGATTCGTGTACTTCGTTGAAACACTAGCGAGGAGCATCTGTGAGAAACTAGAGCAATCTGGGCTCCCTGTTGGAGGGAATGAGAAGAAGGCTAAATTGGCAAATGTTGTGAGAAAAATGATGACTAACTCACAAGATACAGAGCTCTCCTTTACAATTACTGGAGACAACACCAAATG<br>GAATGAGAATCAAAACCCTCGGATGTTTTTGGCAATGATAACATATATCACAAGAAACCAACCTGAATGGTTTAGAAATGTCTTAAGCATTGCCCCTATAATGTTCTCAAACAAAATGGCGAGATTAGGGAAAGGATACATGTTTGAAAGTAAGAGCATGAAGCTAAGAACACAAATACCAGCAGAGATGCTTACAAATATTGATCTGAAGTATTTCAACGAACCAACGAGAA<br>AGAAAATCGAGAAAATAAGACCTCTGCTGATTGATGGCACGGCCTCATTGAGTCCTGGGATGATGATGGGCATGTTCAATATGCTGAGCACAGTATTGGGGGTCTCAATCCTGAATCTCGGGCAAAAGAGGTACACCAAAACCACATACTGGTGGGATGGACTTCAATCCTCTGATGATTTCGCTCTCATAGTGAATGCACCGAATCATGAGGGGATACAAGCAGGAGTGGAT<br>AGATTCTATAGGACCTGCAAACTGGTTGGGATCAACATGAGCAAAAAGAAGTCTTACATAAACCGAACAGGAACATTTGAGTTCACAAGTTTTTTCTACCGCTATGGATTTGTAGCTAACTTCAGTATGGAATTACCCAGCTTTGGAGTGTCTGGAATCAATGAATCAGCTGACATGAGCATTGGAGTTACAGTGATAAAAAACAATATGATAAACAATGATCTTGGACCAGCG<br>ACAGCTCAAATGGCTCTTCAGTTATTCATCAAAGACTATAGGTACACATACCGATGCCACAGGGGTGATACACAAATTCAAACGAGGAGATCATTYGAGCTGAAGAAGCTGTGGGAGCAGACCCGTTCAAAGGCAGGGCTGTTGATATCAGACGGGGGGCCAAACCTATACAACATTCGGAACCTCCACATCCCAGAGGTCTGCTTGAAGTGGGGGCTGATGGATGAAGACT<br>ACCAAGGCAGGCTGTGCAATCCTCTGAATCCATTTGTCAGTCATAAAGAGATTGAGTCCGTAAACAATGCTGTAGTAATGCCCGCCCATGGCCCGGCCAAGAGCATGGAATATGATGCTGTTGCGACCACACATTCGTGGATTCCTAAGAGGAACCGGTCCATTCTCAATACCAGCCAAAGGGGAATTCTTGAGGATGAGCAGATGTACCAAAAGTGCTGTAGTCTATTCGAG<br>AAATTCTTCCCCAGCAGTTCATACAGGAGGCCAGTTGGAATTTCCAGCATGGTGGAGGCCATGGTGTCTAGGGCCCGAATTGATGCACGCATCGACTTCGAATCTGGAAGGATTAAGAAAGAAGAGTTTGCTGAGATCATGAAGATCTGTTCCACCATTGAAGAGCTCAGACGGCAAAAATAG
EPI_ISL_1295639	>A/turkey/Norway/FU496/2020_H5N8_2020-11-30<br>ATGGATGTCAATCCGACTTTACTTTTCTTAAAAGTGCCAGCGCAAAATGCTATAAGTACTACATTCCCTTACACTGGAGATCCTCCATACAGCCATGGAACAGGAACAGGGTATACCATGGACACAGTAAACAGAACACATCAATACTCAGAAAAGGGAAAGTGGACAACAAACACAGAAACCGGAGCACCCCAACTCAACCCAATTGATGGACCATTACCAGAGGACAATGA<br>GCCAAGCGGATATGCACAAACTGATTGCGTGTTGGAAGCAATGGCTTTCCTTGAAGAATCCCACCCAGGGATATTTGAAAACTCTTGTCTTGAAGCGATGGAAATCGTTCAGCAAACAAGAGTGGACAAACTAACCCAAGGTCGCCAGACTTATGACTGGACACTGAACAGAAACCAACCAGCTGCAACCTCTTTGGCCAACACTATAGAGGTGTTCAGATCGAATGGTCTGA<br>CAGCCAATGAATCAGGGAGACTGATAGATTTTCTCAGAGATGTGATGGAATCAATGGATAAAGAAGAGATGGAAGTAACAACACATTTCCAGAGAAAAAGAAGAGTGAGGGACAACATGACTAAGAAGATGGTCACACAAAGAACAATAGGGAAGAAGAAGCAGAGGCTGAACAAGAGGAGTTACTTAATAAGAGCACTGACATTGAATACAATGACCAAAGATGCAGA<br>AAGAGGCAAGTTGAAGAGACGGGCAATTGCAACACCCGGGATGCAGATTAGAGGATTCGTGTACTTCGTTGAAACACTAGCGAGGAGCATCTGTGAGAAACTAGAGCAATCTGGGCTCCCTGTTGGAGGGAATGAGAAGAAGGCTAAATTGGCAAATGTTGTGAGAAAAATGATGACTAACTCACAAGATACAGAGCTCTCCTTTACAATTACTGGAGACAACACCAAATG<br>GAATGAGAATCAAAACCCTCGGATGTTTTTGGCAATGATAACATATATCACAAGAAACCAACCTGAATGGTTTAGAAATGTCTTAAGCATCGCCCCTATAATGTTCTCAAACAAAATGGCGAGATTAGGGAAAGGATACATGTTTGAAAGTAAGAGCATGAAGCTAAGAACACAAATACCAGCAGAGATGCTTACAAATATCGATCTGAAGTATTTCAACGAACCAACGAGAA<br>AGAAAATCGAGAAAATAAGACCTCTGCTGATTGATGGCACGGCCTCATTGAGTCCTGGGATGATGATGGGCATGTTCAATATGCTGAGCACAGTATTAGGGGTCTCAATCCTGAATCTCGGGCAAAAAAGGTACACCAAAACCACATACTGGTGGGATGGACTTCAATCCTCTGATGATTTCGCTCTCATAGTGAATGCACCGAATCATGAGGGGATACAAGCAGGAGTGGAT<br>AGATTCTATAGGACCTGCAAACTGGTTGGGATCAACATGAGCAAAAAGAAGTCTTACATAAACCGAACAGGAACATTTGAGTTCACAAGTTTTTTCTACCGCTATGGATTTGTAGCTAACTTCAGTATGGAATTACCCAGCTTTGGAGTGTCTGGAATCAATGAATCAGCTGACATGAGCATTGGAGTTACAGTGATAAAAAACAATATGATAAACAATGATCTTGGACCAGCA<br>ACAGCTCAAATGGCTCTTCAGTTATTCGTCAAAGACTATAGGTACACATACCGATGCCACAGGGGTGATACACAAATTCAAACGAGGAGATCATTCGAGCTGAAGAAGCTGTGGGAGCAGACCCGTTCAAAGGCAGGGCTGTTGATATCAGACGGGGGGCCAAACCTATACAACATTCGGAATCTCCACATCCCAGAGGTCTGCTTGAAGTGGGAGCTGATGGATGAAGACT<br>ATCAAGGCAGGCTGTGCAATCCTCTGAATCCATTTGTCAGTCATAAAGAGATTGAGTCCGTAAACAATGCTGTAGTAATGCCCGCCCATGGCCCGGCCAAGAGCATGGAATATGATGCTGTTGCGACCACACACTCGTGGATTCCTAAGAGGAACCGTTCCATTCTCAATACCAGCCAAAGGGGAATTCTTGAGGATGAGCAGATGTACCAAAAGTGCTGTAGTCTATTCGAGA<br>AATTCTTCCCCAGCAGTTCATACAGGAGGCCAGTTGGAATTTCCAGCATGGTGGAGGCCATGGTGTCTAGGGCCCGAATTGATGCACGCATCGACTTCGAATCTGGAAGGATTAAGAAAGAAGAGTTTGCTGAGATCATGAAGATCTGTTCCACCATTGAAGAGCTCAGACGGCAAAAATAG
EPI_ISL_14760588	>A/turkey/Italy/21VIR9647-2/2021_H5N1_2021-11-15<br>ATGGATGTCAATCCGACTTTACTTTTCTTAAAAGTGCCAGCGCAAGATGCCATAAGTACCACATTCCCTTACACTGGAGATCCTCCATACAGCCATGGAACAGGGACAGGATACACAATGGACACAGTCAACAGAACACATCAATACTCAGAGAAGGGAAAATGGACAACAAACACAGAAACCGGAGCACCTCAACTCAACCCAATTGATGGGCCACTACCTGAGGACAACGA<br>ACCGAGCGGATATGCACAAACAGATTGCGTGTTGGAAGCAATGGCTTTCCTTGAAGAGTCCCACCCAGGGATCTTTGAAAACTCTTGTCTTGAAACGATGGAAGTCGTTCAGCAAACAAGAGTGGACAAACTAACTCAAGGTCGCCAGACATATGACTGGACACTGAATAGAAACCAACCAGCTGCAACTGCCCTGGCCAACACTATAGAGGTCTTCAGATCAAACGGTCTAA<br>CAGCCAATGAATCGGGGAGACTAATAGATYTCCTCAAGGATGTGATGGACTCAATGGATAAAGAAGAAATGGAAATAACAACACATTTCCAGAGAAAGAGAAGAGTAAGGGACAACATGACCAAGAAAATGGTCACACAAAGAACAATAGGAAAGAAGAAACAAAGGCTAAACAAGAGGAGCTACTTAATAAGAGCACTGACACTGAATACAATGACAAAAGATGCAGA<br>AAGAGGCAAATTGAAGAGACGGGCGATTGCAACACCAGGGATGCAGATTAGAGGATTTGTGTACTTTGTCGAAACACTGGCAAGGAGCATCTGTGAAAAACTTGAGCAATCTGGACTCCCCGTTGGAGGGAATGAGAAGAAGGCTAAATTGGCAAATGTCGTGAGAAAAATGATGACTAACTCACAAGATACAGAGCTCTCCTTCACAATTACTGGAGACAACACCAAATG<br>GAATGAGAATCAAAATCCTCGGATGTTTCTGGCAATGATAACATACATTACAAGAAACCAACCTGAATGGTTTAGAAATGTCTTAAGTATTGCCCCTATAATGTTCTCGAACAAAATGGCGAGATTGGGAAAAGGGTACATGTTTGAAAGTAAGAGCATGAAGTTACGGACACAAATACCTGCAGAAATGCTTGCAAACATTGACTTAAAATACTTCAATGAATCAACAAGAAA<br>GAAAATCGAAAAAATAAGGCCTCTACTAATAGATGGCACTGCCTCATTGAGTCCTGGAATGATGATGGGCATGTTCAATATGCTGAGTACAGTATTAGGAGTTTCAATCCTAAATCTTGGGCAAAAGAATTACACCAAAACCACATACTGGTGGGATGGACTCCAATCCTCTGATGATTTCGCCCTCATAGTAAATGCACCGAATCATGAGGGAATACAAGCAGGAGTGGATAG<br>GTTCTATAGGACCTGCAAACTGGTCGGGATCAATATGAGCAAAAAGAAGTCTTACATAAACCGGACTGGAACATTTGAGTTCACAAGCTTTTTCTATCGCTATGGATTTGTGGCTAACTTCAGTATGGAGCTGCCCAGCTTTGGGGTTTCTGGGATCAATGAATCAGCTGACATGAGCATTGGCGTCACAGTGATAAAGAACAACATGATAAACAATGACCTTGGACCAGCAAC<br>AGCTCAAATGGCCCTTCAACTATTCATCAAAGATTACAGGTACACGTACCGATGCCACAGAGGTGACACACAAATTCAAACGAGGAGATCATTCGAGCTGAAGAAGCTGTGGGAACAGACCCGTTCAAAGGCAGGACTGTTGGTGTCAGATGGAGGACCAAATCTATACAACATTCGGAATCTCCATATCCCAGAGGTCTGCCTGAAGTGGGAGCTGATGGACGAAGATTAC<br>CAGGGCAGGTTGTGTAATCCTCTGAACCCATTTGTCAGCCATAAAGAAATTGAGTCCGTAAACAATGCTGTGGTGATGCCAGCTCACGGTCCAGCCAAAAGCATGGAATATGATGCCGTTGCGACTACACACTCATGGACTCCTAAAAGGAATCGTTCCATTCTCAATACCAGTCAAAGGGGAATTCTTGAGGATGAACAGATGTACCAGAAATGCTGCAGTCTATTCGAGAAA<br>TTCTTCCCCAGTAGTTCATACAGGAGACCAGTTGGAATTTCCAGCATGGTGGAGGCCATGGTGTCTAGGGCCCGAATCGATGCACGCATTGATTTCGAATCTGGAAGGATCAAGAAGGAAGAGTTTGCTGAGATCATGAAGATCTGTTCCACCATTGAAGAGCTCAGACGGCAAAAATAG
EPI_ISL_14760999	>A/turkey/Italy/21VIR11504/2021_H5N1_2021-12-21<br>ATGGATGTCAATCCGACTTTACTTTTCTTAAAAGTGCCAGCGCAAGATGCCATAAGTACCACATTCCCTTACACTGGAGATCCTCCATACAGCCATGGAACAGGGACAGGATACACAATGGACACAGTCAACAGAACACATCAATACTCAGAGAAGGGAAAATGGACAACAAACACAGAAACCGGAGCACCTCAACTCAACCCCATTGATGGGCCACTACCTGAGGACAACGA<br>ACCGAGCGGATATGCACAAACAGATTGCGTGTTGGAAGCAATGGCTTTCCTTGAAGAGTCCCACCCAGGGATCTTTGAAAACTCTTGTCTTGAAACGATGGAAGTCGTTCAGCAAACAAGAGTGGACAAACTAACTCAAGGTCGCCAGACTTATGACTGGACACTGAATAGAAACCAACCAGCTGCAACTGCCCTGGCCAACACTATAGAGGTCTTCAGATCAAACGGTCTAA<br>CAGCCAATGAATCGGGGAGACTAATAGATTTCCTCAAGGATGTGATGGACTCAATGGATAAAGAAGAAATGGAAATAACAACACATTTCCAGAGAAAGAGAAGAGTAAGGGACAACATGACCAAGAAAATGGTCACACAAAGAACAATAGGAAAGAAGAAACAAAGGCTAAACAAGAGGAGCTACTTAATAAGAGCACTGACACTGAATACAATGACAAAAGATGCAGA<br>AAGAGGCAAATTGAAGAGACGGGCGATTGCAACACCAGGGATGCAGATTAGAGGATTTGTGTACTTTGTCGAAACACTGGCAAGGAGCATCTGTGAAAAACTTGAGCAATCTGGACTCCCCGTTGGAGGGAATGAGAAGAAGGCTAAATTGGCAAATGTCGTGAGAAAAATGATGACTAACTCACAAGATACAGAGCTCTCCTTCACAATCACTGGAGATAACACCAAATG<br>GAATGAGAATCAAAATCCTCGGATGTTTYTGGCAATGATAACATACATTACCAGAAACCAACCTGAATGGTTTAGAAATGTCTTGAGTATTGCCCCTATAATGTTCTCGAACAAAATGGCGAGATTGGGAAAAGGGTACATGTTTGAAAGTAAGAGCATGAAGTTACGGACACAAATACCTGCAGAAATGCTTGCAAACATTGACTTAAAATACTTCAATGAATCAACAAGAAA<br>GAAAATCGAAAAAATAAGGCCTCTACTAATAGATGGCACAGCCTCATTGAGTCCTGGAATGATGATGGGCATGTTCAATATGCTGAGTACAGTATTAGGAGTTTCAATCCTAAATCTTGGGCAAAAGAAGTACACCAAAACCACATACTGGTGGGATGGACTCCAATCCTCTGATGATTTCGCCCTCATAGTAAATGCACCGAATCATGAGGGAATACAAGCAGGAGTGGATA<br>GGTTCTATAGGACCTGCAAACTGGTCGGGATCAATATGAGCAAAAAGAAGTCTTACATAAACCGGACTGGAACATTTGAGTTCACAAGCTTTTTCTATCGCTATGGATTTGTGGCTAACTTCAGTATGGAGCTGCCCAGCTTTGGAGTTTCTGGGATCAATGAATCAGCTGACATGAGCATTGGCGTCACAGTGATAAAGAACAACATGATAAACAACGACCTTGGACCAGCAA<br>CAGCTCAAATGGCCCTTCAACTATTCATCAAAGATTACAGGTACACGTACCGATGCCACAGAGGTGACACACAAATTCAAACGAGGAGATCATTCGAGCTGAAGAAGCTGTGGGAACAGACCCGTTCAAAGGCAGGACTGTTGGTGTCAGATGGAGGACCAAATCTATACAACATTCGGAATCTCCATATCCCAGAGGTCTGCCTGAAGTGGGAGCTGATGGACGAAGATTA<br>CCAGGGCAGGTTGTGTAATCCTCTGAACCCATTTGTCAGCCATAAAGAAATTGAGTCCGTAAACAATGCTGTGGTGATGCCAGCCCACGGTCCAGCCAAAAGCATGGAATATGATGCCGTTGCGACTACACACTCATGGATTCCTAAAAGGAATCGTTCCATTCTCAATACCAGTCAAAGGGGGATTCTTGAGGATGAACAGATGTACCAGAAATGCTGCAGTCTATTCGAGAA<br>ATTCTTCCCCAGTAGTTCATACAGAAGACCAGTTGGAATTTCCAGCATGGTGGAGGCCATGGTGTCTAGGGCCCGAATCGATGCACGCATTGATTTCGAATCTGGAAGGATCAAGAAGGAAGAGTTTGCTGAGATCATGAAGATCTGTTCCACCATTGAAGAGCTCAGACGGCAAAAATAG
EPI_ISL_12917757	>A/turkey/Israel/564/2021__H5N1_2021-10-29<br>ATGGATGTCAATCCGACTTTACTTTTCTTAAAAGTGCCAGCGCAAGATGCCATAAGTACCACATTCCCTTACACTGGAGATCCTCCATACAGCCATGGAACAGGGACAGGATACACAATGGACACAGTCAACAGAACACATCAATACTCAGAGAAGGGAAAATGGACAACAAACACAGAAACCGGAGCACCTCAACTCAACCCAATTGATGGGCCACTACCTGAGGACAACGA<br>ACCGAGCGGATATGCACAAACAGATTGCGTGTTGGAAGCAATGGCTTTCCTTGAAGAGTCCCACCCAGGGATCTTTGAAAACTCTTGTCTTGAAACGATGGAAGTCGTTCAGCAAACAAGAGTGGACAAACTAACTCAAGGTCGCCAGACATATGACTGGACACTGAATAGAAACCAACCAGCTGCAACTGCCCTGGCCAACACTATAGAGGTCTTCAGATCAAACGGTCTAA<br>CAGCCAATGAATCGGGGAGACTAATAGATTTCCTCAAGGATGTGATGGACTCAATGGATAAAGAAGAAATGGAAATAACAACACATTTCCAGAGAAAGAGAAGAGTAAGGGACAACATGACCAAGAAAATGGTCACACAAAGAACAATAGGAAAGAAGAAACAAAGGCTAAACAAGAGGAGCTACTTAATAAGAGCACTGACACTGAATACAATGACAAAAGATGCAGA<br>AAGAGGCAAATTGAAAAGACGGGCGATTGCAACACCAGGGATGCAGATTAGAGGATTTGTGTACTTTGTCGAAACACTGGCAAGGAGCATCTGTGAAAAACTTGAGCAATCTGGACTCCCCGTTGGAGGGAATGAGAAGAAGGCTAAATTGGCAAATGTCGTGAGAAAAATGATGACTAACTCACAAGATACAGAGCTCTCCTTCACAATTACTGGAGATAACACCAAATG<br>GAATGAGAATCAAAATCCTCGGATGTTTCTGGCAATGATAACATACATTACAAGAAACCAACCTGAATGGTTTAGAAATGTCTTGAGTATTGCCCCTATAATGTTCTCGAACAAAATGGCGAGATTGGGAAAAGGGTACATGTTTGAAAGTAAGAGCATGAAGTTACGGACACAAATACCTGCAGAAATGCTTGCAAACATTGACTTAAAATACTTCAATGAATCAACAAGAAA<br>GAAAATCGAAAAAATAAGGCCTCTACTAATAGATGGCACTGCCTCATTGAGTCCTGGAATGATGATGGGCATGTTCAATATGCTGAGTACAGTATTAGGAGTTTCAATCCTAAATCTTGGGCAAAAGAAGTACACCAAAACCACATACTGGTGGGATGGACTCCAATCCTCTGATGATTTCGCCCTCATAGTAAATGCACCGAATCATGAGGGAATACAAGCAGGAGTGGATA<br>GGTTCTATAGGACCTGCAAACTGGTCGGGATCAATATGAGCAAAAAGAAGTCTTACATAAACCGGACTGGAACATTTGAGTTCACAAGCTTTTTCTATCGCTATGGATTTGTGGCTAACTTCAGTATGGAGCTGCCCAGCTTTGGAGTTTCTGGGATCAATGAATCAGCTGACATGAGCATTGGCGTCACAGTGATAAAGAACAACATGATAAACAATGACCTTGGACCAGCAA<br>CAGCTCAAATGGCCCTTCAACTATTCATCAAAGATTACAGGTACACGTACCGATGCCACAGAGGTGACACACAAATTCAAACTAGGAGATCATTCGAGCTGAAGAAGCTGTGGGAACAGACCCGTTCAAAGGCAGGACTGTTGGTGTCAGATGGAGGACCAAATCTATACAACATTCGGAATCTCCATATCCCAGAGGTCTGCCTGAAGTGGGAGCTGATGGACGAAGATTAC<br>CAGGGCAGGTTGTGTAATCCTCTGAACCCATTTGTCAGCCATAGAGAGATTGAGTCCGTAAACAATGCTGTGGTGATGCCAGCCCACGGTCCAGCCAAAAGCATGGAATATGATGCCGTTGCGACTACACACTCATGGATTCCTAAAAGGAATCGTTCCATTCTCAATACCAGTCAAAGGGGAATTCTTGAGGATGAACAGATGTACCAGAAATGCTGCAGTCTATTCGAGAAA<br>TTCTTCCCCAGTAGTTCATACAGGAGACCAGTTGGAATTTCCAGCATGGTGGAGGCCATGGTGTCTAGGGCCCGAATCGATGCACGAATTGATTTCGAATCTGGAAGGATCAAGAAGGAAGAGTTTGCTGAGATCATGAAGATCTGTTCCACCATTGAAGAGCTCAGACGGCAAAAATAG
EPI_ISL_3135897	>A/Turkey/Hungary/16603/2021_H5N1_2021-04-13<br>ATGGATGTCAATCCGACTTTACTTTTCTTAAAAGTGCCAGCGCAAAATGCCATAAGTACCACATTCCCTTATACTGGAGATCCTCCATACAGCCATGGAACAGGGACAGGATACACAATGGACACAGTCAACAGAACACATCAATACTCAGAGAAGGGAAAATGGACAACAAACACAGAAACCGGAGCACCTCAACTCAACCCAATTGAAGGACCACTGCCTGAGGACGACGA<br>ACCGAGCGGATATGCACAAACAGATTGCGTGTTGGAAGCAATGGCTTTCCTTGAAGAGTCCCACCCAGGGATCTTTGGAAACTCTTGTCTTGAAACGATGGAAGTCGTTCAGCAAACAAGAGTGGACAAACTAACTCAAGGTCGCCAGACTTATGACTGGACACTGAATAGAAACCAACCAGCTGCAACTGCCCTGGCCAACACTATAGAGGTCTTCAGATCAAACGGTCTAA<br>CAGCCAATGAATCGGGGAGACTAATAGATTTCCTCAAGGATGTGATGGACTCAATGGATAAAGAAGAAATGGAAATAACAACACATTTCCAGAGAAAGAGAAGAGTAAGGGACAACATGACCAAGAAAATGGTCACACAAAGAACAATAGGAAAGAAGAAACAAAGGCTAAACAAGAGGAGCTACTTAATAAGAGCACTGACACTGAATACAATGACAAAAGATGCAGA<br>AAGAGGCAAATTGAAGAGACGGGCGATTGCAACACCAGGGATGCAGATTAGAGGATTTGTGTACTTTGTCGAAACACTGGCAAGGAGCATCTGTGAAAAACTTGAGCAATCTGGACTCCCCGTTGGAGGGAATGAGAAGAAGGCTAAATTGGCAAATGTCGTGAGAAAAATGATGACTAACTCACAAGATACAGAGCTCTCCTTCACAATTACTGGAGATAACACCAAATG<br>GAATGAGAATCAAAATCCTCGGATGTTTCTGGCAATGATAACATACATTACAAGAAACCAACCTGAATGGTTTAGAAATGTCTTGAGTATTGCCCCTATAATGTTCTCGAACAAAATGGCGAGATTGGGAAAAGGGTACATGTTTGAAAGTAAGAGCATGAAGTTACGGACACAAATACCTGCAGAAATGCTTGCGAACATTGACTTAAAATACTTCAATGAATCAACAAGAAA<br>GAAAATCGAAAAAATAAGGCCTCTACTAATAGATGGCACTGCCTCATTGAGTCCTGGAATGATGATGGGCATGTTCAATATGCTGAGTACAGTATTAGGAGTTTCAATCCTAAATCTTGGGCAAAAGAGGTACACCAAAACCACATACTGGTGGGATGGACTCCAATCCTCTGATGATTTCGCCCTCATAGTAAATGCACCGAATCATGAGGGAATACAAGCAGGAGTGGATA<br>GGTTCTATAGGACCTGCAAACTGGTCGGGATCAATATGAGCAAAAAGAAGTCTTACATAAACCGGACTGGAACATTTGAGTTCACAAGCTTTTTCTATCGCTATGGATTTGTGGCTAACTTCAGTATGGAGCTGCCCAGCTTTGGAGTTTCTGGGATCAATGAATCAGCTGACATGAGCATTGGCGTAACAGTGATAAAGAACAACATGATAAACAATGACCTTGGACCAGCAA<br>CAGCTCAAATGGCCCTTCAACTATTCATCAAAGATTACAGGTACACGTACCGATGCCACAGAGGTGACACACAAATTCAAACGAGGAGATCATTCGAGCTGAAGAAGCTGTGGGAACAGACCCGTTCAAAGGCAGGACTGTTGGTGTCAGATGGAGGACCAAATCTATACAACATTCGGAATCTCCATATCCCAGAGGTCTGCCTGAAGTGGGAGCTGATGGACGAAGATTA<br>CCAGGGCAGGTTGTGTAATCCTCTGAACCCATTTGTCAGTCATAAAGAAATTGAGTCCGTAAACAATGCTGTGGTGATGCCAGCCCACGGTCCAGCCAAAAGCATGGAATATGATGCCGTTGCGACTACACACTCATGGATTCCTAAAAGGAATCGTTCCATTCTCAATACCGGTCAAAGGGGAATTCTTGAGGATGAACAGATGTACCAGAAATGCTGCAGTCTATTCGAAAA<br>ATTCTTCCCCAGTAGTTCATACAGGAGACCAGTTGGAATTTCCAGCATGGTGGAGGCCATGGTGTCTAGGGCCCGAATCGATGCACGCATTGATTTCGAATCTGGAAGGATCAAGAAGGAAGAGTTTGCTGAGATCATGAAGATCTGTTCCACCATTGAAGAGCTCAGACGGCAAAAATAG

EPI_ISL_5134658	>A/turkey/Germany-NI/AI02943/2021_H5N8_2021-03-15<br>ATGGATGTCAATCCGACTTTACTTTTCTTAAAAGTGCCAGCGCAAAATGCTATAAGTACTACATTCCCTTACACTGGAGATCCCCCATACAGCCATGGAACAGGAACAGGGTATACCATGGACACAGTAAACAGAACACATCAATACTCAGAAAAGGGAAAGTGGACAACAAACACAGAAACCGGAGCACCCYAACTCAACCCAATTGATGGACCATTACCAGAGGACAATGA<br>GCCAAGCGGATATGCACAAACTGATTGCGTGTTGGAAGCAATGGCTTTTCTTGAAGAATCCCACCCAGGGATATTTGAAAACTCTTGTCTTGAAGCGATGGAAATCGTTCAGCAAACAAGAGTGGACAAACTAACCCAAGGTCGCCAGACTTATGACTGGACACTGAACAGAAACCAACCAGCTGCAACCTCTTTGGCCAACACTATAGAGGTGTTCAGATCGAATGGTCTGA<br>CAGCCAATGAATCAGGGAGACTGATAGATTTTCTCAGGGATGTGATGGAATCAATGGATAAAGAAGAGATGGAAGTAACAACACATTTCCAGAGAAAAAGAAGAGTGAGGGACAACATGACTAAGAAGATGGTCACACAAAGAACAATAGGGAAGAAGAAGCAGAGGCTGAACAAGAGGAGTTACTTAATAAGAGCACTGACATTGAATACAATGACCAAAGATGCAGA<br>AAGAGGCAAGTTGAAGAGACGGGCAATTGCAACACCCGGGATGCAGATTAGAGGATTCGTGTACTTCGTTGAAACACTAGCGAGGAGCATCTGTGAGAAACTAGAGCAATCTGGGCTCCCTGTTGGAGGGAATGAGAAGAAGGCTAAATTGGCAAATGTTGTGAGAAAAATGATGACTAACTCACAAGATACAGAGCTCTCCTTTACAATTACTGGAGACAACACCAAATG<br>GAATGAGAATCAAAACCCTCGGATGTTTTTGGCAATGATAACATATATCACAAGAAACCAACCTGAATGGTTTAGAAATGTCTTAAGCATTGCCCCTATAATGTTCTCAAACAAAATGGCGAGATTAGGGAAAGGATACATGTTTGAAAGTAAGAGCATGAAGCTAAGAACACAAATACCAGCAGAGATGCTTACAAATATTGATCTGAAGTATTTCAACGAACCAACGAGAA<br>AGAAAATCGAGAAAATAAGACCTCTGCTGATTGATGGCACGGCCTCATTGAGTCCTGGGATGATGATGGGCATGTTCAATATGCTGAGCACAGTATTAGGGGTCTCAATCCTGAATCTCGGGCAAAAGAGGTACACCAAAACCACATACTGGTGGGATGGACTTCAATCCTCTGATGATTTCGCTCTCATAGTGAATGCACCGAATCATGAGGGGATACAAGCAGGAGTGGAT<br>AGATTCTATAGGACCTGCAAACTGGTTGGGATCAACATGAGCAAAAAGAAGTCTTACATAAACCGAACAGGAACATTTGAGTTCACAAGTTTTTTCTACCGCTATGGATTTGTAGCCAACTTCAGTATGGAATTACCCAGCTTTGGAGTGTCTGGAATCAATGAATCAGCTGACATGAGCATTGGAGTTACAGTGATAAAAAACAATATGATAAACAATGATCTTGGACCAGCA<br>ACAGCTCAAATGGCTCTTCAGTTATTCATCAAAGACTATAGGTACACATACCGATGCCACAGGGGTGATACACAAATTCAAACGAGGAGATCATTCGAGCTGAAGAAGCTGTGGGAGCAGACCCGTTCAAAGGCAGGGCTGTTGATATCAGACGGGGGGCCAAATCTATACAACATTCGGAATCTCCACATCCCAGAGGTCTGCTTGAAGTGGGAGCTGATGGATGAAGACT<br>ACCAAGGCAGGCTGTGCAATCCTCTGAATCCATTTGTCAGTCATAAAGAGATTGAGTCCGTAAACAATGCTGTAGTAATGCCCGCCCATGGCCCGGCCAAGAGCATGGAATATGATGCTGTTGCGACCACACACTCGTGGATTCCTAAGAGGAACCGTTCCATTCTCAATACCAGCCAAAGGGGAATTCTTGAGGATGAGCAGATGTACCAAAAGTGCTGTAGTCTATTCGAGA<br>AATTCTTCCCCAGCAGTTCATACAGGAGGCCAGTTGGAATTTCCAGCATGGTGGAGGCCATGGTGTCTAGGGCCCGAATTGATGCACGCATCGACTTCGAATCTGGAAGGATTAAGAAAGAAGAGTTTGCTGAGATCATGAAGATCTGTTCCACCATTGAAGAGCTCAGACGGCAAAAATAG
EPI_ISL_5098132	>A/turkey/Germany-NI/AI00616/2021_H5N8_2021-01-15<br>ATGGATGTCAATCCGACTTTACTTTTCTTAAAAGTGCCAGCGCAAAATGCTATAAGTACTACATTCCCTTACACTGGAGATCCCCCATACAGCCATGGAACAGGAACAGGGTATACCATGGACACAGTAAACAGAACACATCAATACTCAGAAAAGGGAAAGTGGACAACAAACACAGAAACCGGAGCACCCCAACTCAACCCAATTGATGGACCATTACCAGAGGACAATGA<br>GCCAAGCGGATATGCACAAACTGATTGCGTGTTGGAAGCAATGGCTTTCCTTGAAGAATCCCACCCAGGGATATTTGAAAACTCTTGTCTTGAAGCGATGGAAATCGTTCAGCAAACAAGAGTGGACAAACTAACCCAAGGTCGCCAGACTTATGACTGGACACTGAACAGAAACCAACCAGCTGCAACCTCTTTGGCCAACACTATAGAGGTGTTCAGATCGAATGGTCTGA<br>CAGCCAATGAATCAGGGAGACTGATAGATTTTCTCAGGGATGTGATGGAATCAATGGATAAAGAAGAGATGGAAGTAACAACACATTTCCAGAGAAAAAGAAGAGTGAGGGACAACATGACTAAGAAGATGGTCACACAAAGAACAATAGGGAAGAAGAAGCAGAGGCTGAACAAGAGGAGTTACTTAATAAGAGCACTGACATTGAATACAATGACCAAAGATGCAGA<br>AAGAGGCAAGTTGAAGAGACGGGCAATTGCAACACCCGGGATGCAGATTAGAGGATTCGTGTACTTCGTTGAAACACTAGCGAGGAGCATCTGTGAGAAACTAGAGCAATCTGGGCTCCCTGTTGGAGGGAATGAGAAAAAGGCTAAATTGGCAAATGTTGTGAGAAAAATGATGACTAACTCACAAGATACAGAGCTCTCCTTTACAATTACTGGAGACAACACCAAATG<br>GAATGAGAATCAAAACCCTCGGATGTTTTTGGCAATGATAACATATATCACAAGAAACCAACCTGAATGGTTTAGAAATGTCTTAAGCATTGCCCCTATAATGTTCTCAAACAAAATGGCGAGATTAGGGAAAGGATACATGTTTGAAAGTAAGAGCATGAAGCTAAGAACACAAATACCAGCAGAGATGCTTACAAATATTGATCTGAAGTATTTCAACGAACCAACGAGAA<br>AGAAAATCGAGAAAATAAGACCTCTGCTGATTGATGGCACGGCCTCATTGAGTCCTGGGATGATGATGGGCATGTTCAATATGCTGAGCACAGTATTAGGGGTCTCAATCCTGAATCTCGGGCAAAAGAGGTACACCAAAACCACATACTGGTGGGATGGACTCCAATCCTCTGATGATTTCGCTCTCATAGTGAATGCACCGAATCATGAGGGGATACAAGCAGGAGTGGAT<br>AGATTCTATAGGACCTGCAAACTGGTTGGGATCAACATGAGCAAAAAGAAGTCTTACATAAACCGAACAGGAACATTTGAGTTCACAAGTTTTTTCTACCGCTATGGATTTGTAGCCAACTTCAGTATGGAATTACCCAGCTTTGGAGTGTCTGGAATCAATGAATCAGCTGACATGAGCATTGGAGTTACAGTGATAAAAAACAATATGATAAACAATGATCTTGGACCAGCA<br>ACAGCTCAAATGGCTCTTCAGTTATTCATCAAAGACTATAGGTACACATACCGATGCCACAGGGGTGATACACAAATTCAAACGAGGAGATCATTCGAGCTGAAGAAGCTGTGGGAGCAGACCCGTTCAAAGGCAGGGCTGTTGATATCAGACGGGGGGCCAAACCTATACAACATTCGGAATCTCCACATCCCAGAGGTCTGCTTGAAGTGGGAGCTGATGGATGAAGACT<br>ACCAAGGCAGGCTGTGCAATCCTCTGAATCCATTTGTCAGTCATAAAGAGATTGAGTCCGTAAACAATGCTGTAGTAATGCCCGCCCATGGCCCGGCCAAGAGCATGGAATATGATGCTGTTGCGACCACACACTCGTGGATTCCTAAGAGGAACCGTTCCATCCTCAATACCAGCCAAAGGGGAATTCTTGAGGATGAGCAGATGTACCAAAAGTGCTGTAGTCTATTCGAGA<br>AATTCTTCCCCAGCAGTTCATACAGGAGGCCAGTTGGAATTTCCAGCATGGTGGAGGCCATGGTGTCTAGGGCCCGAATTGATGCACGCATCGACTTCGAATCTGGAAGGATTAAGAAAGAAGAGTTTGCTGAGATCATGAAGATCTGTTCCACCATTGAAGAGCTCAGACGGCAAAAATAG
EPI_ISL_5098133	>A/turkey/Germany-NI/AI00591/2021_H5N8_2021-01-14<br>ATGGATGTCAATCCGACTTTACTTTTCTTAAAAGTGCCAGCGCAAAATGCTATAAGTACTACATTCCCTTACACTGGAGATCCCCCATACAGCCATGGAACAGGAACAGGGTATACCATGGACACAGTAAACAGAACACATCAATACTCAGAAAAGGGAAAGTGGACAACAAACACAGAAACCGGAGCACCCCAACTCAACCCAATTGATGGACCATTACCAGAGGACAATGA<br>GCCAAGCGGATATGCACAAACTGATTGCGTGTTGGAAGCAATGGCTTTCCTTGAAGAATCCCACCCAGGGATATTTGAAAACTCTTGTCTTGAAGCGATGGAAATCGTTCAGCAAACAAGAGTGGACAAACTAACCCAAGGTCGCCAGACTTATGACTGGACACTGAACAGAAACCAACCAGCTGCAACCTCTTTGGCCAACACTATAGAGGTGTTCAGATCGAATGGTCTGA<br>CAGCCAATGAATCAGGGAGACTGATAGATTTTCTCAGGGATGTGATGGAATCAATGGATAAAGAAGAGATGGAAGTAACAACACATTTCCAGAGAAAAAGAAGAGTGAGGGACAACATGACTAAGAAGATGGTCACACAAAGAACAATAGGGAAGAAGAAGCAGAGGCTGAACAAGAGGAGTTACTTAATAAGAGCACTGACATTGAATACAATGACCAAAGATGCAGA<br>AAGAGGCAAGTTGAAGAGACGGGCAATTGCAACACCCGGGATGCAGATTAGAGGATTCGTGTACTTCGTTGAAACACTAGCGAGGAGCATCTGTGAGAAACTAGAGCAATCTGGGCTCCCTGTTGGAGGGAATGAGAAAAAGGCTAAATTGGCAAATGTTGTGAGAAAAATGATGACTAACTCACAAGATACAGAGCTCTCCTTTACAATTACTGGAGACAACACCAAATG<br>GAATGAGAATCAAAACCCTCGGATGTTTTTGGCAATGATAACATATATCACAAGAAACCAACCTGAATGGTTTAGAAATGTCTTAAGCATTGCCCCTATAATGTTCTCAAACAAAATGGCGAGATTAGGGAAAGGATACATGTTTGAAAGTAAGAGCATGAAGCTAAGAACACAAATACCAGCAGAGATGCTTACAAATATTGATCTGAAGTATTTCAACGAACCAACGAGAA<br>AGAAAATCGAGAAAATAAGACCTCTGCTGATTGATGGCACGGCCTCATTGAGTCCTGGGATGATGATGGGCATGTTCAATATGCTGAGCACAGTATTAGGGGTCTCAATCCTGAATCTCGGGCAAAAGAGGTACACCAAAACCACATACTGGTGGGATGGACTCCAATCCTCTGATGATTTCGCTCTCATAGTGAATGCACCGAATCATGAGGGGATACAAGCAGGAGTGGAT<br>AGATTCTATAGGACCTGCAAACTGGTTGGGATCAACATGAGCAAAAAGAAGTCTTACATAAACCGAACAGGAACATTTGAGTTCACAAGTTTTTTCTACCGCTATGGATTTGTAGCCAACTTCAGTATGGAATTACCCAGCTTTGGAGTGTCTGGAATCAATGAATCAGCTGACATGAGCATTGGAGTTACAGTGATAAAAAACAATATGATAAACAATGATCTTGGACCAGCA<br>ACAGCTCAAATGGCTCTTCAGTTATTCATCAAAGACTATAGGTACACATACCGATGCCACAGGGGTGATACACAAATTCAAACGAGGAGATCATTCGAGCTGAAGAAGCTGTGGGAGCAGACCCGTTCAAAGGCAGGGCTGTTGATATCAGACGGGGGGCCAAACCTATACAACATTCGGAATCTCCACATCCCAGAGGTCTGCTTGAAGTGGGAGCTGATGGATGAAGACT<br>ACCAAGGCAGGCTGTGCAATCCTCTGAATCCATTTGTCAGTCATAAAGAGATTGAGTCCGTAAACAATGCTGTAGTAATGCCCGCCCATGGCCCGGCCAAGAGCATGGAATATGATGCTGTTGCGACCACACACTCGTGGATTCCTAAGAGGAACCGTTCCATCCTCAATACCAGCCAAAGGGGAATTCTTGAGGATGAGCAGATGTACCAAAAGTGCTGTAGTCTATTCGAGA<br>AATTCTTCCCCAGCAGTTCATACAGGAGGCCAGTTGGAATTTCCAGCATGGTGGAGGCCATGGTGTCTAGGGCCCGAATTGATGCACGCATCGACTTCGAATCTGGAAGGATTAAGAAAGAAGAGTTTGCTGAGATCATGAAGATCTGTTCCACCATTGAAGAGCTCAGACGGCAAAAATAG
EPI_ISL_5098154	>A/turkey/Germany-MV/AI01127/2021_H5N8_2021-02-06<br>ATGGATGTCAATCCGACTTTACTTTTCTTAAAAGTGCCAGCGCAAAATGCTATAAGTACTACATTCCCTTACACTGGAGATCCCCCATACAGCCATGGAACAGGAACAGGGTATACCATGGACACAGTAAACAGAACACATCAATACTCAGAAAAGGGAAAGTGGACAACAAACACAGAAACCGGAGCACCCYAACTCAACCCAATTGATGGACCATTACCAGAGGACAATGA<br>GCCAAGCGGATATGCACAAACTGATTGCGTGTTGGAAGCCATGGCTTTCCTTGAAGAATCCCACCCAGGGATATTTGAAAACTCTTGTCTTGAAGCGATGGAAATCGTTCAGCAAACAAGAGTGGACAAACTAACCCAAGGTCGCCAGACTTATGACTGGACACTGAACAGAAACCAACCAGCTGCAACCTCTTTGGCCAACACTATAGAGGTGTTCAGATCGAATGGTCTGA<br>CAGCCAATGAATCAGGGAGACTGATAGATTTTCTCAGGGATGTGATGGAATCAATGGATAAAGAAGAGATGGAAGTAACAACACATTTCCAGAGAAAAAGAAGAGTGAGGGACAACATGACTAAGAAGATGGTCACACAAAGAACAATAGGGAAGAAGAAGCAGAGGCTGAACAAGAGGAGTTACTTAATAAGAGCACTGACATTGAATACAATGACCAAAGATGCAGA<br>AAGAGGCAAGTTGAAGAGACGGGCAATTGCAACACCCGGGATGCAGATTAGAGGATTCGTGTACTTCGTTGAAACACTAGCGAGGAGCATCTGTGAGAAACTAGAGCAATCTGGGCTCCCTGTTGGAGGGAATGAGAAAAAGGCTAAATTGGCAAATGTTGTGAGAAAAATGATGACTAACTCACAAGATACAGAGCTCTCCTTTACAATTACTGGAGACAACACCAAATG<br>GAACGAGAATCAAAACCCTCGGATGTTTTTGGCAATGATAACATATATCACAAGAAACCAACCTGAATGGTTTAGAAATGTCTTAAGCATTGCCCCTATAATGTTCTCAAACAAAATGGCGAGATTAGGGAAAGGATACATGTTTGAAAGTAAGAGCATGAAGCTAAGAACACAAATACCAGCAGAGATGCTTACAAATATTGATCTGAAGTATTTCAACGAACCAACGAGAA<br>AGAAAATCGAGAAAATAAGACCTCTGCTGATTGATGGCACAGCCTCATTGAGTCCTGGGATGATGATGGGCATGTTCAATATGCTGAGCACAGTATTAGGGGTCTCAATCCTGAATCTCGGGCAAAAGAGGTACACCAAAACCACATACTGGTGGGATGGACTTCAATCCTCTGATGATTTCGCTCTCATAGTGAATGCACCGAATCATGAGGGGATACAAGCAGGAGTGGAT<br>AGATTCTATAGGACCTGCAAATTGGTTGGGATCAACATGAGCAAAAAGAAGTCTTACATAAACCGAACAGGAACATTTGAGTTCACAAGTTTTTTCTACCGCTATGGATTTGTAGCCAACTTCAGTATGGAATTACCCAGCTTTGGAGTGTCTGGAATCAATGAATCAGCTGACATGAGCATTGGAGTTACAGTGATAAAAAACAATATGATAAACAATGATCTTGGACCAGCA<br>ACAGCTCAAATGGCTCTTCAGTTATTCATCAAAGACTATAGGTACACATACCGATGCCACAGGGGTGATACACAAATTCAAACGAGGAGATCATTCGAGCTGAAGAAGCTGTGGGAGCAGACCCGTTCAAAGGCAGGGCTGTTGATATCAGACGGGGGGCCAAACCTATACAACATTCGGAATCTCCACATCCCAGAGGTCTGCTTGAAGTGGGAGCTGATGGATGAAGACT<br>ACCAAGGCAGGCTGTGCAATCCTCTGAATCCATTTGTCAGTCATAAAGAGATTGAGTCCGTAAACAATGCTGTAGTAATGCCCGCCCATGGCCCGGCCAAGAGCATGGAATATGATGCTGTTGCGACCACACACTCGTGGATTCCTAAGAGGAACCGTTCCATCCTCAATACCAGCCAAAGGGGAATTCTTGAGGATGAGCAGATGTACCAAAAGTGCTGTAGTCTATTCGAGA<br>AATTCTTCCCCAGCAGTTCATACAGGAGGCCAGTTGGAATTTCCAGCATGGTGGAGGCCATGGTGTCTAGGGCCCGAATTGATGCACGCATCGACTTCGAATCTGGAAGGATTAAGAAAGAAGAGTTTGCTGAGATCATGAAGATCTGTTCCACCATTGAAGAGCTCAGACGGCAAAAATAG
EPI_ISL_5143417	>A/turkey/Germany-BB/AI02434/2021_H5N8_2021-03-10<br>ATGGATGTCAATCCGACTTTACTTTTCTTAAAAGTGCCAGCGCAAAATGCTATAAGTACTACATTCCCTTACACTGGAGATCCTCCATACAGCCATGGAACAGGAACAGGGTATACCATGGACACAGTAAACAGAACACATCAATACTCAGAAAAGGGAAAGTGGACAACAAACACAGAAACCGGAGCACCCCAACTCAACCCAATTGATGGACCATTACCAGAGGACAATGA<br>GCCAAGCGGATATGCACAAACTGATTGCGTGTTGGAAGCAATGGCTTTCCTTGAAGAATCCCACCCAGGGATATTTGAAAACTCTTGTCTTGAAGCGATGGAAATCGTTCAGCAAACAAGAGTGGACAAACTAACCCAAGGTCGCCAGACTTATGACTGGACACTGAACAGAAACCAACCAGCTGCAACCTCTTTGGCCAACACTATAGAGGTGTTCAGATCGAATGGTCTGA<br>CAGCCAATGAATCAGGAAGACTGATAGATTTTCTCAGGGATGTGATGGAATCAATGGATAAAGAAGAGATGGAAGTAACAACACACTTCCAGAGAAAAAGAAGAGTGAGGGACAACATGACTAAGAAGATGGTCACACAAAGAACAATAGGGAAGAAGAAGCAGAGGCTGAACAAGAGGAGTTACTTAATAAGAGCACTGACATTGAATACAATGACCAAAGATGCAGA<br>AAGAGGCAAGTTGAAGAGACGGGCAATTGCAACACCCGGGATGCAGATTAGAGGATTCGTGTACTTCGTTGAAACACTAGCAAGGAGCATCTGTGAGAAACTAGAGCAATCTGGGCTCCCTGTTGGAGGGAATGAGAAGAAGGCTAAATTGGCAAATGTTGTGAGAAAAATGATGACTAACTCACAAGATACAGAGCTCTCCTTTACAATTACTGGAGACAACACCAAATG<br>GAATGAGAATCAAAACCCTCGGATGTTTTTGGCAATGGTAACATATATCACAAGAAACCAACCTGAATGGTTTAGAAATGTCTTAAGCATTGCCCCTATAATGTTCTCAAACAAAATGGCGAGATTAGGGAAAGGATACATGTTTGAAAGTAAGAGCATGAAGCTAAGAACACAAATACCAGCAGAGATGCTCACAAATATTGATCTGAAGTATTTCAACGAACCAACGAGAA<br>AGAAAATCGAGAAAGTAAGACCTCTGCTGATTGATGGCACGGCCTCATTGAGTCCTGGGATGATGATGGGCATGTTCAATATGCTGAGCACAGTATTGGGGGTCTCAATCCTGAATCTCGGGCAAAAGAGGTACACCAAAACCACATACTGGTGGGATGGACTTCAATCCTCTGATGATTTTGCTCTCATAGTGAATGCACCGAATCATGAGGGGATACAAGCAGGAGTGGAT<br>AGATTCTATAGAACCTGCAAACTGGTTGGGATCAACATGAGCAAAAAGAAGTCTTACATAAACCGAACAGGAACATTTGAGTTCACAAGTTTTTTCTACCGCTATGGATTTGTAGCTAACTTCAGTATGGAATTACCCAGCTTTGGAGTGTCTGGAATCAATGAATCAGCTGACATGAGCATTGGAGTTACAGTGATAAAAAACAATATGATAAACAATGATCTTGGACCAGCAA<br>CAGCTCAAATGGCTCTTCAGTTATTCATCAAAGACTATAGGTACACATACCGATGCCACAGGGGTGATACACAAATTCAAACGAGGAGATCATTCGAGCTGAAGAAGCTGTGGGAGCAGACCCGTTCAAAGGCAGGGCTGTTGATATCAGACGGGGGGCCAAACCTATACAACATTCGGAACCTCCACATCCCAGAGGTCTGCTTGAAGTGGGAGCTGATGGATGAAGACTA<br>CCAAGGCAGGCTGTGCAATCCTCTGAATCCATTTGTCAGTCATAAAGAGATTGAGTCCGTAAACAATGCTGTAGTAATGCCCGCCCATGGCCCGGCCAAGAGCATGGAATATGATGCTGTTGCGACCACACATTCGTGGATTCCAAAGAGGAACCGCTCCATTCTCAATACCAGCCAAAGGGGAATTCTTGAGGATGAGCAGATGTACCAAAAGTGCTGTAGTCTATTCGAGA<br>AATTCTTCCCCAGCAGTTCATACAGGAGGCCAGTTGGAATTTCCAGCATGGTGGAGGCCATGGTGTCTAGGGCCCGAATTGATGCACGCATCGACTTCGAATCTGGAAGGATTAAGAAAGAAGAGTTTGCTGAGATCATGAAGATCTGTTCCACCATTGAAGAGCTCAGACGGCAAAAATAG
EPI_ISL_5098145	>A/turkey/Germany-BB/AI01120/2021_H5N8_2021-02-04<br>ATGGATGTCAATCCGACTTTACTTTTCTTAAAAGTGCCAGCGCAAAATGCTATAAGTACTACATTCCCTTACACTGGAGATCCTCCATACAGCCATGGAACAGGAACAGGGTATACCATGGACACAGTAAACAGAACACATCAATACTCAGAAAAGGGAAAGTGGACAACAAACACAGAAACCGGAGCACCCCAACTCAACCCAATTGATGGACCATTACCAGAGGACAATGA<br>GCCAAGCGGATATGCACAAACTGATTGCGTGTTGGAAGCAATGGCTTTCCTTGAAGAATCCCACCCAGGGATATTTGAAAACTCTTGTCTTGAAGCGATGGAAATCGTTCAGCAAACAAGAGTGGACAAACTAACCCAAGGTCGCCAGACTTATGACTGGACACTGAACAGAAACCAACCAGCTGCAACCTCTTTGGCCAACACTATAGAGGTGTTCAGATCGAATGGTCTGA<br>CAGCCAATGAATCAGGGAGACTGATAGATTTTCTCAGGGATGTGATGGAATCAATGGATAAAGAAGAGATGGAAGTAACAACACATTTCCAGAGAAAAAGAAGAGTGAGAGACAACATGACTAAGAAGATGGTCACACAAAGAACAATAGGGAAGAAGAAGCAGAGGCTGATCAAGAGGAGTTACTTAATAAGAGCACTGACATTGAATACAATGACCAAAGATGCAGA<br>AAGAGGCAAGTTGAAGAGACGGGCAATTGCAACACCCGGGATGCAGATTAGAGGATTCGTGTACTTCGTTGAAACACTAGCGAGGAGCATCTGTGAGAAACTAGAGCAATCTGGGCTCCCTGTTGGAGGGAATGAGAAGAAGGCTAAATTGGCAAATGTTGTGAGAAAAATGATGACTAACTCACAAGATACAGAGCTCTCCTTTACAATTACTGGAGACAACACCAAATG<br>GAATGAGAATCAAAACCCTCGGATGTTTTTGGCAATGATAACATATATCACAAGAAACCAACCTGAATGGTTTAGAAATGTCTTAAGCATTGCCCCTATAATGTTCTCAAACAAAATGGCGAGATTAGGGAAAGGATACATGTTTGAAAGTAAGAGCATGAAGCTAAGAACACAAATACCAGCAGAGATGCTTACAAATATCGATCTGAAGTATTTCAACGAACCAACGAGAA<br>AGAAAATCGAGAAAATAAGACCTCTGCTGATTGATGGCACGGCCTCATTGAGTCCTGGGATGATGATGGGCATGTTCAATATGCTGAGCACAGTATTAGGGGTCTCAATCCTGAATCTCGGGCAAAAAAGGTACACCAAAACCACATACTGGTGGGATGGACTTCAATCCTCTGATGATTTCGCTCTCATAGTGAATGCACCGAATCATGAGGGGATACAAGCAGGAGTGGAT<br>AGATTCTATAGGACCTGCAAACTGGTTGGGATCAACATGAGCAAAAAGAAGTCTTACATAAACCGAACAGGAACATTTGAGTTCACAAGTTTTTTCTACCGCTATGGATTTGTAGCTAACTTCAGTATGGAATTACCCAGCTTTGGAGTGTCTGGAATCAATGAATCAGCTGACATGAGCATTGGAGTTACAGTGATAAAAAACAATATGATAAACAATGATCTTGGACCAGCA<br>ACAGCTCAAATGGCTCTTCAGTTATTCATCAAAGACTATAGGTACACATACCGATGCCACAGGGGTGATACACAAATTCAAACGAGGAGATCATTCGAGCTGAAGAAGCTGTGGGAGCAGACCCGTTCAAAGGCAGGGCTGTTGATATCAGACGGGGGGCCAAACCTATACAACATTCGGAATCTCCACATCCCAGAGGTCTGCTTGAAGTGGGAGCTGATGGATGAAGACT<br>ACCAAGGCAGGCTGTGCAATCCTCTGAATCCATTTGTCAGTCATAAAGAGATTGAGTCCGTAAACAATGCTGTAGTAATGCCCGCCCATGGCCCGGCCAAGAGCATGGAATATGATGCTGTTGCGACCACACACTCGTGGATTCCTAAGAGGAACCGTTCCATTCTCAATACCAGCCAAAGGGGAATTCTTGAGGATGAGCAGATGTACCAAAAGTGCTGTAATCTATTCGAGA<br>AATTCTTCCCCAGCAGTTCATACAGGAGGCCAGTTGGAATTTCCAGCATGGTGGAGGCCATGGTGTCTAGGGCCCGAATTGATGCACGCATCGACTTCGAATCTGGAAGGATTAAGAAAGAAGAGTTTGCTGAGATCATGAAGATCTGTTCCACCATTGAAGAGCTCAGACGGCAAAAATAG
EPI_ISL_5095644	>A/turkey/Germany-BB/AI00868/2021_H5N8_2021-01-22<br>ATGGATGTCAATCCGACTTTACTTTTCTTAAAAGTGCCAGCGCAAAATGCTATAAGTACTACATTCCCTTACACTGGAGATCCTCCATACAGCCATGGAACAGGAACAGGGTATACCATGGACACAGTAAACAGAACACATCAATACTCAGAAAAGGGAAAGTGGACAACAAACACAGAAACCGGAGCACCCCAACTCAACCCAATTGATGGACCATTACCAGAGGACAATGA<br>GCCAAGCGGATATGCACAAACTGATTGCGTGTTGGAAGCAATGGCTTTCCTTGAAGAATCCCACCCAGGGATATTTGAAAACTCTTGTCTTGAAGCGATGGAAATCGTTCAGCAAACAAGAGTGGACAAACTAACCCAAGGTCGCCAGACTTATGACTGGACACTGAACAGGAACCAACCAGCTGCAACCTCTTTGGCCAACACTATAGAGGTGTTCAGATCGAATGGTCTGA<br>CAGCCAATGAATCAGGGAGACTGATAGATTTTCTCAGGGATGTGATGGAATCAATGGACAAAGAAGAGATGGAAGTAACAACACATTTCCAGAGAAAAAGAAGAGTGAGGGACAACATGACTAAGAAGATGGTCACACAAAGAACAATAGGGAAGAAGAAGCAGAGGCTGAACAAGAGGAGTTACTTAATAAGAGCACTGACATTGAATACAATGACCAAAGATGCAGA<br>AAGAGGCAAGTTGAAGAGACGGGCAATTGCAACACCCGGGATGCAGATTAGAGGATTCGTGTACTTCGTTGAAACACTAGCGAGGAGCATCTGTGAGAAACTAGAGCAATCTGGGCTCCCTGTTGGAGGGAATGAGAAGAAGGCTAAATTGGCAAATGTTGTGAGAAAAATGATGACTAACTCACAAGATACAGAGCTCTCCTTTACAATTACTGGAGACAACACCAAATG<br>GAATGAGAATCAAAACCCTCGGATGTTTTTGGCAATGATAACATATATCACAAGAAACCAACCTGAATGGTTTAGAAATGTCTTAAGCATTGCCCCTATAATGTTCTCAAACAAAATGGCGAGATTAGGGAAAGGATACATGTTTGAAAGTAAGAGCATGAAGCTAAGAACACAAATACCAGCAGAGATGCTTACAAATATCGATCTGAAGTATTTCAACGAACCAACGAGAA<br>AGAAAATCGAGAAAATAAGACCTCTGCTGATTGATGGCACGGCCTCATTGAGTCCTGGGATGATGATGGGCATGTTCAATATGCTGAGCACAGTATTAGGGGTCTCAATCCTGAATCTCGGGCAAAAAAGGTACACCAAAACCACATACTGGTGGGATGGACTTCAATCCTCTGATGATTTCGCTCTCATAGTGAATGCACCGAATCATGAGGGGATACAAGCAGGAGTGGAT<br>AGATTCTATAGGACCTGCAAACTGGTTGGGATCAACATGAGCAAAAAGAAGTCTTACATAAACCGAACAGGAACATTTGAGTTCACAAGTTTTTTCTACCGCTATGGATTTGTAGCTAACTTCAGTATGGAATTACCCAGCTTTGGAGTGTCTGGAATCAATGAATCAGCTGACATGAGCATTGGAGTTACAGTGATAAAAAACAATATGATAAACAATGATCTTGGACCAGCA<br>ACAGCTCAAATGGCTCTTCAGTTATTCATCAAAGACTATAGGTACACATACCGATGCCACAGGGGTGATACACAAATTCAAACGAGGAGATCATTCGAGCTGAAGAAGCTGTGGGAGCAGACCCGTTCAAAGGCAGGGCTGTTGATATCAGACGGGGGGCCAAACCTATACAACATTCGGAATCTCCACATCCCAGAGGTCTGCTTGAAGTGGGAGCTGATGGATGAAGACT<br>ACCAAGGCAGGCTGTGCAATCCTCTGAATCCATTTGTCAGTCATAAAGAGATTGAGTCCGTAAACAATGCTGTAGTAATGCCCGCCCATGGCCCGGCCAAGAGCATGGAATATGATGCTGTTGCGACCACACACTCGTGGATTCCTAAGAGGAACCGTTCCATTCTCAATACCAGCCAAAGGGGAATTCTTGAGGATGAGCAGATGTACCAAAAGTGCTGTAGTCTATTCGAGA<br>AATTCTTCCCCAGCAGTTCATACAGGAGGCCAGTTGGAATTTCCAGCATGGTGGAGGCCATGGTGTCTAGGGCCCGAATTGATGCACGCATCGACTTCGAATCTGGAAGGATTAAGAAAGAAGAGTTTGCTGAGATCATGAAGATCTGTTCCACCATTGAAGAGCTCAGACGGCAAAAATAG
EPI_ISL_9029957	>A/turkey/England/057679/2021_H5N1_2021-11-16<br>ATGGATGTCAATCCGACTTTACTTTTCTTAAAAGTGCCAGCGCAAGATGCCATAAGTACCACATTCCCTTACACTGGAGATCCTCCATACAGCCATGGAACAGGGACAGGATACACAATGGACACAGTCAACAGAACACATCAATACTCAGAGAAGGGAAAATGGACAACAAACACAGAAACCGGAGCACCTCAACTCAACCCAATTGATGGGCCACTACCTGAGGACAACGA<br>ACCGAGCGGATATGCACAAACAGATTGCGTGTTGGAAGCAATGGCTTTCCTTGAAGAGTCCCACCCAGGGATCTTTGAAAACTCTTGTCTTGAAACGATGGAAGTCGTTCAGCAAACAAGAGTGGACAAACTAACTCAAGGTCGCCAGACATATGACTGGACACTGAATAGAAACCAACCAGCTGCAACTGCCCTGGCCAACACTATAGAGGTCTTCAGATCAAACGGTCTAA<br>CAGCCAATGAATCGGGGAGACTAATAGATTTCCTCAAGGATGTGATGGACTCAATGGATAAAGAAGAAATGGAAATAACAACACATTTCCAGAGAAAGAGAAGAGTAAGGGACAACATGACCAAGAAAATGGTCACACAAAGAACAATAGGAAAGAAGAAACAAAGGCTAAACAAGAGGAGCTACTTAATAAGAGCACTGACACTGAATACAATGACAAAAGATGCAGA<br>AAGAGGCAAATTGAAAAGAGGGCGATTGCAACACCAGGGATGCAGATTAGAGGATTTGTGTACTTTGTCGAAACACTGGCAAGGAGCATCTGTGAAAAACTTGAGCAATCTGGACTCCCCGTTGGAGGGAATGAGAAGAAGGCTAAATTGGCAAATGTCGTGAGAAAAATGATGACTAACTCACAAGATACAGAGCTCTCCTTCACAATTACTGGAGATAACACCAAATG<br>GAATGAGAATCAAAATCCTCGGATGTTTCTGGCAATGATAACATACATTACAAGAAACCAACCTGAATGGTTTAGAAATGTCTTGAGTATTGCCCCTATAATGTTCTCGAACAAAATGGCGAGATTGGGAAAAGGGTACATGTTTGAAAGTAAGAGCATGAAGTTACGGACACAAATACCTGCAGAAATGCTTGCAAACATTGACTTAAAATACTTCAATGAATCAACAAGAAA<br>GAAAATCGAAAAAATAAGGCCTCTACTAATAGATGGCACTGCCTCATTGAGTCCTGGAATGATGATGGGCATGTTCAATATGCTGAGTACAGTATTAGGAGTTTCAATCCTAAATCTTGGGCAAAAGAAGTACACCAAAACCACATACTGGTGGGATGGGCTCCAATCCTCTGATGATTTCGCCCTCATAGTAAATGCACCGAATCATGAGGGAATACAAGCAGGAGTGGATA<br>GGTTCTATAGGACCTGCAAACTGGTCGGGATCAATATGAGCAAAAAGAAGTCTTACATAAACCGGACTGGAACATTTGAGTTCACAAGCTTTTTCTATCGCTATGGATTTGTGGCTAACTTCAGTATGGAGCTGCCCAGCTTTGGAGTTTCTGGGATCAATGAATCAGCTGACATGAGCATTGGCGTCACAGTGATAAAGAACAACATGATAAACAATGACCTTGGACCAGCAA<br>CAGCTCAAATGGCCCTTCAACTATTCATCAAAGATTACAGGTACACGTACCGATGCCACAGAGGTGACACACAAATTCAAACGAGGAGATCATTCGAGCTGAAGAAGCTGTGGGAACAGACCCGTTCAAAGGCAGGACTGTTGGTGTCAGATGGAGGACCAAATCTATACAACATTCGGAATCTCCATATCCCAGAGGTCTGCCTGAAGTGGGAGCTGATGGACGAAGATTA<br>CCAGGGCAGGTTGTGTAATCCTCTGAACCCATTTGTCAGCCATAGAGAAATTGAGTCCGTAAACAATGCTGTGGTGATGCCAGCCCACGGTCCAGCCAAAAGCATGGAATATGATGCCGTTGCGACTACACACTCATGGATTCCTAAAAGGAATCGTTCCATTCTCAATACCAGTCAAAGGGGAATTCTTGAGGATGAACAGATGTACCAGAAATGCTGCAGTCTATTCGAGAA<br>ATTCTTCCCCAGTAGTTCATACAGGAGACCAGTTGGAATTTCCAGCATGGTGGAGGCCATGGTGTCTAGGGCCCGAATCGATGCACGAATTGATTTCGAATCTGGAAGGATCAAGAAGGAAGAGTTTGCTGAGATCATGAAGATCTGTTCCACCATTGAAGAGCTCAGACGGCAAAAATAG
EPI_ISL_710504	>A/turkey/England/037784/2020_H5N8_2020-11-28<br>ATGGATGTCAATCCGACTTTACTTTTCTTAAAAGTGCCAGCGCAAAATGCTATAAGTACTACATTCCCTTACACTGGAGATCCTCCATACAGCCATGGAACAGGAACAGGGTATACCATGGACACAGTAAACAGAACACATCAATACTCAGAAAAGGGAAAGTGGACAACAAACACAGAAACCGGAGCACCCCAACTCAACCCAATTGATGGACCATTACCAGAGGACAATGA<br>GCCAAGCGGATATGCACAAACTGATTGCGTGTTGGAAGCAATGGCTTTCCTTGAAGAATCCCACCCAGGGATATTTGAAAACTCTTGTCTTGAAGCGATGGAAATCGTTCAGCAAACAAGAGTGGACAAACTAACCCAAGGTCGCCAGACTTATGACTGGACACTGAACAGAAACCAACCAGCTGCAACTTCTTTGGCCAACACTATAGAGGTGTTCAGATCGAATGGTCTGA<br>CAGCCAATGAATCAGGGAGACTGATAGATTTTCTCAGAGATGTGATGGAATCAATGGATAAAGAAGAGATGGAAGTAACAACACATTTCCAGAGAAAAAGAAGAGTGAGGGACAACATGACTAAGAAGATGGTCACACAAAGAACAATAGGGAAGAAGAAGCAGAGGCTGAACAAGAGGAGTTACTTAATAAGAGCACTGACATTGAATACAATGACCAAAGATGCAGA<br>AAGAGGCAAGTTGAAGAGACGGGCAATTGCAACACCCGGGATGCAGATTAGAGGATTCGTGTACTTCGTTGAAACACTAGCGAGGAGCATCTGTGAGAAACTAGAGCAATCTGGGCTCCCTGTTGGAGGGAATGAGAAGAAGGCTAAATTGGCAAATGTTGTGAGAAAAATGATGACTAACTCACAAGATACAGAGCTCTCCTTTACAATTACTGGAGACAACACCAAATG<br>GAATGAGAATCAAAACCCTCGGATGTTTTTGGCAATGATAACATATATCACAAGAAACCAACCTGAATGGTTTAGAAATGTCTTAAGCATTGCCCCTATAATGTTCTCAAACAAAATGGCGAGATTAGGGAAAGGATACATGTTTGAAAGTAAGAGCATGAAGCTAAGAACACAAATACCAGCAGAGATGCTTACAAATATCGATCTGAAGTATTTCAACGAACCAACGAGAA<br>AGAAAATCGAGAAAATAAGACCTCTGCTGATTGATGGCACGGCCTCATTGAGTCCTGGGATGATGATGGGCATGTTCAATATGCTGAGCACAGTATTAGGGGTCTCAATCCTGAATCTCGGGCAAAAAAGGTACACCAAAACCACATACTGGTGGGATGGACTTCAATCCTCTGATGATTTCGCTCTCATAGTGAATGCACCGAATCATGAGGGGATACAAGCAGGAGTGGAT<br>AGATTCTATAGGACCTGCAAACTGGTTGGGATCAACATGAGCAAAAAGAAGTCTTACATAAACCGAACAGGAACATTTGAGTTCACAAGTTTTTTCTACCGCTATGGATTTGTAGCTAACTTCAGTATGGAATTACCCAGCTTTGGAGTGTCTGGAATCAATGAATCAGCTGACATGAGCATTGGAGTTACAGTGATAAAAAACAATATGATAAACAATGATCTTGGACCAGCA<br>ACAGCTCAAATGGCTCTTCAGTTATTCATCAAAGACTATAGGTACACATACCGATGCCACAGGGGTGATACACAAATTCAAACGAGGAGATCATTCGAGCTGAAGAAGCTGTGGGAGCAGACCCGTTCAAAGGCAGGGCTGTTGATATCAGACGGGGGGCCAAACCTATACAACATTCGGAATCTCCACATCCCAGAGGTCTGCTTGAAGTGGGAGCTGATGGATGAAGACT<br>ACCAAGGCAGGCTGTGCAATCCTCTGAATCCATTTGTCAGTCATAAAGAGATTGAGTCCGTAAACAATGCTGTAGTAATGCCCGCCCATGGCCCGGCCAAGAGCATGGAATATGATGCTGTTGCGACCACACACTCGTGGATTCCTAAGAGGAACCGTTCCATTCTCAATACCAGCCAAAGGGGAATTCTTGAGGATGAGCAGATGTACCAAAAGTGCTGTAGTCTATTCGAGA<br>AATTCTTCCCCAGCAGTTCATACAGGAGGCCAGTTGGAATTTCCAGCATGGTGGAGGCCATGGTGTCTAGGGCCCGAATTGATGCACGCATCGACTTCGAATCTGGAAGGATTAAGAAAGAAGAGTTTGCTGAGATCATGAAGATCTGTTCCACCATTGAAGAGCTCAGACGGCAAAAATAG
EPI_ISL_666890	>A/turkey/Croatia/104/2020_H5N8_2020-11-17<br>ATGGATGTCAATCCGACTTTACTTTTCTTAAAAGTGCCAGCGCAAAATGCTATAAGTACTACATTCCCTTACACTGGAGATCCCCCATACAGCCATGGAACAGGAACAGGGTATACCATGGACACAGTAAACAGAACACATCAATACTCAGAAAAGGGAAAGTGGACAACAAACACAGAAACCGGAGCACCCCAACTCAACCCAATTGATGGACCATTACCAGAGGACAATGA<br>GCCAAGCGGATATGCACAAACTGATTGCGTGTTGGAAGCAATGGCTTTCCTTGAAGAATCCCACCCAGGGATATTTGAAAACTCTTGTCTTGAAGCGATGGAAATCGTTCAGCAAACAAGAGTGGACAAACTAACCCAAGGTCGCCAGACTTATGACTGGACACTGACCAGAAACCAACCAGCTGCAACCTCTTTGGCCAACACTATAGAGGTGTTCAGATCGAATGGTCTGA<br>CAGCCAATGAATCAGGGAGACTGATAGATYTTCTCAGGGATGTGATGGAATCAATGGATAAAGAAGAGATGGAAGTAACAACACATTTCCAGAGAAAAAGAAGAGTGAGGGACAACATGACTAAGAAGATGGTCACACAAAGAACAATAGGGAAGAAGAAGCAGAGGCTGAACAAGAGGAGTTACTTAATAAGAGCACTGACATTGAATACAATGACCAAAGATGCAGA<br>AAGAGGCAAGTTGAAGAGACGGGCAATTGCAACACCCGGGATGCAGATTAGAGGATTCGTGTACTTCGTTGAAACACTAGCGAGGAGCATCTGTGAGAAACTAGAGCAATCTGGGCTCCCTGTTGGAGGGAATGAGAAGAAGGCTAAATTGGCAAATGTTGTGAGAAAAATGATGACTAACTCACAAGATACAGAGCTCTCCTTTACAATTACTGGAGACAACACCAAATG<br>GAATGAGAATCAAAACCCTCGGATGTTTTTGGCAATGATAACATATATCACAAGAAACCAACCTGAATGGTTTAGAAATGTCTTGAGCATTGCCCCTATAATGTTCTCAAACAAAATGGCGAGATTAGGGAAAGGATACATGTTTGAAAGTAAGAGCATGAAGCTAAGAACACAAATACCAGCAGAGATGCTTACAAATATTGATCTGAAGTATTTCAACGAACCAACGAGAA<br>AGAAAATCGAGAAAATAAGACCTCTGCTGATTGATGGCACGGCCTCATTGAGTCCTGGGATGATGATGGGCATGTTCAATATGCTGAGCACAGTATTAGGGGTCTCAATCCTGAATCTCGGGCAAAAGAGGTACACCAAAACCACATACTGGTGGGATGGACTTCAATCCTCTGATGATTTCGCTCTCATAGTGAATGCACCGAATCATGAGGGGATACAAGCAGGAGTGGAT<br>AGATTCTATAGGACCTGCAAACTGGTTGGGATCAACATGAGCAAAAAGAAGTCTTACATAAACCGAACAGGAACATTTGAGTTCACAAGTTTTTTCTACCGCTATGGATTTGTAGCCAACTTCAGTATGGAATTACCCAGCTTTGGAGTGTCTGGAATCAATGAATCAGCTGACATGAGCATTGGAGTTACAGTGATAAAAAACAATATGATAAACAATGATCTTGGACCAGCA<br>ACAGCTCAAATGGCTCTTCAGTTATTCATCAAAGACTATAGGTACACATACCGATGCCACAGGGGTGATACACAAATTCAAACGAGGAGATCATTCGAGCTGAAGAAGCTGTGGGAGCAGACCCGTTCAAAGGCAGGGCTGTTGATATCAGACGGGGGGCCAAACCTATACAACATTCGGAATCTCCACATCCCAGAGGTCTGCTTGAAGTGGGAGCTGATGGATGAAGACT<br>ACCAAGGCAGGCTGTGCAATCCTCTGAATCCATTTGTCAGTCATAAAGAGATTGAGTCCGTAAACAATGCTGTAGTAATGCCCGCCCATGGCCCGGCCAAGAGCATGGAGTATGATGCTGTTGCGACCACACACTCGTGGATTCCTAAGAGGAACCGTTCCATCCTCAATACCAGCCAAAGGGGAATTCTTGAGGATGAGCAGATGTACCAAAAGTGCTGTAGTCTATTCGAG<br>AAATTCTTCCCCAGCAGTTCATACAGGAGGCCAGTTGGAATTTCCAGCATGGTGGAGGCCATGGTGTCTAGGGCCCGAATTGATGCACGCATCGACTTCGAATCTGGAAGGATTAAGAAAGAAGAGTTTGCTGAGATCATGAAGATCTGTTCCACCATTGAAGAGCTCAGACGGCAAAAATAG
EPI_ISL_1220094	>A/tundra_bean_goose/Poland/MB132/2020_H5N8_2020-12-11<br>ATGGATGTCAATCCGACTTTACTTTTCTTAAAAGTGCCAGCGCAAAATGCTATAAGTACTACATTCCCTTACACTGGAGATCCTCCATACAGCCATGGAACAGGAACAGGGTATACCATGGACACAGTAAACAGAACACATCAATACTCAGAAAAGGGAAAGTGGACAACAAACACAGAAACCGGAGCACCCCAACTCAACCCAATTGATGGACCATTACCAGAGGACAATGA<br>GCCAAGCGGATATGCACAAACTGATTGCGTGTTGGAAGCAATGGCTTTCCTTGAAGAATCCCACCCAGGGATATTTGAAAACTCTTGTCTTGAAGCGATGGAAATCGTTCAGCAAACAAGAGTGGACAAACTAACCCAAGGTCGCCAGACTTATGACTGGACACTGAACAGAAACCAACCAGCTGCAACCTCTTTGGCCAACACTATAGAGGTGTTCAGATCGAATGGTCTGA<br>CAGCCAATGAATCAGGGAGACTGATAGATTTTCTCAGGGATGTGATGGAATCAATGGATAAAGAAGAGATGGAAGTAACAACACATTTCCAGAGAAAAAGAAGAGTGAGGGACAACATGACCAAGAAGATGGTCACACAAAGAACAATAGGGAAGAAGAAGCAGAGGCTGAACAAGAGGAGTTACTTAATAAGAGCACTGACATTGAATACAATGACCAAAGATGCAGA<br>AAGAGGCAAGTTGAAGAGACGGGCAATTGCAACACCCGGGATGCAGATTAGAGGATTCGTGTACTTCGTTGAAACACTAGCGAGGAGCATCTGTGAGAAACTAGAGCAATCTGGGCTCCCTGTTGGAGGGAATGAGAAGAAGGCTAAATTGGCAAATGTTGTGAGAAAAATGATGACTAACTCACAAGATACAGAGCTCTCCTTTACAATTACTGGAGACAACACCAAATG<br>GAATGAGAATCAAAACCCTCGGATGTTTTTGGCAATGATAACATATATCACAAGAAACCAACCTGAATGGTTTAGAAATGTCTTAAGCATTGCCCCTATAATGTTCTCAAACAAAATGGCGAGATTAGGGAAAGGATACATGTTTGAAAGTAAGAGCATGAAGCTAAGAACACAAATACCAGCAGAGTTGCTTACAAATATCGATCTGAAGTATTTCAACGAAACAACGAGAA<br>AGAAAATCGAGAAAATAAGACCTCTGCTGATTGATGGCACGGCCTCATTGAGTCCTGGGATGATGATGGGCATGTTCAATATGCTGAGCACAGTATTAGGGGTCTCAATCCTGAATCTCGGGCAAAAAAGGTACACCAAAACCACATACTGGTGGGATGGACTTCAATCCTCTGATGATTTCGCTCTCATAGTGAATGCACCGAATCATGAGGGGATACAAGCAGGAGTGGAT<br>AGATTCTATAGGACCTGCAAACTGGTTGGGATCAACATGAGCAAAAAGAAGTCTTACATAAACCGAACAGGAACATTTGAGTTCACAAGTTTTTTCTACCGCTATGGATTTGTAGCTAACTTCAGTATGGAATTACCCAGCTTTGGAGTGTCTGGAATCAATGAATCAGCTGACATGAGCATTGGAGTTACAGTGATAAAAAACAATATGATAAACAATGATCTTGGACCAGCA<br>ACAGCTCAAATGGCTCTTCAGTTATTCATCAAAGACTATAGGTACACATACCGATGCCACAGGGGTGATACACAAATTCAAACGAGGAGATCATTCGAGCTGAAGAAGCTGTGGGAGCAGACCCGTTCAAAGGCAGGGCTGTTGATATCAGACGGGGGGCCAAACCTATACAACATTCGGAATCTCCACATCCCAGAGGTCTGCTTGAAGTGGGAGCTGATGGATGAAGACT<br>ACCAAGGCAGGCTGTGCAATCCTCTGAATCCATTTGTCAGTCATAAAGAGATTGAGTCCGTAAACAATGCTGTAGTAATGCCCGCCCATGGCCCGGCCAAGAGCATGGAATATGATGCTGTTGCGACCACACACTCGTGGATTCCTAAGAGGAACCGTTCCATTCTCAATACCAGCCAAAGGGGAATTCTTGAGGATGAGCAGATGTACCAAAAGTGCTGTAGTCTATTCGAGA<br>AATTCTTCCCCAGCAGTTCATACAGGAGGCCAGTTGGAATTTCCAGCATGGTGGAGGCCATGGTGTCTAGGGCCCGAATTGATGCACGCATCGACTTCGAATCTGGAAGGATTAAGAAAGAAGAGTTTGCTGAGATCATGAAGATCTGTTCCACCATTGAAGAGCTCAGACGGCAAAAATAG
EPI_ISL_1255051	>A/tufted_duck/Poland/MB061/2021_H5N5_2021-02-01<br>ATGGATGTCAATCCGACTTTACTTTTCTTAAAAGTGCCAGCGCAAAATGCTATAAGTACTACGTTCCCTTACACTGGAGATCCTCCATACAGCCATGGAACAGGAACAGGATACACCATGGACACAGTCAACAGAACGCATCAATACTCAGAGAAGGGAAAGTGGACAACGAACACAGAGACTGGGGCGCCCCAACTCAACCCAATTGATGGACCATTACCAGAGGACAATGA<br>GCCAAGCGGATATGCACAAACAGATTGCGTGTTGGAAGCAATGGCTTTCCTTGAAGAATCACACCCAGGGATCTTTGAAAACTCTTGTCTTGAAACGATGGAGGTCGTTCAGCAAACAAGAGTGGACAAACTAACCCAAGGTCGCCAGACTTATGATTGGACACTGAATAGAAACCAGCCAGCTGCAACTGCTTTGGCCAATACTATAGAGGTCTTCAGATCGAACGGTCTAA<br>CAGCCAATGAGTCGGGGAGACTAATAGATTTCCTCAAGGATGTGATGGAATCAATGGATAAAGAAGAAATGGAAATAACAACACACTTTCAGAGAAAGAGAAGAGTAAGGGACAACATGACCAAGAAGATGGTCACACAAAGAACGATAGGAAAGAAGAAGCAGAGACTGAACAAGAGGAGCTACTTAATAAGGGCACTGACATTGAACACAATGACCAAAGATGCAGA<br>AAGAGGCAAATTGAAGAGGCGGGCAATTGCAACACCCGGGATGCAGATTAGAGGATTTGTGTACTTTGTCGAAACACTAGCAAGGAGCATCTGTGAGAAACTCGAGCAGTCTGGACTCCCTGTTGGAGGGAATGAGAAGAAGGCCAAATTGGCAAATGTCGTGAGAAAAATGATGACTAACTCACAAGATACAGAGCTCTCCTTTACAATTACCGGAGACAACACCAAATG<br>GAATGAGAATCAAAATCCTCGGATGTTTTTGGCAATGATAACATACATCACAAGAAACCAACCCGAATGGTTTAGAAATGTCTTGAGCATTGCCCCTATAATGTTCTCAAACAAAATGGCGAGATTAGGAAAAGGATACATGTTTGAGAGCAAGAGCATGAAGCTAAGGACACAAATACCGGCAGAAATGCTTGCAAACATTGACTTGAAATACTTCAATGAATCAACGAGAA<br>AGAAAATCGAGAAAATTAGACCTCTGCTAATCGATGGCACAGCCTCACTGAGTCCTGGAATGATGATGGGCATGTTCAATATGCTGAGCACAGTATTGGGGGTCTCAATCCTGAATCTTGGACAAAAGAGGTACACCAAAACCACATACTGGTGGGATGGACTCCAATCCTCTGATGATTTCGCTCTCATAGTGAATGCACCGAATCATGAGGGGATACAAGCAGGGGTGGAT<br>AGGTTCTATAGGACCTGCAAACTGGTTGGGATCAACATGAGTAAGAAGAAGTCTTACATAAACCGGACAGGAACATTTGAGTTCACAAGCTTTTTCTACCGCTATGGATTTGTAGCCAACTTCAGTATGGAGTTACCCAGCTTTGGAGTGTCTGGGATCAATGAATCGGCTGACATGAGCATTGGAGTTACAGTGATAAAGAACAATATGATAAACAATGACCTTGGACCTGCA<br>ACAGCTCAAATGGCTCTTCAGCTATTCATCAAGGACTATAGGTACACGTACCGATGCCACAGAGGTGATACACAAATTCAAACGAGGAGATCATTCGAGCTGAAGAAGCTGTGGGAGCAGACCCGTTCAAAGGCAGGGCTGTTGGTATCAGATGGAGGACCAAACCTATACAACATTCGGAATCTCCACATACCAGAGGTCTGCTTGAAGTGGGAACTAATGGATGAAGACT<br>ACCAGGGCAGGCTGTGTAATCCTCTGAATCCGTTTGTCAATCATAAAGAAATTGAGTCAGTAAACAATGCTGTGGTAATGCCAGCCCATGGCCCAGCCAAGAGCATGGAATATGATGCTGTTGCGACTACACACTCATGGATTCCTAAGAGGAACCGTTCCATTCTCAATACCAGCCAGAGGGGAATTCTCGAGGATGAGCAGATGTACCAGAAGTGCTGCAGTCTATTCGAA<br>AAATTCTTCCCCAGTAGTTCATACAGGAGGCCAGTTGGAATTTCCAGCATGGTGGAGGCCATGGTGTCCAGGGCCCGAATTGATGCACGCATTGATTTCGAATCTGGAAGGATTAAGAAAGAAGAGTTTGCTGAGATCATGAAGATCTGTTCCACCATTGAAGAGCTCAGACGGCAAAAATAG

EPI_ISL_300529	>A/teal/Markeev/13-3-11/2016_H5N2_2016-11-03<br>ATGGATGTCAATCCGACTTTACTTTTCTTAAAAGTGCCAGCGCAAAATGCTATAAGCACTACGTTCCCTTACACTGGAGATCCTCCATACAGCCATGGAACAGGAACAGGATACACCATGGATACAGTCAACAGAACACATCAATACTCAGAGAAGGGAAAGTGGACAACGAACACAGAGACTGGGGCGCCCCAACTCAACCCAATTGATGGACCATTACCAGAGGACAATGA<br>GCCAAGTGGATATGCACAAACAGATTGCGTGTTGGAAGCAATGGCTTTCCTTGAAGAATCCCACCCAGGGATCTTTGAAAACTCTTGTCTTGAAACGATGGAGGTCGTTCAGCAAACAAGAGTGGACAAACTAACCCAAGGTCGCCAGACTTATGATTGGACACTGAATAGAAACCAACCAGCTGCAACTGCTCTGGCCAACACTATAGAGGTCTTCAGATCGAACGGTCTAA<br>CAGCCAATGAGTCGGGGAGACTAATAGATTTCCTCAAGGATGTGATGGAATCAATGGATAAAGAAGAAATGGAAATAACAACACATTTCCAGAGAAAGAGAAGAGTAAGGGACAACATGACCAAGAAGATGATCACACAAAGAACGATAGGAAAGAAGAAGCAGAGACTGAACAAGAGGAGCTACTTAATAAGGGCACTGACATTGAACACAATGACCAAAGATGCAGA<br>AAGAGGCAAGTTGAAGAGGCGGGCAATTGCAACACCCGGGATGCAGATTAGAGGATTCGTATACTTTGTCGAAACACTAGCAAGGAGCATCTGTGAGAAACTCGAGCAGTCTGGACTCCCTGTTGGAGGGAATGAGAAGAAGGCCAAATTGGCAAATGTCGTGAGAAAAATGATGACTAATTCACAAGATACAGAGCTCTCCTTTACAATTACTGGAGACAACACCAAATG<br>GAATGAGAATCAAAATCCTCGGATGTTTTTGGCGATGATAACATACATCACAAGAAACCAACCTGAATGGTTTAGAAATGTCTTGAGCATTGCCCCTATAATGTTCTCAAACAAAATGGCGAGATTAGGAAAAGGATACATGTTTGAGAGTAAGAGCATGAAGCTAAGAACACAAATACCTGCAGAAATGCTTGCAAACATTGACTTGAAATACTTCAACGAATCAACGAGAA<br>AGAAAATCGAGAAAATTAGACCTCTGCTAATTGATGGCACAGCCTCATTGAGCCCTGGAATGATGATGGGCATGTTCAATATGCTGAGCACAGTATTGGGGGTCTCAATCCTGAATCTTGGACAAAAGAAGTACACCAAAACCACATACTGGTGGGATGGACTCCAATCCTCTGATGATTTCGCTCTCATAGTGAATGCACCGAATCATGAGGGGATACAAGCAGGGGTGGAT<br>AGGTTTTATAGGACATGCAAACTGGTTGGGATCAACATGAGCAAAAAGAAATCTTACATAAACCGGACAGGAACATTTGAGTTCACAAGCTTTTTCTACCGCTATGGATTTGTAGCCAACTTCAGTATGGAGTTACCCAGCTTTGGAGTGTCTGGAATCAATGAATCGGCTGACATGAGCATTGGAGTTACAGTGATAAAGAACAATATGATAAACAATGACCTTGGACCTGCA<br>ACAGCTCAAATGGCTCTTCAGCTATTCATCAAGGACTATAGGTACACGTACCGATGCCACAGAGGTGATACACAAATTCAAACGAGGAGATCATTCGAGCTGAAGAAGCTGTGGGAGCAGACCCGTTCAAAGGCAGGGCTGTTGGTATCAGATGGAGGACCAAACCTATACAACATTCGGAATCTCCACATTCCAGAAGTCTGCTTGAAATGGGAACTAATGGATGAAGACT<br>ACCAGGGCAGGCTGTGTAATCCTCTGAATCCGTTTGTCAGTCATAAGGAAATTGAGTCCGTAAACAATGCTGTGGTAATGCCAGCCCATGGCCCAGCCAAGAGCATGGAATATGATGCTGTTGCGACTACACACTCATGGATTCCTAAGAGGAACCGTTCCATTCTCAATACCAGCCAGAGGGGAATTCTTGAGGATGAGCAGATGTACCAGAAGTGCTGCAGTCTATTCGAA<br>AAATTCTTCCCCAGTAGTTCATACAGGAGGCCAGTTGGAATTTCCAGCATGGTGGAGGCCATGGTATCCAGGGCCCGAATTGATGCACGCATTGATTTCGAGTCTGGAAGGATTAAGAAAGAAGAGTTTGCTGAGATCATGAAGATCTGTTCCACCATTGAAGAGCTCAGACGGCAAAAAATAG
EPI_ISL_387970	>A/teal/Egypt/MB-D-698C/2016_H7N3_2016-03-17<br>ATGGATGTCAATCCGACTTTACTTTTCTTGAAAGTGCCAGCGCAAAATGCCATAAGTACCACATTCCCTTATACTGGAGATCCTCCATACAGCCATGGAACAGGAACAGGATACACCATGGACACAGTCAACAGAACACATCAATACTCAGAAAAGGGGAAATGGACAACAAACACAGAGACCGGAGCACCCCAACTCAACCCAATTGATGGACCACTACCTGAGGACAACGA<br>ACCAAGCGGATATGCACAAACAGATTGCGTGTTGGAAGCAATGGCTTTCCTTGAAGAGTCCCACCCAGGGATCTTTGAAAACTCTTGTCTTGAAACGATGGAAGTCGTTCAGCAAACAAGAGTGGACAAACTAACTCAAGGTCGCCAGACTTATGACTGGACACTGAATAGAAACCAACCAGCTGCAACTGCCCTGGCCAACACTATAGAGGTCTTCAGATCGAACGGTCTGA<br>CAGCCAATGAATCGGGGAGACTAATAGATTTCCTCAAGGATGTGATGGACTCAATGGACAAAGAAGAAATGGAAATAACAACACATTTCCAGAGAAAGAGAAGAGTAAGGGACAACATGACCAAGAAAATGGTCACACAAAGAACAATAGGGAAGAAGAAACAAAGGCTAAATAAGAGGAGCTACTTAATAAGAGCATTGACACTGAATACAATGACAAAAGACGCAGA<br>AAGAGGCAAATTGAAGAGACGGGCAATTGCAACACCAGGGATGCAGATTAGAGGATTTGTGTACTTTGTCGAAACACTGGCAAGGAGCATCTGTGAAAAACTTGAGCAATCTGGACTCCCCGTTGGAGGGAATGAGAAGAAGGCTAAATTGGCAAATGTCGTGAGAAAAATGATGACTAACTCACAAGATACAGAGCTCTCCTTCACAATTACTGGAGATAACACCAAATG<br>GAATGAGAATCAAACCCTCGGATGTTTCTGGCAATGATAACATACATCACAAGAAACCAACCTGAATGGTTTAGAAATGTTTTGAGCATTGCCCCTATAATGTTCTCGAACAAAATGGCGAGATTGGGAAAAGGATACATGTTTGAAAGTAAGAGCATGAAGCTACGGACACAAATACCTGCAGAAATGCTTGCAAACATTGACTTAAAATACTTCAATGAGTCAACAAGAAA<br>GAAAATCGAGAAAATAAGACCTCTATTAATAGATGGCACCGCCTCATTGAGTCCTGGAATGATGATGGGCATGTTCAACATGCTGAGTACAGTATTAGGAGTCTCAATCCTGAATCTTGGGCAAAAGAGGTACACCAAAACCACATACTGGTGGGATGGACTCCAATCCTCTGATGATTTTGCTCTCATAGTGAATGCACCGAATCATGAGGGAATACAAGCAGGAGTGGATA<br>GGTTCTATAGGACCTGCAAACTGGTTGGGATCAACATGAGCAAAAAGAAGTCTTACATAAACCGGACAGGAACATTTGAGTTCACAAGCTTTTTCTATCGCTATGGATTTGTGGCTAACTTCAGTATGGAGCTGCCCAGCTTTGGAGTGTCTGGAATCAATGAATCAGCTGACATGAGCATTGGAGTTACAGTGATAAAGAACAACATGATAAACAATGACCTTGGACCAGCAA<br>CAGCTCAAATGGCCCTTCAACTATTCATCAAGGACTATAGGTACACATACCGATGTCACAGGGGTGACACACAAATTCAAACGAGGAGATCATTCGAGCTGAAGAAGCTGTGGGAGCAGACCCGTTCAAAGGCAGGACTGTTGGTGTCAGATGGAGGACCAAATCTATACAACATTCGGAATCTCCATATCCCAGAGGTCTGCCTGAAGTGGGAGCTGATGGACGAAGATTA<br>CCAGGGCAGGTTGTGTAATCCTCTGAACCCATTTGTCAGTCATAAAGAAATTGAGTCCGTAAACAATGCTGTGGTAATGCCAGCCCATGGCCCAGCCAAGAGCATGGAATATGATGCCGTTGCGACTACACACTCATGGATTCCTAAAAGGAATCGTTCCATTCTCAATACCAGCCAAAGGGGAATTCTTGAGGATGAGCAGATGTACCAGAAATGCTGCAGTCTATTCGAGAA<br>ATTCTTCCCCAGTAGTTCATACAGGAGACCAGTTGGAATTTCCAGCATGGTGGAGGCCATGGTGTCTAGGGCCCGAATCGATGCACGCATTGATTTCGAATCTGGAAGGATTAAGAAGGAAGAGTTTGCTGAGATCATGAAGATCTGTTCCACCATTGAAGAGCTCAGACGGCAAAAAATAG
EPI_ISL_387969	>A/teal/Egypt/MB-D-125OP/2015_H7N3_2015-01-27<br>ATGGATGTCAATCCGACTTTACTTTTCTTAAAAGTGCCAGCGCAAAATGCTATAAGTACTACATTCCCTTACACTGGAGATCCTCCATACAGCCATGGAACAGGAACAGGATACACTATGGACACAGTCAACAGAACACATCAATACTCAGAGAAGGGAAAATGGACAACAAACACAGAGACCGGAGCACCCCAACTCAATCCAATTGATGGACCATTACCAGAGGACAACGA<br>GCCAAGCGGATATGCACAAACGGATTGCGTGTTGGAAGCAATGGCTTTCCTTGAAGAATCCCACCCAGGGATCTTTGAAAACTCTTGTCTCGAAACGATGGAAGTCGTTCAGCAAACAAGATTGGACAAACTAACCCAAGGTCGCCAGACTTATGACTGGACATTGAACAGAAATCAACCAGCTGCAACTGCTTTGGCCAACACTATAGAGGTGTTCAGATCGAATGGTCTGA<br>CAGCCAATGAGTCGGGGAGACTAATAGATTTCCTCAAGGATGTGATGGAATCAATGGATAAAGAAGAAATGGAAATAACAACACATTTCCAGAGAAAGAGAAGAGTGAGGGACAACATGACCAAGAAGATGGTCACACAAAGAACAATAGGAAAGAAGAAGCAGAGGCTGAATAAGAGGAGTTACTTAATAAGAGCACTGACATTGAACACAATGACCAAAGATGCAGA<br>AAGAGGCAAACTGAAGAGACGGGCAATTGCAACACCCGGGATGCAGATTAGAGGGTTCGTGTACTTTGTCGAAACACTAGCGAGGAGCATCTGTGAGAAACTCGAGCAATCTGGACTCCCTGTTGGAGGGAATGAGAAGAAGGCTAAATTGGCAAATGTCGTGAGAAAAATGATGACTAACTCACAAGATACAGAGCTCTCCTTTACAATTACTGGAGACAACACCAAATG<br>GAATGAGAATCAAAATCCTCGGATGTTTTTGGCAATGATAACATACATCACAAGAAACCAACCTGAATGGTTTAGGAATGTCTTGAGCATCGCCCCTATAATGTTCTCAAACAAAATGGCGAGATTAGGAAAAGGATACATGTTTGAAAGTAAGAGCATGAAGCTAAGAACACAAATACCGGCAGAAATGCTTGCAAACATTGATTTGAAATATTTCAACGAATCAACGAGAA<br>AGAAAATCGAGAGAATAAGACCTCTGCTAATTGATGGCACAGCCTCATTGAGTCCTGGAATGATGATGGGCATGTTCAACATGCTGAGCACAGTATTAGGGGTCTCAATCCTGAATCTTGGGCAAAAGAGGTACACCAAAACCACATACTGGTGGGACGGACTCCAATCCTCTGATGATTTCGCTCTCATAGTGAATGCACCGAATCATGAGGGGATACAAGCAGGAGTGGAT<br>AGGTTCTATAGGACCTGCAAACTGGTTGGGATCAACATGAGCAAAAAGAAGTCTTACATAAACCGGACAGGGACATTTGAATTCACAAGTTTTTTCTACCGCTATGGATTTGTAGCTAACTTCAGTATGGAATTACCCAGCTTTGGAGTGTCTGGAATCAATGAATCGGCTGACATGAGCATTGGAGTTACAGTGATAAAGAACAATATGATAAACAATGACCTTGGACCAGCA<br>ACAGCTCAAATGGCTCTTCAGCTATTCATCAAGGACTATAGGTACACGTACCGATGCCACAGGGGTGATACACAAATTCAAACGAGGAGATCATTCGAGCTGAAGAAGCTGTGGGAGCAGACCCGTTCAAAGGCAGGGTTGTTGGTATCAGATGGAGGACCAAACCTATACAACATTCGGAATCTCCACATCCCAGAGGTCTGCTTGAAGTGGGAGCTGATGGATGAAGACT<br>ACCAGGGCAGGCTGTGTAATCCTTTGAATCCATTTGTCAGTCATAAGGAAATTGAGTCCGTAAACAATGCTGTGGTAATGCCAGCCCATGGCCCAGCCAAGAGCATGGAATATGATGCTGTTGCGACTACACACTCGTGGATTCCTAAGAGAAACCGTTCCATTCTCAATACCAGCCAAAGGGGAATTCTTGAGGATGAGCAAATGTACCAGAAGTGCTGTAGTCTATTTGAGA<br>AATTCTTCCCCAGCAGTTCATACAGGAGGCCAGTTGGAATTTCCAGCATGGTGGAGGCCATGGTGTCTAGGGCCCGAATTGATGCACGCATTGATTTCGAATCTGGAAGGATTAAGAAAGAAGAGTTTGCTGAGATCATGAAGATCTGTTCCACCATTGAAGAGCTCAGACGGCAAAAAATAG
EPI_ISL_14389203	>A/Tadorna_tadorna/Belgium/1260_0003/2022_H4N6_2022-01-22<br>ATGGATGTCAATCCGACTTTACTTTTCTTAAAGGTGCCAGCGCAAAATGCCATAAGTACCACATTCCCTTATACTGGAGATCCTCCATACAGCCATGGAACAGGAACAGGATACACCATGGACACAGTCAACAGAACACATCAATACTCAGAAAAGGGGAAATGGACAACAAACACAGAGACTGGAGCACCCCAACTCAACCCAATTGATGGACCACTACCTGAGGACAACGA<br>ACCAAGCGGATATGCACAAACAGATTGCGTGTTGGAAGCAATGGCTTTCCTTGAAGAGTCCCACCCAGGGATCTTTGAAAACTCTTGTCTTGAAACGATGGAAGTCGTTCAGCAAACAAGAGTGGACAAACTAACTCAAGGTCGCCAGACTTATGACTGGACACTGAATAGAAACCAACCAGCTGCAACTGCCCTGGCCAACACTATAGAGGTCTTCAGATCGAACGGTCTGA<br>CAGCCAATGAATCCGGGAGACTAATAGATTTCCTCAAGGATGTGATGGACTCAATGGACAAAGAAGAAATGGAAATAACAACACATTTCCAGAGAAAGAGAAGGGTAAGGGACAACATGACCAAGAAAATGGTCACACAAAGAACAATAGGGAAGAAGAAGCAAAGGCTAAATAAGAGGAGCTACTTAATAAGAGCACTGACACTGAATACAATGACAAAGGACGCAGA<br>AAGAGGCAAATTGAAGAGACGGGCAATTGCAACACCAGGGATGCAGATTAGAGGATTTGTGTACTTTGTCGAAACACTGGCAAGGAGCATTTGTGAAAAACTTGAGCAATCTGGACTCCCCGTTGGAGGGAATGAGAAGAAGGCTAAATTGGCAAATGTAGTGAGAAAAATGATGACTAACTCACAGGATACAGAGCTCTCCTTCACAATTACTGGAGATAACACCAAATG<br>GAATGAGAATCAAAATCCTCGGATGTTTCTGGCAATGATAACATACATCACAAGAAACCAACCTGAATGGTTTAGAAATGTCTTGAGCATTGCCCCTATAATGTTCTCGAACAAAATGGCGAGACTGGGAAAAGGATACATGTTTGAAAGCAAAAGCATGAAGCTACGGACACAAATACCTGCAGAAATGCTTGCAAACATTGACTTAAAATACTTCAATGAGTCAACAAGAA<br>AGAAAATCGAGAAAATAAGACCTCTACTAATAGATGGCACCGCCTCATTGAGTCCCGGAATGATGATGGGCATGTTCAATATGCTGAGTACAGTGTTAGGAGTCTCAATCCTGAATCTTGGGCAAAAGAGGTACACCAAGACCACATACTGGTGGGATGGACTCCAATCCTCTGATGATTTCGCCCTCATAGTGAATGCACCGAATCATGAGGGAATACAAGCAGGAGTGGAT<br>AGGTTCTATAGGACCTGCAAACTGGTTGGGATCAACATGAGCAAAAAGAAGTCTTACATAAACCGGACAGGAACATTTGAGTTCACAAGCTTTTTCTATCGCTATGGATTTGTGGCCAACTTCAGTATGGAGCTGCCCAGTTTTGGAGTGTCTGGAATCAATGAATCAGCTGACATGAGCATTGGAGTTACAGTGATAAAGAACAACATGATAAACAATGACCTTGGACCAGCA<br>ACAGCTCAAATGGCCCTTCAACTATTCATCAAGGACTACAGGTACACGTACCGATGTCACAGGGGTGACACACAGATTCAAACGAGGAGATCATTCGAGCTGAAGGAGCTGTGGGAGCAGACCCGTTCCAAGGCAGGACTGTTGGTGTCAGATGGAGGACCAAATCTATACAACATCCGGAATCTCCATATCCCAGAAGTCTGCCTGAAGTGGGAGCTGATGGACGAAGATT<br>ACCAGGGCAGGTTGTGTAATCCTCTGAACCCATTTGTCAGTCATAAAGAAATTGAATCCGTAAACAATGCTGTGGTAATGCCAGCCCATGGCCCAGCCAAGAGCATGGAATATGATGCTGTTGCGACTACACACTCATGGATTCCCAAAAGGAATCGTTCCATTCTCAATACCAGCCAAAGGGGAATTCTTGAGGATGAGCAGATGTACCAGAAATGCTGCAGTCTATTCGAGA<br>AATTCTTCCCCAGTAGTTCATACAGGAGACCAGTTGGAATTTCCAGCATGGTGGAGGCCATGGTGTCTAGGGCCCGAATCGACGCACGCATTGATTTCGAATCTGGAAGGATTAAGAAGGAAGAGTTTGCTGAGATCATGAAGATCTGTTCCACCATTGAAGAGCTCAGACGGCAAAAAATAG
EPI_ISL_11007532	>A/swan/Slovenia/2072_22VIR777-5/2021_H5N1_2021-12-30<br>ATGGATGTCAATCCGACTTTACTTTTCTTAAAAGTGCCAGCGCAAGATGCCATAAGTACCACATTCCCTTACACTGGAGATCCTCCATACAGCCATGGAACAGGGACAGGATACACAATGGACACAGTCAACAGAACACATCAATACTCAGAGAAGGGAAAATGGACAACAAACACAGAAACCGGAGCACCTCAACTCAACCCAATTGATGGGCCACTACCTGAGGACAACGA<br>ACCGAGCGGATATGCACAAACAGATTGCGTGTTGGAAGCAATGGCTTTCCTTGAAGAGTCCCACCCAGGGATCTTTGAGAACTCTTGTCTTGAAACGATGGAAGTCGTTCAGCAAACAAGAGTGGACAAACTAACTCAAGGTCGCCAGACATATGACTGGACACTGAATAGAAACCAACCAGCTGCAACTGCCCTGGCCAACACTATAGAGGTCTTCAGATCAAACGGTCTAA<br>CAGCCAATGAATCGGGGAGACTAATAGATTTCCTCAAGGATGTGATGGACTCAATGGATAAAGAAGAAATGGAAATAACAACACATTTCCAGAGAAAGAGAAGAGTAAGGGACAACATGACCAAGAAAATGGTCACACAAAGAACAATAGGAAAGAAGAAACAAAGGCTAAACAAGAGGAGCTACTTAATAAGAGCACTGACACTGAATACAATGACAAAAGATGCAGA<br>AAGAGGCAAATTGAAGAGACGGGCGATTGCAACACCAGGGATGCAGATTAGAGGATTTGTGTACTTTGTCGAAACACTGGCAAGGAGCATCTGTGAAAAACTTGAGCAATCTGGACTCCCCGTTGGAGGGAATGAGAAGAAGGCTAAATTGGCAAATGTCGTGAGAAAAATGATGACTAACTCACAAGATACAGAGCTCTCCTTCACAATTACTGGAGATAACACCAAATG<br>GAATGAGAATCAAATCCTCGGATGTTTCTGGCAATGATAACATACATTACAAGAAACCAACCTGAATGGTTTAGAAATGTCTTGAGTATTGCCCCTATAATGTTCTCGAACAAAATGGCGAGATTGGGAAAAGGGTACATGTTTGAAAGTAAGAGCATGAAGTTACGGACACAAATACCTGCAGAAATGCTTGCAAACATTGACTTAAAATACTTCAATGAATCAACAAGAAA<br>GAAAATCGAAAAAATAAGGCCTCTACTAATAGATGGCACTGCCTCATTGAGTCCTGGAATGATGATGGGCATGTTCAATATGCTGAGTACAGTATTAGGAGTTTCAATCCTAAATCTTGGGCAAAAGAAGTACACCAAAACCACATACTGGTGGGATGGACTCCAATCCTCTGATGATTTCGCCCTCATAGTAAATGCACCGAATCATGAGGGAATACAAGCAGGAGTGGATA<br>GGTTCTATAGGACCTGCAAACTGGTCGGGATCAATATGAGCAAAAAGAAGTCTTACATGAACCGGACTGGAACATTTGAGTTCACAAGCTTTTTCTATCGCTATGGATTTGTGGCTAACTTCAGTATGGAGCTGCCCAGCTTTGGAGTTTCTGGGATCAATGAATCAGCTGACATGAGCATTGGCGTCACAGTGATAAAGAACAACATGATAAACAATGACCTTGGACCGGCAA<br>CAGCTCAAATGGCCCTTCAACTATTCATCAAAGATTACAGGTACACGTACCGATGCCACAGAGGTGACACACAAATTCAAACGAGGAGATCATTCGAGCTGAAGAAGCTGTGGGAACAGACCCGTTCAAAGGCAGGACTGTTGGTGTCAGATGGAGGACCAAATCTATACAACATTCGGAATCTCCATATCCCAGAGGTCTGCCTGAAGTGGGAGCTGATGGACGAAGATTA<br>CCAGGGCAGGTTGTGTAATCCTCTGAACCCATTTGTCAGCCATAAAGAACTTGAGTCCGTAAACAATGCTGTGGTGATGCCAGCCCACGGTCCAGCCAAAAGCATGGAATATGATGCCGTTGCGACTACACACTCATGGATTCCTAAAAGGAATCGTTCCATTCTCAATACCAGTCAAAGGGGAATTCTTGAGGATGAACAGATGTACCAGAAATGCTGCAGTCTATTCGAGAA<br>ATTCTTCCCCAGTAGTTCATACAGGAGACCAGTTGGAATTTCCAGCATGGTGGAGGCCATGGTGTCTAGGGCCCGAATCGATGCACGCATTGATTTCGAATCTGGAAGGATCAAGAAGGAAGAGTTTGCTGAGATCATGAAGATCTGTTCCACCATTGAAGAGCTCAGACGGCAAAAAATAG
EPI_ISL_11007535	>A/swan/Slovenia/13_22VIR777-8/2022_H5N1_2022-01-03<br>ATGGATGTCAATCCGACTTTACTTTTCTTAAAAGTGCCAGCGCAAGATGCCATAAGTACCACATTCCCTTACACTGGAGATCCTCCATACAGCCATGGAACAGGGACAGGATACACAATGGACACAGTCAACAGAACACATCAATACTCAGAGAAGGGAAAATGGACAACAAACACAGAAACCAGAGCACCTCAACTCAACCCAATTGATGGGCCACTACCTGAGGACAACGA<br>ACCGAGCGGATATGCACAAACAGATTGCGTGTTGGAGCAATGGCTTTCCTTGAAGAGTCCCACCCAGGGATCTTTGAAAACTCTTGTCTTGAAACGATGGAAGTCGTTCAGCAAACAAGAGTGGACAAACTAACTCAAGGTCGCCAGACATATGACTGGACACTGAATAGAAACCAACCAGCTGCAACTGCCCTGGCCAACACTATAGAGGTCTTCAGATCAAACGGTCTAA<br>CAGCCAATGAATCGGGGAGACTAATAGATTTCCTCAAGGATGTGATGGACTCAATGGATAAAGAAGAAATGGAAATAACAACACATTTCCAGAGAAAGAGAAGAGTAAGGGACAACATGACCAAGAAAATGGTCACACAAAGAACAATAGGAAAGAAGAAACAAAGGCTAAACAAGAGGAGCTACTTAATAAGAGCACTGACACTGAATACAATGACAAAAGATGCAGA<br>AAGAGGCAAATTGAAAAGACGGGCGATTGCAACACCAGGGATGCAGATTAGAGGATTTGTGTACTTTGTCGAAACACTGGCAAGGAGCATCTGTGAAAAACTTGAGCAATCTGGACTCCCCGTTGGAGGGAATGAGAAGAAGGCTAAATTGGCAAATGTCGTGAGAAAAATGATGACTAACTCACAAGATACAGAGCTCTCCTTCACAATTACTGGAGATAACACCAAATG<br>GAATGAGAATCAAACCCTCGGATGTTTCTGGCAATGATAACATACATTACAAGAAACCAACCTGAATGGTTTAGAAATGTCTTGAGTGTTGCCCCTATAATGTTCTCGAACAAAATGGCGAGATTGGGAAAAGGGTACATGTTTGAAAGTAAGAGCATGAAGTTACGGACACAAATACCTGCAGAAATGCTTGCAAACATTGACTTAAAATACTTCAATGAATCAACAAGAAA<br>GAAAATCGAAAAAATAAGGCCTCTACTAATAGATGGCACTGCCTCATTGAGTCCTGGAATGATGATGGGCATGTTCAATATGCTGAGTACAGTATTAGGAGTTTCAATCCTAAATCTTGGGCAAAAGAAGTACACCAAAACCACATACTGGTGGGATGGACTCCAATCCTCTGATGATTTCGCCCTCATAGTAAATGCACCGAATCATGAGGGAATACAAGCAGGAGTGGATA<br>GGTTCTATAGGACCTGCAAACTGGTCGGGATCAATATGAGCAAAAAGAAGTCTTACATAAACCGGACTGGAACATTTGAGTTCACAAGCTTTTTCTATCGCTATGGATTTGTGGCTAACTTCAGTATGGAGCTGCCCAGCTTTGGAGTTTCTGGGATCAATGAATCAGCTGACATGAGCATTGGCGTCACAGTGATAAAGAACAACATGATAAACAATGACCTTGGACCAGCAA<br>CAGCTCAAATGGCCCTTCAACTATTCATCAAAGATTACAGGTACACGTACCGATGCCACAGAGGTGATACACAAATTCAAACGAGGAGATCATTCGAGCTGAAGAAGCTGTGGGAACAGACCCGTTCAAAGGCAGGACTGTTGGTGTCAGATGGAGGACCAAATCTATACAACATTCGGAATCTCCATATCCCAGAGGTCTGCCTGAAGTGGGAGCTGATGGACGAAGATTA<br>CCAGGGCAGGTTGTGTAATCCTCTGAACCCATTTGTCAGCCATAGAGAAATTGAGTCCGTAAACAATGCTGTGGTGATGCCAGCCCACGGTCCAGCCAAAAGCATGGAATATGATGCCGTTGCGACTACACACTCATGGATTCCTAAAAGGAATCGTTCCATTCTCAATACCAGTCAAAGGGGAATTCTTGAGGATGAACAGATGTACCAGAAATGCTGCAGTCTATTCGAGAA<br>ATTCTTCCCCAGTAGTTCATACAGGAGACCAGTTGGAATTTCCAGCATGGTGGAGGCCATGGTGTCTAGGGCCCGAATCGATGCACGAATTGATTTCGAATCTGGAAGGATAAAGAAGGAAGAGTTTGCTGAGATCATGAAGATCTGTTCCACCATTGAAGAGCTCAGACGGCAAAAAATAG
EPI_ISL_11922821	>A/swan/Romania/10678_22VIR2749-7/2022_H5N1_2022-02-23<br>ATGGATGTCAATCCGACTTTACTTTTCTTAAAAGTGCCAGCGCAAGATGCCATAAGTACCACATTCCCTTACACTGGAGATCCTCCATACAGCCATGGAACAGGGACAGGATACACAATGGACACAGTCAACAGAACACATCAATACTCAGAGAAGGGAAAATGGACAACAAACACAGAAACTGGAGCACCTCAACTCAACCCAATTGACGGGCCACTACCTGAGGACAACGA<br>ACCGAGCGGATATGCACAAACAGATTGCGTGTTGGAAGCAATGGCTTTCCTTGAAGAGTCCCACCCAGGGATCTTTGAAAACTCTTGTCTTGAAACGATGGAAGTCGTTCAGCAAACAAGAGTGGACAAACTAACTCAAGGTCGCCAGACATATGACTGGACACTGAATAGAAACCAACCAGCTGCAACTGCCCTGGCCAACACTATAGAGGTCTTCAGATCAAACGGTCTAA<br>CAGCCAATGAATCGGGGAGACTAATAGATTTCCTCAAGGATGTGATGGACTCAATGGATAAAGAAGAAATGGAAATAACAACACATTTCCAGAGAAAGAGAAGAGTAAGGGACAACATGACCAAGAAAATGGTCACACAAAGAACAATAGGAAAGAAGAAACAAAGGCTAAACAAGAGGAGCTACTTAATAAGAGCACTGACACTGAATACAATGACAAAAGATGCAGA<br>AAGAGGCAAATTGAAAAGACGGGCGATTGCAACACCAGGGATGCAGATTAGAGGATTTGTGTACTTTGTCGAAACACTGGCAAGGAGCATCTGTGAAAAACTTGAGCAATCTGGACTCCCCGTTGGAGGGAATGAGAAGAAGGCTAAATTGGCAAATGTCGTGAGAAAAATGATGACTAACTCACAAGATACAGAGCTCTCCTTCACAATTACTGGAGATAACACCAAATG<br>GAATGAGAATCAAATCCTCGGATGTTTCTGGCAATGATAACATACATTACAAGAAACCAACCTGAATGGTTTAGAAATGTCTTGAGTATTGCCCCTATAATGTTCTCGAACAAAATGGCGAGATTGGGAAAAGGGTACATGTTTGAAAGTAAGAGCATGAAGTTACGGACACAAATACCTGCAGAAATGCTTGCAAACATTGACTTAAAATACTTCAATGAATCAACAAGAAA<br>GAAAATCGAAAAAATAAGGCCTCTACTAATAGATGGCACTGCCTCATTGAGTCCTGGAATGATGATGGGCATGTTCAATATGCTGAGTACAGTATTAGGAGTTTCAATCCTAAATCTTGGGCAAAAGAAGTACACCAAAACCACATACTGGTGGGATGGACTCCAATCCTCTGATGATTTCGCCCTCATAGTAAATGCACCGAATCATGAGGGAATACAAGCAGGAGTGGATA<br>GGTTCTATAGGACCTGCAAACTGGTCGGGATCAATATGAGCAAAAAGAAGTCTTACATAAACCGGACTGGAACATTTGAGTTCACAAGCTTTTTCTATCGCTATGGATTTGTGGCTAACTTCAGTATGGAGCTGCCCAGCTTTGGAGTTTCTGGGATCAATGAATCAGCTGACATGAGCATTGGCGTCACAGTGATAAAGAACAACATGATAAACAATGACCTTGGACCAGCAA<br>CAGCTCAAATGGCCCTTCAACTATTCATCAAAGATTACAGGTACACGTACCGATGCCACAGAGGTGACACACAAATTCAAACGAGGAGATCATTCGAGCTGAAGAAGCTGTGGGAACAGACCCGTTCAAAGGCAGGACTGTTGGTGTCAGATGGAGGACCAAATCTATACAACATTCGGAATCTCCATATCCCAGAGGTCTGCCTGAAGTGGGAGCTGATGGACGAAGATTA<br>CCAGGGCCGGTTGTGTAATCCTCTGAACCCATTTGTCAGCCATAGAGAAATTGAGTCCGTAAACAATGCTGTGGTGATGCCAGCCCACGGTCCAGCCAAAAGCATGGAATATGATGCCGTTGCGACTACACACTCATGGATTCCTAAAAGGAATCGTTCCATTCTCAATACCAGTCAAAGGGGAATTCTTGAGGATGAACAGATGTACCAGAAATGCTGCAGTCTATTCGAGAA<br>ATTCTTCCCCAGTAGTTCATACAGGAGACCAGTTGGAATTTCCAGCATGGTGGAGGCCATGGTGTCTAGGGCCCGAATCGATGCACGAATTGATTTCGAATCTGGAAGGATCAAGAAGGAAGAGTTTGCTGAGATCATGAAGATCTGTTCCACCATTGAAGAGCTCAGACGGCAAAAAATAG
EPI_ISL_18245819	>A/swan/Poland/MB152/2022_H2N3_2022-07-15<br>ATGGATGTCAATCCGACTTTACTTTTCTTAAAAGTGCCAGCGCAAAATGCCATAAGTACCACATTCCCTTATACTGGAGATCCTCCATACAGCCATGGAACAGGAACAGGATACACCATGGACACAGTCAACAGAACACATCAATACTCGGAAAAGGGGAAATGGACAACAAACACAGAGACTGGAGCACCCCAACTCAACCCAATTGATGGACCACTACCTGAGGACAACGA<br>ACCAAGCGGATATGCACAAACAGATTGCGTGTTGGAAGCAATGGCTTTCCTTGAAGAGTCCCACCCAGGGATCTTTGAAAACTCTTGTCTTGAAACGATGGAAGTCGTTCAGCAAACAAGAGTGGACAAACTAACTCAAGGTCGCCAGACTTATGACTGGACACTGAATAGAAACCAACCAGCTGCAACTGCCCTGGCCAACACTATAGAGGTCTTCAGGTCGAACGGTTTGA<br>CAGCCAATGAATCAGGGAGACTAATAGATTTCCTCAAGGATGTGATGGACTCAATGGACAAAGAAGAAATGGAAATAACAACACATTTCCAGAGAAAGAGAAGAGTAAGGGACAACATGACCAAGAAAATGGTCACACAAAGAACAGTAGGAAAGAAGAAGCAAAGGCTAAATAAGAGGAGCTACTTAATAAGAGCACTGACACTAAATACAATGACAAAGGACGCAGA<br>AAGAGGCAAATTGAAAAGACGGGCAATTGCAACACCAGGGATGCAGATTAGAGGATTTGTGTACTTTGTCGAAACACTGGCAAGGAGCATTTGTGAAAAACTTGAGCAATCTGGACTTCCCGTTGGAGGGAATGAGAAGAAGGCTAAATTGGCAAATGTCGTGAGAAAAATGATGACTAACTCACAAGATACAGAGCTCTCCTTCACAATTACTGGAGATAACACCAAATGG<br>AATGAAAATCAAAATCCTCGGATGTTTCTGGCAATGATAACATACATCACAAGAAACCAACCTGAATGGTTTAGAAATGTCTTGAGCATTGCCCCTATAATGTTCTCGAACAAAATGGCGAGACTGGGAAAAGGATACATGTTTGAAAGCAAAAGCATGAAGCTACGGACACAAATACCTGCAGAAATGCTTGCAAACATTGACTTAAAATACTTCAATGATTCAACAAGAAAG<br>AAAATCGAGAAAATAAGACCTCTAYTAATAGATGGCACCGCCTCATTGAGTCCTGGAATGATGATGGGCATGTTCAATATGCTGAGTACAGTGTTAGGAGTCTCAATCCTGAATCTTGGGCAAAAGAGGTACACCAAGACCACATACTGGTGGGATGGGCTCCAATCCTCTGATGATTTCGCCCTCATAGTGAATGCACCGAATCATGAGGGAATACAAGCAGGAGTGGATAG<br>GTTCTATAGGACCTGCAAACTGGTTGGGATCAACATGAGCAAAAAGAAGTCTTACATAAACCGGACAGGAACATTTGAGTTCACAAGCTTTTTCTATCGCTATGGATTTGTGGCCAATTTCAGTATGGARCTGCCCAGTTTTGGAGTGTCTGGAATCAATGAATCAGCTGACATGAGCATTGGAGTTACAGTGATAAAGAACAACATGATAAACAATGACCTTGGACCAGCAAC<br>AGCTCAAATGGCCTTCAACTATTCATCAAGGACTACAGGTACACGTACCGATGTCACAGGGGTGACACACAGATTCAAACGAGGAGATCATTCGAGCTGAAGAAACTGTGGGAGCAGACCCGTTCCAAGGCAGGACTGTTGGTGTCAGATGGAGGGCCAAATCTATACAACATCCGGAATCTCCATATCCCAGAAGTCTGCCTGAAGTGGGAGCTGATGGACGAAGATTAC<br>CAGGGCAGGTTGTGTAATCCTCTGAACCCATTTGTCAGTCATAAAGAAATTGAATCCGTAAACAATGCTGTGGTAATGCCAGCCCATGGCCCAGCCAAGAGCATGGAATATGATGCTGTTGCGACTACACACTCATGGATTCCCAAAAGGAATCGTTCCATTCTCAATACCAGCCAAAGGGGAATTCTTGAGGATGAGCAGATGTACCAAAAATGCTGCAGTCTATTCGAGAAA<br>TTCTTCCCCAGTAGTTCATACAGGAGACCAGTTGGAATTTCCAGCATGGTGGAGGCCATGGTGTCTAGGGCCCGAATCGACGCACGCATTGATTTCGAGTCTGGAAGGATTAAGAAGGAAGAGTTTGCTGAGATCATGAAGATCTGTTCCACCATTGAAGAGCTCAGACGGCAAAAAATAG
EPI_ISL_846623	>A/swan/Poland/MB141/2020_H5N8_2020-12-17<br>ATGGATGTCAATCCGACTTTACTTTTCTTAAAAGTGCCAGCGCAAAATGCTATAAGTACTACATTCCCTTACACTGGAGATCCCCCATACAGCCATGGAACAGGAACAGGGTATACCATGGACACAGTAAACAGAACACATCAATACTCAGAAAAGGGAAAGTGGACAACAAACACAGAAACCGGAGCACCCCAACTCAACCCAATTGATGGACCATTACCAGAGGACAATGA<br>GCCAAGCGGATATGCACAAACTGATTGCGTGTTGGAAGCAATGGCTTTCCTTGAAGAATCCCACCCAGGGATATTTGAAAACTCTTGTCTTGAAGCGATGGAAATCGTTCAGCAAACAAGAGTGGACAAACTAACCCAAGGTCGCCAGACTTATGACTGGACACTGAACAGAAACCAACCAGCTGCAACCTCTTTGGCCAACACTATAGAGGTGTTCAGATCGAATGGTCTAA<br>CAGCCAATGAATCAGGGAGACTGATAGATTTTCTCAGGGATGTGATGGAATCAATGGATAAAGAAGAGATGGAAGTAACAACACATTTCCAGAGAAAAAGAAGAGTGAGGGACAACATGACTAAGAAGATGGTCACACAAAGAACAATAGGGAAGAAGAAGCAGAGGATGAACAAGAGGAGTTACTTAATAAGAGCACTGACATTGAATACAATGACCAAAGATGCAGA<br>AAGAGGCAAGTTGAAGAGACGGGCAATTGCAACACCCGGGATGCAGATTAGAGGATTCGTGTACTTCGTTGAAACACTAGCGAGGAGCATCTGTGAGAAACTAGAGCAATCTGGGCTCCCTGTTGGAGGGAATGAGAAGAAGGCTAAATTGGCAAATGTTGTGAGAAAAATGATGACTAACTCACAAGATACAGAGCTCTCCTTTACAATTACTGGAGACAACACCAAATG<br>GAATGAGAATCAAACCCTCGGATGTTTTGGCAATGATAACATATATCACAAGAAACCAACCTGAATGGTTTAGAAATGTCTTAAGCATTGCCCCTATAATGTTCTCAAACAAAATGGCGAGATTAGGGAAAGGATACATGTTTGAAAGTAAGAGCATGAAGCTAAGAACACAAATACCAGCAGAGATGCTTACAAATATTGATCTGAAGTATTTCAACGAACCAACGAGAA<br>AGAAAATCGAGAAAATAAGACCTCTGCTGATTGATGGCACGGCCTCATTGAGTCCTGGGATGATGATGGGCATGTTCAATATGCTGAGCACAGTATTAGGGGTCTCAATCCTGAATCTCGGGCAAAAGAGGTACACCAAAACCACATACTGGTGGGATGGACTTCAATCCTCTGATGATTTCGCTCTCATAGTGAATGCACCGAATCATGAGGGGATACAAGCAGGAGTGGAT<br>AGATTCTATAGGACCTGCAAACTGGTTGGGATCAACATGAGCAAAAAGAAGTCTTACATAAACCGAACAGGAACATTTGAGTTCACAAGTTTTTTCTACCGCTATGGATTTGTAGCCAACTTCAGTATGGAATTACCCAGCTTTGGAGTGTCTGGAATCAATGAATCAGCTGACATGAGCATTGGAGTTACAGTGATAAAAAACAATATGATAAACAATGATCTTGGACCAGCA<br>ACAGCTCAAATGGCTCTTCAGTTATTCATCAAAGACTATAGGTACACATACCGATGCCACAGGGGTGATACACAAATTCAAACGAGGAGATCATTCGAGCTGAAGAAGCTGTGGGAGCAGACCCGTTCAAAGGCAGGGCTGTTGATATCAGACGGGGGGCCAAATCTATACAACATTCGGAATCTCCACATCCCAGAGGTCTGCTTGAAGTGGGAGCTGATGGATGAAGACT<br>ACCAAGGCAGGCTGTGCAATCCTCTGAATCCATTTGTCAGTCATAAAGAAATTGAGTCCGTAAACAATGCTGTAGTAATGCCCGCCCATGGCCCGGCCAAGAGCATGGAATATGATGCTGTTGCGACCACACACTCGTGGATTCCTAAGAGGAACCGTTCCATTCTCAATACCAGCCAAAGGGGAATTCTTGAGGACGAGCAGATGTACCAAAAGTGCTGTAGTCTATTCGAGA<br>AATTCTTCCCCAGCAGTTCATACAGGAGGCCAGTTGGAATTTCCAGCATGGTGGAGGCCATGGTGTCTAGGGCCCGAATTGATGCACGCATCGACTTCGAATCTGGAAGGATTAAGAAAGAAGAGTTTGCTGAGATCATGAAGATCTGTTCCACCATTGAAGAGCTCAGACGGCAAAAAATAG
EPI_ISL_18244493	>A/swan/Poland/MB122-21_22VIR5675-9/2021_H5N8_2021-02-18<br>ATGGATGTCAATCCGACTTTGCTTTTCTTAAAAGTGCCAGCGCAAAATGCTATAAGTACTACATTCCCTTACACTGGAGATCCCCCATACAGCCATGGAACAGGAACAGGGTATACCATGGACACAGTAAACAGAACACATCAATATTCAGAAAAGGGAAAGTGGACAACAAACACAGAAACCGGAGCACCCCAACTCAACCCAATTGATGGACCATTACCAGATGACAATGA<br>GCCAAGCGGATATGCACAAACTGATTGCGTGTTGGAAGCAATGGCTTTCCTTGAAGAATCCCACCCAGGGATATTTGAAAACTCTTGTCTTGAAGCGATGGAAATCGTTCAGCAAACAAGAGTGGACAAACTAACCCAAGGTCGCCAGACTTATGACTGGACACTGAACAGAAACCAACCAGCTGCAACCTCTTTGGCCAACACTATAGAGGTGTTCAGATCGAATGGTCTGA<br>CAGCCAATGAATCAGGGAGACTGATAGATTTTCTCAGGGATGTGATGGAATCAATGGATAAAGAAGAGATGGAAGTAACAACACATTTCCAGAGAAAAAGAAGAGTGAGGGACAACATGACTAAGAAGATGGTCACACAAAGAACAATAGGGAAGAAGAAGCAGAGGCTGAACAAGAGGAGTTACTTAATAAGAGCACTGACATTGAATACAATGACCAAAGATGCAGA<br>AAGAGGCAAGTTGAAGAGACGGGCAATTGCAACACCCGGGATGCAGATTAGAGGATTCGTGTACTTCGTTGAAACACTAGCGAGGAGCATCTGTGAGAAACTAGAGCAATCTGGGCTCCCTGTTGGAGGGAATGAGAAAAAGGCTAAATTGGCAAATGTTGTGAGAAAAATGATGACTAACTCACAAGATACAGAGCTCTCCTTTACAATTACTGGAGACAACACCAAATG<br>GAATGAGAATCAAACCCTCGGATGTTTTTGGCAATGATAACATATATCACAAGAAACCAACCTGAATGGTTTAGAAATGTCTTAAGCATTGCCCCTATAATGTTCTCAAACAAAATGGCGAGATTAGGGAAAGGATACATGTTTGAAAGTAAGAGCATGAAGCTAAGAACACAAATACCAGCAGAGATGCTTACAAATATTGATCTGAAGTATTTCAACGAACCAACGAGAA<br>AGAAAATCGAGAAAATAAGACCTCTGCTGATTGATGGCACGGCTTCATTGAGCCCTGGGATGATGATGGGCATGTTCAATATGCTGAGCACAGTATTAGGGGTCTCAATCCTGAATCTCGGGCAAAAGAGGTACACCAAAACCACATACTGGTGGGATGGACTTCAATCCTCTGATGATTTCGCTCTCATAGTGAATGCACCGAATCATGAGGGGATACAAGCAGGAGTGGAT<br>AGATTCTATAGGACCTGCAAACTGGTTGGGATCAACATGAGCAAAAAGAAGTCTTACATAAACCGAACAGGAACATTTGAGTTCACAAGTTTTTTCTACCGCTATGGATTTGTAGCCAACTTCAGTATGGAATTACCCAGCTTTGGAGTGTCTGGAATCAATGAATCAGCTGACATGAGCATTGGAGTTACAGTGATAAAAAACAACATGATAAACAATGATCTTGGACCAGCA<br>ACAGCTCAAATGGCTCTTCAGTTATTCATCAAAGACTATAGGTACACATACCGATGCCACAGGGGTGATACACAAATTCAAACGAGGAGATCATTCGAGCTGAAGAAGCTGTGGGAGCAGACCCGTTCAAAGGCAGGACTGTTGATATCAGACGGGGGGCCAAACCTATACAACATTCGGAATCTCCACATCCCAGAGGTCTGCTTGAAGTGGGAGCTGATGGATGAAGACT<br>ACCAAGGCAGGCTGTGCAATCCTCTGAATCCATTTGTCAGTCATAAAGAGATTGAGTCCGTAAACAATGCTGTAGTAATGCCCGCCCATGGCCCGGCCAAGAGCATGGAATATGATGCTGTTGCGACCACACACTCGTGGATTCCTAAGAGGAACCGTTCCATCCTCAATACCAGCCAAAGGGGAATTCTTGAGGATGAGCAGATGTACCAAAAGTGCTGTAGTCTATTCGAGA<br>AATTCTTCCCCAGCAGTTCATACAGGAGGCCAGTTGGAATTTCCAGCATGGTGGAGGCCATGGTGTCTAGGGCCCGAATTGATGCACGCATCGACTTCGAATCTGGAAGGATTAAGAAAGAAGAGTTTGCTGAGATCATGAAGATCTGTTCCACCATTGAAGAGCTCAGACGGCAAAAAATAG
internal NVRI repository	>A/swan/Poland/MB097/2021_H5N8_2021-02-12<br>ATGGATGTCAATCCGACTTTACTTTTCTTAAAAGTGCCAGCGCAAAATGCTATAAGTACTACATTCCCTTACACTGGAGATCCTCCATACAGCCATGGAACAGGAACAGGGTATACCATGGACACAGTAAACAGAACACATCAATACTCAGAAAAGGGAAAGTGGACAACAAACACAGAAACCGGAGCACCCCAACTCAACCCAATTGATGGACCATTACCAGAGGACAATGA<br>GCCAAGCGGATATGCACAAACTGATTGCGTGTTGGAAGCAATGGCTTTCCTTGAAGAATCCCACCCAGGGATATTTGAAAACTCTTGTCTTGAARCGATGGAAATCGTTCAGCAAACAAGAGTGGACAAACTAACCCAAGGTCGCCAGACTTATGACTGGACACTGAACAGAAACCAACCAGCTGCAACCTCTTTGGCCAACACTATAGAGGTGTTCAGATCGAATGGTCTGAC<br>AGCCAATGAATCAGGGAGACTGATAGATTTTCTCAGGGATGTGATGGAATCAATGGATAAAGAAGAGATGGAAGTAACAACACATTTCCAGAGAAAAAGAAGAGTGAGGGACAACATGACTAAGAAGATGGTCACACAAAGAACAATAGGGAAGAAGAAGCAGAGGCTGAACAAGAGGAGTTACTTAATAAGAGCACTGACATTGAATACAATGACCAAAGATGCAGAA<br>AGAGGCAAGTTGAAGAGACGGGCAATTGCAACACCCGGGATGCAGATTAGAGGATTCGTGTACTTCGTTGAAACACTAGCGAGGAGCATCTGTGAGAAACTAGAGCAATCTGGGCTCCCTGTTGGAGGGAATGAGAAGAAGGCTAAATTGGCAAATGTTGTGAGAAAAATGATGACTAACTCACAAGATACAGAGCTCTCCTTTACAATTACTGGAGACAACACCAAATGG<br>AATGAGAATCAAACCCTCGGATGTTTTTGGCAATGATAACATATATCACAAGAAACCAACCTGAATGGTTTAGAAATGTCTTAAGCATTGCCCCTATAATGTTCTCAAACAAAATGGCGAGATTAGGGAAAGGATACATGTTTGAAAGTAAGAGCATGAAGCTAAGAACACAAATACCAGCAGAGATGCTTACAAATATCGATCTGAAGTATTTCAACGAACCAACGAGAAA<br>GAAAATCGAGAAAATAAGACCTCTGCTGATTGATGGCACGGCCTCATTGAGTCCTGGGATGATGATGGGCATGTTCAATATGCTGAGCACAGTATTAGGGGTCTCAATCCTGAATCTCGGGCAAAAAAGGTACACCAAAACCACATACTGGTGGGATGGACTTCAATCCTCTGATGATTTCGCTCTCATAGTGAATGCACCGAATCATGAGGGGATACAAGCAGGAGTGGATA<br>GATTCTATAGGACCTGCAAACTGGTTGGGATCAACATGAGCAAAAAGAAGTCTTACATAAACCGAACAGGAACATTTGAGTTCACAAGTTTTTTCTACCGCTATGGATTTGTAGCTAACTTCAGTATGGAATTACCCAGCTTTGGAGTGTCTGGAATCAATGAATCAGCTGACATGAGCATTGGAGTTACAGTGATAAAAAACAATATGATAAACAATGATCTTGGACCAGCAAC<br>AGCTCAAATGGCTCTTCAGTTATTCATCAAAGACTATAGGTACACATACCGATGCCACAGGGGTGATACACAAATTCAAACGAGGAGATCATTCGAGCTGAAGAAGCTGTGGGAGCAGACCCGCTCAAAGGCAGGGCTGTTGATATCAGACGGGGGGCCAAACCTATACAACATTCGGAATCTCCACATCCCAGAGGTCTGCTTGAAGTGGGAGTTGATGGATGAAGACTAC<br>CAAGGCAGGCTGTGCAATCCTCTGAATCCATTTGTCAGTCATAAAGAGATTGAGTCCGTAAACAATGCTGTAGTAATGCCCGCCCATGGCCCGGCCAAGAGCATGGAATATGATGCTGTTGCGACCACACACTCGTGGATTCCTAAGAGGAACCGTTCCATTCTCAATACCAGCCAAAGGGGAATTCTTGAGGATGAGCAGATGTACCAAAAGTGCTGTAGTCTATTCGAGAAA<br>TTCTTCCCCAGCAGTTCATACAGGAGGCCAGTTGGAATTTCCAGCATGGTGGAGGCCATGGTGTCTAGGGCCCGAATTGATGCACGCATCGACTTCGAATCTGGAAGGATTAAGAAAGAAGAGTTTGCTGAGATCATGAAGATCTGTTCCACCATTGAAGAGCTCAGACGGCAAAAAATAG
EPI_ISL_11922814	>A/swan/Poland/MB083_22VIR2515-8/2022_H5N1_2022-02-18<br>ATGGATGTCAATCCGACTTTACTTTTCTTAAAAGTGCCAGCGCAAGATGCCATAAGTACCACATTCCCTTACACTGGAGATCCTCCATACAGCCATGGAACAGGGACAGGATACACAATGGACACAGTCAACAGAACACATCAATACTCAGAGAAGGGAAAATGGACAACAAACACAGAAACCGGAGCACCTCAACTCAACCCAATTGATGGGCCACTACCTGAGGACAACGA<br>ACCGAGCGGATATGCACAAACAGATTGCGTGTTGGAAGCAATGGCTTTCCTTGAAGAGTCCCACCCAGGGATCTTTGAAAACTCTTGTCTTGAAACGATGGAAGTCGTTCAGCAAACAAGAGTGGACAAACTAACTCAAGGTCGCCAGACATATGACTGGACACTGAATAGAAACCAACCAGCTGCAACTGCCCTGGCCAACACTATAGAGGTCTTCAGATCAAACGGTCTAA<br>CAACCAATGAATCGGGGAGACTAATAGATTTCCTCAAGGATGTGATGGACTCAATGGATAAAGAAGAAATGGAAATAACAACACATTTCCAGAGAAAGAGAAGAGTAAGGGACAACATGACCAAGAAAATGGTCACACAAAGAACAATAGGAAAGAAGAAACAAAGGCTAAACAAGAGGAGCTACTTAATAAGAGCACTGACACTGAATACAATGACAAAAGATGCAGA<br>AAGAGGCAAATTGAAAAGACGGGCGATTGCAACACCAGGGATGCAGATTAGGGGATTTGTGTACTTTGTCGAAACACTGGCAAGGAGCATCTGTGAAAAACTTGAGCAATCTGGACTCCCCGTTGGAGGGAATGAGAAGAAGGCTAAATTGGCAAATGTCGTGAGAAAAATGATGACTAACTCACAAGATACAGAGCTCTCCTTCACAATTACTGGAGATAACACCAAATG<br>GAATGAGAATCAAATCCTCGGATGTTTCTGGCAATGATAACATACATTACAAGAAACCAACCTGAATGGTTTAGAAATGTCTTGAGTATTGCCCCTATAATGTTCTCGAACAAAATGGCGAGATTGGGAAAAGGGTACATGTTTGAAAGTAAGAGCATGAAGTTACGGACACAAATACCTGCAGAAATGCTTGCAAACATTGACTTAAAATACTTCAATGAATCAACAAGAAA<br>GAAAATCGAAAAAATAAGGCCTCTACTAATAGATGGCACTGCCTCATTGAGTCCTGGAATGATGATGGGCATGTTCAATATGCTGAGTACAGTATTAGGAGTTTCAATCCTAAATCTTGGGCAAAAGAAGTACACCAAAACAACATACTGGTGGGATGGACTCCAATCCTCTGATGATTTCGCCCTCATAGTAAATGCACCGAATCATGAGGGAATACAAGCAGGAGTGGATA<br>GGTTCTATAGGACCTGCAAACTGGTCGGGATCAATATGAGCAAAAAGAAGTCTTACATAAACCGGACTGGAACATTTGAGTTCACAAGCTTTTTCTATCGCTATGGATTTGTGGCTAACTTCAGTATGGAGCTGCCCAGCTTTGGAGTTTCTGGGATCAATGAATCAGCTGACATGAGCATTGGCATCACAGTGATAAAGAACAACATGATAAACAATGACCTTGGACCAGCAA<br>CAGCTCAAATGGCCCTTCAACTATTCATCAAAGATTACAGGTACACGTACCGATGCCACAGAGGTGACACACAAATTCAAACGAGGAGATCATTCGAGCTGAAGAAGCTGTGGGAACAGACCCGTTCAAAGGCAGGACTGTTGGTGTCAGATGGAGGACCAAATCTATACAACATTCGGAATCTCCATATCCCAGAGGTCTGCCTGAAGTGGGAGCTGATGGACGAAGATTA<br>CCAGGGCAGGTTGTGTAATCCTCTGAACCCATTTGTCAGCCATAGAGAAATTGAGTCCGTAAACAATGCTGTGGTGATGCCAGCCCACGGTCCAGCCAAAAGCATGGAATATGATGCCGTTGCGACTACACACTCATGGATTCCTAAAAGGAATCGTTCCATTCTCAATACCAGTCAAAGGGGAATTCTTGAGGATGAACAGATGTACCAGAAATGCTGCAGTCTATTCGAGAA<br>ATTCTTCCCCAGTAGTTCATACAGGAGACCAGTTGGAATTTCCAGCATGGTGGAGGCCATGGTGTCTAGGGCCCGAATCGATGCACGCATTGATTTCGAATCTGGAAGGATCAAGAAGGAAGAGTTTGCTGAGATCATGAAGATCTGTTCCACCATTGAAGAGCTCAGACGGCAAAAAATAG

EPI_ISL_11922813	>A/swan/Poland/MB078_22VIR2515-7/2022_H5N1_2022-02-15<br>ATGGATGTCAATCCGACTTTACTTTTCTTAAAAGTGCCAGCGCAAGATGCCATAAGTACCACATTCCCTTACACTGGAGATCCTCCATACAGCCATGGAACAGGGACAGGATACACAATGGACACAGTCAACAGAACACATCAATACTCAGAGAAGGGAAAATGGACAACAAACACAGAAACCGGAGCACCTCAACTCAACCCAATTGATGGGCCACTACCTGAGGACAACGA<br>ACCGAGCGGATATGCACAAACAGATTGCGTGTTGGAAGCAATGGCTTTCCTTGAAGAGTCCCACCCAGGGATCTTTGAAAACTCTTGTCTTGAAACGATGGAAGTCGTTCAGCAAACAAGAGTGGACAAACTAACTCAAGGTCGCCAGACATATGACTGGACACTGAATAGAAACCAACCAGCTGCAACTGCCCTGGCCAACACTATAGAGGTCTTCAGATCAAACGGTCTAA<br>CAGCCAATGAATCGGGGAGACTAATAGATTTCCTCAAGGATGTGATGGACTCAATGGATAAAGAAGAAATGGAAATAACAACACATTTCCAGAGAAAGAGAAGAGTAAGGGACAACATGACCAAGAAAATGGTCACACAAAGAACAATAGGAAAGAAGAAACAAAGGCTAAACAAGAGGAGCTACTTAATAAGAGCACTGACACTGAATACAATGACAAAAGATGCAGA<br>AAGAGGCAAATTGAAAAGACGGGCGATTGCAACACCAGGGATGCAGATTAGAGGATTTGTGTACTTTGTCGAAACACTGGCAAGGAGCATCTGTGAAAAACTTGAGCAATCTGGACTCCCCGTTGGAGGGAATGAGAAGAAGGCTAAATTGGCAAATGTCGTGAGAAAAATGATGACTAACTCACAAGATACAGAGCTCTCCTTCACAATTACTGGAGACAACACCAAATG<br>GAATGAGAATCAAAATCCTCGGATGTTTCTGGCAATGATAACATACATTACAAGAAACCAACCTGAATGGTTTAGAAATGTCTTGAGTATTGCCCCTATAATGTTCTCGAACAAAATGGCGAGATTGGGAAAAGGGTACATGTTTGAAAGTAAGAGCATGAAGTTACGGACACAAATACCTGCAGAAATGCTTGCAAACATTGACTTAAAATACTTCAATGAATCAACAAGAAA<br>GAAAATCGAAAAAATAAGGCCTCTACTAATAGATGGCACTGCCTCATTGAGTCCTGGAATGATGATGGGCATGTTCAATATGCTGAGTACAGTATTAGGAGTTTCAATCCTAAATCTTGGGCAAAAGAAGTACACCAAAACAACATACTGGTGGGATGGACTCCAATCCTCTGATGATTTCGCCCTCATAGTAAATGCACCGAATCATGAGGGAATACAAGCAGGAGTGGATA<br>GGTTCTATAGGACCTGCAAACTGGTCGGGATCAATATGAGCAAAAAGAAGTCTTACATAAACCGGACTGGAACATTTGAGTTCACAAGCTTTTTCTATCGCTATGGATTTGTGGCTAACTTCAGTATGGAGCTGCCCAGCTTTGGAGTTTCTGGGATCAATGAATCAGCTGACATGAGCATTGGCGTCACAGTGATAAAGAACAACATGATAAACAATGACCTTGGACCAGCAA<br>CAGCTCAAATGGCCCTTCAACTATTCATCAAAGATTACAGGTACACGTACCGATGCCACAGAGGTGACACACAAATTCAAACGAGGAGATCATTCGAGCTGAAGAAGCTGTGGGAACAGACCCGTTCAAAGGCAGGACTGTTGGTGTCAGATGGAGGACCAAATCTATACAACATTCGGAATCTCCATATCCCAGAGGTCTGCCTGAAGTGGGAGCTGATGGACAAAGATTA<br>CCAGGGCAGGTTGTGTAATCCTCTGAACCCATTTGTCAGCCATAGAGAAATTGAGTCCGTAAACAATGCTGTGGTGATGCCAGCCCACGGTCCAGCCAAAAGCATGGAATATGATGCCGTTGCGACTACACACTCATGGATTCCTAAAAGGAATCGTTCCATTCTCAATACCAGTCAAAGGGGAATTCTTGAGGATGAACAGATGTACCAGAAATGCTGCAGTCTATTCGAGAA<br>ATTCTTCCCCAGTAGTTCATACAGGAGACCAGTTGGAATTTCCAGCATGGTGGAGGCCATGGTGTCTAGGGCCCGAATCGATGCACGCATTGATTTCGAATCTGGAAGGATCAAGAAGGAAGAGTTTGCTGAGATCATGAAGATCTGTTCCACCATTGAAGAGCTCAGACGGCAAAAATAG
EPI_ISL_18245790	>A/swan/Poland/MB058-22_22VIR5675-2/2022_H5N1_2022-02-03<br>ATGGATGTCAATCCGACTTTACTTTTCTTAAAAGTGCCAGCGCAAGATGCCATAAGTACCACATTCCCTTACACTGGAGATCCTCCATACAGCCATGGAACAGGGACAGGATACACAATGGACACAGTCAACAGAACACATCAATACTCAGAGAAGGGAAAATGGACAACAAACACAGAAACCGGAGCACCTCAACTCAACCCAATTGATGGGCCACTACCTGAGGACAACGA<br>ACCGAGCGGATATGCACAAACAGATTGCGTGTTGGAAGCAATGGCTTTCCTTGAAGAGTCCCACCCAGGGATCTTTGAAAACTCTTGTCTTGAAACGATGGAAGTCGTTCAGCAAACAAGAGTGGACAAACTAACTCAAGGTCGCCAGACATATGACTGGACACTGAATAGAAACCAACCAGCTGCAACTGCCCTGGCCAACACTATAGAGGTCTTCAGATCAAATGGTCTAA<br>CAGCCAATGAATCGGGGAGACTAATAGATTTCCTCAAGGATGTGATGGACTCAATGGATAAAGAAGAAATGGAAATAACAACACATTTCCAGAGAAAGAGAAGAGTAAGGGACAACATGACCAAGAAAATGGTCACACAAAGAACAATAGGAAAGAAGAAACAAAGGCTAAACAAGAGGAGCTACTTAATAAGAGCACTGACACTGAATACAATGACAAAAGATGCAGA<br>AAGAGGCAAATTGAAAAGACGGGCGATTGCAACACCAGGGATGCAGATTAGAGGATTTGTATACTTTGTCGAAACACTGGCAAGGAGCATCTGTGAAAAACTTGAGCAATCTGGACTCCCCGTTGGAGGGAATGAGAAGAAGGCTAAATTGGCAAATGTCGTGAGAAAAATGATGACTAACTCACAAGATACAGAGCTCTCCTTCACAATTACTGGAGATAACACCAAATG<br>GAATGAGAATCAAAATCCTCGGATGTTTCTGGCAATGATAACATACATTACAAGAAACCAACCTGAATGGTTTAGAAATGTCTTGAGTATTGCCCCTATAATGTTCTCGAACAAAATGGCGAGATTGGGAAAAGGGTACATGTTTGAAAGTAAGAGCATGAAGTTACGGACACAAATACCTGCAGAAATGCTTGCAAACATTGACTTAAAATACTTCAATGAATCAACAAGAAA<br>GAAAATCGAAAAAATAAGGCCTCTACTAATAGATGGCACCGCCTCATTGAGTCCTGGAATGATGATGGGCATGTTCAATATGCTGAGTACAGTATTAGGAGTTTCAATCCTAAATCTTGGGCAAAAGAAGTACACCAAAACCACATACTGGTGGGATGGACTCCAATCCTCTGATGATTTCGCCCTCATAGTAAATGCACCGAATCATGAGGGAATACAAGCAGGAGTGGATA<br>GGTTCTATAGGACCTGCAAACTGGTCGGGATCAATATGAGCAAAAAGAAGTCTTACATAAACCGGACTGGAACATTTGAGTTCACAAGCTTTTTCTATCGCTATGGATTTGTGGCTAACTTCAGTATGGAGCTGCCCAGCTTTGGGGTTTCTGGGATCAATGAATCAGCTGACATGAGCATTGGCGTCACAGTGATAAAGAACAACATGATAAACAATGACCTTGGACCAGCAA<br>CAGCTCAAATGGCCCTTCAACTATTCATCAAAGATTACAGGTACACGTACCGATGCCACAGAGGTGACACACAAATTCAAACGAGGAGATCATTCGAGCTGAAGAAGCTGTGGGAACAGACCCGTTCAAAGGCAGGACTGTTGGTGTCAGATGGAGGACCAAATCTATACAACATTCGGAATCTCCATATCCCAGAGGTCTGCCTGAAGTGGGAGCTGATGGACGAAGATTA<br>CCAGGGCAGGTTGTGTAATCCTCTGAACCCATTTGTCAGCCATAGAGAAATTGAGTCCGTAAACAATGCTGTGGTGATGCCAGCCCACGGTCCAGCCAAAAGCATGGAATATGATGCCGTTGCGACTACACACTCATGGATTCCTAAAAGGAATCGTTCCATTCTCAATACCAGTCAAAGGGGAATTCTTGAGGATGAACAGATGTACCAGAAATGCTGCAGTCTATTCGAGAA<br>ATTCTTCCCCAGTAGTTCATACAGGAGACCAGTTGGAATTTCCAGCATGGTGGAGGCCATGGTGTCTAGGGCCCGAATCGATGCACGCATTGATTTCGAATCTGGAAGGATCAAGAAGGAAGAGTTTGCTGAGATCATGAAGATCTGTTCCACCATTGAAGAGCTCAGACGGCAAAAATAG
EPI_ISL_1719911	>A/swan/Lithuania/1842PG1_21VIR2606-5/2021_H5N8_2021-03-19<br>ATGGATGTCAATCCGACTTTACTTTTCTTAAAAGTGCCAGCGCAAAATGCTATAAGTACTACATTCCCTTACACTGGAGATCCTCCATACAGCCATGGAACAGGAACAGGGTATACCATGGACACAGTAAACAGAACACATCAATACTCAGAAAAGGGAAAGTGGACAACAAACACAGAAACCGGAGCACCCCAACTCAACCCAATTGATGGACCATTACCAGAGGACAATGA<br>GCCAAGCGGATATGCACAAACTGATTGCGTGTTGGAAGCAATGGCTTTCCTTGAAGAATCCCACCCAGGGATATTTGAAAACTCTTGTCTTGAAGCGATGGAAATCGTTCAGCAAACAAGAGTGGACAAACTAACCCAAGGTCGCCAGACTTATGACTGGACACTGAACAGAAACCAACCAGCTGCAACCTCTTTGGCCAACACTATAGAGGTGTTCAGATCGAATGGTCTGA<br>CAGCCAACGAATCAGGGAGACTGATAGATTTTCTCAGGGATGTGATGGAATCAATGGATAAAGAAGAGATGGAAGTAACAACACATTTCCAGAGAAAAAGAAGAGTGAGGGACAACATGACTAAGAAGATGGTCACACAAAGAACAATAGGGAAGAAGAAGCAGAGGCTGAACAAGAGGAGTTACTTAATAAGAGCACTGACATTGAATACAATGACCAAAGATGCAGA<br>AAGAGGCAAGTTGAAGAGACGGGCAATTGCAACACCCGGGATGCAGATTAGAGGATTCGTGTACTTCGTTGAAACACTAGCGAGGAGCATCTGTGAGAAACTAGAGCAATCTGGGCTCCCTGTTGGAGGGAATGAGAAGAAGGCTAAATTGGCAAATGTTGTGAGAAAAATGATGACTAACTCACAAGATACAGAGCTCTCCTTTACAATTACTGGAGACAACACCAAATG<br>GAATGAGAATCAAAACCCTCGGATGTTTTTGGCAATGATAACATATATCACAAGAAACCAACCTGAATGGTTTAGAAATGTCTTAAGCATTGCCCCTATAATGTTCTCAAACAAAATGGCGAGATTAGGGAAAGGATACATGTTTGAAAGTAAGAGCATGAAGCTAAGAACACAAATACCAGCAGAGATGCTTACAAATATCGATCTGAAGTATTTCAACGAACCAACGAGAA<br>AGAAAATCGAGAAAATAAGACCTCTGCTGATTGATGGCACGGCCTCATTGAGTCCTGGGATGATGATGGGCATGTTCAATATGCTGAGCACAGTATTAGGGGTCTCAATCCTGAATCTCGGGCAAAAAAGGTACACCAAAACCACATACTGGTGGGATGGACTTCAATCCTCTGATGATTTCGCTCTCATAGTGAATGCACCGAATCATGAGGGGATACAAGCAGGAGTGGAT<br>AGATTCTATAGGACCTGCAAACTGGTTGGGATCAACATGAGCAAAAAGAAGTCTTACATAAACCGAACAGGAACATTTGAGTTCACAAGTTTTTTCTACCGCTATGGATTTGTAGCTAACTTCAGTATGGAATTACCCAGCTTTGGAGTGTCTGGAATCAATGAATCAGCTGACATGAGCATTGGAGTTACAGTGATAAAAAACAATATGATAAACAATGATCTTGGACCAGCA<br>ACAGCTCAAATGGCTCTTCAGTTATTCATCAAAGACTATAGGTACACATACCGATGCCACAGGGGTGATACACAAATTCAAACGAGGAGATCATTCGAGCTGAAGAAGCTGTGGGAGCAGACCCGTTCAAAGGCAGGGCTGTTGATATCAGACGGGGGGCCAAACCTATACAACATTCGGAATCTCCACATCCCAGAGGTCTGCTTGAAGTGGGAGTTGATGGATGAAGACT<br>ACCAAGGCAGGCTGTGCAATCCTCTGAATCCATTTGTCAGTCATAAAGAGATTGAGTCCGTAAACAATGCTGTAGTAATGCCCGCCCATGGCCCGGCCAAGAGCATGGAATATGATGCTGTTGCGACCACACACTCGTGGATTCCTAAGAGGAACCGTTCCATTCTCAATACCAGCCAAAGGGGAATTCTTGAGGATGAGCAGATGTACCAAAAGTGCTGTAGTCTATTCGAGA<br>AATTCTTCCCCAGCAGTTCATACAGGAGGCCAGTTGGAATTTCCAGCATGGTGGAGGCCATGGTGTCTAGGGCTCGAATTGATGCACGCATCGACTTCGAATCTGGAAGGATTAAGAAAGAAGAGTTTGCTGAGATCATGAAGATCTGTTCCACCATTGAAGAGCTCAGACGGCAAAAATAG
EPI_ISL_10434073	>A/swan/Germany-BW/AI00997/2022_H5N2_2022-01-30<br>ATGGATGTCAATCCGACTTTACTTTTCTTAAAAGTGCCAGCGCAAGATGCCATAAGTACCACATTCCCTTACACTGGAGATCCTCCATACAGCCATGGAACAGGGACAGGATACACAATGGACACAGTCAACAGAACACATCAATACTCAGAGAAGGGAAAATGGACAACAAACACAGAAACCGGAGCACCTCAACTCAACCCAATTGATGGGCCACTACCTGAGGACAACGA<br>ACCGAGCGGATATGCACAAACAGATTGCGTGTTGGAAGCAATGGCTTTCCTTGAAGAGTCCCACCCAGGGATCTTTGAAAACTCTTGTCTTGAAACGATGGAAGTCGTTCAGCAAACAAGAGTGGACAAACTAACTCAAGGTCGCCAGACATATGACTGGACACTGAATAGAAACCAACCAGCTGCAACTGCCCTGGCCAACACTATAGAGGTCTTCAGATCAAACGGTCTAA<br>CAGCCAATGAATCGGGGAGACTAATAGATTTCCTCAAGGATGTGATGGATTCAATGGATAAAGAAGAAATGGAAATAACAACACATTTCCAGAGAAAGAGAAGAGTAAGGGACAACATGACCAAGAAAATGGTCACACAAAGAACAATAGGAAAGAAGAAACAAAGGCTAAACAAGAGGAGCTACTTAATAAGAGCACTGACACTGAATACAATGACAAAAGATGCAGA<br>AAGAGGCAAATTGAAAAGACGGGCGATTGCAACACCAGGGATGCAGATTAGAGGATTTGTGTACTTTGTCGAAACACTGGCAAGGAGCATCTGTGAAAAACTTGAGCAATCTGGACTCCCCGTTGGAGGGAATGAGAAGAAGGCTAAATTGGCAAATGTCGTGAGAAAAATGATGACTAACTCACAAGATACAGAGCTCTCCTTCACAATTACTGGAGATAACACCAAATG<br>GAATGAGAATCAAAATCCTCGGATGTTTCTGGCAATGATAACATACATTACAAGAAACCAACCTGAATGGTTTAGAAATGTCTTGAGTATTGCCCCTATAATGTTCTCGAACAAAATGGCGAGACTGGGAAAAGGGTACATGTTTGAAAGTAAGAGCATGAAGTTACGGACACAAATACCTGCAGAAATGCTTGCAAACATTGACTTAAAATACTTCAATGAATCAACAAGAAA<br>GAAAATCGAAAAAATAAGGCCTCTACTAATAGATGGCACTGCCTCATTGAGTCCTGGAATGATGATGGGCATGTTCAATATGCTGAGTACAGTATTAGGAGTTTCAATCCTAAATCTTGGGCAAAAGAAGTACACCAAAACCACATACTGGTGGGATGGACTCCAATCCTCTGATGATTTCGCCCTCATAGTAAATGCACCGAATCATGAGGGAATACAAGCAGGAGTGGATA<br>GGTTCTATAGGACCTGCAAACTGGTCGGGATCAATATGAGCAAAAAGAAGTCTTACATAAACCGGACTGGAACATTTGAGTTCACAAGCTTTTTCTATCGCTATGGATTTGTGGCTAACTTCAGTATGGAGCTGCCCAGCTTTGGAGTTTCTGGGATCAATGAATCAGCTGACATGAGCATTGGCGTAACAGTGATAAAGAACAACATGATAAACAATGACCTTGGACCAGCAA<br>CAGCTCAAATGGCTCTTCAACTATTCATCAAAGATTACAGGTACACGTACCGATGCCACAGAGGTGACACACAAATTCAAACGAGGAGATCATTCGAGCTGAAGAAGCTGTGGGAACAGACCCGTTCAAAGGCAGGACTGTTGGTGTCAGATGGAGGACCTAATCTATACAACATTCGGAATCTCCATATCCCAGAGGTCTGCCTGAAGTGGGAGCTGATGGACGAAGATTAC<br>CAGGGCAGGTTGTGTAATCCTCTGAACCCATTTGTCAGCCATAGAGAGATTGAGTCCGTAAACAATGCTGTGGTGATGCCAGCCCACGGTCCAGCCAAAAGCATGGAATATGATGCCGTTGCGACTACACACTCATGGATTCCTAAAAGGAATCGTTCCATTCTCAATACCAGTCAAAGAGGAATTCTTGAGGATGAACAGATGTACCAGAAATGCTGCAGTCTATTCGAGAAA<br>TTCTTCCCCAGTAGTTCATACAGGAGACCAGTTGGAATTTCCAGCATGGTGGAGGCCATGGTGTCTAGGGCCCGAATCGATGCACGAATTGATTTCGAATCTGGGAGGATCAAGAAGGAAGAGTTTGCTGAGATCATGAAGATCTGTTCCACCATTGAAGAGCTCAGACGGCAAAAATAG
EPI_ISL_331306	>A/shoveler/Ubinskoe_Lake/43/2017_H12N5_2017-09-02<br>ATGGATGTCAATCCGACTTTACTTTTCTTAAAAGTGCCAGCGCAAAATGCTATAAGTACTACGTTTCCTTACACTGGAGATCCTCCATACAGCCATGGAACAGGAACAGGATACACCATGGACACAGTCAACAGAACACATCAATACTCAGAGAAGGGAAAGTGGACAACAAACACAGAGACTGGGGCGCCCCAACTCAACCCAATTGATGGACCATTGCCAGAGGACAACGA<br>GCCAAGCGGATATGCACAAACAGATTGCGTGTTGGAAGCAATGGCTTTCCTTGAAGAATCCCACCCAGGGATCTTTGAAAACTCTTGTCTTGAAACGATGGAGGTCGTTCAGCAAACAAGAGTGGACAAACTAACCCAAGGTCGCCAGACTTATGATTGGACACTGAATAGAAACCAGCCAGCTGCAACTGCTTTGGCCAATACTATAGAGGTCTTCAGATCGAACGGTCTAA<br>CAGCCAATGAGTCGGGGAGACTAATAGATTTCCTCAAGGATGTGATGGAATCAATGGATAGGGAAGAAATGGAAATAACAACACATTTCCAGAGAAAGAGAAGAGTAAGGGACAACATGACCAAGAAGATGGTCACACAAAGAACGATAGGAAAGAAGAAGCAGAGGCTGAACAAGAGGAGCTACTTAATAAGAGCACTGACATTGAACACAATGACCAAAGATGCAGA<br>AAGAGGCAAATTGAAGAGGCGGGCAATTGCAACACCCGGGATGCAGATTAGAGGATTTGTGTACTTTGTCGAAACACTAGCAAGGAGCATCTGTGAGAAACTCGAGCAATCTGGACTCCCTGTTGGAGGGAATGAGAAAAAGGCCAAATTGGCAAATGTCGTGAGAAAAATGATGACTAACTCGCAAGATACAGAGCTCTCCTTTACAATTACTGGAGACAACACCAAATG<br>GAATGAAAATCAAAATCCTCGGATGTTTTTGGCAATGATAACATACATCACAAGAAACCAACCCGAATGGTTTAGAAATGTCTTGAGCATTGCCCCTATAATGTTCTCAAACAAAATGGCAAGATTGGGAAAAGGATACATGTTTGAAAGTAAGAGCATGAAGCTAAGGACACAAATACCGGCAGAAATGCTTGCAAACATTGACTTGAAATACTTCAATGAATCAACGAGAA<br>AGAAAATCGAGAAAATAAGACCTCTGCTAATTGATGGCACAGCCTCATTGAGTCCTGGAATGATGATGGGCATGTTCAATATGCTGAGCACAGTATTGGGGGTCTCAATCCTGAATCTTGGACAAAAGAGGTACACCAAAACCACATACTGGTGGGATGGACTCCAATCCTCTGATGATTTCGCTCTCATAGTGAATGCACCGAATCATGAGGGGATACAAGCAGGGGTGGAT<br>AGGTTCTATAGGACCTGCAAACTGGTTGGGATCAACATGAGCAAAAAGAAGTCTTACATAAACCGGACAGGAACATTTGAGTTCACAAGCTTTTTCTACCGCTATGGATTTGTAGCCAACTTCAGTATGGAGTTACCCAGCTTTGGAGTGTCTGGAATCAATGAGTCAGCTGACATGAGCATTGGAGTTACAGTGATAAAGAACAATATGATAAACAATGACCTTGGACCAGCA<br>ACAGCTCAAATGGCTCTTCAGCTATTCATCAAAGATTATAGGTACACGTACCGATGTCACAGAGGTGATACACAAATTCAAACGAGGAGATCATTCGAGCTTAAGAAGCTGTGGGAGCAGACCCGTTCAAAGGCAGGGCTGTTGGTATCAGATGGAGGACCAAACCTATACAACATTCGGAATCTCCACATCCCAGAGGTCTGCTTGAAGTGGGAACTAATGGATGAAGATTA<br>CCAGGGCAGGCTGTGTAATCCTCTGAATCCGTTTGTCAGTCATAAGGAAATTGAGTCCGTAAACAATGCTGTGGTAATGCCAGCCCATGGCCCAGCCAAGAGCATGGAATATGATGCTGTTGCGACTACACACTCATGGATTCCTAAGAGGAACCGTTCCATTCTCAATACCAGCCAGAGGGGAATTCTCGAGGATGAGCAGATGTACCAGAAGTGCTGCAGTTTATTCGAGA<br>AATTCTTCCCCAGTAGTTCGTACAGGAGGCCAGTTGGAATTTCCAGCATGGTGGAGGCCATGGTGTCCAGGGCCCGAATTGATGCACGCATTGATTTCGAATCTGGAAGGATTAAGAAAGAAGAGTTTGCTGAGATCATGAAGATCTGTTCCACCATTGAAGAGCTCAGACGGCAAAAATAG
EPI_ISL_240672	>A/shoveler/Chany/82K/2014_H3N8_2014-09-10<br>ATGGATGTCAATCCGACTTTACTTTTCTTAAAAGTGCCAGCGCAAAATGCCATAAGTACTACGTTTCCTTATACTGGAGATCCTCCATACAGCCATGGAACAGGAACAGGATACACCATGGACACAGTCAACAGAACACATCAATACTCAGAAAAGGGGAAATGGACAACAAACACAGAGACCGGAGCACCCCAACTCAACCCAATTGATGGACCACTACCTGAGGACAACGA<br>ACCAAGCGGATATGCACAAACAGATTGTGTGTTGGAAGCAATGGCTTTCCTTGAAGAGTCCCACCCAGGGATCTTTGAAAACTCTTGTCTTGAAACGATGGAAGTTGTTCAGCAAACAAGAGTGGACAAACTAACTCAAGGTCGCCAGACTTATGACTGGACACTGAATAGAAACCAACCAGCTGCAACTGCCCTGGCCAACACTATAGAGGTCTTCAGATCGAACGGTCTGA<br>CAGCCAATGAATCGGGGAGACTAATAGATTTCCTCAAGGATGTGATGGACTCAATGGACAAAGAAGAAATGGAAATAACAACACATTTCCAGAGAAAGAGAAGAGTAAGGGACAACATGACCAAGAAAATGGTCACACAAAGAACAATAGGGAAAAAGAAACAAAGGCTGAATAAGAAGAGCTACCTAATAAGAGCACTGACACTGAACACAATGACAAAAGATGCAGA<br>AAGAGGCAAATTGAAGAGGCGGGCAATTGCAACACCAGGGATGCAGATTAGAGGATTTGTGTACTTTGTCGAAACACTGGCAAGGAGCATCTGTGAGAAACTTGAGCAATCTGGACTCCCCGTTGGAGGGAATGAGAAAAAGGCTAAATTGGCAAATGTCGTGAGAAAAATGATGACTAACTCACAAGATACAGAGCTCTCTTTCACAATTACTGGAGATAACACCAAATG<br>GAATGAGAATCAAAATCCTCGGATGTTCCTGGCAATGATAACATACATCACAAGAAACCAACCTGAATGGTTTAGAAATGTCTTGAGCATTGCCCCTATAATGTTCTCGAACAAAATGGCGAGATTGGGAAAAGGATACATGTTTGAAAGTAAGAGCATGAAGCTACGGACACAAATACCTGCAGAAATGCTTGCAAACATTGACTTAAAATATTTCAATGAATCAACAAGAAA<br>GAAAATCGAGAAAATAAGACCTCTGCTAATAGATGGCACAGCCTCATTGAGTCCTGGAATGATGATGGGCATGTTCAATATGCTGAGTACAGTATTAGGAGTCTCAATCCTGAATCTTGGGCAAAAGAGGTACACCAAAACCACATACTGGTGGGATGGACTCCAATCCTCTGATGATTTCGCCCTCATAGTGAATGCACCGAATCATGAGGGAATACAAGCAGGAGTGGATA<br>GGTTCTATAGGACATGCAAACTGGTTGGAATCAACATGAGCAAAAAGAAGTCTTACATAAACCGGACAGGAACATTTGAGTTCACAAGCTTTTTCTATCGCTATGGATTTGTGGCTAATTTCAGTATGGAGCTGCCCAGCTTTGGAGTGTCTGGAATCAATGAATCAGCTGACATGAGCATTGGAGTTACAGTGATAAAGAATAACATGATAAACAATGACCTTGGACCAGCAA<br>CAGCTCAGATGGCTCTTCAGCTATTCATCAAGGACTACAGGTACACGTACCGATGCCACAGGGGTGACACACAAATTCAAACGAGGAGATCATTCGAGCTGAAGAAGCTGTGGGAGCAGACCCGTTCAAAGGCAGGACTGTTGGTGTCGGATGGAGGACCAAACCTATACAACATTCGGAATCTCCACATCCCAGAGGTCTGCTTGAAGTGGGAGCTGATGGACGAAGATTA<br>CCAGGGCAGGTTGTGTAATCCTCTGAACCCATTTGTCAGTCATAAGGAAATTGAGTCCGTAAACAATGCTGTGGTGATGCCAGCCCATGGCCCAGCCAAGAGCATGGAATATGATGCTGTTGCGACTACACACTCATGGATTCCTAAGAGGAATCGTTCCATTCTCAATACCAGCCAAAGGGGAATTCTTGAGGATGAGCAGATGTACCAGAAGTGCTGTAGTCTATTCGAGAA<br>ATTCTTCCCCAGTAGTTCATACAGGAGGCCAGTTGGAATTTCCAGCATGGTAGAGGCCATGGTATCTAGGGCCCGAATTGATGCACGCATTGATTTCGAATCTGGAAGGATTAAGAAAGAAGAGTTTGCTGAGATCATGAAGATCTGTTCCACCATTGAAGAGCTCAGACGGCAAAAATAG
EPI_ISL_167226	>A/Seal/Sweden/SVA0546/2014_H10N7_2011-09-24<br>ATGGATGTCAATCCGACTTTACTTTTCTTAAAAGTGCCAGTGCAAAATGCTATAAGTACTACGTTCCCTTACACTGGAGATCCTCCATACAGCCATGGAACAGGAACAGGATACACCATGGACACAGTCAACAGAACACATCAATACTCAGAGAAGGGAAAGTGGACAACGAACACAGAGACTAGGGCGCCCCAACTCAACCCAATTGATGGACCATTACCAGAGGACAATGA<br>GCCAAGCGGATATGCACAAACAGATTGCGTGTTGGAAGCAATGGCTTTCCTTGAAGAATCCCACCCAGGGATCTTTGAAAACTCTTGTCTTGAAACGATGGAGGTCGTTCAGCAAACAAGAGTGGACAAACTAACCCAAGGTCGCCAGACTTATGATTGGACACTAAATAGAAACCAACCAGCTGCAACTGCTTTGGCCAACACCATAGAGGTCTTCAGATCGAACGGTCTAA<br>CAGCCAATGAGTCGGGGAGACTAATAGATTTCCTCAAGGATGTGATGGAATCAATGGATAAAGAAGAAATGGAAATAACAACACATTTCCAGAGAAAGAGAAGAGTAAGGGACAACATGACCAAGAAGATGGTCACACAAAGAACGATAGGAAAGAAGAAGCAGAGACTGAACAAGAGGAGCTACTTAATAAGGGCACTGACATTGAACACAATGACCAAAGATGCAGA<br>AAGAGGCAAATTGAAGAGGCGGGCAATTGCAACACCCGGGATGCAGATTAGAGGATTCGTGTACTTTGTCGAAACACTAGCAAGGAGCATCTGTGAGAAACTCGAGCAGTCTGGACTCCCTATTGGAGGGAATGAGAAGAAGGCCAAATTGGCAAATGTCGTGAGAAAAATGATGACTAACTCACAAGATACAGAGCTCTCCTTTACAATTACTGGAGACAACACCAAATG<br>GAATGAGAATCAAAATCCTCGGATGTTTTTGGCAATGATAACATACATCACAAGAAACCAACCCGAATGGTTTAGAAATGTCTTGAGCATTGCCCCTATAATGTTCTCAAACAAAATGGCGAGATTAGGAAAAGGATACATGTTTGAGAGTAAGAGCATGAAGCTAAGGACACAAATACCGGCAGAAATGCTTGCAAACATTGACTTGAAATACTTCAATGAATCAACGAGAA<br>AGAAAATCGAGAAAATTAGACCTCTGCTAATTGATGGCACAGCCTCATTGAGTCCTGGGATGATGATGGGCATGTTCAATATGCTGAGCACAGTATTGGGGGTCTCAATCCTGAATCTTGGACAAAAGAGGTACACCAAAACCACATACTGGTGGGATGGACTTCAATCCTCTGATGATTTCGCTCTCATAGTGAATTCACCGAATCATGAGGGGATACAAGCAGGGGTGGAT<br>AGGTTCTATAGGACCTGCAAACTGGTTGGGATCAACATGAGCAAAAAGAAGTCTTACATAAACCGGACAGGAACATTTGAGTTCACAAGCTTTTTCTACCGCTATGGATTTGTAGCCAACTTCAGTATGGAGTTACCCAGCTTTGGAGTGTCTGGAATCAATGAATCGGCTGACATGAGCATTGGAGTTACAGTGATAAAGAACAATATGATAAACAATGACCTTGGACCTGCA<br>ACAGCTCAAATGGCTCTTCAGCTATTCATCAAGGACTATAGGTACACGTACCGATGCCACAGAGGTGATACACAAATTCAAACGAGGAGATCATTCGAGCTGAGGAAGCTGTGGGAGCAGACCCGTTCAAAGGCAGGGCTATTAGTATCAGATGGAGGACCAAACCTATACAACATTCGGAATCTCCACATCCCAGAGGTCTGCTTGAAGTGGGAACTAATGGATGAAGACT<br>ACCAGGGCAGGCTGTGTAATCCTCTGAATCCGTTTGTCAGTCATAAGGAAATTGAGTCCGTAAACAATGCTGTGGTAATGCCAGCCCATGGTCCAGCCAAGAGCATGGAATATGATGCTGTTGCGACTACACACTCATGGATTCCTAAGAGGAACCGTTCCATTCTCAATACCAGCCAGAGGGGAATTCTCGAGGATGAGCGAATGTACCAGAAGTGCTGCAATCTATTCGAAA<br>AATTCTTCCCCAGTAGTTCATACAGGAGGCCAGTTGGAATTTCCAGCATGGTGGAGGCCATGGTGTCCAGGGCCCGAATTGATGCACGCATTGATTTCGAGTCTGGAAGGATTAAGAAAGAAGAGTTTGCTGAGATCATGAAGATCTGTTCCACCATTGAAGAGCTCAGACGGCAAAAATAG
EPI_ISL_11007534	>A/seagull/Slovenia/2075_22VIR777-7/2021_H5N1_2021-12-30<br>ATGGATGTCAATCCGACTTTACTTTTCTTAAAAGTGCCAGCGCAAGATGCCATAAGTACCACATTCCCTTACACTGGAGATCCTCCATACAGCCATGGAACAGGGACAGGATACACAATGGACACAGTCAACAGAACACATCAATACTCAGAGAAGGGAAAATGGACAACAAACACAGAAACCGGAGCACCTCAACTCAACCCAATTGATGGGCCACTACCTGAGGACAACGA<br>ACCGAGCGGATATGCACAAACAGATTGCGTGTTGGAAGCAATGGCTTTCCTTGAAGAGTCCCACCCAGGGATCTTTGAGAACTCTTGTCTTGAAACGATGGAAGTCGTTCAGCAAACAAGAGTGGACAAACTAACTCAAGGTCGCCAGACATATGACTGGACACTGAATAGAAACCAACCAGCTGCAACTGCCCTGGCCAACACTATAGAGGTCTTCAGATCAAACGGTCTAA<br>CAGCCAATGAATCGGGGAGACTAATAGATTTCCTCAAGGATGTGATGGACTCAATGGATAAAGAAGAAATGGAAATAACAACACATTTCCAGAGAAAGAGAAGAGTAAGGGACAACATGACCAAGAAAATGGTCACACAAAGAACAATAGGAAAGAAGAAACAAAGGCTAAACAAGAGGAGCTACTTAATAAGAGCACTGACACTGAATACAATGACAAAAGATGCAGA<br>AAGAGGCAAATTGAAGAGACGGGCGATTGCAACACCAGGGATGCAGATTAGAGGATTTGTGTACTTTGTCGAAACACTGGCAAGGAGCATCTGTGAAAAACTTGAGCAATCTGGACTCCCCGTTGGAGGGAATGAGAAGAAGGCTAAATTGGCAAATGTCGTGAGAAAAATGATGACTAACTCACAAGATACAGAGCTCTCCTTCACAATTACTGGAGATAACACCAAATG<br>GAATGAGAATCAAAATCCTCGGATGTTTCTGGCAATGATAACATACATTACAAGAAACCAACCTGAATGGTTTAGAAATGTCTTGAGTATTGCCCCTATAATGTTCTCGAACAAAATGGCGAGATTGGGAAAAGGGTACATGTTTGAAAGTAAGAGCATGAAGTTACGGACACAAATACCTGCAGAAATGCTTGCAAACATTGACTTAAAATACTTCAATGAATCAACAAGAAA<br>GAAAATCGAAAAAATAAGGCCTCTACTAATAGATGGCACTGCCTCATTGAGTCCTGGAATGATGATGGGCATGTTCAATATGCTGAGTACAGTATTAGGAGTTTCAATCCTAAATCTTGGGCAAAAGAAGTACACCAAAACCACATACTGGTGGGATGGACTCCAATCCTCTGATGATTTCGCCCTCATAGTAAATGCACCGAATCATGAGGGAATACAAGCAGGAGTGGATA<br>GGTTCTATAGGACCTGCAAACTGGTCGGGATCAATATGAGCAAAAAGAAGTCTTACATGAACCGGACTGGAACATTTGAGTTCACAAGCTTTTTCTATCGCTATGGATTTGTGGCTAACTTCAGTATGGAGCTGCCCAGCTTTGGAGTTTCTGGGATCAATGAATCAGCTGACATGAGCATTGGCGTCACAGTGATAAAGAACAACATGATAAACAATGACCTTGGACCGGCAA<br>CAGCTCAAATGGCCCTTCAACTATTCATCAAAGATTACAGGTACACGTACCGATGCCACAGAGGTGACACACAAATTCAAACGAGGAGATCATTCGAGCTGAAGAAGCTGTGGGAACAGACCCGTTCAAAGGCAGGACTGTTGGTGTCAGATGGAGGACCAAATCTATACAACATTCGGAATCTCCATATCCCAGAGGTCTGCCTGAAGTGGGAGCTGATGGACGAAGATTA<br>CCAGGGCAGGTTGTGTAATCCTCTGAACCCATTTGTCAGCCATAAAGAACTTGAGTCCGTAAACAATGCTGTGGTGATGCCAGCCCACGGTCCAGCCAAAAGCATGGAATATGATGCCGTTGCGACTACACACTCATGGATTCCTAAAAGGAATCGTTCCATTCTCAATACCAGTCAAAGGGGAATTCTTGAGGATGAACAGATGTACCAGAAATGCTGCAGTCTATTCGAGAA<br>ATTCTTCCCCAGTAGTTCATACAGGAGACCAGTTGGAATTTCCAGCATGGTGGAGGCCATGGTGTCTAGGGCCCGAATCGATGCACGCATTGATTTCGAATCTGGAAGGATCAAGAAGGAAGAGTTTGCTGAGATCATGAAGATCTGTTCCACCATTGAAGAGCTCAGACGGCAAAAATAG
EPI_ISL_8882218	>A/seagull/Italy/21VIR11259-12/2021_H5N1_2021-12-15<br>ATGGATGTCAATCCGACTTTACTTTTCTTAAAAGTGCCAGCGCAAAATGCCATAAGTACCACATTCCCTTATACTGGAGATCCTCCATACAGCCATGGAACAGGGACAGGATACACAATGGACACAGTCAACAGAACACATCAATACTCAGAGAAGGGAAAATGGACAACAAACACAGAAACCGGAGCACCTCAACTCAACCCAATTGATGGGCCACTACCTGAGGACAACGA<br>ACCGAGCGGATATGCACAGACAGATTGCGTGTTGGAAGCAATGGCTTTCCTTGAAGAGTCCCACCCAGGGATCTTTGGAAACTCTTGTCTTGAAACGATGGAAGTCGTTCAGCAAACAAGAGTGGACAAACTAACTCAAGGTCGCCAGACTTATGACTGGACACTGAATAGAAACCAACCAGCTGCAACTGCCCTGGCCAACACTATAGAGGTCTTCAGATCAAACGGTCTAA<br>CAGCCAATGAATCGGGGAGACTAATAGATTTCCTCAGGGATGTGATGGACTCAATGGATAAAGAAGAAATGGAAATAACAACACACTTCCAGAGAAAGAGAAGAGTAAGGGACAACATGACCAAGAAAATGGTCACACAAAGAACAATAGGAAAGAAGAAACAAAGGCTAAACAGGAGGAGCTACTTAATAAGAGCACTGACACTGAATACAATGACAAAAGATGCAGA<br>AAGAGGCAAATTGAAGAGACGGGCGATTGCAACACCAGGGATGCAGATTAGAGGATTTGTGTACTTTGTCGAAACACTGGCAAGGAGCATCTGTGAAAAACTTGAGCAATCTGGACTCCCCGTTGGAGGAAATGAGAAGAAGGCTAAATTGGCAAATGTCGTGAGGAAAATGATGACTAACTCACAAGATACAGAGCTCTCCTTCACAATTACTGGAGATAACACCAAATG<br>GAATGAGAATCAAAATCCTCGGATGTTTCTGGCAATGATAACGTACATTACAAGAAACCAACCTGAATGGTTTAGAAATGTCTTGAGTATTGCCCCTATAATGTTCTCGAACAAAATGGCGAGATTGGGAAAAGGGTACATGTTTGAAAGTAAGAGCATGAAGTTACGGACACAAATACCTGCAGAAATACTTGCAGACATTGACTTAAAATACTTCAATGAATCAACAAGAAA<br>GAAAATCGAAAAAATAAGGCCTCTACTAATAGATGGCACTGCCTCATTGAGTCCTGGAATGATGATGGGCATGTTCAATATGCTGAGTACAGTATTAGGAGTTTCAATCCTAAATCTTGGGCAAAAGAGGTACACCAAAACCACATACTGGTGGGATGGACTCCAATCCTCTGATGATTTCGCCCTCATAGTAAATGCACCGAATCATGAGGGAATACAAGCAGGAGTGGATA<br>GGTTCTATAGGACCTGCAAACTGGTCGGGATCAATATGAGCAAAAAGAAGTCTTACATAAACCGGACTGGAACATTTGAGTTCACAAGCTTTTTCTATCGCTATGGATTTGTGGCTAACTTCAGTATGGAGTTGCCCAGCTTTGGAGTTTCTGGAATCAATGAATCAGCTGACATGAGCATTGGCGTCACAGTGATAAAGAACAACATGATAAACAATGACCTTGGACCAGCAA<br>CAGCTCAAATGGCCCTTCAACTATTCATCAAAGATTACAGGTACACGTACCGATGCCACAGAGGTGACACACAAATTCAAACGAGGAGATCATTCGAGCTGAAGAAGCTGTGGGAACAGACCCGTTCAAAGGCAGGACTGTTGGTGTCAGATGGAGGACCAAATCTATACAACATTCGGAATCTCCATATCCCAGAGGTCTGCCTGAAGTGGGAGCTGATGGACGAAGATTA<br>CCAGGGCAGGTTGTGTAATCCTCTGAACCCATTTGTCAGTCATAAAGAAATTGAGTCCGTAAACAATGCTGTGGTGATGCCAGCCCACGGTCCAGCCAAAAGCATGGAATATGATGCCGTTGCAACTACACACTCATGGATTCCTAAAAGGAATCGTTCCATTCTCAATACCAGCCAAAGGGGAATTCTTGAGGATGAACAGATGTACCAGAAATGCTGCAGTCTATTCGAGAA<br>ATTCTTCCCCAGTAGTTCATACAGGAGACCAGTTGGAATTTCCAGCATGGTGGAGGCCATGGTGTCTAGGGCCCGAATCGATGCACGCATTGATTTCGAATCTGGAAGGATCAAGAAGGGAGAGTTTGCTGAGATCATAAAGATCTGTTCCACCATTGAAGAGCTCAGACGGCAAAAATAG
EPI_ISL_8882213	>A/seagull/Italy/21VIR11259-10/2021_H5N1_2021-12-15<br>ATGGATGTCAATCCGACTTTACTTTTCTTAAAAGTGCCAGCGCAAGATGCCATAAGTACCACATTCCCTTACACTGGAGATCCTCCATACAGCCATGGAACAGGGACAGGATACACAATGGACACAGTCAACAGAACACATCAATACTCAGAGAAGGGAAAATGGACAACAAACACAGAAACCGGAGCACCTCAACTCAACCCAATTGATGGGCCACTACCTGAGGACAACGA<br>ACCGAGCGGATATGCACAAACAGATTGCGTGTTGGAAGCAATGGCTTTCCTTGAAGAGTCCCACCCAGGGATCTTTGAAAACTCTTGTCTTGAAACGATGGAAGTCGTTCAGCAAACAAGAGTGGACAAACTAACTCAAGGTCGCCAGACATATGACTGGACACTGAATAGAAACCAACCAGCTGCAACTGCCCTGGCCAACACTATAGAGGTCTTCAGATCAAACGGTCTAA<br>CAGCCAATGAATCGGGGAGACTAATAGATTTCCTCAAGGATGTGATGGACTCAATGGATAAAGAAGAAATGGAAATAACAACACATTTCCAGAGAAAGAGAAGAGTAAGGGACAACATGACCAAGAAAATGGTCACACAAAGAACAATAGGAAAGAAGAAACAAAGGCTAAACAAGAGGAGCTACTTAATAAGAGCACTGACACTGAATACAATGACAAAAGATGCAGA<br>AAGAGGCAAATTGAAAAGACGGGCGATTGCAACACCAGGGATGCAAATTAGAGGATTTGTGTACTTTGTCGAAACACTGGCAAGGAGCATCTGTGAAAAACTTGAGCAATCTGGACTCCCCGTTGGAGGGAATGAGAAGAAGGCTAAATTGGCAAATGTCGTGAGAAAAATGATGACTAACTCACAAGATACAGAGCTCTCCTTCACAATTACTGGAGATAACACCAAATG<br>GAATGAGAATCAAAATCCTCGGATGTTTCTGGCAATGATAACATACATTACAAGAAACCAACCTGAATGGTTTAGAAATGTCTTGAGTATTGCCCCTATAATGTTCTCGAACAAAATGGCGAGATTGGGAAAAGGGTACATGTTTGAAAGTAAGAGCATGAAGTTACGGACACAAATACCTGCAGAAATGCTTGCAAACATTGACTTAAAATACTTCAATGAATCAACAAGAAA<br>GAAAATCGAAAAAATAAGGCCTCTACTAATAGATGGCACTGCCTCATTGAGTCCTGGAATGATGATGGGCATGTTCAATATGCTGAGTACAGTATTAGGAGTTTCAATCCTAAATCTTGGGCAAAAGAAGTACACCAAAACCACATACTGGTGGGATGGACTCCAATCCTCTGATGATTTCGCCCTCATAGTAAATGCACCGAATCATGAGGGAATACAAGCAGGAGTGGATA<br>GGTTCTATAGGACCTGCAAACTGGTCGGGATCAATATGAGCAAAAAGAAGTCTTACATAAACCGGACTGGAACATTTGAGTTCACAAGCTTTTTCTATCGCTATGGATTTGTGGCTAACTTCAGTATGGAGCTGCCCAGCTTTGGAGTTTCTGGGATCAATGAATCAGCTGACATGAGCATTGGCGTTACAGTGATAAAGAACAACATGATAAACAATGACCTTGGACCAGCAA<br>CAGCTCAAATGGCCCTTCAACTATTCATCAAAGATTACAGGTACACGTACCGATGCCACAGAGGTGACACACAAATTCAAACGAGGAGATCATTCGAGCTGAAGAAGCTGTGGGAACAGACCCGTTCAAAGGCAGGACTGTTGGTGTCAGATGGAGGACCAAATCTATACAACATTCGGAATCTCCATATCCCAGAGGTCTGCCTGAAGTGGGAGCTGATGGACGAAGATTA<br>CCAGGGCAGGTTGTGTAATCCTCTGAACCCATTTGTCAGCCATAGAGAAATTGAGTCCGTAAACAATGCTGTGGTGATGCCAGCCCACGGTCCAGCCAAAAGCATGGAATATGATGCCGTTGCAACTACACACTCATGGATTCCTAAAAGGAATCGTTCCATTCTCAATACCAGTCAAAGGGGAATTCTTGAGGATGAACAGATGTACCAGAAATGCTGCAGTCTATTCGAGAA<br>ATTCTTCCCCAGTAGTTCATACAGGAGACCAGTTGGAATTTCCAGCATGGTGGAGGCCATGGTGTCTAGGGCCCGAATCGATGCACGAATTGATTTCGAATCTGGAAGGATCAAGAAGGAAGAGTTTGCTGAGATCATGAAGATCTGTTCCACCATTGAAGAGCTCAGACGGCAAAAATAG
EPI_ISL_13778475	>A/Sandwich_Tern/Netherlands/12/2022_H5N1_2022-06-07<br>ATGGATGTCAATCCGACTTTACTTTTCTTAAAAGTGCCAGCGCAAGATGCCATAAGTACCACATTCCCTTACACTGGAGATCCTCCATACAGCCATGGAACAGGGACAGGATACACAATGGACACGGTCAACAGAACACATCAATACTCAGAGAAGGGAAAATGGACAACAAACACAGAAACCGGAGCACCTCAACTCAACCCAATTGATGGGCCACTACCTGAGGACAACGA<br>ACCGAGCGGATATGCACAAACAGATTGCGTGTTGGAAGCAATGGCTTTCCTTGAAGAGTCCCATCCAGGGATCTTTGAAAACTCTTGTCTTGAAACGATGGAAGTCGTTCAGCAAACAAGAGTGGACAAACTAACTCAAGGTCGCCAGACATATGACTGGACACTGAATAGAAACCAACCAGCTGCAACTGCCTTGGCCAACACTATAGAGGTCTTAAGATCAAACGGTCTAA<br>TAGCCAATGAATCGGGGAGACTAATAGATTTCCTCAAGGATGTGATGGACTCAATGGATAAAGAAGAAATGGAAATAACAACACATTTCCAGAGAAAGAGAAGAGTAAGGGACAACATGACCAAGAAAATGGTCACACAAAGAACAATAGGAAAGAAGAAACAAAGGCTAAACAAGAGGAGCTACTTAATAAGAGCACTGACACTGAATACAATGACAAAAGATGCAGA<br>AAGAGGCAAATTGAAGAGACGGGCGATTGCAACACCAGGGATGCAGATTAGAGGATTTGTGTACTTTGTCGAAACACTGGCAAGGAGCATCTGTGAAAAACTTGAGCAATCTGGACTCCCCGTTGGAGGGAATGAGAAGAAGGCTAAATTGGCAAATGTTGTGAGAAAAATGATGACTAACTCACAAGATACAGAGCTCTCCTTCACAATTACTGGAGATAACACCAAATG<br>GAATGAGAATCAAAATCCTCGGATGTTTCTGGCAATGGTAACATACATTACAAGAAACCAACCTGAATGGTTTAGGAATGTCTTGAGTATTGCCCCTATAATGTTCTCGAACAAAATGGCGAGATTGGGAAAAGGGTACATGTTTGAAAGTAAGAGCATGAAGTTACGGACACAAATACCTGCAGAAATGCTTGCAAACATTGACTTAAAATATTTCAATGAATCAACAAGAAA<br>GAAAATCGAAAAAATAAGGCCTCTACTAATAGATGGCACTGCCTCATTGAGTCCTGGAATGATGATGGGCATGTTCAATATGCTGAGTACAGTATTAGGAGTTTCAATCCTAAATCTTGGGCAAAAGAAGTACACCAAAACCACATACTGGTGGGATGGACTCCAATCCTCTGATGATTTCGCCCTCATAGTAAATGCACCGAATCATGAGGGAATACAAGCAGGAGTGGATA<br>GGTTCTATAGGACCTGCAAACTGGTCGGGATCAATATGAGCAAAAAGAAGTCTTACATAAACCGGACTGGAACATTTGAGTTCACAAGCTTTTTCTATCGCTATGGATTTGTGGCTAATTTCAGTATGGAGCTGCCCAGCTTTGGAGTTTCTGGGATCAATGAATCAGCTGACATGAGCATTGGCATCACAGTGATAAAGAACAACATGATAAACAATGACCTTGGACCAGCAA<br>CAGCTCAAATGGCCCTTCAACTATTCATCAAAGATTACAGGTACACGTACCGATGCCATAGAGGTGACACACAAATTCAAACGAGGAGATCATTCGAGCTGAAGAAGCTGTGGGAACAGACCCGTTCAAAGGCAGGACTGTTGGTGTCAGATGGAGGACCAAATCTATACAACATTCGGAATCTCCATATCCCAGAGGTCTGCCTGAAATGGGAGCTGATGGACGAAGATTA<br>CAAGGGCAGGTTGTGTAATCCTCTGAACCCATTTGTCAGCCATAAAGAAATTGAGTCCGTAAACAATGCTGTGGTGATGCCAGCCCACGGTCCAGCCAAAAGCATGGAATATGATGCCGTTGCGACTACACACTCATGGATTCCTAAAAGGAATCGTTCCATTCTCAATACCAGTCAAAGGGGAATTCTTGAGGATGAACAGATGTACCAGAAATGCTGCGGTCTATTCGAGAA<br>ATTCTTCCCCAGTAGTTCATACAGGAGACCAGTTGGAATTTCCAGCATGGTGGAGGCCATGGTGTCTAGGGCCCGAATCGATGCACGCATTGATTTCGAATCTGGAAGGATCAAGAAGGAAGAGTTTGCTGAGATCATGAAGATCTGTTCCACCATTGAAGAGCTCAGACGGCAAAAATAG
EPI_ISL_13778467	>A/Sandwich_Tern/Netherlands/11/2022_H5N1_2022-06-19<br>ATGGATGTCAATCCGACTTTACTTTTCTTAAAAGTGCCAGCGCAAGATGCCATAAGTACCACATTCCCTTACACTGGAGATCCTCCATACAGCCATGGAACAGGGACAGGATACACAATGGACACGGTCAACAGAACACATCAATACTCAGAGAAGGGAAAATGGACAACAAACACAGAAACCGGAGCACCTCAACTCAACCCAATTGATGGGCCACTACCTGAGGACAACGA<br>ACCGAGCGGATATGCACAAACAGATTGCGTGTTGGAAGCAATGGCTTTCCTTGAAGAGTCCCATCCAGGGATCTTTGAAAACTCTTGTCTTGAAACGATGGAAGTCGTTCAGCAAACAAGAGTGGACAAACTAACTCAAGGTCGCCAGACATATGACTGGACACTGAATAGAAACCAACCAGCTGCAACTGCCTTGGCCAACACTATAGAGGTCTTAAGATCAAACGGTCTAA<br>TAGCCAATGAATCGGGGAGACTAATAGATTTCCTCAAGGATGTGATGGACTCAATGGATAAAGAAGAAATGGAAATAACAACACATTTCCAGAGAAAGAGAAGAGTAAGGGACAACATGACCAAGAAAATGGTCACACAAAGAACAATAGGAAAGAAGAAACAAAGGCTAAACAAGAGGAGCTACTTAATAAGAGCACTGACACTGAATACAATGACAAAAGATGCAGA<br>AAGAGGCAAATTGAAGAGACGGGCGATTGCAACACCAGGGATGCAGATTAGAGGATTTGTGTACTTTGTCGAAACACTGGCAAGGAGCATCTGTGAAAAACTTGAGCAATCTGGACTCCCCGTTGGAGGGAATGAGAAGAAGGCTAAATTGGCAAATGTTGTGAGAAAAATGATGACTAACTCACAAGATACAGAGCTCTCCTTCACAATTACTGGAGATAACACCAAATG<br>GAATGAGAATCAAAATCCTCGGATGTTTCTGGCAATGATAACATACATTACAAGAAACCAACCTGAATGGTTTAGGAATGTCTTGAGTATTGCCCCTATAATGTTCTCGAACAAAATGGCGAGATTGGGAAAAGGGTACATGTTTGAAAGTAAGAGCATGAAGTTACGGACACAAATACCTGCAGAAATGCTTGCAAACATTGACTTAAAATATTTCAATGAATCAACAAGAAA<br>GAAAATCGAAAAAATAAGGCCTCTACTAATAGATGGCACTGCCTCATTGAGTCCTGGAATGATGATGGGCATGTTCAATATGCTGAGTACAGTATTAGGAGTTTCAATCCTAAATCTTGGGCAAAAGAAGTACACCAAAACCACATACTGGTGGGATGGACTCCAATCCTCTGATGATTTCGCCCTCATAGTAAATGCACCGAATCATGAGGGAATACAAGCAGGAGTGGATA<br>GGTTCTATAGGACCTGCAAACTGGTCGGGATCAATATGAGCAAAAAGAAGTCTTACATAAACCGGACTGGAACATTTGAGTTCACAAGCTTTTTCTATCGCTATGGATTTGTGGCTAATTTCAGTATGGAGCTGCCCAGCTTTGGAGTTTCTGGGATCAATGAATCAGCTGACATGAGCATTGGCATCACAGTGATAAAGAACAACATGATAAACAATGACCTTGGACCAGCAA<br>CAGCTCAAATGGCCCTTCAACTATTCATCAAAGATTACAGGTACACGTACCGATGCCATAGAGGTGACACACAAATTCAAACGAGGAGATCATTCGAGCTGAAGAAGCTGTGGGAACAGACCCGTTCAAAGGCAGGACTGTTGGTGTCAGATGGAGGACCAAATCTATACAACATTCGGAATCTCCATATCCCAGAGGTCTGCCTGAAATGGGAGCTGATGGACGAAGATTA<br>CAAGGGCAGGTTGTGTAATCCTCTGAACCCATTTGTCAGCCATAAAGAAATTGAGTCCGTAAACAATGCTGTGGTGATGCCAGCCCACGGTCCAGCCAAAAGCATGGAATATGATGCCGTTGCGACTACACACTCATGGATTCCTAAAAGGAATCGTTCCATTCTCAATACCAGTCAAAGGGGAATTCTTGAGGATGAACAGATGTACCAGAAATGCTGCGGTCTATTCGAGAA<br>ATTCTTCCCCAGTAGTTCATACAGGAGACCAGTTGGAATTTCCAGCATGGTGGAGGCCATGGTGTCTAGGGCCCGAATCGATGCACGCATTGATTTCGAATCTGGAAGGATCAAGAAGGAAGAGTTTGCTGAGATCATGAAGATCTGTTCCACCATTGAAGAGCTCAGACGGCAAAAATAG

EPI_ISL_18245817	>A/sandwich_stern/Poland/MB142/2022_H5N1_2022-06-15<br>ATGGATGTCAATCCGACTTTACTTTTCTTAAAGGTGCCAGCGCAAGATGCCATAAGTACCACATTCCCTTACACTGGAGATCCTCCATACAGCCATGGAACAGGGACAGGATACACAATGGACACGGTCAACAGAACACATCAATACTCAGAGAAGGGAAAATGGACAACAAACACAGAAACCGGAGCACCTCAACTCAACCCAATTGATGGGCCATTACCTGAGGACAACGA<br>ACCGAGCGGATATGCACAAACAGATTGCGTGTTGGAAGCAATGGCTTTCCTTGAAGAGTCCCATCCAGGGATCTTTGAAAACTCTTGTCTTGAAACGATGGAAGTCGTTCAGCAAACAAGAGTGGACAAACTAACTCAAGGTCGCCAGACATATGACTGGACACTGAATAGAAACCAACCAGCTGCAACTGCCTTGGCCAACACTATAGAGGTCTTAAGATCAAACGGTCTAA<br>CAGCCAATGAATCGGGGAGACTAATAGACTTCCTCAAGGATGTGATGGACTCAATGGATAAAGAAGAAATGGAAATAACAACACATTTCCAGAGAAAGAGAAGAGTAAGGGACAACATGACCAAGAAAATGGTCACACAAAGAACAATAGGAAAGAAGAAACAAAGGCTAAACAAAAGGAGCTACTTAATAAGAGCACTGACACTGAATACAATGACAAAAGATGCAGA<br>AAGAGGCAAATTGAAGAGGCGGGCGATTGCAACACCAGGGATGCAGATTAGAGGATTTGTGTACTTTGTCGAAACACTGGCAAGGAGCATCTGTGAAAAACTTGAGCAATCTGGACTCCCCGTTGGAGGGAATGAGAAGAAGGCTAAATTGGCAAATGTTGTGAGAAAAATGATGACTAACTCACAAGATACAGAGCTCTCTTTCACAATTACTGGAGATAACACCAAATG<br>GAATGAGAATCAAAATCCTCGGATGTTTCTGGCAATGATAACATACATTACAAGAAACCAACCTGAATGGTTTAGGAATGTCTTGAGTATTGCCCCTATAATGTTCTCGAACAAAATGGCGAGATTGGGAAAAGGGTACATGTTTGAAAGTAAGAGCATGAAGTTACGGACACAAATACCTGCAGAAATGCTTGCAAACATTGACTTAAAATATTTCAATGAATCAACAAGAAA<br>GAAAATCGAAAAAATAAGGCCTCTACTAATAGATGGCACTGCCTCATTGAGTCCTGGAATGATGATGGGCATGTTCAATATGCTGAGTACAGTATTAGGAGTTTCAATCCTAAATCTTGGGCAAAAGAAGTACACCAAAACCACATACTGGTGGGATGGACTCCAATCCTCTGATGATTTCGCCCTCATAGTAAATGCACCGAATCATGAGGGAATACAAGCAGGAGTGGATA<br>GGTTCTATAGGACCTGCAAACTGGTCGGGATCAATATGAGCAAAAAGAAGTCTTACATAAACCGGACTGGAACATTTGAGTTCACAAGCTTTTTCTATCGCTATGGATTTGTGGCTAATTTCAGTATGGAGCTGCCCAGCTTTGGAGTTTCTGGGATCAATGAATCAGCTGACATGAGCATTGGCATCACAGTGATAAAGAACAACATGATAAACAATGACCTTGGACCAGCAA<br>CAGCTCAAATGGCCCTTCAACTATTCATCAAAGATTACAGGTACACGTACCGATGCCATAGAGGTGACACACAAATTCAAACGAGGAGATCATTCGAGCTGAAGAAGCTGTGGGAACAGACCCGTTCAAAGGCAGGACTGTTGGTGTCAGATGGAGGACCAAATCTATACAACATTCGGAATCTCCATATCCCAGAGGTCTGCCTGAAGTGGGAGCTGATGGACGAAGATTA<br>CCAGGGCAGGTTGTGTAATCCTCTGAACCCATTTGTCAGCCATAAAGAAATTGAGTCCGTAAACAATGCTGTGGTGATGCCAGCCCACGGTCCAGCCAAAAGCATGGAATATGATGCCGTTGCGACTACACACTCATGGATTCCTAAAAGGAATCGTTCCATTCTCAATACCAGTCAAAGGGGAATTCTTGAGGATGAACAGATGTACCAGAAATGCTGCGGTCTATTCGAGAA<br>ATTCTTCCCCAGTAGTTCATACAGGAGACCAGTTGGAATTTCCAGCATGGTGGAGGCCATGGTGTCTAGGGCCCGAATCGATGCACGCATTGATTTCGAATCTGGAAGGATCAAGAAGGAAGAGTTTGCTGAGATCATGAAGATCTGTTCCACCATTGAAGAGCTCAGACGGCAAAAATAG
EPI_ISL_1206285	>A/red_knot/Germany-NI/AI00394/2020_H5N3_2020-12-30<br>ATGGATGTCAATCCGACTTTACTTTTCTTAAAAGTGCCAGCGCAAAATGCCATAAGTACCACATTCCCTTATACTGGAGATCCTCCATACAGCCATGGAACAGGAACAGGATACACCATGGACACAGTCAACAGAACACATCAATACTCGGAAAAGGGGAAATGGACAACAAACACAGAGACCGGAGCACCCCAACTCAACCCAATTGATGGACCACTACCTGAGGACAACGA<br>ACCAAGTGGATATGCACAAACAGATTGCGTGTTGGAAGCAATGGCTTTTCTTGAAGAGTCCCACCCAGGGATCTTTGAAAACTCTTGTCTTGAAACGATGGAAGTCGTTCAGCAAACAAGAATGGACAAACTAACTCAAGGTCGCCAGACTTATGACTGGACACTGAATAGAAACCAACCAGCTGCAACTGCCCTGGCCAACACTATAGAGGTCTTCAGATCGAATGGTCTGAC<br>AGCCAATGAATCAGGGAGACTAATAGATTTCCTCAAGGATGTGATGGACTCAATGGACAAAGAAGAAATGGAAATAACAACACATTTCCAGAGAAAGAGAAGAGTAAGGGACAACATGACCAAGAAAATGGTCACACAAAGAACAATAGGGAAGAAGAAGCAAAGGCTAAATAAGAGGAGCTACTTAATAAGAGCACTGACACTGAATACAATGACAAAAGACGCAGAA<br>AGAGGCAAATTGAAGAGGCGGGCAATTGCAACACCAGGGATGCAGATTAGAGGATTTGTGTACTTTGTTGAAACACTGGCAAGGAGCATCTGTGAAAAACTTGAGCAATCTGGACTCCCCGTTGGAGGGAATGAAAAGAAGGCTAAATTGGCAAATGTCGTGAGAAAAATGATGACTAACTCACAAGATACAGAGCTCTCCTTCACAATTACTGGAGATAACACCAAATGG<br>AATGAGAATCAAAATCCTCGGATGTTTCTGGCAATGATAACATACATCACAAGAAACCAACCTGAATGGTTTAGAAATGTCTTGAGCATTGCCCCCATAATGTTCTCGAACAAAATGGCGAGATTGGGGAAAGGATACATGTTTGAAAGTAAAAGTATGAAGCTACGAACACAAATACCTGCAGAAATGCTTGCAAACATTGACTTAAAATACTTCAATGAGTCAACAAGAAAG<br>AAAATCGAGAAAATAAGACCTCTACTAATAGATGGCACCGCCTCATTGAGTCCTGGAATGATGATGGGCATGTTCAATATGCTGAGTACAGTATTAGGAGTCTCAATCCTGAATCTTGGGCAAAAGAGGTACACCAAAACCACATACTGGTGGGATGGACTCCAATCCTCTGATGATTTCGCTCTCATAGTAAATGCACCGAATCATGAGGGAATACAAGCAGGAGTGGATAG<br>GTTCTATAGGACTTGCAAACTGGTTGGGATCAACATGAGCAAAAAGAAGTCTTACATAAACCGGACAGGAACATTTGAGTTCACAAGCTTTTTCTATCGCTATGGATTTGTGGCCAACTTCAGTATGGAGCTGCCTAGCTTTGGAGTGTCTGGAATCAATGAATCAGCTGACATGAGCATTGGAGTTACAGTGATAAAGAACAACATGATAAACAATGACCTTGGACCAGCAAC<br>AGCTCAAATGGCCCTTCAACTATTCATCAAGGACTACAGGTACACGTACCGATGTCACAGGGGTGACACACAAATTCAAACGAGGAGATCATTCGAGCTGAAGAAGCTGTGGGAGCAGACCCGTTCAAAGGCAGGACTGTTGGTGTCAGATGGAGGACCAAATCTATACAACATCCGGAATCTCCATATCCCAGAAGTCTGCCTGAAGTGGGAGCTGATGGACGAAGATTAC<br>CAGGGCAGGTTGTGCAATCCTCTGAACCCATTTGTCAGTCATAAAGAAATTGAATCCGTAAACAATGCTGTGGTAATGCCAGCCCATGGCCCAGCCAAGAGCATGGAATATGATGCTGTTGCGACTACACACTCATGGATTCCTAAAAGGAATCGTTCCATTCTCAATACCAGCCAAAGGGGAATTCTTGAGGATGAGCAGATGTACCAGAAATGCTGCAGTCTATTCGAAAAA<br>TTCTTCCCCAGTAGTTCATACAGGAGACCAGTTGGAATTTCCAGCATGGTGGAGGCCATGGTGTCTAGGGCCCGAATCGACGCACGCATTGATTTCGAATCTGGAAGGATTAAGAAGGAAGAGTTTGCTGAGATCATGAAGATCTGTTCCACCATTGAAGAGCTCAGACGGCAAAAATAG
EPI_ISL_6959593	>A/quail/Korea/H526/2021_H5N1_2021-11-08<br>ATGGATGTCAATCCGACTTTACTTTTCTTAAAAGTGCCAGCGCAAAATGCTATAAGCACTACGTTCCCTTACACTGGAGACCCTCCATACAGCCATGGAACAGGAACAGGATACACCATGGACACAGTCAACAGAACACATCAATACTCAGAAAAAGGAAAGTGGACAACAAACACAGAGACCGGGGCGCCCCAACTCAACCCAATTGATGGACCATTGCCAGAGGACAACGA<br>CCCAAGCGGATATGCACAAACAGATTGCGTGTTGGAAGCAATGGCTTTCCTTGAAGAATCCCACCCAGGGATCTTTGAAAACTCTTGTCTTGAAACAATGGAGATCGTTCAGCAAACAAGAGTGGACAAACTAACCCAAGGTCGCCAGACCTATGACTGGACACTGAATAGAAACCAACCAGCTGCAACTGCTTTAGCCAATACTATAGAGGTCTTCAGATCGAACGGTCTAAC<br>AGCCAATGAGTCGGGGAGACTAATAGATTTCCTCAAGGATGTGATGGAATCAATGGATAAAGAAGAAATGGAAATAACAACACATTTCCAGAGAAAGAGAAGAGTAAGGGACAACATGACCAAAAAGATGGTCACACAAAGAACGATAGGAAAGAAGAAGCAGAGGCTGAACAAGAGAAGCTACTTAATAAGAGCGCTGACATTGAACACAATGACCAAAGATGCAGAA<br>AGAGGCAAATTGAAGAGGCGGGCAATTGCAACACCCGGGATGCAGATTAGAGGATTTGTGTACTTTGTCGAAACACTAGCGAGGAGCATCTGTGAGAAACTTGAGCAATCCGGACTCCCTGTCGGAGGGAATGAGAAAAAGGCCAAATTGGCAAATGTCGTGAGGAAAATGATGACTAACTCGCAAGATACAGAGCTCTCCTTTACAATCACCGGGGACAACACCAAATGG<br>AATGAGAATCAAAATCCTCGGATGTTTTTGGCAATGATAACATACATCACAAGAAACCAACCCGAATGGTTTAGAAATGTCTTGAGCATTGCCCCCATAATGTTCTCAAACAAAATGGCAAGATTAGGGAAAGGATACATGTTTGAAAGTAAGAGCATGAAGCTAAGGACACAAATACCGGCAGAAATGATTGCAAACATTGACTTAAAATACTTCAATGAATCAACGAGAAA<br>GAAAATCGAGAAAATAAGACCTCTGCTAATTGATGGCACAGCCTCATTGAGTCCTGGAATGATGATGGGCATGTTCAATATGCTGAGCACTGTATTGGGGGTCTCAATCCTGAATCTTGGACAAAAGAGGTACACCAAAACCACATACTGGTGGGATGGACTCCAATCTTCTGATGACTTTGCTCTCATAGTGAATGCACCGAATCATGAGGGGATACAAGCCGGGGTGGATA<br>GGTTCTATAGGACATGCAAACTGGTTGGGATCAACATGAGCAAAAAGAAGTCTTACATAAACCGGACAGGAACATTTGAGTTCACAAGCTTTTTCTACCGCTATGGATTTGTAGCCAACTTCAGTATGGAGTTACCCAGCTTTGGAGTATCTGGAATCAATGAGTCAGCTGACATGAGCATTGGAGTTGCAGTGATAAAGAACAACATGATAAACAATGACCTTGGACCAGCAA<br>CAGCTCAAATGGCTCTTCAGCTATTCATCAAGGATTATAGGTACACGTACCGATGTCACAGAGGTGATACACAAATTCAAACGAGGAGATCATTCGAGCTGAAGAAGCTGTGGGAGCAGACCCGTTCAAAGGCAGGGCTGTTGGTATCAGATGGAGGACCAAACCTATACAACATTCGGAATCTCCACATCCCAGAGGTCTGCTTGAAGTGGGAGCTAATGGATGAAGACTA<br>CCAGGGCAGGCTGTGTAATCCTCTGAATCCATTTGTCAGTCATAAGGAAATTGAATCCGTAAACAATGCTGTAGTAATGCCAGCCCATGGCCCAGCCAAGAGCATGGAATACGATGCTGTTGCGACTACACACTCATGGATTCCTAAGAGGAACCGTTCCATTCTCAATACCAGCCAGAGGGGAATTCTCGAGGATGAGCAGATGTACCAGAAGTGCTGCAGCTTATTCGAGA<br>AGTTCTTCCCCAGTAGTTCATACAGGAGGCCAGTTGGAATTTCCAGCATGGTGGAGGCCATGGTGTCCAGGGCCCGAATTGATGCACGCATTGATTTCGAATCTGGAAGGATTAAGAAAGAAGAGTTTGCTGAGATCATGAAGATCTGTTCCACCATTGAGGAGCTCAGACGGCAAAAATAG
EPI_ISL_337148	>A/pochard/Buryatia/33/2018_H4N6_2018-08-25<br>ATGGATGTCAATCCGACTTTACTTTTCTTGAAAGTGCCAGCGCAAAATGCCATAAGTACCACATTCCCTTATACTGGAGATCCTCCATACAGCCATGGAACAGGAACAGGATACACCATGGACACAGTCAACAGAACACATCAATACTCAGAAAAGGGGAAATGGACAACAAACACAGAGACCGGAGCACCCCAACTCAATCCAATTGATGGACCACTACCTGAGGACAACGA<br>ACCAAGCGGATATGCACAAACAGATTGCGTGTTGGAAGCAATGGCTTTCCTTGAAGAGTCCCACCCAGGGATCTTTGAAAACTCTTGTCTTGAAACGATGGAAGTCGTTCAGCAAACAAGAGTGGACAAACTAACTCAAGGTCGCCAGACTTATGACTGGACACTGAATAGAAACCAACCAGCTGCAACTGCCCTGGCCAACACTATTGAGGTCTTCAGATCGAACGGTCTGA<br>CAGCCAATGAATCAGGGAGACTAATAGATTTCCTCAAGGATGTGATGGACTCAATGGACAAAGAAGAAATGGAAATAACAACACATTTCCAGAGAAAGAGAAGAGTAAGGGACAACATGACCAAGAAAATGGTCACACAAAGAACAATAGGGAAGAAGAAACAAAGGCTAAATAAAAGGAGCTACTTAATAAGAGCATTGACACTGAATACAATGACAAAAGACGCAGA<br>AAGAGGCAAATTGAAGAGACGGGCAATTGCAACACCAGGGATGCAGATTAGAGGATTTGTGTACTTTGTCGAAACACTGGCAAGGAGCATCTGTGAAAAACTTGAGCAATCTGGACTCCCCGTTGGAGGGAATGAGAAGAAGGCTAAATTGGCAAATGTCGTAAGAAAAATGATGACTAACTCACAAGATACAGAGCTCTCCTTCACAATTACTGGAGATAACACCAAATG<br>GAATGAGAATCAAAATCCTCGGATGTTTCTGGCAATGATAACATACATCACAAGAAACCAACCTGAATGGTTTAGAAATGTCTTGAGCATTGCCCCTATAATGTTCTCGAACAAAATGGCGAGATTGGGAAAAGGATACATGTTTGAAAGTAAGAGCATGAAGCTACGGACACAAATACCTGCAGAAATGCTTGCAAACATTGACTTAAAATACTTCAATGAGTCAACAAGAAA<br>GAAAATCGAGAAAATAAGACCTCTACTAATAGATGGCACCGCCTCATTGAGCCCTGGAATGATGATGGGCATGTTCAATATGCTGAGTACAGTATTAGGGGTCTCAATCCTGAATCTTGGGCAAAAGAGGTACACCAAAACCACATACTGGTGGGATGGACTCCAATCCTCTGATGATTTTGCCCTCATAGTGAATGCACCGGATCATGAGGGAATACAAGCAGGAGTGGATA<br>GGTTCTATAGGACCTGCAAACTGGTTGGGATCAACATGAGCAAAAAGAAGTCTTACATAAACCGGACAGGAACATTTGAGTTCACAAGCTTTTTCTATCGCTATGGATTTGTGGCTAACTTCAGTATGGAGCTGCCCAGCTTTGGAGTGTCTGGAATCAACGAATCAGCTGACATGAGCATTGGAGTTACAGTGATAAAGAACAACATGATAAACAATGACCTTGGACCAGCAA<br>CAGCCCAAATGGCCCTTCAACTATTCATCAAGGACTACAGGTACACGTACCGATGTCACAGGGGTGACACACAAATTCAAACGAGGAGATCATTCGAGCTGAAGAAGCTGTGGGAGCAGACCCGTTCAAAGGCAGGACTGTTGGTGTCAGATGGAGGACCAAATCTATACAACATTCGGAATCTCCATATCCCAGAGGTCTGCCTGAAGTGGGAGCTGATGGACGAGGATTA<br>CCAGGGCAGATTATGTAATCCTCTGAACCCATTTGTCAGTCATAAAGAAATTGAGTCCGTAAACAATGCTGTGGTAATGCCAGCCCATGGTCCAGCCAAGAGCATGGAATATGATGCTGTTGCGACTACACACTCATGGATTCCTAAAAGGAATCGTTCCATTCTCAATACCAGCCAAAGGGGAATTCTTGAGGATGAGCAGATGTACCAGAAATGCTGCAGTCTATTCGAGAA<br>ATTCTTCCCCAGTAGCTCATACAGGAGACCAGTTGGAATTTCCAGCATGGTGGAGGCCATGGTGTCTAGGGCCCGAATCGATGCACGCATTGATTTCGAATCTGGAAGGATTAAGAAGGAAGAGTTTGCTGAGATCATGAAGATCTGTTCCACCATTGAAGAGCTCAGACGGCAAAAGTAG
EPI_ISL_337147	>A/pintail/Novosibirsk_region/519k/2018_H3N8_2018-10-01<br>ATGGATGTCAATCCGACTTTACTTTTCTTAAAAGTGCCAGCGCAAAATGCCATAAGTACTACGTTTCCTTATACTGGAGATCCTCCATACAGCCATGGAACAGGAACAGGATACACCATGGACACAGTCAACAGAACACATCAATACTCAGAAAAGGGGAAATGGACAACAAACACAGAGACCGGAGCACCCCAGCTCAACCCAATTGATGGACCACTACCTGAGGACAACGA<br>ACCAAGCGGATATGCACAAACAGATTGTGTGTTGGAAGCAATGGCTTTCCTTGAAGAGTCCCACCCAGGGATCTTTGAAAACTCTTGTCTTGAAACGATGGAAGTTGTTCAGCAAACAAGAGTGGACAAACTAACTCAAGGTCGCCAGACTTATGACTGGACACTGAATAGAAACCAACCAGCTGCAACTGCCCTGGCCAACACTATAGAGGTCTTCAGATCGAACGGTCTGA<br>CAGCCAATGAATCGGGGAGACTGATAGATTTCCTCAAGGATGTGATGGACTCAATGGACAAAGAAGAAATGGAAATAACAACACATTTCCAGAGAAAGAGAAGAGTAAGGGACAACATGACCAAGAAAATGGTCACACAAAGAACAATAGGGAAAAAGAAACAAAGGCTGAATAAGAAGAGCTACTTAATAAGGGCACTGACACTAAACACAATGACAAAAGATGCAGA<br>AAGAGGCAAATTGAAGAGGCGGGCAATTGCAACACCAGGGATGCAGATTAGAGGATTTGTGTACTTTGTCGAAACACTGGCAAGGAGCATCTGTGAGAAACTTGAGCAATCTGGACTCCCCGTTGGAGGGAATGAGAAAAAGGCCAAATTGGCAAATGTCGTGAGAAAAATGATGACCAACTCACAAGATACAGAGCTCTCTTTCACAATTACTGGAGATAACACCAAATG<br>GAATGAAAATCAAAATCCTCGGATGTTTCTGGCAATGATAACATACATCACAAGAAACCAACCTGAATGGTTTAGAAATGTCTTGAGCATTGCCCCTATAATGTTCTCGAACAAAATGGCGAGATTGGGAAAAGGATACATGTTTGAAAGTAAGAGCATGAAGCTACGGACACAAATACCTGCAGACATGCTTGCAAACATTGACTTAAAATATTTCAATGAATCAACAAGAAA<br>GAAAATCGAGAAAATAAGACCTCTGCTAATAGATGGCACAGCCTCATTGAGTCCTGGAATGATGATGGGCATGTTCAATATGCTGAGTACAGTATTAGGAGTCTCAATTCTGAATCTTGGGCAAAAGAGGTACACCAAAACCACATACTGGTGGGATGGACTCCAATCCTCTGATGATTTTGCCCTCATAGTGAATGCACCGAATCATGAGGGAATACAAGCAGGAGTTGATA<br>GGTTCTATAGGACGTGCAAACTGGTTGGAATCAACATGAGCAAAAAGAAGTCTTACATAAATAGGACAGGAACATTTGAGTTCACAAGCTTTTTCTATCGCTATGGATTTGTGGCTAATTTCAGTATGGAGCTGCCCAGCTTTGGAGTGTCTGGAATCAATGAATCAGCTGACATGAGCATTGGAGTTACAGTGATAAAGAATAACATGATAAACAATGACCTTGGACCAGCAA<br>CAGCTCAGATGGCTCTTCAGCTATTCATCAAGGACTACAGGTACACGTACCGATGTCACAGGGGTGACACACAAATTCAAACGAGGAGATCATTCGAGCTGAAGAAGCTGTGGGAGCAGACCCGTTCGAAGGCAGGACTGTTGGTGTCGGATGGAGGACCAAACCTATACAACATTCGGAATCTCCACATCCCAGAGGTCTGCTTGAAGTGGGAGCTGATGGACGAAGATTA<br>CCAGGGCAGGTTGTGTAATCCTCTGAACCCATTTGTCAGTCATAAGGAAATTGAGTCCGTAAACAATGCTGTGGTGATGCCGGCCCATGGCCCAGCCAAGAGCATGGAATATGATGCTGTTGCGACTACACACTCATGGATTCCTAAGAGGAATCGTTCCATTCTCAATACCAGCCAAAGGGGAATTCTTGAGGATGAGCAGATGTACCAGAAGTGCTGTAGTCTATTCGAGAA<br>ATTCTTCCCCAGTAGTTCATACAGGAGGCCAGTTGGAATTTCCAGCATGGTAGAGGCCATGGTATCTAGGGCCCGAATTGATGCACGCATTGATTTCGAATCTGGAAGGATTAAGAAAGAAGAGTTTGCTGAGATCATGAAGATCTGTTCCACCATTGAAGAGCTCAGACGGCAAAAATAG
EPI_ISL_9012572	>A/pheasant/Wales/385129/2021_H5N1_2021-10-27<br>ATGGATGTCAATCCGACTTTACTTTTCTTAAAAGTGCCAGCGCAAAATGCCATAAGTACCACATTCCCTTATACTGGAGATCCTCCATACAGCCATGGAACAGGGACAGGATACACAATGGACACAGTCAACAGAACACATCAATACTCAGAGAAGGGAAAATGGACAACAAACACAGAAACCGGAGCACCTCAACTCAACCCAATTGATGGGCCACTACCTGAGGACAACGA<br>ACCGAGCGGATATGCACAGACAGATTGCGTGTTGGAAGCAATGGCTTTCCTTGAAGAGTCCCACCCAGGGATCTTTGGAAACTCTTGTCTTGAAACGATGGAAGTCGTTCAGCAAACAAGAGTGGACAAACTAACTCAAGGTCGCCAGACTTATGACTGGACACTGAATAGAAACCAACCAGCTGCAACTGCCCTGGCCAACACTATAGAGGTCTTCAGATCAAACGGTCTAA<br>CAGCCAATGAATCGGGGAGACTAATAGATTTCCTCAAGGATGTGATGGACTCAATGGATAAAGAAGAAATGGAAATAACAACACATTTCCAGAGAAAGAGAAGAGTAAGGGACAACATGACCAAGAAAATGGTCACACAAAGAACAATAGGAAAGAAGAAACAAAGGCTAAACAGGAGGAGCTACTTAATAAGAGCACTGACACTGAATACAATGACAAAAGATGCAGA<br>AAGAGGCAAATTGAAGAGACGGGCGATTGCAACACCAGGGATGCAGATTAGAGGATTTGTGTACTTTGTCGAAACACTGGCAAGGAGCATCTGTGAAAAACTTGAGCAATCTGGACTCCCCGTTGGAGGAAATGAGAAGAAGGCTAAATTGGCAAATGTCGTGAGGAAAATGATGACTAACTCACAAGATACAGAGCTCTCCTTCACAATTACTGGAGATAACACCAAATG<br>GAATGAGAATCAGAATCCTCGGATGTTTCTGGCAATGATAACGTACATTACAAGAAACCAACCTGAATGGTTTAGAAATGTCTTGAGTATTGCCCCTATAATGTTCTCGAACAAAATGGCGAGATTGGGAAAAGGGTACATGTTTGAAAGTAAGAGCATGAAGTTACGGACACAAATACCTGCAGAAATACTTGCAAACATTGACTTAAAATACTTCAATGAATCAACAAGAAA<br>GAAAATCGAAAAAATAAGGCCTCTACTAATAGATGGCACTGCCTCATTGAGTCCTGGAATGATGATGGGCATGTTCAATATGCTGAGTACAGTATTAGGAGTTTCAATCCTAAATCTTGGGCAAAAGAGGTACACCAAAACCACATACTGGTGGGATGGACTCCAATCCTCTGATGATTTCGCCCTCATAGTAAATGCACCGAATCATGAGGGAATACAAGCAGGAGTGGATA<br>GGTTCTATAGGACCTGCAAACTGGTCGGGATCAATATGAGCAAAAAGAAGTCTTACATAAACCGGACTGGAACATTTGAGTTCACAAGCTTTTTCTATCGCTATGGATTTGTGGCTAACTTCAGTATGGAGCTGCCCAGCTTTGGAGTTTCTGGAATCAATGAATCAGCTGACATGAGCATTGGCGTCACAGTGATAAAGAACAACATGATAAACAATGACCTTGGACCAGCAA<br>CAGCTCAAATGGCCCTTCAACTATTCATCAAAGATTACAGGTACACGTACCGATGCCACAGAGGTGACACACAAATTCAAACGAGGAGATCATTCGAGCTGAAGAAGCTGTGGGAACAGACCCGTTCAAAGGCAGGACTGTTGGTGTCAGATGGAGGACCAAATCTATACAACATTCGGAATCTCCATATCCCAGAGGTCTGCCTGAAGTGGGAGCTGATGGACGAAGATTA<br>CCAGGGCAGGTTGTGTAATCCTCTGAACCCATTTGTCAGTCATAAAGAAATTGAGTCCGTAAACAATGCTGTGGTGATGCCAGCCCACGGTCCAGCCAAAAGCATGGAATATGATGCCGTTGCAACTACACACTCATGGATTCCTAAAAGGAATCGTTCCATTCTCAATACCAGCCAAAGGGGAATTCTTGAGGATGAACAGATGTACCAGAAATGCTGCAGTCTATTCGAGAA<br>ATTCTTCCCCAGTAGTTCATACAGGAGACCAGTTGGAATTTCCAGCATGGTGGAGGCCATGGTGTCTAGGGCCCGAATCGATGCACGCATTGATTTCGAATCTGGAAGGATCAAGAAGGGAGAGTTTGCTGAGATCATGAAGATCTGTTCCACCATTGAAGAGCTCAGACGGCAAAAATAG
EPI_ISL_1139169	>A/peregrine_falcon/Netherlands/20018821-002/2020_H5N8_2020-11-25<br>ATGGATGTCAATCCGACTTTACTTTTCTTAAAAGTGCCAGCGCAAAATGCTATAAGTACTACATTCCCTTACACTGGAGATCCCCCATACAGCCATGGAACAGGAACAGGGTATACCATGGACACAGTAAACAGAACACATCAATACTCAGAAAAGGGAAAGTGGACAACAAACACAGAAACCGGAGCACCCCAACTCAACCCAATTGATGGACCATTACCAGAGGACAATGA<br>GCCAAGCGGATATGCACAAACTGATTGCGTGTTGGAAGCAATGGCTTTCCTTGAAGAATCCCACCCAGGGATATTTGAAAACTCTTGTCTTGAAGCGATGGAAATCGTTCAGCAAACAAGAGTGGACAAACTAACCCAAGGTCGCCAGACTTATGACTGGACACTGAATAGAAACCAACCAGCTGCAACCTCTTTGGCCAACACTATAGAGGTGTTCAGATCGAATGGTCTGA<br>CAGCCAATGAATCAGGGAGACTGATAGATTTTCTCAGGGATGTGATGGAATCAATGGATAAAGAAGAGATGGAAGTAACAACACATTTCCAGAGAAAAAGAAGAGTGAGGGACAACATGACTAAGAAGATGGTCACACAAAGAACAATAGGGAAGAAGAAGCAGAGGCTGAACAAGAGGAGTTACTTAATAAGAGCACTGACATTGAATACAATGACCAAAGATGCAGA<br>AAGAGGCAAGTTGAAGAGACGGGCAATTGCAACACCCGGGATGCAGATTAGAGGATTCGTGTACTTCGTTGAAACACTAGCGAGGAGCATCTGTGAGAAACTAGAGCAATCTGGGCTCCCTGTTGGAGGGAATGAGAAGAAGGCTAAATTGGCAAATGTTGTGAGAAAAATGATGACTAACTCACAAGATACAGAGCTCTCCTTTACAATTACTGGAGACAACACCAAATG<br>GAATGAGAATCAAAACCCTCGGATGTTTTTGGCAATGATAACATATATCACAAGAAACCAACCTGAATGGTTTAGAAATGTCTTAAGCATTGCCCCTATAATGTTCTCAAACAAAATGGCGAGATTAGGGAAAGGATACATGTTTGAAAGTAAGAGCATGAAGCTAAGAACACAAATACCAGCAGAGATGCTTACAAATATTGATCTGAAGTATTTCAACGAACCAACGAGAA<br>AGAAAATCGAGAAAATAAGACCTCTGCTGATTGATGGCACGGCCTCATTGAGTCCTGGGATGATGATGGGCATGTTCAATATGCTGAGCACAGTATTAGGGGTCTCAATCCTGAATCTCGGGCAAAAGAGGTACACCAAAACCACATACTGGTGGGATGGACTTCAATCCTCTGATGATTTCGCTCTCATAGTGAATGCACCGAATCATGAGGGGATACAAGCAGGAGTGGAT<br>AGATTCTATAGGACCTGCAAACTGGTTGGGATCAACATGAGCAAAAAGAAGTCTTACATAAACCGAACAGGAACATTTGAGTTCACAAGTTTTTTCTACCGCTATGGATTTGTAGCCAACTTCAGTATGGAATTACCCAGCTTTGGAGTGTCTGGAATCAATGAATCAGCTGACATGAGCATTGGAGTTACAGTGATAAAAAACAATATGATAAACAATGATCTTGGACCAGCA<br>ACAGCTCAAATGGCTCTTCAGTTATTCATCAAAGACTATAGGTACACATACCGATGCCACAGGGGTGATACACAAATTCAAACGAGGAGATCATTCGAGCTGAAGAAGCTGTGGGAGCAGACCCGTTCAAAGGCAGGGCTGTTGATATCAGACGGGGGGCCAAATCTATACAACATTCGGAATCTCCACATCCCAGAGGTCTGCTTGAAGTGGGAGCTGATGGATGAAGACT<br>ACCAAGGCAGGCTGTGCAATCCTCTGAATCCATTTGTCAGTCATAAAGAGATTGAGTCCGTAAACAATGCTGTAGTAATGCCCGCCCATGGCCCGGCCAAGAGCATGGAATATGATGCTGTTGCGACCACACACTCGTGGATTCCTAAGAGGAACCGTTCCATTCTCAATACCAGCCAAAGGGGAATTCTTGAGGATGAGCAGATGTACCAAAAGTGCTGTAGTCTATTCGAGA<br>AATTCTTCCCCAGCAGTTCATACAGGAGGCCAGTTGGAATTTCCAGCATGGTGGAGGCCATGGTGTCTAGGGCCCGAATTGATGCACGCATCGACTTCGAATCTGGAAGGATTAAGAAAGAAGAGTTTGCTGAGATCATGAAGATCTGTTCCACCATTGAAGAGCTCAGACGGCAAAAATAG
EPI_ISL_813979	>A/peregrine_falcon/Ireland/20VIR7872-1/2020_H5N8_2020-11<br>ATGGATGTCAATCCGACTTTACTTTTCTTAAAAGTGCCAGCGCAAAATGCTATAAGTACTACATTCCCTTACACTGGAGATCCTCCATACAGCCATGGAACAGGAACAGGGTATACCATGGACACAGTAAACAGAACACATCAATACTCAGAAAAGGGAAAGTGGACAACAAACACAGAAACCGGAGCACCCCAACTCAACCCAATTGATGGACCATTACCAGAGGACAATGA<br>GCCAAGCGGATATGCACAAACTGATTGCGTGTTGGAAGCAATGGCTTTCCTTGAAGAATCCCACCCAGGGATATTTGAAAACTCTTGTCTTGAAGCGATGGAAATCGTTCAGCAAACAAGAGTGGACAAACTAACCCAAGGTCGCCAGACTTATGACTGGACACTGAACAGAAACCAACCAGCTGCAACCTCTTTGGCCAACACTATAGAGGTGTTCAGATCGAATGGTCTGA<br>CAGCCAATGAATCAGGGAGACTGATAGATTTTCTCAGGGATGTGATGGAATCAATGGATAAAGAAGAGATGGAAGTAACAACACATTTCCAGAGAAAAAGAAGAGTGAGGGACAACATGACTAAGAAGATGGTCACACAAAGAACAATAGGGAAGAAGAAGCAGAGGCTGAACAAGAGGAGTTACTTAATAAGAGCACTGACATTGAATACAATGACCAAAGATGCAGA<br>AAGAGGCAAGTTGAAGAGACGGGCAATTGCAACACCCGGGATGCAGATTAGAGGATTCGTGTACTTCGTTGAAACACTAGCGAGGAGCATCTGTGAGAAACTAGAGCAATCTGGGCTCCCTGTTGGAGGGAATGAGAAGAAGGCTAAATTGGCAAATGTTGTGAGAAAAATGATGACTAACTCACAAGATACAGAGCTCTCCTTTACAATTACTGGAGACAACACCAAATG<br>GAATGAGAATCAAAACCCTCGGATGTTTTTGGCAATGATAACATATATCACAAGAAACCAACCTGAATGGTTTAGAAATGTCTTAAGCATTGCCCCTATAATGTTCTCAAACAAAATGGCGAGATTAGGGAAAGGATACATGTTTGAAAGTAAGAGCATGAAGCTAAGAACACAAATACCAGCAGAGATGCTTACAAATATCGATCTGAAGTATTTCAACGAACCAACGAGAA<br>AGAAAATCGAGAAAATAAGACCTCTGCTGATTGATGGCACGGCCTCATTGAGCCCTGGGATGATGATGGGCATGTTCAATATGCTGAGCACAGTATTAGGGGTCTCAATCCTGAATCTCGGGCAAAAAAGGTACACCAAAACCACATACTGGTGGGATGGACTTCAATCCTCTGATGATTTCGCTCTCATAGTGAATGCACCGAATCATGAGGGGATACAAGCAGGAGTGGAT<br>AGATTCTATAGGACCTGCAAACTGGTTGGGATCAACATGAGCAAAAAGAAGTCTTACATAAACCGAACAGGAACATTTGAGTTCACAAGTTTTTTCTACCGCTATGGATTTGTAGCTAACTTCAGTATGGAATTACCCAGCTTTGGAGTGTCTGGAATCAATGAATCAGCTGACATGAGCATTGGAGTTACAGTGATAAAAAACAATATGATAAACAATGATCTTGGACCAGCA<br>ACAGCTCAAATGGCTCTTCAGTTATTCATCAAAGACTATAGGTACACATACCGATGCCACAGGGGTGATACACAAATTCAAACGAGGAGATCATTCGAGCTGAAGAAGCTGTGGGAGCAGACCCGTTCAAAGGCAGGGCTGTTGATATCAGACGGGGGGCCAAACCTATACAACATTCGGAATCTCCACATCCCAGAGGTCTGCTTGAAGTGGGAGCTGATGGATGAAGACT<br>ACCAAGGCAGGCTGTGCAATCCTCTGAATCCATTTGTCAGTCATAAAGAGATTGAGTCCGTAAACAATGCTGTAGTAATGCCCGCCCATGGCCCGGCCAAGAGCATGGAATATGATGCTGTTGCGACCACACACTCGTGGATTCCTAAGAGGAACCGTTCCATCCTCAATACCAGCCAAAGGGGAATTCTTGAGGATGAGCAGATGTACCAAAAGTGCTGTAGTCTATTCGAGA<br>AATTCTTCCCCAGCAGTTCATACAGGAGGCCAGTTGGAATTTCCAGCATGGTGGAGGCCATGGTGTCTAGGGCCCGAATTGATGCACGCATCGACTTCGAATCTGGAAGGATTAAGAAAGAAGAGTTTGCTGAGATCATGAAGATCTGTTCCACCATTGAAGAGCTCAGACGGCAAAAATAG
EPI_ISL_11259276	>A/peregrine_falcon/Ireland/032476_22VIR1325-3/2021_H5N1_2021-11-10<br>ATGGATGTCAATCCGACTTTACTTTTCTTAAAAGTGCCAGCGCAAGATGCCATAAGTACCACATTCCCTTACACTGGAGATCCTCCATACAGCCATGGAACAGGGACAGGATACACAATGGACACAGTCAACAGAACACATCAATACTCAGAGAAGGGAAAATGGACAACAAACACAGAAACCGGAGCACCTCAACTCAACCCAATTGATGGGCCACTACCTGAGGACAACGA<br>ACCGAGCGGATATGCACAAACAGATTGCGTGTTGGAAGCAATGGCTTTCCTTGAAGAGTCCCATCCAGGGATCTTTGAAAACTCTTGTCTTGAAACGATGGAAGTCGTTCAGCAAACAAGAGTGGACAAACTAACTCAAGGTCGCCAGACATATGACTGGACACTGAATAGAAACCAACCGGCTGCAACTGCCCTGGCCAACACTATAGAGGTCTTCAGATCAAACGGTCTAA<br>CAGCCAATGAATCGGGGAGACTAATAGATTTCCTCAAGGATGTGATGGACTCAATGGATAAAGAAGAAATGGAAATAACAACACATTTCCAGAGAAAGAGAAGAGTAAGGGACAACATGACCAAGAAAATGGTCACACAAAGAACAATAGGAAAGAAGAAACAAAGGCTAAACAAGAGGAGCTACTTAATAAGAGCACTGACACTGAATACAATGACAAAAGATGCAGA<br>AAGAGGCAAATTGAAGAGACGGGCGATTGCAACACCAGGGATGCAGATTAGAGGATTTGTGTACTTTGTCGAAACACTGGCAAGGAGCATCTGTGAAAAACTTGAGCAATCTGGACTCCCCGTTGGAGGGAATGAGAAGAAGGCTAAATTGGCAAATGTCGTGAGAAAAATGATGACTAACTCACAAGATACAGAGCTCTCCTTCACAATTACTGGAGATAACACCAAATG<br>GAATGAGAATCAAAATCCTCGGATGTTTCTGGCAATGATAACATACATTACAAGAAACCAACCTGAATGGTTTAGGAATGTCTTGAGTATTGCCCCTATAATGTTCTCGAACAAAATGGCGAGATTGGGAAAAGGGTACATGTTTGAAAGTAAGAGCATGAAGTTACGGACACAAATACCTGCAGAAATGCTTGCAAACATTGACTTAAAATACTTCAATGAATCAACAAGAAA<br>GAAAATCGAAAAAATAAGGCCTCTACTAATAGATGGCACTGCCTCATTGAGTCCTGGAATGATGATGGGCATGTTCAATATGCTGAGTACAGTATTAGGAGTTTCAATCCTAAATCTTGGGCAAAAGAAGTACACCAAAACCACATACTGGTGGGATGGACTCCAATCCTCTGATGATTTCGCCCTCATAGTAAATGCACCGAATCATGAGGGAATACAAGCAGGAGTGGATA<br>GGTTCTATAGGACCTGCAAACTGGTCGGGATCAATATGAGCAAAAAGAAGTCTTACATAAACCGGACTGGAACATTTGAGTTCACAAGCTTTTTCTATCGCTATGGATTTGTGGCTAACTTCAGTATGGAGCTGCCCAGCTTTGGAGTTTCTGGGATCAATGAATCAGCTGACATGAGCATTGGCATCACAGTGATAAAGAACAACATGATAAACAATGACCTTGGACCAGCAA<br>CAGCTCAAATGGCCCTTCAACTATTCATCAAAGATTACAGGTACACGTACCGATGCCATAGAGGTGACACACAAATTCAAACGAGGAGATCATTCGAGCTGAAGAAGCTGTGGGAACAGACCCGTTCAAAGGCAGGACTGTTGGTGTCAGATGGAGGACCAAATCTATACAACATTCGGAATCTCCATATCCCAGAGGTCTGCCTGAAGTGGGAGCTGATGGACGAAGATTA<br>CCAGGGCAGGTTGTGTAATCCTCTGAACCCATTTGTCAGCCATAAAGAAATTGAGTCCGTAAACAATGCTGTGGTGATGCCAGCCCATGGTCCAGCCAAAAGCATGGAATATGATGCCGTTGCGACTACACACTCATGGATTCCTAAAAGGAATCGTTCCATTCTCAATACCAGCCAAAGGGGAATTCTTGAGGATGAACAGATGTACCAGAAATGCTGCGGTCTATTCGAGAA<br>ATTCTTCCCCAGTAGTTCATACAGGAGACCAGTTGGAATTTCCAGCATGGTGGAGGCCATGGTGTCTAGGGCCCGAATCGATGCACGCATTGATTTCGAATCTGGAAGGATCAAGAAGGAAGAGTTTGCTGAGATCATGAAGATCTGTTCCACCATTGAAGAGCTCAGACGGCAAAAATAG
EPI_ISL_9261743	>A/Oystercatcher/Netherlands/1/2022_H5N1_2022-01-11<br>ATGGATGTCAATCCGACTTTACTTTTCTTAAAAGTGCCAGCGCAAGATGCCATAAGTACCACATTCCCTTACACTGGAGATCCTCCATACAGCCATGGAACAGGGACAGGATACACAATGGACACGGTCAACAGAACACATCAATACTCAGAGAAGGGAAAATGGACAACAAACACAGAAACCGGAGCACCTCAACTCAACCCAATTGATGGGCCACTACCTGAGGACAACGA<br>ACCGAGCGGATATGCACAAACAGATTGCGTGTTGGAAGCAATGGCTTTCCTTGAAGAGTCCCATCCAGGGATCTTTGAAAACTCTTGTCTTGAAACGATGGAAGTCGTTCAGCAAACAAGAGTGGACAAACTAACTCAAGGTCGCCAGACATATGACTGGACACTGAATAGAAACCAACCAGCTGCAACTGCCTTGGCCAACACTATAGAGGTCTTCAGATCAAACGGTCTAA<br>CAGCCAATGAATCGGGGAGACTAATAGATTTCCTCAAGGATGTGATGGACTCAATGGATAAAGAAGAAATGGAAATAACAACACATTTCCAGAGAAAGAGAAGAGTAAGGGACAACATGACCAAGAAAATGGTCACACAAAGAACAATAGGAAAGAAGAAACAAAGGCTAAACAAGAGGAGCTACTTAATAAGAGCACTGACACTGAATACAATGACAAAAGATGCAGA<br>AAGAGGCAAATTGAAGAGACGGGCGATTGCAACACCAGGGATGCAGATTAGAGGATTTGTGTACTTTGTCGAAACACTGGCAAGGAGCATCTGTGAAAAACTTGAGCAATCTGGACTCCCCGTTGGAGGGAATGAGAAGAAGGCTAAATTGGCAAATGTTGTGAGAAAAATGATGACTAACTCACAAGATACAGAGCTCTCCTTCACAATTACTGGAGATAACACCAAATG<br>GAATGAGAATCAAAATCCTCGGATGTTTCTGGCAATGATAACATACATTACAAGAAACCAACCTGAATGGTTTAGGAATGTCTTGAGTATTGCCCCTATAATGTTCTCGAACAAAATGGCGAGATTGGGAAAAGGGTACATGTTTGAAAGTAAGAGCATGAAGTTACGGACACAAATACCTGCAGAAATGCTTGCAAACATTGACTTAAAATATTTCAATGAATCAACAAGAAA<br>GAAAATCGAAAAAATAAGGCCTCTACTAATAGATGGCACTGCCTCATTGAGTCCTGGAATGATGATGGGCATGTTCAATATGCTGAGTACAGTATTAGGAGTTTCAATCCTAAATCTTGGGCAAAAGAAGTACACCAAAACCACATACTGGTGGGATGGACTCCAATCCTCTGATGATTTCGCCCTCATAGTAAATGCACCGAATCATGAGGGAATACAAGCAGGAGTGGATA<br>GGTTCTATAGGACCTGCAAACTGGTCGGGATCAATATGAGCAAAAAGAAGTCTTACATAAACCGGACTGGAACATTTGAGTTCACAAGCTTTTTCTATCGCTATGGATTTGTGGCTAATTTCAGTATGGAGCTGCCCAGCTTTGGAGTTTCTGGGATCAATGAATCAGCTGACATGAGCATTGGCATCACAGTGATAAAGAACAACATGATAAACAATGACCTTGGACCAGCAA<br>CAGCTCAAATGGCCCTTCAACTATTCATCAAAGATTACAGGTACACGTACCGATGCCATAGAGGTGACACACAAATTCAAACGAGGAGATCATTCGAGCTGAAGAAGCTGTGGGAACAGACCCGTTCAAAGGCAGGACTGTTGGTGTCAGATGGAGGACCAAATCTATACAACATTCGGAATCTCCATATCCCAGAGGTCTGCCTGAAGTGGGAGCTGATGGACGAAGATTA<br>CCAGGGCAGGTTGTGTAATCCTCTGAACCCATTTGTCAGCCATAAAGAAATTGAGTCCGTAAACAATGCTGTGGTGATGCCAGCCCACGGTCCAGCCAAAAGCATGGAATATGATGCCGTTGCGACTACACACTCATGGATTCCTAAAAGGAATCGTTCCATTCTCAATACCAGTCAAAGGGGAATTCTTGAGGATGAACAGATGTACCAGAAATGCTGCGGTCTATTCGAGAA<br>ATTCTTCCCCAGTAGTTCATACAGGAGACCAGTTGGAATTTCCAGCATGGTGGAGGCCATGGTGTCTAGGGCCCGAATCGATGCACGCATTGATTTCGAATCTGGAAGGATCAAGAAGGAAGAGTTTGCTGAGATCATGAAGATCTGTTCCACCATTGAAGAGCTCAGACGGCAAAAATAG
EPI_ISL_387973	>A/northern_shoveler/Egypt/MB-D-695C/2016_H7N3_2016-03-17<br>ATGGATGTCAATCCGACTTTACTTTTCTTGAAAGTGCCAGCGCAAAATGCCATAAGTACCACATTCCCTTATACTGGAGATCCTCCATACAGCCATGGAACAGGAACAGGATACACCATGGACACAGTCAACAGAACACATCAATACTCAGAAAAGGGGAAATGGACAACAAACACAGAGACCGGAGCACCCCAACTCAACCCAATTGATGGACCACTACCTGAGGACAACGA<br>ACCAAGCGGATATGCACAAACAGATTGCGTGTTGGAAGCAATGGCTTTCCTTGAAGAGTCCCACCCAGGGATCTTTGAAAACTCTTGTCTTGAAACGATGGAAGTCGTTCAGCAAACAAGAGTGGACAAACTAACTCAAGGTCGCCAGACTTATGACTGGACACTGAATAGAAACCAACCAGCTGCAACTGCCCTGGCCAACACTATAGAGGTCTTCAGATCGAACGGTCTGA<br>CAGCCAATGAATCGGGGAGACTAATAGATTTCCTCAAGGATGTGATGGACTCAATGGACAAAGAAGAAATGGAAATAACAACACATTTCCAGAGAAAGAGAAGAGTAAGGGACAACATGACCAAGAAAATGGTCACACAAAGAACAATAGGGAAGAAGAAACAAAGGCTAAATAAGAGGAGCTACTTAATAAGAGCATTGACACTGAATACAATGACAAAAGACGCAGA<br>AAGAGGCAAATTGAAGAGACGGGCAATTGCAACACCAGGGATGCAGATTAGAGGATTTGTGTACTTTGTCGAAACACTGGCAAGGAGCATCTGTGAAAAACTTGAGCAATCTGGACTCCCCGTTGGAGGGAATGAGAAGAAGGCTAAATTGGCAAATGTCGTGAGAAAAATGATGACTAACTCACAAGATACAGAGCTCTCCTTCACAATTACTGGAGATAACACCAAATG<br>GAATGAGAATCAAAACCCTCGGATGTTTCTGGCAATGATAACATACATCACAAGAAACCAACCTGAATGGTTTAGAAATGTTTTGAGCATTGCCCCTATAATGTTCTCGAACAAAATGGCGAGATTGGGAAAAGGATACATGTTTGAAAGTAAGAGCATGAAGCTACGGACACAAATACCTGCAGAAATGCTTGCAAACATTGACTTAAAATACTTCAATGAGTCAACAAGAAA<br>GAAAATCGAGAAAATAAGACCTCTATTAATAGATGGCACCGCCTCATTGAGTCCTGGAATGATGATGGGCATGTTCAATATGCTGAGTACAGTATTAGGAGTCTCAATCCTGAATCTTGGGCAAAAGAGGTACACCAAAACCACATACTGGTGGGATGGACTCCAATCCTCTGATGATTTTGCTCTCATAGTGAATGCACCGAATCATGAGGGAATACAAGCAGGAGTGGATA<br>GGTTCTATAGGACCTGCAAACTGGTTGGGATCAACATGAGCAAAAAGAAGTCTTACATAAACCGGACAGGAACATTTGAGTTCACAAGCTTTTTCTATCGCTATGGATTTGTGGCTAACTTCAGTATGGAGCTGCCCAGCTTTGGAGTGTCTGGAATCAATGAATCAGCTGACATGAGCATTGGAGTTACAGTGATAAAGAACAACATGATAAACAATGACCTTGGACCAGCAA<br>CAGCTCAAATGGCCCTTCAACTATTCATCAAGGACTATAGGTACACATACCGATGTCACAGGGGTGACACACAAATTCAAACGAGGAGATCATTCGAGCTGAAGAAGCTGTGGGAGCAGACCCGTTCAAAGGCAGGACTGTTGGTGTCAGATGGAGGACCAAATCTATACAACATTCGGAATCTCCATATCCCAGAGGTCTGCCTGAAGTGGGAGCTGATGGACGAAGATTA<br>CCAGGGCAGGTTGTGTAATCCTCTGAACCCATTTGTCAGTCATAAAGAAATTGAGTCCGTAAACAATGCTGTGGTAATGCCAGCCCATGGCCCAGCCAAGAGCATGGAATATGATGCCGTTGCGACTACACACTCATGGATTCCTAAAAGGAATCGTTCCATTCTCAATACCAGCCAAAGGGGAATTCTTGAGGATGAGCAGATGTACCAGAAATGCTGCAGTCTATTCGAGAA<br>ATTCTTCCCCAGTAGTTCATACAGGAGACCAGTTGGAATTTCCAGCATGGTGGAGGCCATGGTGTCTAGGGCCCGAATCGATGCACGCATTGATTTCGAATCTGGAAGGATTAAGAAGGAAGAGTTTGCTGAGATCATGAAGATCTGTTCCACCATTGAAGAGCTCAGACGGCAAAAATAG
EPI_ISL_387972	>A/northern_shoveler/Egypt/MB-D-690C/2016_H7N3_2016-03-17<br>ATGGATGTCAATCCGACTTTACTTTTCTTGAAAGTGCCAGCGCAAAATGCCATAAGTACCACATTCCCTTATACTGGAGATCCTCCATACAGCCATGGAACAGGAACAGGATACACCATGGACACAGTCAACAGAACACATCAATACTCAGAAAAGGGGAAATGGACAACAAACACAGAGACCGGAGCACCCCAACTCAACCCAATTGATGGACCACTACCTGAGGACAACGA<br>ACCAAGCGGATATGCACAAACAGATTGCGTGTTGGAAGCAATGGCTTTCCTTGAAGAGTCCCACCCAGGGATCTTTGAAAACTCTTGTCTTGAAACGATGGAAGTCGTTCAGCAAACAAGAGTGGACAAACTAACTCAAGGTCGCCAGACTTATGACTGGACACTGAATAGAAACCAACCAGCTGCAACTGCCCTGGCCAACACTATAGAGGTCTTCAGATCGAACGGTCTGA<br>CAGCCAATGAATCGGGGAGACTAATAGATTTCCTCAAGGATGTGATGGACTCAATGGACAAAGAAGAAATGGAAATAACAACACATTTCCAGAGAAAGAGAAGAGTAAGGGACAACATGACCAAGAAAATGGTCACACAAAGAACAATAGGGAAGAAGAAACAAAGGCTAAATAAGAGGAGCTACTTAATAAGAGCATTGACACTGAATACAATGACAAAAGACGCAGA<br>AAGAGGCAAATTGAAGAGACGGGCAATTGCAACACCAGGGATGCAGATTAGAGGATTTGTGTACTTTGTCGAAACACTGGCAAGGAGCATCTGTGAAAAACTTGAGCAATCTGGACTCCCCGTTGGAGGGAATGAGAAGAAGGCTAAATTGGCAAATGTCGTGAGAAAAATGATGACTAACTCACAAGATACAGAGCTCTCCTTCACAATTACTGGAGATAACACCAAATG<br>GAATGAGAATCAAAACCCTCGGATGTTTCTGGCAATGATAACATACATCACAAGAAACCAACCTGAATGGTTTAGAAATGTTTTGAGCATTGCCCCTATAATGTTCTCGAACAAAATGGCGAGATTGGGAAAAGGATACATGTTTGAAAGTAAGAGCATGAAGCTACGGACACAAATACCTGCAGAAATGCTTGCAAACATTGACTTAAAATACTTCAATGAGTCAACAAGAAA<br>GAAAATCGAGAAAATAAGACCTCTATTAATAGATGGCACCGCCTCATTGAGTCCTGGAATGATGATGGGCATGTTCAATATGCTGAGTACAGTATTAGGAGTCTCAATCCTGAATCTTGGGCAAAAGAGGTACACCAAAACCACATACTGGTGGGATGGACTCCAATCCTCTGATGATTTTGCTCTCATAGTGAATGCACCGAATCATGAGGGAATACAAGCAGGAGTGGATA<br>GGTTCTATAGGACCTGCAAACTGGTTGGGATCAACATGAGCAAAAAGAAGTCTTACATAAACCGGACAGGAACATTTGAGTTCACAAGCTTTTTCTATCGCTATGGATTTGTGGCTAACTTCAGTATGGAGCTGCCCAGCTTTGGAGTGTCTGGAATCAATGAATCAGCTGACATGAGCATTGGAGTTACAGTGATAAAGAACAACATGATAAACAATGACCTTGGACCAGCAA<br>CAGCTCAAATGGCCCTTCAACTATTCATCAAGGACTATAGGTACACATACCGATGTCACAGGGGTGACACACAAATTCAAACGAGGAGATCATTCGAGCTGAAGAAGCTGTGGGAGCAGACCCGTTCAAAGGCAGGACTGTTGGTGTCAGATGGAGGACCAAATCTATACAACATTCGGAATCTCCATATCCCAGAGGTCTGCCTGAAGTGGGAGCTGATGGACGAAGATTA<br>CCAGGGCAGGTTGTGTAATCCTCTGAACCCATTTGTCAGTCATAAAGAAATTGAGTCCGTAAACAATGCTGTGGTAATGCCAGCCCATGGCCCAGCCAAGAGCATGGAATATGATGCCGTTGCGACTACACACTCATGGATTCCTAAAAGGAATCGTTCCATTCTCAATACCAGCCAAAGGGGAATTCTTGAGGATGAGCAGATGTACCAGAAATGCTGCAGTCTATTCGAGAA<br>ATTCTTCCCCAGTAGTTCATACAGGAGACCAGTTGGAATTTCCAGCATGGTGGAGGCCATGGTGTCTAGGGCCCGAATCGATGCACGCATTGATTTCGAATCTGGAAGGATTAAGAAGGAAGAGTTTGCTGAGATCATGAAGATCTGTTCCACCATTGAAGAGCTCAGACGGCAAAAATAG

EPI_ISL_1240717	>A/northern_goshawk/Sweden/SVA210225SZ0302/KN000664/2021_H5N8_2021-02-21<br>ATGGATGTCAATCCGACTTTACTTTTCTTAAAAGTGCCAGCGCAAAATGCTATAAGTACTACATTCCCTTACACTGGAGATCCCCCATACAGCCATGGAACAGGAACAGGGTATACCATGGACACAGTAAACAGAACACATCAATACTCAGAAAAGGGAAAGTGGACAACAAACACAGAAACCGGAGCACCCCAACTCAACCCAATTGATGGACCATTACCAGAGGACAATGA<br>GCCAAGCGGATATGCACAAACTGATTGCGTGTTGGAAGCAATGGCTTTCCTTGAAGAATCCCACCCAGGGATATTTGAAAACTCTTGTCTTGAAGCGATGGAAATCGTTCAGCAAACAAGAGTGGACAAACTAACCCAAGGTCGCCAGACTTATGACTGGACACTGAACAGAAACCAACCAGCTGCAACCTCTTTGGCCAACACTATAGAGGTGTTCAGATCGAATGGTCTGA<br>CAGCCAATGAATCAGGGAGACTGATAGATTTTCTCAGGGATGTGATGGAATCAATGGATAAAGAAGAGATGGAAGTAACAACACATTTCCAGAGAAAAAGAAGAGTGAGGGACAACATGACTAAGAAGATGGTCACACAAAGAACAATAGGGAAGAAGAAGCAGAGGCTGAACAAGAGGAGTTACTTAATAAGAGCACTGACATTGAATACAATGACCAAAGATGCAGA<br>AAGAGGCAAGTTGAAGAGACGGGCAATTGCAACACCCGGGATGCAGATTAGAGGATTCGTGTACTTCGTTGAAACACTAGCGAGGAGCATCTGTGAGAAACTAGAGCAATCTGGGCTCCCTGTTGGAGGGAATGAGAAGAAGGCTAAATTGGCAAATGTTGTGAGAAAAATGATGACTAACTCACAAGATACAGAGCTCTCCTTTACAATTACTGGAGACAACACCAAATG<br>GAATGAGAATCAAAACCCTCGGATGTTTTTGGCAATGATAACATATATCACAAGAAACCAACCTGAATGGTTTAGAAATGTCTTAAGCATTGCCCCTATAATGTTCTCAAACAAAATGGCGAGATTAGGGAAAGGATACATGTTTGAAAGTAAGAGCATGAAGCTAAGAACACAAATACCAGCAGAGATGCTTACAAATATTGATCTGAAGTATTTCAACGAACCAACGAGAA<br>AGAAAATCGAGAAAATAAGACCTCTGCTGATTGATGGCACGGCCTCATTGAGTCCTGGGATGATGATGGGCATGTTCAATATGCTGAGCACAGTATTAGGGGTCTCAATCCTGAATCTCGGGCAAAAGAGGTACACCAAAACCACATACTGGTGGGATGGACTTCAATCCTCTGATGATTTCGCTCTCATAGTGAATGCACCGAATCATGAGGGGATACAAGCAGGAGTGGAT<br>AGATTCTATAGGACCTGCAAACTGGTTGGGATCAACATGAGCAAAAAGAAGTCTTACATAAACCGAACAGGAACATTTGAGTTCACAAGTTTTTTCTACCGCTATGGATTTGTAGCCAACTTCAGTATGGAATTACCCAGCTTTGGAGTGTCTGGAATCAATGAATCAGCTGACATGAGCATTGGAGTTACAGTGATAAAAAACAATATGATAAACAATGATCTTGGACCAGCA<br>ACAGCTCAAATGGCTCTTCAGTTATTCATCAAAGACTATAGGTACACATACCGATGCCACAGGGGTGATACACAAATTCAAACGAGGAGATCATTCGAGCTGAAGAAGCTGTGGGAGCAGACCCGTTCAAAGGCAGGGCTGTTGATATCAGACGGGGGGCCAAATCTATACAACATTCGGAATCTCCACATCCCAGAGGTCTGCTTGAAGTGGGAGCTGATGGATGAAGACT<br>ACCAAGGCAGGCTGTGCAATCCTCTGAATCCATTTGTCAGTCATAAAGAGATTGAGTCCGTAAACAATGCTGTAGTAATGCCCGCCCATGGCCCGGCCAAGAGCATGGAATATGATGCTGTTGCGACCACACACTCGTGGATTCCTAAGAGGAACCGTTCCATTCTCAATACCAGCCAAAGGGGAATTCTTGAGGATGAGCAGATGTACCAAAAGTGCTGTAGTCTATTCGAGA<br>AATTCTTCCCCAGCAGTTCATACAGGAGGCCAGTTGGAATTTCCAGCATGGTGGAGGCCATGGTGTCTAGGGCCCGAATTGATGCACGCATCGACTTCGAATCTGGAAGGATTAAGAAAGAAGAGTTTGCTGAGATCATGAAGATCTGTTCCACCATTGAAGAGCTCAGACGGCAAAAATAG
EPI_ISL_1139164	>A/northern_goshawk/Netherlands/20018560-002/2020_H5N8_2020-11-22<br>ATGGATGTCAATCCGACTTTACTTTTCTTAAAAGTGCCAGCGCAAAATGCTATAAGTACTACATTCCCTTACACTGGAGATCCCCCATACAGCCATGGAACAGGAACAGGGTATACCATGGACACAGTAAACAGAACACATCAATACTCAGAAAAGGGAAAGTGGACAACAAACACAGAAACCGGAGCACCCCAACTCAACCCAATTGATGGACTATTACCAGAGGACAATGA<br>GCCAAGCGGATATGCACAAACTGATTGCGTGTTGGAAGCAATGGCTTTCCTTGAAGAATCCCACCCAGGGATATTTGAAAACTCTTGTCTTGAAGCGATGGAAATCGTTCAGCAAACAAGAGTGGACAAACTAACCCAAGGTCGCCAGACTTATGACTGGACACTGAACAGAAACCAACCAGCTGCAACCTCTTTGGCCAACACTATAGAGGTGTTCAGATCGAATGGTCTGA<br>CAGCCAATGAATCAGGGAGACTGATAGATTTTCTCAGGGATGTGATGGAATCAATGGATAAAGAAGAGATGGAAGTAACAACACATTTCCAGAGAAAAAGAAGAGTGAGGGACAACATGACTAAGAAGATGGTCACACAAAGAACAATAGGGAAGAAGAAGCAGAGGCTGAACAAGAGGAGTTACTTAATAAGAGCACTGACATTGAATACAATGACCAAAGATGCAGA<br>AAGAGGCAAGTTGAAGAGACGGGCAATTGCAACACCCGGGATGCAGATTAGAGGATTCGTGTACTTCGTTGAAACACTAGCGAGGAGCATCTGTGAGAAACTAGAGCAATCTGGGCTCCCTGTTGGAGGGAATGAGAAGAAGGCTAAATTGGCAAATGTTGTGAGAAAAATGATGACTAACTCACAAGATACAGAGCTCTCCTTTACAATTACTGGAGACAACACCAAATG<br>GAATGAGAATCAAAACCCTCGGATGTTTTTGGCAATGATAACATATATCACAAGAAACCAACCTGAATGGTTTAGAAATGTCTTAAGCATTGCCCCTATAATGTTCTCAAACAAAATGGCGAGATTAGGGAAAGGATACATGTTTGAAAGTAAGAGCATGAAGCTAAGAACACAAATACCAGCAGAGATGCTTACAAATATTGATCTGAAGTATTTCAACGAACCAACGAGAA<br>AGAAAATCGAGAAAATAAGACCTCTGCTGATTGATGGCACGGCCTCATTGAGTCCTGGGATGATGATGGGCATGTTCAATATGCTGAGCACAGTATTAGGGGTCTCAATCCTGAATCTCGGGCAAAAGAGGTACACCAAAACCACATACTGGTGGGATGGACTTCAATCCTCTGATGATTTCGCTCTCATAGTGAATGCACCGAATCATGAGGGGATACAAGCAGGAGTGGAT<br>AGATTCTATAGGACCTGCAAACTGGTTGGGATCAACATGAGCAAAAAGAAGTCTTACATAAACCGAACAGGAACATTTGAGTTCACAAGTTTTTTCTACCGCTATGGATTTGTAGCCAACTTCAGTATGGAATTACCCAGCTTTGGAGTGTCTGGAATCAATGAATCAGCTGACATGAGCATTGGAGTTACAGTGATAAAAAACAATATGATAAACAATGATCTTGGACCAGCA<br>ACAGCTCAAATGGCTCTTCAGTTATTCATCAAAGACTATAGGTACACATACAGATGCCACAGGGGTGATACACAAATTCAAACGAGGAGATCATTCGAGCTGAAGAAGCTGTGGGAGCAGACCCGTTCAAAGGCAGGGCTGTTGATATCAGACGGGGGGCCAAATCTATACAACATTCGGAATCTCCACATCCCAGAGGTCTGCTTGAAGTGGGAGCTGATGGATGAAGACT<br>ACCAAGGCAGGCTGTGCAATCCTCTGAATCCATTTGTCAGTCATAAAGAGATTGAGTCCGTAAACAATGCTGTAGTAATGCCCGCCCATGGCCCGGCCAAGAGCATGGAATATGATGCTGTTGCGACCACACACTCGTGGATTCCTAAGAGGAACCGTTCCATTCTCAATACCAGCCAAAGGGGAATTCTTGAGGATGAGCAGATGTACCAAAAGTGCTGTAGTCTATTCGAGA<br>AATTCTTCCCCAGCAGTTCATACAGGAGGCCAGTTGGAATTTCCAGCATGGTGGAGGCCATGGTGTCTAGGGCCCGAATTGATGCACGCATCGACTTCGAATCTGGAAGGATTAAGAAAGAAGAGTTTGCTGAGATCATGAAGATCTGTTCCACCATTGAAGAGCTCAGACGGCAAAAATAG
EPI_ISL_15088298	>A/Northern_Gannet/Netherlands/1/2022_H5N1_2022-08-23<br>ATGGATGTCAATCCGACTTTACTTTTCTTAAAAGTGCCAGCGCAAGATGCCATAAGTACCACATTCCCTTACACTGGAGATCCTCCATACAGCCATGGAACAGGGACAGGATACACAATGGACACAGTCAACAGAACACATCAATACTCAGAGAAGGGAAAATGGACAACAAACACAGAAACCGGAGCACCTCAACTCAACCCAATTGATGGGCCACTACCTGAGGACAACGA<br>ACCGAGCGGATATGCACAAACAGATTGCGTGTTGGAAGCAATGGCTTTCCTTGAAGAGTCCCATCCAGGGATCTTTGAAAACTCTTGTCTTGAAACGATGGAAGTCGTTCAGCAAACAAGAGTGGACAAACTAACTCAAGGTCGCCAGACATATGACTGGACACTGAATAGAAACCAACCAGCTGCAACTGCCCTGGCCAACACTATAGAGGTCTTCAGATCAAACGGTCTAA<br>CAGCCAATGAATCGGGGAGACTAATAGATTTCCTCAAGGATGTGATGGACTCAATGGATAAAGAAGAAATGGAAATAACAACACATTTCCAGAGAAAGAGAAGAGTAAGGGACAACATGACCAAGAAAATGGTCACACAAAGAACAATAGGAAAGAAGAAACAAAGGCTAAACAAGAGGAGCTACTTAATAAGAGCACTGACACTGAATACAATGACAAAAGATGCAGA<br>AAGAGGCAAATTGAAGAGACGGGCGATTGCAACACCAGGGATGCAGATTAGAGGATTTGTGTACTTTGTCGAAACACTGGCAAGGAGCATCTGTGAAAAACTTGAGCAATCTGGACTCCCCGTTGGAGGGAATGAGAAGAAGGCTAAATTGGCAAATGTTGTGAGAAAAATGATGACTAACTCACAAGATACAGAGCTCTCCTTCACAATTACTGGAGATAACACCAAATG<br>GAATGAGAATCAAAATCCTCGGATGTTTCTGGCAATGATAACATACATTACAAGAAACCAACCTGAATGGTTTAGGAATGTCTTGAGTATTGCCCCTATAATGTTCTCGAACAAAATGGCGAGATTGGGAAAAGGGTACATGTTTGAAAGTAAGAGCATGAAGTTACGGACACAAATACCTGCAGAAATGCTTGCAAACATTGACTTAAAATATTTCAATGAATCAACAAGAAA<br>GAAAATCGAAAAAATAAGGCCTCTACTAATAGATGGCACTGCCTCATTGAGTCCTGGAATGATGATGGGCATGTTCAATATGCTGAGTACAGTATTAGGAGTTTCAATCCTAAATCTTGGGCAAAAGAAGTACACCAAAACCACATACTGGTGGGATGGACTCCAATCCTCTGATGATTTCGCCCTCATAGTAAATGCACCGAATCATGAGGGAATACAAGCAGGAGTGGATA<br>GGTTCTATAGGACCTGCAAACTGGTCGGGATCAATATGAGCAAAAAGAAGTCTTACATAAACCGGACTGGAACATTTGAGTTCACAAGCTTTTTCTATCGCTATGGATTTGTGGCTAACTTCAGTATGGAGCTGCCCAGCTTTGGAGTTTCTGGGATCAATGAATCAGCTGACATGAGCATTGGCATCACAGTGATAAAGAACAACATGATAAACAATGACCTTGGACCAGCAA<br>CAGCTCAAATGGCCCTTCAACTATTCATCAAAGATTACAGGTACACGTACCGATGCCATAGAGGTGACACACAAATTCAAACGAGGAGATCATTCGAGCTGAAGAAGCTGTGGGAACAGACCCGTTCAAAGGCAGGACTGTTGGTGTCAGATGGAGGACCAAATCTATACAACATTCGGAATCTCCATATCCCAGAGGTCTGCCTGAAGTGGGAGCTGATGGACGAAGATTA<br>CCAGGGCAGGTTGTGTAATCCTCTGAACCCATTTGTCAGCCATAAAGAAATTGAGTCCGTAAACAATGCTGTGGTGATGCCAGCCCACGGTCCAGCCAAAAGCATGGAATATGATGCCGTTGCGACTACACACTCATGGATTCCTAAAAGGAATCGTTCCATTCTCAATACCAGTCAAAGGGGAATTCTTGAGGATGAACAGATGTACCAGAAATGCTGCGGTCTATTCGAGAA<br>ATTCTTCCCCAGTAGTTCATACAGGAGACCAGTTGGAATTTCCAGCATGGTGGAGGCCATGGTGTCTAGGGCCCGAATCGATGCACGCATTGATTTCGAATCTGGAAGGATCAAGAAGGAAGAGTTTGCTGAGATCATGAAGATCTGTTCCACCATTGAAGAGCTCAGACGGCAAAAATAG
EPI_ISL_1665261	>A/mute_swan/Slovakia/Pah15_21VIR1086-3/2021_H5N5_2021-01-29<br>ATGGATGTCAATCCGACTTTACTTTTCTTAAAAGTGCCAGCGCAAAATGCCATAAGTACCACATTCCCTTATACTGGAGATCCTCCATACAGCCATGGAACAGGGACAGGATACACAATGGACACAGTCAACAGAACACATCAATACTCAGAGAAGGGAAAATGGACAACAAACACAGAAACCGGAGCACCTCAACTCAACCCAATTGATGGACCACTACCTGAGGACAACGA<br>ACCGAGCGGATATGCACAAACAGATTGCGTGTTGGAAGCAATGGCTTTCCTTGAAGAGTCCCACCCAGGGATCTTTGGAAACTCTTGTCTTGAAACGATGGAAGTCGTTCAGCAAACAAGAGTGGACAAACTAACTCAAGGTCGCCAGACTTATGACTGGACACTGAATAGAAACCAACCAGCTGCAACTGCCCTGGCCAACACTATAGAGGTCTTCAGATCAAACGGTCTAA<br>CAGCCAATGAATCGGGGAGACTAATAGATTTCCTCAAGGATGTGATGGACTCAATGGATAAAGAAGAAATGGAAATAACAACACATTTCCAGAGAAAGAGAAGAGTAAGGGACAACATGACCAAGAAAATGGTCACACAAAGAACAATAGGAAAGAAGAAACAAAGGCTAAACAAGAGGAGCTACTTAATAAGAGCACTGACACTGAATACAATGACAAAAGATGCAGA<br>AAGAGGCAAATTGAAGAGACGGGCGATTGCAACACCAGGGATGCAGATTAGAGGATTTGTGTACTTTGTCGAAACACTGGCAAGGAGCATCTGTGAAAAACTTGAGCAATCTGGACTCCCCGTTGGAGGGAATGAGAAGAAGGCTAAATTGGCAAATGTCGTGAGAAAAATGATGACTAACTCACAAGATACAGAGCTCTCCTTCACAATTACTGGAGATAACACCAAATG<br>GAATGAAAATCAAAATCCTCGGATGTTTCTGGCAATGATAACGTACATTACAAGAAACCAACCTGAATGGTTTAGAAATGTCTTGAGTATTGCCCCTATAATGTTCTCGAACAAAATGGCGAGATTGGGAAAAGGGTACATGTTTGAAAGTAAGAGCATGAAGTTACGGACACAAATACCTGCAGAAATGCTTGCAAACATTGACTTAAAATACTTCAATGAATCAACAAGAAA<br>GAAAATCGAAAAAATAAGGCCTCTACTAATAGATGGCACTGCCTCATTGAGTCCTGGAATGATGATGGGCATGTTCAATATGCTGAGTACAGTATTAGGAGTTTCAATCCTAAATCTTGGGCAAAAGAGGTACACCAAAACCACATACTGGTGGGATGGACTCCAATCCTCTGATGATTTCGCCCTCATAGTAAATGCACCGAATCATGAGGGAATACAAGCAGGAGTGGATA<br>GGTTCTATAGGACCTGCAAACTGGTCGGGATCAATATGAGCAAAAAGAAGTCTTACATAAACCGGACTGGAACATTTGAGTTCACAAGCTTTTTCTATCGCTATGGATTTGTGGCTAACTTCAGTATGGAGCTGCCCAGCTTTGGAGTTTCTGGGATCAATGAATCAGCTGACATGAGCATTGGCGTCACAGTGATAAAGAACAACATGATAAACAATGACCTTGGACCAGCAA<br>CAGCTCAAATGGCCCTTCAACTATTCATCAAAGATTACAGGTACACGTACCGATGCCACAGAGGTGACACACAAATTCAAACGAGGAGATCATTCGAGCTGAAGAAGCTGTGGGAACAGACCCGTTCAAAGGCAGGACTGTTGGTGTCAGATGGAGGACCAAATCTATACAACATTCGGAATCTCCATATCCCAGAGGTCTGCCTGAAGTGGGAGCTGATGGATGAAGATTA<br>CCAGGGCAGGTTGTGTAATCCTCTGAACCCATTTGTCAGTCATAAAGAAATTGAGTCCGTAAACAATGCTGTGGTGATGCCAGCCCACGGTCCAGCCAAAAGCATGGAATATGATGCCGTTGCGACTACACACTCATGGATTCCTAAAAGGAATCGTTCCATTCTCAATACCAGCCAAAGGGGAATTCTTGAGGATGAACAGATGTACCAGAAATGCTGCAGTCTATTCGAGAA<br>ATTCTTCCCCAGTAGTTCATACAGGAGACCAGTTGGAATTTCCAGCATGGTGGAGGCCATGGTGTCTAGGGCCCGAATCGATGCACGCATTGATTTCGAATCTGGAAGGATCAAGAAGGAAGAGTTTGCTGAGATCATGAAGATCTGTTCCACCATTGAAGAGCTCAGACGGCAAAAATAG
EPI_ISL_17978927	>A/mute_swan/Poland/P071/2020_H9N2_2020-09-18<br>ATGGATGTCAATCCGACTTTACTTTTCTTAAAAGTGCCAGCGCAAAATGCTATAAGTACTACGTTTCCTTACACTGGAGATCCTCCATACAGCCATGGAACAGGAACAGGATACACCATGGACACAGTCAACAGAACACATCAATACTCAGAGAAAGGAAAGTGGACAACAAACACAGAGACTGGGGCACCCCAACTCAACCCAATTGATGGACCATTACCAGAGGACAACGA<br>GCCAAGCGGATATGCACAAACAGATTGCGTGTTGGAAGCAATGGCTTTCCTTGAAGAATCCCACCCAGGGATCTTTGAAAACTCTTGTCTTGAAACGATGGAGGTCGTTCAACAAACAAGAGTGGACAAACTAACCCAAGGTCGCCAGACTTATGATTGGACACTGAATAGAAACCAACCAGCTGCAACTGCTTTGGCCAATACTATAGAGGTCTTCAGATCGAACGGTCTAG<br>CAGCCAATGAGTCGGGGAGACTAATAGATTTCCTCAAGGATGTGATGGAATCAATGGATAAGGAAGAAATGGAAATAACAACACATTTCCAGAGAAAGAGAAGAGTAAGGGACAACATGACCAAGAAGATGGTCACACAAAGAACGATAGGAAAGAAGAAGCAGAGGCTGAACAAGAGGAGCTACTTAATAAGAGCACTGACATTGAACACAATGACCAAAGATGCAGA<br>AAGAGGCAAATTGAAGAGGCGGGCAATTGCAACACCCGGGATGCAGATTAGAGGATTTGTGTACTTCGTCGAAACACTAGCAAGGAGCATCTGTGAGAAACTCGAGCAATCTGGACTCCCTGTTGGAGGGAATGAGAAAAAGGCCAAATTGGCAAATGTCGTTAGAAAAATGATGACTAACTCACAAGATACGGAGCTCTCCTTTACAATTACTGGAGACAACACCAAATG<br>GAATGAAAATCAAAATCCTCGGATGTTTTTGGCAATGATAACATACATCACAAGAAACCAACCCGAATGGTTTAGAAATGTCTTGAGCATTGCCCCTATAATGTTCTCAAACAAAATGGCAAGATTAGGAAAAGGATACATGTTTGAAAGTAAGAGCATGAAGCTAAGGACACAAATACCGGCAGAAATGCTTGCAAACATTGACTTGAAATACTTCAATGAATCAACGAGAA<br>AGAAAATCGAGAAAATAAGACCTCTGCTAATTGATGGCACAGCCTCATTGAGTCCTGGAATGATGATGGGCATGTTCAATATGCTGAGCACAGTATTGGGAGTTTCAATCCTGAATCTTGGACAAAAGAGGTACACCAAAACCACATACTGGTGGGATGGACTCCAATCCTCTGATGATTTCGCTCTCATAGTGAATGCACCGAATCATGAGGGGATACAAGCAGGGGTGGAT<br>AGGTTCTATAGGACCTGCAAACTGGTTGGGATCAACATGAGCAAAAAGAAGTCTTACATAAACCGGACAGGAACATTTGAGTTCACAAGCTTTTTCTACCGCTATGGGTTTGTAGCCAATTTCAGTATGGAATTACCCAGCTTTGGAGTGTCTGGAATCAATGAGTCAGCTGATATGAGCATTGGAGTTACAGTGATAAAGAACAATATGATAAACAATGACCTTGGACCAGCA<br>ACAGCTCAAATGGCTCTTCAGCTATTCATCAAAGATTATAGGTACACGTACCGATGTCACAGAGGTGATACACAAATTCAAACGAGGAGATCATTCGAGCTTAAGAAGCTGTGGGAGCAGACCCGTTCAAAGGCAGGGCTGTTGGTATCAGATGGTGGACCAAACCTATACAACATTCGGAATCTCCACATCCCAGAGGTCTGCCTGAAGTGGGAACTTATGGATGAAGATTA<br>CCAGGGCAGGCTGTGTAATCCTCTGAATCCGTTTGTCAGTCATAAGGAAATTGAGTCCGTAAACAGTGCTGTGGTAATGCCAGCCCATGGCCCAGCCAAGAGCATGGAATATGATGCTGTTGCGACTACACACTCATGGATTCCTAAGAGGAACCGTTCCATTCTCAATACCAGCCAGAGGGGAATTCTCGAGGATGAGCAGATGTACCAGAAGTGTTGCAATTTATTCGAGA<br>AATTCTTCCCCAGTAGTTCGTACAGGAGGCCAGTTGGAATTTCCAGCATGGTGGAGGCCATGGTGTCCAGGGCCCGAATTGATGCACGCATTGATTTCGAATCTGGAAGGATTAAGAAAGAAGAGTTTGCTGAGATCATGAAGATCTGTTCCACCATTGAAGAGCTCAGACGGCCAAAATAG
EPI_ISL_18245815	>A/mute_swan/Poland/MB551/2021_H5N1_2021-12-23<br>ATGGATGTCAATCCGACTTTACTTTTCTTAAAAGTGCCAGCGCAAGATGCCATAAGTACCACATTCCCTTACACTGGAGATCCTCCATACAGCCATGGAACAGGGACAGGATACACAATGGACACAGTCAACAGAACACATCAATACTCAGAGAAGGGAAAATGGACAACAAACACAGAAACCGGAGCACCTCAACTCAACCCAATTGATGGGCCACTACCTGAGGACAACGA<br>ACCGAGCGGATATGCACAAACAGATTGCGTGTTGGAAGCAATGGCTTTCCTTGAAGAGTCCCACCCAGGGATCTTTGAAAACTCTTGTCTTGAAACGATGGAAGTCGTTCAGCAAACAAGAGTGGACAAACTAACTCAAGGTCGCCAGACATATGACTGGACACTGAATAGAAACCAACCAGCTGCAACTGCCCTGGCCAACACTATAGAGGTCTTCAGATCAAACGGTCTAA<br>CAACCAATGAATCGGGGAGACTAATAGATTTCCTCAAGGATGTGATGGACTCAATGGATAAAGAAGAAATGGAAATAACAACACATTTCCAAAGAAAGAGAAGAGTAAGGGACAACATGACCAAGAAAATGGTCACACAAAGAACAATAGGAAAGAAGAAACAAAGGCTAACCAAGAGGAGCTACTTAATAAGAGCACTGACACTGAATACAATGACAAAAGATGCAGAA<br>AGAGGCAAATTGAAAAGACGGGCGATTGCAACACCAGGGATGCAGATTAGAGGATTTGTGTACTTTGTCGAAACACTGGCAAGGAGCATCTGTGAAAAACTTGAGCAATCTGGACTCCCCGTTGGAGGGAATGAGAAGAAGGCTAAATTGGCAAATGTCGTGAGAAAAATGATGACTAACTCACAAGATACAGAGCTCTCCTTCACAATTACTGGAGATAACACCAAATGG<br>AATGAGAATCAAAATCCTCGGATGTTTCTGGCAATGATAACATACATTACAAGAAACCAACCTGAATGGTTTAGAAATGTCTTGAGTATTGCCCCTATAATGTTCTCGAACAAAATGGCGAGATTGGGAAAAGGGTACATGTTTGAAAGTAAGAGCATGAAGTTACGGACACAAATACCTGCAGAAATGCTTGCAAACATTGACTTAAAATACTTCAATGAATCAACAAGAAAG<br>AAAATCGAAAAAATAAGGCCTCTACTAATAGATGGCACTGCCTCATTGAGTCCTGGAATGATGATGGGCATGTTCAATATGCTGAGTACAGTATTAGGAGTTTCAATCCTAAATCTTGGGCAAAAGAAGTACACCAAAACCACATACTGGTGGGATGGACTCCAATCCTCTGATGATTTCGCCCTCATAGTAAATGCACCGAATCATGAGGGAATACAAGCAGGAGTGGATAG<br>GTTCTATAGGACCTGCAAACTGGTCGGGATCAATATGAGCAAAAAGAAGTCTTACATAAACCGGACTGGAACATTTGAGTTCACAAGCTTTTTCTATCGCTATGGATTTGTGGCTAACTTCAGTATGGAGTTGCCCAGCTTTGGAGTTTCTGGGATCAATGAATCAGCTGACATGAGCATTGGCATCACAGTGATAAAGAACAACATGATAAACAATGACCTTGGACCAGCAAC<br>AGCTCAAATGGCCCTTCAACTATTCATCAAAGATTACAGGTACACGTACCGATGCCACAGAGGTGACACACAAATTCAAACGAGGAGATCATTCGAGCTGAAGAAGCTGTGGGAACAGACCCGTTCAAAGGCAGGACTGTTGGTGTCAGATGGAGGACCAAATCTATACAACATTCGGAATCTCCATATCCCAGAGGTCTGCCTGAAGTGGGAGCTGATGGACGAAGATTAC<br>CAGGGCAGGTTGTGTAATCCTCTGAACCCATTTGTCAGCCATAGAGAAATTGAGTCCGTAAACAATGCTGTGGTGATGCCAGCCCACGGTCCAGCCAAAAGCATGGAATATGATGCCGTTGCGACTACACACTCATGGATTCCTAAAAGGAATCGTTCCATTCTCAATACCAGTCAAAGGGGAATTCTTGAGGATGAACAGATGTACCAGAAATGCTGCAGTCTATTCGAGAAA<br>TTCTTCCCCAGTAGTTCATACAGGAGACCAGTTGGAATTTCCAGCATGGTGGAGGCCATGGTGTCTAGGGCCCGAATCGATGCACGCATTGATTTCGAATCTGGAAGGATCAAGAAGGAAGAGTTTGCTGAGATCATGAAGATCTGTTCCACCATTGAAGAGCTCAGACGGCAAAAATAG
EPI_ISL_18245814	>A/mute_swan/Poland/MB550/2021_H5N1_2021-12-23<br>ATGGATGTCAATCCGACTTTACTTTTCTTAAAAGTGCCAGCGCAAGATGCCATAAGTACCACATTCCCTTACACTGGAGATCCTCCATACAGCCATGGAACAGGGACAGGATACACAATGGACACAGTCAACAGAACACATCAATACTCAGAGAAGGGAAAATGGACAACAAACACAGAAACCAGAGCACCTCAACTCAACCCAATTGATGGGCCACTACCTGAGGACAACGA<br>ACCGAGCGGATATGCACAAACAGATTGCGTGTTGGAAGCAATGGCTTTCCTTGAAGAGTCCCACCCAGGGATCTTTGAAAACTCTTGTCTTGAGACGATGGAAGTCGTTCAGCAAACAAGAGTGGACAAACTAACTCAAGGCCGCCAGACATATGACTGGACACTGAATAGAAACCAACCAGCTGCAACTGCCCTGGCCAACACTATAGAGGTCTTCAGATCAAACGGTCTAA<br>CAGCCAATGAATCGGGGAGACTAATAGATTTCCTCAAGGATGTGATGGACTCAATGGACAAGGAAGAAATGGAAATAACAACACATTTCCAGAGAAAGAGAAGAGTAAGGGACAACATGACCAAGAAAATGGTCACACAAAGAACAATAGGAAAGAAGAAACAAAGGCTAAACAAGAGGAGCTACTTAATAAGAGCACTGACACTGAATACAATGACAAAAGATGCAGA<br>AAGAGGAAAATTGAAAAGACGGGCGATTGCAACACCAGGGATGCAGATTAGAGGATTTGTGTACTTTGTCGAAACACTGGCAAGGAGCATCTGTGAAAAACTTGAGCAATCTGGACTCCCCGTTGGAGGGAATGAGAAGAAGGCTAAATTGGCAAATGTCGTGAGAAAAATGATGACTAACTCACAAGATACAGAGCTCTCCTTCACAATTACTGGAGATAACACCAAATG<br>GAATGAGAATCAAAATCCTCGGATGTTTCTGGCAATGATAACATACATTACAAGAAACCAACCTGAATGGTTTAGAAATGTCTTGAGTATTGCCCCTATAATGTTCTCGAACAAAATGGCGAGATTGGGAAAAGGGTACATGTTTGAAAGTAAGAGCATGAAGTTACGGACACAAATACCTGCAGAAATGCTTGCAAACATTGACTTAAAATACTTCAATGAATCAACAAGAAA<br>GAAAATCGAAAAAATAAGGCCTCTACTAATAGATGGCACTGCCTCATTGAGTCCTGGAATGATGATGGGCATGTTCAATATGCTGAGTACAGTATTAGGAGTTTCAATCCTAAATCTTGGGCAAAAGAAGTACACCAAAACCACATACTGGTGGGATGGACTCCAATCCTCTGATGATTTCGCCCTCATAGTAAATGCACCGAATCATGAGGGAATACAAGCAGGAGTGGATA<br>GGTTCTATAGGACCTGCAAACTGGTCGGGATCAATATGAGCAAAAAGAAGTCTTACATAAACCGGACTGGAACATTTGAGTTCACAAGCTTTTTCTATCGCTATGGATTTGTGGCTAACTTCAGTATGGAGCTGCCCAGCTTTGGAGTTTCTGGGATCAATGAATCAGCTGACATGAGCATTGGCGTCACAGTGATAAAGAACAACATGATAAACAATGACCTTGGACCAGCAA<br>CAGCTCAAATGGCCCTTCAACTATTCATCAAAGATTACAGGTACACGTACCGATGCCACAGAGGTGACACACAAATTCAAACGAGGAGATCATTCGAGCTGAAGAAGCTGTGGGAACAGACCCGTTCAAAGGCAGGACTGTTGGTGTCAGATGGAGGACCAAATCTATACAACATTCGGAATCTCCATATCCCAGAGGTCTGCCTGAAGTGGGAGCTGATGGACGAAGATTA<br>CCAGGGCAGGTTGTGTAATCCTCTGAACCCATTTGTCAGCCATAGAGAAATTGAGTCCGTAAACAATGCTGTGGTGATGCCAGCCCACGGTCCAGCCAAAAGCATGGAATATGATGCCGTTGCGACTACACACTCATGGATTCCTAAAAGGAATCGTTCCATTCTCAATACCAGTCAAAGGGGAATTCTTGAGGATGAACAGATGTACCAGAAATGCTGCAGTCTATTCGAGAA<br>ATTCTTCCCCAGTAGTTCATACAGGAGACCAGTTGGAATTTCCAGCATGGTGGAGGCCATGGTGTCTAGGGCCCGAATCGATGCACGAATTGATTTCGAATCTGGAAGGATAAAGAAGGAAGAGTTTGCTGAGATCATGAAGATCTGTTCCACCATTGAAGAGCTCAGACGGCAAAAATAG
EPI_ISL_6937114	>A/mute_swan/Poland/MB490-L1/2021_H5N1_2021-11-08<br>ATGGATGTCAATCCGACTTTACTTTTCTTAAAAGTGCCAGCGCAAAATGCCATAAGTACCACATTCCCTTATACTGGAGATCCTCCATACAGCCATGGAACAGGGACAGGATACACAATGGACACAGTCAACAGAACACATCAATACTCAGAGAAGGGAAAATGGACAACAAACACAGAAACCGGAGCACCTCAACTCAACCCAATTGATGGGCCACTACCTGAGGACAACGA<br>ACCGAGCGGATATGCACAGACAGATTGCGTGTTGGAAGCAATGGCTTTCCTTGAAGAGTCCCACCCAGGGATCTTTGGAAACTCTTGTCTTGAAACGATGGAAGTCGTTCAGCAAACAAGAGTGGACAAACTAACTCAAGGTCGCCAGACTTATGACTGGACACTGAATAGAAACCAACCAGCTGCAACTGCCCTGGCCAACACTATAGAGGTCTTCAGATCAAACGGTCTAA<br>CAGCCAATGAATCGGGGAGACTAATAGATTTCCTCAAGGATGTGATGGACTCAATGGATAAAGAAGAAATGGAAATAACAACACACTTCCAGAGAAAGAGAAGAGTAAGGGACAACATGACCAAGAAAATGGTCACACAAAGAACAATAGGAAAGAAGAAACAAAGGCTAAACAGGAGGAGCTACTTAATAAGAGCACTGACACTGAATACAATGACAAAAGATGCAGA<br>AAGAGGCAAATTGAAGAGACGGGCGATTGCAACACCAGGGATGCAGATTAGAGGATTTGTGTACTTTGTCGAAACACTGGCAAGGAGCATCTGTGAAAAACTTGAGCAATCTGGACTCCCCGTTGGAGGAAATGAGAAGAAGGCTAAATTGGCAAATGTCGTGAGGAAAATGATGACTAACTCACAAGATACAGAGCTCTCCTTCACAATTACTGGAGATAACACCAAATG<br>GAATGAGAATCAAAATCCTCGGATGTTTCTGGCAATGATAACGTACATTACAAGAAACCAACCTGAATGGTTTAGAAATGTCTTGAGTATTGCCCCTATAATGTTCTCGAACAAAATGGCGAGATTGGGAAAAGGGTACATGTTTGAAAGTAAGAGCATGAAGTTACGGACACAAATACCTGCAGAAATACTTGCAGACATTGACTTAAAATACTTCAATGGATCAACAAGAAA<br>GAAAATCGAAAAAATAAGGCCTCTACTAATAGATGGCACTGCCTCATTGAGTCCTGGAATGATGATGGGCATGTTCAATATGCTGAGTACAGTATTAGGAGTTTCAATCCTAAATCTTGGGCAAAAGAGGTACACCAAAACCACATACTGGTGGGATGGACTCCAATCCTCTGATGATTTCGCCCTCATAGTAAATGCACCGAATCATGAGGGAATACAAGCAGGAGTGGATA<br>GGTTCTATAGGACCTGCAAACTGGTCGGGATCAATATGAGCAAAAAGAAGTCTTACATAAACCGGACTGGAACATTTGAGTTCACAAGCTTTTTCTATCGCTATGGATTTGTGGCTAACTTCAGTATGGAGTTGCCCAGCTTTGGAGTTTCTGGAATCAATGAATCAGCTGACATGAGCATTGGCGTCACAGTGATAAAGAACAACATGATAAACAATGACCTTGGACCAGCAA<br>CAGCTCAAATGGCCCTTCAACTATTCATCAAAGATTACAGGTACACGTACCGATGCCACAGAGGTGACACACAAATTCAAACGAGGAGATCATTCGAGCTGAAGAAGCTGTGGGAACAGACCCGTTCAAAGGCAGGACTGTTGGTGTCAGATGGAGGACCAAATCTATACAACATTCGGAATCTCCATATCCCAGAGGTCTGCCTGAAGTGGGAGCTGATGGACGAAGATTA<br>CCAGGGCAGGTTGTGTAATCCTCTGAACCCATTTGTCAGTCATAAAGAAATTGAGTCCGTAAACAATGCTGTGGTGATGCCAGCCCACGGTCCAGCCAAAAGCATGGAATATGATGCCGTTGCAACTACACACTCATGGATTCCTAAAAGGAATCGTTCCATTCTCAATACCAGCCAAAGGGGAATTCTTGAGGATGAACAGATGTACCAGAAATGCTGCAGTCTATTCGAGAA<br>ATTCTTCCCCAGTAGTTCATACAGGAGACCAGTTGGAATTTCCAGCATGGTGGAGGCCATGGTGTCTAGGGCCCGAATCGATGCACGCATTGATTTCGAATCTGGAAGGATCAAGAAGGGAGAGTTTGCTGAGATCATGAAGATCTGTTCCACCATTGAAGAGCTCAGACGGCAAAAATAG
EPI_ISL_3102078	>A/mute_swan/Poland/MB396_21RS1385-19/2021_H5N8_2021-04-21<br>ATGGATGTCAATCCGACTTTACTTTTCTTAAAAGTGCCAGCGCAAAATGCTATAAGTACTACATTCCCTTACACTGGAGATCCTCCATACAGCCATGGGACAGGAACAGGGTATACCATGGACACAGTAAACAGAACACATCAATACTCAGAAAAGGGAAAGTGGACAACAAACACAGAAACCGGAGCACCCCAACTCAACCCAATTGATGGACCATTACCAGAGGACAATGA<br>GCCAAGCGGATATGCACAAACTGATTGCGTGTTGGAAGCAATGGCTTTCCTTGAAGAATCCCACCCAGGGATATTTGAAAACTCTTGTCTTGAAGCGATGGAAATCGTTCAGCAAACAAGAGTGGACAAACTAACCCAAGGTCGCCAGACTTATGACTGGACACTGAACAGAAACCAACCAGCTGCAACCTCTTTGGCCAACACTATAGAGGTGTTCAGATCGAATGGTCTGA<br>CAGCCAATGAATCAGGGAGACTGATAGATTTTCTCAGGGATGTGATGGAATCAATGGATAAAGAAGAGATGGAAGTAACAACACATTTCCAGAGAAAAAGAAGAGTGAGGGACAACATGACTAAGAAGATGGTCACACAAAGAACAATAGGGAAGAAGAAGCAGAGGCTGAACAAGAGGAGTTACTTGATAAGAGCACTGACATTGAATACAATGACCAAAGATGCAGA<br>AAGAGGCAAGTTGAAGAGACGGGCAATTGCAACACCCGGGATGCAGATTAGAGGATTCGTGTACTTCGTTGAAACACTAGCGAGGAGCATCTGTGAGAAACTAGAGCAATCTGGGCTCCCTGTTGGAGGGAATGAGAAGAAGGCTAAATTGGCAAATGTTGTGAGAAAAATGATGACTAACTCACAAGATACAGAGCTCTCCTTTACAATTACTGGAGACAACACCAAATG<br>GAATGAGAATCAAAACCCTCGGATGTTTTTGGCAATGATAACATATATCACAAGAAACCAACCTGAATGGTTTAGAAATGTCTTAAGCATTGCCCCTATAATGTTCTCAAACAAAATGGCGAGATTAGGAAAAGGATACATGTTTGAAAGTAAGAGCATGAAGCTAAGAACACAAATACCAGCAGAGATGCTTACAAATATTGATCTGAAGTATTTCAACGAACCAACGAGAAA<br>GAAAATCGAGAAAATAAGACCCCTGCTGATTGATGGCACGGCCTCATTGAGTCCTGGGATGATGATGGGCATGTTCAATATGCTGAGCACAGTATTGGGGGTCTCAATCCTGAATCTCGGGCAAAAGAGGTACACCAAAACCACATACTGGTGGGATGGACTTCAATCCTCTGATGATTTCGCTCTCATAGTGAATGCACCGGATCATGAGGGAATACAAGCAGGAGTGGATA<br>GATTCTATAGGACCTGCAAACTGGTTGGGATCAACATGAGCAAAAAGAAGTCTTACATAAACCGAACAGGAACATTTGAGTTCACAAGTTTTTTCTACCGCTATGGATTTGTAGCTAACTTCAGTATGGAATTACCCAGCTTTGGAGTGTCTGGAATCAATGAATCAGCTGACATGAGCATTGGAGTTACAGTGATAAAAAACAATATGATAAACAATGATCTTGGACCAGCAAC<br>AGCTCAAATGGCTCTTCAGTTATTCATCAAAGACTATAGGTACACATACCGATGCCACAGGGGTGATACACAAATTCAAACGAGGAGATCATTCGAGCTGAAGAAGCTGTGGGAGCAGACCCGTTCAAAGGCAGGGCTGTTGATATCAGACGGGGGGCCAAACCTATACAACATTCGGAACCTCCACATCCCAGAGGTCTGCTTGAAGTGGGAGCTGATGGATGAAGACTAC<br>CAAGGCAGGCTGTGCAATCCTCTGAATCCATTTGTCAGTCATAAAGAGATTGAGTCCGTAAACAATGCTGTAGTAATGCCCGCCCATGGCCCGGCCAAGAGCATGGAATATGATGCTGTTGCGACCACACATTCGTGGATTCCTAAGAGGAACCGCTCCATTCTCAATACCAACCAAAGGGGAATTCTTGAGGATGAGCAGATGTACCAAAAGTGCTGTAGTCTATTCGAGAAA<br>TTCTTCCCCAGCAGTTCATACAGGAGGCCAGTTGGAATTTCCAGCATGGTGGAGGCCATGGTGTCTAGGGCCCGAATTGATGCACGCATCGACTTCGAATCTGGAAGGATTAAGAAAGAAGAGTTTGCTGAGATCATGAAGATCTGTTCCACCATTGGAGAGCTCAGACGGCAAAAATAG
EPI_ISL_18245535	>A/mute_swan/Poland/MB372-21_22VIR5675-19/2021_H5N8_2021-04-07<br>ATGGATGTCAATCCGACTTTACTTTTCTTAAAAGTGCCAGCGCAAAATGCTATAAGTACTACATTCCCTTACACTGGAGATCCTCCATACAGCCATGGAACAGGAACAGGGTATACCATGGACACAGTAAACAGAACACATCAATACTCAGAAAGGGGAAAGTGGACAACAAACACAGAAACCGGAGCACCCCAACTCAACCCAATTGATGGACCATTACCAGAGGACAATGA<br>GCCAAGCGGATATGCACAAACTGATTGCGTGTTGGAAGCAATGGCTTTCCTTGAAGAATCCCACCCAGGGATATTTGAAAACTCTTGTCTTGAAGCGATGGAAATTGTTCAGCAAACAAGAGTGGACAAACTAACCCAAGGTCGCCAGACTTATGACTGGACACTGAACAGAAACCAACCAGCTGCAACCTCTTTGGCCAACACTATAGAGGTGTTCAGATCGAATGGTCTGA<br>CAGCCAATGAATCAGGGAGACTGATAGATTTTCTCAGGGATGTGATGGAATCAATGGACAAAGAAGAGATGGAAGTAACAACACATTTCCAGAGAAAAAGAAGAGTGAGGGACAACATGACTAAGAAGATGGTCACACAAAGAACAATAGGGAAGAAGAAGCAGAGGCTGAACAAGAGGAGTTACTTAATAAGAGCACTGACATTGAATACAATGACCAAAGATGCAGA<br>AAGAGGCAAGTTGAAGAGACGGGCAATTGCAACACCCGGGATGCAGATTAGAGGATTCGTGTACTTCGTTGAAACACTAGCGAGGAGCATCTGTGAGAAACTAGAGCAATCTGGGCTCCCTGTTGGAGGGAATGAGAAGAAGGCTAAATTGGCAAATGTTGTGAGAAAAATGATGACTAACTCACAAGATACAGAGCTCTCCTTTACAATTACTGGAGACAACACCAAATG<br>GAATGAGAATCAAAACCCTCGGATGTTTTTGGCAATGATAACATATATCACAAGAAACCAACCTGAATGGTTTAGAAATGTCTTAAGCATTGCCCCTATAATGTTCTCAAACAAAATGGCGAGATTAGGGAAAGGATACATGTTTGAAAGTAAGAGCATGAAGCTAAGAACACAAATACCAGCAGAGATGCTTACAAATATCGATCTGAAGTATTTCAACGAACCAACGAGAA<br>AGAAAATCGAGAAAATAAGACCTCTGCTGATTGATGGCACGGCCTCATTGAGTCCTGGGATGATGATGGGCATGTTCAATATGCTGAGCACAGTATTAGGGGTCTCAATCCTGAATCTCGGGCAAAAAAGGTACACCAAAACCACATACTGGTGGGATGGACTTCAATCCTCTGATGATTTCGCTCTCATAGTGAATGCACCGAATCATGAGGGGATACAAGCAGGAGTGGAT<br>AGATTCTATAGGACCTGCAAACTGGTTGGGATCAACATGAGCAAAAAGAAGTCTTACATAAACCGAACAGGAACATTTGAGTTCACAAGTTTTTTCTACCGCTATGGATTTGTAGCTAACTTCAGTATGGAATTACCCAGCTTTGGAGTGTCTGGAATCAATGAATCAGCTGACATGAGCATTGGAGTTACAGTGATAAAAAACAATATGATAAACAATGATCTTGGACCAGCA<br>ACAGCTCAAATGGCTCTTCAGTTATTCATCAAAGACTATAGGTACACATACCGATGTCACAGGGGTGATACACAAATTCAAACGAGGAGATCATTCGAGCTGAAGAAGCTGTGGGAGCAGACCCGTTCAAAGGCAGGGCTGTTGATATCAGACGGGGGGCCAAACCTATACAACATTCGGAATCTCCACATCCCAGAGGTCTGCTTGAAGTGGGAGCTGATGGATGAAGACT<br>ACCAAGGCAGGCTGTGCAATCCTCTGAATCCATTTGTCAGTCATAAAGAGATTGAGTCCGTAAACAATGCTGTAGTAATGCCCGCCCATGGCCCGGCCAAGAGCATGGAATATGATGCTGTTGCGACCACACACTCGTGGATTCCTAAGAGGAACCGTTCCATTCTCAATACCAGCCAAAGGGGAATTCTTGAGGATGAGCAGATGTACCAAAAGTGCTGTAGTCTATTCGAGA<br>AATTCTTCCCCAGCAGTTCATACAGGAGGCCAGTTGGAATTTCCAGCATGGTGGAGGCCATGGTGTCTAGGGCCCGAATTGATGCACGCATCGACTTCGAATCTGGAAGGATTAAGAAAGAAGAGTTTGCTGAGATCATGAAGATCTGTTCCACCATTGAAGAGCTCAGACGGCAAAAATAG
EPI_ISL_18245531	>A/mute_swan/Poland/MB306-21_22VIR5675-20/2021_H5N8_2021-03-16<br>ATGGATGTCAATCCGACTTTACTTTTCTTAAAAGTGCCAGCGCAAAATGCTATAAGTACTACATTCCCTTACACTGGAGATCCCCCATACAGCCATGGAACAGGAACAGGGTATACCATGGATACAGTAAACAGAACACATCAATACTCAGAAAAGGGAAAGTGGACAACAAACACAGAAACCGGAGCACCCCAACTCAACCCAATTGATGGACCATTACCAGAGGACAATGA<br>GCCAAGCGGGTATGCACAAACTGATTGCGTGTTGGAAGCAATGGCTTTCCTTGAAGAATCCCACCCAGGGATATTTGAAAACTCTTGTCTTGAAGCGATGGAAATCGTTCAGCAAACAAGAGTGGACAAACTAACCCAAGGTCGCCAGACTTATGACTGGACACTGAACAGAAACCAACCAGCTGCAACCTCTTTGGCCAACACTATAGAGGTGTTCAGATCGAATGGTCTGA<br>CAGCCAATGAATCAGGGAGACTGATAGATTTTCTCAGGGATGTGATGGAATCAATGGATAAAGAAGAGATGGAAGTAACAACACATTTCCAGAGAAAAAGAAGAGTGAGGGACAACATGACTAAGAAGATGGTCACACAAAGAACAATAGGGAAGAAGAAGCAGAGGCTGAACAAGAGGAGTTACTTAATAAGAGCACTGACATTGAATACAATGACCAAAGATGCAGA<br>AAGAGGCAAGTTGAAGAGACGGGCAATTGCAACACCCGGGATGCAGATTAGAGGATTCGTGTACTTCGTTGAAACACTAGCGAGGAGCATCTGTGAGAAACTAGAGCAATCTGGGCTCCCTGTTGGAGGGAATGAGAAAAAGGCTAAATTGGCAAATGTTGTGAGAAAAATGATGACTAACTCACAAGATACAGAGCTCTCCTTTACAATTACTGGAGACAACACCAAATG<br>GAATGAGAATCAAAACCCTCGGATGTTTTTGGCAATGATAACATATATCACAAGAAACCAACCTGAATGGTTTAGAAATGTCTTAAGCATTGCCCCTATAATGTTCTCAAACAAAATGGCGAGATTAGGGAAAGGATACATGTTTGAAAGTAAGAGCATGAAGCTAAGAACACAAATACCAGCAGAGATGCTTACAAATATTGATCTGAAGTATTTCAACGAACCAACGAGAA<br>AGAAAATCGAGAAAATAAGACCTCTGCTGATTGATGGCACGGCCTCATTGAGTCCTGGGATGATGATGGGCATGTTCAATATGCTGAGCACAGTATTAGGGGTCTCAATCCTGAATCTCGGGCAAAAGAGGTACACCAAAACCACATACTGGTGGGATGGACTTCAATCCTCTGATGATTTCGCTCTCATAGTGAATGCACCGAATCATGAGGGGATACAAGCAGGAGTGGAT<br>AGATTCTATAGGACCTGCAAACTGGTTGGGATCAACATGAGCAAAAAGAAGTCTTACATAAACCGAACAGGAACATTTGAGTTCACAAGTTTTTTCTACCGCTATGGATTTGTAGCCAACTTCAGTATGGAATTACCCAGCTTTGGAGTGTCTGGAATCAATGAATCAGCCGACATGAGCATTGGAGTTACAGTGATAAAAAACAATATGATAAACAATGATCTTGGACCAGCA<br>ACAGCTCAAATGGCCCTTCAGTTATTCATCAAGGACTATAGGTACACATACCGATGCCACAGGGGTGATACACAAATTCAAACGAGGAGATCATTCGAGCTGAAGAAGCTGTGGGAGCAGACCCGTTCAAAGGCAGGGCTGTTGATATCAGACGGGGGGCCAAACCTATACAACATTCGGAATCTCCACATCCCAGAGGTCTGCTTGAAGTGGGAGCTGATGGATGAAGACT<br>ACCAAGGCAGGCTGTGCAATCCTCTGAATCCATTTGTCAGTCATAAAGAGATTGAGTCCGTAAACAATGCTGTAGTAATGCCCGCCCATGGCCCGGCCAAGAGCATGGAATATGATGCTGTTGCGACCACACACTCGTGGATTCCTAAGAGGAACCGTTCCATCCTCAATACCAGCCAAAGGGGAATTCTTGAGGATGAGCAGATGTACCAAAAGTGCTGTAGTCTATTCGAGA<br>AATTCTTCCCCAGCAGTTCATACAGGAGGCCAGTTGGAATTTCCAGCATGGTGGAGGCCATGGTGTCTAGGGCCAGAATTGATGCACGCATCGACTTCGAATCTGGAAGGATTAAGAAAGAAGAGTTTGCTGAGATCATGAAGATCTGTTCCACCATTGAAGAGCTCAGACGGCAAAAATAG
EPI_ISL_2114013	>A/mute_swan/Poland/MB272/2021_H5N8_2021-03-09<br>ATGGATGTCAATCCGACTTTACTTTTCTTAAAAGTGCCAGCGCAAAATGCTATAAGTACTACATTCCCTTACACTGGAGATCCTCCATACAGCCATGGAACAGGAACAGGGTATACCATGGACACAGTAAACAGAACACATCAATACTCAGAAAAGGGAAAGTGGACAACAAACACAGAAACCGGAGCACCCCAACTCAACCCAATTGATGGACCATTACCAGAGGACAATGA<br>GCCAAGCGGATATGCACAAACTGATTGCGTGTTGGAAGCAATGGCTTTCCTTGAAGAATCCCACCCAGGGATATTTGAAAACTCTTGTCTTGAAGCGATGGAAATCGTTCAGCAAACAAGAGTGGACAAACTAACCCAAGGTCGCCAGACTTATGACTGGACACTGAACAGAAACCAACCAGCTGCAACCTCTTTGGCCAACACTATAGAGGTGTTCAGATCGAATGGTCTGA<br>CAGCCAATGAATCAGGGAGACTGATAGATTTTCTCAGGGATGTGATGGAATCAATGGATAAAGAAGAGATGGAAGTAACAACACATTTCCAGAGAAAAAGAAGAGTGAGAGACAACATGACTAAGAAGATGGTCACACAAAGAACAATAGGGAAGAAGAAGCAGAGGCTGATCAAGAGGAGTTACTTAATAAGAGCACTGACATTGAATACAATGACCAAAGATGCAGA<br>AAGAGGCAAGTTGAAGAGACGGGCAATTGCAACACCCGGGATGCAGATTAGAGGATTCGTGTACTTCGTTGAAACACTAGCGAGGAGCATCTGTGAGAAACTAGAGCAATCTGGGCTCCCTGTTGGAGGGAATGAGAAGAAGGCTAAATTGGCAAATGTTGTGAGAAAAATGATGACTAACTCACAAGATACAGAGCTCTCCTTTACAATTACTGGAGACAACACCAAATG<br>GAATGAGAATCAAAACCCTCGGATGTTTTTGGCAATGATAACATATATCACAAGAAACCAACCTGAATGGTTTAGAAATGTCTTAAGCATTGCCCCTATAATGTTCTCAAACAAAATGGCGAGATTAGGGAAAGGATACATGTTTGAAAGTAAGAGCATGAAGCTAAGAACACAAATACCAGCAGAGATGCTTACAAATATCGATCTGAAGTATTTCAACGAACCAACGAGAA<br>AGAAAATCGAGAAAATAAGACCTCTGCTGATTGATGGCACGGCCTCATTGAGTCCTGGAATGATGATGGGCATGTTCAATATGCTGAGCACAGTATTAGGGGTCTCAATCCTGAATCTCGGGCAAAAAAGGTACACCAAAACCACATACTGGTGGGATGGACTTCAATCCTCTGATGATTTCGCTCTCATAGTGAATGCACCGAATCATGAGGGGATACAAGCAGGAGTGGAT<br>AGATTCTATAGGACCTGCAAACTGGTTGGGATCAACATGAGCAAAAGGAAGTCTTACATAAACCGAACAGGAACATTTGAGTTCACAAGTTTTTTCTACCGCTATGGATTTGTAGCTAACTTCAGTATGGAATTACCCAGCTTTGGAGTGTCTGGAATCAATGAATCAGCTGACATGAGCATTGGAGTTACAGTGATAAAAAACAATATGATAAACAATGATCTTGGACCAGCA<br>ACAGCTCAAATGGCTCTTCAGTTATTCATCAAAGACTATAGGTACACATACCGATGCCACAGGGGTGATACACAAATTCAAACGAGGAGATCATTCGAGCTGAAGAAGCTGTGGGAGCAGACCCGTTCAAAGGCAGGGCTGTTGATATCAGACGGGGGGCCAAACCTATACAACATTCGGAATCTCCACATCCCAGAGGTCTGCTTGAAGTGGGAGCTGATGGATGAAGACT<br>ACCAAGGCAGGCTGTGCAATCCTCTGAATCCATTTGTCAGTCATAAAGAGATTGAGTCCGTAAACAATGCTGTAGTAATGCCCGCCCATGGCCCGGCCAAGAGCATGGAATATGATGCTGTTGCGACCACACACTCGTGGATTCCTAAGAGGAACCGTTCCATTCTCAATACCAGCCAAAGGGGAATTCTTGAGGATGAGCAGATGTACCAAAAGTGCTGTAATCTATTCGAGA<br>AATTCTTCCCCAGCAGTTCATACAGGAGGCCAGTTGGAATTTCCAGCATGGTGGAGGCCATGGTGTCTAGGGCCCGAATTGATGCACGCATCGACTTCGAATCTGGAAGGATTAAGAAAGAAGAGTTTGCTGAGATCATGAAGATCTGTTCCACCATTGAAGAGCTCAGACGGCAAAAATAG

EPI_ISL_2113493	>A/mute_swan/Poland/MB268/2021_H5N8_2021-03-09<br>[illegible]
EPI_ISL_2113167	>A/mute_swan/Poland/MB189/2021_H5N8_2021-02-26<br>[illegible]
EPI_ISL_2199986	>A/mute_swan/Poland/MB185/2021_H5N8_2021-02-26<br>[illegible]
EPI_ISL_2111625	>A/mute_swan/Poland/MB131/2021_H5N8_2021-02-19<br>[illegible]
EPI_ISL_18245816	>A/mute_swan/Poland/MB122/2022_H5N1_2022-04-07<br>[illegible]
EPI_ISL_18245789	>A/mute_swan/Poland/MB042-22_22VIR5675-6/2022_H5N1_2022-01-24<br>[illegible]
EPI_ISL_18245744	>A/mute_swan/Poland/MB040-22_22VIR5675-8/2022_H5N1_2022-01-20<br>[illegible]
EPI_ISL_18245743	>A/mute_swan/Poland/MB034-22_22VIR5675-12/2022_H5N1_2022-01-18<br>[illegible]
EPI_ISL_18244181	>A/mute_swan/Poland/MB021-21_22VIR5675-11/2021_H5N8_2021-01-19<br>[illegible]
EPI_ISL_18245613	>A/mute_swan/Poland/MB020-22_22VIR5675-3/2022_H5N1_2022-01-11<br>[illegible]
EPI_ISL_18245597	>A/mute_swan/Poland/MB008-22_22VIR5675-16/2022_2022-01-05<br>[illegible]
EPI_ISL_1139039	>A/mute_swan/Netherlands/20016960-001/2020_H5N8_2020-11-02<br>[illegible]

EPI_ISL_614401	>A/mute_swan/Kazakhstan/1-267-20-B/2020_H5N8_2020-09-23<br>ATGGATGTCAATCCGACTTTACTTTTCTTAAAAGTGCCAGCGCAAAATGCTATAAGTACTACATTCCCTTACACTGGAGATCCCCCATACAGCCATGGAACAGGAACAGGGTATACCATGGACACAGTAAACAGAACACATCAATACTCAGAAAAGGGAAAGTGGACAACAAACACAGAAACCGGAGCACCCCAACTCAACCCAATTGATGGACCATTACCAGAGGACAATGA<br>GCCAATCGGATATGCACAAACTGATTGCGTGTTGGAAGCAATGGCTTTCCTTGAAGAATCCCACCCAGGGATATTTGAAAACTCTTGTCTTGAAGCGATGGAAATCGTTCAGCAAACAAGAGTGGACAAACTAACCCAAGGTCGCCAGACTTATGACTGGACACTGAACAGAAACCAACCAGCTGCAACCTCTTTGGCCAACACTATAGAGGTGTTCAGATCGAATGGTCTGAC<br>AGCCAATGAATCAGGGAGACTGATAGATTTTCTCAGGGATGTGATGGAATCAATGGATAAAGAAGAGATGGAAGTAACAACACATTTCCAGAGAAAAAGAAGAGTGAGGGACAACATGACTAAGAAGATGGTCACACAAAGAACAATAGGGAAGAAGAAGCAGAGGCTGAACAAGAGGAGTTACTTAATAAGAGCACTGACATTGAATACAATGACCAAAGATGCAGAA<br>AGAGGCAAGTTGAAGAGACGGGCAATTGCAACACCCGGGATGCAGATTAGAGGATTCGTGTACTTCGTTGAAACACTAGCGAGGAGCATCTGTGAGAAACTAGAGCAATCTGGGCTCCCTGTTGGAGGGAATGAGAAGAAGGCTAAATTGGCAAATGTTGTGAGAAAAATGATGACTAACTCACAAGATACAGAGCTCTCCTTTACAATTACTGGAGACAACACCAAATGG<br>AATGAGAATCAAAACCCTCGGATGTTTTTGGCAATGATAACATATATCACAAGAAACCAACCTGAATGGTTTAGAAATGTCTTAAGCATTGCCCCTATAATGTTCTCAAACAAAATGGCGAGATTAGGGAAAGGATACATGTTTGAAAGTAAGAGCATGAAGCTAAGAACACAAATACCAGCAGAGATGCTTACAAATATTGATCTGAAGTATTTCAACGAACCAACGAGAAA<br>GAAAATCGAGAAAATAAGACCTCTGCTGATTGATGGCACGGCCTCATTGAGTCCTGGGATGATGATGGGCATGTTCAATATGCTGAGCACAGTATTAGGGGTCTCAATCCTGAATCTCGGGCAAAAGAGGTACACCAAAACCACATACTGGTGGGATGGACTTCAATCCTCTGATGATTTCGCTCTCATAGTGAATGCACCGAATCATGAGGGGATACAAGCAGGAGTGGATA<br>GATTCTATAGGACCTGCAAACTGGTTGGGATCAACATGAGCAAAAAGAAGTCTTACATAAACCGAACAGGAACATTTGAGTTCACAAGTTTTTTCTACCGCTATGGATTTGTAGCTAACTTCAGTATGGAACTACCCAGCTTTGGAGTGTCTGGAATCAATGAATCAGCTGACATGAGCATTGGAGTTACAGTGATAAAAAACAATATGATAAACAATGATCTTGGACCAGCAA<br>CAGCTCAAATGGCTCTTCAGTTATTCATCAAAGACTATAGGTACACATACCGATGCCACAGGGGTGATACACAAATTCAAACGAGGAGATCATTCGAGCTGAAGAAGCTGTGGGAGCAGACCCGTTCAAAGGCAGGGCTGTTGATATCAGACGGGGGGCCAAACCTATACAACATTCGGAATCTCCACATCCCAGAGGTCTGCTTGAAGTGGGAGCTGATGGATGAAGACTA<br>CCAAGGCAGGCTGTGCAATCCTCTGAATCCATTTGTCAGTCATAAAGAGATTGAGTCCGTAAACAATGCTGTAGTAATGCCCGCCCATGGCCCGGCCAAGAGCATGGAATATGATGCTGTTGCGACCACACACTCGTGGATTCCTAAGAGGAACCGTTCCATTCTCAATACCAGCCAAAGGGGAATTCTTGAGGATGAGCAGATGTACCAAAAGTGCTGTAGTCTATTCGAGAA<br>ATTCTTCCCCAGCAGTTCATACAGGAGGCCAGTTGGAATTTCCAGCATGGTGGAGGCCATGGTGTCTAGGGCCCGAATTGATGCACGCATCGACTTCGAATCTGGAAGGATTAAGAAAGAAGAGTTTGCTGAGATCATGAAGATCTGTTCCACCATTGAAGAGCTCAGACGGCAAAAATAG
EPI_ISL_11259264	>A/mute_swan/Ireland/033169_22VIR1325-14/2021_H5N1_2021-11-15<br>ATGGATGTCAATCCGACTTTACTTTTCTTAAAAGTGCCAGCGCAAGATGCCATAAGTACCACATTCCCTTACACTGGAGATCCTCCATACAGCCATGGAACAGGGACAGGATACACAATGGACACAGTCAACAGAACACATCAATACTCAGAGAAGGGAAAATGGACAACAAACACAGAAACCGGAGCACCTCAACTCAACCCAATTGATGGGCCACTACCTGAGGACAACGA<br>ACCGAGCGGATATGCACAAACAGATTGCGTGTTGGAAGCAATGGCTTTCCTTGAAGAGTCCCACCCAGGGATCTTTGAAAACTCTTGTCTTGAAACGATGGAAGTCGTTCAGCAAACAAGAGTGGACAAACTAACTCAAGGTCGCCAGACATATGACTGGACACTGAATAGAAACCAACCAGCTGCAACTGCCCTGGCCAACACTATAGAGGTCTTCAGATCAAACGGTCTAA<br>CAACCAATGAATCGGGGAGACTAATAGATTTCCTCAAGGATGTGATGGACTCAATGGATAAAGAAGAAATGGAAATAACAACACATTTCCAGAGAAAGAGAAGAGTAAGGGACAACATGACCAAGAAAATGGTCACACAAAGAACAATAGGAAAGAAGAAACAAAGGCTAACCAAGAGGAGCTACTTAATAAGAGCACTGACACTGAATACAATGACAAAAGATGCAGAA<br>AGAGGCAAATTGAAAAGACGGGCGATTGCAACACCAGGGATGCAGATTAGAGGATTTGTGTACTTTGTCGAAACACTGGCAAGGAGCATCTGTGAAAAACTTGAGCAATCTGGACTCCCCGTTGGAGGGAATGAGAAGAAGGCTAAATTGGCAAATGTCGTGAGAAAAATGATGACTAACTCACAAGATACAGAGCTCTCCTTCACAATTACTGGAGATAACACCAAATGG<br>AATGAGAATCAAAATCCTCGGATGTTTCTGGCAATGATAACATACATTACAAGAAACCAACCTGAATGGTTTAGAAATGTCTTGAGTATTGCCCCTATAATGTTCTCGAACAAAATGGCGAGATTGGGAAAAGGGTACATGTTTGAAAGTAAGAGCATGAAGTTACGGACACAAATACCTGCAGAAATGCTTGCAAACATTGACTTAAAATACTTCAATGAATCAACAAGAAAG<br>AAAATCGAAAAAATAAGGCCTCTACTAATAGATGGCACTGCCTCATTGAGTCCTGGAATGATGATGGGCATGTTCAATATGCTGAGTACAGTATTAGGAGTTTCAATCCTAAATCTTGGGCAAAAGAAGTACACCAAAACCACATACTGGTGGGATGGACTCCAATCCTCTGATGATTTCGCCCTCATAGTAAATGCACCGAATCATGAGGGAATACAAGCAGGAGTGGATAG<br>GTTCTATAGGACCTGCAAACTGGTCGGGATCAATATGAGCAAAAAGAAGTCTTACATAAACCGGACTGGAACATTTGAGTTCACAAGCTTTTTCTATCGCTATGGATTTGTGGCTAACTTCAGTATGGAGCTGCCCAGCTTTGGAGTTTCTGGGATCAATGAATCAGCTGACATGAGCATTGGCATCACAGTGATAAAGAACAACATGATAAACAATGACCTTGGACCAGCAAC<br>AGCTCAAATGGCCCTTCAACTATTCATCAAAGATTACAGGTACACGTACCGATGCCACAGAGGTGACACACAAATTCAAACGAGGAGATCATTCGAGCTGAAGAAGCTGTGGGAACAGACCCGTTCAAAGGCAGGACTGTTGGTGTCAGATGGAGGACCAAATCTATACAACATTCGGAATCTCCATATCCCAGAGGTCTGCCTGAAGTGGGAGCTGATGGACGAAGATTAC<br>CAGGGCAGGTTGTGTAATCCTCTGAACCCATTTGTCAGCCATAGAGAAATTGAGTCCGTAAACAATGCTGTGGTGATGCCAGCCCACGGTCCAGCCAAAAGCATGGAATATGATGCCGTTGCAACTACACACTCATGGATTCCTAAAAGGAATCGTTCCATTCTCAATACCAGTCAAAGGGGAATTCTTGAGGATGAACAGATGTACCAGAAATGCTGCAGTCTATTCGAGAAA<br>TTCTTCCCCAGTAGTTCATACAGGAGACCAGTTGGAATTTCCAGCATGGTGGAGGCCATGGTGTCTAGGGCCCGAATCGATGCACGCATTGATTTCGAATCTGGAAGGATCAAGAAGGAAGAGTTTGCTGAGATCATGAAGATCTGTTCCACCATTGAAGAGCTCAGACGGCAAAAATAG
EPI_ISL_256462	>A/Mute_swan/Hungary/5879/2017_H5N5_2017-02-14<br>ATGGATGTCAATCCGACTTTACTTTTCTTAAAAGTGCCAGCGCAAAATGCCATAAGTACCACATTCCCTTATACTGGAGATCCTCCATACAGCCATGGAACAGGAACAGGATACACCATGGACACAGTCAACAGAACACATCAATACTCAGAAAAGGGGAAATGGACAACAAACACAGAGACCGGAGCACCCCAACTCAACCCAATTGATGGACCACTACCTGAGGACAACGA<br>ACCAAGCGGATATGCACAAACAGATTGCGTGTTGGAAGCAATGGCTTTCCTTGAAGAGTCCCACCCAGGGATCTTTGAAAACTCTTGTCTTGAAACGATGGAAGTCGTTCATCAAACAAGAGTGGACAAACTAACTCAAGGTCGCCAGACTTATGACTGGACACTGAATAGAAACCAACCAGCTGCAACTGCCCTGGCCAACACTATAGAGGTCTTCAGATCGAACGGTCTGA<br>CAGCCAATGAATCAGGGAGACTAATAGATTTCCTCAAAGATGTGATGGACTCAATGGACAAAGAAGAAATGGAAATAACAACACATTTCCAGAGAAAGAGAAGAGTAAGGGACAACATGACCAAGAAAATGGTCACACAAAGAACAATAGGGAAGAAGAAACAAAGGCTAAATAAGAGGAGCTACTTAATAAGAGCACTGACACTGAATACAATGACAAAAGACGCAGA<br>AAGAGGCAAATTGAAGAGACGGGCAATTGCAACACCAGGGATGCAGATTAGAGGATTTGTGTACTTTGTCGAAACACTGGCAAGGAGCATCTGTGAAAAACTTGAGCAATCTGGACTCCCCGTTGGAGGGAATGAGAAGAAGGCTAAATTGGCAAATGTCGTGAGAAAAATGATGACTAACTCACAAGATACAGAGCTCTCCTTCACAATTACTGGAGATAACACCAAATG<br>GAATGAGAATCAAAATCCTCGGATGTTTCTAGCAATGATAACATACATCACAAGAAACCAACCTGAATGGTTTAGAAACGTCTTGAGCATTGCCCCTATAATGTTCTCGAACAAGATGGCGAGATTGGGAAAAGGATACATGTTTGAAAGTAAGAGCATGAAGCTACGGACACAAATACCTGCAGAAATGCTTGCAAACATTGACTTAAAATACTTCAATGAGTCAACAAGAA<br>AGAAAATCGAGAAAATAAGACCTCTACTGATAGATGGCACCGCCTCATTGAGTCCTGGAATGATGATGGGCATGTTCAATATGCTGAGTACAGTATTAGGAGTCTCAATCCTGAATCTTGGGCAAAAGAGGTACACCAAAACCACATACTGGTGGGATGGACTCCAATCCTCTGATGATTTCGCCCTCATAGTGAATGCACCGAATCATGAGGGAATACAAGCAGGAGTGGAT<br>AGGTTCTATAGGACCTGCAAACTGGTTGGGATCAACATGAGCAAAAAGAAGTCTTACATAAACCGGACAGGAACATTTGAGTTCACAAGCTTTTTCTATCGCTATGGATTTGTGGCTAACTTCAGTATGGAGCTGCCCAGCTTTGGAGTGTCTGGAATCAATGAATCAGCTGACATGAGCATCGGAGTTACAGTGATAAAGAACAACATGATAAACAATGACCTTGGACCAGCA<br>ACAGCTCAAATGGCCCTTCAACTATTCATCAAGGACTACAGGTACACGTACCGATGCCACAGGGGTGACACACAAATTCAAACGAGGAGATCATTCGAGCTGAAGAAGCTGTGGGAGCAGACCCGTTCAAAGGCAGGACTGTTGGTGTCAGATGGAGGACCAAATCTATACAACATTCGGAATCTCCATATCCCAGAGGTCTGCCTGAAGTGGGAGCTGATGGACGAAGATT<br>ACCAGGGCAGGCTGTGTAATCCTCTGAACCCATTTGTCAGTCATAAAGAAATTGAGTCCGTAAACAATGCTGTGGTAATGCCAGCCCACGGCCCAGCCAAGAGCATGGAATATGATGCTGTTGCGACTACACACTCATGGATTCCTAAAAGGAATCGTTCCATTCTCAATACCAGCCAAAGGGGAATTCTTGAGGATGAGCAGATGTATCAGAAATGCTGCAGCCTATTCGAGA<br>AATTTTTCCCCAGTAGTTCATACAGGAGACCAGTTGGAATTTCCAGCATGGTGGAGGCCATGGTGTCTAGGGCCCGAATCGATGCACGCATTGATTTCGAATCTGGAAGGATTAAGAAGGAAGAGTTTGCTGAGATCATGAAGATCTGTTCCACCATTGAAGAGCTCAGACGGCAAAAATAG
EPI_ISL_7778770	>A/mute_swan/Finland/1325_21VIR7689-2/2021_H5N8_2021-01-01<br>ATGGATGTCAATCCGACTTTACTTTTCTTAAAAGTGCCAGCGCAAAATGCTATAAGTACTACATTCCCTTACACTGGAGATCCCCCATACAGCCATGGAACAGGAACAGGGTATACCATGGACACAGTAAACAGAACACATCAATACTCAGAAAAGGGAAAGTGGACAACAAACACAGAAACCGGAGCACCCCAACTCAACCCAATTGATGGACCATTACCAGAGGACAATGA<br>GCCAAGCGGATATGCACAAACTGATTGCGTGTTGGAAGCAATGGCTTTCCTTGAAGAATCCCACCCAGGGATATTTGAAAACTCTTGTCTTGAAGCGATGGAAATCGTTCAGCAAACAAGAGTGGACAAACTAACCCAAGGTCGCCAGACTTATGACTGGACACTGAACAGAAACCAACCAGCTGCAACCTCTTTGGCCAACACTATAGAGGTGTTCAGATCGAATGGTCTGA<br>CAGCCAATGAATCAGGGAGACTGATAGATTTTCTCAGGGATGTGATGGAATCAATGGATAAAGAAGAGATGGAAGTAACAACACATTTCCAGAGAAAAAGAAGAGTGAGGGACAACATGACTAAGAAGATGGTCACACAAAGAACAATAGGGAAGAAGAAGCAGAGGCTGAACAAGAGGAGTTACTTAATAAGAGCACTGACATTGAATACAATGACCAAAGATGCAGA<br>AAGAGGCAAGTTGAAGAGACGGGCAATTGCAACACCCGGGATGCAGATTAGAGGATTCGTGTACTTCGTTGAAACACTAGCGAGGAGCATCTGTGAGAAACTAGAGCAATCTGGGCTCCCTGTTGGAGGGAATGAGAAGAAGGCTAAATTGGCAAATGTTGTGAGAAAAATGATGACTAACTCACAAGATACAGAGCTCTCCTTTACAATTACTGGAGACAACACCAAATG<br>GAATGAGAATCAAAACCCTCGGATGTTTTTGGCAATGATAACATATATCACAAGAAACCAACCTGAATGGTTTAGAAATGTCTTAAGCATTGCCCCTATAATGTTCTCAAACAAAATGGCGAGATTAGGGAAAGGATACATGTTTGAAAGTAAGAGCATGAAGCTAAGAACACAAATACCAGCAGAGATGCTTACAAATATTGATCTGAAGTATTTCAACGAACCAACGAGAA<br>AGAAAATCGAGAAAATAAGACCTCTGCTGATTGATGGCACGGCCTCATTGAGTCCTGGGATGATGATGGGCATGTTCAATATGCTGAGCACAGTATTAGGGGTCTCAATCCTGAATCTCGGGCAAAAGAGGTACACCAAAACCACATACTGGTGGGATGGACTTCAATCCTCTGATGATTTCGCTCTCATAGTGAATGCACCGAATCATGAGGGGATACAAGCAGGAGTGGAT<br>AGATTCTATAGGACCTGCAAACTGGTTGGGATCAACATGAGCAAAAAGAAGTCTTACATAAACCGAACAGGAACATTTGAGTTCACAAGTTTTTTCTACCGCTATGGATTTGTAGCCAACTTCAGTATGGAATTACCCAGCTTTGGAGTGTCTGGAATCAATGAATCAGCTGACATGAGCATTGGAGTTACAGTGATAAAAAACAATATGATAAACAATGATCTTGGACCAGCA<br>ACAGCTCAAATGGCTCTTCAGTTATTCATCAAAGACTATAGGTACACATACCGATGCCACAGGGGTGATACACAAATTCAAACGAGGAGATCATTCGAGCTGAAGAAGCTGTGGGAGCAGACCCGTTCAAAGGCAGGGCTGTTGATATCAGACGGGGGGCCAAATCTATACAACATTCGGAATCTCCACATCCCAGAGGTCTGCTTGAAGTGGGAGCTGATGGATGAAGACT<br>ACCAAGGCAGGCTGTGCAATCCTCTGAATCCATTTGTCAGTCATAAAGAGATTGAGTCCGTAAACAATGCTGTAGTAATGCCCGCCCATGGCCCGGCCAAGAGCATGGAATATGATGCTGTTGCGACCACACACTCGTGGATTCCTAAGAGGAACCGTTCCATTCTCAATACCAGCCAAAGGGGAATTCTTGAGGATGAGCAGATGTACCAAAAGTGCTGTAGTCTATTCGAGA<br>AATTCTTCCCCAGCAGTTCATACAGGAGGCCAGTTGGAATTTCCAGCATGGTGGAGGCCATGGTGTCTAGGGCCCGAATTGATGCACGCATCGACTTCGAATCTGGAAGGATTAAGAAAGAAGAGTTTGCTGAGATCATGAAGATCTGTTCCACCATTGAAGAGCTCAGACGGCAAAAATAG
EPI_ISL_1033124	>A/mute_swan/Czech_Republic/2600/2021_H5N8_2021-02-04<br>ATGGATGTCAATCCGACTTTACTTTTCTTAAAAGTGCCAGCGCAAAATGCTATAAGTACTACATTCCCTTACACTGGAGATCCCCCATACAGCCATGGAACAGGAACAGGGTATACCATGGACACAGTAAACAGAACACATCAATACTCAGAAAAGGGAAAGTGGACAACAAACACAGAAACCGGAGCACCCCAACTCAACCCAATTGATGGACCATTACCAGAGGACAATGA<br>GCCAAGCGGATATGCACAAACTGATTGCGTGTTGGAAGCAATGGCTTTCCTTGAAGAATCCCACCCAGGGATATTTGAAAACTCTTGTCTTGAAGCAATGGAAATCGTTCAGCAAACAAGAGTGGACAAACTAACCCAAGGTCGCCAGACTTATGACTGGACACTGAACAGAAACCAACCAGCTGCAACCTCTTTGGCCAACACTATAGAGGTGTTCAGATCGAATGGTCTGA<br>CAGCCAATGAATCAGGGAGACTGATAGATTTTCTCAGGGATGTGATGGAATCAATGGATAAAGAAGAGATGGAAATAACAACACATTTCCAGAGAAAAAGAAGAGTGAGGGACAACATGACTAAGAAGATGGTCACACAAAGAACAATAGGGAAGAAGAAGCAGAGGCTGAACAAGAGGAGTTACTTAATAAGAGCACTGACATTGAATACAATGACCAAAGATGCAGA<br>AAGAGGCAAGTTGAAGAGACGGGCAATTGCAACACCCGGGATGCAGATTAGAGGATTCGTGTACTTCGTTGAAACACTAGCGAGGAGCATCTGTGAGAAACTAGAGCAATCTGGGCTCCCTGTTGGAGGGAATGAGAAAAAGGCTAAATTGGCAAATGTTGTGAGAAAAATGATGACTAACTCACAAGATACAGAGCTCTCCTTTACAATTACTGGAGACAACACCAAATG<br>GAATGAGAATCAAAACCCTCGGATGTTTTTGGCAATGATAACATATATCACAAGAAACCAACCTGAATGGTTTAGAAATGTCTTAAGCATTGCCCCTATAATGTTCTCAAACAAAATGGCGAGATTAGGGAAAGGATACATGTTTGAAAGTAAGAGCATGAAGCTAAGAACACAAATACCAGCAGAGATGCTTACAAATATTGATCTGAAGTATTTCAACGAACCAACGAGAA<br>AGAAAATCGAGAAAATAAGACCTCTGCTGATTGATGGCACGGCCTCATTGAGTCCTGGGATGATGATGGGCATGTTCAATATGCTGAGCACAGTATTAGGGGTCTCAATCCTGAATCTCGGGCAAAAGAGGTACACCAAAACCACATACTGGTGGGATGGACTTCAATCCTCTGATGATTTCGCTCTCATAGTGAATGCACCGAATCATGAGGGGATACAAGCAGGAGTGGAT<br>AGATTCTATAGGACCTGCAAACTGGTTGGGATCAACATGAGCAAAAAGAAGTCTTACATAAACCGAACAGGAACATTTGAGTTCACAAGTTTTTTCTACCGCTATGGATTTGTAGCCAACTTCAGTATGGAATTACCCAGCTTTGGAGTGTCTGGAATCAATGAATCAGCTGACATGAGCATTGGAGTTACAGTGATAAAAAACAATATGATAAACAATGATCTTGGACCAGCA<br>ACAGCTCAAATGGCTCTTCAGTTATTCATCAAAGACTATAGGTACACATACCGATGCCACAGGGGTGATACACAAATTCAAACGAGGAGATCATTCGAGCTGAAGAAGCTGTGGGAGCAGACCCGTTCAAAGGCAGGGCTGTTGATATCAGACGGGGGGCCAAACCTATACAACATTCGGAATCTCCACATCCCAGAGGTCTGCTTGAAGTGGGAGCTGATGGATGAAGACT<br>ACCAAGGCAGGCTGTGCAATCCTCTGAATCCATTTGTCAGTCATAAAGAGATTGAGTCCGTAAACAATGCTGTAGTAATGCCCGCCCATGGCCCGGCCAAGAGCATGGAATATGATGCTGTTGCGACCACACACTCGTGGATTCCTAAGAGGAACCGTTCCATCCTCAATACCAGCCAAAGGGGAATTCTTGAGGATGAGCAGATGTACCAAAAGTGCTGTAGTCTATTCGAGA<br>AATTCTTCCCCAGCAGTTCATACAGGAGGCCAGTTGGAATTTCCAGCATGGTGGAGGCCATGGTGTCTAGGGCCCGAATTGATGCACGCATCGACTTCGAATCTGGAAGGATTAAGAAAGAAGAGTTTGCTGAGATCATGAAGATCTGTTCCACCATTGAAGAGCTCAGACGGCAAAAATAG
EPI_ISL_9603927	>A/mute_swan/Czech_Republic/25702-2/2021_H5N1_2021-12-20<br>ATGGATGTCAATCCGACTTTACTTTTCTTAAAAGTGCCAGCGCAAGATGCCATAAGTACCACATTCCCTTACACTGGAGATCCTCCATACAGCCATGGAACAGGGACAGGATACACAATGGACACAGTCAACAGAACACATCAATACTCAGAGAAGGGAAAATGGACAACAAACACAGAAACCGGAGCACCTCAACTCAACCCAATTGATGGGCCACTACCTGAGGACAACGA<br>ACCGAGCGGATATGCACAAACAGATTGCGTGTTGGAAGCAATGGCTTTCCTTGAAGAGTCCCACCCAGGGATCTTTGAAAACTCTTGTCTTGAAACGATGGAAGTCGTTCAGCAAACAAGAGTGGACAAACTAACTCAAGGTCGCCAGACATATGACTGGACACTGAATAGAAACCAACCAGCTGCAACTGCCCTGGCCAACACTATAGAGGTCTTCAGATCAAACGGTCTAA<br>CAACCAATGAATCGGGGAGACTAATAGATTTCCTCAAGGATGTGATGGACTCAATGGATAAAGAAGAAATGGAAATAACAACACATTTCCAGAGAAAGAGAAGAGTAAGGGACAACATGACCAAGAAAATGGTCACACAAAGAACAATAGGAAAGAAGAAACAAAGGCTAAACAAGAGGAGCTACTTAATAAGAGCACTGACACTGAATACAATGACAAAGGATGCAGA<br>AAGAGGCAAATTGAAAAGACGGGCGATTGCAACACCAGGGATGCAGATTAGAGGATTTGTGTACTTTGTCGAAACACTGGCAAGGAGCATCTGTGAAAAACTTGAGCAATCTGGACTCCCCGTTGGAGGGAATGAGAAGAAGGCTAAATTGGCAAATGTCGTGAGAAAAATGATGACTAACTCACAAGATACAGAGCTCTCCTTCACAATTACTGGAGATAACACCAAATG<br>GAATGAGAATCAAAATCCTCGGATGTTTCTGGCAATGATAACATACATTACAAGAAACCAACCTGAATGGTTTAGAAATGTCTTGAGTATTGCCCCTATAATGTTCTCGAACAAAATGGCGAGATTGGGAAAAGGGTACATGTTTGAAAGTAAGAGCATGAAGTTACGGACACAAATACCTGCAGAAATGCTTGCAAACATTGACTTAAAATACTTCAATGAATCAACAAGAAA<br>GAAAATCGAAAAAATAAGGCCTCTACTAATAGATGGCACTGCCTCATTGAGTCCTGGAATGATGATGGGCATGTTCAATATGCTGAGTACAGTATTAGGAGTTTCAATCCTAAATCTTGGGCAAAAGAAGTACACCAAAACCACATACTGGTGGGATGGACTCCAATCCTCTGATGATTTCGCCCTCATAGTAAATGCACCGAATCATGAGGGAATACAAGCAGGAGTGGATA<br>GGTTCTATAGGACCTGCAAACTGGTCGGGATCAATATGAGCAAAAAGAAGTCTTACATAAACCGGACTGGAACATTTGAGTTCACAAGCTTTTTCTATCGCTATGGATTTGTGGCTAACTTCAGTATGGAGCTGCCCAGCTTTGGAGTTTCTGGGATCAATGAATCAGCTGACATGAGCATTGGCATCACAGTGATAAAGAACAACATGATAAACAATGACCTTGGACCAGCAA<br>CTGCTCAAATGGCCCTTCAACTATTCATCAAAGATTACAGGTACACGTACCGATGCCACAGAGGTGACACACAAATTCAAACGAGGAGATCATTCGAGCTGAAGAAGCTGTGGGAACAGACCCGTTCAAAGGCAGGACTGTTGGTGTCAGATGGAGGACCAAATCTATACAACATTCGGAATCTCCATATCCCAGAGGTCTGCCTGAAGTGGGAGCTGATGGACGAAGATTA<br>CCAGGGCAGGTTGTGTAATCCTCTGAACCCATTTGTCAGCCATAGAGAAATTGAGTCCGTAAACAATGCTGTGGTGATGCCAGCCCACGGTCCAGCCAAAAGCATGGAATATGATGCCGTTGCGACTACACACTCATGGATTCCTAAAAGGAATCGTTCCATTCTCAATACCAGTCAAAGGGGAATTCTTGAGGATGAACAGATGTACCAGAAATGCTGCAGTCTATTCGAGAA<br>ATTCTTCCCCAGTAGTTCATACAGGAGACCAGTTGGAATTTCCAGCATGGTGGAGGCCATGGTGTCTAGGGCCCGAATCGATGCACGCATTGATTTCGAATCTGGAAGGATCAAGAAGGAAGAGTTTGCTGAGATCATGAAGATCTGTTCCACCATTGAAGAGCTCAGACGGCAAAAATAG
EPI_ISL_1180234	>A/mute_swan/Czech_Republic/1656-1/2021_H5N8_2021-01-24<br>ATGGATGTCAATCCGACTTTACTTTTCTTAAAAGTGCCAGCGCAAAATGCTATAAGTACTACATTCCCTTACACTGGAGATCCCCCATACAGCCATGGAACAGGAACAGGGTATACCATGGACACAGTAAACAGAACACATCAATACTCAGAAAAGGGAAAGTGGACAACAAACACAGAAACCGGAGCACCCCAACTCAACCCAATTGATGGACCATTACCAGAGGACAATGA<br>GCCAAGCGGATATGCACAAACTGATTGCGTGTTGGAAGCAATGGCTTTCCTTGAAGAATCCCACCCAGGGATATTTGAAAACTCTTGTCTTGAAGCGATGGAAATCGTTCAGCAAACAAGAGTGGACAAACTAACCCAAGGTCGCCAGACTTATGACTGGACACTGAACAGAAACCAACCAGCTGCAACCTCTTTGGCCAACACTATAGAGGTGTTCAGATCGAATGGTCTGA<br>CAGCCAATGAATCAGGGAGACTGATAGATTTTCTCAGGGATGTGATGGAATCAATGGATAAAGAAGAGATGGAAGTAACAACACATTTCCAGAGAAAAAGAAGAGTGAGGGACAACATGACTAAGAAGATGGTCACACAAAGAACAATAGGGAAGAAGAAGCAGAGGCTGAACAAGAGGAGTTACTTAATAAGAGCACTGACATTGAATACAATGACCAAAGATGCAGA<br>AAGAGGCAAGTTGAAGAGACGGGCAATTGCAACACCCGGGATGCAGATTAGAGGATTCGTGTACTTCGTTGAAACACTAGCGAGGAGCATCTGTGAGAAACTAGAGCAATCTGGGCTCCCTGTTGGAGGGAATGAGAAGAAGGCTAAATTGGCAAATGTTGTGAGAAAAATGATGACTAACTCACAAGATACAGAGCTCTCCTTTACAATTACTGGAGACAACACCAAATG<br>GAATGAGAATCAAAACCCTCGGATGTTTTTGGCAATGATAACATATATCACAAGAAACCAACCTGAATGGTTTAGAAATGTCTTAAGCATTGCCCCTATAATGTTCTCAAACAAAATGGCGAGATTAGGGAAAGGATACATGTTTGAAAGTAAGAGCATGAAGCTAAGAACACAAATACCAGCAGAGATGCTTACAAATATTGATCTGAAGTATTTCAACGAACCAACGAGAA<br>AGAAAATCGAGAAAATAAGACCTCTGCTGATTGATGGCACGGCCTCATTGAGTCCTGGGATGATGATGGGCATGTTCAATATGCTGAGCACAGTATTAGGGGTCTCAATCCTGAATCTCGGGCAAAAGAGGTACACCAAAACCACATACTGGTGGGATGGACTTCAATCCTCTGATGATTTCGCTCTCATAGTGAATGCACCGAATCATGAGGGGATACAAGCAGGAGTGGAT<br>AGATTCTATAGGACCTGCAAACTGGTTGGGATCAACATGAGCAAAAAGAAGTCTTACATAAACCGAACAGGAACATTTGAGTTCACAAGTTTTTTCTACCGCTATGGATTTGTAGCCAACTTCAGTATGGAATTACCCAGCTTTGGAGTGTCTGGAATCAATGAATCAGCTGACATGAGCATTGGAGTTACAGTGATAAAAAACAATATGATAAACAATGATCTTGGACCAGCA<br>ACAGCTCAAATGGCTCTTCAGTTATTCATCAAAGACTATAGGTACACATACCGATGCCACAGGGGTGATACACAAATTCAAACGAGGAGATCATTCGAGCTGAAGAAGCTGTGGGAGCAGACCCGTTCAAAGGCAGGGCTGTTGATATCAGACGGGGGGCCAAATCTATACAACATTCGGAATCTCCACATCCCAGAGGTCTGCTTGAAGTGGGAGCTGATGGATGAAGACT<br>ACCAAGGCAGGCTGTGCAATCCTCTGAATCCATTTGTCAGTCATAAAGAGATTGAGTCCGTAAACAATGCTGTAGTAATGCCCGCCCATGGCCCGGCCAAGAGCATGGAATATGATGCTGTTGCGACCACACACTCGTGGATTCCTAAGAGGAACCGTTCCATTCTCAATACCAGCCAAAGGGGAATTCTTGAGGATGAGCAGATGTACCAAAAGTGCTGTAGTCTATTCGAGA<br>AATTCTTCCCCAGCAGTTCATACAGGAGGCCAGTTGGAATTTCCAGCATGGTGGAGGCCATGGTGTCTAGGGCCCGAATTGATGCACGCATCGACTTCGAATCTGGAAGGATTAAGAAAGAAGAGTTTGCTGAGATCATGAAGATCTGTTCCACCATTGAAGAGCTCAGACGGCAAAAATAG
EPI_ISL_7357590	>A/mute_swan/Croatia/101/2021_H5N1_2021-11-12<br>ATGGATGTCAATCCGACTTTACTTTTCTTAAAAGTGCCAGCGCAAGATGCCATAAGTACCACATTCCCTTACACTGGAGATCCTCCATACAGCCATGGAACAGGGACAGGATACACAATGGACACAGTCAACAGAACACATCAATACTCAGAGAAGGGAAAATGGACAACAAACACAGAAACCGGAGCACCTCAACTCAACCCAATTGATGGGCCACTACCTGAGGACAACGA<br>ACCGAGCGGATATGCACAAACAGATTGCGTGTTGGAAGCAATGGCTTTCCTTGAAGAGTCCCATCCAGGGATCTTTGAAAACTCTTGTCTTGAAACGATGGAAGTCGTTCAGCAAACAAGAGTGGACAAACTAACTCAAGGTCGCCAGACATATGACTGGACACTGAATAGAAACCAACCAGCTGCAACTGCCCTGGCCAACACTATAGAGGTCTTCAGATCAAACGGTCTAA<br>CAGCCAATGAATCGGGGAGACTAATAGATTTCCTCAAGGATGTGATGGACTCAATGGATAAAGAAGAAATGGAAATAACAACACATTTCCAGAGAAAGAGAAGAGTAAGGGACAACATGACCAAGAAAATGGTCACACAAAGAACAATAGGAAAGAAGAAACAAAGGCTAAACAAGAGGAGCTACTTAATAAGAGCACTGACACTGAATACAATGACAAAAGATGCAGA<br>AAGAGGCAAATTGAAGAGACGGGCGATTGCAACACCAGGGATGCAGATTAGAGGATTTGTGTACTTTGTCGAAACACTGGCAAGGAGCATCTGTGAAAAACTTGAGCAATCTGGACTCCCCGTTGGAGGGAATGAGAAGAAGGCTAAATTGGCAAATGTCGTGAGAAAAATGATGACTAACTCACAAGATACAGAGCTCTCCTTCACAATTACTGGAGATAACACCAAATG<br>GAATGAGAATCAAAATCCTCGGATGTTTCTGGCAATGATAACATACATTACAAGAAACCAACCTGAATGGTTTAGGAATGTCTTGAGTATTGCCCCTATAATGTTCTCGAACAAAATGGCGAGATTGGGAAAAGGGTACATGTTTGAAAGTAAGAGCATGAAGTTACGGACACAAATACCTGCAGAAATGCTTGCAAACATTGACTTAAAATACTTCAATGAATCAACAAGAAA<br>GAAAATCGAAAAAATAAGGCCTCTACTAATAGATGGCACTGCCTCATTGAGTCCTGGAATGATGATGGGCATGTTCAATATGCTGAGTACAGTATTAGGAGTTTCAATCCTAAATCTTGGGCAAAAGAAGTACACCAAAACCACATACTGGTGGGATGGACTCCAATCCTCTGATGATTTCGCCCTCATAGTAAATGCACCGAATCATGAGGGAATACAAGCAGGAGTGGATA<br>GGTTCTATAGGACCTGCAAACTGGTCGGGATCAATATGAGCAAAAAGAAGTCTTACATAAACCGGACTGGAACATTTGAGTTCACAAGCTTTTTCTATCGCTATGGATTTGTGGCTAACTTCAGTATGGAGCTGCCCAGCTTTGGGGTTTCTGGGATCAATGAATCAGCTGACATGAGCATTGGCATCACAGTGATAAAGAACAACATGATAAACAATGACCTTGGACCAGCAA<br>CAGCTCAAATGGCCCTTCAACTATTCATCAAAGATTACAGGTACACGTACCGATGCCATAGAGGTGACACACAAATTCAAACGAGGAGATCATTCGAGCTGAAGAAGCTGTGGGAACAGACCCGTTCAAAGGCAGGACTGTTGGTGTCAGATGGAGGACCAAATCTGTACAACATTCGGAATCTCCATATCCCAGAGGTCTGCCTGAAGTGGGAGCTGATGGACGAAGATTA<br>CCAGGGCAGGTTGTGTAATCCTCTGAACCCATTTGTCAGCCATAAAGAAATTGAGTCCGTAAACAATGCTGTGGTGATGCCAGCCCACGGTCCAGCCAAAAGCATGGAATATGATGCCGTTGCGACTACACACTCATGGATTCCTAAAAGGAATCGTTCCATTCTCAATACCAGTCAAAGGGGAATTCTTGAGGATGAACAGATGTACCAGAAATGCTGCGGTCTATTCGAGAA<br>ATTCTTCCCCAGTAGTTCATACAGGAGACCAGTTGGAATTTCCAGCATGGTGGAGGCCATGGTGTCTAGGGCCCGAATCGATGCACGCATTGATTTCGAATCTGGAAGGATCAAGAAGGAAGAGTTTGCTGAGATCATGAAGATCTGTTCCACCATTGAAGAGCTCAGACGGCAAAAATAG
EPI_ISL_267438	>A/Mediterranean_gull/Netherlands/1/2012_H13N6_2012-06-20<br>ATGGATGTCAATCCGACTTTACTTTTCTTGAAAGTGCCAGCGCAAAATGCTATAAGTACTACATTCCCTTATACTGGTGACCCTCCATACAGCCATGGAACAGGAACAGGATATACCATGGACACAGTCAACAGAACACACCAGTACTCAGAAAAAGGGACATGGACAACAAACACCGAAACTGGAGCACCTCAGCTTAACCCGATCGATGGACCACTACCGGAGGATAATGA<br>GCCAAGCGGATATGCACAAACGGATTGTGTATTGGAGGCCATGGCTTTTCTTGAAGAATCCCACCCAGGGATCTTTGAGAACTCATGTCTCGAAACGATGGAAGTTGTTCAGCAAACACGAGTGGACAAGCTGACCCAAGGCCGACAAACATATGATTGGACATTGAATAGAAACCAGCCAGCTGCAACGGCTTTAGCCAACACCATAGAGGTCTTCAGATCGAACGGTCTAA<br>CAGCTAATGAATCGGGGAGACTCATTGATTTCCTTAAGGATGTGATGGATTCAATGGATAAAGAAGGAATGGAAATAACAACACATTTCCAAAGAAAAAGAAGAGTAAGGGACAACATGACCAAGAAAATGGTCACACAAAGAACAATAGGGAAAAAGAAACAGAGATTAAGCAAAAAAAGCTACCTAATAAGGGCATTGACATTGAATACAATGACCAAAGACGCAGAA<br>AGAGGTAAATTGAAGAGGAGGGCGATTGCAACACCCGGGATGCAGATCAGAGGTTTCGTCTACTTTGTTGAAACACTTGCAAGGAGCATATGTGAGAAACTTGAACAGTCAGGGCTCCCAGTTGGAGGAAATGAGAAAAAGGCTAAATTGGCAAATGTTGTAAGAAAGATGATGACTAATTCGCAAGACACAGAGCTCTCCTTCACAATTACTGGGGACAACACCAAGTGG<br>AATGAAAATCAAAACCCTCGGATGTTCCTAGCGATGATAACATACATTACACGAAACCAGCCTGAATGGTTCAGAAATGTCTTGAGCATTGCCCCTATAATGTTCTCAAACAAAATGGCAAGACTTGGGAAAGGATATATGTTCGAAAGTAAGAGCATGAAGCTACGAACACAAATACCAGCAGAAATGCTTGCAAGTATTGATCTCAAGTATTTCAATGAGTCAACGAGAAAG<br>AAAATCGAGAGCATAAGACCCCTCTTAATAGATGGCACAGCCTCATTGAGTCCTGGAATGATGATGGGCATGTTCAACATGTTGAGTACAGTCTTAGGAGTCTCAATCTTAAATCTTGGGCAAAAGAGGTACACCAAGACCACATACTGGTGGGATGGACTCCAATCCTCTGATGACTTTGCACTCATAGTGAACGCACCTAATCATGAGGGAATACAAGCAGGAGTGGACAG<br>ATTTTACAGAACCTGCAAATTAGTTGGAATCAACATGAGTAAAAAGAAGTCCTACATAAATAGAACAGGAACATTTGAATTCACAAGCTTTTTCTACCGCTATGGGTTTGTAGCTAACTTCAGTATGGAGCTACCAAGCTTTGGCGTATCAGGGATTAATGAGTCAGCTGACATGAGCATTGGAGTAACAGTGATAAAGAACAACATGATAAACAATGACCTCGGACCAGCAAC<br>AGCCCAAATGGCTCTTCAACTGTTCATCAAGGACTACAGGTACACATATCGATGCCATAGGGGTGATACTCAAATCCAAACAAGGAGATCATTTGAGTTGAAGAAGCTTTGGGAGCAAACCCGTTCAAAGGCAGGGCTGCTGGTTTCAGATGGAGGACCAAACCTATATAATATCAGAAATCTCCATATTCCAGAGGTCTGTCTGAAATGGGAGCTAATGGATGAAGATTATC<br>AGGGCAGGCTGTGCAACCCACTGAATCCATTCGTAAGCCATAAGGAAATTGAGTCCGTAAATAATGCTGTGGTAATGCCAGCCCATGGTCCAGCCAAAAGCATGGAATATGATGCTGTTGCAACTACACATTCCTGGGTCCCCAAGAGGAACCGTTCCATTCTCAACACTAGCCAAAGGGGAATCCTTGAGGATGAACAGATGTATCAAAAATGCTGCAATCTATTCGAAAAAT<br>TCTTCCCTAGCAGTTCGTACAGGAGACCAGTTGGAATTTCCAGCATGGTGGAGGCCATGGTATCCAGGGCCAGAATTGACGCACGGATTGACTTCGAATCTGGAAGGATCAAGAAAGAAGAGTTTGCTGAGATCATGAAGATCTGTTCCACCATTGAAGAGCTCAGACGGCAAAAGTAG
EPI_ISL_6959592	>A/mandarin_duck/Korea/WA585/2021_H5N1_2021-10-26<br>ATGGATGTCAATCCGACTTTACTTTTCTTAAAAGTGCCAGCGCAAAATGCTATAAGCACTACGTTCCCTTACACTGGAGATCCTCCATACAGCCATGGAACAGGAACAGGATACACCATGGACACAGTCAACAGAACACATCAATACTCAGAAAAAGGAAAGTGGACAACAAACACAGAGACCGGGGCGCCCCAACTCAACCCAATTGATGGACCATTGCCAGAGGACAACGA<br>CCCAAGCGGATATGCACAAACAGATTGCGTGTTGGAAGCAATGGCTTTCCTTGAAGAATCCCACCCAGGGATCTTTGAAAACTCTTGTCTTGAAACGATGGAGATCGTTCAGCAAACAAGAGTGGACAAACTAACCCAAGGTCGCCAGACCTATGACTGGACACTGAATAGAAACCAACCAGCTGCAACTGCTTTAGCCAATACTATAGAGGTCTTCAGATCGAACGGTCTAAC<br>AGCCAATGAGTCGGGGAGACTAATAGATTTCCTCAAGGATGTGATGGAATCAATGGATAAAGAAGAAATGGAAATAACAACACATTTCCAGAGAAAGAGAAGAGTAAGGGACAACATGACCAAAAAGATGGTCACACAAAGAACGATAGGAAAGAAGAAGCAGAGGCTGAACAAGAGAAGCTACTTAATAAGAGCGTTGACATTGAACACAATGACCAAAGATGCAGAA<br>AGAGGCAAATTGAAGAGGCGGGCAATTGCAACACCCGGGATGCAGATTAGAGGATTTGTGTACTTTGTCGAAACACTAGCGAGGAGCATCTGTGAGAAACTTGAGCAATCCGGACTCCCTGTCGGAGGGAATGAGAAAAAGGCCAAATTGGCAAATGTCGTGAGGAAAATGATGACTAACTCGCAAGATACAGAGCTCTCTTTTACAATCACCGGGGACAACACCAAATGG<br>AATGAGAATCAAAATCCTCGGATGTTTTTGGCAATGATAACATACATCACAAGAAACCAACCCGAATGGTTTAGAAATGTCTTGAGCATTGCCCCCATAATGTTCTCAAACAAAATGGCAAGATTAGGGAAAGGATACATGTTTGAAAGTAAGAGCATGAAGCTAAGGACACAAATACCGGCAGAAATGATTGCAAACATTGACTTGAAATACTTCAATGAATCAACGAGAAA<br>GAAAATCGAGAAAATAAGACCTCTACTAATTGATGGCACAGCCTCATTGAGTCCTGGAATGATGATGGGCATGTTCAATATGCTGAGCACTGTATTGGGGGTCTCAATCCTGAATCTTGGACAAAAGAGGTACACCAAAACCACATACTGGTGGGATGGACTCCAATCTTCTGATGACTTCGCTCTCATAGTGAATGCACCGAATCATGAGGGGATACAAGCCGGGGTGGATA<br>GGTTCTATAGGACATGCAAACTGGTTGGGATCAACATGAGCAAAAAGAAGTCTTACATAAACCGGACAGGAACATTTGAGTTCACAAGCTTTTTCTACCGCTATGGATTTGTAGCCAACTTCAGTATGGAGTTACCCAGCTTTGGTGTGTCTGGAATCAATGAGTCAGCTGACATGAGCATTGGAGTTACAGTGATAAAGAACAACATGATAAACAATGACCTTGGACCAGCAA<br>CAGCTCAAATGGCTCTTCAGCTATTCATCAAGGATTATAGGTACACGTACCGATGTCACAGAGGTGATACACAAATTCAAACGAGGAGATCATTCGAGCTGAAGAAGCTGTGGGAGCAGACCCGTTCAAAGGCAGGGCTGTTGGTATCAGATGGAGGACCAAACCTATACAACATTCGGAATCTCCACATCCCAGAGGTCTGCTTGAAATGGGAGCTAATGGATGAAGACTA<br>CCAGGGCAGGCTGTGTAATCCTCTGAATCCATTTGTCAGTCATAAGGAAATTGGATCCGTAAACAATGCTGTAGTAATGCCAGCCCATGGCCCAGCCAAGAGCATGGAATACGATGCTGTTGCGACTACACACTCATGGATTCCTAAGAGGAACCGTTCCATTCTCAATACCAGCCAGAGGGGAATTCTCGAGGATGAGCAGATGTACCAGAAGTGCTGCAGCTTATTCGAGA<br>AGTTCTTCCCCAGTAGTTCATACAGGAGGCCAGTTGGAATTTCCAGCATGGTGGAGGCCATGGTGTCCAGGGCCCGAATTGATGCACGCATTGATTTCGAATCTGGAAGGATTAAGAACGAAGAGTTTGCTGATATCATGAAGATCTGTTCCACCATTGAGGAGCTCAGACGGCAAAAATAG
EPI_ISL_267352	>A/mallard_duck/Netherlands/9/2014_H6N2_2014-10-28<br>ATGGATGTCAATCCGACTTTACTTTTCTTAAAAGTGCCAGCGCAAAATGCTATAAGTACTACATTCCCTTACACTGGAGATCCTCCATACAGCCATGGAACAGGAACAGGATACACTATGGACACAGTCAACAGAACACATCAATACTCAGAGAAGGGAAAATGGACAACAAACACAGAGACCGGAGCACCCCAACTCAATCCAATTGATGGACCATTACCAGAGGACAACGA<br>GCCAAGCGGATATGCACAAACGGATTGCGTGTTGGAAGCAATGGCTTTCCTTGAAGAATCCCACCCAGGGATCTTTGAAAACTCTTGTCTTGAAACGATGGAAATCGTTCAGCAAACAAGAGTGGACAAACTAACTCAAGGTCGCCAGACTTATGACTGGACACTGAACAGAAACCAACCAGCTGCAACTGCTTTGGCCAACACTATAGAGGTGTTCAGATCGAATGGTCTGG<br>CAGCCAATGAGTCGGGGAGACTAATAGATTTCCTCAAGGATGTGATGGAATCAATGGATAAAGAAGAAATGGAAATAACAACACATTTCCAGAGAAAGAGAAGAGTGAGGGACAACATGACCAAGAAGATGGTCACACAAAGAACAATAGGAAAGAAGAAGCAGAGGCTGAATAAGAGGAGTTACTTAATAAGAGCACTGACATTGAACACAATGACCAAAGATGCAGA<br>AAGAGGCAAACTGAAGAGACGGGCAATTGCAACACCCGGGATGCAGATTAGAGGATTCGTGTACTTTGTTGAAACACTAGCGAGGAGCATCTGTGAGAAACTCGAGCAATCTGGGCTCCCTGTTGGAGGGAATGAGAAGAAGGCTAAATTGGCAAATGTCGTGAGAAAAATGATGACTAACTCACAAGATACAGAGCTCTCCTTTACAATTACTGGAGACAACACCAAATG<br>GAATGAGAATCAAAATCCTCGGATGTTTTTGGCAATGATAACATACATCACAAGAAACCAACCTGAATGGTTTAGAAACGTCTTGAGCATCGCCCCTATAATGTTCTCAAATAAAATGGCGAGATTAGGAAAAGGATACATGTTTGAAAGTAAGAGCATGAAGCTGAGAACACAAATACCGGCAGAGATGCTTGCAAACATTGATTTGAAATATTTCAACGAATCAACGAGAA<br>AGAAAATCGAGAAAATAAGACCTCTGCTGATTGATGGCACAGCCTCATTGAGTCCTGGAATGATGATGGGCATGTTCAATATGCTGAGCACAGTGTTAGGGGTCTCAATCCTGAATCTTGGGCAAAAGAGGTACACCAAAACCACATACTGGTGGGACGGACTCCAATCCTCTGATGATTTCGCTCTCATAGTGAATGCACCGAATCATGAGGGGATACAAGCAGGAGTGGAT<br>AGGTTCTATAGGACCTGCAAACTGGTTGGGATCAACATGAGCAAAAAGAAATCTTACATAAACCGGACAGGAACATTTGAGTTCACAAGTTTTTTCTACCGCTATGGATTTGTAGCTAACTTCAGTATGGAATTACCCAGCTTTGGAGTATCTGGAATCAATGAATCGGCTGATATGAGCATTGGAGTTACAGTGATAAAGAACAATATGATAAACAATGACCTTGGACCAGCA<br>ACAGCTCAAATGGCCCTCCAGCTATTCATCAAGGACTATAGGTACACGTACCGATGCCACAGGGGTGATACACAAATTCAAACGAGGAGATCATTCGAGCTGAAGAAGCTGTGGGAGCAGACCCGTTCAAAGGCAGGGTTGTTGGTATCAGATGGAGGACCAAACCTATACAACATTCGGAATCTCCACATCCCAGAGGTCTGCTTGAAGTGGGAGCTGATGGATGAAGACT<br>ACCAGGGCAGGCTGTGTAATCCCTTGAATCCGTTTGTCAGTCATAAGGAAATTGAGTCCGTAAACAATGCTGTGGTAATGCCAGCCCATGGCCCAGCCAAGAGCATGGAATATGATGCTGTTGCGACTACACACTCGTGGATTCCTAAGAGGAACCGTTCCATTCTCAATACCAGCCAAAGGGGAATTCTTGAGGATGAGCAAATGTACCAGAAGTGCTGTAGTCTATTTGAGA<br>AATTCTTCCCCAGCAGTTCATACAGGAGGCCAGTTGGAATTTCCAGCATGGTGGAGGCCATGGTGTCTAGGGCCCGAATTGATGCACGCATTGATTTCGAATCTGGAAGGATTAAGAAAGAAGAGTTTGCTGAGATCATGAAGATCTGTTCCACCATTGAAGAGCTCAGACGGCAAAAATAG
EPI_ISL_243588	>A/mallard_duck/Netherlands/20/2011_H6N8_2011-09-24<br>ATGGATGTCAATCCGACTTTACTTTTCTTAAAAGTGCCAGCGCAAAATGCTATAAGTACTACGTTCCCTTACACTGGAGATCCTCCATACAGCCATGGAACAGGAACAGGATACACCATGGACACAGTCAACAGAACACATCAATACTCAGAGAAGGGAAAGTGGACAACGAACACAGAGACTGGGGCGCCCCAACTCAACCCAATTGATGGACCATTACCAGGGGACAATGA<br>GCCAAGCGGATATGCACAAACAGATTGCGTGTTGGAAGCAATGGCTTTCCTTGAAGAATCCCACCCAGGGATCTTTGAAAACTCTTGTCTTGAAACGATGGAGGTCGTTCAGCAAACAAGAGTGGACAAACTAACCCAAGGTCGCCAGACTTATGATTGGACACTAAATAGAAACCAACCAGCTGCAACTGCTTTGGCCAACACTATAGAGGTCTTCAGATCGAACGGTCTAA<br>CGGCCAATGAGTCGGGGAGACTAATAGATTTCCTCAAGGATGTGGTGGAATCAATGGATAAAGAAGAAATGGAAATAACAACACATTTCCAGAGAAAGAGAAGAGTAAGGGACAACATGACCAAGAAGATGGTCACACAAAGAACGATAGGAAAGAAGAAGCAGAGGCTGAACAAGAGGAGCTACTTAATAAGGGCACTGACATTGAACACAATGACCAAAGATGCAGA<br>AAGAGGCAAATTGAAGAGGCGGGCAATTGCAACACCCGGGATGCAGATTAGAGGATTCGTGTACTTTGTCGAAACACTAGCAAGGAGCATCTGTGAGAAACTCGAGCAGTCTGGACTCCCTGTTGGAGGGAATGAGAAGAAGGCCAAATTGGCAAATGTCGTGAGAAAAATGATGACTAACTCGCAAGATACAGAGCTCTCCTTTACAATTACTGGAGACAACACCAAATG<br>GAATGAGAATCAAAATCCTCGGATGTTTTTGGCAATGATAACATACATCACAAGAAACCAACCCGAATGGTTTAGAAATGTCTTGAGCATTGCCCCTATAATGTTCTCAAACAAAATGGCGAGATTAGGAAAAGGATACATGTTTGAGAGTAAGAGCATGAAGCTAAGGACACAAATACCGGCAGAAATGCTTGCAAACATTGACTTGAAATACTTCAATGAATCAACGAGAA<br>AGAAAATCGAGAAAATTAGGCCTCTGCTAATTGATGGCACAGCCTCATTGAGTCCTGGAATGATGATGGGCATGTTCAATATGCTGAGCACAGTATTGGGGGTCTCAATCCTGAATCTTGGACAAAAGAGGTACACCAAAACCACATACTGGTGGGATGGACTCCAATCCTCTGATGATTTCGCTCTCATAGTGAATGCACCGAATCATGAGGGGATACAAGCAGGGGTGGAT<br>AGGTTCTATAGGACCTGCAAACTGGTTGGGATCAACATGAGCAAAAAGAAGTCTTACATAAACCGGACAGGAACATTTGAGTTCACAAGCTTTTTCTACCGCTATGGATTTGTAGCCAACTTCAGTATGGAGTTACCCAGCTTTGGAGTGTCTGGAATCAATGAATCGGCTGACATGAGCATTGGAGTTACAGTGATAAAGAACAATATGATAAACAATGACCTTGGACCTGCA<br>ACAGCTCAAATGGCTCTTCAGCTATTCATCAAGGACTATAGGTACACGTACCGATGCCACAGAGGTGATACACAAATTCAAACGAGGAGATCATTCGAGCTGAAGAAGCTGTGGGAGCAGACCCGTTCAAAGGCAGGGCTGTTGGTATCAGATGGAGGACCAAACCTATACAACATTCGGAATCTCCACATCCCAGAGGTCTGCTTGAAGTGGGAACTAATGGATGAAGACT<br>ACCAGGGCAGGCTGTGTAATCCTCTGAATCCGTTTGTCAGTCATAAGGAAATTGAGTCCGTAAACAATGCTGTGGTAATGCCAGCCCATGGCCCAGCCAAGAGCATGGAATATGATGCTGTTGCGACTACACACTCATGGATTCCTAAGAGGAACCGTTCCATTCTCAATACCAGCCAGAGGGGAATTCTCGAGGATGAGCAGATGTACCAGAAGTGCTGCAGTCTATTCGAA<br>AAATTCTTCCCCAGTAGTTCATACAGGAGGCCAGTTGGAATTTCCAGCATGGTGGAGGCCATGGTGTCCAGGGCCCGAATTGATGCACGCATTGATTTCGAATCTGGAAGGATTAAGAAAGAAGAGTTTGCTGAGATCATGAAGATCTGTTCCACCATTGAAGAGCTCAGACGGCAAAAATAG

EPI_ISL_243659	>A/mallard_duck/Netherlands/10/2012_H6N1_2012-09-05<br>ATGGATGTCAATCCGACTTTACTTTTCTTAAAAGTGCCAGCGCAAAATGCTATAAGTACTACGTTCCCTTACACTGGAGATCCTCCATACAGCCATGGAACAGGAACAGGATACACCATGGACACAGTCAACAGAACACACCAATACTCAGAGAAGGGAAAGTGGACAACGAACACAGAGACTGGGGCGCCCCAACTCAACCCAATTGATGGACCATTACCAGAGGACAATGA<br>GCCAAGCGGATATGCACAAACAGATTGCGTGTTGGAAGCAATGGCTTTCCTTGAAGAATCCCACCCAGGGATCTTTGAAAACTCTTGTCTTGAAACGATGGAGGTCGTTCAGCAAACAAGAGTGGACAAACTAACCCAAGGTCGCCAGACTTATGATTGGACACTAAATAGAAACCAACCAGCTGCAACTGCTTTGGCCAACACTATAGAGGTCTTCAGATCGAACGGTCTAA<br>CAGCCAATGAGTCGGGGAGACTAATAGATTTCCTCAAGGATGTGATGGAATCAATGGATAAAGAAGAAATGGAAATAACAACACATTTCCAGAGAAAGAGAAGAGTAAGGGACAACATGACCAAGAAGATGGTCACACAAAGAACGATAGGAAAGAAGAAGCAGAGACTGAACAAGAGGAGCTACTTAATAAGGGCACTGACATTGAACACAATGACCAAAGATGCAGA<br>AAGAGGCAAATTGAAGAGGCGGGCAATTGCAACACCCGGGATGCAGATTAGAGGATTCGTGTACTTTGTCGAAACACTAGCAAGGAGCATCTGTGAGAAACTCGAGCAGTCTGGACTCCCTGTTGGAGGGAATGAGAAGAAGGCCAAATTGGCAAATGTCGTGAGAAAAATGATGACTAACTCACAAGATACAGAGCTCTCCTTTACAATTACTGGAGACAACACCAAATG<br>GAATGAGAATCAAAATCCTCGGATGTTTTTGGCAATGATAACATACATCACAAGAAACCAACCCGAATGGTTTAGAAATGTCTTGAGCATTGCCCCTATAATGTTCTCAAACAAAATGGCGAGATTAGGAAAAGGATACATGTTTGAGAGTAAGAGCATGAAGCTAAGGACACAAATACCGGCAGAAATGCTTGCAAACATTGACTTGAAATACTTCAATGAATCAACGAGAA<br>AGAAAATCGAGAAAATTAGACCTCTGCTAATTGATGGCACAGCCTCATTGAGTCCTGGAATGATGATGGGCATGTTCAATATGCTGAGCACAGTATTGGGGGTCTCAATCCTGAATCTTGGACAAAAGAGGTACACCAAAACCACATACTGGTGGGATGGACTCCAATCCTCTGATGATTTCGCTCTCATAGTGAATGCACCGAATCATGAGGGGATACAAGCAGGGGTGGAT<br>AGGTTCTATAGGACCTGCAAACTGGTTGGGATCAACATGAGCAAAAAGAAGTCTTACATAAACCGGACAGGAACATTTGAGTTCACAAGCTTTTTCTACCGCTATGGATTTGTAGCCAACTTCAGTATGGAGTTACCCAGCTTTGGAGTGTCTGGAATCAATGAATCGGCTGACATGAGCATTGGAGTTACAGTGATAAAGAACAATATGATAAACAATGACCTTGGACCTGCA<br>ACAGCTCAAATGGCTCTTCAGCTATTCATCAAGGACTATAGGTACACGTACCGATGCCACAGAGGTGATACACAAATTCAAACGAGGAGATCATTCGAGATGAAGAAGCTGTGGGAGCAGACCCGTTCAAAGGCAGGGCTGTTGGTATCAGACGGAGGACCAAACCTATACAACATTCGGAATCTCCACATCCCAGAGGTCTGCTTGAAGTGGGAACTAATGGATGAAGACT<br>ACCAGGGCAGGCTGTGTAATCCTCTGAATCCGTTTGTCAGTCATAAGGAAATTGAGTCCGTAAACAATGCTGTGGTAATGCCAGCCCATGGCCCAGCCAAGAGCATGGAATATGATGCTGTTGCGACTACACACTCATGGATTCCTAAGAGGAACCGTTCCATTCTCAATACCAGCCAGAGGGGAATTCTCGAGGATGAGCAGATGTACCAGAAGTGCTGCAGTCTATTCGAA<br>AAATTCTTCCCCAGTAGTTCATACAGGAGGCCAGTTGGAATTTCCAGCATGGTGGAGGCCATGGTGTCCAGGGCCCGAATTGATGCACGCATTGATTTCGAGTCTGGAAGGATTAAGAAAGAAGAGTTTGCTGAGATCATGAAGATCTGTTCCACCATTGAAGAGCTCAGACGGCAAAAATAG
EPI_ISL_189730	>A/mallard_duck/Georgia/6/2016_H4N6_2016-09-30<br>ATGGATGTCAATCCGACTTTACTTTTCTTAAAAGTGCCAGCGCAAAATGCCATAAGTACTACGTTTCCTTATACTGGAGATCCTCCATACAGCCATGGAACAGGAACAGGATACACCATGGACACAGTCAACAGAACACATCAATACTCAGAAAAGGGGAAATGGACAACAAACACAGAGACCGGAGCACCCCAACTCAACCCAATTGATGGACCACTGCCTGAGGACAACGA<br>ACCAAGCGGATATGCACAAACAGATTGTGTGTTGGAAGCAATGGCTTTCCTTGAAGAGTCCCACCCAGGGATCTTTGAAAACTCTTGTCTTGAAACGATGGAAGTTGTTCAGCAAACAAGAGTGGACAAACTAACTCAAGGTCGCCAGACTTATGACTGGACACTGAATAGAAACCAACCAGCTGCAACTGCCCTGGCCAACACTATAGAGGTCTTCAGATCGAACGGTCTGA<br>CAGCCAATGAATCGGGGAGGCTAATAGATTTCCTCAAGGATGTGATGGACTCAATGGACAAAGAAGAAATGGAAATAACAACACATTTCCAGAGAAAGAGAAGAGTAAGGGACAACATGACCAAGAAAATGGTCACACAAAGAACAATAGGGAAAAAGAAACAAAGGCTGAATAAGAAGAGCTACTTAATAAGGGCACTGACACTGAACACAATGACAAAAGATGCAGA<br>AAGAGGCAAATTGAAGAGGCGTGCAATTGCAACACCAGGGATGCAGATCAGAGGATTTGTGTACTTTGTCGAAACACTGGCAAGGAGCATCTGTGAGAAACTTGAGCAATCTGGACTCCCCGTTGGAGGGAATGAGAAAAAGGCTAAATTGGCAAATGTCGTGAGAAAAATGATGACCAACTCACAAGATACAGAGCTCTCTTTCACAATTACTGGAGATAACACCAAATG<br>GAATGAGAATCAAAATCCTCGGATGTTTCTGGCAATGATAACATACATCACAAGAAACCAACCTGAATGGTTTAGGAATGTCTTGAGCATTGCCCCTATAATGTTCTCGAACAAAATGGCGAGATTGGGAAAAGGATACATGTTTGAAAGTAAGAGCATGAAGCTACGGACACAAATACCTGCAGAAATGCTTGCAAATATTGACTTAAAATATTTCAATGAATCAACAAGAAA<br>GAAAATCGAGAAAATAAGACCTCTGCTGATAGATGGCACAGCCTCATTGAGTCCTGGAATGATGATGGGCATGTTCAATATGCTGAGTACAGTATTAGGAGTCTCAATCCTGAATCTTGGGCAAAAGAGGTACACCAAAACCACATACTGGTGGGATGGACTCCAATCCTCTGATGATTTCGCCCTCATAGTGAATGCACCGAATCATGAGGGAATACAAGCAGGAGTTGATA<br>GGTTCTATAGGACATGCAAACTGGTTGGAATCAACATGAGCAAAAAGAAGTCTTACATAAACAGGACAGGAACATTTGAGTTCACAAGCTTTTTCTATCGCTATGGATTTGTGGCTAATTTCAGTATGGAGCTGCCCAGCTTTGGAGTGTCTGGAATCAATGAATCAGCTGACATGAGTATTGGAGTTACAGTGATAAAGAATAACATGATAAACAATGACCTTGGACCAGCAA<br>CAGCTCAGATGGCTCTTCAGCTATTCATCAAGGACTACAGGTACACGTACCGATGCCACAGGGGTGACACACAAATTCAAACGAGGAGATCATTCGAGCTGAAGAAGCTGTGGGAGCAGACTCGTTCAAAGGCAGGACTGTTGGTGTCGGATGGAGGACCAAACCTATATAACATTCGGAATCTCCACATCCCAGAGGTCTGCTTGAAGTGGGAGCTGATGGACGAAGATTA<br>CCAGGGCAGGTTGTGTAATCCTCTGAACCCATTTGTCAGTCATAAGGAAATTGAGTCCGTAAACAATGCTGTGGTGATGCCGGCCCATGGCCCAGCCAAGAGCATGGAATATGATGCTGTTGCGACTACACACTCATGGGTTCCTAAGAGGAATCGTTCCATTCTCAATACCAGCCAAAGGGGAATTCTTGAGGATGAGCAGATGTACCAGAAGTGCTGTAGTCTATTCGAGA<br>AATTCTTCCCCAGTAGTTCATACAGGAGGCCAGTTGGAATTTCCAGCATGGTAGAGGCCATGGTATCTAGGGCCCGAATTGATGCACGCATTGATTTCGAATCTGGAAGGATTAAGAAAGAAGAGTTTGCTGAGATCATGAAGATCTGTTCCACCATTGAAGAGCTCAGACGGCAAAAATAG
EPI_ISL_328973	>A/mallard_duck/Georgia/3/2016_H4N6_2019-09-08<br>ATGGATGTCAATCCGACTTTACTTTTCTTAAAAGTGCCAGCGCAAAATGCCATAAGTACTACGTTTCCTTATACTGGAGATCCTCCATACAGCCATGGAACAGGAACAGGATACACCATGGACACAGTCAACAGAACACATCAATACTCAGAAAAGGGGAAATGGACAACAAACACAGAGACCGGAGCACCCCAACTCAACCCAATTGATGGACCACTGCCTGAGGACAACGA<br>ACCAAGCGGATATGCACAAACAGATTGTGTGTTGGAAGCAATGGCTTTCCTTGAAGAGTCCCACCCAGGGATCTTTGAAAACTCTTGTCTTGAAACGATGGAAGTTGTTCAGCAAACAAGAGTGGACAAACTAACTCAAGGTCGCCAGACTTATGACTGGACACTGAATAGAAACCAACCAGCTGCAACTGCCCTGGCCAACACTATAGAGGTCTTCAGATCGAACGGTCTGA<br>CAGCCAATGAATCGGGGAGGCTAATAGATTTCCTCAAGGATGTGATGGACTCAATGGACAAAGAAGAAATGGAAATAACAACACATTTCCAGAGAAAGAGAAGAGTAAGGGACAACATGACCAAGAAAATGGTCACACAAAGAACAATAGGGAAAAAGAAACAAAGGCTGAATAAGAAGAGCTACTTAATAAGGGCACTGACACTGAACACAATGACAAAAGATGCAGA<br>AAGAGGCAAATTGAAGAGGCGTGCAATTGCAACACCAGGGATGCAGATCAGAGGATTTGTGTACTTTGTCGAAACACTGGCAAGGAGCATCTGTGAGAAACTTGAGCAATCTGGACTCCCCGTTGGAGGGAATGAGAAAAAGGCTAAATTGGCAAATGTCGTGAGAAAAATGATGACCAACTCACAAGATACAGAGCTCTCTTTCACAATTACTGGAGATAACACCAAATG<br>GAATGAGAATCAAAATCCTCGGATGTTTCTGGCAATGATAACATACATCACAAGAAACCAACCTGAATGGTTTAGGAATGTCTTGAGCATTGCCCCTATAATGTTCTCGAACAAAATGGCGAGATTGGGAAAAGGATACATGTTTGAAAGTAAGAGCATGAAGCTACGGACACAAATACCTGCAGAAATGCTTGCAAATATTGACTTAAAATATTTCAATGAATCAACAAGAAA<br>GAAAATCGAGAAAATAAGACCTCTGCTGATAGATGGCACAGCCTCATTGAGTCCTGGAATGATGATGGGCATGTTCAATATGCTGAGTACAGTATTAGGAGTCTCAATCCTGAATCTTGGGCAAAAGAGGTACACCAAAACCACATACTGGTGGGATGGACTCCAATCCTCTGATGATTTCGCCCTCATAGTGAATGCACCGAATCATGAGGGAATACAAGCAGGAGTTGATA<br>GGTTCTATAGGACATGCAAACTGGTTGGAATCAACATGAGCAAAAAGAAGTCTTACATAAACAGGACAGGAACATTTGAGTTCACAAGCTTTTTCTATCGCTATGGATTTGTGGCTAATTTCAGTATGGAGCTGCCCAGCTTTGGAGTGTCTGGAATCAATGAATCAGCTGACATGAGTATTGGAGTTACAGTGATAAAGAATAACATGATAAACAATGACCTTGGACCAGCAA<br>CAGCTCAGATGGCTCTTCAGCTATTCATCAAGGACTACAGGTACACGTACCGATGCCACAGGGGTGACACACAAATTCAAACGAGGAGATCATTCGAGCTGAAGAAGCTGTGGGAGCAGACTCGTTCAAAGGCAGGACTGTTGGTGTCGGATGGAGGACCAAACCTATATAACATTCGGAATCTCCACATCCCAGAGGTCTGCTTGAAGTGGGAGCTGATGGACGAAGATTA<br>CCAGGGCAGGTTGTGTAATCCTCTGAACCCATTTGTCAGTCATAAGGAAATTGAGTCCGTAAACAATGCTGTGGTGATGCCGGCCCATGGCCCAGCCAAGAGCATGGAATATGATGCTGTTGCGACTACACACTCATGGGTTCCTAAGAGGAATCGTTCCATTCTCAATACCAGCCAAAGGGGAATTCTTGAGGATGAGCAGATGTACCAGAAGTGCTGTAGTCTATTCGAGA<br>AATTCTTCCCCAGTAGTTCATACAGGAGGCCAGTTGGAATTTCCAGCATGGTAGAGGCCATGGTATCTAGGGCCCGAATTGATGCACGCATTGATTTCGAATCTGGAAGGATTAAGAAAGAAGAGTTTGCTGAGATCATGAAGATCTGTTCCACCATTGAAGAGCTCAGACGGCAAAAATAG
EPI_ISL_328976	>A/mallard_duck/Georgia/10/2016_H7N7_2016-09-30<br>ATGGATGTCAATCCGACTTTACTTTTCTTAAAAGTGCCAGCGCAAAATGCTATAAGTACTACGTTCCCCTACACTGGAGATCCCCCATACAGCCATGGAACAGGAACAGGATACACCATGGACACAGTCAACAGAACACATCAATACTCAGAGAAGGGAAAGTGGACAACAAACACAGAGACTGGGGCGCCCCAACTCAACCCAATCGATGGACCATTGCCAGAGGACAACGA<br>GCCAAGCGGATATGCACAAACAGATTGCGTGTTGGAAGCAATGGCTTTCCTTGAAGAATCCCACCCAGGGATCTTTGAAAACTCTTGTCTTGAAACGATGGAGGTCGTTCAGCAAACAAGAGTGGACAAACTAACCCAAGGTCGCCAGACTTATGATTGGACACTGAATAGAAACCAACCAGCTGCAACTGCTTTGGCCAATACTATAGAGGTCTTTAGATCGAACGGTCTAAC<br>AGCCAATGAGTCGGGGAGACTAATAGATTTCCTCAAGGATGTGATGGAATCAATGGATAAAGAAGAAATGGAAATAACAACACATTTCCAGAGAAAGAGAAGAGTAAGGGATAACATGACCAAGAAGATGGTCACACAAAGAACGATAGGAAAGAAGAAGCAGAGGCTGAACAAGAGGAGCTACTTAATAAGAGCACTGACATTGAACACAATGACCAAAGATGCAGAA<br>AGAGGCAAATTGAAGAGGCGGGCAATTGCAACACCCGGGATGCAGATTAGAGGATTTGTGTACTTTGTCGAAACACTAGCGAGGAGCATCTGTGAGAAACTCGAGCAATCTGGACTCCCTGTTGGAGGGAATGAGAAAAAGGCCAAATTGGCAAATGTCGTGAGAAAAATGATGACTAACTCGCAAGATACAGAGCTCTCCTTTACAATTACTGGAGACAACACCAAATGG<br>AATGAGAATCAAAATCCTCGGATGTTTTTGGCAATGATAACATACATCACAAGAAACCAACCCGAATGGTTTAGAAATGTCTTGAGCATTGCCCCTATAATGTTCTCAAACAAAATGGCAAGATTAGGAAAAGGATACATGTTTGAAAGTAAGAGCATGAAGCTAAGGACACAAATACCGGCAGAAATGCTTGCAAACATTGACTTGAAATACTTCAATGAACCAACGAGAAA<br>GAAAATCGAGAAAATAAGGCCTCTGCTAATTGATGGCACAGCCTCATTGAGTCCTGGAATGATGATGGGCATGTTCAATATGCTGAGCACAGTATTGGGGGTCTCAATCCTGAATCTTGGACAAAAGAGGTACACTAAAACCACATACTGGTGGGATGGACTCCAATCCTCTGATGATTTCGCTCTCATCGTGAATGCACCGAATCATGAGGGGATACAAGCAGGGGTGGATA<br>GGTTCTATAGGACCTGCAAACTGGTTGGGATCAACATGAGCAAAAAGAAGTCTTACATAAACCGGACAGGAACATTTGAGTTCACAAGCTTTTTCTACCGCTATGGATTTGTAGCCAACTTCAGTATGGAGTTACCCAGCTTTGGAGTGTCTGGAATCAATGAGTCAGCTGACATGAGCATTGGAGTTACAGTGATAAAGAACAATATGATAAACAATGACCTTGGACCAGCAA<br>CAGCTCAAATGGCTCTTCAGCTATTCATCAAGGATTATAGGTACACGTACCGATGTCACAGAGGTGATACACAAATTCAAACGAGGAGATCATTCGAGCTGAAGAAGCTGTGGGAGCAGACCCGTTCAAAGGCAGGGCTGTTGGTATCAGATGGAGGACCAAACCTATACAACATTCGGAATCTCCACATCCCAGAGGTCTGCTTGAAGTGGGAACTAATGGATGAAGACTA<br>CCAGGGCAGGCTGTGTAATCCTCTGAATCCGTTTGTCAGTCATAAGGAAATTGAGTCCGTAAACAATGCTGTGGTAATGCCAGCCCATGGCCCAGCCAAGAGCATGGAATATGATGCTGTTGCGACTACACACTCATGGATTCCTAAGAGGAACCGTTCCATTCTCAATACCAGCCAGAGGGGAATTCTCGAGGATGAGCAGATGTACCAGAAGTGCTGCAGTTTATTCGAGA<br>AATTCTTCCCCAGTAGTTCATACAGGAGGCCAGTTGGAATTTCCAGCATGGTGGAGGCCATGGTGTCCAGGGCCCGAATTGACGCACGCATTGATTTCGAATCTGGAAGGATTAAGAAAGAAGAGTTTGCTGAGATCATGAAGATCTGTTCCACCATTGAAGAGCTCAGACGGCAAAAATAG
EPI_ISL_17978951	>A/mallard/Poland/P096/2020_H9N2_2020-11-02<br>ATGGATGTCAATCCGACTTTACTTTTCTTAAAAGTGCCAGCGCAAAATGCCATAAGTACCACATTCCCTTATACTGGAGATCCTCCATACAGCCATGGAACAGGAACAGGATACACCATGGACACAGTCAACAGAACACATCAATATTCGGAAAAGGGGAAATGGACAACAAACACAGAGACCGGAGCACCCCAACTCAACCCAATTGATGGACCACTACCTGAGGRCAACGA<br>ACCAAGMGGATATGCACAAACAGATTGCGTGTTGGAAGCAATGGCTTTCCTTGAAGAGTCCCACCCAGGGATCTTTGAAAACTCTTGTCTTGAAACGATGGAAGTCGTTCAGCAAACAAGAGTGGACAAACTAACTCAAGGTCGCCAGACTTATGATTGGACACTGAATAGAAACCAACCAGCTGCAACTGCCCTGGCCAACACTATAGAGGTCTTCAGATCGAACGGTCTGA<br>CAGCCAATGAATCAGGGAGATTAATAGATTTCCTCAAGGATGTGATGGACTCAATGGACAAAGAAGAAATGGAAATAACAACACATTTCCAGAGAAAGAGAAGAGTAAGGGACAACATGACCAAGAAAATGGTCACACAAAGAACAATAGGGAAGAAGAAGCAAAGGCTAAATAAGAGGAGCTACTTAATAAGAGCACTGACACTGAATACAATGACAAAAGACGCAGA<br>AAGAGGCAAATTGAAGAGACGGGCAATTGCAACACCAGGGATGCAGATTAGAGGATTTGTGTACTTTGTCGAAACACTGGCAAGGAGCATCTGTGAAAAACTTGAGCAATCTGGACTACCCGTTGGAGGGAATGAGAAGAAGGCTAAATTGGCAAATGTCGTGAGAAAAATGATGACTAACTCACAAGATACAGAGCTCTCCTTCACAATTACTGGAGACAACACCAAATG<br>GAATGAGAATCAAAATCCTCGGATGTTTCTGGCAATGATAACATACATCACAAGAAACCAACCTGAATGGTTTAGAAATGTTTTGAGCATTGCCCCTATAATGTTCTCGAACAAAATGGCGAGACTGGGAAAAGGATACATGTTTGAAAGTAAAAGCATGAAGCTACGGACACAAATACCTGCAGAAATGCTTGCAAACATTGACTTAAAATACTTCAATGAGTCAACAAGAAA<br>GAAAATCGAGAAAATAAGACCTCTACTAATAGATGGCACCGCCTCATTGAGTCCTGGAATGATGATGGGCATGTTCAATATGCTGAGTACAGTACTAGGAGTCTCAATCCTGAATCTTGGGCAAAAGAGGTACACCAAAACCACATACTGGTGGGATGGACTCCAATCCTCTGATGATTTCGCCCTCATAGTGAATGCACCGAATCATGAGGGAATACAAGCAGGAGTGGATA<br>GGTTCTATAGGACCTGCAAACTGGTTGGGATCAACATGAGCAAAAAGAAGTCTTACATAAACCGGACAGGAACATTTGAGTTCACAAGCTTTTTCTATCGCTATGGATTTGTGGCCAACTTCAGTATGGAGCTGCCCAGCTTTGGAGTGTCTGGAATCAATGAATCAGCTGACATGAGCATTGGAGTTACAGTGATAAAGAACAACATGATAAACAATGACCTTGGACCAGCAA<br>CAGCTCAAATGGCCCTTCAACTATTCATCAAGGACTACAGGTACACGTACCGATGTCACAGGGGTGACACACAAATTCAAACGAGGAGATCATTCGAGCTGAAGAAGCTGTGGGAGCAGACCCGTTCAAAGGTAGGACTGTTGGTGTCAGATGGAGGACCAAATCTATACAACATCCGGAATCTCCATATCCCAGAAGTCTGCCTGAAGTGGGAGCTGATGGACGAAGATTA<br>CCAGGGCAGGTTGTGTAATCCTCTGAACCCATTTGTCAGTCATAAAGAAATTGAATCCGTAAACAATGCTGTGGTAATGCCAGCCCATGGCCCAGCCAAGAGCATGGAATATGATGCTGTTGCGACTACACACTCATGGATTCCTAAAAGGAATCGTTCCATTCTCAATACCAGCCAAAGGGGAATTCTTGAGGACGAGCAGATGTACCAGAAATGCTGCAGTCTATTCGAGAA<br>ATTCTTCCCCAGTAGTTCATACAGGAGACCAGTTGGAATTTCAAGCATGGTGGAGGCCATGGTGTCTAGGGCTCGAATCGACGCACGCATTGATTTCGAATCTGGAAGGATTAAGAAGGAAGAGTTTGCTGAGATCATGAAGATCTGTTCCACCATTGAAGAGCTCAGACGGCAAAAATAG
EPI_ISL_17978925	>A/mallard/Poland/P077w1/2018_H5N2_2018-09-12<br>ATGGATGTCAATCCGACTTTACTTTTCTTAAAAGTGCCAGCGCAAAATGCTATAAGCACTACGTTCCCTTACACTGGAGATCCTCCATACAGCCATGGAACAGGAACAGGATACACCATGGATACAGTCAACAGGACACATCAATACTCAGAGAAGGGAAAGTGGACAACGAACACAGAGACTGGGGCGCCCCAACTCAACCCAATTGATGGACCATTACCAGAGGACAATGA<br>GCCAAGCGGATATGCACAAACAGATTGCGTGTTGGAAGCAATGGCTTTCCTTGAAGAATCCCACCCAGGGATCTTTGAAAACTCTTGTCTTGAAACGATGGAGGTCGTTCAGCAAACAAGAGTGGACAAACTAACCCAAGGTCGCCAGACTTATGATTGGACACTGAACAGAAACCAACCAGCTGCAACTGCTCTGGCCAACACTATAGAGGTCTTCAGATCGAACGGTCTAA<br>CAGCCAATGAGTCGGGGAGACTAATAGATTTCCTCAAGGATGTGATGGAATCAATGGATAAAGAAGAAATGGAAATAACAACACATTTCCAGAGAAAGAGAAGAGTAAGGGACAATATGACCAAGAAGATGATCACACAAAGAACGATAGGAAAGAAAAAGCAGAGACTAAACAAGAAGAGCTACTTAATAAGGGCACTGACATTGAACACAATGACCAAAGATGCGGA<br>AAGAGGCAAGTTGAAGAGGCGGGCAATTGCAACACCCGGGATGCAGATTAGAGGATTCGTATACTTTGTCGAAACACTAGCAAGGAGCATCTGTGAGAAACTCGAGCAGTCTGGACTCCCTGTTGGAGGGAATGAGAAGAAGGCCAAATTGGCAAATGTCGTGAGAAAAATGATGACTAATTCACAAGATACAGAGCTCTCCTTTACAATTACTGGAGACAACACCAAATG<br>GAATGAGAATCAAAATCCTCGGATGTTTTTGGCGATGATAACATACATCACAAGAAACCAACCTGAATGGTTTAGAAATGTCTTGAGCATTGCCCCTATAATGTTCTCAAACAAAATGGCGAGATTAGGAAAAGGATACATGTTTGAGAGTAAGAGCATGAAGCTAAGAACACAAATACCTGCAGAAATGCTTGCAAACATTGACTTGAAATACTTCAACGAATCAACGAGAA<br>AGAAAATCGAGAAAATTAGACCTCTGCTAATTGATGGCACAGCCTCATTGAGCCCTGGAATGATGATGGGCATGTTCAATATGCTGAGCACAGTATTGGGAGTCTCAATCCTGAATCTTGGACAAAAGAGGTACACCAAAACCACATACTGGTGGGATGGACTCCAATCCTCTGATGATTTCGCTCTCATAGTGAATGCACCGAATCATGAGGGGATACAAGCAGGGGTGGAT<br>AGGTTTTATAGGACATGCAAACTGGTTGGGATCAACATGAGCAAAAAGAAATCTTACATAAACCGGACAGGAACATTTGAGTTCACAAGCTTTTTCTACCGCTATGGATTTGTAGCCAACTTCAGTATGGAGTTACCCAGCTTTGGAGTGTCTGGAATCAATGAATCGGCTGACATGAGCATTGGAGTTACAGTAATAAAGAACAATATGATAAACAATGACCTTGGACCTGCA<br>ACAGCTCAAATGGCTCTTCAGCTATTCATCAAGGACTATAGGTACACGTACCGATGCCACAGAGGTGATACACAAATTCAAACGAGGAGATCATTCGAGCTGAAGAAGCTGTGGGAGCAGACCCGTTCAAAGGCAGGGCTGTTGGTATCAGATGGAGGACCAAACCTATACAACATTCGGAATCTCCACATTCCAGAAGTCTGCTTAAAATGGGAACTAATGGATGAAGACTA<br>CCAGGGCAGGCTGTGTAATCCTCTGAATCCGTTTGTCAGTCATAAGGAAATTGAGTCCGTAAACAATGCTGTGGTAATGCCAGCCCATGGTCCAGCCAAGAGCATGGAATATGATGCTGTTGCGACTACACACTCATGGATTCCTAAGAGGAACCGTTCCATCCTCAATACCAGCCAGAGGGGAATTCTCGAGGATGAACAGATGTACCAGAAGTGCTGCAGTCTATTCGAAA<br>AATTCTTCCCCAGTAGTTCATACAGGAGGCCAGTTGGAATTTCCAGCATGGTGGAGGCCATGGTATCCAGGGCCCGAATTGATGCACGCATTGATTTCGAGTCTGGAAGGATTAAGAAAGAAGAGTTTGCTGAGATCATGAAGATCTGTTCCACCATTGAAGAGCTCAGACGGCAAAAATAG
EPI_ISL_17978924	>A/mallard/Poland/P074w5/2018_H3N8_2018-09-05<br>ATGGATGTCAATCCGACTTTACTTTTCTTAAAAGTGCCAGCGCAAAATGCCATAAGTACCACATTCCCTTATACTGGAGATCCTCCATACAGCCATGGAACAGGAACAGGATACACCATGGACACAGTCAACAGAACACATCAATACTCAGAAAAAGGGAAATGGACAACAAACACAGAGACCGGAGCACCCCAACTCAACCCAATTGATGGACCACTACCTGAGGACAACGA<br>ACCAAGCGGATATGCACAAACAGATTGCGTGTTGGAAGCAATGGCTTTCCTTGAAGAGTCCCACCCAGGGATCTTTGAAAACTCTTGTCTTGAAACGATGGAAGTCATTCAGCAAACAAGAGTGGACAAACTAACTCAAGGTCGCCAGACTTATGACTGGACACTGAATAGAAACCAACCAGCTGCAACTGCCCTGGCCAACACTATAGAGGTTTTCAGATCGAACGGTCTGA<br>CAGCCAATGAATCGGGGAGACTAATAGATTTCCTCAAGGATGTGATGGACTCAATGGACAAAGAAGAAATGGAAATAACAACACATTTCCAGAGAAAGAGAAGAGTAAGGGACAACATGACCAAGAAAATGGTCACACAAAGAACAATAGGGAAGAAGAAACAAAGGCTAAATAAGAGGAGCTACTTAATAAGAGCACTGACACTGAATACAATGACAAAAGACGCAGA<br>AAGAGGCAAATTGAAGAGACGGGCAATTGCAACACCAGGGATGCAGATTAGAGGATTTGTGTACTTTGTCGAAACACTGGCAAGGAGCATCTGTGAAAAACTTGAGCAATCTGGACTCCCCGTTGGAGGGAATGAGAAGAAGGCTAAATTGGCAAATGTCGTGAGAAAAATGATGACTAACTCACAAGATACAGAACTCTCCTTCACAATTACTGGAGATAACACCAAATG<br>GAATGAGAATCAAAATCCTCGGATGTTTCTGGCAATGATAACATACATCACAAGAAACCAACCTGAATGGTTTAGAAATGTCTTGAGCATTGCCCCTATAATGTTCTCGAACAAAATGGCGAGATTGGGAAAAGGATACATGTTTGAAAGTAAAAGCATGAAACTACGGACTCAAATACCTGCAGAAATGCTTGTGAACATTGACTTAAAATACTTCAATGAGTCAACAAGAAA<br>GAAAATCGAGAAAATAAGACCTCTACTAATAGATGGCACCGCCTCATTGAGTCCTGGAATGATGATGGGCATGTTCAATATGCTGAGTACAGTATTAGGAGTCTCAATCCTGAATCTTGGGCAAAAGAGGTACACCAAAACCACATACTGGTGGGATGGACTCCAATCCTCTGATGATTTCGCCCTCATAGTGAATGCACCGAATCATGAGGGAATACAAGCAGGAGTAGATA<br>GGTTCTATAGGACCTGCAAACTGGTTGGGATCAACATGAGCAAAAAGAAGTCTTACATAAACCGGACAGGAACATTTGAGTTCACAAGCTTTTTCTATCGCTATGGATTTGTGGCTAACTTCAGTATGGAGCTGCCCAGCTTTGGAGTGTCTGGAATCAATGAATCAGCTGACATGAGCATTGGAGTTACGGTGATAAAGAACAACATGATAAACAATGACCTTGGACCAGCAA<br>CAGCTCAAATGGCCCTTCAACTATTCATCAAGGACTACAGGTACACGTACCGATGTCACAGGGGTGACACACAAATTCAAACGAGGAGATCATTCGAGCTGAAGAAGCTGTGGGAGCAGACCCGTTCAAAGGCAGGACTGTTGGTGTCAGATGGAGGACCAAATCTATACAACATCCGGAATCTCCATATCCCAGAGGTCTGCCTGAAGTGGGAGCTGATGGACGAAGATTA<br>CCAGGGCAGGTTGTGTAATCCTCTGAACCCATTTGTCAGTCATAAAGAGATTGAGTCCGTAAACAATGCTGTGGTAATGCCAGCCCATGGCCCAGCCAAGAGCATGGAGTATGATGCTGTTGCGACTACACACTCATGGATTCCTAAAAGGAATCGTTCCATTCTCAATACCAGCCAAAGGGGAATTCTTGAGGATGAGCAGATGTACCAGAAATGCTGCAGTCTATTCGAGAA<br>ATTCTTCCCCAGTAGTTCATACAGGAGACCAGTTGGAATTTCCAGCATGGTGGAGGCCATGGTGTCTAGGGCCCGAATCGATGCACGCATTGATTTCGAATCTGGAAGGATTAAGAAGGAAGAGTTTGCTGAGATCATGAAGATCTGTTCCACCATTGAAGAGCTCAGACGGCAAAAATAG
EPI_ISL_17978926	>A/mallard/Poland/P060w2/2020_H3N8_2020-08-25<br>ATGGATGTCAATCCGACTTTACTTTTCTTAAAAGTGCCAGCGCAAAATGCCATAAGTACCACATTCCCTTATACTGGAGATCCTCCATACAGCCATGGAACAGGAACAGGGTACACCATGGACACAGTCAACAGAACACATCAATACTCAGAAAAGGGGAAATGGACAACAAACACAGAGACCGGAGCACCCCAACTCAACCCAATTGATGGACCACTACCTGAGGACAACGA<br>ACCAAGCGGATATGCACAAACAGATTGCGTATTGGAAGCAATGGCTTTCCTTGAAGAGTCCCACCCAGGGATCTTTGAAAACTCTTGTCTTGAAACGATGGAAGTCGTTCAGCAAACAAGAGTGGACAAATTAACTCAAGGTCGCCAGACTTATGACTGGACACTGAATAGAAACCAACCAGCCGCAACTGCCCTGGCCAACACTATAGAAGTCTTCAGATCGAACGGCCTGA<br>CAGCCAATGAATCGGGGAGACTAATAGATTTCCTCAAGGATGTGATGGACTCAATGGACAAAGAAGAAATGGAAATAACAACACATTTCCAGAGAAAGAGAAGAGTAAGGGACAACATGACCAAGAAAATGGTCACACAAAGAACAATAGGGAAGAAGAAACAAAGGCTAAATAAGAGGAGCTACTTAATAAGAGCACTGACACTGAATACAATGACAAAAGACGCAGA<br>AAGAGGCAAATTGAAGAGACGGGCAATTGCAACACCAGGGATGCAGATTAGAGGATTTGTGTACTTTGTCGAAACACTGGCAAGGAGCATCTGTGAGAAACTTGAGCAATCTGGGCTCCCCGTTGGAGGGAATGAGAAAAAGGCTAAATTGGCAAATGTTGTGAGAAAAATGATGACTAACTCACAAGATACAGAGCTCTCCTTCACAATTACTGGAGATAATACCAAATG<br>GAATGAGAATCAGAATCCTCGGATGTTTCTGGCAATGATAACATACATCACAAGAAACCAACCTGAATGGTTTAGAAATGTCTTGAGCATTGCCCCTATAATGTTCTCGAACAAAATGGCGAGATTGGGAAAAGGATACATGTTCGAAAGTAAGAGCATGAAGCTACGGACACAAATACCTGCAGAAATGCTTGCAAACATTGACTTAAAATACTTCAACGAGTCAACAAGAA<br>AAAAAATCGAGAAAATAAGACCTCTATTAATAGATGGCACCGCCTCATTGAGTCCTGGAATGATGATGGGCATGTTCAATATGCTGAGTACAGTATTAGGAGTCTCAATCCTGAATCTTGGGCAAAAGAGGTACACCAAAACCACATACTGGTGGGATGGACTCCAATCCTCTGATGATTTCGCCCTCATAGTGAATGCACCGAATCATGAGGGAATACAAGCAGGAGTGGAT<br>AGATTCTATAGGACCTGCAAACTGGTTGGGATCAACATGAGCAAAAAGAAGTCTTACATAAACCGAACAGGAACATTTGAGTTCACAAGCTTCTTCTATCGCTATGGATTTGTGGCCAACTTCAGTATGGAGCTGCCCAGCTTTGGAGTGTCTGGAGTCAATGAATCAGCTGACATGAGCATTGGAGTTACAGTGATAAAGAACAACATGATAAACAATGACCTTGGACCAGCA<br>ACAGCTCAAATGGCCCTTCAACTATTCATCAAGGACTACAGGTACACTTACCGATGCCACAGGGGGGACACACAAATTCAAACGAGGAGATCATTCGAGCTGAAGAAGCTGTGGGAGCAGACCCGTTCAAAGGCAGGGCTGTTGGTGTCAGATGGAGGACCAAATCTATACAACATTCGGAATCTCCATATCCCAGAGGTCTGCCTAAAGTGGGAGCTAATGGACGAAGATT<br>ACCAGGGCAGGTTGTGTAATCCTTTGAACCCATTTGTCAGTCATAAAGAAATTGAGTCCGTAAACAATGCTGTGGTAATGCCAGCCCATGGCCCAGCCAAGAGCATGGAATATGATGCTGTTGCGACTACACACTCATGGATTCCTAAAAGGAATCGTTCTATCCTCAACACCAGCCAAAGGGGAATTCTTGAGGATGAGCAGATGTACCAGAAATGCTGCAGTCTATTCGAGA<br>AATTCTTCCCCAGTAGCTCATACAGGAGACCAGTTGGAATTTCCAGCATGGTGGAGGCCATGGTGTCTAGGGCCCGAATCGATGCACGCATTGATTTCGAATCTGGAAGGATTAAGAAGGAAGAGTTTGCTGAGATCATGAAGATCTGTTCCACCATTGAAGAGCTCAGACGGCAAAAATAG
EPI_ISL_337569	>A/mallard/Novosibirsk_region/963k/2018_H3N2_2018-09-29<br>ATGGATGTCAATCCGACTTTACTTTTCTTAAAAGTGCCAGCGCAAAATGCCATAAGTACTACGTTTCCTTATACTGGAGATCCTCCATACAGCCATGGAACAGGAACAGGATACACCATGGACACAGTCAACAGAACACATCAATACTCAGAAAAGGGGAAATGGACAACAAACACAGAGACCGGAGCACCCCAGCTCAACCCAATTGATGGACCACTACCTGAGGACAACGA<br>ACCAAGCGGATATGCACAAACAGATTGTGTGTTGGAAGCAATGGCTTTCCTTGAAGAGTCCCACCCAGGGATCTTTGAAAACTCTTGTCTTGAAACGATGGAAGTTGTTCAGCAAACAAGAGTGGACAAACTAACTCAAGGTCGCCAGACTTATGACTGGACACTGAATAGAAACCAACCAGCTGCAACTGCCCTGGCCAACACTATAGAGGTCTTCAGATCGAACGGTCTGA<br>CAGCCAATGAATCGGGGAGACTAATAGATTTCCTCAAGGATGTGATGGACTCAATGGACAAAGAAGAAATGGAAATAACAACACATTTCCAGAGAAAGAGAAGAGTAAGGGACAACATGACCAAGAAAATGGTCACACAAAGAACAATAGGGAAAAAGAAACAAAGGCTGAATAAGAAGAGCTACTTGATAAGGGCACTGACACTAAACACAATGACAAAAGATGCAGA<br>AAGAGGCAAATTGAAGAGGCGGGCAATTGCAACACCAGGGATGCAGATCAGAGGATTTGTGTACTTTGTTGAAACACTGGCAAGGAGCATCTGTGAGAAACTTGAGCAATCTGGACTCCCCGTTGGAGGGAATGAGAAAAAGGCCAAATTGGCAAATGTCGTGAGAAAAATGATGACCAACTCACAAGATACAGAGCTCTCTTTCACAATTACTGGAGATAACACCAAATG<br>GAATGAGAATCAAAATCCTCGGATGTTTCTGGCAATGATAACATACATCACAAGAAACCAACCTGAATGGTTTAGAAATGTCTTGAGCATTGCCCCTATAATGTTCTCGAACAAAATGGCGAGATTGGGAAAAGGATACATGTTTGAAAGTAAGAGCATGAAGCTACGGACACAAATACCTGCAGAAATGCTTGCGAACATTGACTTAAAATATTTCAATGAATCAACAAGAAA<br>GAAAATCGAGAAAATAAGACCTCTGCTAATAGATGGCACAGCCTCATTGAGTCCAGGAATGATGATGGGCATGTTCAATATGCTGAGTACAGTATTAGGAGTCTCAATCCTGAATCTTGGGCAAAAGAGGTACACCAAAACCACATACTGGTGGGATGGACTCCAATCCTCTGATGATTTCGCCCTCATAGTGAATGCACCGAATCATGAGGGAATACAAGCAGGAGTTGATA<br>GGTTCTATAGGACGTGCAAACTGGTTGGAATCAACATGAGCAAAAAGAAGTCTTACATAAATAGGACAGGAACATTTGAGTTCACAAGCTTTTTCTATCGCTATGGATTTGTGGCTAATTTCAGTATGGAGCTGCCCAGCTTTGGAGTGTCTGGAATCAATGAATCAGCTGACATGAGCATTGGAGTTACAGTGATAAAGAATAACATGATAAACAATGACCTTGGACCAGCAA<br>CAGCTCAGATGGCTCTTCAGCTATTCATCAAGGACTACAGGTACACGTACCGATGCCACAGGGGTGACACACAAATTCAAACGAGGAGATCATTCGAGCTGAAGAAGCTGTGGGAGCAGACCCGTTCAAAGGCAGGACTGTTGGTGTCGGATGGAGGACCAAACCTATACAACATTCGGAATCTCCACATCCCAGAGGTCTGCTTGAAGTGGGAGCTGATGGACGAAGATTA<br>CCAGGGCAGGTTGTGTAATCCTCTGAACCCATTTGTCAGTCATAAGGAAATTGAGTCCGTAAACAATGCTGTGGTGATGCCAGCCCATGGCCCAGCCAAGAGCATGGAATATGATGCTGTTGCGACTACACACTCATGGATTCCTAAGAGGAATCGTTCTATTCTCAATACCAGCCAAAGGGGAATTCTTGAGGATGAGCAGATGTACCAGAAGTGCTGTAGTCTATTCGAGAA<br>ATTCTTCCCCAGTAGTTCATACAGGAGGCCAGTTGGAATTTCCAGCATGGTAGAGGCCATGGTATCTAGGGCCCGAATTGATGCACGCATTGATTTCGAATCTGGAAGGATTAAGAAAGAAGAGTTTGCTGAGATCATGAAGATCTGTTCCACCATTGAAGAGCTCAGACGGCAAAAATAG
EPI_ISL_400274	>A/mallard/Novosibirsk_region/1894k/2019_H4N6_2019-09-08<br>ATGGATGTCAATCCGACTTTACTTTTCTTAAAAGTGCCAGCGCAAAATGCCATAAGTACTACGTTTCCTTATACTGGGGATCCTCCATACAGCCATGGAACAGGAACAGGATACACCATGGACACAGTCAACAGAACACATCAATACTCAGAAAAGGGGAAATGGACAACAAACACAGAGACCGGAGCACCCCAGCTCAACCCAATTGATGGACCACTACCTGAGGACAACGA<br>ACCAAGCGGATATGCACAAACAGATTGTGTGTTGGAAGCAATGGCTTTCCTTGAAGAGTCCCACCCAGGGATCTTTGAAAACTCTTGTCTTGAAACGATGGAAGTTGTTCAGCAAACAAGAGTGGACAAACTAACTCAAGGTCGCCAGACTTATGACTGGACACTGAATAGAAACCAACCAGCTGCAACTGCCCTGGCCAACACTATAGAGGTCTTCAGATCGAACGGTCTGA<br>CAGCCAATGAATCGGGGAGACTAATAGATTTCCTCAAGGATGTGATGGACTCAATGGACAAAGAAGAAATGGAAATAACAACACATTTCCAGAGAAAGAGAAGAGTAAGGGACAACATGACCAAGAAAATGGTCACACAAAGAACAATAGGGAAAAAGAAACAAAGGCTGAATAAGAAGAGCTACTTAATAAGGGCACTGACACTAAACACAATGACAAAAGATGCAGA<br>AAGAGGCAAATTGAAGAGGCGGGCAATTGCAACACCAGGGATGCAGATTAGAGGATTTGTGTACTTTGTTGAAACACTGGCAAGGAGCATCTGTGAGAAACTTGAGCAATCTGGACTCCCCGTTGGAGGGAATGAGAAAAAGGCCAAATTGGCAAATGTCGTGAGAAAAATGATGACCAACTCACAAGATACAGAGCTCTCATTCACAATTACTGGAGATAACACCAAATG<br>GAATGAGAATCAAAATCCTCGGATGTTTCTGGCAATGATAACATACATCACAAGAAACCAACCTGAATGGTTTAGAAATGTCTTGAGCATTGCCCCTATAATGTTCTCGAACAAAATGGCGAGATTGGGAAAAGGATACATGTTTGAAAGTAAGAGCATGAAGCTACGGACACAAATACCTGCAGAAATGCTTGCAAACATTGACTTGAAATATTTCAATGAATCAACAAGAAA<br>GAAAATCGAGAAAATAAGACCTCTGCTAATAGATGGCACAGCCTCATTGAGTCCAGGAATGATGATGGGCATGTTCAATATGCTGAGTACAGTATTAGGAGTCTCAATCCTGAATCTTGGGCAAAAGAGGTACACCAAAACCACATACTGGTGGGATGGACTCCAATCCTCTGATGATTTCGCCCTCATAGTGAATGCACCGAATCATGAGGGAATACAAGCAGGAGTTGATA<br>GGTTCTATAGGACTTGCAAACTGGTTGGAATCAACATGAGCAAAAAGAAGTCTTACATAAATAGGACAGGAACATTTGAGTTCACAAGTTTTTTCTATCGCTATGGATTTGTGGCTAATTTCAGTATGGAGCTGCCCAGCTTCGGAGTGTCTGGAATCAATGAATCAGCTGACATGAGCATTGGAGTTACAGTGATAAAGAATAACATGATTAACAATGACCTTGGACCAGCAA<br>CAGCTCAGATGGCTCTTCAGCTATTCATCAAGGACTACAGGTACACGTACCGTTGCCACAGGGGTGACACACAAATTCAAACGAGGAGATCATTCGAGCTGAAGAAGCTGTGGGAGCAGACCCGTTCAAAGGCAGGACTGTTGGTGTCGGATGGAGGACCAAACCTATACAACATTCGGAATCTCCACATCCCAGAGGTCTGCTTGAAGTGGGAGCTGATGGACGAAGATTA<br>CCAGGGCAGGTTGTGTAATCCTCTGAACCCATTTGTCAGTCATAAGGAAATCGAGTCCGTAAACAATGCTGTGGTGATGCCGGCCCATGGTCCAGCCAAGAGCATGGAATATGATGCTGTTGCGACTACACACTCATGGATTCCTAAGAGGAACCGTTCCATTCTCAATACCAGCCAAAGGGGAATTCTTGAGGATGAGCAGATGTACCAGAAATGCTGTAGTCTATTCGAGAA<br>ATTCTTCCCCAGTAGTTCATACAGGAGGCCAGTTGGAATTTCCAGCATGGTAGAGGCCATGGTATCTAGGGCCCGAATTGATGCACGCATTGATTTCGAATCTGGAAGGATTAAGAAAGAAGAGTTTGCTGAGATCATGAAGATCTGTTCCACCATTGAAGAGCTCAGACGGCAAAAATAG
EPI_ISL_14989784	>A/mallard/Netherlands/22014777-004/2022_H3N2_2022-08-28<br>ATGGATGTCAATCCGACTTTACTTTTCTTAAAAGTGCCAGCGCAAAATGCCATAAGTACCACATTCCCTTATACTGGAGATCCTCCATACAGCCATGGAACAGGAACAGGATACACCATGGACACAGTCAACAGAACACATCAATACTCGGAAAAGGGGAAATGGACAACAAACACAGAGACTGGAGCACCCCAACTCAACCCAATTGATGGACCACTACCTGAGGACAACGA<br>ACCAAGCGGATATGCACAAACAGATTGCGTGTTGGAAGCAATGGCTTTCCTTGAAGAATCCCACCCAGGGATCTTTGAAAACTCTTGTCTTGAAACGATGGAAGTCGTTCAGCAAACAAGAGTGGACAAACTAACTCAAGGTCGCCAGACTTATGACTGGACACTGAATAGAAACCAACCAGCTGCAACTGCCCTGGCCAACACTATAGAGGTCTTCAGATCGAACGGTCTGA<br>CAGCCAATGAATCAGGGAGACTAATAGATTTCCTCAAGGATGTGATGGATTCAATGGACAAAGAAGAAATGGAAATAACAACACATTTCCAAAGAAAGAGAAGAGTAAGGGACAACATGACCAAGAAAATGGTCACACAAAGAACAATAGGGAAGAAGAAGCAAAGGCTAAATAAGAGGAGCTACTTAATAAGAGCACTGACACTGAATACAATGACAAAGGACGCAGA<br>AAGAGGCAAATTGAAGAGACGAGCAATTGCAACACCAGGGATGCAGATTAGAGGATTTGTGTACTTTGTCGAAACACTGGCAAGGAGCATTTGTGAAAAACTTGAACAATCTGGACTCCCCGTTGGAGGGAATGAGAAGAAGGCTAAATTGGCAAATGTCGTGAGAAAGATGATGACTAACTCACAAGATACAGAGCTCTCCTTCACAATCACTGGAGATAACACCAAATG<br>GAATGAGAATCAAAATCCTCGGATGTTTCTGGCAATGATAACATACATCACAAGAAACCAACCTGAATGGTTTAGGAATGTCTTGAGCATTGCCCCTATAATGTTCTCGAACAAAATGGCGAGACTGGGAAAAGGATACATGTTTGAAAGCAAAAGCATGAAGCTACGGACACAAATACCTGCAGAAATGCTTGCAAACATTGACTTAAAATACTTCAATGAGTCAACAAGAA<br>AGAAAATCGAGAAGATAAGACCTCTACTAATAGATGGCACCGCCTCATTGAGTCCTGGAATGATGATGGGCATGTTCAATATGCTGAGTACAGTGTTAGGAGTCTCAATCCTGAATCTTGGGCAGAAGAGGTACACCAAGACCACATACTGGTGGGATGGACTCCAATCCTCTGATGATTTCGCCCTCATAGTGAATGCACCGAATCATGAGGGAATACAAGCAGGAGTGGAT<br>AGGTTCTATAGGACCTGCAAACTGGTTGGAATCAACATGAGCAAAAAGAAGTCTTACATAAACCGGACAGGAACATTTGAGTTCACAAGCTTTTTCTATCGCTATGGATTTGTGGCCAACTTCAGTATGGAGCTGCCCAGTTTTGGAGTGTCTGGAATCAATGAATCAGCTGACATGAGCATTGGAGTTACAGTGATAAAGAACAACATGATAAACAATGACCTTGGACCAGCA<br>ACAGCTCAAATGGCCCTTCAACTATTCATCAAGGACTACAGGTACACGTACCGATGTCACAGGGGTGACACACAGATTCAAACGAGGAGATCATTCGAGCTGAAGAAGCTGTGGGAGCAGACCCGTTCCAAGGCAGGACTGTTGGTGTCAGATGGAGGACCAAATCTATACAACATCCGGAATCTCCATATCCCGGAAGTCTGCCTGAAATGGGAGCTGATGGACGAAGATT<br>ACCAGGGCAGGTTGTGTAATCCTCTGAACCCATTTGTCAGTCATAAAGAAATCGAATCCGTAAACAATGCTGTGGTAATGCCAGCCCATGGCCCAGCCAAGAGCATGGAATATGATGCTGTTGCGACTACACACTCATGGATTCCCAAAAGGAATCGTTCCATTCTCAATACCAGCCAAAGGGGAATTCTTGAGGATGAGCAGATGTACCAGAAATGCTGCAGTCTATTCGAGA<br>AGTTCTTCCCCAGTAGTTCATACAGGAGACCAGTTGGAATTTCCAGCATGGTGGAGGCCATGGTGTCTAGGGCCCGAATCGATGCACGCATTGATTTCGAATCTGGAAGGACTAAGAAGGAAGAGTTTGCTGAGATCATGAAGATCTGTTCCACCATTGAAGAGCTCAGACGGCAAAAATAG
EPI_ISL_9012576	>A/Mallard/Netherlands/12/2021_H5N2_2021-12-23<br>ATGGATGTCAATCCGACTTTACTTTTCTTAAAAGTGCCAGCGCAAAATGCCATAAGTACCACATTCCCTTATACTGGAGATCCTCCATACAGCCATGGAACAGGAACAGGATACACCATGGACACAGTCAACAGAACACATCAATACTCGGAAAAGGGGAAATGGACAACAAACACAGAGACTGGAGCACCCCAACTCAACCCAATTGATGGACCACTACCTGAGGACAACGA<br>ACCAAGCGGATATGCACAAACAGATTGCGTGTTGGAAGCAATGGCTTTCCTTGAAGAGTCCCACCCAGGGATCTTTGAAAACTCTTGTCTTGAAACGATGGAAGTCGTTCAGCAAACAAGAGTAGACAAACTAACTCAAGGTCGCCAGACTTATGACTGGACACTGAATAGAAACCAACCAGCTGCAACTGCCCTGGCCAACACTATAGAGGTCTTCAGATCGAACGGTCTGA<br>CAGCCAATGAATCTGGGAGACTAATAGATTTCCTCAAGGATGTGATGGACTCAATGGACAAAGAAGAAATGGAAATAACAACACATTTCCAGAGGAAGAGAAGAGTAAGGGACAACATGACCAAGAAAATGGTCACACAAAGAACAATAGGGAAGAAGAAGCAAAGGCTAAATAAGAGGAGCTACTTAATAAGAGCACTGACACTGAATACAATGACAAAGGACGCAGA<br>AAGAGGCAAATTGAAGAGACGGGCAATTGCAACACCAGGGATGCAGATTAGAGGATTTGTGTACTTTGTCGAAACACTGGCAAGGAGCATTTGTGAAAAACTTGAGCAATCTGGACTCCCCGTTGGAGGGAATGAGAAGAAGGCCAAATTGGCAAATGTCGTGAGAAAAATGATGACTAACTCACAAGATACAGAGCTCTCCTTCACAATTACTGGAGATAACACCAAATG<br>GAATGAGAATCAAAATCCTCGGATGTTTCTGGCAATGATAACATACATCACAAGAAACCAACCTGAATGGTTTAGAAATGTCTTGAGCATTGCCCCTATAATGTTCTCGAATAAAATGGCGAGACTGGGAAAAGGATACATGTTTGAAAGCAAAAGCATGAAGCTACGGACACAAATACCTGCAGAAATGCTTGCAAACATTGACTTAAAATACTTCAATGAGTCAACAAGAAA<br>GAAAATCGAGAAAATAAGACCTCTACTAATAGATGGCACCGCCTCATTGAGTCCTGGAATGATGATGGGCATGTTCAATATGCTGAGTACAGTGTTAGGAGTCTCAATCCTGAATCTTGGGCAAAAGAGGTACACCAAGACCACATACTGGTGGGATGGACTCCAGTCCTCTGATGATTTCGCCCTCATAGTGAATGCACCGAATCATGAGGGAATACAAGCAGGAGTGGATA<br>GGTTCTATAGGACCTGCAAACTGGTTGGGATCAACATGAGCAAAAAGAAGTCTTACATAAACCGGACAGGAACATTTGAGTTCACAAGCTTTTTCTATCGCTATGGATTTGTGGCCAACTTCAGTATGGAGCTGCCCAGTTTTGGAGTGTCTGGAATCAATGAATCAGCTGACATGAGCATTGGAGTTACAGTGATAAAGAACAACATGATAAACAATGACCTTGGACCAGCAA<br>CAGCTCAAATGGCCCTTCAACTATTCATCAAGGACTACAGGTACACGTACCGATGTCACAGGGGTGACACACAGATTCAAACGAGGAGATCATTCGAGCTGAAGAAGCTGTGGGAGCAGACCCGTTCCAAGGCAGGACTGTTGGTGTCAGATGGAGGACCAAATCTATACAACATCCGGAATCTCCATATCCCAGAAGTCTGCCTGAAGTGGGAGCTGATGGACGAAGATTA<br>CCAGGGAAGGTTGTGTAATCCTCTGAACCCATTTGTCAGTCATAAAGAAATTGAATCCGTAAACAATGCTGTGGTAATGCCGGCCCATGGCCCAGCCAAGAGCATGGAATATGATGCTGTTGCGACTACACACTCATGGATTCCCAAAAGGAATCGTTCCATTCTCAATACCAGCCAAAGGGGAATTCTTGAGGATGAGCAGATGTACCAGAAATGCTGCAGTCTATTCGAGAA<br>GTTCTTCCCCAGTAGTTCATACAGGAGACCAGTTGGAATTTCCAGCATGGTGGAGGCCATGGTGTCTAGGGCCCGAATCGACGCACGCATTGATTTCGAATCTGGAAGGATTAAGAAGGAAGAGTTTGCTGAGATCATGAAGATCTGTTCCACCATTGAAGAGCTCAGACGGCAAAAATAG

EPI_ISL_683594	>A/mallard/Italy/20VIR7139-124_feather/2020_H5N8_2020-11-14<br>ATGGATGTCAATCCGACTTTACTTTTCTTAAAAGTGCCAGCGCAAAATGCTATAAGTACTACATTCCCTTACACTGGAGATCCCCCATACAGCCATGGAACAGGAACAGGGTATACCATGGACACAGTAAACAGAACACATCAATACTCAGAAAAGGGAAAGTGGACAACAAACACAGAAACCGGAGCACCCCAACTCAACCCAATTGATGGACCATTACCAGAGGACAATGA<br>GCCAAGCGGATATGCACAAACTGATTGCGTGTTGGAAGCAATGGCTTTCCTTGAAGAATCCCACCCAGGGATATTTGAAAACTCTTGTCTTGAAGCGATGGAAATCGTTCAGCAAACAAGAGTGGACAAACTAACCCAAGGTCGCCAGACTTATGACTGGACACTGAACAGAAACCAACCAGCTGCAACTCTTTGGCCAACACTATAGAGGTTTCAGATCGAATGGTCTGA<br>CAGCCAATGAATCAGGGAGACTGATAGATTTTCTCAGGGATGTGATGGAATCAATGGAAAAAGAAGAGATGGAAGTAACAACACATTTCCAGAGAAAAAGAAGAGTGAGGGACAACATGACTAAGAAGATGGTCACACAAAGAACAATAGGGAAGAAGAAGCAGAGGCTGAACAAGAGGAGTTACTTAATAAGAGCACTGACATTGAATACAATGACCAAAGATGCGGA<br>AAGAGGCAAGTTGAAGAGACGGGCAATTGCAACACCCGGGATGCAGATTAGAGGATTCGTGTACTTTGTTGAAACACTAGCGAGGAGCATCTGTGAGAAACTAGAGCAATCTGGGCTCCCTGTTGGAGGGAATGAGAAGAAGGCTAAATTGGCAAATGTTGTGAGAAAAATGATGACTAACTCACAAGATACAGAGCTCTCCTTTACAATTACTGGAGACAACACCAAATG<br>GAATGAGAATCAAAACCCTCGGATGTTTTTGGCAATGATAACATATATCACAAGAAACCAACCTGAATGGTTTAGAAATGTCTTAAGCATTGCCCCTATAATGTTCTCAAACAAAATGGCTAGATTAGGGAAAGGATACATGTTTGAAAGTAAGAGCATGAAGCTAAGAACACAAATACCAGCAGAGATGCTTACAAATATTGATCTGAAGTATTTCAACGAACCAACGAGAAA<br>GAAAATCGAGAAAATAAGACCTCTGCTGATTGATGGCACGGCCTCATTGAGTCCTGGGATGATGATGGGCATGTTCAATATGCTGAGCACAGTATTAGGGGTCTCAATCCTGAATCTCGGGCAAAAGAGGTACACCAAAACCACATACTGGTGGGATGGACTTCAATCCTCTGATGATTTCGCTCTCATAGTGAATGCACCAATCGATGAGGGGATACAAGCAGGAGTGGATA<br>GATTCTATAGGACCTGCAAACTGGTTGGGATCAACATGAGCAAAAAGAAGTCTTACATAAACCGAACAGGAACATTTGAGTTCACAAGTTTTTTCTACCGCTATGGATTTGTAGCCAACTTCAGTATGGAATTACCCAGCTTTGGAGTGTCTGGAATCAATGAATCAGCTGACATGAGCATTGGAGTTACAGTGATAAAAAACAATATGATAAACAATGATCTTGGACCAGCAA<br>CAGCTCAAATGGCTCTTCAGTTATTCATCAAAGACTATAGGTACACATACCGATGCCACAGGGGTGATACACAAATTCAAACGAGGAGATCATTCGAGCTGAAGAAGCTGTGGGAGCAGACCCGTTCAAAGGCAGGCTGTTGATATCAGACGGGGGCCAAACCTATACAACATTCGGAATCTCCATCCCAGAGGTCTGCTTGAAGTGGGAGCTGATGGATGAAGACTA<br>CCAAGGCAGGCTGTGCAATCCTCTGAATCCATTTGTCAGTCATAAAGAGATTGAGTCCGTAAACAATGCTGTAGTAATGCCCGCCCATGGCCCGGCCAAGAGCATGGAATATGATGCTGTTGCGACACACACTCGTGGATTCCTAAGAGGAACCGTTCCATTCTCAATACCAGCCAAAGGGGAATTCTTGAGGATGAGCAGATGTACCAAAGTGCTGTAGTCTATTCGAGAA<br>ATTCTTCCCCAGCAGTTCATACAGGAGGCCAGTTGGAATTTCAGCATGGTGGAGGCCATGGTGTCTAGGGCCCGAATTGATGCACGCATCGACTTCGAATCTGGAAGGATTAAGAAAGAAGAGTTTGCTGAGATCATGAAGATCTGTTCCACCATTGAAGAGCTCAGACGGCAAAAATAG
EPI_ISL_5096070	>A/mallard/Germany-MV/AI00639/2021_H5N8_2021-01-20<br>ATGGATGTCAATCCGACTTTACTTTTCTTAAAAGTGCCAGCGCAAAATGCTATAAGTACTACATTCCCTTACACTGGAGATCCTCCATACAGCCATGGAACAGGAACAGGGTATACCATGGACACAGTAAACAGAACACATCAATACTCAGAAAAGGGAAAGTGGACAACAAACACAGAAACCGGAGCACCCCAACTCAACCCAATTGATGGACCATTACCAGAGGACAATGA<br>GCCAAGCGGATATGCACAAACTGATTGCGTGTTGGAAGCAATGGCTTTCCTTGAAGAATCCCACCCAGGGATATTTGAAAACTCTTGTCTTGAAGCGATGGAAATCGTTCAGCAAACAAGAGTGGACAAACTAACCCAAGGTCGCCAGACTTATGACTGGACACTGAACAGGAACCAACCAGCTGCAACTCTTTGGCCAACACTATAGAGGTTTCAGATCGAATGGTCTGA<br>CAGCCAATGAATCAGGGAGACTGATAGATTTTCTCAGGGATGTGATGGAATCAATGGAAAAAGAAGAGATGGAAGTAACAACACATTTCCAGAGAAAAAGAAGAGTGAGGGACAACATGACTAAGAAGATGGTCACACAAAGAACAATAGGGAAGAAGAAGCAGAGGCTGAACAAGAGGAGTTACTTAATAAGAGCACTGACATTGAATACAATGACCAAAGATGCAGA<br>AAGAGGCAAGTTGAAGAGACGGGCAATTGCAACACCCGGGATGCAGATTAGAGGATTCGTGTACTTTGTTGAAACACTAGCGAGGAGCATCTGTGAGAAACTAGAGCAATCTGGGCTCCCTGTTGGAGGGAATGAGAAGAAGGCTAAATTGGCAAATGTTGTGAGAAAAATGATGACTAACTCACAAGATACAGAGCTCTCCTTTACAATTACTGGAGACAACACCAAATG<br>GAATGAGAATCAAAACCCTCGGATGTTTTTGGCAATGATAACATATATCACAAGAAACCAACCTGAATGGTTTAGAAATGTCTTAAGCATTGCCCCTATAATGTTCTCAAACAAAATGGCGAGATTAGGGAAAGGATACATGTTTGAAAGTAAGAGCATGAAGCTAAGAACACAAATACCAGCAGAGATGCTTACAAATATCGATCTGAAGTATTTCAACGAACCAACGAGAAA<br>AGAAAATCGAGAAAATAAGACCTCTGCTGATTGATGGCACGGCCTCATTGAGTCCTGGGATGATGATGGGCATGTTCAATATGCTGAGCACAGTATTAGGGGTCTCAATCCTGAATCTCGGGCAAAAAAGGTACACCAAAACCACATACTGGTGGGATGGACTCAATCCTCTGATGATTTCGCTCTCATAGTGAATGCACCAATCGATGAGGGGATACAAGCAGGAGTGGAT<br>AGATTCTATAGGACCTGCAAACTGGTTGGGATCAACATGAGCAAAAAGAAGTCTTACATAAACCGAACAGGAACATTTGAGTTCACAAGTTTTTTCTACCGCTATGGATTTGTAGCTAACTTCAGTATGGAATTACCCAGCTTTGGAGTGTCTGGAATCAATGAATCAGCTGACATGAGCATTGGAGTTACAGTGATAAAAAACAATATGATAAACAATGATCTTGGACCAGCA<br>ACAGCTCAAATGGCTCTTCAGTTATTCATCAAAGACTATAGGTACACATACCGATGCCACAGGGGTGATACACAAATTCAAACGAGGAGATCATTCGAGCTGAAGAAGCTGTGGGAGCAGACCCGTTCAAAGGCAGGCTGTTGATATCAGACGGGGGCCAAACCTATACAACATTCGGAATCTCCATCCCAGAGGTCTGCTTGAAGTGGGAGCTGATGGATGAAGACTA<br>ACCAAGGCAGGCTGTGCAATCCTCTGAATCCATTTGTCAGTCATAAAGAGATTGAGTCCGTAAACAATGCTGTAGTAATGCCCGCCCATGGCCCGGCCAAGAGCATGGAATATGATGCTGTTGCGACACACACTCGTGGATTCCTAAGAGGAACCGTTCCATTCTCAATACCAGCCAAAGGGGAATTCTTGAGGATGAGCAGATGTACCAAAGTGCTGTAGTCTATTCGAGA<br>AATTCTTCCCCAGCAGTTCATACAGGAGGCCAGTTGGAATTTCAGCATGGTGGAGGCCATGGTGTCTAGGGCCCGAATTGATGCACGCATCGACTTCGAATCTGGAAGGATTAAGAAAGAAGAGTTTGCTGAGATCATGAAGATCTGTTCCACCATTGAAGAGCTCAGACGGCAAAAATAG
EPI_ISL_5863359	>A/mallard/Georgia/DT-23072/2020_H5N8_2020-10-27<br>ATGGATGTCAATCCGACTTTACTTTTCTTAAAAGTGCCAGCGCAAAATGCTATAAGTACTACATTCCCTTACACTGGAGATCCTCCATACAGCCATGGAACAGGAACAGGGTATACCATGGACACAGTAAACAGAACACATCAATACTCAGAAAAGGGAAAGTGGACAACAAACACAGAAACCGGAGCACCCCAACTCAACCCAATTGATGGACCATTACCAGAGGACAATGA<br>GCCAAGCGGATATGCACAAACTGATTGCGTGTTGGAAGCAATGGCTTTCCTTGAAGAATCCCACCCAGGGATATTTGAAAACTCTTGTCTTGAAGCGATGGAAATCGTTCAGCAAACAAGAGTGGACAAACTAACCCAAGGTCGCCAGACTTATGACTGGACACTGAACAGGAACCAACCAGCTGCAACTCTTTGGCCAACACTATAGAGGTTTCAGATCGAATGGTATGA<br>CAGCCAATGAATCAGGGAGACTGATAGATTTTCTCAGGGATGTGATGGAATCAATGGAAAAAGAAGAGATGGAAGTAACAACACATTTCCAGAGAAAAAGAAGAGTGAGGGACAACATGACTAAGAAGATGGTCACACAAAGAACAATAGGGAAGAAGAAGCAGAGGCTGAACAAGAGGAGTTACTTAATAAGAGCACTGACATTGAATACAATGACCAAAGATGCAGA<br>AAGAGGCAAGTTGAAGAGACGGGCAATTGCAACACCCGGGATGCAGATTAGAGGATTCGTGTACTTTGTTGAAACACTAGCGAGGAGCATCTGTGAGAAACTAGAGCAATCTGGGCTCCCTGTTGGAGGGAATGAGAAGAAGGCTAAATTGGCAAATGTTGTGAGAAAAATGATGACTAACTCACAAGATACAGAGCTCTCCTTTACAATTACTGGAGACAACACCAAATG<br>GAATGAGAATCAAAACCCTCGGATGTTTTTGGCAATGATAACATATATCACAAGAAACCAACCTGAATGGTTTAGAAATGTCTTAAGCATTGCCCCTATAATGTTCTCAAACAAAATGGCGAGATTAGGGAAAGGATACATGTTTGAAAGTAAGAGCATGAAGCTAAGAACACAAATACCAGCAGAGATGCTTACAAATATCGATCTGAAGTATTTCAACGAACCAACGAGAAA<br>AGAAAATCGAGAAAATAAGACCTCTGCTGATTGATGGCACGGCCTCATTGAGTCCTGGGATGATGATGGGCATGTTCAATATGCTGAGCACAGTATTAGGGGTCTCAATCCTGAATCTCGGGCAAAAAAGGTACACCAAAACCACATACTGGTGGGATGGACTTCAATCCTCTGATGATTTCGCTCTCATAGTGAATGCACCAATCGATGAGGGGATACAAGCAGGAGTGGAT<br>AGATTCTATAGGACCTGCAAACTGGTTGGGATCAACATGAGCAAAAAGAAGTCTTACATAAACCGAACAGGAACATTTGAGTTCACAAGTTTTTTCTACCGCTATGGATTTGTAGCTAACTTCAGTATGGAATTACCCAGCTTTGGAGTGTCTGGAATCAATGAATCAGCTGACATGAGCATTGGAGTTACAGTGATAAAAAACAATATGATAAACAATGATCTTGGACCAGCA<br>ACAGCTCAAATGGCTCTTCAGTTATTCATCAAAGACTATAGGTACACATACCGATGCCACAGGGGTGATACACAAATTCAAACGAGGAGATCATTCGAGCTGAAGAAGCTGTGGGAGCAGACCCGTTCAAAGGCAGGCTGTTGATATCAGACGGGGGCCAAACCTATACAACATTCGGAATCTCCATCCCAGAGGTCTGCTTGAAGTGGGAGCTGATGGATGAAGACT<br>ACCAAGGCAGGCTGTGCAATCCTCTGAATCCATTTGTCAGTCATAAAGAGATTGAGTCCGTAAACAATGCTGTAGTAATGCCCGCCCATGGCCCGGCCAAGAGCATGGAATATGATGCTGTTGCGACACACACTCGTGGATTCCTAAGAGGAACCGTTCCATTCTCAATACCAGCCAAAGGGGAATTCTTGAGGATGAGCAGATGTACCAAAGTGCTGTAGTCTATTCGAGA<br>AATTCTTCCCCAGCAGTTCATACAGGAGGCCAGTTGGAATTTCAGCATGGTGGAGGCCATGGTGTCCCGAATTGATGCACGCATCGACTTCGAATCTGGAAGGATTAAGAAAGAAGAGTTTGCTGAGATCATGAAGATCTGTTCCACCATTGAAGAGCTCAGACGGCAAAAATAG
EPI_ISL_1263541	>A/mallard/France/20P017917/2020_H5N3_2020-12-07<br>ATGGATATCAATCCGACTTTACTTTTCTTAAAAGTGCCAGCGCAAAATGCCATAAGTACCACATTCCCTTATACTGGAGATCCTCCATACAGCCATGGAACAGGAACAGGATACACCATGGACACAGTCAACAGAACACATCAATACTCGGAAAAGGGAAAATGGACAACAAACACAGAGACCGGAGCACCCCAACTCAACCCAATTGATGGACCACTACCTGAGGACAACGA<br>ACCAAGYGGATATGCACAAACAGATTGCGTGTTGGAAGCAATGGCTTTTCTTGAAGAGTCCCACCCAGGGATCTTTGAAAACTCTTGTCTTGAAACGATGGAAGTGGTTCAGCAAACAAGAGTGGACAAACTAACTCAAGGTCGCCAGACTTATGACTGGACACTGAATAGAAACCAACCAGCTGCAACTGCCCTGGCCAACACTATAGAGGTCTTCAGATCGAACGGTCTGA<br>CAGCCAATGAATCAGGGAGACTAATAGATTTCCTCAAGGATGTGATGGACTCAATGGACAAAGAAGAAATGGAAATAACAACACATTTCCAGAGAAAGAGAAGAGTAAGGGACAACATGACCAAGAAAATGGTCACACAAAGAACAATAGGGAAGAAGAAGCAAAGGCTAAATAAGAGGAGCTACCTGACAATGACAATGACCAAAGACGCAGA<br>AAGAGGCAAATTGAAGAGGCGGGCAATTGCAACACCAGGGATGCAGATTAGAGGGTTTGTGTACTTTGTCGAAACACTGGCAAGGAGTATCTGTGAAAAACTTGAGCAATCTGGACTCCCCGTTGGAGGGAATGAAAAGAAGGCTAAATTGGCAAATGTCGTGAGAAAAATGATGACTAACTCACAAGATACAGAGCTCTCCTTCACAATTACTGGAGATAACACCAAATG<br>GAATGAGAATCAAAACCCTCGGATGTTTCTGGCAATGATAACATACATCACAAGAAACCAACCAGAATGGTTTAGAAATGTCTTGAGCATTGCCCCAATAATGTTCTCAAACAAAATGGCGAGATTAGGGAAAGGATACATGTTTGAAAGTAAGAGCATGAAGCTACGAACACAAATACCAGCAGAAATGCTTGCAAACATTGATCTTAAATACTTCAATGAATCAACAAGAA<br>GAAAATCGAGAAAATAAGACCTCTACTAATAGATGGCACGCCCTCATTGAGTCCTGGAATGATGATGGGCATGTTCAATATGCTGAGTACAGTATTAGGAGTTCTCAATCCTGAATCTTGGGCAAAAGAGGTACACCAAAACCACATACTGGTGGGATGGACTCCAATCCTCTGATGATTTTGCTCTCATAGTAAATGCACCCGAATTATGAGGGAATACAAGCAGGAGTGGATA<br>GGTTCTATAGGACCTGCAACTGGTTGGGATCAACATGAGCAAAAAGAAGTCTTACATAAACCGGACAGGAACATTTGAGTTCACAAGCTTTTTCTATCGCTATGGATTTGTGGCTAACTTCAGTATGGAGCTGCCCAGCTTTGGAGTGTCTGGAATCAATGAATCAGCTGACATGAGCATTGGAGTTACAGTGATAAAGAACAACATGATAAACAATGACCTTGGACCAGCAA<br>CAGCTCAAATGGCCCTTCAACTATTCATCAAGGACTACAGGTACACGTACCGATGTCACAGGGGTGACACACAAATTCAAACGAGGAGATCATTCGAGCTGAAGAAGCTGTGGGAGCAGACCCGTTCAAAGGCAGGACTGTTGGTGTCAGATGGAGGACCAAATCTATACAACATCCGGAATCTCCATCCCAGAAGTCTGCCTGAAGTGGGAGCTGATGGACGAAGATTA<br>CCAGGGCAGGTTGTGCAATCCTCTGAACCCATTTGTCAGTCATAAAGAAATTGAATCCGTAAACAATGCTGTGGTAATGCCAGCCCATGGCCCAGCCAAGAGCATGGAATATGATGCTGTTGCTACACACTCATGGATTCCTAAAAGGAATCGTTCCATTCTCAATACCAGCCAAGGGGGAATTCTTGAGGATGAGCAGATGTACCAGAATGCTGCAGTCTATTCGAAAA<br>ATTCTTCCCCAGTAGTTCATACAGGAGACCAGTTGGAATTTCAGCATGGTGGAGGCCATGGTGTCTAGGGCCCGAATCGACGCACGCATTGATTTCGAATCTGGAAGGATTAAGAAGGAAGAGTTTGCTGAGATCATGAAGATCTGTTCCACCATTGAAGAGCTCAGACGGCAAAAATAG
EPI_ISL_339038	>A/mallard/Chany_Lake/18/2018_H1N3_2018-09-06<br>ATGGATGTCAATCCGACTTTACTTTTCTTAAAAGTGCCAGCGCAAAATGCTATAAGTACTACGTTCCCTTACACTGGAGATCCTCCATACAGCCATGGAACAGGAACAGGATACACCATGGACACAGTCAACAGAACACATCAATACTCAGAGAAGGGAAAGTGGACAACAAACACAGAGACTGGGGCGCCCCAACTCAACCCAATTGATGGACCATTGCCAGAGGACAACGA<br>GCCGAGCGGATATGCACAAACAGATTGCGTGTTGGAAGCAATGGCTTTCCTTGAAGAATCCCACCCAGGGATCTTTGAAAACTCTTGTCTTGAAACGATGGAGGTCGTTCAGCAAACAAGAGTGGACAAACTAACCCAAGGTCGCCAGACTTATGATTGGACACTGAATAGAAACCAACCAGCTGCAACTGCTTTGGCCAATACTATAGAGGTCTTCAGATCGAACAGTCTAA<br>CAGCCAATGAGTCGGGGAGACTAATAGATTTCCTCAAGGATGTGATGGAATCAATGGATAAGGAAGAAATGGAAATAACAACACATTTCCAGAGAAAGAGAAGAGTAAGGGACAACATGACCAAGAAGATGGTCACACAAAGAACAATAGGGAAGAAGAAGCAGAGGCTGAACAAGAGGAGCTACTTAATAAGAGCATTGACATTGAACACAATGACCAAAGATGCGGA<br>AAGAGGCAAATTGAAAAGGCGGGCAATTGCAACACCCGGGATGCAGATTAGAGGGTTTGTGTATTTTGTCGAAACACTAGCAAGGAGCATCTGTGAGAAACTCGAGCAATCTGGACTCCCTGTTGGAGGGAATGAGAAAAAGGCCAAATTGGCAAATGTCGTGAGAAAAATGATGACTAACTCGCAGATACAGAGCTCTCCTTTACAATTACTGGAGACAACACCAAATG<br>GAATGAAAATCAAAACCCTCGGATGTTTTTGGCAATGATAACATATATCACAAGAAACCAACCCGAATGGTTTAGAAATGTCTTGAGCATTGCCCCTATAATGTTCTCAAACAAAATGGCAAGATTAGGAAAAGGATACATGTTTGAAAGTAAGAGCATGAAGCTAAGGACACAAATACCGGCAGAAATGCTTGCAAACATTGATCTTAAATACTTCAATGAATCAACAAGAA<br>AGAAAATCGAGAAAATAAGACCTCTGCTAATTGATGGCACAGCCTCATTGAGTCCTGGAATGATGATGGGCATGTTCAATATGCTGAGCACAGTATTGGGGGTTCAATCCTGAATCTTGGACAAAAGAGGTACACCAAAACCACATACTGGTGGGATGGACTCCAATCCTCTGATGATTTCGCTCTCATAGTGAATGCACCAATCGATGAGGGGATACAAGCAGGGGTGGAT<br>AGGTTCTATAGGACCTGCAAACTGGTTGGGATCAACATGAGCAAAAAGAAGTCTTACATAAACCGGACAGGAACATTTGAGTTCACAAGCTTTTTCTATCGCTATGGATTTGTAGCCAACTTCAGTATGGAGTTACCCAGCTTTGGAGTGTCTGGAATCAATGAATCAGCTGACATGAGCATTGGAGTTACAGTGATAAAGAACAATATGATAAACAATGACCTTGGACCAGCA<br>ACAGCTCAAATGGCTCTTCAGCTATTCATCAAAGATTATAGGTACACGTACCGATGTCACAGAGGTGATACACAAATTCAAACGAGGAGATCATTCGAGCTTAAGAAGCTGTGGGAGCAGACCCGTTCAAAGGCAGGGCTGTTGGTATCAGATGGTGGACCAAACCTATACAACATTCGGAATCTCCATCCCAGAGGTCTGCTTGAAGTGGGACTTATGGATGAAGATTA<br>CCAGGGCAGGCTGTGTAATCCTCTGAATCCGTTTGTCAGTCATAAGGAAATTGAATCCGTAAACAATGCTGTGGTAATGCCAGCCCATGGCCCAGCCAAGAGCATGGAATATGATGCTGTTGCAACACACTCATGGATTCCTAAGAGGAACCGTTCCATTCTCAATACCAGCCAGGGGGGAATTCTCGAGGATGAGCAGATGTACCAGAGTGCTGCAATTTATTCGAGA<br>AATTCTTCCCCAGTAGTTCGTACAGGAGGCCAGTTGGAATTTCAGCATGGTGGAGGCCATGGTGTCCAGGGCCCGAATTGATGCACGCATTGATTTCGAATCTGGAAGGATTAAGAAAGAAGAGTTTGCTGAGATCATGAAGATCTGTTCCACCATTGAAGAGCTCAGACGGCAAAAATAG
EPI_ISL_11259278	>A/magpie/Ireland/032958_22VIR1325-5/2021_H5N1_2021-11-12<br>ATGGATGTCAATCCGACTTTACTTTTCTTAAAAGTGCCAGCGCAAGATGCTATAAGTACCACATTCCCTTACACTGGAGATCCTCCATACAGCCATGGAACAGGGACAGGATACACAATGGACACAGTCAACAGAACACATCAATACTCAGAGAAGGGAAAATGGACAACAAACACAGAAACCGGAGCACCTCAACTCAACCCAATTGATGGGCCACTACCTGAGGACAACGA<br>ACCGAGCGGATATGCACAAACAGATTGCGTGTTGGAAGCAATGGCTTTCCTTGAAGAGTCCCACCCAGGGATCTTTGAAAACTCTTGTCTTGAAACGATGGAAGTCGTTCAGCAAACAAGAGTGGACAAACTAACTCAAGGTCGCCAGACATATGACTGGACACTGAATAGAAACCAACCAGCTGCAACTGCCCTGGCCAACACTATAGAGGTCTTCAGATCAAACGGTCTAA<br>CAACCAATGAATCGGGGAGACTAATAGATTTTCTCAAGGATGTGATGGACTCAATGGACAAAGAAGAAATGGAAATAACAACACATTTCCAGAGAAAGAGAAGAGTAAGGGACAACATGACCAAGAAAATGGTCACACAAAGAACAATAGGAAAGAAGAAACAAAGGCTCAACCAAAGGAGCTACCTAATAAGAGCACTGACATTGAATACAATGACAAAAGATGCAGAA<br>AGAGGCAAATTGAAAAGACGGGCGATTGCAACACCAGGGATGCAGATTAGAGGATTTGTGTACTTTGTCGAAACACTGGCAAGGAGCATCTGTGAAAAACTTGAGCAATCTGGACTCCCCGTTGGAGGGAATGAGAAGAAGGCTAAATTGGCAAATGTCGTGAGAAAAATGATGACTAACTCACAAGATACAGAGCTCTCCTTCACAATTACTGGAGATAACACCAAATGG<br>AATGAGAATCAAAATCCTCGGATGTTTCTGGCAATGATAACATACATCACAAGAAACCAACCTGAATGGTTTAGAAATGTCTTGAGTATTGCCCCTATAATGTTCTCGAACAAAATGGCGAGATTGGGAAAAGGGTACATGTTTGAAAGTAAGAGCATGAAGTTACGGACACAAATACCTGCAGAAATGCTTGCAAACATTGACTTAAAATACTTCAATGAATCAACAAGAAAG<br>AAAATCGAAAAAATAAGGCCTCTACTAATAGATGGCACTGCCTCATTGAGTCCTGGAATGATGATGGGCATGTTCAATATGCTGAGTACAGTATTAGGAGTTTCAATCCTAAATCTTGGGCAAAAGAGGTACACCAAAACCACATACTGGTGGGATGGACTCCAATCCTCTGATGATTTCGCCCTCATAGTAAATGCACCGAATCATGAGGGAATACAAGCAGGAGTGGATAG<br>GTTCTATAGGACCTGCAAACTGGTCGGGATCAATATGAGCAAAAAGAAGTCTTACATAAACCGGACTGGAACATTTGAATTCACAAGCTTTTTCTATCGCTATGGATTTGTGGCTAACTTCAGTATGGAGCTGCCCAGCTTTGGAGTTTCTGGGATCAATGAATCAGCTGACATGAGCATTGGCATCACAGTGATAAAGAACAACATGATAAACAATGACCTTGGACCAGCAAC<br>AGCTCAAATGGCCCTTCAACTATTCATCAAAGATTACAGGTACACGTACCGATGCCACAGAGGTGACACACAAATTCAAACGAGGAGATCATTCGAGCTGAAGAAGCTGTGGGACAGACCCGTTCAAAGGCAGGACTGTTGGTGTCAGATGGAGGACCAAATCTATACAACATTCGGAATCTCCATCCCAGAGGTCTGCCTGAAGTGGGAGCTGATGGACGAAGATTAC<br>CAGGGCAGGTTGTGTAATCCTCTGAACCCATTTGTCAGCCATAGAGAAATTGAATCCGTAAACAATGCTGTGGTGATGCCAGCCCATGGTCCAGCCAAGAGCATGGAATATGATGCCGTTGCTACTACACACTCATGGATTCCTAAAAGGAATCGTTCCATTCTCAATACCAGCCAAAGGGGAATTCTTGAGGATGAACAGATGTACCAGAAATGCTGCAGTCTATTCGAGAA<br>TTCTTCCCCAGTAGTTCATACAGGAGACCAGTTGGAATTTCAGCATGGTGGAGGCCATGGTGTCTAGGGCCCGAATCGATGCACGCATTGATTTCGAATCTGGAAGGATCAAGAAGGAAGAGTTTGCTGAGATCATGAAGATCTGTTCCACCATTGAAGAGCTCAGACGGCAAAAATAG
EPI_ISL_287565	>A/M_Swan/NL-Groningen/16015826-001/2016_H5N5_2016-12-13<br>ATGGATGTCAATCCGACTTTACTTTTCTTAAAAGTGCCAGCGCAAAATGCTATAAGTACCACATTCCCTTATACTGGAGATCCTCCATACAGCCATGGAACAGGAACAGGATACACCATGGACACAGTCAACAGAACACATCAATACTCAGAAAAGGGGAAATGGACAACAAACACAGAGACCGGAGCACCCCAACTCAACCCAATTGATGGACCACTACCTGAGGACAACGA<br>ACCAAGCGGATATGCACAAACAGATTGCGTGTTGGAAGCAATGGCTTTCCTTGAAGAGTCCCACCCAGGGATCTTTGAAAACTCTTGTCTTGAAACGATGGAAGTCGTTCATCAAACAAGAGTGGACAAACTAACTCAAGGTCGCCAGACTTATGACTGGACACTGAATAGAAACCAACCAGCTGCAACTGCCCTGGCCAACACTATAGAGGTCTTCAGATCGAACGGTCTGA<br>CAGCCAATGAATCGGGGAGACTAATAGATTTCCTCAAAGATGTGATGGACTCAATGGACAAAGAAGAAATGGAAATAACAACACATTTCCAGAGAAAGAGAAGAGTAAGGGACAACATGACCAAGAAAATGGTCACACAAAGAACAATAGGGAAGAAGAAGCAAAGGCTAAATAAGAGGAGCTACCTAATAAGAGCACTGACATTGAATACAATGACCAAAGACGCGGA<br>AAGAGGCAAATTGAAGAGACGGGCAATTGCAACACCAGGGATGCAGATTAGAGGATTTGTGTACTTTGTCGAAACACTGGCAAGGAGCATCTGTGAAAAACTTGAGCAATCTGGACTCCCCGTTGGAGGGAATGAGAAGAAGGCTAAATTGGCAAATGTCGTGAGAAAAATGATGACTAACTCACAAGATACAGAGCTCTCCTTCACAATTACTGGAGATAACACCAAATG<br>GAATGAGAATCAAAACCCTCGGATGTTTCTGGCAATGATAACATACATCACAAGAAACCAACCTGAATGGTTTAGAAATGTCTTGAGCATTGCCCCTATAATGTTCTCAAACAAAATGGCAAGATTAGGGAAAGGATACATGTTTGAAAGTAAGAGCATGAAGCTACGAACACAAATACCAGCAGAAATGCTTGCAAACATTGATCTTAAATACTTCAATGAATCAACAAGAA<br>AGAAAATCGAGAAAATAAGACCTCTACTAATAGATGGCACTGCCTCATTGAGTCCTGGAATGATGATGGGCATGTTCAATATGCTGAGTACAGTATTAGGAGTCTCAATCCTGAATCTTGGGCAAAAGAGGTACACCAAAACCACATACTGGTGGGATGGACTCCAATCCTCTGATGATTTTGCTCTCATAGTGAATGCACCGAATCATGAGGGAATACAAGCAGGAGTGGAT<br>AGGTTCTATAGGACCTGCAAACTGGTTGGGATCAACATGAGCAAAAAGAAGTCTTACATAAACCGGACAGGAACATTTGAGTTCACAAGCTTTTTCTATCGCTATGGATTTGTGGCTAACTTCAGTATGGAGCTGCCCAGCTTTGGAGTGTCTGGAATCAATGAATCAGCTGACATGAGCATCGGAGTTACAGTGATAAAGAACAACATGATAAACAATGACCTTGGACCAGCA<br>ACAGCTCAAATGGCCCTTCAACTATTCATCAAGGACTACAGGTACACGTACCGATGCCACAGGGGTGACACACAAATTCAAACGAGGAGATCATTCGAGCTGAAGAAGCTGTGGGAGCAGACCCGTTCAAAGGCAGGACTGTTGGTGTCAGATGGAGGACCAAATCTATACAACATCCGGAATCTCCATCCCAGAGGTCTGCCTGAAGTGGGAGCTGATGGAGGAAGATT<br>ACCAGGGCAGGCTGTGTAATCCTCTGAACCCATTTGTCAGTCATAAAGAAATTGAGTCCGTAAACAATGCTGTGGTAATGCCAGCCCATGGCCCAGCCAAGAGCATGGAATATGATGCTGTTGCGACTACACACTCATGGATTCCTAAAAGGAATCGTTCCATTCTCAATACCAGCCAAGGGGGAATTCTTGAGGATGAGCAGATGTACCAGAAATGCTGCAGTCTATTCGAGA<br>AATTCTTCCCCAGTAGTTCATACAGGAGACCAGTTGGAATTTCAGCATGGTGGAGGCCATGGTGTCTAGGGCCCGAATCGATGCACGCATTGATTTCGAATCTGGAAGGATTAAGAAGGAAGAGTTTGCTGAGATCATGAAGATCTGTTCCACCATTGAAGAGCTCAGACGGCAAAAATAG
EPI_ISL_7753309	>A/lesser_white-fronted_goose/Germany-MV/AI05973/2021_H5N1_2021-10-20<br>ATGGATGTCAATCCGACTTTACTTTTCTTAAAAGTGCCAGCGCAAGATGCTATAAGTACCACATTCCCTTACACTGGAGATCCTCCATACAGCCATGGAACAGGGACAGGATACACAATGGACACAGTCAACAGAACACATCAATACTCAGAGAAGGGAAAATGGACAACAAACACAGAAACCGGAGCACCTCAACTCAACCCAATTGATGGGCCACTACCTGAGGACAACGA<br>ACCGAGCGGATATGCACAAACAGATTGCGTGTTGGAAGCAATGGCTTTCCTTGAAGAGTCCCACCCAGGGATCTTTGAAAACTCTTGTCTTGAAACGATGGAAGTCGTTCAGCAAACAAGAGTGGACAAACTAACTCAAGGTCGCCAGACATATGACTGGACACTGAATAGAAACCAACCAGCTGCAACTGCCCTGGCCAACACTATAGAGGTCTTCAGATCAAACGGTCTAA<br>CAACCAATGAATCGGGGAGACTAATAGATTTCCTCAAGGATGTGATGGACTCAATGGAAAAAGAAGAAATGGAAATAACAACACATTTCCAGAGAAAGAGAAGAGTAAGGGACAACATGACCAAGAAAATGGTCACACAAAGAACAATAGGAAAGAAGAAACAAAGGCTCAACAAAAGGAGCTACCTAATAAGAGCACTGACATTGAATACAATGACAAAAGATGCAGAA<br>AGAGGCAAATTGAAAAGACGGGCGATTGCAACACCAGGGATGCAGATTAGAGGATTTGTGTACTTTGTCGAAACACTGGCAAGGAGCATCTGTGAAAAACTTGAGCAATCTGGACTCCCCGTTGGAGGGAATGAGAAGAAGGCTAAATTGGCAAATGTCGTGAGAAAAATGATGACTAACTCACAAGATACAGAGCTCTCCTTCACAATTACTGGAGATAACACCAAATGG<br>AATGAGAATCAAAATCCTCGGATGTTTCTGGCAATGATAACATACATTACAAGAAACCAACCTGAATGGTTTAGAAATGTCTTGAGTATTGCCCCTATAATGTTCTCGAACAAAATGGCGAGATTGGGAAAAGGGTACATGTTTGAAAGTAAGAGCATGAAGTTACGGACACAAATACCTGCAGAAATGCTTGCAAACATTGACTTAAAATACTTCAATGAATCAACAAGAAAG<br>AAAATCGAAAAAATAAGGCCTCTACTAATAGATGGCACTGCCTCATTGAGTCCTGGAATGATGATGGGCATGTTCAATATGCTGAGTACAGTATTAGGAGTTTCAATCCTAAATCTTGGGCAAAAGAGGTACACCAAAACCACATACTGGTGGGATGGACTCCAATCCTCTGATGATTTCGCCCTCATAGTAAATGCACCGAATCATGAGGGAATACAAGCAGGAGTGGATAG<br>GTTCTATAGGACCTGCAAACTGGTCGGGATCAATATGAGCAAAAAGAAGTCTTACATAAACCGGACTGGAACATTTGAGTTCACAAGCTTTTTCTATCGCTATGGATTTGTGGCTAACTTCAGTATGGAGCTGCCCAGCTTTGGAGTTTCTGGGATCAATGAATCAGCTGACATGAGCATTGGCATCACAGTGATAAAGAACAACATGATAAACAATGACCTTGGACCAGCAAC<br>AGCTCAAATGGCCCTTCAACTATTCATCAAAGATTACAGGTACACGTACCGATGCCACAGAGGTGACACACAAATTCAAACGAGGAGATCATTCGAGCTGAAGAAGCTGTGGGAGCAGACCCGTTCAAAGGCAGGACTGTTGGTGTCAGATGGAGGACCAAATCTATACAACATTCGGAATCTCCATCCCAGAGGTCTGCCTGAAGTGGGAGCTGATGGACGAAGATTAC<br>CAGGGCAGGTTGTGTAATCCTCTGAACCCATTTGTCAGCCATAGAGAAATTGAGTCCGTAAACAATGCTGTGGTGATGCCAGCCCACGGTCCAGCCAAAGAGCATGGAATATGATGCCGTTGCTACTACACACTCATGGATTCCTAAAAGGAATCGTTCCATTCTCAATACCAGCCAAAGGGGAATTCTTGAGGATGAACAGATGTACCAGAAATGCTGCAGTCTATTCGAGAA<br>TTCTTCCCCAGTAGTTCATACAGGAGACCAGTTGGAATTTCAGCATGGTGGAGGCCATGGTGTCTAGGGCCCGAATCGATGCACGCATTGATTTCGAATCTGGAAGGATCAAGAAGGAAGAGTTTGCTGAGATCATGAAGATCTGTTCCACCATTGAAGAGCTCAGACGGCAAAAATAG
EPI_ISL_13201050	>A/Lesser_Black-backed_Gull/Netherlands/1/2022_H5N1_2022-04-29<br>ATGGATGTCAATCCGACTTTACTTTTCTTAAAAGTGCCAGCGCAAGATGCTATAAGTACCACATTCCCTTACACTGGAGATCCTCCATACAGCCATGGAACAGGGACAGGATACACAATGGACACGGTCAACAGAACACATCAATACTCAGAGAAGGGAAAATGGACAACAAACACAGAAACCGGAGCACCTCAACTCAACCCAATTGATGGGCCACTACCTGAGGACAACGA<br>ACCGAGCGGATATGCACAAACAGATTGCGTGTTGGAAGCAATGGCTTTCCTTGAAGAGTCCCACCCAGGGATCTTTGAAAACTCTTGTCTTGAAACGATGGAAGTCGTTCAGCAAACAAGAGTGGACAAACTAACTCAAGGTCGCCAGACATATGACTGGACACTGAATAGAAACCAACCAGCTGCAACTGCCCTGGCCAACACTATAGAGGTCTTCAGATCAAACGGTCTAA<br>CAGCCAATGAATCGGGGAGACTAATAGATTTCCTCAAGGATGTGATGGACTCAATGGAAAAAGAAGAAATGGAAATAACAACACATTTCCAGAGAAAGAGAAGAGTAAGGGACAACATGACCAAGAAAATGGTCACACAAAGAACAATAGGAAAGAAGAAACAAAGGCTCAACAAAAGGAGTTACCTAATAAGAGCACTGACATTGAATACAATGACAAAAGATGCAGAA<br>AGAGGCAAATTGAAGAGACGGGCGATTGCAACACCAGGGATGCAGATTAGAGGATTTGTGTACTTTGTCGAAACACTGGCAAGGAGCATCTGTGAAAAACTTGAGCAATCTGGACTCCCCGTTGGAGGGAATGAGAAGAAGGCTAAATTGGCAAATGTCGTGAGAAAAATGATGACTAACTCACAAGATACAGAGCTCTCCTTCACAATTACTGGAGATAACACCAAATGG<br>AATGAGAATCAAAATCCTCGGATGTTTCTGGCAATGATAACATACATTACAAGAAACCAACCTGAATGGTTTAGGAATGTCTTGAGTATTGCCCCTATAATGTTCTCGAACAAAATGGCGAGATTGGGAAAAGGGTACATGTTTGAAAGTAAGAGCATGAAGTTACGGACACAAATACCTGCAGAAATGCTTGCAAACATTGACTTAAAATACTTCAATGAATCAACAAGAAAG<br>AAAATCGAAAAAATAAGGCCTCTACTAATAGATGGCACTGCCTCATTGAGTCCTGGAATGATGATGGGCATGTTCAATATGCTGAGTACAGTATTAGGAGTTTCAATCCTAAATCTTGGGCAAAAGAGGTACACCAAAACCACATACTGGTGGGATGGACTCCAATCCTCTGATGATTTCGCCCTCATAGTAAATGCACCGAATCATGAGGGAATACAAGCAGGAGTGGATAG<br>GTTCTATAGGACCTGCAAACTGGTCGGGATCAATATGAGCAAAAAGAAGTCTTACATAAACCGGACTGGAACATTTGAGTTCACAAGCTTTTTCTATCGCTATGGATTTGTGGCTAACTTCAGTATGGAGCTGCCCAGCTTTGGAGTTTCTGGGATCAATGAATCAGCTGACATGAGCATTGGCATCACAGTGATAAAGAACAACATGATAAACAATGACCTTGGACCAGCAAC<br>AGCTCAAATGGCCCTTCAACTATTCATCAAAGATTACAGGTACACGTACCGATGCCACAGAGGTGACACACAAATTCAAACGAGGAGATCATTCGAGCTGAAGAAGCTGTGGGAGCAGACCCGTTCAAAGGCAGGACTGTTGGTGTCAGATGGAGGACCAAATCTATACAACATTCGGAATCTCCATCCCAGAGGTCTGCCTGAAGTGGGAGCTGATGGACGAAGATTAC<br>CAGGGCAGGTTGTGTAATCCTCTGAACCCATTTGTCAGCCATAGAGAAATTGAGTCCGTAAACAATGCTGTGGTGATGCCAGCCCACGGTCCAGCCAAAGAGCATGGAATATGATGCCGTTGCTACTACACACTCATGGATTCCTAAAAGGAATCGTTCCATTCTCAATACCAGCCAAAGGGGAATTCTTGAGGATGAACAGATGTACCAGAAATGCTGCGGTCTATTCGAGAA<br>TTCTTCCCCAGTAGTTCATACAGGAGACCAGTTGGAATTTCAGCATGGTGGAGGCCATGGTGTCTAGGGCCCGAATCGATGCACGCATTGATTTCGAATCTGGAAGGATCAAGAAGGAAGAGTTTGCTGAGATCATGAAGATCTGTTCCACCATTGAAGAGCTCAGACGGCAAAAATAG
internal NVRI repository	>A/laying_hen/Poland/H363/2022_H5N1_2022-07-20<br>ATGGATGTCAATCCGACTTTACTTTTCTTAAAAGTGCCAGCGCAAGATGCTATAAGTACCACATTCCCTTACACTGGAGATCCTCCATACAGCCATGGAACAGGGACAGGATACACAATGGACACAGTCAACAGAACACATCAATACTCAGAGAAGGGAAAATGGACAACAAACACAGAAACCGGAGCACCTCAACTCAACCCAATTGATGGGCCACTACCTGAGGACAACGA<br>ACCGAGCGGATATGCACAAACAGATTGCGTGTTGGAAGCAATGGCTTTCCTTGAAGAGTCCCACCCAGGGATCTTTGAAAACTCTTGTCTTGAAACGATGGAAGTCGTTCAGCAAACAAGAGTGGACAAACTAACTCAAGGTCGCCAGACATATGACTGGACACTGAATAGAAACCAACCAGCTGCAACTGCCCTGGCCAACACTATAGAGGTCTTCAGATCAAACGGTCTAA<br>CAGCCAATGAATCGGGGAGACTAATAGATTTCCTCAAGGATGTGATGGACTCAATGGAAAAAGAAGAAATGGAAATAACAACACATTTCCAGAGAAAGAGAAGAGTAAGGGACAACATGACCAAGAAAATGGTCACACAAAGAACAATAGGAAAGAAGAAACAAAGGCTCAACAAAAGGAGCTACCTAATAAGAGCACTGACATTGAATACAATGACAAAAGATGCAGAA<br>AGAGGCAAATTGAAAAGACGGGCGATTGCAACACCAGGGATGCAGATTAGAGGATTTGTGTACTTTGTCGAAACACTGGCAAGGAGCATCTGTGAAAAACTTGAGCAATCTGGACTCCCCGTTGGAGGGAATGAGAAGAAGGCTAAATTGGCAAATGTCGTGAGAAAAATGATGACTAACTCACAAGATACAGAGCTCTCCTTCACAATTACTGGAGATAACACCAAATGG<br>GAATGAGAATCAAAATCCTCGGATGTTTCTGGCAATGATAACATACATCACAAGAAACCAACCTGAATGGTTTAGAAATGTCTTGAGTATTGCCCCTATAATGTTCTCGAACAAAATGGCGAGATTGGGAAAAGGGTACATGTTTGAAAGTAAGAGCATGAAGTTACGGACACAAATACCTGCAGAAATGCTTGCAAACATTGACTTAAAATACTTCAATGAATCAACAAGAA<br>GAAAATCGAAAAAATAAGGCCTCTACTAATAGATGGCACTGCCTCATTGAGTCCTGGAATGATGATGGGCATGTTCAATATGCTGAGTACAGTATTAGGAGTTTCAATCCTAAATCTTGGGCAAAAGAGGTACACCAAAACCACATACTGGTGGGATGGACTCCAATCCTCTGATGATTTCGCCCTCATAGTAAATGCACCGAATCATGAGGGAATACAAGCAGGAGTGGATA<br>GGTTCTATAGGACCTGCAAACTGGTCGGGATCAATATGAGCAAAAAGAAGTCTTACATAAACCGGACTGGAACATTTGAGTTCACAAGCTTTTTCTATCGCTATGGATTTGTGGCTAACTTCAGTATGGAGCTGCCCAGCTTTGGAGTTTCTGGGATCAATGAATCAGCTGACATGAGCATTGGCATCACAGTGATAAAGAACAACATGATAAACAATGACCTTGGACCAGCAA<br>CAGCTCAAATGGCCCTTCAACTATTCATCAAAGATTACAGGTACACGTACCGATGCCACAGAGGTGACACACAAATTCAAACGAGGAGATCATTCGAGCTGAAGAAGCTGTGGGAGCAGACCCGTTCAAAGGCAGGACTGTTGGTGTCAGATGGAGGACCAAATCTATACAACATTCGGAATCTCCATCCCAGAGGTCTGCCTGAAGTGGGAGCTGATGGACGAAGATTA<br>CCAGGGCAGGTTGTGTAATCCTCTGAACCCATTTGTCAGCCATAGAGAAATTGAGTCCGTAAACAATGCTGTGGTGATGCCAGCCCACGGTCCAGCCAAAGAGCATGGAATATGATGCCGTTGCTACTACACACTCATGGATTCCTAAAAGGAATCGTTCCATTCTCAATACCAGCCAAAGGGGAATTCTTGAGGATGAACAGATGTACCAGAAATGCTGCAGTCTATTCGAGAA<br>ATTCTTCCCCAGTAGTTCATACAGGAGACCAGTTGGAATTTCAGCATGGTGGAGGCCATGGTGTCTAGGGCCCGAATCGATGCACGAATTGATTTCGAATCTGGAAGGATCAAGAAGGAAGAGTTTGCTGAGATCATGAAGATCTGTTCCACCATTGAAGAGCTCAGACGGCAAAAATAG
internal NVRI repository	>A/laying_hen/Poland/H290-T/2022_H5N1_2022-04-20<br>ATGGATGTCAATCCGACTTTACTTTTCTTAAAAGTGCCAGCGCAAGATGCTATAAGTACCACATTCCCTTACACTGGAGATCCTCCATACAGCCATGGAACAGGGACAGGATACACAATGGACACAGTCAACAGAACACATCAATACTCAGAGAAGGGAAAATGGACAACAAACACAGAAACCGGAGCACCTCAACTCAACCCAATTGATGGGCCACTACCTGAGGACAACGA<br>ACCGAGCGGATATGCACAAACAGATTGCGTGTTGGAAGCAATGGCTTTCCTTGAAGAGTCCCACCCAGGGATCTTTGAAAACTCTTGTCTTGAAACGATGGAAGTCGTTCAGCAAACAAGAGTGGACAAACTAACTCAAGGTCGCCAGACATATGACTGGACACTGAATAGAAACCAACCAGCTGCAACTGCCCTGGCCAACACTATAGAGGTCTTCAGATCAAACGGTCTAA<br>CAGCCAATGAATCGGGGAGACTAATAGATTTCCTCAAGGATGTGATGGACTCAATGGAAAAAGAAGAAATGGAAATAACAACACATTTCCAGAGAAAGAGAAGAGTAAGGGACAACATGACCAAGAAAATGGTCACACAAAGAACAATAGGAAAGAAGAAACAAAGGCTCAACAAAAGGAGCTACCTAATAAGAGCACTAACATTGAATACAATGACAAAAGATGCAGAA<br>AGAGGCAAATTGAAGAGACGGGCAATTGCAACACCAGGGATGCAGATTAGAGGATTTGTGTACTTTGTCGAAACACTGGCAAGGAGCATCTGTGAAAAACTTGAGCAATCTGGACTCCCCGTTGGAGGGAATGAGAAGAAGGCTAAATTGGCAAATGTCGTGAGAAAAATGATGACTAACTCACAAGATACAGAGCTCTCCTTCACAATTACTGGAGATAACACCAAATGG<br>GAATGAGAATCAAAATCCTCGGATGTTTCTGGCAATGATAACATACATCACAAGAAACCAACCTGAATGGTTTAGAAATGTCTTGAGTATTGCCCCTATAATGTTCTCGAACAAAATGGCGAGATTGGGAAAAGGGTACATGTTTGAAAGTAAGAGCATGAAGTTACGGACACAAATACCTGCAGAAATGCTTGCAAACATTGACTTAAAATACTTCAATGAATCAACAAGAA<br>GAAAATCGAAAAAATAAGGCCTCTACTAATAGATGGCACTGCCTCATTGAGTCCTGGAATGATGATGGGCATGTTCAATATGCTGAGTACAGTATTAGGAGTTTCAATCCTAAATCTTGGGCAAAAGAGGTACACCAAAACCACATACTGGTGGGATGGACTCCAATCCTCTGATGATTTCGCCCTCATAGTAAATGCACCGAATCATGAGGGAATACAAGCAGGAGTGGATA<br>GGTTCTATAGGACCTGCAAACTGGTCGGGATCAATATGAGCAAAAAGAAGTCTTACATAAACCGGACTGGAACATTTGAGTTCACAAGCTTTTTCTATCGCTATGGATTTGTGGCTAACTTCAGTATGGAGCTGCCCAGCTTTGGAGTTTCTGGGATCAATGAATCAGCTGACATGAGCATTGGCATCACAGTGATAAAGAACAACATGATAAACAATGACCTTGGCCCGGCAA<br>CAGCTCAAATGGCCCTTCAACTATTCATCAAAGATTACAGGTACACGTACCGATGCCACAGAGGTGACACACAAATTCAAACGAGGAGATCATTCGAGCTGAAGAAGCTGTGGGAGCAGACCCGTTCAAAGGCAGGACTGTTGGTGTCAGATGGAGGACCAAATCTATACAACATTCGGAATCTCCATCCCAGAGGTCTGCCTGAAGTGGGAGCTGATGGACGAAGATTA<br>CCAGGGCAGGTTGTGTAATCCTCTGAACCCATTTGTCAGCCATAGAGAAATTGAGTCCGTAAACAATGCTGTGGTGATGCCAGCCCACGGTCCAGCCAAAGAGCATGGAATATGATGCCGTTGCTACTACACACTCATGGATTCCTAAAAGGAATCGTTCCATTCTCAATACCAGCCAAAGGGGAATTCTTGAGGATGAACAGATGTACCAGAAATGCTGCAGTCTATTCGAGAA<br>ATTCTTCCCCAGTAGTTCATACAGGAGACCAGTTGGAATTTCAGCATGGTGGAGGCCATGGTGTCTAGGGCCCGAATCGATGCACGCATTGATTTCGAATCTGGAAGGATTAAGAAGGAAGAGTTTGCTGAGATCATGAAGATCTGTTCCACCATTGAAGAGCTCAGACGGCAAAAATAG
internal NVRI repository	>A/laying_hen/Poland/H283/2022_H5N1_2022-04-12<br>ATGGATGTCAATCCGACTTTACTTTTCTTAAAAGTGCCAGCGCAAGATGCTATAAGTACCACATTCCCTTACACTGGAGATCCTCCATACAGCCATGGAACAGGGACAGGATACACAATGGACACAGTCAACAGAACACATCAATACTCAGAGAAGGGAAAATGGACAACAAACACAGAAACCGGAGCACCTCAACTCAACCCAATTGATGGGCCACTACCTGAGGACAACGA<br>ACCGAGCGGATATGCACAAACAGATTGCGTGTTGGAAGCAATGGCTTTCCTTGAAGAGTCCCACCCAGGGATCTTTGAAAACTCTTGTCTTGAAACGATGGAAGTCGTTCAGCAAACAAGAGTGGACAAACTAACTCAAGGTCGCCAGACATATGACTGGACACTGAATAGAAACCAACCAGCTGCAACTGCCCTGGCCAACACTATAGAGGTCTTCAGATCAAACGGTCTAA<br>CAGCCAATGAATCGGGGAGACTAATAGATTTCCTCAAGGATGTGATGGACTCAATGGAAAAAGAAGAAATGGAAATAACAACACATTTCCAGAGAAAGAGAAGAGTAAGGGACAACATGACCAAGAAAATGGTCACACAAAGAACAATAGGAAAGAAGAAACAAAGGCTCAACAAAAGGAGCTACCTAATAAGAGCACTGACATTGAATACAATGACAAAAGATGCAGAA<br>AGAGGCAAATTGAAGAGACGGGCAATTGCAACACCAGGGATGCAGATTAGAGGATTTGTGTACTTTGTCGAAACACTGGCAAGGAGCATCTGTGAAAAACTTGAGCAATCTGGACTCCCCGTTGGAGGGAATGAGAAGAAGGCTAAATTGGCAAATGTCGTGAGAAAAATGATGACTAACTCACAAGATACAGAGCTCTCCTTCACAATTACTGGAGATAACACCAAATGG<br>GAATGAGAATCAAAATCCTCGGATGTTTCTGGCAATGATAACATACATCACAAGAAACCAACCTGAATGGTTTAGAAATGTCTTGAGTATTGCCCCTATAATGTTCTCGAACAAAATGGCGAGATTGGGAAAAGGGTACATGTTTGAAAGTAAGAGCATGAAGTTACGGACACAAATACCTGCAGAAATGCTTGCAAACATTGACTTAAAATACTTCAATGAATCAACAAGAA<br>GAAAATCGAAAAAATAAGGCCTCTACTAATAGATGGCACTGCCTCATTGAGTCCTGGAATGATGATGGGCATGTTCAATATGCTGAGTACAGTATTAGGAGTTTCAATCCTAAATCTTGGGCAAAAGAGGTACACCAAAACCACATACTGGTGGGATGGACTCCAATCCTCTGATGATTTCGCCCTCATAGTAAATGCACCGAATCATGAGGGAATACAAGCAGGAGTGGATA<br>GGTTCTATAGGACCTGCAAACTGGTCGGGATCAATATGAGCAAAAAGAAGTCTTACATAAACCGGACTGGAACATTTGAGTTCACAAGCTTTTTCTATCGCTATGGATTTGTGGCTAACTTCAGTATGGAGCTGCCCAGCTTTGGAGTTTCTGGGATCAATGAATCAGCTGACATGAGCATTGGCATCACAGTGATAAAGAACAACATGATAAACAATGACCTTGGCCCGGCAA<br>CAGCTCAAATGGCCCTTCAACTATTCATCAAAGATTACAGGTACACGTACCGATGCCACAGAGGTGACACACAAATTCAAACGAGGAGATCATTCGAGCTGAAGAAGCTGTGGGAGCAGACCCGTTCAAAGGCAGGACTGTTGGTGTCAGATGGAGGACCAAATCTATACAACATTCGGAATCTCCATCCCAGAGGTCTGCCTGAAGTGGGAGCTGATGGACGAAGATTA<br>CCAGGGCAGGTTGTGTAATCCTCTGAACCCATTTGTCAGCCATAGAGAAATTGAGTCCGTAAACAATGCTGTGGTGATGCCAGCCCACGGTCCAGCCAAAGAGCATGGAATATGATGCCGTTGCTACTACACACTCATGGATTCCTAAAAGGAATCGTTCCATTCTCAATACCAGCCAAAGGGGAATTCTTGAGGATGAACAGATGTACCAGAAATGCTGCAGTCTATTCGAGAA<br>ATTCTTCCCCAGTAGTTCATACAGGAGACCAGTTGGAATTTCAGCATGGTGGAGGCCATGGTGTCTAGGGCCCGAATCGATGCACGCATTGATTTCGAATCTGGAAGGATYAAGAAGGAAGAGTTTGCTGAGATCATGAAGATCTGTTCCACCATTGAAGAGCTCAGACGGCAAAAATAG

internal NVRI repository	>A/laying_hen/Poland/H2159-T3/2021_H5N1_2021-12-27<br>ATGGATGTCAATCCGACTTTACTTTTCTTAAAAGTGCCAGCGCAAGATGCCATAAGTACCACATTCCCTTACACTGGAGATCCTCCATACAGCCATGGAACAGGGACAGGATACACAATGGACACAGTCAACAGAACACATCAATACTCAGAGAAGGGAAAATGGACAACAAACACAGAAACCGGAGCACCTCAACTCAACCCAATTGATGGGCCACTACCTGAGGACAACGA<br>ACCGAGCGGATATGCACAAACAGATTGCGTGTTGGAAGCAATGGCTTTCCTTGAAGAGTCCCACCCAGGGATCTTTGAAAACTCTTGTCTTGAAACGATGGAAGTCGTTCAGCAAACAAGAGTGGACAAACTAACTCAAGGTCGCCAGACATATGACTGGACACTGAATAGAAACCAACCAGCTGCAACTGCCCTGGCCAACACTATAGAGGTCTTCAGATCAAACGGTCTAA<br>CAGCCAATGAATCGGGGAGACTAATAGATTTCCTCAAGGATGTGATGGACTCAATGGATAAAGAAGAAATGGAAATAACAACACATTTCCAGAGAAAGAGAAGAGTAAGGGACAACATGACCAAGAAAATGGTCACACAAAGAACAATAGGAAAGAAGAAACAAAGGCTAAACAAGAGGAGCTACTTAATAAGAGCACTGACACTGAATACAATGACAAAAGATGCAGA<br>AAGAGGCAAATTGAAGAGACGGGCGATTGCAACACCAGGGATGCAGATTAGAGGATTTGTGTACTTTGTCGAAACACTGGCAAGGAGCATCTGTGAAAAACTTGAGCAATCTGGACTCCCCGTTGGAGGGAATGAGAAGAAGGCTAAATTGGCAAATGTCGTGAGAAAAATGATGACTAACTCACAAGATACAGAGCTCTCCTTCACAATTACTGGAGATAACACCAAATG<br>GAATGAGAATCAAAATCCTCGGATGTTTCTGGCAATGATAACATACATTACAAGAAACCAACCTGAATGGTTTAGAAATGTCTTGAGTATTGCCCCTATAATGTTCTCGAACAAAATGGCGAGATTGGGAAAAGGGTACATGTTTGAAAGTAAGAGCATGAAGTTACGGACACAAATACCTGCAGAAATGCTTGCAAACATTGACTTAAAATACTTCAATGAATCAACAAGAAA<br>GAAAATCGAAAAAATAAGGCCTCTACTAATAGATGGCACTGCCTCATTGAGTCCTGGAATGATGATGGGCATGTTCAATATGCTGAGTACAGTATTAGGAGTTTCAATCCTAAATCTTGGGCAAAAGAAGTACACCAAAACCACATACTGGTGGGATGGACTCCAATCCTCTGATGATTTCGCCCTCATAGTAAATGCACCGAATCATGAGGGAATACAAGCAGGAGTGGATA<br>GGTTCTATAGGACCTGCAAACTGGTCGGGATCAATATGAGCAAAAAGAAGTCTTACATGAACCGGACTGGAACATTTGAGTTCACAAGCTTTTTCTATCGCTATGGATTTGTGGCTAACTTCAGTATGGAGCTGCCCAGCTTTGGAGTTTCTGGGATCAATGAATCAGCTGACATGAGCATTGGCGTCACAGTGATAAAGAACAACATGATAAACAATGACCTTGGACCGGCAA<br>CAGCTCAAATGGCCCTTCAACTATTCATCAAAGATTACAGGTACACGTACCGATGCCACAGAGGTGACACACAAATTCAAACGAGGAGATCATTCGAGCTGAAGAAGCTGTGGGAACAGACCCGTTCAAAGGCAGGACTGTTGGTGTCAGATGGAGGACCAAATCTATACAACATTCGGAATCTCCATATCCCAGAGGTCTGCCTGAAGTGGGAGCTGATGGACGAAGATTA<br>CCAGGGCAGGTTGTGTAATCCTCTGAACCCATTTGTCAGCCATAAAGAACTTGAGTCCGTAAACAATGCTGTGGTGATGCCAGCCCACGGTCCAGCCAAAAGCATGGAATATGATGCCGTTGCGACTACACACTCATGGATTCCTAAAAGGAATCGTTCCATTCTCAATACCAGTCAAAGGGGAATTCTTGAGGATGAACAGATGTACCAGAAATGCTGCAGTCTATTCGAGAA<br>ATTCTTCCCCAGTAGTTCATACAGGAGACCAGTTGGAATTTCCAGCATGGTGGAGGCCATGGTGTCTAGGGCCCGAATCGATGCACGCATTGATTTCGAATCTGGAAGGATCAAGAAGGAAGAGTTTGCTGAGATCATGAAGATCTGTTCCACCATTGAAGAGCTCAGACGGCAAAAATAG
internal NVRI repository	>A/laying_hen/Poland/H2157-N/2021_H5N1_2021-12-27<br>ATGGATGTCAATCCGACTTTACTTTTCTTAAAAGTGCCAGCGCAAGATGCCATAAGTACCACATTCCCTTACACTGGAGATCCTCCATACAGCCATGGAACAGGGACAGGATACACAATGGACACAGTCAACAGAACACATCAATACTCAGAGAAGGGAAAATGGACAACAAACACAGAAACCGGAGCACCTCAACTCAACCCAATTGATGGGCCACTACCTGAGGACAACGA<br>ACCGAGCGGATATGCACAAACAGATTGCGTGTTGGAAGCAATGGCTTTCCTTGAAGAGTCCCACCCAGGGATCTTTGAAAACTCTTGTCTTGAAACGATGGAAGTCGTTCAGCAAACAAGAGTGGACAAACTAACTCAAGGTCGCCAGACATATGACTGGACACTGAATAGAAACCAACCAGCTGCAACTGCCCTGGCCAACACTATAGAGGTCTTCAGATCAAACGGTCTAA<br>CAACCAATGAATCGGGGAGACTAATAGATTTCCTCAAGGATGTGATGGACTCAATGGATAAAGAAGAAATGGAAATAACAACACATTTCCAGAGAAAGAGAAGAGTAAGGGACAACATGACCAAGAAAATGGTCACACAAAGAACAATAGGAAAGAAGAAACAAAGGCTAAACAAGAGGAGCTACTTAATAAGAGCACTGACACTGAATACAATGACAAAAGATGCAGA<br>AAGAGGCAAATTGAAAAGACGGGCGATTGCAACACCAGGGATGCAGATTAGAGGATTTGTGTACTTTGTCGAAACACTGGCAAGGAGCATCTGTGAAAAACTTGAGCAATCTGGACTCCCCGTTGGAGGGAATGAGAAGAAGGCTAAATTGGCAAATGTCGTGAGAAAAATGATGACTAACTCACAAGATACAGAGCTCTCCTTCACAATTACTGGAGATAACACCAAATG<br>GAATGAGAATCAAAATCCTCGGATGTTTCTGGCAATGATAACATACATTACAAGAAACCAACCTGAATGGTTTAGAAATGTCTTGAGTATTGCCCCTATAATGTTCTCGAACAAAATGGCGAGATTGGGAAAAGGGTACATGTTTGAAAGTAAGAGCATGAAGTTACGGACACAAATACCTGCAGAAATGCTTGCAAACATTGACTTAAAATACTTCAATGAATCAACAAGAAA<br>GAAAATCGAAAAAATAAGGCCTCTACTAATAGATGGCACTGCCTCATTGAGTCCTGGAATGATGATGGGCATGTTCAATATGCTGAGTACAGTATTAGGAGTTTCAATCCTAAATCTTGGGCAAAAGAAGTACACCAAAACCACATACTGGTGGGATGGACTCCAATCCTCTGATGATTTCGCCCTCATAGTAAATGCACCGAATCATGAGGGAATACAAGCAGGAGTGGATA<br>GGTTCTATAGGACCTGCAAACTGGTCGGGATCAATATGAGCAAAAAGAAGTCTTACATAAACCGGACTGGAACATTTGAGTTCACAAGCTTTTTCTATCGCTATGGATTTGTGGCTAACTTCAGTATGGAGCTGCCCAGCTTTGGAGTTTCTGGGATCAATGAATCAGCTGACATGAGCATTGGCATCACAGTGATAAAGAACAACATGATAAACAATGACCTTGGACCAGCAA<br>CAGCTCAAATGGCCCTTCAACTATTCATCAAAGATTACAGGTACACGTACCGATGCCACAGAGGTGACACACAAATTCAAACGAGGAGATCATTCGAGCTGAAGAAGCTGTGGGAACAGACCCGTTCAAAGGCAGGGCTGTTGGTGTCAGATGGAGGACCAAATCTATACAACATTCGGAATCTCCATATCCCAGAGGTCTGCCTGAAGTGGGAGCTGATGGACGAAGATTA<br>CCAGGGCAGGTTGTGTAATCCTCTGAACCCATTTGTCAGCCATAGAGAAATTGAGTCCGTAAACAATGCTGTGGTGATGCCAGCCCACGGTCCAGCCAAAAGCATGGAATATGATGCCGTTGCGACTACACACTCATGGATTCCTAAAAGGAATCGTTCCATTCTCAATACCAGTCAAAGGGGAATTCTTGAGGATGAACAGATGTACCAGAAATGCTGCAGTCTATTCGAGAA<br>ATTCTTCCCCAGTAGTTCATACAGGAGACCAGTTGGAATTTCCAGCATGGTGGAGGCCATGGTGTCTAGGGCCCGAATCGATGCACGCATTGATTTCGAATCTGGAAGGATCAAGAAGGAAGAGTTTGCTGAGATCATGAAGATCTGTTCCACCATTGAAGAGCTCAGACGGCAAAAATAG
internal NVRI repository	>A/laying_hen/Poland/H205/2022_H5N1_2022-03-08<br>ATGGATGTCAATCCGACTTTACTTTTCTTAAAAGTGCCAGCGCAAGATGCCATAAGTACCACATTCCCTTACACTGGAGATCCTCCATACAGCCATGGAACAGGGACAGGATACACAATGGACACAGTCAACAGAACACATCAATACTCAGAGAAGGGAAAATGGACAACAAACACAGAAACCGGAGCACCTCAACTCAACCCAATTGATGGGCCACTACCTGAGGACAACGA<br>ACCGAGCGGATATGCACAAACAGATTGCGTGTTGGAAGCAATGGCTTTCCTTGAAGAGTCCCACCCAGGGATCTTTGAAAACTCTTGTCTTGAAACGATGGAAGTCGTTCAGCAAACAAGAGTGGACAAACTAACTCAAGGTCGCCAGACATATGACTGGACACTGAATAGAAACCAACCAGCTGCAACTGCCCTGGCCAACACTATAGAGGTCTTCAGATCAAACGGTCTAA<br>CAGCCAATGAATCGGGGAGACTAATAGATTTCCTCAAGGATGTGATGGACTCAATGGATAAAGAAGAAATGGAAATAACAACACATTTCCAGAGAAAGAGAAGAGTAAGGGACAACATGACCAAGAAAATGGTCACACAAAGAACAATAGGAAAGAAGAAACAAAGGCTAAACAAGAGGAGCTACTTAATAAGAGCACTGACACTGAATACAATGACAAAAGATGCAGA<br>AAGAGGCAAATTGAAGAGACGGGCGATTGCAACACCAGGGATGCAGATTAGAGGATTTGTGTACTTTGTCGAAACACTGGCAAGGAGCATCTGTGAAAAACTTGAGCAATCTGGACTCCCCGTTGGAGGGAATGAGAAGAAGGCTAAATTGGCAAATGTCGTGAGAAAAATGATGACTAACTCACAAGATACAGAGCTCTCCTTCACAATTACTGGAGATAACACCAAATG<br>GAATGAGAATCAAAATCCTCGGATGTTTCTGGCAATGATAACATACATTACAAGAAACCAACCTGAATGGTTTAGAAATGTCTTGAGTATTGCCCCTATAATGTTCTCGAACAAAATGGCGAGATTGGGAAAAGGGTACATGTTTGAAAGTAAGAGCATGAAGTTACGGACACAAATACCTGCAGAAATGCTTGCAAACATTGACTTAAAATACTTCAATGAATCAACAAGAAA<br>GAAAATCGAAAAAATAAGGCCTCTACTAATAGATGGCACTGCCTCATTGAGTCCTGGAATGATGATGGGCATGTTCAATATGCTGAGTACAGTATTAGGAGTTTCAATCCTAAATCTTGGGCAAAAGAAGTACACCAAAACCACATACTGGTGGGATGGACTCCAATCCTCTGATGATTTCGCCCTCATAGTAAATGCACCGAATCATGAGGGAATACAAGCAGGAGTGGATA<br>GGTTCTATAGGACCTGCAAACTGGTCGGGATCAATATGAGCAAAAAGAAGTCTTACATGAACCGGACTGGAACATTTGAGTTCACAAGCTTTTTCTATCGCTATGGATTTGTGGCTAACTTCAGTATGGAGCTGCCCAGCTTTGGAGTTTCTGGGATCAATGAATCAGCTGACATGAGCATTGGCGTCACAGTGATAAAGAACAACATGATAAACAATGACCTTGGACCGGCAA<br>CAGCTCAAATGGCCCTTCAACTATTCATCAAAGATTACAGGTACACGTACCGATGCCACAGAGGTGACACACAAATTCAAACGAGGAGATCATTCGAGCTGAAGAAGCTGTGGGAACAGACCCGTTCAAAGGCAGGACTGTTGGTGTCAGATGGAGGACCAAATCTATACAACATTCGGAATCTCCATATCCCAGAGGTCTGCCTGAAGTGGGAGCTGATGGACGAAGATTA<br>CCAGGGCAGGTTGTGTAATCCTCTGAACCCATTTGTCAGCCATAAAGAACTTGAGTCCGTAAACAATGCTGTGGTGATGCCAGCCCACGGTCCAGCCAAAAGCATGGAATATGATGCCGTTGCGACTACACACTCATGGATTCCTAAAAGGAATCGTTCCATTCTCAATACCAGTCAAAGGGGAATTCTTGAGGATGAACAGATGTACCAGAAATGCTGCAGTCTATTCGAGAA<br>ATTCTTCCCCAGTAGTTCATACAGGAGACCAGTTGGAATTTCCAGCATGGTGGAGGCCATGGTGTCTAGGGCCCGAATCGATGCACGCATTGATTTCGAATCTGGAAGGATCAAGAAGGAAGAGTTTGCTGAGATCATGAAGATCTGTTCCACCATTGAAGAGCTCAGACGGCAAAAATAG
EPI_ISL_14761318	>A/laying_hen/Italy/22VIR204-1/2022_H5N1_2022-01-10<br>ATGGATGTCAATCCGACTTTACTTTTCTTAAAAGTGCCAGCGCAAGATGCCATAAGTACCACATTCCCTTACACTGGAGATCCTCCATACAGCCATGGAACAGGGACAGGATACACAATGGACACAGTCAACAGAACACATCAATACTCAGAGAAGGGAAAATGGACAACAAACACAGAAACCGGAGCACCTCAACTCAACCCCATTGATGGGCCACTACCTGAGGACAACGA<br>ACCGAGCGGATATGCACAAACAGATTGCGTGTTGGAAGCAATGGCTTTCCTTGAAGAGTCCCACCCAGGGATCTTTGAAAACTCTTGTCTTGAAACGATGGAAGTCGTTCAGCAAACAAGAGTGGACAAACTAACTCAAGGTCGCCAGACTTATGACTGGACACTGAATAGAAACCAACCAGCTGCAACTGCCCTGGCCAACACTATAGAGGTCTTCAGATCAAACGGTCTAA<br>CAGCCAATGAATCGGGGAGACTAATAGATTTCCTCAAGGATGTGATGGACTCAATGGATAAAGAAGAAATGGAAATAACAACACATTTCCAGAGAAAGAGAAGAGTAAGGGACAACATGACCAAGAAAATGGTCACACAAAGAACAATAGGAAAGAAGAAACAAAGGCTAAACAAGAGGAGCTACTTAATAAGAGCACTGACACTGAATACAATGACAAAAGATGCAGA<br>AAGAGGCAAATTGAAGAGACGGGCGATTGCAACACCAGGGATGCAGATTAGAGGATTTGTGTACTTTGTCGAAACACTGGCAAGGAGCATCTGTGAAAAACTTGAGCAATCTGGACTCCCCGTTGGAGGGAATGAGAAGAAGGCTAAATTGGCAAATGTCGTGAGAAAAATGATGACTAACTCACAAGATACAGAGCTCTCCTTCACAATCACTGGAGATAACACCAAATG<br>GAATGAGAATCAAAATCCTCGGATGTTTCTGGCAATGATAACATACATTACCAGAAACCAACCTGAATGGTTTAGAAATGTCTTGAGTATTGCCCCTATAATGTTCTCGAACAAAATGGCGAGATTGGGAAAAGGGTACATGTTTGAAAGTAAGAGCATGAAGTTACGGACACAAATACCTGCAGAAATGCTTGCAAACATTGACTTAAAATACTTCAATGAATCAACAAGAAA<br>GAAAATTGAAAAAATAAGGCCTCTATTAATAGATGGCACAGCCTCATTGAGTCCTGGAATGATGATGGGCATGTTCAATATGCTGAGTACAGTATTAGGAGTTTCAATCCTAAATCTTGGGCAAAAGAAGTACACCAAAACCACATATTGGTGGGATGGACTCCAATCCTCTGATGATTTCGCCCTCATAGTAAATGCACCGAATCATGAGGGAATACAAGCAGGAGTGGATA<br>GGTTCTATAGGACCTGCAAACTGGTCGGGATCAATATGAGCAAAAAGAAGTCTTACATAAACCGGACTGGAACATTTGAGTTCACAAGCTTTTTCTATCGCTATGGATTTGTGGCTAACTTCAGTATGGAGCTGCCCAGCTTTGGAGTTTCTGGGATCAATGAATCAGCTGACATGAGCATTGGCGTCACAGTGATAAAGAACAACATGATAAACAACGACCTTGGACCAGCAA<br>CAGCTCAAATGGCCCTTCAACTATTCATCAAAGATTACAGGTACACGTACCGATGCCACAGAGGTGACACACAAATTCAAACGAGGAGATCATTCGAGCTGAAGAAGCTGTGGGAACAGACCCGTTCAAAGGCAGGACTGTTGGTGTCAGATGGAGGACCAAATCTATACAACATTCGGAATCTCCATATCCCAGAGGTCTGCCTGAAGTGGGAGCTGATGGACGAAGATTA<br>CCAGGGCAGGTTGTGTAATCCTCTGAACCCATTTGTCAGCCATAAAGAAATTGAGTCCGTAAACAATGCTGTGGTGATGCCAGCCCACGGTCCAGCCAAAAGCATGGAATATGATGCCGTTGCGACTACACACTCATGGATTCCTAAAAGGAATCGTTCCATTCTCAATACCAGTCAAAGGGGGATTCTTGAGGATGAACAGATGTACCAGAAATGCTGCAGTCTATTCGAGAA<br>ATTCTTCCCCAGTAGTTCATACAGAAGACCAGTTGGAATTTCCAGCATGGTGGAGGCCATGGTGTCTAGGGCCCGAATCGATGCACGCATTGATTTCGAATCTGGAAGGATCAAGAAGGAAGAGTTTGCTGAGATCATGAAGATCTGTTCCACCATTGAAGAGCTCAGACGGCAAAAATAG
EPI_ISL_2234808	>A/knot_wader/Ireland/473_21VIR2956-2/2021_H5N3_2021-01-01<br>ATGGATGTCAATCCGACTTTACTTTTCTTAAAAGTGCCAGCGCAAAATGCCATAAGTACCACATTCCCTTATACTGGAGATCCTCCATACAGCCATGGAACAGGAACAGGATACACCATGGACACAGTCAACAGAACACATCAATACTCGGAAAAAGGGGAAATGGACAACAAACACAGAGACCGGAGCACCCCAACTCAACCCAATTGATGGACCACTACCTGAGGACAACGA<br>ACCAAGTGGATATGCACAAACAGATTGCGTGTTGGAAGCAATGGCTTTTCTTGAAGAGTCCCACCCAGGGATCTTTGAAAACTCTTGTCTTGAAACGATGGAAGTCGTTCAGCAAACAAGAATGGACAAACTAACTCAAGGTCGCCAGACTTATGACTGGACACTGAATAGAAACCAACCAGCTGCAACTGCCCTGGCCAACACTATAGAGGTCTTCAGATCGAATGGTCTGAC<br>AGCCAATGAATCAGGGAGACTAATAGATTTCCTCAAGGATGTGATGGACTCAATGGACAAAGAAGAAATGGAAATAACAACACATTTCCAGAGAAAGAGAAGAGTAAGGGACAACATGACCAAGAAAATGGTCACACAAAGAACAATAGGGAAGAAGAAGCAAAGGCTAAATAAGAGGAGCTACTTAATAAGAGCACTGACACTGAATACAATGACAAAAGACGCAGAA<br>AGAGGCAAATTGAAGAGGCGGGCAATTGCAACACCAGGGATGCAGATTAGAGGATTTGTGTACTTTGTTGAAACACTGGCAAGGAGCATCTGTGAAAAACTTGAGCAATCTGGACTCCCCGTTGGAGGGAATGAAAAGAAGGCTAAATTGGCAAATGTCGTGAGAAAAATGATGACTAACTCACAAGATACAGAGCTCTCCTTCACAATTACTGGAGATAACACCAAATGG<br>AATGAGAATCAAAATCCTCGGATGTTTCTGGCAATGATAACATACATCACAAGAAACCAACCTGAATGGTTTAGAAATGTCTTGAGCATTGCCCCCATAATGTTCTCGAACAAAATGGCGAGATTGGGGAAAGGATACATGTTTGAAAGTAAAAGTATGAAGCTACGAACACAAGTACCTGCAGAAATGCTTGCAAACATTGACTTAAAATACTTCAATGAGTCAACAAGAAAG<br>AAAATCGAGAAAATAAGACCTCTACTAATAGATGGCACCGCCTCATTGAGTCCTGGAATGATGATGGGCATGTTCAATATGCTGAGTACAGTATTAGGAGTCTCAATCCTGAATCTTGGGCAAAAGAGGTACACCAAAACCACATACTGGTGGGATGGACTCCAATCCTCTGATGATTTCGCTCTCATAGTAAATGCACCGAATCATGAGGGAATACAAGCAGGAGTGGATAG<br>GTTCTATAGGACTTGCAAACTGGTTGGGATCAACATGAGCAAAAAGAAGTCTTACATAAACCGGACAGGAACATTTGAGTTCACAAGCTTTTTCTATCGCTATGGATTTGTGGCCAACTTCAGTATGGAGCTGCCTAGCTTTGGAGTGTCTGGAATCAATGAATCAGCTGACATGAGYATTGGAGTTACAGTGATAAAGAACAACATGATAAACAATGACCTTGGACCAGCAAC<br>AGCTCAAATGGCCCTTCAACTATTCATCAAGGACTACAGGTACACGTACCGATGTCACAGGGGTGACACACAAATTCAAACGAGGAGATCATTCGAGCTGAAGAAGCTGTGGGAGCAGACCCGTTCAAAGGCAGGACTGTTGGTGTCAGATGGAGGACCAAATCTATACAACATCCGGAATCTCCATATCCCAGAAGTCTGCCTGAAGTGGGAGCTGATGGACGAAGATTAC<br>CAGGGCAGGTTGTGCAATCCTCTGAACCCATTTGTCAGTCATAAAGAAATTGAATCCGTAAACAATGCTGTGGTAATGCCAGCCCATGGCCCAGCCAAGAGCATGGAATATGATGCTGTTGCGACTACACACTCATGGATTCCTAAAAGGAATCGTTCCATTCTCAATACCAGCCAAAGGGGAATTCTTGAGGATGAGCAGATGTACCAGAAATGCTGCAGTCTATTCGAAAAA<br>TTCTTCCCCAGTAGTTCATACAGGAGACCAGTTGGAATTTCCAGCATGGTGGAGGCCATGGTGTCTAGGGCCCGAATCGACGCACGCATTGATTTCGAATCTGGAAGGATTAAGAAGGAAGAGTTTGCTGAGATCATGAAGATCTGTTCCACCATTGAAGAGCTCAGACGGCAAAAATAG
EPI_ISL_17978928	>A/herring_gull/Poland/P092/2020_H16N3_2020-10-28<br>ATGGATGTCAATCCGACTTTACTTTTCTTGAAAGTGCCAGCGCAAAATGCTATAAGTACTACATTCCCTTATACTGGTGACCCTCCATACAGCCATGGAACAGGAACAGGATATACCATGGACACGGTCAACAGAACACACCAGTACTCAGAAAAAGGAACATGGACAACAAACACCGAAACTGGAGCACCTCAGCTTAACCCGATCGATGGACCACTACCGGAGGATAATGA<br>GCCAAGCGGATATGCACAAACGGATTGTGTGTTAGAGGCAATGGCTTTCCTCGAAGAATCCCATCCAGGGATCTTTGAGAACTCATGTCTTGAAACGATGGAAGTTGTTCAGCAAACACGAGTGGACAAGTTGACCCAAGGCCGACAAACATATGATTGGACATTGAATAGAAATCAGCCAGCTGCAACGGCTTTAGCCAACACCATAGAGGTCTTCAGATCGAACGGTCTAA<br>CAGCTAATGAATCGGGGAGGCTCATTGATTTCCTTAAGGATGTGATGGATTCAATGGATAAAGAAGGAATGGAAATAACAACACATTTCCAAAGGAAAAGAAGAGTAAGGGACAACATGACCAAGAAAATGGTCACGCAAAGAACAATAGGGAAAAAGAAACAGAGATTGAGCAAAAAAAGCTACCTAATAAGGGCATTGACATTGAATACAATGACCAAAGATGCAGAA<br>AGAGGTAAATTGAAAAGGAGAGCGATTGCAACACCTGGGATGCAGATCAGAGGATTCGTCTACTTTGTTGAAACACTTGCAAGGAGCATATGTGAGAAACTTGAACAGTCAGGGCTCCCAGTTGGAGGAAATGAGAAAAAAGCTAAGTTGGCAAACGTTGTAAGAAAGATGATGACTAATTCGCAAGACACAGAGCTCTCCTTCACCATTACTGGGGACAACACCAAGTGG<br>AATGAAAATCAAAACCCTCGGATGTTCCTAGCAATGATAACATACATCACAAGAAACCAGCCTGAATGGTTCAGAAATGTCTTGAGCATTGCCCCCATAATGTTCTCAAACAAAATGGCAAGACTTGGGAAAGGATACATGTTCGAAAGTAAGAGCATGAAGCTACGAACACAAATACCAGCAGAAATGCTTGCAAGTATTGACCTCAAATATTTCAATGAGTCAACGAGAAA<br>GAAAATTGAAAACATAAGACCCCTCTTAATAGATGGCACAGCCTCATTGAGTCCCGGAATGATGATGGGCATGTTCAATATGTTGAGTACAGTCTTAGGGGTCTCAATCTTAAATCTTGGGCAAAAAAGGTACACCAAGACCACATACTGGTGGGATGGGCTCCAATCCTCTGATGACTTTGCACTCATAGTGAACGCACCTAATCATGAGGGAATACAAGCAGGAGTGGACA<br>GATTTTACAGAACCTGCAAATTAGTTGGAATCAACATGAGTAAAAAGAAGTCCTACATCAATAGAACAGGAACATTTGAGTTCACAAGCTTTTTCTATCGCTATGGGTTTGTAGCTAACTTCAGTATGGAGTTGCCAAGCTTTGGTGTATCAGGGATTAATGAGTCAGCTGACATGAGTATTGGAGTAACAGTGATAAAGAACAACATGATAAACAATGACCTTGGACCAGCAA<br>CAGCCCAAATGGCTCTTCAACTGTTCATCAAGGACTACAGGTACACATATCGATGCCATAGAGGTGATACTCAAATCCAAACAAGGAGATCATTTGAGTTGAAGAAACTTTGGGAGCAGACCCGTTCAAAAGCGGGGCTACTGGTTTCAGATGGAGGTCCAAATCTATATAACATCAGAAATCTCCATATTCCAGAGGTCTGTCTGAAATGGGAGCTAATGGATGAAGATTATC<br>AAGGCAGGCTGTGCAATCCTCTGAATCCTTTCGTAAGCCATAAGGAAATTGAGTCTGTAAACAATGCTGTGGTAATGCCAGCCCATGGTCCAGCCAAAAGTATGGAATATGATGCTGTTGCAACTACACATTCCTGGGTCCCCAAGAGGAACCGTTCCATTCTCAACACTAGCCAAAGGGGAATCCTTGAGGATGAACAGATGTATCAAAAATGTTGCAATCTATTCGAAAAAT<br>TCTTCCCTAGCAGTTCGTACAGGAGACCAGTTGGAATTTCCAGCATGGTGGAGGCCATGGTATCCAGGGCCCGAATTGACGCACGGATTGACTTCGAATCTGGAAGGATCAAGAAAGAAGAGTTTGCTGAGATCATGAAGATCTGTTCCACCATTGAAGAGCTCAGACGGCAAAAGTAG
EPI_ISL_14917979	>A/herring_gull/Poland/MB138/2022_H5N1_2022-05-30<br>ATGGATGTCAATCCGACTTTACTCTTCTTAAAAGTGCCAGCGCAAGATGCCATAAGTACCACATTCCCTTACACTGGAGATCCTCCATACAGCCATGGAACAGGGACAGGATACACAATGGACACGGTCAACAGAACACATCAATACTCAGAGAAGGGAAAATGGACAACAAACACAGAAACCGGAGCACCTCAACTCAACCCAATTGATGGGCCACTACCTGAGGACAACGA<br>ACCGAGCGGATATGCACAAACAGATTGCGTGTTGGAAGCAATGGCTTTCCTTGAAGAGTCCCATCCAGGGATCTTTGAAAACTCTTGTCTTGAAACGATGGAAGTCGTTCAGCAAACAAGAGTGGACAAACTAACTCAAGGTCGCCAGACATATGACTGGACACTGAATAGAAACCAACCAGCTGCAACTGCCTTGGCCAACACTATAGAGGTCTTAAGATCAAACGGTCTAA<br>CAGCCAATGAATCGGGGAGACTAATAGATTTCCTCAAGGATGTGATGGACTCAATGGATAAAGAAGAAATGGAAATAACAACACATTTCCAGAGAAAGAGAAGAGTAAGGGACAACATGACCAAGAAAATGGTCACACAAAGAACAATAGGAAAGAAGAAACAAAGGCTAAACAAAAGGAGCTACTTAATAAGAGCACTGACACTGAATACAATGACAAAAGATGCAGA<br>AAGAGGCAAATTGAAGAGGCGGGCGATTGCAACACCAGGGATGCAGATTAGAGGATTTGTGTACTTTGTCGAAACACTGGCAAGGAGCATCTGTGAAAAACTTGAGCAATCTGGACTCCCCGTTGGAGGGAATGAGAAGAAGGCTAAATTGGCAAATGTTGTGAGAAAAATGATGACTAACTCACAAGATACAGAGCTCTCCTTCACAATTACTGGAGATAACACCAAATG<br>GAATGAGAATCAAAATCCTCGGATGTTTCTGGCAATGATAACATACATTACAAGAAACCAACCTGAATGGTTTAGGAATGTCTTGAGTATTGCCCCTATAATGTTCTCGAACAAAATGGCGAGATTGGGAAAAGGGTACATGTTTGAAAGTAAGAGCATGAAGTTACGGACACAAATACCTGCAGAAATGCTTGCAAACATTGACTTAAAATATTTCAATGAATCAACAAGAAA<br>GAAAATCGAAAAAATAAGGCCTCTACTAATAGATGGCACTGCCTCATTGAGTCCTGGAATGATGATGGGCATGTTCAATATGCTGAGTACAGTATTAGGAGTTTCAATCCTAAATCTTGGGCAAAAGAAGTACACCAAAACCACATACTGGTGGGATGGACTCCAATCCTCTGATGATTTCGCCCTCATAGTAAATGCACCGAATCATGAGGGAATACAAGCAGGAGTGGATA<br>GGTTCTATAGGACCTGCAAACTGGTCGGGATCAATATGAGCAAAAAGAAGTCTTACATAAACCGGACTGGAACATTTGAGTTCACAAGCTTTTTCTATCGCTATGGATTTGTGGCTAATTTCAGTATGGAGCTGCCCAGCTTTGGAGTTTCTGGGATCAATGAATCAGCTGACATGAGCATTGGCATCACAGTGATAAAGAACAACATGATAAACAATGACCTTGGACCAGCAA<br>CAGCTCAAATGGCCCTTCAACTATTCATCAAAGATTACAGGTACACGTACCGATGCCATAGAGGTGACACACAAATTCAAACGAGGAGATCATTCGAGCTGAAGAAGCTGTGGGAACAGACCCGTTCAAAGGCAGGACTGTTGGTGTCAGATGGAGGACCAAATCTATACAACATTCGGAATCTCCATATCCCAGAGGTCTGCCTGAAGTGGGAGCTGATGGACGAAGATTA<br>CCAGGGCAGGTTGTGTAATCCTCTGAACCCATTTGTCAGCCATAAAGAAATTGAGTCCGTAAACAATGCTGTGGTGATGCCAGCCCACGGTCCAGCCAAAAGCATGGAATATGATGCCGTTGCGACTACACACTCATGGATTCCTAAAAGGAATCGTTCCATTCTCAATACCAGTCAAAGGGGAATTCTTGAGGATGAACAGATGTACCAGAAATGCTGCGGTCTATTCGAGAA<br>ATTCTTCCCCAGTAGTTCATACAGGAGACCAGTTGGAATTTCCAGCATGGTGGAGGCCATGGTGTCTAGGGCCCGAATCGATGCACGCATTGATTTCGAATCTGGAAGGATCAAGAAGGAAGAGTTTGCTGAGATCATGAAGATCTGTTCCACCATTGAAGAGCTCAGACGGCAAAAATAG
EPI_ISL_18245813	>A/hawk/Poland/MB544/2021_H5N1_2021-12-22<br>ATGGATGTCAATCCGACTTTACTTTTCTTAAAAGTGCCAGCGCAAGATGCCATAAGTACCACATTCCCTTACACTGGAGATCCTCCATACAGCCATGGAACAGGGACAGGATACACAATGGACACAGTCAACAGAACACATCAATACTCAGAGAAGGGAAAATGGACAACAAACACAGAAACCAGAGCACCTCAACTCAACCCAATTGATGGGCCACTACCTGAGGACAACGA<br>ACCGAGCGGATATGCACAAACAGATTGCGTGTTGGAAGCAATGGCTTTCCTTGAAGAGTCCCACCCAGGGATCTTTGAAAACTCTTGTCTTGAAACGATGGAAGTCGTTCAGCAAACAAGAGTGGACAAACTAACTCAAGGTCGCCAGACATATGACTGGACACTGAATAGAAACCAACCAGCTGCAACTGCCCTGGCCAACACTATAGAGGTCTTCAGATCAAACGGTCTAA<br>CAGCCAATGAATCGGGGAGACTAATAGATTTCCTCAAGGATGTGATGGACTCAATGGATAAAGAAGAAATGGAAATAACAACACATTTCCAGAGAAAGAGAAGAGTAAGGGACAACATGACCAAGAAAATGGTCACACAAAGAACAATAGGAAAGAAGAAACAAAGGCTAAACAAGAGGAGCTACTTAATAAGAGCACTGACACTGAATACAATGACAAAAGATGCAGA<br>AAGAGGCAAATTGAAAAGACGGGCGATTGCAACACCAGGGATGCAGATTAGAGGATTTGTGTACTTTGTCGAAACACTGGCAAGGAGCATCTGTGAAAAACTTGAGCAATCTGGACTCCCCGTTGGAGGGAATGAGAAGAAGGCTAAATTGGCAAATGTCGTGAGAAAAATGATGACTAACTCACAAGATACAGAGCTCTCCTTCACAATTACTGGAGATAACACCAAATG<br>GAATGAGAATCAAAATCCTCGGATGTTTCTGGCAATGATAACATACATTACAAGAAACCAACCTGAATGGTTTAGAAATGTCTTGAGTGTTGCCCCTATAATGTTCTCGAACAAAATGGCGAGATTGGGAAAAGGGTACATGTTTGAAAGTAAGAGCATGAAGTTACGGACACAAATACCTGCAGAAATGCTTGCAAACATTGACTTAAAATACTTCAATGAATCAACAAGAAA<br>GAAAATCGAAAAAATAAGGCCTCTACTAATAGATGGCACTGCCTCATTGAGTCCTGGAATGATGATGGGCATGTTCAATATGCTGAGTACAGTATTAGGAGTTTCAATCCTAAATCTTGGGCAAAAGAAGTACACCAAAACCACATACTGGTGGGATGGACTCCAATCCTCTGATGATTTCGCCCTCATAGTAAATGCACCGAATCATGAGGGAATACAAGCAGGAGTGGATA<br>GGTTCTATAGGACCTGCAAACTGGTCGGGATCAATATGAGCAAAAAGAAGTCTTACATAAACCGGACTGGAACATTTGAGTTCACAAGCTTTTTCTATCGCTATGGATTTGTGGCTAACTTCAGTATGGAGCTGCCCAGCTTTGGAGTTTCTGGGATCAATGAATCAGCTGACATGAGCATTGGCGTCACAGTGATAAAGAACAACATGATAAACAATGACCTTGGACCAGCAA<br>CAGCTCAAATGGCCCTTCAACTATTCATCAAAGATTACAGGTACACGTACCGATGCCACAGAGGTGACACACAAATTCAAACGAGGAGATCATTCGAGCTGAAGAAGCTGTGGGAACAGACCCGTTCAAAGGCAGGACTGTTGGTGTCAGATGGAGGACCAAATCTATACAACATTCGGAATCTCCATATCCCAGAGGTCTGCCTGAAGTGGGAGCTGATGGACGAAGATTA<br>CCAGGGCAGGTTGTGTAATCCTCTGAACCCATTTGTCAGCCATAGAGAAATTGAGTCCGTAAACAATGCTGTGGTGGCGCCAGCCCACGGTCCAGCCAAAAGCATGGAATATGATGCCGTTGCGACTACACACTCATGGATTCCTAAAAGGAATCGTTCCATTCTCAATACCAGTCAAAGGGGAATTCTTGAGGATGAACAGATGTACCAGAAATGCTGCAGTCTATTCGAGAA<br>ATTCTTCCCCAGTAGTTCATACAGGAGACCAGTTGGAATTTCCAGCATGGTGGAGGCCATGGTGTCTAGGGCCCGAATCGATGCACGAATTGATTTCGAATCTGGAAGGATAAAGAAGGAAGAGTTTGCTGAGATCATGAAGATCTGTTCCACCATTGAAGAGCTCAGACGGCAAAAATAG
EPI_ISL_661312	>A/guinea_fowl/Germany-NW/AI01184/2020_H5N8_2020-09-11<br>ATGGATGTCAATCCGACTTTACTTTTCTTAAAAGTGCCAGCGCAGAATGCTATAAGTACTACGTTTCCTTACACTGGAGATCCTCCATACAGCCATGGAACAGGAACAGGATACACCATGGACACAGTCAACAGAACACATCAATACTCAGAGAAGGGAAAGTGGACAACAAACACAGAAACTGGAGCGCCCCAACTCAATCCAATTGATGGACCATTGCCAGAGGACAACGA<br>GCCAAGCGGATATGCACAAACAGATTGCGTGTTGGAAGCAATGGCTTTCCTTGAAGAATCCCACCCAGGGATCTTTGAAAACTCTTGTCTTGAAACGATGGAGGTCGTTCAGCAAACAAGAGTGGACAAACYAACCCAAGGTCGCCAGACTTATGATTGGACACTGAATAGAAACCAACCAGCTGCAACTGCTTTGGCCAATACTATAGAGGTCTTCAGATCGAACGGTCTAA<br>CAGCCAATGAGTCGGGGAGACTAATAGACTTCCTCAAGGATGTGATGGAATCAATGGATAAGGAAGAAATGGAAATAACAACACATTTCCAGAGAAAGAGAAGAGTAAGGGACAACATGACCAAGAAGATGGTCACACAAAGAACGATAGGAAAGAAGAAGCAGAGACTGAACAAGAGGAACTACTTAATAAGAGCACTGACATTGAACACAATGACCAAAGATGCGGA<br>AAGAGGCAAATTGAAGAGGCGGGCAATTGCAACACCCGGGATGCAGATTAGAGGATTTGTGTACTTTGTCGAAACACTAGCAAGGAGCATCTGTGAGAAACTCGAGCAATCTGGACTCCCTGTTGGAGGGAATGAGAAAAAGGCCAAGTTGGCAAATGTCGTGAGAAAAATGATGACTAACTCGCAAGATACAGAGCTCTCCTTTACAATTACTGGAGACAACACCAAATG<br>GAATGAAAATCAAAATCCTCGGATGTTTTTGGCAATGATAACATACATCACAAGAAACCAACCCGAATGGTTTAGAAATGTCTTGAGCATTGCCCCTATAATGTTCTCAAACAAAATGGCAAGATTAGGAAAAGGATACATGTTTGAAAGTAAGAGCATGAAGCTAAGGACACAAATACCAGCAGAAATGCTTGCAAACATTGACTTGAAATACTTCAATGAATCAACGAGAAA<br>GAAAATCGAGAAAATAAGACCTCTGCTAATTGATGGCACAGCCTCATTGAGTCCTGGAATGATGATGGGCATGTTCAATATGCTGAGCACAGTATTGGGGGTCTCAATCCTGAATCTTGGACAAAAAAGGTACACCAAAACCACATACTGGTGGGATGGACTCCAATCCTCTGATGATTTCGCTCTCATAGTGAATGCACCGAACCATGAGGGGATACAAGCGGGGGTGGATA<br>GGTTCTATAGGACCTGCAAACTGGTTGGGATCAACATGAGCAAAAAGAAATCTTATATAAACCGGACAGGAACATTTGAGTTCACAAGCTTTTTCTACCGCTATGGATTTGTAGCCAACTTCAGTATGGAGTTACCCAGCTTTGGAGTGTCTGGAATCAATGAGTCTGCTGACATGAGCATTGGAGTTACAGTGATAAAGAACAATATGATAAACAATGACCTTGGGCCAGCAA<br>CAGCTCAAATGGCTCTTCAGCTATTCATCAAAGATTATAGGTACACGTACCGATGTCACAGAGGTGATACACAAATTCAAACGAGGAGATCATTCGAGCTTAAGAAGCTGTGGGAGCAGACCCGTTCAAAGGCAGGGCTGTTGGTATCAGATGGAGGACCAAACCTATACAACATTCGGAATCTCCACATCCCAGAGGTCTGCTTGAAGTGGGAACTAATGGATGAAGATTAC<br>CAGGGCAGACTGTGTAATCCTCTGAATCCGTTTGTCAGTCATAAGGAAATTGAGTCCGTAAACAATGCTGTGGTAATGCCAGCCCATGGCCCAGCCAAGAGCATGGAATATGATGCTGTTGCGACTACACACTCATGGATTCCTAAGAGGAACCGTTCCATTCTCAATACCAGCCAGAGGGGAATCCTCGAAGATGAGCAGATGTACCAGAAGTGTTGCAGTTTATTCGAGAA<br>ATTCTTCCCCAGTAGTTCGTACAGGAGGCCAGTTGGAATTTCCAGCATGGTGGAGGCCATGGTGTCCAGGGCCCGAATTGATGCACGCATTGATTTCGAATCTGGAAGGATTAAGAAAGAAGAGTTTGCTGAGATCATGAAGATCTGTTCCACCATTGAAGAGCTCAGACGGCAAAAATAG
EPI_ISL_1095363	>A/Greylag_goose/Sweden/SVA210209SZ0422/KN000346/2021_H5N8_2021-02-03<br>ATGGATGTCAATCCGACTTTACTTTTCTTAAAAGTGCCAGCGCAAAATGCTATAAGTACTACATTCCCTTACACTGGAGATCCCCCATACAGCCATGGAACAGGAACAGGGTATACCATGGACACAGTAAACAGAACACATCAATACTCAGAAAAGGGAAAGTGGACAACAAACACAGAAACCGGAGCACCCCAACTCAACCCAATTGATGGACCATTACCAGAGGACAATGA<br>GCCAAGCGGATATGCACAAACTGATTGCGTGTTGGAAGCAATGGCTTTCCTTGAAGAATCCCACCCAGGGATATTTGAAAACTCTTGTCTTGAAGCGATGGAAATCGTTCAGCAAACAAGAGTGGACAAACTAACCCAAGGTCGCCAGACTTATGACTGGACACTGAACAGAAACCAACCAGCTGCAACCTCTTTGGCCAACACTATAGAGGTGTTCAGATCGAATGGTCTGA<br>CAGCCAATGAATCAGGGAGACTGATAGATTTTCTCAGGGATGTGATGGAATCAATGGATAAAGAAGAGATGGAAGTAACAACACATTTCCAGAGAAAAAGAAGAGTGAGGGACAACATGACTAAGAAGATGGTCACACAAAGAACAATAGGGAAGAAGAAGCAGAGGCTGAACAAGAGGAGTTACTTAATAAGAGCACTGACATTGAATACAATGACCAAAGATGCAGA<br>AAGAGGCAAGTTGAAGAGACGGGCAATTGCAACACCCGGGATGCAGATTAGAGGATTCGTGTACTTCGTTGAAACACTAGCGAGGAGCATCTGTGAGAAACTAGAGCAATCTGGGCTCCCTGTTGGAGGGAATGAGAAGAAGGCTAAATTGGCAAATGTTGTGAGAAAAATGATGACTAACTCACAAGATACAGAGCTCTCCTTTACAATTACTGGAGACAACACCAAATG<br>GAATGAGAATCAAAACCCTCGGATGTTTTTGGCAATGATAACATATATCACAAGAAACCAACCTGAATGGTTTAGAAATGTCTTAAGCATTGCCCCTATAATGTTCTCAAACAAAATGGCGAGATTAGGGAAAGGATACATGTTTGAAAGTAAGAGCATGAAGCTAAGAACACAAATACCAGCAGAGATGCTTACAAATATTGATCTGAAGTATTTCAACGAACCAACGAGAA<br>AGAAAATCGAGAAAATAAGACCTCTGCTGATTGATGGCACGGCCTCATTGAGTCCTGGGATGATGATGGGCATGTTCAATATGCTGAGCACAGTATTAGGGGTCTCAATCCTGAATCTCGGGCAAAAGAGGTACACCAAAACCACATACTGGTGGGATGGACTTCAATCCTCTGATGATTTCGCTCTCATAGTGAATGCACCGAATCATGAGGGGATACAAGCAGGAGTGGAT<br>AGATTCTATAGGACCTGCAAACTGGTTGGGATCAACATGAGCAAAAAGAAGTCTTACATAAACCGAACAGGAACATTTGAGTTCACAAGTTTTTTCTACCGCTATGGATTTGTAGCCAACTTCAGTATGGAATTACCCAGCTTTGGAGTGTCTGGAATCAATGAATCAGCTGACATGAGCATTGGAGTTACAGTGATAAAAAACAATATGATAAACAATGATCTTGGACCAGCA<br>ACAGCTCAAATGGCTCTTCAGTTATTCATCAAAGACTATAGGTACACATACCGATGCCACAGGGGTGATACACAAATTCAAACGAGGAGATCATTCGAGCTGAAGAAGCTGTGGGAGCAGACCCGTTCAAAGGCAGGGCTGTTGATATCAGACGGGGGGCCAAATCTATACAACATTCGGAATCTCCACATCCCAGAGGTCTGCTTGAAGTGGGAGCTGATGGATGAAGACT<br>ACCAAGGCAGGCTGTGCAATCCTCTGAATCCATTTGTCAGTCATAAAGAGATTGAGTCCGTAAACAATGCTGTAGTAATGCCCGCCCATGGCCCGGCCAAGAGCATGGAATATGATGCTGTTGCGACCACACACTCGTGGATTCCTAAGAGGAACCGTTCCATTCTCAATACCAGCCAAAGGGGAATTCTTGAGGATGAGCAGATGTACCAAAAGTGCTGTAGTCTATTCGAGA<br>AATTCTTCCCCAGCAGTTCATACAGGAGGCCAGTTGGAATTTCCAGCATGGTGGAGGCCATGGTGTCTAGGGCCCGAATTGATGCACGCATCGACTTCGAATCTGGAAGGATTAAGAAAGAAGAGTTTGCTGAGATCATGAAGATCTGTTCCACCATTGAAGAGCTCAGACGGCAAAAATAG
EPI_ISL_18245810	>A/greylag_goose/Poland/MB503_21RS3290-18/2021_H5N1_2021-11-22<br>ATGGATGTCAATCCGACTTTACTTTTCTTAAAAGTGCCAGCGCAAGATGCCATAAGTACCACATTCCCTTACACTGGAGATCCTCCATACAGCCATGGAACAGGGACAGGATACACAATGGACACAGTCAACAGAACACATCAATACTCAGAGAAGGGAAAATGGACAACAAACACAGAAACCGGAGCACCTCAACTCAACCCAATTGATGGGCCACTACCTGAGGACAACGA<br>ACCGAGCGGATATGCACAAACAGATTGCGTGTTGGAAGCAATGGCTTTCCTTGAAGAGTCCCACCCAGGGATCTTTGAAAACTCTTGTCTTGAAACGATGGAAGTCGTTCAGCAAACAAGAGTGGACAAACTAACTCAAGGTCGCCAGACWTATGACTGGACACTGAATAGAAACCAACCAGCTGCAACTGCCCTGGCCAACACTATAGAGGTCTTCAGATCAAACGGTCTAA<br>CAGCCAATGAATCGGGGAGACnnnnnnnnnnCTCAAGGATGTGATGGACTCAATGGATAAAGAAGAAATGGAAATAACAACACATTTCCAGAGAAAGAGAAGAGTAAGGGACAACATGACCAAGAAAATGGTCACACAAAGAACAATAGGAAAGAAGAAACAAAGGCTAAACAAGAGGAGCTACTTAATAAGAGCACTGACACTGAATACAATGACAAAAGAnnnnnnn<br>nnnnnnnnnnnnnnnnnnnnnnnnnnnnnnnCACCAGGGATGCAGATTAGAGGATTTGTGTACTTTGTCGAAACACTGGCAAGGAGCnnnnnnnnnnnnnnnnnnnnnnnnnnnnnnnnnnnnnnnnnnnnnnnnnnnnnnnnnnnnnnnnnnnnnGAGAAAAATGATGACTAACTCACAAGATACAGAGCTCTCCTTCACAATCACTGGAGATAACACCAAATGGAATGAG<br>AATCAAAATCCTCGGATGTTTCTGGCAATGATAACATACATTACCAGAAACCAACCTGAATGGTTTAGAAnnnnnnnnnnnnnnnnnnnnnTGTTCTCGAACAAAATGGCGAGATTGGGAAAAGGGTACATGTTTGnnnnnnnnnGCATGAAGTTACnnnnnCAAATACCTGnnnnnnnnnnnnnnnnnnnnnnnnnnnnnnnnnnnnnnnnnnnnnnnnnnnnnnnnnnnnn<br>nnnnnnnnnnnnnnnnnnnnnnnnnnnnnnnnnnnnnnnnnnnnnnnnnnnnnnnnnnnnnnnnnnnnnnnnnnnnnnnnnnnnnnnnnnnnnnnnnnnnnnnnnnnnnnnnnnnnnnGTACACCAAAACCACATACnnnnnnnnnnnnnnnnnnnnnnnnnnnnnnnnnnnnnnnnnnnnnnnnnnnnnnnnnnnnnnnnnnnnnnnnnnnnGGAGTGGATAGGTTCTATAGGACCTGCAAA<br>CTGGTCGGGATCAATATGAGCAAAAAGAAGTCTTACATAAACCGGACTGGAACATTTGAGTTCACAAGCTTTTTCTATCGCTATGGATTTGTGGCTAACTTCAGTATGGAGCTGCCCAGCTTTGGAGTTTCTGGGATCAATGAATCAGCTGACATGAGCATTGGCGTCACAGTGATAAAGAACAACATGATAAACAACGACCTTGGACCAGCAACAGCTCAAATGGCCCTTCAA<br>CTATTCATCAAAGATTACAGGTACACGTACCGATGCCACAGAGGTGACACACAAATTCAAACGAGGAGATCATTCGAGCTGAAGAAGCTGTGGGAACAGACCCGTTCAAAGGCAGGACTGTTGGTGTCAGATGGAGGACCAAATCTATACAACATTCGGAATCTCCATATCCCAGAGGTCTGCCTGAAGTGGGAGCTGATGGACGAAGATTACCAGGGCAGGTTGTGTAATC<br>CTCTGAACCCATTTGTCAGCCATAAAGAAATTGAGTCCGTAAACAATGCTGTGGTGATGCCAGCCCACGGTCCAGCCAAAAGCATGGAATATGATGCCGTTGCGACTACACACTCATGGATTCCTAAAAGGAATCGTTCCATTCTCAATACCAGTCAAAGGGGGATTCTTGAGGATGAACAGATGTACCAGAAATGCTGCAGTCTATTCGAGAAATTCTTCCCCAGTAGTTCATA<br>CAGAAGACCAGTTGGAATTTCCAGCATGGTGGAGGCCATGGTGTCTAGGGCCCGAATCGATGCACGCATTGATTTCGAATCTGGAAGGATCAAGAAGGAAGAGTTTGCTGAGATCATGAAGATCTGTTCCACCATTGAAGAGCTCAGACGGCAAAAATAG
EPI_ISL_6761000	>A/greylag_goose/Netherlands/21037809-001/2021_H5N1_2021-10-31<br>ATGGATGTCAATCCGACTTTACTTTTCTTAAAAGTGCCAGCGCAAGATGCCATAAGTACCACATTCCCTTACACTGGAGATCCTCCATACAGCCATGGAACAGGGACAGGATACACAATGGACACAGTCAACAGAACACATCAATACTCAGAGAAGGGAAAATGGACAACAAACACAGAAACCGGAGCACCTCAACTCAACCCAATTGATGGGCCACTACCTGAGGACAACGA<br>ACCGAGCGGATATGCACAAACAGATTGCGTGTTGGAAGCAATGGCTTTCCTTGAAGAGTCCCACCCAGGGATCTTTGAAAACTCTTGTCTTGAAACGATGGAAGTCGTTCAGCAAACAAGAGTGGACAAACTAACTCAAGGTCGCCAGACATATGACTGGACACTGAATAGAAACCAACCAGCTGCAACTGCCCTGGCCAACACTATAGAGGTCTTCAGATCAAACGGTCTAA<br>CAGCCAATGAATCGGGGAGACTAATAGATTTCCTCAAGGATGTGATGGACTCAATGGATAAAGAAGAAATGGAAATAACAACACATTTCCAGAGAAAGAGAAGAGTAAGGGACAACATGACCAAGAAAATGGTCACACAAAGAACAATAGGAAAGAAGAAACAAAGGCTAAACAAGAGGAGCTACTTAATAAGAGCACTGACACTGAATACAATGACAAAAGATGCAGA<br>AAGAGGCAAATTGAAAAGACGGGCGATTGCAACACCGGGGATGCAGATTAGAGGATTTGTGTACTTTGTCGAAACACTGGCAAGGAGCATCTGTGAAAAACTTGAGCAATCTGGACTCCCCGTTGGAGGGAATGAGAAGAAGGCTAAATTGGCAAATGTCGTGAGAAAAATGATGACTAACTCACAAGATACAGAGCTCTCCTTCACAATTACTGGAGATAACACCAAATG<br>GAATGAGAATCAAAATCCTCGGATGTTTCTGGCAATGATAACATACATTACAAGAAACCAACCTGAATGGTTTAGAAATGTCTTGAGTATTGCCCCTATAATGTTCTCGAACAAAATGGCGAGATTGGGAAAAGGGTACATGTTTGAAAGTAAGAGCATGAAGTTACGGACACAAATACCTGCAGAAATGCTTGCAAACATTGACTTAAAATACTTCAATGAATCAACAAGAAA<br>GAAAATCGAAAAAATAAGGCCTCTACTAATAGATGGCACTGCCTCATTGAGTCCTGGAATGATGATGGGCATGTTCAATATGCTGAGTACAGTATTAGGAGTTTCAATCCTAAATCTTGGGCAAAAGAAGTACACCAAAACAACATACTGGTGGGATGGACTCCAATCCTCTGATGATTTCGCCCTCATAGTAAATGCACCGAATCATGAGGGAATACAAGCAGGAGTGGATA<br>GGTTCTATAGGACCTGCAAACTGGTCGGGATCAATATGAGCAAAAAGAAGTCTTACATAAACCGGACTGGAACATTTGAGTTCACAAGCTTTTTCTATCGCTATGGATTTGTGGCTAACTTCAGTATGGAGCTGCCCAGCTTTGGAGTTTCTGGGATCAATGAATCAGCTGACATGAGCATTGGCGTCACAGTGATAAAGAACAACATGATAAACAATGACCTTGGACCAGCAA<br>CAGCTCAAATGGCCCTTCAACTATTCATCAAAGATTACAGGTACACGTACCGATGCCACAGAGGTGACACACAAATTCAAACGAGGAGATCATTCGAGCTGAAGAAGCTGTGGGAACAGACCCGTTCAAAGGCAGGACTGTTGGTGTCAGATGGAGGACCAAATCTATACAACATTCGGAATCTCCATATCCCAGAGGTCTGCCTGAAGTGGGAGCTGATGGACGAAGATTA<br>CCAGGGCAGGTTGTGTAATCCTCTGAACCCATTTGTCAGCCATAGAGAAATTGAGTCCGTAAACAATGCTGTGGTGATGCCAGCCCACGGTCCAGCCAAAAGCATGGAATATGATGCCGTTGCGACTACACACTCATGGATTCCTAAAAGGAATCGTTCCATTCTCAATACCAGTCAAAGGGGAATTCTTGAGGATGAACAGATGTACCAGAAATGCTGCAGTCTATTCGAGAA<br>ATTCTTCCCCAGTAGTTCATACAGGAGACCAGTTGGAATTTCCAGCATGGTGGAGGCCATGGTGTCTAGGGCCCGAATCGATGCACGAATTGATTTCGAATCTGGAAGGATCAAGAAGGAAGAGTTTGCTGAGATCATGAAGATCTGTTCCACCATTGAAGAGCTCAGACGGCAAAAATAG

EPI_ISL_7753406	>A/greylag_goose/Germany-SH/AI06144/2021_H5N1_2021-10-25<br>[illegible]
EPI_ISL_11798578	>A/greylag_goose/Denmark/24309-1.01/2021-10-27_H5N1_2021-10-27<br>[illegible]
EPI_ISL_13026236	>A/Grey_heron/Luxembourg/21243177/2021_H5N1_2021-12-14<br>[illegible]
EPI_ISL_8515481	>A/grey_heron/Czech_Republic/23608/2021_H5N1_2021-11-28<br>[illegible]
EPI_ISL_355938	>A/green_sandpiper/Kurgan/1050/2018_H3N8_2018-08-26<br>[illegible]
EPI_ISL_243479	>A/greater_white-fronted_goose/Netherlands/6/2011_H6N8_2011-12-14<br>[illegible]
EPI_ISL_5116065	>A/greater_rhea/Germany-SN/AI02351/2021_H5N8_2021-03-02<br>[illegible]
EPI_ISL_8515483	>A/great_egret/Czech_Republic/23609/2021_H5N1_2021-11-28<br>[illegible]
EPI_ISL_5463806	>A/goose/Tyumen/33-53V/2021_H5N1_2021-10-07<br>[illegible]
EPI_ISL_11259315	>A/goose/Spain/88-3_22VIR2142-4/2022_H5N1_2022-01-10<br>[illegible]
EPI_ISL_11259316	>A/goose/Spain/141-9_22VIR2142-6/2022_H5N1_2022-01-14<br>[illegible]
EPI_ISL_626651	>A/goose/Russian_Federation/Kurgan/1345-25/2020_H5N8_2020-08-20<br>[illegible]

EPI_ISL_11922811	>A/goose/Poland/H124_22VIR2515-5/2022_H5N1_2022-02-05<br>ATGGATGTCAATCCGACTTTACTTTTCTTAAAAGTGCCAGCGCAAGATGCCATAAGTACCACATTCCCTTACACTGGAGATCCTCCATACAGCCATGGAACAGGGACAGGATACACAATGGACACAGTCAACAGAACACATCAATACTCAGAGAAGGGAAAATGGACAACAAACACAGAAACCGGAGCACCTCAACTCAACCCAATTGATGGGCCACTACCTGAGGACAACGA<br>ACCGAGCGGATATGCACAAACAGATTGCGTGTTGGAAGCAATGGCTTTCCTTGAAGAGTCCCATCCAGGGATCTTTGGAAACTCTTGTCTTGAAACGATGGAAGTCGTTCAGCAAACAAGAGTGGACAAACTAACTCAAGGTCGCCAGACATATGACTGGACACTGAATAGAAACCAACCAGCTGCAACTGCCCTGGCCAACACTATAGAGGTCTTCAGATCAAACGGTCTAA<br>CAGCCAATGAATCGGGGAGACTAATAGATTTCCTCAAGGATGTGATGGACTCAATGGATAAAGAAGAAATGGAAATAACAACACATTTCCAGAGAAAGAGAAGAGTAAGGGACAACATGACCAAGAAAATGGTCACACAAAGAACAATAGGAAAGAAGAAACAAAGGCTAAACAAGAGGAGCTACTTAATAAGAGCACTGACACTGAATACAATGACAAAAGATGCAGA<br>AAGAGGCAAATTGAAGAGACGGGCGATTGCAACACCAGGGATGCAGATTAGAGGATTTGTGTACTTTGTCGAAACACTGGCAAGGAGCATCTGTGAAAAACTTGAACAATCTGGACTCCCCGTTGGAGGGAATGAGAAGAAGGCTAAATTGGCAAATGTCGTGAGAAAAATGATGACTAACTCACAAGATACAGAGCTCTCCTTCACAATTACTGGAGATAACACCAAATG<br>GAATGAGAATCAAAATCCTCGGATGTTTCTGGCAATGATAACATACATTACAAGAAACCAACCTGAATGGTTTAGGAATGTCTTGAGTATTGCCCCTATAATGTTCTCGAACAAAATGGCGAGATTGGGAAAAGGGTACATGTTTGAAAGTAAGAGCATGAAGTTACGGACACAAATACCTGCAGAAATGCTTGCAAACATTGACTTAAAATACTTCAATGAATCAACAAGAAA<br>GAAAATCGAAAAAATAAGGCCTCTACTAATAGATGGCACTGCCTCATTGAGTCCTGGAATGATGATGGGCATGTTCAATATGCTGAGTACAGTATTAGGAGTTTCAATCCTAAATCTTGGGCAAAAGAAGTACACCAAAACCACATACTGGTGGGATGGACTCCAATCCTCTGATGATTTCGCCCTCATAGTAAATGCACCGAATCATGAGGGAATACAAGCAGGAGTGGATA<br>GGTTCTATAGGACCTGCAAACTGGTCGGGATCAATATGAGCAAAAAGAAGTCTTACATAAACCGGACTGGAACATTTGAGTTCACAAGCTTTTTCTATCGCTATGGATTTGTGGCTAACTTCAGTATGGAGCTGCCCAGCTTTGGAGTTTCTGGGATCAATGAATCAGCTGACATGAGCATTGGCATCACAGTGATAAAGAACAACATGATAAACAATGACCTTGGACCAGCAA<br>CAGCTCAAATGGCCCTTCAACTATTCATCAAAGATTACAGGTACACGTACCGATGCCATAGAGGTGACACACAAATTCAAACGAGGAGATCATTCGAGCTGAAGAAGCTGTGGGAACAGACCCGTTCAAAGGCAGGGCTGTTGGTGTCAGATGGAGGACCAAATCTATACAACATTCGGAATCTCCATATCCCTGAGGTCTGCCTGAAGTGGGAACTGATGGACGAAGATTAC<br>CAGGGCAGGTTGTGCAATCCTCTGAACCCATTTGTCAGCCATAAAGAAATTGAGTCCGTAAACAATGCTGTGGTGATGCCAGCCCACGGTCCAGCCAAAAGCATGGAATATGATGCCGTTGCGACTACACACTCATGGATTCCTAAAAGGAATCGTTCCATTCTCAATACCAGTCAAAGGGGAATTCTTGAGGATGAACAGATGTACCAGAAATGCTGCGGTCTATTCGAGAAA<br>TTCTTCCCCAGTAGTTCATACAGGAGACCAGTTGGAATTTCCAGCATGGTGGAGGCCATGGTGTCTAGGGCCCGAATCGATGCACGCATTGATTTCGAATCTGGAAGGATCAAGAAGGAAGAGTTTGCTGAGATCATGAAGATCTGTTCCACCATTGAAGAGCTCAGACGGCAAAAATAG
EPI_ISL_3102063	>A/goose/Poland/H1044_21RS1385-4/2021_H5N8_2021-05-05<br>ATGGATGTCAATCCGACTTTGCTTTTCTTAAAAGTGCCAGCGCAAAATGCTATAAGTACTACATTCCCTTACACTGGAGATCCCCCATACAGCCATGGAACAGGAACAGGGTATACCATGGACACAGTAAACAGAACACATCAATATTCAGAAAAGGGAAAGTGGACAACAAACACAGAAACCGGAGCACCCCAACTCAACCCAATTGATGGACCATTACCAGAGGACAATGA<br>GCCAAGCGGATATGCACAAACTGATTGCGTGTTGGAAGCAATGGCTTTCCTTGAAGAATCCCACCCAGGGATATTTGAAAACTCTTGTCTTGAAGCGATGGAAATCGTTCAGCAAACAAGAGTGGACAAACTAACCCAAGGTCGCCAGACTTATGACTGGACACTGAACAGAAACCAACCAGCTGCAACCCTCTTTGGCCAACACTATAGAGGTGTTCAGATCGAATGGTCTGA<br>CAGCCAATGAATCAGGGAGACTGATAGATTTTCTCAGGGATGTGATGGAATCAATGGATAAAGAAGAGATGGAAGTAACAACACATTTCCAGAGAAAAAGAAGAGTGAGGGACAACATGACTAAGAAGATGGTCACACAAAGAACAATAGGGAAGAAGAAGCAGAGGCTGAACAAGAGGAGTTACTTAATAAGAGCACTGACATTGAATACAATGACCAAAGATGCAGA<br>AAGAGGCAAGTTGAAGAGACGGGCAATTGCAACACCCGGGATGCAGATTAGAGGATTCGTGTACTTCGTTGAAACACTAGCGAGGAGCATCTGTGAGAAACTAGAGCAATCTGGGCTCCCTGTTGGAGGGAATGAGAAAAAGGCTAAATTGGCAAATGTTGTGAGAAAAATGATGACTAACTCACAAGATACAGAGCTCTCCTTTACAATTACTGGAGACAACACCAAATG<br>GAATGAGAATCAAAACCCTCGGATGTTTTTGGCAATGATAACATATATCACAAGAAACCAACCTGAATGGTTTAGAAATGTCTTAAGCATTGCCCCTATAATGTTCTCAAACAAAATGGCGAGATTAGGGAAAGGATACATGTTTGAAAGTAAGAGCATGAAGCTAAGAACACAAATACCAGCAGAGATGCTTACAAATATTGATCTGAAGTATTTCAACGAACCAACGAGAA<br>AGAAAATCGAGAATATAAGACCTCTGCTGATTGATGGCACGGCTTCATTGAGCCCTGGGATGATGATGGGCATGTTCAATATGCTGAGCACAGTATTAGGGGTCTCAATCCTGAATCTCGGGCAAAAGAGGTACACCAAAACCACATACTGGTGGGATGGACTTCAATCCTCTGATGATTTCGCTCTCATAGTGAATGCACCGAATCATGAGGGGATACAAGCAGGAGTGGAT<br>AGATTCTATAGGACCTGCAAACTGGTTGGRATCAACATGAGCAAAAAGAAGTCTTACATAAACCGAACAGGAACATTTGAGTTCACAAGTTTTTTCTACCGCTATGGATTTGTAGCCAACTTCAGTATGGAATTACCCAGCTTTGGAGTGTCTGGAATCAATGAATCAGCTGACATGAGCATTGGAGTTACAGTGATAAAAAACAATATGATAAACAATGATCTTGGACCAGCAA<br>CAGCTCAAATGGCTCTTCAGTTATTCATCAAAGACTATAGGTACACATACCGATGCCACAGGGGTGATACACAAATTCAAACGAGGAGATCATTCGAGCTGAAGAAGCTGTGGGAGCAGACCCGTTCAAAGGCAGGACTGTTGATATCAGACGGGGGGCCAAACCTATACAACATTCGGAATCTCCACATCCCAGAGGTCTGCTTGAAGTGGGAGCTGATGGATGAAGACTA<br>CCAAGGCAGGCTGTGCAATCCTCTGAATCCATTTGTCAGTCATAAAGAGATTGAGTCCGTAAACAATGCTGTAGTAATGCCCGCCCATGGCCCGGCCAAGAGCATGGAATATGATGCTGTTGCGACCACACACTCGTGGATTCCTAAGAGGAACCGTTCCATCCTCAATACCAGCCAAAGGGGAATTCTTGAGGATGAGCAGATGTACCAGAAGTGCTGTAGTCTATTCGAGA<br>AATTCTTCCCCAGCAGTTCATACAGGAGGCCAGTTGGAATTTCCAGCATGGTGGAGGCCATGGTGTCTAGGGCCCGAATTGATGCACGCATCGACTTCGAATCTGGAAGGATTAAGAAAGAAGAGCTTGCTGAGATCATGAAGATCTGTTCCACCATTGAAGAGCTCAGACGGCAAAAATAG
EPI_ISL_3102065	>A/goose/Poland/H1036_21RS1385-6/2021_H5N8_2021-05-03<br>ATGGATGTCAATCCGACTTTGCTTTTCTTAAAAGTGCCAGCGCAAAATGCTATAAGTACTACATTCCCTTACACTGGAGATCCCCCATACAGCCATGGAACAGGAACAGGGTATACCATGGACACAGTAAACAGAACACATCAATATTCAGAAAAGGGAAAGTGGACAACAAACACAGAAACCGGAGCACCCCAACTCAACCCAATTGATGGACCATTACCAGAGGACAATGA<br>GCCAAGCGGATATGCACAAACTGATTGCGTGTTGGAAGCAATGGCTTTCCTTGAAGAATCCCACCCAGGGATATTTGAAAACTCTTGTCTTGAAGCGATGGAAATCGTTCAGCAAACAAGAGTGGACAAACTAACCCAAGGTCGCCAGACTTATGACTGGACACTGAACAGAAACCAACCAGCTGCAACCCTCTTTGGCCAACACTATAGAGGTGTTCAGATCGAATGGTCTGA<br>CAGCCAATGAATCAGGGAGACTGATAGATTTTCTCAGGGATGTGATGGAATCAATGGATAAAGAAGAGATGGAAGTAACAACACATTTCCAGAGAAAAAGAAGAGTGAGGGACAACATGACTAAGAAGATGGTCACACAAAGAACAATAGGGAAGAAGAAGCAGAGGCTGAACAAGAGGAGTTACTTAATAAGAGCACTGACATTGAATACAATGACCAAAGATGCAGA<br>AAGAGGCAAGTTGAAGAGACGGGCAATTGCAACACCCGGGATGCAGATTAGAGGATTCGTGTACTTCGTTGAAACACTAGCGAGGAGCATCTGTGAGAAACTAGAGCAATCTGGGCTCCCTGTTGGAGGGAATGAGAAAAAGGCTAAATTGGCAAATGTTGTGAGAAAAATGATGACTAACTCACAAGATACAGAGCTCTCCTTTACAATTACTGGAGACAACACCAAATG<br>GAATGAGAATCAAAACCCTCGGATGTTTTTGGCAATGATAACATATATCACAAGAAACCAACCTGAATGGTTTAGAAATGTCTTAAGCATTGCCCCTATAATGTTCTCAAACAAAATGGCGAGATTAGGGAAAGGATACATGTTTGAAAGTAAGAGCATGAAGCTAAGAACACAAATACCAGCAGAGATGCTTACAAATATTGATCTGAAGTATTTCAACGAACCAACGAGAA<br>AGAAAATCGAGAATATAAGACCTCTGCTGATTGATGGCACGGCTTCATTGAGCCCTGGGATGATGATGGGCATGTTCAATATGCTGAGCACAGTATTAGGGGTCTCAATCCTGAATCTCGGGCAAAAGAGATACACCAAAACCACATACTGGTGGGATGGACTTCAATCCTCTGATGATTTCGCTCTCATAGTGAATGCACCGAATCATGAGGGGATACAAGCAGGAGTGGAT<br>AGATTCTATAGGACCTGCAAACTGGTTGGGATCAACATGAGCAAAAAGAAGTCTTACATAAACCGAACAGGAACATTTGAGTTCACAAGTTTTTTCTACCGCTATGGATTTGTAGCCAACTTCAGTATGGAATTACCCAGCTTTGGAGTGTCTGGAATCAATGAATCAGCTGACATGAGCATTGGAGTTACAGTGATAAAAAACAATATGATAAACAATGATCTTGGACCAGCA<br>ACAGCTCAAATGGCTCTTCAGTTATTCATCAAAGACTATAGGTACACATACCGATGCCACAGGGGTGATACACAAATTCAAACGAGGAGATCATTCGAGCTGAAGAAGCTGTGGGAGCAGACCCGTTCAAAGGCAGGACTGTTGATATCAGACGGGGGGCCAAACCTATACAACATTCGGAATCTCCACATCCCAGAGGTCTGCTTGAAGTGGGAGCTGATGGATGAAGACT<br>ACCAAGGCAGGCTGTGCAATCCTCTGAATCCATTTGTCAGTCATAAAGAGATTGAGTCCGTAAACAATGCTGTAGTAATGCCCGCCCATGGCCCGGCCAAGAGCATGGAATATGATGCTGTTGCGACCACACACTCGTGGATTCCTAAGAGGAACCGTTCCATCCTCAATACCAGCCAAAGGGGAATTCTTGAGGATGAGCAGATGTACCAGAAGTGCTGTAGTCTATTCGAG<br>AAATTCTTCCCCAGCAGTTCATACAGGAGGCCAGTTGGAATTTCCAGCATGGTGGAGGCCATGGTGTCTAGGGCCCGAATTGATGCACGCATCGACTTCGAATCTGGAAGGATTAAGAAAGAAGAGTTTGCTGAGATCATGAAGATCTGTTCCACCATTGAAGAGCTCAGACGGCAAAAATAG
EPI_ISL_644152	>A/goose/Omsk/01161/2020_H5N8_2020-08-17<br>ATGGATGTCAATCCGACTTTACTTTTCTTAAAAGTGCCAGCGCAAAATGCTATAAGTACTACATTCCCTTACACTGGAGATCCCCCATACAGCCATGGAACAGGAACAGGGTATACCATGGACACAGTAAACAGAACACATCAATACTCAGAAAAGGGAAAGTGGACAACAAACACAGAAACCGGAGCACCCCAACTCAACCCAATTGATGGACCATTACCAGAGGACAATGA<br>GCCAAGCGGATATGCACAAACTGATTGCGTGTTGGAAGCAATGGCTTTCCTTGAAGAATCCCACCCAGGGATATTTGAAAACTCTTGTCTTGAAGCGATGGAAATCGTTCAGCAAACAAGAGTGGACAAACTAACCCAAGGTCGCCAGACTTATGACTGGACACTGAACAGAAACCAACCAGCTGCAACCCTCTTTGGCCAACACTATAGAGGTGTTCAGATCGAATGGTCTGA<br>CAGCCAATGAATCAGGGAGACTGATAGATTTTCTCAGGGATGTGATGGAATCAATGGATAAAGAAGAGATGGAAGTAACAACACATTTCCAGAGAAAAAGAAGAGTGAGGGACAACATGACTAAGAAGATGGTCACACAAAGAACAATAGGGAAGAAGAAGCAGAGGCTGAACAAGAGGAGTTACTTAATAAGAGCACTGACATTGAATACAATGACCAAAGATGCAGA<br>AAGAGGCAAGTTGAAGAGACGGGCAATTGCAACACCCGGGATGCAGATTAGAGGGTTCGTGTACTTCGTTGAAACACTAGCGAGGAGCATCTGTGAGAAACTAGAGCAATCTGGGCTCCCTGTTGGAGGGAATGAGAAGAAGGCTAAATTGGCAAATGTTGTGAGAAAAATGATGACTAACTCACAAGATACAGAGCTCTCCTTTACAATTACTGGAGACAACACCAAATG<br>GAATGAGAATCAAAACCCTCGGATGTTTTTGGCAATGATAACATATATCACAAGAAACCAACCTGAATGGTTTAGAAATGTCTTAAGCATTGCCCCTATAATGTTCTCAAACAAAATGGCGAGATTAGGGAAAGGATACATGTTTGAAAGTAAGAGCATGAAGCTAAGAACACAAATACCAGCAGAGATGCTTACAAATATTGATCTGAAGTATTTCAACGAACCAACGAGAA<br>AGAAAATCGAGAAAATAAGACCTCTGCTGATTGATGGCACGGCCTCATTGAGTCCTGGGATGATGATGGGCATGTTCAATATGCTGAGCACAGTATTAGGGGTCTCAATCCTGAATCTCGGGCAAAAGAGGTACACCAAAACCACATACTGGTGGGATGGACTTCAATCCTCTGATGATTTCGCTCTCATAGTGAATGCACCGAATCATGAGGGGATACAAGCAGGAGTGGAT<br>AGATTCTATAGGACCTGCAAACTGGTTGGGATCAACATGAGCAAAAAGAAGTCTTACATAAACCGAACAGGAACATTTGAGTTCACAAGTTTTTTCTACCGCTATGGATTTGTAGCTAACTTCAGTATGGAATTACCCAGCTTTGGAGTGTCTGGAATCAATGAATCAGCTGACATGAGCATTGGAGTTACAGTGATAAAAAACAATATGATAAACAATGATCTTGGACCAGCA<br>ACAGCTCAAATGGCTCTTCAGTTATTCATCAAAGACTATAGGTACACATACCGATGCCACAGGGGTGATACACAAATTCAAACGAGGAGATCATTCGAGCTGAAGAAGCTGTGGGAGCAGACCCGTTCAAAGGCAGGGCTGTTGATATCAGACGGGGGGCCAAACCTATACAACATTCGGAATCTCCACATCCCAGAGGTCTGCTTGAAGTGGGAGCTGATGGATGAAGACT<br>ACCAAGGCAGGCTGTGCAATCCTCTGAATCCATTTGTCAGTCATAAAGAGATTGAGTCCGTAAACAATGCTGTAGTAATGCCCGCCCATGGCCCGGCCAAGAGCATGGAATATGATGCTGTTGCGACCACACACTCGTGGATTCCTAAGAGGAACCGTTCCATTCTCAATACCAGCCAAAGGGGAATTCTTGAGGATGAGCAGATGTACCAAAAGTGCTGTAGTCTATTCGAGA<br>AATTCTTCCCCAGCAGTTCATACAGGAGGCCAGTTGGAATTTCCAGCATGGTGGAGGCCATGGTGTCTAGGGCCCGAATTGATGCACGCATCGACTTCGAATCTGGAAGGATTAAGAAAGAAGAGTTTGCTGAGATCATGAAGATCTGTTCCACCATTGAAGAGCTCAGACGGCAAAAATAG
EPI_ISL_644127	>A/goose/Omsk/0113/2020_H5N8_2020-08-17<br>ATGGATGTCAATCCGACTTTACTTTTCTTAAAAGTGCCAGCGCAAAATGCTATAAGTACTACATTCCCTTACACTGGAGATCCTCCATACAGCCATGGAACAGGAACAGGGTATACCATGGACACAGTAAACAGAACACATCAATACTCAGAAAAGGGAAAGTGGACAACAAACACAGAAACCGGAGCACCCCAACTCAACCCAATTGATGGACCATTACCAGAGGACAATGA<br>GCCAAGCGGATATGCACAAACTGATTGCGTGTTGGAAGCAATGGCTTTCCTTGAAGAATCCCACCCAGGGATATTTGAAAACTCTTGTCTTGAAGCGATGGAAATCGTTCAGCAAACAAGAGTGGACAAACTAACCCAAGGTCGCCAGACTTATGACTGGACACTGAACAGAAACCAACCAGCTGCAACCCTCTTTGGCCAACACTATAGAGGTGTTCAGATCGAATGGTCTGA<br>CAGCCAATGAATCAGGGAGACTGATAGATTTTCTCAGGGATGTGATGGAATCAATGGATAAAGAAGAGATGGAAGTAACAACACATTTCCAGAGAAAAAGAAGAGTGAGGGACAACATGACTAAGAAGATGGTCACACAAAGAACAATAGGGAAGAAGAAGCAGAGGCTGAACAAGAGGAGTTACTTAATAAGAGCACTGACATTGAATACAATGACCAAAGATGCAGA<br>AAGAGGCAAGTTGAAGAGACGGGCAATTGCAACACCCGGGATGCAGATTAGAGGATTCGTGTACTTCGTTGAAACACTAGCGAGGAGCATCTGTGAGAAACTAGAGCAATCTGGGCTCCCTGTTGGAGGGAATGAGAAGAAGGCTAAATTGGCAAATGTTGTGAGAAAAATGATGACTAACTCACAAGATACAGAGCTCTCCTTTACAATTACTGGAGACAACACCAAATG<br>GAATGAGAATCAAAACCCTCGGATGTTTTTGGCAATGATAACATATATCACAAGAAACCAACCTGAATGGTTTAGAAATGTCTTAAGCATTGCCCCTATAATGTTCTCAAACAAAATGGCGAGATTAGGGAAAGGATACATGTTTGAAAGTAAGAGCATGAAGCTAAGAACACAAATACCAGCAGAGATGCTTACAAATATTGATCTGAAGTATTTCAACGAACCAACGAGAA<br>AGAAAATCGAGAAAATAAGACCTCTGCTGATTGATGGCACGGCCTCATTGAGTCCTGGGATGATGATGGGCATGTTCAATATGCTGAGCACAGTATTGGGGGTCTCAATCCTGAATCTCGGGCAAAAGAGGTACACCAAAACCACATACTGGTGGGATGGACTTCAATCCTCTGATGATTTCGCTCTCATAGTGAATGCACCGAATCATGAGGGGATACAAGCAGGAGTGGAT<br>AGATTCTATAGGACCTGCAAACTGGTTGGGATCAACATGAGCAAAAAGAAGTCTTACATAAACCGAACAGGAACATTTGAGTTCACAAGTTTTTTCTACCGCTATGGATTTGTAGCTAACTTCAGTATGGAATTACCCAGCTTTGGAGTGTCTGGAATCAATGAATCAGCTGACATGAGCATTGGAGTTACAGTGATAAAAAACAATATGATAAACAATGATCTTGGACCAGCA<br>ACAGCTCAAATGGCTCTTCAGTTATTCATCAAAGACTATAGGTACACATACCGATGCCACAGGGGTGATACACAAATTCAAACGAGGAGATCATTCGAGCTGAAGAAGCTGTGGGAGCAGACCCGTTCAAAGGCAGGGCTGTTGATATCAGACGGGGGGCCAAACCTATACAACATTCGGAACCTCCACATCCCAGAGGTCTGCTTGAAGTGGGAGCTGATGGATGAAGACT<br>ACCAAGGCAGGCTGTGCAATCCTCTGAATCCATTTGTCAGTCATAAAGAGATTGAGTCCGTAAACAATGCTGTAGTAATGCCCGCCCATGGCCCGGCCAAGAGCATGGAATATGATGCTGTTGCGACCACACATTCGTGGATCCCTAAGAGGAACCGTTCCATTCTCAATACCAGCCAAAGGGGAATTCTTGAGGATGAGCAGATGTACCAAAAGTGCTGTAGTCTATTCGAGA<br>AATTCTTCCCCAGCAGTTCATACAGGAGGCCAGTTGGAATTTCCAGCATGGTGGAGGCCATGGTGTCTAGGGCCCGAATTGATGCACGCATCGACTTCGAATCTGGAAGGATTAAGAAAGAAGAGTTTGCTGAGATCATGAAGATCTGTTCCACCATTGAAGAGCTCAGACGGCAAAAATAG
EPI_ISL_255174	>A/Goose/Hungary/65817/2016_H5N8_2016-12-18<br>ATGGATGTCAATCCGACTTTACTTTTCTTAAAAGTGCCAGCGCAAAATGCCATAAGTACTACGTTTCCTTATACTGGAGATCCTCCATACAGCCATGGAACAGGAACAGGATACACCATGGACACAGTCAACAGAACACATCAATACTCAGAAAAGGGGAAATGGACAACAAACACAGAGACCGGAGCACCCCAACTCAACCCAATTGATGGACCACTGCCTGAGGACAACGA<br>ACCAAGCGGATATGCACAAACAGATTGTGTGTTGGAAGCAATGGCTTTCCTTGAAGAGTCCCACCCAGGGATCTTTGAAAACTCTTGTCTTGAAACGATGGAAGTTGTTCAGCAAACAAGAGTGGACAAACTAACTCAAGGTCGCCAGACTTATGACTGGACACTGAATAGAAACCAACCAGCTGCAACTGCCCTGGCCAACACTATAGAGGTCTTCAGATCGAACGGTCTGA<br>CAGCCAATGAATCGGGGAGGCTAATAGATTTCCTCAAGGATGTGATGGACTCAATGGACAAAGAAGAAATGGAAATAACAACACATTTCCAGAGAAAGAGAAGAGTAAGGGACAACATGACCAAGAAAATGGTCACACAAAGAACAATAGGGAAAAAGAAACAAAGGCTGAATAAGAAGAGCTACTTAATAAGGGCACTGACACTGAACACAATGACAAAAGATGCAGA<br>AAGAGGCAAATTGAAGAGGCGGGCAATTGCAACACCAGGGATGCAGATTAGAGGATTTGTGTACTTTGTCGAAACACTGGCAAGGAGCATCTGTGAGAAACTTGAGCAATCTGGACTCCCCGTTGGAGGGAATGAGAAAAAGGCTAAATTGGCAAATGTCGTGAGAAAAATGATGACCAACTCACAAGATACAGAGCTCTCTTTCACAATTACTGGAGACAACACCAAATG<br>GAATGAGAATCAAAATCCTCGGATGTTTCTGGCAATGATAACATACATCACAAGAAACCAACCTGAATGGTTTAGAAATGTCTTGAGCATTGCCCCTATAATGTTCTCAAACAAAATGGCGAGATTGGGAAAAGGATATATGTTTGAAAGTAAGAGCATGAAGCTACGGACACAAATACCTGCAGAAATGCTTGCAAATATTGACTTAAAATATTTCAATGAATCAACAAGAAA<br>GAAAATCGAGAAAATAAGACCTCTGCTAATAGATGGCACAGCCTCATTGAGTCCTGGAATGATGATGGGCATGTTCAATATGCTGAGTACAGTATTAGGAGTCTCAATCCTGAATCTTGGGCAAAAGAGGTACACCAAAACCACATACTGGTGGGATGGACTCCAATCTTCTGATGATTTCGCCCTCATAGTGAATGCACCGAATCATGAGGGAATACAAGCAGGAGTTGATA<br>GGTTCTATAGGACATGCAAACTGGTTGGAATCAACATGAGCAAAAAGAAGTCTTACATAAACAGGACAGGAACATTTGAGTTCACAAGCTTTTTCTATCGCTATGGATTTGTGGCTAATTTCAGTATGGAGCTGCCCAGCTTTGGAGTGTCTGGAATCAATGAATCAGCTGACATGAGCATTGGAGTTACAGTGATAAAGAATAACATGATAAACAATGACCTTGGACCAGCAA<br>CAGCTCAGATGGCTCTTCAGCTATTCATCAAGGACTACAGGTACACGTACCGATGCCACAGGGGTGACACACAAATTCAAACGAGGAGATCATTCGAGCTGAAGAAGCTGTGGGAGCAGACTCGTTCAAAGGCAGGACTGTTGGTGTCGGATGGAGGACCAAACCTATACAACATTCGGAATCTCCACATCCCAGAGGTCTGCTTGAAGTGGGAGCTGATGGACGAAGATTA<br>CCAGGGCAGGTTGTGTAATCCTCTGAACCCATTTATCAGTCATAAGGAAATTGAGTCCGTAAACAATGCTGTGGTGATGCCGACCCATGGCCCAGCCAAGAGCATGGAATATGATGCTGTTGCGACTACACACTCATGGATTCCTAAGAGGAATCGTTCCATTCTCAATACCAGCCAAAGGGGAATTCTTGAGGATGAGCAGATGTACCAGAAGTGCTGTAGTCTATTCGAGAA<br>ATTCTTCCCCAGTAGTTCATACAGGAGGCCAGTTGGAATTTCCAGCATGGTAGAGGCCATGGTATCTAGGGCCCGAATTGATGCACGCATTGATTTCGAATCTGGAAGGATTAAGAAAGAAGAGTTTGCTGAGATCATGAAGATCTGTTCCACCATTGAAGAGCTCAGACGGCAAAAATAG
EPI_ISL_9377021	>A/goose/France/21P014207/2021_H5N1_2021-12-23<br>ATGGATGTCAATCCGACTTTACTTTTCTTAAAAGTGCCAGCGCAAGATGCCATAAGTACCACATTCCCTTACACTGGAGATCCTCCATACAGCCATGGAACAGGGACAGGATACACAATGGACACAGTCAACAGAACACATCAATACTCAGAGAAGGGAAAATGGACAACAAACACAGAAACCGGAGCACCCCAACTCAACCCAATTGATGGGCCACTACCTGAGGACAACGA<br>ACCGAGCGGATATGCACAAACAGATTGCGTGTTGGAAGCAATGGCTTTCCTTGAAGAGTCCCACCCAGGGATCTTTGAAAACTCTTGTCTTGAAACGATGGAAGTCGTTCAGCAAACAAGAGTGGACAAACTAACTCAAGGTCGCCAGACATATGACTGGACACTGAATAGAAACCAACCAGCTGCAACTGCCCTGGCCAACACTATAGAGGTCTTCAGATCAAATGGTCTAA<br>CAGCCAATGAATCGGGGAGACTAATAGATTTCCTCAAGGATGTGATGGACTCAATGGATAAAGAAGAAATGGAAATAACAACACATTTCCAGAGAAAGAGAAGAGTAAGGGACAACATGACCAAGAAAATGGTCACACAAAGAACAATAGGAAAGAAGAAACAAAGGCTAAACAAGAGGAGCTACTTAATAAGAGCACTGACACTGAATACAATGACAAAAGATGCAGA<br>AAGAGGCAAATTGAAAAGACGGGCGATTGCAACACCAGGGATGCAGATTAGAGGATTTGTATACTTTGTCGAAACACTGGCAAGGAGCATCTGTGAAAAACTTGAGCAATCTGGACTCCCCGTTGGAGGGAATGAGAAGAAGGCTAAATTGGCAAATGTCGTGAGAAAAATGATGACTAACTCACAAGATACAGATCTCTCCTTCACAATTACTGGAGATAACACCAAATGG<br>AATGAGAATCAAAATCCTCGGATGTTTCTGGCAATGATAACATACATTACAAGAAACCAACCTGAATGGTTTAGAAATGTCTTGAGTATTGCCCCTATAATGTTCTCGAACAAAATGGCAAGATTGGGAAAAGGGTACATGTTTGAAAGTAAGAGCATGAAGTTACGGACACAAATACCTGCAGAAATGCTTGCAAACATTGACTTAAAATACTTCAATGAATCAACAAGAAAG<br>AAAATCGAAAAAATAAGGCCTCTACTAATAGATGGCACCGCCTCATTGAGTCCTGGAATGATGATGGGCATGTTCAATATGCTGAGTACAGTATTAGGAGTTTCAATCCTAAATCTTGGGCAAAAGAAGTACACCAAAACCACATACTGGTGGGATGGACTCCAATCCTCTGATGATTTCGCCCTCATAGTAAATGCACCGAATCATGAGGGAATACAAGCAGGAGTGGATAG<br>GTTCTATAGGACCTGCAAACTGGTCGGGATCAATATGAGCAAAAAGAAGTCTTACATAAACCGGACTGGAACATTTGAGTTCACAAGCTTTTTCTATCGCTATGGATTTGTGGCTAACTTCAGTATGGAGCTGCCCAGCTTTGGAGTTTCTGGGATCAATGAATCAGCTGACATGAGCATTGGCGTCACAGTGATAAAGAACAACATGATAAACAATGACCTTGGACCAGCAAC<br>AGCTCAAATGGCCCTTCAACTATTCATCAAAGATTACAGGTACACGTACCGATGCCACAGAGGTGACACACAAATTCAAACGAGGAGATCATTCGAGCTGAAGAAGCTGTGGGAACAGACCCGTTCAAAGGCAGGACTGTTGGTGTCAGATGGAGGACCAAATCTATACAACATTCGGAATCTCCATATCCCTGAGGTCTGCCTGAAGTGGGAGCTGATGGACGAAGATTAC<br>CAGGGCAGGTTGTGTAATCCTCTGAACCCATTTGTCAGCCATAGAGAAATTGAGTCCGTAAACAATGCTGTGGTGATGCCAGCCCACGGTCCAGCCAAAAGCATGGAATATGATGCCGTTGCGACTACACACTCATGGATCCCTAAAAGGAATCGTTCCATTCTCAATACCAGTCAAAGGGGAATTCTTGAGGATGAACAGATGTACCAGAAATGCTGCAGTCTATTCGAGAAA<br>TTCTTCCCCAGTAGTTCATACAGGAGACCAGTTGGAATTTCCAGCATGGTGGAGGCCATGGTGTCTAGGGCCCAAATCGATGCACGCATTGATTTCGAATCTGGAAGGATCAAGAAGGAAGAGTTTGCTGAGATCATGAAGATCTGTTCCACCATTGAAGAGCTCAGACGGCAAAAATAG
EPI_ISL_503509	>A/garganey/Bangladesh/38920/2019_H7N4_2019-02-07<br>ATGGATGTCAATCCGACTTTACTTTTCTTAAAAGTGCCAGCGCAAAATGCTATAAGTACTACATTTCCTTACACTGGAGATCCTCCATACAGCCATGGAACAGGAACAGGATACACCATGGACACAGTCAACAGAACACATCAATACTCAGAGAAGGGAAAGTGGACAACAAACACAGAGACTGGGGCGCCCCAACTCAACCCAATTGATGGACCATTGCCAGAGGACAACGA<br>GCCAAGCGGATATGCACAAACAGATTGCGTGTTGGAAGCAATGGCTTTCCTTGAAGAGTCCCACCCAGGGATCTTTGAAAACTCTTGTCTTGAAACGATGGAGGTCGTTCAGCAAACAAGAGTGGACAAACTAACCCAAGGTCGCCAGACTTATGATTGGACACTGAATAGAAACCAGCCAGCTGCAACTGCTTTGGCCAATACTATAGAGGTCTTCAGATCGAACGGTCTAA<br>CAGCCAATGAGTCGGGGAGACTAATAGATTTCCTCAAGGATGTGATGGAATCAATGGATAAAGAAGAAATGGAAATAACAACACATTTCCAGAGAAAGAGAAGAGTAAGGGACAACATGACCAAGAAGATGGTCACACAAAGAACGATAGGAAAGAAGAAGCAGAGGCTGAACAAGAGGAGCTACTTAATAAGAGCACTGACATTGAACACAATGACCAAAGATGCAGA<br>AAGAGGCAAATTGAAGAGGCGGGCAATTGCAACACCCGGGATGCAGATTAGAGGATTTGTGTACTTTGTCGAAACACTAGCGAGGAGCATCTGTGAAAAACTCGAGCAATCTGGACTCCCCGTTGGAGGGAATGAGAAAAAGGCCAAATTGGCAAATGTCGTGAGAAAAATGATGACTAACTCGCAAGATACAGAGCTCTCCTTTACAATTACTGGAGACAACACCAAATG<br>GAATGAGAATCAAAATCCTCGGATGTTTTTGGCAATGATAACATACATCACAAGAAACCAACCCGAATGGTTTAGAAATGTCTTGAGCATTGCCCCTATAATGTTCTCAAACAAAATGGCAAGATTAGGAAAAGGATATATGTTTGAAAGTAAAAGCATGAAGCTAAGGACACAAATACCGGCAGAAATGCTTGCAAACATTGACTTGAAATACTTCAATGAATCAACGAGAA<br>AGAAAATCGAGAAAATAAGACCTCTGCTAATTGATGGCACAGCCTCATTGAGTCCTGGAATGATGATGGGCATGTTCAATATGCTGAGCACAGTATTGGGGGTCTCAATCCTGAATCTTGGACAAAAGAGGTACACCAAAACCACATACTGGTGGGATGGACTCCAATCCTCTGATGATTTCGCTCTCATAGTGAATGCACCGAATCATGAGGGGATACAAGCAGGGGTGGAT<br>AGGTTCTATAGGACCTGCAAACTGGTTGGGATCAACATGAGCAAAAAGAAGTCTTACATAAACCGGACAGGAACATTTGAGTTCACAAGCTTTTTCTACCGCTATGGATTTGTAGCTAACTTCAGTATGGAGTTACCCAGCTTTGGAGTGTCTGGAATCAATGAGTCAGCTGACATGAGCATTGGAGTTACTGTGATAAAGAACAATATGATAAACAATGACCTTGGACCAGCA<br>ACAGCTCAAATGGCCCTTCAGCTATTCATCAAGGATTATAGGTACACGTACCGATGTCACAGAGGTGATACACAAATTCAAACGAGGAGATCATTCGAGCTGAAGAAGCTGTGGGAGCAGACCCGTTCAAAGGCAGGGCTGTTGGTATCAGATGGAGGACCAAACCTATACAACATTCGGAATCTTCACATCCCAGAGGTCTGCTTGAAGTGGGAGCTAATGGATGAAGACT<br>ACCAGGGCAGGCTGTGTAATCCTCTGAATCCGTTTGTCAGTCATAAGGAAATTGAGTCCGTAAACAATGCTGTGGTAATGCCAGCCCATGGCCCAGCCAAGAGCATGGAATATGATGCTGTTGCGACTACACACTCATGGATTCCCAAGAGGAACCGTTCCATTCTCAATACCAGCCAGAGGGGAATTCTCGAGGATGAGCAGATGTACCAGAAGTGCTGCAGTTTATTCGAG<br>AAATTCTTCCCCAGTAGTTCGTACAGGAGACCAGTTGGAATTTCCAGCATGGTGGAGGCCATGGTGTCCAGGGCCCGAATTGATGCACGCATTGATTTCGAATCTGGAAGGATTAAGAAAGAAGAGTTTGCTGAGATCATGAAGATCTGTTCCACCATTGAAGAGCTCAGACGGCAAAAATAG
EPI_ISL_400283	>A/Gadwall/Omsk_Region/129/2019_H3N8_2019-09-01<br>ATGGATGTCAATCCGACTTTACTTTTCTTAAAAGTGCCAGCACAAAATGCCATAAGTACTACGTTTCCTTATACTGGAGATCCTCCATACAGCCATGGAACAGGAACAGGATACACCATGGACACAGTCAACAGAACACATCAATACTCAGAAAAGGGGAAATGGACAACAAACACAGAGACCGGAGCACCCCAGCTCAACCCAATTGATGGACCACTACCTGAGGACAACGA<br>ACCAAGCGGATATGCACAAACAGATTGTGTGTTGGAAGCAATGGCTTTCCTTGAAGAGTCCCACCCAGGGATCTTTGAAAACTCTTGTCTTGAAACGATGGAAGTTGTTCAGCAAACAAGAGTGGACAAACTAACTCAAGGTCGCCAGACTTATGACTGGACACTGAATAGAAATCAACCAGCTGCAACTGCCCTGGCCAACACTATAGAGGTCTTCAGATCGAACGGTCTGA<br>CAGCCAATGAATCGGGGAGACTAATAGATTTCCTCAAGGATGTGATGGACTCAATGGACAAAGAAGAAATGGAAATAACAACACATTTCCAGAGAAAGAGAAGAGTAAGGGACAACATGACCAAGAAAATGGTCACACAAAGGACAATAGGGAAAAAGAAACAAAGGCTGAATAAGAAGAGCTACTTAATAAGGGCACTGACGCTAAACACAATGACAAAAGATGCAGA<br>AAGAGGCAAATTGAAGAGGCGGGCAATTGCAACACCAGGGATGCAGATTAGAGGATTTGTGTACTTTGTCGAAACACTGGCAAGGAGCATCTGTGAGAAACTTGAGCAATCTGGACTCCCCGTTGGAGGGAATGAGAAAAAGGCCAAATTGGCAAATGTCGTGAGAAAAATGATGACCAACTCACAAGATACAGAGCTCTCTTTCACAATTACTGGAGATAACACCAAATG<br>GAATGAGAATCAAAATCCTCGGATGTTTCTGGCAATGATAACATACATCACAAGAAACCAACCTGAATGGTTTAGAAATGTCTTGAGCATTGCCCCTATAATGTTCTCGAACAAAATGGCGAGATTGGGAAAAGGATACATGTTTGAAAGTAAGAGCATGAAGCTACGGACACAAATACCTGCAGAAATGCTTGCAAACATTGACTTAAAATATTTCAATGAATCAACAAGAAA<br>GAAAATCGAGAAAATAAGACCTCTGCTAATAGATGGCACAGCCTCATTGAGTCCAGGAATGATGATGGGCATGTTCAATATGCTGAGTACAGTATTAGGAGTCTCAATCCTGAATCTTGGGCAAAAGAGGTACACCAAAACCACATACTGGTGGGATGGACTCCAATCCTCTGATGATTTCGCCCTCATAGTGAATGCACCGAATCATGAGGGAATACAAGCAGGAGTTGATA<br>GGTTCTATAGGACGTGCAAACTGGTTGGAATCAACATGAGCAAAAAGAAGTCTTACATAAATAGGACAGGAACATTTGAGTTCACAAGCTTTTTCTATCGCTATGGATTTGTGGCTAATTTCAGTATGGAGCTGCCCAGCTTTGGAGTGTCTGGAATCAATGAATCAGCTGACATGAGCATTGGAGTTACAGTGATAAAGAACAACATGATAAACAATGACCTTGGACCAGCAA<br>CAGCTCAGATGGCTCTTCAGCTATTCATCAAGGACTACAGGTACACGTACCGATGCCACAGGGGTGACACACAAATTCAAACGAGGAGATCATTCGAGCTGAAGAAGCTGTGGGAGCAGACCCGTTCAAAGGCAGGACTGTTGGTGTCGGATGGAGGACCAAACCTATACAACATTCGGAATCTCCACATCCCAGAGGTCTGCTTGAAGTGGGAGCTGATGGACGAAGATTA<br>CCAGGGCAGGTTGTGTAATCCTCTGAACCCATTTGTCAGTCATAAGGAAATTGAGTCCGTAAACAATGCTGTGGTGATGCCGGCCCATGGCCCAGCCAAGAGCATGGAATATGATGCTGTTGCGACTACACACTCATGGATTCCTAAGAGGAATCGTTCCATTCTCAATACCAGCCAAAGGGGAATTCTAGAGGATGAGCAGATGTACCAGAAGTGCTGTATTCTATTCGAGAA<br>ATTCTTCCCCAGTAGTTCATACAGGAGGCCAGTTGGAATTTCCAGCATGGTAGAGGCCATGGTATCTAGGGCCCGAATTGATGCACGCATTGATTTCGAATCTGGAAGGATTAAGAAAGAAGAATTTGCTGAGATCATGAAGATCTGTTCCACCATTGAAGAGCTCAGACGGCAAAAATAG
EPI_ISL_250238	>A/gadwall/Chany/97/2016_H6N8_2016-09-10<br>ATGGATGTCAATCCGACTTTACTTTTCTTAAAAGTGCCAGCGCAAAATGCTATAAGTACTACGTTTCCTTACACTGGAGATCCTCCATACAGCCATGGAACAGGAACAGGATACACCATGGACACAGTCAACAGAACACATCAATACTCAGAGAAGGGAAAGTGGACAACAAACACAGAGACTGGGGCGCCCCAACTCAACCCAATTGATGGACCATTGCCAGGGGACAACGA<br>GCCAAGCGGATATGCACAAACAGATTGCGTGTTGGAAGCAATGGCTTTCCTTGAAGAATCCCACCCAGGGATCTTTGAAAACTCTTGTCTTGAAACGATGGAGGTCGTTCAGCAAACAAGAGTGGACAAACTAACCCAAGGTCGCCAGACTTATGATTGGACACTGAATAGAAACCAACCAGCTGCAACTGCTTTGGCCAATACTATAGAGGTCTTCAGATCGAACGGTCTAA<br>CAGCCAATGAGTCGGGGAGACTAATAGATTTCCTCAAGGATGTGATGGAATCAATGGATAAGGAAGAAATGGAAATAACAACACATTTCCAGAGAAAGAGAAGAGTAAGGGACAACATGACCAAGAAGATGGTCACACAAAGAACGATAGGAAAGAAGAAGCAGAGGCTGAACAAGAGGAGCTACTTAATAAGAGCATTGACATTGAACACAATGACCAAAGATGCGGA<br>AAGAGGCAAATTGAAGAGGCGGGCAATTGCAACACCCGGGATGCAGATTAGAGGATTTGTGTACTTTGTCGAAACACTAGCGAGGAGCATCTGTGAGAAACTCGAGCAATCTGGACTCCCTGTTGGAGGGAATGAGAAAAAGGCCAAATTGGCAAATGTCGTGAGAAAAATGATGACTAACTCGCAAGATACAGAGCTCTCCTTTACAATTACTGGAGACAACACCAAATG<br>GAATGAAAATCAAATCCTCGGATGTTTTTGGCAATGATAACATACATCACAAGAAACCAACCCGAATGGTTTAGAAATGTCTTGAGCATTGCCCCTATAATGTTCTCAAACAAAATGGCAAGATTAGGAAAGGATACATGTTTGAAAGTAAGAGCATGAAGCTGAGGACACAAATACCGGCAGAAATGCTTGCAAACATTGACTTGAAATACTTCAATGAATCAACGAGAA<br>AGAAAATCGAGAAAATAAGACCTCTGCTAATTGATGGCACAGCCTCATTGAGTCCTGGAATGATGATGGGCATGTTCAATATGCTGAGCACAGTATTGGGGGTCTCAATCCTGAATCTTGGACAAAAGAGGTACACCAAAACCACATACTGGTGGGATGGACTCCAATCCTCTGATGATTTCGCTCTCATAGTGAATGCACCGAATCATGAGGGGATACAAGCAGGGGTGGAT<br>AGGTTCTATAGGACCTGCAAACTGGTTGGGATCAACATGAGCAAAAAGAAGTCTTACATAAACCGAACAGGAACATTTGAGTTCACAAGCTTTTTCTACCGCTATGGATTTGTAGCCAACTTCAGTATGGAGTTACCCAGCTTTGGAGTGTCTGGAATCAATGAGTCAGCTGACATGAGCATTGGAGTTACAGTGATAAAGAACAATATGATAAACAATGACCTTGGACCAGCA<br>ACAGCTCAAATGGCTCTTCAGCTATTCATCAAGGATTATAGGTACACGTACCGATGTCACAGAGGTGATACACAAATTCAAACGAGGAGATCATTCGAGCTTAAGAAGCTGTGGGAGCAGACCCGTTCAAAGGCAGGGCTGTTGGTATCAGATGGAGGACCAAACCTATACAACATTCGGAATCTCCACATCCCAGAGGTCTGCTTGAAGTGGGAACTAATGGATGAAGATTA<br>CCAGGGCAGGCTGTGTAATCCTCTGAATCCGTTTGTCAGTCATAAGGAAATTGAGTCCGTAAACAATGCTGTGGTAATGCCAGCCCATGGCCCAGCCAAGAGCATGGAATATGATGCTGTTGCGACTACACACTCATGGATTCCTAAGAGGAACCGTTCCATTCTCAATACCAGCCAGAGGGGAATTCTCGAGGATGAGCAGATGTACCAGAAGTGCTGCAGTTTATTCGAGA<br>AATTCTTCCCCAGTAGTTCGTACAGGAGGCCAGTTGGAATTTCCAGCATGGTGGAGGCCATGGTGTCCAGGGCCCGAATTGATGCACGCATTGATTTCGAATCTGGAAGGATTAAGAAAGAAGAGTTTGCTGAGATCATGAAGATCTGTTACACCATTGAAGAGCTCAGACGGCAAAAATAG
EPI_ISL_12066188	>A/Fox/Netherlands/EMC1/2022_H5N1_2022-01-26<br>ATGGATGTCAATCCGACTTTACTTTTCTTAAAAGTGCCAGCGCAAGATGCCATAAGTACCACATTCCCTTACACTGGAGATCCTCCATACAGCCATGGAACAGGGACAGGATACACAATGGACACGGTCAACAGAACACATCAATACTCAGAGAAGGGAAAATGGACAACAAACACAGAAACCGGAGCACCTCAACTCAACCCAATTGATGGGCCACTACCTGAGGACAACGA<br>ACCGAGCGGATATGCACAAACAGATTGCGTGTTGGAAGCAATGGCTTTCCTTGAAGAGTCCCATCCAGGGATCTTTGAAAACTCTTGTCTTGAAACGATGGAAGTCGTTCAGCAAACAAGAGTGGACAAACTAACTCAAGGTCGCCAGACATATGACTGGACACTGAATAGAAACCAACCAGCTGCAACTGCCTTGGCCAACACTATAGAGGTCTTAAGATCAAACGGTCTAA<br>CAGCCAATGAATCGGGGAGACTAATAGATTTCCTCAAGGATGTGATGGACTCAATGGATAAAGAAGAAATGGAAATAACAACACATTTCCAGAGAAAGAGAAGAGTAAGGGACAACATGACCAAGAAAATGGTCACACAAAGAACAATAGGAAAGAAGAAACAAAGGCTAAACAAGAGGAGCTACTTAATAAGAGCACTGACACTGAATACAATGACAAAAGATGCAGA<br>AAGAGGCAAATTGAAGAGACGGGCGATTGCAACACCAGGGATGCAGATTAGAGGATTTGTGTACTTTGTCGAAACACTGGCAAGGAGCATCTGTGAAAAACTTGAGCAATCTGGACTCCCCGTTGGAGGGAATGAGAAGAAGGCTAAATTGGCAAATGTTGTGAGAAAAATGATGACTAACTCACAAGATACAGAGCTCTCCTTCACAATTACTGGAGATAACACCAAATG<br>GAATGAGAATCAAAATCCTCGGATGTTTCTGGCAATGATAACATACATTACAAGAAACCAACCTGAATGGTTTAGGAATGTCTTGAGTATTGCCCCTATAATGTTCTCGAACAAAATGGCGAGATTGGGAAAAGGGTACATGTTTGAAAGTAAGAGCATGAAGTTACGGACACAAATACCTGCAGAAATGCTTGCAAACATTGACTTAAAATATTTCAATGAATCAACAAGAAA<br>GAAAATCGAAAAAATAAGGCCTCTACTAATAGATGGCACTGCCTCATTGAGTCCTGGAATGATGATGGGCATGTTCAATATGCTGAGTACAGTATTAGGAGTTTCAATCCTAAATCTTGGGCAAAAGAAGTACACCAAAACCACATACTGGTGGGATGGACTCCAATCCTCTGATGATTTCGCCCTCATAGTAAATGCACCGAATCATGAGGGAATACAAGCAGGAGTGGATA<br>GGTTCTATAGGACCTGCAAACTGGTCGGGATCAATATGAGCAAAAAGAAGTCTTACATAAACCGGACTGGAACATTTGAGTTCACAAGCTTTTTCTATCGCTATGGATTTGTGGCTAATTTCAGTATGGAGCTGCCCAGCTTTGGAGTTTCTGGGATCAATGAATCAGCTGACATGAGCATTGGCATCACAGTGATAAAGAACAACATGATAAACAATGACCTTGGACCAGCAA<br>CAGCTCAAATGGCCCTTCAACTATTCATCAAAGATTACAGGTACACGTACCGATGCCATAGAGGTGACACACAAATTCAAACGAGGAGATCATTCGAGCTGAAGAAGCTGTGGGAACAGACCCGTTCAAAGGCAGGACTGTTGGTGTCAGATGGAGGACCAAATCTATACAACATTCGGAATCTCCATATCCCAGAGGTCTGCCTGAAGTGGGAGCTGATGGACGAAGATTA<br>CCAGGGCAGGTTGTGTAATCCTCTGAACCCATTTGTCAGCCATAAAGAAATTGAGTCCGTAAACAATGCTGTGGTGATGCCAGCCCACGGTCCAGCCAAAAGCATGGAATATGATGCCGTTGCGACTACACACTCATGGATTCCTAAAAGGAATCGTTCCATTCTCAATACCAGTCAAAGGGGAATTCTTGAGGATGAACAGATGTACCAGAAATGCTGCGGTCTATTCGAGAA<br>ATTCTTCCCCAGTAGTTCATACAGGAGACCAGTTGGAATTTCCAGCATGGTGGAGGCCATGGTGTCTAGGGCCCGAATCGATGCACGCATTGATTTCGAATCTGGAAGGATCAAGAAGGAAGAGTTTGCTGAGATCATGAAGATCTGTTCCACCATTGAAGAGCTCAGACGGCAAAAATAG
EPI_ISL_7778766	>A/European_herring_gull/Finland/9722_21VIR7689-13/2021_H5N1_2021-08-01<br>ATGGATGTCAATCCGACTTTACTTTTCTTAAAAGTGCCAGCGCAAAATGCCATAAGTACCACATTCCCTTATACTGGAGATCCTCCATACAGCCATGGAACAGGGACAGGATACACAATGGACACAGTCAACAGAACACATCAATACTCAGAGAAGGGAAAATGGACAACAAACACAGAAACCGGAGCACCTCAACTCAACCCAATTGATGGGCCACTACCTGAGGACAACGA<br>ACCGAGCGGATATGCACAGACAGATTGCGTGTTGGAAGCAATGGCTTTCCTTGAAGAGTCCCACCCAGGGATCTTTGGAAACTCTTGTCTTGAAACGATGGAAGTCGTTCAGCAAACAAGAGTGGACAAACTAACTCAAGGTCGCCAGACTTATGACTGGACACTGAATAGAAACCAACCAGCTGCAACTGCCCTGGCCAACACTATAGAGGTCTTCAGATCAAACGGTCTAA<br>CAGCCAATGAATCGGGGAGACTAATAGATTTCCTCAAGGATGTGATGGACTCAATGGATAAAGAAGAAATGGAAATAACAACACATTTCCAGAGAAAGAGAAGAGTAAGGGACAACATGACCAAGAAAATGGTCACACAAAGAACAATAGGAAAGAAGAAACAAAGGCTAAACAGGAGGAGCTACTTAATAAGAGCACTGACACTGAATACAATGACAAAAGATGCAGA<br>AAGAGGCAAATTGAAGAGACGGGCGATTGCAACACCAGGGATGCAGATTAGAGGATTTGTGTACTTTGTCGAAACACTGGCAAGGAGCATCTGTGAAAAACTTGAGCAATCTGGACTCCCCGTTGGAGGAAATGAGAAGAAGGCTAAATTGGCAAATGTCGTGAGGAAAATGATGACTAACTCACAAGATACAGAGCTCTCCTTCACAATTACTGGAGATAACACCAAATG<br>GAATGAGAATCAAAATCCTCGGATGTTTCTGGCAATGATAACGTACATTACAAGAAACCAACCTGAATGGTTTAGAAATGTCTTGAGTATTGCCCCTATAATGTTCTCGAACAAAATGGCGAGATTGGGAAAAGGGTACATGTTTGAAAGTAAGAGCATGAAGTTACGGACACAAATACCTGCAGAAATACTTGCAAACATTGACTTAAAATACTTCAATGAATCAACAAGAAA<br>GAAAATCGAAAAAATAAGGCCTCTACTAATAGATGGCACTGCCTCATTGAGTCCTGGAATGATGATGGGCATGTTCAATATGCTGAGTACAGTATTAGGAGTTTCAATCCTAAATCTTGGGCAAAAGAGGTACACCAAAACCACATACTGGTGGGATGGACTCCAATCCTCTGATGATTTCGCCCTCATAGTAAATGCACCGAATCATGAGGGAATACAAGCAGGAGTGGATA<br>GGTTCTATAGGACCTGCAAACTGGTCGGGATCAATATGAGCAAAAAGAAGTCTTACATAAACCGGACTGGAACATTTGAGTTCACAAGCTTTTTCTATCGCTATGGATTTGTGGCTAACTTCAGTATGGAGCTGCCCAGCTTTGGAGTTTCTGGAATCAATGAATCAGCTGACATGAGCATTGGCGTCACAGTGATAAAGAACAACATGATAAACAATGACCTTGGACCAGCAA<br>CAGCTCAAATGGCCCTTCAACTATTCATCAAAGATTACAGGTACACGTACCGATGCCACAGAGGTGACACACAAATTCAAACGAGGAGATCATTCGAGCTGAAGAAGCTGTGGGAACAGACCCGTTCAAAGGTAGGACTGTTGGTGTCAGATGGAGGACCAAATCTATACAACATTCGGAATCTCCATATCCCAGAGGTCTGCCTGAAGTGGGAGCTGATGGACGAAGATTA<br>CCAGGGCAGGTTGTGTAATCCTCTGAACCCATTTGTCAGTCATAAAGAAATTGAGTCCGTAAACAATGCTGTGGTGATGCCAGCCCACGGTCCAGCCAAAAGCATGGAATATGATGCCGTTGCAACTACACACTCATGGATTCCTAAAAGGAATCGTTCCATTCTCAATACCAGCCAAAGGGGAATTCTTGAGGATGAACAGATGTACCAGAAATGCTGCAGTCTATTCGAGAA<br>ATTCTTCCCCAGTAGTTCATACAGGAGACCAGTTGGAATTTCCAGCATGGTGGAGGCCATGGTGTCTAGGGCCCGAATCGATGCACGCATTGATTTCGAATCTGGAAGGATCAAGAAGGGAGAGTTTGCTGAGATCATGAAGATCTGTTCCACCATTGAAGAGCTCAGACGGCAAAAATAG

EPI_ISL_683593	>A/Eurasian_wigeon/Italy/20VIR7139-121/2020_H5N8_2020-11-14<br>ATGGATGTCAATCCGACTTTACTTTTCTTAAAAGTGCCAGCGCAAAATGCTATAAGTACTACATTCCCTTACACTGGAGATCCCCCATACAGCCATGGAACAGGAACAGGGTATACCATGGACACAGTAAACAGAACACATCAATACTCAGAAAAGGGAAAGTGGACAACAAACACAGAAACCGGAGCACCCCAACTCAACCCAATTGATGGACCATTACCAGAGGACAATGA<br>GCCAAGCGGATATGCACAAACTGATTGCGTGTTGGAAGCAATGGCTTTCCTTGAAGAATCCCACCCAGGGATATTTGAAAACTCTTGTCTTGAAGCGATGGAAATCGTTCAGCAAACAAGAGTGGACAAACTAACCCAAGGTCGCCAGACTTATGACTGGACACTGAACAGAAACCAACCAGCTGCAACCTCTTTGGCCAACACTATAGAGGTGTTCAGATCGAATGGTCTGA<br>CAGCCAATGAATCAGGGAGACTGATAGATTTTCTCAGGGATGTGATGGAATCAATGGATAAAGAAGAGATGGAAGTAACAACACATTTCCAGAGAAAAAGAAGAGTGAGGGACAACATGACTAAGAAGATGGTCACACAAAGAACAATAGGGAAGAAGAAGCAGAGGCTGAACAAGAGGAGTTACTTAATAAGAGCACTGACATTGAATACAATGACCAAAGATGCGGA<br>AAGAGGCAAGTTGAAGAGACGGGCAATTGCAACACCCGGGATGCAGATTAGAGGATTCGTGTACTTCGTTGAAACACTAGCGAGGAGCATCTGTGAGAAACTAGAGCAATCTGGGCTCCCTGTTGGAGGGAATGAGAAGAAGGCTAAATTGGCAATGTTGTGAGAAAAATGATGACTAACTCACAAGATACAGAGCTCTCCTTTACAATTACTGGAGACAACACCAAATG<br>GAATGAGAATCAAAACCCTCGGACGTTTTTGGCAATGATAACATATATCACAAGAAACCAACCTGAATGGTTTAGAAATGTCTTAAGCATTGCCCCTATAATGTTCTCAAACAAAATGGCTAGATTAGGGAAAGGATACATGTTTGAAAGTAAGAGCATGAAGCTAAGAACACAAATACCAGCAGATATGCTTACAAATATTGATCTGAAGTATTTCAACGAACCAACGAGAAA<br>GAAAATCGAGAAAATAAGACCTCTGCTGATTGATGGCACGGCCTCATTGAGTCCTGGGATGATGATGGGCATGTTCAATATGCTGAGCACAGTATTAGGGGTCTCAATCCTGAATCTCGGGCAAAAGAGGTACACCAAAACCACATACTGGTGGGATGGACTTCAATCCTCTGATGATTTCGCTCTCATAGTGAATGCACCGAATCATGAGGGGATACAAGCAGGAGTGGATA<br>GATTCTATAGGACCTGCAAACTGGTTGGGATCAACATGAGCAAAAAGAAGTCTTACATAAACCGAACAGGAACATTTGAGTTCACAAGTTTTTTCTACCGCTATGGATTTGTAGCCAACTTCAGTATGGAATTACCCAGCTTTGGAGTGTCTGGAATCAATGAATCAGCTGACATGAGCATTGGAGTTACAGTGATAAAAAACAATATGATAAACAATGATCTTGGACCAGCAA<br>CAGCTCAAATGGCTCTTCAGTTATTCATCAAAGACTATAGGTACACATACCGATGCCACAGGGGTGATACACAAATTCAAACGAGGAGATCATTCGAGCTGAAGAAGCTGTGGGAGCAGACCCGTTCAAAGGCAGGGCTATTGATATCAGACGGGGGGCCAAACCTATACAACATTCGGAATCTCCACATCCCAGAGGTCTGCTTGAAGTGGGAGCTGATGGATGAAGACTA<br>CCAAGGCAGGCTGTGCAATCCTCTGAATCCATTTGTCAGTCATAAAGAGATTGAGTCCGTAAACAATGCTGTAGTAATGCCCGCCCATGGCCCGGCCAAGAGCATGGAATATGATGCTGTTGCGACCACACACTCGTGGATTCCTAAGAGGAACCGTTCCATTCTCAATACCAGCCAAAGGGGAATTCTTGAGGATGAGCAGATGTACCAAAAGTGCTGTAGTCTATTCGAGAA<br>ATTCTTCCCCAGCAGTTCATACAGGAGGCCAGTTGGAATTTCCAGCATGGTGGAGGCCATGGTGTCTAGGGCCCGAATTGATGCACGCATCGACTTCGAATCTGGAAGGATTAAGAAAGAAGAGTTTGCTGAGATCATGAAGATCTGTTCCACCATTGAAGAGCTCAGACGGCAAAAATAG
EPI_ISL_11798574	>A/Eurasian_wigeon/Denmark/24279-1/2021-10-24_H5N1_2021-10-24<br>ATGGATGTCAATCCGACTTTACTTTTCTTAAAAGTGCCAGCGCAAGATGCCATAAGTACCACATTCCCTTACACTGGAGATCCTCCATACAGCCATGGAACAGGGACAGGATACACAATGGACACAGTCAACAGAACACATCAATACTCAGAGAAGGGAAAATGGACAACAAACACAGAAACCGGAGCACCTCAACTCAACCCAATTGATGGGCCACTACCTGAGGACAACGA<br>ACCGAGCGGATATGCACAAACAGATTGCGTGTTGGAAGCAATGGCTTTCCTTGAAGAGTCCCATCCAGGGATCTTTGAAAACTCTTGTCTTGAAACGATGGAAGTCGTTCAGCAAACAAGAGTGGACAAACTAACTCAAGGTCGCCAGACATATGACTGGACACTGAATAGAAACCAACCAGCTGCAACTGCCCTGGCCAACACTATAGAGGTCTTCAGATCAAACGGTCTAA<br>CAGCCAATGAATCGGGGAGACTAATAGATTTCCTCAAGGATGTGATGGACTCAATGGATAAAGAAGAAATGGAAATAACAACACATTTCCAGAGAAAGAGAAGAGTAAGGGACAACATGACCAAGAAAATGGTCACACAAAGAACAATAGGAAAGAAGAAACAAAGGCTAAACAAGAGGAGCTACTTAATAAGAGCACTGACACTGAATACAATGACAAAAGATGCAGA<br>AAGAGGCAAATTGAAGAGACGGGCGATTGCAACACCAGGGATGCAGATTAGAGGATTTGTGTACTTTGTCGAAACACTGGCAAGGAGCATCTGTGAAAAACTTGAGCAATCTGGACTCCCCGTTGGAGGGGAATGAGAAGAAGGCTAAATTGGCAAATGTCGTGAGAAAAATGATGACTAACTCACAAGATACAGAGCTCTCCTTCACAATTACTGGAGATAACACCAAATG<br>GAATGAGAATCAAAATCCTCGGATGTTTCTGGCAATGATAACATACATTACAAGAAACCAACCTGAATGGTTTAGGAATGTCTTGAGTATTGCCCCTATAATGTTCTCGAACAAAATGGCGAGATTGGGAAAAGGGTACATGTTTGAAAGTAAGAGCATGAAGTTACGGACACAAATACCTGCAGAAATGCTTGCAAACATTGACTTAAAATACTTCAATGAATCAACAAGAAA<br>GAAAATCGAAAAAATAAGGCCTCTACTAATAGATGGCACTGCCTCATTGAGTCCTGGAATGATGATGGGCATGTTCAATATGCTGAGTACAGTATTAGGAGTTTCAATCCTAAATCTTGGGCAAAAGAAGTACACCAAAACCACATACTGGTGGGATGGACTCCAATCCTCTGATGATTTCGCCCTCATAGTAAATGCACCGAATCATGAGGGAATACAAGCAGGAGTGGATA<br>GGTTCTATAGGACCTGCAAACTGGTCGGGATCAATATGAGCAAAAAGAAGTCTTACATAAACCGGACTGGAACATTTGAGTTCACAAGCTTTTTCTATCGCTATGGATTTGTGGCTAACTTCAGTATGGAGCTGCCCAGCTTTGGAGTTTCTGGGATCAATGAATCAGCTGACATGAGCATTGGCATCACAGTGATAAAGAACAACATGATAAACAATGACCTTGGACCAGCAA<br>CAGCTCAAATGGCCCTTCAACTATTCATCAAAGATTACAGGTACACGTACCGATGCCATAGAGGTGACACACAAATTCAAACGAGGAGATCATTCGAGCTGAAGAAGCTGTGGGAACAGACCCGTTCAAAGGCAGGACTGTTGGTGTCAGATGGAGGACCAAATCTATACAACATTCGGAATCTCCATATCCCAGAGGTCTGCCTGAAGTGGGAGCTGATGGACGAAGATTA<br>CCAGGGCAGGTTGTGTAATCCTCTGAACCCATTTGTCAGCCATAAAGAAATTGAGTCCGTAAACAATGCTGTGGTGATGCCAGCCCACGGTCCAGCCAAAAGCATGGAATATGATGCCGTTGCGACTACACACTCATGGATTCCTAAAAGGAATCGTTCCATTCTCAATACCAGCCAAAGGGGAATTCTTGAGGATGAACAGATGTACCAGAAATGCTGCGGTCTATTCGAGAA<br>ATTCTTCCCCAGTAGTTCATACAGGAGACCAGTTGGAATTTCCAGCATGGTGGAGGCCATGGTGTCTAGGGCCCGAATCGATGCACGCATTGATTTCGAATCTGGAAGGATCAAGAAGGAAGAGTTTGCTGAGATCATGAAGATCTGTTCCACCATTGAAGAGCTCAGACGGCAAAAATAG
EPI_ISL_632315	>A/eurasian_teal/Netherlands/20016896-013/2020_H5N1_2020-11-02<br>ATGGATGTCAATCCGACTTTACTTTTCTTAAAAGTGCCAGCGCAAAATGCCATAAGTACCACATTCCCTTATACTGGAGATCCTCCATACAGCCATGGAACAGGGACAGGATACACAATGGACACAGTCAACAGAACACATCAATACTCAGAGAAGGGAAAATGGACAACAAACACAGAAACCGGAGCACCTCAACTCAACCCAATTGATGGACCACTACCTGAGGACAACGA<br>ACCGAGCGGATATGCACAAACAGATTGCGTGTTGGAAGCAATGGCTTTCCTTGAAGAGTCCCACCCAGGGATCTTTGGAAACTCTTGTCTTGAAACGATGGAAGTCGTTCAGCAAACAAGAGTGGACAAACTAACTCAAGGTCGCCAGACTTATGACTGGACACTGAATAGAAACCAACCAGCTGCAACTGCCCTGGCCAACACTATAGAGGTCTTCAGATCAAACGGTCTAA<br>CAGCCAATGAATCGGGGAGACTAATAGATTTCCTCAAGGATGTGATGGACTCAATGGATAAAGAAGAAATGGAAATAACAACACATTTCCAGAGAAAGAGAAGAGTAAGGGACAACATGACCAAGAAAATGGTCACACAAAGAACAATAGGAAAGAAGAAACAAAGGCTAAACAAGAGGAGCTACTTAATAAGAGCACTGACACTGAATACAATGACAAAAGATGCAGA<br>AAGAGGCAAATTGAAGAGACGGGCGATTGCAACACCAGGGATGCAGATTAGAGGATTTGTGTACTTTGTCGAAACACTGGCAAGGAGCATCTGTGAAAAACTTGAGCAATCTGGACTCCCCGTTGGAGGAAATGAGAAGAAGGCTAAATTGGCAAATGTCGTGAGAAAAATGATGACTAACTCACAAGATACAGAGCTCTCCTTCACAATTACTGGAGATAACACCAAATG<br>GAATGAGAATCAAAATCCTCGGATGTTTCTGGCAATGATAACGTACATTACAAGAAACCAACCTGAATGGTTTAGAAATGTCTTGAGTATTGCCCCTATAATGTTCTCGAACAAAATGGCGAGATTGGGAAAAGGGTACATGTTTGAAAGTAAGAGCATGAAGTTACGGACACAAATACCTGCAGAAATACTTGCAAACATTGACTTAAAATACTTCAATGAATCAACAAGAAA<br>GAAAATCGAAAAAATAAGGCCTCTACTAATAGATGGCACTGCCTCATTGAGTCCTGGAATGATGATGGGCATGTTCAATATGCTGAGTACAGTATTAGGAGTTTCAATCCTAAATCTTGGGCAAAAGAAGTACACCAAAACCACATACTGGTGGGATGGACTCCAATCCTCTGATGATTTCGCCCTCATAGTAAATGCACCGAATCATGAGGGAATACAAGCAGGAGTGGATA<br>GGTTCTATAGGACCTGCAAACTGGTCGGGATCAATATGAGCAAAAAGAAGTCTTACATAAACCGGACTGGAACATTTGAGTTCACAAGCTTTTTCTATCGCTATGGATTTGTGGCTAACTTCAGTATGGAGCTGCCCAGCTTTGGAGTTTCTGGGATCAATGAATCAGCTGACATGAGCATTGGCGTCACAGTGATAAAGAACAACATGATAAACAATGACCTTGGACCAGCAA<br>CAGCTCAAATGGCCCTTCAACTATTCATCAAAGATTACAGGTACACGTACCGATGCCACAGAGGTGACACACAAATTCAAACGAGGAGATCATTCGAGCTGAAGAAGCTGTGGGAACAGACCCGTTCAAAGGCAGGACTGTTGGTGTCAGATGGAGGACCAAATCTATACAACATTCGGAATCTCCATATCCCAGAGGTCTGCCTGAAGTGGGAGCTGATGGACGAAGATTA<br>CCAGGGCAGGTTGTGTAATCCTCTGAACCCATTTGTCAGTCATAAAGAAATTGAGTCCGTAAACAATGCTGTGGTGATGCCAGCCCACGGTCCAGCCAAAAGCATGGAATATGATGCCGTTGCGACTACACACTCATGGATTCCTAAAAGGAATCGTTCCATTCTCAATACCAGTCAAAGGGGAATTCTTGAGGATGAACAGATGTACCAGAAATGCTGCAGTCTATTCGAGAA<br>ATTCTTCCCCAGTAGTTCATACAGGAGACCAGTTGGAATTTCCAGCATGGTGGAGGCCATGGTGTCTAGGGCCCGAATCGATGCACGCATTGATTTCGAATCTGGAAGGATCAAGAAGGGAGAGTTTGCTGAGATCATGAAGATCTGTTCCACCATTGAAGAGCTCAGACGGCAAAAATAG
EPI_ISL_243513	>A/Eurasian_teal/Netherlands/1/2011_H3N8_2011-10-15<br>ATGGATGTCAATCCGACTTTACTTTTCTTAAAAGTGCCAGCGCAAAATGCTATAAGTACTACGTTCCCTTACACTGGAGATCCTCCATACAGCCATGGAACAGGAACAGGATACACCATGGACACAGTCAACAGAACACATCAATACTCAGAGAAGGGAAAGTGGACAACGAACACAGAGACTGGGGCGCCCCAACTCAACCCAATTGATGGACCATTACCAGAGGACAATGA<br>GCCAAGCGGATATGCACAAACAGATTGCGTGTTGGAAGCAATGGCTTTCCTTGAAGAATCCCACCCAGGGATCTTTGAAAACTCTTGTCTTGAAACGATGGAGGTCGTTCAGCAAACAAGAGTGGACAAACTAACCCAAGGTCGCCAGACTTATGATTGGACACTAAATAGAAACCAACCAGCTGCAACTGCTTTGGCCAACACTATAGAGGTCTTCAGATTGAACGGTCTAAC<br>AGCCAATGAGTCGGGGAGACTAATAGATTTCCTCAAGGATGTGATGGAATCAATGGATAAAGAAGAAATGGAAATAACAACACATTTCCAGAGAAAGAGAAGAGTAAGGGACAACATGACCAAGAAGATGGTCACACAAAGAACGATAGGAAAGAAGAAACAGAGGCTGAACAAGAGGAGCTACTTAATAAGGGCACTGACATTGAACACAATGACCAAAGATGCAGAA<br>AGAGGCAAATTGAAGAGGCGGGCAATTGCAACACCCGGGATGCAGATTAGAGGATTCGTGTACTTTGTCGAAACACTAGCAAGGAGCATCTGTGAGAAACTCGAGCAGTCTGGACTCCCTGTTGGAGGGAATGAGAAGAAGGCCAAATTGGCAAATGTCGTGAGAAAAATGATGACTAACTCGCAAGATACAGAGCTCTCCTTTACAATTACTGGAGACAACACCAAATGG<br>AATGAGAATCAAAATCCTCGGATGTTTTTGGCAATGATAACATACATCACAAGAAACCAACCCGAATGGTTTAGAAATGTCTTGAGCATTGCCCCTATAATGTTCTCAAACAAAATGGCGAGATTAGGAAAAGGATACATGTTTGAGAGTAAGAGCATGAAGCTAAGGACACAAATACCGGCAGAAATGCTTGCAAACATTGACTTGAAATATTTCAATGAATCAACGAGAAA<br>GAAAATCGAGAAAATTAGACCTCTGCTAATTGATGGCACAGCCTCATTGAGTCCTGGAATGATGATGGGCATGTTCAATATGCTGAGCACAGTATTGGGGGTCTCAATCCTGAATCTTGGACAAAAGAGGTACACCAAAACCACATACTGGTGGGATGGACTCCAATCCTCTGATGATTTCGCTCTCATAGTGAATGCACCGAATCATGAGGGGATACAAGCAGGGGTGGATA<br>GGTTCTATAGGACCTGCAAACTGGTTGGGATCAACATGAGCAAAAAGAAGTCTTACATAAACCGGACAGGAACATTTGAGTTCACAAGTTTTTTCTACCGCTATGGATTTGTAGCCAACTTCAGTATGGAGTTACCCAGCTTTGGAGTGTCTGGAATCAATGAATCGGCTGACATGAGCATTGGAGTTACAGTGATAAAGAACAATATGATAAACAATGACCTTGGACCTGCAA<br>CAGCTCAAATGGCTCTTCAGCTATTCATCAAGGACTATAGGTACACGTACCGATGCCACAGAGGTGATACACAAATTCAAACGAGGAGATCATTCGAGCTGAAGAAGCTGTGGGAGCAGACCCGTTCAAAGGCAGGGCTGTTGGTATCAGATGGAGGACCAAACCTATACAACATTCGGAATCTCCACATCCCAGAGGTCTGCTTGAAGTGGGAACTAATGGATGAAGACTA<br>CCAGGGCAGGCTGTGTAATCCTCTGAATCCGTTTGTCAGTCATAAGGAAATTGAGTCCGTAAACAATGCTGTGGTAATGCCATCCCATGGCCCAGCCAAGAGCATGGAATATGATGCTGTTGCGACTACACACTCATGGATTCCTAAGAGGAACCGTTCCATTCTCAATACCAGCCAGAGGGGAATTCTCGAGGATGAGCAGATGTACCAGAAGTGCTGCAGTCTATTCGAAAA<br>ATTCTTCCCCAGTAGTTCATACAGGAGGCCAGTTGGAATTTCCAGCATGGTGGAGGCCATGGTGTCCAGGGCCCGAATTGATGCACGCATTGATTTCGAATCTGGAAGGATTAAGAAAGAAGAGTTTGCTGAGATCATGAAGATCTGTTCCACCATTGAAGAGCTCAGACGGCAAAAATAG
EPI_ISL_7778768	>A/Eurasian_eagle-owl/Finland/10617_21VIR7689-15/2021_H5N1_2021-08-01<br>ATGGATGTCAATCCGACTTTACTTTTCTTAAAAGTGCCAGCGCAAAATGCCATAAGTACCACATTCCCTTATACTGGAGATCCTCCATACAGCCATGGAACAGGGACAGGATACACAATGGACACAGTCAACAGAACACATCAATACTCAGAGAAGGGAAAATGGACAACAAACACAGAAACCGGAGCACCTCAACTAAACCCAATTGATGGGCCACTACCTGAGGACAACGA<br>ACCGAGCGGATATGCACAGACAGATTGCGTGTTGGAAGCAATGGCTTTCCTTGAAGAGTCCCACCCAGGGATCTTTGGAAACTCTTGTCTTGAAACGATGGAAGTCGTTCAGCAAACAAGAGTGGACAAACTAACTCAAGGTCGCCAGACTTATGACTGGACACTGAATAGAAACCAACCAGCTGCAACTGCCCTGGCCAACACTATAGAGGTCTTCAGATCAAACGGTCTAA<br>CAGCCAATGAATCGGGGAGACTAATAGATTTCCTCAAGGATGTGATGGACTCAATGGATAAAGAAGAAATGGAAATAACAACACATTTCCAGAGAAAGAGAAGAGTAAGGGACAACATGACCAAGAAAATGGTCACACAAAGAACAATAGGAAAGAAGAAACAAAGGCTAAACAGGAGGAGCTACTTAATAAGAGCACTGACACTGAATACAATGACAAAAGATGCAGA<br>AAGAGGCAAATTGAAGAGACGGGCGATTGCAACACCAGGGATGCAGATTAGAGGATTTGTGTACTTTGTCGAAACACTGGCAAGGAGCATCTGTGAAAAACTTGAGCAATCTGGACTCCCCGTTGGAGGAAATGAGAAGAAGGCTAAATTGGCAAATGTCGTGAGGAAAATGATGACTAACTCACAAGATACAGAGCTCTCCTTCACAATTACTGGAGATAACACCAAATG<br>GAATGAGAATCAAAATCCTCGGATGTTTCTGGCAATGATAACGTACATTACAAGAAACCAACCTGAATGGTTTAGAAATGTCTTGAGTATTGCCCCTATAATGTTCTCGAACAAAATGGCGAGATTGGGAAAAGGGTACATGTTTGAAAGTAAGAGCATGAAGTTACGGACACAAATACCTGCAGAAATACTTGCAAACATTGACTTAAAATACTTCAATGAATCAACAAGAAA<br>GAAAATCGAAAAAATAAGGCCTCTACTAATAGATGGCACTGCCTCATTGAGTCCTGGAATGATGATGGGCATGTTCAATATGCTGAGTACAGTATTAGGAGTTTCAATCCTAAATCTTGGGCAAAAGAGGTACACCAAAACCACATACTGGTGGGATGGACTCCAATCCTCTGATGATTTCGCCCTCATAGTAAATGCACCGAATCATGAGGGAATACAAGCAGGAGTGGATA<br>GGTTCTATAGGACCTGCAAACTGGTCGGGATCAATATGAGCAAAAAGAAGTCTTACATAAACCGGACTGGAACATTTGAGTTCACAAGCTTTTTCTATCGCTATGGATTTGTGGCTAACTTCAGTATGGAGCTGCCCAGCTTTGGAGTTTCTGGAATCAATGAATCAGCTGACATGAGCATTGGCGTCACAGTGATAAAGAACAACATGATAAACAATGACCTTGGACCAGCAA<br>CAGCTCAAATGGCCCTTCAACTATTCATCAAAGATTACAGGTACACGTACCGATGCCACAGAGGTGACACACAAATTCAAACGAGGAGATCATTCGAGCTGAAGAAGCTGTGGGAACAGACCCGTTCAAAGGCAGGACTGTTGGTGTCAGATGGAGGACCAAATCTATACAACATTCGGAATCTCCATATCCCAGAGGTCTGCCTGAAGTGGGAGCTGATGGACGAAGATTA<br>CCAGGGCAGGTTGTGTAATCCTCTGAACCCATTTGTCAGTCATAAAGAAATTGAGTCCGTAAACAATGCTGTGGTGATGCCAGCCCACGGTCCAGCCAAAAGCATGGAATATGATGCCGTTGCAACTACACACTCATGGATTCCTAAAAGGAATCGTTCCATTCTCAATACCAGCCAAAGGGGAATTCTTGAGGATGAACAGATGTACCAGAAATGCTGCAGTCTATTCGAGAA<br>ATTCTTCCCCAGTAGTTCATACAGGAGACCAGTTGGAATTTCCAGCATGGTGGAGGCCATGGTGTCTAGGGCCCGAATCGATGCACGCATTGATTTCGAATCTGGAAGGATCAAGAAGGGAGAGTTTGCTGAGATCATGAAGATCTGTTCCACCATTGAAGAGCTCAGACGGCAAAAATAG
EPI_ISL_632316	>A/eurasian_curlew/Netherlands/20016890-001/2020_H5N1_2020-11-01<br>ATGGATGTCAATCCGACTTTACTTTTCTTAAAAGTGCCAGCGCAGAATGCCATAAGTACCACATTCCCTTATACTGGAGATCCTCCATACAGCCATGGAACAGGAACAGGGTACACCATGGACACAGTCAACAGAACACATCAATACTCAGAAAAGGGGAAATGGACAACAAACACAGAGACCGGAGCACCCCAACTCAACCCAATTGATGGACCACTACCTGAGGACAACGA<br>ACCAAGCGGATATGCACAGACAGATTGCGTGTTGGAAGCAATGGCTTTCCTTGAAGAGTCCCACCCAGGGATCTTTGAAAACTCCTGTCTTGAAACGATGGAAGTCGTTCAGCAAACAAGAGTGGACAAACTAACTCAAGGTCGCCAGACTATTGACTGGACACTAAATAGAAACCAACCAGCTGCAACTGCCCTGGCCAACACTATAGAAGTCTTCAGATCGAACGGCCTGA<br>CAGCCAATGAATCGGGGAGACTAATAGATTTCCTCAAGGATGTGATGGATTCAATGGACAAAGAAGAAATGGAAATAACAACACATTTCCAGAGAAAGAGAAGAGTAAGGGACAACATGACCAAGAAAATGGTCACACAAAGAACAATAGGGAAGAAGAAACAAAGGCTAAATAAGAGGAGCTACTTAATAAGAGCACTGACACTGAATACAATGACAAAAGACGCAGA<br>AAGAGGCAAACTGAAGAGACGGGCAATTGCAACACCAGGGATGCAGATTAGAGGATTTGTGTACTTTGTCGAAACACTGGCAAGGAGCATCTGTGAGAAACTTGAGCAATCTGGGCTCCCCGTTGGAGGGAATGAGAAGAAGGCTAAATTGGCAAATGTTGTGAGAAAAATGATGACTAACTCACAAGATACAGAGCTCTCCTTCACAATTACTGGAGATAATACCAAATG<br>GAATGAGAATCAAAATCCTCGGATGTTTCTGGCAATGATAACATACATCACAAGAAACCAACCTGAATGGTTTAGAAATGTCTTGAGCATTGCCCCTATAATGTTCTCGAACAAAATGGCGAGATTGGGAAAAGGATACATGTTCGAAAGTAAGAGCATGAAGCTACGGACACAAATACCTGCAGAAATGCTTGCAAACATTGACTTAAAATACTTCAACGAGTCAACAAGAA<br>AGAAAATCGAGAAAATAAGACCTCTATTAATAGATGGCACAGCCTCATTGAGTCCTGGAATGATGATGGGCATGTTCAATATGCTGAGTACAGTATTAGGAGTCTCAATCCTGAATCTTGGGCAAAAGAGGTACACCAAAACCACATACTGGTGGGATGGACTCCAATCCTCTGATGATTTCGCCCTCATAGTGAATGCACCGAATCATGAGGGAATACAAGCAGGAGTGGAT<br>AGATTCTATAGGACCTGCAAACTGGTTGGGATCAACATGAGCAAAAAGAAGTCTTACATAAACCGGACAGGAACATTTGAGTTCACAAGCTTTTTCTATCGCTATGGATTTGTGGCCAACTTCAGTATGGAGCTGCCCAGCTTTGGAGTGTCTGGAATCAATGAATCAGCTGACATGAGCATTGGAGTTACAGTGATAAAGAACAACATGATAAACAATGACCTTGGACCAGCA<br>ACAGCTCAAATGGCCCTTCAACTATTCATCAAGGACTACAGGTACACTTACCGATGCCACAGGGGTGACACACAAATTCAAACGAGGAGATCATTCGAGCTGAAGAAGCTGTGGGAGCAGACCCGTTCAAAGGCAGGACTGTTGGTGTCAGATGGAGGACCAAATCTATACAACATTCGGAATCTCCATATCCCAGAGGTCTGCCTAAAGTGGGAGCTGATGGACGAAGATT<br>ACCAGGGCAGGTTGTGTAATCCTTTGAACCCATTTGTCAGTCATAAAGAAATTGAGTCCGTAAACAATGCTGTGGTAATGCCAGCCCATGGCCCAGCCAAGAGCATGGAATATGATGCTGTTGCGACTACACACTCATGGATTCCTAAAAGGAATCGTTCTATCCTCAACACCAGCCAAAGGGGAATTCTTGAGGATGAGCAGATGTACCAGAAATGCTGCAGTCTATTCGAGA<br>AATTCTTCCCCAGTAGCTCATACAGGAGACCAGTTGGAATTTCCAGCATGGTGGAGGCCATGGTGTCTAGGGCCCGAATCGATGCACGCATTGATTTCGAATCTGGAAGGATTAAGAAGGAAGAGTTTGCTGAGATCATGAAGATCTGTTCCACCATTGAAGAGCTCAGACGGCAAAAATAG
EPI_ISL_15072539	>A/emu/Hokkaido/A-2/2022_H5N1_2022-04-16<br>ATGGATGTCAATCCGACTTTACTTTTCTTAAAAGTGCCAGCGCAAGATGCCATAAGTACCACATTCCCTTACACTGGAGATCCTCCATACAGCCATGGAACAGGGACAGGATACACAATGGACACAGTCAACAGAACACATCAATACTCAGAGAAGGGAAAATGGACAACAAACACAGAAACCGGAGCACCTCAACTCAACCCAATTGATGGGCCACTACCTGAGGACAACGA<br>ACCGAGCGGATATGCACAAACAGATTGCGTGTTGGAAGCAATGGCTTTCCTTGAGGAGTCCCACCCAGGGATCTTTGAAAACTCTTGTCTTGAAACGATGGAAGTCGTTCAGCAAACAAGAGTGGACAAACTAACTCAAGGTCGCCAGACTTATGATTGGACACTGAATAGAAACCAACCAGCTGCAACTGCCCTGGCCAACACTATAGAGGTCTTCAGATCAAACGGTCTAA<br>CAGCCAATGAATCGGGGAGACTAATAGATTTCCTCAAGGATGTGATGGACTCAATGGATAAAGAAGAAATGGAAATAACAACACATTTCCAGAGAAAGAGAAGAGTAAGGGACAACATGACCAAGAAAATGGTCACACAAAGAACAATAGGAAAGAAGAAACAAAGGCTAAACAAGAGGAGCTACTTAATAAGAGCACTGACACTGAATACAATGACAAAAGATGCAGA<br>AAGAGGCAAATTGAAGAGACGGGCGATTGCAACACCAGGGATGCAGATTAGAGGATTTGTGTACTTTGTCGAAACACTGGCAAGGAGCATCTGTGAAAAACTTGAGCAATCTGGACTCCCCGTTGGAGGGAATGAGAAGAAGGCTAAATTGGCAAATGTCGTGAGAAAAATGATGACTAACTCACAAGATACAGAGCTCTCCTTCACAATTACTGGAGATAACACCAAATG<br>GAATGAGAATCAAAATCCTCGGATGTTTCTGGCAATGATAACATACATTACAAGAAACCAACCTGAATGGTTTAGAAATGTCTTGAGTATTGCCCCTATAATGTTCTCGAACAAAATGGCGAGATTGGGAAAAGGGTACATGTTTGAAAGTAAGAGCATGAAGTTACGGACACAAATACCTGCAGAAATGCTTGCAAACATTGACTTAAAATACTTCAATGAATCAACAAGAAA<br>GAAAATCGAAAAAATAAGGCCTCTACTAATAGATGGCACTGCCTCATTGAGTCCTGGAATGATGATGGGCATGTTCAATATGCTGAGTACAGTATTAGGAGTTTCAATCCTAAATCTTGGGCAAAAGAAGTACACCAAAACCACATACTGGTGGGATGGACTCCAATCCTCTGATGATTTCGCCCTCATAGTAAATGCACCGAATCATGAGGGAATACAAGCAGGAGTGGATA<br>GGTTCTATAGGACCTGCAAACTGGTCGGGATCAATATGAGCAAAAAGAAGTCTTACATAAACCGGACTGGAACATTTGAGTTCACAAGCTTTTTCTATCGCTATGGATTTGTGGCTAACTTCAGTATGGAGCTGCCCAGCTTTGGAGTTTCTGGGATCAATGAATCAGCTGACATGAGCATTGGCGTCACAGTGATAAAGAACAACATGATAAACAATGACCTTGGACCAGCAA<br>CAGCTCAAATGGCCCTTCAACTATTCATCAAAGATTACAGGTACACGTACCGATGCCACAGAGGTGACACACAAATTCAAACGAGGAGATCATTCGAGCTGAAGAAGCTGTGGGAACAGACCCGTTCAAAGGCAGGACTGTTGGTGTCAGATGGAGGACCAAATCTATACAACATTCGGAATCTCCATATCCCAGAGGTCTGCCTGAAGTGGGAGCTGATGGACGAAGATTA<br>CCAGGGCAGGTTGTGTAATCCTCTGAACCCATTTGTCAGCCATAAAGAAATTGAGTCCGTAAACAATGCTGTGGTGATGCCAGCCCACGGTCCAGCCAAAAGCATGGAATATGATGCCGTTGCGACTACACACTCATGGATTCCTAAAAGGAATCGTTCCATTCTCAATACCAGTCAAAGGGGAATTCTTGAGGATGAACAGATGTACCAGAAATGCTGCAGTCTATTCGAGAA<br>ATTCTTCCCCAGTAGTTCATACAGGAGACCAGTTGGAATTTCCAGCATGGTGGAGGCCATGGTGTCTAGGGCCCGAATCGATGCACGCATTGATTTCGAATCTGGAAGGATCAAGAAGGAAGAGTTTGCTGAGATCATGAAGATCTGTTCCACCATTGAAGAGCTCAGACGGCAAAAATAG
EPI_ISL_654824	>A/duck/Saratov/29804/2020_H5N8_2020-09-15<br>ATGGATGTCAATCCGACTTTACTTTTCTTAAAAGTGCCAGCGCAAAATGCTATAAGTACTACATTCCCTTACACTGGAGATCCCCCATACAGCCATGGAACAGGAACAGGGTATACCATGGACACAGTAAACAGAACACATCAATACTCAGAAAAGGGAAAGTGGACAACAAACACAGAAACCGGAGCACCCCAACTCAACCCAATTGATGGACCATTACCAGAGGACAATGA<br>GCCAAGCGGATATGCACAAACTGATTGCGTGTTGGAAGCAATGGCTTTCCTTGAAGAATCCCACCCAGGGATATTTGAAAACTCTTGTCTTGAAGCGATGGAAATCGTTCAGCAAACAAGAGTGGACAAACTAACCCAAGGTCGCCAGACTTATGACTGGACACTGAACAGAAACCAACCAGCTGCAACCTCTTTGGCCAACACTATAGAGGTGTTCAGATCGAATGGTCTGA<br>CAGCCAATGAATCAGGGAGACTGATAGATTTTCTCAGGGATGTGATGGAATCAATGGATAAAGAAGAGATGGAAGTAACAACACATTTCCAGAGAAAAAGAAGAGTGAGGGACAACATGACTAAGAAGATGGTCACACAAAGAACAATAGGGAAGAAGAAGCAGAGGCTGAACAAGAGGAGTTACTTAATAAGAGCACTGACATTGAATACAATGACCAAAGATGCAGA<br>AAGAGGCAAGTTGAAGAGACGGGCAATTGCAACACCCGGGATGCAGATTAGAGGATTCGTGTACTTCGTTGAAACACTAGCGAGGAGCATCTGTGAGAAACTAGAGCAATCTGGGCTCCCTGTTGGAGGGAATGAGAAGAAGGCTAAATTGGCAAATGTTGTGAGAAAAATGATGACTAACTCACAAGATACAGAGCTCTCCTTTACAATTACTGGAGACAACACCAAATG<br>GAATGAGAATCAAAACCCTCGGATGTTTTTGGCAATGATAACATATATCACAAGAAACCAACCTGAATGGTTTAGAAATGTCTTAAGCATTGCCCCTATAATGTTCTCAAACAAAATGGCGAGATTAGGGAAAGGATACATGTTTGAAAGTAAGAGCATGAAGCTAAGAACACAAATACCAGCAGAGATGCTTACAAATATTGATCTGAAGTATTTCAACGAACCAACGAGAAA<br>AGAAAATCGAGAAAATAAGACCTCTGCTGATTGATGGCACGGCCTCATTGAGTCCTGGGATGATGATGGGCATGTTCAATATGCTGAGCACAGTATTAGGGGTCTCAATCCTGAATCTCGGGCAAAAGAGGTACACCAAAACCACATACTGGTGGGATGGACTTCAATCCTCTGATGATTTCGCTCTCATAGTGAATGCACCGAATCATGAGGGGATACAAGCAGGAGTGGAT<br>AGATTCTATAGGACCTGCAAACTGGTTGGGATCAACATGAGCAAAAAGAAGTCTTACATAAACCGAACAGGAACATTTGAGTTCACAAGTTTTTTCTACCGCTATGGATTTGTAGCCAACTTCAGTATGGAATTACCCAGCTTTGGAGTGTCTGGAATCAATGAATCAGCTGACATGAGCATTGGAGTTACAGTGATAAAAAACAATATGATAAACAATGATCTTGGACCAGCA<br>ACAGCTCAAATGGCTCTTCAGTTATTCATCAAAGACTATAGGTACACATACCGATGCCACAGGGGTGATACACAAATTCAAACGAGGAGATCATTCGAGCTGAAGAAGCTGTGGGAGCAGACCCGTTCAAAGGCAGGGCTGTTGATATCAGACGGGGGGCCAAACCTATACAACATTCGGAATCTCCACATCCCAGAGGTCTGCTTGAAGTGGGAGCTGATGGATGAAGACT<br>ACCAAGGCAGGCTGTGCAATCCTCTGAATCCATTTGTCAGTCATAAAGAGATTGAGTCCGTAAACAATGCTGTAGTAATGCCCGCCCATGGCCCGGCCAAGAGCATGGAATATGATGCTGTTGCGACCACACACTCGTGGATTCCTAAGAGGAACCGTTCCGTTCTCAATACCAGCCAAAGGGGAATTCTTGAGGATGAGCAGATGTACCAAAAGTGCTGTAGTCTATTCGAGA<br>AATTCTTCCCCAGCAGTTCATACAGGAGGCCAGTTGGAATTTCCAGCATGGTGGAGGCCATGGTGTCTAGGGCCCGAATTGATGCACGCATCGACTTCGAATCTGGAAGGATTAAGAAAGAAGAGTTTGCTGAGATCATGAAGATCTGTTCCACCATTGAAGAGCTCAGACGGCAAAAATAG
EPI_ISL_5463803	>A/duck/Saratov/29-11V/2021_H5N1_2021-09-30<br>ATGGATGTCAATCCGACTTTACTTTTCTTAAAAGTGCCAGCGCAAGATGCCATAAGTACCACATTCCCTTACACTGGAGATCCTCCATACAGCCATGGAACAGGGACAGGATACACAATGGACACAGTCAACAGAACACATCAATACTCAGAGAAGGGAAAATGGACAACAAACACAGAAACCGGAGCACCTCAACTCAACCCAATTGATGGGCCACTACCTGAGGACAACGA<br>ACCGAGCGGATATGCACAAACAGATTGCGTGTTGGAAGCAATGGCTTTCCTTGAAGAGTCCCATCCAGGGATCTTTGAAAACTCTTGTCTTGAAACGATGGAAGTCGTTCAGCAAACAAGAGTGGACAAACTAACTCAAGGTCGCCAGACATATGACTGGACACTGAATAGAAACCAACCAGCTGCAACTGCCCTGGCCAACACTATAGAGGTCTTCAGATCAAACGGTCTAA<br>CAGCCAATGAATCGGGGAGACTAATAGATTTCCTCAAGGATGTGATGGACTCAATGGATAAAGAAGAAATGGAAATAACAACACATTTCCAGAGAAAGAGAAGAGTAAGGGACAACATGACCAAGAAAATGGTCACACAAAGAACAATAGGAAAGAAGAAACAAAGGCTAAACAAGAGGAGCTACTTAATAAGAGCACTGACACTGAATACAATGACAAAAGATGCAGA<br>AAGAGGCAAATTGAAGAGACGGGCGATTGCAACACCAGGGATGCAGATTAGAGGATTTGTGTACTTTGTCGAAACACTGGCAAGGAGCATCTGTGAAAAACTTGAGCAATCTGGACTCCCCGTTGGAGGGAATGAGAAGAAGGCTAAATTGGCAAATGTCGTGAGAAAAATGATGACTAACTCACAAGATACAGAGCTCTCCTTCACAATTACTGGAGATAACACCAAATG<br>GAATGAGAATCAAAATCCTCGGATGTTTCTGGCAATGATAACATACATTACAAGAAACCAACCTGAATGGTTTAGGAATGTCTTGAGTATTGCCCCTATAATGTTCTCGAACAAAATGGCGAGATTGGGAAAAGGGTACATGTTTGAAAGTAAGAGCATGAAGTTACGGACACAAATACCTGCAGAAATGCTTGCAAACATTGACTTAAAATACTTCAATGAATCAACAAGAAA<br>GAAAATCGAAAAAATAAGGCCTCTACTAATAGATGGCACTGCCTCATTGAGTCCTGGAATGATGATGGGCATGTTCAATATGCTGAGTACAGTATTAGGAGTTTCAATCCTAAATCTTGGGCAAAAGAAGTACACCAAAACCACATACTGGTGGGATGGACTCCAATCCTCTGATGATTTCGCCCTCATAGTAAATGCACCGAATCATGAGGGAATACAAGCAGGAGTGGATA<br>GGTTCTATAGGACCTGCAAACTGGTCGGGATCAATATGAGCAAAAAGAAGTCTTACATAAACCGGACTGGAACATTTGAGTTCACAAGCTTTTTCTATCGCTATGGATTTGTGGCTAACTTCAGTATGGAGCTGCCCAGCTTTGGGGTTTCTGGGATCAATGAATCAGCTGACATGAGCATTGGCATCACAGTGATAAAGAACAACATGATAAACAATGACCTTGGACCAGCAA<br>CAGCTCAAATGGCCCTTCAACTATTCATCAAAGATTACAGGTACACGTACCGATGCCATAGAGGTGACACACAAATTCAAACGAGGAGATCATTCGAGCTGAAGAAGCTGTGGGAACAGACCCGTTCAAAGGCAGGACTGTTGGTGTCAGATGGAGGACCAAATCTATACAACATTCGGAATCTCCATATCCCAGAGGTCTGCCTGAAGTGGGAGCTGATGGACGAAGATTA<br>CCAGGGCAGGTTGTGTAATCCTCTGAACCCATTTGTCAGCCATAAAGAAATTGAGTCCGTAAACAATGCTGTGGTGATGCCAGCCCACGGTCCAGCCAAAAGCATGGAATATGATGCCGTTGCGACTACACACTCATGGATTCCTAAAAGGAATCGTTCCATTCTCAATACCAGTCAAAGGGGAATTCTTGAGGATGAACAGATGTACCAGAAATGCTGCGGTCTATTCGAGAA<br>ATTCTTCCCCAGTAGTTCATACAGGAGACCAGTTGGAATTTCCAGCATGGTGGAGGCCATGGTGTCTAGGGCCCGAATCGATGCACGCATTGATTTCGAATCTGGAAGGATCAAGAAGGAAGAGTTTGCTGAGATCATGAAGATCTGTTCCACCATTGAAGAGCTCAGACGGCAAAAATAG
EPI_ISL_3102061	>A/duck/Poland/H542_21RS1385-2/2021_H5N8_2021-04-09<br>ATGGATGTCAATCCGACTTTGCTTTTCTTAAAAGTGCCAGCGCAAAATGCTATAAGTACTACATTCCCTTACACTGGAGATCCCCCATACAGCCATGGAACAGGAACAGGGTATACCATGGACACAGTAAACAGAACACATCAATATTCAGAAAAGGGAAAGTGGACAACAAACACAGAAACCGGAGCACCCCAACTCAACCCAATTGATGGACCATTACCAGAGGACAATGA<br>GCCAAGCGGATATGCACAAACTGATTGCGTGTTGGAAGCAATGGCTTTCCTTGAAGAATCCCACCCAGGGATATTTGAAAACTCTTGTCTTGAAGCGATGGAAATCGTTCAGCAAACAAGAGTGGACAAACTAACCCAAGGTCGCCAGACTTATGACTGGACACTGAACAGAAACCAACCAGCTGCAACCTCTTTGGCCAACACTATAGAGGTGTTCAGATCGAATGGTCTGA<br>CAGCCAATGAATCAGGGAGACTGATAGATTTTCTCAGGGATGTGATGGAATCAATGGATAAAGAAGAGATGGAAGTAACAACACATTTCCAGAGAAAAAGAAGAGTGAGGGACAACATGACTAAGAAGATGGTCACACAAAGAACAATAGGGAAGAAGAAGCAGAGGCTGAACAAGAGGAGTTACTTAATAAGAGCACTGACATTGAATACAATGACCAAAGATGCAGA<br>AAGAGGCAAGTTGAAGAGACGGGCAATTGCAACACCCGGGATGCAGATTAGAGGATTCGTGTACTTCGTTGAAACACTAGCGAGGAGCATCTGTGAGAAACTAGAGCAATCTGGGCTCCCTGTTGGAGGGAATGAGAAAAAGGCTAAATTGGCAAATGTTGTGAGAAAAATGATGACTAACTCACAAGATACAGAGCTCTCCTTTACAATTACTGGAGACAACACCAAATG<br>GAATGAGAATCAAAACCCTCGGATGTTTTTGGCAATGATAACATATATCACAAGAAACCAACCTGAATGGTTTAGAAATGTCTTAAGCATTGCCCCTATAATGTTCTCAAACAAAATGGCGAGATTAGGGAAAGGATACATGTTTGAAAGTAAGAGCATGAAGCTAAGAACACAAATACCAGCAGAGATGCTTACAAATATTGATCTGAAGTATTTCAACGAACCAACGAGAAA<br>AGAAAATCGAGAATATAAGACCTCTGCTGATTGATGGCACGGCTTCATTGAGCCCTGGGATGATGATGGGCATGTTCAATATGCTGAGCACAGTATTAGGGGTCTCAATCCTGAATCTCGGGCAAAAGAGGTACACCAAAACCACATACTGGTGGGATGGACTTCAATCCTCTGATGATTTCGCTCTCATAGTGAATGCACCGAATCATGAGGGGATACAAGCAGGAGTGGAT<br>AGATTCTATAGGACCTGCAAACTGGTTGGGATCAACATGAGCAAAAAGAAGTCTTACATAAACCGAACAGGAACATTTGAGTTCACAAGTTTTTTCTACCGCTATGGATTTGTAGCCAACTTCAGTATGGAATTACCCAGCTTTGGAGTGTCTGGAATCAATGAATCAGCTGACATGAGCATTGGAGTTACAGTGATAAAAAACAATATGATAAACAATGATCTTGGACCAGCA<br>ACAGCTCAAATGGCTCTCCAGTTATTCATCAAAGACTATAGGTACACATACCGATGCCACAGGGGTGATACACAAATTCAAACGAGGAGATCATTCGAGCTGAAGAAGCTGTGGGAGCAGACCCGTTCAAAGGCAGGACTGTTGATATCAGACGGGGGGCCAAACCTATACAACATTCGGAATCTCCACATCCCAGAGGTCTGCTTGAAGTGGGAGCTGATGGATGAAGACT<br>ACCAAGGCAGGCTGTGCAATCCTCTGAATCCATTTGTCAGTCATAAAGAGATTGAGTCCGTAAACAATGCTGTAGTAATGCCCGCCCATGGCCCGGCCAAGAGCATGGAATATGATGCTGTTGCGACCACACACTCGTGGATTCCTAAGAGGAACCGTTCCATCCTCAATACCAGCCAAAGGGGAATTCTTGAGGATGAGCAGATGTACCAGAAGTGCTGTAGTCTATTCGAG<br>AAATTCTTCCCCAGCAGTTCATACAGGAGGCCAGTTGGAATTTCCAGCATGGTGGAGGCCATGGTGTCTAGGGCCCGAATTGATGCACGCATCGACTTCGAATCTGGAAGGATTAAGAAAGAAGAGTTTGCTGAGATCATGAAGATCTGTTCCACCATTGAAGAGCTCAGACGGCAAAAATAG
EPI_ISL_3102076	>A/duck/Poland/H515_21RS1385-17/2021_H5N8_2021-04-07<br>ATGGATGTCAATCCGACTTTGCTTTTCTTAAAAGTGCCAGCGCAAAATGCTATAAGTACTACATTCCCTTACACTGGAGATCCCCCATACAGCCATGGAACAGGAACAGGGTATACCATGGACACAGTAAACAGAACACATCAATATTCAGAAAAGGGAAAGTGGACAACAAACACAGAAACCGGAGCACCCCAACTCAACCCAATTGATGGACCATTACCAGAGGACAATGA<br>GCCAAGCGGATATGCACAAACTGATTGCGTGTTGGAAGCAATGGCTTTCCTTGAAGAATCCCACCCAGGGATATTTGAAAACTCTTGTCTTGAAGCGATGGAAATCGTTCAGCAAACAAGAGTGGACAAACTAACCCAAGGTCGCCAGACTTATGACTGGACACTGAACAGAAACCAACCAGCTGCAACCTCTTTGGCCAACACTATAGAGGTGTTCAGATCGAATGGTCTGA<br>CAGCCAATGAATCAGGGAGACTGATAGATTTTCTCAGGGATGTGATGGAATCAATGGATAAAGAAGAGATGGAAGTAACAACACATTTCCAGAGAAAAAGAAGAGTGAGGGACAACATGACTAAGAAGATGGTCACACAAAGAACAATAGGGAAGAAGAAGCAGAGGCTGAACAAGAGGAGTTACTTAATAAGAGCACTGACATTGAATACAATGACCAAAGATGCAGA<br>AAGAGGCAAGTTGAAGAGACGGGCAATTGCAACACCCGGGATGCAGATTAGAGGATTCGTGTACTTCGTTGAAACACTAGCGAGGAGCATCTGTGAGAAACTAGAGCAATCTGGGCTCCCTGTTGGAGGGAATGAGAAAAAGGCTAAATTGGCAAATGTTGTGAGAAAAATGATGACTAACTCACAAGATACAGAGCTCTCCTTTACAATTACTGGAGACAACACCAAATG<br>GAATGAGAATCAAAACCCTCGGATGTTTTTGGCAATGATAACATATATCACAAGAAACCAACCTGAATGGTTTAGAAATGTCTTAAGCATTGCCCCTATAATGTTCTCAAACAAAATGGCGAGATTAGGGAAAGGATACATGTTTGAAAGTAAGAGCATGAAGCTAAGAACACAAATACCAGCAGAGATGCTTACAAATATTGATCTGAAGTATTTCAACGAACCAACGAGAAA<br>AGAAAATCGAGAATATAAGACCTCTGCTGATTGATGGCACGGCTTCATTGAGCCCTGGGATGATGATGGGCATGTTCAATATGCTGAGCACAGTATTAGGGGTCTCAATCCTGAATCTCGGGCAAAAGAGGTACACCAAAACCACATACTGGTGGGATGGACTTCAATCCTCTGATGATTTCGCTCTCATAGTGAATGCACCGAATCATGAGGGGATACAAGCAGGAGTGGAT<br>AGATTCTATAGGACCTGCAAACTGGTTGGGATCAACATGAGCAAAAAGAAGTCTTACATAAACCGAACAGGAACATTTGAGTTCACAAGTTTTTTCTACCGCTATGGATTTGTAGCCAACTTCAGTATGGAATTACCCAGCTTTGGAGTGTCTGGAATCAATGAATCAGCTGACATGAGCATTGGAGTTACAGTGATAAAAAACAATATGATAAACAATGATCTTGGACCAGCA<br>ACAGCTCAAATGGCTCTTCAGTTATTCATCAAAGACTATAGGTACACATACCGATGCCACAGGGGTGATACACAAATTCAAACGAGGAGATCATTCGAGCTGAAGAAGCTGTGGGAGCAGACCCGTTCAAAGGCAGGACTGTTGATATCTGACGGGGGGCCAAACCTATACAACATTCGGAATCTCCACATCCCAGAGGTCTGCTTGAAGTGGGAGCTGATGGATGAAGACT<br>ACCAAGGCAGGCTGTGCAATCCTCTGAATCCATTTGTCAGTCATAAAGAGATTGAGTCCGTAAACAATGCTGTAGTAATGCCCGCCCATGGCCCGGCCAAGAGCATGGAATATGATGCTGTTGCGACCACACACTCGTGGATTCCTAAGAGGAACCGTTCCATCCTCAATACCAGCCAAAGGGGAATTCTTGAGGATGAGCAGATGTACCAGAAGTGCTGTAGTCTATTCGAG<br>AAATTCTTCCCCAGCAGTTCATACAGGAGGCCAGTTGGAATTTCCAGCATGGTGGAGGCCATGGTGTCTAGGGCCCGAATTGATGCACGCATCGACTTCGAATCTGGAAGGATTAAGAAAGAAGAGTTTGCTGAGATCATGAAGATCTGTTCCACCATTGAAGAGCTCAGACGGCAAAAATAG
EPI_ISL_3102077	>A/duck/Poland/H514_21RS1385-18/2021_H5N8_2021-04-07<br>ATGGATGTCAATCCGACTTTACTTTTCTTAAAAGTGCCAGCGCAAAATGCTATAAGTACTACATTCCCTTACACTGGAGATCCTCCATACAGCCATGGGACAGGAACAGGGTATACCATGGACACAGTAAACAGAACACATCAATACTCAGAAAAGGGAAAGTGGACAACAAACACAGAAACCGGAGCACCCCAACTCAACCCAATTGATGGACCATTACCAGAGGACAATGA<br>GCCAAGCGGATATGCACAAACTGATTGCGTGTTGGAAGCAATGGCTTTCCTTGAAGAATCCCACCCAGGGATATTTGAAAACTCTTGTCTTGAAGCGATGGAAATCGTTCAGCAAACAAGAGTGGACAAACTAACCCAAGGTCGCCAGACTTATGACTGGACACTGAACAGAAACCAACCAGCTGCAACCTCTTTGGCCAACACTATAGAGGTGTTCAGATCGAATGGTCTGA<br>CAGCCAATGAATCAGGGAGACTGATAGATTTTCTCAGGGATGTGATGGAATCAATGGATAAAGAAGAGATGGAAGTAACAACACATTTCCAGAGAAAAAGAAGAGTGAGGGACAACATGACTAAGAAGATGGTCACACAAAGAACAATAGGGAAGAAGAAGCAGAGGCTGAACAAGAGGAGTTACTTGATAAGAGCACTGACATTGAATACAATGACCAAAGATGCAGA<br>AAGAGGCAAGTTGAAGAGACGGGCAATTGCAACACCCGGGATGCAGATTAGAGGATTCGTGTACTTCGTTGAAACACTAGCGAGGAGCATCTGTGAGAAACTAGAGCAATCTGGGCTCCCTGTTGGAGGGAATGAGAAGAAGGCTAAATTGGCAAATGTTGTGAGAAAAATGATGACTAACTCACAAGATACAGAGCTCTCCTTTACAATTACTGGAGACAACACCAAATG<br>GAATGAGAATCAAAACCCTCGGATGTTTTTGGCAATGATAACATATATCACAAGAAACCAACCTGAATGGTTTAGAAATGTCTTAAGCATTGCCCCTATAATGTTCTCAAACAAAATGGCGAGATTAGGGAAAGGATACATGTTTGAAAGTAAGAGCATGAAGCTAAGAACACAAATACCAGCAGAGATGCTTACAAATATTGATCTGAAGTATTTCAACGAACCAACGAGAAA<br>GAAAATCGAGAAAATAAGACCCCTGCTGATTGATGGCACGGCCTCATTGAGTCCTGGGATGATGATGGGCATGTTCAATATGCTGAGCACAGTATTGGGGGTCTCAATCCTGAATCTCGGGCAAAAGAGGTACACCAAAACCACATACTGGTGGGATGGACTTCAATCCTCTGATGATTTCGCTCTCATAGTGAATGCACCGAATCATGAGGGGATACAAGCAGGAGTGGATA<br>GATTCTATAGGACCTGCAAACTGGTTGGGATCAACATGAGCAAAAAGAAGTCTTACATAAACCGAACAGGAACATTTGAGTTCACAAGTTTTTTCTACCGCTATGGATTTGTAGCTAACTTCAGTATGGAATTACCCAGCTTTGGAGTGTCTGGAATCAATGAATCAGCTGACATGAGCATTGGAGTTACAGTGATAAAAAACAATATGATAAACAATGATCTTGGACCAGCAAC<br>AGCTCAAATGGCTCTTCAGTTATTCATCAAAGACTATAGGTACACATACCGATGCCACAGGGGTGATACACAAATTCAAACGAGGAGATCATTCGAGCTGAAGAAGCTGTGGGAGCAAACCCGTTCAAAGGCAGGGCTGTTGATATCAGACGGGGGGCCAAACCTATACAACATTCGGAACCTCCACATCCCAGAGGTCTGCTTGAAGTGGGAGCTGATGGATGAAGACTAC<br>CAAGGCAGGCTGTGCAATCCTCTGAATCCATTTGTCAGTCATAAAGAGATTGAGTCCGTAAACAATGCTGTAGTAATGCCCGCCCATGGCCCGGCCAAGAGCATGGAATATGATGCTGTTGCGACCACACATTCGTGGATTCCTAAGAGGAACCGCTCCATTCTCAATACCAACCAAAGGGGAATTCTTGAGGATGAGCAGATGTACCAAAAGTGCTGTAGTCTATTCGAGAAA<br>TTCTTCCCCAGCAGTTCATACAGGAGGCCAGTTGGAATTTCCAGCATGGTGGAGGCCATGGTGTCTAGGGCCCGAATTGATGCACGCATCGACTTCGAATCTGGAAGGATTAAGAAAGAAGAGTTTGCTGAGATCATGAAGATCTGTTCCACCATTGGAGAGCTCAGACGGCAAAAATAG

internal NVRI repository	>A/duck/Poland/H2014_21RS3290-17/2021_H5N1_2021-11-20<br>ATGGATGTCAATCCGACTTTACTTTTCTTAAAAGTGCCAGCGCAAGATGCCATAAGTACCACATTCCCTTACACTGGAGATCCTCCATACAGCCATGGAACAGGGACAGGATACACAATGGACACAGTCAACAGAACACATCAATACTCAGAGAAGGGAAAATGGACAACAAACACAGAAACCAGAGCACCTCAACTCAACCCAATTGATGGGCCACTACCTGAGGACAACGA<br>ACCGAGCGGATATGCACAAACAGATTGCGTGTTGGAAGCAATGGCTTTCCTTGAAGAGTCCCACCCAGGGATCTTTGAAAACTCTTGTCTTGAAACGATGGAAGTCGTTCAGCAAACAAGAGTGGACAAACTAACTCAAGGTCGCCAGACATATGACTGGACACTGAATAGAAACCAACCAGCTGCAACTGCCCTGGCCAACACTATAGAGGTCTTCAGATCAAACGGTCTAA<br>CAGCCAATGAATCGGGGAGACTAATAGATTTCCTCAAGGATGTGATGGACTCAATGGATAAAGAAGAAATGGAAATAACAACACATTTCCAGAGAAAGAGAAGAGTAAGGGACAACATGACCAAGAAAATGGTCACACAAAGAACAATAGGAAAGAAGAAACAAAGGCTAAACAAGAGGAGCTACTTAATAAGAGCACTGACACTGAATACAATGACAAAAGATGCAGA<br>AAGAGGCAAATTGAAAAGACGGGCGATTGCAACACCAGGGATGCAGATTAGAGGATTTGTGTACTTTGTCGAAACACTGGCAAGGAGCATCTGTGAAAAACTTGAGCAATCTGGACTCCCCGTTGGAGGGAATGAGAAGAAGGCTAAATTGGCAAATGTCGTGAGAAAAATGATGACTAACTCACAAGATACAGAGCTCTCCTTCACAATTACTGGAGATAACACCAAATG<br>GAATGAGAATCAAAATCCTCGGATGTTTCTGGCAATGATAACATACATTACAAGAAACCAACCTGAATGGTTTAGAAATGTCTTGAGTGTTGCCCCTATAATGTTCTCGAACAAAATGGCGAGATTGGGAAAAGGGTACATGTTTGAAAGTAAGAGCATGAAGTTACGGACACAAATACCTGCAGAAATGCTTGCAAACATTGACTTAAAATACTTCAATGAATCAACAAGAAA<br>GAAAATCGAAAAAATAAGGCCTCTACTAATAGATGGCACTGCCTCATTGAGTCCTGGAATGATGATGGGCATGTTCAATATGCTGAGTACAGTATTAGGAGTTTCAATCCTAAATCTTGGGCAAAAGAAGTACACCAAAACCACATACTGGTGGGATGGACTCCAATCCTCTGATGATTTCGCCCTCATAGTAAATGCACCGAATCATGAGGGAATACAAGCAGGAGTGGATA<br>GGTTCTATAGGACCTGCAAACTGGTCGGGATCAATATGAGCAAAAAGAAGTCTTACATAAACCGGACTGGAACATTTGAGTTCACAAGCTTTTTCTATCGCTATGGATTTGTGGCTAACTTCAGTATGGAGCTGCCCAGCTTTGGAGTTTCTGGGATCAATGAATCAGCTGACATGAGCATTGGCGTCACAGTGATAAAGAACAACATGATAAACAATGACCTTGGACCAGCAA<br>CAGCTCAAATGGCCCTTCAACTATTCATCAAAGATTACAGGTACACGTACCGATGCCACAGAGGTGACACACAAATTCAAACGAGGAGATCATTCGAGCTGAAGAAGCTGTGGGAACAGACCCGTTCAAAGGCAGGACTGTTGGTGTCAGATGGAGGACCAAATCTATACAACATTCGGAATCTCCATATCCCAGAGGTCTGCCTGAAGTGGGAGCTGATGGACGAAGATTA<br>CCAGGGCAGGTTGTGTAATCCTCTGAACCCATTTGTCAGCCATAGAGAAATTGAGTCCGTAAACAATGCTGTGGTGGTGCCAGCCCACGGTCCAGCCAAAAGCATGGAATATGATGCCGTTGCGACTACACACTCATGGATTCCTAAAAGGAATCGTTCCATTCTCAATACCAGTCAAAGGGGAATTCTTGAGGATGAACAGATGTACCAGAAATGCTGCAGTCTATTCGAGAA<br>ATTCTTCCCCAGTAGTTCATACAGGAGACCAGTTGGAATTTCCAGCATGGTGGAGGCCATGGTGTCTAGGGCCCGAATCGATGCACGAATTGATTTCGAATCTGGAAGGATAAAGAAGGAAGAGTTTGCTGAGATCATGAAGATCTGTTCCACCATTGAAGAGCTCAGACGGCAAAAATAG
internal NVRI repository	>A/duck/Poland/H2008_21RS3290-16/2021_H5N1_2021-11-18<br>ATGGATGTCAATCCGACTTTACTTTTCTTAAAAGTGCCAGCGCAAGATGCCATAAGTACCACATTCCCTTACACTGGAGATCCTCCATACAGCCATGGAACAGGGACAGGATACACAATGGACACAGTCAACAGAACACATCAATACTCAGAGAAGGGAAAATGGACAACAAACACAGAAACCAGAGCACCTCAACTCAACCCAATTGATGGGCCACTACCTGAGGACAACGA<br>ACCGAGCGGATATGCACAAACAGATTGCGTGTTGGAAGCAATGGCTTTCCTTGAAGAGTCCCACCCAGGGATCTTTGAAAACTCTTGTCTTGAAACGATGGAAGTCGTTCAGCAAACAAGAGTGGACAAACTAACTCAAGGTCGCCAGACATATGACTGGACACTGAATAGAAACCAACCAGCTGCAACTGCCCTGGCCAACACTATAGAGGTCTTCAGATCAAACGGTCTAA<br>CAGCCAATGAATCGGGGAGACTAATAGATTTCCTCAAGGATGTGATGGACTCAATGGATAAAGAAGAAATGGAAATAACAACACATTTCCAGAGAAAGAGAAGAGTAAGGGACAACATGACCAAGAAAATGGTCACACAAAGAACAATAGGAAAGAAGAAACAAAGGCTAAACAAGAGGAGCTACTTAATAAGAGCACTGACACTGAATACAATGACAAAAGATGCAGA<br>AAGAGGCAAATTGAAAAGACGGGCGATTGCAACACCAGGGATGCAGATTAGAGGATTTGTGTACTTTGTCGAAACACTGGCAAGGAGCATCTGTGAAAAACTTGAGCAATCTGGACTCCCCGTTGGAGGGAATGAGAAGAAGGCTAAATTGGCAAATGTCGTGAGAAAAATGATGACTAACTCACAAGATACAGAGCTCTCCTTCACAATTACTGGAGATAACACCAAATG<br>GAATGAGAATCAAAATCCTCGGATGTTTCTGGCAATGATAACATACATTACAAGAAACCAACCTGAATGGTTTAGAAATGTCTTGAGTGTTGCCCCTATAATGTTCTCGAACAAAATGGCGAGATTGGGAAAAGGGTACATGTTTGAAAGTAAGAGCATGAAGTTACGGACACAAATACCTGCAGAAATGCTTGCAAACATTGACTTAAAATACTTCAATGAATCAACAAGAAA<br>GAAAATCGAAAAAATAAGGCCTCTACTAATAGATGGCACTGCCTCATTGAGTCCTGGAATGATGATGGGCATGTTCAATATGCTGAGTACAGTATTAGGAGTTTCAATCCTAAATCTTGGGCAAAAGAAGTACACCAAAACCACATACTGGTGGGATGGACTCCAATCCTCTGATGATTTCGCCCTCATAGTAAATGCACCGAATCATGAGGGAATACAAGCAGGAGTGGATA<br>GGTTCTATAGGACCTGCAAACTGGTCGGGATCAATATGAGCAAAAAGAAGTCTTACATAAACCGGACTGGAACATTTGAGTTCACAAGCTTTTTCTATCGCTATGGATTTGTGGCTAACTTCAGTATGGAGCTGCCCAGCTTTGGAGTTYCTGGGATCAATGAATCAGCTGACATGAGCATTGGCGTCACAGTGATAAAGAACAACATGATAAACAATGACCTTGGACCAGCAA<br>CAGCTCAAATGGCCCTTCAACTATTCATCAAAGATTACAGGTACACGTACCGATGCCACAGAGGTGACACACAAATTCAAACGAGGAGATCATTCGAGCTGAAGAAGCTGTGGGAACAGACCCGTTCAAAGGCAGGACTGTTGGTGTCAGATGGAGGACCAAATCTATACAACATTCGGAATCTCCATATCCCAGAGGTCTGCCTGAAGTGGGAGCTGATGGACGAAGATTA<br>CCAGGGCAGGTTGTGTAATCCTCTGAACCCATTTGTCAGCCATAGAGAAATTGAGTCCGTAAACAATGCTGTGGTGATGCCAGCCCACGGTCCAGCCAAAAGCATGGAATATGATGCCGTTGCGACTACACACTCATGGATTCCTAAAAGGAATCGTTCCATTCTCAATACCAGTCAAAGGGGAATTCTTGAGGATGAACAGATGTACCAGAAATGCTGCAGTCTATTCGAGAA<br>ATTCTTCCCCAGTAGTTCATACAGGAGACCAGTTGGAATTTCCAGCATGGTGGAGGCCATGGTGTCTAGGGCCCGAATCGATGCACGAATTGATTTCGAATCTGGAAGGATAAAGAAGGAAGAGTTTGCTGAGATCATGAAGATCTGTTCCACCATTGAAGAGCTCAGACGGCAAAAATAG
internal NVRI repository	>A/duck/Poland/H2007_21RS3290-15/2021_H5N1_2021-11-17<br>ATGGATGTCAATCCGACTTTACTTTTCTTAAAAGTGCCAGCGCAAGATGCCATAAGTACCACATTCCCTTACACTGGAGATCCTCCATACAGCCATGGAACAGGGACAGGATACACAATGGACACAGTCAACAGAACACATCAATACTCAGAGAAGGGAAAATGGGCAACAAACACAGAAACCGGAGCACCTCAACTCAACCCAATTGATGGGCCACTACCTGAGGACAACGA<br>ACCGAGCGGATATGCACAAACAGATTGCGTGTTGGAAGCAATGGCTTTCCTTGAAGAGTCCCATCCAGGGATCTTTGAAAACTCTTGTCTTGAAACGATGGAAGTCGTTCAGCAAACAAGAGTGGACAAACTAACTCAAGGTCGCCAGACATATGACTGGACACTGAATAGAAACCAACCAGCTGCAACTGCCCTGGCCAACACTATAGAGGTCTTCAGATCAAACGGTCTAA<br>CAGCCAATGAATCGGGGAGACTAATAGATTTCCTCAAGGATGTGATGGACTCAATGGATAAAGAAGAAATGGAAATAACAACACATTTCCAGAGAAAGAGAAGAGTAAGGGACAACATGACCAAGAAAATGGTCACACAAAGAACAATAGGAAAGAAGAAACAAAGGCTAAACAAGAGGAGCTACTTAATAAGAGCACTGACACTGAATACAATGACAAAAGATGCAGA<br>AAGAGGCAAATTGAAGAGACGGGCGATTGCAACACCAGGGATGCAGATTAGAGGATTTGTGTACTTTGTCGAAACACTGGCAAGGAGCATCTGTGAAAAACTTGAGCAATCTGGACTCCCCGTTGGAGGGAATGAGAAGAAGGCTAAATTGGCAAATGTTGTGAGAAAAATGATGACTAACTCACAAGATACAGAGCTCTCCTTCACAATTACTGGAGATAACACCAAATG<br>GAATGAGAATCAAAATCCTCGGATGTTTCTGGCAATGATAACATACATTACAAGAAACCAACCTGAATGGTTTAGGAATGTCTTGAGTATTGCCCCTATAATGTTCTCGAACAAAATGGCGAGATTGGGAAAAGGGTACATGTTTGAAAGTAAGAGCATGAAGTTACGGACACAAATACCTGCAGAAATGCTTGCAAACATTGACTTAAAATACTTCAATGAATCAACAAGAAA<br>GAAAATCGAAAAAATAAGGCCTCTACTAATAGATGGCACTGCCTCATTGAGTCCTGGAATGATGATGGGCATGTTCAATATGCTGAGTACAGTATTGGGAGTTTCAATCCTAAATCTTGGGCAAAAGAAGTACACCAAAACCACATACTGGTGGGATGGACTCCAATCCTCTGATGATTTCGCCCTCATAGTAAATGCACCGAATCATGAGGGAATACAAGCAGGAGTGGATA<br>GGTTCTATAGGACCTGCAAACTGGTCGGGATCAATATGAGCAAAAAGAAGTCTTACATAAACCGGACTGGAACATTTGAGTTCACAAGCTTTTTCTATCGCTATGGATTTGTGGCTAACTTCAGTATGGAGCTGCCCAGCTTTGGGGTTTCTGGGATCAATGAATCAGCTGACATGAGCATTGGCATCACAGTGATAAAGAACAACATGATAAACAATGACCTTGGACCAGCAA<br>CAGCTCAAATGGCCCTTCAACTATTCATCAAAGATTACAGGTACACGTACCGATGCCATAGAGGTGACACACAAATTCAAACGAGGAGATCATTCGAGCTGAAGAAGCTGTGGGAACAGACCCGTTCAAAGGCAGGACTGTTGGTGTCAGATGGAGGACCAAATCTATACAACATTCGGAATCTCCATATCCCAGAGGTCTGCCTGAAGTGGGAGCTGATGGACGAAGATTA<br>CCAGGGCAGGTTGTGTAATCCTCTGAACCCATTTGTCAGCCATAAAGAAATTGAGTCCGTAAACAATGCTGTGGTGATGCCAGCCCACGGTCCAGCCAAAAGCATGGAATATGATGCCGTTGCGACTACACACTCATGGATTCCTAAAAGGAATCGTTCCATTCTCAATACCAGTCAAAGGGGAATTCTTGAGGATGAACAGATGTACCAGAAATGCTGCGGTCTATTCGAGAA<br>ATTCTTCCCCAGTAGTTCATACAGGAGACCAGTTGGAATTTCCAGCATGGTGGAGGCCATGGTGTCTAGGGCCCGAATCGATGCACGCATTGATTTCGAATCTGGAAGGATCAAGAAGGAAGAGTTTGCTGAGATCATGAAGATCTGTTCCACCATTGAAGAGCTCAGACGGCAAAAATAG
internal NVRI repository	>A/duck/Poland/H1993_21RS3290-14/2021_H5N1_2021-11-17<br>ATGGATGTCAATCCGACTTTACTTTTCTTAAAAGTGCCAGCGCAAGATGCCATAAGTACCACATTCCCTTACACTGGAGATCCTCCATACAGCCATGGAACAGGGACAGGATACACAATGGACACAGTCAACAGAACACATCAATACTCAGAGAAGGGAAAATGGACAACAAACACAGAAACCAGAGCACCTCAACTCAACCCAATTGATGGGCCACTACCTGAGGACAACGA<br>ACCGAGCGGATATGCACAAACAGATTGCGTGTTGGAAGCAATGGCTTTCCTTGAAGAGTCCCACCCAGGGATCTTTGAAAACTCTTGTCTTGAAACGATGGAAGTCGTTCAGCAAACAAGAGTGGACAAACTAACTCAAGGTCGCCAGACATATGACTGGACACTGAATAGAAACCAACCAGCTGCAACTGCCCTGGCCAACACTATAGAGGTCTTCAGATCAAACGGTCTAA<br>CAGCCAATGAATCGGGGAGACTAATAGATTTCCTCAAGGATGTGATGGACTCAATGGATAAAGAAGAAATGGAAATAACAACACATTTCCAGAGAAAGAGAAGAGTAAGGGACAACATGACCAAGAAAATGGTCACACAAAGAACAATAGGAAAGAAGAAACAAAGGCTAAACAAGAGGAGCTACTTAATAAGAGCACTGACACTGAATACAATGACAAAAGATGCAGA<br>AAGAGGCAAATTGAAAAGACGGGCGATTGCAACACCAGGGATGCAGATTAGAGGATTTGTGTACTTTGTCGAAACACTGGCAAGGAGCATCTGTGAAAAACTTGAGCAATCTGGACTCCCCGTTGGAGGGAATGAGAAGAAGGCTAAATTGGCAAATGTCGTGAGAAAAATGATGACTAACTCACAAGATACAGAGCTCTCCTTCACAATTACTGGAGATAACACCAAATG<br>GAATGAGAATCAAAATCCTCGGATGTTTCTGGCAATGATAACATACATTACAAGAAACCAACCTGAATGGTTTAGAAATGTCTTGAGTGTTGCCCCTATAATGTTCTCGAACAAAATGGCGAGATTGGGAAAAGGGTACATGTTTGAAAGTAAGAGCATGAAGTTACGGACACAAATACCTGCAGAAATGCTTGCAAACATTGACTTAAAATACTTCAATGAATCAACAAGAAA<br>GAAAATCGAAAAAATAAGGCCTCTACTAATAGATGGCACTGCCTCATTGAGTCCTGGAATGATGATGGGCATGTTCAATATGCTGAGTACAGTATTAGGAGTTTCAATCCTAAATCTTGGGCAAAAGAAGTACACCAAAACCACATACTGGTGGGATGGACTCCAATCCTCTGATGATTTCGCCCTCATAGTAAATGCACCGAATCATGAGGGAATACAAGCAGGAGTGGATA<br>GGTTCTATAGGACCTGCAAACTGGTCGGGATCAATATGAGCAAAAAGAAGTCTTACATAAACCGGACTGGAACATTTGAGTTCACAAGCTTTTTCTATCGCTATGGATTTGTGGCTAACTTCAGTATGGAGCTGCCCAGCTTTGGAGTTTCTGGGATCAATGAATCAGCTGACATGAGCATTGGCGTCACAGTGATAAAGAACAACATGATAAACAATGACCTTGGACCAGCAA<br>CAGCTCAAATGGCCCTTCAACTATTCATCAAAGATTACAGGTACACGTACCGATGCCACAGAGGTGACACACAAATTCAAACGAGGAGATCATTCGAGCTGAAGAAGCTGTGGGAACAGACCCGTTCAAAGGCAGGACTGTTGGTGTCAGATGGAGGACCAAATCTATACAACATTCGGAATCTCCATATCCCAGAGGTCTGCCTGAAGTGGGAGCTGATGGACGAAGATTA<br>CCAGGGCAGGTTGTGTAATCCTCTGAACCCATTTGTCAGCCATAGAGAAATTGAGTCCGTAAACAATGCTGTGGTGGTGCCAGCCCACGGTCCAGCCAAAAGCATGGAATATGATGCCGTTGCGACTACACACTCATGGATTCCTAAAAGGAATCGTTCCATTCTCAATACCAGTCAAAGGGGAATTCTTGAGGATGAACAGATGTACCAGAAATGCTGCAGTCTATTCGAGAA<br>ATTCTTCCCCAGTAGTTCATACAGGAGACCAGTTGGAATTTCCAGCATGGTGGAGGCCATGGTGTCTAGGGCCCGAATCGATGCACGAATTGATTTCGAATCTGGAAGGATAAAGAAGGAAGAGTTTGCTGAGATCATGAAGATCTGTTCCACCATTGAAGAGCTCAGACGGCAAAAATAG
internal NVRI repository	>A/duck/Poland/H1982_21RS3290-12/2021_H5N1_2021-11-16<br>ATGGATGTCAATCCGACTTTACTTTTCTTAAAAGTGCCAGCGCAAGATGCCATAAGTACCACATTCCCTTACACTGGAGATCCTCCATACAGCCATGGAACAGGGACAGGATACACAATGGACACAGTCAACAGAACACATCAATACTCAGAGAAGGGAAAATGGACAACAAACACAGAAACCAGAGCACCTCAACTCAACCCAATTGATGGGCCACTACCTGAGGACAACGA<br>ACCGAGCGGATATGCACAAACAGATTGCGTGTTGGAAGCAATGGCTTTCCTTGAAGAGTCCCACCCAGGGATCTTTGAAAACTCTTGTCTTGAAACGATGGAAGTCGTTCAGCAAACAAGAGTGGACAAACTAACTCAAGGTCGCCAGACATATGACTGGACACTGAATAGAAACCAACCAGCTGCAACTGCCCTGGCCAACACTATAGAGGTCTTCAGATCAAACGGTCTAA<br>CAGCCAATGAATCGGGGAGACTAATAGATTTCCTCAAGGATGTGATGGACTCAATGGATAAAGAAGAAATGGAAATAACAACACATTTCCAGAGAAAGAGAAGAGTAAGGGACAACATGACCAAGAAAATGGTCACACAAAGAACAATAGGAAAGAAGAAACAAAGGCTAAACAAGAGGAGCTACTTAATAAGAGCACTGACACTGAATACAATGACAAAAGATGCAGA<br>AAGAGGCAAATTGAAAAGACGGGCGATTGCAACACCAGGGATGCAGATTAGAGGATTTGTGTACTTTGTCGAAACACTGGCAAGGAGCATCTGTGAAAAACTTGAGCAATCTGGACTCCCCGTTGGAGGGAATGAGAAGAAGGCTAAATTGGCAAATGTCGTGAGAAAAATGATGACTAACTCACAAGATACAGAGCTCTCCTTCACAATTACTGGAGATAACACCAAATG<br>GAATGAGAATCAAAATCCTCGGATGTTTCTGGCAATGATAACATACATTACAAGAAACCAACCTGAATGGTTTAGAAATGTCTTGAGTGTTGCCCCTATAATGTTCTCGAACAAAATGGCGAGATTGGGAAAAGGGTACATGTTTGAAAGTAAGAGCATGAAGTTACGGACACAAATACCTGCAGAAATGCTTGCAAACATTGACTTAAAATACTTCAATGAATCAACAAGAAA<br>GAAAATCGAAAAAATAAGGCCTCTACTAATAGATGGCACTGCCTCATTGAGTCCTGGAATGATGATGGGCATGTTCAATATGCTGAGTACAGTATTAGGAGTTTCAATCCTAAATCTTGGGCAAAAGAAGTACACCAAAACCACATACTGGTGGGATGGACTCCAATCCTCTGATGATTTCGCCCTCATAGTAAATGCACCGAATCATGAGGGAATACAAGCAGGAGTGGATA<br>GGTTCTATAGGACCTGCAAACTGGTCGGGATCAATATGAGCAAAAAGAAGTCTTACATAAACCGGACTGGAACATTTGAGTTCACAAGCTTTTTCTATCGCTATGGATTTGTGGCTAACTTCAGTATGGAGCTGCCCAGCTTTGGAGTTTCTGGGATCAATGAATCAGCTGACATGAGCATTGGCGTCACAGTGATAAAGAACAACATGATAAACAATGACCTTGGACCAGCAA<br>CAGCTCAAATGGCCCTTCAACTATTCATCAAAGATTACAGGTACACGTACCGATGCCACAGAGGTGACACACAAATTCAAACGAGGAGATCATTCGAGCTGAAGAAGCTGTGGGAACAGACCCGTTCAAAGGCAGGACTGTTGGTGTCAGATGGAGGACCAAATCTATACAACATTCGGAATCTCCATATCCCAGAGGTCTGCCTGAAGTGGGAGCTGATGGACGAAGATTA<br>CCAGGGCAGGTTGTGTAATCCTCTGAACCCATTTGTCAGCCATAGAGAAATTGAGTCCGTAAACAATGCTGTGGTGATGCCAGCCCACGGTCCAGCCAAAAGCATGGAATATGATGCCGTTGCGACTACACACTCATGGATTCCTAAAAGGAATCGTTCCATTCTCAATACCAGTCAAAGGGGAATTCTTGAGGATGAACAGATGTACCAGAAATGCTGCAGTCTATTCGAGAA<br>ATTCTTCCCCAGTAGTTCATACAGGAGACCAGTTGGAATTTCCAGCATGGTGGAGGCCATGGTGTCTAGGGCCCGAATCGATGCACGAATTGATTTCGAATCTGGAAGGATAAAGAAGGAAGAGTTTGCTGAGATCATGAAGATCTGTTCCACCATTGAAGAGCTCAGACGGCAAAAATAG
internal NVRI repository	>A/duck/Poland/H1980_21RS3290-11/2021_H5N1_2021-11-16<br>ATGGATGTCAATCCGACTTTACTTTTCTTAAAAGTGCCAGCGCAAGATGCCATAAGTACCACATTCCCTTACACTGGAGATCCTCCATACAGCCATGGAACAGGGACAGGATACACAATGGACACAGTCAACAGAACACATCAATACTCAGAGAAGGGAAAATGGACAACAAACACAGAAACCAGAGCACCTCAACTCAACCCAATTGATGGGCCACTACCTGAGGACAACGA<br>ACCGAGCGGATATGCACAAACAGATTGCGTGTTGGAAGCAATGGCTTTCCTTGAAGAGTCCCACCCAGGGATCTTTGAAAACTCTTGTCTTGAAACGATGGAAGTCGTTCAGCAAACAAGAGTGGACAAACTAACTCAAGGTCGCCAGACATATGACTGGACACTGAATAGAAACCAACCAGCTGCAACTGCCCTGGCCAACACTATAGAGGTCTTCAGATCAAACGGTCTAA<br>CAGCCAATGAATCGGGGAGACTAATAGATTTCCTCAAGGATGTGATGGACTCAATGGATAAAGAAGAAATGGAAATAACAACACATTTCCAGAGAAAGAGAAGAGTAAGGGACAACATGACCAAGAAAATGGTCACACAAAGAACAATAGGAAAGAAGAAACAAAGGCTAAACAAGAGGAGCTACTTAATAAGAGCACTGACACTGAATACAATGACAAAAGATGCAGA<br>AAGAGGCAAATTGAAAAGACGGGCGATTGCAACACCAGGGATGCAGATTAGAGGATTTGTGTACTTTGTCGAAACACTGGCAAGGAGCATCTGTGAAAAACTTGAGCAATCTGGACTCCCCGTTGGAGGGAATGAGAAGAAGGCTAAATTGGCAAATGTCGTGAGAAAAATGATGACTAACTCACAAGATACAGAGCTCTCCTTCACAATTACTGGAGATAACACCAAATG<br>GAATGAGAATCAAAATCCTCGGATGTTTCTGGCAATGATAACATACATTACAAGAAACCAACCTGAATGGTTTAGAAATGTCTTGAGTGTTGCCCCTATAATGTTCTCGAACAAAATGGCGAGATTGGGAAAAGGGTACATGTTTGAAAGTAAGAGCATGAAGTTACGGACACAAATACCTGCAGAAATGCTTGCAAACATTGACTTAAAATACTTCAATGAATCAACAAGAAA<br>GAAAATCGAAAAAATAAGGCCTCTACTAATAGATGGCACTGCCTCATTGAGTCCTGGAATGATGATGGGCATGTTCAATATGCTGAGTACAGTATTAGGAGTTTCAATCCTAAATCTTGGGCAAAAGAAGTACACCAAAACCACATACTGGTGGGATGGACTCCAATCCTCTGATGATTTCGCCCTCATAGTAAATGCACCGAATCATGAGGGAATACAAGCAGGAGTGGATA<br>GGTTCTATAGGACCTGCAAACTGGTCGGGATCAATATGAGCAAAAAGAAGTCTTACATAAACCGGACTGGAACATTTGAGTTCACAAGCTTTTTCTATCGCTATGGATTTGTGGCTAACTTCAGTATGGAGCTGCCCAGCTTTGGAGTTTCTGGGATCAATGAATCAGCTGACATGAGCATTGGCGTCACAGTGATAAAGAACAACATGATAAACAATGACCTTGGACCAGCAA<br>CAGCTCAAATGGCCCTTCAACTATTCATCAAAGATTACAGGTACACGTACCGATGCCACAGAGGTGACACACAAATTCAAACGAGGAGATCATTCGAGCTGAAGAAGCTGTGGGAACAGACCCGTTCAAAGGCAGGACTGTTGGTGTCAGATGGAGGACCAAATCTATACAACATTCGGAATCTCCATATCCCAGAGGTCTGCCTGAAGTGGGAGCTGATGGACGAAGATTA<br>CCAGGGCAGGTTGTGTAATCCTCTGAACCCATTTGTCAGCCATAGAGAAATTGAGTCCGTAAACAATGCTGTGGTGGTGCCAGCCCACGGTCCAGCCAAAAGCATGGAATATGATGCCGTTGCGACTACACACTCATGGATTCCTAAAAGGAATCGTTCCATTCTCAATACCAGTCAAAGGGGAATTCTTGAGGATGAACAGATGTACCAGAAATGCTGCAGTCTATTCGAGAA<br>ATTCTTCCCCAGTAGTTCATACAGGAGACCAGTTGGAATTTCCAGCATGGTGGAGGCCATGGTGTCTAGGGCCCGAATCGATGCACGAATTGATTTCGAATCTGGAAGGATAAAGAAGGAAGAGTTTGCTGAGATCATGAAGATCTGTTCCACCATTGAAGAGCTCAGACGGCAAAAATAG
internal NVRI repository	>A/duck/Poland/H1971_21RS3290-8/2021_H5N1_2021-11-13<br>ATGGATGTCAATCCGACTTTACTTTTCTTAAAAGTGCCAGCGCAAGATGCCATAAGTACCACATTCCCTTACACTGGAGATCCTCCATACAGCCATGGAACAGGGACAGGATACACAATGGACACAGTCAACAGAACACATCAATACTCAGAGAAGGGAAAATGGACAACAAACACAGAAACCAGAGCACCTCAACTCAACCCAATTGATGGGCCACTACCTGAGGACAACGA<br>ACCGAGCGGATATGCACAAACAGATTGCGTGTTGGAAGCAATGGCTTTCCTTGAAGAGTCCCACCCAGGGATCTTTGAAAACTCTTGTCTTGAAACGATGGAAGTCGTTCAGCAAACAAGAGTGGACAAACTAACTCAAGGTCGCCAGACATATGACTGGACACTGAATAGAAACCAACCAGCTGCAACTGCCCTGGCCAACACTATAGAGGTCTTCAGATCAAACGGTCTAA<br>CAGCCAATGAATCGGGGAGACTAATAGATTTCCTCAAGGATGTGATGGACTCAATGGATAAAGAAGAAATGGAAATAACAACACATTTCCAGAGAAAGAGAAGAGTAAGGGACAACATGACCAAGAAAATGGTCACACAAAGAACAATAGGAAAGAAGAAACAAAGGCTAAACAAGAGGAGCTACTTAATAAGAGCACTGACACTGAATACAATGACAAAAGATGCAGA<br>AAGAGGCAAATTGAAAAGACGGGCGATTGCAACACCAGGGATGCAGATTAGAGGATTTGTGTACTTTGTCGAAACACTGGCAAGGAGCATCTGTGAAAAACTTGAGCAATCTGGACTCCCCGTTGGAGGGAATGAGAAGAAGGCTAAATTGGCAAATGTCGTGAGAAAAATGATGACTAACTCACAAGATACAGAGCTCTCCTTCACAATTACTGGAGATAACACCAAATG<br>GAATGAGAATCAAAATCCTCGGATGTTTCTGGCAATGATAACATACATTACAAGAAACCAACCTGAATGGTTTAGAAATGTCTTGAGTGTTGCCCCTATAATGTTCTCGAACAAAATGGCGAGATTGGGAAAAGGGTACATGTTTGAAAGTAAGAGCATGAAGTTACGGACACAAATACCTGCAGAAATGCTTGCAAACATTGACTTAAAATACTTCAATGAATCAACAAGAAA<br>GAAAATCGAAAAAATAAGGCCTCTACTAATAGATGGCACTGCCTCATTGAGTCCTGGAATGATGATGGGCATGTTCAATATGCTGAGTACAGTATTAGGAGTTTCAATCCTAAATCTTGGGCAAAAGAAGTACACCAAAACCACATACTGGTGGGATGGACTCCAATCCTCTGATGATTTCGCCCTCATAGTAAATGCACCGAATCATGAGGGAATACAAGCAGGAGTGGATA<br>GGTTCTATAGGACCTGCAAACTGGTCGGGATCAATATGAGCAAAAAGAAGTCTTACATAAACCGGACTGGAACATTTGAGTTCACAAGCTTTTTCTATCGCTATGGATTTGTGGCTAACTTCAGTATGGAGCTGCCCAGCTTTGGAGTTTCTGGGATCAATGAATCAGCTGACATGAGCATTGGCGTCACAGTGATAAAGAACAACATGATAAACAATGACCTTGGACCAGCAA<br>CAGCTCAAATGGCCCTTCAACTATTCATCAAAGATTACAGGTACACGTACCGATGCCACAGAGGTGACACACAAATTCAAACGAGGAGATCATTCGAGCTGAAGAAGCTGTGGGAACAGACCCGTTCAAAGGCAGGACTGTTGGTGTCAGATGGAGGACCAAATCTATACAACATTCGGAATCTCCATATCCCAGAGGTCTGCCTGAAGTGGGAGCTGATGGACGAAGATTA<br>CCAGGGCAGGTTGTGTAATCCTCTGAACCCATTTGTCAGCCATAGAGAAATTGAGTCCGTAAACAATGCTGTGGTGGTGCCAGCCCACGGTCCAGCCAAAAGCATGGAATATGATGCCGTTGCGACTACACACTCATGGATTCCTAAAAGGAATCGTTCCATTCTCAATACCAGTCAAAGGGGAATTCTTGAGGATGAACAGATGTACCAGAAATGCTGCAGTCTATTCGAGAA<br>ATTCTTCCCCAGTAGTTCATACAGGAGACCAGTTGGAATTTCCAGCATGGTGGAGGCCATGGTGTCTAGGGCCCGAATCGATGCACGAATTGATTTCGAATCTGGAAGGATAAAGAAGGAAGAGTTTGCTGAGATCATGAAGATCTGTTCCACCATTGAAGAGCTCAGACGGCAAAAATAG
internal NVRI repository	>A/duck/Poland/H1970_21RS3290-7/2021_H5N1_2021-11-13<br>ATGGATGTCAATCCGACTTTACTTTTCTTAAAAGTGCCAGCGCAAGATGCCATAAGTACCACATTCCCTTACACTGGAGATCCTCCATACAGCCATGGAACAGGGACAGGATACACAATGGACACAGTCAACAGAACACATCAATACTCAGAGAAGGGAAAATGGACAACAAACACAGAAACCAGAGCACCTCAACTCAACCCAATTGATGGGCCACTACCTGAGGACAACGA<br>ACCGAGCGGATATGCACAAACAGATTGCGTGTTGGAAGCAATGGCTTTCCTTGAAGAGTCCCACCCAGGGATCTTTGAAAACTCTTGTCTTGAAACGATGGAAGTCGTTCAGCAAACAAGAGTGGACAAACTAACTCAAGGTCGCCAGACATATGACTGGACACTGAATAGAAACCAACCAGCTGCAACTGCCCTGGCCAACACTATAGAGGTCTTCAGATCAAACGGTCTAA<br>CAGCCAATGAATCGGGGAGACTAATAGATTTCCTCAAGGATGTGATGGACTCAATGGATAAAGAAGAAATGGAAATAACAACACATTTCCAGAGAAAGAGAAGAGTAAGGGACAACATGACCAAGAAAATGGTCACACAAAGAACAATAGGAAAGAAGAAACAAAGGCTAAACAAGAGGAGCTACTTAATAAGAGCACTGACACTGAATACAATGACAAAAGATGCAGA<br>AAGAGGCAAATTGAAAAGACGGGCGATTGCAACACCAGGGATGCAGATTAGAGGATTTGTGTACTTTGTCGAAACACTGGCAAGGAGCATCTGTGAAAAACTTGAGCAATCTGGACTCCCCGTTGGAGGGAATGAGAAGAAGGCTAAATTGGCAAATGTCGTGAGAAAAATGATGACTAACTCACAAGATACAGAGCTCTCCTTCACAATTACTGGAGATAACACCAAATG<br>GAATGAGAATCAAAATCCTCGGATGTTTCTGGCAATGATAACATACATTACAAGAAACCAACCTGAATGGTTTAGAAATGTCTTGAGTGTTGCCCCTATAATGTTCTCGAACAAAATGGCGAGATTGGGAAAAGGGTACATGTTTGAAAGTAAGAGCATGAAGTTACGGACACAAATACCTGCAGAAATGCTTGCAAACATTGACTTAAAATACTTCAATGAATCAACAAGAAA<br>GAAAATCGAAAAAATAAGGCCTCTACTAATAGATGGCACTGCCTCATTGAGTCCTGGAATGATGATGGGCATGTTCAATATGCTGAGTACAGTATTAGGAGTTTCAATCCTAAATCTTGGGCAAAAGAAGTACACCAAAACCACATACTGGTGGGATGGACTCCAATCCTCTGATGATTTCGCCCTCATAGTAAATGCACCGAATCATGAGGGAATACAAGCAGGAGTGGATA<br>GGTTCTATAGGACCTGCAAACTGGTCGGGATCAATATGAGCAAAAAGAAGTCTTACATAAACCGGACTGGAACATTTGAGTTCACAAGCTTTTTCTATCGCTATGGATTTGTGGCTAACTTCAGTATGGAGCTGCCCAGCTTTGGAGTTTCTGGGATCAATGAATCAGCTGACATGAGCATTGGCGTCACAGTGATAAAGAACAACATGATAAACAATGACCTTGGACCAGCAA<br>CAGCTCAAATGGCCCTTCAACTATTCATCAAAGATTACAGGTACACGTACCGATGCCACAGAGGTGACACACAAATTCAAACGAGGAGATCATTCGAGCTGAAGAAGCTGTGGGAACAGACCCGTTCAAAGGCAGGACTGTTGGTGTCAGATGGAGGACCAAATCTATACAACATTCGGAATCTCCATATCCCAGAGGTCTGCCTGAAGTGGGAGCTGATGGACGAAGATTA<br>CCAGGGCAGGTTGTGTAATCCTCTGAACCCATTTGTCAGCCATAGAGAAATTGAGTCCGTAAACAATGCTGTGGTGGTGCCAGCCCACGGTCCAGCCAAAAGCATGGAATATGATGCCGTTGCGACTACACACTCATGGATTCCTAAAAGGAATCGTTCCATTCTCAATACCAGTCAAAGGGGAATTCTTGAGGATGAACAGATGTACCAGAAATGCTGCAGTCTATTCGAGAA<br>ATTCTTCCCCAGTAGTTCATACAGGAGACCAGTTGGAATTTCCAGCATGGTGGAGGCCATGGTGTCTAGGGCCCGAATCGATGCACGAATTGATTTCGAATCTGGAAGGATAAAGAAGGAAGAGTTTGCTGAGATCATGAAGATCTGTTCCACCATTGAAGAGCTCAGACGGCAAAAATAG
internal NVRI repository	>A/duck/Poland/H1969_21RS3290-6/2021_H5N1_2021-11-12<br>ATGGATGTCAATCCGACTTTACTTTTCTTAAAAGTGCCAGCGCAAGATGCCATAAGTACCACATTCCCTTACACTGGAGATCCTCCATACAGCCATGGAACAGGGACAGGATACACAATGGACACAGTCAACAGAACACATCAATACTCAGAGAAGGGAAAATGGACAACAAACACAGAAACCAGAGCACCTCAACTCAACCCAATTGATGGGCCACTACCTGAGGACAACGA<br>ACCGAGCGGATATGCACAAACAGATTGCGTGTTGGAAGCAATGGCTTTCCTTGAAGAGTCCCACCCAGGGATCTTTGAAAACTCTTGTCTTGAAACGATGGAAGTCGTTCAGCAAACAAGAGTGGACAAACTAACTCAAGGTCGCCAGACATATGACTGGACACTGAATAGAAACCAACCAGCTGCAACTGCCCTGGCCAACACTATAGAGGTCTTCAGATCAAACGGTCTAA<br>CAGCCAATGAATCGGGGAGACTAATAGATTTCCTCAAGGATGTGATGGACTCAATGGATAAAGAAGAAATGGAAATAACAACACATTTCCAGAGAAAGAGAAGAGTAAGGGACAACATGACCAAGAAAATGGTCACACAAAGAACAATAGGAAAGAAGAAACAAAGGCTAAACAAGAGGAGCTACTTAATAAGAGCACTGACACTGAATACAATGACAAAAGATGCAGA<br>AAGAGGCAAATTGAAAAGACGGGCGATTGCAACACCAGGGATGCAGATTAGAGGATTTGTGTACTTTGTCGAAACACTGGCAAGGAGCATCTGTGAAAAACTTGAGCAATCTGGACTCCCCGTTGGAGGGAATGAGAAGAAGGCTAAATTGGCAAATGTCGTGAGAAAAATGATGACTAACTCACAAGATACAGAGCTCTCCTTCACAATTACTGGAGATAACACCAAATG<br>GAATGAGAATCAAAATCCTCGGATGTTTCTGGCAATGATAACATACATTACAAGAAACCAACCTGAATGGTTTAGAAATGTCTTGAGTGTTGCCCCTATAATGTTCTCGAACAAAATGGCGAGATTGGGAAAAGGGTACATGTTTGAAAGTAAGAGCATGAAGTTACGGACACAAATACCTGCAGAAATGCTTGCAAACATTGACTTAAAATACTTCAATGAATCAACAAGAAA<br>GAAAATCGAAAAAATAAGGCCTCTACTAATAGATGGCACTGCCTCATTGAGTCCTGGAATGATGATGGGCATGTTCAATATGCTGAGTACAGTATTAGGAGTTTCAATCCTAAATCTTGGGCAAAAGAAGTACACCAAAACCACATACTGGTGGGATGGACTCCAATCCTCTGATGATTTCGCCCTCATAGTAAATGCACCGAATCATGAGGGAATACAAGCAGGAGTGGATA<br>GGTTCTATAGGACCTGCAAACTGGTCGGGATCAATATGAGCAAAAAGAAGTCTTACATAAACCGGACTGGAACATTTGAGTTCACAAGCTTTTTCTATCGCTATGGATTTGTGGCTAACTTCAGTATGGAGCTGCCCAGCTTTGGAGTTTCTGGGATCAATGAATCAGCTGACATGAGCATTGGCGTCACAGTGATAAAGAACAACATGATAAACAATGACCTTGGACCAGCAA<br>CAGCTCAAATGGCCCTTCAACTATTCATCAAAGATTACAGGTACACGTACCGATGCCACAGAGGTGACACACAAATTCAAACGAGGAGATCATTCGAGCTGAAGAAGCTGTGGGAACAGACCCGTTCAAAGGCAGGACTGTTGGTGTCAGATGGAGGACCAAATCTATACAACATTCGGAATCTCCATATCCCAGAGGTCTGCCTGAAGTGGGAGCTGATGGACGAAGATTA<br>CCAGGGCAGGTTGTGTAATCCTCTGAACCCATTTGTCAGCCATAGAGAAATTGAGTCCGTAAACAATGCTGTGGTGGTGCCAGCCCACGGTCCAGCCAAAAGCATGGAATATGATGCCGTTGCGACTACACACTCATGGATTCCTAAAAGGAATCGTTCCATTCTCAATACCAGTCAAAGGGGAATTCTTGAGGATGAACAGATGTACCAGAAATGCTGCAGTCTATTCGAGAA<br>ATTCTTCCCCAGTAGTTCATACAGGAGACCAGTTGGAATTTCCAGCATGGTGGAGGCCATGGTGTCTAGGGCCCGAATCGATGCACGAATTGATTTCGAATCTGGAAGGATAAAGAAGGAAGAGTTTGCTGAGATCATGAAGATCTGTTCCACCATTGAAGAGCTCAGACGGCAAAAATAG
internal NVRI repository	>A/duck/Poland/H1968_21RS3290-5/2021_H5N1_2021-11-12<br>ATGGATGTCAATCCGACTTTACTTTTCTTAAAAGTGCCAGCGCAAGATGCCATAAGTACCACATTCCCTTACACTGGAGATCCTCCATACAGCCATGGAACAGGGACAGGATACACAATGGACACAGTCAACAGAACACATCAATACTCAGAGAAGGGAAAATGGACAACAAACACAGAAACCAGAGCACCTCAACTCAACCCAATTGATGGGCCACTACCTGAGGACAACGA<br>ACCGAGCGGATATGCACAAACAGATTGCGTGTTGGAAGCAATGGCTTTCCTTGAAGAGTCCCACCCAGGGATCTTTGAAAACTCTTGTCTTGAAACGATGGAAGTCGTTCAGCAAACAAGAGTGGACAAACTAACTCAAGGTCGCCAGACATATGACTGGACACTGAATAGAAACCAACCAGCTGCAACTGCCCTGGCCAACACTATAGAGGTCTTCAGATCAAACGGTCTAA<br>CAGCCAATGAATCGGGGAGACTAATAGATTTCCTCAAGGATGTGATGGACTCAATGGATAAAGAAGAAATGGAAATAACAACACATTTCCAGAGAAAGAGAAGAGTAAGGGACAACATGACCAAGAAAATGGTCACACAAAGAACAATAGGAAAGAAGAAACAAAGGCTAAACAAGAGGAGCTACTTAATAAGAGCACTGACACTGAATACAATGACAAAAGATGCAGA<br>AAGAGGCAAATTGAAAAGACGGGCGATTGCAACACCAGGGATGCAGATTAGAGGATTTGTGTACTTTGTCGAAACACTGGCAAGGAGCATCTGTGAAAAACTTGAGCAATCTGGACTCCCCGTTGGAGGGAATGAGAAGAAGGCTAAATTGGCAAATGTCGTGAGAAAAATGATGACTAACTCACAAGATACAGAGCTCTCCTTCACAATTACTGGAGATAACACCAAATG<br>GAATGAGAATCAAAATCCTCGGATGTTTCTGGCAATGATAACATACATTACAAGAAACCAACCTGAATGGTTTAGAAATGTCTTGAGTGTTGCCCCTATAATGTTCTCGAACAAAATGGCGAGATTGGGAAAAGGGTACATGTTTGAAAGTAAGAGCATGAAGTTACGGACACAAATACCTGCAGAAATGCTTGCAAACATTGACTTAAAATACTTCAATGAATCAACAAGAAA<br>GAAAATCGAAAAAATAAGGCCTCTACTAATAGATGGCACTGCCTCATTGAGTCCTGGAATGATGATGGGCATGTTCAATATGCTGAGTACAGTATTAGGAGTTTCAATCCTAAATCTTGGGCAAAAGAAGTACACCAAAACCACATACTGGTGGGATGGACTCCAATCCTCTGATGATTTCGCCCTCATAGTAAATGCACCGAATCATGAGGGAATACAAGCAGGAGTGGATA<br>GGTTCTATAGGACCTGCAAACTGGTCGGGATCAATATGAGCAAAAAGAAGTCTTACATAAACCGGACTGGAACATTTGAGTTCACAAGCTTTTTCTATCGCTATGGATTTGTGGCTAACTTCAGTATGGAGCTGCCCAGCTTTGGAGTTTCTGGGATCAATGAATCAGCTGACATGAGCATTGGCGTCACAGTGATAAAGAACAACATGATAAACAATGACCTTGGACCAGCAA<br>CAGCTCAAATGGCCCTTCAACTATTCATCAAAGATTACAGGTACACGTACCGATGCCACAGAGGTGACACACAAATTCAAACGAGGAGATCATTCGAGCTGAAGAAGCTGTGGGAACAGACCCGTTCAAAGGCAGGACTGTTGGTGTCAGATGGAGGACCAAATCTATACAACATTCGGAATCTCCATATCCCAGAGGTCTGCCTGAAGTGGGAGCTGATGGACGAAGATTA<br>CCAGGGCAGGTTGTGTAATCCTCTGAACCCATTTGTCAGCCATAGAGAAATTGAGTCCGTAAACAATGCTGTGGTGGTGCCAGCCCACGGTCCAGCCAAAAGCATGGAATATGATGCCGTTGCGACTACACACTCATGGATTCCTAAAAGGAATCGTTCCATTCTCAATACCAGTCAAAGGGGAATTCTTGAGGATGAACAGATGTACCAGAAATGCTGCAGTCTATTCGAGAA<br>ATTCTTCCCCAGTAGTTCATACAGGAGACCAGTTGGAATTTCCAGCATGGTGGAGGCCATGGTGTCTAGGGCCCGAATCGATGCACGAATTGATTTCGAATCTGGAAGGATAAAGAAGGAAGAGTTTGCTGAGATCATGAAGATCTGTTCCACCATTGAAGAGCTCAGACGGCAAAAATAG
EPI_ISL_11922808	>A/duck/Poland/H188_22VIR2515-2/2022_H5N1_2022-03-02<br>ATGGATGTCAATCCGACTTTACTTTTCTTAAAAGTGCCAGCGCAARATGCCATAAGTACCACATTCCCTTACACTGGAGATCCTCCATACAGCCATGGAACAGGGACAGGATACACAATGGACACAGTCAACAGAACACATCAATACTCAGAGAAGGGAAAATGGACAACAAACACAGAAACCGGAGCACCTCAACTCAACCCAATTGATGGGCCACTACCTGAGGACAACGA<br>ACCGAGCGGATATGCACAAACAGATTGCGTGTTGGAAGCAATGGCTTTCCTTGAAGAGTCCCACCCAGGGATCTTTGAAAACTCTTGTCTTGAAACGATGGAAGTCGTTCAGCAAACAAGAGTGGACAAACTAACTCAAGGTCGCCAGACATATGACTGGACACTGAATAGAAACCAACCAGCTGCAACTGCCCTGGCCAACACTATAGAGGTCTTCAGATCAAACGGTCTAA<br>CAGCCAATGAATCGGGGAGACTAATAGATTTCCTCAAGGATGTGATGGACTCAATGGATAAAGAAGAAATGGAAATAACAACACATTTCCAGAGAAAGAGAAGAGTAAGGGACAACATGACCAAGAAAATGGTCACACAAAGAACAATAGGAAAGAAGAAACAAAGGCTAAACAAGAGGAGCTACTTAATAAGAGCACTGACACTGAATACAATGACAAAAGATGCAGA<br>AAGAGGCAAATTGAAAAGACGGGCGATTGCAACACCAGGGATGCAGATTAGAGGATTTGTGTACTTTGTCGAAACACTGGCAAGGAGCATCTGTGAAAAACTTGAGCAATCTGGACTCCCCGTTGGAGGGAATGAGAAGAAGGCTAAATTGGCAAATGTCGTGAGAAAAATGATGACTAACTCACAAGATACAGAGCTCTCCTTCACAATTACTGGAGACAACACCAAATG<br>GAATGAGAATCAAAATCCTCGGATGTTTCTGGCAATGATAACATACATTACAAGAAACCAACCTGAATGGTTTAGAAATGTCTTGAGTATTGCCCCTATAATGTTCTCGAACAAAATGGCGAGATTGGGAAAAGGGTACATGTTTGAAAGTAAGAGCATGAAGTTACGGACACAAATACCTGCAGAAATGCTTGCAAACATTGACTTAAAATACTTCAATGAATCAACAAGAAA<br>GAAAATCGAAAAAATAAGGCCTCTACTAATAGATGGCACTGCCTCATTGAGTCCTGGAATGATGATGGGCATGTTCAATATGCTGAGTACAGTATTAGGAGTTTCAATCCTAAATCTTGGGCAAAAGAAGTACACCAAAACAACATACTGGTGGGATGGACTCCAATCCTCTGATGATTTCGCCCTCATAGTAAATGCACCGAATCATGAGGGAATACAAGCAGGAGTGGATA<br>GGTTCTATAGGACCTGCAAACTGGTCGGGATCAATATGAGCAAAAAGAAGTCTTACATAAACCGGACTGGAACATTTGAGTTCACAAGCTTTTTCTATCGCTATGGATTTGTGGCTAACTTCAGTATGGAGCTGCCCAGCTTTGGAGTTTCTGGGATCAATGAATCAGCTGACATGAGCATTGGCGTCACAGTGATAAAGAACAACATGATAAACAATGACCTTGGACCAGCAA<br>CAGCTCAAATGGCCCTTCAACTATTCATCAAAGATTACAGGTACACGTACCGATGCCACAGAGGTGACACACAAATTCAAACGAGGAGATCATTCGAGCTGAAGAAGCTGTGGGAACAGACCCGTTCAAAGGCAGGACTATTGGTGTCAGATGGAGGACCAAATCTATACAACATTCGGAATCTCCATATCCCAGAAGTCTGCCTGAAGTGGGAGCTGATGGACAAAGATTAC<br>CAGGGCAGGTTGTGTAATCCTCTGAACCCATTTGTCAGCCATAGAGAAATTGAGTCCGTAAACAATGCTGTGGTGATGCCAGCCCACGGTCCAGCCAAAAGCATGGAATATGATGCCGTTGCGACTACACACTCATGGATTCCTAAAAGGAATCGTTCCATTCTCAATACCAGTCAAAGGGGAATTCTTGAGGATGAACAGATGTACCAGAAATGCTGCAGTCTATTCGAGAAA<br>TTCTTCCCCAGTAGTTCATACAGGAGACCAGTTGGAATTTCCAGCATGGTGGAGGCCATGGTGTCTAGGGCCCGAATCGATGCACGAATTGATTTCGAATCTGGAAGGATCAAGAAGGAAGAGTTTGCTGAGATCATGAAGATCTGTTCCACCATTGAAGAGCTCAGACGGCAAAAATAG
EPI_ISL_3102060	>A/duck/Poland/H160_21RS1385-1/2021_H5N8_2021-03-05<br>ATGGATGTCAATCCGACTTTGCTTTTCTTAAAAGTGCCAGCGCAAAATGCTATAAGTACTACATTCCCTTACACTGGAGATCCCCCATACAGCCATGGAACAGGAACAGGGTATACCATGGACACAGTAAACAGAACACATCAATATTCAGAAAAGGGAAAGTGGACAACAAACACAGAAACCGGAGCACCCCAACTCAACCCAATTGATGGACCATTACCAGAGGACAATGA<br>GCCAAGCGGATATGCACAAACTGATTGCGTGTTGGAAGCAATGGCTTTCCTTGAAGAATCCCACCCAGGGATATTTGAAAACTCTTGTCTTGAAGCGATGGAAATCGTTCAGCAAACAAGAGTGGACAAACTAACCCAAGGTCGCCAGACTTATGACTGGACACTGAACAGAAACCAACCAGCTGCAACCTCTTTGGCCAACACTATAGAGGTGTTCAGATCGAATGGTCTGA<br>CAGCCAATGAATCAGGGAGACTGATAGATTTTCTCAGGGATGTGATGGAATCAATGGATAAAGAAGAGATGGAAGTAACAACACATTTCCAGAGAAAAAGAAGAGTGAGGGACAACATGACTAAGAAGATGGTCACACAAAGAACAATAGGGAAGAAGAAGCAGAGGCTGAACAAGAGGAGTTACTTAATAAGAGCACTGACATTGAATACAATGACCAAAGATGCAGA<br>AAGAGGCAAGTTGAAGAGACGGGCAATTGCAACACCCGGGATGCAGATTAGAGGATTCGTGTACTTCGTTGAAACACTAGCGAGGAGCATCTGTGAGAAACTAGAGCAATCTGGGCTCCCTGTTGGAGGGAATGAGAAAAAGGCTAAATTGGCAAATGTTGTGAGAAAAATGATGACTAACTCACAAGATACAGAGCTCTCCTTTACAATTACTGGAGACAACACCAAATG<br>GAATGAGAATCAAAACCCTCGGATGTTTTTGGCAATGATAACATATATCACAAGAAACCAACCTGAATGGTTTAGAAATGTCTTAAGCATTGCCCCTATAATGTTCTCAAACAAAATGGCGAGATTAGGGAAAGGATACATGTTTGAAAGTAAGAGCATGAAGCTAAGAACACAAATACCAGCAGAGATGCTTACAAATATTGATCTGAAGTATTTCAACGAACCAACGAGAA<br>AGAAAATCGAGAAAATAAGACCTCTGCTGATTGATGGCACGGCTTCATTGAGCCCTGGGATGATGATGGGCATGTTCAATATGCTGAGCACAGTATTAGGGGTCTCAATCCTGAATCTCGGGCAAAAGAGGTACACCAAAACCACATACTGGTGGGATGGACTTCAATCCTCTGATGATTTCGCTCTCATAGTGAATGCACCGAATCATGAGGGGATACAAGCAGGAGTGGAT<br>AGATTCTATAGGACCTGCAAACTGGTTGGGATCAACATGAGCAAAAAGAAGTCTTACATAAACCGAACAGGAACATTTGAGTTCACAAGTTTTTTCTACCGCTATGGATTTGTAGCCAACTTCAGTATGGAATTACCCAGCTTTGGAGTGTCTGGAATCAATGAATCAGCTGACATGAGCATTGGAGTTACAGTGATAAAAAACAATATGATAAACAATGATCTTGGACCAGCA<br>ACAGCTCAAATGGCTCTTCAGTTATTCATCAAAGACTATAGGTACACATACCGATGCCACAGGGGTGATACACAAATTCAAACGAGGAGATCATTCGAGCTGAAGAAGCTGTGGGAGCAGACCCGTTCAAAGGCAGGACTGTTGATATCAGACGGGGGGCCAAACCTATACAACATTCGGAATCTCCACATCCCAGAGGTCTGCTTGAAGTGGGAGCTGATGGATGAAGACT<br>ACCAAGGCAGGCTGTGCAATCCTCTGAATCCATTTGTCAGTCATAAAGAGATTGAGTCCGTAAACAATGCTGTAGTAATGCCCGCCCATGGCCCGGCCAAGAGCATGGAATATGATGCTGTTGCGACCACACACTCGTGGATTCCTAAGAGGAACCGTTCCATCCTCAATACCAGCCAAAGGGGAATTCTTGAGGATGAGCAGATGTACCAAAAGTGCTGTAGTCTATTCGAGA<br>AATTCTTCCCCAGCAGTTCATACAGGAGGCCAGTTGGAATTTCCAGCATGGTGGAGGCCATGGTGTCTAGGGCCCGAATTGATGCACGCATCGACTTCGAATCTGGAAGGATTAAGAAAGAAGAGTTTGCTGAGATCATGAAGATCTGTTCCACCATTGAAGAGCTCAGACGGCAAAAATAG

EPI_ISL_11922810	>A/duck/Poland/H126_22VIR2515-4/2022_H5N1_2022-02-08<br>ATGGATGTCAATCCGACTTTACTTTTCTTAAAAGTGCCAGCGCAAGATGCCATAAGTACCACATTCCCTTACACTGGAGATCCTCCATACAGCCATGGAACAGGGACAGGATACACAATGGACACAGTCAACAGAACACATCAATACTCAGAGAAGGGAAAATGGACAACAAACACAGAAACCGGAGCACCTCAACTCAACCCAATTGATGGGCCACTACCTGAGGACAACGA<br>ACCGAGCGGATATGCACAAACAGATTGCGTGTTGGAAGCAATGGCTTTCCTTGAAGAGTCCCATCCAGGGATCTTTGGAAACTCTTGTCTTGAAACGATGGAAGTCGTTCAGCAAACAAGAGTGGACAAACTAACTCAAGGTCGCCAGACATATGACTGGACACTGAATAGAAACCAACCAGCTGCAACTGCCCTGGCCAACACTATAGAGGTCTTCAGATCAAACGGTCTAA<br>CAGCCAATGAATCGGGGAGACTAATAGATTTCCTCAAGGATGTGATGGACTCAATGGATAAAGAAGAAATGGAAATAACAACACATTTCCAGAGAAAGAGAAGAGTAAGGGACAACATGACCAAGAAAATGGTCACACAAAGAACAATAGGAAAGAAGAAACAAAGGCTAAACAAGAGGAGCTACTTAATAAGAGCACTGACACTGAATACAATGACAAAAGATGCAGA<br>AAGAGGCAAATTGAAGAGACGGGCGATTGCAACACCAGGGATGCAGATTAGAGGATTTGTGTACTTTGTCGAAACACTGGCAAGGAGCATCTGTGAAAAACTTGAACAATCTGGACTCCCCGTTGGAGGGAATGAGAAGAAGGCTAAATTGGCAATGTCGTGAGAAAAATGATGACTAACTCACAAGATACAGAGCTCTCCTTCACAATTACTTGGAGATAACACCAAATG<br>GAATGAGAATCAAAATCCTCGGATGTTTCTGGCAATGATAACATACATTACAAGAAACCAACCTGAATGGTTTAGGAATGTCTTGAGTATTGCCCCTATAATGTTCTCGAACAAAATGGCGAGATTGGGAAAAGGGTACATGTTTGAAAGTAAGAGCATGAAGTTACGGACACAAATACCTGCAGAAATGCTTGCAAACATTGACTTAAAATACTTCAATGAATCAACAAGAAA<br>GAAAATCGAAAAAATAAGGCCTCTACTAATAGATGGCACTGCCTCATTGAGTCCTGGAATGATGATGGGCATGTTCAATATGCTGAGTACAGTATTAGGAGTTTCAATCCTAAATCTTGGGCAAAAGAAGTACACCAAAACCACATACTGGTGGGATGGACTCCAATCCTCTGATGATTTCGCCCTCATAGTAAATGCACCGAATCATGAGGGAATACAAGCAGGAGTGGATA<br>GGTTCTATAGGACCTGCAAACTGGTCGGGATCAATATGAGCAAAAAGAAGTCTTACATAAACCGGACTGGAACATTTGAGTTCACAAGCTTTTTCTATCGCTATGGATTTGTGGCTAACTTCAGTATGGAGCTGCCCAGCTTTGGAGTTTCTGGGATCAATGAATCAGCTGACATGAGCATTGGCATCACAGTGATAAAGAACAACATGATAAACAATGACCTTGGACCAGCAA<br>CAGCTCARATGGCCCTTCAACTATTCATCAAAGATTACAGGTACACGTACCGATGCCATAGAGGTGACACACAAATTCAAACGAGGAGATCATTCGAGCTGAAGAAGCTGTGGGAACAGACCCGTTCAAAGGCAGGGCTGTTGGTGTCAGATGGAGGACCAAATCTATACAACATTCGGAATCTCCATATCCCTGAGGTCTGCCTGAAGTGGGAACTGATGGACRAAGATTAC<br>CAGGGCAGGTTGTGCAATCCTCTGAACCCATTTGTCAGCCATAAAGAAATTGAGTCCGTAAACAATGCTGTGGTGATGCCAGCCCACGGTCCAGCCAAAAGCATGGAATATGATGCCGTTGCGACTACACACTCATGGATTCCTAAAAGGAATCGTTCCATTCTCAATACCAGTCAAAGGGGAATTCTTGAGGATGAACAGATGTACCAGAAATGCTGCGGTCTATTCGAGAAA<br>TTCTTCCCCAGTAGTTCATACAGGAGACCAGTTGGAATTTCCAGCATGGTGGAGGCCATGGTGTCTAGGGCCCGAATCGATGCACGCATTGATTTCGAATCTGGAAGGATCAAGAAGGAAGAGTTTGCTGAGATCATGAAGATCTGTTCCACCATTGAAGAGCTCAGACGGCAAAAATAG
EPI_ISL_3102064	>A/duck/Poland/H1029_21RS1385-5/2021_H5N8_2021-05-01<br>ATGGATGTCAATCCGACTTTGCTTTTCTTAAAAGTGCCAGCGCAAAATGCTATAAGTACTACATTCCCTTACACTGGAGATCCCCCATACAGCCATGGAACAGGAACAGGGTATACCATGGACACAGTAAACAGAACACATCAATATTCAGAAAAGGGAAAGTGGACAACAAACACAGAAACCGGAGCACCCCAACTCAACCCAATTGATGGACCATTACCAGAGGACAATGA<br>GCCAAGCGGATATGCACAAACTGATTGCGTGTTGGAAGCAATGGCTTTCCTTGAAGAATCCCACCCAGGGATATTTGAAAACTCTTGTCTTGAAGCGATGGAAATCGTTCAGCAAACAAGAGTGGACAAACTAACCCAAGGTCGCCAGACTTATGACTGGACACTGAACAGAAACCAACCAGCTGCAACCTCTTTGGCCAACACTATAGAGGTGTTCAGATCGAATGGTCTGA<br>CAGCCAATGAATCAGGGAGACTGATAGATTTTCTCAGGGATGTGATGGAATCAATGGATAAAGAAGAGATGGAAGTAACAACACATTTCCAGAGAAAAAGAAGAGTGAGGGACAACATGACTAAGAAGATGGTCACACAAAGAACAATAGGGAAGAAGAAGCAGAGGCTGAACAAGAGGAGTTACTTAATAAGAGCACTGACATTGAATACAATGACCAAAGATGCAGA<br>AAGAGGCAAGTTGAAGAGACGGGCAATTGCAACACCCGGGATGCAGATTAGAGGATTCGTGTACTTCGTTGAAACACTAGCGAGGAGCATCTGTGAGAAACTAGAGCAATCTGGGCTCCCTGTTGGAGGGAATGAGAAAAAGGCTAAATTGGCAAATGTTGTGAGAAAAATGATGACTAACTCACAAGATACAGAGCTCTCCTTTACAATTACTGGAGACAACACCAAATG<br>GAATGAGAATCAAAACCCTCGGATGTTTTTGGCAATGATAACATATATCACAAGAAACCAACCTGAATGGTTTAGAAATGTCTTAAGCATTGCCCCTATAATGTTCTCAAACAAAATGGCGAGATTAGGGAAAGGATACATGTTTGAAAGTAAGAGCATGAAGCTAAGAACACAAATACCAGCAGAGATGCTTACAAATATTGATCTGAAGTATTTCAACGAACCAACGAGAA<br>AGAAAATCGAGAATATAAGACCTCTGCTGATTGATGGCACGGCTTCATTGAGCCCTGGGATGATGATGGGCATGTTCAATATGCTGAGCACAGTATTAGGGGTCTCAATCCTGAATCTCGGGCAAAAGAGGTACACCAAAACCACATACTGGTGGGATGGACTTCAATCCTCTGATGATTTCGCTCTCATAGTGAATGCACCGAATCATGAGGGGATACAAGCAGGAGTGGAT<br>AGATTCTATAGGACCTGCAAACTGGTTGGGATCAACATGAGCAAAAAGAAGTCTTACATAAACCGAACAGGAACATTTGAGTTCACAAGTTTTTTCTACCGCTATGGATTTGTAGCCAACTTCAGTATGGAATTACCCAGCTTTGGAGTGTCTGGAATCAATGAATCAGCTGACATGAGCATTGGAGTTACAGTGATAAAAAACAATATGATAAACAATGATCTTGGACCAGCA<br>ACAGCTCAAATGGCTCTTCAGTTATTCATCAAAGACTATAGGTACACATACCGATGCCACAGGGGTGATACACAAATTCAAACGAGGAGATCATTCGAGCTGAAGAAGCTGTGGGAGCAGACCCGTTCAAAGGCAGGACTGTTGATATCAGACGGGGGGCCAAACCTATACAACATTCGGAATCTCCACATCCCAGAGGTCTGCTTGAAGTGGGAGCTGATGGATGAAGACT<br>ACCAAGGCAGGCTGTGCAATCCTCTGAATCCATTTGTCAGTCATAAAGAGATTGAGTCCGTAAACAATGCTGTAGTAATGCCCGCCCATGGCCCGGCCAAGAGCATGGAATATGATGCTGTTGCGACCACACACTCGTGGATTCCTAAGAGGAACCGTTCCATCCTCAATACCAGCCAAAGGGGAATTCTTGAGGATGAGCAGATGTACCAGAAGTGCTGTAGTCTATTCGAG<br>AAATTCTTCCCCAGCAGTTCATACAGGAGGCCAGTTGGAATTTCCAGCATGGTGGAGGCCATGGTGTCTAGGGCCCGAATTGATGCACGCATCGACTTCGAATCTGGAAGGATTAAGAAAGAAGAGYTTGCTGAGATCATGAAGATCTGTTCCACCATTGAAGAGCTCAGACGGCAAAAATAG
EPI_ISL_697686	>A/duck/Moscow/5743/2019_H1N1_2019-10-15<br>ATGGATGTCAATCCGACTTTACTTTTCTTAAAAGTGCCAGCGCAAAATGCTATAAGTACTACATTCCCTTACACTGGAGATCCTCCATACAGCCATGGAACAGGAACAGGATACACTATGGACACAGTCAACAGAACGCATCAATACTCAGAGAAGGGAAAATGGACAACAAACACAGAGACCGGAGCACCCCAACTCAATCCAATTGATGGACCATTACCAGAAGACAACGA<br>GCCAAGCGGATATGCACAAACGGATTGCGTGTTGGAGCAATGGCTTTCCTTGAAGAATCCCACCCAGGGATCTTTGAAAACTCTTGTCTTGAAACGATGGAAGTCGTTCAGCAAACAAGAGTGGACAAACTAACCCAAGGTCGCCAGACTTATGACTGGACACTGAACAGAAATCAACCAGCTGCAACTGCTTTGGCCAACACTATAGAGGTGTTCAGATCGAATGGTCTGA<br>CAGCCAATGAGTCGGGGAGATTAATAGATTTCCTCAAGGATGTGATGGAATCAATGGATAAAGAAGAAATGGAAATAACAACACATTTTCAGAGAAAGAGAAGAGTGAGGGACAACATGACCAAGAAGATGGTCACACAAAGAACAATAGGAAAGAAGAAGCAGAGGCTGAATAAGAGGAGTTACTTAATAAGAGCACTGACATTGAACACAATGACCAAAGATGCGGA<br>AAGAGGCAAACTGAAGAGACGGGCAATTGCAACACCCGGGATGCAGATTAGAGGATTCGTGTACTTTGTCGAAACACTAGCGAGGAGCATCTGTGAGAAACTCGAACAATCTGGACTCCCTGTTGGAGGGAATGAGAAAAAGGCTAAATTGGCGAATGTCGTGAGAAAAATGATGACTAACTCACAAGATACAGAACTCTCCTTTACAATTACTGGAGACAACACCAAATG<br>GAATGAGAATCAAATCCTCGGATGTTTTTGGCAATGATAACATACATCACAAGAAACCAACCTGAATGGTTTAGAAATGTCTTGAGCATTGCCCCTATAATGTTCTCAAACAAAATGGCAAGATTAGGAAAAGGATACATGTTTGAAAGTAAGAGCATGAAGCTGAGAACACAAATACCGGCAGAGATGCTTGCAAACATTGATTTGAAATATTTCAACGAATCAACGAAAAA<br>GAAAATCGAGAAAATAAGACCTCTGCTGATTGATGGCACAGCCTCATTGAGTCCTGGAATGATGATGGGCATGTTCAATATGCTGAGCACAGTATTAGGGGTCTCAATTCTGAATCTTGGGCAAAAGAAGTACACCAAAACCACATACTGGTGGGACGGACTCCAATCCTCTGATGATTTCGCTCTCATAGTGAATGCACCGAATCATGAGGGGATACAGGCAGGAGTGGATA<br>GGTTCTATAGGACCTGCAAACTGGTTGGGATCAACATGAGCAAAAAGAAGTCTTACATAAACCGGACAGGAACATTTGAGTTCACAAGTTTTTTCTACCGTTATGGATTTGTAGCTAACTTCAGTATGGAATTACCCAGCTTTGGAGTGTCTGGAATCAATGAATCGGCTGACATGAGCATTGGAGTTACAGTGATAAAGAACAACATGATAAACAATGACCTTGGACCAGCAA<br>CAGCTCAAATGGCTCTTCAACTATTCATCAAGGACTATAGGTACACGTACCGATGCCACAGAGGTGATACACAAATTCAAACGAGGAGATCATTCGAGCTGAAGAAGCTGTGGGAGCAAACCCGCTCAAAGGCAGGGTTGTTGGTATCAGATGGAGGACCAAACCTATACAACATTCGGAATCTACACATCCCAGAGGTCTGCTTGAAGTGGGAGCTGATGGATGAAGACTA<br>CCAGGGCAGGCTGTGTAATCCTTTGAATCCGTTTGTCAGTCATAAGGAAATTGAGTCCGTAAACAATGCTGTGGTAATGCCAGCCCATGGCCCAGCCAAGAGCATGGAATATGATGCTGTTGCGACTACACACTCGTGGGTTCCTAAGAGAAACCGTTCTATTCTCAATACCAGCCAAAGGGGAATTCTTGAGGATGAGCAAATGTACCAGAAGTGCTGTAGTCTATTTGAGAA<br>ATTCTTCCCCAGCAGTTCATACAGGAGGCCAGTTGGGATTTCCAGCATGGTGGAGGCCATGGTGTCTAGGGCCCGAATTGATGCACGCATTGATTTCGAATCTGGAAGGATTAAGAAAGAAGAGTTTGCTGAGATCATGAAGATCTGTTCCACCATTGAAGAGCTCAGACGGCAAAAATAG
EPI_ISL_697685	>A/duck/Moscow/5712U/2019_H11N6_019-10-21<br>ATGGATGTCAATCCGACTTTACTTTTCTTAAAAGTGCCAGCGCAAAATGCCATAAGTACCACATTCCCTTATACTGGAGATCCTCCATACAGCCATGGAACAGGAACAGGATACACCATGGACACAGTCAACAGAACACATCAATACTCGGAAAAGGGGAAATGGACAACAAACACAGAGACTGGAGCACCCCAACTCAACCCAATTGATGGACCGCTACCTGAGGACAACGA<br>ACCAAGCGGATATGCACAAACAGATTGCGTGTTGGAAGCAATGGCTTTCCTTGAAGAGTCCCACCCAGGGATCTTTGAAAACTCTTGTCTTGAAACGATGGAAGTCGTTCAGCAAACAAGAGTGGACAAACTAACTCAAGGTCGCCAGACTTATGACTGGACACTGAATAGAAACCAACCAGCTGCAACTGCCCTGGCCAACACTATAGAGGTCTTCAGATCGAACGGTCTGA<br>CAGCCAATGAATCAGGGAGACTAATAGATTTCCTCAAGGATGTGATGGACTCAATGGACAAAGAAGAAATGGAAATAACAACACATTTCCAGAGAAAGAGAAGAGTAAGGGACAACATGACCAAGAAAATGGTCACACAAAGAACAATAGGGAAGAAGAAGCAAAGGCTAAATAAGAGGAGCTACTTAATAAGAGCACTGACACTGAATACAATGACAAAAGACGCAGA<br>AAGAGGCAAATTGAAGAGACGGGCAATTGCAACACCAGGGATGCAGATTAGAGGATTTGTGTACTTTGTCGAAACACTGGCAAGGAGCATCTGTGAAAAACTTGAGCAATCTGGACTCCCCGTTGGAGGGAATGAGAAGAAGGCTAAATTGGCAAATGTCGTGAGAAAAATGATGACTAACTCACAAGATACAGAGCTCTCCTTCACAATTACTGGAGATAACACCAAATG<br>GAATGAGAATCAAAATCCTCGGATGTTTCTGGCAATGATAACATACATCACAAGAAACCAACCTGAATGGTTTAGAAATGTCTTGAGCATTGCCCCTATAATGTTCTCGAACAAAATGGCGAGACTGGGAAAAGGATACATGTTTGAAAGTAAAAGCATGAAGCTACGGACACAAATACCTGCAGAAATGCTTGCAAACATTGACTTAAAATACTTCAATGAGTCAACAAGAAA<br>GAAAATCGAGAAAATAAGACCTCTACTAATAGATGGCACCGCCTCATTGAGTCCTGGAATGATGATGGGCATGTTCAACATGCTGAGTACAGTATTAGGAGTCTCAATCCTGAATCTTGGGCAAAAGAGGTACACCAAAACCACATACTGGTGGGATGGACTCCAATCCTCTGATGATTTCGCCCTCATAGTGAACGCACCGAATCATGAGGGAATACAAGCAGGAGTGGATA<br>GGTTCTATAGGACCTGCAAACTGGTTGGGATCAACATGAGCAAAAAGAAGTCTTACATAAACCGGACAGGAACATTTGAGTTCACAAGCTTTTTCTATCGCTATGGATTTGTGGCCAACTTCAGTATGGAGCTGCCCAGTTTTGGAGTGTCTGGAATCAATGAATCAGCTGACATGAGCATTGGAGTTACAGTGATAAAGAACAACATGATAAACAATGACCTTGGACCAGCAA<br>CAGCTCAAATGGCCCTTCAACTATTCATCAAGGACTACAGGTACACGTACCGATGTCACAGGGGTGACACACAAATTCAAACGAGGAGATCATTCGAGCTGAAGAAGCTGTGGGAGCAGACCCGTTCAAAGGCAGGACTGTTGGTGTCAGATGGAGGACCAAATCTATACAACATCCGGAATCTCCATATCCCAGAAGTCTGCCTGAAGTGGGAGCTGATGGACGAAGATTA<br>CCAGGGCAGGTTGTGTAATCCTCTGAACCCATTTGTCAGTCATAAAGAAATTGAATCCGTAAACAATGCTGTGGTAATGCCAGCCCATGGCCCAGCCAAGAGTATGGAATATGATGCTGTTGCGACTACACACTCATGGATTCCTAAAAGGAATCGTTCCATTCTCAATACCAGCCAAAGGGGAATTCTTGAGGATGAGCAGATGTACCAGAAATGCTGCAGTCTATTCGAGAA<br>GTTCTTCCCCAGTAGTTCATACAGGAGACCAGTTGGAATTTCCAGCATGGTGGAGGCCATGGTGTCTAGGGCCCGAATCGACGCACGCATTGATTTCGAATCTGGAAGGATTAAGAAGGAAGAGTTTGCTGAGATCATGAAGATCTGTTCCACCATTGAAGAGCTCAGACGGCAAAAATAG
EPI_ISL_504944	>A/duck/Moscow/4203/2010_H3N8_2010-11<br>ATGGATGTCAATCCGACTTTACTTTTCTTAAAAGTGCCAGCGCAAAATGCTATAAGTACTACGTTCCCTTACACTGGAGATCCTCCATACAGCCATGGAACAGGAACAGGATACACCATGGACACAGTCAACAGAACACATCAATACTCAGAGAAGGGAAAGTGGACAACGAACACAGAGACTGGGGCGCCCCAACTCAACCCAATTGATGGACCATTACCAGAGGACAATGA<br>GCCAAGCGGATATGCACAAACAGATTGCGTGTTGGAAGCAATGGCTTTCCTTGAAGAATCCCACCCAGGGATCTTTGAAAACTCTTGTCTTGAAACGATGGAGGTCGTTCAGCAAACAAGAGTGGACAAACTAACCCAAGGTCGCCAGACTTATGATTGGACACTGAATAGAAACCAACCAGCTGCAACTGCTTTGGCCAACACTATAGAGGTCTTCAGATCGAACGGTCTAA<br>CAGCCAATGAGTCGGGGAGACTAATAGATTTCCTCAAGGATGTGATGGAATCAATGGATAAAGAAGAAATGGAAATAACAACACATTTCCAGAGAAAGAGAAGAGTAAGGGACAACATGACCAAGAAGATGGTCACACAAAGAACGATAGGAAAGAAGAAGCAGAGGCTGAACAAGAGGAGCTACTTAATAAGAGCACTGACATTGAACACAATGACCAAAGATGCAGA<br>AAGAGGCAAATTGAAGAGGCGGGCAATTGCAACACCCGGGATGCAGATTAGAGGATTCGTGTACTTTGTCGAAACACTAGCAAGGAGCATCTGTGAGAAACTCGAGCAATCCGGACTCCCTGTTGGAGGGAATGAGAAGAAGGCCAAATTGGCAAATGTCGTGAGAAAAATGATGACTAACTCGCAAGATACAGAGCTCTCCTTTACAATTACTGGAGACAACACCAAATG<br>GAATGAGAATCAAAATCCTCGGATGTTTTTGGCAATGATAACATACATCACAAGAAACCAACCCGAATGGTTTAGAAATGTCTTGAGCATTGCCCCTATAATGTTCTCAAACAAAATGGCGAGATTAGGAAAAGGATACATGTTTGAAAGTAAGAGCATGAAGCTAAGGACACAAATACCGGCAGAAATGCTTGCAAACATTGACTTGAAATACTTCAATGAATCAACGAGAA<br>AGAAAATCGAGAAAATAAGACCTCTGCTAATTGATGGCACAGCCTCATTGAGTCCTGGAATGATGATGGGCATGTTCAATATGCTGAGCACAGTATTGGGGGTCTCAATCCTGAATCTTGGACAAAAGAGGTACACCAAAACCACATACTGGTGGGATGGACTCCAATCCTCTGATGATTTCGCTCTCATAGTGAATGCACCGAATCATGAGGGGATACAAGCAGGGGTGGAT<br>AGGTTCTATAGGACCTGCAAACTGGTTGGGATCAACATGAGCAAAAAGAAGTCTTACATAAACCGGACAGGAACATTTGAGTTCACAAGCTTTTTCTACCGCTATGGATTTGTAGCCAACTTCAGTATGGAGTTACCCAGCTTTGGAGTGTCTGGAATCAATGAATCGGCTGACATGAGCATTGGAGTTACAGTGATAAAGAACAATATGATAAACAATGACCTTGGACCAGCA<br>ACAGCTCAAATGGCTCTTCAGCTATTCATCAAGGACTATAGGTACACGTACCGATGCCACAGAGGTGATACACAAATTCAAACGAGGAGATCATTCGAGCTGAAGAAGCTGTGGGAGCAGACCCGTTCAAAGGCAGGGCTGTTGGTATCAGATGGAGGACCAAACCTATACAACATTCGGAATCTCCACATCCCAGAGGTCTGCCTGAAGTGGGAACTAATGGATGAAGACT<br>ACCAGGGCAGGCTGTGTAATCCTCTGAATCCGTTTGTCAGTCATAAGGAAATTGAGTCCGTAAACAATGCTGTGGTAATGCCAGCCCATGGCCCAGCCAAGAGCATGGAATATGATGCTGTTGCGACTACACACTCATGGATTCCTAAGAGGAACCGTTCCATTCTCAATACCAGCCAGAGGGGAATTCTCGAGGATGAGCAGATGTACCAGAAGTGCTGCAGTCTATTCGAG<br>AAATTCTTCCCCAGTAGTTCATACAGGAGGCCAGTTGGAATTTCCAGCATGGTGGAGGCCATGGTGTCCAGGGCCCGAATTGATGCACGCATTGATTTCGAATCTGGAAGGATTAAGAAAGAAGAGTTTGCTGAGATCATGAAGATCTGTTCCACCATTGAAGAGCTCAGACGGCAAAAATAG
EPI_ISL_327474	>A/Duck/Mongolia/786/2017_H7N3_2017-09<br>ATGGATGTCAATCCGACTTTACTTTTCTTAAAAGTGCCAGCGCAAAATGCTATAAGTACTACGTTTCCTTACACTGGAGATCCTCCATACAGCCATGGAACAGGAACAGGATACACCATGGACACAGTCAACAGAACACATCAATACTCAGAGAAAGGAAAGTGGACAACAAACACAGAGACTGGGGCGCCCCAACTCAACCCAATTGATGGACCATTGCCAGAGGACAACGA<br>ACCAAGCGGATATGCACAAACAGATTGCGTGTTGGAAGCAATGGCTTTCCTTGAAGAATCCCACCCAGGGATCTTTGAAAACTCTTGTCTTGAAACGATGGAGGTCGTTCAGCAAACAAGAGTGGACAAACTAACCCAAGGTCGCCAGACTTATGATTGGACACTGAATAGAAACCAACCAGCTGCAACTGCTTTGGCCAATACTATAGAGGTCTTCAGATCAAACGGTCTAAC<br>AGCCAATGAGTCGGGGAGACTAATAGATTTCCTCAAGGATGTGATGGAATCAATGGATAAGGAAGAAATGGAAATAACAACACATTTCCAGAGAAAGAGAAGAGTAAGGGACAACATGACCAAGAAGATGGTCACACAAAGAACGATAGGAAAGAAGAAGCAGAGGCTGAACAAGAGGAGCTACTTAATAAGAGCACTGACATTGAACACAATGACCAAAGATGCGGAA<br>AGAGGCAAATTGAAGAGGCGGGCAATTGCAACACCCGGGATGCAGATTAGAGGATTTGTGTACTTTGTCGAAACACTAGCGAGGAGCATCTGTGAGAAACTCGAGCAATCTGGACTCCCTGTTGGAGGGAATGAGAAAAAGGCCAAATTGGCAAATGTCGTGAGAAAAATGATGACTAACTCGCAAGATACAGAGCTCTCCTTTACAATTACTGGAGACAACACCAAATGG<br>AATGAAAATCAAAATCCTCGGATGTTTTTGGCAATGATAACATACATTACAAGAAACCAACCCGAATGGTTTAGAAATGTCTTGAGCATTGCCCCTATAATGTTCTCAAACAAAATGGCAAGATTAGGAAAAGGATACATGTTTGAAAGTAAGAGCATGAAGCTAAGGACACAAATACCGGCAGAAATGCTTGCAAACATTGACTTGAAATACTTCAATGAATCAACGAGAAAG<br>AAAATCGAGAAAATAAGACCTCTGCTAATTGATGGCACAGCCTCATTGAGTCCTGGAATGATGATGGGCATGTTCAATATGCTGAGCACAGTATTGGGGGTCTCAATCCTGAATCTTGGACAAAAGAGATACACCAAAACCACATACTGGTGGGATGGACTCCAATCCTCTGATGATTTCGCTCTCATAGTGAATGCACCGAATCATGAGGGGATACAAGCAGGGGTGGATAG<br>GTTCTATAGAACCTGCAAACTGGTTGGAATAAACATGAGCAAAAAGAAGTCTTACATAAATCGGACAGGAACATTTGAGTTCACAAGCTTCTTCTACCGCTATGGATTTGTAGCCAACTTCAGTATGGAGTTACCCAGCTTTGGAGTGTCTGGAATCAATGAGTCAGCTGACATGAGCATTGGAGTTACAGTGATAAAGAACAATATGATAAACAATGACCTTGGACCAGCAAC<br>AGCTCAAATGGCTCTTCAGCTATTCATCAAGGATTATAGGTACACGTACCGATGTCACAGAGGTGATACACAAATTCAAACGAGGAGATCATTCGAGCTTAAGAAGCTGTGGGAGCAGACCCGTTCAAAGGCAGGGCTGTTGGTATCAGATGGAGGACCAAACCTATACAACATTCGGAATCTCCACATCCCAGAGGTATGCTTGAAGTGGGAACTAATGGATGAAGATTACC<br>AGGGCAGGCTGTGTAATCCTCTGAATCCGTTTGTCAGTCATAAGGAAATTGAGTCCGTAAACAATGCTGTGGTAATGCCAGCCCATGGCCCAGCCAAGAGCATGGAATATGATGCTGTTGCGACTACACACTCATGGATTCCTAAGAGGAACCGTTCCATTCTCAATACCAGCCAGAGGGGAATTCTCGAGGATGAGCAGATGTACCAGAAGTGCTGCAGTTTATTCGAGAAA<br>TTCTTCCCCAGTAGTTCGTACAGGAGGCCAGTTGGAATTTCCAGCATGGTGGAGGCCATGGTGTCCAGGGCCCGAATTGATGCACGCATTGATTTCGAATCTGGAAGGATTAAGAAAGAAGAGTTTGCTGAGATCATGAAGATCTGTTCCACCATTGAAGAGCTCAGACGGCAAAAATAG
EPI_ISL_697696	>A/duck/Mongolia/451/2018_H4N1_2018-09-02<br>ATGGATGTCAATCCGACTTTACTTTTCTTAAAAGTGCCAGCGCAAAATGCCATAAGTACCACATTCCCTTATACTGGAGATCCTCCATACAGCCATGGAACAGGAACAGGATACACCATGGACACAGTCAACAGAACACATCAATACTCAGAAAAGGGGAAATGGACAACAAACACAGAGACCGGAGCACCCCAACTCAACCCAATTGATGGACCACTACCTGAGGACAACGA<br>ACCAAGCGGATATGCACAAACAGATTGCGTGTTGGAAGCAATGGCTTTCCTTGAAGAGTCCCACCCAGGGATCTTTGAAAACTCTTGTCTTGAAACGATGGAAGTCGTTCAGCAAACAAGAGTGGACAAACTAACTCAAGGTCGCCAGACTTATGACTGGACACTGAATAGAAACCAACCAGCTGCAACTGCCCTGGCCAACACTATAGAGGTCTTCAGATCGAACGGTCTGA<br>CAGCCAATGAATCGGGAAGACTAATAGATTTCCTCAAGGATGTGATGGACTCAATGGACAAAGAAGAGATGGAAATAACAACACATTTCCAGAGAAAGAGAAGAGTAAGGGACAACATGACCAAGAAAATGGTCACACAAAGAACAATAGGGAAGAAGAAGCAAAGGCTAAATAAGAGGAGCTACTTAATAAGAGCACTGACACTGAATACAATGACAAAAGACGCAGA<br>AAGAGGCAAATTGAAGAGACGGGCAATTGCAACACCAGGGATGCAGATTAGAGGATTTGTGTACTTTGTCGAAACACTGGCAAGGAGCATCTGTGAAAAACTTGAGCAATCTGGACTCCCCGTTGGAGGGAATGAGAAGAAAGCTAAATTGGCAAATGTCGTGAGAAAAATGATGACTAACTCACAAGATACAGAGCTCTCCTTCACAATTACTGGAGATAACACCAAATG<br>GAATGAGAATCAAAATCCTCGGATGTTTCTGGCAATGATAACATACATCACAAGAAACCAACCTGAATGGTTTAGAAACGTCTTGAGCATTGCCCCTATAATGTTCTCGAACAAAATGGCGAGACTGGGAAAAGGATACATGTTTGAAAGTAAAAGCATGAAGCTACGGACACAAATACCTGCAGAAATGCTTGCAAACATTGACTTAAAATACTTCAATGAGTCAACAAGAA<br>AGAAAATCGAGAAAATAAGACCTCTACTAATAGATGGCACCGCCTCATTGAGTCCTGGAATGATGATGGGCATGTTCAATATGCTGAGTACAGTATTAGGAGTCTCAATCCTGAATCTTGGGCAAAAGAGGTACACCAAAACCACATACTGGTGGGATGGACTCCAATCCTCTGATGATTTCGCCCTCATAGTGAATGCACCGAATCATGAGGGAATACAAGCTGGAGTGGAT<br>AGGTTCTATAGGACTTGCAAGCTGGTTGGGATCAACATGAGCAAAAAGAAGTCTTACATAAACCGGACAGGAACATTTGAGTTCACAAGCTTTTTCTATCGCTATGGATTTGTGGCCAACTTCAGTATGGAGCTGCCCAGCTTTGGAGTGTCTGGAATCAATGAATCAGCTGACATGAGCATTGGAGTTACAGTGATAAAGAACAACATGATAAACAATGACCTTGGACCAGCA<br>ACAGCTCAAATGGCCCTTCAACTATTCATCAAGGACTACAGGTACACGTATCGATGTCACAGGGGTGACACACAAATTCAAACGAGGAGATCATTCGAGCTGAAGAAGCTGTGGGAGCAGACCCGTTCAAAGGCAGGACTGTTGGTGTCAGATGGAGGACCAAATCTGTACAACATCCGGAATCTCCATATCCCAGAGGTCTGCCTGAAGTGGGAGCTGATGGACGAAGATT<br>ACCAGGGCAGGTTGTGTAATCCTCTGAACCCATTTGTCAGTCATAAAGAAATTGAATCCGTAAACAATGCTGTGGTAATGCCAGCCCATGGCCCAGCCAAGAGCATGGAATATGATGCTGTTGCGACTACACACTCATGGATTCCTAAAAGGAATCGTTCCATTCTCAATACCAGCCAAAGGGGAATTCTTGAGGATGAGCAGATGTATCAGAAATGCTGCAGTCTATTCGAGA<br>AATTCTTCCCCAGTAGTTCATACAGGAGACCAGTTGGAATTTCCAGCATGGTGGAGGCCATGGTGTCTAGGGCCCGAATCGACGCACGCATTGATTTCGAATCTGGAAGGATTAAGAAGGAAGAGTTTGCTGAGATCATGAAGATCTGTTCCACCATTGAAGAGCTCAGACGGCAAAAATAG
EPI_ISL_237166	>A/duck/Kumamoto/431119/2014_H4N6_2014-11<br>ATGGATGTCAATCCGACTTTACTTTTCTTAAAAGTGCCAGCGCAAAATGCCATAAGTACTACGTTTCCTTATACTGGAGATCCTCCATACAGCCATGGAACAGGAACAGGATACACCATGGACACAGTCAACAGAACACATCAATACTCAGAAAAGGGGAAATGGACAACAAACACAGAGACCGGAGCACCTCAACTCAACCCAATTGATGGACCACTACCTGAGGACAACGA<br>ACCAAGCGGATATGCACAAACAGATTGTGTGTTGGAAGCAATGGCTTTCCTTGAAGAGTCCCACCCAGGGATCTTTGAAAACTCTTGTCTTGAAACGATGGAAGTTGTTCAGCAAACAAGAGTGGACAAACTAACTCAAGGTCGCCAGACTTATGACTGGACACTGAATAGAAACCAACCAGCTGCAACTGCCCTGGCCAACACTATAGAGGTCTTCAGATCGAACGGTCTGA<br>CATCCAATGAATCGGGGAGACTAATAGATTTTCTCAAGGATGTGATGGACTCAATGGACAAAGAAGAAATGGAAATAACAACACATTTCCAGAGAAAGAGAAGAGTAAGGGACAACATGACCAAGAAGATGGTCACACAAAGAACAATAGGGAAAAAGAAACAAAGGCTGAATAAGAAGAGCTACTTAATAAGAGCACTGACACTGAACACAATGACAAAAGATGCAGAA<br>AGAGGCAAATTGAAGAGGCGAGCAATTGCAACACCAGGGATGCAGATTAGAGGATTTGTGTACTTTGTCGAAACACTGGCAAGGAGCATCTGTGAGAAACTTGAGCAGTCTGGACTCCCCGTTGGAGGGAATGAGAAAAAGGCTAAATTGGCAAATGTCGTGAGAAAAATGATGACTAACTCACAAGATACAGAGCTCTCTTTCACAATTACTGGAGATAACACCAAATGG<br>AATGAGAATCAAAATCCTCGGATGTTTCTGGCAATGATAACATACATCACAAGAAACCAACCTGAATGGTTTAGAAATGTCTTGAGCATTGCCCCTATAATGTTCTCGAACAAAATGGCGAGATTGGGAAAAGGATACATGTTTGAAAGTAAGAGCATGAAGCTACGGACACAAATACCTGCAGAAATGCTTGCAAACATTGACTTAAAATATTTCAATGAATCAACAAGAAAG<br>AAAATCGAGAAAATAAGACCTCTGCTAATAGATGGCACAGCCTCATTGAGTCCTGGAATGATGATGGGCATGTTCAATATGCTTAGTACAGTATTAGGAGTCTCAATCCTGAATCTTGGGCAAAAGAGGTACACCAAAACCACATACTGGTGGGATGGACTCCAATCCTCTGATGATTTCGCCCTCATAGTGAATGCACCGAATCATGAGGGAATACAAGCAGGAGTGGATAG<br>GTTCTATAGAACATGCAAACTGGTTGGAATCAACATGAGCAAAAAGAAGTCCTACATAAACCGGACAGGAACATTTGAGTTCACAAGCTTTTTCTATCGCTATGGATTTGTGGCTAATTTCAGTATGGAGCTGCCCAGCTTTGGAGTGTCTGGAATCAATGAATCAGCTGACATGAGCATTGGAGTTACAGTGATAAAGAATAACATGATAAACAATGACCTTGGACCAGCAAC<br>AGCTCAGATGGCTCTTCAGCTATTCATCAAGGACTACAGGTACACGTACCGATGCCACAGGGGTGACACACAAATTCAAACGAGGAGATCATTCGAGCTGAAGAAGCTGTGGGAGCAGACCCGTTCAAAGGCAGGACTGTTGGTGTCGGATGGAGGACCAAACCTATACAACATTCGGAATCTCCACATTCCAGAGGTCTGCTTGAAGTGGGAGCTGATGGACGAAGATTAC<br>CAGGGCAGGTTGTGTAATCCTCTGAACCCATTTGTCAGTCATAAGGAAATTGAGTCCGTAAACAATGCTGTGGTGATGCCAGCCCATGGCCCAGCCAAGAGCATGGAATATGATGCTGTTGCGACTACACACTCATGGATTCCTAAGAGGAATCGTTCCATTCTCAATACCAGCCAAAGGGGAATTCTTGAGGATGAGCAGATGTACCAGAAGTGCTGTAGTCTATTCGAGAAA<br>TTCTTCCCCAGTAGTTCATACAGGAGGCCAGTTGGAATTTCCAGCATGGTAGAGGCCATGGTATCTAGGGCCCGAATTGATGCACGCATTGATTTCGAATCTGGAAGGATTAAGAAAGAAGAGTTTGCTGAGATCATGAAGATCTGTTCCACCATTGAAGAGCTCAGACGGCAAAAATAG
EPI_ISL_7987336	>A/duck/Italy/21VIR8024-4/2021_H5N3_2021-09-23<br>ATGGATGTCAATCCGACTTTACTTTTCTTAAAAGTGCCAGCGCAAAATGCCATAAGTACCACATTCCCTTATACTGGAGATCCTCCATACAGCCATGGAACAGGAACAGGATACACCATGGACACAGTCAACAGAACACATCAATACTCGGAAAAGGGGAAATGGACAACAAACACAGAGACTGGAGCACCCCAACTCAACCCAATTGATGGACCACTACCTGAGGACAACGA<br>ACCAAGCGGATATGCACAAACAGATTGCGTGTTGGAAGCAATGGCTTTCCTTGAAGAGTCCCACCCAGGGATCTTTGAAAACTCTTGTCTTGAAACGATGGAAGTCGTTCAGCAAACAAGAGTGGACAAACTAACTCAAGGTCGCCAGACTTATGACTGGACACTGAATAGAAACCAACCAGCTGCAACTGCCCTGGCCAACACTATAGAGGTCTTCAGATCGAACGGTCTGA<br>CAGCCAATGAATCAGGGAGACTAATAGATTTCCTCAAGGATGTGATGGACTCAATGGACAAAGAAGAAATGGAAATAACAACACATTTCCAGAGAAAGAGAAGAGTAAGGGACAACATGACCAAGAAAATGGTCACACAAAGAACAATAGGGAAGAAGAAGCAAAGGCTAAATAAAAGGAGCTACTTAATAAGAGCACTGACACTGAATACAATGACAAAAGACGCAGA<br>AAGAGGCAAATTGAAGAGACGGGCAATTGCAACACCAGGGATGCAGATTAGAGGATTTGTGTACTTTGTCGAAACACTGGCAAGGAGCATTTGTGAAAAACTTGAGCAATCTGGACTCCCCGTTGGAGGGAATGAGAAGAAGGCTAAATTGGCAAATGTCGTGAGAAAAATGATGACTAACTCACAAGATACAGAGCTCTCCTTCACAATTACTGGAGATAACACCAAATG<br>GAATGAAAATCAAAATCCTCGGATGTTTCTGGCAATGATAACATACATCACAAGAAACCAACCTGAATGGTTTAGAAATGTCTTGAGCATTGCCCCTATAATGTTCTCGAACAAAATGGCGAGACTGGGAAAAGGATACATGTTTGAAAGCAAAAGCATGAAGCTACGGACACAAATACCTGCAGAAATGCTTGCAAACATTGACTTAAAATACTTCAATGAGTCAACAAGAAA<br>GAAAATTGAGAAAATAAGACCTCTACTAATAGATGGCACCGCCTCATTGAGTCCTGGAATGATGATGGGCATGTTCAATATGCTGAGTACAGTGTTAGGAGTCTCAATCCTGAATCTTGGGCAAAAGAGGTACACCAAGACCACATACTGGTGGGATGGACTCCAATCCTCTGATGATTTCGCCCTCATAGTGAATGCACCGAATCATGAGGGAATACAAGCAGGAGTGGATA<br>GGTTCTATAGGACCTGCAAACTGGTTGGGATCAACATGAGCAAAAAGAAGTCTTACATAAACCGAACAGGAACATTTGAATTCACAAGCTTTTTCTATCGCTATGGATTTGTGGCCAACTTCAGTATGGAGCTGCCCAGTTTTGGAGTGTCTGGAATCAATGAATCAGCTGACATGAGCATTGGAGTTACAGTGATAAAGAACAACATGATAAACAATGACCTTGGACCAGCAA<br>CAGCTCAAATGGCCCTTCAACTATTCATCAAGGACTACAGGTACACGTACCGATGTCACAGGGGTGACACACAGATTCAAACGAGGAGATCATTCGAGCTGAAGAAGCTGTGGGAGCAGACCCGTTCCAAGGCAGGACTGTTGGTGTCAGATGGAGGACCAAATCTATACAACATCCGGAATCTCCATATCCCAGAAGTCTGCCTGAAGTGGGAGCTGATGGACGAAGATTA<br>CCAGGGCAGGTTGTGTAATCCTCTGAACCCATTTGTCAGTCATAAAGAAATTGAATCCGTAAACAATGCTGTGGTAATGCCAGCCCATGGCCCAGCCAAGAGCATGGAATATGATGCTGTTGCGACTACACACTCATGGATTCCCAAAAGGAATCGTTCCATTCTCAATACCAGCCAAAGGGGAATTCTTGAGGATGAGCAGATGTACCAGAAATGCTGCAGTCTATTCGAGAA<br>GTTCTTCCCCAGTAGTTCATACAGGAGACCAGTTGGAATTTCCAGCATGGTGGAGGCCATGGTGTCTAGGGCCCGAATCGACGCACGCATTGATTTCGAATCTGGAAGGATTAAGAAGGAAGAGTTTGCTGAGATCATGAAGATCTGTTCCACCATTGAAGAGCTCAGACGGCAAAAATAG
EPI_ISL_7987335	>A/duck/Italy/21VIR8024-3/2021_H5N3_2021-09-23<br>ATGGATGTCAATCCGACTTTACTTTTCCTAAAAGTGCCAGCGCAAAATGCCATAAGTACCACATTCCCTTATACTGGAGATCCTCCATACAGCCATGGAACAGGAACAGGATACACCATGGACACAGTCAACAGAACACATCAATACTCGGAAAAGGGGAAATGGACAACAAACACAGAGACTGGAGCACCCCAACTCAACCCAATTGATGGACCACTACCTGAGGACAACGA<br>ACCAAGCGGATATGCACAAACAGATTGCGTGTTGGAAGCAATGGCTTTCCTTGAAGAGTCCCACCCAGGGATCTTTGAAAACTCTTGTCTTGAAACGATGGAAGTCGTTCAGCAAACAAGAGTGGACAAACTAACTCAAGGTCGCCAGACTTATGACTGGACACTGAATAGAAACCAACCAGCTGCAACTGCCCTGGCCAACACTATAGAGGTCTTCAGATCGAACGGTCTGA<br>CAGCCAATGAATCAGGGAGACTAATAGATTTCCTCAAGGATGTGATGGACTCAATGGACAAAGAAGAAATGGAAATAACAACACATTTCCAGAGAAAGAGAAGAGTAAGGGACAACATGACCAAGAAAATGGTCACACAAAGAACAATAGGGAAGAAGAAGCAAAGGCTAAATAAAAGGAGCTACTTAATAAGAGCACTGACACTGAATACAATGACAAAAGACGCAGA<br>AAGAGGCAAATTGAAGAGACGGGCAATTGCAACACCAGGGATGCAGATTAGAGGATTTGTGTACTTTGTCGAAACACTGGCAAGGAGCATTTGTGAAAAACTTGAGCAATCTGGACTCCCCGTTGGAGGGAATGAGAAGAAGGCTAAATTGGCAAATGTCGTGAGAAAAATGATGACTAACTCACAAGATACAGAGCTCTCCTTCACAATTACTGGAGATAACACCAAATG<br>GAATGAAAATCAAAATCCTCGGATGTTTCTGGCAATGATAACATACATCACAAGAAACCAACCTGAATGGTTTAGAAATGTCTTGAGCATTGCCCCTATAATGTTCTCGAACAAAATGGCGAGACTGGGAAAAGGATACATGTTTGAAAGCAAAAGCATGAAGCTACGGACACAAATACCTGCAGAAATGCTTGCAAACATTGACTTAAAATACTTCAATGAGTCAACAAGAAA<br>GAAAATTGAGAAAATAAGACCTCTACTAATAGATGGCACCGCCTCATTGAGTCCTGGAATGATGATGGGCATGTTCAATATGCTGAGTACAGTGTTAGGAGTCTCAATCCTGAATCTTGGGCAAAAGAGGTACACCAAGACCACATACTGGTGGGATGGACTCCAATCCTCTGATGATTTCGCCCTCATAGTGAATGCACCGAATCATGAGGGAATACAAGCAGGAGTGGATA<br>GGTTCTATAGGACCTGCAAACTGGTTGGGATCAACATGAGCAAAAAGAAGTCTTACATAAACCGAACAGGAACATTTGAATTCACAAGCTTTTTCTATCGCTATGGATTTGTGGCCAACTTCAGTATGGAGCTGCCCAGTTTTGGAGTGTCTGGAATCAATGAATCAGCTGACATGAGCATTGGAGTTACAGTGATAAAGAACAACATGATAAACAATGACCTTGGACCAGCAA<br>CAGCTCAAATGGCCCTTCAACTATTCATCAAGGACTACAGGTACACGTACCGATGTCACAGGGGTGACACACAGATTCAAACGAGGAGATCATTCGAGCTGAAGAAGCTGTGGGAGCAGACCCGTTCCAAGGCAGGACTGTTGGTGTCAGATGGAGGACCAAATCTATACAACATCCGGAATCTCCATATCCCAGAAGTCTGCCTGAAGTGGGAGCTGATGGACGAAGATTA<br>CCAGGGCAGGTTGTGTAATCCTCTGAACCCATTTGTCAGTCATAAAGAAATTGAATCCGTAAACAATGCTGTGGTAATGCCAGCCCATGGCCCAGCCAAGAGCATGGAATATGATGCTGTTGCGACTACACACTCATGGATTCCCAAAAGGAATCGTTCCATTCTCAATACCAGCCAAAGGGGAATTCTTGAGGATGAGCAGATGTACCAGAAATGCTGCAGTCTATTCGAGAA<br>GTTCTTCCCCAGTAGTTCATACAGGAGACCAGTTGGAATTTCCAGCATGGTGGAGGCCATGGTGTCTAGGGCCCGAATCGACGCACGCATTGATTTCGAATCTGGAAGGATTAAGAAGGAAGAGTTTGCTGAGATCATGAAGATCTGTTCCACCATTGAAGAGCTCAGACGGCAAAAATAG
EPI_ISL_252817	>A/duck/Hubei/ZYSYG3/2015_H6N2_2015-11-21<br>ATGGATGTCAATCCGACTTTACTTTTCTTAAAAGTGCCAGCGCAAAATGCCATAAGTACCACATTCCCTTATACTGGAGATCCTCCATACAGCCATGGAACAGGAACAGGGTACACCATGGACACAGTCAACAGAACACATCAATACTCAGAAAAGGGGAAATGGACAACAAACACAGAGACCGGAGCACCCCAACTCAACCCAATTGATGGACCACTACCTGAGGACAACGA<br>ACCAAGCGGATATGCACAAACAGATTGCGTGTTGGAAGCAATGGCTTTCCTTGAAGAGTCCCACCCAGGGATCTTTGAAAACTCTTGTCTTGAAACGATGGAAGTCGTTCAGCAAACAAGAGTGGACAAACTAACTCAAGGTCGCCAGACTTATGACTGGACACTGAATAGAAACCAACCAGCTGCAACTGCCCTGGCCAACACCATAGAGGTCTTCAGATCGAATGGTCTGA<br>CAGCAAATGAATCGGGGAGACTAATAGATTTCCTCAAGGATGTGATGGACTCAATGGACAAAGAAGAAATGGAAATAACAACACATTTCCAGAGAAAGAGAAGAGTAAGGGACAACATGACCAAGAAAATGGTCACACAAAGAACAATAGGGAAGAAGAAACAAAGGCTAAATAAAAGGAGCTACTTAATAAGAGCACTGACACTGAATACAATGACAAAAGACGCAGA<br>AAGAGGCAAATTGAAGAGACGGGCAATTGCAACACCAGGGATGCAGATTAGAGGATTTGTGTACTTTGTCGAAACACTGGCAAGGAGCATCTGTGAAAAGCTTGAGCAATCTGGACTCCCCGTTGGAGGGAATGAAAAGAAGGCTAAATTGGCAAATGTCGTGAGAAAAATGATGACTAACTCACAAGATACAGAGCTCTCCTTCACAATTACTGGAGATAACACCAAATG<br>GAATGAGAATCAAAATCCTCGAATGTTTCTGGCAATGATAACATACATCACAAGAAACCAACCTGAATGGTTTAGAAATGTCTTGAGCATTGCCCCTATAATGTTCTCGAACAAAATGGCGAGATTAGGAAAAGGATACATGTTTGAAAGTAAGAGCATGAAACTACGGACACAAATACCTGCAGAAATGCTTGCAAACATTGACTTAAAATACTTCAATGAGTCAACAAGAAA<br>GAAAATCGAGAAAATAAGACCTCTACTAATAGATGGCACCGCCTCATTGAGTCCTGGAATGATGATGGGCATGTTCAATATGCTGAGTACAGTATTAGGAGTCTCAATCCTGAATCTTGGGCAAAAGAGGTACACCAAAACCACATACTGGTGGGATGGACTCCAATCCTCTGATGATTTCGCCCTCATAGTGAACGCACCGAATCATGAGGGAATACAAGCAGGAGTGGATA<br>GGTTCTATAGGACCTGCAAACTGGTTGGGATCAACATGAGCAAAAAGAAGTCTTACATAAACCGGACAGGAACATTTGAATTCACAAGCTTTTTCTATCGCTATGGATTTGTGGCTAACTTCAGTATGGAGCTGCCCAGCTTTGGAGTGTCTGGAATCAATGAATCAGCTGACATGAGCATTGGAGTTACAGTGATAAAGAACAACATGATAAACAATGACCTTGGACCAGCAA<br>CAGCTCAAATGGCCCTTCAACTATTCATCAAGGACTACAGGTACACGTACCGATGTCACAGGGGTGACACACAAATTCAAACGAGGAGATCATTCGAGCTGAAGAAGCTGTGGGAGCAGACCCGTTCAAAGGTAGGACTGTTGGTGTCAGATGGAGGACCAAATCTATACAACATTCGGAATCTCCACATCCCAGAGGTCTGCCTGAAGTGGGAGCTGATGGACGAAGATTA<br>CCAGGGCAGGTTGTGTAATCCTCTGAACCCATTTGTCAGTCACAAAGAAATTGAGTCCGTAAACAATGCTGTGGTAATGCCAGCCCATGGCCCAGCCAAGAGCATGGAATATGATGCTGTTGCGACTACACACTCATGGATTCCTAAAAGGAATCGTTCCATTCTCAATACCAGCCAAAGGGGAATTCTTGAGGATGAGCAGATGTACCAGAAATGCTGCAGTCTATTCGAGAA<br>ATTCTTCCCCAGTAGTTCATACAGGAGACCAGTTGGAATTTCCAGCATGGTGGAGGCCATGGTGTCTAGGGCCCGAATCGATGCACGCATTGATTTCGAATCTGGAAGGATTAAGAAGGAAGAGTTTGCTGAGATCATGAAGATCTGTTCCACCATTGAAGAGCTCAGACGGCAAAAATAG
EPI_ISL_12572656	>A/duck/Guizhou/S1321/2022_H5N1_2022-02-22<br>ATGGATGTCAATCCGACTTTACTTTTCTTAAAAGTGCCAGCGCAAAATGCTATAAGCACTACGTTCCCTTACACTGGAGATCCTCCATACAGCCATGGAACAGGAACAGGATACACCATGGACACAGTCAACAGAACACATCAATACTCAGAAAAAGGAAAGTGGACAACAAACACAGAGACCGGGGCGCCCCAACTCAACCCAATTGATGGACCATTGCCAGAGGACAACGA<br>CCCAAGCGGATATGCACAAACAGATTGCGTGTTGGAAGCAATGGCTTTCCTTGAAGAATCCCACCCAGGGATCTTTGAAAACTCTTGTCTTGAAACGATGGAGATCGTTCAGCAAACAAGAGTGGACAAACTAACCCAAGGTCGCCAGACCTATGACTGGACACTGAATAGAAACCAACCAGCTGCAACTGCTTTAGCCAATACTATAGAGGTCTTCAGATCGAACGGTCTAAC<br>AGCCAATGAGTCGGGGAGACTAATAGATTTCCTCAAGGATGTGATGGAATCAATGGATAAAGAAGAAATGGAAATAACAACACATTTCCAGAGAAAGAGAAGAGTAAGGGACAACCTGACCAAAAAGATGGTCACACAAAGAACGATAGGAAAGAAGAAGCAGAGGCTGAACAAGAGAAGCTACTTAATAAGAGCGTTGACATTGAACACAATGACCAAAGATGCAGAA<br>AGAGGCAAATTGAAGAGGCGGGCAATTGCAACACCCGGGATGCAGATTAGAGGATTTGTGTACTTTGTCGAAACACTAGCGAGGAGCATCTGTGAGAAACTTGAGCAATCCGGACTCCCTGTCGGAGGGAATGAGAAAAAGGCCAAATTGGCAAATGTCGTGAGGAAAATGATGACTAACTCGCAAGATACAGAGCTCTCTTTTACAATCACCGGGGACAACACCAAATGG<br>AATGAGAATCAAAATCCTCGGATGTTTTTGGCAATGATAACATACATCACAAGAAACCAACCCGAATGGTTTAGAAATGTCTTGAGCATTGCCCCCATAATGTTCTCAAACAAAATGGCAAGATTAGGGAAAGGATACATGTTTGAAAGTAAGAGCATGAAGCTAAGGACACAAATACCGGCAGAAATGATTGCAAACATTGACTTGAAATACTTCAATGAATCAACGAGAAA<br>GAAAATCGAGAAAATAAGACCTCTACTAATTGATGGCACAGCCTCATTGAGTCCTGGAATGATGATGGGCATGTTCAATATGCTGAGCACTGTATTGGGGGTCTCAATCCTGAATCTTGGACAAAAGAGGTACACCAAAACCACATACTGGTGGGATGGACTCCAATCTTCTGATGACTTCGCTCTCATAGTGAATGCACCGAATCATGAGGGGATACAAGCCGGGGTGGATA<br>GGTTCTATAGGACATGCAAACTGGTTGGGATCAACATGAGCAAAAAGAAGTCTTACATAAACCGGACAGGAACATTTGAGTTCACAAGCTTTTTCTACCGCTATGGATTTGTAGCCAACTTCAGTATGGAGTTACCCAGCTTTGGTGTGTCTGGAATCAATGAGTCAGCTGACATGAGCATTGGAGTTACAGTGATAAAGAACAACATGATAAACAATGACCTTGGACCAGCAA<br>CAGCTCAAATGGCTCTTCAGCTATTCATCAAGGATTATAGGTACACGTACCGATGTCACAGAGGTGATACACACATTCAAACGAGGAGATCATTCGAGCTGAAGAAGCTGTGGGAGCAGACCCGTTCAAAGGCAGGGCTGTTGGTATCAGATGGAGGACCAAACCTATACAACATTCGGAATCTCCACATCCCAGAGGTCTGCTTGAAATGGGAGCTAATGGATGAAGACTAC<br>CAGGGCAGGCTGTGTAATCCTCTGAATCCATTTGTCAGTCATAAGGAAATTGAATCCGTAAACAATGCTGTAGTAATGCCAGCCCATGGCCCAGCCAAGAGCATGGAATACGATGCTGTTGCGACTACACACTCATGGATTCCTAAGAGGAACCGTTCCATTCTCAATACCAGCCAGAGGGGAATTCTCGAGGATGAGCAGATGTACCAGAAGTGCTGCAGCTTATTCGAGAA<br>GTTCTTCCCCAGTAGTTCATACAGGAGGCCAGTTGGAATTTCCAGCATGGTGGAGGCCATGGTGTCCAGGGCCCGAATTGATGCACGCATTGATTTCGAATCTGGAAGGATTAAGAAAGAAGAGTTTGCTGAGATCATGAAGATCTGTTCCACCATTGAGGAGCTCAGACGGCAAAAATAG

EPI_ISL_12572655	>A/duck/Guangdong/S4525/2021_H5N1_2021-12-08<br>ATGGATGTCAATCCGACTTTACTTTTCTTAAAAGTGCCAGCGCAAAATGCTATAAGCACTACGTTCCCTTACACTGGAGATCCTCCATACAGCCATGGAACAGGAACAGGATACACCATGGACACAGTCAACAGAACACATCAATACTCAGAAAAAGGAAAGTGGACAACAAACACAGAGACCGGGGCGCCCCAACTCAACCCAATTGATGGACCATTGCCAGAGGACAACGA<br>CCCAAGCGGATATGCACAAACAGATTGCGTGTTGGAAGCAATGGCTTTCCTTGAAGAATCCCACCCAGGGATCTTTGAAAACTCTTGTCTTGAAACGATGGAGATCGTTCAGCAAACAAGAGTGGACAAACTAACCCAAGGTCGCCAGACCTATGACTGGACACTGAATAGAAACCAACCAGCTGCAACTGCTTTAGCCAATACTATAGAGGTCTTCAGATCGAATGGTCTAAC<br>AGCCAATGAGTCGGGGAGACTAATAGATTTCCTCAAGGATGTGATGGAATCAATGGATAAAGAAGAAATGGAAATAACAACACATTTCCAGAGAAAGAGAAGAGTAAGGGACAACATGACCAAAAAGATGGTCACACAAAGAACGATAGGAAAGAAGAAGCAGAGGCTGAACAAGAGAAGCTACTTAATAAGAGCGTTGACATTGAACACAATGACCAAAGATGCAGAA<br>AGAGGCAAATTGAAGAGGCGGGCAATTGCAACACCCGGGATGCAGATTAGAGGATTTGTGTACTTTGTCGAAACACTAGCGAGGAGCATCTGTGAGAAACTTGAGCAATCCGGACTCCCTGTCGGAGGGAATGAGAAAAAGGCCAAATTGGCAAATGTCGTGAGGAAAATGATGACTAACTCGCAAGATACAGAGCTCTCTTTTACAATCACCGGGGACAACACCAAATGG<br>AATGAGAATCAAAATCCTCGGATGTTTTTGGCAATGATAACATACATCACAAGAAACCAACCCGAATGGTTTAGAAATGTCTTGAGCATTGCCCCCATAATGTTCTCAAACAAAATGGCAAGATTAGGGAAAGGATACATGTTTGAAAGTAAGAGCATGAAGCTAAGGACACAAATACCGGCAGAAATGATTGCAAACATTGACTTGAAATACTTCAATGAATCAACGAAAA<br>GAAAATCGAGAAAATAAGACCTCTACTAATTGATGGCACAGCCTCATTGAGTCCTGGAATGATGATGGGCATGTTCAATATGCTGAGCACTGTATTGGGGGTCTCAATCCTGAATCTTGGACAAAAGAGATACACCAAAACCACATACTGGTGGGATGGACTCCAATCTTCTGATGACTTCGCTCTCATAGTGAATGCACCGAATCATGAGGGGATACAAGCCGGGGTGGATA<br>GGTTCTATAGGACATGCAAACTGGTTGGGATCAACATGAGCAAAAAGAAGTCTTACATAAACCGGACAGGAACATTTGAGTTCACAAGCTTTTTCTACCGCTATGGATTTGTAGCCAACTTCAGTATGGAGTTACCCAGCTTTGGTGTGTCTGGAATCAATGAGTCAGCTGACATGAGCATTGGAGTTACAGTGATAAAGAACAACATGATAAACAATGACCTTGGACCAGCAA<br>CAGCTCAAATGGCTCTTCAGCTATTCATCAAGGATTATAGGTACACGTACCGATGTCACAGAGGTGATACACAAATTCAAACGAGGAGATCATTCGAGCTGAAGAAGCTGTGGGAGCAGACCCGTTCAAAGGCAGGGCTGTTGGTATCAGATGGAGGACCAAACCTATACAACATTCGGAATCTCCACATCCCAGAGGTCTGCTTGAAATGGGAGCTAATGGATGAAGACTA<br>CCAGGGCAGGCTGTGTAATCCTCTGAATCCATTTGTCAGTCATAAGGAAATTGAATCCGTAAACAATGCTGTAGTAATGCCAGCCCATGGCCCAGCCAAGAGCATGGAATACGATGCTGTTGCGACTACACACTCATGGATTCCTAAGAGGAACCGTTCCATTCTCAATACCAGCCAGAGGGGAATTCTCGAGGATGAGCAGATGTACCAGAAGTGCTGCAGCTTATTCGAGA<br>AGTTCTTCCCCAGTAGTTCATACAGGAGGCCAGTTGGAATTTCCAGCATGGTGGAGGCCATGGTGTCCAGGGCCCGAATTGATGCACGCATTGATTTCGAATCTGGAAGGATTAAGAAAGAAGAGTTTGCTGAGATCATGAAGATCTGTTCCACCATTGAGGAGCTCAGACGGCAAAAATAG
EPI_ISL_14933724	>A/duck/France/22P020165/2022_H5N1_2022-08-24<br>ATGGATGTCAATCCGACTTTACTTTTCTTAAAAGTGCCAGCGCAAAATGCCATAAGTACCACATTCCCTTATACTGGAGATCCTCCATACAGCCATGGAACAGGAACAGGATACACCATGGACACAGTCAACAGAACACATCAATACTCGGAAAAGGGAAAATGGACAACAAACACAGAGACCGGAGCACCCCAACTCAACCCAATTGATGGACCACTACCTGAGGACAACGA<br>ACCAAGCGGATATGCACAAACAGATTGCGTGTTGGAAGCAATGGCTTTCCTTGAAGAGTCCCACCCAGGGATCTTTGAAAACTCTTGTCTTGAAACGATGGAAGTCGTTCAGCAAATAAGAGTGGACAAACTAACTCAAGGTCGCCAGACTTATGACTGGACACTGAATAGAAACCAACCAGCTGCAACTGCCCTGGCCAACACTATAGAGGTCTTCAGATCGAACGGTCTGA<br>CAGCCAATGAATCAGGGAGACTAATAGATTTCCTCAAGGATGTGATGGACTCAATGGACAAAGAAGAAATGGAAATAACAACACATTTCCAGAGAAAGAGAAGAGTAAGGGACAACATGACCAAGAAAATGGTCACACAAAGAACAATAGGGAAGAAGAAGCAAAGGCTAAATAAGAGGAGCTACTTAATAAGAGCACTGACACTGAATACAATGACAAAAGACGCAGA<br>AAGAGGCAAATTGAAGAGGCGGGCAATTGCAACACCAGGGATGCAGATTAGAGGATTTGTGTACTTTGTCGAAACACTGGCAAGGAGCATCTGTGAAAAACTTGAGCAATCTGGACTACCCGTTGGGGGGAATGAGAAGAAGGCTAAATTGGCAAATGTCGTGAGAAAAATGATGACTAACTCACAAGATACAGAGCTCTCATTCACAATTACTGGAGATAACACCAAATG<br>GAATGAGAATCAAAATCCTCGGATGTTTCTGGCAATGATAACATACATCACAAGGAACCAACCTGAATGGTTTAGAAATGTCTTGAGCATTGCCCCTATAATGTTCTCGAACAAAATGGCTAGACTGGGAAAAGGATACATGTTTGAAAGTAAAAGCATGAAGCTACGGACACAAATACCTGCCGAAATGCTTGCAAACATTGACTTAAAATACTTCAATGAGTCAACAAGAAA<br>GAAAATCGAGAAAATAAGACCTCTACTAATAGATGGCACCGCCTCATTGAGTCCTGGAATGATGATGGGCATGTTCAATATGTTGAGTACAGTATTAGGAGTCTCAATCCTGAATCTTGGGCAAAAGAGGTACACCAAAACCACATACTGGTGGGATGGACTCCAATCCTCTGATGATTTCGCCCTCATAGTGAATGCACCAAATCATGAGGGAATACAAGCAGGAGTGGATA<br>GGTTCTATAGGACCTGCAAACTGGTTGGAATCAACATGAGCAAAAAGAAGTCTTACATAAACCGAACTGGAACATTTGAGTTCACAAGCTTTTTCTATCGCTATGGATTTGTGGCCAACTTCAGTATGGAGCTGCCCAGCTTTGGAGTGTCTGGAATCAATGAATCAGCTGACATGAGCATTGGAGTTACAGTGATAAAGAACAACATGATAAACAATGACCTTGGACCAGCAA<br>CAGCTCAAATGGCCCTTCAACTATTCATCAAGGACTACAGGTACACGTACCGATGCCACAGGGGTGACACACAAATTCAAACGAGGAGATCATTCGAGATGAAGAAGCTGTGGGAGCAGACCCGTTCAAAGGCAGGACTGTTGGTGTCAGATGGAGGACCAAATCTATACAACATCCGGAATCTCCATATCCCAGAAGTCTGCCTGAAGTGGGAGCTGATGGACGAAGATTA<br>CCAGGGCAGGTTGTGTAATCCTCTGAACCCATTTGTCAGTCATAAAGAAATTGAATCCGTAAACAATGCTGTGGTAATGCCAGCCCATGGCCCAGCCAAAAGCATGGAATATGATGCTGTTGCGACTACACACTCATGGATTCCTAAAAGGAATCGTTCCATTCTCAATACCAGCCAAAGGGGAATTCTTGAGGATGAGCAGATGTACCAGAAATGCTGCAGTCTATTCGAGAA<br>ATTCTTCCCCAGTAGTTCATACAGGAGACCAGTTGGAATTTCCAGCATGGTGGAGGCCATGGTGTCTAGGGCCCGAATTGACGCACGCATTGATTTCGAATCTGGAAGGATTAAGAAGGAAGAGTTTGCTGAGATCATGAAGATCTGTTCCACCATTGAAGAGCTCAGACGGCAAAAATAG
EPI_ISL_333102	>A/duck/Bangladesh/17D747/2016_H3N5_2016-12-18<br>ATGGATGTCAATCCGACTTTACTTTTCTTAAAAGTGCCAGCGCAAAATGCCATAAGTACCACATTCCCTTATACTGGAGATCCTCCATACAGCCATGGAACAGGAACAGGATACACCATGGACACAGTCAACAGAACACATCAATACTCAGAAAAGGGGAAATGGACAACAAACACAGAGACCGGAGCACCCCAACTCAACCCAATTGATGGACCACTACCTGAGGACAACGA<br>ACCAAGTGGATATGCACAAACAGATTGCGTATTGGAAGCAATGGCTTTCCTTGAAGAGTCCCACCCAGGGATCTTTGAAAACTCTTGTCTTGAAACGATGGAAGTCGTTCAGCAAACAAGAGTGGACAAACTAACTCAAGGTCGCCAGACTTATGACTGGACACTGAATAGAAACCAACCAGCTGCAACTGCCCTGGCCAACACTATAGAGGTCTTCAGATCGAACGGTCTGA<br>CAGCCAATGAATCGGGGAGACTAATAGATTTCCTCAAGGATGTGATGGACTCAATGGACAAAGAAGAAATGGAAATAACAACACATTTCCAGAGAAAGAGAAGAGTAAGGGACAACATGACCAAGAAAATGGTCACACAAAGGACAATAGGGAAGAAGAAACAAAGGCTAAATAGGAGGAGCTACTTAATAAGAGCACTGACACTGAATACAATGACAAAAGACGCAGA<br>AAGAGGCAAATTGAAGAGACGGGCAATTGCAACACCAGGGATGCAGATTAGAGGATTTGTGTACTTTGTCGAAACACTGGCAAGGAGCATCTGTGAAAAACTTGAGCAATCTGGACTCCCCGTTGGAGGGAATGAGAAGAAGGCTAAATTGGCAAATGTCGTGAGAAAAATGATGACTAACTCACAAGATACAGAGCTCTCCTTCACAATTACTGGAGATAACACCAAATG<br>GAATGAGAATCAAAATCCTCGGATGTTTCTGGCAATGATAACATACATCACAAGAAACCAACCTGAATGGTTTAGAAATGTCTTGAGCATTGCCCCTATAATGTTCTCGAACAAAATGGCGAGATTGGGAAAAGGATACATGTTTGAAAGTAAGAGCATGAAGCTACGGACACAAATACCTGCAGAAATGCTTGCAAACATTGACTTAAAATACTTCAATGAGTCAACAAGAAA<br>GAAAATCGAGAAGATAAGACCTCTACTAATAGATGGCACCGCCTCATTGAGTCCTGGAATGATGATGGGCATGTTCAATATGCTGAGTACAGTATTAGGAGTCTCAATCCTGAATCTTGGGCAAAAGAGGTACACCAAAACCACATACTGGTGGGATGGACTCCAATCCTCTGATGATTTCGCCCTCATAGTGAATGCACCGAATCATGAGGGAATACAGGCAGGAGTGGATA<br>GATTCTATAGGACCTGCAAACTGGTTGGGATCAACATGAGCAAAAAGAAGTCTTACATAAACCGGACAGGAACATTTGAGTTCACAAGCTTTTTCTATCGCTATGGATTTGTGGCCAACTTCAGTATGGAGCTGCCCAGCTTTGGAGTGTCTGGAATCAATGAATCAGCTGACATGAGCATTGGAGTTACAGTGATAAAGAACAACATGATAAACAATGACCTTGGACCAGCAA<br>CAGCTCAAATGGCCCTTCAACTATTCATCAAGGACTACAGATACACGTACCGATGCCACAGGGGTGACACACAAATTCAAACGAGGAGATCATTCGAGCTGAAGAAGCTGTGGGAGCAGACCCGTTCAAAGGCAGGACTGTTGGTGTCAGATGGAGGACCAAATCTATACAACATTCGGAATCTCCATATCCCAGAGGTCTGCCTGAAGTGGGAGCTGATGGACGAAGATTA<br>CCAGGGCAGGTTGTGTAATCCTCTGAACCCATTTGTCAGTCATAAAGAAATTGAGTCCGTAAACAATGCTGTGGTAATGCCAGCCCATGGCCCAGCCAAGAGCATGGAATATGATGCTGTTGCGACTACACACTCATGGATCCCTAAAAGGAATCGTTCCATTCTCAATACCAGCCAAAGGGGAATTCTTGAGGATGAGCAGATGTACCAGAAATGCTGCAGCCTATTCGAGAA<br>ATTCTTCCCCAGTAGTTCATACAGGAGACCAGTTGGAATTTCCAGCATGGTGGAGGCCATGGTGTCTAGGGCCCGAATCGATGCACGTATTGATTTCGAATCTGGAAGGATTAAGAAGGAAGAGTTTGCTGAGATCATGAAGATCTGTTCCACCATTGAAGAGCTCAGACGGCAAAAATAG
EPI_ISL_15535272	>A/domestic_goose/Poland/H397-N/2022_H5N1_2022-09-20<br>ATGGATGTCAATCCGACTTTACTTTTCTTAAAAGTGCCAGCGCAAGATGCCATAAGTACCACATTCCCTTACACTGGAGATCCTCCATACAGCCATGGAACAGGGACAGGATACACAATGGACACGGTCAACAGAACACATCAATACTCAGAGAAGGGAAAATGGACAACAAACACAGAAACCGGAGCACCTCAACTCAACCCAATTGATGGGCCACTACCTGAGGACAACGA<br>ACCGAGCGGATATGCACAAACAGATTGCGTGTTGGAAGCAATGGCTTTCCTTGAAGAGTCCCATCCAGGGATCTTTGAAAACTCTTGTCTTGAAACGATGGAAGTCGTTCAGCAAACAAGAGTGGACAAACTAACTCAAGGTCGCCAGACATATGACTGGACACTGAATAGAAACCAACCAGCTGCAACTGCCTTGGCCAACACTATAGAGGTCTTAAGATCAAACGGTCTAA<br>TAGCCAATGAATCGGGGAGACTAATAGATTTCCTCAAGGATGTGATGGACTCAATGGATAAAGAAGAAATGGAAATAACAACACATTTCCAGAGAAAGAGAAGAGTAAGGGACAACATGACCAAGAAAATGGTCACACAAAGAACAATAGGAAAGAAGAAACAAAGGCTAAACAAGAGGAGCTACTTAATAAGAGCACTGACACTGAATACAATGACAAAAGATGCAGA<br>AAGAGGCAAATTGAAGAGACGGGCGATTGCAACACCAGGGATGCAGATTAGAGGATTTGTGTACTTTGTCGAAACACTGGCAAGGAGCATCTGTGAAAAACTTGAGCAATCTGGACTCCCCGTTGGAGGGAATGAGAAGAAGGCTAAATTGGCAAATGTTGTGAGAAAAATGATGACTAACTCACAAGATACAGAGCTCTCCTTCACAATTACTGGAGATAACACCAAATG<br>GAATGAGAATCAAAATCCTCGGATGTTTCTGGCAATGATAACATACATTACAAGAAACCAACCTGAATGGTTTAGGAATGTCTTGAGTATTGCCCCTATAATGTTCTCGAACAAAATGGCAAGATTGGGAAAAGGGTACATGTTTGAAAGTAAGAGCATGAAGTTACGGACACAAATACCTGCAGAAATGCTTGCAAACATTGACTTAAAATATTTCAATGAATCAACAAGAAA<br>GAAAATCGAAAAAATAAGGCCTCTACTAATAGATGGCACTGCCTCATTGAGTCCTGGAATGATGATGGGCATGTTCAATATGCTGAGTACAGTATTAGGAGTTTCAATCCTAAATCTTGGGCAAAAGAAGTACACCAAAACCATATACTGGTGGGATGGACTCCAATCCTCTGATGATTTCGCCCTCATAGTAAATGCACCGAATCATGAGGGAATACAAGCAGGAGTGGATA<br>GGTTCTATAGGACCTGCAAACTGGTCGGGATCAATATGAGCAAAAAGAAGTCTTACATAAACCGGACTGGAACATTTGAGTTCACAAGCTTTTTCTATCGCTATGGGTTTGTGGCTAATTTCAGTATGGAGCTGCCCAGCTTTGGAGTTTCTGGGATCAATGAATCAGCTGACATGAGCATTGGCATCACAGTGATAAAGAACAACATGATAAACAATGACCTTGGACCAGCAA<br>CAGCTCAAATGGCCCTTCAACTATTCATCAAAGATTACAGGTACACGTACCGATGCCATAGAGGTGACACACAAATTCAAACGAGGAGATCATTCGAGCTGAAGAAGCTGTGGGAACAGACCCGTTCAAAGGCAGGACTGTTGGTGTCAGATGGAGGACCAAATCTATACAACATTCGGAATCTCCATATCCCAGAGGTCTGCCTGAAGTGGGAGCTGATGGACGAAGATTA<br>CCAGGGCAGGTTGTGTAATCCTCTGAACCCATTTGTCAGCCATAAAGAAATTGAGTCCGTAAACAATGCTGTGGTGATGCCAGCCCACGGTCCAGCCAAAAGCATGGAATATGATGCCGTTGCGACTACACACTCATGGATTCCTAAAAGGAATCGTTCCATTCTCAATACCAGTCAAAGGGGAATTCTTGAGGATGAACAGATGTACCAGAAATGCTGCGGTCTATTCGAGAA<br>ATTTTTCCCCAGTAGTTCATACAGGAGACCAGTTGGAATTTCCAGCATGGTGGAGGCCATGGTGTCTAGGGCCCGAATCGATGCACGCATTGATTTCGAATCTGGAAGGATCAAGAAGGAAGAGTTTGCTGAGATCATGAAGATCTGTTCCACCATTGAAGAGCTCAGACGGCAAAAATAG
EPI_ISL_14917951	>A/domestic_goose/Poland/H371/2022_H5N1_2022-07-21<br>ATGGATGTCAATCCGACTTTACTTTTCTTAAAAGTGCCAGCGCAAGATGCCATAAGTACCACATTCCCTTACACTGGAGATCCTCCATACAGCCATGGAACAGGGACAGGATACACAATGGACACAGTCAACAGAACACATCAATACTCAGAGAAGGGAAAATGGACAACAAACACAGAAACCGGAGCACCTCAACTCAACCCAATTGATGGGCCACTACCTGAGGACAACGA<br>ACCGAGCGGATATGCACAAACAGATTGCGTGTTGGAAGCAATGGCTTTCCTTGAAGAGTCCCACCCAGGGATCTTTGAAAACTCTTGTCTTGAAACGATGGAAGTCGTTCAGCAAACAAGAGTGGACAAACTAACTCAAGGTCGCCAGACATATGACTGGACACTGAATAGAAACCAACCAGCTGCAACTGCCCTGGCCAACACTATAGAGGTCTTCAGATCAAACGGTCTAA<br>CAGCCAATGAATCGGGGAGACTAATAGATTTCCTCAAGGATGTGATGGACTCAATGGATAAAGAAGAAATGGAAATAACAACACATTTCCAGAGAAAGAGAAGAGTAAGGGACAACATGACCAAGAAAATGGTCACACAAAGAACAATAGGAAAGAAGAAACAAAGGCTAAACAAGAGGAGCTACTTAATAAGAGCACTGACACTGAATACAATGACAAAAGATGCAGA<br>AAGAGGCAAATTGAAAAGACGGGCGATTGCAACACCAGGGATGCAGATTAGAGGATTTGTGTACTTTGTCGAAACACTGGCAAGGAGCATCTGTGAAAAACTTGAGCAATCTGGACTCCCCGTTGGAGGGAATGAGAAGAAGGCTAAATTGGCAAATGTCGTGAGAAAAATGATGACTAACTCACAAGATACAGAGCTCTCCTTCACAATTACTGGAGACAACACCAAATG<br>GAATGAGAATCAAAATCCTCGGATGTTTCTGGCAATGATAACATACATTACAAGAAACCAACCTGACTGGTTTAGAAATGTCTTGAGTATTGCCCCTATAATGTTCTCGAACAAAATGGCGAGATTGGGAAAAGGGTACATGTTTGAAAGTAAGAGCATGAAGTTACGGACACAAATACCTGCAGAAATGCTTGCAAACATTGACTTAAAATACTTCAATGAATCAACAAGAAA<br>GAAAATCGAAAAAATAAGGCCTCTACTAATAGATGGCACTGCCTCATTGAGTCCTGGAATGATGATGGGCATGTTCAATATGCTGAGTACAGTATTAGGAGTTTCAATCCTAAATCTTGGGCAAAAGAAGTACACCAAAACAACATACTGGTGGGATGGACTCCAATCCTCTGATGATTTCGCCCTCATAGTAAATGCACCGAATCATGAGGGAATACAAGCAGGAGTGGATA<br>GGTTCTATAGGACCTGCAAACTGGTCGGGATCAATATGAGCAAAAAGAAGTCTTACATAAACCGGACTGGAACATTCGAGTTCACAAGCTTTTTCTATCGCTATGGATTTGTGGCTAACTTCAGTATGGAGCTGCCCAGCTTTGGAGTTTCTGGGATCAATGAATCAGCTGACATGAGCATTGGCGTCACAGTGATAAAGAACAACATGATAAACAATGACCTTGGACCAGCAA<br>CAGCYCAAATGGCCCTTCAACTATTCATCAAAGATTACAGGTACACGTACCGATGCCACAGAGGTGACACACAAATTCAAACGAGGAGATCATTCGAGCTGAAGAAGCTGTGGGAACAGACCCGTTCAAAGGCAGGACTGTTGGTGTCAGATGGAGGACCAAATCTATACAACATTCGGAATCTCCATATCCCAGAGGTCTGCCTGAAGTGGGAGCTGATGGACAAAGATTA<br>CCAGGGCAGGTTGTGTAATCCTCTGAACCCATTTGTCAGCCATAGAGAAATTGAGTCCGTAAACAATGCTGTGGTGATGCCAGCCCACGGTCCAGCCAAAAGCATGGAATATGATGCCGTTGCGACTACACACTCATGGATTCCTAAAAGGAATCGTTCCATTCTCAATACCAGTCAAAGGGGAATTCTTGAGGATGAACAGATGTACCAGAAATGCTGCAGTCTATTCGAGAA<br>ATTCTTCCCCAGTAGTTCATACAGGAGACCAGTTGGAATTTCCAGCATGGTGGAGGCCATGGTGTCTAGGGCCCGAATCGATGCACGAATTGATTTCGAATCTGGAAGGATCAAGAAGGAAGAGTTTGCTGAGATCATGAAGATCTGTTCCACCATTGAAGAGCTCAGACGGCAAAAATAG
internal NVRI repository	>A/domestic_goose/Poland/H28-T/2022_H5N1_2022-01-05<br>ATGGATGTCAATCCGACTTTACTTTTCTTAAAAGTGCCAGCGCAAGATGCCATAAGTACCACATTCCCTTACACTGGAGATCCTCCATACAGCCATGGAACAGGGACAGGATACACAATGGACACAGTCAACAGAACACATCAATACTCAGAGAAGGGAAAATGGACAACAAACACAGAAACCGGAGCACCTCAACTCAACCCAATTGATGGGCCACTACCTGAGGACAACGA<br>ACCGAGCGGATATGCACAAACAGATTGCGTGTTGGAAGCAATGGCTTTCCTTGAAGAGTCCCATCCAGGGATCTTTGGAAACTCTTGTCTTGAAACGATGGAAGTCGTTCAGCAAACAAGAGTGGACAAACTAACTCAAGGTCGCCAGACATATGACTGGACACTGAATAGAAACCAACCAGCTGCAACTGCCCTGGCCAACACTATAGAGGTCTTCAGATCAAACGGTCTAA<br>CAGCCAATGAATCGGGGAGACTAATAGATTTCCTCAAGGATGTGATGGACTCAATGGATAAAGAAGAAATGGAAATAACAATACATTTCCAGAGAAAGAGAAGAGTAAGGGACAACATGACCAAGAAAATGGTCACACAAAGAACAATAGGAAAGAAGAAACAAAGGCTAAACAAGAGGAGCTACTTAATAAGAGCACTGACACTGAATACAATGACAAAAGATGCAGA<br>AAGAGGCAAATTGAAGAGACGGGCGATTGCAACACCAGGGATGCAGATTAGAGGATTTGTGTACTTTGTCGAAACACTGGCAAGGAGCATCTGTGAAAAACTTGAGCAATCTGGACTCCCCGTTGGAGGGAATGAGAAGAAGGCTAAATTGGCAAATGTCGTGAGAAAAATGATGACTAACTCACAAGATACAGAGCTCTCCTTCACAATTACTGGAGATAACACCAAATG<br>GAATGAGAATCAAAATCCTCGGATGTTTCTGGCAATGATAACATACATTACAAGAAACCAACCTGAATGGTTTAGGAATGTCTTGAGTATTGCCCCTATAATGTTCTCGAACAAAATGGCGAGATTGGGAAAAGGGTACATGTTTGAAAGTAAGAGCATGAAGTTACGGACACAAATACCTGCAGAAATGCTTGCAAACATTGACTTAAAATACTTCAATGAATCAACAAGAAA<br>GAAAATCGAAAAAATAAGGCCTCTACTAATAGATGGCACTGCCTCATTGAGTCCTGGAATGATGATGGGCATGTTCAATATGCTGAGTACAGTATTAGGAGTTTCAATCCTAAATCTTGGGCAAAAGAAGTACACCAAAACCACATACTGGTGGGATGGACTCCAATCCTCTGATGATTTCGCCCTCATAGTAAATGCACCGAATCATGAGGGAATACAAGCAGGAGTGGATA<br>GGTTCTATAGGACCTGCAAACTGGTCGGGATCAATATGAGCAAAAAGAAGTCTTACATAAACCGGACTGGAACATTTGAGTTCACAAGCTTTTTCTATCGCTATGGATTTGTGGCTAACTTCAGTATGGAGCTGCCCAGCTTTGGAGTTTCTGGGATCAATGAATCAGCTGACATGAGCATTGGCATCACAGTGATAAAGAACAACATGATAAACAATGACCTTGGACCAGCAA<br>CAGCTCAAATGGCCCTTCAACTATTCATCAAAGATTACAGGTACACGTACCGATGCCATAGAGGTGACACACAAATTCAAACGAGGAGATCATTCGAGCTGAAGAAGCTGTGGGAACAGACCCGTTCAAAGGCAGGGCTGTTGGTGTCAGATGGAGGACCAAATCTATACAACATTCGGAATCTCCATATCCCTGAGGTCTGCCTGAAGTGGGAGCTGATGGACGAAGATTA<br>CCAGGGCAGGTTGTGCAATCCTCTGAACCCATTTGTCAGCCATAAAGAAATTGAGTCCGTAAACAATGCTGTGGTGATGCCAGCCCACGGTCCAGCCAAAAGCATGGAATATGATGCCGTTGCGACTACACACTCATGGATTCCTAAAAGGAATCGTTCCATTCTCAATACCAGTCAAAGGGGAATTCTTGAGGATGAACAGATGTACCAGAAATGCTGCGGTCTATTCGAGAA<br>ATTCTTCCCCAGTAGTTCATACAGGAGACCAGTTGGAATTTCCAGCATGGTGGAGGCCATGGTGTCTAGGGCCCGAATCGATGCACGCATTGATTTCGAATCTGGAAGGATCAAGAAGGAAGAGTTTGCTGAGATCATGAAGATCTGTTCCACCATTGAAGAGCTCAGACGGCAAAAATAG
EPI_ISL_6931008	>A/domestic_goose/Poland/H1931-T1/2021_H5N1_2021-11-03<br>ATGGATGTCAATCCGACTTTACTTTTCTTAAAAGTGCCAGCGCAAGATGCCATAAGTACCACATTCCCTTACACTGGAGATCCTCCATACAGCCATGGAACAGGGACAGGATACACAATGGACACGGTCAACAGAACACATCAATACTCAGAGAAGGGAAAGTGGACAACAAACACAGAAACCGGAGCACCTCAACTCAACCCAATTGATGGGCCACTACCTGAGGACAACGA<br>ACCGAGCGGATATGCACAAACAGATTGCGTGTTGGAAGCAATGGCTTTCCTTGAAGAGTCCCACCCAGGGATCTTTGAAAACTCTTGTCTTGAAACGATGGAAGTCGTTCAGCAAACAAGAGTGGACAAACTAACTCAAGGTCGCCAGACATATGACTGGACACTGAATAGAAACCAACCAGCTGCAACTGCCCTGGCCAACACTATAGAGGTCTTCAGATCAAACGGTCTAA<br>CAGCCAATGAATCGGGGAGACTAATAGATTTCCTCAAGGATGTGATGGACTCAATGGATAAAGAAGAAATGGAAATAACAACACATTTCCAGAGAAAGAGAAGAGTAAGGGACAACATGACCAAGAAAATGGTCACACAAAGAACAATAGGAAAGAAGAAACAAAGGCTAAACAAGAGGAGCTACTTAATAAGAGCACTGACACTGAATACAATGACAAAAGATGCAGA<br>AAGAGGCAAATTGAAGAGACGGGCGATTGCAACACCAGGGATGCAGATTAGAGGATTTGTGTACTTTGTCGAAACACTGGCAAGGAGCATCTGTGAAAAACTTGAGCAATCTGGACTCCCCGTTGGAGGGAATGAGAAGAAGGCTAAATTGGCAAATGTCGTGAGAAAAATGATGACTAACTCACAAGATACAGAGCTCTCCTTCACAATTACTGGAGATAACACCAAATG<br>GAATGAGAATCAAAATCCTCGGATGTTTCTGGCAATGATAACATACATTACAAGAAACCAACCTGAATGGTTTAGAAATGTCTTGAGTATTGCCCCTATAATGTTCTCGAACAAAATGGCGAGATTGGGAAAAGGGTACATGTTTGAAAGTAAGAGCATGAAGTTACGGACACAAATACCTGCAGAAATGCTTGCAAACATTGACCTAAAATACTTCAATGAATCAACAAGAAA<br>GAAAATCGAAAAAATAAGGCCTCTACTAATAGATGGCACTGCCTCATTGAGTCCTGGAATGATGATGGGCATGTTCAATATGCTGAGTACAGTATTAGGGGTTTCAATCCTAAATCTTGGACAAAAGAAGTACACCAAAACCACATACTGGTGGGATGGACTCCAATCCTCTGATGATTTCGCCCTCATAGTAAATGCACCGAATCATGAGGGAATACAAGCAGGAGTGGATA<br>GGTTCTATAGGACCTGCAAACTGGTCGGGATCAATATGAGCAAAAAGAAGTCTTACATAAACCGGACTGGAACATTTGAGTTCACAAGCTTTTTCTATCGCTATGGATTTGTGGCTAACTTCAGTATGGAGCTGCCCAGCTTTGGAGTTTCTGGGATCAATGAATCAGCTGATATGAGCATTGGCGTCACAGTGATAAAGAACAACATGATAAACAATGACCTTGGACCAGCAA<br>CAGCTCAAATGGCCCTTCAACTATTCATCAAAGATTACAGGTACACGTACCGATGCCACAGAGGTGACACACAAATTCAAACGAGGAGATCATTCGAGCTGAAGAAGCTGTGGGAACAGACCCGTTCAAAGGCAGGACTGTTGGTGTCAGATGGAGGACCAAATCTATACAACATTCGGAATCTCCATATCCCAGAGGTCTGCCTGAAGTGGGAGCTGATGGACGAAGATTA<br>CCAGGGCAGGTTGTGTAATCCTCTGAACCCATTTGTCAGCCATAAAGAAATTGAGTCCGTAAACAATGCTGTGGTGATGCCAGCCCACGGTCCAGCCAAAAGCATGGAATATGATGCCGTTGCGACTACACACTCATGGATTCCTAAAAGGAATCGTTCCATTCTCAATACCAGTCAAAGGGGAATTCTTGAGGATGAACAGATGTACCAGAAATGTTGCAGTCTATTCGAGAA<br>ATTCTTCCCCAGTAGTTCATACAGGAGACCAGTTGGAATTTCCAGCATGGTGGAGGCCATGGTGTCTAGGGCCCGAATCGATGCACGCATTGATTTCGAATCTGGAAGGATCAAGAAGGAAGAGTTTGCTGAGATCATGAAGATCTGTTCCACCATTGAAGAGCTCAGACGGCAAAAATAG
internal NVRI repository	>A/domestic_goose/Poland/53/2022_H5N1_2022-01-12<br>ATGGATGTCAATCCGACTTTACTTTTCTTAAAAGTGCCAGCGCAAGATGCCATAAGTACCACATTCCCTTACACTGGAGATCCTCCATACAGCCATGGAACAGGGACAGGATACACAATGGACACAGTCAACAGAACACATCAATACTCAGAGAAGGGAAAATGGACAACAAACACAGAAACCGGAGCACCTCAACTCAACCCAATTGATGGGCCACTACCTGAGGACAACGA<br>ACCGAGCGGATATGCACAAACAGATTGCGTGTTGGAAGCAATGGCTTTCCTTGAAGAGTCCCACCCAGGGATCTTTGAAAACTCTTGTCTTGAAACGATGGAAGTCGTTCAGCAAACAAGAGTGGACAAACTAACTCAAGGTCGCCAGACATATGACTGGACACTGAATAGAAACCAACCAGCTGCAACTGCCCTGGCCAACACTATAGAGGTCTTCAGATCAAACGGTCTAA<br>CAGCCAATGAATCGGGGAGACTAATAGATTTCCTCAAGGATGTGATGGACTCAATGGATAAAGAAGAAATGGAAATAACAACACATTTCCAGAGAAAGAGAAGAGTAAGGGACAACATGACCAAGAAAATGGTCACACAAAGAACAATAGGAAAGAAGAAACAAAGGCTAAACAAGAGGAGCTACTTAATAAGAGCACTGACACTGAATACAATGACAAAAGATGCAGA<br>AAGAGGCAAATTGAAGAGACGGGCGATTGCAACACCAGGGATGCAGATTAGAGGATTTGTGTACTTTGTCGAAACACTGGCAAGGAGCATCTGTGAAAAACTTGAGCAATCTGGACTCCCCGTTGGAGGGAATGAGAAGAAGGCTAAATTGGCAAATGTCGTGAGAAAAATGATGACTAACTCACAAGATACAGAGCTCTCCTTCACAATTACTGGAGATAACACCAAATG<br>GAATGAGAATCAAAATCCTCGGATGTTTCTGGCAATGATAACATACATTACAAGAAACCAACCTGAATGGTTTAGAAATGTCTTGAGTATTGCCCCTATAATGTTCTCGAACAAAATGGCGAGATTGGGAAAAGGGTACATGTTTGAAAGTAAGAGCATGAAGTTACGGACACAAATACCTGCAGAAATGCTTGCAAACATTGACTTAAAATACTTCAATGAATCAACAAGAAA<br>GAAAATCGAAAAAATAAGGCCTCTACTAATAGATGGCACTGCCTCATTGAGTCCTGGAATGATGATGGGCATGTTCAATATGCTGAGTACAGTATTAGGAGTTTCAATCCTAAATCTTGGGCAAAAGAAGTACACCAAAACCACATACTGGTGGGATGGACTCCAATCCTCTGATGATTTCGCCCTCATAGTAAATGCACCGAATCATGAGGGAATACAAGCAGGAGTGGATA<br>GGTTCTATAGGACCTGCAAACTGGTCGGGATCAATATGAGCAAAAAGAAGTCTTACATAAACCGGACTGGAACATTTGAGTTCACAAGCTTTTTCTATCGCTATGGATTTGTGGCTAACTTCAGTATGGAGCTGCCCAGCTTTGGAGTTTCTGGGATCAATGAATCAGCTGACATGAGCATTGGCGTCACAGTGATAAAGAACAACATGATAAACAATGACCTTGGACCGGCAA<br>CAGCTCAAATGGCCCTTCAACTATTCATCAAAGATTACAGGTACACGTACCGATGCCACAGAGGTGACACACAAATTCAAACGAGGAGATCATTCGAGCTGAAGAAGCTGTGGGAACAGACCCGTTCAAAGGCAGGACTGTTGGTGTCAGATGGAGGACCAAATCTATACAACATTCGGAATCTCCATATCCCAGAGGTCTGCCTGAAGTGGGAGCTGATGGACGAAGATTA<br>CCAGGGCAGGTTGTGTAATCCTCTGAACCCATTTGTCAGCCATAAAGAAATTGAGTCCGTAAACAATGCTGTGGTGATGCCAGCCCATGGTCCAGCCAAAAGCATGGAATATGATGCCGTTGCGACTACACACTCATGGATTCCTAAAAGGAATCGTTCCATTCTCAATACCAGTCAAAGGGGAATTCTTGAGGATGAACAGATGTACCAGAAATGCTGCAGTCTATTCGAGAA<br>ATTCTTCCCCAGTAGTTCATACAGGAGACCAGTTGGAATTTCCAGCATGGTGGAGGCCATGGTGTCTAGGGCCCGAATCGATGCACGCATTGATTTCGAATCTGGAAGGATCAAGAAGGAAGAGTTTGCTGAGATCATGAAGATCTGTTCCACCATTGAAGAGCTCAGACGGCAAAAATAG
internal NVRI repository	>A/domestic_goose/Poland/33/2022_H5N1_2022-01-09<br>ATGGATGTCAATCCGACTTTACTTTTCTTAAAAGTGCCAGCGCAAGATGCCATAAGTACCACATTCCCTTACACTGGAGATCCTCCATACAGCCATGGAACAGGGACAGGATACACAATGGACACAGTCAACAGAACACATCAATACTCAGAGAAGGGAAAATGGACAACAAACACAGAAACCGGAGCACCTCAACTCAACCCAATTGATGGGCCACTACCTGAGGACAACGA<br>ACCGAGCGGATATGCACAAACAGATTGCGTGTTGGAAGCAATGGCTTTCCTTGAAGAGTCCCACCCAGGGATCTTTGAAAACTCTTGTCTTGAAACGATGGAAGTCGTTCAGCAAACAAGAGTGGACAAACTAACTCAAGGTCGCCAGACATATGACTGGACACTGAATAGAAACCAACCAGCTGCAACTGCCCTGGCCAACACTATAGAGGTCTTCAGATCAAACGGTCTAA<br>CAGCCAATGAATCGGGGAGACTAATAGATTTCCTCAAGGATGTGATGGACTCAATGGATAAAGAAGAAATGGAAATAACAACACATTTCCAGAGAAAGAGAAGAGTAAGGGACAACATGACCAAGAAAATGGTCACACAAAGAACAATAGGAAAGAAGAAACAAAGGCTAAACAAGAGGAGCTACTTAATAAGAGCACTGACACTGAATACAATGACAAAAGATGCAGA<br>AAGAGGCAAATTGAAGAGACGGGCGATTGCAACACCAGGGATGCAGATTAGAGGATTTGTGTACTTTGTCGAAACACTGGCAAGGAGCATCTGTGAAAAACTTGAGCAATCTGGACTCCCCGTTGGAGGGAATGAGAAGAAGGCTAAATTGGCAAATGTCGTGAGAAAAATGATGACTAACTCACAAGATACAGAGCTCTCCTTCACAATTACTGGAGATAACACCAAATG<br>GAATGAGAATCAAAATCCTCGGATGTTTCTGGCAATGATAACATACATTACAAGAAACCAACCTGAATGGTTTAGAAATGTCTTGAGTATTGCCCCTATAATGTTCTCGAACAAAATGGCGAGATTGGGAAAAGGGTACATGTTTGAAAGTAAGAGCATGAAGTTACGGACACAAATACCTGCAGAAATGCTTGCAAACATTGACTTAAAATACTTCAATGAATCAACAAGAAA<br>GAAAATCGAAAAAATAAGGCCTCTACTAATAGATGGCACTGCCTCATTGAGTCCTGGAATGATGATGGGCATGTTCAATATGCTGAGTACAGTATTAGGAGTTTCAATCCTAAATCTTGGGCAAAAGAAGTACACCAAAACCACATACTGGTGGGATGGACTCCAATCCTCTGATGATTTCGCCCTCATAGTAAATGCACCGAATCATGAGGGAATACAAGCAGGAGTGGATA<br>GGTTCTATAGGACCTGCAAACTGGTCGGGATCAATATGAGCAAAAAGAAGTCTTACATAAACCGGACTGGAACATTTGAGTTCACAAGCTTTTTCTATCGCTATGGATTTGTGGCTAACTTCAGTATGGAGCTGCCCAGCTTTGGAGTTTCTGGGATCAATGAATCAGCTGACATGAGCATTGGCGTCACAGTGATAAAGAACAACATGATAAACAATGACCTTGGACCGGCAA<br>CAGCTCAAATGGCCCTTCAACTATTCATCAAAGATTACAGGTACACGTACCGATGCCACAGAGGTGACACACAAATTCAAACGAGGAGATCATTCGAGCTGAAGAAGCTGTGGGAACAGACCCGTTCAAAGGCAGGACTGTTGGTGTCAGATGGAGGACCAAATCTATACAACATTCGGAATCTCCATATCCCAGAGGTCTGCCTGAAGTGGGAGCTGATGGACGAAGATTA<br>CCAGGGCAGGTTGTGTAATCCTCTGAACCCATTTGTCAGCCATAAAGAAATTGAGTCCGTAAACAATGCTGTGGTGATGCCAGCCCATGGTCCAGCCAAAAGCATGGAATATGATGCCGTTGCGACTACACACTCATGGATTCCTAAAAGGAATCGTTCCATTCTCAATACCAGTCAAAGGGGAATTCTTGAGGATGAACAGATGTACCAGAAATGCTGCAGTCTATTCGAGAA<br>ATTCTTCCCCAGTAGTTCATACAGGAGACCAGTTGGAATTTCCAGCATGGTGGAGGCCATGGTGTCTAGGGCCCGAATCGATGCACGCATTGATTTCGAATCTGGAAGGATCAAGAAGGAAGAGTTTGCTGAGATCATGAAGATCTGTTCCACCATTGAAGAGCTCAGACGGCAAAAATAG
EPI_ISL_9012700	>A/domestic_duck/Scotland/054469/2021_H5N1_2021-11-01<br>ATGGATGTCAATCCGACTTTACTTTTCTTAAAAGTGCCAGCGCAAGATGCCATAAGTACCACATTCCCTTACACTGGAGATCCTCCATACAGCCATGGAACAGGGACAGGATACACAATGGACACAGTCAACAGAACACATCAATACTCAGAGAAGGGAAAATGGACAACAAACACAGAAACCGGAGCACCTCAACTCAACCCAATTGATGGGCCACTACCTGAGGACAACGA<br>ACCGAGCGGATATGCACAAACAGATTGCGTGTTGGAAGCAATGGCTTTCCTTGAAGAGTCCCATCCAGGGATCTTTGAAAACTCTTGTCTTGAAACGATGGAAGTCGTTCAGCAAACAAGAGTGGACAAACTAACTCAAGGTCGCCAGACATATGACTGGACACTGAATAGAAACCAACCAGCTGCAACTGCCCTGGCCAACACTATAGAGGTCTTCAGATCAAACGGTCTAA<br>CAGCCAATGAATCGGGGAGACTAATAGATTTCCTCAAGGATGTGATGGACTCAATGGATAAAGAAGAAATGGAAATAACAACACATTTCCAGAGAAAGAGAAGAGTAAGGGACAACATGACCAAGAAAATGGTCACACAAAGAACAATAGGAAAGAAGAAACAAAGGCTAAACAAGAGGAGCTACTTAATAAGAGCACTGACACTGAATACAATGACAAAAGATGCAGA<br>AAGAGGCAAATTGAAGAGACGGGCGATTGCAACACCAGGGATGCAGATTAGAGGATTTGTGTACTTTGTCGAAACACTGGCAAGGAGCATCTGTGAAAAACTTGAGCAATCTGGACTCCCCGTTGGAGGGAATGAGAAGAAGGCTAAATTGGCAAATGTCGTGAGAAAAATGATGACTAACTCACAAGATACAGAGCTCTCCTTCACAATTACTGGAGATAACACCAAATG<br>GAATGAGAATCAAAATCCTCGGATGTTTCTGGCAATGATAACATACATTACAAGAAACCAACCTGAATGGTTTAGGAATGTCTTGAGTATTGCCCCTATAATGTTCTCGAACAAAATGGCGAGATTGGGAAAAGGGTACATGTTTGAAAGTAAGAGCATGAAGTTACGGACACAAATACCTGCAGAAATGCTTGCAAACATTGACTTAAAATACTTCAATGAATCAACAAGAAA<br>GAAAATCGAAAAAATAAGGCCTCTACTAATAGATGGCACTGCCTCATTGAGTCCTGGAATGATGATGGGCATGTTCAATATGCTGAGTACAGTATTAGGAGTTTCAATCCTAAATCTTGGGCAAAAGAAGTACACCAAAACCACATACTGGTGGGATGGACTCCAATCCTCTGATGATTTCGCCCTCATAGTAAATGCACCGAATCATGAGGGAATACAAGCAGGAGTGGATA<br>GGTTCTATAGGACCTGCAAACTGGTCGGGATCAATATGAGCAAAAAGAAGTCTTACATAAACCGGACTGGAACATTTGAGTTCACAAGCTTTTTCTATCGCTATGGATTTGTGGCTAACTTCAGTATGGAGCTGCCCAGCTTTGGAGTTTCTGGGATCAATGAATCAGCTGACATGAGCATTGGCATCACAGTGATAAAGAACAACATGATAAACAATGACCTTGGACCAGCAA<br>CAGCTCAAATGGCCCTTCAACTATTCATCAAAGATTACAGGTACACGTACCGATGCCATAGAGGTGACACACAAATTCAAACGAGGAGATCATTCGAGCTGAAGAAGCTGTGGGAACAGACCCGTTCAAAGGCAGGACTGTTGGTGTCAGATGGAGGACCAAATCTATACAACATTCGGAATCTCCATATCCCAGAGGTCTGCCTGAAGTGGGAGCTGATGGACGAAGATTA<br>CCAGGGCAGGTTGTGTAATCCTCTGAACCCATTTGTCAGCCATAAAGAAATTGAGTCCGTAAACAATGCTGTGGTGATGCCAGCCCACGGTCCAGCCAAAAGCATGGAATATGATGCCGTTGCGACTACACACTCATGGATTCCTAAAAGGAATCGTTCCATTCTCAATACCAGCCAAAGGGGAATTCTTGAGGATGAACAGATGTACCAGAAATGCTGCGGTCTATTCGAGAA<br>ATTCTTCCCCAGTAGTTCATACAGGAGACCAGTTGGAATTTCCAGCATGGTGGAGGCCATGGTGTCTAGGGCCCGAATCGATGCACGCATTGATTTCGAATCTGGAAGGATCAAGAAGGAAGAGTTTGCTGAGATCATGAAGATCTGTTCCACCATTGAAGAGCTCAGACGGCAAAAATAG
internal NVRI repository	>A/domestic_duck/Poland/H2154_N/2021_H5N1_2021-12-24<br>ATGGATGTCAATCCGACTTTACTTTTCTTAAAAGTGCCAGCGCAAGATGCCATAAGTACCACATTCCCTTACACTGGAGATCCTCCATACAGCCATGGAACAGGGACAGGATACACAATGGACACAGTCAACAGAACACATCAATACTCAGAGAAGGGAAAATGGACAACAAACACAGAAACCGGAGCACCTCAACTCAACCCAATTGACGGGCCACTACCTGAGGACAACGA<br>ACCGAGCGGATATGCACAAACAGATTGCGTGTTGGAAGCAATGGCTTTCCTTGAAGAGTCCCACCCAGGGATCTTTGAAAACTCTTGTCTTGAAACGATGGAAGTCGTTCAGCAAACAAGAGTGGACAAACTAACTCAAGGTCGCCAGACATATGACTGGACACTGAATAGAAACCAACCAGCTGCAACTGCCCTGGCCAACACTATAGAGGTCTTCAGATCAAACGGTCTAA<br>CAGCCAATGAATCGGGGAGACTAATAGATTTCCTCAAGGATGTGATGGACTCAATGGATAAAGAAGAAATGGAAATAACAACACATTTCCAGAGAAAGAGAAGAGTAAGGGACAACATGACCAAGAAAATGGTCACACAAAGAACAATAGGAAAGAAGAAACAAAGGCTAAACAAGAGGAGCTACTTAATAAGAGCACTGACACTGAATACAATGACAAAAGATGCAGA<br>AAGAGGCAAATTGAAAAGACGGGCGATTGCAACACCAGGGATGCAGATTAGAGGATTTGTGTACTTTGTCGAAACACTGGCAAGGAGCATCTGTGAAAAACTTGAGCAATCTGGACTCCCCGTTGGAGGGAATGAGAAGAAGGCTAAATTGGCAAATGTCGTGAGAAAAATGATGACTAACTCACAAGATACAGAGCTCTCCTTCACAATTACTGGAGATAACACCAAATG<br>GAATGAGAATCAAAATCCTCGGATGTTTCTGGCAATGATAACATACATTACAAGAAACCAACCTGAATGGTTTAGAAATGTCTTGAGTATTGCCCCTATAATGTTCTCGAACAAAATGGCGAGATTGGGAAAAGGGTACATGTTTGAAAGTAAGAGCATGAAGTTACGGACACAAATACCTGCAGAAATGCTTGCAAACATTGACTTAAAATACTTCAATGAATCAACAAGAAA<br>GAGAATCGAAAAAATAAGGCCTCTACTAATAGATGGCACTGCCTCATTGAGTCCTGGAATGATGATGGGCATGTTCAATATGCTGAGTACAGTATTAGGAGTTTCAATCCTAAATCTTGGGCAAAAGAAGTACACCAAAACCACATACTGGTGGGATGGACTCCAATCCTCTGATGATTTCGCCCTCATAGTAAATGCACCGAATCATGAGGGAATACAAGCAGGAGTGGATA<br>GGTTCTATAGGACCTGCAAACTGGTCGGGATCAATATGAGCAAAAAGAAGTCTTACATAAACCGGACTGGAACATTTGAGTTCACAAGCTTTTTCTATCGCTATGGATTTGTGGCTAACTTCAGTATGGAGCTGCCCAGCTTTGGAGTTTCTGGGATCAATGAATCAGCTGACATGAGCATTGGCGTCACAGTGATAAAGAACAACATGATAAACAATGACCTTGGACCAGCAA<br>CAGCTCAAATGGCCCTTCAACTATTCATCAAAGATTACAGGTACACGTACCGATGCCACAGAGGTGACACACAAATTCAAACGAGGAGATCATTCGAGCTGAAGAAGCTGTGGGAACAGACCCGTTCAAAGGCAGGACTGTTGGTGTCAGATGGAGGACCAAATCTATACAACATTCGGAATCTCCATATCCCAGAGGTCTGCCTGAAGTGGGAGCTGATGGACGAAGATTA<br>CCAGGGCCGGTTGTGTAATCCCCTGAACCCATTTGTCAGCCATAGAGAAATTGAGTCCGTAAACAATGCTGTGGTGATGCCAGCCCACGGTCCAGCCAAAAGCATGGAATATGATGCCGTTGCGACTACACACTCATGGATTCCTAAAAGGAATCGTTCCATTCTCAATACCAGTCAAAGGGGAATTCTTGAGGATGAACAGATGTACCAGAAATGCTGCAGTCTATTCGAGAA<br>ATTCTTCCCCAGTAGTTCATACAGGAGACCAGTTGGAATTTCCAGCATGGTGGAGGCCATGGTGTCTAGGGCCCGAATCGATGCACGAATTGATTTCGAATCTGGAAGGATCAAGAAGGAAGAGTTTGCTGAGATCATGAAGATCTGTTCCACCATTGAAGAGCTCAGACGGCAAAAATAG
internal NVRI repository	>A/domestic_duck/Poland/H2153_T3/2021_H5N1_2021-12-25<br>ATGGATGTCAATCCGACTTTACTTTTCTTAAAAGTGCCAGCGCAAGATGCCATAAGTACCACATTCCCTTACACTGGAGATCCTCCATACAGCCATGGAACAGGGACAGGATACACAATGGACACAGTCAACAGAACACATCAATACTCAGAGAAGGGAAAATGGGCAACAAACACAGAAACCGGAGCACCTCAACTCAACCCAATTGATGGGCCACTACCTGAGGACAACGA<br>ACCGAGCGGATATGCACAAACAGATTGCGTGTTGGAAGCAATGGCTTTCCTTGAAGAGTCCCATCCAGGGATCTTTGAAAACTCTTGTCTTGAAACGATGGAAGTCGTTCAGCAAACAAGAGTGGACAAACTAACTCAAGGTCGCCAGACATATGACTGGACACTGAATAGAAACCAACCAGCTGCAACTGCCCTGGCCAACACTATAGAGGTCTTCAGATCAAACGGTCTAA<br>CAGCCAATGAATCGGGGAGACTAATAGATTTCCTCAAGGATGTGATGGACTCAATGGATAAAGAAGAAATGGAAATAACAACACATTTCCAGAGAAAGAGAAGAGTAAGGGACAACATGACCAAGAAAATGGTCACACAAAGAACAATAGGAAAGAAGAAACAAAGGCTAAACAAGAGGAGCTACTTAATAAGAGCACTGACACTGAATACAATGACAAAAGATGCAGA<br>AAGAGGCAAATTGAAGAGACGGGCGATTGCAACACCAGGGATGCAGATTAGAGGATTTGTGTACTTTGTCGAAACACTGGCAAGGAGCATCTGTGAAAAACTTGAGCAATCTGGACTCCCTGTTGGAGGGAATGAGAAGAAGGCTAAATTGGCAAATGTTGTGAGAAAAATGATGACTAACTCACAAGATACAGAGCTCTCCTTCACAATTACTGGAGATAACACCAAATG<br>GAATGAGAATCAAAATCCTCGGATGTTTCTGGCAATGATAACATACATTACAAGAAACCAACCTGAATGGTTTAGGAATGTCTTGAGTATTGCCCCTATAATGTTCTCGAACAAAATGGCGAGATTGGGAAAAGGGTACATGTTTGAAAGTAAGAGCATGAAGTTACGGACACAAATACCTGCAGAAATGCTTGCAAACATTGACTTAAAATACTTCAATGAATCAACAAGAAA<br>GAAAATCGAAAAAATAAGGCCTCTACTAATAGATGGCACTGCCTCATTGAGTCCTGGAATGATGATGGGCATGTTCAATATGCTGAGTACAGTATTGGGAGTTTCAATCCTAAATCTTGGGCAAAAGAAGTACACCAAAACCACATACTGGTGGGATGGACTCCAATCCTCTGATGATTTCGCCCTCATAGTAAATGCACCGAATCATGAGGGAATACAAGCAGGAGTGGATA<br>GGTTCTATAGGACCTGCAAACTGGTCGGGATCAATATGAGCAAAAAGAAGTCTTACATAAACCGGACTGGAACATTTGAGTTCACAAGCTTTTTCTATCGCTATGGATTTGTGGCTAACTTCAGTATGGAGCTGCCCAGCTTTGGGGTTTCTGGGATCAATGAATCAGCTGACATGAGCATTGGCATCACAGTGATAAAGAACAACATGATAAACAATGACCTTGGACCAGCAA<br>CAGCTCAAATGGCCCTTCAACTATTCATCAAAGATTACAGGTACACGTACCGATGCCATAGAGGTGACACACAAATTCAAACGAGGAGATCATTCGAGCTGAAGAAGCTGTGGGAACAGACCCGTTCAAAGGCAGGACTGTTGGTGTCAGATGGAGGACCAAATCTATACAACATTCGGAATCTCCATATCCCAGAGGTCTGCCTGAAGTGGGAGCTGATGGACGAAGATTA<br>CCAGGGCAGGTTGTGTAATCCTCTGAACCCATTTGTCAGCCATAAAGAAATTGAGTCCGTAAACAATGCTGTGGTGATGCCAGCCCACGGTCCAGCCAAAAGCATGGAATATGATGCCGTTGCGACTACACACTCATGGATTCCTAAAAGGAATCGTTCCATTCTCAATACCAGTCAAAGGGGAATTCTTGAGGATGAACAGATGTACCAGAAATGCTGCGGTCTATTCGAGAA<br>ATTCTTCCCCAGTAGTTCATACAGGAGACCAGTTGGAATTTCCAGCATGGTGGAGGCCATGGTGTCTAGGGCCCGAATCGATGCACGCATTGATTTCGAATCTGGAAGGATCAAGAAGGAAGAGTTTGCTGAGATCATGAAGATCTGTTCCACCATTGAAGAGCTCAGACGGCAAAAATAG

internal NVRI repository	>A/domestic_duck/Poland/H2126-N/2021_H5N1_2021-12-20<br>ATGGATGTCAATCCGACTTTACTTTTCTTAAAAGTGCCAGCGCAAGATGCCATAAGTACCACATTCCCTTACACTGGAGATCCTCCATACAGCCATGGAACAGGGACAGGATACACAATGGACACAGTCAACAGAACACATCAATACTCAGAGAAGGGAAAATGGGCAACAAACACAGAAACCGGAGCACCTCAACTCAACCCAATTGATGGGCCACTACCTGAGGACAACGA<br>ACCGAGCGGATATGCACAAACAGATTGCGTGTTGGAAGCAATGGCTTTCCTTGAAGAGTCCCATCCAGGGATCTTTGAAAACTCTTGTCTTGAAACGATGGAAGTCGTTCAGCAAACAAGAGTGGACAAACTAACTCAAGGTCGCCAGACATATGACTGGACACTGAATAGAAACCAACCAGCTGCAACTGCCCTGGCCAACACTATAGAGGTCTTCAGATCAAACGGTCTAA<br>CAGCCAATGAATCGGGGAGACTAATAGATTTCCTCAAGGATGTAATGGACTCAATGGATAAAGAAGAAATGGAAATAACAACACATTTCCAGAGAAAGAGAAGAGTAAGGGACAACATGACCAAGAAAATGGTCACACAAAGAACAATAGGAAAGAAGAAACAAAGGCTAAACAAGAGGAGCTACTTAATAAGAGCACTGACACTGAATACAATGACAAAAGATGCAGA<br>AAGAGGCAAATTGAAGAGACGGGCGATTGCAACACCAGGGATGCAGATTAGAGGATTTGTGTACTTTGTCGAAACACTGGCAAGGAGCATCTGTGAAAAACTTGAGCAATCTGGACTCCCTGTTGGAGGGAATGAGAAGAAGGCTAAATTGGCAAATGTTGTGAGAAAAATGATGACTAACTCACAAGATACAGAGCTCTCCTTCACAATTACTGGAGATAACACCAAATG<br>GAATGAGAATCAAAATCCTCGGATGTTTCTGGCAATGATAACATACATTACAAGAAACCAACCTGAATGGTTTAGGAATGTCTTGAGTATTGCCCCTATAATGTTCTCGAACAAAATGGCGAGATTGGGAAAAGGGTACATGTTTGAAAGTAAGAGCATGAAGTTACGGACACAAATACCTGCAGAAATGCTTGCAAACATTGACTTAAAATACTTCAATGAATCAACAAGAAA<br>GAAAATCGAAAAAATAAGGCCTCTACTAATAGATGGCACTGCCTCATTGAGTCCTGGAATGATGATGGGCATGTTCAATATGCTGAGTACAGTATTGGGAGTTTCAATCCTAAATCTTGGGCAAAAGAAGTACACCAAAACCACATACTGGTGGGATGGACTCCAATCCTCTGATGATTTCGCCCTCATAGTAAATGCACCGAATCATGAGGGAATACAAGCAGGAGTGGATA<br>GGTTCTATAGGACCTGCAAACTGGTCGGGATCAATATGAGCAAAAAGAAGTCTTACATAAACCGGACTGGAACATTTGAGTTCACAAGCTTTTTCTATCGCTATGGATTTGTGGCTAACTTCAGTATGGAGCTGCCCAGCTTTGGGGTTTCTGGGATCAATGAATCAGCTGACATGAGCATTGGCATCACAGTGATAAAGAACAACATGATAAACAATGACCTTGGACCAGCAA<br>CAGCTCAAATGGCCCTTCAACTATTCATCAAAGATTACAGGTACACGTACCGATGCCATAGAGGTGACACACAAATTCAAACGAGGAGATCATTCGAGCTGAAGAAGCTGTGGGAACAGACCCGTTCAAAGGCAGGACTGTTGGTGTCAGATGGAGGACCAAATCTATACAACATTCGGAATCTCCATATCCCAGAGGTCTGCCTGAAGTGGGAGCTGATGGACGAAGATTA<br>CCAGGGCAGGTTGTGTAATCCTCTGAACCCATTTGTCAGCCATAAAGAAATTGAGTCCGTAAACAATGCTGTGGTGATGCCAGCCCACGGTCCAGCCAAAAGCATGGAATATGATGCCGTTGCGACTACACACTCATGGATTCCTAAAAGGAATCGTTCCATTCTCAATACCAGTCAAAGGGGAATTCTTGAGGATGAACAGATGTACCAGAAATGCTGCGGTCTATTCGAGAA<br>ATTCTTCCCCAGCAGTTCATACAGGAGACCAGTTGGAATTTCCAGCATGGTGGAGGCCATGGTGTCTAGGGCCCGAATCGATGCACGCATTGATTTCGAATCTGGAAGGATCAAGAAGGAAGAGTTTGCTGAGATCATGAAGATCTGTTCCACCATTGAAGAGCTCAGACGGCAAAAATAG
internal NVRI repository	>A/domestic_duck/Poland/H2117-T1/2021_H5N1_2021-12-16<br>ATGGATGTCAATCCGACTTTACTTTTCTTAAAAGTGCCAGCGCAAGATGCCATAAGTACCACATTCCCTTACACTGGAGATCCTCCATACAGCCATGGAACAGGGACAGGATACACAATGGACACAGTCAACAGAACACATCAATACTCAGAGAAGGGAAAATGGACAACAAACACAGAAACCAGAGCACCTCAACTCAACCCAATTGATGGGCCACTACCTGAGGACAACGA<br>ACCGAGCGGATATGCACAAACAGATTGCGTGTTGGAAGCAATGGCTTTCCTTGAAGAGTCCCACCCAGGGATCTTTGAAAACTCTTGTCTTGAAACGATGGAAGTCGTTCAGCAAACAAGAGTGGACAAACTAACTCAAGGTCGCCAGACATATGACTGGACACTGAATAGAAACCAACCAGCTGCAACTGCCCTGGCCAACACTATAGAGGTCTTCAGATCAAACGGTCTAA<br>CAGCCAATGAATCGGGGAGACTAATAGATTTCCTCAAGGATGTGATGGACTCAATGGATAAAGAAGAAATGGAAATAACAACACATTTCCAGAGAAAGAGAAGAGTAAGGGACAACATGACCAAGAAAATGGTCACACAAAGAACAATAGGAAAGAAGAAACAAAGGCTAAACAAGAGGAGCTACTTAATAAGAGCACTGACACTGAATACAATGACAAAAGATGCAGA<br>AAGAGGCAAATTGAAAAGACGGGCGATTGCAACACCAGGGATGCAGATTAGAGGATTTGTGTACTTTGTCGAAACACTGGCAAGGAGCATCTGTGAAAAACTTGAGCAATCTGGACTCCCCGTTGGAGGGAATGAGAAGAAGGCTAAATTGGCAAATGTCGTGAGAAAAATGATGACTAACTCACAAGATACAGAGCTCTCCTTCACAATTACTGGAGATAACACCAAATG<br>GAATGAGAATCAAAATCCTCGGATGTTTCTGGCAATGATAACATACATTACAAGAAACCAACCTGAATGGTTTAGAAATGTCTTGAGTGTTGCCCCTATAATGTTCTCGAACAAAATGGCGAGATTGGGAAAAGGGTACATGTTTGAAAGTAAGAGCATGAAGTTACGGACACAAATACCTGCAGAAATGCTTGCAAACATTGACTTAAAATACTTCAATGAATCAACAAGAAA<br>GAAAATCGAAAAAATAAGGCCTCTACTAATAGATGGCACTGCCTCATTGAGTCCTGGAATGATGATGGGCATGTTCAATATGCTGAGTACAGTATTAGGAGTTTCAATCCTAAATCTTGGGCAAAAGAAGTACACCAAAACCACATACTGGTGGGATGGACTCCAATCCTCTGATGATTTCGCCCTCATAGTAAATGCACCGAATCATGAGGGAATACAAGCAGGAGTGGATA<br>GGTTCTATAGGACCTGCAAACTGGTCGGGATCAATATGAGCAAAAAGAAGTCTTACATAAACCGGACTGGAACATTTGAGTTCACAAGCTTTTTCTATCGCTATGGATTTGTGGCTAACTTCAGTATGGAGCTGCCCAGCTTTGGAGTTTCTGGGATCAATGAATCAGCTGACATGAGCATTGGCGTCACAGTGATAAAGAACAACATGATAAACAATGACCTTGGACCAGCAA<br>CAGCTCAAATGGCCCTTCAACTATTCATCAAAGATTACAGGTACACGTACCGATGCCACAGAGGTGACACACAAATTCAAACGAGGAGATCATTCGAGCTGAAGAAGCTGTGGGAACAGACCCGTTCAAAGGCAGGACTGTTGGTGTCAGATGGAGGACCAAATCTATACAACATTCGGAATCTCCATATCCCAGAGGTCTGCCTGAAGTGGGAGCTGATGGACGAAGATTA<br>CCAGGGCAGGTTGTGTAATCCTCTGAACCCATTTGTCAGCCATAGAGAAATTGAGTCCGTAAACAATGCTGTGGTGGCGCCAGCCCACGGTCCAGCCAAAAGCATGGAATATGATGCCGTTGCGACTACACACTCATGGATTCCTAAAAGGAATCGTTCCATTCTCAATACCAGTCAAAGGGGAATTCTTGAGGATGAACAGATGTACCAGAAATGCTGCAGTCTATTCGAGAA<br>ATTCTTCCCCAGTAGTTCATACAGGAGACCAGTTGGAATTTCCAGCATGGTGGAGGCCATGGTGTCTAGGGCCCGAATCGATGCACGAATTGATTTCGAATCTGGAAGGATAAAGAAGGAAGAGTTTGCTGAGATCATGAAGATCTGTTCCACCATTGAAGAGCTCAGACGGCAAAAATAG
internal NVRI repository	>A/domestic_duck/Poland/H2102-N/2021_H5N1_2021-12-13<br>ATGGATGTCAATCCGACTTTACTTTTCTTAAAAGTGCCAGCGCAAGATGCCATAAGTACCACATTCCCTTACACTGGAGATCCTCCATACAGCCATGGAACAGGGACAGGATACACAATGGACACAGTCAACAGAACACATCAATACTCAGAGAAGGGAAAATGGACAACAAACACAGAAACCGGAGCACCTCAACTCAACCCAATTGATGGGCCACTACCTGAGGACAACGA<br>ACCGAGCGGATATGCACAAACAGATTGCGTGTTGGAAGCAATGGCTTTCCTTGAAGAGTCCCACCCAGGGATCTTTGAAAACTCTTGTCTTGAAACGATGGAAGTCGTTCAGCAAACAAGAGTGGACAAACTAACTCAAGGTCGCCAGACATATGACTGGACACTGAATAGAAACCAACCAGCTGCAACTGCCCTGGCCAACACTATAGAGGTCTTCAGATCAAACGGTCTAA<br>CAGCCAATGAATCGGGGAGACTAATAGATTTCCTCAAGGATGTGATGGACTCAATGGATAAAGAAGAAATGGAAATAACAACACATTTCCAGAGAAAGAGAAGAGTAAGGGACAACATGACCAAGAAAATGGTCACACAAAGAACAATAGGAAAGAAGAAACAAAGGCTAAACAAGAGGAGCTACTTAATAAGAGCACTGACACTGAATACAATGACAAAAGATGCAGA<br>AAGAGGCAAATTGAAGAGACGGGCGATTGCAACACCAGGGATGCAGATTAGAGGATTTGTGTACTTTGTCGAAACACTGGCAAGGAGCATCTGTGAAAAACTTGAGCAATCTGGACTCCCCGTTGGAGGGAATGAGAAGAAGGCTAAATTGGCAAATGTCGTGAGAAAAATGATGACTAACTCACAAGATACAGAGCTCTCCTTCACAATTACTGGAGACAACACCAAATG<br>GAATGAGAATCAAAATCCTCGGATGTTTCTGGCAATGATAACATACATTACAAGAAACCAACCTGAATGGTTTAGAAATGTCTTAAGTATTGCCCCTATAATGTTCTCGAACAAAATGGCGAGATTGGGAAAAGGGTACATGTTTGAAAGTAAGAGCATGAAGTTACGGACACAAATACCTGCAGAAATGCTTGCAAACATTGACTTAAAATACTTCAATGAATCAACAAGAAA<br>GAAAATCGAAAAAATAAGGCCTCTACTAATAGATGGCACTGCCTCATTGAGTCCTGGAATGATGATGGGCATGTTCAATATGCTGAGTACAGTATTAGGAGTTTCAATCCTAAATCTTGGGCAAAAGAAGTACACCAAAACCACATACTGGTGGGATGGACTCCAATCCTCTGATGATTTCGCCCTCATAGTAAATGCACCGAATCATGAGGGAATACAAGCAGGAGTGGATA<br>GGTTCTATAGGACCTGCAAACTGGTCGGGATCAATATGAGCAAAAAGAAGTCTTACATAAACCGGACTGGAACATTTGAGTTCACAAGCTTTTTCTATCGCTATGGATTTGTGGCTAACTTCAGTATGGAGCTGCCCAGCTTTGGAGTTTCTGGGATCAATGAATCAGCTGACATGAGCATTGGCGTCACAGTGATAAAGAACAACATGATAAACAATGACCTTGGACCAGCAA<br>CAGCTCAAATGGCCCTTCAACTATTCATCAAAGATTACAGGTACACGTACCGATGCCACAGAGGTGACACACAAATTCAAACGAGGAGATCATTCGAGCTGAAGAAGCTGTGGGAACAGACCCGTTCAAAGGCAGGACTGTTGGTGTCAGATGGAGGACCAAATCTATACAACATTCGGAATCTCCATATCCCAGAGGTCTGCCTGAAGTGGGAGCTGATGGACGAAGATTA<br>CCAGGGCAGGTTGTGTAATCCTCTGAACCCATTTGTCAGCCATAAAGAAATTGAGTCCGTAAACAATGCTGTGGTGATGCCAGCTCACGGTCCAGCCAAAAGCATGGAATATGATGCCGTTGCGACTACACACTCATGGACTCCTAAAAGGAATCGTTCCATTCTCAATACCAGTCAAAGGGGAATTCTTGAGGATGAACAGATGTACCAGAAATGCTGCAGTCTATTCGAGAA<br>ATTCTTCCCCAGTAGTTCATACAGGAGACCAGTTGGAATTTCCAGCATGGTGGAGGCCATGGTGTCTAGGGCCCGAATCGATGCACGCATTGATTTCGAATCTGGAAGGATCAAGAAGGAAGAGTTTGCTGAGATCATGAAGATCTGTTCCACCATTGAAGAGCTCAGACGGCAAAAATAG
internal NVRI repository	>A/domestic_duck/Poland/H2099-N/2021_H5N1_2021-12-11<br>ATGGATGTCAATCCGACTTTACTTTTCTTAAAAGTGCCAGCGCAAGATGCCATAAGTACCACATTCCCTTACACTGGAGATCCCCCATACAGCCATGGAACAGGGACAGGATACACAATGGACACAGTCAACAGAACACATCAATACTCAGAGAAGGGGAAAATGGACAACAAACACAGAAACCAGAGCACCTCAACTCAACCCAATTGATGGGCCACTACCTGAGGACAACGA<br>ACCGAGCGGATATGCACAAACAGATTGCGTGTTGGAAGCAATGGCTTTCCTTGAAGAGTCCCACCCAGGGATCTTTGAAAACTCTTGTCTTGAAACGATGGAAGTCGTTCAGCAAACAAGAGTGGACAAACTAACTCAAGGTCGCCAGACATATGACTGGACACTGAATAGAAACCAACCAGCTGCAACTGCCCTGGCCAACACTATAGAGGTCTTCAGATCAAACGGTCTAA<br>CAGCCAATGAATCGGGGAGACTAATAGATTTCCTCAAGGATGTGATGGACTCAATGGATAAAGAAGAAATGGAAATAACAACACATTTCCAGAGAAAGAGAAGAGTAAGGGACAACATGACCAAGAAAATGGTCACACAAAGAACAATAGGAAAGAAGAAACAAAGGCTAAACAAGAGGAGCTACTTAATAAGAGCACTGACACTGAATACAATGACAAAAGATGCAGA<br>AAGAGGCAAATTGAAAAGACGGGCGATTGCAACACCAGGGATGCAGATTAGAGGATTTGTGTACTTTGTCGAAACACTGGCAAGGAGCATCTGTGAAAAACTTGAGCAATCTGGACTCCCCGTTGGAGGGAATGAGAAGAAGGCTAAATTGGCAAATGTCGTGAGAAAAATGATGACTAACTCACAAGATACAGAGCTCTCCTTCACAATTACTGGAGATAACACCAAATG<br>GAATGAGAATCAAAATCCTCGGATGTTTCTGGCAATGATAACATACATTACAAGAAACCAACCTGAATGGTTTAGAAATGTCTTGAGTGTTGCCCCTATAATGTTCTCGAACAAAATGGCGAGATTGGGAAAAGGGTACATGTTTGAAAGTAAGAGCATGAAGTTACGGACACAAATACCTGCAGAAATGCTTGCAAACATTGACTTAAAATACTTCAATGAATCAACAAGAAA<br>GAAAATCGAAAAAATAAGGCCTCTACTAATAGATGGCACTGCCTCATTGAGTCCTGGAATGATGATGGGCATGTTCAATATGCTGAGTACAGTATTAGGAGTTTCAATCCTAAATCTTGGGCAAAAGAAGTACACCAAAACCACATACTGGTGGGATGGACTCCAATCCTCTGATGATTTCGCCCTCATAGTAAATGCACCGAATCATGAGGGAATACAAGCAGGAGTGGATA<br>GGTTCTATAGGACCTGCAAACTGGTCGGGATCAATATGAGCAAAAAGAAGTCTTACATAAACCGGACTGGAACATTTGAGTTCACAAGCTTTTTCTATCGCTATGGATTTGTGGCTAACTTCAGTATGGAGCTGCCCAGCTTTGGAGTTTCTGGGATCAATGAATCAGCTGACATGAGCATTGGCGTCACAGTGATAAAGAACAACATGATAAACAATGACCTTGGACCAGCAA<br>CAGCTCAAATGGCCCTTCAACTATTCATCAAAGATTACAGGTACACGTACCGATGCCACAGAGGTGACACACAAATTCAAACGAGGAGATCATTCGAGCTGAAGAAGCTGTGGGAACAGACCCGTTCAAAGGCAGGACTGTTGGTGTCAGATGGAGGACCAAATCTATACAACATTCGGAATCTCCATATCCCAGAGGTCTGCCTGAAGTGGGAGCTGATGGACGAAGATTA<br>CCAGGGCAGGTTGTGTAATCCTCTGAACCCATTTGTCAGCCATAGAGAAATTGAGTCCGTAAACAATGCTGTGGTGGCGCCAGCCCACGGTCCAGCCAAAAGCATGGAATATGATGCCGTTGCGACTACACACTCATGGATTCCTAAAAGGAATCGTTCCATTCTCAATACCAGTCAAAGGGGAATTCTTGAGGATGAACAGATGTACCAGAAATGCTGCAGTCTATTCGAGAA<br>ATTCTTCCCCAGTAGTTCATACAGGAGACCAGTTGGAATTTCCAGCATGGTGGAGGCCATGGTGTCTAGGGCCCGAATCGATGCACGAATTGATTTCGAATCTGGAAGGATAAAGAAGGAAGAGTTTGCTGAGATCATGAAGATCTGTTCCACCATTGAAGAGCTCAGACGGCAAAAATAG
internal NVRI repository	>A/domestic_duck/Poland/H2085-T2/2021_H5N1_2021-12-06<br>ATGGATGTCAATCCGACTTTACTTTTCTTAAAAGTGCCAGCGCAAGATGCCATAAGTACCACATTCCCTTACACTGGAGATCCTCCATACAGCCATGGAACAGGGACAGGATACACAATGGACACAGTCAACAGAACACATCAATACTCAGAGAAGGGAAAATGGACAACAAACACAGAAACCAGAGCACCTCAACTCAACCCAATTGATGGGCCACTACCTGAGGACAACGA<br>ACCGAGCGGATATGCACAAACAGATTGCGTGTTGGAAGCAATGGCTTTCCTTGAAGAGTCCCACCCAGGGATCTTTGAAAACTCTTGTCTTGAAACGATGGAAGTCGTTCAGCAAACAAGAGTGGACAAACTAACTCAAGGTCGCCAGACATATGACTGGACACTGAATAGAAACCAACCAGCTGCAACTGCCCTGGCCAACACTATAGAGGTCTTCAGATCAAACGGTCTAA<br>CAGCCAATGAATCGGGGAGACTAATAGATTTCCTCAAGGATGTGATGGACTCAATGGATAAAGAAGAAATGGAAATAACAACACATTTCCAGAGAAAGAGAAGAGTAAGGGACAACATGACCAAGAAAATGGTCACACAAAGAACAATAGGAAAGAAGAAACAAAGGCTAAACAAGAGGAGCTACTTAATAAGAGCACTGACACTGAATACAATGACAAAAGATGCAGA<br>AAGAGGCAAATTGAAAAGACGGGCGATTGCAACACCAGGGATGCAGATTAGAGGATTTGTGTACTTTGTCGAAACACTGGCAAGGAGCATCTGTGAAAAACTTGAGCAATCTGGACTCCCCGTTGGAGGGAATGAGAAGAAGGCTAAATTGGCAAATGTCGTGAGAAAAATGATGACTAACTCACAAGATACAGAGCTCTCCTTCACAATTACTGGAGATAACACCAAATG<br>GAATGAGAATCAAAATCCTCGGATGTTTCTGGCAATGATAACATACATTACAAGAAACCAACCTGAATGGTTTAGAAATGTCTTGAGTGTTGCCCCTATAATGTTCTCGAACAAAATGGCGAGATTGGGAAAAGGGTACATGTTTGAAAGTAAGAGCATGAAGTTACGGACACAAATACCTGCAGAAATGCTTGCAAACATTGACTTAAAATACTTCAATGAATCAACAAGAAA<br>GAAAATCGAAAAAATAAGGCCTCTACTAATAGATGGCACTGCCTCATTGAGTCCTGGAATGATGATGGGCATGTTCAATATGCTGAGTACAGTATTAGGAGTTTCAATCCTAAATCTTGGGCAAAAGAAGTACACCAAAACCACATACTGGTGGGATGGACTCCAATCCTCTGATGATTTCGCCCTCATAGTAAATGCACCGAATCATGAGGGAATACAAGCAGGAGTGGATA<br>GGTTCTATAGGACCTGCAAACTGGTCGGGATCAATATGAGCAAAAAGAAGTCTTACATAAACCGGACTGGAACATTTGAGTTCACAAGCTTTTTCTATCGCTATGGATTTGTGGCTAACTTCAGTATGGAGCTGCCCAGCTTTGGAGTTTCTGGGATCAATGAATCAGCTGACATGAGCATTGGCGTCACAGTGATAAAGAACAACATGATAAACAATGACCTTGGACCAGCAA<br>CAGCTCAAATGGCCCTTCAACTATTCATCAAAGATTACAGGTACACGTACCGATGCCACAGAGGTGACACACAAATTCAAACGAGGAGATCATTCGAGCTGAAGAAGCTGTGGGAACAGACCCGTTCAAAGGCAGGACTGTTGGTGTCAGATGGAGGACCAAATCTATACAACATTCGGAATCTCCATATCCCAGAGGTCTGCCTGAAGTGGGAGCTGATGGACGAAGATTA<br>CCAGGGCAGGTTGTGTAATCCTCTGAACCCATTTGTCAGCCATAGAGAAATTGAGTCCGTAAACAATGCTGTGGTGGCGCCAGCCCACGGTCCAGCCAAAAGCATGGAATATGATGCCGTTGCGACTACACACTCATGGATTCCTAAAAGGAATCGTTCCATTCTCAATACCAGTCAAAGGGGAATTCTTGAGGATGAACAGATGTACCAGAAATGCTGCAGTCTATTCGAGAA<br>ATTCTTCCCCAGTAGTTCATACAGGAGACCAGTTGGAATTTCCAGCATGGTGGAGGCCATGGTGTCTAGGGCCCGAATCGATGCACGAATTGATTTCGAATCTGGAAGGATAAAGAAGGAAGAGTTTGCTGAGATCATGAAGATCTGTTCCACCATTGAAGAGCTCAGACGGCAAAAATAG
internal NVRI repository	>A/domestic_duck/Poland/H2078-N/2021_H5N1_2022-12-05<br>ATGGATGTCAATCCGACTTTACTTTTCTTAAAAGTGCCAGCGCAAGATGCCATAAGTACCACATTCCCTTACACTGGAGATCCTCCATACAGCCATGGAACAGGGACAGGATACACAATGGACACAGTCAACAGAACACATCAATACTCAGAGAAGGGAAAATGGACAACAAACACAGAAACCAGAGCACCTCAACTCAACCCAATTGATGGGCCACTACCTGAGGACAACGA<br>ACCGAGCGGATATGCACAAACAGATTGCGTGTTGGAAGCAATGGCTTTCCTTGAAGAGTCCCACCCAGGGATCTTTGAAAACTCTTGTCTTGAAACGATGGAAGTCGTTCAGCAAACAAGAGTGGACAAACTAACTCAAGGTCGCCAGACATATGACTGGACACTGAATAGAAACCAACCAGCTGCAACTGCCCTGGCCAACACTATAGAGGTCTTCAGATCAAACGGTCTAA<br>CAGCCAATGAATCGGGGAGACTAATAGATTTCCTCAAGGATGTGATGGACTCAATGGATAAAGAAGAAATGGAAATAACAACACATTTCCAGAGAAAGAGAAGAGTAAGGGACAACATGACCAAGAAAATGGTCACACAAAGAACAATAGGAAAGAAGAAACAAAGGCTAAACAAGAGGAGCTACTTAATAAGAGCACTGACACTGAATACAATGACAAAAGATGCAGA<br>AAGAGGCAAATTGAAAAGACGGGCGATTGCAACACCAGGGATGCAGATTAGAGGATTTGTGTACTTTGTCGAAACACTGGCAAGGAGCATCTGTGAAAAACTTGAGCAATCTGGACTCCCCGTTGGAGGGAATGAGAAGAAGGCTAAATTGGCAAATGTCGTGAGAAAAATGATGACTAACTCACAAGATACAGAGCTCTCCTTCACAATTACTGGAGATAACACCAAATG<br>GAATGAGAATCAAAATCCTCGGATGTTTCTGGCAATGATAACATACATTACAAGAAACCAACCTGAATGGTTTAGAAATGTCTTGAGTGTTGCCCCTATAATGTTCTCGAACAAAATGGCGAGATTGGGAAAAGGGTACATGTTTGAAAGTAAGAGCATGAAGTTACGGACACAAATACCTGCAGAAATGCTTGCAAACATTGACTTAAAATACTTCAATGAATCAACAAGAAA<br>GAAAATCGAAAAAATAAGGCCTCTACTAATAGATGGCACTGCCTCATTGAGCCCTGGAATGATGATGGGCATGTTCAATATGCTGAGTACAGTATTAGGAGTTTCAATCCTAAATCTTGGGCAAAAGAAGTACACCAAAACCACATACTGGTGGGATGGACTCCAATCCTCTGATGATTTCGCCCTCATAGTAAATGCACCGAATCATGAGGGAATACAAGCAGGAGTGGATA<br>GGTTCTATAGGACCTGCAAACTGGTCGGGATCAATATGAGCAAAAAGAAGTCTTACATAAACCGGACTGGAACATTTGAGTTCACAAGCTTTTTCTATCGCTATGGATTTGTGGCTAACTTCAGTATGGAGCTGCCCAGCTTTGGAGTTTCTGGGATCAATGAATCAGCTGACATGAGCATTGGCGTCACAGTGATAAAGAACAACATGATAAACAATGACCTTGGACCAGCAA<br>CAGCTCAAATGGCCCTTCAACTATTCATCAAAGATTACAGGTACACGTACCGATGCCACAGAGGTGACACACAAATTCAAACGAGGAGATCATTCGAGCTGAAGAAGCTGTGGGAACAGACCCGTTCAAAGGCAGGACTGTTGGTGTCAGATGGAGGACCAAATCTATACAACATTCGGAATCTCCATATCCCAGAGGTCTGCCTGAAGTGGGAGCTGATGGACGAAGATTA<br>TCAGGGCAGGTTGTGTAATCCTCTGAACCCATTTGTCAGCCATAGAGAAATTGAGTCCGTAAACAATGCTGTGGTGGCGCCAGCCCACGGTCCAGCCAAAAGCATGGAATATGATGCCGTTGCGACTACACACTCATGGATTCCTAAAAGGAATCGTTCCATTCTCAATACCAGTCAAAGGGGAATTCTTGAGGATGAACAGATGTACCAGAAATGCTGCAGTCTATTCGAGAA<br>ATTCTTCCCCAGTAGTTCATACAGGAGACCAGTTGGAATTTCCAGCATGGTGGAGGCCATGGTGTCTAGGGCCCGAATCGATGCACGAATTGATTTCGAATCTGGAAGGATAAAGAAGGAAGAGTTTGCTGAGATCATGAAGATCTGTTCCACCATTGAAGAGCTCAGACGGCAAAAATAG
internal NVRI repository	>A/domestic_duck/Poland/H2077-J/2021_H5N1_2021-12-04<br>ATGGATGTCAATCCGACTTTACTTTTCTTAAAAGTGCCAGCGCAAGATGCCATAAGTACCACATTCCCTTACACTGGAGATCCTCCATACAGCCATGGAACAGGGACAGGATACACAATGGACACAGTCAACAGAACACATCAATACTCAGAGAAGGGAAAATGGACAACAAACACAGAAACCAGAGCACCTCAACTCAACCCAATTGATGGGCCACTACCTGAGGACAACGA<br>ACCGAGCGGATATGCACAAACAGATTGCGTGTTGGAAGCAATGGCTTTCCTTGAAGAGTCCCACCCAGGGATCTTTGAAAACTCTTGTCTTGAAACGATGGAAGTCGTTCAGCAAACAAGAGTGGACAAACTAACTCAAGGTCGCCAGACATATGACTGGACACTGAATAGAAACCAACCAGCTGCAACTGCCCTGGCCAACACTATAGAGGTCTTCAGATCAAACGGTCTAA<br>CAGCTAATGAATCGGGGAGACTAATAGATTTCCTCAAGGATGTGATGGACTCAATGGATAAAGAAGAAATGGAAATAACAACACATTTCCAGAGAAAGAGAAGAGTAAGGGACAACATGACCAAGAAAATGGTCACACAAAGAACAATAGGAAAGAAGAAACAAAGGCTAAACAAGAGGAGCTACTTAATAAGAGCACTGACACTGAATACAATGACAAAAGATGCAGA<br>AAGAGGCAAATTGAAAAGACGGGCGATTGCAACACCAGGGATGCAGATTAGAGGATTTGTGTACTTTGTCGAAACACTGGCAAGGAGCATCTGTGAAAAACTTGAGCAATCTGGACTCCCCGTTGGAGGGAATGAGAAGAAGGCTAAATTGGCAAATGTCGTGAGAAAAATGATGACTAACTCACAAGATACAGAGCTCTCCTTCACAATTACTGGAGATAACACCAAATG<br>GAATGAGAATCAAAATCCTCGGATGTTTCTGGCAATGATAACATACATTACAAGAAACCAACCTGAATGGTTTAGAAATGTCTTGAGTGTTGCCCCTATAATGTTCTCGAACAAAATGGCGAGATTGGGAAAAGGGTACATGTTTGAAAGTAAGAGCATGAAGTTACGGACACAAATACCTGCAGAAATGCTTGCAAACATTGACTTAAAATACTTCAATGAATCAACAAGAAA<br>GAAAATCGAAAAAATAAGGCCTCTACTAATAGATGGCACTGCCTCATTGAGTCCTGGAATGATGATGGGCATGTTCAATATGCTGAGTACAGTATTAGGAGTTTCAATCCTAAATCTTGGGCAAAAGAAGTACACCAAAACCACATACTGGTGGGATGGACTCCAATCCTCTGATGATTTCGCCCTCATAGTAAATGCACCGAATCATGAGGGAATACAAGCAGGAGTGGATA<br>GGTTCTATAGGACCTGCAAACTGGTCGGGATCAATATGAGCAAAAAGAAGTCTTACATAAACCGGACTGGAACATTTGAGTTCACAAGCTTTTTCTATCGCTATGGATTTGTGGCTAACTTCAGTATGGAGCTGCCCAGCTTTGGAGTTTCTGGGATCAATGAATCAGCTGACATGAGCATTGGCGTCACAGTGATAAAGAACAACATGATAAACAATGACCTTGGACCAGCAA<br>CAGCTCAAATGGCCCTTCAACTATTCATCAAAGATTACAGGTACACGTACCGATGCCACAGAGGTGACACACAAATTCAAACGAGGAGATCATTCGAGCTGAAGAAGCTGTGGGAACAGACCCGTTCAAAGGCAGGACTGTTGGTGTCAGATGGAGGACCAAATCTATACAACATTCGGAATCTCCATATCCCAGAGGTCTGCCTGAAGTGGGAGCTGATGGACGAAGATTA<br>CCAGGGCAGGTTGTGTAATCCTCTGAACCCATTTGTCAGCCATAGAGAAATTGAGTCCGTAAACAATGCTGTGGTGGCGCCAGCCCACGGTCCAGCCAAAAGCATGGAATATGATGCCGTTGCGACTACACACTCATGGATTCCTAAAAGGAATCGTTCCATTCTCAATACCAGTCAAAGGGGAATTCTTGAGGATGAACAGATGTACCAGAAATGCTGCAGTCTATTCGAGAA<br>ATTCTTCCCCAGTAGTTCATACAGGAGACCAGTTGGAATTTCCAGCATGGTGGAGGCCATGGTGTCTAGGGCCCGAATCGATGCACGAATTGATTTCGAATCTGGAAGGATAAAGAAGGAAGAGTTTGCTGAGATCATGAAGATCTGTTCCACCATTGAAGAGCTCAGACGGCAAAAATAG
internal NVRI repository	>A/domestic_duck/Poland/H2045-T1/2021_H5N1_2021-11-26<br>ATGGATGTCAATCCGACTTTACTTTTCTTAAAAGTGCCAGCGCAAGATGCCATAAGTACCACATTCCCTTACACTGGAGATCCTCCATACAGCCATGGAACAGGGACAGGATACACAATGGACACAGTCAACAGAACACATCAATACTCAGAGAAGGGAAAATGGACAACAAACACAGAAACCAGAGCACCTCAACTCAACCCAATTGATGGGCCACTACCTGAGGACAACGA<br>ACCGAGCGGATATGCACAAACAGATTGCGTGTTGGAAGCAATGGCTTTCCTTGAAGAGTCCCACCCAGGGATCTTTGAAAACTCTTGTCTTGAAACGATGGAAGTCGTTCAGCAAACAAGAGTGGACAAACTAACTCAAGGTCGCCAGACATATGACTGGACACTGAATAGAAACCAACCAGCTGCAACTGCCCTGGCCAACACTATAGAGGTCTTCAGATCAAACGGTCTAA<br>CAGCCAATGAATCGGGGAGACTAATAGATTTCCTCAAGGATGTGATGGACTCAATGGATAAAGAAGAAATGGAAATAACAACACATTTCCAGAGAAAGAGAAGAGTAAGGGACAACATGACCAAGAAAATGGTCACACAAAGAACAATAGGAAAGAAGAAACAAAGGCTAAACAAGAGGAGCTACTTAATAAGAGCACTGACACTGAATACAATGACAAAAGATGCAGA<br>AAGAGGCAAATTGAAAAGACGGGCGATTGCAACACCAGGGATGCAGATTAGAGGATTTGTGTACTTTGTCGAAACACTGGCAAGGAGCATCTGTGAAAAACTTGAGCAATCTGGACTCCCCGTTGGAGGGAATGAGAAGAAGGCTAAATTGGCAAATGTCGTGAGAAAAATGATGACTAACTCACAAGATACAGAGCTCTCCTTCACAATTACTGGAGATAACACCAAATG<br>GAATGAGAATCAAAATCCTCGGATGTTTCTGGCAATGATAACATACATTACAAGAAACCAACCTGAATGGTTTAGAAATGTCTTGAGTGTTGCCCCTATAATGTTCTCGAACAAAATGGCGAGATTGGGAAAAGGGTACATGTTTGAAAGTAAGAGCATGAAGTTACGGACACAAATACCTGCAGAAATGCTTGCAAACATTGACTTAAAATACTTCAATGAATCAACAAGAAA<br>GAAAATCGAAAAAATAAGGCCTCTACTAATAGATGGCACTGCCTCATTGAGTCCTGGAATGATGATGGGCATGTTCAATATGCTGAGTACAGTATTAGGAGTTTCAATCCTAAATCTTGGGCAAAAGAAGTACACCAAAACCACATACTGGTGGGATGGACTCCAATCCTCTGATGATTTCGCCCTCATAGTAAATGCACCGAATCATGAGGGAATACAAGCAGGAGTGGATA<br>GGTTCTATAGGACCTGCAAACTGGTCGGGATCAATATGAGCAAAAAGAAGTCTTACATAAACCGGACTGGAACATTTGAGTTCACAAGCTTTTTCTATCGCTATGGATTTGTGGCTAACTTCAGTATGGAGCTGCCCAGCTTTGGAGTTTCTGGGATCAATGAATCAGCTGACATGAGCATTGGCGTCACAGTGATAAAGAACAACATGATAAACAATGACCTTGGACCAGCAA<br>CAGCTCAAATGGCCCTTCAACTATTCATCAAAGATTACAGGTACACGTACCGATGCCACAGAGGTGACACACAAATTCAAACGAGGAGATCATTCGAGCTGAAGAAGCTGTGGGAACAGACCCGTTCAAAGGCAGGACTGTTGGTGTCAGATGGAGGACCAAATCTATACAACATTCGGAATCTCCATATCCCAGAGGTCTGCCTGAAGTGGGAGCTGATGGACGAAGATTA<br>CCAGGGCAGGTTGTGTAATCCTCTGAACCCATTTGTCAGCCATAGAGAAATTGAGTCCGTAAACAATGCTGTGGTGGCGCCAGCCCACGGTCCAGCCAAAAGCATGGAATATGATGCCGTTGCGACTACACACTCATGGATTCCTAAAAGGAATCGTTCCATTCTCAATACCAGTCAAAGGGGAATTCTTGAGGATGAACAGATGTACCAGAAATGCTGCAGTCTATTCGAGAA<br>ATTCTTCCCCAGTAGTTCATACAGGAGACCAGTTGGAATTTCCAGCATGGTGGAGGCCATGGTGTCTAGGGCCCGAATCGATGCACGAATTGATTTCGAATCTGGAAGGATAAAGAAGGAAGAGTTTGCTGAGATCATGAAGATCTGTTCCACCATTGAAGAGCTCAGACGGCAAAAATAG
internal NVRI repository	>A/domestic_duck/Poland/H2020-N/2021_H5N1_2021-11-23<br>ATGGATGTCAATCCGACTTTACTTTTCTTAAAAGTGCCAGCGCAAGATGCCATAAGTACCACATTCCCTTACACTGGAGATCCTCCATACAGCCATGGAACAGGGACAGGATACACAATGGACACAGTCAACAGAACACATCAATACTCAGAGAAGGGAAAATGGACAACAAACACAGAAACCAGAGCACCTCAACTCAACCCAATTGATGGGCCACTACCTGAGGACAACGA<br>ACCGAGCGGATATGCACAAACAGATTGCGTGTTGGAAGCAATGGCTTTCCTTGAAGAGTCCCACCCAGGGATCTTTGAAAACTCTTGTCTTGAAACGATGGAAGTCGTTCAGCAAACAAGAGTGGACAAACTAACTCAAGGTCGCCAGACATATGACTGGACACTGAATAGAAACCAACCAGCTGCAACTGCCCTGGCCAACACTATAGAGGTCTTCAGATCAAACGGTCTAA<br>CAGCCAATGAATCGGGGAGACTAATAGATTTCCTCAAGGATGTGATGGACTCAATGGATAAAGAAGAAATGGAAATAACAACACATTTCCAGAGAAAGAGAAGAGTAAGGGACAACATGACCAAGAAAATGGTCACACAAAGAACAATAGGAAAGAAGAAACAAAGGCTAAACAAGAGGAGCTACTTAATAAGAGCACTGACACTGAATACAATGACAAAAGATGCAGA<br>AAGAGGCAAATTGAAAAGACGGGCGATTGCAACACCAGGGATGCAGATTAGAGGATTTGTGTACTTTGTCGAAACACTGGCAAGGAGCATCTGTGAAAAACTTGAGCAATCTGGACTCCCCGTTGGAGGGAATGAGAAGAAGGCTAAATTGGCAAATGTCGTGAGAAAAATGATGACTAACTCACAAGATACAGAGCTCTCCTTCACAATTACTGGAGATAACACCAAATG<br>GAATGAGAATCAAAATCCTCGGATGTTTCTGGCAATGATAACATACATTACAAGAAACCAACCTGAATGGTTTAGAAATGTCTTGAGTGTTGCCCCTATAATGTTCTCGAACAAAATGGCGAGATTGGGAAAAGGGTACATGTTTGAAAGTAAGAGCATGAAGTTACGGACACAAATACCTGCAGAAATGCTTGCAAACATTGACTTAAAATACTTCAATGAATCAACAAGAAA<br>GAAAATCGAAAAAATAAGGCCTCTACTAATAGATGGCACTGCCTCATTGAGTCCTGGAATGATGATGGGCATGTTCAATATGCTGAGTACAGTATTAGGAGTTTCAATCCTAAATCTTGGGCAAAAGAAGTACACCAAAACCACATACTGGTGGGATGGACTCCAATCCTCTGATGATTTCGCCCTCATAGTAAATGCACCGAATCATGAGGGAATACAAGCAGGAGTGGATA<br>GGTTCTATAGGACCTGCAAACTGGTCGGGATCAATATGAGCAAAAAGAAGTCTTACATAAACCGGACTGGAACATTTGAGTTCACAAGCTTTTTCTATCGCTATGGATTTGTGGCTAACTTCAGTATGGAGCTGCCCAGCTTTGGAGTTTCTGGGATCAATGAATCAGCTGACATGAGCATTGGCGTCACAGTGATAAAGAACAACATGATAAACAATGACCTTGGACCAGCAA<br>CAGCTCAAATGGCCCTTCAACTATTCATCAAAGATTACAGGTACACGTACCGATGCCACAGAGGTGACACACAAATTCAAACGAGGAGATCATTCGAGCTGAAGAAGCTGTGGGAACAGACCCGTTCAAAGGCAGGACTGTTGGTGTCAGATGGAGGACCAAATCTATACAACATTCGGAATCTCCATATCCCAGAGGTCTGCCTGAAGTGGGAGCTGATGGACGAAGATTA<br>CCAGGGCAGGTTGTGTAATCCTCTGAACCCATTTGTCAGCCATAGAGAAATTGAGTCCGTAAACAATGCTGTGGTGGTGCCAGCCCACGGTCCAGCCAAAAGCATGGAATATGATGCCGTTGCGACTACACACTCATGGATTCCTAAAAGGAATCGTTCCATTCTCAATACCAGTCAAAGGGGAATTCTTGAGGATGAACAGATGTACCAGAAATGCTGCAGTCTATTCGAGAA<br>ATTCTTCCCCAGTAGTTCATACAGGAGACCAGTTGGAATTTCCAGCATGGTGGAGGCCATGGTGTCTAGGGCCCGAATCGATGCACGAATTGATTTCGAATCTGGAAGGATAAAGAAGGAAGAGTTTGCTGAGATCATGAAGATCTGTTCCACCATTGAAGAGCTCAGACGGCAAAAATAG
internal NVRI repository	>A/domestic_duck/Poland/H1942-Nd/2021_H5N1_2021-11-07<br>ATGGATGTCAATCCGACTTTACTTTTCTTAAAAGTGCCAGCGCAAGATGCCATAAGTACCACATTCCCTTACACTGGAGATCCTCCATACAGCCATGGAACAGGGACAGGATACACAATGGACACAGTCAACAGAACACATCAATACTCAGAGAAGGGAAAATGGACAACAAACACAGAAACCAGAGCACCTCAACTCAACCCAATTGATGGGCCACTACCTGAGGACAACGA<br>ACCGAGCGGATATGCACAAACAGATTGCGTGTTGGAAGCAATGGCTTTCCTTGAAGAGTCCCACCCAGGGATCTTTGAAAACTCTTGTCTTGAAACGATGGAAGTCGTTCAGCAAACAAGAGTGGACAAACTAACTCAAGGTCGCCAGACATATGACTGGACACTGAATAGAAACCAACCAGCTGCAACTGCCCTGGCCAACACTATAGAGGTCTTCAGATCAAACGGTCTAA<br>CAGCCAATGAATCGGGGAGACTAATAGATTTCCTCAAGGATGTGATGGACTCAATGGATAAAGAAGAAATGGAAATAACAACACATTTCCAGAGAAAGAGAAGAGTAAGGGACAACATGACCAAGAAAATGGTCACACAAAGAACAATAGGAAAGAAGAAACAAAGGCTAAACAAGAGGAGCTACTTAATAAGAGCACTGACACTGAATACAATGACAAAAGATGCAGA<br>AAGAGGCAAATTGAAAAGACGGGCGATTGCAACACCAGGGATGCAGATTAGAGGATTTGTGTACTTTGTCGAAACACTGGCAAGGAGCATCTGTGAAAAACTTGAGCAATCTGGACTCCCCGTTGGAGGGAATGAGAAGAAGGCTAAATTGGCAAATGTCGTGAGAAAAATGATGACTAACTCACAAGATACAGAGCTCTCCTTCACAATTACTGGAGATAACACCAAATG<br>GAATGAGAATCAAAATCCTCGGATGTTTCTGGCAATGATAACATACATTACAAGAAACCAACCTGAATGGTTTAGAAATGTCTTGAGTGTTGCCCCTATAATGTTCTCGAACAAAATGGCGAGATTGGGAAAAGGGTACATGTTTGAAAGTAAGAGCATGAAGTTACGGACACAAATACCTGCAGAAATGCTTGCAAACATTGACTTAAAATACTTCAATGAATCAACAAGAAA<br>GAAAATCGAAAAAATAAGGCCTCTACTAATAGATGGCACTGCCTCATTGAGTCCTGGAATGATGATGGGCATGTTCAATATGCTGAGTACAGTATTAGGAGTTTCAATCCTAAATCTTGGGCAAAAGAAGTACACCAAAACCACATACTGGTGGGATGGACTCCAATCCTCTGATGATTTCGCCCTCATAGTAAATGCACCGAATCATGAGGGAATACAAGCAGGAGTGGATA<br>GGTTCTATAGGACCTGCAAACTGGTCGGGATCAATATGAGCAAAAAGAAGTCTTACATAAACCGGACTGGAACATTTGAGTTCACAAGCTTTTTCTATCGCTATGGATTTGTGGCTAACTTCAGTATGGAGCTGCCCAGCTTTGGAGTTTCTGGGATCAATGAATCAGCTGACATGAGCATTGGCGTCACAGTGATAAAGAACAACATGATAAACAATGACCTTGGACCAGCAA<br>CAGCTCAAATGGCCCTTCAACTATTCATCAAAGATTACAGGTACACGTACCGATGCCACAGAGGTGACACACAAATTCAAACGAGGAGATCATTCGAGCTGAAGAAGCTGTGGGAACAGACCCGTTCAAAGGCAGGACTGTTGGTGTCAGATGGAGGACCAAATCTATACAACATTCGGAATCTCCATATCCCAGAGGTCTGCCTGAAGTGGGAGCTGATGGACGAAGATTA<br>CCAGGGCAGGTTGTGTAATCCTCTGAACCCATTTGTCAGCCATAGAGAAATTGAGTCCGTAAACAATGCTGTGGTGGTGCCAGCCCACGGTCCAGCCAAAAGCATGGAATATGATGCCGTTGCGACTACACACTCATGGATTCCTAAAAGGAATCGTTCCATTCTCAATACCAGTCAAAGGGGAATTCTTGAGGATGAACAGATGTACCAGAAATGCTGCAGTCTATTCGAGAA<br>ATTCTTCCCCAGTAGTTCATACAGGAGACCAGTTGGAATTTCCAGCATGGTGGAGGCCATGGTGTCTAGGGCCCGAATCGATGCACGAATTGATTTCGAATCTGGAAGGATAAAGAAGGAAGAGTTTGCTGAGATCATGAAGATCTGTTCCACCATTGAAGAGCTCAGACGGCAAAAATAG
EPI_ISL_6934175	>A/domestic_duck/Poland/H1942-N/2021_H5N1_2021-11-07<br>ATGGATGTCAATCCGACTTTACTTTTCTTAAAAGTGCCAGCGCAAGATGCCATAAGTACCACATTCCCTTACACTGGAGATCCTCCATACAGCCATGGAACAGGGACAGGATACACAATGGACACAGTCAACAGAACACATCAATACTCAGAGAAGGGAAAATGGACAACAAACACAGAAACCAGAGCACCTCAACTCAACCCAATTGATGGGCCACTACCTGAGGACAACGA<br>ACCGAGCGGATATGCACAAACAGATTGCGTGTTGGAAGCAATGGCTTTCCTTGAAGAGTCCCACCCAGGGATCTTTGAAAACTCTTGTCTTGAAACGATGGAAGTCGTTCAGCAAACAAGAGTGGACAAACTAACTCAAGGTCGCCAGACATATGACTGGACACTGAATAGAAACCAACCAGCTGCAACTGCCCTGGCCAACACTATAGAGGTCTTCAGATCAAACGGTCTAA<br>CAGCCAATGAATCGGGGAGACTAATAGATTTCCTCAAGGATGTGATGGACTCAATGGATAAAGAAGAAATGGAAATAACAACACATTTCCAGAGAAAGAGAAGAGTAAGGGACAACATGACCAAGAAAATGGTCACACAAAGAACAATAGGAAAGAAGAAACAAAGGCTAAACAAGAGGAGCTACTTAATAAGAGCACTGACACTGAATACAATGACAAAAGATGCAGA<br>AAGAGGCAAATTGAAAAGACGGGCGATTGCAACACCAGGGATGCAGATTAGAGGATTTGTGTACTTTGTCGAAACACTGGCAAGGAGCATCTGTGAAAAACTTGAGCAATCTGGACTCCCCGTTGGAGGGAATGAGAAGAAGGCTAAATTGGCAAATGTCGTGAGAAAAATGATGACTAACTCACAAGATACAGAGCTCTCCTTCACAATTACTGGAGATAACACCAAATG<br>GAATGAGAATCAAAATCCTCGGATGTTTCTGGCAATGATAACATACATTACAAGAAACCAACCTGAATGGTTTAGAAATGTCTTGAGTGTTGCCCCTATAATGTTCTCGAACAAAATGGCGAGATTGGGAAAAGGGTACATGTTTGAAAGTAAGAGCATGAAGTTACGGACACAAATACCTGCAGAAATGCTTGCAAACATTGACTTAAAATACTTCAATGAATCAACAAGAAA<br>GAAAATCGAAAAAATAAGGCCTCTACTAATAGATGGCACTGCCTCATTGAGTCCTGGAATGATGATGGGCATGTTCAATATGCTGAGTACAGTATTAGGAGTTTCAATCCTAAATCTTGGGCAAAAGAAGTACACCAAAACCACATACTGGTGGGATGGACTCCAATCCTCTGATGATTTCGCCCTCATAGTAAATGCACCGAATCATGAGGGAATACAAGCAGGAGTGGATA<br>GGTTCTATAGGACCTGCAAACTGGTCGGGATCAATATGAGCAAAAAGAAGTCTTACATAAACCGGACTGGAACATTTGAGTTCACAAGCTTTTTCTATCGCTATGGATTTGTGGCTAACTTCAGTATGGAGCTGCCCAGCTTTGGAGTTTCTGGGATCAATGAATCAGCTGACATGAGCATTGGCGTCACAGTGATAAAGAACAACATGATAAACAATGACCTTGGACCAGCAA<br>CAGCTCAAATGGCCCTTCAACTATTCATCAAAGATTACAGGTACACGTACCGATGCCACAGAGGTGACACACAAATTCAAACGAGGAGATCATTCGAGCTGAAGAAGCTGTGGGAACAGACCCGTTCAAAGGCAGGACTGTTGGTGTCAGATGGAGGACCAAATCTATACAACATTCGGAATCTCCATATCCCAGAGGTCTGCCTGAAGTGGGAGCTGATGGACGAAGATTA<br>CCAGGGCAGGTTGTGTAATCCTCTGAACCCATTTGTCAGCCATAGAGAAATTGAGTCCGTAAACAATGCTGTGGTGGTGCCAGCCCACGGTCCAGCCAAAAGCATGGAATATGATGCCGTTGCGACTACACACTCATGGATTCCTAAAAGGAATCGTTCCATTCTCAATACCAGTCAAAGGGGAATTCTTGAGGATGAACAGATGTACCAGAAATGCTGCAGTCTATTCGAGAA<br>ATTCTTCCCCAGTAGTTCATACAGGAGACCAGTTGGAATTTCCAGCATGGTGGAGGCCATGGTGTCTAGGGCCCGAATCGATGCACGAATTGATTTCGAATCTGGAAGGATAAAGAAGGAAGAGTTTGCTGAGATCATGAAGATCTGTTCCACCATTGAAGAGCTCAGACGGCAAAAATAG
internal NVRI repository	>A/domestic_duck/Poland/H189/2022_H5N1_2022-03-04<br>ATGGATGTCAATCCGACTTTACTTTTCTTAAAAGTGCCAGCGCAAGATGCCATAAGTACCACATTCCCTTACACTGGAGATCCTCCATACAGCCATGGAACAGGGACAGGATACACAATGGACACAGTCAACAGAACACATCAATACTCAGAGAAGGGAAAATGGACAACAAACACAGAAACCGGAGCACCTCAACTCAACCCAATTGATGGGCCACTACCTGAGGACAACGA<br>ACCGAGCGGATATGCACAAACAGATTGCGTGTTGGAAGCAATGGCTTTCCTTGAAGAGTCCCACCCAGGGATCTTTGAAAACTCTTGTCTTGAAACGATGGAAGTCGTTCAGCAAACAAGAGTGGACAAACTAACTCAAGGTCGCCAGACATATGACTGGACACTGAATAGAAACCAACCAGCTGCAACTGCCCTGGCCAACACTATAGAGGTCTTCAGATCAAACGGTCTAA<br>CAGCCAATGAATCGGGGAGACTAATAGATTTCCTCAAGGATGTGATGGACTCAATGGATAAAGAAGAAATGGAAATAACAACACATTTCCAGAGAAAGAGAAGAGTAAGGGACAACATGACCAAGAAAATGGTCACACAAAGAACAATAGGAAAGAAGAAACAAAGGCTAAACAAGAGGAGCTACTTAATAAGAGCACTGACACTGAATACAATGACAAAAGATGCAGA<br>AAGAGGCAAATTGAAAAGACGGGCGATTGCAACACCAGGGATGCAGATTAGAGGATTTGTGTACTTTGTCGAAACACTGGCAAGGAGCATCTGTGAAAAACTTGAGCAATCTGGACTCCCCGTTGGAGGGAATGAGAAGAAGGCTAAATTGGCAAATGTCGTGAGAAAAATGATGACTAACTCACAAGATACAGAGCTCTCCTTTACAATTACTGGAGACAACACCAAATG<br>GAATGAGAATCAAAATCCTCGGATGTTTCTGGCAATGATAACATACATTACAAGAAACCAACCTGAATGGTTTAGAAATGTCTTGAGTATTGCCCCTATAATGTTCTCGAACAAAATGGCGAGATTGGGAAAAGGGTACATGTTTGAAAGTAAGAGCATGAAGTTACGGACACAAATACCTGCAGAAATGCTTGCAAACATTGACTTAAAATACTTCAATGAATCAACAAGAAA<br>GAAAATCGAAAAAATAAGGCCTCTACTAATAGATGGCACTGCCTCATTGAGTCCTGGAATGATGATGGGCATGTTCAATATGCTGAGTACAGTATTAGGAGTTTCAATCCTAAATCTTGGGCAAAAGAAGTACACCAAAACAACATACTGGTGGGATGGACTCCAATCCTCTGATGATTTCGCCCTCATAGTAAATGCACCGAATCATGAGGGAATACAAGCAGGAGTGGATA<br>GGTTCTATAGGACCTGCAAACTGGTCGGGATCAATATGAGCAAAAAGAAGTCTTACATAAACCGGACTGGAACATTTGAGTTCACAAGCTTTTTCTATCGCTATGGATTTGTGGCTAACTTCAGTATGGAGCTGCCCAGCTTTGGAGTTTCTGGGATCAATGAATCAGCTGACATGAGCATTGGCGTCACAGTGATAAAGAACAACATGATAAACAATGACCTTGGACCAGCAA<br>CAGCTCAAATGGCCCTTCAACTATTCATCAAAGATTACAGGTACACGTACCGATGCCACAGAGGTGACACACAAATTCAAACGAGGAGATCATTCGAGCTGAAGAAGCTGTGGGAACAGACCCGTTCAAAGGCAGGACTGTTGGTGTCAGATGGAGGACCAAATCTATACAACATTCGGAATCTCCATATCCCAGAAGTCTGCCTGAAGTGGGAGCTGATGGACAAAGATTA<br>CCAGGGCAGGTTGTGTAATCCTCTGAACCCATTTGTCAGCCATAGAGAAATTGAGTCCGTAAACAATGCTGTGGTGATGCCAGCCCACGGTCCAGCCAAAAGCATGGAATATGATGCCGTTGCGACTACACACTCATGGATTCCTAAAAGGAATCGTTCCATTCTCAATACCAGTCAAAGGGGAATTCTTGAGGATGAACAGATGTACCAGAAATGCTGCAGTCTATTCGAGAA<br>ATTCTTCCCCAGTAGTTCATACAGGAGACCAGTTGGAATTTCCAGCATGGTGGAGGCCATGGTGTCTAGGGCCCGAATCGATGCACGAATTGATTTCGAATCTGGAAGGATCAAGAAGGAAGAGTTTGCTGAGATCATGAAGATCTGTTCCACCATTGAAGAGCTCAGACGGCAAAAATAG

internal NVRI repository	>A/domestic_duck/Poland/H158/2022_H5N1_2022-02-19<br>[illegible]
internal NVRI repository	>A/domestic_duck/Poland/H146/2022_H5N1_2022-02-14<br>[illegible]
internal NVRI repository	>A/domestic_duck/Poland/H144/2022_H5N1_2022-02-13<br>[illegible]
internal NVRI repository	>A/domestic_duck/Poland/2177/2021_H5N1_2021-12-29<br>[illegible]
internal NVRI repository	>A/domestic_duck/Poland/2160/2021_H5N1_2021-12-27<br>[illegible]
internal NVRI repository	>A/domestic_duck/Poland/01/2022_H5N1_2021-12-31<br>[illegible]
EPI_ISL_5067957	>A/domestic_duck/Germany-NI/AI00079/2021_H5N8_2021-01-04<br>[illegible]
EPI_ISL_5123590	>A/domestic_duck/Germany-BB/AI02599/2021_H5N8_2021-03-11<br>[illegible]
EPI_ISL_9029959	>A/domestic_duck/England/058612/2021_H5N1_2021-11-18<br>[illegible]
EPI_ISL_11406402	>A/domestic_duck/England/007588/2022_H5N1_2022-01-23<br>[illegible]
EPI_ISL_309195	>A/Cygnus_olor/Belgium/2967/2017_H5N8_2017-03-21<br>[illegible]
EPI_ISL_18245811	>A/crane/Poland/MB528/2021_H5N1_2021-12-10<br>[illegible]

EPI_ISL_18245818	>A/common_tern/Poland/MB143/2022_H5N1_2022-06-15<br>ATGGATGTCAATCCGACTTTACTTTTCTTAAAAGTGCCAGCGCAAGATGCCATAAGTACCACATTCCCTTACACTGGAGATCCTCCATACAGCCATGGAACAGGGACAGGATACACAATGGACACGGTCAACAGAACACATCAATACTCAGAGAAGGGAAAATGGACAACAAACACAGAAACCGGAGCACCTCAACTCAACCCAATTGATGGGCCATTACCTGAGGACAACGA<br>ACCGAGCGGATATGCACAAACAGATTGCGTGTTGGAAGCAATGGCTTTCCTTGAAGAGTCCCATCCAGGGATCTTTGAAAACTCTTGTCTTGAAACGATGGAAGTCGTTCAGCAAACAAGAGTGGACAAACTAACTCAAGGTCGCCAGACATATGACTGGACACTGAATAGAAACCAACCAGCTGCAACTGCCTTGGCCAACACTATAGAGGTCTTAAGATCAAACGGTCTAA<br>CAGCCAATGAATCGGGGAGACTAATAGACTTCCTCAAGGATGTGATGGACTCAATGGATAAAGAAGAAATGGAAATAACAACACATTTCCAGAGAAAGAGAAGAGTAAGGGACAACATGACCAAGAAAATGGTCACACAAAGAACAATAGGAAAGAAGAAACAAAGGCTAAACAAAAGGAGCTACTTAATAAGAGCACTGACACTGAATACAATGACAAAAGATGCAGA<br>AAGAGGCAAATTGAAGAGGCGGGCGATTGCAACACCAGGGATGCAGATTAGAGGATTTGTGTACTTTGTCGAAACACTGGCAAGGAGCATCTGTGAAAAACTTGAGCAATCTGGACTCCCCGTTGGAGGGAATGAGAAGAAGGCTAAATTGGCAAATGTTGTGAGAAAAATGATGACTAACTCACAAGATACAGAGCTCTCCTTCACAATTACTGGAGATAACACCAAATG<br>GAATGAGAATCAAAATCCTCGGATGTTTCTGGCAATGATAACATACATTACAAGAAACCAACCTGAATGGTTTAGGAATGTCTTGAGTATTGCCCCTATAATGTTCTCGAACAAAATGGCGAGATTGGGAAAAGGGTACATGTTTGAAAGTAAGAGCATGAAGTTACGGACACAAATACCTGCAGAAATGCTTGCAAACATTGACTTAAAATATTTCAATGAATCAACAAGAAA<br>GAAAATCGAAAAAATAAGGCCTCTACTAATAGATGGCACTGCCTCATTGAGTCCTGGAATGATGATGGGCATGTTCAATATGCTGAGTACAGTATTAGGAGTTTCAATCCTAAATCTTGGGCAAAAGAAGTACACCAAAACCACATACTGGTGGGATGGACTCCAATCCTCTGATGATTTCGCCCTCATAGTAAATGCACCGAATCATGAGGGAATACAAGCAGGAGTGGATA<br>GGTTCTATAGGACCTGCAAACTGGTCGGGATCAATATGAGCAAAAAGAAGTCTTACATAAACCGGACTGGAACATTTGAGTTCACAAGCTTTTTCTATCGCTATGGATTTGTGGCTAATTTCAGTATGGAGCTGCCCAGCTTTGGAGTTTCTGGGATCAATGAATCAGCTGACATGAGCATTGGCATCACAGTGATAAAGAACAACATGATAAACAATGACCTTGGACCAGCAA<br>CAGCTCAAATGGCCCTTCAACTATTCATCAAAGATTACAGGTACACGTACCGATGCCATAGAGGTGACACACAAATTCAAACGAGGAGATCATTCGAGCTGAAGAAGCTGTGGGAACAGACCCGTTCAAAGGCAGGACTGTTGGTGTCAGATGGAGGACCAAATCTATACAACATTCGGAATCTCCATATCCCAGAGGTCTGCCTGAAGTGGGAGCTGATGGACGAAGATTA<br>CCAGGGCAGGTTGTGTAATCCTCTGAACCCATTTGTCAGCCATAAAGAAATTGAGTCCGTAAACAATGCTGTGGTGATGCCAGCCCACGGTCCAGCCAAAAGCATGGAATATGATGCCGTTGCGACTACACACTCATGGATTCCTAAAAGGAATCGTTCCATTCTCAATACCAGTCAAAGGGGAATTCTTGAGGATGAACAGATGTACCAGAAATGCTGCGGTCTATTCGAGAA<br>ATTCTTCCCCAGTAGTTCATACAGGAGACCAGTTGGAATTTCCAGCATGGTGGAGGCCATGGTGTCTAGGGCCCGAATCGATGCACGCATTGATTTCGAATCTGGAAGGATCAAGAAGGAAGAGTTTGCTGAGATCATGAAGATCTGTTCCACCATTGAAGAGCTCAGACGGCAAAAATAG
EPI_ISL_1005678	>A/common_teal/Chany_Lake/4/2020_H10N7_2020-08-29<br>ATGGATGTCAATCCGACTTTACTTTTCTTAAAAGTGCCAGCGCAAAATGCTATAAGCACTACGTTCCCTTACACTGGAGATCCTCCATACAGCCATGGAACAGGAACAGGATACACCATGGACACAGTCAACAGAACACATCAATACTCAGAAAAAGGAAAGTGGACAACAAACACAGAGACAGGGGCGCCCCAACTCAACCCAATTGATGGACCATTGCCAGAGGACAACGA<br>TCCAAGCGGATATGCACAAACAGATTGCGTGTTGGAGGCAATGGCTTTCCTTGAAGAATCCCACCCAGGGATCTTTGAAAACTCTTGTCTTGAAACGATGGAGGTTGTTCAGCAAACAAGAGTGGACAAACTAACCCAAGGTCGCCAGACCTATGACTGGACACTGAATAGAAACCAACCAGCTGCAACTGCTTTGGCCAATACTATAGAGGTCTTCAGATCGAACGGTCTAAC<br>AGCCAATGAGTCGGGAAGACTAATAGATTTCCTCAAGGATGTGATGGAATCAATGGATAAAGAAGAAATGGAAATAACAACACATTTCCAGAGAAAGAGAAGAGTAAGGGACAACATGACCAAAAAGATGGTCACACAAAGAACGATAGGAAAGAAGAAGCAGAGGCTGAACAAGAGAAGCTACTTAATAAGAGCACTGACATTGAACACAATGACCAAAGATGCAGAA<br>AGAGGCAAATTGAAGAGGCGGGCAATTGCAACACCCGGGATGCAGATTAGAGGATTTGTGTACTTTGTCGAAACACTAGCGAGAAGCATCTGTGAGAAACTTGAGCAATCCGGACTCCCTGTCGGAGGGAATGAGAAAAAGGCCAAATTGGCAAATGTCGTGAGGAAAATGATGACTAACTCGCAAGATACAGAGCTCTCCTTTACAATCACCGGAGACAACACCAAATGG<br>AATGAGAATCAAAATCCTCGGATGTTTTTGGCAATGATAACATACATCACAAGAAACCAACCCGAATGGTTTAGAAATGTCTTGAGCATTGCCCCTATAATGTTCTCAAACAAAATGGCAAGACTAGGGAAAGGATACATGTTTGAAAGTAAGAGCATGAAGCTAAGGACACAAATACCGGCAGAAATGCTTGCAAACATTGACTTGAAATACTTCAATGAATCAACGAGAAA<br>GAAAATCGAGAAAATAAGACCTCTGCTAATTGATGGCACAGCCTCATTGAGTCCTGGAATGATGATGGGCATGTTCAATATGCTGAGCACCGTATTGGGGGTCTCAATCCTGAATCTTGGACAAAAGAGGTACACCAAAACCACATACTGGTGGGATGGACTCCAATCTTCTGATGACTTCGCTCTCATAGTGAATGCACCGAATCATGAGGGGATACAAGCCGGGGTGGATA<br>GGTTCTATAGGACCTGCAAACTGGTTGGGATCAACATGAGCAAAAAGAAGTCTTACATAAACCGGACAGGAACATTTGAGTTCACAAGCTTTTTCTACCGCTATGGATTTGTAGCCAACTTCAGTATGGAGTTACCCAGCTTTGGAGTGTCTGGAATCAATGAGTCAGCTGACATGAGCATTGGAGTTACAGTGATAAAGAACAATATGATAAACAATGACCTTGGACCAGCAA<br>CAGCTCAAATGGCTCTTCAGCTATTCATCAAGGATTATAGGTACACGTACCGATGTCACAGAGGTGATACACAAATTCAAACGAGGAGATCATTCGAGCTGAAGAAGCTGTGGGAGCAGACCCGTTCAAAGGCAGGGCTGTTGGTATCAGATGGAGGACCAAACCTATACAACATTCGGAATCTCCACATCCCAGAGGTCTGCTTGAAGTGGGAGCTAATGGATGAAGACTA<br>CCAGGGCAGGCTGTGTAATCCTCTGAATCCGTTTGTCAGTCATAAGGAAATTGAGTCCGTAAACAATGCTGTAGTAATGCCAGCCCATGGCCCAGCCAAGAGCATGGAATACGATGCTGTTGCGACTACACACTCATGGATTCCTAAGAGGAACCGTTCCATTCTCAATACCAGCCAGAGGGGAATTCTCGAGGATGAGCAAATGTATCAGAAGTGCTGCAGCTTATTCGAGA<br>AATTCTTCCCCAGTAGTTCATACAGGAGGCCAGTTGGAATTTCCAGCATGGTGGAGGCCATGGTATCCAGGGCCCGAATTGATGCACGCATTGATTTCGAATCTGGAAGGATTAAGAAAGAAGAGTTTGCTGAGATCATGAAGATCTGTTCCACCATTGAAGAGCTCAGACGGCAAAAATAG
EPI_ISL_400267	>A/Common_Teal/Chany_Lake/29/2019_H14N3_2019-09-07<br>ATGGATGTCAATCCGACTTTACTTTTCTTAAAAGTGCCAGCGCAAAATGCTATAAGCACTACGTTCCCTTACACTGGAGATCCTCCATACAGCCATGGAACAGGAACAGGATACACCATGGACACAGTCAACAGAACACATCAATACTCAGAGAAAGGAAAGTGGACAACAAACACAGAGACCGGGGCGCCCCAACTCAACCCAATTGATGGACCATTGCCAGAGGACAACGA<br>GCCAAGCGGATATGCACAAACAGATTGCGTGTTGGAAGCAATGGCTTTCCTTGAAGAATCCCACCCAGGGATCTTTGAAAACTCTTGTCTTGAAACGATGGAGGTCGTTCAGCAAACAAGAGTGGACAAACTAACCCAAGGTCGCCAGACCTATGACTGGACACTGAATAGAAACCAACCAGCTGCAACTGCTTTGGCCAATACTATAGAGGTCTTCAGATCGAACGGTCTAA<br>CAGCCAATGAGTCGGGGAGACTAATAGATTTCCTCAAGGATGTGATGGAATCAATGGATAAAGAAGAAATGGAAATAACAACACATTTCCAGAGAAAGAGAAGAGTAAGGGACAACATGACCAAAAAGATGGTCACACAAAGAACGATAGGAAAGAAGAAGCAGAGGCTGAATAAGAGAAGCTACTTAATAAGAGCACTGACATTGAACACAATGACCAAAGATGCAGA<br>AAGAGGCAAATTGAAGAGGCGGGCAATTGCAACACCCGGAATGCAGATTAGAGGATTTGTGTACTTTGTCGAAACACTAGCGAGGAGCATCTGTGAGAAACTTGAGCAATCTGGACTCCCTGTTGGAGGGAATGAGAAAAAGGCCAAATTGGCAAATGTCGTGAGGAAAATGATGACTAACTCGCAAGATACAGAGCTCTCCTTTACAATCACCGGAGACAACACCAAATG<br>GAATGAGAATCAAAATCCTCGGATGTTTTTGGCAATGATAACATACATCACAAGAAACCAACCCGAATGGTTTAGAAATGTCTTGAGCATTGCCCCTATAATGTTCTCAAACAAAATGGCAAGATTAGGAAAAGGATACATGTTTGAAAGTAAGAGCATGAAGCTAAGGACACAAATACCGGCAGAAATGCTTGCAAACATTGACTTGAAATACTTCAATGAATCAACGAGAA<br>AGAAAATCGAGAAAATAAGACCTCTGCTAATTGATGGCACAGCCTCATTGAGTCCTGGAATGATGATGGGCATGTTCAATATGCTGAGCACTGTATTGGGGGTCTCAATCCTGAATCTTGGACAAAAGAGGTACACCAAAACCACATACTGGTGGGATGGACTCCAATCTTCTGATGACTTCGCTCTCATAGTGAATGCACCGAATCATGAGGGGATACAAGCCGGGGTGGAT<br>AGGTTCTATAGGACCTGCAAACTGGTTGGGATCAACATGAGCAAAAAGAAGTCTTACATAAACCGGACAGGAACATTTGAGTTCACAAGCTTTTTCTACCGCTATGGATTTGTAGCCAACTTCAGTATGGAGTTACCCAGCTTTGGAGTGTCTGGAATCAATGAGTCAGCTGACATGAGCATTGGAGTTACAGTGATAAAGAACAATATGATAAACAATGACCTTGGACCAGCA<br>ACAGCTCAAATGGCTCTTCAGCTATTCATCAAGGATTATAGGTACACGTACCGATGTCACAGAGGTGATACGCAAATTCAAACGAGGAGATCATTCGAGCTGAAGAAGCTGTGGGAGCAGACCCGTTCAAAGGCAGGGCTGTTGGTATCAGATGGAGGACCAAACCTATACAACATTCGGAATCTCCACATCCCAGAGGTCTGCTTGAAGTGGGAGCTAATGGATGAAGACT<br>ACCAGGGCAGGCTGTGTAATCCTCTGAATCCGTTTGTCAGTCATAAGGAAATTGAGTCCGTAAACAATGCTGTGGTAATGCCAGCCCATGGCCCAGCCAAGAGCATGGAATATGATGCTGTTGCGACTACACACTCATGGATTCCTAAGAGGAACCGTTCCATTCTCAATACCAGCCAGAGGGGAATTCTCGAGGATGAGCAGATGTACCAGAAGTGCTGCAGCTTATTCGAG<br>AAATTCTTCCCCAGTAGTTCATACAGGAGGCCAGTTGGAATTTCCAGCATGGTGGAGGCCATGGTGTCCAGGGCCCGAATTGATGCACGCATTGATTTCGAATCTGGAAGGATTAAGAAAGAAGAGTTTGCTGAGATCATGAAGATCTGTTCCACCATTGAAGAGCTCAGACGGCAAAAATAG
EPI_ISL_189732	>A/common_snipe/Republic_of_Georgia/1/2012_H13N2_2012-10-05<br>ATGGATGTCAATCCGACTTTACTTTTCTTGAAAGTGCCAGCGCAAAATGCTATAAGTACTACATTCCCTTATACTGGTGACCCTCCATACAGCCATGGAACAGGAACAGGATATACCATGGACACAGTCAACAGAACACACCAGTACTCAGAAAAAGGAACATGGACAACAAACACCGAAACTGGAGCACCTCAGCTTAACCCGATCGATGGACCACTACCGGAGGATAATGA<br>GCCAAGCGGATATGCACAAACGGATTGTGTATTGGAGGCCATGGCTTTCCTCGAAGAATCCCATCCAGGGATCTTTGAGAACTCATGTCTTGAAACGATGGAAGTTGTTCAGCAAACACGAGTGGACAAGCTGACCCAAGGCCGACAAACATATGATTGGACATTGAATAGAAACCAGCCAGCTGCAACGGCTTTAGCCAACACCATAGAGGTCTTCAGATCGAACGGTCTAA<br>CAGCTAATGAATCGGGGAGACTCATTGATTTCCTTAAGGATGTGATGGATTCAATGGATAAAGAAGGAATGGAAATAACAACACATTTCCAAAGAAAAAGAAGAGTAAGGGACAACATGACCAAGAAAATGGTCACACAAAGAACAATAGGGAAAAAGAAACAGAGATTGAGCAAAAAAAGCTACCTAATAAGGGCATTGACATTGAATACAATGACCAAAGATGCAGAA<br>AGAGGTAAATTGAAAAGGAGAGCGATTGCAACACCCGGGATGCAGATCAGAGGTTTCGTCTACTTCGTTGAAACACTTGCAAGGAGCATATGTGAGAAACTTGAACAGTCAGGGCTCCCAGTTGGAGGAAATGAGAAAAAGGCTAAATTGGCAAACGTTGTAAGAAAGATGATGACTAATTCGCAAGACACAGAGCTCTCCTTCACAATTACTGGGGACAACACCAAGTGG<br>AATGAAAATCAAAACCCTCGGATGTTCCTAGCGATGATAACATACATCACAAGAAACCAGCCTGAATGGTTCAGAAATGTCTTGAGCATTGCCCCTATAATGTTCTCAAACAAAATGGCAAGACTTGGGAAAGGATATATGTTCGAAAGTAAGAGCATGAAGCTACGAACACAAATACCAGCAGAAATGCTTGCAAGTATTGACCTCAAATATTTCAACGAGTCAACGAGAAA<br>GAAAATCGAGAACATAAGACCCCTCTTAATAGATGGCACAGCCTCATTGAGTCCTGGAATGATGATGGGCATGTTCAACATGTTGAGTACAGTCTTAGGAGTCTCAATCTTAAATCTTGGGCAAAAGAGGTACACCAAGACCACATACTGGTGGGATGGACTCCAATCCTCTGATGACTTTGCACTCATAGTGAACGCACCTAATCATGAGGGAATACAAGCAGGAGTAGACA<br>GATTTTACAGAACCTGCAAATTAGTTGGGATCAACATGAGTAAAAAGAAGTCCTACATCAATAGAACAGGAACATTTGAATTCACAAGCTTTTTCTATCGCTATGGGTTTGTAGCTAACTTCAGTATGGAGCTACCAAGCTTTGGCGTATCAGGGATTAATGAGTCAGCTGACATGAGCATTGGAGTAACAGTGATAAAGAACAACATGATAAACAACGACCTTGGACCAGCAA<br>CAGCCCAAATGGCTCTTCAACTGTTCATCAAGGACTACAGGTACACATATCGATGCCATAGAGGTGATACTCAAATCCAAACAAGGAGATCATTTGAGTTGAAGAAGCTTTGGGAGCAAACCCGTTCAAAGGCGGGGCTGCTGGTTTCAGATGGAGGACCAAACCTATATAATATCAGAAATCTCCATATTCCAGAGGTCTGTCTGAAATGGGAGCTAATGGATGAAGATTAT<br>CAGGGCAGGCTGTGCAACCCTCTGAATCCATTCGTAAGCCATAAGGAAATTGAGTCCGTAAATAATGCTGTGGTAATGCCAGCCCATGGTCCAGCCAAAAGTATGGAATATGATGCTGTTGCAACTACACATTCCTGGGTCCCCAAGAGGAACCGTTCCATTCTCAACACTAGCCAAAGGGGAATCCTTGAGGATGAACAGATGTATCAAAAATGCTGCAATCTATTCGAAAAA<br>TTCTTCCCTAGCAGTTCGTACAGGAGACCAGTTGGAATTTCCAGCATGGTGGAGGCCATGGTATCCAGGGCCCGAATTGACGCACGGATTGACTTCGAATCTGGAAGGATCAAGAAAGAAGAGTTTGCTGAGATCATGAAGATCTGTTCCACCATTGAAGAGCTCAGACGGCAAAAGTAG
EPI_ISL_4653177	>A/common_pheasant_/Sweden/SVA210923SZ0341/KN000366/M-2021_H5N1_2021-09-22<br>ATGGATGTCAATCCGACTTTACTTTTCTTAAAAGTGCCAGCGCAAAATGCCATAAGTACCACATTCCCTTATACTGGAGATCCTCCATACAGCCATGGAACAGGGACAGGATACACAATGGACACAGTCAACAGAACACATCAATACTCAGAGAAGGGAAAATGGACAACAAACACAGAAACCGGAGCACCTCAACTCAACCCAATTGATGGGCCACTACCTGAGGACAACGA<br>ACCGAGCGGATATGCACAGACAGATTGCGTGTTGGAAGCAATGGCTTTCCTTGAAGAGTCCCACCCAGGGATCTTTGGAAACTCTTGTCTTGAAACGATGGAAGTCGTTCAGCAAACAAGAGTGGACAAACTAACTCAAGGTCGCCAGACTTATGACTGGACACTGAATAGAAACCAACCAGCTGCAACTGCCCTGGCCAACACTATAGAGGTCTTCAGATCAAACGGTCTAA<br>CAGCCAATGAATCGGGGAGACTAATAGATTTCCTCAAGGATGTGATGGACTCAATGGATAAAGAAGAAATGGAAATAACAACACATTTCCAGAGAAAGAGAAGAGTAAGGGACAACATGACCAAGAAAATGGTCACACAAAGAACAATAGGAAAGAAGAAACAAAGGCTAAACAGGAGGAGCTACTTAATAAGAGCACTGACACTGAATACAATGACAAAAGATGCAGA<br>AAGAGGCAAATTGAAGAGACGGGCGATTGCAACACCAGGGATGCAGATTAGAGGATTTGTGTACTTTGTCGAAACACTGGCAAGGAGCATCTGTGAAAAACTTGAGCAATCTGGACTCCCCGTTGGAGGAAATGAGAAGAAGGCTAAATTGGCAAATGTCGTGAGGAAAATGATGACTAACTCACAAGATACAGAGCTCTCCTTCACAATTACTGGAGATAACACCAAATG<br>GAATGAGAATCAAAATCCTCGGATGTTTCTGGCAATGATAACGTACATTACAAGAAACCAACCTGAGTGGTTTAGAAATGTCTTGAGTATTGCCCCTATAATGTTCTCGAACAAAATGGCGAGATTGGGAAAAGGGTACATGTTTGAAAGTAAGAGCATGAAGTTACGGACACAAATACCTGCAGAAATACTTGCAAACATTGACTTAAAATACTTCAATGAATCAACAAGAAA<br>GAAAATCGAAAAAATAAGGCCTCTACTAATAGATGGCACTGCCTCATTGAGTCCTGGAATGATGATGGGCATGTTCAATATGCTGAGTACAGTATTAGGAGTTTCAATCCTAAATCTTGGGCAAAAGAGGTACACCAAAACCACATACTGGTGGGATGGACTCCAATCCTCTGATGATTTCGCCCTCATAGTAAATGCACCGAATCATGAGGGAATACAAGCAGGAGTGGATA<br>GGTTCTATAGGACCTGCAAACTGGTCGGGATCAATATGAGCAAAAAGAAGTCTTACATAAACCGGACTGGAACATTTGAGTTCACAAGCTTTTTCTATCGCTATGGATTTGTGGCTAACTTCAGTATGGAGCTGCCCAGCTTTGGAGTTTCTGGAATCAATGAATCAGCTGACATGAGCATTGGCGTCACAGTGATAAAGAACAACATGATAAACAATGACCTTGGACCAGCAA<br>CAGCTCAAATGGCCCTTCAACTATTCATCAAAGATTACAGGTACACGTACCGATGCCACAGAGGTGACACACAAATTCAAACGAGGAGATCATTCGAGCTGAAGAAGCTGTGGGAACAGACCCGTTCAAAGGCAGGACTGTTGGTGTCAGATGGAGGACCAAATCTATACAACATTCGGAATCTCCATATCCCAGAGGTCTGCCTGAAGTGGGAGCTGATGGACGAAGATTA<br>CCAGGGCAGGTTGTGTAATCCTCTGAACCCATTTGTCAGTCATAAAGAAATTGAGTCCGTAAACAATGCTGTGGTGATGCCAGCCCACGGTCCAGCCAAAAGCATGGAATATGATGCCGTTGCAACTACACACTCATGGATTCCTAAAAGGAATCGTTCCATTCTCAATACCAGCCAAAGGGGAATTCTTGAGGATGAACAGATGTACCAGAAATGCTGCAGTCTATTCGAGAA<br>ATTCTTCCCCAGTAGTTCATACAGGAGACCAGTTGGAATTTCCAGCATGGTGGAGGCCATGGTGTCTAGGGCCCGAATCGATGCACGCATTGATTTCGAATCTGGAAGGATCAAGAAGGGAGAGTTTGCTGAGATCATGAAGATCTGTTCCACCATTGAAGAGCTCAGACGGCAAAAATAG
EPI_ISL_14917968	>A/common_murre/Poland/MB151/2022_H5N1_2022-07-13<br>ATGGATGTCAATCCGACTTTACTTTTCTTAAAAGTGCCAGCGCAAGATGCCATAAGTACCACATTCCCTTACACTGGAGATCCTCCATACAGCCATGGAACAGGGACAGGATACACAATGGACACGGTCAACAGAACACATCAATACTCAGAGAAGGGAAAATGGACAACAAACACAGAAACCGGAGCACCTCAACTCAACCCAATTGATGGGCCACTACCTGAGGACAACGA<br>ACCGAGCGGATATGCACAAACAGATTGCGTGTTGGAAGCAATGGCTTTCCTTGAAGAGTCCCATCCAGGGATCTTTGAAAACTCTTGTCTTGAAACGATAGAAGTCGTTCAGCAAACAAGAGTGGACAAACTAACTCAAGGTCGCCAGACATATGACTGGACACTGAATAGAAACCAACCAGCTGCAACTGCCTTGGCCAACACTATAGAGGTCTTAAGATCAAACGGTCTAA<br>CAGCCAATGAATCGGGGAGACTAATAGATTTCCTCAAGGATGTGATGGACTCAATGGATAAAGAAGAAATGGAAATAACAACACATTTCCAGAGAAAGAGAAGGGTAAGGGACAACATGACCAAGAAAATGGTCACACAAAGAACAATAGGAAAGAAGAAACAAAGGCTAAACAAAAGGAGCTACTTAATAAGAGCACTGACACTGAATACAATGACAAAAGATGCAGA<br>AAGAGGCAAATTGAAGAGACGGGCGATTGCAACACCAGGGATGCAGATTAGAGGATTTGTGTACTTTGTCGAAACACTGGCAAGGAGCATCTGTGAAAAACTTGAGCAATCTGGACTCCCCGTTGGAGGGAATGAGAAGAAGGCTAAATTGGCAAATGTTGTGAGAAAAATGATGACTAACTCACAAGATACAGAGCTCTCCTTCACAATTACTGGAGATAACACCAAATG<br>GAATGAGAATCAAAATCCTCGGATGTTTCTGGCAATGATAACATACATTACAAGAAACCAACCTGAATGGTTTAGGAATGTCTTGAGTATTGCCCCTATAATGTTCTCGAACAAAATGGCGAGATTGGGAAAAGGGTACATGTTTGAAAGTAAGAGCATGAAGTTACGGACACAAATACCTGCAGAAATGCTTGCAAACATTGACTTAAAATATTTCAATGAATCAACAAGAAA<br>GAAAATCGAAAAAATAAGGCCTCTACTAATAGATGGCACTGCCTCATTGAGTCCTGGAATGATGATGGGCATGTTCAATATGCTGAGTACAGTATTAGGAGTTTCAATCCTAAATCTTGGGCAAAAGAAGTACACCAAAACCACATACTGGTGGGATGGACTCCAATCCTCTGATGATTTCGCCCTCATAGTAAATGCACCGAATCATGAGGGAATACAAGCAGGAGTGGATA<br>GGTTCTATAGGACCTGCAAACTGGTCGGGATCAATATGAGCAAAAAGAAGTCTTACATAAACCGGACTGGAACATTTGAGTTCACAAGCTTTTTCTATCGCTATGGATTTGTGGCTAATTTCAGTATGGAGCTGCCCAGCTTTGGAGTTTCTGGGATCAATGAATCAGCTGACATGAGCATTGGCATCACAGTGATAAAGAACAACATGATAAACAATGACCTTGGACCAGCAA<br>CAGCTCAAATGGCCCTTCAACTATTCATCAAAGATTACAGGTACACGTACCGATGCCATAGAGGTGACACACAAATTCAAACGAGGAGATCATTCGAGCTGAAGAAGCTGTGGGAACAGACCCGTTCAAAGGCAGGACTGTTGGTGTCAGATGGAGGACCAAATCTATACAACATTCGGAATCTCCACATCCCAGAGGTCTGCCTGAAGTGGGAGCTGATGGACGAAGATTA<br>CCAGGGCAGGTTGTGTAATCCTCTGAACCCATTTGTCAGCCATAAAGAAATTGAGTCCGTAAACAATGCTGTGGTGATGCCAGCCCACGGTCCAGCCAAAAGCATGGAATATGATGCCGTTGCGACTACACACTCATGGATTCCTAAAAGGAATCGTTCCATCCTCAATACCAGTCAAAGGGGAATTCTTGAGGATGAACAGATGTACCAGAAATGCTGCGGTCTATTCGAGAA<br>ATTCTTCCCCAGTAGTTCATACAGGAGACCAGTTGGAATTTCCAGCATGGTGGAGGCCATGGTGTCTAGGGCCCGAATCGATGCACGCATTGATTTCGAATCTGGAAGGATCAAGAAGGAAGAGTTTGCTGAGATCATGAAGATCTGTTCCACCATTGAAGAGCTCAGACGGCAAAAATAG
EPI_ISL_1063993	>A/common_kestrel/Denmark/16023-01/2021-01-01_H5N3_2021-01-01<br>ATGGATGTCAATCCGACTTTACTTTTCTTAAAAGTGCCAGCGCAAAATGCCATAAGTACCACATTCCCTTATACTGGAGATCCTCCATACAGCCATGGAACAGGAACAGGATACACCATGGACACAGTCAACAGAACACATCAATACTCGGAAAAGGGGAAATGGACAACAAACACAGAGACCGGAGCACCCCAACTCAACCCAATTGATGGACCACTACCTGAGGACAACGA<br>ACCAAGTGGATATGCACAAACAGATTGCGTGTTGGAAGCAATGGCTTTTCTTGAAGAGTCCCACCCAGGGATCTTTGAAAACTCTTGTCTTGAAACGATGGAAGTCGTTCAGCAAACAAGAATGGACAAACTAACTCAAGGTCGCCAGACTTATGACTGGACACTGAATAGAAACCAACCAGCTGCAACTGCCCTGGCCAACACTATAGAGGTCTTCAGATCGAATGGTCTGAC<br>AGCCAATGAATCAGGGAGACTAATAGATTTCCTCAAGGATGTGATGGACTCAATGGACAAAGAAGAAATGGAAATAACAACACATTTCCAGAGAAAGAGAAGAGTAAGGGACAACATGACCAAGAAAATGGTCACACAAAGAACAATAGGGAAGAAGAAGCAAAGGCTAAATAAGAGGAGCTACTTAATAAGAGCACTGACACTGAATACAATGACAAAAGACGCAGAA<br>AGAGGCAAATTGAAGAGGCGGGCAATTGCAACACCAGGGATGCAGATTAGAGGATTTGTGTACTTTGTTGAAACACTGGCAAGGAGCATCTGTGAAAAACTTGAGCAATCTGGACTCCCCGTTGGAGGGAATGAAAAGAAGGCTAAATTGGCAAATGTCGTGAGAAAAATGATGACTAACTCACAAGATACAGAGCTCTCCTTCACAATTACTGGAGATAACACCAAATGG<br>AATGAGAATCAAAATCCTCGGATGTTTCTGGCAATGATAACATACATCACAAGAAACCAACCTGAATGGTTTAGAAATGTCTTGAGCATTGCCCCCATAATGTTCTCGAACAAAATGGCGAGATTGGGGAAAGGATACATGTTTGAAAGTAAAAGTATGAAGCTACGAACACAAATACCTGCAGAAATGCTTGCAAACATTGACTTAAAATACTTCAATGAGTCAACAAGAAAG<br>AAAATCGAGAAAATAAGACCTCTACTAATAGATGGCACCGCCTCATTGAGTCCTGGAATGATGATGGGCATGTTCAATATGCTGAGTACAGTATTAGGAGTCTCAATCCTGAATCTTGGGCAAAAGAGGTACACCAAAACCACATACTGGTGGGATGGACTCCAATCCTCTGATGATTTCGCTCTCATAGTAAATGCACCGAATCATGAGGGAATACAAGCAGGAGTGGATAG<br>GTTCTATAGGACTTGCAAACTGGTTGGGATCAACATGAGCAAAAAGAAGTCTTACATAAACCGGACAGGAACATTTGAGTTCACAAGCTTTTTCTATCGCTATGGATTTGTGGCCAACTTCAGTATGGAGCTGCCTAGCTTTGGAGTGTCTGGAATCAATGAATCAGCTGACATGAGCATTGGAGTTACAGTGATAAAGAACAACATGATAAACAATGACCTTGGACCAGCAAC<br>AGCTCAAATGGCCCTTCAACTATTCATCAAGGACTACAGGTACACGTACCGATGTCACAGGGGTGACACACAAATTCAAACGAGGAGATCATTCGAGCTGAAGAAGCTGTGGGAGCAGACCCGTTCAAAGGCAGGACTGTTGGTGTCAGATGGAGGACCAAATCTATACAACATCCGGAATCTCCATATCCCAGAAGTCTGCCTGAAGTGGGAGCTGATGGACGAAGATTAC<br>CAGGGCAGGTTGTGCAATCCTCTGAACCCATTTGTCAGTCATAAAGAAATTGAATCCGTAAACAATGCTGTGGTAATGCCAGCCCATGGCCCAGCCAAGAGCATGGAATATGATGCTGTTGCGACTACACACTCATGGATTCCTAAAAGGAATCGTTCCATTCTCAATACCAGCCAAAGGGGAATTCTTGAGGATGAGCAGATGTACCAGAAATGCTGCAGTCTATTCGAAAAA<br>TTCTTCCCCAGTAGTTCATACAGGAGACCAGTTGGAATTTCCAGCATGGTGGAGGCCATGGTGTCTAGGGCCCGAATCGACGCACGCATTGATTTCGAATCTGGAAGGATTAAGAAGGAAGAGTTTGCTGAGATCATGAAGATCTGTTCCACCATTGAAGAGCTCAGACGGCAAAAATAG
EPI_ISL_1080579	>A/common_coot/Czech_Republic/3160-2/2021_H5N8_2021-02-14<br>ATGGATGTCAATCCGACTTTACTTTTCTTAAAAGTGCCAGCGCAAAATGCTATAAGTACTACATTCCCTTACACTGGAGATCCTCCATACAGCCATGGAACAGGAACAGGGTATACCATGGACACAGTAAACAGAACACATCAATACTCAGAAAAGGGAAAGTGGACAACAAACACAGAAACCGGAGCACCCCAACTCAACCCAATTGATGGACCATTACCAGAGGACAATGA<br>GCCAAGCGGATATGCACAAACTGATTGCGTGTTGGAAGCAATGGCTTTCCTTGAAGAATCCCACCCAGGGATATTTGAAAACTCTTGTCTTGAAGCGATGGAAATCGTTCAGCAAACAAGAGTGGACAGACTAACCCAAGGTCGCCAGACTTATGACTGGACACTGAACAGAAACCAACCAGCTGCAACCTCTTTGGCCAACACTATAGAGGTGTTCAGATCGAATGGTCTGA<br>CAGCCAATGAATCAGGAAGACTGATAGATTTTCTCAGGGATGTGATGGAATCAATGGATAAAGAAGAGATGGAAGTAACAACACACTTCCAGAGAAAAAGAAGAGTGAGGGACAACATGACTAAGAAGATGGTCACACAAAGAACAATAGGGAAGAAGAAGCAGAGGCTGAACAAGAGGAGTTACTTAATAAGAGCACTGACATTGAATACAATGACCAAAGATGCAGA<br>AAGAGGCAAGTTGAAGAGACGGGCAATTGCAACACCCGGGATGCAGATTAGAGGATTCGTGTACTTCGTTGAAACACTAGCAAGGAGCATCTGTGAGAAACTAGAGCAATCTGGGCTCCCTGTTGGAGGGAATGAGAAGAAGGCTAAATTGGCAAATGTTGTGAGAAAAATGATGACTAACTCACAAGATACAGAGCTCTCCTTTACAATTACTGGAGACAACACCAAATG<br>GAATGAGAATCAAAACCCTCGGATGTTTTTGGCAATGGTAACATATATCACAAGAAACCAACCTGAATGGTTTAGAAATGTCTTAAGCATTGCCCCTATAATGTTCTCAAACAAAATGGCGAGATTAGGGAAAGGATACATGTTTGAAAGTAAGAGCATGAAGCTAAGAACACAAATACCAGCAGAGATGCTCACAAATATTGATCTGAAGTATTTCAACGAACCAACGAGAA<br>AGAAAATCGAGAAAATAAGACCTCTGCTGATTGATGGCACGGCCTCATTGAGTCCTGGGATGATGATGGGCATGTTCAATATGCTGAGCACAGTATTGGGGGTCTCAATCCTGAATCTCGGGCAAAAGAGGTACACCAAAACCACATACTGGTGGGATGGACTTCAATCCTCTGATGATTTTGCTCTCATAGTGAATGCACCGAATCATGAGGGGATACAAGCAGGAGTGGAT<br>AGATTCTATAGGACCTGCAAACTGGTTGGGATCAACATGAGCAAAAAGAAGTCTTACATAAACCGAACAGGAACATTTGAGTTCACAAGTTTTTTCTACCGCTATGGATTTGTAGCTAACTTCAGTATGGAATTACCCAGCTTTGGAGTGTCTGGAATCAATGAATCAGCTGACATGAGCATTGGAGTTACAGTGATAAAAAACAATATGATAAACAATGATCTTGGACCAGCA<br>ACAGCTCAAATGGCTCTTCAGTTATTCATCAAAGACTATAGGTACACATACCGATGCCACAGGGGTGATACACAAATTCAAACGAGGAGATCATTCGAGCTGAAGAAGCTGTGGGAGCAGACCCGTTCAAAGGCAGGGCTGTTGATATCAGACGGGGGGCCAAACCTATACAACATTCGGAACCTCCACATCCCAGAGGTCTGCTTGAAGTGGGAGCTGATGGATGAAGACT<br>ACCAAGGCAGGCTGTGCAATCCTCTGAATCCATTTGTCAGTCATAAAGAGATTGAGTCCGTAAACAATGCTGTAGTAATGCCCGCCCATGGCCCGGCCAAGAGCATGGAATATGATGCTGTTGCGACCACACATTCGTGGATTCCAAAGAGGAACCGCTCCATTCTCAATACCAGCCAAAGGGGAATTCTTGAGGATGAGCAGATGTACCAAAAGTGCTGTAGTCTATTCGAG<br>AAATTCTTCCCCAGCAGTTCATACAGGAGGCCAGTTGGAATTTCCAGCATGGTGGAGGCCATGGTGTCTAGGGCCCGAATTGATGCACGCATCGACTTCGAATCTGGAAGGATTAAGAAAGAAGAGTTTGCTGAGATCATGAAGATCTGTTCCACCATTGAAGAGCTCAGACGGCAAAAATAG
EPI_ISL_7267244	>A/common_buzzard/Netherlands/21038793-001/2021_H5N1_2021-11-12<br>ATGGATGTCAATCCGACTTTACTTTTCTTAAAAGTGCCAGCGCAAAATGCCATAAGTACCACATTCCCTTATACTGGAGATCCTCCATACAGCCATGGAACAGGGACAGGATACACAATGGACACAGTCAACAGAACACATCAATACTCAGAGAAGGGAAAATGGACAACAAACACAGAAACCGGAGCACCTCAACTCAACCCAATTGATGGGCCACTACCTGAGGACAACGA<br>ACCGAGCGGATATGCACAGACAGATTGCGTGTTGGAAGCAATGGCTTTCCTTGAAGAGTCCCACCCAGGGATCTTTGGAAACTCTTGTCTTGAAACGATGGAAGTCGTTCAGCAAACAAGAGTGGACAAACTAACTCAAGGTCGCCAGACTTATGACTGGACACTGAATAGAAACCAACCAGCTGCAACTGCCCTGGCCAACACTATGGAGGTCTTCAGATCAAACGGTCTAA<br>CAGCCAATGAATCGGGGAGACTAATAGATTTCCTCAAGGATGTGATGGACTCAATGGATAAAGAAGAAATGGAAATAACAACACATTTCCAGAGAAAGAGAAGAGTAAGGGACAACATGACCAAGAAAATGGTCACACAAAGAACAATAGGAAAGAAGAAACAAAGGCTAAACAGGAGGAGCTACTTAATAAGAGCACTGACACTGAATACAATGACAAAAGATGCAGA<br>AAGAGGCAAATTGAAGAGACGGGCGATTGCAACACCAGGGATGCAGATTAGAGGATTTGTGTACTTTGTCGAAACACTGGCAAGGAGCATCTGTGAAAAACTTGAGCAATCTGGACTCCCCGTTGGAGGAAATGAGAAGAAGGCTAAATTGGCAAATGTCGTGAGGAAAATGATGACTAACTCACAAGATACAGAGCTCTCCTTCACAATTACTGGAGATAACACCAAATG<br>GAATGAGAATCAGAATCCTCGGATGTTTCTGGCAATGATAACGTACATTACAAGAAACCAACCTGAATGGTTTAGAAATGTCTTGAGTATTGCCCCTATAATGTTCTCGAACAAAATGGCGAGATTGGGAAAAGGGTACATGTTTGAAAGTAAGAGCATGAAGTTACGGACACAAATACCTGCAGAAATACTTGCAAACATTGACTTAAAATACTTCAATGAATCAACAAGAAA<br>GAAAATCGAAAAAATAAGGCCTCTACTAATAGATGGCACTGCCTCATTGAGTCCTGGAATGATGATGGGCATGTTCAATATGCTGAGTACAGTATTAGGAGTTTCAATCCTAAATCTTGGGCAAAAGAGGTACACCAAAACCACATACTGGTGGGATGGACTCCAATCCTCTGATGATTTCGCCCTCATAGTAAATGCACCGAATCATGAGGGAATACAAGCAGGAGTGGATA<br>GGTTCTATAGGACCTGCAAACTGGTCGGGATCAATATGAGCAAAAAGAAGTCTTACATAAACCGGACTGGAACATTTGAGTTCACAAGCTTTTTCTATCGCTATGGATTTGTGGCTAACTTCAGTATGGAGCTGCCCAGCTTTGGAGTTTCTGGAATCAATGAATCAGCTGACATGAGCATTGGCGTCACAGTGATAAAGAACAACATGATAAACAATGACCTTGGACCAGCAA<br>CAGCTCAAATGGCCCTTCAACTATTCATCAAAGATTACAGGTACACGTACCGATGCCACAGAGGTGACACACAAATTCAAACGAGGAGATCATTCGAGCTGAAGAAGCTGTGGGAACAGACCCGTTCAAAGGCAGGACTGTTGGTGTCAGATGGAGGACCAAATCTATACAACATTCGGAATCTCCATATCCCAGAGGTCTGCCTGAAGTGGGAGCTGATGGACGAAGATTA<br>CCAGGGCAGGTTGTGTAATCCTCTGAACCCATTTGTCAGTCATAAAGAAATTGAGTCCGTAAACAATGCTGTGGTGATGCCAGCCCACGGTCCAGCCAAAAGCATGGAATATGATGCCGTTGCAACTACACACTCATGGATTCCTAAAAGGAATCGTTCCATTCTCAATACCAGCCAAAGGGGAATTCTTGAGGATGAACAGATGTACCAGAAATGCTGCAGTCTATTCGAGAA<br>ATTCTTCCCCAGTAGTTCATACAGGAGACCAGTTGGAATTTCCAGCATGGTGGAGGCCATGGTGTCTAGGGCCCGAATCGATGCACGCATTGATTTCGAATCTGGAAGGATCAAGAAGGGAGAGTTTGCTGAGATCATGAAGATCTGTTCCACCATTGAAGAGCTCAGACGGCAAAAATAG
EPI_ISL_2172526	>A/common_buzzard/Netherlands/21023939-001/2021_H5N8_2021-02-21<br>ATGGATGTCAATCCGACTTTACTTTTCTTAAAAGTGCCAGCGCAAAATGCTATAAGTACTACATTCCCTTACACTGGAGATCCTCCATACAGCCACGGAACAGGAACAGGGTATACCATGGACACAGTAAACAGAACACATCAATACTCAGAAAAGGGAAAGTGGACAACAAACACAGAAACCGGAGCACCCCAACTCAACCCAATTGATGGACCATTACCAGAGGACAATGA<br>GCCAAGCGGATATGCACAAACTGATTGCGTGTTGGAAGCAATGGCTTTCCTTGAAGAATCCCACCCAGGGATATTTGAAAACTCTTGTCTTGAAGCGATGGAAATCGTTCAGCAAACAAGAGTGGACAAACTAACCCAAGGTCGCCAGACTTATGACTGGACACTGAACAGAAACCAACCAGCTGCAACCTCTTTGGCCAACACTATAGAGGTGTTCAGATCGAATGGTCTGA<br>CAGCCAATGAATCAGGAAGACTGATAGATTTTCTCAGGGATGTGATGGAATCAATGGATAAAGAAGAGATGGAAGTAACAACACATTTCCAGAGAAAAAGAAGAGTGAGGGACAACATGACTAAGAAGATGGTCACACAAAGAACAATAGGGAAGAAGAAGCAGAGGCTGAACAAGAGGAGTTACTTAATAAGAGCACTGACATTGAATACAATGACCAAAGATGCAGA<br>AAGAGGCAAGTTGAAGAGACGGGCAATTGCAACACCCGGGATGCAGATTAGAGGATTCGTGTACTTCGTTGAAACACTAGCAAGGAGCATCTGTGAGAAACTAGAGCAATCTGGGCTCCCTGTTGGAGGGAATGAGAAGAAGGCTAAATTGGCAAATGTTGTGAGAAAAATGATGACTAACTCACAAGATACAGAGCTCTCCTTTACAATTACTGGAGACAACACCAAATG<br>GAATGAGAATCAAAACCCTCGGATGTTTTTGGCAATGGTAACATATATCACAAGAAACCAACCTGAATGGTTTAGAAATGTCTTAAGCATTGCCCCTATAATGTTCTCAAACAAAATGGCGAGATTAGGGAAAGGATACATGTTTGAAAGTAAGAGCATGAAGCTAAGAACACAAATACCAGCAGAGATGCTCACAAATATTGATCTGAAGTATTTCAACGAACCAACGAGAA<br>AGAAAATCGAGAAAATAAGACCTCTGCTGATTGATGGCACGGCCTCATTGAGTCCTGGGATGATGATGGGCATGTTCAATATGCTGAGCACAGTATTGGGGGTCTCAATCCTGAATCTCGGGCAAAAGAGGTACACCAAAACCACATACTGGTGGGATGGACTTCAATCCTCTGATGATTTCGCTCTCATAGTGAATGCACCGAATCATGAGGGGATACAAGCAGGAGTGGAT<br>AGATTCTATAGGACCTGCAAACTGGTTGGGATCAACATGAGCAAAAAGAAGTCTTACATAAACCGAACAGGAACATTTGAGTTCACAAGTTTTTTCTACCGCTATGGATTTGTAGCTAACTTCAGTATGGAATTACCCAGCTTTGGAGTGTCTGGAATCAATGAATCAGCTGACATGAGCATTGGAGTTACAGTGATAAAAAACAATATGATAAACAATGATCTTGGACCAGCA<br>ACAGCTCAAATGGCTCTTCAGTTATTCATCAAAGACTATAGGTACACATACCGATGCCACAGGGGTGATACACAAATTCAAACGAGGAGATCATTCGAGCTGAAGAAGCTGTGGGAGCAGACCCGTTCAAAGGCAGGGCTGTTGATATCAGACGGGGGGCCAAACCTATACAACATTCGGAACCTCCACATCCCAGAGGTCTGCTTGAAGTGGGAGCTGATGGATGAAGACT<br>ACCAAGGCAGGCTGTGCAATCCTCTGAATCCATTTGTCAGTCATAAAGAGATTGAGTCCGTAAACAATGCTGTAGTAATGCCCGCCCATGGCCCGGCCAAGAGCATGGAATATGATGCTGTTGCGACCACACATTCGTGGATTCCAAAGAGGAACCGCTCCATTCTCAATACCAGCCAAAGGGGAATTCTTGAGGATGAGCAGATGTACCAAAAGTGCTGTAGTCTATTTGAGA<br>AATTCTTCCCCAGCAGTTCATACAGGAGGCCAGTTGGAATTTCCAGCATGGTGGAGGCCATGGTGTCTAGGGCCCGAATTGATGCACGCATCGACTTCGAATCTGGAAGGATTAAGAAAGAAGAGTTTGCTGAGATCATGAAGATCTGTTCCACCATTGAAGAGCTCAGACGGCAAAAATAG
EPI_ISL_2175628	>A/common_buzzard/Netherlands/21021187-001/2021_H5N8_2021-01-04<br>ATGGATGTCAATCCGACTTTACTTTTCTTAAAAGTGCCAGCGCAAAATGCTATAAGTACTACATTCCCTTACACTGGAGATCCCCCATACAGCCATGGAACAGGAACAGGGTATACCATGGACACAGTAAACAGAACACATCAATACTCAGAAAAGGGAAAGTGGACAACAAACACAGAAACCGGAGCACCCCAACTCAACCCAATTGATGGACCATTACCAGAGGACAATGA<br>GCCAAGCGGATATGCACAAACTGATTGCGTGTTGGAAGCAATGGCTTTCCTTGAAGAATCCCACCCAGGGATATTTGAAAACTCTTGTCTTGAAGCGATGGAAATCGTTCAGCAAACAAGAGTGGACAAACTAACCCAAGGTCGCCAGACTTATGACTGGACACTGAACAGAAACCAACCAGCTGCAACCTCTTTGGCCAACACTATAGAGGTGTTCAGATCGAATGGTCTGA<br>CAGCCAATGAATCAGGGAGACTGATAGATTTTCTCAGGGATGTGATGGAATCAATGGATAAAGAAGAGATGGAAGTAACAACACATTTCCAGAGAAAAAGAAGAGTGAGGGACAACATGACTAAGAAGATGGTCACACAAAGAACAATAGGGAAGAAGAAGCAGAGGCTGAACAAGAGGAGTTACTTAATAAGAGCACTGACATTGAATACAATGACCAAAGATGCAGA<br>AAGAGGCAAGTTGAAGAGACGGGCAATTGCAACACCCGGGATGCAGATTAGAGGATTCGTGTACTTAGTTGAAACACTAGCGAGGAGCATCTGTGAGAAACTAGAGCAATCTGGGCTCCCTGTTGGAGGGAATGAGAAGAAGGCTAAATTGGCAAATGTTGTGAGAAAAATGATGACTAACTCACAAGATACAGAGCTCTCCTTTACAATTACTGGAGACAACACCAAATG<br>GAATGAGAATCAAAACCCTCGGATGTTTTTGGCAATGATAACATATATCACAAGAAACCAACCTGAATGGTTTAGAAATGTCTTAAGCATTGCCCCTATAATGTTCTCAAACAAAATGGCGAGATTAGGGAAAGGATACATGTTTGAAAGTAAGAGCATGAAGCTAAGAACACAAATACCAGCAGAGATGCTTACAAATATTGATCTGAAGTATTTCAACGAACCAACGAGAA<br>AGAAAATCGAGAAAATAAGACCTCTGCTGATTGATGGCACGGCCTCATTGAGTCCTGGGATGATGATGGGCATGTTCAATATGCTGAGCACAGTATTAGGGGTCTCAATCCTGAATCTCGGGCAAAAGAGGTACACCAAAACCACATACTGGTGGGATGGACTTCAATCCTCTGATGATTTCGCTCTCATAGTGAATGCACCGAATCATGAGGGGATACAAGCAGGAGTGGAT<br>AGATTCTATAGGACCTGCAAACTGGTTGGGATCAACATGAGCAAAAAGAAGTCTTACATAAACCGAACAGGAACATTTGAGTTCACAAGTTTTTTCTACCGCTATGGATTTGTAGCCAACTTCAGTATGGAATTACCCAGCTTTGGAGTGTCTGGAATCAATGAATCAGCTGACATGAGCATTGGAGTTACAGTGATAAAAAACAATATGATAAACAATGATCTTGGACCAGCA<br>ACAGCTCAAATGGCTCTTCAGTTATTCATCAAAGACTATAGGTACACATACCGATGCCACAGGGGTGATACACAAATTCAAACGAGGAGATCATTCGAGCTGAAGAAGCTGTGGGAGCAGACCCGTTCAAAGGCAGGGCTGTTGATATCAGACGGGGGGCCAAATCTATACAACATTCGGAATCTCCACATCCCAGAGGTCTGCTTGAAGTGGGAGCTGATGGATGAAGACT<br>ACCAAGGCAGGCTGTGCAATCCTCTGAATCCATTTGTCAGTCATAAAGAAATTGAGTCCGTAAACAATGCTGTAGTAATGCCCGCCCATGGCCCGGCCAAGAGCATGGAATATGATGCTGTTGCGACCACACACTCGTGGATTCCTAAGAGGAACCGTTCCATTCTCAATACCAGCCAAAGGGGAATTCTTGAGGACGAGCAGATGTACCAAAAGTGCTGTAGTCTATTCGAGA<br>AATTCTTCCCCAGCAGTTCATACAGGAGGCCAGTTGGAATTTCCAGCATGGTGGAGGCCATGGTGTCTAGGGCCCGAATTGATGCACGCATCGACTTCGAATCTGGAAGGATTAAGAAAGAAGAGTTTGCTGAGATCATGAAGATCTGTTCCACCATTGAAGAGCTCAGACGGCAAAAATAG
EPI_ISL_11798571	>A/common_buzzard/Denmark/24271-1.02/2021-10-25_H5N1_2021-10-25<br>ATGGATGTCAATCCGACTTTACTTTTCTTAAAAGTGCCAGCGCAAGATGCCATAAGTACCACATTCCCTTACACTGGAGATCCTCCATACAGCCATGGAACAGGGACAGGATACACAATGGACACAGTCAACAGAACACATCAATACTCAGAGAAGGGAAAATGGACAACAAACACAGAAACCGGAGCACCTCAACTCAACCCAATTGATGGGCCACTACCTGAGGACAACGA<br>ACCGAGCGGATATGCACAAACAGATTGCGTGTTGGAAGCAATGGCTTTCCTTGAAGAGTCCCACCCAGGGATCTTTGAAAACTCTTGTCTTGAAACGATGGAAGTCGTTCAGCAAACAAGAGTGGACAAACTAACTCAAGGTCGCCAGACATATGACTGGACACTGAATAGAAACCAACCAGCTGCAACTGCCCTGGCCAACACTATAGAGGTCTTCAGATCAAACGGTCTAA<br>CAACCAATGAATCGGGGAGACTAATAGATTTCCTCAAGGATGTGATGGACTCAATGGATAAAGAAGAAATGGAAATAACAACACATTTCCAGAGAAAGAGAAGAGTAAGGGACAACATGACCAAGAAAATGGTCACACAAAGAACAATAGGAAAGAAGAAACAAAGGCTAACCAAGAGGAGCTACTTAATAAGAGCACTGACACTGAATACAATGACAAAAGATGCAGAA<br>AGAGGCAAATTGAAAAGACGGGCGATTGCAACACCAGGGATGCAGATTAGAGGATTTGTGTACTTTGTCGAAACACTGGCAAGGAGCATCTGTGAAAAACTTGAGCAATCTGGACTCCCCGTTGGAGGGAATGAGAAGAAGGCTAAATTGGCAAATGTCGTGAGAAAAATGATGACTAACTCACAAGATACAGAGCTCTCCTTCACAATTACTGGAGATAACACCAAATGG<br>AATGAGAATCAAAATCCTCGGATGTTTCTGGCAATGATAACATACATTACAAGAAACCAACCTGAATGGTTTAGAAATGTCTTGAGTATTGCCCCTATAATGTTCTCGAACAAAATGGCGAGATTGGGAAAAGGGTACATGTTTGAAAGTAAGAGCATGAAGTTACGGACACAAATACCTGCAGAAATGCTTGCAAACATTGACTTAAAATACTTCAATGAATCAACAAGAAAG<br>AAAATCGAAAAAATAAGGCCTCTACTAATAGATGGCACTGCCTCATTGAGTCCTGGAATGATGATGGGCATGTTCAATATGCTGAGTACAGTATTAGGAGTTTCAATCCTAAATCTTGGGCAAAAGAAGTACACCAAAACCACATACTGGTGGGATGGACTCCAATCCTCTGATGATTTCGCCCTCATAGTAAATGCACCGAATCATGAGGGAATACAAGCAGGAGTGGATAG<br>GTTCTATAGGACCTGCAAACTGGTCGGGATCAATATGAGCAAAAAGAAGTCTTACATAAACCGGACTGGAACATTTGAGTTCACAAGCTTTTTCTATCGCTATGGATTTGTGGCTAACTTCAGTATGGAGCTGCCCAGCTTTGGAGTTTCTGGGATCAATGAATCAGCTGACATGAGCATTGGCATCACAGTGATAAAGAACAACATGATAAACAATGACCTTGGACCAGCAAC<br>AGCTCAAATGGCCCTTCAACTATTCATCAAAGATTACAGGTACACGTACCGATGCCACAGAGGTGACACACAAATTCAAACGAGGAGATCATTCGAGCTGAAGAAGCTGTGGGAACAGACCCGTTCAAAGGCAGGACTGTTGGTGTCAGATGGAGGACCAAATCTATACAACATTCGGAATCTCCATATCCCAGAGGTCTGCCTGAAGTGGGAGCTGATGGACGAAGATTAC<br>CAGGGCAGGTTGTGTAATCCTCTGAACCCATTTGTCAGCCATAGAGAAATTGAGTCCGTAAACAATGCTGTGGTGATGCCAGCCCACGGTCCAGCCAAAAGCATGGAATATGATGCCGTTGCGACTACACACTCATGGATTCCTAAAAGGAATCGTTCCATTCTCAATACCAGTCAAAGGGGAATTCTTGAGGATGAACAGATGTACCAGAAATGCTGCAGTCTATTCGAGAAA<br>TTCTTCCCCAGTAGTTCATACAGGAGACCAGTTGGAATTTCCAGCATGGTGGAGGCCATGGTGTCTAGGGCCCGAATCGATGCACGCATTGATTTCGAATCTGGAAGGATCAAGAAGGAAGAGTTTGCTGAGATCATGAAGATCTGTTCCACCATTGAAGAGCTCAGACGGCAAAAATAG

EPI_ISL_984691	>A/common_buzzard/Denmark/14600-1/2020_H5N8_2020-11-06<br>[illegible]
EPI_ISL_7622539	>A/Chroicocephalus_ridibundus/Belgium/13464/2020_H13N8_2021-12-09<br>[illegible]
EPI_ISL_9012618	>A/chicken/Wales/053969/2021_H5N1_2021-10-30<br>[illegible]
EPI_ISL_943553	>A/Chicken/Sweden/SVA210117SZ0004/KN011326-IP3/2021_H5N5_2021-01-16<br>[illegible]
EPI_ISL_11259312	>A/chicken/Spain/649-6_22VIR2142-38/2022_H5N1_2022-02-16<br>[illegible]
EPI_ISL_1665250	>A/chicken/Slovakia/Pah10_21VIR1086-5/2021_H5N5_2021-01-22<br>[illegible]
EPI_ISL_9012696	>A/chicken/Scotland/054477/2021_H5N1_2021-11-01<br>[illegible]
EPI_ISL_654821	>A/chicken/Saratov/29801/2020_H5N8_2020-09-15<br>[illegible]
EPI_ISL_5463801	>A/chicken/Saratov/29-07V/2021_H5N1_2021-09-30<br>[illegible]
EPI_ISL_297234	>A/chicken/Rostov-on-Don/1321/2017_H5N8_2017-10-18<br>[illegible]
EPI_ISL_1665249	>A/chicken/Romania/10101_21VIR2044-1/2021_H5N5_2021-02-23<br>[illegible]
EPI_ISL_3102068	>A/chicken/Poland/H984_21RS1385-9/2021_H5N8_2021-04-28<br>[illegible]

EPI_ISL_3102075	>A/chicken/Poland/H928_21RS1385-16/2021_H5N8_2021-04-25<br>ATGGATGTCAATCCGACTTTACTTTTCTTAAAAGTGCCAGCGCAAAATGCTATAAGTACTACATTCCCTTACACTGGAGATCCTCCATACAGCCATGGAACAGGAACAGGGTATACCATGGACACAGTAAACAGAACACATCAATACTCAGAAAAGGGAAAGTGGACAACAAACACAGAAACCGGAGCACCCCAACTCAACCCAATTGATGGACCATTACCAGAGGACAATGA<br>GCCAAGCGGATATGCACAAACGGATTGCGTGTTGGAAGCAATGGCTTTCCTTGAAGAATCCCACCCAGGGATATTTGAAAACTCTTGTCTTGAAGCGATGGAAATCGTTCAGCAAACAAGAGTGGACAAACTAACCCAAGGTCGCCAGACTTATGACTGGACACTGAACAGAAACCAACCAGCTGCAACCCTCTTTGGCCAACACTATAGAGGTGTTCAGATCGAATGGTCTGA<br>CAGCCAATGAATCAGGGAGACTGATAGATTTTCTCAGGGATGTGATGGAATCAATGGATAAAGAAGAGATGGAAGTAACAACACATTTCCAGAGAAAAAGAAGAGTGAGGGACAACATGACTAAGAAGATGGTCACACAAAGAACAATAGGGAAGAAGAAGCAGAGGCTGATCAAGAGGAGTTACTTAATAAGAGCACTGACATTGAATACAATGACCAAAGATGCAGA<br>AAGAGGCAAGTTGAAGAGACGGGCAATTGCAACACCCGGGATGCAGATTAGAGGATTCGTGTACTTCGTTGAAACACTAGCGAGGAGCATCTGTGAGAAACTAGAGCAATCTGGGCTCCCTGTTGGAGGGAATGAGAAGAAGGCTAAATTGGCAAATGTTGTGAGAAAAATGATGACTAACTCACAAGATACAGAGCTCTCCTTTACAATTACTGGAGACAACACCAAATG<br>GAATGAGAATCAAAACCCTCGGATGTTTTTGGCAATGATAACATATATCACAAGAAACCAACCTGAATGGTTTAGAAATGTCTTAAGCATTGCCCCTATAATGTTCTCAAACAAAATGGCGAGATTAGGGAAAGGATACATGTTTGAAAGTAAGAGCATGAAGCTAAGAACACAAATATCAGCAGAGATGCTTACAAATATCGATCTGAAGTATTTCAACGAACCAACGAGAA<br>AGAAAATCGAGAAAATAAGACCTCTGCTGATTGATGGCACGGCTTCATTGAGTCCTGGGATGATGATGGGCATGTTCAATATGCTGAGCACAGTATTAGGGGTCTCAATCCTGAATCTCGGGCAAAAAAGGTACACCAAAACCACATACTGGTGGGATGGACTTCAATCCTCTGATGATTTCGCTCTCATAGTGAATGCACCGAATCATGAGGGGATACAAGCAGGAGTGGAT<br>AGATTCTATAGGACCTGCAAACTGGTTGGGATCAACATGAGCAAAAAGAAGTCTTACATAAACCGAACAGGAACATTTGAGTTCACAAGTTTTTTCTACCGCTATGGATTTGTAGCTAACTTCAGTATGGAATTACCCAGCTTTGGAGTGTCTGGAATCAATGAATCAGCTGACATGAGCATTGGAGTTACAGTGATAAAAAACAATATGATAAACAATGATCTTGGACCAGCA<br>ACAGCTCAAATGGCTCTTCAGTTATTTATCAAAGACTATAGGTACACATACCGATGCCACAGGGGTGATACACAAATTCAAACGAGGAGATCATTCGAGCTGAAGAAGCTGTGGGAGCAGACCCGTTCAAAGGCAGGGCTGTTGATATCAGATGGGGGGCCAAACCTATACAACATTCGGAATCTCCACATCCCAGAGGTCTGCTTGAAGTGGGAGCTGATGGATGAAGACT<br>ACCAAGGCAGGCTGTGCAATCCTCTGAATCCATTTGTCAGTCATAAAGAGATTGAGTCCGTAAACAATGCTGTAGTAATGCCCGCCCATGGCCCGGCCAAGAGCATGGAATATGATGCTGTTGCGACCACACACTCGTGGATTCCTAAGAGGAACCGTTCCATTCTCAATACCAGCCAAAGGGGAATTCTTGAGGATGAGCAGATGTACCAAAAGTGCTGTAGTCTATTCGAGA<br>AATTCTTCCCCAGCAGTTCATACAGGAGGCCAGTTGGAATTTCCAGCATGGTGGAGGCCATGGTGTCTAGGGCCCGAATTGATGCACGCATCGACTTCGAATCTGGAAGGATTAAGAAGAAGAGTTTGCTGAGATCATGAAGATCTGTTCCACCATTGAAGAGCTCAGACGGCAAAAATAG
EPI_ISL_3102066	>A/chicken/Poland/H812_21RS1385-7/2021_H5N8_2021-04-20<br>ATGGATGTCAATCCGACTTTACTTTTCTTAAAAGTGCCAGCGCAAAATGCTATAAGTACTACATTCCCTTACACTGGAGATCCTCCATACAGCCATGGAACAGGAACAGGGTATACCATGGACACAGTAAACAGAACACATCAATACTCAGAAAAGGGAAAGTGGACAACAAACACAGAAACCGGAGCACCCCAACTCAACCCAATTGATGGACCATTACCAGAGGACAATGA<br>GCCAAGCGGATATGCACAAACTGATTGCGTGTTGGAAGCAATGGCTTTCCTTGAAAAATCCCACCCAGGGATATTTGAAAACTCTTGTCTTGAAGCAATGGAAATCGTTCAGCAAACAAGAGTGGACAAACTAACCCAAGGTCGCCAGACTTATGACTGGACACTGAACAGAAACCAACCAGCTGCAACCCTCTTTGGCCAACACTATAGAGGTGTTCAGATCGAATGGTCTGAC<br>AGCCAATGAATCAGGGAGACTGATAGATTTTCTCAGGGATGTGATGGAATCAATGGACAAAGAAGAGATGGAAGTAACAACACATTTCCAGAGAAAAAGAAGAGTGAGGGACAACATGACTAAGAAGATGGTCACACAAAGAACAATAGGGAAGAAGAAGCAGAGGCTGAACAAGAGAAGTTACTTAATAAGAGCACTGACATTGAATACAATGACCAAAGATGCAGAA<br>AGAGGCAAGTTGAAGAGACGGGCAATTGCAACACCCGGGATGCAGATTAGAGGATTCGTGTACTTCGTTGAAACACTAGCGAGGAGCATCTGTGAGAAACTAGAGCAATCTGGGCTCCCTGTTGGAGGGAATGAGAAGAAGGCTAAATTGGCAAATGTTGTGAGAAAAATGATGACTAACTCACAAGATACAGAGCTCTCCTTTACAATTACTGGAGACAACACCAAATGG<br>AATGAGAATCAAAACCCTCGGATGTTTTTGGCAATGATAACATATATCACAAGAAACCAACCTGAATGGTTTAGAAATGTCTTAAGCATTGCCCCTATAATGTTCTCAAACAAAATGGCGAGATTAGGGAAAGGATACATGTTTGAAAGTAAGAGCATGAAGCTAAGAACACAAATACCAGCAGAGATGCTTACAAATATCGATCTGAAGTATTTCAACGAACCAACGAGAAA<br>GAAAATCGAGAAAATAAGACCTCTGCTGATTGATGGCACGGCCTCATTGAGTCCTGGGATGATGATGGGCATGTTCAATATGCTGAGCACAGTATTAGGGGTCTCAATCCTGAATCTCGGGCAAAAAAGGTACACCAAAACCACATACTGGTGGGATGGACTTCAATCCTCTGATGATTTCGCTCTCATAGTGAATGCACCGAATCATGAGGGGATACAAGCAGGAGTGGATA<br>GGTTCTATAGGACCTGCAAACTGGTTGGGATCAACATGAGCAAAAAGAAGTCTTACATAAACCGAACAGGAACATTTGAGTTCACAAGTTTTTTCTACCGCTATGGATTTGTAGCTAACTTCAGTATGGAATTACCCAGTTTTGGAGTGTCTGGAATCAATGAATCAGCTGACATGAGCATTGGAGTTACAGTGATAAAAAACAATATGATAAACAATGATCTTGGACCAGCAAC<br>AGCTCAAATGGCTCTTCAGTTATTCATCAAAGACTATAGGTACACATACCGATGCCACAGGGGTGATACACAAATTCAAACGAGGAGATCATTCGAGCTGAAGAAGCTGTGGGAGCAGACCCGTTCAAAGGCAGGGCTGTTGATATCAGACGGGGGGCCAAACCTATACAACATTCGGAATCTCCACATCCCAGAGGTCTGCTTGAAGTGGGAGCTGATGGATGAAGACTAC<br>CAAGGCAGGCTGTGCAATCCTCTGAATCCATTTGTCAGTCATAAAGAGATTGAGTCCGTAAACAATGCTGTAGTAATGCCCGCCCATGGCCCGGCCAAGAGCATGGAATATGATGCTGTTGCGACCACACACTCGTGGATTCCTAAGAGGAACCGTTCCATTCTCAATACCAGCCAAAGGGGAATTCTTGAGGATGAGCAGATGTACCAAAAGTGCTGTAGTCTATTCGAGAAA<br>TTCTTCCCCAGCAGTTCATACAGGAGGCCAGTTGGAATTTCCAGCATGGTGGAGGCCATGGTGTCTAGGGCCCGAATTGATGCACGCATCGACTTCGAATCTGGAAGGATTAAGAAAGAAGAGTTTGCTGAGATCATGAAGATCTGTTCCACCATTGAAGAGCTCAGACGGCAAAAATAG
EPI_ISL_3102071	>A/chicken/Poland/H712_21RS1385-12/2021_H5N8_2021-04-16<br>ATGGATGTCAATCCGACTTTACTTTTCTTAAAAGTGCCAGCGCAAAATGCTATAAGTACTACATTCCCTTACACTGGAGATCCTCCATACAGCCATGGAACAGGAACAGGGTATACCATGGACACAGTAAACAGAACACATCAATACTCAGAAAAGGGAAAGTGGACAACAAACACAGAAACCGGAGCACCCCAACTCAACCCAATTGATGGACCATTACCAGAGGACAATGA<br>GCCAAGCGGATATGCACAAACGGATTGCGTGTTGGAAGCAATGGCTTTCCTTGAAGAATCCCACCCAGGGATATTTGAAAACTCTTGTCTTGAAGCGATGGAAATCGTTCAGCAAACAAGAGTGGACAAACTAACCCAAGGTCGCCAGACTTATGACTGGACACTGAACAGAAACCAACCAGCTGCAACCCTCTTTGGCCAACACTATAGAGGTGTTCAGATCGAATGGTCTGA<br>CAGCCAATGAATCAGGGAGACTGATAGATTTTCTCAGGGATGTGATGGAATCAATGGATAAAGAAGAGATGGAAGTAACAACACATTTCCAGAGAAAAAGAAGAGTGAGGGACAACATGACTAAGAAGATGGTCACACAAAGAACAATAGGGAAGAAGAAGCAGAGGCTGATCAAGAGGAGTTACTTAATAAGAGCACTGACATTGAATACAATGACCAAAGATGCAGA<br>AAGAGGCAAGTTGAAGAGACGGGCAATTGCAACACCCGGGATGCAGATTAGAGGATTCGTGTACTTCGTTGAAACACTAGCGAGGAGCATCTGTGAGAAACTAGAGCAATCTGGGCTCCCTGTTGGAGGGAATGAGAAGAAGGCTAAATTGGCAAATGTTGTGAGAAAAATGATGACTAACTCACAAGATACAGAGCTCTCCTTTACAATTACTGGAGACAACACCAAATG<br>GAATGAGAATCAAAACCCTCGGATGTTTTTGGCAATGATAACATATATCACAAGAAACCAACCTGAATGGTTTAGAAATGTCTTAAGCATTGCCCCTATAATGTTCTCAAACAAAATGGCGAGATTAGGGAAAGGATACATGTTTGAAAGTAAGAGCATGAAGCTAAGAACACAAATATCAGCAGAGATGCTTACAAATATCGATCTGAAGTATTTCAACGAACCAACGAGAA<br>AGAAAATCGAGAAAATAAGACCTCTGCTGATTGATGGCACGGCTTCATTGAGTCCTGGGATGATGATGGGCATGTTCAATATGCTGAGCACAGTATTAGGGGTCTCAATCCTGAATCTCGGGCAAAAAAGGTACACCAAAACCACATACTGGTGGGATGGACTTCAATCCTCTGATGATTTCGCTCTCATAGTGAATGCACCGAATCATGAGGGGATACAAGCAGGAGTGGAT<br>AGATTCTATAGGACCTGCAAACTGGTTGGGATCAACATGAGCAAAAAGAAGTCTTACATAAACCGAACAGGAACATTTGAGTTCACAAGTTTTTTCTACCGCTATGGATTTGTAGCTAACTTCAGTATGGAATTACCCAGCTTTGGAGTGTCTGGAATCAATGAATCAGCTGACATGAGCATTGGAGTTACAGTGATAAAAAACAATATGATAAACAATGATCTTGGACCAGCA<br>ACAGCTCAAATGGCTCTTCAGTTATTTATCAAAGACTATAGGTACACATACCGATGCCACAGGGGTGATACACAAATTCAAACGAGGAGATCATTCGAGCTGAAGAAGCTGTGGGAGCAGACCCGTTCAAAGGCAGGGCTGTTGATATCAGATGGGGGGCCAAACCTATACAACATTCGGAATCTCCACATCCCAGAGGTCTGCTTGAAGTGGGAGCTGATGGATGAAGACT<br>ACCAAGGCAGGCTGTGCAATCCTCTGAATCCATTTGTCAGTCATAAAGAGATTGAGTCCGTAAACAATGCTGTAGTAATGCCCGCCCATGGCCCGGCCAAGAGCATGGAATATGATGCTGTTGCGACCACACACTCGTGGATTCCTAAGAGGAACCGTTCCATTCTCAATACCAGCCAAAGGGGAATTCTTGAGGATGAGCAGATGTACCAAAAGTGCTGTAGTCTATTCGAGA<br>AATTCTTCCCCAGCAGTTCATACAGGAGGCCAGTTGGAATTTCCAGCATGGTGGAGGCCATGGTGTCTAGGGCCCGAATTGATGCACGCATCGACTTCGAATCTGGAAGGATTAAGAAGAAGAGTTTGCTGAGATCATGAAGATCTGTTCCACCATTGAAGAGCTCAGACGGCAAAAATAG
internal NVRI repository	>A/chicken/Poland/H62-N/2022_H5N1_2022-01-17<br>ATGGATGTCAATCCGACTTTACTTTTCTTAAAAGTGCCAGCGCAAGATGCCATAAGTACCACATTCCCTTACACTGGAGATCCTCCATACAGCCATGGAACAGGGACAGGATACACAATGGACACAGTCAACAGAACACATCAATACTCAGAGAAGGGAAAATGGACAACAAACACAGAAACCAGAGCACCTCAACTCAACCCAATTGATGGGCCACTACCTGAGGACAACGA<br>ACCGAGCGGATATGCACAAACAGATTGCGTGTTGGAAGCAATGGCTTTCCTTGAAGAGTCCCACCCAGGGATCTTTGAAAACTCTTGTCTTGAAACGATGGAAGTCGTTCAGCAAACAAGAGTGGACAAACTAACTCAAGGTCGCCAGACATATGACTGGACACTGAATAGAAACCAACCAGCTGCAACTGCCCTGGCCAACACTATAGAGGTCTTCAGATCAAACGGTCTAA<br>CAGCCAATGAATCGGGGAGACTAATAGATTTCCTCAAGGATGTGATGGACTCAATGGATAAAGAAGAAATGGAAATAACAACACATTTCCAGAGAAAGAGAAGAGTAAGGGACAACATGACCAAGAAAATGGTCACACAAAGAACAATAGGAAAGAAGAAACAAAGGCTAAACAAGAGGAGCTACTTAATAAGAGCACTGACACTGAATACAATGACAAAAGATGCAGA<br>AAGAGGCAAATTGAAAAGACGGGCGATTGCAACACCAGGGATGCAGATTAGAGGATTTGTGTACTTTGTCGAAACACTGGCAAGGAGCATCTGTGAAAAACTTGAGCAATCTGGACTCCCCGTTGGAGGGAATGAGAAGAAGGCTAAATTGGCAAATGTCGTGAGAAAAATGATGACTAACTCACAAGATACAGAGCTCTCCTTCACAATTACTGGAGATAACACCAAATG<br>GAATGAGAATCAAAATCCTCGGATGTTTCTGGCAATGATAACATACATTACAAGAAACCAACCTGAATGGTTTAGAAATGTCTTGAGTGTTGCCCCTATAATGTTCTCGAACAAAATGGCGAGATTGGGAAAAGGGTACATGTTTGAAAGTAAGAGCATGAAGTTACGGACACAAATACCTGCAGAAATGCTTGCAAACATTGACTTAAAATACTTCAATGAAWCAACAAGAA<br>AGAAAATCGAAAAAATAAGGCCTCTACTAATAGATGGCACTGCCTCATTGAGTCCTGGAATGATGATGGGCATGTTCAATATGCTGAGTACAGTATTAGGAGTTTCAATCCTAAATCTTGGGCAAAAGAAGTACACCAAAACCACATACTGGTGGGATGGACTCCAATCCTCTGATGATTTCGCCCTCATAGTAAATGCACCGAATCATGAGGGAATACAAGCAGGAGTGGAT<br>AGGTTCTATAGGACCTGCAAACTGGTCGGGATCAATATGAGCAAAAAGAAGTCTTACATAAACCGGACTGGAACATTTGAGTTCACAAGCTTTTTCTATCGCTATGGATTTGTGGCTAACTTCAGTATGGAGCTGCCCAGCTTTGGAGTTTCTGGGATCAATGAATCAGCTGACATGAGCATTGGCGTCACAGTGATAAAGAACAACATGATAAACAATGACCTTGGACCAGCA<br>ACAGCTCAAATGGCCCTTCAACTATTCATCAAAGATTACAGGTACACGTACCGATGCCACAGAGGTGACACACAAATTCAAACGAGGAGATCATTCGAGCTGAAGAAGCTGTGGGAACAGACCCGTTCAAAGGCAGGACTGTTGGTGTCAGATGGAGGACCAAATCTATACAACATTCGGAATCTCCATATCCCAGAGGTCTGCCTGAAGTGGGAGCTGATGGACGAAGATT<br>ACCAGGGCAGGTTGTGTAATCCTCTGAACCCATTTGTCAGCCATAGAGAAATTGAGTCCGTAAACAATGCTGTGGTGGCGCCAGCCCACGGTCCAGCCAAAAGCATGGAATATGATGCCGTTGCGACTACACACTCATGGATTCCTAAAAGGAATCGTTCCATTCTCAATACCAGTCAAAGGGGAATTCTTGAGGATGAACAGATGTACCAGAAATGCTGCAGTCTATTCGAGA<br>AATTCTTCCCCAGTAGTTCATACAGGAGACCAGTTGGAATTTCCAGCATGGTGGAGGCCATGGTGTCTAGGGCCCGAATCGATGCACGAATTGATTTCGAATCTGGAAGGATAAAGAAGGAAGAGTTTGCTGAGATCATGAAGATCTGTTCCACCATTGAAGAGCTCAGACGGCAAAAATAG
internal NVRI repository	>A/chicken/Poland/H36/2022_H5N1_2022-01-08<br>ATGGATGTCAATCCGACTTTACTTTTCTTAAAAGTGCCAGCGCAAGATGCCATAAGTACCACATTCCCTTACACTGGAGATCCTCCATACAGCCATGGAACAGGGACAGGATACACAATGGACACAGTCAACAGAACACATCAATACTCAGAGAAGGGAAAATGGGCAACAAACACAGAAACCGGAGCACCTCAACTCAACCCAATTGATGGGCCACTACCTGAGGACAACGA<br>ACCGAGCGGATATGCACAAACAGATTGCGTGTTGGAAGCAATGGCTTTCCTTGAAGAGTCCCATCCAGGGATCTTTGAAAACTCTTGTCTTGAAACGATGGAAGTCGTTCAGCAAACAAGAGTGGACAAACTAACTCAAGGTCGCCAGACATATGACTGGACACTGAATAGAAACCAACCAGCTGCAACTGCCCTGGCCAACACTATAGAGGTCTTCAGATCAAACGGTCTAA<br>CAGCCAATGAATCGGGGAGACTAATAGATTTCCTCAAGGATGTGATGGACTCAATGGATAAAGAAGAAATGGAAATAACAACACATTTCCAGAGAAAGAGAAGAGTAAGGGACAACATGACCAAGAAAATGGTCACACAAAGAACAATAGGAAAGAAGAAACAAAGGCTAAACAAGAGGAGCTACTTACTAAGAGCACTGACACTGAATACAATGACAAAAGATGCAGA<br>AAGAGGCAAATTGAAGAGACGGGCGATTGCAACACCAGGGATGCAGATTAGAGGATTTGTGTACTTTGCCGAAACACTGGCAAGGAGCATCTGTGAAAAACTTGAGCAATCTGGACTCCCTGTTGGAGGGAATGAGAAGAAGGCTAAATTGGCAAATGTTGTGAGAAAAATGATGACTAACTCACAAGATACAGAGCTCTCCTTCACAATTACTGGAGATAACACCAAATG<br>GAATGAGAATCAAAATCCTCGGATGTTTCTGGCAATGGTAACATACATTACAAGAAACCAACCTGAATGGTTTAGGAATGTCTTGAGTATTGCCCCTATAATGTTCTCGAACAAAATGGCGAGATTGGGAAAAGGGTACATGTTTGAAAGTAAGAGCATGAAGTTACGGACACAAATACCTGCAGAAATGCTTGCAAACATTGACTTAAAATACTTCAATGAATCAACAAGAAA<br>GAAAATCGAAAAAATAAGGCCTCTACTAATAGATGGCACTGCCTCATTGAGTCCTGGAATGATGATGGGCATGTTCAATATGCTGAGTACAGTATTGGGAGTTTCAATCCTAAATCTTGGGCAAAAGAAGTACACCAAAACCACATACTGGTGGGATGGACTCCAATCCTCTGATGATTTCGCCCTCATAGTAAATGCACCGAATCATGAGGGAATACAAGCAGGAGTGGATA<br>GGTTCTATAGGACCTGCAAACTGGTCGGGATCAATATGAGCAAAAAGAAGTCTTACATAAACCGGACTGGAACATTTGAGTTCACAAGCTTTTTCTATCGCTATGGATTTGTGGCTAACTTCAGTATGGAGCTGCCCAGCTTTGGGGTTTCTGGGATCAATGAATCAGCTGACATGAGCATTGGCATCACAGTGATAAAGAACAACATGATAAACAATGACCTTGGACCAGCAA<br>CAGCTCAAATGGCCCTTCAACTATTCATCAAAGATTACAGGTACACGTACCGATGCCATAGAGGTGACACACAAATTCAAACGAGGAGATCATTCGAGCTGAAGAAGCTGTGGGAACAGACCCGTTCAAAGGCAGGACTGTTGGTGTCAGATGGAGGACCAAATCTATACAACATTCGGAATCTCCATATCCCAGAGGTCTGCCTGAAGTGGGAGCTGATGGACGAAGATTA<br>CCAGGGCAGGTTGTGTAATCCTCTGAACCCATTTGTCAGCCATAAAGAAATTGAGTCCGTAAACAATGCTGTGGTGATGCCAGCCCACGGTCCAGCCAAAAGCATGGAATATGATGCCGTTGCGACTACACACTCATGGATTCCTAAAAGGAATCGTTCCATTCTCAATACCAGTCAAAGGGGAATTCTTGAGGATGAACAGATGTACCAGAAATGCTGCGGTCTATTCGAGAA<br>ATTCTTCCCCAGTAGTTCATACAGGAGACCAGTTGGAATTTCCAGCATGGTGGAGGCCATGGTGTCTAGGGCCCGAATCGATGCACGCATTGATTTCGAATCTGGAAGGATCAAGAAGGAAGAGTTTGCTGAGATCATGAAGATCTGTTCCACCATTGAAGAGCTCAGACGGCAAAAATAG
EPI_ISL_3102067	>A/chicken/Poland/H293_21RS1385-8/2021_H5N8_2021-03-23<br>ATGGATGTCAATCCGACTTTACTTTTCTTAAAAGTGCCAGCGCAAAATGCTATAAGTACTACATTCCCTTACACTGGAGATCCTCCATACAGCCATGGAACAGGAACAGGGTATACCATGGACACAGTAAACAGAACACATCAATACTCAGAAAAGGGAAAGTGGACAACAAACACAGAAACCGGAGCACCCCAACTCAACCCAATTGATGGACCATTACCAGAGGACAATGA<br>GCCAAGCGGATATGCACAAACGGATTGCGTGTTGGAAGCAATGGCTTTCCTTGAAGAATCCCACCCAGGGATATTTGAAAACTCTTGTCTTGAAGCGATGGAAATCGTTCAGCAAACAAGAGTGGACAAACTAACCCAAGGTCGCCAGACTTATGACTGGACACTGAACAGAAACCAACCAGCTGCAACCCTCTTTGGCCAACACTATAGAGGTGTTCAGATCGAATGGTCTGA<br>CAGCCAATGAATCAGGGAGACTGATAGATTTTCTCAGGGATGTGATGGAATCAATGGATAAAGAAGAGATGGAAGTAACAACACATTTCCAGAGAAAAAGAAGAGTGAGGGACAACATGACTAAGAAGATGGTCACACAAAGAACAATAGGGAAGAAGAAGCAGAGGCTGATCAAGAGGAGTTACTTAATAAGAGCACTGACATTGAATACAATGACCAAAGATGCAGA<br>AAGAGGCAAGTTGAAGAGACGGGCAATTGCAACACCCGGGATGCAGATTAGAGGATTCGTGTACTTCGTTGAAACACTAGCGAGGAGCATCTGTGAGAAACTAGAGCAATCTGGGCTCCCTGTTGGAGGGAATGAGAAGAAGGCTAAATTGGCAAATGTTGTGAGAAAAATGATGACTAACTCACAAGATACAGAGCTCTCCTTTACAATTACTGGAGACAACACCAAATG<br>GAATGAGAATCAAAACCCTCGGATGTTTTTGGCAATGATAACATATATCACAAGAAACCAACCTGAATGGTTTAGAAATGTCTTAAGCATTGCCCCTATAATGTTCTCAAACAAAATGGCGAGATTAGGGAAAGGATACATGTTTGAAAGTAAGAGCATGAAGCTAAGAACACAAATATCAGCAGAGATGCTTACAAATATCGATCTGAAGTATTTCAACGAACCAACGAGAA<br>AGAAAATCGAGAAAATAAGACCTCTGCTGATTGATGGCACGGCTTCATTGAGTCCTGGGATGATGATGGGCATGTTCAATATGCTGAGCACAGTATTAGGGGTCTCAATCCTGAATCTCGGGCAAAAAAGGTACACCAAAACCACATACTGGTGGGATGGACTTCAATCCTCTGATGATTTCGCTCTCATAGTGAATGCACCGAATCATGAGGGGATACAAGCAGGAGTGGAT<br>AGATTCTATAGGACCTGCAAACTGGTTGGGATCAACATGAGCAAAAAGAAGTCTTACATAAACCGAACAGGAACATTTGAGTTCACAAGTTTTTTCTACCGCTATGGATTTGTAGCTAACTTCAGTATGGAATTACCCAGCTTTGGAGTGTCTGGAATCAATGAATCAGCTGACATGAGCATTGGAGTTACAGTGATAAAAAACAATATGATAAACAATGATCTTGGACCAGCA<br>ACAGCTCAAATGGCTCTTCAGTTATTTATCAAAGACTATAGGTACACATACCGATGCCACAGGGGTGATACACAAATTCAAACGAGGAGATCATTCGAGCTGAAGAAGCTGTGGGAGCAGACCCGTTCAAAGGCAGGGCTGTTGATATCAGATGGGGGGCCAAACCTATACAACATTCGGAATCTCCACATCCCAGAGGTCTGCTTGAAGTGGGAGCTGATGGATGAAGACT<br>ACCAAGGCAGGCTGTGCAATCCTCTGAATCCATTTGTCAGTCATAAAGAGATTGAGTCCGTAAACAATGCTGTAGTAATGCCCGCCCATGGCCCGGCCAAGAGCATGGAATATGATGCTGTTGCGACCACACACTCGTGGATTCCTAAGAGGAACCGTTCCATTCTCAATACCAGCCAAAGGGGAATTCTTGAGGATGAGCAGATGTACCAAAAGTGCTGTAGTCTATTCGAGA<br>AATTCTTCCCCAGCAGTTCATACAGGAGGCCAGTTGGAATTTCCAGCATGGTGGAGGCCATGGTGTCTAGGGCCCGAATTGATGCACGCATCGACTTCGAATCTGGAAGGATTAAGAAGAAGAGTTTGCTGAGATCATGAAGATCTGTTCCACCATTGAAGAGCTCAGACGGCAAAAATAG
internal NVRI repository	>A/chicken/Poland/H2175-N/2021_H5N1_2021-12-18<br>ATGGATGTCAATCCGACTTTACTTTTCTTAAAAGTGCCAGCGCAAGATGCCATAAGTACCACATTCCCTTACACTGGAGATCCTCCATACAGCCATGGAACAGGGACAGGATACACAATGGACACAGTCAACAGAACACATCAATACTCAGAGAAGGGAAAATGGACAACAAACACAGAAACCAGAGCACCTCAACTCAACCCAATTGATGGGCCACTACCTGAGGACAACGA<br>ACCGAGCGGATATGCACAAACAGATTGCGTGTTGGAAGCAATGGCTTTCCTTGAAGAGTCCCACCCAGGGATCTTTGAAAACTCTTGTCTTGAAACGATGGAAGTCGTTCAGCAAACAAGAGTGGACAAACTAACTCAAGGTCGCCAGACATATGACTGGACACTGAATAGAAACCAACCAGCTGCAACTGCCCTGGCCAACACTATAGAGGTCTTCAGATCAAACGGTCTAA<br>CAGCCAATGAATCGGGGAGACTAATAGATTTCCTCAAGGATGTGATGGACTCAATGGATAAAGAAGAAATGGAAATAACAACACATTTCCAGAGAAAGAGAAGAGTAAGGGACAACATGACCAAGAAAATGGTCACACAAAGAACAATAGGAAAGAAGAAACAAAGGCTAAACAAGAGGAGCTACTTAATAAGAGCACTGACACTGAATACAATGACAAAAGATGCAGA<br>AAGAGGCAAATTGAAAAGACGGGCGATTGCAACACCAGGGATGCAGATTAGAGGATTTGTGTACTTTGTCGAAACACTGGCAAGGAGCATCTGTGAAAAACTTGAGCAATCTGGACTCCCCGTTGGAGGGAATGAGAAGAAGGCTAAATTGGCAAATGTCGTGAGAAAAATGATGACTAACTCACAAGATACAGAGCTCTCCTTCACAATTACTGGAGATAACACCAAATG<br>GAATGAGAATCAAAATCCTCGGATGTTTCTGGCAATGATAACATACATTACAAGAAACCAACCTGAATGGTTTAGAAATGTCTTGAGTGTTGCCCCTATAATGTTCTCGAACAAAATGGCGAGATTGGGAAAAGGGTACATGTTTGAAAGTAAGAGCATGAAGTTACGGACACAAATACCTGCAGAAATGCTTGCAAACATTGACTTAAAATACTTCAATGAATCAACAAGAAA<br>GAAAATCGAAAAAATAAGGCCTCTACTAATAGATGGCACTGCCTCATTGAGTCCTGGAATGATGATGGGCATGTTCAATATGCTGAGTACAGTATTAGGAGTTTCAATCCTAAATCTTGGGCAAAAGAAGTACACCAAAACCACATACTGGTGGGATGGACTCCAATCCTCTGATGATTTCGCCCTCATAGTAAATGCACCGAATCATGAGGGAATACAAGCAGGAGTGGATA<br>GGTTCTATAGGACCTGCAAACTGGTCGGGATCAATATGAGCAAAAAGAAGTCTTACATAAACCGGACTGGAACATTTGAGTTCACAAGCTTTTTCTATCGCTATGGATTTGTGGCTAACTTCAGTATGGAGCTGCCCAGCTTTGGAGTTTCTGGGATCAATGAATCAGCTGACATGAGCATTGGCGTCACAGTGATAAAGAACAACATGATAAACAATGACCTTGGACCAGCAA<br>CAGCTCAAATGGCCCTTCAACTATTCATCAAAGATTACAGGTACACGTACCGATGCCACAGAGGTGACACACAAATTCAAACGAGGAGATCATTCGAGCTGAAGAAGCTGTGGGAACAGACCCGTTCAAAGGCAGGACTGTTGGTGTCAGATGGAGGACCAAATCTATACAACATTCGGAATCTCCATATCCCAGAGGTCTGCCTGAAGTGGGAGCTGATGGACGAAGATTA<br>CCAGGGCAGGTTGTGTAATCCTCTGAACCCATTTGTCAGCCATAGAGAAATTGAGTCCGTAAACAATGCTGTGGTGGCGCCAGCCCACGGTCCAGCCAAAAGCATGGAATATGATGCCGTTGCGACTACACACTCATGGATTCCTAAAAGGAATCGTTCCATTCTCAATACCAGTCAAAGGGGAATTCTTGAGGATGAACAGATGTACCAGAAATGCTGCAGTCTATTCGAGAA<br>ATTCTTCCCCAGTAGTTCATACAGGAGACCAGTTGGAATTTCCAGCATGGTGGAGGCCATGGTGTCTAGGGCCCGAATCGATGCACGAATTGATTTCGAATCTGGAAGGATAAAGAAGGAAGAGTTTGCTGAGATCATGAAGATCTGTTCCACCATTGAAGAGCTCAGACGGCAAAAATAG
internal NVRI repository	>A/chicken/Poland/H2130-T1c/2021_H5N1_2021-12-20<br>ATGGATGTCAATCCGACTTTACTTTTCTTAAAAGTGCCAGCGCAAGATGCCATAAGTACCACATTCCCTTACACTGGAGATCCTCCATACAGCCATGGAACAGGGACAGGATACACAATGGACACAGTCAACAGAACACATCAATACTCAGAGAAGGGAAAATGGACAACAAACACAGAAACCGGAGCACCTCAACTCAACCCAATTGATGGGCCACTACCTGAGGACAACGA<br>ACCGAGCGGATATGCACAAACAGATTGCGTGTTGGAAGCAATGGCTTTCCTTGAAGAGTCCCACCCAGGGATCTTTGAAAACTCTTGTCTTGAAACGATGGAAGTCGTTCAGCAAACAAGAGTGGACAAACTAACTCAAGGTCGCCAGACATATGACTGGACACTGAATAGAAACCAACCAGCTGCAACTGCCCTGGCCAACACTATAGAGGTCTTCAGATCAAACGGTCTAA<br>CAGCCAATGAATCGGGGAGACTAATAGATTTCCTCAAGGATGTGATGGACTCAATGGATAAAGAAGAAATGGAAATAACAACACATTTCCAGAGAAAGAGAAGAGTAAGGGACAACATGACCAAGAAAATGGTCACACAAAGAACAATAGGAAAGAAGAAACAAAGGCTAAACAAGAGGAGCTACTTAATAAGAGCACTGACACTGAATACAATGACAAAAGATGCAGA<br>AAGAGGCAAATTGAAGAGACGGGCGATTGCAACACCAGGGATGCAGATTAGAGGATTTGTGTACTTTGTCGAAACACTGGCAAGGAGCATCTGTGAAAAACTTGAGCAATCTGGACTCCCCGTTGGAGGGAATGAGAAGAAGGCTAAATTGGCAAATGTCGTTAGAAAAATGATGACTAACTCACAAGACACAGAGCTCTCCTTCACAATTACTGGAGATAACACCAAATG<br>GAATGAGAATCAAAATCCTCGGATGTTTCTGGCAATGATAACATACATTACAAGAAACCAACCTGAATGGTTTAGAAATGTCTTGAGTATTGCCCCTATAATGTTCTCGAACAAAATGGCGAGATTGGGAAAAGGTTACATGTTTGAAAGTAAGAGCATGAAGTTACGGACACAAATACCTGCAGAAATGCTTGCAAACATTGACTTAAAATACTTCAATGAATCAACAAGAAA<br>GAAAATCGAAAAAATAAGGCCTCTACTAATAGATGGCACTGCCTCATTGAGTCCTGGAATGATGATGGGCATGTTCAATATGCTAAGTACAGTATTAGGAGTTTCAATCCTAAATCTTGGGCAAAAGAAGTACACCAAAACCACATACTGGTGGGATGGACTCCAATCCTCTGATGATTTCGCCCTCATAGTAAATGCACCGAACCATGAGGGAATACAAGCAGGAGTGAATA<br>GGTTCTATAGAACCTGCAAACTGGTCGGGATCAATATGAGCAAAAAGAAGTCTTACATAAACCGGACTGGAACATTTGAGTTCACAAGCTTTTTCTATCGCTATGGATTTGTGGCTAACTTCAGTATGGAGCTGCCCAGCTTTGGAGTTTCTGGGATCAATGAATCAGCTGACATGAGCATTGGCGTCACAGTGATAAAGAACAACATGATAAACAATGACCTTGGACCAGCAA<br>CAGCTCAAATGGCCCTTCAACTATTCGTCAAAGATTACAGGTACACGTACCGATGCCACAGAGGTGACACACAAATTCAAACGAGGAGATCATTCGAGCTGAAGAAGCTGTGGGAACAGACCCGTTCAAAGGCAGGACTGTTGGTGTCAGATGGAGGACCAAATCTATACAACATTCGGAATCTCCATATCCCAGAGGTCTGCCTGAAGTGGGAGCTGATGGACGAAGATTA<br>CCAGGGCAGGTTGTGTAATCCTCTGAACCCATTTGTCAGCCATAAAGAAATTGAGTCTGTAAACAATGCTGTGGTGATGCCAGCCCACGGTCCAGCCAAAAGCATGGAATATGATGCCGTTGCGACTACACACTCATGGATTCCWAAAAGGAATCGTTCCATTCTCAATACCAGTCAAAGGGGAATTCTTGAGGATGAACAGATGTACCAGAAATGCTGCAGTCTATTCGAGA<br>AATTCTTCCCCAGTAGTTCATACAGGAGACCAGTTGGAATTTCCAGCATGGTGGAGGCCATGGTGTCTAGGGCCCGAATCGATGCACGCATTGATTTCGAATCTGGAAGGATCAAGAAGGAAGAGTTTGCTGAGATCATGAAGATCTGTTCCACCATTGAAGAGCTCAGACGGCAAAAATAG
internal NVRI repository	>A/chicken/Poland/H1983_21RS3290-13/2021_H5N1_2021-11-16<br>ATGGATGTCAATCCGACTTTACTTTTCTTAAAAGTGCCAGCGCAAGATGCCATAAGTACCACATTCCCTTACACTGGAGATCCTCCATACAGCCATGGAACAGGGACAGGATACACAATGGACACAGTCAACAGAACACATCAATACTCAGAGAAGGGAAAATGGACAACAAACACAGAAACCAGAGCACCTCAACTCAACCCAATTGATGGGCCACTACCTGAGGACAACGA<br>ACCGAGCGGATATGCACAAACAGATTGCGTGTTGGAAGCAATGGCTTTCCTTGAAGAGTCCCACCCAGGGATCTTTGAAAACTCTTGTCTTGAAACGATGGAAGTCGTTCAGCAAACAAGAGTGGACAAACTAACTCAAGGTCGCCAGACATATGACTGGACACTGAATAGAAACCAACCAGCTGCAACTGCCCTGGCCAACACTATAGAGGTCTTCAGATCAAACGGTCTAA<br>CAGCCAATGAATCGGGGAGACTAATAGATTTCCTCAAGGATGTGATGGACTCAATGGATAAAGAAGAAATGGAAATAACAACACATTTCCAGAGAAAGAGAAGAGTAAGGGACAACATGACCAAGAAAATGGTCACACAAAGAACAATAGGAAAGAAGAAACAAAGGCTAAACAAGAGGAGCTACTTAATAAGAGCACTGACACTGAATACAATGACAAAAGATGCAGA<br>AAGAGGCAAATTGAAAAGACGGGCGATTGCAACACCAGGGATGCAGATTAGAGGATTTGTGTACTTTGTCGAAACACTGGCAAGGAGCATCTGTGAAAAACTTGAGCAATCTGGACTCCCCGTTGGAGGGAATGAGAAGAAGGCTAAATTGGCAAATGTCGTGAGAAAAATGATGACTAACTCACAAGATACAGAGCTCTCCTTCACAATTACTGGAGATAACACCAAATG<br>GAATGAGAATCAAAATCCTCGGATGTTTCTGGCAATGATAACATACATTACAAGAAACCAACCTGAATGGTTTAGAAATGTCTTGAGTGTTGCCCCTATAATGTTCTCGAACAAAATGGCGAGATTGGGAAAAGGGTACATGTTTGAAAGTAAGAGCATGAAGTTACGGACACAAATACCTGCAGAAATGCTTGCAAACATTGACTTAAAATACTTCAATGAATCAACAAGAAA<br>GAAAATCGAAAAAATAAGGCCTCTACTAATAGATGGCACTGCCTCATTGAGTCCTGGAATGATGATGGGCATGTTCAATATGCTGAGTACAGTATTAGGAGTTTCAATCCTAAATCTTGGGCAAAAGAAGTACACCAAAACCACATACTGGTGGGATGGACTCCAATCCTCTGATGATTTCGCCCTCATAGTAAATGCACCGAATCATGAGGGAATACAAGCAGGAGTGGATA<br>GGTTCTATAGGACCTGCAAACTGGTCGGGATCAATATGAGCAAAAAGAAGTCTTACATAAACCGGACTGGAACATTTGAGTTCACAAGCTTTTTCTATCGCTATGGATTTGTGGCTAACTTCAGTATGGAGCTGCCCAGCTTTGGAGTTTCTGGGATCAATGAATCAGCTGACATGAGCATTGGCGTCACAGTGATAAAGAACAACATGATAAACAATGACCTTGGACCAGCAA<br>CAGCTCAAATGGCCCTTCAACTATTCATCAAAGATTACAGGTACACGTACCGATGCCACAGAGGTGACACACAAATTCAAACGAGGAGATCATTCGAGCTGAAGAAGCTGTGGGAACAGACCCGTTCAAAGGCAGGACTGTTGGTGTCAGATGGAGGACCAAATCTATACAACATTCGGAATCTCCATATCCCAGAGGTCTGCCTGAAGTGGGAGCTGATGGACGAAGATTA<br>CCAGGGCAGGTTGTGTAATCCTCTGAACCCATTTGTCAGCCATAGAGAAATTGAGTCCGTAAACAATGCTGTGGTGGTGCCAGCCCACGGTCCAGCCAAAAGCATGGAATATGATGCCGTTGCGACTACACACTCATGGATTCCTAAAAGGAATCGTTCCATTCTCAATACCAGTCAAAGGGGAATTCTTGAGGATGAACAGATGTACCAGAAATGCTGCAGTCTATTCGAGAA<br>ATTCTTCCCCAGTAGTTCATACAGGAGACCAGTTGGAATTTCCAGCATGGTGGAGGCCATGGTGTCTAGGGCCCGAATCGATGCACGAATTGATTTCGAATCTGGAAGGATAAAGAAGGAAGAGTTTGCTGAGATCATGAAGATCTGTTCCACCATTGAAGAGCTCAGACGGCAAAAATAG
internal NVRI repository	>A/chicken/Poland/H1976_21RS3290-9/2021_H5N1_2021-11-15<br>ATGGATGTCAATCCGACTTTACTTTTCTTAAAAGTGCCAGCGCAAGATGCCATAAGTACCACATTCCCTTACACTGGAGATCCTCCATACAGCCATGGAACAGGGACAGGATACACAATGGACACAGTCAACAGAACACATCAATACTCAGAGAAGGGAAAATGGACAACAAACACAGAAACCAGAGCACCTCAACTCAACCCAATTGATGGGCCACTACCTGAGGACAACGA<br>ACCGAGCGGATATGCACAAACAGATTGCGTGTTGGAAGCAATGGCTTTCCTTGAAGAGTCCCACCCAGGGATCTTTGAAAACTCTTGTCTTGAAACGATGGAAGTCGTTCAGCAAACAAGAGTGGACAAACTAACTCAAGGTCGCCAGACATATGACTGGACACTGAATAGAAACCAACCAGCTGCAACTGCCCTGGCCAACACTATAGAGGTCTTCAGATCAAACGGTCTAA<br>CAGCCAATGAATCGGGGAGACTAATAGATTTCCTCAAGGATGTGATGGACTCAATGGATAAAGAAGAAATGGAAATAACAACACATTTCCAGAGAAAGAGAAGAGTAAGGGACAACATGACCAAGAAAATGGTCACACAAAGAACAATAGGAAAGAAGAAACAAAGGCTAAACAAGAGGAGCTACTTAATAAGAGCACTGACACTGAATACAATGACAAAAGATGCAGA<br>AAGAGGCAAATTGAAAAGACGGGCGATTGCAACACCAGGGATGCAGATTAGAGGATTTGTGTACTTTGTCGAAACACTGGCAAGGAGCATCTGTGAAAAACTTGAGCAATCTGGACTCCCCGTTGGAGGGAATGAGAAGAAGGCTAAATTGGCAAATGTCGTGAGAAAAATGATGACTAACTCACAAGATACAGAGCTCTCCTTCACAATTACTGGAGATAACACCAAATG<br>GAATGAGAATCAAAATCCTCGGATGTTTCTGGCAATGATAACATACATTACAAGAAACCAACCTGAATGGTTTAGAAATGTCTTGAGTGTTGCCCCTATAATGTTCTCAAACAAAATGGCGAGATTGGGAAAAGGGTACATGTTTGAAAGTAAGAGCATGAAGTTACGGACACAAATACCTGCAGAAATGCTTGCAAACATTGACTTAAAATACTTCAATGAATCAACAAGAAA<br>GAAAATCGAAAAAATAAGGCCTCTACTAATAGATGGCACTGCCTCATTGAGTCCTGGAATGATGATGGGCATGTTCAATATGCTGAGTACAGTATTAGGAGTTTCAATCCTAAATCTTGGGCAAAAGAAGTACACCAAAACCACATACTGGTGGGATGGACTCCAATCCTCTGATGATTTCGCCCTCATAGTAAATGCACCGAATCATGAGGGAATACAAGCAGGAGTGGATA<br>GGTTCTATAGGACCTGCAAACTGGTCGGGATCAATATGAGCAAAAAGAAGTCTTACATAAACCGGACTGGAACATTTGAGTTCACAAGCTTTTTCTATCGCTATGGATTTGTGGCTAACTTCAGTATGGAGCTGCCCAGCTTTGGAGTTTCTGGGATCAATGAATCAGCTGACATGAGCATTGGCGTCACAGTGATAAAGAACAACATGATAAACAATGACCTTGGACCAGCAA<br>CAGCTCAAATGGCCCTTCAACTATTCATCAAAGATTACAGGTACACGTACCGATGCCACAGAGGTGACACACAAATTCAAACGAGGAGATCATTCGAGCTGAAGAAGCTGTGGGAACAGACCCGTTCAAAGGCAGGACTGTTGGTGTCAGATGGAGGACCAAATCTATACAACATTCGGAATCTCCATATCCCAGAGGTCTGCCTGAAGTGGGAGCTGATGGACGAAGATTA<br>CCAGGGCAGGTTGTGTAATCCTCTGAACCCATTTGTCAGCCATAGAGAAATTGAGTCCGTAAACAATGCTGTGGTGGTGCCAGCCCACGGTCCAGCCAAAAGCATGGAATATGATGCCGTTGCGACTACACACTCATGGATTCCTAAAAGGAATCGTTCCATTCTCAATACCAGTCAAAGGGGAATTCTTGAGGATGAACAGATGTACCAGAAATGCTGCAGTCTATTCGAGAA<br>ATTCTTCCCCAGTAGTTCATACAGGAGACCAGTTGGAATTTCCAGCATGGTGGAGGCCATGGTGTCTAGGGCCCGAATCGATGCACGAATTGATTTCGAATCTGGAAGGATAAAGAAGGAAGAGTTTGCTGAGATCATGAAGATCTGTTCCACCATTGAAGAGCTCAGACGGCAAAAATAG
internal NVRI repository	>A/chicken/Poland/H1962_21RS3290-4/2021_H5N1_2021-11-11<br>ATGGATGTCAATCCGACTTTACTTTTCTTAAAAGTGCCAGCGCAAGATGCCATAAGTACCACATTCCCTTACACTGGAGATCCTCCATACAGCCATGGAACAGGGACAGGATACACGATGGACACAGTCAACAGAACACATCAATACTCAGAGAAGGGAAAATGGACAACAAACACAGAAACCGGAGCACCTCAACTCAACCCAATTGATGGGCCACTACCTGAGGACAACGA<br>ACCGAGCGGATATGCACAAACAGATTGCGTGTTGGAAGCAATGGCTTTCCTTGAAGAGTCCCACCCAGGGATCTTTGAAAACTCTTGTCTTGAAACGATGGAAGTCGTTCAGCAAACAAGAGTGGACAAACTAACTCAAGGTCGCCAGACATATGACTGGACACTGAATAGAAACCAACCAGCTGCAACTGCCCTGGCCAACACTATAGAGGTCTTCAGATCAAACGGTCTAA<br>CAGCCAATGAATCGGGGAGACTAATAGATTTCCTCAAGGATGTGATGGACTCAATGGATAAAGAAGAAATGGAAATAACAACACATTTCCAGAGAAAGAGAAGAGTAAGGGACAACATGACCAAGAAAATGGTCACACAAAGAACAATAGGAAAGAAGAAACAAAGGCTAAACAAGAGGAGCTACTTAATAAGAGCACTGACACTGAATACAATGACAAAAGATGCAGA<br>AAGAGGCAAATTGAAGAGACGGGCGATTGCAACACCAGGGATGCAGATTAGAGGATTTGTGTACTTTGTCGAAACACTGGCAAGGAGCATCTGTGAAAAACTTGAGCAATCTGGACTCCCCGTTGGAGGGAATGAGAAGAAGGCTAAATTGGCAAATGTCGTGAGAAAAATGATGACTAACTCACAAGATACAGAGCTCTCCTTCACAATTACTGGAGACAACACCAAATG<br>GAATGAGAATCAAAATCCTCGGATGTTTCTGGCAATGATAACATACATTACAAGAAACCAACCTRAATGGTTTAGAAATGTCTTAAGTATTGCCCCTATAATGTTCTCGAACAAAATGGCGAGATTGGGAAAAGGGTACATGTTTGAAAGTAAGAGCATGAAGTTACGGACACAAATACCTGCAGAAATGCTTGCAAACATTGACTTAAAATACTTCAATGAATCAACAAGAAA<br>GAAAATCGAAAAAATAAGGCCTCTACTAATAGATGGCACTGCCTCATTGAGTCCTGGAATGATGATGGGCATGTTCAATATGCTGAGTACAGTATTAGGAGTTTCAATCCTAAATCTTGGGCAAAAGAAGTACACCAAAACCACATACTGGTGGGATGGACTCCAATCCTCTGATGATTTCGCCCTCATAGTAAATGCACCGAATCATGAGGGAATACAAGCAGGAGTGGATA<br>GGTTCTATAGGACCTGCAAACTGGTCGGGATCAATATGAGCAAAAAGAAGTCTTACATAAACCGGACTGGAACATTTGAGTTCACAAGCTTTTTCTATCGCTATGGATTTGTGGCTAACTTCAGTATGGAGCTGCCCAGCTTTGGGGTTTCTGGGATCAATGAATCAGCTGACATGAGCATTGGCGTCACAGTGATAAAGAACAACATGATAAACAATGACCTTGGACCAGCAA<br>CAGCTCAAATGGCCCTTCAACTATTCATCAAAGATTACAGGTACACGTACCGATGCCACAGAGGTGACACACAAATTCAAACGAGGAGATCATTCGAGCTGAAGAAGCTGTGGGAACAGACCCGTTCAAAGGCAGGACTGTTGGTGTCAGATGGAGGACCAAATCTATACAACATTCGGAATCTCCATATCCCAGAGGTCTGCCTGAAGTGGGAGCTGATGGACGAAGATTA<br>CCAGGGCAGGTTGTGTAATCCTCTGAACCCATTTGTCAGCTATAAAGAAATTGAGTCCGTAAACAATGCTGTGGTGATGCCAGCTCACGGTCCAGCCAAAAGCATGGAATATGATGCCGTTGCGACTACACACTCATGGACTCCTAAAAGGAATCGTTCCATTCTCAATACCAGTCAAAGGGGAATTCTTGAGGATGAACAGATGTACCAGAAATGCTGCAGTCTATTCGAGAA<br>ATTCTTCCCCAGTAGTTCATACAGGAGACCAGTTGGAATTTCCAGCATGGTGGAGGCCATGGTGTCTAGGGCCCGAATCGATGCACGCATTGATTTCGAATCTGGAAGGATCAAGAAGGAGGAGTTTGCTGAGATCATGAAGATCTGTTCCACCATTGAAGAGCTCAGACGGCAAAAATAG
EPI_ISL_6931288	>A/chicken/Poland/H1940-N/2021_H5N1_2021-11-05<br>ATGGATGTCAATCCGACTTTACTTTTCTTAAAAGTGCCAGCTCAAGATGCCATAAGTACCACATTCCCTTACACTGGAGATCCTCCATACAGCCATGGAACAGGGACAGGATACACAATGGACACAGTCAACAGAACACATCAATACTCAGAGAAGGGAAAATGGACAACAAACACAGAAACCGGAGCACCTCAACTCAACCCAATTGATGGGCCACTACCTGAGGACAACGA<br>ACCGAGCGGATATGCACAAACAGATTGCGTGTTGGAAGCAATGGCTTTCCTTGAAGAGTCCCACCCAGGGATCTTTGAAAACTCTTGTCTTGAAACGATGGAAGTCGTTCAGCAAACAAGAGTGGACAAACTAACTCAAGGTCGCCAGACATATGACTGGACACTGAATAGAAACCAACCAGCTGCAACTGCCCTGGCCAACACTATAGAGGTCTTCAGATCAAACGGTCTAA<br>CAGCCAATGAATCGGGGAGACTAATAGATTTCCTCAAGGATGTGATGGACTCAATGGATAAAGAAGAAATGGAAATGACAACACATTTCCAGAGAAAGAGAAGAGTAAGGGACAACATGACCAAGAAAATGGTCACACAAAGAACAATAGGAAAGAAGAAACAAAGGCTAAACAAGAGGAGCTACTTAATAAGAGCACTGACACTGAATACAATGACAAAAGATGCAGA<br>AAGAGGCAAATTGAAGAGACGGGCGATTGCAACACCAGGGATGCAGATTAGAGGATTTGTGTACTTTGTCGAAACACTGGCAAGGAGCATCTGTGAAAAACTTGAGCAATCTGGACTCCCCGTTGGAGGGAATGAGAAGAAGGCTAAATTGGCAAATGTCGTGAGAAAAATGATGACTAACTCACAAGATACAGAGCTCTCCTTCACAATTACTGGAGATAACACCAAATG<br>GAATGAGAATCAAAATCCTCGGATGTTTCTGGCAATGATAACATACATTACAAGAAACCAACCTGAATGGTTTAGAAATGTCTTGAGTATTGCCCCTATAATGTTCTCGAACAAAATGGCGAGATTGGGAAAAGGGTACATGTTTGAAAGTAAGAGCATGAAGTTACGGACACAAATACCTGCAGAAATGCTTGCAAACATTGACTTAAAATACTTCAATGAATCAACAAGAAA<br>GAAAATCGAAAAAATAAGGCCTCTACTAATAGATGGCACTGCCTCATTGAGTCCTGGAATGATGATGGGCATGTTCAATATGCTGAGTACAGTATTAGGAGTTTCAATCCTAAATCTTGGGCAAAAGAAGTACACCAAAACCACATACTGGTGGGATGGACTCCAATCCTCTGATGATTTCGCCCTCATAGTAAATGCACCGAATCATGAGGGAATACAAGCAGGAGTGGATA<br>GGTTCTATAGGACCTGCAAACTGGTCGGGATCAATATGAGCAAAAAGAAGTCTTACATAAACCGGACTGGAACATTTGAGTTCACAAGCTTTTTCTATCGCTATGGATTTGTGGCTAACTTCAGTATGGAGCTGCCCAGCTTTGGAGTTTCTGGGATCAATGAATCAGCTGACATGAGCATTGGCGTCACAGTGATAAAGAACAACATGGTAAACAATGACCTTGGACCAGCAA<br>CAGCTCAAATGGCCCTTCAACTATTCATCAAAGATTACAGGTACACGTACCGATGCCACAGAGGTGACACACAAATTCAAACGAGGAGATCATTCGAGCTGAAGAAGCTGTGGGAACAGACCCGTTCAAAGGCAGGACTGTTGGTGTCAGATGGAGGACCAAATCTATACAACATTCGGAATCTCCATATCCCAGAGGTCTGCCTGAAGTGGGAGCTGATGGACGAAGATTA<br>CCAGGGCAGGTTGTGTAATCCTCTGAACCCATTTGTCAGCCATAAAGAAATTGAGTCCGTAAACAATGCTGTGGTGATGCCAGCCCACGGTCCAGCCAAAAGCATGGAATATGATGCCGTTGCGACTACACACTCATGGATTCCTAAAAGGAATCGTTCCATTCTCAATACCAGTCAAAGGGGAATTCTTGAGGATGAACAGATGTACCAGAAATGCTGCAGTCTATTCGAGAA<br>ATTCTTCCCCAGTAGTTCATACAGGAGACCAGTTGGAATTTCCAGCATGGTGGAGGCCATGGTGTCTAGGGCCCGAATCGATGCACGCATTGATTTCGAATCTGGAAGGATCAAGAAGGAGGAGTTTGCTGAGATCATGAAGATCTGTTCCACCATTGAAGAGCTCAGACGGCAAAAATAG

internal NVRI repository	>A/chicken/Poland/H1928_21RS3290-2/2021_H5N1_2021-11-03<br>[illegible]
EPI_ISL_11922807	>A/chicken/Poland/H182_22VIR2515-1/2022_H5N2_2022-02-28<br>[illegible]
EPI_ISL_11922809	>A/chicken/Poland/H157_22VIR2515-3/2022_H5N1_2022-02-18<br>[illegible]
EPI_ISL_3102069	>A/chicken/Poland/H1161_21RS1385-10/2021_H5N8_2021-05-13<br>[illegible]
EPI_ISL_3102062	>A/chicken/Poland/H1124_21RS1385-3/2021_H5N8_2021-05-11<br>[illegible]
EPI_ISL_3102072	>A/chicken/Poland/H1084_21RS1385-13/2021_H5N8_2021-05-07<br>[illegible]
EPI_ISL_11922812	>A/chicken/Poland/H071_22VIR2515-6/2022_H5N1_2022-01-23<br>[illegible]
EPI_ISL_846603	>A/chicken/Poland/476/2020_H5N8_2020-12-04<br>[illegible]
EPI_ISL_846601	>A/chicken/Poland/474/2020_H5N8_2020-12-03<br>[illegible]
EPI_ISL_661177	>A/chicken/Poland/448/2020_H5N8_2020-11-24<br>[illegible]
EPI_ISL_644150	>A/chicken/Omsk/0112/2020_H5N8_2020-08-17<br>[illegible]
EPI_ISL_4061485	>A/chicken/Nigeria/VRD21-109_21VIR2370-425/2021_H5N1_2021-03-01<br>[illegible]

EPI_ISL_6101848	>A/chicken/Netherlands/21038165-006010/2021_H5N1_2021-11-07<br>ATGGATGTCAATCCGACTTTACTTTTCTTAAAAGTGCCAGCGCAAGATGCCATAAGTACCACATTCCCTTACACTGGAGATCCTCCATACAGCCATGGAACAGGGACAGGATACACAATGGACACAGTCAACAGAACACATCAATACTCAGAGAAGGGAAAATGGACAACAAACACAGAAACCAGAGCACCTCAACTCAACCCAATTGATGGGCCACTACCTGAGGACAACGA<br>ACCGAGCGGATATGCACAAACAGATTGCGTGTTGGAAGCAATGGCTTTCCTTGAAGAGTCCCACCCAGGGATCTTTGAAAACTCTTGTCTTGAAACGATGGAAGTCGTTCAGCAAACAAGAGTGGACAAACTAACTCAAGGTCGCCAGACATATGACTGGACACTGAATAGAAACCAACCAGCTGCAACTGCCCTGGCCAACACTATAGAGGTCTTCAGATCAAACGGTCTAA<br>CAGCCAATGAATCGGGGAGACTAATAGATTTCCTCAAGGATGTGATGGACTCAATGGATAAAGAAGAAATGGAAATAACAACACATTTCCAGAGAAAGAGAAGAGTAAGGGACAACATGACCAAGAAAATGGTCACACAAAGAACAATAGGAAAGAAGAAACAAAGGCTAAACAAGAGGAGCTACTTAATAAGAGCACTGACACTGAATACAATGACAAAAGATGCAGA<br>AAGAGGCAAATTGAAAAGACGGGCGATTGCAACACCAGGGATGCAGATTAGAGGATTTGTGTACTTTGTCGAAACACTGGCAAGGAGCATCTGTGAAAAACTTGAGCAATCTGGACTCCCCGTTGGAGGGAATGAGAAGAAGGCTAAATTGGCAAATGTCGTGAGAAAAATGATGACTAACTCACAAGATACAGAGCTCTCCTTCACAATTACTGGAGATAACACCAAATG<br>GAATGAGAATCAAAATCCTCGGATGTTTCTGGCAATGATAACATACATTACAAGAAACCAACCTGAATGGTTTAGAAATGTCTTGAGTATTGCCCCTATAATGTTCTCGAACAAAATGGCGAGATTGGGAAAAGGGTACATGTTTGAAAGTAAGAGCATGAAGTTACGGACACAAATACCTGCAGAAATGCTTGCAAACATTGACTTAAAATACTTCAATGAATCAACAAGAAA<br>GAAAATCGAAAAAATAAGGCCTCTACTAATAGATGGCACTGCCTCATTGAGTCCTGGAATGATGATGGGCATGTTCAATATGCTGAGTACAGTATTAGGAGTTTCAATCCTAAATCTTGGGCAAAAGAAGTACACCAAAACCACATACTGGTGGGATGGGCTCCAATCCTCTGATGATTTCGCCCTCATAGTAAATGCACCGAATCATGAGGGAATACAAGCAGGAGTGGATA<br>GGTTCTATAGGACCTGCAAACTGGTCGGGATCAATATGAGCAAAAAGAAGTCTTACATAAACCGGACTGGAACATTTGAGTTCACAAGCTTTTTCTATCGCTATGGATTTGTGGCTAACTTCAGTATGGAGCTGCCCAGCTTTGGAGTTTCTGGGATCAATGAATCAGCTGACATGAGCATTGGCGTCACAGTGATAAAGAACAACATGATAAACAATGACCTTGGACCAGCAA<br>CAGCTCAAATGGCCCTTCAACTATTCATCAAAGATTACAGGTACACGTACCGATGCCACAGAGGTGACACACAAATTCAAACGAGGAGATCATTCGAGCTGAAGAAGCTGTGGGAACAGACCCGTTCAAAGGCAGGACTGTTGGTGTCAGATGGAGGACCAAATCTATACAACATTCGGAATCTCCATATCCCAGAGGTCTGCCTGAAGTGGGAGCTGATGGACGAAGATTA<br>CCAGGGCAGGTTGTGTAATCCTCTGAACCCATTTGTCAGCCATAGAGAAATTGAGTCCGTAAACAATGCTGTGGTGATGCCAGCCCACGGTCCAGCCAAAAGCATGGAATATGATGCCGTTGCGACTACACACTCATGGATTCCTAAAAGGAATCGTTCCATTCTCAATACCAGTCAAAGGGGAATTCTTGAGGATGAACAGATGTACCAGAAATGCTGCAGTCTATTCGAGAA<br>ATTCTTCCCCAGTAGTTCATACAGGAGACCAGTTGGAATTTCCAGCATGGTGGAGGCCATGGTGTCTAGGGCCCGAATCGATGCACGAATTGATTTCGAATCTGGAAGGATAAAGAAGGAAGAGTTTGCTGAGATCATGAAGATCTGTTCCACCATTGAAGAGCTCAGACGGCAAAAATAG
EPI_ISL_641395	>A/chicken/Netherlands/20017694-004/2020_H5N8_2020-11-11<br>ATGGATGTCAATCCGACTTTACTTTTCTTAAAAGTGCCAGCGCAAAATGCTATAAGTACTACATTCCCTTACACTGGAGATCCTCCATACAGCCATGGAACAGGAACAGGGTATACCATGGACACAGTAAACAGAACACATCAATACTCAGAAAAGGGAAAGTGGACAACAAACACAGAAACCGGAGCACCCCAACTCAACCCAATTGATGGACCATTACCAGAGGACAATGA<br>GCCAAGCGGATATGCACAAACTGATTGCGTGTTGGAAGCAATGGCTTTCCTTGAAGAATCCCACCCAGGGATATTTGAAAACTCTTGTCTTGAAGCGATGGAAATCGTTCAGCAAACAAGAGTGGACAAACTAACCCAAGGTCGCCAGACTTATGACTGGACACTGAACAGAAACCAACCAGCTGCAACCTCTTTGGCCAACACTATAGAGGTGTTCAGATCGAATGGTCTGA<br>CAGCCAATGAATCAGGGAGACTGATAGATTTTCTCAGAGATGTGATGGAATCAATGGATAAAGAAGAGATGGAAGTAACAACACATTTCCAGAGAAAAAGAAGAGTGAGGGACAACATGACTAAGAAGATGGTCACACAAAGAACAATAGGGAAGAAGAAGCAGAGGCTGAACAAGAGGAGTTACTTAATAAGAGCACTGACATTGAATACAATGACCAAAGATGCAGA<br>AAGAGGCAAGTTGAAGAGACGGGCAATTGCAACACCCGGGATGCAGATTAGAGGATTCGTGTACTTCGTTGAAACACTAGCGAGGAGCATCTGTGAGAAACTAGAGCAATCTGGGCTCCCTGTTGGAGGGAATGAGAAGAAGGCTAAATTGGCAAATGTTGTGAGAAAAATGATGACTAACTCACAAGATACAGAGCTCTCCTTTACAATTACTGGAGACAACACCAAATG<br>GAATGAGAATCAAAACCCTCGGATGTTTTTGGCAATGATAACATATATCACAAGAAACCAACCTGAATGGTTTAGAAATGTCTTAAGCATTGCCCCTATAATGTTCTCAAACAAAATGGCGAGATTAGGGAAAGGATACATGTTTGAAAGTAAGAGCATGAAGCTAAGAACACAAATACCAGCAGAGATGCTTACAAATATCGATCTGAAGTATTTCAACGAACCAACGAGAA<br>AGAAAATCGAGAAAATAAGACCTCTGCTGATTGATGGCACGGCCTCATTGAGTCCTGGGATGATGATGGGCATGTTCAATATGCTGAGCACAGTATTAGGGGTCTCAATCCTGAATCTCGGGCAAAAAAGGTACACCAAAACCACATACTGGTGGGATGGACTTCAATCCTCTGATGATTTCGCTCTCATAGTGAATGCACCGAATCATGAGGGGATACAAGCAGGAGTGGAT<br>AGATTCTATAGGACCTGCAAACTGGTTGGGATCAACATGAGCAAAAAGAAGTCTTACATAAACCGAACAGGAACATTTGAGTTCACAAGTTTTTTCTACCGCTATGGATTTGTAGCTAACTTCAGTATGGAATTACCCAGCTTTGGAGTGTCTGGAATCAATGAATCAGCTGACATGAGCATTGGAGTTACAGTGATAAAAAACAATATGATAAACAATGATCTTGGACCAGCA<br>ACAGCTCAAATGGCTCTTCAGTTATTCATCAAAGACTATAGATACACATACCGATGCCACAGGGGTGATACACAAATTCAAACGAGGAGATCATTCGAGCTGAAGAAGCTGTGGGAGCAGACCCGTTCAAAGGCAGGGCTGTTGATATCAGACGGGGGGCCAAACCTATACAACATTCGGAATCTCCACATCCCAGAGGTCTGCTTGAAGTGGGAGCTGATGGATGAAGACT<br>ACCAAGGCAGGCTGTGCAATCCTCTGAATCCATTTGTCAGTCATAAAGAGATTGAGTCCGTAAACAATGCTGTAGTAATGCCCGCCCATGGCCCGGCCAAGAGCATGGAATATGATGCTGTTGCGACCACACACTCGTGGATTCCTAAGAGGAACCGTTCCATTCTCAATACCAGCCAAAGGGGAATTCTTGAGGATGAGCAGATGTACCAAAAGTGCTGTAGTCTATTCGAGA<br>AATTCTTCCCCAGCAGTTCATACAGGAGGCCAGTTGGAATTTCCAGCATGGTGGAGGCCATGGTGTCTAGGGCCCGAATTGATGCACGCATCGACTTCGAATCTGGAAGGATTAAGAAAGAAGAGTTTGCTGAGATCATGAAGATCTGTTCCACCATTGAAGAGCTCAGACGGCAAAAATAG
EPI_ISL_1114745	>A/chicken/Kostroma/304-10/2020_H5N8_2020-10-17<br>ATGGATGTCAATCCGACTTTACTTTTCTTAAAAGTGCCAGCGCAAAATGCTATAAGTACTACATTCCCTTACACTGGAGATCCTCCATACAGCCATGGAACAGGAACAGGGTATACCATGGACACAGTAAACAGAACACATCAATACTCAGAAAAGGGAAAGTGGACAACAAACACAGAAACCGGAGCACCCCAACTCAACCCAATTGATGGACCATTACCAGAGGACAATGA<br>GCCAAGCGGATATGCACAAACTGATTGCGTGTTGGAAGCAATGGCTTTCCTTGAAGAATCCCACCCAGGGATATTTGAAAACTCTTGTCTTGAAGCGATGGAAATCGTTCAGCAAACAAGAGTGGACAAACTAACCCAAGGTCGCCAGACTTATGACTGGACACTGAACAGAAACCAACCAGCTGCAACCTCTTTGGCCAACACTATAGAGGTGTTCAGATCGAATGGTCTGA<br>CAGCCAATGAATCAGGAAGACTGATAGATTTTCTCAGGGATGTGATGGAATCAATGGATAAAGAAGAGATGGAAGTAACAACACATTTCCAGAGAAAAAGAAGAGTGAGGGACAACATGACTAAGAAGATGGTCACACAAAGAACAATAGGGAAGAAGAAGCAGAGGCTGAACAAGAGGAGTTACTTAATAAGAGCACTGACATTGAATACAATGACCAAAGATGCAGA<br>AAGAGGCAAGTTGAAGAGACGGGCAATTGCAACACCCGGGATGCAGATTAGAGGATTCGTGTACTTCGTTGAAACACTAGCAAGGAGCATCTGTGAGAAACTAGAGCAATCTGGGCTCCCTGTTGGAGGGAATGAGAAGAAGGCTAAATTGGCAAATGTTGTGAGAAAAATGATGACTAACTCACAAGATACAGAGCTCTCCTTTACAATTACTGGAGACAACACCAAATG<br>GAATGAGAATCAAAACCCTCGGATGTTTTTGGCAATGGTAACATATATCACAAGAAACCAACCTGAATGGTTTAGAAATGTCTTAAGCATTGCCCCTATAATGTTCTCAAACAAAATGGCGAGATTAGGGAAAGGATACATGTTTGAAAGTAAGAGCATGAAGCTAAGAACACAAATACCAGCAGAGATGCTCACAAATATTGATCTGAAGTATTTCAACGAACCAACGAGAA<br>AGAAAATCGAGAAAATAAGACCTCTGCTGATTGATGGCACGGCCTCATTGAGTCCTGGGATGATGATGGGCATGTTCAATATGCTGAGCACAGTATTGGGGGTCTCAATCCTGAATCTCGGGCAAAAGAGGTACACCAAAACCACATACTGGTGGGATGGACTTCAATCCTCTGATGATTTCGCTCTCATAGTGAATGCACCGAATCATGAGGGGATACAAGCAGGAGTGGAT<br>AGATTCTATAGGACCTGCAAACTGGTTGGGATCAACATGAGCAAAAAGAAGTCTTACATAAACCGAACAGGAACATTTGAGTTCACAAGTTTTTTCTACCGCTATGGATTTGTAGCTAACTTCAGTATGGAATTACCCAGCTTTGGAGTGTCTGGAATCAATGAATCAGCTGACATGAGCATTGGAGTTACAGTGATAAAAAACAATATGATAAACAATGATCTTGGACCAGCA<br>ACAGCTCAAATGGCTCTTCAGTTATTCATCAAAGACTATAGGTACACATACCGATGCCACAGGGGTGATACACAAATTCAAACGAGGAGATCATTCGAGCTGAAGAAGCTGTGGGAGCAGACCCGTTCAAAGGCAGGGCTGTTGATATCAGACGGGGGGCCAAACCTATACAACATTCGGAACCTCCACATCCCAGAGGTCTGCTTGAAGTGGGAGCTGATGGATGAAGACT<br>ACCAAGGCAGGCTGTGCAATCCTCTGAATCCATTTGTCAGTCATAAAGAGATTGAGTCCGTAAACAATGCTGTAGTAATGCCCGCCCATGGCCCGGCCAAGAGCATGGAATATGATGCTGTTGCGACCACACATTCGTGGATTCCAAAGAGGAACCGCTCCATTCTCAATACCAGCCAAAGGGGAATTCTTGAGGATGAGCAGATGTACCAAAAGTGCTGTAGTCTATTCGAG<br>AAATTCTTCCCCAGCAGTTCATACAGGAGGCCAGTTGGAATTTCCAGCATGGTGGAGGCCATGGTGTCTAGGGCCCGAATTGATGCACGCATCGACTTCGAATCTGGAAGGATTAAGAAAGAAGAGTTTGCTGAGATCATGAAGATCTGTTCCACCATTGAAGAGCTCAGACGGCAAAAATAG
EPI_ISL_6829533	>A/chicken/Kagoshima/21A6T/2021_H5N1_2021-11-12<br>ATGGATGTCAATCCGACTTTACTTTTCTTAAAAGTGCCAGCGCAAAATGCTATAAGCACTACGTTCCCTTACACTGGAGATCCTCCATACAGCCATGGAACAGGAACAGGATACACCATGGACACAGTCAACAGAACACATCAATACTCAGAAAAAGGAAAGTGGACAACAAACACAGAGACCGGGGCGCCCCAACTCAACCCAATTGATGGACCATTGCCAGAGGACAACGA<br>CCCAAGCGGATATGCACAAACAGATTGCGTGTTGGAAGCAATGGCTTTCCTTGAAGAATCCCACCCAGGGATCTTTGAAAACTCTTGTCTTGAAACGATGGAGATCGTTCAGCAAACAAGAGTGGACAAACTAACCCAAGGTCGCCAGACCTATGACTGGACACTGAATAGAAACCAACCAGCTGCAACTGCTTTAGCCAATACTATAGAGGTCTTCAGATCGAACGGTCTAAC<br>AGCCAATGAGTCGGGGAGACTAATAGATTTCCTCAAGGATGTGATGGAATCAATGGATAAAGAAGAAATGGAAATAACAACACATTTCCAGAGAAAGAGAAGAGTAAGGGACAACATGACCAAAAAGATGGTCACACAAAGAACGATAGGAAAGAAGAAGCAGAGGCTGAACAAGAGAAGCTACTTAATAAGAGCGTTGACATTGAACACAATGACCAAAGATGCAGAA<br>AGAGGCAAATTGAAGAGGCGGGCAATTGCAACACCCGGGATGCAGATTAGAGGATTTGTGTACTTTGTCGAAACACTAGCGAGGAGCATCTGTGAGAAACTTGAGCAATCCGGACTCCCTGTCGGAGGGAATGAGAAAAAGGCCAAATTGGCAAATGTCGTGAGGAAAATGATGACTAACTCGCAAGATACAGAGCTCTCTTTTACAATCACCGGGGACAACACCAAATGG<br>AATGAGAATCAAAATCCTCGGATGTTTTTGGCAATGATAACATACATCACAAGAAACCAACCCGAATGGCTTAGAAATGTCTTGAGCATTGCCCCCATAATGTTCTCAAACAAAATGGCAAGATTAGGGAAAGGATACATGTTTGAAAGTAAGAGCATGAAGCTAAGGACACAAATACCGGCAGAAATGATTGCAAACATTGACTTGAAATACTTCAATGAATCAACGAGAAA<br>GAAAATCGAGAAAATAAGACCTCTACTAATTGATGGCACAGCCTCATTGAGTCCTGGAATGATGATGGGCATGTTCAATATGCTGAGCACTGTATTGGGGGTCTCAATCCTGAATCTTGGACAAAAGAGGTACACCAAAACCACATACTGGTGGGATGGACTCCAATCTTCTGATGACTTCGCTCTCATAGTGAATGCACCGAATCATGAGGGGATACAAGCCGGGGTGGATA<br>GGTTCTATAGGACATGCAAACTGGTTGGGATCAACATGAGCAAAAAGAAGTCTTACATAAACCGGACAGGAACATTTGAGTTCACAAGCTTTTTCTACCGCTATGGATTTGTAGCCAACTTCAGTATGGAGTTACCCAGCTTTGGTGTGTCTGGAATCAATGAGTCAGCTGACATGAGCATTGGAGTTACAGTGATAAAGAACAACATGATAAACAATGACCTTGGACCAGCAA<br>CAGCTCAAATGGCTCTTCAGCTATTCATCAAGGATTATAGGTACACGTACCGATGTCACAGAGGTGATACACAAATTCAAACGAGGAGATCATTCGAGCTGAAGAAGCTGTGGGAGCAGACCCGTTCAAAGGCAGGGCTGTTGGTATCAGATGGAGGACCAAACCTATACAACATTCGGAATCTCCACATCCCAGAGGTCTGCTTGAAATGGGAGCTAATGGATGAAGACTA<br>CCAGGGCAGGCTGTGTAATCCTCTGAATCCATTTGTCAGTCATAAGGAAATTGAATCCGTAAACAATGCTGTAGTAATGCCAGCCCATGGCCCAGTCAAGAGCATGGAATACGATGCTGTTGCGACTACACACTCATGGATTCCTAAGAGGAACCGTTCCATTCTCAATACCAGCCAGAGGGGAATTCTCGAGGATGAGCAGATGTACCAGAAGTGCTGCAGCTTATTCGAGAA<br>GTTCTTCCCCAGTAGTTCATACAGGAGGCCAGTTGGAATTTCCAGCATGGTGGAGGCCATGGTGTCCAGGGCCCGAATTGATGCACGCATTGATTTCGAATCTGGAAGGATTAAGAAAGAAGAGTTTGCTGAGATCATGAAGATCTGTTCCACCATTGAGGAGCTCAGACGGCAAAAATAG
EPI_ISL_14761220	>A/chicken/Italy/21VIR11774-2/2021_H5N1_2021-12-28<br>ATGGATGTCAATCCGACTTTACTTTTCTTAAAAGTGCCAGCGCAAGATGCCATAAGTACCACATTCCCTTACACTGGAGATCCTCCATACAGCCATGGAACAGGGACAGGATACACAATGGACACAGTCAACAGAACACATCAATACTCAGAGAAGGGAAAATGGACAACAAACACAGAAACCGGAGCACCTCAACTCAACCCCATTGATGGGCCACTACCTGAGGACAACGA<br>ACCGAGCGGATATGCACAAACAGATTGCGTGTTGGAAGCAATGGCTTTCCTTGAAGAGTCCCACCCAGGGATCTTTGAAAACTCTTGTCTTGAAACGATGGAAGTCGTTCAGCAAACAAGAGTGGACAAACTAACTCAAGGTCGCCAGACTTATGACTGGACACTGAATAGAAACCAACCAGCTGCAACTGCCCTGGCCAACACTATAGAGGTCTTCAGATCAAACGGTCTAA<br>CAGCCAATGAATCGGGGAGACTAATAGATTTCCTCAAGGATGTGATGGACTCAATGGATAAAGAAGAAATGGAAATAACAACACATTTCCAGAGAAAGAGAAGAGTAAGGGACAACATGACCAAGAAAATGGTCACACAAAGAACAATAGGAAAGAAGAAACAAAGGCTAAACAAGAGGAGCTACTTAATAAGAGCACTGACACTGAATACAATGACAAAAGATGCAGA<br>AAGAGGCAAATTGAAGAGACGGGCGATTGCAACGCCAGGGATGCAGATTAGAGGATTTGTGTACTTTGTCGAAACACTGGCAAGGAGCATCTGTGAAAAACTTGAGCAATCTGGACTCCCCGTTGGAGGGAATGAGAAGAAGGCTAAATTGGCAAATGTCGTGAGAAAAATGATGACTAACTCACAAGATACAGAGCTCTCCTTCACAATCACTGGAGATAACACCAAATG<br>GAATGAGAATCAAAATCCTCGGATGTTTCTGGCAATGATAACATACATTACCAGAAACCAACCTGAATGGTTTAGAAATGTCTTGAGTATTGCCCCTATAATGTTCTCGAACAAAATGGCGAGATTGGGAAAAGGGTACATGTTTGAAAGTAAGAGCATGAAGTTACGGACACAAATACCTGCAGAAATGCTTGCAAACATTGACTTAAAATACTTCAATGAATCAACAAGAAA<br>GAAAATCGAAAAAATAAGGCCTCTACTAATAGATGGCACAGCCTCATTGAGTCCTGGAATGATGATGGGCATGTTCAATATGCTGAGTACAGTATTAGGAGTTTCAATCCTAAATCTTGGGCAAAAGAAGTACACCAAAACCACATACTGGTGGGATGGACTCCAATCCTCTGATGATTTCGCCCTCATAGTAAATGCACCGAATCATGAGGGAATACAAGCAGGAGTGGATA<br>GGTTCTATAGGACCTGCAAACTGGTCGGGATCAATATGAGCAAAAAGAAGTCTTACATAAACCGGACTGGAACATTTGAGTTCACAAGCTTTTTCTATCGCTATGGATTTGTGGCTAACTTCAGTATGGAGCTGCCCAGCTTTGGAGTTTCTGGGATCAATGAATCAGCTGACATGAGCATTGGCGTCACAGTGATAAAGAACAACATGATAAACAACGACCTTGGACCAGCAA<br>CAGCTCAAATGGCCCTTCAACTATTCATCAAAGATTACAGGTACACGTACCGATGCCACAGAGGTGACACACAAATTCAAACGAGGAGATCATTCGAGCTGAAGAAGCTGTGGGAACAGACCCGTTCAAAGGCAGGACTGTTGGTGTCAGATGGAGGACCAAATCTATACAACATTCGGAATCTCCATATCCCAGAGGTCTGCCTGAAGTGGGAGCTGATGGACGAAGATTA<br>CCAGGGCAGGTTGTGTAATCCTCTGAACCCATTTGTCAGCCATAAAGAAATTGAGTCCGTAAACAATGCTGTGGTGATGCCAGCCCACGGTCCAGCCAAAAGCATGGAATATGATGCCGTTGCGACTACACACTCATGGATTCCTAAAAGGAATCGTTCCATTCTCAATACCAGTCAAAGGGGGATTCTTGAGGATGAACAGATGTACCAGAAATGCTGCAGTCTATTCGAGAA<br>ATTCTTCCCCAGTAGTTCATACAGAAGACCAGTTGGAATTTCCAGCATGGTGGAGGCCATGGTGTCTAGGGCCCGAATCGATGCACGCATTGATTTCGAATCTGGAAGGATCAAGAAGGAAGAGTTTGCTGAGATCATGAAGATCTGTTCCACCATTGAAGAGCTCAGACGGCAAAAATAG
EPI_ISL_5143586	>A/chicken/Germany-SN/AI03496/2021_H5N8_2021-03-24<br>ATGGATGTCAATCCGACTTTGCTTTTCTTAAAAGTGCCAGCGCAAAATGCTATAAGTACTACATTCCCTTACACTGGAGATCCCCCATACAGCCATGGAACAGGAACAGGGTATACCATGGACACAGTAAACAGAACACATCAATATTCAGAAAAGGGAAAGTGGACAACAAACACAGAAACCGGAGCACCCYAACTCAACCCAATTGATGGACCATTACCAGAGGACAATGA<br>GCCAAGCGGATATGCACAAACTGATTGCGTGTTGGAAGCAATGGCTTTCCTTGAAGAATCCCACCCAGGGATATTTGAAAACTCTTGTCTTGAAGCGATGGAAATCGTTCAGCAAACAAGAGTGGACAAACTAACCCAAGGTCGCCAGACTTATGACTGGACACTGAACAGAAACCAACCAGCTGCAACCTCTTTGGCCAACACTATAGAGGTGTTCAGATCGAATGGTCTGA<br>CAGCCAATGAATCAGGGAGACTGATAGATTTTCTCAGGGATGTGATGGAATCAATGGATAAAGAAGAGATGGAAGTAACAACACATTTCCAGAGAAAAAGAAGAGTGAGGGACAACATGACTAAGAAGATGGTCACACAAAGAACAATAGGGAAGAAGAAGCAGAGGCTGAACAAGAGGAGTTACTTAATAAGAGCACTGACATTGAATACAATGACCAAAGATGCAGA<br>AAGAGGCAAGTTGAAGAGACGGGCAATTGCAACACCCGGGATGCAGATTAGAGGATTCGTGTACTTCGTTGAAACACTAGCGAGGAGCATCTGTGAGAAACTAGAGCAATCTGGGCTCCCTGTTGGAGGGAATGAGAAAAAGGCTAAATTGGCAAATGTTGTGAGAAAAATGATGACTAACTCACAAGATACAGAGCTCTCCTTTACAATTACTGGAGACAACACCAAATG<br>GAATGAGAATCAAAACCCTCGGATGTTTTTGGCAATGATAACATATATCACAAGAAACCAACCTGAATGGTTTAGAAATGTCTTAAGCATTGCCCCTATAATGTTCTCAAACAAAATGGCGAGATTAGGGAAAGGATACATGTTTGAAAGTAAGAGCATGAAGCTAAGAACACAAATACCAGCAGAGATGCTTACAAATATTGATCTGAAGTATTTCAACGAACCAACGAGAA<br>AGAAAATCGAGAAAATAAGACCTCTGCTGATTGATGGCACGGCTTCATTGAGCCCTGGGATGATGATGGGCATGTTCAATATGCTGAGCACAGTATTAGGGGTCTCAATCCTGAATCTCGGGCAAAAGAGGTACACCAAAACCACATACTGGTGGGATGGACTTCAATCCTCTGATGATTTCGCTCTCATAGTGAATGCACCGAACCATGAGGGGATACAAGCAGGAGTGGAT<br>AGATTCTATAGGACCTGCAAACTGGTTGGGATCAACATGAGCAAAAAGAAGTCTTACATAAACCGAACAGGAACATTTGAGTTCACAAGTTTTTTCTACCGCTATGGATTTGTAGCCAACTTCAGTATGGAATTACCCAGCTTTGGAGTGTCTGGAATCAATGAATCAGCTGACATGAGCATTGGAGTTACAGTGATAAAAAACAATATGATAAACAATGATCTTGGACCAGCA<br>ACAGCTCAAATGGCTCTTCAGTTATTCATCAAAGACTATAGGTACACATACCGATGCCACAGGGGTGATACACAAATTCAAACGAGGAGATCATTCGAGCTGAAGAAGCTGTGGGAGCAGACCCGTTCAAAGGCAGGACTGTTGATATCAGACGGGGGGCCAAACCTATACAACATTCGGAATCTCCACATCCCAGAGGTCTGCTTGAAGTGGGAACTGATGGATGAAGACT<br>ACCAAGGCAGGCTGTGCAATCCTCTGAATCCATTTGTCAGTCATAAAGAGATTGAGTCCGTAAACAATGCTGTAGTAATGCCCGCCCATGGCCCGGCCAAGAGCATGGAATATGATGCTGTTGCGACCACACACTCGTGGATTCCTAAGAGGAACCGTTCCATCCTCAATACCAGCCAAAGGGGAATTCTTGAGGATGAGCAGATGTACCAAAAGTGCTGTAGTCTATTCGAGA<br>AATTCTTCCCCAGCAGTTCATACAGGAGGCCAGTTGGAATTTCCAGCATGGTGGAGGCCATGGTGTCTAGGGCCCGAATTGATGCACGCATCGACTTCGAATCTGGAAGGATTAAGAAAGAAGAGTTTGCTGAGATCATGAAGATCTGTTCCACCATTGAAGAGCTCAGACGGCAAAAATAG
EPI_ISL_5098143	>A/chicken/Germany-NI/AI00887/2021_H5N8_2021-01-22<br>ATGGATGTCAATCCGACTTTACTTTTCTTAAAAGTGCCAGCGCAAAATGCTATAAGTACTACATTCCCTTACACTGGAGATCCCCCATACAGCCATGGAACAGGAACAGGGTATACCATGGACACAGTAAACAGAACACATCAATACTCAGAAAAGGGAAAGTGGACAACAAACACAGAAACCGGAGCACCCCAACTCAACCCAATTGATGGACCATTACCAGAGGACAATGA<br>GCCAAGCGGATATGCACAAACTGATTGCGTGTTGGAAGCAATGGCTTTCCTTGAAGAATCCCACCCAGGGATATTTGAAAACTCTTGTCTTGAAGCGATGGAAATCGTTCAGCAAACAAGAGTGGACAAACTAACCCAAGGTCGCCAGACTTATGACTGGACACTGAACAGAAACCAACCAGCTGCAACCTCTTTGGCCAACACTATAGAGGTGTTCAGATCGAATGGTCTGA<br>CAGCCAATGAATCAGGGAGACTGATAGATTTTCTCAGGGATGTGATGGAATCAATGGATAAAGAAGAGATGGAAGTAACAACACATTTCCAGAGAAAAAGAAGAGTGAGGGACAACATGACTAAGAAGATGGTCACACAAAGAACAATAGGGAAGAAGAAGCAGAGGCTGAACAAGAGGAGTTACTTAATAAGAGCACTGACATTGAATACAATGACCAAAGATGCAGA<br>AAGAGGCAAGTTGAAGAGACGGGCAATTGCAACACCCGGGATGCAGATTAGAGGATTCGTGTACTTCGTTGAAACACTAGCGAGGAGCATCTGTGAGAAACTAGAGCAATCTGGGCTCCCTGTTGGAGGGAATGAGAAAAAGGCTAAATTGGCAAATGTTGTGAGAAAAATGATGACTAACTCACAAGATACAGAGCTCTCCTTTACAATTACTGGAGACAACACCAAATG<br>GAATGAGAATCAAAACCCTCGGATGTTTTTGGCAATGATAACATATATCACAAGAAACCAACCTGAATGGTTTAGAAATGTCTTAAGCATTGCCCCTATAATGTTCTCAAACAAAATGGCGAGATTAGGGAAAGGATACATGTTTGAAAGTAAGAGCATGAAGCTAAGAACACAAATACCAGCAGAGATGCTTACAAATATTGATCTGAAGTATTTCAACGAACCAACGAGAA<br>AGAAAATCGAGAAAATAAGACCTCTGCTGATTGATGGCACGGCCTCATTGAGTCCTGGGATGATGATGGGCATGTTCAATATGCTGAGCACAGTATTAGGGGTCTCAATCCTGAATCTCGGGCAAAAGAGGTACACCAAAACCACATACTGGTGGGATGGACTCCAATCCTCTGATGATTTCGCTCTCATAGTGAATGCACCGAATCATGAGGGGATACAAGCAGGAGTGGAT<br>AGATTCTATAGGACCTGCAAACTGGTTGGGATCAACATGAGCAAAAAGAAGTCTTACATAAACCGAACAGGAACATTTGAGTTCACAAGTTTTTTCTACCGCTATGGATTTGTAGCCAACTTCAGTATGGAATTACCCAGCTTTGGAGTGTCTGGAATCAATGAATCAGCTGACATGAGCATTGGAGTTACAGTGATAAAAAACAATATGATAAACAATGATCTTGGACCAGCA<br>ACAGCTCAAATGGCTCTTCAGTTATTCATCAAAGACTATAGGTACACATACCGATGCCACAGGGGTGATACACAAATTCAAACGAGGAGATCATTCGAGCTGAAGAAGCTGTGGGAGCAGACCCGTTCAAAGGCAGGGCTGTTGATATCAGACGGGGGGCCAAACCTATACAACATTCGGAATCTCCACATCCCAGAGGTCTGCTTGAAGTGGGAGCTGATGGATGAAGACT<br>ACCAAGGCAGGCTGTGCAATCCTCTGAATCCATTTGTCAGTCATAAAGAGATTGAGTCCGTAAACAATGCTGTAGTAATGCCCGCCCATGGCCCGGCCAAGAGCATGGAATATGATGCTGTTGCGACCACACACTCGTGGATTCCTAAGAGGAACCGTTCCATCCTCAATACCAGCCAAAGGGGAATTCTTGAGGATGAGCAGATGTACCAAAAGTGCTGTAGTCTATTCGAGA<br>AATTCTTCCCCAGCAGTTCATACAGGAGGCCAGTTGGAATTTCCAGCATGGTGGAGGCCATGGTGTCTAGGGCCCGAATTGATGCACGCATCGACTTCGAATCTGGAAGGATTAAGAAAGAAGAGTTTGCTGAGATCATGAAGATCTGTTCCACCATTGAAGAGCTCAGACGGCAAAAATAG
EPI_ISL_5095555	>A/chicken/Germany-NI/AI00547/2021_H5N8_2021-01-12<br>ATGGATGTCAATCCGACTTTACTTTTCTTAAAAGTGCCAGCGCAAAATGCTATAAGTACTACATTCCCTTACACTGGAGATCCCCCATACAGCCATGGAACAGGAACAGGGTATACCATGGACACAGTAAACAGAACACATCAATACTCAGAAAAGGGAAAGTGGACAACAAACACAGAAACCGGAGCACCCCAACTCAACCCAATTGATGGACCATTACCAGAGGACAATGA<br>GCCAAGCGGATATGCACAAACTGATTGCGTGTTGGAAGCAATGGCTTTCCTTGAAGAATCCCACCCAGGGATATTTGAAAACTCTTGTCTTGAAGCGATGGAAATCGTTCAGCAAACAAGAGTGGACAAACTAACCCAAGGTCGCCAGACTTATGACTGGACACTGAACAGAAACCAACCAGCTGCAACCTCTTTGGCCAACACTATAGAGGTGTTCAGATCGAATGGTCTGA<br>CAGCCAATGAATCAGGGAGACTGATAGATTTTCTCAGGGATGTGATGGAATCAATGGATAAAGAAGAGATGGAAGTAACAACACATTTCCAGAGAAAAAGAAGAGTGAGGGACAACATGACTAAGAAGATGGTCACACAAAGAACAATAGGGAAGAAGAAGCAGAGGCTGAACAAGAGGAGTTACTTAATAAGAGCACTGACATTGAATACAATGACCAAAGATGCAGA<br>AAGAGGCAAGTTGAAGAGACGGGCAATTGCAACACCCGGGATGCAGATTAGAGGATTCGTGTACTTCGTTGAAACACTAGCGAGGAGCATCTGTGAGAAACTAGAGCAATCTGGGCTCCCTGTTGGAGGGAATGAGAAAAAGGCTAAATTGGCAAATGTTGTGAGAAAAATGATGACTAACTCACAAGATACAGAGCTCTCCTTTACAATTACTGGAGACAACACCAAATG<br>GAATGAGAATCAAAACCCTCGGATGTTTTTGGCAATGATAACATATATCACAAGAAACCAACCTGAATGGTTTAGAAATGTCTTAAGCATTGCCCCTATAATGTTCTCAAACAAAATGGCGAGATTAGGGAAAGGATACATGTTTGAAAGTAAGAGCATGAAGCTAAGAACACAAATACCAGCAGAGATGCTTACAAATATTGATCTGAAGTATTTCAACGAACCAACGAGAA<br>AGAAAATCGAGAAAATAAGACCTCTGCTGATTGATGGCACGGCCTCATTGAGTCCTGGGATGATGATGGGCATGTTCAATATGCTGAGCACAGTATTAGGGGTCTCAATCCTGAATCTCGGGCAAAAGAGGTACACCAAAACCACATACTGGTGGGATGGACTCCAATCCTCTGATGATTTCGCTCTCATAGTGAATGCACCGAATCATGAGGGGATACAAGCAGGAGTGGAT<br>AGATTCTATAGGACCTGCAAACTGGTTGGGATCAACATGAGCAAAAAGAAGTCTTACATAAACCGAACAGGAACATTTGAGTTCACAAGTTTTTTCTACCGCTATGGATTTGTAGCCAACTTCAGTATGGAATTACCCAGCTTTGGAGTGTCTGGAATCAATGAATCAGCTGACATGAGCATTGGAGTTACAGTGATAAAAAACAATATGATAAACAATGATCTTGGACCAGCA<br>ACAGCTCAAATGGCTCTTCAGTTATTCATCAAAGACTATAGGTACACATACCGATGCCACAGGGGTGATACACAAATTCAAACGAGGAGATCATTCGAGCTGAAGAAGCTGTGGGAGCAGACCCGTTCAAAGGCAGGGCTGTTGATATCAGACGGGGGGCCAAACCTATACAACATTCGGAATCTCCACATCCCAGAGGTCTGCTTGAAGTGGGAGCTGATGGATGAAGACT<br>ACCAAGGCAGGCTGTGCAATCCTCTGAATCCATTTGTCAGTCATAAAGAGATTGAGTCCGTAAACAATGCTGTAGTAATGCCCGCCCATGGCCCGGCCAAGAGCATGGAATATGATGCTGTTGCGACCACACACTCGTGGATTCCTAAGAGGAACCGTTCCATCCTCAATACCAGCCAAAGGGGAATTCTTGAGGATGAGCAGATGTACCAAAAGTGCTGTAGTCTATTCGAGA<br>AATTCTTCCCCAGCAGTTCATACAGGAGGCCAGTTGGAATTTCCAGCATGGTGGAGGCCATGGTGTCTAGGGCCCGAATTGATGCACGCATCGACTTCGAATCTGGAAGGATTAAGAAAGAAGAGTTTGCTGAGATCATGAAGATCTGTTCCACCATTGAAGAGCTCAGACGGCAAAAATAG
EPI_ISL_5102132	>A/chicken/Germany-BY/AI02064/2021_H5N8_2021-03-01<br>ATGGATGTCAATCCGACTTTACTTTTCTTAAAAGTGCCAGCGCAAAATGCTATAAGTACTACATTCCCTTACACTGGAGATCCTCCATACAGCCATGGAACAGGAACAGGGTATACCATGGACACAGTAAACAGAACACATCAATACTCAGAAAAGGGAAAGTGGACAACAAACACAGAAACCGGAGCACCCYAACTCAACCCAATTGATGGACCATTACCAGAGGACAATGA<br>GCCAAGCGGATATGCACAAACTGATTGCGTGTTGGAAGCAATGGCTTTCCTTGAAGAATCCCACCCAGGGATATTTGAAAACTCTTGTCTTGAAGCGATGGAAATCGTTCAGCAAACAAGAGTGGACAAACTAACCCAAGGTCGCCAGACTTATGACTGGACACTGAACAGAAACCAACCAGCTGCAACCTCTTTGGCCAACACTATAGAGGTGTTCAGATCGAATGGTCTGA<br>CAGCCAATGAATCAGGGAGACTGATAGATTTTCTCAGGGATGTGATGGAATCAATGGATAAAGAAGAGATGGAAGTAACAACACATTTCCAGAGAAAAAGAAGAGTGAGGGACAATATGACTAAGAAGATGGTCACACAAAGAACAATAGGGAAGAAGAAGCAGAGGCTGATCAAGAGGAGTTACTTAATAAGAGCACTGACATTGAATACAATGACCAAAGATGCAGA<br>AAGAGGCAAGTTGAAGAGACGGGCAATTGCAACACCCGGGATGCAGATTAGAGGATTCGTGTACTTCGTTGAAACACTAGCGAGGAGCATCTGTGAGAAACTAGAGCAATCTGGGCTCCCTGTTGGAGGGAATGAGAAGAAGGCTAAATTGGCAAATGTTGTGAGAAAAATGATGACTAACTCACAAGATACAGAGCTCTCCTTTACAATTACTGGAGACAACACCAAATG<br>GAATGAGAATCAAAACCCTCGGATGTTTTTGGCAATGATAACATATATCACAAGAAACCAACCTGAATGGTTTAGAAATGTCTTAAGCATTGCCCCTATAATGTTCTCAAACAAAATGGCGAGATTAGGGAAAGGATACATGTTTGAAAGTAAGAGCATGAAGCTAAGAACACAAATACCAGCAGAGATGCTTACAAATATCGATCTGAAGTATTTCAACGAACCAACGAGAA<br>AGAAAATCGAGAAAATAAGACCTCTGCTGATTGATGGCACGGCCTCATTGAGTCCTGGGATGATGATGGGCATGTTCAATATGCTGAGCACAGTATTAGGGGTCTCAATCCTGAATCTCGGGCAAAAAAGGTACACCAAAACCACATACTGGTGGGATGGACTTCAATCCTCTGATGATTTCGCTCTCATAGTGAATGCACCGAATCATGAGGGGATACAAGCAGGAGTGGAT<br>AGATTCTATAGGACCTGCAAACTGGTTGGGATCAACATGAGCAAAAAGAAGTCTTACATAAACCGAACAGGAACATTTGAGTTCACAAGTTTTTTCTACCGCTATGGATTTGTAGCTAACTTCAGTATGGAATTACCCAGCTTTGGAGTGTCTGGAATCAATGAATCAGCTGACATGAGCATTGGAGTTACAGTGATAAAAAACAATATGATAAACAATGATCTTGGACCAGCA<br>ACAGCTCAAATGGCTCTTCAGTTATTCATCAAAGACTATAGGTACACATACCGATGCCACAGGGGTGATACACAAATTCAAACGAGGAGATCATTCGAGCTGAAGAAGCTGTGGGAGCAGACCCGTTCAAAGGCAGGGCTGTTGATATCAGACGGGGGGCCAAACCTATACAACATTCGGAATCTCCACATCCCAGAGGTCTGCTTGAAGTGGGAGCTGATGGATGAAGACT<br>ACCAAGGCAGGCTGTGCAATCCTCTGAATCCATTTGTCAGTCATAAAGAGATTGAGTCCGTAAACAATGCTGTAGTAATGCCCGCCCATGGCCCGGCCAAGAGCATGGAATATGATGCTGTTGCGACCACACACTCGTGGATTCCTAAGAGGAACCGTTCCATTCTCAATACCAGCCAAAGGGGAATTCTTGAGGATGAGCAGATGTACCAAAAGTGCTGTAGTCTATTCGAGA<br>AATTCTTCCCCAGCAGTTCATACAGGAGGCCAGTTGGAATTTCCAGCATGGTGGAGGCCATGGTGTCTAGGGCCCGAATTGATGCACGCATCGACTTCGAATCTGGAAGGATTAAGAAAGAAGAGTTTGCTGAGATCATGAAGATCTGTTCCACCATTGAAGAGCTCAGACGGCAAAAATAG
EPI_ISL_5145613	>A/chicken/Germany-BY/AI02060/2021_H5N8_2021-03-01<br>ATGGATGTCAATCCGACTTTACTTTTCTTAAAAGTGCCAGCGCAAAATGCTATAAGTACTACATTCCCTTACACTGGAGATCCTCCATACAGCCATGGAACAGGAACAGGGTATACCATGGACACAGTAAACAGAACACATCAATACTCAGAAAAGGGAAAGTGGACAACAAACACAGAAACCGGAGCACCCYAACTCAACCCAATTGATGGACCATTACCAGAGGACAATGA<br>GCCAAGCGGATATGCACAAACTGATTGCGTGTTGGAAGCAATGGCTTTCCTTGAAGAATCCCACCCAGGGATATTTGAAAACTCTTGTCTTGAAGCGATGGAAATCGTTCAGCAAACAAGAGTGGACAAACTAACCCAAGGTCGCCAGACTTATGACTGGACACTGAACAGAAACCAACCAGCTGCAACCTCTTTGGCCAACACTATAGAGGTGTTCAGATCGAATGGTCTGA<br>CAGCCAATGAATCAGGGAGACTGATAGATTTTCTCAGGGATGTGATGGAATCAATGGATAAAGAAGAGATGGAAGTAACAACACATTTCCAGAGAAAAAGAAGAGTGAGGGACAATATGACTAAGAAGATGGTCACACAAAGAACAATAGGGAAGAAGAAGCAGAGGCTGATCAAGAGGAGTTACTTAATAAGAGCACTGACATTGAATACAATGACCAAAGATGCAGA<br>AAGAGGCAAGTTGAAGAGACGGGCAATTGCAACACCCGGGATGCAGATTAGAGGATTCGTGTACTTCGTTGAAACACTAGCGAGGAGCATCTGTGAGAAACTAGAGCAATCTGGGCTCCCTGTTGGAGGGAATGAGAAGAAGGCTAAATTGGCAAATGTTGTGAGAAAAATGATGACTAACTCACAAGATACAGAGCTCTCCTTTACAATTACTGGAGACAACACCAAATG<br>GAATGAGAATCAAAACCCTCGGATGTTTTTGGCAATGATAACATATATCACAAGAAACCAACCTGAATGGTTTAGAAATGTCTTAAGCATTGCCCCTATAATGTTCTCAAACAAAATGGCGAGATTAGGGAAAGGATACATGTTTGAAAGTAAGAGCATGAAGCTAAGAACACAAATACCAGCAGAGATGCTTACAAATATCGATCTGAAGTATTTCAACGAACCAACGAGAA<br>AGAAAATCGAGAAAATAAGACCTCTGCTGATTGATGGCACGGCCTCATTGAGTCCTGGGATGATGATGGGCATGTTCAATATGCTGAGCACAGTATTAGGGGTCTCAATCCTGAATCTCGGGCAAAAAAGGTACACCAAAACCACATACTGGTGGGATGGACTTCAATCCTCTGATGATTTCGCTCTCATAGTGAATGCACCGAATCATGAGGGGATACAAGCAGGAGTGGAT<br>AGATTCTATAGGACCTGCAAACTGGTTGGGATCAACATGAGCAAAAAGAAGTCTTACATAAACCGAACAGGAACATTTGAGTTCACAAGTTTTTTCTACCGCTATGGATTTGTAGCTAACTTCAGTATGGAATTACCCAGCTTTGGAGTGTCTGGAATCAATGAATCAGCTGACATGAGCATTGGAGTTACAGTGATAAAAAACAATATGATAAACAATGATCTTGGACCAGCA<br>ACAGCTCAAATGGCTCTTCAGTTATTCATCAAAGACTATAGGTACACATACCGATGCCACAGGGGTGATACACAAATTCAAACGAGGAGATCATTCGAGCTGAAGAAGCTGTGGGAGCAGACCCGTTCAAAGGCAGGGCTGTTGATATCAGACGGGGGGCCAAACCTATACAACATTCGGAATCTCCACATCCCAGAGGTCTGCTTGAAGTGGGAGCTGATGGATGAAGACT<br>ACCAAGGCAGGCTGTGCAATCCTCTGAATCCATTTGTCAGTCATAAAGAGATTGAGTCCGTAAACAATGCTGTAGTAATGCCCGCCCATGGCCCGGCCAAGAGCATGGAATATGATGCTGTTGCGACCACACACTCGTGGATTCCTAAGAGGAACCGTTCCATTCTCAATACCAGCCAAAGGGGAATTCTTGAGGATGAGCAGATGTACCAAAAGTGCTGTAGTCTATTCGAGA<br>AATTCTTCCCCAGCAGTTCATACAGGAGGCCAGTTGGAATTTCCAGCATGGTGGAGGCCATGGTGTCTAGGGCCCGAATTGATGCACGCATCGACTTCGAATCTGGAAGGATTAAGAAAGAAGAGTTTGCTGAGATCATGAAGATCTGTTCCACCATTGAAGAGCTCAGACGGCAAAAATAG
EPI_ISL_5099462	>A/chicken/Germany-BY/AI01617/2021_H5N8_2021-02-24<br>ATGGATGTCAATCCGACTTTACTTTTCTTAAAAGTGCCAGCGCAAAATGCTATAAGTACTACATTCCCTTACACTGGAGATCCTCCATACAGCCATGGAACAGGAACAGGGTATACCATGGACACAGTAAACAGAACACATCAATACTCAGAAAAGGGAAAGTGGACAACAAACACAGAAACCGGAGCACCCYAACTCAACCCAATTGATGGACCATTACCAGAGGACAATGA<br>GCCAAGCGGATATGCACAAACTGATTGCGTGTTGGAAGCAATGGCTTTCCTTGAAGAATCCCACCCAGGGATATTTGAAAACTCTTGTCTTGAAGCGATGGAAATCGTTCAGCAAACAAGAGTGGACAAACTAACCCAAGGTCGCCAGACTTATGACTGGACACTGAACAGAAACCAACCAGCTGCAACCTCTTTGGCCAACACTATAGAGGTGTTCAGATCGAATGGTCTGA<br>CAGCCAATGAATCAGGGAGACTGATAGATTTTCTCAGGGATGTGATGGAATCAATGGATAAAGAAGAGATGGAAGTAACAACACATTTCCAGAGAAAAAGAAGAGTGAGGGACAATATGACTAAGAAGATGGTCACACAAAGAACAATAGGGAAGAAGAAGCAGAGGCTGATCAAGAGGAGTTACTTAATAAGAGCACTGACATTGAATACAATGACCAAAGATGCAGA<br>AAGAGGCAAGTTGAAGAGACGGGCAATTGCAACACCCGGGATGCAGATTAGAGGATTCGTGTACTTCGTTGAAACACTAGCGAGGAGCATCTGTGAGAAACTAGAGCAATCTGGGCTCCCTGTTGGAGGGAATGAGAAGAAGGCTAAATTGGCAAATGTTGTGAGAAAAATGATGACTAACTCACAAGATACAGAGCTCTCCTTTACAATTACTGGAGACAACACCAAATG<br>GAATGAGAATCAAAACCCTCGGATGTTTTTGGCAATGATAACATATATCACAAGAAACCAACCTGAATGGTTTAGAAATGTCTTAAGCATTGCCCCTATAATGTTCTCAAACAAAATGGCGAGATTAGGGAAAGGATACATGTTTGAAAGTAAGAGCATGAAGCTAAGAACACAAATACCAGCAGAGATGCTTACAAATATCGATCTGAAGTATTTCAACGAACCAACGAGAA<br>AGAAAATCGAGAAAATAAGACCTCTGCTGATTGATGGCACGGCCTCATTGAGTCCTGGGATGATGATGGGCATGTTCAATATGCTGAGCACAGTATTAGGGGTCTCAATCCTGAATCTCGGGCAAAAAAGGTACACCAAAACCACATACTGGTGGGATGGACTTCAATCCTCTGATGATTTCGCTCTCATAGTGAATGCACCGAATCATGAGGGGATACAAGCAGGAGTGGAT<br>AGATTCTATAGGACCTGCAAACTGGTTGGGATCAACATGAGCAAAAAGAAGTCTTACATAAACCGAACAGGAACATTTGAGTTCACAAGTTTTTTCTACCGCTATGGATTTGTAGCTAACTTCAGTATGGAATTACCCAGCTTTGGAGTGTCTGGAATCAATGAATCAGCTGACATGAGCATTGGAGTTACAGTGATAAAAAACAATATGATAAACAATGATCTTGGACCAGCA<br>ACAGCTCAAATGGCTCTTCAGTTATTCATCAAAGACTATAGGTACACATACCGATGCCACAGGGGTGATACACAAATTCAAACGAGGAGATCATTCGAGCTGAAGAAGCTGTGGGAGCAGACCCGTTCAAAGGCAGGGCTGTTGATATCAGACGGGGGGCCAAACCTATACAACATTCGGAATCTCCACATCCCAGAGGTCTGCTTGAAGTGGGAGCTGATGGATGAAGACT<br>ACCAAGGCAGGCTGTGCAATCCTCTGAATCCATTTGTCAGTCATAAAGAGATTGAGTCCGTAAACAATGCTGTAGTAATGCCCGCCCATGGCCCGGCCAAGAGCATGGAATATGATGCTGTTGCGACCACACACTCGTGGATTCCTAAGAGGAACCGTTCCATTCTCAATACCAGCCAAAGGGGAATTCTTGAGGATGAGCAGATGTACCAAAAGTGCTGTAGTCTATTCGAGA<br>AATTCTTCCCCAGCAGTTCATACAGGAGGCCAGTTGGAATTTCCAGCATGGTGGAGGCCATGGTGTCTAGGGCCCGAATTGATGCACGCATCGACTTCGAATCTGGAAGGATTAAGAAAGAAGAGTTTGCTGAGATCATGAAGATCTGTTCCACCATTGAAGAGCTCAGACGGCAAAAATAG
EPI_ISL_11798570	>A/chicken/Denmark/24357-11/2021-11-02_H5N1_2021-11-02<br>ATGGATGTCAATCCGACTTTACTTTTCTTAAAAGTGCCAGCGCAAGATGCCATAAGTACCACATTCCCTTACACTGGAGATCCTCCATACAGCCATGGAACAGGGACAGGATACACAATGGACACAGTCAACAGAACACATCAATACTCAGAGAAGGGAAAATGGACAACAAACACAGAAACCGGAGCACCTCAACTCAACCCAATTGATGGGCCACTACCTGAGGACAACGA<br>ACCGAGCGGATATGCACAAACAGATTGCGTGTTGGAAGCAATGGCTTTCCTTGAAGAGTCCCACCCAGGGATCTTTGAAAACTCTTGTCTTGAAACGATGGAAGTCGTTCAGCAAACAAGAGTGGACAAACTAACTCAAGGTCGCCAGACATATGACTGGACACTGAATAGAAACCAACCAGCTGCAACTGCCCTGGCCAACACTATAGAGGTCTTCAGATCAAACGGTCTAA<br>CAACCAATGAATCGGGGAGACTAATAGATTTCCTCAAGGATGTGATGGACTCAATGGATAAAGAAGAAATGGAAATAACAACACATTTCCAGAGAAAGAGAAGAGTAAGGGACAACATGACCAAGAAAATGGTCACACAAAGAACAATAGGAAAGAAGAAACAAAGGCTAACCAAGAGGAGCTACTTAATAAGAGCACTGACACTGAATACAATGACAAAAGATGCAGAA<br>AGAGGCAAATTGAAAAGACGGGCGATTGCAACACCAGGGATGCAGATTAGAGGATTTGTGTACTTTGTCGAAACACTGGCAAGGAGCATCTGTGAAAAACTTGAGCAATCTGGACTCCCCGTTGGAGGGAATGAGAAGAAGGCTAAATTGGCAAATGTCGTGAGAAAAATGATGACTAACTCACAAGATACAGAGCTCTCCTTCACAATTACTGGAGATAACACCAAATGG<br>AATGAGAATCAAAATCCTCGGATGTTTCTGGCAATGATAACATACATTACAAGAAACCAACCTGAATGGTTTAGAAATGTCTTGAGTATTGCCCCTATAATGTTCTCGAACAAAATGGCGAGATTGGGAAAAGGGTACATGTTTGAAAGTAAGAGCATGAAGTTACGGACACAAATACCTGCAGAAATGCTTGCAAACATTGACTTAAAATACTTCAATGAATCAACAAGAAAG<br>AAAATCGAAAAAATAAGGCCTCTACTAATAGATGGCACTGCCTCATTGAGTCCTGGAATGATGATGGGCATGTTCAATATGCTGAGTACAGTATTAGGAGTTTCAATCCTAAATCTTGGGCAAAAGAAGTACACCAAAACCACATACTGGTGGGATGGACTCCAATCCTCTGATGATTTCGCCCTCATAGTAAATGCACCGAATCATGAGGGAATACAAGCAGGAGTGGATAG<br>GTTCTATAGGACCTGCAAACTGGTCGGGATCAATATGAGCAAAAAGAAGTCTTACATAAACCGGACTGGAACATTTGAGTTCACAAGCTTTTTCTATCGCTATGGATTTGTGGCTAACTTCAGTATGGAGCTGCCCAGCTTTGGAGTTTCTGGGATCAATGAATCAGCTGACATGAGCATTGGCATCACAGTGATAAAGAACAACATGATAAACAATGACCTTGGACCAGCAAC<br>AGCTCAAATGGCCCTTCAACTATTCATCAAAGATTACAGGTACACGTACCGATGCCACAGAGGTGACACACAAATTCAAACGAGGAGATCATTCGAGCTGAAGAAGCTGTGGGAACAGACCCGTTCAAAGGCAGGACTGTTGGTGTCAGATGGAGGACCAAATCTATACAACATTCGGAATCTCCATATCCCAGAGGTCTGCCTGAAGTGGGAGCTGATGGACGAAGATTAC<br>CAGGGCAGGTTGTGTAATCCTCTGAACCCATTTGTCAGCCATAGAGAAATTGAGTCCGTAAACAATGCTGTGGTGATGCCAGCCCACGGTCCAGCCAAAAGCATGGAATATGATGCCGTTGCGACTACACACTCATGGATTCCTAAAAGGAATCGTTCCATTCTCAATACCAGTCAAAGGGGAATTCTTGAGGATGAACAGATGTACCAGAAATGCTGCAGTCTATTCGAGAAA<br>TTCTTCCCCAGTAGTTCATACAGGAGACCAGTTGGAATTTCCAGCATGGTGGAGGCCATGGTGTCTAGGGCCCGAATCGATGCACGCATTGATTTCGAATCTGGAAGGATCAAGAAGGAAGAGTTTGCTGAGATCATGAAGATCTGTTCCACCATTGAAGAGCTCAGACGGCAAAAATAG

EPI_ISL_1941582	>A/chicken/Czech_Republic/7100/2021_H5N8_2021-04-08<br>ATGGATGTCAATCCGACTTTGCTTTTCTTAAAAGTGCCAGCGCAAAATGCTATAAGTACTACATTCCCTTACACTGGAGATCCCCCATACAGCCATGGAACAGGAACAGGGTATACCATGGACACAGTAAACAGAACACATCAATATTCAGAAAAGGGAAAGTGGACAACAAACACAGAAACCGGAGCACCCCAACTCAACCCAATTGATGGACCATTACCAGAGGACAATGA<br>GCCAAGCGGATATGCACAAACTGATTGCGTGTTGGAAGCAATGGCTTTCCTTGAAGAATCCCACCCAGGGATATTTGAAAACTCTTGTCTTGAAGCGATGGAAATCGTTCAGCAAACAAGAGTGGACAAACTAACCCAAGGTCGCCAGACTTATGACTGGACACTGAACAGAAACCAACCAGCTGCAACCTCTTTGGCCAACACTATAGAGGTGTTCAGATCGAATGGTCTGA<br>CAGCCAATGAATCAGGGAGACTGATAGATTTTCTCAGGGATGTGATGGAATCAATGGATAAAGAAGAGATGGAAGTAACAACACATTTCCAGAGAAAAAGAAGAGTGAGGGACAACATGACTAAGAAGATGGTCACACAAAGAACAATAGGGAAGAAGAAGCAGAGGCTGAACAAGAGGAGTTACTTAATAAGAGCACTGACATTGAATACAATGACCAAAGATGCAGA<br>AAGAGGCAAGTTGAAGAGACGGGCAATTGCAACACCCGGGATGCAGATTAGAGGATTCGTGTACTTCGTTGAAACACTAGCGAGGAGCATCTGTGAGAAACTAGAGCAATCTGGGCTCCCTGTTGGAGGGAATGAGAAAAAGGCTAAATTGGCAATGTTGTGAGAAAAATGATGACTAACTCACAAGATACAGAGCTCTCCTTTACAATTACTGGAGACAACACCAAATG<br>GAATGAGAATCAAAACCCTCGGATGTTTTTGGCAATGATAACATATATCACAAGAAACCAACCTGAATGGTTTAGAAATGTCTTAAGCATTGCCCCTATAATGTTCTCAAACAAAATGGCGAGATTAGGGAAAGGATACATGTTTGAAAGTAAGAGCATGAAGCTAAGAACACAAATACCAGCAGAGATGCTTACAAATATTGATCTGAAGTATTTCAACGAACCAACGAGAA<br>AGAAAATCGAGAATATAAGACCTCTGCTGATTGATGGCACGGCTTCATTGAGCCCTGGGATGATGATGGGCATGTTCAATATGCTGAGCACAGTATTAGGGGTCTCAATCCTGAATCTCGGGCAAAAGAGGTACACCAAAACCACATACTGGTGGGATGGACTTCAATCCTCTGATGATTTCGCTCTCATAGTGAATGCACCGAATCATGAGGGGATACAAGCAGGAGTGGAT<br>AGATTCTATAGGACCTGCAAACTGGTTGGGATCAACATGAGCAAAAAGAAGTCTTACATAAACCGAACAGGAACATTTGAGTTCACAAGTTTTTTCTACCGCTATGGATTTGTAGCCAACTTCAGTATGGAATTACCCAGCTTTGGAGTGTCTGGAATCAATGAATCAGCTGACATGAGCATTGGAGTTACAGTGATAAAAAACAATATGATAAACAATGATCTTGGACCAGCA<br>ACAGCTCAAATGGCTCTTCAGTTATTCATCAAAGACTATAGGTACACATACCGATGCCACAGGGGTGATACACAAATTCAAACGAGGAGATCATTCGAGCTGAAGAAGCTGTGGGAGCAGACCCGTTCAAAGGCAGGACTGTTGATATCAGACGGGGGGCCAAACCTATACAACATTCGGAATCTCCACATCCCAGAGGTCTGCTTGAAGTGGGAGCTGATGGATGAAGACT<br>ACCAAGGCAGGCTGTGCAATCCTCTGAATCCATTTGTCAGTCATAAAGAGATTGAGTCCGTAAACAATGCTGTAGTAATGCCCGCCCATGGCCCGGCCAAGAGCATGGAATATGATGCTGTTGCGACCACACACTCGTGGATTCCTAAGAGGAACCGTTCCATCCTCAATACCAGCCAAAGGGGAATTCTTGAGGATGAGCAGATGTACCAGAAGTGCTGTAGTCTATTCGAG<br>AAATTCTTCCCCAGCAGTTCATACAGGAGGCCAGTTGGAATTTCCAGCATGGTGGAGGCCATGGTGTCTAGGGCCCGAATTGATGCACGCATCGACTTCGAATCTGGAAGGATTAAGAAAGAAGAGTTTGCTGAGATCATGAAGATCTGTTCCACCATTGAAGAGCTCAGACGGCAAAAATAG
EPI_ISL_11327090	>A/chicken/Czech_Republic/63/2022_H5N1_2022-01-03<br>ATGGATGTCAATCCGACTTTACTTTTCTTAAAAGTGCCAGCGCAAGATGCCATAAGTACCACATTCCCTTACACTGGAGATCCTCCATACAGCCATGGAACAGGGACAGGATACACAATGGACACAGTCAACAGAACACATCAATACTCAGAGAAGGGAAAATGGACAACAAACACAGAAACCAGAGCACCTCAACTCAACCCAATTGATGGGCCACTACCTGAGGACAACGA<br>ACCGAGCGGATATGCACAAACAGATTGCGTGTTGGAAGCAATGGCTTTCCTTGAAGAGTCCCACCCAGGGATCTTTGAAAACTCTTGTCTTGAAACGATGGAAGTCGTTCAGCAAACAAGAGTGGACAAACTAACTCAAGGTCGCCAGACATATGACTGGACACTGAATAGAAACCAACCAGCTGCAACTGCCCTGGCCAACACTATAGAGGTCTTCAGATCAAACGGTCTAA<br>CAGCCAATGAATCGGGGAGACTAATAGATTTCCTCAAGGATGTGATGGACTCAATGGATAAAGAAGAAATGGAAATAACAACACATTTCCAGAGAAAGAGAAGAGTAAGGGACAACATGACCAAGAAAATGGTCACACAAAGAACAATAGGAAAGAAGAAACAAAGGCTAAACAAGAGGAGCTACTTAATAAGAGCACTGACACTGAATACAATGACAAAAGATGCAGA<br>AAGAGGCAAATTGAAAAGACGGGCGATTGCAACACCAGGGATGCAGATTAGAGGATTTGTGTACTTTGTCGAAACACTGGCAAGGAGCATCTGTGAAAAACTTGAGCAATCTGGACTCCCCGTTGGAGGGAATGAGAAGAAGGCTAAATTGGCAAATGTCGTGAGAAAAATGATGACTAACTCACAAGATACAGAGCTCTCCTTCACAATTACTGGAGATAACACCAAATG<br>GAATGAGAATCAAAATCCTCGGATGTTTCTGGCAATGATAACATACATTACAAGAAACCAACCTGAATGGTTTAGAAATGTCTTGAGTGTTGCCCCTATAATGTTCTCGAACAAAATGGCGAGATTGGGAAAAGGGTACATGTTTGAAAGTAAAAGCATGAAGTTACGGACACAAATACCTGCAGAAATGCTTGCAAACATTGACTTAAAATACTTCAATGAATCAACAAGAAA<br>GAAAATCGAAAAAATAAGGCCTCTACTAATAGATGGCACTGCCTCATTGAGTCCTGGAATGATGATGGGCATGTTCAATATGCTGAGTACAGTATTAGGAGTTTCAATCCTAAATCTTGGGCAAAAGAAGTACACCAAAACCACATACTGGTGGGATGGACTCCAATCCTCTGATGATTTCGCCCTCATAGTAAATGCACCGAATCATGAGGGAATACAAGCAGGAGTGGATA<br>GGTTCTATAGGACCTGCAAACTGGTCGGGATCAATATGAGCAAAAAGAAGTCTTACATAAACCGGACTGGAACATTTGAGTTCACAAGCTTTTTCTATCGCTATGGATTTGTGGCTAACTTCAGTATGGAGCTGCCCAGCTTTGGAGTTTCTGGGATCAATGAATCAGCTGACATGAGCATTGGCGTCACAGTGATAAAGAACAACATGATAAACAATGACCTTGGACCAGCAA<br>CAGCTCAAATGGCCCTTCAACTATTCATCAAAGATTACAGGTACACGTACCGATGCCACAGAGGTGACACACAAATTCAAACGAGGAGATCATTCGAGCTGAAGAAGCTGTGGGAACAGACCCGTTCAAAGGCAGGACTGTTGGTGTCAGATGGAGGACCAAATCTATACAACATTCGGAATCTCCATATCCCAGAGGTCTGCCTGAAGTGGGAGCTGATGGACGAAGATTA<br>CCAGGGCAGGTTGTGTAATCCTCTGAACCCATTTGTCAGCCATAGAGAAATTGAGTCCGTAAACAATGCTGTGGTGATGCCAGCCCACGGTCCAGCCAAAAGCATGGAATATGATGCCGTTGCGACTACACACTCATGGATTCCTAAAAGGAATCGTTCCATTCTCAATACCAGTCAAAGGGGAATTCTTGAGGATGAACAGATGTACCAGAAATGCTGCAGTCTATTCGAGAA<br>ATTCTTCCCCAGTAGTTCATACAGGAGACCAGTTGGAATTTCCAGCATGGTGGAGGCCATGGTGTCTAGGGCCCGAATCGATGCACGAATTGATTTCGAATCTGGAAGGATAAAGAAGGAAGAGTTTGCTGAGATCATGAAGATCTGTTCCACCATTGAAGAGCTCAGACGGCAAAAATAG
EPI_ISL_1697194	>A/chicken/Czech_Republic/4980/2021_H5N8_2021-03-12<br>ATGGATGTCAATCCGACTTTACTTTTCTTAAAAGTGCCAGCGCAAAATGCTATAAGTACTACATTCCCTTACACTGGAGATCCTCCATACAGCCATGGAACAGGAACAGGGTATACCATGGACACAGTAAACAGAACACATCAATACTCAGAAAAGGGAAAGTGGACAACAAACACAGAAACCGGAGCACCCCAACTCAACCCAATTGATGGACCATTACCAGAGGACAATGA<br>GCCAAGCGGATATGCACAAACTGATTGCGTGTTGGAAGCAATGGCTTTCCTTGAAGAATCCCACCCAGGGATATTTGAAAACTCTTGTCTTGAAGCGATGGAAATCGTTCAGCAAACAAGAGTGGACAAACTAACCCAAGGTCGCCAGACTTATGACTGGACACTGAACAGAAACCAACCAGCTGCAACCTCTTTGGCCAACACTATAGAGGTGTTCAGATCGAATGGTCTGA<br>CAGCCAATGAATCAGGGAGACTGATAGATTTTCTCAGGGATGTGATGGAATCAATGGATAAAGAAGAGATGGAAGTAACAACACATTTCCAGAGAAAAAGAAGAGTGAGGGACAACATGACTAAGAAGATGGTCACACAAAGAACAATAGGGAAGAAGAAGCAGAGGCTGAACAAGAGGAGTTACTTAATAAGAGCACTGACATTGAATACAATGACCAAAGATGCAGA<br>AAGAGGCAAGTTGAAGAGACGGGCAATTGCAACACCCGGGATGCAGATTAGAGGATTCGTGTACTTCGTTGAAACACTAGCGAGGAGCATCTGTGAGAAACTAGAGCAATCTGGGCTCCCTGTTGGAGGGAATGAGAAGAAGGCTAAATTGGCAATGTTGTGAGAAAAATGATGACTAACTCACAAGATACAGAGCTCTCCTTTACAATTACTGGAGACAACACCAAATG<br>GAATGAGAATCAAAACCCTCGGATGTTTTTGGCAATGATAACATATATCACAAGAAACCAACCTGAATGGTTTAGAAATGTCTTAAGCATTGCCCCTATAATGTTCTCAAACAAAATGGCGAGATTAGGGAAAGGATACATGTTTGAAAGTAAGAGCATGAAGCTAAGAACACAAATACCAGCAGAGATGCTTACAAATATCGATCTGAAGTATTTCAACGAACCAACGAGAA<br>AGAAAATCGAGAAAATAAGACCTCTGCTGATTGATGGCACGGCCTCATTGAGTCCTGGGATGATGATGGGCATGTTCAATATGCTGAGCACAGTATTAGGGGTCTCAATCCTGAATCTCGGGCAAAAAAGGTACACCAAAACCACATACTGGTGGGATGGACTTCAATCCTCTGATGATTTCGCTCTCATAGTGAATGCACCGAATCATGAGGGGATACAAGCAGGAGTGGAT<br>AGATTCTATAGGACCTGCAAACTGGTTGGGATCAACATGAGCAAAAGGAAGTCTTACATAAACCGAACAGGAACATTTGAGTTCACAAGTTTTTTCTACCGCTATGGATTTGTAGCTAACTTCAGTATGGAATTACCCAGCTTTGGAGTGTCTGGAATCAATGAATCAGCTGACATGAGCATTGGAGTTACAGTGATAAAAAACAATATGATAAACAATGATCTTGGACCAGCA<br>ACAGCTCAAATGGCTCTTCAGTTATTCATCAAAGACTACAGGTACACATACCGATGCCACAGGGGTGATACACAAATTCAAACGAGGAGATCATTCGAGCTGAAGAAGCTGTGGGAGCAGACCCGCTCAAAGGCAGGGCTGTTGATATCAGACGGGGGGCCAAACCTATACAACATTCGGAATCTCCACATCCCAGAGGTCTGCTTGAAGTGGGAGTTGATGGATGAAGACT<br>ACCAAGGCAGGCTGTGCAATCCTCTGAATCCATTTGTCAGTCATAAAGAGATTGAGTCCGTAAACAATGCTGTAGTAATGCCCGCCCATGGCCCGGCCAAGAGCATGGAATATGATGCTGTTGCGACCACACACTCGTGGATTCCTAAGAGGAACCGTTCCATTCTCAATACCAGCCAAAGGGGAATTCTTGAAGATGAGCAGATGTACCAAAAGTGCTGTAGTCTATTCGAGA<br>AATTCTTCCCCAGCAGTTCATACAGGAGGCCAGTTGGAATTTCCAGCATGGTGGAGGCCATGGTGTCTAGGGCCCGAATTGATGCACGCATCGACTTCGAATCTGGAAGGATTAAGAAAGAAGAGTTTGCTGAGATCATGAAGATCTGTTCCACCATTGAAGAGCTCAGACGGCAAAAATAG
EPI_ISL_1058022	>A/chicken/Czech_Republic/3099-2/2021_H5N8_2021-02-12<br>ATGGATGTCAATCCGACTTTACTTTTCGTAAAAGTGCCAGCGCAAAATGCTATAAGTACTACATTCCCTTACACTGGAGATCCCCCATACAGCCATGGAACAGGAACAGGGTATACCATGGACACAGTAAACAGAACACATCAATACTCAGAAAAGGGAAAGTGGACAACAAACACAGAAACCGGAGCACCCCAACTCAACCCAATTGATGGACCATTACCAGAGGACAATGA<br>GCCAAGCGGATATGCACAAACTGATTGCGTGTTGGAAGCAATGGCTTTCCTTGAAGAATCCCACCCAGGGATATTTGAAAACTCTTGTCTTGAAGCAATGGAAATCGTTCAGCAAACAAGAGTGGACAAACTAACCCAAGGTCGCCAGACTTATGACTGGACACTGAACAGAAACCAACCAGCTGCAACCTCTTTGGCCAACACTATAGAGGTGTTCAGATCGAATGGTCTGA<br>CAGCCAATGAATCAGGGAGACTGATAGATTTTCTCAGGGATGTGATGGAATCAATGGATAAAGAAGAGATGGAAATAACAACACATTTCCAGAGAAAAAGAAGAGTGAGGGACAACATGACTAAGAAGATGGTCACACAAAGAACAATAGGGAAGAAGAAGCAGAGGCTGAACAAGAGGAGTTACTTAATAAGAGCACTGACATTGAATACAATGACCAAAGATGCAGA<br>AAGAGGCAAGTTGAAGAGACGGGCAATTGCAACACCCGGGATGCAGATTAGAGGATTCGTGTACTTCGTTGAAACACTAGCGAGGAGCATCTGTGAGAAACTAGAGCAATCTGGGCTCCCTGTTGGAGGGAATGAGAAAAAGGCTAAATTGGCAATGTTGTGAGAAAAATGATGACTAACTCACAAGATACAGAGCTCTCCTTTACAATTACTGGAGACAACACCAAATG<br>GAATGAGAATCAAAACCCTCGGATGTTTTTGGCAATGATAACATATATCACAAGAAACCAACCTGAATGGTTTAGAAATGTCTTAAGCATTGCCCCTATAATGTTCTCAAACAAAATGGCGAGATTAGGGAAAGGATACATGTTTGAAAGTAAGAGCATGAAGCTAAGAACACAAATACCAGCAGAGATGCTTACAAATATTGATCTGAAGTATTTCAACGAACCAACGAGAA<br>AGAAAATCGAGAAAATAAGACCTCTGCTGATTGATGGCACGGCCTCATTGAGTCCTGGGATGATGATGGGCATGTTCAATATGCTGAGCACAGTATTAGGGGTCTCAATCCTGAATCTCGGGCAAAAGAGGTACACCAAAACCACATACTGGTGGGATGGACTTCAATCCTCTGATGATTTCGCTCTCATAGTGAATGCACCGAATCATGAGGGGATACAAGCAGGAGTGGAT<br>AGATTCTATAGGACCTGCAAACTGGTTGGGATCAACATGAGCAAAAAGAAGTCTTACATAAACCGAACAGGAACATTTGAGTTCACAAGTTTTTTCTACCGCTATGGATTTGTAGCCAACTTCAGTATGGAATTACCCAGCTTTGGAGTGTCTGGAATCAATGAATCAGCTGACATGAGCATTGGAGTTACAGTGATAAAAAACAATATGATAAACAATGATCTTGGACCAGCA<br>ACAGCTCAAATGGCTCTTCAGTTATTCATCAAAGACTATAGGTACACATACCGATGCCACAGGGGTGATACACAAATTCAAACGAGGAGATCATTCGAGCTGAAGAAGCTGTGGGAGCAGACCCGTTCAAAGGCAGGGCTGTTGATATCAGACGGGGGGCCAAACCTATACAACATTCGGAATCTCCACATCCCAGAGGTCTGCTTGAAGTGGGAGCTGATGGATGAAGACT<br>ACCAAGGCAGGCTGTGCAATCCTCTGAATCCATTTGTCAGTCATAAAGAGATTGAGTCCGTAAACAATGCTGTAGTAATGCCCGCCCATGGCCCGGCCAAGAGCATGGAATATGATGCTGTTGCGACCACACACTCGTGGATTCCTAAGAGGAACCGTTCCATCCTCAATACCAGCCAAAGGGGAATTCTTGAGGATGAGCAGATGTACCAAAAGTGCTGTAGTCTATTCGAGA<br>AATTCTTCCCCAGCAGTTCATACAGGAGGCCAGTTGGAATTTCCAGCATGGTGGAGGCCATGGTGTCTAGGGCCCGAATTGATGCACGCATCGACTTCGAATCTGGAAGGATTAAGAAAGAAGAGTTTGCTGAGATCATGAAGATCTGTTCCACCATTGAAGAGCTCAGACGGCAAAAATAG
EPI_ISL_1058020	>A/chicken/Czech_Republic/29392021_H5N8_2021-02-10<br>ATGGATGTCAATCCGACTTTACTTTTCGTAAAAGTGCCAGCGCAAAATGCTATAAGTACTACATTCCCTTACACTGGAGATCCCCCATACAGCCATGGAACAGGAACAGGGTATACCATGGACACAGTAAACAGAACACATCAATACTCAGAAAAGGGAAAGTGGACAACAAACACAGAAACCGGAGCACCCCAACTCAACCCAATTGATGGACCATTACCAGAGGACAATGA<br>GCCAAGCGGATATGCACAAACTGATTGCGTGTTGGAAGCAATGGCTTTCCTTGAAGAATCCCACCCAGGGATATTTGAAAACTCTTGTCTTGAAGCAATGGAAATCGTTCAGCAAACAAGAGTGGACAAACTAACCCAAGGTCGCCAGACTTATGACTGGACACTGAACAGAAACCAACCAGCTGCAACCTCTTTGGCCAACACTATAGAGGTGTTCAGATCGAATGGTCTGA<br>CAGCCAATGAATCAGGGAGACTGATAGATTTTCTCAGGGATGTGATGGAATCAATGGATAAAGAAGAGATGGAAATAACAACACATTTCCAGAGAAAAAGAAGAGTGAGGGACAACATGACTAAGAAGATGGTCACACAAAGAACAATAGGGAAGAAGAAGCAGAGGCTGAACAAGAGGAGTTACTTAATAAGAGCACTGACATTGAATACAATGACCAAAGATGCAGA<br>AAGAGGCAAGTTGAAGAGACGGGCAATTGCAACACCCGGGATGCAGATTAGAGGATTCGTGTACTTCGTTGAAACACTAGCGAGGAGCATCTGTGAGAAACTAGAGCAATCTGGGCTCCCTGTTGGAGGGAATGAGAAAAAGGCTAAATTGGCAATGTTGTGAGAAAAATGATGACTAACTCACAAGATACAGAGCTCTCCTTTACAATTACTGGAGACAACACCAAATG<br>GAATGAGAATCAAAACCCTCGGATGTTTTTGGCAATGATAACATATATCACAAGAAACCAACCTGAATGGTTTAGAAATGTCTTAAGCATTGCCCCTATAATGTTCTCAAACAAAATGGCGAGATTAGGGAAAGGATACATGTTTGAAAGTAAGAGCATGAAGCTAAGAACACAAATACCAGCAGAGATGCTTACAAATATTGATCTGAAGTATTTCAACGAACCAACGAGAA<br>AGAAAATCGAGAAAATAAGACCTCTGCTGATTGATGGCACGGCCTCATTGAGTCCTGGGATGATGATGGGCATGTTCAATATGCTGAGCACAGTATTAGGGGTCTCAATCCTGAATCTCGGGCAAAAGAGGTACACCAAAACCACATACTGGTGGGATGGACTTCAATCCTCTGATGATTTCGCTCTCATAGTGAATGCACCGAATCATGAGGGGATACAAGCAGGAGTGGAT<br>AGATTCTATAGGACCTGCAAACTGGTTGGGATCAACATGAGCAAAAAGAAGTCTTACATAAACCGAACAGGAACATTTGAGTTCACAAGTTTTTTCTACCGCTATGGATTTGTAGCCAACTTCAGTATGGAATTACCCAGCTTTGGAGTGTCTGGAATCAATGAATCAGCTGACATGAGCATTGGAGTTACAGTGATAAAAAACAATATGATAAACAATGATCTTGGACCAGCA<br>ACAGCTCAAATGGCTCTTCAGTTATTCATCAAAGACTATAGGTACACATACCGATGCCACAGGGGTGATACACAAATTCAAACGAGGAGATCATTCGAGCTGAAGAAGCTGTGGGAGCAGACCCGTTCAAAGGCAGGGCTGTTGATATCAGACGGGGGGCCAAACCTATACAACATTCGGAATCTCCACATCCCAGAGGTCTGCTTGAAGTGGGAGCTGATGGATGAAGACT<br>ACCAAGGCAGGCTGTGCAATCCTCTGAATCCATTTGTCAGTCATAAAGAGATTGAGTCCGTAAACAATGCTGTAGTAATGCCCGCCCATGGCCCGGCCAAGAGCATGGAATATGATGCTGTTGCGACCACACACTCGTGGATTCCTAAGAGGAACCGTTCCATCCTCAATACCAGCCAAAGGGGAATTCTTGAGGATGAGCAGATGTACCAAAAGTGCTGTAGTCTATTCGAGA<br>AATTCTTCCCCAGCAGTTCATACAGGAGGCCAGTTGGAATTTCCAGCATGGTGGAGGCCATGGTGTCTAGGGCCCGAATTGATGCACGCATCGACTTCGAATCTGGAAGGATTAAGAAAGAAGAGTTTGCTGAGATCATGAAGATCTGTTCCACCATTGAAGAGCTCAGACGGCAAAAATAG
EPI_ISL_405391	>A/chicken/Czech_Republic/1175-1/2020_H5N8|2020-01-17<br>ATGGATGTCAATCCGACTTTACTTTTCTTAAAAGTGCCAGCGCAAAATGCTATAAGTACTACGTTCCCTTACACTGGAGATCCTCCATACAGCCATGGAACAGGAACAGGATACACCATGGACACAGTCAACAGAACACATCAATACTCAGAGAAGGGAAAGTGGACAACAAACACAGAGACCGGGGCGCCCCAACTCAACCCAATTGATGGACCATTGCCAGAGGACAACGA<br>GCCAAGCGGATATGCACAAACAGATTGCGTGTTGGAAGCAATGGCTTTCCTTGAAGAATCCCACCCAGGGATCTTTGAAAACTCTTGTCTTGAAACGATGGAGGTCGTTCAGCAAACAAGAGTGGACAAACTAACCCAAGGTCGCCAGACCTATGACTGGACACTGAATAGAAACCAACCAGCTGCAACTGCTTTGGCCAATACTATAGAGGTCTTCAGATCGAACGGTCTAA<br>CAGCCAATGAGTCGGGGAGACTAATAGATTTCCTCAAGGATGTGATGGAATCAATGGATAAAGAAGAAATGGAAATAACAACACATTTCCAGAGAAAGAGAAGAGTAAGGGACAACATGACCAAAAAGATGGTCACACAAAGAACGATAGGGAAGAAGAAGCAGAGGCTGAACAAGAGAAGCTACTTAATAAGAGCACTGACATTGAACACAATGACCAAAGATGCAGA<br>AAGAGGCAAATTGAAGAGGCGGGCAATTGCAACACCCGGGATGCAGATTAGAGGATTTGTGTACTTTGTCGAAACACTAGCGAGGAGCATCTGTGAGAAACTCGAGCAATCTGGACTCCCTGTTGGAGGGAATGAGAAAAAGGCCAAATTGGCAAATGTCGTGAGGAAAATGATGACTAACTCGCAAGATACAGAGCTCTCCTTTACAATCACCGGAGACAACACCAAATG<br>GAATGAGAATCAAAATCCTCGGATGTTTTTGGCAATGATAACATACATCACAAGAAACCAACCCGAATGGTTTAGAAATGTCTTGAGCATTGCCCCTATAATGTTCTCAAACAAAATGGCAAGATTAGGAAAAGGATACATGTTTGAAAGTAAGAGCATGAAGCTAAGGACACAAATACCGGCAGAAATGCTTGCAAACATTGACTTGAAATACTTCAATGAATCAACGAGAA<br>AGAAAATCGAGAAAATAAGACCTCTGCTAATTGATGGCACAGCCTCATTGAGTCCTGGAATGATGATGGGCATGTTCAATATGCTGAGCACAGTATTGGGGGTCTCAATCCTGAATCTTGGACAAAAGAGGTACACCAAAACCACATACTGGTGGGATGGACTCCAATCCTCTGATGATTTCGCTCTCATAGTGAATGCACCGAATCATGAGGGGATACAAGCTGGGGTGGAT<br>AGGTTCTATAGGACCTGCAAATTGGTTGGGATCAACATGAGCAAGAAGAAGTCTTACATAAACCGGACAGGAACATTTGAGTTCACAAGCTTTTTCTACCGCTATGGATTTGTAGCCAACTTCAGTATGGAGTTACCCAGCTTTGGAGTGTCTGGAATCAATGAGTCAGCTGACATGAGCATTGGAGTTACAGTGATAAAGAACAATATGATAAACAATGACCTTGGACCAGCA<br>ACAGCTCAAATGGCTCTTCAGCTATTCATCAAGGATTATAGGTACACGTACCGATGTCACAGAGGTGATACACAAATTCAAACGAGGAGATCATTCGAGCTGAAGAAGCTGTGGGAGCAGACCCGTTCAAAGGCAGGGCTGTTGGTATCAGATGGAGGACCAAACCTATACAACATTCGGAATCTCCACATCCCAGAGGTCTGCTTGAAGTGGGAGCTAATGGATGAAGACT<br>ACCAGGGCAGGCTGTGTAATCCTCTGAATCCGTTTGTCAGTCATAAGGAAATTGAGTCCGTAAACAATGCTGTGGTAATGCCAGCCCATGGCCCAGCCAAGAGCATGGAATATGATGCTGTTGCGACTACACACTCATGGATTCCCAAGAGGAACCGTTCCATTCTCAATACCAGCCAGAGGGGAATTCTCGAGGATGAGCAGATGTACCAGAAGTGCTGCAGTTTATTCGAG<br>AAATTCTTCCCCAGTAGTTCATACAGGAGGCCAGTTGGAATTTCCAGCATGGTGGAGGCCATGGTGTCCAGGGCCCGAATTGATGCACGTATTGATTTCGAATCTGGAAGGATTAAGAAAGAAGAGTTTGCCGAGATCATGAAGATCTGTTCCACCATTGAAGAGCTCAGACGGCAAAAATAG
EPI_ISL_3102059	>A/chicken/Bulgaria/298-1_21VIR4270-9/2021_H5N8_2021-05-05<br>ATGGATGTCAATCCGACTTTACTTTTCTTAAAAGTGCCAGCGCAAAATGCTATAAGTACTACATTCCCTTACACTGGAGATCCTCCATACAGCCATGGAACAGGAACAGGGTATACCATGGACACAGTAAACAGAACACATCAGTACTCAGAAAAGGGAAAGTGGACAACAAACACAGAAACCGGAGCACCCCAACTCAACCCAATTGATGGACCATTACCAGAGGACAATGA<br>GCCAAGCGGATATGCACAAACTGATTGCGTGTTGGAAGCAATGGCTTTCCTTGAAGAATCCCACCCAGGGATATTTGAAAACTCTTGTCTTGAAGCGATGGAAATCGTTCAGCAAACAAGAGTGGACAAACTAACCCAAGGTCGCCAGACTTATGACTGGACACTGAACAGAAACCAACCAGCTGCAACCTCTTTGGCCAACACTATAGAGGTGTTCAGATCGAATGGTCTGA<br>CAGCCAATGAATCAGGGAGACTGATAGATTTTCTCAGGGATGTGATGGAATCAATGGATAAAGAAGAGATGGAAGTAACAACACATTTCCAGAGAAAAAGAAGAGTGAGGGACAACATGACTAAGAAGATGGTCACACAAAGAACAATAGGGAAGAAGAAGCAGAGGCTGAACAAGAGGAGTTACTTAATAAGAGCACTGACATTGAATACAATGACCAAAGATGCAGA<br>AAGAGGCAAGTTGAAGAGACGGGCAATTGCAACACCCGGGATGCAGATTAGAGGATTCGTGTACTTCGTTGAAACACTAGCGAGGAGCATCTGTGAGAAACTAGAGCAATCTGGGCTCCCTGTTGGAGGGAATGAGAAGAAGGCTAAATTGGCAATGTTGTGAGAAAAATGATGACTAACTCACAAGATACAGAGCTCTCCTTTACAATTACTGGAGACAACACCAAATG<br>GAATGAGAATCAAAACCCTCGGATGTTTTTGGCAATGATAACATATATCACAAGAAACCAACCTGAATGGTTTAGAAATGTCTTAAGCATTGCCCCTATAATGTTCTCAAACAAAATGGCGAGATTAGGGAAAGGATACATGTTTGAAAGTAAGAGCATGAAGCTAAGAACACAAATACCAGCAGAGATGCTTACAAATATCGATCTGAAGTATTTCAACGAACCAACGAGAA<br>AGAAAATCGAGAAAATAAGACCTCTGCTGATTGATGGCACGGCCTCATTGAGTCCTGGGATGATGATGGGCATGTTCAATATGCTGAGCACAGTATTAGGGGTCTCAATCCTGAATCTCGGGCAAAAAAGGTACACCAAAACCACATACTGGTGGGATGGACTTCAATCCTCTGATGATTTCGCTCTCATAGTGAATGCACCGAATCATGAGGGGATACAAGCAGGAGTGGAT<br>AGATTCTATAGGACCTGCAAACTGGTTGGGATCAACATGAGCAAAAAGAAGTCTTACATAAACCGAACAGGAACATTTGAGTTCACAAGTTTTTTCTACCGCTATGGATTTGTAGCTAACTTCAGTATGGAATTACCCAGCTTTGGAGTGTCTGGAATCAATGAATCAGCTGACATGAGCATTGGAGTTACAGTGATAAAAAACAATATGATAAACAATGATCTTGGACCAGCA<br>ACAGCTCAAATGGCTCTTCAGTTATTCATCAAAGATTATAGGTACACATACCGATGCCACAGGGGTGATACACAAATTCAAACGAGGAGATCATTCGAGCTGAAGAAGCTGTGGGAGCAGACCCGTTCAAAGGCAGGGCTGTTGATATCAGACGGGGGGCCAAACCTATACAACATTCGGAATCTCCACATCCCAGAGGTCTGCTTGAAGTGGGAGCTGATGGATGAAGACT<br>ACCAAGGCAGGCTGTGCAATCCTCTGAATCCATTTGTCAGTCATAAAGAGATTGAGTCCGTAAACAATGCTGTAGTAATGCCCGCCCATGGCCCGGCCAAGAGCATGGAATATGATGCTGTTGCGACCACACACTCGTGGATTCCTAAGAGGAACCGTTCCATTCTCAATACCAGCCAAAGGGGAATTCTTGAGGATGAGCAGATGTACCAAAAGTGCTGTAGTCTATTCGAGA<br>AATTCTTCCCCAGCAGTTCATACAGGAGGCCAGTTGGAATTTCCAGCATGGTGGAGGCCATGGTGTCTAGGGCCCGAATTGATGCACGCATCGACTTCGAATCTGGAAGGATTAAGAAAGAAGAGTTTGCTGAGATCATGAAGATCTGTTCCACCATTGAAGAGCTCAGACGGCAAAAATAG
EPI_ISL_3102057	>A/chicken/Bulgaria/275-4_21VIR4270-6/2021_H5N8_2021-04-26<br>ATGGATGTCAATCCGACTTTACTTTTCTTAAAAGTGCCAGCGCAAAATGCTATAAGTACTACATTCCCTTACACTGGAGATCCTCCATACAGCCATGGAACAGGAACAGGGTATACCATGGACACAGTAAACAGAACACATCAGTACTCAGAAAAGGGAAAGTGGACAACAAACACAGAAACCGGAGCACCCCAACTCAACCCAATTGATGGACCATTACCAGAGGACAATGA<br>GCCAAGCGGATATGCACAAACTGATTGCGTGTTGGAAGCAATGGCTTTCCTTGAAGAATCCCACCCAGGGATATTTGAAAACTCTTGTCTTGAAGCGATGGAAATCGTTCAGCAAACAAGAGTGGACAAACTAACCCAAGGTCGCCAGACTTATGACTGGACACTGAACAGAAACCAACCAGCTGCAACCTCTTTGGCCAACACTATAGAGGTGTTCAGATCGAATGGTCTGA<br>CAGCCAATGAATCAGGGAGACTGATAGATTTTCTCAGGGATGTGATGGAATCAATGGATAAAGAAGAGATGGAAGTAACAACACATTTCCAGAGAAAAAGAAGAGTGAGGGACAACATGACTAAGAAGATGGTCACACAAAGAACAATAGGGAAGAAGAAGCAGAGGCTGAACAAGAGGAGTTACTTAATAAGAGCACTGACATTGAATACAATGACCAAAGATGCAGA<br>AAGAGGCAAGTTGAAGAGACGGGCAATTGCAACACCCGGGATGCAGATTAGAGGATTCGTGTACTTCGTTGAAACACTAGCGAGGAGCATCTGTGAGAAACTAGAGCAATCTGGGCTCCCTGTTGGAGGGAATGAGAAGAAGGCTAAATTGGCAATGTTGTGAGAAAAATGATGACTAACTCACAAGATACAGAGCTCTCCTTTACAATTACTGGAGACAACACCAAATG<br>GAATGAGAATCAAAACCCTCGGATGTTTTTGGCAATGATAACATATATCACAAGAAACCAACCTGAATGGTTTAGAAATGTCTTAAGCATTGCCCCTATAATGTTCTCAAACAAAATGGCGAGATTAGGGAAAGGATACATGTTTGAAAGTAAGAGCATGAAGCTAAGAACACAAATACCAGCAGAGATGCTTACAAATATCGATCTGAAGTATTTCAACGAACCAACGAGAA<br>AGAAAATCGAGAAAATAAGACCTCTGCTGATTGATGGCACGGCCTCATTGAGTCCTGGGATGATGATGGGCATGTTCAATATGCTGAGCACAGTATTAGGGGTCTCAATCCTGAATCTCGGGCAAAAAAGGTACACCAAAACCACATACTGGTGGGATGGACTTCAATCCTCTGATGATTTCGCTCTCATAGTGAATGCACCGAATCATGAGGGGATACAAGCAGGAGTGGAT<br>AGATTCTATAGGACCTGCAAACTGGTTGGGATCAACATGAGCAAAAAGAAGTCTTACATAAACCGAACAGGAACATTTGAGTTCACAAGTTTTTTCTACCGCTATGGATTTGTAGCTAACTTCAGTATGGAATTACCCAGCTTTGGAGTGTCTGGAATCAATGAATCAGCTGACATGAGCATTGGAGTTACAGTGATAAAAAACAATATGATAAACAATGATCTTGGACCAGCA<br>ACAGCTCAAATGGCTCTTCAGTTATTCATCAAAGATTATAGGTACACATACCGATGCCACAGGGGTGATACACAAATTCAAACGAGGAGATCATTCGAGCTGAAGAAGCTGTGGGAGCAGACCCGTTCAAAGGCAGGGCTGTTGATATCAGACGGGGGGCCAAACCTATACAACATTCGGAATCTCCACATCCCAGAGGTCTGCTTGAAGTGGGAGCTGATGGATGAAGACT<br>ACCAAGGCAGGCTGTGCAATCCTCTGAATCCATTTGTCAGTCATAAAGAGATTGAGTCCGTAAACAATGCTGTAGTAATGCCCGCCCATGGCCCGGCCAAGAGCATGGAATATGATGCTGTTGCGACCACACACTCGTGGATTCCTAAGAGGAACCGTTCCATTCTCAATACCAGCCAAAGGGGAATTCTTGAGGATGAGCAGATGTACCAAAAGTGCTGTAGTCTATTCGAGA<br>AATTCTTCCCCAGCAGTTCATACAGGAGGCCAGTTGGAATTTCCAGCATGGTGGAGGCCATGGTGTCTAGGGCCCGAATTGATGCACGCATCGACTTCGAATCTGGAAGGATTAAGAAAGAAGAGTTTGCTGAGATCATGAAGATCTGTTCCACCATTGAAGAGCTCAGACGGCAAAAATAG
EPI_ISL_9261749	>A/Caspian_gull/Netherlands/1/2022_H5N1_2022-01-14<br>ATGGATGTCAATCCGACTTTACTTTTCTTAAAAGTGCCAGCGCAAGATGCCATAAGTACCACATTCCCTTACACTGGAGATCCTCCATACAGCCATGGAACAGGGACAGGATACACAATGGACACAGTCAACAGAACACATCAATACTCAGAGAAGGGAAAATGGACAACAAACACAGAAACCGGAGCACCTCAACTCAACCCAATTGATGGGCCACTACCTGAGGACAACGA<br>ACCGAGCGGATATGCACAAACAGATTGCGTGTTGGAAGCAATGGCTTTCCTTGAAGAGTCCCACCCAGGGATCTTTGAAAACTCTTGTCTTGAAACGATGGAAGTCGTTCAGCAAACAAGAGTGGACAAACTAACTCAAGGTCGCCAGACATATGACTGGACACTGAATAGAAACCAACCAGCTGCAACTGCCCTGGCCAACACTATAGAGGTCTTCAGATCAAACGGTCTAA<br>CAACCAATGAATCGGGGAGACTAATAGATTTCCTCAAGGATGTGGTGGACTCAATGGATAAAGAAGAAATGGAAATAACAACACATTTCCAAAGAAAGAGAAGAGTAAGGGACAACATGACCAAGAAAATGGTCACACAAAGAACAATAGGAAAGAAGAAACAAAGGCTAACCAAGAGGAGCTACTTAATAAGAGCACTGACACTGAATACAATGACAAAAGATGCAGAA<br>AGAGGCAAATTGAAAAGACGGGCGATTGCAACACCAGGGATGCAGATTAGAGGATTTGTGTACTTTGTCGAAACACTGGCAAGGAGCATCTGTGAAAAACTTGAGCAATCTGGACTCCCCGTTGGAGGGAATGAGAAGAAGGCTAAATTGGCAAATGTCGTGAGAAAAATGATGACTAACTCACAAGATACAGAGCTCTCCTTCACAATTACTGGAGATAACACCAAATGG<br>AATGAGAATCAAAATCCTCGGATGTTTCTGGCAATGATAACATACATTACAAGAAACCAACCTGAATGGTTTAGAAATGTCTTGAGTATTGCCCCTATAATGTTCTCGAACAAAATGGCGAGATTGGGAAAAGGGTACATGTTTGAAAGTAAGAGCATGAAGTTACGGACACAAATACCTGCAGAAATGCTTGCAAACATTGACTTAAAATACTTCAATGAATCAACAAGAAAG<br>AAAATCGAAAAAATAAGGCCTCTACTAATAGATGGCACTGCCTCATTGAGTCCTGGAATGATGATGGGCATGTTCAATATGCTGAGTACAGTATTAGGAGTTTCAATCCTAAATCTTGGGCAAAAGAAGTACACCAAAACCACATACTGGTGGGATGGACTCCAATCCTCTGATGATTTCGCCCTCATAGTAAATGCACCGAATCATGAGGGAATACAAGCAGGAGTGGATAG<br>GTTCTATAGGACCTGCAAACTGGTCGGGATCAATATGAGCAAAAAGAAGTCTTACATAAACCGGACTGGAACATTTGAGTTCACAAGCTTTTTCTATCGCTATGGATTTGTGGCTAACTTCAGTATGGAGCTGCCCAGCTTTGGAGTTTCTGGGATCAATGAATCAGCTGACATGAGCATTGGCATCACAGTGATAAAGAACAACATGATAAACAATGACCTTGGACCAGCAAC<br>AGCTCAAATGGCCCTTCAACTATTCATCAAAGATTACAGGTACACGTACCGATGCCACAGAGGTGACACACAAATTCAAACGAGGAGATCATTCGAGCTGAAGAAGCTGTGGGAACAGACCCGTTCAAAGGCAGGACTGTTGGTGTCAGATGGAGGACCAAATCTATACAACATTCGGAATCTCCATATCCCAGAGGTCTGCCTGAAGTGGGAGCTGATGGACGAAGATTAC<br>CAGGGCAGGTTGTGTAATCCTCTGAACCCATTTGTCAGCCATAGAGAAATTGAGTCCGTAAACAATGCTGTGGTGATGCCAGCCCACGGTCCAGCCAAAAGCATGGAATATGATGCCGTTGCGACTACACACTCATGGATTCCTAAAAGGAATCGTTCCATTCTCAATACCAGTCAAAGGGGAATTCTTGAGGATGAACAGATGTACCAGAAATGCTGCAGTCTATTCGAGAAA<br>TTCTTCCCCAGTAGTTCATACAGGAGACCAGTTGGAATTTCCAGCATGGTGGAGGCCATGGTGTCTAGGGCCCGAATCGATGCACGCATTGATTTCGAATCTGGAAGGATCAAGAAGGAAGAGTTTGCTGAGATCATGAAGATCTGTTCCACCATTGAAGAGCTCAGACGGCAAAAATAG
EPI_ISL_13026106	>A/Canada_goose/Luxembourg/21239614/2021_H5N1_2021-12-10<br>ATGGATGTCAATCCGACTTTACTTTTCTTAAAAGTGCCAGCGCAAGATGCCATAAGTACCACATTCCCTTACACTGGAGATCCTCCATACAGCCATGGAACAGGGACAGGATACACGATGGACACAGTCAACAGAACACATCAATACTCAGAGAAGGGAAAATGGACAACAAACACAGAAACCGGAGCACCTCAACTCAACCCAATTGATGGGCCACTACCTGAGGACAACGA<br>ACCGAGCGGATATGCACAAACAGATTGCGTGTTGGAAGCAATGGCTTTCCTTGAAGAGTCCCACCCAGGGATCTTTGAAAACTCTTGTCTTGAAACGATGGAAGTCGTTCAGCAAACAAGAGTGGACAAACTAACTCAAGGTCGCCAGACATATGACTGGACACTGAATAGAAACCAACCAGCTGCAACTGCCCTGGCCAACACTATAGAGGTCTTCAGATCAAACGGTCTAA<br>CAGCCAATGAATCGGGGAGACTAATAGATTTCCTCAAGGATGTGATGGACTCAATGGATAAAGAAGAAATGGAAATAACAACACATTTCCAGAGAAAGAGAAGAGTAAGGGACAACATGACCAAGAAAATGGTCACACAAAGAACAATAGGAAAGAAGAAACAAAGGCTAAACAAGAGGAGCTACTTAATAAGAGCACTGACACTGAATACAATGACAAAAGATGCAGA<br>AAGAGGCAAATTGAAGAGACGGGCGATTGCAACACCAGGGATGCAGATTAGAGGATTTGTGTACTTTGTCGAAACACTGGCAAGGAGCATCTGTGAAAAACTTGAGCAATCTGGACTCCCCGTTGGAGGGAATGAGAAGAAGGCTAAATTGGCAAATGTCGTGAGAAAAATGATGACTAACTCACAAGATACAGAGCTCTCCTTCACAATTACTGGAGACAACACCAAATG<br>GAATGAGAATCAAAATCCTCGGATGTTTCTGGCAATGATAACATACATTACAAGAAACCAACCTGAATGGTTTAGAAATGTCTTAAGTATTGCCCCTATAATGTTCTCGAACAAAATGGCGAGATTGGGAAAAGGGTACATGTTTGAAAGTAAGAGCATGAAGTTACGGACACAAATACCTGCAGAAATGCTTGCAAACATTGACTTAAAATACTTCAATGAATCAACAAGAAA<br>GAAAATCGAAAAAATAAGGCCTCTACTAATCGATGGCACTGCCTCATTGAGTCCTGGAATGATGATGGGCATGTTCAATATGCTGAGTACAGTATTAGGAGTTTCAATCCTAAATCTTGGGCAAAAGAAGTACACCAAAACCACATACTGGTGGGATGGACTCCAATCCTCTGATGATTTCGCCCTCATAGTAAATGCACCGAATCATGAGGGAATACAAGCAGGAGTGGATA<br>GGTTCTATAGGACCTGCAAACTGGTCGGGATCAATATGAGCAAAAAGAAGTCTTACATAAACCGGACTGGAACATTTGAGTTCACAAGCTTTTTCTATCGCTATGGATTTGTGGCTAACTTCAGTATGGAGCTGCCCAGCTTTGGGGTTTCTGGGATCAATGAATCAGCTGACATGAGCATTGGCGTCACAGTGATAAAGAACAACATGATAAACAATGACCTTGGACCAGCAA<br>CAGCTCAAATGGCCCTTCAACTATTCATCAAAGATTACAGGTACACGTACCGATGCCACAGAGGTGACACACAAATTCAAACGAGGAGATCATTCGAGCTGAAGAAGCTGTGGGAACAGACCCGTTCAAAGGCAGGACTGTTGGTGTCAGATGGAGGACCAAATCTATACAACATTCGGAATCTCCATATCCCAGAGGTCTGCCTGAAGTGGGAGCTGATGGACGAAGATTA<br>CCAGGGCAGGTTGTGTAATCCTCTGAACCCATTTGTCAGCCATAAAGAAATTGAGTCCGTAAACAATGCTGTGGTGATGCCAGCTCACGGTCCAGCCAAAAGCATGGAATATGATGCCGTTGCGACTACACACTCATGGACTCCTAAAAGGAATCGTTCCATTCTCAATACCAGTCAAAGGGGAATTCTTGAGGATGAACAGATGTACCAGAAATGCTGCAGTCTATTCGAGAA<br>ATTCTTCCCCAGTAGTTCATACAGGAGACCAGTTGGAATTTCCAGCATGGTGGAGGCCATGGTGTCTAGGGCCCGAATCGATGCACGCATTGATTTCGAATCTGGAAGGATCAAGAAGGAAGAGTTTGCTGAGATCATGAAGATCTGTTCCACCATTGAAGAGCTCAGACGGCAAAAATAG
EPI_ISL_710506	>A/Canada_goose/England/032697/2020_H5N8_2020-11-03<br>ATGGATGTCAATCCGACTTTACTTTTCTTAAAAGTGCCAGCGCAAAATGCTATAAGTACTACATTCCCTTACACTGGAGATCCCCCATACAGCCATGGAACAGGAACAGGGTATACCATGGACACAGTAAACAGAACACATCAATACTCAGAAAAGGGAAAGTGGACAACAAACACAGAAACCGGAGCACCCCAACTCAACCCAATTGATGGACCATTACCAGAGGACAATGA<br>GCCAAGCGGATATGCACAAACTGATTGCGTGTTGGAAGCAATGGCTTTCCTTGAAGAATCCCACCCAGGGATATTTGAAAACTCTTGTCTTGAAGCGATGGAAATCGTTCAGCAAACAAGAGTGGACAAACTAACCCAAGGTCGCCAGACTTATGACTGGACACTGAACAGAAACCAACCAGCTGCAACCTCTTTGGCCAACACTATAGAGGTGTTCAGATCGAATGGTCTGA<br>CAGCCAATGAATCAGGAAGACTGATAGATTTTCTCAGGGATGTGATGGAATCAATGGATAAAGAAGAGATGGAAGTAACAACACATTTCCAGAGAAAAAGAAGAGTGAGGGACAACATGACTAAGAAGATGGTCACACAAAGAACAATAGGGAAGAAGAAGCAGAGGCTGAACAAGAGGAGTTACTTAATAAGAGCACTGACATTGAATACAATGACCAAAGATGCAGA<br>AAGAGGCAAGTTGAAGAGACGGGCAATTGCAACACCCGGGATGCAGATTAGAGGATTCGTGTACTTCGTTGAAACACTAGCGAGGAGCATCTGTGAGAAACTAGAGCAATCTGGGCTCCCTGTTGGAGGGAATGAGAAGAAGGCTAAATTGGCAATGTTGTGAGAAAAATGATGACTAACTCACAAGATACAGAGCTCTCCTTTACAATTACTGGAGACAACACCAAATG<br>GAATGAGAATCAAAACCCTCGGATGTTTTTGGCAATGATAACGTATATCACAAGAAACCAACCTGAATGGTTTAGAAATGTCTTAAGCATTGCCCCTATAATGTTCTCAAACAAAATGGCGAGATTAGGGAAAGGATACATGTTTGAAAGTAAGAGCATGAAGCTAAGAACACAAATACCAGCAGAGATGCTTACAAATATTGATCTGAAGTATTTCAACGAACCAACGAGAA<br>AGAAAATCGAGAAAATAAGACCTCTGCTGATTGATGGCACGGCCTCATTGAGTCCTGGGATGATGATGGGCATGTTCAATATGCTGAGCACAGTATTAGGGGTCTCAATCCTGAATCTCGGGCAAAAGAGGTACACCAAAACCACATACTGGTGGGATGGACTTCAATCCTCTGATGATTTCGCTCTCATAGTGAATGCACCGAATCATGAGGGGATACAAGCAGGAGTGGAT<br>AGATTCTATAGGACCTGCAAACTGGTTGGGATCAACATGAGCAAAAAGAAGTCTTACATAAACCGAACAGGAACATTTGAGTTCACAAGTTTTTTCTACCGCTATGGATTTGTAGCTAACTTCAGTATGGAATTACCCAGCTTTGGAGTGTCTGGAATCAATGAATCAGCTGACATGAGCATTGGAGTTACAGTGATAAAAAACAATATGATAAACAATGATCTTGGACCAGCA<br>ACAGCTCAAATGGCTCTTCAGTTATTCATCAAAGACTATAGGTACACATACCGATGCCACAGGGGTGATACACAAATTCAAACGAGGAGATCATTCGAGCTGAAGAAGCTGTGGGAGCAGACCCGTTCAAAGGCAGGGCTGTTGATATCAGACGGGGGGCCAAACCTATACAACATTCGGAATCTCCACATCCCAGAGGTCTGCTTGAAGTGGGAGCTGATGGATGAAGACT<br>ACCAAGGCAGGCTGTGCAATCCTCTGAATCCATTTGTCAGTCATAAAGAGATTGAGTCCGTAAACAATGCTGTAGTAATGCCCGCCCACGGCCCGGCCAAGAGCATGGAATATGATGCTGTTGCGACCACACACTCGTGGATTCCTAAGAGGAACCGTTCCATTCTCAATACCAGCCAAAGGGGAATTCTTGAGGATGAGCAGATGTACCAAAAGTGCTGTAGTCTATTCGAGA<br>AATTCTTCCCCAGCAGTTCATACAGGAGGCCAGTTGGAATTTCCAGCATGGTGGAGGCCATGGTGTCTAGGGCCCGAATTGATGCACGCATCGACTTCGAATCTGGAAGGATTAAGAAAGAAGAGTTTGCTGAGATCATGAAGATCTGTTCCACCATTGAAGAGCTCAGACGGCAAAAATAG
EPI_ISL_18244823	>A/buzzard/Poland/MB277-21_22VIR5675-18/2021_H5N8_2021-03-09<br>ATGGATGTCAATCCGACTTTACTTTTCTTAAAAGTGCCAGCGCAAAATGCTATAAGTACTACATTCCCTTACACTGGAGATCCCCCATACAGCCATGGAACAGGAACAGGGTATACCATGGACACAGTAAACAGAACACATCAATACTCAGAAAAGGGAAAGTGGACAACAAACACAGAAACCGGAGCACCCCAACTCAACCCAATTGATGGACCATTACCAGAGGATAATGA<br>GCCAAGCGGATATGCACAAACTGATTGCGTGTTGGAAGCCATGGCTTTCCTTGAAGAATCCCACCCAGGGATATTTGAAAACTCTTGTCTTGAAGCGATGGAAATCGTTCAGCAAACAAGAGTGGACAAACTAACCCAAGGTCGCCAGACTTATGACTGGACACTGAACAGAAACCAACCAGCAGCAACCTCTTTGGCCAACACTATAGAGGTGTTCAGATCGAATGGTCTGA<br>CAGCCAATGAATCAGGGAGACTGATAGATTTTCTCAGGGATGTGATGGAATCAATGGATAAAGAAGAGATGGAAGTAACAACACATTTCCAGAGAAAAAGAAGAGTGAGGGACAACATGACTAAGAAGATGGTCACACAAAGAACAATAGGGAAGAAGAAGCAGAGGCTGAACAAGAGGAGTTACTTAATAAGAGCACTGACATTGAATACAATGACCAAAGATGCAGA<br>AAGAGGCAAGTTGAAGAGACGGGCAATTGCAACACCCGGGATGCAGATTAGAGGATTCGTGTACTTCGTTGAAACACTAGCGAGGAGCATCTGTGAGAAACTAGAGCAATCTGGGCTCCCTGTTGGAGGGAATGAGAAAAAGGCTAAATTGGCAATGTTGTGAGAAAAATGATGACTAACTCACAAGATACAGAGCTCTCCTTTACAATTACTGGAGACAACACCAAATG<br>GAACGAGAATCAAAACCCTCGGATGTTTTTGGCAATGATAACATATATCACAAGAAACCAACCTGAATGGTTTAGAAATGTCTTAAGCATTGCCCCTATAATGTTCTCAAACAAAATGGCGAGATTAGGGAAAGGATACATGTTTGAAAGTAAGAGCATGAAGCTAAGAACACAAATACCAGCAGAGATGCTTACAAATATTGATCTGAAGTATTTCAACGAACCAACGAGAA<br>AGAAAATCGAGAAAATAAGACCTCTGCTGATTGATGGCACGGCCTCATTGAGTCCTGGGATGATGATGGGCATGTTCAATATGCTGAGCACAGTATTAGGGGTCTCAATCCTGAATCTCGGGCAAAAGAGGTACACCAAAACCACATACTGGTGGGATGGACTTCAATCCTCTGATGATTTCGCTCTCATAGTGAATGCACCGAATCATGAGGGGATACAAGCAGGAGTGGAT<br>AGATTCTATAGGACCTGCAAATTGGTTGGGATCAACATGAGCAAAAAGAAGTCTTACATAAACCGAACAGGAACATTTGAGTTCACAAGTTTTTTCTACCGCTATGGATTTGTAGCCAACTTCAGTATGGAATTACCCAGCTTTGGAGTGTCTGGAATCAATGAATCAGCTGACATGAGCATTGGAGTTACAGTGATAAAAAACAATATGATAAACAATGATCTTGGACCAGCA<br>ACAGCTCAAATGGCTCTTCAGTTATTCATCAAAGACTATAGGTACACATACCGATGCCACAGGGGTGATACACAAATTCAAACGAGGAGATCATTCGAGCTGAAGAAGCTGTGGGAGCAGACCCGTTCAAAGGCAGGGCTGTTGATATCAGACGGGGGGCCAAACCTATACAACATTCGGAATCTCCACATCCCAGAGGTCTGCTTGAAGTGGGAGCTGATGGATGAAGACT<br>ACCAAGGCAGGCTGTGCAATCCTCTGAATCCATTTGTCAGTCATAAAGAGATTGAGTCCGTAAACAATGCTGTAGTAATGCCCGCCCATGGCCCGGCCAAGAGCATGGAATATGATGCTGTTGCGACCACACACTCGTGGATTCCTAAGAGGAACCGTTCCATCCTCAATACCAGCCAAAGGGGAATTCTTGAGGATGAGCAGATGTACCAAAAGTGCTGTAGTCTATTCGAGA<br>AATTCTTCCCCAGCAGTTCATACAGGAGGCCAGTTGGAATTTCCAGCATGGTGGAGGCCATGGTGTCTAGGGCCCGAATTGATGCACGCATCGACTTCGAATCTGGAAGGATTAAGAAAGAAGAGTTTGCTGAGATCATGAAGATCTGTTCCACCATTGAAGAGCTCAGACGGCAAAAATAG

EPI_ISL_18244810	>A/buzzard/Poland/MB129-21_22VIR5675-7/2021_2021-02-19<br>ATGGATGTCAATCCGACTTTACTTTTCTTAAAAGTGCCAGCGCAAAATGCTATAAGTACTACATTCCCTTACACTGGAGATCCCCCATACAGCCATGGAACAGGAACAGGGTATACCATGGACACAGTAAACAGAACACATCAATATTCAGAAAAGGGAAAGTGGACAACAAACACAGAAACCGGAGCACCCCAACTCAACCCAATTGATGGACCATTACCAGAGGACAATGA<br>GCCAAGCGGATATGCACAAACTGATTGCGTGTTGGAAGCAATGGCTTTCCTTGAAGAATCCCACCCAGGGATATTTGAAAACTCTTGTCTTGAAGCGATGGAAATCGTTCAGCAAACAAGAGTGGACAAACTAACCCAAGGTCGCCAGACTTATGACTGGACATTGAACAGAAACCAACCAGCTGCAACCTCTTTGGCCAACACTATAGAGGTGTTCAGATCGAATGGTCTGA<br>CAGCCAATGAATCAGGGAGACTGATAGATTTTCTCAGGGATGTGATGGAATCAATGGATAAAGAAGAGATGGAAGTAACAACACATTTCCAGAGAAAAAGAAGAGTGAGGGACAACATGACTAAGAAGATGGTCACACAAAGAACAATAGGGAAGAAGAAGCAGAGGCTGAACAAGAGGAGTTACTTAATAAGAGCACTGACATTGAATACAATGACCAAAGATGCAGA<br>AAGAGGCAAGTTGAAGAGACGGGCAATTGCAACACCCGGGATGCAGATTAGAGGATTCGTGTACTTCGTTGAAACACTAGCGAGGAGCATCTGTGAGAAACTAGAGCAATCTGGGCTCCCTGTTGGGGGGAATGAGAAAAAGGCTAAATTGGCAATGTTGTGAGAAAAATGATGACTAACTCACAAGATACARAGCTCTCCTTTACAATTACTGGAGACAACACCAAATG<br>GAATGAGAATCAAAACCCTCGGATGTTTTGGCAATGATAACATATATCACAAGAAACCAACCTGAATGGTTTAGAAATGTCTTAAGCATTGCCCCTATAATGTTCTCAAACAAAATGGCGAGATTAGGGAAAGGATACATGTTTGAAAGTAAGARCATGAAGCTAARAACACAAATACCAGCAGAGATGCTTACAAATATTGATCTRAARTATTTCAACRAACCAACGAGAAA<br>GAAAATCGAGAAAATAAGACCTCTGCTGATTGATGGCACGGCCTCATTGAGCCCTGGGATGATGATGGGCATGTTCAATATGCTGAGCACAGTATTAGGGGTCTCAATCCTGAATCTCGGGCAAAAGAGGTACACCAAAACCACATACTGGTGGGATGGACTTCAATCCTCTGATGATTTCGCTCTCATAGTGAATGCACCGAATCATGAGGGGATACAAGCAGGAGTGGATA<br>GATTCTATAGGACCTGCAAACTGGTTGGGATCAACATGAGCAAAAAGAAGTCTTACATAAACCGAACAGGAACATTTGAGTTCACAAGTTTTTCTACCGCTATGGATTTGTAGCCAACTTCAGTATGGAATTACCCAGCTTTGGAGTGTCTGGAATCAATGAATCAGCTGACATGAGCATTGGAGTTACAGTGATAAAAAACAATATGATAAACAATGATCTTGGACCAGCAA<br>CAGCTCAAATGGCTCTTCAGTTATTCATCAAAGACTATAGGTACACATACCGATGCCACAGGGGTGATACACAAATTCAAACGAGGAGATCATTCGAGCTGAAGAAGCTGTGGGAGCAGACCCGTTCAAAGGCAGGACTGTTGATATCAGACGGGGGGCCAAACCTATACAACATTCGGAATCTCCACATCCCAGAGGTCTGCTTGAAGTGGGAGCTGATGGATGAAGATTA<br>CCAAGGCAGGCTGTGCAATCCTCTGAATCCATTTGTCAGTCATAAAGAGATTGAGTCCGTAAACAATGCTGTAGTAATGCCCGCCCATGGCCCGGCCAAGAGCATGGAATATGATGCTGTTGCGACCACACACTCGTGGATTCCTAAGAGGAACCGTTCCATCCTCAATACCAGCCAAAGGGGAATTCTTGAGGATGAGCAGATGTACCAAAAGTGCTGTAGTCTATTCGAGAA<br>ATTCTTCCCCAGCAGTTCATACAGGAGGCCAGTTGGAATTTCCAGCATGGTGGAGGCCATGGTGTCTAGGGCCCGAATTGATGCACGCATCGACTTCGAATCTGGAAGGATTAAGAAAGAAGAGTTTGCTGAGATCATGAAGATCTGTTCCACCATTGAAGAGCTCAGACGGCAAAAATAG
EPI_ISL_417414	>A/buzzard/Germany-SN/AI00285/2020_H5N8_2020-03-20<br>ATGGATGTCAATCCGACTTTACTTTTCTTAAAAGTGCCAGCGCAAAATGCTATAAGTACTACGTTCCCTTACACTGGAGATCCTCCATACAGCCATGGAACAGGAACAGGATACACCATGGACACAGTCAACAGAACACATCAATACTCAGAGAAGGGAAAGTGGACAACAAACACAGAGACCGGGGCGCCCCAACTCAACCCAATTGATGGACCATTGCCAGAGGACAACGA<br>GCCAAGCGGATATGCACAAACAGATTGCGTGTTGGAAGCAATGGCTTTCCTTGAAGAATCCCACCCAGGGATCTTTGAAAACTCTTGTCTTGAAACGATGGAGGTCGTTCAGCAAACAAGAGTGGACAAACTAACCCAAGGTCGCCAGACCTATGACTGGACACTGAATAGAAACCAACCAGCTGCAACTGCTTTGGCCAATACTATAGAGGTCTTCAGATCGAACGGTCTAA<br>CAGCCAATGAGTCGGGGAGACTAATAGATTTCCTCAAGGATGTGATGGAATCAATGGATAAAGAAGAAATGGAAATAACAACACATTTCCAGAGAAAGAGAAGAGTAAGGGACAACATGACCAAAAAGATGGTCACACAAAGAACGATAGGGAAGAAAAAGCAGAGGCTGAACAAGAGAAGCTACTTAATAAGAGCACTGACATTGAACACAATGACCAAAGATGCAGA<br>AAGAGGCAAATTGAAGAGGCGGGCAATTGCAACACCCGGGATGCAGATTAGAGGATTTGTGTACTTTGTCGAAACACTAGCGAGGAGCATCTGTGAGAAACTCGAGCAATCTGGACTCCCTGTTGGAGGGAATGAGAAAAAGGCCAAATTGGCAAATGTCGTGAGGAAAATGATGACTAACTCGCAAGATACAGAGCTCTCCTTTACAATCACCGGAGACAACACCAAATG<br>GAATGAGAATCAAAATCCTCGGATGTTTTGGCAATGATAACATACATCACAAGAAACCAACCCGAATGGTTTAGAAATGTCTTGAGCATTGCCCCTATAATGTTCTCAAACAAAATGGCAAGATTAGGAAAAGGATACATGTTTGAAAGTAAGAGCATGAAGCTAAGGACACAAATACCGGCAGAAATGCTTGCAAACATTGACTTGAAATACTTCAATGAATCAACGAGAA<br>AGAAAATCGAGAAAATAAGACCTCTGCTAATTGATGGCACAGCCTCATTGAGCCCTGGAATGATGATGGGCATGTTCAATATGCTGAGCACAGTATTGGGGGTCTCAATCCTGAATCTTGGACAAAAGAGGTACACCAAAACCACATACTGGTGGGATGGACTCCAATCCTCTGATGATTTCGCTCTCATAGTGAATGCACCGAATCATGAGGGGATACAAGCTGGGGTGGAT<br>AGGTTCTATAGGACCTGCAAATTGGTTGGGATCAACATGAGCAAGAAGAAGTCTTACATAAACCGGACAGGAACATTTGAGTTCACAAGCTTTTTCTACCGCTATGGATTTGTAGCCAACTTCAGTATGGAGTTACCCAGCTTTGGAGTGTCTGGAATCAATGAGTCAGCTGACATGAGCATTGGAGTTACAGTGATAAAGAACAATATGATAAACAATGACCTTGGACCAGCA<br>ACAGCTCAAATGGCTCTTCAGCTATTCATCAAGGATTATAGGTACACGTACCGATGTCACAGAGGTGATACACAAATTCAAACGAGGAGATCATTCGAGCTGAAGAAGCTGTGGGAGCAGACCCGTTCAAAGGCAGGGCTGTTGGTATCAGATGGAGGACCAAACCTATACAACATTCGGAATCTCCACATCCCAGAGGTCTGCTTGAAGTGGGAGCTAATGGATGAAGACT<br>ACCAGGGCAGGCTGTGTAATCCTCTGAATCCGTTTGTCAGTCATAAGGAAATTGAGTCCGTAAACAATGCTGTGGTAATGCCAGCCCATGGCCCAGCCAAGAGCATGGAATATGATGCTGTTGCGACTACACACTCATGGATTCCCAAGAGGAACCGTTCCATTCTCAATACCAGCCAGAGGGGAATTCTCGAGGATGAGCAGATGTACCAGAAGTGCTGCAGTTTATTCGAG<br>AAATTCTTCCCCAGTAGTTCATATAGGAGGCCAGTTGGAATTTCCAGCATGGTGGAGGCCATGGTGTCCAGGGCCCGAATTGATGCACGTATTGATTTCGAATCTGGAAGGATTAAGAAAGAAGAGTTTGCTGAGATCATGAAGATCTGTTCCACCATTGATGAGCTCAGACGGCAAAAATAG
EPI_ISL_614399	>A/buzzard/Germany-MV/AI02166/2020_H5N5_2020-10-29<br>ATGGATGTCAATCCGACTTTACTTTTCTTAAAAGTGCCAGCGCAAAATGCTATAAGTACTACATTCCCTTACACTGGAGATCCTCCATACAGCCATGGAACAGGAACAGGGTATACCATGGACACAGTAAACAGAACACATCAATACTCAGAAAAGGGAAAGTGGACAACAAACACAGAAACCGGAGCGCCCCAACTCAACCCAATTGATGGACCATTACCAGAGGACAATGA<br>GCCAAGCGGATATGCACAAACTGATTGCGTGTTGGAAGCAATGGCTTTCCTTGAAGAATCCCACCCAGGGATATTTGAAAACTCTTGTCTTGAAGCGATGGAAATCGTTCAGCAAACAAGAGTGGACAAACTAACCCAAGGTCGCCAGACTTATGACTGGACACTGAACAGAAACCAACCAGCTGCAACCTCTTTGGCCAACACTATAGAGGTGTTCAGATCGAATGGTCTGA<br>CAGCCAATGAATCAGGGAGACTGATAGATTTTCTCAGGGATGTGATGGAATCAATGGATAAAGAAGAGATGGAAGTAACAACACATTTCCAGAGAAAAAGAAGAGTGAGGGACAACATGACTAAAAAGATGGTCACACAAAGAACAATAGGGAAGAAGAAGCAGAGGCTGAACAAGAGGAGTTACTTAATAAGAGCACTGACATTGAATACAATGACCAAAGATGCAGA<br>AAGAGGCAAGTTGAAGAGACGGGCAATTGCAACACCCGGGATGCAGATTAGAGGATTCGTGTACTTCGTTGAAACACTAGCGAGGAGCATCTGTGAGAAACTAGAGCAATCTGGGCTCCCTGTTGGAGGGAATGAGAAGAAGGCTAAATTGGCAATGTTGTGAGAAAAATGATGACTAACTCACAAGATACAGAGCTCTCCTTTACAATTACTGGAGACAACACCAAATG<br>GAATGAGAATCAAAACCCTCGGATGTTTTGGCAATGATAACATATATCACAAGAAACCAACCTGAATGGTTTAGAAATGTCTTAAGCATTGCCCCTATAATGTTCTCAAACAAAATGGCGCGATTAGGGAAAGGATACATGTTTGAAAGTAAGAGCATGAAGCTAAGAACACAAATACCAGCAGAGATGCTTACAAATATTGATCTGAAGTATTTCAACGAACCAACGAGAAA<br>GAAAATCGAGAAAATAAGACCTCTGCTGATTGATGGCACGGCCTCATTGAGTCCTGGGATGATGATGGGCATGTTCAATATGCTGAGCACAGTATTAGGGGTCTCAATCCTGAATCTCGGGCAAAAAAGGTACACCAAAACCACATACTGGTGGGATGGACTTCAATCCTCTGATGATTTCGCTCTCATAGTGAATGCACCGAATCATGAGGGGATACAAGCAGGAGTGGATA<br>GATTCTATAGGACCTGCAAACTGGTTGGGATCAACATGAGCAAAAAGAAGTCTTACATAAACCGAACAGGAACATTTGAGTTCACAAGTTTTTCTACCGCTATGGATTTGTAGCTAACTTCAGTATGGAATTACCCAGCTTTGGAGTGTCTGGAATCAATGAATCAGCTGACATGAGCATTGGAGTTACAGTGATAAAAAACAATATGATAAACAATGATCTTGGACCAGCAAC<br>AGCTCAAATGGCTCTTCAGTTATTCATCAAAGACTATAGGTACACATACCGATGCCACAGGGGTGATACACAAATTCAAACGAGGAGATCATTCGAGCTGAAGAAGCTGTGGGAGCAGACCCGTTCAAAGGCAGGGCTGTTGATATCAGACGGGGGGCCAAACCTATACAACATTCGGAATCTCCACATCCCAGAGGTCTGCTTGAAGTGGGAGCTGATGGATGAAGACTAC<br>CAAGGCAGGCTGTGCAATCCTCTGAATCCATTTGTCAGTCATAAAGAGATTGAGTCCGTAAACAATGCTGTAGTAATGCCCGCCCATGGCCCGGCCAAGAGCATGGAATATGATGCTGTTGCGACCACACACTCGTGGATTCCTAAGAGGAACCGTTCCATTCTCAATACCAGCCAAAGGGGAATTCTTGAGGATGAGCAGATGTACCAAAAGTGCTGTAGTCTATTCGAGAAA<br>TTCTTCCCCAGCAGTTCATACAGGAGGCCAGTTGGAATTTCCAGCATGGTGGAGGCCATGGTGTCTAGGGCCCGAATTGATGCACGCATCGACTTCGAATCTGGAAGGATTAAGAAAGAAGAGTTTGCTGAGATCATGAAGATCTGTTCCACCATTGAAGAGCTCAGACGGCAAAAATAG
EPI_ISL_11259282	>A/brent_goose/Ireland/033257_22VIR1325-9/2021_H5N1_2021-11-16<br>ATGGATGTCAATCCGACTTTACTTTTCTTAAAAGTGCCAGCGCAAGATGCCATAAGTACCACATTCCCTTACACTGGAGATCCTCCATACAGCCATGGAACAGGGACAGGATACACAATGGACACAGTCAACAGAACACATCAATACTCAGAGAAGGGAAAATGGACAACAAACACAGAAACCGGAGCACCTCAACTCAACCCAATTGATGGGCCACTACCTGAGGACAACGA<br>ACCGAGCGGATATGCACAAACAGATTGCGTGTTGGAAGCAATGGCTTTCCTTGAAGAGTCCCATCCAGGGATCTTTGAAAACTCTTGTCTTGAAACGATGGAAGTCGTTCAGCAAACAAGAGTGGACAAACTAACTCAAGGTCGCCAGACATATGACTGGACACTGAATAGAAACCAACCGGCTGCAACTGCCCTGGCCAACACTATAGAGGTCTTCAGATCAAACGGTCTAA<br>CAGCCAATGAATCGGGGAGACTAATAGATTTCCTCAAGGATGTGATGGACTCAATGGATAAAGAAGAAATGGAAATAACAACACATTTCCAGAGAAAGAGAAGAGTAAGGGACAACATGACCAAGAAAATGGTCACACAAAGAACAATAGGAAAGAAGAAACAAAGGCTAAACAAGAGGAGCTACTTAATAAGAGCACTGACACTGAATACAATGACAAAAGATGCAGA<br>AAGAGGCAAATTGAAGAGACGGGCGATTGCAACACCAGGGATGCAGATTAGAGGATTTGTGTACTTTGTCGAAACACTGGCAAGGAGCATCTGTGAAAAACTTGAGCAATCTGGACTCCCCGTTGGAGGGAATGAGAAGAAGGCTAAATTGGCAAATGTCGTGAGAAAAATGATGACTAACTCACAAGATACAGAGCTCTCCTTCACAATTACTGGAGATAACACCAAATG<br>GAATGAGAATCAAAATCCTCGGATGTTTCTGGCAATGATAACATACATTACAAGAAACCAACCTGAATGGTTTAGGAATGTCTTGAGTATTGCCCCTATAATGTTCTCGAACAAAATGGCGAGATTGGGAAAAGGGTACATGTTTGAAAGTGAGAGCATGAAGTTACGGACACAAATACCTGCAGAAATGCTTGCAAACATTGACTTAAAATACTTCAATGAATCAACAAGAAA<br>GAAAATCGAAAAAATAAGGCCTCTACTAATAGATGGCACTGCCTCATTGAGTCCTGGAATGATGATGGGCATGTTCAATATGCTGAGTACAGTATTAGGAGTTTCAATCCTAAATCTTGGGCAAAAGAAGTACACCAAAACCACATACTGGTGGGATGGACTCCAATCCTCTGATGATTTCGCCCTCATAGTAAATGCACCGAATCATGAGGGAATACAAGCAGGAGTGGATA<br>GGTTCTATAGGACCTGCAAACTGGTCGGGATCAATATGAGCAAAAAGAAGTCTTACATAAACCGGACTGGAACATTTGAGTTCACAAGCTTTTTCTATCGCTATGGATTTGTGGCTAACTTCAGTATGGAGCTGCCCAGCTTTGGAGTTTCTGGGATCAATGAATCAGCTGACATGAGCATTGGCATCACAGTGATAAAGAACAACATGATAAACAATGACCTTGGACCAGCAA<br>CAGCTCAAATGGCCCTTCAACTATTCATCAAAGATTACAGGTACACGTACCGATGCCATAGAGGTGACACACAAATTCAAACGAGGAGATCATTCGAGCTGAAGAAGCTGTGGGAACAGACCCGTTCAAAGGCAGGACTGTTGGTGTCAGATGGAGGACCAAATCTATACAACATTCGGAATCTCCATATCCCAGAGGTCTGCCTGAAGTGGGAGCTGATGGACGAAGATTA<br>CCAGGGCAGGTTGTGTAATCCTCTGAACCCATTTGTCAGCCATAAAGAAATTGAGTCCGTAAACAATGCTGTGGTGATGCCAGCCCACGGTCCAGCCAAAAGCATGGAATATGATGCCGTTGCGACTACACACTCATGGATTCCTAAAAGGAATCGTTCCATTCTCAATACCAGCCAAAGGGGAATTCTTGAGGATGAACAGATGTACCAGAAATGCTGCGGTCTATTCGAGAA<br>ATTCTTCCCCAGTAGTTCATACAGGAGACCAGTTGGAATTTCCAGCATGGTGGAGGCCATGGTGTCTAGGGCCCGAATCGATGCACGCATTGATTTCGAATCTGGAAGGATCAAGAAGGAAGAGTTTGCTGAGATCATGAAGATCTGTTCCACCATTGAAGAGCTCAGACGGCAAAAATAG
EPI_ISL_1123357	>A/brent_goose/England/233339/2020_H5N8_2020-11-08<br>ATGGATGTCAATCCGACTTTACTTTTCTTAAAAGTGCCAGCGCAAAATGCTATAAGTACTACATTCCCTTACACTGGAGATCCTCCATACAGCCATGGAACAGGAACAGGGTACACCATGGACACAGTAAACAGAACACATCAATACTCAGAAAAGGGAAAGTGGACAACAAACACAGAAACCGGAGCACCCCAACTCAACCCAATTGATGGACCATTACCAGAGGACAATGA<br>GCCAAGCGGATATGCACAAACTGATTGCGTGTTGGAAGCAATGGCTTTCCTTGAAGAATCCCACCCAGGGATATTTGAAAACTCTTGTCTTGAAGCGATGGAAATCGTTCAGCAAACAAGAGTGGACAAACTAACCCAAGGTCGCCAGACTTATGACTGGACACTGAACAGAAACCAACCAGCTGCAACCTCTTTGGCCAACACTATAGAGGTGTTCAGATCGAATGGTCTGA<br>CAGCCAATGAATCAGGGAGACTGATAGATTTTCTCAGAGATGTGATGGAATCAATGGATAAAGAAGAGATGGAAGTAACAACACATTTCCAGAGAAAAAGAAGAGTGAGGGACAACATGACTAAGAAGATGGTCACACAAAGAACAATAGGGAAGAAGAAGCAGAGGCTGAACAAGAGGAGTTACTTAATAAGAGCACTGACATTGAATACAATGACCAAAGATGCAGA<br>AAGAGGCAAGTTGAAGAGACGGGCAATTGCAACACCCGGGATGCAGATTAGAGGATTCGTGTACTTCGTTGAAACACTAGCGAGGAGCATCTGTGAGAAACTAGAGCAATCTGGGCTCCCTGTTGGAGGGAATGAGAAGAAGGCTAAATTGGCAATGTTGTGAGAAAAATGATGACTAACTCACAAGATACAGAGCTCTCCTTTACAATTACTGGAGACAACACCAAATG<br>GAATGAGAATCAAAACCCTCGGATGTTTTGGCAATGATAACATATATCACAAGAAACCAACCTGAATGGTTTAGAAATGTCTTAAGCATTGCCCCTATAATGTTCTCAAACAAAATGGCGAGATTAGGGAAAGGATACATGTTTGAAAGTAAGAGCATGAAGCTAAGAACACAAATACCAGCAGAGATGCTTACAAATATCGATCTGAAGTATTTCAACGAACCAACGAGAA<br>AGAAAATCGAGAAAATAAGACCTCTGCTGATTGATGGCACGGCCTCATTGAGTCCTGGGATGATGATGGGCATGTTCAATATGCTGAGCACAGTATTAGGGGTCTCAATCCTGAATCTCGGGCAAAAAAGGTACACCAAAACCACATACTGGTGGGATGGACTTCAATCCTCTGATGATTTCGCTCTCATAGTGAATGCACCGAATCATGAGGGGATACAAGCAGGAGTGGAT<br>AGATTCTATAGGACCTGCAAACTGGTTGGGATCAACATGAGCAAAAAGAAGTCTTACATAAACCGAACAGGAACATTTGAGTTCACAAGTTTTTCTACCGCTATGGATTTGTAGCTAACTTCAGTATGGAATTACCCAGCTTTGGAGTGTCTGGAATCAATGAATCAGCTGACATGAGCATTGGAGTTACAGTGATAAAAAACAATATGATAAACAATGATCTTGGACCAGCA<br>ACAGCTCAAATGGCTCTTCAGTTATTCATCAAAGACTATAGGTACACATACCGATGCCACAGGGGTGATACACAAATTCAAACGAGGAGATCATTCGAGCTGAAGAAGCTGTGGGAGCAGACCCGTTCAAAGGCAGGGCTGTTGATATCAGACGGGGGGCCAAACCTATACAACATTCGGAATCTCCACATCCCAGAGGTCTGCTTGAAGTGGGAGCTGATGGATGAAGACT<br>ACCAAGGCAGGCTGTGCAATCCTCTGAACCCATTTGTCAGTCATAAAGAGATTGAGTCCGTAAACAATGCTGTAGTAATGCCCGCCCATGGCCCGGCCAAGAGCATGGAATATGATGCTGTTGCGACCACACACTCGTGGATTCCTAAGAGGAACCGTTCCATTCTCAATACCAGCCAAAGGGGAATTCTTGAGGATGAGCAGATGTACCAAAAGTGCTGTAGTCTATTCGAGA<br>AATTCTTCCCCAGCAGTTCATACAGGAGGCCAGTTGGAATTTCCAGCATGGTGGAGGCCATGGTGTCTAGGGCCCGAATTGATGCACGCATCGACTTCGAATCTGGAAGGATTAAGAAAGAAGAGTTTGCTGAGATCATGAAGATCTGTTCCACCATTGAAGAGCTCAGACGGCAAAAATAG
EPI_ISL_14917942	>A/breeding_hen/Poland/H364/2022_H5N1_2022-07-21<br>ATGGATGTCAATCCGACTTTACTTTTCTTAAAAGTGCCAGCGCAAGATGCCATAAGTACCACATTCCCTTACACTGGAGATCCTCCATACAGCCATGGAACAGGGACAGGATACACAATGGACACAGTCAACAGAACACATCAATACTCAGAGAAGGGAAAATGGACAACAAACACAGAAACCGGAGCACCTCAACTCAACCCAATTGATGGGCCACTACCTGAGGACAACGA<br>ACCGAGCGGATATGCACAAACAGATTGCGTGTTGGAAGCAATGGCTTTCCTTGAAGAGTCCCACCCAGGGATCTTTGAAAACTCTTGTCTTGAAACGATGGAAGTCGTTCAGCAAACAAGAGTGGACAAACTAACTCAAGGTCGCCAGACATATGACTGGACACTGAATAGAAACCAACCAGCTGCAACTGCCCTGGCCAACACTATAGAGGTCTTCAGATCAAACGGTCTAA<br>CAGCCAATGAATCGGGGAGACTAATAGATTTCCTCAAGGATGTGATGGACTCAATGGATAAAGAAGAAATGGAAATAACAACACATTTCCAGAGAAAGAGAAGAGTAAGGGACAACATGACCAAGAAAATGGTCACACAAAGAACAATAGGAAAGAAGAAACAAAGGCTAAACAAGAGGAGCTACTTAATAAGAGCACTGACACTGAATACAATGACAAAAGATGCAGA<br>AAGAGGCAAATTGAAAAGACGGGCGATTGCAACACCAGGGATGCAGATTAGAGGATTTGTGTACTTTGTCGAAACACTGGCAAGGAGCATCTGTGAAAAACTTGAGCAATCTGGACTCCCCGTTGGAGGGAATGAGAAGAAGGCTAAATTGGCAAATGTCGTGAGAAAAATGATGACTAACTCACAAGATACAGAGCTCTCCTTCACAATTACTGGAGACAACACCAAATG<br>GAATGAGAATCAAAATCCTCGGATGTTTCTGGCAATGATAACATACATTACAAGAAACCAACCTGACTGGTTTAGAAATGTCTTGAGTATTGCCCCTATAATGTTCTCGAACAAAATGGCGAGATTGGGAAAAGGGTACATGTTTGAAAGTAAGAGCATGAAGTTACGGACACAAATACCTGCAGAAATGCTTGCAAACATTGACTTAAAATACTTCAATGAATCAACAAGAAA<br>GAAAATCGAAAAAATAAGGCCTCTACTAATAGATGGCACTGCCTCATTGAGTCCTGGAATGATGATGGGCATGTTCAATATGCTGAGTACAGTATTAGGAGTTTCAATCCTAAATCTTGGGCAAAAGAAGTACACCAAAACAACATACTGGTGGGATGGACTCCAATCCTCTGATGATTTCGCCCTCATAGTAAATGCACCGAATCATGAGGGAATACAAGCAGGAGTGGATA<br>GGTTCTATAGGACCTGCAAACTGGTCGGGATCAATATGAGCAAAAAGAAGTCTTACATAAACCGGACTGGAACATTCGAGTTCACAAGCTTTTTCTATCGCTATGGATTTGTGGCTAACTTCAGTATGGAGCTGCCCAGCTTTGGAGTTTCTGGGATCAATGAATCAGCTGACATGAGCATTGGCGTCACAGTGATAAAGAACAACATGATAAACAATGACCTTGGACCAGCAA<br>CAGCTCAAATGGCCCTTCAACTATTCATCAAAGATTACAGGTACACGTACCGATGCCACAGAGGTGACACACAAATTCAAACGAGGAGATCATTCGAGCTGAAGAAGCTGTGGGAACAGACCCGTTCAAAGGCAGGACTGTTGGTGTCAGATGGAGGACCAAATCTATACAACATTCGGAATCTCCATATCCCAGAAGTCTGCCTGAAGTGGGAGCTGATGGACAAAGATTA<br>CCAGGGCAGGTTGTGTAATCCTCTGAACCCATTTGTCAGCCATAGAGAAATTGAGTCCGTAAACAATGCTGTGGTGATGCCAGCCCACGGTCCAGCCAAAAGCATGGAATATGATGCCGTTGCGACTACACACTCATGGATTCCTAAAAGGAATCGTTCCATTCTCAATACCAGTCAAAGGGGAATTCTTGAGGATGAACAGATGTACCAGAAATGCTGCAGTCTATTCGAGAA<br>ATTCTTCCCCAGTAGTTCATACAGGAGACCAGTTGGAATTTCCAGCATGGTGGAGGCCATGGTGTCTAGGGCCCGAATCGATGCACGAATTGATTTCGAATCTGGAAGGATCAAGAAGGAAGAGTTTGCTGAGATCATGAAGATCTGTTCCACCATTGAAGAGCTCAGACGGCAAAAATAG
EPI_ISL_14917915	>A/breeding_hen/Poland/H361/2022_H5N1_2022-07-18<br>ATGGATGTCAATCCGACTTTACTTTTCTTAAAAGTGCCAGCGCAAGATGCCATAAGTACCACATTCCCTTACACTGGAGATCCTCCATACAGCCATGGAACAGGGACAGGATACACAATGGACACAGTCAACAGAACACATCAATACTCAGAGAAGGGAAAATGGACAACAAACACAGAAACCGGAGCACCTCAACTCAACCCAATTGATGGGCCACTACCTGAGGACAACGA<br>ACCGAGCGGATATGCACAAACAGATTGCGTGTTGGAAGCAATGGCTTTCCTTGAAGAGTCCCACCCAGGGATCTTTGAAAACTCTTGTCTTGAAACGATGGAAGTCGTTCAGCAAACAAGAGTGGACAAACTAACTCAAGGTCGCCAGACATATGACTGGACACTGAATAGAAACCAACCAGCTGCAACTGCCCTGGCCAACACTATAGAGGTCTTCAGATCAAACGGTCTAA<br>CAGCCAATGAATCGGGGAGACTAATAGATTTCCTCAAGGATGTGATGGACTCAATGGATAAAGAAGAAATGGAAATAACAACACATTTCCAGAGAAAGAGAAGAGTAAGGGACAACATGACCAAGAAAATGGTCACACAAAGAACAATAGGAAAGAAGAAACAAAGGCTAAACAAGAGGAGCTACTTAATAAGAGCACTGACACTGAATACAATGACAAAAGATGCAGA<br>AAGAGGCAAATTGAAAAGACGGGCGATTGCAACACCAGGGATGCAGATTAGAGGATTTGTGTACTTTGTCGAAACACTGGCAAGGAGCATCTGTGAAAAACTTGAGCAATCTGGACTCCCCGTTGGAGGGAATGAGAAGAAGGCTAAATTGGCAAATGTCGTGAGAAAAATGATGACTAACTCACAAGATACAGAGCTCTCCTTCACAATTACTGGAGACAACACCAAATG<br>GAATGAGAATCAAAATCCTCGGATGTTTCTGGCAATGATAACATACATTACAAGAAACCAACCTGACTGGTTTAGAAATGTCTTGAGTATTGCCCCTATAATGTTCTCGAACAAAATGGCGAGATTGGGAAAAGGGTACATGTTTGAAAGTAAGAGCATGAAGTTACGGACACAAATACCTGCAGAAATGCTTGCAAACATTGACTTAAAATACTTCAATGAATCAACAAGAAA<br>GAAAATCGAAAAAATAAGGCCTCTACTAATAGATGGCACTGCCTCATTGAGTCCTGGAATGATGATGGGCATGTTCAATATGCTGAGTACAGTATTAGGAGTTTCAATCCTAAATCTTGGGCAAAAGAAGTACACCAAAACAACATACTGGTGGGATGGACTCCAATCCTCTGATGATTTCGCCCTCATAGTAAATGCACCGAATCATGAGGGAATACAAGCAGGAGTGGATA<br>GGTTCTATAGGACCTGCAAACTGGTCGGGATCAATATGAGCAAAAAGAAGTCTTACATAAACCGGACTGGAACATTCGAGTTCACAAGCTTTTTCTATCGCTATGGATTTGTGGCTAACTTCAGTATGGAGCTGCCCAGCTTTGGAGTTTCTGGGATCAATGAATCAGCTGACATGAGCATTGGCGTCACAGTGATAAAGAACAACATGATAAACAATGACCTTGGACCAGCAA<br>CAGCTCAAATGGCCCTTCAACTATTCATCAAAGATTACAGGTACACGTACCGATGCCACAGAGGTGACACACAAATTCAAACGAGGAGATCATTCGAGCTGAAGAAGCTGTGGGAACAGACCCGTTCAAAGGCAGGACTGTTGGTGTCAGATGGAGGACCAAATCTATACAACATTCGGAATCTCCATATCCCAGAAGTCTGCCTGAAGTGGGAGCTGATGGACAAAGATTA<br>CCAGGGCAGGTTGTGTAATCCTCTGAACCCATTTGTCAGCCATAGAGAAATTGAGTCCGTAAACAATGCTGTGGTGATGCCAGCCCACGGTCCAGCCAAAAGCATGGAATATGATGCCGTTGCGACTACACACTCATGGATTCCTAAAAGGAATCGTTCCATTCTCAATACCAGTCAAAGGGGAATTCTTGAGGATGAACAGATGTACCAGAAATGCTGCAGTCTATTCGAGAA<br>ATTCTTCCCCAGTAGTTCATACAGGAGACCAGTTGGAATTTCCAGCATGGTGGAGGCCATGGTGTCTAGGGCCCGAATCGATGCACGAATTGATTTCGAATCTGGAAGGATCAAGAAGGAAGAGTTTGCTGAGATCATGAAGATCTGTTCCACCATTGAAGAGCTCAGACGGCAAAAATAG
EPI_ISL_17978956	>A/black-headed_gull/Poland/P075/2021_H9N7_2021-02-09<br>ATGGATGTCAATCCGACTTTACTTTTCTTAAAAGTGCCAGCGCAAAATGCCATAAGTACTACGTTTCCTTATACTGGAGATCCTCCATACAGCCATGGAACAGGAACAGGATACACCATGGACACAGTCAACAGAACACATCAATACTCAGAAAAGGGGAAATGGACAACAAACACAGAAACCGGAGCGCCCCAGCTCAACCCAATTGATGGACCACTACCTGAAGACAACGA<br>ACCAAGCGGATATGCACAAACAGATTGTGTGTTGGAAGCAATGGCTTTCCTTGAAGAGTCCCACCCAGGGATCTTTGAAAACTCTTGTCTTGAAACGATGGAAATTGTTCAGCAAACAAGAGTGGACAAACTAACTCAAGGTCGCCAGACTTATGACTGGACACTGAATAGAAACCAACCAGCTGCAACTGCCCTGGCCAACACTATAGAGGTCTTCAGATCAAACGGTCTGAC<br>AGCCAATAAATCGGGGAGACTAATAGATTTCCTCAAGGACGTGATGGACTCAATGGACAAAGAAGAAATGGAAATAACAACACATTTCCAGAGAAAGAGAAGAGTAAGGGACAACATGACCAAGAAAATGGTCACACAAAGAACAATAGGGAAAAAGAAACAAAGGCTGAATAAGAAGAGCTACTTAATAAGGGCACTGACACTAAACACAATGACAAAAGATGCGGAA<br>AGAGGCAAATTGAAGAGGCGGGCAATTGCAACACCAGGGATGCAGATTAGAGGATTTGTGTACTTTGTCGAAACACTGGCAAGGAGCATCTGTGAGAAACTTGAGCAATCTGGACTCCCCGTTGGAGGGAATGAAAAAAAGGCCAAATTGGCAAATGTCGTGAGAAAAATGATGACCAACTCACAAGATACAGAGCTCTCTTTCACAATTACTGGAGATAACACCAAATGG<br>AATGAGAATCAAAATCCTCGGATGTTTCTGGCAATGATAACATACATCACAAGAAACCAACCTGAATGGTTTAGAAATGTCTTGAGCATTGCCCCTATAATGTTCTCGAACAAAATGGCGAGATTGGGAAAGGATACATGTTTGAAAGCAAGAGCATGAAGCTACGGACACAAATACCTGCAGAAATGCTTGCAAACATTGACTTAAAATATTTCAATGAATCAACAAGAAAG<br>AAAATCGAGAAAATAAGACCTCTGCTAATAGATGGCACAGCCTCATTGAGTCCAGGAATGATGATGGGCATGTTCAACATGCTGAGCACAGTATTAGGAGTCTCAATCCTGAATCTTGGGCAAAAGAGGTACACCAAAACCACATACTGGTGGGATGGACTCCAATCCTCTGATGATTTCGCCCTCATAGTGAATGCACCAAATCATGAGGGAATACAAGCAGGAGTTGATAG<br>GTTCTATAGGACGTGCAAACTGGTTGGAATCAACATGAGCAAAAAGAAGTCTTACATAAATAAGACAGGAACATTTGAGTTCACAAGCTTTTTCTATCGCTATGGATTTGTGGCTAATTTCAGTATGGAGCTGCCCAGCTTTGGAGTGTCTGGAATCAATGAATCAGCTGACATGAGCATTGGAGTTACAGTGATAAAGAATAACATGATAAACAATGACCTTGGGCCAGCAAC<br>AGCTCAGATGGCTCTTCAGCTATTCATCAAGGACTACAGGTACACGTACCGATGCCACAGGGGTGACACACAAATTCAAACGAGGAGATCATTCGAGCTGAAGAAGCTGTGGGAGCAGACCCGCTCAAAGGCAGGATTGTTGGTGTCGGATGGAGGACCAAACCTATACAACATTCGGAATCTCCACATCCCAGAGGTCTGCTTGAAGTGGGAGCTGATGGACGAAGATTAT<br>CAGGGCAGGTTGTGTAATCCTCTGAACCCATTTGTCAGTCATAAGGAAATTGAGTCCGTGAACAATGCTGTGGTGATGCCGGCCCATGGCCCAGCCAAGAGCATGGAATATGATGCTGTTGCGACTACACACTCATGGATTCCTAAGAGGAATCGTTCCATTCTCAATACCAGCCAAAGGGGAATTCTTGAGGATGAGCAAATGTATCAGAAGTGCTGTAGTCTATTCGAGAAA<br>TTCTTCCCCAGTAGTTCATACAGGAGGCCAGTTGGAATTTCCAGCATGGTAGAGGCCATGGTATCTAGGGCCCGAATTGATGCACGCATTGATTTCGAATCTGGAAGGATTAAGAAAGAAGAGTTTGCTGAGATCATGAAGATCTGTTCCACCATTGAAGAGCTCAGACGGCAAAAATAG
EPI_ISL_14917999	>A/black-headed_gull/Poland/MB139/2022_H5N1_2022-05-30<br>ATGGATGTCAATCCGACTTTACTCTTCTTAAAAGTGCCAGCGCAAGATGCCATAAGTACCACATTCCCTTACACTGGAGATCCTCCATACAGCCATGGAACAGGGACAGGATACACAATGGACACGGTCAACAGAACACATCAATACTCAGAGAAGGGAAAATGGACAACAAACACAGAAACCGGAGCACCTCAACTCAACCCAATTGATGGGCCACTACCTGAGGACAACGA<br>ACCGAGCGGATATGCACAAACAGATTGCGTGTTGGAAGCAATGGCTTTCCTTGAAGAGTCCCATCCAGGGATCTTTGAAAACTCTTGTCTTGAAACGATGGAAGTCGTTCAGCAAACAAGAGTGGACAAACTAACTCAAGGTCGCCAGACATATGACTGGACACTGAATAGAAACCAACCAGCTGCAACTGCCTTGGCCAACACTATAGAGGTCTTAAGATCAAACGGTCTAA<br>CAGCCAATGAATCGGGGAGACTAATAGATTTCCTCAAGGATGTGATGGACTCAATGGATAAAGAAGAAATGGAAATAACAACACATTTCCAGAGAAAGAGAAGAGTAAGGGACAACATGACCAAGAAAATGGTCACACAAAGAACAATAGGAAAGAAGAAACAAAGGCTAAACAAAAGGAGCTACTTAATAAGAGCACTGACACTGAATACAATGACAAAAGATGCAGA<br>AAGAGGCAAATTGAAGAGGCGGGCGATTGCAACACCAGGGATGCAGATTAGAGGATTTGTGTACTTTGTCGAAACACTGGCAAGGAGCATCTGTGAAAAACTTGAGCAATCTGGACTCCCCGTTGGAGGGAATGAGAAGAAGGCTAAATTGGCAAATGTTGTGAGAAAAATGATGACTAACTCACAAGATACAGAGCTCTCCTTCACAATTACTGGAGATAACACCAAATG<br>GAATGAGAATCAAAATCCTCGGATGTTTCTGGCAATGATAACATACATTACAAGAAACCAACCTGAATGGTTTAGGAATGTCTTGAGTATTGCCCCTATAATGTTCTCGAACAAAATGGCGAGATTGGGAAAAGGGTACATGTTTGAAAGTAAGAGCATGAAGTTACGGACACAAATACCTGCAGAAATGCTTGCAAACATTGACTTAAAATATTTCAATGAATCAACAAGAAA<br>GAAAATCGAAAAAATAAGGCCTCTACTAATAGATGGCACTGCCTCATTGAGTCCTGGAATGATGATGGGCATGTTCAATATGCTGAGTACAGTATTAGGAGTTTCAATCCTAAATCTTGGGCAAAAGAAGTACACCAAAACCACATACTGGTGGGATGGACTCCAATCCTCTGATGATTTCGCCCTCATAGTAAATGCACCGAATCATGAGGGAATACAAGCAGGAGTGGATA<br>GGTTCTATAGGACCTGCAAACTGGTCGGGATCAATATGAGCAAAAAGAAGTCTTACATAAACCGGACTGGAACATTTGAGTTCACAAGCTTTTTCTATCGCTATGGATTTGTGGCTAATTTCAGTATGGAGCTGCCCAGCTTTGGAGTTTCTGGGATCAATGAATCAGCTGACATGAGCATTGGCATCACAGTGATAAAGAACAACATGATAAACAATGACCTTGGACCAGCAA<br>CAGCTCAAATGGCCCTTCAACTATTCATCAAAGATTACAGGTACACGTACCGATGCCATAGAGGTGACACACAAATTCAAACGAGGAGATCATTCGAGCTGAAGAAGCTGTGGGAACAGACCCGTTCAAAGGCAGGACTGTTGGTGTCAGATGGAGGACCAAATCTATACAACATTCGGAATCTCCATATCCCAGAGGTCTGCCTGAAGTGGGAGCTGATGGACGAAGATTA<br>CCAGGGCAGGTTGTGTAATCCTCTGAACCCATTTGTCAGCCATAAAGAAATTGAGTCCGTAAACAATGCTGTGGTGATGCCAGCCCACGGTCCAGCCAAAAGCATGGAATATGATGCCGTTGCGACTACACACTCATGGATTCCTAAAAGGAATCGTTCCATTCTCAATACCAGTCAAAGGGGAATTCTTGAGGATGAACAGATGTACCAGAAATGCTGCGGTCTATTCGAGAA<br>ATTCTTCCCCAGTAGTTCATACAGGAGACCAGTTGGAATTTCCAGCATGGTGGAGGCCATGGTGTCTAGGGCCCGAATCGATGCACGCATTGATTTCGAATCTGGAAGGATCAAGAAGGAAGAGTTTGCTGAGATCATGAAGATCTGTTCCACCATTGAAGAGCTCAGACGGCAAAAATAG
EPI_ISL_243502	>A/black-headed_gull/Netherlands/17/2014_H13N6_2014-07-11<br>ATGGATGTCAATCCGACTTTACTTTTCTTGAAAGTGCCAGCGCAAAATGCTATAAGTACTACATTCCCTTATACTGGTGACCCTCCATACAGCCATGGAACAGGAACAGGATATACCATGGACACAGTCAACAGAACACACCAGTACTCAGAAAAAGGAACATGGACAACAAACACCGAAACTGGAGCACCTCAGCTTAACCCGATCGATGGACCACTACCGAAGGATAATGAG<br>CCAAGCGGATATGCACAAACGGATTGTGTATTGGAGGCCATGGCTTTCCTCGAAGAATCCCATCCAGGGATCTTTGAGAACTCATGTCTTGAAACGATGGAAGTTGTTCAGCAAACACGAGTGGACAAGCTGACCCAAGGCCGACAAACATATGATTGGACATTGAATAGAAACCAGCCAGCTGCAACGGCTTTAGCCAACACCATAGAGGTATTCAGATCGAACGGTCTAAC<br>AGCTAATGAATCGGGGAGACTCATTGATTTCCTTAAGGATGTGATGGATTCAATGGATAAAGAAGGAATGGAAATAACAACACATTTCCAAAGAAAAAGGAGAGTAAGGGACAACATGACCAAGAAAATGGTCACACAAAGAACAATAGGGAAAAAGAAACAGAGATTGAGCAAAAAAAGCTACCTAATAAGGGCATTGACATTGAATACAATGACCAAAGATGCAGAAA<br>GAGGTAAATTGAAAAGGAGAGCGATTGCAACACCCGGGATGCAGATCAGAGGTTTCGTCTACTTCGTTGAAACACTTGCAAGGAGCATATGTGAGAAACTTGAACAGTCGGGGCTCCCAGTTGGAGGAAATGAGAAAAAGGCTAAATTGGCAAACGTTGTAAGAAAGATGATGACTAATTCGCAAGACACAGAGCTCTCCTTCACAATTACTGGGGACAACACCAAGTGGA<br>ATGAAAATCAAAACCCTCGGATGTTCCTAGCGATGATAACATACATCACAAGAAACCAGCCTGAATGGTTCAGAAATGTCTTGAGCATTGCCCCTATAATGTTCTCAAACAAAATGGCAAGACTTGGGAAAGGATATATGTTCGAAAGTAAGAGCATGAAGCTACGAACACAAATACCAGCAGAAATGCTTGCAAGTATTGACCTCAAATATTTCAACGAGTCAACGAGAAAG<br>AAAATCGAGAACATAAGACCCCTCTTAATAGATGGCACAGCCTCATTGAGTCCTGGAATGATGATGGGCATGTTCAACATGTTGAGTACAGTCTTAGGAGTCTCAATCTTAAATCTTGGGCAAAAGAGGTACACCAAGACCACATACTGGTGGGATGGACTCCAATCCTCTGATGACTTTGCACTCATAGTGAATGCACCTAATCATGAGGGAATACAAGCAGGAGTGGACAG<br>ATTTTACAGAACCTGCAAATTAGTTGGAATCAACATGAGTAAAAAGAAGTCCTACATCAATAGAACAGGAACATTTGAATTCACAAGCTTTTTCTATCGCTATGGGTTTGTAGCTAACTTCAGTATGGAGCTACCAAGCTTTGGCGTATCAGGGATTAATGAGTCAGCTGACATGAGCATTGGAGTAACAGTGATAAAGAACAACATGATAAACAACGACCTTGGACCAGCAAC<br>AGCCCAAATGGCTCTTCAACTGTTCATCAAGGACTACAGGTACACATATCGATGCCATAGAGGTGATACTCAAATCCAAACAAGGAGATCATTTGAGTTGAAGAAGCTTTGGGAGCAAACCCGTTCAAAGGCGGGGCTGCTGGTTTCAGATGGAGGACCAAACCTATATAATATCAGAAATCTCCATATTCCAGAGGTCTGTCTGAAATGGGAGCTAATGGATGAAGATTATC<br>AGGGCAGGCTGTGCAACCCTCTGAATCCATTCGTAAGCCATAAGGAAATTGAGTCCGTAAATAATGCTGTGGTAATGCCAGCCCATGGTCCAGCCAAAAGTATGGAATATGATGCTGTTGCAACTACACATTCCTGGGTCCCCAAGAGGAACCGTTCCATCCTCAACACTAGCCAAAGGGGAATCCTTGAGGATGAACAGATGTATCAAAAATGCTGCAACCTATTCGAAAAAT<br>TCTTCCCTAGCAGTTCGTACCGGAGACCAGTTGGAATTTCCAGCATGGTGGAGGCCATGGTATCCAGGGCCCGAATTGACGCACGGATTGACTTCGAATCTGGAAGGATCAAGAAAGAAGAGTTTGCTGAGATCATGAAGATCTGTTCCACCATTGAAGAGCTCAGACGGCAAAGTAG
EPI_ISL_267201	>A/black-headed_gull/Netherlands/1/2015_H16N3_2015-01-27<br>ATGGATGTCAATCCGACTTTACTTTTCTTGAAAGTGCCAGCGCAAAATGCTATAAGTACTACATTCCCTTATACTGGTGACCCTCCATACAGCCATGGAACAGGAACAGGATATACCATGGACACAGTCAACAGAACACACCAGTACTCAGAAAAAGGAACATGGACAACAAACACCGAAACTGGAGCACCTCAACTTAACCCGATCGATGGACCACTACCGGAGGATAATGAG<br>CCAAGCGGATATGCACAAACGGATTGTGTATTGGAGGCAATGGCTTTCCTTGAAGAATCCCATCCAGGGATCTTTGAGAACTCATGTCTTGAAACGATGGAAGTTGTTCAGCAAACACGAGTGGACAAGCTGACCCAAGGCCGACAAACATATGATTGGACATTGAATAGAAACCAGCCAGCTGCAACGGCTTTAGCCAACACCATAGAGGTCTTCAGATCGAACGGTCTAAC<br>AGCTAATGAATCGGGGAGACTCATTGATTTCCTTAAGGATGTGATGGATTCAATGGATAAAGAAGGAATGGAAATAACAACACATTTCCAAAGAAAAAGAAGAGTAAGGGACAACATGACCAAGAAAATGGTCACGCAAAGAACAATAGGGAAAAAGAAACAGAGATTGAGCAAAAAAAGCTACCTAATAAGGGCATTGACATTGAATACAATGACCAAAGATGCAGAAA<br>GAGGTAAATTGAAAAGGAGAGCGATTGCAACACCCGGGATGCAGATCAGAGGTTTCGTCTACTTCGTTGAAACGCTTGCAAGGAGCATATGTGAGAAACTTGAACAGTCAGGGCTCCCAGTTGGAGGAAATGAGAAAAAGGCTAAATTGGCAAACGTTGTAAGAAAGATGATGACTAATTCGCAAGACACAGAGCTCTCCTTCACAATTACTGGGGACAACACCAAGTGGA<br>ATGAAAATCAAAACCCTCGGATGTTCCTAGCGATGATAACATACATCACAAGAAACCAGCCTGAATGGTTCAGAAATGTCTTGAGCATTGCCCCTATAATGTTCTCAAACAAAATGGCAAGACTTGGGAAAGGATATATGTTCGAAAGTAAGAGCATGAAGCTACGAACACAAATACCAGCAGAAATGCTTGCAAGTATTGACCTCAAATATTTCAATGAGTCAACGAGAAAG<br>AAAATCGAGAACATAAGACCCCTCTTAATAGATGGCACAGCCTCATTGAGTCCTGGAATGATGATGGGCATGTTCAACATGTTGAGTACAGTCTTAGGGGTCTCAATCTTAAATCTTGGGCAAAAGAGGTACACCAAGACCACATACTGGTGGGATGGACTCCAATCCTCTGATGACTTTGCACTCATAGTGAACGCACCTAACCATGAGGGGATACAAGCAGGAGTGGACAG<br>ATTTTACAGAACCTGCAAATTAGTTGGAATCAACATGAGTAAAAAGAAGTCCTACATCAATAGAACAGGAACATTTGAATTCACAAGCTTTTTCTATCGCTATGGGTTTGTAGCTAACTTCAGTATGGAGCTACCAAGCTTTGGCGTATCAGGGATTAATGAGTCAGCTGACATGAGCATTGGAGTAACAGTGATAAAGAACAACATGATAAACAATGACCTTGGACCAGCAAC<br>AGCCCAAATGGCTCTTCAACTGTTCATCAAGGACTACAGGTACACATATCGATGCCATAGAGGTGATACTCAAATCCAAACAAGGAGATCATTTGAGTTGAAGAAGCTTTGGGAGCAAACCCGTTCAAAGGCGGGGCTGCTGGTTTCAGATGGAGGACCAAACCTATATAATATCAGAAATCTCCATATTCCAGAGGTCTGTCTGAAATGGGAGCTAATGGATGAAGATTATC<br>AGGGCAGGCTGTGCAACCCTCTGAATCCATTCGTAAGCCACAAGGAAATTGAGTCCGTAAATAATGCTGTGGTAATGTCAGCCCATGGTCCAGCCAAAAGTATGGAATATGATGCTGTTGCAACTACACATTCCTGGGTCCCCAAGAGGAACCGTTCCATTCTCAACACTAGCCAAAGGGGAATCCTTGAGGATGAACAGATGTATCAAAAATGCTGCAATCTATTCGAAAAAT<br>TCTTCCCTAGCAGTTCGTACAGGAGACCAGTTGGAATTTCCAGCATGGTGGAGGCCATGGTATCCAGGGCCCGAATTGACGCACGGATTGACTTCGAATCTGGAAGGATCAAGAAAGAAGAGTTTGCTGAGATCATGAAGATCTGTTCCACCATTGAAGAGCTCAGACGGCAAAGTAG
EPI_ISL_12177629	>A/black-backed_gull/Netherlands/22006711-001/2022_H5N1_2022-04-05<br>ATGGATGTCAATCCGACTTTACTTTTCTTAAAAGTGCCAGCGCAAGATGCCATAAGTACCACATTCCCTTACACTGGAGATCCTCCATACAGCCATGGAACAGGGACAGGATACACAATGGACACGGTCAACAGAACACATCAATACTCAGAGAAGGGAAAATGGACAACAAACACAGAAACCGGAGCACCTCAACTCAACCCAATTGATGGGCCACTACCTGAGGACAACGA<br>ACCGAGCGGATATGCACAAACAGATTGCGTGTTGGAAGCAATGGCTTTCCTTGAAGAGTCCCATCCAGGGATCTTTGAAAACTCTTGTCTTGAAACGATGGAAGTCGTTCAGCAAACAAGAGTGGACAAACTAACTCAAGGTCGCCAGACATATGACTGGACACTGAATAGAAACCAACCAGCTGCAACTGCCTTGGCCAACACTATAGAGGTCTTAAGATCAAACGGTCTAA<br>CAGCCAATGAATCGGGGAGACTAATAGATTTCCTCAAGGATGTGATGGACTCAATGGATAAAGAAGAAATGGAAATAACAACACATTTCCAGAGAAAGAGAAGAGTAAGGGACAACATGACCAAGAAAATGGTCACACAAAGAACAATAGGAAAGAAGAAACAAAGGCTAAACAAGAGGAGCTACTTAATAAGAGCACTGACACTGAATACAATGACAAAAGATGCAGA<br>AAGAGGCAAATTGAAGAGACGGGCGATTGCAACACCAGGGATGCAGATTAGAGGATTTGTGTACTTTGTCGAAACACTGGCAAGGAGCATCTGTGAAAAACTTGAGCAATCTGGACTCCCCGTTGGAGGGAATGAGAAGAAGGCTAAATTGGCAAATGTTGTGAGAAAAATGATGACTAACTCACAAGATACAGAGCTCTCCTTCACAATTACTGGAGATAACACCAAATG<br>GAATGAGAATCAAAATCCTCGGATGTTTCTGGCAATGATAACATACATTACAAGAAACCAACCTGAATGGTTTAGGAATGTCTTGAGTATTGCCCCTATAATGTTCTCGAACAAAATGGCGAGATTGGGAAAAGGGTACATGTTTGAAAGTAAGAGCATGAAGTTACGGACACAAATACCTGCAGAAATGCTTGCAAACATTGACTTAAAATATTTCAATGAATCAACAAGAAA<br>GAAAATCGAAAAAATAAGGCCTCTACTAATAGATGGCACTGCCTCATTGAGTCCTGGAATGATGATGGGCATGTTCAATATGCTGAGTACAGTATTAGGAGTTTCAATCCTAAATCTTGGGCAAAAGAAGTACACCAAAACCACATACTGGTGGGATGGACTCCAATCCTCTGATGATTTCGCCCTCATAGTAAATGCACCGAATCATGAGGGAATACAAGCAGGAGTGGATA<br>GGTTCTATAGGACCTGCAAACTGGTCGGGATCAATATGAGCAAAAAGAAGTCTTACATAAACCGGACTGGAACATTTGAGTTCACAAGCTTTTTCTATCGCTATGGATTTGTGGCTAATTTCAGTATGGAGCTGCCCAGCTTTGGAGTTTCTGGGATCAATGAATCAGCTGACATGAGCATTGGCATCACAGTGATAAAGAACAACATGATAAACAATGACCTTGGACCAGCAA<br>CAGCTCAAATGGCCCTTCAACTATTCATCAAAGATTACAGGTACACGTACCGATGCCATAGAGGTGACACACAAATTCAAACGAGGAGATCATTCGAGCTGAAGAAGCTGTGGGAACAGACCCGTTCAAAGGCAGGACTGTTGGTGTCAGATGGAGGACCAAATCTATACAACATTCGGAATCTCCATATCCCAGAGGTCTGCCTGAAGTGGGAGCTGATGGACGAAGATTA<br>CCAGGGCAGGTTGTGTAATCCTCTGAACCCATTTGTCAGCCATAAAGAAATTGAGTCCGTAAACAATGCTGTGGTGATGCCAGCCCACGGTCCAGCCAAAAGCATGGAATATGATGCCGTTGCGACTACACACTCATGGATTCCTAAAAGGAATCGTTCCATTCTCAATACCAGTCAAAGGGGAATTCTTGAGGATGAACAGATGTACCAGAAATGCTGCGGTCTATTCGAGAA<br>ATTCTTCCCCAGTAGTTCATACAGGAGACCAGTTGGAATTTCCAGCATGGTGGAGGCCATGGTGTCTAGGGCCCGAATCGATGCACGCATTGATTTCGAATCTGGAAGGATCAAGAAAGAAGAGTTTGCTGAGATCATGAAGATCTGTTCCACCATTGAAGAGCTCAGACGGCAAAAATAG

EPI_ISL_217877	>A/Bean_Goose/Hubei/chenhu_XVI270-1/2016_H11N5_2016-02-26<br>ATGGATGTCAATCCGACTTTACTTTTCTTAAAAGTGCCAGCGCAAAATGCCATAAGTACCACATTCCCTTATACTGGAGATCCTCCATACAGCCATGGAACAGGAACAGGGTACACCATGGACACAGTCAACAGAACACATCAATACTCAGAAAAGGGGAAATGGACAACAAACACAGAGACCGGAGCACCCCAACTCAACCCAATTGATGGACCACTACCTGATGACAACGA<br>ACCAAGCGGATATGCACAAACAGATTGCGTGTTGGAAGCAATGGCTTTCCTTGAAGAGTCCCACCCAGGGATCTTTGAAAACTCTTGTCTTGAAACGATGGAAGTCGTTCAGCAAACAAGAGTGGACAAACTAGCTCAAGGTCGCCAGACTTATGACTGGACACTGAATAGAAACCAACCGGCTGCAACTGCCCTGGCCAACACTATAGAAGTCTTCAGATCGAACGGTCTGA<br>CAGCCAACGAATCGGGGAGACTAATAGATTTCCTCAAGGATGTGATGGACTCAATGGACAAAGAAGAAATGGAAATAACAACACATTTCCAGAGAAAGAGAAGAGTAAGGGACAACATGACCAAGAAAATGGTCACACAAAGAACAATAGGGAAGAAGAAACAAAGGCTAAATAAGAGGAGCTACTTAATAAGAGCACTGACACTGAATACAATGACAAAAGACGCAGA<br>AAGAGGCAAATTGAAGAGACGGGCAATTGCAACACCAGGGATGCAGATTAGAGGATTTGTGTACTTTGTTGAAACACTGGCAAGGAGCATCTGTGAAAAACTTGAGCAATCTGGACTCCCCGTTGGAGGGAATGAGAAGAAGGCTAAATTGGCAAATGTCGTGAGAAAAATGATGACTAACTCACAAGATACAGAGCTCTCCTTCACAATTACTGGAGATAACACCAAATG<br>GAATGAGAATCAAAATCCTAGGATGTTTCTGGCAATGATAACATACATCACAAGAAACCAACCTGAATGGTTTAGAAATGTCTTGAGCATTGCCCCTATAATGTTCTCGAACAAAATGGCGAGATTGGGAAAAGGATACATGTTTGAAAGTAAGAGCATGAAGCTACGGACACAAATACCTGCAGAAATGCTTGCAAACATTGACTTAAAATACTTCAATGAGTCAACAAGAA<br>AGAAAATCGAGAAAATAAGACCTTTACTAATAGATGGCACCGCCTCATTGAGTCCTGGAATGATGATGGGCATGTTCAATATGCTGAGTACAGTATTAGGAGTCTCAATCTTGAATCTTGGGCAAAAGAGGTACACCAAAACCACATACTGGTGGGATGGACTCCAATCCTCTGATGATTTCGCCCTCATAGTGAATGCACCGAATCATGAGGGAATACAAGCAGGAGTGGAT<br>AGGTTCTACAGGACCTGCAAACTGGTTGGGATCAACATGAGCAAAAAGAAGTCTTATATAAACCGGACAGGAACATTTGAGTTCACAAGCTTTTTCTATCGCTATGGATTTGTGGCTAACTTCAGTATGGAGCTGCCCAGCTTTGGAGTGTCTGGAATCAATGAATCAGCTGACATGAGTATTGGAGTTACAGTGATAAAGAACAACATGATAAACAATGACCTTGGACCAGCA<br>ACAGCTCAAATGGCCCTTCAACTATTCATCAAAGACTACAGGTACACATACCGATGTCACAGGGGTGACACACAAATTCAAACGAGGAGATCATTCGAGCTGAAGAAGCTGTGGGAGCAGACCCGTTCAAAGGCAGGACTGTTGGTGTCAGATGGAGGACCAAATCTATACAACATTCGGAATCTCCATATCCCAGAGGCCTGCCTGAAGTGGGAGCTGATGGACGAAGATT<br>ACCAGGGCAGGTTGTGTAATCCTCTGAACCCATTTGTCAGTCATAAAGAAATTGAGTCCGTAAACAATGCTGTGGTAATGCCAGCCCATGGCCCAGCCAAGAGCATGGAATATGATGCTGTCGCAACTACACACTCATGGATTCCTAAAAGGAATCGTTCCATTCTCAATACCAGCCAAAGGGGAATTCTTGAGGATGAGCAGATGTACCAGAAATGCTGCAGTCTATTCGAGA<br>AATTCTTCCCCAGTAGTTCATACAGGAGACCAGTTGGAATTTCCAGCATGGTGGAGGCCATGGTGTCTAGGGCCCGAATCGATGCACGCATTGATTTCGAATCTGGAAGGATTAAGAAGGAAGAGTTTGCTGAGATCATGAAGATCTGTTCCACCATTGAAGAGCTCAGACGGCAAAAATAG
EPI_ISL_3102208	>A/barnacle_goose/Sweden/SVA210210SZ0372/FB000374/M-2021_H5N8_2021-02-01<br>ATGGATGTCAATCCGACTTTACTTTTCTTAAAAGTGCCAGCGCAAAATGCTATAAGTACTACATTCCCTTACACTGGAGATCCCCCATACAGCCATGGAACAGGAACAGGGTATACCATGGACACAGTAAACAGAACACATCAATACTCAGAAAAGGGAAAGTGGACAACAAACACAGAAACCGGAGCACCCCAACTCAACCCAATTGATGGACCATTACCAGAGGACAATGA<br>GCCAAGCGGATATGCACAAACTGATTGCGTGTTGGAAGCAATGGCTTTCCTTGAAGAATCCCACCCAGGGATATTTGAAAACTCTTGTCTTGAAGCGATGGAAATCGTTCAGCAAACAAGAGTGGACAAACTAACCCAAGGTCGCCAGACTTATGACTGGACACTGAACAGAAACCAACCAGCTGCAACCTCTTTGGCCAACACTATAGAGGTGTTCAGATCGAATGGTCTGA<br>CAGCCAATGAATCAGGGAGACTGATAGATTTTCTCAGGGATGTGATGGAATCAATGGATAAAGAAGAGATGGAAGTAACAACACATTTCCAGAGAAAAAGAAGAGTGAGGGACAACATGACTAAGAAGATGGTCACACAAAGAACAATAGGGAAGAAGAAGCAGAGGCTGAACAAGAGGAGTTACTTAATAAGAGCACTGACATTGAATACAATGACCAAAGATGCAGA<br>AAGAGGCAAGTTGAAGAGACGGGCAATTGCAACACCCGGGATGCAGATTAGAGGATTCGTGTACTTCGTTGAAACACTAGCGAGGAGCATCTGTGAGAAACTAGAGCAATCTGGGCTCCCTGTTGGAGGGAATGAGAAGAAGGCTAAATTGGCAAATGTTGTGAGAAAAATGATGACTAACTCACAAGATACAGAGCTCTCCTTTACAATTACTGGAGACAACACCAAATG<br>GAATGAGAATCAAAACCCTCGGATGTTTTTGGCAATGATAACATATATCACAAGAAACCAACCTGAATGGTTTAGAAATGTCTTAAGCATTGCCCCTATAATGTTCTCAAACAAAATGGCGAGATTAGGGAAAGGATACATGTTTGAAAGTAAGAGCATGAAGCTAAGAACACAAATACCAGCAGAGATGCTTACAAATATTGATCTGAAGTATTTCAACGAACCAACGAGAA<br>AGAAAATCGAGAAAATAAGACCTCTGCTGATTGATGGCACGGCCTCATTGAGTCCTGGGATGATGATGGGCATGTTCAATATGCTGAGCACAGTATTAGGGGTCTCAATCCTGAATCTCGGGCAAAAGAGGTACACCAAAACCACATACTGGTGGGATGGACTTCAATCCTCTGATGATTTCGCTCTCATAGTGAATGCACCGAATCATGAGGGGATACAAGCAGGAGTGGAT<br>AGATTCTATAGGACCTGCAAACTGGTTGGGATCAACATGAGCAAAAAGAAGTCTTACATAAACCGAACAGGAACATTTGAGTTCACAAGTTTTTTCTACCGCTATGGATTTGTAGCCAACTTCAGTATGGAATTACCCAGCTTTGGAGTGTCTGGAATCAATGAATCAGCTGACATGAGCATTGGAGTTACAGTGATAAAAAACAATATGATAAACAATGATCTTGGACCAGCA<br>ACAGCTCAAATGGCTCTTCAGTTATTCATCAAAGACTATAGGTACACATACCGATGCCACAGGGGTGATACACAAATTCAAACGAGGAGATCATTCGAGCTGAAGAAGCTGTGGGAGCAGACCCGTTCAAAGGCAGGGCTGTTGATATCAGACGGGGGGCCAAATCTATACAACATTCGGAATCTCCACATCCCAGAGGTCTGCTTGAAGTGGGAGCTGATGGATGAAGACT<br>ACCAAGGCAGGCTGTGCAATCCTCTGAATCCATTTGTCAGTCATAAAGAGATTGAGTCCGTAAACAATGCTGTAGTAATGCCCGCCCATGGCCCGGCCAAGAGCATGGAATATGATGCTGTTGCGACCACACACTCGTGGATTCCTAAGAGGAACCGTTCCATTCTCAATACCAGCCAAAGGGGAATTCTTGAGGACGAGCAGATGTACCAAAAGTGCTGTAGTCTATTCGAGA<br>AATTCTTCCCCAGCAGTTCATACAGGAGGCCAGTTGGAATTTCCAGCATGGTGGAGGCCATGGTGTCTAGGGCCCGAATTGATGCACGCATCGACTTCGAATCTGGAAGGATTAAGAAAGAAGAGTTTGCTGAGATCATGAAGATCTGTTCCACCATTGAAGAGCTCAGACGGCAAAAATAG
EPI_ISL_9261747	>A/Barnacle_goose/Netherlands/5/2022_H5N1_2022-01-14<br>ATGGATGTCAATCCGACTTTACTTTTCTTAAAAGTGCCAGCGCAAGATGCCATAAGTACCACATTCCCTTACACTGGAGATCCTCCATACAGCCATGGAACAGGGACAGGATACACAATGGACACGGTCAACAGAACACATCAATACTCAGAGAAGGGAAAATGGACAACAAACACAGAAACCGGAGCACCTCAACTCAACCCAATTGATGGGCCACTACCTGAGGACAACGA<br>ACCGAGCGGATATGCACAAACAGATTGCGTGTTGGAAGCAATGGCTTTCCTTGAAGAGTCCCATCCAGGGATCTTTGAAAACTCTTGTCTTGAAACGATGGAAGTCGTTCAGCAAACAAGAGTGGACAAACTAACTCAAGGTCGCCAGACATATGACTGGACACTGAATAGAAACCAACCAGCTGCAACTGCCTTGGCCAACACTATAGAGGTCTTAAGATCAAACGGTCTAA<br>CAGCCAATGAATCGGGGAGACTAATAGATTTCCTCAAGGATGTGATGGACTCAATGGATAAAGAAGAAATGGAAATAACAACACATTTCCAGAGAAAGAGAAGAGTAAGGGACAACATGACCAAGAAAATGGTCACACAAAGAACAATAGGAAAGAAGAAACAAAGGCTAAACAAGAGGAGCTACTTAATAAGAGCACTGACACTGAATACAATGACAAAAGATGCAGA<br>AAGAGGCAAATTGAAGAGACGGGCGATTGCAACACCAGGGATGCAGATTAGAGGATTTGTGTACTTTGTCGAAACACTGGCAAGGAGCATCTGTGAAAAACTTGAGCAATCTGGACTCCCCGTTGGAGGGAATGAGAAGAAGGCTAAATTGGCAAATGTTGTGAGAAAAATGATGACTAACTCACAAGATACAGAGCTCTCCTTCACAATTACTGGAGATAACACCAAATG<br>GAATGAGAATCAAAATCCTCGGATGTTTCTGGCAATGATAACATACATTACAAGAAACCAACCTGAATGGTTTAGGAATGTCTTGAGTATTGCCCCTATAATGTTCTCGAACAAAATGGCGAGATTGGGAAAAGGGTACATGTTTGAAAGTAAGAGCATGAAGTTACGGACACAAATACCTGCAGAAATGCTTGCAAACATTGACTTAAAATATTTCAATGAATCAACAAGAAA<br>GAAAATCGAAAAAATAAGGCCTCTACTAATAGATGGCACTGCCTCATTGAGTCCTGGAATGATGATGGGCATGTTCAATATGCTGAGTACAGTATTAGGAGTTTCAATCCTAAATCTTGGGCAAAAGAAGTACACCAAAACCACATACTGGTGGGATGGACTCCAATCCTCTGATGATTTCGCCCTCATAGTAAATGCACCGAATCATGAGGGAATACAAGCAGGAGTGGATA<br>GGTTCTATAGGACCTGCAAACTGGTCGGGATCAATATGAGCAAAAAGAAGTCTTACATAAACCGGACTGGAACATTTGAGTTCACAAGCTTTTTCTATCGCTATGGATTTGTGGCTAATTTCAGTATGGAGCTGCCCAGCTTTGGAGTTTCTGGGATCAATGAATCAGCTGACATGAGCATTGGCATCACAGTGATAAAGAACAACATGATAAACAATGACCTTGGACCAGCAA<br>CAGCTCAAATGGCCCTTCAACTATTCATCAAAGATTACAGGTACACGTACCGATGCCATAGAGGTGACACACAAATTCAAACGAGGAGATCATTCGAGCTGAAGAAGCTGTGGGAACAGACCCGTTCAAAGGCAGGACTGTTGGTGTCAGATGGAGGACCAAATCTATACAACATTCGGAATCTCCATATCCCAGAGGTCTGCCTGAAGTGGGAGCTGATGGACGAAGATTA<br>CCAGGGCAGGTTGTGTAATCCTCTGAACCCATTTGTCAGCCATAAAGAAATTGAGTCCGTAAACAATGCTGTGGTGATGCCAGCCCACGGTCCAGCCAAAAGCATGGAATATGATGCCGTTACGACTACACACTCATGGATTCCTAAAAGGAATCGTTCCATTCTCAATACCAGTCAAAGGGGAATTCTTGAGGATGAACAGATGTACCAGAAATGCTGCGGTCTATTCGAGAA<br>ATTCTTCCCCAGTAGTTCATACAGGAGACCAGTTGGAATTTCCAGCATGGTGGAGGCCATGGTGTCTAGGGCCCGAATCGATGCACGCATTGATTTCGAATCTGGAAGGATCAAGAAGGAAGAGTTTGCTGAGATCATGAAGATCTGTTCCACCATTGAAGAGCTCAGACGGCAAAAATAG
EPI_ISL_2172531	>A/barnacle_goose/Netherlands/21024358-001/2021_H5N8_2021-02-21<br>ATGGATGTCAATCCGACTTTACTTTTCTTAAAAGTGCCAGCGCAAAATGCTATAAGTACTACATTCCCTTACACTGGAGATCCCCCATACAGCCATGGAACAGGAACAGGGTATACCATGGATACAGTAAACAGAACACATCAATACTCAGAAAAGGGAAAGTGGACAACAAACACAGAAACCGGAGCACCCCAACTCAACCCAATTGATGGACCATTACCAGAGGACAATGA<br>GCCAAGCGGATATGCACAAACTGATTGCGTGTTGGAAGCAATGGCTTTCCTTGAAGAATCCCACCCAGGGATATTTGAAAACTCTTGTCTTGAAGCGATGGAAATCGTTCAGCAAACAAGAGTGGACAAACTAACCCAAGGTCGCCAGACTTATGACTGGACACTGAACAGAAACCAACCAGCTGCAACCTCTTTGGCCAACACTATAGAGGTGTTCAGATCGAATGGTCTGA<br>CAGCCAATGAATCAGGGAGACTGATAGATTTTCTCAGGGATGTGATGGAATCAATGGATAAAGAAGAGATGGAAGTAACAACACATTTCCAGAGAAAAAGAAGAGTGAGGGACAACATGACTAAGAAGATGGTCACACAAAGAACAATAGGGAAGAAGAAGCAGAGGCTGAACAAGAGGAGTTACTTAATAAGAGCACTGACATTGAATACAATGACCAAAGATGCAGA<br>AAGAGGCAAGTTGAAGAGACGGGCAATTGCAACACCCGGGATGCAGATTAGAGGATTCGTGTACTTCGTTGAAACACTAGCGAGGAGCATCTGTGAGAAACTAGAGCAATCTGGGCTCCCTGTTGGAGGGAATGAGAAAAAGGCTAAATTGGCAAATGTTGTGAGAAAAATGATGACTAACTCACAAGATACAGAGCTCTCCTTTACAATTACTGGAGACAACACCAAATG<br>GAATGAGAATCAAAACCCTCGGATGTTTTTGGCAATGATAACATATATCACAAGAAACCAACCTGAATGGTTTAGAAATGTCTTAAGCATTGCCCCTATAATGTTCTCAAACAAAATGGCGAGATTAGGGAAAGGATACATGTTTGAAAGTAAGAGCATGAAGCTAAGAACACAAATACCAGCAGAGATGCTTACAAATATTGATCTGAAGTATTTCAACGAACCAACGAGAA<br>AGAAAATCGAGAAAATAAGACCTCTGCTGATTGATGGCACGGCCTCATTGAGTCCTGGGATGATGATGGGCATGTTCAATATGCTGAGCACAGTATTAGGGGTCTCAATCCTGAATCTCGGGCAAAAGAGGTACACCAAAACCACATACTGGTGGGATGGACTTCAATCCTCTGATGATTTCGCTCTCATAGTGAATGCACCGAATCATGAGGGGATACAAGCAGGAGTGGAT<br>AGATTCTATAGGACCTGCAAACTGGTTGGGATCAACATGAGCAAAAAGAAGTCTTACATAAACCGAACAGGAACATTTGAGTTCACAAGTTTTTTCTACCGCTATGGATTTGTAGCCAACTTCAGTATGGAATTACCCAGCTTTGGAGTGTCTGGAATCAATGAATCAGCTGACATGAGCATTGGAGTTACAGTGATAAAAAACAATATGATAAACAATGATCTTGGACCAGCA<br>ACAGCTCAAATGGCCCTCCAGTTATTCATCAAGGACTATAGGTACACATACCGATGCCACAGGGGTGATACACAAATTCAAACGAGGAGATCATTCGAGCTGAAGAAGCTGTGGGAGCAGACCCGTTCAAAGGCAGGGCTGTTGATATCAGACGGGGGGCCAAACCTATACAACATTCGGAATCTCCACATCCCAGAGGTCTGCTTGAAGTGGGAGCTGATGGATGAAGACT<br>ACCAAGGCAGGCTGTGCAATCCTCTGAATCCATTTGTCAGTCATAAAGAGATTGAGTCCGTAAACAATGCTGTAGTAATGCCCGCCCATGGCCCGGCCAAGAGCATGGAATATGATGCTGTTGCGACCACACACTCGTGGATTCCTAAGAGGAACCGTTCCATTCTCAATACCAGCCAAAGGGGAATTCTTGAGGATGAGCAGATGTACCAAAAGTGCTGTAGTCTATTCGAGA<br>AATTCTTCCCCAGCAGTTCATACAGGAGGCCAGTTGGAATTTCCAGCATGGTGGAGGCCATGGTGTCTAGGGCCAGAATTGATGCACGCATCGACTTCGAATCTGGAAGGATTAAGAAAGAAGAGTTTGCTGAGATCATGAAGATCTGTTCCACCATTGAAGAGCTCAGACGGCAAAAATAG
EPI_ISL_11112346	>A/Barnacle_Goose/Netherlands/13/2022_H5N1_2022-02-23<br>ATGGATGTCAATCCGACTTTACTTTTCTTAAAAGTGCCAGCGCAAGATGCCATAAGTACCACATTCCCTTACACTGGAGATCCTCCATACAGCCATGGAACAGGGACAGGATACACAATGGACACAGTCAACAGAACACATCAATACTCAGAGAAGGGAAAATGGACAACAAACACAGAAACCGGAGCACCTCAACTCAACCCAATTGATGGGCCACTACCTGAGGACAACGA<br>ACCGAGCGGATATGCACAAACAGATTGCGTGTTGGAAGCAATGGCTTTCCTTGAAGAGTCCCATCCAGGGATCTTTGAAAACTCTTGTCTTGAAACGATGGAAGTCGTTCAGCAAACAAGAGTGGACAAACTAACTCAAGGTCGCCAGACATATGACTGGACACTGAATAGAAACCAACCGGCTGCAACTGCCCTGGCCAACACTATAGAGGTCTTCAGATCAAACGGTCTAA<br>CAGCCAATGAATCGGGGAGACTAATAGATTTCCTCAAGGATGTGATGGACTCAATGGATAAAGAAGAAATGGAAATAACAACACATTTCCAGAGAAAGAGAAGAGTAAGGGACAACATGACCAAGAAAATGGTCACACAAAGAACAATAGGAAAGAAGAAACAAAGGCTAAACAAGAGGAGCTACTTAATAAGAGCACTGACACTGAATACAATGACAAAAGATGCAGA<br>AAGAGGCAAATTGAAGAGACGGGCGATTGCAACACCAGGGATGCAGATTAGAGGATTTGTGTACTTTGTCGAAACACTGGCAAGGAGCATCTGTGAAAAACTTGAGCAATCTGGACTCCCCGTTGGAGGGAATGAGAAGAAGGCTAAATTGGCAAATGTCGTGAGAAAAATGATGACTAACTCACAAGATACAGAGCTCTCCTTCACAATTACTGGAGATAACACCAAATG<br>GAATGAGAATCAAAATCCTCGGATGTTTCTGGCAATGATAACATACATTACAAGAAACCAACCTGAATGGTTTAGGAATGTCTTGAGTATTGCCCCTATAATGTTCTCGAACAAAATGGCGAGATTGGGAAAAGGGTACATGTTTGAAAGTAAGAGCATGAAGTTACGGACACAAATACCTGCAGAAATGCTTGCAAACATTGACTTAAAATACTTCAATGAATCAACAAGAAA<br>GAAAATCGAAAAAATAAGGCCTCTACTAATAGATGGCACTGCCTCATTGAGTCCTGGAATGATGATGGGCATGTTCAATATGCTGAGTACAGTATTAGGAGTTTCAATCCTAAATCTTGGGCAAAAGAAGTACACCAAAACCACATACTGGTGGGATGGACTCCAATCCTCTGATGATTTCGCCCTCATAGTAAATGCACCGAATCATGAGGGAATACAAGCAGGAGTGGATA<br>GGTTCTATAGGACCTGCAAACTGGTCGGGATCAATATGAGCAAAAAGAAGTCTTACATAAACCGGACTGGAACATTTGAGTTCACAAGCTTTTTCTATCGCTATGGATTTGTGGCTAACTTCAGTATGGAGCTGCCCAGCTTTGGAGTTTCTGGGATCAATGAATCAGCTGACATGAGCATTGGCATCACAGTGATAAAGAACAACATGATAAACAATGACCTTGGACCAGCAA<br>CAGCTCAAATGGCCCTTCAACTATTCATCAAAGATTACAGGTACACGTACCGATGCCATAGAGGTGACACACAAATTCAAACGAGGAGATCATTCGAGCTGAAGAAGCTGTGGGAACAGACCCGTTCAAAGGCAGGACTGTTGGTGTCAGATGGAGGACCAAATCTATACAACATTCGGAATCTCCATATCCCAGAGGTCTGCCTGAAGTGGGAGCTGATGGACGAAGATTA<br>CCAGGGCAGGTTATGTAATCCTCTGAACCCATTTGTCAGCCATAAAGAAATTGAGTCCGTAAACAATGCTGTGGTGATGCCAGCCCACGGTCCAGCCAAAAGCATGGAATATGATGCCGTTGCGACTACACACTCATGGATTCCTAAAAGGAATCGTTCCATTCTCAATACCAGCCAAAGGGGAATTCTTGAGGATGAACAGATGTACCAGAAATGCTGCGGTCTATTCGAGAA<br>ATTCTTCCCCAGTAGTTCATACAGGAGACCAGTTGGAATTTCCAGCATGGTGGAGGCCATGGTGTCTAGGGCCCGAATCGATGCACGCATTGATTTCGAATCTGGAAGGATCAAGAAGGAAGAGTTTGCTGAGATCATGAAGATCTGTTCCACCATTGAAGAGCTCAGACGGCAAAAATAG
EPI_ISL_13026246	>A/Barnacle_goose/Luxembourg/22033922/2022_H5N1_2022-02-04<br>ATGGATGTCAATCCGACTTTACTTTTCTTAAAAGTGCCAGCGCAAGATGCCATAAGTACCACATTCCCTTACACTGGAGATCCTCCATACAGCCATGGAACAGGGACAGGATACACAATGGACACGGTCAACAGAACACATCAATACTCAGAGAAGGGAAAATGGACAACAAACACAGAAACCGGAGCACCTCAACTCAACCCAATTGATGGGCCACTACCTGAGGACAACGA<br>ACCGAGCGGATATGCACAAACAGATTGCGTGTTGGAAGCAATGGCTTTCCTTGAAGAGTCCCATCCAGGGATCTTTGAAAACTCTTGTCTTGAAACGATGGAAGTCGTTCAGCAAACAAGAGTGGACAAACTAACTCAAGGTCGCCAGACATATGACTGGACACTGAATAGAAACCAACCAGCTGCAACTGCCTTGGCCAACACTATAGAGGTCTTAAGATCAAACGGTCTAA<br>CAGCCAATGAATCGGGGAGACTAATAGATTTCCTCAAGGATGTGATGGACTCAATGGATAAAGAAGAAATGGAAATAACAACACATTTCCAGAGAAAGAGAAGAGTAAGGGACAACATGACCAAGAAAATGGTCACACAAAGAACAATAGGAAAGAAGAAACAAAGGCTAAACAAGAGGAGCTACTTAATAAGAGCACTGACACTGAATACAATGACAAAAGATGCAGA<br>AAGAGGCAAATTGAAGAGACGGGCGATTGCAACACCAGGGATGCAGATTAGAGGATTTGTGTACTTTGTCGAAACACTGGCAAGGAGCATCTGTGAAAAACTTGAGCAATCTGGACTCCCCGTTGGAGGGAATGAGAAGAAGGCTAAATTGGCAAATGTTGTGAGAAAAATGATGACTAACTCACAAGATACAGAGCTCTCCTTCACAATTACTGGAGATAACACCAAATG<br>GAATGAGAATCAAAATCCTCGGATGTTTCTGGCAATGATAACATACATTACAAGAAACCAACCTGAATGGTTTAGGAATGTCTTGAGTATTGCCCCTATAATGTTCTCGAACAAAATGGCGAGATTGGGAAAAGGGTACATGTTTGAAAGTAAGAGCATGAAGTTACGGACACAAATACCTGCAGAAATGCTTGCAAACATTGACTTAAAATATTTCAATGAATCAACAAGAAA<br>GAAAATCGAAAAAATAAGGCCTCTACTAATAGATGGCACTGCCTCATTGAGTCCTGGAATGATGATGGGCATGTTCAATATGCTGAGTACAGTATTAGGAGTTTCAATCCTAAATCTTGGGCAAAAGAAGTACACCAAAACCACATACTGGTGGGATGGACTCCAATCCTCTGATGATTTCGCCCTCATAGTAAATGCACCGAATCATGAGGGAATACAAGCAGGAGTGGATA<br>GGTTCTATAGGACCTGCAAACTGGTCGGGATCAATATGAGCAAAAAGAAGTCTTACATAAACCGGACTGGAACATTTGAGTTCACAAGCTTTTTCTATCGCTATGGATTTGTGGCTAATTTCAGTATGGAGCTGCCCAGCTTTGGAGTTTCTGGGATCAATGAATCAGCTGACATGAGCATTGGCATCACAGTGATAAAGAACAACATGATAAACAATGACCTTGGACCAGCAA<br>CAGCTCAAATGGCCCTTCAACTATTCATCAAAGATTACAGGTACACGTACCGATGCCATAGAGGTGACACACAAATTCAAACGAGGAGATCATTCGAGCTGAAGAAGCTGTGGGAACAGACCCGTTCAAAGGCAGGACTGTTGGTGTCAGATGGAGGACCAAATCTATACAACATTCGGAATCTCCATATCCCAGAGGTCTGCCTGAAGTGGGAGCTGATGGACGAAGATTA<br>CCAGGGCAGGTTGTGTAATCCTCTGAACCCATTTGTCAGCCATAAAGAAATTGAGTCCGTAAACAATGCTGTGGTGATGCCAGCCCACGGTCCAGCCAAAAGCATGGAATATGATGCCGTTGCGACTACACACTCATGGATTCCTAAAAGGAATCGTTCCATTCTCAATACCAGTCAAAGGGGAATTCTTGAGGATGAACAGATGTACCAGAAATGCTGCGGTCTATTCGAGAA<br>ATTCTTCCCCAGTAGTTCATACAGGAGACCAGTTGGAATTTCCAGCATGGTGGAGGCCATGGTGTCTAGGGCCCGAATCGATGCACGCATTGATTTCGAATCTGGAAGGATCAAGAAGGAAGAGTTTGCTGAGATCATGAAGATCTGTTCCACCATTGAAGAGCTCAGACGGCAAAAATAG
EPI_ISL_5099456	>A/barnacle_goose/Germany-NI/AI01605/2021_H5N1_2021-02-17<br>ATGGATGTCAATCCGACTTTACTTTTCTTAAAAGTGCCAGCGCAAAATGCCATAAGTACCACATTCCCTTATACTGGAGATCCTCCATACAGCCATGGAACAGGGACAGGATACACAATGGACACAGTCAACAGAACACATCAATACTCAGAGAAGGGAAAATGGACAACAAACACAGAAACCGGAGCACCTCAACTCAACCCAATTGATGGACCACTACCTGAGGACAACGA<br>ACCGAGCGGATATGCACAAACAGATTGCGTGTTGGAAGCAATGGCTTTCCTTGAAGAGTCCCACCCAGGGATCTTTGGAAACTCTTGTCTTGAAACGATGGAAGTCGTTCAGCAAACAAGAGTGGACAAACTAACTCAAGGTCGCCAGACTTATGACTGGACACTGAATAGAAACCAACCAGCTGCAACTGCCCTGGCCAACACTATAGAGGTCTTCAGATCAAACGGTCTAA<br>CAGCCAATGAATCGGGGAGACTAATAGATTTCCTCAAGGATGTGATGGACTCAATGGATAAAGAAGAAATGGAAATAACAACACATTTCCAGAGAAAGAGAAGAGTAAGGGACAACATGACCCAGAAAATGGTCACACAAAGAACAATAGGAAAGAAGAAACAAAGGCTAAACAAGAGGAGCTACTTAATAAGAGCACTGACACTGAATACAATGACAAAAGATGCAGA<br>AAGAGGCAAATTGAAGAGACGGGCGATTGCAACACCAGGGATGCAGATTAGAGGATTTGTGTACTTTGTCGAAACACTGGCAAGGAGCATCTGTGAAAAACTTGAGCAATCTGGACTCCCCGTTGGAGGGAATGAGAAGAAGGCTAAATTGGCAAATGTCGTGAGGAAAATGATGACTAACTCACAAGATACAGAGCTCTCCTTCACAATTACTGGAGATAACACCAAATG<br>GAATGAGAATCAAAATCCTCGGATGTTTCTGGCAATGATAACGTACATTACAAGAAACCAACCTGAATGGTTTAGAAATGTCTTGAGTATTGCCCCTATAATGTTCTCGAACAAAATGGCGAGATTGGGAAAAGGGTACATGTTTGAAAGTAAGAGCATGAAGTTACGGACACAAATACCTGCAGAAATACTTGCAAACATTGACTTAAAATACTTCAATGAATCAACAAGAAA<br>GAAAATCGAAAAAATAAGGCCTCTACTAATAGATGGCACTGCCTCATTGAGTCCTGGAATGATGATGGGCATGTTCAATATGCTGAGTACAGTATTAGGAGTTTCAATCCTAAATCTTGGGCAAAAGAGGTACACCAAAACCACATACTGGTGGGATGGACTCCAATCCTCTGATGATTTCGCCCTCATAGTAAATGCACCGAATCATGAGGGAATACAAGCAGGAGTGGATA<br>GGTTCTATAGGACCTGCAAACTGGTCGGGATCAATATGAGCAAAAAGAAGTCTTACATAAACCGGACTGGAACATTTGAGTTCACAAGCTTTTTCTATCGCTATGGATTTGTGGCTAACTTCAGTATGGAGCTGCCCAGCTTTGGAGTTTCTGGGATCAATGAATCAGCTGACATGAGCATTGGCGTCACAGTGATAAAGAACAACATGATAAACAATGACCTTGGACCAGCAA<br>CAGCTCAAATGGCCCTTCAACTATTCATCAAAGATTACAGGTACACGTACCGATGCCACAGAGGTGACACACAAATTCAAACGAGGAGATCATTCGAGCTGAAGAAGCTGTGGGAACAGACCCGTTCAAAGGCAGGACTGTTGGTGTCAGATGGAGGACCAAATCTATACAACATTCGGAATCTCCATATCCCAGAGGTCTGCCTGAAGTGGGAGCTGATGGACGAAGATTA<br>CCAGGGCAGGTTGTGTAATCCTCTGAACCCATTTGTCAGTCATAAAGAAATTGAGTCCGTAAACAATGCTGTGGTGATGCCAGCCCACGGTCCAGCCAAAAGCATGGAATATGATGCCGTTGCGACTACACACTCATGGATTCCTAAAAGGAATCGTTCCATTCTCAATACCAGTCAAAGGGGAATTCTTGAGGATGAACAGATGTACCAGAAATGCTGCAGTCTATTCGAGAA<br>ATTCTTCCCCAGTAGTTCATACAGGAGACCAGTTGGAATTTCCAGCATGGTGGAGGCCATGGTGTCTAGGGCCCGAATCGATGCACGCATTGATTTCGAATCTGGAAGGATCAAGAAGGGAGAGTTTGCTGAGATCATGAAGATCTGTTCCACCATTGAAGAGCTCAGACGGCAAAAATAG
EPI_ISL_11725991	>A/barnacle_goose/Germany-MV/AI06175/2021_H5N1_2021-10-26<br>ATGGATGTCAATCCGACTTTACTTTTCTTAAAAGTGCCAGCGCAAGATGCCATAAGTACCACATTCCCTTACACTGGAGATCCTCCATACAGCCATGGAACAGGGACAGGATACACAATGGACACAGTCAACAGAACACATCAATACTCAGAGAAGGGAAAATGGACAACAAACACAGAAACCGGAGCACCTCAACTCAACCCAATTGATGGGCCACTACCTGAGGACAACGA<br>ACCGAGCGGATATGCACAAACAGATTGCGTGTTGGAAGCAATGGCTTTCCTTGAAGAGTCCCATCCAGGGATCTTTGAAAACTCTTGTCTTGAAACGATGGAAGTCGTTCAGCAAACAAGAGTGGACAAACTAACTCAAGGTCGCCAGACATATGACTGGACACTGAATAGAAACCAACCAGCTGCAACTGCCCTGGCCAACACTATAGAGGTCTTCAGATCAAACGGTCTAA<br>CAGCCAATGAATCGGGGAGACTAATAGATTTCCTCAAGGATGTGATGGACTCAATGGATAAAGAAGAAATGGAAATAACAACACATTTCCAGAGAAAGAGAAGAGTAAGGGACAACATGACCAAGAAAATGGTCACACAAAGAACAATAGGAAAGAAGAAACAAAGGCTAAACAAGAGGAGCTACTTAATAAGAGCACTGACACTGAATACAATGACAAAAGATGCAGA<br>AAGAGGCAAATTGAAGAGACGGGCGATTGCAACACCAGGGATGCAGATTAGAGGATTTGTGTACTTTGTCGAAACACTGGCAAGGAGCATCTGTGAAAAACTTGAGCAATCTGGACTCCCCGTTGGAGGGAATGAGAAGAAGGCTAAATTGGCAAATGTCGTGAGAAAAATGATGACTAACTCACAAGATACAGAGCTCTCCTTCACAATTACTGGAGATAACACCAAATG<br>GAATGAGAATCAAAATCCTCGGATGTTTCTGGCAATGATAACATACATTACAAGAAACCAACCTGAATGGTTTAGGAATGTCTTGAGTATTGCCCCTATAATGTTCTCGAACAAAATGGCGAGATTGGGAAAAGGGTACATGTTTGAAAGTAAGAGCATGAAGTTACGGACACAAATACCTGCAGAAATGCTTGCAAACATTGACTTAAAATACTTCAATGAATCAACAAGAAA<br>GAAAATCGAAAAAATAAGGCCTCTACTAATAGATGGCACTGCCTCATTGAGTCCTGGAATGATGATGGGCATGTTCAATATGCTGAGTACAGTATTAGGAGTTTCAATCCTAAATCTTGGGCAAAAGAAGTACACCAAAACCACATACTGGTGGGATGGACTCCAATCCTCTGATGATTTCGCCCTCATAGTAAATGCACCGAATCATGAGGGAATACAAGCAGGAGTGGATA<br>GGTTCTATAGGACCTGCAAACTGGTCGGGATCAATATGAGCAAAAAGAAGTCTTACATAAACCGGACTGGAACATTTGAGTTCACAAGCTTTTTCTATCGCTATGGATTTGTGGCTAACTTCAGTATGGAGCTGCCCAGCTTTGGAGTTTCTGGGATCAATGAATCAGCTGACATGAGCATTGGCATCACAGTGATAAAGAACAACATGATAAACAATGACCTTGGACCAGCAA<br>CAGCTCAAATGGCCCTTCAACTATTCATCAAAGATTACAGGTACACGTACCGATGCCATAGAGGTGACACACAAATTCAAACGAGGAGATCATTCGAGCTGAAGAAGCTGTGGGAACAGACCCGTTCAAAGGCAGGACTGTTGGTGTCAGATGGAGGACCAAATCTATACAACATTCGGAATCTCCATATCCCAGAGGTCTGCCTGAAGTGGGAGCTGATGGACGAAGATTA<br>CCAGGGCAGGTTGTGTAATCCTCTGAACCCATTTGTCAGCCATAAAGAAATTGAGTCCGTAAACAATGCTGTGGTGATGCCAGCCCACGGTCCAGCCAAAAGCATGGAATATGATGCCGTTGCGACTACACACTCATGGATTCCTAAAAGGAATCGTTCCATTCTCAATACCAGCCAAAGGGGAATTCTTGAGGATGAACAGATGTACCAGAAATGCTGCGGTCTATTCGAGAA<br>ATTCTTCCCCAGTAGTTCATACAGGAGACCAGTTGGAATTTCCAGCATGGTGGAGGCCATGGTGTCTAGGGCCCGAATCGATGCACGCATTGATTTCGAATCTGGAAGGATCAAGAAGGAAGAGTTTGCTGAGATCATGAAGATCTGTTCCACCATTGAAGAGCTCAGACGGCAAAAATAG
EPI_ISL_984684	>A/barnacle_goose/Denmark/14537-1/2020_H5N8_2020-11-04<br>ATGGATGTCAATCCGACTTTACTTTTCTTAAAAGTGCCAGCGCAAAATGCTATAAGTACTACATTCCCTTACACTGGAGATCCTCCATACAGCCATGGAACAGGAACAGGGTATACCATGGACACAGTAAACAGAACACATCAATACTCAGAAAAGGGAAAGTGGACAACAAACACAGAAACCGGAGCACCCCAACTCAACCCAATTGATGGACCATTACCAGAGGACAATGA<br>GCCAAGCGGATATGCACAAACTGATTGCGTGTTGGAAGCAATGGCTTTCCTTGAAGAATCCCACCCAGGGATATTTGAAAACTCTTGTCTTGAAGCGATGGAAATCGTTCAGCAAACAAGAGTGGACAAACTAACCCAAGGTCGCCAGACTTATGACTGGACACTGAACAGAAACCAACCAGCTGCAACCTCTTTGGCCAACACTATAGAGGTGTTCAGATCGAATGGTCTGA<br>CAGCCAATGAATCAGGGAGACTGATAGATTTTCTCAGAGATGTGATGGAATCAATGGATAAAGAAGAGATGGAAGTAACAACACATTTCCAGAGAAAAAGAAGAGTGAGGGACAACATGACTAAGAAGATGGTCACACAAAGAACAATAGGGAAGAAGAAGCAGAGGCTGAACAAGAGGAGTTACTTAATAAGAGCACTGACATTGAATACAATGACCAAAGATGCAGA<br>AAGAGGCAAGTTGAAGAGACGGGCAATTGCAACACCCGGGATGCAGATTAGAGGATTCGTGTACTTCGTTGAAACACTAGCGAGGAGCATCTGTGAGAAACTAGAGCAATCTGGGCTCCCTGTTGGAGGGAATGAGAAGAAGGCTAAATTGGCAAATGTTGTGAGAAAAATGATGACTAACTCACAAGATACAGAGCTCTCCTTTACAATTACTGGAGACAACACCAAATG<br>GAATGAGAATCAAAACCCTCGGATGTTTTTGGCAATGATAACATATATCACAAGAAACCAACCTGAATGGTTTAGAAATGTCTTAAGCATTGCCCCTATAATGTTCTCAAACAAAATGGCGAGATTAGGGAAAGGATACATGTTTGAAAGTAAGAGCATGAAGCTAAGAACACAAATACCAGCAGAGATGCTTACAAATATCGATCTGAAGTATTTCAACGAACCAACGAGAA<br>AGAAAATCGAGAAAATAAGACCTCTGCTGATTGATGGCACGGCCTCATTGAGTCCTGGGATGATGATGGGCATGTTCAATATGCTGAGCACAGTATTAGGGGTCTCAATCCTGAATCTCGGGCAAAAAAGGTACACCAAAACCACATACTGGTGGGATGGACTTCAATCCTCTGATGATTTCGCTCTCATAGTGAATGCACCGAATCATGAGGGGATACAAGCAGGAGTGGAT<br>AGATTCTATAGGACCTGCAAACTGGTTGGGATCAACATGAGCAAAAAGAAGTCTTACATAAACCGAACAGGAACATTTGAGTTCACAAGTTTTTTCTACCGCTATGGATTTGTAGCTAACTTCAGTATGGAATTACCCAGCTTTGGAGTGTCTGGAATCAATGAATCAGCTGACATGAGCATTGGAGTTACAGTGATAAAAAACAATATGATAAACAATGATCTTGGACCAGCA<br>ACAGCTCAAATGGCTCTTCAGTTATTCATCAAAGACTATAGGTACACATACCGATGCCACAGGGGTGATACACAAATTCAAACGAGGAGATCATTCGAGCTGAAGAAGCTGTGGGAGCAGACCCGTTCAAAGGCAGGGCTGTTGATATCAGACGGGGGGCCAAACCTATACAACATTCGGAATCTCCACATCCCAGAGGTCTGCTTGAAGTGGGAGCTGATGGATGAAGACT<br>ACCAAGGCAGGCTGTGCAATCCTCTGAATCCATTTGTCAGTCATAAAGAGATTGAGTCCGTAAACAATGCTGTAGTAATGCCCGCCCATGGCCCGGCCAAGAGCATGGAATATGATGCTGTTGCGACCACACACTCGTGGATTCCTAAGAGGAACCGTTCCATTCTCAATACCAGCCAAAGGGGAATTCTTGAGGATGAGCAGATGTACCAAAAGTGCTGTAGTCTATTCGAGA<br>AATTCTTCCCCAGCAGTTCATACAGGAGGCCAGTTGGAATTTCCAGCATGGTGGAGGCCATGGTGTCTAGGGCCCGAATTGATGCACGCATCGACTTCGAATCTGGAAGGATTAAGAAAGAAGAGTTTGCTGAGATCATGAAGATCTGTTCCACCATTGAAGAGCTCAGACGGCAAAAATAG
EPI_ISL_644824	>A/barnacle_goose/Denmark/14138-1/2020-11-04_H5N8_2020-11-04<br>ATGGATGTCAATCCGACTTTACTTTTCTTAAAAGTGCCAGCGCAAAATGCTATAAGTACTACATTCCCTTACACTGGAGATCCTCCATACAGCCATGGAACAGGAACAGGGTATACCATGGACACAGTAAACAGAACACATCAATACTCAGAAAAGGGAAAGTGGACAACAAACACAGAAACCGGAGCACCCCAACTCAACCCAATTGATGGACCATTACCAGAGGACAATGA<br>GCCAAGCGGATATGCACAAACTGATTGCGTGTTGGAAGCAATGGCTTTCCTTGAAGAATCCCACCCAGGGATATTTGAAAACTCTTGTCTTGAAGCGATGGAAATCGTTCAGCAAACAAGAGTGGACAAACTAACCCAAGGTCGCCAGACTTATGACTGGACACTGAACAGAAACCAACCAGCTGCAACCTCTTTGGCCAACACTATAGAGGTGTTCAGATCGAATGGTCTGA<br>CAGCCAATGAATCAGGGAGACTGATAGATTTTCTCAGAGATGTGATGGAATCAATGGATAAAGAAGAGATGGAAGTAACAACACATTTCCAGAGAAAAAGAAGAGTGAGGGACAACATGACTAAGAAGATGGTCACACAAAGAACAATAGGGAAGAAGAAGCAGAGGCTGAACAAGAGGAGTTACTTAATAAGAGCACTGACATTGAATACAATGACCAAAGATGCAGA<br>AAGAGGCAAGTTGAAGAGACGGGCAATTGCAACACCCGGGATGCAGATTAGAGGATTCGTGTACTTCGTTGAAACACTAGCGAGGAGCATCTGTGAGAAACTAGAGCAATCTGGGCTCCCTGTTGGAGGGAATGAGAAGAAGGCTAAATTGGCAAATGTTGTGAGAAAAATGATGACTAACTCACAAGATACAGAGCTCTCCTTTACAATTACTGGAGACAACACCAAATG<br>GAATGAGAATCAAAACCCTCGGATGTTTTTGGCAATGATAACATATATCACAAGAAACCAACCTGAATGGTTTAGAAATGTCTTAAGCATTGCCCCTATAATGTTCTCAAACAAAATGGCGAGATTAGGGAAAGGATACATGTTTGAAAGTAAGAGCATGAAGCTAAGAACACAAATACCAGCAGAGATGCTTACAAATATCGATCTGAAGTATTTCAACGAACCAACGAGAA<br>AGAAAATCGAGAAAATAAGACCTCTGCTGATTGATGGCACGGCCTCATTGAGTCCTGGGATGATGATGGGCATGTTCAATATGCTGAGCACAGTATTAGGGGTCTCAATCCTGAATCTCGGGCAAAAAAGGTACACCAAAACCACATACTGGTGGGATGGACTTCAATCCTCTGATGATTTCGCTCTCATAGTGAATGCACCGAATCATGAGGGGATACAAGCAGGAGTGGAT<br>AGATTCTATAGGACCTGCAAACTGGTTGGGATCAACATGAGCAAAAAGAAGTCTTACATAAACCGAACAGGAACATTTGAGTTCACAAGTTTTTTCTACCGCTATGGATTTGTAGCTAACTTCAGTATGGAATTACCCAGCTTTGGAGTGTCTGGAATCAATGAATCAGCTGACATGAGCATTGGAGTTACAGTGATAAAAAACAATATGATAAACAATGATCTTGGACCAGCA<br>ACAGCTCAAATGGCTCTTCAGTTATTCATCAAAGACTATAGGTACACATACCGATGCCACAGGGGTGATACACAAATTCAAACGAGGAGATCATTCGAGCTGAAGAAGCTGTGGGAGCAGACCCGTTCAAAGGCAGGGCTGTTGATATCAGACGGGGGGCCAAACCTATACAACATTCGGAATCTCCACATCCCAGAGGTCTGCTTGAAGTGGGAGCTGATGGATGAAGACT<br>ACCAAGGCAGGCTGTGCAATCCTCTGAATCCATTTGTCAGTCATAAAGAGATTGAGTCCGTAAACAATGCTGTAGTAATGCCCGCCCATGGCCCGGCCAAGAGCATGGAATATGATGCTGTTGCGACCACACACTCGTGGATTCCTAAGAGGAACCGTTCCATTCTCAATACCAGCCAAAGGGGAATTCTTGAGGATGAGCAGATGTACCAAAAGTGCTGTAGTCTATTCGAGA<br>AATTCTTCCCCAGCAGTTCATACAGGAGGCCAGTTGGAATTTCCAGCATGGTGGAGGCCATGGTGTCTAGGGCCCGAATTGATGCACGCATCGACTTCGAATCTGGAAGGATTAAGAAAGAAGAGTTTGCTGAGATCATGAAGATCTGTTCCACCATTGAAGAGCTCAGACGGCAAAAATAG
EPI_ISL_18245197	>A/mute_swan/Poland/MB292-21_22VIR5675-14/2021_H5N8_2021-03-11<br>ATGGATGTCAATCCGACTTTACTTTTCTTAAAAGTGCCAGCGCAAAATGCTATAAGTACTACATTCCCTTACACTGGAGATCCTCCATACAGCCATGGAACAGGAACAGGGTATACCATGGACACAGTAAACAGAACACATCAATACTCAGAAAAGGGAAAGTGGACAACAAACACAGAAACCGGAGCACCCCAACTCAACCCAATTGATGGACCATTACCAGAGGACAATGA<br>GCCAAGCGGATATGCACAAACTGATTGCGTGTTGGAAGCAATGGCTTTCCTTGAAGAATCCCACCCAGGGATATTTGAAAACTCTTGTCTTGAAGCGATGGAAATCGTTCAGCAAACAAGAGTGGACAAACTAACCCAAGGTCGCCAGACTTATGACTGGACACTGAACAGAAACCAACCAGCTGCAACCTCTTTGGCCAACACTATAGAGGTGTTCAGATCGAATGGTCTGA<br>CAGCCAATGAATCAGGGAGACTGATAGATTTTCTCAGGGATGTGATGGAATCAATGGACAAAGAAGATATGGAAGTAACAACACATTTCCAGAGAAAAAGAAGAGTGAGGGACAACATGACTAAGAARATGGTCACACAAAGAACAATAGGGAAGAAGAAGCAGAGGCTGAACAAGAAGAGTTACTTAATAAGAGCACTGACATTGAATACAATGACCAAAGATGCAGA<br>AAGAGGCAAGTTGAAGAGACGGGCAATTGCAACACCCGGGATGCAGATTAGAGGATTCGTATACTTCGTTGAAACACTAGCGAGGAGCATCTGTGAGAAACTAGAGCAATCTGGGCTCCCTGTTGGAGGGAATGAGAAGAAGGCTAAATTGGCAAATGTTGTGAGAAAAATGATGACTAACTCACAAGATACAGAGCTCTCCTTTACAATTACTGGAGACAACACCAAATG<br>GAATGAGAATCAAAACCCTCGGATGTTTTTGGCAATGATAACATATATCACAAGAAACCAACCTGAATGGTTTAGAAATGTCTTAAGCATTGCCCCTATAATGTTCTCAAACAAAATGGCGAGATTAGGGAAAGGATACATGTTTGAAAGTAAGAGCATGAAGCTAAGAACACAAATACCAGCAGAGATGCTTACAAATATCGATCTGAAGTATTTCAACGAACCAACGAGAA<br>AGAAAATCGAGAAAATAAGACCTCTGCTGATTGATGGCACGGCCTCATTGAGTCCTGGGATGATGATGGGCATGTTCAATATGCTGAGCACAGTATTAGGGGTCTCAATCCTGAATCTCGGGCAAAAAAGGTACACCAAAACCACATACTGGTGGGATGGACTTCAATCCTCTGATGATTTCGCTCTCATAGTGAATGCACCGAATCATGAGGGGATACAAGCAGGAGTGGAT<br>AGATTCTATAGGACCTGCAAACTGGTTGGGATCAACATGAGCAAAAAGAAGTCTTACATAAACCGAACAGGAACATTTGAGTTCACAAGTTTTTTCTACCGCTATGGATTTGTAGCTAACTTCAGTATGGAATTACCCAGCTTTGGAGTGTCTGGAATCAATGAATCAGCTGACATGAGCATTGGAATTACAGTGATAAAAAACAATATGATAAACAATGATCTTGGACCAGCAA<br>CAGCTCAAATGGCTCTTCAGTTATTCATCAAAGACTATAGGTACACATACCGATGCCACAGGGGTGATACACAAATTCAAACGAGGAGATCATTCGAGCTGAAGAAGCTGTGGGAGCAGACCCGTTCAAAGGCAGGGCTGTTGATATCAGACGGGGGGCCAAACCTATACAACATTCGGAATCTCCACATCCCAGAGGTCTGCTTGAAGTGGGAGCTGATGGATGAAGACTA<br>CCAAGGCAGGCTGTGCAATCCTCTGAATCCATTTGTCAGTCATAAAGAGATTGAGTCCGTAAACAATGCTGTAGTAATGCCCGCCCATGGCCCGGCCAAGAGCATGGAATATGATGCTGTTGCGACCACACACTCGTGGATTCCTAAGAGGAACCGTTCCATTCTCAATACCAGCCAAAGGGGAATTCTTGAGGATGAGCAGATGTACCAAAAGTGCTGTAGTCTATTCGAGAA<br>ATTCTTCCCCAGCAGTTCATACAGGAGGCCAGTTGGAATTTCCAGCATGRTGGAGGCCATGGTGTCTAGGGCCCGAATTGATGCACGCATCGACTTCGAATCTGGAAGGATTAAGAAAGAAGAGTTTGCTGAGATCATGAAGATCTGTTCCACCATTGAAGAGCTCAGACGGCAAAAATAG
EPI_ISL_1697197	>A/australian_brushturkey/Czech_Republic/5904/2021_H5N8_2021-03-25<br>ATGGATGTCAATCCGACTTTACTTTTCTTAAAAGTGCCAGCGCAAAATGCTATAAGTACTACATTCCCTTACACTGGAGATCCCCCATACAGCCATGGAACAGGAACAGGGTATACCATGGACACAGTAAACAGAACACATCAATACTCAGAAAAGGGAAAGTGGACAACAAACACAGAAACCGGAGCACCCCAACTCAACCCAATTGATGGACCATTACCAGAGGACAATGA<br>GCCAAGCGGATATGCACAAACTGATTGCGTGTTGGAAGCCATGGCTTTCCTTGAAGAATCCCACCCAGGGATATTTGAAAACTCTTGTCTTGAAGCGATGGAAATCGTTCAGCAAACGAGAGTGGACAAACTAACCCAAGGTCGCCAGACTTATGACTGGACACTGAACAGAAACCAACCAGCTGCAACCTCTTTGGCCAACACTATAGAGGTGTTCAGATCGAATGGTCTGA<br>CAGCCAATGAATCAGGGAGACTGATAGATTTTCTCAGGGATGTGATGGAATCAATGGATAAAGAAGAGATGGAAGTAACAACACATTTCCAGAGAAAAAGAAGAGTGAGGGACAACATGACTAAGAAGATGGTCACACAAAGAACAATAGGGAAGAAGAAGCAGAGGCTGAACAAGAGGAGTTACTTAATAAGAGCACTGACATTGAATACAATGACCAAAGATGCAGA<br>AAGAGGCAAGTTGAAGAGACGGGCAATTGCAACACCCGGGATGCAGATTAGAGGATTCGTGTACTTCGTTGAAACACTAGCGAGGAGCATCTGTGAGAAACTAGAGCAATCTGGGCTCCCTGTTGGAGGGAATGAGAAAAAGGCTAAATTGGCAAATGTTGTGAGAAAAATGATGACTAACTCACAAGATACAGAGCTCTCCTTTACAATTACTGGAGACAACACCAAATG<br>GAACGAGAATCAAAACCCTCGGATGTTTTTGGCAATGATAACATATATCACAAGAAACCAACCTGAATGGTTTAGAAATGTCTTAAGCATTGCCCCTATAATGTTCTCAAACAAAATGGCGAGATTAGGGAAAGGATACATGTTTGAAAGTAAGAGCATGAAGCTAAGAACACAAATACCAGCAGAAATGCTTACAAATATTGATCTGAAGTATTTCAACGAACCAACGAGAA<br>AGAAAATCGAGAAAATAAGACCTCTGCTGATTAATGGCACGGCCTCATTGAGTCCTGGGATGATGATGGGCATGTTCAATATGCTGAGCACAGTATTAGGGGTCTCAATCCTGAATCTCGGGCAAAAGAGGTACACCAAAACCACATACTGGTGGGATGGACTTCAATCCTCTGATGATTTCGCTCTCATAGTGAATGCACCGAATCATGAGGGGATACAAGCAGGAGTGGAT<br>AGATTCTATAGGACCTGCAAATTGGTTGGGATCAACATGAGCAAAAAGAAGTCTTACATAAACCGAACAGGAACATTTGAGTTCACAAGTTTTTTCTACCGCTATGGATTTGTAGCCAACTTCAGTATGGAATTACCCAGCTTTGGAGTGTCTGGAATCAATGAATCAGCTGACATGAGCATTGGAGTTACAGTGATAAAAAACAATATGATAAACAATGATCTTGGACCAGCA<br>ACAGCTCAAATGGCTCTTCAGTTATTCATCAAAGACTATAGGTACACATACCGATGCCACAGGGGTGATACACAAATTCAAACGAGGAGATCATTCGAGCTGAAGAAGCTGTGGGAGCAGACCCGTTCAAAGGCAGGGCTGTTGATATCAGACGGGGGGCCAAACCTATACAACATTCGGAATCTCCACATCCCAGAGGTCTGCTTGAAGTGGGAGCTGATGGATGAAGACT<br>ACCAAGGCAGGCTGTGCAATCCTCTGAATCCATTTGTCAGTCATAAAGAGATTGAGTCCGTAAACAATGCTGTAGTAATGCCCGCCCATGGCCCGGCCAAGAGCATGGAATATGATGCTGTTGCGACCACACACTCGTGGATTCCTAAGAGGAACCGTTCCATCCTCAATACCAGCCAAAGGGGAATTCTTGAGGATGAGCAGATGTACCAAAAGTGCTGTAGTCTATTCGAGA<br>AATTCTTCCCCAGCAGTTCATACAGGAGGCCAGTTGGAATTTCCAGCATGGTGGAGGCCATGGTGTCTAGGGCCCGAATTGATGCACGCATCGACTTCGAATCTGGAAGGATTAAGAAAGAAGAGTTTGCTGAGATCATGAAGATCTGTTCCACCATTGAAGAGCTCAGACGGCAAAAATAG

EPI_ISL_189730	>A/Armenian_gull/Republic_of_Georgia/2/2012_H13N2_2012-11-01<br>ATGGATGTCAATCCGACTTTACTTTTCTTGAAAGTGCCAGCGCAAAATGCTATAAGTACTACATTCCCTTATACTGGTGACCCTCCATACAGCCATGGAACAGGAACAGGATATACCATGGACACAGTCAACAGAACACACCAGTACTCAGAAAAAGGAACATGGACAACAAACACCGAAACTGGAGCACCTCAGCTTAACCCGATCGATGGACCACTACCGGAGGATAATGA<br>GCCAAGCGGATATGCACAAACGGATTGTGTATTGGAGGCCATGGCTTTCCTCGAAGAATCCCATCCAGGGATCTTTGAGAACTCATGTCTTGAAACGATGGAAGTTGTTCAGCAAACACGAGTGGACAAGCTGACCCAAGGCCGACAAACATATGATTGGACATTGAATAGAAACCAGCCAGCTGCAACGGCTTTAGCCAACACCATAGAGGTCTTCAGATCGAACGGTCTAA<br>CAGCTAATGAATCGGGGAGACTCATTGATTTCCTTAAGGATGTGATGGATTCAATGGATAAAGAAGGAATGGAAATAACAACACATTTCCAAAGAAAAAGAAGAGTAAGGGACAACATGACCAAGAAAATGGTCACACAAAGAACAATAGGGAAAAAGAAACAGAGATTGAGCAAAAAAAGCTACCTAATAAGGGCATTGACATTGAATACAATGACCAAAGATGCAGAA<br>AGAGGTAAATTGAAAAGGAGAGCGATTGCAACACCCGGGATGCAGATCAGAGGTTTCGTCTACTTCGTTGAAACACTTGCAAGGAGCATATGTGAGAAACTTGAACAGTCAGGGCTCCCAGTTGGAGGAAATGAGAAAAAGGCTAAATTGGCAAACGTTGTAAGAAAGATGATGACTAATTCGCAAGACACAGAGCTCTCCTTCACAATTACTGGGGACAACACCAAGTGG<br>AATGAAAATCAAAACCCTCGGATGTTCCTAGCGATGATAACATACATCACAAGAAACCAGCCTGAATGGTTCAGAAATGTCTTGAGCATTGCCCCTATAATGTTCTCAAACAAAATGGCAAGACTTGGGAAAGGATATATGTTCGAAAGTAAGAGCATGAAGCTACGAACACAAATACCAGCAGAAATGCTTGCAAGTATTGACCTCAAATATTTCAACGAGTCAACGAGAAA<br>GAAAATCGAGAACATAAGACCCCTCTTAATAGATGGCACAGCCTCATTGAGTCCTGGAATGATGATGGGCATGTTCAACATGTTGAGTACAGTCTTAGGAGTCTCAATCTTAAATCTTGGGCAAAAGAGGTACACCAAGACCACATACTGGTGGGATGGACTCCAATCCTCTGATGACTTTGCACTCATAGTGAACGCACCTAATCATGAGGGAATACAAGCAGGAGTAGACA<br>GATTTTACAGAACCTGCAAATTAGTTGGGATCAACATGAGTAAAAAGAAGTCCTACATCAATAGAACAGGAACATTTGAATTCACAAGCTTTTTCTATCGCTATGGGTTTGTAGCTAACTTCAGTATGGAGCTACCAAGCTTTGGCGTATCAGGGATTAATGAGTCAGCTGACATGAGCATTGGAGTAACAGTGATAAAGAACAACATGATAAACAACGACCTTGGACCAGCAA<br>CAGCCCAAATGGCTCTTCAACTGTTCATCAAGGACTACAGGTACACATATCGATGCCATAGAGGTGATACTCAAATCCAAACAAGGAGATCATTTGAGTTGAAGAAGCTTTGGGAGCAAACCCGTTCAAAGGCGGGGCTGCTGGTTTCAGATGGAGGACCAAACCTATATAATATCAGAAATCTCCATATTCCAGAGGTCTGTCTGAAATGGGAGCTAATGGATGAAGATTAT<br>CAGGGCAGGCTGTGCAACCCTCTGAATCCATTCGTAAGCCATAAGGAAATTGAGTCCGTAAATAATGCTGTGGTAATGCCAGCCCATGGTCCAGCCAAAAGTATGGAATATGATGCTGTTGCAACTACACATTCCTGGGTCCCCAAGAGGAACCGTTCCATTCTCAACACTAGCCAAAGGGGAATCCTTGAGGATGAACAGATGTATCAAAAATGCTGCAATCTATTCGAAAAA<br>TTCTTCCCTAGCAGTTCGTACAGGAGACCAGTTGGAATTTCCAGCATGGTGGAGGCCATGGTATCCAGGGCCCGAATTGACGCACGGATTGACTTCGAATCTGGAAGGATCAAGAAAGAAGAGTTTGCTGAGATCATGAAGATCTGTTCCACCATTGAAGAGCTCAGACGGCAAAAGTAG
EPI_ISL_397334	>A/Anser_Fabalis/Jiangsu/J746/2017_H6N1_2017-01-19<br>ATGGATGTCAATCCGACTTTACTTTTCTTAAAAGTGCCAGCGCAAAATGCCATAAGTACCACATTCCCTTATACTGGAGATCCTCCATACAGCCATGGAACAGGAACAGGGTACACCATGGACACAGTCAACAGAACACATCAATACTCAGAAAAGGGGAAATGGACAACAAACACAGAGACCGGAGCACCCCAACTCAACCCAATTGATGGACCACTACCTGATGACAACGA<br>ACCAAGCGGATATGCACAAACAGATTGCGTGTTGGAAGCAATGGCTTTCCTTGAAGAGTCCCACCCAGGGATCTTTGAAAACTCTTGTCTTGAAACGATGGAAGTCGTTCAGCAAACAAGAGTGGACAAACTAACTCAAGGTCGCCAGACTTATGACTGGACACTGAATAGAAACCAACCGGCTGCAACTGCCCTGGCCAACACTATAGAAGTCTTCAGATCGAACGGTCTGA<br>CAGCCAACGAATCGGGGAGACTAATAGATTTCCTCAAGGATGTGATGGACTCAATGGACAAAGAAGAAATGGAAATAACAACACATTTCCAGAGAAAGAGAAGAGTAAGGGACAACATGACCAAGAAAATGGTCACACAAAGAACAATAGGGAAGAAGAAACAAAGGCTAAATAAGAGGAGCTACTTAATAAGAGCACTGACACTGAATACAATGACAAAAGACGCAGA<br>AAGAGGCAAATTGAAGAGACGGGCAATTGCAACACCAGGGATGCAGATTAGAGGATTTGTGTACTTTGTTGAAACACTGGCAAGGAGCATCTGTGAAAAACTTGAGCAATCTGGACTCCCCGTTGGAGGGAATGAGAAGAAGGCTAAATTGGCAAATGTCGTGAGAAAAATGATGACTAACTCACAAGATACAGAGCTCTCCTTCACAATTACTGGAGATAACACCAAATG<br>GAATGAGAATCAAAATCCTAGGATGTTTCTGGCAATGATAACATACATCACAAGAAACCAACCTGAATGGTTTAGAAATGTCTTGAGCATTGCCCCTATAATGTTCTCGAACAAAATGGCGAGATTGGGAAAAGGATACATGTTTGAAAGTAAGAGCATGAAGCTACGGACACAAATACCTGCAGAAATGCTTGCAAACATTGACTTAAAATACTTCAATGAGTCAACAAGAA<br>AGAAAATCGAGAAAATAAGACCTTTACTAATAGATGGCACCGCCTCATTGAGTCCTGGAATGATGATGGGCATGTTCAATATGCTGAGTACAGTATTAGGAGTCTCAATCYTGAATCTTGGGCAAAAGAGGTACACCAAAACCACATACTGGTGGGATGGACTCCAATCCTCTGATGATTTCGCCCTCATAGTGAATGCACCGAATCATGAGGGAATACAAGCAGGAGTGGAT<br>AGGTTCTACAGGACCTGCAAACTGGTTGGGATCAACATGAGCAAAAAGAAGTCTTATATAAACCGGACAGGAACATTTGAGTTCACAAGCTTTTTCTATCGCTATGGATTTGTGGCTAACTTCAGTATGGAGCTGCCCAGCTTTGGAGTGTCTGGAATCAATGAATCAGCTGACATGAGTATTGGAGTTACAGTGATAAAGAACAACATGATAAACAATGACCTTGGACCAGCA<br>ACAGCTCAAATGGCCCTTCAACTATTCATCAAAGACTACAGGTACACATACCGATGTCACAGGGGTGACACACAAATTCAAACGAGGAGATATTCGAGCTGAAGAAGCTGTGGGAGCAGACCCGTTCAAAGGCAGGACTGTTGGTGTCAGATGGAGGACCAAATCTATACAACATTCGGAATCTCCATATCCCAGAGGTCTGCCTGAAGTGGGAGCTGATGGACGAAGATT<br>ACCAGGGCAGGTTGTGTAATCCTCTGAACCCATTTGTCAGTCATAAAGAAATTGAGTCCGTAAACAATGCTGTGGTAATGCCAGCCCATGGCCCAGCCAAGAGCATGGAATATGATGCTGTCGCAACTACACACTCATGGATTCCTAAAAGGAATCGTTCCATTCTCAATACCAGCCAAAGGGGAATTCTTGAGGATGAGCAGATGTACCAGAAATGCTGCAGTCTATTCGAGA<br>AATTCTTCCCCAGTAGTTCATACAGGAGACCAGTTGGAATTTCCAGCATGGTGGAGGCCATGGTGTCTAGGGCCCGAATCGATGCACGCATTGATTTCGAATCTGGAAGGATTAAGAAGGAAGAGTTTGCTGAGATCATGAAGATCTGTTCCACCATTGAAGAGCTCAGACGGCAAAAATAG
EPI_ISL_2234811	>A/anser_anser/Spain/102-8_21VIR1230-4/2021_H5N8_2021-01-07<br>ATGGATGTCAATCCGACTTTACTTTTCTTAAAAGTGCCAGCGCAAAATGCTATAAGTACTACATTCCCTTACACTGGAGATCCCCCATACAGCCATGGAACAGGAACAGGGTATACCATGGACACAGTAAACAGAACACATCAATACTCAGAAAAGGGAAAGTGGACAACAAACACAGAAACCGGAGCACCCCAACTCAACCCAATTGATGGACCATTACCAGAGGACAATGA<br>GCCAAGCGGATATGCACAAACTGATTGCGTGTTGGAAGCAATGGCTTTCCTTGAAGAATCCCACCCAGGGATATTTGAAAACTCTTGTCTTGAAGCGATGGAAATCGTTCAGCAAACAAGAGTGGACAAACTAACCCAAGGTCGCCAGACTTATGACTGGACACTGAACAGAAACCAACCAGCTGCAACCTCTTTGGCCAACACTATAGAGGTGTTCAGATCGAATGGTCTGA<br>CAGCCAATGAATCAGGGAGACTGATAGATTTTCTCAGGGATGTGATGGAATCAATGGATAAAGAAGAGATGGAAGTAACAACACATTTCCAGAGAAAAAGAAGAGTGAGGGACAACATGACTAAGAAGATGGTCACACAAAGAACAATAGGGAAGAAGAAGCAGAGGCTGAACAAGAGGAGTTACTTAATAAGAGCACTGACATTGAATACAATGACCAAAGATGCAGA<br>AAGAGGCAAGTTGAAGAGACGGGCAATTGCAACACCCGGGATGCAGATTAGAGGATTCGTGTACTTCGTTGAAACACTAGCGAGGAGCATCTGTGAGAAACTAGAGCAATCTGGGCTCCCTGTTGGAGGGAATGAGAAAAAGGCTAAATTGGCAAATGTTGTGAGAAAAATGATGACTAACTCACAAGATACAGAGCTCTCCTTTACAATTACTGGAGACAACACCAAATG<br>GAATGAGAATCAAACCCTCGGATGTTTTTGGCAATGATAACATATATCACAAGAAACCAACCTGAATGGTTTAGAAATGTCTTAAGCATTGCCCCTATAATGTTCTCAAACAAAATGGCGAGATTAGGGAAAGGATACATGTTTGAAAGTAAGAGCATGAAGCTAAGAACACAAATACCAGCAGAGATGCTTACAAATATTGATCTGAAGTATTTCAACGAACCAACGAGAA<br>AGAAAATCGAGAAAATAAGACCTCTGCTGATTGATGGCACGGCCTCATTGAGTCCTGGGATGATGATGGGCATGTTCAATATGCTGAGCACAGTATTAGGGGTCTCAATCCTGAATCTCGGGCAAAAGAGGTACACCAAAACCACATACTGGTGGGATGGACTTCAATCCTCTGATGATTTCGCTCTCATAGTGAATGCACCGAATCATGAGGGGATACAAGCAGGAGTGGAT<br>AGATTCTATAGGACCTGCAAACTGGTTGGGATCAACATGAGCAAAAAGAAGTCTTACATAAACCGAACAGGAACATTTGAGTTCACAAGTTTTTTCTACCGCTATGGATTTGTAGCCAACTTCAGTATGGAATTACCCAGCTTTGGAGTGTCTGGAATCAATGAATCAGCTGACATGAGCATTGGAGTTACAGTGATAAAAAACAATATGATAAACAATGATCTTGGACCAGCA<br>ACAGCTCAAATGGCTCTTCAGTTATTCATCAAAGACTATAGGTACACATACCGATGCCACAGGGGTGATACACAAATTCAAACGAGGAGATCATTCGAGCTGAAGAAGCTGTGGGAGCAGACCCGTTCAAAGGCAGGGCTGTTGATATCAGACGGGGGGCCAAACCTATACAACATTCGGAATCTCCACATCCCAGAGGTCTGCTTGAAGTGGGAGCTGATGGATGAAGACT<br>ACCAAGGCAGACTGTGCAATCCTCTGAATCCATTTGTCAGTCATAAAGAGATTGAGTCCGTAAACAATGCTGTAGTAATGCCCGCCCATGGCCCGGCCAAGAGCATGGAATATGATGCTGTTGCGACCACACACTCGTGGATTCCTAAGAGGAACCGTTCCATCCTCAATACCAGCCAAAGGGGAATTCTTGAGGATGAGCAGATGTACCAAAAGTGCTGTAGTCTATTCGAGA<br>AATTCTTCCCCAGCAGTTCATACAGGAGGCCAGTTGGAATTTCCAGCATGGTGGAGGCCATGGTGTCTAGGGCCCGAATTGATGCACGCATCGACTTCGAATCTGGAAGGATTAAGAAAGAAGAGTTTGCTGAGATCATGAAGATCTGTTCCACCATTGAAGAGCTCAGACGGCAAAAATAG
EPI_ISL_502614	>A/Anas_platyrhynchos/Belgium/7976/2018_H4N2_2018-04-09<br>ATGGATGTCAATCCGACTTTACTTTTCTTAAAAGTGCCAGCGCAAAATGCTATAAGTACTACATTCCCTTACACTGGAGATCCTCCATACAGCCATGGAACAGGAACAGGATACACTATGGACACAGTCAACAGAACACATCAATACTCAGAGAAGGGAAAATGGACAACAAACACAGAAACCGGAGCACCCCAACTCAATCCAATTGATGGACCATTACCAGAGGATAACGA<br>GCCAAGCGGATATGCACAAACGGATTGCGTGTTGGAAGCAATGGCTTTTCTTGAAGAATCCCACCCAGGGATCTTTGAAAACTCTTGTCTTGAAACGATGGAAGTCGTTCAGCAAACAAGAGTGGACAAACTAACCCAAGGTCGCCAGACTTATGACTGGACACTGAACAGAAACCAACCAGCTGCAACTGCTTTGGCCAACACTATAGAGGTGTTCAGATCGAATGGTCTGA<br>CATCCAATGAATCGGGGAGACTAATAGATTTCCTTAAGGATGTGATGGAATCAATGGATAAAGAAGAAATGGAAATAACAACACATTTTCAGAGAAAGAGAAGAGTGAGGGACAACATGACCAAGAAGATGGTCACACAAAGAACAATAGGAAAGAAGAAGCAGAGGCTGAATAAAAGGAGTTACTTAATAAGAGCACTGACATTGAACACAATGACCAAAGATGCAGAA<br>AGAGGCAAACTGAAGAGACGGGCAATTGCAACACCCGGGATGCAGATTAGAGGATTCGTGTACTTTGTTGAAACACTAGCGAGAAGCATCTGTGAGAAACTCGAGCAATCTGGACTCCCTGTTGGAGGGAATGAGAAGAAGGCTAAGTTGGCAAATGTCGTGAGAAAAATGATGACTAACTCACAAGATACAGAGCTCTCCTTTACAATTACTGGAGACAACACCAAATGG<br>AATGAGAATCAAAACCCTCGGATGTTTTTGGCAATGATAACATACATCACAAGAAACCAACCTGAATGGTTTAGAAATGTCTTGAGCATCGCCCCTATAATGTTCTCAAACAAAATGGCGAGATTAGGAAAAGGATACATGTTTGAAAGTAAGAGCATGAAGCTGAGAACACAAATACCGGCAGAGATGCTTGCAAACATTGATTTGAAATATTTCAACGAATCAACGAGAAA<br>GAAAATCGAGAAAATAAGACCTCTGCTGATTGATGGCACAGCCTCATTGAGTCCTGGAATGATGATGGGCATGTTCAATATGCTGAGCACAGTATTAGGGGTCTCAATCCTGAATCTTGGGCAAAAGAAGTACACCAAAACCACATACTGGTGGGACGGACTCCAATCCTCTGATGATTTCGCTCTCATAGTGAATGCACCGAATCATGAGGGGATACAGGCAGGAGTGAACA<br>GGTTCTATAGGACCTGCAAACTGGTTGGGATCAACATGAGCAAAAAGAAGTCTTACATAAACCGGACAGGAACATTTGAGTTCACAAGTTTTTTCTACCGCTATGGATTTGTAGCTAACTTCAGTATGGAATTACCCAGCTTTGGAGTGTCTGGAATCAATGAATCGGCTGACATGAGCATTGGAGTTACAGTGATAAAGAACAATATGATAAACAATGACCTTGGACCAGCAA<br>CAGCTCAAATGGCTCTTCAGCTATTCATCAAGGACTATAGGTACACGTACCGATGCCACAGGGGTGATACACAAATTCAAACGAGGAGATCATTCGAGCTGAAGAAGCTGTGGGAGCAAACCCGTTCAAAGGCAGGATTGTTGGTATCAGATGGAGGACCAAACCTATACAACATTCGGAATCTCCACATCCCAGAGGTCTGCTTGAAGTGGGAGCTGATGGATGAAGACTA<br>CCAGGGCAGGCTGTGTAATCCTTTGAATCCGTTTGTCAGCCATAAGGAAATTGAGTCCGTAAACAATGCTGTGGTAATGCCAGCCCATGGCCCAGCCAAGAGCATGGAATATGATGCTGTTGCGACTACACACTCGTGGATTCCTAAGAGGAACCGTTCCATTCTCAATACCAGCCAAAGGGGAATTCTTGAGGATGAGCAAATGTACCAGAAGTGCTGTAGTCTATTTGAGAA<br>ATTCTTCCCCAGCAGTTCATACAGGAGGCCAGTTGGAATTTCCAGCATGGTGGAGGCCATGGTGTCTAGGGCCCGAATTGATGCACGCATTGATTTCGAATCTGGAAGGATTAAGAAAGAAGAGTTTGCTGAGATCATGAAGATCTGTTCCACCATTGAAGAGCTCAGACGGCAAAAATAG
EPI_ISL_502613	>A/Anas_platyrhynchos/Belgium/7828/2018_H12N5_2018-08-29<br>ATGGATGTCAATCCGACTTTACTTTTCTTAAAAGTGCCAGCGCAAAATGCTATAAGTACTACGTTTCCTTACACTGGAGATCCTCCATACAGCCATGGAACAGGAACAGGATACACCATGGACACAGTCAACAGAACACATCAATACTCAGAGAAGGGAAAGTGGACAACAAACACAGAAACTGGGGCGCCCCAACTCAACCCAATTGATGGACCATTGCCAGAGGACAACGA<br>GCCAAGCGGATATGCACAAACAGATTGCGTGTTGGAAGCAATGGCTTTCCTTGAAGAATCCCACCCAGGGATATTTGAAAACTCTTGTCTTGAAACGATGGAGGTCGTTCAGCAAACAAGAGTGGACAAACTAACCCAAGGTCGCCAGACTTATGATTGGACACTGAATAGAAACCAACCAGCTGCAACTGCTTTGGCCAATACTATAGAGGTCTTCAGATCGAACGGTCTAA<br>CAGCCAATGAGTCGGGGAGACTAATAGATTTCCTCAAGGATGTGATGGAATCAATGGATAAGGAAGAAATGGAAATAACAACACATTTCCAGAGAAAGAGAAGAGTAAGGGACAACATGACCAAGAAGATGGTCACACAAAGAACGATAGGAAAGAAGAAGCAGAGGCTGAACAAGAGGAACTACTTAATAAGAGCACTGACATTGAACACAATGACCAAAGATGCGGA<br>AAGAGGCAAATTGAAGAGGCGGGCAATTGCAACACCCGGGATGCAGATTAGAGGATTTGTGTACTTTGTCGAAACACTAGCAAGGAGCATCTGTGAGAAACTCGAGCAATCTGGACTCCCTGTTGGAGGGAATGAGAAAAAGGCCAAGTTGGCAAATGTCGTGAGAAAAATGATGACTAACTCGCAAGATACAGAGCTCTCCTTTACAATTACTGGAGACAACACCAAATG<br>GAATGAAAATCAAAATCCTCGGATGTTTTTGGCAATGATAACGTACATCACAAGAAACCAACCCGAATGGTTTAGAAATGTCTTGAGCATTGCCCCTATAATGTTCTCAAACAAAATGGCAAGATTAGGAAAAGGATACATGTTTGAAAGTAAGAGCATGAAGCTAAGGACACAAATACCAGCAGAAATGCTTGCAAACATTGACTTGAAATACTTCAACGAATCAACGAGAA<br>AGAAAATCGAGAAAATAAGACCTCTGCTAATTGATGGCACAGCCTCATTGAGTCCTGGAATGATGATGGGCATGTTCAATATGCTGAGCACAGTATTGGGGGTCTCAATCCTGAATCTTGGACAAAAGAGGTACACCAAAACCACATACTGGTGGGATGGACTCCAATCCTCTGATGATTTCGCTCTCATAGTGAATGCACCGAACCATGAGGGGATACAAGCAGGGGTGGAT<br>AGGTTCTATAGGACCTGCAAACTGGTTGGGATCAACATGAGCAAAAAGAAGTCTTACATAAACCGGACAGGAACATTTGAGTTCACAAGCTTTTTCTACCGCTATGGATTTGTAGCCAACTTCAGTATGGAGTTACCCAGCTTTGGAGTGTCTGGAATCAATGAGTCAGCTGACATGAGCATTGGAGTTACAGTGATAAAGAACAATATGATAAACAATGACCTTGGCCAGCA<br>ACAGCTCAAATGGCTCTTCAGCTATTCATCAAAGATTATAGGTACACGTACCGATGTCACAGAGGTGATACACAAATTCAAACGAGGAGATCATTCGAGCTTAAGAAGCTGTGGGAGCAGACCCGTTCAAAGGCAGGGCTGTTGGTATCAGATGGAGGACCAAACCTATACAACATTCGGAATCTCCACATCCCAGAGGTCTGCTTGAAGTGGGAACTAATGGATGAAGATTA<br>CCAGGGCAGGCTGTGTAATCCTCTGAATCCGTTTGTCAGTCATAAGGAAATTGAGTCCGTAAACAATGCTGTGGTAATGCCAGCCCATGGCCCAGCCAAGAGCATGGAATATGATGCTGTTGCGACTACACACTCATGGATTCCTAAGAGGAACCGTTCCATTCTCAATACCAGCCAGAGGGGAATTCTCGAAGATGAGCAGATGTACCAGAAGTGCTGCAGTTTATTCGAGA<br>AATTCTTCCCCAGTAGTTCGTACAGGAGGCCAGTTGGAATTTCCAGCATGGTGGAGGCCATGGTGTCCAGGGCCCGAATTGATGCACGCATTGATTTCGAATCTGGAAGGATTAAGAAAGAAGAGTTTGCTGAGATCATGAAGATCTGTTCCACCATTGAAGAGCTCAGACGGCAAAAATAG
EPI_ISL_14390393	>A/Anas_platyrhynchos/Belgium/2214_0001/2021_H3N8_2021-09-02<br>ATGGATGTCAATCCGACTTTACTTTTCTTAAAGGTGCCAGCGCAAAATGCCATAAGTACCACATTCCCTTATACTGGAGATCCTCCATACAGCCATGGAACAGGAACAGGATACACCATGGACACAGTCAACAGAACACATCAATACTCGGAAAAGGGGAAATGGACAACAAACACAGAGACTGGAGCACCCCAACTCAACCCAATTGATGGACCACTACCTGAGGACAACGA<br>ACCAAGCGGATATGCACAAACAGATTGCGTGTTGGAAGCAATGGCTTTCCTTGAAGAGTCCCACCCAGGGATCTTTGAAAGCTCTTGTCTTGAAACGATGGAAGTCGTTCAGCAAACAAGAGTGGACAAACTAACTCAAGGTCGCCAGACTTATGACTGGACACTGAATAGAAACCAACCAGCTGCAACTGCCTTGGCCAACACTATAGAGGTCTTCAGATCAAACGGTCTGA<br>CAGCCAATGAATCAGGGAGACTAATAGATTTCCTCAAGGATGTGATGGACTCAATGGACAAAGAAGAAATGGAAATAACAACACATTTCCAGAGAAAGAGAAGAGTAAGGGATAACATGACCAAGAAAATGGTCACACAAAGAACAATAGGGAAGAAGAAGCAAAGGCTAAATAAGAGGAGCTACTTAATAAGAGCACTGACACTGAATACAATGACAAAGGACGCAGA<br>AAGAGGCAAATTGAAGAGACGGGCAATTGCAACACCAGGGATGCAGATTAGAGGATTTGTGTACTTTGTCGAAACACTGGCAAGGAGCATTTGTGAAAAACTTGAGCAATCTGGACTCCCCGTTGGAGGGAATGAGAAGAAGGCTAAATTGGCAAATGTCGTGAGAAAAATGATGACTAACTCACAAGATACAGAGCTCTCCTTCACAATTACTGGAGATAACACCAAATG<br>GAATGAGAATCAAAATCCTCGGATGTTTCTGGCAATGATAACATACATCACAAGAAACCAACCTGAATGGTTTAGAAATGTCTTGAGCATTGCCCCTATAATGTTCTCGAACAAAATGGCGAGACTGGGAAAAGGATACATGTTTGAAAGCAAAAGCATGAAGCTACGGACACAAATACCTGCAGAAATGCTTGCAAACATTGACTTAAAATACTTCAATGAGTCAACAAGAA<br>AGAAAATCGAGAAAATAAGACCTCTACTAATAGATGGCACCGCCTCATTGAGTCCTGGAATGATGATGGGCATGTTCAATATGCTGAGTACAGTGTTAGGAGTATCAATCCTGAATCTTGGGCAAAAGAGGTACACCAAGACCACATACTGGTGGGATGGACTCCAATCCTCTGATGATTTCGCCCTCATAGTGAATGCACCGAATCATGAGGGAATACAAGCAGGAGTGGAT<br>AGGTTCTATAGGACCTGCAAACTGGTTGGGATCAACATGAGCAAAAAGAAGTCTTACATAAACCGGACAGGAACATTTGAGTTCACAAGTTTTTTCTATCGCTATGGATTTGTGGCCAACTTCAGTATGGAGCTGCCCAGTTTTGGAGTGTCTGGAATCAATGAATCAGCTGACATGAGCATTGGAGTTACAGTGATAAAGAACAACATGATAAACAATGACCTTGGACCAGCA<br>ACAGCTCAAATGGCCCTTCAACTATTCATCAAGGACTACAGGTACACGTACCGATGTCACAGGGGTGACACACAGATTCAAACGAGGAGATCATTCGAGCTGAAGAAGCTGTGGGAGCAGACCCGTTCCAAGGCAGGACTGTTGGTATCAGATGGAGGACCAAATCTATACAACATCCGGAATCTCCATATCCCAGAAGTCTGCCTGAAGTGGGAGCTGATGGACGAAGATT<br>ATCAGGGCAGGTTGTGTAATCCTCTGAACCCATTTGTCAGTCATAAAGAAATTGAATCCGTAAACAATGCTGTGGTAATGCCAGCCCATGGCCCAGCCAAGAGCATGGAATATGATGCTGTTGCGACTACACACTCATGGATTCCCAAAAGGAATCGTTCCATTCTCAATACCAGCCAAAGGGGAATTCTTGAGGATGAGCAGATGTACCAGAAATGCTGCAGTCTATTCGAGA<br>AGTTCTTCCCCAGTAGTTCATACAGGAGACCAGTTGGAATTTCCAGCATGGTGGAGGCCATGGTGTCTAGGGCCCGAATCGACGCACGCATTGATTTCGAATCTGGAAGGATTAAGAAGGAAGAGTTTGCTGAGATCATGAAGATCTGTTCCACCATTGAAGAGCTCAGACGGCAAAAATAG
EPI_ISL_502605	>A/Anas_platyrhynchos/Belgium/11958/2018_H1N1_2018-11-26<br>ATGGATGTCAATCCGACTTTACTTTTCTTAAAAGTGCCAGCGCAAAATGCCATAAGTACCACATTCCCTTATACTGGAGATCCTCCATACAGCCATGGAACAGGAACAGGATACACCATGGACACAGTCAACAGAACACATCAATACTCAGAAAAAGGGAAATGGACAACAAACACAGAGACCGGAGCACCCCAACTCAACCCAATTGATGGACCACTACCTGAGGACAACGA<br>ACCAAGCGGATATGCACAAACAGAATGCGTGTTGGAAGCAATGGCTTTCCTTGAAGAGTCCCACCCAGGGATCTTTGAAAACTCTTGTCTTGAAACGATGGAARTCGTTCAGCAAACAAGAGTGGACAAACTAACTCAAGGTCGCCAGACTTATGACTGGACACTGAATAGAAACCAACCAGCTGCAACTGCCCTGGCCAACACTATAGAGGTCTTCAGATCGAACAGTCTGA<br>CAGCCAATGAATCGGGGAGACTAATAGATTTCCTCAAGGATGTGATGGACTCAATGGACAAAGAAGAAATGGAAATAACAACACATTTCCAGAGAAAGAGAAGAGTAAGGGACAACATGACCAAGAAAATGGTCACACAAAGAACAATAGGGAAGAAGAAACAAAGGCTAAATAAGAGGAGCTACTTAATAAGAGCACTGACACTGAATACAATGACAAAAGACGCAGA<br>AAGAGGCAAATTGAAGAGACGGGCAATTGCAACACCAGGGATGCAGATTAGAGGATTTGTGTACTTTGTCGAAACACTGGCAAGGAGCATCTGTGAAAAACTTGAGCAATCTGGACTCCCCGTTGGAGGGAATGAGAAGAAGGCTAAATTGGCAAATGTCGTGAGAAAAATGATGACTAACTCACAAGATACAGAACTCTCCTTCACAATTACTGGAGATAACACCAAATG<br>GAATGAGAATCAAAATCCTCGGATGTTTCTGGCAATGATAACATACATCACAAGAAACCAACCTGAATGGTTTAGAAATGTCTTGAGCATTGCCCCTATAATGTTCTCGAACAAAATGGCGAGATTGGGAAAAGGCTACATGTTTGAAAGTAAAAGCATGAAACTACGGACACAAATACCTGCAGAAATGCTTGCAAACATTGACTTAAAATACTTCAATGAGTCAACAAGAAA<br>GAAAATCGAGAAAATAAGACCTCTACTAATAGATGGCACCGCCTCATTGAGTCCTGGAATGATGATGGGCATGTTCAATATGCTGAGTACAGTATTAGGAGTCTCAATCCTGAATCTTGGGCAAAAGAGGTACACCAAGACCACATACTGGTGGGATGGACTCCAATCCTCTGATGATTTCGCCCTCATAGTGAATGCACCGAACCATGAGGGAATACAAGCAGGAGTGGATA<br>GGTTCTATAGGACCTGCAAACTGGTTGGGATCAACATGAGCAAAAAGAAGTCTTACATAAACCGGACAGGAACATTTGAGTTCACAAGCTTTTTCTATCGCTATGGATTTGTGGCTAACTTCAGTATGGAGCTGCCCAGCTTTGGAGTGTCTGGAATCAATGAATCAGCTGATATGAGCATTGGAGTTACAGTGATAAAGAACAACATGATAAACAATGACCTTGGACCAGCAA<br>CAGCTCAAATGGCCCTTCAACTATTCATTAAGGACTACAGGTACACGTACCGATGTCACAGGGGTGACACACAAATTCAAACGAGGAGATCATTCGAGCTGAAGAAGCTGTGGGAGCAGACCCGTTCAAAGGCAGGACTGTTAGTGTCAGATGGAGGACCAAATCTATACAACATCCGGAATCTCCATATCCCAGAGGTCTGCCTGAAGTGGGAGCTGATGGACGAAGATTA<br>CCAGGGCAGGTTGTGTAATCCTCTGAACCCATTTGTCAGTCATAAAGAGATTGAGTCCGTAAACAATGCTGTGGTAATGCCAGCCCATGGCCCAGCCAAGAGCATGGAGTATGATGCTGTTGCGACTACACACTCATGGATTCCTAAAAGGAATCGTTCCATTCTCAATACCAGCCAAAGGGGAATTCTTGAGGATGAGCAGATGTACCAGAAATGCTGCAGTCTATTCGAGAA<br>ATTCTTCCCCAGTAGTTCATACAGGAGACCAGTTGGAATTTCCAGCATGGTGGAGGCCATGGTGTCTAGGGCCCGAATCGATGCACGCATTGATTTCGAATCTGGAAGGATTAAGAAGGAAGAGTTTGCTGAGATCATGAAGATCTGTTCCACCATTGAAGAGCTCAGACGGCAAAAATAG
EPI_ISL_399475	>A/Anas_platyrhynchos/Belgium/11089/2016_H11N2_2016-11-26<br>ATGGATGTCAATCCGACTTTACTTTTCTTAAAAGTGCCAGCGCAAAATGCTATAAGTACTACATTCCCTTACACTGGAGATCCTCCATACAGCCATGGAACAGGAACAGGATACACTATGGACACAGTCAACAGAACACATCAATACTCAGAGAAGGGAAAATGGACAACAAACACAGAGACCGGAGCACCCCAACTCAATCCAATTGATGGACCATTACCAGAGGACAACGA<br>GCCAAGCGGATATGCACAAACGGATTGCGTGTTGGAAGCAATGGCTTTCCTTGAAGAATCCCACCCAGGGATCTTTGAAAACTCTTGTCTTGAAACGATGGAAGTCGTTCAGCAAACAAGAGTGGACAAACTAACCCAAGGTCGCCAGACTTATGACTGGACACTGAACAGAAACCAACCAGCTGCAACTGCTTTGGCCAACACTATAGAGGTGTTCAGATCGAATGGTCTGA<br>CAGCCAATGAGTCGGGGAGACTAATAGATTTCCTCAAGGATGTGATGGAATCAATGGATAAAGAAGAAATGGAAATAACAACACATTTCCAGAGAAAGAGAAGAGTGAGGGACAACATGACCAAGAAGATGGTCACACAAAGAACAATAGGAAAGAAGAAGCAGAGGCTGAATAAGAGGAGTTACTTAATAAGAGCACTGACATTGAACACAATGACCAAAGATGCAGA<br>AAGGGGCAAACTGAAGAGACGGGCAATTGCAACACCCGGGATGCAGATTAGAGGATTCGTGTATTTTGTTGAAACACTAGCGAGGAGCATCTGTGAGAAACTCGAGCAATCTGGACTCCCTGTTGGAGGGAATGAGAAGAAGGCTAAATTGGCAAATGTCGTGAGAAAAATGATGACTAACTCACAAGATACAGAACTCTCCTTTACAATTACTGGAGACAACACCAAATG<br>GAATGAGAATCAAAATCCTCGAATGTTTTTGGCAATGATAACATACATCACAAGAAACCAACCTGAATGGTTTAGAAATGTCTTGAGCATCGCCCCTATAATGTTCTCAAATAAAATGGCGAGATTAGGAAAAGGATACATGTTTGAAAGTAAGAGCATGAAGCTGAGAACACAAATACCGGCAGAGATGCTTGCAAACATTGATTTGAAATATTTCAACGAATCAACGAGAA<br>AGAAAATCGAGAAAATAAGACCTCTGCTGATTGATGGCACAGCCTCATTGAGTCCTGGAATGATGATGGGCATGTTCAATATGCTGAGCACAGTATTAGGGGTCTCAATCCTGAATCTTGGGCAAAAGAGGTACACCAAAACCACATACTGGTGGGATGGACTCCAATCCTCTGATGATTTCGCTCTCATAGTGAATGCACCGAATCATGAGGGGATACAAGCAGGAGTGGAT<br>AGGTTCTATAGGACCTGCAAACTGGTTGGGATCAACATGAGCAAAAAGAAGTCTTACATAAACCGGACAGGAACATTTGAGTTCACAAGTTTTTTCTACCGCTATGGATTTGTAGCTAACTTCAGTATGGAATTACCCAGCTTTGGAGTGTCTGGAATCAATGAATCGGCTGATATGAGCATTGGAGTTACAGTGATAAAGAACAATATGATAAACAATGACCTTGGACCAGCA<br>ACAGCTCAAATGGCTCTTCAGCTATTCATCAAGGACTATAGGTACACGTACCGATGCCACAGGGGTGATACACAAATTCAAACGAGGAGATCATTCGAGCTGAAGAAGCTGTGGGAGCAGACCCGTTCAAAAGCAGGGTTGTTGGTATCAGATGGAGGACCAAACCTATATAACATTCGGAATCTCCACATCCCAGAGGTCTGCTTGAAGTGGGAGCTGATGGATGAAGACT<br>ACCAGGGCAGGCTGTGTAATCCCTTGAATCCATTTGTCAGTCATAAGGAAATTGAGTCCGTAAACAATGCTGTGGTAATGCCAGCCCATGGCCCAGCCAAGAGCATGGAATATGATGCTGTTGCGACTACACACTCGTGGATTCCTAAGAGGAACCGTTCCATTCTCAATACCAGCCAAAGGGGAATTCTTGAGGATGAGCAAATGTACCAGAAGTGCTGTAGTCTATTTGAGA<br>AATTCTTCCCCAGCAGTTCATACAGGAGGCCAGTTGGAATTTCCAGCATGGTGGAGGCCATGGTGTCTAGGGCCCGAATTGATGCACGCATTGATTTCGAATCTGGAAGGATTAAGAAAGAAGAGTTTGCTGAGATCATGAAGATCTGTTCCACCATTGAAGAGCTCAGACGGCAAAAATAG

PA

Isolate ID	Sequence
EPI_ISL_14823405	>A/Wild_geese/Hubei/H358/2021_H5N8_2021-01-27<br>ATGGAAGACTTTGTGCGACAATGCTTCAATCCAATGATTGTCGAGCTTGCGGAAAAGGCAATGAAAGAATATGGGGAAGATCCTAAGATCGAAACAAACAAGTTTGCCGCAATATGTACACACTTAGAAGTCTGCTTCATGTATTCGGATTTCCATTTCATTGATGAACGAGGCGAATCAATAATCGTAGAATCTGGCGACCCGAATGCATTATTGAAGCACCGATTTGAGA<br>TAATTGAAGGGAGAGACCGAACAATGGCCTGGACAGTGGTGAATAGTATATGCAACACTACAGGAGTCGAAAAGCCCAAGTTCCTTCCTGATTTGTATGACTACAGAGAAAACCGATTCATTGAAATTGGAGTAACGCGAAGGGAAGTTCACATATACTATCTTGAAAAAGCCAACAAGATAAAATCAGAGAAAACACACATTCACATATTCTCATTCACTGGGGAGGAA<br>ATGGCCACCAAGGCAGATTACACTCTTGATGAAGAGAGCAGAGCAAGAATAAAAACCAGGCTATTCACTATAAGACAAGAGATGGCCAATAGGGGTCTATGGGATTCCTTTCGTCAGTCCGAGAGAGGCGAAGAGACAATTGAAGAAAGATTTGAAATCACAGGAACCATGCGCAGGCTTGCTGACCAAAGTCTCCCACCGAACTTCGCCAGCCTTGAAAACTTTAGAG<br>CCTATGTGGATGGATTTGAACCGAACGGCTGCATTGAGGGCAAGCTTTCTCAAATGTCAAAAGAAGTGAATGCCAGAATTGAGCCATTTCTGAAGACAACACCACGCCCTCTCAGATTACCTGATGGACCTCCCTGTTCCCAGCGGTCGAAGTTCTTGCTAATGGATGCCCTTAAATTGAGCATTGAAGACCCGAGCCATGAGGGGGAGGGTATACCGCTGTACGATGCAA<br>CCAAATGCATGAAGACATTTTTTGGCTGGAAGGAGCCCAACATCGTGAAACCACATGAAAAGGGCATAAACCCTAATTACCTCCTGGCTTGGAAGCAGGTGCTATCAGAACTCCAAGATATTGAAAACGAGGAGAAAATCCCAAAAACAAAGAACATGAAGAAGACGAGCCAACTGAAGTGGGCACTTGGTGAAAACATGGCACCAGAGAAAGTGGACTTTGAGGACT<br>GTAAAGATGTTAGCGATCTAAGACAGTACGACAGTGACGAACCAGAATCTAGATCACTAGCAAGTTGGATCCAGAGTGAATTTAACAAGGCTTGCGAACTGACAGATTCGAGTTGGATTGAACTTGATGAAATAGGGGAAGACGTTGCTCCGATTGAACACATTGCGAGTATGAGGAGGAACTATTTCACAGCGGAAGTATCCCATTGCAGGGCTACAGAATACATAATG<br>AAAGGAGTATACATAAACACAGCTTTATTGAATGCATCCTGTGCAGCCATGGATGACTTCCAACTGATTCCAATGATAAGCAAATGCAGAACCAAAGAAGGGAGACGGAAGACAAATCTGTATGGCTTCATTATAAAAGGAAGATCCCATTTGAGGAATGACACCGATGTGGTAAACTTTGTGAGCATGGAATTCTCTCTCACTGACCCAAGGCTGGAGCCACACAAATGG<br>GAAAAGTACTGTGTGCTTGAGGTAGGAGACATGCTCCTACGGACTGCAATAGGCCAAGTGTCAAGGCCCATGTTCCTGTATGTGAGAACCAATGGGACTTCTAAGATCAAAATGAAATGGGGTATGGAGATGAGGCGATGCCTTCTTCAATCCCTTCAACAAATTGAGAGCATGATTGAAGCCGAGTCTTCTGTCAAAGAAAAAGACATGACCAAGGAATTCTTTGAAAAC<br>AGATCAGAAACATGGCCAATTGGAGAGTCACCCAAAGGAGTGGAGGAAGGCTCCATTGGGAAGGTGTGCAGAACATTGCTAGCAAAGTCTGTGTTCAACAGCCTATATGCATCTCCACAACTCGAGGGGTTTTCAGCTGAATCAAGAAAGTTGCTTCTCATTGTTCAGGCACTTAGGGACAACCTGGAACCTGGGACCTTCGATCTTGGGGGGCTATATGAAGCAATTGA<br>GGAGTGCCTGATTAACGATCCCTGGGTTTTGCTTAATGCGTCTTGGTTCAACTCCTTCCTCACACATGCACTGAAATAG
EPI_ISL_977513	>A/chicken/Czech_Republic/1566-1/2021_H5N8_2021-01-22<br>ATGGAAGACTTTGTGCGACAATGCTTCAATCCAATGATTGTCGAGCTTGCGGAAAAGGCAATGAAAGAATATGGGGAAGATCCTAAGATCGAAACAAACAAGTTTGCCGCAATATGTACGCACTTAGAAGTCTGCTTCATGTATTCGGATTTCCATTTCATTGATGAACGAGGCGAATCAATAATCGTAGAATCTGGCGACCCGAATGCATTATTGAAGCACCGATTTGAGA<br>TAATTGAAGGGAGAGACCGAACAATGGCCTGGACAGTGGTGAATAGTATATGCAACACTACAGGAGTCGAAAAGCCCAAGTTCCTTCCTGATTTGTATGACTACAGAGAAAACCGATTCATTGAAATTGGAGTAACGCGAAGGGAAGTTCACATATACTATCTTGAAAAAGCCAACAAGATAAAATCAGAGAAAACACACATTCACATATTCTCATTCACTGGGGAGGAA<br>ATGGCCACCAAGGCAGATTACACTCTTGATGAAGAGAGCAGAGCAAGAATAAAAACCAGGCTATTCACTATAAGACAAGAGATGGCCAATAGGGGTCTATGGGATTCCTTTCGTCAGTCCGAGAGAGGCGAAGAGACAATTGAAGAAAGATTTGAAATCACAGGAACCATGCGCAGGCTTGCTGACCAAAGTCTCCCACCGAACTTCGCCAGCCTTGAAAACTTTAGAG<br>CCTATGTGGATGGATTTGAACCGAACGGCTGCATTGAGGGCAAGCTTTCTCAAATGTCAAAAGAAGTGAATGCCAGAATTGAGCCATTTCTGAAGACAACACCACGCCCTCTCAGATTACCTGATGGACCTCCCTGTTCCCAGCGGTCGAAGTTCTTGCTAATGGATGCCCTTAAATTGAGCATTGAAGACCCGAGCCATGAGGGGGAGGGTATACCGCTGTACGATGCAA<br>CCAAATGCATGAAGACATTTTTTGGCTGGAAGGAGCCCAACATCGTGAAACCACATGAAAAGGGCATAAACCCTAATTACCTCCTGGCTTGGAAGCAGGTGCTATCAGAACTCCAAGATATTGAAAACGAGGAGAAAATCCCAAAAACAAAGAACATGAAGAAGACGAGCCAACTGAAGTGGGCACTTGGTGAAAACATGGCACCAGAGAAAGTGGACTTTGAGGACT<br>GTAAAGATGTTAGCGATCTAAGACAGTACGACAGTGACGAACCAGAATCTAGATCACTAGCAAGTTGGATCCAGAGTGAATTTAACAAGGCTTGCGAACTGACAGATTCGAGTTGGATTGAACTTGATGAAATAGGGGAAGACGTTGCTCCGATTGAACACATTGCGAGTATGAGGAGGAACTATTTCACAGCGGAAGTATCCCATTGCAGGGCTACAGAATACATAATG<br>AAAGGAGTATACATAAACACAGCTTTATTGAATGCATCCTGTGCTGCCATGGATGACTTCCAACTGATTCCAATGATAAGCAAATGCAGAACCAAAGAAGGAAGACGGAAGACAAATCTGTATGGCTTCATTATAAAAGGAAGATCCCATTTGAGGAATGACACCGATGTGGTAAACTTTGTGAGCATGGAATTCTCTCTCACTGACCCAAGGCTGGAGCCACACAAATGG<br>GAAAAGTACTGTGTGCTTGAGGTAGGAGACATGCTCCTACGGACTGCAATAGGCCAAGTGTCAAGGCCCATGTTCCTGTATGTGAGAACCAATGGGACTTCTAAGATCAAAATGAAATGGGGTATGGAGATGAGGCGATGCCTTCTTCAATCCCTTCAACAAATTGAGAGCATGATTGAAGCCGAGTCTTCTGTCAAAGAAAAAGACATGACCAAGGAATTCTTTGAAAAC<br>AGATCAGAAACATGGCCAATTGGAGAGTCACCCAAAGGAGTGGAGGAAGGCTCCATTGGGAAGGTGTGCAGAACATTGCTAGCAAAGTCTGTGTTCAACAGCCTATATGCATCTCCACAACTCGAGGGGTTTTCAGCTGAATCAAGAAAGTTGCTTCTCATTGTTCAGGCACTTAGGGACAACCTGGAACCTGGGACCTTCGATCTTGGGGGGCTATATGAAGCAATTGA<br>GGAGTGCCTGATTAACGATCCCTGGGTTTTGCTTAATGCGTCTTGGTTCAACTCCTTCCTCACACATGCACTGAAATAG
EPI_ISL_14836082	>A/Whooper_swan/Sanmenxia/Y27/2020_H5N8_2020-11-16<br>ATGGAAGACTTTGTGCGACAATGCTTCAATCCAATGATTGTCGAGCTTGCGGAAAAGGCAATGAAAGAATATGGGGAAGATCCTAAGATCGAAACAAACAAGTTTGCCGCAATATGTACACACTTAGAAGTCTGCTTCATGTATTCGGATTTCCATTTCATTGATGAACGAGGCGAATCAATAATCGTAGAATCTGGCGACCCGAATGCATTATTGAAGCACCGATTTGAGA<br>TAATTGAAGGGAGAGACCGAACAATGGCCTGGACAGTGGTGAATAGTATATGCAACACTACAGGAGTCGAAAAGCCCAAGTTCCTTCCTGATTTGTATGACTACAGAGAAAACCGATTCATTGAAATTGGAGTAACGCGAAGGGAAGTTCACATATACTATCTTGAAAAAGCCAACAAGATAAAATCAGAGAAAACACACATTCACATATTCTCATTCACTGGGGAGGAA<br>ATGGCCACCAAGGCAGATTACACTCTTGATGAAGAGAGCAGAGCAAGAATAAAAACCAGGCTATTCACTATAAGACAAGAGATGGCCAATAGGGGTCTATGGGATTCCTTTCGTCAGTCCGAGAGAGGCGAAGAGACAATTGAAGAAAGATTTGAAATCACAGGAACCATGCGCAGGCTTGCTGACCAAAGTCTCCCACCGAACTTCGCCAGCCTTGAAAACTTTAGAG<br>CCTATGTGGATGGATTTGAACCGAACGGCTGCATTGAGGGCAAGCTTTCTCAAATGTCAAAAGAAGTGAATGCCAGAATTGAGCCATTTCTGAAGACAACACCACGCCCTCTCAGATTACCTGATGGACCTCCCTGTTCCCAGCGGTCGAAGTTCTTGCTAATGGATGCCCTTAAATTGAGCATTGAAGACCCGAGCCATGAGGGGGAGGGTATACCGCTGTACGATGCAA<br>CCAAATGCATGAAGACATTTTTTGGCTGGAAGGAGCCCAACATCGTGAAACCACATGAAAAGGGCATAAACCCTAATTACCTCCTGGCTTGGAAGCAGGTGCTATCAGAACTCCAAGATATTGAAAACGAGGAGAAAATCCCAAAAACAAAGAACATGAAGAAGACGAGCCAACTGAAGTGGGCACTTGGTGAAAACATGGCACCAGAGAAAGTGGACTTTGAGGACT<br>GTAAAGATGTTAGCGATCTAAGACAGTACGACAGTGACGAACCAGAATCTAGATCACTAGCAAGTTGGATCCAGAGTGAATTTAACAAGGCTTGCGAACTGACAGATTCGAGTTGGATTGAACTTGATGAAATAGGGGAAGACGTTGCTCCGATTGAACACATTGCGAGTATGAGGAGGAACTATTTCACAGCGGAAGTATCCCATTGCAGGGCTACAGAATACATAATG<br>AAAGGAGTATACATAAACACAGCTTTATTGAATGCATCCTGTGCAGCCATGGATGACTTCCAACTGATTCCAATGATAAGCAAATGCAGAACCAAAGAAGGGAGACGGAAGACAAATCTGTATGGCTTCATTATAAAAGGAAGATCCCATTTGAGGAATGACACCGATGTGGTAAACTTTGTGAGCATGGAATTCTCTCTCACTGACCCAAGGCTGGAGCCACACAAATGG<br>GAAAAGTACTGTGTGCTTGAGGTAGGAGACATGCTCCTACGGACTGCAATAGGCCAAGTGTCAAGGCCCATGTTCCTGTATGTGAGAACCAATGGGACTTCTAAGATCAAAATGAAATGGGGTATGGAGATGAGGCGATGCCTTCTTCAATCCCTTCAACAAATTGAGAGCATGATTGAAGCCGAGTCTTCTGTCAAAGAAAAAGACATGACCAAGGAATTCTTTGAAAAC<br>AGATCAGAAACATGGCCAATTGGAGAGTCACCCAAAGGAGTGGAGGAAGGCTCCATTGGGAAGGTGTGCAGAACATTGCTAGCAAAGTCTGTGTTCAACAGCCTATATGCATCTCCACAACTCGAGGGGTTTTCAGCTGAATCAAGAAAGTTGCTTCTCATTGTTCAGGCACTTAGGGACAACCTGGAACCTGGGACCTTCGATCTTGGGGGGCTATATGAAGCAATTGA<br>GGAGTGCCTGATTAACGATCCCTGGGTTTTGCTTAATGCGTCTTGGTTCAACTCCTTCCTCACACATGCACTGAAATAG
EPI_ISL_7880696	>A/Gallus_gallus/Belgium/15977/2021_H5N1_2021-12-06<br>ATGGAAGACTTTGTGCGACAATGCTTCAATCCAATGATTGTCGAGCTTGCGGAAAAGGCAATGAAAGAGTATGGGGAAGATCCGAAAATCGAAACAAACAAATTTGCCGCAATATGCACACACTTAGAAGTCTGTTTCATGTATTCGGATTTCCACTTTATTGATGAACGAGGCGAATCAATAATTGTAGAATCTGGCGATCCGAATGCATTATTGAAACACCGATTCGAGA<br>TAATTGAAGGGAGAGACCGAACTATGGCCTGGACAGTGGTGAATAGTATCTGCAACACCACAGGAGTCGAAAAGCCCAAATTCCTCCCTGATTTGTATGACTACAAAGAGAACCGATTCATTGAAATTGGAGTAACGCGAAGGGAAATTCACATATACTATCTAGAGAAAGCCAACAAGATAAAATCAGAGAAGACACACATTCATATATTCTCATTCACTGGAGAGGAA<br>ATGGCCACCAAGGCGGACTACACCCTTGATGAAGAGAGTAGAGCAAGAATAAAAACCAGGCTGTTCACTATAAGACAAGAAATGGCCAGTAGGGGTCTATGGGATTCCTTTCGTCAGTCCGAGAGAGGCGAAGAGACAATTGAAGAAAGATTTGAAATCACAGGAACCATGCGCAGGCTTGCCGACCAAAGTCTCCCACCGAACTTCTCCAGCCTTGAAAACTTTAGAG<br>CCTATGTGGATGGATTCGAACCGAACGGCTGCATTGAGGGCAAGCTTTCTCAAATGTCAAAAGAAGTGAATGCCAGAATTGAGCCATTTCTGAAGACAACACCACGCCCTCTCAGATTACCTGATGGGCCTCCCTGTTCTCAGCGGTCGAAGTTCTTGCTGATGGATGCCCTCAAATTGAGCATCGAAGACCCGAGCCATGAGGGGGAGGGTATACCGCTGTATGATGCAA<br>TCAAATGCATGAAGACATTTTTTGGCTGGAAAGAGCCTAACATCGTAAAACCGCATGAAAAAGGCATAAACCCTAATTACCTTCTGGCTTGGAAACAGGTGCTGGCAGAACTCCAAGATATTGAAAATGAGGAGAAAATCCCAAAAACAAAGAACATGAAGAAAACAAGCCAATTGAAGTGGGCACTTGGTGAGAACATGGCACCAGAGAAAGTGGACTTTGAGGACTG<br>TAAAGATGTTAGTGATCTAAGACAGTACGACAGTGACGAACCAGAGTCTAGATCACTAGCAAGCTGGATCCAGAGTGAATTCAACAAGGCATGCGAATTGACAGATTCGAGTTGGATTGAACTTGATGAAATAGGGGAAGACATTGCTCCAATTGAACACATTGCGAGTATGAGGAGAAACTATTTCACAGCGGAGGTGTCCCATTGCAGGGCTACTGAATACATAATGA<br>AGGGAGTATACATAAACACAGCCCTGTTGAATGCATCCTGTGCAGCGATGGATGACTTTCAACTGATTCCTATGATAAGCAAGTGCAGAACCAAAGAAGGAAGACGGAAGACAAATTTATATGGATTCATTATAAAAGGAAGATCCCATTTGAGGAATGACACCGATGTGGTGAACTTTGTGAGCATGGAATTCTCTCTCACTGACCCGAGGCTGGAGCCACACAAATGG<br>GAAAAGTACTGTGTTCTTGAGATAGGAGACATGCTCCTACGGACTGCAATAGGCCAAGTGTCAAGGCCCATGTTCCTGTATGTGAGAACCAATGGAACTTCCAAGATCAAAATGAAATGGGGCATGGAGATGAGACGATGCCTTCTTCAGTCTCTTCAACAAATTGAGAGCATGATTGAGGCCGAGTCTTCTGTCAAAGAGAAGGACATGACCAAGGAATTCTTTGAAAAC<br>AAATCAGAAACATGGCCAATTGGGGAATCACCCAAAGGGGTGGAAGAAGGCTCCATTGGGAAGGTGTGCAGAACATTGCTAGCAAAGTCTGTGTTCAACAGCCTATATGCATCTCCACAACTCGAGGGGTTTTCAGCTGAATCAAGAAAATTGCTTCTCATTGTTCAGGCACTTAGGGACAACCTGGAACCTGGAACCTTCGATCTTGGGGGGCTATATGAAGCAATTGA<br>GGAGTGCCTGATTAACGATCCCTGGGTTTTGCTTAATGCGTCTTGGTTCAACTCCTTCCTCACACATGCACTGAAATAG
EPI_ISL_8377418	>A/goose/France/21P013228/2021_H5N1_2021-11-25<br>ATGGAAGACTTTGTGCGACAATGCTTCAATCCAATGATTGTCGAGCTTGCGGAAAAGGCAATGAAAGAGTATGGGGAAGATCCGAAAATCGAAACAAACAAATTTGCCGCAATATGCACACACTTAGAAGTCTGTTTCATGTATTCGGATTTCCACTTTATTGATGAACGAGGCGAATCAATAATTGTAGAATCTGGCGATCCGAATGCATTATTGAAACACCGATTCGAGA<br>TAATTGAAGGGAGAGACCGAACTATGGCCTGGACAGTGGTGAATAGTATCTGCAACACCACAGGAGTCGAAAAGCCCAAATTCCTCCCTGATTTGTATGACTACAAAGAGAACCGATTCATTGAAATTGGAGTAACGCGAAGGGAAATTCACATATACTATCTAGAGAAAGCCAACAAGATAAAATCAGAGAAGACACACATTCATATATTCTCATTCACTGGAGAGGAA<br>ATGGCCACCAAGGCGGACTACACCCTTGATGAAGAGAGTAGAGCAAGAATAAAAACCAGGCTGTTCACTATAAGACAAGAAATGGCCAGTAGGGGTCTATGGGATTCCTTTCGTCAGTCCGAGAGAGGCGAAGAGACAATTGAAGAAAGATTTGAAATCACAGGAACCATGCGCAGGCTTGCCGACCAAAGTCTCCCACCGAACTTCTCCAGCCTTGAAAACTTTAGAG<br>CCTATGTGGATGGATTCGAACCGAACGGCTGCATTGAGGGCAAGCTTTCTCAAATGTCAAAAGAAGTGAATGCCAGAATTGAGCCATTTCTGAAGACAACACCACGCCCTCTCAGATTACCTGATGGGCCTCCCTGTTCTCAGCGGTCGAAGTTCTTGCTGATGGATGCCCTCAAATTGAGCATCGAAGACCCGAGCCATGAGGGGGAGGGTATACCGCTGTATGATGCAA<br>TCAAATGCATGAAGACATTTTTTGGCTGGAAAGAGCCTAACATCGTAAAACCGCATGAAAAAGGCATAAACCCTAATTACCTTCTGGCTTGGAAACAGGTGCTGGCAGAACTCCAAGATATTGAAAATGAGGAGAAAATCCCAAAAACAAAGAACATGAAGAAAACAAGCCAATTGAAGTGGGCACTTGGTGAGAACATGGCACCAGAGAAAGTGGACTTTGAGGACTG<br>TAAAGATGTTAGTGATCTAAGACAGTACGACAGTGACGAACCAGAGTCTAGATCACTAGCAAGCTGGATCCAGAGTGAATTCAACAAGGCATGCGAATTGACAGATTCGAGTTGGATTGAACTTGATGAAATAGGGGAAGACATTGCTCCAATTGAACACATTGCGAGTATGAGGAGAAACTATTTCACAGCGGAGGTGTCCCATTGCAGGGCTACTGAATACATAATGA<br>AGGGAGTATACATAAACACAGCCCTGTTGAATGCATCCTGTGCAGCGATGGATGACTTTCAACTGATTCCTATGATAAGCAAGTGCAGAACCAAAGAAGGAAGACGGAAGACAAATTTATATGGATTCATTATAAAAGGAAGATCCCATTTGAGGAATGACACCGATGTGGTGAACTTTGTGAGCATGGAATTCTCTCTCACTGACCCGAGGCTGGAGCCACACAAATGG<br>GAAAAGTACTGTGTTCTTGAGATAGGAGACATGCTCCTACGGACTGCAATAGGCCAAGTGTCAAGGCCCATGTTCCTGTATGTGAGAACCAATGGAACTTCCAAGATCAAAATGAAATGGGGCATGGAGATGAGACGATGCCTTCTTCAGTCTCTTCAACAAATTGAGAGCATGATTGAGGCCGAGTCTTCTGTCAAAGAGAAGGACATGACCAAGGAATTCTTTGAAAAC<br>AAATCAGAAACATGGCCAATTGGGGAATCACCCAAAGGGGTGGAAGAAGGCTCCATTGGGAAGGTGTGCAGAACATTGCTAGCAAAGTCTGTGTTCAACAGCCTATATGCATCTCCACAACTCGAGGGGTTTTCAGCTGAATCAAGAAAATTGCTTCTCATTGTTCAGGCACTTAGGGACAACCTGGAACCTGGAACCTTCGATCTTGGGGGGCTATATGAAGCAATTGA<br>GGAGTGCCTGATTAACGATCCCTGGGTTTTGCTTAATGCGTCTTGGTTCAACTCCTTCCTCACACATGCACTGAAATAG
EPI_ISL_8440976	>A/mute_swan/Romania/16790_21VIR11355/2021_H5N1_2021-11-26<br>ATGGAAGACTTTGTGCGACAATGCTTCAATCCAATGATTGTCGAGCTTGCGGAAAAGGCAATGAAAGAGTATGGGGAAGATCCGAAAATCGAAACAAACAAATTTGCCGCAATATGCACACACTTAGAAGTCTGTTTCATGTATTCGGATTTCCACTTTATTGATGAACGAGGCGAATCAATAATTGTAGAATCTGGCGATCCGAATGCATTATTGAAACACCGATTCGAGA<br>TAATTGAAGGGAGAGACCGAACTATGGCCTGGACAGTGGTGAATAGTATCTGCAACACCACAGGAGTCGAAAAGCCCAAATTCCTCCCTGATTTGTATGACTACAAAGAGAACCGATTCATTGAAATTGGAGTAACGCGAAGGGAAGTTCACATATACTATCTAGAGAAAGCCAACAAGATAAAATCAGAGAAGACACACATTCATATATTCTCATTCACTGGAGAGGAA<br>ATGGCCACCAAAGCGGACTACACCCTTGATGAAGAGAGTAGAGCAAGAATAAAAACCAGGCTGTTCACTATAAGACAAGAAATGGCCAGTAGGGGTCTATGGGATTCCTTTCGTCAGTCCGAGAGAGGCGAAGAGACAATTGAAGAAAGATTTGAAATCACAGGAACCATGCGCAGGCTTGCCGACCAAAGTCTCCCACCGAACTTCTCCAGCCTTGAAAACTTTAGAG<br>CCTATGTGGATGGATTCGAACCGAACGGCTGCATTGAGGGCAAGCTTTCTCAAATGTCAAAAGAAGTGAATGCCAGAATTGAGCCATTTCTGAAGACAACACCACGCCCTCTCAGATTACCTGATGGGCCTCCCTGTTCTCAGCGGTCGAAGTTCTTGCTGATGGATGCCCTCAAATTGAGCATCGAAGACCCGAGCCATGAGGGGGAGGGTATACCGCTGTATGATGCAA<br>TCAAATGCATGAAGACATTTTTTGGCTGGAAAGAGCCTAACATCGTAAAACCGCATGAAAAAGGCATAAACCCTAATTACCTTCTGGCTTGGAAACAGGTGCTGGCAGAACTCCAAGATATTGAAAATGAGGAGAAAATCCCAAAAACAAAGAACATGAAGAAAACAAGCCAATTGAAGTGGGCACTTGGTGAGAACATGGCACCAGAGAAAGTGGACTTTGAGGACTG<br>TAAAGATGTTAGTGATCTAAGACAGTACGACAGTGACGAACCAGAGTCTAGATCACTAGCAAGCTGGATCCAGAGTGAATTCAACAAGGCATGCGAATTGACAGATTCGAGTTGGATTGAACTTGATGAAATAGGGGAAGACATTGCTCCAATTGAACACATTGCGAGTATGAGGAGAAACTATTTCACAGCGGAGGTGTCCCATTGCAGGGCTACTGAATACATAATGA<br>AGGGAGTATACATAAACACAGCCCTGTTGAATGCATCCTGTGCAGCGATGGATGACTTTCAACTGATTCCTATGATAAGCAAGTGCAGAACCAAAGAAGGAAGACGGAAGACAAATTTATATGGATTCATTATAAAAGGAAGATCCCATTTGAGGAATGACACCGATGTGGTGAACTTTGTGAGCATGGAATTCTCTCTCACTGACCCGAGGCTGGAGCCACACAAATGG<br>GAAAAGTACTGTGTTCTTGAGATAGGAGACATGCTCCTACGGACTGCAATAGGCCAAGTGTCAAGGCCCATGTTCCTGTATGTGAGAACCAATGGAACTTCCAAGATCAAAATGAAATGGGGCATGGAGATGAGACGATGCCTTCTTCAGTCCCTTCAACAAATTGAGAGCATGATTGAGGCCGAGTCTTCTGTCAAAGAGAAGGACATGACCAAGGAATTCTTTGAAAAC<br>AAATCAGAAACATGGCCAATTGGGGAATCACCCAAAGGGGTGGAAGAAGGCTCCATTGGGAAGGTGTGCAGAACATTGCTAGCAAAGTCTGTGTTCAACAGCCTATATGCATCTCCACAACTCGAGGGGTTTTCAGCTGAATCAAGAAAATTGCTTCTCATTGTTCAGGCACTTAGGGACAACCTGGAACCTGGAACCTTCGATCTTGGGGGGCTATATGAAGCAATTGA<br>GGAGTGCCTGATTAACGATCCCTGGGTTTTGCTTAATGCGTCTTGGTTCAACTCCTTCCTCACACATGCACTGAAATAG
EPI_ISL_7053817	>A/barnacle_goose/Sweden/SVA211111SZ0376/FB004496/2021_H5N1_2021-11-01<br>ATGGAAGACTTTGTGCGACAATGCTTCAATCCAATGATTGTCGAGCTTGCGGAAAAGGCAATGAAAGAGTATGGGGAAGATCCGAAAATCGAAACAAACAAATTTGCCGCAATATGCACACACTTAGAAGTCTGTTTCATGTATTCGGATTTCCACTTTATTGATGAACGAGGCGAATCAATAATTGTAGAATCTGGCGATCCGAATGCATTATTGAAACACCGATTCGAGA<br>TAATTGAAGGGAGAGACCGAACTATGGCCTGGACAGTGGTGAATAGTATCTGCAACACCACAGGAGTCGAAAAGCCCAAATTCCTCCCTGATTTGTATGACTACAAAGAGAACCGATTCATTGAAATTGGAGTAACGCGAAGGGAAGTTCACATATACTATCTAGAGAAAGCCAACAAGATAAAATCAGAGAAGACACACATTCATATATTCTCATTCACTGGAGAGGAA<br>ATGGCCACCAAGGCGGACTACACCCTTGATGAAGAGAGTAGAGCAAGAATAAAAACCAGGCTGTTCACTATAAGACAAGAAATGGCCAGTAGGGGTCTATGGGATTCCTTTCGTCAGTCCGAGAGAGGCGAAGAGACAATTGAAGAAAGATTTGAAATCACAGGAACCATGCGCAGGCTTGCCGACCAAAGTCTCCCACCGAACTTCTCCAGCCTTGAAAACTTTAGAG<br>CCTATGTGGATGGATTCGAACCGAACGGCTGCATTGAGGGCAAGCTTTCTCAAATGTCAAAAGAAGTGAATGCCAGAATTGAGCCATTTCTGAAGACAACACCACGCCCTCTCAGATTACCTGATGGGCCTCCCTGTTCTCAGCGGTCGAAGTTCTTGCTGATGGATGCCCTCAAATTGAGCATCGAAGACCCGAGCCATGAGGGGGAGGGTATACCGCTGTATGATGCAA<br>TCAAATGCATGAAGACATTTTTTGGCTGGAAAGAGCCTAACATCGTAAAACCGCATGAAAAAGGCATAAACCCTAATTACCTTCTGGCTTGGAAACAGGTGCTGGCAGAACTCCAAGATATTGAAAATGAGGAGAAAATCCCAAAAACAAAGAACATGAAGAAAACAAGCCAATTGAAGTGGGCACTTGGTGAGAACATGGCACCAGAGAAAGTGGACTTTGAGGACTG<br>TAAAGATGTTAGTGATCTAAGACAGTACGACAGTGACGAACCAGAGTCTAGATCACTAGCAAGCTGGATCCAGAGTGAATTCAACAAGGCATGCGAATTGACAGATTCGAGTTGGATTGAACTTGATGAAATAGGGGAAGACATTGCTCCAATTGAACACATTGCGAGTATGAGGAGAAACTATTTCACAGCGGAGGTGTCCCATTGCAGGGCTACTGAATACATAATGA<br>AGGGAGTATACATAAACACAGCCCTGTTGAATGCATCCTGTGCAGCGATGGATGACTTTCAACTGATTCCTATGATAAGCAAGTGCAGAACCAAAGAAGGAAGACGGAAGACAAATTTATATGGATTCATTATAAAAGGAAGATCCCATTTGAGGAATGACACCGATGTGGTGAACTTTGTGAGCATGGAATTCTCTCTCACTGACCCGAGGCTGGAGCCACACAAATGG<br>GAAAAGTACTGTGTTCTTGAGATAGGAGACATGCTCCTACGGACTGCAATAGGCCAAGTGTCAAGGCCCATGTTCCTGTATGTGAGAACCAATGGAACTTCCAAGATCAAAATGAAATGGGGCATGGAGATGAGACGATGCCTTCTTCAGTCTCTTCAACAAATTGAGAGCATGATTGAGGCCGAGTCTTCTGTCAAAGAGAAGGACATGACCAAGGAATTCTTTGAAAAC<br>AAATCAGAAACATGGCCAATTGGGGAATCACCCAAAGGGGTGGAAGAAGGCTCCATTGGGAAGGTGTGCAGAACATTGCTAGCAAAGTCTGTGTTCAACAGCCTATATGCATCTCCACAACTCGAGGGGTTTTCAGCTGAATCAAGAAAATTGCTTCTCATTGTTCAGGCACTTAGGGACAACCTGGAACCTGGAACCTTCGATCTTGGGGGGCTATATGAAGCAATTGA<br>GGAGTGCCTGATTAACGATCCCTGGGTTTTGCTTAATGCGTCTTGGTTCAACTCCTTCCTCACACATGCACTGAAATAG
EPI_ISL_14761395	>A/herring_gull/Italy/22VIR1710-3/2022_H5N1_2022-02-01<br>ATGGAAGACTTTGTGCGACAATGCTTCAATCCAATGATTGTCGAGCTTGCGGAAAAGGCAATGAAAGAGTATGGGGAAGATCCGAAAATCGAAACAAACAAATTTGCCGCAATATGCACACACTTAGAAGTCTGTTTCATGTATTCGGATTTCCACTTTATTGATGAACGAGGCGAATCAATAATTGTAGAATCTGGCGATCCGAATGCATTATTGAAACACCGATTCGAGA<br>TAATTGAAGGGAGAGACCGAACTATGGCCTGGACAGTGGTGAATAGTATCTGCAACACCACAGGAGTCGAAAAGCCCAAATTCCTCCCTGATTTGTATGACTACAAAGAGAACCGATTCATTGAAATTGGAGTAACGCGAAGGGAAGTTCACATATACTATCTGGAGAAAGCCAACAAGATAAAATCAGAGAAGACACACATTCATATATTCTCATTCACTGGAGAGGAA<br>ATGGCCACCAAGGCGGACTACACCCTTGATGAAGAGAGTAGAGCAAGAATAAAAACCAGGCTGTTCACTATAAGACAAGAAATGGCCAGTAGGGGTCTATGGGATTCCTTTCGTCAGTCCGAGAGAGGCGAAGAGACAATTGAAGAAAGATTTGAAATCACAGGAACCATGCGCAGGCTTGCCGACCAAAGTCTCCCACCGAACTTCTCCAGCCTTGAAAACTTTAGAG<br>CCTATGTGGATGGATTCGAACCGAACGGCTGCATTGAGGGCAAGCTTTCTCAAATGTCAAAAGAAGTGAATGCCAGAATTGAGCCATTTCTGAAGACAACACCACGCCCTCTCAGATTACCTGATGGGCCTCCCTGTTCTCAGCGGTCGAAGTTCTTGCTGATGGATGCCCTCAAATTGAGCATCGAAGACCCGAGCCATGAGGGGGAGGGTATACCGCTGTATGATGCAA<br>TCAAATGCATGAAGACATTTTTTGGCTGGAAAGAGCCTAACATCGTAAAACCGCATGAAAAAGGCATAAACCCTAATTACCTTCTGGCTTGGAAACAGGTGCTGGCAGAACTCCAAGATATTGAAAATGAGGAGAAAATCCCAAAAACAAAGAACATGAAAAAAACAAGCCAATTGAAGTGGGCACTTGGTGAGAACATGGCACCAGAGAAAGTGGACTTTGAGGACTG<br>TAAAGATGTTAGTGATCTAAGACAGTACGACAGTGACGAACCAGAGTCTAGATCACTAGCAAGCTGGATCCAGAGTGAATTCAACAAGGCATGCGAATTGACAGATTCGAGTTGGATTGAACTTGATGAAATAGGGGAAGACATTGCTCCAATTGAACACATTGCGAGTATGAGGAGAAACTATTTCACAGCGGAGGTGTCCCATTGCAGGGCTACTGAATACATAATGA<br>AGGGAGTATACATAAACACAGCCCTGTTGAATGCATCCTGTGCAGCGATGGATGACTTTCAACTGATTCCTATGATAAGCAAGTGCAGAACCAAAGAAGGAAGACGGAAGACAAATTTATATGGATTCATTATAAAAGGAAGATCCCATTTGAGGAATGACACCGATGTGGTGAACTTTGTGAGCATGGAATTCTCTCTCACTGACCCGAGGCTGGAGCCACACAAATGG<br>GAAAAGTACTGTGTTCTTGAGATAGGAGACATGCTCCTACGGACTGCAATAGGCCAAGTGTCAAGGCCCATGTTCCTGTATGTGAGAACCAATGGAACTTCCAAGATCAAAATGAAATGGGGCATGGAGATGAGACGATGCCTTCTTCAGTCCCTTCAACAAATTGAGAGCATGATTGAGGCCGAGTCTTCTGTCAAAGAGAAGGACATGACCAAGGAATTCTTTGAAAAC<br>AAATCAGAAACATGGCCAATTGGGGAATCACCCAAAGGGGTGGAAGAAGGCTCCATTGGGAAGGTGTGCAGAACATTGCTAGCAAAGTCTGTGTTCAACAGCCTATATGCATCTCCACAACTCGAGGGGTTTTCAGCTGAATCAAGAAAATTGCTTCTCATTGTTCAGGCACTTAGGGACAACCTGGAACCTGGAACCTTCGATCTTGGGGGGCTATATGAAGCAATTGA<br>GGAGTGCCTGATTAACGATCCCTGGGTTTTGCTTAATGCGTCTTGGTTCAACTCCTTCCTCACACATGCACTGAAATAG
EPI_ISL_13658359	>A/common_kestel/Israel/49-2/2022_H5N1_2022-01-02<br>ATGGAAGACTTTGTGCGACAATGCTTCAATCCAATGATTGTCGAGCTTGCGGAAAAGGCAATGAAAGAGTATGGGGAAGATCCGAAAATCGAAACAAACAAATTTGCCGCAATATGCACACACTTAGAAGTCTGTTTCATGTATTCGGATTTCCACTTTATTGATGAACGAGGCGAATCAATAATTGTAGAATCTGGCGATCCGAATGCATTATTGAAACACCGATTCGAGA<br>TAATTGAAGGGAGAGACCGAACTATGGCCTGGACAGTGGTGAATAGTATCTGCAACACCACAGGAGTCGAAAAGCCCAAATTCCTCCCTGATTTGTATGACTACAAAGAGAACCGATTCATTGAAATTGGAGTAACGCGAAGGGAAGTTCACATATACTATCTAGAGAAAGCCAACAAGATAAAATCAGAGAAGACACACATTCATATATTCTCATTCACTGGAGAGGAA<br>ATGGCCACCAAAGCGGACTACACCCTTGATGAAGAGAGTAGAGCAAGAATAAAAACCAGGCTGTTCACTATAAGACAAGAAATGGCCAGTAGGGGTCTATGGGATTCCTTTCGTCAGTCCGAGAGAGGCGAAGAGACAATTGAAGAAAGATTTGAAATCACAGGAACCATGCGCAGGCTTGCCGACCAAAGTCTCCCACCGAACTTCTCCAGCCTTGAAAACTTTAGAG<br>CCTATGTGGATGGATTCGAACCGAACGGCTGCATTGAGGGCAAGCTTTCTCAAATGTCAAAAGAAGTGAATGCCAGAATTGAGCCATTTCTGAAGACAACACCACGCCCTCTCAGATTACCTGATGGGCCTCCCTGTTCTCAGCGGTCGAAGTTCTTGCTGATGGATGCCCTAAAATTGAGCATCGAAGACCCGAGCCATGAGGGGGAGGGTATACCGCTGTATGATGCAA<br>TCAAATGCATGAAGACATTTTTTGGCTGGAAAGAGCCTAACATCGTAAAACCGCATGAAAAAGGCATAAACCCTAATTACCTTCTGGCTTGGAAACAGGTGCTGGCAGAACTCCAAGATATTGAAAATGAGGAGAAAATCCCAAAAACAAAGAACATGAAGAAAACAAGCCAATTGAAGTGGGCACTTGGTGAGAACATGGCACCAGAGAAAGTGGACTTTGAGGACTG<br>TAAAGATGTTAGTGATCTAAGACAGTACGACAGTGACGAACCAGAGTCTAGATCACTAGCAAGCTGGATCCAGAGTGAATTCAACAAGGCATGCGAATTGACAGATTCGAGTTGGATTGAACTTGATGAAATAGGGGAAGACATTGCTCCAATTGAACACATTGCGAGTATGAGGAGAAACTATTTCACAGCGGAGGTGTCCCATTGCAGGGCTACTGAATACATAATGA<br>AGGGAGTATACATAAACACAGCCCTGTTGAATGCATCCTGTGCAGCGATGGATGACTTTCAACTGATTCCTATGATAAGCAAGTGCAGAACCAAAGAAGGAAGACGGAAGACAAATTTATATGGATTCATTATAAAAGGAAGATCCCATTTGAGGAATGACACCGATGTGGTGAACTTTGTGAGCATGGAATTCTCTCTCACTGACCCGAGGCTGGAGCCACACAAATGG<br>GAAAAGTACTGTGTTCTTGAGATAGGAGACATGCTCCTACGGACTGCAATAGGCCAAGTGTCAAGGCCCATGTTCCTGTATGTGAGAACCAATGGAACTTCCAAGATCAAAATGAAATGGGGCATGGAGATGAGACGATGCCTTCTTCAGTCCCTTCAACAAATTGAGAGCATGATTGAGGCCGAGTCTTCTGTCAAAGAGAAGGACATGACCAAGGAATTCTTTGAAAAC<br>AAATCAGAAACATGGCCAATTGGGGAATCACCCAAAGGGGTGGAAGAAGGCTCCATTGGGAAGGTGTGCAGAACATTGCTAGCAAAGTCTGTGTTCAACAGCCTATATGCATCTCCACAACTCGAGGGGTTTTCAGCTGAATCAAGAAAATTGCTTCTCATTGTTCAGGCACTTAGGGACAACCTGGAACCTGGAACCTTCGATCTTGGGGGGCTATATGAAGCAATTGA<br>GGAGTGCCTGATTAACGATCCCTGGGTTTTGCTTAATGCGTCTTGGTTCAACTCCTTCCTCACACATGCACTGAAATAG
EPI_ISL_5463802	>A/duck/Saratov/29-08V/2021_H5N1_2021-09-30<br>ATGGAAGACTTTGTGCGACAATGCTTCAATCCAATGATTGTCGAGCTTGCGGAAAAGGCAATGAAAGAGTATGGGGAAGATCCGAAAATCGAAACAAACAAATTTGCCGCAATATGCACACACTTAGAAGTCTGTTTCATGTATTCGGATTTCCACTTTATTGATGAACGAGGCGAATCAATAATTGTAGAATCTGGCGATCCGAATGCATTATTGAAACACCGATTCGAGA<br>TAATTGAAGGGAGAGACCGAACTATGGCCTGGACAGTGGTGAATAGTATCTGCAACACCACAGGAGTCGAAAAGCCCAAATTCCTCCCTGATTTGTATGACTACAAAGAGAACCGATTCATTGAAATTGGAGTAACGCGAAGGGAAGTTCACATATACTATCTAGAGAAAGCCAACAAGATAAAATCAGAGAAGACACACATTCATATATTCTCATTCACTGGAGAGGAA<br>ATGGCCACCAAGGCGGACTACACCCTTGATGAAGAGAGTAGAGCAAGAATAAAAACCAGGCTGTTCACTATAAGACAAGAAATGGCCAGTAGGGGTCTATGGGATTCCTTTCGTCAGTCCGAGAGAGGCGAAGAGACAATTGAAGAAAGATTTGAAATCACAGGAACCATGCGCAGGCTTGCCGACCAAAGTCTCCCACCGAACTTCTCCAGCCTTGAAAACTTTAGAG<br>CCTATGTGGATGGATTCGAACCGAACGGCTGCATTGAGGGCAAGCTTTCTCAAATGTCAAAAGAAGTGAATGCCAGAATTGAGCCATTTCTGAAGACAACACCACGCCCTCTCAGATTACCTGATGGGCCTCCCTGTTCTCAGCGGTCGAAGTTCTTGCTGATGGATGCCCTCAAATTGAGCATCGAAGACCCGAGCCATGAGGGGGAGGGTATACCGCTGTATGATGCAA<br>TCAAATGCATGAAGACATTTTTTGGCTGGAAAGAGCCTAACATCGTAAAACCGCATGAAAAAGGCATAAACCCTAATTACCTTCTGGCTTGGAAACAGGTGCTGGCAGAACTCCAAGATATTGAAAATGAGGAGAAAATCCCAAAAACAAAGAACATGAAGAAAACAAGCCAATTGAAGTGGGCACTTGGTGAGAACATGGCACCAGAGAAAGTGGACTTTGAGGACTG<br>TAAAGATGTTAGTGATCTAAGACAGTACGACAGTGACGAACCAGAGTCTAGATCACTAGCAAGCTGGATCCAGAGTGAATTCAACAAGGCATGCGAATTGACAGATTCGAGTTGGATTGAACTTGATGAAATAGGGGAAGACATTGCTCCAATTGAACACATTGCGAGTATGAGGAGAAACTATTTCACAGCGGAGGTGTCCCATTGCAGGGCTACTGAATACATAATGA<br>AGGGAGTATACATAAACACAGCCCTGTTGAATGCATCCTGTGCAGCGATGGATGACTTTCAACTGATTCCTATGATAAGCAAGTGCAGAACCAAAGAAGGAAGACGGAAGACAAATTTATATGGATTCATTATAAAAGGAAGATCCCATTTGAGGAATGACACCGATGTGGTGAACTTTGTGAGCATGGAATTCTCTCTCACTGACCCGAGGCTGGAGCCACACAAATGG<br>GAAAAGTACTGTGTTCTTGAGATAGGAGACATGCTCCTACGGACTGCAATAGGCCAAGTGTCAAGGCCCATGTTCCTGTATGTGAGAACCAATGGAACTTCCAAGATCAAAATGAAATGGGGCATGGAGATGAGACGATGCCTTCTTCAGTCCCTTCAACAAATTGAGAGCATGATTGAGGCCGAGTCTTCTGTCAAAGAGAAGGACATGACCAAGGAATTCTTTGAAAAC<br>AAATCAGAAACATGGCCAATTGGGGAATCACCCAAAGGGGTGGAAGAAGGCTCCATTGGGAAGGTGTGCAGAACATTGCTAGCAAAGTCTGTGTTCAACAGCCTATATGCATCTCCACAACTCGAGGGGTTTTCAGCTGAATCAAGAAAATTGCTTCTCATTGTTCAGGCACTTAGGGACAACCTGGAACCTGGAACCTTCGATCTTGGGGGGCTATATGAAGCAATTGA<br>GGAGTGCCTGATTAACGATCCCTGGGTTTTGCTTAATGCGTCTTGGTTCAACTCCTTCCTCACACATGCACTGAAATAG
EPI_ISL_5463801	>A/chicken/Saratov/29-07V/2021_H5N1_2021-09-30<br>ATGGAAGACTTTGTGCGACAATGCTTCAATCCAATGATTGTCGAGCTTGCGGAAAAGGCAATGAAAGAGTATGGGGAAGATCCGAAAATCGAAACAAACAAATTTGCCGCAATATGCACACACTTAGAAGTCTGTTTCATGTATTCGGATTTCCACTTTATTGATGAACGAGGCGAATCAATAATTGTAGAATCTGGCGATCCGAATGCATTATTGAAACACCGATTCGAGA<br>TAATTGAAGGGAGAGACCGAACTATGGCCTGGACAGTGGTGAATAGTATCTGCAACACCACAGGAGTCGAAAAGCCCAAATTCCTCCCTGATTTGTATGACTACAAAGAGAACCGATTCATTGAAATTGGAGTAACGCGAAGGGAAGTTCACATATACTATCTAGAGAAAGCCAACAAGATAAAATCAGAGAAGACACACATTCATATATTCTCATTCACTGGAGAGGAA<br>ATGGCCACCAAGGCGGACTACACCCTTGATGAAGAGAGTAGAGCAAGAATAAAAACCAGGCTGTTCACTATAAGACAAGAAATGGCCAGTAGGGGTCTATGGGATTCCTTTCGTCAGTCCGAGAGAGGCGAAGAGACAATTGAAGAAAGATTTGAAATCACAGGAACCATGCGCAGGCTTGCCGACCAAAGTCTCCCACCGAACTTCTCCAGCCTTGAAAACTTTAGAG<br>CCTATGTGGATGGATTCGAACCGAACGGCTGCATTGAGGGCAAGCTTTCTCAAATGTCAAAAGAAGTGAATGCCAGAATTGAGCCATTTCTGAAGACAACACCACGCCCTCTCAGATTACCTGATGGGCCTCCCTGTTCTCAGCGGTCGAAGTTCTTGCTGATGGATGCCCTCAAATTGAGCATCGAAGACCCGAGCCATGAGGGGGAGGGTATACCGCTGTATGATGCAA<br>TCAAATGCATGAAGACATTTTTTGGCTGGAAAGAGCCTAACATCGTAAAACCGCATGAAAAAGGCATAAACCCTAATTACCTTCTGGCTTGGAAACAGGTGCTGGCAGAACTCCAAGATATTGAAAATGAGGAGAAAATCCCAAAAACAAAGAACATGAAGAAAACAAGCCAATTGAAGTGGGCACTTGGTGAGAACATGGCACCAGAGAAAGTGGACTTTGAGGACTG<br>TAAAGATGTTAGTGATCTAAGACAGTACGACAGTGACGAACCAGAGTCTAGATCACTAGCAAGCTGGATCCAGAGTGAATTCAACAAGGCATGCGAATTGACAGATTCGAGTTGGATTGAACTTGATGAAATAGGGGAAGACATTGCTCCAATTGAACACATTGCGAGTATGAGGAGAAACTATTTCACAGCGGAGGTGTCCCATTGCAGGGCTACTGAATACATAATGA<br>AGGGAGTATACATAAACACAGCCCTGTTGAATGCATCCTGTGCAGCGATGGATGACTTTCAACTGATTCCTATGATAAGCAAGTGCAGAACCAAAGAAGGAAGACGGAAGACAAATTTATATGGATTCATTATAAAAGGAAGATCCCATTTGAGGAATGACACCGATGTGGTGAACTTTGTGAGCATGGAATTCTCTCTCACTGACCCGAGGCTGGAGCCACACAAATGG<br>GAAAAGTACTGTGTTCTTGAGATAGGAGACATGCTCCTACGGACTGCAATAGGCCAAGTGTCAAGGCCCATGTTCCTGTATGTGAGAACCAATGGAACTTCCAAGATCAAAATGAAATGGGGCATGGAGATGAGACGATGCCTTCTTCAGTCCCTTCAACAAATTGAGAGCATGATTGAGGCCGAGTCTTCTGTCAAAGAGAAGGACATGACCAAGGAATTCTTTGAAAAC<br>AAATCAGAAACATGGCCAATTGGGGAATCACCCAAAGGGGTGGAAGAAGGCTCCATTGGGAAGGTGTGCAGAACATTGCTAGCAAAGTCTGTGTTCAACAGCCTATATGCATCTCCACAACTCGAGGGGTTTTCAGCTGAATCAAGAAAATTGCTTCTCATTGTTCAGGCACTTAGGGACAACCTGGAACCTGGAACCTTCGATCTTGGGGGGCTATATGAAGCAATTGA<br>GGAGTGCCTGATTAACGATCCCTGGGTTTTGCTTAATGCGTCTTGGTTCAACTCCTTCCTCACACATGCACTGAAATAG
EPI_ISL_5463803	>A/duck/Saratov/29-11V/2021_H5N1_2021-09-30<br>ATGGAAGACTTTGTGCGACAATGCTTCAATCCAATGATTGTCGAGCTTGCGGAAAAGGCAATGAAAGAGTATGGGGAAGATCCGAAAATCGAAACAAACAAATTTGCCGCAATATGCACACACTTAGAAGTCTGTTTCATGTATTCGGATTTCCACTTTATTGATGAACGAGGCGAATCAATAATTGTAGAATCTGGCGATCCGAATGCATTATTGAAACACCGATTCGAGA<br>TAATTGAAGGGAGAGACCGAACTATGGCCTGGACAGTGGTGAATAGTATCTGCAACACCACAGGAGTCGAAAAGCCCAAATTCCTCCCTGATTTGTATGACTACAAAGAGAACCGATTCATTGAAATTGGAGTAACGCGAAGGGAAGTTCACATATACTATCTAGAGAAAGCCAACAAGATAAAATCAGAGAAGACACACATTCATATATTCTCATTCACTGGAGAGGAA<br>ATGGCCACCAAGGCGGACTACACCCTTGATGAAGAGAGTAGAGCAAGAATAAAAACCAGGCTGTTCACTATAAGACAAGAAATGGCCAGTAGGGGTCTATGGGATTCCTTTCGTCAGTCCGAGAGAGGCGAAGAGACAATTGAAGAAAGATTTGAAATCACAGGAACCATGCGCAGGCTTGCCGACCAAAGTCTCCCACCGAACTTCTCCAGCCTTGAAAACTTTAGAG<br>CCTATGTGGATGGATTCGAACCGAACGGCTGCATTGAGGGCAAGCTTTCTCAAATGTCAAAAGAAGTGAATGCCAGAATTGAGCCATTTCTGAAGACAACACCACGCCCTCTCAGATTACCTGATGGGCCTCCCTGTTCTCAGCGGTCGAAGTTCTTGCTGATGGATGCCCTCAAATTGAGCATCGAAGACCCGAGCCATGAGGGGGAGGGTATACCGCTGTATGATGCAA<br>TCAAATGCATGAAGACATTTTTTGGCTGGAAAGAGCCTAACATCGTAAAACCGCATGAAAAAGGCATAAACCCTAATTACCTTCTGGCTTGGAAACAGGTGCTGGCAGAACTCCAAGATATTGAAAATGAGGAGAAAATCCCAAAAACAAAGAACATGAAGAAAACAAGCCAATTGAAGTGGGCACTTGGTGAGAACATGGCACCAGAGAAAGTGGACTTTGAGGACTG<br>TAAAGATGTTAGTGATCTAAGACAGTACGACAGTGACGAACCAGAGTCTAGATCACTAGCAAGCTGGATCCAGAGTGAATTCAACAAGGCATGCGAATTGACAGATTCGAGTTGGATTGAACTTGATGAAATAGGGGAAGACATTGCTCCAATTGAACACATTGCGAGTATGAGGAGAAACTATTTCACAGCGGAGGTGTCCCATTGCAGGGCTACTGAATACATAATGA<br>AGGGAGTATACATAAACACAGCCCTGTTGAATGCATCCTGTGCAGCGATGGATGACTTTCAACTGATTCCTATGATAAGCAAGTGCAGAACCAAAGAAGGAAGACGGAAGACAAATTTATATGGATTCATTATAAAAGGAAGATCCCATTTGAGGAATGACACCGATGTGGTGAACTTTGTGAGCATGGAATTCTCTCTCACTGACCCGAGGCTGGAGCCACACAAATGG<br>GAAAAGTACTGTGTTCTTGAGATAGGAGACATGCTCCTACGGACTGCAATAGGCCAAGTGTCAAGGCCCATGTTCCTGTATGTGAGAACCAATGGAACTTCCAAGATCAAAATGAAATGGGGCATGGAGATGAGACGATGCCTTCTTCAGTCCCTTCAACAAATTGAGAGCATGATTGAGGCCGAGTCTTCTGTCAAAGAGAAGGACATGACCAAGGAATTCTTTGAAAAC<br>AAATCAGAAACATGGCCAATTGGGGAATCACCCAAAGGGGTGGAAGAAGGCTCCATTGGGAAGGTGTGCAGAACATTGCTAGCAAAGTCTGTGTTCAACAGCCTATATGCATCTCCACAACTCGAGGGGTTTTCAGCTGAATCAAGAAAATTGCTTCTCATTGTTCAGGCACTTAGGGACAACCTGGAACCTGGAACCTTCGATCTTGGGGGGCTATATGAAGCAATTGA<br>GGAGTGCCTGATTAACGATCCCTGGGTTTTGCTTAATGCGTCTTGGTTCAACTCCTTCCTCACACATGCACTGAAATAG
EPI_ISL_14393097	>A/Fox/Netherlands/EMC4/2022_H5N1_2022-04-02<br>ATGGAAGACTTTGTGCGACAATGCTTCAATCCAATGATTGTCGAGCTTGCGGAAAAGGCAATGAAAGAGTATGGGGAAGATCCGAAAATCGAAACAAACAAATTTGCCGCAATATGCACACACTTAGAAGTCTGTTTCATGTATTCGGATTTCCACTTTATTGATGAACGAGGCGAATCAACAATTGTAGAATCTGGCGATCCGAATGCATTATTGAAACACCGATTCGAGA<br>TAATTGAAGGGAGAGACCGAACTATGGCCTGGACAGTGGTGAATAGTATCTGCAACACCACAGGAGTCGAAAAGCCCAAATTCCTCCCTGATTTGTATGACTACAAAGAGAACCGATTCATTGAAATTGGAGTAACGCGAAGGGAAGTTCACATATACTATCTAGAGAAAGCCAACAAGATAAAATCAGAGAAGACACACATTCATATATTCTCATTCACTGGAGAGGAA<br>ATGGCCACCAAGGCGGACTACACCCTTGATGAAGAGAGTAGAGCAAGAATAAAAACCAGGCTGTTCACTATAAGACAAGAAATGGCCAGTAGGGGTCTATGGGATTCCTTTCGTCAGTCCGAGAGAGGCGAAGAGACAATTGAAGAAAGATTTGAAATCACAGGAACCATGCGCAGGCTTGCCGACCAAAGTCTCCCACCGAACTTCTCCAGCCTTGAAAACTTTAGAG<br>CCTATGTGGATGGATTCGAACCGAACGGCTGCATTGAGGGCAAGCTTTCTCAAATGTCAAAAGAAGTGAATGCCAGAATTGAGCCATTTCTGAAGACAACACCACGCCCTCTCAGATTACCTGATGGGCCTCCCTGTTCTCAGCGGTCGAAGTTCTTGCTGATGGATGCCCTCAAATTGAGCATCGAAGACCCGAGCCATGAGGGGGAGGGTATACCGCTGTATGATGCAA<br>TCAAATGCATGAAGACATTTTTTGGCTGGAAAGAGCCTAACATCGTAAAACCGCATGAAAAAGGCATAAACCCTAATTACCTTCTGGCTTGGAAACAGGTGCTGGCAGAACTCCAAGATATTGAAAATGAGGAGAAAATCCCAAAAACAAAGAACATGAAGAAAACAAGCCAATTGAAGTGGGCACTTGGTGAGAACATGGCACCAGAGAAAGTGGACTTTGAGGACTG<br>TAAAGATGTTAGTGATCTAAGACAGTACGACAGTGACGAACCAGAGTCTAGATCACTAGCAAGCTGGATCCAGAGTGAATTCAACAAGGCATGCGAATTGACAGATTCGAGTTGGATTGAACTTGATGAAATAGGGGAAGACATTGCTCCAATTGAACACATTGCGAGTATGAGGAGAAACTATTTCACAGCGGAGGTGTCCCATTGCAGGGCTACTGAATACATAATGA<br>AGGGAGTATACATAAACACAGCCCTGTTGAATGCATCCTGTGCAGCGATGGATGACTTTCAACTGATTCCTATGATAAGCAAGTGCAGAACCAAAGAAGGAAGACGGAAGACAAATTTATATGGATTCATTATAAAAGGAAGATCCCATTTGAGGAATGACACCGATGTGGTGAACTTTGTGAGCATGGAATTCTCTCTCACTGACCCGAGGCTGGAGCCACACAAATGG<br>GAAAAGTACTGTGTTCTTGAGATAGGAGACATGCTCCTACGGACTGCAATAGGCCAAGTGTCAAGGCCCATGTTCTTGTATGTGAGAACCAATGGAACTTCCAAGATCAAAATGAAATGGGGCATGGAGATGAGACGATGCCTTCTTCAGTCCCTTCAACAAATTGAGAGCATGATTGAGGCCGAGTCTTCTGTCAAAGAGAAGGACATGACCAAGGAATTCTTTGAAAAC<br>AAATCAGAAACATGGCCAATTGGGGAATCACCCAAAGGGGTGGAAGAAGGCTCCATTGGGAAGGTGTGCAGAACATTGCTAGCAAAGTCTGTGTTCAACAGCCTATATGCATCTCCACAACTCGAGGGGTTTTCAGCTGAATCAAGAAAATTGCTTCTCATTGTTCAGGCACTTAGGGACAACCTGGAACCTGGAACCTTCGATCTTGGGGGGCTATATGAAGCAATTGA<br>GGAGTGCCTGATTAACGATCCCTGGGTTTTGCTTAATGCGTCTTGGTTCAACTCCTTCCTCACACATGCACTGAAATAG

EPI_ISL_12514573	>A/Song_Thrush/Netherlands/1/2022_H5N1_2022-04-13<br>ATGGAAGACTTTGTGCGACAATGCTTCAATCCAATGATTGTCGAGCTTGCGGAAAAGGCAATGAAAGAGTATGGGGAAGATCCGAAAATCGAAACAAACAAATTTGCCGCAATATGCACACACTTAGAAGTCTGTTTCATGTATTCGGATTTCCACTTTATTGATGAACGAGGCGAATCAATAATTGTAGAATCTGGCGATCCGAATGCATTATTGAAACACCGATTCGAGA<br>TAATTGAAGGGAGAGACCGAACTATGGCCTGGACAGTGGTGAATAGTATCTGCAACACCACAGGAGTCGAAAAGCCCAAATTCCTCCCTGATTTGTATGACTACAAAGAGAACCGATTCATTGAAATTGGAGTAACGCGAAGGGAAGTTCACATATACTATCTAGAGAAAGCCAACAAGATAAAATCAGAGAAGACACACATTCATATATTCTCATTCACTGGAGAGGAA<br>ATGGCCACCAAGGCGGACTACACCCTTGATGAAGAGAGTAGAGCAAGAATAAAAACCAGGCTGTTCACTATAAGACAAGAAATGGCCAGTAGGGGTCTATGGGATTCCTTTCGTCAGTCCGAGAGAGGCGAAGAGACAATTGAAGAAAGATTTGAAATCACAGGAACCATGCGCAGGCTTGCCGACCAAAGTCTCCCACCGAACTTCTCCAGCCTTGAAAACTTTAGAG<br>CCTATGTGGATGGATTCGAACCGAACGGCTGCATTGAGGGCAAGCTTTCTCAAATGTCAAAAGAAGTGAATGCCAGAATTGAGCCATTTCTGAAGACAACACCACGCCCTCTCAGATTACCTGATGGGCCTCCCTGTTCTCAGCGGTCGAAGTTCTTGCTGATGGATGCCCTCAAATTGAGCATTGAAGACCCGAGCCATGAGGGGGAGGGTATACCGCTGTATGATGCAA<br>TCAAATGCATGAAGACATTTTTTGGCTGGAAAGAGCCTAACATCGTAAAACCGCATGAAAAGGGCATAAACCCTAATTACCTTCTGGCTTGGAAACAGGTGCTGGCAGAACTCCAAGATATTGAAAATGAGGAGAAAATCCCAAAAACAAAGAACATGAAGAAAACAAGCCAATTGAAGTGGGCACTTGGTGAGAACATGGCACCAGAGAAAGTGGACTTTGAGGACTG<br>TAAAGATGTTAGTGATCTAAGACAGTACGACAGTGACGAACCAGAGTCTAGATCACTAGCAAGCTGGATCCAGAGTGAATTCAACAAGGCATGCGAATTGACAGATTCGAGTTGGATTGAACTTGATGAAATAGGGGAAGACATTGCTCCAATTGAACACATTGCGAGTATGAGGAGAAACTATTTCACAGCGGAGGTGTCCCATTGCAGGGCTACTGAATACATAATGA<br>AGGGAGTATACATAAACACAGCCCTGTTGAATGCATCCTGTGCAGCGATGGATGACTTTCAACTGATTCCTATGATAAGCAAGTGCAGAACCAAAGAAGGAAGACGGAAGACAAATTTATATGGATTCATTATAAAAGGAAGATCCCATTTGAGGAATGACACCGATGTGGTGAACTTTGTGAGCATGGAATTCTCTCTCACTGACCCGAGGCTGGAGCCACACAAATGG<br>GAAAAGTACTGTGTTCTTGAGATAGGAGACATGCTCCTACGGACTGCAATAGGCCAAGTGTCAAGGCCCATGTTCTTGTATGTGAGAACCAATGGAACTTCCAAGATCAAAATGAAATGGGGCATGGAGATGAGACGATGCCTTCTTCAGTCCCTTCAACAAATTGAGAGCATGATTGAGGCCGAGTCTTCTGTCAAAGAGAAGGACATGACCAAGGAATTCTTTGAAAAC<br>AAATCAGAAACATGGCCAATTGGGGAATCACCCAAAGGGGTGGAAGAAGGCTCCATTGGGAAGGTGTGCAGAACATTGCTAGCAAAGTCTGTGTTCAACAGCCTATATGCATCTCCACAACTCGAGGGGTTTTCAGCTGAATCAAGAAAATTGCTTCTCATTGTTCAGGCACTTAGGGACAACCTGGAACCTGGAACCTTCGATCTTGGGGGGCTATATGAAGCAATTGA<br>GGAGTGCCTGATTAACGATCCCTGGGTTTTGCTTAATGCGTCTTGGTTCAACTCCTTCCTCACACATGCACTGAAATAG
EPI_ISL_9690841	>A/great_black-backed_gull/Netherlands/22000090-002/2022_H5N1_2022-01-03<br>ATGGAAGACTTTGTGCGACAATGCTTCAATCCAATGATTGTCGAGCTTGCGGAAAAGGCAATGAAAGAGTATGGGGAAGATCCGAAAATCGAAACAAACAAATTTGCCGCAATATGCACACACTTAGAAGTCTGTTTCATGTATTCGGATTTCCACTTTATTGATGAACGAGGCGAATCAACAATTGTAGAATCTGGCGATCCGAATGCATTATTGAAACACCGATTCGAGA<br>TAATTGAAGGGAGAGACCGAACTATGGCCTGGACAGTGGTGAATAGTATCTGCAACACCACAGGAGTCGAAAAGCCCAAATTCCTCCCTGATTTGTATGACTACAAAGAGAACCGATTCATTGAAATTGGAGTAACGCGAAGGGAAGTTCACATATACTATCTAGAGAAAGCCAACAAGATAAAATCAGAGAAGACACACATTCATATATTCTCATTCACTGGAGAGGAA<br>ATGGCCACCAAGGCGGACTACACCCTTGATGAAGAGAGTAGAGCAAGAATAAAAACCAGGCTGTTCACTATAAGACAAGAAATGGCCAGTAGGGGTCTATGGGATTCCTTTCGTCAGTCCGAGAGAGGCGAAGAGACAATTGAAGAAAGATTTGAAATCACAGGAACCATGCGCAGGCTTGCCGACCAAAGTCTCCCACCGAACTTCTCCAGCCTTGAAAACTTTAGAG<br>CCTATGTGGATGGATTCGAACCGAACGGCTGCATTGAGGGCAAGCTTTCTCAAATGTCAAAAGAAGTGAATGCCAGAATTGAGCCATTTCTGAAGACAACACCACGCCCTCTCAGATTACCTGATGGGCCTCCCTGTTCTCAGCGGTCGAAGTTCTTGCTGATGGATGCCCTCAAATTGAGCATCGAAGACCCGAGCCATGAGGGGGAGGGTATACCGCTGTATGATGCAA<br>TCAAATGCATGAAGACATTTTTGGCTGGAAAGAGCCTAACATCGTAAAACCGCATGAAAAGGGCATAAACCCTAATTACCTTCTGGCTTGGAAACAGGTGCTGGCAGAACTCCAAGATATTGAAAATGAGGAGAAAATCCCAAAAACAAAGAACATGAAGAAAACAAGCCAATTGAAGTGGGCACTTGGTGAGAACATGGCACCAGAGAAAGTGGACTTTGAGGACTG<br>TAAAGATGTTAGTGATCTAAGACAGTACGACAGTGACGAACCAGAGTCTAGATCACTAGCAAGCTGGATCCAGAGTGAATTCAACAAGGCATGCGAATTGACAGATTCGAGTTGGATTGAACTTGATGAAATAGGGGAAGACATTGCTCCAATTGAACACATTGCGAGTATGAGGAGAAACTATTTCACAGCGGAGGTGTCCCATTGCAGGGCTACTGAATACATAATGA<br>AGGGAGTATACATAAACACAGCCCTGTTGAATGCATCCTGTGCAGCGATGGATGACTTTCAACTGATTCCTATGATAAGCAAGTGCAGAACCAAAGAAGGAAGACGGAAGACAAATTTATATGGATTCATTATAAAAGGAAGATCCCATTTGAGGAATGACACCGATGTGGTGAACTTTGTGAGCATGGAATTCTCTCTCACTGACCCGAGGCTGGAGCCACACAAATGG<br>GAAAAGTACTGTGTTCTTGAGATAGGAGACATGCTCCTACGGACTGCAATAGGCCAAGTGTCAAGGCCCATGTTCTTGTATGTGAGAACCAATGGAACTTCCAAGATCAAAATGAAATGGGGCATGGAGATGAGACGATGCCTTCTTCAGTCCCTTCAACAAATTGAGAGCATGATTGAGGCCGAGTCTTCTGTCAAAGAGAAGGACATGACCAAGGAATTCTTTGAAAAC<br>AAATCAGAAACATGGCCAATTGGGGAATCACCCAAAGGGGTGGAAGAAGGCTCCATTGGGAAGGTGTGCAGAACATTGCTAGCAAAGTCTGTGTTCAACAGCCTATATGCATCTCCACAACTCGAGGGGTTTTCAGCTGAATCAAGAAAATTGCTTCTCATTGTTCAGGCACTTAGGGACAACCTGGAACCTGGAACCTTCGATCTTGGGGGGCTATATGAAGCAATTGA<br>GGAGTGCCTGATTAACGATCCCTGGGTTTTGCTTAATGCGTCTTGGTTCAACTCCTTCCTCACACATGCACTGAAATAG
EPI_ISL_6959593	>A/quail/Korea/H526/2021_H5N1_2021-11-08<br>ATGGAAGACTTTGTGCGACAATGCTTCAATCCAATGATTGTCGAGCTTGCGGAAAAGGCAATGAAAGAATATGGGGAAGATCCGAAAATCGAGACAAACAAATTTGCCGCAATATGCACACACTTAGAAGTCTGTTTCATGTACTCGGATTTCCATTTTATTGATGAACGAGGCGAATCAGTAATTGTAGAATCTGGCGATCCGAATGCATTATTGAAACACCGATTTGAGA<br>TAATCGAAGGGAGAGACCGAACAATGGCCTGGACAGTGGTGAATAGTATCTGCAACACCACAGGAGTCGAAAAGCCCAAATTCCTCCCTGATTTGTATGACTACAAAGAGAACCGATTCATTGAGATTGGAGTAACGCGAAGGGAGGTCCACATATACTATTTAGAAAAAGCCAACAAGATAAAATCAGAGAAGACACACATTCACATATTCTCATTCACTGGAGAGGAA<br>ATGGCCACCAAGGCGGACTACACCCTTGACGAAGAGAGTAGAGCAAGAATAAAAACCAGGCTATTCACTATAAGACAGGAAATGGCCAGTAGAGGTCTATGGGATTCCTTTCGTCAGTCTGAGAGAGGCGAAGAGACAATTGAAGAAAGATTTGAAATCACAGGAACCATGCGCAGGCTTGCCGACCAAAGTCTCCCACCGAACTTCTCCAGCCTTGAAAACTTTAGAG<br>CCTATGTGGATGGATTCGAACCGAACGGCTGCATTGAGGGCAAGCTTTCTCAAATGTCAAAAGAAGTGAACGCCAGAATTGAACCATTTCTGAAGACAACATCACGCCCTCTCAGATTGCCTGATGGGCCTCCCTGTTCTCAGCGGTCGAAGTTCTTGCTGATGGATGCTCTTAAATTGAGCATCGAAGACCCTAGTCATGAGGGGGAGGGCATACCGCTGTATGACGCAAT<br>CAAATGCATGAAGACATTTTTGGCTGGAAAGAGCCCAACATCGTAAAACCGCATGGGAAAGGCGTAAACCCTAATTACCTCCTGGCTTGGAAGCAGGTGCTGGCAGAACTTCAAGATATTGAAAATGAGGAGAAAATTCCAAAAACAAAGAACATGAAGAAAACAAGCCAATTGAAGTGGGCACTTGGTGAGAACATGGCACCAGAAAAAGTGGACTTTGAGGACTG<br>CAAAGATGTTAGCGATCTAAGACAGTACGACAGTGACGAACCAGAGTCTAGATCACTAGCAAGCTGGATTCAGAGTGAATTCAACAAGGCCTGCGAATTGACAGATTCGAGTTGGACTGAACTTGATGAGATAGGGGAAGACGTTGCTCCAATCGAACACATTGCGAGTATGAGGAGGAACTATTTCACGGCGGAGATATCCCATTGCAGGGCCACTGAGTACATAATG<br>AAGGGAGTATACATAAACACAGCCCTATTGAATGCATCCTGTGCAGCCATGGATGACTTCCAGCTGATTCCAATGATAAGCAAGTGCAGAACCAAAGAAGGAAGACGGAAGACAAATCTGTATGGATTCATTATAAAAGGAAGATCCCATTTGAGGAATGACACCGATGTGGTAAACTTTGTGAGCATGGAATTCTCTCTAACTGACCCGAGGCTAGAGCCACACAAATG<br>GGAAAAGTACTGTGTTCTTGAGGTAGGAGATATGCTCCTACGGACTGCAATAGGCCAAGTGTCGAGGCCCATGTTCCTGTATGTGAGAACCAATGGGACTTCCAAGATCAAAATGAAATGGGGCATGGAGATGAGACGATGCCTTCTACAGTCCCTTCAACAAATTGAGAGCATGATTGAGGCCGAGTCTTCTGTCAAAGAAAAGGACATGACCAAGGAATTCTTTGAAA<br>ACAAATCAGAAACATGGCCAATTGGAGAATCACCCAAAGGGGTGGAGGAAGGCTCCATTGGGAAAGTATGCAGAACATTGCTAGCAAAATCTGTGTTCAACAGCCTATATGCATCTCCACAACTCGAGGGGTTTTCAGCTGAATCAAGAAAATTGCTTCTCATTGTTCAGGCACTTAGGGACAACCTGGAACCTGGAACCTTCGATCTTGGGGGGCTATATGAAGCAATTG<br>AGGAGTGCCTAATTAACGATCCCTGGGTTTTGCTTAATGCGTCTTGGTTCAACTCCTTCCTCACACATGCACTGAAATAG
EPI_ISL_12572659	>A/duck/Hubei/SE220/2022_H5N1_2022-01-10<br>ATGGAAGACTTTGTGCGACAATGCTTCAATCCAATGATTGTCGAGCTTGCGGAAAAGGCAATGAAAGAATATGGGGAAGATCCGAAAATCGAGACAAACAAATTTGCCGCAATATGCACACACTTAGAAGTCTGTTTCATGTATTCGGATTTCCATTTTATTGATGAACGAGGCGAATCAATAATTGTAGAATCTGGCGATCCGAATGCATTATTGAAACACCGATTTGAGA<br>TAATCGAAGGGAGAGACCGAACAATGGCCTGGACAGTGGTGAATAGTATCTGCAACACCACAGGAGTCGGAAAGCCCAAATTCCTCCCTGATTTGTATGACTACAAAGAGAACCGATTCATTGAGATTGGAGTAACGCGAAGGGAGGTTCACATATACTATTTAGAAAAAGCCAACAAGATAAAATCAGAGAAGACACACATTCACATATTCTCATTCACTGGAGAGGAA<br>ATGGCCACCAAGGCGGACTACACCCTTGATGAAGAGAGTAGAGCAAGAATAAAAACCAGGCTATTCACTATAAGACAAGAAATGGCCAGTAGAGGTCTATGGGATTCCTTTCGTCAGTCTGAGAGAGGCGAAGAGACAATTGAAGAAAGATTTGAAATCACAGGAACCATGCGCAGGCTTGCCGACCAAAGTCTCCCACCGAACTTCTCCAGCCTTGAAAACTTTAGAG<br>CCTATGTGGATGGATTCGAACCGAACGGCTGCATTGAGGGCAAGCTTTCTCAAATGTCAAAAGAAGTGAACGCCAGAATTGAACCATTTCTGAAGACAACATCACGCCCTCTCAGATTGCCTGATGGGCCTCCCTGTTCTCAGCGGTCGAAGTTCTTGCTGATGGATGCTCTTAAATTAAGCATCGAAGACCCTAGTCATGAGGGGGAGGGCATACCGCTGTATGACGCAAT<br>CAAATGCATGAAGACATTTTTGGCTGGAAAGAGCCCAACATCGTAAAACCGCATGGGAAGGGCGTAAACCCTAATTACCTCCTGGCTTGGAAGCAGGTGCTGGCAGAACTTCAAGATATTGAAAATGAGGAGAAAATTCCAAAAACAAAGAACATGAAGAAAACAAGCCAATTGAAGTGGGCACTTGGTGAGAACATGGCACCAGAAAAAGTGGACTTTGAGGACTG<br>CAAAGATGTTAGCGATCTAAAACAGTACGACAGTGACGAACCAGAGTCTAGATCACTAGCAAGCTGGATTCAGAGTGAATTCAACAAGGCATGCGAATTGACAGATTCGAGTTGGACTGAACTTGATGAGATAGGGGAAGACGTTGCTCCAATCGAACACATTGCAAGTATGAGGAGGAACTATTTCACGGCGGAGATATCCCATTGCAGGGCCACTGAGTACATAATG<br>AAGGGAGTATACATAAACACAGCCCTATTGAATGCATCCTGTGCAGCCATGGATGACTTCCAGCTGATTCCAATGATAAGCAAGTGCAGAACCAAAGAAGGAAGACGGAAGACAAACCTGTATGGATTCATTATAAAGGGAAGATCCCATTTGAGGAATGACACCGATGTGGTAAACTTTGTGAGCATGGAATTCTCTCTAACTGACCCGAGGCTAGAGCCACACAAATG<br>GGAAAAGTACTGTGTTCTTGAGGTAGGAGATATGCTCCTACGGACTGCAATAGGCCAAGTGTCGAGGCCCATGTTCCTGTATGTGAGAACCAATGGGACTTCCAAGATCAAAATGAAATGGGGCATGGAGATGAGACGATGCCTTCTACAGTCCCTTCAACAAATTGAGAGCATGATTGAGGCCGAGTCTTCAGTCAAAGAAAAGGACATGACCAAGGAATTCTTTGAAA<br>ACAAATCAGAAACATGGCCAATTGGAGAATCACCCAAAGGGGTGGAGGAAGGCTCCATTGGGAAAGTATGCAGAACATTGCTAGCAAAGTCTGTGTTCAACAGCCTATATGCATCTCCACAACTCGAGGGGTTTTCAGCTGAATCAAGAAAATTGCTTCTCATTGTTCAGGCACTTAGGGACAACCTGGAACCTGGAACCTTCGATCTTGGGGGGCTATATGAAGCAATTG<br>AGGAGTGCCTAATTAACGATCCCTGGGTTTTGCTTAATGCGTCTTGGTTCAACTCCTTCCTCACACATGCACTGAAATAG
EPI_ISL_12572655	>A/duck/Guangdong/S4525/2021_H5N1_2021-12-08<br>ATGGAAGACTTTGTGCGACAATGCTTCAATCCAATGATTGTCGAGCTTGCGGAAAAGGCAATGAAAGAATATGGGGAAGATCCGAAAATCGAGACAAACAAATTTGCCGCAATATGCACACACTTAGAAGTCTGTTTCATGTATTCGGATTTCCATTTTATTGATGAACGAGGCGAATCAATAATTGTAGAATCTGGCGATCCGAATGCATTATTGAAACACCGATTTGAGA<br>TAATCGAAGGGAGAGACCGAACAGTGGCCTGGACAGTGGTGAATAGTGTCTGCAACACCACAGGAGTCGAAAAGCCCAAATTCCTCCCTGATTTGTATGACTACAAAGAGAACCGATTCATTGAGATTGGAGTAACGCGAAGGGAGGTTCACATATACTATTTAGAAAAAGCCAACAAGATAAAATCAGAGAAGACACACATTCACATATTCTCATTCACTGGAGAGGAA<br>ATGGCCACCAAGGCGGACTACACCCTTGATGAAGAGAGTAGAGCAAGAATAAAAACCAGGCTATTCACTATAAGACAAGAAATGGCCAGTAGAGGTCTATGGGATTCCTTTCGTCAGTCTGAGAGAGGCGAAGAGACAATTGAAGAAAGATTTGAAATCACAGGAACCATGCGCAGGCTTGCCGACCAAAGTCTCCCACCGAACTTCTCCAGCCTTGAAAACTTTAGAG<br>CCTATGTGGATGGATTCGAACCGAACGGCTGCATTGAGGGCAAGCTTTCTCAAATGTCAAAAGAAGTGAACGCCAGAATTGAACCATTTCTGAAGACAACATCACGCCCTCTCAGATTGCCTGATGGGCCTCCCTGTTCTCAGCGGTCGAAGTTCTTGCTGATGGATGCTCTTAAATTGAGCATCGAAGACCCTAGTCATGAGGGGGAGGGCATACCGCTGTATGACGCAAT<br>CAAATGCATGAAGACATTTTTGGCTGGAAAGAGCCCAACATCGTAAAACCGCATGGGAAAGGCGTAAACCATAATTACCTCCTGGCTTGGAAGCAGGTGCTGGCAGAACTTCAAGATATTGAAAATGAGGAGAAAATTCCAAAAACAAAGAACATGAAGAAAACAAGCCAATTGAAGTGGGCACTTGGTGAGAACATGGCACCAGAAAAAGTGGACTTTGAGGACTG<br>CAAAGATGTTAGCGATTTAAGACAGTACGACAGTGACGAACCAGAGTCTAGATCACTAGCAAGCTGGATTCAGAGTGAATTCAACAAGGCATGCGAATTGACAGATTCGAGTTGGACTGAACTTGATGAGATAGGGGAAGACGTTGCTCCAATCGAACACATTGCAAGTATGAGGAGGAACTATTTCACGGCGGAGATATCCCATTGCAGGGCCACTGAGTACATAATG<br>AAGGGAGTATACATAAACACAGCCCTACTGAATGCATCCTGTGCAGCCATGGATGACTTCCAGCTGATTCCAATGATAAGCAAGTGCAGAACCAAAGAAGGAAGACGGAAGACAAATCTGTATGGATTCATTATAAAAGGAAGATCCCATTTGAGGAATGACACCGATGTGGTAAACTTTGTGAGCATGGAATTCTCTCTAACTGACCCGAGGCTAGAGCCACACAAATG<br>GGAAAAGTACTGTGTTCTTGAGGTAGGAGATATGCTCCTACGGACTGCAATAGGCCAAGTGTCGAGGCCCATGTTCCTGTATGTGAGAACCAATGGGACTTCCAAGATCAAAATGAAATGGGGCATGGAGATGAGACGATGCCTTCTACAGTCCCTTCAACAAATTGAGAGCATGATTGAGGCCGAGTCTTCAGTCAAAGAAAAGGACATGACCAAGGAATTCTTTGAAA<br>ACAAATCAGAAACATGGCCAATTGGAGAATCACCCAAAGGGGTGGAGGAAGGCTCCATTGGGAAAGTATGCAGAACATTGCTAGCAAAGTCTGTGTTCAACAGCCTATATGCATCTCCACAACTCGAGGGGTTTTCAGCTGAATCAAGAAAGTTGCTTCTCATTGTTCAGGCACTTAGGGACAACCTGGAACCTGGAACCTTCGATCTTGGGGGGCTATATGAAGCAATTG<br>AGGAGTGCCTAATTAACGATCCCTGGGTTTTGCTTAATGCGTCTTGGTTCAACTCCTTCCTCACACATGCACTGAAATAG
EPI_ISL_6829533	>A/chicken/Kagoshima/21A6T/2021_H5N1_2021-11-12<br>ATGGAAGACTTTGTGCGACAATGCTTCAATCCAATGATTGTCGAGCTTGCGGAAAAGGCAATGAAAGAATATGGGGAAGATCCGAAAATCGAGACAAACAAATTTGCCGCAATATGCACACACTTAGAAGTCTGTTTCATGTATTCGGATTTCCATTTTATTGATGAACGAGGCGAATCAATAATTGTAGAATCTGGCGATCCGAATGCATTATTGAAACACCGATTTGAGA<br>TAATCGAAGGGAGAGACCGAACAATGGCCTGGACAGTGGTGAATAGTATCTGCAACACCACAGGAGTCGAAAAGCCCAAATTCCTCCCTGATTTGTATGACTACAAAGAGAACCGATTCATTGAGATTGGAGTAACGCGAAGGGAGGTTCACATATACTATTTAGAAAAAGCCAACAAGATAAAATCAGAGAAGACACACATTCACATATTCTCATTCACTGGAGAGGAA<br>ATGGCCACCAAGGCGGACTACACCCTTGATGAAGAGAGTAGAGCAAGAATAAAAACCAGGCTATTCACTATAAGACAAGAAATGGCCAGTAGAGGTCTATGGGATTCCTTTCGTCAGTCTGAGAGAGGCGAAGAGACAATTGAAGAAAGATTTGAAATCACAGGAACCATGCGCAGGCTTGCCGACCAAAGTCTCCCACCGAACTTCTCCAGCCTTGAAAACTTTAGAG<br>CCTATGTGGATGGATTCGAACCGAACGGCTGCATTGAGGGCAAGCTTTCTCAAATGTCAAAAGAAGTGAACGCCAGAATTGAACCATTTCTGAAGACAACATCACGCCCTCTCAGATTGCCTGATGGGCCTCCCTGTTCTCAGCGGTCGAAGTTCTTGCTGATGGATGCTCTTAAATTGAGCATCGAAGACCCTAGTCATGAGGGGGAGGGCATACCGCTGTATGACGCAAT<br>CAAATGCATGAAGACATTTTTGGCTGGAAAGAGCCCAACATCGTAAAACCGCATGGGAAAGGCGTAAACCCTAATTACCTCCTGGCTTGGAAGCAGGTGCTGGCAGAACTTCAAGATATTGAAAATGAGGAGAAAATTCCAAAAACAAAGAACATGAAGAAAACAAGCCAATTGAAGTGGGCACTTGGTGAGAACATGGCACCAGAAAAAGTGGACTTTGAGGACTG<br>CAAAGATGTTAGCGATCTAAGACAGTACGACAGTGACGAACCAGAGTCTAGATCACTAGCAAGCTGGATTCAGAGTGAATTCAACAAGGCATGCGAATTGACAGATTCGAGTTGGACTGAACTTGATGAGATAGGGGAAGACGTTGCTCCAATCGAACACATTGCAAGTATGAGGAGGAACTATTTCACGGCGGAGATATCCCATTGCAGGGCCACTGAGTACATAATG<br>AAGGGAGTATACATAAACACAGCCCTATTGAATGCATCCTGTGCAGCCATGGATGACTTCCAGCTGATCCCAATGATAAGCAAGTGCAGAACCAAAGAAGGAAGACGGAAGACAAATCTGTATGGATTCATTATAAAAGGAAGATCCCATTTGAGGAATGACACCGATGTGGTAAACTTTGTGAGCATGGAATTCTCTCTAACTGACCCGAGGCTAGAGCCACACAAATG<br>GGAAAAGTACTGTGTTCTTGAGGTAGGAGATATGCTCCTACGGACTGCAATAGGCCAAGTGTCGAGGCCCATGTTCCTGTATGTGAGAACCAATGGGACTTCCAAGATCAAAATGAAATGGGGCATGGAGATGAGACGATGCCTTCTACAGTCCCTTCAACAAATTGAGAGCATGATTGAGGCCGAGTCTTCAGTCAAAGAAAAGGACATGACCAAGGAATTCTTTGAAA<br>ACAAATCAGAAACATGGCCAATTGGAGAATCACCCAAAGGGGTGGAGGAAGGCTCCATTGGGAAAGTATGCAGAACATTGCTAGCAAAGTCTGTGTTCAACAGCCTATATGCATCTCCACAACTCGAGGGGTTTTCAGCTGAATCAAGAAAATTGCTTCTCATTGTTCAGGCACTTAGGGACAACCTGGAACCTGGAACCTTCGATCTTGGGGGGCTATATGAAGCAATTG<br>AGGAGTGCCTAATTAACGATCCCTGGGTTTTGCTTAATGCGTCTTGGTTCAACTCCTTCCTCACACATGCACTGAAATAG
EPI_ISL_6959592	>A/mandarin_duck/Korea/WA585/2021_H5N1_2021-10-26<br>ATGGAAGACTTTGTGCGACAATGCTTCAATCCAATGATTGTCGAGCTTGCGGAAAAGGCAATGAAAGAATATGGGGAAGATCCGAAAATCGAGACAAACAAATTTGCCGCAATATGCACACACTTAGAAGTCTGTTTCATGTATTCGGATTTCCATTTTATTGATGAACGAGGCGAATCAATAATTGTAGAATCTGGCGATCCGAATGCATTATTGAAACACCGATTTGAGA<br>TAATCGAAGGGAGAGACCGAACAATGGCCTGGACAGTGGTGAATAGTATCTGCAACACCACAGGAGTCGAAAAGCCCAAATTCCTCCCTGATTTGTATGACTACAAAGAGAACCGATTCATTGAGATTGGAGTAACGCGAAGGGAGGTTCACATATACTATTTAGAAAAAGCCAACAAGATAAAATCAGAGAAGACACACATTCACATATTCTCATTCACTGGAGAGGAA<br>ATGGCCACCAAGGCGGACTACACCCTTGATGAAGAGAGTAGAGCAAGAATAAAAACCAGGCTATTCACTATAAGACAAGAAATGGCCAGTAGAGGTCTATGGGATTCCTTTCGTCAGTCTGAGAGAGGCGAAGAGACAATTGAAGAAAGATTTGAAATCACAGGAACCATGCGCAGGCTTGCCGACCAAAGTCTCCCACCGAACTTCTCCAGCCTTGAAAACTTTAGAG<br>CCTATGTGGATGGATTCGAACCGAACGGCTGCATTGAGGGCAAGCTTTCTCAAATGTCAAAAGAAGTGAACGCCAGAATTGAACCATTTCTGAAGACAACATCACGCCCTCTCAGATTGCCTGATGGGCCTCCCTGTTCTCAGCGGTCGAAGTTCTTGCTGATGGATGCTCTTAAATTAAGCATCGAAGACCCTAGTCATGAGGGGGAGGGCATACCGCTGTATGACGCAAT<br>CAAATGCATGAAGACATTTTTGGCTGGAAAGAGCCCAACATCGTAAAACCGCATGGGAAAGGCGTAAACCCTAATTACCTCCTGGCTTGGAAGCAGGTGCTGGCAGAACTTCAAGATATTGAAAATGAGGAGAAAATTCCAAAAACAAAGAACATGAAGAAAACAAGCCAATTGAAGTGGGCACTTGGTGAGAACATGGCACCAGAAAAAGTGGACTTTGAGGACTG<br>CAAAGATGTTAGCGATCTAAGACAGTACGACAGTGACGAACCAGAGTCTAGATCACTAGCAAGCTGGATTCAGAGTGAATTCAACAAGGCATGCGAATTGACAGATTCGAGTTGGACTGAACTTGATGAGATAGGGGAAGACGTTGCTCCAATCGAACACATTGCAAGTATGAGGAGGAACTATTTCACGGCGGAGATATCCCATTGCAGGGCCACTGAGTACATAATG<br>AAGGGAGTATACATAAACACAGCCCTATTGAATGCATCCTGTGCAGCCATGGATGACTTCCAGCTGATTCCAATGATAAGCAAGTGCAGAACCAAAGAAGGAAGACGGAAGACAAATCTGTATGGATTCATTATAAAAGGAAGATCCCATTTGAGGAATGACACCGATGTGGTAAACTTTGTGAGCATGGAATTCTCTCTAACTGACCCGAGGCTAGAGCCACACAAATG<br>GGAAAAGTACTGTGTTCTTGAGGTAGGAGATATGCTCCTACGGACTGCAATAGGCCAAGTGTCGAGGCCCATGTTCCTGTATGTGAGAACCAATGGGACTTCCAAGATCAAAATGAAATGGGGCATGGAGATGAGACGATGCCTTCTACAGTCCCTTCAACAAATTGAGAGCATGATTGAGGCCGAGTCTTCAGTCAAAGAAAAGGACATGACCAAGGAATTCTTTGAAA<br>ACAAATCAGAAACATGGCCAATTGGAGAATCACCCAAAGGGGTGGAGGAAGGCTCCATTGGGAAAGTATGCAGAACATTGCTAGCAAAGTCTGTGTTCAACAGCCTATATGCATCTCCACAACTCGAGGGGTTTTCAGCTGAATCAAGAAAATTGCTTCTCATTGTTCAGGCACTTAGGGACAACCTGGAACCTGGAACCTTCGATCTTGGGGGGCTATATGAAGCAATTG<br>AGGAGTGCCTAATTAACGATCCCTGGGTTTTGCTTAATGCGTCTTGGTTCAACTCCTTCCTCACACATGCACTGAAATAG
EPI_ISL_12572656	>A/duck/Guizhou/S1321/2022_H5N1_2022-02-22<br>ATGGAAGACTTTGTGCGACAATGCTTCAATCCAATGATTGTCGAGCTTGCGGAAAAGGCAATGAAAGAATATGGGGAAGATCCGAAAATCGAGACAAACAAATTTGCCGCAATATGCACACACTTAGAAGTCTGTTTCATGTATTCGGATTTCCATTTTATTGATGAACGAGGCGAATCAATAATTGTAGAATCTGGCGATCCGAATGCATTATTGAAACACCGATTTGAGA<br>TAATCGAAGGGAGAGACCGAACAATGGCCTGGACAGTGGTGAATAGTATCTGCAACACCACAGGAGTCGAAAAGCCCAAATTCCTCCCTGATTTGTATGACTACAAAGAGAACCGATTCATTGAGATTGGAGTAACGCGAAGGGAGGTTCACATATACTATTTAGAAAAAGCCAACAAGATAAAATCAGAGAAGACACACATTCACATATTCTCATTCACTGGAGAGGAA<br>ATGGCCACCAAGGCGGACTACACCCTTGATGAAGAGAGTAGAGCAAGAATAAAAACCAGGCTATTCACTATAAGACAAGAAATGGCCAGTAGAGGTCTATGGGATTCCTTTCGTCAGTCTGAGAGAGGCGAAGAGACAATTGAAGAAAGATTTGAAATCACAGGAACCATGCGCAGGCTTGCCGACCAAAGTCTCCCACCGAACTTCTCCAGCCTTGAAAACTTTAGAG<br>CCTATGTGGATGGATTCGAACCGAACGGCTGCATTGAGGGCAAGCTTTCTCAAATGTCAAAAGAAGTGAACGCCAGAATTGAACCATTTCTGAAGACAACATCACGCCCTCTCAGATTGCCTGATGGGCCTCCCTGTTCTCAGCGGTCGAAGTTCTTGCTGATGGATGCTCTTAAATTGAGCATCGAAGACCCTAGTCATGAGGGGGAGGGCATACCGCTGTATGACGCAAT<br>CAAATGCATGAAGACATTTTTGGCTGGAAAGAGCCCAACATCGTAAAACCGCATGGGAAAGGCGTAAACCCTAATTACCTCCTGGCTTGGAAGCAGGTGCTGGCAGAACTTCAAGATATTGAAAATGAGGAGAAAATTCCAAAAACCAAGAACATGAAGAAAACAAGCCAATTGAAGTGGGCACTTGGTGAGAACATGGCACCAGAAAAAGTGGACTTTGAGGACTG<br>CAAAGATGTTAGCGATCTAAGACAGTACGACAGTGACGAACCAGAGTCTAGATCACTAGCAAGCTGGATTCAGAGTGAATTCAACAAGGCATGCGAATTGACAGATTCGAGTTGGACTGAACTTGATGAGATAGGGGAAGACGTTGCTCCAATCGAACACATTGCAAGTATGAGGAGGAACTATTTCACGGCGGAGATATCCCATTGCAGGGCCACTGAGTACATAATG<br>AAGGGAGTATACATAAACACAGCCCTATTGAATGCATCCTGTGCAGCCATGGATGACTTCCAGCTGATTCCAATGATAAGCAAGTGCAGAACCAAAGAAGGAAGACGGAAGACAAATCTGTATGGATTCATTATAAAAGGAAGATCCCATTTGAGGAATGACACCGATGTGGTAAACTTTGTGAGCATGGAATTCTCTCTAACTGACCCGAGGCTAGAGCCACACAAATG<br>GGAAAAGTACTGTGTTCTTGAGGTAGGAGATATGCTCCTACGGACTGCAATAGGCCAAGTGTCGAGGCCCATGTTCCTGTATGTGAGAACCAATGGGACTTCCAAGATCAAAATGAAATGGGGCATGGAGATGAGACGATGCCTTCTACAGTCCCTTCAACAAATTGAGAGTATGATTGAGGCCGAGTCTTCAGTCAAAGAAAAGGACATGACCAAGGAATTCTTTGAAA<br>ACAAATCAGAAACATGGCCAATTGGAGAATCACCCAAAGGGGTGGAGGAAGGCTCCATTGGGAAAGTATGCAGAACATTGCTAGCAAAGTCTGTGTTCAACAGCCTATATGCATCTCCACAACTCGAGGGGTTTTCAGCTGAATCAAGAAAATTGCTTCTCATTGTTCAGGCACTTAGGGACAACCTGGAACCTGGAACCTTCGATCTTGGGGGGCTATATGAAGCAATTG<br>AGGAGTGCCTAATTAACGATCCCTGGGTTTTGCTTAATGCGTCTTGGTTCAACTCCTTCCTCACACATGCACTGAAATAG
EPI_ISL_11007532	>A/swan/Slovenia/2072_22VIR777-5/2021_H5N1_2021-12-30<br>ATGGAAGACTTTGTGCGACAATGCTTCAATCCAATGATTGTCGAGCTTGCGGAAAAGGCAATGAAAGAATATGGGGAAGATCCGAAAATCGAGACAAACAAATTTGCCGCAATATGCACACACTTAGAAGTCTGTTTCATGTATTCGGATTTCCATTTTATTGATGAACGAGGCGAATCAATAATTGTAGAATCTGGCGATCCGAATGCATTATTGAAACACCGATTTGAAA<br>TAATCGAAGGGAGAGACCGAACAATGGCCTGGACAGTGGTGAATAGTATCTGCAACACCACAGGAGTCGAAAAGCCCAAATTCCTCCCTGATTTGTATGACTACAAAGAAAACCGATTCATTGAGATTGGAGTAACGCGGAGGGAAGTTCACATATACTATTTAGAAAAAGCCAACAAGATAAAATCAGAGAAGACACACATTCACATATTCTCATTCACTGGAGAGGAA<br>ATGGCCACCAAGGCGGACTACACCCTTGATGAAGAGAGCAGAGCAAGAATAAAAACCAGACTGTTCACTATAAGACAAGAAATGGCCAGTAGAGGTCTATGGGATTCCTTTCGTCAGTCCGAGAGAGGCGAAGAGACAATTGAAGAAAGATTTGAAATCACAGGAACCATGCGCAGGCTTGCCGACCAAAGTCTCCCACCGAACTTCTCCAGCCTTGAAAACTTTAGAG<br>CCTATGTGGATGGATTCGAACCGAACGGCTGCATTGAGGGCAAGCTTTCTCAAATGTCAAAAGAAGTGAACGCCAGAATTGAGCCATTTCTGAAGACAACACCACGCCCTCTCAAATTACCTGATGGGCCTCCCTGTTCTCAGCGGTCGAAGTTCTTGCTAATGGATGCCCTTAAACTGAGCATCGAAGACCCTAGCCATGAGGGGGAGGGCATACCGCTGTATGATGCAA<br>TCAAATGCATGAAGACATTTTTGGCTGGAAAGAGCCCAACATCGTAAAAACGCATGAGAAAGGCATAAACCCTAATTACCTCCTGGCTTGGAAGCAGGTGCTGGCAGAACTTCAAGATATTGAAAATGAGGAGAAAATTCCAAAAACAAAGAACATGAAGAAAACAAGTCAATTGAAGTGGGCACTTGGTGAGAACATGGCACCAGAAAAAGTGGACTTTGAGGACTG<br>CAAAGATGTTAGCGATCTAAGACAGTATGACAGTGACGAACCAGAGTCTAGATCACTAGCAAGCTGGATTCAGAGTGAATTCAACAAGGCATGCGAATTGACAGATTCGAGTTGGATTGAGCTTGATGAGATAGGGGAAGACGTTGCTCCAATCGAACACATTGCGAGTATGAGGAGGAACTATTTCACAGCAGAGGTATCCCATTGCAGGGCCACTGAGTACATAATG<br>AAGGGAGTATACATAAACACAGCCCTATTGAATGCATCCTGTGCAGCCATGGATGACTTCCAACTGATTCCAATGATAAGCAAGTGCAGGACCAAAGAAGGAAGACGGAAGACAAATCTGTATGGGTTCATTATAAAAGGAAGATCCCACTTGAGGAATGACACCGATGTGGTAAACTTTGTGAGCATGGAATTCTCTCTAACTGACCCGAGGCTAGAGCCACACAAATG<br>GGAAAAGTACTGTGTTCTTGAGATAGGAGACATGCTCCTACGGACTGCAATAGGCCAAGTGTCGAGGCCCATGTTCCTATATGTGAGAACCAATGGGACTTCCAAGATCAAAATGAAATGGGGCATGGAGATGAGACGATGCCTTCTTCAGTCCCTTCAACAAATTGAGAGCATGATTGAGGCCGAGTCTTCTGTCAAAGAGAAGGACATGACCAAGGAATTCTTTGAAA<br>ACAAATCAGAGACATGGCCAATTGGAGAATCACCCAAAGGGGTGGAGGAAGGCTCCATTGGAAAAGTATGCAGAACATTGCTAGCAAAGTCTGTGTTCAACAGCCTATATGCATCTCCACAACTCGAGGGGTTTTCAGCTGAGTCAAGAAAATTGCTTCTCATTGTTCAGGCACTTAGGGACAACCTGGAACCTGGAACCTTCGATCTTGGGGGGCTATATGAAGCAATTG<br>AGGAGTGCCTGATTAATGATCCCTGGGTTTTGCTTAATGCGTCTTGGTTCAACTCCTTCCTCACACATGCACTGAAATAG
EPI_ISL_11007534	>A/seagull/Slovenia/2075_22VIR777-7/2021_H5N1_2021-12-30<br>ATGGAAGACTTTGTGCGACAATGCTTCAATCCAATGATTGTCGAGCTTGCGGAAAAGGCAATGAAAGAATATGGGGAAGATCCGAAAATCGAGACAAACAAATTTGCCGCAATATGCACACACTTAGAAGTCTGTTTCATGTATTCGGATTTCCATTTTATTGATGAACGAGGCGAATCAATAGTTGTAGAATCTGGCGATCCGAATGCATTATTGAAACACCGATTTGAAA<br>TAATCGAAGGGAGAGACCGAACAATGGCCTGGACAGTGGTGAATAGTATCTGCAACACCACAGGAGTCGAAAAGCCCAAATTCCTCCCTGATTTGTATGACTACAAAGAAAACCGATTCATTGAGATTGGAGTAACGCGGAGGGAAGTTCACATATACTATTTAGAAAAAGCCAACAAGATAAAATCAGAGAAGACACACATTCACATATTCTCATTCACTGGAGAGGAA<br>ATGGCCACCAAGGCGGACTACACCCTTGATGAAGAGAGCAGAGCAAGAATAAAAACCAGACTGTTCACTATAAGACAAGAAATGGCCAGTAGAGGTCTATGGGATTCCTTTCGTCAGTCCGAGAGAGGCGAAGAGACAATTGAAGAAAGATTTGAAATCACAGGAACCATGCGCAGGCTTGCCGACCAAAGTCTCCCACCGAACTTCTCCAGCCTTGAAAACTTTAGAG<br>CCTATGTGGATGGATTCGAACCGAACGGCTGCATTGAGGGCAAGCTTTCTCAAATGTCAAAAGAAGTGAACGCCAGAATTGAGCCATTTCTGAAGACAACACCACGCCCTCTCAAATTACCTGATGGGCCTCCCTGTTCTCAGCGGTCGAAGTTCTTGCTAATGGATGCCCTTAAACTGAGCATCGAAGACCCTAGCCATGAGGGGGAGGGCATACCGCTGTATGATGCAA<br>TCAAATGCATGAAGACATTTTTGGCTGGAAAGAGCCCAACATCGTAAAAACGCATGAGAAAGGCATAAACCCTAATTACCTCCTGGCTTGGAAGCAGGTGCTGGCAGAACTTCAAGATATTGAAAATGAGGAGAAAATTCCAAAAACAAAGAACATGAAGAAAACAAGTCAATTGAAGTGGGCACTTGGTGAGAACATGGCACCAGAAAAAGTGGACTTTGAGGACTG<br>CAAAGATGTTAGCGATCTAAGACAGTATGACAGTGACGAACCAGAGTCTAGATCACTAGCAAGCTGGATTCAGAGTGAATTCAACAAGGCATGCGAATTGACAGATTCGAGTTGGATTGAGCTTGATGAGATAGGGGAAGACGTTGCTCCAATCGAACACATTGCGAGTATGAGGAGGAACTATTTCACAGCAGAGGTATCCCATTGCAGGGCCACTGAGTACATAATG<br>AAGGGAGTATACATAAACACAGCCCTATTGAATGCATCCTGTGCAGCCATGGATGACTTCCAACTGATTCCAATGATAAGCAAGTGCAGGACCAAAGAAGGAAGACGGAAGACAAATCTGTATGGGTTCATTATAAAAGGAAGATCCCACTTGAGGAATGACACCGATGTGGTAAACTTTGTGAGCATGGAATTCTCTCTAACTGACCCGAGGCTAGAGCCACACAAATG<br>GGAAAAGTACTGTGTTCTTGAGATAGGAGACATGCTCCTACGGACTGCAATAGGCCAAGTGTCGAGGCCCATGTTCCTATATGTGAGAACCAATGGGACTTCCAAGATCAAAATGAAATGGGGCATGGAGATGAGACGATGCCTTCTTCAGTCCCTTCAACAAATTGAGAGCATGATTGAGGCCGAGTCTTCTGTCAAAGAGAAGGACATGACCAAGGAATTCTTTGAAA<br>ACAAATCAGAGACATGGCCAATTGGAGAATCACCCAAAGGGGTGGAGGAAGGCTCCATTGGAAAAGTATGCAGAACATTGCTAGCAAAGTCTGTGTTCAACAGCCTATATGCATCTCCACAACTCGAGGGGTTTTCAGCTGAGTCAAGAAAATTGCTTCTCATTGTTCAGGCACTTAGGGACAACCTGGAACCTGGAACCTTCGATCTTGGGGGGCTATATGAAGCAATTG<br>AGGAGTGCCTGATTAATGATCCCTGGGTTTTGCTTAATGCGTCTTGGTTCAACTCCTTCCTCACACATGCACTGAAATAG
EPI_ISL_14760636	>A/chicken/Italy/21VIR10146/2021_H5N1_2021-11-25<br>ATGGAAGACTTTGTGCGACAATGCTTCAATCCAATGATTGTCGAGCTTGCGGAAAAAGCAATGAAAGAATATGGGGAAGATCCAAAAATCGAGACAAACAAATTTGCCGCAATATGCACACACTTAGAAGTCTGTTTCATGTATTCGGATTTCCATTTTATTGATGAACGAGGCGAATCAATGATTGTAGAATCTGGCGATCCAAATGCATTATTGAAACACCGATTTGAGA<br>TAATCGAAGGGAGAGACCGAGCAATGGCCTGGACAGTGGTGAATAGTATCTGCAACACCACAGGAGTCGAAAAGCCCAAATTCCTCCCTGATTTGTATGACTACAAAGAGAACCGATTCATTGAAATTGGAGTAACGCGAAGGGAAGTTCACATATACTATTTAGAAAAAGCCAACAAGATAAAATCGGAGAAAACACACATTCACATATTCTCATTCACTGGAGAGGAA<br>ATGGCCACCAAGGCGGAATACACCCTTGATGAAGAGAGCAGAGCAAGAATAAAAACCAGACTGTTCACTATAAGACAAGAAATGGCCAGTAGAGGTCTATGGGATTCCTTTCGTCAGTCCGAGAGAGGCGAAGAGACAATTGAAGAAAGATTTGAAATCACAGGAACCATGCGCAGGCTTGCCGACCAAAGTCTCCCACCGAACTTCTCCAGCCTTGAAAACTTTAGAG<br>CCTATGTGGATGGATTCGAACCGAACGGCTGCATTGAGGGCAAGCTTTCTCAAATGTCAAAAGAAGTGAACGCCAGAATTGAGCCATTCCTGAAGACAACACCACGCCCTCTCAGATTACCTGATGGGCCTCCTTGTTCTCAGCGGTCGAAGTTCTTGCTGATGGATGCCCTTAAGTTGAGCATCGAAGACCCTAGCCATGAGGGGGAGGGCATACCGCTGTATGATGCAA<br>TCAAATGCATGAAGACATTTTTGGCTGGAAAGAGCCCAACCTCGTAAAGCCGCATGAGAAAGGCATAAACCCTAATTACCTCCTGGCTTGGAAGCAGGTGCTGGCAGAACTTCAAGATATTGAGAATGAGGAGAAATTTCCAAAAACAAAGAACATGAAGAAAACAAGCCAATTGAAGTGGGCACTTGGTGAGAACATGGCACCAGAAAAAGTGGACTTTGAGGACTG<br>CAAAGATGTTAGCGATCTAAGACAGTACGACAGTGACGAACCAGAGCCTAGATCACTAGCAAGCTGGATTCAGAGTGAATTCAACAAGGCATGCGAATTGACAGATTCGAGTTGGATTGAACTTGATGAGATAGGGGAAGACGTTGCTCCAATCGAACACATTGCGAGTATGAGGAGGAACTATTTCACAGCGGAGGTATCCCATTGCAGGGCCACTGAATACATAATG<br>AAGGGAGTATACATAAACACAGCCCTATTGAATGCATCCTGTGCAGCCATGGATGACTTCCAACTGATTCCAATGATAAGCAAGTGCAGAACCAAAGAAGGAAGACGGAAGACAAATCTGTATGGATTCATTATAAAAGGAAGATCCCATTTGAGGAATGACACCGATGTGGTAAACTTTGTGAGCATGGAATTCTCTCTAACTGACCCGAGGCTAGAGCCACACAAATG<br>GGAAAAGTACTGTGTTCTTGAGATAGGAGACATGCTCCTACGGACTGCAATAGGCCAAGTGTCGAGGCCCATGTTCCTGTATGTGAGAACCAATGGGACTTCCAAGATCAAAATGAAATGGGGCATGGAGATGAGACGATGCCTTCTTCAGTCCCTTCAACAAATTGAGAGCATGATTGAGGCCGAATCTTCTGTCAAAGAGAAGGACATGACCAAGGAATTCTTTGAAA<br>ACAAATCAGAAACATGGCCAATTGGAGAATCACCCAAAGGGGTTGAGGAAGGCTCTATTGGGAAAGTATGCAGAACATTGCTAGCAAAGTCTGTGTTCAACAGCCTATATGCATCTCCACAACTCGAGGGATTTTCAGCTGAATCAAGAAAATTGCTTCTCATTGTTCAGGCACTTAGGGACAACCTGGAACCTGGAACCTTCGATCTTGGGGGGCTATATGAAGCAATTG<br>AGGAGTGCCTGATTAACGATCCCTGGGTTTTGCTTAATGCGTCTTGGTTCAACTCCTTCCTCACACATGCACTGAAATAG
EPI_ISL_14760633	>A/Guinea_fowl/Italy/21VIR10231/2021_H5N1_2021-11-24<br>ATGGAAGACTTTGTGCGACAATGCTTCAATCCAATGATTGTCGAGCTTGCGGAAAAAGCAATGAAAGAATATGGGGAAGATCCGAAAATCGAGACAAACAAATTTGCCGCAATATGCACACACTTAGAAGTCTGTTTCATGTATTCGGATTTCCATTTTATTGATGAACGAGGCGAATCAATGATTGTAGAATCTGGCGATCCAAATGCATTATTGAAACACCGATTTGAGA<br>TAATCGAAGGGAGAGACCGAGCAATGGCCTGGACAGTGGTGAATAGTATCTGCAACACCACAGGAGTCGAAAAGCCCAAATTCCTCCCTGATTTGTATGACTACAAAGAGAACCGATTCATTGAAATTGGAGTAACGCGAAGGGAAGTTCACATATACTATTTAGAAAAAGCCAACAAGATAAAATCGGAGAAAACACACATTCACATATTCTCATTCACTGGAGAGGAA<br>ATGGCCACCAAGGCGGAATACACCCTTGATGAAGAGAGCAGAGCAAGAATAAAAACCAGACTGTTCACTATAAGACAAGAAATGGCCAGTAGAGGTCTATGGGATTCCTTTCGTCAGTCCGAGAGAGGCGAAGAGACAACTGAAGAAAGATTTGAAATCACAGGAACCATGCGCAGGCTTGCCGACCAAAGTCTCCCACCGAACTTCTCCAGCCTTGAAAACTTTAGAG<br>CCTATGTGGATGGATTCGAACCGAACGGCTGCATTGAGGGCAAGCTTTCTCAAATGTCAAAAGAAGTGAACGCCAGAATTGAGCCATTCCTGAAGACAACACCACGCCCTCTCAGATTACCTGATGGGCCTCCTTGTTCTCAGCGGTCGAAGTTCTTGCTGATGGATGCCCTTAAGTTGAGCATCGAAGACCCTAGCCATGAGGGGGAGGGCATACCGCTGTATGATGCAA<br>TCAAATGCATGAAGACATTTTTGGCTGGAAAGAGCCCAACCTCGTAAAGCCGCATGAGAAAGGCATAAACCCTAATTACCTCCTGGCTTGGAAGCAGGTGCTGGCAGAACTTCAAGATATTGAGAATGAGGAGAAATTTCCAAAAACAAAGAACATGAAGAAAACAAGCCAATTGAAGTGGGCACTTGGTGAGAACATGGCACCAGAAAAAGTGGACTTTGAGGACTG<br>CAAAGATGTTAGCGATCTAAGACAGTACGACAGTGACGAACCAGAGCCTAGATCACTAGCAAGCTGGATTCAGAGTGAATTCAACAAGGCATGCGAATTGACAGATTCGAGTTGGATTGAACTTGATGAGATAGGGGAAGACGTTGCTCCAATCGAACACATTGCGAGTATGAGGAGGAACTATTTCACAGCGGAGGTATCCCATTGCAGGGCCACTGAATACATAATG<br>AAGGGAGTATACATAAACACAGCCCTATTGAATGCATCCTGTGCAGCCATGGATGACTTCCAACTGATTCCAATGATAAGCAAGTGCAGAACCAAAGAAGGAAGACGGAAGACAAATCTGTATGGATTCATTATAAAAGGAAGATCCCATTTGAGGAATGACACCGATGTGGTAAACTTTGTGAGCATGGAATTCTCTCTAACTGACCCGAGGCTAGAGCCACACAAATG<br>GGAAAAGTACTGTGTTCTTGAGATAGGAGACATGCTCCTACGGACTGCAATAGGCCAAGTGTCGAGGCCCATGTTCCTGTATGTGAGAACCAATGGGACTTCCAAGATCAAAATGAAATGGGGCATGGAGATGAGACGATGCCTTCTTCAGTCCCTTCAACAAATTGAGAGCATGATTGAGGCCGAATCTTCTGTCAAAGAGAAGGACATGACCAAGGAATTCTTTGAAA<br>ACAAATCAGAAACATGGCCAATTGGAGAATCACCCAAAGGGGTTGAGGAAGGCTCTATTGGGAAAGTATGCAGAACATTGCTAGCAAAGTCTGTGTTCAACAGCCTATATGCATCTCCACAACTCGAGGGATTTTCAGCTGAATCAAGAAAATTGCTTCTCATTGTTCAGGCACTTAGGGACAACCTGGAACCTGGAACCTTCGATCTTGGGGGGCTATATGAAGCAATTG<br>AGGAGTGCCTGATTAACGATCCCTGGGTTTTGCTTAATGCGTCTTGGTTCAACTCCTTCCTCACACATGCACTGAAATAG
EPI_ISL_14761152	>A/turkey/Italy/21VIR11661-7/2021_H5N1_2021-12-26<br>ATGGAAGACTTTGTGCGACAATGCTTCAATCCAATGATTGTCGAGCTTGCGGAAAAAGCAATGAAAGAATATGGGGAAGATCCGAAAATCGAGACAAACAAATTTGCCGCAATATGCACACACTTAGAAGTCTGTTTCATGTATTCGGATTTCCATTTTATTGATGAACGAGGCGAATCAATGATTGTAGAATCTGGCGATCCAAATGCATTATTGAAACACCGATTTGAGA<br>TAATCGAAGGGAGAGACCGAGCAATGGCCTGGACAGTGGTGAATAGTATCTGCAACACCACAGGAGTCGAAAAGCCCAAATTCCTCCCTGATTTGTATGACTACAAAGAGAACCGATTCATTGAAATTGGAGTAACGCGAAGGGAAGTTCACATATACTATTTAGAAAAAGCCAACAAGATAAAATCGGAGAAAACACACATTCACATATTCTCATTCACTGGAGAGGAA<br>ATGGCCACCAAGGCGGAATACACCCTTGATGAAGAGAGCAGAGCAAGAATAAAAACCAGACTGTTCACTATAAGACAAGAAATGGCCAGTAGAGGTCTATGGGATTCCTTTCGTCAGTCCGAGAGAGGCGAAGAGACAACTGAAGAAAGATTTGAAATCACAGGAACCATGCGCAGGCTTGCCGACCAAAGTCTCCCACCGAACTTCTCCAGCCTTGAAAACTTTAGAG<br>CCTATGTGGATGGATTCGAACCGAACGGCTGCATTGAGGGCAAGCTTTCTCAAATGTCAAAAGAAGTGAACGCCAGAATTGAGCCATTCCTGAAGACAACACCACGCCCTCTCAGATTACCTGATGGGCCTCCTTGTTCTCAGCGGTCGAAGTTCTTGCTGATGGATGCCCTTAAGTTGAGCATCGAAGACCCTAGCCATGAGGGGGAGGGCATACCGCTGTATGATGCAA<br>TCAAATGCATGAAGACATTTTTGGCTGGAAAGAGCCCAACCTCGTAAAGCCGCATGAGAAAGGCATAAACCCTAATTACCTCCTGGCTTGGAAGCAGGTGCTGGCAGAACTTCAAGATATTGAGAATGAGGAGAAATTTCCAAAAACAAAGAACATGAAGAAAACAAGCCAATTGAAGTGGGCACTTGGTGAGAACATGGCACCAGAAAAAGTGGACTTTGAGGACTG<br>CAAAGATGTTAGCGATCTAAGACAGTACGACAGTGACGAACCAGAGCCTAGATCACTAGCAAGCTGGATTCAGAGTGAATTCAACAAGGCATGCGAATTGACAGATTCGAGTTGGATTGAACTTGATGAGATAGGGGAAGACGTTGCTCCAATCGAACACATTGCGAGTATGAGGAGGAACTATTTCACAGCGGAGGTATCCCATTGCAGGGCCACTGAATACATAATG<br>AAGGGAGTATACATAAACACAGCCCTATTGAATGCATCCTGTGCAGCCATGGATGACTTCCAACTGATTCCAATGATAAGCAAGTGCAGAACCAAAGAAGGAAGACGGAAGACAAATCTGTATGGATTCATTATAAAAGGAAGATCCCATTTGAGGAATGACACCGATGTGGTAAACTTTGTGAGCATGGAATTCTCTCTAACTGACCCGAGGCTAGAGCCACACAAATG<br>GGAAAAGTACTGTGTTCTTGAGATAGGAGACATGCTCCTACGGACTGCAATAGGCCAAGTGTCGAGGCCCATGTTCCTGTATGTGAGAACCAATGGGACTTCCAAGATCAAAATGAAATGGGGCATGGAGATGAGACGATGCCTTCTTCAGTCCCTTCAACAAATTGAGAGCATGATTGAGGCCGAATCTTCTGTCAAAGAGAAGGACATGACCAAGGAATTCTTTGAAA<br>ACAAATCAGAAACATGGCCAATTGGAGAATCACCCAAAGGGGTTGAGGAAGGCTCTATTGGGAAAGTATGCAGAACATTGCTAGCAAAGTCTGTGTTCAACAGCCTATATGCATCTCCACAACTCGAGGGATTTTCAGCTGAATCAAGAAAATTGCTTCTCATTGTTCAGGCACTTAGGGACAACCTGGAACCTGGAACCTTCGATCTTGGGGGGCTATATGAAGCAATTG<br>AGGAGTGCCTGATTAACGATCCCTGGGTTTTGCTTAATGCGTCTTGGTTCAACTCCTTCCTCACACATGCACTGAAATAA

EPI_ISL_9603922	>A/mute_swan/Czech_Republic/22684/2021_H5N1_2021-11-15<br>ATGGAAGACTTTGTGCGACAATGCTTCAATCCAATGATTGTCGAGCTTGCGGAAAAAGCAATGAAAGAATATGGGGAAGATCCGAAAATCGAGACAAACAAATTTGCCGCAATATGCACACACTTAGAAGTCTGTTTCATGTATTCGGATTTCCATTTTATTGATGAACGAGGCGAATCAACGATTGTAGAATCTGGCGATCCAAATGCATTATTGAAACACCGATTTGAGA<br>TAATCGAAGGGAGAGATCGAGCAATGGCCTGGACAGTGGTGAATAGTATCTGCAACACCACAGGAGTCGAAAAGCCCAAATTCCTCCCTGATTTGTATGACTACAAAGAGAACCGATTCATTGAAATTGGAGTAACGCGAAGGGAAGTTCACATATACTATTTAGAAAAAGCCAACAAGATAAAATCAGAGAAAACACACATTCACATATTCTCATTCACTGGAGAGGAA<br>ATGGCCACCAAGGCGGAATACACCCTTGATGAAGAGAGCAGAGCAAGAATAAAAACCAGACTGTTCACTATAAGACAAGAAATGGCCAGTAGAGGTCTATGGGATTCCTTTCGTCAGTCCGAAAGAGGCGAAGAGACAACTGAAGAAAGATTTGAAATCACAGGAACCATGCGCAGGCTTGCCGACCAAAGCCTCCCACCGAACTTCTCCAGCCTTGAAAACTTTAGAG<br>CCTATGTGGATGGATTCGAACCGAACGGCTGCATTGAGGGCAAGCTTTCTCAAATGTCAAAAGAAGTGAACGCCAGAATTGAGCCATTCCTGAAGACAACACCACGCCCTCTCAGATTACCTGATGGGCCTCCTTGTTCTCAGCGGTCGAAGTTCTTGCTGATGGATGCCCTTAAGTTGAGCATCGAAGACCCTAGCCATGAGGGGGAGGGCATACCGCTGTATGATGCAA<br>TCAAATGCATGAAGACATTTTTTGGCTGGAAAGAGCCCAACCTCGTAAAACCGCATGAGAAAGGCATAAACCCTAATTACCTCCTGGCTTGGAAGCAGGTGCTGGCAGAACTTCAAGATATTGAGAATGAGGAGAAATTTCCAAAAACAAAGAACATGAAGAAAACAAGCCAATTGAAGTGGGCACTTGGTGAGAACATGGCACCAGAAAAAGTGGACTTTGAGGACTG<br>CAAAGATGTTAGCGATCTAAGACAGTACGACAGTGACGAACCAGAGCCTAGATCACTGGCAAGCTGGATTCAGAGTGAATTCAACAAGGCATGCGAATTGACAGATTCGAGTTGGATTGAACTTGATGAGATAGGGGAAGACGTTGCTCCAATCGAACACATTGCGAGTATGAGGAGGAACTATTTCACAGCGGAGGTATCCCATTGCAGGGCCACTGAATACATAATG<br>AAGGGAGTATACATAAACACAGCCCTATTGAATGCATCCTGTGCAGCCATGGATGACTTCCAACTGATTCCAATGATAAGCAAGTGCAGAACCAAAGAGGGAAGACGGAAGACAAATCTGTATGGATTCATTATAAAAGGAAGATCCCATTTGAGGAATGACACCGATGTGGTGAACTTTGTGAGCATGGAATTCTCTCTAACTGACCCGAGGCTAGAGCCACACAAATG<br>GGAAAAGTACTGTGTTCTTGAGATAGGAGACATGCTCCTACGGACTGCAATAGGCCAAGTGTCGAGGCCCATGTTCCTGTATGTGAGAACCAATGGGACTTCCAAGATCAAAATGAAATGGGGCATGGAGATGAGACGATGCCTTCTTCAGTCCCTTCAACAAATTGAGAGCATGATTGAGGCCGAATCTTCTGTCAAAGAGAAGGACATGACCAAGGAATTCTTTGAAA<br>ACAAATCAGAAACATGGCCAATTGGAGAATCACCCAAAGGGGTTGAGGAAGGCTCTATTGGGAAAGTATGCAGAACATTGCTAGCAAAGTCTGTGTTCAACAGCCTATATGCTTCTCCACAACTCGAGGGATTTTCAGCTGAATCAAGAAAATTGCTTCTCATTGTTCAGGCACTTAGGGACAACCTGGAACCTGGAACCTTCGATCTTGGGGGGCTATATGAAGCAATTG<br>AGGAGTGCCTGATTAACGATCCCTGGGTTTTGCTTAATGCGTCTTGGTTCAACTCCTTCATCACACATGCACTGAAATAG
EPI_ISL_9603939	>A/pheasant/Czech_Republic/25827-3/2021_H5N1_2021-12-25<br>ATGGAAGACTTTGTGCGACAATGCTTCAATCCAATGATTGTCGAGCTTGCGGAAAAAGCAATGAAAGAATATGGGGAAGATCCGAAAATCGAGACAAACAAATTTGCCGCAATATGCACACACTTAGAAGTCTGTTTCATGTATTCGGATTTCCATTTTATTGATGAACGAGGCGAATCAACGATTGTAGAATCTGGCGATCCAAATGCATTATTGAAACACCGATTTGAGA<br>TAATCGAAGGGAGAGATCGAGCAATGGCCTGGACAGTGGTGAATAGTATCTGCAACACCACAGGAGTCGAAAAGCCCAAATTCCTCCCTGATTTGTATGACTACAAAGAGAACCGATTCATTGAAATTGGAGTAACGCGAAGGGAAGTTCACATATACTATTTAGAAAAAGCCAACAAGATAAAATCAGAGAAAACACACATTCACATATTCTCATTCACTGGAGAGGAA<br>ATGGCCACCAAGGCGGAATACACCCTTGATGAAGAGAGCAGAGCAAGAATAAAAACCAGACTGTTCACTATAAGACAAGAAATGGCCAGTAGAGGTCTATGGGATTCCTTTCGTCAGTCCGAAAGAGGCGAAGAGACAACTGAAGAAAGATTTGAAATCACAGGAACCATGCGCAGGCTTGCCGACCAAAGCCTCCCACCGAACTTCTCCAGCCTTGAAAACTTTAGAG<br>CCTATGTGGATGGATTCGAACCGAACGGCTGCATTGAGGGCAAGCTTTCTCAAATGTCAAAAGAAGTGAACGCCAGAATTGAGCCATTCCTGAAGACAACACCACGCCCTCTCAGATTACCTGATGGGCCTCCTTGTTCTCAGCGGTCGAAGTTCTTGCTGATGGATGCCCTTAAGTTGAGCATCGAAGACCCTAGCCATGAGGGGGAGGGCATACCGCTGTATGATGCAA<br>TCAAATGCATGAAGACATTTTTTGGCTGGAAAGAGCCCAACCTCGTAAAACCGCATGAGAAAGGCATAAACCCTAATTACCTCCTGGCTTGGAAGCAGGTGCTGGCAGAACTTCAAGATATTGAGAATGAGGAGAAATTTCCAAAAACAAAGAACATGAAGAAAACAAGCCAATTGAAGTGGGCACTTGGTGAGAACATGGCACCAGAAAAAGTGGACTTTGAGGACTG<br>CAAAGATGTTAGCGATCTAAGACAGTACGACAGTGACGAACCAGAGCCTAGATCACTGGCAAGCTGGATTCAGAGTGAATTCAACAAGGCATGCGAATTGACAGATTCGAGTTGGATTGAACTTGATGAGATAGGGGAAGACGTTGCTCCAATCGAACACATTGCGAGTATGAGGAGGAACTATTTCACAGCGGAGGTATCCCATTGCAGGGCCACTGAATACATAATG<br>AAGGGAGTATACATAAACACAGCCCTATTGAATGCATCCTGTGCAGCCATGGATGACTTCCAACTGATTCCAATGATAAGCAAGTGCAGAACCAAAGAGGGAAGACGGAAGACAAATCTGTATGGATTCATTATAAAAGGAAGATCCCATTTGAGGAATGACACCGATGTGGTGAACTTTGTGAGCATGGAATTCTCTCTAACTGACCCGAGGCTAGAGCCACACAAATG<br>GGAAAAGTACTGTGTTCTTGAGATAGGAGACATGCTCCTACGGACTGCAATAGGCCAAGTGTCGAGGCCCATGTTCCTGTATGTGAGAACCAATGGGACTTCCAAGATCAAAATGAAATGGGGCATGGAGATGAGACGATGCCTTCTTCAGTCCCTTCAACAAATTGAGAGCATGATTGAGGCCGAATCTTCTGTCAAAGAGAAGGACATGACCAAGGAATTCTTTGAAA<br>ACAAATCAGAAACATGGCCAATTGGAGAATCACCCAAAGGGGTTGAGGAAGGCTCTATTGGGAAAGTATGCAGAACATTGCTAGCAAAGTCTGTGTTCAACAGCCTATATGCTTCTCCACAACTCGAGGGATTTTCAGCTGAATCAAGAAAATTGCTTCTCATTGTTCAGGCACTTAGGGACAACCTGGAACCTGGAACCTTCGATCTTGGGGGGCTATATGAAGCAATTG<br>AGGAGTGCCTGATTAACGATCCCTGGGTTTTGCTTAATGCGTCTTGGTTCAACTCCTTCATCACACATGCACTGAAATAG
EPI_ISL_6101848	>A/chicken/Netherlands/21038165-006010/2021_H5N1_2021-11-07<br>ATGGAAGACTTTGTGCGACAATGCTTCAATCCAATGATTGTCGAGCTTGCGGAAAAAGCAATGAAAGAATATGGGGAAGATCCGAAAATCGAGACAAACAAATTTGCCGCAATATGCACACACTTAGAAGTCTGTTTCATGTATTCGGATTTCCATTTTATTGATGAACGAGGCGAATCAATGATTGTAGAATCTGGCGATCCAAATGCATTATTGAAACACCGATTTGAGA<br>TAATCGAAGGGAGAGACCGAGCAATGGCCTGGACAGTGGTGAATAGTATCTGCAACACTACAGGAGTCGAAAAGCCCAAATTCCTCCCTGATTTGTATGACTACAAAGAGAACCGATTCATTGAAATTGGAGTAACGCGAAGGGAAGTTCACATATACTATTTAGAAAAAGCCAACAAGATAAAATCAGAGAAAACACACATTCACATATTCTCATTCACTGGAGAGGAA<br>ATGGCCACCAAGGCGGAATACACCCTTGATGAAGAGAGCAGAGCAAGAATAAAAACCAGACTGTTCACTATAAGACAAGAAATGGCCAGTAGAGGTCTATGGGATTCCTTTCGTCAGTCCGAGAGAGGCGAAGAGACAACTGAAGAAAGATTTGAAATCACAGGAACCATGCGCAGGCTTGCCGACCAAAGTCTCCCACCGAACTTCTCCAGCCTTGAAAACTTTAGAG<br>CCTATGTGGATGGATTCGAACCGAACGGCTGCATTGAGGGCAAGCTTTCTCAAATGTCAAAAGAAGTGAACGCCAGAATTGAGCCATTCCTGAAGACAACACCACGCCCTCTCAGATTACCTGATGGGCCTCCTTGTTCTCAGCGGTCGAAGTTCTTGCTGATGGATGCCCTTAAGTTGAGCATCGAAGATCCTAGCCATGAGGGGGAGGGCATACCGCTGTATGATGCAA<br>TCAAATGCATGAAGACATTTTTTGGCTGGAAAGAGCCCAACCTCGTAAAGCCGCATGAGAAAGGCATAAACCCTAATTACCTCCTGGCTTGGAAGCAGGTGCTGGCAGAACTTCAAGATATTGAGAATGAGGAGAAATTCCCAAAAACAAAGAACATGAAGAAAACAAGCCAATTGAAGTGGGCACTTGGTGAGAACATGGCACCAGAAAAAGTGGACTTTGAGGACTG<br>CAAAGATGTTAGCGATCTAAGACAGTACGACAGTGACGAACCAGAGCCTAGATCACTAGCAAGCTGGATTCAGAGTGAATTCAACAAGGCATGCGAATTGACAGATTCGAGTTGGATTGAACTTGATGAGATAGGGGAAGACGTTGCTCCAATCGAACACATTGCGAGTATGAGGAGGAACTATTTCACAGCGGAGGTATCCCATTGCAGGGCCACTGAATACATAATG<br>AAGGGAGTATACATAAACACAGCCCTATTGAATGCATCCTGTGCAGCCATGGATGACTTCCAACTGATTCCAATGATAAGCAAGTGCAGAACCAAAGAGGGAAGACGGAAGACAAATCTGTATGGATTCATTATAAAAGGAAGATCCCATTTGAGGAATGACACCGATGTGGTAAACTTTGTGAGCATGGAATTCTCTCTAACTGACCCGAGGCTAGAGCCACACAAATG<br>GGAAAAGTACTGTGTTCTTGAGATAGGAGACATGCTCCTACGGACTGCAATAGGCCAAGTGTCGAGGCCCATGTTCCTGTATGTGAGAACCAATGGGACTTCCAAGATCAAAATGAAATGGGGCATGGAGATGAGACGATGCCTTCTTCAGTCCCTTCAACAAATTGAGAGCATGATTGAGGCCGAATCTTCTGTCAAAGAGAAGGACATGACCAAGGAATTCTTTGAAA<br>ACAAATCAGAAACATGGCCAATTGGAGAATCACCCAAAGGGGTTGAGGAAGGCTCTATTGGGAAAGTATGCAGAACATTGCTAGCAAAGTCTGTGTTCAACAGCCTATATGCATCTCCACAACTCGAGGGATTTTCAGCTGAATCAAGAAAATTGCTTCTCATTGTTCAGGCACTTAGGGACAACCTGGAACCTGGAACCTTCGATCTTGGGGGGCTATATGAAGCAATTG<br>AGGAGTGCCTGATTAACGATCCCTGGGTTTTGCTTAATGCGTCTTGGTTCAACTCCTTCATCACACATGCACTGAAATAG
EPI_ISL_14388434	>A/Buteo_buteo/belgium/334_0013/2021_H5N1_2021-12-24<br>ATGGAAGACTTTGTGCGACAATGCTTCAATCCAATGATTGTCGAGCTTGCGGAAAAAGCAATGAAAGAATATGGGGAAGATCCGAAAATCGAGACAAACAAATTTGCCGCAATATGCACACACTTAGAAGTCTGTTTCATGTATTCGGATTTCCATTTTATTGATGAACGAGGCGAATCAATGATTGTAGAATCTGGCGATCCAAATGCATTATTGAAACACCGATTTGAGA<br>TAATCGAAGGGAGAGACCGAGCAATGGCCTGGACAGTGGTGAATAGTATCTGCAACACTACAGGAGTCGAAAAGCCCAAATTCCTCCCTGATTTGTATGACTACAAAGAGAACCGATTCATTGAAATTGGAGTAACGCGAAGGGAAGTTCACATATACTATTTAGAAAAAGCCAACAAGATAAAATCAGAGAAAACACACATTCACATATTCTCATTCACTGGAGAGGAA<br>ATGGCCACCAAGGCGGAATACACCCTTGATGAAGAGAGCAGAGCAAGAATAAAAACCAGACTGTTCACTATAAGACAAGAAATGGCCAGTAGAGGTCTATGGGATTCCTTTCGTCAGTCCGAGAGAGGCGAAGAGACAACTGAAGAAAGATTTGAAATCACAGGAACCATGCGCAGGCTTGCCGACCAAAGTCTCCCACCGAACTTCTCCAGCCTTGAAAACTTTAGAG<br>CCTATGTGGATGGATTCGAACCGAACGGCTGCATTGAGGGCAAGCTTTCTCAAATGTCAAAAGAAGTGAACGCCAGAATTGAGCCATTCCTGAAGACAACACCACGCCCTCTCAGATTACCTGATGGGCCTCCTTGTTCTCAGCGGTCGAAGTTCTTGCTGATGGATGCCCTTAAGTTGAGCATCGAAGATCCTAGCCATGAGGGGGAGGGCATACCGCTGTATGATGCAA<br>TCAAATGCATGAAGACATTTTTTGGCTGGAAAGAGCCCAACCTCGTAAAGCCGCATGAGAAAGGCATAAACCCTAATTACCTCCTGGCTTGGAAGCAGGTGCTGGCAGAACTTCAAGATATTGAGAATGAGGAGAAATTCCCAAAAACAAAGAACATGAAGAAAACAAGCCAATTGAAGTGGGCACTTGGTGAGAACATGGCACCAGAAAAAGTGGACTTTGAGGACTG<br>CAAAGATGTTAGCGATCTAAGACAGTACGACAGTGACGAACCAGAGCCTAGATCACTAGCAAGCTGGATTCAGAGTGAATTCAACAAGGCATGCGAATTGACAGAGTCGAGTTGGATTGAACTTGATGAGATAGGGGAAGACGTTGCTCCAATCGAACACATTGCGAGTATGAGGCGGAACTATTTCACAGCGGAGGTATCCCATTGCAGGGCCACTGAATACATAATG<br>AAGGGAGTATACATAAACACAGCCCTATTGAATGCATCCTGTGCAGCCATGGATGACTTCCAACTGATTCCAATGATAAGCAAGTGCAGAACCAAAGAGGGAAGACGGAAGACAAATCTGTATGGATTCATTATAAAAGGAAGATCCCATTTGAGGAATGACACCGATGTGGTAAACTTTGTGAGCATGGAATTCTCTCTAACTGACCCGAGGCTAGAGCCACACAAATG<br>GGAAAAGTACTGTGTTCTTGAGATAGGAGACATGCTCCTACGGACTGCAATAGGCCAAGTGTCGAGGCCCATGTTCCTGTATGTGAGAACCAATGGGACTTCCAAGATCAAAATGAAATGGGGCATGGAGATGAGACGATGCCTTCTTCAGTCCCTTCAACAAATTGAGAGCATGATTGAGGCCGAATCTTCTGTCAAAGAGAAGGACATGACCAAGGAATTCTTTGAAA<br>ACAAATCAGAAACATGGCCAATTGGAGAATCACCCAAAGGGGTTGAGGAAGGCTCTATTGGGAAAGTATGCAGAACATTGCTAGCAAAGTCTGTGTTCAACAGCCTATATGCATCTCCACAACTCGAGGGATTTTCAGCTGAATCAAGAAAATTGCTTCTCATTGTTCAGGCACTTAGGGACAACCTGGAACCTGGAACCTTCGATCTTGGGGGGCTATATGAAGCAATTG<br>AGGAGTGCCTGATTAACGATCCCTGGGTTTTGCTTAATGCGTCTTGGTTCAACTCCTTCATCACACATGCACTGAAATAG
EPI_ISL_14388346	>A/Tyto_alba/Belgium/334_0012/2021_H5N1_2021-12-23<br>ATGGAAGACTTTGTGCGACAATGCTTCAATCCAATGATTGTCGAGCTTGCGGAAAAAGCAATGAAAGAATATGGGGAAGATCCGAAAATCGAGACAAACAAATTTGCCGCAATATGCACACACTTAGAAGTCTGTTTCATGTATTCGGATTTCCATTTTATTGATGAACGAGGCGAATCAATGATTGTAGAATCTGGCGATCCAAATGCATTATTGAAACACCGATTTGAGA<br>TAATCGAAGGGAGAGACCGAGCAATGGCCTGGACAGTGGTGAATAGTATCTGCAACACTACAGGAGTCGAAAAGCCCAAATTCCTCCCTGATTTGTATGACTACAAAGAGAACCGATTCATTGAAATTGGAGTAACGCGAAGGGAAGTTCACATATACTATTTAGAAAAAGCCAACAAGATAAAATCAGAGAAAACACACATTCACATATTCTCATTCACTGGAGAGGAA<br>ATGGCCACCAAGGCGGAATACACCCTTGATGAAGAGAGCAGAGCAAGAATAAAAACCAGACTGTTCACTATAAGACAAGAAATGGCCAGTAGAGGTCTATGGGATTCCTTTCGTCAGTCCGAGAGAGGCGAAGAGACAACTGAAGAAAGATTTGAAATCACAGGAACCATGCGCAGGCTTGCCGACCAAAGTCTCCCACCGAACTTCTCCAGCCTTGAAAACTTTAGAG<br>CCTATGTGGATGGATTCGAACCGAACGGCTGCATTGAGGGCAAGCTTTCTCAAATGTCAAAAGAAGTGAACGCCAGAATTGAGCCATTCCTGAAGACAACACCACGCCCTCTCAGATTACCTGATGGGCCTCCTTGTTCTCAGCGGTCGAAGTTCTTGCTGATGGATGCCCTTAAGTTGAGCATCGAAGATCCTAGCCATGAGGGGGAGGGCATACCGCTGTATGATGCAA<br>TCAAATGCATGAAGACATTTTTTGGCTGGAAAGAGCCCAACCTCGTAAAGCCGCATGAGAAAGGCATAAACCCTAATTACCTCCTGGCTTGGAAGCAGGTGCTGGCAGAACTTCAAGATATTGAGAATGAGGAGAAATTCCCAAAAACAAAGAACATGAAGAAAACAAGCCAATTGAAGTGGGCACTTGGTGAGAACATGGCACCAGAAAAAGTGGACTTTGAGGACTG<br>CAAAGATGTTAGCGATCTAAGACAGTACGACAGTGACGAACCAGAGCCTAGATCACTAGCAAGCTGGATTCAGAGTGAATTCAACAAGGCATGCGAATTGACAGAGTCGAGTTGGATTGAACTTGATGAGATAGGGGAAGACGTTGCTCCAATCGAACACATTGCGAGTATGAGGCGGAACTATTTCACAGCGGAGGTATCCCATTGCAGGGCCACTGAATACATAATG<br>AAGGGAGTATACATAAACACAGCCCTATTGAATGCATCCTGTGCAGCCATGGATGACTTCCAACTGATTCCAATGATAAGCAAGTGCAGAACCAAAGAGGGAAGACGGAAGACAAATCTGTATGGATTCATTATAAAAGGAAGATCCCATTTGAGGAATGACACCGATGTGGTAAACTTTGTGAGCATGGAATTCTCTCTAACTGACCCGAGGCTAGAGCCACACAAATG<br>GGAAAAGTACTGTGTTCTTGAGATAGGAGACATGCTCCTACGGACTGCAATAGGCCAAGTGTCGAGGCCCATGTTCCTGTATGTGAGAACCAATGGGACTTCCAAGATCAAAATGAAATGGGGCATGGAGATGAGACGATGCCTTCTTCAGTCCCTTCAACAAATTGAGAGCATGATTGAGGCCGAATCTTCTGTCAAAGAGAAGGACATGACCAAGGAATTCTTTGAAA<br>ACAAATCAGAAACATGGCCAATTGGAGAATCACCCAAAGGGGTTGAGGAAGGCTCTATTGGGAAAGTATGCAGAACATTGCTAGCAAAGTCTGTGTTCAACAGCCTATATGCATCTCCACAACTCGAGGGATTTTCAGCTGAATCAAGAAAATTGCTTCTCATTGTTCAGGCACTTAGGGACAACCTGGAACCTGGAACCTTCGATCTTGGGGGGCTATATGAAGCAATTG<br>AGGAGTGCCTGATTAACGATCCCTGGGTTTTGCTTAATGCGTCTTGGTTCAACTCCTTCATCACACATGCACTGAAATAG
EPI_ISL_11327090	>A/chicken/Czech_Republic/63/2022_H5N1_2022-01-03<br>ATGGAAGACTTTGTGCGACAATGCTTCAATCCAATGATTGTCGAGCTTGCGGAAAAAGCAATGAAAGAATATGGGGAAGATCCGAAAATCGAGACAAACAAATTTGCCGCAATATGCACACACTTAGAAGTCTGTTTCATGTATTCGGATTTCCATTTTATTGATGAACGAGGCGAATCAATGATTGTAGAATCTGGCGATCCAAATGCATTATTGAAACACCGATTTGAGA<br>TAATCGAAGGGAGAGACCGAGCAATGGCCTGGACAGTGGTGAATAGTATCTGCAACACTACAGGAGTCGAAAAGCCCAAATTCCTCCCTGATTTGTATGACTACAAAGAGAACCGATTCATTGAAATTGGAGTAACGCGAAGGGAAGTTCACATATACTATTTAGAAAAAGCCAACAAGATAAAATCAGAGAAAACACACATTCACATATTCTCATTCACTGGAGAGGAA<br>ATGGCCACCAAGGCGGAATACACCCTTGATGAAGAGAGCAGAGCAAGAATAAAAACCAGACTGTTCACTATAAGACAAGAAATGGCCAGTAGAGGTCTATGGGATTCCTTTCGTCAGTCCGAGAGAGGCGAAGAGACAACTGAAGAAAGATTTGAAATCACAGGAACCATGCGCAGGCTTGCCGACCAAAGTCTCCCACCGAACTTCTCCAGCCTTGAAAACTTTAGAG<br>CCTATGTGGATGGATTCGAACCGAACGGCTGCATTGAGGGCAAGCTTTCTCAAATGTCAAAAGAAGTGAACGCCAGAATTGAGCCATTCCTGAAGACAACACCACGCCCTCTCAGATTACCTGATGGGCCTCCTTGTTCTCAGCGGTCGAAGTTCTTGCTGATGGATGCCCTTAAGTTGAGCATCGAAGATCCTAGCCATGAGGGGGAGGGCATACCGCTGTATGATGCAA<br>TCAAATGCATGAAGACATTTTTTGGCTGGAAAGAGCCCAACCTCGTAAAGCCGCATGAGAAAGGCATAAACCCTAATTACCTCCTGGCTTGGAAGCAGGTGCTGGCAGAACTTCAAGATATTGAGAATGAGGAGAAATTCCCAAAAACAAAGAACATGAAGAAAACAAGCCAATTGAAGTGGGCACTTGGTGAGAACATGGCACCAGAAAAAGTGGACTTTGAGGACTG<br>CAAAGATGTTAGCGATCTAAGACAGTACGACAGTGACGAACCAGAGCCTAGATCACTAGCAAGCTGGATTCAGAGTGAATTCAACAAGGCATGCGAATTGACAGAGTCGAGTTGGATTGAACTTGATGAGATAGGGGAAGACGTTGCTCCAATCGAACACATTGCGAGTATGAGGCGGAACTATTTCACAGCGGAGGTATCCCATTGCAGGGCCACTGAATACATAATG<br>AAGGGAGTATACATAAACACAGCCCTATTGAATGCATCCTGTGCAGCCATGGATGACTTCCAACTGATTCCAATGATAAGCAAGTGCAGAACCAAAGAGGGAAGACGGAAGACAAATCTGTATGGATTCATTATAAAAGGAAGATCCCATTTGAGGAATGACACCGATGTGGTAAACTTTGTGAGCATGGAATTCTCTCTAACTGACCCGAGGCTAGAGCCACACAAATG<br>GGAAAAGTACTGTGTTCTTGAGATAGGAGACATGCTCCTACGGACTGCAATAGGCCAAGTGTCGAGGCCCATGTTCCTGTATGTGAGAACCAATGGGACTTCCAAGATCAAAATGAAATGGGGCATGGAGATGAGACGATGCCTTCTTCAGTCCCTTCAACAAATTGAGAGCATGATTGAGGCCGAATCTTCTGTCAAAGAGAAGGACATGACCAAGGAATTCTTTGAAA<br>ACAAATCAGAAACATGGCCAATTGGAGAATCACCCAAAGGGGTTGAGGAAGGCTCTATTGGGAAAGTATGCAGAACATTGCTAGCAAAGTCTGTGTTCAACAGCCTATATGCATCTCCACAACTCGAGGGATTTTCAGCTGAATCAAGAAAATTGCTTCTCATTGTTCAGGCACTTAGGGACAACCTGGAACCTGGAACCTTCGATCTTGGGGGGCTATATGAAGCAATTG<br>AGGAGTGCCTGATTAACGATCCCTGGGTTTTGCTTAATGCGTCTTGGTTCAACTCCTTCATCACACATGCACTGAAATAG
EPI_ISL_12754531	>A/laying_hen/Romania/11343_22VIR4106-1/2022_H5N1_2022-03-18<br>ATGGAAGACTTTGTGCGACAATGCTTCAATCCAATGATTGTCGAGCTTGCGGAAAAAGCAATGAAAGAATATGGGGAAGATCCGAAAATCGAGACAAACAAATTTGCCGCAATATGCACACACTTAGAAGTCTGTTTCATGTATTCGGATTTCCATTTTATTGATGAACGAGGCGAATCAATGATTGTAGAATCTGGCAATCCAAATGCATTATTGAAACACCGATTTGAGA<br>TAATCGAAGGGAGAGACCGAGCAATGGCCTGGACAGTGGTGAATAGTATCTGCAACACCACAGGAGTCGAAAAGCCCAAATTCCTCCCTGATTTGTATGACTACAAAGAGAACCGATTCATTGAAATTGGAGTAACGCGAAGGGAAGTTCACATATACTATTTAGAAAAAGCCAACAAGATAAAATCAGAGAAAACACACATTCACATATTCTCATTCACTGGAGAGGAA<br>ATGGCCACCAAGGCGGAATACACCCTTGATGAAGAGAGCAGAGCAAGAATAAAAACCAGACTGTTCACTATAAGACAAGAAATGGCCAGTAGAGGTCTATGGGATTCCTTTCGTCAGTCCGAGAGAGGCGAAGAGACAACTGAAGAAAGATTTGAAATCACAGGAACCATGCGCAGGCTTGCCGACCAAAGTCTCCCACCGAACTTCTCCAGCCTTGAAAACTTTAGAG<br>CCTATGTGGATGGATTCGAACCGAACGGCTGCATTGAGGGCAAGCTTTCTCAAATGTCAAAAGAAGTGAACGCCAGAATTGAGCCATTCCTGAAGACAACACCACGCCCTCTCAGATTACCTGATGGGCCTCCTTGTTCTCAGCGGTCGAAGTTCTTGCTGATGGATGCCCTTAAGTTGAGCATCGAAGATCCTAGCCATGAGGGGGAGGGCATACCGCTGTATGATGCAA<br>TCAAATGCATGAAGACATTTTTTGGCTGGAAAGAGCCCAACCTCGTAAAGCCGCATGAGAAAGGCATAAACCCTAATTACCTCCTGGCTTGGAAGCAGGTGCTGGCAGAACTTCAAGATATTGAGAATGAGGAGAAATTTCCAAAAACAAAGAACATGAAGAAAACAAGCCAATTGAAGTGGGCACTTGGTGAGAACATGGCACCAGAAAAAGTGGACTTTGAGGACTG<br>CAAAGATGTTAGCGATCTAAGACAGTACGACAGTGACGAACCAGAGCCTAGATCACTAGCAAGCTGGATTCAGAGTGAATTCAACAAGGCATGCGAATTGACAGATTCGAGTTGGATTGAACTTGATGAGATAGGGGAAGACGTTGCTCCAATCGAACACATTGCGAGTATGAGGAGGAACTATTTCACAGCGGAGGTATCCCATTGCAGGGCCACTGAATACATAATG<br>AAGGGAGTATACATAAACACAGCCCTATTGAATGCATCCTGTGCAGCCATGGATGACTTCCAACTGATTCCAATGATAAGCAAGTGCAGAACCAAAGAGGGAAGACGGAAGACAAATCTGTATGGATTCATTATAAAAGGAAGATCCCATTTGAGGAATGACACCGATGTGGTAAACTTTGTGAGCATGGAATTCTCTCTAACTGACCCGAGGCTAGAGCCACACAAATG<br>GGAAAAGTACTGTGTTCTTGAGATAGGAGACATGCTCCTACGGACTGCAATAGGCCAAGTGTCGAGGCCCATGTTCCTGTATGTGAGAACCAATGGGACTTCCAAGATCAAAATGAAATGGGGCATGGAGATGAGACGATGCCTTCTTCAGTCCCTTCAACAAATTGAGAGCATGATTGAGGCCGAATCTTCTGTCAAAGAGAAGGACATGACCAAGGAATTCTTTGAAA<br>ACAAATCAGAAACATGGCCAATTGGAGAATCACCCAAAGGGGTTGAGGAAGGCTCTATTGGGAAAGTATGCAGAACATTGCTAGCAAAGTCTGTGTTCAACAGCCTATATGCATCTCCACAACTCGAGGGATTTTCAGCTGAATCAAGAAAATTGCTTCTCATTGTTCAGGCACTTAGGGACAACCTGGAACCTGGAACCTTCGATCTTGGGGGGCTATATGAAGCAATTG<br>AGGAGTGCCTGATTAACGATCCCTGGGTTTTGCTTAATGCGTCTTGGTTCAACTCCTTCATCACACATGCACTGAAATAG
EPI_ISL_12749687	>A/turkey/Israel/537/2021_H5N1_2021-10-12<br>ATGGAAGACTTTGTGCGACAATGCTTCAATCCAATGATTGTCGAGCTTGCGGAAAAAGCAATGAAAGAATATGGGGAAGATCCGAAAATCGAGACAAACAAATTTGCCGCAATATGCACACACTTAGAAGTCTGTTTCATGTATTCGGATTTCCATTTTATTGATGAACGAGGCGAATCAATGATTGTAGAATCTGGCGATCCAAATGCATTATTGAAACACCGGTTTGAGA<br>TAATCGAAGGGAGAGACCGAGCAATGGCCTGGACAGTGGTGAATAGTATCTGCAACACCACAGGAGTCGAAAAGCCCAAATTCCTCCCTGATTTGTATGACTACAAAGAGAACCGATTCATTGAAATTGGAGTAACGCGAAGGGAAGTTCACATATACTATTTAGAAAAAGCCAACAAGATAAAATCAGAGAAAACACACATTCACATATTCTCATTCACTGGAGAGGAA<br>ATGGCCACCAAGGCGGAATACACCCTTGATGAAGAGAGCAGAGCAAGAATAAAAACCAGACTGTTCACTATAAGACAAGAAATGGCCAGTAGAGGTCTATGGGATTCCTTTCGTCAGTCCGAGAGAGGCGAAGAGACAACTGAAGAAAGATTTGAAATCACAGGAACCATGCGCAGGCTTGCCGACCAAAGTCTCCCACCGAACTTCTCCAGCCTTGAAAACTTTAGAG<br>CCTATGTGGATGGATTCGAACCGAACGGCTGCATTGAGGGCAAGCTTTCTCAAATGTCAAAAGAAGTGAACGCCAGAATTGAGCCATTCCTGAAGACAACACCACGCCCTCTCAGATTACCTGATGGGCCTCCTTGTTCTCAGCGGTCGAAGTTCTTGCTGATGGATGCCCTTAAGTTGAGCATCGAAGATCCTAGCCATGAGGGGGAGGGCATACCGCTGTATGATGCAA<br>TCAAATGCATGAAGACATTTTTTGGCTGGAAAGAGCCCAACCTCGTAAAGCCGCATGAGAAAGGCATAAACCCTAATTACCTCCTGGCTTGGAAGCAGGTGCTGGCAGAACTTCAAGATATTGAGAATGAGGAGAAATTTCCAAAAACAAAGAACATGAAGAAAACAAGCCAATTGAAGTGGGCACTTGGTGAGAACATGGCACCAGAAAAAGTGGACTTTGAGGACTG<br>CAAAGATGTTAGCGATCTAAGACAGTACGACAGTGACGAACCAGAGCCTAGATCACTAGCAAGCTGGATTCAGAGTGAATTCAACAAGGCATGCGAATTGACAGATTCGAGTTGGATTGAACTTGATGAGATAGGGGAAGACGTTGCTCCAATCGAACACATTGCGAGTATGAGGAGGAACTATTTCACAGCGGAGGTATCCCATTGCAGGGCCACTGAATACATAATG<br>AAGGGAGTATACATAAACACAGCCCTATTGAATGCATCCTGTGCAGCCATGGATGACTTCCAACTGATTCCAATGATAAGCAAGTGCAGAACCAAAGAGGGAAGACGGAAGACAAATCTGTATGGATTCATTATAAAAGGAAGATCCCATTTGAGGAATGACACCGATGTGGTAAACTTTGTGAGCATGGAATTCTCTCTAACTGACCCGAGGCTAGAGCCACACAAATG<br>GGAAAAGTACTGTGTTCTTGAGATAGGAGACATGCTCCTACGGACTGCAATAGGCCAAGTGTCGAGGCCCATGTTCCTGTATGTGAGAACCAATGGGACTTCCAAGATCAAAATGAAATGGGGCATGGAGATGAGACGATGCCTTCTTCAGTCCCTTCAACAAATTGAGAGCATGATTGAGGCCGAATCTTCTGTCAAAGAGAAGGACATGACCAAGGAATTCTTTGAAA<br>ACAAATCAGAAACATGGCCAATTGGAGAATCACCCAAAGGGGTTGAGGAAGGCTCTATTGGGAAAGTATGCAGAACATTGCTAGCAAAGTCTGTGTTCAACAGCCTATATGCATCTCCACAACTCGAGGGATTTTCAGCTGAATCAAGAAAATTGCTTCTCATTGTTCAGGCACTTAGGGACAACCTGGAACCTGGAACCTTCGATCTTGGGGGGCTATATGAAGCAATTG<br>AGGAGTGCCTGATTAACGATCCCTGGGTTTTGCTTAATGCGTCTTGGTTCAACTCCTTCATCACACATGCACTGAAATAG
EPI_ISL_11561592	>A/turkey/England/016515/2022_H5N1_2022-02-20<br>ATGGAAGACTTTGTGCGACAATGCTTCAATCCAATGATTGTCGAGCTTGCGGAAAAAGCAATGAAAGAATATGGGGAAGATCCGAAAATCGAGACAAACAAATTTGCCGCAATATGCACACACTTAGAAGTCTGTTTCATGTATTCGGATTTCCATTTTATTGATGAACGAGGCGAATCAATGATTGTAGAATCTGGCGATCCCAATGCATTATTGAAACACCGATTTGAGA<br>TAATCGAAGGGAGAGACCGAGCAATGGCCTGGACAGTGGTGAATAGTATCTGCAACACTACAGGAGTCGAAAAGCCTAAATTCCTCCCTGATTTGTATGACTACAAAGAGAACCGATTCATTGAAATTGGAGTAACGCGAAGGGAAGTTCACATATACTATTTAGAAAAAGCCAACAAGATAAAATCAGAGAAAACACACATTCACATATTCTCATTCACTGGAGAGGAA<br>ATGGCCACCAAGGCGGAATACACCCTTGATGAAGAGAGCAGAGCAAGAATAAAAACCAGACTGTTCACTATAAGACAAGAAATGGCCAGTAGAGGTCTATGGGATTCCTTTCGTCAGTCCGAGAGAGGCGAAGAGACAACTGAAGAAAGATTTGAAATCACAGGAACCATGCGCAGGCTTGCCGACCAAAGTCTCCCACCGAACTTCTCCAGCCTTGAAAACTTTAGAG<br>CCTATGTGGATGGATTCGAACCGAACGGCTGCATTGAGGGCAAGCTTTCTCAAATGTCAAAAGAAGTGAACGCCAGAATTGAGCCATTCCTGAAGACAACACCACGCCCTCTCAGATTACCTGATGGGCCTCCTTGTTCTCAGCGGTCGAAGTTCTTGCTGATGGATGCCCTTAAGTTGAGCATCGAAGATCCTAGCCATGAGGGGGAGGGCATACCGCTGTATGATGCAA<br>TCAAATGCATGAAGACATTTTTTGGCTGGAAAGAGCCCAACCTCGTAAAGCCGCATGAGAAAGGCATAAACCCTAATTACCTCCTGGCTTGGAAGCAGGTGCTGGCAGAACTTCAAGATATTGAGAATGAGGAGAAATTCCCAAAAACAAAGAACATGAAGAAAACAAGCCAATTGAAGTGGGCACTTGGTGAGAACATGGCACCAGAAAAAGTGGACTTTGAGGACTG<br>CAAAGATGTTAGCGATCTAAGACAGTACGACAGTGACGAACCAGAGCCTAGATCACTAGCAGGCTGGATTCAGAGTGAATTCAACAAGGCATGCGAATTGACAGATTCGAGTTGGATTGAACTTGATGAGATAGGGGAAGACGTTGCTCCAATCGAACACATTGCGAGTATGAGGAGGAACTATTTCACAGCGGAGGTATCCCATTGCAGGGCCACTGAATACATAATG<br>AAGGGAGTATACATAAACACAGCCCTATTGAATGCATCCTGTGCAGCCATGGATGACTTCCAACTGATTCCAATGATAAGCAAGTGCAGAACCAAAGAGGGAAGACGGAAGACAAATCTGTATGGATTCATTATAAAAGGAAGATCCCATTTGAGGAATGACACCGATGTGGTAAACTTTGTGAGCATGGAATTCTCTCTAACTGACCCGAGGCTAGAGCCACACAAATG<br>GGAAAAGTACTGTGTTCTTGAGATAGGAGACATGCTCCTACGGACTGCAATAGGCCAAGTGTCGAGGCCCATGTTCCTGTATGTGAGAACCAATGGGACTTCCAAGATCAAAATGAAATGGGGCATGGAGATGAGACGATGCCTTCTTCAGTCCCTTCAACAAATTGAGAGCATGATTGAGGCCGAATCTTCTGTCAAAGAGAAGGACATGACCAAGGAATTCTTTGAAA<br>ACAAATCAGAAACATGGCCAATTGGAGAATCACCCAAAGGGGTTGAGGAAGGCTCTATTGGGAAAGTATGCAGAACATTGCTAGCAAAGTCTGTGTTCAACAGCCTATATGCATCTCCACAACTCGAGGGATTTTCAGCTGAATCAAGAAAATTGCTTCTCATTGTTCAGGCACTTAGGGACAACCTGGAACCTGGAACCTTCGATCTTGGGGGGCTATATGAAGCAATTG<br>AGGAGTGCCTGATTAACGATCCCTGGGTTTTGCTTAATGCGTCTTGGTTCAACTCCTTCATCACACATGCACTGAAATAG
EPI_ISL_7778754	>A/Withe-tiled_eagle/Estonia/TA2124126-1_21VIR10433-11/2021_H5N1_2021-10-12<br>ATGGAAGACTTTGTGCGACAATGCTTCAATCCAATGATTGTCGAGCTTGCGGAAAAAGCAATGAAAGAATATGGGGAAGATCCGAAAATCGAGACAAACAAATTTGCCGCAATATGCACACACTTAGAAGTCTGTTTCATGTATTCGGATTTCCATTTTATTGATGAACGAGGCGAATCAATGATTGTAGAATCTGGCGATCCAAATGCATTATTGAAACACCGATTTGAGA<br>TAATCGAAGGGAGAGACCGAGCAATGGCCTGGACAGTGGTGAATAGTATCTGCAACACTACAGGAGTCGAAAAGCCCAAATTCCTCCCTGATTTGTATGACTACAAAGAGAACCGATTCATTGAAATTGGAGTAACGCGAAGGGAAGTTCACATATACTATTTAGAAAAAGCCAACAAGATAAAATCAGAGAAAACACACATTCACATATTCTCATTCACTGGAGAGGAA<br>ATGGCCACCAAGGCGGAATACACCCTTGATGAAGAGAGCAGAGCAAGAATAAAAACCAGACTGTTCACTATAAGACAAGAAATGGCCAGTAGAGGTCTATGGGATTCCTTTCGTCAGTCCGAGAGAGGCGAAGAGACAACTGAAGAAAGATTTGAAATCACAGGAACCATGCGCAGGCTTGCCGACCAAAGTCTCCCACCGAACTTCTCCAGCCTTGAAAACTTTAGAG<br>CCTATGTGGATGGATTCGAACCGAACGGCTGCATTGAGGGCAAGCTTTCTCAAATGTCAAAAGAAGTGAACGCCAGAATTGAGCCATTCCTGAAGACAACACCACGCCCTCTCAGATTACCTGATGGGCCTCCTTGTTCTCAGCGGTCGAAGTTCTTGCTGATGGATGCCCTTAAGTTGAGCATCGAAGATCCTAGCCATGAGGGGGAGGGCATACCGCTGTATGATGCAA<br>TCAAATGCATGAAGACATTTTTTGGCTGGAAAGAGCCCAACCTCGTAAAGCCGCATGAGAAAGGCATAAACCCTAATTACCTCCTGGCTTGGAAGCAGGTGCTGGCAGAACTTCAAGATATTGAGAATGAGGAGAAATTCCCAAAAACAAAGAACATGAAGAAAACAAGCCAATTGAAGTGGGCACTTGGTGAGAACATGGCACCAGAAAAAGTGGACTTTGAGGACTG<br>CAAAGATGTTAGCGATCTAAGACAGTACGACAGTGACGAACCAGAGCCTAGATCACTAGCAAGCTGGATTCAGAGTGAATTCAACAAGGCATGCGAATTGACAGATTCGAGTTGGATTGAACTTGATGAGATAGGGGAAGACGTTGCTCCAATCGAACACATTGCGAGTATGAGGAGGAACTATTTCACAGCGGAGGTATCCCATTGCAGGGCCACTGAATACATAATG<br>AAGGGAGTATACATAAACACAGCCCTATTGAATGCATCCTGTGCAGCCATGGATGACTTCCAACTGATTCCAATGATAAGCAAGTGCAGAACCAAAGAGGGAAGACGGAAGACAAATCTGTATGGATTCATTATAAAAGGAAGATCCCATTTGAGGAATGACACCGATGTGGTAAACTTTGTGAGCATGGAATTCTCTCTAACTGACCCGAGGCTAGAGCCACACAAATG<br>GGAAAAGTACTGTGTTCTTGAGATAGGAGACATGCTCCTACGGACTGCAATAGGCCAAGTGTCGAGGCCCATGTTCCTGTATGTGAGAACCAATGGGACTTCCAAGATCAAAATGAAATGGGGCATGGAGATGAGACGATGCCTTCTTCAGTCCCTTCAACAAATTGAGAGCATGATTGAGGCCGAATCTTCTGTCAAAGAGAAGGACATGACCAAGGAATTCTTTGAAA<br>ACAAATCAGAAACATGGCCAATTGGAGAATCACCCAAAGGGGTTGAGGAAGGCTCTATTGGGAAAGTATGCAGAACATTGCTAGCAAAGTCTGTGTTCAACAGCCTATATGCATCTCCACAACTCGAGGGATTTTCAGCTGAATCAAGAAAATTGCTTCTCATTGTTCAGGCACTTAGGGACAACCTGGAACCTGGAACCTTCGATCTTGGGGGGCTATATGAAGCAATTG<br>AGGAGTGCCTGATTAACGATCCCTGGGTTTTGCTTAATGCGTCTTGGTTCAACTCCTTCATCACACATGCACTGAAATAG
EPI_ISL_328975	>A/mallard_duck/Georgia/2/2016_H7N7_2016-01-04<br>ATGGAAGACTTTGTGCGACAATGCTTCAATCCAATGATTGTCGAGCTTGCGGAAAAGGCAATGAAAGAATATGGGGAAGATCCGAAAATCGAAACGAACAAATTTGCCGCAATATGCACACACTTAGAAGTCTGTTTCATGTATTCGGATTTTCACTTTATTGATGAACGGGGCGAATCAATAATTGTAGAATCTGGCGATCCGAATGCATTATTGAAACACCGATTTGAGA<br>TAATTGAAGGGAGAGACCGAACAATGGCCTGGACAGTGGTAAATAGTATCTGCAACACCACAGGAGTCGACAAGCCTAAATTCCTCCCAGATTTGTATGACTACAAAGAGAACCGATTCATTGAAATTGGAGTAACACGAAGGGAAGTTCACATATACTATCTAGAAAAAGCCAACAAGATAAAATCAGAGAAGACACACATTCACATATTCTCATTCACTGGAGAGGAA<br>ATGGCCACCAAAGCGGACTATACCCTTGACGAGGAGAGCAGAGCAAGAATCAAGACCAGGCTGTTCACTATAAGGCAAGAAATGGCCAGTAGAGGTCTGTGGGATTCCTTTCGTCAGTCCGAGAGAGGCGAAGAGACAATTGAAGAAAGATTTGAAATCACAGGAACCATGCGCAGGCTTGCCGACCAAAGTCTCCCACCGAACTTCTCCAGCCTTGAAAACTTTAGAG<br>CCTATGTGGATGGATTCGAACCGAACGGCTGCATTGAGGGCAAGCTTTCTCAAATGTCAAAGGAAGTGAACGCCAGGATTGAGCCATTTCTGAAGACAACACCACGCCCTCTCAGATTACCTGATGGGCCTCCCTGCTCTCAGCGGTCAAAGTTCTTACTGATGGATGCCCTTAAATTAAGCATCGAAGACCCGAGCCATGAAGGAGAAGGCATACCGCTATATGATGCAA<br>TCAAATGCATGAAGACATTTTTCGGCTGGAAAGAGCCCAACATCGTGAAACCACATGAAAAAGGCATAAACCCCAATTACCTCCTGGCTTGGAAGCAGGTGCTAGCAGAGCTCCAAGATATTGAAAATGAGGAGAAAATTCCAAAAACAAAGAACATGAAGAAAACAAGTCAATTGAAGTGGGCACTTGGTGAGAACATGGCACCAGAGAAAGTAGACTTTGAGGACT<br>GCAAAGACGTTAGCGATCTAAGACAATATGATAGTGATGAACCAGAATCTAGATCGCTAGCAAGCTGGATCCAGAGCGAATTCAACAAGGCATGCGAATTGACAGATTCAAGTTGGATTGAACTTGATGAAATAGGGGAAGACGTTGCTCCAATTGAGCACATTGCGAGTATGAGAAGAAACTACTTCACAGCGGAAGTATCCCATTGCAGGGCTACTGAATACATAATG<br>AAGGGAGTGTACATAAACACAGCCCTGTTGAATGCATCCTGTGCAGCCATGGATGACTTCCAACTGATTCCAATGATAAGCAAGTGCAGAACCAAAGAAGGAAGACGGAAGACAAACCTGTATGGATTCATCATAAAAGGAAGATCCCATTTGAGGAATGACACCGATGTGGTAAACTTTGTGAGCATGGAATTCTCTCTTACGGACCCGAGGCTGGAGCCACACAAGTG<br>GGAAAAGTACTGTGTTCTCGAGATAGGAGACATGCTCCTGCGGACTGCAATAGGCCAAGTGTCAAGGCCTATGTTCCTGTATGTGAGAACCAATGGGACTTCCAAGATCAAGATGAAATGGGGCATGGAGATGAGGCGATGCCTTCTTCAATCCCTTCAACAAATTGAGAGCATGATTGAGGCCGAGTCTTCTGTCAAAGAGAAAGACATGACCAAAGAATTCTTTGAAA<br>ACAAATCAGAAACATGGCCAATTGGAGAATCACCTAAAGGGGTGGAGGAAGGCTCCATTGGGAAGGTGTGCAGAACATTACTAGCAAAATCTGTATTCAACAGCCTATATGCATCTCCACAACTCGAGGGGTTTTCGGCTGAATCAAGAAAATTGCTTCTCATTGTTCAGGCACTTAGGGACAACCTGGAACCTGGGACCTTCGATCTTGGGGGGCTATATGAAGCAATTG<br>AGGAGTGCCTGATTAACGATCCCTGGGTTTTGCTTAATGCGTCTTGGTTCAACTCCTTCCTCACACATGCACTGAAATA-
EPI_ISL_309836	>A/Chicken/Netherlands/16007311-037-041/2016_H7N9_2016-06-08<br>ATGGAAGACTTTGTGCGACAATGCTTCAATCCAATGATTGTCGAGCTTGCGGAAAAGGCAATGAAAGAATATGGGGAAGATCCGAAAATCGAAACGAACAAATTTGCCGCAATATGCACACATTTAGAAGTCTGTTTCATGTATTCGGATTTTCATTTTATTGATGAACGGGGCGAATCAATAATTGTAGAATCTGGCGATCCGAATGCATTATTGAAACACCGATTTGAGA<br>TAATTGAAGGGAGAGACCGAACAATGGCCTGGACAGTGGTAAATAGTATCTGCAACACCACAGGAGTCGATAAGCCTAAATTCCTCCCAGATTTGTATGACTACAAAGAGAACCGATTCATTGAAATTGGAGTAACGCGAAGGGAAGTTCACATATACTATCTAGAAAAAGCCAACAAAATAAAATCAGAGAAGACACACATTCACATATTCTCATTCACTGGAGAGGAA<br>ATGGCCACCAAAGCGGACTATACCCTTGACGAGGAGAGTAGAGCAAGAATCAAAACCAGGCTGTTCACTATAAGGCAAGAGATGGCCAGTAGGGGTCTATGGGATTCCTTTCGTCAGTCCGAGAGAGGCGAAGAGACAATTGAAGAAAGATTTGAAATCACAGGAACCATGCGCAGGCTTGCCGACCAAAGTCTCCCACCGAACTTCTCCAGCCTTGAAAACTTTAGAG<br>CCTATGTGGATGGATTCGAACCGAACGGCTGCATTGAGGGCAAGCTTTCTCAAATGTCAAAAGAAGTGAACGCCAGGATTGAACCATTTCTGAAGACAACACCACGCCCTCTCAGATTACCTGATGGGCCTCCCTGCTCTCAGCGGTCGAAGTTCTTACTGATGGATGCCCTTAAATTAAGCATCGAAGACCCGAACCATGAAGGAGAAGGCATACCGCTATATGATGCAA<br>TCAAATGCATGAAGACATTTTTCGGCTGGAAAGAACCCAACATCGTGAAACCACATGAAAAAGGCATAAACCCCAATTACCTCCTGGCTTGGAAGCAGGTGCTGGCAGAACTCCAAGATATTGAAAATGAGGAGAAAATTCCAAAAACAAAGAACATGAAGAAAACAAGTCAATTGAAGTGGGCACTTGGTGAGAACATGGCACCAGAGAAAGTAGACTTTGAGGACTG<br>CAAAGACGTCAGCGATCTAAGACAATATGATAGTGATGAACCAGAGTCTAGATCGCTAGCAAGCTGGATCCAGAGCGAATTTAACAAAGCATGCGAATTGACAGATTCAAGTTGGATTGAACTTGATGAAATAGGGGAAGACGTTGCTCCAATTGAGCACATTGCGAGTATGAGAAGAAACTACTTCACAGCGGAAGTATCCCATTGCAGGGCTACTGAATACATAATGA<br>AGGGAGTGTACATAAACACAGCCCTGTTGAATGCATCCTGTGCAGCCATGGATGACTTCCAACTGATTCCAATGATAAGCAAGTGCAGAACCAAAGAAGGAAGACGAAAGACAAATCTGTATGGATTCATCATAAAAGGAAGATCCCATTTGAGGAATGACACCGATGTGGTAAACTTTGTGAGCATGGAATTCTCTCTTACGGACCCGAGGCTGGAGCCACACAAGTGG<br>GAAAAGTACTGTGTTCTCGAGATAGGAGACATGCTCCTACGGACTGCAATAGGCCAAGTGTCAAGGCCCATGTTCCTGTATGTGAGAACCAATGGGACTTCCAAGATCAAGATGAAATGGGGCATGGAGATGAGGCGGTGCCTTCTTCAATCCCTTCAACAAATTGAGAGCATGATTGAGGCCGAGTCTTCTGTCAAAGAGAAAGACATGACCAAAGAATTCTTTGAAAA<br>CAAATCAGAAACATGGCCAATTGGAGAATCACCTAAAGGGGTGGAGGAAGGCTCCATTGGGAAGGTGTGCAGAACATTACTAGCAAAATCTGTATTCAACAGCCTATATGCATCTCCACAACTCGAGGGGTTTTCGGCTGAATCAAGAAAATTGCTTCTCATTGTTCAGGCACTTAGGGACAACCTGGAACCTGGGACCTTCGATCTTGGGGGGCTATATGAAGCAATTGA<br>GGAGTGCCTGATTAACGATCCCTGGGTTTTGCTTAATGCGTCTTGGTTCAACTCCTTCCTCACACATGCACTGAAATA-
EPI_ISL_2234825	>A/chicken/Ireland/005930_20VIR5487-1/2020_H6N1_2021-03-01<br>ATGGAAGACTTTGTGCGACAATGCTTCAATCCAATGATTGTCGAGCTTGCGGAAAAGGCAATGAAAGAATATGGGGAAGATCCGAAAATCGAAACGAACAAATTTGCCGCAATATGCACACACTTAGAAGTCTGTTTCATGTATTCGGATTTTCACTTTATTAATGAACGGGGCGAATCAATAATTGTAGAATCTGGCGATCCGAATGCATTACTGAAACACCGATTTGAGA<br>TAATTGAAGGGAGAGACCGAACAATGGCCTGGACAGTGGTAAATAGTATCTGCAACACCACAGGAGTCGATAAGCCTAAATTCCTCCCAGATTTGTATGACTACAAAGAGAACCGATTCATTGAAATTGGAGTAACACGAAGGGAAGTCCACATATACTATCTAGAAAAAGCCAACAAGATAAAATCAGAGAAGACACACATTCACATATTCTCATTCACTGGAGAGGAA<br>ATGGCCACCAAAGCGGACTATACCCTTGACGAGGAGAGCAGAGCAAGAATCAAAACCAGGCTGTTCACTATAAGACAAGAAATGGCCAGTAGGGGTCTATGGGATTCCTTTCGTCAGTCCGAGAGAGGCGAAGAGACAATTGAAGAAAGATTTGAAATCACAGGAACCATGCGCAGGCTTGCCGACCAAAGTCTCCCACCGAACTTCTCCAGCCTTGAAAACTTTAGAG<br>CCTATGTGGATGGATTCGAACCGAACGGCTGCATTGAGGGCAAGCTTTCTCAAATGTCAAAAGAAGTGAACGCCAGGATTGAGCCATTTCTGAAGACAACACCACGCCCTCTCAGATTACCTGATGGGCCTCCCTGCTCTCAGCGGTCGAAGTTCTTACTGATGGATGCCCTTAAATTAAGCATCGAAGACCCGAGCCATGAAGGAGAAGGCATACCGCTATATGATGCAA<br>TCAAATGCATGAAGACATTTTTCGGCTGGAAAGAGCCCAACATCGTGAAACCACATGAAAAAGGCATAAACCCCAATTACCTCCTGGCTTGGAAGCAGGTGCTGGCAGAACTCCAAGATATTGAAAATGAGGAGAAAATTCCAAAAACAAAGAATATGAAGAAAACAAGTCAATTAAAGTGGGCACTTGGTGAGAACAGGGCACCAGAGAAAGTAGACTTTGAGGACT<br>GCAAAGACGTCAGCGATCTAAGACAATATGATAGTGATGAACCAGAGTCTAGGTCGCTAGCAAGCTGGATCCAGAGCGAATTCAACAAAGCATGCGAATTGACAGATTCAAGTTGGATTGAACTTGATGAAATAGGAGAGGACGTTGCTCCAATTGARCACATTGCGAGTATGAGAAGAAACTACTTCACAGCAGAAGTATCCCATTGTAGGGCTACTGAATACATAATG<br>AAGGGAGTGTACATAAACACAGCCCTGTTGAATGCATCCTGTGCAGCCATGGATGACTTCCAATTGATTCCAATGATAAGCAAGTGCAGAACCAAAGAAGGAAGACGGAAGACAAATCTGTATGGATTCATCATAAAAGGAAGATCCCATTTGAGGAATGACACCGATGTAGTAAACTTTGTGAGCATGGAATTCTCCCTTACGGACCCGAGGCTGGAGCCACACAAATG<br>GGAAAAGTACTGTGTTCTCGAGATAGGAGACATGCTCCTACGGACTGCAATAGGCCAAGTGTCAAGGCCCATGTTCCTGTATGTGAGAACCAATGGGACTTCCAAGATCAAGATGAAATGGGGCATGGAGATGAGGCGGTGCCTTCTTCAATCCCTTCAACAAATTGAGAGCATGATTGAGGCCGAGTCCTCTGTCAAAGAGAAAGACATGACCAAAGAATTCTTTGAAA<br>ACAAATCAGAAACATGGCCAATTGGAGAATCACCTAAAGGGGTGGAGGAAGGCTCCATTGGGAAGGTGTGCAGAACATTACTAGCAAAATCTGTATTCAACAGCCTATATGCATCTCCACAACTCGAGGGGTTTTCGGCTGAATCAAGAAAATTGCTTCTCATTGTTCAGGCACTTAGGGACAACCTGGAACCTGGGACCTTCGATCTTGGAGGGCTATATGAAGCAATTG<br>AGGAGTGCCTGATTAACGATCCCTGGGTTTTGCTTAATGCGTCTTGGTTCAACTCCTTCCTCACACATGCACTGAAATA-

EPI_ISL_399548	>A/Anas_platyrhynchos/Belgium/33_2016/2015_H6N2_2015-11-24<br>ATGGAAGACTTTGTGCGACAATGCTTCAATCCAATGATTGTCGAGCTTGCGGAAAAGGCAATGAAAGAATATGGGGAAGATCCGAAAATCGAAACGAACAAATTTGCCGCAATATGCACACACTTAGAAGTCTGTTTCATGTATTCGGATTTTCACTTTATTGATGAGCGGGGCGAATCAATAATTGTAGAATCTGGCGATCCGAATGCTTTATTGAAACACCGATTTGAGA<br>TAATTGAAGGGAGAGACCGAACAATGGCCTGGACAGTGGTAAATAGTATCTGCAACACCACAGGAGTCGATAAGCCTAAATTCCTCCCAGATTTGTATGACTACAAAGAGAACCGATTCATTGAAATTGGAGTAACACGAAGGGAAGTTCACATATACTATCTAGAAAAAGCCAACAAGATAAAATCAGAGAAGACACACATTCACATATTCTCATTCACTGGAGAGGAA<br>ATGGCCACCAAAGCGGACTATACCCTTGACGAGGAGAGCAGAGCAAGAATCAAAACCAGGCTGTTCACTATAAGGCAAGAAATGGCCAGTAGGGGTCTATGGGATTCCTTTCGTCAGTCCGAGAGAGGCGAAGAGACAATTGAAGAAAGATTTGAAATCACAGGAACCATGCGCAGGCTTGCCGACCAAAGTCTCCCACCGAACTTCTCCAGCCTTGAAAACTTTAGAG<br>CCTATGTGGATGGATTCGAACCGAACGGCTGCATTGAGGGCAAGCTTTCTCAAATGTCAAAAGAAGTGAACGCCAGGATTGAGCCATTTCTGAAGACAACGCCACGCCCTCTCAGATTACCTGATGGGCCTCCCTGCTCTCAGCGGTCGAAGTTCTTACTGATGGATGCCCTTAAATTAAGCATCGAAGACCCGAGCCATGAAGGAGAAGGCATACCGCTATATGATGCAA<br>TCAAATGCATGAAGACATTTTTCGGCTGGAAAGAGCCCAGCATCGTGAAACCACATGAAAAAGGCATAAACCCCAATTACCTCCTGGCTTGGAAGCAGGTGCTGGCAGAACTCCAAGATATTGAAAATGAGGAGAAAATTCCAAAAACAAAGAACATGAAGAAAACAAGTCAATTGAAGTGGGCACTTGGTGAGAACATGGCACCAGAGAAAGTAGACTTTGAGGACT<br>GCAAAGACGTTAGCGATCTAAGACAATATGATAGTGATGAACCAGAGTCCAGATCGCTAGCAAGCTGGATCCAGAGCGAATTCAACAAAGCATGCGAATTGACAGATTCAAGTTGGATTGAACTTGATGAAATAGGGGAAGACGTTGCTCCAATTGAGCACATTGCGAGTATGAGAAGAAACTACTTCACAGCGGAAGTATCCCATTGCAGGGCTACTGAATACATAATG<br>AAGGGAGTGTACATAAACACAGCCCTGTTGAATGCATCCTGTGCAGCCATGGATGACTTCCAACTGATTCCAATGATAAGCAAGTGCAGAACCAAAGAAGGAAGACGGAAGACAAATCTGTATGGATTCATCATAAAAGGAAGATCCCATTTGAGGAATGACACCGATGTGGTAAACTTTGTGAGCATGGAATTCTCTCTTACGGACCCGAGGCTGGAGCCACACAAGTG<br>GGAAAAGTACTGTGTTCTCGAGATAGGAGACATGCTCCTACGGACTGCAATAGGCCAAGTGTCAAGGCCCATGTTCCTGTATGTGAGAACCAATGGGACTTCCAAGATCAAGATGAAATGGGGCATGGAGATGAGGCGATGCCTTCTTCAATCCCTTCAACAAATTGAGAGCATGATTGAGGCCGAGTCCTCTGTCAAAGAGAAAGACATGACCAAAGAATTCTTTGAGA<br>ACAAATCAGAAACATGGCCAATTGGAGAATCACCTAAAGGGGTGGAGGAAGGCTCCATTGGGAAGGTGTGCAGAACATTACTAGCAAAATCTGTATTCAACAGCCTATATGCATCTCCACAGCTCGAGGGGTTTTCGGCTGAATCAAGAAAATTGCTTCTCATTGTTCAGGCACTTAGGGACAACCTGGAACCTGGGACCTTCGATCTTGGGGGGCTATATGAAGCAATTG<br>AGGAGTGCCTGATTAACGATCCCTGGGTTTTGCTTAATGCGTCTTGGTTCAACTCCTTCCTCACACATGCACTGAAATA-
EPI_ISL_5115763	>A/turkey/Germany-NW/AI02290/2021_H5N8_2021-03-02<br>ATGGAAGACTTTGTGCGACAATGCTTCAATCCAATGATTGTCGAGCTTGCGGAAAAGGCAATGAAAGAATATGGGGAAGATCCGAAAATCGAAACGAACAAATTTGCCGCAATATGCACACACTTAGAAGTCTGTTTCATGTATTCGGATTTTCACTTTATTGATGAACGGGGCGAATCAATAATTGTAGAATCTGGCGATCCGAATGCATTACTGAAACACCGATTTGAGA<br>TAATTGAAGGGAGAGACCGAACAATGGCCTGGACAGTGGTAAATAGTATCTGCAACACCACAGGAGTCGATAAGCCTAAATTCCTCCCAGATTTGTATGACTACAAAGAGAACCGATTCATTGAAATTGGAGTAACACGAAGGGAAGTCCACATATACTATCTAGAAAAGGCCAACAAGATAAAATCAGAGAAGACACACATTCACATATTCTCATTCACTGGAGAGGAA<br>ATGGCCACCAAAGCGGACTATACCCTTGACGAGGAGAGCAGAGCAAGAATCAAAACCAGGCTGTTCACTATAAGACAAGAAATGGCCAGTAGGGGTCTATGGGATTCCTTTCGTCAGTCCGAGAGAGGCGAAGAGACAATTGAAGAAAGATTTGAAATCACAGGAACCATGCGCAGGCTTGCCGACCAAAGTCTCCCACCGAACTTCTCCAGCCTTGAAAACTTTAGAG<br>CCTATGTGGATGGATTCGAACCGAACGGCTGCATTGAGGGCAAGCTTTCTCAAATGTCAAAAGAAGTGAACGCCAGGATTGAGCCATTTCTGAAGACAACGCCACGCCCTCTCAAATTACCTGATGGGCCTCCCTGCTCTCAGCGGTCGAAGTTCTTACTGATGGATGCCCTTAAATTAAGCATCGAAGACCCGAGCCATGAAGGAGAAGGCATACCGCTATATGATGCAA<br>TCAAATGCATGAAGACATTTTTCGGTTGGAAAGAGCCCAACATCGTGAAACCACATGAAAAAGGCATAAACCCCAATTACCTCCTATCTTGGAAGCAGGTACTGGCAGAACTCCAAGATATTGAAAATGAGGAGAAAATTCCAAAAACAAAAAACATGAAGAAAACAAGTCAATTAAAGTGGGCACTTGGTGAGAACATGGCACCAGAGAAAGTAGACTTTGAGGACTG<br>CAAAGATGTTAGCGATCTAAGACAATATGATAGTGATGAACCAGAGTCTAGATCGCTAGCAAGCTGGATCCAGAGCGAATTCAACAAAGCATGCGAATTGACAGATTCAAGTTGGATTGAACTTGATGAAATAGGAGAGGACGTTGCTCCAATTGAGCACATTGCGAGTATGAGAAGGAACTACTTCACAGCGGAAGTATCCCATTGTAGGGCCACTGAATACATAATGA<br>AGGGAGTGTACATAAACACAGCCCTGTTGAATGCATCCTGTGCAGCCATGGATGATTTCCAACTGATTCCAATGATAAGCAAGTGCAGAACCAAAGAAGGAAGACGGAAGACAAATCTGTATGGATTCATCATAAAAGGAAGATCCCATTTGAGGAATGACACCGATGTGGTAAACTTTGTGAGCATGGAATTCTCTCTTACGGACCCGAGGCTGGAGCCACACAAGTGG<br>GAAAAGTACTGTGTTCTCGAGATAGGAGACATGCTCCTACGGACTGCAATAGGCCAAGTGTCAAGGCCCATGTTCCTGTATGTGAGAACCAATGGGACTTCCAAGATCAAGATGAAATGGGGCATGGAGATGAGGCGGTGCCTTCTTCAATCCCTTCAACAAATTGAGAGCATGATTGAGGCCGAGTCCTCTGTCAAAGAGAAAGACATGACCAAAGAATTCTTTGAAAA<br>CAAATCAGAAACATGGCCAATTGGAGAATCACCTAAAGGGGTGGAGGAAGGCTCCATTGGGAAGGTGTGCAGAACATTACTAGCAAAATCTGTATTCAACAGCCTATATGCATCTCCACAACTCGAGGGGTTTTCGGCTGAATCAAGAAAATTGCTTCTCATTGTTCAGGCACTTAGGGACAACCTGGAACCTGGGACCTTCGATCTTGGRGGGCTATATGAAGCAATTGA<br>GGAGTGCCTGATTAACGATCCCTGGGTTTTGCTTAATGCGTCTTGGTTCAACTCCTTCCTCACACATGCACTGAAATA-
EPI_ISL_15364796	>A/Mallard/Netherlands/9/2022_H5N1_2022-09-11<br>ATGGAGGACTTTGTGCGACAATGCTTCAATCCAATGATTGTCGAGCTTGCGGAAAAGGCAATGAAAGAGTATGGGGAAGATCCGAAAATCGAAACAAACAAATTTGCCGCAATATGCACACACTTAGAAGTCTGTTTCATGTATTCGGATTTCCACTTTATTGATGAACGAGGCGAATCAACAATTGTAGAATCTGGCGATCCGAATGCATTATTGAAACACCGATTCGAGA<br>TAATTGAAGGGAGAGACCGAACTATGGCCTGGACAGTGGTGAATAGTATCTGCAACACCACAGGAGTCGAAAAGCCCAAATTCCTCCCTGATTTGTATGACTACAAAGAGAACCGATTCATTGAAATTGGAGTAACGCGAAGGGAAGTTCACATATACTATCTAGAGAAAGCCAACAAGATAAAATCAGAGAAGACTCACATTCATATATTCTCATTCACTGGAGAGGAA<br>ATGGCCACCAAGGCGGACTACACCCTTGATGAAGAGAGTAGAGCAAGAATAAAAACCAGGCTGTTCACTATAAGACAAGAAATGGCCAGTAAGGGTCTATGGGATTCCTTTCGTCAGTCCGAGAGAGGCGAAGAGACAATTGAAGAAAGATTTGAAATCACAGGAACCATGCGCAGGCTTGCCGACCAAAGTCTCCCACCGAACTTCTCCAGCCTTGAAAACTTTAGAG<br>CCTATGTGGATGGATTCGAACCGAACGGCTGCATTGAGGGCAAGCTTTCTCAAATGTCAAAAGAAGTGAATGCCAGAATTGAGCCATTTCTGAAGAAAACACCACGCCCTCTCAGATTACCTGATGGGCCTCCCTGTTCTCAGCGGTCGAAGTTCTTGCTGATGGATGCCCTCAAATTGAGCATCGAAGACCCGAGCCATGAGGGGGAGGGTATACCGCTGTATGATGCAA<br>TCAAATGCATGAAGACATTTTTGGCTGGAAAGAGCCTAACATCGTAAAACCGCATGAAAAGGGCATAAACCCTAATTACCTTCTGGCCTGGAAACAGGTGCTGGCAGAACTCCAAGATATTAAAAATGAGGAGAAAATCCCAAAAACAAAGAACATGAAGAAAACAAGCCAATTGAAGTGGGCACTTGGTGAGAACATGGCACCAGAGAAAGTGGACTTTGAGGACTG<br>TAAAGGTGTTAGTGATCTAAGACAGTACGACAGTGACGAACCAGAGTCTAGATCACTAGCAAGCTGGATCCAGAGTGAATTCAACAAGGCATGCGAATTGACAGATTCGAGTTGGATTGAACTTGATGAAATAGGGGAAGACATTGCTCCAATTGAACACATTGCGAGTATGAGGAGAAACTATTTCACAGCGGAGGTGTCCCATTGCAGGGCTACTGAATACATAATGA<br>AGGGAGTATACATAAACACAGCCCTGTTGAATGCATCCTGTGCAGCGATGGATGACTTTCAACTGATTCCTATGATAAGCAAGTGCAGAACCAAAGAAGGAAGACGGAAGACAAATTTATATGGATTCATTATAAAAGGAAGATCCCATTTGAGGAATGACACCGATGTGGTGAACTTTGTGAGCATGGAATTCTCTCTCACTGACCCGAGGCTGGAGCCACACAAATGG<br>GAAAAGTACTGTGTTCTTGAGATAGGAGACATGCTCCTACGGACTGCAATAGGCCAAGTGTCAAGGCCCATGTTCTTGTATGTGAGAACCAATGGAACTTCCAAGATCAAAATGAAGTGGGGCATGGAGATGAGACGATGCCTTCTTCAGTCCCTTCAACAAATTGAGAGCATGATTGAGGCCGAGTCTTCTGTCAAAGAGAAGGACATGACCAAGGAATTCTTTGAAAAC<br>AAATCAGAAACATGGCCAATTGGGGAGTCACCCAAAGGGGTGGAAGAAGGCTCCATTGGGAAGGTGTGCAGAACATTGCTAGCAAAGTCTGTGTTCAACAGCCTATATGCATCTCCACAACTCGAGGGGTTTTCAGCTGAATCAAGAAAATTGCTTCTCATTGTTCAGGCACTTAGGGACAACCTGGAACCTGGAACCTTCGATCTTGGGGGGCTATATGAAGCAATTGA<br>GGAGTGCCTGATTAACGATCCCTGGGTTTTGCTTAATGCGTCTTGGTTCAACTCCTTCCTCACACATGCACTGAAATA-
EPI_ISL_13778466	>A/Oystercatcher/Netherlands/2/2022_H5N1_2022-06-19<br>ATGGAGGACTTTGTGCGACAATGCTTCAATCCAATGATTGTCGAGCTTGCGGAAAAGGCAATGAAAGAGTATGGGGAAGATCCGAAAATCGAAACAAACAAATTTGCCGCAATATGCACACACTTAGAAGTCTGTTTCATGTATTCGGATTTCCACTTTATTGATGAACGAGGCGAATCAATAATTGTAGAATCTGGCGATCCGAATGCATTATTGAAACACCGATTCGAGA<br>TAATTGAAGGGAGAGACCGAACTATGGCCTGGACAGTGGTGAATAGTATCTGCAACACCACAGGAGTCGAAAAGCCCAAATTCCTCCCTGATTTGTATGACTACAAAGAGAACCGATTCATTGAAATTGGAGTAACGCGAAGGGAAGTTCACATATACTATCTAGAGAAAGCCAACAAGATAAAATCAGAGAAGACACACATTCATATATTCTCATTCACTGGAGAGGAA<br>ATGGCCACCAAGGCGGACTACACCCTTGATGAAGAGAGTAGAGCAAGAATAAAAACCAGGCTGTTCACTATAAGACAAGAAATGGCCAGTAGGGGTCTATGGGATTCCTTTCGTCAGTCCGAGAGAGGCGAAGAGACAATTGAAGAAAGATTTGAAATCACAGGAACCATGCGCAGGCTTGCCGACCAAAGTCTCCCACCGAACTTCTCCAGCCTTGAAAACTTTAGAG<br>CCTATGTGGATGGATTCGAACCGAACGGCTGCATTGAGGGCAAGCTTTCTCAAATGTCAAAAGAAGTGAATGCCAGAATTGAGCCATTTCTGAAGACAACACCACGCCCTCTCAGATTACCTGATGGGCCTCCCTGTTCTCAGCGGTCGAAGTTCTTGCTGATGGATGCCCTCAAATTGAGCATCGAAGACCCGAGCCATGAGGGGGAGGGTATACCGCTGTATGATGCAA<br>TCAAATGCATGAAGACATTTTTGGCTGGAAAGAGCCTAACATCGTAAAACCGCATGAAAAGGGCATAAACCCTAATTACCTTCTGGCTTGGAAACAGGTGCTGGCAGAACTCCAAGATATTGAAAATGAGGAGAAAATCCCAAAAACAAAGAACATGAAGAAAACAAGCCAATTGAAGTGGGCACTTGGTGAGAACATGGCACCAGAGAAAGTGGACTTTGAGGACTG<br>TAAAGATGTTAGTGATCTAAGACAGTACGACAGTGACGAACCAGAGTCTAGATCACTAGCAAGCTGGATCCAGAGTGAATTCAACAAGGCATGCGAATTGACAGATTCGAGTTGGATTGAACTTGATGAAATAGGGGAAGACATTGCTCCAATTGAACACATTGCGAGTATGAGGAGAAACTATTTCACAGCGGAGGTGTCCCATTGCAGGGCTACTGAATACATAATGA<br>AGGGAGTATACATAAACACAGCCCTGTTGAATGCATCCTGTGCAGCGATGGATGACTTTCAACTGATTCCTATGATAAGCAAGTGCAGAACCAAAGAAGGAAGACGGAAGACAAATTTATATGGATTCATTATAAAAGGAAGATCCCATTTGAGGAATGACACCGATGTGGTGAACTTTGTGAGCATGGAATTCTCTCTCACTGACCCGAGGCTGGAGCCACACAAATGG<br>GAAAAGTACTGTGTTCTTGAGATAGGAGACATGCTCCTACGGACTGCAATAGGCCAAGTGTCAAGGCCCATGTTCTTGTATGTGAGAACCAATGGAACTTCCAAGATCAAAATGAAATGGGGCATGGAGATGAGACGATGCCTTCTTCAGTCCCTTCAACAAATTGAGAGCATGATTGAGGCCGAGTCTTCTGTCAAAGAGAAGGACATGACCAAGGAATTCTTTGAAAAC<br>AAATCAGAAACATGGCCAATTGGGGAGTCACCCAAAGGGGTGGAAGAAGGCTCCATTGGGAAGGTGTGCAGAACATTGCTAGCAAAGTCTGTGTTCAACAGCCTATATGCATCTCCACAACTCGAGGGGTTTTCAGCTGAATCAAGAAAATTGCTTCTCATTGTTCAGGCACTTAGGGACAACCTGGAACCTGGAACCTTCGATCTTGGGGGGCTATATGAAGCAATTGA<br>GGAGTGCCTGATTAACGATCCCTGGGTTTTGCTTAATGCGTCTTGGTTCAACTCCTTCCTCACACATGCACTGAAATA-
EPI_ISL_14393115	>A/Fox/Netherlands/EMC5/2022_H5N1_2022-02-09<br>ATGGAGGACTTTGTGCGACAATGCTTCAATCCAATGATTGTCGAGCTTGCGGAAAAGGCAATGAAAGAGTATGGGGAAGATCCGAAAATCGAAACAAACAAATTTGCCGCAATATGCACACACTTAGAAGTCTGTTTCATGTATTCGGATTTCCACTTTATTGATGAACGAGGCGAATCAATAATTGTAGAATCTGGCGATCCGAATGCATTATTGAAACACCGATTCGAGA<br>TAATTGAAGGGAGAGACCGAACTATGGCCTGGACAGTGGTGAATAGTATCTGCAACACCACAGGAGTCGAAAAGCCCAAATTCCTCCCTGATTTGTATGACTACAAAGAGAACCGATTCATTGAAATTGGAGTAACGCGAAGGGAAGTTCACATATACTATCTAGAGAAAGCCAACAAGATAAAATCAGAGAAGACTCACATTCATATATTCTCATTCACTGGAGAGGAA<br>ATGGCCACCAAGGCGGACTACACCCTTGATGAAGAGAGTAGAGCAAGAATAAAAACCAGGCTGTTCACTATAAGACAAGAAATGGCCAGTAGGGGTCTATGGGATTCCTTTCGTCAGTCCGAGAGAGGCGAAGAGACAATTGAAGAAAGATTTGAAATCACAGGAACCATGCGCAGGCTTGCCGACCAAAGTCTCCCACCGAACTTCTCCAGCCTTGAAAACTTTAGAG<br>CCTATGTGGATGGATTCGAACCGAACGGCTGCATTGAGGGCAAGCTTTCTCAAATGTCAAAAGAAGTGAATGCCAGAATTGAGCCATTTCTGAAGACAACACCACGCCCTCTCAGATTACCTGATGGGCCTCCCTGTTCTCAGCGGTCGAAGTTCTTGCTGATGGATGCCCTCAAATTGAGCATCGAAGACCCGAGCCATGAGGGGGAGGGTATACCGCTGTATGATGCAA<br>TCAAATGCATGAAGACATTTTTGGCTGGAAAGAGCCTAACATCGTAAAACCGCATGAAAAGGGCATAAACCCTAATTACCTTCTGGCTTGGAAACAGGTGCTGGCAGAACTCCAAGATATTGAAAATGAGGAGAAAATCCCAAAAACAAAGAACATGAAGAAAACAAGCCAATTGAAGTGGGCACTTGGTGAGAACATGGCACCAGAGAAAGTGGACTTTGAGGACTG<br>TAAAGATGTTAGTGATCTAAGACAGTACGACAGTGACGAACCAGAGTCTAGATCACTAGCAAGCTGGATCCAGAGTGAATTCAACAAGGCATGCGAATTGACAGATTCGAGTTGGATTGAACTTGATGAAATAGGGGAAGACATTGCTCCAATTGAACACATTGCGAGTATGAGGAGAAACTATTTCACAGCGGAGGTGTCCCATTGCAGGGCTACTGAATACATAATGA<br>AGGGAGTATACATAAACACAGCCCTGTTGAATGCATCCTGTGCAGCGATGGATGACTTTCAACTGATTCCTATGATAAGCAAGTGCAGAACCAAAGAAGGAAGACGGAAGACAAATTTATATGGATTCATTATAAAAGGAAGATCCCATTTGAGGAATGACACCGATGTGGTGAACTTTGTGAGCATGGAATTCTCTCTCACTGACCCGAGGCTGGAGCCACACAAATGG<br>GAAAAGTACTGTGTTCTTGAGATAGGAGACATGCTCCTACGGACTGCAATAGGCCAAGTGTCAAGGCCCATGTTCTTGTATGTGAGAACCAATGGAACTTCCAAGATCAAAATGAAATGGGGCATGGAGATGAGACGATGCCTTCTTCAGTCCCTTCAACAAATTGAGAGCATGATTGAGGCCGAGTCTTCTGTCAAAGAGAAGGACATGACCAAGGAATTCTTTGAAAAC<br>AAATCAGAAACATGGCCAATTGGGGAGTCACCCAAAGGGGTGGAAGAAGGCTCCATTGGGAAGGTGTGCAGAACATTGCTAGCAAAGTCTGTGTTCAACAGCCTATATGCATCTCCACAACTCGAGGGGTTTTCAGCTGAATCAAGAAAATTGCTTCTCATTGTTCAGGCACTTAGGGACAACCTGGAACCTGGAACCTTCGATCTTGGGGGGCTATATGAAGCAATTGA<br>GGAGTGCCTGATTAACGATCCCTGGGTTTTGCTTAATGCGTCTTGGTTCAACTCCTTCCTCACACATGCACTGAAATA-
EPI_ISL_14233919	>A/Eurasian_Spoonbill/Netherlands/3/2022_H5N1_2022-07-24<br>ATGGAGGACTTTGTGCGACAATGCTTCAATCCAATGATTGTCGAGCTTGCGGAAAAGGCAATGAAAGAGTATGGGGAAGATCCGAAAATCGAAACAAACAAATTTGCCGCAATATGCACACACTTAGAAGTCTGTTTCATGTACTCGGATTTCCACTTTATTGATGAACGAGGCGAATCAATAATTGTAGAATCTGGCGATCCGAATGCATTATTGAAACACCGATTCGAGA<br>TAATTGAAGGGAGAGACCGAACTATGGCCTGGACAGTGGTGAATAGTATCTGCAACACCACAGGAGTCGAAAAGCCCAAATTCCTCCCTGATTTGTATGACTACAAAGAGAACCGATTCATTGAAATTGGAGTAACGCGAAGGGAAGTTCACATATACTATCTAGAGAAAGCCAACAAGATAAAATCAGAGAAGACTCACATTCATATATTCTCATTCACTGGAGAGGAA<br>ATGGCCACCAAGGCGGACTACACCCTTGATGAAGAGAGTAGAGCAAGAATAAAAACCAGGCTGTTCACTATAAGACAAGAAATGGCCAGTAAGGGTCTATGGGATTCCTTTCGTCAGTCCGAGAGAGGCGAAGAGACAATTGAAGAAAGATTTGAAATCACAGGAACCATGCGCAGGCTTGCCGACCAAAGTCTCCCACCGAACTTCTCCAGCCTTGAAAACTTTAGAG<br>CCTATGTGGATGGATTCAAACCGAACGGCTGCATTGAGGGCAAGCTTTCTCAAATGTCAAAAGAAGTGAATGCCAGAATTGAGCCATTTCTGAAGAAAACACCACGCCCTCTCAGATTACCTGATGGGCCTCCCTGTTCTCAGCGGTCGAAGTTCTTGCTGATGGATGCCCTCAAATTGAGCATCGAAGACCCGAGCCATGAGGGGGAGGGTATACCGCTGTATGATGCAA<br>TCAAATGCATGAAGACATTTTTGGCTGGAAAGAGCCTAACATCGTAAAACCGCATGAAAAGGGCATAAACCCTAATTACCTTCTGGCCTGGAAACAGGTGCTGGCAGAACTCCAAGATATTGAAAATGAGGAGAAAATCCCAAAAACAAAGAACATGAAGAAAACAAGCCAATTGAAGTGGGCACTTGGTGAGAACATGGCACCAGAGAAAGTGGACTTTGAGGACTG<br>TAAAGATGTTAGTGATCTAAGACAGTACGACAGTGACGAACCAGAGTCTAGATCACTAGCAAGCTGGATCCAGAGTGAATTCAACAAGGCATGCGAATTGACAGATTCGAGTTGGATTGAACTTGATGAAATAGGGGAAGACATTGCTCCAATTGAACACATTGCGAGTATGAGGAGAAACTATTTCACAGCGGAGGTGTCCCATTGCAGGGCTACTGAATACATAATGA<br>AGGGAGTATACATAAACACAGCCCTGTTGAATGCATCCTGTGCAGCGATGGATGACTTTCAACTGATTCCTATGATAAGCAAGTGCAGAACCAAAGAAGGAAGACGGAAGACAAATTTATATGGATTCATTATAAAAGGAAGATCCCATTTGAGGAATGACACCGATGTGGTGAACTTTGTGAGCATGGAATTCTCTCTCACTGACCCGAGGCTGGAGCCACACAAATGG<br>GAAAAGTACTGTGTTCTTGAGATAGGAGACATGCTCCTACGGACTGCAATAGGCCAAGTGTCAAGGCCCATGTTCTTGTATGTGAGAACCAATGGAACTTCCAAGATCAAAATGAAATGGGGCATGGAGATGAGACGATGCCTTCTTCAGTCCCTTCAACAAATTGAGAGCATGATTGAGGCCGAGTCTTCTGTCAAAGAGAAGGACATGACCAAGGAATTCTTTGAAAAC<br>AAATCAGAAACATGGCCAATTGGGGAGTCACCCAAAGGGGTGGAAGAAGGCTCCATTGGGAAGGTGTGCAGAACATTGCTAGCAAAGTCTGTGTTCAACAGCCTATATGCATCTCCACAACTCGAGGGGTTTTCAGCTGAATCAAGAAAATTGCTTCTCATTGTTCAGGCACTTAGGGACAACCTGGAACCTGGAACCTTCGATCTTGGGGGGCTATATGAAGCAATTGA<br>GGAGTGCCTTATTAACGATCCCTGGGTTTTGCTTAATGCGTCTTGGTTCAACTCCTTCCTCACACATGCACTGAAATA-
EPI_ISL_13429296	>A/Sandwich_Tern/Netherlands/2/2022_H5N1_2022-06-03<br>ATGGAGGACTTTGTGCGACAATGCTTCAATCCAATGATTGTCGAGCTTGCGGAAAAGGCAATGAAAGAGTATGGGGAAGATCCGAAAATCGAAACAAACAAATTTGCCGCAATATGCACACACTTAGAAGTCTGTTTCATGTATTCGGATTTCCACTTTATTGATGAACGAGGCGAATCAATAATTGTAGAATCTGGCGATCCGAATGCATTATTGAAACACCGATTCGAGA<br>TAATTGAAGGGAGAGACCGAACTATGGCCTGGACAGTGGTGAATAGTATCTGCAACACCACAGGAGTCGAAAAGCCCAAATTCCTCCCTGATTTGTATGACTACAAAGAGAACCGATTCATTGAAATTGGAGTAACGCGAAGGGAAGTTCACATATACTATCTAGAGAAAGCCAACAAGATAAAATCAGAGAAGACTCACATTCATATATTCTCATTCACTGGAGAGGAA<br>ATGGCCACCAAGGCGGACTACACCCTTGATGAAGAGAGTAGAGCAAGAATAAAAACCAGGCTGTTCACTATAAGACAAGAAATGGCCAGTAAGGGTCTATGGGATTCCTTTCGTCAGTCCGAGAGAGGCGAAGAGACAATTGAAGAAAGATTTGAAATCACAGGAACCATGCGCAGGCTTGCCGACCAAAGTCTCCCACCGAACTTCTCCAGCCTTGAAAACTTTAGAG<br>CCTATGTGGATGGATTCGAACCGAACGGCTGCATTGAGGGCAAGCTTTCTCAAATGTCAAAAGAAGTGAATGCCAGAATTGAGCCATTTCTGAAGAAAACACCACGCCCTCTCAGATTACCTGATGGGCCTCCCTGTTCTCAGCGGTCGAAGTTCTTGCTGATGGATGCCCTCAAATTGAGCATCGAAGACCCGAGCCATGAGGGGGAGGGTATACCGCTGTATGATGCAA<br>TCAAATGCATGAAGACATTTTTGGCTGGAAAGAGCCTAACATCGTAAAACCGCATGAAAAGGGCATAAACCCTAATTACCTTCTGGCCTGGAAACAGGTGCTGGCAGAACTCCAAGATATTGAAAATGAGGAGAAAATCCCAAAAACAAAGAACATGAAGAAAACAAGCCAATTGAAGTGGGCACTTGGTGAGAACATGGCACCAGAGAAAGTGGACTTTGAGGACTG<br>TAAAGATGTTAGTGATCTAAGACAGTACGACAGTGACGAACCAGAGTCTAGATCACTAGCAAGCTGGATCCAGAGTGAATTCAACAAGGCATGCGAATTGACAGATTCGAGTTGGATTGAACTTGATGAAATAGGGGAAGACATTGCTCCAATTGAACACATTGCGAGTATGAGGAGAAACTATTTCACAGCGGAGGTGTCCCATTGCAGGGCTACTGAATACATAATGA<br>AGGGAGTATACATAAACACAGCCCTGTTGAATGCATCCTGTGCAGCGATGGATGACTTTCAACTGATTCCTATGATAAGCAAGTGCAGAACCAAAGAAGGAAGACGGAAGACAAATTTATATGGATTCATTATAAAAGGAAGATCCCATTTGAGGAATGACACCGATGTGGTGAACTTTGTGAGCATGGAATTCTCTCTCACTGACCCGAGGCTGGAGCCACACAAATGG<br>GAAAAGTACTGTGTTCTTGAGATAGGAGACATGCTCCTACGGACTGCAATAGGCCAAGTGTCAAGGCCCATGTTCTTGTATGTGAGAACCAATGGAACTTCCAAGATCAAAATGAAATGGGGCATGGAGATGAGACGATGCCTTCTTCAGTCCCTTCAACAAATTGAGAGCATGATTGAGGCCGAGTCTTCTGTCAAAGAGAAGGACATGACCAAGGAATTCTTTGAAAAC<br>AAATCAGAAACATGGCCAATTGGGGAGTCACCCAAAGGGGTGGAAGAAGGCTCCATTGGGAAGGTGTGCAGAACATTGCTAGCAAAGTCTGTGTTCAACAGCCTATATGCATCTCCACAACTCGAGGGGTTTTCAGCTGAATCAAGAAAATTGCTTCTCATTGTTCAGGCACTTAGGGACAACCTGGAACCTGGAACCTTCGATCTTGGGGGGCTATATGAAGCAATTGA<br>GGAGTGCCTGATTAACGATCCCTGGGTTTTGCTTAATGCGTCTTGGTTCAACTCCTTCCTCACACATGCACTGAAATA-
EPI_ISL_14233926	>A/European_Herring_Gull/Netherlands/10/2022_H5N1_2022-07-14<br>ATGGAGGACTTTGTGCGACAATGCTTCAATCCAATGATTGTCGAGCTTGCGGAAAAGGCAATGAAAGAGTATGGGGAAGATCCGAAAATCGAAACAAACAAATTTGCCGCAATATGCACACACTTAGAAGTCTGTTTCATGTATTCGGATTTCCACTTTATTGATGAACGAGGCGAATCAATAATTGTAGAATCTGGCGATCCGAATGCATTATTGAAACACCGATTCGAGA<br>TAATTGAAGGGAGAGACCGAACTATGGCCTGGACAGTGGTGAATAGTATCTGCAACACCACAGGAGTCGAAAAGCCCAAATTCCTCCCTGATTTGTATGACTACAAAGAGAACCGATTCATTGAAATTGGAGTAACGCGAAGGGAAGTTCACATATACTATCTAGAGAAAGCCAACAAGATAAAATCAGAGAAGACTCACATTCATATATTCTCATTCACTGGAGAGGAA<br>ATGGCCACCAAGGCGGACTACACCCTTGATGAAGAGAGTAGAGCAAGAATAAAAACCAGGCTGTTCACTATAAGACAAGAAATGGCCAGTAAGGGTCTATGGGATTCCTTTCGTCAGTCCGAGAGAGGCGAAGAGACAATTGAAGAAAGATTTGAAATCACAGGAACCATGCGCAGGCTTGCCGACCAAAGTCTCCCACCGAACTTCTCCAGCCTTGAAAACTTTAGAG<br>CCTATGTGGATGGATTCGAACCGAACGGCTGCATTGAGGGCAAGCTTTCTCAAATGTCAAAAGAAGTGAATGCCAGAATTGAGCCATTTCTGAAGAAAACACCACGCCCTCTCAGATTACCTGATGGGCCTCCCTGTTCTCAGCGGTCGAAGTTCTTGCTGATGGATGCCCTCAAATTGAGCATCGAAGACCCGAGCCATGAGGGGGAGGGTATACCGCTGTATGATGCAA<br>TCAAATGCATGAAGACATTTTTGGCTGGAAAGAGCCTAACATCGTAAAACCGCATGAAAAGGGCATAAACCCTAATTACCTTCTGGCCTGGAAACAGGTGCTGGCAGAACTCCAAGATATTGAAAATGAGGAGAAAATCCCAAAAACAAAGAACATGAAGAAAACAAGCCAATTGAAGTGGGCACTTGGTGAGAACATGGCACCAGAGAAAGTGGACTTTGAGGACTG<br>TAAAGATGTTAGTGATCTAAGACAGTACGACAGTGACGAACCAGAGTCTAGATCACTAGCAAGCTGGATCCAGAGTGAATTCAACAAGGCATGCGAATTGACAGATTCGAGTTGGATTGAACTTGATGAAATAGGGGAAGACATTGCTCCAATTGAACACATTGCGAGTATGAGGAGAAACTATTTCACAGCGGAGGTGTCCCATTGCAGGGCTACTGAATACATAATGA<br>AGGGAGTATACATAAACACAGCCCTGTTGAATGCATCCTGTGCAGCGATGGATGACTTTCAACTGATTCCTATGATAAGCAAGTGCAGAACCAAAGAAGGAAGACGGAAGACAAATTTATATGGATTCATTATAAAAGGAAGATCCCATTTGAGGAATGACACCGATGTGGTGAACTTTGTGAGCATGGAATTCTCTCTCACTGACCCGAGGCTGGAGCCACACAAATGG<br>GAAAAGTACTGTGTTCTTGAGATAGGAGACATGCTCCTACGGACTGCAATAGGCCAAGTGTCAAGGCCCATGTTCTTGTATGTGAGAACCAATGGAACTTCCAAGATCAAAATGAAATGGGGCATGGAGATGAGACGATGCCTTCTTCAGTCCCTTCAACAAATTGAGAGCATGATTGAGGCCGAGTCTTCTGTCAAAGAGAAGGACATGACCAAGGAATTCTTTGAAAAC<br>AAATCAGAAACATGGCCAATTGGGGAGTCACCCAAAGGGGTGGAAGAAGGCTCCATTGGGAAGGTGTGCAGAACATTGCTAGCAAAGTCTGTGTTCAACAGCCTATATGCATCTCCACAACTCGAGGGGTTTTCAGCTGAATCAAGAAAATTGCTTCTCATTGTTCAGGCACTTAGGGACAACCTGGAACCTGGAACCTTCGATCTTGGGGGGCTATATGAAGCAATTGA<br>GGAGTGCCTGATTAACGATCCCTGGGTTTTGCTTAATGCGTCTTGGTTCAACTCCTTCCTCACACATGCACTGAAATA-
EPI_ISL_13778526	>A/Common_Tern/Netherlands/7/2022_H5N1_2022-06-20<br>ATGGAGGACTTTGTGCGACAATGCTTCAATCCAATGATTGTCGAGCTTGCGGAAAAGGCAATGAAAGAGTATGGGGAAGATCCGAAAATCGAAACAAACAAATTTGCCGCAATATGCACACACTTAGAAGTCTGTTTCATGTATTCGGATTTCCACTTTATTGATGAACGAGGCGAATCAATAATTGTAGAATCTGGCGATCCGAATGCATTATTGAAACACCGATTCGAGA<br>TAATTGAAGGGAGAGACCGAACTATGGCCTGGACAGTGGTGAATAGTATCTGCAACACCACAGGAGTCGAAAAGCCCAAATTCCTCCCTGATTTGTATGACTACAAAGAGAACCGATTCATTGAAATTGGAGTAACGCGAAGGGAAGTTCACATATACTATCTAGAGAAAGCCAACAAGATAAAATCAGAGAAGACTCACATTCATATATTCTCATTCACTGGAGAGGAA<br>ATGGCCACCAAGGCGGACTACACCCTTGATGAAGAGAGTAGAGCAAGAATAAAAACCAGGCTGTTCACTATAAGACAAGAAATGGCCAGTAAGGGTCTATGGGATTCCTTTCGTCAGTCCGAGAGAGGCGAAGAGACAATTGAAGAAAGATTTGAAATCACAGGAACCATGCGCAGGCTTGCCGACCAAAGTCTCCCACCGAACTTCTCCAGCCTTGAAAACTTTAGAG<br>CCTATGTGGATGGATTCGAACCGAACGGCTGCATTGAGGGCAAGCTTTCTCAAATGTCAAAAGAAGTGAATGCCAGAATTGAGCCATTTCTGAAGAAAACACCACGCCCTCTCAGATTACCTGATGGGCCTCCCTGTTCTCAGCGGTCGAAGTTCTTGCTGATGGATGCCCTCAAATTGAGCATCGAAGACCCGAGCCATGAGGGGGAGGGTATACCGCTGTATGATGCAA<br>TCAAATGCATGAAGACATTTTTGGCTGGAAAGAGCCTAACATCGTAAAACCGCATGAAAAGGGCATAAACCCTAATTACCTTCTGGCCTGGAAACAGGTGCTGGCAGAACTCCAAGATATTGAAAATGAGGAGAAAATCCCAAAAACAAAGAACATGAAGAAAACAAGCCAATTGAAGTGGGCACTTGGTGAGAACATGGCACCAGAGAAAGTGGACTTTGAGGACTG<br>TAAAGATGTTAGTGATCTAAGACAGTACGACAGTGACGAACCAGAGTCTAGATCACTAGCAAGCTGGATCCAGAGTGAATTCAACAAGGCATGCGAATTGACAGATTCGAGTTGGATTGAACTTGATGAAATAGGGGAAGACATTGCTCCAATTGAACACATTGCGAGTATGAGGAGAAACTATTTCACAGCGGAGGTGTCCCATTGCAGGGCTACTGAATACATAATGA<br>AGGGAGTATACATAAACACAGCCCTGTTGAATGCATCCTGTGCAGCGATGGATGACTTTCAACTGATTCCTATGATAAGCAAGTGCAGAACCAAAGAAGGAAGACGGAAGACAAATTTATATGGATTCATTATAAAAGGAAGATCCCATTTGAGGAATGACACCGATGTGGTGAACTTTGTGAGCATGGAATTCTCTCTCACTGACCCGAGGCTGGAGCCACACAAATGG<br>GAAAAGTACTGTGTTCTTGAGATAGGAGACATGCTCCTACGGACTGCAATAGGCCAAGTGTCAAGGCCCATGTTCTTGTATGTGAGAACCAATGGAACTTCCAAGATCAAAATGAAATGGGGCATGGAGATGAGACGATGCCTTCTTCAGTCCCTTCAACAAATTGAGAGCATGATTGAGGCCGAGTCTTCTGTCAAAGAGAAGGACATGACCAAGGAATTCTTTGAAAAC<br>AAATCAGAAACATGGCCAATTGGGGAGTCACCCAAAGGGGTGGAAGAAGGCTCCATTGGGAAGGTGTGCAGAACATTGCTAGCAAAGTCTGTGTTCAACAGCCTATATGCATCTCCACAACTCGAGGGGTTTTCAGCTGAATCAAGAAAATTGCTTCTCATTGTTCAGGCACTTAGGGACAACCTGGAACCTGGAACCTTCGATCTTGGGGGGCTATATGAAGCAATTGA<br>GGAGTGCCTGATTAACGATCCCTGGGTTTTGCTTAATGCGTCTTGGTTCAACTCCTTCCTCACACATGCACTGAAATA-
internal NVRI repository	>A/domestic_goose/Poland/H397-N/2022_H5N1_2022-09-20<br>ATGGAGGACTTTGTGCGACAATGCTTCAATCCAATGATTGTCGAGCTTGCGGAAAAGGCAATGAAAGAGTATGGGGAAGATCCGAAAATCGAAACAAACAAATTTGCCGCAATATGCACACACTTAGAAGTCTGTTTCATGTATTCGGATTTCCACTTTATTGATGAACGAGGCGAATCAATAATGGTAGAATCTGGCGATCCGAATGCATTATTGAAACACCGATTCGAG<br>ATAATTGAAGGGAGAGACCGAACTATGGCCTGGACAGTGGTGAATAGTATCTGCAGCACCACAGGAGTCGAAAAGCCCAAATTCCTCCCTGATTTGTATGACTACAAAGAGAACCGATTCATTGAAATTGGTGTAACGCGAAGGGAAGTTCACATATACTATCTAGAGAAAGCCAACAAGATAAAATCAGAGAAGACTCACATTCATATATTCTCATTCACTGGAGAGGA<br>AATGGCCACCAAGGCGGACTACACCCTTGATGAAGAGAGTAGAGCAAGAATAAAAACCAGGCTGTTCACTATAAGACAAGAAATGGCCAGTAAGGGTCTATGGGATTCCTTTCGTCAGTCCGAGAGAGGCGAAGAGACAATTGAAGAAAGATTTGAAATCACAGGAACCATGCGCAGGCTTGCCGACCAAAGTCTCCCACCGAACTTCTCCAGCCTTGAAAACTTTAGA<br>GCCTATGTGGATGGATTCGAACCGAACGGCTGCATTGAGGGCAAGCTTTCTCAAATGTCAAAAGAAGTGAATGCCAGAATTGAGCCATTTCTGAAGAAAACACCACGCCCTCTCAGATTACCTGATGGGCCTCCCTGTTCTCAGCGGTCGAAGTTCTTGCTGATGGATGCCCTCAAATTGAGCATCGAAGACCCGAGCCATGAGGGGGAGGGTATACCGCTGTATGATGCA<br>ATCAAATGCATGAAGACATTTTTGGCTGGAAAGAGCCTAACATCGTAAAACCGCATGAAAAGGGCATAAACCCTAATTACCTTCTGGCCTGGAAACAGGTGCTGGCAGAACTCCAAGATATTGAAAATGAGGAGAAAATCCCAAAAACAAAGAACATGAAGAAAACAAGCCAATTGAAGTGGGCACTTGGTGAGAATATGGCACCAGAGAAAGTGGACTTTGAGGACT<br>GTAAAGATGTTAGTGATCTAAGACAGTACGACAGTGACGAACCAGAGTCTAGATCACTAGCAAGCTGGATCCAGAGTGAATTCAACAAGGCATGCGAATTGACAGATTCGAGTTGGATTGAACTTGATGAAATAGGGGAAGACATTGCTCCAATTGAACACATTGCGAGTATGAGGAGAAACTATTTCACAGCGGAGGTGTCCCATTGCAGGGCTACTGAATACATAATG<br>AAGGGAGTATACATAAACACAGCCCTGTTGAATGCATCCTGTGCAGCGATGGATGACTTTCAACTGATTCCTATGATAAGCAAGTGCAGAACCAAAGAAGGAAGACGGAAGACAAATTTATATGGATTCATTATAAAAGGAAGATCCCATTTGAGGAATGACACCGATGTGGTGAACTTTGTGAGCATGGAATTCTCTCTCACTGACCCGAGGCTGGAGCCACACAAATG<br>GGAAAAGTACTGTGTTCTTGAGATAGGAGACATGCTCCTACGGACTGCAATAGGCCAAGTGTCAAGGCCCATGTTCTTGTATGTRAGAACCAATGGAACTTCCAAGATCAAAATGAAATGGGGCATGGAGATGAGACGATGCCTTCTTCAGTCCCTTCAACAAATTGAGAGCATGATTGAGGCCGAGTCTTCTGTCAAAGAGAAGGACATGACCAAGGAATTCTTTGAAAA<br>CAAATCAGAAACATGGCCAATTGGGGAGTCACCCAAAGGGGTGGAAGAAGGCTCCATTGGGAAGGTGTGCAGAACATTGCTAGCAAAGTCTGTGTTCAACAGCCTATATGCATCTCCACAACTCGAGGGGTTTTCAGCTGAATCAAGAAAATTGCTTCTCATTGTTCAGGCACTTAGGGACAACCTGGAACCTGGAACCTTCGATCTTGGGGGGCTATATGAAGCAATTG<br>AGGAGTGCCTGATTAACGATCCCTGGGTTTTGCTTAATGCGTCTTGGTTCAACTCCTTCCTCACACATGCACTGAAATA-
EPI_ISL_18245819	>A/swan/Poland/MB152/2022_H2N3_2022-07-15<br>ATGGAAGACTTTGTGCGACAATACTTCAATCCAATGATTGTCGAGCTTGCGGAAAAGGCAATGAAAGAATATGGGGAAGACCCGAAAATCGAAACGAACAAATTTGCCGCAATATGCACACACTTAGAAGTCTGTTTCATGTATTCGGATTTTCACTTTATTGATGAACGGGGAGAATCAATAATTGTAGAATCTGGCGATCCGAATGCATTAnTGAAACACCGATTTGAGA<br>TAATTGAAGGGAGAGACCGAACAATGGCTTGGACAGTGGTAAATAGTATCTGCAACACCACGGGAGTCGATAAGCCTAAATTCCTCCCAGATTTGTATGACTACAAAGAGAACCGATTCATTGAAATTGGAATAACACGAAGGGAAGTCCACATATACTATCTAGAAAAAGCCAACAAGATAAAATCAGAGAAGACACACATTCACATATTCTCATTCACTGGAGAGGAA<br>ATGGCCACCAAAGCGGACTATACCCTTGACGAGGAGAGCAGAGCAAGAATCAAAACCAGGCTGTTCACTATAAGACAAGAAATGGCCAGTAGGGGTCTATGGGATTCCTTTCGTCAGTCCGAGAGAGGCGAAGAGACAATTGAAGAAAGATTTGAAATCACAGGAACCATGCGCAGGCTTGCCGACCAAAGTCTCCCACCGAACTTCTCCAGCCTTGAAAACTTTAGAG<br>CCTATGTGGATGGATTCGAACCGAACGGCTGCATTGAGGGCAAGCTTTCTCAAATGTCAAAAGAAGTGAACGCCAGGATTGAGCCATTTCTGAAGACAACGCCACGCCCTCTCAGATTACCTGATGGGCCTCCCTGCTCTCAGCGGTCGAAGTTCTTACTGATGGATGCCCTTAAATTAAGCATCGAAGACCCGAGCCATGAAGGAGAAGGCATACCGCTATATGATGCAA<br>TCAAATGCATGAAGACATTTTTCGGCTGGAAAGAGCCCAACATCGTGAAACCACATGAGAAAGGCATAAACCCCAATTACCTCCTGGCTTGGAAGCAGGTGCTGGCAGAACTCCAAGATATTGAAAATGAGGAGAAAATTCCAAAAACAAAAAACATGAAGAAAACAAGTCAATTAAAGTGGGCACTTGGTGAGAACATGGCACCAGAGAAAGTAGACTTTGAGGACTG<br>TAAAGACATTAGCGATCTAAGACAATATGATRGTGATGAACCAGAGTCTAGATCGCTAGCAAGTTGGATCCAGAGTGAATTCAACAAAGCATGCGAATTGACAGATTCAAGTTGGATTGAACTTGATGAAATAGGAGAGGACGTTGCTCCAATTGAGCACATTGCGAGTATGAGAAGAAACTACTTCACAGCGGAAGTATCCCATTGTAGGGCCACTGAATACATAATGA<br>AGGGAGTGTACATAAACACAGCCCTGTTGAATGCATCCTGTGCAGCCATGGATGATTTCCAACTGATTCCAATGATAAGCAAGTGCAGAACCAAAGAAGGAAGACGGAAGACAAATCTGTATGGATTCATCATAAAAGGAAGATCCCATTTGAGGAATGACACCGATGTGGTAAACTTTGTGAGCATGGAATTCTCTCTTACGGACCCGAGGCTGGAGCCACACAAGTGG<br>GAAAAGTACTGTGTTCTCGAGATAGGAGACATGCTCCTACGGGCTGCAATAGGCCAAGTGTCAAGGCCCATGTTCCTGTATGTGAGAACCAATGGGACTTCCAAGATCAAGATGAAATGGGGCATGGAGATGAGGCGATGCCTTCTTCAATCCCTTCAACAAATTGAGAGCATGATTGAGGCCGAGTCCTCTGTCAAAGAGAAAGACATGACCAAAGAATTCTTTGAAAA<br>CAAATCAGAAACATGGCCAATTGGAGAATCACCTAAAGGGGTGGAGGAAGGCTCCATTGGGAAAGTGTGCAGAACATTACTAGCAAAATCTGTATTCAACAGCCTATATGCATCTCCACAACTCGAGGGGTTTTCGGCTGAATCAAGAAAATTGCTTCTCATTGTTCAGGCACTTAGGGACAACCTGGAACCTGGGACTTTCGATCTTGGAGGGCTATATGAAGCAATTGA<br>GGAGTGCCTGATTAATGATCCCTGGGTTTTGCTTAATGCGTCTTGGTTCAACTCCTTCCTCACACATGCACTGAAATA-
internal NVRI repository	>A/turkey/Poland/H02-T1/2022_H5N1_2022-01-01<br>ATGGAAGACTTTGTGCGACAATGCTTCAATCCAATGATTGTCGAGCTTGCGGAAAAAGCAATGAAAGAATATGGGGAAGATCCGAAAATCGAGACAAACAAATTTGCCGCAATATGCACACACTTAGAAGTCTGTTTCATGTATTCGGATTTCCATTTTATCGATGAACGAGGCGAATCAACGATTGTAGAATCTGGCGATCCAAATGCATTATTGAAACACCGATTTGAGA<br>TAATCGAAGGGAGAGATCGAGCAATGGCCTGGACAGTGGTGAATAGTATCTGCAACACCACAGGAGTCGAAAAGCCCAAATTCCTCCCTGATTTGTATGACTACAAAGAGAACCGATTCATTGAAATTGGAGTAACGCGAAGGGAAGTTCACATATACTATTTAGAAAAAGCCAACAAGATAAAATCAGAGAAAACACACATTCACATATTCTCATTCACTGGAGAGGAA<br>ATGGCCACCAAGGCGGAATACACCCTTGATGAAGAGAGCAGAGCAAGAATAAAAACCAGACTGTTCACTATAAGACAAGAAATGGCCAGTAGAGGTCTATGGGATTCCTTTCGTCAGTCCGAAAGAGGCGAAGAGACAACTGAAGAAAGATTTGAAATCACAGGAACCATGCGCAGGCTTGCCGACCAAAGCCTCCCACCGAACTTCTCCAGCCTTGAAAACTTTAGAG<br>CCTATGTGGATGGATTCGAACCGAACGGCTGCATTGAGGGCAAGCTTTCTCAAATGTCAAAAGAAGTGAACGCCAGAATTGAGCCATTCCTGAAGACAACACCACGCCCTCTCAGATTACCTGATGGGCCTCCTTGTTCTCAGCGGTCGAAGTTCTTGCTGATGGATGCCCTTAAGTTGAGCATCGAAGACCCTAGCCATGAGGGGGAGGGCATACCGCTGTATGATGCAA<br>TCAAATGCATGAAGACATTTTTGGCTGGAAAGAGCCCAACCTCGTAAAACCGCATGAGAAAGGCATAAACCCTAATTACCTCCTGGCTTGGAAGCAGGTGCTGGCAGAACTTCAAGATATTGAGAATGAGGAGAAATTTCCAAAAACAAAGAACATGAAGAAAACAAGCCAATTGAAGTGGGCACTTGGTGAGAACATGGCACCAGAAAAAGTGGACTTTGAGGACTG<br>CAAAGATGTTAGCGATCTAAGACAGTACGACAGTGACGAACCAGAGCCTAGATCACTGGCAAGCTGGATTCAGAGTGAATTCAACAAGGCATGCGAATTGACAGATTCGAGTTGGATTGAACTTGATGAGATAGGGGAAGACGTTGCTCCAATCGAACACATTGCGAGTATGAGGAGGAACTATTTCACAGCGGAGGTATCCCATTGCAGGGCCACTGAATACATAATG<br>AAGGGAGTATACATAAACACAGCCCTATTGAATGCATCCTGTGCAGCCATGGATGACTTCCAACTGATTCCAATGATAAGCAAGTGCAGAACCAAAGAGGGAAGACGGAAGACAAATCTGTATGGATTCATTATAAAAGGAAGATCCCATTTGAGGAATGACACCGATGTGGTGAACTTTGTGAGCATGGAATTCTCTCTAACTGACCCGAGGCTAGAGCCACACAAATG<br>GGAAAAGTACTGTGTTCTTGAGATAGGAGACATGCTCCTACGGACTGCAATAGGCCAAGTGTCGAGGCCCATGTTCCTGTATGTGAGAACCAATGGGACTTCCAAGATCAAAATGAAATGGGGCATGGAGATGAGACGATGCCTTCTTCAGTCCCTTCAACAAATTGAGAGCATGATTGAGGCCGAATCTTCTGTCAAAGAGAAGGACATGACCAAGGAATTCTTTGAAA<br>ACAAATCAGAAACATGGCCAATTGGAGAATCACCCAAAGGGGTTGAGGAAGGCTCTATTGGGAAAGTATGCAGAACATTGCTAGCAAAGTCTGTGTTCAACAGCCTATATGCTTCTCCACAACTCGAGGGATTTTCAGCTGAATCAAGAAAATTGCTTCTCATTGTTCAGGCACTTAGGGACAACCTGGAACCTGGAACCTTCGATCTTGGGGGGCTATATGAAGCAATTG<br>AGGAGTGCCTGATTAACGATCCCTGGGTTTTGCTTAATGCGTCTTGGTTCAACTCCTTCATCACACATGCACTGAAATA-
EPI_ISL_6929958	>A/turkey/Poland/H1910-T3/2021_H5N1_2021-11-01<br>ATGGAAGACTTTGTGCGACAATGCTTCAATCCAATGATTGTCGAGCTTGCGGAAAAAGCAATGAAAGAATATGGGGAAGATCCGAAAATCGAGACAAACAAATTTGCCGCAATATGCACACACTTAGAAGTCTGTTTCATGTATTCGGATTTCCATTTTATTGATGAACGAGGCGAATCAATGATTGTAGAATCTGGCGATTCAAATGCATTATTGAAACACCGATTTGAGA<br>TAATCGAAGGGAGAGACCGAGCAATGGCCTGGACAGTGGTGAATAGTATCTGCAACACCACAGGAGTCGAAAAGCCCAAATTCCTCCCTGATTTGTATGACTACAAAGAGAACCGATTCATTGAAATTGGAGTAACGCGAAGGGAAGTTCACATATACTATTTAGAAAAAGCCAACAAGATAAAATCGGAGAAAACACACATTCACATATTCTCATTCACTGGAGAGGAA<br>ATGGCCACCAAGGCGGAATACACCCTTGATGAAGAGAGCAGAGCAAGAATAAAAACCAGACTGTTCACTATAAGACAAGAAATGGCCAGTAGAGGTCTATGGGATTCCTTTCGTCAGTCCGAGAGAGGCGAAGAGACAACTGAAGAAAGATTTGAAATCACAGGAACCATGCGCAGGCTTGCCGACCAAAGTCTCCCACCGAACTTCTCCAGCCTTGAAAACTTTAGAG<br>CCTATGTGGATGGATTCGAACCGAACGGCTGCATTGAGGGCAAGCTTTCTCAAATGTCAAAAGAAGTGAACGCCAGAATTGAGCCATTCCTGAAGACAACACCACGCCCTCTCAGATTACCTGATGGGCCTCCTTGTTCTCAGCGGTCGAAGTTCTTGCTGATGGATGCCCTTAAGTTGAGCATCGAAGACCCTAGCCATGAGGGGGAGGGCATACCGCTGTATGATGCAA<br>TCAAATGCATGAAGACATTTTTGGCTGGAAAGAGCCCAACCTCGTAAAGCCGCATGAGAAAGGCATAAACCCTAATTACCTCCTGGCTTGGAAGCAGGTGCTGGCAGAACTTCAAGATATTGAGAATGAGGAGAAATTTCCAAAAACAAAGAACATGAAGAAAACAAGCCAATTGAAGTGGGCACTTGGTGAGAACATGGCACCAGAAAAAGTGGACTTTGAGGACTG<br>CAAAGATGTTAGCGATCTAAGACAGTACGACAGTGACGAACCAGAGCCTAGATCACTAGCAAGCTGGATTCAGAGTGAATTCAACAAGGCATGCGAATTGACAGATTCGAGTTGGATTGAACTTGATGAGATAGGGGAAGACGTTGCTCCAATCGAACACATTGCGAGTATGAGGAGGAACTATTTCACAGCGGAGGTATCCCATTGCAGGGCCACTGAATACATAATG<br>AAGGGAGTATACATAAACACAGCCCTATTGAATGCATCCTGTGCAGCCATGGATGACTTCCAACTGATTCCAATGATAAGCAAGTGCAGAACCAAAGAAGGAAGACGGAAGACAAATCTGTATGGATTCATTATAAAAGGAAGATCCCATTTGAGGAATGACACCGATGTGGTAAACTTTGTGAGCATGGAATTCTCTCTAACTGACCCGAGGCTAGAGCCACACAAATG<br>GGAAAAGTACTGTGTTCTTGAGATAGGAGACATGCTCCTACGGACTGCAATAGGCCAAGTGTCGAGGCCCATGTTCCTGTATGTGAGAACCAATGGGACTTCCAAGATCAAAATGAAATGGGGCATGGAGATGAGACGATGCCTTCTTCAGTCCCTTCAACAAATTGAGAGCATGATTGAGGCCGAATCTTCTGTCAAAGAGAAGGACATGACCAAGGAATTCTTTGAAA<br>ACAAATCAGAAACATGGCCAATTGGAGAATCACCCAAAGGGGTTGAGGAAGGCTCTATTGGGAAAGTATGCAGAACATTGCTAGCAAAGTCTGTGTTCAACAGCCTATATGCATCTCCACAACTCGAGGGATTTTCAGCTGAATCAAGAAAATTGCTTCTCATTGTTCAGGCACTTAGGGACAACCTGGAGCCTGGAACCTTCGATCTTGGGGGGCTATATGAAGCAATTG<br>AGGAGTGCCTGATTAACGATCCCTGGGTTTTGCTTAATGCGTCTTGGTTCAACTCCTTCCTCACACATGCACTGAAATA-

EPI_ISL_6929970	>A/turkey/Poland/H1911-N/2021_H5N1_2021-11-01<br>ATGGAAGACTTTGTGCGACAATGCTTCAATCCAATGATTGTCGAGCTTGCGGAAAAAGCAATGAAAGAATATGGGGAAGATCCGAAAATCGAGACAAACAAATTTGCCGCAATATGCACACACTTAGAAGTCTGTTTCATGTATTCGGATTTCCATTTTATTGATGAACGAGGCGAATCAATGATTGTAGAATCTGGCGATTCAAATGCATTATTGAAACACCGATTTGAGA<br>TAATCGAAGGGAGAGACCGAGCAATGGCCTGGACAGTGGTGAATAGTATCTGCAACACCACAGGAGTCGAAAAGCCCAAATTCCTCCCTGATTTGTATGACTACAAAGAGAACCGATTCATTGAAATTGGAGTAACGCGAAGGGAAGTTCACATATACTATTTAGRAAAAGCCAACAAGATAAAATCGGAGAAAACACACATTCACATATTCTCATTCACTGGAGAGGAA<br>ATGGCCACCAAGGCGGAATACACCCTTGATGAAGAGAGCAGAGCAAGAATAAAAACCAGACTGTTCACTATAAGACAAGAAATGGCCAGTAGAGGTCTATGGGATTCCTTTCGTCAGTCCGAGAGAGGCGAAGAGACAACTGAAGAAAGATTTGAAATCACAGGAACCATGCGCAGGCTTGCCGACCAAAGTCTCCCACCGAACTTCTCCAGCCTTGAAAACTTTAGAG<br>CCTATGTGGATGGATTCGAACCGAACGGCTGCATTGAGGGCAAGCTTTCTCAAATGTCAAAAGAAGTGAACGCCAGAATTGAGCCATTCCTGAAGACAACACCACGCCCTCTCAGATTACCTGATGGGCCTCCTTGTTCTCAGCGGTCGAAGTTCTTGCTGATGGATGCCCTTAAGTTGAGCATCGAAGACCCTAGCCATGAGGGGGAGGGCATACCGCTGTATGATGCAA<br>TCAAATGCATGAAGACATTTTTTGGCTGGAAAGAGCCCAACCTCGTAAAGCCGCATGAGAAAGGCATAAACCCTAATTACCTCCTGGCTTGGAAGCAGGTGCTGGCAGAACTTCAAGATATTGAGAATGAGGAGAAATTTCCAAAAACAAAGAACATGAAGAAAACAAGCCAATTGAAGTGGGCACTTGGTGAGAACATGGCACCAGAAAAAGTGGACTTTGAGGACTG<br>CAAAGATGTTAGCGATCTAAGACAGTACGACAGTGACGAACCAGAGCCTAGATCACTAGCAAGCTGGATTCAGAGTGAATTCAACAAGGCATGCGAATTGACAGATTCGAGTTGGATTGAACTTGATGAGATAGGGGAAGACGTTGCTCCAATCGAACACATTGCGAGTATGAGGAGGAACTATTTCACAGCGGAGGTATCCCATTGCAGGGCCACTGAATACATAATG<br>AAGGGAGTATACATAAACACAGCCCTATTGAATGCATCCTGTGCAGCCATGGATGACTTCCAACTGATTCCAATGATAAGCAAGTGCAGAACCAAAGAAGGAAGACGGAAGACAAATCTGTATGGATTCATTATAAAAGGAAGATCCCATTTGAGGAATGACACCGATGTGGTAAACTTTGTGAGCATGGAATTCTCTCTAACTGACCCGAGGCTAGAGCCACACAAATG<br>GGAAAAGTACTGTGTTCTTGAGATAGGAGACATGCTCCTACGGACTGCAATAGGCCAAGTGTCGAGGCCCATGTTCCTGTATGTGAGAACCAATGGGACTTCCAAGATCAAAATGAAATGGGGCATGGAGATGAGACGATGCCTTCTTCAGTCCCTTCAACAAATTGAGAGCATGATTGAGGCCGAATCTTCTGTCAAAGAGAAGGACATGACCAAGGAATTCTTTGAAA<br>ACAAATCAGAAACATGGCCAATTGGAGAATCACCCAAAGGGGTTGAGGAAGGCTCTATTGGGAAAGTATGCAGAACATTGCTAGCAAAGTCTGTGTTCAACAGCCTATATGCATCTCCACAACTCGAGGGATTTTCAGCTGAATCAAGAAAATTGCTTCTCATTGTTCAGGCACTTAGGGACAACCTGGAGCCTGGAACCTTCGATCTTGGGGGGCTATATGAAGCAATTG<br>AGGAGTGCCTGATTAACGATCCCTGGGTTTTGCTTAATGCGTCTTGGTTCAACTCCTTCCTCACACATGCACTGAAATA-
EPI_ISL_6930238	>A/turkey/Poland/H1913-T1/2021_H5N1_2021-11-02<br>ATGGAAGACTTTGTGCGACAATGCTTCAATCCAATGATTGTCGAGCTTGCGGAAAAAGCAATGAAAGAATATGGGGAAGATCCGAAAATCGAGACAAACAAATTTGCCGCAATATGCACACACTTAGAAGTCTGTTTCATGTATTCGGATTTCCATTTTATTGATGAACGAGGCGAATCAATGATTGTAGAATCTGGCGATTCAAATGCATTATTGAAACACCGATTTGAGA<br>TAATCGAAGGGAGAGACCGAGCAATGGCCTGGACAGTGGTGAAYAGTATCTGCAACACCACAGGAGTCGAAAAGCCCAAATTCCTCCCTGATTTGTATGACTACAAAGAGAACCGATTCATTGAAATTGGAGTAACGCGAAGGGAAGTTCACATATACTATTTAGAAAAAGCCAACAAGATAAAATCGGAGAAAACACACATTCACATATTCTCATTCACTGGAGAGGAA<br>ATGGCCACCAAGGCGGAATACACCCTTGATGAAGAGAGCAGAGCAAGAATAAAAACCAGACTGTTCACTATAAGACAAGAAATGGCCAGTAGAGGTCTATGGGATTCCTTTCGTCAGTCCGAGAGAGGCGAAGAGACAACTGAAGAAAGATTTGAAATCACAGGAACCATGCGCAGGCTTGCCGACCAAAGTCTCCCACCGAACTTCTCCAGCCTTGAAAACTTTAGAG<br>CCTATGTGGATGGATTCGAACCGAACGGCTGCATTGAGGGCAAGCTTTCTCAAATGTCAAAAGAAGTGAACGCCAGAATTGAGCCATTCCTGAAGACAACACCACGCCCTCTCAGATTACCTGATGGGCCTCCTTGTTCTCAGCGGTCGAAGTTCTTGCTGATGGATGCCCTTAAGTTGAGCATCGAAGACCCTAGCCATGAGGGGGAGGGCATACCGCTGTATGATGCAA<br>TCAAATGCATGAAGACATTTTTTGGCTGGAAAGAGCCCAACCTCGTAAAGCCGCATGAGAAAGGCATAAACCCTAATTACCTCCTGGCTTGGAAGCAGGTGCTGGCAGAACTTCAAGATATTGAGAATGAGGAGAAATTTCCAAAAACAAAGAACATGAAGAAAACAAGCCAATTGAAGTGGGCACTTGGTGAGAACATGGCACCAGAAAAAGTGGACTTTGAGGACTG<br>CAAAGATGTTAGCGATCTAAGACAGTACGACAGTGACGAACCAGAGCCTAGATCACTAGCAAGCTGGATTCAGAGTGAATTCAACAAGGCATGCGAATTGACAGATTCGAGTTGGATTGAACTTGATGAGATAGGGGAAGACGTTGCTCCAATCGAACACATTGCGAGTATGAGGAGGAACTATTTCACAGCGGAGGTATCCCATTGCAGGGCCACTGAATACATAATG<br>AAGGGAGTATACATAAACACAGCCCTATTGAATGCATCCTGTGCAGCCATGGATGACTTCCAACTGATTCCAATGATAAGCAAGTGCAGAACCAAAGAAGGAAGACGGAAGACAAATCTGTATGGATTCATTATAAAAGGAAGATCCCATTTGAGGAATGACACCGATGTGGTAAACTTTGTGAGCATGGAATTCTCTCTAACTGACCCGAGGCTAGAGCCACACAAATG<br>GGAAAAGTACTGTGTTCTTGAGATAGGAGACATGCTCCTACGGACTGCAATAGGCCAAGTGTCGAGGCCCATGTTCCTGTATGTGAGAACCAATGGGACTTCCAAGATCAAAATGAAATGGGGCATGGAGATGAGACGATGCCTTCTTCAGTCCCTTCAACAAATTGAGAGCATGATTGAGGCCGAATCTTCTGTCAAAGAGAAGGACATGACCAAGGAATTCTTTGAAA<br>ACAAATCAGAAACATGGCCAATTGGAGAATCACCCAAAGGGGTTGAGGAAGGCTCTATTGGGAAAGTATGCAGAACATTGCTAGCAAAGTCTGTGTTCAACAGCCTATATGCATCTCCACAACTCGAGGGATTTTCAGCTGAATCAAGAAAATTGCTTCTCATTGTTCAGGCACTTAGGGACAACCTGGAGCCTGGAACCTTCGATCTTGGGGGGCTATATGAAGCAATTG<br>AGGAGTGCCTGATTAACGATCCCTGGGTTTTGCTTAATGCGTCTTGGTTCAACTCCTTCCTCACACATGCACTGAAATA-
internal NVRI repository	>A/turkey/Poland/H1923_21RS3290-1/2021_H5N1_2021-11-03<br>ATGGAAGACTTTGTGCGACAATGCTTCAATCCAATGATTGTCGAGCTTGCGGAAAAAGCAATGAAAGAATATGGGGAAGATCCGAAAATCGAGACAAACAAATTTGCCGCAATATGCACACACTTAGAAGTCTGTTTCATGTATTCGGATTTCCATTTTATTGATGAACGAGGCGAATCAATGATTGTAGAATCTGGCGATTCAAATGCATTATTGAAACACCGATTTGAGA<br>TAATCGAAGGGAGAGACCGAGCAATGGCCTGGACAGTGGTGAATAGTATCTGCAACACCACAGGAGTCGAAAAGCCCAAATTCCTCCCTGATTTGTATGACTACAAAGAGAACCGATTCATTGAAATTGGAGTAACGCGAAGGGAAGTTCACATATACTATTTAGAAAAAGCCAACAAGATAAAATCGGAGAAAACACACATTCACATATTCTCATTCACTGGAGAGGAA<br>ATGGCCACCAAGGCGGAATACACCCTTGATGAAGAGAGCAGAGCAAGAATAAAAACCAGACTGTTCACTATAAGACAAGAAATGGCCAGTAGAGGTCTATGGGATTCCTTTCGTCAGTCCGAGAGAGGCGAAGAGACAACTGAAGAAAGATTTGAAATCACAGGAACCATGCGCAGGCTTGCCGACCAAAGTCTCCCACCGAACTTCTCCAGCCTTGAAAACTTTAGAG<br>CCTATGTGGATGGATTCGAACCGAACGGCTGCATTGAGGGCAAGCTTTCTCAAATGTCAAAAGAAGTGAACGCCAGAATTGAGCCATTCCTGAAGACAACACCACGCCCTCTCAGATTACCTGATGGGCCTCCTTGTTCTCAGCGGTCGAAGTTCTTGCTGATGGATGCCCTTAAGTTGAGCATCGAAGACCCTAGCCATGAGGGGGAGGGCATACCGCTGTATGATGCAA<br>TCAAATGCATGAAGACATTTTTTGGCTGGAAAGAGCCCAACCTCGTAAAGCCGCATGAGAAAGGCATAAACCCTAATTACCTCCTGGCTTGGAAGCAGGTGCTGGCAGAACTTCAAGATATTGAGAATGAGGAGAAATTTCCAAAAACAAAGAACATGAAGAAAACAAGCCAATTGAAGTGGGCACTTGGTGAGAACATGGCACCAGAAAAAGTGGACTTTGAGGACTG<br>CAAAGATGTTAGCGATCTAAGACAGTACGACAGTGACGAACCAGAGCCTAGATCACTAGCAAGCTGGATTCAGAGTGAATTCAACAAGGCATGCGAATTGACAGATTCGAGTTGGATTGAACTTGATGAGATAGGGGAAGACGTTGCTCCAATCGAACACATTGCGAGTATGAGGAGGAACTATTTCACAGCGGAGGTATCCCATTGCAGGGCCACTGAATACATAATG<br>AAGGGAGTATACATAAACACAGCCCTATTGAATGCATCCTGTGCAGCCATGGATGACTTCCAACTGATTCCAATGATAAGCAAGTGCAGAACCAAAGAAGGAAGACGGAAGACAAATCTGTATGGATTCATTATAAAAGGAAGATCCCATTTGAGGAATGACACCGATGTGGTAAACTTTGTGAGCATGGAATTCTCTCTAACTGACCCGAGGCTAGAGCCACACAAATG<br>GGAAAAGTACTGTGTTCTTGAGATAGGAGACATGCTCCTACGGACTGCAATAGGCCAAGTGTCGAGGCCCATGTTCCTGTATGTGAGAACCAATGGGACTTCCAAGATCAAAATGAAATGGGGCATGGAGATGAGACGATGCCTTCTTCAGTCCCTTCAACAAATTGAGAGCATGATTGAGGCCGAATCTTCTGTCAAAGAGAAGGACATGACCAAGGAATTCTTTGAAA<br>ACAAATCAGAAACATGGCCAATTGGAGAATCACCCAAAGGGGTTGAGGAAGGCTCTATTGGGAAAGTATGCAGAACATTGCTAGCAAAGTCTGTGTTCAACAGCCTATATGCATCTCCACAACTCGAGGGATTTTCAGCTGAATCAAGAAAATTGCTTCTCATTGTTCAGGCACTTAGGGACAACCTGGAGCCTGGAACCTTCGATCTTGGGGGGCTATATGAAGCAATTG<br>AGGAGTGCCTGATTAACGATCCCTGGGTTTTGCTTAATGCGTCTTGGTTCAACTCCTTCCTCACACATGCACTGAAATA-
EPI_ISL_6930564	>A/turkey/Poland/H1924-T1/2021_H5N1_2021-11-03<br>ATGGAAGACTTTGTGCGACAATGCTTCAATCCAATGATTGTCGAGCTTGCGGAAAAAGCAATGAAAGAATATGGGGAAGATCCGAAAATCGAGACAAACAAATTTGCCGCAATATGCACACACTTAGAAGTCTGTTTCATGTATTCGGATTTCCATTTTATTGATGAACGAGGCGAATCAATGATTGTAGAATCTGGCGATTCAAATGCATTATTGAAACACCGATTTGAGA<br>TAATCGAAGGGAGAGACCGAGCAATGGCCTGGACAGTGGTGAATAGTATCTGCAACACCACAGGAGTCGAAAAGCCCAAATTCCTCCCTGATTTGTATGACTACAAAGAGAACCGATTCATTGAAATTGGAGTAACGCGAAGGGAAGTTCACATATACTATTTAGAAAAAGCCAACAAGATAAAATCGGAGAAAACACACATTCACATATTCTCATTCACTGGAGAGGAA<br>ATGGCCACCAAGGCGGAATACACCCTTGATGAAGAGAGCAGAGCAAGAATAAAAACCAGACTGTTCACTATAAGACAAGAAATGGCCAGTAGAGGTCTATGGGATTCCTTTCGTCAGTCCGAGAGAGGCGAAGAGACAACTGAAGAAAGATTTGAAATCACAGGAACCATGCGCAGGCTTGCCGACCAAAGTCTCCCACCGAACTTCTCCAGCCTTGAAAACTTTAGAG<br>CCTATGTGGATGGATTCGAACCGAACGGCTGCATTGAGGGCAAGCTTTCTCAAATGTCAAAAGAAGTGAACGCCAGAATTGAGCCATTCCTGAAGACAACACCACGCCCTCTCAGATTACCTGATGGGCCTCCTTGTTCTCAGCGGTCGAAGTTCTTGCTGATGGATGCCCTTAAGTTGAGCATCGAAGACCCTAGCCATGAGGGGGAGGGCATACCGCTGTATGATGCAA<br>TCAAATGCATGAAGACATTTTTTGGCTGGAAAGAGCCCAACCTCGTAAAGCCGCATGAGAAAGGCATAAACCCTAATTACCTCCTGGCTTGGAAGCAGGTGCTGGCAGAACTTCAAGATATTGAGAATGAGGAGAAATTTCCAAAAACAAAGAACATGAAGAAAACAAGCCAATTGAAGTGGGCACTTGGTGAGAACATGGCACCAGAAAAAGTGGACTTTGAGGACTG<br>CAAAGATGTTAGCGATCTAAGACAGTACGACAGTGACGAACCAGAGCCTAGATCACTAGCAAGCTGGATTCAGAGTGAATTCAACAAGGCATGCGAATTGACAGATTCGAGTTGGATTGAACTTGATGAGATAGGGGAAGACGTTGCTCCAATCGAACACATTGCGAGTATGAGGAGGAACTATTTCACAGCGGAGGTATCCCATTGCAGGGCCACTGAATACATAATG<br>AAGGGAGTATACATAAACACAGCCCTATTGAATGCATCCTGTGCAGCCATGGATGACTTCCAACTGATTCCAATGATAAGCAAGTGCAGAACCAAAGAAGGAAGACGGAAGACAAATCTGTATGGATTCATTATAAAAGGAAGATCCCATTTGAGGAATGACACCGATGTGGTAAACTTTGTGAGCATGGAATTCTCTCTAACTGACCCGAGGCTAGAGCCACACAAATG<br>GGAAAAGTACTGTGTTCTTGAGATAGGAGACATGCTCCTACGGACTGCAATAGGCCAAGTGTCGAGGCCCATGTTCCTGTATGTGAGAACCAATGGGACTTCCAAGATCAAAATGAAATGGGGCATGGAGATGAGACGATGCCTTCTTCAGTCCCTTCAACAAATTGAGAGCATGATTGAGGCCGAATCTTCTGTCAAAGAGAAGGACATGACCAAGGAATTCTTTGAAA<br>ACAAATCAGAAACATGGCCAATTGGAGAATCACCCAAAGGGGTTGAGGAAGGCTCTATTGGGAAAGTATGCAGAACATTGCTAGCAAAGTCTGTGTTCAACAGCCTATATGCATCTCCACAACTCGAGGGATTTTCAGCYGAATCAAGAAAATTGCTTCTCATTGTTCAGGCACTTAGGGACAACCTGGAGCCTGGAACCTTCGATCTTGGGGGGCTATATGAAGCAATTG<br>AGGAGTGCCTGATTAACGATCCCTGGGTTTTGCTTAATGCGTCTTGGTTCAACTCCTTCCTCACACATGCACTGAAATA-
internal NVRI repository	>A/turkey/Poland/H1941_21RS3290-3/2021_H5N1_2021-11-06<br>ATGGAAGACTTTGTGCGACAATGCTTCAATCCAATGATTGTCGAGCTTGCGGAAAAAGCAATGAAAGAATATGGGGAAGATCCGAAAATCGAGACAAACAAATTTGCCGCAATATGCACACACTTAGAAGTCTGTTTCATGTATTCGGATTTCCATTTTATTGATGAACGAGGCGAATCAATGATTGTAGAATCTGGCGATCCAAATGCATTATTGAAACACCGATTTGAGA<br>TAATCGAAGGGAGAGACCGAGCAATGGCCTGGACAGTGGTGAATAGTATCTGCAACACCGCAGGAGTCGAAAAGCCCAAATTCCTCCCTGATTTGTATGACTACAAAGAGAACCGATTCATTGAAATTGGAGTAACGCGAAGGGAAGTTCACATATACTATTTAGAAAAAGCCAACAAGATAAAATCAGAGAAAACACACATTCACATATTCTCATTCACTGGAGAGGAA<br>ATGGCCACCAAGGCGGAATACACCCTTGATGAAGAGAGCAGAGCAAGAATAAAAACCAGACTGTTCACTATAAGACAAGAAATGGCCAGTAGAGGTCTATGGGATTCCTTTCGTCAGTCCGAGAGAGGCGAAGAGACAACTGAAGAAAGATTTGAAATCACAGGAACCATGCGCAGGCTTGCCGACCAAAGTCTCCCACCGAACTTCTCCAGCCTTAAAAACTTTAGAG<br>CCTATGTGGATGGATTCGAACCGAACGGCTACATTGAGGGCAAGCTTTCTCAAATGTCAAAAGAAGTGAACGCCAGAATTGAGCCATTCCTGAAGACAACACCACGCCCTCTCAGATTACCTGATGGGCCTCCTTGTTCTCAGCGGTCGAAGTTCTTGCTGATGGATGCCCTTAAGTTGAGCATCGAAGACCCTAGCCATGAGGGGGAGGGCATACCGCTGTATGATGCAA<br>TCAAATGCATGAAGACATTTTTTGGCTGGAAAGAGCCAAACCTCGTAAAGCCGCATGAGAAAGGCATAAACCTTAATTACCTCCTGGCTTGGAAGCAGGTGCTGGCAGAACTTCAAGATATTGAGAATGAGGAGAAATTTCCAAAAACAAAGAACATGAAGAAAACAAGCCAATTGAAGTGGGCACTTGGTGAGAACATGGCACCAGAAAAAGTGGACTTTGAGGATTG<br>CAAAGATGTTAGCGATCTAAGACAGTACGACAGTGACGAACCAGAGCCTAGATCACTAGCAAGCTGGATTCAGAGTGAATTCAACAAGGCATGCGAATTGACAGATTCGAGTTGGATTGAACTTGATGAGATAGGGGAAGACGTTGCTCCAATCGAACACATTGCGAGTATGAGGAGGAACTATTTCACAGCGGAGGTATCCCATTGCAGGGCCACTGAATACATAATG<br>AAGGGAGTATACATAAACACAGCCCTATTGAATGCATCCTGTGCAGCCATGGATGACTTCCAACTGATTCCAATGATAAGCAAGTGCAGAACCAAAGAAGGAAGACGGAAGACAAATCTGTATGGATTCATTATAAAAGGAAGATCCCATTTGAGGAATGACACCGATGTGGTAAACTTTGTGAGCATGGAATTCTCTCTAACTGACCCGAGGCTAGAGCCACACAAATG<br>GGAAAAGTACTGTGTTCTTGAGATAGGAGACATGCTCCTACGGACTGCAATAGGCCAAGTGTCGAGGCCCATGTTCCTGTATGTGAGAACCAATGGGACTTCCAAGATCAAAATGAAATGGGGCATGGAGATGAGACGATGCCTTCTTCAGTCCCTTCAACAAATTGAGAGCATGATTGAGGCCGAATCTTCTGTCAAAGAGAAGGACATGACCAAGGATTTCTTTGAAAA<br>CAAATCAGAAACATGGCCAATTGGAGAATCACCCAAAGGGGTTGAGGAAGGCTCTATTGGGAAAGTATGCAGAACATTGCTAGCAAAGTCTGTGTTCAACAGCCTATATGCATCTCCACAACTCGAGGGATTTTCAGCTGAATCAAGAAAATTGCTTCTCATTGTTCAGGCACTTAGGGACAACCTGGAACCTGGAACCTTCGATCTTGGGGGGCTATATGAAGCAATTGA<br>GGAGTGCCTGATTAACGATCCCTGGGTTTTGCTTAATGCGTCTTGGTTCAACTCCTTCCTCACACATGCACTGAAATA-
EPI_ISL_6935584	>A/turkey/Poland/H1944-N/2021_H5N1_2021-11-08<br>ATGGAAGACTTTGTGCGACAATGCTTCAATCCAATGATTGTCGAGCTTGCGGAAAAAGCAATGAAAGAATATGGGGAAGATCCGAAAATCGAGACAAACAAATTTGCCGCAATATGCACACACTTAGAAGTCTGTTTCATGTATTCGGATTTCCATTTTATTGATGAACGAGGCGAATCAATGATTGTAGAATCTGGCGATTCAAATGCATTATTGAAACACCGATTTGAGA<br>TAATCGAAGGGAGAGACCGAGCAATGGCCTGGACAGTGGTGAATAGTATCTGCAACACCACAGGAGTCGAAAAGCCCAAATTCCTCCCTGATTTGTATGACTACAAAGAGAACCGATTCATTGAAATTGGAGTAACGCGAAGGGAAGTTCACATATACTATTTAGAAAAAGCCAACAAGATAAAATCGGAGAAAACACACATTCACATATTCTCATTCACTGGAGAGGAA<br>ATGGCCACCAAGGCGGAATACACCCTTGATGAAGAGAGCAGAGCAAGAATAAAAACCAGACTGTTCACTATAAGACAAGAAATGGCCAGTAGAGGTCTATGGGATTCCTTTCGTCAGTCCGAGAGAGGCGAAGAGACAACTGAAGAAAGATTTGAAATCACAGGAACCATGCGCAGGCTTGCCGACCAAAGTCTCCCACCGAACTTCTCCAGCCTTGAAAACTTTAGAG<br>CCTATGTGGATGGATTCGAACCGAACGGCTGCATTGAGGGCAAGCTTTCTCAAATGTCAAAAGAAGTGAACGCCAGAATTGAGCCATTCCTGAAGACAACACCACGCCCTCTCAGATTACCTGATGGGCCTCCTTGTTCTCAGCGGTCGAAGTTCTTGCTGATGGATGCCCTTAAGTTGAGCATCGAAGACCCTAGCCATGAGGGGGAGGGCATACCGCTGTATGATGCAA<br>TCAAATGCATGAAGACATTTTTTGGCTGGAAAGAGCCCAACCTCGTAAAGCCGCATGAGAAAGGCATAAACCCTAATTACCTCCTGGCTTGGAAGCAGGTGCTGGCAGAACTTCAAGATATTGAGAATGAGGAGAAATTTCCAAAAACAAAGAACATGAAGAAAACAAGCCAATTGAAGTGGGCACTTGGTGAGAACATGGCACCAGAAAAAGTGGACTTTGAGGACTG<br>CAAAGATGTTAGCGATCTAAGACAGTACGACAGTGACGAACCAGAGCCTAGATCACTAGCAAGCTGGATTCAGAGTGAATTCAACAAGGCATGCGAATTGACAGATTCGAGTTGGATTGAACTTGATGAGATAGGGGAAGACGTTGCTCCAATCGAACACATTGCGAGTATGAGGAGGAACTATTTCACAGCGGAGGTATCCCATTGCAGGGCCACTGAATACATAATG<br>AAGGGAGTATACATAAACACAGCCCTATTGAATGCATCCTGTGCAGCCATGGATGACTTCCAACTGATTCCAATGATAAGCAAGTGCAGAACCAAAGAAGGAAGACGGAAGACAAATCTGTATGGATTCATTATAAAAGGAAGATCCCATTTGAGGAATGACACCGATGTGGTAAACTTTGTGAGCATGGAATTCTCTCTAACTGACCCGAGGCTAGAGCCACACAAATG<br>GGAAAAGTACTGTGTTCTTGAGATAGGAGACATGCTCCTACGGACTGCAATAGGCCAAGTGTCGAGGCCCATGTTCCTGTATGTGAGAACCAATGGGACTTCCAAGATCAAAATGAAATGGGGCATGGAGATGAGACGATGCCTTCTTCAGTCCCTTCAACAAATTGAGAGCATGATTGAGGCCGAATCTTCTGTCAAAGAGAAGGACATGACCAAGGAATTCTTTGAAA<br>ACAAATCAGAAACATGGCCAATTGGAGAATCACCCAAAGGGGTTGAGGAAGGCTCTATTGGGAAAGTATGCAGAACATTGCTAGCAAAGTCTGTGTTCAACAGCCTATATGCATCTCCACAACTCGAGGGATTTTCAGCTGAATCAAGAAAATTGCTTCTCATTGTTCAGGCACTTAGGGACAACCTGGAGCCTGGAACCTTCGATCTTGGGGGGCTATATGAAGCAATTG<br>AGGAGTGCCTGATTAACGATCCCTGGGTTTTGCTTAATGCGTCTTGGTTCAACTCCTTCCTCACACATGCACTGAAATA-
internal NVRI repository	>A/turkey/Poland/H1977_21RS3290-10/2021_H5N1_2021-11-16<br>ATGGAAGACTTTGTGCGACAATGCTTCAATCCAATGATTGTCGAGCTTGCGGAAAAAGCAATGAAAGAATATGGGGAAGATCCGAAAATCGAGACAAACAAATTTGCCGCAATATGCACACACTTAGAAGTCTGTTTCATGTATTCGGATTTCCATTTTATTGATGAACGAGGCGAATCAATGATTGTAGAATCTGGCGATTCAAATGCATTATTGAAACACCGATTTGAGA<br>TAATCGAAGGGAGAGACCGAGCAATGGCCTGGACAGTGGTGAATAGTATCTGCAACACCACAGGAGTCGAAAAGCCCAAATTCCTCCCTGATTTGTATGACTACAAAGAGAACCGATTCATTGAAATTGGAGTAACGCGAAGGGAAGTTCACATATACTATTTAGAAAAAGCCAACAAGATAAAATCGGAGAAAACACACATTCACATATTCTCATTCACTGGAGAGGAA<br>ATGGCCACCAAGGCGGAATACACCCTTGATGAAGAGAGCAGAGCAAGAATAAAAACCAGACTGTTCACTATAAGACAAGAAATGGCCAGTAGAGGTCTATGGGATTCCTTTCGTCAGTCCGAGAGAGGCGAAGAGACAACTGAAGAAAGATTTGAAATCACAGGAACCATGCGCAGGCTTGCCGACCAAAGTCTCCCACCGAACTTCTCCAGCCTTGAAAACTTTAGAG<br>CCTATGTGGATGGATTCGAACCGAACGGCTGCATTGAGGGCAAGCTTTCTCAAATGTCAAAAGAAGTGAACGCCAGAATTGAGCCATTCCTGAAGACAACACCACGCCCTCTCAGATTACCTGATGGGCCTCCTTGTTCTCAGCGGTCGAAGTTCTTGCTGATGGATGCCCTTAAGTTGAGCATCGAAGACCCTAGCCATGAGGGGGAGGGCATACCGCTGTATGATGCAA<br>TCAAATGCATGAAGACATTTTTTGGCTGGAAAGAGCCCAACCTCGTAAAGCCGCATGAGAAAGGCATAAACCCTAATTACCTCCTGGCTTGGAAGCAGGTGCTGGCAGAACTTCAAGATATTGAGAATGAGGAGAAATTTCCAAAAACAAAGAACATGAAGAAAACAAGCCAATTGAAGTGGGCACTTGGTGAGAACATGGCACCAGAAAAAGTGGACTTTGAGGACTG<br>CAAAGATGTTAGCGATCTAAGACAGTACGACAGTGACGAACCAGAGCCTAGATCACTAGCAAGCTGGATTCAGAGTGAATTCAACAAGGCATGCGAATTGACAGATTCGAGTTGGATTGAACTTGATGAGATAGGGGAAGACGTTGCTCCAATCGAACACATTGCGAGTATGAGGAGGAACTATTTCACAGCGGAGGTATCCCATTGCAGGGCCACTGAATACATAATG<br>AAGGGAGTATACATAAACACAGCCCTATTGAATGCATCCTGTGCAGCCATGGATGACTTCCAACTGATTCCAATGATAAGCAAGTGCAGAACCAAAGAAGGAAGACGGAAGACAAATCTGTATGGATTCATTATAAAAGGAAGATCCCATTTGAGGAATGACACCGATGTGGTAAACTTTGTGAGCATGGAATTCTCTCTAACTGACCCGAGGCTAGAGCCACACAAATG<br>GGAAAAGTACTGTGTTCTTGAGATAGGAGACATGCTCCTACGGACTGCAATAGGCCAAGTGTCGAGGCCCATGTTCCTGTATGTGAGAACCAATGGGACTTCCAAGATCAAAATGAAATGGGGCATGGAGATGAGACGATGCCTTCTTCAGTCCCTTCAACAAATTGAGAGCATGATTGAGGCCGAATCTTCTGTCAAAGAGAAGGACATGACCAAGGAATTCTTTGAAA<br>ACAAATCAGAAACATGGCCAATTGGAGAATCACCCAAAGGGGTTGAGGAAGGCTCTATTGGGAAAGTATGCAGAACATTGCTAGCAAAGTCTGTGTTCAACAGCCTATATGCATCTCCACAACTCGAGGGATTTTCAGCTGAATCAAGAAAATTGCTTCTCATTGTTCAGGCACTTAGGGACAACCTGGAGCCTGGAACCTTCGATCTTGGGGGGCTATATGAAGCAATTG<br>AGGAGTGCCTGATTAACGATCCCTGGGTTTTGCTTAATGCGTCTTGGTTCAACTCCTTCCTCACACATGCACTGAAATA-
internal NVRI repository	>A/turkey/Poland/H2043-T2/2021_H5N1_2021-11-27<br>ATGGAAGACTTTGTGCGACAATGCTTCAATCCAATGATTGTCGAGCTTGCGGAAAAAGCAATGAAAGAATATGGGGAAGATCCGAAAATCGAGACAAACAAATTTGCCGCAATATGCACACACTTAGAAGTCTGTTTCATGTATTCGGATTTCCATTTTATTGATGAACGAGGCGAATCAATGATTGTAGAATCTGGCGATCCAAATGCATTATTGAAACACCGATTTGAGA<br>TAATCGAAGGGAGAGACCGAGCAATGGCCTGGACAGTGGTGAATAGTATCTGCAACACCACAGGAGTCGAAAAGCCCAAATTCCTCCCTGATTTGTATGACTACAAAGAGAACCGATTCATTGAAATTGGAGTAACGCGAAGGGAAGTTCACATATACTATTTAGAAAAAGCCAACAAGATAAAATCAGAGAAAACACACATTCACATATTCTCATTCACTGGAGAGGAA<br>ATGGCCACCAAGGCGGAATACACCCTTGATGAAGAGAGCAGAGCAAGAATAAAAACCAGACTGTTCACTATAAGACAAGAAATGGCCAGTAGAGGTCTATGGGATTCCTTTCGTCAGTCCGAGAGAGGCGAAGAGACAACTGAAGAAAGATTTGAAATCACAGGAACCATGCGCAGGCTTGCCGACCAAAGTCTCCCACCGAACTTCTCCAGCCTTGAAAACTTTAGAG<br>CCTATGTGGATGGATTCGAACCGAACGGCTGCATTGAGGGCAAGCTTGCTCAAATGTCAAAAGAAGTGAACGCCAGAATTGAGCCGTTCCTGAAGACAACACCACGCCCTCTCAGATTGCCTGATGGGCCTCCTTGTTCTCAGCGGTCGAAGTTCTTGCTGATGGATGCCCTTAAGTTGAGCATCGAAGACCCTAGCCATGAGGGGGAGGGCATACCGCTGTATGATGCAA<br>TCAAATGCATGAAGACATTTTTTGGCTGGAAAGAGCCCAACCTCGTAAAGCCGCATGAGAAAGGCATAAACCTTAATTACCTCCTGGCTTGGAAGCAGGTGCTGGCAGAACTTCAAGATATTGAGAATGAGGAGAAATTTCCAAAAACAARGAACATGAAGAAAACAAGCCAATTGAAGTGGGCACTTGGTGAGAACATGGCACCAGAAAAAGTGGACTTTGAGGACTG<br>CAAAGATGTTAGCGATCTAAGACAGTACGACAGTGACGAACCAGAGCCTAGATCACTAGCAAGCTGGATTCAGAGTGAATTCAACAAGGCATGCGAATTGACAGATTCGAGTTGGATTGAACTTGATGAGATAGGGGAAGACGTTGCTCCAATCGAACACATTGCGAGTATGAGGAGGAACTATTTCACAGCGGAGGTATCCCATTGCAGGGCCACTGAATACATAATG<br>AAGGGAGTATACATAAACACAGCCCTATTGAATGCATCCTGTGCAGCCATGGATGACTTCCAACTGATTCCAATGATAAGCAAGTGCAGAACCAAAGAAGGAAGACGGAAGACAAATCTGTATGGATTCATTATAAAAGGAAGATCCCATTTGAGGAATGACACCGATGTGGTAAACTTTGTGAGCATGGAATTCTCTCTAACTGACCCGAGGCTAGAGCCACACAAATG<br>GGAAAAGTACTGTGTTCTTGAGATAGGAGACATGCTCCTACGGACTGCAATAGGCCAAGTGTCGAGGCCCATGTTCCTGTATGTGAGAACCAATGGGACTTCCAAGATCAAAATGAAATGGGGCATGGAGATGAGACGATGCCTTCTTCAGTCCCTTCAACAAATTGAGAGCATGATTGAGGCCGAATCTTCTGTCAAAGAGAAGGACATGACCAAGGAATTCTTTGAAA<br>ACAAATCAGAAACATGGCCAATTGGAGAATCACCCAAAGGGGTTGAGGAAGGCTCTATTGGGAAAGTATGCAGAACATTGCTAGCAAAGTCTGTGTTCAACAGCCTATATGCATCTCCACAACTCGAGGGATTTTCAGCTGAATCAAGAAAATTGCTTCTCATTGTTCAGGCACTTAGGGACAACCTGGAACCTGGAACCTTCGATCTTGGGGGGCTATATGAAGCAATTG<br>AGGAGTGCCTGATTAACGATCCCTGGGTTTTGCTTAATGCGTCTTGGTTCAACTCCTTCCTCACACATGCACTGAAATA-
internal NVRI repository	>A/turkey/Poland/H2113-T1/2021_H5N1_2021-12-15<br>ATGGAAGACTTTGTGCGACAATGCTTCAATCCAATGATTGTCGAGCTTGCAGAAAAAGCAATGAAAGAATATGGGGAAGATCCGAAAATCGAAACAAACAAATTTGCCGCAATATGCACACACTTAGAAGTCTGTTTCATGTATTCGGATTTCCATTTTATTGATGAACGAGGCGAATCAATGATTGTAGAATCTGGCGATCCAAATGCATTATTGAAACACCGATTTGAGA<br>TAATCGAAGGGAGAGACCGAGCAATGGCCTGGACAGTGGTGAATAGTATCTGCAACACCACAGGAGTCGAAAAGCCCAAATTCCTCCCTGATTTGTATGACTACAAAGAGAACCGATTCATTGAAATTGGAGTAACGCGAAGGGAAGTTCACATATACTATTTAGAAAAAGCCAACAAGATAAAATCAGAGAAAACACACATTCACATATTCTCATTCACTGGAGAGGAA<br>ATGGCCACCAGGGCGGAATACACCCTTGATGAAGAGAGCAGAGCAAGAATAAAAACCAGACTGTTCACTATAAGACAAGAAATGGCCAGTAGAGGTCTATGGGATTCCTTTCGTCAGTCCGAGAGAGGCGAAGAGACAACTGAAGAAAGATTTGAAATCACAGGAACCATGCGCAGGCTTGCCGACCAAAGTCTCCCACCGAACTTCTCCAGCCTTGAAAACTTTAGAG<br>CCTATGTGGATGGATTCGAACCGAACGGCTGCATTGAGGGCAAGCTTTCTCAAATGTCAAAAGAAGTGAACGCCAGAATTGAGCCATTCCTGAAGACAACACCACGCCCTCTCAGATTACCTGATGGGCCTCCTTGTTCTCAGCGGTCGAAGTTCTTGCTGATGGATGCCCTTAAGTTGAGCATCGAAGACCCTAGCCATGAGGGGGAGGGCATACCGCTGTATGATGCAA<br>TCAAATGTATGAAGACATTTTTTGGCTGGAAAGAGCCCAACCTCGTAAAGCCGCATGAGAAGGGCATAAACCCTAATTACCTCCTGGCTTGGAAGCAGGTGCTGGCAGAACTTCAAGATATTGAGAATGAGGAGAAATTTCCAAAAACAAAGAACATGAAGAAAACAAGCCAATTGAAGTGGGCACTTGGTGAGAACATGGCACCAGAAAAAGTGGACTTTGAGGACTG<br>CAAAGATGTTAGCGATCTAAGACAGTACGACAGTGACGAACCAGAGCCTAGATCACTAGCAAGCTGGATTCAGAGTGAATTCAACAAGGCATGCGAATTGACAGATTCGAGTTGGATTGAACTTGATGAGATAGGGGAAGACGTTGCTCCAATCGAACACATTGCGAGTATGAGGAGGAACTATTTCACAGCGGAGGTATCCCATTGCAGGGCCACTGAATACATAATG<br>AAGGGAGTATACATAAACACAGCCCTATTGAATGCATCCTGTGCAGCCATGGATGACTTCCAACTGATTCCAATGATAAGCAAGTGCAGAACCAAAGAGGGAAGACGGAAGACAAATCTGTATGGATTCATTATAAAAGGAAGATCCCATTTGAGGAATGACACCGATGTGGTAAACTTTGTGAGCATGGAATTCTCTCTAACTGACCCGAGGCTAGAGCCACACAAATG<br>GGAAAAGTACTGTGTTCTTGAGATAGGAGACATGCTCCTACGGACTGCAATAGGCCAAGTGTCGAGGCCCATGTTCCTGTATGTGAGAACCAATGGGACTTCCAAGATCAAAATGAAATGGGGCATGGAGATGAGACGATGCCTTCTTCAGTCCCTTCAACAAATTGAGAGCATGATTGAGGCCGAATCTTCTGTCAAAGAGAAGGACATGACCAAGGAATTCTTTGAAA<br>ACAAATCAGAAACATGGCCAATTGGAGAATCACCCAAAGGGGTTGAGGAAGGCTCTATTGGGAAAGTATGCAGAACATTGCTAGCAAAGTCTGTGTTCAACAGCCTATATGCATCTCCACAACTCGAGGGATTTTCAGCTGAATCAAGAAAATTGCTTCTCATTGTTCAGGCACTTAGGGACAACCTGGAACCTGGAACCTTCGATCTTGGGGGGCTATATGAAGCAATTG<br>AGGAGTGCCTGATTAACGATCCCTGGGTTTTGCTTAATGCGTCTTGGTTCAACTCCTTCATCACACATGCACTGAAATA-
internal NVRI repository	>A/turkey/Poland/H2118-N/2021_H5N1_2021-12-16<br>ATGGAAGACTTTGTGCGACAATGCTTCAATCCAATGATTGTCGAGCTTGCGGAAAAAGCAATGAAAGAATATGGGGAAGATCCGAAAATCGAGACAAACAAATTTGCCGCAATATGCACACACTTAGAAGTCTGTTTCATGTATTCGGATTTCCATTTTATTGATGAACGAGGCGAATCAACGATTGTAGAATCTGGCGATCCAAATGCATTATTGAAACACCGATTTGAGA<br>TAATCGAAGGGAGAGACCGAGCAATGGCCTGGACAGTGGTGAATAGTATCTGCAACACCACAGGAGTCGAAAAGCCCAAATTCCTCCCTGATTTGTATGACTACAAAGAGAACCGATTCATTGAAATTGGAGTAACGCGAAGGGAAGTTCACATATACTATTTAGAAAAAGCCAACAAGATAAAATCAGAGAAAACACACATTCACATATTCTCATTCACTGGAGAGGAA<br>ATGGCCACCAAGGCGGAATACACCCTTGATGAAGAGAGCAGAGCAAGAATAAAAACCAGACTGTTCACTATAAGACAAGAAATGGCCAGTAGAGGTCTATGGGATTCCTTTCGTCAGTCCGAAAGAGGCGAAGAGACAATTGAAGAAAAAATTTGAAATCACAGGAACCATGCGCAGGCTTGCCGACCAAAGCCTCCCACCGAACTTCTCCAGCCTTGAAAACTTTAGAG<br>CCTATGTGGATGGATTCGAACCGAACGGCTGCATTGAGGGCAAGCTTTCTCAAATGTCAAAAGAAGTGAACGCCAGAATTGAGCCATTCCTGAAGACAACACCACGCCCTCTCAGATTACCTGATGGGCCTCCTTGTTCTCAGCGGTCGAAGTTCTTGCTGATGGATGCCCTTAAGTTGAGCATCGAAGACCCTAGCCATGAGGGGGAGGGCATACCGCTGTATGATGCAA<br>TCAAATGCATGAAGACATTTTTTGGCTGGAAAGAGCCCAACCTCGTAAAGCCGCATGAGAAAGGCATAAACCCTAATTACCTCCTGGCTTGGAAGCAGGTGCTGGCAGAACTTCAAGATATTGAGAATGAGGAGAAATTTCCAAAAACAAAGAACATGAAGAAAACAAGCCAATTGAAGTGGGCACTTGGTGAGAACATGGCACCAGAAAAAGTGGACTTTGAGGACTG<br>CAAAGATGTTAGCGATCTAAGACAGTACGACAGTGACGAACCAGAGCCTAGATCACTGGCAAGCTGGATTCAGAGTGAATTCAACAAGGCATGCGAATTGACAGATTCGAGTTGGATTGAACTTGATGAGATAGGGGAAGACGTTGCTCCAATCGAACACATTGCGAGTATGAGGAGGAACTATTTCACAGCGGAGGTATCCCATTGCAGGGCCACTGAATACATAATG<br>AAGGGAGTATACATAAACACAGCCCTATTGAATGCATCCTGTGCAGCCATGGATGACTTCCAACTGATTCCAATGATAAGCAAGTGCAGAACCAAAGAGGGAAGACGGAAGACAAATCTGTATGGATTCATTATAAAAGGAAGATCCCATTTGAGGAATGACACCGATGTGGTGAACTTTGTGAGCATGGAATTCTCTCTAACTGACCCGAGGCTAGAGCCACACAAATG<br>GGAAAAGTACTGTGTTCTTGAGATAGGAGACATGCTCCTACGGACTGCAATAGGCCAAGTGTCGAGGCCCATGTTCCTGTATGTGAGAACCAATGGGACTTCCAAGATCAAAATGAAATGGGGCATGGAGATGAGACGATGCCTTCTTCAGTCCCTTCAACAAATTGAGAGCATGATTGAGGCCGAATCTTCTGTCAAAGAGAAGGACATGACCAAGGAATTCTTTGAAA<br>ACAAATCAGAAACATGGCCAATTGGAGAATCACCCAAAGGGGTTGAGGAAGGCTCTATTGGGAAAGTATGCAGAACATTGCTAGCAAAGTCTGTGTTCAACAGCCTATATGCTTCTCCACAACTCGAGGGATTTTCAGCTGAATCAAGAAAATTGCTTCTCATTGTTCAGGCACTTAGGGACAACCTGGAACCTGGAACCTTCGATCTTGGGGGGCTATATGAAGCAATTG<br>AGGAGTGCCTGATTAACGATCCCTGGGTTTTGCTTAATGCGTCTTGGTTCAACTCCTTCATCACACATGCACTGAAATA-
internal NVRI repository	>A/turkey/Poland/H2179-T4/2021_H5N1_2021-12-29<br>ATGGAAGACTTTGTGCGACAATGCTTCAATCCAATGATTGTCGAGCTTGCGGAAAAGGCAATGAAAGAATATGGGGAAGATCCGAAAATCGAGACAAACAAATTTGCCGCAATATGCACACACTTAGAAGTCTGTTTCATGTATTCGGATTTCCATTTTATTGATGAACGAGGCGAATCAATAATTGTAGAATCTGGCGATCCGAATGCATTATTGAAACACCGATTTGAAA<br>TAATCGAGGGGAGAGACCGAACAATGGCCTGGACAGTGGTGAATAGTATCTGCAACACCACAGGAGTCGAAAAGCCCAAATTCCTCCCTGATTTGTATGACTACAAAGAAAACCGATTCATTGAGATTGGAGTAACGCGGAGGGAAGTTCACATATACTATTTAGAAAAAGCCAACAAGATAAAATCAGAGAAGACACACATTCACATATTCTCATTCACTGGAGAGGAA<br>ATGGCCACCAAGGCGGACTACACCCTTGATGAAGAGAGCAGAGCAAGAATAAAAACCAGACTGTTCACTATAAGACAAGAAATGGCCAGTAGAGGTCTATGGGATTCCTTTCGTCAGTCCGAGAGAGGCGAAGAGACAATTGAAGAAAGATTTGAAATCACAGGAACCATGCGCAGGCTTGCCGACCAAAGTCTCCCACCGAACTTCTCCAGCCTTGAAAACTTTAGAG<br>CCTATGTGGATGGATTCGAACCGAACGGCTGCATTGAGGGCAAGCTTTCTCAAATGTCAAAAGAAGTGAACGCCAGAATTGAGCCATTTCTGAAGACAACACCACGCCCTCTCAAATTACCTGATGGGCCTCCCTGTTCTCAGCGGTCGAAGTTCTTGCTAATGGATGCCCTTAAACTGAGCATCGAAGACCCTAGCCATGAGGGGGAGGGCATACCGCTGTATGATGCAA<br>TCAAATGCATGAAGACATTTTTTGGCTGGAAAGAGCCCAACATCGTAAAAACGCATGAGAAAGGCATAAACCCTAATTACCTCCTGGCTTGGAAGCAGGTGCTGGCAGAACTTCAAGATATTGAAAATGAGGAGAAAATTCCAAAAACAAAGAACATGAAGAAAACAAGTCAATTGAAGTGGGCACTTGGTGAGAACATGGCACCAGAAAAAGTGGACTTTGAGGACTG<br>CAAAGATGTTAGCGATCTAAGACAGTATGACAGTGACGAACCAGAGTCTAGATCACTAGCAAGCTGGATTCAGAGTGAATTCAACAAGGCATGCGAATTGACAGATTCGAGTTGGATTGAGCTTGATGAGATAGGGGAAGACGTTGCTCCAATCGAACACATTGCGAGTATGAGGAGGAACTATTTCACAGCAGAGGTATCCCATTGCAGGGCCACTGAGTACATAATG<br>AAGGGAGTATACATAAACACAGCCCTATTGAATGCATCCTGTGCAGCCATGGATGACTTCCAACTGATTCCAATGATAAGCAAGTGCAGGACCAAAGAAGGAAGACGGAAGACAAATCTGTATGGGTTCATTATAAAAGGAAGATCCCACTTGAGGAATGACACCGATGTGGTAAACTTTGTGAGCATGGAATTCTCTCTAACTGACCCGAGGCTAGAGCCACACAAATG<br>GGAAAAGTACTGTGTTCTTGAGATAGGAGACATGCTCCTACGGACTGCAATAGGCCAAGTGTCGAGGCCCATGTTCCTATATGTGAGAACCAATGGGACTTCCAAGATCAAAATGAAATGGGGCATGGAGATGAGACGATGCCTTCTTCAGTCCCTTCAACAAATTGAGAGCATGATTGAGGCCGAGTCTTCTGTCAAAGAGAAGGACATGACCAAGGAATTCTTTGAAA<br>ACAAATCAGAGACATGGCCAATTGGAGAATCACCCAAAGGGGTGGAGGAAGGCTCCATTGGAAAAGTATGCAGAACATTGCTAGCAAAGTCTGTGTTCAACAGCCTATATGCATCTCCACAACTCGAGGGGTTTTCAGCTGAGTCAAGAAAATTGCTTCTCATTGTTCAGGCACTTAGGGACAACCTGGAACCTGGAACCTTCGATCTTGGGGGGCTATATGAAGCAATTG<br>AGGAGTGCCTGATTAATGATCCCTGGGTTTTGCTTAATGCGTCTTGGTTCAACTCCTTCCTCACACATGCACTGAAATA-
internal NVRI repository	>A/turkey/Poland/H2188-Ni/2021_H5N1_2021-12-30<br>ATGGAAGACTTTGTGCGACAATGCTTCAATCCAATGATTGTCGAGCTTGCGGAAAAGGCAATGAAAGAGTATGGGGAAGATCCGAAAATCGAAACAAACAAATTTGCCGCAATATGCACACACTTAGAAGTCTGTTTCATGTATTCGGATTTCCACTTTATTGATGAACGCGGCGAATCAATAATTGTAGAATCTGGCGATCCGAATGCATTATTGAAACACCGATTCGAGA<br>TAATTGAAGGGAGAGACCGAACTATGGCCTGGACAGTGGTGAATAGTATCTGCAACACCACAGGAGTCGAAAAGCCCAAATTCCTCCCTGATTTGTATGACTACAAAGAGAACCGATTCATTGAAATTGGAGTAACGCGAAGGGAAGTTCACATATACTATCTAGAGAAAGCCAACAAGATAAAATCAGAGAAGACACACATTCATATATTCTCATTCACTGGAGAGGAA<br>ATGGCCACCAAGGCGGACTACACCCTTGATGAAGAGAGTAGAGCAAGAATAAAAACCAGGCTATTCACTATAAGACAAGAAATGGCCAGTAGGGGTCTATGGGATTCCTTTCGTCAGTCCGAGAGAGGCGAAGAGACAATTGAAGAAAGATTTGAAATCACAGGAACCATGCGCAGGCTTGCCGACCAAAGTCTCCCACCGAACTTCTCCAGCCTTGAAAACTTTAGAG<br>CCTATGTGGATGGATTCGAACCGAACGGCTGCATTGAGGGCAAGCTTTCTCAAATGTCAAAAGAAGTGAATGCCAGAATTGAGCCATTTCTGAAGACAACACCACGCCCTCTCAGATTACCTGATGGGCCTCCCTGTTCTCAGCGGTCGAAGTTCTTGCTGATGGATGCCCTCAAATTGAGCATCGAAGACCCGAGCCATGAGGGGGAGGGTATACCGCTGTATGATGCAA<br>TCAAATGCATGAAGACATTTTTTGGCTGGAAAGAGCCTAACATCGTAAAACCGCATGAAAAGGGCATAAACCCTAATTACCTYCTGGCTTGGAAACAGGTGCTGGCAGAACTCCAAGATATTGAAAATGAGGAGAAAATCCCAAAAACAAAGAACATGAAGAAAACAAGCCAATTGAAGTGGGCACTTGGTGAGAACATGGCACCAGAGAAAGTGGACTTTGAGGACTG<br>TAAAGATRTTAGTGATCTAAGACAGTACGACAGTGACGAACCAGAGTCTAGATCACTAGCAAGCTGGATCCAGAGTGAATTCAACAAGGCATGCGAATTGACAGATTCGAGTTGGATTGAACTTGATGAAATAGGGGAAGACATTGCTCCAATTGAACACATTGCGAGTATGAGGAGAAACTATTTCACAGCGGAGGTGTCCCATTGCAGGGCTACTGAATACATAATGA<br>AGGGAGTATACATAAACACAGCCCTGTTGAATGCATCCTGTGCAGCGATGGATGACTTCCAACTGATTCCTATGATAAGCAAATGCAGAACCAAAGAAGGAAGACGGAAGACAAATTTATATGGATTCATTATAAAAGGAAGATCCCATTTGAGGAATGACACCGATGTGGTGAACTTTGTGAGCATGGAATTCTCTCTCACTGACCCGAGGCTGGAGCCACACAAATGG<br>GAAAAGTACTGTGTTCTTGAGATAGGAGACATGCTCCTACGGACTGCAATAGGCCATGTGTCAAGGCCCATGTTCCTGTATGTGAGAACCAATGGAACTTCCAAGATCAAAATGAAATGGGGCATGGAGATGAGACGATGCCTTCTTCAGTCCCTTCAACAAATTGAGAGCATGATTGAGGCCGAGTCTTCTGTCAAAGAGAAGGACATRACCAAGGAATTCTTTGAAAAC<br>AAATCAGAAACATGGCCAATTGGGGAATCACCCAAAGGGGTGGAAGAAGGCTCCATTGGGAAGGTGTGCAGAACATTGCTAGCAAAGTCTGTGTTCAACAGCCTATATGCATCTCCACAACTCGAGGGGTTTTCAGCTGAATCAAGAAAATTGCTTCTCATTGTTCAGGCACTTAGGGACAACCTGGAACCTGGAACCTTCGATCTTGGGGGGCTATATGAAGCAATTGA<br>GGAGTGCCTGATTAACGATCCCTGGGTTTTGCTTAATGCGTCTTGGTTCAACTCCTTCCTCACACATGCACTGAAATA-
internal NVRI repository	>A/turkey/Poland/H296/2022_H5N1_2022-04-21<br>ATGGAAGACTTTGTGCGACAATGCTTCAATCCAATGATTGTCGAGCTTGCGGAAAAGGCAATGAAAGAATATGGGGARGATCCGAAAATCGAGACAAACAAATTTGCCGCAATATGCACACACTTAGAAGTCTGTTTCATGTATTCGGATTTCCATTTTATTGATGAACGAGGCGAATCAATAATTGTAGAATCTGGCGATCCGAATGCATTATTAAAACACCGATTTGAAA<br>TAATCGAGGGGAGAGACCGAACAATGGCCTGGACAGTGGTGAATAGTATCTGCAACACCACAGGAGTCGAAAAGCCCAAATTCCTCCCTGATTTGTATGACTACAAAGAAAACCGATTCATTGAGATTGGAGTAACGCGGAGGGAAGTTCACATATACTATTTAGAAAAAGCCAACAAGATAAAATCAGAGAAGACACACATTCACATATTCTCATTCACTGGAGAGGAA<br>ATGGCCACCAAGGCGGACTACACCCTTGATGAAGAGAGCAGAGCAAGAATAAAAACCAGACTGTTCACTATAAGACAAGAAATGGCCAGTAGAGGTCTATGGGATTCCTTTCGTCAGTCCGAGAGAGGCGAAGAGACAATTGAAGAAAGATTTGAAATCACAGGAACCATGCGCAGGCTTGCCGACCAAAGTCTCCCACCGAACTTCTCCAGCCTTGAAAACTTTAGAG<br>CCTATGTGGATGGATTCGAACCGAACGGCTGCATTGAGGGCAAGCTTTCTCAAATGTCAAAAGAAGTGAACGCCAGAATTGAGCCATTTCTGAAGACAACACCACGCCCTCTCAAATTACCTGATGGGCCTCCCTGTTCTCAGCGGTCGAAGTTCTTGCTAATGGATGCCCTTAAACTGAGCATCGAAGACCCTAGCCATGAGGGGGAGGGCATACCGCTGTATGATGCAA<br>TCAAATGCATGAAGACATTTTTTGGCTGGAAAGAGCCCAACATCGTAAAAACGCATGAGAAAGGCATAAACCCTAATTACCTCCTGGCTTGGAAGCAGGTGCTGGCAGAACTTCAAGATATTGAAAATGAGGAGAAAATTCCAAAAACAAAGAACATGAAGAAAACAAGTCAATTGAAGTGGGCACTTGGTGAGAACATGGCACCAGAAAAAGTGGACTTTGAGGACTG<br>CAAAGATGTTAGCGATCTAAGACAGTATGACAGTGACGAACCAGAGTCTAGATCACTAGCAAGCTGGATTCAGAGTGAATTCAACAAGGCATGCGAATTGACAGATTCGAGTTGGATTGAGCTTGATGAGATAGGGGAAGACGTTGCTCCAATCGAGCACATTGCGAGTATGAGGAGGAACTATTTCACAGCAGAGGTATCCCATTGCAGGGCCACTGAGTACATAATG<br>AAGGGAGTTTACATAAACACAGCCCTATTGAATGCATCCTGTGCAGCCATGGATGACTTCCAACTGATTCCAATGATAAGCAAGTGCAGGACCAAAGAAGGAAGACGGAAGACAAATCTGTATGGGTTCATTATAAAAGGAAGATCCCACTTGAGGAATGACACCGATGTGGTAAACTTTGTGAGCATGGAATTCTCTCTAACTGACCCGAGGCTAGAGCCACACAAATG<br>GGAAAAGTACTGTGTTCTTGAGATAGGAGACATGCTCCTACGGACTGCAATAGGCCAAGTGTCGAGGCCCATGTTCCTATATGTGAGAACCAATGGGACTTCCAAGATCAAAATGAAATGGGGCATGGAGATGAGACGATGCCTTCTTCAGTCCCTTCAACAAATTGAGAGCATGATTGAGGCCGAGTCTTCTGTCAAAGAGAAGGACATGACCAAGGAATTCTTTGAAA<br>ACAAATCAGAGACATGGCCAATTGGAGAATCACCCAAAGGGGTGGAGGAAGGCTCCATTGGAAAAGTATGCAGAACATTGCTAGCAAAGTCTGTGTTCAACAGCCTATATGCATCTCCACAACTCGAGGGGTTTTCAGCTGAGTCAAGAAAATTGCTTCTCATTGTTCAGGCACTTAGGGACAACCTGGAACCTGGAACCTTCGATCTTGGGGGGCTATATGAAGCAATTG<br>AGGAGTGCCTGATTAATGATCCCTGGGTTTTGCTTAATGCGTCTTGGTTCAACTCCTTCCTCACACATGCACTGAAATA-

internal NVRI repository	>A/turkey/Poland/H297/2022_H5N1_2022-04-21<br>ATGGAAGACTTTGTGCGACAATGCTTCAATCCAATGATTGTCGAGCTTGCGGAAAAGGCAATGAAAGAATATGGGGAAGATCCGAAAATCGAGACAAACAAATTTGCCGCAATATGCACACACTTAGAAGTCTGTTTCATGTATTCGGATTTCCATTTTATTGATGAACGAGGCGAATCAATAATTGTAGAATCTGGCGATCCGAATGCATTATTAAAACACCGATTTGAAA<br>TAATCGAAGGGAGAGACCGAACAATGGCCTGGACAGTGGTGAATAGTATCTGCAACACCACAGGAGTCGAAAAGCCCAAATTCCTCCCTGATTTGTATGACTACAAAGAAAACCGATTCATTGAGATTGGAGTAACGCGGAGGGAAGTTCACATATACTATTTAGAAAAAGCCAACAAGATAAAATCAGAGAAGACACACATTCACATATTCTCATTCACTGGAGAGGAA<br>ATGGCCACCAAGGCGGACTACACCCTTGATGAAGAGAGCAGAGCAAGAATAAAAACCAGACTGTTCACTATAAGACAAGAAATGGCCAGTAGAGGTCTATGGGATTCCTTTCGTCAGTCCGAGAGAGGCGAAGAGACAATTGAAGAAAGATTTGAAATCACAGGAACCATGCGCAGGCTTGCCGACCAAAGTCTCCCACCGAACTTCTCCAGCCTTGAAAACTTTAGAG<br>CCTATGTGGATGGATTCGAACCGAACGGCTGCATTGAGGGCAAGCTTTCTCAAATGTCAAAAGAAGTGAACGCCAGAATTGAGCCATTTCTGAAGACAACACCACGCCCTCTCAAATTACCTGATGGGCCTCCCTGTTCTCAGCGGTCGAAGTTCTTGCTAATGGATGCCCTTAAACTGAGCATCGAAGACCCTAGCCATGAGGGGGAGGGCATACCGCTGTATGATGCAA<br>TCAAATGCATGAAGACATTTTTTGGCTGGAAAGAGCCCAACATCGTAAAAACGCATGAGAAAGGCATAAACCCTAATTACCTCCTGGCTTGGAAGCAGGTGCTGGCAGAACTTCAAGATATTGAAAATGAGGAGAAAATTCCAAAAACAAAGAACATGAAGAAAACAAGTCAATTGAAGTGGGCACTTGGTGAGAACATGGCACCAGAAAAAGTGGACTTTGAGGACTG<br>CAAAGATGTTAGCGATCTAAGACAGTATGACAGTGACGAACCAGAGTCTAGATCACTAGCAAGCTGGATTCAGAGTGAATTCAACAAGGCATGCGAATTGACAGATTCGAGTTGGATTGAGCTTGATGAGATAGGGGAAGACGTTGCTCCAATCGAGCACATTGCGAGTATGAGGAGGAACTATTTCACAGCAGAGGTATCCCATTGCAGGGCCACTGAGTACATAATG<br>AAGGGAGTTTACATAAACACAGCCCTATTGAATGCATCCTGTGCAGCCATGGATGACTTCCAACTGATTCCAATGATAAGCAAGTGCAGGACCAAAGAAGGAAGACGGAAGACAAATCTGTATGGGTTCATTATAAAAGGAAGATCCCACTTGAGGAATGACACCGATGTGGTAAACTTTGTGAGCATGGAATTCTCTCTAACTGACCCGAGGCTAGAGCCACACAAATG<br>GGAAAAGTACTGTGTTCTTGAGATAGGAGACATGCTCCTACGGACTGCAATAGGCCAAGTGTCGAGGCCCATGTTCCTATATGTGAGAACCAATGGGACTTCCAAGATCAAAATGAAATGGGGCATGGAGATGAGACGATGCCTTCTTCAGTCCCTTCAACAAATTGAGAGCATGATTGAGGCCGAGTCTTCTGTCAAAGAGAAGGACATGACCAAGGAATTCTTTGAAA<br>ACAAATCAGAGACATGGCCAATTGGAGAATCACCCAAAGGGGTGGAGGAAGGCTCCATTGGAAAAGTATGCAGAACATTGCTAGCAAAGTCTGTGTTCAACAGCCTATATGCATCTCCACAACTCGAGGGGTTTTCAGCTGAGTCAAGAAAATTGCTTCTCATTGTTCAGGCACTTAGGGACAACCTGGAACCTGGAACCTTCGATCTTGGGGGGCTATATGAAGCAATTG<br>AGGAGTGCCTGATTAATGATCCCTGGGTTTTGCTTAATGCGTCTTGGTTCAACTCCTTCCTCACACATGCACTGAAATA-
internal NVRI repository	>A/turkey/Poland/H303/2022_H5N1_2022-04-26<br>ATGGAAGACTTTGTGCGACAATGCTTCAATCCAATGATTGTCGAGCTTGCGGAAAAGGCAATGAAAGAATATGGGGAAGATCCGAAAATCGAGACAAACAAATTTGCCGCAATATGCACACACTTAGAAGTCTGTTTCATGTATTCGGATTTCCATTTTATTGATGAACGAGGCGAATCAATAATTGTAGAATCTGGCGATCCGAATGCATTATTAAAACACCGATTTGAAA<br>TAATCGAAGGGAGAGACCGAACAATGGCCTGGACAGTGGTGAATAGTATCTGCAACACCACAGGAGTCGAAAAGCCCAAATTCCTCCCTGATTTGTATGACTACAAAGAAAACCGATTCATTGAGATTGGAGTAACGCGGAGGGAAGTTCACATATACTATTTAGAAAAAGCCAACAAGATAAAATCAGAGAAGACACACATTCACATATTCTCATTCACTGGAGAGGAA<br>ATGGCCACCAAGGCGGACTACACCCTTGATGAAGAGAGCAGAGCAAGAATAAAAACCAGACTGTTCACTATAAGACAAGAAATGGCCAGTAGAGGTCTATGGGATTCCTTTCGTCAGTCCGAGAGAGGCGAAGAGACAATTGAAGAAAGATTTGAAATCACAGGAACCATGCGCAGGCTTGCCGACCAAAGTCTCCCACCGAACTTCTCCAGCCTTGAAAACTTTAGAG<br>CCTATGTGGATGGATTCGAACCGAACGGCTGCATTGAGGGCAAGCTTTCTCAAATGTCAAAAGAAGTGAACGCCAGAATTGAGCCATTTCTGAAGACAACACCACGCCCTCTCAAATTACCTGATGGGCCTCCCTGTTCTCAGCGGTCGAAGTTCTTGCTAATGGATGCCCTTAAACTGAGCATCGAAGACCCTAGCCATGAGGGGGAGGGCATACCGCTGTATGATGCAA<br>TCAAATGCATGAAGACATTTTTTGGCTGGAAAGAGCCCAACATCGTAAAAACGCATGAGAAAGGCATAAACCCTAATTACCTCCTGGCTTGGAAGCAGGTGCTGGCAGAACTTCAAGATATTGAAAATGAGGAGAAAATTCCAAAAACAAAGAACATGAAGAAAACAAGTCAATTGAAGTGGGCACTTGGTGAGAACATGGCACCAGAAAAAGTGGACTTTGAGGACTG<br>CAAAGATGTTAGCGATCTAAGACAGTATGACAGTGACGAACCAGAGTCTAGATCACTAGCAAGCTGGATTCAGAGTGAATTCAACAAGGCATGCGAATTGACAGATTCGAGTTGGATTGAGCTTGATGAGATAGGGGAAGACGTTGCTCCAATCGAGCACATTGCGAGTATGAGGAGGAACTATTTCACAGCAGAGGTATCCCATTGCAGGGCCACTGAGTACATAATG<br>AAGGGAGTTTACATAAACACAGCCCTATTGAATGCATCCTGTGCAGCCATGGATGACTTCCAACTGATTCCAATGATAAGCAAGTGCAGGACCAAAGAAGGAAGACGGAAGACAAATCTGTATGGGTTCATTATAAAAGGAAGATCCCACTTGAGGAATGACACCGATGTGGTAAACTTTGTGAGCATGGAATTCTCTCTAACTGACCCGAGGCTAGAGCCACACAAATG<br>GGAAAAGTACTGTGTTCTTGAGATAGGAGACATGCTCCTACGGACTGCAATAGGCCAAGTGTCGAGGCCCATGTTCCTATATGTGAGAACCAATGGGACTTCCAAGATCAAAATGAAATGGGGCATGGAGATGAGACGATGCCTTCTTCAGTCCCTTCAACAAATTGAGAGCATGATTGAGGCCGAGTCTTCTGTCAAAGAGAAGGACATGACCAAGGAATTCTTTGAAA<br>ACAAATCAGAGACATGGCCAATTGGAGAATCACCCAAAGGGGTGGAGGAAGGCTCCATTGGAAAAGTATGCAGAACATTGCTAGCAAAGTCTGTGTTCAACAGCCTATATGCATCTCCACAACTCGAGGGGTTTTCAGCTGAGTCAAGAAAATTGCTTCTCATTGTTCAGGCACTTAGGGACAACCTGGAACCTGGAACCTTCGATCTTGGGGGGCTATATGAAGCAATTG<br>AGGAGTGCCTGATTAATGATCCCTGGGTTTTGCTTAATGCGTCTTGGTTCAACTCCTTCCTCACACATGCACTGAAATA-
internal NVRI repository	>A/turkey/Poland/H32-T/2022_H5N1_2022-01-09<br>ATGGAAGACTTTGTGCGACAATGCTTCAATCCAATGATTGTCGAGCTTGCGGAAAAAGCAATGAAAGAATATGGGGAAGATCCGAAAATCGAGAYAAACAAATTTGCCGCAATATGCACACACTTAGAAGTCTGTTTCATGTATTCGGATTTCCATTTTATTGATGAACGAGGCGAATCAATGATTGTAGAATCTGGCGATCCAAATGCATTATTGAAACACCGATTTGAGA<br>TAATCGAAGGGAGAGACCGAGCAATGGCCTGGACAGTGGTGAATAGTATCTGCAACACTACAGGAGTCGAAAAGCCCAAATTCCTCCCTGATTTGTATGACTACAAAGAGAACCGATTCATTGAAATTGGAGTAACGCGAAGGGAAGTTCACATATACTATTTAGAAAAAGCCAACAAGATAAAATCAGAGAAAACACACATTCACATATTCTCATTCACTGGAGAGGAA<br>ATGGCCACAAAGGCGGAATACACCCTTGATGAAGAGAGTAGAGCAAGAATAAAAACCAGACTGTTCACTATAAGACAAGAAATGGCCAGTAGAGGTCTATGGGATTCCTTTCGTCAGTCCGAGAGAGGCGAAGAGACAACTGAAGAAAGATTTGAAATCACAGGAACCATGCGCAGGCTTGCCGACCAAAGTCTCCCACCGAACTTCTCCAGCCTTGAAAACTTTAGAG<br>CCTATGTGGATGGATTCGAACCGAACGGCTGCATTGAGGGCAAGCTTTCTCAAATGTCAAAAGAAGTGAACGCCAGAATTGAGCCATTCCTGAAGACAACACCACGCCCTCTCAGATTACCTGATGGGCCTCCTTGTTCTCAGCGGTCTAAGTTCTTGCTGATGGATGCCCTTAAGTTGAGCATCGAAGATCCTAGCCATGAGGGGGAGGGCATACCGCTGTATGATGCAAT<br>CAAATGCATGAAAACATTTTTTGGCTGGAAAGAGCCCAACCTCGTAAAGCCGCATGAGAAAGGCATAAACCCTAATTACCTCCTGGCTTGGAAGCAGGTGCTGGCAGAACTTCAAGATATTGAGAATGAGGAGAAATTCCCAAAAACAAAGAACATGAAGAAAACAAGCCAATTGAAGTGGGCACTTGGTGAGAACATGGCACCAGAAAAAGTGGACTTTGAGGACTGC<br>AAAGATGTTAGCGATCTAAGACAGTACGACAGTGACGAACCAGAGCCTAGATCACTAGCAAGCTGGATTCAGAGTGAATTCAACAAGGCATGCGAATTGACAGATTCGAGTTGGATTGAACTTGATGAGATAGGGGAAGACGTTGCTCCAATCGAACACATTGCGAGTATGAGGAGGAACTATTTCACAGCGGAGGTATCCCATTGCAGGGCCACTGAATACATAATGA<br>AGGGAGTATACATAAACACAGCCCTATTGAATGCATCCTGTGCAGCCATGGATGACTTCCAACTGATTCCAATGATAAGCAAGTGCAGAACCAAAGAGGGAAGACGGAAGACAAATCTGTATGGATTCATTATAAAAGGAAGATCCCATTTGAGGAATGACACCGATGTGGTAAACTTTGTGAGCATGGAATTCTCTCTAACTGACCCGAGGCTAGAGCCACACAAATGG<br>GAAAAGTACTGTGTTCTTGAGATAGGAGACATGCTCCTACGGACTGCAATAGGCCAAGTGTCGAGGCCCATGTTCCTGTATGTGAGAACCAATGGGACTTCCAAGATCAAAATGAAATGGGGCATGGAGATGAGACGATGCCTTCTTCAGTCCCTTCAACAAATTGAGAGCATGATTGAGGCCGAATCTTCTGTCAAAGAGAAGGACATGACCAAGGAATTCTTTGAAAA<br>CAAATCAGAAACATGGCCAATTGGAGAATCACCCAAAGGGGTTGAGGAAGGCTCTATTGGGAAAGTATGCAGAACATTGCTAGCAAAGTCTGTGTTCAACAGCCTATATGCATCTCCACAACTCGAGGGATTTTCAGCTGAATCAAGAAAATTGCTTCTCATTGTTCAGGCACTTAGGGACAACCTGGAACCTGGAACCTTCGATCTTGGGGGGCTATATGAAGCAATTGA<br>GGAGTGCCTGATTAACGATCCCTGGGTTTTGCTTAATGCGTCTTGGTTCAACTCCTTCATCACACATGCACTGAAATA-
internal NVRI repository	>A/turkey/Poland/H35-T/2022_H5N1_2022-01-09<br>ATGGAAGACTTTGTGCGACAATGCTTCAATCCAATGATTGTCGAGCTTGCGGAAAAAGCAATGAAAGAATATGGGGAAGATCCGAAAATCGAGACAAACAAATTTGCCGCAATATGCACACACTTAGAAGTCTGTTTCATGTATTCGGATTTCCATTTTATCGATGAACGAGGCGAATCAACGATTGTAGAATCTGGCGATCCAAATGCATTATTGAAACACCGATTTGAGA<br>TAATCGAAGGGAGAGATCGAGCAATGGCCTGGACAGTGGTGAATAGTATCTGCAACACCACAGGAGTCGAAAAGCCCAAATTCCTCCCTGATTTGTATGACTACAAAGAGAACCGATTCATTGAAATTGGAGTAACGCGAAGGGAAGTTCACATATACTATTTAGAAAAAGCCAACAAGATAAAATCAGAGAAAACACACATTCACATATTCTCATTCACTGGAGAGGAA<br>ATGGCCACCAAGGCGGAATACACCCTTGATGAAGAGAGCAGAGCAAGAATAAAAACCAGACTGTTCACTATAAGACAAGAAATGGCCAGTAGAGGTCTATGGGATTCCTTTCGTCAGTCCGAAAGAGGCGAAGAGACAACTGAAGAAAGATTTGAAATCACAGGAACCATGCGCAGGCTTGCCGACCAAAGCCTCCCRCCGAACTTCTCCAGCCTTGAAAACTTTAGAG<br>CCTATGTGGATGGATTCGAACCGAACGGCTGCATTGAGGGCAAGCTTTCTCAAATGTCAAAAGAAGTGAACGCCAGAATTGAGCCATTCCTGAAGACAACACCACGCCCTCTCAGATTACCAGATGGGCCTCCTTGTTCTCAGCGGTCGAAGTTCTTGCTGATGGATGCCCTTAAGTTGAGCATCGAAGACCCTAGCCATGAGGGGGAGGGCATACCGCTGTATGATGCAA<br>TCAAATGCATGAAGACATTTTTTGGCTGGAAAGAGCCCAACCTCGTAAAACCGCATGAGAAAGGCATAAACCCTAATTACCTCCTGGCTTGGAAGCAGGTGCTGGCAGAACTTCAAGATATTGAGAATGAGGAGAAATTTCCAAAAACAAAGAACATGAAGAAAACAAGCCAATTGAAGTGGGCACTTGGTGAGAACATGGCACCAGAAAAAGTGGACTTTGAGGACTG<br>CAAAGATGTTAGCGATCTAAGACAGTACGACAGTGACGAACCAGAGCCTAGATCACTGGCAAGCTGGATTCAGAGTGAATTCAACAAGGCATGCGAATTGACAGATTCGAGTTGGATTGAACTTGATGAGATAGGGGAAGACGTTGCTCCAATCGAACACATTGCGAGTATGAGGAGGAACTATTTCACAGCGGAGGTATCCCATTGCAGGGCCACTGAATACATAATG<br>AAGGGAGTATACATAAACACAGCCCTATTGAATGCATCCTGTGCAGCCATGGATGACTTCCAACTGATTCCAATGATAAGCAAGTGCAGAACCAAAGAGGGAAGACGGAAGACAAATCTGTATGGATTCATTATAAAAGGAAGATCCCATTTGAGGAATGACACCGATGTGGTGAACTTTGTGAGCATGGAATTCTCTCTAACTGACCCGAGGCTAGAGCCACACAAATG<br>GGAAAAGTACTGTGTTCTTGAGATAGGAGACATGCTCCTACGGACTGCAATAGGCCAAGTGTCGAGGCCCATGTTCCTGTATGTGAGAACCAATGGGACTTCCAAGATCAAAATGAAATGGGGCATGGAGATGAGACGATGCCTTCTTCAGTCCCTTCAACAAATTGAGAGCATGATTGAGGCCGAATCTTCTGTCAAAGAGAAGGACATGACCAAGGAATTCTTTGAAA<br>ACAAATCAGAAACATGGCCAATTGGAGAATCACCCAAAGGGGTTGAGGAAGGCTCTATTGGGAAAGTATGCAGAACATTGCTAGCAAAGTCTGTGTTCAACAGCCTATATGCTTCTCCACAACTCGAGGGATTTTCAGCTGAATCAAGAAAATTGCTTCTCATTGTTCAGGCACTTAGGGACAACCTGGAACCTGGAACCTTCGATCTTGGGGGGCTATATGAAGCAATTG<br>AGGAGTGCCTGATTAACGATCCCTGGGTTTTGCTTAATGCGTCTTGGTTCAACTCCTTCATCACACATGCACTGAAATA-
EPI_ISL_18245817	>A/sandwich_stern/Poland/MB142/2022_H5N1_2022-06-15<br>ATGGAGGACTTTGTGCGACAATGTTTCAATCCAATGATTGTCGAGCTTGCGGAAAAGGCAATGAAAGAGTATGGGGAAGATCCGAAAATCGAAACAAACAAATTTGCCGCAATATGCACACACTTAGAAGTCTGTTTCATGTATTCGGATTTCCACTTTATTGATGAACGAGGCGAATCAACAATTGTAGAATCTGGCGATCCGAATGCATTATTGAAACACCGATTCGAGA<br>TAATTGAAGGGAGAGATCGAACTATGGCCTGGACAGTGGTGAATAGTATCTGCAACACCACAGGAGTCGAAAAGCCCAAATTCCTCCCTGATTTGTATGACTACAAAGAGAACCGATTCATTGAAATTGGAGTAACGCGAAGGGAAGTTCACATATACTATCTAGAGAAAGCCAACAAGATAAAATCAGAGAAGACTCACATTCATATATTCTCATTCACTGGAGAGGAA<br>ATGGCCACCAAGGCGGACTACACCCTTGATGAAGAGAGTAGAGCAAGAATAAAAACCAGGCTGTTCACTATAAGACAAGAAATGGCCAGTAGGGGTCTATGGGATTCCTTTCGTCAGTCCGAGAGAGGCGAAGAGACAATTGAAGAAAGATTTGAAATCACAGGAACCATGCGCAGGCTTGCCGACCAAAGTCTCCCACCGAACTTCTCCAGCATTGAAAACTTTAGAG<br>CCTATGTGGATGGATTCGAACCGAACGGCTGCATTGAGGGCAAGCTTTCTCAAATGTCAAAAGAAGTGAATGCCAGAATTGAGCCATTTCTGAAGACAACACCACGCCCTCTCAGATTACCTGATGGGCCTCCCTGTTCTCAGCGGTCGAAGTTCTTGCTGATGGATGCCCTTAAATTGAGCATCGAAGACCCGAGCCATGAGGGGGAGGGTATACCGCTGTATGATGCAA<br>TCAAATGCATGAAGACATTTTTGGCTGGAAAGAGCCTAACATCGTAAAACCGCATGAAAAGGGCATAAACCCTAATTACCTTTTGGCTTGGAAACAGGTGCTGACAGAACTCCAAGATATTGAAAATGAGGAGAAAATCCCAAAAACAAAGAACATGAGGAAAACAAGCCAATTGAAGTGGGCACTTGGTGAGAACATGGCACCAGAGAAAGTGGACTTTGAGGACTG<br>TAAAGATGTTAGTGATCTAAGACAGTACGACAGTGACGAGCCAGAGTCTAGATCACTAGCAAGCTGGATCCAGAGTGAATTCAACAAGGCATGCGAATTGACAGATTCGAGTTGGATTGAACTTGATGAAATAGGGGAAGACATTGCTCCAATTGAACACATTGCGAGTATGAGGAGAAACTATTTCACAGCGGAGGTGTCCCATTGCAGGGCTACTGAATACATAATGA<br>AGGGAGTATACATAAACACAGCCCTGTTGAATGCATCCTGTGCAGCGATGGATGACTTTCAACTGATTCCTATGATAAGCAAGTGCAGAACCAAAGAAGGAAGACGGAAGACAAATTTATATGGATTCATTATAAAAGGAAGATCCCATTTGAGGAATGATACCGATGTGGTGAACTTTGTGAGCATGGAATTCTCTCTCACTGACCCGAGGCTGGAGCCACACAAATGG<br>GAAAAGTACTGTGTTCTTGAGATAGGAGACATGCTCCTACGGACTGCAATAGGCCAAGTGTCAAGGCCCATGTTCTTGTATGTGAGAACCAATGGAACTTCCAAGATCAAAATGAAATGGGGCATGGAGATGAGACGATGCCTTCTTCAGTCCCTTCAACAAATTGAGAGCATGATTGAGGCCGAGTCTTCTGTCAAAGAGAAGGACATGACCAAGGAATTCTTTGAAAAC<br>AAATCAGAAACATGGCCAATTGGGGAGTCACCCAAGGGGTGGAAGAAGGCTCCATTGGGAAGGTGTGCAGAACATTGCTAGCAAAGTCTGTGTTCAACAGCCTATATGCATCTCCACAACTCGAGGGGTTTTCAGCTGAATCAAGAAAATTGCTTCTCATTGTTCAGGCACTTAGGGACAACCTGGAACCTGGAACCTTCGATCTTGGGGGGCTATATGGAGCAATTGA<br>GGAGTGCCTGATTAACGATCCCTGGGTTTTGCTTAATGCGTCTTGGTTCAACTCCTTCCTCACACATGCACTGAAATA-
internal NVRI repository	>A/laying_hen/Poland/H205/2022_H5N1_2022-03-08<br>ATGGAAGACTTTGTGCGACAATGCTTCAATCCAATGATTGTCGAGCTTGCGGAAAAGGCAATGAAAGAATATGGGGAAGRTCCGAAAATCGAGACAAACAAATTTGCCGCAATATGCACACACTTAGAAGTCTGTTTCATGTATTCGGATTTCCATTTTATTGATGAACGAGGCGAATCAATAATTGTAGAATCTGGCGATCCGAATGCATTATTAAAACACCGATTTGAAA<br>TAATCGAAGGGAGAGACCGAACAATGGCCTGGACAGTGGTGAATAGTATCTGCAACACCACAGGAGTCGAAAAGCCCAAATTCCTCCCTGATTTGTATGACTACAAAGAAAACCGATTCATTGAGATTGGAGTAACGCGGAGGGAAGTTCACATATACTATTTAGAAAAAGCCAACAAGATAAAATCAGAGAAGACACACATTCACATATTCTCATTCACTGGAGAGGAA<br>ATGGCCACCAAGGCGGACTACACCCTTGATGAAGAGAGCAGAGCAAGAATAAAAACCAGACTGTTCACTATAAGACAAGAAATGGCCAGTAGAGGTCTATGGGATTCCTTTCGTCAGTCCGAGAGAGGCGAAGAGACAATTGAAGAAAGATTTGAAATCACAGGAACCATGCGCAGGCTTGCCGACCAAAGTCTCCCACCGAACTTCTCCAGCCTTGAAAACTTTAGAG<br>CCTATGTRGATGGATTCGAACCGAACGGCTGCATTGAGGGCAAGCTTTCTCAAATGTCAAAAGAAGTGAACGCCAGAATTGAGCCATTTCTGAAGACAACACCACGCCCTCTCAAATTACCTGATGGGCCTCCCTGTTCTCAGCGGTCGAAGTTCTTGCTAATGGATGCCCTTAAACTGAGCATCGAAGACCCTAGCCATGAGGGGGAGGGCATACCGCTGTATGATGCAA<br>TCAAATGCATGAAGACATTTTTTGGCTGGAAAGAGCCCAACATCGTAAAAACGCATGAGAAAGGCATAAACCCTAATTACCTCCTGGCTTGGAAGCAGGTGCTGGCAGAACTTCAAGATATTGAAAATGAGGAGAAAATTCCAAAAACAAAGAACATGAAGAAAACAAGYCAATTGAAGTGGGCACTTGGTGAGAACATGGCACCAGAAAAAGTGGACTTTGAGGACTG<br>CAAAGATGTTAGCGATCTAAGACAGTATGACAGTGACGAACCAGAGTCTAGATCACTAGCAAGCTGGATTCAGAGTGAATTCAACAAGGCATGCGAATTGACAGATTCGAGTTGGATTGAGCTTGATGAGATAGGGGAAGACGTTGCTCCAATCGAGCACATTGCGAGTATGAGGAGGAACTATTTCACAGCAGAGGTATCCCATTGCAGGGCCACTGAGTACATAATG<br>AAGGGAGTWTACATAAACACAGCCCTATTGAATGCATCCTGTGCAGCCATGGATGACTTCCAACTGATTCCAATGATAAGCAAGTGCAGGACCAAAGAAGGAAGACGGAAGACAAATCTGTATGGGTTCATTATAAAAGGAAGATCCCACTTGAGGAATGACACCGATGTGGTAAACTTTGTGAGCATGGAATTCTCTCTAACTGACCCGAGGCTAGAGCCACACAAATG<br>GGAAAAGTACTGTGTTCTTGAGATAGGAGACATGCTCCTACGGACTGCAATAGGCCAAGTGTCGAGGCCCATGTTCCTATATGTGAGAACCAATGGGACTTCCAAGATCAAAATGAAATGGGGCATGGAGATGAGACGATGCCTTCTTCAGTCCCTTCAACAAATTGAGAGCATGATTGAGGCCGAGTCTTCTGTCAAAGAGAAGGACATGACCAAGGAATTCTTTGAAA<br>ACAAATCAGAGACATGGCCAATTGGAGAATCACCCAAAGGGGTGGAGGAAGGCTCCATTGGAAAAGTATGCAGAACATTGCTAGCAAAGTCTGTGTTCAACAGCCTATATGCATCTCCACAACTCGAGGGGTTTTCAGCTGAGTCAAGAAAATTGCTTCTCATTGTTCAGGCACTTAGGGACAACCTGGAACCTGGAACCTTCGATCTTGGGGGGCTATATGAAGCAATTG<br>AGGAGTGCCTGATTAATGATCCCTGGGTTTTGCTTAATGCGTCTTGGTTCAACTCCTTCCTCACACATGCACTGAAATA-
internal NVRI repository	>A/laying_hen/Poland/H2157-N/2021_H5N1_2021-12-27<br>ATGGAAGACTTTGTGCGACAATGCTTCAATCCAATGATTGTCGAGCTTGCGGAAAAAGCAATGAAAGAATATGGGGAAGATCCGAAAATCGAGACAAACAAATTTGCCGCAATATGCACACACTTAGAAGTCTGTTTCATGTATTCGGATTTCCATTTTATTGATGAACGAGGCGAATCAATGATTGTAGAATCTGGCGATCCAAATGCATTATTGAAACACCGATTTGAGA<br>TAATCGAAGGGAGAGACCGAGCAATGGCCTGGACAGTGGTGAATAGCATCTGCAACACCACAGGAGTCGAAAAGCCCAAATTCCTCCCTGATTTGTATGACTACAAAGAGAACCGATTCATTGAAATTGGAGTAACGCGAAGGGAAGTTCACATATACTATTTAGAAAAAGCCAACAAGATAAAATCAGAGAAAACACACATTCACATATTCTCATTCACTGGAGAGGAA<br>ATGGCCACCAAGGCGGAATACACCCTTGATGAAGAGAGCAGAGCAAGAATAAAAACCAGACTGTTCACTATAAGACAAGAAATGGCCAGTAGAGGTCTATGGGATTCCTTTCGTCAGTCCGAGAGAGGCGAAGAGACAACTGAAGAAAGATTTGAAATCACAGGAACCATGCGCAGGCTTGCCGACCAAAGTCTCCCACCGAACTTCTCCAGCCTTGAAAACTTTAGAG<br>CCTATGTGGATGGATTCGAACCGAACGGCTGCATTGAGGGCAAGCTTTCTCAAATGTCAAAAGAAGTGAACGCCAGAATTGAGCCATTCCTGAAGACAACACCACGCCCTCTCAGATTACCTGATGGGCCTCCTTGTTCTCAGCGGTCGAAGTTCTTGCTGATGGATGCCCTTAAGTTGAGCATCGAAGACCCTAGCCATGAGGGGGAGGGCATACCGCTGTATGATGCAA<br>TCAAATGCATGAAGACATTTTTTGGCTGGAAAGAGCCCAACCTCGTAAAGCCGCATGAGAAAGGCATAAACCCTAATTACCTCCTGGCTTGGAAGCAGGTGCTGGCAGAACTTCAAGATATTGAGAATGAGGAGAAATTTCCAAAAACAAAGAACATGAAGAAAACAAGCCAATTGAAGTGGGCACTTGGTGAGAACATGGCACCAGAAAAAGTGGACTTTGAGGACTG<br>CAAAGATGTTAGCGATCTAAGACAGTACGACAGTGACGAACCAGAGCCTAGATCACTAGCAAGCTGGATTCAGAGTGAATTCAACAAGGCATGCGAATTGACAGATTCGAGTTGGATTGAACTTGATGAGATAGGGGAAGACGTTGCTCCAATCGAACACATTGCGAGTATGAGGAGGAACTATTTCACAGCGGAGGTATCCCATTGCAGGGCCACTGAATACATAATG<br>AAGGGAGTATACATAAACACAGCCCTATTGAATGCATCCTGTGCAGCCATGGATGACTTCCAACTGATTCCAATGATAAGCAAGTGCAGAACCAAAGAGGGAAGACGGAAGACAAATCTGTATGGATTCATTATAAAAGGAAGATCCCATTTGAGGAATGACACCGATGTGGTAAACTTTGTGAGCATGGAATTCTCTCTAACTGACCCGAGGCTAGAGCCACACAAATG<br>GGAAAAGTACTGTGTTCTTGAGATAGGAGACATGCTCCTACGGACTGCAATAGGCCAAGTGTCGAGGCCCATGTTCCTGTATGTGAGAACCAATGGGACTTCCAAGATCAAAATGAAATGGGGCATGGAGATGAGACGATGCCTTCTTCAGTCCCTTCAACAAATTGAGAGCATGATTGAGGCCGAATCTTCTGTCAAAGAGAAGGACATGACCAAGGAATTCTTTGAAA<br>ACAAATCAGAAACATGGCCAATTGGAGAATCACCCAAAGGGGTTGAGGAAGGCTCTATTGGGAAAGTATGCAGAACATTGCTAGCAAAGTCTGTGTTCAACAGCCTATATGCTTCTCCACAACTCGAGGGATTTTCAGCTGAATCAAGAAAATTGCTTCTCATTGTTCAGGCACTTAGGGACAACCTGGAACCTGGAACCTTCGATCTTGGGGGGCTATATGAAGCAATTG<br>AGGAGTGCCTGATTAACGATCCCTGGGTTTTGCTTAATGCGTCTTGGTTCAACTCCTTCATCACACATGCACTGAAATA-
internal NVRI repository	>A/laying_hen/Poland/H2159-T3/2021_H5N1_2021-12-27<br>ATGGAAGACTTTGTGCGACAATGCTTCAATCCAATGATTGTCGAGCTTGCGGAAAAGGCAATGAAAGAATATGGGGAAGATCCGAAAATCGAGACAAACAAATTTGCCGCAATATGCACACACTTAGAAGTCTGTTTCATGTATTCGGATTTCCATTTTATTGATGAACGAGGCGAATCAATAATTGTAGAATCTGGCGATCCGAATGCATTATTAAAACACCGATTTGAAA<br>TAATCGAAGGGAGAGACCGAACAATGGCCTGGACAGTGGTGAATAGTATCTGCAACACCACAGGAGTCGAAAAGCCCAAATTCCTCCCTGATTTGTATGACTACAAAGAAAACCGATTCATTGAGATTGGAGTAACGCGGAGGGAAGTTCACATATACTATTTAGAAAAAGCCAACAAGATAAAATCAGAGAAGACACACATTCACATATTCTCATTCACTGGAGAGGAA<br>ATGGCCACCAAGGCGGACTACACCCTTGATGAAGAGAGCAGAGCAAGAATAAAAACCAGACTGTTCACTATAAGACAAGAAATGGCCAGTAGAGGTCTATGGGATTCCTTTCGTCAGTCCGAGAGAGGCGAAGAGACAATTGAAGAAAGATTTGAAATCACAGGAACCATGCGCAGGCTTGCCGACCAAAGTCTCCCACCGAACTTCTCCAGCCTTGAAAACTTTAGAG<br>CCTATGTGGATGGATTCGAACCGAACGGCTGCATTGAGGGCAAGCTTTCTCAAATGTCAAAAGAAGTGAACGCCAGAATTGAGCCATTTCTGAAGACAACACCACGCCCTCTCAAATTACCTGATGGGCCYCCCTGTTCTCAGCGGTCGAAGTTCTTGCTAATGGATGCCCTTAAACTGAGCATCGAAGACCCTAGCCATGAGGGGGAGGGCATACCGCTGTATGATGCAA<br>TCAAATGCATGAAGACATTTTTTGGCTGGAAAGAGCCCAACATCGTAAAAACGCATGAGAAAGGCATAAACCCTAATTACCTCCTGGCTTGGAAGCAGGTGCTGGCAGAACTTCAAGATATTGAAAATGAGGAGAAAATTCCAAAAACAAAGAACATGAAGAAAACAAGTCAATTGAAGTGGGCACTTGGTGAGAACATGGCACCAGAAAAAGTGGACTTTGAGGACTG<br>CAAAGATGTTAGCGATCTAAGACAGTATGACAGTGACGAACCAGAGTCTAGATCACTAGCAAGCTGGATTCAGAGTGAATTCAACAAGGCATGCGAATTGACAGATTCGAGTTGGATTGAGCTTGATGAGATAGGGGAAGACGTTGCTCCAATCGAGCACATTGCGAGTATGAGGAGGAACTATTTCACAGCAGAGGTATCCCATTGCAGGGCCACTGAGTACATAATG<br>AAGGGAGTATACATAAACACAGCCCTATTGAATGCATCCTGTGCAGCCATGGATGACTTCCAACTGATTCCAATGATAAGCAAGTGCAGGACCAAAGAAGGAAGACGGAAGACAAATCTGTATGGGTTCATTATAAAAGGAAGATCCCACTTGAGGAATGACACCGATGTGGTAAACTTTGTGAGCATGGAATTCTCTCTAACTGACCCGAGGCTAGAGCCACACAAATG<br>GGAAAAGTACTGTGTTCTTGAGATAGGAGACATGCTCCTACGGACTGCAATAGGCCAAGTGTCGAGGCCCATGTTCCTATATGTGAGAACCAATGGGACTTCCAAGATCAAAATGAAATGGGGCATGGAGATGAGACGATGCCTTCTTCAGTCCCTTCAACAAATTGAGAGCATGATTGAGGCCGAGTCTTCTGTCAAAGAGAAGGACATGACCAAGGAATTCTTTGAAA<br>ACAAATCAGAGACATGGCCAATTGGAGAATCACCCAAAGGGGTGGAGGAAGGCTCCATTGGAAAAGTATGCAGAACATTGCTAGCAAAGTCTGTGTTCAACAGCCTATATGCATCTCCACAACTCGAGGGGTTTTCAGCTGAGTCAAGAAAATTGCTTCTCATTGTTCAGGCACTTAGGGACAACCTGGAACCTGGAACCTTCGATCTTGGGGGGCTATATGAAGCAATTG<br>AGGAGTGCCTGATTAATGATCCCTGGGTTTTGCTTAATGCGTCTTGGTTCAACTCCTTCCTCACACATGCACTGAAATA-
internal NVRI repository	>A/laying_hen/Poland/H283/2022_H5N1_2022-04-12<br>ATGGAAGACTTTGTGCGACAATGCTTCAATCCAATGATTGTCGAGCTTGCGGAAAAGGCAATGAAAGAATATGGGGAAGATCCGAAAATCGAGACAAACAAATTTGCCGCAATATGCACACACTTAGAAGTCTGTTTCATGTATTCGGATTTCCATTTTATTGATGAACGAGGCGAATCAATAATTGTAGAATCTGGCGATCCGAATGCATTATTAAAACACCGATTTGAAA<br>TAATCGAAGGGAGAGACCGAACAATGGCCTGGACAGTGGTGAATAGTATCTGCAACACCACAGGAGTCGAAAAGCCCAAATTCCTCCCTGATTTGTATGACTACAAAGAAAACCGATTCATTGAGATTGGAGTAACGCGGAGGGAAGTTCACATATACTATTTAGAAAAAGCCAACAAGATAAAATCAGAGAAGACACACATTCACATATTCTCATTCACTGGAGAGGAA<br>ATGGCCACCAAGGCGGACTACACCCTTGATGAAGAGAGCAGAGCAAGAATAAAAACCAGACTGTTCACTATAAGACAAGAAATGGCCAGTAGAGGTCTATGGGATTCCTTTCGTCAGTCCGAGAGAGGCGAAGAGACAATTGAAGAAAGATTTGAAATCACAGGAACCATGCGCAGGCTTGCCGACCAAAGTCTCCCACCGAACTTCTCCAGCCTTGAAAACTTTAGAG<br>CCTATGTGGATGGATTCGAACCGAACGGCTGCATTGAGGGCAAGCTTTCTCAAATGTCAAAAGAAGTGAACGCCAGAATTGAGCCATTTCTGAAGACAACACCACGCCCTCTCAAATTACCTGATGGGCCTCCCTGTTCTCAGCGGTCGAAGTTCTTGCTAATGGATGCCCTTAAACTGAGCATCGAAGACCCTAGCCATGAGGGGGAGGGCATACCGCTGTATGATGCAA<br>TCAAATGCATGAAGACATTTTTTGGCTGGAAAGAGCCCAACATCGTAAAAACGCATGAGAAAGGCATAAACCCTAATTACCTCCTGGCTTGGAAGCAGGTGCTGGCAGAACTTCAAGATATTGAAAATGAGGAGAAAATTCCAAAAACAAAGAACATGAAGAAAACAAGTCAATTGAAGTGGGCACTTGGTGAGAACATGGCACCAGAAAAAGTGGACTTTGAGGACTG<br>CAAAGATGTTAGCGATCTAAGACAGTATGACAGTGACGAACCAGAGTCTAGATCACTAGCAAGCTGGATTCAGAGTGAATTCAACAAGGCATGCGAATTGACAGATTCGAGTTGGATTGAGCTTGATGAGATAGGGGAAGACGTTGCTCCAATCGAGCACATTGCGAGTATGAGGAGGAACTATTTCACAGCAGAGGTATCCCATTGCAGGGCCACTGAGTACATAATG<br>AAGGGAGTTTACATAAACACAGCCCTATTGAATGCATCCTGTGCAGCCATGGATGACTTCCAACTGATTCCAATGATAAGCAAGTGCAGGACCAAAGAAGGAAGACGGAAGACAAATCTGTATGGGTTCATTATAAAAGGAAGATCCCACTTGAGGAATGACACCGATGTGGTAAACTTTGTGAGCATGGAATTCTCTCTAACTGACCCGAGGCTAGAGCCACACAAATG<br>GGAAAAGTACTGTGTTCTTGAGATAGGAGACATGCTCCTACGGACTGCAATAGGCCAAGTGTCGAGGCCCATGTTCCTATATGTGAGAACCAATGGGACTTCCAAGATCAAAATGAAATGGGGCATGGAGATGAGACGATGCCTTCTTCAGTCCCTTCAACAAATTGAGAGCATGATTGAGGCCGAGTCTTCTGTCAAAGAGAAGGACATGACCAAGGAATTCTTTGAAA<br>ACAAATCAGAGACATGGCCAATTGGAGAATCACCCAAAGGGGTGGAGGAAGGCTCCATTGGAAAAGTATGCAGAACATTGCTAGCAAAGTCTGTGTTCAACAGCCTATATGCATCTCCACAACTCGAGGGGTTTTCAGCTGAGTCAAGAAAATTGCTTCTCATTGTTCAGGCACTTAGGGACAACCTGGAACCTGGAACCTTCGATCTTGGGGGGCTATATGAAGCAATTG<br>AGGAGTGCCTGATTAATGATCCCTGGGTTTTGCTTAATGCGTCTTGGTTCAACTCCTTCCTCACACATGCACTGAAATA-
internal NVRI repository	>A/laying_hen/Poland/H290-T/2022_H5N1_2022-04-20<br>ATGGAAGACTTTGTGCGACAATGCTTCAATCCAATGATTGTCGAGCTTGCGGAAAAGGCAATGAAAGAATATGGGGAAGATCCGAAAATCGAGACAAACAAATTTGCCGCAATATGCACACACTTAGAAGTCTGTTTCATGTATTCGGATTTCCATTTTATTGATGAACGAGGCGAATCAATAATTGTAGAATCTGGCGATCCGAATGCATTATTAAAACACCGATTTGAAA<br>TAATCGAAGGGAGAGACCGAACAATGGCCTGGACAGTGGTGAATAGTATCTGCAACACCACAGGAGTCGAAAAGCCCAAATTCCTCCCTGATTTGTATGACTACAAAGAAAACCGATTCATTGAGATTGGAGTAACGCGGAGGGAAGTTCACATATACTATTTAGAAAAAGCCAACAAGATAAAATCAGAGAAGACACACATTCACATATTCTCATTCACTGGAGAGGAA<br>ATGGCCACCAAGGCGGACTACACCCTTGATGAAGAGAGCAGAGCAAGAATAAAAACCAGACTGTTCACTATAAGACAAGAAATGGCCAGTAGAGGTCTATGGGATTCCTTTCGTCAGTCCGAGAGAGGCGAAGAGACAATTGAAGAAAGATTTGAAATCACAGGAACCATGCGCAGGCTTGCCGACCAAAGTCTCCCACCGAACTTCTCCAGCCTTGAAAACTTTAGAG<br>CCTATGTGGATGGATTCGAACCGAACGGCTGCATTGAGGGCAAGCTTTCTCAAATGTCAAAAGAAGTGAACGCCAGAATTGAGCCATTTCTGAAGACAACACCACGCCCTCTCAAATTACCTGATGGGCCTCCCTGTTCTCAGCGGTCGAAGTTCTTGCTAATGGATGCCCTTAAACTGAGCATCGAAGACCCTAGCCATGAGGGGGAGGGCATACCGCTGTATGATGCAA<br>TCAAATGCATGAAGACATTTTTTGGCTGGAAAGAGCCCAACATCGTAAAAACGCATGAGAAAGGCATAAACCCTAATTACCTCCTGGCTTGGAAGCAGGTGCTGGCAGAACTTCAAGATATTGAAAATGAGGAGAAAATTCCAAAAACAAAGAACATGAAGAAAACAAGTCAATTGAAGTGGGCACTTGGTGAGAACATGGCACCAGAAAAAGTGGACTTTGAGGACTG<br>CAAAGATGTTAGCGATCTAAGACAGTATGACAGTGACGAGCCAGAGTCTAGATCACTAGCAAGCTGGATTCAGAGTGAATTCAACAAGGCATGCGAATTGACAGATTCGAGTTGGATTGAGCTTGATGAGATAGGGGAAGACGTTGCTCCAATCGAGCACATTGCGAGTATGAGGAGGAACTATTTCACAGCAGAGGTATCCCATTGCAGGGCCACTGAGTACATAATG<br>AAGGGAGTTTACATAAACACAGCCCTATTGAATGCATCCTGTGCAGCCATGGATGACTTCCAACTGATTCCAATGATAAGCAAGTGCAGGACCAAAGAAGGAAGACGGAAGACAAATCTGTATGGGTTCATTATAAAAGGAAGATCCCACTTGAGGAATGACACCGATGTGGTAAACTTTGTGAGCATGGAATTCTCTCTAACTGACCCGAGGCTAGAGCCACACAAATG<br>GGAAAAGTACTGTGTTCTTGAGATAGGAGACATGCTCCTACGGACTGCAATAGGCCAAGTGTCGAGGCCCATGTTCCTATATGTGAGAACCAATGGGACTTCCAAGATCAAAATGAAATGGGGCATGGAGATGAGACGATGCCTTCTTCAGTCCCTTCAACAAATTGAGAGCATGATTGAGGCCGAGTCTTCTGTCAAAGAGAAGGACATGACCAAGGAATTCTTTGAAA<br>ACAAATCAGAGACATGGCCAATTGGAGAATCACCCAAAGGGGTGGAGGAAGGCTCCATTGGAAAAGTATGCAGAACATTGCTAGCAAAGTCTGTGTTCAACAGCCTATATGCATCTCCACAACTCGAGGGGTTTTCAGCTGAGTCAAGAAAATTGCTTCTCATTGTTCAGGCACTTAGGGACAACCTGGAACCTGGAACCTTCGATCTTGGGGGGCTATATGAAGCAATTG<br>AGGAGTGCCTGATTAATGATCCCTGGGTTTTGCTTAATGCGTCTTGGTTCAACTCCTTCCTCACACATGCACTGAAATA-
internal NVRI repository	>A/laying_hen/Poland/H363/2022_H5N1_2022-07-20<br>ATGGAAGACTTTGTGCGACAATGCTTCAATCCAATGATTGTCGAGCTTGCGGAAAAGGCAATGAAAGAATATGGGGAAGACCCGAAAATCGAGACAAACAAATTTGCCGCAATATGCACACACTTAGAAGTCTGTTTCATGTATTCGGATTTCCACTTTATTGATGAACGAGGAGAATCAATAATTGTAGAATCCGGCGATCCGAATGCATTATTGAAACATCGATTTGAGA<br>TAATTGAAGGGAGAGACCGAACAATGGCTTGGACAGTGGTAAATAGTATATGCAACACTACAGGAGTCGAAAAGCCCAAATTCCTTCCTGATCTGTATGACTACAAAGAGAACCGGTTCATTGAAATTGGAGTAACGCGAAGGGAGGTTCACATATATTATCTAGAGAAAGCCAACAAGATAAAGTCAGAGAAGACACACATTCACATATTCTCATTCACTGGAGAGGAA<br>ATGGCCACCAAGGCGGACTACACTCTTGATGAAGAGAGCAGAGCAAGAATAAAAACAAGGCTATTCACCATAAGACAGGAAATGGCCAGTAGGGGTCTATGGGATTCCTTTCGTCAGTCCGAGAGAGGCGAAGAGACAATTGAAGAAAGATTTGAAATCACAGGAACCATGCGCAGGCTTGCCGACCAAAGTCTCCCACCGAACTTCTCCAGCCTTGAAAACTTTAGAG<br>CCTATGTGGATGGATTCGAACCGAACGGCTGCATTGAGGGCAAGCTTTCTCAAATGTCAAAAGAAGTGAATGCAAGAATTGAGCCATTTCTGAAGACAACACCACGCCCTCTCAGATTACCTGATGGGCCTCCCTGTTCTCAGCGCTCGAAATTCTTGCTGATGGATGCCCTTAAATTGAGTATTGAAGACCCGAGCCATGAGGGGGAGGGCATACCGCTGTATGATGCAAT<br>CAAATGCATGAAGACCTTCTTTGGCTGGAAAGAGCCCAACATCGTGAAACCACATGAAAAGGGCATAAACCCTAATTACCTTCTGGCTTGGAAGCAGGTGCTAGCAGAACTCCAAGATATTGAAAATGAGGAGAAATCCCAAAAACAAAGAATATGAAGAAAACAAGCCAACTGAAGTGGGCACTTGGTGAGAACATGGCACCAGAGAAAGTGGACTTTGAGGACTG<br>TAAAGATGTTAGTGATCTAAAACAGTACAACAGTGACGAACCAGAGTCTAGATCACTAGCAAGCTGGATCCAGAGTGAATTTAACAAAGCATGCGAATTGACAGATTCAAGTTGGATTGAACTTGATGAAATAGGAGAAGATGTTGCTCCAATTGAACACATTGCGAGTATGAGGAGGAACTATTTCACAGCGGAAGTATCTCATTGCAGGGCTACTGAATACATAATGAA<br>GGGGGTATACATAAACACAGCCCTACTGAATGCATCCTGTGCAGCCATGGATGACTTCCAACTGATCCCAATGATAAGCAAGTGCAGAACCAAAGAAGGGGAGGCGGAAGACAAATCTGTATGGATTCATTATAAAAGGAAGATCCCATTTGAGGAATGACACCGATGTGGTAAACTTTGTGAGCATGGAGTTCTCTCTCACTGATCCAAGGCTAGAGCCACACAAATGGG<br>AAAGTACTGTGTTCTTGAGATAGGAGACATGCTCCTACGGACTGCAATAGGCCAAGTGTCAAGGCCCATGTTCCTGTATGTGAGAACCAATGGGACTTCCAAGATCAAAATGAAATGGGGAATGGAGATGAGACGATGCCTTCTTCAGTCTCTTCAACAAATTGAGAGCATGATTGAGGCCGAGTCTTCTGTCAAAGAGAAGGACATGACCAAGGAATTCTTTGAAAACA<br>AATCAGAAACATGGCCAATTGGAGAATCACCCAAAGGGGTGGAAGAAGGCTCCATCGGGAAGGTATGCAGAACACTGTTAGCAAAGTCTGTGTTCAATAGCCTATATGCATCTCCACAACTTGAGGGATTTTCAGCTGAATCAAGAAAATTACTTCTCATTGTTCAGGCACTTAGGGACAACCTGGAACCTGGAACCTTCGATCTTGGGGGGCTATATGAAGCAATTGAGG<br>AGTGCCTGATTAACGATCCCTGGGTTTTGCTTAATGCGTCTTGGTTCAACTCCTTCCTCACACATGCACTGAAATA-
internal NVRI repository	>A/domestic_duck/Poland/01/2022_H5N1_2021-12-31<br>ATGGAAGACTTTGTGCGACAATGCTTCAATCCAATGATTGTCGAGCTTGCGGAAAAGGCAATGAAAGAGTATGGGGAAGATCCGAAAATCGAAACAAACAAATTTGCCGCAATATGCACACACTTAGAAGTCTGTTTCATGTATTCGGATTTCCACTTTATTGATGAACGCGGCGAATCAATAATTGTAGAATCTGGCGATCCGAATGCATTATTGAAACACCGATTCGAGA<br>TAATTGAAGGGAGAGACCGAACTATGGCCTGGACAGTGGTGAATAGTATCTGCAACACCACAGGAGTCGAAAAGCCCAAATTCCTCCCTGATTTGTATGACTACAAAGAGAACCGATTCATTGAAATTGGAGTAACGCGAAGGGAAGTTCACATATACTATCTAGAGAAAGCCAACAAGATAAAATCAGAGAAGACACACATTCATATATTCTCATTCACTGGAGAGGAA<br>ATGGCCACCAAGGCGGACTACACCCTTGATGAAGAGAGTAGAGCAAGAATAAAAACCAGGCTATTCACTATAAGACAAGAAATGGCCAGTAGGGGTCTATGGGATTCCTTTCGTCAGTCCGAGAGAGGCGAAGAGACAATTGAAGAAAGATTTGAAATCACAGGAACCATGCGCAGGCTTGCCGACCAAAGTCTCCCACCGAACTTCTCCAGCCTTGAAAACTTTAGAG<br>CCTATGTGGATGGATTCGAACCGAACGGCTGCATTGAGGGCAAGCTTTCTCAAATGTCAAAAGAAGTGAATGCCAGAATTGAGCCATTTCTGAAGACAACACCACGCCCTCTCAGATTACCTGATGGGCCTCCCTGTTCTCAGCGGTCGAAGTTCTTGCTGATGGATGCCCTCAAATTGAGCATCGAAGACCCGAGCCATGAGGGGGAGGGTATACCGCTGTATGATGCAA<br>TCAAATGCATGAAGACATTTTTGGCTGGAAAGAGCCTAACATCGTAAAACCGCATGAAAAGGGCATAAACCCTAATTACCTTCTGGCTTGGAAACAGGTGCTGGCAGAACTCCAAGATATTGAAAATGAGGAGAAAATCCCAAAAACAAAGAACATGAAGAAAACAAGCCAATTGAAGTGGGCACTTGGTGAGAACATGGCACCAGAGAAAGTGGACTTTGAGGACTG<br>TAAAGATGTTAGTGATCTAAGACAGTACGACAGTGACGAACCAGAGTCTAGATCACTAGCAAGCTGGATCCAGAGTGAATTCAACAAGGCATGCGAATTGACAGATTCGAGTTGGATTGAACTTGATGAAATAGGGGAAGACATTGCTCCAATTGAACACATTGCGAGTATGAGGAGAAACTATTTCACAGCGGAGGTGTCCCATTGCAGGGCTACTGAATACAYAATGA<br>AGGGAGTATACATAAACACAGCCCTGTTGAATGCATCCTGTGCAGCGATGGATGACTTCCAACTGATTCCTATGATAAGCAAATGCAGAACCAAAGAAGGAAGACGGAAGACAAATTTATATGGATTCATTATAAAAGGAAGATCCCATTTGAGGAATGACACCGATGTGGTGAACTTTGTGAGCATGGAATTCTCTCTCACTGACCCGAGGCTGGAGCCACACAAATGG<br>GAAAAGTACTGTGTTCTTGAGATAGGAGACATGCTCCTACGGACTGCAATAGGCCATGTGTCAAGGCCCATGTTCTTGTATGTGAGAACCAATGGAACTTCCAAGATCAAAATGAAATGGGGCATGGAGATGAGACGATGCCTTCTTCAGTCCCTTCAACAAATTGAGAGCATGATTGAGGCCGAGTCTTCTGTCAAAGAGAAGGACATGACCAAGGAATTCTTTGAAAAC<br>AAATCAGAAACATGGCCAATTGGGGAATCACCCAAAGGGGTGGAAGAAGGCTCCATTGGGAAGGTGTGCAGAACATTGCTAGCAAAGTCTGTGTTCAACAGCCTATATGCATCTCCACAACTCGAGGGGTTTTCAGCTGAATCAAGAAAATTGCTTCTCATTGTTCAGGCACTTAGGGACAACCTGGAACCTGGAACCTTCGATCTTGGGGGGCTATATGAAGCAATTGA<br>GGAGTGCCTGATTAACGATCCCTGGGTTTTGCTTAATGCGTCTTGGTTCAACTCCTTCCTCACACATGCACTGAAATA-
internal NVRI repository	>A/domestic_duck/Poland/2160/2021_H5N1_2021-12-27<br>ATGGAAGACTTTGTGCGACAATGCTTCAATCCAATGATTGTCGAGCTTGCGGAAAAGGCAATGAAAGAGTATGGGGAAGATCCGAAAATCGAAACAAACAAATTTGCCGCAATATGCACACACTTAGAAGTCTGTTTCATGTATTCGGATTTCCACTTTATTGATGAACGCGGCGAATCAATAATTGTAGAATCTGGCGATCCGAATGCATTATTGAAACACCGATTCGAGA<br>TAATTGAAGGGAGAGACCGAACTATGGCCTGGACAGTGGTGAATAGTATCTGCAACACCACAGGAGTCGAAAAGCCCAAATTCCTCCCTGATTTGTATGACTACAAAGAGAACCGATTCATTGAAATTGGAGTAACGCGAAGGGAAGTTCACATATACTATCTAGAGAAAGCCAACAAGATAAAATCAGAGAAGACACACATTCATATATTCTCATTCACTGGAGAGGAA<br>ATGGCCACCAAGGCGGACTACACCCTTGATGAAGAGAGTAGAGCAAGAATAAAAACCAGGCTATTCACTATAAGACAAGAAATGGCCAGTAGGGGTCTATGGGATTCCTTTCGTCAGTCCGAGAGAGGCGAAGAGACAATTGAAGAAAGATTTGAAATCACAGGAACCATGCGCAGGCTTGCCGACCAAAGTCTCCCACCGAACTTCTCCAGCCTTGAAAACTTTAGAG<br>CCTATGTGGATGGATTCGAACCGAACGGCTGCATTGAGGGCAAGCTTTCTCAAATGTCAAAAGAAGTGAATGCCAGAATTGAGCCATTTCTGAAGACAACACCACGCCCTCTCAGATTACCTGATGGGCCTCCCTGTTCTCAGCGGTCGAAGTTCTTGCTGATGGATGCCCTCAAATTGAGCATCGAAGACCCGAGCCATGAGGGGGAGGGTATACCGCTGTATGATGCAA<br>TCAAATGCATGAAGACATTTTTGGCTGGAAAGAGCCTAACATCGTAAAACCGCATGAAAAGGGCATAAACCCTAATTACCTTCTGGCTTGGAAACAGGTGCTGGCAGAACTCCAAGATATTGAAAATGAGGAGAAAATCCCAAAAACAAAGAACATGAAGAAAACAAGCCAATTGAAGTGGGCACTTGGTGAGAACATGGCACCAGAGAAAGTGGACTTTGAGGACTG<br>TAAAGATGTTAGTGATCTAAGACAGTACGACAGTGACGAACCAGAGTCTAGATCACTAGCAAGCTGGATCCAGAGTGAATTCAACAAGGCATGCGAATTGACAGATTCGAGTTGGATTGAACTTGATGAAATAGGGGAAGACATTGCTCCAATTGAACACATTGCGAGTATGAGGAGAAACTATTTCACAGCGGAGGTGTCCCATTGCAGGGCTACTGAATACATAATGA<br>AGGGAGTATACATAAACACAGCCCTGTTGAATGCATCCTGTGCAGCGATGGATGACTTCCAACTGATTCCTATGATAAGCAAATGCAGAACCAAAGAAGGAAGACGGAAGACAAATTTATATGGATTCATTATAAAAGGAAGATCCCATTTGAGGAATGACACCGATGTGGTGAACTTTGTGAGCATGGAATTCTCTCTCACTGACCCGAGGCTGGAGCCACACAAATGG<br>GAAAAGTACTGTGTTCTTGAGATAGGAGACATGCTCCTACGGACTGCAATAGGCCATGTGTCAAGGCCCATGTTCTTGTATGTGAGAACCAATGGAACTTCCAAGATCAAAATGAAATGGGGCATGGAGATGAGACGATGCCTTCTTCAGTCCCTTCAACAAATTGAGAGCATGATTGAGGCCGAGTCTTCTGTCAAAGAGAAGGACATGACCAAGGAATTCTTTGAAAAC<br>AAATCAGAAACATGGCCAATTGGGGAATCACCCAAAGGGGTGGAAGAAGGCTCCATTGGGAAGGTGTGCAGAACATTGCTAGCAAAGTCTGTGTTCAACAGCCTATATGCATCTCCACAACTCGAGGGGTTTTCAGCTGAATCAAGAAAATTGCTTCTCATTGTTCAGGCACTTAGGGACAACCTGGAACCTGGAACCTTCGATCTTGGGGGGCTATATGAAGCAATTGA<br>GGAGTGCCTGATTAACGATCCCTGGGTTTTGCTTAATGCGTCTTGGTTCAACTCCTTCCTCACACATGCACTGAAATA-

internal NVRI repository	>A/domestic_duck/Poland/2177/2021_H5N1_2021-12-29<br>ATGGAAGACTTTGTGCGACAATGCTTCAATCCAATGATTGTCGAGCTTGCGGAAAAGGCAATGAAAGAGTATGGGGAAGATCCGAAAATCGAAACAAACAAATTTGCCGCAATATGCACACACTTAGAAGTCTGTTTCATGTATTCGGATTTCCACTTTATTGATGAACGCGGCGAATCAATAATTGTAGAATCTGGCGATCCGAATGCATTATTGAAACACCGATTCGAGA<br>TAATTGAAGGGAGAGACCGAACTATGGCCTGGACAGTGGTGAATAGTATCTGCAACACCACAGGAGTCGAAAAGCCCAAATTCCTCCCTGATTTGTATGACTACAAAGAGAACCGATTCATTGAAATTGGAGTAACGCGAAGGGAAGTTCACATATACTATCTAGAGAAAGCCAACAAGATAAAATCAGAGAAGACACACATTCATATATTCTCATTCACTGGAGAGGAA<br>ATGGCCACCAAGGCGGACTACACCCTTGATGAAGAGAGTAGAGCAAGAATAAAAACCAGGCTATTCACTATAAGACAAGAAATGGCCAGTAGGGGTCTATGGGATTCCTTTCGTCAGTCCGAGAGAGGCGAAGAGACAATTGAAGAAAGATTTGAAATCACAGGAACCATGCGCAGGCTTGCCGACCAAAGTCTCCCACCGAACTTCTCCAGCCTTGAAAACTTTAGAG<br>CCTATGTGGATGGATTCGAACCGAACGGCTGCATTGAGGGCAAGCTTTCTCAAATGTCAAAAGAAGTGAATGCCAGAATTGAGCCATTTCTGAAGACAACACCACGCCCTCTCAGATTACCTGATGGGCCTCCCTGTTCTCAGCGGTCGAAGTTCTTGCTGATGGATGCCCTCAAATTGAGCATCGAAGACCCGAGCCATGAGGGGGAGGGTATACCGCTGTATGATGCAA<br>TCAAATGCATGAAGACATTTTTTGGCTGGAAAGAGCCTAACATCGTAAAACCGCATGAAAAGGGCATAAACCCTAATTACCTTCTGGCTTGGAAACAGGTGCTGGCAGAACTCCAAGATATTGAAAATGAGGAGAAAATCCCAAAAACAAAGAACATGAAGAAAACAAGCCAATTGAAGTGGGCACTTGGTGAGAACATGGCACCAGAGAAAGTGGACTTTGAGGACTG<br>TAAAGATGTTAGTGATCTAAGACAGTACGACAGTGACGAACCAGAGTCTAGATCACTAGCAAGCTGGATCCAGAGTGAATTCAACAAGGCATGCGAATTGACAGATTCGAGTTGGATTGAACTTGATGAAATAGGGGAAGACATTGCTCCAATTGAACACATTGCGAGTATGAGGAGAAACTATTTCACAGCGGAGGTGTCCCATTGCAGGGCTACTGAATACATAATGA<br>AGGGAGTATACATAAACACAGCCCTGTTGAATGCATCCTGTGCAGCGATGGATGACTTCCAACTGATTCCTATGATAAGCAAGTGCAGAACCAAAGAAGGAAGACGGAAGACAAATTTATATGGATTCATTATAAAAGGAAGATCCCATTTGAGGAATGACACCGATGTGGTGAACTTTGTGAGCATGGAATTCTCTCTCACTGACCCGAGGCTGGAGCCACACAAATGG<br>GAAAAGTACTGTGTTCTTGAGATAGGAGACATGCTCCTACGGACTGCAATAGGCCATGTGTCAAGGCCCATGTTCTTGTATGTGAGAACCAATGGAACTTCCAAGATCAAAATGAAATGGGGCATGGAGATGAGACGATGCCTTCTTCAGTCCCTTCAACAAATTGAGAGCATGATTGAGGCCGAGTCTTCTGTCAAAGAGAAGGACATGACCAAGGAATTCTTTGAAAAC<br>AAATCAGAAACATGGCCAATTGGGGAATCACCCAAAGGGGTGGAAGAAGGCTCCATTGGGAAGGTGTGCAGAACATTGCTAGCAAAGTCTGTGTTCAACAGCCTATATGCATCTCCACAACTCGAGGGGGTTTTCAGCTGAATCAAGAAAATTGCTTCTCATTGTTCAGGCACTTAGGGACAACCTGGAACCTGGAACCTTCGATCTTGGGGGGCTATATGAAGCAATTGA<br>GGAGTGCCTGATTAACGATCCCTGGGTTTTGCTTAATGCGTCTTGGTTCAACTCCTTCCTCACACATGCACTGAAATA-
internal NVRI repository	>A/domestic_duck/Poland/H144/2022_H5N1_2022-02-13<br>ATGGAAGACTTTGTGCGACAATGCTTCAATCCAATGATTGTCGAGCTTGCGGAAAAAGCAATGAAAGAATATGGGGAAGATCCGAAAATCGAGACAAACAAATTTGCCGCAATATGCACACACTTAGAAGTCTGTTTCATGTATTCGGATTTCCATTTTATTGATGAACGAGGCGAATCAATGATTGTAGAATCTGGCGATCCAAATGCATTATTGAAACACCGATTTGAGA<br>TAATCGAAGGGAGAGACCGAGCAATGGCCTGGACAGTGGTGAATAGTATCTGCAACACTACAGGAGTCGAAAAGCCCAAATTCCTCCCTGATTTGTATGACTACAAAGAGAACCGATTCATTGAARTTGGAGTAACGCGAAGGGAAGTTCACATATACTATTTAGAAAAAGCCAACAAGATAAAATCAGAGAAAACACACATTCACATATTCTCATTCACTGGAGAGGAA<br>ATGGCCACCAAGGCGGAATACACCCTTGATGAAGAGAGCAGAGCAAGAATAAAAACCAGACTGTTCACTATAAGACAAGAAATGGCCAGTAGAGGTCTATGGGATTCCTTTCGTCAGTCCGAGAGAGGCGAAGAGACAACTGAAGAAAGATTTGAAATCACAGGAACCATGCGCAGGCTTGCCGACCAAAGTCTCCCACCGAACTTCTCCAGCCTTGAAAACTTTAGAG<br>CCTATGTGGATGGATTCGAACCGAACGGCTGCATTGAGGGCAAGCTTTCTCAAATGTCAAAAGAAGTGAACGCCAGAATTGAGCCATTCCTGAAGACAACACCACGCCCTCTCAGATTACCTGATGGGCCTCCTTGTTCTCAGCGGTCGAAGTTCTTGCTGATGGATGCCCTTAAGTTGAGCATCGAAGATCCTAGCCATGAGGGGGAGGGCATACCGCTGTATGATGCAA<br>TCAAATGCATGAAGACATTTTTTGGCTGGAAAGAGCCCAACCTCGTAAAGCCGCATGAGAAAGGCATAAACCCTAATTACCTCCTGGCTTGGAAGCAGGTGCTGGCAGAACTTCAAGATATTGAGAATGAGGAGAAATTCCCAAAAACAAAGAACATGAAGAAAACAAGCCAATTGAAGTGGGCACTTGGTGAGAACATGGCACCAGAAAAAGTGGACTTTGAGGACTG<br>CAAAGATGTTAGCGATCTAAGACAGTACGACAGTGACGAACCAGAGCCTAGATCACTAGCAAGCTGGATTCAGAGTGAATTCAACAAGGCATGCGAATTGACAGATTCGAGTTGGATTGAACTTGATGAGATAGGGGAAGACGTTGCTCCAATCGAACACATTGCGAGTATGAGGAGGAACTATTTCACAGCGGAGGTATCCCATTGCAGGGCCACTGAATACATAATG<br>AAGGGAGTATACATAAACACAGCCCTATTGAATGCATCCTGTGCAGCCATGGATGACTTCCAACTGATTCCAATGATAAGCAAGTGCAGAACCAAAGAGGGAAGACGGAAGACAAATCTGTATGGATTCATTATAAAAGGAAGATCCCATTTGAGGAATGACACCGATGTGGTAAACTTTGTGAGCATGGAATTCTCTCTAACTGACCCGAGGCTAGAGCCACACAAATG<br>GGAAAAGTACTGTGTTCTTGAGATAGGAGACATGCTCCTACGGACTGCAATAGGCCAAGTGTCGAGGCCCATGTTCCTGTATGTGAGAACCAATGGGACTTCCAAGATCAAAATGAAATGGGGCATGGAGATGAGACGATGCCTTCTTCAGTCCCTTCAACAAATTGAGAGCATGATTGAGGCCGAATCTTCTGTCAAAGAGAAGGACATGACCAAGGAATTCTTTGAAA<br>ACAAATCAGAAACATGGCCAATTGGAGAATCACCCAAAGGGGTTGAGGAAGGCTCTATTGGGAAAGTATGCAGAACATTGCTAGCAAAGTCTGTGTTCAACAGCCTATATGCATCTCCACAACTCGAGGGATTTTCAGCTGAATCAAGAAAATTGCTTCTCATTGTTCAGGCACTTAGGGACAACCTGGAACCTGGAACCTTCGATCTTGGGGGGCTATATGAAGCAATTG<br>AGGAGTGCCTGATTAACGATCCCTGGGTTTTGCTTAATGCGTCTTGGTTCAACTCCTTCATCACACATGCACTGAAATA-
internal NVRI repository	>A/domestic_duck/Poland/H146/2022_H5N1_2022-02-14<br>ATGGAAGACTTTGTGCGACAATGCTTCAATCCAATGATTGTCGAGCTTGCGGAAAAAGCAATGAAAGAATATGGGGAAGATCCGAAAATCGAGACAAACAAATTTGCCGCAATATGCACACACTTAGAAGTCTGTTTCATGTATTCGGATTTCCATTTTATTGATGAACGAGGCGAATCAATGATTGTAGAATCTGGCGATCCAAATGCATTATTGAAACACCGATTTGAGA<br>TAATCGAAGGGAGAGACCGAGCAATGGCCTGGACAGTGGTGAATAGTATCTGCAACACTACAGGAGTCGAAAAGCCCAAATTCCTCCCTGATTTGTATGACTACAAAGAGAACCGATTCATTGAAATTGGAGTAACGCGAAGGGAAGTTCACATATACTATTTAGAAAAAGCCAACAAGATAAAATCAGAGAAAACACACATTCACATATTCTCATTCACTGGAGAGGAA<br>ATGGCCACCAAGGCGGAATACACCCTTGATGAAGAGAGCAGAGCAAGAATAAAAACCAGACTGTTCACTATAAGACAAGAAATGGCCAGTAGAGGTCTATGGGATTCCTTTCGTCAGTCCGAGAGAGGCGAAGAGACAACTGAAGAAAGATTTGAAATCACAGGAACCATGCGCAGGCTTGCCGACCAAAGTCTCCCACCGAACTTCTCCAGCCTTGAAAACTTTAGAG<br>CCTATGTGGATGGATTCGAACCGAACGGCTGCATTGAGGGCAAGCTTTCTCAAATGTCAAAAGAAGTGAACGCCAGAATTGAGCCATTCCTGAAGACAACACCACGCCCTCTCAGATTACCTGATGGGCCTCCTTGTTCTCAGCGGTCGAAGTTCTTGCTGATGGATGCCCTTAAGTTGAGCATCGAAGATCCTAGCCATGAGGGGGAGGGCATACCGCTGTATGATGCAA<br>TCAAATGCATGAAGACATTTTTTGGCTGGAAAGAGCCCAACCTCGTAAAGCCGCATGAGAAAGGCATAAACCCTAATTACCTCCTGGCTTGGAAGCAGGTGCTGGCAGAACTTCAAGATATTGAGAATGAGGAGAAATTCCCAAAAACAAAGAACATGAAGAAAACAAGCCAATTGAAGTGGGCACTTGGTGAGAACATGGCACCAGAAAAAGTGGACTTTGAGGACTG<br>CAAAGATGTTAGCGATCTAAGACAGTACGACAGTGACGAACCAGAGCCTAGATCACTAGCAAGCTGGATTCAGAGTGAATTCAACAAGGCATGCGAATTGACAGATTCGAGTTGGATTGAACTTGATGAGATAGGGGAAGACGTTGCTCCAATCGAACACATTGCGAGTATGAGGAGGAACTATTTCACAGCGGAGGTATCCCATTGCAGGGCCACTGAATACATAATG<br>AAGGGAGTATACATAAACACAGCCCTATTGAATGCATCCTGTGCAGCCATGGATGACTTCCAACTGATTCCAATGATAAGCAAGTGCAGAACCAAAGAGGGAAGACGGAAGACAAATCTGTATGGATTCATTATAAAAGGAAGATCCCATTTGAGGAATGACACCGATGTGGTAAACTTTGTGAGCATGGAATTCTCTCTAACTGACCCGAGGCTAGAGCCACACAAATG<br>GGAAAAGTACTGTGTTCTTGAGATAGGAGACATGCTCCTACGGACTGCAATAGGCCAAGTGTCGAGGCCCATGTTCCTGTATGTGAGAACCAATGGGACTTCCAAGATCAAAATGAAATGGGGCATGGAGATGAGACGATGCCTTCTTCAGTCCCTTCAACAAATTGAGAGCATGATTGAGGCCGAATCTTCTGTCAAAGAGAAGGACATGACCAAGGAATTCTTTGAAA<br>ACAAATCAGAAACATGGCCAATTGGAGAATCACCCAAAGGGGTTGAGGAAGGCTCTATTGGGAAAGTATGCAGAACATTGCTAGCAAAGTCTGTGTTCAACAGCCTATATGCATCTCCACAACTCGAGGGATTTTCAGCTGAATCAAGAAAATTGCTTCTCATTGTTCAGGCACTTAGGGACAACCTGGAACCTGGAACCTTCGATCTTGGGGGGCTATATGAAGCAATTG<br>AGGAGTGCCTGATTAACGATCCCTGGGTTTTGCTTAATGCGTCTTGGTTCAACTCCTTCATCACACATGCACTGAAATA-
internal NVRI repository	>A/domestic_duck/Poland/H158/2022_H5N1_2022-02-19<br>ATGGAAGACTTTGTGCGACAATGCTTCAATCCAATGATTGTCGAGCTTGCGGAAAAGGCAATGAAAGAGTATGGGGAAGATCCGAAAATCGAAACAAACAAATTTGCCGCAATATGCACACACTTAGAAGTCTGTTTCATGTATTCGGATTTCCACTTTATTGATGAACGCGGCGAATCAATAATTGTAGAATCTGGCGATCCGAATGCATTATTGAAACACCGATTCGAGA<br>TAATTGAAGGGAGAGACCGAACTATGGCCTGGACAGTGGTGAATAGTATCTGCAACACCACAGGAGTCGAAAAGCCCAAATTCCTCCCTGATTTGTATGACTACAAAGAGAACCGATTCATTGAAATTGGAGTAACGCGAAGGGAAGTTCACATATACTATCTAGAGAAAGCCAACAAGATAAAATCAGAGAAGACACACATTCATATATTCTCATTCACTGGAGAGGAA<br>ATGGCCACCAAGGCGGACTACACCCTTGATGAAGAGAGTAGAGCAAGAATAAAAACCAGGCTATTCACTATAAGACAAGAAATGGCCAGTAGGGGTCTATGGGATTCCTTTCGTCAGTCCGAGAGAGGCGAAGAGACAATTGAAGAAAGATTTGAAATCACAGGAACCATGCGCAGGCTTGCCGACCAAAGTCTCCCACCGAACTTCTCCAGCCTTGAAAACTTTAGAG<br>CCTATGTGGATGGATTCGAACCGAACGGCTGCATTGAGGGCAAGCTTTCTCAAATGTCAAAAGAAGTGAATGCCAGAATTGAGCCATTTCTGAAGACAACACCACGCCCTCTCAGATTACCTGATGGGCCTCCCTGTTCTCAGCGGTCGAAGTTCTTGCTGATGGATGCCCTCAAATTGAGCATCGAAGACCCGAGCCATGAGGGGGAGGGTATACCGCTGTATGATGCAA<br>TCAAATGCATGAAGACATTTTTTGGCTGGAAAGAGCCTAACATCGTAAAACCGCATGAAAAGGGCATAAACCCTAATTACCTTCTGGCTTGGAAACAGGTGCTGGCAGAACTCCAAGATATTGAAAATGAGGAGAAAATCCCAAAAACAAAGAACATGAAGAAAACAAGCCAATTGAAGTGGGCACTTGGTGAGAACATGGCACCAGAGAAAGTGGACTTTGAGGACTG<br>TAAAGATGTTAGTGATCTAAGACAGTACGACAGTGACGAACCAGAGTCTAGATCACTAGCAAGCTGGATCCAGAGTGAATTCAACAAGGCATGCGAATTGACAGATTCGAGTTGGATTGAACTTGATGAAATAGGGGAAGACATTGCTCCAATTGAACACATTGCGAGTATGAGGAGAAACTATTTCACAGCGGAGGTGTCCCATTGCAGGGCTACTGAATACATAATGA<br>AGGGAGTATACATAAACACAGCCCTGTTGAATGCATCCTGTGCAGCGATGGATGACTTCCAACTGATTCCTATGATAAGCAAGTGCAGAACCAAAGAAGGAAGACGGAAGACAAATTTATATGGATTCATTATAAAAGGAAGATCCCATTTGAGGAATGACACCGATGTGGTGAACTTTGTGAGCATGGAATTCTCTCTCACTGACCCGAGGCTGGAGCCACACAAATGG<br>GAAAAGTACTGTGTTCTTGAGATAGGAGACATGCTCCTACGGACTGCAATAGGCCATGTGTCAAGGCCCATGTTCTTGTATGTGAGAACCAATGGAACTTCCAAGATCAAAATGAAATGGGGCATGGAGATGAGACGATGCCTTCTTCAGTCCCTTCAACAAATTGAGAGCATGATTGAGGCCGAGTCTTCTGTCAAAGAGAAGGACATGACCAAGGAATTCTTTGAAAAC<br>AAATCAGAAACATGGCCAATTGGGGAATCACCCAAAGGGGTGGAAGAAGGCTCCATTGGGAAGGTGTGCAGAACATTGCTAGCAAAGTCTGTGTTCAACAGCCTATATGCATCTCCACAACTCGAGGGGGTTTTCAGCTGAATCAAGAAAATTGCTTCTCATTGTTCAGGCACTTAGGGACAACCTGGAACCTGGAACCTTCGATCTTGGGGGGCTATATGAAGCAATTGA<br>GGAGTGCCTGATTAACGATCCCTGGGTTTTGCTTAATGCGTCTTGGTTCAACTCCTTCCTCACACATGCACTGAAATA-
internal NVRI repository	>A/domestic_duck/Poland/H189/2022_H5N1_2022-03-04<br>ATGGAAGACTTTGTGCGACAATGCTTCAATCCAATGATTGTCGAGCTTGCGGAAAAGGCAATGAAAGAATATGGGGAAGACCCGAAAATCGAGACAAACAAATTTGCCGCAATATGCACACACTTAGAAGTCTGTTTCATGTATTCGGATTTCCACTTTATTGATGAACGAGGAGAATCAAYAATTGTAGAATCCGGCGATCCGAATGCATTATTGAAACATCGATTTGAGA<br>TAATTGAAGGGAGAGACCGAACAATGGCTTGGACAGTGGTAAATAGTATATGCAACACTACAGGAGTCGAAAAGCCCAAATTCCTTCCTGATCTGTATGACTACAAAGAGAACCGGTTCATTGAAATTGGAGTAACGCGAAGGGAGGTTCACATATATTATCTAGAGAAAGCCAACAAGATAAAGTCAGAGAAGACACACATTCACATATTCTCATTCACTGGAGAGGAA<br>ATGGCCACCAAGGCGGACTACACTCTTGATGAAGAGAGCAGAGCAAGAATAAAAACAAGGCTATTCACCATAAGACAGGAAATGGCCAGTAGGGGTCTATGGGATTCCTTTCGTCAGTCCGAGAGAGGCGAAGAGACAATTGAAGAAAGATTTGAAATCACAGGAACCATGCGCAGGCTTGCCGACCAAAGTCTCCCACCGAACTTCTCCAGCCTTGAAAACTTTAGAG<br>CCTATGTGGATGGATTCGAACCGAACGGCTGCATTGAGGGCAAGCTTTCTCAAATGTCAAAAGAAGTGAATGCAAGAATTGAGCCATTTCTGAAGACAACACCACGCCCTCTCAGATTACCTGATGGGCCTCCCTGTTCTCAGCGCTCGAAATTCTTGCTGATGGATGCCCTTAAATTGAGTATTGAAGACCCGAGCCATGAGGGGGAGGGCATACCGCTGTATGATGCAAT<br>CAAATGCATGAAGACCTTCTTTGGCTGGAAAGAGCCCAACATCGTGAAACCACATGAAAAGGGCATAAACCCTAATTACCTTCTGGCTTGGAAGCAGGTGCTAGCAGAACTCCAAGATATTGAAAATGAGGAGAAAATCCCAAAAACAAAGAATATGAAGAAAACAAGCCAACTGAAGTGGGCACTTGGTGAGAACATGGCACCAGAGAAAGTGGACTTTGAGGACTG<br>TAAAGATGTTAGTGATCTAAAACAGTACAACAGTGACGAACCAGAGTCTAGATCACTAGCAAGCTGGATCCAGAGTGAATTCAACAAAGCATGCGAATTGACAGATTCAAGTTGGATTGAACTTGATGAAATAGGAGAAGATGTTGCTCCAATTGAACACATTGCGAGTATGAGGAGGAACTATTTCACAGCGGAAGTATCTCATTGCAGGGCTACTGAATACATAATGA<br>AGGGGGTAAACATAAACACAGCCCTACTGAATGCATCCTGTGCAGCCATGGATGACTTCCAACTGATCCCAATGATAAGCAAGTGCAGAACCAAAGAAGGGAGGCGGAAGACAAATCTGTATGGATTCATTATAAAAGGAAGATCCCATTTGAGGAATGACACCGATGTGGTAAACTTTGTGAGCATGGAGTTCTCTCTCACTGATCCAAGGCTAGAGCCACACAAATGG<br>GAAAAGTACTGTGTTCTTGAGATAGGAGACATGCTCCTACGGACTGCAATAGGCCAAGTGTCAAGGCCCATGTTCCTGTATGTGAGAACCAATGGGACTTCCAAGATCAAAATGAAATGGGGAATGGAGATGAGACGATGCCTTCTTCAGTCTCTTCAACAAATTGAGAGCATGATTGAGGCCGAGTCTTCTGTCAAAGAGAAGGACATGACCAAGGAATTCTTTGAAAAC<br>AAATCAGAAACATGGCCAATTGGAGAATCACCCAAAGGGGTGGAAGAAGGCTCCATCGGGAAGGTATGCAGAACACTGTTAGCAAAGTCTGTGTTCAATAGCCTATATGCATCTCCACAACTTGAGGGATTTTCAGCTGAATCAAGAAAATTACTTCTCATTGTTCAGGCACTTAGGGACAACCTGGAACCTGGAACCTTCGATCTTGGGGGGCTATATGAAGCAATTGAG<br>GAGTGCCTGATTAACGATCCCTGGGTTTTGCTTAATGCGTCTTGGTTCAACTCCTTCCTCACACATGCACTGAAATA-
internal NVRI repository	>A/domestic_duck/Poland/H1942-Nd/2021_H5N1_2021-11-07<br>ATGGAAGACTTTGTGCGACAATGCTTCAATCCAATGATTGTCGAGCTTGCGGAAAAAGCAATGAAAGAATATGGGGAAGATCCGAAAATCGAGACAAACAAATTTGCCGCAATATGCACACACTTAGAAGTCTGTTTCATGTATTCGGATTTCCATTTTATTGATGAACGAGGCGAATCAATGATTGTAGAATCTGGCGATCCAAATGCATTATTGAAACACCGATTTGAGA<br>TAATCGAAGGGAGAGACCGAGCAATGGCCTGGACAGTGGTGAATAGTATCTGCAACACTACAGGAGTCGAAAAGCCCAAATTCCTCCCTGATTTGTATGACTACAAAGAGAACCGATTCATTGAAATTGGAGTAACGCGAAGGGAAGTTCACATATACTATTTAGAAAAAGCCAACAAGATAAAATCAGAGAAAACACACATTCACATATTCTCATTCACTGGAGAGGAA<br>ATGGCCACCAAGGCGGAATACACCCTTGATGAAGAGAGCAGAGCAAGAATAAAAACCAGACTGTTCACTATAAGACAAGAAATGGCCAGTAGAGGTCTATGGGATTCCTTTCGTCAGTCCGAGAGAGGCGAAGAGACAACTGAAGAAAGATTTGAAATCACAGGAACCATGCGCAGGCTTGCCGACCAAAGTCTCCCACCGAACTTCTCCAGCCTTGAAAACTTTAGAG<br>CCTATGTGGATGGATTCGAACCGAACGGCTGCATTGAGGGCAAGCTTTCTCAAATGTCAAAAGAAGTGAACGCCAGAATTGAGCCATTCCTGAAGACAACACCACGCCCTCTCAGATTACCTGATGGGCCTCCTTGTTCTCAGCGGTCGAAGTTCTTGCTGATGGATGCCCTTAAGTTGAGCATCGAAGATCCTAGCCATGAGGGGGAGGGCATACCGCTGTATGATGCAA<br>TCAAATGCATGAAGACATTTTTTGGCTGGAAAGAGCCCAACCTCGTAAAGCCGCATGAGAAAGGCATAAACCCTAATTACCTCCTGGCTTGGAAGCAGGTGCTGGCAGAACTTCAAGATATTGAGAATGAGGAGAAATTCCCAAAAACAAAGAACATGAAGAAAACAAGCCAATTGAAGTGGGCACTTGGTGAGAACATGGCACCAGAAAAAGTGGACTTTGAGGACTG<br>CAAAGATGTTAGCGATCTAAGACAGTACGACAGTGACGAACCAGAGCCTAGATCACTAGCAAGCTGGATTCAGAGTGAATTCAACAAGGCATGCGAATTGACAGATTCGAGTTGGATTGAACTTGATGAGATAGGGGAAGACGTTGCTCCAATCGAACACATTGCGAGTATGAGGAGGAACTATTTCACAGCGGAGGTATCCCATTGCAGGGCCACTGAATACATAATG<br>AAGGGAGTATACATAAACACAGCCCTATTGAATGCATCCTGTGCAGCCATGGATGACTTCCAACTGATTCCAATGATAAGCAAGTGCAGAACCAAAGAGGGAAGACGGAAGACAAATCTGTATGGATTCATTATAAAAGGAAGATCCCATTTGAGGAATGACACCGATGTGGTAAACTTTGTGAGCATGGAATTCTCTCTAACTGACCCGAGGCTAGAGCCACACAAATG<br>GGAAAAGTACTGTGTTCTTGAGATAGGAGACATGCTCCTACGGACTGCAATAGGCCAAGTGTCGAGGCCCATGTTCCTGTATGTGAGAACCAATGGGACTTCCAAGATCAAAATGAAATGGGGCATGGAGATGAGACGATGCCTTCTTCAGTCCCTTCAACAAATTGAGAGCATGATTGAGGCCGAATCTTCTGTCAAAGAGAAGGACATGACCAAGGAATTCTTTGAAA<br>ACAAATCAGAAACATGGCCAATTGGAGAATCACCCAAAGGGGTTGAGGAAGGCTCTATTGGGAAAGTATGCAGAACATTGCTAGCAAAGTCTGTGTTCAACAGCCTATATGCATCTCCACAACTCGAGGGATTTTCAGCTGAATCAAGAAAATTGCTTCTCATTGTTCAGGCACTTAGGGACAACCTGGAACCTGGAACCTTCGATCTTGGGGGGCTATATGAAGCAATTG<br>AGGAGTGCCTGATTAACGATCCCTGGGTTTTGCTTAATGCGTCTTGGTTCAACTCCTTCATCACACATGCACTGAAATA-
internal NVRI repository	>A/domestic_duck/Poland/H2020-N/2021_H5N1_2021-11-23<br>ATGGAAGACTTTGTGCGACAATGCTTCAATCCAATGATTGTCGAGCTTGCGGAAAAAGCAATGAAAGAATATGGGGAAGATCCGAAAATCGAGACAAACAAATTTGCCGCAATATGCACACACTTAGAAGTCTGTTTCATGTATTCGGATTTCCATTTTATTGATGAACGAGGCGAATCAATGATTGTAGAATCTGGCGATCCAAATGCATTATTGAAACACCGATTTGAGA<br>TAATCGAAGGGAGAGACCGAGCAATGGCCTGGACAGTGGTGAATAGTATCTGCAACACTACAGGAGTCGAAAAGCCCAAATTCCTCCCTGATTTGTATGACTACAAAGAGAACCGATTCATTGAAATTGGAGTAACGCGAAGGGAAGTTCACATATACTATTTAGAAAAAGCCAACAAGATAAAATCAGAGAAAACACACATTCACATATTCTCATTCACTGGAGAGGAA<br>ATGGCCACCAAGGCGGAATACACCCTTGATGAAGAGAGCAGAGCAAGAATAAAAACCAGACTGTTCACTATAAGACAAGAAATGGCCAGTAGAGGTCTATGGGATTCCTTTCGTCAGTCCGAGAGAGGCGAAGAGACAACTGAAGAAAGATTTGAAATCACAGGAACCATGCGCAGGCTTGCCGACCAAAGTCTCCCACCGAACTTCTCCAGCCTTGAAAACTTTAGAG<br>CCTATGTGGATGGATTCGAACCGAACGGCTGCATTGAGGGCAAGCTTTCTCAAATGTCAAAAGAAGTGAACGCCAGAATTGAGCCATTCCTGAAGACAACACCACGCCCTCTCAGATTACCTGATGGGCCTCCTTGTTCTCAGCGGTCGAAGTTCTTGCTGATGGATGCCCTTAAGTTGAGCATCGAAGATCCTAGCCATGAGGGGGAGGGCATACCGCTGTATGATGCAA<br>TCAAATGCATGAAGACATTTTTTGGCTGGAAAGAGCCCAACCTCGTAAAGCCGCATGAGAAAGGCATAAACCCTAATTACCTCCTGGCTTGGAAGCAGGTGCTGGCAGAACTTCAAGATATTGAGAATGAGGAGAAATTCCCAAAAACAAAGAACATGAAGAAAACAAGCCAATTGAAGTGGGCACTTGGTGAGAACATGGCACCAGAAAAAGTGGACTTTGAGGACTG<br>CAAAGATGTTAGCGATCTAAGACAGTACGACAGTGACGAACCAGAGCCTAGATCACTAGCAAGCTGGATTCAGAGTGAATTCAACAAGGCATGCGAATTGACAGATTCGAGTTGGATTGAACTTGATGAGATAGGGGAAGACGTTGCTCCAATCGAACACATTGCGAGTATGAGGAGGAACTATTTCACAGCGGAGGTATCCCATTGCAGGGCCACTGAATACATAATG<br>AAGGGAGTATACATAAACACAGCCCTATTGAATGCATCCTGTGCAGCCATGGATGACTTCCAACTGATTCCAATGATAAGCAAGTGCAGAACCAAAGAGGGAAGACGGAAGACAAATCTGTATGGATTCATTATAAAAGGAAGATCCCATTTGAGGAATGACACCGATGTGGTAAACTTTGTGAGCATGGAATTCTCTCTAACTGACCCGAGGCTAGAGCCACACAAATG<br>GGAAAAGTACTGTGTTCTTGAGATAGGAGACATGCTCCTACGGACTGCAATAGGCCAAGTGTCGAGGCCCATGTTCCTGTATGTGAGAACCAATGGGACTTCCAAGATCAAAATGAAATGGGGCATGGAGATGAGACGATGCCTTCTTCAGTCCCTTCAACAAATTGAGAGCATGATTGAGGCCGAATCTTCTGTCAAAGAGAAGGACATGACCAAGGAATTCTTTGAAA<br>ACAAATCAGAAACATGGCCAATTGGAGAATCACCCAAAGGGGTTGAGGAAGGCTCTATTGGGAAAGTATGCAGAACATTGCTAGCAAAGTCTGTGTTCAACAGCCTATATGCATCTCCACAACTCGAGGGATTTTCAGCTGAATCAAGAAAATTGCTTCTCATTGTTCAGGCACTTAGGGACAACCTGGAACCTGGAACCTTCGATCTTGGGGGGCTATATGAAGCAATTG<br>AGGAGTGCCTGATTAACGATCCCTGGGTTTTGCTTAATGCGTCTTGGTTCAACTCCTTCATCACACATGCACTGAAATA-
internal NVRI repository	>A/domestic_duck/Poland/H2045-T1/2021_H5N1_2021-11-26<br>ATGGAAGACTTTGTGCGACAATGCTTCAATCCAATGATTGTCGAGCTTGCGGAAAAAGCAATGAAAGAATATGGGGAAGATCCGAAAATCGAGACAAACAAATTTGCCGCAATATGCACACACTTAGAAGTCTGTTTCATGTATTCGGATTTCCATTTTATTGATGAACGAGGCGAATCAATGATTGTAGAATCTGGCGATCCAAATGCATTATTGAAACACCGATTTGAGA<br>TAATCGAAGGGAGAGACCGAGCAATGGCCTGGACAGTGGTGAATAGTATCTGCAACACTACAGGAGTCGAAAAGCCCAAATTCCTCCCTGATTTGTATGACTACAAAGAGAACCGATTCATTGAAATTGGAGTAACGCGAAGGGAAGTTCACATATACTATTTAGAAAAAGCCAACAAGATAAAATCAGAGAAAACACACATTCACATATTCTCATTCACTGGAGAGGAA<br>ATGGCCACCAAGGCGGAATACACCCTTGATGAAGAGAGCAGAGCAAGAATAAAAACCAGACTGTTCACTATAAGACAAGAAATGGCCAGTAGAGGTCTATGGGATTCCTTTCGTCAGTCCGAGAGAGGCGAAGAGACAACTGAAGAAAGATTTGAAATCACAGGAACCATGCGCAGGCTTGCCGACCAAAGTCTCCCACCGAACTTCTCCAGCCTTGAAAACTTTAGAG<br>CCTATGTGGATGGATTCGAACCGAACGGCTGCATTGAGGGCAAGCTTTCTCAAATGTCAAAAGAAGTGAACGCCAGAATTGAGCCATTCCTGAAGACAACACCACGCCCTCTCAGATTACCTGATGGGCCTCCTTGTTCTCAGCGGTCGAAGTTCTTGCTGATGGATGCCCTTAAGTTGAGCATCGAAGATCCTAGCCATGAGGGGGAGGGCATACCGCTGTATGATGCAA<br>TCAAATGCATGAAGACATTTTTTGGCTGGAAAGAGCCCAACCTCGTAAAGCCGCATGAGAAAGGCATAAACCCTAATTACCTCCTGGCTTGGAAGCAGGTGCTGGCAGAACTTCAAGATATTGAGAATGAGGAGAAATTCCCAAAAACAAAGAACATGAAGAAAACAAGCCAATTGAAGTGGGCACTTGGTGAGAACATGGCACCAGAAAAAGTGGACTTTGAGGACTG<br>CAAAGATGTTAGCGATCTAAGACAGTACGACAGTGACGAACCAGAGCCTAGATCACTAGCAAGCTGGATTCAGAGTGAATTCAACAAGGCATGCGAATTGACAGATTCGAGTTGGATTGAACTTGATGAGATAGGGGAAGACGTTGCTCCAATCGAACACATTGCGAGTATGAGGAGGAACTATTTCACAGCGGAGGTATCCCATTGCAGGGCCACTGAATACATAATG<br>AAGGGAGTATACATAAACACAGCCCTATTGAATGCATCCTGTGCAGCCATGGATGACTTCCAACTGATTCCAATGATAAGCAAGTGCAGAACCAAAGAGGGAAGACGGAAGACAAATCTGTATGGATTCATTATAAAAGGAAGATCCCATTTGAGGAATGACACCGATGTGGTAAACTTTGTGAGCATGGAATTCTCTCTAACTGACCCGAGGCTAGAGCCACACAAATG<br>GGAAAAGTACTGTGTTCTTGAGATAGGAGACATGCTCCTACGGACTGCAATAGGCCAAGTGTCGAGGCCCATGTTCCTGTATGTGAGAACCAATGGGACTTCCAAGATCAAAATGAAATGGGGCATGGAGATGAGACGATGCCTTCTTCAGTCCCTTCAACAAATTGAGAGCATGATTGAGGCCGAATCTTCTGTCAAAGAGAAGGACATGACCAAGGAATTCTTTGAAA<br>ACAAATCAGAAACATGGCCAATTGGAGAATCACCCAAAGGGGTTGAGGAAGGCTCTATTGGGAAAGTATGCAGAACATTGCTGGCAAAGTCTGTGTTCAACAGCCTATATGCATCTCCACAACTCGAGGGATTTTCAGCTGAATCAAGAAAATTGCTTCTCATTGTTCAGGCACTTAGGGACAACCTGGAACCTGGAACCTTCGATCTTGGGGGGCTATATGAAGCAATTG<br>AGGAGTGCCTGATTAACGATCCCTGGGTTTTGCTTAATGCGTCTTGGTTCAACTCCTTCATCACACATGCACTGAAATA-
internal NVRI repository	>A/domestic_duck/Poland/H2077-J/2021_H5N1_2021-12-04<br>ATGGAAGACTTTGTGCGACAATGCTTCAATCCAATGATTGTCGAGCTTGCGGAAAAAGCAATGAAAGAATATGGGGAAGATCCGAAAATCGAGACAAACAAATTTGCCGCAATATGCACACACTTAGAAGTCTGTTTCATGTATTCGGATTTCCATTTTATTGATGAACGAGGCGAATCAATGATTGTAGAATCTGGCGATCCAAATGCATTATTGAAACACCGATTTGAGA<br>TAATCGAAGGGAGAGACCGAGCAATGGCCTGGACAGTGGTGAATAGTATCTGCAACACTACAGGAGTCGAAAAGCCCAAATTCCTCCCTGATTTGTATGACTACAAAGAGAACCGATTCATTGAAATTGGAGTAACGCGAAGGGAAGTTCACATATACTATTTAGAAAAAGCCAACAAGATAAAATCAGAGAAAACACACATTCACATATTCTCATTCACTGGAGAGGAA<br>ATGGCCACCAAGGCGGAATACACCCTTGATGAAGAGAGCAGAGCAAGAATAAAAACCAGACTGTTCACTATAAGACAAGAAATGGCCAGTAGAGGTCTATGGGATTCCTTTCGTCAGTCCGAGAGAGGCGAAGAGACAACTGAAGAAAGATTTGAAATCACAGGAACCATGCGCAGGCTTGCCGACCAAAGTCTCCCACCGAACTTCTCCAGCCTTGAAAACTTTAGAG<br>CCTATGTGGATGGATTCGAACCGAACGGCTGCATTGAGGGCAAGCTTTCTCAAATGTCAAAAGAAGTGAACGCCAGAATTGAGCCATTCCTGAAGACAACACCACGCCCTCTCAGATTACCTGATGGGCCTCCTTGTTCTCAGCGGTCGAAGTTCTTGCTGATGGATGCCCTTAAGTTGAGCATCGAAGATCCTAGCCATGAGGGGGAGGGCATACCGCTGTATGATGCAA<br>TCAAATGCATGAAGACATTTTTTGGCTGGAAAGAGCCCAACCTCGTAAAGCCGCATGAGAAAGGCATAAACCCTAATTACCTCCTGGCTTGGAAGCAGGTGCTGGCAGAACTTCAAGATATTGAGAATGAGGAGAAATTCCCAAAAACAAAGAACATGAAGAAAACAAGCCAATTGAAGTGGGCACTTGGTGAGAACATGGCACCAGAAAAAGTGGACTTTGAGGACTG<br>CAAAGATGTTAGCGATCTAAGACAGTACGACAGTGACGAACCAGAGCCTAGATCACTAGCAAGCTGGATTCAGAGTGAATTCAACAAGGCATGCGAATTGACAGATTCGAGTTGGATTGAACTTGATGAGATAGGGGAAGACGTTGCTCCAATCGAACACATTGCGAGTATGAGGAGGAACTATTTCACAGCGGAGGTATCCCATTGCAGGGCCACTGAATACATAATG<br>AAGGGAGTATACATAAACACAGCCCTATTGAATGCATCCTGTGCAGCCATGGATGACTTCCAACTGATTCCAATGATAAGCAAGTGCAGAACCAAAGAGGGAAGACGGAAGACAAATCTGTATGGATTCATTATAAAAGGAAGATCCCATTTGAGGAATGACACCGATGTGGTAAACTTTGTGAGCATGGAATTCTCTCTAACTGACCCGAGGCTAGAGCCACACAAATG<br>GGAAAAGTACTGTGTTCTTGAGATAGGAGACATGCTCCTACGGACTGCAATAGGCCAAGTGTCGAGGCCCATGTTCCTGTATGTGAGAACCAATGGGACTTCCAAGATCAAAATGAAATGGGGCATGGAGATGAGACGATGCCTTCTTCAGTCCCTTCAACAAATTGAGAGCATGATTGAGGCCGAATCTTCTGTCAAAGAGAAGGACATGACCAAGGAATTCTTTGAAA<br>ACAAATCAGAAACATGGCCAATTGGAGAATCACCCAAAGGGGTTGAGGAAGGCTCTATTGGGAAAGTATGCAGAACATTGCTGGCAAAGTCTGTGTTCAACAGCCTATATGCATCTCCACAACTCGAGGGATTTTCAGCTGAATCAAGAAAATTGCTTCTCATTGTTCAGGCACTTAGGGACAACCTGGAACCTGGAACCTTCGATCTTGGGGGGCTATATGAAGCAATTG<br>AGGAGTGCCTGATTAACGATCCCTGGGTTTTGCTTAATGCGTCTTGGTTCAACTCCTTCATCACACATGCACTGAAATA-
internal NVRI repository	>A/domestic_duck/Poland/H2078-N/2021_H5N1_2022-12-05<br>ATGGAAGACTTTGTGCGACAATGCTTCAATCCAATGATTGTCGAGCTTGCGGAAAAAGCAATGAAAGAATATGGGGAAGATCCGAAAATCGAGACAAACAAATTTGCCGCAATATGCACACACTTAGAAGTCTGTTTCATGTATTCGGATTTCCATTTTATTGATGAACGAGGCGAATCAATGATTGTAGAATCTGGCGATCCAAATGCATTATTGAAACACCGATTTGAGA<br>TAATCGAAGGGAGAGACCGAGCAATGGCCTGGACAGTGGTGAATAGTATCTGCAACACTACAGGAGTCGAAAAGCCCAAATTCCTCCCTGATTTGTATGACTACAAAGAGAACCGATTCATTGAAATTGGAGTAACGCGAAGGGAAGTTCACATATACTATTTAGAAAAAGCCAACAAGATAAAATCAGAGAAAACACACATTCACATATTCTCATTCACTGGAGAGGAA<br>ATGGCCACCAAGGCGGAATACACCCTTGATGAAGAGAGCAGAGCAAGAATAAAAACCAGACTGTTCACTATAAGACAAGAAATGGCCAGTAGAGGTCTATGGGATTCCTTTCGTCAGTCCGAGAGAGGCGAAGAGACAACTGAAGAAAGATTTGAAATCACAGGAACCATGCGCAGGCTTGCCGACCAAAGTCTCCCACCGAACTTCTCCAGCCTTGAAAACTTTAGAG<br>CCTATGTGGATGGATTCGAACCGAACGGCTGCATTGAGGGCAAGCTTTCTCAAATGTCAAAAGAAGTGAACGCCAGAATTGAGCCATTCCTGAAGACAACACCACGCCCTCTCAGATTACCTGATGGGCCTCCTTGTTCTCAGCGGTCGAAGTTCTTGCTGATGGATGCCCTTAAGTTGAGCATCGAAGATCCTAGCCATGAGGGGGAGGGCATACCGCTGTATGATGCAA<br>TCAAATGCATGAAGACATTTTTTGGCTGGAAAGAGCCCAACCTCGTAAAGCCGCATGAGAAAGGCATAAACCCTAATTACCTCCTGGCTTGGAAGCAGGTGCTGGCAGAACTTCAAGATATTGAGAATGAGGAGAAATTCCCAAAAACAAAGAACATGAAGAAAACAAGCCAATTGAAGTGGGCACTTGGTGAGAACATGGCACCAGAAAAAGTGGACTTTGAGGACTG<br>CAAAGATGTTAGCGATCTAAGACAGTACGACAGTGACGAACCAGAGCCTAGATCACTAGCAAGCTGGATTCAGAGTGAATTCAACAAGGCATGCGAATTGACAGATTCGAGTTGGATTGAACTTGATGAGATAGGGGAAGACGTTGCTCCAATCGAACACATTGCGAGTATGAGGAGGAACTATTTCACAGCGGAGGTATCCCATTGCAGGGCCACTGAATACATAATG<br>AAGGGAGTATACATAAACACAGCCCTATTGAATGCATCCTGTGCAGCCATGGATGACTTCCAACTGATTCCAATGATAAGCAAGTGCAGAACCAAAGAGGGAAGACGGAAGACAAATCTGTATGGATTCATTATAAAAGGAAGATCCCATTTGAGGAATGACACCGATGTGGTAAACTTTGTGAGCATGGAATTCTCTCTAACTGACCCGAGGCTAGAGCCACACAAATG<br>GGAAAAGTACTGTGTTCTTGAGATAGGAGACATGCTCCTACGGACTGCAATAGGCCAAGTGTCGAGGCCCATGTTCCTGTATGTGAGAACCAATGGGACTTCCAAGATCAAAATGAAATGGGGCATGGAGATGAGACGATGCCTTCTTCAGTCCCTTCAACAAATTGAGAGCATGATTGAGGCCGAATCTTCTGTCAAAGAGAAGGACATGACCAAGGAATTCTTTGAAA<br>ACAAATCAGAAACATGGCCAATTGGAGAATCACCCAAAGGGGTTGAGGAAGGCTCTATTGGGAAAGTATGCAGAACATTGCTGGCAAAGTCTGTGTTCAACAGCCTATATGCATCTCCACAACTCGAGGGATTTTCAGCTGAATCAAGAAAATTGCTTCTCATTGTTCAGGCACTTAGGGACAACCTGGAACCTGGAACCTTCGATCTTGGGGGGCTATATGAAGCAATTG<br>AGGAGTGCCTGATTAACGATCCCTGGGTTTTGCTTAATGCGTCTTGGTTCAACTCCTTCATCACACATGCACTGAAATA-
internal NVRI repository	>A/domestic_duck/Poland/H2085-T2/2021_H5N1_2021-12-06<br>ATGGAAGACTTTGTGCGACAATGCTTCAATCCAATGATTGTCGAGCTTGCGGAAAAAGCAATGAAAGAATATGGGGAAGATCCGAAAATCGAGACAAACAAATTTGCCGCAATATGCACACACTTAGAAGTCTGTTTCATGTATTCGGATTTCCATTTTATTGATGAACGAGGCGAATCAATGATTGTAGAATCTGGCGATCCAAATGCATTATTGAAACACCGATTTGAGA<br>TAATCGAAGGGAGAGACCGAGCAATGGCCTGGACAGTGGTGAATAGTATCTGCAACACTACAGGAGTCGAAAAGCCCAAATTCCTCCCTGATTTGTATGACTACAAAGAGAACCGATTCATTGAAATTGGAGTAACGCGAAGGGAAGTTCACATATACTATTTAGAAAAAGCCAACAAGATAAAATCAGAGAAAACACACATTCACATATTCTCATTCACTGGAGAGGAA<br>ATGGCCACCAAGGCGGAATACACCCTTGATGAAGAGAGCAGAGCAAGAATAAAAACCAGACTGTTCACTATAAGACAAGAAATGGCCAGTAGAGGTCTATGGGATTCCTTTCGTCAGTCCGAGAGAGGCGAAGAGACAACTGAAGAAAGATTTGAAATCACAGGAACCATGCGCAGGCTTGCCGACCAAAGTCTCCCACCGAACTTCTCCAGCCTTGAAAACTTTAGAG<br>CCTATGTGGATGGATTCGAACCGAACGGCTGCATTGAGGGCAAGCTTTCTCAAATGTCAAAAGAAGTGAACGCCAGAATTGAGCCATTCCTGAAGACAACACCACGCCCTCTCAGATTACCTGATGGGCCTCCTTGTTCTCAGCGGTCGAAGTTCTTGCTGATGGATGCCCTTAAGTTGAGCATCGAAGATCCTAGCCATGAGGGGGAGGGCATACCGCTGTATGATGCAA<br>TCAAATGCATGAAGACATTTTTTGGCTGGAAAGAGCCCAACCTCGTAAAGCCGCATGAGAAAGGCATAAACCCTAATTACCTCCTGGCTTGGAAGCAGGTGCTGGCAGARCTTCAAGATATTGAGAATGAGGAGAAATTCCCAAAAACAAAGAACATGAAGAAAACAAGCCAATTGAAGTGGGCACTTGGTGAGAACATGGCACCAGAAAAAGTGGACTTTGAGGACTG<br>CAAAGATGTTAGCGATCTAAGACAGTACGACAGTGACGAACCAGAGCCTAGATCACTAGCAAGCTGGATTCAGAGTGAATTCAACAAGGCATGCGAATTGACAGATTCGAGTTGGATTGAACTTGATGAGATAGGGGAAGACGTTGCTCCAATCGAACACATTGCGAGTATGAGGAGGAACTATTTCACAGCGGAGGTATCCCATTGCAGGGCCACTGAATACATAATG<br>AAGGGAGTATACATAAACACAGCCCTATTGAATGCATCCTGTGCAGCCATGGATGACTTCCAACTGATTCCAATGATAAGCAAGTGCAGAACCAAAGAGGGAAGACGGAAGACAAATCTGTATGGATTCATTATAAAAGGAAGATCCCATTTGAGGAATGACACCGATGTGGTAAACTTTGTGAGCATGGAATTCTCTCTAACTGACCCGAGGCTAGAGCCACACAAATG<br>GGAAAAGTACTGTGTTCTTGAGATAGGAGACATGCTCCTACGGACTGCAATAGGCCAAGTGTCGAGGCCCATGTTCCTGTATGTGAGAACCAATGGGACTTCCAAGATCAAAATGAAATGGGGCATGGAGATGAGACGATGCCTTCTTCAGTCCCTTCAACAAATTGAGAGCATGATTGAGGCCGAATCTTCTGTCAAAGAGAAGGACATGACCAAGGAATTCTTTGAAA<br>ACAAATCAGAAACATGGCCAATTGGAGAATCACCCAAAGGGGTTGAGGAAGGCTCTATTGGGAAAGTATGCAGAACATTGCTGGCAAAGTCTGTGTTCAACAGCCTATATGCATCTCCACAACTCGAGGGATTTTCAGCTGAATCAAGAAAATTGCTTCTCATTGTTCAGGCACTTAGGGACAACCTGGAACCTGGAACCTTCGATCTTGGGGGGCTATATGAAGCAATTG<br>AGGAGTGCCTGATTAACGATCCCTGGGTTTTGCTTAATGCGTCTTGGTTCAACTCCTTCATCACACATGCACTGAAATA-
internal NVRI repository	>A/domestic_duck/Poland/H2099-N/2021_H5N1_2021-12-11<br>ATGGAAGACTTTGTGCGACAATGCTTCAATCCAATGATTGTCGAGCTTGCGGAAAAAGCAATGAAAGAATATGGGGAAGATCCGAAAATCGAGACAAACAAATTTGCCGCAATATGCACACACTTAGAAGTCTGTTTCATGTATTCGGATTTCCATTTTATTGATGAACGAGGCGAATCAATGATTGTAGAATCTGGCGATCCAAATGCATTATTGAAACACCGATTTGAGA<br>TAATCGAAGGGAGAGACCGAGCAATGGCCTGGACAGTGGTGAATAGTATCTGCAACACTACAGGAGTCGAAAAGCCCAAATTCCTCCCTGATTTGTATGACTACAAAGAGAACCGATTCATTGAAATTGGAGTAACGCGAAGGGAAGTTCACATATACTATTTAGAAAAAGCCAACAAGATAAAATCAGAGAAAACACACATTCACATATTCTCATTCACTGGAGAGGAA<br>ATGGCCACCAAGGCAGAATACACCCTTGATGAAGAGAGCAGAGCAAGAATAAAAACCAGACTGTTCACTATAAGACAAGAAATGGCCAGTAGAGGTCTATGGGATTCCTTTCGTCAGTCCGAGAGAGGCGAAGAGACAACTGAAGAAAGATTTGAAATCACAGGAACCATGCGCAGGCTTGCCGACCAAAGTCTCCCACCGAACTTCTCCAGCCTTGAAAACTTTAGAG<br>CCTATGTGGATGGATTCGAACCGAACGGCTGCATTGAGGGCAAGCTTTCTCAAATGTCAAAAGAAGTGAACGCCAGAATTGAGCCATTCCTGAAGACAACACCACGCCCTCTCAGATTACCTGATGGGCCTCCTTGTTCTCAGCGGTCGAAGTTCTTGCTGATGGATGCCCTTAAGTTGAGCATCGAAGATCCTAGCCATGAGGGGGAGGGCATACCGCTGTATGATGCAA<br>TCAAATGCATGAAGACATTTTTTGGCTGGAAAGAGCCCAACCTCGTAAAGCCGCATGAGAAAGGCATAAACCCTAATTACCTCCTGGCTTGGAAGCAGGTGCTGGCAGAACTTCAAGATATTGAGAATGAGGAGAAATTCCCAAAAACAAAGAACATGAAGAAAACAAGCCAATTGAAGTGGGCACTTGGTGAGAACATGGCACCAGAAAAAGTGGACTTTGAGGACTG<br>CAAAGATGTTAGCGATCTAAGACAGTACGACAGTGACGAACCAGAGCCTAGATCACTAGCAAGCTGGATTCAGAGTGAATTCAACAAGGCATGCGAATTGACAGATTCGAGTTGGATTGAACTTGATGAGATAGGGGAAGACGTTGCTCCAATCGAACACATTGCGAGTATGAGGAGGAACTATTTCACAGCGGAGGTATCCCATTGCAGGGCCACTGAATACATAATG<br>AAGGGAGTATACATAAACACAGCCCTATTGAATGCATCCTGTGCAGCCATGGATGACTTCCAACTGATTCCAATGATAAGCAAGTGCAGAACCAAAGAGGGAAGACGGAAGACAAATCTGTATGGATTCATTATAAAAGGAAGATCCCATTTGAGGAATGACACCGATGTGGTAAACTTTGTGAGCATGGAATTCTCTCTAACTGACCCGAGGCTAGAGCCACACAAATG<br>GGAAAAGTACTGTGTTCTTGAGATAGGAGACATGCTCCTACGGACTGCAATAGGCCAAGTGTCGAGGCCCATGTTCCTGTATGTGAGAACCAATGGGACTTCCAAGATCAAAATGAAATGGGGCATGGAGATGAGACGATGCCTTCTTCAGTCCCTTCAACAAATTGAGAGCATGATTGAGGCCGAATCTTCTGTCAAAGAGAAGGACATGACCAAGGAATTCTTTGAAA<br>ACAAATCAGAAACATGGCCAATTGGAGAATCACCCAAAGGGGTTGAGGAAGGCTCTATTGGGAAAGTATGCAGAACATTGCTGGCAAAGTCTGTGTTCAACAGCCTATATGCATCTCCACAACTCGAGGGATTTTCAGCTGAATCAAGAAAATTGCTTCTCATTGTTCAGGCACTTAGGGACAACCTGGAACCTGGAACCTTCGATCTTGGGGGGCTATATGAAGCAATTG<br>AGGAGTGCCTGATTAACGATCCCTGGGTTTTGCTTAATGCGTCTTGGTTCAACTCCTTCATCACACATGCACTGAAATA-
internal NVRI repository	>A/domestic_duck/Poland/H2102-N/2021_H5N1_2021-12-13<br>ATGGAAGACTTTGTGCGACAATGCTTCAATCCAATGATTGTCGAGCTTGCGGAAAAAGCAATGAAAGAATATGGGGAAGATCCGAAAGTCGAGACAAACAAATTTGCCGCAATATGCACACACTTAGAAGTCTGTTTCATGTATTCGGATTTCCATTTTATTGATGAACGAGGCGAATCAATGATTGTAGAATCTGGCGATCCAAATGCATTATTGAAACACCGATTTGAGA<br>TAATCGAAGGGAGAGACCGAGCAATGGCCTGGACAGTGGTGAATAGTATCTGCAACACCACAGGAGTCGAAAAGCCCAAATTCCTCCCTGATTTGTATGACTACAAAGAGAACCGATTCATTGAAATTGGAGTAACGCGAAGGGAAGTTCACATATACTATTTAGAAAAAGCCAACAAGATAAAATCGGAGAAAACACACATTCACATATTCTCATTCACTGGAGAGGAA<br>ATGGCCACCAAGGCGGAATACACCCTTGATGAAGAGAGCAGAGCAAGAATAAAAACCAGACTGTTCACTATAAGACAAGAAATGGCCAGTAGAGGTCTATGGGATTCCTTTCGTCAGTCCGAGAGAGGCGAAGAGACAACTGAAGAAAGATTTGAAATCACAGGAACCATGCGCAGGCTTGCCGACCAAAGTCTCCCACCGAACTTCTCCAGCCTTGAAAACTTTAGAG<br>CCTATGTGGATGGATTCGAACCGAACGGCTGCATTGAGGGCAAGCTTTCTCAAATGTCAAAAGAAGTGAACGCCAGAATTGAGCCATTCCTGAAGACAACACCACGCCCTCTCAGATTACCTGATGGGCCTCCTTGTTCTCAGCGGTCGAAGTTCCTGCTGATGGATGCCCTTAAGTTGAGCATCGAAGACCCTAGCCATGAGGGGGAGGGCATACCGCTGTATGATGCAA<br>TCAAATGCATGAAGACATTTTTTGGCTGGAAAGAGCCCAACCTCGTAAAGCCGCATGAGAAAGGCATAAACCCTAATTACCTCCTGGCTTGGAAGCAGGTGCTGGCAGAACTTCAAGATATTGAGAATGAGGAGAAATTTCCAAAAACAAAGAACATGAAGAAAACAAGCCAATTGAAGTGGGCACTTGGTGAGAACATGGCACCAGAAAAAGTGGACTTTGAGGACTG<br>CAAAGATGTTAGCGATCTAAGACAGTACGACAGTGACGAACCAGAGCCTAGATCACTAGCAAGCTGGATTCAGAGTGAATTCAACAAGGCATGCGAATTGACAGATTCGAGTTGGATTGAACTTGATGAGATAGGGGAAGACGTTGCTCCAATCGAACACATTGCGAGTATGAGGAGGAACTATTTCACAGCGGAGGTATCCCATTGTAGGGCCACTGAATACATAATG<br>AAGGGAGTATACATAAACACAGCCCTATTGAATGCATCCTGTGCAGCCATGGATGACTTCCAACTGATTCCAATGATAAGCAAGTGCAGAACCAAAGAAGGAAGACGGAAGACAAATCTGTATGGATTCATTATAAAAGGAAGATCCCATTTGAGGAATGACACCGATGTGGTAAACTTTGTGAGCATGGAATTCTCTCTAACTGACCCGAGGCTAGAGCCACACAAATG<br>GGAAAAGTACTGTGTTCTTGAGATAGGAGACATGCTCCTACGGACTGCAATAGGCCAAGTGTCGAGGCCCATGTTCCTGTATGTGAGAACCAATGGGACTTCCAAGATCAAAATGAAATGGGGCATGGAGATGAGACGATGCCTTCTTCAGTCCCTTCAACAAATTGAGAGCATGATTGAGGCCGAATCTTCTGTCAAAGAGAAGGACATGACCAAGGAATTCTTTGAAA<br>ACAAAACAGAAACATGGCCAATTGGAGAATCACCCAAAGGGGTTGAGGAAGGCTCTATTGGGAAAGTATGCAGGACATTGCTAGCAAAGTCTGTGTTCAACAGCCTATATGCATCTCCACAACTCGAGGGATTTTCAGCTGAATCAAGAAAATTGCTTCTCATTGTTCAGGCACTTAGGGACAACCTGGAACCTGGAACCTTCGATCTTGGGGGGCTATATGAAGCAATTG<br>AGGAGTGCCTGATTAACGATCCCTGGGTTTTGCTTAATGCGTCTTGGTTCAACTCCTTCCTCACACATGCACTGAAATA-

internal NVRI repository	>A/domestic_duck/Poland/H2117-T1/2021_H5N1_2021-12-16<br>[illegible]
internal NVRI repository	>A/domestic_duck/Poland/H2126-N/2021_H5N1_2021-12-20<br>[illegible]
internal NVRI repository	>A/domestic_duck/Poland/H2153_T3/2021_H5N1_2021-12-25<br>[illegible]
internal NVRI repository	>A/domestic_duck/Poland/H2154_N/2021_H5N1_2021-12-24<br>[illegible]
internal NVRI repository	>A/domestic_goose/Poland/33/2022_H5N1_2022-01-09<br>[illegible]
internal NVRI repository	>A/domestic_goose/Poland/53/2022_H5N1_2022-01-12<br>[illegible]
EPI_ISL_6931008	>A/domestic_goose/Poland/H1931-T1/2021_H5N1_2021-11-03<br>[illegible]
internal NVRI repository	>A/domestic_goose/Poland/H28-T/2022_H5N1_2022-01-05<br>[illegible]
EPI_ISL_14917951	>A/domestic_goose/Poland/H371/2022_H5N1_2022-07-21<br>[illegible]
EPI_ISL_11922810	>A/duck/Poland/H126_22VIR2515-4/2022_H5N1_2022-02-08<br>[illegible]
EPI_ISL_11922808	>A/duck/Poland/H188_22VIR2515-2/2022_H5N1_2022-03-02<br>[illegible]
internal NVRI repository	>A/duck/Poland/H1968_21RS3290-5/2021_H5N1_2021-11-12<br>[illegible]
internal NVRI repository	>A/duck/Poland/H1969_21RS3290-6/2021_H5N1_2021-11-12<br>[illegible]

internal NVRI repository	>A/duck/Poland/H1970_21RS3290-7/2021_H5N1_2021-11-13<br>ATGGAAGACTTTGTGCGACAATGCTTCAATCCAATGATTGTCGAGCTTGCGGAAAAAGCAATGAAAGAATATGGGGAAGATCCGAAAATCGAGACAAACAAATTTGCCGCAATATGCACACACTTAGAAGTCTGTTTCATGTATTCGGATTTCCATTTTATTGATGAACGAGGCGAATCAATGATTGTAGAATCTGGCGATCCAAATGCATTATTGAAACACCGATTTGAGA<br>TAATCGAAGGGAGAGACCGAGCAATGGCCTGGACAGTGGTGAATAGTATCTGCAACACTACAGGAGTCGAAAAGCCCAAATTCCTCCCTGATTTGTATGACTACAAAGAGAACCGATTCATTGAAATTGGAGTAACGCGAAGGGAAGTTCACATATACTATTTAGAAAAAGCCAACAAGATAAAATCAGAGAAAACACACATTCACATATTCTCATTCACTGGAGAGGAA<br>ATGGCCACCAAGGCGGAATACACCCTTGATGAAGAGAGCAGAGCAAGAATAAAAACCAGACTGTTCACTATAAGACAAGAAATGGCCAGTAGAGGTCTATGGGATTCCTTTCGTCAGTCCGAGAGAGGCGAAGAGACAACTGAAGAAAGATTTGAAATCACAGGAACCATGCGCAGGCTTGCCGACCAAAGTCTCCCACCGAACTTCTCCAGCCTTGAAAACTTTAGAG<br>CCTATGTGGATGGATTCGAACCGAACGGCTGCATTGAGGGCAAGCTTTCTCAAATGTCAAAAGAAGTGAACGCCAGAATTGAGCCATTCCTGAAGACAACACCACGCCCTCTCAGATTACCTGATGGGCCTCCTTGTTCTCAGCGGTCGAAGTTCTTGCTGATGGATGCCCTTAAGTTGAGCATCGAAGATCCTAGCCATGAGGGGGAGGGCATACCGCTGTATGATGCAA<br>TCAAATGCATGAAGACATTTTTTGGCTGGAAAGAGCCCAACCTCGTAAAGCCGCATGAGAAAGGCATAAACCCTAATTACCTCCTGGCTTGGAAGCAGGTGCTGGCAGAACTTCAAGATATTGAGAATGAGGAGAAATTCCCAAAAACAAAGAACATGAAGAAAACAAGCCAATTGAAGTGGGCACTTGGTGAGAACATGGCACCAGAAAAAGTGGACTTTGAGGACTG<br>CAAAGATGTTAGCGATCTAAGACAGTACGACAGTGACGAACCAGAGCCTAGATCACTAGCAAGCTGGATTCAGAGTGAATTCAACAAGGCATGCGAATTGACAGATTCGAGTTGGATTGAACTTGATGAGATAGGGGAAGACGTTGCTCCAATCGAACACATTGCGAGTATGAGGAGGAACTATTTCACAGCGGAGGTATCCCATTGCAGGGCCACTGAATACATAATG<br>AAGGGAGTATACATAAACACAGCCCTATTGAATGCATCCTGTGCAGCCATGGATGACTTCCAACTGATTCCAATGATAAGCAAGTGCAGAACCAAAGAGGGAAGACGGAAGACAAATCTGTATGGATTCATTATAAAAGGAAGATCCCATTTGAGGAATGACACCGATGTGGTAAACTTTGTGAGCATGGAATTCTCTCTAACTGACCCGAGGCTAGAGCCACACAAATG<br>GGAAAAGTACTGTGTTCTTGAGATAGGAGACATGCTCCTACGGACTGCAATAGGCCAAGTGTCGAGGCCCATGTTCCTGTATGTGAGAACCAATGGGACTTCCAAGATCAAAATGAAATGGGGCATGGAGATGAGACGATGCCTTCTTCAGTCCCTTCAACAAATTGAGAGCATGATTGAGGCCGAATCTTCTGTCAAAGAGAAGGACATGACCAAGGAATTCTTTGAAA<br>ACAAATCAGAAACATGGCCAATTGGAGAATCACCCAAAGGGGTTGAGGAAGGCTCTATTGGGAAAGTATGCAGAACATTGCTAGCAAAGTCTGTGTTCAACAGCCTATATGCATCTCCACAACTCGAGGGATTTTCAGCTGAATCAAGAAAATTGCTTCTCATTGTTCAGGCACTTAGGGACAACCTGGAACCTGGAACCTTCGATCTTGGGGGGCTATATGAAGCAATTG<br>AGGAGTGCCTGATTAACGATCCCTGGGTTTTGCTTAATGCGTCTTGGTTCAACTCCTTCATCACACATGCACTGAAATA-
internal NVRI repository	>A/duck/Poland/H1971_21RS3290-8/2021_H5N1_2021-11-13<br>ATGGAAGACTTTGTGCGACAATGCTTCAATCCAATGATTGTCGAGCTTGCGGAAAAAGCAATGAAAGAAYATGGGGAAGATCCGAAAATCGAGACAAACAAATTTGCCGCAATATGCACACACTTAGAAGTCTGTTTCATGTATTCGGATTTCCATTTTATTGATGAACGAGGCGAATCAATGATTGTAGAATCTGGCGATCCAAATGCATTATTGAAACACCGATTTGAGA<br>TAATCGAAGGGAGAGACCGAGCAATGGCCTGGACAGTGGTGAATAGTATCTGCAACACTACAGGAGTCGAAAAGCCCAAATTCCTCCCTGATTTGTATGACTACAAAGAGAACCGATTCATTGAAATTGGAGTAACGCGAAGGGAAGTTCACATATACTATTTAGAAAAAGCCAACAAGATAAAATCAGAGAAAACACACATTCACATATTCTCATTCACTGGAGAGGAA<br>ATGGCCACCAAGGCGGAATACACCCTTGATGAAGAGAGCAGAGCAAGAATAAAAACCAGACTGTTCACTATAAGACAAGAAATGGCCAGTAGAGGTCTATGGGATTCCTTTCGTCAGTCCGAGAGAGGCGAAGAGACAACTGAAGAAAGATTTGAAATCACAGGAACCATGCGCAGGCTTGCCGACCAAAGTCTCCCACCGAACTTCTCCAGCCTTGAAAACTTTAGAG<br>CCTATGTGGATGGATTCGAACCGAACGGCTGCATTGAGGGCAAGCTTTCTCAAATGTCAAAAGAAGTGAACGCCAGAATTGAGCCATTCCTGAAGACAACACCACGCCCTCTCAGATTACCTGATGGGCCTCCTTGTTCTCAGCGGTCGAAGTTCTTGCTGATGGATGCCCTTAAGTTGAGCATCGAAGATCCTAGCCATGAGGGGGAGGGCATACCGCTGTATGATGCAA<br>TCAAATGCATGAAGACATTTTTTGGCTGGAAAGAGCCCAACCTCGTAAAGCCGCATGAGAAAGGCATAAACCCTAATTACCTCCTGGCTTGGAAGCAGGTGCTGGCAGAACTTCAAGATATTGAGAATGAGGAGAAATTCCCAAAAACAAAGAACATGAAGAAAACAAGCCAATTGAAGTGGGCACTTGGTGAGAACATGGCACCAGAAAAAGTGGACTTTGAGGACTG<br>CAAAGATGTTAGCGATCTAAGACAGTACGACAGTGACGAACCAGAGCCTAGATCACTAGCAAGCTGGATTCAGAGTGAATTCAACAAGGCATGCGAATTGACAGATTCGAGTTGGATTGAACTTGATGAGATAGGGGAAGACGTTGCTCCAATCGAACACATTGCGAGTATGAGGAGGAACTATTTCACAGCGGAGGTATCCCATTGCAGGGCCACTGAATACATAATG<br>AAGGGAGTATACATAAACACAGCCCTATTGAATGCATCCTGTGCAGCCATGGATGACTTCCAACTGATTCCAATGATAAGCAAGTGCAGAACCAAAGAGGGAAGACGGAAGACAAATCTGTATGGATTCATTATAAAAGGAAGATCCCATTTGAGGAATGACACCGATGTGGTAAACTTTGTGAGCATGGAATTCTCTCTAACTGACCCGAGGCTAGAGCCACACAAATG<br>GGAAAAGTACTGTGTTCTTGAGATAGGAGACATGCTCCTACGGACTGCAATAGGCCAAGTGTCGAGGCCCATGTTCCTGTATGTGAGAACCAATGGGACTTCCAAGATCAAAATGAAATGGGGCATGGAGATGAGACGATGCCTTCTTCAGTCCCTTCAACAAATTGAGAGCATGATTGAGGCCGAATCTTCTGTCAAAGAGAAGGACATGACCAAGGAATTCTTTGAAA<br>ACAAATCAGAAACATGGCCAATTGGAGAATCACCCAAAGGGGTTGAGGAAGGCTCTATTGGGAAAGTATGCAGAACATTGCTAGCAAAGTCTGTGTTCAACAGCCTATATGCATCTCCACAACTCGAGGGATTTTCAGCTGAATCAAGAAAATTGCTTCTCATTGTTCAGGCACTTAGGGACAACCTGGAACCTGGAACCTTCGATCTTGGGGGGCTATATGAAGCAATTG<br>AGGAGTGCCTGATTAACGATCCCTGGGTTTTGCTTAATGCGTCTTGGTTCAACTCCTTCATCACACATGCACTGAAATA-
internal NVRI repository	>A/duck/Poland/H1980_21RS3290-11/2021_H5N1_2021-11-16<br>ATGGAAGACTTTGTGCGACAATGCTTCAATCCAATGATTGTCGAGCTTGCGGAAAAAGCAATGAAAGAATATGGGGAAGATCCGAAAATCGAGACAAACAAATTTGCCGCAATATGCACACACTTAGAAGTCTGTTTCATGTATTCGGATTTCCATTTTATTGATGAACGAGGCGAATCAATGATTGTAGAATCTGGCGATCCAAATGCATTATTGAAACACCGATTTGAGA<br>TAATCGAAGGGAGAGACCGAGCAATGGCCTGGACAGTGGTGAATAGTATCTGCAACACTACAGGAGTCGAAAAGCCCAAATTCCTCCCTGATTTGTATGACTACAAAGAGAACCGATTCATTGAAATTGGAGTAACGCGAAGGGAAGTTCACATATACTATTTAGAAAAAGCCAACAAGATAAAATCAGAGAAAACACACATTCACATATTCTCATTCACTGGAGAGGAA<br>ATGGCCACCAAGGCGGAATACACCCTTGATGAAGAGAGCAGAGCAAGAATAAAAACCAGACTGTTCACTATAAGACAAGAAATGGCCAGTAGAGGTCTATGGGATTCCTTTCGTCAGTCCGAGAGAGGCGAAGAGACAACTGAAGAAAGATTTGAAATCACAGGAACCATGCGCAGGCTTGCCGACCAAAGTCTCCCACCGAACTTCTCCAGCCTTGAAAACTTTAGAG<br>CCTATGTGGATGGATTCGAACCGAACGGCTGCATTGAGGGCAAGCTTTCTCAAATGTCAAAAGAAGTGAACGCCAGAATTGAGCCATTCCTGAAGACAACACCACGCCCTCTCAGATTACCTGATGGGCCTCCTTGTTCTCAGCGGTCGAAGTTCTTGCTGATGGATGCCCTTAAGTTGAGCATCGAAGATCCTAGCCATGAGGGGGAGGGCATACCGCTGTATGATGCAA<br>TCAAATGCATGAAGACATTTTTTGGCTGGAAAGAGCCCAACCTCGTAAAGCCGCATGAGAAAGGCATAAACCCTAATTACCTCCTGGCTTGGAAGCAGGTGCTGGCAGAACTTCAAGATATTGAGAATGAGGAGAAATTCCCAAAAACAAAGAACATGAAGAAAACAAGCCAATTGAAGTGGGCACTTGGTGAGAACATGGCACCAGAAAAAGTGGACTTTGAGGACTG<br>CAAAGATGTTAGCGATCTAAGACAGTACGACAGTGACGAACCAGAGCCTAGATCACTAGCAAGCTGGATTCAGAGTGAATTCAACAAGGCATGCGAATTGACAGATTCGAGTTGGATTGAACTTGATGAGATAGGGGAAGACGTTGCTCCAATCGAACACATTGCGAGTATGAGGAGGAACTATTTCACAGCGGAGGTATCCCATTGCAGGGCCACTGAATACATAATG<br>AAGGGAGTATACATAAACACAGCCCTATTGAATGCATCCTGTGCAGCCATGGATGACTTCCAACTGATTCCAATGATAAGCAAGTGCAGAACCAAAGAGGGAAGACGGAAGACAAATCTGTATGGATTCATTATAAAAGGAAGATCCCATTTGAGGAATGACACCGATGTGGTAAACTTTGTGAGCATGGAATTCTCTCTAACTGACCCGAGGCTAGAGCCACACAAATG<br>GGAAAAGTACTGTGTTCTTGAGATAGGAGACATGCTCCTACGGACTGCAATAGGCCAAGTGTCGAGGCCCATGTTCCTGTATGTGAGAACCAATGGGACTTCCAAGATCAAAATGAAATGGGGCATGGAGATGAGACGATGCCTTCTTCAGTCCCTTCAACAAATTGAGAGCATGATTGAGGCCGAATCTTCTGTCAAAGAGAAGGACATGACCAAGGAATTCTTTGAAA<br>ACAAATCAGAAACATGGCCAATTGGAGAATCACCCAAAGGGGTTGAGGAAGGCTCTATTGGGAAAGTATGCAGAACATTGCTAGCAAAGTCTGTGTTCAACAGCCTATATGCATCTCCACAACTCGAGGGATTTTCAGCTGAATCAAGAAAATTGCTTCTCATTGTTCAGGCACTTAGGGACAACCTGGAACCTGGAACCTTCGATCTTGGGGGGCTATATGAAGCAATTG<br>AGGAGTGCCTGATTAACGATCCCTGGGTTTTGCTTAATGCGTCTTGGTTCAACTCCTTCATCACACATGCACTGAAATA-
internal NVRI repository	>A/duck/Poland/H1982_21RS3290-12/2021_H5N1_2021-11-16<br>ATGGAAGACTTTGTGCGACAATGCTTCAATCCAATGATTGTCGAGCTTGCGGAAAAAGCAATGAAAGAATATGGGGAAGATCCGAAAATCGAGACAAACAAATTTGCCGCAATATGCACACACTTAGAAGTCTGTTTCATGTATTCGGATTTCCATTTTATTGATGAACGAGGCGAATCAATGATTGTAGAATCTGGCGATCCAAATGCATTATTGAAACACCGATTTGAGA<br>TAATCGAAGGGAGAGACCGAGCAATGGCCTGGACAGTGGTGAATAGTATCTGCAACACTACAGGAGTCGAAAAGCCCAAATTCCTCCCTGATTTGTATGACTACAAAGAGAACCGATTCATTGAAATTGGAGTAACGCGAAGGGAAGTTCACATATACTATTTAGAAAAAGCCAACAAGATAAAATCAGAGAAAACACACATTCACATATTCTCATTCACTGGAGAGGAA<br>ATGGCCACCAAGGCGGAATACACCCTTGATGAAGAGAGCAGAGCAAGAATAAAAACCAGACTGTTCACTATAAGACAAGAAATGGCCAGTAGAGGTCTATGGGATTCCTTTCGTCAGTCCGAGAGAGGCGAAGAGACAACTGAAGAAAGATTTGAAATCACAGGAACCATGCGCAGGCTTGCCGACCAAAGTCTCCCACCGAACTTCTCCAGCCTTGAAAACTTTAGAG<br>CCTATGTGGATGGATTCGAACCGAACGGCTGCATTGAGGGCAAGCTTTCTCAAATGTCAAAAGAAGTGAACGCCAGAATTGAGCCATTCCTGAAGACAACACCACGCCCTCTCAGATTACCTGATGGGCCTCCTTGTTCTCAGCGGTCGAAGTTCTTGCTGATGGATGCCCTTAAGTTGAGCATCGAAGATCCTAGCCATGAGGGGGAGGGCATACCGCTGTATGATGCAA<br>TCAAATGCATGAAGACATTTTTTGGCTGGAAAGAGCCCAACCTCGTAAAGCCGCATGAGAAAGGCATAAACCCTAATTACCTCCTGGCTTGGAAGCAGGTGCTGGCAGAACTTCAAGATATTGAGAATGAGGAGAAATTCCCAAAAACAAAGAACATGAAGAAAACAAGCCAATTGAAGTGGGCACTTGGTGAGAACATGGCACCAGAAAAAGTGGACTTTGAGGACTG<br>CAAAGATGTTAGCGATCTAAGACAGTACGACAGTGACGAACCAGAGCCTAGATCACTAGCAAGCTGGATTCAGAGTGAATTCAACAAGGCATGCGAATTGACAGAGTCGAGTTGGATTGAACTTGATGAGATAGGGGAAGACGTTGCTCCAATCGAACACATTGCGAGTATGAGGCGGAACTATTTCACAGCGGAGGTATCCCATTGCAGGGCCACTGAATACATAATG<br>AAGGGAGTATACATAAACACAGCCCTATTGAATGCATCCTGTGCAGCCATGGATGACTTCCAACTGATTCCAATGATAAGCAAGTGCAGAACCAAAGAGGGAAGACGGAAGACAAATCTGTATGGATTCATTATAAAAGGAAGATCCCATTTGAGGAATGACACCGATGTGGTAAACTTTGTGAGCATGGAATTCTCTCTAACTGACCCGAGGCTAGAGCCACACAAATG<br>GGAAAAGTACTGTGTTCTTGAGATAGGAGACATGCTCCTACGGACTGCAATAGGCCAAGTGTCGAGGCCCATGTTCCTGTATGTGAGAACCAATGGGACTTCCAAGATCAAAATGAAATGGGGCATGGAGATGAGACGATGCCTTCTTCAGTCCCTTCAACAAATTGAGAGCATGATTGAGGCCGAATCTTCTGTCAAAGAGAAGGACATGACCAAGGAATTCTTTGAAA<br>ACAAATCAGAAACATGGCCAATTGGAGAATCACCCAAAGGGGTTGAGGAAGGCTCTATTGGGAAAGTATGCAGAACATTGCTAGCAAAGTCTGTGTTCAACAGCCTATATGCATCTCCACAACTCGAGGGATTTTCAGCTGAATCAAGAAAATTGCTTCTCATTGTTCAGGCACTTAGGGACAACCTGGAACCTGGAACCTTCGATCTTGGGGGGCTATATGAAGCAATTG<br>AGGAGTGCCTGATTAACGATCCCTGGGTTTTGCTTAATGCGTCTTGGTTCAACTCCTTCATCACACATGCACTGAAATA-
internal NVRI repository	>A/duck/Poland/H1993_21RS3290-14/2021_H5N1_2021-11-17<br>ATGGAAGACTTTGTGCGACAATGCTTCAATCCAATGATTGTCGAGCTTGCGGAAAAAGCAATGAAAGAATATGGGGAAGATCCGAAAATCGAGACAAACAAATTTGCCGCAATATGCACACACTTAGAAGTCTGTTTCATGTATTCGGATTTCCATTTTATTGATGAACGAGGCGAATCAATGATTGTAGAATCTGGCGATCCAAATGCATTATTGAAACACCGATTTGAGA<br>TAATCGAAGGGAGAGACCGAGCAATGGCCTGGACAGTGGTGAATAGTATCTGCAACACTACAGGAGTCGATAAGCCCAAATTCCTCCCTGATTTGTATGACTACAAAGAGAACCGATTCATTGAAATTGGAGTAACGCGAAGGGAAGTTCACATATACTATTTAGAAAAAGCCAACAAGATAAAATCAGAGAAAACACACATTCACATATTCTCATTCACTGGAGAGGAA<br>ATGGCCACCAAGGCGGAATACACCCTTGATGAAGAGAGCAGAGCAAGAATAAAAACCAGACTGTTCACTATAAGACAAGAAATGGCCAGTAGAGGTCTATGGGATTCCTTTCGTCAGTCCGAGAGAGGCGAAGAGACAACTGAAGAAAGATTTGAAATCACAGGAACCATGCGCAGGCTTGCCGACCAAAGTCTCCCACCGAACTTCTCCAGCCTTGAAAACTTTAGAG<br>CCTATGTGGATGGATTCGAACCGAACGGCTGCATTGAGGGCAAGCTTTCTCAAATGTCAAAAGAAGTGAACGCCAGAATTGAGCCATTCCTGAAGACAACACCACGCCCCCTCAGATTACCTGATGGGCCTCCTTGTTCTCAGCGGTCGAAGTTCTTGCTGATGGATGCCCTTAAGTTGAGCATCGAAGATCCTAGCCATGAGGGGGAGGGCATACCGCTGTATGATGCAA<br>TCAAATGCATGAAGACATTTTTTGGCTGGAAAGAGCCCAACCTCGTAAAGCCGCATGAGAAAGGCATAAACCCTAATTACCTCCTGGCTTGGAAGCAGGTGCTGGCAGAACTTCAAGATATTGAGAATGAGGAGAAATTCCCAAAAACAAAGAACATGAAGAAAACAAGCCAATTGAAGTGGGCACTTGGTGAGAACATGGCACCAGAAAAAGTGGACTTTGAGGACTG<br>CAAAGATGTTAGCGATCTAAGACAGTACGACAGTGGCGAACCAGAGCCTAGATCACTAGCAAGCTGGATTCAGAGTGAATTCAACAAGGCATGCGAATTGACAGATTCGAGTTGGATTGAACTTGATGAGATAGGGGAAGACGTTGCTCCAATCGAACACATTGCGAGTATGAGGAGGAACTATTTCACAGCGGAGGTATCCCATTGCAGGGCCACTGAATACATAATG<br>AAGGGAGTATACATAAACACAGCCCTATTGAATGCATCCTGTGCAGCCATGGATGACTTCCAACTGATTCCAATGATAAGCAAGTGCAGAACCAAAGAGGGAAGACGGAAGACAAATCTGTATGGATTCATTATAAAAGGAAGATCCCATTTGAGGAATGACACCGATGTGGTAAACTTTGTGAGCATGGAATTCTCTCTAACTGACCCGAGGCTAGAGCCACACAAATG<br>GGAAAAGTACTGTGTTCTTGAGATAGGAGACATGCTCCTACGGACTGCAATAGGCCAAGTGTCGAGGCCCATGTTCCTGTATGTGAGAACCAATGGGACTTCCAAGATCAAAATGAAATGGGGCATGGAGATGAGACGATGCCTTCTTCAGTCCCTTCAACAAATTGAGAGCATGATTGAGGCCGAATCTTCTGTCAAAGAGAAGGACATGACCAAGGAATTCTTTGAAA<br>ACAAATCAGAAACATGGCCAATTGGAGAATCACCCAAAGGGGTTGAGGAAGGCTCTATTGGGAAAGTATGCAGAACATTGCTAGCAAAGTCTGTGTTCAACAGCCTATATGCATCTCCACAACTCGAGGGATTTTCAGCTGAATCAAGAAAATTGCTTCTCATTGTTCAGGCACTTAGGGACAACCTGGAACCTGGAACCTTCGATCTTGGGGGGCTATATGAAGCAATTG<br>AGGAGTGCCTGATTAACGATCCCTGGGTTTTGCTTAATGCGTCTTGGTTCAACTCCTTCATCACACATGCACTGAAATA-
internal NVRI repository	>A/duck/Poland/H2007_21RS3290-15/2021_H5N1_2021-11-17<br>ATGGAAGACTTTGTGCGACAATGCTTCAATCCAATGATTGTCGAGCTTGCGGAAAAGGCAATGAAAGAGTATGGGGAAGATCCGAAAATCGAAACAAACAAATTTGCCGCAATATGCACACACTTAGAAGTCTGTTTCATGTATTCGGATTTCCACTTTATTGATGAACGCGGCGAATCAATAATTGTAGAATCTGGCGATCCGAATGCATTATTGAAACACCGATTCGAGA<br>TAATTGAAGGGAGAGACCGAACTATGGCCTGGACAGTGGTGAATAGTATCTGCAACACCACAGGAGTCGAAAAGCCCAAATTCCTCCCTGATTTGTATGACTACAAAGAGAACCGATTCATTGAAATTGGAGTAACGCGAAGGGAAGTTCACATATACTATCTAGAGAAAGCCAACAAGATAAATCAGAGAAGACACACATTCATATATTCTCATTCACTGGAGAGGAA<br>ATGGCCACCAAGGCGGACTACACCCTTGATGAAGAGAGTAGAGCAAGAATAAAAACCAGGCTATTCACTATAAGACAAGAAATGGCCAGTAGGGGTCTATGGGATTCCTTTCGTCAGTCCGAGAGAGGCGAAGAGACAATTGAAGAAAGATTTGAAATCACAGGAACCATGCGCAGGCTTGCCGACCAAAGTCTCCCACCGAACTTCTCCAGCCTTGAAAACTTTAGAG<br>CCTATGTGGATGGATTCGAACCGAACGGCTGCATTGAGGGCAAGCTTTCTCAAATGTCAAAAGAAGTGAATGCCAGAATTGAGCCATTTCTGAAGACAACACCACGCCCTCTCAGATTACCTGATGGGCCTCCCTGTTCTCAGCGGTCGAAGTTCTTGCTGATGGATGCCCTCAAATTGAGCATCGAAGACCCGAGCCATGAGGGGGAGGGTATACCGCTGTATGATGCAA<br>TCAAATGCATGAAGACATTTTTGGCTGGAAAGAGCCTAACATCGTAAAACCGCATGAAAAGGGCATAAACCCTAATTACCTTCTGGCTTGGAAACAGGTGCTGGCAGAACTCCAAGATATTGAAAATGAGGAGAAAATCCCAAAAACAAAGAACATGAAGAAAACAAGCCAATTGAAGTGGGCACTTGGTGAGAACATGGCACCAGAGAAAGTGGACTTTGAGGACTG<br>TAAAGATGTTAGTGATCTAAGACAGTACGACAGTGACGAACCAGAGTCTAGATCACTAGCAAGCTGGATCCAGAGTGAATTCAACAAGGCATGCGAATTGACAGATTCGAGTTGGATTGAACTTGATGAAATAGGGGAAGACATTGCTCCAATTGAACACATTGCGAGTATGAGGAGAAACTATTTCACAGCGGAGGTGTCCCATTGCAGGGCTACTGAATACATAATGA<br>AGGGAGTATACATAAACACAGCCCTGTTGAATGCATCCTGTGCAGCGATGGATGACTTCCAACTGATTCCTATGATAAGCAAGTGCAGAACCAAAGAAGGAAGACGGAGACAAATTTATATGGATTCATTATAAAAGGAAGATCCCATTTGAGGAATGACACCGATGTGGTGAACTTTGTGAGCATGGAATTCTCTCTCACTGACCCGAGGCTGGAGCCACACAAATGG<br>GAAAAGTACTGTGTTCTTGAGATAGGAGACATGCTCCTACGGACTGCAATAGGCCATGTGTCAAGGCCCATGTTCTTGTATGTGAGAACCAATGGAACTTCCAAGATCAAAATGAAATGGGGCATGGAGATGAGACGATGCCTTCTTCAGTCCCTTCAACAAATTGAGAGCATGATTGAGGCCGAGTCTTCTGTCAAAGAGAAGGACATGACCAAGGAATTCTTTGAAAAC<br>AAATCAGAAACATGGCCAATTGGGGAATCACCCAAAGGGGTGGAAGAAGGCTCCATTGGGAAGGTGTGCAGAACATTGCTAGCAAAGTCTGTGTTCAACAGCCTATATGCATCTCCACAACTCGAGGGGTTTTCAGCTGAATCAAGAAAATTGCTTCTCATTGTTCAGGCACTTAGGGACAACCTGGAACCTGGAACCTTCGATCTTGGGGGGCTATATGAAGCAATTGA<br>GGAGTGCCTGATTAACGATCCCTGGGTTTTGCTTAATGCGTCTTGGTTCAACTCCTTCCTCACACATGCACTGAAATA-
internal NVRI repository	>A/duck/Poland/H2008_21RS3290-16/2021_H5N1_2021-11-18<br>ATGGAAGACTTTGTGCGACAATGCTTCAATCCAATGATTGTCGAGCTTGCGGAAAAAGCAATGAAAGAATATGGGGAAGATCCGAAAATCGAGACAAACAAATTTGCCGCAATATGCACACACTTAGAAGTCTGTTTCATGTATTCGGATTTCCATTTTATTGATGAACGAGGCGAATCAATGATTGTAGAATCTGGCGATCCAAATGCATTATTGAAACACCGATTTGAGA<br>TAATCGAAGGGAGAGACCGAGCAATGGCCTGGACAGTGGTGAATAGTATCTGCAACACTACAGGAGTCGAAAAGCCCAAATTCCTCCCTGATTTGTATGACTACAAAGAGAACCGATTCATTGAAATTGGAGTAACGCGAAGGGAAGTTCACATATACTATTTAGAAAAAGCCAACAAGATAAAATCAGAGAAAACACACATTCACATATTCTCATTCACTGGAGAGGAA<br>ATGGCCACCAAGGCGGAATACACCCTTGATGAAGAGAGCAGAGCAAGAATAAAAACCAGACTGTTCACTATAAGACAAGAAATGGCCAGTAGAGGTCTATGGGATTCCTTTCGTCAGTCCGAGAGAGGCGAAGAGACAACTGAAGAAAGATTTGAAATCACAGGAACCATGCGCAGGCTTGCCGACCAAAGTCTCCCACCGAACTTCTCCAGCCTTGAAAACTTTAGAG<br>CCTATGTGGATGGATTCGAACCGAACGGCTGCATTGAGGGCAAGCTTTCTCAAATGTCAAAAGAAGTGAACGCCAGAATTGAGCCATTCCTGAAGACAACACCACGCCCTCTCAGATTACCTGATGGGCCTCCTTGTTCTCAGCGGTCGAAGTTCTTGCTGATGGATGCCCTTAAGTTGAGCATCGAAGATCCTAGCCATGAGGGGGAGGGCATACCGCTGTATGATGCAA<br>TCAAATGCATGAAGACATTTTTTGGCTGGAAAGAGCCCAACCTCGTAAAGCCGCATGAGAAAGGCATAAACCCTAATTACCTCCTGGCTTGGAAGCAGGTGCTGGCAGAACTTCAAGATATTGAGAATGAGGAGAAATTCCCAAAAACAAAGAACATGAAGAAAACAAGCCAATTGAAGTGGGCACTTGGTGAGAACATGGCACCAGAAAAAGTGGACTTTGAGGACTG<br>CAAAGATGTTAGCGATCTAAGACAGTACGACAGTGACGAACCAGAGCCTAGATCACTAGCAAGCTGGATTCAGAGTGAATTCAACAAGGCATGCGAATTGACAGAGTCGAGTTGGATTGAACTTGATGAGATAGGGGAAGACGTTGCTCCAATCGAACACATTGCGAGTATGAGGCGGAACTATTTCACAGCGGAGGTATCCCATTGCAGGGCCACTGAATACATAATG<br>AAGGGAGTATACATAAACACAGCCCTATTGAATGCATCCTGTGCAGCCATGGATGACTTCCAACTGATTCCAATGATAAGCAAGTGCAGAACCAAAGAGGGAAGACGGAAGACAAATCTGTATGGATTCATTATAAAAGGAAGATCCCATTTGAGGAATGACACCGATGTGGTAAACTTTGTGAGCATGGAATTCTCTCTAACTGACCCGAGGCTAGAGCCACACAAATG<br>GGAAAAGTACTGTGTTCTTGAGATAGGAGACATGCTCCTACGGACTGCAATAGGCCAAGTGTCGAGGCCCATGTTCCTGTATGTGAGAACCAATGGGACTTCCAAGATCAAAATGAAATGGGGCATGGAGATGAGACGATGCCTTCTTCAGTCCCTTCAACAAATTGAGAGCATGATTGAGGCCGAATCTTCTGTCAAAGAGAAGGACATGACCAAGGAATTCTTTGAAA<br>ACAAATCAGAAACATGGCCAATTGGAGAATCACCCAAAGGGGTTGAGGAAGGCTCTATTGGGAAAGTATGCAGAACATTGCTAGCAAAGTCTGTGTTCAACAGCCTATATGCATCTCCACAACTCGAGGGATTTTCAGCTGAATCAAGAAAATTGCTTCTCATTGTTCAGGCACTTAGGGACAACCTGGAACCTGGAACCTTCGATCTTGGGGGGCTATATGAAGCAATTG<br>AGGAGTGCCTGATTAACGATCCCTGGGTTTTGCTTAATGCGTCTTGGTTCAACTCCTTCATCACACATGCACTGAAATA-
internal NVRI repository	>A/duck/Poland/H2014_21RS3290-17/2021_H5N1_2021-11-20<br>ATGGAAGACTTTGTGCGACAATGCTTCAATCCAATGATTGTCGAGCTTGCGGAAAAAGCAATGAAAGAATATGGGGAAGATCCGAAAATCGAGACAAACAAATTTGCCGCAATATGCACACACTTAGAAGTCTGTTTCATGTATTCGGATTTCCATTTTATTGATGAACGAGGCGAATCAATGATTGTAGAATCTGGCGATCCAAATGCATTATTGAAACACCGATTTGAGA<br>TAATCGAAGGGAGAGACCGAGCAATGGCCTGGACAGTGGTGAATAGTATCTGCAACACTACAGGAGTCGAAAAGCCCAAATTCCTCCCTGATTTGTATGACTACAAAGAGAACCGATTCATTGAAATTGGAGTAACGCGAAGGGAAGTTCACATATACTATTTAGAAAAAGCCAACAAGATAAAATCAGAGAAAACACACATTCACATATTCTCATTCACTGGAGAGGAA<br>ATGGCCACCAAGGCGGAATACACCCTTGATGAAGAGAGCAGAGCAAGAATAAAAACCAGACTGTTCACTATAAGACAAGAAATGGCCAGTAGAGGTCTATGGGATTCCTTTCGTCAGTCCGAGAGAGGCGAAGAGACAACTGAAGAAAGATTTGAAATCACAGGAACCATGCGCAGGCTTGCCGACCAAAGTCTCCCACCGAACTTCTCCAGCCTTGAAAACTTTAGAG<br>CCTATGTGGATGGATTCGAACCGAACGGCTGCATTGAGGGCAAGCTTTCTCAAATGTCAAAAGAAGTGAACGCCAGAATTGAGCCATTCCTGAAGACAACACCACGCCCTCTCAGATTACCTGATGGGCCTCCTTGTTCTCAGCGGTCGAAGTTCTTGCTGATGGATGCCCTTAAGTTGAGCATCGAAGATCCTAGCCATGAGGGGGAGGGCATACCGCTGTATGATGCAA<br>TCAAATGCATGAAGACATTTTTTGGCTGGAAAGAGCCCAACCTCGTAAAGCCGCATGAGAAAGGCATAAACCCTAATTACCTCCTGGCTTGGAAGCAGGTGCTGGCAGAACTTCAAGATATTGAGAATGAGGAGAAATTCCCAAAAACAAAGAACATGAAGAAAACAAGCCAATTGAAGTGGGCACTTGGTGAGAACATGGCACCAGAAAAAGTGGACTTTGAGGACTG<br>CAAAGATGTTAGCGATCTAAGACAGTACGACAGTGACGAACCAGAGCCTAGATCACTAGCAAGCTGGATTCAGAGTGAATTCAACAAGGCATGCGAATTGACAGATTCGAGTTGGATTGAACTTGATGAGATAGGGGAAGACGTTGCTCCAATCGAACACATTGCGAGTATGAGGAGGAACTATTTCACAGCGGAGGTATCCCATTGCAGGGCCACTGAATACATAATG<br>AAGGGAGTATACATAAACACAGCCCTATTGAATGCATCCTGTGCAGCCATGGATGACTTCCAACTGATTCCAATGATAAGCAAGTGCAGAACCAAAGAGGGAAGACGGAAGACAAATCTGTATGGATTCATTATAAAAGGAAGATCCCATTTGAGGAATGACACCGATGTGGTAAACTTTGTGAGCATGGAATTCTCTCTAACTGACCCGAGGCTAGAGCCACACAAATG<br>GGAAAAGTACTGTGTTCTTGAGATAGGAGACATGCTCCTACGGACTGCAATAGGCCAAGTGTCGAGGCCCATGTTCCTGTATGTGAGAACCAATGGGACTTCCAAGATCAAAATGAAATGGGGCATGGAGATGAGACGATGCCTTCTTCAGTCCCTTCAACAAATTGAGAGCATGATTGAGGCCGAATCTTCTGTCAAAGAGAAGGACATGACCAAGGAATTCTTTGAAA<br>ACAAATCAGAAACATGGCCAATTGGAGAATCACCCAAAGGGGTTGAGGAAGGCTCTATTGGGAAAGTATGCAGAACATTGCTRGCAAAGTCTGTGTTCAACAGCCTATATGCATCTCCACAACTCGAGGGATTTTCAGCTGAATCAAGAAAATTGCTTCTCATTGTTCAGGCACTTAGGGACAACCTGGAACCTGGAACCTTCGATCTTGGGGGGCTATATGAAGCAATTG<br>AGGAGTGCCTGATTAACGATCCCTGGGTTTTGCTTAATGCGTCTTGGTTCAACTCCTTCATCACACATGCACTGAAATA-
EPI_ISL_11922811	>A/goose/Poland/H124_22VIR2515-5/2022_H5N1_2022-02-05<br>ATGGAAGACTTTGTGCGACAATGCTTCAATCCAATGATTGTCGAGCTTGCGGAAAAGGCAATGAAAGAGTATGGGGAAGATCCGAAAATCGAAACAAACAAATTTGCCGCAATATGCACACACTTAGAAGTCTGTTTCATGTATTCGGATTTCCACTTTATTGATGAACGAGGCGAATCAATAATTGTAGAATCTGGCGATCCGAATGCATTATTGAAACACCGATTCGAGA<br>TAATTGAAGGGAGAGACCGAACTACGGCCTGGACAGTGGTGAATAGTATCTGCAACACCACAGGAGTCGAAAAGCCCAAATTCCTCCCTGATTTGTATGACTACAAAGAGAACCGATTCATTGAAATTGGAGTAACGCGAAGGGAAGTTCACATATACTATCTAGAGAAAGCCAACAAGATAAATCAGAGAAGACACACATTCATATATTCTCATTCACTGGAGAGGAA<br>ATGGCCACCAAGGCGGACTACACCCTTGATGAAGAGAGTAGAGCAAGAATAAAAACCAGGCTGTTCACTATAAGACAAGAAATGGCCAGTAGGGGTCTATGGGATTCCTTTCGTCAGTCCGAGAGAGGCGAAGAGACAATTGAAGAAAGATTTGAAATCACAGGAACCATGCGCAGGCTTGCCGACAAAAGTCTCCCACCGAACTTCTCCAGCCTTGAAAACTTTAGAG<br>CCTATGTGGATGGATTCGAACCGAACGGCTGCATTGAGGGCAAGCTTTCTCAAATGTCAAAAGAAGTGAATGCCAGAATTGAGCCATTTCTGAAGACAACACCACGCCCTCTCAGATTACCTGATGGGCATCCCTGTTCTCAGCGGTCGAAGTTCTTGCTGATGGATGCCCTCAAATTGAGCATCGAAGACCCGAGCCATGAGGGGGAGGGTATACCGCTGTATGATGCAA<br>TCAAATGCATGAAGACATTTTTGGCTGGAAAGAGCCTAACATCGTAAAACCGCATGAAAAGGGCATAAACCCTAATTACCTTCTGGCTTGGAAACAGGTGCTGGCAGAACTCCAAGATATTGAAAATGAGGAGAAAATCCCAAAAACAAAGAACATGAAGAAAACAAGCCAATTGAAGTGGGCACTTGGTGAGAACATGGCACCAGAGAAAGTGGACTTTGAGGACTG<br>TAAAGATGTTAGTGATCTAAGACAGTACGACAGTGACGAACCAGAGTCTAGATCACTAGCAAGCTGGATCCAGAGTGAATTCAACAAGGCATGCGAATTGACAGATTCGAGTTGGATTGAACTTGATGAAATAGGGGAAGACATTGCTCCAATTGAACACATTGCGAGTATGAGGAGAAACTATTTCACAACGGAGGTGTCCCATTGCAGGGCTACTGAATACATAATGA<br>AGGGAGTATACATAAACACAGCCCTGTTGAATGCATCCTGTGCAGCGATGGATGACTTTCAACTGATTCCTATGATAAGCAAGTGCAGAACCAAAGAAGGAAGACGGAGACAAATTTATATGGATTCATTATAAAAGGAAGATCCCATTTGAGGAATGACACCGATGTGGTGAACTTTGTGAGCATGGAATTCTCTCTCACTGACCCGAGGCTGGAGCCACACAAATGG<br>GAAAAGTACTGTGTTCTTGAGATAGGAGACATGCTCCTACGGACTGCAATAGGCCAAGTGTCAAGGCCCATGTTCCTGTATGTGAGAACCAATGGAACTTCCAAGATCAAAATGAAATGGGGCATGGAGATGAGACGATGCCTTCTTCAGTCCCTTCAACAAATTGAGAGCATGATTGAGGCCGAGTCTTCTGTCAAAGAGAAGGACATGACCAAGGAATTCTTTGAAAAC<br>AAATCAGAAACATGGCCAATTGGGGAATCACCCAAAGGGGTGGAAGAAGGCTCCATTGGGAAGGTGTGCAGAACATTACTAGCAAAGTCTGTGTTCAACAGCCTATATGCATCTCCACAACTCGAGGGGTTTTCAGCTGAATCAAGAAAATTGCTTCTCATTGTTCAGGCACTTAGGGACAACCTGGAACCTGGAACCTTCGATCTTGGGGGGCTATATGAAGCAATTGAG<br>GAGTGCCTGATTAACGATCCCTGGGTTTTGCTTAATGCGTCTTGGTTCAACTCCTTCCTCACACATGCACTGAAATA-
EPI_ISL_18245818	>A/common_tern/Poland/MB143/2022_H5N1_2022-06-15<br>ATGGAGGACTTTGTGCGACAATGTTTCAATCCAATGATTGTCGAGCTTGCGGAAAAGGCAATGAAAGAGTATGGGGAAGATCCGAAAATCGAAACAAACAAATTTGCCGCAATATGCACACACTTAGAAGTCTGTTTCATGTATTCGGATTTCCACTTTATTGATGAACGAGGCGAATCAATAATTGTAGAATCTGGCGATCCGAATGCATTATTGAAACACCGATTCGAGA<br>TAATTGAAGGGAGAGATCGAACTATGGCCTGGACAGTGGTGAATAGTATCTGCAACACCACAGGAGTCGAAAAGCCCAAATTCCTCCCTGATTTGTATGACTACAAAGAGAACCGATTCATTGAAATTGGAGTAACGCGAAGGGAAGTTCACATATACTATCTAGAGAAAGCCAACAAGATAAATCAGAGAAGACTCACATTCATATATTCTCATTCACTGGAGAGGAA<br>ATGGCCACCAAGGCGGACTACACCCTTGATGAAGAGAGTAGAGCAAGAATAAAAACCAGGCTGTTCACTATAAGACAAGAAATGGCCAGTAGGGGTCTATGGGATTCCTTTCGTCAGTCCGAGAGAGGCGAAGAGACAATTGAAGAAAGATTTGAAATCACAGGAACCATGCGCAGGCTTGCCGACCAAAGTCTCCCACCGAACTTCTCCAGCATTGAAAACTTTAGAG<br>CCTATGTGGATGGATTCGAACCGAACGGCTGCATTGAGGGCAAGCTTTCTCAAATGTCAAAAGAAGTGAATGCCAGAATTGAGCCATTTCTGAAGACAACACCACGCCCTCTCAGATTACCTGATGGGCCTCCCTGTTCTCAGCGGTCGAAGTTCTTGCTGATGGATGCCCTAAATTGAGCATCGAAGACCCGAGCCATGAGGGGGAGGGTATACCGCTGTATGATGCAA<br>TCAAATGCATGAAGACATTTTTGGCTGGAAAGAGCCTAACATCGTAAAACCGCATGAAAAGGGCATAAACCCTAATTACCTTTTGGCTTGGAAACAGGTGCTGACAGAACTCCAAGATATTGAAAATGAGGAGAAAATCCCAAAAACAAAGAACATGAGGAAAACAAGCCAATTGAAGTGGGCACTTGGTGAGAACATGGCACCAGAGAAAGTGGACTTTGAGGACTG<br>TAAAGATGTTAGTGATCTAAGACAGTACGACAGTGACGAGCCAGAGTCTAGATCACTAGCAAGCTGGATCCAGAGTGAATTCAACAAGGCATGCGAATTGACAGATTCGAGTTGGATTGAACTTGATGAAATAGGGGAAGACATTGCTCCAATTGAACACATTGCGAGTATGAGGAGAAACTATTTCACAGCGGAGGTGTCCCATTGCAGGGCTACTGAATACATAATGA<br>AGGGAGTATACATAAACACAGCCCTGTTGAATGCATCCTGTGCAACGATGGATGACTTTCAACTGATTCCTATGATAAGCAAGTGCAGAACCAAAGAAGGAAGACGGAAGACAAATTTATATGGATTCATTATAAAAGGAAGATCCCATTTGAGGAATGACACCGATGTGGTGAACTTTGTGAGCATGGAATTCTCTCTCACTGACCCGAGGCTGGAGCCACACAAATGG<br>GAAAAGTACTGTGTTCTTGAGATAGGAGACATGCTCCTACGGACTGCAATAGGCCAAGTGTCAAGGCCCATGTTCTTGTATGTGAGAACCAATGGAACTTCCAAGATCAAAATGAAATGGGGCATGGAGATGAGACGATGCCTTCTTCAGTCCCTTCAACAAATTGAGAGCATGATTGAGGCCGAGTCTTCTGTCAAAGAGAAGGACATGACCAAGGAATTCTTTGAAAAC<br>AAATCAGAAACATGGCCAATTGGGGAGTCACCCAAAGGGGTGGAAGAAGGCTCCATTGGGAAGGTGTGCAGAACATTGCTAGCAAAGTCTGTGTTCAACAGCCTATATGCATCTCCACAACTCGAGGGGTTTTCAGCTGAATCAAGAAAATTGCTTCTCATTGTTCAGGCACTTAGGGACAACCTGGAACCTGGAACCTTCGATCTTGGGGGGCTATATGGAGCAATTGA<br>GGAGTGCCTGATTAACGATCCCTGGGTTTTGCTTAATGCGTCTTGGTTCAACTCCTTCCTCACACATGCACTGAAATA-
EPI_ISL_14917915	>A/breeding_hen/Poland/H361/2022_H5N1_2022-07-18<br>ATGGAAGACTTTGTGCGACAATGCTTCAATCCAATGATTGTCGAGCTTGCGGAAAAGGCAATGAAAGAATATGGGGAAGACCCGAAAATCGAGACAAACAAATTTGCCGCAATATGCACACACTTAGAAGTCTGTTTCATGTATTCGGATTTCCACTTTATTGATGAACGAGGAGAATCAATAATTGTAGAATCCGGCGATCCGAATGCATTATTGAAACATCGATTTGAGA<br>TAATTGAAGGGAGAGACCGAACAATGGCTTGGACAGTGGTAAATAGTATATGCAACACTACAGGAGTCGAAAAGCCCAAATTCCTTCCTGATCTGTATGACTACAAAGAGAACCGGTTCATTGAAATTGGAGTAACGCGAAGGGAGGTTCACATATATTATCTAGAGAAAGCCAACAAGATAAAGTCAGAGAAGACACACATTCACATATTCTCATTCACTGGAGAGGAA<br>ATGGCCACCAAGGCGGACTACACTCTTGATGAAGAGAGCAGAGCAAGAATAAAAACAAGGCTATTCACCATAAGACAGGAAATGGCCAGTAGGGGTCTATGGGATTCCTTTCGTCAGTCCGAGAGAGGCGAAGAGACAATTGAAGAAAGATTTGAAATCACAGGAACCATGCGCAGGCTTGCCGACCAAAGTCTCCCACCGAACTTCTCCAGCCTTGAAAACTTTAGAG<br>CCTATGTGGATGGATTCGAACCGAACGGCTGCATTGAGGGCAAGCTTTCTCAAATGTCAAAAGAAGTGAATGCAAGAATTGAGCCATTTCTGAAGACAACACCACGCCCTCTCAGATTACCTGATGGGCCTCCCTGTTCTCAGCGCTCGAAATTCTTGCTGATGGATGCCCTTAAATTGAGTATTGAAGACCCGAGCCATGAGGGGGAGGGCATACCGCTGTATGATGCAAT<br>CAAATGCATGAAGACCTTCTTTGGCTGGAAAGAGCCCAACATCGTGAAACCACATGAAAAGGGCATAAACCCTAATTACCTTCTGGCTTGGAAGCAGGTGCTAGCAGAACTCCAAGATATTGAAAATGAGGAGAAAATCCCAAAAACAAAGAATATGAAGAAAACAAGCCAACTGAAGTGGGCACTTGGTGAGAACATGGCACCAGAGAAAGTGGACTTTGAGGACTG<br>TAAAGATGTTAGTGATCTAAAACAGTACAACAGTGACGAACCAGAGTCTAGATCACTAGCAAGCTGGATCCAGAGTGAATTTAACAAAGCATGCGAATTGACAGATTCAAGTTGGATTGAACTTGATGAAATAGGAGAAGATGTTGCTCCAATTGAACACATTGCGAGTATGAGGAGGAACTATTTCACAGCGGAAGTATCTCATTGCAGGGCTACTGAATACATAATGAA<br>GGGGGTATACATAAACACAGCCCTACTGAATGCATCCTGTGCAGCCATGGATGACTTCCAACTGATCCCAATGATAAGCAAGTGCAGAACCAAAGAAGGGGAGGCGGAAGACAAATCTGTATGGATTCATTATAAAAGGAAGATCCCATTTGAGGAATGACACCGATGTGGTAAACTTTGTGAGCATGGAGTTCTCTCTCACTGATCCAAGGCTAGAGCCACACAAATGGG<br>AAAAGTACTGTGTTCTTGAGATAGGAGACATGCTCCTACGGACTGCAATAGGCCAAGTGTCAAGGCCCATGTTCCTGTATGTGAGAACCAATGGGACTTCCAAGATCAAAATGAAATGGGGAATGGAGATGAGACGATGCCTTCTTCAGTCTCTTCAACAAATTGAGAGCATGATTGAGGCCGAGTCTTCTGTCAAAGAGAAGGACATGACCAAGGAATTCTTTGAAAACA<br>AATCAGAAACATGGCCAATTGGAGAATCACCCAAAGGGGTGGAAGAAGGCTCCATCGGGAAGGTATGCAGAACACTGTTAGCAAAGTCTGTGTTCAATAGCCTATATGCATCTCCACAACTTGAGGGATTTTCAGCTGAATCAAGAAAATTACTTCTCATTGTTCAGGCACTTAGGGACAACCTGGAACCTGGAACCTTCGATCTTGGGGGGCTATATGAAGCAATTGAGG<br>AGTGCCTGATTAACGATCCCTGGGTTTTGCTTAATGCGTCTTGGTTCAACTCCTTCCTCACACATGCACTGAAATA-
EPI_ISL_14917942	>A/breeding_hen/Poland/H364/2022_H5N1_2022-07-21<br>ATGGAAGACTTTGTGCGACAATGCTTCAATCCAATGATTGTCGAGCTTGCGGAAAAGGCAATGAAAGAATATGGGGAAGACCCGAAAATCGAGACAAACAAATTTGCCGCAATATGCACACACTTAGAAGTCTGTTTCATGTATTCGGATTTCCACTTTATTGATGAACGAGGAGAATCAATAATTGTAGAATCCGGCGATCCGAATGCATTATTGAAACATCGATTTGAGA<br>TAATTGAAGGGAGAGACCGAACAATGGCTTGGACAGTGGTAAATAGTATATGCAACACTACAGGAGTCGAAAAGCCCAAATTCCTTCCTGATCTGTATGACTACAAAGAGAACCGGTTCATTGAAATTGGAGTAACGCGAAGGGAGGTTCACATATATTATCTAGAGAAAGCCAACAAGATAAAGTCAGAGAAGACACACATTCACATATTCTCATTCACTGGAGAGGAA<br>ATGGCCACCAAGGCGGACTACACTCTTGATGAAGAGAGCAGAGCAAGAATAAAAACAAGGCTATTCACCATAAGACAGGAAATGGCCAGTAGGGGTCTATGGGATTCCTTTCGTCAGTCCGAGAGAGGCGAAGAGACAATTGAAGAAAGATTTGAAATCACAGGAACCATGCGCAGGCTTGCCGACCAAAGTCTCCCACCGAACTTCTCCAGCCTTGAAAACTTTAGAG<br>CCTATGTGGATGGATTCGAACCGAACGGCTGCATTGAGGGCAAGCTTTCTCAAATGTCAAAAGAAGTGAATGCAAGAATTGAGCCATTTCTGAAGACAACACCACGCCCTCTCAGATTACCTGATGGGCCTCCCTGTTCTCAGCGCTCGAAATTCTTGCTGATGGATGCCCTTAAATTGAGTATTGAAGACCCGAGCCATGAGGGGGAGGGCATACCGCTGTATGATGCAAT<br>CAAATGCATGAAGACCTTCTTTGGCTGGAAAGAGCCCAACATCGTGAAACCACATGAAAAGGGCATAAACCCTAATTACCTTCTGGCTTGGAAGCAGGTGCTAGCAGAACTCCAAGATATTGAAAATGAGGAGAAAATCCCAAAAACAAAGAATATGAAGAAAACAAGCCAACTGAAGTGGGCACTTGGTGAGAACATGGCACCAGAGAAAGTGGACTTTGAGGACTG<br>TAAAGATGTTAGTGATCTAAAACAGTACAACAGTGACGAACCAGAGTCTAGATCACTAGCAAGCTGGATCCAGAGTGAATTTAACAAAGCATGCGAATTGACAGATTCAAGTTGGATTGAACTTGATGAAATAGGAGAAGATGTTGCTCCAATTGAACACATTGCGAGTATGAGGAGGAACTATTTCACAGCGGAAGTATCTCATTGCAGGGCTACTGAATACATAATGAA<br>GGGGGTATACATAAACACAGCCCTACTGAATGCATCCTGTGCAGCCATGGATGACTTCCAACTGATCCCAATGATAAGCAAGTGCAGAACCAAAGAAGGGGAGGCGGAAGACAAATCTGTATGGATTCATTATAAAAGGAAGATCCCATTTGAGGAATGACACCGATGTGGTAAACTTTGTGAGCATGGAGTTCTCTCTCACTGATCCAAGGCTAGAGCCACACAAATGGG<br>AAAAGTACTGTGTTCTTGAGATAGGAGACATGCTCCTACGGACTGCAATAGGCCAAGTGTCAAGGCCCATGTTCCTGTATGTGAGAACCAATGGGACTTCCAAGATCAAAATGAAATGGGGAATGGAGATGAGACGATGCCTTCTTCAGTCTCTTCAACAAATTGAGAGCATGATTGAGGCCGAGTCTTCTGTCAAAGAGAAGGACATGACCAAGGAATTCTTTGAAAACA<br>AATCAGAAACATGGCCAATTGGAGAATCACCCAAAGGGGTGGAAGAAGGCTCCATCGGGAAGGTATGCAGAACACTGTTAGCAAAGTCTGTGTTCAATAGCCTATATGCATCTCCACAACTTGAGGGATTTTCAGCTGAATCAAGAAAATTACTTCTCATTGTTCAGGCACTTAGGGACAACCTGGAACCTGGAACCTTCGATCTTGGGGGGCTATATGAAGCAATTGAGG<br>AGTGCCTGATTAACGATCCCTGGGTTTTGCTTAATGCGTCTTGGTTCAACTCCTTCCTCACACATGCACTGAAATA-
EPI_ISL_11922812	>A/chicken/Poland/H071_22VIR2515-6/2022_H5N1_2022-01-23<br>ATGGAAGACTTTGTGCGACAATGCTTCAATCCAATGATTGTCGAGCTTGCGGAAAAGGCAATGAAAGAGTATGGGGAAGATCCGAAAATCGAAACAAACAAATTTGCCGCAATATGCACACACTTAGAAGTCTGTTTCATGTATTCGGATTTCCACTTTATTGATGAACGAGGCGAATCAATAATTGTAGAATCTGGCGATCCGAATGCATTATTGAAACACCGATTCGAGA<br>TAATTGAAGGGAGAGACCGAACTATGGCCTGGACAGTGGTGAATAGTATCTGCAACACCACAGGAGTCGAAAAGCCCAAATTCCTCCCTGATTTGTATGACTACAAAGAGAACCGATTCATTGAAATTGGAGTAACGCGAAGGGAAGTTCACATATACTATCTAGAGAAAGCCAACAAGATAAATCAGAGAAGACACACATTCATATATTCTCATTCACTGGAGAGGAA<br>ATGGCCACCAAGGCGGACTACACCCTTGATGAAGAGAGTAGAGCAAGAATAAAAACCAGGCTGTTCACTATAAGACAAGAAATGGCCAGTAGGGGTCTATGGGATTCCTTTCGTCAGTCCGAGAGAGGCGAAGAGACAATTGAAGAAAGATTTGAAATCACAGGAACCATGCGCAGGCTTGCCGACCAAAGTCTCCCACCGAACTTCTCCAGCCTTGAAAACTTTAGAG<br>CCTATGTGGATGGATTCGAACCGAACGGCTGCATTGAGGGCAAGCTTTCTCAAATGTCAAAAGAAGTGAATGCCAGAATTGAGCCATTTCTGAAGACAACACCACGTCCTCTCAGATTACCTGATGGGCCTCCCTGTTCTCAGCGGTCGAAGTTCTTGCTGATGGATGCCCTCAAATTGAGCATCGAAGACCCGAGCCATGAGGGGGAGGGTATACCGCTGTATGATGCAA<br>TCAAATGCATGAAGACATTTTTGGCTGGAAAGAGCCTAACATCGTAAAACCGCATGAAAAAGGCATAAACCCTAATTACCTTCTGGCTTGGAAACAGGTGCTGGCAGAACTCCAAGATATTGAAAATGAGGAGAAAATCCCAAAAACAAAGAACATGAAGAAAACAAGCCAATTGAAGTGGGCACTTGGTGAGAACATGGCACCAGAGAAAGTGGACTTTGAGGACTG<br>TAAAGATGTTAGTGATCTAAGACAGTACGACAGTGACGAACCAGAGTCTAGATCACTAGCAAGCTGGATCCAGAGTGAATTCAACAAGGCATGCGAATTGACAGATTCGAGTTGGATTGAACTTGATGAAATAGGGGAAGACATTGCTCCAATTGAACACATTGCGAGTATGAGGAGAAACTATTTCACAGCAGAGGTGTCCCATTGCAGGGCTACTGAATACATAATGA<br>AGGGAGTATACATAAACACAGCCCTGTTGAATGCATCCTGTGCAGCGATGGATGACTTTCAACTGATTCCTATGATAAGCAAGTGCAGAACCAAAGAAGGAAGACGGAAGACAAATTTATATGGATTCATTATAAAAGGAAGATCCCATTTGAGGAATGACACCGATGTGGTGAACTTTGTGAGCATGGAATTCTCTCTCACTGACCCGAGGCTGGAGCCACACAAATGG<br>GAAAAGTACTGTGTTCTTGAGATAGGAGACATGCTCCTACGGACTGCAATAGGCCAAGTGTCAAGGCCCATGTTCCTGTATGTGAGAACCAATGGAACTTCCAAGATCAAAATGAAATGGGGCATGGAGATGAGACGATGCCTTCTTCAGTCTCTTCAACAAATTGAGAGCATGATTGAGGCCGAGTCTTCTGTCAAAGAGAAGGACATGACCAAGGAATTCTTTGAAAAC<br>AAATCAGAAACATGGCCAATTGGGGAATCACCCAAAGGGGTGGAAGAAGGCTCCATTGGGAAGGTGTGCAGAACATTGCTAGCAAAGTCTGTGTTCAACAGCCTATATGCATCTCCACAACTCGAGGGGTTTTCAGCTGAATCAAGAAAATTACTTCTCATTGTTCAGGCACTTAGGGACAACCTGGAACCTGGAACCTTCGATCTTGGGGGGCTATATGAAGCAATTGAG<br>GAGTGCCTGATTAACGATCCCTGGGTTTTGCTTAATGCGTCTTGGTTCAACTCCTTCCTCACACATGCACTGAAATA-

EPI_ISL_11922809	>A/chicken/Poland/H157_22VIR2515-3/2022_H5N1_2022-02-18 [illegible]
EPI_ISL_11922807	>A/chicken/Poland/H182_22VIR2515-1/2022_H5N2_2022-02-28 [illegible]
internal NVRI repository	>A/chicken/Poland/H1928_21RS3290-2/2021_H5N1_2021-11-03 [illegible]
EPI_ISL_6931288	>A/chicken/Poland/H1940-N/2021_H5N1_2021-11-05 [illegible]
internal NVRI repository	>A/chicken/Poland/H1962_21RS3290-4/2021_H5N1_2021-11-11 [illegible]
internal NVRI repository	>A/chicken/Poland/H1976_21RS3290-9/2021_H5N1_2021-11-15 [illegible]
internal NVRI repository	>A/chicken/Poland/H1983_21RS3290-13/2021_H5N1_2021-11-16 [illegible]
internal NVRI repository	>A/chicken/Poland/H2130-T1c/2021_H5N1_2021-12-20 [illegible]
internal NVRI repository	>A/chicken/Poland/H2175-N/2021_H5N1_2021-12-18 [illegible]
internal NVRI repository	>A/chicken/Poland/H36/2022_H5N1_2022-01-08 [illegible]
internal NVRI repository	>A/chicken/Poland/H62-N/2022_H5N1_2022-01-17 [illegible]
EPI_ISL_7778768	>A/Eurasian_eagle-owl/Finland/10617_21VIR7689-15/2021_H5N1_2021-08-01 [illegible]
EPI_ISL_4653177	>A/common_pheasant_/Sweden/SVA210923SZ0341/KN000366/M-2021_H5N1_2021-09-22 [illegible]

EPI_ISL_7778766	>A/European_herring_gull/Finland/9722_21VIR7689-13/2021_H5N1_2021-08-01<br>ATGGAAGACTTTGTGCGACAATGCTTCAATCCAATGATTGTCGAGCTTGCGGAAAAAGCAATGAAAGAATATGGGGAAGATCCGAAAATCGAGACAAACAAATTTGCCGCAATATGCACACACTTAGAAGTCTGTTTCATGTATTCGGATTTCCATTTTATCGATGAACGAGGCGAATCAATGATTGTAGAATCTGGCGATCCAAATGCATTATTGAAACACCGATTTGAGA<br>TAATCGAAGGGAGAGACCGAGCAATGGCCTGGACAGTGGTGAATAGTATCTGCAACACCACAGGGGTCGAAAAGCCCAAATTCCTCCCTGATTTGTATGACTACAAAGAGAACCGATTCATTGAAATTGGAGTAACGCGAAGGGAAGTTCACATATACTATTTAGAAAAAGCCAACAAGATAAAATCAGAGAAAACACACATTCACATATTCTCATTCACTGGAGAGGAA<br>ATGGCCACCAAGGCGGACTATACCCTTGATGAAGAGAGCAGAGCAAGAATAAAAACCAGACTGTTCACTATAAGACAAGAAATGGCCAGTAGAGGTCTATGGGATTCCTTTCGTCAGTCCGAGAGAGGCGAAGAGACAATTGAAGAAAGATTTGAAATCACAGGAACCATGCGCAGGCTTGCCGACCAAAGTCTCCCACCGAGCTTCTCCAGCCTTGAAAACTTTAGAG<br>CCTATGTGGATGGATTCGAACCGAACGGCTACATTGAGGGCAAGCTTTCTCAAATGTCAAAAGAAGTGAAAGCCAGAATTGAGCCATTTGTGAAGACAACACCACGCCCTCTCAGATTACCTGATGGGCCTCCCTGTTCTCAGCGGTCGAAGTTCTTGCTGATGGATGCCCTTAAGTTGAGCATCGAAGACCCTAGCCATGAGGGGGAGGGCATACCGCTGTATGATGCAA<br>TCAAATGCATGAAGACATTTTTTGGCTGGAAAGAGCCCAACATCGTAAAGCCGCATGAGAAAGGCATAAACCCTAATTACCTCCTGGCTTGGAAGCAGGTGCTGGCAGAACTTCAAGACATTGAAAATGAGGAGAAATTTCCAAAAACAAAGAACATGAAGAAAACAAGCCAATTGAAGTGGGCACTTGGTGAGAACATGGCTCCAGAGAAAGTGGACTTTGAGGACTG<br>CAAAGATGTTAGCGATCTAAGACAGTACGACAGTGACGAACCAGAGTCTAGATCACTAGCAAGCTGGATTCAGAGTGAATTCAACAAGGCATGCGAACTGACAGATTCGAGTTGGATTGAACTTGATGAGATAGGGGAAGACGTTGCTCCAATCGAACACATTGCGAGTATGAGGAGGAACTATTTCACAGCGGAGGTATCCCATTGCAGGGCCACTGAATACATAATG<br>AAGGGAGTATACATAAACACAGCCCTATTGAATGCATCCTGTGCAGCCATGGATGACTTCCAACTGATTCCAATGATAAGCAAGTGCAGAACTAAAGAAGGAAGACGGAAGACAAATCTGTATGGATTCATTATAAAAGGAAGATCCCATTTGAGGAATGACACCGATGTGGTAAACTTTGTGAGCATGGAATTCTCTCTAACTGACCCGAGGCTAGAGCCACACAAATG<br>GGAAAAGTACTGTGTTCTTGAGATAGGAGACATGCTCCTACGGACTGCAATAGGCCAAGTGTCGAGGCCCATGTTCCTGTATGTGAGAACCAATGGGACTTCCAAGATCAAAATGAAATGGGGCATGGAGATGAGACGATGCCTTCTTCAGTCCCTTCAACAAATTGAGAGCATGATTGAGGCCGAATCTTCTGTCAAAGAGAAGGACATGTCCAAGGAATTCTTTGAAAA<br>CAAATCAGAAACATGGCCAATTGGAGAATCACCCAAAGGGGTGGAGGAAGGCTCTATTGGGAAAGTATGCAGAACATTGCTAGCAAAGTCTGTGTTCAACAGCCTATATGCATCTCCACAACTCGAGGGGTTTTCAGCTGAATCAAGAAAATTGCTTCTCATTGTTCAGGCACTTAGGGACAACCTGGAACCTGGAACCTTCGATCTTGGGGGGCTATATGAAGCAATTGA<br>GGAGTGCCTGATTAACGATCCCTGGGTTTTGCTTAATGCGTCTTGGTTCAACTCCTTCCTCACACATGCACTGAAATA-
EPI_ISL_5804708	>A/mute_swan/England/053054/2021_H5N1_2021-10-24<br>ATGGAAGACTTTGTGCGACAATGCTTCAATCCAATGATTGTCGAGCTTGCGGAAAAAGCAATGAAAGAATATGGGGAAGATCCGAAAATCGAGACAAACAAATTTGCCGCAATATGCACACACTTAGAAGTCTGTTTCATGTATTCGGATTTCCATTTTATCGATGAACGAGGCGAATCAATGATTGTAGAATCTGGCGATCCAAATGCATTATTGAAACACCGATTTGAGA<br>TAATCGAAGGGAGAGACCGAGCAATGGCCTGGACAGTGGTGAATAGTATCTGCAACACCACAGGGGTCGAAAAGCCCAAATTCCTCCCTGATTTGTATGACTACAAAGAGAACCGATTCATTGAAATTGGAGTAACGCGAAGGGAAGTTCACATATACTATTTAGAAAAAGCCAACAAGATAAAATCAGAGAAAACACACATTCACATATTCTCATTCACTGGAGAGGAA<br>ATGGCCACCAAGGCGGACTATACCCTTGATGAAGAGAGCAGAGCAAGAATAAAAACCAGACTGTTCACTATAAGACAAGAAATGGCCAGTAGAGGTCTATGGGATTCCTTTCGTCAGTCCGAGAGAGGCGAAGAGACAATTGAAGAAAGATTTGAAATCACAGGAACCATGCGCAGGCTTGCCGACCAAAGTCTCCCACCGAGCTTCTCCAGCCTTGAAAACTTTAGAG<br>CCTATGTGGATGGATTCGAACCGAACGGCTACATTGAGGGCAAGCTTTCTCAAATGTCAAAAGAAGTGAAAGCCAGAATTGAGCCATTTGTGAAGACAACACCACGCCCTCTCAGATTACCTGATGGGCCTCCCTGTTCTCAGCGGTCGAAGTTCTTGCTGATGGATGCCCTTAAGTTGAGCATCGAAGACCCTAGCCATGAGGGGGAGGGCATACCGCTGTATGATGCAA<br>TCAAATGCATGAAGACATTTTTTGGCTGGAAAGAGCCCAACATCGTAAAGCCGCATGAGAAAGGCATAAACCCTAATTACCTCCTGGCTTGGAAGCAGGTGCTGGCAGAACTTCAAGACATTGAAAATGAGGAGAAATTTCCAAAAACAAAGAACATGAAGAAAACAAGCCAATTGAAGTGGGCACTTGGTGAGAACATGGCTCCAGAGAAAGTGGACTTTGAGGACTG<br>CAAAGATGTTAGCGATCTAAGACAGTACGACAGTGACGAACCAGAGTCTAGATCACTAGCAAGCTGGATTCAGAGTGAATTCAACAAGGCATGCGAACTGACAGATTCGAGTTGGATTGAACTTGATGAGATAGGGGAAGACGTTGCTCCAATCGAACACATTGCGAGTATGAGGAGGAACTATTTCACAGCGGAGGTATCCCATTGCAGGGCCACTGAATACATAATG<br>AAGGGAGTATACATAAACACAGCCCTATTGAATGCATCCTGTGCAGCAATGGATGACTTCCAACTGATTCCAATGATAAGCAAGTGCAGAACTAAAGAAGGAAGACGGAAGACAAATCTGTATGGATTCATTATAAAAGGAAGATCCCATTTGAGGAATGACACCGATGTGGTAAACTTTGTGAGCATGGAATTCTCTCTAACTGACCCGAGGCTAGAGCCACACAAATG<br>GGAAAAGTACTGTGTTCTTGAGATAGGAGACATGCTCCTACGGACTGCAATAGGCCAAGTGTCGAGGCCCATGTTCCTGTATGTGAGAACCAATGGGACTTCCAAGATCAAAATGAAATGGGGCATGGAGATGAGACGATGCCTTCTTCAGTCCCTTCAACAAATTGAGAGCATGATTGAGGCCGAATCTTCTGTCAAAGAGAAGGACATGTCCAAGGAATTCTTTGAAAA<br>CAAATCAGAAACATGGCCAATTGGAGAATCACCCAAAGGGGTGGAGGAAGGCTCTATTGGGAAAGTATGCAGAACATTGCTAGCAAAGTCTGTGTTCAACAGCCTATATGCATCTCCACAACTCGAGGGGTTTTCAGCTGAATCAAGAAAATTGCTTCTCATTGTTCAGGCACTTAGGGACAACCTGGAACCTGGAACCTTCGATCTTGGGGGGCTATATGAAGCAATTGA<br>GGAGTGCCTGATTAACGATCCCTGGGTTTTGCTTAATGCGTCTTGGTTCAACTCCTTCCTCACACATGCACTGAAATA-
EPI_ISL_7753347	>A/herring_gull/Germany-SH/AI06141/2021_H5N1_2021-10-21<br>ATGGAAGACTTTGTGCGACAATGCTTCAATCCAATGATTGTCGAGCTTGCGGAAAAAGCAATGAAAGAATATGGGGAAGATCCGAAAATCGAGACAAACAAATTTGCCGCAATATGCACACACTTAGAAGTCTGTTTCATGTATTCGGATTTCCATTTTATCGATGAACGAGGCGAATCAATGATTGTAGAATCTGGCGATCCAAATGCATTATTGAAACACCGATTTGAGA<br>TAATCGAAGGGAGAGACCGAGCAATGGCCTGGACAGTGGTGAATAGTATCTGCAACACCACAGGAGTCGAAAAGCCCAAATTCCTCCCTGATTTGTATGACTACAAAGAGAACCGATTCATTGAAATTGGAGTAACGCGAAGGGAAGTTCACATATACTATTTAGAAAAAGCCAACAAGATAAAATCAGAGAAAACACACATTCACATATTCTCATTCACTGGAGAGGAA<br>ATGGCCACCAAGGCGGACTATACCCTTGATGAAGAGAGCAGAGCAAGAATAAAAACCAGACTGTTCACTATAAGACAAGAAATGGCCAGTAGAGGTCTATGGGATTCCTTTCGTCAGTCCGAGAGAGGCGAAGAGACAATTGAAGAAAGATTTGAAATCACAGGAACCATGCGCAGGCTTGCCGACCAAAGTCTCCCACCGAGCTTCTCCAGCCTTGAAAACTTTAGAG<br>CCTATGTGGATGGATTCGAACCGAACGGCTACATTGAGGGCAAGCTTTCTCAAATGTCAAAAGAAGTGAAAGCCAGAATTGAGCCATTTGTGAAGACAACACCACGCCCTCTCAGATTACCTGATGGGCCTCCCTGTTCTCAGCGGTCGAAGTTCTTGCTGATGGATGCCCTTAAGTTGAGCATCGAAGACCCTAGCCATGAGGGGGAGGGCATACCGCTGTATGATGCAA<br>TCAAATGCATGAAGACATTTTTTGGCTGGAAAGAGCCCAACATCGTAAAGCCGCATGAGAAAGGCATAAACCCTAATTACCTCCTGGCTTGGAAGCAGGTGCTGGCAGAACTTCAAGACATTGAAAATGAGGAGAAATTTCCAAAAACAAAGAACATGAAGAAAACAAGCCAATTGAAGTGGGCACTTGGTGAGAACATGGCTCCAGAGAAAGTGGACTTTGAGGACTG<br>CAAAGATGTTAGCGATCTAAGACAGTACGACAGTGACGAACCAGAGTCTAGATCACTAGCAAGCTGGATTCAGAGTGAATTCAACAAGGCATGCGAACTGACAGATTCGAGTTGGATTGAACTTGATGAGATAGGGGAAGACGTTGCTCCAATCGAACACATTGCGAGTATGAGGAGGAACTATTTCACAGCGGAGGTATCCCATTGCAGGGCCACTGAATACATAATG<br>AAGGGAGTATACATAAACACAGCCCTATTGAATGCATCCTGTGCAGCCATGGATGACTTCCAACTGATTCCAATGATAAGCAAGTGCAGAACTAAAGAAGGAAGACGGAAGACAAATCTGTATGGATTCATTATAAAAGGAAGATCCCATTTGAGGAATGACACCGATGTGGTAAACTTTGTGAGCATGGAATTCTCTCTAACTGACCCGAGGCTAGAGCCACACAAATG<br>GGAAAAGTACTGTGTTCTTGAGATAGGAGACATGCTCCTACGGACTGCAATAGGCCAAGTGTCGAGGCCCATGTTCCTGTATGTGAGAACCAATGGGACTTCCAAGATCAAAATGAAATGGGGCATGGAGATGAGACGATGCCTTCTTCAGTCCCTTCAACAAATTGAGAGCATGATTGAGGCCGAATCTTCTGTCAAAGAGAAGGACATGTCCAAGGAATTCTTTGAAAA<br>CAAATCAGAAACATGGCCAATTGGAGAATCACCCAAAGGGGTGGAGGAAGGTTCTATTGGGAAAGTATGCAGAACATTGCTAGCAAAGTCTGTGTTCAACAGCCTATATGCATCTCCACAACTCGAGGGGTTTTCAGCTGAATCAAGAAAATTGCTTCTCATTGTTCAGGCACTTAGGGACAACCTGGAACCTGGAACCTTCGATCTTGGGGGGCTATATGAAGCAATTGA<br>GGAGTGCCTGATTAACGATCCCTGGGTTTTGCTTAATGCGTCTTGGTTCAACTCCTTCCTCACACATGCACTGAAATA-
EPI_ISL_8882218	>A/seagull/Italy/21VIR11259-12/2021_H5N1_2021-12-15<br>ATGGAAGACTTTGTGCGACAATGCTTCAATCCAATGATTGTCGAGCTTGCGGAAAAAGCAATGAAAGAATATGGGGAAGATCCGAAAATCGAGACAAACAAATTTGCCGCAATATGCACACACTTAGAAGTCTGTTTCATGTATTCGGATTTCCATTTTATCGATGAACGAGGCGAATCAATGATTGTAGAATCTGGCGATCCAAATGCATTATTGAAACACCGATTTGAGA<br>TAATCGAAGGGAGAGACCGGGCAATGGCCTGGACAGTGGTGAATAGTATTTGCAACACCACAGGGGTCGAAAAGCCCAAATTCCTCCCTGATTTGTATGACTACAAAGAGAACCGATTCATTGAAATTGGAGTAACGCGAAGGGAAGTTCACATATACTATTTAGAAAAAGCCAACAAGATAAAATCAGAGAAAACACACATTCACATATTCTCATTCACTGGAGAGGAA<br>ATGGCCACCAAGGCGGACTATACCCTTGATGAAGAGAGCAGAGCAAGAATAAAAACCAGACTGTTCACTATAAGACAAGAAATGGCCAGTAGAGGTCTATGGGATTCCTTTCGTCAGTCCGAAAGAGGCGAAGAGACAATTGAAGAAAGATTTGAAATCACAGGAACCATGCGCAGGCTTGCCGACCAAAGTCTCCCACCGAGCTTCTCCAGCCTTGAGAACTTTAGAG<br>CCTATGTGGATGGATTCGAACCGAACGGCTACATTGAGGGCAAGCTTTCTCAAATGTCAAAAGAAGTGAAAGCCAGAATTGAGCCATTTGTGAAGACAACACCACGCCCTCTCAGATTACCTGATGGGCCTCCCTGTTCTCAGCGGTCGAAGTTCTTGCTGATGGATGCCCTTAAGTTGAGCATCGAAGACCCTAGCCATGAGGGGGAGGGCATACCGCTGTATGATGCAA<br>TCAAATGCATGAAGACATTTTTTGGCTGGAAAGAGCCCAACATCGTAAAGCCGCATGAGAAAGGCATAAACCCTAATTACCTCCTGGCTTGGAAGCAGGTGCTGGCAGAACTTCAAGACATTGAAAATGAGGAGAAATTTCCAAAAACAAAGAACATGAAGAAAACAAGCCAGTTGAAGTGGGCACTTGGTGAGAACATGGCTCCAGAGAAAGTGGACTTTGAGGACTG<br>CAAAGATGTTAGCGATCTAAGACAGTACGACAGTGACGAACCAGAGTCTAGATCACTAGCAAGCTGGATTCAGAGTGAATTCAACAAGGCATGCGAACTGACAGATTCGAGTTGGATTGAACTTGATGAGATAGGGGAAGACGTTGCTCCAATCGAACACATAGCGAGTATGAGGAGGAACTATTTCACAGCGGAGGTATCCCATTGCAGGGCCACTGAATACATAATG<br>AAGGGAGTATACATAAACACAGCCCTATTGAATGCATCCTGTGCAGCCATGGATGACTTCCAACTGATTCCAATGATAAGCAAGTGCAGAACTAAAGAAGGAAGACGGAAGACAAATCTGTATGGATTCATTATAAAAGGAAGATCCCATTTGAGGAATGACACCGATGTGGTAAACTTTGTGAGCATGGAATTCTCTCTAACTGACCCGAGGCTAGAGCCACACAAATG<br>GGAAAAGTACTGTGTTCTTGAGATAGGAGACATGCTCCTACGGACTGCAATAGGCCAAGTGTCGAGGCCCATGTTCCTGTATGTGAGAACCAATGGGACTTCCAAGATCAAAATGAAATGGGGCATGGAGATGAGACGATGCCTTCTTCAGTCCCTTCAACAAATTGAGAGCATGATTGAGGCCGAATCTTCTGTCAAAGAGAAGGACATGTCCAAGGAATTCTTTGAAAA<br>CAAATCAGAAACATGGCCAATTGGAGAATCACCCAAAGGGGTGGAGGAAGGCTCTATTGGGAAAGTATGCAGAACATTGCTAGCAAAGTCTGTGTTCAACAGCCTATATGCATCTCCACAACTCGAGGGGTTTTCAGCTGAATCAAGGAAATTGCTCCTCATTGTTCAGGCACTTAGGGACAACCTGGAACCTGGAACCTTCGATCTTGGGGGGCTATATGAAGCAATTGA<br>GGAGTGCCTGATTAACGATCCCTGGGTTTTGCTTAATGCGTCTTGGTTCAACTCCTTCCTCACACATGCACTGAAATA-
EPI_ISL_6937114	>A/mute_swan/Poland/MB490-L1/2021_H5N1_2021-11-08<br>ATGGAAGACTTTGTGCGACAATGCTTCAATCCAATGATTGTCGAGCTTGCGGAAAAAGCAATGAAAGAATATGGGGAAGATCCGAAAATCGAGACAAACAAATTTGCCGCAATATGCACACACTTAGAAGTCTGTTTCATGTATTCGGATTTCCATTTTATCGATGAACGAGGCGAATCAATGATTGTAGAATCTGGCGATCCAAATGCATTATTGAAACACCGATTTGAGA<br>TAATCGAAGGGAGAGACCGGGCAATGGCCTGGACAGTGGTGAATAGTATCTGCAACACCACAGGGGTCGAAAAGCCCAAATTCCTCCCTGATTTGTATGACTACAAAGAGAACCGATTCATTGAAATTGGAGTAACGCGAAGGGAAGTTCACATATACTATTTAGAAAAAGCCAACAAGATAAAATCAGAGAAAACACACATTCACATATTCTCATTCACTGGAGAGGAA<br>ATGGCCACCAAGGCGGACTATACCCTTGATGAAGAGAGCAGAGCAAGAATAAAAACCAGACTGTTCACTATAAGACAAGAAATGGCCAGTAGAGGTCTATGGGATTCCTTTCGTCAGTCCGAAAGAGGCGAAGAGACAATTGAAGAAAGATTTGAAATCACAGGAACCATGCGCAGGCTTGCCGACCAAAGTCTCCCACCGAGCTTCTCCAGCCTTGAGAACTTTAGAG<br>CCTATGTGGATGGGTTCGAACCGAACGGCTACATTGAGGGCAAGCTTTCTCAAATGTCAAAAGAAGTGAAAGCCAGAATTGAGCCATTTGTGAAGACAACACCACGCCCTCTCAGATTACCTGATGGGCCTCCCTGTTCTCAGCGGTCGAAGTTCTTGCTGATGGATGCCCTTAAGTTGAGCATCGAAGACCCTAGCCATGAGGGGGAGGGCATACCGCTGTATGATGCAA<br>TCAAATGCATGAAGACATTTTTTGGCTGGAAAGAGCCCAACATCGTAAAGCCGCATGAGAAAGGCATAAACCCTAATTACCTCCTGGCTTGGAAGCAGGTGCTGGCAGAACTTCAAGACATTGAAAATGAGGAGAAATTTCCAAAAACAAAGAACATGAAGAAAACAAGCCAGTTGAAGTGGGCACTTGGTGAGAACATGGCTCCAGAGAAAGTGGACTTTGAGGACTG<br>CAAAGATGTTAGCGATCTAAGACAGTACGACAGTGACGAACCAGAGTCTAGATCACTAGCAAGCTGGATTCAGAGTGAATTCAACAAGGCATGCGAACTGACAGATTCGAGTTGGATTGAACTTGATGAGATAGGGGAAGACGTTGCTCCAATCGAACACATAGCGAGTATGAGGAGGAACTATTTCACAGCGGAGGTATCCCATTGCAGGGCCACTGAATACATAATG<br>AAGGGAGTATACATAAACACAGCCCTATTGAATGCATCCTGTGCAGCCATGGATGACTTCCAACTGATTCCAATGATAAGCAAGTGCAGAACTAAAGAAGGAAGACGGAAGACAAATCTGTATGGATTCATTATAAAAGGAAGATCCCATTTGAGGAATGACACCGATGTGGTAAACTTTGTGAGCATGGAATTCTCTCTAACTGACCCGAGGCTAGAGCCACACAAATG<br>GGAAAAGTACTGTGTTCTTGAGATAGGAGACATGCTCCTACGGACTGCAATAGGCCAAGTGTCGAGGCCCATGTTCCTGTATGTGAGAACCAATGGGACTTCCAAGATCAAAATGAAATGGGGCATGGAGATGAGACGATGCCTTCTTCAGTCCCTTCAACAAATTGAGAGCATGATTGAGGCYGAATCTTCTGTCAAAGAGAAGGACATGTCCAAGGAATTCTTTGAAAA<br>CAAATCAGAAACATGGCCAATTGGAGAATCACCCAAAGGGGTGGAGGAAGGCTCTATTGGGAAAGTATGCAGAACATTGCTAGCAAAGTCTGTGTTCAACAGCCTATATGCATCTCCACAACTCGAGGGGTTTTCAGCTGAATCAAGGAAATTGCTTCTCATTGTTCAGGCACTTAGGGACAACCTGGAACCTGGAACCTTCGATCTTGGGGGGCTATATGAAGCAATTGA<br>GGAGTGCCTGATTAACGATCCCTGGGTTTTGCTTAATGCGTCTTGGTTCAACTCCTTCCTCACACATGCACTGAAATA-
EPI_ISL_3135897	>A/Turkey/Hungary/16603/2021_H5N1_2021-04-13<br>ATGGAAGACTTTGTGCGACAATGCTTCAATCCAATGATTGTCGAGCTTGCGGAAAAAGCAATGAAAGAATATGGGGAAGATCCGAAAATCGAGACAAACAAATTTGCCGCAATATGCACACACTTAGAAGTCTGTTTCATGTATTCGGATTTCCATTTTATTGATGAACGAGGCGAATCAATGATTGTAGAATCTGGCGATCCAAATGCATTATTGAAACACCGATTTGAGA<br>TAATCGAAGGGAGAGACCGAGCAATGGCCTGGACAGTGGTGAATAGTATCTGCAACACCACAGGAGTCGAAAAGCCCAAATTCCTCCCTGATTTGTATGACTACAAAGAGAACCGATTCATTGAAATTGGAGTAACGCGAAGGGAAGTTCACATATACTATTTAGAAAAAGCCAACAAGATAAAATCAGAGAAAACACACATTCACATATTCTCGTTCACTGGAGAGGAA<br>ATGGCCACCAAGGCGGAATACACCCTTGATGAAGAGAGCAGAGCAAGAATAAAAACCAGACTGTTCACTATAAGACAAGAAATGGCCAGTAGAGGTCTATGGGATTCCTTTCGTCAGTCCGAGAGAGGCGAAGAGACAATTGAAGAAAGATTTGAAATCACAGGAACCATGCGCAGGCTTGCCGACCAAAGTCTCCCACCGAACTTCTCCAGCCTTGAAAACTTTAGAG<br>CCTATGTGGATGGATTCGAACCGAACGGCTGCATTGAGGGCAAGCTTTCTCAAATGTCAAAAGAAGTGAACGCCAGAATTGAGCCATTTCTGAAGACAACACCACGCCCTCTCAGATTACCTGATGGGCCTCCCTGTTCTCAGCGGTCGAAGTTCTTGCTGATGGATGCCCTTAAGTTGAGCATCGAAGACCCTAGCCATGAGGGGGAGGGCATACCGCTGTATGATGCAA<br>TCAAATGCATGAAGACATTTTTTGGCTGGAAAGAGCCCAACATCGTAAAGCCGCATGAGAAAGGCATAAACCCTAATTACCTCCTGGCTTGGAAGCAGGTGCTGGCAGAACTTCAAGATATTGAAAATGAGGAGAAAATTCCAAAAACAAAGAACATGAAGAAAACAAGCCAATTGAAGTGGGCACTTGGTGAGAACATGGCACCAGAAAAAGTGGACTTTGGGGATTG<br>CAAAGATGTTAGCGATCTAAGACAGTACGACAGTGATGAACCAGAGTCTAGATCACTAGCAAGCTGGATTCAGAGTGAATTCAACAAGGCATGCGAATTGACAGATTCGAGTTGGATTGAACTTGATGAAATAGGGGAAGACGTTGCTCCAATCGAACACATTGCGAGTATGAGGAGGAACTATTTCACAGCGGAGGTATCCCATTGCAGGGCCACTGAATACATAATGA<br>AGGGAGTATACATAAACACAGCCCTATTGAATGCATCCTGTGCAGCCATGGATGACTTCCAACTGATTCCAATGATAAGCAAATGCAGAACCAAAGAAGGAAGACGGAAGACAAATCTGTATGGATTCATTATAAAAGGAAGATCCCATTTGAGGAATGACACCGATGTGGTAAACTTTGTGAGCATGGAATTCTCTCTAACTGACCCGAGGCTAGAGCCACACAAATGG<br>GAAAAGTACTGTGTTCTTGAGATAGGAGACATGCTCCTACGGACTGCAATAGGCCAAGTGTCGAGGCCCATGTTCCTGTATGTGAGAACCAATGGAACTTCCAAGATCAAAATGAAATGGGGCATGGAGATGAGACGATGCCTTCTTCAGTCCCTTCAACAAATTGAGAGCATGATTGAGGCCGAATCTTCTGTCAAAGAGAAGGACATGACCAAGGAATTCTTTGAAAAC<br>AAATCAGAAACATGGCCAATTGGAGAATCACCCAAAGGGGTGGAGGAAGGCTCTATTGGGAAAGTATGCAGAACATTGCTAGCAAAGTCTGTGTTCAACAGCCTATATGCATCTCCACAACTCGAGGGGTTTTCAGCTGAATCAAGAAAATTGCTTCTCATTGTTCAGGCACTTAGGGACAACCTGGAACCTGGAACCTTCGATCTTGGGGGGCTATATGAAGCAATTGAG<br>GAGTGCCTGATTAACGATCCCTGGGTTTTGCTTAATGCGTCTTGGTTCAACTCCTTCCTCACACATGCACTGAAATA-
EPI_ISL_2681045	>A/white_stork/Poland/MB391/2021_H5N1_2021-04-20<br>ATGGAAGACTTTGTGCGACAATGCTTCAATCCAATGATTGTCGAGCTTGCGGAAAAAGCAATGAAAGAATATGGGGAAGATCCGAAAATCGAGACAAACAAATTTGCCGCAATATGCACACACTTAGAAGTCTGTTTCATGTATTCGGATTTCCATTTTATTGATGAACGAGGCGAATCAATGATTGTAGAATCTGGCGATCCAAATGCATTATTGAAACACCGATTTGAGA<br>TAATCGAAGGGAGAGACCGAGCAATGGCCTGGACAGTGGTGAATAGTATCTGCAACACCACAGGAGTCGAAAAGCCCAAATTCCTCCCTGATTTGTATGACTACAAAGAGAACCGATTCATTGAAATTGGAGTAACGCGAAGGGAAGTTCACATATACTATTTAGAAAAAGCCAACAAGATAAAATCAGAGAAAACACACATTCACATATTCTCATTCACTGGAGAGGAA<br>ATGGCCACCAAGGCGGAATACACCCTTGATGAAGAGAGCAGAGCAAGAATAAAAACCAGACTGTTCACTATAAGACAAGAAATGGCCAGTAGAGGTCTATGGGATTCCTTTCGTCAGTCCGAGAGAGGCGAAGAGACAATTGAAGAAAGATTTGAAATCACAGGAACCATGCGCAGGCTTGCCGACCAAAGTCTCCCACCGAACTTCTCCAGCCTTGAAAACTTTAGAG<br>CCTATGTGGATGGATTCGAACCGAACGGCTGCATTGAGGGCAAGCTTTCTCAAATGTCAAAAGAAGTGAACGCCAGAATTGAGCCATTTCTGAAGACAACACCACGCCCTCTCAGATTACCTGATGGGCCTCCCTGTTCTCAGCGGTCGAAGTTCTTGCTGATGGATGCCCTTAAGTTGAGCATCGAAGACCCTAGCCATGAGGGGGAGGGCATACCGCTGTATGATGCAA<br>TCAAATGCATGAAGACATTTTTTGGCTGGAAAGAGCCCAACATCGTAAAGCCGCATGAGAAAGGCATAAACCCTAATTACCTCCTGGCTTGGAAGCAGGTGCTGGCAGAACTTCAAGATATTGAAAATGAGGAGAAAATTCCAAAAACAAAGAACATGAAGAAAACAAGCCAATTGAAGTGGGCACTTGGTGAGAACATGGCACCAGAAAAAGTGGACTTTGGGGATTG<br>CAAAGATGTTAGCGATCTAAGACAGTACGACAGTGATGAACCAGAGTCTAGATCACTAGCAAGCTGGATTCAGAGTGAATTCAACAAGGCATGCGAATTGACAGATTCGAGTTGGATTGAACTTGATGAGATAGGGGAAGACGTTGCTCCAATCGAACACATTGCGAGTATGAGGAGGAACTATTTCACAGCGGAGGTATCCCATTGCAGGGCCACTGAATACATAATG<br>AAGGGAGTATACATAAACACAGCCCTATTGAATGCATCCTGTGCAGCCATGGATGACTTCCAACTGATTCCAATGATAAGCAAATGCAGAACCAAAGAAGGAAGACGGAAGACAAATCTGTATGGATTCATTATAAAAGGAAGATCCCATTTGAGGAATGACACCGATGTGGTAAACTTTGTGAGCATGGAATTCTCTCTAACTGACCCGAGGCTAGAGCCACACAAATG<br>GGAAAAGTACTGTGTTCTTGAGATAGGAGACATGCTCCTACGGACTGCAATAGGCCAAGTGTCGAGGCCCATGTTCCTGTATGTGAGAACCAATGGAACTTCCAAGATCAAAATGAAATGGGGCATGGAGATGAGACGATGCCTTCTTCAGTCCCTTCAACAAATTGAGAGCATGATTGAGGCCGAATCTTCTGTCAAAGAGAAGGACATGACCAAGGAATTCTTTGAAA<br>ACAAATCAGAAACATGGCCAATTGGAGAATCACCCAAAGGGGTGGAGGAAGGCTCTATTGGGAAAGTATGCAGAACATTGCTAGCAAAGTCTGTGTTCAACAGCCTATATGCATCTCCACAACTCGAGGGGTTTTCAGCTGAATCAAGAAAATTGCTTCTCATTGTTCAGGCACTTAGGGACAACCTGGAACCTGGAACCTTCGATCTTGGGGGGCTATATGAAGCAATTG<br>AGGAGTGCCTGATTAACGATCCCTGGGTTTTGCTTAATGCGTCTTGGTTCAACTCCTTCCTCACACATGCACTGAAATA-
EPI_ISL_1048240	>A/common_buzzard/Netherlands/21021396-002/2021_H5N1_2021-01-09<br>ATGGAAGACTTTGTGCGACAATGCTTCAATCCAATGATTGTCGAGCTTGCGGAAAAAGCAATGAAAGAATATGGGGAAGATCCGAAAATCGAGACAAACAAATTTGCCGCAATATGCACACACTTAGAAGTCTGTTTCATGTATTCGGATTTCCATTTTATTGATGAACGAGGCGAATCAATGATTGTAGAATCTGGCGATCCAAATGCATTATTGAAACACCGATTTGAGA<br>TAATCGAAGGGAGAGACCGAGCAATGGCCTGGACAGTGGTGAATAGTATCTGCAACACCACAGGAGTCGAAAAGCCCAAATTCCTCCCTGATTTGTATGACTACAAAGAGAACCGATTCATTGAAATTGGAGTAACGCGAAGGGAAGTTCACATATACTATTTAGAAAAAGCCAACAAGATAAAATCAGAGAAAACACACATTCACATATTCTCATTCACTGGAGAGGAA<br>ATGGCCACCAAGGCGGACTACACCCTTGATGAAGAGAGCAGAGCAAGAATAAAAACCAGACTGTTCACTATAAGACAAGAAATGGCCAGTAGAGGTCTATGGGATTCCTTTCGTCAGTCCGAGAGAGGCGAAGAGACAATTGAAGAAAGATTTGAAATCACAGGAACCATGCGCAGGCTTGCCGACCAAAGTCTCCCACCGAACTTCTCCAGCCTTGAAAACTTTAGAG<br>CCTATGTGGATGGATTCGAACCGAACGGCTGCATTGAGGGCAAGCTTTCTCAAATGTCAAAAGAAGTGAGCGCCAGAATTGAGCCATTTCTGAAGACAACACCACGCCCTCTCAGATTACCTGATGGGCCTCCCTGTTCTCAGCGGTCGAAGTTCTTGCTGATGGATGCCCTTAAGTTGAGCATCGAAGACCCTAGCCATGAGGGGGAGGGCATACCGCTGTATGATGCAA<br>TCAAATGCATGAAGACATTTTTTGGCTGGAAAGAGCCCAACATCGTAAAGCCGCATGAGAAAGGCATAAACCCTAATTACCTCCTGGCTTGGAAGCAGGTGCTGGCAGAACTTCAAGACATTGAAAATGAGGAGAAAATTCCAAAAACAAAGAACATGAAGAAAACAAGCCAATTGAAGTGGGCACTTGGTGAGAACATGGCACCAGAAAAAGTGGACTTTGAGGACT<br>GCAAAGATGTTAGCGATCTAAGACAGTACGACAGTGATGAACCAGAGTCTAGATCACTAGCAAGCTGGATTCAGAGTGAATTCAACAAGGCATGCGAACTGACAGATTCGAGTTGGATTGAACTTGATGAGATAGGGGAAGACGTTGCTCCAATCGAACACATTGCGAGTATGAGGAGGAACTATTTCACAGCGGAGGTATCCCATTGCAGGGCCACTGAATACATAAT<br>GAAGGGAGTATACATAAACACAGCCCTATTGAATGCATCCTGTGCAGCCATGGATGACTTCCAACTGATTCCAATGATAAGCAAGTGCAGAACCAAAGAAGGAAGACGGAAGACAAATCTGTATGGATTCATTATAAAAGGAAGATCCCATTTGAGGAATGACACCGATGTGGTAAACTTTGTGAGCATGGAATTCTCTCTAACTGACCCGAGGCTAGAGCCACACAAAT<br>GGGAAAAGTACTGTGTTCTTGAGATAGGAGACATGCTCCTACGGACTGCAATAGGCCAAGTGTCGAGGCCCATGTTCCTGTATGTGAGAACCAATGGGACTTCCAAGATCAAAATGAAATGGGGCATGGAGATGAGACGATGCCTTCTTCAGTCCCTTCAACAAATTGAGAGCATGATTGAGGCCGAATCTTCTGTCAAAGAGAAGGACATGTCCAAGGAATTCTTTGAA<br>AACAAATCAGAAACATGGCCAATTGGAGAATCACCCAAAGGGGTGGAGGAAGGCTCTATTGGGAAAGTATGCAGAACATTGCTAGCAAAGTCTGTGTTCAACAGCCTATATGCATCTCCACAACTCGAGGGGTTTTCAGCTGAATCAAGAAAATTGCTTCTCATTGTTCAGGCACTTAGGGACAACCTGGAACCTGGAACCTTCGATCTTGGGGGGCTATATGAAGCAATT<br>GAGGAGTGCCTGATTAACGATCCCTGGGTTTTGCTTAATGCGTCTTGGTTCAACTCCTTCCTCACACATGCACTGAAATA-
EPI_ISL_632315	>A/eurasian_teal/Netherlands/20016896-013/2020_H5N1_2020-11-02<br>ATGGAAGACTTTGTGCGACAATGCTTCAATCCAATGATTGTCGAGCTTGCGGAAAAAGCAATGAAAGAATATGGGGAAGATCCGAAAATCGAGACAAACAAATTTGCCGCAATATGCACACACTTAGAAGTCTGTTTCATGTATTCGGATTTCCATTTTATTGATGAACGAGGCGAATCAATGATTGTAGAATCTGGCGATCCAAATGCATTATTGAAACACCGATTTGAGA<br>TAATCGAAGGGAGAGACCGAGCAATGGCCTGGACAGTGGTGAATAGTATCTGCAACACCACAGGAGTCGAAAAGCCCAAATTCCTCCCTGATTTGTATGACTACAAAGAGAACCGATTCATTGAAATTGGAGTAACGCGAAGGGAAGTTCACATATACTATTTAGAAAAAGCCAACAAGATAAAATCAGAGAAAACACACATTCACATATTCTCATTCACTGGAGAGGAA<br>ATGGCCACCAAGGCGGACTACACCCTTGATGAAGAGAGCAGAGCAAGAATAAAAACCAGACTGTTCACTATAAGACAAGAAATGGCCAGTAGAGGTCTATGGGATTCCTTTCGTCAGTCCGAGAGAGGCGAAGAGACAATTGAAGAAAGATTTGAAATCACAGGAACCATGCGCAGGCTTGCCGACCAAAGTCTCCCACCGAACTTCTCCAGCCTTGAAAACTTTAGAG<br>CCTATGTGGATGGATTCGAACCGAACGGCTGCATTGAGGGCAAGCTTTCTCAAATGTCAAAAGAAGTGAACGCCAGAATTGAGCCATTTCTGAAGACAACACCACGCCCTCTCAGATTACCTGATGGGCCTCCCTGTTCTCAGCGGTCGAAGTTCTTGCTGATGGATGCCCTTAAGTTGAGCATCGAAGACCCTAGCCATGAGGGGGAGGGCATACCGCTGTATGATGCAA<br>TCAAATGCATGAAGACATTTTTTGGCTGGAAAGAGCCCAACATCGTAAAGCCGCATGAGAAAGGCATAAACCCTAATTACCTCCTGGCTTGGAAGCAGGTGCTGGCAGAACTTCAAGACATTGAAAATGAGGAGAAAATTCCAAAAACAAAGAACATGAAGAAAACAAGCCAATTGAAGTGGGCACTTGGTGAGAACATGGCACCAGAAAAAGTGGACTTTGAGGACT<br>GCAAAGATGTTAGCGATCTAAGACAGTACGACAGTGACGAACCAGAGTCTAGATCACTAGCAAGCTGGATTCAGAGTGAATTCAACAAGGCATGCGAACTGACAGATTCGAGTTGGATTGAACTTGATGAGATAGGGGAAGACGTTGCTCCAATCGAACACATTGCGAGTATGAGGAGGAACTATTTCACAGCGGAGGTATCCCATTGCAGGGCCACTGAATACATAAT<br>GAAGGGAGTATACATAAACACAGCCCTATTGAATGCATCCTGTGCAGCCATGGATGACTTCCAACTGATTCCAATGATAAGCAAGTGCAGAACCAAAGAAGGAAGACGGAAGACAAATCTGTATGGATTCATTATAAAAGGAAGATCCCATTTGAGGAATGACACCGATGTGGTAAACTTTGTGAGCATGGAATTCTCTCTAACTGACCCGAGGCTAGAGCCACACAAAT<br>GGGAAAAGTACTGTGTTCTTGAGATAGGAGACATGCTCCTACGGACTGCAATAGGCCAAGTGTCGAGGCCCATGTTCCTGTATGTGAGAACCAATGGGACTTCCAAGATCAAAATGAAATGGGGCATGGAGATGAGACGATGCCTTCTTCAGTCCCTTCAACAAATTGAGAGCATGATTGAGGCCGAATCTTCTGTCAAAGAGAAGGACATGTCCAAGGAATTCTTTGAA<br>AACAAATCAGAAACATGGCCAATTGGAGAATCACCCAAAGGGGTGGAGGAAGGCTCTATTGGGAAAGTATGCAGAACATTGCTAGCAAAGTCTGTGTTCAACAGCCTATATGCATCTCCACAACTCGAGGGGTTTTCAGCTGAATCAAGAAAATTGCTTCTCATTGTTCAGGCACTTAGGGACAACCTGGAACCTGGAACCTTCGATCTTGGGGGGCTATATGAAGCAATT<br>GAGGAGTGCCTGATTAACGATCCCTGGGTTTTGCTTAATGCGTCTTGGTTCAACTCCTTCCTCACACATGCACTGAAATA-
EPI_ISL_632314	>A/greylag_goose/Netherlands/20016582-004/2020_H5N1_2020-10-28<br>ATGGAAGACTTTGTGCGACAATGCTTCAATCCAATGATTGTCGAGCTTGCGGAAAAAGCAATGAAAGAATATGGGGAAGATCCGAAAATCGAGACAAACAAATTTGCCGCAATATGCACACACTTAGAAGTCTGTTTCATGTATTCGGATTTCCATTTTATTGATGAACGAGGCGAATCAATGATTGTAGAATCTGGCGATCCAAATGCATTATTGAAACACCGATTTGAGA<br>TAATCGAAGGGAGAGACCGAGCAATGGCCTGGACAGTGGTGAATAGTATCTGCAACACCACAGGAGTCGAAAAGCCCAAATTCCTCCCTGATTTGTATGACTACAAAGAGAACCGATTCATTGAAATTGGAGTAACGCGAAGGGAAGTTCACATATACTATTTAGAAAAAGCCAACAAGATAAAATCAGAGAAAACACACATTCACATATTCTCATTCACTGGAGAGGAA<br>ATGGCCACCAAGGCGGACTACACCCTTGATGAAGAGAGCAGAGCAAGAATAAAAACCAGACTGTTCACTATAAGACAAGAAATGGCCAGTAGAGGTCTATGGGATTCCTTTCGTCAGTCCGAGAGAGGCGAAGAGACAATTGAAGAAAGATTTGAAATCACAGGAACCATGCGCAGGCTTGCCGACCAAAGTCTCCCACCGAACTTCTCCAGCCTTGAAAACTTTAGAG<br>CCTATGTGGATGGATTCGAACCGAACGGCTGCATTGAGGGCAAGCTTTCTCAAATGTCAAAAGAAGTGAACGCCAGAATTGAGCCATTTCTGAAGACAACACCACGCCCTCTCAGATTACCTGATGGGCCTCCCTGTTCTCAGCGGTCGAAGTTCTTGCTGATGGATGCCCTTAAGTTGAGCATCGAAGACCCTAGCCATGAGGGGGAGGGCATACCGCTGTATGATGCAA<br>TCAAATGCATGAAGACATTTTTTGGCTGGAAAGAGCCCAACATCGTAAAGCCGCATGAGAAAGGCATAAACCCTAATTACCTCCTGGCTTGGAAGCAGGTGCTGGCAGAACTTCAAGACATTGAAAATGAGGAGAAAATTCCAAAAACAAAGAACATGAAGAAAACAAGCCAATTGAAGTGGGCACTTGGTGAGAACATGGCACCAGAAAAAGTGGACTTTGAGGACT<br>GCAAAGATGTTAGCGATCTAAGACAGTACGACAGTGACGAACCAGAGTCTAGATCACTAGCAAGCTGGATTCAGAGTGAATTCAACAAGGCATGCGAACTGACAGATTCGAGTTGGATTGAACTTGATGAGATAGGGGAAGACGTTGCTCCAATCGAACACATTGCGAGTATGAGGAGGAACTATTTCACAGCGGAGGTATCCCATTGCAGGGCCACTGAATACATAAT<br>GAAGGGAGTATACATAAACACAGCCCTATTGAATGCATCCTGTGCAGCCATGGATGACTTCCAACTGATTCCAATGATAAGCAAGTGCAGAACCAAAGAAGGAAGACGGAAGACAAATCTGTATGGATTCATTATAAAAGGAAGATCCCATTTGAGGAATGACACCGATGTGGTAAACTTTGTGAGCATGGAATTCTCTCTAACTGACCCGAGGCTAGAGCCACACAAAT<br>GGGAAAAGTACTGTGTTCTTGAGATAGGAGACATGCTCCTACGGACTGCAATAGGCCAAGTGTCGAGGCCCATGTTCCTGTATGTGAGAACCAATGGGACTTCCAAGATCAAAATGAAATGGGGCATGGAGATGAGACGATGCCTTCTTCAGTCCCTTCAACAAATTGAGAGCATGATTGAGGCCGAATCTTCTGTCAAAGAGAAGGACATGTCCAAGGAATTCTTTGAA<br>AACAAATCAGAAACATGGCCAATTGGAGAATCACCCAAAGGGGTGGAGGAAGGCTCTATTGGGAAAGTATGCAGAACATTGCTAGCAAAGTCTGTGTTCAACAGCCTATATGCATCTCCACAACTCGAGGGGTTTTCAGCTGAATCAAGAAAATTGCTTCTCATTGTTCAGGCACTTAGGGACAACCTGGAACCTGGAACCTTCGATCTTGGGGGGCTATATGAAGCAATT<br>GAGGAGTGCCTGATTAACGATCCCTGGGTTTTGCTTAATGCGTCTTGGTTCAACTCCTTCCTCACACATGCACTGAAATA-
EPI_ISL_1665262	>A/mute_swan/Slovakia/Pah6_21VIR1086-2/2021_H5N5_2021-01-15<br>ATGGAAGACTTTGTGCGACAATGCTTCAATCCAATGATTGTCGAGCTTGCGGAAAAAGCAATGAAAGAATATGGGGAAGATCCGAAAATCGAGACAAACAAATTTGCCGCAATATGCACACACTTAGAAGTCTGTTTCATGTATTCGGATTTCCATTTTATTGATGAACGAGGCGAATCAATGATTGTAGAATCTGGCGATCCAAATGCATTATTGAAACACCGATTTGAGA<br>TAATCGAAGGGAGAGACCGAGCAATGGCCTGGACAGTGGTGAATAGTATCTGCAACACCACAGGAGTCGAAAAGCCCAAATTCCTCCCTGATTTGTATGACTATAAAGAGAACCGATTCATTGAAATTGGAGTAACGCGAAGGGAAGTTCACATATACTATTTAGAAAAAGCCAACAAGATAAAATCAGAGAAAACACACATTCACATATTCTCATTCACTGGAGAGGAA<br>ATGGCCACCAAGGCGGACTACACCCTTGATGAAGAGAGCAGAGCAAGAATAAAAACCAGACTGTTCACTATAAGACAAGAAATGGCCAGTAGAGGTCTATGGGATTCCTTTCGTCAGTCCGAGAGAGGCGAAGAGACAATTGAAGAAAGATTTGAAATCACAGGAACCATGCGCAGGCTTGCCGACCAAAGTCTCCCACCGAACTTCTCCAGCCTTGAAAACTTTAGAG<br>CCTATGTGGATGGATTCGAACCGAACGGCTGCATTGAGGGCAAGCTTTCTCAAATGTCAAAAGAAGTGAACGCCAGAATTGAGCCATTTCTGAAGACAACACCACGCCCTCTCAGATTACCTGATGGGCCTCCCTGTTCTCAGCGGTCGAAGTTCTTGCTGATGGATGCCCTTAAGTTGAGCATCGAAGACCCTAGCCATGAGGGGGAGGGCATACCGCTGTATGATGCAA<br>TCAAATGCATGAAGACATTTTTTGGCTGGAAAGAGCCCAACATCGTAAAGCCGCATGAGAAAGGCATAAACCCTAATTACCTCCTGGCTTGGAAGCAGGTGCTGGCAGAACTTCAAGACATTGAAAATGAGGAGAAAATTCCAAAAACAAAGAACATGAAGAAAACAAGCCAATTGAAGTGGGCACTTGGTGAGAACATGGCACCAGAAAAAGTGGACTTTGAGGACT<br>GCAAAGATGTTAGCGATCTAAGACAGTACGACAGTGACGAACCAGAGTCTAGATCACTAGCAAGCTGGATTCAGAGTGAATTCAACAAGGCATGCGAACTGACAGATTCGGGTTGGATTGAACTTGATGAGATAGGGGAAGACGTTGCTCCAATCGAACACATTGCGAGTATGAGGAGGAACTATTTCACAGCGGAGGTATCCCATTGCAGGGCCACTGAATACATAAT<br>GAAGGGAGTATACATAAACACAGCCCTATTGAATGCATCCTGTGCAGCCATGGATGACTTCCAACTGATTCCAATGATAAGCAAGTGCAGAACCAAAGAAGGAAGACGGAAGACAAATCTGTATGGATTCATTATAAAAGGAAGATCCCATTTGAGGAATGACACCGATGTGGTAAACTTTGTGAGCATGGAATTCTCTCTAACTGACCCGAGGCTAGAGCCACACAAAT<br>GGGAAAAGTACTGTGTTCTTGAGATAGGAGACATGCTCCTACGGACTGCAATAGGCCAAGTGTCGAGGCCCATGTTCCTGTATGTGAGAACCAATGGGACTTCCAAGATCAAAATGAAATGGGGCATGGAGATGAGACGATGCCTTCTTCAGTCCCTTCAACAAATTGAGAGCATGATTGAGGCCGAATCTTCTGTCAAAGAGAAGGACATGACCAAGGAATTCTTTGAA<br>AACAAATCAGAAACATGGCCAATTGGAGAATCACCCAAAGGGGTGGAGGAAGGCTCTATTGGGAAAGTATGCAGAACATTGCTAGCAAAGTCTGTGTTCAACAGCCTATATGCATCTCCACAACTCGAGGGGTTTTCAGCTGAATCAAGAAAATTGCTTCTCATTGTTCAGGCACTTAGGGACAACCTGGAACCTGGAACCTTCGATCTTGGGGGGCTATATGAAGCAATT<br>GAGGAGTGCCTGATTAACGATCCCTGGGTTTTGCTTAATGCGTCTTGGTTCAACTCCTTCCTCACACATGCACTGAAATA-
EPI_ISL_3102081	>A/mute_swan/Romania/11981-1_21VIR3163-5/2021_H5N5_2021-04-08<br>ATGGAAGACTTTGTGCGACAATGCTTCAATCCAATGATTGTCGAGCTTGCGGAAAAAGCAATGAAAGAATATGGGGAAGATCCGAAAATCGAGACAAACAAATTTGCCGCAATATGCACACACTTAGAAGTCTGTTTCATGTATTCGGATTTCCATTTTATTGATGAACGAGGCGAATCAATGATTGTAGAATCTGGCGATCCAAATGCATTATTGAAACACCGATTTGAGA<br>TAATCGAGGGGAGAGACCGAGCAATGGCCTGGACAGTGGTGAATAGTATCTGCAACACCACAGGAGTCGAAAAGCCCAAATTCCTCCCTGATTTGTATGACTACAAAGAGAACCGATTCATTGAAATTGGAGTAACGCGAAGGGAAGTTCACATATACTATTTAGAAAAAGCCAACAAGATAAAATCAGAGAAAACACACATTCACATATTCTCATTCACTGGAGAGGAA<br>ATGGCCACCAAGGCGGACTACACCCTTGATGAAGAGAGCAGAGCAAGAATAAAAACCAGACTGTTCACTATAAGACAAGAAATGGCCAGTAGAGGTCTATGGGATTCCTTTCGTCAGTCCGAGAGAGGCGAAGAGACAATTGAAGAAAGATTTGAAATCACAGGAACCATGCGCAGGCTTGCCGACCAAAGTCTCCCACCGAACTTCTCCAGCCTTGAAAACTTTAGAG<br>CCTATGTGGATGGATTCGAACCGAACGGCTGCATTGAGGGCAAGCTTTCTCAAATGTCAAAAGAAGTGAACGCCAGAATTGAGCCATTTCTGAAGACAACACCACGCCCTCTCAGATTACCTGATGGGCCTCCCTGTTCTCAGCGGTCGAAGTTCTTGCTGATGGATGCCCTTAAGTTGAGCATCGAAGACCCTAGCCATGAGGGGGAGGGCATACCGCTGTATGATGCAA<br>TCAAATGCATGAAGACATTTTTTGGCTGGAAAGAGCCCAACATCGTAAAGCCGCATGAGAAAGGCATAAACCCTAATTACCTCCTGGCTTGGAAGCAGGTGCTGGCAGAACTTCAAGACATTGAAAATGAGGAGAAAATTCCAAAAACAAAGAACATGAAGAAAACAAGCCAATTGAAGTGGGCACTTGGTGAGAACATGGCACCAGAAAAAGTGGACTTTGAGGACT<br>GCAAAGATGTTAGCGATCTAAGACAGTACGACAGTGACGAACCAGAGTCTAGATCACTAGCAAGCTGGATTCAGAGTGAATTCAACAAGGCATGCGAACTGACAGATTCGGGTTGGATTGAACTTGATGAGATAGGGGAAGACGTTGCTCCAATCGAACACATTGCGAGTATGAGGAGGAACTATTTCACAGCGGAGGTATCCCATTGCAGGGCCACTGAATACATAAT<br>GAAGGGAGTATACATAAACACAGCCCTATTGAATGCATCCTGTGCAGCCATGGATGACTTCCAACTGATTCCAATGATAAGCAAGTGCAGAACCAAAGAAGGAAGACGGAAGACAAATCTGTATGGATTCATTATAAAAGGAAGATCCCATTTGAGGAATGACACCGATGTGGTAAACTTTGTGAGCATGGAATTCTCTCTAACTGACCCGAGGCTAGAGCCACACAAAT<br>GGGAAAAGTACTGTGTTCTTGAGATAGGAGACATGCTCCTACGGACTGCAATAGGCCAAGTGTCGAGGCCCATGTTCCTGTATGTTAGAACCAATGGGACTTCCAAGATCAAAATGAAATGGGGCATGGAGATGAGACGATGCCTTCTTCAGTCCCTTCAACAAATTGAGAGCATGATTGAGGCCGAATCTTCTGCCAAAGAGAAGGACATGACCAAGGAATTCTTTGAA<br>AACAAATCAGAAACATGGCCAATTGGAGAATCACCCAAAGGGGTGGAGGAAGGCTCTATTGGGAAAGTATGCAGAACATTGCTAGCAAAGTCTGTGTTCAACAGCCTATATGCATCTCCACAACTCGAGGGGTTTTCAGCTGAATCAAGAAAATTGCTTCTCATTGTTCAGGCACTTAGGGACAACCTGGAACCTGGAACCTTCGATCTTGGGGGGCTATATGAAGCAATT<br>GAGGAGTGCCTGATTAACGATCCCTGGGTTTTGCTTAATGCGTCTTGGTTCAACTCCTTCCTCACACATGCACTGAAATA-
EPI_ISL_6328409	>A/mute_swan/Czech_Republic/21312/2021_H5N1_2021-11-01<br>ATGGAAGACTTTGTGCGACAATGCTTCAATCCAATGATTGTCGAGCTTGCGGAAAAAGCAATGAAAGAATATGGGGAAGATCCGAAAATCGAGACAAACAAATTTGCCGCAATATGCACACACTTAGAAGTCTGTTTCATGTATTCGGATTTCCATTTTATTGATGAACGAGGCGAATCAATGATTGTAGAATCTGGCGATCCAAATGCATTATTGAAACACCGATTTGAGA<br>TAATCGAAGGGAGAGACCGAGCAATGGCCTGGACAGTGGTGAATAGTATCTGCAACACCACAGGAGTCGAAAAGCCCAAATTCCTCCCTGATTTGTATGACTACAAAGAGAACCGATTCATTGAAATTGGAGTAACGCGAAGGGAAGTTCACATATACTATTTAGAAAAAGCCAACAAGATAAAATCAGAGAAAACACACATTCACATATTCTCATTCACTGGAGAGGAA<br>ATGGCCACCAAGGCGGAATACACCCTTGATGAAGAGAGCAGAGCAAGAATAAAAACCAGACTGTTCACTATAAGACAAGAAATGGCCAGTAGAGGTCTATGGGATTCCTTTCGTCAGTCCGAGAGAGGCGAAGAGACAACTGAAGAAAGATTTGAAATCACAGGAACCATGCGCAGGCTTGCCGACCAAAGTCTCCCACCGAACTTCTCCAGCCTTGAAAACTTTAGAG<br>CCTATGTGGATGGATTCGAACCGAACGGCTGCATTGAGGGCAAGCTTTCTCAAATGTCAAAAGAAGTGAACGCCAGAATTGAGCCATTCCTGAAGACAACACCACGCCCTCTCAGATTACCTGATGGGCCTCCTTGTTCTCAGCGGTCGAAGTTCTTGCTGATGGATGCCCTTAAGTTGAGCATCGAAGACCCTAGCCATGAGGGGGAGGGCATACCGCTGTATGATGCAA<br>TCAAATGCATGAAGACATTTTTTGGCTGGAAAGAGCCCAACCTCGTAAAGCCGCATGAGAAAGGCATAAACCCTAATTACCTCCTGGCTTGGAAGCAGGTGCTGGCAGAACTTCAAGATATTGAGAATGAGGAGAAATTTCCAAAAACAAAGAACATGAAGAAAACAAGCCAATTGAAGTGGGCACTTGGTGAGAACATGGCACCAGAAAAAGTGGACTTTGAGGACTG<br>CAAAGATGTTAGCGATCTAAGACAGTACGACAGTGACGAACCAGAGCCTAGATCACTAGCAAGCTGGATTCAGAGTGAATTCAACAAGGCATGCGAATTGACAGATTCGAGTTGGATTGAACTTGATGAGATAGGGGAAGACGTTGCTCCAATCGAACACATTGCGAGTATGAGGAGGAACTATTTCACAGCGGAGGTATCCCATTGCAGGGCCACTGAATACATAATG<br>AAGGGAGTATACATAAACACAGCCCTATTGAATGCATCCTGTGCAGCCATGGATGACTTCCAACTGATTCCAATGATAAGCAAGTGCAGAACCAAAGAGGGAAGACGGAAGACAAATCTGTATGGATTCATTATAAAAGGAAGATCCCATTTGAGGAATGACACCGATGTGGTAAACTTTGTGAGCATGGAATTCTCTCTAACTGACCCGAGGCTAGAGCCACACAAATG<br>GGAAAAGTACTGTGTTCTTGAGATAGGAGACATGCTCCTACGGACTGCAATAGGCCAAGTGTCGAGGCCCATGTTCCTGTATGTGAGAACCAATGGGACTTCCAAGATCAAAATGAAATGGGGCATGGAGATGAGACGATGCCTTCTTCAGTCCCTTCAACAAATTGAGAGCATGATTGAGGCCGAATCTTCTGTCAAAGAGAAGGACATGACCAAGGAATTCTTTGAAA<br>ACAAATCAGAAACATGGCCAATTGGAGAATCACCCAAAGGGGTTGAGGAAGGCTCTATTGGGAAAGTATGCAGAACATTGCTAGCAAAGTCTGTGTTCAACAGCCTATATGCTTCTCCACAACTCGAGGGATTTTCAGCTGAATCAAGAAAATTGCTTCTCATTGTTCAGGCACTTAGGGACAACCTGGAACCTGGAACCTTCGATCTTGGGGGGCTATATGAAGCAATTG<br>AGGAGTGCCTGATTAACGATCCCTGGGTTTTGCTTAATGCGTCTTGGTTCAACTCCTTCATCACACATGCACTGAAATA-

EPI_ISL_9029957	>A/turkey/England/057679/2021_H5N1_2021-11-16<br>ATGGAAGACTTTGTGCGACAATGCTTCAATCCAATGATTGTCGAGCTTGCGGAAAAAGCAATGAAAGAATATGGGGAAGATCCGAAAATCGAGACAAACAAATTTGCCGCAATATGCACACACTTAGAAGTCTGTTTCATGTATTCGGATTTCCATTTTATTGATGAACGAGGCGAATCAATGATTGTAGAATCTGGCGATCCAAATGCATTATTGAAACACCGATTTGAGA<br>TAATCGAAGGGAGAGACCGAGCAATGGCCTGGACAGTGGTGAATAGTATCTGCAACACCACAGGAGTCGAAAAGCCCAAATTCCTCCCTGATTTGTATGACTACAAAGAGAACCGATTCATTGAAATTGGAGTAACGCGAAGGGAAGTTCACATATACTATTTAGAAAAAGCCAACAAGATAAAATCAGAGAAAACACACATTCACATATTCTCATTCACTGGAGAGGAA<br>ATGGCCACCAAGGCGGAATACACCCTTGATGAAGAGAGCAGAGCAAGAATAAAAACCAGACTGTTCACTATAAGACAAGAAATGGCCAGTAGAGGTCTATGGGATTCCTTTCGTCAGTCCGAGAGAGGCGAAGAGACAACTGAAGAAAGATTTGAAATCACAGGAACCATGCGCAGGCTTGCCGACCAAAGTCTCCCACCGAACTTCTCCAGCCTTGAAAACTTTAGAG<br>CCTATGTGGATGGATTCGAACCGAACGGCTGCATTGAGGGCAAGCTTTCTCAAATGTCAAAAGAAGTGAACGCCAGAATTGAGCCATTCCTGAAGACAACACCACGCCCTCTCAGATTACCTGATGGGCCTCCTTGTTCTCAGCGGTCGAAGTTCTTGCTGATGGATGCCCTTAAGTTGAGCATCGAAGACCCTAGCCATGAGGGGGAGGGCATACCGCTGTATGATGCAA<br>TCAAATGCATGAAGACATTTTTTGGCTGGAAAGAGCCCAACCTCGTAAAGCCGCATGAGAAAGGCATAAACCCTAATTACCTCCTGGCTTGGAAGCAGGTGCTGGCAGAACTTCAAGATATTGAGAATGAGGAGAAATTTCCAAAAACAAAGAACATGAAGAAAACAAGCCAATTGAAGTGGGCACTTGGTGAGAACATGGCACCAGAAAAAGTGGACTTTGAGGACTG<br>CAAAGATGTTAGCGATCTAAGACAGTACGACAGTGACGAACCAGAGCCTAGATCACTAGCAAGCTGGATTCAGAGTGAATTCAACAAGGCATGCGAATTGACAGATTCGAGTTGGATTGAACTTGATGAGATAGGGGAAGACGTTGCTCCAATCGAACACATTGCGAGTATGAGGAGGAACTATTTCACAGCGGAGGTATCCCATTGCAGGGCCACTGAATACATAATG<br>AAGGGAGTATACATAAACACAGCCCTATTGAATGCATCCTGTGCAGCCATGGATGACTTCCAACTGATTCCAATGATAAGCAAGTGCAGAACCAAAGAGGGAAGACGGAAGACAAATCTGTATGGATTCATTATAAAAGGAAGATCCCATTTGAGGAATGACACCGATGTGGTAAACTTTGTGAGCATGGAATTCTCTCTAACTGACCCGAGGCTAGAGCCACACAAATG<br>GGAAAAGTACTGTGTTCTTGAGATAGGAGACATGCTCCTACGGACTGCAATAGGCCAAGTGTCGAGGCCCATGTTCCTGTATGTGAGAACCAATGGGACTTCCAAGATCAAAATGAAATGGGGCATGGAGATGAGACGATGCCTTCTTCAGTCCCTTCAACAAATTGAGAGCATGATTGAGGCCGAATCTTCTGTCAAAGAGAAGGACATGACCAAGGAATTCTTTGAAA<br>ACAAATCAGAAACATGGCCAATTGGAGAATCACCCAAAGGGGTTGAGGAAGGCTCTATTGGGAAAGTATGCAGAACATTGCTAGCAAAGTCTGTGTTCAACAGCCTATATGCATCTCCACAACTCGAGGGATTTTCAGCTGAATCAAGAAAATTGCTTCTCATTGTTCAGGCACTTAGGGACAACCTGGAACCTGGAACCTTCGATCTTGGGGGGCTATATGAAGCAATTG<br>AGGAGTGCCTGATTAACGATCCCTGGGTTTTGCTTAATGCGTCTTGGTTCAACTCCTTCATCACACATGCACTGAAATA-
EPI_ISL_11798577	>A/gray_heron/Denmark/24326-1.02/2021-10-28_H5N1_2021-10-28<br>ATGGAAGACTTTGTGCGACAATGCTTCAATCCAATGATTGTCGAGCTTGCGGAAAAAGCAATGAAAGAATATGGGGAAGATCCGAAAATCGAGACAAACAAATTTGCCGCAATATGCACACACTTAGAAGTCTGTTTCATGTATTCGGATTTCCATTTTATTGATGAACGAGGCGAATCAATGATTGTAGAATCTGGCGATCCAAATGCATTATTGAAACACCGATTTGAGA<br>TAATCGAAGGGAGAGACCGAGCAATGGCCTGGACAGTGGTGAATAGTATCTGCAACACCACAGGAGTCGAAAAGCCCAAATTCCTCCCTGATTTGTATGACTACAAAGAGAACCGATTCATTGAAATTGGAGTAACGCGAAGGGAAGTTCACATATACTATTTAGAAAAAGCCAACAAGATAAAATCAGAGAAAACACACATTCACATATTCTCATTCACTGGAGAGGAA<br>ATGGCCACCAAGGCGGAATACACCCTTGATGAAGAGAGCAGAGCAAGAATAAAAACCAGACTGTTCACTATAAGACAAGAAATGGCCAGTAGAGGTCTATGGGATTCCTTTCGTCAGTCCGAGAGAGGCGAAGAGACAACTGAAGAAAGATTTGAAATCACAGGAACCATGCGCAGGCTTGCCGACCAAAGTCTCCCACCGAACTTCTCCAGCCTTGAAAACTTTAGAG<br>CCTATGTGGATGGATTCGAACCGAACGGCTGCATTGAGGGCAAGCTTTCTCAAATGTCAAAAGAAGTGAACGCCAGAATTGAGCCATTCCTGAAGACAACACCACGCCCTCTCAGATTACCTGATGGGCCTCCTTGTTCTCAGCGGTCGAAGTTCTTGCTGATGGATGCCCTTAAGTTGAGCATCGAAGACCCTAGCCATGAGGGGGAGGGCATACCGCTGTATGATGCAA<br>TCAAATGCATGAAGACATTTTTTGGCTGGAAAGAGCCCAACCTCGTAAAGCCGCATGAGAAAGGCATAAACCCTAATTACCTCCTGGCTTGGAAGCAGGTGCTGGCAGAACTTCAAGATATTGAGAATGAGGAGAAATTTCCAAAAACAAAGAACATGAAGAAAACAAGCCAATTGAAGTGGGCACTTGGTGAGAACATGGCACCAGAAAAAGTGGACTTTGAGGACTG<br>CAAAGATGTTAGCGATCTAAGACAGTACGACAGTGACGAACCAGAGCCTAGATCACTAGCAAGCTGGATTCAGAGTGAATTCAACAAGGCATGCGAATTGACAGATTCGAGTTGGATTGAACTTGATGAGATAGGGGAAGACGTTGCTCCAATCGAACACATTGCGAGTATGAGGAGGAACTATTTCACAGCGGAGGTATCCCATTGCAGGGCCACTGAATACATAATG<br>AAGGGAGTATACATAAACACAGCCCTATTGAATGCATCCTGTGCAGCCATGGATGACTTCCAACTGATTCCAATGATAAGCAAGTGCAGAACCAAAGAGGGAAGACGGAAGACAAATCTGTATGGATTCATTATAAAAGGAAGATCCCATTTGAGGAATGACACCGATGTGGTAAACTTTGTGAGCATGGAATTCTCTCTAACTGACCCGAGGCTAGAGCCACACAAATG<br>GGAAAAGTACTGTGTTCTTGAGATAGGAGACATGCTCCTACGGACTGCAATAGGCCAAGTGTCGAGGCCCATGTTCCTGTATGTGAGAACCAATGGGACTTCCAAGATCAAAATGAAATGGGGCATGGAGATGAGACGATGCCTTCTTCAGTCCCTTCAACAAATTGAGAGCATGATTGAGGCCGAATCTTCTGTCAAAGAGAAGGACATGACCAAGGAATTCTTTGAAA<br>ACAAATCAGAAACATGGCCAATTGGAGAATCACCCAAAGGGGTTGAGGAAGGCTCTATTGGGAAAGTATGCAGAACATTGCTAGCAAAGTCTGTGTTCAACAGCCTATATGCATCTCCACAACTCGAGGGATTTTCAGCTGAATCAAGAAAATTGCTTCTCATTGTTCAGGCACTTAGGGACAACCTGGAACCTGGAACATTCGATCTTGGGGGGCTATATGAAGCAATTG<br>AGGAGTGCCTGATTAACGATCCCTGGGTTTTGCTTAATGCGTCTTGGTTCAACTCCTTCATCACACATGCACTGAAATA-
EPI_ISL_5463805	>A/goose/Tyumen/33-52V/2021_H5N1_2021-10-07<br>ATGGAAGACTTTGTGCGACAATGCTTCAATCCAATGATTGTCGAGCTTGCGGAAAAAGCAATGAAAGAATATGGGGAAGATCCGAAAATCGAGACAAACAAATTTGCCGCAATATGCACACACTTAGAAGTCTGTTTCATGTATTCGGATTTCCATTTTATTGATGAACGAGGCGAATCAATGATTGTAGAATCTGGCGATCCAAATGCATTATTGAAACACCGATTTGAGA<br>TAATCGAAGGGAGAGACCGAGCAATGGCCTGGACAGTGGTGAATAGTATCTGCAACACCACAGGAGTCGAAAAGCCCAAATTCCTCCCTGATTTGTATGACTACAAAGAGAACCGATTCATTGAAATTGGAGTAACGCGAAGGGAAGTTCACATATACTATTTAGAAAAAGCCAACAAGATAAAATCAGAGAAAACACACATTCACATATTCTCATTCACTGGAGAGGAA<br>ATGGCCACCAAGGCGGAATACACCCTTGATGAAGAGAGCAGAGCAAGAATAAAAACCAGACTGTTCACTATAAGACAAGAAATGGCCAGTAGAGGTCTATGGGATTCCTTTCGTCAGTCCGAGAGAGGCGAAGAGACAACTGAAGAAAGATTTGAAATCACAGGAACCATGCGCAGGCTTGCCGACCAAAGTCTCCCACCGAACTTCTCCAGCCTTGAAAACTTTAGAG<br>CCTATGTGGATGGATTCGAACCGAACGGCTGCATTGAGGGCAAGCTTTCTCAAATGTCAAAAGAAGTGAACGCCAGAATTGAGCCATTCCTGAAGACAACACCACGCCCTCTCAGATTACCTGATGGGCCTCCTTGTTCTCAGCGGTCGAAGTTCTTGCTGATGGATGCCCTTAAGTTGAGCATCGAAGACCCTAGCCATGAGGGGGAGGGCATACCGCTGTATGATGCAA<br>TCAAATGCATGAAGACATTTTTTGGCTGGAAAGAGCCCAACCTCGTAAAGCCGCATGAGAAAGGCATAAACCCTAATTACCTCCTGGCTTGGAAGCAGGTGCTGGCAGAACTTCAAGATATTGAGAATGAGGAGAAATTTCCAAAAACAAAGAACATGAAGAAAACAAGCCAATTGAAGTGGGCACTTGGTGAGAACATGGCACCAGAAAAAGTGGACTTTGAGGACTG<br>CAAAGATGTTAGCGATCTAAGACAGTACGACAGTGACGAACCAGAGCCTAGATCACTAGCAAGCTGGATTCAGAGTGAATTCAACAAGGCATGCGAATTGACAGATTCGAGTTGGATTGAACTTGATGAGATAGGGGAAGACGTTGCTCCAATCGAACACATTGCGAGTATGAGGAGGAACTATTTCACAGCGGAGGTATCCCATTGCAGGGCCACTGAATACATAATG<br>AAGGGAGTATACATAAATACAGCCCTATTGAATGCATCCTGTGCAGCCATGGATGACTTCCAACTGATTCCAATGATAAGCAAGTGCAGAACCAAAGAGGGAAGACGGAAGACAAATCTGTATGGATTCATTATAAAAGGAAGATCCCATTTGAGGAATGACACCGATGTGGTAAACTTTGTGAGCATGGAATTCTCTCTAACTGACCCGAGGCTAGAGCCACACAAATG<br>GGAAAAGTACTGTGTTCTTGAGATAGGAGACATGCTCCTACGGACTGCAATAGGCCAAGTGTCGAGGCCCATGTTCCTGTATGTGAGAACCAATGGGACTTCCAAGATCAAAATGAAATGGGGCATGGAGATGAGACGATGCCTTCTTCAGTCCCTTCAACAAATTGAGAGCATGATTGAGGCCGAATCTTCTGTCAAAGAGAAGGACATGACCAAGGAATTCTTTGAAA<br>ACAAATCAGAAACATGGCCAATTGGAGAATCACCCAAAGGGGTTGAGGAAGGCTCTATTGGGAAAGTATGCAGAACATTGCTAGCAAAGTCTGTGTTCAACAGCCTATATGCATCTCCACAACTCGAGGGATTTTCAGCTGAATCAAGAAAATTGCTTCTCATTGTTCAGGCACTTAGGGACAACCTGGAACCTGGAACCTTCGATCTTGGGGGGCTATATGAAGCAATTG<br>AGGAGTGCCTGATTAACGATCCCTGGGTTTTGCTTAATGCGTCTTGGTTCAACTCCTTCATCACACATGCACTGAAATA-
EPI_ISL_7753251	>A/barnacle_goose/Germany-SH/AI06005/2021_H5N1_2021-10-19<br>ATGGAAGACTTTGTGCGACAATGCTTCAATCCAATGATTGTCGAGCTTGCGGAAAAAGCAATGAAAGAATATGGGGAAGATCCGAAAATCGAGACAAACAAATTTGCCGCAATATGCACACACTTAGAAGTCTGTTTCATGTATTCGGATTTCCATTTTATTGATGAACGAGGCGAATCAATGATTGTAGAATCTGGCGATCCAAATGCATTATTGAAACACCGATTTGAGA<br>TAATCGAAGGGAGAGACCGAGCAATGGCCTGGACAGTGGTGAATAGTATCTGCAACACCACAGGAGTCGAAAAGCCCAAATTCCTCCCTGATTTGTATGACTACAAAGAGAACCGATTCATTGAAATTGGAGTAACGCGAAGGGAAGTTCACATATACTATTTAGAAAAAGCCAACAAGATAAAATCAGAGAAAACACACATTCACATATTCTCATTCACTGGAGAGGAA<br>ATGGCCACCAAGGCGGAATACACCCTTGATGAAGAGAGCAGAGCAAGAATAAAAACCAGACTGTTCACTATAAGACAAGAAATGGCCAGTAGAGGTCTATGGGATTCCTTTCGTCAGTCCGAGAGAGGCGAAGAGACAACTGAAGAAAGATTTGAAATCACAGGAACCATGCGCAGGCTTGCCGACCAAAGTCTCCCACCGAACTTCTCCAGCCTTGAAAACTTTAGAG<br>CCTATGTGGATGGATTCGAACCGAACGGCTGCATTGAGGGCAAGCTTTCTCAAATGTCAAAAGAAGTGAACGCCAGAATTGAGCCATTCCTGAAGACAACACCACGCCCTCTCAGATTACCTGATGGGCCTCCTTGTTCTCAGCGGTCGAAGTTCTTGCTGATGGATGCCCTTAAGTTGAGCATCGAAGACCCTAGCCATGAGGGGGAGGGCATACCGCTGTATGATGCAA<br>TCAAATGCATGAAGACATTTTTTGGCTGGAAAGAGCCCAACCTCGTAAAGCCGCATGAGAAAGGCATAAACCCTAATTACCTCCTGGCTTGGAAGCAGGTGCTGGCAGAACTTCAAGATATTGAGAATGAGGAGAAATTTCCAAAAACAAAGAACATGAAGAAAACAAGCCAATTGAAGTGGGCACTTGGTGAGAACATGGCACCAGAAAAAGTGGACTTTGAGGACTG<br>CAAAGATGTTAGCGATCTAAGACAGTACGACAGTGACGAACCAGAGCCTAAATCACTAGCAAGCTGGATTCAGAGTGAATTCAACAAGGCATGCGAATTGACAGATTCGAGTTGGATTGAACTTGATGAGATAGGGGAAGACGTTGCTCCAATCGAACACATTGCGAGTATGAGGAGGAACTATTTCACAGCGGAGGTATCCCATTGCAGGGCCACTGAATACATAATG<br>AAGGGAGTATACATAAACACAGCCCTATTGAATGCATCCTGTGCAGCCATGGATGACTTCCAACTGATTCCAATGATAAGCAAGTGCAGAACCAAAGAGGGAAGACGGAAGACAAATCTGTATGGATTCATTATAAAAGGAAGATCCCATTTGAGGAATGACACCGATGTGGTAAACTTTGTGAGCATGGAATTCTCTCTAACTGACCCGAGGCTAGAGCCACACAAATG<br>GGAAAAGTACTGTGTTCTTGAGATAGGAGACATGCTCCTACGGACTGCAATAGGCCAAGTGTCGAGGCCCATGTTCCTGTATGTGAGAACCAATGGGACTTCCAAGATCAAAATGAAATGGGGCATGGAGATGAGACGATGCCTTCTTCAGTCCCTTCAACAAATTGAGAGCATGATTGAGGCCGAATCTTCTGTCAAAGAGAAGGACATGACCAAGGAATTCTTTGAAA<br>ACAAATCAGAAACATGGCCAATTGGAGAATCACCCAAAGGGGTTGAGGAAGGCTCTATTGGGAAAGTATGCAGAACATTGCTAGCAAAGTCTGTGTTCAACAGCCTATATGCATCTCCACAACTCGAGGGATTTTCAGCTGAATCAAGAAAATTGCTTCTCATTGTTCAGGCACTTAGGGACAACCTGGAACCTGGAACCTTCGATCTTGGGGGGCTATATGAAGCAATTG<br>AGGAGTGCCTGATTAACGATCCCTGGGTTTTGCTTAATGCGTCTTGGTTCAACTCCTTCATCACACATGCACTGAAATA-
EPI_ISL_15022831	>A/swan/Lithuania/1220PG1_22VIR7255-1/2022_H5N1_2022-02-09<br>ATGGAAGACTTTGTGCGACAATGCTTCAATCCAATGATTGTCGAGCTTGCGGAAAAAGCAATGAAAGAATATGGGGAAGATCCGAAAGTCGAGACAAACAAATTTGCCGCAATATGCACACACTTAGAAGTCTGTTTCATGTATTCGGATTTCCATTTTATTGATGAACGAGGCGAATCAATGATTGTAGAATCTGGCGATCCAAATGCATTATTGAAACACCGATTTGAGA<br>TAATCGAAGGGAGAGACCGAGCAATGGCCTGGACAGTGGTGAATAGTATCTGCAACACCACAGGAGTCGAAAAGCCCAAATTCCTCCCTGATTTGTATGACTACAAAGAGAACCGATTCATTGAAATTGGAGTAACGCGAAGGGAAGTTCACATATACTATTTAGAAAAAGCCAACAAGATAAAATCAGAGAAAACACACATTCACATATTCTCATTCACTGGAGAGGAA<br>ATGGCCACCAAGGCGGAATACACCCTTGATGAAGAGAGCAGAGCAAGAATAAAAACCAGACTGTTCACTATAAGACAAGAAATGGCCAGTAGAGGTCTATGGGATTCCTTTCGTCAGTCCGAGAGAGGCGAAGAGACAACTGAAGAAAGATTTGAAATCACAGGAACCATGCGCAGGCTTGCCGACCAAAGTCTCCCACCGAACTTCTCCAGCCTTGAAAACTTTAGAG<br>CCTATGTGGATGGATTCGAACCGAACGGCTGCATTGAGGGCAAGCTTTCTCAAATGTCAAAAGAAGTGAACGCCAGAATTGAGCCATTCCTGAAGACAACACCACGCCCTCTCAGATTACCTGATGGGCCTCCTTGTTCTCAGCGGTCGAAGTTCTTGCTGATGGATGCCCTTAAGTTGAGCATCGAAGACCCTAGCCATGAGGGGGAGGGCATACCGCTGTATGATGCAA<br>TCAAATGCATGAAGACATTTTTTGGCTGGAAAGAGCCCAACCTCGTGAAGCCGCATGAGAAAGGCATAAACCCTAATTACCTCCTGGCTTGGAAGCAGGTGCTGGCAGAACTTCAAGATATTGAGAATGAGGAGAAATTTCCAAAAACAAAGAACATGAAGAAAACAAGCCAATTGAAGTGGGCACTTGGTGAGAACATGGCACCAGAAAAAGTGGACTTTGAGGACTG<br>CAAAGATGTTAGCGATCTAAGACAGTACGACAGTGACGAACCAGAGCCTAGATCACTAGCAAGCTGGATTCAGAGTGAATTCAACAAGGCATGCGAATTGACAGATTCGAGTTGGATTGAACTTGATGAGATAGGGGAAGACGTTGCTCCAATCGAACACATTGCGAGTATGAGGAGGAACTATTTCACAGCGGAGGTATCCCATTGCAGGGCCACTGAATACATAATG<br>AAGGGAGTATACATAAACACAGCCCTATTGAATGCATCCTGTGCAGCCATGGATGACTTCCAACTGATTCCAATGATAAGCAAGTGCAGAACCAAAGAGGGAAGACGGAAGACAAATCTGTATGGATTCATTATAAAAGGAAGATCCCATTTGAGGAATGACACCGATGTGGTAAACTTTGTGAGCATGGAATTCTCTCTAACTGACCCGAGGCTAGAGCCACACAAATG<br>GGAAAAGTACTGTGTTCTTGAGATAGGAGACATGCTCCTACGGACTGCAATAGGCCAAGTGTCGAGGCCCATGTTCCTGTATGTGAGAACCAATGGGACTTCCAAGATCAAAATGAAATGGGGCATGGAGATGAGACGATGCCTTCTTCAGTCCCTTCAACAAATTGAGAGCATGATTGAGGCCGAATCTTCTGTCAAAGAGAAGGACATGACCAAGGAATTCTTTGAAA<br>ACAAATCAGAAACATGGCCAATTGGAGAATCACCCAAAGGGGTTGAGGAAGGCTCTATTGGGAAAGTATGCAGAACATTGCTAGCAAAGTCTGTGTTCAACAGCCTATATGCATCTCCACAACTCGAGGGATTTTCAGCTGAATCAAGAAAATTGCTTCTCATTGTTCAGGCACTTAGGGACAACCTGGAACCTGGAACCTTCGATCTTGGGGGGCTATATGAAGCAATTG<br>AGGAGTGCCTGATTAACGATCCCTGGGTTTTGCTTAATGCGTCTTGGTTCAACTCCTTCATCACACATGCACTGAAATA-
EPI_ISL_14760543	>A/turkey/Italy/21VIR8816-1/2021_H5N1_2021-10-25<br>ATGGAAGACTTTGTGCGACAATGCTTCAATCCAATGATTGTCGAGCTTGCGGAAAAAGCAATGAAAGAATATGGGGAAGATCCAAAAATCGAGACAAACAAATTTGCCGCAATATGCACACACTTAGAAGTCTGTTTCATGTATTCGGATTTCCATTTTATTGATGAACGAGGCGAATCAATGATTGTAGAATCTGGCGATCCAAATGCATTATTGAAACACCGATTTGAGA<br>TAATCGAAGGGAGAGACCGAGCAATGGCCTGGACAGTGGTGAATAGTATCTGCAACACCACAGGAGTCGAAAAGCCCAAATTCCTCCCTGATTTGTATGACTACAAAGAGAACCGATTCATTGAAATTGGAGTAACGCGAAGGGAAGTTCACATATACTATTTAGAAAAAGCCAACAAGATAAAATCGGAGAAAACACACATTCACATATTCTCATTCACTGGAGAGGAA<br>ATGGCCACCAAGGCGGAATACACCCTTGATGAAGAGAGCAGAGCAAGAATAAAAACCAGACTGTTCACTATAAGACAAGAAATGGCCAGTAGAGGTCTATGGGATTCCTTTCGTCAGTCCGAGAGAGGCGAAGAGACAACTGAAGAAAGATTTGAAATCACAGGAACCATGCGCAGGCTTGCCGACCAAAGTCTCCCACCGAACTTCTCCAGCCTTGAAAACTTTAGAG<br>CCTATGTGGATGGATTCGAACCGAACGGCTGCATTGAGGGCAAGCTTTCTCAAATGTCAAAAGAAGTGAACGCCAGAATTGAGCCATTCCTGAAGACAACACCACGCCCTCTCAGATTACCTGATGGGCCTCCTTGTTCTCAGCGGTCGAAGTTCTTGCTGATGGATGCCCTTAAGTTGAGCATCGAAGACCCTAGCCATGAGGGGGAGGGCATACCGCTGTATGATGCAA<br>TCAAATGCATGAAGACATTTTTTGGCTGGAAAGAGCCCAACCTCGTAAAGCCGCATGAGAAAGGCATAAACCCTAATTACCTCCTGGCTTGGAAGCAGGTGCTGGCAGAACTTCAAGATATTGAGAATGAGGAGAAATTTCCAAAAACAAAGAACATGAAGAAAACAAGCCAATTGAAGTGGGCACTTGGTGAGAACATGGCACCAGAAAAAGTGGACTTTGAGGACTG<br>CAAAGATGTTAGCGATCTAAGACAGTACGACAGTGACGAACCAGAGCCTAGATCACTAGCAAGCTGGATTCAGAGTGAATTCAACAAGGCATGCGAATTGACAGATTCGAGTTGGATTGAACTTGATGAGATAGGGGAAGACGTTGCTCCAATCGAACACATTGCGAGTATGAGGAGGAACTATTTCACAGCGGAGGTATCCCATTGCAGGGCCACTGAATACATAATG<br>AAGGGAGTATACATAAACACAGCCCTATTGAATGCATCCTGTGCAGCCATGGATGACTTCCAACTGATTCCAATGATAAGCAAGTGCAGAACCAAAGAAGGAAGACGGAAGACAAATCTGTATGGATTCATTATAAAAGGAAGATCCCATTTGAGGAATGACACCGATGTGGTAAACTTTGTGAGCATGGAATTCTCTCTAACTGACCCGAGGCTAGAGCCACACAAATG<br>GGAAAAGTACTGTGTTCTTGAGATAGGAGACATGCTCCTACGGACTGCAATAGGCCAAGTGTCGAGGCCCATGTTCCTGTATGTGAGAACCAATGGGACTTCCAAGATCAAAATGAAATGGGGCATGGAGATGAGACGATGCCTTCTTCAGTCCCTTCAACAAATTGAGAGCATGATTGAGGCCGAATCTTCTGTCAAAGAGAAGGACATGACCAAGGAATTCTTTGAAA<br>ACAAATCAGAAACATGGCCAATTGGAGAATCACCCAAAGGGGTTGAGGAAGGCTCTATTGGGAAAGTATGCAGAACATTGCTAGCAAAGTCTGTGTTCAACAGCCTATATGCATCTCCACAACTCGAGGGATTTTCAGCTGAATCAAGAAAATTGCTTCTCATTGTTCAGGCACTTAGGGACAACCTGGAACCTGGAACCTTCGATCTTGGGGGGCTATATGAAGCAATTG<br>AGGAGTGCCTGATTAACGATCCCTGGGTTTTGCTTAATGCGTCTTGGTTCAACTCCTTCCTCACACATGCACTGAAATA-
EPI_ISL_14760632	>A/Guinea_fowl/Italy/21VIR10216/2021_H5N1_2021-11-24<br>ATGGAAGACTTTGTGCGACAATGCTTCAATCCAATGATTGTCGAGCTTGCGGAAAAAGCAATGAAAGAATATGGGGAAGATCCGAAAATCGAGACAAACAAATTTGCCGCAATATGCACACACTTAGAAGTCTGTTTCATGTATTCGGATTTCCATTTTATTGATGAACGAGGCGAATCAATGATTGTAGAATCTGGCGATCCAAATGCATTATTGAAACACCGATTTGAGA<br>TAATCGAAGGGAGAGACCGAGCAATGGCCTGGACAGTGGTGAATAGTATCTGCAACACCACAGGAGTCGAAAAGCCCAAATTCCTCCCTGATTTGTATGACTACAAAGAGAACCGATTCATTGAAATTGGAGTAACGCGAAGGGAAGTTCACATATACTATTTAGAAAAAGCCAACAAGATAAAATCGGAGAAAACACACATTCACATATTCTCATTCACTGGAGAGGAA<br>ATGGCCACCAAGGCGGAATACACCCTTGATGAAGAGAGCAGAGCAAGAATAAAAACCAGACTGTTCACTATAAGACAAGAAATGGCCAGTAGAGGTCTATGGGATTCCTTTCGTCAGTCCGAGAGAGGCGAAGAGACAACTGAAGAAAGATTTGAAATCACAGGAACCATGCGCAGGCTTGCCGACCAAAGTCTCCCACCGAACTTCTCCAGCCTTGAAAACTTTAGAG<br>CCTATGTGGATGGATTCGAACCGAACGGCTGCATTGAGGGCAAGCTTTCTCAAATGTCAAAAGAAGTGAACGCCAGAATTGAGCCATTCCTGAAGACAACACCACGCCCTCTCAGATTACCTGATGGGCCTCCTTGTTCTCAGCGGTCGAAGTTCTTGCTGATGGATGCCCTTAAGTTGAGCATCGAAGACCCTAGCCATGAGGGGGAGGGCATACCGCTGTATGATGCAA<br>TCAAATGCATGAAGACATTTTTTGGCTGGAAAGAGCCCAACCTCGTAAAGCCGCATGAGAAAGGCATAAACCCTAATTACCTCCTGGCTTGGAAGCAGGTGCTGGCAGAACTTCAAGATATTGAGAATGAGGAGAAATTTCCAAAAACAAAGAACATGAAGAAAACAAGCCAATTGAAGTGGGCACTTGGTGAGAACATGGCACCAGAAAAAGTGGACTTTGAGGACTG<br>CAAAGATGTTAGCGATCTAAGACAGTACGACAGTGACGAACCAGAGCCTAGATCACTAGCAAGCTGGATTCAGAGTGAATTCAACAAGGCATGCGAATTGACAGATTCGAGTTGGATTGAACTTGATGAGATAGGGGAAGACGTTGCTCCAATCGAACACATTGCGAGTATGAGGAGGAACTATTTCACAGCGGAGGTATCCCATTGCAGGGCCACTGAATACATAATG<br>AAGGGAGTATACATAAACACAGCCCTATTGAATGCATCCTGTGCAGCCATGGATGACTTCCAACTGATTCCAATGATAAGCAAGTGCAGAACCAAAGAAGGAAGACGGAAGACAAATCTGTATGGATTCATTATAAAAGGAAGATCCCATTTGAGGAATGACACCGATGTGGTAAACTTTGTGAGCATGGAATTCTCTCTAACTGACCCGAGGCTAGAGCCACACAAATG<br>GGAAAAGTACTGTGTTCTTGAGATAGGAGACATGCTCCTACGGACTGCAATAGGCCAAGTGTCGAGGCCCATGTTCCTGTATGTGAGAACCAATGGGACTTCCAAGATCAAAATGAAATGGGGCATGGAGATGAGACGATGCCTTCTTCAGTCCCTTCAACAAATTGAGAGCATGATTGAGGCCGAATCTTCTGTCAAAGAGAAGGACATGACCAAGGAATTCTTTGAAA<br>ACAAATCAGAAACATGGCCAATTGGAGAATCACCCAAAGGGGTTGAGGAAGGCTCTATTGGGAAAGTATGCAGAACATTGCTAGCAAAGTCTGTGTTCAACAGCCTATATGCATCTCCACAACTCGAGGGATTTTCAGCTGAATCAAGAAAATTGCTTCTCATTGTTCAGGCACTTAGGGACAACCTGGAACCTGGAACCTTCGATCTTGGGGGGCTATATGAAGCAATTG<br>AGGAGTGCCTGATTAACGATCCCTGGGTTTTGCTTAATGCGTCTTGGTTCAACTCCTTCCTCACACATGCACTGAAATA-
EPI_ISL_13026106	>A/Canada_goose/Luxembourg/21239614/2021_H5N1_2021-12-10<br>ATGGAAGACTTTGTGCGACAATGCTTCAATCCAATGATTGTCGAGCTTGCGGAAAAAGCAATGAAAGAATATGGGGAAGATCCGAAAATCGAGACAAACAAATTTGCCGCAATATGCACACACTTAGAAGTCTGTTTCATGTATTCGGATTTCCATTTTATTGATGAACGAGGCGAATCAATGATTGTAGAATCTGGCGATCCAAATGCATTATTGAAACACCGATTTGAGA<br>TAATCGAAGGGAGAGACCGAGCAATGGCCTGGACAGTGGTGAATAGTATCTGCAACACCACAGGAGTCGAAAAGCCCAAATTCCTCCCTGATTTGTATGACTACAAAGAGAACCGATTCATTGAAATTGGAGTAACGCGAAGGGAAGTTCACATATACTATTTAGAAAAAGCCAACAAGATAAAATCGGAGAAAACACACATTCACATATTCTCATTCACTGGAGAGGAA<br>ATGGCCACCAAGGCGGAATACACCCTTGATGAAGAGAGCAGAGCAAGAATAAAAACCAGACTGTTCACTATAAGACAAGAAATGGCCAGTAGAGGTCTATGGGATTCCTTTCGTCAGTCCGAGAGAGGCGAAGAGACAACTGAAGAAAGATTTGAAATCACAGGAACCATGCGCAGGCTTGCCAACCAAAGTCTCCCACCGAACTTCTCCAGCCTTGAAAACTTTAGAG<br>CCTATGTGGATGGATTCGAACCGAACGGCTGCATTGAGGGCAAGCTTTCTCAAATGTCAAAAGAAGTGAACGCCAGAATTGAGCCATTCCTGAAGACAACACCACGCCCTCTCAGATTACCTGATGGGCCTCCTTGTTCTCAGCGGTCGAAGTTCTTGCTGATGGATGCCCTTAAGTTGAGCATCGAAGACCCTAGCCATGAAGGGGAGGGCATACCGCTGTATGATGCAA<br>TCAAATGCATGAAGACATTTTTTGGCTGGAAAGAGCCCAACCTCGTAAAGCCGCATGAGAAAGGCATAAACCCTAATTACCTCCTGGCTTGGAAGCAGGTGCTGGCAGAACTTCAAGATATTGAGAATGAGGAGAAATTTCCAAAAACAAAGAACATGAAGAAAACAAGCCAATTGAAGTGGGCACTTGGTGAGAACATGGCACCAGAAAAAGTGGACTTTGAGGACTG<br>CAAAGATGTTAGCGATCTAAGACAGTACGACAGTGACGAACCAGAGCCTAGATCACTAGCAAGCTGGATTCAGAGTGAATTCAACAAGGCATGCGAATTGACAGATTCGAGTTGGATTGAACTTGATGAGATAGGGGAAGACGTTGCTCCAATCGAACACATTGCGAGTATGAGGAGGAACTATTTCACAGCGGAGGTATCCCATTGCAGGGCCACTGAATACATAATG<br>AAGGGAGTATACATAAACACAGCCCTATTGAATGCATCCTGTGCAGCCATGGATGACTTCCAACTGATTCCAATGATAAGCAAGTGCAGAACCAAAGAAGGAAGACGGAAGACAAATCTGTATGGATTCATTATAAAAGGAAGATCCCATTTGAGGAATGACACCGATGTGGTAAACTTTGTGAGCATGGAATTCTCTCTAACTGACCCGAGGCTAGAGCCACACAAATG<br>GGAAAAGTACTGTGTTCTTGAGATAGGAGACATGCTCCTACGGACTGCAATAGGCCAAGTGTCGAGGCCCATGTTCCTGTATGTGAGAACCAATGGGACTTCCAAGATCAAAATGAAATGGGGCATGGAGATGAGACGATGCCTTCTTCAGTCCCTTCAACAAATTGAGAGCATGATTGAGGCCGAATCTTCTGTCAAAGAGAAGGACATGACCAAGGAATTCTTTGAAA<br>ACAAATCAGAAACATGGCCAATTGGAGAATCACCCAAAGGGGTTGAGGAAGGCTCTATTGGGAAAGTATGCAGAACATTGCTAGCAAAGTCTGTGTTCAACAGCCTATATGCATCTCCACAACTCGAGGGATTTTCAGCTGAATCAAGAAAATTGCTTCTCATTGTTCAGGCACTTAGGGACAACCTGGAGCCTGGAACCTTCGATCTTGGGGGGCTATATGAAGCAATTG<br>AGGAGTGTCTGATTAACGATCCCTGGGTTTTGCTTAATGCGTCTTGGTTCAACTCCTTCCTCACACATGCACTGAAATA-
EPI_ISL_13026099	>A/Greylag_goose/Luxembourg/21217773/2021_H5N1_2021-11-24<br>ATGGAAGACTTTGTGCGACAATGCTTCAATCCAATGATTGTCGAGCTTGCGGAAAAAGCAATGAAAGAATATGGGGAAGATCCGAAAATCGAGACAAACAAATTTGCCGCAATATGCACACACTTAGAAGTCTGTTTCATGTATTCGGATTTCCATTTTATTGATGAACGAGGCGAATCAATGATTGTAGAATCTGGCGATCCAAATGCATTATTGAAACACCGATTTGAGA<br>TAATCGAAGGGAGAGACCGAGCAATGGCCTGGACAGTGGTGAATAGTATCTGCAACACCACAGGAGTCGAAAAGCCCAAATTCCTCCCTGATTTGTATGACTACAAAGAGAACCGATTCATTGAAATTGGAGTAACGCGAAGGGAAGTTCACATATACTATTTAGAAAAAGCCAACAAGATAAAATCGGAGAAAACACACATTCACATATTCTCATTCACTGGAGAGGAA<br>ATGGCCACCAAGGCGGAATACACCCTTGATGAAGAGAGCAGAGCAAGAATAAAAACCAGACTGTTCACTATAAGACAAGAAATGGCCAGTAGAGGTCTATGGGATTCCTTTCGTCAGTCCGAGAGAGGCGAAGAGACAACTGAAGAAAGATTTGAAATCACAGGAACCATGCGCAGGCTTGCCAACCAAAGTCTCCCACCGAACTTCTCCAGCCTTGAAAACTTTAGAG<br>CCTATGTGGATGGATTCGAACCGAACGGCTGCATTGAGGGCAAGCTTTCTCAAATGTCAAAAGAAGTGAACGCCAGAATTGAGCCATTCCTGAAGACAACACCACGCCCTCTCAGATTACCTGATGGGCCTCCTTGTTCTCAGCGGTCGAAGTTCTTGCTGATGGATGCCCTTAAGTTGAGCATCGAAGACCCTAGCCATGAAGGGGAGGGCATACCGCTGTATGATGCAA<br>TCAAATGCATGAAGACATTTTTTGGCTGGAAAGAGCCCAACCTCGTAAAGCCGCATGAGAAAGGCATAAACCCTAATTACCTCCTGGCTTGGAAGCAGGTGCTGGCAGAACTTCAAGATATTGAGAATGAGGAGAAATTTCCAAAAACAAAGAACATGAAGAAAACAAGCCAATTGAAGTGGGCACTTGGTGAGAACATGGCACCAGAAAAAGTGGACTTTGAGGACTG<br>CAAAGATGTTAGCGATCTAAGACAGTACGACAGTGACGAACCAGAGCCTAGATCACTAGCAAGCTGGATTCAGAGTGAATTCAACAAGGCATGCGAATTGACAGATTCGAGTTGGATTGAACTTGATGAGATAGGGGAAGACGTTGCTCCAATCGAACACATTGCGAGTATGAGGAGGAACTATTTCACAGCGGAGGTATCCCATTGCAGGGCCACTGAATACATAATG<br>AAGGGAGTATACATAAACACAGCCCTATTGAATGCATCCTGTGCAGCCATGGATGACTTCCAACTGATTCCAATGATAAGCAAGTGCAGAACCAAAGAAGGAAGACGGAAGACAAATCTGTATGGATTCATTATAAAAGGAAGATCCCATTTGAGGAATGACACCGATGTGGTAAACTTTGTGAGCATGGAATTCTCTCTAACTGACCCGAGGCTAGAGCCACACAAATG<br>GGAAAAGTACTGTGTTCTTGAGATAGGAGACATGCTCCTACGGACTGCAATAGGCCAAGTGTCGAGGCCCATGTTCCTGTATGTGAGAACCAATGGGACTTCCAAGATCAAAATGAAATGGGGCATGGAGATGAGACGATGCCTTCTTCAGTCCCTTCAACAAATTGAGAGCATGATTGAGGCCGAATCTTCTGTCAAAGAGAAGGACATGACCAAGGAATTCTTTGAAA<br>ACAAATCAGAAACATGGCCAATTGGAGAATCACCCAAAGGGGTTGAGGAAGGCTCTATTGGGAAAGTATGCAGAACATTGCTAGCAAAGTCTGTGTTCAACAGCCTATATGCATCTCCACAACTCGAGGGATTTTCAGCTGAATCAAGAAAATTGCTTCTCATTGTTCAGGCACTTAGGGACAACCTGGAGCCTGGAACCTTCGATCTTGGGGGGCTATATGAAGCAATTG<br>AGGAGTGTCTGATTAACGATCCCTGGGTTTTGCTTAATGCGTCTTGGTTCAACTCCTTCCTCACACATGCACTGAAATA-
EPI_ISL_11798571	>A/common_buzzard/Denmark/24271-1.02/2021-10-25_H5N1_2021-10-25<br>ATGGAAGACTTTGTGCGACAATGCTTCAATCCAATGATTGTCGAGCTTGCGGAAAAAGCAATGAAAGAATATGGGGAAGATCCGAAAATCGAGACAAACAAATTTGCCGCAATATGCACACACTTAGAAGTCTGTTTCATGTATTCGGATTTCCATTTTATTGATGAACGAGGCGAATCAACGATTGTAGAATCTGGCGATCCAAATGCATTATTGAAACACCGATTTGAGA<br>TAATCGAAGGGAGAGACCGAGCAATGGCCTGGACAGTGGTGAATAGTATCTGCAACACCACAGGAGTCGAAAAGCCCAAATTCCTCCCTGATTTGTATGACTACAAAGAGAACCGATTCATTGAAATTGGAGTAACGCGAAGGGAAGTTCACATATACTATTTAGAAAAAGCCAACAAGATAAAATCAGAGAAAACACACATTCACATATTCTCATTCACTGGAGAGGAA<br>ATGGCCACCAAGGCGGAATACACCCTTGATGAAGAGAGCAGAGCAAGAATAAAAACCAGACTGTTCACTATAAGACAAGAAATGGCCAGTAGAGGTCTATGGGATTCCTTTCGTCAGTCCGAAAGAGGCGAAGAGACAACTGAAGAAAGATTTGAAATCACAGGAACCATGCGCAGGCTTGCCGACCAAAGTCTCCCACCGAACTTCTCCAGCCTTGAAAACTTTAGAG<br>CCTATGTGGATGGATTCGAACCGAACGGCTGCATTGAGGGCAAGCTTTCTCAAATGTCAAAAGAAGTGAACGCCAGAATTGAGCCATTCCTGAAGACAACACCACGCCCTCTCAGATTACCTGATGGGCCTCCTTGTTCTCAGCGGTCGAAGTTCTTGCTGATGGATGCCCTTAAGTTGAGCATCGAAGACCCTAGCCATGAGGGGGAGGGCATACCGCTGTATGATGCAA<br>TCAAATGCATGAAGACATTTTTTGGCTGGAAAGAGCCCAACCTCGTAAAGCCGCATGAGAAAGGCATAAACCCTAATTACCTCCTGGCTTGGAAGCAGGTGCTGGCAGAACTTCAAGATATTGAGAATGAGGAGAAATTTCCAAAAACAAAGAACATGAAGAAAACAAGCCAATTGAAGTGGGCACTTGGTGAGAACATGGCACCAGAAAAAGTGGACTTTGAGGACTG<br>CAAAGATGTTAGCGATCTAAGACAGTACGACAGTGACGAACCAGAGCCTAGATCACTAGCAAGCTGGATTCAGAGTGAATTCAACAAGGCATGCGAATTGACAGATTCGAGTTGGATTGAACTTGATGAGATAGGGGAAGACGTTGCTCCAATCGAACACATTGCGAGTATGAGGAGGAACTATTTCACAGCGGAGGTATCCCATTGCAGGGCCACTGAATACATAATG<br>AAGGGAGTATACATAAACACAGCCCTATTGAATGCATCCTGTGCAGCCATGGATGACTTCCAACTGATTCCAATGATAAGCAAGTGCAGAACCAAAGAGGGAAGACGGAAGACAAATCTGTATGGATTCATTATAAAAGGAAGATCCCATTTGAGGAATGACACCGATGTGGTGAACTTTGTGAGCATGGAATTCTCTCTAACTGACCCGAGGCTAGAGCCACACAAATG<br>GGAAAAGTACTGTGTTCTTGAGATAGGAGACATGCTCCTACGGACTGCAATAGGCCAAGTGTCGAGGCCCATGTTCCTGTATGTGAGAACCAATGGGACTTCCAAGATCAAAATGAAATGGGGCATGGAGATGAGACGATGCCTTCTTCAGTCCCTTCAACAAATTGAGAGCATGATTGAGGCCGAATCTTCTGTCAAAGAGAAGGACATGACCAAGGAATTCTTTGAAA<br>ACAAATCAGAAACATGGCCAATTGGAGAATCACCCAAAGGGGTTGAGGAAGGCTCTATTGGGAAAGTATGCAGAACATTGCTAGCAAAGTCTGTGTTCAACAGCCTATATGCTTCTCCACAACTCGAGGGATTTTCAGCTGAATCAAGAAAATTGCTTCTCATTGTTCAGGCACTTAGGGACAACCTGGAACCTGGAACCTTCGATCTTGGGGGGCTATATGAAGCAATTG<br>AGGAGTGCCTGATTAACGATCCCTGGGTTTTGCTTAATGCGTCTTGGTTCAACTCCTTCATCACACATGCACTGAAATA-
EPI_ISL_11259280	>A/greylag_goose/Ireland/033062_22VIR1325-7/2021_H5N1_2021-11-15<br>ATGGAAGACTTTGTGCGACAATGCTTCAATCCAATGATTGTCGAGCTTGCGGAAAAAGCAATGAAAGAATATGGGGAAGATCCGAAAATCGAGACAAACAAATTTGCCGCAATATGCACACACTTAGAAGTCTGTTTCATGTATTCGGATTTCCATTTTATTGATGAACGAGGCGAATCAACGATTGTAGAATCTGGCGATCCAAATGCATTATTGAAACACCGATTTGAGA<br>TAATCGAAGGGAGAGACCGAGCAATGGCCTGGACAGTGGTGAATAGTATCTGCAACACCACAGGAGTCGAAAAGCCCAAATTCCTCCCTGATTTGTATGACTACAAAGAGAACCGATTCATTGAAATTGGAGTAACGCGAAGGGAAGTTCACATATACTATTTAGAAAAAGCCAACAAGATAAAATCAGAGAAAACACACATTCACATATTCTCATTCACTGGAGAGGAA<br>ATGGCCACCAAGGCGGAATACACCCTTGATGAAGAGAGCAGAGCAAGAATAAAAACCAGACTGTTCACTATAAGACAAGAAATGGCCAGTAGAGGTCTATGGGATTCCTTTCGTCAGTCCGAAAGAGGCGAAGAGACAACTGAAGAAAGATTTGAAATCACAGGAACCATGCGCAGGCTTGCCGACCAAAGTCTCCCACCGAACTTCTCCAGCCTTGAAAACTTTAGAG<br>CCTATGTGGATGGATTCGAACCGAACGGCTGCATTGAGGGCAAGCTTTCTCAAATGTCAAAAGAAGTGAACGCCAGAATTGAGCCATTCCTGAAGACAACACCACGCCCTCTCAGATTACCTGATGGGCCTCCTTGTTCTCAGCGGTCGAAGTTCTTGCTGATGGATGCCCTTAAGTTGAGCATCGAAGACCCTAGCCATGAGGGGGAGGGCATACCGCTGTATGATGCAA<br>TCAAATGCATGAAGACATTTTTTGGCTGGAAAGAGCCCAACCTCGTAAAGCCGCATGAGAAAGGCATAAACCCTAATTACCTCCTGGCTTGGAAGCAGGTGCTGGCAGAACTTCAAGATATTGAGAATGAGGAGAAATTTCCAAAAACAAAGAACATGAAGAAAACAAGCCAATTGAAGTGGGCACTTGGTGAGAACATGGCACCAGAAAAAGTGGACTTTGAGGACTG<br>CAAAGATGTTAGCGATCTAAGACAGTACGACAGTGACGAACCAGAGCCTAGATCACTAGCAAGCTGGATTCAGAGTGAATTCAACAAGGCATGCGAATTGACAGATTCGAGTTGGATTGAACTTGATGAGATAGGGGAAGACGTTGCTCCAATCGAACACATTGCGAGTATGAGGAGGAACTATTTCACAGCGGAGGTATCCCATTGCAGGGCCACTGAATACATAATG<br>AAGGGAGTATACATAAACACAGCCCTATTGAATGCATCCTGTGCAGCCATGGATGACTTCCAACTGATTCCAATGATAAGCAAGTGCAGAACCAAAGAGGGAAGACGGAAGACAAATCTGTATGGATTCATTATAAAAGGAAGATCCCATTTGAGGAATGACACCGATGTGGTGAACTTTGTGAGCATGGAATTCTCTCTAACTGACCCGAGGCTAGAGCCACACAAATG<br>GGAAAAGTACTGTGTTCTTGAGATAGGAGACATGCTCCTACGGACTGCAATAGGCCAAGTGTCGAGGCCCATGTTCCTGTATGTGAGAACCAATGGGACTTCCAAGATCAAAATGAAATGGGGCATGGAGATGAGACGATGCCTTCTTCAGTCCCTTCAACAAATTGAGAGCATGATTGAGGCCGAATCTTCTGTCAAAGAGAAGGACATGACCAAGGAATTCTTTGAAA<br>ACAAATCAGAAACATGGCCAATTGGAGAATCACCCAAAGGGGTTGAGGAAGGCTCTATTGGGAAAGTATGCAGAACATTGCTAGCAAAGTCTGTGTTCAACAGCCTATATGCTTCTCCACAACTCGAGGGATTTTCAGCTGAATCAAGAAAATTGCTTCTCATTGTTCAGGCACTTAGGGACAACCTGGAACCTGGAACCTTCGATCTTGGGGGGCTATATGAAGCAATTG<br>AGGAGTGCCTGATTAACGATCCCTGGGTTTTGCTTAATGCGTCTTGGTTCAACTCCTTCATCACACATGCACTGAAATA-
EPI_ISL_9029962	>A/Whooper_swan/Scotland/056219/2021_H5N1_2021-11-09<br>ATGGAAGACTTTGTGCGACAATGCTTCAATCCAATGATTGTCGAGCTTGCGGAAAAAGCAATGAAAGAATATGGGGAAGATCCGAAAATCGAGACAAACAAATTTGCCGCAATATGCACACACTTAGAAGTCTGTTTCATGTATTCGGATTTCCATTTTATTGATGAACGAGGCGAATCAACGATTGTAGAATCTGGCGATCCAAATGCATTATTGAAACACCGATTTGAGA<br>TAATCGAAGGGAGAGACCGAGCAATGGCCTGGACAGTGGTGAATAGTATCTGCAACACCACAGGAGTCGAAAAGCCCAAATTCCTCCCTGATTTGTATGACTACAAAGAGAACCGATTCATTGAAATTGGAGTAACGCGAAGGGAAGTTCACATATACTATTTAGAAAAAGCCAACAAGATAAAATCAGAGAAAACACACATTCACATATTCTCATTCACTGGAGAGGAA<br>ATGGCCACCAAGGCGGAATACACCCTTGATGAAGAGAGCAGAGCAAGAATAAAAACCAGACTGTTCACTATAAGACAAGAAATGGCCAGTAGAGGTCTATGGGATTCCTTTCGTCAGTCCGAAAGAGGCGAAGAGACAACTGAAGAAAGATTTGAAATCACAGGAACCATGCGCAGGCTTGCCGACCAAAGTCTCCCACCGAACTTCTCCAGCCTTGAAAACTTTAGAG<br>CCTATGTGGATGGATTCGAACCGAACGGCTGCATTGAGGGCAAGCTTTCTCAAATGTCAAAAGAAGTGAACGCCAGAATTGAGCCATTCCTGAAGACAACACCACGCCCTCTCAGATTACCTGATGGGCCTCCTTGTTCTCAGCGGTCGAAGTTCTTGCTGATGGATGCCCTTAAGTTGAGCATCGAAGACCCTAGCCATGAGGGGGAGGGCATACCGCTGTATGATGCAA<br>TCAAATGCATGAAGACATTTTTTGGCTGGAAAGAGCCCAACCTCGTAAAGCCGCATGAGAAAGGCATAAACCCTAATTACCTCCTGGCTTGGAAGCAGGTGCTGGCAGAACTTCAAGATATTGAGAATGAGGAGAAATTTCCAAAAACAAAGAACATGAAGAAAACAAGCCAATTGAAGTGGGCACTTGGTGAGAACATGGCACCAGAAAAAGTGGACTTTGAGGACTG<br>CAAAGATGTTAGCGATCTAAGACAGTACGACAGTGACGAACCAGAGCCTAGATCACTAGCAAGCTGGATTCAGAGTGAATTCAACAAGGCATGCGAATTGACAGATTCGAGTTGGATTGAACTTGATGAGATAGGGGAAGACGTTGCTCCAATCGAACACATTGCGAGTATGAGGAGGAACTATTTCACAGCGGAGGTATCCCATTGCAGGGCCACTGAATACATAATG<br>AAGGGAGTATACATAAACACAGCCCTATTGAATGCATCCTGTGCAGCCATGGATGACTTCCAACTGATTCCAATGATAAGCAAGTGCAGAACCAAAGAGGGAAGACGGAAGACAAATCTGTATGGATTCATTATAAAAGGAAGATCCCATTTGAGGAATGACACCGATGTGGTGAACTTTGTGAGCATGGAATTCTCTCTAACTGACCCGAGGCTAGAGCCACACAAATG<br>GGAAAAGTACTGTGTTCTTGAGATAGGAGACATGCTCCTACGGACTGCAATAGGCCAAGTGTCGAGGCCCATGTTCCTGTATGTGAGAACCAATGGGACTTCCAAGATCAAAATGAAATGGGGCATGGAGATGAGACGATGCCTTCTTCAGTCCCTTCAACAAATTGAGAGCATGATTGAGGCCGAATCTTCTGTCAAAGAGAAGGACATGACCAAGGAATTCTTTGAAA<br>ACAAATCAGAAACATGGCCAATTGGAGAATCACCCAAAGGGGTTGAGGAAGGCTCTATTGGGAAAGTATGCAGAACATTGCTAGCAAAGTCTGTGTTCAACAGCCTATATGCTTCTCCACAACTCGAGGGATTTTCAGCTGAATCAAGAAAATTGCTTCTCATTGTTCAGGCACTTAGGGACAACCTGGAACCTGGAACCTTCGATCTTGGGGGGCTATATGAAGCAATTG<br>AGGAGTGCCTGATTAACGATCCCTGGGTTTTGCTTAATGCGTCTTGGTTCAACTCCTTCATCACACATGCACTGAAATA-
EPI_ISL_11406402	>A/domestic_duck/England/007588/2022_H5N1_2022-01-23<br>ATGGAAGACTTTGTGCGACAATGCTTCAATCCAATGATTGTCGAGCTTGCGGAAAAAGCAATGAAAGAATATGGGGAAGATCCGAAAATCGAGACAAACAAATTTGCCGCAATATGCACACACTTAGAAGTCTGTTTCATGTATTCGGATTTCCATTTTATTGATGAACGAGGCGAATCAACGATTGTAGAATCTGGCGATCCAAATGCATTATTGAAACACCGATTTGAGA<br>TAATCGAAGGGAGAGACCGAGCAATGGCCTGGACAGTGGTGAATAGTATCTGCAACACCACAGGAGTCGAAAAGCCCAAATTCCTCCCTGATTTGTATGACTACAAAGAGAACCGATTCATTGAAATTGGAGTAACGCGAAGGGAAGTTCACATATACTATTTAGAAAAAGCCAACAAGATAAAATCAGAGAAAACACACATTCACATATTCTCATTCACTGGAGAGGAA<br>ATGGCCACCAAGGCGGAATACACCCTTGATGAAGAGAGCAGAGCAAGAATAAAAACTAGACTGTTCACTATAAGACAAGAAATGGCCAGTAGAGGTCTATGGGATTCCTTTCGTCAGTCCGAAAGAGGCGAAGAGACAACTGAAGAAAGATTTGAAATCACAGGAACCATGCGCAGGCTTGCCGACCAAAGTCTCCCACCGAACTTCTCCAGCCTTGAAAACTTTAGAG<br>CCTATGTGGATGGATTCGAACCGAACGGCTGCATTGAGGGCAAGCTTTCTCAAATGTCAAAAGAAGTGAACGCCAGAATTGAGCCATTCCTGAAGACAACACCACGCCCTCTCAGATTACCTGATGGGCCTCCTTGTTCTCAGCGGTCGAAGTTCTTGCTGATGGATGCCCTTAAGTTGAGCATCGAAGACCCTAGCCATGAGGGGGAGGGCATACCGCTGTATGATGCAA<br>TCAAATGCATGAAGACATTTTTTGGCTGGAAAGAGCCCAACCTCGTAAAGCCGCATGAGAAAGGCATAAACCCTAATTACCTCCTGGCTTGGAAGCAGGTGCTGGCAGAACTTCAAGATATTGAGAATGAGGAGAAATTTCCAAAAACAAAGAACATGAAGAAAACAAGCCAATTGAAGTGGGCACTTGGTGAGAACATGGCACCAGAAAAAGTGGACTTTGAGGACTG<br>CAAAGATGTTAGCGATCTAAGACAGTACGACAGTGACGAACCAGAGCCTAGATCACTAGCAAGCTGGATTCAGAGTGAATTCAACAAGGCATGCGAATTGACAGATTCGAGTTGGATTGAACTTGATGAGATAGGGGAAGACGTTGCTCCAATCGAACACATTGCGAGTATGAGGAGGAACTATTTCACAGCGGAGGTATCCCATTGCAGGGCCACTGAATACATAATG<br>AAGGGAGTATACATAAACACAGCCCTATTGAATGCATCCTGTGCAGCCATGGATGACTTCCAACTGATTCCAATGATAAGCAAGTGCAGAACCAAAGAGGGAAGACGGAAGACAAATCTGTATGGATTCATTATAAAAGGAAGATCCCATTTGAGGAATGACACCGATGTGGTGAACTTTGTGAGCATGGAATTTTCTCTAACTGACCCGAGGCTAGAGCCACACAAATG<br>GGAAAAGTACTGTGTTCTTGAGATAGGAGACATGCTCCTACGGACTGCAATAGGCCAAGTGTCGAGGCCCATGTTCCTGTATGTGAGAACCAATGGGACTTCCAAGATCAAAATGAAATGGGGCATGGAGATGAGACGATGCCTTCTTCAGTCCCTTCAACAAATTGAGAGCATGATTGAGGCCGAATCTTCTGTCAAAGAGAAGGACATGACCAAGGAATTCTTTGAAA<br>ACAAATCAGAAACATGGCCAATTGGAGAATCACCCAAAGGGGTTGAGGAAGGCTCTATTGGGAAAGTATGCAGAACATTGCTAGCAAAGTCTGTGTTCAACAGCCTATATGCTTCTCCACAACTCGAGGGATTTTCAGCTGAATCAAGAAAATTGCTTCTCATTGTTCAGGCACTTAGGGACAACCTGGAACCTGGAACCTTCGATCTTGGGGGGCTATATGAAGCAATTG<br>AGGAGTGCCTGATTAACGATCCCTGGGTTTTGCTTAATGCGTCTTGGTTCAACTCCTTCATCACACATGCACTGAAATA-

EPI_ISL_11922816	>A/swan/Romania/10324_22VIR2749-2/2022_H5N1_2022-02-01<br>ATGGAAGACTTTGTGCGACAATGCTTCAATCCAATGATTGTCGAGCTTGCGGAAAAAGCAATGAAAGAATATGGGGAAGATCCGAAAATCGAGACAAACAAATTTGCCGCAATATGCACACACTTAGAAGTCTGTTTCATGTATTCGGATTTCCATTTTATTGATGAACGAGGCGAATCAACGATTGTAGAATCTGGCGATCCAAATGCATTATTGAAACACCGATTTGAGA<br>TAATCGAAGGGAGAGACCGAGCAATGGCCTGGACAGTGGTGAATAGTATCTGCAACACCACAGGAGTCGAAAAGCCCAAATTCCTCCCTGATTTGTATGACTACAAAGAGAACCGATTCATTGAAATTGGAGTAACGCGAAGGGAAGTTCACATATACTATTTAGAAAAAGCCAACAAGATAAAATCAGAGAAAACACACATTCACATATTCTCATTCACTGGAGAGGAA<br>ATGGCCACCAAGGCGGAATACACCCTTGATGAAGAGAGCAGAGCAAGAATAAAAACCAGACTGTTCACTATAAGACAAGAAATGGCCAGTAGAGGTCTATGGGATTCCTTTCGTCAGTCCGAAAGAGGCGAAGAGACAACTGAAGAAAGATTTGAAATCACAGGAACCATGCGCAGGCTTGCCGACCAAAGTCTCCCACCGAACTTCTCCAGCCTTGAAAACTTTAGAG<br>CCTATGTGGATGGATTCGAACCGAACGGCTGCATTGAGGGCAAGCTTTCTCAAATGTCAAAAGAAGTGAACGCCAGAATTGAGCCATTCCTGAAGACAACACCACGCCCTCTCAGATTACCTGATGGGCCTCCTTGTTCTCAGCGGTCGAAGTTCTTGCTGATGGATGCCCTTAAGTTGAGCATCGAAGACCCTAGCCATGAGGGGGAGGGCATACCGCTGTATGATGCAA<br>TCAAATGCATGAAGACATTTTTTGGCTGGAAAGAGCCCAACCTCGTAAAGCCGCATGAGAAAGGCATAAACCCTAATTACCTCCTGGCTTGGAAGCAGGTGCTGGCAGAACTTCAAGATATTGAGAATGAGGAGAAATTTCCAAAAACAAAGAACATGAAGAAAACAAGCCAATTGAAGTGGGCACTTGGTGAGAACATGGCACCAGAAAAAGTGGACTTTGAGGACTG<br>CAAAGATGTTAGCGATCTAAGACAGTACGACAGTGACGAACCAGAGCCTAGATCACTGGCAAGCTGGATTCAGAGTGAATTCAACAAGGCATGCGAATTGACAGATTCGAGTTGGATTGAACTTGATGAGATAGGGGAAGACGTTGCTCCAATCGAACACATTGCGAGTATGAGGAGGAACTATTTCACAGCGGAGGTATCCCATTGCAGGGCCACTGAATACATAATG<br>AAGGGAGTATACATAAACACAGCCCTATTGAATGCATCCTGTGCAGCCATGGATGACTTCCAGCTGATTCCAATGATAAGCAAGTGCAGAACCAAAGAGGGAAGACGGAAGACAAATCTGTATGGATTCATTATAAAAGGAAGATCCCATTTGAGGAATGACACCGATGTGGTGAACTTTGTGAGCATGGAATTCTCTCTAACTGACCCGAGGCTAGAGCCACACAAATG<br>GGAAAAGTACTGTGTTCTTGAGATAGGAGACATGCTCCTACGGACTGCAATAGGCCAAGTGTCGAGGCCCATGTTCCTGTATGTGAGAACCAATGGGACTTCCAAGATCAAAATGAAATGGGGCATGGAGATGAGACGATGCCTTCTTCAGTCCCTTCAACAAATTGAGAGCATGATTGAGGCCGAATCTTCTGTCAAAGAGAAGGACATGACCAAGGAATTCTTTGAAA<br>ACAAATCAGAAACATGGCCAATTGGAGAATCACCCAAAGGGGTTGAGGAAGGCTCTATTGGGAAAGTATGCAGAACATTGCTAGCAAAGTCTGTGTTCAACAGCCTATATGCTTCTCCACAACTCGAGGGATTTTCAGCTGAATCAAGAAAATTGCTTCTCATTGTTCAGGCACTTAGGGACAACCTGGAACCTGGAACCTTCGATCTTGGGGGGCTATATGAAGCAATTG<br>AGGAGTGCCTGATTAACGATCCCTGGGTTTTGCTTAATGCGTCTTGGTTCAACTCCTTCATCACACATGCACTGAAATA-
EPI_ISL_11007535	>A/swan/Slovenia/13_22VIR777-8/2022_H5N1_2022-01-03<br>ATGGAAGACTTTGTGCGACAATGCTTCAATCCAATGATTGTCGAGCTTGCGGAAAAAGCAATGAAAGAATATGGGGAAGATCCGAAAATCGAGACAAACAAATTTGCCGCAATATGCACACACTTAGAAGTCTGTTTCATGTATTCGGATTTCCATTTTATTGATGAACGAGGCGAATCAAGGATTGTAGAATCTGGCGATCCAAATGCATTATTGAAACACCGATTTGAGA<br>TAATCGAAGGGAGAGACCGAGCAATGGCCTGGACAGTGGTGAATAGTATCTGCAACACTACAGGAGTCGAAAAGCCCAAATTCCTCCCTGATTTGTATGACTACAAAGAGAACCGATTCATTGAAATTGGAGTAACGCGAAGGGAAGTTCACATATACTATTTAGAAAAAGCCAACAAGATAAAATCAGAGAAAACACACATTCACATATTCTCATTCACTGGAGAGGAA<br>ATGGCCACCAAGGCGGAATACACCCTTGATGAAGAGAGCAGAGCAAGAATAAAAACCAGACTGTTCACTATAAGACAAGAAATGGCCAGTAGAGGTCTATGGGATTCCTTTCGTCAGTCCGAGAGAGGCGAAGAGACAACTGAAGAAAGATTTGAAATCACAGGAACCATGCGCAGGCTTGCCGACCAAAGTCTCCCACCGAACTTCTCCAGCCTTGAAAACTTTAGAG<br>CCTATGTGGATGGATTCGAACCGAACGGCTGCATTGAGGGCAAGCTTTCTCAAATGTCAAAAGAAGTGAACGCCAGAATTGAGCCATTCCTGAAGACAACACCACGCCCTCTCAGATTACCTGATGGGCCTCCTTGTTCTCAGCGGTCGAAGTTCTTGCTGATGGATGCCCTTAAGTTGAGCATCGAAGATCCTAGCCATGAGGGGGAGGGCATACCGCTGTATGATGCAA<br>TCAAATGCATGAAGACATTTTTTGGCTGGAAAGAGCCCAACCTCGTAAAGCCGCATGAGAAAGGCATAAACCCTAATTACCTCCTGGCTTGGAAGCAGGTGCTGGCAGAACTTCAAGATATTGAGAATGAGGAGAAATTCCCAAAAACAAAGAACATGAAGAAAACAAGCCAATTGAAGTGGGCACTTGGTGAGAACATGGCACCAGAAAAAGTGGACTTTGAGGACTG<br>CAAAGATGTTAGCGATCTAAGACAGTACGACAGTGACGAACCAGAGCCTAGATCACTAGCAAGCTGGATTCAGAGTGAATTCAACAAGGCATGCGAATTGACAGATTCTAGTTGGATTGAACTTGATGAGATAGGGGAAGACGTTGCTCCAATCGAACACATTGCGAGTATGAGGAGGAACTATTTCACAGCGGAGGTATCCCATTGCAGGGCCACTGAATACATAATGA<br>AGGGAGTATACATAAACACAGCCCTATTGAATGCATCCTGTGCAGCCATGGATGACTTCCAACTGATTCCAATGATAAGCAAGTGCAGAACCAAAGAGGGAAGACGGAAGACAAATCTGTATGGATTCATTATAAAAGGAAGATCCCATTTGAGGAATGACACCGATGTGGTAAATTTTGTGAGCATGGAATTCTCTCTAACTGACCCGAGGCTAGAGCCACACAAATGG<br>GAAAAGTACTGTGTTCTTGAGATAGGAGACATGCTCCTACGGACTGCAATAGGCCAAGTGTCGAGGCCCATGTTCCTGTATGTGAGAACCAATGGGACTTCCAAGATCAAAATGAAATGGGGCATGGAGATGAGACGATGCCTTCTTCAGTCCCTTCAACAAATTGAGAGCATGATTGAGGCCGAATCTTCTGTCAAAGAGAAGGACATGACCAAGGAATTCTTTGAAAA<br>CAAATCAGAAACATGGCCAATTGGAGAATCACCCAAAGGGGTTGAGGAAGGCTCTATTGGGAAAGTATGCAGAACATTGCTAGCAAAGTCTGTGTTCAACAGCCTATATGCATCTCCACAACTCGAGGGATTTTCAGCTGAATCAAGAAAATTGCTTCTCATTGTTCAGGCACTTAGGGACAACCTGGAACCTGGAACCTTCGATCTTGGGGGGCTATATGAAGCAATTGA<br>GGAGTGCCTGATTAACGATCCCTGGGTTTTGCTTAATGCGTCTTGGTTCAACTCCTTCATCACACATGCACTGAAATA-
EPI_ISL_14761507	>A/broiler/Italy/22VIR1892-3/2022_H5N1_2022-02-23<br>ATGGAAGACTTTGTGCGACAATGCTTCAATCCAATGATTGTCGAGCTTGCGGAAAAAGCAATGAAAGAATATGGGGAAGATCCGAAAGTCGAGACAAACAAATTTGCCGCAATATGCACACACTTAGAAGTCTGTTTCATGTATTCGGATTTCCATTTTATTGATGAACGAGGCGAATCAATGATTGTAGAATCTGGCGATCCAAATGCATTATTGAAACACCGATTTGAGA<br>TAATCGAAGGGAGAGACCGAGCAATGGCCTGGACAGTGGTGAATAGTATCTGCAACACCACAGGAGTCGAAAAGCCCAAATTCCTCCCTGATTTGTATGACTACAAAGAGAACCGATTCATTGAAATTGGAGTAACGCGAAGGGAAGTTCACATATACTATTTAGAAAAAGCCAACAAGATAAAATCGGAGAAAACACACATTCACATATTCTCATTCACTGGAGAGGAA<br>ATGGCCACCAAGGCGGAATACACCCTTGATGAAGAGAGCAGAGCAAGAATAAAAACCAGACTATTCACTATAAGACAAGAAATGGCCAGTAGAGGTCTATGGGATTCCTTTCGTCAGTCCGAGAGAGGCGAAGAGACAACTGAAGAAAGATTTGAAATCACAGGAACCATGCGCAGGCTTGCCGACCAAAGTCTCCCACCGAACTTCTCCAGCCTTGAAAACTTTAGAG<br>CCTATGTGGATGGATTCGAACCGAACGGCTGCATTGAGGGCAAGCTTTCTCAAATGTCAAAAGAAGTGAACGCCAGAATTGAGCCATTCCTGAAGACAACACCACGCCCTCTCAGATTACCTGATGGGCCTCCTTGYTCTCAGCGGTCGAAGTTCCTGCTGATGGATGCCCTTAAGTTGAGCATCGAAGACCCTAGCCATGAGGGGGAGGGCATACCGCTGTATGATGCAA<br>TCAAATGCATGAAGACATTTTTTGGCTGGAAAGAGCCCAACCTCGTAAAGCCACATGAGAAAGGCATAAACCCTAATTACCTCCTGGCTTGGAAGCAGGTGCTAGCAGAACTTCAAGATATTGAGAATGAGGAGAAATTTCCAAAAACAAAGAACATGAAGAAAACAAGCCAATTGAAGTGGGCACTTGGTGAGAACATGGCACCAGAAAAAGTGGACTTTGAGGACTG<br>CAAAGATGTTAGCGATCTAAGACAGTACGACAGTGACGAACCAGAGCCTAGATCACTAGCAAGCTGGATTCAGAGTGAATTCAACAAGGCATGCGAATTGACAGATTCGAGTTGGATTGAACTTGATGAGATAGGGGAAGACGTTGCTCCAATCGAACACATTGCGAGTATGAGGAGGAACTATTTCACAGCGGAGGTATCCCATTGCAGGGCCACTGAATACATAATG<br>AAGGGAGTATACATAAACACAGCCCTATTGAATGCATCCTGTGCAGCCATGGATGACTTCCAACTGATTCCAATGATAAGCAAGTGCAGAACCAAAGAAGGAAGACGGAAGACAAATCTGTATGGATTCATTATAAAAGGAAGATCCCATTTGAGGAATGACACCGATGTGGTAAACTTTGTGAGCATGGAATTCTCTCTAACTGACCCGAGGCTAGAGCCACACAAATG<br>GGAAAAGTACTGTGTTCTTGAGATAGGAGACATGCTCCTACGGACTGCAATAGGCCAAGTGTCGAGGCCCATGTTCCTGTATGTGAGAACCAATGGGACTTCCAAGATCAAAATGAAATGGGGCATGGAGATGAGACGATGCCTTCTTCAGTCCCTTCAACAAATTGAGAGCATGATTGAGGCCGAATCTTCTGTCAAAGAGAAGGACATGACCAAGGAATTCTTTGAAA<br>ACAAATCAGAAACATGGCCAATTGGAGAATCACCCAAAGGGGTTGAGGAAGGCTCTATTGGGAAAGTATGCAGAACATTGCTAGCAAAGTCTGTGTTCAACAGCCTATATGCATCTCCACAACTCGAGGGATTTTCAGCTGAATCAAGAAAATTGCTTCTCATTGTTCAGGCACTTAGGGACAACCTGGAACCTGGAACCTTCGATCTTGGGGGGCTATATGAAGCAATTG<br>AGGAGTGCCTGATTAACGATCCCTGGGTTTTGCTTAATGCGTCTTGGTTCAACTCCTTCCTCACACATGCACTGAAATA-
EPI_ISL_7733591	>A/mallard/Italy/21VIR8919-2/2021_H5N1_2021-10-27<br>ATGGAAGACTTTGTGCGACAATGCTTCAATCCAATGATTGTCGAGCTTGCGGAAAAAGCAATGAAAGAATATGGGGAAGATCCGAAAATCGAGACAAACAAATTTGCCGCAATATGCACACACTTAGAAGTCTGTTTCATGTATTCGGATTTCCATTTTATTGATGAACGAGGCGAATCAATGATTGTAGAATCTGGCGATCCAAATGCATTATTGAAACACCGATTTGAGA<br>TAATCGAAGGGAGAGACCGAGCAATGGCCTGGACAGTGGTGAATAGTATCTGCAACACCACAGGAGTCGAAAAGCCCAAATTCCTCCCTGATTTGTATGACTACAAAGAGAACCGATTCATTGAAATTGGAGTAACGCGAAGAGAAGTTCACATATACTATTTAGAAAAGGCCAACAAGATAAAATCAGAGAAAACACACATTCACATATTCTCGTTCACTGGAGAGGAA<br>ATGGCCACCAAGGCGGAATACACCCTTGATGAAGAGAGCAGAGCAAGAATAAAAACCAGACTGTTCACTATAAGACAAGAAATGGCCAGTAGAGGTCTATGGGATTCCTTTCGTCAGTCCGAGAGAGGCGAAGAGACAACTGAAGAAAGATTTGAAATCACAGGAACCATGCGCAGGCTTGCCGACCAAAGTCTCCCACCGAACTTCTCCAGCCTTGAAAACTTTAGAG<br>CCTATGTGGATGGATTCGAACCGAACGGCTGCATTGAGGGCAAGCTTTCTCAAATGTCAAAAGAAGTGAACGCCAGAATTGAGCCATTCCTGAAGACAACACCACGCCCTCTCAGATTACCTGATGGGCCTCCTTGTTCTCAGCGGTCGAAGTTCTTGCTGATGGATGCCCTTAAGTTGAGCATCGAAGACCCTAGCCATGAGGGGGAGGGCATACCGCTGTATGATGCAA<br>TCAAATGCATGAAGACATTTTTTGGCTGGAAAGAGCCCAACCTCGTAAAGCCGCATGAGAAAGGCATAAACCCTAATTACCTCCTGGCTTGGAAGCAGGTGCTGGCAGAACTTCAAGATATTGAGAATGAGGAGAAATTTCCAAAAACAAAGAACATGAAGAAAACAAGCCAATTGAAGTGGGCACTTGGTGAGAACATGGCACCAGAAAAAGTGGACTTTGAGGACTG<br>CAAAGATGTTAGCGATCTAAGACAGTACGACAGTGACGAACCAGAGCCTAGATCACTAGCAAGCTGGATTCAGAGTGAATTCAACAAGGCATGCGAATTGACAGATTCGAGTTGGATTGAACTTGACGAGATAGGGGAAGACGTTGCTCCAATCGAACACATTGCGAGTATGAGGAGGAACTATTTCACAGCGGAGGTATCCCATTGCAGGGCCACTGAATACATAATG<br>AAGGGAGTATACATAAACACAGCCCTATTGAATGCATCCTGTGCAGCCATGGATGACTTCCAACTGATTCCAATGATAAGCAAGTGCAGAACCAAAGAAGGAAGACGGAAGACAAATCTGTATGGATTCATTATAAAAGGAAGATCCCATTTGAGGAATGACACCGATGTGGTAAACTTTGTGAGCATGGAATTCTCTCTAACTGACCCGAGGCTAGAGCCACACAAATG<br>GGAAAAGTACTGTGTTCTTGAGATAGGAGACATGCTCCTACGGACTGCAATAGGCCAAGTGTCGAGGCCCATGTTCCTGTATGTGAGAACCAATGGGACTTCCAAGATCAAAATGAAATGGGGCATGGAGATGAGACGATGCCTTCTTCAGTCCCTTCAACAAATTGAGAGCATGATTGAGGCCGAATCTTCTGTCAAAGAGAAGGACATGACCAAGGAATTCTTTGAAA<br>ACAAATCAGAAACATGGCCAATTGGAGAATCACCCAAAGGGGTTGAGGAAGGCTCTATTGGGAAAGTATGCAGAACATTGCTAGCAAAGTCTGTGTTCAACAGCCTATATGCATCTCCACAACTCGAGGGATTTTCAGCTGAATCAAGAAAATTGCTTCTCATTGTTCAGGCACTTAGGGACAACCTGGAACCTGGAACCTTCGATCTTGGGGGGCTATATGAAGCAATTG<br>AGGAGTGCCTGATTAACGATCCCTGGGTTTTGCTTAATGCGTCTTGGTTCAACTCCTTCCTCACACATGCACTGAAATA-
EPI_ISL_14761349	>A/hawk/Italy/22VIR428-7/2022_H5N1_2022-01-14<br>ATGGAAGACTTTGTGCGACAATGCTTCAATCCAATGATTGTCGGGCTTGCGGAAAAAGCAATGAAAGAATATGGGGAAGATCCGAAAATCGAGACAAACAAATTTGCCGCAATATGCACACACTTAGAAGTCTGTTTCATGTATTCGGATTTCCATTTTATTGATGAACGAGGCGAATCAATGATTGTAGAATCTGGCGATCCAAATGCATTATTGAAACACCGATTTGAGA<br>TAATCGAAGGGAGAGACCGAGCAATGGCCTGGACAGTGGTGAATAGTATCTGCAACACCACAGGAGTCGAAAAGCCCAAATTCCTCCCTGATTTGTATGACTACAAAGAGAACCGATTCATTGAAATTGGAGTAACGCGAAGAGAAGTTCACATATACTATTTAGAAAAGGCCAACAAGATAAAATCAGAGAAAACACACATTCACATATTCTCGTTCACTGGAGAGGAA<br>ATGGCCACCAAGGCGGAATACACCCTTGATGAAGAGAGCAGAGCAAGAATAAAAACCAGACTGTTCACTATAAGACAAGAAATGGCCAGTAGAGGTCTATGGGATTCCTTTCGTCAGTCCGAGAGAGGCGAAGAGACAACTGAAGAAAGATTTGAAATCACAGGAACCATGCGCAGGCTTGCCGACCAAAGTCTCCCACCGAACTTCTCCAGCCTTGAAAACTTTAGAG<br>CCTATGTGGATGGATTCGAACCGAACGGCTGCATTGAGGGCAAGCTTTCTCAAATGTCAAAAGAAGTGAACGCCAGAATTGAGCCATTCCTGAAGACAACACCACGCCCTCTCAGATTACCTGATGGGCCTCCTTGTTCTCAGCGGTCGAAGTTCTTGCTGATGGATGCCCTTAAGTTGAGCATCGAAGACCCTAGCCATGAGGGGGAGGGCATACCGCTGTATGATGCAA<br>TCAAATGCATGAAGACATTTTTTGGCTGGAAAGAGCCCAACCTCGTAAAGCCGCATGAGAAAGGCATAAACCCTAATTACCTCCTGGCTTGGAAGCAGGTGCTGGCAGAACTTCAAGATATTGAGAATGAGGAGAAATTTCCAAAAACAAAGAACATGAAGAAAACAAGCCAATTGAAGTGGGCACTTGGTGAGAACATGGCACCAGAAAAAGTGGACTTTGAGGACTG<br>CAAAGATGTTAGCGATCTAAGACAGTACGACAGTGACGAACCAGAGCCTAGATCACTAGCAAGCTGGATTCAGAGTGAATTCAACAAGGCATGCGAATTGACAGATTCGAGTTGGATTGAACTTGACGAGATAGGGGAAGACGTTGCTCCAATCGAACACATTGCGAGTATGAGGAGGAACTATTTCACAGCGGAGGTATCCCATTGCAGGGCCACTGAATACATAATG<br>AAGGGAGTATACATAAACACAGCCCTATTGAATGCATCCTGTGCAGCCATGGATGACTTCCAACTGATTCCAATGATAAGCAAGTGCAGAACCAAAGAAGGAAGACGGAAGACAAATCTGTATGGATTCATTATAAAAGGAAGATCCCATTTGAGGAATGACACCGATGTGGTAAACTTTGTGAGCATGGAATTCTCTCTAACTGACCCGAGGCTAGAGCCACACAAATG<br>GGAAAAGTACTGTGTTCTTGAGATAGGAGACATGCTCCTACGGACTGCAATAGGCCAAGTGTCGAGGCCCATGTTCCTGTATGTGAGAACCAATGGGACTTCCAAGATCAAAATGAAATGGGGCATGGAGATGAGACGATGCCTTCTTCAGTCCCTTCAACAAATTGAGAGCATGATTGAGGCCGAATCTTCTGTCAAAGAGAAGGACATGACCAAGGAATTCTTTGAAA<br>ACAAATCAGAAACATGGCCAATTGGAGAATCACCCAAAGGGGTTGAGGAAGGCTCTATTGGGAAAGTATGCAGAACATTGCTAGCAAAGTCTGTGTTCAACAGCCTATATGCATCTCCACAACTCGAGGGATTTTCAGCTGAATCAAGAAAATTGCTTCTCATTGTTCAGGCACTTAGGGACAACCTGGAACCTGGAACCTTCGATCTTGGGGGGCTATATGAAGCAATTG<br>AGGAGTGCCTGATTAACGATCCCTGGGTTTTGCTTAATGCGTCTTGGTTCAACTCCTTCCTCACACATGCACTGAAATA-
EPI_ISL_14760736	>A/chicken/Italy/21VIR11054-1/2021_H5N1_2021-12-13<br>ATGGAAGACTTTGTGCGACAATGCTTCAATCCAATGATTGTCGGGCTTGCGGAAAAAGCAATGAAAGAATATGGGGAAGATCCGAAAATCGAGACAAACAAATTTGCCGCAATATGCACACACTTAGAAGTCTGTTTCATGTATTCGGATTTCCATTTTATTGATGAACGAGGCGAATCAATGATTGTAGAATCTGGCGATCCAAATGCATTATTGAAACACCGATTTGAGA<br>TAATCGAAGGGAGAGACCGAGCAATGGCCTGGACAGTGGTGAATAGTATCTGCAACACCACAGGAGTCGAAAAGCCCAAATTCCTCCCTGATTTGTATGACTACAAAGAGAACCGATTCATTGAAATTGGAGTAACGCGAAGAGAAGTTCACATATACTATTTAGAAAAGGCCAACAAGATAAAATCAGAGAAAACACACATTCACATATTCTCGTTCACTGGAGAGGAA<br>ATGGCCACCAAGGCGGAATACACCCTTGATGAAGAGAGCAGAGCAAGAATAAAAACCAGACTGTTCACTATAAGACAAGAAATGGCCAGTAGAGGTCTATGGGATTCCTTTCGTCAGTCCGAGAGAGGCGAAGAGACAACTGAAGAAAGATTTGAAATCACAGGAACCATGCGCAGGCTTGCCGACCAAAGTCTCCCACCGAACTTCTCCAGCCTTGAAAACTTTAGAG<br>CCTATGTGGATGGATTCGAACCGAACGGCTGCATTGAGGGCAAGCTTTCTCAAATGTCAAAAGAAGTGAACGCCAGAATTGAGCCATTCCTGAAGACAACACCACGCCCTCTCAGATTACCTGATGGGCCTCCTTGTTCTCAGCGGTCGAAGTTCTTGCTGATGGATGCCCTTAAGTTGAGCATCGAAGACCCTAGCCATGAGGGGGAGGGCATACCGCTGTATGATGCAA<br>TCAAATGCATGAAGACATTTTTTGGCTGGAAAGAGCCCAACCTCGTAAAGCCGCATGAGAAAGGCATAAACCCTAATTACCTCCTGGCTTGGAAGCAGGTGCTGGCAGAACTTCAAGATATTGAGAATGAGGAGAAATTTCCAAAAACAAAGAACATGAAGAAAACAAGCCAATTGAAGTGGGCACTTGGTGAGAACATGGCACCAGAAAAAGTGGACTTTGAGGACTG<br>CAAAGATGTTAGCGATCTAAGACAGTACGACAGTGACGAACCAGAGCCTAGATCACTAGCAAGCTGGATTCAGAGTGAATTCAACAAGGCATGCGAATTGACAGATTCGAGTTGGATTGAACTTGACGAGATAGGGGAAGACGTTGCTCCAATCGAACACATTGCGAGTATGAGGAGGAACTATTTCACAGCGGAGGTATCCCATTGCAGGGCCACTGAATACATAATG<br>AAGGGAGTATACATAAACACAGCCCTATTGAATGCATCCTGTGCAGCCATGGATGACTTCCAACTGATTCCAATGATAAGCAAGTGCAGAACCAAAGAAGGAAGACGGAAGACAAATCTGTATGGATTCATTATAAAAGGAAGATCCCATTTGAGGAATGACACCGATGTGGTAAACTTTGTGAGCATGGAATTCTCTCTAACTGACCCGAGGCTAGAGCCACACAAATG<br>GGAAAAGTACTGTGTTCTTGAGATAGGAGACATGCTCCTACGGACTGCAATAGGCCAAGTGTCGAGGCCCATGTTCCTGTATGTGAGAACCAATGGGACTTCCAAGATCAAAATGAAATGGGGCATGGAGATGAGACGATGCCTTCTTCAGTCCCTTCAACAAATTGAGAGCATGATTGAGGCCGAATCTTCTGTCAAAGAGAAGGACATGACCAAGGAATTCTTTGAAA<br>ACAAATCAGAAACATGGCCAATTGGAGAATCACCCAAAGGGGTTGAGGAAGGCTCTATTGGGAAAGTATGCAGAACATTGCTAGCAAAGTCTGTGTTCAACAGCCTATATGCATCTCCACAACTCGAGGGATTTTCAGCTGAATCAAGAAAATTGCTTCTCATTGTTCAGGCACTTAGGGACAACCTGGAACCTGGAACCTTCGATCTTGGGGGGCTATATGAAGCAATTG<br>AGGAGTGCCTGATTAACGATCCCTGGGTTTTGCTTAATGCGTCTTGGTTCAACTCCTTCCTCACACATGCACTGAAATA-
EPI_ISL_9261750	>A/Black-headed_gull/Netherlands/1/2022_H5N1_2022-01-14<br>ATGGAAGACTTTGTGCGACAATGCTTCAATCCAATGATTGTCGAGCTTGCGGAAAAAGCAATGAAAGAATATGGGGAAGATCCGAAAATCGAGACAAACAAATTTGCCGCAATATGCACACACTTAGAAGTCTGTTTCATGTATTCGGATTTCCATTTTATTGATGAACGAGGCGAATCAATGATTGTAGAATCTGGCGATCCAAATGCATTATTGAAACACCGATTTGAGA<br>TAATCGAAGGGAGAGACCGAGCAATGGCCTGGACAGTGGTGAATAGTATCTGCAACACCACAGGAGTCGAAAAGCCCAAATTCCTCCCTGATTTGTATGACTACAAAGAGAACCGATTCATTGAAATTGGAGTAACGCGAAGAGAAGTTCACATATACTATTTAGAAAAGGCCAACAAGATAAAATCAGAGAAAACACACATTCACATATTCTCGTTCACTGGAGAGGAA<br>ATGGCCACCAAGGCGGAATACACCCTTGATGAAGAGAGCAGAGCAAGAATAAAAACCAGACTGTTCACTATAAGACAAGAAATGGCCAGTAGAGGTCTATGGGATTCCTTTCGTCAGTCCGAGAGAGGCGAAGAGACAACTGAAGAAAGATTTGAAATCACAGGAACCATGCGCAGGCTTGCCGACCAAAGTCTCCCACCGAACTTCTCCAGCCTTGAAAACTTTAGAG<br>CCTATGTGGATGGATTCGAACCGAACGGCTGCATTGAGGGCAAGCTTTCTCAAATGTCAAAAGAAGTGAATGCCAGAATTGAGCCATTCCTGAAGACAACACCACGCCCTCTCAGATTACCTGATGGGCCTCCTTGTTCTCAGCGGTCGAAGTTCTTGCTGATGGATGCCCTTAAGTTGAGCATCGAAGACCCTAGCCATGAGGGGGAGGGCATACCGCTGTATGATGCAA<br>TCAAATGCATGAAGACATTTTTTGGCTGGAAAGAGCCCAACCTCGTAAAGCCGCATGAGAAAGGCATAAACCCTAATTACCTCCTGGCTTGGAAGCAGGTGCTGGCAGAACTTCAAGATGTTGAGAATGAGGAGAAATTTCCAAAAACAAAGAACATGAAGAAAACAAGCCAATTGAAGTGGGCACTTGGTGAGAACATGGCACCAGAAAAAGTGGACTTTGAGGACTG<br>CAAAGATGTTAGCGATCTAAGACAGTACGACAGTGACGAACCAGAGCCTAGATCACTAGCAAGCTGGATTCAGAGTGAATTCAACAAGGCATGCGAATTGACAGATTCGAGTTGGATTGAACTTGACGAGATAGGGGAAGACGTTGCTCCAATCGAACACATTGCGAGTATGAGGAGGAACTATTTCACAGCGGAGGTATCCCATTGCAGGGCCACTGAATACATAATG<br>AAGGGAGTATACATAAACACAGCCCTATTGAATGCATCCTGTGCAGCCATGGATGACTTCCAACTGATTCCAATGATAAGCAAGTGCAGAACCAAAGAAGGAAGACGGAAGACAAATCTGTATGGATTCATTATAAAAGGAAGATCCCATTTGAGGAATGACACCGATGTGGTAAACTTTGTGAGCATGGAATTCTCTCTAACTGACCCGAGGCTAGAGCCACACAAATG<br>GGAAAAGTACTGTGTTCTTGAGATAGGAGACATGCTCCTACGGACTGCAATAGGCCAAGTGTCGAGGCCCATGTTCCTGTATGTGAGAACCAATGGGACTTCCAAGATCAAAATGAAATGGGGCATGGAGATGAGACGATGCCTTCTTCAGTCCCTTCAACAAATTGAGAGCATGATTGAGGCCGAATCTTCTGTCAAAGAGAAGGACATGACCAAGGAATTCTTTGAAA<br>ACAAATCAGAAACATGGCCAATTGGAGAATCACCCAAAGGGGTTGAGGAAGGCTCTATTGGGAAAGTATGCAGAACATTGCTAGCAAAGTCTGTGTTCAACAGCCTATATGCATCTCCACAACTCGAGGGATTTTCAGCTGAATCAAGAAAATTGCTTCTCATTGTTCAGGCACTTAGGGACAACCTGGAACCTGGAACCTTCGATCTTGGGGGGCTATATGAAGCAATTG<br>AGGAGTGCCTGATTAACGATCCCTGGGTTTTGCTTAATGCGTCTTGGTTCAACTCCTTCCTCACACATGCACTGAAATA-
EPI_ISL_14389133	>A/Larus_argentatus/Belgium/595_0008/2022_H5N1_2022-01-06<br>ATGGAAGACTTTGTGCGACAATGCTTCAATCCAATGATTGTCGAGCTTGCGGAAAAAGCAATGAAAGAATATGGGGAAGATCCGAAAATCGAGACAAACAAATTTGCCGCAATATGCACACACTTAGAAGTCTGTTTCATGTATTCGGATTTCCATTTTATTGATGAACGAGGCGAATCAATGATTGTAGAATCTGGCGATCCAAATGCATTATTGAAACACCGATTTGAGA<br>TAATCGAAGGGAGAGACCGAGCAATGGCCTGGACAGTGGTGAATAGTATCTGCAACACCACAGGAGTCGAAAAGCCCAAATTCCTCCCTGATTTGTATGACTACAAAGAGAACCGATTCATTGAAATTGGAGTAACGCGAAGAGAAGTTCACATATACTATTTAGAAAAGGCCAACAAGATAAAATCAGAGAAAACACACATTCACATATTCTCGTTCACTGGAGAGGAA<br>ATGGCCACCAAGGCGGAATACACCCTTGATGAAGAGAGCAGAGCAAGAATAAAAACCAGACTGTTCACTATAAGACAAGAAATGGCCAGTAGAGGTCTATGGGATTCCTTTCGTCAGTCCGAGAGAGGCGAAGAGACAACTGAAGAAAGATTTGAAATCACAGGAACCATGCGCAGGCTTGCCGACCAAAGTCTCCCACCGAACTTCTCCAGCCTTGAAAACTTTAGAG<br>CCTATGTGGATGGATTCGAACCGAACGGCTGCATTGAGGGCAAGCTTTCTCAAATGTCAAAAGAAGTGAACGCCAGAATTGAGCCATTCCTGAAGACAACACCACGCCCTCTCAGATTACCTGATGGGCCTCCTTGTTCTCAGCGGTCGAAGTTCTTGCTGATGGATGCCCTTAAGTTGAGCATCGAAGACCCTAGCCATGAGGGGGAGGGCATACCGCTGTATGATGCAA<br>TCAAATGCATGAAGACATTTTTTGGCTGGAAAGAGCCCAACCTCGTAAAGCCGCATGAGAAAGGCATAAACCCTAATTACCTCCTGGCTTGGAAGCAGGTGCTGGCAGAACTTCAAGATATTGAGAATGAGGAGAAATTTCCAAAAACAAAGAACATGAAGAAAACAAGCCAATTGAAGTGGGCACTTGGTGAGAACATGGCACCAGAAAAAGTGGACTTTGAGGACTG<br>CAAAGATGTTAGCGATCTAAGACAGTACGACAGTGACGAACCAGAGCCTAGATCACTAGCAAGCTGGATTCAGAGTGAATTCAACAAGGCATGCGAATTGACAGATTCGAGTTGGATTGAACTTGATGAGATAGGGGAAGACGTTGCTCCAATCGAACATATTGCGAGTATGAGGAGGAACTATTTCACAGCGGAGGTATCTCATTGCAGGGCCACTGAATACATAATG<br>AAGGGAGTATACATAAACACAGCCCTATTGAATGCATCCTGTGCAGCCATGGATGACTTCCAACTGATTCCAATGATAAGCAAGTGCAGAACCAAAGAAGGAAGACGGAAGACAAATCTGTATGGATTCATTATAAAAGGAAGATCCCATTTGAGGAATGACACCGATGTGGTAAACTTTGTGAGCATGGAATTCTCTCTAACTGACCCGAGGCTAGAGCCACACAAATG<br>GGAAAAGTACTGTGTTCTTGAGATAGGAGACATGCTCCTACGGACTGCAATAGGCCAAGTGTCGAGGCCCATGTTCCTGTATGTGAGAACCAATGGGACTTCCAAGATCAAAATGAAATGGGGCATGGAGATGAGACGATGCCTTCTTCAGTCCCTTCAACAAATTGAGAGCATGATTGAGGCCGAATCTTCTGTCAAAGAGAAGGACATGACCAAGGAATTCTTTGAAA<br>ACAAATCAGAAACATGGCCAATTGGAGAATCACCCAAAGGGGTTGAGGAAGGCTCTATTGGGAAAGTATGCAGAACATTGCTAGCAAAGTCTGTGTTCAACAGCCTATATGCATCTCCACAACTCGAGGGATTTTCAGCTGAATCAAGAAAATTGCTTCTCATTGTTCAGGCACTTAGGGACAACCTGGAACCTGGAACCTTCGATCTTGGGGGGCTATATGAAGCAATTG<br>AGGAGTGCCTGATTAACGATCCCTGGGTTTTGCTTAATGCGTCTTGGTTCAACTCCTTCCTCACACATGCACTGAAATA-
EPI_ISL_18245810	>A/greylag_goose/Poland/MB503_21RS3290-18/2021_H5N1_2021-11-22<br>ATGGAAGACTTTGTGCGACAATGCTTCAATCCAATGATTGTCGAGCTTGCGGAAAAAGCAATGAAAGAATATGGGGAAGATCCGAAAATCGAGACAAACAAATTTGCCGCAATATGCACACACTTAGAAGTCTGTTTCATGTATTCGGATTTCCATTTTATTGATGAACGAGGCGAATCAATGATTGTAGAATCTGGCGATCCAAATGCATTATTGAAACACCGATTTGAGA<br>TAATCGAAGGGAGAGACCGAGCAATGGCCTGGACAGTGGTGAATAGTATCTGCAACACCACAGGAGTCGAAAAGCCCAAATTCCTCCCTGATTTGTATGACTACAAAGAGAACCGATTCATTGAAATTGGAGTAACGCGAAGAGAAGTTCACATATACTATTTAGAAAAGGCCAACAAGATAAAATCAGAGAAAACACACATTCACATATTCTCGTTCACTGGAGAGGAA<br>ATGGCCACCAAGGCGGAATACACCCTTGATGAAGAGAGCAGAGCAAGAATAAAAACCAGACTGTTCACTATAAGACAAGAAATGGCCAGTAGAGGTCTATGGGATTCCTTTCGTCAGTCCGAGAGAGGCGAAGAGACAACTGAAGAAAGATTTGAAATCACAGGAACCATGCGCAGGCTTGCCGACCAAAGTCTCCCACCGAACTTCTCCAGCCTTGAAAACTTTAGAG<br>CCTATGTGGATGGATTCGAACCGAACGGCTGCATTGAGGGCAAGCTTTCTCAAATGTCAAAAGAAGTGAACGCCAGAATTGAGCCATTCCTGAAGACAACACCACGCCCTCTCAGATTACCTGATGGGCCTCCTTGTTCTCAGCGGTCGAAGTTCTTGCTGATGGATGCCCTTAAGTTGAGCATCGAAGACCCTAGCCATGAGGGGGAGGGCATACCGCTGTATGATGCAA<br>TCAAATGCATGAAGACATTTTTTGGCTGGAAAGAGCCCAACCTCGTAAAGCCGCATGAGAAAGGCATAAACCCTAATTACCTCCTGGCTTGGAAGCAGGTGCTGGCAGAACTTCAAGATATTGAGAATGAGGAGAAATTTCCAAAAACAAAGAACATGAAGAAAACAAGCCAATTGAAGTGGGCACTTGGTGAGAACATGGCACCAGAAAAAGTGGACTTTGAGGACTG<br>CAAAGATGTTAGCGATCTAAGACAGTACGACAGTGACGAACCAGAGCCTAGATCACTAGCAAGCTGGATTCAGAGTGAATTCAACAAGGCATGCGAATTGACAGATTCGAGTTGGATTGAACTTGACGAGATAGGGGAAGACGTTGCTCCAATCGAACACATTGCGAGTATGAGGAGGAACTATTTCACAGCGGAGGTATCCCATTGCAGGGCCACTGAATACATAATG<br>AAGGGAGTATACATAAACACAGCCCTATTGAATGCATCCTGTGCAGCCATGGATGACTTCCAACTGATTCCAATGATAAGCAAGTGCAGAACCAAAGAAGGAAGACGGAAGACAAATCTGTATGGATTCATTATAAAAGGAAGATCCCATTTGAGGAATGACACCGATGTGGTAAACTTTGTGAGCATGGAATTCTCTCTAACTGACCCGAGGCTAGAGCCACACAAATG<br>GGAAAAGTACTGTGTTCTTGAGATAGGAGACATGCTCCTACGGACTGCAATAGGCCAAGTGTCGAGGCCCATGTTCCTGTATGTGAGAACCAATGGGACTTCCAAGATCAAAATGAAATGGGGCATGGAGATGAGACGATGCCTTCTTCAGTCCCTTCAACAAATTGAGAGCATGATTGAGGCCGAATCTTCTGTCAAAGAGAAGGACATGACCAAGGAATTCTTTGAAA<br>ACAAATCAGAAACATGGCCAATTGGAGAATCACCCAAAGGGGTTGAGGAAGGCTCTATTGGGAAAGTATGCAGAACATTGCTAGCAAAGTCTGTGTTCAACAGCCTATATGCATCTCCACAACTCGAGGGATTTTCAGCTGAATCAAGAAAATTGCTTCTCATTGTTCAGGCACTTAGGGACAACCTGGAACCTGGAACCTTCGATCTTGGGGGGCTATATGAAGCAATTG<br>AGGAGTGCCTGATTAACGATCCCTGGGTTTTGCTTAATGCGTCTTGGTTCAACTCCTTCCTCACACATGCACTGAAATA-
EPI_ISL_18245597	>A/mute_swan/Poland/MB008-22_22VIR5675-16/2022_2022-01-05<br>ATGGAAGACTTTGTGCGACAATGCTTCAATCCAATGATTGTCGAGCTTGCGGAAAAAGCAATGAAAGAATATGGGGAAGATCCAAAAATCGAGACAAACAAATTTGCCGCAATATGCACACACTTAGAAGTCTGTTTCATGTATTCGGATTTCCATTTTATTGATGAACGAGGCGAATCAATGATTGTAGAATCTGGCGATCCAAATGCATTATTGAAACACCGATTTGAGA<br>TAATCGAAGGGAGAGACCGAGCAATGGCCTGGACAGTGGTGAATAGTATCTGCAACACCACAGGAGTCGAAAAGCCCAAATTCCTCCCTGATTTGTATGACTACAAAGAGAACCGATTCATTGAAATTGGAGTAACGCGAAGGGAAGTTCACATATACTATTTAGAAAAAGCCAACAAGATAAAATCGGAGAAAACACACATTCACATATTCTCATTCACTGGAGAGGAA<br>ATGGCCACCAAGGCGGAATACACCCTTGATGAAGAGAGCAGAGCAAGAATAAAAACCAGACTGTTCACTATAAGACAAGAAATGGCCAGTAGAGGTCTATGGGATTCCTTTCGTCAGTCCGAGAGAGGCGAAGAGACAACTGAAGAAAGATTTGAAATCACAGGAACCATGCGCAGGCTTGCCAACCAAAGTCTCCCACCGAACTTCTCCAGCCTTGAAAACTTTAGAG<br>CCTATGTGGATGGATTCGAACCGAACGGCTGCATTGAGGGCAAGCTTTCTCAAATGTCAAAAGAAGTGAACGCCAGAATTGAGCCATTCCTGAAGACAACACCACGCCCTCTCAGATTACCTGATGGGCCTCCTTGTTCTCAGCGGTCGAAGTTCTTGCTGATGGATGCCCTTAAGTTGAGCATCGAAGACCCTAGCCATGAGGGGGAGGGCATACCGCTGTATGATGCAA<br>TCAAATGCATGAAGACATTTTTTGGCTGGAAAGAGCCCAACCTCGTAAAGCCGCATGAGAAAGGCATAAACCCTAATTACCTCCTGGCTTGGAAGCAGGTGCTGGCAGAACTTCAAGATATTGAGAATGAGGAGAAATTTCCAAAAACAAAGAACATGAAGAAAACAAGCCAATTGAAATGGGCACTTGGTGAGAACATGGCACCAGAAAAAGTGGACTTTGAGGACTG<br>CAAAGATGTTAGCGATCTAAGACAGTACTACAGTGACGAACCAGAGCCTAGATCACTAGCAAGCTGGATTCAGAGTGAATTCAACAAGGCATGCGAATTGACAGATTCGAGTTGGATTGAACTTGATGAGATAGGGGAAGACGTTGCTCCAATCGAACACATTGCGAGTATGAGGAGGAACTATTTCACAGCGGAGGTATCCCATTGCAGGGCCACTGAATACATAATGA<br>AGGGAGTATACATAAACACAGCCCTATTGAATGCATCCTGTGCAGCCATGGATGACTTCCAACTGATTCCAATGATAAGCAAGTGCAGAACCAAAGAAGGAAGACGGAAGACAAATCTGTATGGATTCATTATAAAAGGAAGATCCCATTTGAGGAATGACACCGATGTGGTAAACTTTGTGAGCATGGAATTCTCTCTAACTGACCCGAGGCTAGAGCCACACAAATGG<br>GAAAAGTACTGTGTTCTTGAGATAGGAGACATGCTCCTACGGACTGCAATAGGCCAAGTGTCGAGGCCCATGTTCCTGTATGTGAGAACCAATGGGACTTCCAAGATCAAAATGAAATGGGGCATGGAGATGAGACGATGCCTTCTTCAGTCCCTTCAACAAATTGAGAGCATGATTGAAGCCGAATCTTCTGTCAAAGAGAAGGACATGACCAAGGAATTCTTTGAAAAC<br>AAATCAGAAACATGGCCAATTGGAGAATCACCCAAAGGGGTTGAGGAAGGCTCTATTGGGAAAGTATGCAGAACATTGCTAGCAAAGTCTGTGTTCAACAGCCTATATGCATCTCCACAACTCGAGGGATTTTCAGCTGAATCAAGAAAATTGCTTCTCATTGTTCAGGCACTTAGGGACAACCTGGAGCCTGGAACCTTCGATCTTGGGGGGCTATATGAAGCAATTGAG<br>GAGTGCCTGATTAACGATCCCTGGGTTTTGCTTAATGCGTCTTGGTTCAACTCCTTCCTCACACATGCACTGAAATA-
EPI_ISL_18245815	>A/mute_swan/Poland/MB551/2021_H5N1_2021-12-23<br>ATGGAAGACTTTGTGCGACAATGCTTCAATCCAATGATTGTCGAGCTTGCGGAAAAAGCAATGAAAGAATATGGGGAAGATCCGAAAATCGAGACAAACAAATTTGCCGCAATATGCACACACTTAGAAGTCTGTTTCATGTATTCGGATTTCCATTTTATTGATGAACGAGGCGAATCAACGATTGTAGAATCTGGCGATCCAAATGCATTATTGAAACACCGATTTGAGA<br>TAATCGAAGGGAGAGACCGAGCAATGGCCTGGACAGTGGTGAATAGTATCTGCAACACCACAGGAGTCGAAAAGCCCAAATTCCTCCCTGATTTGTATGACTACAAAGAGAACCGATTCATTGAAATTGGAGTAACGCGAAGGGAAGTTCACATATACTATTTAGAAAAAGCCAACAAGATAAAATCAGAGAAAACACACATTCACATATTCTCATTCACTGGAGAGGAA<br>ATGGCCACCAAGGCGGAATACACCCTTGATGAAGAGAGCAGAGCAAGAATAAAAACCAGACTGTTCACTATAAGACAAGAAATGGCCAGTAGAGGTCTATGGGATTCCTTTCGTCAATCCGAAAGAGGCGAAGAGACAACTGAAGAAAGATTTGAAATCACAGGAACCATGCGCAGGCTTGCCGACCAAAGTCTCCCACCGAACTTCTCCAGCCTTGAAAACTTTAGAG<br>CCTATGTGGATGGATTCGAACCGAACGGCTGCATTGAGGGCAAGCTTTCTCAAATGTCAAAAGAAGTGAACGCCAGAATTGAGCCATTCCTGAAGACAACACCACGCCCTCTCAGATTACCTGATGGGCCTCCTTGTTCTCAGCGGTCGAAGTTCTTGCTGATGGATGCCCTTAAGTTAAGCATCGAAGACCCTAGCCATGAGGGGGAGGGCATACCGCTGTATGATGCAA<br>TCAAATGCATGAAGACATTTTTTGGCTGGAAAGAGCCCAACCTCGTAAAGCCGCATGAGAAAGGCATAAACCCTAATTACCTCCTGGCTTGGAAGCAGGTGCTGGCAGAACTTCAAGATATTGAGAATGAGGAGAAATTTCCAAAAACAAAGAACATGAAGAAAACAAGCCAATTGAAGTGGGCACTTGGTGAGAACATGGCACCAGAAAAAGTGGACTTTGAGGACTG<br>CAAAGATGTTAGCGATCTAAGACAGTACGACAGTGACGAACCAGAGCCTAGATCACTAGCAAGCTGGATTCAGAGTGAATTCAACAAGGCATGCGAATTGACAGATTCGAGTTGGATTGAACTTGATGAGATAGGGGAAGACGTTGCTCCAATCGAACACATTGCGAGTATGAGGAGGAACTATTTCACAGCGGAGGTATCCCATTGCAGGGCCACTGAATACATAATG<br>AAGGGAGTATACATAAACACAGCCCTATTGAATGCATCCTGTGCAGCCATGGATGACTTCCAACTGATTCCAATGATAAGCAAGTGCAGAACCAAAGAGGGAAGACGGAAGACAAATCTGTATGGATTCATTATAAAAGGAAGATCCCATTTGAGGAATGACACCGATGTGGTGAACTTTGTGAGCATGGAATTCTCTCTAACTGACCCGAGGCTAGAGCCACACAAATG<br>GGAAAAGTACTGTGTTCTTGAGATAGGAGACATGCTCCTACGGACTGCAATAGGCCAAGTGTCGAGGCCCATGTTCCTGTATGTGAGAACCAATGGGACTTCCAAGATCAAAATGAAATGGGGCATGGAGATGAGACGATGCCTTCTTCAGTCCCTTCAACAAATTGAGAGCATGATTGAGGCCGAATCTTCTGTCAAAGAGAAGGACATGACCAAGGAATTCTTTGAAA<br>ACAAATCAGAAACATGGCCAATTGGAGAATCACCCAAAGGGGTTGAGGAAGGCTCTATTGGGAAAGTATGCAGAACATTGCTAGCAAAGTCTGTGTTCAACAGCCTATATGCTTCTCCACAACTCGAGGGATTTTCAGCTGAATCAAGAAAATTGCTTCTCATTGTTCAGGCACTTAGGGACAACCTGGAACCTGGAACCTTCGATCTTGGGGGGCTATATGAAGCAATTG<br>AGGAGTGCCTGATTAACGATCCCTGGGTTTTGCTTAATGCGTCTTGGTTCAACTCCTTCATCACACATGCACTGAAATA-
EPI_ISL_18245613	>A/mute_swan/Poland/MB020-22_22VIR5675-3/2022_H5N1_2022-01-11<br>ATGGAAGACTTTGTGCGACAATGCTTCAATCCAATGATTGTCGAGCTTGCGGAAAAAGCAATGAAAGAATATGGGGAAGATCCGAAAATCGAGACAAACAAATTTGCCGCAATATGCACACACTTAGAAGTCTGTTTCATGTATTCGGATTTCCATTTTATTGATGAACGAGGCGAATCAACGATTGTAGAATCTGGCGATCCAAATGCATTATTGAAACACCGATTTGAGA<br>TAATCGAAGGGAGAGACCGAGCAATGGCCTGGACAGTGGTGAATAGTATCTGCAACACCACAGGAGTCGAAAAGCCCAAATTCCTCCCTGATTTGTATGACTACAAAGAGAACCGATTCATTGAAATTGGAGTAACGCGAAGGGAAGTTCACATATACTATTTAGAAAAAGCCAACAAGATAAAATCAGAGAAAACACACATTCACATATTCTCATTCACTGGAGAGGAA<br>ATGGCCACCAAGGCGGAATACACCCTTGATGAAGAGAGCAGAGCAAGAATAAAAACCAGACTGTTCACTATAAGACAAGAAATGGCCAGTAGAGGTCTATGGGATTCCTTTCGTCAGTCCGAAAGAGGCGAAGAGACAACTGAAGAAAAATTTGAAATCACAGGAACCATGCGCAGGCTTGCCGACCAAAGTCTCCCACCGAACTTCTCCAGCCTTGAAAACTTTAGAG<br>CCTATGTGGATGGATTCGAACCGAACGGCTGCATTGAGGGCAAGCTTTCTCAAATGTCAAAAGAAGTGAACGCCAGAATTGAGCCATTCCTGAAGACAACACCACGCCCTCTCAGATTACCTGATGGGCCTCCTTGTTCTCAGCGGTCGAAGTTCTTGCTGATGGATGCCCTTAAGTTGAGCATCGAAGACCCTAGCCATGAGGGGGAGGGCATACCGCTGTATGATGCAA<br>TCAAATGCATGAAGACATTTTTTGGCTGGAAAGAGCCCAACCTCGTAAAGCCGCATGAGAAAGGCATAAACCCTAATTACCTCCTGGCTTGGAAGCAGGTGCTGGCAGAACTTCAAGATATTGAGAATGAGGAGAAATTTCCAAAAACAAAGAACATGAAGAAAACAAGCCAATTGAAGTGGGCACTTGGTGAGAACATGGCACCAGAAAAAGTGGACTTTGAGGACTG<br>CAAAGATGTTAGCGATCTAAGACAGTACGACAGTGACGAACCAGAGCCTAGATCACTAGCAAGCTGGATTCAGAGTGAATTCAACAAGGCATGCGAATTGACAGATTCGAGTTGGATTGAACTTGATGAGATAGGGGAAGACGTTGCTCCAATCGAACACATTGCGAGTATGAGGAGGAACTATTTCACAGCGGAGGTATCCCATTGCAGGGCCACTGAATACATAATG<br>AAGGGAGTATACATAAACACAGCCCTATTGAATGCATCCTGTGCAGCCATGGATGACTTCCAACTGATTCCAATGATAAGCAAGTGCAGAACCAAAGAGGGAAGACGGAAGACAAATCTGTATGGATTCATTATAAAAGGAAGATCCCATTTGAGGAATGACACCGATGTGGTGAACTTTGTGAGCATGGAATTCTCTCTAACTGACCCGAGGCTAGAGCCACACAAATG<br>GGAAAAGTACTGTGTTCTTGAGATAGGAGACATGCTCCTACGGACTGCAATAGGCCAAGTGTCGAGGCCCATGTTCCTGTATGTGAGAACCAATGGGACTTCCAAGATCAAAATGAAATGGGGCATGGAGATGAGACGATGCCTTCTTCAGTCCCTTCAACAAATTGAGAGCATGATTGAGGCCGAATCTTCTGTCAAAGAGAAGGACATGACCAAGGAATTCTTTGAAA<br>ACAAATCAGAAACATGGCCAATTGGAGAATCACCCAAAGGGGTTGAGGAAGGCTCTATTGGGAAAGTATGCAGAACATTGCTAGCAAAGTCTGTGTTCAACAGCCTATATGCTTCTCCACAACTCGAGGGATTTTCAGCTGAATCAAGAAAATTGCTTCTCATTGTTCAGGCACTTAGGGACAACCTGGAACCTGGAACCTTCGATCTTGGGGGGCTATATGAAGCAATTG<br>AGGAGTGCCTGATTAACGATCCCTGGGTTTTGCTTAATGCGTCTTGGTTCAACTCCTTCATCACACATGCACTGAAATA-
EPI_ISL_18245811	>A/crane/Poland/MB528/2021_H5N1_2021-12-10<br>ATGGAAGACTTTGTGCGACAATGCTTCAATCCAATGATTGTCGAGCTTGCGGAAAAAGCAATGAAAGAATATGGGGAAGATCCGAAAATCGAGACAAACAAATTTGCCGCAATATGCACACACTTAGAAGTCTGTTTCATGTATTCGGATTTCCATTTTATTGATGAACGAGGCGAATCAACGATTGTAGAATCTGGCGATCCAAATGCATTATTGAAACACCGATTTGAGA<br>TAATCGAAGGGAGAGACCGAGCAATGGCCTGGACAGTGGTGAATAGTATCTGCAACACCACAGGAGTCGAAAAGCCCAAATTCCTCCCTGATTTGTATGACTACAAAGAGAACCGATTCATTGAAATTGGAGTAACGCGAAGGGAAGTTCACATATACTATTTAGAAAAAGCCAACAAGATAAAATCAGAGAAAACACACATTCACATATTCTCATTCACTGGAGAGGAA<br>ATGGCCACCAAGGCGGAATACACCCTTGATGAAGAGAGCAGAGCAAGAATAAAAACCAGACTGTTCACTATAAGACAAGAAATGGCCAGTAGAGGTCTATGGGATTCCTTTCGTCAGTCCGAAAGAGGCGAAGAGACAACTGAAGAAAGATTTGAAATCACAGGAACCATGCGCAGGCTTGCCGACCAAAGTCTCCCACCGAACTTCTCCAGCCTTGAAAACTTTAGAG<br>CCTATGTGGATGGATTCGAACCGAACGGCTGCATTGAGGGCAAGCTTTCTCAAATGTCAAAAGAAGTGAACGCCAGAATTGAGCCATTCCTGAAGACAACACCACGCCCTCTCAGATTACCTGATGGACCTCCTTGTTCTCAGCGGTCGAAGTTCTTGCTGATGGATGCCCTTAAGTTGAGCATCGAAGACCCTAGCCATGAGGGGGAGGGCATACCGCTGTATGATGCAA<br>TCAAATGCATGAAGACATTTTTTGGCTGGAAAGAGCCCAACCTCGTAAAGCCGCATGAGAAAGGCATAAACCCTAATTACCTCCTGGCTTGGAAGCAGGTGCTGGCAGAACTTCAAGATATTGAGAATGAGGAGAAATTTCCAAAAACAAAGAACATGAAGAAAACAAGCCAATTGAAGTGGGCACTTGGTGAGAACATGGCACCAGAAAAAGTGGACTTTGAGGACTG<br>CAAAGATGTTAGCGATCTAAGACAGTACGACAGTGACGAACCAGAGCCTAGATCACTAGCAAGCTGGATTCAGAGTGAATTCAACAAGGCATGCGAATTGACAGATTCGAGTTGGATTGAACTTGATGAGATAGGGGAAGACGTTGCTCCAATCGAACACATTGCGAGTATGAGGAGGAACTATTTCACAGCGGAGATATCCCATTGCAGGGCCACTGAATACATAATG<br>AAGGGAGTATACATAAACACAGCCCTATTGAATGCATCCTGTGCAGCCATGGATGACTTCCAACTGATTCCAATGATAAGCAAGTGCAGAACCAAAGAGGGAAGACGGAAGACAAATCTGTATGGATTCATTATAAAAGGAAGATCCCATTTGAGGAATGACACCGATGTGGTAAACTTTGTGAGCATGGAATTCTCTCTAACTGACCCGAGGCTAGAGCCACACAAATG<br>GGAAAAGTACTGTGTTCTTGAGATAGGAGACATGCTCCTACGGACTGCAATAGGCCAAGTGTCGAGGCCCATGTTCCTGTATGTGAGAACCAATGGGACTTCCAAGATCAAAATGAAATGGGGCATGGAGATGAGACGATGCCTTCTTCAGTCCCTTCAACAAATTGAGAGCATGATTGAGGCCGAATCTTCTGTCAAAGAGAAGGACATGACCAAGGAATTCTTTGAAA<br>ACAAATCAGAAACATGGCCAATTGGAGAATCACCCAAAGGGGTTGAGGAAGGCTCTATTGGGAAAGTATGCAGAACATTGCTAGCAAAGTCTGTGTTCAACAGCCTATATGCTTCTCCACAACTCGAGGGATTTTCAGCTGAATCAAGAAAATTGCTTCTCATTGTTCAGGCACTTAGGGACAACCTGGAACCTGGAACCTTCGATCTTGGGGGGCTATATGAAGCAATTG<br>AGGAGTGCCTGATTAACGATCCCTGGGTTTTGCTTAATGCGTCTTGGTTCAACTCCTTCATCACACATGCACTGAAATA-

EPI_ISL_11922814	>A/swan/Poland/MB083_22VIR2515-8/2022_H5N1_2022-02-18<br>ATGGAAGACTTTGTGCGACAATGCTTCAATCCAATGATTGTCGAGCTTGCGGAAAAAGCAATGAAAGAATATGGGGAAGATCCGAAAATCGAGACAAACAAATTTGCCGCAATATGCACACACTTAGAAGTCTGTTTCATGTATTCGGATTTCCATTTTATTGATGAACGAGGCGAATCAATGATTGTAGAATCTGGCGATCCAAATGCATTATTGAAACACCGATTTGAGA<br>TAATCGAAGGGAGAGACCGAGCAATGGCCTGGACAGTGGTGAATAGTATCTGCAACACCACAGGAGTCGAAAAGCCCAAATTCCTCCCTGATTTGTATGACTACAAAGAGAACCGATTCATTGAAATTGGAGTAACGCGAAGGGAAGTTCACATATACTATTTAGAAAAAGCCAACAAGATAAAATCAGAGAAAACACACATTCACATATTCTCATTCACTGGAGAGGAA<br>ATGGCCACCAAGGCGGAATACACCCTTGATGAAGAGAGCAGAGCAAGAATAAAAACCAGACTGTTCACTATAAGACAAGAAATGGCCAGTAGAGGTCTATGGGATTCCTTTCGTCAGTCCGAGAGAGGCGAAGAGACAACTGAAGAAAGATTTGAAATCACAGGAACCATGCGCAGGCTTGCCGACCAAAGTCTCCCACCGAACTTCTCCAGCCTTGAAAACTTTAGAG<br>CCTATGTGGATGGATTCGAACCGAACGGCTGCATTGAGGGCAAGCTTTCTCAAATGTCAAAAGAAGTGAACGCCAGAATTGAGCCATTCCTGAAGACAACACCACGCCCTCTCAGATTACCTGATGGCCTCCTTGTTCTCAGCGGTCGAAGTTCTTGCTGATGGATGCCCTTAAGTTGAGCATCGAAGACCTAGCCATGAGGGGGAGGGATACCGCTGTATGATGCAA<br>TCAAATGCATGAAGACATTTTTTGGCTGGAAAGAGCCCAACCTCGTAAAGCCGCATGAGAAAGGCATAAACCCTAATTACCTCCTGGCTTGGAAGCAGGTGCTGGCAGAACTTCAAGATATTGAGAATGAGGAGAAAATTCCCAAAAACAAAGAACATGAAGAAAACAAGCCAATTGAAGTGGGCACTTGGTGAGACATGGCACCAGAAAAAGTGGACTTTGAGGACTG<br>CAAAGATGTTAGCGATCTAAGACAGTACGACAGTGACGAACCAGAGCCTAGATCACTAGCAAGCTGGATTCAGAGTGAATTCAACAAGGCATGCGAATTGACAGATTCGAGTTGGATTGAACTTGATGAGATAGGGGAAGACGTTGCTCCAATCGAACACATTGCGAGTATGAGGAGGAACTATTTCACAGCGGAGGTATCCCATTGCAGGGCCACTGAATATACAATATG<br>AAGGGAGTATACATAAACACAGCCCTATTGAATGCATCCTGTGCAGCCATGGATGACTTCCAACTGATTCCAATGATAAGCAAGTGCAGAACCAAAGAGGGAAGACGGAAGACAAATCTGTATGGATTCATTATAAAAGGAAGATCCCATTTGAGGAATGACACCGATGTGGTAAACTTTGTGAGCATGGAATTCTCTCTAACTGACCCGAGGCTAGAGCCACACAAATG<br>GGAAAAGTACTGTGTTCTTGAGATAGGAGACATGCTCCTACGGACTGCAATAGGCCAAGTGTCGAGGCCCATGTTCCTGTATGTGAGAACCAATGGGACTTCCAAGATCAAAATGAAATGGGGCATGGAGATGAGACGATGCCTTCTTCAGTCCCTTCAACAATTGAGAGCATGATTGAGGCCGAATCTTCTGTCAAAGAGAAGGACATGACCAAGGAATTCTTTGAAA<br>ACAAATCAGAAACATGGCCAATTGGAGAATCACCCAAAGGGGTTGAGGAAGGCTCTATTGGGAAAGTGTGCAGAACATTGCTAGCAAAGTCTGTGTTCAACAGCCTATATGCTTCTCCACAACTCGAGGGATTTTCAGCTGAATCAAGAAAATTGCTTCTCATTGTTCAGGCACTTAGGGACAACCTGGAACCTGGAACCTTCGATCTTGGGGGGCTATATGAAGCAATTG<br>AGGAGTGCCTGATTAACGATCCCTGGGTTTTGCTTAATGCGTCTTGGTTCAACTCCTTCATCACACATGCACTGAAATA-
EPI_ISL_18245812	>A/wild_bird/Poland/MW542/2021_H5N1_2021-12-20<br>ATGGAAGACTTTGTGCGACAATGCTTCAATCCAATGATTGTCGAGCTTGCGGAAAAAGCAATGAAAGAATATGGGGAAGATCCGAAAATCGAGACAAACAAATTTGCCGCAATATGCACACACTTAGAAGTCTGTTTCATGTATTCGGATTTCCATTTTATTGATGAACGAGGCGAATCAATGATTGTAGAATCTGGCGATCCAAATGCATTATTGAAACACCGATTTGAGA<br>TAATCGAAGGGAGAGACCGAGCAATGGCCTGGACAGTGGTGAATAGTATCTGCAACACTACAGGAGTCGAAAAGCCCAAATTCCTCCCTGATTTGTATGACTACAAAGAGAACCGATTCATTGAAATTGGAGTAACGCGAAGGGAAGTTCACATATACTATTTAGAAAAAGCCAACAAGATAAAATCAGAGAAAACACACATTCACATATTCTCATTCACTGGAGAGGAA<br>ATGGCCACCAAGGCGGAATACACCCTTGATGAAGAGAGCAGAGCAAGAATAAAAACCAGACTGTTCACTATAAGACAAGAAATGGCCAGTAGAGGTCTATGGGATTCCTTTCGTCAGTCCGAGAGAGGCGAAGAGACAACTGAAGAAAGATTTGAAATCACAGGAACCATGCGCAGGCTTGCCGACCAAAGTCTCCCACCGAACTTCTCCAGCCTTGAAAACTTTAGAG<br>CCTATGTGGATGGATTCGAACCGAACGGCTGCATTGAGGGCAAGCTTTCTCAAATGTCAAAAGAAGTGAACGCCAGAATTGAGCCATTCCTGAAGACAACACCACGCCCTCTCAGATTACCTGATGGCCTCCTTGTTCTCAGCGGTCGAAGTTCTTGCTGATGGATGCCCTTAAGTTGAGCATCGAAGACCTAGCCATGAGGGGGAGGGATACCGCTGTATGATGCAA<br>TCAAATGCATGAAGACATTTTTTGGCTGGAAAGAGCCCAACCTCGTAAAGCCGCATGAGAAAGGCATAAACCCTAATTACCTCCTGGCTTGGAAGCAGGTGCTGGCAGAACTTCAAGATATTGAGAATGAGGAGAAAATTCCCAAAAACAAAGAACATGAAGAAAACAAGCCAATTGAAGTGGGCACTTGGTGAGACATGGCACCAGAAAAAGTGGACTTTGAGGACTG<br>CAAAGATGTTAGCGATCTAAGACAGTACGACAGTGACGAACCAGAGCCTAGATCACTAGCAAGCTGGATTCAGAGTGAATTCAACAAGGCATGCGAATTGACAGATTCGAGTTGGATTGAACTTGATGAGATAGGGGAAGACGTTGCTCCAATCGAACACATTGCGAGTATGAGGAGGAACTATTTCACAGCGGAGGTATCCCATTGCAGGGCCACTGAATACATAATG<br>AAGGGAGTATACATAAACACAGCCCTATTGAATGCATCCTGTGCAGCCATGGATGACTTCCAACTGATTCCAATGATAAGCAAGTGCAGAACCAAAGAGGGAAGACGGAAGACAAATCTGTATGGATTCATTATAAAAGGAAGATCCCATTTGAGGAATGACACCGATGTGGTAAACTTTGTGAGCATGGAATTCTCTCTAACTGACCCGAGGCTAGAGCCACACAAATG<br>GGAAAAGTACTGTGTTCTTGAGATAGGAGACATGCTCCTACGGACTGCAATAGGCCAAGTGTCGAGGCCCATGTTCCTGTATGTGAGAACCAATGGGACTTCCAAGATCAAAATGAAATGGGGCATGGAGATGAGACGATGCCTTCTTCAGTCCCTTCAACAATTGAGAGCATGATTGAGGCCGAATCTTCTGTCAAAGAGAAGGACATGACCAAGGAATTCTTTGAAA<br>ACAAATCAGAAACATGGCCAATTGGAGAATCACCCAAAGGGGTTGAGGAAGGCTCTATTGGGAAAGTATGCAGAACATTGCTGGCAAAGTCTGTGTTCAACAGCCTATATGCATCTCCACAACTCGAGGGATTTTCAGCTGAATCAAGAAAATTGCTTCTCATTGTTCAGGCACTTAGGGACAACCTGGAACCTGGAACCTTCGATCTTGGGGGGCTATATGAAGCAATTG<br>AGGAGTGCCTGATTAACGATCCCTGGGTTTTGCTTAATGCGTCTTGGTTCAACTCCTTCATCACACATGCACTGAAATA-
EPI_ISL_6934175	>A/domestic_duck/Poland/H1942-N/2021_H5N1_2021-11-07<br>ATGGAAGACTTTGTGCGACAATGCTTCAATCCAATGATTGTCGAGCTTGCGGAAAAAGCAATGAAAGAATATGGGGAAGATCCGAAAATCGAGACAAACAAATTTGCCGCAATATGCACACACTTAGAAGTCTGTTTCATGTATTCGGATTTCCATTTTATTGATGAACGAGGCGAATCAATGATTGTAGAATCTGGCGATCCAAATGCATTATTGAAACACCGATTTGAGA<br>TAATCGAAGGGAGAGACCGAGCAATGGCCTGGACAGTGGTGAATAGTATCTGCAACACCACAGGAGTCGAAAAGCCCAAATTCCTCCCTGATTTGTATGACTACAAAGAGAACCGATTCATTGAAATTGGAGTAACGCGAAGGGAAGTTCACATATACTATTTAGAAAAAGCCAACAAGATAAAATCAGAGAAAACACACATTCACATATTCTCATTCACTGGAGAGGAA<br>ATGGCCACCAAGGCGGAATACACCCTTGATGAAGAGAGCAGAGCAAGAATAAAAACCAGACTGTTCACTATAAGACAAGAAATGGCCAGTAGAGGTCTATGGGATTCCTTTCGTCAGTCCGAGAGAGGCGAAGAGACAACTGAAGAAAGATTTGAAATCACAGGAACCATGCGCAGGCTTGCCGACCAAAGTCTCCCACCGAACTTCTCCAGCCTTGAAAACTTTAGAG<br>CCTATGTGGATGGATTCGAACCGAACGGCTGCATTGAGGGCAAGCTTTCTCAAATGTCAAAAGAAGTGAACGCCAGAATTGAGCCATTCCTGAAGACAACACCACGCCCTCTCAGATTACCTGATGGCCTCCTTGTTCTCAGCGGTCGAAGTTCTTGCTGATGGATGCCCTTAAGTTGAGCATCGAAGACCTAGCCATGAGGGGGAGGGATACCGCTGTATGATGCAA<br>TCAAATGCATGAAGACATTTTTTGGCTGGAAAGAGCCCAACCTCGTAAAGCCGCATGAGAAAGGCATAAACCCTAATTACCTCCTGGCTTGGAAGCAGGTGCTGGCAGAACTTCAAGATATTGAGAATGAGGAGAAAATTCCCAAAAACAAAGAACATGAAGAAAACAAGCCAATTGAAGTGGGCACTTGGTGAGACATGGCACCAGAAAAAGTGGACTTTGAGGACTG<br>CAAAGATGTTAGCGATCTAAGACAGTACGACAGTGACGAACCAGAGCCTAGATCACTAGCAAGCTGGATTCAGAGTGAATTCAACAAGGCATGCGAATTGACAGATTCGAGTTGGATTGAACTTGATGAGATAGGGGAAGACGTTGCTCCAATCGAACACATTGCGAGTATGAGGAGGAACTATTTCACAGCGGAGGTATCCCATTGCAGGGCCACTGAATACATAATG<br>AAGGGAGTATACATAAACACAGCCCTATTGAATGCATCCTGTGCAGCCATGGATGACTTCCAACTGATTCCAATGATAAGCAAGTGCAGAACCAAAGAGGGAAGACGGAAGACAAATCTGTATGGATTCATTATAAAAGGAAGATCCCATTTGAGGAATGACACCGATGTGGTAAACTTTGTGAGCATGGAATTCTCTCTAACTGACCCGAGGCTAGAGCCACACAAATG<br>GGAAAAGTACTGTGTTCTTGAGATAGGAGACATGCTCCTACGGACTGCAATAGGCCAAGTGTCGAGGCCCATGTTCCTGTATGTGAGAACCAATGGGACTTCCAAGATCAAAATGAAATGGGGCATGGAGATGAGACGATGCCTTCTTCAGTCCCTTCAACAATTGAGAGCATGATTGAGGCCGAATCTTCTGTCAAAGAGAAGGACATGACCAAGGAATTCTTTGAAA<br>ACAAATCAGAAACATGGCCAATTGGAGAATCACCCAAAGGGGTTGAGGAAGGCTCTATTGGGAAAGTATGCAGAACATTGCTAGCAAAGTCTGTGTTCAACAGCCTATATGCATCTCCACAACTCGAGGGATTTTCAGCTGAATCAAGAAAATTGCTTCTCATTGTTCAGGCACTTAGGGACAACCTGGAACCTGGAACCTTCGATCTTGGGGGGCTATATGAAGCAATTG<br>AGGAGTGCCTGATTAACGATCCCTGGGTTTTGCTTAATGCGTCTTGGTTCAACTCCTTCATCACACATGCACTGAAATA-
EPI_ISL_18245813	>A/hawk/Poland/MB544/2021_H5N1_2021-12-22<br>ATGGAAGACTTTGTGCGACAATGCTTCAATCCAATGATTGTCGAGCTTGCGGAAAAAGCAATGAAAGAATATGGGGAAGATCCAAAAATCGAGACAAACAAATTTGCCGCAATATGCACACACTTAGAAGTCTGTTTCATGTATTCGGATTTCCATTTTATTGATGAACGAGGCGAATCAATGATTGTAGAATCTGGCGATCCAAATGCATTATTGAAACACCGATTTGAGA<br>TAATCGAAGGGAGAGACCGAGCAATGGCCTGGACAGTGGTGAATAGTATCTGCAACACTACAGGAGTCGAAAAGCCCAAATTCCTCCCTGATTTGTATGACTACAAAGAGAACCGATTCATTGAAATTGGAGTAACGCGAAGGGAAGTTCACATATACTATTTAGAAAAAGCCAACAAGATAAAATCAGAGAAAACACACATTCACATATTCTCATTCACTGGAGAGGAA<br>ATGGCCACCAAGGCGGAATACACCCTTGATGAAGAGAGCAGAGCAAGAATAAAAACCAGACTGTTCACTATAAGACAAGAAATGGCCAGTAGAGGTCTATGGGATTCCTTTCGTCAGTCCGAGAGAGGCGAAGAGACAACTGAAGAAAGATTTGAAATCACAGGAACCATGCGCAGGCTTGCCGACCAAAGTCTCCCACCGAACTTCTCCAGCCTTGAAAACTTTAGAG<br>CCTATGTGGATGGATTCGAACCGAACGGCTGCATTGAGGGCAAGCTTTCTCAAATGTCAAAAGAAGTGAACGCCAGAATTGAGCCATTCCTGAAGACAACACCACGCCCTCTCAGATTACCTGATGGCCTCCTTGTTCTCAGCGGTCGAAGTTCTTGCTGATGGATGCCCTTAAGTTGAGCATCGAAGACCTAGCCATGAGGGGGAGGGATACCGCTGTATGATGCAA<br>TCAAATGCATGAAGACATTTTTTGGCTGGAAAGAGCCCAACCTCGTAAAGCCGCATGAGAAAGGCATAAACCCTAATTACCTCCTGGCTTGGAAGCAGGTGCTGGCAGAACTTCAAGATATTGAGAATGAGGAGAAAATTCCCAAAAACAAAGAACATGAAGAAAACAAGCCAATTGAAGTGGGCACTTGGTGAGACATGGCACCAGAAAAAGTGGACTTTGAGGACTG<br>CAAAGATGTTAGCGATCTAAGACAGTACGACAGTGACGAACCAGAGCCTAGATCACTAGCAAGCTGGATTCAGAGTGAATTCAACAAGGCATGCGAATTGACAGATTCGAGTTGGATTGAACTTGATGAGATAGGGGAAGACGTTGCTCCAATCGAACACATTGCGAGTATGAGGAGGAACTATTTCACAGCGGAGGTATCCCATTGCAGGGCCACTGAATACATAATG<br>AAGGGAGTATACATAAACACAGCCCTATTGAATGCATCCTGTGCAGCCATGGATGACTTCCAACTGATTCCAATGATAAGCAAGTGCAGAACCAAAGAGGGAAGACGGAAGACAAATCTGTATGGATTCATTATAAAAGGAAGATCCCATTTGAGGAATGACACCGATGTGGTAAACTTTGTGAGCATGGAATTCTCTCTAACTGACCCGAGGCTAGAGCCACACAAATG<br>GGAAAAGTACTGTGTTCTTGAGATAGGAGACATGCTCCTACGGACTGCAATAGGCCAAGTGTCGAGGCCCATGTTCCTGTATGTGAGAACCAATGGGACTTCCAAGATCAAAATGAAATGGGGCATGGAGATGAGACGATGCCTTCTTCAGTCCCTTCAACAATTGAGAGCATGATTGAGGCCGAATCTTCTGTCAAAGAGAAGGACATGACCAAGGAATTCTTTGAAA<br>ACAAATCAGAAACATGGCCAATTGGAGAATCACCCAAAGGGGTTGAGGAAGGCTCTATTGGGAAAGTATGCAGAACATTGCTGGCAAAGTCTGTGTTCAACAGCCTATATGCATCTCCACAACTCGAGGGATTTTCAGCTGAATCAAGAAAATTGCTTCTCATTGTTCAGGCACTTAGGGACAACCTGGAACCTGGAACCTTCGATCTTGGGGGGCTATATGAAGCAATTG<br>AGGAGTGCCTGATTAACGATCCCTGGGTTTTGCTTAATGCGTCTTGGTTCAACTCCTTCATCACACATGCACTGAAATA-
EPI_ISL_18245790	>A/swan/Poland/MB058-22_22VIR5675-2/2022_H5N1_2022-02-03<br>ATGGAAGACTTTGTGCGACAATGCTTCAATCCAATGATTGTCGAGCTTGCGGAAAAAGCAATGAAAGAATATGGGGAAGATCCGAAAATCGAGACAAACAAATTTGCCGCAATATGCACACACTTAGAAGTCTGTTTCATGTATTCGGATTTCCATTTTATTGATGAACGAGGCGAATCAATGATTGTAGAATCTGGCGATCCAAATGCATTATTGAAACACCGATTTGAGA<br>TAATCGAAGGGAGAGACCGAGCAATGGCCTGGACAGTGGTGAATAGTATCTGCAACACCACAGGAGTCGAAAAGCCCAAATTCCTCCCTGATTTGTATGACTACAAAGAGAACCGATTCATTGAAATTGGAGTAACGCGAAGGGAAGTTCACATATACTATTTAGAAAAAGCCAACAAGATAAAATCAGAGAAAACACACATTCACATATTCTCATTCACTGGAGAGGAA<br>ATGGCCACCAGGGCGGAATACACCCTTGATGAAGAGAGCAGAGCAAGAATAAAAACCAGACTGTTCACTATAAGACAAGAAATGGCCAGTAGAGGTCTATGGGATTCCTTTCGTCAGTCCGAGAGAGGCGAAGAGACAAATGAAGAAAGATTTGAAATCACAGGAACCATGCGCAGGCTTGCCGACCAAAGTCTCCCACCGAACTTCTCCAGCCTTGAAAACTTTAGAG<br>CCTATGTGGATGGATTCGAACCGAACGGCTGCATTGAGGGCAAGCTTTCTCAAATGTCAAAAGAAGTGAACGCCAGAATTGAGCCATTCCTGAAGACAACACCACGCCCTCTCAGATTACCTGATGGCCTCCTTGTTCTCAGCGGTCGAAGTTCTTGCTGATGGATGCCCTTAAATTGAGCATCGAAGACCCTAGCCATGAGGGGGAGGGCATACCGCTGTATGATGCAA<br>TCAAATGCATGAAGACATTTTTTGGCTGGAAAGAGCCCAACCTCGTAAAGCCGCATGAGAAGGGCATAAACCCTAATTACCTCCTGGCTTGGAAGCAGGTGCTGGCAGAACTTCAAGATATTGAGAATGAGGAGAAATTTCCAAAAACAAAGAACATGAAGAAAACAAGCCAATTGAAGTGGGCACTTGGTGAGAACATGGCACCAGAAAAAGTGGACTTTGAGGACTG<br>CAAAGATGTTAGCGATCTAAGACAGTACGACAGTGACGAACCAGAGCCTAGATCACTAGCAAGCTGGATTCAGAGTGAATTCAACAAGGCATGCGAATTGACAGATTCGAGTTGGATTGAACTTGATGAGATAGGGGAAGACGTTGCTCCAATCGAACACATTGCGAGTATGAGGAGGAACTATTTCACAGCGGAGGTATCCCATTGCAGGGCCACTGAATACATAATG<br>AAGGGAGTATACATAAACACAGCCCTATTGAATGCATCCTGTGCAGCCATGGATGACTTCCAACTGATTCCAATGATAAGCAAGTGCAGAACCAAAGAGGGAAGACGGAAGACAAATCTGTATGGATTCATTATAAAAGGAAGATCCCATTTGAGGAATGACACCGATGTGGTAAACTTTGTGAGCATGGAATTCTCTCTAACTGACCCGAGGCTAGAGCCACACAAATG<br>GGAAAAGTACTGTGTTCTTGAGATAGGAGACATGCTCCTACGGACTGCAATAGGCCAAGTGTCGAGGCCCATGTTCCTATATGTGAGAACCAATGGGACTTCCAAGATCAAAATGAAATGGGGCATGGAGATGAGACGATGCCTTCTTCAGTCCCTTCAACAAATTGAGAGCATGATTGAGGCCGAATCTTCTGTCAAAGAGAAGGACATGACCAAGGAATTCTTTGAAA<br>ACAAATCAGAAACATGGCCAATTGGAGAATCACCCAAAGGGGTTGAGGAAGGCTCTATTGGGAAAGTATGCAGAACATTGCTAGCAAAGTCTGTGTTCAACAGCCTATATGCATCTCCACAACTCGAGGGATTTTCAGCTGAATCAAGAAAATTGCTTCTCATTGTTCAGGCACTTAGGGACAACCTGGAACCTGGAACCTTCGATCTTGGGGGGCTATATGAAGCAATTG<br>AGGAGTGCCTGATTAACGATCCCTGGGTTTTGCTTAATGCGTCTTGGTTCAACTCCTTCATCACACATGCACTGAAATA-
EPI_ISL_18245742	>A/white-fronted_goose/Poland/MB028-22_22VIR5675-1/2022_2022-01-14<br>ATGGAAGACTTTGTGCGACAATGCTTCAATCCAATGATTGTCGAGCTTGCGGAAAAAGCAATGAAAGAATATGGGGAAGATCCGAAAATCGAGACAAACAAATTTGCCGCAATATGCACACACTTAGAAGTCTGTTTCATGTATTCGGATTTCCATTTTATTGATGAACGAGGCGAATCAATGATTGTAGAATCTGGCGATCCAAATGCATTATTGAAACACCGATTTGAGA<br>TAATCGAAGGGAGAGACCGAGCAATGGCCTGGACAGTGGTGAATAGTATCTGCAACACCACAGGAGTCGAAAAGCCCAAATTCCTCCCTGATTTGTATGACTACAAAGAGAACCGATTCATTGAAATTGGAGTAACGCGAAGGGAAGTTCACATATACTATTTAGAAAAAGCCAACAAGATAAAATCAGAGAAAACACACATTCACATATTCTCATTCACTGGAGAGGAA<br>ATGGCCACCAGGGCGGAATACACCCTTGATGAAGAGAGCAGAGCAAGAATAAAAACCAGACTGTTCACTATAAGACAAGAAATGGCCAGTAGAGGTCTATGGGATTCCTTTCGTCAGTCCGAGAGAGGCGAAGAGACAAATGAAGAAAGATTTGAAATCACAGGAACCATGCGCAGGCTTGCCGACCAAAGTCTCCCACCGAACTTCTCCAGCCTTGAAAACTTTAGAG<br>CCTATGTGGATGGATTCGAACCGAACGGCTGCATTGAGGGCAAGCTTTCTCAAATGTCAAAAGAAGTGAACGCCAGAATTGAGCCATTCCTGAAGACAACACCACGCCCTCTCAGATTACCTGATGGCCTCCTTGTTCTCAGCGGTCGAAGTTCTTGCTGATGGATGCCCTTAAATTGAGCATCGAAGACCCTAGCCATGAGGGGGAGGGCATACCGCTGTATGATGCAA<br>TCAAATGCATGAAGACATTTTTTGGCTGGAAAGAGCCCAACCTCGTAAAGCCGCATGAGAAGGGCATAAACCCTAATTACCTCCTGGCTTGGAAGCAGGTGCTGGCAGAACTTCAAGATATTGAGAATGAGGAGAAATTTCCAAAAACAAAGAACATGAAGAAAACAAGCCAATTGAAGTGGGCACTTGGTGAGAACATGGCACCAGAAAAAGTGGACTTTGAGGACTG<br>CAAAGATGTTAGCGATCTAAGACAGTACGACAGTGACGAACCAGAGCCTAGATCACTAGCAAGCTGGATTCAGAGTGAATTCAACAAGGCATGCGAATTGACAGATTCGAGTTGGATTGAACTTGATGAGATAGGGGAAGACGTTGCTCCAATCGAACACATTGCGAGTATGAGGAGGAACTATTTCACAGCGGAGGTATCCCATTGCAGGGCCACTGAATACATAATG<br>AAGGGAGTATACATAAACACAGCCCTATTGAATGCATCCTGTGCAGCCATGGATGACTTCCAACTGATTCCAATGATAAGCAAGTGCAGAACCAAAGAGGGAAGACGGAAGACAAATCTGTATGGATTCATTATAAAAGGAAGATCCCATTTGAGGAATGACACCGATGTGGTAAACTTTGTGAGCATGGAATTCTCTCTAACTGACCCGAGGCTAGAGCCACACAAATG<br>GGAAAAGTACTGTGTTCTTGAGATAGGAGACATGCTCCTACGGACTGCAATAGGCCAAGTGTCGAGGCCCATGTTCCTATATGTGAGAACCAATGGGACTTCCAAGATCAAAATGAAATGGGGCATGGAGATGAGACGATGCCTTCTTCAGTCCCTTCAACAAATTGAGAGCATGATTGAGGCCGAATCTTCTGTCAAAGAGAAGGACATGACCAAGGAATTCTTTGAAA<br>ACAAATCAGAAACATGGCCAATTGGAGAATCACCCAAAGGGGTTGAGGAAGGCTCTATTGGGAAAGTATGCAGAACATTGCTAGCAAAGTCTGTGTTCAACAGCCTATATGCATCTCCACAACTCGAGGGATTTTCAGCTGAATCAAGAAAATTGCTTCTCATTGTTCAGGCACTTAGGGACAACCTGGAACCTGGAACCTTCGATCTTGGGGGGCTATATGAAGCAATTG<br>AGGAGTGCCTGATTAACGATCCCTGGGTTTTGCTTAATGCGTATTGGTTCAACTCCTTCATCACACATGCACTGAAATA-
EPI_ISL_18245814	>A/mute_swan/Poland/MB550/2021_H5N1_2021-12-23<br>ATGGAAGACTTTGTGCGACAATGCTTCAATCCAATGATTGTCGAGCTTGCGGAAAAAGCAATGAAAGAATATGGGGAAGATCCGAAAATCGAGACAAACAAATTTGCCGCAATATGCACACACTTAGAAGTCTGTTTCATGTATTCGGATTTCCATTTTATTGATGAACGAGGCGAATCAATGATTGTAGAATCTGGCGATCCAAATGCATTATTGAAACACCGATTTGAGA<br>TAATCGAAGGGAGAGACCGAGCAATGGCCTGGACAGTGGTGAATAGTATCTGCAACACCACAGGAGTCGAAAAGCCCAAATTCCTCCCTGATTTGTATGACTACAAAGAGAACCGATTCATTGAAATTGGAGTAACGCGAAGAGAAGTTCACATATACTATTTAGAAAAAGCCAACAAGATAAAATCAGAGAAAACACACATTCACATATTCTCATTCACTGGAGAGGAA<br>ATGGCCACCAAGGCGGAATACACCCTTGATGAAGAGAGCAGAGCAAGAATAAAAACCAGACTGTTCACTATAAGACAAGAAATGGCCAGTAGAGGTCTATGGGATTCCTTTCGTCAGTCCGAGAGAGGCGAAGAGACAACTGAAGAAAGATTTGAAATCACAGGAACCATGCGCAGGCTTGCCGACCAAAGTCTCCCACCGAACTTCTCCAGCCTTGAAAACTTTAGAG<br>CCTATGTGGATGGATTCGAACCGAACGGCTGCATTGAGGGCAAGCTTTCTCAAATGTCAAAAGAAGTGAACGCCAGAATTGAGCCATTCCTGAAGACAACACCACGCCCTCTCAGATTACCTGATGGCCTCCTTGTTCTCAGCGGTCGAAGTTCTTGCTGATGGATGCCCTTAAGTTGAGCATCGAAGACCTAGCCATGAGGGGGAGGGATACCGCTGTATGATGCAA<br>TCAAATGCATGAAGACATTTTTTGGCTGGAAAGAGCCCAACCTCGTAAAGCCGCATGAGAAAGGCATAAACCCTAATTACCTCCTGGCTTGGAAGCAGGTGCTGGCAGAACTTCAAGATATTGAGAATGAGGAGAAAATTCCCAAAAACAAAGAACATGAAGAAAACAAGCCAATTGAAGTGGGCACTTGGTGAGACATGGCACCAGAAAAAGTGGACTTTGAGGACTG<br>CAAAGATGTTAGCGATCTAAGACAGTACGACAGTGACGAACCAGAGCCTAGATCACTAGCAAGCTGGATTCAGAGTGAATTCAACAAGGCATGCGAATTGACAGATTCGAGTTGGATTGAACTTGATGAGATAGGGGAAGACGTTGCTCCAATCGAACACATTGCGAGTATGAGGAGGAACTATTTCACAGCGGAGGTATCCCATTGCAGGGCCACTGAATACATAATG<br>AAGGGAGTATACATAAACACAGCCCTATTGAATGCATCCTGTGCAGCCATGGATGACTTCCAACTGATTCCAATGATAAGCAAGTGCAGAACCAAAGAGGGAAGACGGAAGACAAATCTGTATGGATTCATTATAAAAGGAAGATCCCATTTGAGGAATGACACCGATGTGGTAAACTTTGTGAGCATGGAATTCTCTCTAACTGACCCGAGGCTAGAGCCACACAAATG<br>GGAAAAGTACTGTGTTCTTGAGATAGGAGACATGCTCCTACGGACTGCAATAGGCCAAGTGTCGAGGCCCATGTTCCTGTATGTGAGAACCAATGGGACTTCCAAGATCAAAATGAAATGGGGCATGGAGATGAGACGATGCCTTCTTCAGTCCCTTCAACAATTGAGAGCATGATTGAGGCCGAATCTTCTGTCAAAGAGAAGGACATGACCAAGGAATTCTTTGAAA<br>ACAAATCAGAAACATGGCCAATTGGAGAATCACCCAAAGGGGTTGAGGAAGGCTCTATTGGGAAAGTATGCAGAACATTGCTAGCAAAGTCTGTGTTCAACAGCCTATATGCATCTCCACAACTCGAGGGATTTTCAGCTGAATCAAGAAAATTGCTTCTCATTGTTCAGGCACTTAGGGACAACCTGGAACCTGGAACCTTCGATCTTGGGGGGCTATATGAAGCAATTG<br>AGGAGTGCCTGATTAACGATCCCTGGGTTTTGCTTAATGCGTCTTGGTTCAACTCCTTCATCACACATGCACTGAAATA-
EPI_ISL_18245789	>A/mute_swan/Poland/MB042-22_22VIR5675-6/2022_H5N1_2022-01-24<br>ATGGAAGACTTTGTGCGACAATGCTTCAATCCAATGATTGTCGAGCTTGCGGAAAAAGCAATGAAAGAATATGGGGAAGATCCGAAAATCGAGACAAACAAATTTGCCGCAATATGCACACACTTAGAAGTCTGTTTCATGTATTCGGATTTCCATTTTATTGATGAACGAGGCGAATCAATGATTGTAGAATCTGGCGATCCAAATGCATTATTGAAACACCGATTTGAGA<br>TAATCGAAGGGAGAGACCGAGCAATGGCCTGGACAGTGGTGAATAGTATCTGCAACACCACAGGAGTCGAAAAGCCCAAATTCCTCCCTGATTTGTATGACTACAAAGAGAACCGATTCATTGAAATTGGAGTAACGCGAAGGGAAGTTCACATATACTATTTAGAAAAAGCCAACAAGATAAAATCAGAGAAAACACACATTCACATATTCTCATTCACTGGAGAGGAA<br>ATGGCCACCAAGGCGGAATACACCCTTGATGAAGAGAGCAGAGCAAGAATAAAAACCAGACTGTTCACTATAAGACAAGAAATGGCCAGTAGAGGTCTATGGGATTCCTTTCGTCAGTCCGAGAGAGGCGAAGAGACAAATGAAGAAAGATTTGAAATCACAGGAACCATGCGCAGGCTTGCCGACCAAAGTCTCCCACCGAACTTCTCCAGCCTTGAAAACTTTAGAG<br>CCTATGTGGATGGATTCGAACCGAACGGCTGCATTGAGGGCAAGCTTTCTCAAATGTCAAAAGAAGTGAACGCCAGAATTGAGCCATTCCTGAAGACAACACCACGCCCTCTCAGATTACCTGATGGCCTCCTTGTTCTCAGCGGTCGAAGTTCTTGCTGATGGATGCCCTTAAATTGAGCATCGAAGACCCTAGCCATGAGGGGGAGGGCATACCGCTGTATGATGCAA<br>TCAAATGCATGAAGACATTTTTTGGCTGGAAAGAGCCCAACCTCGTAAAGCCGCATGAGAAGGGCATAAACCCTAATTACCTCCTGGCTTGGAAGCAGGTGCTGGCAGAACTTCAAGATATTGAGAATGAGGAGAAATTTCCAAAAACAAAGAACATGAAGAAAACAAGCCAATTGAAGTGGGCACTTGGTGAGAACATGGCACCAGAAAAAGTGGACTTTGAGGACTG<br>CAAAGATGTTAGCGATCTAAGACAGTACGACAGTGACGAACCAGAGCCTAGATCACTAGCAAGCTGGATTCAGAGTGAATTCAACAAGGCATGCGAATTGACAGATTCGAGTTGGATTGAACTTGATGAGATAGGGGAAGACGTTGCTCCAATCGAACACATTGCGAGTATGAGGAGGAACTATTTCACAGCGGAGGTATCCCATTGCAGGGCCACTGAATACATAATG<br>AAGGGAGTATACATAAACACAGCCCTATTGAATGCATCCTGTGCAGCCATGGATGACTTCCAACTGATTCCAATGATAAGCAAGTGCAGAACCAAAGAGGGAAGACGGAAGACAAATCTGTATGGATTCATTATAAAAGGAAGATCCCATTTGAGGAATGACACCGATGTGGTAAACTTTGTGAGCATGGAATTCTCTCTAACTGACCCGAGGCTAGAGCCACACAAATG<br>GGAAAAGTACTGTGTTCTTGAGATAGGAGACATGCTCCTACGGACTGCAATAGGCCAAGTGTCGAGGCCCATGTTCCTATATGTGAGAACCAATGGGACTTCCAAGATCAAAATGAAATGGGGCATGGAGATGAGACGATGCCTTCTTCAGTCCCTTCAACAAATTGAGAGCATGATTGAGGCCGAATCTTCTGTCAAAGAGAAGGACATGACCAAGGAATTCTTTGAAA<br>ACAAATCAGAAACATGGCCAATTGGAGAATCACCCAAAGGGGTTGAGGAAGGCTCTATTGGGAAAGTATGCAGAACATTGCTAGCAAAGTCTGTGTTCAACAGCCTATATGCATCTCCACAACTCGAGGGATTTTCAGCTGAATCAAGAAAATTGCTTCTCATTGTTCAGGCACTTAGGGACAACCTGGAACCTGGAACCTTCGATCTTGGGGGGCTATATGAAGCAATTG<br>AGGAGTGCCTGATTAACGATCCCTGGGTTTTGCTTAATGCGTATTGGTTCAACTCCTTCATCACACATGCACTGAAATA-
EPI_ISL_18245744	>A/mute_swan/Poland/MB040-22_22VIR5675-8/2022_H5N1_2022-01-20<br>ATGGAAGACTTTGTGCGACAATGCTTCAATCCAATGATTGTCGAGCTTGCGGAAAAAGCAATGAAAGAATATGGGGAAGATCCAAAAATCGAGACAAACAAATTTGCCGCAATATGCACACACTTAGAAGTCTGTTTCATGTATTCGGATTTCCATTTTATTGATGAACGAGGCGAATCAATGATTGTAGAATCTGGCGATCCAAATGCATTATTGAAACACCGATTTGAGA<br>TAATCGAAGGGAGAGACCGAGCAATGGCCTGGACAGTGGTGAATAGTATCTGCAACACCACAGGAGTCGAAAAGCCCAAATTCCTCCCTGATnnnnnnnnnnnnnnnnnnnnnnnnnnnnnnnnnnnnnnnnnnnnnnnnnnnnnnnnnnnnnnnnnnnnnnnnnnnnnnnnnnnnnnnnnnnnnnnnnnnnnnnnnnnnnnnnnnnnnn<br>nnnnnnnnnnnnnnTGATGAAGAGAGCAGAGCAAGAATAAAAACCAGACTGTTCACTATAAGACAAGAAATGGnnnnnnnnnnnnnnnnnnnnnnnnnnnnnnnnnnnnnnnnnnnnnnnnnnnnnnnnnnnnnnnnnnnnnnnnnnnnnnnnnnnnnnnnnnnnnnnnnnnnnnnnnnnnnnnnnnnnnnnnnnnnnnnnnnnnnnnnnnnnnn<br>nn<br>nnn<br>nnnnnnnnnnnnnnnnnnnnnnnnnnnnnnnnnnnnnnnnnnnnnnnnnnnnnnnnnnnnnnnnnnnnnnnnnnnnnnnnnnnnnnnnnnnnnnnnnnnnnnnnnnnnnnnnnnnnnnnnnnnnnnnnnnnnnnnnnnnnnnnnnnnnnnnnnnCAGTACGACAGTGACGAACCAGAATCTAGATCACTAGC<br>AAGTTGGATCCAGAGTGAATTTAACAAGGCTTGCGAACTGACAGATTCGAGTTGGATTGAACTTGATGAAATAGGGGAAGACGTTGCTCCAATTGAACACATTGCGAGTATGAGGAGGAACTATTTCACAGCGGAAGTATCCCATTGCAGGGCTnnnnnnnnnnnnnnnnnnGAGTATACATAAACACAGCTTTATTGAATGCATCCTGTGCAGCCATGGATGACTTCCAAC<br>TGATTCCAATGATAAGCAAATGCAGAACCAAAGAAGGGAGACGGAAGACAAATCTGTATGGCTTCATTATAAAAGGAAGATCCCATTTGAGGAATGACACCGATGTGGTAAACTTTGTGAGCATGGAATTCTCTCTMACTGACCCRAGGCTRGAGCCACACAAATGGGAAAAGTACTGTGTKCTTGAGRTAnnnGACATGCTCCTACGGACTGCAATAGGCnnnnnnnnnn<br>nnnnnnnnnnnnnnnnnnnnnnnnnnnnnnnnnnnnAGATCAAAATGAAATGGGGYATGGAGATGAGACGATGCCTTCTTCARTCCCTTCAACAAATnnnnnnnnnnnnnnnGCCGAATCTTCTGTCAAAGAGAAGGACATGACCAAGGAATTCTTTGAAAACAAATCAGAAACATGGCCAATTGGAGAATCACCCAAAGGGGTTGAGGAAGGCTCTATTGGGAAAGTAT<br>GCAGAACATTGCTAGCAAAGTCTGTGTTCAACAGCCTATATGCATCTCCACAACTCGAGGGATTTTCAGCTGAATCAAGAAAATTGCTTCTCATTGTTCAGGCACTTAGGGACAACCTGGAGCCTGGAACCTTCGATCTTGGGGGGCTATATGAAGCAATTGAGGAGTGCCTGATTAACGATCCCTGGGTTTTGCTTAATGCGTCTTGGTTCAACTCCTTCCTCACACATGCAC<br>TGAAATA-
EPI_ISL_7753387	>A/Eurasian_wigeon/Germany-SH/AI06143/2021_H5N1_2021-10-22<br>ATGGAAGACTTTGTGCGACAATGCTTCAATCCAATGATTGTCGAGCTTGCGGAAAAGGCAATGAAAGAGTATGGGGAAGATCCGAAAATCGAAACAAACAAATTTGCCGCAATATGCACACACTTAGAAGTCTGTTTCATGTATTCGGATTTCCACTTTATTGATGAACGAGGCGAATCAATAATTGTAGAATCTGGCGATCCGAATGCATTATTGAAACACCGATTCGAGA<br>TAATTGAAGGGAGAGACCGAACTATGGCCTGGACAGTGGTGAATAGTATCTGCAACACCACAGGAGTCGAAAAGCCCAAATTCCTCCCTGATTTGTATGACTACAAAGAGAACCGATTCATTGAAATTGGAGTAACGCGAAGGGAAATTCACATATACTATCTAGAGAAAGCCAACAAGATAAAATCAGAGAAGACACACATTCATATATTCTCATTCACTGGAGAGGAA<br>ATGGCCACCAAGGCGGACTACACCCTTGATGAAGAGAGTAGAGCAAGAATAAAAACCAGGCTGTTCACTATAAGACAAGAAATGGCCAGTAGGGGTCTATGGGATTCCTTTCGTCAGTCCGAGAGAGGCGAAGAGACAATTGAAGAAAGATTTGAAATCACAGGAACCATGCGCAGGCTTGCCGACCAAAGTCTCCCACCGAACTTCTCCAGCCTTGAAAACTTTAGAG<br>CCTATGTGGATGGATTCGAACCGAACGGCTGCATTGAGGGCAAGCTTTCTCAAATGTCAAAAGAAGTGAATGCCAGAATTGAGCCATTTCTGAAGACAACACCACGCCCTCTCAGATTACCTGATGGGCCTCCCTGTTCTCAGCGGTCGAAGTTCTTGCTGATGGATGCCCTCAAATTGAGCATCGAAGACCCGAGCCATGAGGGGGAGGGTATACCGCTGTATGATGCAA<br>TCAAATGCATGAAGACATTTTTTGGCTGGAAAGAGCCTAACATCGTAAAACCGCATGAAAAAGGCATAAACCCTAATTACCTTCTGGCTTGGAAACAGGTGCTGGCAGAACTCCAAGATATTGAAAATGAGGAGAAAATCCCAAAAACAAAGAACATGAAGAAAACAAGCCAATTGAAGTGGGCACTTGGTGAGAACATGGCACCAGAGAAAGTGGACTTTGAGGACTG<br>TAAAGATGTTAGTGATCTAAGACAGTACGACAGTGACGAACCAGAGTCTAGATCACTAGCAAGCTGGATCCAGAGTGAATTCAACAAGGCATGCGAATTGACAGATTCGAGTTGGATTGAACTTGATGAAATAGGGGAAGACATTGCTCCAATTGAACACATTGCGAGTATGAGGAGAAACTATTTCACAGCGGAGGTGTCCCATTGCAGGGCTACTGAATACATAATGA<br>AGGGAGTATACATAAACACAGCCCTGTTGAATGCATCCTGTGCAGCGATGGATGACTTTCAACTGATTCCTATGATAAGCAAGTGCAGAACCAAAGAAGGAAGACGGAAGACAAATTTATATGGATTCATTATAAAAGGAAGATCCCATTTGAGGAATGACACCGATGTGGTGAACTTTGTGAGCATGGAATTCTCTCTCACTGACCCGAGGCTGGAGCCACACAAATGG<br>GAAAAGTACTGTGTTCTTGAGATAGGAGACATGCTCCTACGGACTGCAATAGGCCAAGTGTCAAGGCCCATGTTCCTGTATGTGAGAACCAATGGAACTTCCAAGATCAAAATGAAATGGGGCATGGAGATGAGACGATGCCTTCTTCAGTCTCTTCAACAAATTGAGAGCATGATTGAGGCCGAGTCTTCTGTCAAAGAGAAGGACATGACCAAGGAATTCTTTGAAAAC<br>AAATCAGAAACATGGCCAATTGGGGAATCACCCAAAGGGGTGGAAGAAGGCTCCATTGGGAAGGTGTGCAGAACATTGCTAGCAAAGTCTGTGTTCAACAGCCTATATGCATCTCCACAACTCGAGGGGTTTTCAGCTGAATCAAGAAAATTGCTTCTCATTGTTCAGGCACTTAGGGACAACCTGGAACCTGGAACCTTCGATCTTGGGGGGCTATATGAAGCAATTGA<br>GGAGTGCCTGATTAACGATCCCTGGGTTTTGCTTAATGCGTCTTGGTTCAACTCCTTCCTCACACATGCACTGAAATA-
EPI_ISL_5588100	>A/chicken/Netherlands/21037287-006010/2021_H5N1_2021-10-25<br>ATGGAAGACTTTGTGCGACAATGCTTCAATCCAATGATTGTCGAGCTTGCGGAAAAGGCAATGAAAGAGTATGGGGAAGATCCGAAAATCGAAACAAACAAATTTGCCGCAATATGCACACACTTAGAAGTCTGTTTCATGTATTCGGATTTCCACTTTATTGATGAACGAGGCGAATCAATAATTGTAGAATCTGGCGATCCGAATGCATTATTGAAACACCGATTCGAGA<br>TAATTGAAGGGAGAGACCGAACTATGGCCTGGACAGTGGTGAATAGTATCTGCAACACCACAGGAGTCGAAAAGCCCAAATTCCTCCCTGATTTGTATGACTACAAAGAGAACCGATTCATTGAAATTGGAGTAACGCGAAGGGAAATTCACATATACTATCTAGAGAAAGCCAACAAGATAAAATCAGAGAAGACACACATTCATATATTCTCATTCACTGGAGAGGAA<br>ATGGCCACCAAGGCGGACTACACCCTTGATGAAGAGAGTAGAGCAAGAATAAAAACCAGGCTGTTCACTATAAGACAAGAAATGGCCAGTAGGGGTCTATGGGATTCCTTTCGTCAGTCCGAGAGAGGCGAAGAGACAATTGAAGAAAGATTTGAAATCACAGGAACCATGCGCAGGCTTGCCGACCAAAGTCTCCCACCGAACTTCTCCAGCCTTGAAAACTTTAGAG<br>CCTATGTGGATGGATTCGAACCGAACGGCTGCATTGAGGGCAAGCTTTCTCAAATGTCAAAAGAAGTGAATGCCAGAATTGAGCCATTTCTGAAGACAACACCACGCCCTCTCAGATTACCTGATGGGCCTCCCTGTTCTCAGCGGTCGAAGTTCTTGCTGATGGATGCCCTCAAATTGAGCATCGAAGACCCGAGCCATGAGGGGGAGGGTATACCGCTGTATGATGCAA<br>TCAAATGCATGAAGACATTTTTTGGCTGGAAAGAGCCTAACATCGTAAAACCGCATGAAAAAGGCATAAACCCTAATTACCTTCTGGCTTGGAAACAGGTGCTGGCAGAACTCCAAGATATTGAAAATGAGGAGAAAATCCCAAAAACAAAGAACATGAAGAAAACAAGCCAATTGAAGTGGGCACTTGGTGAGAACATGGCACCAGAGAAAGTGGACTTTGAGGACTG<br>TAAAGATGTTAGTGATCTAAGACAGTACGACAGTGACGAACCAGAGTCTAGATCACTAGCAAGCTGGATCCAGAGTGAATTCAACAAGGCATGCGAATTGACAGATTCGAGTTGGATTGAACTTGATGAAATAGGGGAAGACATTGCTCCAATTGAACACATTGCGAGTATGAGGAGAAACTATTTCACAGCGGAGGTGTCCCATTGCAGGGCTACTGAATACATAATGA<br>AGGGAGTATACATAAACACAGCCCTGTTGAATGCATCCTGTGCAGCGATGGATGACTTTCAACTGATTCCTATGATAAGCAAGTGCAGAACCAAAGAAGGAAGACGGAAGACAAATTTATATGGATTCATTATAAAAGGAAGATCCCATTTGAGGAATGACACCGATGTGGTGAACTTTGTGAGCATGGAATTCTCTCTCACTGACCCGAGGCTGGAGCCACACAAATGG<br>GAAAAGTACTGTGTTCTTGAGATAGGAGACATGCTCCTACGGACTGCAATAGGCCAAGTGTCAAGGCCCATGTTCCTGTATGTGAGAACCAATGGAACTTCCAAGATCAAAATGAAATGGGGCATGGAGATGAGACGATGCCTTCTTCAGTCTCTTCAACAAATTGAGAGCATGATTGAGGCCGAGTCTTCTGTCAAAGAGAAGGACATGACCAAGGAATTCTTTGAAAAC<br>AAATCAGAAACATGGCCAATTGGGGAATCACCCAAAGGGGTGGAAGAAGGCTCCATTGGGAAGGTGTGCAGAACATTGCTAGCAAAGTCTGTGTTCAACAGCCTATATGCATCTCCACAACTCGAGGGGTTTTCAGCTGAATCAAGAAAATTGCTTCTCATTGTTCAGGCACTTAGGGACAACCTGGAACCTGGAACCTTCGATCTTGGGGGGCTATATGAAGCAATTGA<br>GGAGTGCCTGATTAACGATCCCTGGGTTTTGCTTAATGCGTCTTGGTTCAACTCCTTCCTCACACATGCACTGAAATA-
EPI_ISL_7753405	>A/Eurasian_wigeon/Germany-SH/AI06142/2021_H5N1_2021-10-22<br>ATGGAAGACTTTGTGCGACAATGCTTCAATCCAATGATTGTCGAGCTTGCGGAAAAGGCAATGAAAGAGTATGGGGAAGATCCGAAAATCGAAACAAACAAATTTGCCGCAATATGCACACACTTAGAAGTCTGTTTCATGTATTCGGATTTCCACTTTATTGATGAACGAGGCGAATCAATAATTGTAGAATCTGGCGATCCGAATGCATTATTGAAACACCGATTCGAGA<br>TAATTGAAGGGAGAGACCGAACTATGGCCTGGACAGTGGTGAATAGTATCTGCAACACCACAGGAGTCGAAAAGCCCAAATTCCTCCCTGATTTGTATGACTACAAAGAGAACCGATTCATTGAAATTGGAGTAACGCGAAGGGAAATTCACATATACTATCTAGAGAAAGCCAACAAGATAAAATCAGAGAAGACACACATTCATATATTCTCATTCACTGGAGAGGAA<br>ATGGCCACCAAGGCGGACTACACCCTTGATGAAGAGAGTAGAGCAAGAATAAAAACCAGGCTGTTCACTATAAGACAAGAAATGGCCAGTAGGGGTCTATGGGATTCCTTTCGTCAGTCCGAGAGAGGCGAAGAGACAATTGAAGAAAGATTTGAAATCACAGGAACCATGCGCAGGCTTGCCGACCAAAGTCTCCCACCGAACTTCTCCAGCCTTGAAAACTTTAGAG<br>CCTATGTGGATGGATTCGAACCGAACGGCTGCATTGAGGGCAAGCTTTCTCAAATGTCAAAAGAAGTGAATGCCAGAATTGAGCCATTTCTGAAGACAACACCACGCCCTCTCAGATTACCTGATGGGCCTCCCTGTTCTCAGCGGTCGAAGTTCTTGCTGATGGATGCCCTCAAATTGAGCATCGAAGACCCGAGCCATGAGGGGGAGGGTATACCGCTGTATGATGCAA<br>TCAAATGCATGAAGACATTTTTTGGCTGGAAAGAGCCTAACATCGTAAAACCGCATGAAAAAGGCATAAACCCTAATTACCTTCTGGCTTGGAAACAGGTGCTGGCAGAACTCCAAGATATTGAAAATGAGGAGAAAATCCCAAAAACAAAGAACATGAAGAAAACAAGCCAATTGAAGTGGGCACTTGGTGAGAACATGGCACCAGAGAAAGTGGACTTTGAGGACTG<br>TAAAGATGTTAGTGATCTAAGACAGTACGACAGTGACGAACCAGAGTCTAGATCACTAGCAAGCTGGATCCAGAGTGAATTCAACAAGGCATGCGAATTGACAGATTCGAGTTGGATTGAACTTGATGAAATAGGGGAAGACATTGCTCCAATTGAACACATTGCGAGTATGAGGAGAAACTATTTCACAGCGGAGGTGTCCCATTGCAGGGCTACTGAATACATAATGA<br>AGGGAGTATACATAAACACAGCCCTGTTGAATGCATCCTGTGCAGCGATGGATGACTTTCAACTGATTCCTATGATAAGCAAGTGCAGAACCAAAGAAGGAAGACGGAAGACAAATTTATATGGATTCATTATAAAAGGAAGATCCCATTTGAGGAATGACACCGATGTGGTGAACTTTGTGAGCATGGAATTCTCTCTCACTGACCCGAGGCTGGAGCCACACAAATGG<br>GAAAAGTACTGTGTTCTTGAGATAGGAGACATGCTCCTACGGACTGCAATAGGCCAAGTGTCAAGGCCCATGTTCCTGTATGTGAGAACCAATGGAACTTCCAAGATCAAAATGAAATGGGGCATGGAGATGAGACGATGCCTTCTTCAGTCTCTTCAACAAATTGAGAGCATGATTGAGGCCGAGTCTTCTGTCAAAGAGAAGGACATGACCAAGGAATTCTTTGAAAAC<br>AAATCAGAAACATGGCCAATTGGGGAATCACCCAAAGGGGTGGAAGAAGGCTCCATTGGGAAGGTGTGCAGAACATTGCTAGCAAAGTCTGTGTTCAACAGCCTATATGCATCTCCACAACTCGAGGGGTTTTCAGCTGAATCAAGAAAATTGCTTCTCATTGTTCAGGCACTTAGGGACAACCTGGAACCTGGAACCTTCGATCTTGGGGGGCTATATGAAGCAATTGA<br>GGAGTGCCTGATTAACGATCCCTGGGTTTTGCTTAATGCGTCTTGGTTCAACTCCTTCCTCACACATGCACTGAAATA-
EPI_ISL_11259281	>A/white-fronted_goose/Ireland/033181_22VIR1325-8/2021_H5N1_2021-11-15<br>ATGGAAGACTTTGTGCGACAATGCTTCAATCCAATGATTGTCGAGCTTGCGGAAAAGGCAATGAAAGAGTATGGGGAAGATCCGAAAATCGAAACAAACAAATTTGCCGCAATATGCACACACTTAGAAGTCTGTTTCATGTATTCGGATTTCCACTTTATTGATGAACGAGGCGAATCAATAATTGTAGAATCTGGCGATCCGAATGCATTATTGAAACACCGATTCGAGA<br>TAATTGAAGGGAGAGACCGAACTATGGCCTGGACAGTGGTGAATAGTATCTGCAACACCACAGGAGTCGAAAAGCCCAAATTCCTCCCTGATTTGTATGACTACAAAGAGAACCGATTCATTGAAATTGGAGTAACGCGAAGGGAAATTCACATATACTATCTAGAGAAAGCCAACAAGATAAAATCAGAGAAGACACACATTCATATATTCTCATTCACTGGAGAGGAA<br>ATGGCCACCAAGGCGGACTACACCCTTGATGAAGAGAGTAGAGCAAGAATAAAAACCAGGCTGTTCACTATAAGACAAGAAATGGCCAGTAGGGGTCTATGGGATTCCTTTCGTCAGTCCGAGAGAGGCGAAGAGACAATTGAAGAAAGATTTGAAATCACAGGAACCATGCGCAGGCTTGCCGACCAAAGTCTCCCACCGAACTTCTCCAGCCTTGAAAACTTTAGAG<br>CCTATGTGGATGGATTCGAACCGAACGGCTGCATTGAGGGCAAGCTTTCTCAAATGTCAAAAGAAGTGAATGCCAGAATTGAGCCATTTCTGAAGACAACACCACGCCCTCTCAGATTACCTGATGGGCCTCCCTGTTCTCAGCGGTCGAAGTTCTTGCTGATGGATGCCCTCAAATTGAGCATCGAAGACCCGAGCCATGAGGGGGAGGGTATACCGCTGTATGATGCAA<br>TCAAATGCATGAAGACATTTTTTGGCTGGAAAGAGCCTAACATCGTAAAACCGCATGAAAAAGGCATAAACCCTAATTACCTTCTGGCTTGGAAACAGGTGCTGGCAGAACTCCAAGATATTGAAAATGAGGAGAAAATCCCAAAAACAAAGAACATGAAGAAAACAAGCCAATTGAAGTGGGCACTTGGTGAGAACATGGCACCAGAGAAAGTGGACTTTGAGGACTG<br>TAAAGATGTTAGTGATCTAAGACAGTACGACAGTGACGAACCAGAGTCTAGATCACTAGCAAGCTGGATCCAGAGTGAATTCAACAAGGCATGCGAATTGACAGATTCGAGTTGGATTGAACTTGATGAAATAGGGGAAGACATTGCTCCAATTGAACACATTGCGAGTATGAGGAGAAACTATTTCACAGCGGAGGTGTCCCATTGCAGGGCTACTGAATACATAATGA<br>AGGGAGTATACATAAACACAGCCCTGTTGAATGCATCCTGTGCAGCGATGGATGACTTTCAACTGATTCCTATGATAAGCAAGTGCAGAACCAAAGAAGGAAGACGGAAGACAAATTTATATGGATTCATTATAAAAGGAAGATCCCATTTGAGGAATGACACCGATGTGGTGAACTTTGTGAGCATGGAATTCTCTCTCACTGACCCGAGGCTGGAGCCACACAAATGG<br>GAAAAGTACTGTGTTCTTGAGATAGGAGACATGCTCCTACGGACTGCAATAGGCCAAGTGTCAAGGCCCATGTTCCTGTATGTGAGAACCAATGGAACTTCCAAGATCAAAATGAAATGGGGCATGGAGATGAGACGATGCCTTCTTCAGTCTCTTCAACAAATTGAGAGCATGATTGAGGCCGAGTCTTCTGTCAAAGAGAAGGACATGACCAAGGAATTCTTTGAAAAC<br>AAATCAGAAACATGGCCAATTGGGGAATCACCCAAAGGGGTGGAAGAAGGCTCCATTGGGAAGGTGTGCAGAACATTGCTAGCAAAGTCTGTGTTCAACAGCCTATATGCATCTCCACAACTCGAGGGGTTTTCAGCTGAATCAAGAAAATTACTTCTCATTGTTCAGGCACTTAGGGACAACCTGGAACCTGGAACCTTCGATCTTGGGGGGCTATATGAAGCAATTGAG<br>GAGTGCCTGATTAACGATCCCTGGGTTTTGCTTAATGCGTCTTGGTTCAACTCCTTCCTCACACATGCACTGAAATA-

EPI_ISL_11112484	>A/Caspian_Gull/Netherlands/2/2022_H5N1_2022-02-23<br>ATGGAGGACTTTGTGCGACAATGCTTCAATCCAATGATTGTCGAGCTTGCGGAAAAGGCAATGAAAGAGTATGGGGAAGATCCGAAAATCGAAACAAACAAATTTGCCGCAATATGCACACACTTAGAAGTCTGTTTCATGTATTCGGATTTCCACTTTATTGATGAACGAGGCGAATCAATAACTGTAGAATCTGGCGATCCGAATGCATTATTGAAACACCGATTCGAGA<br>TAATTGAAGGGAGAGACCGAACTATGGCCTGGACAGTGGTGAATAGTATCTGCAACACCACAGGAGTCGAAAAGCCCAAATTCCTCCCTGATTTGTATGACTACAAAGAGAACCGATTCATTGAAATTGGAGTAACGCGAAGGGAAGTTCACATATACTATCTAGAGAAAGCCAACAAGATAAAATCAGAGAAGACTCACATTCATATATTCTCATTCACTGGAGAGGAA<br>ATGGCCACCAAGGCGGACTACACCCTTGATGAAGAGAGTAGAGCAAGAATAAAAACCAGGCTGTTCACTATAAGACAAGAAATGGCCAGTAGGGGTCTATGGGATTCCTTTCGTCAGTCCGAGAGAGGCGAAGAGACAATTGAAGAAAGATTTGAAATCACAGGAACCATGCGCAGGCTTGCCGACCAAAGTCTCCCACCGAACTTCTCCAGCCTTGAAAACTTTAGAG<br>CCTATGTGGATGGATTCGAACCGAACGGCTGCATTGAGGGCAAGCTTTCTCAAATGTCAAAAGAAGTGAATGCCAGAATTGAGCCATTTCTGAAGACAACACCACGCCCTCTCAGATTACCTGATGGGCCTCCCTGTTCTCAGCGGTCGAAGTTCTTGCTGATGGATGCCCTCAAATTGAGCATCGAAGACCCGAGCCATGAGGGGGAGGGTATACCGCTGTATGATGCAA<br>TCAAATGCATGAAGACATTTTTTGGCTGGAAAGAGCCTAACATCGTAAAACCGCATGAAAAGGGCATAAACCCTAATTACCTTCTGGCTTGGAAACAGGTGCTGGCAGAACTCCAAGATATTGAAAATGAGGAGAAAATCCCAAAAACAAAGAACATGAAGAAAACAAGCCAATTGAAGTGGGCACTTGGTGAGAACATGGCACCAGAGAAAGTGGACTTTGAGGACTG<br>TAAAGATGTTAGTGATCTAAGACAGTACGACAGTGACGAGCCAGAGTCTAGATCACTAGCAAGCTGGATCCAGAGTGAATTCAACAAGGCATGCGAATTGACAGATTCGAGTTGGATTGAACTTGATGAAATAGGGGAAGACATTGCTCCAATTGAACACATTGCGAGTATGAGGAGAAACTATTTCACAGCGGAGGTGTCCCATTGCAGGGCTACTGAATACATAATGA<br>AGGGAGTATACATAAACACAGCCCTGTTGAATGCATCCTGTGCAGCGATGGATGACTTTCAACTGATTCCTATGATAAGCAAGTGCAGAACCAAAGAAGGAAGACGGAAGACAAATTTATATGGATTCATTATAAAAGGAAGATCCCATTTGAGGAATGACACCGATGTGGTGAACTTTGTGAGCATGGAATTCTCTCTCACTGACCCGAGGCTGGAGCCACACAAATGG<br>GAAAAGTACTGTGTTCTTGAGATAGGAGACATGCTCCTACGGACTGCAATAGGCCAAGTGTCAAGGCCCATGTTCTTGTATGTGAGAACCAATGGAACTTCCAAGATCAAAATGAAATGGGGCATGGAGATGAGACGATGCCTTCTTCAGTCCCTTCAACAAATTGAGAGCATGATTGAGGCCGAGTCTTCTGTCAAAGAGAAGGACATGACCAAGGAATTCTTTGAAAAC<br>AAATCAGAAACATGGCCAATTGGGGAGTCACCCAAAGGGGTGGAAGAAGGCTCCATTGGGAAGGTGTGCAGAACATTGCTAGCAAAGTCTGTGTTCAACAGCCTATATGCATCTCCACAACTCGAGGGGTTTTCAGCTGAATCAAGAAAATTGCTTCTCATTGTTCAGGCACTTAGGGACAACCTGGAACCTGGAACCTTCGATCTTGGGGGGCTATATGAAGCAATTGA<br>GGAGTGCCTGATTAACGATCCCTGGGTTTTGCTTAATGCGTCTTGGTTCAACTCCTTCCTCACACATGCACTGAAATA-
EPI_ISL_13026246	>A/Barnacle_goose/Luxembourg/22033922/2022_H5N1_2022-02-04<br>ATGGAGGACTTTGTGCGACAATGCTTCAATCCAATGATTGTCGAGCTTGCGGAAAAGGCAATGAAAGAGTATGGGGAAGATCCGAAAATCGAAACAAACAAATTTGCCGCAATATGCACACACTTAGAAGTCTGTTTCATGTATTCGGATTTCCACTTTATTGATGAACGAGGCGAATCAATAATTGTAGAATCTGGCGATCCGAATGCATTATTGAAACACCGATTCGAGA<br>TAATTGAAGGGAGAGACCGAACTATGGCCTGGACAGTGGTGAATAGTATCTGCAACACCACAGGAGTCGAAAAGCCCAAATTCCTCCCTGATCTGTATGACTACAAAGAGAACCGATTCATTGAAATTGGAGTAACGCGAAGGGAAGTTCACATATACTATCTAGAGAAAGCCAACAAGATAAAATCAGAGAAGACTCACATTCATATATTCTCATTCACTGGAGAGGAA<br>ATGGCCACCAAGGCGGACTACACCCTTGATGAAGAGAGTAGAGCAAGAATAAAAACCAGGCTGTTCACTATAAGACAAGAAATGGCCAGTAGGGGTCTATGGGATTCCTTTCGTCAGTCCGAGAGAGGCGAAGAGACAATTGAAGAAAGATTTGAAATCACAGGAACCATGCGCAGGCTTGCCGACCAAAGTCTCCCACCGAACTTCTCCAGCCTTGAAAACTTTAGAG<br>CCTATGTGGATGGATTCGAACCGAACGGCTGCATTGAGGGCAAGCTTTCTCAAATGTCAAAAGAAGTGAATGCCAGAATTGAGCCATTTCTGAAGACAACACCACGCCCTCTCAGATTACCTGATGGGCCTCCCTGTTCTCAGCGGTCGAAGTTCTTGCTGATGGATGCCCTCAAATTGAGCATCGAAGACCCGAGCCATGAGGGGGAGGGTATACCGCTGTATGATGCAA<br>TCAAATGCATGAAGACATTTTTTGGCTGGAAAGAGCCTAACATCGTAAAACCGCATGAAAAGGGCATAAACCCTAATTACCTTCTGGCTTGGAAACAGGTGCTGGCAGAACTCCAAGATATTGAAAATGAGGAGAAAATCCCAAAAACAAAGAACATGAAGAAAACAAGCCAATTGAAGTGGGCACTTGGTGAGAACATGGCACCAGAGAAAGTGGACTTTGAGGACTG<br>TAAAGATGTTAGTGATCTAAGACAGTACGACAGTGACGAACCAGAGTCTAGATCACTAGCAAGCTGGATCCAGAGTGAATTCAACAAGGCATGCGAATTGACAGATTCGAGTTGGATTGAACTTGATGAAATAGGGGAAGACATTGCTCCAATTGAACACATTGCGAGTATGAGGAGAAACTATTTCACAGCGGAGGTGTCCCATTGCAGGGCTACTGAATACATAATGA<br>AGGGAGTATACATAAACACAGCCCTGTTGAATGCATCCTGTGCAGCGATGGATGACTTTCAACTGATTCCTATGATAAGCAAGTGCAGAACCAAAGAAGGAAGACGGAAGACAAATTTATATGGATTCATTATAAAAGGAAGATCCCATTTGAGGAATGACACCGATGTGGTGAACTTTGTGAGCATGGAATTCTCTCTCACTGACCCGAGGCTGGAGCCACACAAATGG<br>GAAAAGTACTGTGTTCTTGAGATAGGAGACATGCTCCTACGGACTGCAATAGGCCAAGTGTCAAGGCCCATGTTCTTGTATGTGAGAACCAATGGAACTTCCAAGATCAAAATGAAATGGGGCATGGAGATGAGACGATGCCTTCTTCAGTCCCTTCAACAAATTGAGAGCATGATTGAGGCCGAGTCTTCTGTCAAAGAGAAGGACATGACCAAGGAATTCTTTGAAAAC<br>AAATCAGAAACATGGCCAATTGGGGAGTCACCCAAAGGGGTGGAAGAAGGCTCCATTGGGAAGGTGTGCAGAACATTGCTAGCAAAGTCTGTGTTCAACAGCCTATATGCATCTCCACAACTCGAGGGGTTTTCAGCTGAATCAAGAAAATTGCTTCTCATTGTTCAGGCACTTAGGGACAACCTGGAACCTGGAACCTTCGATCTTGGGGGGCTATATGAAGCAATTGA<br>GGAGTGCCTGATTAACGATCCCTGGGTTTTGCTTAATGCGTCTTGGTTCAACTCCTTCCTCACACATGCACTGAAATA-
EPI_ISL_12177629	>A/black-backed_gull/Netherlands/22006711-001/2022_H5N1_2022-04-05<br>ATGGAGGACTTTGTGCGACAATGCTTCAATCCAATGATTGTCGAGCTTGCGGAAAAGGCAATGAAAGAGTATGGGGAAGATCCGAAAATCGAAACAAACAAATTTGCCGCAATATGCACACACTTAGAAGTCTGTTTCATGTATTCGGATTTCCACTTTATTGATGAACGAGGCGAATCAATAATTGTAGAATCTGGCGATCCGAATGCATTATTGAAACACCGATTCGAGA<br>TAATTGAAGGGAGAGACCGAACTATGGCCTGGACAGTGGTGAATAGTATCTGCAACACCACAGGAGTCGAAAAGCCCAAATTCCTCCCTGATTTGTATGACTACAAAGAGAACCGATTCATTGAAATTGGAGTAACGCGAAGGGAAGTTCACATATACTATCTAGAGAAAGCCAACAAGATAAAATCAGAGAAGACTCACATTCATATATTCTCATTCACTGGAGAGGAA<br>ATGGCCACCAAGGCGGACTACACCCTTGATGAAGAGAGTAGAGCAAGAATAAAAACCAGGCTGTTCACTATAAGACAAGAAATGGCCAGTAGGGGTCTATGGGATTCCTTTCGTCAGTCCGAGAGAGGCGAAGAGACAATTGAAGAAAGATTTGAAATCACAGGAACCATGCGCAGGCTTGCCGACCAAAGTCTCCCACCGAACTTCTCCAGCCTTGAAAACTTTAGAG<br>CCTATGTGGATGGATTCGAACCGAACGGCTGCATTGAGGGCAAGCTTTCTCAAATGTCAAAAGAAGTGAATGCCAGAATTGAGCCATTTCTGAAGAAAACACCACGCCCTCTCAGATTACCTGATGGGCCTCCCTGTTCTCAGCGGTCGAAGTTCTTGCTGATGGATGCCCTCAAATTGAGCATCGAAGACCCGAGCCATGAGGGGGAGGGTATACCGCTGTATGATGCAA<br>TCAAATGCATGAAGACATTTTTTGGCTGGAAAGAGCCTAACATCGTAAAACCGCATGAAAAGGGCATAAACCCTAATTACCTTCTGGCTTGGAAACAGGTGCTGGCAGAACTCCAAGATATTGAAAATGAGGAGAAAATCCCAAAAACAAAGAACATGAAGAAAACAAGCCAATTGAAGTGGGCACTTGGTGAGAACATGGCACCAGAGAAAGTGGACTTTGAGGACTG<br>TAAAGATGTTAGTGATCTAAGACAGTACGACAGTGACGAACCAGAGTCTAGATCACTAGCAAGCTGGATCCAGAGTGAATTCAACAAGGCATGCGAATTGACAGATTCGAGTTGGATTGAACTTGATGAAATAGGGGAAGACATTGCTCCAATTGAACACATTGCGAGTATGAGGAGAAACTATTTCACAGCGGAGGTGTCCCATTGCAGGGCTACTGAATACATAATGA<br>AGGGAGTATACATAAACACAGCCCTGTTGAATGCATCCTGTGCAGCGATGGATGACTTTCAACTGATTCCTATGATAAGCAAGTGCAGAACCAAAGAAGGAAGACGGAAGACAAATTTATATGGATTCATTATAAAAGGAAGATCCCATTTGAGGAATGACACCGATGTGGTGAACTTTGTGAGCATGGAATTCTCTCTCACTGACCCGAGGCTGGAGCCACACAAATGG<br>GAAAAGTACTGTGTTCTTGAGATAGGAGACATGCTCCTACGGACTGCAATAGGCCAAGTGTCAAGGCCCATGTTCTTGTATGTGAGAACCAATGGAACTTCCAAGATCAAAATGAAATGGGGCATGGAGATGAGACGATGCCTTCTTCAGTCCCTTCAACAAATTGAGAGCATGATTGAGGCCGAGTCTTCTGTCAAAGAGAAGGACATGACCAAGGAATTCTTTGAAAAC<br>AAATCAGAAACATGGCCAATTGGGGAGTCACCCAAAGGGGTGGAAGAAGGCTCCATTGGGAAGGTGTGCAGAACATTGCTAGCAAAGTCTGTGTTCAACAGCCTATATGCATCTCCACAACTCGAGGGGTTTTCAGCTGAATCAAGAAAATTGCTTCTCATTGTTCAGGCACTTAGGGACAACCTGGAACCTGGAACCTTCGATCTTGGGGGGCTATATGAAGCAATTGA<br>GGAGTGCCTGATTAACGATCCCTGGGTTTTGCTTAATGCGTCTTGGTTCAACTCCTTCCTCACACATGCACTGAAATA-
EPI_ISL_18245743	>A/mute_swan/Poland/MB034-22_22VIR5675-12/2022_H5N1_2022-01-18<br>ATGGAAGACTTTGTGCGACAATGCTTCAATCCAATGATTGTCGAGCTTGCGGAAAAGGCAATGAAAGAGTATGGGGAAGATCCGAAAATCGAAACAAACAAATTTGCCGCAATATGCACACACTTAGAAGTCTGTTTCATGTATTCGGATTTCCACTTTATTGATGAACGAGGCGAATCAATAATTGTAGAATCTGGCGATCCGAATGCATTATTGAAACACCGATTCGAGA<br>TAATTGAAGGGAGAGACCGAACTATGGCCTGGACAGTGGTGAATAGTATCTGCAACACCACAGGAGTCGAAAAGCCCAAATTCCTCCCTGATTTGTATGACTACAAAGAGAACCGATTCATTGAAATTGGAGTAACGCGAAGGGAAGTTCACATATACTATCTAGAGAAAGCCAACAAGATAAAATCAGAGAAGACACACATTCATATATTCTCATTCACTGGAGAGGAA<br>ATGGCCACCAAGGCGGACTACACCCTTGATGAAGAGAGTAGAGCAAGAATAAAAACCAGGCTGTTCACTATAAGACAAGAAATGGCCAGTAGGGGTCTATGGGATTCCTTTCGTCAGTCCGAGAGAGGCGAAGAGACAATTGAAGAAAGATTTGAAATCACAGGAACCATGCGCAGGCTTGCCGACCAAAGTCTCCCACCGAACTTCTCCAGCCTTGAAAACTTTAGAG<br>CCTATGTGGATGGATTCGAACCGAACGGCTGCATTGAGGGCAAGCTTTCTCAAATGTCAAAAGAAGTGAATGCCAGAATTGAGCCATTTCTGAAGACAACACCACGCCCTCTCAGATTACCTGATGGGCCTCCCTGTTCTCAGCGGTCGAAGTTCTTGCTGATGGATGCCCTCAAATTGAGCATCGAAGACCCGAGCCATGAGGGGGAGGGTATACCGCTGTATGATGCAA<br>TCAAATGCATGAAGACATTTTTTGGCTGGAAAGAGCCTAACATCGTAAAACCGCATGAAAAAGGCATAAACCCTAATTACCTTCTGGCTTGGAAACAGGTGCTGGCAGAACTCCAAGATATTGAAAATGAGGAGAAAATCCCAAAAACAAAGAACATGAAGAAAACAAGCCAATTGAAGTGGGCACTTGGTGAGAACATGGCACCAGAGAAAGTGGACTTTGAGGACTG<br>TAAAGATGTTAGTGATCTAAGACAGTACGACAGTGACGAACCAGAGTCTAGATCACTAGCAAGCTGGATCCAGAGTGAATTCAACAAGGCATGCGAATTGACAGATTCGAGTTGGATTGAACTTGATGAAATAGGGGAAGACATTGCTCCAATTGAACACATTGCGAGTATGAGGAGAAACTATTTCACAGCGGAGGTGTCCCATTGCAGGGCTACTGAATACATAATGA<br>AGGGAGTATACATAAACACAGCCCTGTTGAATGCATCCTGTGCAGCGATGGATGACTTTCAACTGATTCCTATGATAAGCAAGTGCAGAACCAAAGAAGGAAGACGGAAGACAAATTTATATGGATTCATTATAAAAGGAAGATCCCATTTGAGGAATGACACCGATGTGGTGAACTTTGTGAGCATGGAATTCTCTCTCACTGACCCGAGGCTGGAGCCACACAAATGG<br>GAAAAGTACTGTGTTCTTGAGATAGGAGACATGCTACTACGGACTGCAATAGGCCAAGTGTCAAGGCCCATGTTCCTGTATGTGAGAACCAATGGAACTTCCAAGATCAAAATGAAATGGGGCATGGAGATGAGACGATGCCTTCTTCAGTCTCTTCAACAAATTGAGAGCATGATTGAGGCCGAATCTTCTGTCAAAGAGAAGGACATGACCAAGGAATTCTTTGAAAAC<br>AAATCAGAAACATGGCCAATTGGGGAATCACCCAAAGGGGTGGAAGAAGGCTCCATTGGGAAGGTGTGCAGAACATTGCTAGCAAAGTCTGTGTTCAACAGCCTATATGCATCTCCACAACTCGAGGGGTTTTCAGCTGAATCAAGAAAATTACTTCTCATTGTTCAGGCACTTAGGGACAACCTGGAACCTGGAACCTTCGATCTTGGGGGGCTATATGAAGCAATTGAG<br>GAGTGCCTGATTAACGATCCCTGGGTTTTGCTTAATGCGTCTTGGTTCAACTCCTTCCTCACACATGCACTGAAATA-
EPI_ISL_14917999	>A/black-headed_gull/Poland/MB139/2022_H5N1_2022-05-30<br>ATGGAGGACTTTGTGCGACAATGTTTCAATCCAATGATTGTCGAGCTTGCGGAAAAGGCAATGAAAGAGTATGGGGAAGATCCGAAAATCGAAACAAACAAATTTGCCGCAATATGCACACACTTAGAAGTCTGTTTCATGTATTCGGATTTCCACTTTATTGATGAACGAGGCGAATCAATAATTGTAGAATCTGGCGATCCGAATGCATTATTGAAACACCGATTCGAGA<br>TAATTGAAGGGAGAGATCGAACTATGGCCTGGACAGTGGTGAATAGTATCTGCAACACCACAGGAGTCGAAAAGCCCAAATTCCTCCCTGATTTGTATGACTACAAAGAGAACCGATTCATTGAAATTGGAGTAACGCGAAGGGAAGTTCACATATACTATCTAGAGAAAGCCAACAAGATAAAATCAGAGAAGACTCACATTCATATATTCTCATTCACTGGAGAGGAA<br>ATGGCCACCAAGGCGGACTACACCCTTGATGAAGAGAGTAGAGCAAGAATAAAAACCAGGCTGTTCACTATAAGACAAGAAATGGCCAGTAGGGGTCTATGGGATTCCTTTCGTCAGTCCGAGAGAGGCGAAGAGACAATTGAAGAAAGATTTGAAATCACAGGAACCATGCGCAGGCTTGCCGACCAAAGTCTCCCACCGAACTTCTCCAGCATTGAAAACTTTAGAG<br>CCTATGTGGATGGATTCGAACCGAACGGCTGCATTGAGGGCAAGCTTTCTCAAATGTCAAAAGAAGTGAATGCCAGAATTGAGCCATTTCTGAAGACAACACCACGCCCTCTCAGATTACCTGATGGGCCTCCCTGTTCTCAGCGGTCGAAGTTCTTGCTGATGGATGCCCTTAAATTGAGCATCGAAGACCCGAGCCATGAGGGGGAGGGTATACCGCTGTATGATGCAA<br>TCAAATGCATGAAGACATTTTTTGGCTGGAAAGAGCCTAACATCGTAAAACCGCATGAAAAGGGCATAAACCCTAATTACCTTCTGGCTTGGAAACAGGTGCTGGCAGAACTCCAAGATATTGAAAATGAGGAGAAAATCCCAAAAACAAAGAACATGAGGAAAACAAGCCAATTGAAGTGGGCACTTGGTGAGAACATGGCACCAGAGAAAGTGGACTTTGAGGACTG<br>TAAAGATGTTAGTGATCTAAGACAGTACGACAGTGACGAGCCAGAGTCTAGATCACTAGCAAGCTGGATCCAGAGTGAATTCAACAAGGCATGCGAATTGACAGATTCGAGTTGGATTGAACTTGATGAAATAGGGGAAGACATTGCTCCAATTGAACACATTGCGAGTATGAGGAGAAACTATTTCACAGCGGAGGTGTCCCATTGCAGGGCTACTGAATACATAATGA<br>AGGGAGTATACATAAACACAGCCCTGTTGAATGCATCCTGTGCAGCGATGGATGACTTTCAACTGATTCCTATGATAAGCAAGTGCAGAACCAAAGAAGGAAGACGGAAGACAAATTTATATGGATTCATTATAAAAGGAAGATCCCATTTGAGGAATGACACCGATGTGGTGAACTTTGTGAGCATGGAATTCTCTCTCACTGACCCGAGGCTGGAGCCACACAAATGG<br>GAAAAGTACTGTGTTCTTGAGATAGGAGACATGCTCCTACGGACTGCAATAGGCCAAGTGTCAAGGCCCATGTTCTTGTATGTGAGAACCAATGGAACTTCCAAGATCAAAATGAAATGGGGCATGGAGATGAGACGATGCCTTCTTCAGTCCCTTCAACAAATTGAGAGCATGATTGAGGCCGAGTCTTCTGTCAAAGAGAAGGACATGACCAAGGAATTCTTTGAAAAC<br>AAATCAGAAACATGGCCAATTGGGGAGTCACCCAAAGGGGTGGAAGAAGGCTCCATTGGGAAGGTGTGCAGAACATTGCTAGCAAAGTCTGTGTTCAACAGCCTATATGCATCTCCACAACTCGAGGGGTTTTCAGCTGAATCAAGAAAATTGCTTCTCATTGTTCAGGCACTTAGGGACAACCTGGAACCTGGAACCTTCGATCTTGGGGGGCTATATGGAGCAATTGA<br>GGAGTGCCTGATTAACGATCCCTGGGTTTTGCTTAATGCGTCTTGGTTCAACTCCTTCCTCACACATGCACTGAAATA-
EPI_ISL_14917979	>A/herring_gull/Poland/MB138/2022_H5N1_2022-05-30<br>ATGGAGGACTTTGTGCGACAATGTTTCAATCCAATGATTGTCGAGCTTGCGGAAAAGGCAATGAAAGAGTATGGGGAAGATCCGAAAATCGAAACAAACAAATTTGCCGCAATATGCACACACTTAGAAGTCTGTTTCATGTATTCGGATTTCCACTTTATTGATGAACGAGGCGAATCAATAATTGTAGAATCTGGCGATCCGAATGCATTATTGAAACACCGATTCGAGA<br>TAATTGAAGGGAGAGATCGAACTATGGCCTGGACAGTGGTGAATAGTATCTGCAACACCACAGGAGTCGAAAAGCCCAAATTCCTCCCTGATTTGTATGACTACAAAGAGAACCGATTCATTGAAATTGGAGTAACGCGAAGGGAAGTTCACATATACTATCTAGAGAAAGCCAACAAGATAAAATCAGAGAAGACTCACATTCATATATTCTCATTCACTGGAGAGGAA<br>ATGGCCACCAAGGCGGACTACACCCTTGATGAAGAGAGTAGAGCAAGAATAAAAACCAGGCTGTTCACTATAAGACAAGAAATGGCCAGTAGGGGTCTATGGGATTCCTTTCGTCAGTCCGAGAGAGGCGAAGAGACAATTGAAGAAAGATTTGAAATCACAGGAACCATGCGCAGGCTTGCCGACCAAAGTCTCCCACCGAACTTCTCCAGCATTGAAAACTTTAGAG<br>CCTATGTGGATGGATTCGAACCGAACGGCTGCATTGAGGGCAAGCTTTCTCAAATGTCAAAAGAAGTGAATGCCAGAATTGAGCCATTTCTGAAGACAACACCACGCCCTCTCAGATTACCTGATGGGCCTCCCTGTTCTCAGCGGTCGAAGTTCTTGCTGATGGATGCCCTTAAATTGAGCATCGAAGACCCGAGCCATGAGGGGGAGGGTATACCGCTGTATGATGCAA<br>TCAAATGCATGAAGACATTTTTTGGCTGGAAAGAGCCTAACATCGTAAAACCGCATGAAAAGGGCATAAACCCTAATTACCTTCTGGCTTGGAAACAGGTGCTGGCAGAACTCCAAGATATTGAAAATGAGGAGAAAATCCCAAAAACAAAGAACATGAGGAAAACAAGCCAATTGAAGTGGGCACTTGGTGAGAACATGGCACCAGAGAAAGTGGACTTTGAGGACTG<br>TAAAGATGTTAGTGATCTAAGACAGTACGACAGTGACGAGCCAGAGTCTAGATCACTAGCAAGCTGGATCCAGAGTGAATTCAACAAGGCATGCGAATTGACAGATTCGAGTTGGATTGAACTTGATGAAATAGGGGAAGACATTGCTCCAATTGAACACATTGCGAGTATGAGGAGAAACTATTTCACAGCGGAGGTGTCCCATTGCAGGGCTACTGAATACATAATGA<br>AGGGAGTATACATAAACACAGCCCTGTTGAATGCATCCTGTGCAGCGATGGATGACTTTCAACTGATTCCTATGATAAGCAAGTGCAGAACCAAAGAAGGAAGACGGAAGACAAATTTATATGGATTCATTATAAAAGGAAGATCCCATTTGAGGAATGACACCGATGTGGTGAACTTTGTGAGCATGGAATTCTCTCTCACTGACCCGAGGCTGGAGCCACACAAATGG<br>GAAAAGTACTGTGTTCTTGAGATAGGAGACATGCTCCTACGGACTGCAATAGGCCAAGTGTCAAGGCCCATGTTCTTGTATGTGAGAACCAATGGAACTTCCAAGATCAAAATGAAATGGGGCATGGAGATGAGACGATGCCTTCTTCAGTCCCTTCAACAAATTGAGAGCATGATTGAGGCCGAGTCTTCTGTCAAAGAGAAGGACATGACCAAGGAATTCTTTGAAAAC<br>AAATCAGAAACATGGCCAATTGGGGAGTCACCCAAAGGGGTGGAAGAAGGCTCCATTGGGAAGGTGTGCAGAACATTGCTAGCAAAGTCTGTGTTCAACAGCCTATATGCATCTCCACAACTCGAGGGGTTTTCAGCTGAATCAAGAAAATTGCTTCTCATTGTTCAGGCACTTAGGGACAACCTGGAACCTGGAACCTTCGATCTTGGGGGGCTATATGGAGCAATTGA<br>GGAGTGCCTGATTAACGATCCCTGGGTTTTGCTTAATGCGTCTTGGTTCAACTCCTTCCTCACACATGCACTGAAATA-
EPI_ISL_14917968	>A/common_murre/Poland/MB151/2022_H5N1_2022-07-13<br>ATGGAGGACTTTGTGCGACAATGCTTCAATCCAATGATTGTCGAGCTTGCGGAAAAGGCAATGAAAGAGTATGGGGAAGATCCGAAAATCGAAACAAACAAATTTGCCGCAATATGCACACACTTAGAAGTCTGTTTCATGTATTCGGATTTCCACTTTATTGATGAACGAGGCGAATCAATAATTGTAGAATCTGGCGATCCGAATGCATTATTGAAACACCGATTCGAGA<br>TAATTGAAGGGAGAGACCGAACTATGGCCTGGACAGTGGTGAATAGTATCTGCAACACCACAGGAGTCGAAAAGCCCAAATTCCTCCCTGATTTGTATGACTACAAAGAGAACCGATTCATTGAAATTGGAGTAACGCGAAGGGAAGTTCACATATACTATCTAGAGAAAGCCAACAAGATAAAATCAGAGAAGACTCACATTCATATATTCTCATTCACTGGAGAGGAA<br>ATGGCCACCAAGGCGGACTACACCCTTGATGAAGAGAGTAGAGCAAGAATAAAAACCAGGCTGTTCACTATAAGACAAGAAATGGCCAGTAGGGGTCTATGGGATTCCTTTCGTCAGTCCGAGAGAGGCGAAGAGACAATTGAAGAAAGATTTGAAATCACAGGAACCATGCGCAGGCTTGCCGACCAAAGTCTCCCACCGAACTTCTCCGGCCTTGAAAACTTTAGAG<br>CCTATGTGGATGGATTCGAACCGAACGGCTGCATTGAGGGCAAGCTTTCTCAAATGTCAAAAGGAGTGAATGCCAGAATTGAGCCATTTCTGAAGACAACACCACGCCCTCTCAGATTACCTGATGGGCCTCCCTGTTCTCAGCGGTCGAAGTTCTTGCTGATGGATGCCCTCAAATTGAGCATCGAAGACCCGAGCCATGAGGGGGAGGGTATACCGCTGTATGATGCAA<br>TCAAATGCATGAAGACATTTTTTGGCTGGAAAGAGCCTAACATCGTAAAACCGCATGAAAAGGGCATAAACCCTAATTACCTTCTGGCTTGGAAACAGGTGCTGGCAGAACTCCAAGATATTGAAAATGAGGAGAAAATCCCAAAAACAAAGAACATGAGGAAAACAAGCCAATTGAAGTGGGCACTTGGTGAGAACATGGCACCAGAGAAAGTGGACTTTGAGGACTG<br>TAAAGATGTTAGTGATCTAAGACAGTACGACAGTGACGAGCCAGAGTCTAGATCACTAGCAAGCTGGATCCAGAGTGAATTCAACAAGGCATGCGAATTGACAGATTCGAGTTGGATTGAACTTGATGAAATAGGGGAAGACATTGCTCCAATTGAACACATTGCGAGTATGAGGAGAAACTATTTCACAGCGGAGGTGTCCCATTGCAGGGCTACTGAATACATAATGA<br>AGGGAGTATACATAAACACAGCCCTGTTGAATGCATCCTGTGCAGCGATGGATGACTTTCAACTGATTCCTATGATAAGCAAGTGCAGAATCAAAGAAGGAAGACGGAAGACAAATTTATATGGATTCATTATAAAAGGAAGATCCCATTTGAGGAATGACACCGATGTGGTGAACTTTGTGAGCATGGAATTCTCTCTCACTGACCCGAGGCTGGAGCCACACAAGTGG<br>GAAAAGTACTGTGTTCTTGAGATAGGAGACATGCTCCTACGGACTGCAATAGGCCAAGTGTCAAGGCCCATGTTCTTGTATGTGAGAACCAATGGAACTTCCAAGATCAAAATGAAATGGGGCATGGAGATGAGACGATGCCTTCTTCAGTCCCTTCAACAAATTGAGAGCATGATTGAGGCCGAGTCTTCTGTCAAAGAGAAGGACATGACCAAGGAATTCTTTGAAAAC<br>AAATCAGAAACATGGCCAATTGGGGAGTCACCCAAAGGGGTGGAAGAAGGCTCCATTGGGAAGGTGTGCAGAACATTGCTAGCAAAGTCTGTGTTCAACAGCCTATATGCATCTCCACAACTCGAGGGGTTTTCAGCTGAATCAAGAAAATTGCTTCTCATTGTTCAGGCACTTAGGGACAACCTGGAACCTGGAACCTTCGATCTTGGGGGGCTATATGAAGCAATTGA<br>GGAGTGCCTGATTAACGATCCCTGGGTTTTGCTTAATGCGTCTTGGTTCAACTCCTTCCTCACACATGCACTGAGATA-
EPI_ISL_502736	>A/Anas_platyrhynchos/Belgium/10402_H195386/2017_H1N1_2017-11-19<br>ATGGAAGACTTTGTGCGACAATGCTTCAATCCAATGATTGTCGAGCTTGCGGAAAAGGCAATGAAAGAATATGGGGAAGATCCGAAAATCGAAACAAACAAATTTGCCGCAATATGCACACACTTAGAAGTCTGTTTCATGTATTCGGATTTCCACTTTATTGATGAACGAGGCGAATCAATAATTGTAGAATCTGGCGATCCAAATGCATTATTGAAACACCGATTTGAGA<br>TAATTGAAGGGAGAGACCGAACTATGGCCTGGACGGTGGTGAATAGTATCTGCAACACCACAGGAGTCGAAAAGCCCAAATTCCTCCCTGATCTGTATGACTACAAGGAGAACCGATTCATTGAAATTGGAGTAACGCGAAGGGAAGTTCACATATACTATCTAGAAAAGGCCAACAAGATAAAATCAGAGAAGACACACATTCACATATTCTCATTCACTGGAGAGGAA<br>ATGGCCACCAAGGCGGACTACACCCTTGATGAAGAGAGCAGAGCAAGAATAAAAACCAGGCTGTTCACTATAAGACAAGAAATGGCCAGTAGGGGTCTATGGGATTCCTTTCGTCAGTCCGAGAGAGGCGAAGAGACAATTGAAGAAAGATTTGAAATCACAGGAACCATGCGCAGGCTTGCCGACCAAAGTCTCCCACCGAACTTCTCCAGCCTTGAAAACTTTAGAG<br>CCTATGTGGATGGATTCGAACCGAACGGCTGCATTGAGGGCAAGCTTTCTCAAATGTCAAAAGAAGTGAATGCCAGAATTGAGCCATTTCTGAAGACAACACCACGCCCTCTCAGATTACCTGATGGGCCTCCCTGTTCTCAGCGGTCGAAGTTCTTGCTGATGGATGCCCTCAAATTGAGCATCGAAGACCCGAGCCATGAGGGGGAGGGTATACCGCTGTATGATGCAA<br>TCAAATGCATGAAGACATTTTTTGGCTGGAAAGAGCCCAACATCGTAAAACCACATGAAAAAGGCATAAACCCTAATTACCTCCTGGCTTGGAAGCAGGTGCTGGCAGAACTCCAAGATATTGAAAATGAGGAGAAAATCCCAAAAACAAAGAACATGAAGAAAACAAGCCAATTGAAGTGGGCACTTGGTGAGAACATGGCACCAGAGAAAGTGGACTTTGAGGACT<br>GTAAAGATGTTAGCGATCTAAAACAGTACGACAGTGACGAACCAGAGTCTAGATCACTAGCAAGCTGGATCCAGAGTGAATTCAACAAGGCATGCGAATTGACAGATTCGAGTTGGATTGAACTTGATGAAATAGGGGAAGACATTGCTCCAATTGAACACATTGCGAGTATGAGGAGGAACTATTTCACAGCGGAGGTGTCCCATTGCAGGGCTACTGAATACATAATG<br>AAGGGAGTATACATAAACACAGCCCTATTGAATGCATCCTGTGCAGCCATGGATGACTTCCAACTGATTCCAATGATAAGCAAGTGCAGAACCAAAGAAGGAAGACGGAAGACAAATTTGTATGGATTCATTATAAAGGGAAGATCCCATTTGAGGAATGACACCGATGTGGTAAACTTTGTGAGCATGGAATTCTCTCTCACTGACCCGAGGCTGGAGCCACACAAATG<br>GGAAAAGTACTGTGTTCTTGAGATAGGAGACATGCTCCTACGGACTGCAATAGGCCAAGTGTCAAGGCCCATGTTCCTGTATGTGAGAACCAATGGGACTTCCAAGATCAAGATGAAATGGGGCATGGAGATGAGGCGATGCCTTCTTCAGTCCCTTCAACAAATTGAGAGCATGATTGAGGCCGAGTCTTCTGTCAAAGAGAAGGACATGACCAAGGAATTCTTTGAAA<br>ACAAATCAGAAACATGGCCAATTGGAGAATCACCCAAAGGGGTGGAGGAAGGCTCCATTGGGAAGGTGTGCAGAACATTGCTAGCAAAGTCTGTGTTCAACAGCCTATATGCATCTCCACAACTCGAGGGGTTTTCAGCTGAATCAAGAAAATTGCTTCTCATTGTTCAGGCACTTAGGGACAACCTGGAACCTGGAACCTTCGATCTTGGGGGGCTATATGAAGCAATTG<br>AGGAGTGCCTGATTAACGATCCCTGGGTTTTGCTTAATGCGTCTTGGTTCAACTCCTTCCTCACACATGCACTGAAATA-
EPI_ISL_250238	>A/gadwall/Chany/97/2016_H6N8_2016-09-10<br>ATGGAAGACTTTGTGCGACAATGCTTCAATCCAATGATTGTCGAGCTTGCGGAAAAGGCAATGAAAGAATATGGGGAAGATCCGAAAATCGAAACAAACAAATTTGCCGCAATATGCACACACTTAGAAGTCTGTTTCATGTATTCGGATTTCCACTTTATTGATGAACGAGGCGAATCAATAATTGTAGAATCTGGCGATCCGAATGCATTATTGAAACACCGATTTGAGA<br>TAATTGAAGGGAGAGACCGAACTATGGCCTGGACGGTGGTGAATAGTATCTGCAACACCACAGGAGTCGAAAAGCCCAAATTCCTCCCTGATTTGTATGACTACAAAGAGAACCGATTCATTGAAATTGGAGTAACGCGAAGGGAAGTTCACATATACTATCTAGAAAAAGCCAACAAGATAAAATCAGAGAAGACACACATTCACATATTCTCATTCACTGGAGAGGAA<br>ATGGCCACCAAGGCGGACTACACCCTTGATGAAGAGAGCAGAGCAAGAATAAAAACCAGGCTGTTCACTATAAGACAAGAAATGGCCAGTAGGGGTCTATGGGATTCCTTTCGTCAGTCCGAGAGAGGCGAAGAGACAATTGAAGAAAGATTTGAAATCACAGGAACCATGCGCAGGCTTGCCGACCAAAGTCTCCCACCGAACTTCTCCAGCCTTGAAAACTTTAGAG<br>CCTATGTGGATGGATTCGAACCGAACGGCTGCATTGAGGGCAAGCTTTCTCAAATGTCAAAAGAGGTGAATGCCAGAATTGAGCCATTTCTGAAGACAACACCACGCCCTCTCAGATTACCTGATGGGCCTCCCTGTTCTCAGCGGTCGAAGTTCTTGCTGATGGATGCCCTCAAATTGAGCATCGAAGACCCGAGCCATGAGGGGGAGGGTATACCGCTGTATGATGCAA<br>TCAAATGCATGAAGACATTTTTTGGCTGGAAAGAGCCCAACATCGTAAAACCACATGAAAAAGGCATAAACCCTAATTACCTCCTGGCTTGGAAGCAGGTGCTGGCAGAACTCCAAGATATTGAGAATGAGGAGAAAATCCCAAAAACAAAGAACATGAAGAAAACAAGCCAATTGAAGTGGGCACTTGGTGAGAACATGGCACCAGAGAAAGTGGACTTTGAGGACT<br>GTAAGGATGTTAGCGATCTAAGACAGTACGACAGTGACGAACCAGAGTCTAGATCACTAGCAAGCTGGATCCAGAGTGAATTCAACAAGGCATGCGAATTGACAGATTCGAGTTGGATTGAACTTGATGAAATAGGGGAAGACATTGCTCCAATTGAACACATTGCGAGTATGAGGAGGAACTATTTCACAGCGGAGGTGTCCCATTGCAGGGCTACTGAATACATAATG<br>AAGGGAGTATACATAAACACAGCCCTATTGAATGCATCCTGTGCAGCCATGGATGACTTCCAACTGATTCCAATGATAAGCAAGTGCAGAACCAAAGAAGGAAGACGGAAGACAAATCTGTATGGATTCATTATAAAGGGAAGATCCCATTTGAGGAATGACACTGATGTGGTAAACTTTGTGAGCATGGAATTCTCTCTCACTGACCCGAGGCTGGAGCCACACAAATG<br>GGAGAAGTACTGTGTTCTTGAGATAGGAGACATGCTCCTACGGACTGCGATAGGCCAAGTGTCAAGGCCCATGTTCCTGTATGTGAGAACCAATGGGACCTCCAAAATCAAAATGAAATGGGGCATGGAGATGAGGCGATGCCTTCTTCAGTCCCTTCAACAAATTGAGAGCATGATTGAGGCCGAGTCTTCTGTCAAAGAGAAGGACATGACCAAGGAATTCTTTGAAA<br>ACAAATCAGAAACATGGCCAATTGGAGAATCACCCAAAGGGGTGGAGGAAGGCTCCATTGGGAAGGTGTGCAGAACATTGCTAGCAAAGTCTGTGTTCAACAGCCTATATGCATCTCCACAACTCGAGGGGTTTTCAGCTGAATCAAGAAAATTGCTTCTCATTGTTCAGGCACTTAGGGACAACCTGGAACCTGGAACCTTCGATCTTGGGGGGCTATATGAAGCAATTG<br>AGGAGTGCCTGATTAACGATCCCTGGGTTTTGCTTAATGCGTCTTGGTTCAACTCCTTCCTCACACATGCACTGAAATA-
EPI_ISL_355938	>A/green_sandpiper/Kurgan/1050/2018_H3N8_2018-08-26<br>ATGGAAGACTTTGTGCGACAATGCTTCAATCCAATGATTGTCGAGCTTGCGGAAAAGGCAATGAAAGAATATGGGGAAGATCCGAAAATCGAAACAAACAAATTTGCCGCAATATGCACACACTTAGAAGTCTGTTTCATGTATTCGGATTTCCACTTTATTGATGAACGAGGCGAATCAATAATTGTAGAATCTGGCGATCCGAATGCATTATTGAAACACCGATTTGAGA<br>TAATTGAAGGGAGAGACCGAACTATGGCCTGGACGGTGGTGAATAGTATCTGCAACACCACAGGAGTCGAAAAGCCCAAATTCCTCCCTGATTTGTATGACTACAAAGAGAACCGATTCATTGAAATTGGAGTAACGCGAAGGGAAGTTCACATATACTATCTAGAAAAAGCCAACAAGATAAAATCAGAGAAGACACACATTCACATATTCTCATTCACTGGAGAGGAA<br>ATGGCCACCAAGGCGGACTACACCCTTGATGAAGAGAGCAGAGCAAGAATAAAAACCAGGCTGTTCACTATAAGACAAGAAATGGCCAGTAGGGGTCTATGGGATTCCTTTCGTCAGTCCGAGAGAGGCGAAGAGACAATTGAAGAAAGATTTGAAATCACAGGAACCATGCGCAGGCTTGCCGACCAAAGTCTCCCACCGAACTTCTCCAGCCTTGAAAACTTTAGAG<br>CCTATGTGGATGGATTCGAACCGAACGGCTGCATTGAGGGCAAGCTTTCTCAAATGTCAAAAGAAGTGAATGCCAGAATTGAGCCATTTCTGAAGACAACACCACGCCCTCTCAGATTACCTGATGGGCCTCCCTGTTCTCAGCGGTCGAAGTTCTTGCTGATGGATGCCCTCAAATTGAGCATCGAAGACCCGAGCCATGAGGGGGAGGGTATACCGCTGTATGATGCAA<br>TCAAATGCATGAAGACATTTTTTGGCTGGAAAGAGCCCAACATCGTAAAACCACATGAAAAAGGCATAAACCCTAATTACCTCCTGGCTTGGAAGCAGGTACTGGCAGAACTCCAAGATATTGAAAATGAGGAGAAAATCCCAAAAACAAAGAACATGAAGAAAACAAGCCAATTGAAGTGGGCACTTGGTGAGAACATGGCACCAGAGAAAGTGGACTTTGAGGACTG<br>TAAAGATGTTAGCGATCTAAGACAGTACGACAGTGACGAACCAGAGTCTAGATCACTAGCAAGCTGGATCCAGAGTGAATTCAACAAGGCATGCGAATTGACAGATTCGAGTTGGGTTGAACTTGATGAAATAGGGGAAGACATTGCTCCAATTGAACACATTGCGAGTATGAGGAGGAACTATTTCACAGCGGAGGTGTCCCATTGCAGGGCTACTGAATACATAATGA<br>AGGGAGTATACATAAACACAGCCCTATTGAATGCATCCTGTGCAGCCATGGATGACTTCCAACTGATTCCAATGATAAGCAAGTGCAGAACCAAAGAAGGAAGACGGAAGACAAATCTGTATGGATTCATTATAAAGGGAAGATCCCATTTGAGGAATGACACCGATGTGGTAAACTTTGTGAGCATGGAATTCTCTCTCACTGACCCGAGGCTGGAACCACACAAATGG<br>GAAAAGTACTGTGTTCTTGAGATAGGAGACATGCTCCTACGGACTGCAATAGGCCAAGTGTCAAGGCCCATGTTCCTGTATGTGAGAACCAATGGGACTTCCAAGATCAAAATGAAATGGGGCATGGAGATGAGGCGATGCCTTCTTCAGTCCCTTCAACAAATTGAGAGCATGATTGAGGCCGAGTCTTCTGTCAAAGAGAAGGACATGACCAAGGAATTCTTTGAAAA<br>CAAATCAGAAACATGGCCAATTGGAGAATCACCCAAAGGGGTGGAGGAAGGCTCCATTGGGAAGGTGTGCAGGACATTGCTAGCGAAGTCTGTGTTCAACAGCCTATATGCATCTCCACAACTCGAGGGGTTTTCAGCTGAATCAAGAAAATTGCTTCTCATTGTTCAGGCACTTAGGGACAACCTGGAACCTGGGACCTTCGATCTTGGGGGGTTATATGAAGCAATTGA<br>GGAGTGCCTGATTAACGATCCCTGGGTTTTGCTTAATGCGTCTTGGTTCAACTCCTTCCTCACACATGCACTGAAATA-
EPI_ISL_697696	>A/duck/Mongolia/451/2018_H4N1_2018-09-02<br>ATGGAAGACTTTGTGCGACAATGCTTCAATCCAATGATTGTCGAGCTTGCGGAAAAGGCAATGAAAGAATATGGGGAAGATCCGAAAATCGAAACAAACAAATTTGCCGCAATATGCACACACTTAGAAGTCTGTTTCATGTATTCGGATTTCCACTTTATTGATGAGCGAGGCGAATCAATAATTGTAGAATCTGGCGATCCGAATGCATTATTGAAACACCGATTTGAGA<br>TAATTGAAGGGAGAGACCGAACTATGGCCTGGACGGTGGTGAATAGTATCTGCAACACCACAGGAGTCGAAAAGCCCAAATTCCTCCCTGATTTGTATGACTACAAAGAGAACCGATTCATTGAAATTGGAGTAACGCGAAGGGAAGTTCACATATACTATCTAGAAAAAGCCAACAAGATAAAATCAGAGAAGACACACATTCACATATTCTCATTCACTGGAGAGGAA<br>ATGGCCACCAAGGCGGACTACACCCTTGATGAAGAGAGCAGAGCAAGAATAAAAACCAGGCTGTTCACTATAAGACAAGAAATGGCCAGTAGGGGTCTATGGGATTCCTTTCGTCAGTCCGAGAGAGGCGAAGAGACAATTGAAGAAAGATTTGAAATCACAGGAACCATGCGCAGGCTTGCCGACCAAAGTCTCCCACCGAACTTCTCCAGCCTTGAAAACTTTAGAG<br>CCTATGTGGATGGATTCGAACCGAACGGCTGCATTGAGGGCAAGCTTTCTCAAATGTCAAAAGAAGTGAATGCCAGAATTGAGCCATTTCTGAAGACAACACCACGCCCTCTCAGATTACCTGATGGGCCTCCCTGCTCTCAGCGGTCGAAGTTCTTGCTGATGGATGCCCTCAAATTGAGCATCGAAGACCCGAGCCATGAGGGGGAGGGTATACCGCTGTATGATGCAA<br>TCAAATGCATGAAGACATTTTTTGGCTGGAAAGAGCCCAACATCGTAAAACCACATGAAAAAGGCATAAACCCTAATTACCTCCTGGCTTGGAAGCAGGTGCTGGCAGAACTCCAAGATATTGAAAATGAGGAGAAAATCCCAAAAACAAAGAACATGAAGAAAACAAGCCAATTGAAGTGGGCACTTGGCGAGAACATGGCACCAGAGAAAGTGGACTTTGAGGACT<br>GCAAAGATGTTAGTGATCTAAAACAATACGACAGTGACGAACCAGAGTCTAGATCACTAGCAAACTGGATCCAGAGTGAATTCAACAAAGCATGCGAATTGACAGATTCGAGTTGGATTGAACTTGATGAAATAGGGGAAGACATTGCTCCAATTGAACACATTGCGAGTATGAGGAGGAACTATTTCACAGCGGAGGTGTCCCATTGCAGGGCTACTGAATACATAATG<br>AAGGGAGTATACATAAACACAGCCCTATTGAATGCATCCTGTGCAGCCATGGATGACTTCCAACTGATTCCAATGATAAGCAAGTGCAGAACCAAGGAAGGAAGACGGAAGACAAATCTGTATGGATTCATTATAAAGGGAAGATCCCATTTGAGGAATGACACCGATGTGGTAAACTTTGTGAGCATGGAATTCTCTCTCACTGACCCGAGGCTGGAGCCACACAAATG<br>GGAAAAGTACTGTGTTCTTGAGATAGGAGACATGCTCCTACGGACTGCAATAGGCCAAGTGTCAAGGCCCATGTTCCTGTATGTGAGAACCAATGGGACTTCCAAGATCAAAATGAAATGGGGCATGGAGATGAGGCGATGCCTTCTTCAGTCCCTTCAACAAATTGAGAGCATGATTGAGGCCGAGTCTTCTGTCAAAGAGAAGGACATGACCAAGGAATTCTTTGAAA<br>ACAAATCAGAAACATGGCCAATTGGAGAATCACCCAAAGGGGTGGAGGAAGGCTCCATTGGGAAGGTGTGCAGAACATTGCTAGCAAAGTCTGTGTTCAACAGCCTATATGCATCTCCACAACTCGAGGGGTTTTCAGCTGAATCAAGAAAATTGCTTCTCATTGTTCAGGCACTTAGGGACAACCTGGAACCTGGAACCTTCGATCTTGGGGGGCTATATGAAGCAATTG<br>AGGAGTGCCTGATTAACGATCCCTGGGTTTTGCTTAATGCGTCTTGGTTCAACTCCTTCCTCACACATGCACTGAAATA-
EPI_ISL_644150	>A/chicken/Omsk/0112/2020_H5N8_2020-08-17<br>ATGGAAGACTTTGTGCGACAATGCTTCAATCCAATGATTGTCGAGCTTGCGGAAAAGGCAATGAAAGAATATGGGGAAGATCCGAAAATCGAAACAAACAAATTTGCCGCAATATGCACACACTTAGAAGTCTGTTTCATGTATTCGGATTTCCACTTTATTGATGAACGGGGCGAATCAATAATTGTAGAATCTGGCGATCCGAATGCATTATTGAAACACCGATTTGAGA<br>TAATTGAGGGGAGAGACCGAACTATGGCCTGGACGGTGGTGAATAGTATCTGCAACACCACAGGAGTCGAAAAGCCCAAATTCCTCCCTGATTTGTATGACTACAAAGAGAACCGATTCATTGAAATTGGAGTAACGCGAAGGGAAGTTCACATATACTATCTAGAAAAAGCCAACAAGATAAAATCAGAGAAGACACACATTCACATATTCTCATTCACTGGAGAGGAA<br>ATGGCCACCAAGGCTGACTACACCCTTGATGAAGAGAGCAGAGCAAGAATAAAAACCAGGCTGTTCACTATAAGACAAGAAATGGCCAGTAGGGGTCTATGGGATTCCTTTCGTCAGTCCGAGAGAGGCGAAGAGACAATTGAAGAAAGATTTGAAATCACAGGAACCATGCGCAGGCTTGCCGACCAAAGTCTCCCACCGAACTTCTCCAGCCTTGAAAACTTTAGAG<br>CCTATGTGGATGGATTCGAACCGAACGGCTGCATTGAGGGCAAGCTTTCTCAAATGTCAAAAGAAGTGAATGCCAGAATTGAGCCATTTCTGAAGACAACACCACGCCCTCTCAGATTACCTGATGGGCCTCCCTGTTCTCAGCGGTCGAAGTTTTTGCTGATGGATGCCCTCAAATTGAGCATCGAAGACCCGAGCCATGAGGGGGAGGGTATACCGCTGTATGATGCAA<br>TCAAATGCATGAAGACATTTTTTGGCTGGAAAGAGCCCAACATCGTAAAACCACATGAAAAAGGCATAAACCCTAATTACCTCCTGGCTTGGAAGCAGGTGCTGGCAGAACTCCAAGATATTGAAAATGAGGAGAAAATCCCAAAAACAAAGAACATGAAGAAAACAAGCCAATTGAAGTGGGCACTTGGTGAGAACATGGCACCAGAGAAAGTGGACTTTGAGGACT<br>GTAAAGATGTTAGCGATCTAAGACAGTACGACAGTGATGAGCCAGAGTCTAGATCACTAGCAAGCTGGATCCAGAGTGAGTTCAACAAGGCATGCGAATTGACAGATTCGAGTTGGATTGAACTTGATGAAATAGGGGAAGACATTGCTCCAATTGAACACATTGCGAGCATGAGGAGGAACTATTTCACAGCGGAAGTGTCCCATTGCAGGGCTACTGAATACATAATG<br>AAGGGAGTATACATAAACACAGCCCTATTGAATGCATCCTGTGCAGCCATGGATGACTTCCAACTGATTCCAATGATAAGCAAGTGCAGAACCAAAGAAGGAAGACGAAAGACAAATCTGTATGGATTCATCATAAAGGGAAGATCCCATTTGAGGAATGACACCGATGTGGTAAACTTTGTGAGCATGGAATTCTCTCTCACTGACCCGAGGCTGGAGCCACACAAATG<br>GGAAAAGTACTGTGTTCTTGAGATAGGAGACATGCTCCTACGGACTGCAATAGGCCAAGTGTCAAGGCCCATGTTCCTGTATGTGAGAACAAACGGGACTTCCAAGATCAAAATGAAATGGGGCATGGAGATGAGGCGATGTCTTCTTCAGTCCCTTCAACAAATTGAGAGCATGATTGAGGCCGAGTCTTCTGTCAAAGAGAAGGACATGACCAAGGAATTCTTTGAAA<br>ATAAATCAGAAACATGGCCAATTGGGGAATCACCCAAAGGGGTGGAGGAAGGCTCCATTGGGAAGGTATGCAGAACATTGCTAGCAAAGTCTGTGTTCAACAGCCTATATGCATCTCCACAACTCGAGGGGTTTTCAGCTGAATCAAGAAAATTGCTTCTCATTGTTCAGGCACTTAGGGACAACCTGGAACCTGGAACTTTCGATCTTGGGGGGCTATATGAAGCAATTG<br>AGGAGTGCCTGATTAACGATCCCTGGGTTTTGCTTAATGCGTCTTGGTTCAACTCCTTCCTCACACATGCACTGAAATA-
EPI_ISL_17978924	>A/mallard/Poland/P074w5/2018_H3N8_2018-09-05<br>ATGGAAGACTTTGTGCGACAATGCTTCAATCCAATGATTGTCGAGCTTGCGGAAAAGGCAATGAAAGAATATGGGGAAGATCCGAAAATCGAAACAAACAAATTTGCCGCAATATGCACACACTTAGAAGTCTGTTTCATGTATTCGGATTTCCACTTTATTGATGAACGAGGCGAATCAATAATTGTAGAATCTGGCGATCCAAATGCATTATTGAAACACCGATTTGAGA<br>TAATTGAAGGGAGAGACCGAACAATGGCCTGGACGGTGGTGAATAGTATCTGCAACACCACAGGAGTCGAAAAGCCTAAATTCCTCCCTGATTTGTATGACTACAAAGAGAACCGATTCATTGAAATTGGGGTAACGCGAAGGGAAGTTCACATATACTATCTAGAAAAAGCCAACAAGATAAAATCAGAGAAGACACACATTCACATATTTTCATTCACTGGAGAGGAA<br>ATGGCCACCAAGGCGGACTACACCCTTGATGAAGAGAGCAGAGCAAGAGTAAAAACCAGGCTGTTCACTATCAGACAAGAAATGGCCAGTAGGGGTCTATGGGATTCCTTTCGTCAGTCCGAGAGAGGCGAAGAGACAATTGAAGAAAGATTTGAAATCACAGGAACCATGCGCAGGCTTGCCGACCAAAGTCTCCCACCGAACTTCTCCAGCCTTGAAAACTTTAGAG<br>CCTATGTGGATGGATTCGAACCGAACGGCTGCATTGAGGGCAAGCTTTCTCAAATGTCAAAAGAAGTGAATGCCAGAATTGAGCCATTTCTGAAGACAACACCACGCCCTCTCAGATTACCTGATGGGCCTCCCTGTTCTCAGCGGTCGAAGTTCTTACTGATGGATGCCCTCAAATTGAGCATCGAAGATCCAAGCCATGAGGGGGAGGGTATACCGCTGTACGATGCAA<br>TCAAATGCATGAAGACATTTTTTGGCTGGAAAGAGCCCAACATCATAAAACCGCATGAAAAAGGCATAAACCCTAATTACCTCCTGGCTTGGAAGCAGGTGCTGGCAGAACTCCAAGATATTGAAAATGAAGAGAAAATCCCAAAAACAAAGAATATGAAGAAAACAAGCCAATTGAAGTGGGCACTTGGTGAGAACATGGCACCAGAGAAGGTGGACTTTGAGGACTG<br>TAAAGATGTTGGCGATTTAAAACAGTACAACAGTGACGAACCAGAGTCTAGATCACTAGCAAGCTGGATCCAGAGTGAGTTCAACAAGGCATGCGAATTGACAGATTCGAGTTGGATTGAACTTGATGAAATAGGGGAAGACATTGCTCCAATTGAACACATTGCAAGTATGAGGAGGAACTATTTCACAGCGGAGGTATCCCATTGCAGGGCTACTGAATACATAATGA<br>AGGGAGTATACATGAACACAGCCCTATTGAATGCATCCTGTGCAGCCATGGATGACTTCCAACTGATTCCAATGATAAGCAAGTGCAGAACCAAAGAAGGAAGACGGAAGACAAATCTGTATGGATTCATTATAAAAGGAAGATCCCATTTGAGGAATGACACTGATGTGGTAAACTTTGTGAGCATGGAATTCTCTCTCACTGACCCGAGGCTGGAGCCACACAAATGG<br>GAAAAGTACTGTGTCCTTGAGATAGGAGACATGCTCCTACGGACTGCAATAGGCCAAGTGTCAAGGCCCATGTTCCTGTATGTGAGAACCAATGGGACTTCCAAGATCAAAATGAAATGGGGTATGGAGATGAGGCGATGCCTTCTTCAGTCCCTTCAACAAATCGAGAGCATGATTGAGGCCGAGTCTTCTGTCAAAGAGAAGGATATGACCAAAGAATTCTTTGAAAG<br>CAAATCAGAAACTTGGCCAATTGGAGAATCACCCAAAGGGGTGGAGGAAGGCTCCATTGGGAAGGTGTGCAGAACATTGCTAGCAAAGTCTGTGTTCAACAGCCTATATGCATCTCCACAACTCGAGGGGTTTTCAGCTGAATCAAGAAAATTGCTTCTCATTGTTCAGGCACTTAGGGACAACCTGGAACCTGGAACCTTCGATCTTGGGGGGCTATATGAAGCAATTGA<br>GGAGTGCCTGATTAATGATCCCTGGGTTTTGCTTAATGCGTCTTGGTTCAACTCCTTCCTCACACATGCACTGAAATA-

EPI_ISL_18245816	>A/mute_swan/Poland/MB122/2022_H5N1_2022-04-07<br>[illegible]
EPI_ISL_815400	>A/mallard/Netherlands/18015513-001/2018_H1N1_2018-10-11<br>[illegible]
EPI_ISL_502605	>A/Anas_platyrhynchos/Belgium/11958/2018_H1N1_2018-11-26<br>[illegible]
EPI_ISL_9012576	>A/Mallard/Netherlands/12/2021_H5N2_2021-12-23<br>[illegible]
EPI_ISL_331295	>A/teal/Chany/324/2017_H12N5_2017-09-03<br>[illegible]
EPI_ISL_503498	>A/duck/Bangladesh/37607/2019_H10N3_2019-01-17<br>[illegible]
EPI_ISL_337148	>A/pochard/Buryatia/33/2018_H4N6_2018-08-25<br>[illegible]
EPI_ISL_337396	>A/pintail/Novosibirsk_region/504k/2018_H4N6_2018-10-01<br>[illegible]
EPI_ISL_503482	>A/duck/Bangladesh/38803/2019_H11N3_2019-02-09<br>[illegible]
EPI_ISL_697693	>A/duck/Mongolia/398/2018_H3N8_2018-09-02<br>[illegible]
EPI_ISL_17978927	>A/mute_swan/Poland/P071/2020_H9N2_2020-09-18<br>[illegible]
EPI_ISL_17978925	>A/mallard/Poland/P077w1/2018_H5N2_2018-09-12<br>[illegible]
EPI_ISL_2234824	>A/whooper_swan/Romania/10362_21VIR2593-20/2021_H5N5_2021-01-22<br>[illegible]

EPI_ISL_3102055	>A/grey_heron/Bulgaria/223_21VIR4270-2/2021_H5N5_2021-03-30<br>[illegible]
EPI_ISL_3102054	>A/European_herring_gull/Bulgaria/222_21VIR4270-1/2021_H5N5_2021-03-30<br>[illegible]
EPI_ISL_12223688	>A/grey_heron/Czech_Republic/25338-1/2021_H5N1_2021-12-18<br>[illegible]
EPI_ISL_8515483	>A/great_egret/Czech_Republic/23609/2021_H5N1_2021-11-28<br>[illegible]
EPI_ISL_11922813	>A/swan/Poland/MB078_22VIR2515-7/2022_H5N1_2022-02-15<br>[illegible]
EPI_ISL_1697189	>A/chicken/Czech_Republic/6532-2/2021_H5N8_2021-04-01<br>[illegible]
EPI_ISL_1080579	>A/common_coot/Czech_Republic/3160-2/2021_H5N8_2021-02-14<br>[illegible]
EPI_ISL_1114745	>A/chicken/Kostroma/304-10/2020_H5N8_2020-10-17<br>[illegible]
EPI_ISL_5115614	>A/chicken/Germany-BE/AI01974/2021_H5N8_2021-02-26<br>[illegible]
EPI_ISL_2685900	>A/tundra_swan/Hubei/BQ7/2020_H5N8_2020-11-16<br>[illegible]
EPI_ISL_14836369	>A/Whooper_swan/Sanmenxia/Y48B/2020_H5N8_2020-11-17<br>[illegible]
EPI_ISL_1937890	>A/whooper_swan/Henan/SM31/2020_H5N8_2020-11-10<br>[illegible]
EPI_ISL_14819899	>A/Wild_Duck/Ningxia/Y99/2021_H5N8_2021-03-09<br>[illegible]

EPI_ISL_2932616	>A/chicken/Kazakhstan/12-20-B-Talg-45/2020_H5N8_2020-09-19<br>ATGGAAGACTTTGTGCGACAATGCTTCAATCCAATGATTGTCGAGCTTGCGGAAAAGGCAATGAAAGAATATGGGGAAGATCCTAAGATCGAAACAAACAAGTTTGCCGCAATATGTACACACTTAGAAGTCTGCTTCATGTATTCGGATTTCCATTTCATTGATGAACGAGGCGAATCAATAATCGTAGAATCTGGCGACCCGAATGCATTATTGAAGCACCGATTTGAGA<br>TAATTGAAGGGAGAGACCGAACAATGGCCTGGACAGTGGTGAATAGTATATGCAACACTACAGGAGTCGAAAAGCCCAAGTTCCTTCCTGATTTGTATGACTACAGAGAAAACCGATTCATTGAAATTGGAGTAACGCGAAGGGAAGTTCACATATACTATCTTGAAAAAGCCAACAAGATAAAATCAGAGAAAACACACATTCACATATTCTCATTCACTGGGGAGGAA<br>ATGGCCACCAAGGCAGATTACACTCTTGATGAAGAGAGCAGAGCAAGAATAAAAACCAGGCTATTCACTATAAGACAAGAGATGGCCAATAGGGGTCTATGGGATTCCTTTCGTCAGTCCGAGAGAGGCGAAGAGACAATTGAAGAAAGATTTGAAATCACAGGAACCATGCGCAGGCTTGCTGACCAAAGTCTCCCACCGAACTTCGCCAGCCTTGAAAACTTTAGAG<br>CCTATGTGGATGGATTTGAACCGAACGGCTGCATTGAGGGCAAGCTTTCTCAAATGTCAAAAGAAGTGAATGCCAGAATTGAGCCATTTCTGAAGACAACACCACGCCCTCTCAGATTACCTGATGGACCTCCCTGTTCCCAGCGGTCGAAGTTCTTGCTAATGGATGCCCTTAAATTGAGCATTGAAGACCCGAGCCATGAGGGGGAGGGTATACCGCTGTACGATGCAA<br>CCAAATGCATGAAGACATTTTTTGGCTGGAAGGAGCCCAACATCGTGAAACCACATGAAAAGGGCATAAACCCTAATTACCTCCTGGCTTGGAAGCAGGTGCTATCAGAACTCCAAGATATTGAAAACGAGGAGAAAATCCCAAAAACAAAGAACATGAAGAAGACGAGCCAACTGAAGTGGGCACTTGGTGAAAACATGGCACCAGAGAAAGTGGACTTTGAGGACT<br>GTAAAGATGTTAGCGATCTAAGACAGTACGACAGTGACGAACCAGAATCTAGATCACTAGCAAGTTGGATCCAGAGTGAATTTAACAAGGCTTGCGAACTGACAGATTCGAGTTGGATTGAACTTGATGAAATAGGGGAAGACGTTGCTCCGATTGAACACATTGCGAGTATGAGGAGGAACTATTTCACAGCGGAAGTATCCCATTGCAGGGCTACAGAATACATAATG<br>AAAGGAGTATACATAAACACAGCTTTATTGAATGCATCCTGTGCAGCCATGGATGACTTCCAACTGATTCCAATGATAAGCAAATGCAGAACCAAAGAAGGGAGACGGAAGACAAATCTGTATGGCTTCATTATAAAAGGAAGATCCCATTTGAGGAATGACACCGATGTGGTAAACTTTGTGAGCATGGAATTCTCTCTCACTGACCCAAGGCTGGAGCCACACAAATGG<br>GAAAAGTACTGTGTGCTTGAGGTAGGAGACATGCTCCTACGGACTGCAATAGGCCAAGTGTCAAGGCCCATGTTCCTGTATGTGAGAACCAATGGGACTTCTAAGATCAAAATGAAATGGGGTATGGAGATGAGGCGATGCCTTCTTCAATCCCTTCAACAAATTGAGAGCATGATTGAAGCCGAGTCTTCTGTCAAAGAAAAAGACATGACCAAGGAATTCTTTGAAAAC<br>AGATCAGAAACATGGCCAATTGGAGAGTCACCCAAAGGAGTGGAGGAAGGCTCCATTGGGAAGGTGTGCAGAACATTGCTAGCAAAGTCTGTGTTCAACAGCCTATATGCATCTCCACAACTCGAGGGGTTTCAGCTGAATCAAGAAAGTTGCTTCTCATTGTTCAGGCACTTAGGGACAACCTGGAACCTGGGACCTTCGATCTTGGGGGGCTATATGAAGCAATTGA<br>GGAGTGCCTGATTAACGATCCCTGGGTTTTGCTTAATGCGTCTTGGTTCAACTCCTTCCTCACACATGCACTGAAATA-
EPI_ISL_644155	>A/chicken/Omsk/0119/2020_H5N8_2020-08-17<br>ATGGAAGACTTTGTGCGACAATGCTTCAATCCAATGATTGTCGAGCTTGCGGAAAAGGCAATGAAAGAATATGGGGAAGATCCTAAGATCGAAACAAACAAGTTTGCCGCAATATGTACACACTTAGAAGTCTGCTTCATGTATTCGGATTTCCATTTCATTGATGAACGAGGCGAATCAATAATCGTAGAATCTGGCGACCCGAATGCATTATTGAAGCACCGATTTGAGA<br>TAATTGAAGGGAGAGACCGAACAATGGCCTGGACAGTGGTGAATAGTATATGCAACACTACAGGAGTCGAAAAGCCCAAGTTCCTTCCTGATTTGTATGACTACAGAGAAAACCGATTCATTGAAATTGGAGTAACGCGAAGGGAAGTTCACATATACTATCTTGAAAAAGCCAACAAGATAAAATCAGAGAAAACACACATTCACATATTCTCATTCACTGGGGAGGAA<br>ATGGCCACCAAGGCAGATTACACTCTTGATGAAGAGAGCAGAGCAAGAATAAAAACCAGGCTATTCACTATAAGACAAGAGATGGCCAATAGGGGTCTATGGGATTCCTTTCGTCAGTCCGAGAGAGGCGAAGAGACAATTGAAGAAAGATTTGAAATCACAGGAACCATGCGCAGGCTTGCTGACCAAAGTCTCCCACCGAACTTCGCCAGCCTTGAAAACTTTAGAG<br>CCTATGTGGATGGATTTGAACCGAACGGCTGCATTGAGGGCAAGCTTTCTCAAATGTCAAAAGAAGTGAATGCCAGAATTGAGCCATTTCTGAAGACAACACCACGCCCTCTCAGATTACCTGATGGACCTCCCTGTTCCCAGCGGTCGAAGTTCTTGCTAATGGATGCCCTTAAATTGAGCATTGAAGACCCGAGCCATGAGGGGGAGGGTATACCGCTGTACGATGCAA<br>CCAAATGCATGAAGACATTTTTTGGCTGGAAGGAGCCCAACATCGTGAAACCACATGAAAAGGGCATAAACCCTAATTACCTCCTGGCTTGGAAGCAGGTGCTATCAGAACTCCAAGATATTGAAAACGAGGAGAAAATCCCAAAAACAAAGAACATGAAGAAGACGAGCCAACTGAAGTGGGCACTTGGTGAAAACATGGCACCAGAGAAAGTGGACTTTGAGGACT<br>GTAAAGATGTTAGCGATCTAAGACAGTACGACAGTGACGAACCAGAATCTAGATCACTAGCAAGTTGGATCCAGAGTGAATTTAACAAGGCTTGCGAACTGACAGATTCGAGTTGGATTGAACTTGATGAAATAGGGGAAGACGTTGCTCCGATTGAACACATTGCGAGTATGAGGAGGAACTATTTCACAGCGGAAGTATCCCATTGCAGGGCTACAGAATACATAATG<br>AAAGGAGTATACATAAACACAGCTTTATTGAATGCATCCTGTGCAGCCATGGATGACTTCCAACTGATTCCAATGATAAGCAAATGCAGAACCAAAGAAGGGAGACGGAAGACAAATCTGTATGGCTTCATTATAAAAGGAAGATCCCATTTGAGGAATGACACCGATGTGGTAAACTTTGTGAGCATGGAATTCTCTCTCACTGACCCAAGGCTGGAGCCACACAAATGG<br>GAAAAGTACTGTGTGCTTGAGGTAGGAGACATGCTCCTACGGACTGCAATAGGCCAAGTGTCAAGGCCCATGTTCCTGTATGTGAGAACCAATGGGACTTCTAAGATCAAAATGAAATGGGGTATGGAGATGAGGCGATGCCTTCTTCAATCCCTTCAACAAATTGAGAGCATGATTGAAGCCGAGTCTTCTGTCAAAGAAAAAGACATGACCAAGGAATTCTTTGAAAAC<br>AGATCAGAAACATGGCCAATTGGAGAGTCACCCAAAGGAGTGGAGGAAGGCTCCATTGGGAAGGTGTGCAGAACATTGCTAGCAAAGTCTGTGTTCAACAGCCTATATGCATCTCCACAACTCGAGGGGTTTCAGCTGAATCAAGAAAGTTGCTTCTCATTGTTCAGGCACTTAGGGACAACCTGGAACCTGGGACCTTCGATCTTGGGGGGCTATATGAAGCAATTGA<br>GGAGTGCCTGATTAACGATCCCTGGGTTTTGCTTAATGCGTCTTGGTTCAACTCCTTCCTCACACATGCACTGAAATA-
EPI_ISL_644132	>A/goose/Omsk/01171/2020_H5N8_2020-08-17<br>ATGGAAGACTTTGTGCGACAATGCTTCAATCCAATGATTGTCGAGCTTGCGGAAAAGGCAATGAAAGAATATGGGGAAGATCCTAAGATCGAAACAAACAAGTTTGCCGCAATATGTACACACTTAGAAGTCTGCTTCATGTATTCGGATTTCCATTTCATTGATGAACGAGGCGAATCAATAATCGTAGAATCTGGCGACCCGAATGCATTATTGAAGCACCGATTTGAGA<br>TAATTGAAGGGAGAGACCGAACAATGGCCTGGACAGTGGTGAATAGTATATGCAACACTACAGGAGTCGAAAAGCCCAAGTTCCTTCCTGATTTGTATGACTACAGAGAAAACCGATTCATTGAAATTGGAGTAACGCGAAGGGAAGTTCACATATACTATCTTGAAAAAGCCAACAAGATAAAATCAGAGAAAACACACATTCACATATTCTCATTCACTGGGGAGGAA<br>ATGGCCACCAAGGCAGATTACACTCTTGATGAAGAGAGCAGAGCAAGAATAAAAACCAGGCTATTCACTATAAGACAAGAGATGGCCAATAGGGGTCTATGGGATTCCTTTCGTCAGTCCGAGAGAGGCGAAGAGACAATTGAAGAAAGATTTGAAATCACAGGAACCATGCGCAGGCTTGCTGACCAAAGTCTCCCACCGAACTTCGCCAGCCTTGAAAACTTTAGAG<br>CCTATGTGGATGGATTTGAACCGAACGGCTGCATTGAGGGCAAGCTTTCTCAAATGTCAAAAGAAGTGAATGCCAGAATTGAGCCATTTCTGAAGACAACACCACGCCCTCTCAGATTACCTGATGGACCTCCCTGTTCCCAGCGGTCGAAGTTCTTGCTAATGGATGCCCTTAAATTGAGCATTGAAGACCCGAGCCATGAGGGGGAGGGTATACCGCTGTACGATGCAA<br>CCAAATGCATGAAGACATTTTTTGGCTGGAAGGAGCCCAACATCGTGAAACCACATGAAAAGGGCATAAACCCTAATTACCTCCTGGCTTGGAAGCAGGTGCTATCAGAACTCCAAGATATTGAAAACGAGGAGAAAATCCCAAAAACAAAGAACATGAAGAAGACGAGCCAACTGAAGTGGGCACTTGGTGAAAACATGGCACCAGAGAAAGTGGACTTTGAGGACT<br>GTAAAGATGTTAGCGATCTAAGACAGTACGACAGTGACGAACCAGAATCTAGATCACTAGCAAGTTGGATCCAGAGTGAATTTAACAAGGCTTGCGAACTGACAGATTCGAGTTGGATTGAACTTGATGAAATAGGGGAAGACGTTGCTCCGATTGAACACATTGCGAGTATGAGGAGGAACTATTTCACAGCGGAAGTATCCCATTGCAGGGCTACAGAATACATAATG<br>AAAGGAGTATACATAAACACAGCTTTATTGAATGCATCCTGTGCAGCCATGGATGACTTCCAACTGATTCCAATGATAAGCAAATGCAGAACCAAAGAAGGGAGACGGAAGACAAATCTGTATGGCTTCATTATAAAAGGAAGATCCCATTTGAGGAATGACACCGATGTGGTAAACTTTGTGAGCATGGAATTCTCTCTCACTGACCCAAGGCTGGAGCCACACAAATGG<br>GAAAAGTACTGTGTGCTTGAGGTAGGAGACATGCTCCTACGGACTGCAATAGGCCAAGTGTCAAGGCCCATGTTCCTGTATGTGAGAACCAATGGGACTTCTAAGATCAAAATGAAATGGGGTATGGAGATGAGGCGATGCCTTCTTCAATCCCTTCAACAAATTGAGAGCATGATTGAAGCCGAGTCTTCTGTCAAAGAAAAAGACATGACCAAGGAATTCTTTGAAAAC<br>AGATCAGAAACATGGCCAATTGGAGAGTCACCCAAAGGAGTGGAGGAAGGCTCCATTGGGAAGGTGTGCAGAACATTGCTAGCAAAGTCTGTGTTCAACAGCCTATATGCATCTCCACAACTCGAGGGGTTTCAGCTGAATCAAGAAAGTTGCTTCTCATTGTTCAGGCACTTAGGGACAACCTGGAACCTGGGACCTTCGATCTTGGGGGGCTATATGAAGCAATTGA<br>GGAGTGCCTGATTAACGATCCCTGGGTTTTGCTTAATGCGTCTTGGTTCAACTCCTTCCTCACACATGCACTGAAATA-
EPI_ISL_2111625	>A/mute_swan/Poland/MB131/2021_H5N8_2021-02-19<br>ATGGAAGACTTTGTGCGACAATGCTTCAATCCAATGATTGTCGAGCTTGCGGAAAAGGCAATGAAAGAATATGGGGAAGATCCTAAGATCGAAACAAACAAGTTTGCCGCAATATGTACACACTTAGAAGTCTGCTTCATGTATTCGGATTTCCATTTCATTGATGAACGAGGCGAATCAATAATCGTAGAATCTGGCGACCCGAATGCATTATTGAAGCACCGATTTGAGA<br>TAATTGAAGGGAGAGACCGAACAATGGCCTGGACAGTGGTGAATAGTATATGCAACACTACAGGAATCGAAAAGCCCAAGTTCCTTCCTGATTTGTATGACTACAGAGAAAACCGATTCATTGAAATTGGAGTAACGCGAAGGGAAGTTCACATATACTATCTTGAAAAAGCCAACAAGATAAAATCAGGGAAAACACACATTCACATATTCTCATTCACTGGGGAGGAA<br>ATGGCCACCAAGGCAGATTACACTCTTGATGAAGAGAGCAGAGCAAGAATAAAAACCAGGCTATTCACTATAAGACAAGAGATGGCCAATAGGGGTCTATGGGATTCCTTTCGTCAGTCCGAGAGAGGCGAAGAGACAATTGAAGAAAGATTTGAAATCACAGGAACCATGCGCAGGCTTGCTGACCAAAGTCTCCCACCGAACTTCGCCAGCCTTGAAAACTTTAGAG<br>CCTATGTGGATGGATTTGAACCGAACGGCTGCATTGAGGGCAAGCTTTCTCAAATGTCAAAAGAAGTGAATGCCAGAATTGAGCCATTTCTGAAGACAACACCACGCCCTCTCAGATTACCTGATGGACCTCCCTGTTCCCAGCGGTCGAAGTTCTTGCTAATGGATGCCCTTAAATTGAGCATTGAAGACCCGAGCCATGAGGGGGAGGGTATACCGCTGTACGATGCAA<br>CCAAATGCATGAAGACATTTTTTGGCTGGAAGGAGCCCAACATCGTGAAACCACATGAAAAGGGCATAAACCCTAATTACCTCCTGGCTTGGAAGCAGGTGCTATCAGAACTCCAAGATATTGAAAACGAGGAGAAAATCCCAAAAACAAAGAACATGAAGAAGACGAGCCAACTGAAGTGGGCACTTGGTGAAAACATGGCACCAGAGAAAGTGGACTTTGAGGACT<br>GTAAAGATGTTAGCGATCTAAGACAGTACGACAGTGACGAACCAGAATCTAGATCACTAGCAAGTTGGATCCAGAGTGAATTTAACAAGGCTTGCGAACTGACAGATTCGAGTTGGATTGAACTTGATGAAATAGGGGAAGACGTTGCTCCGATTGAACACATTGCGAGTATGAGGAGGAACTATTTCACAGCGGAAGTATCCCATTGCAGGGCTACAGAATACATAATG<br>AAAGGAGTATACATAAACACAGCTTTATTGAATGCATCCTGTGCAGCCATGGATGACTTCCAACTGATTCCAATGATAAGCAAATGCAGAACCAAAGAAGGGAGACGGAAGACAAATCTGTATGGCTTCATTATAAAAGGAAGATCCCATTTGAGGAATGACACCGATGTGGTAAACTTTGTGAGCATGGAATTCTCTCTCACTGACCCAAGGCTGGAGCCACACAAATGG<br>GAAAAGTACTGTGTGCTTGAGGTAGGAGACATGCTCCTACGGACTGCAATAGGCCAAGTGTCAAGGCCTATGTTCCTGTATGTGAGAACCAATGGGACTTCTAAGATCAAAATGAAATGGGGTATGGAGATGAGGCGATGCCTTCTTCAATCCCTTCAACAAATTGAGAGCATGATTGAAGCTGAATCTTCTGTCAAAGAAAAAGACATGACCAAGGAATTCTTTGAAAAC<br>AGATCAGAAACATGGCCAATTGGAGAGTCACCCAAAGGAGTGGAGGAAGGCTCCATTGGGAAGGTGTGCAGAACATTGCTAGCAAAGTCTGTGTTCAACAGCCTATATGCATCTCCACAACTCGAGGGGTTTCAGCTGAATCAAGAAAGTTGCTTCTCATTGTTCAGGCACTTAGGGACAACCTGGAACCTGGGACCTTCGATCTTGGGGGGCTATATGAAGCAATTGA<br>GGAGTGCCTGATTAACGATCCCTGGGTTTTGCTTAATGCGTCTTGGTTCAACTCCTTCCTCACACATGCACTGAAATA-
EPI_ISL_1039238	>A/chicken/Astrakhan/321-09/2020_H5N8_2020-12-12<br>ATGGAAGACTTTGTGCGACAATGCTTCAATCCAATGATTGTCGAGCTTGCGGAAAAGGCAATGAAAGAATATGGGGAAGATCCTAAGATCGAAACAAACAAGTTTGCCGCAATATGTACACACTTAGAAGTCTGCTTCATGTATTCGGATTTCCATTTCATTGATGAACGAGGCGAATCAATAATCGTAGAATCTGGCGACCCGAATGCATTATTGAAGCACCGATTTGAGA<br>TAATTGAAGGGAGAGACCGAACAATGGCCTGGACAGTGGTGAATAGTATATGCAACACTACAGGAGTCGAAAAGCCCAAGTTCCTTCCTGATTTGTATGACTACAGAGAAGACCGATTCATTGAAATTGGAGTAACGCGAAGGGAAGTTCACATATACTATCTTGAAAAAGCCAACAAGATAAAATCAGAGAAAACACACATTCACATATTCTCATTCACTGGGGAGGAA<br>ATGGCCACCAAGGCAGATTACACTCTTGATGAAGAGAGCAGAGCAAGAATAAAAACCAGGCTATTCACTATAAGACAAGAGATGGCCAATAGGGGTCTATGGGATTCCTTTCGTCAGTCCGAGAGAGGCGAAGAGACAATTGAAGAAAGATTTGAAATCACAGGAACCATGCGCAGGCTTGCTGACCAAAGTCTCCCACCGAACTTCGCCAGCCTTGAAAACTTTAGAG<br>CCTATGTGGATGGATTTGAACCGAACGGCTGCATTGAGGGCAAGCTTTCTCAAATGTCAAAAGAAGTGAATGCCAGAATTGAGCCATTTCTGAAGACAACACCACGCCCTCTCAGATTACCTGATGGACCTCCCTGTTCCCAGCGGTCGAAGTTCTTGCTAATGGATGCCCTTAAATTGAGCATTGAAGACCCGAGCCATGAGGGGGAGGGTATACCGCTGTACGATGCAA<br>CCAAATGCATGAAGACATTTTTTGGCTGGAAGGAGCCCAACATCGTGAAACCACATGAAAAGGGCATAAACCCTAATTACCTCCTGGCTTGGAAGCAGGTGCTATCAGAACTCCAAGATATTGAAAACGAGGAGAAAATCCCAAAAACAAAGAACATGAAGAAGACGAGCCAACTGAAGTGGGCACTTGGTGAAAACATGGCACCAGAGAAAGTGGACTTTGAGGACT<br>GTAAAGATGTTAGCGATCTAAGACAGTACGACAGTGACGAACCAGAATCTAGATCACTAGCAAGTTGGATCCAGAGTGAATTTAACAAGGCTTGCGAACTGACAGATTCGAGTTGGATTGAACTTGATGAAATAGGGGAAGACGTTGCTCCGATTGAACACATTGCGAGTATGAGGAGGAACTATTTCACAGCGGAAGTATCCCATTGCAGGGCTACAGAATACATAATG<br>AAAGGAGTATACATAAACACAGCTTTATTGAATGCATCCTGTGCAGCCATGGATGACTTCCAACTGATTCCAATGATAAGCAAATGCAGAACCAAAGAAGGGAGACGGAAGACAAATCTGTATGGCTTCATTATAAAAGGAAGATCCCATTTGAGGAATGACACCGATGTGGTAAACTTTGTGAGCATGGAATTCTCTCTCACTGACCCAAGGCTGGAGCCACACAAATGG<br>GAAAAGTACTGTGTGCTTGAGGTAGGAGACATGCTCCTACGGACTGCAATAGGCCAAGTGTCAAGGCCCATGTTCCTGTATGTGAGAACCAATGGGACTTCTAAGATCAAAATGAAATGGGGTATGGAGATGAGGCGATGCCTTCTTCAATCCCTTCAACAAATTGAGAGCATGATTGAAGCCGAGTCTTCTGTCAAAGAAAAAGACATGACCAAGGAATTCTTTGAAAAC<br>AGATCAGAAACCTGGCCAATTGGAGAGTCACCCAAAGGAGTGGAGGAAGGCTCCATTGGGAAGGTGTGCAGAACATTGCTAGCAAAGTCTGTGTTCAACAGCCTATATGCATCTCCACAACTCGAGGGGTTTCAGCTGAATCAAGAAAGTTGCTTCTCATTGTTCAGGCACTTAGGGACAACCTGGAACCTGGGACCTTCGATCTTGGGGGGCTATATGAAGCAATTGA<br>GGAGTGCCTGATTAACGATCCCTGGGTTTTGCTTAATGCGTCTTGGTTCAACTCCTTCCTCACACATGCACTGAAATA-
EPI_ISL_5095555	>A/chicken/Germany-NI/AI00547/2021_H5N8_2021-01-12<br>ATGGAAGACTTTGTGCGACAATGCTTCAATCCAATGATTGTCGAGCTTGCGGAAAAGGCAATGAAAGAATATGGGGAAGATCCTAAGATCGAAACAAACAAGTTTGCCGCAATATGTACACACTTAGAAGTCTGCTTCATGTATTCGGATTTCCATTTCATTGATGAACGAGGCGAATCAATAATCGTAGAATCTGGCGACCCGAATGCATTATTGAAGCACCGATTTGAGA<br>TAATTGAAGGGAGAGACCGAACAATGGCCTGGACAGTGGTGAATAGTATATGCAACACTACAGGAGTCGAAAAGCCCAAGTTCCTTCCTGATTTGTATGACTACAGAGAAAACCGATTCATTGAAATTGGAGTAACGCGAAGGGAAGTTCACATATACTATCTTGAAAAAGCCAACAAGATAAAGTCAGAGAAAACACACATTCACATATTCTCATTCACTGGGGAGGAA<br>ATGGCCACCAAGGCAGATTACACTCTTGATGAAGAGAGCAGAGCAAGAATAAAAACCAGGCTATTCACTATAAGACAAGAGATGGCCAATAGGGGTCTATGGGATTCCTTTCGTCAGTCCGAGAGAGGCGAAGAGACAATTGAAGAAAGATTTGAAATCACAGGAACCATGCGCAGGCTTGCTGACCAAAGTCTCCCACCGAACTTCGCCAGCCTTGAAAACTTTAGAG<br>CCTATGTGGATGGATTTGAACCGAACGGCTGCATTGAGGGCAAGCTTTCTCAAATGTCAAAAGAAGTGAATGCCAGAATTGAGCCATTTCTGAAGACAACACCACGCCCTCTCAGATTACCTGATGGACCTCCCTGTTCCCAGCGGTCGAAGTTCTTGCTAATGGATGCCCTTAAATTGAGCATTGAAGACCCGAGCCATGAGGGGGAGGGTATACCGCTGTACGATGCAA<br>CCAAATGCATGAAGACATTTTTTGGCTGGAAGGAGCCCAACATCGTGAAACCACATGAAAAGGGCATAAACCCTAATTACCTCCTGGCTTGGAAGCAGGTGCTATCAGAACTCCAAGATATTGAAAACGAGGAGAAAATCCCAAAAACAAAGAACATGAAGAAGACGAGCCAACTGAAGTGGGCACTTGGTGAAAACATGGCACCAGAGAAAGTGGACTTTGAGGACT<br>GTAAAGATGTTAGCGATCTAAAACAGTACGACAGTGACGAACCAGAATCTAGATCACTAGCAAGTTGGATCCAGAGTGAATTTAACAAGGCTTGCGAACTGACAGATTCGAGTTGGATTGAACTTGATGAAATAGGGGAAGACATTGCTCCAATTGAACACATTGCGAGTATGAGGAGGAACTATTTCACAGCGGAAGTATCCCATTGCAGGGCTACAGAATACATAATG<br>AAAGGAGTATACATAAACACAGCTTTATTGAATGCATCCTGTGCAGCCATGGATGACTTCCAACTGATTCCAATGATAAGCAAATGCAGAACCAAAGAAGGGAGACGGAAGACAAATCTGTATGGCTTCATTATAAAAGGAAGATCCCATTTGAGGAATGACACCGATGTGGTAAACTTTGTGAGCATGGAATTCTCTCTCACTGACCCAAGGCTGGAGCCACACAAATGG<br>GAAAAGTACTGTGTGCTTGAGGTAGGAGACATGCTCCTACGGACTGCAATAGGCCAAGTGTCAAGGCCCATGTTCCTGTATGTGAGAACCAATGGGACTTCTAAGATCAAAATGAAATGGGGTATGGAGATGAGGCGATGCCTTCTTCAATCCCTTCAACAAATTGAGAGCATGATTGAAGCCGAGTCTTCTGTCAAAGAAAAAGACATGACCAAGGAATTCTTTGAAAAC<br>AGATCAGAAACATGGCCAATTGGAGAGTCACCCAAAGGAGTGGAGGAAGGCTCCATTGGGAAGGTGTGCAGAACATTGCTAGCAAAGTCTGTGTTCAACAGCCTATATGCATCTCCACAACTCGAGGGGTTTCAGCTGAATCAAGAAAGTTGCTTCTCATTGTTCAGGCACTTAGGGACAACCTGGAACCTGGAACCTTCGATCTTGGGGGGCTATATGAAGCAATTGA<br>GGAGTGCCTGATTAACGATCCCTGGGTTTTGCTTAATGCGTCTTGGTTCAACTCCTTCCTCACACATGCACTGAAATA-
EPI_ISL_5067957	>A/domestic_duck/Germany-NI/AI00079/2021_H5N8_2021-01-04<br>ATGGAAGACTTTGTGCGACAATGCTTCAATCCAATGATTGTCGAGCTTGCGGAAAAGGCAATGAAAGAATATGGGGAAGATCCTAAGATCGAAACAAACAAGTTTGCCGCAATATGTACACACTTAGAAGTCTGCTTCATGTATTCGGATTTCCATTTCATTGATGAACGAGGCGAATCAATAATCGTAGAATCTGGCGACCCGAATGCATTATTGAAGCACCGATTTGAGA<br>TAATTGAAGGGAGAGACCGAACAATGGCCTGGACAGTGGTGAATAGTATATGCAACACTACAGGAGTCGAAAAGCCCAAGTTCCTTCCTGATTTGTATGACTACAGAGAAAACCGATTCATTGAAATTGGAGTAACGCGAAGGGAAGTTCACATATACTATCTTGAAAAAGCCAACAAGATAAAGTCAGAGAAAACACACATTCACATATTCTCATTCACTGGGGAGGAA<br>ATGGCCACCAAGGCAGATTACACTCTTGATGAAGAGAGCAGAGCAAGAATAAAAACCAGGCTATTCACTATAAGACAAGAGATGGCCAATAGGGGTCTATGGGATTCCTTTCGTCAGTCCGAGAGAGGCGAAGAGACAATTGAAGAAAGATTTGAAATCACAGGAACCATGCGCAGGCTTGCTGACCAAAGTCTCCCACCGAACTTCGCCAGCCTTGAAAACTTTAGAG<br>CCTATGTGGATGGATTTGAACCGAACGGCTGCATTGAGGGCAAGCTTTCTCAAATGTCAAAAGAAGTGAATGCCAGAATTGAGCCATTTCTGAAGACAACACCACGCCCTCTCAGATTACCTGATGGACCTCCCTGTTCCCAGCGGTCGAAGTTCTTGCTAATGGATGCCCTTAAATTGAGCATTGAAGACCCGAGCCATGAGGGGGAGGGTATACCGCTGTACGATGCAA<br>CCAAATGCATGAAGACATTTTTTGGCTGGAAGGAGCCCAACATCGTGAAACCACATGAAAAGGGCATAAACCCTAATTACCTCCTGGCTTGGAAGCAGGTGCTATCAGAACTCCAAGATATTGAAAACGAGGAGAAAATCCCAAAAACAAAGAACATGAAGAAGACGAGCCAACTGAAGTGGGCACTTGGTGAAAACATGGCACCAGAGAAAGTGGACTTTGAGGACT<br>GTAAAGATGTTAGCGATCTAAAACAGTACGACAGTGACGAACCAGAATCTAGATCACTAGCAAGTTGGATCCAGAGTGAATTTAACAAGGCTTGCGAACTGACAGATTCGAGTTGGATTGAACTTGATGAAATAGGGGAAGACATTGCTCCAATTGAACACATTGCGAGTATGAGGAGGAACTATTTCACAGCGGAAGTATCCCATTGCAGGGCTACAGAATACATAATG<br>AAAGGAGTATACATAAACACAGCTTTATTGAATGCATCCTGTGCAGCCATGGATGACTTCCAACTGATTCCAATGATAAGCAAATGCAGAACCAAAGAAGGGAGACGGAAGACAAATCTGTATGGCTTCATTATAAAAGGAAGATCCCATTTGAGGAATGACACCGATGTGGTAAACTTTGTGAGCATGGAATTCTCTCTCACTGACCCAAGGCTGGAGCCACACAAATGG<br>GAAAAGTACTGTGTGCTTGAGGTAGGAGACATGCTCCTACGGACTGCAATAGGCCAAGTGTCAAGGCCCATGTTCCTGTATGTGAGAACCAATGGGACTTCTAAGATCAAAATGAAATGGGGTATGGAGATGAGGCGATGCCTTCTTCAATCCCTTCAACAAATTGAGAGCATGATTGAAGCCGAGTCTTCTGTCAAAGAAAAAGACATGACCAAGGAATTCTTTGAAAAC<br>AGATCAGAAACATGGCCAATTGGAGAGTCACCCAAAGGAGTGGAGGAAGGCTCCATTGGGAAGGTGTGCAGAACATTGCTAGCAAAGTCTGTGTTCAACAGCCTATATGCATCTCCACAACTCGAGGGGTTTCAGCTGAATCAAGAAAGTTGCTTCTCATTGTTCAGGCACTTAGGGACAACCTGGAACCTGGAACCTTCGATCTTGGGGGGCTATATGAAGCAATTGA<br>GGAGTGCCTGATTAACGATCCCTGGGTTTTGCTTAATGCGTCTTGGTTCAACTCCTTCCTCACACATGCACTGAAATA-
EPI_ISL_5066996	>A/turkey/Germany-NI/AI03432/2020_H5N8_2020-12-20<br>ATGGAAGACTTTGTGCGACAATGCTTCAATCCAATGATTGTCGAGCTTGCGGAAAAGGCAATGAAAGAATATGGGGAAGATCCTAAGATCGAAACAAACAAGTTTGCCGCAATATGTACACACTTAGAAGTCTGCTTCATGTATTCGGATTTCCATTTCATTGATGAACGAGGCGAATCAATAATCGTAGAATCTGGCGACCCGAATGCATTATTGAAGCACCGATTTGAGA<br>TAATTGAAGGGAGAGACCGAACAATGGCCTGGACAGTGGTGAATAGTATATGCAACACTACAGGAGTCGAAAAGCCCAAGTTCCTTCCTGATTTGTATGACTACAGAGAAAACCGATTCATTGAAATTGGAGTAACGCGAAGGGAAGTTCACATATACTATCTTGAAAAAGCCAACAAGATAAAGTCAGAGAAAACACACATTCACATATTCTCATTCACTGGGGAGGAA<br>ATGGCCACCAAGGCAGATTACACTCTTGATGAAGAGAGCAGAGCAAGAATAAAAACCAGGCTATTCACTATAAGACAAGAGATGGCCAATAGGGGTCTATGGGATTCCTTTCGTCAGTCCGAGAGAGGCGAAGAGACAATTGAAGAAAGATTTGAAATCACAGGAACCATGCGCAGGCTTGCTGACCAAAGTCTCCCACCGAACTTCGCCAGCCTTGAAAACTTTAGAG<br>CCTATGTGGATGGATTTGAACCGAACGGCTGCATTGAGGGCAAGCTTTCTCAAATGTCAAAAGAAGTGAATGCCAGAATTGAGCCATTTCTGAAGACAACACCACGCCCTCTCAGATTACCTGATGGACCTCCCTGTTCCCAGCGGTCGAAGTTCTTGCTAATGGATGCCCTTAAATTGAGCATTGAAGACCCGAGCCATGAGGGGGAGGGTATACCGCTGTACGATGCAA<br>CCAAATGCATGAAGACATTTTTTGGCTGGAAGGAGCCCAACATCGTGAAACCACATGAAAAGGGCATAAACCCTAATTACCTCCTGGCTTGGAAGCAGGTGCTATCAGAACTCCAAGATATTGAAAACGAGGAGAAAATCCCAAAAACAAAGAACATGAAGAAGACGAGCCAACTGAAGTGGGCACTTGGTGAAAACATGGCACCAGAGAAAGTGGACTTTGAGGACT<br>GTAAAGATGTTAGCGATCTAAAACAGTACGACAGTGACGAACCAGAATCTAGATCACTAGCAAGTTGGATCCAGAGTGAATTTAACAAGGCTTGCGAACTGACAGATTCGAGTTGGATTGAACTTGATGAAATAGGGGAAGACATTGCTCCAATTGAACACATTGCGAGTATGAGGAGGAACTATTTCACAGCGGAAGTATCCCATTGCAGGGCTACAGAATACATAATG<br>AAAGGAGTATACATAAACACAGCTTTATTGAATGCATCCTGTGCAGCCATGGATGACTTCCAACTGATTCCAATGATAAGCAAATGCAGAACCAAAGAAGGGAGACGGAAGACAAATCTGTATGGCTTCATTATAAAAGGAAGATCCCATTTGAGGAATGACACCGATGTGGTAAACTTTGTGAGCATGGAATTCTCTCTCACTGACCCAAGGCTGGAGCCACACAAATGG<br>GAAAAGTACTGTGTGCTTGAGGTAGGAGACATGCTCCTACGGACTGCAATAGGCCAAGTGTCAAGGCCCATGTTCCTGTATGTGAGAACCAATGGGACTTCTAAGATCAAAATGAAATGGGGTATGGAGATGAGGCGATGCCTTCTTCAATCCCTTCAACAAATTGAGAGCATGATTGAAGCCGAGTCTTCTGTCAAAGAAAAAGACATGACCAAGGAATTCTTTGAAAAC<br>AGATCAGAAACATGGCCAATTGGAGAGTCACCCAAAGGAGTGGAGGAAGGCTCCATTGGGAAGGTGTGCAGAACATTGCTAGCAAAGTCTGTGTTCAACAGCCTATATGCATCTCCACAACTCGAGGGGTTTCAGCTGAATCAAGAAAGTTGCTTCTCATTGTTCAGGCACTTAGGGACAACCTGGAACCTGGAACCTTCGATCTTGGGGGGCTATATGAAGCAATTGA<br>GGAGTGCCTGATTAACGATCCCTGGGTTTTGCTTAATGCGTCTTGGTTCAACTCCTTCCTCACACATGCACTGAAATA-
EPI_ISL_1399245	>A/mute_swan/Czech_Republic/4799/2021_H5N8_2021-03-06<br>ATGGAAGACTTTGTGCGACAATGCTTCAATCCAATGATTGTCGAGCTTGCGGAAAAGGCAATGAAAGAATATGGGGAAGATCCTAAGATCGAAACAAACAAGTTTGCCGCAATATGTACACACTTAGAAGTCTGCTTCATGTATTCGGATTTCCATTTCATTGATGAACGAGGCGAATCAATAATCGTAGAATCTGGCGACCCGAATGCATTATTGAAGCACCGATTTGAGA<br>TAATTGAAGGGAGAGACCGAACAATGGCCTGGACAGTGGTGAATAGTATATGCAACACTACAGGAGTCGAAAAGCCCAAGTTCCTTCCTGATTTGTATGACTACAGAGAAAACCGATTCATTGAAATTGGAGTAACGCGAAGGGAAGTTCACATATACTATCTTGAAAAAGCCAACAAGATAAAGTCAGAGAAAACACACATTCACATATTCTCATTCACTGGGGAGGAA<br>ATGGCCACCAAGGCAGATTACACTCTTGATGAAGAGAGCAGAGCAAGAATAAAAACCAGGCTATTCACTATAAGACAAGAGATGGCCAATAGGGGTCTATGGGATTCCTTTCGTCAGTCCGAGAGAGGCGAAGAGACAATTGAAGAAAGATTTGAAATCACAGGAACCATGCGCAGGCTTGCTGACCAAAGTCTCCCACCGAACTTCGCCAGCCTTGAAAACTTTAGAG<br>CCTATGTGGATGGATTTGAACCGAACGGCTGCATTGAGGGCAAGCTTTCTCAAATGTCAAAAGAAGTGAATGCCAGAATTGAGCCATTTCTGAAGACAACACCACGCCCTCTCAGATTACCTGATGGACCTCCCTGTTCCCAGCGGTCGAAGTTCTTGCTAATGGATGCCCTTAAATTGAGCATTGAAGACCCGAGCCATGAGGGGGAGGGTATACCGCTGTACGATGCAA<br>CCAAATGCATGAAGACATTTTTTGGCTGGAAGGAGCCCAACATCGTGAAACCACATGAAAAGGGCATAAACCCTAATTACCTCCTGGCTTGGAAGCAGGTGCTATCAGAACTCCAAGATATTGAAAACGAGGAGAAAATCCCAAAAACAAAGAACATGAAGAAGACGAGCCAACTGAAGTGGGCACTTGGTGAAAACATGGCACCAGAGAAAGTGGACTTTGAGGACT<br>GTAAAGATGTTAGCGATCTAAAACAGTACGACAGTGACGAACCAGAATCTAGATCACTAGCAAGTTGGATCCAGAGTGAATTTAACAAGGCTTGCGAACTGACAGATTCGAGTTGGATTGAACTTGATGAAATAGGGGAAGACGTTGCTCCAATTGAACACATTGCGAGTATGAGGAGGAACTATTTCACAGCGGAAGTATCCCATTGCAGGGCTACAGAATACATAATG<br>AAAGGAGTATACATAAACACAGCTTTATTGAATGCATCCTGTGCAGCCATGGATGACTTCCAACTGATTCCAATGATAAGCAAATGCAGAACCAAAGAAGGGAGACGGAAGACAAATCTGTATGGCTTCATTATAAAAGGAAGATCCCATTTGAGGAATGACACCGATGTGGTAAACTTTGTGAGCATGGAATTCTCTCTCACTGACCCAAGGCTGGAGCCACACAAATGG<br>GAAAAGTACTGTGTGCTTGAGGTAGGAGACATGCTCCTACGGACTGCAATAGGCCAAGTGTCAAGGCCCATGTTCCTGTATGTGAGAACCAATGGGACTTCTAAGATCAAAATGAAATGGGGTATGGAGATGAGGCGATGCCTTCTTCAATCCCTTCAACAAATTGAGAGCATGATTGAAGCCGAGTCTTCTGTCAAAGAAAAAGACATGACCAAGGAATTCTTTGAAAAC<br>AGATCAGAAACATGGCCAATTGGAGAGTCACCCAAAGGAGTGGAGGAAGGCTCCATTGGGAAGGTGTGCAGAACATTGCTAGCAAAGTCTGTGTTCAACAGCCTATATGCATCTCCACAACTCGAGGGGTTTCAGCTGAATCAAGAAAGTTGCTTCTCATTGTTCAGGCACTTAGGGACAACCTGGAACCTGGAACCTTCGATCTTGGGGGGCTATATGAAGCAATTGA<br>GGAGTGCCTGATTAACGATCCCTGGGTTTTGCTTAATGCGTCTTGGTTCAACTCCTTCCTCACACATGCACTGAAATA-
EPI_ISL_18244181	>A/mute_swan/Poland/MB021-21_22VIR5675-11/2021_H5N8_2021-01-19<br>ATGGAAGACTTTGTGCGACAATGCTTCAATCCAATGATTGTCGAGCTTGCGGAAAAGGCAATGAAAGAATATGGGGAAGATCCTAAGATCGAAACAAACAAGTTTGCCGCAATATGTACACACTTAGAAGTCTGCTTCATGTATTCGGATTTCCATTTCATTGATGAACGAGGCGAATCAATAATCGTAGAATCTGGCGACCCGAATGCATTATTGAAGCACCGATTTGAGA<br>TAATTGAAGGGAGAGACCGAACAATGGCCTGGACAGTGGTGAATAGTATATGCAACACTACAGGAGTCGAAAAGCCCAAGTTCCTTCCTGATTTGTATGACTACAGAGAAAACCGATTCATTGAAATTGGAGTAACGCGAAGGGAAGTTCACATATACTATCTTGAAAAAGCCAACAAGATAAAGTCAGAGAAAACACACATTCACATATTCTCATTCACTGGGGAGGAA<br>ATGGCCACCAAGGCAGATTACACTCTTGATGAAGAGAGCAGAGCAAGAATAAAAACCAGGCTATTCACTATAAGACAAGAGATGGCCAATAGGGGTCTATGGGATTCCTTTCGTCAGTCCGAGAGAGGCGAAGAGACAATTGAAGAAAGATTTGAAATCACAGGAACCATGCGCAGGCTTGCTGACCAAAGTCTCCCACCGAACTTCGCCAGCCTTGAAAACTTTAGAG<br>CCTATGTGGATGGATTTGAACCGAACGGCTGCATTGAGGGCAAGCTTTCTCAAATGTCAAAAGAAGTGAATGCCAGAATTGAGCCATTTCTGAAGACAACACCACGCCCTCTCAGATTACCTGATGGACCTCCCTGTTCCCAGCGGTCGAAGTTCTTGCTAATGGATGCCCTTAAATTGAGCATTGAAGACCCGAGCCATGAGGGGGAGGGTATACCGCTGTACGATGCAA<br>CCAAATGCATGAAGACATTTTTTGGCTGGAAGGAGCCCAACATCGTGAAACCACATGAAAAGGGCATAAACCCTAATTACCTCCTGGCTTGGAAGCAGGTGCTATCAGAACTCCAAGATATTGAAAACGAGGAGAAAATCCCAAAAACAAAGAACATGAAGAAGACGAGCCAACTGAAGTGGGCACTTGGTGAAAACATGGCACCAGAGAAAGTGGACTTTGAGGACT<br>GTAAAGATGTTAGCGATCTAAAACAGTACGACAGTGACGAACCAGAATCTAGATCACTAGCAAGTTGGATCCAGAGTGAATTTAACAAGGCTTGCGAACTGACAGATTCGAGTTGGATTGAACTTGATGAAATAGGGGAAGACGTTGCTCCAATTGAACACATTGCGAGTATGAGGAGGAACTATTTCACAGCGGAAGTATCCCATTGCAGGGCTACAGAATACATAATG<br>AAAGGAGTATACATAAACACAGCTTTATTGAATGCATCCTGTGCAGCCATGGATGACTTCCAACTGATTCCAATGATAAGCAAATGCAGAACCAAAGAAGGGAGACGGAAGACAAATCTGTATGGCTTCATTATAAAAGGAAGATCCCATTTGAGGAATGACACCGATGTGGTAAACTTTGTGAGCATGGAATTCTCTCTCACTGACCCAAGGCTGGAGCCACACAAATGG<br>GAAAAGTACTGTGTGCTTGAGGTAGGAGACATGCTCCTACGGACTGCAATAGGCCAAGTGTCAAGGCCCATGTTCCTGTATGTRAGAACCAATGGGACTTCTAAGATCAAAATGAAATGGGGTATGGAGATGAGGCGATGCCTTCTTCAATCCCTTCAACAAATTGAGAGCATGATTGAAGCCGAGTCTTCTGTCAAAGAAAAAGACATGACCAAGGAATTCTTTGAAAAC<br>AGATCAGAAACATGGCCAATTGGAGAGTCACCCAAAGGAGTGGAGGAAGGCTCCATTGGGAAGGTGTGCAGAACATTGCTAGCAAAGTCTGTGTTCAACAGCCTATATGCATCTCCACAACTCGAGGGGTTTCAGCTGAATCAAGAAAGTTGCTTCTCATTGTTCAGGCACTTAGGGACAACCTGGAACCTGGAACCTTCGATCTTGGGGGGCTATATGAAGCAATTGA<br>GGAGTGCCTGATTAACGATCCCTGGGTTTTGCTTAATGCGTCTTGGTTCAACTCCTTCCTCACACATGCACTGAAATA-
EPI_ISL_2234811	>A/anser_anser/Spain/102-8_21VIR1230-4/2021_H5N8_2021-01-07<br>ATGGAAGACTTTGTGCGACAATGCTTCAATCCAATGATTGTCGAGCTTGCGGAAAAGGCAATGAAAGAATATGGGGAAGATCCTAAGATCGAAACAAACAAGTTTGCCGCAATATGTACACACTTAGAAGTCTGCTTCATGTATTCGGATTTCCATTTCATTGATGAACGAGGCGAATCAATAATCGTAGAATCTGGCGACCCGAATGCATTATTGAAGCACCGATTTGAGA<br>TAATTGAAGGGAGAGACCGAACAATGGCCTGGACAGTGGTGAATAGTATATGCAACACTACAGGAGTCGAAAAGCCCAAGTTCCTTCCTGATTTGTATGACTACAGAGAAAACCGATTCATTGAAATTGGAGTAACGCGAAGGGAAGTTCACATATACTATCTTGAAAAAGCCAACAAGATAAAGTCAGAGAAAACACACATTCACATATTCTCATTCACTGGGGAGGAA<br>ATGGCCACCAAGGCAGATTACACTCTTGATGAAGAGAGCAGAGCAAGAATAAAAACCAGGCTATTCACTATAAGACAAGAGATGGCCAATAGGGGTCTATGGGATTCCTTTCGTCAGTCCGAGAGAGGCGAAGAGACAATTGAAGAAAGATTTGAAATCACAGGAACCATGCGCAGGCTTGCTGACCAAAGTCTCCCACCGAACTTCGCCAGCCTTGAAAACTTTAGAG<br>CCTATGTGGATGGATTTGAACCGAACGGCTGCATTGAGGGCAAGCTTTCTCAAATGTCAAAAGAAGTGAATGCCAGAATTGAGCCATTTCTGAAGACAACACCACGCCCTCTCAGATTACCTGATGGACCTCCCTGTTCCCAGCGGTCGAAGTTCTTGCTAATGGATGCCCTTAAATTGAGCATTGAAGACCCGAGCCATGAGGGGGAGGGTATACCGCTGTACGATGCAA<br>CCAAATGCATGAAGACATTTTTTGGCTGGAAGGAGCCCAACATCGTGAAACCACATGAAAAGGGCATAAACCCTAATTACCTCCTGGCTTGGAAGCAGGTGCTATCAGAACTCCAAGATATTGAAAACGAGGAGAAAATCCCAAAAACAAAGAACATGAAGAAGACGAGCCAACTGAAGTGGGCACTTGGTGAAAACATGGCACCAGAGAAAGTGGACTTTGAGGACT<br>GTAAAGATGTTAGCGATCTAAAACAGTACGACAGTGACGAACCAGAATCTAGATCACTAGCAAGTTGGATCCAGAGTGAATTTAACAAGGCTTGCGAACTGACAGATTCGAGTTGGATTGAACTTGATGAAATAGGGGAAGACGTTGCTCCAATTGAACACATTGCGAGTATGAGGAGGAACTATTTCACAGCGGAAGTATCCCATTGCAGGGCTACAGAATACATAATG<br>AAAGGAGTATACATAAACACAGCTTTATTGAATGCATCCTGTGCAGCCATGGATGACTTCCAACTGATTCCAATGATAAGCAAATGCAGAACCAAAGAAGGGAGACGGAAGACAAATCTGTATGGCTTCATTATAAAAGGAAGATCCCATTTGAGGAATGACACCGATGTGGTAAACTTTGTGAGCATGGAATTCTCTCTCACTGACCCAAGGCTGGAGCCACACAAATGG<br>GAAAAGTACTGTGTGCTTGAGGTAGGAGACATGCTCCTACGGACTGCAATAGGCCAAGTGTCAAGGCCCATGTTCCTGTATGTGAGAACCAATGGGACTTCTAAGATCAAAATGAAATGGGGTATGGAGATGAGGCGATGCCTTCTTCAATCCCTTCAACAAATTGAGAGCATGATTGAAGCCGAGTCTTCTGTCAAAGAAAAAGACATGACCAAGGAATTCTTTGAAAAC<br>AGATCAGAAACATGGCCAATTGGAGAGTCACCCAAAGGAGTGGAGGAAGGCTCCATTGGGAAGGTGTGCAGAACATTGCTAGCAAAGTCTGTGTTCAACAGCCTATATGCATCTCCACAACTCGAGGGGTTTCAGCTGAATCAAGAAAGTTGCTTCTCATTGTTCAGGCACTTAGGGACAACCTGGAACCTGGAACCTTCGATCTTGGGGGGCTATATGAAGCAATTGA<br>GGAGTGCCTGATTAACGATCCCTGGGTTTTGCTTAATGCGTCTTGGTTCAACTCCTTCCTCACACATGCACTGAAATA-
EPI_ISL_647969	>A/Turkey/Sweden/SVA201114SZ0001/20KN303106/2020_H5N8_2020-11-13<br>ATGGAAGACTTTGTGCGACAATGCTTCAATCCAATGATTGTCGAGCTTGCGGAAAAGGCAATGAAAGAATATGGGGAAGATCCTAAGATCGAAACAAACAAGTTTGCCGCAATATGTACACACTTAGAAGTCTGCTTCATGTATTCGGATTTCCATTTCATTGATGAACGAGGCGAATCAATAATCGTAGAATCTGGCGACCCGAATGCATTATTGAAGCACCGATTTGAGA<br>TAATTGAAGGGAGAGACCGAACAATGGCCTGGACAGTGGTGAATAGTATATGCAACACTACAGGAGTCGAAAAGCCCAAGTTCCTTCCTGATTTGTATGACTACAGAGAAAACCGATTCATTGAAATTGGAGTAACGCGAAGGGAAGTTCACATATACTATCTTGAAAAAGCCAACAAGATAAAGTCAGAGAAAACACACATTCACATATTCTCATTCACTGGGGAGGAA<br>ATGGCCACCAAGGCAGATTACACTCTTGATGAAGAGAGCAGAGCAAGAATAAAAACCAGGCTATTCACTATAAGACAAGAGATGGCCAATAGGGGTCTATGGGATTCCTTTCGTCAGTCCGAGAGAGGCGAAGAGACAATTGAAGAAAGATTTGAAATCACAGGAACCATGCGCAGGCTTGCTGACCAAAGTCTCCCACCGAACTTCGCCAGCCTTGAAAACTTTAGAG<br>CCTATGTGGATGGATTTGAACCGAACGGCTGCATTGAGGGCAAGCTTTCTCAAATGTCAAAAGAAGTGAATGCCAGAATTGAGCCATTTCTGAAGACAACACCACGCCCTCTCAGATTACCTGATGGACCTCCCTGTTCCCAGCGGTCGAAGTTCTTGCTAATGGATGCCCTTAAATTGAGCATTGAAGACCCGAGCCATGAGGGGGAGGGTATACCGCTGTACGATGCAA<br>CCAAATGCATGAAGACATTTTTTGGCTGGAAGGAGCCCAACATCGTGAAACCACATGAAAAGGGCATAAACCCTAATTACCTCCTGGCTTGGAAGCAGGTGCTATCAGAACTCCAAGATATTGAAAACGAGGAGAAAATCCCAAAAACAAAGAACATGAAGAAGACGAGCCAACTGAAGTGGGCACTTGGTGAAAACATGGCACCAGAGAAAGTGGACTTTGAGGACT<br>GTAAAGATGTTAGCGATCTAAAACAGTACGACAGTGACGAACCAGAATCTAGATCACTAGCAAGTTGGATCCAGAGTGAATTTAACAAGGCTTGCGAACTGACAGATTCGAGTTGGATTGAACTTGATGAAATAGGGGAAGACGTTGCTCCAATTGAACACATTGCGAGTATGAGGAGGAACTATTTCACAGCGGAAGTATCCCATTGCAGGGCTACAGAATACATAATG<br>AAAGGAGTATACATAAACACAGCTTTATTGAATGCATCCTGTGCAGCCATGGATGACTTCCAACTGATTCCAATGATAAGCAAATGCAGAACCAAAGAAGGGAGACGGAAGACAAATCTGTATGGCTTCATTATAAAAGGAAGATCCCATTTGAGGAATGACACCGATGTGGTAAACTTTGTGAGCATGGAATTCTCTCTCACTGACCCAAGGCTGGAGCCACACAAATGG<br>GAAAAGTACTGTGTGCTTGAGGTAGGAGACATGCTCCTACGGACTGCAATAGGCCAAGTGTCAAGGCCCATGTTCCTGTATGTGAGAACCAATGGGACTTCTAAGATCAAAATGAAATGGGGTATGGAGATGAGGCGATGCCTTCTTCAATCCCTTCAACAAATTGAGAGCATGATTGAAGCCGAGTCTTCTGTCAAAGAAAAAGACATGACCAAGGAATTCTTTGAAAAC<br>AGATCAGAAACATGGCCAATTGGAGAGTCACCCAAAGGAGTGGAGGAAGGCTCCATTGGGAAGGTGTGCAGAACATTGCTAGCAAAGTCTGTGTTCAACAGCCTATATGCATCTCCACAACTCGAGGGGTTTCAGCTGAATCAAGAAAGTTGCTTCTCATTGTTCAGGCACTTAGGGACAACCTGGAACCTGGAACCTTCGATCTTGGGGGGCTATATGAAGCAATTGA<br>GGAGTGCCTGATTAACGATCCCTGGGTTTTGCTTAATGCGTCTTGGTTCAACTCCTTCCTCACACATGCACTGAAATA-
EPI_ISL_846604	>A/turkey/Poland/477/2020_H5N8_2020-12-07<br>ATGGAAGACTTTGTGCGACAATGCTTCAATCCAATGATTGTCGAGCTTGCGGAAAAGGCAATGAAAGAATATGGGGAAGATCCTAAGATCGAAACAAACAAGTTTGCCGCAATATGTACACACTTAGAAGTCTGCTTCATGTATTCGGATTTCCATTTCATTGATGAACGAGGCGAATCAATAATCGTAGAATCTGGCGACCCGAATGCATTATTGAAGCACCGATTTGAGA<br>TAATTGAAGGGAGAGACCGAACAATGGCCTGGACAGTGGTGAATAGTATATGCAACACTACAGGAGTCGAAAAGCCCAAGTTCCTTCCTGATTTGTATGACTACAGAGAAAACCGATTCATTGAAATTGGAGTAACGCGAAGGGAAGTTCACATATACTATCTTGAAAAAGCCAACAAGATAAAGTCAGAGAAAACACACATTCACATATTCTCATTCACTGGGGAGGAA<br>ATGGCCACCAAGGCAGATTACACTCTTGATGAAGAGAGCAGAGCAAGAATAAAAACCAGGCTATTCACTATAAGACAAGAGATGGCCAATAGGGGTCTATGGGATTCCTTTCGTCAGTCCGAGAGAGGCGAAGAGACAATTGAAGAAAGATTTGAAATCACAGGAACCATGCGCAGGCTTGCTGACCAAAGTCTCCCACCGAACTTCGCCAGCCTTGAAAACTTTAGAG<br>CCTATGTGGATGGATTTGAACCGAACGGCTGCATTGAGGGCAAGCTTTCTCAAATGTCAAAAGAAGTGAATGCCAGAATTGAGCCATTTCTGAAGACAACACCACGCCCTCTCAGATTACCTGATGGACCTCCCTGTTCCCAGCGGTCGAAGTTCTTGCTAATGGATGCCCTTAAATTGAGCATTGAAGACCCGAGCCATGAGGGGGAGGGTATACCGCTGTACGATGCAA<br>CCAAATGCATGAAGACATTTTTTGGCTGGAAGGAGCCCAACATCGTGAAACCACATGAAAAGGGCATAAACCCTAATTACCTCCTGGCTTGGAAGCAGGTGCTATCAGAACTCCAAGATATTGAAAACGAGGAGAAAATCCCAAAAACAAAGAACATGAAGAAGACGAGCCAACTGAAGTGGGCACTTGGTGAAAACATGGCACCAGAGAAAGTGGACTTTGAGGACT<br>GTAAAGATGTTAGCGATCTAAAACAGTACGACAGTGACGAACCAGAATCTAGATCACTAGCAAGTTGGATCCAGAGTGAATTTAACAAGGCTTGCGAACTGACAGATTCGAGTTGGATTGAACTTGATGAAATAGGGGAAGACGTTGCTCCAATTGAACACATTGCGAGTATGAGGAGGAACTATTTCACAGCGGAAGTATCCCATTGCAGGGCTACAGAATACATAATG<br>AAAGGAGTATACATAAACACAGCTTTATTGAATGCATCCTGTGCAGCCATGGATGACTTCCAACTGATTCCAATGATAAGCAAATGCAGAACCAAAGAAGGGAGACGGAAGACAAATCTGTATGGCTTCATTATAAAAGGAAGATCCCATTTGAGGAATGACACCGATGTGGTAAACTTTGTGAGCATGGAATTCTCTCTCACTGACCCAAGGCTGGAGCCACACAAATGG<br>GAAAAGTACTGTGTGCTTGAGGTAGGAGACATGCTCCTACGGACTGCAATAGGCCAAGTGTCAAGGCCCATGTTCCTGTATGTGAGAACCAATGGGACTTCTAAGATCAAAATGAAATGGGGTATGGAGATGAGGCGATGCCTTCTTCAATCCCTTCAACAAATTGAGAGCATGATTGAAGCCGAGTCTTCTGTCAAAGAAAAAGACATGACCAAGGAATTCTTTGAAAAC<br>AGATCAGAAACATGGCCAATTGGAGAGTCACCCAAAGGAGTGGAGGAAGGCTCCATTGGGAAGGTGTGCAGAACATTGCTAGCAAAGTCTGTGTTCAACAGCCTATATGCATCTCCACAACTCGAGGGGTTTCAGCTGAATCAAGAAAGTTGCTTCTCATTGTTCAGGCACTTAGGGACAACCTGGAACCTGGAACCTTCGATCTTGGGGGGCTATATGAAGCAATTGA<br>GGAGTGCCTGATTAACGATCCCTGGGTTTTGCTTAATGCGTCTTGGTTCAACTCCTTCCTCACACATGCACTGAAATA-

EPI_ISL_5096629	>A/wild_goose/Germany-NI/AI00625/2021_H5N8_2021-01-12<br>ATGGAAGACTTTGTGCGACAATGCTTCAATCCAATGATTGTCGAGCTTGCGGAAAAGGCAATGAAAGAATATGGGGAAGATCCTAAGATCGAAACAAACAAGTTTGCCGCAATATGTACACACTTAGAAGTCTGCTTCATGTATTCGGATTTCCATTTCATTGATGAACGAGGCGAATCAATAATCGTAGAATCTGGCGACCCGAATGCATTATTGAAGCACCGATTTGAGA<br>TAATTGAAGGGAGAGACCGAACAATGGCCTGGACAGTGGTGAATAGTATATGCAACACTACAGGAGTCGAAAAGCCCAAGTTCCTTCCTGATTTGTATGACTACAGAGAAAACCGATTCATTGAAATTGGAGTAACGCGAAGGGAAGTTCACATATACTATCTTGAAAAAGCCAACAAGATAAAGTCAGAGAAAACACACATTCACATATTCTCATTCACTGGGGAGGAA<br>ATGGCCACCAAGGCAGATTACACTCTTGATGAAGAGAGCAGAGCAAGAATAAAAACCAGGCTATTCACTATAAGACAAGAGATGGCCAATAGGGGTCTATGGGATTCCTTTCGTCAGTCCGAGAGAGGCGAAGAGACAATTGAAGAAAGATTTGAAATCACAGGAACCATGCGCAGGCTTGCTGACCAAAGTCTCCCACCGAACTTCGCCAGCCTTGAAAACTTTAGAG<br>CCTATGTGGATGGATTTGAACCGAACGGCTGCATTGAGGGCAAGCTTTCTCAAATGTCAAAAGAAGTGAATGCCAGAATTGAGCCATTTCTGAAGACAACACCACGCCCTCTCAGATTACCTGATGGACCTCCCTGTTCCCAGCGGTCGAAGTTCTTGCTAATGGATGCCCTTAAATTGAGCATTGAAGACCCGAGCCATGAGGGGGAGGGTATACCGCTGTACGATGCAA<br>CCAAATGCATGAAGACATTTTTTGGCTGGAAGGAGCCCAACATCGTGAAACCACATGAAAAGGGCATAAACCCTAATTACCTCCTGGCTTGGAAGCAGGTGCTATCAGAACTCCAAGATATTGAAAACGAGGAGAAAATCCCAAAAACAAAGAACATGAAGAAGACGAGCCAACTGAAGTGGGCACTTGGTGAAAACATGGCACCAGAGAAAGTGGACTTTGAGGACT<br>GTAAAGATGTTAGCGATCTAAAACAGTACGACAGTGACGAACCAGAATCTAGATCACTAGCAAGTTGGATCCAGAGTGAATTTAACAAGGCTTGCGAACTGACAGATTCGAGTTGGATTGAACTTGATGAAATAGGGGAAGACATTGCTCCAATTGAACACATTGCGAGTATGAGGAGGAACTATTTCACAGCGGAAGTATCCCATTGCAGGGCTACAGAATACATAATG<br>AAAGGAGTATACATAAACACAGCTTTATTGAATGCATCCTGTGCAGCCATGGATGACTTCCAACTGATTCCAATGATAAGCAAATGCAGAACCAAAGAAGGGAGACGGAAGACAAATCTGTATGGCTTCATTATAAAAGGAAGATCCCATTTGAGGAATGACACCGATGTGGTAAACTTTGTGAGCATGGAATTCTCTCTCACTGACCCAAGGCTGGAGCCACACAAATGG<br>GAAAAGTACTGTGTGCTTGAGGTAGGGGACATGCTCCTACGGACTGCAATAGGCCAAGTGTCAAGGCCCATGTTCCTGTATGTGAGAACCAATGGGACTTCTAAGATCAAAATGAAATGGGGTATGGAGATGAGGCGATGCCTTCTTCAATCCCTTCAACAAATTGAGAGCATGATTGAAGCCGAGTCTTCTGTCAAAGAAAAAGACATGACCAAGGAATTCTTTGAAAAC<br>AGATCAGAAACATGGCCAATTGGAGAGTCACCCAAAGGAGTGGAGGAAGGCTCCATTGGGAAGGTGTGCAGAACATTGCTAGCAAAGTCTGTGTTCAACAGCCTATATGCATCTCCACAACTCGAGGGGTTTTCAGCTGAATCAAGAAAGTTGCTTCTCATTGTTCAGGCACTTAGGGACAACCTGGAACCTGGAACCTTCGATCTTGGGGGGCTATATGAAGCAATTGA<br>GGAGTGCCTGATTAACGATCCCTGGGTTTTGCTTAATGCGTCTTGGTTCAACTCCTTCCTCACACATGCACTGAAATA-
EPI_ISL_666890	>A/turkey/Croatia/104/2020_H5N8_2020-11-17<br>ATGGAAGACTTTGTGCGACAATGCTTCAATCCAATGATTGTCGAGCTTGCGGAAAAGGCAATGAAAGAATATGGGGAAGATCCTAAGATCGAAACAAACAAGTTTGCCGCAATATGTACACACTTAGAAGTCTGCTTCATGTATTCGGATTTCCATTTCATTGATGAACGAGGCGAATCAATAATCGTAGAATCTGGCGACCCGAATGCATTATTGAAGCACCGATTTGAGA<br>TAATTGAAGGGAGAGACCGAACAATGGCCTGGACAGTGGTGAATAGTATATGCAACACTACAGGAGTCGAAAAGCCCAAGTTCCTTCCTGATTTGTATGACTACAGAGAAAACCGATTCATTGAAATTGGAGTAACGCGAAGGGAAGTTCACATATACTATCTTGAAAAAGCCAACAAGATAAAGTCAGAGAAAACACACATTCACATATTCTCATTCACTGGGGAGGAA<br>ATGGCCACCAAGGCAGATTACACTCTTGATGAAGAGAGCAGAGCAAGAATAAAAACCAGGCTATTCACTATAAGACAAGAGATGGCCAATAGGGGTCTATGGGATTCCTTTCGTCAGTCCGAGAGAGGCGAAGAGACAATTGAAGAAAGATTTGAAATCACAGGAACCATGCGCAGGCTTGCTGACCAAAGTCTCCCACCGAACTTCGCCAGCCTTGAAAACTTTAGAG<br>CCTATGTGGATGGATTTGAACCGAACGGCTGCATTGAGGGCAAGCTTTCTCAAATGTCAAAAGAAGTGAATGCCAGAATTGAGCCATTTCTGAAGACAACACCACGCCATCTCAGATTACCTGATGGACCTCCCTGTTCCCAGCGGTCGAAGTTCTTGCTAATGGATGCCCTTAAATTGAGCATTGAAGACCCGAGCCATGAGGGGGAGGGTATACCGCTGTACGATGCAA<br>CCAAATGCATGAAGACATTTTTTGGCTGGAAGGAGCCCAACATCGTGAAACCACATGAAAAGGGCATAAACCCTAATTACCTCCTGGCTTGGAAGCAGGTGCTATCAGAACTCCAAGATATTGAAAACGAGGAGAAAATCCCAAAAACAAAGAACATGAAGAAGACGAGCCAACTGAAGTGGGCACTTGGTGAAAACATGGCACCAGAGAAAGTGGACTTTGAGGACT<br>GTAAAGATGTTAGCGATCTAAAACAGTACGACAGTGACGAACCAGAATCTAGATCACTAGCAAGTTGGATCCAGAGTGAATTTAACAAGGCTTGCGAACTGACAGATTCGAGTTGGATTGAACTTGATGAAATAGGGGAAGACGTTGCTCCAATTGAACACATTGCGAGTATGAGGAGGAACTATTTCACAGCGGAAGTATCCCATTGCAGGGCTACAGAATACATAATG<br>AAAGGAGTATACATAAACACAGCTTTATTGAATGCATCCTGTGCAGCCATGGATGACTTCCAACTGATTCCAATGATAAGCAAATGCAGAACCAAAGAAGGGAGACGGAAGACAAATCTGTATGGCTTCATTATAAAAGGAAGATCCCATTTGAGGAATGACACCGATGTGGTAAACTTTGTGAGCATGGAATTCTCTCTCACTGACCCAAGGCTGGAGCCACACAAATGG<br>GAAAAGTACTGTGTGCTTGAGGTAGGAGACATGCTCCTACGGACTGCAATAGGCCAAGTGTCAAGGCCCATGTTCCTGTATGTGAGAACCAATGGGACTTCTAAGATCAAAATGAAATGGGGTATGGAGATGAGGCGATGCCTTCTTCAATCCCTTCAACAAATTGAGAGCATGATTGAAGCCGAGTCTTCTGTCAAAGAAAAAGACATGACCAAGGAATTCTTTGAAAAC<br>AGATCAGAAACATGGCCAATTGGAGAGTCACCCAAAGGAGTGGAGGAAGGCTCCATTGGGAAGGTGTGCAGAACATTGCTAGCAAAGTCTGTGTTCAACAGCCTATATGCATCTCCACAACTCGAGGGGTTTTCAGCTGAATCAAGAAAGTTGCTTCTCATTGTTCAGGCACTTAGGGACAACCTGGAACCTGGAACCTTCGATCTTGGGGGGCTATATGAAGCAATTGA<br>GGAGTGCCTGATTAACGATCCCTGGGTTTTGCTTAATGCGTCTTGGTTCAACTCCTTCCTCACACATGCACTGAAATA-
EPI_ISL_5116065	>A/greater_rhea/Germany-SN/AI02351/2021_H5N8_2021-03-02<br>ATGGAAGACTTTGTGCGACAATGCTTCAATCCAATGATTGTCGAGCTTGCGGAAAAGGCAATGAAAGAATATGGGGAAGATCCTAAGATCGAAACAAACAAGTTTGCCGCAATATGTACACACTTAGAAGTCTGCTTCATGTATTCGGATTTCCATTTCATTGATGAACGAGGCGAATCAATAATCGTAGAATCTGGCGACCCGAATGCATTATTGAAGCACCGATTTGAGA<br>TAATTGAAGGGAGAGACCGAACAATGGCCTGGACAGTGGTGAATAGTATATGCAACACTACAGGAGTCGAAAAGCCCAAGTTCCTTCCTGATTTGTATGACTACAGAGAAAACCGATTCATTGAAATTGGAGTAACGCGAAGGGAAGTTCACATATACTATCTTGAAAAAGCCAACAAGATAAAGTCAGAGAAAACACACATTCACATATTCTCATTCACTGGGGAGGAA<br>ATGGCCACCAAGGTAGATTACACTCTTGATGAAGAGAGCAGAGCAAGAATAAAAACCAGGCTATTCACTATAAGACAAGAGATGGCCAATAGGGGTCTATGGGATTCCTTTCGTCAGTCCGAGAGAGGCGAAGAGACAATTGAAGAAAGATTTGAAATCACAGGAACCATGCGCAGGCTTGCTGACCAAAGTCTCCCACCGAACTTCGCCAGCCTTGAAAACTTTAGAG<br>CCTATGTGGATGGATTTGAACCGAACGGCTGCATTGAGGGCAAGCTTTCTCAAATGTCAAAAGAAGTGAATGCCAGAATTGAGCCATTTCTGAAGACAACACCACGCCCTCTCAGATTACCTGATGGACCTCCCTGTTCCCAGCGGTCGAAGTTCTTGCTAATGGATGCCCTTAAATTGAGCATTGAAGACCCGAGCCATGAGGGGGAGGGTATACCGCTGTACGATGCAA<br>CCAAATGCATGAAGACATTTTTTGGCTGGAAGGAGCCCAACATCGTGAAACCACATGAAAAGGGCATAAACCCTAATTACCTCCTGGCTTGGAAGCAGGTGCTATCAGAACTCCAAGATATTGAAAACGAGGAGAAAATCCCAAAAACAAAGAACATGAAGAAGACGAGCCAACTGAAGTGGGCACTTGGTGAAAACATGGCACCAGAGAAAGTGGACTTTGAGGACT<br>GTAAAGATGTTAGCGATCTAAAACAGTACGACAGTGACGAACCAGAATCTAGATCACTAGCAAGTTGGATCCAGAGTGAATTTAACAAGGCTTGCGAACTGACAGATTCGAGTTGGATTGAACTTGATGAAATAGGGGAAGACGTTGCTCCAATTGAACACATTGCGAGTATGAGGAGGAACTATTTCACAGCGGAAGTATCCCATTGCAGGGCTACAGAATACATAATG<br>AAAGGAGTATACATAAACACAGCTTTATTGAATGCATCCTGTGCAGCCATGGATGACTTCCAACTGATTCCAATGATAAGCAAATGCAGAACCAAAGAAGGGAGACGGAAGACAAATCTGTATGGCTTCATTATAAAAGGAAGATCCCATTTGAGGAATGACACCGATGTGGTAAACTTTGTGAGCATGGAATTCTCTCTCACTGACCCAAGGCTGGAGCCACACAAATGG<br>GAAAAGTACTGTGTGCTTGAGGTAGGAGACATGCTCCTACGGACTGCAATAGGCCAAGTGTCAAGGCCCATGTTCCTGTATGTGAGAACCAATGGGACTTCTAAGATCAAAATGAAATGGGGTATGGAGATGAGGCGATGCCTTCTTCAATCCCTTCAACAAATTGAGAGCATGATTGAAGCCGAGTCTTCTGTCAAAGAAAAAGACATGACCAAGGAATTCTTTGAAAAC<br>AGATCAGAAACATGGCCAATTGGAGAGTCACCCAAAGGAGTGGAGGAAGGCTCCATTGGGAAGGTGTGCAGAACATTGCTAGCAAAGTCTGTGTTCAACAGCCTATATGCATCTCCACAACTCGAGGGGTTTTCAGCTGAATCAAGGAAGTTGCTTCTCATTGTTCAGGCACTTAGGGACAACCTGGAACCTGGAACCTTCGATCTTGGGGGGCTATATGAAGCAATTGA<br>GGAGTGCCTGATTAACGATCCCTGGGTTTTGCTTAATGCGTCTTGGTTCAACTCCTTCCTCACACATGCACTGAAATA-
EPI_ISL_1399241	>A/chicken/Czech_Republic/4526/2021_H5N8_2021-03-06<br>ATGGAAGACTTTGTGCGACAATGCTTCAATCCAATGATTGTCGAGCTTGCGGAAAAGGCAATGAAAGAATATGGGGAAGATCCTAAGATCGAAACAAACAAGTTTGCCGCAATATGTACACACTTAGAAGTCTGCTTCATGTATTCGGATTTCCATTTCATTGATGAACGAGGCGAATCGATAATCGTAGAATCTGGCGACCCGAATGCATTATTGAAGCACCGATTTGAGA<br>TAATTGAAGGGAGAGACCGAACAATGGCCTGGACAGTGGTGAATAGTATATGCAACACTACAGGAGTCGAAAAGCCCAAGTTCCTTCCTGATTTGTATGACTACAGAGAAAACCGATTCATTGAAATTGGAGTAACGCGAAGGGAAGTTCACATATACTATCTTGAAAAAGCCAACAAGATAAAGTCAGAGAAAACACACATTCACATATTCTCATTCACTGGGGAGGAA<br>ATGGCCACCAAGGCAGATTACACTCTTGATGAAGAGAGCAGAGCAAGAATAAAAACCAGGCTATTCACTATAAGACAAGAGATGGCCAATAGGGGTCTATGGGATTCCTTTCGTCAGTCCGAGAGAGGCGAAGAGACAATTGAAGAAAGATTTGAAATCACAGGAACCATGCGCAGGCTTGCTGACCAAAGTCTCCCACCGAACTTCGCCAGCCTTGAAAACTTTAGAG<br>CCTATGTGGATGGATTTGAACCGAACGGCTGCATTGAGGGCAAGCTTTCTCAAATGTCAAAAGAAGTGAATGCCAGAATTGAGCCATTTCTGAAGACAACACCACGCCCTCTCAGATTACCTGATGGACCTCCCTGTTCCCAGCGGTCGAAGTTCTTGCTAATGGATGCCCTTAAATTGAGCATTGAAGACCCGAGCCATGAGGGGGAGGGTATACCGCTGTACGATGCAA<br>CCAAATGCATGAAGACATTTTTTGGCTGGAAGGAGCCCAACATCGTGAAACCACATGAAAAGGGCATAAACCCTAATTACCTCCTGACTTGGAAGCAGGTGCTATCAGAACTCCAAGATATTGAAAACGAGGAGAAAATCCCAAAAACAAAGAACATGAAGAAGACGAGCCAACTGAAGTGGGCACTTGGTGAAAACATGGCACCAGAGAAAGTGGACTTTGAGGACT<br>GTAAAGATGTTAGCGATCTAAAACAGTACGACAGTGACGAACCAGAATCTAGATCACTAGCAAGTTGGATCCAGAGTGAATTTAACAAGGCTTGCGAACTGACAGATTCGAGTTGGATTGAACTTGATGAAATAGGGGAAGACGTTGCTCCAATTGAACACATTGCGAGTATGAGGAGGAACTATTTCACAGCGGAAGTATCCCATTGCAGGGCTACAGAATACATAATG<br>AAAGGAGTATACATAAACACAGCTTTATTGAATGCATCCTGTGCAGCCATGGATGACTTCCAACTGATTCCAATGATAAGCAAATGCAGAACCAAAGAAGGGAGACGGAAGACAAATCTGTATGGCTTCATTATAAAAGGAAGATCCCATTTGAGGAATGACACCGATGTGGTAAACTTTGTGAGCATGGAATTCTCTCTCACTGACCCAAGGCTGGAGCCACACAAATGG<br>GAAAAGTACTGTGTGCTTGAGGTAGGAGACATGCTCCTACGGACTGCAATAGGCCAAGTGTCAAGGCCCATGTTCCTGTATGTGAGAACCAATGGGACTTCTAAGATCAAAATGAAATGGGGTATGGAGATGAGGCGATGCCTTCTTCAATCCCTTCAACAAATTGAGAGCATGATTGAAGCCGAGTCTTCTGTCAAAGAAAAAGACATGACCAAGGAATTCTTTGAAAAC<br>AGATCAGAAACATGGCCAATTGGAGAGTCACCCAAAGGAGTGGAGGAAGGCTCCATTGGGAAGGTGTGCAGAACATTGCTAGCAAAGTCTGTGTTCAACAGCCTATATGCATCTCCACAACTCGAGGGGTTTTCAGCTGAATCAAGAAAGTTGCTTCTCATTGTTCAGGCACTTAGGGACAACCTGGAACCTGGAACCTTCGATCTTGGGGGGCTATATGAAGCAATTGA<br>GGAGTGCCTGATTAACGATCCCTGGGTTTTGCTTAATGCGTCTTGGTTCAACTCCTTCCTCACACATGCACTGAAATA-
EPI_ISL_18244810	>A/buzzard/Poland/MB129-21_22VIR5675-7/2021_2021-02-19<br>ATGGAAGACTTTGTGCGACAATGCTTCAATCCAATGATTGTCGAGCTTGCGGAAAAGGCAATGAAAGAATATGGGGAAGATCCTAAGATCGAAACAAACAAGTTTGCCGCAATATGTACACACTTAGAAGTCTGCTTCATGTATTCGGATTTCCATTTCATTGATGAACGAGGCGAATCGATAATCGTAGAATCTGGCGACCCGAATGCATTATTGAAGCACCGATTTGAGA<br>TAATTGAAGGGAGAGACCGAACAATGGCCTGGACAGTGGTGAATAGTATATGCAACACTACAGGAGTCGAAAAGCCCAAGTTCCTTCCTGATTTGTATGACTACAGAGAAAACCGATTCATTGAAATTGGAGTAACGCGAAGGGAAGTTCACATATACTATCTTGAAAAAGCCAACAAGATAAAGTCAGAGAAAACACACATTCACATATTCTCATTCACTGGGGAGGAA<br>ATGGCCACCAAGGCAGATTACACTCTTGATGAAGAGAGCAGAGCAAGAATAAAAACCAGGCTATTCACTATAAGACAAGAGATGGCCAATAGGGGTCTATGGGATTCCTTTCGTCAGTCCGAGAGAGGCGAAGAGACAATTGAAGAAAGATTTGAAATCACAGGAACCATGCGCAGGCTTGCTGACCAAAGTCTCCCACCGAACTTCGCCAGCCTTGAAAACTTTAGAG<br>CCTATGTGGATGGATTTGAACCGAACGGCTGCATTGAGGGCAAGCTTTCTCAAATGTCAAAAGAAGTGAATGCCAGAATTGAGCCATTTCTGAAGACAACACCACGCCCTCTCAGATTACCTGATGGACCTCCCTGTTCCCAGCGGTCGAAGTTCTTGCTAATGGATGCCCTTAAATTGAGCATTGAAGACCCGAGCCATGAGGGGGAGGGTATACCGCTGTACGATGCAA<br>CCAAATGCATGAAGACATTTTTTGGCTGGAAGGAGCCCAACATCGTGAAACCACATGAAAAGGGCATAAACCCTAATTACCTCCTGACTTGGAAGCAGGTGCTATCAGAACTCCAAGATATTGAAAACGAGGAGAAAATCCCAAAAACAAAGAACATGAAGAAGACGAGCCAACTGAAGTGGGCACTTGGTGAAAACATGGCACCAGAGAAAGTGGACTTTGAGGACT<br>GTAAAGATGTTAGCGATCTAAAACAGTACGACAGTGACGAACCAGAATCTAGATCACTAGCAAGTTGGATCCAGAGTGAATTTAACAAGGCTTGCGAACTGACAGATTCGAGTTGGATTGAACTTGATGAAATAGGGGAAGACGTTGCTCCAATTGAACACATTGCGAGTATGAGGAGGAACTATTTCACAGCGGAAGTATCCCATTGCAGGGCTACAGAATACATAATG<br>AAAGGAGTATACATAAACACAGCTTTATTGAATGCATCCTGTGCAGCCATGGATGACTTCCAACTGATTCCAATGATAAGCAAATGCAGAACCAAAGAAGGGAGACGGAAGACAAATCTGTATGGCTTCATTATAAAAGGAAGATCCCATTTGAGGAATGACACCGATGTGGTAAACTTTGTGAGCATGGAATTCTCTCTCACTGACCCAAGGCTGGAGCCACACAAATGG<br>GAAAAGTACTGTGTGCTTGAGGTAGGAGACATGCTCCTACGGACTGCAATAGGCCAAGTGTCAAGGCCCATGTTCCTGTATGTGAGAACCAATGGGACTTCTAAGATCAAAATGAAATGGGGTATGGAGATGAGGCGATGCCTTCTTCAATCCCTTCAACAAATTGAGAGCATGATTGAAGCCGAGTCTTCTGTCAAAGAAAAAGACATGACCAAGGAATTCTTTGAAAAC<br>AGATCAGAAACATGGCCAATTGGAGAGTCACCCAAAGGAGTGGAGGAAGGCTCCATTGGGAAGGTGTGCAGAACATTGCTAGCAAAGTCTGTGTTCAACAGCCTATATGCATCTCCACAACTCGAGGGGTTTTCAGCTGAATCAAGAAAGTTGCTTCTCATTGTTCAGGCACTTAGGGACAACCTGGAACCTGGAACCTTCGATCTTGGGGGGCTATATGAAGCAATTGA<br>GGAGTGCCTGATTAACGATCCCTGGGTTTTGCTTAATGCGTCTTGGTTCAACTCCTTCCTCACACATGCACTGAAATA-
EPI_ISL_984690	>A/peregrine_falcon/Denmark/14596-1/2020_H5N8_2020-11-06<br>ATGGAAGACTTTGTGCGACAATGCTTCAATCCAATGATTGTCGAGCTTGCGGAAAAGGCAATGAAAGAATATGGGGAAGATCCTAAGATCGAAACAAACAAGTTTGCCGCAATATGTACACACTTAGAAGTCTGCTTCATGTATTCGGATTTCCATTTCATTGATGAACGAGGCGAATCAATAATCGTAGAATCTGGCGACCCGAATGCATTATTGAAGCACCGATTTGAGA<br>TAATTGAAGGGAGAGACCGAACAATGGCCTGGACAGTGGTGAATAGTATATGCAACACTACAGGAGTCGAAAAGCCCAAGTTCCTTCCTGATTTGTATGACTACAGAGAAAACCGATTCATTGAAATTGGAGTAACGCGAAGGGAAGTTCACATATACTATCTTGAAAAAGCCAACAAGATAAAATCAGAGAAAACACACATTCACATATTCTCATTCACTGGGGAGGAA<br>ATGGCCACCAAGGCAGATTACACTCTTGATGAAGAGAGCAGAGCAAGAATAAAAACCAGGCTATTCACTATAAGACAAGAGATGGCCAATAGGGGTCTATGGGATTCCTTTCGTCAGTCCGAGAGAGGCGAAGAGACAATTGAAGAAAGATTTGAAATCACAGGAACCATGCGCAGGCTTGCTGACCAAAGTCTCCCACCGAACTTCGCCAGCCTTGAAAACTTTAGAG<br>CCTATGTGGATGGATTTGAACCGAACGGCTGCATTGAGGGCAAGCTTTCTCAAATGTCAAAAGAAGTGAATGCCAGAATTGAGCCATTTCTGAAGACAACACCACGCCCTCTCAGATTACCTGATGGACCTCCCTGTTCCCAGCGGTCGAAATTCTTGCTAATGGATGCCCTTAAATTGAGCATTGAAGACCCGAGCCATGAGGGGGAGGGTATACCGCTGTACGATGCAA<br>CCAAATGCATGAAGACATTTTTTGGCTGGAAGGAGCCCAACATCGTGAAACCACATGAAAAGGGCATAAACCCTAATTACCTCCTGGCTTGGAAGCAGGTGCTATCAGAACTCCAAGATATTGAAAACGAGGAGAAAATCCCAAAAACAAAGAACATGAAGAAGACGAGCCAACTGAAGTGGGCACTTGGTGAAAACATGGCACCAGAGAAAGTGGACTTTGAGGACT<br>GTAAAGATGTTAGCGATCTAAAACAGTACGACAGTGACGAACCAGAATCTAGATCACTAGCAAGTTGGATCCAGAGTGAATTTAACAAGGCTTGCGAACTGACAGATTCGAGTTGGATTGAACTTGATGAAATAGGGGAAGACGTTGCTCCAATTGAACACATTGCGAGTATGAGGAGGAACTATTTCACAGCGGAAGTATCCCATTGCAGGGCTACAGAATACATAATG<br>AAAGGAGTATACATAAACACAGCTTTATTGAATGCATCCTGTGCAGCCATGGATGACTTCCAACTGATTCCAATGATAAGCAAATGCAGAACCAAAGAAGGGAGACGGAAGACAAATCTGTATGGCTTCATTATAAAAGGAAGATCCCATTTGAGGAATGACACCGATGTGGTAAACTTTGTGAGCATGGAATTCTCTCTCACTGACCCAAGGCTGGAGCCACACAAATGG<br>GAAAAGTACTGTGTGCTTGAGGTAGGAGACATGCTCCTACGGACTGCAATAGGCCAAGTGTCAAGGCCCATGTTCCTGTATGTGAGAACCAATGGGACTTCTAAGATCAAAATGAAATGGGGTATGGAGATGAGGCGATGCCTTCTTCAATCCCTTCAACAAATTGAGAGCATGATTGAAGCCGAGTCTTCTGTCAAAGAAAAAGACATGACCAAGGAATTCTTTGAAAAC<br>AGATCAGAAACATGGCCAATTGGAGAGTCACCCAAAGGAGTGGAGGAAGGCTCCATTGGGAAGGTGTGCAGAACATTGCTAGCAAAGTCTGTGTTCAACAGCCTATATGCATCTCCACAACTCGAGGGGTTTTCAGCTGAATCAAGAAAGTTGCTTCTCATTGTTCAGGCACTTAGGGACAACCTGGAACCTGGAACCTTCGATCTTGGGGGGCTATATGAAGCAATTGA<br>GGAGTGCCTGATTAACGATCCCTGGGTTTTGCTTAATGCGTCTTGGTTCAACTCCTTCCTCACACATGCACTGAAATA-
EPI_ISL_644138	>A/wild_duck/Omsk/01111/2020_H5N8_2020-08-17<br>ATGGAAGACTTTGTGCGACAATGCTTCAATCCAATGATTGTCGAGCTTGCGGAAAAGGCAATGAAAGAATATGGGGAAGATCCTAAGATCGAAACAAACAAGTTTGCCGCAATATGTACACACTTAGAAGTCTGCTTCATGTATTCGGATTTCCATTTCATTGATGAACGAGGCGAATCAATAATCGTAGAATCTGGCGACCCGAATGCATTATTGAAGCACCGATTTGAGA<br>TAATTGAAGGGAGAGACCGAACAATGGCCTGGACGGTGGTGAATAGTATATGCAACACTACAGGAGTCGAAAAGCCCAAGTTCCTTCCTGATTTGTATGACTACAGAGAAAACCGATTCATTGAAATTGGAGTAACGCGAAGGGAAGTTCACATATACTATCTTGAAAAAGCCAACAAGATAAAATCAGAGAAAACACACATTCACATATTCTCATTCACTGGGGAGGAA<br>ATGGCCACCAAGGCAGATTACACTCTTGATGAAGAGAGCAGAGCAAGAATAAAAACCAGGCTATTCACTATAAGACAAGAGATGGCCAATAGGGGTCTATGGGATTCCTTTCGTCAGTCCGAGAGAGGCGAAGAGACAATTGAAGAAAGATTTGAAATCACAGGAACCATGCGCAGGCTTGCTGACCAAAGTCTCCCACCGAACTTCGCCAGCCTTGAAAACTTTAGAG<br>CCTATGTGGATGGATTTGAACCGAACGGCTGCATTGAGGGCAAGCTTTCTCAAATGTCAAAAGAAGTGAATGCCAGAATTGAGCCATTTCTGAAGACAACACCACGCCCTCTCAGATTACCTGATGGACCTCCCTGTTCCCAGCGGTCGAAATTCTTGCTAATGGATGCCCTTAAATTGAGCATTGAAGACCCGAGCCATGAGGGGGAGGGTATACCGCTGTACGATGCAA<br>CCAAATGCATGAAGACATTTTTTGGCTGGAAGGAGCCCAACATCGTGAAACCACATGAAAAGGGCATAAACCCTAATTACCTCCTGGCTTGGAAGCAGGTGCTATCAGAACTCCAAGATATTGAAAACGAGGAGAAAATCCCAAAAACAAAGAACATGAAGAAGACGAGCCAACTGAAGTGGGCACTTGGTGAAAACATGGCACCAGAGAAAGTGGACTTTGAGGACT<br>GTAAAGATGTTAGCGATCTAAAACAGTACGACAGTGACGAACCAGAATCTAGATCACTAGCAAGTTGGATCCAGAGTGAATTTAACAAGGCTTGCGAACTGACAGATTCGAGTTGGATTGAACTTGATGAAATAGGGGAAGACGTTGCTCCAATTGAACACATTGCGAGTATGAGGAGGAACTATTTCACAGCGGAAGTATCCCATTGCAGGGCTACAGAATACATAATG<br>AAAGGAGTATACATAAACACAGCTTTATTGAATGCATCCTGTGCAGCCATGGATGACTTCCAACTGATTCCAATGATAAGCAAATGCAGAACCAAAGAAGGGAGACGGAAGACAAATCTGTATGGCTTCATTATAAAAGGAAGATCCCATTTGAGGAATGACACCGATGTGGTAAACTTTGTGAGCATGGAATTCTCTCTCACTGACCCAAGGCTGGAGCCACACAAATGG<br>GAAAAGTACTGTGTGCTTGAGGTAGGAGACATGCTCCTACGGACTGCAATAGGCCAAGTGTCAAGGCCCATGTTCCTGTATGTGAGAACCAATGGGACTTCTAAGATCAAAATGAAATGGGGTATGGAGATGAGGCGATGCCTTCTTCAATCCCTTCAACAAATTGAGAGCATGATTGAAGCCGAGTCTTCTGTCAAAGAAAAAGACATGACCAAGGAATTCTTTGAAAAC<br>AGATCAGAAACATGGCCAATTGGAGAGTCACCCAAAGGAGTGGAGGAAGGCTCCATTGGGAAGGTGTGCAGAACATTGCTAGCAAAGTCTGTGTTCAACAGCCTATATGCATCTCCACAACTCGAGGGGTTTTCAGCTGAATCAAGAAAGTTGCTTCTCATTGTTCAGGCACTTAGGGACAACCTGGAACCTGGAACCTTCGATCTTGGGGGGCTATATGAAGCAATTGA<br>GGAGTGCCTGATTAACGATCCCTGGGTTTTGCTTAATGCGTCTTGGTTCAACTCCTTCCTCACACATGCACTGAAATA-
EPI_ISL_668456	>A/Peregrine_falcon/Sweden/SVA201117SZ0467/20KN003345/2020_H5N8_2020-11-10<br>ATGGAAGACTTTGTGCGACAATGCTTCAATCCAATGATTGTCGAGCTTGCGGAAAAGGCAATGAAAGAATATGGGGAAGATCCTAAGATCGAAACAAACAAGTTTGCCGCAATATGTACACACTTAGAAGTCTGCTTCATGTATTCGGATTTCCATTTCATTGATGAACGAGGCGAATCAATAATCGTAGAATCTGGCGACCCGAATGCATTATTGAAGCACCGATTTGAGA<br>TAATTGAAGGGAGAGACCGAACAATGGCCTGGACAGTGGTGAATAGTATATGCAACACTACAGGAGTCGAAAAGCCCAAGTTCCTTCCTGATTTGTATGACTACAGAGAAAACCGATTCATTGAAATTGGAGTAACGCGAAGGGAAGTTCACATATACTATCTTGAAAAAGCCAACAAGATAAAATCAGAGAAAACACACATTCACATATTCTCATTCACTGGGGAGGAA<br>ATGGCCACCAAGGCAGATTACACTCTTGATGAAGAGAGCAGAGCAAGAATAAAAACCAGGCTATTCACTATAAGACAAGAGATGGCCAATAGGGGTCTATGGGATTCCTTTCGTCAGTCCGAGAGAGGCGAAGAGACAATTGAAGAAAGATTTGAAATCACAGGAACCATGCGCAGGCTTGCTGACCAAAGTCTCCCACCGAACTTCGCCAGCCTTGAAAACTTTAGAG<br>CCTATGTGGATGGATTTGAACCGAACGGCTGCATTGAGGGCAAGCTTTCTCAAATGTCAAAAGAAGTGAATGCCAGAATTGAGCCATTTCTGAAGACAACACCACGCCCTCTCAGATTACCTGATGGACCCCCCTGTTCCCAGCGGTCGAAGTTCTTGCTAATGGATGCCCTTAAATTGAGCATTGAAGACCCGAGCCATGAGGGGGAGGGTATACCGCTGTACGATGCAA<br>CCAAATGCATGAAGACATTTTTTGGCTGGAAGGAGCCCAACATCGTGAAACCACATGAAAAGGGCATAAACCCTAATTACCTCCTGGCTTGGAAGCAGGTGCTATCAGAACTCCAAGATATTGAAAACGAGGAGAAAATCCCAAAAACAAAGAACATGAAGAAGACGAGCCAACTGAAGTGGGCACTTGGTGAAAACATGGCACCAGAGAAAGTGGATTTTGAGGACT<br>GTAAAGATGTTAGCGATCTAAAACAGTACGACAGTGACGAACCAGAATCTAGATCACTAGCAAGTTGGATCCAGAGTGAATTTAACAAGGCTTGCGAACTGACAGATTCGAGTTGGATTGAACTTGATGAAATAGGGGAAGACGTTGCTCCAATTGAACACATTGCGAGTATGAGGAGGAACTATTTCACAGCGGAAGTATCCCATTGCAGGGCTACAGAATACATAATG<br>AAAGGAGTATACATAAACACAGCTTTATTGAATGCATCCTGTGCAGCCATGGATGACTTCCAACTGATTCCAATGATAAGCAAATGCAGAACCAAAGAAGGGAGACGGAAGACAAATCTGTATGGCTTCATTATAAAAGGAAGATCCCATTTGAGGAATGACACCGATGTGGTAAACTTTGTGAGCATGGAATTCTCTCTCACTGACCCAAGGCTGGAGCCACACAAATGG<br>GAAAAGTACTGTGTGCTTGAGGTAGGAGACATGCTCCTACGGACTGCAATAGGCCAAGTGTCAAGGCCCATGTTCCTGTATGTGAGAACCAATGGGACTTCTAAGATCAAAATGAAATGGGGTATGGAGATGAGGCGATGCCTTCTTCAATCCCTTCAACAAATTGAGAGCATGATTGAAGCCGAGTCTTCTGTCAAAGAAAAAGACATGACCAAGGAATTCTTTGAAAAC<br>AGATCAGAAACATGGCCAATTGGAGAGTCACCCAAAGGAGTGGAGGAAGGCTCCATTGGGAAGGTGTGCAGAACATTGCTAGCAAAGTCTGTGTTCAACAGCCTATATGCATCTCCACAACTCGAGGGGTTTTCAGCTGAATCAAGAAAGTTGCTTCTCATTGTTCAGGCACTTAGGGACAACCTGGAACCTGGAACCTTCGATCTTGGGGGGCTATATGAAGCAATTGA<br>GGAGTGCCTGATTAACGATCCCTGGGTTTTGCTTAATGCGTCTTGGTTCAACTCCTTCCTCACACATGCACTGAAATA-
EPI_ISL_2932614	>A/swan/Kazakhstan/9-20-B-Talg-39/2020_H5N8_2020-09-21<br>ATGGAAGACTTTGTGCGACAATGCTTCAATCCAATGATTGTCGAGCTTGCGGAAAAGGCAATGAAAGAATATGGGGAAGATCCTAAGATCGAAACAAACAAGTTTGCCGCAATATGTACACACTTAGAAGTCTGCTTCATGTATTCGGATTTCCATTTCATTGATGAACGAGGCGAATCAATAATCGTAGAATCTGGCGACCCGAATGCATTATTGAAGCACCGATTTGAGA<br>TAATTGAAGGGAGAGACCGAACAATGGCCTGGACAGTGGTGAATAGTATATGCAACACTACAGGAGTCGAAAAGCCCAAGTTCCTTCCTGATTTGTATGACTACAGAGAAAACCGATTCATTGAAATTGGAGTAACGCGAAGGGAAGTTCACATATACTATCTTGAAAAAGCCAACAAGATAAAATCAGAGAAAACACACATTCACATATTCTCATTCACTGGGGAGGAA<br>ATGGCCACCAAGGCAGATTACACTCTTGATGAAGAGAGCAGAGCAAGAATAAAAACCAGGCTATTCACTATAAGACAAGAGATGGCCAATAGGGGTCTATGGGATTCCTTTCGTCAGTCCGAGAGAGGCGAAGAGACAATTGAAGAAAGATTTGAAATCACAGGAACCATGCGCAGGCTTGCTGACCAAAGTCTCCCACCGAACTTCGCCAGCCTTGAAAACTTTAGAG<br>CCTATGTGGATGGATTTGAACCGAACGGCTGCATTGAGGGCAAGCTTTCTCAAATGTCAAAAGAAGTGAATGCCAGAATTGAGCCATTTCTGAAGACAACACCACGCCCTCTCAGATTGCCTGATGGACCTCCCTGTTCCCAGCGGTCGAAGTTCTTGCTAATGGATGCCCTTAAATTGAGCATTGAAGACCCGAGCCATGAGGGGGAGGGTATACCGCTGTACGATGCAA<br>CCAAATGCATGAAGACATTTTTTGGCTGGAAGGAGCCCAACATCGTGAAACCACATGAAAAGGGCATAAACCCTAATTACCTCCTGGCTTGGAAGCAGGTGCTATCAGAACTCCAAGATATTGAAAACGAGGAGAAAATCCCAAAAACAAAGAACATGAAGAAGACGAGCCAACTGAAGTGGGCACTTGGTGAAAACATGGCACCAGAGAAAGTGGACTTTGAGGACT<br>GTAAAGATGTTAGCGATCTAAAACAGTACGACAGTGACGAACCAGAATCTAGATCACTAGCAAGTTGGATCCAGAGTGAATTTAACAAGGCTTGCGAACTGACAGATTCGAGTTGGATTGAACTTGATGAAATAGGGGAAGACGTTGCTCCAATTGAACACATTGCGAGTATGAGGAGGAACTATTTCACAGCGGAAGTATCCCATTGCAGGGCTACAGAATACATAATG<br>AAAGGAGTATACATAAACACAGCTTTATTGAATGCATCCTGTGCAGCCATGGATGACTTCCAACTGATTCCAATGATAAGCAAATGCAGAACCAAAGAAGGGAGACGGAAGACAAATCTGTATGGCTTCATTATAAAAGGAAGATCCCATTTGAGGAATGACACCGATGTGGTAAACTTTGTGAGCATGGAATTCTCTCTCACTGACCCAAGGCTGGAGCCACACAAATGG<br>GAAAAGTACTGTGTGCTTGAGGTAGGAGACATGCTCCTACGGACTGCAATAGGCCAAGTGTCAAGGCCCATGTTCCTGTATGTGAGAACCAATGGGACTTCTAAGATCAAAATGAAATGGGGTATGGAGATGAGGCGATGCCTTCTTCAATCCCTTCAACAAATTGAGAGCATGATTGAAGCCGAGTCTTCTGTCAAAGAAAAAGACATGACCAAGGAATTCTTTGAAAAC<br>AGATCAGAAACATGGCCAATTGGAGAGTCACCCAAAGGAGTGGAGGAAGGCTCCATTGGGAAGGTGTGCAGAACATTGCTAGCAAAGTCTGTGTTCAATAGCCTATATGCATCTCCACAACTCGAGGGGTTTTCAGCTGAATCAAGAAAGTTGCTTCTCATTGTTCAGGCACTTAGGGACAACCTGGAACCTGGAACCTTCGATCTTGGGGGGCTATATGAAGCAATTGA<br>GGAGTGCCTGATTAACGATCCCTGGGTTTTGCTTAATGCGTCTTGGTTCAACTCCTTCCTCACACATGCACTGAAATA-
EPI_ISL_603132	>A/chicken/Netherlands/20016597-026030/2020_H5N8_2020-10-28<br>ATGGAAGACTTTGTGCGACAATGCTTCAATCCAATGATTGTCGAGCTTGCGGAAAAGGCAATGAAAGAATATGGGGAAGATCCTAAGATCGAAACAAACAAGTTTGCCGCAATATGTACACACTTAGAAGTCTGCTTCATGTATTCGGATTTCCATTTCATTGATGAACGAGGCGAATCAATAATCGTGGAATCTGGCGACCCGAATGCATTATTGAAGCACCGATTTGAGA<br>TAATTGAAGGGAGAGACCGAACAATGGCCTGGACAGTGGTGAATAGTATATGCAACACTACAGGAGTCGAAAAGCCCAAGTTCCTTCCTGATTTGTATGACTACAGAGAAAACCGATTCATTGAAATTGGAGTAACGCGAAGGGAAGTTCACATATACTATCTTGAAAAAGCCAACAAGATAAAATCAGAGAAAACACACATTCACATATTCTCATTCACTGGGGAGGAA<br>ATGGCCACCAAGGCAGATTACACTCTTGATGAAGAGAGCAGAGCAAGAATAAAAACCAGGCTATTCACTATAAGACAAGAGATGGCCAATAGGGGTCTATGGGATTCCTTTCGTCAGTCCGAGAGAGGCGAAGAGACAATTGAAGAAAGATTTGAAATCACAGGAACCATGCGCAGGCTTGCTGACCAAAGTCTCCCACCGAACTTCGCCAGCCTTGAAAACTTTAGAG<br>CCTATGTGGATGGATTTGAACCGAACGGCTGCATTGAGGGCAAGCTTTCTCAAATGTCAAAAGAAGTGAATGCCAGAATTGAGCCATTTCTGAAGACAACACCACGCCCTCTCAAATTACCTGATGGACCTCCCTGTTCCCAGCGGTCGAAGTTCTTGCTAATGGATGCCCTTAAATTGAGCATTGAAGACCCGAGCCATGAGGGGGAGGGTATACCGCTGTACGATGCAA<br>CCAAATGCATGAAGACATTTTTTGGCTGGAAGGAGCCCAACATCGTGAAACCACATGAAAAGGGCATAAACCCTAATTACCTCCTGGCTTGGAAGCAGGTGCTATCAGAACTCCAAGATATTGAAAACGAGGAGAAAATCCCAAAAACAAAGAACATGAAGAAGACGAGCCAACTGAAGTGGGCACTTGGTGAAAACATGGCACCAGAGAAAGTGGACTTTGAGGACT<br>GTAAAGATGTTAGCGATCTAAAACAGTACGACAGTGACGAACCAGAATCTAGATCACTAGCAAGTTGGATCCAGAGTGAATTTAACAAGGCTTGCGAACTGACAGATTCGAGTTGGATTGAACTTGATGAAATAGGGGAAGACGTTGCTCCAATTGAACACATTGCGAGTATGAGGAGGAACTATTTCACAGCGGAAGTATCCCATTGCAGGGCTACAGAATACATAATG<br>AAAGGAGTATACATAAACACAGCTTTATTGAATGCATCCTGTGCAGCCATGGATGACTTCCAACTGATTCCAATGATAAGCAAATGCAGAACCAAAGAAGGGAGACGGAAGACAAATCTGTATGGCTTCATTATAAAAGGAAGATCCCATTTGAGGAATGACACCGATGTGGTAAACTTTGTGAGCATGGAATTCTCTCTCACTGACCCAAGGCTGGAGCCACACAAATGG<br>GAAAAGTACTGTGTGCTTGAGGTAGGAGACATGCTCCTACGGACTGCAATAGGCCAAGTGTCAAGGCCCATGTTCCTGTATGTGAGAACCAATGGGACTTCTAAGATCAAAATGAAATGGGGTATGGAGATGAGGCGATGCCTTCTTCAATCCCTTCAACAAATTGAGAGCATGATTGAAGCCGAGTCTTCTGTCAAAGAAAAAGACATGACCAAGGAATTCTTTGAAAAC<br>AGATCAGAAACATGGCCAATTGGAGAGTCACCCAAAGGAGTGGAGGAAGGCTCCATTGGGAAGGTGTGCAGAACATTGCTAGCAAAGTCTGTGTTCAACAGCCTATATGCATCTCCACAACTCGAGGGGTTTTCAGCTGAATCAAGAAAGTTGCTTCTCATTGTTCAGGCACTTAGGGACAACCTGGAACCTGGAACCTTCGATCTTGGGGGGCTATATGAAGCAATTGA<br>GGAGTGCCTGATTAACGATCCCTGGGTTTTGCTTAATGCGTCTTGGTTCAACTCCTTCCTCACACATGCACTGAAATA-
EPI_ISL_1123353	>A/duck/England/046311/2020_H5N8_2020-12-27<br>ATGGAAGACTTTGTGCGACAATGCTTCAATCCAATGATTGTCGAGCTTGCGGAAAAGGCAATGAAAGAATATGGGGAAGATCCTAAGATCGAAACAAACAAGTTTGCCGCAATATGTACACACTTAGAAGTCTGCTTCATGTATTCGGATTTCCATTTCATTGATGAACGAGGCGAATCAATAATCGTGGAATCTGGCGACCCGAATGCATTATTGAAGCACCGATTTGAGA<br>TAATTGAAGGGAGAGACCGAACAATGGCCTGGACAGTGGTGAATAGTATATGCAACACTACAGGAGTCGAAAAGCCCAAGTTCCTTCCTGATTTGTATGACTACAGAGAAAACCGATTCATTGAAATTGGAGTAACGCGAAGGGAAGTTCACATATACTATCTTGAAAAAGCCAACAAGATAAAATCAGAGAAAACACACATTCACATATTCTCATTCACTGGGGAGGAA<br>ATGGCCACCAAGGCAGATTACACTCTTGATGAAGAGAGCAGAGCAAGAATAAAAACCAGGCTATTCACTATAAGACAAGAGATGGCCAATAGGGGTCTATGGGATTCCTTTCGTCAGTCCGAGAGAGGCGAAGAGACAATTGAAGAAAGATTTGAAATCACAGGAACCATGCGCAGGCTTGCTGACCAAAGTCTCCCACCGAACTTCGCCAGCCTTGAAAACTTTAGAG<br>CCTATGTGGATGGATTTGAACCGAACGGCTGCATTGAGGGCAAGCTTTCTCAAATGTCAAAAGAAGTGAATGCCAGAATTGAGCCATTTCTGAAGACAACACCACGCCCTCTCAAATTACCTGATGGACCTCCCTGTTCCCAGCGGTCGAAGTTCTTGCTAATGGATGCCCTTAAATTGAGCATTGAAGACCCGAGCCATGAGGGGGAGGGTATACCGCTGTACGATGCAA<br>CCAAATGCATGAAGACATTTTTTGGCTGGAAGGAGCCCAACATCGTGAAACCACATGAAAAGGGCATAAACCCTAATTACCTCCTGGCTTGGAAGCAGGTGCTATCAGAACTCCAAGATATTGAAAACGAGGAGAAAATCCCAAAAACAAAGAACATGAAGAAGACGAGCCAACTGAAGTGGGCACTTGGTGAAAACATGGCACCAGAGAAAGTGGACTTTGAGGACT<br>GTAAAGATGTTAGCGATCTAAAACAGTACGACAGTGACGAACCAGAATCTAGATCACTAGCAAGTTGGATCCAGAGTGAATTTAACAAGGCTTGCGAACTGACAGATTCGAGTTGGATTGAACTTGATGAAATAGGGGAAGACGTTGCTCCAATTGAACACATTGCGAGTATGAGGAGGAACTATTTCACAGCGGAAGTATCCCATTGCAGGGCTACAGAATACATAATG<br>AAAGGAGTATACATAAACACAGCTTTATTGAATGCATCCTGTGCAGCCATGGATGACTTCCAACTGATTCCAATGATAAGCAAATGCAGAACCAAAGAAGGGAGACGGAAGACAAATCTGTATGGCTTCATTATAAAAGGAAGATCCCATTTGAGGAATGACACCGATGTGGTAAACTTTGTGAGCATGGAATTCTCTCTCACTGACCCAAGGCTGGAGCCACACAAATGG<br>GAAAAGTACTGTGTGCTTGAGGTAGGAGACATGCTCCTACGGACTGCAATAGGCCAAGTGTCAAGGCCCATGTTCCTGTATGTGAGAACCAATGGGACTTCTAAGATCAAAATGAAATGGGGTATGGAGATGAGGCGATGCCTTCTTCAATCCCTTCAACAAATTGAGAGCATGATTGAAGCCGAGTCTTCTGTCAAAGAAAAAGACATGACCAAGGAATTCTTTGAAAAC<br>AGATCAGAAACATGGCCAATTGGAGAGTCACCCAAAGGAGTGGAGGAAGGCTCCATTGGGAAGGTGTGCAGAACATTGCTAGCAAAGTCTGTGTTCAACAGCCTATATGCATCTCCACAACTCGAGGGGTTTTCAGCTGAATCAAGAAAGTTGCTTCTCATTGTTCAGGCACTTAGGGACAACCTGGAACCTGGAACCTTCGATCTTGGGGGGCTATATGAAGCAATTGA<br>GGAGTGCCTGATTAACGATCCCTGGGTTTTGCTTAATGCGTCTTGGTTCAACTCCTTCCTCACACATGCACTGAAATA-
EPI_ISL_710506	>A/Canada_goose/England/032697/2020_H5N8_2020-11-03<br>ATGGAAGACTTTGTGCGACAATGCTTCAATCCAATGATTGTCGAGCTTGCGGAAAAGGCAATGAAAGAATATGGGGAAGATCCTAAGATCGAAACAAACAAGTTTGCCGCAATATGTACACACTTAGAAGTCTGCTTCATGTATTCGGATTTCCATTTCATTGATGAACGAGGCGAATCAATAATCGTGGAATCTGGCGACCCGAATGCATTATTGAAGCACCGATTTGAGA<br>TAATTGAAGGGAGAGACCGAACAATGGCCTGGACAGTGGTGAATAGTATATGCAACACTACAGGAGTCGAAAAGCCCAAGTTCCTTCCTGATTTGTATGACTACAGAGAAAACCGATTCATTGAAATTGGAGTAACGCGAAGGGAAGTTCACATATACTATCTTGAAAAAGCCAACAAGATAAAATCAGAGAAAACACACATTCACATATTCTCATTCACTGGGGAGGAA<br>ATGGCCACCAAGGCAGATTACACTCTTGATGAAGAGAGCAGAGCAAGAATAAAAACCAGGCTATTCACTATAAGACAAGAGATGGCCAATAGGGGTCTATGGGATTCCTTTCGTCAGTCCGAGAGAGGCGAAGAGACAATTGAAGAAAGATTTGAAATCACAGGAACCATGCGCAGGCTTGCTGACCAAAGTCTCCCACCGAACTTCGCCAGCCTTGAAAACTTTAGAG<br>CCTATGTGGATGGATTTGAACCGAACGGCTGCATTGAGGGCAAGCTTTCTCAAATGTCAAAAGAAGTGAATGCCAGAATTGAGCCATTTCTGAAGACAACACCACGCCCTCTCAAATTACCTGATGGACCTCCCTGTTCCCAGCGGTCGAAGTTCTTGCTAATGGATGCCCTTAAATTGAGCATTGAAGACCCGAGCCATGAGGGGGAGGGTATACCGCTGTACGATGCAA<br>CCAAATGCATGAAGACATTTTTTGGCTGGAAGGAGCCCAACATCGTGAAACCACATGAAAAGGGCATAAACCCTAATTACCTCCTGGCTTGGAAGCAGGTGCTATCAGAACTCCAAGATATTGAAAACGAGGAGAAAATCCCAAAAACAAAGAACATGAAGAAGACGAGCCAACTGAAGTGGGCACTTGGTGAAAACATGGCACCAGAGAAAGTGGACTTTGAGGACT<br>GTAAAGATGTTAGCGATCTAAAACAGTACGACAGTGACGAACCAGAATCTAGATCACTAGCAAGTTGGATCCAGAGTGAATTTAACAAGGCTTGCGAACTGACAGATTCGAGTTGGATTGAACTTGATGAAATAGGGGAAGACGTTGCTCCAATTGAACACATTGCGAGTATGAGGAGGAACTATTTCACAGCGGAAGTATCCCATTGCAGGGCTACAGAATACATAATG<br>AAAGGAGTATACATAAACACAGCTTTATTGAATGCATCCTGTGCAGCCATGGATGACTTCCAACTGATTCCAATGATAAGCAAATGCAGAACCAAAGAAGGGAGACGGAAGACAAATCTGTATGGCTTCATTATAAAAGGAAGATCCCATTTGAGGAATGACACCGATGTGGTAAACTTTGTGAGCATGGAATTCTCTCTCACTGACCCAAGGCTGGAGCCACACAAATGG<br>GAAAAGTACTGTGTGCTTGAGGTAGGAGACATGCTCCTACGGACTGCAATAGGCCAAGTGTCAAGGCCCATGTTCCTGTATGTGAGAACCAATGGGACTTCTAAGATCAAAATGAAATGGGGTATGGAGATGAGGCGATGCCTTCTTCAATCCCTTCAACAAATTGAGAGCATGATTGAAGCCGAGTCTTCTGTCAAAGAAAAAGACATGACCAAGGAATTCTTTGAAAAC<br>AGATCAGAAACATGGCCAATTGGAGAGTCACCCAAAGGAGTGGAGGAAGGCTCCATTGGGAAGGTGTGCAGAACATTGCTAGCAAAGTCTGTGTTCAACAGCCTATATGCATCTCCACAACTCGAGGGGTTTTCAGCTGAATCAAGAAAGTTGCTTCTCATTGTTCAGGCACTTAGGGACAACCTGGAACCTGGAACCTTCGATCTTGGGGGGCTATATGAAGCAATTGA<br>GGAGTGCCTGATTAACGATCCCTGGGTTTTGCTTAATGCGTCTTGGTTCAACTCCTTCCTCACACATGCACTGAAATA-
EPI_ISL_632318	>A/greylag_goose/Netherlands/20016879-001/2020_H5N8_2020-11-01<br>ATGGAAGACTTTGTGCGACAATGCTTCAATCCAATGATTGTCGAGCTTGCGGAAAAGGCAATGAAAGAATATGGGGAAGATCCTAAGATCGAAACAAACAAGTTTGCCGCAATATGTACACACTTAGAAGTCTGCTTCATGTATTCGGATTTCCATTTCATTGATGAACGAGGCGAATCAATAATCGTGGAATCTGGCGACCCGAATGCATTATTGAAGCACCGATTTGAGA<br>TAATTGAAGGGAGAGACCGAACAATGGCCTGGACAGTGGTGAATAGTATATGCAACACTACAGGAGTCGAAAAGCCCAAGTTCCTTCCTGATTTGTATGACTACAGAGAAAACCGATTCATTGAAATTGGAGTAACGCGAAGGGAAGTTCACATATACTATCTTGAAAAAGCCAACAAGATAAAATCAGAGAAAACACACATTCACATATTCTCATTCACTGGGGAGGAA<br>ATGGCCACCAAGGCAGATTACACTCTTGATGAAGAGAGCAGAGCAAGAATAAAAACCAGGCTATTCACTATAAGACAAGAGATGGCCAATAGGGGTCTATGGGATTCCTTTCGTCAGTCCGAGAGAGGCGAAGAGACAATTGAAGAAAGATTTGAAATCACAGGAACCATGCGCAGGCTTGCTGACCAAAGTCTCCCACCGAACTTCGCCAGCCTTGAAAACTTTAGAG<br>CCTATGTGGATGGATTTGAACCGAACGGCTGCATTGAGGGCAAGCTTTCTCAAATGTCAAAAGAAGTGAATGCCAGAATTGAGCCATTTCTGAAGACAACACCACGCCCTCTCAAATTACCTGATGGACCTCCCTGTTCCCAGCGGTCGAAGTTCTTGCTAATGGATGCCCTTAAATTGAGCATTGAAGACCCGAGCCATGAGGGGGAGGGTATACCGCTGTACGATGCAA<br>CCAAATGCATGAAGACATTTTTTGGCTGGAAGGAGCCCAACATCGTGAAACCACATGAAAAGGGCATAAACCCTAATTACCTCCTGGCTTGGAAGCAGGTGCTATCAGAACTCCAAGATATTGAAAACGAGGAGAAAATCCCAAAAACAAAGAACATGAAGAAGACGAGCCAACTGAAGTGGGCACTTGGTGAAAACATGGCACCAGAGAAAGTGGACTTTGAGGACT<br>GTAAAGATGTTAGCGATCTAAAACAGTACGACAGTGACGAACCAGAATCTAGATCACTAGCAAGTTGGATCCAGAGTGAATTTAACAAGGCTTGCGAACTGACAGATTCGAGTTGGATTGAACTTGATGAAATAGGGGAAGACGTTGCTCCAATTGAACACATTGCGAGTATGAGGAGGAACTATTTCACAGCGGAAGTATCCCATTGCAGGGCTACAGAATACATAATG<br>AAAGGAGTATACATAAACACAGCTTTATTGAATGCATCCTGTGCAGCCATGGATGACTTCCAACTGATTCCAATGATAAGCAAATGCAGAACCAAAGAAGGGAGACGGAAGACAAATCTGTATGGCTTCATTATAAAAGGAAGATCCCATTTGAGGAATGACACCGATGTGGTAAACTTTGTGAGCATGGAATTCTCTCTCACTGACCCAAGGCTGGAGCCACACAAATGG<br>GAAAAGTACTGTGTGCTTGAGGTAGGAGACATGCTCCTACGGACTGCAATAGGCCAAGTGTCAAGGCCCATGTTCCTGTATGTGAGAACCAATGGGACTTCTAAGATCAAAATGAAATGGGGTATGGAGATGAGGCGATGCCTTCTTCAATCCCTTCAACAAATTGAGAGCATGATTGAAGCCGAGTCTTCTGTCAAAGAAAAAGACATGACCAAGGAATTCTTTGAAAAC<br>AGATCAGAAACATGGCCAATTGGAGAGTCACCCAAAGGAGTGGAGGAAGGCTCCATTGGGAAGGTGTGCAGAACATTGCTAGCAAAGTCTGTGTTCAACAGCCTATATGCATCTCCACAACTCGAGGGGTTTTCAGCTGAATCAAGAAAGTTGCTTCTCATTGTTCAGGCACTTAGGGACAACCTGGAACCTGGAACCTTCGATCTTGGGGGGCTATATGAAGCAATTGA<br>GGAGTGCCTGATTAACGATCCCTGGGTTTTGCTTAATGCGTCTTGGTTCAACTCCTTCCTCACACATGCACTGAAATA-

EPI_ISL_7597827	>A/Podiceps_cristatus/Belgium/12659_0015/2020_H5N8_2020-11-24<br>ATGGAAGACTTTGTGCGACAATGCTTCAATCCAATGATTGTCGAGCTTGCGGAAAAGGCAATGAAAGAATATGGGGAAGATCCTAAGATCGAAACAAACAAGTTTGCCGCAATATGTACACACTTAGAAGTCTGCTTCATGTATTCGGATTTCCATTTCATTGATGAACGAGGCGAATCAATAATCGTGGAATCTGGCGACCCGAATGCATTATTGAAGCACCGATTTGAGA<br>TAATTGAAGGGAGAGACCGAACAATGGCCTGGACAGTGGTGAATAGTATATGCAACACTACAGGAGTCGAAAAGCCCAAGTTCCTTCCTGATTTGTATGACTACAGAGAAAACCGATTCATTGAAATTGGAGTAACGCGAAGGGAAGTTCACATATACTATCTTGAAAAAGCCAACAAGATAAAATCAGAGAAAACACACATTCACATATTCTCATTCACTGGGGAGGAA<br>ATGGCCACCAAGGCAGATTACACTCTTGATGAAGAGAGCAGAGCAAGAATAAAAACCAGGCTATTCACTATAAGACAAGAGATGGCCAATAGGGGTCTATGGGATTCCTTTCGTCAGTCCGAGAGAGGCGAAGAGACAATTGAAGAAAGATTTGAAATCACAGGAACCATGCGCAGGCTTGCTGACCAAAGTCTCCCACCGAACTTCGCCAGCCTTGAAAACTTTAGAG<br>CCTATGTGGATGGATTTGAACCGAACGGCTGCATTGAGGGCAAGCTTTCTCAAATGTCAAAAGAAGTGAATGCCAGAATTGAGCCATTTCTGAAGACTACACCACGCCCTCTCAAATTACCTGATGGACCTCCCTGTTCCCAGCGGTCGAAGTTCTTGCTAATGGATGCCCTTAAATTGAGCATTGAAGACCCGAGCCATGAGGGGGAGGGTATACCGCTGTACGATGCAAC<br>CAAATGCATGAAGACATTTTTTGGCTGGAAGGAGCCCAACATCGTGAAACCACATGAAAAGGGCATAAACCCTAATTACCTCCTGGCTTGGAAGCAGGTGCTATCAGAACTCCAAGATATTGAAAACGAGGAGAAAATCCCAAAAACAAAGAACATGAAGAAGACGAGCCAACTGAAGTGGGCACTTGGTGAAAACATGGCACCAGAGAAAGTGGACTTTGAGGACTG<br>TAAAGATGTTAGCGATCTAAAACAGTACGACAGTGACGAACCAGAATCTAGATCACTAGCAAGTTGGATCCAGAGTGAATTTAACAAGGCTTGCGAACTGACAGATTCGAGTTGGATTGAACTTGATGAAATAGGGGAAGACGTTGCTCCAATTGAACACATTGCGAGTATGAGGAGGAACTATTTCACAGCGGAAGTATCCCATTGCAGGGCTACAGAATACATAATGA<br>AAGGAGTATACATAAACACAGCTTTATTGAATGCATCCTGTGCAGCCATGGATGACTTCCAACTGATTCCAATGATAAGCAAATGCAGAACCAAAGAAGGGAGACGGAAGACAAATCTGTATGGCTTCATTATAAAAGGAAGATCCCATTTGAGGAATGACACCGATGTGGTAAACTTTGTGAGCATGGAATTCTCTCTCACTGACCCAAGGCTGGAGCCACACAAATGGG<br>AAAAGTACTGTGTGCTTGAGGTAGGAGACATGCTCCTACGGACTGCAATAGGCCAAGTGTCAAGGCCCATGTTCCTGTATGTGAGAACCAATGGGACTTCTAAGATCAAAATGAAATGGGGTATGGAGATGAGGCGATGCCTTCTTCAATCCCTTCAACAAATTGAGAGCATGATTGAAGCCGAGTCTTCTGTCAAAGAAAAAGACATGACCAAGGAATTCTTTGAAAACA<br>GATCAGAAACATGGCCAATTGGAGAGTCACCCAAAGGAGTGGAGGAAGGCTCCATTGGGAAGGTGTGCAGAACATTGCTAGCAAAGTCTGTGTTCAACAGCCTATATGCATCTCCACAACTCGAGGGGTTTTCAGCTGAATCAAGAAAGTTGCTTCTCATTGTTCAGGCACTTAGGGACAACCTGGAACCTGGAACCTTCGATCTTGGGGGGCTATATGAAGCAATTGAG<br>GAGTGCCTGATTAACGATCCCTGGGTTTTGCTTAATGCGTCTTGGTTCAACTCCTTCCTCACACATGCACTGAAATA-
EPI_ISL_2172531	>A/barnacle_goose/Netherlands/21024358-001/2021_H5N8_2021-02-21<br>ATGGAAGACTTTGTGCGACAATGCTTCAATCCAATGATTGTCGAGCTTGCGGAAAAGGCAATGAAAGAATATGGGGAAGATCCTAAGATCGAAACAAACAAGTTTGCCGCAATATGTACACACTTAGAAGTCTGCTTCATGTATTCGGATTTCCATTTCATTGATGAACGAGGCGAATCAATAATCGTAGAATCTGGCGACCCGAATGCATTATTGAAGCACCGATTTGAGA<br>TAATTGAAGGGAGAGACCGAACAATGGCCTGGACAGTGGTGAATAGTATATGCAACACTACAGGAGTCGAAAAGCCCAAGTTCCTTCCTGATTTGTATGACTACAGAGAAAACCGATTCATTGAAATTGGAGTAACGCGAAGGGAAGTTCACATATACTATCTTGAAAAAGCCAACAAGATAAAGTCAGAGAAAACACACATTCACATATTCTCATTCACTGGGGAGGAA<br>ATGGCCACCAAGGCAGATTACACTCTTGATGAAGAGAGCAGAGCAAGAATAAAAACCAGGCTATTCACTATAAGACAAGAGATGGCCAATAGGGGTCTATGGGATTCCTTTCGTCAGTCCGAGAGAGGCGAAGAGACAATTGAAGAAAGATTTGAAATCACAGGAACCATGCGCAGGCTTGCTGACCAAAGTCTTCCACCGAACTTCGCCAGCCTTGAAAACTTTAGAG<br>CCTATGTGGATGGATTTGAACCGAACGGCTGCATTGAGGGCAAGCTTTCTCAAATGTCAAAAGAAGTGAATGCCAGAATTGAGCCATTTCTGAAGACAACACCACGCCCTCTCAGATTACCTGATGGACCTCCCTGTTCCCAGCGGTCGAAGTTCTTGCTAATGGATGCCCTTAAATTAAGCATTGAAGACCCGAGCCATGAGGGGGAGGGTATACCGCTGTACGATGCAA<br>CCAAATGCATGAAGACATTTTTTGGCTGGAAGGAGCCCAACATCGTGAAACCACATGAAAAGGGCATAAACCCTAATTACCTCCTGGCTTGGAAGCAGGTGCTATCAGAACTCCAAGATATTGAAAACGAGGAGAAAATCCCAAAAACAAAGAACATGAAGAAGACGAGCCAACTGAAGTGGGCACTTGGTGAAAACATGGCACCAGAGAAAGTGGACTTTGAGGACT<br>GTAAAGATGTTAGCGATCTAAAACAGTACGACAGTGACGAACCAGAATCTAGATCACTAGCAAGTTGGATCCAGAGTGAATTTAACAAGGCTTGCGAACTGACAGATTCGAGTTGGATTGAACTTGATGAAATAGGGGAAGACGTTGCTCCAATTGAACACATTGCGAGTATGAGGAGGAACTATTTCACAGCGGAAGTATCCCATTGCAGGGCTACAGAATACATAATG<br>AAAGGAGTATACATAAACACAGCTTTATTGAATGCATCCTGTGCAGCCATGGATGACTTCCAACTGATTCCAATGATAAGTAAATGCAGAACCAAAGAAGGGAGACGGAAGACAAATCTGTATGGCTTCATTATAAAAGGAAGATCCCATTTGAGGAATGACACCGATGTGGTAAACTTTGTGAGCATGGAATTCTCTCTCACTGACCCAAGGCTGGAGCCACACAAATGG<br>GAAAAGTACTGTGTGCTTGAGGTAGGAGACATGCTCCTACGGACTGCAATAGGCCAAGTGTCAAGGCCCATGTTTCTGTATGTGAGAACCAATGGGACTTCTAAGATCAAAATGAAATGGGGTATGGAGATGAGGCGATGCCTTCTTCAATCCCTTCAACAAATTGAGAGCATGATTGAAGCCGAGTCTTCTGTCAAAGAAAAAGACATGACCAAGGAATTCTTTGAAAAC<br>AGATCAGAAACATGGCCAATTGGAGAGTCACCCAAAGGAGTGGAGGAAGGCTCCATTGGGAAGGTGTGCAGAACATTGCTAGCAAAGTCTGTGTTCAACAGCCTATATGCATCTCCACAACTCGAGGGGTTTTCAGCTGAATCAAGAAAGTTGCTTCTCATTGTTCAGGCACTTAGGGACAACCTGGAACCTGGAACCTTCGATCTTGGGGGGCTATATGAAGCAATTGA<br>GGAGTGCCTGATTAACGATCCCTGGGTTTTGCTTAATGCGTCTTGGTTCAACTCCTTCCTCACACATGCACTGAAATA-
EPI_ISL_18245531	>A/mute_swan/Poland/MB306-21_22VIR5675-20/2021_H5N8_2021-03-16<br>ATGGAAGACTTTGTGCGACAATGCTTCAATCCAATGATTGTCGAGCTTGCGGAAAAGGCAATGAAAGAATATGGGGAAGATCCTAAGATCGAAACAAACAAGTTTGCCGCAATATGTACACACTTAGAAGTCTGCTTCATGTATTCGGATTTCCATTTCATTGATGAACGAGGCGAATCAATAATCGTAGAATCTGGCGACCCGAATGCATTATTGAAGCACCGATTTGAGA<br>TAATTGAAGGGAGAGACCGAACAATGGCCTGGACAGTGGTGAATAGTATATGCAACACTACAGGAGTCGAAAAGCCCAAGTTCCTTCCTGATTTGTATGACTACAGAGAAAACCGATTCATTGAAATTGGAGTAACGCGAAGGGAAGTTCACATATACTATCTTGAAAAAGCCAACAAGATAAAGTCAGAGAAAACACACATTCACATATTCTCATTCACTGGGGAGGAA<br>ATGGCCACCAAGGCAGATTACACTCTTGATGAAGAGAGCAGAGCAAGAATAAAAACCAGGCTATTCACTATAAGACAAGAGATGGCCAATAGGGGTCTATGGGATTCCTTTCGTCAGTCCGAGAGAGGCGAAGAGACAATTGAAGAAAGATTTGAAATTACAGGAACCATGCGCAGGCTTGCTGACCAAAGTCTTCCACCGAACTTCGCCAGCCTTGAAAACTTTAGAGC<br>CTATGTGGATGGATTTGAACCGAACGGCTGCATTGAGGGCAAGCTTTCTCAAATGTCAAAAGAAGTGAATGCCAGAATTGAGCCATTTCTGAAGACAACACCACGCCCTCTCAGATTACCTGATGGACCTCCCTGTTCCCAGCGGTCGAAGTTCTTGCTAATGGATGCCCTTAAATTGAGCATTGAAGACCCGAGCCATGAGGGGGAGGGTATACCGCTGTACGATGCAAC<br>CAAATGCATGAAGACATTTTTTGGCTGGAAGGAGCCCAACATCGTGAAACCACATGAAAAGGGCATAAACCCTAATTACCTCCTGGCTTGGAAGCAGGTACTATCAGAACTCCAAGATATTGAAAACGAGGAGAAAATCCCAAAAACAAAGAACATGAAGAAGACGAGCCAACTGAAGTGGGCACTTGGTGAAAACATGGCACCAGAGAAAGTGGACTTTGAGGACTG<br>TAAAGATGTTAGCGATCTAAAACAGTACGACAGTGACGAACCAGAATCTAGATCACTAGCAAGTTGGATCCAGAGTGAATTTAACAAGGCTTGCGAACTGACAGATTCGAGTTGGATTGAACTTGATGAAATAGGGGAAGACGTTGCTCCAATTGAACACATTGCGAGTATGAGGAGGAACTATTTCACAGCGGAAGTATCCCATTGCAGGGCTACAGAATACATAATGA<br>AAGGAGTATACATAAACACAGCTTTATTGAATGCATCCTGTGCAGCCATGGATGACTTCCAACTGATTCCAATGATAAGCAAATGCAGAACCAAAGAAGGGAGACGGAAGACAAATCTGTATGGCTTCATTATAAAAGGAAGATCCCATTTGAGGAATGACACCGATGTGGTAAACTTTGTGAGCATGGAATTCTCTCTCACTGACCCAAGGCTGGAGCCACACAAATGGG<br>AAAAGTACTGTGTGCTTGAGGTAGGAGACATGCTCCTACGGACTGCAATAGGCCAAGTGTCAAGGCCCATGTTTCTGTATGTGAGAACCAATGGGACTTCTAAGATCAAAATGAAATGGGGTATGGAGATGAGGCGATGCCTTCTTCAATCCCTTCAACAAATTGAGAGCATGATTGAAGCCGAGTCTTCTGTCAAAGAAAAAGACATGACCAAGGAATTCTTTGAAAACA<br>GATCAGAAACATGGCCAATTGGAGAGTCACCCAAAGGAGTGGAGGAAGGCTCCATTGGGAAGGTGTGCAGAACATTGCTAGCAAAGTCTGTGTTCAACAGCCTATATGCATCTCCACAACTCGAGGGGTTTTCAGCTGAATCAAGAAAGTTGCTTCTCATTGTTCAGGCACTTAGGGACAACCTGGAACCTGGAACCTTCGATCTTGGGGGGCTATATGAAGCAATTGAG<br>GAGTGCCTGATTAACGATCCCTGGGTTTTGCTTAATGCGTCTTGGTTCAACTCCTTCCTCACACATGCACTGAAATA-
EPI_ISL_2113167	>A/mute_swan/Poland/MB189/2021_H5N8_2021-02-26<br>ATGGAAGACTTTGTGCGACAATGCTTCAATCCAATGATTGTCGAGCTTGCGGAAAAGGCAATGAAAGAATATGGGGAAGATCCTAAGATCGAAACAAACAAGTTTGCCGCAATATGTACACACTTAGAAGTCTGCTTCATGTATTCGGATTTCCATTTCATTGATGAACGAGGCGAATCAATAATCGTAGAATCTGGCGACCCGAATGCATTATTGAAGCACCGATTTGAGA<br>TAATTGAAGGGAGAGACCGAACAATGGCCTGGACAGTGGTGAATAGTATATGCAACACTACAGGAGTCGAAAAGCCCAAGTTCCTTCCTGATTTGTATGACTACAGAGAAAACCGATTCATTGAAATTGGAGTAACGCGAAGGGAAGTTCACATATACTATCTTGAAAAAGCCAACAAGATAAAGTCAGAGAAAACACACATTCACATATTCTCATTCACTGGGGAGGAA<br>ATGGCCACCAAGGCAGATTACACTCTTGATGAAGAGAGCAGAGCAAGAATAAAAACCAGGCTATTCACTATAAGACAAGAGATGGCCAATAGGGGTCTATGGGATTCCTTTCGTCAGTCCGAGAGAGGCGAAGAGACAATTGAAGAAAGATTTGAAATCACAGGAACCATGCGCAGGCTTGCTGACCAAAGTCTTCCACCGAACTTCGCCAGCCTTGAAAACTTTAGAG<br>CCTATGTGGATGGATTTGAACCGAACGGCTGCATTGAGGGCAAGCTTTCTCAAATGTCAAAAGAAGTGAATGCCAGAATTGAGCCATTTCTGAAGACAACACCACGCCCTCTCAGATTACCTGATGGACCTCCCTGTTCCCAGCGGTCGAAGTTCTTGCTAATGGATGCCCTTAAATTGAGCATTGAAGACCCGAGCCATGAGGGGGAGGGTATACCGCTGTACGATGCAA<br>CCAAATGCATGAAGACATTTTTTGGCTGGAAGGAGCCCAACATCGTGAAACCACATGAAAAGGGCATAAACCCTAATTACCTCCTGGCTTGGAAGCAGGTACTATCAGAACTCCAAGATATTGAAAACGAGGAGAAAATCCCAAAAACAAAGAACATGAAGAAGACGAGCCAACTGAAGTGGGCACTTGGTGAAAACATGGCACCAGAGAAAGTGGACTTTGAGGACT<br>GTAAAGATGTTAGCGATCTAAAACAGTACGACAGTGACGAACCAGAATCTAGATCACTAGCAAGTTGGATCCAGAGTGAATTTAACAAGGCTTGCGAACTGACAGATTCGAGTTGGATTGAACTTGATGAAATAGGGGAAGACGTTGCTCCAATTGAACACATTGCGAGTATGAGGAGGAACTATTTCACAGCGGAAGTATCCCATTGCAGGGCTACAGAATACATAATG<br>AAAGGAGTATACATAAACACAGCTTTATTGAATGCATCCTGTGCAGCCATGGATGACTTCCAACTGATTCCAATGATAAGCAAATGCAGAACCAAAGAAGGGAGACGGAAGACAAATCTGTATGGCTTCATTATAAAAGGAAGATCCCATTTGAGGAATGACACCGATGTGGTAAACTTTGTGAGCATGGAATTCTCTCTCACTGACCCAAGGCTGGAGCCACACAAATGG<br>GAAAAGTACTGTGTGCTTGAGGTAGGAGACATGCTCCTACGGACTGCAATAGGCCAAGTGTCAAGGCCCATGTTTCTGTATGTGAGAACCAATGGGACTTCTAAGATCAAAATGAAATGGGGTATGGAGATGAGGCGATGCCTTCTTCAATCCCTTCAACAAATTGAGAGCATGATTGAAGCCGAGTCTTCTGTCAAAGAAAAAGACATGACCAAGGAATTCTTTGAAAAC<br>AGATCAGAAACATGGCCAATTGGAGAGTCACCCAAAGGAGTGGAGGAAGGCTCCATTGGGAAGGTGTGCAGAACATTGCTAGCAAAGTCTGTGTTCAACAGCCTATATGCATCTCCACAACTCGAGGGGTTTTCAGCTGAATCAAGAAAGTTGCTTCTCATTGTTCAGGCACTTAGGGACAACCTGGAACCTGGAACCTTCGATCTTGGGGGGCTATATGAAGCAATTGA<br>GGAGTGCCTGATTAACGATCCCTGGGTTTTGCTTAATGCGTCTTGGTTCAACTCCTTCCTCACACATGCACTGAAATA-
EPI_ISL_3102061	>A/duck/Poland/H542_21RS1385-2/2021_H5N8_2021-04-09<br>ATGGAAGACTTTGTGCGACAATGCTTCAATCCAATGATTGTCGAGCTTGCGGAAAAGGCAATGAAAGAATATGGGGAAGATCCTAAGATCGAAACAAACAAGTTTGCCGCAATATGTACACACTTAGAAGTCTGCTTCATGTATTCGGATTTCCATTTCATTGATGAACGAGGCGAATCAATAATCGTAGAATCTGGCGACCCGAATGCATTATTGAAGCACCGATTTGAGA<br>TAATTGAAGGGAGAGACCGAACAATGGCCTGGACAGTGGTGAATAGTATATGCAACACTACAGGAGTCGAAAAGCCCAAGTTCCTTCCTGATTTGTATGACTACAGAGAAAACCGATTCATTGAAATTGGAGTAACGCGAAGGGAAGTTCACATATACTATCTTGAAAAAGCCAACAAGATAAAGTCAGAGAAAACACACATTCACATATTCTCATTCACTGGGGAGGAA<br>ATGGCCACCAAGGCAGATTACACTCTTGATGAAGAGAGCAGAGCAAGAATAAAAACCAGGCTATTCACTATAAGACAAGAGATGGCCAATAGGGGTCTATGGGATTCCTTTCGTCAGTCCGAGAGAGGCGAAGAGACAATTGAAGAAAGATTTGAAATCACAGGAACCATGCGCAGGCTTGCTGACCAAAGTCTCCCACCGAACTTCACCAGCCTTGAAAACTTTAGAG<br>CCTATGTGGATGGATTTGAACCGAACGGCTGCATTGAGGGCAAGCTTTCTCAAATGTCAAAAGAAGTGAATGCCAGAATTGAGCCATTTCTGAAGACAACACCACGCCCTCTCAGATTACCTGATGGACCTCCCTGTTCCCAGCGGTCGAAGTTCTTGCTAATGGATGCCCTTAAATTGAGCATTGAAGACCCGAGCCATGAGGGGGAGGGTATACCGCTGTACGATGCAA<br>CCAAATGCATGAAGACATTTTTTGGCTGGAAGGAGCCCAACATCGTGAAACCACATGAAAAGGGCATAAACCCTAATTACCTCCTGGCTTGGAAGCAGGTGCTATCAGAACTCCAAGATATTGAAAACGAGGAGAAAATCCCAAAAACAAAGAACATGAAGAAGACGAGCCAACTGAAGTGGGCACTTGGTGAAAACATGGCACCAGAGAAAGTGGACTTTGAGGACT<br>GTAAAGATGTTAGCGATCTAAAACAGTACGACAGTGACGAACCAGAATCTAGATCACTAGCAAGTTGGATCCAGAGTGAATTTAACAAGGCTTGCGAACTGACAGATTCGAGTTGGATTGAACTTGATGAAATAGGGGAAGACGTTGCTCCAATTGAACACATTGCGAGTATGAGGAGGAACTATTTCACAGCGGAAGTATCCCATTGCAGGGCTACAGAATACATAATG<br>AAAGGAGTATACATAAACACAGCTTTATTGAATGCATCCTGTGCAGCCATGGATGACTTCCAACTGATTCCAATGATAAGCAAATGCAGAACCAAAGAAGGGAGGCGGAAGACAAATCTGTATGGCTTCATTATAAAAGGAAGATCCCATTTGAGGAATGACACCGATGTGGTAAACTTTGTGAGCATGGAATTCTCTCTCACTGACCCAAGGCTGGAGCCACACAAATGG<br>GAAAAGTACTGTGTGCTTGAGGTAGGAGACATGCTCCTACGGACTGCAATAGGCCAAGTGTCAAGGCCCATGTTCCTGTATGTGAGAACCAATGGGACTTCTAAGATCAAAATGAAATGGGGTATGGAGATGAGGCGATGCCTTCTTCAATCCCTTCAACAAATTGAGAGCATGATTGAAGCCGAGTCTTCTGTCAAAGAAAAAGACATGACCAAGGAATTCTTTGAAAAC<br>AGATCAGAAACATGGCCAATTGGAGAGTCACCCAAAGGAGTGGAGGAAGGCTCCATTGGGAAGGTGTGCAGAACATTGCTAGCAAAGTCTGTGTTCAACAGCCTATATGCATCTCCACAACTCGAGGGGTTTTCAGCTGAATCAAGAAAGTTGCTTCTCATTGTTCAGGCACTTAGGGACAACCTGGAACCTGGAACCTTCGATCTTGGGGGGCTATATGAAGCAATTGA<br>GGAGTGCCTGATTAACGATCCCTGGGTTTTGCTTAATGCGTCTTGGTTTAACTCCTTCCTCACACATGCACTGAAATA-
EPI_ISL_3102062	>A/chicken/Poland/H1124_21RS1385-3/2021_H5N8_2021-05-11<br>ATGGAAGACTTTGTGCGACAATGCTTCAATCCAATGATTGTCGAGCTTGCGGAAAAGGCAATGAAAGAACATGGGGAAGATCCTAAGGTCGAAACAAACAAGTTTGCCGCAATATGTACACACTTAGAAGTCTGCTTCATGTATTCGGATTTCCATTTCATTGATGAACGAGGCGAATCAATAATCGTAGAATCTGGCGACCCGAATGCATTATTGAAGCACCGATTTGAGA<br>TAATTGAAGGGAGAGACCGAACGATGGCCTGGACAGTGGTGAATAGTATATGCAACACTACAGGAGTCGAAAAGCCCAAGTTCCTTCCTGATTTGTATGACTACAGAGAAAACCGATTCATTGAAATTGGAGTAACGCGAAGGGAAGTTCACATATACTATCTTGAAAAAGCCAACAAGATAAAGTCAGAGAAAACACACATTCACATATTCTCATTCACTGGGGAGGAA<br>ATGGCCACCAAGGCAGATTACACTCTTGATGAAGAGAGCAGAGCAAGAATAAAAACCAGGCTATTCACTATAAGACAAGAGATGGCCAATAGGGGTCTATGGGATTCCTTTCGTCAGTCCGAGAGAGGCGAAGAGACAATTGAAGAAAGATTTGAAATCACAGGAACCATGCGCAGGCTTGCTGACCAAAGTCTCCCACCGAACTTCACCAGCCTTGAAAACTTTAGAG<br>CCTATGTGGATGGATTTGAACCGAACGGCTGCATTGAGGGCAAGCTTTCTCAAATGTCAAAAGAAGTGAATGCCAGAATTGAGCCATTTCTGAAGACAACACCACGCCCTCTCAGATTACCTGATGGACCTCCCTGTTCCCAGCGGTCGAAGTTCTTGCTAATGGATGCCCTTAAATTGAGCATTGAAGACCCGAGCCATGAGGGGGAGGGTATACCGCTGTACGATGCAA<br>CCAAATGCATGAAGACATTTTTTGGCTGGAAGGAGCCCAACATCGTGAAACCACATGAAAAGGGCATAAACCCTAATTACCTCCTGGCTTGGAAGCAGGTGCTATCAGAACTCCAAGATATTGAAAACGAGGAGAAAATCCCAAAAACAAAGAACATGAAGAAGACGAGCCAACTGAAGTGGGCACTTGGTGAAAACATGGCACCAGAGAAAGTGGACTTTGAGGACT<br>GTAAAGATGTTAGCGATCTAAAACAGTACGACAGTGACGAACCAGAATCTAGATCACTAGCAAGTTGGATCCAGAGTGAATTTAACAAGGCTTGCGAACTGACAGATTCGAGTTGGATTGAACTTGATGAAATAGGGGAAGACGTTGCTCCAATTGAACACATTGCGAGTATGAGGAGGAACTATTTCACAGCGGAAGTATCCCATTGCAGGGCTACAGAATACATAATG<br>AAAGGAGTATACATAAACACAGCTTTATTGAATGCATCCTGTGCAGCCATGGATGACTTCCAACTGATTCCAATGATAAGCAAATGCAGAACCAAAGAAGGGAGGCGGAAGACAAATCTGTATGGCTTCATTATAAAAGGAAGATCCCATTTGAGGAATGACACCGATGTGGTAAACTTTGTGAGCATGGAATTCTCTCTCACTGACCCAAGGCTGGAGCCACACAAATGG<br>GAAAAGTACTGTGTGCTTGAGGTAGGAGACATGCTCCTACGGACTGCAATAGGCCAAGTGTCAAGGCCCATGTTCCTGTATGTGAGAACCAATGGGACTTCTAAGATCAAAATGAAATGGGGTATGGAGATGAGGCGATGCCTTCTTCAATCCCTTCAACAAATTGAGAGCATGATTGAAGCCGAGTCTTCTGTCAAAGAAAAAGACATGACCAAGGAATTCTTTGAAAAC<br>AGATCAGAAACATGGCCAATTGGAGAGTCACCCAAAGGAGTGGAGGAAGGCTCCATTGGGAAGGTGTGCAGAACATTGCTAGCAAAGTCTGTGTTCAACAGCCTATATGCATCTCCACAACTCGAGGGGTTTTCAGCTGAATCAAGAAAGTTGCTTCTCATTGTTCAGGCACTTAGGGACAACCTGGAACCTGGAACCTTCGATCTTGGGGGGCTATATGAAGCAATTGA<br>GGAGTGCCTGATTAACGATCCCTGGGTTTTGCTTAATGCGTCTTGGTTCAACTCCTTCCTCACACATGCACTGAAATA-
EPI_ISL_3102076	>A/duck/Poland/H515_21RS1385-17/2021_H5N8_2021-04-07<br>ATGGAAGACTTTGTGCGACAATGCTTCAATCCAATGATTGTCGAGCTTGCGGAAAAGGCAATGAAAGAACATGGGGAAGATCCTAAGGTCGAAACAAACAAGTTTGCCGCAATATGTACACACTTAGAAGTCTGCTTCATGTATTCGGATTTCCATTTCATTGATGAACGAGGCGAATCAATAATCGTAGAATCTGGCGACCCGAATGCATTATTGAAGCACCGATTTGAGA<br>TAATTGAAGGGAGAGACCGAACAATGGCCTGGACAGTGGTGAATAGTATATGCAACACTACAGGAGTCGAAAAGCCCAAGTTCCTTCCTGATTTGTATGACTACAGAGAAAACCGATTCATTGAAATTGGAGTAACGCGAAGGGAAGTTCACATATACTATCTTGAAAAAGCCAACAAGATAAAGTCAGAGAAAACACACATTCACATATTCTCATTCACTGGGGAGGAA<br>ATGGCCACCAAGGCAGATTACACTCTTGATGAAGAGAGCAGAGCAAGAATAAAAACCAGGCTATTCACTATAAGACAAGAGATGGCCAATAGGGGTCTATGGGATTCCTTTCGTCAGTCCGAGAGAGGCGAAGAGACAATTGAAGAAAGATTTGAAATCACAGGAACCATGCGCAGGCTTGCTGACCAAAGTCTCCCACCGAACTTCACCAGCCTTGAAAACTTTAGAG<br>CCTATGTGGATGGATTTGAACCGAACGGCTGCATTGAGGGCAAGCTTTCTCAAATGTCAAAAGAAGTGAATGCCAGAATTGAGCCATTTCTGAAGACAACACCACGCCCTCTCAGATTACCTGATGGACCTCCCTGTTCCCAGCGGTCGAAGTTCTTGCTAATGGATGCCCTTAAATTGAGCATTGAAGACCCGAGCCATGAGGGGGAGGGTATACCGCTGTACGATGCAA<br>CCAAATGCATGAAGACATTTTTTGGCTGGAAGGAGCCCAACATCGTGAAACCACATGAAAAGGGCATAAACCCTAATTACCTCCTGGCTTGGAAGCAGGTGCTATCAGAACTCCAAGATATTGAAAACGAGGAGAAAATCCCAAAAACAAAGAACATGAAGAAGACGAGCCAACTGAAGTGGGCACTTGGTGAAAACATGGCACCAGAGAAAGTGGACTTTGAGGACT<br>GTAAAGATGTTAGCGATCTAAAACAGTACGACAGTGACGAACCAGAATCTAGATCACTAGCAAGTTGGATCCAGAGTGAATTTAACAAGGCTTGCGAACTGACAGATTCGAGTTGGATTGAACTTGATGAAATAGGGGAAGACGTTGCTCCAATTGAACACATTGCGAGTATGAGGAGGAACTATTTCACAGCGGAAGTATCCCATTGCAGGGCTACAGAATACATAATG<br>AAAGGAGTATACATAAACACAGCTTTATTGAATGCATCCTGTGCAGCCATGGATGACTTCCAACTGATTCCAATGATAAGCAAATGCAGAACCAAAGAAGGGAGGCGGAAGACAAATCTGTATGGCTTCATTATAAAAGGAAGATCCCATTTGAGGAATGACACCGATGTGGTAAACTTTGTGAGCATGGAATTCTCTCTCACTGACCCAAGGCTGGAGCCACACAAATGG<br>GAAAAGTACTGTGTGCTTGAGGTAGGAGACATGCTCCTACGGACTGCAATAGGCCAAGTGTCAAGGCCCATGTTCCTGTATGTGAGAACCAATGGGACTTCTAAGATCAAAATGAAATGGGGTATGGAGATGAGGCGATGCCTTCTTCAATCCCTTCAACAAATTGAGAGCATGATTGAAGCCGAGTCTTCTGTCAAAGAAAAAGACATGACCAAGGAATTCTTTGAAAAC<br>AGATCAGAAACATGGCCAATTGGAGAGTCACCCAAAGGAGTGGAGGAAGGCTCCATTGGGAAGGTGTGCAGAACATTGCTAGCAAAGTCTGTGTTCAACAGCCTATATGCATCTCCACAACTCGAGGGGTTTTCAGCTGAATCAAGAAAGTTGCTTCTCATTGTTCAGGCACTTAGGGACAACCTGGAACCTGGAACCTTCGATCTTGGGGGGCTATATGAAGCAATTGA<br>GGAGTGCCTGATTAACGATCCCTGGGTTTTGCTTAATGCGTCTTGGTTCAACTCCTTCCTCACACATGCACTGAAATA-
EPI_ISL_3102065	>A/goose/Poland/H1036_21RS1385-6/2021_H5N8_2021-05-03<br>ATGGAAGACTTTGTGCGACAATGCTTCAATCCAATGATTGTCGAGCTTGCGGAAAAGGCAATGAAAGAACATGGGGAAGATCCTAAGGTCGAAACAAACAAGTTTGCCGCAATATGTACACACTTAGAAGTCTGCTTCATGTATTCGGATTTCCATTTCATTGATGAACGAGGCGAATCAATAATCGTAGAATCTGGCGACCCGAATGCATTATTGAAGCACCGATTTGAGA<br>TAATTGAAGGGAGAGACCGAACAATGGCCTGGACAGTGGTGAATAGTATATGCAACACTACAGGAGTCGAAAAGCCCAAGTTCCTTCCTGATTTGTATGACTACAGAGAAAACCGATTCATTGAAATTGGAGTAACGCGAAGGGAAGTTCACATATACTATCTTGAAAAAGCCAACAAGATAAAGTCAGAGAAAACACACATTCACATATTCTCATTCACTGGGGAGGAA<br>ATGGCCACCAAGGCAGATTACACTCTTGATGAAGAGAGCAGAGCAAGAATAAAAACCAGGCTATTCACTATAAGACAAGAGATGGCCAATAGGGGTCTATGGGATTCCTTTCGTCAGTCCGAGAGAGGCGAAGAGACAATTGAAGAAAGATTTGAAATCACAGGAACCATGCGCAGGCTTGCTGACCAAAGTCTCCCACCGAACTTCACCAGCCTTGAAAACTTTAGAG<br>CCTATGTGGATGGATTTGAACCGAACGGCTGCATTGAGGGCAAGCTTTCTCAAATGTCAAAAGAAGTGAATGCCAGAATTGAGCCATTTCTGAAGACAACACCACGCCCTCTCAGATTACCTGATGGACCTCCCTGTTCCCAGCGGTCGAAGTTCTTGCTAATGGATGCCCTTAAATTGAGCATTGAAGACCCGAGCCATGAGGGGGAGGGTATACCGCTGTACGATGCAA<br>CCAAATGCATGAAGACATTTTTTGGCTGGAAGGAGCCCAACATCGTGAAACCACATGAAAAGGGCATAAACCCTAATTACCTCCTGGCTTGGAAGCAGGTGCTATCAGAACTCCAAGATATTGAAAACGAGGAGAAAATCCCAAAAACAAAGAACATGAAGAAGACGAGCCAACTGAAGTGGGCACTTGGTGAAAACATGGCACCAGAGAAAGTGGACTTTGAGGACT<br>GTAAAGATGTTAGCGATCTAAAACAGTACGACAGTGACGAACCAGAATCTAGATCACTAGCAAGTTGGATCCAGAGTGAATTTAACAAGGCTTGCGAACTGACAGATTCGAGTTGGATTGAACTTGATGAAATAGGGGAAGACGTTGCTCCAATTGAACACATTGCGAGTATGAGGAGGAACTATTTCACAGCGGAAGTATCCCATTGCAGGGCTACAGAATACATAATG<br>AAAGGAGTATACATAAACACAGCTTTATTGAATGCATCCTGTGCAGCCATGGATGACTTCCAACTGATTCCAATGATAAGCAAATGCAGAACCAAAGAAGGGAGGCGGAAGACAAATCTGTATGGCTTCATTATAAAAGGAAGATCCCATTTGAGGAATGACACCGATGTGGTAAACTTTGTGAGCATGGAATTCTCTCTCACTGACCCAAGGCTGGAGCCACACAAATGG<br>GAAAAGTACTGTGTGCTTGAGGTAGGAGACATGCTCCTACGGACTGCAATAGGCCAAGTGTCAAGGCCCATGTTCCTGTATGTGAGAACCAATGGGACTTCTAAGATCAAAATGAAATGGGGTATGGAGATGAGGCGATGCCTTCTTCAATCCCTTCAACAAATTGAGAGCATGATTGAAGCCGAGTCTTCTGTCAAAGAAAAAGACATGACCAAGGAATTCTTTGAAAAC<br>AGATCAGAAACATGGCCAATTGGAGAGTCACCCAAAGGAGTGGAGGAAGGCTCCATTGGGAAGGTGTGCAGAACATTGCTAGCAAAGTCTGTGTTCAACAGCCTATATGCATCTCCACAACTCGAGGGGTTTTCAGCTGAATCAAGAAAGTTGCTTCTCATTGTTCAGGCGCTTAGGGACAACCTGGAACCTGGAACCTTCGATCTTGGGGGGCTATATGAAGCAATTGA<br>GGAGTGCCTGATTAACGATCCCTGGGTTTTGCTTAATGCGTCTTGGTTCAACTCCTTCCTCACACATGCACTGAAATA-
EPI_ISL_3102063	>A/goose/Poland/H1044_21RS1385-4/2021_H5N8_2021-05-05<br>ATGGAAGACTTTGTGCGACAATGCTTCAATCCAATGATTGTCGAGCTTGCGGAAAAGGCAATGAAAGAATATGGGGAAGATCCTAAGATCGAAACAAACAAGTTTGCCGCAATATGTACACACTTAGAAGTCTGCTTCATGTATTCGGATTTCCATTTCATTGATGAACGAGGCGAATCAATAATCGTAGAATCTGGCGACCCGAATGCATTATTGAAGCACCGATTTGAGA<br>TAATTGAAGGGAGAGACCGAACAATGGCCTGGACAGTGGTGAATAGTATATGCAACACTACAGGAGTCGAAAAGCCCAAGTTCCTTCCTGATTTGTATGACTACAGAGAAAACCGATTCATTGAAATTGGAGTAACGCGAAGGGAAGTTCACATATACTATCTTGAAAAAGCCAACAAGATAAAGTCAGAGAAAACACACATTCACATATTCTCATTCACTGGGGAGGAA<br>ATGGCCACCAAGGCAGATTACACTCTTGATGAAGAGAGCAGAGCAAGAATAAAAACCAGGCTATTCACTATAAGACAAGAGATGGCCAATAGGGGTCTATGGGATTCCTTTCGTCAGTCCGAGAGAGGCGAAGAGACAATTGAAGAAAGATTTGAAATCACAGGAACCATGCGCAGGCTTGCTGACCAAAGTCTCCCACCGAACTTCACCAGCCTTGAAAACTTTAGAG<br>CCTATGTGGATGGATTTGAACCGAACGGCTGCATTGAGGGCAAGCTTTCTCAAATGTCAAAAGAAGTGAATGCCAGAATTGAGCCATTTCTGAAGACAACACCACGCCCTCTCAGATTACCTGATGGACCTCCCTGTTCCCAGCGGTCGAAGTTCTTGCTAATGGATGCCCTTAAATTGAGCATTGAAGACCCGAGCCATGAGGGGGAGGGTATACCGCTGTACGATGCAA<br>CCAAATGCATGAAGACATTTTTTGGCTGGAAGGAGCCCAACATCGTGAAACCACATGAAAAGGGCATAAACCCTAATTACCTCCTGGCTTGGAAGCAGGTGCTATCAGAACTCCAAGATATTGAAAACGAGGAGAAAATCCCAAAAACAAAGAACATGAATAAGACGAGCCAACTGAAGTGGGCACTTGGTGAAAACATGGCACCAGAGAAAGTGGACTTTGAGGACT<br>GTAAAGATGTTAGCGATCTAAAACAGTACGACAGTGACGAACCAGAATCTAGATCACTAGCAAGTTGGATCCAGAGTGAATTTAACAAGGCTTGCGAACTGACAGATTCGAGTTGGATTGAACTTGATGAAATAGGGGAAGACGTTGCTCCAATTGAACACATTGCGAGTATGAGGAGGAACTATTTCACAGCGGAAGTATCCCATTGCAGGGCTACAGAATACATAATG<br>AAAGGAGTATACATAAACACAGCTTTATTGAATGCGTCCTGTGCAGCCATGGATGACTTCCAACTGATTCCAATGATAAGCAAATGCAGAACCAAAGAAGGGAGGCGGAAGACAAATCTGTATGGCTTCATTATAAAAGGAAGATCCCATTTGAGGAATGACACCGATGTGGTAAACTTTGTGAGCATGGAATTCTCTCTCACTGACCCAAGGCTGGAGCCACACAAATGG<br>GAAAAGTACTGTGTGCTTGAGGTAGGAGACATGCTCCTACGGACTGCAATAGGCCAAGTGTCAAGGCCCATGTTCCTGTATGTGAGAACCAATGGGACTTCTAAGATCAAAATGAAATGGGGTATGGAGATGAGGCGATGCCTTCTTCAATCCCTTCAACAAATTGAGAGCATGATTGAAGCCGAGTCTTCTGTCAAAGAAAAAGACATGACCAAGGAATTCTTTGAAAAC<br>AGATCAGAAACATGGCCAATTGGAGAGTCACCCAAAGGAGTGGAGGAAGGCTCCATTGGGAAGGTGTGCAGAACATTGCTAGCAAAGTCTGTGTTCAACAGCCTATATGCATCTCCACAACTCGAGGGGTTTTCAGCTGAATCAAGAAAGTTGCTTCTCATTGTTCAGGCACTTAGGGACAACCTGGAACCTGGAACCTTCGATCTTGGGGGGCTATATGAAGCAATTGA<br>GGAGTGCCTGATTAACGATCCCTGGGTTTTGCTTAATGCGTCTTGGTTTAACTCCTTCCTCACACATGCACTGAAATA-
EPI_ISL_3102064	>A/duck/Poland/H1029_21RS1385-5/2021_H5N8_2021-05-01<br>ATGGAAGACTTTGTGCGACAATGCTTCAATCCAATGATTGTCGAGCTTGCGGAAAAGGCAATGAAAGAATATGGGGAAGATCCTAAGATCGAAACAAACAAGTTTGCCGCAATATGTACACACTTAGAAGTCTGCTTCATGTATTCGGATTTCCATTTCATTGATGAACGAGGCGAATCAATAATCGTAGAATCTGGCGACCCGAATGCATTATTGAAGCACCGATTTGAGA<br>TAATTGAAGGGAGAGACCGAACAATGGCCTGGACAGTGGTGAATAGTATATGCAACACTACAGGAGTCGAAAAGCCCAAGTTCCTTCCTGATTTGTATGACTACAGAGAAAACCGATTCATTGAAATTGGAGTAACGCGAAGGGAAGTTCACATATACTATCTTGAAAAAGCCAACAAGATAAAGTCAGAGAAAACACACATTCACATATTCTCATTCACTGGGGAGGAA<br>ATGGCCACCAAGGCAGATTACACTCTTGATGAAGAGAGCAGAGCAAGAATAAAAACCAGGCTATTCACTATAAGACAAGAGATGGCCAATAGGGGTCTATGGGATTCCTTTCGTCAGTCCGAGAGAGGCGAAGAGACAATTGAAGAAAGATTTGAAATCACAGGAACCATGCGCAGGCTTGCTGACCAAAGTCTCCCACCGAACTTCACCAGCCTTGAAAACTTTAGAG<br>CCTATGTGGATGGATTTGAACCGAACGGCTGCATTGAGGGCAAGCTTTCTCAAATGTCAAAAGAAGTGAATGCCAGAATTGAGCCATTTCTGAAGACAACACCACGCCCTCTCAGATTACCTGATGGACCTCCCTGTTCCCAGCGGTCGAAGTTCTTGCTAATGGATGCCCTTAAATTGAGCATTGAAGACCCGAGCCATGAGGGGGAGGGTATACCGCTGTACGATGCAA<br>CCAAATGCATGAAGACATTTTTTGGCTGGAAGGAGCCCAACATCGTGAAACCACATGAAAAGGGCATAAACCCTAATTACCTCCTGGCTTGGAAGCAGGTGCTATCAGAACTCCAAGATATTGAAAACGAGGAGAAAATCCCAAAAACAAAGAACATGAATAAGACGAGCCAACTGAAGTGGGCACTTGGTGAAAACATGGCACCAGAGAAAGTGGACTTTGAGGACT<br>GTAAAGATGTTAGCGATCTAAAACAGTACGACAGTGACGAACCAGAATCTAGATCACTAGCAAGTTGGATCCAGAGTGAATTTAACAAGGCTTGCGAACTGACAGATTCGAGTTGGATTGAACTTGATGAAATAGGGGAAGACGTTGCTCCAATTGAACACATTGCGAGTATGAGGAGGAACTATTTCACAGCGGAAGTATCCCATTGCAGGGCTACAGAATACATAATG<br>AAAGGAGTATACATAAACACAGCTTTATTGAATGCGTCCTGTGCAGCCATGGATGACTTCCAACTGATTCCAATGATAAGCAAATGCAGAACCAAAGAAGGGAGGCGGAAGACAAATCTGTATGGCTTCATTATAAAAGGAAGATCCCATTTGAGGAATGACACCGATGTGGTAAACTTTGTGAGCATGGAATTCTCTCTCACTGACCCAAGGCTGGAGCCACACAAATGG<br>GAAAAGTACTGTGTGCTTGAGGTAGGAGACATGCTCCTACGGACTGCAATAGGCCAAGTGTCAAGGCCCATGTTCCTGTATGTGAGAACCAATGGGACTTCTAAGATCAAAATGAAATGGGGTATGGAGATGAGGCGATGCCTTCTTCAATCCCTTCAACAAATTGAGAGCATGATTGAAGCCGAGTCTTCTGTCAAAGAAAAAGACATGACCAAGGAATTCTTTGAAAAC<br>AGATCAGAAACATGGCCAATTGGAGAGTCACCCAAAGGAGTGGAGGAAGGCTCCATTGGGAAGGTGTGCAGAACATTGCTAGCAAAGTCTGTGTTCAACAGCCTATATGCATCTCCACAACTCGAGGGGTTTTCAGCTGAATCAAGAAAGTTGCTTCTCATTGTTCAGGCACTTAGGGACAACCTGGAACCTGGAACCTTCGATCTTGGGGGGCTATATGAAGCAATTGA<br>GGAGTGCCTGATTAACGATCCCTGGGTTTTGCTTAATGCGTCTTGGTTTAACTCCTTCCTCACACATGCACTGAAATA-
EPI_ISL_18244493	>A/swan/Poland/MB122-21_22VIR5675-9/2021_H5N8_2021-02-18<br>ATGGAAGACTTTGTGCGACAATGCTTCAATCCAATGATTGTCGAGCTTGCGGAAAAGGCAATGAAAGAATATGGGGAAGATCCTAAGATCGAAACAAACAAGTTTGCCGCAATATGTACACACTTAGAAGTCTGCTTCATGTATTCGGATTTCCATTTCATTGATGAACGAGGCGAATCAATAATCGTAGAATCTGGCGACCCGAATGCATTATTGAAGCACCGATTTGAGA<br>TAATTGAAGGGAGAGACCGAACAATGGCCTGGACAGTGGTGAATAGTATATGCAACACTACAGGAGTCGAAAAGCCCAAGTTCCTTCCTGATTTGTATGACTACAGAGAAAACCGATTCATTGAAATTGGAGTAACGCGAAGGGAAGTTCACATATACTATCTTGAAAAAGCCAACAAGATAAAGTCAGAGAAAACACACATTCACATATTCTCATTCACTGGGGAGGAA<br>ATGGCCACCAAGGCAGATTACACTCTTGATGAAGAGAGCAGAGCAAGAATAAAAACCAGGCTATTCACTATAAGACAAGAGATGGCCAATAGGGGTCTATGGGATTCCTTTCGTCAGTCCGAGAGAGGCGAAGAGACAATTGAAGAAAGATTTGAAATCACAGGAACCATGCGTAGGCTTGCTGACCAAAGTCTCCCACCGAACTTCACCAGCCTTGAAAACTTTAGAG<br>CCTATGTGGATGGATTTGAACCGAACGGCTGCATTGAGGGCAAGCTTTCTCAAATGTCAAAAGAAGTGAATGCCAGAATTGAGCCATTTCTGAAGACAACACCACGCCCTCTCAGATTACCTGATGGACCTCCCTGTTCCCAGCGGTCGAAGTTCTTGCTAATGGATGCCCTTAAATTGAGCATTGAAGACCCGAGCCATGAGGGGGAGGGTATACCGCTGTACGATGCAA<br>CCAAATGCATGAAGACATTTTTTGGCTGGAAGGAGCCCAACATCGTGAAACCACATGAAAAGGGCATAAACCCTAATTACCTCCTGGCTTGGAAGCAGGTGCTATCAGAACTCCAAGATATTGAAAACGAGGAGAAAATCCCAAAAACAAAGAACATGAAGAAGACGAGCCAACTGAAGTGGGCACTTGGTGAAAACATGGCACCAGAGAAAGTGGACTTTGAGGACT<br>GTAAAGATGTTAGCGATCTAAAACAGTACGACAGTGACGAACCAGAATCTAGATCACTAGCAAGTTGGATCCAGAGTGAATTTAACAAGGCTTGCGAACTGACAGATTCGAGTTGGATTGAACTTGATGAAATAGGGGAAGACGTTGCTCCAATTGAACACATTGCGAGTATGAGGAGGAACTATTTCACAGCGGAAGTATCCCATTGCAGGGCTACAGAATACATAATG<br>AAAGGAGTATACATAAACACAGCTTTATTGAATGCATCCTGTGCAGCCATGGATGACTTCCAACTGATTCCAATGATAAGCAAATGCAGAACCAAAGAAGGGAGACGGAAGACAAATCTGTATGGCTTCATTATAAAAGGAAGATCCCATTTGAGGAATGACACCGATGTGGTAAACTTTGTGAGCATGGAATTCTCTCTCACTGACCCAAGGCTGGAGCCACACAAATGG<br>GAAAAGTACTGTGTGCTTGAGGTAGGAGACATGCTCCTACGGACTGCAATAGGCCAAGTGTCAAGGCCCATGTTCCTGTATGTGAGAACCAATGGGACTTCTAAGATCAAAATGAAATGGGGTATGGAGATGAGGCGATGCCTTCTTCAATCCCTTCAACAAATTGAGAGCATGATTGAAGCCGAGTCTTCAGTCAAAGAAAAAGACATGACCAAGGAATTCTTTGAAAA<br>CAGATCAGAAACATGGCCAATTGGAGAGTCACCCAAAGGAGTGGAGGAAGGCTCCATTGGGAAGGTGTGCAGAACATTGCTAGCAAAGTCTGTGTTCAACAGCCTATATGCATCTCCACAACTCGAGGGGTTTTCAGCTGAATCAAGAAAGTTGCTTCTCATTGTTCAGGCACTTAGGGACAACCTGGAACCTGGAACCTTCGATCTTGGGGGGCTATATGAAGCAATTG<br>AGGAGTGCCTGATTAACGATCCCTGGGTTTTGCTTAATGCGTCTTGGTTCAACTCCTTCCTCACACATGCACTGAAATA-
EPI_ISL_3102060	>A/duck/Poland/H160_21RS1385-1/2021_H5N8_2021-03-05<br>ATGGAAGACTTTGTGCGACAATGCTTCAATCCAATGATTGTCGAGCTTGCGGAAAAGGCAATGAAAGAATATGGGGAAGATCCTAAGATCGAAACAAACAAGTTTGCCGCAATATGTACACACTTAGAAGTCTGCTTCATGTATTCGGATTTCCATTTCATTGATGAACGAGGCGAATCAATAATCGTAGAATCTGGCGACCCGAATGCATTATTGAAGCACCGATTTGAGA<br>TAATTGAAGGGAGAGACCGAACAATGGCCTGGACAGTGGTGAATAGTATATGCAACACTACAGGAGTCGAAAAGCCCAAGTTCCTTCCTGATTTGTATGACTACAGAGAAAACCGATTCATTGAAATTGGAGTAACGCGAAGGGAAGTTCACATATACTATCTTGAAAAAGCCAACAAGATAAAGTCAGAGAAAACACACATTCACATATTCTCATTCACCGGGGAGGAA<br>ATGGCCACCAAGGCAGATTACACTCTTGATGAAGAGAGCAGAGCCAGAATAAAAACCAGGCTATTCACTATAAGACAAGAGATGGCCAATAGGGGTCTATGGGATTCCTTTCGTCAGTCCGAGAGAGGCGAAGAGACAATTGAAGAAAGATTTGAAATCACAGGAACCATGCGCAGGCTTGCTAACCAAAGTCTCCCACCGAACTTCACCAGCCTTGAAAACTTTAGAGC<br>CTATGTGGATGGATTTGAACCGAACGGCTGCATTGAGGGCAAGCTTTCTCAAATGTCAAAAGAAGTGAATGCCAGAATTGAGCCATTTCTGAAGACAACACCACGCCCTCTCAGATTACCTGATGGACCTCCCTGTTCCCAGCGGTCGAAGTTCTTGCTAATGGATGCCCTTAAATTGAGCATTGAAGACCCGAGCCATGAGGGGGAGGGTATACCGCTGTACGATGCAAC<br>CAAATGCATGAAGACATTTTTTGGCTGGAAGGAGCCCAACATCGTGAAACCACATGAAAAGGGCATAAACCCTAATTACCTCCTGGCTTGGAAGCAGGTGCTATCAGAACTCCAAGATATTGAAAACGAGGAGAAAATCCCAAAAACAAAGAACATGAAGAAGACGAGCCAACTGAAGTGGGCACTTGGTGAAAACATGGCACCAGAGAAAGTGGACTTTGAGGACTG<br>TAAAGATGTTAGCGATCTAAAACAGTACGACAGTGACGAACCAGAATCTAGATCACTAGCAAGTTGGATCCAGAGTGAATTTAACAAGGCTTGCGAACTGACAGATTCGAGTTGGATTGAACTTGATGAAATAGGGGAAGACGTTGCTCCAATTGAACACATTGCGAGTATGAGGAGGAACTATTTCACAGCGGAAGTATCCCATTGCAGGGCTACAGAATACATAATGA<br>AAGGAGTATACATAAACACAGCTTTATTGAATGCATCCTGTGCAGCCATGGATGACTTCCAACTGATTCCAATGATAAGCAAATGCAGAACCAAAGAAGGGAGGCGGAAGACAAATCTGTATGGCTTCATTATAAAAGGAAGATCCCATTTGAGGAATGACACCGATGTGGTAAACTTTGTGAGCATGGAATTCTCTCTCACTGACCCAAGGCTGGAGCCACACAAATGGG<br>AAAAGTACTGTGTGCTTGAGGTAGGAGACATGCTCCTACGGACTGCAATAGGCCAAGTGTCAAGGCCCATGTTCCTGTATGTGAGAACCAATGGGACTTCTAAGATCAAAATGAAATGGGGTATGGAGATGAGGCGATGCCTTCTTCAATCCCTTCAACAAATTGAGAGCATGATTGAAGCCGAGTCTTCTGTCAAAGAAAAAGACATGACCAAGGAATTCTTTGAAAACA<br>GATCAGAAACATGGCCAATTGGAGAGTCACCCAAAGGAGTGGAGGAAGGCTCCATTGGGAAGGTGTGCAGAACATTGCTAGCAAAGTCTGTGTTCAACAGCCTATATGCATCTCCACAACTCGAGGGGTTTTCAGCTGAATCAAGAAAGTTGCTTCTCATTGTTCAGGCACTTAGGGACAACCTGGAACCTGGAACCTTCGATCTTGGGGGGCTATATGAAGCAATTGAG<br>GAGTGCCTGATTAACGATCCCTGGGTTTTGCTTAATGCGTCTTGGTTCAACTCCTTCCTCACACATGCACTGAAATA-
EPI_ISL_1093426	>A/black-headed_gull/Sweden/SVA210216SZ0398/KN000437/2021_H5N8_2021-02-07<br>ATGGAAGACTTTGTGCGACAATGCTTCAATCCAATGATTGTCGAGCTTGCGGAAAAGGCAATGAAAGAATATGGGGAAGATCCTAAGATCGAAACAAACAAGTTTGCCGCAATATGTACACACTTAGAAGTCTGCTTCATGTATTCGGATTTCCATTTCATTGATGAACGAGGCGAATCAATAATCGTAGAATCTGGCGACCCGAATGCATTATTGAAGCACCGATTTGAGA<br>TAATTGAAGGGAGAGACCGAACAATGGCCTGGACAGTGGTGAATAGTATATGCAACACTACAGGAGTCGAAAAGCCCAAGTTCCTTCCTGATTTGTATGACTACCGAGAAAACCGATTCATTGAAATTGGAGTAACGCGAAGGGAAGTTCACATATACTATCTTGAAAAAGCCAACAAGATAAAATCAGAGAAAACACACATTCACATATTCTCATTCACTGGGGAGGAA<br>ATGGCCACCAAGGCAGATTACACTCTTGATGAAGAGAGCAGAGCAAGAATAAAAACCAGGCTATTCACTATAAGACAAGAGATGGCCAATAGGGGTCTATGGGATTCCTTTCGTCAGTCCGAGAGAGGCGAAGAGACAATTGAAGAAAGATTTGAAATCACAGGAACCATGCGCAGGCTTGCTGACCAAAGTCTCCCACCGAACTTCGCCAGCCTTGAAAACTTTAGAG<br>CCTATGTGGATGGATTTGAACCGAACGGCTGCATTGAGGGCAAGCTTTCTCAAATGTCAAAAGAAGTGAATGCCAGAATTGAGCCATTTCTGAAGACGACACCACGCCCTCTCAGATTACCTGATGGACCTCCCTGTTCCCAGCGGTCGAAGTTCTTGCTAATGGATGCCCTTAAATTGAGCATTGAAGACCCGAGCCATGAGGGGGAGGGTATACCGCTGTACGATGCAA<br>CCAAATGCATGAAGACATTTTTTGGCTGGAAGGAGCCCAACATCGTGAAACCACATGAAAAGGGCATAAACCCTAATTACCTCCTGGCTTGGAAGCAGGTGCTATCAGAACTCCAAGATATTGAAAACGAGGAGAAAATCCCAAAAACAAAGAACATGAAGAAGACGAGCCAACTGAAGTGGGCACTTGGTGAAAACATGGCACCAGAGAAAGTGGACTTTGAGGACT<br>GTAAAGATGTTAGCGATCTAAGACAGTACGACAGTGACGAACCAGAGTCTAGATCACTAGCAAGTTGGATCCAGAGTGAATTTAACAAGGCTTGCGAACTGACAGATTCGAGTTGGATTGAACTTGATGAAATAGGGGAAGACGTTGCTCCAATTGAACACATTGCGAGTATGAGGAGGAACTATTTCACAGCGGAAGTATCCCATTGCAGGGCTACAGAATACATAATG<br>AAAGGAGTATACATAAACACAGCTTTATTGAATGCATCCTGTGCAGCCATGGATGACTTCCAACTGATTCCAATGATAAGCAAATGCAGAACCAAAGAAGGGAGACGGAAGACAAATCTGTATGGCTTCATTATAAAAGGAAGATCCCATTTGAGGAATGACACCGATGTGGTAAACTTTGTGAGCATGGAATTCTCTCTCACTGACCCAAGGCTGGAGCCACACAAATGG<br>GAAAAGTACTGTGTACTTGAGGTAGGAGACATGCTCCTACGGACTGCAATAGGCCAAGTGTCAAGGCCCATGTTCCTGTATGTGAGAACCAATGGGACTTCTAAGATCAAAATGAAATGGGGTATGGAGATGAGGCGATGCCTTCTTCAATCCCTTCAACAAATTGAGAGCATGATTGAAGCCGAGTCTTCTGTCAAAGAAAAAGACATGACCAAGGAATTCTTTGAAAAC<br>AGATCAGAAACATGGCCAATTGGAGAGTCACCCAAAGGAGTGGAGGAATGCTCCATTGGGAAGGTGTGCAGAACATTGCTAGCAAAGTCTGTGTTCAACAGCCTATATGCATCTCCACAACTCGAGGGGTTTTCAGCTGAATCAAGAAAGTTGCTTCTCATTGTTCAGGCACTTAGGGACAACCTGGAACCTGGAACCTTCGATCTTGGGGGGCTATATGAAGCAATTGAG<br>GAGTGCCTGATTAACGATCCCTGGGTTTTGCTTAATGCGTCTTGGTTCAACTCCTTCCTCACACATGCACTGAAATA-

EPI_ISL_5126214	>A/eagle_owl/Germany-ST/AI02542/2021_H5N8_2021-03-10<br>ATGGAAGACTTTGTGCGACAATGCTTCAATCCAATGATTGTCGAGCTTGCGGAAAAGGCAATGAAAGAATATGGGGAAGATCCTAAGATCGAAACAAACAAGTTTGCCGCAATATGTACACACTTAGAAGTCTGCTTCATGTATTCGGATTTCCATTTCATTGATGAACGAGGCGAATCAATAATCGTAGAATCTGGCGACCCGAATGCATTATTGAAGCACCGGTTTGAGA<br>TAATTGAAGGGAGAGACCGAACAATGGCCTGGACAGTGGTGAATAGTATATGCAACACTACAGGAGTCGAAAAGCCCAAGTTCCTTCCTGATTTGTATGACTACCGGGAAAACCGATTCATTGAAATTGGAGTAACGCGAAGGGAAGTTCACATATACTATCTTGAAAAAGCCAACAAGATAAAATCAGAGAAAACACACATTCACATATTCTCATTCACTGGGGAGGAA<br>ATGGCCACCAAGGCAGATTACACTCTTGATGAAGAGAGCAGAGCAAGAATAAAAACCAGGCTATTCACTATAAGACAAGAGATGGCCAATAGGGGTCTATGGGATTCCTTTCGTCAGTCCGAGAGAGGCGAAGAGACAATTGAAGAAAGATTTGAAATCACAGGAACCATGCGCAGGCTTGCTGACCAAAGTCTCCCACCGAACTTCGCCAGCCTTGAAAACTTTAGAG<br>CCTATGTGGATGGATTTGAACCGAACGGCTGCATTGAGGGCAAGCTTTCTCAAATGTCAAAAGAAGTGAATGCCAGAATTGAGCCATTTCTGAAGACGACACCACGCCCTCTCAGATTACCTGATGGACCTCCCTGTTCCCAGCGGTCGAAGTTCTTGCTAATGGATGCCCTTAAATTGAGCATTGAAGACCCGAGCCATGAGGGGGAGGGTATACCGCTGTACGATGCAA<br>CCAAATGCATGAAGACATTTTTTGGCTGGAAGGAGCCCAACATCGTGAAACCACATGAAAAGGGCATAAACCCTAATTACCTCCTGGCTTGGAAGCAGGTGCTATCAGAACTCCAAGATATTGAAAACGAGGAGAAAATCCCAAAAACAAAGAACATGAAGAAGACGAGCCAACTGAAGTGGGCACTTGGTGAAAACATGGCACCAGAGAAAGTGGACTTTGAGGACT<br>GTAAAGATGTTAGCGATCTAAGACAGTACGACAGTGACGAACCAGAGTCTAGATCACTAGCAAGTTGGATCCAGAGTGAATTTAACAAGGCTTGCGAACTGACAGATTCGAGTTGGATTGAACTTGATGAAATAGGGGAAGACGTTGCTCCAATTGAACACATTGCGAGTATGAGGAGGAACTATTTCACAGCGGAAGTATCCCATTGCAGGGCTACAGAATACATAATG<br>AAAGGAGTATACATAAACACAGCTTTATTGAATGCATCCTGTGCAGCCATGGATGACTTCCAACTGATTCCAATGATAAGCAAATGCAGAACCAAAGAAGGGAGACGGAAGACAAATCTGTATGGCTTCATTATAAAAGGAAGATCCCATTTGAGGAATGACACCGATGTGGTAAACTTTGTGAGCATGGAATTCTCTCTCACTGACCCAAGGCTGGAGCCACACAAATGG<br>GAAAAGTACTGTGTACTTGAGGTAGGAGACATGCTCCTACGGACTGCAATAGGCCAAGTGTCAAGGCCCATGTTCCTGTATGTGAGAACCAATGGGACTTCTAAGATCAAAATGAAATGGGGTATGGAGATGAGGCGATGCCTTCTTCAATCCCTTCAACAAATTGAGAGCATGATTGAAGCCGAGTCTTCTGTCAAAGAAAAAGACATGACCAAGGAATTCTTTGAAAAC<br>AGATCAGAAACATGGCCAATTGGAGAGTCACCCAAAGGAGTGGAGGAAGGCTCCATTGGGAAGGTGTGCAGAACATTGCTAGCAAAGTCTGTGTTCAACAGCCTATATGCATCTCCACAACTCGAGGGGTTTTCAGCTGAATCAAGAAAGTTGCTTCTCATTGTTCAGGCACTTAGGGACAACCTGGAACCTGGAACCTTCGATCTTGGGGGGCTATATGAAGCAATTGA<br>GGAGTGCCTGATTAACGATCCCTGGGTTTTGCTTAATGCGTCTTGGTTCAACTCCTTCCTCACACATGCACTGAAATA-
EPI_ISL_5098144	>A/pomeranian_goose/Germany-MV/AI01100/2021_H5N8_2021-02-01<br>ATGGAAGACTTTGTGCGACAATGCTTCAATCCAATGATTGTCGAGCTTGCGGAAAAGGCAATGAAAGAATATGGGGAAGATCCTAAGATCGAAACAAACAAGTTTGCCGCAATATGTACACACTTAGAAGCCTGCTTCATGTATTCGGATTTCCATTTCATTGATGAACGAGGCGAATCAATAATCGTAGAATCTGGCGACCCGAATGCATTATTGAAGCACCGATTTGAGA<br>TAATTGAAGGGAGAGACCGAACAATGGCCTGGACAGTGGTGAATAGTATATGCAACACTACAGGAGTCGAAAAGCCCAAGTTCCTTCCTGATTTGTATGACTACCGAGAAAACCGATTCATTGAAATTGGAGTAACGCGAAGGGAAGTTCACATATACTATCTTGAAAAAGCCAACAAGATAAAATCAGAGAAAACACACATTCACATATTCTCATTCACTGGGGAGGAA<br>ATGGCCACCAAGGCAGATTACACTCTTGATGAAGAGAGCAGAGCAAGAATAAAAACCAGGCTATTCACTATAAGACAAGAGATGGCCAATAGGGGTCTATGGGATTCCTTTCGTCAGTCCGAGAGAGGCGAAGAGACAATTGAAGAAAGATTTGAAATCACAGGAACCATGCGCAGGCTTGCTGACCAAAGTCTCCCACCGAACTTCGCCAGCCTTGAAAACTTTAGAG<br>CCTATGTGGATGGATTTGAACCGAACGGCTGCATTGAGGGCAAGCTTTCTCAAATGTCAAAAGAAGTGAATGCCAGAATTGAGCCATTTCTGAAGACGACACCACGCCCTCTCAGATTACCTGATGGACCTCCCTGTTCCCAGCGGTCGAAGTTCTTGCTAATGGATGCCCTTAAATTGAGCATTGAAGACCCGAGCCATGAGGGGGAGGGTATACCGCTGTACGATGCAA<br>CCAAATGCATGAAGACATTTTTTGGCTGGAAGGAGCCCAACATCGTGAAACCACATGAAAAGGGCATAAACCCTAATTACCTCCTGGCTTGGAAGCAGGTGCTATCAGAACTCCAAGATATTGAAAACGAGGAGAAAATCCCAAAAACAAAGAACATGAAGAAGACGAGCCAACTGAAGTGGGCACTTGGTGAAAACATGGCACCAGAGAAAGTGGACTTTGAGGACT<br>GTAAAGATGTTAGCGATCTAAGACAGTACGACAGTGACGAACCAGAGTCTAGATCACTAGCAAGTTGGATCCAGAGTGAATTTAACAAGGCTTGCGAACTGACAGATTCGAGTTGGATTGAACTTGATGAAATAGGGGAAGACGTTGCTCCAATTGAACACATTGCGAGTATGAGGAGGAACTATTTCACAGCGGAAGTATCCCATTGCAGGGCTACAGAATACATAATG<br>AAAGGAGTATACATAAACACAGCTTTATTGAATGCATCCTGTGCAGCCATGGATGACTTCCAACTGATTCCAATGATAAGCAAATGCAGAACCAAAGAAGGGAGACGGAAGACAAATCTGTATGGCTTCATTATAAAAGGAAGATCCCATTTGAGGAATGACACCGATGTGGTAAACTTTGTGAGCATGGAATTCTCTCTCACTGACCCAAGGCTGGAGCCACACAAATGG<br>GAAAAGTACTGTGTACTTGAGGTAGGAGACATGCTCCTACGGACTGCAATAGGCCAAGTGTCAAGGCCCATGTTCCTGTATGTGAGAACCAATGGGACTTCTAAGATCAAAATGAAATGGGGTATGGAGATGAGGCGATGCCTTCTTCAATCCCTTCAACAAATTGAGAGCATGATTGAAGCCGAGTCTTCTGTCAAAGAAAAAGACATGACCAAGGAATTCTTTGAAAAC<br>AGATCAGAAACATGGCCAATTGGAGAGTCACCCAAAGGAGTGGAGGAAGGCTCCATTGGGAAGGTGTGCAGAACATTGCTAGCAAAGTCTGTGTTCAACAGCCTATATGCATCTCCACAACTCGAGGGGTTTTCAGCTGAATCAAGAAAGTTGCTTCTCATTGTTCAGGCACTTAGGGACAACCTGGAACCTGGAACCTTCGATCTTGGGGGGCTATATGAAGCAATTGA<br>GGAGTGCCTGATTAACGATCCCTGGGTTTTGCTTAATGCGTCTTGGTTCAACTCCTTCCTCACACATGCACTGAAATA-
EPI_ISL_846624	>A/wild_goose/Poland/MB142/2020_H5N8_2020-12-17<br>ATGGAAGACTTTGTGCGACAATGCTTCAATCCAATGATTGTCGAGCTTGCGGAAAAGGCAATGAAAGAATATGGGGAAGATCCTAAGATCGAAACAAACAAGTTTGCCGCAATATGTACACACTTAGAAGTCTGCTTCATGTATTCGGATTTCCATTTCATTGATGAACGAGGCGAATCAATAATCGTAGAATCTGACGACCCGAATGCATTATTGAAGCACCGATTTGAGA<br>TAATTGAAGGGAGAGACCGAACAATGGCCTGGACAGTGGTGAATAGTATATGCAACACTACAGGAGTCGAAAAGCCCAAGTTCCTTCCTGATTTGTATGACTACCGAGAAAACCGATTCATTGAAATTGGAGTAACGCGAAGGGAAGTTCACATATACTATCTTGAAAAAGCCAACAAGATAAAATCAGAGAAAACACACATTCACATATTCTCATTCACTGGGGAGGAA<br>ATGGCCACCAAGGCAGATTACACTCTTGATGAAGAGAGCAGAGCAAGAATAAAAACCAGGCTATTCACTATAAGACAAGAGATGGCCAATAGGGGTCTATGGGATTCCTTTCGTCAGTCCGAGAGAGGCGAAGAGACAATTGAAGAAAGATTTGAAATCACAGGAACCATGCGCAGGCTTGCTGACCAAAGTCTCCCACCGAACTTCGCCAGCCTTGAAAACTTTAGAG<br>CCTATGTGGATGGATTTGAACCGAACGGCTGCATTGAGGGCAAGCTTTCTCAAATGTCAAAAGAAGTGAATGCCAGAATTGAGCCATTTCTGAAGACGACACCACGCCCTCTCAGATTACCTGATGGACCTCCCTGTTCCCAGCGGTCGAAGTTCTTGCTAATGGATGCCCTTAAATTGAGCATTGAAGACCCGAGCCATGAGGGGGAGGGTATACCGCTGTACGATGCAA<br>CCAAATGCATGAAGACATTTTTTGGCTGGAAGGAGCCCAACATCGTGAAACCACATGAAAAGGGCATAAACCCTAATTACCTCCTGGCTTGGAAGCAGGTGCTATCAGAACTCCAAGATATTGAAAACGAGGAGAAAATCCCAAAAACAAAGAACATGAAGAAGACGAGCCAACTGAAGTGGGCACTTGGTGAAAACATGGCACCAGAGAAAGTGGACTTTGAGGACT<br>GTAAAGATGTTAGCGATCTAAGACAGTACGACAGTGACGAACCAGAGTCTAGATCACTAGCAAGTTGGATCCAGAGTGAATTTAACAAGGCTTGCGAACTGACAGATTCGAGTTGGATTGAACTTGATGAAATAGGGGAAGACGTTGCTCCAATTGAACACATTGCGAGTATGAGGAGGAACTATTTCACAGCGGAAGTATCCCATTGCAGGGCTACAGAATACATAATG<br>AAAGGAGTATACATAAACACAGCTTTATTGAATGCATCCTGTGCAGCCATGGATGACTTCCAACTGATTCCAATGATAAGCAAATGCAGAACCAAAGAAGGGAGACGGAAGACAAATCTGTATGGCTTCATTATAAAAGGAAGATCCCATTTGAGGAATGACACCGATGTGGTAAACTTTGTGAGCATGGAATTCTCTCTCACTGACCCAAGGCTGGAGCCACACAAATGG<br>GAAAAGTACTGTGTACTTGAGGTAGGAGACATGCTCCTACGGACTGCAATAGGCCAAGTGTCAAGGCCCATGTTCCTGTATGTGAGAACCAATGGGACTTCTAAGATCAAAATGAAATGGGGTATGGAGATGAGGCGATGCCTTCTTCAATCCCTTCAACAAATTGAGAGCATGATTGAAGCCGAGTCTTCTGTCAAAGAAAAAGACATGACCAAGGAATTCTTTGAAAAC<br>AGATCAGAAACATGGCCAATTGGAGAGTCACCCAAAGGAGTGGAGGAAGGCTCCATTGGGAAGGTGTGCAGAACATTGCTAGCAAAGTCTGTGTTCAACAGCCTATATGCATCTCCACAACTCGAGGGGTTTTCAGCTGAATCAAGAAAGTTGCTTCTCATTGTTCAGGCACTTAGGGACAACCTGGAACCTGGAACCTTCGATCTTGGGGGGCTATATGAAGCAATTGA<br>GGAGTGCCTGATTAACGATCCCTGGGTTTTGCTTAATGCGTCTTGGTTCAACTCCTTCCTCACACATGCACTGAAATA-
EPI_ISL_2114013	>A/mute_swan/Poland/MB272/2021_H5N8_2021-03-09<br>ATGGAAGACTTTGTGCGACAATGCTTCAATCCAATGATTGTCGAGCTTGCGGAAAAGGCAATGAAAGAATATGGGGAAGATCCTAAGATCGAAACAAACAAGTTTGCCGCAATATGTACACACTTAGAAGTCTGCTTCATGTATTCGGATTTCCATTTCATTGATGAACGAGGCGAATCAATAATCGTAGAATCTGGCGACCCGAATGCATTATTGAAGCACCGATTTGAGA<br>TAATTGAAGGGAGAGACCGAACAATGGCCTGGACAGTGGTGAATAGTATATGCAACACTACAGGAGTCGAAAAGCCCAAGTTCCTTCCTGATTTGTATGACTACCGAGAAAACCGATTCATTGAAATTGGAGTAACGCGAAGGGAAGTTCACATATACTATCTTGAAAAAGCCAACAAGATAAAATCAGAGAAAACACACATTCACATATTCTCATTCACTGGGGAGGAA<br>ATGGCCACCAAGGCAGATTACACTCTTGATGAAGAGAGCAGAGCAAGAATAAAAACCAGGCTATTCACTATAAGACAAGAGATGGCCAATAGGGGTCTATGGGATTCCTTTCGTCAGTCCGAGAGAGGCGAAGAGACAATTGAAGAAAGATTTGAAATCACAGGAACCATGCGCAGGCTTGCTGACCAAAGTCTCCCACCGAACTTCGCCAGCCTTGAAAACTTTAGAG<br>CCTATGTGGATGGATTTGAACCGAACGGCTGCATTGAGGGCAAGCTTTCTCAAATGTCAAAAGAAGTGAATGCCAGAATTGAGCCATTTCTGAAGACGACACCACGCCCTCTCAGATTACCTGATGGACCTCCCTGTTCCCAGCGGTCGAAGTTCTTGCTAATGGATGCCCTTAAATTGAGCATTGAAGACCCGAGCCATGAGGGGGAGGGTATACCGCTGTACGATGCAA<br>CCAAATGCATGAAGACATTTTTTGGCTGGAAGGAGCCCAACATCGTGAAACCACATGAAAAGGGCATAAACCCTAATTACCTCCTGGCTTGGAAGCAGGTGCTATCAGAACTCCAAGATATTGAAAACGAGGAGAAAATCCCAAAAACAAAGAACATGAAGAAGACGAGCCAACTGAAGTGGGCACTTGGTGAAAACATGGCACCAGAGAAAGTGGACTTTGAGGACT<br>GTAAAGATGTTAGCGATCTAAGACAGTACGACAGTGACGAACCAGAGTCTAGATCACTAGCAAGTTGGATCCAGAGTGAATTTAACAAGGCTTGCGAACTGACAGATTCGAGTTGGATTGAACTTGATGAAATAGGGGAAGACGTTGCTCCAATTGAACACATTGCGAGTATGAGGAGGAACTATTTCACAGCGGAAGTATCCCATTGCAGGGCTACAGAATACATAATG<br>AAAGGAGTATACATAAACACAGCTTTATTGAATGCATCCTGTGCAGCCATGGATGACTTCCAACTGATTCCAATGATAAGCAAATGCAGAACCAAAGAAGGGAGACGGAAGACAAATCTGTATGGCTTCATTATAAAAGGAAGATCCCATTTGAGGAATGACACCGATGTGGTAAACTTTGTGAGCATGGAATTCTCTCTCACTGACCCAAGGCTGGAGCCACACAAATGG<br>GAAAAGTACTGTGTACTTGAGGTAGGAGACATGCTCCTACGGACTGCAATAGGCCAAGTGTCAAGGCCCATGTTCCTGTATGTGAGAACCAATGGGACTTCTAAGATCAAAATGAAATGGGGTATGGAGATGAGGCGATGCCTTCTTCAATCCCTTCAACAAATTGAGAGCATGATTGAAGCCGAGTCTTCTGTCAAAGAAAAAGACATGACCAAGGAATTCTTTGAAAAC<br>AGATCAGAAACATGGCCAATTGGAGAATCACCCAAAGGAGTGGAGGAAGGCTCCATTGGGAAGGTGTGCAGAACATTGCTAGCAAAGTCTGTGTTCAACAGCCTATATGCATCTCCACAACTCGAGGGGTTTTCAGCTGAATCAAGAAAGTTGCTTCTCATTGTTCAGGCACTTAGGGACAACCTGGAACCTGGAACCTTCGATCTTGGGGGGCTATATGAAGCAATTGA<br>GGAGTGCCTGATTAACGATCCCTGGGTTTTGCTTAATGCGTCTTGGTTCAACTCCTTCCTCACACATGCACTGAAATA-
EPI_ISL_661177	>A/chicken/Poland/448/2020_H5N8_2020-11-24<br>ATGGAAGACTTTGTGCGACAATGCTTCAATCCAATGATTGTCGAGCTTGCGGAAAAGGCAATGAAAGAATATGGGGAAGATCCTAAGATCGAAACAAACAAGTTTGCCGCAATATGTACACACTTAGAAGTCTGCTTCATGTATTCGGATTTCCATTTCATTGATGAACGAGGCGAATCAATAATCGTAGAATCTGGCGACCCGAATGCATTATTGAAGCACCGATTTGAGA<br>TAATTGAAGGGAGAGACCGAACAATGGCCTGGACAGTGGTGAATAGTATATGCAACACTACAGGAGTCGAAAAGCCCAAGTTCCTTCCTGATTTGTATGACTACCGAGAAAACCGATTCATTGAAATTGGAGTAACGCGAAGGGAAGTTCACATATACTATCTTGAAAAAGCCAACAAGATAAAATCAGAGAAAACACACATTCACATATTCTCATTCACTGGGGAGGAA<br>ATGGCCACCAAGGCAGATTACACTCTTGATGAAGAGAGCAGAGCAAGAATAAAAACCAGGCTATTCACTATAAGACAAGAGATGGCCAATAGGGGTCTATGGGATTCCTTTCGTCAGTCCGAGAGAGGCGAAGAGACAATTGAAGAAAGATTTGAAATCACAGGAACCATGCGCAGGCTTGCTGACCAAAGTCTCCCACCGAACTTCGCCAGCCTTGAAAACTTTAGAG<br>CCTATGTGGATGGATTTGAACCGAACGGCTGCATTGAGGGCAAGCTTTCTCAAATGTCAAAAGAAGTGAATGCCAGAATTGAGCCATTTCTGAAGACGACACCACGCCCTCTCAGATTACCTGATGGACCTCCCTGTTCCCAGCGGTCGAAGTTCTTGCTAATGGATGCCCTTAAATTGAGCATTGAAGACCCGAGCCATGAGGGGGAGGGTATACCGCTGTACGATGCAA<br>CCAAATGCATGAAGACATTTTTTGGCTGGAAGGAGCCCAACATCGTGAAACCACATGAAAAGGGCATAAACCCTAATTACCTCCTGGCTTGGAAGCAGGTGCTATCAGAACTCCAAGATATTGAAAACGAGGAGAAAATCCCAAAAACAAAGAACATGAAGAAGACGAGCCAACTGAAGTGGGCACTTGGTGAAAACATGGCACCAGAGAAAGTGGACTTTGAGGACT<br>GTAAAGATGTTAGCGATCTAAGACAGTACGACAGTGACGAACCAGAGTCTAGATCACTAGCAAGTTGGATCCAGAGTGAATTTAACAAGGCTTGCGAACTGACAGATTCGAGTTGGATTGAACTTGATGAAATAGGGGAAGACGTTGCTCCAATTGAACACATTGCGAGTATGAGGAGGAACTATTTCACAGCGGAAGTATCCCATTGCAGGGCTACAGAATACATAATG<br>AAGGGAGTATACATAAACACAGCTTTATTGAATGCATCCTGTGCAGCCATGGATGACTTCCAACTGATTCCAATGATAAGCAAATGCAGAACCAAAGAAGGGAGACGGAAGACAAATCTGTATGGCTTCATTATAAAAGGAAGATCCCATTTGAGGAATGACACCGATGTGGTAAACTTTGTGAGCATGGAATTCTCTCTCACTGACCCAAGGCTGGAGCCACACAAATGG<br>GAAAAGTACTGTGTACTTGAGGTAGGAGACATGCTCCTACGGACTGCAATAGGCCAAGTGTCAAGGCCCATGTTCCTGTATGTGAGAACCAATGGGACTTCTAAGATCAAAATGAAATGGGGTATGGAGATGAGGCGATGCCTTCTTCAATCCCTTCAACAAATTGAGAGCATGATTGAAGCCGAGTCTTCTGTCAAAGAAAAAGACATGACCAAGGAATTCTTTGAAAAC<br>AGATCAGAAACATGGCCAATTGGAGAGTCACCCAAAGGAGTGGAGGAAGGCTCCATTGGGAAGGTGTGCAGAACATTGCTAGCAAAGTCTGTGTTCAACAGCCTATATGCATCTCCACAACTCGAGGGGTTTTCAGCTGAATCAAGAAAGTTGCTTCTCATTGTTCAGGCACTTAGGGACAACCTGGAACCTGGAACCTTCGATCTTGGGGGGCTATATGAAGCAATTGA<br>GGAGTGCCTGATTAACGATCCCTGGGTTTTGCTTAATGCGTCTTGGTTCAACTCCTTCCTCACACATGCACTGAAATA-
EPI_ISL_846602	>A/turkey/Poland/475/2020_H5N8_2020-12-03<br>ATGGAAGACTTTGTGCGACAATGCTTCAATCCAATGATTGTCGAGCTTGCGGAAAAGGCAATGAAAGAATATGGGGAAGATCCTAAGATCGAAACAAACAAGTTTGCCGCAATATGTACACACTTAGAAGTCTGCTTCATGTATTCGGATTTCCATTTCATTGATGAACGAGGCGAATCAATAATCGTAGAATCTGGCGACCCGAATGCATTATTGAAGCACCGATTTGAGA<br>TAATTGAAGGGAGAGACCGAACAATGGCCTGGACAGTGGTGAATAGTATATGCAACACTACAGGAGTCGAAAAGCCCAAGTTCCTTCCTGATTTGTATGACTACCGAGAAAACCGATTCATTGAAATTGGAGTAACGCGAAGGGAAGTTCACATATACTATCTTGAAAAAGCCAACAAGATAAAATCAGAGAAAACACACATTCACATATTCTCATTCACTGGGGAGGAA<br>ATGGCCACCAAGGCAGATTACACTCTTGATGAAGAGAGCAGAGCAAGAATAAAAACCAGGCTATTCACTATAAGACAAGAGATGGCCAATAGGGGTCTATGGGATTCCTTTCGTCAGTCCGAGAGAGGCGAAGAGACAATTGAAGAAAGATTTGAAATCACAGGAACCATGCGCAGGCTTGCTGACCAAAGTCTCCCACCGAACTTCGCCAGCCTTGAAAACTTTAGAG<br>CCTATGTGGATGGATTTGAACCGAACGGCTGCATTGAGGGCAAGCTTTCTCAAATGTCAAAAGAAGTGAATGCCAGAATTGAGCCATTTCTGAAGACGACACCACGCCCTCTCAGATTACCTGATGGACCTCCCTGTTCCCAGCGGTCGAAGTTCTTGCTAATGGATGCCCTTAAATTGAGCATTGAAGACCCGAGCCATGAGGGGGAGGGTATACCGCTGTACGATGCAA<br>CCAAATGCATGAAGACATTTTTTGGCTGGAAGGAGCCCAACATCGTGAAACCACATGAAAAGGGCATAAACCCTAATTACCTCCTGGCTTGGAAGCAGGTGCTATCAGAACTCCAAGATATTGAAAACGAGGAGAAAATCCCAAAAACAAAGAACATGAAGAAGACGAGCCAACTGAAGTGGGCACTTGGTGAAAACATGGCACCAGAGAAAGTGGACTTTGAGGACT<br>GTAAAGATGTTAGCGATCTAAGACAGTACGACAGTGACGAACCAGAGTCTAGATCACTAGCAAGTTGGATCCAGAGTGAATTTAACAAGGCTTGCGAACTGACAGATTCGAGTTGGATTGAACTTGATGAAATAGGGGAAGACGTTGCTCCAATTGAACACATTGCGAGTATGAGGAGGAACTATTTCACAGCGGAAGTATCCCATTGCAGGGCTACAGAATACATAATG<br>AAGGGAGTATACATAAACACAGCTTTATTGAATGCATCCTGTGCAGCCATGGATGACTTCCAACTGATTCCAATGATAAGCAAATGCAGAACCAAAGAAGGGAGACGGAAGACAAATCTGTATGGCTTCATTATAAAAGGAAGATCCCATTTGAGGAATGACACCGATGTGGTAAACTTTGTGAGCATGGAATTCTCTCTCACTGACCCAAGGCTGGAGCCACACAAATGG<br>GAAAAGTACTGTGTACTTGAGGTAGGAGACATGCTCCTACGGACTGCAATAGGCCAAGTGTCAAGGCCCATGTTCCTGTATGTGAGAACCAATGGGACTTCTAAGATCAAAATGAAATGGGGTATGGAGATGAGGCGATGCCTTCTTCAATCCCTTCAACAAATTGAGAGCATGATTGAAGCCGAGTCTTCTGTCAAAGAAAAAGACATGACCAAGGAATTCTTTGAAAAC<br>AGATCAGAAACATGGCCAATTGGAGAGTCACCCAAAGGAGTGGAGGAAGGCTCCATTGGGAAGGTGTGCAGAACATTGCTAGCAAAGTCTGTGTTCAACAGCCTATATGCATCTCCACAACTCGAGGGGTTTTCAGCTGAATCAAGAAAGTTGCTTCTCATTGTTCAGGCACTTAGGGACAACCTGGAACCTGGAACCTTCGATCTTGGGGGGCTATAYGAAGCAATTGA<br>GGAGTGCCTGATTAACGATCCCTGGGTTTTGCTTAATGCGTCTTGGTTCAACTCCTTCCTCACACATGCACTGAAATA-
EPI_ISL_846603	>A/chicken/Poland/476/2020_H5N8_2020-12-04<br>ATGGAAGACTTTGTGCGACAATGCTTCAATCCAATGATTGTCGAGCTTGCGGAAAAGGCAATGAAAGAATATGGGGAAGATCCTAAGATCGAAACAAACAAGTTTGCCGCAATATGTACACACTTAGAAGTCTGCTTCATGTATTCGGATTTCCATTTCATTGATGAACGAGGCGAATCAATAATCGTAGAATCTGGCGACCCGAATGCATTATTGAAGCACCGATTTGAGA<br>TAATTGAAGGGAGAGACCGAACAATGGCCTGGACAGTGGTGAATAGTATATGCAACACTACAGGAGTCGAAAAGCCCAAGTTCCTTCCTGATTTGTATGACTACCGAGAAAACCGATTCATTGAAATTGGAGTAACGCGAAGGGAAGTTCACATATACTATCTTGAAAAAGCCAACAAGATAAAATCAGAGAAAACACACATTCACATATTCTCATTCACTGGGGAGGAA<br>ATGGCCACCAAGGCAGATTACACTCTTGATGAAGAGAGCAGAGCAAGAATAAAAACCAGGCTATTCACTATAAGACAAGAAATGGCCAATAGGGGTCTATGGGATTCCTTTCGTCAGTCCGAGAGAGGCGAAGAGACAATTGAAGAAAGATTTGAAATCACAGGAACCATGCGCAGGCTTGCTGACCAAAGTCTCCCACCGAACTTCGCCAGCCTTGAAAACTTTAGAG<br>CCTATGTGGATGGATTTGAACCGAACGGCTGCATTGAGGGCAAGCTTTCTCAAATGTCAAAAGAAGTGAATGCCAGAATTGAGCCATTTCTGAAGACGACACCACGCCCTCTCAGATTACCTGATGGACCTCCCTGTTCCCAGCGGTCGAAGTTCTTGCTAATGGATGCCCTTAAATTGAGCATTGAAGACCCGAGCCATGAGGGGGAGGGTATACCGCTGTACGATGCAA<br>CCAAATGCATGAAGACATTTTTTGGCTGGAAGGAGCCCAACATCGTGAAACCACATGAAAAGGGCATAAACCCTAATTACCTCCTGGCTTGGAAGCAGGTGCTATCAGAACTCCAAGATATTGAAAACGAGGAGAAAATCCCAAAAACAAAGAACATGAAGAAGACGAGCCAACTGAAGTGGGCACTTGGTGAAAACATGGCACCAGAGAAAGTGGACTTTGAGGACT<br>GTAAAGATGTTAGCGATCTAAGACAGTACGACAGTGACGAACCAGAGTCTAGATCACTAGCAAGTTGGATCCAGAGTGAATTTAACAAGGCTTGCGAACTGACAGATTCGAGTTGGATTGAACTTGATGAAATAGGGGAAGACGTTGCTCCAATTGAACACATTGCGAGTATGAGGAGGAACTATTTCACAGCGGAAGTATCCCATTGCAGGGCTACAGAATACATAATG<br>AAGGGAGTATACATAAACACAGCTTTATTGAATGCATCCTGTGCAGCCATGGATGACTTCCAACTGATTCCAATGATAAGCAAATGCAGAACCAAAGAAGGGAGACGGAAGACAAATCTGTATGGCTTCATTATAAAAGGAAGATCCCATTTGAGGAATGACACCGATGTGGTAAACTTTGTGAGCATGGAATTCTCTCTCACTGACCCAAGGCTGGAGCCACACAAATGG<br>GAAAAGTACTGTGTACTTGAGGTAGGAGACATGCTCCTACGGACTGCAATAGGCCAAGTGTCAAGGCCCATGTTCCTGTATGTGAGAACCAATGGGACTTCTAAGATCAAAATGAAATGGGGTATGGAGATGAGGCGATGCCTTCTTCAATCCCTTCAACAAATTGAGAGCATGATTGAAGCCGAGTCTTCTGTCAAAGAAAAAGACATGACCAAGGAATTCTTTGAAAAC<br>AGATCAGAAACATGGCCAATTGGAGAGTCACCCAAAGGAGTGGAGGAAGGCTCCATTGGGAAGGTGTGCAGAACATTGCTAGCAAAGTCTGTGTTCAACAGCCTATATGCATCTCCACAACTCGAGGGGTTTTCAGCTGAATCAAGAAAGTTGCTTCTCATTGTTCAGGCACTTAGGGACAACCTGGAACCTGGAACCTTCGATCTTGGGGGGCTATATGAAGCAATTGA<br>GGAGTGCCTGATTAACGATCCCTGGGTTTTGCTTAATGCGTCTTGGTTCAACTCCTTCCTCACACATGCACTGAAATA-
EPI_ISL_3102066	>A/chicken/Poland/H812_21RS1385-7/2021_H5N8_2021-04-20<br>ATGGAAGACTTTGTGCGACAATGCTTCAATCCAATGATTGTCGAGCTTGCGGAAAAGGCAATGAAAGAATATGGGGAAGATCCTAAGATCGAAACAAACAAGTTTGCCGCAATATGTACACACTTAGAAGTCTGCTTCATGTATTCGGATTTCCATTTCATTGATGAACGAGGCGAATCAATAATCGTAGAATCTGGCGACCCGAATGCATTATTGAAGCACCGATTTGAGA<br>TAATTGAAGGGAGAGACCGAACAATGGCCTGGACAGTGGTGAATAGTATATGCAACACTACAGGAGTCGAAAAGCCCAAGTTCCTTCCTGATTTGTATGACTACCGAGAAAACCGATTCATTGAAATTGGAGTAACGCGAAGGGAAGTTCACATATACTATCTTGAAAAAGCCAACAAGATAAAATCAGAGAAAACACACATTCACATATTCTCATTCACTGGGGAGGAA<br>ATGGCCACCAAGGCAAATTACACTCTTGATGAAGAGAGCAGAGCAAGAATAAAAACCAGGCTATTCACTATAAGACAAGAGATGGCCAATAGGGGTCTATGGGATTCCTTTCGTCAGTCCGAGAGAGGCGAAGAGACAATTGAAGAAAGATTTGAAATCACAGGAACCATGCGCAGGCTTGCTGACCAAAGTCTCCCACCGAACTTCGCCAGCCTTGAAAACTTTAGAG<br>CCTATGTGGATGGATTTGAACCGAACGGCTGCATTGAGGGCAAGCTTTCTCAAATGTCAAAAGAAGTGAATGCCAGAATTGAGCCATTTCTGAAGACGACACCACGCCCTCTCAGATTACCTGATGGACCTCCCTGTTCCCAGCGGTCGAAGTTCTTGCTAATGGATGCCCTTAAATTGAGCATTGAAGACCCGAGCCATGAGGGGGAGGGTATACCGCTGTACGATGCAA<br>CCAAATGCATGAAGACATTTTTTGGCTGGAAGGAGCCCAACATCGTGAAACCACATGAAAAGGGCATAAACCCTAATTACCTCCTGGCTTGGAAGCAGGTGCTATCAGAACTCCAAGATATTGAAAACGAGGAGAAAATCCCAAAAACAAAGAACATGAAGAAGACGAGCCAACTGAAGTGGGCACTTGGTGAAAACATGGCACCAGAGAAAGTGGACTTTGAGGACT<br>GTAAAGATGTTAGCGATCTAAGACAGTACGACAGTGACGAACCAGAGTCTAGATCACTAGCAAGTTGGATCCAGAGTGAATTTAACAAGGCTTGCGAACTGACAGATTCGAGTTGGATTGAACTTGATGAAATAGGGGAAGACGTTGCTCCAATTGAACACATTGCGAGTATGAGGAGGAACTATTTCACAGCGGAAGTATCCCATTGCAGGGCTACAGAATACATAATG<br>AAGGGAGTATACATAAACACAGCTTTATTGAATGCATCCTGTGCAGCCATGGATGACTTCCAACTGATTCCAATGATAAGCAAATGCAGAACCAAAGAAGGGAGACGGAAGACAAATCTGTATGGCTTCATTATAAAAGGAAGATCCCATTTGAGGAATGACACCGATGTGGTAAACTTTGTGAGCATGGAATTCTCTCTCACTGACCCAAGGCTGGAGCCACACAAATGG<br>GAAAAGTACTGTGTACTTGAGGTAGGAGACATGCTCCTACGGACTGCAATAGGCCAAGTGTCAAGGCCCATGTTCCTGTATGTGAGAACCAATGGGACTTCTAAGATCAAAATGAAATGGGGTATGGAGATGAGGCGATGCCTTCTTCAATCCCTTCAACAAATTGAGAGCATGATTGAAGCCGAGTCTTCTGTCAAAGAAAAAGACATGACCAAGGAGTTCTTTGAAAAC<br>AGATCAGAAACATGGCCAATTGGAGAGTCACCCAAAGGAGTGGAGGAAGGCTCCATTGGGAAGGTGTGCAGAACATTGCTAGCAAAGTCTGTGTTCAACAGCCTATATGCATCTCCACAACTCGAGGGGTTTTCAGCTGAATCAAGAAAGTTGCTTCTCATTGTTCAGGCACTTAGGGACAACCTGGAACCTGGAACCTTCGATCTTGGGGGGCTATATGAAGCAATTGA<br>GGAGTGCCTGATTAACGATCCCTGGGTTTTGCTTAATGCGTCTTGGTTCAACTCCTTCCTCACACATGCACTGAAATA-
EPI_ISL_5144529	>A/domestic_goose/Germany-NI/AI03142/2021_H5N8_2021-03-20<br>ATGGAAGACTTTGTGCGACAATGCTTCAATCCAATGATTGTCGAGCTTGCGGAAAAGGCAATGAAAGAATATGGGGAAGATCCTAAGATCGAAACAAACAAGTTTGCCGCAATATGTACACACTTAGAAGTCTGCTTCATGTATTCGGATTTCCATTTCATTGATGAACGAGGCGAATCAATAATCGTAGAATCTGGMGACCCGAATGCATTATTGAAGCACCGATTTGAG<br>ATAATTGAAGGGAGAGACCGAACAATGGCCTGGACAGTGGTGAATAGTATATGCAACACTACAGGAGTCGAAAAGCCCAAGTTCCTTCCTGATTTGTATGACTACCGAGAAAACCGATTCATTGAAATTGGAGTAACGCGAAGGGAAGTTCACATATACTATCTTGAAAAAGCCAACAAGATAAAATCAGAGAAAACACACATTCACATATTCTCATTCACTGGGGAGGA<br>AATGGCCACCAAGGCAGATTACACTCTTGATGAAGAGAGCAGAGCAAGAATAAAAACCAGGCTATTCACTATAAGACAAGAGATGGCCAATAGGGGTCTATGGGATTCCTTTCGTCAGTCCGAGAGAGGCGAAGAGACAATTGAAGAAAGATTTGAAATCACAGGAACCATGCGCAGGCTTGCTGACCAAAGTCTCCCACCGAACTTCGCCAGCCTTGAAAACTTTAGA<br>GCCTATGTGGATGGATTTGAACCGAACGGCTGCATTGAGGGCAAGCTTTCTCAAATGTCAAAAGAAGTGAATGCCAGAATTGAGCCATTTCTGAAGACGACACCACGCCCTCTCAGATTACCTGATGGACCTCCCTGTTCCCAGCGGTCGAAGTTCTTGCTAATGGATGCCCTTAAATTGAGCATTGAAGACCCGAGCCATGAGGGGGAGGGTATACCGCTGTACGATGCA<br>ACCAAATGCATGAAGACATTTTTTGGCTGGAAGGAGCCCAACATCGTGAAACCACATGAAAAGGGCATAAACCCTAATTACCTCCTGGCTTGGAAGCAGGTGCTATCAGAACTCCAAGATATTGAAAACGAGGAGAAAATCCCAAAAACAAAGAACATGAAGAAGACGAGCCAACTGAAGTGGGCACTTGGTGAAAACATGGCACCAGAGAAAGTGGACTTTGAGGAC<br>TGTAAAGATGTTAGCGATCTAAGACAGTACGACAGTGACGAACCAGAGTCTAGATCACTAGCAAGTTGGATCCAGAGTGAATTTAACAAGGCTTGCGAACTGACAGATTCGAGTTGGATTGAACTTGATGAAATAGGGGAAGACGTTGCTCCAATTGAACACATTGCGAGTATGAGGAGGAACTATTTCACAGCGGAAGTATCCCATTGCAGGGCTACAGAATACATAAT<br>GAAAGGAGTATACATAAACACAGCTTTATTGAATGCATCCTGTGCAGCCATGGATGACTTCCAACTGATTCCAATGATAAGCAAATGCAGAACCAAAGAAGGGAGACGGAAGACAAATCTGTATGGCTTCATTATAAAAGGAAGATCCCATTTGAGGAATGACACCGATGTGGTAAACTTTGTGAGCATGGAATTCTCTCTCACTGACCCAAGGCTGGAGCCACACAAATG<br>GGAAAAGTACTGTGTACTTGAGGTAGGAGACATGCTCCTACGGACTGCAATAGGCCAAGTGTCAAGGCCCATGTTCCTGTATGTGAGAACCAATGGGACTTCTAAGATCAAAATGAAATGGGGTATGGAGATGAGGCGATGCCTTCTTCAATCCCTTCAACAAATTGAGAGCATGATTGAAGCYGAGTCTTCTGTCAAAGAAAAAGACATGACCAAGGAATTCTTTGAAAA<br>CAGATCAGAAACATGGCCAATTGGAGAATCACCCAAAGGAGTGGAGGAAGGCTCCATTGGGAAGGTGTGCAGAACATTGCTAGCAAAGTCTGTGTTCAACAGCCTATATGCATCTCCACAACTCGAGGGGTTTTCAGCTGAATCAAGAAAGTTGCTTCTCATTGTTCAGGCACTTAGGGACAACCTGGAACCTGGAACCTTCGATCTTGGGGGGCTATATGAAGCAATTGA<br>GGAGTGCCTGATTAACGATCCCTGGGTTTTGCTTAATGCGTCTTGGTTCAACTCCTTCCTCACACATGCACTGAAATA-
EPI_ISL_5095645	>A/turkey/Germany-MV/AI00909/2021_H5N8_2021-01-26<br>ATGGAAGACTTTGTGCGACAATGCTTCAATCCAATGATTGTCGAGCTTGCGGAAAAGGCAATGAAAGAATATGGGGAAGATCCTAAGATCGAAACAAACAAGTTTGCCGCAATATGTACACACTTAGAAGTCTGCTTCATGTATTCGGATTTCCATTTCATTGATGAACGAGGCGAATCAATAATCGTAGAATCTGGCGACCCGAATGCATTATTGAAGCACCGATTTGAGA<br>TAATTGAAGGGAGAGACCGAACAATGGCCTGGACAGTGGTGAATAGTATATGCAACACTACAGGAGTCGAAAAGCCCAAGTTCCTTCCTGATTTGTATGACTACMGAGAAAACCGATTCATTGAAATTGGAGTAACGCGAAGGGAAGTTCACATATACTATCTTGAAAAAGCCAACAAGATAAAATCAGAGAAAACACACATTCACATATTCTCATTCACTGGGGAGGAA<br>ATGGCCACCAAGGCAGATTACACTCTTGATGAAGAGAGCAGAGCAAGAATAAAAACCAGGCTATTCACTATAAGACAAGAGATGGCCAATAGGGGTCTATGGGATTCCTTTCGTCAGTCCGAGAGAGGCGAAGAGACAATTGAAGAAAGATTTGAAATCACAGGAACCATGCGCAGGCTTGCTGACCAAAGTCTCCCACCGAACTTCGCCAGCCTTGAAAACTTTAGAG<br>CCTATGTGGATGGATTTGAACCGAACGGCTGCATTGAGGGCAAGCTTTCTCAAATGTCAAAAGAAGTGAATGCCAGAATTGAGCCATTTCTGAAGACGACACCACGCCCTCTCAGATTACCTGATGGACCTCCCTGTTCCCAGCGGTCGAAGTTCTTGCTAATGGATGCCCTTAAATTGAGCATTGAAGACCCGAGCCATGAGGGGGAGGGTATACCGCTGTACGATGCAA<br>CCAAATGCATGAAGACATTTTTTGGCTGGAAGGAGCCCAACATCGTGAAACCACATGAAAAGGGCATAAACCCTAATTACCTCCTGGCTTGGAAGCAGGTGCTATCAGAACTCCAAGATATTGAAAACGAGGAGAAAATCCCAAAAACAAAGAACATGAAGAAGACGAGCCAACTGAAGTGGGCACTTGGTGAAAACATGGCACCAGAGAAAGTGGACTTTGAGGACT<br>GTAAAGATGTTAGCGATCTAAGACAGTACGACAGTGACGAACCAGAGTCTAGATCACTAGCAAGTTGGATCCAGAGTGAATTTAACAAGGCTTGCGAACTGACAGATTCGAGTTGGATTGAACTTGATGAAATAGGGGAAGACGTTGCTCCAATTGAACACATTGCGAGTATGAGGAGGAACTATTTCACAGCGGAAGTATCCCATTGCAGGGCTACAGAATACATAATG<br>AAAGGAGTATACATAAACACAGCTTTATTGAATGCATCCTGTGCAGCCATGGATGACTTCCAACTGATTCCAATGATAAGCAAATGCAGAACCAAAGAAGGGAGACGGAAGACAAATCTGTATGGCTTCATTATAAAAGGAAGATCCCATTTGAGGAATGACACCGATGTGGTAAACTTTGTGAGCATGGAATTCTCTCTCACTGACCCAAGGCTGGAGCCACACAAATGG<br>GAAAAGTACTGTGTACTTGAGGTAGGAGACATGCTCCTACGGACTGCAATAGGCCAAGTGTCAAGGCCCATGTTCCTGTATGTGAGAACCAATGGGACTTCTAAGATCAAAATGAAATGGGGTATGGAGATGAGGCGATGCCTTCTTCAATCCCTTCAACAAATTGAGAGCATGATTGAAGCCGAGTCTTCTGTCAAAGAAAAAGACATGACCAAGGAATTCTTTGAAAAC<br>AGATCAGAAACATGGCCAATTGGAGAATCACCCAAAGGAGTGGAGGAAGGCTCCATTGGGAAGGTGTGCAGAACATTGCTAGCAAAGTCTGTGTTCAACAGCCTATATGCATCTCCACAACTCGAGGGGTTTTCAGCTGAATCAAGAAAGTTGCTTCTCATTGTTCAGGCACTTAGGGACAACCTGGAACCTGGAACCTTCGATCTTGGGGGGCTATATGAAGCAATTGA<br>GGAGTGCCTGATTAACGATCCCTGGGTTTTGCTTAATGCGTCTTGGTTCAACTCCTTCCTCACACATGCACTGAAATA-
EPI_ISL_5102132	>A/chicken/Germany-BY/AI02064/2021_H5N8_2021-03-01<br>ATGGAAGACTTTGTGCGACAATGCTTCAATCCAATGATTGTCGAGCTTGCGGAAAAGGCAATGAAAGAATATGGGGAAGATCCTAAGATCGAAACAAACAAGTTTGCCGCAATATGTACACACTTAGAAGTCTGCTTCATGTATTCGGATTTTCATTTCATTGATGAACGAGGCGAATCAATAATCGTAGAATCTGGCGACCCGAATGCATTATTGAAGCACCGATTTGAGA<br>TAATTGAAGGGAGAGACCGAACAATGGCCTGGACAGTGGTGAATAGTATATGCAACACTACAGGAGTCGAAAAGCCCAAGTTCCTTCCTGATTTGTATGACTACCGAGAAAACCGATTCATTGAAATTGGAGTAACGCGAAGGGAAGTTCACATATACTATCTTGAAAAAGCCAACAAGATAAAATCAGAGAAAACACACATTCACATATTCTCATTCACTGGGGAGGAA<br>ATGGCCACCAAGGCAGATTACACTCTTGATGAAGAGAGCAGAGCAAGAATAAAAACCAGGCTATTCACTATAAGACAAGAGATGGCCAATAGGGGTCTATGGGATTCCTTTCGTCAGTCCGAGAGAGGCGAAGAGACAATTGAAGAAAGATTTGAAATCACAGGAACCATGCGCAGGCTTGCTGACCAAAGTCTCCCACCGAACTTCGCCAGCCTTGAAAACTTTAGAG<br>CCTATGTGGATGGATTTGAACCGAACGGCTGCATTGAGGGCAAGCTTTCTCAAATGTCAAAAGAAGTGAATGCCAGAATTGAGCCATTTCTGAAGACGACACCACGCCCTCTCAGATTACCTGATGGACCTCCCTGTTCCCAGCGGTCGAAGTTCTTGCTAATGGATGCCCTTAAATTGAGCATTGAAGACCCGAGCCATGAGGGGGAGGGTATACCGCTGTACGATGCAA<br>CCAAATGCATGAAGACATTTTTTGGCTGGAAGGAGCCCAACATCGTGAAACCACATGAAAAGGGCATAAACCCTAATTACCTCCTGGCTTGGAAGCAGGTGCTATCAGAACTCCAAGATATTGAAAACGAGGAGAAAATCCCAAAAACAAAGAACATGAAGAAGACGAGCCAACTGAAGTGGGCACTTGGTGAAAACATGGCACCAGAGAAAGTGGACTTTGAGGACT<br>GTAAAGATGTTAGCGATCTAAGACAGTACGACAGTGACGAACCAGAGTCTAGATCACTAGCAAGTTGGATCCAGAGTGAATTTAACAAGGCTTGCGAACTGACAGATTCGAGTTGGATTGAACTTGATGAAATAGGGGAAGACGTTGCTCCAATTGAACACATTGCGAGTATGAGGAGGAACTATTTCACAGCGGAAGTATCCCATTGCAGGGCTACAGAATACATAATG<br>AAAGGAGTATACATAAACACAGCTTTATTGAATGCATCCTGTGCAGCCATGGATGACTTCCAACTGATTCCAATGATAAGCAAATGCAGAACCAAAGAAGGGAGACGGAAGACAAATCTGTATGGCTTCATTATAAAAGGAAGATCCCATTTGAGGAATGACACCGATGTGGTAAACTTTGTGAGCATGGAATTCTCTCTCACTGACCCAAGGCTGGAGCCACACAAATGG<br>GAAAAGTACTGTGTACTTGAGGTAGGAGACATGCTCCTACGGACTGCAATAGGCCAAGTGTCAAGGCCCATGTTCCTGTATGTGAGAACCAATGGGACTTCTAAGATCAAAATGAAATGGGGTATGGAGATGAGGCGATGCCTTCTTCAATCCCTTCAACAAATTGAGAGCATGATTGAAGCCGAGTCTTCTGTCAAAGAAAAAGACATGACCAAGGAATTCTTTGAAAAC<br>AGATCAGAAACATGGCCAATTGGAGAATCACCCAAAGGAGTGGAGGAAGGCTCCATTGGGAAGGTGTGCAGAACATTGCTAGCAAAGTCTGTGTTCAACAGCCTATATGCATCTCCACAACTCGAGGGGTTTTCAGCTGAATCAAGAAAGTTGCTTCTCATTGTTCAGGCACTTAGGGACAACCTGGAACCTGGAACCTTCGATCTTGGGGGGCTATATGAAGCAATTGA<br>GGAGTGCCTGATTAACGATCCCTGGGTTTTGCTTAATGCGTCTTGGTTCAACTCCTTCCTCACACATGCACTGAAATA-
EPI_ISL_3102070	>A/white_stork/Poland/MB412_21RS1385-11/2021_H5N8_2021-05-02<br>ATGGAAGACTTTGTGCGACAATGCTTCAATCCAATGATTGTCGAGCTTGCGGAAAAGGCAATGAAAGAATATGGGGAAGATCCTAAGATCGAAACAAACAAGTTTGCCGCAATATGTACACACTTAGAAGTCTGCTTCATGTATTCGGATTTCCATTTCATTGATGAACGAGGCGAATCAATAATCGTAGAATCTGGCGACCCGAATGCATTATTGAAGCACCGATTTGAGA<br>TAATTGAAGGGAGAGACCGAACAATGGCCTGGACAGTGGTGAATAGTATATGCAACACTACAGGAGTCGAAAAGCCCAAGTTCCTTCCTGATTTGTATGACTACCGAGAAAACCGATTCATTGAAATTGGAGTAACGCGAAGGGAAGTTCACATATACTATCTTGAAAAAGCCAACAAGATAAAATCAGAGAAAACACACATTCACATATTCTCATTCACTGGGGAGGAA<br>ATGGCCACCAAGGCAGATTACACTCTTGATGAAGAGAGCAGAGCAAGAATAAAAACCAGGCTATTCACTATAAGACAAGAGATGGCCAATAGGGGTCTATGGGATTCCTTTCGTCAGTCCGAGAGAGGCGAAGAGACAATTGAAGAAAGATTTGAAATCACAGGAACCATGCGCAGGCTTGCTGACCAAAGTCTCCCACCGAACTTCGCCAGCCTTGAAAACTTTAGAG<br>CCTATGTGGATGGATTTGAACCGAACGGCTGCATTGAGGGCAAGCTTTCTCAAATGTCAAAAGAAGTGAATGCCAGAATTGAGCCATTTCTGAAGACGACACCACGCCCTCTCAGATTACCTGATGGACCTCCCTGTTCCCAGCGGTCGAAGTTCTTGCTAATGGATGCCCTTAAATTAAGCATTGAAGACCCGAGCCATGAGGGGGAGGGTATACCGCTGTACGATGCAA<br>CCAAATGCATGAAGACATTTTTTGGCTGGAAGGAGCCCAACATCGTGAAACCACATGAAAAGGGCATAAACCCTAATTACCTCCTGGCTTGGAAGCAGGTGCTATCAGAACTCCAAGATATTGAAAACGAGGAGAAAATCCCAAAAACAAAGAACATGAAGAAGACGAGCCAACTGAAGTGGGCACTTGGTGAAAACATGGCACCAGAGAAAGTGGACTTTGAGGACT<br>GTAAAGATGTTAGCGATCTAAGACAGTACGACAGTGACGAACCAGAGTCTAGATCACTAGCAAGTTGGATCCAGAGTGAATTTAACAAGGCTTGCGAACTGACAGATTCGAGTTGGATTGAACTTGATGAAATAGGGGAAGACGTTGCTCCAATTGAACACATTGCGAGTATGAGGAGGAACTATTTCACAGCGGAAGTATCCCATTGCAGGGCTACAGAATACATAATG<br>AAAGGAGTATACATAAACACAGCTTTATTGAATGCATCCTGTGCAGCCATGGATGACTTCCAACTGATTCCAATGATAAGCAAATGCAGAACCAAAGAAGGGAGACGGAAGACAAATCTGTATGGCTTCATTATAAAAGGAAGATCCCATTTGAGGAATGACACCGATGTGGTAAACTTTGTGAGCATGGAATTCTCTCTCACTGACCCAAGGCTGGAGCCACACAAATGG<br>GAAAAGTACTGTGTACTTGAGGTAGGAGACATGCTCCTACGGACTGCAATAGGCCAAGTGTCAAGGCCCATGTTCCTGTATGTGAGAACCAATGGGACTTCTAAGATCAAAATGAAATGGGGTATGGAGATGAGGCGATGCCTTCTTCAATCCCTTCAACAAATTGAGAGCATGATTGAAGCCGAGTCTTCTGTCAAAGAAAAAGACATGACCAAGGAATTCTTTGAAAAC<br>AGATCAGAAACATGGCCAATTGGAGAATCACCCAAAGGAGTGGAGGAAGGCTCCATTGGGAAGGTGTGCAGAACATTGCTAGCAAAGTCTGTGTTCAACAGCCTATATGCATCTCCACAACTCGAGGGGTTTTCAGCTGAATCAAGAAAGTTGCTTCTCATTGTTCAGGCACTTAGGGACAACCTGGAACCTGGAACCTTCGATCTTGGGGGGCTATATGAAGCAATTGA<br>GGAGTGCCTGATTAACGATCCCTGGGTTTTGCTTAATGCGTCTTGGTTCAACTCCTTCCTCACACATGCACTGAAATA-
EPI_ISL_3102067	>A/chicken/Poland/H293_21RS1385-8/2021_H5N8_2021-03-23<br>ATGGAAGACTTTGTGCGACAATGCTTCAATCCAATGATTGTCGAGCTTGCGGAAAAGGCAATGAAAGAATATGGGGAAGATCCTAAGATCGAAACAAACAAGTTTGCCGCAATATGTACACACTTAGAAGTCTGCTTCATGTATTCGGATTTCCATTTCATTGATGAACGAGGCGAATCAATAATCGTAGAATCTGGCGACCCGAATGCATTATTGAAGCACCGATTTGAGA<br>TAATTGAAGGGAGAGACCGAACAATGGCCTGGACAGTGGTGAATAGTATATGCAACACTACAGGAGTCGAAAAGCCCAAGTTCCTTCCTGATTTGTATGACTACCGAGAAAACCGATTCATTGAAATTGGAGTAACGCGAAGGGAAGTTCACATATACTATCTTGAAAAAGCCAACAAGATAAAATCAGAGAAAACACACATTCACATATTCTCATTCACTGGGGAGGAA<br>ATGGCCACCAAGGCAGATTACACTCTTGATGAAGAGAGCAGAGCAAGAATAAAAACCAGGCTATTCACTATAAGACAAGAGATGGCCAATAGGGGTCTATGGGATTCCTTTCGTCAGTCCGAGAGAGGCGAAGAGACAATTGAAGAAAGATTTGAAATCACAGGAACCATGCGCAGGCTTGCTGACCAAAGTCTCCCACCGAACTTCGCCAGCCTTGAAAACTTTAGAG<br>CCTATGTGGATGGATTTGAACCGAACGGCTGCATTGAGGGCAAGCTTTCTCAAATGTCAAAAGAAGTGAATGCCAGAATTGAGCCATTTCTGAAGACGACACCACGCCCTCTCAGATTACCTGATGGACCTCCCTGTTCCCAGCGGTCGAAGTTCTTGCTAATGGATGCCCTTAAATTAAGCATTGAAGACCCGAGCCATGAGGGGGAGGGTATACCGCTGTACGATGCAA<br>CCAAATGCATGAAGACATTTTTTGGCTGGAAGGAGCCCAACATCGTGAAACCACATGAAAAGGGCATAAACCCTAATTACCTCCTGGCTTGGAAGCAGGTGCTATCAGAACTCCAAGATATTGAAAACGAGGAGAAAATCCCAAAAACAAAGAACATGAAGAAGACGAGYCAACTGAAGTGGGCACTTGGTGAAAACATGGCACCAGAGAAAGTGGACTTTGAGGACT<br>GTAAAGATGTTAGCGATCTAAGACAGTACGACAGTGACGAACCAGAGTCTAGATCACTAGCAAGTTGGATCCAGAGTGAATTTAACAAGGCTTGCGAACTGACAGATTCGAGTTGGATTGAACTTGATGAAATAGGGGAAGACGTTGCTCCAATTGAACACATTGCGAGTATGAGGAGGAACTATTTCACAGCGGAAGTATCCCATTGCAGGGCTACAGAATACATAATG<br>AAAGGAGTATACATAAACACAGCTTTATTGAATGCATCCTGTGCAGCCATGGATGACTTCCAACTGATTCCAATGATAAGCAAATGCAGAACCAAAGAAGGGAGACGGAAGACAAATCTGTATGGCTTCATTATAAAAGGAAGATCCCATTTGAGGAATGACACCGATGTGGTAAACTTTGTGAGCATGGAATTCTCTCTCACTGACCCAAGGCTGGAGCCACACAAATGG<br>GAAAAGTACTGTGTACTTGAGGTAGGAGACATGCTCCTACGGACTGCAATAGGCCAAGTGTCAAGGCCCATGTTCCTGTATGTGAGAACCAATGGGACTTCTAAGATCAAAATGAAATGGGGTATGGAGATGAGGCGATGCCTTCTTCAATCCCTTCAACAAATTGAGAGCATGATTGAAGCCGAGTCTTCTGTCAAAGAAAAAGACATGACCAAGGAATTCTTTGAAAAC<br>AGATCAGAAACATGGCCAATTGGAGAATCACCCAAAGGAGTGGAGGAAGGCTCCATTGGGAAGGTGTGCAGAACATTGCTAGCAAAGTCTGTGTTCAACAGCCTATATGCATCTCCACAACTCGAGGGGTTTTCAGCTGAATCAAGAAAGTTGCTTCTCATTGTTCAGGCACTTAGGGACAACCTGGAACCTGGAACCTTCGATCTTGGGGGGCTATATGAAGCAATTGA<br>GGAGTGCCTGATTAACGATCCCTGGGTTTTGCTTAATGCGTCTTGGTTCAACTCCTTCCTCACACATGCACTGAAATA-

EPI_ISL_3102071

>A/chicken/Poland/H712_21RS1385-12/2021_H5N8_2021-04-16
ATGGAAGACTTTGTGCGACAATGCTTCAATCCAATGATTGTCGAGCTTGCGGAAAAGGCAATGAAAGAATATGGGGAAGATCCTAAGATCGAAACAAACAAGTTTGCCGCAATATGTACACACTTAGAAGTCTGCTTCATGTATTCGGATTTCCATTTCATTGATGAACGAGGCGAATCAATAATCGTAGAATCTGGCGACCCGAATGCATTATTGAAGCACCGATTTGAGA
TAATTGAAGGGAGAGACCGAACAATGGCCTGGACAGTGGTGAATAGTATATGCAACACTACAGGAGTCGAAAAGCCCAAGTTCCTTCCTGATTTGTATGACTACCGAGAAAACCGATTCATTGAAATTGGAGTAACGCGAAGGGAAGTTCACATATACTATCTTGAAAAAGCCAACAAGATAAAATCAGAGAAAACACACATTCACATATTCTCATTCACTGGGGAGGAA
ATGGCCACCAAGGCAGATTACACTCTTGATGAAGAGAGCAGAGCAAGAATAAAAACCAGGCTATTCACTATAAGACAAGAGATGGCCAATAGGGGTCTATGGGATTCCTTTCGTCAGTCCGAGAGAGGCGAAGAGACAATTGAAGAAAGATTTGAAATCACAGGAACCATGCGCAGGCTTGCTGACCAAAGTCTCCCACCGAACTTCGCCAGCCTTGAAAACTTTAGAG
CCTATGTGGATGGATTTGAACCGAACGGCTGCATTGAGGGCAAGCTTTCTCAAATGTCAAAAGAAGTGAATGCCAGAATTGAGCCATTTCTGAAGACGACACCACGCCCTCTCAGATTACCTGATGGACCTCCCTGTTCCCAGCGGTCGAAGTTCTTGCTAATGGATGCCCTTAAATTAAGCATTGAAGACCCGAGCCATGAGGGGGAGGGTATACCGCTGTACGATGCAA
CCAAATGCATGAAGACATTTTTTGGCTGGAAGGAGCCCAACATCGTGAAACCACATGAAAAGGGCATAAACCCTAATTACCTCCTGGCTTGGAAGCAGGTGCTATCAGAACTCCAAGATATTGAAAACGAGGAGAAAATCCCAAAAACAAAGAACATGAAGAAGACGAGCCAACTGAAGTGGGCACTTGGTGAAAACATGGCACCAGAGAAAGTGGACTTTGAGGACT
GTAAAGATGTTAGCGATCTAAGACAGTACGACAGTGACGAACCAGAGTCTAGATCACTAGCAAGTTGGATCCAGAGTGAATTTAACAAGGCTTGCGAACTGACAGATTCGAGTTGGATTGAACTTGATGAAATAGGGGAAGACGTTGCTCCAATTGAACACATTGCGAGTATGAGGAGGAACTATTTCACAGCGGAAGTATCCCATTGCAGGGCTACAGAATACATAATG
AAAGGAGTATACATAAACACAGCTTTATTGAATGCATCCTGTGCAGCCATGGATGACTTCCAACTGATTCCAATGATAAGCAAATGCAGAACCAAAGAAGGGAGACGGAAGACAAATCTGTATGGCTTCATTATAAAAGGAAGATCCCATTTGAGGAATGACACCGATGTGGTAAACTTTGTGAGCATGGAATTCTCTCTCACTGACCCAAGGCTGGAGCCACACAAATGG
GAAAAGTACTGTGTACTTGAGGTAGGAGACATGCTCCTACGGACTGCAATAGGCCAAGTGTCAAGGCCCATGTTCCTGTATGTGAGAACCAATGGGACTTCTAAGATCAAAATGAAATGGGGTATGGAGATGAGGCGATGCCTTCTTCAATCCCTTCAACAAATTGAGAGCATGATTGAAGCCGAGTCTTCTGTCAAAGAAAAAGACATGACCAAGGAATTCTTTGAAAAC
AGATCAGAAACATGGCCAATTGGAGAATCACCCAAAGGAGTGGAGGAAGGCTCCATTGGGAAGGTGTGCAGAACATTGCTAGCAAAGTCTGTGTTCAACAGCCTATATGCATCTCCACAACTCGAGGGGTTTTCAGCTGAATCAAGAAAGTTGCTTCTCATTGTTCAGGCACTTAGGGACAACCTGGAACCTGGAACCTTCGATCTTGGGGGGCTATATGAAGCAATTGA
GGAGTGCCTGATTAACGATCCCTGGGTTTTGCTTAATGCGTCTTGGTTCAACTCCTTCCTCACACATGCACTGAAATA-

EPI_ISL_3102072

>A/chicken/Poland/H1084_21RS1385-13/2021_H5N8_2021-05-07
ATGGAAGACTTTGTGCGACAATGCTTCAATCCAATGATTGTCGAGCTTGCGGAAAAGGCAATGAAAGAATATGGGGAAGATCCTAAGATCGAAACAAACAAGTTTGCCGCAATATGTACACACTTAGAAGTCTGCTTCATGTATTCGGATTTCCATTTCATTGATGAACGAGGCGAATCAATAATCGTAGAATCTGGCGACCCGAATGCATTATTGAAGCACCGATTTGAGA
TAATTGAAGGGAGAGACCGAACAATGGCCTGGACAGTGGTGAATAGTATATGCAACACTACAGGAGTCGAAAAGCCCAAGTTCCTTCCTGATTTGTATGACTACCGAGAAAACCGATTCATTGAAATTGGAGTAACGCGAAGGGAAGTTCACATATACTATCTTGAAAAAGCCAACAAGATAAAATCAGAGAAAACACACATTCACATATTCTCATTCACTGGGGAGGAA
ATGGCCACCAAGGCAGATTACACTCTTGATGAAGAGAGCAGAGCAAGAATAAAAACCAGGCTATTCACTATAAGACAAGAGATGGCCAATAGGGGTCTATGGGATTCCTTTCGTCAGTCCGAGAGAGGCGAAGAGACAATTGAAGAAAGATTTGAAATCACAGGAACCATGCGCAGGCTTGCTGACCAAAGTCTCCCACCGAACTTCGCCAGCCTTGAAAACTTTAGAG
CCTATGTGGATGGATTTGAACCGAACGGCTGCATTGAGGGCAAGCTTTCTCAAATGTCAAAAGAAGTGAATGCCAGAATTGAGCCATTTCTGAAGACGACACCACGCCCTCTCAGATTACCTGATGGACCTCCCTGTTCCCAGCGGTCGAAGTTCTTGCTAATGGATGCCCTTAAATTAAGCATTGAAGACCCGAGCCATGAGGGGGAGGGTATACCGCTGTACGATGCAA
CCAAATGCATGAAGACATTTTTTGGCTGGAAGGAGCCCAACATCGTGAAACCACATGAAAAGGGCATAAACCCTAATTACCTCCTGGCTTGGAAGCAGGTGCTATCAGAACTCCAAGATATTGAAAACGAGGAGAAAATCCCAAAAACAAAGAACATGAAGAAGACGAGCCAACTGAAGTGGGCACTTGGTGAAAACATGGCACCAGAGAAAGTGGACTTTGAGGACT
GTAAAGATGTTAGCGATCTAAGACAGTACGACAGTGACGAACCAGAGTCTAGATCACTAGCAAGTTGGATCCAGAGTGAATTTAACAAGGCTTGCGAACTGACAGATTCGAGTTGGATTGAACTTGATGAAATAGGGGAAGACGTTGCTCCAATTGAACACATTGCGAGTATGAGGAGGAACTATTTCACAGCGGAAGTATCCCATTGCAGGGCTACAGAATACATAATG
AAAGGAGTATACATAAACACAGCTTTATTGAATGCATCCTGTGCAGCCATGGATGACTTCCAACTGATTCCAATGATAAGCAAATGCAGAACCAAAGAAGGGAGACGGAAGACAAATCTGTATGGCTTCATTATAAAAGGAAGATCCCATTTGAGGAATGACACCGATGTGGTAAACTTTGTGAGCATGGAATTCTCTCTCACTGACCCAAGGCTGGAGCCACACAAATGG
GAAAAGTACTGTGTACTTGAGGTAGGAGACATGCTCCTACGGACTGCAATAGGCCAAGTGTCAAGGCCCATGTTCCTGTATGTGAGAACCAATGGGACTTCTAAGATCAAAATGAAATGGGGTATGGAGATGAGGCGATGCCTTCTTCAATCCCTTCAACAAATTGAGAGCATGATTGAAGCCGAGTCTTCTGTCAAAGAAAAAGACATGACCAAGGAATTCTTTGAAAAC
AGATCAGAAACATGGCCAATTGGAGAATCACCCAAAGGAGTGGAGGAAGGCTCCATTGGGAAGGTGTGCAGAACATTGCTAGCAAAGTCTGTGTTCAACAGCCTATATGCATCTCCACAACTCGAGGGGTTTTCAGCTGAATCAAGAAAGTTGCTTCTCATTGTTCAGGCACTTAGGGACAACCTGGAACCTGGAACCTTCGATCTTGGGGGGCTATATGAAGCAATTGA
GGAGTGCCTGATTAACGATCCCTGGGTTTTGCTTAATGCGTCTTGGTTCAACTCCTTCCTCACACATGCACTGAAATA-

EPI_ISL_3102073

>A/turkey/Poland/H1184_21RS1385-14/2021_H5N8_2021-05-14
ATGGAAGACTTTGTGCGACAATGCTTCAATCCAATGATTGTCGAGCTTGCGGAAAAGGCAATGAAAGAATATGGGGAAGATCCTAAGATCGAAACAAACAAGTTTGCCGCAATATGTACACACTTAGAAGTCTGCTTCATGTATTCGGATTTCCATTTCATTGATGAACGAGGCGAATCAATAATCGTAGAATCTGGCGACCCGAATGCATTATTGAAGCACCGATTTGAGA
TAATTGAAGGGAGAGACCGAACAATGGCCTGGACAGTGGTGAATAGTATATGCAACACTACAGGAGTCGAAAAGCCCAAGTTCCTTCCTGATTTGTATGACTACCGAGAAAACCGATTCATTGAAATTGGAGTAACGCGAAGGGAAGTTCACATATACTATCTTGAAAAAGCCAACAAGATAAAATCAGAGAAAACACACATTCACATATTCTCATTCACTGGGGAGGAA
ATGGCCACCAAGGCAGATTACACTCTTGATGAAGAGAGCAGAGCAAGAATAAAAACCAGGCTATTCACTATAAGACAAGAGATGGCCAATAGGGGTCTATGGGATTCCTTTCGTCAGTCCGAGAGAGGCGAAGAGACAATTGAAGAAAGATTTGAAATCACAGGAACCATGCGCAGGCTTGCTGACCAAAGTCTCCCACCGAACTTCGCCAGCCTTGAAAACTTTAGAG
CCTATGTGGATGGATTTGAACCGAACGGCTGCATTGAGGGCAAGCTTTCTCAAATGTCAAAAGAAGTGAATGCCAGAATTGAGCCATTTCTGAAGACGACACCACGCCCTCTCAGATTACCTGATGGACCTCCCTGTTCCCAGCGGTCGAAGTTCTTGCTAATGGATGCCCTTAAATTAAGCATTGAAGACCCGAGCCATGAGGGGGAGGGTATACCGCTGTACGATGCAA
CCAAATGCATGAAGACATTTTTTGGCTGGAAGGAGCCCAACATCGTGAAACCACATGAAAAGGGCATAAACCCTAATTACCTCCTGGCTTGGAAGCAGGTGCTATCAGAACTCCAAGATATTGAAAACGAGGAGAAAATCCCAAAAACAAAGAACATGAAGAAGACGAGCCAACTGAAGTGGGCACTTGGTGAAAACATGGCACCAGAGAAAGTGGACTTTGAGGACT
GTAAAGATGTTAGCGATCTAAGACAGTACGACAGTGACGAACCAGAGTCTAGATCACTAGCAAGTTGGATCCAGAGTGAATTTAACAAGGCTTGCGAACTGACAGATTCGAGTTGGATTGAACTTGATGAAATAGGGGAAGACGTTGCTCCAATTGAACACATTGCGAGTATGAGGAGGAACTATTTCACAGCGGAAGTATCCCATTGCAGGGCTACAGAATACATAATG
AAAGGAGTATACATAAACACAGCTTTATTGAATGCATCCTGTGCAGCCATGGATGACTTCCAACTGATTCCAATGATAAGCAAATGCAGAACCAAAGAAGGGAGACGGAAGACAAATCTGTATGGCTTCATTATAAAAGGAAGATCCCATTTGAGGAATGACACCGATGTGGTAAACTTTGTGAGCATGGAATTCTCTCTCACTGACCCAAGGCTGGAGCCACACAAATGG
GAAAAGTACTGTGTACTTGAGGTAGGAGACATGCTCCTACGGACTGCAATAGGCCAAGTGTCAAGGCCCATGTTCCTGTATGTGAGAACCAATGGGACTTCTAAGATCAAAATGAAATGGGGTATGGAGATGAGGCGATGCCTTCTTCAATCCCTTCAACAAATTGAGAGCATGATTGAAGCCGAGTCTTCTGTCAAAGAAAAAGACATGACCAAGGAATTCTTTGAAAAC
AGATCAGAAACATGGCCAATTGGAGAATCACCCAAAGGAGTGGAGGAAGGCTCCATTGGGAAGGTGTGCAGAACATTGCTAGCAAAGTCTGTGTTCAACAGCCTATATGCATCTCCACAACTCGAGGGGTTTTCAGCTGAATCAAGAAAGTTGCTTCTCATTGTTCAGGCACTTAGGGACAACCTGGAACCTGGAACCTTCGATCTTGGGGGGCTATATGAAGCAATTGA
GGAGTGCCTGATTAACGATCCCTGGGTTTTGCTTAATGCGTCTTGGTTCAACTCCTTCCTCACACATGCACTGAAATA-

EPI_ISL_3102074

>A/turkey/Poland/H1168_21RS1385-15/2021_H5N8_2021-05-13
ATGGAAGACTTTGTGCGACAATGCTTCAATCCAATGATTGTCGAGCTTGCGGAAAAGGCAATGAAAGAATATGGGGAAGATCCTAAGATCGAAACAAACAAGTTTGCCGCAATATGTACACACTTAGAAGTCTGCTTCATGTATTCGGATTTCCATTTCATTGATGAACGAGGCGAATCAATAATCGTAGAATCTGGCGACCCGAATGCATTATTGAAGCACCGATTTGAGA
TAATTGAAGGGAGAGACCGAACAATGGCCTGGACAGTGGTGAATAGTATATGCAACACTACAGGAGTCGAAAAGCCCAAGTTCCTTCCTGATTTGTATGACTACCGAGAAAACCGATTCATTGAAATCGGAGTAACGCGAAGGGAAGTTCACATATACTATCTTGAAAAAGCCAACAAGATAAAATCAGAGAAAACACACATTCACATATTCTCATTCACTGGGGAGGAA
ATGGCCACCAAGGCAGATTACACTCTTGATGAAGAGAGCAGAGCAAGAATAAAAACCAGGCTATTCACTATAAGACAAGAGATGGCCAATAGGGGTCTATGGGATTCCTTTCGTCAGTCCGAGAGAGGCGAAGAGACAATTGAAGAAAGATTTGAAATCACAGGAACCATGCGCAGGCTTGCTGACCAAAGTCTCCCACCGAACTTCGCCAGCCTTGAAAACTTTAGAG
CCTATGTGGATGGATTTGAACCGAACGGCTGCATTGAGGGCAAGCTTTCTCAAATGTCAAAAGAAGTGAATGCCAGAATTGAGCCATTTCTGAAGACGACACCACGCCCTCTCAGATTACCTGATGGACCTCCCTGTTCCCAGCGGTCGAAGTTCTTGCTAATGGATGCCCTTAAATTAAGCATTGAAGACCCGAGCCATGAGGGGGAGGGTATACCGCTGTACGATGCAA
CCAAATGCATGAAGACATTTTTTGGCTGGAAGGAGCCCAACATCGTGAAACCACATGAAAAGGGCATAAACCCTAATTACCTCCTGGCTTGGAAGCAGGTGCTATCAGAACTCCAAGATATTGAAAACGAGGAGAAAATCCCAAAAACAAAGAACATGAAGAAGACGAGCCAACTGAAGTGGGCACTTGGTGAAAACATGGCACCAGAGAAAGTGGACTTTGAGGACT
GTAAAGATGTTAGCGATCTAAGACAGTACGACAGTGACGAACCAGAGTCTAGATCACTAGCAAGTTGGATCCAGAGTGAATTTAACAAGGCTTGCGAACTGACAGATTCGAGTTGGATTGAACTTGATGAAATAGGGGAAGACGTTGCTCCAATTGAACACATTGCGAGTATGAGGAGGAACTATTTCACAGCGGAAGTATCCCATTGCAGGGCTACAGAATACATAATG
AAAGGAGTATACATAAACACAGCTTTATTGAATGCATCCTGTGCAGCCATGGATGACTTCCAACTGATTCCAATGATAAGCAAATGCAGAACCAAAGAAGGGAGACGGAAGACAAATCTGTATGGCTTCATTATAAAAGGAAGATCCCATTTGAGGAATGACACCGATGTGGTAAACTTTGTGAGCATGGAATTCTCTCTCACTGACCCAAGGCTGGAGCCACACAAATGG
GAAAAGTACTGTGTACTTGAGGTAGGAGACATGCTCCTACGGACTGCAATAGGCCAAGTGTCAAGGCCCATGTTCCTGTATGTGAGAACCAATGGGACTTCTAAGATCAAAATGAAATGGGGTATGGAGATGAGGCGATGCCTTCTTCAATCCCTTCAACAAATTGAGAGCATGATTGAAGCCGAGTCTTCTGTCAAAGAAAAAGACATGACCAAGGAATTCTTTGAAAAC
AGATCAGAAACATGGCCAATTGGAGAATCACCCAAAGGAGTGGAGGAAGGCTCCATTGGGAAGGTGTGCAGAACATTGCTAGCAAAGTCTGTGTTCAACAGCCTATATGCATCTCCACAACTCGAGGGGTTTTCAGCTGAATCAAGAAAGTTGCTTCTCATTGTTCAGGCACTTAGGGACAACCTGGAACCTGGAACCTTCGATCTTGGGGGGCTATATGAAGCAATTGA
GGAGTGCCTGGTTAACGATCCCTGGGTTTTGCTTAATGCGTCTTGGTTCAACTCCTTCCTCACACATGCACTGAAATA-

EPI_ISL_3102075

>A/chicken/Poland/H928_21RS1385-16/2021_H5N8_2021-04-25
ATGGAAGACTTTGTGCGACAATGCTTCAATCCAATGATTGTCGAGCTTGCGGAAAAGGCAATGAAAGAATATGGGGAAGATCCTAAGATCGAAACAAACAAGTTTGCCGCAATATGTACACACTTAGAAGTCTGCTTCATGTATTCGGATTTCCATTTCATTGATGAACGAGGCGAATCAATAATCGTAGAATCTGGTGACCCGAATGCATTATTGAAGCACCGATTTGAGA
TAATTGAAGGGAGAGACCGAACAATGGCCTGGACAGTGGTGAATAGTATATGCAACACTACAGGAGTCGAAAAGCCCAAGTTCCTTCCTGATTTGTATGACTACCGAGAAAACCGATTCATTGAAATTGGAGTAACGCGAAGGGAAGTTCACATATACTATCTTGAAAAAGCCAACAAGATAAAATCAGAGAAAACACACATTCACATATTCTCATTCACTGGGGAGGAA
ATGGCCACCAAGGCAGATTACACTCTTGATGAAGAGAGCAGAGCAAGAATAAAAACCAGGCTATTCACTATAAGACAAGAGATGGCCAATAGGGGTCTATGGGATTCCTTTCGTCAGTCCGAGAGAGGCGAAGAGACAATTGAAGAAAGATTTGAAATCACAGGAACCATGCGCAGGCTTGCTGACCAAAGTCTCCCACCGAACTTCGCCAGCCTTGAAAACTTTAGAG
CCTATGTGGATGGATTTGAACCGAACGGCTGCATTGAGGGCAAGCTTTCTCAAATGTCAAAAGAAGTGAATGCCAGAATTGAGCCATTTCTGAAGACGACACCACGCCCTCTCAGATTACCTGATGGACCTCCCTGTTCCCAGCGGTCGAAGTTCTTGCTAATGGATGCCCTTAAATTAAGCATTGAAGACCCGAGCCATGAGGGGGAGGGTATACCGCTGTACGATGCAA
CCAAATGCATGAAGACATTTTTTGGCTGGAAGGAGCCCAACATCGTGAAACCACATGAAAAGGGCATAAACCCTAATTACCTCCTGGCTTGGAAGCAGGTGCTATCAGAACTCCAAGATATTGAAAACGAGGAGAAAATCCCAAAAACAAAGAACATGAAGAAGACGAGCCAACTGAAGTGGGCACTTGGTGAAAACATGGCACCAGAGAAAGTGGACTTTGAGGACT
GTAAAGATGTTAGCGATCTAAGACAGTACGACAGTGACGAACCAGAGTCTAGATCACTAGCAAGTTGGATCCAGAGTGAATTTAACAAGGCTTGCGAACTGACAGATTCGAGTTGGATTGAACTTGATGAAATAGGGGAAGACGTTGCTCCAATTGAACACATTGCGAGTATGAGGAGGAACTATTTCACAGCGGAAGTATCCCATTGCAGGGCTACAGAATACATAATG
AAAGGAGTATACATAAACACAGCTTTATTGAATGCATCCTGTGCAGCCATGGATGACTTCCAACTGATTCCAATGATAAGCAAATGCAGAACCAAAGAAGGGAGACGGAAGACAAATCTGTATGGCTTCATTATAAAAGGAAGATCCCATTTGAGGAATGACACCGATGTGGTAAACTTTGTGAGCATGGAATTCTCTCTCACTGACCCAAGGCTGGAGCCACACAAATGG
GAAAAGTACTGTGTACTTGAGGTAGGAGACATGCTCCTACGGACTGCAATAGGCCAAGTGTCAAGGCCCATGTTCCTGTATGTGAGAACCAATGGGACTTCTAAGATCAAAATGAAATGGGGTATGGAGATGAGGCGATGCCTTCTTCAATCCCTTCAACAAATTGAGAGCATGATTGAAGCCGAGTCTTCTGTCAAAGAAAAAGACATGACCAAGGAATTCTTTGAAAAC
AGATCAGAAACATGGCCAATTGGGGAATCACCCAAAGGAGTGGAGGAAGGCTCCATTGGGAAGGTGTGCAGAACATTGCTAGCAAAGTCTGTGTTCAACAGCCTATATGCATCTCCACAACTCGAGGGGTTTTCAGCTGAATCAAGAAAGTTGCTTCTCATTGTTCAGGCACTTAGGGACAACCTGGAACCTGGAACCTTCGATCTTGGGGGGCTATATGAAGCAATTGA
GGAGTGCCTGATTAACGATCCCTGGGTTTTGCTTAATGCGTCTTGGTTCAACTCCTTCCTCACACATGCACTGAAATA-

EPI_ISL_3102068

>A/chicken/Poland/H984_21RS1385-9/2021_H5N8_2021-04-28
ATGGAAGACTTTGTGCGACAATGCTTCAATCCAATGATTGTCGAGCTTGCGGAAAAGGCAATGAAAGAATATGGGGAAGATCCTAAGATCGAAACAAACAAGTTTGCCGCAATATGTACACACTTAGAAGTCTGCTTCATGTATTCGGATTTCCATTTCATTGATGAACGAGGCGAATCAATAATCGTAGAATCTGGCGACCCGAATGCATTATTGAAGCACCGATTTGAGA
TAATTGAAGGGAGAGACCGAACAATGGCCTGGACAGTGGTGAATAGTATATGCAACACTACAGGAGTCGAAAAGCCCAAGTTCCTTCCTGATTTGTATGACTACCGAGAAAACCGATTCATTGAAATTGGAGTAACGCGAAGGGAAGTTCACATATACTATCTTGAAAAAGCCAACAAGATAAAATCAGAGAAAACACACATTCACATATTCTCATTCACTGGGGAGGAA
ATGGCCACCAAGGCAGATTACACTCTTGATGAAGAGAGCAGAGCAAGAATAAAAACCAGGCTATTCACTATAAGACAAGAGATGGCCAATAGGGGTCTATGGGATTCCTTTCGTCAGTCCGAGAGAGGCGAAGAGACAATTGAAGAAAGATTTGAAATCACAGGAACCATGCGCAGGCTTGCTGACCAAAGTCTCCCACCGAACTTCGCCAGCCTTGAAAACTTTAGAG
CCTATGTGGATGGATTTGAACCGAACGGCTGCATTGAGGGCAAGCTTTCTCAAATGTCAAAAGAAGTGAATGCCAGAATTGAGCCATTTCTGAAGACGACACCACGCCCTCTCAGATTACCTGATGGACCTCCCTGTTCCCAGCGGTCGAAGTTCTTGCTAATGGATGCCCTTAAATTAAGCATTGAAGACCCGAGCCATGAGGGGGAGGGTATACCGCTGTACGATGCAA
CCAAATGCATGAAGACATTTTTTGGCTGGAAGGAGCCCAACATCGTGAAACCACATGAAAAGGGCATAAACCCTAATTACCTCCTGGCTTGGAAGCAGGTGCTATCAGAACTCCAAGATATTGAAAACGAGGAGAAAATCCCAAAAACAAAGAACATGAAGAAGACGAGCCAACTGAAGTGGGCACTTGGTGAAAACATGGCACCAGAGAAAGTGGACTTTGAGGACT
GTAAAGATGTTAGCGATCTAAGACAGTACGACAGTGACGAACCAGAGTCTAGATCACTAGCAAGTTGGATCCAGAGTGAATTTAACAAGGCTTGCGAACTGACAGATTCGAGTTGGATTGAACTTGATGAAATAGGGGAAGACGTTGCTCCAATTGAACACATTGCGAGTATGAGGAGGAACTATTTCACAGCGGAAGTATCCCATTGCAGGGCTACAGAATACATAATG
AAAGGAGTATACATAAATACAGCTTTATTGAATGCATCCTGTGCAGCCATGGATGACTTCCAACTGATTCCAATGATAAGCAAATGCAGAACCAAAGAAGGGAGACGGAAGACAAATCTGTATGGCTTCATTATAAAAGGAAGATCCCATTTGAGGAATGACACCGATGTGGTAAACTTTGTGAGCATGGAATTCTCTCTCACTGACCCAAGGCTGGAGCCACACAAATGG
GAAAAGTACTGTGTACTTGAGGTAGGAGACATGCTCCTACGGACTGCAATAGGCCAAGTGTCAAGGCCCATGTTCCTGTATGTGAGAACCAATGGGACTTCTAAGATCAAAATGAAATGGGGTATGGAGATGAGGCGATGCCTTCTTCAATCCCTTCAACAAATTGAGAGCATGATTGAAGCCGAGTCTTCTGTCAAAGAAAAAGACATGACCAAGGAATTCTTTGAAAAC
AGATCAGAAACATGGCCAATTGGAGAATCACCCAAAGGAGTGGAGGAAGGCTCCATTGGGAAGGTGTGCAGAACATTGCTAGCAAAGTCTGTGTTCAACAGCCTATATGCATCTCCACAACTCGAGGGGTTTTCAGCTGAATCAAGAAAGTTGCTTCTCATTGTTCAGGCACTTAGGGACAACCTGGAACCTGGAACCTTCGATCTTGGGGGGCTATATGAAGCAATTGA
GGAGTGCCTGATTAACGATCCCTGGGTTTTGCTTAATGCGTCTTGGTTCAACTCCTTCCTCACACATGCACTGAAATA-

EPI_ISL_3102069

>A/chicken/Poland/H1161_21RS1385-10/2021_H5N8_2021-05-13
ATGGAAGACTTTGTGCGACAATGCTTCAATCCAATGATTGTCGAGCTTGCGGAAAAGGCAATGAAAGAATATGGGGAAGATCCTAAGATCGAAACAAACAAGTTTGCCGCAATATGTACACACTTAGAAGTCTGCTTCATGTATTCGGATTTCCATTTCATTGATGAACGAGGCGAATCAATAATCGTAGAATCTGGCGACCCGAATGCATTATTGAAGCACCGATTTGAGA
TAATTGAAGGGAGAGACCGAACAATGGCCTGGACAGTGGTGAATAGTATATGCAACACTACAGGAGTCGAAAAGCCCAAGTTCCTTCCTGATTTGTATGACTACCGAGAAAACCGATTCATTGAAATTGGAGTAACGCGAAGGGAAGTTCACATATACTATCTTGAAAAAGCCAACAAGATAAAATCAGAGAAAACACACATTCACATATTCTCATTCACTGGGGAGGAA
ATGGCCACCAAGGCAGATTACACTCTTGATGAAGAGAGCAGAGCAAGAATAAAAACCAGGCTATTCACTATAAGACAAGAGATGGCCAATAGGGGTCTATGGGATTCCTTTCGTCAGTCCGAGAGAGGCGAAGAGACAATTGAAGAAAGATTTGAAATCACAGGAACCATGCGCAGGCTTGCTGACCAAAGTCTCCCACCGAACTTCGCCAGCCTTGAAAACTTTAGAG
CCTATGTGGATGGATTTGAACCGAACGGCTGCATTGAGGGCAAGCTTTCTCAAATGTCAAAAGAAGTGAATGCCAGAATTGAGCCATTTCTGAAGACGACACCACGCCCTCTCAGATTACCTGATGGACCTCCCTGTTCCCAGCGGTCGAAGTTCTTGCTAATGGATGCCCTTAAATTAAGCATTGAAGACCCGAGCCATGAGGGGGAGGGTATACCGCTGTACGATGCAA
CCAAATGCATGAAGACATTTTTTGGCTGGAAGGAGCCCAACATCGTGAAACCACATGAAAAGGGCATAAACCCTAATTACCTCCTGGCTTGGAAGCAGGTGCTATCAGAACTCCAAGATATTGAAAACGAGGAGAAAATCCCAAAAACAAAGAACATGAAGAAGACGAGCCAACTGAAGTGGGCACTTGGTGAAAACATGGCACCAGAGAAAGTGGACTTTGAGGACT
GTAAAGATGTTAGCGATCTAAGACAGTACGACAGTGACGAACCAGAGTCTAGATCACTAGCAAGTTGGATCCAGAGTGAATTTAACAAGGCTTGCGAACTGACAGATTCGAGTTGGATTGAACTTGATGAAATAGGGGAAGACGTTGCTCCAATTGAACACATTGCGAGTATGAGGAGGAACTATTTCACAGCGGAAGTATCCCATTGCAGGGCTACAGAATACATAATG
AAAGGAGTATACATAAACACAGCTTTATTGAATGCATCCTGTGCAGCCATGGATGACTTCCAACTGATTCCAATGATAAGCAAATGCAGAACCAAAGAAGGGAGACGGAAGACAAATCTGTATGGCTTCATTATAAAAGGAAGATCCCATTTGAGGAATGACACCGATGTGGTAAACTTTGTGAGCATGGAATTCTCTCTCACTGACCCAAGGCTGGAGCCACACAAATGG
GAAAAGTACTGTGTACTTGAGGTAGGAGACATGCTCCTACGGACTGCAATAGGCCAAGTGTCAAGGCCCATGTTCCTGTATGTGAGAACCAATGGGACTTCTAAGATCAAAATGAAATGGGGTATGGAGATGAGGCGATGCCTTCTTCAATCCCTTCAACAAATTGAGAGCATGATTGAAGCCGAGTCTTCTGTCAAAGAAAAAGACATGACCAAGGAATTCTTTGAAAAC
AGATCAGAAACATGGCCAATTGGAGAATCACCCAAAGGAGTGGAGGAAGGCTCCATTGGGAAGGTGTGCAGAACATTGCTAGCAAAGTCTGTGTTCAACAGCCTATATGCATCTCCACAACTCGAGGGGTTTTCAGCTGAATCAAGAAAGTTGCTTCTCATTGTTCAGGCACTTAGGGACAACCTGGAACCTGGAACCTTCGATCTTGGGGGGCTATATGAAGCAATTGA
GGAGTGCCTGATTAACGATCCCTGGGTTTTGCTTAATGCGTCTTGGTTCAACTCCTTCCTCACACATGCACTTAAATA-

EPI_ISL_1719911

>A/swan/Lithuania/1842PG1_21VIR2606-5/2021_H5N8_2021-03-19
ATGGAAGACTTTGTGCGACAATGCTTCAATCCAATGATTGTCGAGCTTGCGGAAAAGGCAATGAAAGAATATGGGGAAGATCCTAAGATCGAAACAAACAAGTTTGCCGCAATATGTACACACTTAGAAGTCTGCTTCATGTATTCGGATTTCCATTTCATTGATGAACGAGGCGAATCAATAATCGTAGAATCTGGCGACCCGAATGCATTATTGAAGCACCGATTTGAGA
TAATTGAAGGGAGAGACCGAACAATGGCCTGGACAGTGGTGAATAGTATATGCAACACTACAGGAGTCGAAAAGCCCAAGTTCCTTCCTGATTTGTATGACTACCGAGAAAACCGATTCATTGAAATTGGAGTAACGCGAAGGGAAGTTCACATATACTATCTTGAAAAAGCCAACAAGATAAAATCAGAGAAAACACACATTCACATATTCTCATTCACTGGGGAGGAA
ATGGCCACCAAGGCAGATTACACTCTTGATGAAGAGAGCAGAGCAAGAATAAAAACCAGGCTATTCACTATAAGACAAGAGATGGCCAATAGGGGTCTATGGGATTCCTTTCGTCAGTCCGAGAGAGGCGAAGAGACAATTGAAGAAAGATTTGAAATCACAGGAACCATGCGCAGGCTTGCTGACCAAAGTCTCCCACCGAACTTCGCCAGCCTTGAAAACTTTAGAG
CCTATGTGGATGGATTTGAACCGAACGGCTGCATTGAGGGCAAGCTTTCTCAAATGTCAAAAGAAGTGAATGCCAGAATTGAGTCATTTCTGAAGACGACACCACGCCCTCTCAGATTACCTGATGGACCTCCCTGTTCCCAGCGGTCGAAGTTCTTGCTAATGGATGCCCTTAAATTGAGCATTGAAGACCCGAGCCATGAGGGGGAGGGTATACCGCTGTACGATGCAA
CCAAATGCATGAAGACATTTTTTGGCTGGAAGGAGCCCAACATCGTGAAACCACATGAAAAGGGCATAAACCCTAATTACCTCCTGGCTTGGAAGCAGGTGCTATCAGAACTCCAAGATATTGAAAACGAGGAGAAAATCCCAAAAACAAAGAACATGAAGAAGACGAGCCAACTGAAGTGGGCACTTGGTGAAAACATGGCACCAGAGAAAGTGGACTTTGAGGACT
GTAAAGATGTTAGCGATCTAAGACAGTACGACAGTGACGAACCAGAGTCTAGATCACTAGCAAGTTGGATCCAGAGTGAATTTAACAAGGCTTGCGAACTGACAGATTCGAGTTGGATTGAACTTGATGAAATAGGGGAAGACGTTGCTCCAATTGAACACATTGCGAGTATGAGGAGGAACTATTTCACAGCGGAAGTATCCCATTGCAGGGCTACAGAATACATAATG
AAAGGAGTATACATAAACACAGCTTTATTGAATGCATCCTGTGCAGCCATGGATGACTTCCAACTGATTCCAATGATAAGCAAATGCAGAACCAAAGAAGGGAGACGGAAGACAAATCTGTATGGCTTCATTATAAAAGGAAGATCCCATTTGAGGAATGACACCGATGTGGTAAACTTTGTGAGCATGGAATTCTCTCTCACTGACCCAAGGTTGGAGCCACACAAATGG
GAAAAGTACTGTGTACTTGAGGTAGGAGACATGCTCCTACGGACTGCAATAGGCCAAGTGTCAAGGCCCATGTTCCTGTATGTGAGAACCAATGGGACTTCTAAGATCAAAATGAAATGGGGTATGGAGATGAGGCGATGCCTTCTTCAATCCCTTCAACAAATTGAGAGCATGATTGAAGCCGAGTCTTCTGTCAAAGAAAAAGACATGACCAAGGAATTCTTTGAAAAC
AGATCAGAAACATGGCCAATTGGAGAATCACCCAAAGGAGTGGAGGAAGGCTCCATTGGGAAGGTGTGCAGAACATTGCTAGCAAAGTCTGTGTTCAACAGCCTATATGCATCTCCACAACTCGAGGGGTTTTCAGCTGAATCAAGAAAGTTGCTTCTCATTGTTCAGGCACTTAGGGACAACCTGGAACCTGGAACCTTCGATCTTGGGGGGCTATATGAAGCAATTGA
GGAGTGCCTGATTAACGATCCCTGGGTTTTGCTTAATGCGTCTTGGTTCAACTCCTTCCTCACACATGCACTGAAATA-

EPI_ISL_3102079

>A/turkey/Poland/H1289_21RS1385-20/2021_H5N8_2021-05-24
ATGGAAGACTTTGTGCGACAATGCTTCAATCCAATGATTGTCGAGCTTGCGGAAAAGGCAATGAAAGAATATGGGGAAGATCCTAAGATCGAAACAAACAAGTTTGCCGCAATATGTACACACTTAGAAGTCTGCTTCATGTATTCGGATTTCCATTTCATTGATGAACGAGGCGAATCAATAATCGTAGAATCAGGCGACCCGAATGCATTATTGAAGCACCGATTTGAGA
TAATTGAAGGGAGAGACCGAACAATGGCCTGGACAGTGGTGAATAGTATATGCAACACTACAGGAGTCGAAAAGCCCAAGTTCCTTCCTGATTTGTATGACTACCGAGAAAACCGATTCATTGAAATTGGAGTAACGCGAAGGGAAGTTCACATATACTATCTTGAAAAAGCCAACAAGATAAAATCAGAGAAAACACACATTCACATATTCTCATTCACTGGGGAGGAA
ATGGCCACCAAGGCAGATTACACTCTTGATGAAGAGAGCAGAGCAAGAATAAAAACCAGGCTATTCACTATAAGACAAGAGATGGCCAATAGGGGTCTATGGGATTCCTTTCGTCAGTCCGAGAGAGGCGAAGAGACAATTGAAGAAAGATTTGAAATCACAGGAACCATGCGCAGGCTTGCTGACCAAAGTCTCCCACCGAACTTCGCCAGCCTTGAAAACTTTAGAG
CCTATGTGGATGGATTTGAACCGAACGGCTGCATTGAGGGCAAGCTTTCTCAAATGTCAAAAGAAGTGAATGCCAGAATTGAGCCATTTCTGAAGACGACACCACGCCCTCTCAGATTACCTGATGGACCTCCCTGTTCCCAGCGGTCGAAGTTCTTGCTAATGGATGCCCTTAAATTAAGCATTGAAGACCCGAGCCATGAGGGGGAGGGTATACCGCTGTACGATGCAA
CCAAATGCATGAAGACATTTTTTGGCTGGAAGGAGCCCAACATCGTGAAACCACATGAAAAGGGCATAAACCCTAATTACCTCCTGGCTTGGAAGCAGGTACTATCAGAACTCCAAGATATTGAAAACGAGGAGAAAATCCCAAAAACAAAGAACATGAAGAAGACGAGCCAACTGAAGTGGGCACTTGGTGAAAACATGGCACCAGAGAAAGTGGACTTTGAGGACT
GTAAAGATGTTAGCGATCTAAGACAGTACGACAGTGACGAACCAGAGTCTAGATCACTAGCAAGTTGGATCCAGAGTGAATTTAACAAGGCTTGCGAACTGACAGATTCGAGTTGGATTGAACTTGATGAAATAGGGGAAGACGTTGCTCCAATTGAACACATTGCGAGTATGAGGAGGAACTATTTCACAGCGGAAGTATCCCATTGCAGGGCTACAGAATACATAATG
AAAGGAGTATACATAAACACAGCTTTATTGAATGCATCCTGTGCAGCCATGGATGACTTCCAACTGATTCCAATGATAAGCAAATGCAGAACCAAAGAAGGGAGACGGAAGACAAATCTGTATGGCTTCATTATAAAAGGAAGATCCCATTTGAGGAATGACACCGATGTGGTAAACTTTGTGAGCATGGAATTCTCTCTCACTGACCCAAGGCTGGAGCCACACAAATGG
GAAAAGTACTGTGTACTTGAGGTAGGAGACATGCTCCTACGGACTGCAATAGGCCAAGTGTCAAGGCCCATGTTCCTGTATGTGAGAACCAATGGGACTTCTAAGATCAAAATGAAATGGGGTATGGAGATGAGGCGATGCCTTCTTCAATCCCTTCAACAAATTGAGAGCATGATTGAAGCCGAGTCTTCTGTCAAAGAAAAAGACATGACCAAGGAATTCTTTGAAAAC
AGATCAGAAACATGGCCAATTGGAGAATCACCCAAAGGAGTGGAGGAAGGCTCCATTGGGAAGGTGTGCAGAACATTGCTAGCAAAGTCTGTGTTCAACAGCCTATATGCATCTCCACAACTCGAGGGGTTTTCAGCTGAATCAAGAAAGTTGCTTCTCATTGTTCAGGCACTTAGGGACAACCTGGAACCTGGAACCTTCGATCTTGGGGGGCTATATGAAGCAATTGA
GGAGTGCCTGATTAACGATCCCTGGGTTTTGCTTAATGCGTCTTGGTTCAACTCCTTCCTCACACATGCACTGAAATA-

EPI_ISL_984691

>A/common_buzzard/Denmark/14600-1/2020_H5N8_2020-11-06
ATGGAAGACTTTGTGCGACAATGCTTCAATCCAATGATTGTCGAGCTTGCGGAAAAGGCAATGAAAGAATATGGGGAAGATCCTAAGATCGAAACAAACAAGTTTGCCGCAATATGTACACACTTAGAAGTCTGCTTCATGTATTCGGATTTCCATTTCATTGATGAACGAGGCGAATCAATAATCGTAGAATCTGGCGACCCGAATGCATTATTGAAGCACCGATTTGAGA
TAATTGAAGGGAGAGACCGAACAATGGCCTGGACAGTGGTGAATAGTATATGCAACACTACAGGAGTCGAAAAGCCCAAGTTCCTTCCTGATTTGTATGACTACAGAGAAAACCGATTCATTGAAATTGGAGTAACGCGAAGGGAAGTTCACATATACTATCTTGAAAAAGCCAACAAGATAAAATCAGAGAAAACACACATTCACATATTCTCATTCACTGGGGAGGAA
ATGGCCACCAAGGCCGATTACACTCTTGATGAAGAGAGCAGAGCAAGAATAAAAACCAGGCTATTCACTATAAGACAAGAGATGGCCAATAGGGGTCTATGGGATTCCTTTCGTCAGTCCGAGAGAGGCGAAGAGACAATTGAAGAAAGATTTGAAATCACAGGAACCATGCGCAGGCTTGCTGACCAAAGTCTCCCACCGAACTTCGCCAGCCTTGAAAACTTTAGAG
CCTATGTGGATGGATTTGAACCGAACGGCTGCATTGAGGGCAAGCTTTCTCAAATGTCAAAAGAAGTGAATGCCAGAATTGAGCCATTTCTGAAGACGACACCACGCCCTCTCAGATTACCTGATGGACCTCCCTGTTCCCAGCGGTCGAAGTTCTTGCTAATGGATGCCCTTAAATTGAGCATTGAAGACCCGAGCCATGAGGGGGAGGGTATACCGCTGTACGATGCAA
CCAAATGCATGAAGACATTTTTTGGCTGGAAGGAGCCCAACATCGTGAAACCACATGAAAAGGGCATAAACCCTAATTACCTCCTGGCTTGGAAGCAGGTGCTATCAGAACTCCATGATATTGAAAACGAGGAGAAAATCCCAAAAACAAAGAACATGAAGAAGACGAGCCAACTGAAGTGGGCACTTGGTGAAAACATGGCACCAGAGAAAGTGGACTTTGAGGACT
GTAAAGATGTTAGCGATCTAAGACAGTACGACAGTGACGAACCAGAGTCTAGATCACTAGCAAGTTGGATCCAGAGTGAATTTAACAAGGCTTGCGAACTGACAGATTCGAGTTGGATTGAACTTGATGAAATAGGGGAAGACGTTGCTCCAATTGAACACATTGCGAGTATGAGGAGGAACTATTTCACAGCGGAAGTATCCCATTGCAGGGCTACAGAATACATAATG
AAAGGAGTATACATAAACACAGCTTTATTGAATGCATCCTGTGCAGCCATGGATGACTTCCAACTGATTCCAATGATAAGCAAATGCAGAACCAAAGAAGGGAGACGGAAGACAAATCTGTATGGCTTCATTATAAAAGGAAGATCCCATTTGAGGAATGACACCGATGTGGTAAACTTTGTGAGCATGGAATTCTCTCTCACTGACCCAAGGCTGGAGCCACACAAATGG
GAAAAGTACTGTGTGCTTGAGGTAGGAGACATGCTCCTACGGACTGCAATAGGCCAAGTGTCAAGGCCCATGTTCCTGTATGTGAGAACCAATGGGACTTCTAAGATCAAAATGAAATGGGGTATGGAGATGAGGCGATGCCTTCTTCAATCCCTTCAACAAATTGAGAGCATGATTGAAGCCGAGTCTTCTGTCAAAGAAAAAGACATGACCAAGGAATTCTTTGAAAAC
AGATCAGAAACATGGCCAATTGGAGAGTCACCCAAAGGAGTGGAGGAAGGCTCCATTGGGAAGGTGTGCAGAACATTGCTAGCAAAGTCTGTGTTCAACAGCCTATATGCATCTCCACAACTCGAGGGGTTTTCAGCTGAATCAAGAAAGTTGCTTCTCATTGTTCAGGCACTTAGGGACAACCTGGAACCTGGAACCTTCGATCTTGGGGGGCTATATGAAGCAATTGA
GGAGTGCCTGATTAACGATCCCTGGGTTTTGCTTAATGCGTCTTGGTTCAACTCCTTCCTCACACATGCACTGAAATA-

EPI_ISL_5062851

>A/herring_gull/Germany-MV/AI02300/2020_H5N8_2020-11-02
ATGGAAGACTTTGTGCGACAATGCTTCAATCCAATGATTGTCGAGCTTGCGGAAAAGGCAATGAAAGAATATGGGGAAGATCCTAAGATCGAAACAAACAAGTTTGCCGCAATATGTACACACTTAGAAGTCTGCTTCATGTATTCGGATTTCCATTTCATTGATGAACGAGGCGAATCAATAATCGTAGAATCTGGCGACCCGAATGCATTATTGAAGCACCGATTTGAGA
TAATTGAAGGGAGAGACCGAACAATGGCCTGGACAGTGGTGAATAGTATATGCAACACTACAGGAGTCGAAAAGCCCAAGTTCCTTCCTGATTTGTATGACTACAGAGAAAACCGATTCATTGAAATTGGAGTAACGCGAAGGGAAGTTCACATATACTATCTTGAAAAAGCCAACAAGATAAAATCAGAGAAAACACACATTCACATATTCTCATTCACTGGGGAGGAA
ATGGCCACCAAGGCCGATTACACTCTTGATGAAGAGAGCAGAGCAAGAATAAAAACCAGGCTATTCACTATAAGACAAGAGATGGCCAATAGGGGTCTATGGGATTCCTTTCGTCAGTCCGAGAGAGGCGAAGAGACAATTGAAGAAAGATTTGAAATCACAGGAACCATGCGCAGGCTTGCTGACCAAAGTCTCCCACCGAACTTCGCCAGCCTTGAAAACTTTAGAG
CCTATGTGGATGGATTTGAACCGAACGGCTGCATTGAGGGCAAGCTTTCTCAAATGTCAAAAGAAGTGAATGCCAGAATTGAGCCATTTCTGAAGACGACACCACGCCCTCTCAGATTACCTGATGGACCTCCCTGTTCCCAGCGGTCGAAGTTCTTGCTAATGGATGCCCTTAAATTGAGCATTGAAGACCCGAGCCATGAGGGGGAGGGTATACCGCTGTACGATGCAA
CCAAATGCATGAAGACATTTTTTGGCTGGAAGGAGCCCAACATCGTGAAACCACATGAAAAGGGCATAAACCCTAATTACCTCCTGGCTTGGAAGCAGGTGCTATCAGAACTCCATGATATTGAAAACGAGGAGAAAATCCCAAAAACAAAGAACATGAAGAAGACGAGCCAACTGAAGTGGGCACTTGGTGAAAACATGGCACCAGAGAAAGTGGACTTTGAGGACT
GTAAAGATGTTAGCGATCTAAGACAGTACGACAGTGACGAACCAGAGTCTAGATCACTAGCAAGTTGGATCCAGAGTGAATTTAACAAGGCTTGCGAACTGACAGATTCGAGTTGGATTGAACTTGATGAAATAGGGGAAGACGTTGCTCCAATTGAACACATTGCGAGTATGAGGAGGAACTATTTCACAGCGGAAGTATCCCATTGCAGGGCTACAGAATACATAATG
AAAGGAGTATACATAAACACAGCTTTATTGAATGCATCCTGTGCAGCCATGGATGACTTCCAACTGATTCCAATGATAAGCAAATGCAGAACCAAAGAAGGGAGACGGAAGACAAATCTGTATGGCTTCATTATAAAAGGAAGATCCCATTTGAGGAATGACACCGATGTGGTAAACTTTGTGAGCATGGAATTCTCTCTCACTGACCCAAGGCTGGAGCCACACAAATGG
GAAAAGTACTGTGTGCTTGAGGTAGGAGACATGCTCCTACGGACTGCAATAGGCCAAGTGTCAAGGCCCATGTTCCTGTATGTGAGAACCAATGGGACTTCTAAGATCAAAATGAAATGGGGTATGGAGATGAGGCGATGCCTTCTTCAATCCCTTCAACAAATTGAGAGCATGATTGAAGCCGAGTCTTCTGTCAAAGAAAAAGACATGACCAAGGAATTCTTTGAAAAC
AGATCAGAAACATGGCCAATTGGAGAGTCACCCAAAGGAGTGGAGGAAGGCTCCATTGGGAAGGTGTGCAGAACATTGCTAGCAAAGTCTGTGTTCAACAGCCTATATGCATCTCCACAACTCGAGGGGTTTTCAGCTGAATCAAGAAAGTTGCTTCTCATTGTTCAGGCACTTAGGGACAACCTGGAACCTGGAACCTTCGATCTTGGGGGGCTATATGAAGCAATTGA
GGAGTGCCTGATTAACGATCCCTGGGTTTTGCTTAATGCGTCTTGGTTCAACTCCTTCCTCACACATGCACTGAAATA-

EPI_ISL_1123358

>A/brent_goose/England/095684/2020_H5N5_2020-11-12
ATGGAAGACTTTGTGCGACAATGCTTCAATCCAATGATTGTCGAGCTTGCGGAAAAGGCAATGAAAGAATATGGGGAAGATCCTAAGATCGAAACAAACAAGTTTGCCGCAATATGTACACACTTAGAAGTCTGCTTCATGTATTCGGATTTCCATTTCATTGATGAACGAGGCGAATCAATAATCGTAGAATCTGGCGACCCGAATGCATTATTGAAGCACCGATTTGAGA
TAATTGAAGGGAGAGACCGAACAATGGCCTGGACAGTGGTGAATAGTATATGCAACACTACAGGAGTCGAAAAGCCCAAGTTCCTTCCTGATTTGTATGACTACAGAGAAAACCGATTCATTGAAATTGGAGTAACGCGAAGGGAAGTTCACATATACTATCTTGAAAAAGCCAACAAGATAAAATCAGAGAAAACACACATTCACATATTCTCATTCACTGGGGAGGAA
ATGGCCACCAAGGCCGATTACACTCTTGATGAAGAGAGCAGAGCAAGAATAAAAACCAGGCTATTCACTATAAGACAAGAGATGGCCAATAGGGGTCTATGGGATTCCTTTCGTCAGTCCGAGAGAGGCGAAGAGACAATTGAAGAAAGATTTGAAATCACAGGAACCATGCGCAGGCTTGCTGACCAAAGTCTCCCACCGAACTTCGCCAGCCTTGAAAACTTTAGAG
CCTATGTGGATGGATTTGAACCGAACGGCTGCATTGAGGGCAAGCTTTCTCAAATGTCAAAAGAAGTGAATGCCAGAATTGAGCCATTTCTGAAGACGACACCACGCCCTCTCAGATTACCTGATGGACCTCCCTGTTCCCAGCGGTCGAAGTTCTTGCTAATGGATGCCCTTAAATTGAGCATTGAAGACCCGAGCCATGAGGGGGAGGGTATACCGCTGTACGATGCAA
CCAAATGCATGAAGACATTTTTTGGCTGGAAGGAGCCCAACATCGTGAAACCACATGAAAAGGGCATAAACCCTAATTACCTCCTGGCTTGGAAGCAGGTGCTATCAGAACTCCATGATATTGAAAACGAGGAGAAAATCCCAAAAACAAAGAACATGAAGAAGACGAGCCAACTGAAGTGGGCACTTGGTGAAAACATGGCACCAGAGAAAGTGGACTTTGAGGACT
GTAAAGATGTTAGCGATCTAAGACAGTACGACAGTGACGAACCAGAGTCTAGATCACTAGCAAGTTGGATCCAGAGTGAATTTAACAAGGCTTGCGAACTGACAGATTCGAGTTGGATTGAACTTGATGAAATAGGGGAAGACGTTGCTCCAATTGAACACATTGCGAGTATGAGGAGGAACTATTTCACAGCGGAAGTATCCCATTGCAGGGCTACAGAATACATAATG
AAAGGAGTATACATAAACACAGCTTTATTGAATGCATCCTGTGCAGCCATGGATGACTTCCAACTGATTCCAATGATAAGCAAATGCAGAACCAAAGAAGGGAGACGGAAGACAAATCTGTATGGCTTCATTATAAAAGGAAGATCCCATTTGAGGAATGACACCGATGTGGTAAACTTTGTGAGCATGGAATTCTCTCTCACTGACCCAAGGCTGGAGCCACACAAATGG
GAAAAGTACTGTGTGCTTGAGGTAGGAGACATGCTCCTACGGACTGCAATAGGCCAAGTGTCAAGGCCCATGTTCCTGTATGTGAGAACCAATGGGACTTCTAAGATCAAAATGAAATGGGGTATGGAGATGAGGCGATGCCTTCTTCAATCCCTTCAACAAATTGAGAGCATGATTGAAGCCGAGTCTTCTGTCAAAGAAAAAGACATGACCAAGGAATTCTTTGAAAAC
AGATCAGAAACATGGCCAATTGGAGAGTCACCCAAAGGAGTGGAGGAAGGCTCCATTGGGAAGGTGTGCAGAACATTGCTAGCAAAGTCTGTGTTCAACAGCCTATATGCATCTCCACAACTCGAGGGGTTTTCAGCTGAATCAAGAAAGTTGCTTCTCATTGTTCAGGCACTTAGGGACAACCTGGAACCTGGAACCTTCGATCTTGGGGGGCTATATGAAGCAATTGA
GGAGTGCCTGATTAACGATCCCTGGGTTTTGCTTAATGCGTCTTGGTTCAACTCCTTCCTCACACATGCACTGAAATA-

EPI_ISL_603136

>A/Eurasian_Wigeon/Netherlands/7/2020_H5N8_2020-10-16
ATGGAAGACTTTGTGCGACAATGCTTCAATCCAATGATTGTCGAGCTTGCGGAAAAGGCAATGAAAGAATATGGGGAAGATCCTAAGATCGAAACAAACAAGTTTGCCGCAATATGTACACACTTAGAAGTCTGCTTCATGTATTCGGATTTCCATTTCATTGATGAACGAGGCGAATCAATAATCGTAGAATCTGGCGACCCGAATGCATTATTGAAGCACCGATTTGAGA
TAATTGAAGGGAGAGACCGAACAATGGCCTGGACAGTGGTGAATAGTATATGCAACACTACAGGAGTCGAAAAGCCCAAGTTCCTTCCTGATTTGTATGACTACAGAGAAAACCGATTCATTGAAATTGGAGTAACGCGAAGGGAAGTTCACATATACTATCTTGAAAAAGCCAACAAGATAAAATCAGAGAAAACACACATTCACATATTCTCATTCACTGGGGAGGAA
ATGGCCACCAAGGCCGATTACACTCTTGATGAAGAGAGCAGAGCAAGAATAAAAACCAGGCTATTCACTATAAGACAAGAGATGGCCAATAGGGGTCTATGGGATTCCTTTCGTCAGTCCGAGAGAGGCGAAGAGACAATTGAAGAAAGATTTGAAATCACAGGAACCATGCGCAGGCTTGCTGACCAAAGTCTCCCACCGAACTTCGCCAGCCTTGAAAACTTTAGAG
CCTATGTGGATGGATTTGAACCGAACGGCTGCATTGAGGGCAAGCTTTCTCAAATGTCAAAAGAAGTGAATGCCAGAATTGAGCCATTTCTGAAGACGACACCACGCCCTCTCAGATTACCTGATGGACCTCCCTGTTCCCAGCGGTCGAAGTTCTTGCTAATGGATGCCCTTAAATTGAGCATTGAAGACCCGAGCCATGAGGGGGAGGGTATACCGCTGTACGATGCAA
CCAAATGCATGAAGACATTTTTTGGCTGGAAGGAGCCCAACATCGTGAAACCACATGAAAAGGGCATAAACCCTAATTACCTCCTGGCTTGGAAGCAGGTGCTATCAGAACTCCATGATATTGAAAACGAGGAGAAAATCCCAAAAACAAAGAACATGAAGAAGACGAGCCAACTGAAGTGGGCACTTGGTGAAAACATGGCACCAGAGAAAGTGGACTTTGAGGACT
GTAAAGATGTTAGCGATCTAAGACAGTACGACAGTGACGAACCAGAGTCTAGATCACTAGCAAGTTGGATCCAGAGTGAATTTAACAAGGCTTGCGAACTGACAGATTCGAGTTGGATTGAACTTGATGAAATAGGGGAAGACGTTGCTCCAATTGAACACATTGCGAGTATGAGGAGGAACTATTTCACAGCGGAAGTATCCCATTGCAGGGCTACAGAATACATAATG
AAAGGAGTATACATAAACACAGCTTTATTGAATGCATCCTGTGCAGCCATGGATGACTTCCAACTGATTCCAATGATAAGCAAATGCAGAACCAAAGAAGGGAGACGGAAGACAAATCTGTATGGCTTCATTATAAAAGGAAGATCCCATTTGAGGAATGACACCGATGTGGTAAACTTTGTGAGCATGGAATTCTCTCTCACTGACCCAAGGCTGGAGCCACACAAATGG
GAAAAGTACTGTGTGCTTGAGGTAGGAGACATGCTCCTACGGACTGCAATAGGCCAAGTGTCAAGGCCCATGTTCCTGTATGTGAGAACCAATGGGACTTCTAAGATCAAAATGAAATGGGGTATGGAGATGAGGCGATGCCTTCTTCAATCCCTTCAACAAATTGAGAGCATGATTGAAGCCGAGTCTTCTGTCAAAGAAAAAGACATGACCAAGGAATTCTTTGAAAAC
AGATCAGAAACATGGCCAATTGGAGAGTCACCCAAAGGAGTGGAGGAAGGCTCCATTGGGAAGGTGTGCAGAACATTGCTAGCAAAGTCTGTGTTCAACAGCCTATATGCATCTCCACAACTCGAGGGGTTTTCAGCTGAATCAAGAAAGTTGCTTCTCATTGTTCAGGCACTTAGGGACAACCTGGAACCTGGAACCTTCGATCTTGGGGGGCTATATGAAGCAATTGA
GGAGTGCCTGATTAACGATCCCTGGGTTTTGCTTAATGCGTCTTGGTTCAACTCCTTCCTCACACATGCACTGAAATA-

EPI_ISL_591075

>A/mute_swan/Netherlands/20015931-001/2020_H5N8_2020-10-17
ATGGAAGACTTTGTGCGACAATGCTTCAATCCAATGATTGTCGAGCTTGCGGAAAAGGCAATGAAAGAATATGGGGAAGATCCTAAGATCGAAACAAACAAGTTTGCCGCAATATGTACACACTTAGAAGTCTGCTTCATGTATTCGGATTTCCATTTCATTGATGAACGAGGCGAATCAATAATCGTAGAATCTGGCGACCCGAATGCATTATTGAAGCACCGATTTGAGA
TAATTGAAGGGAGAGACCGAACAATGGCCTGGACAGTGGTGAATAGTATATGCAACACTACAGGAGTCGAAAAGCCCAAGTTCCTTCCTGATTTGTATGACTACAGAGAAAACCGATTCATTGAAATTGGAGTAACGCGAAGGGAAGTTCACATATACTATCTTGAAAAAGCCAACAAGATAAAATCAGAGAAAACACACATTCACATATTCTCATTCACTGGGGAGGAA
ATGGCCACCAAGGCCGATTACACTCTTGATGAAGAGAGCAGAGCAAGAATAAAAACCAGGCTATTCACTATAAGACAAGAGATGGCCAATAGGGGTCTATGGGATTCCTTTCGTCAGTCCGAGAGAGGCGAAGAGACAATTGAAGAAAGATTTGAAATCACAGGAACCATGCGCAGGCTTGCTGACCAAAGTCTCCCACCGAACTTCGCCAGCCTTGAAAACTTTAGAG
CCTATGTGGATGGATTTGAACCGAACGGCTGCATTGAGGGCAAGCTTTCTCAAATGTCAAAAGAAGTGAATGCCAGAATTGAGCCATTTCTGAAGACGACACCACGCCCTCTCAGATTACCTGATGGACCTCCCTGTTCCCAGCGGTCGAAGTTCTTGCTAATGGATGCCCTTAAATTAAGCATTGAAGACCCGAGCCATGAGGGGGAGGGTATACCGCTGTACGATGCAA
CCAAATGCATGAAGACATTTTTTGGCTGGAAGGAGCCCAACATCGTGAAACCACATGAAAAGGGCATAAACCCTAATTACCTCCTGGCTTGGAAGCAGGTGCTATCAGAACTCCATGATATTGAAAACGAGGAGAAAATCCCAAAAACAAAGAACATGAAGAAGACGAGCCAACTGAAGTGGGCACTTGGTGAAAACATGGCACCAGAGAAAGTGGACTTTGAGGACT
GTAAAGATGTTAGCGATCTAAGACAGTACGACAGTGACGAACCAGAGTCTAGATCACTAGCAAGTTGGATCCAGAGTGAATTTAACAAGGCTTGCGAACTGACAGATTCGAGTTGGATTGAACTTGATGAAATAGGGGAAGACGTTGCTCCAATTGAACACATTGCGAGTATGAGGAGGAACTATTTCACAGCGGAAGTATCCCATTGCAGGGCTACAGAATACATAATG
AAAGGAGTATACATAAACACAGCTTTATTGAATGCATCCTGTGCAGCCATGGATGACTTCCAACTGATTCCAATGATAAGCAAATGCAGAACCAAAGAAGGGAGACGGAAGACAAATCTGTATGGCTTCATTATAAAAGGAAGATCCCATTTGAGGAATGACACCGATGTGGTAAACTTTGTGAGCATGGAATTCTCTCTCACTGACCCAAGGCTGGAGCCACACAAATGG
GAAAAGTACTGTGTGCTTGAGGTAGGAGACATGCTCCTACGGACTGCAATAGGCCAAGTGTCAAGGCCCATGTTCCTGTATGTGAGAACCAATGGGACTTCTAAGATCAAAATGAAATGGGGTATGGAGATGAGGCGATGCCTTCTTCAATCCCTTCAACAAATTGAGAGCATGATTGAAGCCGAGTCTTCTGTCAAAGAAAAAGACATGACCAAGGAATTCTTTGAAAAC
AGATCAGAAACATGGCCAATTGGAGAGTCACCCAAAGGAGTGGAGGAAGGCTCCATTGGGAAGGTGTGCAGAACATTGCTAGCAAAGTCTGTGTTCAACAGCCTATATGCATCTCCACAACTCGAGGGGTTTTCAGCTGAATCAAGAAAGTTGCTTCTCATTGTTCAGGCACTTAGGGACAACCTGGAACCTGGAACCTTCGATCTTGGGGGGCTATATGAAGCAATTGA
GGAGTGCCTGATTAACGATCCCTGGGTTTTGCTTAATGCGTCTTGGTTCAACTCCTTCCTCACACATGCACTGAAATA-

EPI_ISL_1399231

>A/chicken/Czech_Republic/3531-1/2021_H5N8_2021-02-18
ATGGAAGACTTTGTGCGACAATGCTTCAATCCAATGATTGTCGAGCTTGCGGAAAAGGCAATGAAAGAATATGGGGAAGATCCTAAGATCGAAACAAACAAGTTTGCCGCAATATGTACACACTTAGAAGTCTGCTTCATGTATTCGGATTTCCATTTCATTGATGAACGAGGCGAATCAATAATCGTAGAATCTGGCGACCCGAATGCATTATTGAAGCACCGATTTGAGA
TAATTGAAGGGAGAGACCGAACAATGGCCTGGACAGTGGTGAATAGTATATGCAACACTACAGGAGTCGAAAAGCCCAAGTTCCTTCCTGATTTGTATGACTACAGAGAAAACCGATTCATTGAAATTGGAGTAACGCGAAGGGAAGTTCACATATACTATCTTGAAAAAGCCAACAAGATAAAATCAGAGAAAACACACATTCACATATTCTCATTCACTGGGGAGGAA
ATGGCCACCAAGGCCGATTACACTCTTGATGAAGAGAGCAGAGCAAGAATAAAAACCAGGCTATTCACTATAAGACAAGAGATGGCCAATAGGGGTCTATGGGATTCCTTTCGTCAGTCCGAGAGAGGCGAAGAGACAATTGAAGAAAGATTTGAAATCACAGGAACCATGCGCAGGCTTGCTGACCAAAGTCTCCCACCGAACTTCGCCAGCCTTGAAAACTTTAGAG
CCTATGTGGATGGATTTGAACCGAACGGCTGCATTGAGGGCAAGCTTTCTCAAATGTCAAAAGAAGTGAATGCCAGAATTGAGCCATTTCTGAAGACGACACCACGCCCTCTCAGATTACCTGATGGACCTCCCTGTTCCCAGCGGTCGAAGTTCTTGCTAATGGATGCCCTTAAATTAAGCATTGAAGACCCGAGCCATGAGGGGGAGGGTATACCGCTGTACGATGCAA
CCAAATGCATGAAGACATTTTTTGGCTGGAAGGAGCCCAACATCGTGAAACCACATGAAAAGGGCATAAACCCTAATTACCTCCTGGCTTGGAAGCAGGTGCTATCAGAACTCCAAGATATTGAAAACGAGGAGAAAATCCCAAAAACAAAGAACATGAAGAAGACGAGCCAACTGAAGTGGGCACTTGGTGAAAACATGGCACCAGAGAAAGTGGACTTTGAGGACT
GTAAAGATGTTAGCGATCTAAGACAGTACGACAGTGACGAACCAGAGTCTAGATCACTAGCAAGTTGGATCCAGAGTGAATTTAACAAGGCTTGCGAACTGACAGATTCGAGTTGGATTGAACTTGATGAAATAGGGGAAGACGTTGCTCCAATTGAACACATTGCGAGTATGAGGAGGAACTATTTCACAGCGGAAGTATCCCATTGCAGGGCTACAGAATACATAATG
AAAGGAGTATACATAAACACAGCTTTATTGAATGCATCCTGTGCAGCCATGGATGACTTCCAACTGATTCCAATGATAAGCAAATGCAGAACCAAAGAAGGGAGACGGAAGACAAATCTGTATGGCTTCATTATAAAAGGAAGATCCCATTTGAGGAATGACACCGATGTGGTAAACTTTGTGAGCATGGAATTCTCTCTCACTGACCCAAGGCTGGAGCCACACAAATGG
GAAAAGTACTGTGTGCTTGAGGTAGGAGACATGCTCCTACGGACTGCAATAGGCCAAGTGTCAAGGCCCATGTTCCTGTATGTGAGAACCAATGGGACTTCTAAGATCAAAATGAAATGGGGTATGGAGATGAGGCGATGCCTTCTTCAATCCCTTCAACAAATTGAGAGCATGATTGAAGCCGAGTCTTCTGTCAAAGAAAAAGACATGACCAAGGAATTCTTTGAAAAC
AGATCAGAAACATGGCCAATTGGAGAGTCACCCAAAGGAGTGGAGGAAGGCTCCATTGGGAAGGTGTGCAGAACATTGCTAGCAAAGTCTGTGTTCAACAGCCTATATGCATCTCCACAACTCGAGGGGTTTTCAGCTGAATCAAGAAAGTTGCTTCTCATTGTTCAGGCACTTAGGGACAACCTGGAACCTGGAACCTTCGATCTTGGGGGGCTATATGAAGCAATTGA
GGAGTGCCTGATTAACGATCCCTGGGTTTTGCTTAATGCGTCTTGGTTCAACTCCTTCCTCACACATGCACTGAAATA-

EPI_ISL_1220094

>A/tundra-bean-goose/Poland/MB132/2020_H5N8_2020-12-11
ATGGAAGACTTTGTGCGACAATGCTTCAATCCAATGATTGTCGAGCTTGCGGAAAAGGCAATGAAAGAATATGGGGAAGATCCTAAGATCGAAACAAACAAGTTTGCCGCAATATGTACACACTTAGAAGTCTGCTTCATGTATTCGGATTTCCATTTCATTGATGAACGAGGCGAATCAATAATCGTAGAATCTGGCGACCCGAATGCATTATTGAAGCACCGATTTGAGA
TAATTGAAGGGAGAGACCGAACAATGGCCTGGACAGTGGTGAATAGTATATGCAACACTACAGGAGTCGAAAAGCCCAAGTTCCTTCCTGATTTGTATGACTACAGAGAAAACCGATTCATTGAAATTGGAGTAACGCGAAGGGAAGTTCACATATACTATCTTGAAAAAGCCAACAAGATAAAATCAGAGAAAACACACATTCACATATTCTCATTCACTGGGGAGGAA
ATGGCCACCAAGGCCGATTACACTCTTGATGAAGAGAGCAGAGCAAGAATAAAAACCAGGCTATTCACTATAAGACAAGAGATGGCCAATAGGGGTCTATGGGATTCCTTTCGTCAGTCCGAGAGAGGCGAAGAGACAATTGAAGAAAGATTTGAAATCACAGGAACCATGCGCAGGCTTGCTGACCAAAGTCTCCCACCGAACTTCGCCAGCCTTGAAAACTTTAGAG
CCTATGTGGATGGATTTGAACCGAACGGCTGCATTGAGGGCAAGCTTTCTCAAATGTCAAAAGAAGTGAATGCCAGAATTGAGCCATTTCTGAAGACGACACCACGCCCTCTCAGATTACCTGATGGACCTCCCTGTTCCCAGCGGTCGAAGTTCTTGCTAATGGATGCCCTTAAATTAAGCATTGAAGACCCGAGCCATGAGGGGGAGGGTATACCGCTGTACGATGCAA
CCAAATGCATGAAGACATTTTTTGGCTGGAAGGAGCCCAACATCGTGAAACCACATGAAAAGGGCATAAACCCTAATTACCTCCTGGCTTGGAAGCAGGTGCTATCAGAACTCCAAGATATTGAAAACGAGGAGAAAATCCCAAAAACAAAGAACATGAAGAAGACGAGCCAACTGAAGTGGGCACTTGGTGAAAACATGGCACCAGAGAAAGTGGACTTTGAGGACT
GTAAAGATGTTAGCGATCTAAGACAGTACGACAGTGACGAACCAGAGTCTAGATCACTAGCAAGTTGGATCCAGAGTGAATTTAACAAGGCTTGCGAACTGACAGATTCGAGTTGGATTGAACTTGATGAAATAGGGGAAGACGTTGCTCCAATTGAACACATTGCGAGTATGAGGAGGAACTATTTCACAGCGGAAGTATCCCATTGCAGGGCTACAGAATACATAATG
AAAGGAGTATACATAAACACAGCTTTATTGAATGCATCCTGTGCAGCCATGGATGACTTCCAACTGATTCCAATGATAAGCAAATGCAGAACCAAAGAAGGGAGACGGAAGACAAATCTGTATGGCTTCATTATAAAAGGAAGATCCCATTTGAGGAATGACACCGATGTGGTAAACTTTGTGAGCATGGAATTCTCTCTCACTGACCCAAGGCTGGAGCCACACAAATGG
GAAAAGTACTGTGTGCTTGAGGTAGGAGACATGCTCCTACGGACTGCAATAGGCCAAGTGTCAAGGCCCATGTTCCTGTATGTGAGAACCAATGGGACTTCTAAGATCAAAATGAAATGGGGTATGGAGATGAGGCGATGCCTTCTTCAATCCCTTCAACAAATTGAGAGCATGATTGAAGCCGAGTCTTCTGTCAAAGAAAAAGACATGACCAAGGAATTCTTTGAAAAC
AGATCAGAAACATGGCCAATTGGAGAGTCACCCAAAGGAGTGGAGGAAGGCTCCATTGGGAAGGTGTGCAGAACATTGCTAGCAAAGTCTGTGTTCAACAGCCTATATGCATCTCCACAACTCGAGGGGTTTTCAGCTGAATCAAGAAAGTTGCTTCTCATTGTTCAGGCACTTAGGGACAACCTGGAACCTGGAACCTTCGATCTTGGGGGGCTATATGAAGCAATTGA
GGAGTGCCTGATTAACGATCCCTGGGTTTTGCTTAATGCGTCTTGGTTCAACTCCTTCCTCACACATGCACTGAAATA-

EPI_ISL_813979

>A/peregrine_falcon/Ireland/20VIR7872-1/2020_H5N8_2020-11
ATGGAAGACTTTGTGCGACAATGCTTCAATCCAATGATTGTCGAGCTTGCGGAAAAGGCAATGAAAGAATATGGGGAAGATCCTAAGATCGAAACAAACAAGTTTGCCGCAATATGTACACACTTAGAAGTCTGCTTCATGTATTCGGATTTCCATTTCATTGACGAACGAGGCGAATCAATAATCGTAGAATCTGGCGACCCGAATGCATTATTGAAGCACCGATTTGAGA
TAATTGAAGGGAGAGACCGAACAATGGCCTGGACAGTGGTGAATAGTATATGCAACACTACAGGAGTCGAAAAGCCCAAGTTCCTTCCTGATTTGTATGACTACAGAGAAAACCGATTCATTGAAATTGGAGTAACGCGAAGGGAAGTTCACATATACTATCTTGAAAAAGCCAACAAGATAAAATCAGAGAAAACACACATTCACATATTCTCATTCACTGGGGAGGAA
ATGGCCACCAAGGCCGATTACACTCTTGATGAAGAGAGCAGAGCAAGAATAAAAACCAGGCTATTCACTATAAGACAAGAGATGGCCAATAGGGGTCTATGGGATTCCTTTCGTCAGTCCGAGAGAGGCGAAGAGACAATTGAAGAAAGATTTGAAATCACAGGAACCATGCGCAGGCTTGCTGACCAAAGTCTCCCACCGAACTTCGCCAGCCTTGAAAACTTTAGAG
CCTATGTGGATGGATTTGAACCGAACGGCTGCATTGAGGGCAAGCTTTCTCAAATGTCAAAAGAAGTGAATGCCAGAATTGAGCCATTTCTGAAGACGACACCACGCCCTCTCAGATTACCTGATGGACCTCCCTGTTCCCAGCGGTCGAAGTTCTTGCTAATGGATGCCCTTAAATTGAGCATTGAAGACCCGAGCCATGAGGGGGAGGGTATACCGCTGTACGATGCAA
CCAAATGCATGAAGACATTTTTTGGCTGGAAGGAGCCCAACATCGTGAAACCACATGAAAAGGGCATAAACCCTAATTACCTCCTGGCTTGGAAGCAGGTGCTATCAGAACTCCAAGATATTGAAAACGAGGAGAAAATCCCAAAAACAAAGAACATGAAGAAGACGAGCCAACTGAAGTGGGCACTTGGTGAAAACATGGCACCAGAGAAAGTGGACTTTGAGGACT
GTAAAGATGTTAGCGATCTAAGACAGTACGACAGTGACGAACCAGAGTCTAGATCACTAGCAAGTTGGATCCAGAGTGAATTTAACAAGGCTTGCGAACTGACAGATTCGAGTTGGATTGAACTTGATGAAATAGGGGAAGACGTTGCTCCAATTGAACACATTGCGAGTATGAGGAGGAACTATTTCACAGCGGAAGTATCCCATTGCAGGGCTACAGAATACATAATG
AAAGGAGTATACATAAACACAGCTTTATTGAATGCATCCTGTGCAGCCATGGATGACTTCCAACTGATTCCAATGATAAGCAAATGCAGAACCAAAGAAGGGAGACGGAAGACAAATCTGTATGGCTTCATTATAAAAGGAAGATCCCATTTGAGGAATGACACCGATGTGGTAAACTTTGTGAGCATGGAATTCTCTCTCACTGACCCAAGGCTGGAGCCACACAAATGG
GAAAAGTACTGTGTGCTTGAGGTAGGAGACATGCTCCTACGGACTGCAATAGGCCAAGTGTCAAGGCCCATGTTCCTGTATGTGAGAACCAATGGGACTTCTAAGATCAAAATGAAATGGGGTATGGAGATGAGGCGATGCCTTCTTCAATCCCTTCAACAAATTGAGAGCATGATTGAAGCCGAGTCTTCTGTCAAAGAAAAAGACATGACCAAGGAATTCTTTGAAAAC
AGATCAGAAACATGGCCAATTGGAGAGTCACCCAAAGGAGTGGAGGAAGGCTCCATTGGGAAGGTGTGCAGAACATTGCTAGCAAAGTCTGTGTTCAACAGCCTATATGCATCTCCACAACTCGAGGGGTTTTCAGCTGAATCAAGAAAGTTGCTTCTCATTGTTCAGGCACTTAGGGACAACCTGGAACCTGGAACCTTCGATCTTGGGGGGCTATATGAAGCAATTGA
GGAGTGCCTGATTAACGATCCCTGGGTTTTGCTTAATGCGTCTTGGTTCAACTCCTTCCTCACACATGCACTGAAATA-

EPI_ISL_626652

>A/chicken/England/030720/2020_H5N8_2020-11-02
ATGGAAGACTTTGTGCGACAATGCTTCAATCCAATGATTGTCGAGCTTGCGGAAAAGGCAATGAAAGAATATGGGGAAGATCCTAAGATCGAAACAAACAAGTTTGCCGCAATATGTACACACTTAGAAGTCTGCTTCATGTATTCGGATTTCCATTTCATTGATGAACGAGGCGAATCAATAATCGTAGAATCTGGCGACCCGAATGCATTATTGAAGCACCGATTTGAGA
TAATTGAAGGGAGAGACCGAACAATGGCCTGGACAGTGGTGAATAGTATATGCAACACTACAGGAGTCGAAAAGCCCAAGTTCCTTCCTGATTTGTATGACTACAGAGAAAACCGATTCATTGAAATTGGAGTAACGCGAAGGGAAGTTCACATATACTATCTTGAAAAAGCCAACAAGATAAAATCAGAGAAAACACACATTCACATATTCTCATTCACTGGGGAGGAA
ATGGCCACCAAGGCCGATTACACTCTTGATGAAGAGAGCAGAGCAAGAATAAAAACCAGGCTATTCACTATAAGACAAGAGATGGCCAATAGGGGTCTATGGGATTCCTTTCGTCAGTCCGAGAGAGGCGAAGAGACAATTGAAGAAAGATTTGAAATCACAGGAACCATGCGCAGGCTTGCTGACCAAAGTCTCCCACCGAACTTCGCCAGCCTTGAAAACTTTAGAG
CCTATGTGGATGGATTTGAACCGAACGGCTGCATTGAGGGCAAGCTTTCTCAAATGTCAAAAGAAGTGAATGCCAGAATTGAGCCATTTCTGAAGACGACACCACGCCCTCTCAGATTACCTGATGGACCTCCCTGTTCCCAGCGGTCGAAGTTCTTGCTAATGGATGCCCTTAAATTGAGCATTGAAGACCCGAGCCATGAGGGGGAGGGTATACCGCTGTACGATGCAA
CCAAATGCATGAAGACATTTTTTGGCTGGAAGGAGCCCAACATCGTGAAACCACATGAAAAGGGCATAAACCCTAATTACCTCCTGGCTTGGAAGCAGGTGCTATCAGAACTCCAAGATATTGAAAACGAGGAGAAAATCCCAAAAACAAAGAACATGAAGAAGACGAGCCAACTGAAGTGGGCACTTGGTGAAAACATGGCACCAGAGAAAGTGGACTTTGAGGACT
GTAAAGATGTTAGCGATCTAAGACAGTACGACAGTGACGAACCAGAGTCTAGATCACTAGCAAGTTGGATCCAGAGTGAATTTAACAAGGCTTGCGAACTGACAGATTCGAGTTGGATTGAACTTGATGAAATAGGGGAAGACGTTGCTCCAATTGAACACATTGCGAGTATGAGGAGGAACTATTTCACAGCGGAAGTATCCCATTGCAGGGCTACAGAATACATAATG
AAAGGAGTATACATAAACACAGCTTTATTGAATGCATCCTGTGCAGCCATGGATGACTTCCAACTGATTCCAATGATAAGCAAATGCAGAACCAAAGAAGGGAGACGGAAGACAAATCTGTATGGCTTCATTATAAAAGGAAGATCCCATTTGAGGAATGACACCGATGTGGTAAACTTTGTGAGCATGGAATTCTCTCTCACTGACCCAAGGCTGGAGCCACACAAATGG
GAAAAGTACTGTGTGCTTGAGGTAGGAGACATGCTCCTACGGACTGCAATAGGCCAAGTGTCAAGGCCCATGTTCCTGTATGTGAGAACCAATGGGACTTCTAAGATCAAAATGAAATGGGGTATGGAGATGAGGCGATGCCTTCTTCAATCCCTTCAACAAATTGAGAGCATGATTGAAGCCGAGTCTTCTGTCAAAGAAAAAGACATGACCAAGGAATTCTTTGAAAAC
AGATCAGAAACATGGCCAATTGGAGAGTCACCCAAAGGAGTGGAGGAAGGCTCCATTGGGAAGGTGTGCAGAACATTGCTAGCAAAGTCTGTGTTCAACAGCCTATATGCATCTCCACAACTCGAGGGGTTTTCAGCTGAATCAAGAAAGTTGCTTCTCATTGTTCAGGCACTTAGGGACAACCTGGAACCTGGAACCTTCGATCTTGGGGGGCTATATGAAGCAATTGA
GGAGTGCCTGATTAACGATCCCTGGGTTTTGCTTAATGCGTCTTGGTTCAACTCCTTCCTCACACATGCACTGAAATA-

EPI_ISL_626651

>A/goose/Russian_Federation/Kurgan/1345-25/2020__H5N8_2020-08-20
ATGGAAGACTTTGTGCGACAATGCTTCAATCCAATGATTGTCGAGCTTGCGGAAAAGGCAATGAAAGAATATGGGGAAGATCCTAAGATCGAAACAAACAAGTTTGCCGCAATATGTACACACTTAGAAGTCTGCTTCATGTATTCGGATTTCCATTTCATTGATGAACGAGGCGAATCAATAATCGTAGAATCTGGCGACCCGAATGCATTATTGAAGCACCGATTTGAGA
TAATTGAAGGGAGAGACCGAACAATGGCCTGGACAGTGGTGAATAGTATATGCAACACTACAGGAGTCGAAAAGCCCAAGTTCCTTCCTGATTTGTATGACTACAGAGAAAACCGATTCATTGAAATTGGAGTAACGCGAAGGGAAGTTCACATATACTATCTTGAAAAAGCCAACAAGATAAAATCAGAGAAAACACACATTCACATATTCTCATTCACTGGGGAGGAA
ATGGCCACCAAGGCAGATTACACTCTTGATGAAGAGAGCAGAGCAAGAATAAAAACCAGGCTATTCACTATAAGACAAGAGATGGCCAATAGGGGTCTATGGGATTCCTTTCGTCAGTCCGAGAGAGGCGAAGAGACAATTGAAGAAAGATTTGAAATCACAGGAACCATGCGCAGGCTTGCTGACCAGAGTCTCCCACCGAACTTCGCCAGCCTTGAAAACTTTAGAG
CCTATGTGGATGGATTTGAACCGAACGGCTGCATTGAGGGCAAGCTTTCTCAAATGTCAAAAGAAGTGAATGCCAGAATTGAGCCATTTCTGAAGACGACACCACGCCCTCTCAGATTACCTGATGGACCTCCCTGTTCCCAGCGGTCGAAGTTCTTGCTAATGGATGCCCTTAAATTGAGCATTGAAGACCCGAGCCATGAGGGGGAGGGTATACCGCTGTACGATGCAA
CCAAATGCATGAAGACATTTTTTGGCTGGAAGGAGCCCAACATCGTGAAACCACATGAAAAGGGCATAAACCCTAATTACCTCCTGGCTTGGAAGCAGGTGCTATCAGAACTCCAAGATATTGAAAACGAGGAGAAAATCCCAAAAACAAAGAACATGAAGAAGACGAGCCAACTGAAGTGGGCACTTGGTGAAAACATGGCACCAGAGAAAGTGGACTTTGAGGACT
GTAAAGATGTTAGCGATCTAAGACAGTACGACAGTGACGAACCAGAGTCTAGATCACTAGCAAGTTGGATCCAGAGTGAATTTAACAAGGCTTGCGAACTGACAGATTCGAGTTGGATTGAACTTGATGAAATAGGGGAAGACGTTGCTCCAATTGAACACATTGCGAGTATGAGGAGGAACTATTTCACAGCGGAAGTATCCCATTGCAGGGCTACAGAATACATAATG
AAAGGAGTATACATAAACACAGCTTTATTGAATGCATCCTGTGCAGCCATGGATGACTTCCAACTGATTCCAATGATAAGCAAATGCAGAACCAAAGAAGGGAGACGGAAGACAAATCTGTATGGCTTCATTATAAAAGGAAGATCCCATTTGAGGAATGACACCGATGTGGTAAACTTTGTGAGCATGGAATTCTCTCTCACTGACCCAAGGCTGGAGCCACACAAATGG
GAAAAGTACTGTGTGCTTGAGGTAGGAGACATGCTCCTACGGACTGCAATAGGCCAAGTGTCAAGGCCCATGTTCCTGTATGTGAGAACCAATGGGACTTCTAAGATCAAAATGAAATGGGGTATGGAGATGAGGCGATGCCTTCTTCAATCCCTTCAACAAATTGAGAGCATGATTGAAGCCGAGTCTTCTGTCAAAGAAAAAGACATGACCAAGGAATTCTTTGAAAAC
AGATCAGAAACATGGCCAATTGGAGAGTCACCCAAAGGAGTGGAGGAAGGCTCCATTGGGAAGGTGTGCAGAACATTGCTAGCAAAGTCTGTGTTCAACAGCCTATATGCATCTCCACAACTCGAGGGGTTTTCAGCTGAATCAAGAAAGTTGCTTCTCATTGTTCAGGCACTTAGGGACAACCTGGAACCTGGAACCTTCGATCTTGGGGGGCTATATGAAGCAATTGA
GGAGTGCCTGATTAACGATCCCTGGGTTTTGCTTAATGCGTCTTGGTTCAACTCCTTCCTCACACATGCACTGAAATA-

EPI_ISL_1114763

>A/chicken/Krasnodar/334-03/2021_H5N8_2021-01-05
ATGGAAGACTTTGTGCGACAATGCTTCAATCCAATGATTGTCGAGCTTGCGGAAAAGGCAATGAAAGAATATGGGGAAGATCCTAAGATCGAAACAAACAAGTTTGCCGCAATATGTACACACTTAGAAGTCTGCTTCATGTATTCGGATTTCCATTTCATTGATGAACGAGGCGAATCAATAATCGTAGAATCTGGCGACCCGAATGCATTATTGAAGCACCGATTTGAGA
TAATTGAAGGGAGAGACCGAACAATGGCCTGGACAGTGGTGAATAGTATATGCAACACTACAGGAGTCGAAAAGCCCAAGTTCCTTCCTGATTTGTATGACTACAGAGAAAACCGATTCATTGAAATTGGAGTAACGCGAAGGGAAGTTCACATATACTATCTTGAAAAAGCCAACAAGATAAAATCAGAGAAAACACACATTCACATATTCTCATTCACTGGGGAGGAA
ATGGCCACCAAGGCAGATTACACTCTTGATGAAGAGAGCAGAGCAAGAATAAAAACCAGGCTATTCACTATAAGACAAGAGATGGCCAATAGGGGTCTATGGGATTCCTTTCGTCAGTCCGAGAGAGGCGAAGAGACAATTGAAGAAAGATTTGAAATCACAGGAACCATGCGCAGGCTTGCTGACCAAAGTCTCCCACCGAACTTCGCCAGCCTTGAAAACTTTAGAG
CCTATGTGGATGGATTTGAACCGAACGGCTGCATTGAGGGCAAGCTTTCTCAAATGTCAAAAGAAGTGAATGCCAGAATTGAGCCATTTCTGAAGACGACACCACGCCCTCTCAGATTACCTGATGGACCTCCCTGTTCCCAGCGGTCGAAGTTCTTGCTAATGGATGCCCTTAAATTGAGCATTGAAGACCCGAGCCATGAGGGGGAGGGTATACCGCTGTACGATGCAA
CCAAATGCATGAAGACATTTTTTGGCTGGAAGGAGCCCAACATCGTGAAACCACATGAAAAGGGCATAAACCCTAATTACCTCCTGGCTTGGAAGCAGGTGCTATCAGAACTCCAAGATATTGAAAACGAGGAGAAAATCCCAAAAACAAAGAACATGAAGAAGACGAGCCAACTGAAGTGGGCACTTGGTGAAAACATGGCACCAGAGAAATTGGACTTTGAGGACT
GTAAAGATGTTAGCGATCTAAGACAGTACGACAGTGACGAACCAGAGTCTAGATCACTAGCAAGTTGGATCCAGAGTGAATTTAACAAGGCTTGCGAACTGACAGATTCGAGTTGGATCGAACTTGATGAAATAGGGGAAGACGTTGCTCCAATTGAACACATTGCGAGTATGAGGAGGAACTATTTCACAGCGGAAGTATCCCATTGCAGGGCTACAGAATACATAATG
AAAGGAGTATACATAAACACAGCTTTATTGAATGCATCCTGTGCAGCCATGGATGACTTCCAACTGATTCCAATGATAAGCAAATGCAGAACCAAAGAAGGGAGACGGAAGACAAATCTGTATGGCTTCATTATAAAAGGAAGATCCCATTTGAGGAATGACACCGATGTGGTAAACTTTGTGAGCATGGAATTCTCTCTCACTGACCCAAGGCTGGAGCCACACAAATGG
GAAAAGTACTGTGTGCTTGAGGTAGGAGACATGCTCCTACGGACTGCAATAGGCCAAGTGTCAAGGCCCATGTTCCTGTATGTGAGAACCAATGGGACTTCTAAGATCAAAATGAAATGGGGTATGGAGATGAGGCGATGCCTTCTTCAATCCCTTCAACAAATTGAGAGCATGATTGAAGCCGAGTCTTCTGTCAAAGAAAAAGACATGACCAAGGAATTCTTTGAAAAC
AGATCAGAAACATGGCCAATTGGAGAGTCACCCAAAGGAGTGGAGGAAGGCTCCATTGGGAAGGTGTGCAGAACATTGCTAGCAAAGTCTGTGTTCAACAGCCTATATGCATCTCCACAACTCGAGGGGTTTTCAGCTGAATCAAGAAAGTTGCTTCTCATTGTTCAGGCACTTAGGGACAACCTGGAACCTGGAACCTTCGATCTTGGGGGGCTATATGAAGCAATTGA
GGAGTGCCTGATTAACGATCCCTGGGTTTTGCTTAATGCGTCTTGGTTCAACTCCTTCCTCACACATGCACTGAAATA-

EPI_ISL_18245197

>A/mute_swan/Poland/MB292-21_22VIR5675-14/2021_H5N8_2021-03-11
ATGGAAGACTTTGTGCGACAATGCTTCAATCCAATGATTGTCGAGCTTGCGGAAAAGGCAATGAAAGAATATGGGGAAGATCCTAAGATCGAAACAAACAAGTTTACCGCAATATGTACACACTTAGAAGTCTGCTTCATGTATTCGGATTTCCATTTCATTGATGAACGAGGCGAATCAATAATCGTAGAATCTGGCGACCCGAATGCATTATTGAAGCACCGATTTGAGA
TAATTGAAGGGAGAGACCGAACAATGGCCTGGACAGTGGTGAATAGTATATGCAACACTACAGGAGTCGAAAAGCCCAAGTTCCTTCCTGATTTGTATGACTACCGAGAAAACCGATTCATTGAAATTGGAGTAACGCGAAGGGAAGTTCACATATACTATCTTGAAAAAGCCAACAAGATAAAATCAGAGAAAACACACATTCACATATTCTCATTCACTGGGGAGGAA
ATGGCCACCAAGGCAGATTACACTCTTGATGAAGAGAGCAGAGCAAGAATAAAAACCAGGCTATTCACTATAAGACAAGAGATGGCCAATAGGGGTCTATGGGATTCCTTTCGTCAATCCGAGAGAGGCGAAGAGACAATTGAAGAAAGATTTGAAATCACAGGAACCATGCGCAGGCTTGCTGACCAAAGTCTCCCACCGAACTTCGCCAGCCTTGAAAACTTTAGAG
CCTATGTGGATGGATTTGAACCGAACGGCTGCATTGAGGGCAAGCTTTCTCAAATGTCAAAAGAAGTGAATGCCAGAATTGAGCCATTTCTGAAGACGACACCACGCCCTCTCAGATTACCTGATGGACCTCCCTGTTCCCAGCGGTCGAAGTTCTTGCTAATGGATGCCCTTAAATTGAGCATTGAAGACCCGAGCCATGAGGGGGAGGGTATACCGCTGTACGATGCAA
CCAAATGCATGAAGACATTTTTTGGCTGGAAGGAGCCCAACATCGTGAAACCACATGAAAAGGGCATAAACCCTAATTACCTCCTGGCTTGGAAGCAGGTGCTATCAGAACTCCAAGATATTGAAAACGAGGAGAAAATCCCAAAAACAAAGAACATGAAGAAGACGAGCCAACTGAAGTGGGCACTTGGTGAAAACATGGCACCAGAGAAAGTGGACTTTGAGGATT
GTAAAGATGTTAGCGATCTAAGACAGTACGACAGTGACGAACCAGAGTCTAGATCACTAGCAAGTTGGATCCAGAGTGAATTTAACAAGGCTTGCGAACTGACAGATTCGAGTTGGATTGAACTTGATGAAATAGGGGAAGACGTTGCTCCAATTGAACACATTGCGAGTATGAGGAGGAACTATTTCACAGCGGAAGTATCCCATTGCAGGGCTACAGAATACATAATG
AAAGGAGTATACATAAACACAGCTTTATTGAATGCATCCTGTGCAGCCATGGATGACTTCCAACTGATTCCAATGATAAGCAAATGCAGAACCAAAGAAGGGAGACGGAAGACAAATCTGTATGGCTTCATTATAAAAGGAAGATCCCATTTGAGGAATGACACCGATGTGGTAAACTTTGTGAGCATGGAATTCTCTCTCACTGACCCAAGGCTGGAGCCACACAAATGG
GAAAAGTACTGTGTACTTGAGGTAGGAGACATGCTCCTACGGACTGCAATAGGCCAAGTGTCAAGGCCCATGTTCCTGTATGTGAGAACCAATGGGACTTCTAAGATCAAAATGAAATGGGGTATGGAGATGAGGCGATGCCTTCTTCAATCCCTTCAACAAATTGAGAGCATGATTGAAGCCGAGTCTTCTGTCAAAGAAAAAGACATGACCAAGGAATTCTTTGAAAAC
AGATCAGAAACATGGCCAATTGGAGAGTCACCCAAAGGAGTGGAGGAAAGCTCCATTGGGAAGGTGTGCAGAACATTGCTAGCAAAGTCTGTGTTCAACAGCCTATATGCATCTCCACAACTCGAGGGGTTTTCAGCTGAATCAAGAAAGTTGCTTCTCATTGTTCAGGCACTTAGGGACAACCTGGAACCTGGAACCTTCGATCTTGGGGGGCTATATGAAGCAATTGA
GGAGTGCCTGATTAACGATCCCTGGGTTTTGCTTAATGCGTCTTGGTTCAACTCCTTCCTCACACATGCACTGAAATA-

EPI_ISL_2113493

>A/mute_swan/Poland/MB268/2021_H5N8_2021-03-09
ATGGAAGACTTTGTGCGACAATGCTTCAATCCAATGATTGTCGAGCTTGCGGAAAAGGCAATGAAAGAATATGGGGAAGATCCTAAGATCGAAACAAACAAGTTTGCCGCAATATGTACACACTTAGAAGTCTGCTTCATGTATTCGGATTTCCATTTCATTGATGAACGAGGCGAATCAATAATCGTAGAATCTGGCGACCCGAATGCATTATTGAAGCACCGATTTGAGA
TAATTGAAGGGAGAGACCGAACAATGGCCTGGACAGTGGTGAATAGTATATGCAACACTACAGGAGTCGAAAAGCCCAAGTTCCTTCCTGATTTGTATGACTACAGAGAAAACCGATTCATTGAAATTGGAGTAACGCGAAGGGAAGTTCACATATACTATCTTGAAAAAGCCAACAAGATAAAGTCAGAGAAAACACACATTCACATATTCTCATTCACTGGGGAGGAA
ATGGCCACCAAGGCAGATTACACTCTTGATGAAGAGAGCAGAGCAAGAATAAAAACCAGGCTATTCACTATAAGACAAGAGATGGCCAATAGGGGTCTATGGGATTCCTTTCGTCAGTCCGAGAGAGGCGAAGAGACAATTGAAGAAAGATTTGAAATCACAGGAACCATGCGCAGGCTTGCTGACCAAAGTCTCCCACCGAACTTCGCCAGCCTTGAAAACTTTAGAG
CCTATGTGGATGGATTTGAACCGAACGGCTGCATTGAGGGCAAGCTTTCTCAAATGTCAAAAGAAGTGAATGCCAGAATTGAGCCATTTCTGAAGACAACACCACGCCCTCTCAGATTACCTGATGGACCTCCCTGTTCCCAGCGGTCGAAGTTCTTGCTAATGGATGCCCTTAAATTGAGCATTGAAGACCCGAGCCATGAGGGGGAGGGTATACCGCTGTACGATGCAA
CCAAATGCATGAAGACATTTTTTGGCTGGAAGGAGCCCAACATCGTGAAACCACATGAAAAGGGCATAAACCCTAATTACCTCCTGGCTTGGAAGCAGGTGCTATCAGAACTCCAAGATATTGAAAACGAGGAGAAAATCCCTAAAACAAAGAACATGAAGAAGACGAGCCAACTGAAGTGGGCACTTGGTGAAAACATGGCACCAGAGAAAGTGGACTTTGAGGACT
GTAAAGATGTTAGCGATCTAAAACAGTACGACAGTGACGAACCAGAATCTAGATCACTAGCAAGTTGGATCCAGAGTGAATTTAACAAGGCTTGCGAACTGACAGATTCGAGTTGGATTGAACTTGATGAAATAGGGGAAGACGTTGCTCCAATTGAACACATTGCGAGTATGAGGAGGAACTATTTCACAGCGGAAGTATCCCATTGCAGGGCTACAGAATACATAATG
AAAGGAGTATACATAAACACAGCTTTATTGAATGCATCCTGTGCAGCCATGGATGACTTCCAACTGATTCCAATGATAAGCAAATGCAGAACCAAAGAAGGGAGACGGAAGACAAATCTGTATGGCTTCATTATAAAAGGAAGATCCCATTTGAGGAATGACACCGATGTGGTAAACTTTGTGAGCATGGAATTCTCTCTCACTGACCCAAGGCTGGAGCCACACAAATGG
GAAAAGTACTGTGTGCTTGAGGTAGGAGACATGCTCCTACGGACTGCAATAGGCCAAGTGTCAAGGCCCATGTTCCTGTATGTGAGAACCAATGGGACTTCTAAGATCAAAATGAAATGGGGTATGGAGATGAGGCGGTGCCTTCTTCAATCCCTTCAACAAATTGAGAGCATGATTGAAGCCGAGTCTTCTGTCAAAGAAAAAGACATGACCAAGGAATTCTTTGAAAAC
AGATCAGAAACATGGCCAATTGGAGAGTCACCCAAAGGAGTGGAGGAAGGCTCCATTGGGAAGGTATGCAGAACATTGCTAGCAAAGTCTGTGTTCAACAGCCTATATGCATCTCCACAACTCGAGGGGTTTTCAGCTGAATCAAGAAAATTGCTTCTCATTGTTCAGGCACTTAGGGACAACCTGGAACCTGGAACCTTCGATCTTGGGGGGCTATATGAAGCAATTGA
GGAGTGCCTGATTAACGATCCCTGGGTTTTGCTTAATGCGTCTTGGTTCAACTCCTTCCTCACACATGCACTGAAATA-

EPI_ISL_779129

>A/turkey/Poland/464/2020_H5N8_2020-12-01
ATGGAAGACTTTGTGCGACAATGCTTCAATCCAATGATTGTCGAGCTTGCGGAAAAGGCAATGAAAGAATATGGGGAAGATCCTAAGATCGAAACAAACAAGTTTGCCGCAATATGTACGCACTTAGAAGTCTGCTTCATGTATTCGGATTTCCATTTCATTGATGAACGAGGCGAATCAATAATCGTAGAATCTGGCGACCCGAATGCATTATTGAAGCACCGATTTGAGA
TAATTGAAGGGAGAGACCGAACAATGGCCTGGACAGTGGTGAATAGTATATGCAACACTACAGGAGTCGAAAAGCCCAAGTTCCTTCCTGATTTGTATGACTACAGAGAAAACCGATTCATTGAAATTGGAGTAACGCGAAGGGAAGTTCACATATACTATCTTGAAAAAGCCAACAAGATAAAATCAGAGAAAACACACATTCACATATTCTCATTCACTGGGGAGGAA
ATGGCCACCAAGGCAGATTACACTCTTGATGAAGAGAGCAGAGCAAGAATAAAAACCAGGCTATTCACTATAAGACAAGAGATGGCCAATAGGGGTCTATGGGATTCCTTTCGTCAGTCCGAGAGAGGCGAAGAGACAATTGAAGAAAGATTTGAAATCACAGGAACCATGCGCAGGCTTGCTGACCAAAGTCTCCCACCGAACTTCGCCAGCCTTGAAAACTTTAGAG
CCTATGTGGATGGATTTGAACCGAACGGCTGCATTGAGGGCAAGCTTTCTCAAATGTCAAAAGAAGTGAATGCCAGAATTGAGCCATTTCTGAAGACAACACCACGCCCTCTCAGATTACCTGATGGACCTCCCTGTTCCCAGCGGTCGAAGTTCTTGCTAATGGATGCCCTTAAATTGAGCATTGAAGACCCGAGCCATGAGGGGGAGGGTATACCGCTGTACGATGCAA
CYAAATGCATGAAGACATTTTTTGGCTGGAAGGAGCCCAACATCGTGAAACCACATGAAAAGGGCATAAACCCTAATTACCTCCTGGCTTGGAAGCAGGTGCTATCAGACCTCCAAGATATTGAAAACGAGGAGAAAATCCCAAAAACAAAGAACATGAAGAAGACGAGCCAACTGAAGTGGGCACTTGGTGAAAACATGGCACCAGAGAAAGTGGACTTTGAGGACT
GTAAAGATGTTAGCGATCTAAGACAGTACGACAGTGACGAACCAGAATCTAGATCACTAGCAAGTTGGATCCAGAGTGAATTTAACAAGGCTTGCGAACTGACAGATTCGAGTTGGATTGAACTTGATGAAATAGGGGAAGACGTTGCTCCGATTGAACACATTGCGAGTATGAGGAGGAACTATTTCACAGCGGAAGTATCCCATTGCAGGGCTACAGAATACATAATG
AAAGGAGTATACATAAACACAGCTTTATTGAATGCATCCTGTGCTGCCATGGATGACTTCCAACTGATTCCAATGATAAGCAAATGCAGAACCAAAGAAGGGAGACGGAAGACAAATCTGTATGGCTTCATTATAAAAGGAAGATCCCATTTGAGGAATGACACCGATGTGGTAAACTTTGTGAGCATGGAATTCTCTCTCACTGACCCAAGGCTGGAGCCACACAAATGG
GAAAAGTACTGTGTGCTTGAGGTAGGAGACATGCTCCTACGGACTGCAATAGGCCAAGTGTCAAGGCCCATGTTCCTGTATGTGAGAACCAATGGGACTTCTAAGATCAAAATGAAATGGGGTATGGAGATGAGGCGATGCCTTCTTCAATCCCTTCAACAAATTGAGAGCATGATTGAAGCCGAGTCTTCTGTCAAAGAAAAAGACATGACCAAGGAATTCTTTGAAAAC
AGATCAGAAACATGGCCAATTGGAGAGTCACCCAAAGGAGTGGAGGAAGGCTCCATTGGGAAGGTGTGCAGAACATTGCTAGCAAAGTCTGTGTTCAACAGCCTATATGCATCTCCACAACTCGAGGGGTTTTCAGCTGAATCAAGAAAGTTGCTTCTCATTGTTCAGGCACTTAGGGACAACCTGGAACCTGGGACCTTCGATCTTGGGGGGCTATATGAAGCAATTGA
GGAGTGCCTGATTAACGATCCCTGGGTTTTGCTTAATGCGTCTTGGTTCAACTCCTTCCTCACACATGCACTGAAATA-

EPI_ISL_18244823

>A/buzzard/Poland/MB277-21_22VIR5675-18/2021_H5N8_2021-03-09
ATGGAAGATTTTGTGCGACAATGCTTCAATCCAATGATTGTCGAGCTTGCGGAAAAGGCAATGAAAGAATATGGGGAAGATCCTAAGATCGAAACAAACAAGTTTGCCGCAATATGTACACACTTAGAAGTCTGCTTCATGTATTCGGATTTCCATTTTATTGATGAACGAGGCGAATCAATAATCGTAGAATCTGGCGACCCGAATGCATTATTGAAGCACCGATTTGAGA
TAATTGAAGGGAGAGACCGAACAATGGCCTGGACAGTGGTGAATAGTATATGCAACACTACAGGAGTCGAAAAGCCCAAGTTCCTTCCTGATTTGTATGACTACAGAGAAAACCGATTCATTGAAATTGGAGTAACGCGAAGGGAAGTTCACATATACTATCTTGAAAAAGCCAACAAGATAAAGTCAGAGAAAACACACATTCACATATTCTCATTCACTGGGGAGGAA
ATGGCCACCAAAGCAGATTACACTCTTGATGAAGAGAGCAGAGCAAGAATAAAAACCAGGCTATTCACTATAAGACAAGAGATGGCCAATAGGGGTCTATGGGATTCCTTTCGTCAATCCGAGAGAGGCGAAGAGACAATTGAAGAAAGATTTGAAATCACAGGAACCATGCGCAGGCTTGCTGACCAAAGTCTCCCACCGAACTTCGCCAGCCTTGAAAACTTTAGAG
CCTATGTGGATGGATTTGAACCGAACGGCTGCATTGAGGGCAAGCTTTCTCAAATGTCAAAAGAAGTGAATGCCAGAATTGAGCCATTTCTGAAGACAACACCACGCCCTCTCAGATTACCTGATGGACCTCCCTGTTCCCAGCGGTCGAAGTTCTTGCTAATGGATGCCCTTAAATTGAGCATTGAAGACCCGAGCCATGAGGGGGAGGGTATACCGCTGTACGATGCAA
CCAAATGCATGAAGACATTTTTTGGCTGGAAGGAACCCAACATCGTGAAACCACATGAAAAGGGCATAAACCCTAATTACCTCCTGGCTTGGAAGCAGGTGCTATCAGAACTCCAAGATATTGAAAACGAGGAGAAAATCCCAAAAACAAAGAACATGAAGAAGACGAGCCAACTGAAGTGGGCACTTGGTGAAAACATGGCACCAGAGAAAGTGGACTTTGAGGACT
GTAAAGATGTTAGCGATCTAAAACAGTACGACAGTGACGAACCAGAATCTAGATCACTAGCAAGTTGGATCCAGAGTGAATTTAACAAGGCTTGCGAACTGACAGATTCGAGTTGGATTGAACTTGATGAAATAGGGGAAGACGTTGCTCCAATTGAACACATTGCGAGTATGAGGAGGAACTATTTCACAGCGGAAGTATCCCATTGCAGGGCTACAGAATACATAATG
AAAGGAGTATACATAAACACAGCTTTATTGAATGCATCCTGTGCAGCCATGGATGACTTCCAACTGATTCCAATGATAAGCAAATGCAGAACCAAAGAAGGGAGACGGAAGACAAATCTGTATGGCTTCATTATAAAAGGAAGATCCCATTTGAGGAATGACACCGATGTGGTAAACTTTGTGAGCATGGAATTCTCTCTCACTGACCCAAGGCTGGAGCCACACAAATGG
GAAAAGTACTGTGTGCTTGAGGTAGGAGACATGCTCCTACGGACTGCAATAGGCCAAGTGTCAAGGCCCATGTTCCTGTATGTGAGAACCAATGGGACTTCCAAGATCAAAATGAAATGGGGTATGGAGATGAGGCGATGCCTTCTTCAATCCCTTCAACAAATTGAGAGCATGATTGAAGCCGAGTCTTCTGTCAAAGAAAAAGACATGACCAAGGAATTCTTTGAAAA
CAGATCAGAAACATGGCCAATTGGAGAGTCACCCAAAGGAGTGGAGGAAGGCTCCATTGGGAAGGTGTGCAGAACATTGCTAGCAAAGTCTGTGTTCAACAGCCTATATGCATCTCCACAACTCGAGGGGTTTTCAGCTGAATCAAGAAAGTTGCTTCTCATTGTTCAGGCACTTAGGGACAACCTGGAACCTGGAACCTTCGATCTTGGGGGGCTATATGAAGCAATTG
AGGAGTGCCTGATTAACGATCCCTGGGTTTTGCTTAATGCGTCTTGGTTCAACTCCTTCCTCACACATGCACTGAAATA-

EPI_ISL_18245535

>A/mute_swan/Poland/MB372-21_22VIR5675-19/2021_H5N8_2021-04-07
ATGGAAGACTTTGTGCGACAATGCTTCAATCCAATGATTGTCGAGCTTGCGGAAAAGGCAATGAAAGAATATGGGGAAGGTCCTAAGATCGAAACAAACAAGTTTGCCGCAATATGTACACACTTAGAAGTCTGCTTCATGTATTCGGATTTCCATTTCATTGATGAACGAGGCGAATCAATAATCGTAGAATCTGGCGACCCGAATGCATTATTGAAGCACCGATTTGAGA
TAATTGAAGGGAGAGACCGAACAATGGCCTGGACAGTGGTGAATAGTATATGCAACACTACAGGAGTCGAAAAGCCCAAGTTCCTTCCTGATTTGTATGACTACCGAGAAAACCGATTCATTGAAATTGGAGTAACGCGAAGGGAAGTTCACATATACTATCTTGAAAAAGCCAACAAGATAAAATCAGAGAAAACACACATTCACATATTCTCATTCACTGGGGAGGAA
ATGGCCACCAAGGCAGATTACACTCTTGATGAAGAGAGCAGAGCAAGAATAAAAACCAGGCTATTCACTATAAGACAAGAGATGGCCAATAGGGGTCTATGGGATTCCTTTCGTCAGTCCGAGAGAGGCGAAGAGACAATTGAAGAAAGATTTGAAATCACAGGAACCATGCGCAGGCTTGCTGACCAAAGCCTCCCACCGAACTTCGCCAGCCTTGAAAACTTTAGAG
CCTATGTGGATGGATTTGAACCGAACGGCTGCATTGAGGGCAAGCTTTCTCAAATGTCAAAAGAAGTGAATGCCAGAATTGAGCCATTTCTGAAGACGACACCACGCCCTCTCAGATTACCTGATGGACCTCCCTGTTCCCAGCGGTCGAAGTTCTTACTAATGGATGCCCTTAAATTGAGCATTGAAGACCCGAGCCATGAGGGGGAGGGTATACCGCTGTACGATGCAA
CCAAATGCATGAAGACATTTTTTGGCTGGAAGGAGCCCAACATCGTGAAGCCACATGAAAAGGGCATAAACCCTAATTACCTCCTGGCTTGGAAGCAGGTGCTATCAGAACTCCAAGATATTGAAAACGAGGAGAAAATCCCAAAAACAAAGAACATGAAGAAGACGAGCCAACTGAAGTGGGCACTTGGTGAAAACATGGCACCAGAGAAAGTGGACTTTGAGGACT
GTAAAGATGTTAGCGATCTAAGACAGTACGACAGTGACGAACCAGAGTCTAGATCACTAGCAAGTTGGATCCAGAGTGAATTTAACAAGGCTTGCGAACTGACAGATTCGAGTTGGATTGAACTTGATGAAATAGGGGAAGACGTTGCTCCAATTGAACACATTGCGAGTATGAGGAGGAACTATTTCACAGCGGAAGTATCCCATTGCAGGGCTACAGAATACATAATG
AAAGGAGTATACATAAACACAGCTTTATTGAATTCATCCTGTGCAGCCATGGATGACTTCCAACTGATTCCAATGATAAGCAAATGCAGAACCAAAGAAGGGAGACGGAAGACAAATCTGTATGGCTTCATTATAAAAGGAAGATCCCATTTGAGGAATGACACCGATGTGGTAAACTTTGTGAGCATGGAATTCTCTCTCACTGACCCAAGGCTGGAGCCACACAAATGG
GAAAAGTACTGTGTACTTGAGGTAGGAGACATGCTCCTACGGACTGCAATAGGCCAAGTGTCAAGGCCCATGTTCCTGTATGTGAGAACCAATGGGACTTCTAAGATCAAAATGAAATGGGGTATAGAGATGAGGCGATGCCTTCTTCAATCCCTTCAACAAATTGAGAGCATGATTGAAGCCGAGTCTTCTGTCAAAGAAAAAGACATGACCAAGGAATTCTTTGAAAAC
AGATCAGAAACATGGCCAATTGGAGAGTCACCCAAAGGAGTGGAGGAAGGCTCCATTGGGAAGGTGTGCAGAACATTGCTAGCAAAGTCTGTGTTCAACAGCCTATATGCATCTCCACAACTCGAGGGGTTTTCAGCTGAATCAAGAAAGTTGCTTCTCATTGTTCAGGCACTTAGGGACAACCTGGAACCTGGAACCTTCGATCTTGGGGGGCTATATGAAGCAATTGA
GGAGTGCCTGATTAACGATCCCTGGGTTTTGCTTAATGCGTCTTGGTTCAACTCCTTCCTCACACATGCACTGAAATA-

EPI_ISL_18245534

>A/white_stork/Poland/MB363-21_22VIR5675-15/2021_H5N8_2021-04-01
ATGGAAGACTTTGTGCGACAATGCTTCAATCCAATGATTGTCGAGCTTGCGGAAAAGGCAATGAAAGAATATGGGGAAGATCCTAAGATCGAAACAAACAAGTTTGCCGCAATATGTACACACTTAGAAGTCTGCTTCATGTATTCGGATTTCCATTTCATTGATGAACGAGGCGAATCAATAATCGTAGAATCTGGCGACCCGAATGCATTATTGAAGCACCGATTTGAGA
TAATTGAAGGGAGAGACCGAACAATGGCATGGACAGTGGTGAATAGTATATGCAACACTACAGGAGTCGAAAAGCCCAAGTTCCTTCCTGATTTGTATGACTACAGAGARAACCGATTCATTGAAATTGGAGTAACGCGAAGGGAAGTTCACATATACTATCTTGAAAAAGCCAACAAGATAAAATCAGAGAAAACACACATTCACATATTCTCATTCACTGGGGAGGAA
ATGGCCACCAAGGCAGATTACACTCTTGATGAAGAGAGCAGAGCAAGAATAAAAACCAGGCTATTCACTATAAGACAAGAGATGGCCAATAGGGGTCTATGGGATTCCTTTCGTCAGTCCGAGAGAGGCGAAGAGACAATTGAAGAAAGATTTGAAATCACAGGAACCATGCGCAGGCTTGCTGACCAAAGTCTCCCACCGAACTTCGCCAGCCTTGAAAACTTTAGAG
CCTATGTGGATGGATTTGAACCGAACGGCTGCATTGAGGGCAAGCTTTCTCAAATGTCAAAAGAAGTGAATGCCAGAATTGAGCCATTTCTGAAGACGACACCACGCCCTCTCAGATTACCTGATGGACCTCCCTGTTCCCAGCGGTCGAAGTTCTTGCTAATGGATGCCCTTAAATTGAGCATTGAAGACCCGAGCCATGAGGGGGAGGGTATACCGCTGTACGATGCAA
CCAAATGCATGAAGACATTTTTTGGCTGGAAGGAGCCCAACATCGTGAAACCACATGAAAAGGGCATAAACCCTAATTACCTCCTGGCTTGGAAGCAGGTGCTATCAGAACTCCAAGATATTGAAAACGAGGAGAAAATCCCAAAAACAAAGAACATGAAGAAGACGAGCCAACTGAAGTGGGCACTTGGTGAAAACATGGCACCAGAGAAAGTGGACTTTGAGGACT
GTAAAGATATTAGCGATCTAAGACAGTACGACAGTGACAAACCAGAGTCTAGATCACTAGCAAGTTGGGTCCAGAGTGAATTTAACAAGGCTTGCGAACTGACAGATTCGAGTTGGGTTGAACTTGATGAAATAGGGGAAGACGTTGCTCCAATTGAACACATTGCGAGTATGAGGAGGAACTATTTCACAGCGGAAGTATCCCATTGCAGGGCTACAGAATACATAATG
AAAGGAGTATACATAAACACAGCTTTATTGAATGCATCCTGTGCAGCCATGGATGACTTCCAACTGATTCCAATGATAAGCAAATGCAGAACCAAAGAAGGGAGACGGAAGACAAATCTGTATGGCTTCATTATAAAAGGAAGATCCCATTTGAGGAATGACACCGATGTGGTAAACTTTGTGAGCATGGAATTCTCTCTCACTGACCCAAGGCTGGAGCCACACAAATGG
GAAAAGTACTGTGTACTTGAGGTAGGAGACATGCTCCTACGGACTGCAATAGGCCAAGTGTCAAGGCCCATGTTCCTGTATGTGAGAACCAATGGGACTTCTAAGATCAAAATGAAATGGGGTATGGAGATGAGGCGATGCCTTCTTCAATCCCTTCAACAAATTGAGAGCATGATTGAAGCCGAGTCTTCTGTCAAAGAAAAAGACATGACCAAGGAATTCTTTGAAAAC
AGATCAGAAACATGGCCAATTGGAGAGTCACCCAAAGGAGTGGAGGAAGGCTCCATTGGGAAGGTGTGCAGAACATTGCTAGCAAAGTCTGTGTTCAACAGCCTATATGCATCTCCACAACTCGAGGGGTTTTCAGCTGAATCAAGAAAGTTGCTTCTCATTGTTCAGGCACTTAGGGACAACCTGGAACCTGGAACCTTCGATCTTGGGGGGCTATATGAAGCAATTGA
GGAGTGCCTGATTAACGATCCCTGGGTTTTGCTTAATGCGTCTTGGTTCAACTCCTTCCTCACACATGCACTGAAATA-

EPI_ISL_3102078	>A/mute_swan/Poland/MB396_21RS1385-19/2021_H5N8_2021-04-21<br>ATGGAAGACTTTGTGCGACAATGCTTCAATCCAATGATTGTCGAGCTTGCGGAAAAGGCAATGAAAGAATATGGGGAAGATCCTAAGATCGAAACAAACAAGTTTGCCGCAATATGTACACACTTAGAAGTCTGCTTCATGTATTCGGATTTCCATTTCATTAATGAACGAGGCGAATCAATAATCGTAGAATCCGGCGACCCGAATGCATTATTGAAGCACCGATTTGAGA<br>TAATTGAAGGGAGAGACCGAACAATGGCCTGGACAGTGGTGAATAGTATATGCAACACTACAGGAGTCGAAAAGCCCAAGTTCCTTCCTGATTTGTATGACTACAGAGAAAACCGATTCATTGAGATTGGAGTAACGCGAAGGGAAGTTCACATATACTATCTTGAAAAAGCCAACAAGATAAAATCAGAGAAAACACACATTCACATATTCTCATTCACTGGGGAGGAA<br>ATGGCCACCAAGGCAGATTACACTCTTGATGAAGAGAGCAGAGCAAGAATAAAAACCAGGCTATTCACTATAAGACAAGAGATGGCCAATAGGGGTCTATGGGATTCCTTTCGTCAGTCCGAGAGAGGCGAAGAGACAATTGAAGAAAGATTTGAAATCACAGGAACCATGCGCAGGCTTGCTGACCAAAGTCTCCCACCGAACTTCGCCAGCCTTGAAAACTTTAGAG<br>CCTATGTGGATGGATTTGAACCGAACGGCTGCATTGAGGGCAAGCTTTCTCAAATGTCAAAAGAAGTGAATGCCAGAATTGAGCCATTTCTGAAGACAACACCACGCCCTCTCAGATTACCTGATGGACCTCCCTGTTCCCAGCGGTCGAAGTTCTTGCTAATGGATGCCCTTAAATTGAGCATTGAAGACCCGAGCCATGAGGGGGAGGGTATACCGCTGTACGATGCAA<br>CCAAATGCATGAAGACATTTTTTGGCTGGAAGGAGCCCAACATCGTGAAACCACATGAAAAGGGCATAAACCCTAATTACCTCCTGGCTTGGAAGCAGGTGCTATCAGAACTCCAAGATATTGAAACCGAGGAGAAAATCCCAAAAACAAAGAACATGAAGAAGACGAGCCAACTGAAGTGGGCACTTGGTGAAAACATGGCACCAGAGAAAGTGGACTTTGAGGACT<br>GTAAAGATGTTAGCGATCTAAGACAGTACGACAGTGACGAACCAGAATCTAGATCACTAGCAAGTTGGATCCAGAGTGAATTTAACAAGGCTTGCGAACTGACAGATTCGAGTTGGATTGAACTTGATGAAATAGGGGAAGACGTTGCTCCGATTGAACACATTGCGAGTATGAGGAGGAACTATTTCACAGCGGAAGTATCCCATTGCAGGGCTACAGAATACATAATG<br>AAAGGAGTATACATAAACACAGCTTTATTGAATGCATCCTGTGCAGCCATGGATGACTTCCAACTGATTCCAATGATAAGCAAATGCAGAACCAAAGAAGGGAGACGGAAGACAAATCTGTATGGCTTCATTATAAAAGGAAGATCCCATTTGAGGAATGACACCGATGTGGTAAACTTTGTGAGCATGGAATTCTCTCTCACTGACCCAAGACTGGAGCCACACAAATGG<br>GAAAAGTACTGTGTGCTTGAAGTAGGAGACATGCTCCTACGGACTGCAATAGGCCAAGTGTCAAGGCCCATGTTCCTGTATGTGAGAACCAATGGGACTTCTAAGATCAAAATGAAATGGGGTATGGAGATGAGGCGATGCCTTCTCCAATCCCTTCAACAAATTGAGAGCATGATTGAAGCCGAGTCTTCTGTCAAAGAAAAAGACATGACCAAGGAATTCTTTGAAAAC<br>AGATCAGAAACATGGCCAATTGGAGAGTCACCCAAAGGAGTGGAGGAAGGCTCCATTGGGAAGGTGTGCAGAACATTGCTAGCAAAGTCTGTGTTCAACAGCCTATATGCATCCCCACAACTCGAGGGGTTTTCAGCTGAATCAAGAAAGTTGCTTCTCATTGTTCAGGCACTTAGGGACAACCTGGAACCTGGGACCTTCGATCTTGGGGGGCTATATGAAGCAATTGA<br>GGAGTGCCTGATTAACGATCCCTGGGTTTTGCTTAATGCGTCTTGGTTCAACTCCTTCCTCACACATGCACTGAAATA-
EPI_ISL_3102077	>A/duck/Poland/H514_21RS1385-18/2021_H5N8_2021-04-07<br>ATGGAAGACTTTGTGCGACAATGCTTCAATCCAATGATTGTCGAGCTTGCGGAAAAGGCAATGAAAGAATATGGGGAAGACCCTAAGATCGAAACAAACAAGTTTGCCGCAATATGTACACACTTAGAAGTCTGCTTCATGTATTCGGATTTCCATTTCATTAATGAACGAGGCGAATCAATAATCGTAGAATCTGGCGACCCGAATGCATTATTGAAGCACCGATTTGAGA<br>TAATTGAAGGGAGAGACCGAACAATGGCCTGGACAGTGGTGAATAGTATATGCAACACTACAGGAGTCGAAAAGCCCAAGTTCCTTCCTGATTTGTATGACTACAGAGAAAACCGATTCATTGAGATTGGAGTAACGCGAAGGGAAGTTCACATATACTATCTTGAAAAAGCCAACAAGATAAAATCAGAGAAAACACACATTCACATATTCTCATTCACTGGGGAGGAA<br>ATGGCCACCAAGGCAGATTACACTCTTGATGAAGAGAGCAGAGCAAGAATAAAAACCAGGCTATTCACTATAAGACAAGAGATGGCCAATAGGGGTCTATGGGATTCCTTTCGTCAGTCCGAGAGAGGCGAAGAGACAATTGAAGAAAGATTTGAAATCACAGGAACCATGCGCAGGCTTGCTGACCAAAGTCTCCCACCGAACTTCGCCAGCCTTGAAAACTTTAGAG<br>CCTATGTGGATGGATTTGAACCGAACGGCTGCATTGAGGGCAAGCTTTCTCAAATGTCAAAAGAAGTGAATGCCAGAATTGAGCCATTTCTGAAGACAACACCACGCCCTCTCAGATTACCTGATGGACCTCCCTGTTCCCAGCGGTCGAAGTTCTTGCTAATGGATGCCCTTAAATTGAGCATTGAAGACCCGAGCCATGAGGGGGAGGGTATACCGCTGTACGATGCAA<br>CCAAATGCATGAAGACATTTTTTGGCTGGAAGGAGCCCAACATCGTGAAACCACATGAAAAGGGCATAAACCCTAATTACCTCCTGGCTTGGAAGCAGGTGCTATCAGAACTCCAAGATATTGAAACCGAGGAGAAAATCCCAAAAACAAAGAACATGAAGAAGACGAGCCAACTGAAGTGGGCACTTGGTGAAAACATGGCACCAGAGAAAGTGGACTTTGAGGACT<br>GTAAAGATGTTAGCGATCTAAGACAGTACGACAGTGACGAACCAGAATCTAGATCACTAGCAAGTTGGATCCAGAGTGAATTTAACAAGGCTTGCGAACTGACAGATTCGAGTTGGATTGAACTTGATGAAATAGGGGAAGACGTTGCTCCGATTGAACACATTGCGAGTATGAGGAGGAACTATTTCACAGCGGAAGTATCCCATTGCAGGGCTACAGAATACATAATG<br>AAAGGAGTATACATAAACACAGCTTTATTGAATGCATCCTGTGCAGCCATGGATGACTTCCAACTGATTCCAATGATAAGCAAATGCAGAACCAAAGAAGGGAGACGGAAGACAAATCTGTATGGCTTCATTATAAAAGGAAGATCTCATTTGAGGAATGACACCGATGTGGTAAATTTTGTGAGCATGGAATTCTCTCTCACTGACCCAAGACTGGAGCCACACAAATGG<br>GAAAAGTACTGTGTGCTTGAGGTAGGAGACATGCTCCTACGGACTGCAATAGGCCAAGTGTCAAGGCCCATGTTCCTGTATGTGAGAACCAATGGGACTTCTAAGATCAAAATGAAATGGGGTATGGAGATGAGGCGATGCCTTCTTCAATCCCTTCAACAAATTGAGAGCATGATTGAAGCCGAGTCTTCTGTCAAAGAAAAGGACATGACCAAGGAATTCTTTGAAAAC<br>AGATCAGAAACATGGCCAATTGGAGAGTCACCCAAAGGAATGGAGGAAGGCTCCATTGGGAAGGTGTGCAGAACATTGCTAGCAAAGTCTGTGTTCAACAGCCTATATGCATCTCCACAACTCGAGGGGTTTTCAGCTGAATCAAGAAAGTTGCTTCTCATTGTTCAGGCACTTAGGGACAACCTGGAACCTGGGACCTTCGATCTTGGGGGGCTATATGAAGCAATTGA<br>GGAGTGCCTGATTAACGATCCCTGGGTTTTGCTTAATGCGTCTTGGTTCAACTCCTTCCTCACACATGCACTGAAATA-
EPI_ISL_1139169	>A/peregrine_falcon/Netherlands/20018821-002/2020_H5N8_2020-11-25<br>ATGGAAGACTTTGTGCGACAATGCTTCAATCCAATGATTGTCGAGCTTGCGGAAAAGGCAATGAAAGAATATGGGGAAGATCCTAAGATCGAAACAAACAAGTTTGCCGCAATATGTACACACTTAGAAGTCTGCTTCATGTATTCGGATTTCCATTTCATTGATGAACGAGGCGAATCAATAATCGTAGAATCTGGCGACCCGAATGCATTATTGAAGCACCGATTTGAGA<br>TAATTGAAGGGAGAGACCGAACAATGGCCTGGACAGTGGTGAATAGTATATGCAACACTACAGGAGTCGAAAAGCCCAAGTTCCTTCCTGATTTGTATGACTACAGAGAAAACCGATTCATTGAAATTGGAGTAACGCGAAGGGAAGTTCACATATACTATCTTGAAAAAGCCAACAAAATAAAATCAGAGAAAACACACATTCACATATTCTCATTCACTGGGGAGGAA<br>ATGGCCACCAAGGCAGATTACACTCTTGATGAAGAGAGCAGAGCAAGAATAAAAACCAGGCTATTCACTATAAGACAAGAGATGGCCAATAGGGGTCTATGGGATTCCTTTCGTCAGTCCGAGAGAGGCGAAGAGACAATTGAAGAAAGATTTGAAATCACAGGAACCATGCGCAGGCTTGCTGACCAAAGTCTCCCACCGAACTTCGCCAGCCTTGAAAATTTTAGAG<br>CCTATGTGGATGGATTTGAACCGAACGGCTGCATTGAGGGCAAGCTTTCTCAAATGTCAAAAGAAGTGAATGCCAGAATTGAGCCATTTCTGAAGACTACACCACGCCCTCTCAGATTGCCTGATGGACCTCCCTGTTCCCAGCGGTCGAAGTTCTTGCTAATGGATGCCCTTAAATTAAGCATTGAAGACCCGAGCCATGAGGGGGAGGGTATACCGCTGTACGATGCAA<br>CCAAATGCATGAAGACATTTTTTGGCTGGAAGGAGCCCAACATCGTGAAACCACATGAAAAGGGCATAAACCCTAATTACCTCCTGGCTTGGAAGCAGGTGCTATCAGAACTCCAAGATATTGAAAACGAGGAGAAAATCCCAAAAACAAAGAACATGAAGAAGACGAGCCAACTGAAGTGGGCACTTGGTGAAAATATGGCACCAGAGAAAGTGGACTTTGAGGACT<br>GTAAAGATGTTAGCGATCTAAAACAGTACGACAGTGACGAACCAGAATCCAGATCACTAGCAAGTTGGATCCAGAGTGAATTTAACAAGGCTTGCGAACTGACAGATTCGAGTTGGATTGAACTTGATGAAATAGGGGAAGACGTTGCTCCAATTGAACACATTGCGAGTATGAGGAGGAACTATTTCACAGCGGAAGTATCCCATTGCAGGGCTACAGAATACATAATG<br>AAAGGAGTATACATAAACACAGCTTTATTGAATGCATCCTGTGCAGCCATGGATGACTTCCAACTGATTCCAATGATAAGCAAATGCAGAACCAAAGAAGGGAGACGGAAGACAAATCTGTATGGCTTCATTATAAAAGGAAGATCCCATTTGAGGAATGACACCGATGTGGTAAACTTTGTGAGCATGGAATTCTCTCTCACTGACCCAAGGCTGGAGCCACACAAATGG<br>GAAAAGTACTGTGTGCTTGAGGTAGGAGACATGCTCCTACGGACTGCAATAGGCCAAGTGTCAAGGCCCATGTTCCTGTATGTGAGAACCAATGGGACTTCTAAGATCAAAATGAAATGGGGTATGGAGATGAGGCGATGCCTTCTTCAATCCCTTCAACAAATTGAGAGCATGATTGAAGCCGAGTCTTCTGTCAAAGAAAAAGACATGACCAAGGAATTCTTTGAAAAC<br>AGATCAGAAACATGGCCAATTGGAGAGTCACCCAAAGGAGTGGAGGAAGGCTCCATTGGGAAGGTGTGCAGAACATTGCTAGCAAAGTCTGTGTTCAATAGCCTATATGCATCTCCACAACTCGAGGGGTTTTCAGCTGAATCAAGAAAGTTGCTTCTCATTGTTCAGGCACTTAGGGACAACCTGGAACCTGGAACCTTCGATCTTGGGGGGCTATATGAAGCAATTGA<br>GGAGTGCCTGATTAACGATCCCTGGGTTTTGCTTAATGCGTCTTGGTTCAACTCCTTCCTCACACATGCACTGAAATA-
EPI_ISL_1095451	>A/barnacle_goose/Sweden/SVA210126SZ0462/KN000202/2021_H5N8_2021-01-22<br>ATGGAAGACTTTGTGCGACAATGCTTCAATCCAATGATTGTCGAGCTTGCGGAAAAGGCAATGAAAGAATATGGGGAAGATCCTAAGATCGAAACAAACAAGTTTGCCGCAATATGTACACACTTAGAAGTCTGCTTCATGTATTCGGATTTCCATTTCATTGATGAACGAGGCGAATCAATAATCGTAGAATCTGGCGACCCGAATGCATTATTGAAGCACCGATTTGAGA<br>TAATTGAAGGGAGAGACCGAACAATGGCCTGGACAGTGGTGAATAGTATATGCAACACTACAGGAGTCGAAAAGCCCAAGTTCCTTCCTGATTTGTATGACTACAGAGAAAACCGATTCATTGAAATTGGAGTAACGCGAAGGGAAGTTCACATATACTATCTTGAAAAAGCCAACAAAATAAAATCAGAGAAAACACACATTCACATATTCTCATTCACTGGGGAGGAA<br>ATGGCCACCAAGGCAGATTACACTCTTGATGAAGAGAGCAGAGCAAGAATAAAAACCAGGCTATTCACTATAAGACAAGAGATGGCCAATAGGGGTCTATGGGATTCCTTTCGTCAGTCCGAGAGAGGCGAAGAGACAATTGAAGAAAGATTTGAAATCACAGGAACCATGCGCAGGCTTGCTGACCAAAGTCTCCCACCGAACTTCGCCAGCCTTGAAAATTTTAGAG<br>CCTATGTGGATGGATTTGAACCGAACGGCTGCATTGAGGGCAAGCTTTCTCAAATGTCAAAAGAAGTGAATGCCAGAATTGAGCCATTTCTGAAGACTACACCACGCCCTCTCAGATTGCCTGATGGACCTCCCTGTTCCCAGCGGTCGAAGTTCTTGCTAATGGATGCCCTTAAATTAAGCATTGAAGACCCGAGCCATGAGGGGGAGGGTATACCGCTGTACGATGCAA<br>CCAAATGCATGAAGACATTTTTTGGCTGGAAGGAGCCCAACATCGTGAAACCACATGAAAAGGGCATAAACCCTAATTACCTCCTGGCTTGGAAGCAGGTGCTATCAGAACTCCAAGATATTGAAAACGAGGAGAAAATCCCAAAAACAAAGAACATGAAGAAGACGAGCCAACTGAAGTGGGCACTTGGTGAAAATATGGCACCAGAGAAAGTGGACTTTGAGGACT<br>GTAAAGATGTTAGCGATCTAAAACAGTACGACAGTGACGAACCAGAATCCAGATCACTAGCAAGTTGGATCCAGAGTGAATTTAACAAGGCTTGCGAACTGACAGATTCGAGTTGGATTGAACTTGATGAAATAGGGGAAGACGTTGCTCCAATTGAACACATTGCGAGTATGAGGAGGAACTATTTCACAGCGGAAGTATCCCATTGCAGGGCTACAGAATACATAATG<br>AAAGGAGTATACATAAACACAGCTTTATTGAATGCATCCTGTGCAGCCATGGATGACTTCCAACTGATTCCAATGATAAGCAAATGCAGAACCAAAGAAGGGAGACGGAAGACAAATCTGTATGGCTTCATTATAAAAGGAAGATCCCATTTGAGGAATGACACCGATGTGGTAAACTTTGTGAGCATGGAATTCTCTCTCACTGACCCAAGGCTGGAGCCACACAAATGG<br>GAAAAGTACTGTGTGCTTGAGGTAGGAGACATGCTCCTACGGACTGCAATAGGCCAAGTGTCAAGGCCCATGTTCCTGTATGTGAGAACCAATGGGACTTCTAAGATCAAAATGAAATGGGGTATGGAGATGAGGCGATGCCTTCTTCAATCCCTTCAACAAATTGAGAGCATGATTGAAGCCGAGTCTTCTGTCAAAGAAAAAGACATGACCAAGGAATTCTTTGAAAAC<br>AGATCAGAAACATGGCCAATTGGAGAGTCACCCAAAGGAGTGGAGGAAGGCTCCATTGGGAAGGTGTGCAGAACATTGCTAGCAAAGTCTGTGTTCAATAGCCTATATGCATCTCCACAACTCGAGGGGTTTTCAGCTGAATCAAGAAAGTTGCTTCTCATTGTTCAGGCACTTAGGGACAACCTGGAACCTGGAACCTTCGATCTTGGGGGGCTATATGAAGCAATTGA<br>GGAGTGCCTGATTAACGATCCCTGGGTTTTGCTTAATGCGTCTTGGTTCAACTCCTTCCTCACACATGCACTGAAATA-
EPI_ISL_5115613	>A/chicken/Germany-MV/AI01965/2021_H5N8_2021-03-01<br>ATGGAAGACTTTGTGCGACAATGCTTCAATCCAATGATTGTCGAGCTTGCGGAAAAGGCAATGAAAGAATATGGGGAAGATCCTAAGATCGAAACAAACAAGTTTGCCGCAATATGTACACACTTAGAAGTCTGCTTCATGTATTCGGATTTCCATTTCATTGATGAACGAGGCGAATCAATAATCGTAGAATCTGGCGACCCGAATGCATTATTGAAGCACCGATTTGAGA<br>TAATTGAAGGGAGAGACCGAACAATGGCCTGGACAGTGGTGAATAGTATATGCAACACTACAGGAGTCGAAAAGCCCAAGTTCCTTCCTGATTTGTATGACTACAGAGAAAACCGATTCATTGAAATTGGAGTAACGCGAAGGGAAGTTCACATATACTATCTTGAAAAAGCCAACAAAATAAAATCAGAGAAAACACACATTCACATATTCTCATTCACTGGGGAGGAA<br>ATGGCCACCAAGGCAGATTACACTCTTGATGAAGAGAGCAGAGCAAGAATAAAAACCAGGCTATTCACTATAAGACAAGAGATGGCCAATAGGGGTCTATGGGATTCCTTTCGTCAGTCCGAGAGAGGCGAAGAGACAATTGAAGAAAGATTTGAAATCACAGGAACCATGCGCAGGCTTGCTGACCAAAGTCTCCCACCGAACTTCGCCAGCCTTGAAAATTTTAGAG<br>CCTATGTGGATGGATTTGAACCGAACGGCTGCATTGAGGGCAAGCTTTCTCAAATGTCAAAAGAAGTGAATGCCAGAATTGAGCCATTTCTGAAGACTACACCACGCCCTCTCAGATTGCCTGATGGACCTCCCTGTTCCCAGCGGTCGAAGTTCTTGCTAATGGATGCCCTTAAATTAAGCATTGAAGACCCGAGCCATGAGGGGGAGGGTATACCGCTGTACGATGCAA<br>CCAAATGCATGAAGACATTTTTTGGCTGGAAGGAGCCCAACATCGTGAAACCACATGAAAAGGGCATAAACCCTAATTACCTCCTGGCTTGGAAGCAGGTGCTATCAGAACTCCAAGATATTGAAAACGAGGAGAAAATCCCAAAAACAAAGAACATGAAGAAGACGAGCCAACTGAAGTGGGCACTTGGTGAAAATATGGCACCAGAGAAAGTGGACTTTGAGGACT<br>GTAAAGATGTTAGCGATCTAAAACAGTACGACAGTGACGAACCAGAATCCAGATCACTAGCAAGTTGGATCCAGAGTGAATTTAACAAGGCTTGCGAACTGACAGATTCGAGTTGGATTGAACTTGATGAAATAGGGGAAGACGTTGCTCCAATTGAACACATTGCGAGTATGAGGAGGAACTATTTCACAGCGGAAGTATCCCATTGCAGGGCTACAGAATACATAATG<br>AAAGGAGTATACATAAACACAGCTTTATTGAATGCATCCTGTGCAGCCATGGATGACTTCCAACTGATTCCAATGATAAGCAAATGCAGAACCAAAGAAGGGAGACGGAAGACAAATCTGTATGGCTTCATTATAAAAGGAAGATCCCATTTGAGGAATGACACCGATGTGGTAAACTTTGTGAGCATGGAATTCTCTCTCACTGACCCAAGGCTGGAGCCACACAAATGG<br>GAAAAGTACTGTGTGCTTGAGGTAGGAGACATGCTCCTACGGACTGCAATAGGCCAAGTGTCAAGGCCCATGTTCCTGTATGTGAGAACCAATGGGACTTCTAAGATCAAAATGAAATGGGGTATGGAGATGAGGCGATGCCTTCTTCAATCCCTTCAACAAATTGAGAGCATGATTGAAGCCGAGTCTTCTGTCAAAGAAAAAGACATGACCAAGGAATTCTTTGAAAAC<br>AGATCAGAAACATGGCCAATTGGAGAGTCACCCAAAGGAGTGGAGGAAGGCTCCATTGGGAAGGTGTGCAGAACATTGCTAGCAAAGTCTGTGTTCAATAGCCTATATGCATCTCCACAACTCGAGGGGTTTTCAGCTGAATCAAGAAAGTTGCTTCTCATTGTTCAGGCACTTAGGGACAACCTGGAACCTGGAACCTTCGATCTTGGGGGGCTATATGAAGCAATTGA<br>GGAGTGCCTGATTAACGATCCCTGGGTTTTGCTTAATGCGTCTTGGTTCAACTCCTTCCTCACACATGCACTGAAATA-
EPI_ISL_1719912	>A/swan/Lithuania/2306PG1_21VIR2606-7/2021_H5N8_2021-03-19<br>ATGGAAGACTTTGTGCGACAATGCTTCAATCCAATGATTGTCGAGCTTGCGGAAAAGGCAATGAAAGAATATGGGGAAGATCCTAAGATCGAAACAAACAAGTTTGCCGCAATATGTACACACTTAGAAGTCTGCTTCATGTATTCGGATTTCCATTTCATTGATGAACGAGGCGAATCAATAATCGTAGAATCTGGCGACCCGAATGCATTATTGAAGCACCGATTTGAGA<br>TAATTGAAGGGAGAGACCGAACAATGGCCTGGACAGTGGTGAATAGTATATGCAACACTACAGGAGTCGAAAAGCCCAAGTTCCTTCCTGATTTGTATGACTACAGAGAAAACCGATTCATTGAAATTGGAGTAACGCGAAGGGAAGTTCACATATACTATCTTGAAAAAGCCAACAAAATAAAATCAGAGAAAACACACATTCACATATTCTCATTCACTGGGGAGGAA<br>ATGGCCACCAAGGCAGATTACACTCTTGATGAAGAGAGCAGAGCAAGAATAAAAACCAGGCTATTCACTATAAGACAAGAGATGGCCAATAGGGGTCTATGGGATTCCTTTCGTCAGTCCGAGAGAGGCGAAGAGACAATTGAAGAAAGATTTGAAATCACAGGAACCATGCGCAGGCTTGCTGACCAAAGTCTCCCACCGAACTTCGCCAGCCTTGAAAATTTTAGAG<br>CCTATGTGGATGGATTTGAACCGAACGGCTGCATTGAGGGCAAGCTTTCTCAAATGTCAAAAGAAGTGAATGCCAGAATTGAGCCATTTCTGAAGACTACACCACGCCCTCTCAGATTGCCTGATGGACCTCCCTGTTCCCAGCGGTCGAAGTTCTTGCTAATGGATGCCCTTAAATTAAGCATTGAAGACCCGAGCCATGAGGGGGAGGGTATACCGCTGTACGATGCAA<br>CCAAATGCATGAAGACATTTTTTGGCTGGAAGGAGCCCAACATCGTGAAACCACATGAAAAGGGCATAAACCCTAATTACCTCCTGGCTTGGAAGCAGGTGCTATCAGAACTCCAAGATATTGAAAACGAGGAGAAAATCCCAAAAACAAAGAACATGAAGAAGACGAGCCAACTGAAGTGGGCACTTGGTGAAAATATGGCACCAGAGAAAGTGGACTTTGAGGACT<br>GTAAAGATGTTAGCGATCTAAAACAGTACGACAGTGACGAACCAGAATCCAGATCACTAGCAAGTTGGATCCAGAGTGAATTTAACAAGGCTTGCGAACTGACAGATTCGAGTTGGATTGAACTTGATGAAATAGGGGAAGACGTTGCTCCAATTGAACACATTGCGAGTATGAGGAGGAACTATTTCACAGCGGAAGTATCCCATTGCAGGGCTACAGAATACATAATG<br>AAAGGAGTATACATAAACACAGCTTTATTGAATGCATCCTGTGCAGCCATGGATGACTTCCAACTGATTCCAATGATAAGCAAATGCAGAACCAAAGAAGGGAGACGGAAGACAAATCTGTATGGCTTCATTATAAAAGGAAGATCCCATTTGAGGAATGACACCGATGTGGTAAACTTTGTGAGCATGGAATTCTCTCTCACTGACCCAAGGCTGGAGCCACACAAATGG<br>GAAAAGTACTGTGTGCTTGAGGTAGGAGACATGCTCCTACGGACTGCAATAGGCCAAGTGTCAAGGCCCATGTTCCTGTATGTGAGAACCAATGGGACTTCTAAGATCAAAATGAAATGGGGTATGGAGATGAGGCGATGCCTTCTTCAATCCCTTCAACAAATTGAGAGCATGATTGAAGCCGAGTCTTCTGTCAAAGAAAAAGACATGACCAAGGAATTCTTTGAAAAC<br>AGATCAGAAACATGGCCAATTGGAGAGTCACCCAAAGGAGTGGAGGAAGGCTCCATTGGGAAGGTGTGCAGAACATTGCTAGCAAAGTCTGTGTTCAATAGCCTATATGCATCTCCACAACTCGAGGGGTTTTCAGCTGAATCAAGAAAGTTGCTTCTCATTGTTCAGGCACTTAGGGACAACCTGGAACCTGGAACCTTCGATCTTGGGGGGCTATATGAAGCAATTGA<br>GGAGTGCCTGATTAACGATCCCTGGGTTTTGCTTAATGCGTCTTGGTTCAACTCCTTCCTCACACATGCACTGAAATA-
EPI_ISL_1255051	>A/tufted_duck/Poland/MB061/2021_H5N5_2021-02-01<br>ATGGAAGACTTTGTGCGACAATGCTTCAATCCAATGATTGTCGAGCTTGCGGAAAAGGCAATGAAAGAATATGGGGAAGATCCTAAGATCGAAACAAACAAGTTTGCCGCAATATGTACACACTTAGAAGTCTGCTTCATGTATTCGGATTTCCATTTCATTGATGAACGAGGCGAATCAATAATCGTAGAATCTGGCGACCCGAATGCATTATTGAAGCACCGATTTGAGA<br>TAATTGAAGGGAGAGACCGAACAATGGCCTGGACAGTGGTGAATAGTATATGCAACACTACAGGAGTCGAAAAGCCCAAGTTCCTTCCTGATTTGTATGACTACAGAGAAAACCGATTCATTGAAATTGGAGTAACGCGAAGGGAAGTTCACATATACTATCTTGAAAAAGCCAACAAAATAAAATCAGAGAAAACACACATTCACATATTCTCATTCACTGGGGAGGAA<br>ATGGCCACCAAGGCAGATTACACTCTTGATGAAGAGAGCAGAGCAAGAATAAAAACCAGGCTATTCACTATAAGACAAGAGATGGCCAATAGGGGTCTATGGGATTCCTTTCGTCAGTCCGAGAGAGGCGAAGAGACAATTGAAGAAAGATTTGAAATCACAGGAACCATGCGCAGGCTTGCTGACCAAAGTCTCCCACCGAACTTCGCCAGCCTTGAAAATTTTAGAG<br>CCTATGTGGATGGATTTGAACCGAACGGCTGCATTGAGGGCAAGCTTTCTCAAATGTCAAAAGAAGTGAATGCCAGAATTGAGCCATTTCTGAAGACTACACCACGCCCTCTCAGATTGCCTGATGGACCTCCCTGTTCCCAGCGGTCGAAGTTCTTGCTAATGGATGCCCTTAAATTAAGCATTGAAGACCCGAGCCATGAGGGGGAGGGTATACCGCTGTACGATGCAA<br>CCAAATGCATGAAGACATTTTTTGGCTGGAAGGAGCCCAACATCGTGAAACCACATGAAAAGGGCATAAACCCTAATTACCTCCTGGCTTGGAAGCAGGTGCTATCAGAACTCCAAGATATTGAAAACGAGGAGAAAATCCCAAAAACAAAGAACATGAAGAAGACGAGCCAACTGAAGTGGGCACTTGGTGAAAATATGGCACCAGAGAAAGTGGACTTTGAGGACT<br>GTAAAGATGTTAGCGATCTAAAACAGTACGACAGTGACGAACCAGAATCCAGATCACTAGCAAGTTGGATCCAGAGTGAATTTAACAAGGCTTGCGAACTGACAGATTCGAGTTGGATTGAACTTGATGAAATAGGGGAAGACGTTGCTCCAATTGAACACATTGCGAGTATGAGGAGGAACTATTTCACAGCGGAAGTATCCCATTGCAGGGCTACAGAATACATAATG<br>AAAGGAGTATACATAAACACAGCTTTATTGAATGCATCCTGTGCAGCCATGGATGACTTCCAACTGATTCCAATGATAAGCAAATGCAGAACCAAAGAAGGGAGACGGAAGACAAATCTGTATGGCTTCATTATAAAAGGAAGATCCCATTTGAGGAATGACACCGATGTGGTAAACTTTGTGAGCATGGAATTCTCTCTCACTGACCCAAGGCTGGAGCCACACAAATGG<br>GAAAAGTACTGTGTGCTTGAGGTAGGAGACATGCTCCTACGGACTGCAATAGGCCAAGTGTCAAGGCCCATGTTCCTGTATGTGAGAACCAATGGGACTTCTAAGATCAAAATGAAATGGGGTATGGAGATGAGGCGATGCCTTCTTCAATCCCTTCAACAAATTGAGAGCATGATTGAAGCCGAGTCTTCTGTCAAAGAAAAAGACATGACCAAGGAATTCTTTGAAAAC<br>AGATCAGAAACATGGCCAATTGGAGAGTCACCCAAAGGAGTGGAGGAAGGCTCCATTGGGAAGGTGTGCAGAACATTGCTAGCAAAGTCTGTGTTCAATAGCCTATATGCATCTCCACAACTCGAGGGGTTTTCAGCTGAATCAAGAAAGTTGCTTCTCATTGTTCAGGCACTTAGGGACAACCTGGAACCTGGAACCTTCGATCTTGGGGGGCTATATGAAGCAATTGA<br>GGAGTGCCTGATTAACGATCCCTGGGTTTTGCTTAATGCGTCTTGGTTCAACTCCTTCCTCACACATGCACTGAAATA-
EPI_ISL_846623	>A/swan/Poland/MB141/2020_H5N8_2020-12-17<br>ATGGAAGACTTTGTGCGACAATGCTTCAATCCAATGATTGTCGAGCTTGCGGAAAAGGCAATGAAAGAATATGGGGAAGATCCTAAGATCGAAACAAACAAGTTTGCCGCAATATGTACACACTTAGAAGTCTGCTTCATGTATTCGGATTTCCATTTCATTGATGAACGAGGCGAATCAATAATCGTAGAATCTGGCGACCCGAATGCATTATTGAAGCACCGATTTGAGA<br>TAATTGAAGGGAGAGACCGAACAATGGCCTGGACAGTGGTGAATAGTATATGCAACACTACAGGAGTCGAAAAGCCCAAGTTCCTTCCTGATTTGTATGACTACAGAGAAAACCGATTCATTGAAATTGGAGTAACGCGAAGGGAAGTTCACATATACTATCTTGAAAAAGCCAACAAAATAAAATCAGAGAAAACACACATTCACATATTCTCATTCACTGGGGAGGAA<br>ATGGCCACCAAGGCAGATTACACTCTTGATGAAGAGAGCAGAGCAAGAATAAAAACCAGGCTATTCACTATAAGACAAGAGATGGCCAATAGGGGTCTATGGGATTCCTTTCGTCAGTCCGAGAGAGGCGAAGAGACAATTGAAGAAAGATTTGAAATCACAGGAACCATGCGCAGGCTTGCTGACCAAAGTCTCCCACCGAACTTCGCCAGCCTTGAAAATTTTAGAG<br>CCTATGTGGATGGATTTGAACCGAACGGCTGCATTGAGGGCAAGCTTTCTCAAATGTCAAAAGAAGTGAATGCCAGAATTGAGCCATTTCTGAAGACTACACCACGCCCTCTCAGATTGCCTGATGGACCTCCCTGTTCCCAGCGGTCGAAGTTCTTGCTAATGGATGCCCTTAAATTAAGCATTGAAGACCCGAGCCATGAGGGGGAGGGTATACCGCTGTACGATGCAA<br>CCAAATGCATGAAGACATTTTTTGGCTGGAAGGAGCCCAACATCGTGAAACCACATGAAAAGGGCATAAACCCTAATTACCTCCTGGCTTGGAAGCAGGTGCTATCAGAACTCCAAGATATTGAAAACGAGGAGAAAATCCCAAAAACAAAGAACATGAAGAAGACGAGCCAACTGAAGTGGGCACTTGGTGAAAATATGGCACCAGAGAAAGTGGACTTTGAGGACT<br>GTAAAGATGTTAGCGATCTAAAACAGTACGACAGTGACGAACCAGAATCCAGATCACTAGCAAGTTGGATCCAGAGTGAATTTAACAAGGCTTGCGAACTGACAGATTCGAGTTGGATTGAACTTGATGAAATAGGGGAAGACGTTGCTCCAATTGAACACATTGCGAGTATGAGGAGGAACTATTTCACAGCGGAAGTATCCCATTGCAGGGCTACAGAATACATAATG<br>AAAGGAGTATACATAAACACAGCTTTATTGAATGCATCCTGTGCAGCCATGGATGACTTCCAACTGATTCCAATGATAAGCAAATGCAGAACCAAAGAAGGGAGACGGAAGACAAATCTGTATGGCTTCATTATAAAAGGAAGATCCCATTTGAGGAATGACACCGATGTGGTAAACTTTGTGAGCATGGAATTCTCTCTCACTGACCCAAGGCTGGAGCCACACAAATGG<br>GAAAAGTACTGTGTGCTTGAGGTAGGAGACATGCTCCTACGGACTGCAATAGGCCAAGTGTCAAGGCCCATGTTCCTGTATGTGAGAACCAATGGGACTTCTAAGATCAAAATGAAATGGGGTATGGAGATGAGGCGATGCCTTCTTCAATCCCTTCAACAAATTGAGAGCATGATTGAAGCCGAGTCTTCTGTCAAAGAAAAAGACATGACCAAGGAATTCTTTGAAAAC<br>AGATCAGAAACATGGCCAATTGGAGAGTCACCCAAAGGAGTGGAGGAAGGCTCCATTGGGAAGGTGTGCAGAACATTGCTAGCAAAGTCTGTGTTCAATAGCCTATATGCATCTCCACAACTCGAGGGGTTTTCAGCTGAATCAAGAAAGTTGCTTCTCATTGTTCAGGCACTTAGGGACAACCTGGAACCTGGAACCTTCGATCTTGGGGGGCTATATGAAGCAATTGA<br>GGAGTGCCTGATTAACGATCCCTGGGTTTTGCTTAATGCGTCTTGGTTCAACTCCTTCCTCACACATGCACTGAAATA-
EPI_ISL_7778771	>A/pheasant/Finland/1589_21VIR7689-3/2021_H5N8_2021-02-01<br>ATGGAAGACTTTGTGCGACAATGCTTCAATCCAATGATTGTCGAGCTTGCGGAAAAGGCAATGAAAGAATATGGGGAAGATCCTAAGATCGAAACAAACAAGTTTGCCGCAATATGTACACACTTAGAAGTCTGCTTCATGTATTCGGATTTCCATTTCATTGATGAACGAGGCGAATCAATAATCGTAGAATCTGGCGACCCGAATGCATTATTGAAGCACCGATTTGAGA<br>TAATTGAAGGGAGAGACCGAACAATGGCCTGGACAGTGGTGAATAGCATATGCAACACTACAGGAGTCGAAAAGCCCAAGTTCCTTCCTGATTTGTATGACTACAGAGAAAACCGATTCATTGAAATTGGAGTAACGCGAAGGGAAGTTCACATATACTATCTTGAAAAAGCCAACAAAATAAAATCAGAGAAAACACACATTCACATATTCTCATTCACTGGGGAGGAA<br>ATGGCCACCAAGGCAGATTACACTCTTGATGAAGAGAGCAGAGCAAGAATAAAAACCAGGCTATTCACTATAAGACAAGAGATGGCCAATAGGGGTCTATGGGATTCCTTTCGTCAGTCCGAGAGAGGCGAAGAGACAATTGAAGAAAGATTTGAAATCACAGGAACCATGCGCAGGCTTGCTGACCAAAGTCTCCCACCGAACTTCGCCAGCCTTGAAAATTTTAGAG<br>CCTATGTGGATGGATTTGAACCGAACGGCTGCATTGAGGGCAAGCTTTCTCAAATGTCAAAAGAAGTGAATGCCAGAATTGAGCCATTTCTGAAGACTACACCACGCCCTCTCAGATTGCCTGATGGACCTCCCTGTTCCCAGCGGTCGAAGTTCTTGCTAATGGATGCCCTTAAATTAAGCATTGAAGACCCGAGCCATGAGGGGGAGGGTATACCGCTGTACGATGCAA<br>CCAAATGCATGAAGACATTTTTTGGCTGGAAGGAGCCCAACATCGTGAAACCACATGAAAAGGGCATAAACCCTAATTACCTCCTGGCTTGGAAGCAGGTGCTATCAGAACTCCAAGATATTGAAAACGAGGAGAAAATCCCAAAAACAAAGAACATGAAGAAGACGAGCCAACTGAAGTGGGCACTTGGTGAAAATATGGCACCAGAGAAAGTGGACTTTGAGGACT<br>GTAAAGATGTTAGCGATCTAAAACAGTACGACAGTGACGAACCAGAATCCAGATCACTAGCAAGTTGGATCCAGAGTGAATTTAACAAGGCTTGCGAACTGACAGATTCGAGTTGGATTGAACTTGATGAAATAGGGGAAGACGTTGCTCCAATTGAACACATTGCGAGTATGAGGAGGAACTATTTCACAGCGGAAGTATCCCATTGCAGGGCTACAGAATACATAATG<br>AAAGGAGTATACATAAACACAGCTTTATTGAATGCATCCTGTGCAGCCATGGATGACTTCCAACTGATTCCAATGATAAGCAAATGCAGAACCAAAGAAGGGAGACGGAAGACAAATCTGTATGGCTTCATTATAAAAGGAAGATCCCATTTGAGGAATGACACCGATGTGGTAAACTTTGTGAGCATGGAATTCTCTCTCACTGACCCAAGGCTGGAGCCACACAAATGG<br>GAAAAGTACTGTGTGCTTGAGGTAGGAGACATGCTCCTACGGACTGCAATAGGCCAAGTGTCAAGGCCCATGTTCCTGTATGTGAGAACCAATGGGACTTCTAAGATCAAAATGAAATGGGGTATGGAGATGAGGCGATGCCTTCTTCAATCCCTTCAACAAATTGAGAGCATGATTGAAGCCGAGTCTTCTGTCAAAGAAAAAGACATGACCAAGGAATTCTTTGAAAAC<br>AGATCAGAAACATGGCCAATTGGAGAGTCACCCAAAGGAGTGGAGGAAGGCTCCATTGGGAAGGTGTGCAGAACATTGCTAGCAAAGTCTGTGTTCAATAGCCTATATGCATCTCCACAACTCGAGGGGTTTTCAGCTGAATCAAGAAAGTTGCTTCTCATTGTTCAGGCACTTAGGGACAACCTGGAACCTGGAACCTTCGATCTTGGGGGGCTATATGAAGCAATTGA<br>GGAGTGCCTGATTAACGATCCCTGGGTTTTGCTTAATGCGTCTTGGTTCAACTCCTTCCTCACACATGCACTGAAATA-
EPI_ISL_5102033	>A/domestic_duck/Germany-NW/AI02049/2021_H5N8_2021-03-01<br>ATGGAAGACTTTGTGCGACAATGCTTCAATCCAATGATTGTCGAGCTTGCGGAAAAGGCAATGAAAGAATATGGGGAAGATCCCAAGATCGAAACAAACAAGTTTGCCGCAATATGTACACACTTAGAAGTCTGCTTCATGTATTCGGATTTCCATTTCATTGATGAACGAGGCGAATCAATAATCGTAGAATCTGGCGACCCGAATGCATTATTGAAGCACCGATTTGAGA<br>TAATTGAAGGGAGAGACCGAACAATGGCCTGGACAGTGGTGAATAGTATATGCAACACTACAGGAGTCGAAAAGCCCAAGTTCCTTCCTGATTTGTATGACTACAGAGAAAACCGATTCATTGAAATTGGAGTAACGCGAAGGGAAGTTCACATATACTATCTTGAAAAAGCCAACAAAATAAAATCAGAGAAAACACACATTCACATATTCTCATTCACTGGGGAGGAA<br>ATGGCCACCAAGGCAGATTACACTCTTGATGAAGAGAGCAGAGCAAGAATAAAAACCAGGCTATTCACTATAAGACAAGAGATGGCCAATAGGGGTCTATGGGATTCCTTTCGTCAGTCCGAGAGAGGCGAAGAGACAATTGAAGAAAGATTTGAAATCACAGGAACCATGCGCAGGCTTGCTGACCAAAGTCTCCCACCGAACTTCGCCAGCCTTGAAAATTTTAGAG<br>CCTATGTGGATGGATTTGAACCGAACGGCTGCATTGAGGGCAAGCTTTCTCAAATGTCAAAAGAAGTGAATGCCAGAATTGAGCCATTTCTGAAGACTACACCACGCCCTCTCAGATTGCCTGATGGACCTCCCTGTTCCCAGCGGTCGAAGTTCTTGCTAATGGATGCCCTTAAATTAAGCATTGAAGACCCGAGCCATGAGGGGGAGGGTATACCGCTGTACGATGCAA<br>CCAAATGCATGAAGACATTTTTTGGCTGGAAGGAGCCCAACATCGTGAAACCACATGAAAAGGGCATAAACCCTAATTACCTCCTGGCTTGGAAGCAGGTGCTATCAGAACTCCAAGATATTGAAAACGAGGAGAAAATCCCAAAAACAAAGAACATGAAGAAGACGAGCCAACTGAAGTGGGCACTTGGTGAAAATATGGCACCAGAGAAAGTGGACTTTGAGGACT<br>GTAAAGATGTTAGCGATCTAAAACAGTACGACAGTGACGAACCAGAATCCAGATCACTAGCAAGTTGGATCCAGAGTGAATTTAACAAGGCTTGCGAACTGACAGATTCGAGTTGGATTGAACTTGATGAAATAGGGGAAGACGTTGCTCCAATTGAACACATTGCGAGTATGAGGAGGAACTATTTCACAGCGGAAGTATCCCATTGCAGGGCTACAGAATACATAATG<br>AAAGGAGTATACATAAACACAGCTTTATTGAATGCATCCTGTGCAGCCATGGATGACTTCCAACTGATTCCAATGATAAGCAAATGCAGAACCAAAGAAGGGAGACGGAAGACAAATCTGTATGGCTTCATTATAAAAGGAAGATCCCATTTGAGGAATGACACCGATGTGGTAAACTTTGTGAGCATGGAATTCTCTCTCACTGACCCAAGGCTGGAGCCACACAAATGG<br>GAAAAGTACTGTGTGCTTGAGGTAGGAGACATGCTCCTACGGACTGCAATAGGCCAAGTGTCAAGGCCCATGTTCCTGTATGTGAGAACCAATGGGACTTCTAAGATCAAAATGAAATGGGGTATGGAGATGAGGCGATGCCTTCTTCAATCCCTTCAACAAATTGAGAGCATGATTGAAGCCGAGTCTTCTGTCAAAGAAAAAGACATGACCAAGGAATTCTTTGAAAAC<br>AGATCAGAAACATGGCCAATTGGAGAGTCACCCAAAGGAGTGGAGGAAGGCTCCATTGGGAAGGTGTGCAGAACATTGCTAGCAAAGTCTGTGTTCAATAGCCTATATGCATCTCCACAACTCGAGGGGTTTTCAGCTGAATCAAGAAAGTTGCTTCTCATTGTTCAGGCACTTAGGGACAACCTGGAACCTGGAACCTTCGATCTTGGGGGGCTATATGAAGCAATTGA<br>GGAGTGCCTGATTAACGATCCCTGGGTTTTGCTTAATGCGTCTTGGTTCAACTCCTTCCTCACACATGCACTGAAATA-
EPI_ISL_7778761	>A/mute_swan/Estonia/TA2108545-1_21VIR7512-5/2021_H5N8_2021-03-26<br>ATGGAAGACTTTGTGCGACAATGCTTCAATCCAATGATTGTCGAGCTTGCGGAAAAGGCAATGAAAGAATATGGGGAAGATCCTAAGATCGAAACAAACAAGTTTGCCGCAATATGTACACACTTAGAAGTCTGCTTCATGTATTCGGATTTCCATTTCATTGATGAACGAGGCGAATCAATAATCGTAGAATCTGGCGACCCGAATGCATTATTGAAGCACCGATTTGAGA<br>TAATTGAAGGGAGAGACCGAACAATGGCCTGGACAGTGGTGAATAGTATATGCAACACTACAGGAGTCGAAAAGCCCAAGTTCCTTCCTGATTTGTATGACTACAGAGAAAACCGATTCATTGAAATTGGAGTAACGCGAAGGGAAGTTCACATATACTATCTTGAAAAAGCCAACAAAATAAAATCAGAGAAAACACACATTCACATATTCTCATTCACTGGGGAGGAA<br>ATGGCCACCAAGGCAGATTACACTCTTGATGAAGAGAGCAGAGCAAGAATAAAAACCAGGCTATTCACTATAAGACAAGAGATGGCCAATAGGGGTCTATGGGATTCCTTTCGTCAGTCAGAGAGAGGCGAAGAGACAATTGAAGAAAGATTTGAAATCACAGGAACCATGCGCAGGCTTGCTGACCAAAGTCTCCCACCGAACTTCGCCAGCCTTGAAAATTTTAGAG<br>CCTATGTGGATGGATTTGAACCGAACGGCTGCATTGAGGGCAAGCTTTCTCAAATGTCAAAAGAAGTGAATGCCAGAATTGAGCCATTTCTGAAGACTACACCACGCCCTCTCAGATTGCCTGATGGACCTCCCTGTTCCCAGCGGTCGAAGTTCTTGCTAATGGATGCCCTTAAATTAAGCATTGAAGACCCGAGCCATGAGGGGGAGGGTATACCGCTGTACGATGCAA<br>CCAAATGCATGAAGACATTTTTTGGCTGGAAGGAGCCCAACATCGTGAAACCACATGAAAAGGGCATAAACCCTAATTACCTCCTGGCTTGGAAGCAGGTGCTATCAGAACTCCAAGATATTGAAAACGAGGAGAAAATCCCAAAAACAAAGAACATGAAGAAGACGAGCCAACTGAAGTGGGCACTTGGTGAAAATATGGCACCAGAGAAAGTGGACTTTGAGGACT<br>GTAAAGATGTTAGCGATCTAAAACAGTACGACAGTGACGAACCAGAATCCAGATCACTAGCAAGTTGGATCCAGAGTGAATTTAACAAGGCTTGCGAACTGACAGATTCGAGTTGGATTGAACTTGATGAAATAGGGGAAGACGTTGCTCCAATTGAACACATTGCGAGTATGAGGAGGAACTATTTCACAGCGGAAGTATCCCATTGCAGGGCTACAGAATACATAATG<br>AAAGGAGTATACATAAACACAGCTTTATTGAATGCATCCTGTGCAGCCATGGATGACTTCCAACTGATTCCAATGATAAGCAAATGCAGAACCAAAGAAGGGAGACGGAAGACAAATCTGTATGGCTTCATTATAAAAGGAAGATCCCATTTGAGGAATGACACCGATGTGGTAAACTTTGTGAGCATGGAATTCTCTCTCACTGACCCAAGGCTGGAGCCACACAAATGG<br>GAAAAGTACTGTGTGCTTGAGGTAGGAGACATGCTCCTACGGACTGCAATAGGCCAAGTGTCAAGGCCCATGTTCCTGTATGTGAGAACCAATGGGACTTCTAAGATCAAAATGAAATGGGGTATGGAGATGAGGCGATGCCTTCTTCAATCCCTTCAACAAATTGAGAGCATGATTGAAGCCGAGTCTTCTGTCAAAGAAAAAGACATGACCAAGGAATTCTTTGAAAAC<br>AGATCAGAAACATGGCCAATTGGAGAGTCACCCAAAGGAGTGGAGGAAGGCTCCATTGGGAAGGTGTGCAGAACATTGCTAGCAAAGTCTGTGTTCAATAGCCTATATGCATCTCCACAACTCGAGGGGTTTTCAGCTGAATCAAGAAAGTTGCTTCTCATTGTTCAGGCACTTAGGGACAACCTGGAACCTGGAACCTTCGATCTTGGGGGGCTATATGAAGCAATTGA<br>GGAGTGCCTGATTAACGATCCCTGGGTTTTGCTTAATGCGTCTTGGTTCAACTCCTTCCTCACACATGCACTGAAATA-
EPI_ISL_2175628	>A/common_buzzard/Netherlands/21021187-001/2021_H5N8_2021-01-04<br>ATGGAAGACTTTGTGCGACAATGCTTCAATCCAATGATTGTCGAGCTTGCGGAAAAGGCAATGAAAGAATATGGGGAAGATCCTAAGATCGAAACAAACAAGTTTGCCGCAATATGTACACACTTAGAAGTCTGCTTCATGTATTCGGATTTCCATTTCATTGATGAACGAGGCGAATCAATAATCGTAGAATCTGGCGACCCGAATGCATTATTGAAGCACCGATTTGAGA<br>TAATTGAAGGGAGAGACCGAACAATGGCCTGGACAGTGGTGAATAGTATATGCAACACTACAGGAGTCGAAAAGCCCAAGTTCCTTCCTGATTTGTATGACTACAGAGAAAACCGATTCATTGAAATTGGAGTAACGCGAAGGGAAGTTCACATATACTATCTTGAAAAAGCCAACAAAATAAAATCAGAGAAAACACACATTCACATATTCTCATTCACTGGGGAGGAA<br>ATGGCCACCAAGGCAGATTACACTCTTGATGAAGAGAGCAGAGCAAGAATAAAAACCAGGCTATTCACTATAAGACAAGAGATGGCCAATAGGGGTCTATGGGATTCCTTTCGTCAGTCCGAGAGAGGCGAAGAGACAATTGAAGAAAGATTTGAAATCACAGGAACCATGCGCAGGCTTGCTGACCAAAGTCTCCCACCGAACTTCGCCAGCCTTGAAAATTTTAGAG<br>CCTATGTGGATGGATTTGAACCGAACGGCTGCATTGAGGGCAAGCTTTCTCAAATGTCAAAAGAAGTGAATGCCAGAATTGAGCCATTTCTGAAGACTACACCACGCCCTCTCAGATTGCCTGATGGACCTCCCTGTTCCCAGCGGTCGAAGTTCTTGCTAATGGATGCCCTTAAATTAAGCATTGAAGACCCGAGCCATGAGGGGGAGGGTATACCGCTGTACGATGCAA<br>CCAAATGCATGAAGACATTTTTTGGCTGGAAGGAGCCCAACATCGTGAAACCACATGAAAAGGGCATAAACCCTAATTACCTCCTGGCTTGGAAGCAGGTGCTATCAGAACTCCAAGATATTGAAAACGAGGAGAAAATCCCAAAAACAAAGAACATGAAGAAGACGAGCCAACTGAAGTGGGCACTTGGTGAAAATATGGCACCAGAGAAAGTGGACTTTGAGGACT<br>GTAAAGATGTTAGCGATCTAAAACAGTACGACAGTGACGAACCAGAATCCAGATCACTAGCAAGTTGGATCCAGAGTGAATTTAACAAGGCTTGCGAACTGACAGATTCGAGTTGGATTGAACTTGATGAAATAGGGGAAGACGTTGCTCCAATTGAACACATTGCGAGTATGAGGAGGAACTATTTCACAGCGGAAGTATCCCATTGCAGGGCTACAGAATACATAATG<br>AAAGGAGTATACATAAACACCGCTTTATTGAATGCATCCTGTGCAGCCATGGATGACTTCCAACTGATTCCAATGATAAGCAAATGCAGAACCAAAGAAGGGAGACGGAAGACAAATCTGTATGGCTTCATTATAAAAGGAAGATCCCATTTGAGGAATGACACCGATGTGGTAAACTTTGTGAGCATGGAATTCTCTCTCACTGACCCAAGGCTGGAGCCACACAAATGG<br>GAAAAGTACTGTGTGCTTGAGGTAGGAGACATGCTCCTACGGACTGCAATAGGCCAAGTGTCAAGGCCCATGTTCCTGTATGTGAGAACCAATGGGACTTCTAAGATCAAAATGAAATGGGGTATGGAGATGAGGCGATGCCTTCTTCAATCCCTTCAACAAATTGAGAGCATGATTGAAGCCGAGTCTTCTGTCAAAGAAAAAGACATGACCAAGGAATTCTTTGAAAAC<br>AGATCAGAAACATGGCCAATTGGAGAGTCACCCAAAGGAGTGGAGGAAGGCTCCATTGGGAAGGTGTGCAGAACATTGCTAGCAAAGTCTGTGTTCAATAGCCTATATGCATCTCCACAACTCGAGGGGTTTTCAGCTGAATCAAGAAAGTTGCTTCTCATTGTTCAGGCACTTAGGGACAACCTGGAACCTGGAACCTTCGATCTTGGGGGGCTATATGAAGCAATTGA<br>GGAGTGCCTGATTAACGATCCCTGGGTTTTGCTTAATGCGTCTTGGTTCAACTCCTTCCTCACACATGCACTGAAATA-
EPI_ISL_5142961	>A/turkey/Germany-MV/AI02458/2021_H5N8_2021-03-10<br>ATGGAAGACTTTGTGCGACAATGCTTCAATCCAATGATTGTCGAGCTTGCGGAAAAGGCAATGAAAGAATATGGGGAAGATCCTAAGATCGAAACAAACAAGTTTGCCGCAATATGTACACACTTAGAAGTCTGCTTCATGTATTCGGATTTCCATTTCATTGATGAACGAGGCGAATCAATAATCGTAGAATCTGGCGACCCGAATGCATTATTGAAGCACCGATTTGAGA<br>TAATTGAAGGGAGAGACCGAACAATGGCCTGGACAGTGGTGAATAGTATATGCAACACTACAGGAGTCGAAAAGCCCAAGTTCCTTCCTGATTTGTATGACTACAGAGAAAACCGATTCATTGAAATTGGAGTAACGCGAAGGGAAGTTCACATATACTATCTTGAAAAAGCCAACAAAATAAAATCAGAGAAAACACACATTCACATATTCTCATTCACTGGGGAGGAA<br>ATGGCCACCAAGGCAGATTACACTCTTGATGAAGAGAGCAGAGCAAGAATAAAAACCAGGCTATTCACTATAAGACAAGAGATGGCCAATAGGGGTCTATGGGATTCCTTTCGTCAGTCCGAGAGAGGCGAAGAGACAATTGAAGAAAGATTTGAAATCACAGGAACCATGCGCAGGCTTGCTGACCAAAGTCTCCCACCGAACTTCGCCAGCCTTGAAAATTTTAGAG<br>CCTATGTGGATGGATTTGAACCGAACGGCTGCATTGAGGGCAAGCTTTCTCAAATGTCAAAAGAAGTGAATGCCAGAATTGAGCCATTTCTGAAGACTACACCACGCCCTCTCAGATTGCCTGATGGACCTCCCTGTTCCCAGCGGTCGAAGTTCTTGCTAATGGATGCCCTTAAATTAAGCATTGAAGACCCGAGCCATGAGGGGGAGGGTATACCGCTGTACGATGCAA<br>CCAAATGCATGAAGACATTTTTTGGCTGGAAGGAGCCCAACATCGTGAAACCACATGAAAAGGGCATAAACCCTAATTACCTCCTGGCTTGGAAGCAGGTGCTATCAGAACTCCAAGATATTGAAAACGAGGAGAAAATCCCAAAAACAAAGAACATGAAGAAGACGAGCCAACTGAAGTGGGCACTTGGTGAAAATATGGCACCAGAGAAAGTGGACTTTGAGGACT<br>GTAAAGATGTTAGCGATCTAAAACAGTACGACAGTGACGAACCAGAATCCAGATCACTAGCAAGTTGGATCCAGAGTGAATTTAACAAGGCTTGCGAACTGACAGATTCGAGTTGGATTGAACTTGATGAAATAGGGGAAGACGTTGCTCCAATTGAACACATTGCGAGTATGAGGAGGAACTATTTCACAGCGGAAGTATCCCATTGCAGGGCTACAGAATACATAATG<br>AAAGGAGTATATATAAACACAGCTTTATTGAATGCATCCTGTGCAGCCATGGATGACTTCCAACTGATTCCAATGATAAGCAAATGCAGAACCAAAGAAGGGAGACGGAAGACAAATCTGTATGGCTTCATTATAAAAGGAAGATCCCATTTGAGGAATGACACGGATGTGGTAAACTTTGTGAGCATGGAATTCTCTCTCACTGACCCAAGGCTGGAGCCACACAAATGG<br>GAAAAGTACTGTGTGCTTGAGGTAGGAGACATGCTCCTACGGACTGCAATAGGCCAAGTGTCAAGGCCCATGTTCCTGTATGTGAGAACCAATGGGACTTCTAAGATCAAAATGAAATGGGGTATGGAGATGAGGCGATGCCTTCTTCAATCCCTTCAACAAATTGAGAGCATGATTGAAGCCGAGTCTTCTGTCAAAGAAAAAGACATGACCAAGGAATTCTTTGAAAAC<br>AGATCAGAAACATGGCCAATTGGAGAGTCACCCAAAGGAGTGGAGGAAGGCTCCATTGGGAAGGTGTGCAGAACATTGCTAGCAAAGTCTGTGTTCAATAGCCTATATGCATCTCCACAACTCGAGGGGTTTTCAGCTGAATCAAGAAAGTTGCTTCTCATTGTTCAGGCACTTAGGGACAACCTGGAACCTGGAACCTTCGATCTTGGGGGGCTATATGAAGCAATTGA<br>GGAGTGCCTGATTAACGATCCCTGGGTTTTGCTTAATGCGTCTTGGTTCAACTCCTTCCTCACACATGCACTGAAATA-

EPI_ISL_7624142	>A/Gallus_gallus/Belgium/11644/2021_H5N8_2021-09-09<br>[illegible]
EPI_ISL_3102029	>A/barnacle_goose/Sweden/SVA210423SZ0252/FB001647/M2021_H5N8_2021-01-06<br>[illegible]
EPI_ISL_5123847	>A/chicken/Germany-SH/AI02562/2021_H5N8_2021-03-11<br>[illegible]
EPI_ISL_2199986	>A/mute_swan/Poland/MB185/2021_H5N8_2021-02-26<br>[illegible]
EPI_ISL_1139164	>A/northern_goshawk/Netherlands/20018560-002/2020_H5N8_2020-11-22<br>[illegible]
EPI_ISL_846601	>A/chicken/Poland/474/2020_H5N8_2020-12-03<br>[illegible]
EPI_ISL_1420637	>A/wigeon/Latvia/23903/2021_H5N8_2021-02-04<br>[illegible]
EPI_ISL_2177398	>A/peacock/Netherlands/21026542-001/2021_H5N8_2021-04-07<br>[illegible]
EPI_ISL_243439	>A/mallard_duck/Netherlands/13/2013_H7N7_2013-12-10<br>[illegible]
EPI_ISL_378366	>A/Eurasian_Wigeon/Netherlands/5/2007_H7N7_2007-10-22<br>[illegible]
EPI_ISL_394155	>A/chicken/Netherlands/17013178-006010/2017_H10N7_2017-09-27<br>[illegible]
EPI_ISL_17978926	>A/mallard/Poland/P060w2/2020_H3N8_2020-08-25<br>[illegible]
EPI_ISL_4808840	>A/mallard/Denmark/12947-12/2020-10-26_H7N5_2020-10-26<br>[illegible]

EPI_ISL_17978951	>A/mallard/Poland/P096/2020_H9N2_2020-11-02<br>ATGGAAGACTTTGTGCGACAATGCTTCAATCCAATGATTGTCGAGCTTGCGGAAAAGGCAATGAAAGAATATGGGGAAGATCCGAAAATCGAAACGAACAAATTTGCCGCAATATGCACACACTTAGAAGTCTGTTTCATGTATTCGGATTTTCACTTTATTGATGAACGGGGCGAATCAATAATTGTAGAATCTGGCGATCCGAATGCATTACTGAAACACCGATTTGAGA<br>TAATTGAAGGGAGGGACCGAACAATGGCCTGGACAGTGGTAAATAGTATCTGCAACACCACAGGAGTCGATAAGCCTAAATTCCTCCCAGATTTGTATGACTACAAAGAGAACCGATTCATTGAAATTGGAGTAACACGAAGGGAAGTCCACATATACTATCTAGAAAAAGCCAACAAGATAAAATCAGAGAAGACACACATTCACATATTCTCATTCACTGGAGAGGAA<br>ATGGCCACCAAGGCGGACTATACCCTTGACGAGGAAAGCAGAGCAAGAATCAAAACCAGGCTGTTCACTATAAGACAAGAAATGGCCAGTAGGGGTCTATGGGATTCCTTTCGTCAGTCCGAGAGAGGCGAAGAGACAATTGAAGAAAGATTTGAAATCACAGGAACCATGCGCAGGCTTGCCGACCAAAGTCTCCCACCGAACTTCTCCAGCCTTGAAAACTTTAGAG<br>CCTATGTGGATGGATTCGAACCGAACGGCTGCATTGAGGGCAAGCTTTCTCAAATGTCAAAAGAAGTGAACGCCAGGATTGAGCCATTTCTGAAGACAACGCCACGCCCTCTCAGATTACCTGATGGGCCTCCTTGCTCTCAGCGGTCGAAGTTCTTACTGATGGATGCCCTTAAATTAAGCATCGAAGACCCGAGCCATGAAGGAGAAGGCATACCGCTATATGATGCAA<br>TCAAATGCATGAAGACATTTTTCGGCTGGAAAGAGCCCAACATCGTGAAACCACATGAAAAAGGCATAAACCCCAATTACCTCCTGGCTTGGAAGCAGGTGCTGGCAGAACTCCAAGATATTGAAAATGAGGAGAAAATTCCAAAAACAAAAAACATGAAGAAAACAAGTCAATTAAAGTGGGCACTTGGTGAGAACATGGCACCAGAGAAAGTAGACTTTGAGGACTG<br>CAAAGACGTTAGCGATCTAAGACAATATGATAGTGATGAACCAGAGTCTAGATCGCTAGCAAGCTGGATCCAGAGCGAATTCAACAAAGCATGCGAATTGACAGATTCAAGTTGGATTGAACTTGATGAAATAGGAGAGGACGTTGCTCCAATTGAGCACATTGCGAGTATGAGGAGAAACTACTTCACAGCGGAAGTATCCCATTGTAGGGCCACTGAATACATAATGA<br>AGGGAGTGTACATAAACACAGCCCTGTTGAATGCATCCTGTGCAGCCATGGATGACTTCCAACTGATTCCAATGATAAGCAAGTGCAGAACCAAAGAAGGAAGACGGAAGACAAATCTGTATGGATTCATCATAAAGGGAAGATCCCATTTGAGGAATGACACCGATGTGGTAAACTTTGTGAGCATGGAATTCTCTCTTACGGACCCGAGGCTGGAGCCACACAAGTGG<br>GAAAAGTACTGTGTTCTCGAGATAGGAGACATGCTCCTACGGACTGCAATAGGCCAAGTGTCAAGGCCCATGTTCCTGTATGTGAGAACCAATGGGACTTCCAAGATCAAGATGAAATGGGGCATGGAGATGAGGCGGTGCCTTCTTCAATCCCTTCAACAAATTGAGAGCATGATTGAGGCCGAGTCCTCTGTCAAAGAGAAAGACATGASCARAGAATTCTTTGAAAA<br>CAAATCAGAAACATGGCCAATTGGAGAATCACCTAAAGGGGTGGAGGAAGGCTCCATTGGGAAGGTGTGCAGAACATTACTAGCAAAATCTGTATTCAACAGCCTATATGCATCTCCACAACTCGAGGGGTTTTCGGCTGAATCAAGAAAATTGCTTCTCATTGTTCAGGCACTTAGGGACAACCTGGAACCTGGGACCTTCGATCTTGGAGGGCTATATGAAGCAATTGA<br>GGAGTGCCTGATTAACGATCCCTGGGTTTTGCTTAATGCGTCTTGGTTCAACTCCTTCCTCACACATGCACTGAAATA-
EPI_ISL_5095416	>A/turkey/Germany-BB/AI00432/2021_H6N1_2021-02-01<br>ATGGAAGACTTTGTGCGACAATGCTTCAATCCAATGATTGTCGAGCTTGCGGAAAAGGCAATGAAAGAATATGGGGAAGATCCGAAAATCGAAACGAACAAATTTGCCGCAATATGCACACACTTAGAAGTCTGTTTCATGTATTCGGATTTTCACTTTATTGATGAACGGGGCGAATCAATAATTGTAGAATCTGGCGATCCGAATGCATTACTGAAACACCGATTTGAGA<br>TAATTGAAGGGAGAGACCGAACAATGGCCTGGACAGTGGTAAATAGTATCTGCAACACCACAGGAGTCGATAAGCCTAAATTCCTCCCAGATTTRTATGACTACAAARAGAACCGATTCATTGAAATTGGAGTAACACGAAGGGAAGTCCACATATACTATCTAGAAAAGGCCAACAAGATAAAATCAGAGAAGACACACATTCACATATTCTCATTCACTGGAGAGGAA<br>ATGGCCACCAAAGCGGACTATACCCTTGACGAGGAGAGCAGAGCAAGAATCAAAACCAGGCTGTTCACTATAAGACAAGAAATGGCCAGTAGGGGTCTATGGGATTCCTTTCGTCAGTCCGAGAGAGGCGAAGAGACAATTGAAGAAAGATTTGAAATCACAGGAACCATGCGCAGGCTTGCCGACCAGAGTCTCCCACCGAACTTCTCCAGCCTTGAAAACTTTAGAG<br>CCTATGTGGATGGATTCGAACCGAACGGCTGCATTGAGGGCAAGCTTTCTCAAATGTCAAAAGAAGTGAACGCCAGGATTGAGCCATTTCTGAAGACAACGCCACGCCCTCTCAAATTACCTGATGGGCCTCCCTGCTCTCAGCGGTCGAAGTTCTTACTGATGGATGCCCTTAAATTAAGCATCGAAGACCCGAGCCATGAAGGAGAAGGCATACCGCTATATGATGCAA<br>TCAAATGCATGAAGACATTTTTCGGTTGGAAAGAGCCCAACATCGTGAAACCACATGAAAAAGGCATAAACCCCAATTACCTCCTATCTTGGAAGCAGGTGCTGGCAGAACTCCAAGATATTGAAAATGAGGAGAAAATTCCAAAAACAAAAAACATGAAGAAAACAAGTCAATTAAAGTGGGCACTTGGTGAGAACATGGCACCAGAGAAAGTAGACTTTGAGGACTG<br>CAAAGACGTTAGCGATCTAAGACAATATGATAGTGATGAACCAGAGTCTAGATCGCTAGCAAGCTGGATCCAGAGCGAATTCAACAAAGCATGCGAATTGACAGATTCAAGTTGGATTGAACTTGATGAAATAGGAGAGGACGTTGCTCCAATTGAGCACATTGCGAGTATGAGAAGAAACTACTTCACAGCGGAAGTATCCCATTGTAGGGCCACTGAATACATAATGA<br>AGGGAGTGTACATAAACACAGCCCTGTTGAATGCATCCTGTGCAGCCATGGATGACTTCCAACTGATTCCAATGATAAGCAAGTGCAGAACCAAAGAAGGAAGACGGAAGACAAATCTGTATGGATTCATCATAAAAGGAAGATCCCATTTGAGGAATGACACCGATGTGGTAAACTTTGTGAGCATGGAATTCTCTCTTACGGACCCGAGGCTGGAGCCACACAAGTGG<br>GAAAAGTACTGTGTTCTCGAGATAGGAGACATGCTCCTACGGACTGCAATAGGCCAAGTGTCAAGGCCCATGTTCCTGTATGTGAGAACCAATGGGACTTCCAAGATCAAGATGAAATGGGGCATGGAGATGAGGCGGTGCCTTCTTCAATCCCTTCAACAAATTGAGAGCATGATTGAGGCCGAGTCCTCTGTCAAAGAGAAAGACATGACCAAAGAATTCTTTGAAAA<br>CAAATCAGAAACATGGCCAATTGGAGAATCACCTAAAGGGGTGGAGGAAGGCTCCATTGGGAAGGTGTGCAGAACATTACTGGCAAAATCTGTATTCAACAGCCTATATGCATCTCCACAACTCGAGGGGTTTTCGGCTGAATCAAGAAAATTGCTTCTCATTGTTCAGGCACTTAGGGACAACCTGGAACCTGGGACCTTCGATCTTGGAGGGCTATATGAAGCAATTGA<br>GGAGTGCCTGATTAACGATCCCTGGGTTTTGCTTAATGCGTCTTGGTTCAACTCCTTCCTCACACATGCACTGAAATA-
EPI_ISL_2234830	>A/turkey/Ireland/020140_20VIR5487-6/2020_H6N1_2021-03-01<br>ATGGAAGACTTTGTGCGACAATGCTTCAATCCAATGATTGTCGAGCTTGCGGAAAAGGCAATGAAAGAATATGGGGAAGATCCGAAAATCGAAACGAACAAATTTGCCGCAATATGCACACACTTAGAAGTCTGTTTCATGTATTCGGATTTTCACTTTATTAATGAACGGGGCGAATCAATAATTGTAGAATCTGGCGATCCGAATGCATTACTGAAACACCGATTTGAGA<br>TAATTGAAGGGAGAGACCGAACAATGGCCTGGACAGTGGTAAATAGTATCTGCAACACCACAGGAGTCGATAAGCCTAAATTCCTCCCAGATTTGTATGACTACAAAGAGAACCGATTCATTGAAATTGGAGTAACACGAAGGGAAGTCCATATCTACTATCTAGAAAAAGCCAACAAGATAAAATCAGAGAAGACACACATTCACATATTCTCATTCACTGGAGAGGAA<br>ATGGCCACCAAAGCGGACTATACCCTTGACGAGGAGAGCAGAGCAAGAATCAAAACCAGGCTGTTCACTATAAGACAAGAAATGGCCAGTAGGGGTCTATGGGATTCCTTTCGTCAGTCCGAGAGAGGCGAAGAGACAATTGAAGAAAGATTTGAAATCACAGGAACCATGCGCAGGCTTGCCGACCAAAGTCTCCCACCGAACTTCTCCAGCCTTGAAAACTTTAGAG<br>CCTATGTGGATGGATTCGAACCGAACGGCTGCATTGAGGGCAAGCTTTCTCAAATGTCAAAAGAAGTGAACGCCAGGATTGAGCCATTTCTGAAGACAACACCACGCCCTCTCAGATTACCTGATGGGCCTCCCTGCTCTCAGCGGTCGAAGTTCTTACTGATGGATGCCCTTAAATTAAGCATCGAAGACCCGAGCCATGAAGGAGAAGGCATACCGCTATATGATGCAA<br>TCAAATGCATGAAGACATTTTTCGGCTGGAAAGAGCCCAACATCGTGAAACCACATGAAAAAGGCATAAACCCCAATTACCTCCTGGCTTGGAAGCAGGTGCTGGCAGAACTCCAAGATATTGAAAATGAGGAGAAAATTCCAAAAACAAAGAATATGAAGAAAACAAGTCAATTAAAGTGGGCACTTGGTGAGAACAGGGCACCAGAGAAAGTAGACTTTGAGGACT<br>GCAAAGACGTCAGCGATCTAAGACAATATGATAGTGATGAACCAGAGTCTAGGTCGCTAGCAAGCTGGATCCAGAGCGAATTCAACAAAGCATGCGAATTGACAGATTCAAGTTGGATTGAACTTGATGAAATAGGAGAGGACGTTGCTCCAATTGAGCACATTGCGAGTATGAGAAGAAACTACTTCACAGCAGAAGTATCCCATTGTAGGGCTACTGAATACATAATG<br>AAGGGAGTGTACATAAACACAGCCCTGTTGAATGCATCCTGTGCAGCCATGGATGACTTTCAATTGATTCCAATGATAAGCAAGTGCAGAACCAAAGAAGGAAGACGGAAGACAAATCTGTATGGATTCATCATAAAAGGAAGATCCCATTTGAGGAATGACACCGATGTAGTAAACTTTGTGAGCATGGAATTCTCCCTTACGGACCCGAGGCTGGAGCCACACAAATG<br>GGAAAAGTACTGTGTTCTCGAGATAGGAGACATGCTCCTACGGACTGCAATAGGCCAAGTGTCAAGGCCCATGTTCCTGTATGTGAGAACCAATGGGACTTCCAAGATCAAGATGAAATGGGGCATGGAGATGAGGCGGTGCCTTCTTCAATCCCTTCAACAAATTGAGAGCATGATTGAGGCCGAGTCCTCTGTCAAAGAGAAAGACATGACCAAAGAATTCTTTGAAA<br>ACAAATCAGAAACATGGCCAATTGGAGAATCACCTAAAGGGGTGGAGGAAGGCTCCATTGGGAAGGTGTGCAGAACATTACTAGCAAAATCTGTATTCAACAGCCTATATGCATCTCCACAACTCGAGGGGTTTTCGGCTGAATCAAGAAAATTGCTTCTCATTGTTCAGGCACTTAGGGACAACCTGGAACCTGGGACCTTCGATCTTGGAGGGCTATATGAAGCAATTG<br>AGGAGTGCCTGATTAACGATCCCTGGGTTTTGCTTAATGCGTCTTGGTTCAACTCCTTCCTCACACATGCACTGAAATA-
EPI_ISL_13778474	>A/European_Herring_Gull/Netherlands/7/2022_H5N1_2022-06-07<br>ATGGAAGACTTTGTGCGACAATGCTTCAATCCGATGATTGTCGAGCTTGCGGAAAAGGCAATGAAAGAATATGGGGAGGATCCGAAAATTGAAACAAATAAGTTTGCTGCAATATGCACACACTTAGAAGTCTGTTTCATGTATTCGGATTTCCACTTCATTGATGAGCGAGGAGAATCAATAATTGTAGAATCTGGCGACCCGAATGTATTGCTGAAACACCGATTTGAAA<br>TAATCGAAGGGAGAGACCGAACAATGGCCTGGACAGTGGTGAATAGTATTTGCAACACCACAGGAGTCGACAAACCCAAATTTCTTCCAGATTTGTATGACTACAAAGAAAACCGGTTCATCGAAATCGGAGTGACACGAAGGGAAGTTCACATATACTATCTGGAAAAAGCCAACAAGATAAAATCAGAGAAAACACACATTCACATATTCTCATTCACCGGGGAGGAA<br>ATGGCCACTAAAGCAGACTATACCCTTGATGAAGAGAGCAGAGCAAGAATCAAAACCAGGCTGTTCACTATAAGGCAAGAAATGGCCAGTAGGGGTTTATGGGATTCCTTTCGTCAGTCCGAGAGAGGCGAAGAGACAATTGAAGAAAGATTTGAAATCACAGGAACCATGCGCAGGCTTGCCGACCAAAGTCTCCCACCGAACTTCTCCAGCCTTGAAAACTTTAGAGC<br>CTATGTGGATGGATTCGAACCGAACGGCTGCATTGAGGGCAAGCTTTCTCAAATGTCAAAGGAAGTAAACGCCAGAATTGAGCCATTCTTGAAGACAACCCCACGGCCTCTTAGACTACCTGATGGCCCTCCTTGTTCTCAGCGGTCGAAATTCTTGCTGATGGATGCCCTTAAATTAAGCATAGAAGACCCAAGTCATGAGGGGGAAGGTATACCACTATATGATGCAAT<br>AAATGCATGAAGACATTTTTTGGCTGGAAAGAGCCCAATATTGTAAAACCACATGAAAAGGGCATAAACCCCAATTACCTCCTAGCTTGGAAGCAGGTGCTGTCAGAGCTCCAAGACCTTGAAAGTGAGGATAAAATCCCAAAAACAAAGAACATGAAGAAAACAAGCCAGTTGAAGTGGGCACTTGGTGAGAATATGGCACCAGAGAAAGTAGATTTTGAGGACTGCA<br>AAGATGTTAGCGATCTAAGACAGTATGACAGTGATGAACCAGAGTCTAGATCGTTGGCAAGCTGGATCCAGAGTGAATTCAACAAAGCATGCGAATTAACAGATTCAAGTTGGATTGAACTTGATGAAATAGGGGAAGATGTTGCTCCAATTGAGCACATTGCAAGCATGAGAAGGAACTATTTCACAGCGGAAGTATCCCATTGCAGGGCTACTGAGTATATAATGAAG<br>GGAGTGTATATAAATACAGCCCTGTTGAATGCATCCTGTGCAGCCATGGATGACTTCCAACTGATCCCGATGATAAGCAAGTGCAGAACCAAAGAAGGAAGACGGAAAACAAATCTGTATGGATTCATTATAAAAGGAAGATCTCATTTGAGAAATGATACAGATGTAGTAAACTTTGTAAGCATGGAATTCTCTCTCACTGACCCGAGGCTGGAGCCACACAAATGGGAA<br>AAGTACTGTGTTCTCGAGATAGGAGACATGCTCCTACGGACTGCAATAGGCCAAGTGTCAAGGCCCATGTTCCTTTATGTGAGAACCAATGGTACTTCCAAGATCAAAATGAAATGGGGTATGGAGATGAGAAGATGCCTCCTTCAATCTCTTCAACAAATTGAGAGCATGATTGAGGCTGAATCTTCTGTCAAAGAAAAGGACATGACCAAGGAGTTCTTTGAGAACAAA<br>TCTGAAACATGGCCAATTGGAGAGTCGCCCAAAGGGGTAGAGGAAGGATCCATTGGAAAGGTGTGCAGGACATTACTAGCAAAATCTGTGTTTAATAGTCTATATGCATCTCCACAACTCGAGGGGTTTTCAGCCGAGTCAAGAAAATTGCTTCTCATTGTTCAGGCACTTAGGGACAACCTGGAACCTGGGACCTTCGATCTTGGGGGGCTATATGAAGCAATTGAGGAG<br>TGCCTGATTAATGATCCCTGGGTTTTGCTTAATGCGTCTTGGTTCAACTCCTTCCTCACACATGCACTGAAATA-
EPI_ISL_14841913	>A/mallard/22P019377/France/2022_H5N1_2022-07-06<br>ATGGAAGACTTTGTGCGACAATGCTTCAATCCGATGATTGTCGAGCTTGCGGAAAAGGCAATGAAAGAATATGGGGAGGATCCGAAAATTGAAACAAATAAGTTTGCTGCAATATGCACACACTTAGAAGTCTGTTTCATGTATTCGGATTTCCACTTCATTGATGAGCGAGGAGAATCAATAATTGTAGAATCTGGCGACCCGAATGTATTGCTGAAACACCGATTTGAAA<br>TAATCGAAGGGAGAGACCGAACAATGGCCTGGACAGTGGTGAATAGTATTTGCAACACCACAGGAGTCGACAAACCCAAATTTCTTCCAGATTTGTATGACTACAAAGAAAACCGGTTCATCGAAATCGGAGTGACACGAAGGGAAGTTCACATATACTATCTGGAAAAAGCCAACAAGATAAAATCAGAGAAAACACACATTCACATATTCTCATTCACCGGGGAGGAA<br>ATGGCCACTAAAGCAGACTATACCCTTGATGAAGAGAGCAGAGCAAGAATCAAAACCAGGCTGTTCACTATAAGGCAAGAAATGGCCAGTAGGGGTTTATGGGATTCCTTTCGTCAGTCCGAGAGAGGCGAAGAGACAATTGAAGAAAGATTTGAAATCACAGGAACCATGCGCAGGCTTGCCGACCAAAGTCTCCCACCGAACTTCTCCAGCCTTGAAAACTTTAGAGC<br>CTATGTGGATGGATTCGAACCGAACGGCTGCATTGAGGGCAAGCTTTCTCAAATGTCAAAGGAAGTAAACGCCAGAATTGAGCCATTCTTGAAGACAACCCCACGGCCTCTTAGACTACCTGATGGCCCTCCTTGTTCTCAGCGGTCGAAATTCTTGCTGATGGATGCCCTTAAATTAAGCATAGAAGACCCGAGTCATGAGGGGGAAGGTATACCACTATATGATGCAATC<br>AAATGCATGAAGACATTTTTTGGCTGGAAAGAGCCCAATATTGTAAAACCACATGAAAAGGGCATAAACCCCAATTACCTCCTAGCTTGGAAGCAGGTGCTGTCAGAGCTCCAAGACCTTGAAAGTGAGGATAAAATCCCAAAAACAAAGAACATGAAGAAAACAAGCCAGTTGAAGTGGGCACTTGGTGAGAATATGGCACCAGAGAAAGTAGATTTTGAGGACTGCA<br>AAGATGTTAGCGATCTAAGACAGTATGACAGTGATGAACCAGAGTCTAGATCGTTGGCAAGCTGGATCCAGAGTGAATTCAACAAAGCATGCGAATTAACAGATTCAAGTTGGATTGAACTTGATGAAATAGGGGAAGATGTTGCTCCAATTGAGCACATTGCAAGCATGAGAAGGAACTATTTCACAGCGGAAGTATCCCATTGCAGGGCTACTGAGTATATAATGAAG<br>GGAGTGTATATAAATACAGCCCTGTTGAATGCATCCTGTGCAGCCATGGATGACTTCCAACTGATCCCGATGATAAGCAAGTGCAGAACCAAAGAAGGAAGACGGAAAACAAATCTGTATGGATTCATTATAAAAGGAAGATCTCATTTGAGAAATGATACAGATGTAGTAAACTTTGTAAGCATGGAATTCTCTCTCACTGACCCGAGGCTGGAGCCACACAAATGGGAA<br>AAGTACTGTGTTCTCGAGATAGGAGACATGCTCCTACGGACTGCAATAGGCCAAGTGTCAAGGCCCATGTTCCTTTATGTGAGAACCAATGGTACTTCCAAGATCAAAATGAAATGGGGTATGGAGATGAGAAGATGCCTCCTTCAATCTCTTCAACAAATTGAGAGCATGATTGAGGCTGAATCTTCTGTCAAAGAAAAGGACATGACCAAGGAGTTCTTTGAGAACAAA<br>TCTGAAACATGGCCAATTGGAGAGTCGCCCAAAGGGGTAGAGGAAGGATCCATTGGAAAGGTGTGCAGGACATTACTAGCAAAATCTGTGTTTAATAGTCTATATGCATCTCCACAACTCGAGGGGTTTTCAGCCGAGTCAAGAAAATTGCTTCTCATTGTTCAGGCACTTAGGGACAACCTGGAACCTGGGACCTTCGATCTTGGGGGGCTATATGAAGCAATTGAGGAG<br>TGCCTGATTAATGATCCCTGGGTTTTGCTTAATGCGTCTTGGTTCAACTCCTTCCTCACACATGCACTGAAATA-
EPI_ISL_14494796	>A/Vulpes_vulpes/Belgium/9031_0008/2022_H5N1_2022-06-16<br>ATGGAAGACTTTGTGCGACAATGCTTCAATCCGATGATTGTCGAGCTTGCGGAAAAGGCAATGAAAGAATATGGGGAGGATCCGAAAATTGAAACAAATAAGTTTGCTGCAATATGCACACACTTAGAAGTCTGTTTCATGTATTCGGATTTCCACTTCATTGATGAGCGAGGAGAATCAATAATTGTAGAATCTGGCGACCCGAATGTATTGCTGAAACACCGATTTGAAA<br>TAATCGAAGGGAGAGACCGAACAATGGCCTGGACAGTGGTGAATAGTATTTGCAACACCACAGGAGTCGACAAACCCAAATTTCTTCCAGATTTGTATGACTACAAAGAAAACCGGTTCATCGAAATCGGAGTGACACGAAGGGAAGTTCACATATACTATCTGGAAAAAGCCAACAAGATAAAATCAGAGAAAACACACATTCACATATTCTCATTCACCGGGGAGGAA<br>ATGGCCACTAAAGCAGACTATACCCTTGATGAAGAGAGCAGAGCAAGGATCAAAACCAGGCTGTTCACTATAAGGCAAGAAATGGCCAGTAGGGGTTTATGGGATTCCTTTCGTCAGTCCGAGAGAGGCGAAGAGACAATTGAAGAAAGATTTGAAATCACAGGAACCATGCGCAGGCTTGCCGACCAAAGTCTCCCACCGAACTTCTCCAGCCTTGAAAACTTTAGAG<br>CCTATGTGGATGGATTCGAACCGAACGGCTGCATTGAGGGCAAGCTTTCTCAAATGTCAAAGGAAGTAAACGCCAGAATTGAGCCATTCTTGAAGACAACCCCACGGCCTCTTAGACTACCTGATGGCCCTCCTTGTTCTCAGCGGTCGAAATTCTTGCTGATGGATGCCCTTAAATTAAGCATAGAAGACCCAAGTCATGAGGGGGAAGGTATACCACTATATGATGCAAT<br>CAAATGCATGAAGACATTTTTTGGCTGGAAAGAGCCCAATATTGTAAAACCACATGAAAAGGGCATAAACCCCAATTACCTCCTAGCTTGGAAGCAGGTGCTGTCAGAGCTCCAAGACCTTGAAAGTGAGGATAAAATCCCAAAAACAAAGAACATGAAGAAAACAAGCCAGTTGAAGTGGGCACTTGGTGAGAATATGGCACCAGAGAAAGTAGATTTTGAGGACTGC<br>AAAGATGTTAGCGATCTAAGACAGTATGACAGTGATGAACCAGAGTCTAGATCGTTGGCAAGCTGGATCCAGAGTGAATTCAACAAAGCATGCGAATTAACAGATTCAAGTTGGATTGAACTTGATGAAATAGGGGAAGATGTTGCTCCAATTGAGCACATTGCAAGCATGAGAAGGAACTATTTCACAGCGGAAGTATCCCATTGCAGGGCTACTGAGTATATAATGAA<br>GGGAGTGTATATAAATACAGCCCTGTTGAATGCATCCTGTGCAGCCATGGATGACTTCCAACTGATCCCGATGATAAGCAAGTGCAGAACCAAAGAAGGAAGACGGAAAACAAATCTGTATGGATTCATTATAAAAGGAAGATCTCATTTGAGAAATGATACAGATGTAGTAAACTTTGTAAGCATGGAATTCTCTCTCACTGACCCGAGGCTGGAGCCACACAAATGGGA<br>AAAGTACTGTGTTCTCGAGATAGGAGACATGCTCCTACGGACTGCAATAGGCCAAGTGTCAAGGCCCATGTTCCTTTATGTGAGAACCAATGGTACTTCCAAGATCAAAATGAAATGGGGTATGGAGATGAGAAGATGCCTCCTTCAATCTCTTCAACAAATTGAGAGCATGATTGAGGCTGAATCTTCTGTCAAAGAAAAGGACATGACCAAGGAGTTCTTTGAGAACAA<br>ATCTGAAACATGGCCAATTGGAGAGTCGCCCAAAGGGGTAGAGGAAGGATCCATTGGAAAGGTGTGCAGGACATTACTAGCAAAATCTGTGTTTAATAGTCTATATGCATCTCCACAACTCGAGGGGTTTTCAGCCGAGTCAAGAAAATTGCTTCTCATTGTTCAGGCACTTAGGGACAACCTGGAACCTGGGACCTTCGATCTTGGGGGGCTATATGAAGCAATTGAGGA<br>GTGCCTGATTAATGATCCCTGGGTTTTGCTTAATGCGTCTTGGTTCAACTCCTTCCTCACACATGCACTGAAATA-
EPI_ISL_13778469	>A/European_Herring_Gull/Netherlands/6/2022_H5N1_2022-06-07<br>ATGGAAGACTTTGTGCGACAATGCTTCAATCCGATGATTGTCGAGCTTGCGGAAAAGGCAATGAAAGAATATGGGGAGGATCCGAAAATTGAAACAAATAAGTTTGCTGCAATATGCACACACTTAGAAGTCTGTTTCATGTATTCGGATTTCCACTTCATTGATGAGCGAGGAGAATCAATAATTGTAGAATCTGGCGACCCGAATGTATTGCTGAAACACCGATTTGAAA<br>TAATCGAAGGGAGAGACCGAACAATGGCCTGGACAGTGGTGAATAGTATTTGCAACACCACAGGAGTCGACAAACCCAAATTTCTTCCAGATTTGTATGACTACAAAGAAAACCGGTTCATCGAAATCGGAGTGACACGAAGGGAAGTTCACATATACTATCTGGAAAAAGCCAACAAGATAAAATCAGAGAAAACACACATTCACATATTCTCATTCACCGGGGAGGAA<br>ATGGCCACTAAAGCAGACTATACCCTTGATGAAGAGAGCAGAGCAAGGATCAAAACCAGGCTGTTCACTATAAGGCAAGAAATGGCCAGTAGGGGTTTATGGGATTCCTTTCGTCAGTCCGAGAGAGGCGAAGAGACAATTGAAGAAAGATTTGAAATCACAGGAACCATGCACAGGCTTGCCGACCAAAGTCTCCCACCGAACTTCTCCAGCCTTGAAAACTTTAGAGC<br>CTATGTGGATGGATTCGAACCGAACGGCTGCATTGAGGGCAAGCTTTCTCAAATGTCAAAGGAAGTAAACGCCAGAATTGAGCCATTCTTGAAGACAACCCCACGGCCTCTTAGACTACCTGATGGCCCTCCTTGTTCTCAGCGGTCGAAATTCTTGCTGATGGATGCCCTTAAATTAAGCATAGAAGACCCAAGTCATGAGGGGGAAGGTATACCACTATATGATGCAATC<br>AAATGCATGAAGACATTTTTTGGCTGGAAAGAGCCCAATATTGTAAAACCACATGAAAAGGGCATAAACCCCAATTACCTCCTAGCTTGGAAGCAGGTGCTGTCAGAGCTCCAAGACCTTGAAAGTGAGGATAAAATCCCAAAAACAAAGAACATGAAGAAAACAAGCCAGTTGAAGTGGGCACTTGGTGAGAATATGGCACCAGAGAAAGTAGATTTTGAGGACTGCA<br>AAGATGTTAGCGATCTAAGACAGTATGACAGTGATGAACCAGAGTCTAGATCGTTGGCAAGCTGGATCCAGAGTGAATTCAACAAAGCATGCGAATTAACAGATTCAAGTTGGATTGAACTTGATGAAATAGGGGAAGATGTTGCTCCAATTGAGCACATTGCAAGCATGAGAAGGAACTATTTCACAGCGGAAGTATCCCATTGCAGGGCTACTGAGTATATAATGAAG<br>GGAGTGTATATAAATACAGCCCTGTTGAATGCATCCTGTGCAGCCATGGATGACTTCCAACTGATCCCGATGATAAGCAAGTGCAGAACCAAAGAAGGAAGACGGAAAACAAATCTGTATGGATTCATTATAAAAGGAAGATCTCATTTGAGAAATGATACAGATGTAGTAAACTTTGTAAGCATGGAATTCTCTCTCACTGACCCGAGGCTGGAGCCACACAAATGGGAA<br>AAGTACTGTGTTCTCGAGATAGGAGACATGCTCCTACGGACTGCAATAGGCCAAGTGTCAAGGCCCATGTTCCTTTATGTGAGAACCAATGGTACTTCCAAGATCAAAATGAAATGGGGTATGGAGATGAGAAGATGCCTCCTTCAATCTCTTCAACAAATTGAGAGCATGATTGAGGCTGAATCTTCTGTCAAAGAAAAGGACATGACCAAGGAGTTCTTTGAGAACAAA<br>TCTGAAACATGGCCAATTGGAGAGTCGCCCAAAGGGGTAGAGGAAGGATCCATTGGAAAGGTGTGCAGGACATTACTAGCAAAATCTGTGTTTAATAGTCTATATGCATCTCCACAACTCGAGGGGTTTTCAGCCGAGTCAAGAAAATTGCTTCTCATTGTTCAGGCACTTAGGGACAACCTGGAACCTGGGACCTTCGATCTTGGGGGGCTATATGAAGCAATTGAGGAG<br>TGCCTGATTAATGATCCCTGGGTTTTGCTTAATGCGTCTTGGTTCAACTCCTTCCTCACACATGCACTGAAATA-
EPI_ISL_7622539	>A/Chroicocephalus_ridibundus/Belgium/13464/2020_H13N8_2021-12-09<br>ATGGAAGACTTTGTGCGACAATGCTTCAATCCGATGATTGTCGAGCTTGCGGAAAAGGCAATGAAAGAATATGGGGAAGATCCGAAAATTGAAACAAATAAGTTTGCTGCAATATGCACACACTTAGAAGTCTGTTTCATGTATTCGGATTTCCACTTCATTGATGAGCGAGGCGAATCAATAATTGTAGAATCTGGCGACCCGAATGTTTTGCTGAAACACCGATTTGAAA<br>TAATCGAAGGGAGAGACCGAACAATGGCCTGGACAGTGGTGAATAGTATCTGCAACACCACAGGAGTCGACAAACCCAAATTCCTTCCAGATTTGTATGACTACAAAGAAAACCGATTCATTGAAATCGGAGTGACACGAAGAGAAGTTCACATATACTATCTGGAAAAAGCCAACAAGATAAAATCAGAAAAAACACACATTCACATATTCTCATTCACCGGGGAGGAA<br>ATGGCCACTAAAGCAGACTATACCCTTGATGAAGAGAGCAGAGCAAGAATCAAAACCAGACTGTTCACTATAAGGCAAGAAATGGCCAGTAGGGGTTTATGGGATTCCTTTCGTCAGTCCGAGAGAGGCGAAGAGACAATTGAAGAAAGATTTGAAATTACAGGAACCATGCGCAGGCTTGCCGACCAAAGTCTCCCACCGAACTTCTCCAGCCTTGAAAACTTTAGAGC<br>CTATGTGGATGGATTCGAACCGAACGGCTGCATTGAGGGCAAGCTTTCTCAAATGTCAAAGGAAGTAAACGCCAGAATTGAGCCATTCTTGAAGACAACCCCACGGCCTCTTAGACTACCTGAAGGCCCTCCTTGTTCTCAGCGGTCGAAATTTTTGCTGATGGATGCCCTTAAATTAAGCATAGAAGACCCAAGTCATGAGGGGGAAGGGATACCACTATATGATGCAAT<br>CAAATGCATGAAGACGTTTTTTGGCTGGAAAGAGCCCAATATCGTAAAACCACATGAAAAGGGCATAAACCCCAATTACCTCCTGGCTTGGAAGCAGGTGCTGGCAGAGCTCCAAGACCTTGAAAGTGAGGATAAAATCCCAAAAACAAAGAACATGAAGAAAACAAGCCAGTTGAAGTGGGCACTTGGTGAGAATATGGCACCAGAGAAAGTAGACTTTGAGGACTG<br>CAAAGATGTTAGCGATCTGAGACAGTATGACAGTGATGAGCCAGAGTCTAGATCGTTGGCAAGCTGGATCCAGAGTGAATTCAACAAAGCATGCGAATTAACAGATTCAAGTTGGATTGAACTTGATGAAATAGGGGAAGATGTTGCTCCAATTGAGCACATTGCAAGCATGAGAAGGAACTATTTCACAGCGGAAGTATCCCATTGCAGGGCTACTGAGTATATAATGA<br>AGGGAGTGTATATAAATACAGCCCTGTTGAATGCATCCTGTGCAGCCATGGATGACTTCCAACTGATCCCGATGATAAGCAAGTGCAGAACCAAAAGAAGGAAGACGGAAAACAAATCTGTATGGATTCATTATAAAAGGAAGATCTCATTTGAGAAATGATACAGATGTAGTAAACTTTGTGAGCATGGAATTCTCTCTCACTGACCCGAGGCTGGAGCCACACAAATGG<br>GAAAAGTACTGTGTTCTCGAGATAGGAGACATGCTCCTACGGACTGCAATAGGCCAAGTGTCAAGGCCCATGTTCCTTTATGTGAGAACCAATGGTACTTCCAAGATCAAAATGAAATGGGGTATGGAGATGAGAAGATGCCTCCTTCAATCCCTTCAACAAATTGAGAGCATGATCGAGGCTGAGTCTTCTGTCAAAGAAAAGGACATGACCAAGGAGTTCTTTGAAAAC<br>AAATCTGAAACATGGCCAATTGGAGAGTCGCCCAAAGGGGTAGAGGAAGGATCCATTGGAAAGGTGTGCAGGACATTACTAGCAAAATCTGTGTTTAATAGTCTATATGCATCTCCACAACTCGAGGGGTTTTCAGCTGAGTCAAGAAAATTGCTTCTCATTGTTCAGGCACTTAGGGACAACCTGGAACCTGGGACCTTCGATCTTGGGGGGCTATATGAAGCAATTGAG<br>GAGTGCCTGATTAATGATCCCTGGGTTTTGCTTAATGCGTCTTGGTTCAACTCCTTCCTCACACATGCACTGAAATA-
EPI_ISL_3319119	>A/Common_guillemot/Sweden/SVA210729SZ0317/FB002610/I-2021_H13N8_2021-07-13<br>ATGGAAGACTTTGTGCGACAATGCTTCAATCCGATGATTGTCGAGCTTGCGGAAAAGGCAATGAAAGAATATGGGGAAGATCCGAAAATTGAAACAAATAAGTTTGCTGCAATATGCACACACTTAGAAGTCTGTTTCATGTATTCGGATTTCCACTTCATTGATGAACGAGGCGAATCAATAATTGTAGAATCTGGCGACCCGAATGTATTGCTGAAACACCGATTTGAAA<br>TAATCGAAGGGAGAGACCGAACAATGGCCTGGACAGTGGTGAATAGTATCTGCAACACCACAGGAGTAGACAAACCCAAATTCCTCCCAGATTTGTATGACTACAAAGAAAACCGATTCATTGAAATTGGGGTGACACGAAGAGAAGTTCACATATACTATCTGGAAAAAGCCAACAAGATAAAGTCAGAGAAAACACACATTCACATATTCTCATTCACCGGGGAGGAA<br>ATGGCCACTAAAGCAGACTATACCCTTGATGAAGAGAGCAGAGCGAGAATCAAAACCAGGCTGTTCACTATAAGGCAAGAAATGGCCAGTAGGGGTTTATGGGATTCCTTTCGTCAGTCCGAGAGAGGCGAAGAGACAATTGAAGAAAGATTTGAAATCACAGGAACCATGCGCAGGCTTGCCGACCAAAGTCTCCCACCGAACTTCTCCAGCCTTGAAAACTTTAGAG<br>CCTATGTGGATGGATTCGAACCGAACGGCTGCATTGAGGGCAAGCTTTCTCAAATGTCAAAAGAAGTAAACGCCAGAATTGAGCCATTCTTGAAAACAACCCCACGGCCTCTTAGACTACCTGATGGCCCTCCTTGTTCTCAGCGGTCGAAATTCTTGCTGATGGATGCCCTTAAATTAAGCATAGAAGACCCAAGTCATGAGGGGGAAGGTATACCACTATATGATGCAAT<br>CAAATGCATGAAGACATTTTTTGGCTGGAAAGAGCCCAATATCGTAAAACCACATGAAAAAGGCATAAACCCCAATTACCTCCTGGCTTGGAAGCAGGTGCTGGCAGAGCTCCAAGACCTTGAAAGTGAGGATAAAATCCCAAAAACAAAGAACATGAAGAAAACAAGCCAATTGAAGTGGGCACTTGGTGAGAATATGGCACCAGAGAAAGTAGACTTTGAGGACTGC<br>AAAGATGTTAGCGATCTAAGACAGTATGACAGTGATGAACCAGAGTCTAGATCGTTGGCAAGCTGGATCCAGAGTGAATTCAACAAAGCATGCGAATTAACTGATTCAAGTTGGATTGAACTTGATGAAATAGGGGAAGATGTTGCTCCAATTGAGCACATTGCAAGCATGAGAAGGAACTATTTCACAGCGGAAGTATCCCATTGCAGGGCTACTGAGTATATAATGAA<br>GGGAGTGTATATAAATACAGCCCTGTTGAATGCATCCTGTGCAGCCATGGATGACTTCCAACTGATCCCGATGATAAGCAAGTGCAGAACCAAAGAAGGAAGACGGAAAACAAATCTGTATGGATTCATTATAAAAGGAAGATCTCATTTGAGAAATGATACCGATGTAGTAAACTTTGTGAGCATGGAGTTCTCTCTCACTGACCCGAGGCTAGAGCCACACAAATGGGA<br>AAAGTACTGTGTTCTCGAGATAGGAGACATGCTCCTACGGACTGCAATAGGCCAAGTGTCAAGGCCCATGTTCCTTTATGTGAGAACCAATGGCACTTCCAAGATCAAAATGAAATGGGGTATGGAGATGAGAAGATGCCTCCTTCAATCCCTTCAACAAATTGAGAGCATGATTGAGGCTGAGTCTTCTGTCAAAGAAAAGGACATGACCAAGGATTTCTTTGAAAACAA<br>ATCTGAAACATGGCCAATTGGAGAATCGCCCAAAGGGGTAGAGGAAGGATCCATTGGAAAGGTGTGCAGGACATTACTAGCAAAATCTGTGTTTAACAGTCTATATGCATCTCCACAACTTGAGGGGTTTTCAGCTGAATCAAGAAAATTGCTTCTCATTGTTCAGGCACTTAGGGACAACCTGGAACCTGGAACTTTCGATCTTGGGGGGCTATATGAAGCAATTGAGGA<br>GTGCCTGATTAATGATCCCTGGGTTTTGCTTAATGCGTCTTGGTTCAACTCCTTCCTCACACATGCATTGAGATA-
EPI_ISL_17978928	>A/herring_gull/Poland/P092/2020_H16N3_2020-10-28<br>ATGGAAGACTTTGTGCGACAATGCTTCAATCCGATGATTGTCGAGCTTGCGGAAAAGGCAATGAAAGAATATGGGGAAGATCCGAAAATTGAAACAAATAAGTTTGCTGCAATATGCACACACTTAGAAGTCTGTTTCATGTATTCGGATTTCCACTTCATTGATGAACGAGGCGAATCAATAATTGTAGAATCTGGCGACCCGAATGTATTGCTGAAACACCGATTTGAAA<br>TAATTGAAGGGAGAGACCGAACAATGGCCTGGACAGTGGTGAATAGTATCTGCAACACCACAGGAGTAGACAAACCCAAATTCCTCCCAGATTTGTATGACTACAAAGAAAACCGATTCATTGAAATTGGGGTGACACGAAGAGAAGTTCACATATACTATCTGGAAAAAGCCAACAAGATAAAGTCAGAGAAAACACACATTCACATATTCTCATTCACCGGGGAGGAA<br>ATGGCCACTAAAGCAGACTATACCCTTGATGAAGAGAGCAGAGCAAGAATCAAAACCAGGCTGTTCACTATAAGGCAAGAAATGGCCAGTAGGGGTTTATGGGATTCCTTTCGTCAGTCCGAGAGAGGCGAAGAGACAATTGAAGAAAGATTTGAAATCACAGGAACCATGCGCAGGCTTGCCGACCAAAGTCTCCCACCGAACTTCTCCAGCCTTGAAAACTTTAGAGC<br>CTATGTGGATGGATTCGAACCGAACGGCTGCATTGAGGGCAAGCTTTCTCAAATGTCAAAAGAAGTAAACGCCAGAATTGAGCCATTCTTGAAAACAACCCCACGGCCTCTTAGACTACCTGATGGCCCTCCTTGTTCTCAGCGGTCGAAATTCTTGCTGATGGATGCCCTTAAATTAAGCATAGAAGACCCAAGTCATGAGGGGGAAGGTATACCACTATATGATGCAATC<br>AAATGCATGAAGACATTTTTTGGCTGGAAAGAGCCCAATATCGTAAAACCACATGAAAAAGGCATAAACCCCAATTACCTCCTGGCTTGGAAGCAGGTGCTGGCAGAGCTCCAAGACCTTGAAAGTGAGGATAAAATCCCAAAAACAAAGAACATGAAGAAAACAAGCCAATTGAAGTGGGCACTTGGTGAAAATATGGCACCAGAGAAAGTAGACTTTGAGGACTGCA<br>AAGATGTTAGCGATCTAAGACAGTATGACAGTGATGAACCAGAGTCTAGATCGTTGGCAAGCTGGATCCAGAGTGAATTCAACAAAGCATGCGAATTAACTGATTCAAGTTGGATTGAACTTGATGAAATAGGGGAAGATGTTGCTCCAATTGAGCACATTGCAAGCATGAGAAGGAACTATTTCACAGCGGAAGTATCCCATTGCAGGGCTACTGAGTATATAATGAAG<br>GGAGTGTATATAAATACAGCCCTGTTGAATGCATCCTGTGCAGCCATGGATGACTTCCAACTGATCCCGATGATAAGCAAGTGCAGAACCAAAGAAGGAAGACGGAAAACAAATCTGTATGGATTCATTATAAAAGGAAGATCTCATTTGAGAAATGATACAGATGTAGTAAACTTTGTGAGCATGGAGTTCTCTCTCACTGACCCGAGGCTGGAGCCACACAAATGGGA<br>AAAGTACTGTGTTCTCGAGATAGGAGACATGCTCCTACGGACTGCAATAGGCCAAGTGTCAAGGCCCATGTTCCTTTATGTGAGAACCAATGGCACTTCCAAGATCAAAATGAAATGGGGTATGGAGATGAGAAGATGCCTCCTTCAATCCCTTCAACAAATTGAGAGCATGATTGAGGCTGAGTCTTCTGTCAAAGAAAAGGACATGACCAAGGAGTTCTTTGAAAACAA<br>ATCTGAAACATGGCCAATTGGAGAGTCGCCCAAAGGGGTAGAGGAAGGATCCATTGGAAAGGTGTGCAGGACATTACTAGCAAAATCTGTGTTTAACAGTCTATATGCATCTCCACAACTCGAGGGATTTTCAGCTGAATCAAGAAAATTGCTTCTCATTGTTCAGGCACTTAGGGACAACCTGGAACCTGGAACTTTCGATCTTGGGGGGCTATATGAAGCAATTGAGGA<br>GTGCCTGATTAATGATCCCTGGGTTTTGCTTAATGCGTCTTGGTTCAACTCCTTCCTCACACATGCATTGAGATA-
EPI_ISL_17978956	>A/black-headed_gull/Poland/P075/2021_H9N7_2021-02-09<br>ATGGAAGACTTTGTGCGACAATGCTTCAATCCGATGATTGTCGAGCTTGCGGAAAAGGCAATGAAAGAATATGGGGAAGATCCGAAAATTGAAACAAATAAGTTTGCTGCAATATGCACACACTTGGAAGTCTGTTTCATGTATTCGGATTTCCACTTCATTGATGAGCGAGGCGAATCAATAATTGTAGAATCTGGCGACCCGAATGTATTGCTGAAACACCGATTTGAAA<br>TAATCGAAGGGAGAGACCGAACAATGGCCTGGACAGTGGTGAATAGTATCTGCAACACCACAGGAGTAGACAAACCCAAATTCCTTCCAGATTTGTATGACTACAAAGAAAGCCGATTCATTGAAATTGGAGTGACACGAAGAGAAGTTCACATATACTATCTGGAAAAAGCCAACAAGATAAAATCAGAGAAAACACACATTCACATATTCTCATTCACCGGGGAGGAA<br>ATGGCAACTAAAGCAGACTATACCCTTGATGAAGAGAGCAGAGCAAGAATCAAAACCAGGCTGTTCACTATAAGGCAAGAAATGGCCAGTAGGGGTTTGTGGGATTCCTTTCGTCAGTCCGAGAGAGGCGAAGAGACAATTGAAGAAAGATTTGAAATCACAGGAACCATGCGCAGGCTTGCCGACCAAAGTCTCCCACCGAACTTCTCCAGCCTTGAAAACTTTAGAG<br>CCTATGTGGATGGATTCGAACCGAACGGCTGCATTGAGGGCAAGCTTTCTCAAATGTCAAAGGAAGTAAACGCCAGAATTGAGCCATTCTTGAAGACAACCCCACGGCCTCTTAGACTACCTGATGGCCCTCCTTGTTCTCAACGGTCAAAATTCTTGCTGATGGATGCCCTTAAATTAAGTATAGAAGACCCAAGTCATGAGGGGGAAGGTATACCACTATATGATGCAAT<br>CAAATGCATGAAGACATTTTTTGGCTGGAAAGAGCCCAATATCGTAAAACCACATGAAAAAGGCATAAACCCCAATTACCTCCTTGCTTGGAAGCAGGTACTGGCAGAGCTCCAAGACCTTGAAAGTGAGGATAAAATCCCAAAAACAAAGAACATGAAGAAAACAAGCCAATTGAAGTGGGCCCTTGGTGAGAACATGGCACCAGAGAAAGTAGACTTTGAGGACTGC<br>AAAGATGTTAGCGATCTAAGACAGTATGACAGCGATGAACCAGAATCTAGATCGCTGGCAAGCTGGATCCAGAGTGAGTTCAACAAAGCATGCGAATTAACGGATTCAAGTTGGATTGAACTTGATGAAATAGGGGAGGATGTTGCTCCAATTGAGCACATTGCAAGCATGAGAAGGAACTATTTCACAGCAGAAGTATCCCATTGCAGGGCTACTGAGTATATAATGAA<br>GGGAGTGTATATAAATACAGCCCTGTTGAATGCATCCTGTGCAGCCATGGATGACTTCCAACTGATCCCGATGATAAGCAAGTGCAGAACCAAAGAAGGAAGAGGCGGAAAACAAATCTGTATGGATTCATCATAAAAGGAAGATCTCATTTGAGAAATGATACAGATGTAGTAAACTTTGTGAGCATGGAATTCTCTCTCACTGACCCGAGGCTGGAGCCACACAAATGGG<br>AAAAGTACTGTGTTATCGAGATAGGAGACATGCTCCTACGGACTGCAATAGGCCAAGTGTCAAGGCCCATGTTCCTTTATGTGAGAACCAATGGTACTTCAAAGATCAAAATGAAATGGGGTATGGAGATGAGAAGATGCCTCCTTCAATCCCTTCAACAAATTGAGAGCATGATTGAGGCTGAGTCTTCTGTCAGAGAAAAGGACATGACCAAAGAGTTCTTTGAAAACA<br>AATCTGAAACATGGCCAATTGGAGAGTCGCCCAAAGGGGTAGAGGAAGGATCTATTGGAAAGGTGTGCAGGACATTACTAGCAAAATCTGTGTTTAACAGTCTATATGCATCTCCACAACTCGAGGGGTTTTCAGCTGAGTCAAGAAAATTGCTTCTCATTGTTCAGGCACTTAGGGACAACCTGGAACCTGGAACCTTCGATCTTGGGGGGCTATATGAAGCAATTGAGG<br>AGTGCCTGATTAATGATCCCTGGGTTTTGCTTAATGCGTCTTGGTTCAACTCCTTCCTCACACATGCACTGAAATA-
EPI_ISL_267201	>A/black-headed_gull/Netherlands/1/2015_H16N3_2015-01-27<br>ATGGAAGACTTTGTGCGACAATGCTTCAATCCAATGATTGTCGAGCTTGCGGAAAAGGCAATGAAAGAATATGGGGAAGATCCGAAAATCGAAACAAACAAATTTGCCGCAATATGCACACACTTAGAAGTCTGTTTCATGTACTCGGATTTCCACTTCATTGATGAGCGAGGCGAATCAATAATTGTAGAATCTGGCGACCCGAATGTATTACTGAAACACCGATTTGAAA<br>TAATTGAAGGGAGAGACCGAACAATGGCCTGGACAGTGGTAAATAGTATCTGCAACACCACAGGAGTCGACAAACCCAAATTCCTTCCAGATTTGTATGACTACAAAGAGAACCGATTCATTGAAATTGGAGTGACAAGAAGGGAAGTTCACATATACTATCTGGAAAAAGCCAACAAGATAAAATCAGAGAAAACACACATTCACATATTCTCATTCACCGGGGAGGAA<br>ATGGCCACCAAAGCGGACTATACCCTTGATGAAGAGAGCAGAGCAAGAATCAAAACCAGGCTATTCACTATAAGACAAGAAATGGCCAGTAGGGGTCTATGGGATTCCTTTCGTCAGTCCGAGAGAGGCGAAGAGACAATTGAAGAAAGATTTGAAATCACAGGAACCATGCGCAGGCTTGCCGACCAAAGTCTCCCACCGAACTTCTCCAGCCTTGAAAACTTTAGAG<br>CCTATGTGGATGGATTCGAACCGAACGGCTGCATTGAGGGCAAGCTTTCTCAAATGTCAAAAGAAGTAAACGCCAGAATTGAGCCATTCTTGAAGACAACCCCACGGCCTCTTAGACTGCCTGATGGCCCTCCCTGTTCTCAGCGGTCGAAGTTCTTGCTGATGGATGCCCTTAAATTAAGCATAGAAGACCCGAGTCATGAGGGGGAAGGTATACCACTATATGATGCAAT<br>CAAATGCATGAAGACATTTTTTGGCTGGAAAGAGCCCAATATCGTAAAACCACATGAAAAAGGCATAAACCCCAATTACCTCCTGGCTTGGAAGCAGGTGCTGGCAGAACTCCAAGATCTTGAAAGTGAGGAGAAAATTCCAAAAACAAAGAACATGAAGAAAACAAGCCAATTGAAGTGGGCACTTGGTGAGAATATGGCACCAGAGAAAGTAGACTTTGAGGACTGC<br>AAAGATGTCAGCGATCTAAGACAGTATGAAAGTGATGAACCAGAGTCTAGATCGCTGGCAAGCTGGATCCAGAGTGAATTCAACAAAGCATGCGAATTAACGGATTCGAGTTGGATTGAACTTGATGAAATAGGGGAAGATGTTGCTCCAATTGAGCACATTGCAAGCATGAGAAGGAACTATTTCACAGCGGAAGTATCCCATTGCAGGGCTACTGAGTATATAATGAA<br>GGGAGTGTATATAAATACAGCCCTGTTGAATGCATCCTGTGCAGCCATGGATGACTTCCAACTGATCCCGATGATAAGCAAATGCAGAACCAAAGAAGGAAGACGGAAGACAAATCTGTATGGATTCATTATAAAAGGAAGATCTCATTTGAGAAATGACACAGATGTAGTAAACTTTGTGAGCATGGAATTCTCTCTCACTGACCCGAGGCTGGAGCCACACAAATGGG<br>AAAAGTACTGTGTTCTCGAGATAGGAGACATGCTTCTACGGACTGCAATAGGCCAAGTGTCAAGGCCCATGTTCCTGTATGTGAGAACCAATGGGACTTCCAAGATCAAAATGAAATGGGGTATGGAGATGAGGCGATGCCTTCTTCAATCCCTTCAACAAATTGAGAGCATGATTGAGGCTGAGTCTTCTGTCAAAGAAAAGGACATGACCAAGGAATTCTTTGAAACCA<br>AATCAGAAACATGGCCAATTGGAGAATCGCCCAAAGGGGTAGAAGAAGGATCCATTGGAAAGGTGTGCAGAACATTACTAGCAAAATCTGTGTTCAACAGTCTATATGCATCTCCACAACTCGAGGGGTTTCAGCTGAATCAAGAAAATTGCTTCTCATTGTTCAGGCACTTAGGGACAACCTGGAACCTGGAACCTTTGATCTTGGGGGGCTATATGAAGCAATTGAGG<br>AGTGCCTGATTAATGATCCCTGGGTTTTGCTTAATGCGTCTTGGTTCAACTCCTTCCTCACACATGCACTGAAATA-

HA (H2)

Isolate ID	Sequence
EPI_ISL_18245819	>A/swan/Poland/MB152/2022_H2N3_2022-07-15<br>ATGGCAATCATTTATCTGATCCTTCTGTTTACAGTAGTGAGAGGGGACCAGATATGCATTGGATACCACTCCAACAATTCCACAGAAAAGGTCGACACAATTCTAGAAAGGAATGTCACTGTGACTCACGCTCAGGACATTCTTGAGAAGACTCACAATGGAAAATTATGCAAACTAAATGGAATCCCTCCACTTGAACTGGGGGATTGCAGCATCGCTGGATGGCTCCTTGG<br>AAATCCAGAATGTGATAGACTTCTAACTGTACCAGAATGGTCATATATAATGGAGAAAGAAAACCCAAGGAATGGTTTGTGCTACCCAGGCAGTTTCAATGACTATGAGGAATTGAAACACCTCCTTAGTAGTGTAACACATTTCGAAAAAGTGAAGATTCTGCCTAAAGATAGGTGGACACAGCATACAACAACTGGAGGTTCACGAGCCTGCGCAGTGTCTGGTAATCC<br>GTCATTCTTCAGAAACATGGTCTGGTTGACAAAGAAAGGGTCAAATTATCCAGTTGCCAAAGGATCATACAATAATACAAGTGGGGAACAAATGCTGGTCATTTGGGGAGTACACCACCCCAATGATGAAGCTGAGCAAAGAGCACTGTATCAGAATGTTGAGACCTATGTGTCAGTGGGAACATCGACACTCAACAAAAGATCAATTCCGGAAATAGCCACAAGACCTAA<br>AGTAAATGGACAAGGAGGCAGAATGGAATTCTCATGGACCATATTGGACATGTTGGACACCATAAATTTCGAGAGCACTGGTAATCTAATTGCACCGGAATATGGCTTTAAAATATCTAAACGGGGTAGTTCAGGAATCATGAAAACGGAAGGGACACTTGAAAATTGTGAAACCAAATGCCAAACTCCCTTAGGAGCAATAAATACAACATTGCCCTTTCATAATATCCAC<br>CCATTGACCATTGGTGAGTGCCCCAAATATGTAAAATCGGAGAAGCTAGTCTTGGCAACAGGATTAAGAAATGTCCCTCAGATTGAATCAAGAGGGCTGTTTGGGGCAATAGCTGGTTTCATAGAAGGAGGGTGGCAAGGAATGGTTGATGGTTGGTATGGATATCATCATAGTAATGATCAAGGATCTGGGTATGCAGCAGATAAAGAGTCTACTCAAAAGGCAATTGA<br>TGGAATCACCAACAAGGTAAACTCCGTAATTGAGAAAATGAACACTCAATTCGAGGCTGTGGGGAAAGAATTCAGCAATTTGGAGAAAAGGCTGGAAAACCTGAACAAAAAGATGGAAGACGGATTCCTAGATGTTTGGACATACAATGCTGAGCTTCTAGTCCTAATGGAAAATGAGAGGACACTTGACTTCCATGATGCCAATGTCAAGAACCTATATGACAAAGTCA<br>GGATGCAATTGAGGGACAATGCGAAAGAACTAGGAAATGGGTGTTTTGAATTTTACCATAAATGTGATGATGAATGCATGAACAGCGTAAAGAATGGGACATATGATTATCCCAAGTATGAAGAGGAATCTAAACTAAACAGGAATGAAATCAAAGGAGTAAAATTGAGCAACATGGGGGTTTATCAAATACTTGCAATTTACGCCACAGTAGCAGGCTCTCTGTCACTG<br>GCAATCATGATAGCTGGGATCTCTTTATGGATGTGCTCGAATGGGTCTCTGCAATGCAGAATCTGCATATGATTATCAGTCATTTTATAATTA
EPI_ISL_502595	>A/Anas_platyrhynchos/Belgium/10409/2018_H2N3_2018-10-09<br>ATGGCAATCATTTATCTGATCCTTTTGTTTACAGTAGTGAGAGGAGACCAGATATGCATTGGATACCACTCCAACAATTCCACAGAAAAGGTCGACACAATTCTAGAAAGGAATGTCACTGTGACTCATGCTCAGGACATTCTTGAGAAGACTCACAATGGAAAATTATGCAAACTAAATGGAATCCCTCCACTTGAACTGGGAGATTGCAGCATCGCTGGATGGCTCCTCGG<br>AAATCCAGAATGTGATAGACTTCTAACTGTACCAGAATGGTCATATATAATGGAGAGAGAAAATCCAAGGAATGGTTTGTGCTACCCAGGCAGTTTCAATGACTATGAGGAGTTAAAACACCTCATTAGTAGTGTGACACACTTCGAAAAAGTGAAGATTCTGCCTAAAGATAGGTGGACACAGCATACAACAACTGGAGGTTCACGGGCCTGCGCAGTGTCTGGTAATCC<br>GTCATTCTTCAGAAACATGGTCTGGTTGACAAAGAAAGGGTCAAATTACCCAATTGCCAAAGGATCATACAATAATACAAGTGGGGAACAAATGCTGGTCATTTGGGGAGTACATCATCCCAATGATGAAGCTGAACAAAGAACACTGTACCAGAATGTCGGGACTTATGTGTCAATAGGAACATCGACACTTAACAAAAGATCAATTCCGGAAATAGCCACAAGACCTAA<br>AGTGAATGGACAAGGAGGCAGAATGGAATTCTCATGGACCATATTGGACATGTTGGACACCATAAATTTCGAGAGCACTGGGAATCTAATTGCACCGGAATATGGCTTTAAAATATCCAAACGGGGTAGTTCAGGAATCATGAAAACGGAAGGGACACTTGAAAATTGCGAAACCAAATGCCAAACTCCCTTAGGTGCAATAAATACAACATTGCCCTTTCATAATATCCAC<br>CCGTTAACCATTGGTGAGTGCCCCAAATATGTAAAATCGAAGAAGCTAGTCTTGGCAACAGGATTAAGAAATGTCCCTCAGATTGAACCAAGAGGATTGTTTGGGGCAATAGCTGGTTTCATAGAAGGAGGGTGGCAAGGAATGGTTGATGGTTGGTATGGATATCATCATAGTAATGATCAAGGATCCGGGTATGCAGCAGACAAAGAGTCTACTCAAAAGGCAATTGA<br>CGGAATCACCAACAAGGTAAACTCCGTAATTGAGAAAATGAACACTCAATTCGAGGCTGTGGGGAAAGAATTCAGCAATTTGGAGAAAAGACTGGAAAACCTGAACAAAAAGATGGAAGACGGATTCCTAGATGTGTGGACATACAATGCTGAGCTTCTAGTCCTAATGGAAAATGAGAGGACACTTGACTTCCATGACTCCAATGTCAAGAACCTATATGACAAAGTCA<br>GGATGCAATTGAGGGACAATGCGAAAGAACTAGGGAATGGGTGTTTTGAATTTTACCATAAGTGTGATGATGAATGCATGAACAGCGTAAAGAATGGGACATATGATTATCCCAAGTATGAAGAGGAGTCTAAACTAAACAGGAATGAAATCAAAGGAGTAAAATTGAGCAACATGGGGGTTTATCAAATACTAGCAATTTATGCCACAGTAGCAGGCTCTCTGTCACTG<br>GCAATCATGATAGCTGGGATCTCTTTATGGATGTGCTCTAACGGGTCTCTGCAATGCAGAATCTGCATATGATTATCAGTCGTTTTATAATTA
EPI_ISL_338361	>A/shoveler/Novosibirsk_region/998k/2018_H2N1_2018-10-01<br>ATGGCAATCATTTATCTGATTCTTCTGTTTACAGTAGTGAGAGGAGACCAGATATGCATTGGATACCACTCCAACAATTCCACAGAAAAGGTCGACACAATTCTAGAAAGGAATGTCACTGTGACTCATGCTCAAGACATCCTTGAGAAGACTCACAATGGGAAATTATGCAAACTAAATGGAATCCCTCCACTTGAATTGGGAGATTGCAGCATCGCTGGATGGCTCCTTGG<br>AAATCCAGAATGTGATAGACTTCTAACTGTACCAGAATGGTCATATATAATGGAGAAAGAAAACCCAAGGAATGGCTTGTGCTACCCAGGCAGTTTCAATGACTACGAAGAATTGAAACACCTCCTTAGTAGTGTAACACACTTCGAAAAAGTGAAGATTCTGCCTAAAGATAGGTGGACACAGCATACAACAACTGGAGGTTCACGGGCCTGCGCAGTGTCTGGTAATCC<br>GTCATTCTTCAGAAACATGGTCTGGTTGACAAAGAAAGGGTCAAATTATCCAGTTGCCAAAGGATCATACAATAATACAAGTGGGGAACAAATGCTGGTCATTTGGGGAGTACATCATCCCAATGATGAAGCTGAACAAAGGACATTGTATCAGAATGTCGGGACCTATGTGTCAGTGGGAACATCGACGCTTAACAAAAGATCAATTCCGGAAATAGCCACAAGACCTAA<br>AGTGAATGGACAAGGAGGCAGAATGGAATTCTCATGGACCATATTGGACATGTTGGACACCATAAATTTCGAGAGCACTGGTAATTTAATTGCACCGGAATATGGCTTTAAAATATCCAAACGGGGTAGTTCAGGAATTATGAAAACGGAAGGGACACTTGAGAATTGCGAAACCAAATGCCAAACTCCCTTAGGAGCAATAAATACAACATTGCCCTTTCATAATATCCAC<br>CCGTTAACCATTGGTGAGTGCCCCAAATACGTAAAATCGGAGAAGCTAGTCTTGGCAACAGGATTAAGAAATGTCCCTCAGATTGAATCAAGAGGATTGTTTGGGGCAATAGCTGGTTTCATAGAAGGAGGGTGGCAAGGAATGGTTGATGGTTGGTATGGATATCATCATAGTAATGATCAAGGATCCGGGTATGCAGCAGACAAAGAGTCCACTCAAAAGGCAATTGA<br>TGGAATCACCAACAAAGTAAACTCTGTAATTGAGAAAATGAACACTCAATTCGAGGCTGTTGGGAAAGAATTCAGTAATTTGGAGAAAAGGCTGGAAAACCTGAACAAAAAGATGGAAGACGGATTCCTAGATGTGTGGACATACAATGCTGAGCTCCTAGTCCTAATGGAAAATGAGAGGACACTTGACTTCCATGACTCCAATGTCAAGAACCTGTATGACAAAGTCAG<br>GATGCAATTGAGGGACAATGCGAAAGAACTAGGGAATGGGTGTTTTGAATTTTACCATAAATGTGATGATGAATGCATGAACAGCGTAAAGAATGGGACATATGATTATCCCAAGTATGAAGAGGAATCTAAACTAAACAGGAATGAAATCAAAGGAATAAAATTGAGCAACATGGGGGTTTATCAAATACTTGCAATTTATGCTACAGTAGCAGGTTCTCTGTCACTGGC<br>AATCATGATAGCTGGGATCTCTTTATGGATGTGCTCTAACGGGTCTCTGCAATGCAGAATCTGCATATGATTATCAGTCATTTTATAATTA
EPI_ISL_328982	>A/black-headed_gull/Netherlands/4/2016_A_2016-07-15<br>ATGGCAATCATTTATCTGATTCTTCTGTTTACTGTTGTGAGAGGAGACCAGATATGCATTGGATACCACTCCAACAATTCCACAGAAAAGGTCGACACAATTCTAGAGAGGAATGTCACTGTGACTCATGCTCAGGACATTCTTGAGAAGACTCACAATGGAAAATTATGCAAACTAAATGGAATCCCTCCACTTGAATTGGGAGATTGCAGCATCGCCGGATGGCTCCTTGG<br>AAATCCAGAATGTGATAGACTTCTAACTGTACCAGAATGGTCATATATAATGGAGAAAGAAAACCCAAGGAATGGTTTGTGCTACCCAGGCAGTTTCAATGATTATGAAGAATTGAAACACCTCCTTAGTAGTGTAACACACTTCGAGAAAGTGAAGATTTTGCCCAAAGATAGGTGGACACAGCATACAACAACTGGAGGTTCACGGGCCTGTGCAGTATCTGGTAATCCG<br>TCATTCTTCAGAAACATGGTCTGGTTGACAAAGAAAGGGTCAAATTATCCAGTTGCCAAAGGATCATACAATAATACAAGTGGGGAACAAATGCTGGTCATTTGGGGAGTACATCACCCCAATGATGAAACTGAACAAAGGGCATTGTACCAGAATGTCGGGACCTATGTATCAGTGGGAACATCGACACTTAACAAAAGATCAGTTCCAGAAATAGCCACAAGACCTAAA<br>GTGAATGGACAAGGAGGCAGAATGGAATTCTCATGGACCATATTGGACATGTTGGACACCATAAATTTCGAGAGCACTGGTAACCTAATTGCACCGGAATATGGCTTTAAAATATCCAAACGGGGTAGTTCAGGAATCATGAAAACGGAAGGGACACTTGAAAATTGCGAAACTAAATGCCAAACTCCCTTAGGAGCAATAAATACAACATTGCCCTTTCATAATATCCACC<br>CATTAACCATTGGTGAGTGCCCCAAATATGTAAAATCGGAGAGGTTAGTCTTGGCCACAGGATTAAGAAATGTCCCTCAGATTGAATCAAGAGGATTGTTTGGGGCAATAGCTGGTTTCATAGAAGGAGGATGGCAAGGAATGGTTGATGGTTGGTATGGATATCATCACAGTAATGATCAAGGATCCGGGTATGCAGCAGACAAAGAGTCCACTCAAAAGGCAATTGAT<br>GGAATCACCAACAAAGTAAACTCCGTAATTGAGAAAATGAACACTCAATTCGAGGCTGTTGGGAAAGAATTCAGTAATTTGGAGAAAAGGCTGGAAAACCTGAACAAAAAGATGGAAGACGGATTCCTAGATGTGTGGACATACAATGCTGAGCTTCTAGTCCTAATGGAAAATGAGAGGACACTTGACTTCCATGACTCCAATGTCAAGAACCTATATGACAAAGTCAG<br>GATGCAATTGAGGGACAATGCGAAAGAACTAGGGAATGGATGTTTTGAATTTTATCATAAATGTGATGATGAATGCATGAACAGCGTAAAGAATGGGACATATGATTATCCCAAGTATGAAGAGGAATCTAAACTAAACAGGAATGAAATCAAAGGAGTAAAATTGAGCAACATGGGGGTTTATCAAATACTTGCAATTTATGCTACAGTAGCAGGCTCCCTGTCACTGGC<br>AATCATGATAGCTGGGATCTCTTTATGGATGTGCTCTAACGGGTCTCTGCAATGCAGAATCTGCATATGATTATCAGTCGTTTTATAATTA
EPI_ISL_267293	>A/tufted_duck/Georgia/1/2012_H2N3_2012-12-22<br>ATGGCAATCATTTATCTGATTCTTCTGTTTACTGTTGTGAGAGGAGACCAGATATGCATTGGATACCACTCCAACAATTCCACAGAAAAGGTCGACACAATTCTAGAGAGGAATGTCACTGTGACTCATGCTCAGGACATTCTTGAGAAGACTCACAATGGAAAATTATGCAAACTAAATGGAATCCCTCCACTTGAATTGGGAGATTGCAGCATCGCCGGATGGCTCCTTGG<br>AAATCCAGAATGTGATAGACTTCTAACTGTACCAGAATGGTCATATATAATGGAGAAAGAAAACCCAAGGAATGGTTTGTGCTACCCAGGCAGTTTCAATGATTATGAAGAATTGAAACACCTCCTTAGTAGTGTAACACACTTCGAGAAAGTGAAGATTTTGCCCAAAGATAGGTGGACACAGCATACAACAACTGGAGGTTCACGGGCCTGTGCAGTATCTGGTAATCCG<br>TCATTCTTCAGAAACATGGTCTGGTTGACAAAGAAAGGGTCAAATTATCCAGTTGCCAAAGGATCATACAATAATACAAGTGGGGAACAAATGCTGGTCATTTGGGGAGTACATCACCCCAATGATGAAACTGAACAAAGGGCATTGTACCAGAATGTCGGGACCTATGTATCAGTGGGAACATCGACACTTAACAAAAGATCAGTTCCAGAAATAGCCACAAGACCTAAA<br>GTGAATGGACAAGGAGGCAGAATGGAATTCTCATGGACCATATTGGACATGTTGGACACCATAAATTTCGAGAGCACTGGTAACCTAATTGCACCGGAATATGGCTTTAAAATATCCAAACGGGGTAGTTCAGGAATCATGAAAACGGAAGGGACACTTGAAAATTGCGAAACTAAATGCCAAACTCCCTTAGGAGCAATAAATACAACATTGCCCTTTCATAATATCCACC<br>CATTAACCATTGGTGAGTGCCCCAAATATGTAAAATCGGAGAGGTTAGTCTTGGCCACAGGATTAAGAAATGTCCCTCAGATTGAATCAAGAGGATTGTTTGGGGCAATAGCTGGTTTCATAGAAGGAGGATGGCAAGGAATGGTTGATGGTTGGTATGGATATCATCACAGTAATGATCAAGGATCCGGGTATGCAGCAGACAAAGAGTCCACTCAAAAGGCAATTGAT<br>GGAATCACCAACAAAGTAAACTCCGTAATTGAGAAAATGAACACTCAATTCGAGGCTGTTGGGAAAGAATTCAGTAATTTGGAGAAAAGGCTGGAAAACCTGAACAAAAAGATGGAAGACGGATTCCTAGATGTGTGGACATACAATGCTGAGCTTCTAGTCCTAATGGAAAATGAGAGGACACTTGACTTCCATGACTCCAATGTCAAGAACCTATATGACAAAGTCAG<br>GATGCAATTGAGGGACAATGCGAAAGAACTAGGGAATGGATGTTTTGAATTTTATCATAAATGTGATGATGAATGCATGAACAGCGTAAAGAATGGGACATATGATTATCCCAAGTATGAAGAGGAATCTAAACTAAACAGGAATGAAATCAAAGGAGTAAAATTGAGCAACATGGGGGTTTATCAAATACTTGCAATTTATGCTACAGTAGCAGGCTCCCTGTCACTGGC<br>AATCATGATAGCTGGGATCTCTTTATGGATGTGCTCTAACGGGTCTCTGCAATGCAGAATCTGCATATGATTATCAGTCGTTTTATAATTA
EPI_ISL_267171	>A/mallard_duck/Netherlands/22/2013_H2N3_2013-01-07<br>ATGGCAGTCATTTATCTGATTCTTTTGTTTACAGTAGTGAGAGGAGACCAGATATGCATTGGATACCACTCCAACAATTCCACAGAAAAGGTCGACACAATTCTAGAGAGGAATGTCACTGTGACTCATGCTCAGGACATTCTTGAGAAGACTCACAATGGAAAACTATGCAAACTAAATGGAATCCCTCCACTTGAATTGGGAGATTGCAGCATCGCTGGATGGCTCCTTGG<br>AAATCCAGAATGTGATAGACTTCTAACTGTACCAGAATGGTCATATATAATGGAGAAAGAAAACCCAAGAAATGGTTTGTGCTACCCAGGCAGTTTCAATGACTATGAAGAATTGAAACACCTCCTTAGTAGCGTAACACACTTCGAGAAAGTGAAGATTTTGCCCAAAGATAGGTGGACACAGCATACAACAACTGGAGGTTCACGGGCCTGCGCAGTATCTGGTAATCC<br>GTCATTCTTCAGGAACATGGTCTGGTTGACAAAGAAAGGATCAAATTATCCAGTTGCCAAAGGATCATACAATAATACAAGTGGGGAACAAATGCTGGTCATTTGGGGAGTACATCACCCCAATGATGAAGCTGAACAAAGGAATTTGTACCAGAATGTCGGGACCTATGTATCAGTGGGAACATCGACACTTAACAAAAGATCAGTTCCAGAGATAGCCACAAGACCTAA<br>AGTGAATGGACAAGGAGGCAGAATGGAATTCTCATGGACCATATTGGACATGTTGGACATCATAAATTTCGAGAGCACTGGTAATCTAATTGCACCGGAATATGGCTTTAAAATATCCAAACGGGGTAGTTCAGGAATCATGAAAACAGAAGGGACGCTTGAAAATTGCGAAACTAAATGCCAAACTCCCTTAGGAGCAATAAATACAACATTGCCCTTTCATAATATCCAC<br>CCATTAACCATTGGTGAGTGCCCCAAATATGTAAAATCGGAGAGGTTAGTCTTGGCAACAGGATTAAGAAATGTCCCTCAGATTGAATCAAGAGGATTGTTTGGGGCAATAGCTGGTTTCATAGAAGGAGGATGGCAAGGAATGGTTGATGGTTGGTATGGATATCATCACAGTAATGATCAAGGATCCGGGTATGCAGCAGACAAAGAGTCCACTCAAAAGGCAATTGA<br>TGGAATCACCAACAAAGTAAACTCCGTAATTGAGAAAATGAACACTCAATTCGAGGCTGTTGGGAAAGAATTCAGTAATTTGGAGAAAAGGCTGGAAAACCTGAACAAAAAGATGGAAGACGGATTCCTAGATGTGTGGACATACAATGCTGAGCTTCTAGTCCTAATGGAAAATGAGAGGACACTTGACTTCCATGATTCCAATGTCAAGAACCTGTATGACAAAGTTAG<br>GATGCAATTGAGGGACAATGCGAAAGAACTGGGGAATGGATGTTTTGAATTTTACCATAAATGTGATGATGAATGCATGAACAGTGTAAAGAACGGGACATATGATTATCCCAAGTATGAAGAGGAATCTAAACTAAACAGGAATGAAATCAAAGGAGTAAAATTGAGCAACATGGGGGTTTATCAGATACTTGCAATTTATGCTACAGTAGCAGGCTCCCTGTCACTGG<br>CAATCATGATAGCTGGGATCTCTTTATGGATGTGCTCTAACGGGTCTCTGCAATGCAGAATCTGCATATGATTATCAGTCGTTTTATAATTA
EPI_ISL_373068	>A/ruddy_turnstone/Delaware_Bay/184/2018_A_2018-05-21<br>ATGGCAATCATTTATCTGATTCTTCTGTTTACAGTAGTGAGAGGAGACCAGATATGCATTGGATATCACTCCAACAATTCCACCGAAAAAGTCGACACAATTCTAGAAAGGAATGTCACTGTGACTCATGCTCAGGACATTCTTGAGAAGACTCACAATGGAAAATTATGCAAACTAAATGGAATTCCTCCACTTGAATTGGGAGATTGCAGCATCGCCGGATGGCTCCTTGG<br>AAATCCACAATGTGATAGACTTCTGACTGTGCCAGAATGGTCATATATAATGGAGAAAGAAAACCCAAGGAATGGCTTGTGCTACCCAGGCAGTTTCAATGACTATGAAGAATTGAAACACCTCCTTAGTAGTGTAACACAATTCGAAAAAGTGAAGATTCTGCCTAGGAATAGGTGGACACAGCATACAACAACTGGAGGTTCACAGGCCTGCGCAGTGTATGGTGGTCC<br>GTCATTCTTCAGAAACATGGTCTGGTTGACAAAGAAAGAGTCAAATTATCCAGTTGCCAAAGGATCATACAATAATACAAGTGGGGAACAAATGCTGGTCATTTGGGGAATACATCACCCCAATGATGAAACTGAACAAAGGACATTGTATCAGAATGTCGGGACCTATGTGTCAGTGGGAACATCGACACTTAACAAAAGATCAATTCCGGAAATAGCCACAAGACCTAA<br>AGTGAATGGACAAGGAGGCAGAATGGAATTCTCATGGACCATATTGGACATGTTGGACACCATAAATTTCGAGAGCACTGGCAATCTAATTGCACCGGAATATGGCTTTAAAATATCCAAACGGGGTAGTTCAGGGATCATGAAAACGGAAGGGACACTTGAAAATTGCGAAACCAAATGCCAAACTCCCTTAGGAGCAATAAATACAACATTGCCCTTTCATAATATCCAC<br>CCGTTAACCATTGGTGAGTGTCCCAAATATGTAAAATCGGAGAAGCTAGTCTTGGCAACAGGATTAAGAAATGTCCCTCAGATTGAATCAAGAGGATTGTTTGGGGCAATAGCTGGTTTCATAGAAGGAGGGTGGCAAGGAATGGTTGATGGTTGGTATGGATATCATCATAGTAATGATCAAGGATCCGGATATGCAGCAGACAAAGAGTCTACTCAAAAGGCAATTGA<br>TGGAATCACCAACAAGGTAAACTCCATAATTGAGAAAATGAACACTCAATTCGAAGCTGTTGGGAAAGAATTCAGTAATTTGGAGAAAAGGATGGAAAACCTGAACAAAAAGATGGAAGACGGATTCCTAGATGTGTGGACATACAATGCTGAGCTTCTAGTCCTAATGGAAAATGAGAGGACACTTGACTTCCATGACTCCAATGTCAAGAACCTATATGACAAAGTCAG<br>GATGCAATTGAGGGACAATGCGAAAGAACTAGGGAATGGATGTTTTGAATTTTACCATAAATGTGATAATGAATGCATGAACAGCGTAAAGAATGGGACATATGATTATCCCAAGTATGAAGAGGAATCTAAACTAAACAGGAATGAAATCAAAGGAGTAAAATTGAGCAACATGGGGGTTTACCAAATACTTGCAATTTATGCCACAGTAGCAGGCTCTCTGTCACTGGC<br>AATCATGATAGCTGGGATCTCTTTATGGATGTGCTCTAACGGGTCTCTGCAATGCAGAATCTGCATATGATTATCAGTCGTTTTATAATTA
EPI_ISL_399485	>A/Anas_platyrhynchos/Belgium/11127/2016_H2N3_2016-11-29<br>ATGGCAATCATTTATCTGATTCTTCTGTTCACAGTAGTGAGAGGAGACCAGATATGCATTGGATACCACTCCAATAATTCCACGGAAAAGGTAGACACAATTCTAGAGAGGAATGTCACTGTGACCCATGCTCAGGACATCCTTGAGAAGACTCATAATGGAAAGTTATGCAAACTAAATGGAATCCCTCCACTTGAATTGGGAGATTGCAGCATTGCTGGATGGCTCCTTGG<br>AAATCCAGAATGTGATAGACTTCTAACTGTACCAGAATGGTCATATATAATGGAGAAAGAAAACCCAAGGAATGGTTTGTGCTACCCAGGCAGTTTCAATGACTATGAAGAATTGAAACACCTCCTTAGTAGTGTAACACACTTCGAAAAAGTGAAGATTTTGCCTAAAGATAGGTGGACACAGCATACAACAACTGGAGGTTCACGGGCCTGCGCAGTATCTGGCAATCCG<br>TCATTCTTCAGAAACATGGTCTGGCTGACAAAGAAAGGGTCAAATTATCCAGTTGCCAAAGGATCATACAATAATACAAGTGGGGAACAAATGCTGGTCATTTGGGGAGTACATCACCCCAATGATGAAACTGAACAAAGGACACTGTATCAAAATGTCGGGACCTATGTGTCAGTAGGAACATCGACACTTAACAAAAGATCAGTTCCAGAAATAGCCACAAGACCTAAA<br>GTGAATGGACAAGGAGGCAGAATGGAATTCTCATGGACCATATTGGACATATTGGACACCATAAATTTCGAGAGCACTGGTAATCTAATTGCACCGGAATATGGCTTTAAAATATCCAAACGGGGTAGTTCAGGAATCATGAAAACGGAAGGGACACTTGAAAATTGCGAAACCAAATGCCAAACTCCCTTAGGAGCAATAAACACAACATTGCCCTTTCACAATATCCACC<br>CGTTAACCATTGGTGAGTGCCCCAAATATGTAAAATCGGAGAGGTTAGTCTTGGCAACAGGATTAAGAAATGTCCCTCAGATTGAATCAAGAGGATTGTTTGGGGCAATAGCTGGTTTCATAGAAGGAGGGTGGCAAGGAATGGTTGATGGTTGGTATGGATATCATCATAGTAATGATCAAGGATCCGGGTATGCAGCAGACAAAGAGTCCACTCAAAATGCAATTGAT<br>GGAATCACCAACAAAGTAAACTCCATAATTGAGAAAATGAACACCCAATTCGAGGCTGTTGGGAAAGAATTCAGTAATTTGGAGAAAAGGCTGGAAAATCTGAACAAAAAGATGGAAGACGGATTCCTAGATGTGTGGACATACAATGCTGAGCTTCTAGTTCTAATGGAAAATGAGAGGACACTTGACTTCCATGACTCCAATGTCAAGAACCTATATGACAAAGTCAGG<br>ATGCAATTGAGAGACAATGCAAAAGAAATAGGAAATGGGTGTTTTGAATTTTACCATAAATGTGATGATGAATGCATGAACAGCGTAAAGAATGGGACATATGATTATCCCAAGTATGAAGAGGAATCTAAACTAAACAGGAATGAAATCAAAGGAGTAAAATTGAACAACATGGGGGTCTATCAAATACTTGCAATTTATGCTACAGTAGCAGGCTCCCTGTCACTGGCA<br>ATCATGATAGCTGGGATCTCTTTATGGATGTGCTCTAACGGGTCTCTGCAATGCAGAATCTGCATATGATTATCAGTCTTCTTATAATTA
EPI_ISL_373058	>A/ruddy_turnstone/Delaware_Bay/326/2018_H2N1_2018-05-22<br>ATGGCAATCATTTATCTGATTCTTCTGTTTACAGTAGTGAGAGGAGACCAGATATGCATTGGATATCACTCCAACAATTCCACCGAAAAAGTCGACACAATTCTAGAAAGGAATGTCACTGTGACTCATGCTCAGGACATTCTTGAGAAGACTCACAATGGAAAATTATGCAAACTAAATGGAATCCCTCCACTTGAATTGGGAGATTGCAGCATCGCCGGATGGCTCCTTGG<br>AAATCCACAATGTGATAGACTTCTGACTGTGCCAGAATGGTCATATATAATGGAGAAAGAAAACCCAAGGAATGGCTTGTGCTACCCAGGCAGTTTCAATGACTATGAAGAATTGAAACACCTCCTTAGTAGTGTAACACACTTCGAAAAAGTGAAGATTCTGCCTAGGAATAGGTGGACACAGCATACAACAACTGGAGGTTCACAGGCCTGCGCAGTGTATGGTGGTCC<br>GTCATTCTTCAGAAACATGGTCTGGTTGACAAAGAAAGAGTCAAATTATCCAGTTGCCAAAGGATCATACAATAATACAAGTGGGGAACAAATGCTGGTCATTTGGGGAATACATCACCCCAATGATGAAACTGAACAGAGGACATTGTATCAGAATGTCGGGACCTATGTGTCAGTGGGAACATCGACACTTAACAAAAGATCAATTCCGGAAATAGCCACAAGACCTAA<br>AGTGAATGGACAAGGAGGCAGAATGGAATTCTCATGGACCATATTGGACATGTTGGACACCATAAATTTCGAGAGCACTGGCAATCTAATTGCACCGGAATATGGCTTTAAAATATCCAAACGGGGTAGTTCAGGGATCATGAAAACGGAAGGGACATTTGAAAACTGCGAAACCAAATGCCAAACTCCCTTAGGAGCAATAAATACAACATTGCCCTTTCATAATATCCAC<br>CCGTTAACCATTGGTGAGTGTCCCAAATATGTAAAATCGGAGAAGCTAGTCTTGGCAACAGGATTAAGAAATGTCCCTCAGATTGAATCAAGAGGATTGTTTGGGGCAATAGCTGGTTTCATAGAAGGAGGGTGGCAAGGAATGGTTGATGGTTGGTATGGATATCATCATAGTAATGATCAAGGATCCGGATATGCAGCAGACAAAGAGTCTACTCAAAAGGCAATTGA<br>TGGAATCACCAACAAGGTAAACTCCATAATTGAGAAAATGAACACTCAATTCGAAGCTGTTGGGAAAGAATTCAGTAATTTGGAGAAAAGGCTGGAAAACCTGAACAAAAAGATGGAAGACGGATTCCTAGATGTGTGGACATACAATGCTGAGCTTCTAGTCCTAATGGAAAATGAGAGGACACTTGACTTCCATGACTCCAATGTCAAGAACCTATATGACAAAGTCAG<br>GATGCAATTGAGGGACAATGCGAAAGAACTAGGGAATGGATGTTTTGAATTTTACCATAAATGTGATAATGAATGCATGAACAGCGTAAAGAATGGGACATATGATTATCCCAAGTATGAAGAGGAATCTAAACTAAACAGGAATGAAATCAAAGGAGTAAAATTGAGCAACATGGGGGTTTATCAAATACTTGCAATTTATGCCACAGTAGCAGGCTCTCTGTCACTGGC<br>AATCATGATAGCTGGGATCTCTTTATGGATGTGCTCTAACGGGTCTCTGCAATGCAGAATCTGCATATGATTACCAGTCGTTTTATAATTA
EPI_ISL_373062	>A/semipalmated_sandpiper/Delaware_Bay/506/2018_H2N8_2018-05-23<br>ATGGCAATCATTTATCTGATTCTTCTGTTTACAGTAGTGAGAGGAGACCAGATATGCATTGGATATCACTCCAACAATTCCACCGAAAAAGTCGACACAATTCTAGAAAGGAATGTCACTGTGACTCATGCTCAGGACATTCTTGAGAAGACTCACAATGGAAAATTATGCAAACTAAATGGAATTCCTCCACTTGAATTGGGAGATTGCAGCATCGCCGGATGGCTCCTTGG<br>AAATCCACAATGTGATAGTCTTCTGACTGTGCCAGAATGGTCATATATAATGGAGAAAGAAAACCCAAGGAATGGCTTGTGCTACCCAGGCAGTTTCAATGACTATGAAGAATTGAAACACCTCCTTAGTAGTGTAACACACTTCGAAAAAGTGAAGATTCTGCCTAGGAATAGGTGGACACAGCATACAACAACTGGAGGTTCACAGGCCTGCGCAGTGTATGGTGGTCC<br>GTCATTCTTCAGAAACATGGTCTGGTTGACAAAGAAAGAGTCAAATTATCCAGTTGCCAAAGGATCATACAATAATACAAGTGGGGAACAAATGCTGGTCATTTGGGGAATACATCACCCCAATGATGAAACTGAACAAAGGACATTGTATCAGAATGTCGGGACCTATGTGTCAGTGGGAACATCGACACTTAACAAAAGATCAATTCCGGAAATAGCCACAAGACCTAA<br>AGTGAATGGACAAGGAGGCAGAATGGAATTCTCATGGACCATATTGGACATGTTGGACACCATAAATTTCGAGAGCACTGGCAATCTAATTGCACCGGAATATGGCTTTAAAATATCCAAACGGGGTAGTTCAGGGATCATGAAAACGGAAGGGACACTTGAAAATTGCGAAACCAAATGCCAAACTCCCTTAGGAGCAATAAATACAACATTGCCCTTTCATAATATCCAC<br>CCGTTAACCATTGGTGAGTGTCCCAAATATGTAAAATCGGAGAAGCTAGTCTTGGCAACAGGATTAAGAAATGTCCCTCAGATTGAATCAAGAGGATTGTTTGGGGCAATAGCTGGTTTCATAGAAGGAGGGTGGCAAGGAATGGTTGATGGTTGGTATGGATATCATCATAGTAATGATCAAGGATCCGGATATGCAGCAGACAAAGAGTCTACTCAAAAGGCAATTGA<br>TGGAATCACCAACAAGGTAAACTCCATAATTGAGAAAATGAACACTCAATTCGAAGCTGTTGGGAAAGAATTCAGTAATTTGGAGAAAAGGATGGAAAACCTGAACAAAAAGATGGAAGACGGATTCCTAGGTGTGTGGACATACAATGCTGAGCTTCTAGTCCTAATGGAAAATGAGAGGACACTTGACTTCCATGACTCCAATGTCAAGAACCTATATGACAAAGTCAG<br>GATGCAATTGAGGGACAATGCGAAAGAACTAGGGAATGGATGTTTTGAATTTTACCATAAATGTGATAATGAATGCATGAACAGCGTAAAGAATGGGACATATGATTATCCCAAGTATGAAGAGGAATCTAAACTAAACAGGAATGAAATCAAAGGAGTAAAATTGAGCAACATGGGGGTTTATCAAATACTTGCAATTTATGCCACAGTAGCAGGCTCTCTGTCACTGGC<br>AATCATGATAGCTGGGATCTCTTTATGGATGTGCTCTAACGGGTCTCTGCAATGCAGAATCTGCATATGATTATCAGTCGTTTTATAATTA
EPI_ISL_373029	>A/ruddy_turnstone/Delaware_Bay/143/2018_H2N7_2018-05-21<br>ATGGCAATCATTTATCTGATTCTTCTGTTTACAGTAGTGAGAGGAGACCAGATATGCATTGGATATCACTCCAACAATTCCACCGAAAAAGTCGACACAATTCTAGAAAGGAATGTCACTGTGACTCATGCTCAGGACATTCTTGAGAAGACTCACAATGGAAAATTATGCAAACTAAATGGAATCCCTCCACTTGAATTGGGAGATTGCAGCATCGCCGGATGGCTCCTTGG<br>AAATCCACAATGTGATAGACTTCTGACTGTGCAAGAATGGTCATATATAATGGAGAAAGAAAACCCAAGGAATGGCTTGTGCTACCCAGGCAGTTTCAATGACTATGAAGAATTGAAACACCTCCTTAGTAGTGTAACACACTTCGAAAAAGTGAAGATTCTGCCTAGGAATAGGTGGACACAGCATACAACAACTGGAGGTTCACAGGCCTGCGCAGTGTATGGTGGTCC<br>GTCATTCTTCAGAAACATGGTCTGGTTGACAAAGAAAGAGTCAAATTATCCAGTTGCCAAAGGATCATACAATAATACAAGTGGGGAACAAATGCTGGTCATTTGGGGAATACATCACCCCAATGATGAAACTGAACAGAGGACATTGTATCAGAATGTCGGGACCTATGTGTCAGTGGGAACATCGACACTTAACAAAAGATCAATTCCAGAAATAGCCACAAGACCTAA<br>AGTGAATGGACAAGGAGGCAGAATGGAATTCTCATGGACCATATTGGACATGTTGGACACCATAAATTTCGAGAGCACTGGCAATCTAATTGCACCGGAATATGGCTTTAAAATATCCAAACGGGGTAGTTCAGGGATCATAAAAACGGAAGGGACACTTGAAAACTGCGAAACCAAATGCCAAACTCCCTTAGGAGCAATAAATACAACATTGCCCTTTCATAATATCCAC<br>CCGTTAACCATTGGTGAGTGTCCCAAATATGTAAAATCGGAGAAGCTAGTCTTGGCAACAGGATTAAGAAATGTCCCTCAGATTGAATCAAGAGGATTGTTTGGGGCAATAGCTGGTTTCATAGAAGGAGGGTGGCAAGGAATGGTTGATGGTTGGTATGGATATCATCATAGTAATGATCAAGGATCCGGATATGCAGCAGACAAAGAGTCTACTCAAAAGGCAATTGA<br>TGGAATCACCAACAAGGTAAACTCCATAATTGAGAAAATGAACACTCAATTCGAAGCTGTTGGGAAAGAATTCAGTAATTTGGAGAAAAGGCTGGAAAACCTGAACAAAAAGATGGAAGACGGATTCCTAGATGTGTGGACATACAATGCTGAGCTTCTAGTCCTAATGGAAAATGAGAGGACACTTGACTTCCATGACTCCAATGTCAAGAACCTATATGACAAAGTCAG<br>GATGCAATTGAGGGACAATGCGAAAGAACTAGGGAATGGATGTTTTGAATTTTACCATAAATGTGATAATGAATGCATGAACAGCGTAAAGAATGGGACATATGATTATCCCAAGTATGAAGAGGAATCTAAACTAAACAGGAATGAAATCAAAGGAGTAAAATTGAGCAACATGGGGGTTTATCAAATACTTGCAATTTATGCCACAGTAGCAGGCTCTCTGTCACTGGC<br>AATCATGATAGCTGGGATCTCTTTATGGATGTGCTCTAACGGGTCTCTGCAATGCAGAATCTGCATATGATTACCAGTCGTTTTATAATTA
EPI_ISL_373043	>A/ruddy_turnstone/Delaware_Bay/587/2018_H2N7_2018-05-24<br>ATGGCAATCATTTATCTGATTCTTCTGTTTACAGTAGTGAGAGGAGACCAGATATGCATTGGATATCACTCCAACAATTCCACCGAAAAAGTCGACACAATTCTAGAAAGGAATGTCACTGTGACTCATGCTCAGGACATTCTTGAGAAGACTCACAATGGAAAATTATGCAAACTAAATGGAATCCCTCCACTTGAATTGGGAGATTGCAGCATCGCCGGATGGCTCCTTGG<br>AAATCCACAATGTGATGGACTTCTGACTGTGCCAGAATGGTCATATATAATGGAGAAAGAAAACCCAAGGAATGGCTTGTGCTACCCAGGCAGTTTCAATGACTATGAAGAATTGAAACACCTCCTTAGTAGTGTAACACACTTCGAAAAAGTGAAGATTCTGCCTAGGAATAGGTGGACACAGCATACAACAACTGGAGGTTCACAGGCCTGCGCAGTGTATGGTGGTCC<br>GTCATTCTTCAGAAACATGGTCTGGTTGACAAAGAAAGAGTCAAATTATCCAGTTGCCAAAGGATCATACAATAATACAAGTGGGGAACAAATGCTGGTCATTTGGGGAATACATCACCCCAATGATGAAACTGAACAGAGGACATTGTATCAGAATGTCGGGACCTATGTGTCAGTGGGAACATCGACACTTAACAAAAGATCAATTCCGGAAATAGCCACAAGACCTAA<br>AGTGAATGGACAAGGAGGCAGAATGGAATTCTCATGGACCATATTGGACATGTTGGACACCATAAATTTCGAGAGCACTGGCAATCTAATTGCACCGGAATATGGCTTTAAAATATCCAAACGGGGTAGTTCAGGGATCATGAAAACGGAAGGGACACTTGAAAACTGCGAAACCAAATGCCAAACTCCCTTAGGAGCAATAAATACAACATTGCCCTTTCATAATATCCA<br>CCCGTTAACCATTGGTGAGTGTCCCAAATATGTAAAATCGGAGAAGCTAGTCTTGGCAACAGGATTAAGAAATGTCCCTCAGATTGAATCAAGAGGATTGTTTGGGGCAATAGCTGGTTTCATAGAAGGAGGGTGGCAAGGAATGGTTGATGGTTGGTATGGATATCATCATAGTAATGATCAAGGATCCGGATATGCAGCAGACAAAGAGTCTACTCAAAAGGCAATTG<br>ATGGAATCACAAACAAGGTAAACTCCATAATTGAGAAAATGAACACTCAATTCGAAGCTGTTGGGAAAGAATTCAGTAATTTGGAGAAAAGGCTGGAAAACCTGAACAAAAAGATGGAAGACGGATTCCTAGATGTGTGGACATACAATGCTGAGCTTCTAGTCCTAATGGAAAATGAGAGGACACTTGACTTCCATGACTCCAATGTCAAGAACCTATATGACAAAGTCA<br>GGATGCAATTGAGGGACAATGCGAAAGAACTAGGGAATGGATGTTTTGAATTTTACCATAAATGTGATAATGAATGCATGAACAGCGTAAAGAATGGGACATATGATTATTCCAAGTATGAAGAGGAATCTAAACTAAACAGGAATGAAATCAAAGGAGTAAAATTGAGCAACATGGGGGTTTATCAAATACTTGCAATTTATGCCACAGTAGCAGGCTCTCTGTCACTGG<br>CAATCATGATAGCTGGGATCTCTTTATGGATGTGCTCTAACGGGTCTCTGCAATGCAGAATCTGCATATGATTACCAGTCGTTTTATAATTA
EPI_ISL_697588	>A/White-fronted_Goose/Ukraine/Kurganskoe-2-16-11/2011_H2N3_2011-11-16<br>ATGGCAATCATTTATCTGATTCTTCTGTTTACAGTAGTGAGGGGAGACCAGATATGCATTGGATACCACTCCAACAATTCCACAGAAAAGGTCGACACAATTCTAGAGAGGAATGTCACTGTGACTCATGCTCAGGACATTCTTGAGAAGACTCACAATGGGAAATTATGCAAACTAAATGGAATCCCTCCACTTGAATTGGGAGATTGCAGCATCGCCGGATGGCTCCTTGG<br>AAATCCAGAATGTGATAGACTTCTAACTGTACCAGAATGGTCATATATAATGGAGAAAGAAAACCCAAGGAATGGTTTGTGCTACCCAGGCAGTTTCAATGACTATGAAGAATTGAAACACCTACTCAGTAGTGTAACACACTTCGAGAAAGTGAAGATTTTGCCCAAAGATAGGTGGACACAGCATACAACAACTGGAGGTTCACGGGCCTGCGCAGTATCTGGTAATCC<br>GTCATTCTTCAGAAACATGGTCTGGTTGACAAAGAAAGGGTCAAATTATCCAGTTGCCAAAGGATCATACAATAATACAAGTGGGGAACAAATGCTGGTCATTTGGGGAGTACATCACCCCAATGATGAAGCTGAACAAAGGGCATTGTATCAGAATGTCGGGACCTATGTATCAGTGGGAACATCGACACTTAACAAAAGATCAGTTCCAGAAATAGCCACAAGACCTAA<br>AGTGAATGGACAAGGAGGCAGAATGGAATTCTCATGGACCATATTGGACATGTTGGACACCATAAATTTCGAGAGCACTGGTAATCTAATTGCACCGGAATATGGCTTTAAAATATCCAAACAGGGTAGTTCAGGAATCATGAAAACGGAAGGGACACTTGAAAATTGCGAAACTAAATGCCAAACTCCCTTAGGAGCAATAAACACAACATTGCCATTTCATAATATCCAC<br>CCATTAACCATTGGTGAGTGCCCCAAATATGTAAAATCGGAGAGGTTAGTCTTGGCAACAGGATTAAGAAATGTCCCTCAGATTGAGTCAAGAGGATTGTTTGGGGCAATAGCTGGTTTCATAGAAGGAGGATGGCAAGGAATGGTTGATGGTTGGTATGGATATCATCACAGTAATGATCAAGGATCCGGGTACGCAGCAGACAAGGAGTCCACTCAAAAGGCAATTG<br>ATGGAATCACCAACAAAGTAAACTCCGTAATTGAGAAAATGAACACTCAATTCGAGGCTGTTGGGAAAGAATTCAGTAATTTGGAGAAGAGGCTGGAAAACCTGAGCAAAAAGATGGAAGACGGATTCCTAGATGTGTGGACATACAATGCTGAGCTTCTAGTCCTAATGGAAAATGAGAGGACACTTGACTTCCATGACTCCAATGTCAAGAACCTATATGACAAAGTCA<br>GGATGCAATTGAGGGACAATGCGAAAGAACTAGGGAATGGATGTTTTGAATTTTACCATAAATGTGATGATGAATGCATGAACAGCGTAAAGAATGGAACATATGATTATCCCAAGTATGAAGAGGAATCTAAACTAAACAGGAATGAAATCAAAGGAGTAAAATTGAGCAACATGGGGGTTTATCAAATACTTGCAATTTATGCTACAGTAGCAGGCTCCCTGTCACTG<br>GCAATCATGATAGCTGGGATCTCTTTATGGATGTGCTCTAACGGGTCTCTGCAATGCAGAATCTGCATATGATTGTCAGTCGTTTTATAATTA
EPI_ISL_373055	>A/ruddy_turnstone/Delaware_Bay/413/2018_H2N1_2018-05-22<br>ATGGCAATCATTTATCTGATTCTTCTGTTTACAGTAGTGAGAGGAGACCAGATATGCATTGGATATCACTCCAACAATTCCACCGAAAAAGTCGACACAATTCTAGAAAGGAATGTCACTGTGACTCATGCTCAGGACATTCTTGAGAAGACTCACAATGGAAAATTATGCAAACTAAATGGAATCCCTCCACTTGAATTGGGAGATTGCAGCATCGCCGGATGGCTCCTTGG<br>AAATCCACAATGTGATGGACTTCTGACTGTGCCAGAATGGTCATATATAATGGAGAAAGAAAACCCAAGGAATGGCTTGTGCTACCCAGGCAGTTTCAATGACTATGAAGAATTGAAACACCTCCTTAGTAGTGTAACACACTTCGAAAAAGTGAAGATTCTGCCTAGGAATAGGTGGACACAGCATACAACAACTGGAGGTTCACAGGCCTGCGCAGTGTATGGTGGTCC<br>GTCATTCTTCAGAAACATGGTCTGGTTGACAAAGAAAGAGTCAAATTATCCAGTTGCCAAAGGATCATACAATAATACAAGTGGGGAACAAATGCTGGTCATTTGGGGAATACATCACCCCAATGATGAAACTGAACAGAGGACATTGTATCAGAATGTCGGGACCTATGTGTCAGTGGGAACATCGACACTTAACAAAAGATCAATTCCGGAAATAGCCACAAGACCTAA<br>AGTGAATGGACAAGGAGGCAGAATGGAATTCTCATGGACCATATTGGACATGTTGGACACCATAAATTTCGAGAGCACTGGCAATCTAATTGCACCGGAATATGGCTTTAAAATATCCAAACGGGGTAGTTCAGGGATCATGAAAACGGAAGGGACACTTGAAAACTGCGAAACCAAATGCCAAACTCCCTTAGGAGCAATAAATACAACATTGCCCTTTCATAATATCCA<br>CCCGTTAACCATTGGTGAGTGTCCCAAATATGTAAAATCGGAGAAGCTAGTCTTGGCAACAGGATTAAGAAATGTCCCTCAGATTGAATCAAGAGGATTGTTTGGGGCAATAGCTGGTTTCATAGAAGGAGGGTGGCAAGGAATGGTTGATGGTTGGTATGGATATCATCATAGTAATGATCAAGGATCCGGATATGCAGCAGACAAAGAGTCTACTCAAAAGGCAATTG<br>ATGGAATCACAAACAAGGTAAACTCCATAATTGAGAAAATGAACACTCAACTCGAAGCTGTTGGGAAAGAATTCAGTAATTTGGAGAAAAGGCTGGAAAACCTGAACAAAAAGATGGAAGACGGATTCCTAGATGTGTGGACATACAATGCTGAGCTTCTAGTCCTAATGGAAAATGAGAGGACACTTGACTTCCATGACTCCAATGTCAAGAACCTATATGACAAAGTCA<br>GGATGCAATTGAGGGACAATGCGAAAGAACTAGGGAATGGATGTTTTGAATTTTACCATAAATGTGATAATGAATGCATGAACAGCGTAAAGAATGGGACATATGATTATTCCAAGTATGAAGAGGAATCTAAACTAAACAGGAATGAAATCAAAGGAGTAAAATTGAGCAACATGGGGGTTTATCAAATACTTGCAATTTATGCCACAGTAGCAGGCTCTCTGTCACTGG<br>CAATCATGATAGCTGGGATCTCTTTATGGATGTGCTCTAACGGGTCTCTGCAATGCAGAATCTGCATATGATTACCAGTCGTTTTATAATTA
EPI_ISL_373070	>A/ruddy_turnstone/Delaware_Bay/246/2018_H2N7_2018-05-21<br>ATGGCAATCATTTATCTGATTCTTCTGTTTACAGTAGTGAGAGGAGACCAGATATGCATTGGATATCACTCCAACAATTCCACCGAAAAAGTCGACACAATTCTAGAAAGGAATGTCACTGTGACTCATGCTCAGGACATTCTTGAGAAGACTCACAATGGAAAATTATGCAAACTAAATGGAATCCCTCCACTTGAATTGGGAGATTGCAGCATCGCCGGATGGCTCCTTGG<br>AAATCCACAATGTGATGGACTTCTGACTGTGCCAGAATGGTCATATATAATGGAGAAAGAAAACCCAAGGAATGGCTTGTGCTACCCAGGCAGTTTCAATGACTATGAAGAATTGAAACACCTCCTTAGTAGTGTAACACACTTCGAAAAAGTGAAGATTCTGCCTAGGAATAGGTGGACACAGCATACAACAACTGGAGGTTCACAGGCCTGCGCAGTGTATGGTGGTCC<br>GTCATTCTTCAGAAACATGGTCTGGTTGACAAAGAAAGAGTCAAATTATCCAGTTGCCAAAGGATCATACAATAATACAAGTGGGGAACAAATGCTGGTCATTTGGGGAATACATCACCCCAATGATGAAACTGAACAGAGGACATTGTATCAGAATGTCGGGACCTATGTGTCAGTGGGAACATCGACACTTAACAAAAGATCAATTCCGGAAATAGCCACAAGACCTAA<br>AGTGAATGGACAAGGAGGCAGAATGGAATTCTCATGGACCATATTGGACATGTTGGACACCATAAATTTCGAGAGCACTGGCAATCTAATTGCACCGGAATATGGCTTTAAAATATCCAAACGGGGTAGTTCAGGGATCATGAAAACGGAAGGGACATTTGAAAACTGCGAAACCAAATGCCAAACTCCCTTAGGAGCAATAAATACAACATTGCCCTTTCATAATATCCAC<br>CCGTTAACCATTGGTGAGTGTCCCAAATATGTAAAATCGGAGAAGCTAGTCTTGGCAACAGGACCAAGAAATGTCCCTCAGATTGAATCAAGAGGATTGTTTGGGGCAATAGCTGGTTTCATAGAAGGAGGGTGGCAAGGAATGGTTGATGGTTGGTATGGATATCATCATAGTAATGATCAAGGATCCGGATATGCAGCAGACAAAGAGTCTACTCAAAAGGCAATTGA<br>TGGAATCACCAACAAGGTAAACTCCATAATTGAGAAAATGAACACTCAATTCGAAGCTGTTGGGAAAGAATTCAGTAATTTGGAGAGAAGGCTGGAAAACCTGAACAAAAAGATGGAAGACGGATTCCTAGATGTGTGGACATACAATGCTGAGCTTCTAGTCCTAATGGAAAATGAGAGGACACTTGACTTCCATGACTCCAATGTCAAGAACCTATATGACAAAGTCAG<br>GATGCAATTGAGGGACAATGCGAAAGAACTAGGGAATGGATGTTTTGAATTTTACCATAAATGTGATAATGAATGCATGAACAGCGTAAAGAATGGGACATATGATTATCCCAAGTATGAAGAGGAATCTAAACTAAACAGGAATGAAATCAAAGGAGTAAAATTGAGCAACATGGGGGTTTATCAAATACTTGCAATTTATGCCACAGTAGCAGGCTCTCTGTCACTGGC<br>AATCATGATAGCTGGGATCTCTTTATGGATGTGCTCTAACGGGTCTCTGCAATGCAGAATCTGCATATGATTACCAGTCGTTTTATAATTA
EPI_ISL_397752	>A/ruddy_turnstone/South_Carolina/UGAI17-4979/2017_H2N5_2017-12-09<br>ATGGCAATCATTTATCTGATTCTTCTGTTTACAGTAGTGAGAGGAGACCAGATATGCATTGGATATCACTCCAACAATTCCACCGAAAAAGTCGACACAATTCTAGAAAGGAATGTCACTGTGACTCATGCTCAGGACATTCTTGAGAAGACTCACAATGGAAAATTATGCAAACTAAATGGAATCCCTCCACTTGAATTGGGAGATTGCAGCATCGCCGGATGGCTCCTTGG<br>AAATCCAGAATGTGATAGACTTCTAACTGTGCCAGAATGGTCATATATAATGGAGAAAGAAAACCCAAGGAATGGTTTGTGCTACCCAGGCAGTTTCAATGACTATGAAGAATTGAAACACCTCCTTAGTAGTGTAACACACTTCGAAAAAGTGAAGATTCTGCCTAGGAATGGGTGGACACAGCATACAACAACTGGAGGTTCACAGGCCTGCGCAGTGTATGGTGGTCC<br>GTCATTCTTCAGAAACATGGTCTGGTTGACAAAGAAAGGGTCAAATTATCCAGTTGCCAAAGGATCATACAATAATACAAGTGGGGAACAAATGCTGGTCATTTGGGGAATACATCACCCCAATGATGAAACTGAACAGAGGACATTGTATCAGAATGTCGGGACCTATGTGTCAGTGGGAACATCGACACTTAACAAAAGATCAATTCCGGAAATAGCCACAAGACCTAA<br>AGTGAATGGACAAGGAGGCAGAATGGAATTCTCATGGACCATATTGGACATGTTGGACACCATAAATTTCGAGAGCACTGGCAATCTAATTGCACCGGAATATGGCTTTAAAATATCCAAACGGGGTAGTTCAGGGATCATGAAAACGGAAGGGGCACTTGAAAATTGCGAAACCAAATGCCAAACTCCCTTAGGAGCAATAAATACAACATTGCCCTTTCATAATATCCA<br>CCCGTTAACCATTGGTGAGTGCCCCAAATATGTAAAATCGGAGAAGCTAGTCTTGGCAACAGGATTAAGAAATGTCCCTCAGATTGAATCAAGAGGATTGTTTGGGGCAATAGCTGGTTTCATAGAAGGAGGGTGGCAAGGAATGGTTGATGGTTGGTATGGATATCATCATAGTAATGATCAAGGATCCGGATATGCAGCAGACAAAGAGTCTACTCAAAAGGCAATTG<br>ATGGAATCACCAACAAGGTAAACTCCATAATTGAGAAAATGAACACTCAATTCGAAGCTGTTGGGAAAGAATTCAGTAATTTGGAGAAAAGGCTGGAAAACCTGAACAAAAAGATGGAAGACGGATTCCTAGATGTGTGGACATACAATGCTGAGCTTCTAGTCCTAATGGAAAATGAGAGGACACTTGACTTCCATGACTCCAATGTCAAGAACCTATATGACAAAGTCA<br>GGATGCAATTGAGGGACAATGCGAAAGAACTAGGGAATGGATGTTTTGAATTTTACCATAAATGTGATAATGAATGCATGAACAGCGTAAAGAATGGGACATATGATTATCCCAAGTATGAAGAGGAATCTAAACTAAACAGGAATGAAATCAAAGGAGTAAAATTGAGCAACATGGGGGTTTATCAAATACTTGCAATTTATGCCACAGTAGCAGGCTCTCTGTCACTG<br>GCAATCATGATAGCTGGGATCTCTTTATGGATGTGCTCTAACGGGTCTCTGCAATGCAGAATCTGCATATGATTATCAGTCGTTTTATAATTA

EPI_ISL_122034	>A/northern_shoveler/Georgia/1/2010_H2N3_2010-01-01<br>ATGGCAATCATTTATCTGATTCTTCTGTTTACAGTAGTGAGAGGAGACCAGATATGCATTGGATACCACTCCAACAATTCCACAGAAAAGGTCGACACAATTCTAGAGAGGAATGTCACTGTGACTCATGCTCAGGACATTCTTGAGAAGACTCACAATGGAAAATTATGCAAACTAAATGGAATCCCTCCACTTGAATTGGGAGATTGCAGCATCGCCGGATGGCTCCTTGG<br>AAATCCAGAATGTGATAGACTTCTAACTGTACCAGAATGGTCATATATAATGGAGAAAGAAAACCCAAGGAATGGTTTGTGCTATCCAGGCAGTTTCAATGACTATGAAGAATTGAAACACCTCCTTAGCAGTGTAACACACTTCGAGAAAGTGAAGATTTTGCCCAAAGATAGGTGGACACAGCATACAACAACTGGAGGTTCACGGGCCTGCGCAGTATCTGGTAATCC<br>GTCATTCTTCAGAAACATGGTCTGGTTGACAAAGAAAGGGTCAAATTATCCAGTTGCCAAAGGATCATACAATAATACAAGTGGGGAACAAATGCTGGTCATTTGGGGAGTACATCACCCCAATGATGAAGCTGAACAAAGGGCATTGTATCAGAATGTCGGGACCTATGTATCAGTGGGAACATCGACACTTAACAAAAGATCAGTTCCAGAAATAGCCACAAGACCTAA<br>AGTGAATGGACAAGGAGGCAGAATGGAATTCTCATGGACCATATTGGACATGTTGGACACCATAAATTTCGAGAGCACTGGTAATCTAATTGCACCGGAATATGGCTTTAAAATATCCAAACGAGGTAGTTCAGGAATCATGAAAACGGAAGGGACACTTGAAAATTGCGAAACTAAATGCCAAACTCCCTTAGGAGCAATAAATACAACATTGCCCTTTCATAATATCCAC<br>CCATTAACCATTGGTGAGTGCCCCAAATATGTAAAATCGGAGAGGTTAGTCTTGGCAACAGGATTAAGAAATGTCCCTCAGATTGAATCGAGAGGATTGTTTGGGGCAATAGCTGGTTTCATAGAAGGAGGATGGCAAGGAATGGTTGATGGTTGGTATGGATATCATCACAGTAATGATCAAGGATCCGGGTATGCAGCAGACAAGGAGTCCACTCAAAAGGCAATTGA<br>TGGAATCACCAACAAAGTAAACTCAGTAATTGAGAAAATGAACACTCAATTCGAGGCTGTTGGGAAAGAATTCAGTAATTTGGAGAAAAGGCTGGAAAACCTGAACAAAAAGATGGAAGACGGATTCCTAGATGTGTGGACATACAATGCTGAGCTTCTAGTCCTAATGGAAAATGAGAGGACACTTGACTTCCATGACTCCAATGTCAAGAACCTATATGACAAAGTCAG<br>GATGCAATTGAGGGACAATGCGAAAGAACTAGGGAATGGATGTTTTGAATTTTATCATAAATGTGATGATGAATGCATGAACAGCGTAAAGAATGGGACATATGATTATCCCAAGTATGAAGAGGAATCTAAACTAAACAGGAATGAAATCAAAGGAGTAAAATTGAGCAACATGGGGGTTTATCAAATACTTGCAATTTATGCTACAGTAGCAGGCTCCCTGTCACTGGC<br>AATCATGATAGCTGGGATCTCTTTATGGATGTGCTCTAACGGGTCTCTGCAATGCAGAATCTGCATATGATTATCAGTCGTTTTATAATTA
EPI_ISL_267297	>A/mallard_duck/Netherlands/16/2014_H2N9_2014-08-29<br>ATGGCAGTCATTTACCTGATTCTTCTGTTTACAGTAGTGAGAGGAGACCAGATATGCATTGGATACCACTCCAACAATTCCACAGAAAAGGTCGACACAATTCTAGAGAGGAATGTCACTGTGACTCATGCTCAGGACATTCTTGAGAAGACTCACAATGGAAAATTATGCAAACTAAATGGAATCCCTCCACTTGAATTGGGAGATTGCAGCATCGCCGGATGGCTCCTTGG<br>AAATCCAGAATGTGATAGACTTCTGACTGTACCAGAATGGTCATATATAATGGAGAAAGAAAACCCAAGAAATGGTTTGTGCTACCCAGGCAGTTTCAATGACTATGAAGAATTGAAACACCTCCTTAGTAGCGTAACACACTTCGAGAAAGTGAAGATTTTGCCCAAAGATAGATGGACACAGCATACAACAACTGGAGGTTCACGGGCCTGCGCAGTATTTGGTAATCCG<br>TCATTCTTCAGAAACATGGTCTGGTTGACAAAGAAAGGGTCAAATTATCCAGTTGCTAAAGGATCATACAATAATACGAGTGGGGAACAAATGCTGGTCATTTGGGGAGTACATCACCCCAATGATGAAAATGAGCAAAGGAATTTGTACCAGAATGTCGGGACCTATGTATCAGTGGGAACATCGACACTTAACAAAAGATCAATTCCAGAAATAGCCACAAGACCTAAA<br>GTGAATGGACAAGGAGGCAGAATGGAATTCTCATGGACCATATTGGACATGTTGGACACCATAAATTTCGAGAGCACTGGTAATCTAATTGCACCGGAATATGGCTTTAAAATATCCAAACGGGGTAGTTCAGGAATCATGAAAACGGAAGGGACACTTGAAAACTGCGAGACTAAATGTCAAACTCCCTTAGGAGCAATAAATACAACATTGCCCTTTCATAATATCCACC<br>CATTAACCATTGGTGAGTGCCCCAAATATGTAAAATCGGAGAGGTTAGTCTTGGCAACAGGATTAAGAAATGTCCCTCAGATTGAATCAAGAGGATTGTTTGGGGCAATAGCTGGTTTCATAGAAGGAGGATGGCAAGGAATGGTTGATGGTTGGTATGGATATCATCACAGTAATGATCAAGGATCCGGGTATGCAGCAGACAAAGAGTCCACTCAGAAGGCAATTGAT<br>GGAATCACCAACAAAGTAAACTCCGTAATTGAGAAAATGAACACTCAATTCGAGGCTGTTGGGAAAGAATTCAGTAATTTGGAGAAAAGGCTGGAAAACCTGAACAAAAAGATGGAAGACGGATTCCTAGATGTGTGGACATACAATGCTGAGCTTCTAGTCCTAATGGAAAATGAGAGGACACTTGACTTCCATGACTCCAATGTCAAGAACCTGTATGACAAAGTTAG<br>GATGCAATTGAGGGACAATGCAAAAGAACTGGGGAATGGATGTTTTGAATTTTACCATAAATGTGATGATGAGTGCATGAACAGCGTAAAAAATGGAACATATGATTATCCCAAGTATGAGGAGGAATCTAAACTAAACAGGAATGAAATCAAAGGAGTAAAATTGAGCAACATGGGGATTTATCAGATACTTGCAATTTATGCTACAGTAGCAGGCTCCCTGTCACTGGC<br>AATCATGATAGCTGGGATCTCTTTATGGATGTGCTCTAACGGGTCTCTGCAATGCAGAATCTGCATATGATTATCAGTCGTTTTGTAATTA
EPI_ISL_267194	>A/mallard_duck/Netherlands/14/2014_H2N9_2014-08-27<br>ATGGCAGTCATTTACCTGATTCTTCTGTTTACAGTAGTGAGAGGAGACCAGATATGCATTGGATACCACTCCAACAATTCCACAGAAAAGGTCGACACAATTCTAGAGAGGAATGTCACTGTGACTCATGCTCAGGACATTCTTGAGAAGACTCACAATGGAAAATTATGCAAACTAAATGGAATCCCTCCACTTGAATTGGGAGATTGCAGCATCGCCGGATGGCTCCTTGG<br>AAATCCAGAATGTGATAGACTTCTGACTGTACCAGAATGGTCATATATAATGGAGAAAGAAAACCCAAGAAATGGTTTGTGCTACCCAGGCAGTTTCAATGACTATGAAGAATTGAAACACCTCCTTAGTAGCGTAACACACTTCGAGAAAGTGAAGATTTTGCCCAAAGATAGATGGACACAGCATACAACAACTGGAGGTTCACGGGCCTGCGCAGTATTTGGTAATCCG<br>TCATTCTTCAGAAACATGGTCTGGTTGACAAAGAAAGGGTCAAATTATCCAGTTGCTAAAGGATCATACAATAATACGAGTGGGGAACAAATGCTGGTCATTTGGGGAGTACATCACCCCAATGATGAAAATGAGCAAAGGAATTTGTACCAGAATGTCGGGACCTATGTATCAGTGGGAACATCGACACTTAACAAAAGATCAATTCCAGAAATAGCCACAAGACCTAAA<br>GTGAATGGACAAGGAGGCAGAATGGAATTCTCATGGACCATATTGGACATGTTTGACACCATAAATTTCGAGAGCACTGGTAATCTAATTGCACCGGAATATGGCTTTAAAATATCCAAACGGGGTAGTTCAGGAATCATGAAAACGGAAGGGACACTTGAAAATTGCGAGACTAAATGTCAAACTCCCTTAGGAGCAATAAATACAACACTGCCCTTTCATAATATCCACC<br>CATTAACCATTGGTGAGTGCCCCAAATATGTAAAATCGGAGAGGTTAGTCTTGGCAACAGGATTAAGAAATGTCCCTCAGATTGAATCAAGAGGATTGTTTGGGGCAATAGCTGGTTTCATAGAAGGAGGATGGCAAGGAATGGTTGATGGTTGGTATGGATATCATCACAGTAATGATCAAGGATCCGGGTATGCAGCAGACAAAGAGTCCACTCAGAAGGCAATTGAT<br>GGAATCACCAACAAAGTAAACTCCGTAATTGAGAAAATGAACACTCAATTCGAGGCTGTTGGGAAAGAATTCAGTAATTTGGAGAAAAGGCTGGAAAACCTGAACAAAAAGATGGAAGACGGATTCCTAGATGTGTGGACATACAATGCTGAGCTTCTAGTCCTAATGGAAAATGAGAGGACACTTGACTTCCATGACTCCAATGTCAAGAACCTGTATGACAAAGTTAG<br>GATGCAATTGAGGGACAATGCAAAAGAACTGGGGAATGGATGTTTTGAATTTTACCATAAATGTGATGATGAGTGCATGAACAGCGTAAAAAATGGAACATATGATTATCCCAAGTATGAGGAGGAATCTAAACTAAACAGGAATGAAATCAAAGGAGTAAAATTGAGCAACATGGGGATTTATCAGATACTTGCAATTTATGCTACAGTAGCAGGCTCCCTGTCACTGGC<br>AATCATGATAGCTGGGATCTCTTTATGGATGTGCTCTAACGGGTCTCTGCAATGCAGAATCTGCATATGATTATCAGTCGTTTTGTAATTA

HA (H3)

Isolate ID	Sequence
EPI_ISL_337569	>A/mallard/Novosibirsk_region/963k/2018_H3N2_2018-09-29<br>ATGAAGACCATTATCGCTTTAAGCTACGTTCTCTGTCTGGCCTTCAGTCAGGACCTTCCAGGGAGCGACAATAGTACAGCAACACTGTGCCTGGGACACCATGCAGTGCCGAATGGGACAATAGTGAAGACCATTACAGATGATCAGATTGAGGTGACTAATGCTACCGAGCTAGTTCAAAGCTCTTCAACAGGAAAGATATGCAACAATCCTCACAGGATTCTTGATGGAA<br>GGGACTGCACACTAATAGATGCTCTACTGGGGGATCCCCATTGTGATGTCTTTCAAGATGAGACATGGGATCTTTTTGTGGAGCGAAGCAATGCTTTCAGCAATTGCTACCCTTACGATGTACCAGACTATGCATCCCTTCGATCCCTAGTTGCATCATCAGGCACACTGGAGTTCATCACTGAAGGTTTCACTTGGACAGGAGTAACTCAGAATGGAGGGAGCGGTGCTTGC<br>AAAAGGGGACCTGCTAACGGTTTCTTCAGTAGACTGAACTGGTTGACTAAGTCAGGAAGCGCATACCCAGTGTTGAACGTGACTATGCCAAACAATGACAATTTTGACAAACTGTACATATGGGGAGTTCACCACCCGAGTACAAACCAAGAACAAACCAACCTGTATGTTCAAGTCTCAGGAAGAGTCACAGTCTCTACCAGGAGAAGCCAGCAGACTATAATCCCTAACA<br>TTGGGTCTAGACCCTGGGTAAGGGGGCAGTCTGGCAGAATAAGCATCTACTGGACAATAGTCAAACCTGGGGACGTGCTGGTGATCAATAGTAATGGAAACCTAATCGCTCCTCGGGGCTACTTCAAGATGCGAACTGGGAAAAGCTCAATAATGAGGTCAGATGCACCTATTGACACTTGCATTTCTGAATGCATCACCCCAAATGGAAGCATCCCCAATGACAAGCCCT<br>TTCAAAATGTGAACAAGATCACATACGGAGCATGTCCCAAATATGTGAAGCAGAACACCCTGAAGTTGGCAACAGGAATGCGAAATGTACCAGAAAAGCAAACCAGAGGCCTATTCGGTGCAATAGCAGGCTTTATAGAAAATGGATGGGAAGGAATGATAGATGGTTGGTACGGCTTCAGGCATCAAAATTCCGAAGGCACCGGACAAGCAGCAGACCTTAAAAGCAC<br>TCAGGCAGCCATTGATCAGATCAATGGGAAATTGAACAGAGTGATTGAAAAGACGAATGAGAAATTCCATCAAATTGAAAAGGAATTCTCTGAGGTAGAAGGGAGGATTCAGGACCTTGAGAAATACGTTGAAGACACAAAAATAGATCTTTGGTCTTATAATGCGGAACTCCTTGTCGCCCTAGAGAATCAGCATACAATTGATTTGACTGATTCAGAAATGAACAAATT<br>GTTTGAAAAGACCAGAAGGCAACTGAGGGAAAATGCTGAAGACATGGGCAATGGTTGTTTCAAAATATACCACAAGTGTGACAATGCCTGCATAGAGTCAATTAGGAACGGGACTTATGACCATGACATATACAGAGATGAGGCATTGAATAACCGGTTCCAGATCAAGGGTGTCGAGCTAAAGTCTGGATATAAAGACTGGATCCTGTGGATTTCCTTTGCCATATCATG<br>CTTTTTGCTTTGTGTTGTTTTGCTGGGGTTCATTATGTGGCCTGCCAGAGAGGCAACATTAGGTGCAACATTTGC
EPI_ISL_333615	>A/gadwall/Chany/893/2018_H3N8_2018-10-19<br>ATGAAGACCATTATCGCTTTAAGCTACGTTTTCTGTCTGGCCCTCAGTCAGGACCTTCCAGGGAGCGACAATAGTACAGCAACACTGTGCCTGGGACACCATGCAGTGCCGAATGGGACAATAGTGAAGACCATTACAGATGATCAGATTGAGGTGACTAATGCTACCGAGCTAGTTCAAAGCTCTTCAACAGGAAAGATATGCAACAATCCTCACAGGATTCTTGATGGAA<br>GGGACTGCACACTAATAGATGCCCTACTGGGGGATCCCCATTGTGATGTCTTTCAAGATGAGACATGGGATCTTTTTGTGGAGCGAAGCAATGCTTTCAGCAATTGCTACCCTTACGATGTACCAGACTATGCATCCCTTCGATCCCTAGTTGCATCATCAGGCACATTGGAGTTCATCACTGAAGGTTTCACTTGGACAGGAGTAACTCAGAATGGAGGGAGCGGTGCTTGC<br>AAAAGGGGACCTGCTAACGGTTTCTTCAGTAGACTGAACTGGTTGACTAAGTCAGGAAGCGCATACCCAGTGTTGAACGTGACTATGCCAAACAATGACAATTTTGACAAATTGTACATATGGGGAGTTCACCACCCGAGCACAAACCAAGAACAAACCAACCTGTATGTTCAAGCCTCAGGAAGAGTCACAGTCTCTACCAGGAGAAGCCAGCAGACTATAATCCCTAACA<br>TTGGGTCTAGACCCTGGGTAAGGGGCCAGTCTGGCAGAATAAGCATCTACTGGACAATAGTCAAACCTGGGGACGTGCTGGTGATCAATAGTAATGGAAACCTAATCGCTCCTCGGGGCTACTTCAAGATGCGAACTGGGAAAAGCTCAATAATGAGGTCAGATGCACCTATTGACACTTGCATTTCTGAATGCATCACCCCAAATGGAAGCATCCCCAATGACAAGCCCT<br>TTCAAAATGTGAACAAGATCACATACGGAGCATGTCCCAAATATGTGAAGCAGAACACCCTGAAGTTGGCAACAGGAATGCGAAATGTACCAGAAAAGCAAACCAGAGGCCTATTCGGTGCAATAGCAGGCTTTATAGAAAATGGATGGGAAGGAATGATAGATGGTTGGTACGGCTTCAGGCACCAAAATTCCGAAGGCACCGGACAAGCAGCAGACCTTAAAAGCAC<br>TCAGGCAGCCATTGATCAGATCAATGGGAAATTGAACAGAGTGATTGAAAAGACGAATGAGAAATTCCATCAAATTGAAAAGGAATTCTCTGAGGTAGAAGGGAGGATTCAGGACCTTGAGAAATACGTTGAAGACACAAAAATAGATCTTTGGTCTTATAATGCGGAACTCCTTGTCGCCCTAGAGAATCAGCATACAATTGATTTGACTGATTCAGAAATGAACAAATT<br>GTTTGAAAAGACCAGAAGGCAACTGAGGGAAAATGCTGAAGACATGGGCAATGGTTGTTTCAAAATATACCACAAGTGTGACAATGCCTGCATAGAGTCAATTAGGAACGGGACTTATGACCATGACATATACAGAGATGAGGCATTGAATAACCGGTTTCAGATCAAGGGTGTCGAGCTAAAGTCTGGATATAAAGACTGGATCCTGTGGATTTCCTTTGCCATATCATG<br>CTTTTTGCTTTGTGTTGTTTTGCTGGGGTTCATTATGTGGCCTGCCAGAGAGGCAACATTAGGTGCAACATTTGC
EPI_ISL_503358	>A/duck/Mongolia/876/2019_H3N8_2019-09-20<br>ATGAAGACCATTATCGCTTTAAGCTACGTTCTCTGTCTGGCCTTCAGTCAGGACCTTCCAGGGAGCGACAATAGTACAGCAACACTGTGTCTGGGACACCATGCAGTGCCGAATGGGACAATAGTGAAGACCATTACAGATGATCAGATTGAGGTGACTAATGCTACCGAGCTAGTTCAAAGCTCTTCAACAGGAAAGATATGCAACAATCCTCACAGGATTGTTGATGGAA<br>GGGACTGCACACTAATAGATGCCCTACTGGGGGATCCCCATTGTGATGTCTTTCAAGATGAGACATGGGATCTTTTTGTGGAGCGAAGCAATGCTTTCAGCAATTGCTACCCTTACGATGTACCAGACTATGCATCCCTTCGATCCCTAGTTGCATCATCAGGCACACTGGAGTTCATCACTGAAGGTTTCACTTGGACAGGAGTAACTCAGAATGGAGGGAGCGGTGCTTG<br>CAAAAGGGGACCTGCTAACGGTTTCTTCAGTAGACTGAACTGGTTGACTAAGTCAGGAAGCGCATACCCAGTGTTGAACGTGACTATGCCAAACAATGACAATTTTGACAAACTGTACATATGGGGAGTTCACCACCCGAGTACAAACCAAGAACAAACCAACCTGTATGTTCAAGCCTCAGGAAGAGTCACAGTCTCTACCAGAAGAAGCCAGCAGACTATAATCCCTAAC<br>ATTGGGTCTAGACCCTGGGTAAGGGGGCAGTCTGGCAGAATAAGCATCTACTGGACAATAGTCAAACCTGGGGACGTGCTGGTGATCAATAGTAATGGAAACCTAATCGCTCCTCGGGGCTACTTCAAGATGCGAACTGGGAAAAGCTCAATAATGAGGTCAGATGCACCTATTGACACTTGCATTTCTGAATGCATCACCCCAAATGGAAGCATCCCCAATGACAAGCCC<br>TTTCAAAATGTGAACAAGATCACATACGGAGCATGTCCCAAATATGTGAAGCAAAACACCCTGAAGTTGGCAACAGGAATGCGAAATGTACCAGAAAAGCAAACCAGAGGCCTATTCGGTGCAATAGCAGGCTTTATAGAAAATGGATGGGAAGGAATGATAGATGGTTGGTACGGCTTCAGGCATCAAAATTCCGAAGGCACCGGACAAGCAGCAGACCTTAAAAGCA<br>CTCAGGCAGCCATTGATCAGATCAATGGGAAATTGAACAGAGTGATTGAAAAGACGAATGAGAAATTCCATCAAATTGAAAAGGAATTCTCTGAGGTAGAAGGGAGGATTCAGGACCTTGAGAAATACGTTGAAGACACAAAAATAGATCTTTGGTCTTATAATGCGGAACTCCTTGTCGCCCTAGAGAATCAGCATACAATTGATTTGACTGATTCAGAAATGAACAAAT<br>TGTTTGAAAAGACCAGAAGGCAACTGAGGGAAAATGCTGAAGACATGGGCAATGGTTGTTTCAAAATATACCACAAGTGTGACAATGCCTGCATAGAGTCAATTAGGAACGGGACTTATGACCATGACATATACAGAGATGAGGCATTGAATAACCGGTTCCAGATCAAGGGTGTCGAGCTAAAGTCTGGATATAAAGACTGGATCCTGTGGATTTCCTTTGCCATATCAT<br>GCTTTTTGCTTTGTGTTGTTTTGCTGGGGTTCATTATGTGGCCTGCCAGAGAGGCAACATTAGGTGCAACATTTGC
EPI_ISL_338357	>A/Tufted_Duck/Novosibirsk_region/989k/2018_H3N8_2018-10-01<br>ATGAAGACCATTATCGCTTTAAGCTACGTTTTCTGTCTGGCCTTCAGTCAGGACCTTCCAGGGAGCGACAATAGTACAGCAACACTGTGCCTGGGACACCATGCAGTGCCGAATGGGACAATAGTGAAGACCATTACAGATGATCAGATTGAGGTGACTAATGCTACCGAGCTAGTTCAAAGCTCTTCAACAGGAAAGATATGCAACAATCCTCACAGGATTCTTGATGGAA<br>GGGACTGCACACTAATAGATGCCCTACTGGGGGATCCCCATTGTGATGTCTTTCAAGATGAGACATGGGATCTTTTTGTGGAGCGAAGCAATGCTTTCAGCAATTGCTACCCTTACGATGTACCAGACTATGCATCCCTTCGATCCCTAGTTGCATCATCAGGCACATTGGAGTTCATCACTGAAGGTTTCACTTGGACAGGAGTAACTCAGAATGGAGGGAGCGGTGCTTGC<br>AAAAGGGGACCTGCTAACGGTTTCTTCAGTAGACTGAACTGGTTGACTAAGTCAGGAAGCGCATACCCAGTGTTGAACGTGACTATGCCAAACAATGACAATTTTGACAAATTGTACATATGGGGAGTTCACCACCCGAGCACAAACCAAGAACAAACCAACCTGTATGTTCAAGCCTCAGGAAGAGTCACAGTCTCTACCAGGAGAAGCCAGCAGACTATAATCCCTAACA<br>TTGGGTCTAGACCCTGGGTAAGGGGCCAGTCTGGCAGAATAAGCATCTACTGGACAATAGTCAAACCTGGGGACGTGCTGGTGATCAATAGTAATGGAAACCTAATCGCTCCTCGGGGCTACTTCAAGATGCGAACTGGGAAAAGCTCAATAATGAGGTCAGATGCACCTATTGACACTTGCATTTCTGAATGCATCACCCCAAATGGAAGCATTCCCAATGACAAGCCCT<br>TTCAAAATGTGAACAAGATCACATACGGAGCATGTCCCAAATATGTGAAGCAGAACACCCTGAAGTTGGCAACAGGAATGCGAAATGTACCAGAAAAGCAAACCAGAGGCCTATTCGGTGCAATAGCAGGCTTTATAGAAAATGGATGGGAAGGAATGATAGATGGTTGGTACGGCTTCAGGCACCAAAATTCCGAAGGCACCGGACAAGCAGCAGACCTTAAAAGCAC<br>TCAGGCAGCCATTGATCAGATCAATGGGAAATTGAACAGAGTGATTGAAAAGACGAATGAGAAATTCCATCAAATTGAAAAAGAATTCTCTGAGGTAGAAGGGAGGATTCAGGACCTTGAGAAATACGTTGAAGACACAAAAATAGATCTTTGGTCTTATAATGCGGAACTCCTTGTCGCCCTAGAGAATCAGCATACAATTGATTTGACTGATTCAGAAATGAACAAATT<br>GTTTGAAAAGACCAGAAGGCAACTGAGGGAAAATGCTGAAGACATGGGCAATGGTTGTTTCAAAATATACCACAAGTGTGACAATGCCTGCATAGAGTCAATTAGGAACGGGACTTATGACCATGACATATACAGAGATGAGGCATTGAATAACCGGTTTCAGATCAAGGGTGTCGAGCTAAAGTCTGGATATAAAGACTGGATCCTGTGGATTTCCTTTGCCATATCATG<br>CTTTTTGCTTTGTGTTGCTTTGCTGGGGTTCATTATGTGGCCTGCCAGAGAGGCAACATTAGGTGCAACATTTGC
EPI_ISL_502612	>A/Anas_platyrhynchos/Belgium/7827/2018_H3N8_2018-01-09<br>ATGAAGACCATCATCGCTTTAAGCTACATTTTCTGTCTGGCCTTCGGTCAGGACCTTTCAGGGGGCGACAATAGTACAGCAACACTGTGCCTGGGACACCATGCAGTACCGAATGGGACAATAGTGAAGACCATTACAGATGATCAGATTGAGGTGACTAATGCTACCGAGCTAGTTCAAAGCTCTTCAACAGGGAAGATATGCAACAATCCTCACAGGATTCTTGATGGAA<br>GGGATTGCACACTAATAGATGCCTTACTGGGGGATCCTCATTGCGATGTCTTTCAAGATGAGACATGGGATCTTTTTGTGGAACGAAGCAATGCTTTCAGCAATTGCTACCCTTACGATGTACCAGACTACGCATCCCTTCGATCCCTAGTTGCATCATCAGGCACATTGGAGTTCATCACTGAAGGTTTCACTTGGACAGGAGTAACTCAGAATGGAGGGAGCGGTGCTTGC<br>AAAAGGGGACCTGCTAATGGTTTCTTCAGTAGACTGAATTGGTTGACTAAGTCAGGGAGCGCATACCCAGTGTTGAACGTGACTATGCCAAACAATGACGGTTTTGACAAACTGTACATATGGGGAGTTCACCACCCGAGCACAAACCAAGAACAAACCAGCCTGTATGTTCAAGCCTCAGGAAGAGTCACAGTCTCTACCAGGAGAAGCCAGCAGACTATAATCCCTAAC<br>ATTGGATCTAGACCCTGGGTAAGGGGCCAGTCTGGCAGAATAAGCATCTACTGGACAATAGTCAAACCTGGGGACATACTGGTGATCAATAGTAATGGAAACCTAATCGCTCCTCGGGGCTACTTCAAGATGCGAACTGGGAAAAGCTCAATAATGAGGTCAGATGCACCTATTGACACCTGCATTTCTGAATGCATCACCCCAAATGGGAGCATCCCCAACGACAAGCCC<br>TTTCAAAATGTGAACAAGATCACATATGGAGCATGTCCCAAATATGTGAAGCAGAACACCCTGAAGTTGGCAACAGGAATGCGAAATGTACCAGAAAAGCAAACCAGAGGCCTATTCGGTGCAATAGCAGGCTTTATAGAAAATGGATGGGAAGGAATGATAGACGGCTGGTACGGCTTCAGGCATCAAAATTCCGAAGGCACCGGACAAGCAGCAGACCTTAAAAGCA<br>CTCAGGCAGCCATTGATCAGATCAATGGGAAATTGAACAGAGTGATTGAAAAGACGAATGAGAAGTTCCATCAAATTGAAAAGGAATTCTCTGAGGTAGAAGGGAGGATTCAGGACCTTGAGAAATACGTTGAAGACACAAAAATAGATCTTTGGTCTTATAATGCGGAACTCCTTGTTGCCCTAGAGAATCAGCATACAATTGAATTGACTGATTCAGAAATGAACAAAT<br>TGTTYGAAAAGACCAGAAGGCAATTGAGGGAAAATGCTGAAGACATGGGCAATGGTTGTTTCAAAATATACCACAAGTGTGACAATGCCTGCATAGAATCAATTAGGAACGGGACTTATGACCATGACATATACAGAGATGAAGCATTGAATAACCGGTTCCAGATCAAGGGTGTCGAACTAAAATCTGGGTATAAAGATTGGATCCTGTGGATTTCCTTTGCCATATCAT<br>GCTTTTTGCTTTGTGTTGTTTTGCTGGGGTTCATTATGTGGGCCTGCCAGAGAGGCAACATCAGGTGCAACATTTGC
EPI_ISL_14989784	>A/mallard/Netherlands/22014777-004/2022_H3N2_2022-08-28<br>ATGAAGACCATCATCGCTTTAAGCTACATTTTCTGTCTGGCCTTCGGTCAGGACCTTTCAGGGAGCGACAATAGTACAGCAACACTGTGCCTGGGACACCATGCAGTACCGAATGGGACAATAGTGAAGACCATTACAGATGATCAGATTGAGGTGACTAATGCTACCGAGCTAGTTCAAAGCTCTTCAACAGGGAAGATATGCAACAATCCTCACAGGATTCTTGATGGAA<br>GGGATTGCACACTAATAGATGCCTTACTGGGGGATCCTCATTGCGATGTCTTTCAAGATGAGACTTGGGATCTTTTTGTGGAGCGAAGTAATGCTTTCAGCAATTGCTACCCTTACGATGTACCAGACTACGCATCCCTTCGATCCCTAGTTGCATCATCAGGCACATTGGAGTTCATCACCGAAGGTTTCACTTGGACAGGAGTAACTCAGAATGGAGGGAGTGTTGCTTGC<br>AAAAGGGGACCTGCTAATGGTTTCTTCAGTAGACTGAATTGGTTGACTAAGTCAGGGAGCGCATACCCAGTGTTGAACGTGACTATGCCAAACAATGACGGTTTTGACAAACTGTACATATGGGGAGTTCACCACCCGAGCACAAACCAAGAACAAACCAGCCTGTATGTTCAAGCCTCAGGAAGAGTCACAGTCTCTACCAGGAGAAGCCAGCAAACTATAATCCCTAAC<br>ATTGGGTCTAGACCCTGGGTAAGAGGCCAGTCTGGCAGAATAAGCATCTACTGGACAATAGTCAAACCTGGGGACATACTGGTGATCAATAGTAATGGAAACCTAATTGCTCCTCGGGGTTACTTCAAGATGCGTACTGGGAAAAGCTCAATAATGAGATCAGATGCACCTATTGACACCTGCATTTCTGAATGCATCACCCCAAATGGGAGCATCCCCAACGACAAGCCCT<br>TTCAAAATGTGAACAAAATCACATATGGAGCATGCCCCAAATATGTGAAGCAGAACACCCTGAAGTTGGCAACAGGAATGCGGAATGTACCAGAAAAGCAAACCAGAGGCCTATTCGGTGCAATAGCAGGCTTTATAGAAAATGGATGGGAAGGAATGATAGATGGCTGGTACGGCTTCAGGCATCAAAATTCCGAAGGCACCGGACAAGCAGCAGACCTTAAAAGCAC<br>TCAGGCAGCCATTGATCAGATCAATGGGAAATTGAACAGAGTGATAGAAAAGACGAATGAGAAGTTCCATCAAATTGAAAAGGAATTCTCCGAGGTAGAAGGGAGGATTCAGGACCTTGAGAAATACGTTGAAGACACAAAAATAGATCTTTGGTCTTATAATGCGGAACTCCTTGTTGCCCTAGAGAATCAGCATACAATTGATTTGACTGATTCAGAAATGAACAAATT<br>GTTTGAAAAGACCAGAAGGCAACTGAGGGAAAATGCTGAAGACATGGGCAATGGTTGTTTCAAAATATACCACAAGTGTGACAATGCCTGCATAGAATCAATTAGGAACGGGACTTATGACCATGGCATATACAGAGATGAAGCATTGAATAACAGGTTCCAGATCAAGGGTGTCGAACTAAAATCTGGGTACAAAGATTGGATCCTGTGGATTTCCTTTGCCATATCATG<br>CTTTTTGCTTTGTGTTGTTTTGCTGGGGTTCATTATGTGGGCTTGCCAGAGAGGCAACATCAGGTGCAACATTTGC
EPI_ISL_149873	>A/common_shelduck/Mongolia/2076/2011_H3N8_2011-10-08<br>ATGAAGACCATTATCGCTTTAAGCTACGTTTTCTGTCTGGCCTTCGGTCAGGACCTTCCAGGGAGCGACAATAGTACAGCAACACTGTGCCTGGGACACCATGCAGTGCCGAATGGGACAATAGTGAAGACCATTACAGATGATCAGATTGAGGTGACTAATGCTACCGAGCTAGTTCAAAGCTCTTCAACAGGGAAGATATGCAACAATCCTCACAGGATTCTTGATGGAA<br>GGGACTGCACACTAATAGATGCCCTATTGGGGGATCCTCATTGCGATGTCTTTCAAGATGAGACATGGGATCTTTTTGTGGAGCGAAGCAATGCTTTCAGCAATTGCTACCCTTACGATGTACCAGACTATGCATCCCTTCGATCCCTAGTTGCATCATCAGGCACATTGGAGTTCATCACTGAAGGTTTCACTTGGACAGGAGTAACTCAGAATGGAGGGAGCGGTGCTTGC<br>AAAAGGGGACCTGCTAACGGTTTCTTCAGTAGACTGAACTGGTTGACTAAGTCAGGAAGCGCATACCCAGTGTTGAACGTGACTATGCCAAACAATGACAATTTTGACAAACTGTACATATGGGGAGTTCACCACCCGAGCACAAACCAAGAACAAACCAACCTGTATGTTCAAGCCTCAGGAAGAGTCACAGTCTCTACCAGGAGAAGCCAGCAGACTATAATCCCTAACA<br>TTGGATCTAGACCCTGGGTAAGGGGCCAGTCTGGCAGAATAAGCATCTACTGGACAATAGTCAAACCTGGGGACGTACTGGTGATCAACAGTAATGGAAACCTAATCGCTCCTCGGGGCTACTTCAAGATGCGAACTGGGAAAAGCTCGATAATGAGGTCAGATGCACCTATTGACACCTGCATTTCTGAATGCATCACCCCAAATGGAAGCATCCCCAATGACAAGCCCT<br>TTCAAAATGTGAACAAGATCACATACGGAGCATGTCCCAAATATGTGAAGCAGAACACCCTGAAGTTGGCAACAGGAATGCGAAATGTACCAGAAAAGCAAACCAGAGGCCTATTCGGTGCAATAGCAGGCTTTATAGAAAATGGATGGGAAGGAATGATAGATGGTTGGTACGGCTTCAGGCATCAAAATTCCGAAGGCACCGGACAAGCAGCAGACCTTAAAAGCAC<br>TCAGGCAGCCATTGATCAGATCAATGGGAAATTGAACAGAGTGATTGAAAAGACGAATGAGAAGTTCCATCAAATTGAAAAGGAATTCTCTGAGGTAGAAGGGAGGATTCAGGACCTTGAGAAATACGTTGAAGACACAAAAATAGATCTTTGGTCTTATAATGCGGAACTCCTTGTCGCCCTAGAGAATCAGCATACAATTGATTTGACTGATTCAGAAATGAACAAATT<br>GTTTGAAAAGACCAGAAGGCAACTGAGGGAAAATGCTGAAGACATGGGCAATGGTTGTTTCAAAATATACCACAAGTGTGACAATGCCTGCATAGAATCAATTAGGAACGGGACTTATGACCATGACATATACAGAGATGAGGCATTGAATAACCGGTTCCAGATCAAGGTGTCGAACTAAAATCTGGGTATAAAGACTGGATCCTGTGGATTTCCTTTGCCATATCATG<br>CTTTTTGCTTTGTGTTGTTTTGCTGGGATTCATTATGTGGGCCTGCCAGAGAGGCAACATTAGGTGCAACATTTGC
EPI_ISL_373089	>A/Mallard/Netherlands/37/2015_H3N8_2015-08-29<br>ATGAAGACCATTATCGCTTTAAGCTACGTTTTCTGTCTGGCCTTCGGTCAGGACCTTCCAAGGAGCGACAATAGTACGGCAACACTGTGCCTGGGACACCATGCAGTGCCGAATGGGACAATAGTGAAGACCATTACAGATGATCAGATTGAGGTGACTAATGCTACCGAGCTAGTTCAAAGCTCTTCAACAGGGAAGATATGCAATAATCCTCACAGGATTCTTGATGGAA<br>GGGACTGCACACTAATAGATGCCTTACTGGGGGATCCTAATTGCGATGTCTTTCAAGATGAGACATGGGATCTTTTTGTGGAGCGAAGCAATGCTTTCAGCAATTGCTACCCTTACGATGTGCCAGACTACGCATCCCTTCGATCCCTAGTTGCATCATCAGGCACATTGGAGTTCATCACTGAAGGTTTCACTTGGACAGGAGTAACTCAGAATGGAGGGAGCAGTGCTTG<br>CAAAAGGGGACCTGCTAACGGTTTCTTCAGTAGACTGAACTGGTTGACTAAGTCAGGAAGCGCATACCCAGTGTTGAACGTGACTATGCCAAACAATGACAATTTTGACAAACTGTACATATGGGGAGTTCACCACCCGAGCACAAACCAAGAACAAACCAACCTGTATGTTCAAGCCTCAGGAAGAGTCACAGTCTCTACCAGGAGAAGCCAGCAGACTATAATCCCTAAC<br>ATTGGATCTAGACCCTGGGTAAGGGGCCAGTCTGGCAGGATAAGCATCTACTGGACAATAGTCAAACCTGGGGACGTACTGGTGATCAATAGTAATGGAAACCTAATCGCTCCTCGGGGCTACTTCAAGATGCGAACTGGGAAAAGCTCGATAATGAGGTCAGATGCACCTATTGACACCTGCATTTCTGAATGCATCACCCCAAACGGAAGCATCCCCAACGACAAGCCC<br>TTTCAAAATGTGAACAAGATCACATACGGAGCATGTCCCAAATATGTGAAGCAGAACACCTTGAAGTTGGCAACAGGAATGCGAAATGTACCAGAAAAGCAAACCAGAGGCCTATTCGGTGCAATAGCAGGCTTTATAGAAAATGGATGGGAAGGAATGATAGATGGTTGGTACGGCTTCAGGCATCAAAACTCCGAAGGCACCGGACAAGCAGCAGACCTTAAAAGCA<br>CTCAGGCAGCCATTGATCAGATCAATGGGAAATTGAACAGAGTGATTGAAAAGACGAATGAGAAGTTCCATCAAATTGAAAAGGAATTCTCTGAGGTAGAAGGGAGGATTCAGGACCTTGAGAAATACGTTGAAGACACAAAAATAGATCTTTGGTCTTATAATGCGGAACTCCTTGTCGCCCTAGAGAATCAGCATACGATTGATTTAACTGATTCAGAAATGAACAAAT<br>TGTTTGAAAAGACCAGAAGGCAACTGAGGGAAAATGCTGAAGACATGGGCAATGGTTGTTTCAAAATATACCACAAGTGTGACAATGCCTGCATAGAATCAATCAGGAACGGGACTTATGACCATGACATATACAGAGATGAGGCATTGAATAACCGGTTCCAGATCAAGGGTGTCGAACTAAAATCTGGGTATAAGGACTGGATCCTGTGGATTTCCTTTGCCATATCAT<br>GCTTTTTGCTTTGTGTTGTTTTGCTGGGGTTCATTATGTGGGCCTGCCAGAGAGGCAACATCAGGTGCAACATTTGC
EPI_ISL_240672	>A/shoveler/Chany/82K/2014_H3N8_2014-09-10<br>ATGAAGACCATTATCGCTTTAAGCTACGTTTTCTGTCTGGCCTTCGGTCAGGACCTTCCAGGGAGCGACAATAGTACAGCAACACTGTGCCTGGGACACCATGCAGTGCCGAATGGGACAATAGTGAAGACCATTACAGATGATCAGATTGAGGTGACTAATGCTACCGAGCTAGTTCAAAGCTCTTCAACAGGGAAGATATGCAACAATCCTCACAGGATTCTTGATGGAA<br>GGGACTGCACACTAATAGATGCCCTACTGGGGGATCCTCATTGCGATGTCTTTCAAGATGAGACATGGGATCTTTTTGTGGAGCGAAGCAATGCTTTCAGCAATTGCTACCCTTACGATGTACCAGACTATGCATCCCTTCGATCCCTAGTTGCATCATCAGGCACATTGGAGTTCATCACTGAAGGTTTCACTTGGACAGGAGTAACTCAGAATGGAGGGAGCAGTGCTTGC<br>AAAAGGGGACCTGCTAACGGTTTCTTCAGTAGACTGAACTGGTTGACTAAGTCAGGAAGCGCATACCCAGTGTTGAATGTGACTATGCCAAACAATGACAATTTTGACAAACTGTACATATGGGGAGTTCACCACCCAAGCACAAACCAAGAACAAACCAACCTGTATGTTCAAGCCTCAGGAAGAGTCACAGTCTCTACCAGGAGAAGCCAGCAGACTATAATCCCTAACA<br>TTGGATCTAGACCCTGGGTAAGGGGCCAGTCTGGCAGAATAAGCATCTATTGGACAATAGTCAAACCTGGGGACGTACTGGTGATCAATAGTAATGGAAACCTAATCGCTCCTCGGGGCTACTTCAAGATGCGAACTGGGAAAAGCTCGATAATGAGATCAGATGCACCTATTGACACCTGCATTTCTGAATGCATCACCCCAAATGGAAGCATCCCCAATGACAAGCCCTT<br>TCAAAATGTGAACAAGATCACATACGGAGCATGTCCCAAATATGTGAAGCAGAACACCCTGAAGTTGGCAACAGGAATGCGAAATGTACCAGAAAAGCAAGCCAGAGGCCTATTCGGTGCAATAGCAGGCTTTATAGAAAATGGATGGGAAGGAATGATAGATGGTTGGTACGGCTTCAGGCATCAAAATTCCGAAGGCACCGGACAAGCAGCAGACCTTAAAAGCACT<br>CAGGCAGCCATTGATCAGATCAATGGAAAATTGAACAGAGTGATTGAAAAGACGAATGAGAAGTTCCATCAAATTGAAAAGGAATTCTCTGAGGTAGAAGGGAGGATTCAGGACCTTGAGAAATACGTTGAAGACACAAAAATAGATCTTTGGTCTTATAATGCGGAACTACTTGTCGCCCTAGAGAATCAGCATACAATTGATTTGACTGATTCAGAAATGAACAAATTG<br>TTTGAAAAGACCAGAAGGCAACTGAGGGAAAATGCTGAAGACATGGGCAATGGTTGTTTCAAAATATACCACAAGTGTGACAATGCCTGCATAGAATCAATTAGGAACGGGACTTATGACCATGACATATACAGAGATGAGGCATTGAATAACCGGTTCCAGATCAAGGGTGTCGAACTAAAATCTGGGTATAAAGACTGGATCCTGTGGATTTCCTTTGCCATATCATGC<br>TTTTTGCTTTGTGTTGTTTTGCTGGGGTTCATTATGTGGGCCTGCCAGAGAGGCAACATTAGGTGCAACATTTGC
EPI_ISL_337413	>A/teal/Dagestan/44d/2018_H3N8_2018-09-15<br>ATGAAGACCATTATCGCTTTAAGCTACGTTCTCTGTCTGGCCTTCGGTCAGGACCTCCCAGGGAGCGACAATAGTACAGCAACACTGTGCCTGGGACACCATGCAGTGCCGAATGGGACGATAGTGAAGACCATTACAGATGATCAGATTGAGGTGACTAATGCTACCGAGCTAGTTCAAAGCTCTTCAACAGGGAAGATATGCAACAATCCTCACAAGATTCTTGATGGAA<br>GGGACTGCACACTAATAGATGCCTTACTGGGGGATCCTCATTGCGATGTCTTTCAAGATGAGACATGGGATCTTTTTGTGGAGCGAAGCAATGCTTTCAGCAATTGCTACCCTTACGATGTACCAGACTACGCATCCCTTCGATCCCTAGTTGCATCATCAGGCACATTGGAGTTCATCACTGAAGGTTTCACTTGGACAGGAGTAACTCAGAATGGAGGGAGCGGTGCTTGC<br>AAAAGGGGACCCGCTAACGGTTTCTTCAGTAGACTGAACTGGTTGACTAAGTCAGGGAGCGCATACCCAGTGTTGAACGTGACTATGCCAAACAATGACAATTTTGACAAACTGTACATATGGGGGGTTCACCACCCGAGCACAAACCAAGAACAAACCAACCTGTATGTTCAAGCCTCAGGAAGAGTCACAGTCTCTACCAGGAGGAGCCAGCAGACTATAATCCCTAAC<br>ATTGGATCCAGACCCTGGGTAAGGGGCCAGTCTGGCAGAATAAGCATCTACTGGACAATAGTCAAACCTGGGGACGTACTGGTGATCAATAGTAATGGAAACCTAATCGCTCCTCGGGGCTACTACAAGATGCGAACTGGGAAAAGCTCGATAATGAGGTCAGATGCACCTATTGACACCTGCATTTCTGAATGCATCACCCCAAATGGAAGCATCCCCAACGACAAGCCC<br>TTTCAAAATGTGAACAAGATCACATACGGGGCATGTCCCAAATATGTGAAGCAGAACACCCTGAAGTTGGCAACAGGAATGCGAAATGTACCAGAAAAGCAAACCAGAGGCCTATTCGGTGCAATAGCAGGCTTTATAGAAAATGGATGGGAAGGAATGATAGATGGTTGGTACGGCTTCAGGCATCAAAATTCCGAAGGCACCGGACAAGCAGCAGACCTTAAAAGCA<br>CTCAGGCAGCCATTGATCAGATCAACGGGAAATTGAACAGAGTGATTGAAAAGACGAATGAGAAGTTCCATCAAATTGAAAAGGAATTCTCTGAGGTAGAAGGGAGGATTCAGGACCTTGAGAAATACGTTGAAGACACAAAAATAGATCTTTGGTCTTATAATGCGGAACTCCTTGTCGCCCTAGAGAATCAGCATACAATTGATTTGACTGATTCAGAAATGAACAAAT<br>TGTTTGAAAAGACCAGAAGGCAACTGAGGGAAAATGCTGAGGACATGGGCAATGGTTGTTTCAAAATATACCACAAGTGTGACAATGCCTGCATAGAGTCAATTAGGAACGGGACTTATGACCATGACATATACAGAGATGAGGCATTGAACAACCGGTTCCAGATCAAGGGTGTCGAACTAAAATCTGGGTATAAAGACTGGATCCTGTGGATTTCCTTTGCCATATCAT<br>GCTTTTTGCTTTGTGTTGTTTTGCTGGGGTTCATTATGTGGGCCTGCCAGAGAGGCAACATCAGGTGCAACATTTGC
EPI_ISL_7591585	>A/Anas_platyrhynchos/Belgium/827/2020_H3N8_2020-01-19<br>ATGAAGACCATTATCGCTTTAAGCTACGTTCTCTGTCTGGCCTTCAGTCAGGACCTCCCAGGGAGCGACAATAGTACAGCAACACTGTGCCTGGGACACCATGCGGTGCCGAATGGGACGATAGTGAAGACCATTACAGATGATCAGATTGAGGTGACTAATGCTACCGAGCTAGTTCAAAGCTCTTCAACAGGGAAGATATGCAACAATCCTCACAAGATTCTTGATGGAA<br>GGGACTGCACACTAATAGATGCCTTACTGGGGGATCCTCATTGCGATGTCTTTCAAGATGAGACATGGGATCTTTTTGTGGAGCGAAGCAATGCTTTCAGCAATTGCTACCCTTACGATGTACCAGACTACGCATCCCTTCGATCCCTAGTTGCATCATCAGGCACATTAGAGTTCATCACTGAAGGTTTCACTTGGACAGGAGTAACTCAGAATGGAGGGAGCGGTGCTTGC<br>AAAAGGGGACCCGCTAACGGTTTCTTCAGTAGACTGAACTGGTTGACTAAGTCAGGGAGCGCATACCCAGTGTTGAACGTGACTATGCCAAACAATGACAATTTTGACAAACTGTACATATGGGGGGTTCACCACCCGAGCACAAACCAAGAACAAACCAACCTGTATGTTCAAGCCTCAGGAAGAGTCACAGTCTCTACCAGGAGGAGCCAGCAGACTATAATCCCTAAC<br>ATTGGATCCAGACCCTGGGTAAGGGGCCAGTCTGGCAGAATAAGCATCTACTGGACAATAGTCAAACCTGGGGACGTACTGGTGATCAATAGTAATGGAAACCTAATCGCTCCTCGGGGCTACTTCAAGATGCGAACTGGGAAAAGCTCGATAATGAGGTCAGATGCACCTATTGACACCTGCATTTCTGAATGCATCACTCCAAATGGAAGCATCCCCAACGACAAGCCC<br>TTTCAAAATGTGAACAAGATCACATACGGGGCATGTCCCAAATATGTGAAGCAGAACACCCTGAAGTTGGCAACAGGAATGCGAAATGTACCAGAAAAGCAAACCAGAGGCCTATTCGGTGCAATAGCAGGCTTTATAGAAAATGGATGGGAAGGAATGATCGATGGTTGGTACGGCTTCAGGCATCAAAATTCCGAAGGCACCGGACAAGCAGCAGACCTTAAAAGCA<br>CTCAGGCAGCCATTGATCAGATCAACGGGAAATTGAACAGAGTGATTGAAAAGACGAATGAGAAGTTCCATCAAATTGAAAAGGAATTCTCTGAGGTAGAAGGGAGGATTCAGGACCTTGAGAAATACGTTGAAGACACAAAAATAGATCTTTGGTCTTATAATGCGGAACTCCTTGTCGCCCTAGAGAATCAGCATACAATTGATTTGACTGATTCAGAAATGAACAAAT<br>TGTTTGAAAAGACCAGAAGGCAACTGAGGGAAAATGCTGAGGACATGGGCAATGGTTGTTTCAAAATATACCACAAGTGTGACAATGCATGCATAGAGTCAATTAGGAACGGGACTTATGACCATGACATATACAGAGATGAGGCATTGAACAACCGGTTCCAGATCAAGGGTGTCGAACTAAAATCTGGGTATAAAGACTGGATCCTGTGGATTTCCTTTGCCATATCAT<br>GCTTTTTGCTTTGTGTTGTTTTGCTGGGGTTCATTATGTGGGCCTGCCAGAGAGGCAACATCAGGTGCAACATTTGC
EPI_ISL_697689	>A/duck/Mongolia/217/2018_H3N8_2018-09-02<br>ATGAAGACCATTATCGCTTTAAGCTACGTTTTCTGTCTGGCCTTCGGTCAGGACCTTCCAGGGAGCGACAATAGTACAGCAACACTGTGCCTGGGACACCATGCAGTGCCGAATGGGACAATAGTGAAGACCATTACAGATGATCAGATTGAGGTGACTAATGCCACCGAGCTAGTTCAAAGCTCTTCAACAGGGAAGATATGCAACAATCCTCACAGGATTCTTGATGGAA<br>GGGACTGCACACTAATAGATGCCCTACTGGGGGATCCTCATTGCGATGTCTTTCAAGATGAGACATGGGATCTTTTTGTGGAGCGAAGCAATGCTTTCAGCAATTGCTACCCTTACGATGTACCAGACTATGCATCCCTTCGATCCCTAGTTGCATCATCAGGCACATTGGAGTTCATCACTGAAGGTTTCACTTGGACAGGAGTAACTCAAAATGGAGGGAGCGGTGCTTGC<br>AAAAGGGGACCTGCTAACGGTTTCTTCAGTAGACTGAACTGGTTGACTAAGTCAGGAAGCGCATACCCAGTGTTGAATGTGACTATGCCAAACAATGACAATTTTGACAAACTGTACATATGGGGAGTTCACCACCCGAGCACAAACCAAGAACAAACCAACCTGTATGTTCAAGCCTCAGGAAGAGTCACAGTCTCCACCAGGAGGAGCCAGCAGACTATAATCCCTAAC<br>ATTGGATCTAGACCCTGGGTAAGGGGCCAGTCTGGCAGAATAAGCATCTACTGGACAATAGTCAAACCTGGGGACGTACTGGTGATCAATAGTAATGGAAACCTAATCGCTCCTCGGGGCTACTTCAAGATGCGAACTGGAAAAAGCTCGATAATGAGGTCAGATGCACCTATTGACACCTGCATTTCTGAATGCATCACCCCAAATGGAAGCATCCCCAATGACAAGCCC<br>TTTCAAAATGTGAACAAAGTCACATACGGAGCATGTCCCAAATATGTGAAGCAGAACACCCTGAAGTTGGCAACAGGAATGCGAAATGTACCGGAAAAGCAAACCAGAGGCCTATTCGGTGCAATAGCAGGCTTTATAGAAAATGGATGGGAAGGAATGATAGATGGTTGGTACGGCTTCAGGCATCAAAATTCCGAAGGCACCGGACAAGCAGCAGACCTTAAAAGCA<br>CTCAAGCAGCCATTGATCAGATCAATGGGAAATTGAACAGAGTGATTGAAAAAACGAATGAGAAGTTCCATCAAATTGAAAAAGAATTCTCTGAGGTAGAAGGGAGGATTCAGGACCTTGAGAAATACGTTGAAGACACAAAAATAGATCTTTGGTCTTATAATGCGGAACTCCTTGTCGCCCTAGAGAATCAGCATACAATTGATTTGACTGATTCAGAAATGAACAAATT<br>GTTTGAAAAGACCAGAAGGCAACTGAGGGAAAATGCTGAAGACATGGGAAATGGTTGTTTCAAAATATACCACAAGTGTGACAATGCCTGCATAGAATCAATTAGGAACGGGACTTATGATCATGACATATACAGAGATGAGGCATTGAATAACCGGTTCCAGATCAAGGGTGTCGAACTAAAATCTGGGTATAAAGACTGGATCCTGTGGATTTCCTTTGCCATATCATG<br>CTTTTTGCTTTGTGTTGTTTTGCTGGGGTTCATTATGTGGGCCTGCCAGAGAGGCAACATTAGGTGCAACATTTGC
EPI_ISL_1184522	>A/mallard/Novosibirsk_region/3314k/2020_H3N8_2020-08-29<br>ATGAAGACCGTCATTGCTCTAAGCTACATTTTCTGTCTGGCTTTTAGTCAGGACCTTTCAGGGAATGACAACAGCACAGCAACACTGTGCCTGGGACACCATGCAGTGCCGAATGGAACAATAGTGAAGACAATTACAGATGATCAGATTGAGGTGACTAATGCTACCGAGCTAGTCCAAAGCTCTTCAACAGGGAAAATATGCAACAACCCTCACAGAATCCTTGATGGAA<br>GGGACTGCACACTAATAGATGCCCTACTGGGGGATCCTCATTGTGATGTCTTTCAAGATGAAACATGGGACCTCTTTGTAGAGCGGAGCAATGCGTTCAGTAATTGTTACCCTTACGATGTACCAGACTATGCATCCCTTCGATCCCTAGTTGCATCATCAGGCACATTGGAATTCATCACTGAAGGTTTCACTTGGACAGGAGTTACTCAGAATGGAGGGAGCAATGCTTGC<br>AAAAGAGGACCTGCCAGCGGTTTCTTTAGCAGATTGAACTGGTTGACTAAGTCAGGAAACGCATACCCAGTGTTGAACGTGACTATGCCAAACAATGACAATTTTGACAAACTATACATCTGGGGAGTTCACCACCCGAGCACGAATCAAGAACAAACCAACTTGTATGTTCAAGCCTCAGGAAGAGTCACAGTCTCCACCAGGAGAAGCCAACAGACTATAATTCCAAACA<br>TTGGATCTAGACCCTGGGTAAGGGGTCAATCTGGCAGGATAAGCATCTACTGGACAATAGTCAAACCTGGGGACGTACTGGTAATCAATAGCAATGGAAACCTAATCGCTCCTCGGGGCTACTTCAAAATGCGCACTGGGAAAAGCTCAATAATGAGATCAGATGCACCTATTGACACCTGCATTTCTGAATGCATCACTCCAAATGGAAGCATCCCCAATGACAAGCCTTT<br>CCAAAATGTAAACAAAATCACATACGGAGCATGTCCCAAATATGTGAAGCAGAACACCCTAAAGTTGGCAACAGGAATGCGGAATGTACCTGAGAAACAAACCAGAGGACTGTTCGGTGCAATAGCAGGGTTTATAGAAAACGGATGGGAAGGAATGATAGATGGTTGGTATGGCTTCAGACATCAAAATTCCGAAGGCACAGGACAAGCAGCAGACCTTAAAAGCACT<br>CAGGCAGCCATTGACCAGATCAATGGGAAGCTAAACAGAGTAATTGAAAAGACGAATGAGAAGTTCCATCAAATCGAAAAGGAATTCTCTGAAGTAGAAGGGAGGATTCAGGACCTTGAGAAATACGTTGAAGACACAAAGATAGATCTCTGGTCTTATAATGCGGAGCTTCTTGTTGCCCTAGAGAATCAGCATACGATTGATTTGACGGACTCAGAAATGAACAAATT<br>GTTTGAGAAGACCAGGAGGCAACTGAGAGAAAATGCTGAGGACATGGGCAATGGTTGTTTCAAGATATACCACAAATGTGATAATGCTTGTATAGAGTCAATTAGAAACGGGACTTATGACCATGACATATACAGAGATGAGGCATTGAACAACCGGTTTCAGATCAAAGGTGTTGAGCTAAAATCTGGATACAAAGACTGGATCCTGTGGATTTCCTTTGCCATATCATG<br>CTTTTTGCTTTGTGTTGTTTTGCTGGGGTTCATTATGTGGGCCTGCCAGAGAGGCAACATTAGGTGCAACATTTGC
EPI_ISL_506011	>A/greylag_goose/North-Kazakhstan/62/2019_H3N8_2019-10-20<br>ATGAAGACCGTCATTGCTTTAAGCTACATTTTCTGCCTGGCTTTTAGTCAGGACCTTTCAGGGAATGACAACAGCACAGCAACACTGTGCCTGGGACACCATGCAGTGCCGAATGGAACAATAGTGAAGACAATTACAGATGATCAGATTGAGGTGACTAATGCTACCGAGCTAGTCCAAAGCTCTTCAACAGGGAAAATATGCAACAACCCTCACAAAATCCTTGATGGAA<br>GGGACTGCACACTAATAGATGCCCTACTGGGGGATCCTCATTGTGATGTCTTTCAAGATGAAACATGGGACCTCTTTGTAGAGCGGAGCAATGCGTTCAGCAGTTGTTACCCTTACGATGTACCAGACTATGCATCCCTTCGATCCCTAGTTGCATCATCAGGCACATTGGAATTCATCACTGAAGGTTTCACTTGGACAGGAGTTACTCAGAATGGAGGGAGCAATGCTTGC<br>AAAAGAGGACCTGCCAGCGGTTTCTTTAGTAGATTGAACTGGTTGACTAAGTCAGGAAACGCATACCCAGTGTTGAACGTGACTATGCCAAACAATGACAATTTTGACAAACTATACATCTGGGGAGTTCACCACCCGAGCACGAATCAAGAACAAACCAACTTGTATGTTCAAGCCTCAGGAAGAGTCACAGTCTCCACCAGGAGAAGCCAACAGACTATAATTCCGAACA<br>TTGGATCTAGACCCTGGGTAAGGGGCCAATCTGGCAGGATAAGCATCTACTGGACAATAGTCAAACCTGGGGACGTACTGGTAATCAATAGCAATGGAAACCTAATCGCTCCTCGGGGCTACTTCAAAATGCGCACTGGGAAAAGCTCAATAATGAGATCAGATGCACCTATTGACACCTGCATTTCTGAATGCATCACTCCAAATGGAAGCATCCCCAATGACAAGCCCTT<br>CCAAAATGTAAACAAAATCACATACGGAGCATGTCCCAAATATGTGAAACAGAACACTCTAAAGTTGGCAACAGGATTGCGGAATGTACCTGAGAAACAAACCAGAGGACTGTTCGGTGCAATAGCAGGGTTTATAGAAAACGGATGGGAAGGAATGATAGATGGTTGGTATGGCTTCAGACATCAAAATTCCGAAGGCACAGGACAAGTAGCAGACCTTAAAAGCACT<br>CAGGCAGCCATTGACCAGATCAATGGGAAGCTGAACAGAGTAATTGAAAAGACGAATGAGAAGTTCCATCAAATCGAAAAGGAATTCTCCGAAGTAGAAGGGAGGATTCAGGACCTTGAGAAATACGTTGAAGACACAAAGATAGATCTCTGGTCTTATAATGCGGAGCTTCTTGTTGCCCTAGAGAATCAGCATACGATTGATTTGACAGACTCAGAAATGAACAAATT<br>GTTTGAGAAGACCAGGAGGCAACTGAGGGAAAATGCTGAGGACATGGGCAATGGTTGTTTCAAGATATACCACAAATGTGACAATGCTTGTATAGAGTCAATTAGAAACGGGACTTATGACCATGACATATACAGAGATGAGGCATTGAACAACCGGTTCCAGATCAAAGGTGTTGAGCTAAAATCTGGATACAAAGACTGGATCCTGTGGATTTCCTTTGCCATATCATG<br>CTTTTTGCTTTGTGTTGTTTTGCTGGGGTTCATTATGTGGGCCTGCCAGAGAGGCAACATTAGGTGCAACATTTGC
EPI_ISL_506010	>A/white_wagtail/Shackpack/49/2019_H3N8_2019-10-29<br>ATGAAGACCGTCATTGCTTTAAGCTACATTTTCTGCCTGGCTTTTAGTCAGGACCTTTCAGGGAATGACAACAGCACAGCAACACTGTGCCTGGGACACCATGCAGTGCCGAATGGAACAATAGTGAAGACAATTACAGATGATCAGATTGAGGTGACTAATGCTACCGAGCTAGTCCAAAGCTCTTCAACAGGGAAAATATGCAACAACCCTCACAAAATCCTTGATGGAA<br>GGGACTGCACACTAATAGATGCCCTACTGGGGGATCCTCATTGTGATGTCTTTCAAGATGAAACATGGGACCTCTTTGTAGAGCGGAGCAATGCGTTCAGCAGTTGTTACCCTTACGATGTACCAGACTATGCATCCCTTCGATCCCTAGTTGCATCATCAGGCACATTGGAATTCATCACTGAAGGTTTCACTTGGACAGGAGTTACTCAGAATGGAGGGAGCAATGCTTGC<br>AAAAGAGGACCTGCCAGCGGTTTCTTTAGTAGATTGAACTGGTTGACTAAGTCAGGAAACGCATACCCAGTGTTGAACGTGACTATGCCAAACAATGACAATTTTGACAAACTATACATCTGGGGAGTTCACCACCCGAGCACGAATCAAGAACAAACCAACTTGTATGTTCAAGCCTCAGGAAGAGTCACAGTCTCCACCAGGAGAAGCCAACAGACTATAATTCCGAACA<br>TTGGATCTAGACCCTGGGTAAGGGGCCAATCTGGCAGGATAAGCATCTACTGGACAATAGTCAAACCTGGGGACGTACTGGTAATCAATAGCAATGGAAACCTAATCGCTCCTCGGGGCTACTTCAAAATGCGCACTGGGAAAAGCTCAATAATGAGATCAGATGCACCTATTGACACCTGCATTTCTGAATGCATCACTCCAAATGGAAGCATCCCCAATGACAAGCCCTT<br>CCAAAATGTAAACAAAATCACATACGGAGCATGTCCCAAATATGTGAAACAGAACACTCTAAAGTTGGCAACAGGAATGCGGAATGTACCTGAGAAACAAACCAGAGGACTGTTCGGTGCAATAGCAGGGTTTATAGAAAACGGATGGGAAGGAATGATAGATGGTTGGTATGGCTTCAGACATCAAAATTCCGAAGGCACAGGACAAGTAGCAGACCTTAAAAGCACT<br>CAGGCAGCCATTGACCAGATCAATGGGAAGCTGAACAGAGTAATTGAAAAGACGAATGAGAAGTTCCATCAAATCGAAAAGGAATTCTCCGAAGTAGAAGGGAGGATTCAGGACCTTGAGAAATACGTTGAAGACACAAAGATAGATCTCTGGTCTTATAATGCGGAGCTTCTTGTTGCCCTAGAGAATCAGCATACGATTGATTTGACAGACTCAGAAATGAACAAATT<br>GTTTGAGAAGACCAGGAGGCAACTGAGGGAAAATGCTGAGGACATGGGCAATGGTTGTTTCAAGATATACCACAAATGTGACAATGCTTGTATAGAGTCAATTAGAAACGGGACTTATGACCATGACATATACAGAGATGAGGCATTGAACAACCGGTTCCAGATCAAAGGTGTTGAGCTAAAATCTGGATACAAAGACTGGATCCTGTGGATTTCCTTTGCCATATCATG<br>CTTTTTGCTTTGTGTTGTTTTGCTGGGGTTCATTATGTGGGCCTGCCAGAGAGGCAACATTAGGTGCAACATTTGC
EPI_ISL_337147	>A/pintail/Novosibirsk_region/518k/2018_H3N8_2018-10-01<br>ATGAAGACCGTCATTGCTCTAAGCTACATTTTCTGTCTGGCTTTTAGTCAGGACCTTTCAGGGAATGACAACAGCACAGCAACACTGTGCCTGGGACACCATGCAGTGCCGAATGGAACAATAGTGAAGACAATTACAGATGATCAGATTGAGGTGACTAATGCTACCGAGCTAGTCCAAAGCTCTTCAACAGGGAAAATATGCAACAACCCTCACAGAATCCTTGATGGAA<br>GGGACTGCACACTAATAGATGCCCTACTGGGGGATCCTCATTGTGATGTCTTTCAAGATGAAACATGGGACCTCTTTGTAGAGCGGAGCAATGCGTTCAGTAATTGTTACCCTTACGATGTACCAGACTATGCATCCCTTCGATCCCTAGTTGCATCATCAGGCACATTGGAATTCATCACCGAAGGTTTCACTTGGACAGGAGTTACTCAGAATGGAGGGAGCAATGCTTGC<br>AAAAGAGGACCTGCCAGCGGTTTCTTTAGTAGATTGAACTGGTTGACTAAGTCAGGAAACGCATACCCAGTGTTGAACGTGACTATGCCAAACAATGACAATTTTGACAAACTATACATCTGGGGAGTTCACCACCCGAGCACGAATCAGGAACAAACCAACTTGTATGTTCAAGCCTCAGGAAGAGTCACAGTCTCCACCAGGAGAAGCCAACAGACTATAATTCCGAACA<br>TTGGATCTAGACCCTGGGTAAGGGGTCAATCTGGCAGGATAAGCATCTACTGGACAATAGTCAAACCTGGGGACGTACTGGTAATCAATAGCAATGGAAACCTAATCGCTCCTCGGGGCTACTTCAAAATGCGCACTGGGAAAAGCTCAATAATGAGATCAGATGCACCTATTGACACCTGCATTTCTGAATGCATCACTCCAAATGGAAGCATCCCCAATGACAAGCCCTT<br>CCAAAATGTAAACAAAATCACATACGGAGCATGTCCCAAATATGTGAAGCAGAACACCCTAAAGTTGGCAACAGGAATGCGGAATGTACCTGAGAAACAAACCAGAGGACTGTTCGGTGCAATAGCAGGGTTTATAGAAAACGGATGGGAAGGAATGATAGATGGTTGGTATGGCTTCAGACATCAAAATTCTGAAGGCACAGGACAAGCAGCAGACCTTAAAAGCACT<br>CAGGCAGCCATTGACCAGATCAATGGGAAGCTAAACAGAGTAATTGAAAAGACGAATGAGAAGTTCCATCAAATCGAAAAGGAATTCTCCGAAGTAGAAGGGAGGATTCAGGACCTTGAGAAATACGTTGAAGACACAAAGATAGATCTCTGGTCTTATAATGCGGAGCTTCTTGTTGCCCTAGAGAATCAGCATACGATTGATTTGACGGACTCAGAAATGAACAAATT<br>GTTTGAGAAGACCAGGAGGCAACTGAGAGAAAATGCTGAGGACATGGGCAATGGTTGTTTCAAGATATACCACAAATGTGATAATGCTTGTATAGAGTCAATTAGAAACGGGACTTATGACCATGACATATACAGAGATGAGGCATTGAACAACCGGTTTCAGATCAAAGGTGTTGAGCTAAAATCTGGATACAAAGACTGGATCCTGTGGATTTCCTTTGCCATATCATG<br>CTTTTTGCTTTGTGTTGTTTTGCTGGGGTTCATTATGTGGGCCTGCCAGAGAGGCAACATTAGGTGCAACATTTGC

EPI_ISL_333105	>A/duck/Bangladesh/20D678/2016_H3N8_2016-07-22<br>ATGAAGACCGTTATTGCTTTAAGCTACATTTTCTGCCTGGCTTTTAGTCAGGACCTTTCAGGGAATGACAACAGTACGGCAACACTGTGCCTGGGACATCATGCAGTGCCGAATGGAACAATAGTGAAGACAATTACAGATGATCAGATAGAGGTAACTAATGCTACCGAGCTAGTTCAAAGCTCTTCAACAGGGAAAATATGCAACAACCCTCACAGAATCCTTGATGGAA<br>GGGACTGCACACTAATAGATGCCCTACTGGGGGATCCTCATTGTGATGTCTTTCAAGATGAAACATGGGACCTCTTTGTAGAGAGGAGCAATGCGTTCAGTAATTGTTACCCTTACGATGTACCAGACTATGCATCCCTTCGATCCCTAGTTGCATCATCAGGCACATTGGAATTCATCACTGAAGGCTTCACTTGGACAGGAGTTACTCAGAATGGAGGGAGCAATGCTTGC<br>AAAAGGGGACCTGCCAGCGGTTTCTTTAGTAGGTTGAACTGGTTGACTAAGTCAGGAAACGCATACCCAGTGTTGAACGTGACTATGCCAAACAATGACAATTTTGACAAACTATACATCTGGGGAGTTCACCACCCGAGCACAAATCAAGAACAAACCACCTTGTATGTTCAAGCCTCAGGAAGAGTCACAGTCTCCACCAGGAGAAGCCAACAGACTATAATTCCGAACA<br>TTGGATCTAGACCCTGGGTAAGGGGCCAATCTGGCAGAATAAGCATCTACTGGACAATAGTCAAACCTAGGGACGTACTGGTAATCAATAGTAATGGAAACCTAATCGCTCCTCGGGGCTACTTCAAAATGCGCACTGGGAAAAGCTCAATAATGAGATCAGATGCACCTATTGACACCTGCATTTCTGAATGCATCACTCCAAATGGAAGCATCCCMAATGACAAGCCCTT<br>CCAAAATGTAAACAAAATCACATACGGATCATGTCCCAAATATGTGAAGCAGAACACCCTAAAGTTGGCAACAGGAATGCGGAATGTACCTGAGAAACAAACCAGAGGCCTGTTCGGTGCAATAGCAGGGTTTATAGAAAATGGATGGGAAGGAATGATAGATGGTTGGTATGGCTTCAGACATCAAAATTCCGAAGGCACAGGACAAGCAGCAGACCTTAAAAGCACT<br>CAGGCAGCCATTGACCAGATCGATGGGAAGCTGAACAGAGTAATTGAAAAGACGAATGAGAAGTTCCACCAAATCGAAAAGGAATTCTCCGAAGTAGAAGGGAGGATTCAGGACCTTGAGAAATATGTTGAAGACACAAAGATAGATCTCTGGTCTTATAATGCAGAGCTTCTTGTTGCCCTAGAGAATCAGCATACGATTGATTTGACGGACTCAGAAATGAACAAATT<br>GTTTGAGAAGACCAGGAGGCAACTGAGGGAAAATGCTGAGGACATGGGCAATGGTTGTTTCAAGATATATCACAAATGTGACAATGCTTGTATAGAGTCAATTAGAAACGGGACTTATGACCATGACATATACAGAGATGAGGCATTGAACAACCGATTCCAGATCAAAGGTGTTGAGCTAAAATCTGGATACAAAGACTGGATCCTGTGGATTTCCTTTGCCATATCATG<br>CTTTTTGCTTTGTGTTGTTTTGCTGGGGTTCATTATGTGGGCCTGCCAGAGAGGCAACATTAGGTGCAACATTTGC
EPI_ISL_231643	>A/pintail/Chany_Lake/37/2012_H3N8_2012-08-26<br>ATGAAGACTGTTATTGCTTTAAGCTTCATTTTCTGTCTGGCTTTTAGTCAGGACCTTTCAGGGAATGACAACAGTACAGCAACACTGTGCCTGGGACACCATGCAGTGCCGAACGGAACAATAGTTAAGACAATTACAGATGATCAGATCGAGGTGACTAATGCTACCGAGCTAGTTCAAAGCTCTTCAACAGGGAAAATATGCAACAATCCCCACAGAATCCTTGATGGAAG<br>GGACTGCACCCTAATAGATGCCCTACTGGGGGATCCTCATTGTGATGTCTTTCAAGATGAAACATGGGACCTCTTTGTAGAGCGGAGCAATGCTTTCAGCAATTGTTACCCTTACGATGTACCAGACTATGCATCCCTTCGATCCCTAGTTGCATCATCAGGCACATTGGAATTCATCACTGAAGGTTTCACTTGGACAGGAGTTACTCAGAATGGAGGGAGCAATGCTTGCA<br>AAAGGGGACCTGCTAGCGGTTTCTTTAGTAGATTGAACTGGTTGACTAAGTCAGGAAACGCATACCCAGTGTTGAACGTGACTATGCCAAACAATGACAATTTTGACAAACTATACATCTGGGGAGTTCATCACCCGAGCACAAATCAAGAACAAACCAACCTGTATGTTCAAGCCTCAGGAAGAGTCACAGTCTCCACCAGGAGAAGCCAACAGACTATAATTCCTAACAT<br>TGGATCTAGACCCTGGGTAAGGGGCCAATCGGGCAGAATAAGCATCTACTGGACAATAGTCAAACCTGGGGACGTACTAGTAATCAATAGTAATGGAAACCTAATCGCTCCTCGGGGCTACTTCAAAATGCGCACTGGGAAAAGCTCAATAATGAGATCAGATGCACCTATTGACACATGCATCTCTGAATGCATCACTCCAAATGGAAGCATCCCCAATGACAAGCCCTTC<br>CAAAATGTAAACAAGATCACATACGGAGCATGTCCCAAATATGTGAAGCAGAACACCCTAAAGTTGGCAACAGGAATGCGGAATGTACCTGAGAAACAAACCAGAGGCCTGTTCGGTGCAATAGCAGGGTTTATAGAAAATGGGTGGGAAGGGATGATAGATGGTTGGTATGGCTTCAGACATCAAAATTCCGAAGGCACAGGACAAGCAGCGGACCTTAAAAGTACTC<br>AGGCAGCCATTGACCAGATCAATGGGAAGCTGAACAGAGTAATTGAAAAGACGAATGAGAAGTTCCATCAAATCGAAAAAGAATTCTCCGAAGTAGAAGGGAGGATTCAAGACCTTGAGAAATACGTTGAAGACACAAAGATAGATCTCTGGTCTTATAATGCGGAGCTTCTTGTTGCCCTAGAGAATCAGCACACAATTGATTTGACGGACTCAGAAATGAACAAATTG<br>TTTGAGAAGACCAGGAGGCAACTGAGGGAAAATGCTGAGGACATGGGCAATGGTTGTTTCAAGATATACCACAAATGTGACAATGCCTGTATAGAGTCAATTAGAAACGGGACTTATGACCATGACATATACAGAGATGAGGCATTGAACAACCGGTTCCAGATCAAAGGTGTTGAGCTAAAATCTGGCTACAAAGACTGGATCCTGTGGATTTCCTTTGCCATATCATGC<br>TTTTTGCTTTGTGTTGTTTTGCTGGGGTTCATTATGTGGGCCTGCCAGAGAGGCAACATTAGGTGCAACATTTGC
EPI_ISL_7624143	>A/Anas_platyrhynchos/Belgium/11294_004/2021_H3N8_2021-08-28<br>ATGAAGACCGTTATTGCTTTAAGCTACATTTTCTGTCTGGCTTTTAGTCAGGACCTTTCAGGGAATGACAACAGTACAGCAACACTGTGCCTGGGACACCATGCAGTGCCGAACGGAACAATAGTAAAGACAATTACAGATGATCAGATTGAGGTGACTAATGCTACCGAGCTCGTTCAAAGCTCTTCAACAGGGAAAATATGTAACAATCCCCACAGAATCCTTGATGGAA<br>GGGACTGCACACTAATAGATGCCCTACTGGGGGATCCCCATTGTGATGTCTTTCAAGATGAAACATGGGACCTCTTTGTAGAGCGGAGCAATGCTTACAGTAATTGTTACCCTTACGATGTACCAGACTATGCATCCCTTCGATCCCTAGTTGCATCATCAGGCACATTGGAATTCATCACTGAAGGCTTCACTTGGACAGGAGTTACTCAGAATGGAGGGAGCAATGCTTGC<br>AAAAGGGGACCTGCTAGCGGTTTCTTTAGTAGATTGAACTGGTTGACTAAGTCAGGAAACACATACCCAGTGTTGAACGTGACTATGCCAAACAATGACAATTTTGACAAACTATACATCTGGGGAGTTCACCACCCGAGCACAAACCAAGAACAAACCAACCTGTATGTTCAAGCTTCAGGAAGAGTCACAGTCTCCACCAGGAGAAGCCAACAGACTATAATTCCAAACA<br>TTGGATCTAGACCCTGGGTAAGGGGTCAATCTGGCAGAATAAGCATCTACTGGACAATAGTCAAACCTGGGGACGTACTAGTAATCAATAGTAATGGAAACCTAATTGCTCCTCGGGGCTACTTCAAAATGCGCACTGGGAAAAGCTCAATCATGAGATCAGATGCACCTATTGACACCTGCATTTCTGAATGCATCACCCCAAATGGAAGCATCCCCAATGACAAGCCCTT<br>CCAAAATGTAAACAAGATCACATACGGAGCATGCCCCAAATATGTGAAGCAGAACACCCTGAAGTTGGCAACAGGAATGCGGAATGTCCCTGAGAAACAGACCAGAGGCCTGTTCGGTGCAATAGCAGGGTTTATAGAAAATGGATGGGAAGGGATGATAGATGGTTGGTATGGCTTCAGGCATCAAAATTCCGAAGGCACAGGACAAGCAGCAGACCTTAAAAGCAC<br>TCAGGCAGCCATTGACCAGATCAATGGGAAGCTGAACAGAGTAATTGAAAAGACGAATGAAAAGTTTCACCAAATCGAAAAGGAATTCTCCGAAGTAGAAGGGAGGATTCAGGACCTTGAGAAATACGTTGAAGACACAAAGATAGATCTCTGGTCTTATAATGCGGAGCTTCTTGTTGCCCTAGAGAACCAGCATACAATTGATTTGACGGACTCAGAAATGAACAAAT<br>TGTTTGAGAAGACCAGGAGGCAACTTAGGGAAAATGCTGAGGACATGGGCAATGGTTGTTTCAAGATATACCACAAATGCGACAATGCTTGTATAGAGTCAATTAGAAACGGGACTTATGACCATGACATATACAGAGATGAGGCATTGAACAACCGGTTCCAGATCAAAGGTGTTGAGCTAAAATCTGGATACAAGGACTGGATCCTGTGGATTTCCTTTGCCATATCAT<br>GCTTTTTGCTTTGTGTTGTTTTGCTGGGATTCATTATGTGGGCCTGCCAGAAAGGCAACATTAGGTGCAACATTTGC
EPI_ISL_209121	>A/duck/Mongolia/179/2015_H3N8_2015-08-30<br>ATGAAGACCGTTATTGCTTTAAGCTACATTTTCTGCCTGGCTTTTAGTCAGGACCTTTCAGGGAATGACAACAGTACAGCAACACTGTGCCTGGGACACCATGCAGTGCCGAATGGAACAATAGTGAAGACAATTACAGATGATCAGATAGAGGTAACTAATGCTACCGAGCTAGTTCAAAGCTCTTCAACAGGGAAAATATGCAACAACCCTCACAGAATCCTTGATGGAA<br>GGGACTGCACACTAATAGATGCCCTACTGGGGGATCCTCATTGTGATGTCTTTCAAGATGAAACATGGGACCTCTTTGTAGAGAGGAGCAATGCGTTCAGCAATTGTTACCCTTACGATGTACCAGACTATGCATCCCTTCGATCCCTAGTTGCATCATCAGGCACATTGGAATTCATCACTGAAGGCTTCACTTGGACAGGAGTTACTCAGAATGGAGGGAGCAATGCTTGC<br>AAAAGGGGACCTGCCAGCGGTTTCTTTAGTAGATTGAACTGGTTGACTAAGTCAGGGAACGCATACCCAGTGTTGAACGTGACTATGCCAAACAATGACAATTTTGACAAACTATACATCTGGGGAGTTCACCACCCGAGCACAAATCAAGAACAAACCAACTTGTATGTTCAAGCCTCAGGAAGAGTCACAGTCTCCACCAGGAGAAGCCAACAGACTATAATTCCGAACA<br>TTGGATCTAGACCCTGGGTAAGGGGCCAATCTGGCAGAATAAGCATCTACTGGACAATAGTCAAACCTGGGGACGTACTGGTAATCAATAGTAATGGAAACCTAATCGCTCCTCGGGGCTACTTCAAAATGCGCACTGGGAAAAGCTCAATAATGAGATCAGATGCACCTATTGACACCTGCATTTCTGAATGCATCACTCCAAATGGAAGCATCCCCAATGACAAGCCCTT<br>CCAAAATGTAAACAAAATCACATACGGATCATGTCCCAAATATGTGAAGCAGAACACCCTAAAGTTGGCAACAGGAATGCGGAATGTACCTGAGAAACAAACCAGAGGCCTGTTCGGTGCAATAGCAGGGTTTATAGAAAATGGATGGGAAGGAATGATAGATGGTTGGTATGGCTTCAGACATCAAAATTCCGAAGGCACAGGACAAGCAGCAGACCTTAAAAGCACT<br>CAGGCAGCCATTGACCAGATCAATGGGAAGCTGAACAGAGTAATTGAAAAGACGAATGAGAAGTTCCATCAAATCGAAAAAGAATTCTCCGAAGTAGAAGGGAGGATTCAGGACCTTGAGAAATACGTTGAAGACACAAAGATAGATCTCTGGTCTTATAATGCAGAGCTTCTTGTTGCCCTAGAGAATCAGCATACAATTGATTTGACGGACTCAGAAATGAACAAATT<br>GTTTGAGAAGACCAGGAGGCAACTGAGGGAAAATGCTGAGGACATGGGCAATGGTTGTTTCAAGATATACCACAAATGTGACAATGCTTGTATAGAGTCAATTAGAAACGGGACTTATGACCATGACATATACAGAGATGAGGCATTGAACAACCGGTTCCAGATCAGAGGTGTTGAGCTAAAATCTGGATACAAAGACTGGATCCTGTGGATTTCCTTTGCCATATCATG<br>CTTTTTGCTTTGTGTTGTTTTACTGGGGTTCATTATGTGGGCCTGCCAGAGAGGCAACATTAGGTGCAACATTTGC
EPI_ISL_376206	>A/mallard/Netherlands/29/2013_H3N8_2013-10-14<br>ATGAAGACCATTATTGCTTTAAGCTACATTTTCTGTCTGGCTTTTAGTCAGGACCTTTCAGGGAATGACAACAGTACAGCAACACTGTGCCTGGGACACCATGCAGTGCCGAACGGAACAATAGTAAAGACAATTACAGATGATCAGATTGAGGTGACTAATGCTACCGAGCTCGTTCAAAGCTCTTCAACAGGGAAAATATGCAACAATCCCCACAGAATCCTTGATGGAA<br>GGGACTGCACACTAATAGATGCCCTATTGGGGGATCCTCATTGTGATGTCTTTCAAGATGAAACATGGGACCTCTTTGTAGAGCGGAGCAATGCTTTCAGTAATTGTTACCCTTACGATGTACCAGACTATGCATCCCTTCGATCCCTAGTTGCATCATCAGGCACATTGGAATTCATCACTGAAGGCTTCACTTGGACAGGAGTTACTCAGAATGGAGGGAGCAATGCTTGC<br>AAAAGGGGACCTGCTAGCGGTTTCTTTAGTAGATTGAACTGGTTGACTAAGTCAGGAAACGCATACCCAGTGTTGAACGTGACTATGCCAAACAATGACAATTTTGACAAACTATACATCTGGGGAGTTCACCACCCGAGCACAAATCAAGAACAAACCAACCTGTATGTTCAAGCCTCAGGAAGAGTTACAGTCTCCACCAGGAGAAGCCAACAGACTATAATTCCGAACA<br>TTGGATCTAGACCCTGGGTAAGGGGCCAATCTGGCAGAATAAGCATCTACTGGACAATAGTTAAACCTGGGGACGTACTAGTAATCAATAGTAATGGAAACCTAATCGCTCCTCGGGGCTACTTCAAAATGCGCACTGGGAAAAGCTCAATAATGAGATCAGATGCACCCATTGACACCTGCATTTCTGAATGCATCACTCCAAATGGAAGCATCCCCAATGACAAGCCCTT<br>CCAAAATGTAAACAAGATCACATACGGAGCATGTCCCAAATATGTGAAGCAGAACACCCTAAAGTTGGCAACAGGAATGCGGAATGTACCTGAGAAACAGACCAGAGGCCTGTTCGGTGCAATAGCAGGGTTTATAGAAAATGGATGGGAAGGGATGATAGATGGTTGGTATGGCTTCAGACATCAAAATTCCGAAGGCACAGGACAAGCAGCAGACCTTAAAAGCACT<br>CAGGCAGCCATTGACCAGATCAATGGGAAGCTGAACAGAGTAATTGAAAAGACGAATGAGAAGTTCCATCAAATCGAGAAGGAATTTTCCGAAGTAGAAGGGAGGATTCAGGACCTTGAGAAATACGTTGAAGACACAAAGATAGATCTCTGGTCTTATAATGCGGAGCTTCTTGTTGCCCTAGAGAATCAGCATACAATTGATTTGACGGACTCAGAAATGAACAAATT<br>GTTTGAGAAGACCAGGAGGCAACTGAGGGAAAATGCTGAGGACATGGGCAATGGTTGTTTCAAGATATACCACAAATGTGACAATGCTTGTATAGAGTCAATTAGAAACGGGACTTACGACCATGACATATACAGAGATGAGGCATTGAACAACCGGTTCCAGATCAAAGGTGTTGAGCTAAAATCTGGATACAAAGACTGGATCCTGTGGATTTCCTTTGCCATATCATG<br>CTTTTTGCTTTGTGTTGTTTTGCTGGGGTTCATTATGTGGGCCTGCCAGAGAGGCAACATTAGGTGCAACATTTGC
EPI_ISL_14393858	>A/Anas_platyrhynchos/Belgium/2499_0006/2021_(MIXED)_H3_2021-09-04<br>ATGAAGACCGTTATTGCTTTAAGCTACATTTTCTGTCTGGCTTTTAGTCAGGACCTTTCGGGGAATGACAACAGTACAGCAACACTGTGCCTGGGACACCATGCAGTGCCGAACGGAACAATAGTAAAGACAATTACAGATGATCAGATTGAGGTGACTAATGCTACCGAGCTCGTTCAAAGCTCTTCAACAGGGAAAATATGCAACAACCCCCACAGAATCCTTGATGGAA<br>GAGACTGCACACTAATAGATGCCCTGCTGGGGGATCCTCATTGTGATGTCTTTCAAGATGAAACATGGGACCTCTTTGTAGAGCGGAGCAATGCTTTCAGCAATTGTTACCCTTACGATGTACCGGACTATGCATCCCTTCGATCCCTAGTTGCATCATCAGGCACATTGGAATTCATCACTGAGGGCTTCACTTGGACAGGAGTCACTCAGARTGGAGGGAGCAGTGCTTGC<br>AAAAGGGGACCTGCTAGCGGTTTCTTTAGTAGACTGAACTGGTTGACTAAGTCAGGAAACGCATACCCAGTGTTAAATGTGACCATGCCAAACAATGACAATTTTGACAAATTATACATCTGGGGAGTTCACCACCCGAGCACAAATCAAGAACAAACCAACCTGTATGTTCAAGCCTCAGGAAGAGTTACAGTCTCCACCAGAAGAAGCCAACAGACTATAATTCCGAACA<br>TCGGATCCAGACCCTGGGTAAGGGGGCAATCTGGCAGAATAAGCATCTACTGGACAATAGTCAAACCCGGAGACGTACTAGTAATCAATAGTAATGGAAACCTAATCGCTCCTCGGGGCTATTTCAAAATGCGCACTGGGAAAAGCTCAATAATGAGATCAGATGCACCTATTGACACCTGCATTTCTGAATGCATCACTCCAAATGGAAGCATCCCCAATGATAAGCCCTT<br>CCAAAATGTAAACAAGATCACATACGGAGCATGTCCCAAATATGTGAAGCAGAACACCCTAAAGTTGGCAACAGGAATGCGGAATATACCTGAGAAACAGACCAGAGGCCTGTTCGGTGCAATAGCAGGGTTTATAGAAAATGGATGGGAAGGAATGATAGATGGTTGGTATGGCTTCAGACATCAAAATTCCGAGGGCACAGGACAAGCAGCAGACCTTAAAAGCACT<br>CAGGCAGCCATTGACCAGATCAATGGGAAGCTGAACAGAGTAATTGAAAAGACAAATGAGAAGTTCCATCAAATCGAAAAGGAGTTTTCTGAAGTAGAAGGGAGGATTCAGGACCTTGAGAAATACGTTGAAGACACAAAGATAGATCTCTGGTCTTATAATGCGGAGCTTCTTGTTGCCCTAGAAAACCAGCATACAATTGATTTGACGGACTCAGAAATGAACAAATT<br>GTTTGAGAAGACCAGGAGGCAACTGAGGGAAAATGCTGAGGACATGGGCAATGGTTGTTTCAAGATATACCACAAATGTGACAATGCTTGTATAGAGTCAATTAGAAACGGGACTTATGACCATGACATATACAGAGATGAGGCATTGAACAACCGGTTTCAGATCAAAGGTGTTGAGCTAAAATCTGGGTACAAAGACTGGATCCTGTGGATTTCCTTTGCCATATCATG<br>CTTTTTGCTTTGTGTTGTTTTGCTGGGGTTCATTATGTGGGCCTGCCAGAGAGGCAACATTAGGTGCAACATTTGC
EPI_ISL_1184519	>A/euraisan_coot/Novosibirsk_region/3493k/2020_H3N8_2020-08-29<br>ATGAAGACCGTTATTGCTTTAAGCTACATTTTCTGTCTGGCTTTTAGTCAGGACCTTTCAGGGAATGACAACAGTACAGCAACACTGTGCCTGGGACACCATGCAGTGCCGAACGGAACAATAGTAAAGACAATTACAGATGATCAGATTGAGGTGACTAATGCTACCGAGCTCGTTCAAAGCTCTTCAACAGGGAAAATATGCAACAATCCCCACAGAATCCTTGATGGAA<br>GGGACTGCACACTAATAGATGCCCTACTGGGGGATCCTCATTGTGATGTCTTTCAAGATGAAACATGGGACCTCTTTGTAGAGCGGAGCAATGCTTACAGTAATTGTTACCCTTACGATGTACCAGACTATGCATCCCTTCGATCCCTAGTTGCATCATCAGGCACATTGGAATTCATCACTGAAGGCTTCACTTGGACAGGAGTTACTCAGAATGGAGGGAGCAATGCTTGC<br>AAAAGGGGACCTGCTAGCGGTTTCTTTAGTAGATTGAACTGGTTAACTAAGTCAGGAAACGCATACCCAGTGTTGAACGTGACTAAGCCAAACAATGACAATTTTGACAAACTATACATCTGGGGAGTTCACCACCCGAGTACAAACCAAGAACAAACCAACCTGTATGTTCAAGCTTCAGGAAGAGTTACAGTCTCCACCAGGAGAAGCCAACAGACTATAATTCCGAACA<br>TTGGATCTAGACCCTGGGTAAGGGGTCAATCTGGCAGAATAAGCATCTACTGGACAATAGTCAAACCTGGGGACGTACTAGTAATCAATAGTAATGGAAACCTAATTGCTCCTCGGGGCTACTTCAAAATGCGCACTGGGAAAAGCTCAATAATGAGATCAGATGCACCTATTGACACCTGCATTTCTGAATGCATCACTCCAAATGGGAGCATCCCCAATGACAAGCCCTT<br>CCAAAATGTAAACAAGATCACATACGGAGCATGTCCCAAATATGTGAAGCAGAACACCCTAAAGTTGGCAACAGGAATGCGGAATGTCCCTGAGAAACAGACCAGAGGCCTGTTCGGTGCAATAGCAGGGTTTATAGAAAATGGATGGGAAGGGATGATAGATGGTTGGTATGGCTTCAGACATCAAAATTCCGAAGGCACAGGACAAGCAGCAGACCTTAAAAGCACT<br>CAGGCAGCCATTGACCAGATCAATGGGAAGCTGAACAGAGTAATTGAAAAGACGAATGAGAAGTTCCATCAAATCGAAAAGGAATTCTCCGAAGTAGAAGGGAGGATTCAGGACCTTGAGAAATACGTTGAAGACACAAAGATAGATCTCTGGTCTTATAATGCGGAGCTTCTTGTTGCCCTAGAGAATCAGCATACAATTGATTTGACGGACTCAGAAATGAACAAATT<br>GTTTGAGAAGACCAGGAGGCAACTGAGGGAAAATGCTGAGGACATGGGCAATGGTTGTTTCAAGATATACCACAAATGTGACAATGCTTGTATAGAGTCAATTAGAAACGGGACTTATGACCATGACATATACAGAGATGAGGCATTGAACAACCGGTTCCAGATCAAAGGTGTTGAGCTAAAATCTGGATACAAGGACTGGATCCTGTGGATTTCCTTTGCCATATCATG<br>CTTTTTGCTTTGTGTTGTTTTGCTGGGATTCATTATGTGGGCCTGCCAGAGAGGCAACATTAGGTGCAACATTTGC
EPI_ISL_121363	>A/duck/Moscow/3806/2009_H3_2009-09-01<br>ATGAAGACCGTTATTGCTTTAAGCTATATTTTCTGTCTGGCTTTTAGTCAGGACCTTTCAGGGAATGACAACAGTACAGCAACACTGTGCTTGGGACACCATGCAGTGCCGAACGGAACAATAGTGAAGACAATTACAGATGATCAGATTGAGGTGACTAATGCTACCGAACTAGTTCAAAGCTCTTCAACAGGGAAAATATGCAACAATCCCCACAGAATCCTTGATGGAA<br>GAGACTGCACACTAATAGATGCCCTACTGGGAGATCCTCATTGTGATGTCTTTCAAGATGAAACATGGGACCTCTTTGTAGAGCGGAGCAATGCTTTCAGCAATTGTTACCCTTACGATGTACCAGACTATGCATCCCTTCGATCCCTAGTTGCATCATCAGGCACATTGGAATTCATCACTGAAGGTTTCACTTGGACAGGAGTTACTCAGAATGGAGGGAGCAATGCTTGC<br>AAAAGGGGACCTGCTAGCGGTTTCTTTAGTAGATTGAACTGGTTGACTAAGTCAGGAAACGCATACCCAGTGTTGAACGTGACTATGCCAAACAACGACAATTTTGACAAACTTTACATTTGGGGAGTTCACCACCCGAGCACAAATCAAGAACAAACCAACCTGTATGTTCAAGCCTCAGGAAGAGTCACAGTCTCCACCAGGAGAAGCCAACAGACTATAATTCCGAACA<br>TTGGATCTAGACCCTGGGTAAGGGGCCAATCTGGCAGAATAAGCATCTACTGGACAATAGTCAAACCTGGGGACGTACTAGTAATCAATAGTAATGGAAACCTAATCGCTCCTCGGGGCTACTTCAAAATGCGCACTGGGAAAAGCTCAATAATGAGATCAGATGCACCTATTGACACCTGCATTTCTGAATGCATCACTCCAAATGGAAGCATCCCCAATGACAAGCCCTT<br>CCAAAATGTAAACAAGATCACATACGGAGCATGTCCCAAATATGTGAAGCAGAACACCCTAAAGTTGGCAACAGGAATGCGGAATGTACCTGAGAAACAAACCAGAGGCCTGTTCGGTGCAATAGCAGGGTTTATAGAAAATGGATGGGAAGGGATGATAGATGGTTGGTATGGCTTCAGACATCAAAATTCCGAAGGCACAGGACAAGCAGCAGACCTTAAAAGCACT<br>CAGGCAGCCATTGACCAGATCAATGGGAAGCTGAACAGAGTAATTGAAAAGACGAATGAGAAGTTCCATCAAATCGAAAAGGAATTCTCCGAAGTAGAAGGGAGGATTCAGGACCTTGAGAAATACGTTGAAGACACAAAGATAGATCTCTGGTCTTATAATGCGGAGCTTCTTGTTGCCCTAGAGAATCAGCATACAATTGATTTGACGGACTCAGAGATGAACAAATT<br>GTTTGAGAAGACCAGGAGGCAACTGAGAGAAAATGCTGAGGACATGGGCAATGGTTGTTTCAAGATATACCACAAATGTGACAATGCTTGTATAGAGTCAATTAGAAACGGGACTTATGACCATGACATATACAGAGATGAGGCATTGAACAACCGGTTCCAGATCAAAGGTGTTGAGCTAAAATCTGGATACAAAGACTGGATCCTGTGGATTTCCTTTGCCATATCATG<br>CTTTTTGCTTTGTGTTGTTTTGCTGGGATTCATTATGTGGGCCTGCCAGAGAGGCAACATTAGGTGCAACATTTGC
EPI_ISL_502599	>A/Anas_platyrhynchos/Belgium/11027_19/2017_H3N8_2017-10-04<br>ATGAAGACCGTTATTGCTTTAAGCTACATTTTCTGTCTGGCTTTTAGTCAGGACCTTTCAGGGAATGACAACAGTACAGCAACACTGTGCCTGGGACACCATGCAGTGCCAAACGGAACAATAGTAAAGACAATTACAGATGATCAAGTTGAGGTGACTAATGCTACCGAGCTCGTTCAGAGCTCTTCAACAGGGAAAATATGCAACAATCCCCACAGAATCCTTGATGGAA<br>GGGACTGCACACTAATAGATGCCCTACTGGGGGATCCTCATTGTGATGTCTTTCAAGATGAAACATGGGACCTCTTTGTAGAGCGGAGCAATGCTTACAGTAATTGTTACCCTTACGATGTACCAGACTATGCATCCCTTCGATCCCTAGTTGCATCATCAGGCACATTGGAATTCATCACTGAAGGTTTCACTTGGACAGGAGTTACTCAGAATGGAGGGAGCAATGCTTGC<br>AAAAGGGGGCCTGCTAGCGGTTTCTTTAGTAGATTGAACTGGTTGACTAAGTCAGGAAACGCATACCCAGTGTTGAACGTGACTATGCCAAACAATGACAATTTTGACAAACTATACATCTGGGGAGTTCACCACCCGAGCACAAACCAAGAACAAACCAACCTGTATGTTCAAGCTTCAGGAAGAGTTACAGTCTCCACCAGGAGAAGCCAACAGACTATAATTCCGAACA<br>TTGGATCTAGACCCTGGGTAAGGGGTCAATCTGGCAGAATAAGCATCTACTGGACAATAGTCAAACCTGGGGACGTATTAGTAATCAATAGTAATGGAAACCTAATTGCTCCTCGGGGCTACTTCAAAATGCGCACTGGGAAAAGCTCAATAATGAGATCAGATGCACCTATTGACACCTGCATTTCTGAATGCATCACTCCAAATGGAAGCATCCCCAATGACAAGCCCTTC<br>CAAAATGTAAACAAGATCACATACGGAGCATGTCCCAAATATGTGAAGCAGAGCACCCTAAAGTTGGCAACAGGAATGCGGAATGTCCCCGAGAAACAGACCAGAGGCCTGTTCGGTGCAATAGCAGGGTTTATAGAAAATGGATGGGAAGGGATGATAGATGGTTGGTATGGCTTCAGACATCAAAATTCCGAAGGCACAGGACAAGCAGCAGATCTTAAAAGCACTC<br>AGGCAGCCATTGACCAGATCAATGGGAAGCTGAACAGAGTAATTGAAAAGACGAATGAGAAGTTCCATCAAATTGAAAAGGAATTCTCCGAAGTAGAAGGGAGGATTCAGGACCTTGAGAAATACGTTGAAGACACAAAGATAGATCTCTGGTCTTATAATGCGGAGCTTCTTGTTGCCCTAGAGAATCAGCATACAATTGATTTGACGGACTCAGAAATGAACAAATTG<br>TTTGAGAAGACCAGGAGGCAACTGAGGGAAAATGCTGAGGACATGGGCAATGGTTGTTTCAAGATATACCACAAATGTGACAATGCTTGTATAGAGTCAATTAGAAACGGGACTTATGACCATGACATATACAGAGATGAGGCATTGAACAACCGGTTCCAGATCAAAGGTGTTGAGCTAAAATCTGGATACAAGGACTGGATCCTGTGGATTTCCTTTGCCATATCATGC<br>TTTTTGCTTTGTGTTGTTTTGCTGGGATTCATTATGTGGGCCTGCCAGAGAGGCAACATTAGGTGCAACATTTGC
EPI_ISL_63509	>A/gadwall/Altai/1328/2007_H3N8_2007-09-01<br>ATGAAGACCGTTATTGCTTTAAGCTACATTTTCTGTCTGGCTTTCAGTCAGGACCTTCCAGGGAATGACAACAGTACAGCAACACTGTGCCTGGGACACCATGCAGTGCCGAACGGAACAATAGTGAAGACAATCACAGATGATCAGATTGAGGTGACTAATGCTACTGAGCTAGTTCAAGGCTCGTCAACAGGGAAAATATGCAACAATCCCCACAGAATCCTTGATGGAA<br>GGGACTGCACACTAATAGATGCCCTACTGGGGGATCCTCATTGTGATGTCTTTCAAGATGAAACATGGGACCTCTTTGTAGAGCGGAGCAATGCTTTCAGCAATTGTTACCCTTATGATGTACCAGACTATGCATCCCTTCGATCCCTAGTTGCATCATCAGGCACATTGGAATTCATCACTGAAGGTTTCACTTGGACAGGAGTTACTCAGAATGGAGGGAGCAGTGCTTGC<br>AAAAGGGGACCTGCTAGCGGTTTCTTTAGTAGATTGAACTGGTTGACTAAGTCAGGAAACGCATACCCAGTGTTGAACGTGACTATGCCCAACAATGACAATTTTGACAAACTATACATCTGGGGAGTTCACCACCCGAGCACAAATCAAGAACAAACCAACCTGTATGTTCAAGCCTCAGGAAGAGTCACAGTCTCCACCAGGAGAAGCCAACAGACTATAATTCCGAACA<br>TTGGGTCTAGACCCTGGGTAAGGGGCCAATCTGGCAGAATAAGCATCTACTGGACAATAGTCAAACCTGGGGACGTACTAGTAATCAATAGTAATGGAAACCTAATCGCTCCTCGGGGCTACTTCAAAATGCGCACTGGGAAAAGCTCAATAATGAGATCAGATGCACCTATTGACACCTGCATTTCTGAATGCATCACTCCAAATGGAAGCATCCCCAATGACAAGCCCTT<br>CCAAAATGTAAACAAGATCACATACGGAGCATGTCCCAAATATGTGAAGCAGAACACCCTAAAGTTGGCAACAGGAATGCGGAATGTACCTGAGAAACAAACCAGAGGCCTGTTCGGTGCAATAGCAGGGTTTATAGAAAATGGATGGGAAGGGATGATAGATGGTTGGTATGGCTTCAGACATCAAAATTCCGAAGGCACAGGACAAGCAGCAGACCTTAAAAGCACT<br>CAGGCAGCCATTGACCAGATCAATGGGAAGTTGAACAGAGTAATTGAAAAGACGAATGAGAAGTTCCATCAAATCGAAAAGGAATTCTCCGAAGTAGAAGGGAGGATTCAGGACCTTGAGAAATACGTTGAAGACACAAAGATAGATCTATGGTCTTATAATGCGGAACTTCTTGTTGCCCTAGAGAATCAGCATACAATTGATTTGACGGACTCAGAAATGAACAAATT<br>GTTTGAAAAGACCAGGAGGCAACTGAGGGAAAATGCTGAGGACATGGGCAATGGTTGTTTCAAGATATACCACAAATGTGACAATGCTTGTATAGAGTCAATTAGAAATGGGACTTATGACCATGACATATACAGAGATGAGGCATTGAACAACCGGTTCCAGATCAAAGGTGTTGAGCTAAAATCTGGATACAAAGACTGGATCCTGTGGATTTCCTTTGCCATATCATG<br>CTTTTTGCTTTGTGTTGTTTTGCTGGGGTTCATTATGTGGGCCTGCCAGAGAGGCAACATTAGGTGCAACATTTGC
EPI_ISL_243402	>A/dunlin/Netherlands/2/2007_H3N8_2007-07-31<br>ATGAAGACCATTATCGCTTTAAGCTACGTTTTCTGTCTGGCCTTCGGTCAGGACCTTTCAGGGAGCGACAATAGTACAGCAACACTGTGCCTGGGACACCATGCAGTGCCGAATGGGACAATAGTGAAGACCATTACAGATGATCAGATTGAGGTGACTAATGCTACCGAGCTAGTCCAAAGCTCTTCGACAGGGAAAATATGCAACAATCCTCACAGGATTCTTGATGGAA<br>GGGACTGCACACTAATAGATGCCCTACTGGGAGATCCTCATTGCGATGTCTTTCAATATGAGACATGGGATCTTTTTGTGGAGCGAAGCAATGCTTTCAGCAATTGCTACCCTTACGATGTACCAGACTATGCATCCCTTCGATCCCTAGTTGCATCATCAGGCACATTGGAGTTCATCACTGAAGGTTTCACTTGGACAGGAGTAACTCAGAATGGAGGGAGCAATGCCTGC<br>AAAAGGGGACCTGCTAGTGGTTTCTTCAGTAGACTGAACTGGTTGACTAAGTCAGGAAGCACATACCCAGTGTTGAACGTGACTATGCCAAACAATGACAATTTTGACAAACTATACATCTGGGGAGTTCACCACCCGAGCACAAACCAAGAACAAACCAACCTGTATGTTCAAGCCTCAGGAAGAGTCACAGTCTCTACCAGGAGAAGCCAGCAGACTATAATCCCTAACA<br>TTGGGTCTAGACCCTGGGTAAGGGGCCAATCTGGCAGAATAAGCATCTACTGGACAATAGTCAAACCTGGGGACGTACTGGTGATCAATAGTAATGGAAATCTAATCGCTCCTCGGGGCTACTTCAAGATGCGCACTGGGAAAAGCTCGATAATGAGGTCAGATGCACCTATTGACACCTGTATTTCTGAATGCATCACTCCAAATGGAAGCATCCCAAATGACAAGCCCTT<br>CCAAAATGTGAACAAGATCACATACGGAGCATGTCCCAAATATGTGAAGCAGAACACCCTGAAGTTAGCAACAGGAATGCGGAATGTACCAGAGAAGCAAACCAGAGGCCTATTCGGTGCAATAGCAGGTTTTATAGAAAATGGATGGGAAGGAATGATAGATGGTTGGTATGGCTTCAGGCATCAAAATTCCGAAGGTACAGGACAAGCAGCAGACCTTAAAAGCACT<br>CAGACAGCCATTGACCAGATCAATGGGAAATTGAACAGAGTGATTGAAAAGACGAATGAAAAGTTCCATCAAATCGAAAAGGAATTCTCCGAGGTAGAAGGGAGGATTCAGGACCTTGAGAAATACGTTGAAGACACAAAAATAGATCTCTGGTCTTATAATGCGGAACTCCTTGTTGCCCTAGAGAATCAGCATACAATTGATTTGACTGATTCAGAAATGAACAAATTG<br>TTTGAAAAGACCAGAAGGCAACTGAGGGAAAATGCTGAAGACATGGGCAATGGTTGTTTCAAAATATACCACAAGTGTGACAATGCTTGCATAGAGTCAATTAGGAACGGGACTTATGACCATGACATATACAGAGATGAAGCATTGAACAACCGGTTTCAGATCAAGGGTGTCGAGCTGAGATCTGGGTACAAAGACTGGATCCTGTGGATTTCCTTTGCCATATCATGC<br>TTTTTGCTTTGTGTTGTTTTGCTGGGGTTCATTATGTGGGCCTGCCAGAGAGGCAACATTAGGTGCAACATTTGC
EPI_ISL_169777	>A/mallard/Italy/3342/2006_H3N6_2006-01-01<br>ATGAAGACCATTATCGCTTTAAGCTACGTTTTCTGTCTGGCCTTCGGTCAGGACCTTTCAGGGAGCGACAATAGCACAGCAACACTGTGCCTGGGACACCATGCAGTGCCGAATGGGACAATAGTGAAGACCATTACAGATGATCAGATTGAGGTGACTAATGCTACCGAGCTAGTCCAAAGCTCTTCGACAGGGAAAATATGCAACAATCCTCACAGGATTCTTGATGGAA<br>GGGACTGCACACTAATAGATGCCCTACTGGGAGATCCTCATTGCGATGTTTTTCAAGATGAGACATGGGATCTTTTTGTGGAGCGAAGCAATGCTTTCAGCAATTGCTACCCTTACGATGTACCAGACTATGCATCCCTTCGATCCCTAGTTGCATCATCAGGCACATTGGAATTCATCACTGAAGGTTTCACTTGGACAGGAGTAACTCAGAATGGAGGGAGCAATGCCTGC<br>AAAAGGGGACCTGCTAGTGGTTTCTTCAGTAGACTGAACTGGCTGACTAAGTCAGGAAGCACATACCCAGTGTTGAACGTGACTATGCCAAACAATGACAATTTTGACAAACTATACATCTGGGGAGTTCACCACCCGAGCACAAACCAAGAACAAACCAACCTGTATGTTCAAGCCTCAGGAAGAGTCACAGTCTCTACCAGGAGAAGCCAGCAGACTATAATCCCTAACA<br>TTGGATCTAGACCCTGGGTAAGGGGCCAATCTGGCAGAATAAGCATCTACTGGACAATAGCCAAACCTGGGGACGTACTGGTGATCAATAGTAATGGAAATCTAATCGCTCCTCGGGGCTACTTCAAGATGCGCACTGGGAAAAGCTCGATAATGAGGTCAGATGCACCTATTGACACCTGTATTTCTGAATGCATCACTCCAAATGGAAGCATCCCAAATGACAAGCCCTT<br>CCAAAATGTGAACAAGATCACATACGGAGCATGTCCCAAATATGTGAAGCAGAACACCCTGAAGTTGGCAACAGGAATGCGGAATGTACCAGAGAAGCAAACCAGAGGCCTATTCGGTGCAATAGCAGGTTTTATAGAAAATGGATGGGAAGGAATGATAGATGGTTGGTATGGCTTCAGGCATCAAAATTCCGAAGGTACAGGACAAGCAGCAGACCTTAAAAGCACT<br>CAGGCAGCCATTGACCAGATCAATGGGAAATTGAACAGAGTGATTGAAAGGACGAATGAAAAGTTCCATCAAATCGAAAAGGAATTCTCCGAGGTGGAAGGGAGGATTCAGGACCTTGAGAAATACGTTGAAGACACAAAAATAGATCTCTGGTCTTATAATGCGGAACTCCTTGTTGCCCTAGAGAATCAGCATACAATTGATTTGACTGATTCAGAAATGAACAAATTG<br>TTTGAAAAGACCAGAAGGAAACTGAGGGAAAATGCTGAAGACATGGGCAATGGTTGTTTCAAAATATACCACAAGTGTGACAATGCTTGCATAGAGTCAATTAGGAACGGGACTTATGACCATGACATATACAGAGATGAGGCATTGAACAACCGGTTTCAGATCAAGGGTGTCGAGCTGAAATCTGGGTACAAAGACTGGATCCTGTGGATTTCCTTTGCCATATCATGC<br>TTTTTGCTTTGTGTTGTTTTGCTGGGGTTCATTATGTGGGCCTGCCAGAGAGGCAACATTAGGTGCAACATTTGC
EPI_ISL_399510	>A/Anas_platyrhynchos/Belgium/8295/2016_H3N1_2016-08-28<br>ATGAAGACCATTATCGCTTTAAGCTATGTTCTCTGTCTGGCCTTCGGTCAGGACCTTTCAGGGAGCGACAATAGTACGGCAACACTGTGCCTGGGACACCATGCAGTGCCGAATGGGACAATAGTGAAGACCATTACAGATGATCAGATTGAAGTGACTAATGCTACTGAGCTAGTCCAAAGCACTTCGACAGGGAAAATATGCAACAATCCTCACAGGATTCTTGACGGAA<br>GGGACTGCACACTAATAGATGCCCTACTGGGAGATCCTCATTGCGATGTCTTTCAAGATGAGACATGGGATCTCTTTGTAGAGCGAAGCAATGCTTTCAGCAATTGCTACCCTTACGATGTACCAGACTATGCATCCCTTCGATCCCTAGTTGCATCATCAGGCACATTGGAGTTCATCACTGAAGGTTTCACTTGGACAGGAGTAACTCAGAATGGGGGGAGCAATGCCTGC<br>AAAAGGGGACCTGCTAGTGGTTTCTTCAGTAGACTGAACTGGTTGACTAAGTCAGGAAGCACATACCCAGTGTTGAACGTGACTATGCCAAATAATGACAATTTTGACAAACTATACATCTGGGGAGTTCACCACCCGAGCACAAACCAAGAACAAACCAGCCTGTATGTTCAAGCCTCAGGAAGAGTCACAGTCTCTACCAGGAGAAGCCAGCAGACTATAATCCCTAACA<br>TTGGATCTAGACCCTGGGTAAGGGGCCAATCTGGTAGAATAAGCATCTACTGGACAATAGTCAAACCCGGGGACGTACTGGTGATCAATAGTAATGGAAACCTAATCGCTCCTCGGGGCTACTTCAAAATGCGCACTGGGAAGAGCTCAATAATGAGGTCAGATGCACCTATTGACACCTGTATTTCTGAATGCATCACTCCGAATGGAAGCATCCCAAATGACAAGCCCTT<br>CCAAAATGTGAATAAGATCACATACGGAGCATGTCCCAAATATGTGAAGCAGAACACCCTGAAGTTGGCAACAGGAATGCGGAATGTACCAGAGAAGCAAACCAGAGGCCTATTCGGTGCAATAGCAGGTTTTATAGAAAATGGATGGGAAGGAATGATAGAYGGTTGGTATGGCTTCAGGCATCAAAATTCCGAAGGTACAGGACAAGCAGCAGACCTTAAAAGCACT<br>CAGGCAGCCATTGACCAGATCAATGGGAAATTAAACAGAGTGATTGAAAAGACGAATGAAAAGTTCCATCAAATCGAAAAGGAATTCTCTGAGGTAGAAGGGAGAATTCAGGACCTTGAGAAATACGTTGAAGACACAAAAATAGACCTCTGGTCTTATAATGCGGAACTCCTTGTTGCCCTAGAGAATCAGCATACAATCGATTTGACTGATTCAGAAATGAACAAATTG<br>TTTGAAAAGACCGGGAGGCAACTAAGGGAAAATGCTGAAGACATGGGCAATGGTTGCTTCAAAATATACCACAAATGTGACAATGCTTGCATAGAGTCAATTAGGAACGGGACTTATGACCATGACATATACAGAGATGAGGCATTGAACAACCGGTTTCAGATCAAGGGTGTCGAGCTGAAATCTGGGTACAAAGACTGGATCCTATGGATTTCCTTTGCCATATCATGC<br>TTTTTGCTTTGTGTTGTTTTGCTGGGGTTCATTATGTGGGCCTGCCAGAGAGGCAACATTAGATGCAACATTTGC
EPI_ISL_30805	>A/turnstone/Netherlands/1/2007_H3N8_2007-07-14<br>ATGAAGACCATTATCGCTTTAAGCTACGTTTTCTGTCTGGCCTTCGGTCAGGACCTTTCAGGGAGCGACAATAGTACAGCAACACTGTGCCTGGGACACCATGCAGTGCCGAATGGGACAATAGTGAAGACCATTACAGATGATCAGATTGAGGTGACTAATGCTACCGAGCTAGTCCAAAGCTCTTCGACAGGGAAAATATGCAACAATCCTCACAGGATTCTTGATGGAA<br>GGGACTGCACACTAATAGATGCCCTACTGGGAGATCCTCATTGCGATGTCTTTCAAGATGAGACATGGGATCTTTTTGTGGAGCGAAGCAATGCTTTCAGCAATTGCTACCCTTACGATGTACCAGACTATGCATCCCTTCGATCCCTAGTTGCATCATCAGGCACATTGGAGTTCATCACTGAAGGTTTCACTTGGACAGGAGTAACTCAGAATGGAGGGAGCAATGCCTGC<br>AAAAGGGGACCTGCTAGTGGTTTCTTCAGTAGACTGAACTGGTTGACTAAGTCAGGAAGCACATACCCAGTGTTGAACGTGACTATGCCAAACAATGACAATTTTGACAAACTATACATCTGGGGAGTTCACCACCCGAGCACAAACCAAGAACAAACCAACCTGTATGTTCAAGCCTCAGGAAGAGTCACAGTCTCTACCAGGAGAAGCCAGCAGACTATAATCCCTAACA<br>TTGGGTCTAGACCCTGGGTAAGGGGCCAATCTGGCAGAATAAGCATCTACTGGACAATAGTCAAACCTGGGGACGTACTGGTGATCAATAGTAATGGAAATCTAATCGCTCCTCGGGGCTACTTCAAGATGCGCACTGGGAAAAGCTCGATAATGAGGTCAGATGCACCTATTGACACCTGTATTTCTGAATGCATCACTCCAAATGGAAGCATCCCAAATGACAAGCCCTT<br>CCAAAATGTGAACAAGATCACATACGGAGCATGTCCCAAATATGTGAAGCAGAACACCCTGAAGTTAGCAACAGGAATGCGGAATGTACCAGAGAAGCAAACCAGAGGCCTATTCGGTGCAATAGCAGGTTTTATAGAAAATGGATGGGAAGGAATGATAGATGGTTGGTATGGCTTCAGGCATCAAAATTCCGAAGGTACAGGACAAGCAGCAGACCTTAAAAGCACT<br>CAGACAGCCATTGACCAGATCAATGGGAAATTGAACAGAGTGATTGAAAAGACGAATGAAAAGTTCCATCAAATCGAAAAGGAATTCTCCGAGGTAGAAGGGAGGATTCAGGACCTTGAGAAATACGTTGAAGACACAAAAATAGATCTCTGGTCTTATAATGCGGAACTCCTTGTTGCCCTAGAGAATCAGCATACAATTGATTTGACTGATTCAGAAATGAACAAATTG<br>TTTGAAAAGACCAGAAGGCAACTGAGGGAAAATGCTGAAGACATGGGCAATGGTTGTTTCAAAATATACCACAAGTGTGACAATGCTTGCATAGAGTCAATTAGGAACGGGACTTATGACCATGACATATACAGAGATGAAGCATTGAACAACCGGTTTCAGATCAAGGGTGTCGAGCTGAGATCTGGGTACAAAGACTGGATCCTGTGGATTTCCTTTGCCATATCATGC<br>TTTTTGCTTTGTGTTGTTTTGCTGGGGTTCATTATGTGGGCCTGCCAGAGAGGCAACATTAGGTGCAACATTTGC
EPI_ISL_333104	>A/duck/Bangladesh/20D677/2016_H3N8_2016-07-22<br>ATGAAGACCGTTATTGCTTTAAGCTACATTTTCTGCCTGGCTTTTAGTCAGGACCTTTCAGGGAATGACAACAGTACGGCAACACTGTGCCTGGGACATCATGCAGTGCCGAATGGAACAATAGTGAAGACAATTACAGATGATCAGATAGAGGTAACTAATGCTACCGAGCTAGTTCAAAGCTCTTCAACAGGGAAAATATGCAACAACCCTCACAGAATCCTTGATGGAA<br>GGGACTGCACACTAATAGATGCCCTACTGGGGGATCCTCATTGTGATGTCTTTCAAGATGAAACATGGGACCTCTTTGTAGAGAGGAGCAATGCGTTCAGTAATTGTTACCCTTACGATGTACCAGACTATGCATCCCTTCGATCCCTAGTTGCATCATCAGGCACATTGGAATTCATCACTGAAGGCTTCACTTGGACAGGAGTTACTCAGAATGGAGGGAGCAATGCTTGC<br>AAAAGGGGACCTGCCAGCGGTTTCTTTAGTAGGTTGAACTGGTTGACTAAGTCAGGAAACGCATACCCAGTGTTGAACGTGACTATGCCAAACAATGACAATTTTGACAAACTATACATCTGGGGAGTTCACCACCCGAGCACAAATCAAGAACAAACCACCTTGTATGTTCAAGCCTCAGGAAGAGTCACAGTCTCCACCAGGAGAAGCCAACAGACTATAATTCCGAACA<br>TTGGATCTAGACCCTGGGTAAGGGGCCAATCTGGCAGAATAAGCATCTACTGGACAATAGTCAAACCTAGGGACGTACTGGTAATCAATAGTAATGGAAACCTAATCGCTCCTCGGGGCTACTTCAAAATGCGCACTGGGAAAAGCTCAATAATGAGATCAGATGCACCTATTGACACCTGCATTTCTGAATGCATCACTCCAAATGGAAGCATCCCCAATGACAAGCCCTT<br>CCAAAATGTAAACAAAATCACATACGGATCATGTCCCAAATATGTGAAGCAGAACACCCTAAAGTTGGCAACAGGAATGCGGAATGTACCTGAGAAACAAACCAGAGGCCTGTTCGGTGCAATAGCAGGGTTTATAGAAAATGGATGGGAAGGAATGATAGATGGTTGGTATGGCTTCAGACATCAAAATTCCGAAGGCACAGGACAAGCAGCAGACCTTAAAAGCACT<br>CAGGCAGCCATTGACCAGATCGATGGGAAGCTGAACAGAGTAATTGAAAAGACGAATGAGAAGTTCCATCAAATCGAAAAGGAATTCTCCGAAGTAGAAGGGAGGATTCAGGACCTTGAGAAATATGTTGAAGACACAAAGATAGATCTCTGGTCTTATAATGCAGAGCTTCTTGTTGCCCTAGAGAATCAGCATACGATTGATTTGACGGACTCAGAAATGAACAAATT<br>GTTTGAGAAGACCAGGAGGCAACTGAGGGAAAATGCTGAGGACATGGGCAATGGTTGTTTCAAGATATATCACAAATGTGACAATGCTTGTATAGAGTCAATTAGAAACGGGACTTATGACCATGACATATACAGAGATGAGGCATTGAACAACCGATTCCAGATCAAAGGTGTTGAGCTAAAATCTGGATACAAAGACTGGATCCTGTGGATTTCCTTTGCCATATCATG<br>CTTTTTGCTTTGTGTTGTTTTGCTGGGGTTCATTATGTGGGCCTGCCAGAGAGGCAACATTAGGTGCAACATTTGC
EPI_ISL_333615	>A/common_teal/Chany/893/2018_H3N6_2018-09-01<br>ATGAAGACCGTTATTGCCTTAAGCTACATTTTCTGCCTGGCTTTTAGTCAGGACCTTTCAGGGAATGACAACAGTACAGCAACACTGTGCCTGGGACATCATGCAGTGCCGAATGGAACAATAGTGAAGACAATTACAGATGATCAGATAGAGGTAACTAATGCTACCGAGCTAGTTCAAAGCTCTTCAACAGGGAAAATATGCAACAACCCTCACAGAATCCTTGATGGAA<br>GGGACTGCACACTAATAGATGCCCTACTGGGGGATCCTCATTGTGATGTCTTTCAAGATGAAACATGGGACCTCTTTGTAGAGAGGAGCAATGCGTTCAGCAATTGTTACCCTTACGATGTACCAGACTATGCATCCCTTCGATCCCTAGTTGCATCATCAGGCACATTGGAATTCATCACTGAAGGCTTCACTTGGACAGGAGTTACTCAGAATGGAGGGAGCAATGCTTGC<br>AAAAGGGGACCTACCAGCGGTTTCTTTAGTAGATTGAACTGGTTGACTAAGTCAGGAAACGCATACCCAGTGTTGAACGTGACTATGCCAAACAATGACAATTTTGACAAACTATACATCTGGGGAGTTCACCACCCGAGCACAAATCAAGAACAAACCAACTTGTATGTTCAAGCCTCAGGAAGAGTCACAGTCTCCACCAGGAGAAGCCAACAGACTATAATTCCGAACA<br>TTGGATCTAGACCCTGGGTAAGGGGCCAATCTGGCAGAATAAGCATCTACTGGACAATAGTCAAACCTGGGGACGTACTGGTAATCAATAGTAATGGAAACCTAATCGCTCCTCGGGGCTACTTCAAAATGCGCACTGGGAAAAGCTCAATAATGAGATCAGATGCACCTATTGACACCTGCATTTCTGAATGCATCACTCCAAATGGAAGCATCCCCAATGACAAGCCCTT<br>CCAAAATGTAAACAAAATCACATACGGATCATGTCCCAAATATGTGAAGCAGAACACCCTAAAGTTGGCAACAGGAATGCGGAATGTACCTGAGAAACAAACCAGAGGCCTGTTCGGTGCAATAGCAGGGTTTATAGAAAATGGATGGGAAGGAATGATAGATGGTTGGTATGGCTTCAGACATCAAAATTCCGAAGGCACAGGACAAGCAGCAGACCTTAAAAGCACT<br>CAGGCAGCCATTGACCAGATCAATGGGAAGCTGAACAGAGTAATTGAAAAGACGAATGAGAAGTTCCATCAAATCGAAAAGGAATTCTCCGAAGTAGAAGGGAGGATTCAGGACCTTGAGAAATACGTTGAAGACACTAAGATAGATCTCTGGTCTTATAATGCAGAGCTTCTTGTTGCCCTAGAGAATCAGCATACGATTGATTTGACGGACTCAGAAATGAACAAATT<br>GTTTGAGAAGACCAGGAGGCAACTGAGGGAAAATGCTGAGGACATGGGCAACGGTTGTTTCAAGATATACCACAAATGTGACAATGCTTGTATAGAGTCAATTAGAAACGGGACTTATGACCATGACATATACAGAGATGAGGCATTGAACAACCGGTTCCAGATCAAAGGTGTTGAGCTAAAATCTGGATACAAAGACTGGATCCTGTGGATTTCCTTTGCCATATCATG<br>CTTTTTGCTTTGTGTTGTTTTGCTGGGATTCATTATGTGGGCCTGCCAGAGAGGCAACATTAGGTGCAACATTTGC

EPI_ISL_14931849	>A/duck/Chernogolovka/5897/2021_H3N8_2021-11-07<br>ATGAAGACCGTCATTGCTCTAAGCTACATTTTCTGTCTGGCTTTTAGTCAGGACCTCTCAGGGAATGACAACAGCACAGCAACACTGTGCCTGGGACACCATGCAGTGCCGAATGGAACAATAGTGAAGACAATTACAGATGATCAGATTGAGGTGACTAATGCTACCGAGCTAGTCCAAAGCTCTTCAACAGGGAAAATATGCAACAACCCTCACAGAATCCTTGATGGGA<br>GGGACTGCACACTAATAGATGCCCTACTGGGGATCCTCATTGTGATGTCTTTCAAGATGAAACATGGGACCTCTTTGTAGAGCGGAGCAATGCGTTCAGTAATTGTTACCCTTACGATGTACCAGACTATGCATCCCTCCGATCCCTAGTTGCATCATCAGGCACATTGGAATTCATCACTGAAGGTTTCACTTGGACAGGAGTTACTCAGAATGGAGGGAGCAATGCTTGC<br>AAAAGAGGACCTGCCAGCGGTTTCTTTAGTAGATTGAACTGGTTGACTAAGTCAGGAAACGCATACCCAGTGTTGAACGTGACCATGCCAAACAATGACAATTTTGACAAACTATACATCTGGGGAGTTCATCACCCGAGCACGAACCAAGAACAAACCAACTTGTATGTTCAAGCCTCAGGAAGAGTCACAGTCTCCACCAGAAGGAGCCAACAGACTATAATTCCGAACA<br>TTGGATCTAGACCCTGGGTAAGGGGCCAATCTGGCAGGATAAGCATCTACTGGACAATAGTCAAACCTGGGGACGTACTGGTAATCAATAGCAATGGAAACCTAATCGCTCCTCGGGGCTACTTCAAAATGCGCACTGGGAAAAGCTCAATAATGAGATCAGATGCACCTATTGACACCTGCATTTCTGAATGCATCACTCCAATGGAAGCATCCCCAATGACAAGCCCTT<br>CCAAAATGTAAACAAAATCACATATGGAGCATGTCCCAAATATGTGAAGCAGAACACTCTAAAGTTGGCAACAGGAATGCGAAATGTGCCTGAGAAACAAACCAGAGGACTGTTCGGTGCAATAGCAGGGTTTATAGAAAACGGATGGGAGGGGATGATAGATGGTTGGTATGGCTTCCGACATCAAAATTCCGAAGGCACAGGACAAGCAGCAGACCTTAAAAGCACT<br>CAGGCAGCCATTGACCAGATCAATGGGAAGCTGAACAGAGTAATTGAGAAGACGAATGAGAAGTTCCATCAAATCGAAAAGGAATTCTCTGAAGTAGAAGGGAGGATTCAGGACCTTGAGAAATACGTTGAAGACACAAAGATAGATCTCTGGTCTTATAATGCGGAGCTTCTTGTTGCCCTAGAGAATCAGCATACGATTGATTTGACGGACTCAGAAATGAACAAATT<br>GTTTGAGAAGACCAGGAGGCAACTGAGAGAAAATGCTGAAGACATGGGCAATGGTTGTTTCAAGATCTACCACAAATGTGACAATGCTTGTATAGAGTCAATTAGAAACGGGACTTATGACCATGACATATACAGAGATGAGGCATTGAACAATCGGTTCCAGATCAAAGGTGTTGAGCTAAAATCTGGATACAAAGACTGGATCCTGTGGATTTCCTTTGCCATATCATG<br>CTTTTTGCTTTGTGTTGTTTTGCTGGGGTTCATTATGTGGGCCTGCCAGAGAGGCAACATTAGGTGCAACATTTGC
EPI_ISL_14931847	>A/duck/Moscow/5881/2021_H3N2_2021-10-06<br>ATGAAGACCGTCATTGCTCTAAGCTACATTTTCTGTCTGGCTTTTAGTCAGGACCTCCCAGGGAATGACAACAGCACAGCAACACTGTGCCTGGGACACCATGCAGTGCCGAATGGAACAATAGTGAAGACAATTACAGATGATCAGATTGAGGTGACTAATGCTACCGAGCTAGTCCAAAGCTCTTCAACAGGGAAAATATGCAACAACCCTCACAGAATCCTTGATGGGA<br>GGGACTGCACACTAATAGATGCCCTACTGGGGATCCTCATTGTGATGTCCTTCAAGATGAAACATGGGACCTCTTTGTAGAGCGGAGCAATGCGTTCAGTAATTGTTACCCTTACGATGTACCAGACTATGCATCCCTTCGATCCCTAGTTGCATCATCAGGCACATTGGAATTCATCACTGAAGGTTTCACTTGGACAGGAGTTACTCAGAATGGAGGGAGTAATGCTTGC<br>AAAAGAGGACCTGCCAGCGGTTTCTTTAGTAGATTGAACTGGTTGACTAAGTCAGGAAACGCATACCCAGTGTTGAACGTGACCATGCCAAACAATGACAATTTTGACAAACTATACATCTGGGGAGTTCATCACCCGAGCACGAATCAAGAACAAACCAACTTGTATGTTCAAGCCTCAGGAAGAGTCACAGTCTCCACCAGAAGGAGCCAACAGACTATAATTCCGAACA<br>TTGGATCTAGACCCTGGGTAAGGGGCCAATCTGGCAGGATAAGCATCTACTGGACAATAGTCAAACCTGGGGACGTACTGGTAATCAATAGCAATGGGAACCTAATCGCTCCTCGGGGCTACTTCAAAATGCGCACTGGGAAAAGCTCAATAATGAGATCAGATGCACCTATTGACACCTGCATTTCTGAATGCATCACTCCAATGGAAGCATCCCCAATGACAAGCCCTT<br>CCAAAATGTAAACAAAATCACATATGGAGCATGTCCCAAATATGTGAAGCAGAACACTCTAAAGTTGGCAACAGGAATGCGGAATGTGCCTGAGAAACAAACCAGAGGACTGTTCGGTGCAATAGCAGGGTTTATAGAAAACGGATGGGAGGGGATGATAGATGGTTGGTATGGCTTCAGACATCAAAATTCCGAAGGCACAGGACAAGCAGCAGACCTCAAAAGCACT<br>CAGGCAGCCATTGACCAGATCAATGGGAAGCTGAACAGAGTAATTGAGAAGACGAATGAGAAGTTCCATCAAATCGAAAAGGAATTCTCCGAAGTAGAAGGGAGGATTCAGGACCTTGAGAAATACGTTGAAGACACAAAGATAGATCTCTGGTCTTATAATGCGGAGCTTCTTGTTGCCCTAGAGAATCAGCATACGATTGATTTGACGGACTCAGAAATGAACAAATT<br>GTTTGAGAAGACCAGGAGGCAACTGAGGGAAAATGCTGAGGACATGGGCAATGGTTGTTTCAAGATCTACCACAAATGTGACAATGCTTGTATAGAGTCAATTAGAAACGGGACTTATGACCATGACATATACAGAGATGAGGCATTGAACAATCGGTTCCAGATCAAAGGTGTTGAGCTAAAATCTGGATACAAAGACTGGATCCTGTGGATTTCCTTTGCCATATCATG<br>CTTTTTGCTTTGTGTTGTTTTGCTGGGGTTCATTATGTGGGCCTGCCAGAGAGGCAACATTAGGTGCAATATTTGC
EPI_ISL_14392759	>A/Anas_platyrhynchos/Belgium/2987_0001/2021_H3N8_2021-09-10<br>ATGAAGACCGTCATTGTTCTAAGCTACATTTTCTGTCTGGCTTTTAGTCAGGACCTCTCAGGGAATGACAACAGCACAGCAACACTGTGCCTGGGACACCATGCAGTGCCGAATGGAACAATAGTGAAGACAATTACAGATGATCAGATTGAGGTGACTAATGCTACCGAGCTAGTCCAAAGCTCTTCAACAGGGAAAATATGCAACAACCCTCACAGAATCCTTGATGGGA<br>GGGACTGCACACTAATAGACGCTCTACTGGGGATCCTCATTGTGATGTCTTTCAAGATGAAACATGGGACCTCTTTGTAGAGCGGAGCAATGCGTTCAGTAATTGTTACCCTTACGATGTACCAGACTATGCATCCCTTCGATCCCTAGTTGCATCATCAGGCACATTGGAATTCATCACTGAAGGTTTCACTTGGACAGGAGTTACTCAGAATGGAGGGAGCAATGCTTGC<br>AAAAGAGGGCCTGCCAGCGGTTTCTTTAGTAGATTGAACTGGTTGACTAAGTCAGGAAACGCATACCCAGTGTTGAACGTGACCATGCCAAACAATGACAATTTTGACAAACTATACATCTGGGGAGTTCACCACCCGAGCACGAATCAAGAACAAACCAACTTGTATGTTCAAGCCTCAGGAAGAGTCACAGTCTCCACCAGGAGGAGCCAACAGACTATAATTCCGAACA<br>TTGGATCTAGACCCTGGGTAAGGGGCCAGTCTGGCAGGATAAGCATCTACTGGACAATAGTCAAACCTGGGGACGTACTGGTAATCAATAGCAATGGAAACCTAATCGCTCCTCGGGGCTACTTCAAAATGCGCACTGGGAAAAGCTCAATAATGAGATCAGATGCACCTATTGACACCTGCATTTCTGAATGCATCACTCCAATGGAAGCATCCCCAATGACAAGCCCTT<br>CCAAAATGTAAACAAAATCACATACGGAGCATGCCCCAAATATGTGAAGCAGAACACTCTAAAGTTGGCAACAGGAATGCGGAATGTGCCTGAGAAACAAACCAGAGGACTGTTCGGTGCAATAGCAGGGTTTATAGAAAACGGATGGGAGGGGATGATAGATGGTTGGTATGGCTTCAGACATCAAAATTCCGAAGGCACAGGACAAGCAGCAGACCTTAAAAGCACT<br>CAGGCAGCCATTGACCAGATCAATGGGAAGCTGAACAGAGTAATTGAGAAGACGAATGAGAAGTTCCATCAAATCGAAAAGGAATTCTCCGAAGTAGAAGGAAGGATTCAGGACCTTGAGAAATACGTTGAAGACACAAAGATAGATCTCTGGTCTTATAATGCGGAGCTTCTTGTTGCCCTAGAGAATCAGCATACGATTGATTTGACGGACTCAGAAATGAACAAATT<br>GTTTGAGAAGACCAGGAGGCAACTGAGGGAAAATGCTGAGGACATGGGTAATGGTTGTTTCAAGATCTACCACAAATGTGACAATGCTTGTATAGAGTCAATTAGAAACGGGACTTATGACCATGACATATACAGAGATGAGGCATTGAACAATCGGTTCCAGATCAAAGGTGTTGAGCTAAAATCTGGATACAAAGACTGGATCCTGTGGATTTCCTTTGCCATATCATG<br>CTTTTTGCTTTGTGTTGTTTTGCTGGGGTTCATTATGTGGGCCTGCCAGAGAGGCAACATTAGGTGCAACATTTGC
EPI_ISL_339040	>A/teal/Dagestan/23d/2018_H3N8_2018-09-15<br>ATGAAGACCGTCATTGCTCTAAGCTACATTTTCTGTCTGGCTTTTAGTCAGGACCTTTCAGGGAATGACAACAGCACAGCAACACTGTGCCTGGGACACCATGCAGTGCCGAATGGAACAATAGTGAAGACAATTACAGATGATCAGATTGAGGTGACTAATGCTACCGAGCTAGTCCAAAGCTCTTCAACAGGGAAAATATGCAACAACCCTCACAGGATCCTTGATGGAA<br>GGGACTGCACACTAATAGATGCCCTACTGGGGATCCTCATTGTGATGTCTTTCAAGATGAAACATGGGACCTCTTTGTAGAGCGGAGCAATGCGTTCAGTAATTGTTACCCTTACGATGTACCAGACTATGCATCCCTTCGATCCCTAGTTGCATCATCAGGCACATTGGAATTCATTACTGAAGGTTTCACTTGGACAGGAGTTACTCAGAATGGAGGGAGCAATGCTTGC<br>AAAAGAGGACCTGCCAGCGGTTTCTTTAGTAGATTGAACTGGTTGACTAAGTCAGGAAACGCATACCCAGTGTTGAACGTGACTATGCCAAACACTGACAATTTTGACAAACTATACATCTGGGGAGTTCACCACCCGAGCACGAATCAAGAACAAACCAACTTGTATGTTCAGGCCTCAGGAAGAGTCACAGTCTCCACCAGGAGAAGCCAACAGACTATAATTCCGAACA<br>TTGGATCTAGACCCTGGGTAAGGGGCCAATCTGGCAGGATAAGCATCTACTGGACAATAGTCAAACCTGGGGACGTACTGGTAATAAATAGCAATGGAAACCTAATCGCTCCTCGGGGCTACTTCAAAATGCGCACTGGGAAAAGCTCAATAATGAGATCAGATGCACCTATTGACACCTGCATTTCTGAATGCATCACTCCAATGGAAGCATCCCCAATGACAAGCCCTT<br>CCAAAATGTAAACAAAATCACATACGGAGCATGTCCCAAATATGTGAAGCAGAACACCCTAAAGTTGGCAACAGGAATGCGGAATGTACCTGAGAAACAAACCAGAGGACTGTTCGGTGCAATAGCAGGGTTCATAGAAAACGGATGGGAAGGAATGATAGATGGTTGGTATGGCTTCAGACATCAAAATTCCGAAGGCACAGGACAAGCAGCAGACCTTAAAAGCACT<br>CAGGCAGCCATTGACCAGATCAATGGGAAGCTGAACAGAGTAATTGAAAAGACGAATGAGAAGTTCCATCAAATCGAAAAGGAATTCTCCGAAGTAGAAGGGAGGATTCAGGACCTTGAGAAATACGTTGAAGACACAAAGATAGATCTCTGGTCTTATAATGCGGAGCTTCTTGTTGCCCTAGAGAATCAGCATACGATCGATTTAACGGACTCAGAAATGAACAAATT<br>GTTTGAGAAGACCAGGAGGCAACTGAGGGAAAATGCTGAGGACATGGGCAATGGTTGTTTCAAGATATACCACAAATGTGACAATGCTTGTATAGAGTCAATTAGAAACGGGACTTATGACCATGACATATACAGAGATGAGGCATTGAACAACCGGTTCCAGATCAAAGGTGTTGAGCTAAAATCTGGATACAAAGACTGGATCCTGTGGATTTCCTTTGCCATATCATG<br>CTTTTTGCTTTGTGTTGTTTTGCTGGGGTTTATTATGTGGGCCTGCCAGAGAGGCAACATTAGGTGCAACATTTGC
EPI_ISL_355938	>A/green_sandpiper/Kurgan/1050/2018_H3N8_2018-08-26<br>ATGAAGACCGTCATTGCTCTAAGCTACATTTTCTGTCTGGCTTTTAGTCAGGACCTTTCAGGGAATGACAACAGCACAGCAACACTGTGCCTGGGACACCATGCAGTGCCGAATGGAACAATAGTGAAGACAATTACAGATGATCAGATTGAGGTGACTAATGCTACCGAGCTAGTCCAAAGCTCTTCAACAGGGAAAATATGCAACAACCCTCACAGAATCCTTGATGGAA<br>GGGACTGCACACTAATAGATGCCCTACTGGGGATCCTCATTGTGATGTCTTTCAAGATGAAACATGGGACCTCTTTGTAGAGCGGAGCAATGCGTTCAGTAATTGTTACCCTTACGACGTACCAGACTATGCATCCCTTCGATCCCTAGTTGCATCATCAGGCACATTGGAATTCATCACTGAAGGTTTCACTTGGACAGGAGTTACTCAGAATGGAGGGAGCAATGCTTGC<br>AAAAGAGGACCTGCCAGTGGTTTCTTTAGTAGATTGAACTGGTTGACTAAGTCAGGAAACGCATACCCAGTGTTGAACGTGACTATGCCAAACAATGACAATTTTGACAAACTATACATCTGGGGAGTTCACCACCCGAGCACGAATCAAGAACAAACCAACTTGTATGTTCAAGCCTCGGGAAGAGTCACAGTCTCCACCAGGAGAAGCCAACAGACCATAATTCCGAACA<br>TTGGATCTAGACCCTGGGTAAGAGGCCAATCTGGCAGGATAAGCATCTACTGGACAATAGTCAAACCTGGGGACGTGCTGGTAATCAATAGCAATGGAAACCTAATCGCCCCTCGAGGCTACTTCAAAATGCGCACTGGGAAAAGCTCAATAATGAGATCAGATGCACCTATTGACACCTGCATTTCTGAATGCATCACTCCAATGGAAGCATCCCCAATGACAAGCCCTT<br>CCAAAATGTAAACAAAATCACATACGGAGCATGTCCCAAATATGTGAAGCAGAACACCCTAAAGTTGGCAACAGGAATGCGGAATGTACCTGAGAAACAAACCAGAGGACTGTTCGGTGCAATAGCAGGGTTTATAGAAAACGGATGGGAAGGAATGATAGATGGTTGGTATGGCTTCAGACATCAAAATTCCGAAGGCACAGGACAAGCAGCAGACCTTAAAAGCACC<br>CAGGCAGCCATTGACCAGATCAATGGGAAGCTGAACAGAGTAATTGAAAAGACGAATGAGAAGTTCCATCAAATCGAAAAGGAATTCTCCGAAGTAGAAGGGAGGATTCAGGACCTTGAGAAATATGTTGAAGACACAAAGATAGATCTCTGGTCTTATAATGCGGAGCTTCTTGTTGCCCTAGAGAACCAGCATACGATTGATTTGACGGACTCAGAAATGAACAAATT<br>GTTTGAGAAGACCAGGAGGCAACTGAGGGAAAATGCTGAGGACATGGGCAATGGTTGTTTCAAGATATACCACAAATGTGACAATGCTTGTATAGAGTCAATTAGAAACGGGACTTATGACCATGACATATACAGAGATGAGGCATTGAACAACCGGTTCCAGATCAAAGGTGTTGAGCTAAAATCTGGATACAAAGACTGGATCCTGTGGATTTCCTTTGCCATATCATG<br>CTTTTTGCTTTGTGTTGTTTTGCTGGGGTTCATTATGTGGGCCTGCCAGAGAGGCAACATTAGGTGCAACATTTGC
EPI_ISL_337401	>A/mallard/Chany_Lake/48/2018_H3N8_2018-09-30<br>ATGAAAACCGTCATTGCTCTAAGCTACATTTTCTGTCTGGCTTTTAGTCAGGACCTCTCAGGGAATGACAACAGCACAGCAACACTGTGCCTGGGACACCATGCAGTGCCGAATGGAACAATAGTGAAGACAATTACAGATGATCAGATTGAGGTGACTAATGCTACCGAGCTAGTCCAAAGCTCTTCAACAGGGAAAATATGCAACAACCCTCACAGAATCCTTGATGGAA<br>GGGACTGCACACTAATAGATGCCCTACTGGGGATCCTCATTGTGATGTCTTTCAAGATGAAACATGGGACCTCTTTGTAGAGCGGAGCAATGCGTTCAGTAATTGTTACCCTTACGATGTACCAGACTATGCATCCCTTCGATCCCTAATTGCATCATCAGGCACATTGGAATTCATCACTGAAGGTTTCACTTGGACAGGAGTTACTCAGAATGGAGGGAGCAATGCTTGC<br>AAAAGAGGACCTGCCAGCGGTTTCTTTAGTAGATTGAACTGGTTGACTAAGTCAGGAAATGCATACCCAGTGTTGAACGTGACTATGCCAAACAATGACAATTTTGACAAACTATACATCTGGGGAATTCACCACCCGAGCACGAATCAAGAACAAACCAACTTGTATGTTCAAGCCTCAGGAAGAGTCACAGTCTCCACCAGGAGAAGCCAACAGACTATAATTCCGAACA<br>TTGGATCTAGACCCTGGGTAAGGGGCCAATCTGGCAGGATAAGCATCTACTGGACAATAGTCAAACCTGGGGACGTACTGGTAATCAATAGCAATGGAAACCTAATCGCTCCTCGGGGCTACTTCAAAATGCGCGCTGGGAAAAGCTCAATAATGAGATCAGATGCACCTATTGACACCTGCATTTCTGAATGCATCACTCCAATGGAAGCATCCCCAATGACAAGCCCTT<br>CCAAAATGTAAACAAAATCACATACGGAGCATGTCCCAAATATGTGAAGCAGAACACTCTAAAGTTGGCAACAGGAATGCGGAATGTACCTGAGAAACAAACCAGAGGACTGTTCGGTGCAATAGCAGGGTTTATAGAAAACGGATGGGAAGGAATGATAGATGGTTGGTATGGCTTCAGACATCAAAATTCCGAAGGCACAGGACAAGCAGCAGACCTTAAAAGCACT<br>CAGGCAGCCATTGACCAGATCAATGGGAAGCTGAACAGAGTAATTGAAAAGACGAATGAGAAGTTCCATCAAATCGAAAAGGAATTCTCCGAAGTAGAAGGGAGGATTCAGGACCTTGAGAAATACGTTGAAGACACAAAGATAGACCTCTGGTCTTATAATGCGGAGCTTCTTGTTGCCCTAGAGAATCAGCATACGATTGATTTGACGGACTCAGAAATGAACAAATT<br>GTTTGAGAAGACCAGGAGGCAACTGAGGGAAAATGCTGAGGACATGGGCAATGGTTGTTTCAAGATATACCACAAATGTGACAATGCTTGTATAGAGTCAATTAGAAACGGGACTTATGACCATGACATATACAGAGATGAGGCATTGAACAACCGGTTCCAGATCAAAGGTGTTGAGCTAAAATCTGGATACAAAGACTGGATCCTGTGGATTTCCTTTGCCATATCATG<br>CTTTTTGCTTTGTGTTGTTTTGCTGGGGTTCATTATGTGGGCCTGCCAGAGAGGCAACATTAGGTGCAACATTTGC
EPI_ISL_337397	>A/teal/Buryatia/63/2018_H3N8_2018-08-26<br>ATGAAAACCGTCATTGCTCTAAGCTACATTTTCTGTCTGGCTTTTAGTCAGGACCTCTCAGGGAATGACAACAGCACAGCAACACTGTGCCTGGGACACCATGCAGTGCCGAATGGAACAATAGTGAAGACAATTACAGATGATCAGATTGAGGTGACTAATGCTACCGAGCTAGTCCAAAGCTCTTCAACAGGGAAAATATGCAACAACCCTCACAGAATCCTTGATGGAA<br>GGGACTGCACACTAATAGATGCCCTACTGGGGATCCTCATTGTGATGTCTTTCAAGATGAAACATGGGACCTCTTTGTAGAGCGGAGCAATGCGTTCAGTAATTGTTACCCTTACGATGTACCAGACTATGCATCCCTTCGATCCCTAATTGCATCATCAGGCACATTGGAATTCATCACTGAAGGTTTCACTTGGACAGGAGTTACTCAGAATGGAGGGAGCAATGCTTGC<br>AAAAGAGGACCTGCCAGCGGTTTCTTTAGTAGATTGAACTGGTTGACTAAGTCAGGAAATGCATACCCAGTGTTGAACGTGACTATGCCAAACAATGACAATTTTGACAAACTATACATCTGGGGAATTCACCACCCGAGCACGAATCAAGAACAAACCAACTTGTATGTTCAAGCCTCAGGAAGAGTCACAGTCTCCACCAGGAGAAGCCAACAGACTATAATTCCGAACA<br>TTGGATCTAGACCCTGGGTAAGGGGCCAATCTGGCAGGATAAGCATCTACTGGACAATAGTCAAACCTGGGGACGTACTGGTAATCAATAGCAATGGAAACCTAATCGCTCCTCGGGGCTACTTCAAAATGCGCGCTGGGAAAAGCTCAATAATGAGATCAGATGCACCTATTGACACCTGCATTTCTGAATGCATCACTCCAATGGAAGCATCCCCAATGACAAGCCCTT<br>CCAAAATGTAAACAAAATCACATACGGAGCATGTCCCAAATATGTGAAGCAGAACACTCTAAAGTTGGCAACAGGAATGCGGAATGTACCTGAGAAACAAACCAGAGGACTGTTCGGTGCAATAGCAGGGTTTATAGAAAACGGATGGGAAGGAATGATAGATGGTTGGTATGGCTTCAGACATCAAAATTCCGAAGGCACAGGACAAGCAGCAGACCTTAAAAGCACT<br>CAGGCAGCCATTGACCAGATCAATGGGAAGCTGAACAGAGTAATTGAAAAGACGAATGAGAAGTTCCATCAAATCGAAAAGGAATTCTCCGAAGTAGAAGGGAGGATTCAGGACCTTGAGAAATACGTTGAAGACACAAAGATAGACCTCTGGTCTTATAATGCGGAGCTTCTTGTTGCCCTAGAGAATCAGCATACGATTGATTTGACGGACTCAGAAATGAACAAATT<br>GTTTGAGAAGACCAGGAGGCAACTGAGGGAAAATGCTGAGGACATGGGCAATGGTTGTTTCAAGATATACCACAAATGTGACAATGCTTGTATAGAGTCAATTAGAAACGGGACTTATGACCATGACATATACAGAGATGAGGCATTGAACAACCGGTTCCAGATCAAAGGTGTTGAGCTAAAATCTGGATACAAAGACTGGATCCTGTGGATTTCCTTTGCCATATCATG<br>CTTTTTGCTTTGTGTTGTTTTGCTGGGGTTCATTATGTGGGCCTGCCAGAGAGGCAACATTAGGTGCAACATTTGC
EPI_ISL_337149	>A/coot/Novosibirsk_region/563/2018_H3N8_2018-10-03<br>ATGAAGACCGTCATTGCTCTAAGCTACATTTTCTGTCTGGCTTTTAGTCAGGACCTTTCAGGGAATGACAACAGCACAGCAACACTGTGCCTGGGACACCATGCAGTGCCGAATGGAACAATAGTGAAGACAATTACAGATGATCAGATTGAGGTGACTAATGCTACCGAGCTAGTCCAAAGCTCTTCAACAGGGAAAATATGCAACAACCCTCACAGAATCCTTGATGGAA<br>GGGACTGCACACTAATAGATGCCCTACTGGGGATCCTCATTGTGATGTCTTTCAAGATGAAACATGGGACCTCTTTGTAGAGCGGAGCAATGCGTTCAGTAATTGTTACCCTTACGATGTACCAGACTATGCATCCCTTCGATCCCTAGTTGCATCATCAGGCACATTGGAATTCATCACTGAAGGTTTCACTTGGACAGGAGTTACTCAGAATGGAGGGAGCAATGCTTGC<br>AAAAGAGGACCTGCCAGCGGTTTCTTTAGTAGATTGAACTGGTTGACTAAGTCAGGAAACGCATACCCAGTGTTGAACGTGACTATGCCAAACAATGACAATTTTGACAAACTATACATCTGGGGAGTTCACCACCCGAGCACGGATCAAGAACAAACCAACTTGTATGTTCAAGCCTCAGGAAGAGTCACAGTCTCCACCAGGAGAAGCCAACAGACTATAATTCCGAACA<br>TTGGATCTAGACCCTGGGTAAGGGGTCAATCTGGCAGGATAAGCATCTACTGGACAATAGTCAAACCTGGGGACGTACTGGTAATCAATAGCAATGGAAACCTAATCGCTCCTCGGGGCTACTTCAAAATGCGCACTGGGAAAAGCTCAATAATGAGATCAGATGCACCTATTGACACCTGCATTTCTGAATGCATCACTCCAATGGAAGCATCCCCAATGACAAGCCCTT<br>CCAAAATGTAAACAAAATCACATACGGAGCATGTCCCAAATATGTGAAGCAGAACACCCTAAAGTTGGCAACAGGAATGCGAAATGTACCTGAGAAACAAACCAGAGGACTATTCGGTGCAATAGCAGGGTTTATAGAAAACGGATGGGAAGGAATGATAGATGGTTGGTATGGCTTCAGACATCAAAATTCCGAAGGCACAGGACAAGCAGCAGACCTTAAAAGCACT<br>CAGGCAGCCATTGACCAGATCAATGGGAAGCTAAACAGAGTAATTGAAAAGACGAATGAGAAGTTCCATCAAATCGAAAAGGAATTCTCCGAAGTAGAAGGGAGGATTCAGGACCTTGAGAAATACGTTGAAGACACAAAGATAGATCTCTGGTCTTATAATGCGGAGCTTCTTGTTGCCCTAGAGAATCAGCATACGATTGATTTGACGGACTCAGAAATGAACAAATT<br>GTTTGAGAAGACCAGGAGGCAACTGAGGGAAAATGCTGAGGACATGGGCAATGGTTGTTTCAAGATATACCACAAATGTGATAATGCTTGTATAGAGTCAATTAGAAACGGGACTTATGACCATGACATATACAGAGATGAGGCATTGAACAACCGGTTTCAGATCAAAGGTGTTGAGCTAAAATCTGGATACAAAGACTGGATCCTGTGGATTTCCTTTGCCATATCATG<br>CTTTTTGCTTTGTGTTGTTTTGCTGGGGTTCATTATGTGGGCCTGCCAGAGAGGCAACATTAGGTGCAACATTTGC
EPI_ISL_4031115	>A/duck/Assam/DUOR1512100004/2015_H3N8_2015-12-02<br>ATGAAGACCGTCATTGCTCTAAGCTACATTTTCTGTCTGGCTTTTAGTCAGGACCTCTCAGGGAATGACAACAGCACAGCAACACTGTGCCTGGGACACCATGCAGTGCCGAATGGAACAATAGTGAAGACAATTACAGATGATCAGATTGAGGTGACTAATGCTACCGAGCTAGTCCAAAGCTCTTCAACAGGGAAAATATGCAACAACCCTCACAGAATCCTTGATGGAA<br>GGGACTGCACACTAATAGATGCCCTACTGGGGATCCTCACTGTGATGTCTTTCAAGATGAAACATGGGACCTCTTTGTAGAGCGGAGCAATGCGTTCAGTAATTGTTACCCTTACGATGTACCAGACTATGCATCCCTTCGATCCCTAGTTGCATCAGCAGGCACATTGGAATTCATCACTGAAGGTTTCACTTGGACAGGAGTTACTCAGAATGGAGGGAGCAATGCTTGC<br>AAAAGAGGACCTGCCAGTGGTTTCTTTAGTAGATTGAACTGGTTGACTAAGTCAGGAAACGCATACCCAGTGTTGAACGTGACTATGCCAAACAATGACAATTTTGACAAACTATACATCTGGGGAGTTCACCACCCGAGCACGAATCAAGAACAAACCAACTTGTATGTTCAAGCCTCAGGAAGAGTCACAGTCTCCACCAGGAGAAGCCAACAGACTATAATTCCGAACA<br>TTGGATCTAGACCCTGGGTAAGGGGCCAATCTGGCAGGATAAGCATCTACTGGACAATAGTCAAACCTGGGGACGTACTGGTAATCAATAGCAATGGAAACCTAATCGCTCCTCGGGGCTACTTCAAAATGCGCACTGGGAAAAGCTCAATAATGAGATCAGATGCACCTATTGACACCTGCATTTCTGAATGCATCACTCCAATGGAAGCATCCCCAATGACAAGCCCTT<br>CCAAAATGTAAACAAAATCACATACGGAGCATGTCCCAAATATGTGAAGCAGAACACTCTAAAGTTGGCAACAGGAATGCGGAATGTACCTGAGAAACAAACCAGAGGACTGTTCGGTGCAATAGCAGGGTTTATAGAAAACGGATGGGAAGGAATGATAGATGGTTGGTATGGCTTCAGACATCAAAATTCCGAAGGCACAGGACAAGCAGCAGACCTTAAAAGCACT<br>CAGGCAGCCATTGACCAGATCAATGGGAAGCTGAACAGAGTAATTGAAAAGACGAATGAGAAGTTCCATCAAATCGAAAAGGAATTCTCCGAAGTAGAAGGGAGGATTCAGGACCTTGAGAAATACGTTGAAGACACAAAGATAGATCTCTGGTCTTATAATGCAGAGCTTCTTGTTGCCCTAGAGAATCAGCATACGATTGATTTGACGGACTCAGAAATGAACAAATT<br>GTTTGAGAAGACCAGGAGGCAACTGAGGGAAAATGCTGAGGACATGGGCAATGGTTGTTTCAAGATATACCACAAATGTGACAATGCTTGTATAGAGTCAATTAGAAACGGGACTTATGACCATGACATATACAGAGATGAGGCATTGAACAACCGGTTCCAGATCAAAGGTGTTGAGCTAAAATCTGGATACAAAGACTGGATCCTGTGGATTTCCTTTGCCATATCATG<br>CTTTTTGCTTTGTGTTGTTTTGCTGGGGTTCATTATGTGGGCCTGCCAGAGAGGCAACATTAGGTGCAACATTTGC
EPI_ISL_17978924	>A/mallard/Poland/P074w5/2018_H3N8_2018-09-05<br>ATGAAGACCGTCATTGCTCTAAGCTACATTTTCTGTCTGGCTTTTAGTCAGGACCTTTCAGGGAATGACAACAGCACAGCAACACTGTGCCTGGGACACCATGCAGTGCCGAACGGAACAATAGTGAAGACAATTACAGATGATCAGATTGAGGTGACTAATGCTACCGAGCTAGTCCAAAGCTCTTCAACAGGGAAAATATGCAACAACCCTCACAGAATCCTTGATGGAA<br>GGGACTGCACACTAATAGATGCCCTACTGGGGATCCTCATTGTGATGTCTTTCAAGATGAAACATGGGACCTCTTTGTAGAGCGGAGCAATGCGTTCAGTAATTGTTACCCTTACGATGTACCAGACTATGCATCCCTTCGATCCCTAGTTGCATCATCAGGCACATTGGAATTCATCACTGAAGGTTTCACTTGGACAGGAGTTACTCAAAATGGAGGGAGCAATGCTTGC<br>AAAAGAGGACCTGCCAGCGGTTTCTTTAGTAGATTGAACTGGTTGACGAAGTCAGGAAACGCATACCCAGTGTTGAACGTGACTATGCCAAACAATGACAATTTTGACAAACTATACATCTGGGGAGTTCACCACCCGAGCACGAATCAAGAACAAACCAACTTGTATGTTCAAGCCTCAGGAAGAGTCACAGTCTCCACCAGGAGAAGCCAACAGACTATAATTCCGAACA<br>TTGGATCTAGACCCTGGGTGAGGGGCCAATCTGGCAGGATAAGCATCTACTGGACAATAGTCAAACCTGGGGACGTACTGGTAATCAATAGCAATGGAAACCTAATTGCTCCTCGGGGCTACTTCAAAATGCGCACTGGGAAAAGCTCAATAATGAGATCAGATGCACCTATTGACACCTGCATTTCTGAATGCATCACTCCAATGGAAGCATCCCCAATGACAAGCCCTT<br>CCAAAATGTAAACAAAATCACATACGGAGCATGTCCCAAATATGTGAAGCAGAACACCCTAAAGTTGGCAACAGGGATGCGGAATGTACCTGAGAAACAAACCAGAGGACTGTTCGGTGCAATAGCAGGGTTTATAGAAAACGGATGGGAAGGAATGATAGATGGTTGGTATGGCTTCAGACATCAAAATTCCGAAGGCACAGGACAAGCAGCAGACCTTAAAAGCACT<br>CAGGCAGCCATTGACCAGATCAATGGGAAGCTGAACAGAGTAATTGAAAAGACGAATGAGAAGTTCCATCAAATCGAAAAGGAATTCTCCGAAGTAGAAGGGAGGATTCAGGACCTTGAGAAATATGTTGAAGACACAAAGATAGATCTCTGGTCTTATAATGCGGAGCTTCTTGTTGCCCTAGAGAATCAGCATACGATTGATTTGACGGACTCAGAAATGAACAAATT<br>GTTTGAGAAGACCAGGARGCAACTGAGGGAAAATGCTGAGGACATGGGCAATGGTTGTTTCAAGATATACCACAAATGTGACAATGCCTGTATAGAGTCAATTAGAAACGGGACTTATGACCATGACATATACAGAGATGAGGCATTGAACAACCGGTTCCAAATCAAAGGTGTTGAGCTAAAATCTGGATACAAAGACTGGATCCTGTGGATTTCCTTTGCCATATCATG<br>CTTTTTGCTTTGTGTTGTTTTGCTGGGGTTCATTATGTGGGCCTGCCAGAGAGGCAACATTAGGTGCAACATTTGC
EPI_ISL_17978926	>A/mallard/Poland/P060w2/2020_H3N8_2020-08-25<br>ATGAAGACCATTATCGCTTTAAGCTATGTTCTCTGTCTGGCCTTCGGTCAGGACCTTTCAGGGAGCGACAATAGTACAGCAACACTGTGCCTGGGACACCATGCAGTGCCGAATGGGACAATGGTGAAGACCATTACAGATGATCAGATTGAAGTGACTAATGCTACCGAGCTAGTCCAAAGCTCTTCGACAGGGAAGATATGCAACAATCCTCACAGGATTCTTGACGGG<br>AGGGACTGCACACTAATAGATGCCCTACTGGGAGATCCTCATTGCGATGTCTTTCAAGATGAGACATGGGATCTCTTTGTGGAGCGGAGCAATGCTTTCAGCAATTGCTACCCTTACGATGTACCAGACTATGCATCCCTTCGATCCCTAGTTGCATCATCGGGCACATTGGAGTTCATCACTGAAGGTTTCACTTGGACAGGAGTAACTCAGAATGGGGGGAGCAATGCCT<br>GCAAGAGGGGACCTGCTAGTGGTTTTTTCAGTAGACTGAACTGGTTGACTAAGTCAGGAAGCACATACCCAGTGTTGAACGTGACTATGCCAAACAATGACAATTTTGACAAACTATACATCTGGGGAGTTCACCACCCGAGCACAAACCAAGAACAAACCAACCTGTATGTTCAAGCCTCAGGAAGAGTCACGGTCTCTACCAGGAGAAGCCAGCAGACTATAATCCCTAA<br>CATTGGATCTAGACCCTGGGTAAGGGGCCAAYCTGGTAGAATAAGCATCTACTGGACAATAGTCAAACCCGGGGACGTACTGGTGATCAATAGTRATGGAAACCTAATCGCTCCTCGGGGCTACTTCAAAATGCGCACTGGGAAAAGCTCGATAATGAGGTCAGATGCACCTATTGACACCTGTATTTCTGAATGCATCACTCCGAATGGAAGCATCCCAAATGACAAACCC<br>TTCCAAAATGTAAACAAGATCACATACGGAGCATGTCCCAAATATGTGAAGCAGAACACCCTGAAGCTGGCAACAGGAATGCGGAATGTGCCAGAGAAGCAAACCAGAGGCCTATTCGGTGCAATAGCAGGTTTTATAGAAAATGGATGGGAAGGAATGGTAGATGGTTGGTATGGCTTCAGGCATCAAAATTCCGAAGGCACAGGACAAGCAGCAGACCTTAAAAGCA<br>CTCAGGCAGCCATTGACCAGATCAATGGGAAATTGAACAGAGTGATTGAAAGGACTAATGAAAAGTTCCATCAAATCGAAAAGGAATTCTCTGAGGTAGAAGGGAGAATTCAGGACCTTGAGAAATACGTTGAAGACACAAAAATAGACCTCTGGTCTTATAATGCGGAACTCCTTGTTGCCCTAGAGAATCAGCATACAATCGATTTGACTGACTCAGAAATGAACAAAT<br>TGTTTGAAAAGACCAGGAGACAACTAAGGGAAAATGCTGAAGACATGGGCAATGGTTGTTTCAAAATATATCACAAGTGTGACAATGCTTGCATAGAGTCAATTAGGAACGGGACTTATGACCATGACATATACAGAGATGAGGCATTGAACAACCGGTTTCAGATCAAGGGTGTTGAGCTGAAATCTGGGTACAAAGACTGGATCCTATGGATTTCCTTTGCCATATCAT<br>GCTTTTTGCTTTGTGTTGTTTTGCTGGGGTTCATTATGTGGGCCTGCCAGAGAGGCAACATTAGATGCAACATTTGC
EPI_ISL_17982396	>A/common_teal/Poland/P079w24_mixed_H3N8/2018_2018-09-14<br>ATGAAGACCGTTATTGCTTTAAGCTACATTTTCTGTCTGGCTTTTAGTCAGGACCTTTCGGGGAATGACAACAGTACAGCAACACTGTGCCTGGGACACCATGCAGTGCCGAACGGAACAATAGTAAAGACAATTACAGATGATCAGATTGAGGTGACTAATGCTACCGAGCTCGTTCAAAGCTCTTCAACAGGGAAAATATGCAACAATCCCCACAGAATCCTTGATGGAA<br>GGGACTGCACACTAATAGATGCCCTACTGGGGATCCTCATTGTGATGTCTTTCAAGATGAAACATGGGATCTCTTTGTAGAGCGGAGCAATGCTTTCAGCAATTGTTACCCTTACGATGTACCGGACTATGCATCCCTTCGATCCCTAGTTGCATCATCAGGCACATTGGAATTCATCACTGAAGGCTTCACTTGGACAGGAGTTACTCAGAATGGAGGGAGCAATGCTTGC<br>AAAAGGGGACCTGCTAGCGGTTTCTTTAGTAGACTGAACTGGTTGACTAAGTCAGGAAACGCATACCCAGTGTTGAACGTGACTATGCCAAACAATGACAATTTTGACAAATTATACATCTGGGGAGTTCACCACCCGAGCACAAATCAAGAACAAACCAACCTGTATGTTCAAGCCTCAGGAAGAGTTACAGTCTCCACCAGGAGAAGCCAACAGACTATAATTCCGAACA<br>TTGGATCCAGACCCTGGGTAAGGGGGCAATCTGGCAGAATAAGCATCTACTGGACAATAGTCAAACCCGGGGACGTACTAGTAATCAATAGTAATGGAAACCTAATCGCTCCTCGGGGCTACTTCAAAATGCGCACTGGGAAAAGCTCAATAATGAGATCAGATGCACCTATTGACACCTGCATTTCTGAATGCATCACTCCAATGGAAGCATCCCCAATGACAAGCCCTT<br>CCAAAATGTAAACAAGATCACATACGGAGCATGTCCCAAATATGTGAAGCAGAACACCCTAAAGTTGGCAACAGGAATGCGGAATATACCTGAGAAACAGACCAGAGGCCTGTTCGGTGCAATAGCAGGGTTTATAGAAAATGGATGGGAAGGGATGATAGATGGTTGGTATGGCTTCAGACATCAAAATTCCGAGGGCACAGGACAAGCAGCAGACCTTAAAAGCACT<br>CAGGCAGCCATTGACCAGATCAATGGGAAGCTGAACAGAGTAATTGAAAAGACGAATGAGAAGTTCCATCAAATCGAAAAGGAATTTTCCGAAGTAGAAGGGAGGATTCAGGACCTTGAGAAATACGTTGAAGACACAAAGATAGATCTCTGGTCTTATAATGCGGAGCTTCTCGTTGCCCTAGAGAACCAGCATACAATTGATTTGACGGACTCAGAAATGAACAAATT<br>GTTTGAAAAGACCAGGAGGCAACTGAGGGAAAATGCTGAGGACATGGGCAATGGTTGTTTCAAGATATACCACAAATGTGACAATGCTTGTATAGAGTCAATTAGAAACGGGACTTATGACCATGACATATACAGAGATGAGGCATTGAACAACCGGTTTCAGATCAAAGGTGTTGAGCTAAAATCTGGATACAAAGACTGGATCCTGTGGATTTCCTTTGCCATATCATG<br>CTTTTTGCTTTGTGTTGTTTTGCTGGGGTTCATTATGTGGGCCTGCCAGAGAGGCAACATTAGGTGCAGCATTTGC
EPI_ISL_17982400	>A/common_teal/Poland/P079w25_mixed_H3N8/2018_2018-09-14<br>ATGAAGACCATCATCGCTTTAAGCTACATTTTCTGTCTGGCCTTCGGTCAGGACCTTTCAGGGAGCGACAATAGTACAGCAACACTGTGCCTGGGACACCATGCAGTACCGAATGGGACAATAGTGAAGACCATTACAGATGATCAGATTGAGGTGACTAATGCTACCGAGCTAGTTCAAAGCTCTTCAACAGGGAAGATATGCAACAATCCTCACAGGATTCTTGATGGAA<br>GGGATTGCACACTAATAGATGCCTTACTGGGGATCCTCATTGCGATGTCTTTCAAGATGAGACGTGGGATCTTTTTGTGGAGCGAAGCAATGCTTTCAGCAATTGCTACCCTTACGATGTACCAGACTACGCATCCCTTCGATCCCTAGTTGCATCATCAGGCACATTGGAGTTCATCACTGAAGGTTTCACTTGGACAGGAGTAACTCAGAATGGAGGGAGCGGTGCTTGC<br>AAAAGGGGACCTGCTAATGGTTTCTTCAGTAGACTGAATTGGTTGACTAAGTCAGGGAGCGCATACCCAGTGTTGAACGTGACTATGCCAAACAATGACGGTTTTGACAAACTGTACATATGGGGAGTTCACCACCCGAGTACAAACCAAGAACAAACCAGCCTGTATGTTCAAGCCTCAGGAAGAGTCACAGTCTCTACCAGGAGAAGCCAACAGACTATAATCCCTAAC<br>ATTGGATCTAGACCCTGGGTAAGGGGCCAGTCTGGCAGGATAAGCATCTACTGGACAATAGTCAAACCTGGGGACATACTGGTGATCAATAGTAATGGAAACCTAATCGCTCCTCGGGGCTATTTCAAGATGCGAACTGGGAAAAGCTCAATAATGAGGTCAGATGCACCTATTGACACCTGCATTTCTGAATGCATCACCCCAATGGGAGCATCCCCAACGACAAGCCC<br>TTTCAAAATGTGAACAAGATCACATATGGAGCATGTCCCAAATATGTGAAGCAGAACACCCTGAAGTTGGCAACAGGAATGCGAAATGTACCAGAAAAGCAAACCAGAGGCCTATTCGGTGCAATAGCAGGCTTTATAGAAAATGGATGGGAAGGAATGATAGATGGCTGGTACGGCTTCAGGCATCAAAATTCCGAAGGCACCGGACAAGCAGCAGACCTTAAAAGCA<br>CTCAGGCAGCCATTGATCAGATCAATGGGAAATTGAACAGAGTGATTGAAAAGACGAATGAAAAGTTCCATCAAATTGAAAAGGAATTCTCTGAGGTAGAAGGGAGGATTCAGGACCTTGAGAAATACGTTGAAGACACAAAAATAGATCTTTGGTCTTATAATGCGGAACTCCTTGTTGCCCTAGAGAATCAGCATACAATTGATTTGACTGATTCAGAAATGAACAAAT<br>TGTTTGAAAAGACCAGAAGGCAACTGAGGGAAAATGCTGAAGACATGGGCAATGGTTGTTTCAAAATATACCACAAGTGTGACAATGCCTGCATAGAATCAATTAGGAACGGGACTTATGACCATGACATATACAGAGATGAAGCATTGAATAACCGGTTCCAGATCAAGGGTGTCGAACTAAAATCTGGGTATAAAGATTGGATCCTGTGGATTTCCTTTGCCATATCAT<br>GCTTTTTGCTTTGTGTTGTTTTGCTGGGGTTCATTATGTGGGCATGCCAGAGAGGCAACATCAGGTGCAACATTTGC

HA (H5)

Isolate ID	Sequence
EPI_ISL_2234811	>A/anser_anser/Spain/102-8_21VIR1230-4/2021_H5N8_2021-01-07<br>ATGGAGAACATAGTACTTCTTCTTGCAATAGTTAGCCTTGTTAAAAGTGATCAGATTTGCATTGGTTACCATGCAAACAATTCGACAGAGCAAGTTGACACGATAATGGAAAAGAACGTCACTGTTACACATGCCCAAGACATACTGGAAAAAACACACAACGGGAAGCTCTGTGATCTAAATGGGGTGAAGCCTCTGATTTTAAAGGATTGTAGTGTAGCTGGATGGCTCC<br>TCGGAAACCCAATGTGCGACGAATTCATCAGAGTGCCGGAATGGTCCTACATAGTGGAGAGGGCTAATCCGGCTAATGACCTCTGTTACCCAGGGAGCCTCAATGACTATGAAGAACTGAAACACCTGTTGAGCAGAATAAATCATTTTGAGAAGATTATGATCATCCCCAAGAGTTCCTGGCCAAATCATGAAACATCACTAGGGGTGAGCGCAGCTTGTCCATACCAGG<br>GAGCGCCCTCCTTTTTCAGAAATGTGGTGTGGCTTATCAAAAAGAACGATGCATACCCAACAATAAAGATAAGCTACAATAATACCAATCGGGAAGATCTCTTGATACTGTGGGGGATTCATCATTCCAACAATGCAGAAGAGCAGACAAATCTCTATAAAAACCCAACCACCTACATTTCAGTTGGAACATCAACTTTAAACCAGAGGTTGGTACCAAAAATAGCTACTAG<br>ATCCCAAGTAAACGGGCAACGTGGGAGAATGGACTTCTTCTGGACAATTTTAAAACCGGATGATGCAATCCATTTCGAGAGTAATGGAAATTTCATTGCTCCAGAATATGCATACAAAATTGTCAAGAAAGGGGACTCAACAATTATGAAAAGTGGAGTGGAATATGGCCACTGCAACACCAAATGTCAAACCCCAGTAGGAGCGATAAATTCTAGTATGCCATTCCACAA<br>CATACATCCTCTCACCATTGGGGAATGCCCCAAATACGTGAAGTCAAACAAGTTGGTCCTTGCGACTGGGCTCAGAAATAGCCCTCTAAGAGAAAAGAGAAGAAAAAGAGGCCTGTTTGGGGCGATAGCAGGGTTTATAGAGGGAGGATGGCAGGGAATGGTTGATGGTTGGTATGGGTACCACCATAGCAATGAGCAGGGGAGTGGGTACGCTGCAGACAAAGAATC<br>CACCCAAAAGGCAATAGATGGAGTTACCAATAAGGTCAACTCAATAATTGACAAAATGAACACTCAATTTGAGGCAGTTGGAAGGGAGTTTAATAACTTAGAAAGGAGGATAGAGAATTTGAACAAGAAAATGGAAGACGGATTCCTAGATGTTTGGACCTATAATGCTGAACTTCTAGTTCTCATGGAAAACGAGAGGACTCTAGATTTCCATGATTCAAATGTCAAGAA<br>CCTTTACGACAAGGTCAGACTACAGCTTAGGGATAATGCAAAGGAGCTGGGTAACGGCTGTTTCGAATTCTATCACAAATGCGATAATGAATGTATGGAAAGTGTGAGAAATGGGACGTATGACTACCCTCAGTATTCAGAAGAAGCAAGATTAAAAAGAGAAGAAATAAGCGGAGTGAAATTAGAATCAATAGGAACTTACCAGATACTGTCAATTTATTCAACAGCGG<br>CGAGTTCCCTAGCACTGGCAATCATGATGGCTGGTCTATCTTTATGGATGTGCTCCAATGGGTCGTTACAGTGCAGAATTTGCATTTAA
EPI_ISL_2172522	>A/common_buzzard/Netherlands/21021497-001/2021_H5N8_2021-01-10<br>ATGGAGAACATAGTACTTCTTCTTGCAATAGTTAGCCTTGTTAAAAGTGATCAGATTTGCATTGGTTACCATGCAAACAATTCGACAGAGCAAGTTGACACGATAATGGAAAAGAACGTCACTGTTACACATGCCCAAGACATACTGGAAAAAACACACAACGGGAAGCTCTGTGATCTAAATGGGGTGAAGCCTCTGATTTTAAAGGATTGTAGTGTAGCTGGATGGCTCC<br>TCGGAAACCCAATGTGCGACGAATTCATCAGAGTGCCGGAATGGTCCTACATAGTGGAGAGGGCTAATCCAGCTAATGACCTCTGTTACCCAGGGAGCCTCAATGACTATGAAGAACTGAAACACCTGTTGAGCAGAATAAATCATTTTGAGAAGATTCTGATCATCCCCAAGAGTTCCTGGCCAAATCATGAAACATCACTAGGGGTGAGCGCAGCTTGTCCATACCAGG<br>GAGCGCCCTCCTTTTTCAGAAATGTGGTGTGGCTTATCAAAAAGAACGATGCATACCCAACAATAAAGATAAGCTACAATAATACCAATCGGGAAGATCTCTTGATACTGTGGGGGATTCATCATTCCAACAATGCAGAAGAGCAGACAAATCTCTATAAAAACCCAACCACCTACATTTCAGTTGGAACATCAACTTTAAACCAGAGGTTGGTACCAAAAATAGCTACTAG<br>ATCCCAAGTAAACGGGCAACGTGGGAGAATGGACTTCTTCTGGACAATTTTAAAACCGGATGATGCAATCCATTTCGAGAGTAATGGAAATTTCATTGCTCCAGAATATGCATACAAAATTGTCAAGAAAGGGGACTCAACAATTATGAAAAGTGGAGTGGAATATGGCCACTGCAACACCAAATGTCAAACCCCAGTAGGAGCGATAAATTCTAGTATGCCATTCCACAA<br>CATACATCCTCTCACCATTGGGGAATGCCCCAAATACGTGAAGTCAAACAAGTTGGTCCTTGCGACTGGGCTCAGAAATAGTCCTCTAAGAGAAAAGAGAAGAAAAAGAGGCCTGTTTGGGGCGATAGCAGGGTTTATAGAGGGAGGATGGCAGGGAATGGTTGATGGTTGGTATGGGTACCACCATAGCAATGAGCAGGGGAGTGGGTACGCTGCAGACAAAGAATC<br>CACCCAAAAGGCAATAGATGGAGTTACCAATAAGGTCAACTCAATAATTGACAAAATGAACACTCAATTTGAGGCAGTTGGAAGGGAGTTTAATAACTTAGAAAGGAGGATAGAGAATTTGAACAAGAAAATGGAAGACGGATTCCTAGATGTCTGGACCTATAATGCTGAACTTCTAGTTCTCATGGAAAACGAGAGGACTCTAGATTTCCATGATTCAAATGTCAAGAA<br>CCTTTACGACAAGGTCAGACTACAGCTTAGGGATAATGCAAAGGAGCTGGGTAACGGCTGTTTCGAATTCTATCACAAATGCGATAATGAATGTATGGAAAGTGTGAGAAATGGGACGTATGACTACCCTCAGTATTCAGAAGAAGCAAGATTAAAAAGAGAAGAAATAAGCGGAGTGAAATTAGAATCAATAGGAACTTACCAGATACTGTCAATTTATTCAACAGCGG<br>CGAGTTCCCTAGCACTGGCAATCATGATGGCTGGTCTATCTTTATGGATGTGCTCCAATGGGTCGTTACAGTGCAGAATTTGCATTTAA
EPI_ISL_2681044	>A/chicken/Romania/12448_21VIR3734-3/2021_H5N8_2021-05-05<br>ATGGAGAACATAGTACTTCTTCTTGCAATAGTTAGCCTTGTTAAAAGTGATCAGATTTGCATTGGTTACCATGCAAACAATTCGACAGAGCAAGTTGACACGATAATGGAAAAGAACGTCACTGTTACACATGCCCAAGACATACTGGAAAAAACACACAACGGGAAGCTCTGTGATCTAAATGGGGTGAAGCCTCTGATTTTAAAGGATTGTAGTGTAGCTGGATGGCTCC<br>TCGGAAACCCAATGTGCGACGAATTCATCAGAGTGCCGGAATGGTCCTACATAGTGGAGAGGGCTAATCCAGCTAATGACCTCTGTTACCCAGGGAGCCTCAATGACTATGAAGAACTGAAACACCTGTTGAGCAGAATAAATCATTTTGAGAAGATTATGATCATCCCCAAGAGTTCCTGGCCAAATCATGAAACATCACTAGGGGTGAGCGCAGCTTGTCCATACCAGG<br>GAGCGCCCTCCTTTTTCAGAAATGTGGTGTGGCTTATCAAAAAGGACGATGCATACCCAACAATAAAGATAAGCTACAATAATACCAATCGGGAAGATCTCTTGATACTGTGGGGGATTCATCATTCCAACAATGCAGAAGAGCAGACAAATCTCTATACAAACCCAACCACCTACATTTCAGTTGGAACATCAACTTTAAACCAGAGGTTGGTACCAAAAATAGCTACTAG<br>ATCCCAAGTAAACGGGCAACGTGGGAGAATGGACTTCTTCTGGACAATTTTAAAACCAGATGATGCAATCCATTTCGAGAGTAATGGAAATTTCATTGCTCCAGAATATGCATACAAAATTATCAAGAAAGGGGACTCAACAATTATGAAAAGTGAAGTGGAATATGGCCACTGCAACACCAAATGTCAAACCCCAGTAGGAGCGATAAATTCTAGTATGCCATTCCACAA<br>CATACATCCTCTCACCATTGGGGAATGCCCCAAATACGTGAAGTCAAACAAGTTGGTCCTTGCGACTGGGCTCAGAAATAGCCCTCTAAGAGAAAAGAGAAGAAAAAGAGGCCTGTTTGGGGCGATAGCAGGGTTTATAGAGGGAGGATGGCAGGGAATGGTTGATGGTTGGTATGGGTACCACCATAGCAATGAGCAGGGGAGTGGGTACGCTGCAGACAAAGAATC<br>CACCCAAAAGGCAATAGATGGAGTTACCAATAAGGTCAACTCAATAATTGACAAAATGAACACTCAATTTGAGGCAGTTGGAAGGGAGTTTAATAACTTAGAAAGGAGGATAGAGAATTTGAACAAGAAAATGGAAGACGGATTCCTAGATGTCTGGACCTATAATGCTGAACTTCTAGTTCTCATGGAAAACGAGAGGACTCTAGATTTCCATGATTCAAATGTCAAGAA<br>CCTTTACGACAAGGTCAGACTACAGCTTAGGGATAATGCAAAGGAGCTGGGTAACGGCTGTTTCGAATTCTATCACAAATGCGATAATGAATGTATGGAAAGTGTGAGAAATGGGACGTATGACTACCCTCAGTATTCAGAAGAAGCAAGATTAAAAAGAGAAGAAATAAGCGGAGTGAAATTAGAATCAATAGGAACTTACCAGATACTGTCAATTTATTCAACAGCGG<br>CGAGTTCCCTAGCACTGGCAATCATGATGGCTGGTCTATCTTTATGGATGTGCTCCAATGGGTCGTTACAGTGCAGAATTTGCATTTAA
EPI_ISL_647969	>A/Turkey/Sweden/SVA201114SZ0001/20KN303106/2020_H5N8_2020-11-13<br>ATGGAGAACATAGTACTTCTTCTTGCAATAGTTAGCCTTGTTAAAAGTGATCAGATTTGCATTGGTTACCATGCAAACAATTCGACAGAGCAAGTTGACACGATAATGGAAAAGAACGTCACTGTTACACATGCCCAAGACATACTGGAAAAAACACACAACGGGAAGCTCTGTGATCTAAATGGGGTGAAGCCTCTGATTTTAAAGGATTGTAGTGTAGCTGGATGGCTCC<br>TCGGAAACCCAATGTGCGACGAATTCATCAGAGTGCCGGAATGGTCCTACATAGTGGAGAGGGCTAATCCAGCTAATGACCTCTGTTACCCAGGGAGCCTCAATGACTATGAAGAACTGAAACACCTGTTGAGCAGAATAAATCATTTTGAGAAGATTATGATCATCCCCAAGAGTTCCTGGCCAAATCATGAAACATCACTAGGGGTGAGCGCAGCTTGTCCATACCAGG<br>GAGCGCCCTCCTTTTTCAGAAATGTGGTGTGGCTTATCAAAAAGAACGATGCATACCCAACAATAAAGATAAGCTACAATAATACCAATCGGGAAGATCTCTTGATACTGTGGGGGATTCATCATTCCAACAATGCAGAAGAGCAGACAAATCTCTATAAAAACCCAACCACCTACATTTCAGTTGGAACATCAACTTTAAACCAGAGGTTGGTACCAAAAATAGCTACTAG<br>ATCCCAAGTAAACGGGCAACGTGGGAGAATGGACTTCTTCTGGACAATTTTAAAACCGGATGATGCAATCCATTTCGAGAGTAATGGAAATTTCATTGCTCCAGAATATGCATACAAAATTGTCAAGAAAGGGGACTCAACAATTATGAAAAGTGGAGTGGAATATGGCCACTGCAACACCAAATGTCAAACCCCAGTAGGAGCGATAAATTCTAGTATGCCATTCCACAA<br>CATACATCCTCTCACCATTGGGGAATGCCCCAAATACGTGAAGTCAAACAAGTTGGTCCTTGCGACTGGGCTCAGAAATAGCCCTCTAAGAGAAAAGAGAAGAAAAAGAGGCCTGTTTGGGGCGATAGCAGGGTTTATAGAGGGAGGATGGCAGGGAATGGTTGATGGTTGGTATGGGTACCACCATAGCAATGAGCAGGGGAGTGGGTACGCTGCAGACAAAGAATC<br>CACCCAAAAGGCAATAGATGGAGTTACCAATAAGGTCAACTCAATAATTGACAAAATGAACACTCAATTTGAGGCAGTTGGAAGGGAGTTTAATAACTTAGAAAGGAGGATAGAGAATTTGAACAAGAAAATGGAAGACGGATTCCTAGATGTCTGGACCTATAATGCTGAACTTCTAGTTCTCATGGAAAACGAGAGGACTCTAGATTTCCATGATTCAAATGTCAAGAA<br>CCTTTACGACAAGGTCAGACTACAGCTTAGGGATAATGCAAAGGAGCTGGGTAACGGCTGTTTCGAATTCTATCACAAATGCGATAATGAATGTATGGAAAGTGTGAGAAATGGGACGTATGACTACCCTCAGTATTCAGAAGAAGCAAGATTAAAAAGAGAAGAAATAAGCGGAGTGAAATTAGAATCAATAGGAACTTACCAGATACTGTCAATTTATTCAACAGCGG<br>CGAGTTCCCTAGCACTGGCAATCATGATGGCTGGTCTATCTTTATGGATGTGCTCCAATGGGTCGTTACAGTGCAGAATTTGCATTTAA
EPI_ISL_5115615	>A/turkey/Germany-BB/AI01980/2021_H5N8_2021-02-28<br>ATGGAGAACATAGTACTTCTTCTTGCAATAGTTAGCCTTGTTAAAAGTGATCAGATTTGCATTGGTTACCATGCAAACAATTCGACAGAGCAAGTTGACACGATAATGGAAAAGAACGTCACTGTTACACATGCCCAAGACATACTGGAAAAAACACACAACGGGAAGCTCTGTGATCTAAATGGGGTGAAGCCTCTGATTTTAAAGGATTGTAGTGTAGCTGGATGGCTCC<br>TCGGAAACCCAATGTGCGACGAATTCATCAGAGTGCCGGAATGGTCCTACATAGTGGAGAGGGCTAATCCAGCTAATGACCTCTGTTACCCAGGGAGCCTCAATGACTATGAAGAACTGAAACACCTGTTGAGCAGAATAAATCATTTTGAGAAGATTATGATCATCCCCAAGAGTTCCTGGCCAAATCATGAAACATCACTAGGGGTGAGCGCAGCTTGTCCATACCAGG<br>GAGCGCCCTCCTTTTTCAGAAATGTGGTGTGGCTTATCAAAAAGAACGATGCATACCCAACAATAAAGATAAGCTACAATAATACCAATCGGGAAGATCTCTTGATACTATGGGGGATTCATCATTCCAACAATGCAGAAGAGCAGACAAATCTCTATAAAAACCCAACCACCTACATTTCAGTTGGAACATCAACTTTAAACCAGAGGTTGGTACCAAAAATAGCTACTAG<br>ATCCCAAGTAAACGGGCAACGTGGGAGAATGGACTTCTTCTGGACAATTTTAAAACCAGATGATGCAATCCATTTCGAGAGTAATGGAAATTTCATTGCTCCAGAATATGCATACAAAATTATCAAGAAAGGGGACTCAACAATTATGAAAAGTGGAGTGGAATATGGCCACTGCAACACCAAATGTCAAACCCCAGTAGGAGCGATAAATTCTAGTATGCCATTCCACAA<br>CATACATCCTCTCACCATTGGGGAATGCCCCAAATACGTGAAGTCAAACAAGTTGGTCCTTGCGACTGGGCTCAGAAATAGCCCTCTAAGAGAAAAGAGAAGAAAAAGAGGCCTGTTTGGGGCGATAGCAGGGTTTATAGAGGGAGGATGGCAGGGAATGGTTGATGGTTGGTATGGGTACCACCATAGCAATGAGCAGGGGAGTGGGTACGCTGCAGACAAAGAATC<br>CACCCAAAAGGCAATAGATGGAGTTACCAATAAGGTCAACTCAATAATTGACAAAATGAACACTCAATTTGAGGCAGTTGGAAGGGAGTTTAATAACTTAGAAAGGAGGATAGAGAATTTGAACAAGAAAATGGAAGACGGATTCCTAGATGTCTGGACCTATAATGCTGAACTTCTAGTTCTCATGGAAAACGAGAGGACTCTAGATTTCCATGATTCAAATGTCAAGAA<br>CCTTTACGACAAGGTCAGACTACAGCTTAGGGATAATGCAAAGGAGCTGGGTAACGGCTGTTTCGAATTCTATCACAAATGCGATAATGAATGTATGGAAAGTGTGAGAAATGGGACGTATGACTACCCTCAGTATTCAGAAGAAGCAAGATTAAAAAGAGAAGAAATAAGCGGAGTGAAATTAGAATCAATAGGAACTTACCAGATACTGTCAATTTATTCAACAGCGG<br>CGAGTTCCCTAGCACTGGCAATCATGATGGCTGGTCTATCTTTATGGATGTGCTCCAATGGGTCGTTACAGTGCAGAATTTGCATTTAA
EPI_ISL_5099367	>A/chicken/Germany-BY/AI01355/2021_H5N8_2021-02-07<br>ATGAAGAACATAGTACTTCTTCTTGCAATAGTTAGCCTTGTTAAAAGTGATCAGATTTGCATTGGTTACCATGCAAACAATTCGACAGAGCAAGTTGACACGATAATGGAAAAGAACGTCACTGTTACACATGCCCAAGACATACTGGAAAAAACACACAACGGGAAGCTCTGTGATCTAAATGGGGTGAAGCCTCTGATTTTAAAGGATTGTAGTGTAGCTGGATGGCTTC<br>TCRGAAACCCAATGTGCGACGAATTCATCAGAGTGCCGGAATGGTCCTACATAGTGGAGAGGGCTAATCCAGCTAATGACCTCTGTTACCCAGGGAGCCTCAATGACTATGAAGAACTGAAACACCTGTTGAGCAGAATAAATCATTTTGAGAAGATTATGATCATCCCCAAGAGTTCCTGGCCAAATCATGAAACATCACTAGGGGTGAGCGCAGCTTGTCCATACCAGG<br>GAGCGCCCTCCTTTTTCAGAAATGTGGTGTGGCTTATCAAAAAGAACGATGCATACCCAACAATAAAGATAAGCTACAATAATACCAATCRGGAAGATCTCTTGATACTGTGGGGGATTCATCATTCCAACAATGCAGAAGAGCAGACAAATCTCTATAAAAACCCAACCACCTACATTTCAGTTGGAACATCAACTTTAAACCAGAGGTTGGTACCAAAAATAGCTACTAG<br>ATCCCAAGTAAACGGGCAACGTGGGAGAATGGACTTCTTCTGGACAATTTTAAAACCAGATGATGCAATCCATTTCGAGAGTAATGGAAATTTCATTGCTCCAGAATATGCATACAAAATTATCAAGAAAGGGGACTCAACAATTATGAAAAGTGGAGTGGAATATGGCCACTGCAACACCAAATGTCAAACCCCAGTAGGAGCGATAAATTCTAGTATGCCATTCCACAA<br>CATACATCCTCTCACCATTGGGGAATGCCCCAAATACGTGAAGTCAAACAAGTTGGTCCTTGCGACTGGGCTCAGAAATAGCCCTCTAAGAGAAAAGAGAAGAAAAAGAGGCCTGTTTGGGGCGATAGCAGGGTTTATAGAGGGAGGATGGCAGGGAATGGTTGATGGTTGGTATGGGTACCACCATAGCAATGAGCAGGGGAGTGGGTACGCTGCAGACAAAGAATC<br>CACCCAAAAGGCAATAGATGGAGTTACCAATAAGGTCAACTCAATAATTGACAAAATGAACACTCAATTTGAGGCAGTTGGAAGGGAGTTTAATAACTTAGAAAGGAGGATAGAGAATTTGAACAAGAAAATGGAAGACGGATTCCTAGATGTCTGGACCTATAATGCTGAACTTCTAGTTCTCATGGAAAACGAGAGGACTCTAGATTTCCATGATTCAAATGTCAAGAA<br>CCTTTACGACAAGGTCAGACTACAGCTTAGGGATAATGCAAAGGAGCTGGGTAACGGCTGTTTCGAATTCTATCACAAATGCGATAATGAATGTATGGAAAGTGTGAGAAATGGGACGTATGACTACCCTCAGTATTCAGAAGAAGCAAGATTAAAAAGAGAAGAAATAAGCGGAGTGAAATTAGAATCAATAGGAACTTACCAGATACTGTCAATTTATTCAACAGCGG<br>CGAGTTCCCTAGCACTGGCAATCATGATGGCTGGTCTATCTTTATGGATGTGCTCCAATGGGTCGTTACAGTGCAGAATTTGCATTTAA
EPI_ISL_1399241	>A/chicken/Czech_Republic/4526/2021_H5N8_2021-03-06<br>ATGGAGAACATAGTACTTCTTCTTGCAATAGTTAGCCTTGTTAAAAGTGATCAGATTTGCATTGGTTACCATGCAAACAATTCGACAGAGCAAGTTGACACGATAATGGAAAAGAACGTCACTGTTACACATGCCCAAGACATACTGGAAAAAACACACAACGGGAAGCTCTGTGATCTAAATGGGGTGAAGCCTCTGATTTTAAAGGATTGTAGTGTAGCTGGATGGCTTC<br>TCGGAAACCCAATGTGCGACGAATTCATCAGAGTGCCGGAATGGTCCTACATAGTGGAGAGGGCTAATCCAGCTAATGACCTCTGTTACCCAGGGAGCCTCAATGACTATGAAGAACTGAAACACCTGTTGAGCAGAATAAATCATTTTGAGAAGATTATGATCATCCCCAAGAGTTCCTGGCCAAATCATGAAACATCACTAGGGGTGAGCGCAGCTTGTCCATACCAGG<br>GAGCGCCCTCCTTTTTCAGAAATGTGGTGTGGCTTATCAAAAAGAACGATGCATACCCAACAATAAAGATAAGCTACAATAATACCAATCGGGAAGATCTCTTGATACTGTGGGGGATTCATCATTCCAACAATGCAGAAGAGCAGACAAATCTCTATAAAAACCCAACCACCTACATTTCAGTTGGAACATCAACTTTAAACCAGAGGTTGGTACCAAAAATAGCTACTAG<br>ATCCCAAGTAAACGGGCAACGTGGGAGAATGGACTTCTTCTGGACAATTTTAAAACCAGATGATGCAATCCATTTCGAGAGTAATGGAAATTTCATTGCTCCAGAATATGCATACAAAATTATCAAGAAAGGGGACTCAACAATTATGAAAAGTGGAGTGGAATATGGCCACTGCAACACCAAATGTCAAACCCCAGTAGGAGCGATAAATTCTAGTATGCCATTCCACAA<br>CATACATCCTCTCACCATTGGGGAATGCCCCAAATACGTGAAGTCAAACAAGTTGGTCCTTGCGACTGGGCTCAGAAATAGCCCTCTAAGAGAAAAGAGAAGAAAAAGAGGCCTGTTTGGGGCGATAGCAGGGTTTATAGAGGGAGGATGGCAGGGAATGGTTGATGGTTGGTATGGGTACCACCATAGCAATGAGCAGGGGAGTGGGTACGCTGCAGACAAAGAATC<br>CACCCAAAAGGCAATAGATGGAGTTACCAATAAGGTCAACTCAATAATTGACAAAATGAACACTCAATTTGAGGCAGTTGGAAGGGAGTTTAATAACTTAGAAAGGAGGATAGAGAATTTGAACAAGAAAATGGAAGACGGATTCCTAGATGTCTGGACCTATAATGCTGAACTTCTAGTTCTCATGGAAAACGAGAGGACTCTAGATTTCCATGATTCAAATGTCAAGAA<br>CCTTTACGACAAGGTCAGACTACAGCTTAGGGATAATGCAAAGGAGCTGGGTAACGGCTGTTTCGAATTCTATCACAAATGCGATAATGAATGTATGGAAAGTGTGAGAAATGGGACGTATGACTACCCTCAGTATTCAGAAGAAGCAAGATTAAAAAGAGAAGAAATAAGCGGAGTGAAATTAGAATCAATAGGAACTTACCAGATACTGTCAATTTATTCAACAGCGG<br>CGAGTTCCCTAGCACTGGCAATCATGATGGCTGGTCTATCTTTATGGATGTGCTCCAATGGGTCGTTACAGTGCAGRATTTGCATTTAA
EPI_ISL_12437700	>A/duck/Czech_Republic/6017/2021_H5N8_2021-03-26<br>ATGGAGAACATAGTACTTCTTCTTGCAATAGTTAGCCTTGTTAAAAGTGATCAGATTTGCATTGGTTACCATGCAAACAATTCGACAGAGCAAGTTGACACGATAATGGAAAAGAACGTCACTGTTACACATGCCCAAGACATACTGGAAAAAACACACAACGGGAAGCTCTGTGATCTAAATGGGGTGAAGCCTCTGATTTTAAAGGATTGTAGTGTAGCTGGATGGCTCC<br>TCGGAAACCCAATGTGCGACGAATTCATCAGAGTGCCGGAATGGTCCTACATAGTGGAGAGGGCTAATCCAGCTAATGACCTCTGTTACCCAGGGAGCCTCAATGACTATGAAGAACTGAAACACCTGTTGAGCAGAATAAATCATTTTGAGAAGATTATGATCATCCCCAAGAGTTCCTGGCCAAATCATGAAACATCACTAGGGGTGAGCGCAGCTTGTCCATACCAGG<br>GAGCGCCCTCCTTTTTCAGAAATGTGGTGTGGCTTATCAAAAAGGACGATGCATACCCAACAATAAAGATAAGCTACAATAATACCAATCGGGAAGATCTCTTGATACTGTGGGGGATTCATCATTCCAACAATGCAGAAGAGCAGACAAATCTCTATAAAAACCCAACCACCTACATTTCAGTTGGAACATCAACTTTAAACCAGAGGTTGGTACCAAAAATAGCTACTAG<br>ATCCCAAGTAAACGGGCAACGTGGGAGAATGGACTTCTTCTGGACAATTTTAAAACCAGATGATGCAATCCATTTCGAGAGTAATGGAAATTTCATTGCTCCAGAATATGCATACAAAATTATCAAGAAAGGGGACTCAACAATTATGAAAAGTGGAGTGGAATATGGCCACTGCAACACCAAATGTCAAACCCCAGTAGGAGCGATAAATTCTAGTATGCCATTCCACAA<br>CATACATCCTCTCACCATTGGGGAATGCCCCAAATACGTGAAGTCAAACAAGTTGGTCCTTGCGACTGGGCTCAGAAATAGCCCTCTAAGAGAAAAGAGAAGAAAAAGAGGCCTGTTTGGGGCGATAGCAGGGTTTATAGAGGGAGGATGGCAGGGAATGGTTGATGGTTGGTATGGGTACCACCATAGCAATGAGCAGGGGAGTGGGTACGCTGCAGACAAAGAATC<br>CACCCAAAAGGCAATAGATGGAGTTACCAATAAGGTCAACTCAATAATTGACAAAATGAACACTCAATTTGAGGCAGTTGGAAGGGAGTTTAATAACTTAGAAAGGAGGATAGAGAATTTGAACAAGAAAATGGAAGACGGATTCCTAGATGTCTGGACCTATAATGCTGAACTTCTAGTTCTCATGGAAAACGAGAGGACTCTAGATTTCCATGATTCAAATGTCAAGAA<br>CCTTTACGACAAGGTCAGACTACAGCTTAGGGATAATGCAAAGGAGCTGGGTAACGGCTGTTTCGAATTCTATCACAAATGCGATAATGAATGTATGGAAAGTGTGAGAAATGGGACGTATGACTACCCTCAGTATTCAGAAGAAGCAAGATTAAAAAGAGAAGAAATAAGCGGAGTGAAATTAGAATCAATAGGAACTTACCAGATACTGTCAATTTATTCAACAGCGG<br>CGAGTTCCCTAGCACTGGCAATCATGATGGCTGGTCTATCTTTATGGATGTGCTCCAATGGGTCGTTACAGTGCAGAATTTGCATTTAA
EPI_ISL_18244493	>A/swan/Poland/MB122-21_22VIR5675-9/2021_H5N8_2021-02-18<br>ATGGAGAACATAGTACTTCTTCTTGCAATAGTTAGCCTTGTTAAAAGTGATCAGATTTGCATTGGTTACCATGCAAACAATTCGACAGAGCAAGTTGACACGATAATGGAAAAGAACGTCACTGTTACACATGCCCAAGACATACTGGAAAAAACACACAACGGGAAGCTTTGTGATCTAAATGGGGTGAAGCCTCTGATTTTAAAGGATTGTAGTGTAGCTGGATGGCTTC<br>TCGGAAACCCAATGTGCGACGAATTCATCAGAGTGCCGGAATGGTCCTACATAGTGGAGAGGGCTAATCCAGCTAATGACCTCTGTTACCCAGGGAGCCTCAATGACTATGAAGAACTGAAACACCTGTTGAGCAGAATAAATCATTTTGAGAAGATTATGATCATCCCCAAGAGTTCCTGGCCAAATCATGAAACATCACTAGGGGTGAGCGCAGCTTGTCCATACCAGG<br>GAGCGCCCTCCTTTTTCAGAAATGTGGTGTGGCTTATCAAGAAGAACGATGCATACCCAACAATAAAGATAAGCTACAATAATACCAATCGGGAAGATCTCTTGATACTGTGGGGGATTCATCATTCCAACAATGCAGAAGAGCAGACAAATCTCTATAAAAACCCAACCACCTACATTTCAGTTGGAACATCAACTTTAAACCAGAGGTTGGTACCAAAAATAGCTACTAG<br>ATCCCAAGTAAACGGGCAACGTGGGAGAATGGACTTCTTCTGGACAATTTTAAAACCAGATGATGCAATCCATTTCGAGAGTAATGGAAATTTCATTGCTCCAGAATATGCATACAAAATTATCAAGAAAGGGGACTCAACAATTATGAAAAGTGGAGTGGAATATGGCCACTGCAACACCAAATGTCAAACCCCAGTAGGAGCGATAAATTCTAGTATGCCATTCCACAA<br>CATACATCCTCTCACCATTGGGGAATGCCCCAAATACGTGAAGTCAAACAAGTTGGTCCTTGCGACTGGGCTCAGAAATAGCCCTCTAAGAGAAAAGAGAAGAAAAAGAGGCCTGTTTGGGGCGATAGCAGGGTTTATAGAGGGAGGATGGCAGGGAATGGTTGATGGTTGGTATGGGTACCACCATAGCAATGAGCAGGGGAGTGGGTACGCTGCAGACAAAGAATC<br>CACCCAAAAGGCAATAGATGGAGTTACCAATAAGGTCAACTCAATAATTGACAAAATGAACACTCAATTTGAGGCAGTTGGAAGGGAGTTTAATAACTTAGAAAGGAGGATAGAGAATTTGAACAAGAAAATGGAAGACGGATTCCTAGATGTCTGGACCTATAATGCTGAACTTCTAGTTCTCATGGAAAACGAGAGGACTCTAGATTTCCATGATTCAAATGTCAAGAA<br>CCTTTACGACAAGGTCAGACTACAGCTTAGGGATAATGCAAAGGAGCTGGGTAACGGCTGTTTCGAATTCTATCACAAATGCGATAATGAATGTATGGAAAGTGTGAGAAATGGGACGTATGACTACCCTCAGTATTCAGAAGAAGCAAGATTAAAAAGAGAAGAAATAAGCGGAGTGAAATTAGAATCAATAGGAACTTACCAGATACTGTCAATTTATTCAACAGCGG<br>CGAGTTCCCTAGCACTGGCAATCATGATGGCTGGTCTATCTTTATGGATGTGCTCCAATGGGTCGTTACAGTGCAGAATTTGCATTTAA
EPI_ISL_644150	>A/chicken/Omsk/0112/2020_H5N8_2020-08-17<br>ATGGAGAACATAGTACTTCTTCTTGCAATAGTTAGCCTTGTTAAAAGTGATCAGATTTGCATTGGTTACCATGCAAACAATTCGACAGAGCAAGTTGACACGATAATGGAAAAGAACGTCACTGTTACACATGCCCAAGACATACTGGAAAAAACACACAACGGGAAGCTCTGTGATCTAAATGGGGTGAAGCCTCTGATTTTAAAGGATTGTAGTGTAGCTGGATGGCTCC<br>TCGGAAACCCAATGTGCGACGAATTCATCAGAGTGCCGGAATGGTCCTACATAGTGGAGAGGGCTAATCCAGCTAATGACCTCTGTTACCCAGGGAGCCTCAATGACTATGAAGAACTGAAACACCTGTTGAGCAGAATAAATCATTTTGAGAAGATTCTGATCATCCCCAAGAGTTCCTGGCCAAACCATGAAACATCACTAGGGGTGAGCGCAGCTTGTCCATACCAGG<br>GAGCGCCCTCCTTTTTCAGAAATGTGGTGTGGCTTATCAAAAAGAACGATGCATACCCAACAATAAAGATAAGCTACAATAATACCAATCGGGAAGATCTCTTGATACTGTGGGGGATTCATCATTCCAACAATGCAGAAGAGCAGACAAATCTCTATAAAAACCCAACCACCTACATTTCAGTTGGAACATCAACTTTAAACCAGAGGTTGGTACCAAAAATAGCTACTAG<br>ATCCCAAGTAAACGGGCAACGTGGAAGAATGGACTTCTTCTGGACAATTTTAAAACCGGATGATGCAATCCATTTCGAGAGTAATGGAAATTTCATTGCTCCAGAATATGCATACAAAATTGTCAAGAAAGGGGACTCAACAATTATGAAAAGTGGAGTGGAATATGGCCACTGCAACACCAAATGTCAAACCCCAGTAGGAGCGATAAATTCTAGTATGCCATTCCACAA<br>CATACATCCTCTCACCATTGGGGAATGCCCCAAATACGTGAAGTCAAACAAGTTGGTCCTTGCGACTGGGCTCAGAAATAGTCCTCTAAGAGAAAAGAGAAGAAAAAGAGGCCTGTTTGGGGCGATAGCAGGGTTTATAGAGGGAGGATGGCAGGGAATGGTTGATGGTTGGTATGGGTACCACCATAGCAATGAGCAGGGGAGTGGGTACGCTGCAGACAAAGAATC<br>CACCCAAAAGGCAATAGATGGAGTTACCAATAAGGTCAACTCAATCATTGACAAAATGAACACTCAATTTGAGGCAGTTGGAAGGGAGTTTAATAACTTAGAAAGGAGGATAGAGAATTTGAACAAGAAAATGGAAGACGGATTCCTAGATGTCTGGACCTATAATGCTGAACTTCTAGTTCTCATGGAAAACGAGAGGACTCTAGATTTCCATGATTCAAATGTCAAGAA<br>CCTTTACGACAAAGTCAGACTACAGCTTAGGGATAATGCAAAGGAGCTGGGTAACGGCTGTTTCGAATTCTATCACAAATGCGATAATGAATGTATGGAAAGTGTGAGAAATGGGACGTATGACTACCCTCAGTATTCAGAAGAAGCAAGATTAAAAAGAGAAGAAATAAGCGGAGTGAAATTAGAATCAATAGGAACTTACCAGATACTGTCAATTTATTCAACAGCGG<br>CGAGTTCCCTAGCACTGGCAATCATGATGGCTGGTCTATCTTTATGGATGTGCTCCAATGGGTCGTTACAGTGCAGAATTTGCATTTAA
EPI_ISL_644127	>A/goose/Omsk/0113/2020_H5N8_2020-08-17<br>ATGGAGAACATAGTACTTCTTCTTGCAATAGTTAGCCTTGTTAAAAGTGATCAGATTTGCATTGGTTACCATGCAAACAATTCGACAGAGCAAGTTGACACGATAATGGAAAAGAACGTCACTGTTACACATGCCCAAGACATACTGGAAAAAACACACAACGGGAAGCTCTGTGATCTAAATGGGGTGAAGCCTCTGATTTTAAAGGATTGTAGTGTAGCTGGATGGCTCC<br>TCGGAAACCCAATGTGCGACGAATTCATCAGAGTGCCGGAATGGTCCTACATAGTGGAGAGGGCTAATCCAGCTAATGACCTCTGTTACCCAGGGAGCCTCAATGACTATGAAGAACTGAAACACCTGTTGAGCAGAATAAATCATTTTGAGAAGATTCTGATCATCCCCAAGAGTTCCTGGCCAAACCATGAAACATCACTAGGGGTGAGCGCAGCTTGTCCATACCAGG<br>GAGCGCCCTCCTTTTTCAGAAATGTGGTGTGGCTTATCAAAAAGAACGATGCATACCCAACAATAAAGATAAGCTACAATAATACCAATCGGGAAGATCTCTTGATACTGTGGGGGATTCATCATTCCAACAATGCAGAAGAGCAGACAAATCTCTATAAAAACCCAACCACCTACATTTCAGTTGGAACATCAACTTTAAACCAGAGGTTGGTACCAAAAATAGCTACTAG<br>ATCCCAAGTAAACGGGCAACGTGGAAGAATGGACTTCTTCTGGACAATTTTAAAACCGGATGATGCAATCCATTTCGAGAGTAATGGAAATTTCATTGCTCCAGAATATGCATACAAAATTGTCAAGAAAGGGGACTCAACAATTATGAAAAGTGGAGTGGAATATGGCCACTGCAACACCAAATGTCAAACCCCAGTAGGAGCGATAAATTCTAGTATGCCATTCCACAA<br>CATACATCCTCTCACCATTGGGGAATGCCCCAAATACGTGAAGTCAAACAAGTTGGTCCTTGCGACTGGGCTCAGAAATAGTCCTCTAAGAGAAAAGAGAAGAAAAAGAGGCCTGTTTGGGGCGATAGCAGGGTTTATAGAGGGAGGATGGCAGGGAATGGTTGATGGTTGGTATGGGTACCACCATAGCAATGAGCAGGGGAGTGGGTACGCTGCAGACAAAGAATC<br>CACCCAAAAGGCAATAGATGGAGTTACCAATAAGGTCAACTCAATCATTGACAAAATGAACACTCAATTTGAGGCAGTTGGAAGGGAGTTTAATAACTTAGAAAGGAGGATAGAGAATTTGAACAAGAAAATGGAAGACGGATTCCTAGATGTCTGGACCTATAATGCTGAACTTCTAGTTCTCATGGAAAACGAGAGGACTCTAGATTTCCATGATTCAAATGTCAAGAA<br>CCTTTACGACAAAGTCAGACTACAGCTTAGGGATAATGCAAAGGAGCTGGGTAACGGCTGTTTCGAATTCTATCACAAATGCGATAATGAATGTATGGAAAGTGTGAGAAATGGGACGTATGACTACCCTCAGTATTCAGAAGAAGCAAGATTAAAAAGAGAAGAAATAAGCGGAGTGAAATTAGAATCAATAGGAACTTACCAGATACTGTCAATTTATTCAACAGCGG<br>CGAGTTCCCTAGCACTGGCAATCATGATGGCTGGTCTATCTTTATGGATGTGCTCCAATGGGTCGTTACAGTGCAGAATTTGCATTTAA
[truncated: 2,372,295 more chars]
