# Supplementary material for: Phylogenetic and Molecular Characteristics of Wild Bird-Origin Avian Influenza Viruses Circulating in Poland in 2018−2022: Reassortment, Multiple Introductions, and Wild Bird–Poultry Epidemiological Links
Source: Transbound Emerg Dis. 2024 Apr 12;2024:6661672. doi: 10.1155/2024/6661672 (PMC12017110; doi:10.1155/2024/6661672)

# Phylogenetic trees of all eight AIV gene segments

HPAI H5Nx 2020/2021 – colored as follows:

H5N8 sequences (one genotype) – red

H5N1 sequence (single event) – red ▲

H5N5 sequence (single event) – red ●

HPAI H5Nx 2021/2022 – colored as follows:

<u>H5N1 genotypes:</u>

G1 – pink

G2 – dark blue

G3 – salmon

G4 – orange

G5 – light blue

G6 – dark yellow

G7 – purple

LPAIVs from active surveillance + MB152/22 - green

Wild bird-poultry connections – marked as follows:

H5N8 introductions - marked as follows: ★

# PB2

Best-fit model: GTR+F+G4

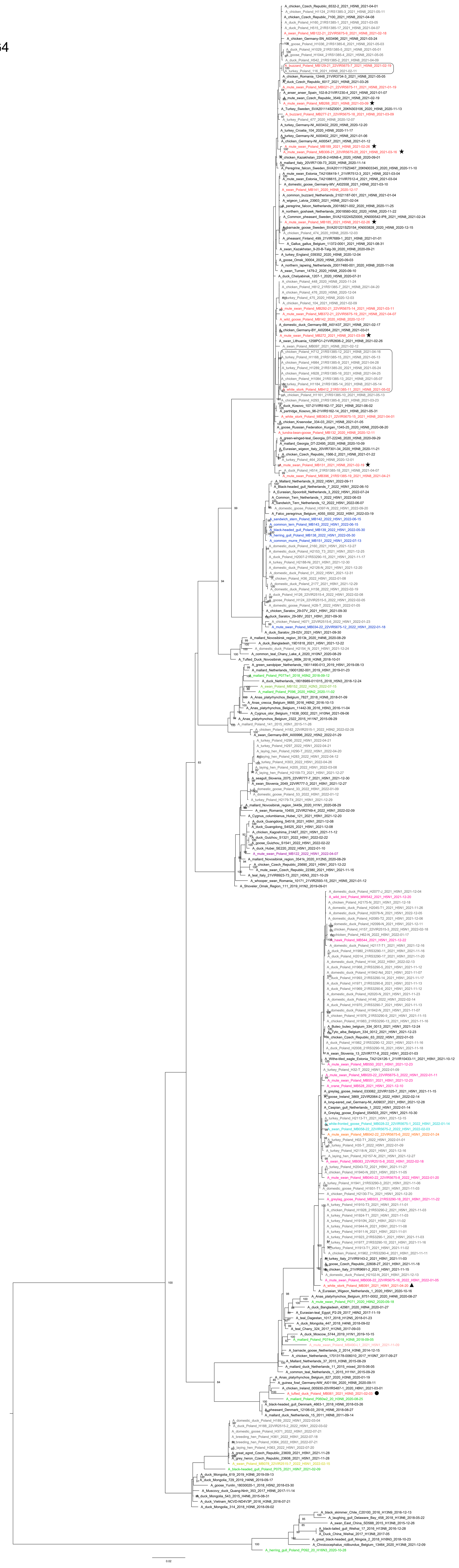

# PB1

Best-fit model: GTR+F+G4

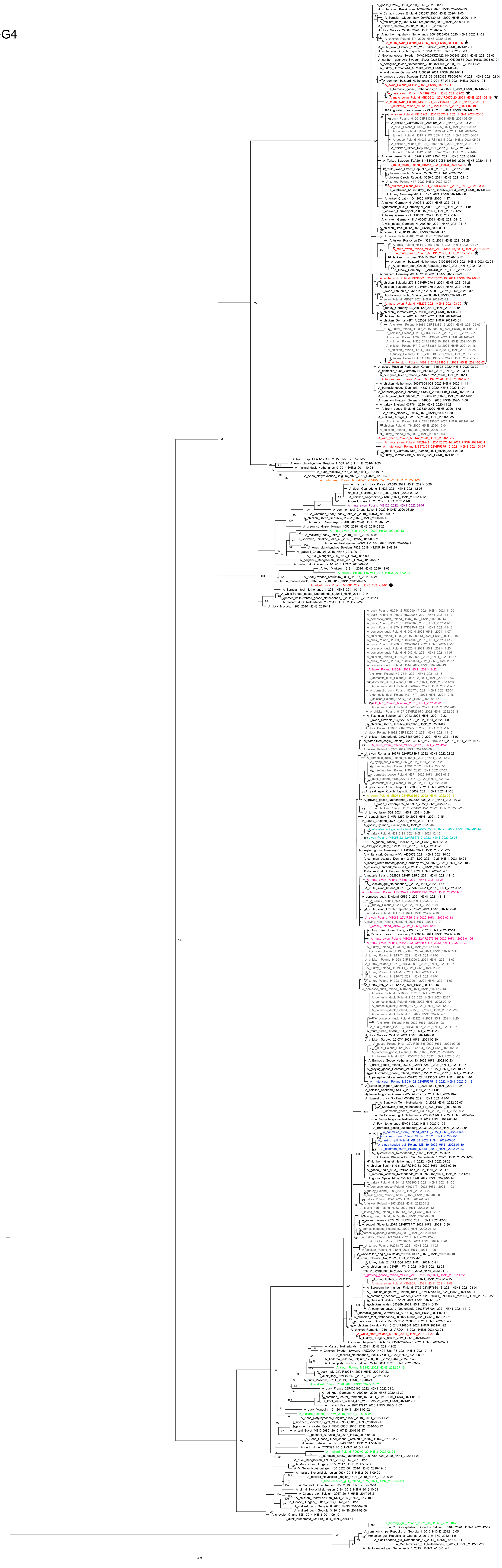

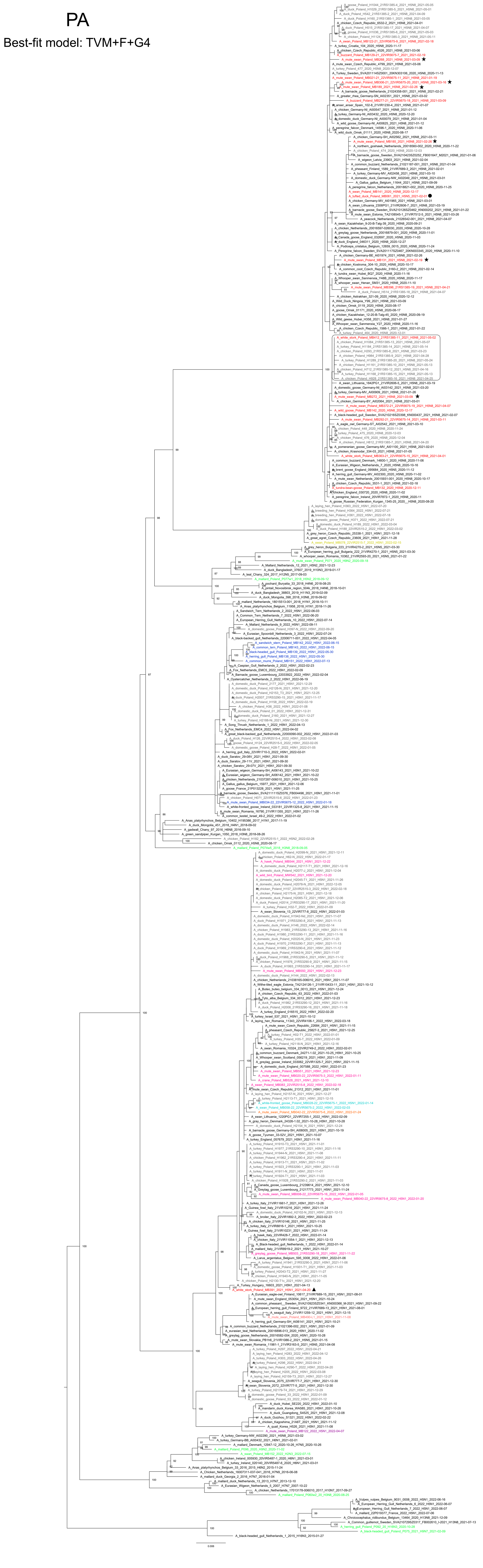
PA
Best-fit model: TVM+F+G4

# HA - H2

## Best-fit model: TIM+F+G4

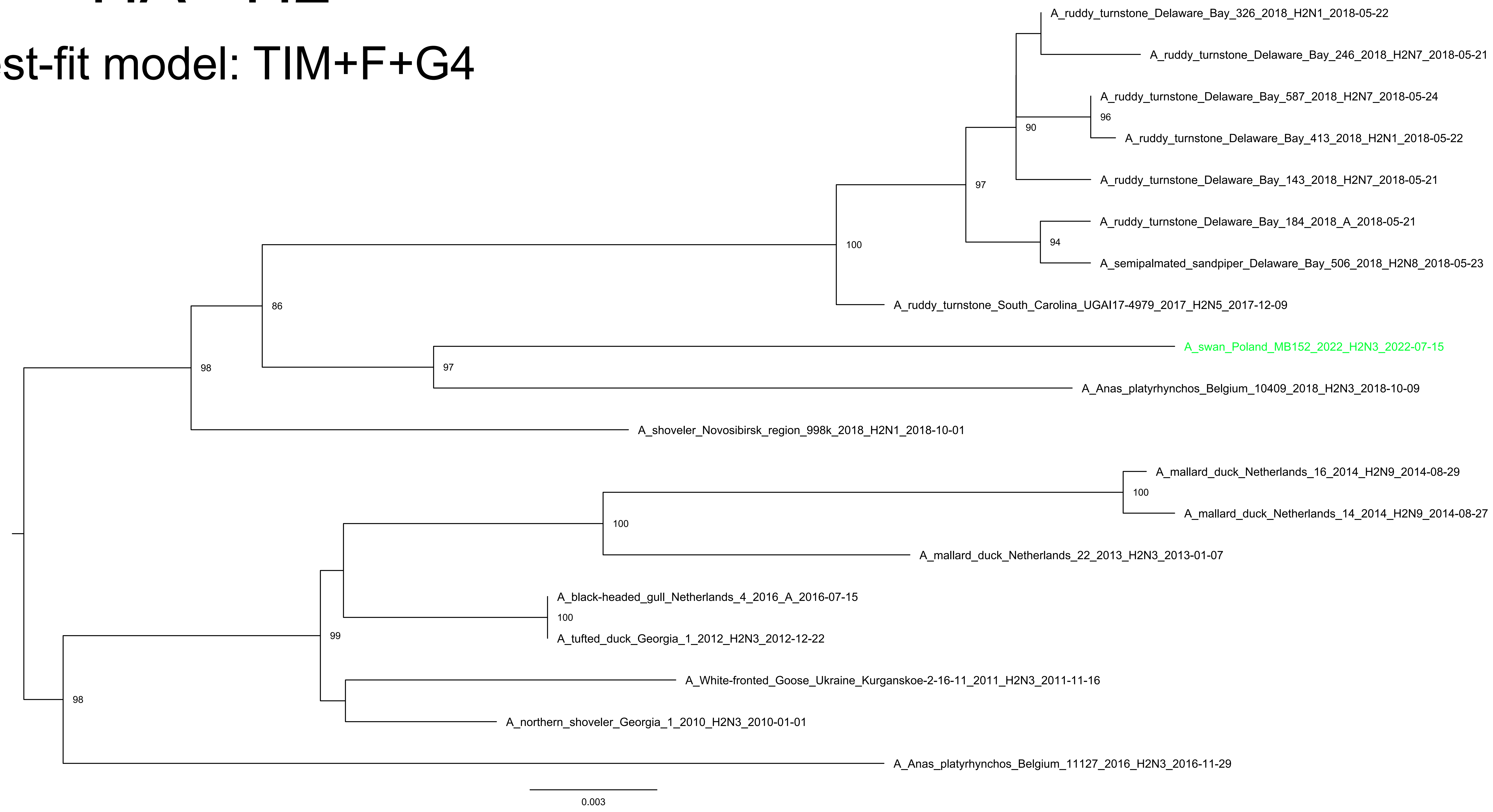

# HA - H3

## Best-fit model: TIM+F+G4

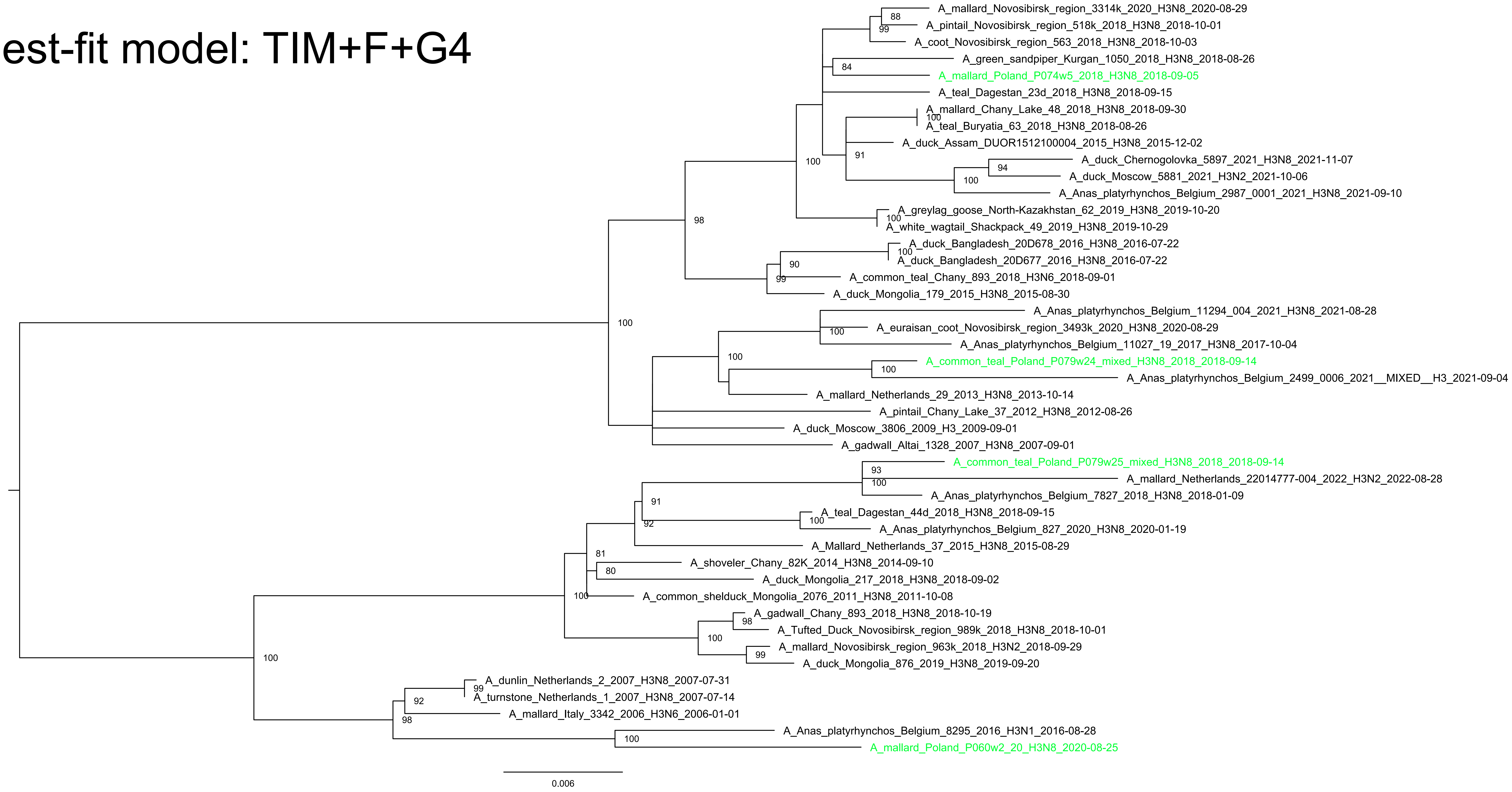

# HA - H5

Best-fit model: GTR+F+G4

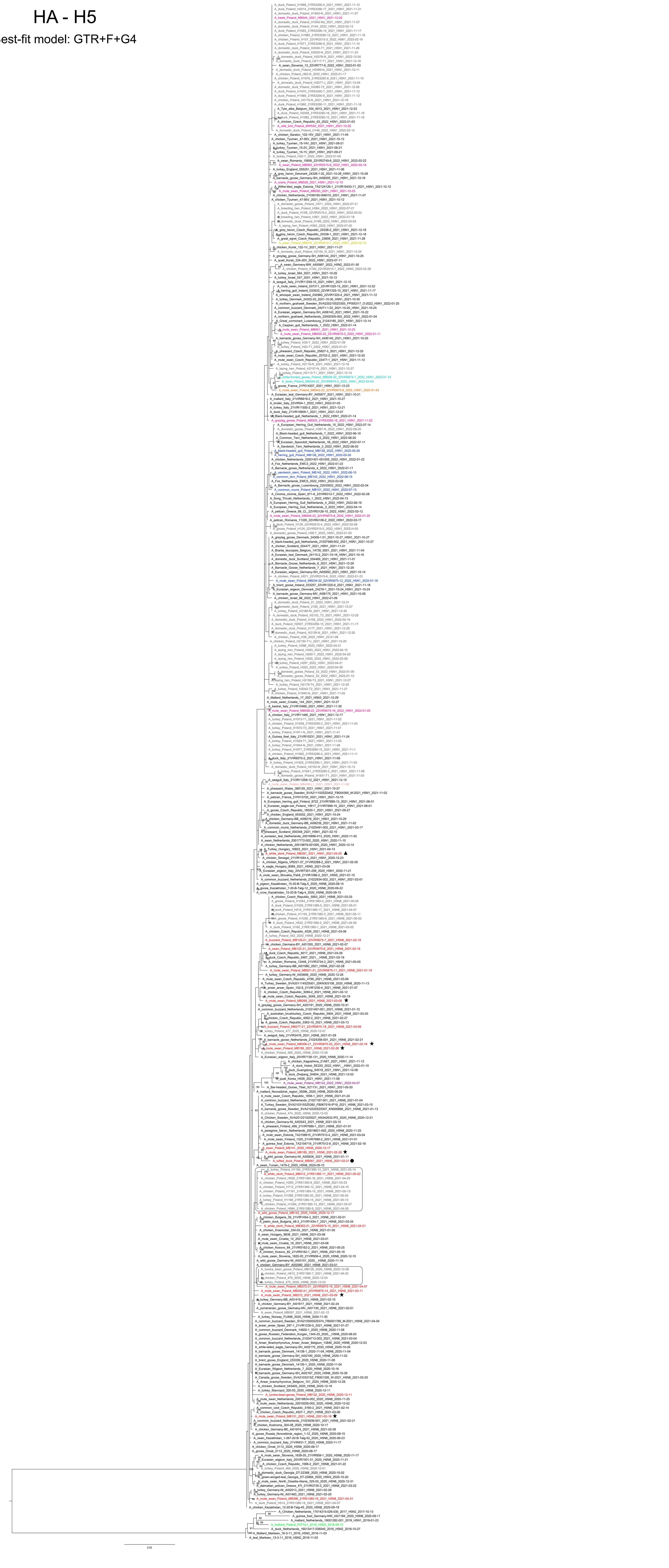

# HA - H9

## Best-fit model: TIM+F+G4

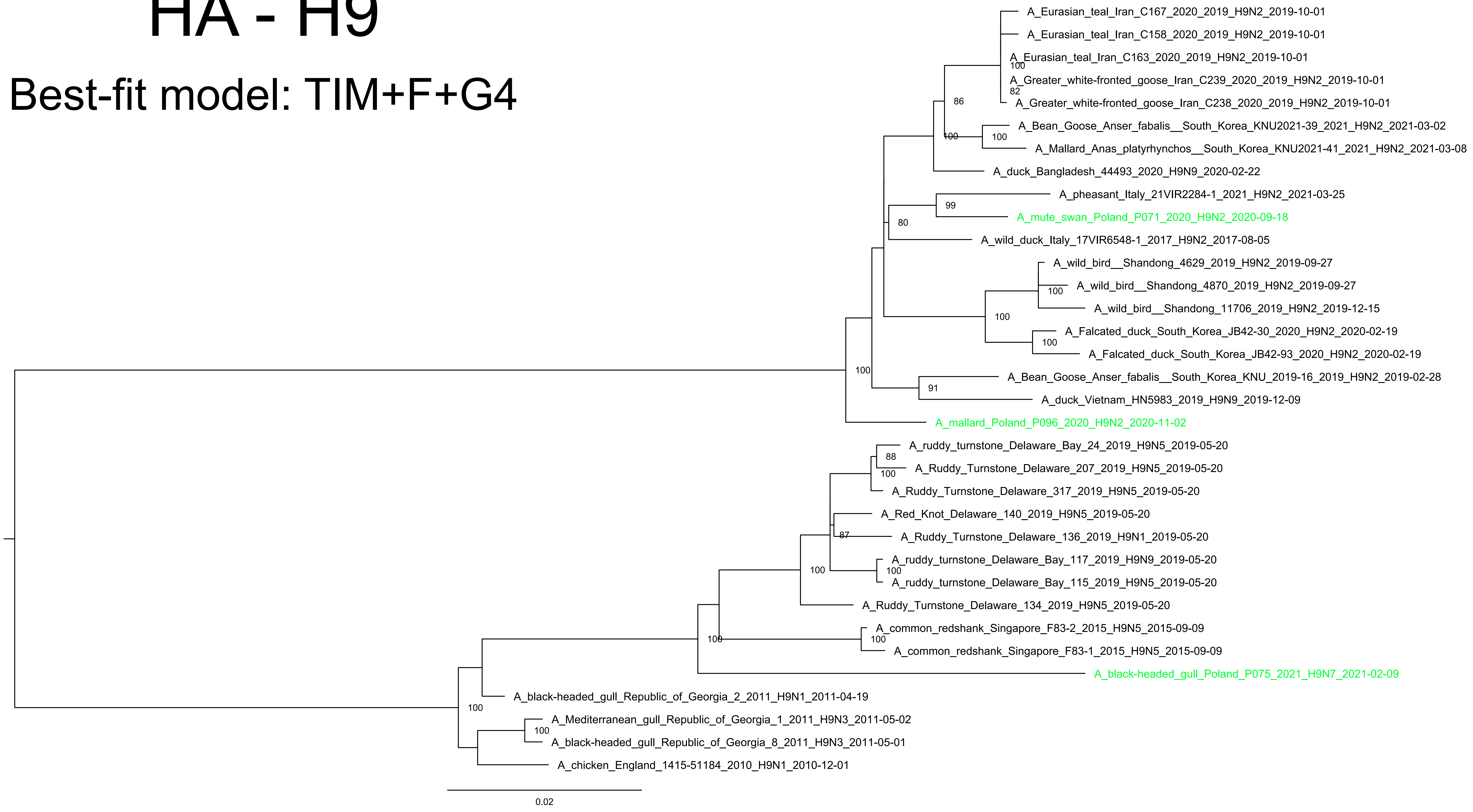

# HA - H12

Best-fit model: TPM3u+F+G4

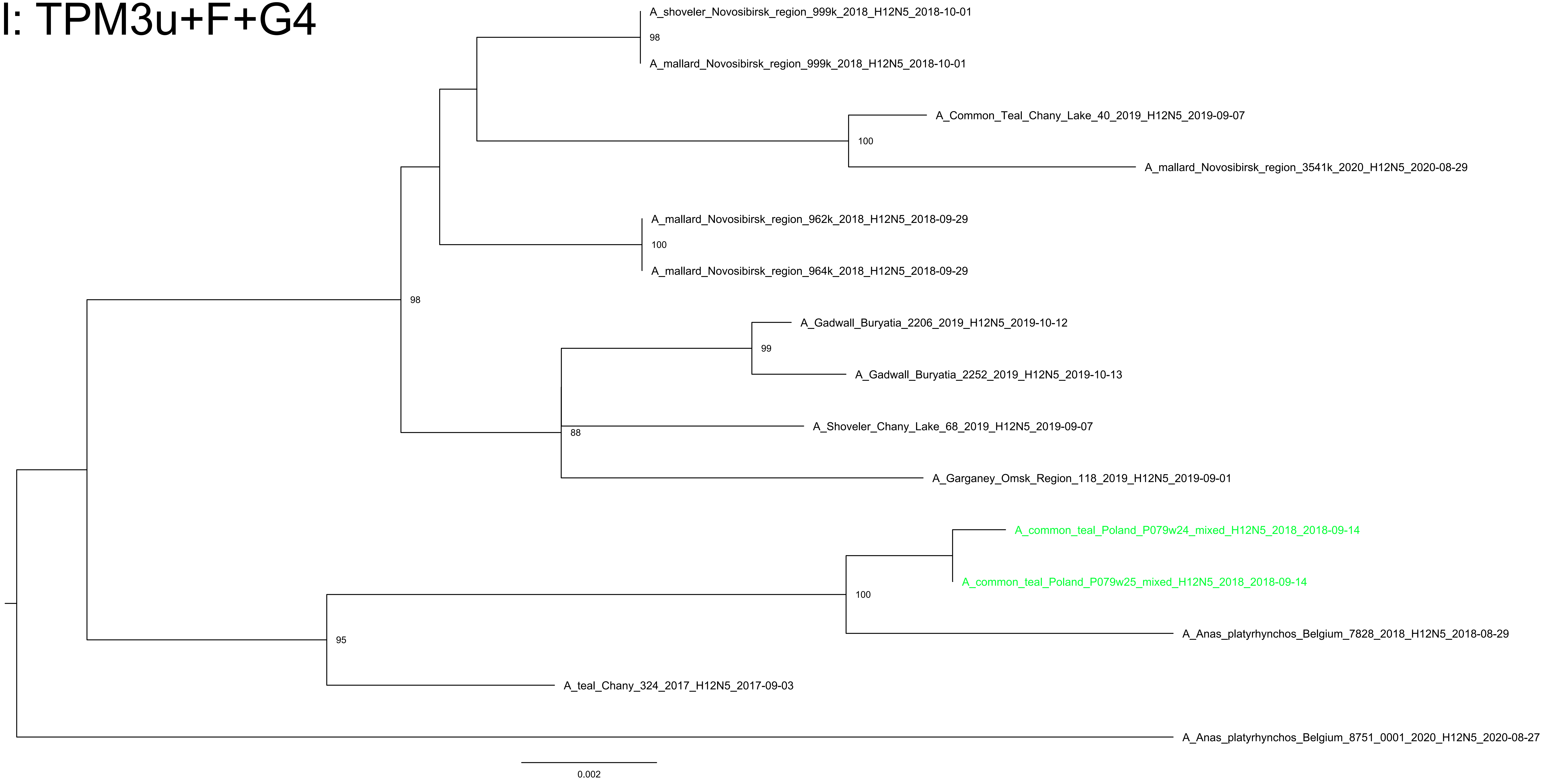

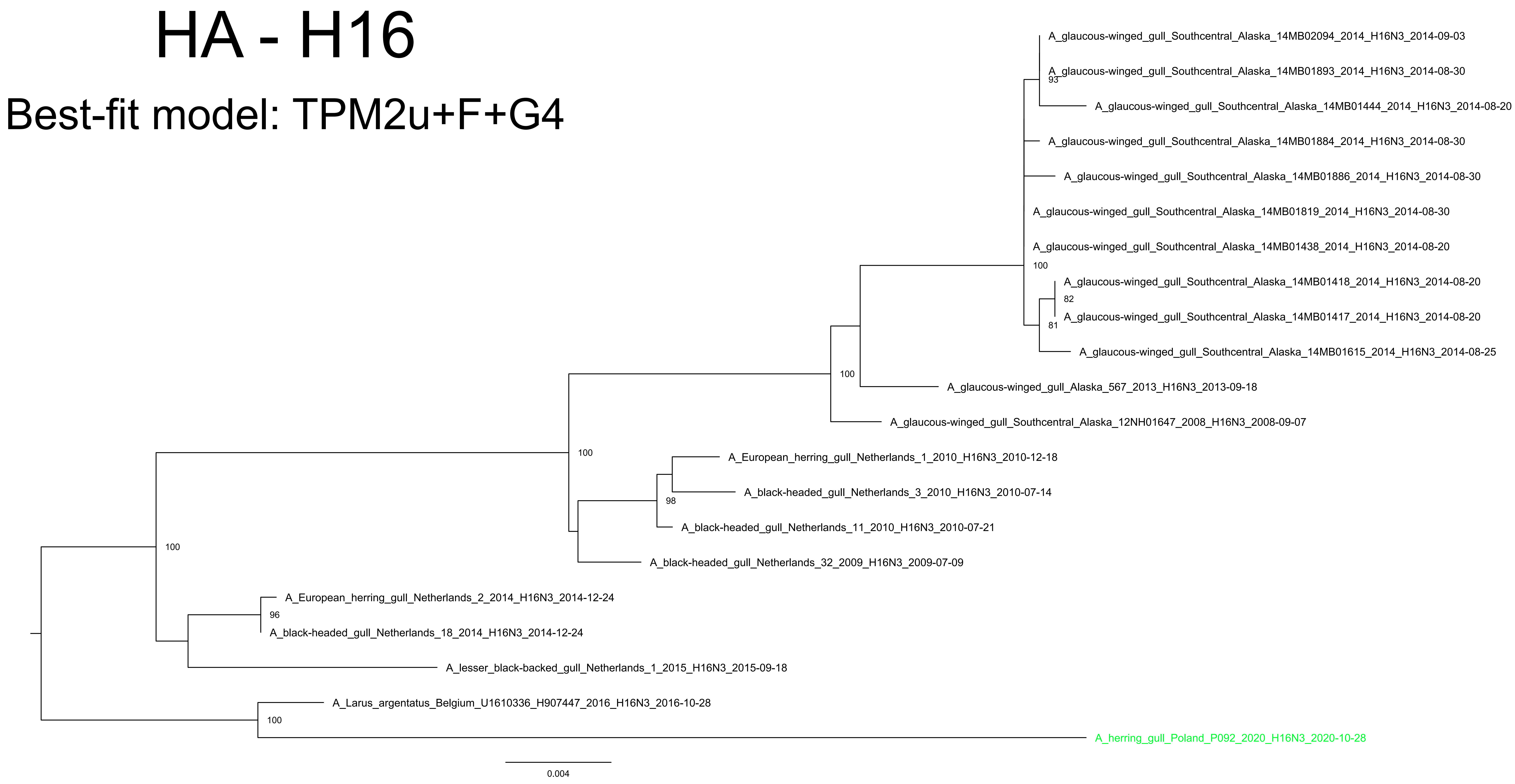
HA - H16
Best-fit model: TPM2u+F+G4
A_glaucous-winged_gull_Southcentral_Alaska_14MB02094_2014_H16N3_2014-09-03
A_glaucous-winged_gull_Southcentral_Alaska_14MB01893_2014_H16N3_2014-08-30
93
A_glaucous-winged_gull_Southcentral_Alaska_14MB01444_2014_H16N3_2014-08-20
A_glaucous-winged_gull_Southcentral_Alaska_14MB01884_2014_H16N3_2014-08-30
A_glaucous-winged_gull_Southcentral_Alaska_14MB01886_2014_H16N3_2014-08-30
A_glaucous-winged_gull_Southcentral_Alaska_14MB01819_2014_H16N3_2014-08-30
A_glaucous-winged_gull_Southcentral_Alaska_14MB01438_2014_H16N3_2014-08-20
100
A_glaucous-winged_gull_Southcentral_Alaska_14MB01418_2014_H16N3_2014-08-20
82
A_glaucous-winged_gull_Southcentral_Alaska_14MB01417_2014_H16N3_2014-08-20
81
A_glaucous-winged_gull_Southcentral_Alaska_14MB01615_2014_H16N3_2014-08-25
100
A_glaucous-winged_gull_Alaska_567_2013_H16N3_2013-09-18
A_glaucous-winged_gull_Southcentral_Alaska_12NH01647_2008_H16N3_2008-09-07
100
A_European_herring_gull_Netherlands_1_2010_H16N3_2010-12-18
A_black-headed_gull_Netherlands_3_2010_H16N3_2010-07-14
98
A_black-headed_gull_Netherlands_11_2010_H16N3_2010-07-21
A_black-headed_gull_Netherlands_32_2009_H16N3_2009-07-09
100
A_European_herring_gull_Netherlands_2_2014_H16N3_2014-12-24
96
A_black-headed_gull_Netherlands_18_2014_H16N3_2014-12-24
A_lesser_black-backed_gull_Netherlands_1_2015_H16N3_2015-09-18
A_Larus_argentatus_Belgium_U1610336_H907447_2016_H16N3_2016-10-28
100
A_herring_gull_Poland_P092_2020_H16N3_2020-10-28
0.004

# NP

Best-fit model: TVM+F+I+G4

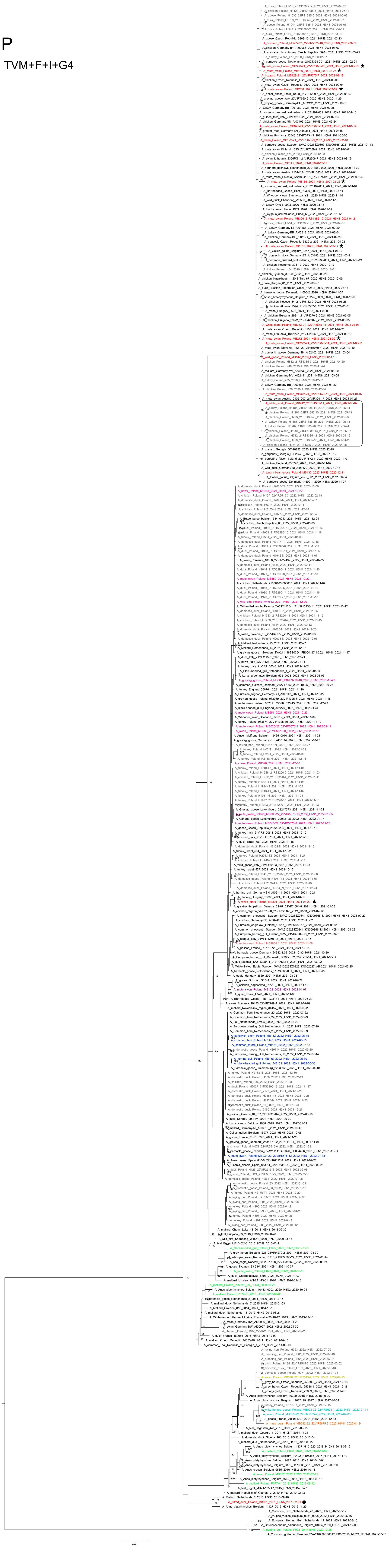

# NA - N1

Best-fit model: HKY+F+I

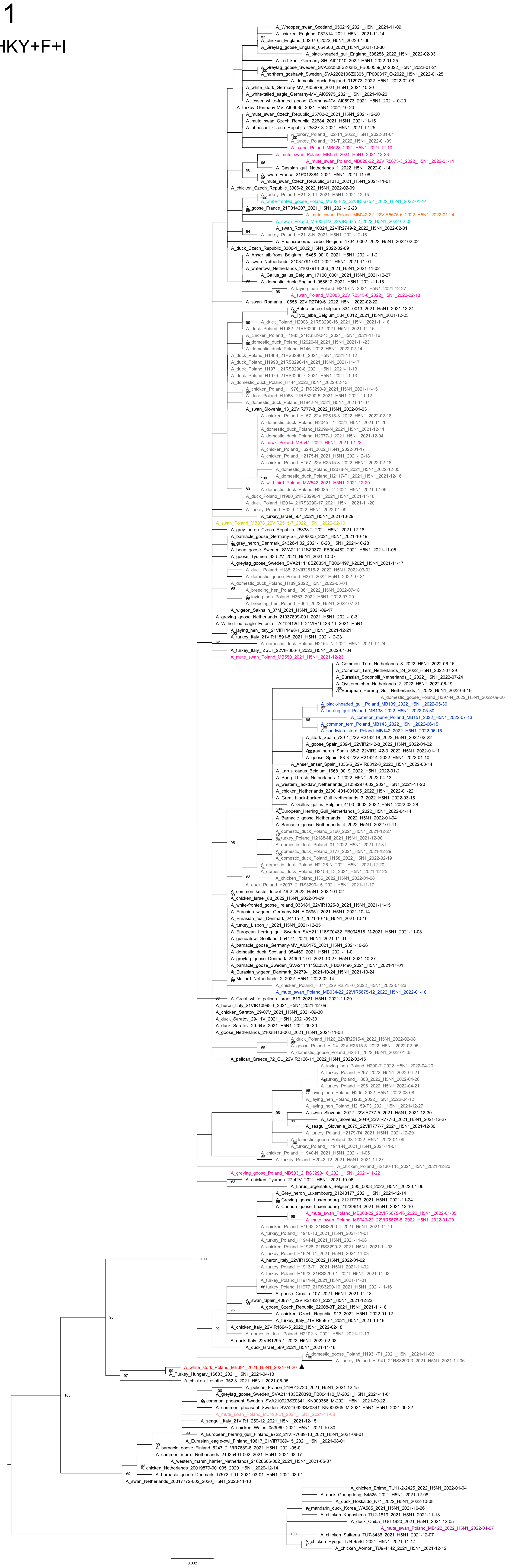

# NA - N2

## Best-fit model: HKY+F+I

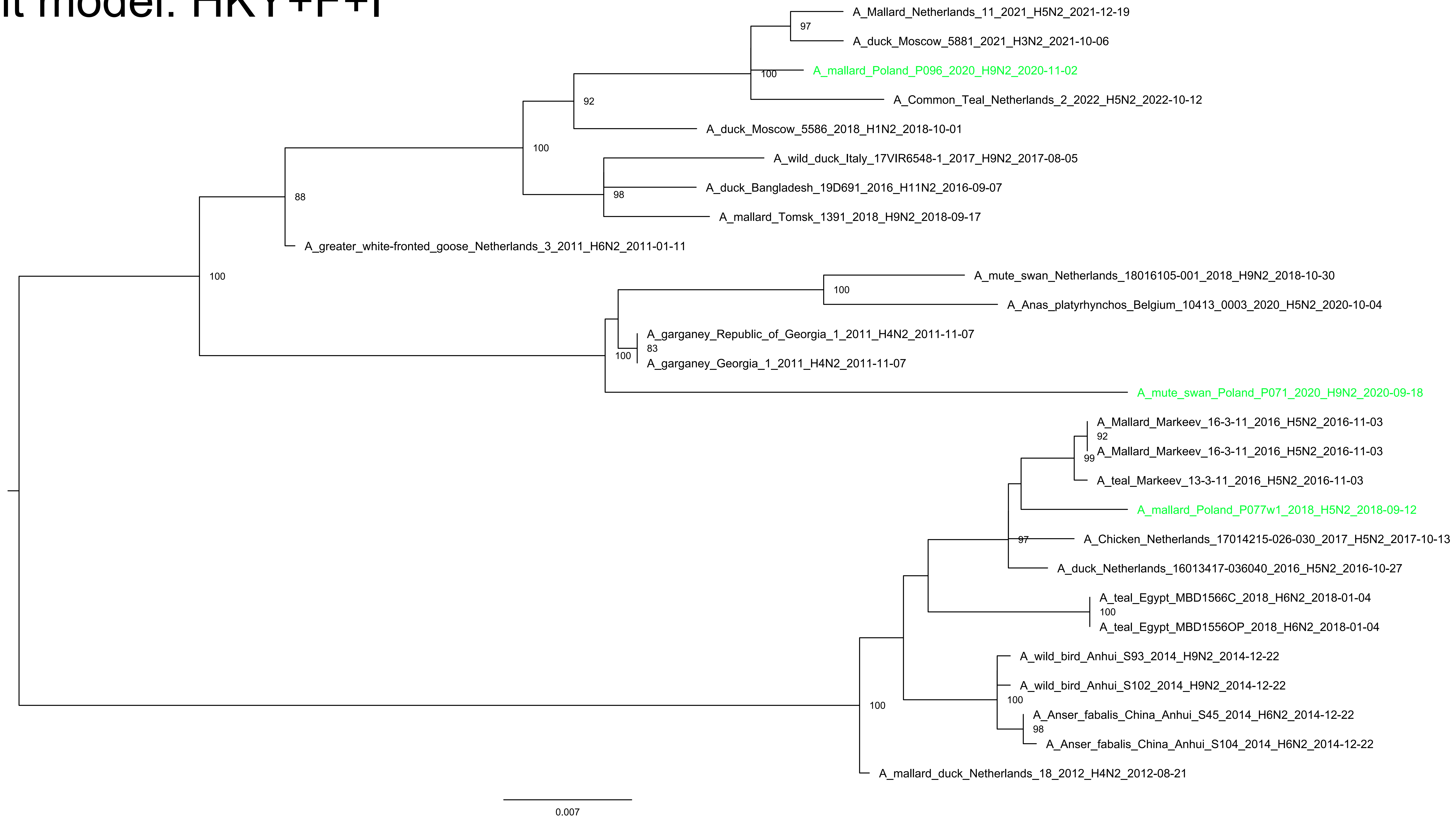

# NA - N3

## Best-fit model: HKY+F+I

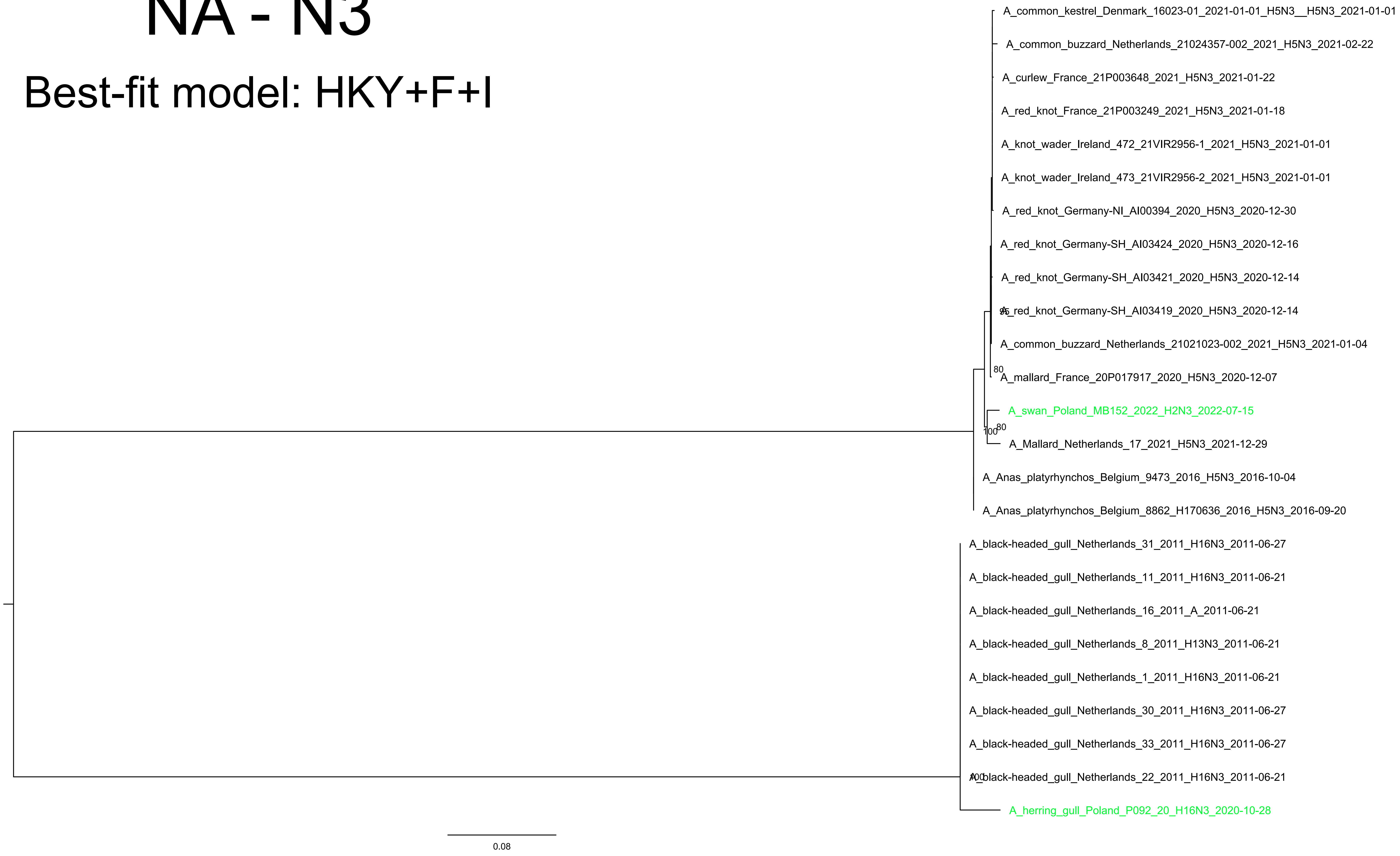

# NA - N5

Best-fit model: HKY+F+G4

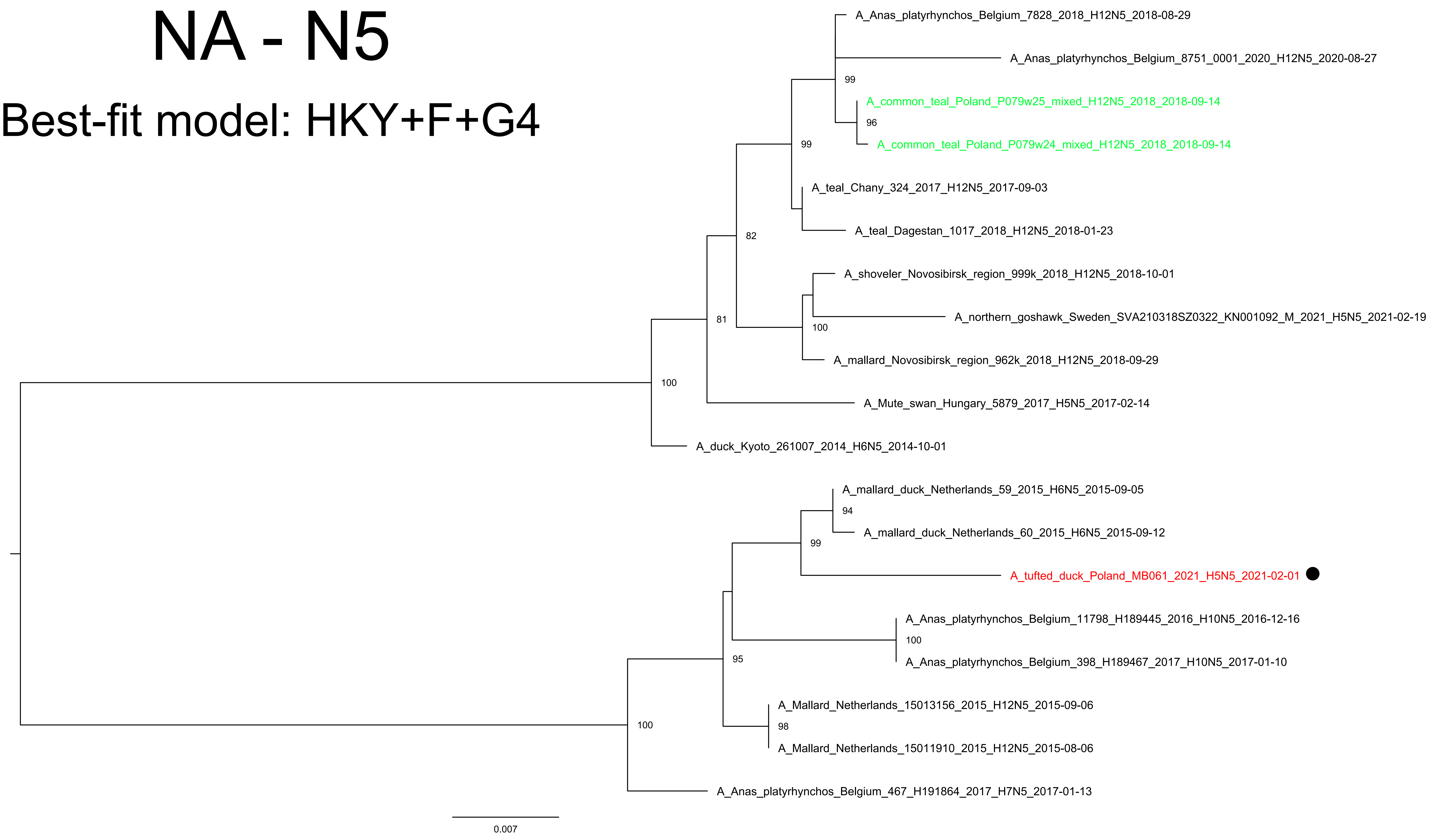

# NA - N7

## Best-fit model: TN+F

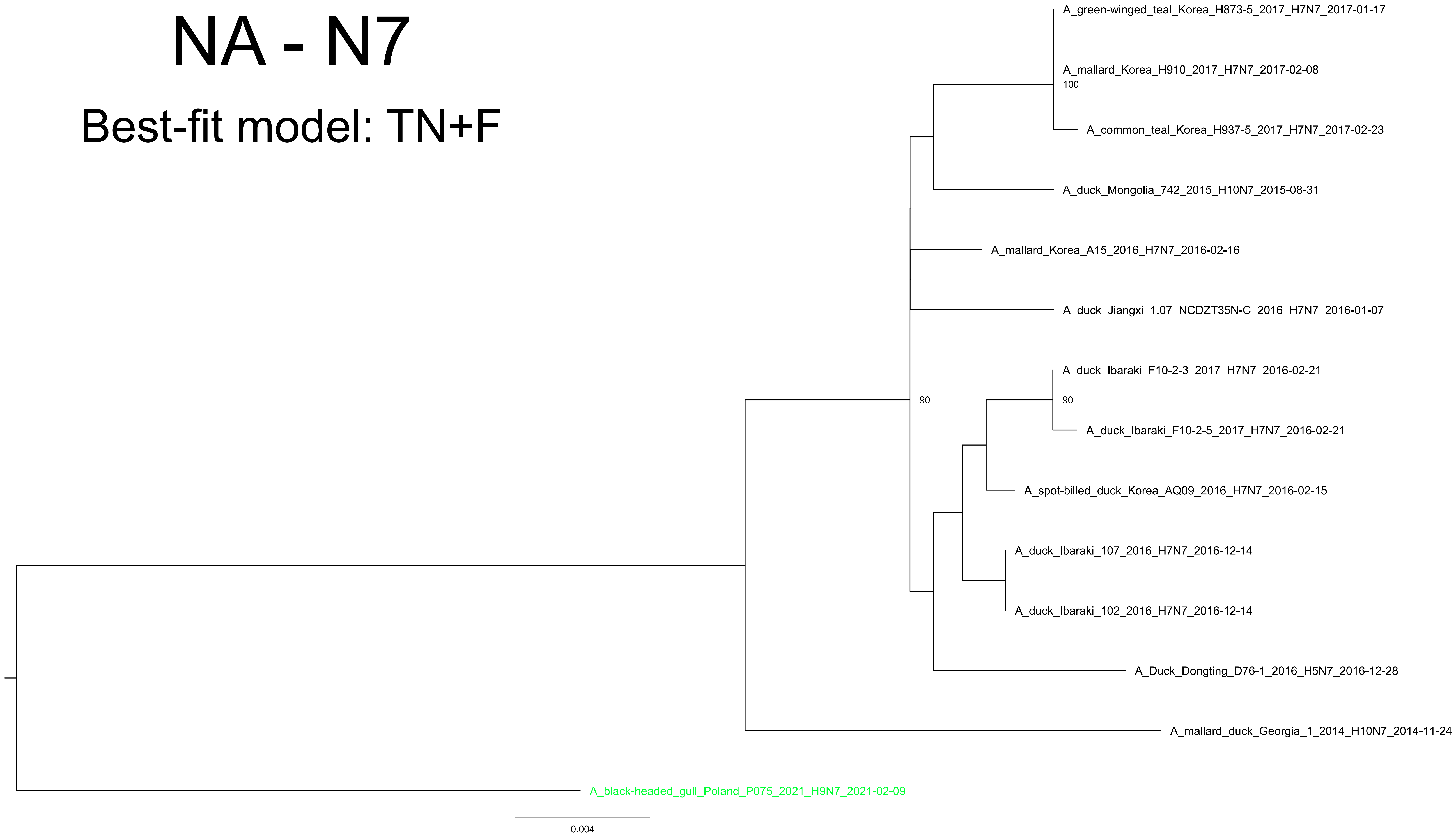

# NA - N8

Best-fit model: K3Pu+F+G4

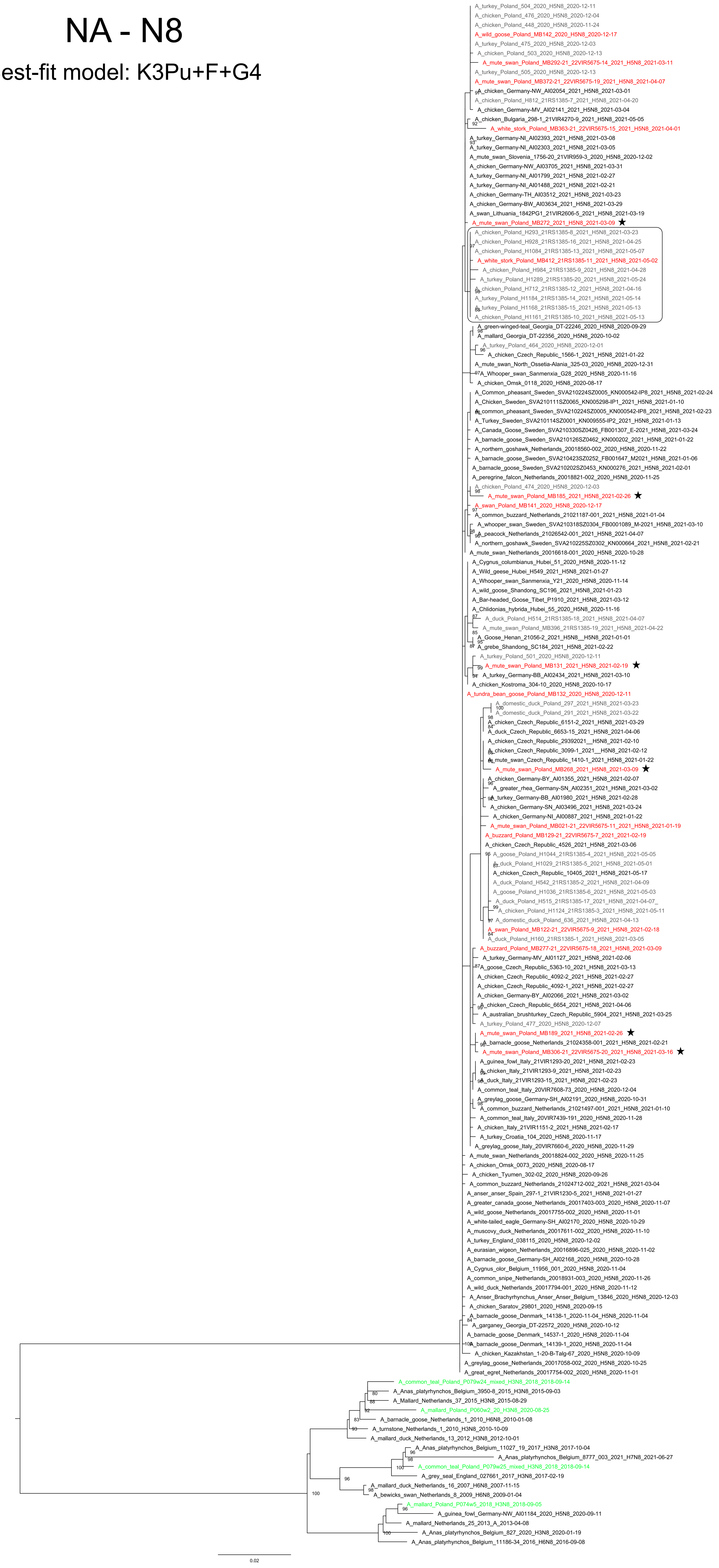

# MP

Best-fit model: TIMe+I+G4

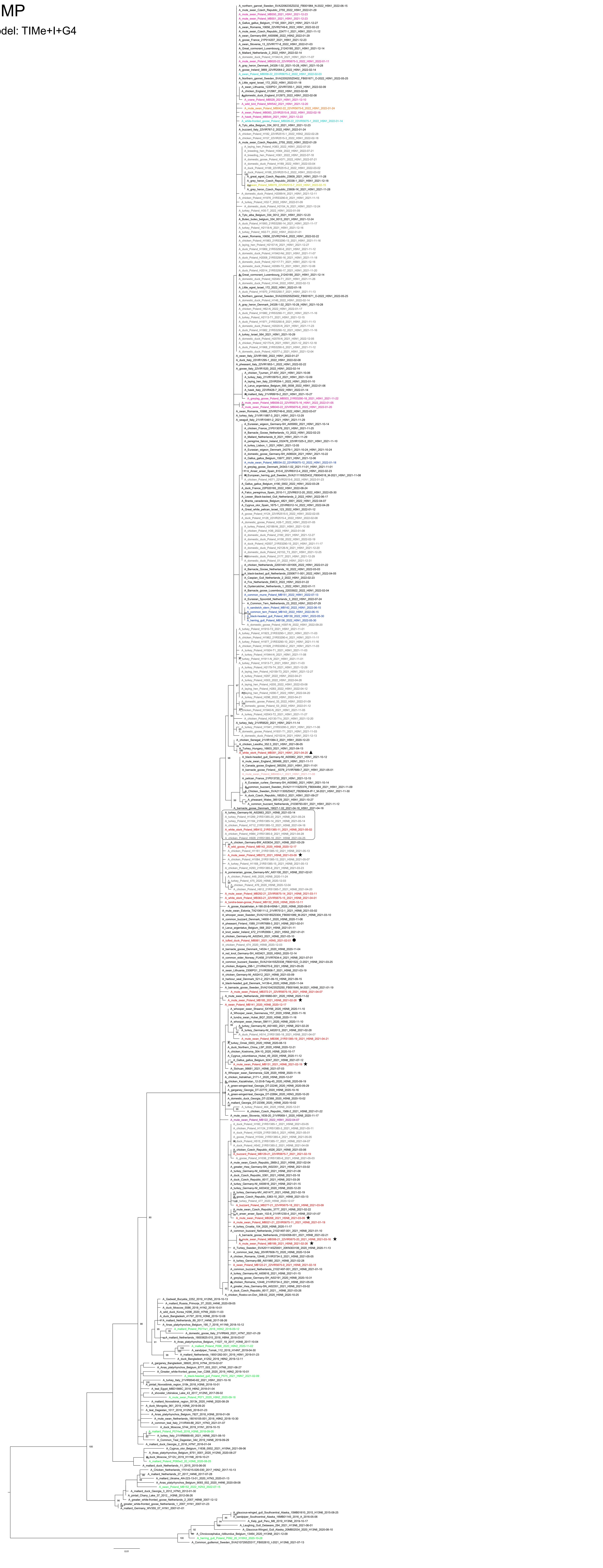

# NS

Best-fit model: K3Pu+F+G4

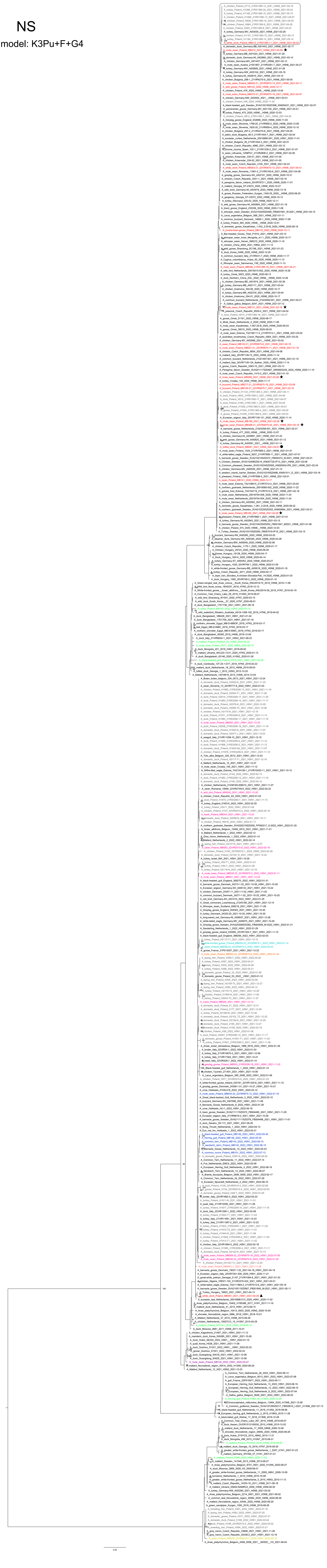

Supplement: Supplementary 3 — Phylogenetic trees. [file 6661672.f3.pdf]
