# Supplementary figures and images for: Phylogenetic and Molecular Characteristics of Wild Bird-Origin Avian Influenza Viruses Circulating in Poland in 2018−2022: Reassortment, Multiple Introductions, and Wild Bird–Poultry Epidemiological Links
Source: Transbound Emerg Dis. 2024 Apr 12;2024:6661672. doi: 10.1155/2024/6661672 (PMC12017110; doi:10.1155/2024/6661672)

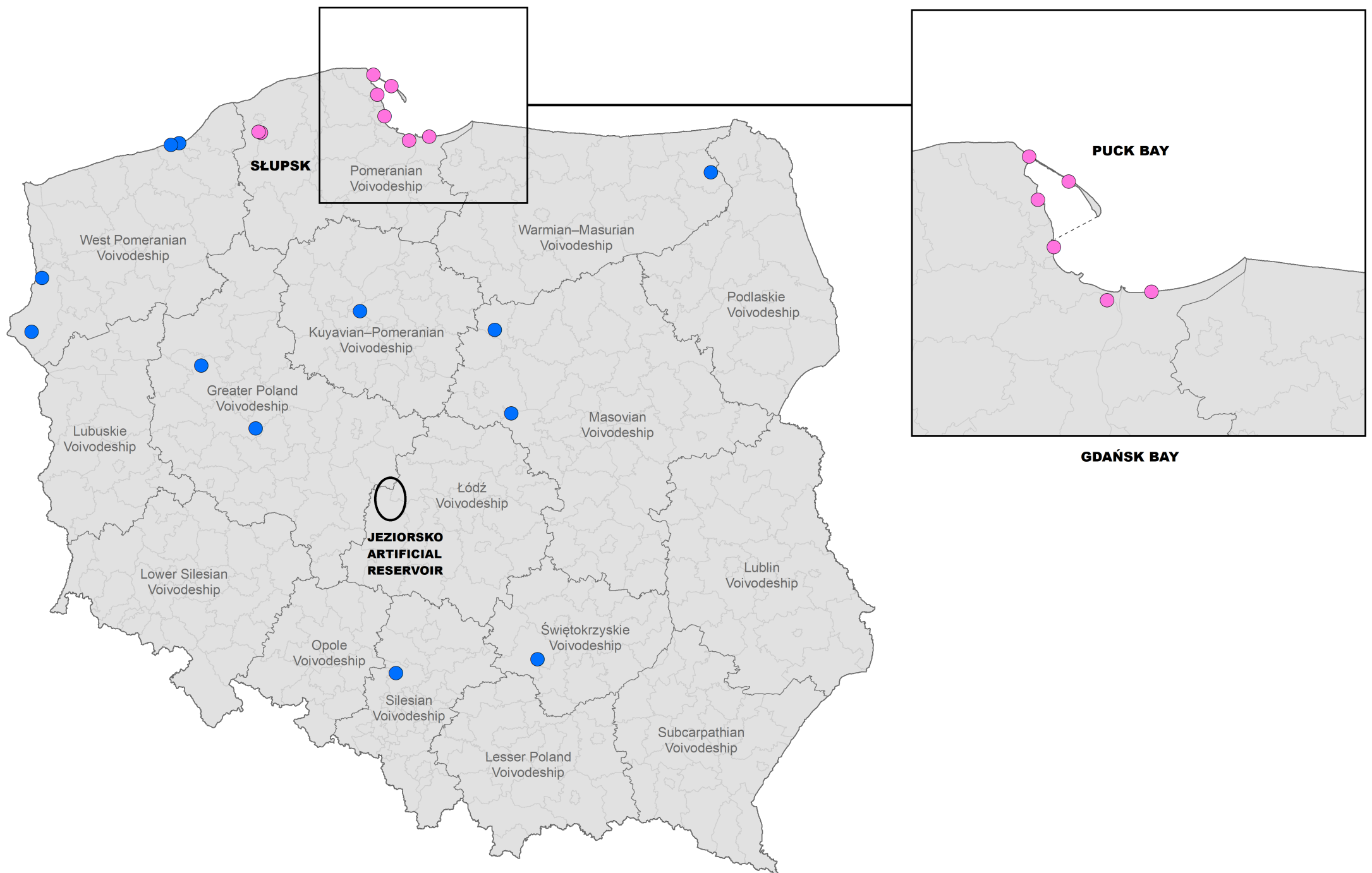

## Different virus introductions during HPAI 2020-2021

- HPAIV H5N8 introductions
- HPAIV H5Nx 2020-2021

Supplement: Supplementary 4 — Different H5N8 introductions. [file 6661672.f4.pdf]
