# Supplementary material for: Phylogenetic and Molecular Characteristics of Wild Bird-Origin Avian Influenza Viruses Circulating in Poland in 2018−2022: Reassortment, Multiple Introductions, and Wild Bird–Poultry Epidemiological Links
Source: Transbound Emerg Dis. 2024 Apr 12;2024:6661672. doi: 10.1155/2024/6661672 (PMC12017110; doi:10.1155/2024/6661672)
Supplement: Supplementary 5 — HACS motifs. [file 6661672.f5.pdf]

		HA CLEAVAGE SITE
ACTIVE SURVEILLANCE 2018-2021 (LPAIVs)	A/mallard/Poland/P074w5/2018_H3N8_2018-09-05	LP H3: PEKQTR/GLF
	A/mallard/Poland/P077w1/2018_H5N2_2018-09-12	LP H5: PQRETR/GLF
	A/common_teal/P079w24/2018_H3-H12-N8-N5_2018_09_14	LP H3: PEKQTR/GLF, LP H12: PQVQDR/GLF
	A/common_teal/P079w24/2018_H3-H12-N8-N5_2018_09_14	
	A/mallard/Poland/P060w2/2020_H3N8_2020-08-25	LP H3: PEKQTR/GLF
	A/mute_swan/Poland/P071/2020_H9N2_2020-09-18	LP H9: PAVSDR/GLF
	A/herring_gull/Poland/P092/2020_H16N3_2020-10-28	LP H16: PSINER/GLF
	A/mallard/Poland/P096/2020_H9N2_2020-11-02	LP H9: PAASDR/GLF
	A/black-headed_gull/Poland/P075/2021_H9N7_2021-02-09	LP H9: PATSDK/GLF
PASSIVE SURVEILLANCE HPAI 2020/2021 (H5N8, H5N1, H5N5)	A/tundra-bean-goose/Poland/MB132/2020_H5N8_2020-12-11	HP H5: PLREKRRKR/GLF
	A/swan/Poland/MB141/2020_H5N8_2020-12-17	
	A/wild_goose/Poland/MB142/2020_H5N8_2020-12-17	
	A/mute_swan/Poland/MB021-21_22VIR5675-11/2021_H5N8_2021-01-19	
	A/tufted_duck/Poland/MB061/2021_H5N5_2021-02-01	
	A/swan/Poland/MB122-21_22VIR5675-9/2021_H5N8_2021-02-18	
	A/buzzard/Poland/MB129-21_22VIR5675-7/2021_2021-02-19	
	A/mute_swan/Poland/MB131/2021_H5N8_2021-02-19	
	A/mute_swan/Poland/MB185/2021_H5N8_2021-02-26	
	A/mute_swan/Poland/MB189/2021_H5N8_2021-02-26	
	A/mute_swan/Poland/MB268/2021_H5N8_2021-03-09	
	A/mute_swan/Poland/MB272/2021_H5N8_2021-03-09	
	A/buzzard/Poland/MB277-21_22VIR5675-18/2021_H5N8_2021-03-09	
	A/mute_swan/Poland/MB292-21_22VIR5675-14/2021_H5N8_2021-03-11	
	A/mute_swan/Poland/MB306-21_22VIR5675-20/2021_H5N8_2021-03-16	
	A/white_stork/Poland/MB363-21_22VIR5675-15/2021_H5N8_2021-04-01	
	A/mute_swan/Poland/MB372-21_22VIR5675-19/2021_H5N8_2021-04-07	
	A/white_stork/Poland/MB391/2021_H5N1_2021-04-20	
	A/mute_swan/Poland/MB396_21RS1385-19/2021_H5N8_2021-04-21	
	A/white_stork/Poland/MB412_21RS1385-11/2021_H5N8_2021-05-02	
PASSIVE SURVEILLANCE HPAI 2021/2022 (H5N1) + LPAIV H2N3	A/mute_swan/Poland/MB490-L1/2021_H5N1_2021-11-08	HP H5: **S**LREKRRKR/GLF
	A/greylag_goose/Poland/MB503_21RS3290-18/2021_H5N1_2021-11-22	HP H5: PLREKRRKR/GLF
	A/crane/Poland/MB528/2021_H5N1_2021-12-10	
	A/wild_bird/Poland/MW542/2021_H5N1_2021-12-20	
	A/hawk/Poland/MB544/2021_H5N1_2021-12-22	
	A/mute_swan/Poland/MB550/2021_H5N1_2021-12-23	
	A/mute_swan/Poland/MB551/2021_H5N1_2021-12-23	
	A/mute_swan/Poland/MB008-22_22VIR5675-16/2022_2022-01-05	
	A/mute_swan/Poland/MB020-22_22VIR5675-3/2022_H5N1_2022-01-11	
	A/white-fronted_goose/Poland/MB028-22_22VIR5675-1/2022_2022-01-14	
	A/mute_swan/Poland/MB034-22_22VIR5675-12/2022_H5N1_2022-01-18	
	A/mute_swan/Poland/MB040-22_22VIR5675-8/2022_H5N1_2022-01-20	
	A/mute_swan/Poland/MB042-22_22VIR5675-6/2022_H5N1_2022-01-24	
	A/swan/Poland/MB058-22_22VIR5675-2/2022_H5N1_2022-02-03	
	A/swan/Poland/MB078_22VIR2515-7/2022_H5N1_2022-02-15	
	A/swan/Poland/MB083_22VIR2515-8/2022_H5N1_2022-02-18	
	A/mute_swan/Poland/MB122/2022_H5N1_2022-04-07	HP H5: PLRE**R**RRKR/GLF
	A/herring_gull/Poland/MB138/2022_H5N1_2022-05-30	HP H5: PLREKRRKR/GLF
	A/black-headed_gull/Poland/MB139/2022_H5N1_2022-05-30	
	A/sandwich_stern/Poland/MB142/2022_H5N1_2022-06-15	
	A/common_tern/Poland/MB143/2022_H5N1_2022-06-15	
	A/common_murre/Poland/MB151/2022_H5N1_2022-07-13	
	A/swan/Poland/MB152/2022_H2N3_2022-07-15	LP H2: PQIESR/GLF
