# Supplementary material for: Phylogenetic and Molecular Characteristics of Wild Bird-Origin Avian Influenza Viruses Circulating in Poland in 2018−2022: Reassortment, Multiple Introductions, and Wild Bird–Poultry Epidemiological Links
Source: Transbound Emerg Dis. 2024 Apr 12;2024:6661672. doi: 10.1155/2024/6661672 (PMC12017110; doi:10.1155/2024/6661672)
Supplement: Supplementary 6 — Genetic markers. [file 6661672.f6.pdf]

		PB2								
		D9N	I292V	K389R	K526R	Q591K	V598T	S715N	L89V + G309D	L89V + G309D + T339K + R477G + I495V + K627E + A676T
ACTIVE SURVEILLANCE 2018-2021 (LPAIVs)	A/mallard/Poland/P074w5/2018_H3N8_2018-09-05			X			X	X	X	X
	A/mallard/Poland/P077w1/2018_H5N2_2018-09-12			X			X	X	X	X
	A/common_teal/P079w24/2018_H3-H12-N8-N5_2018_09_14			X			X	X	X	X
	A/common_teal/P079w24/2018_H3-H12-N8-N5_2018_09_14			X			X	X	X	X
	A/mallard/Poland/P060w2/2020_H3N8_2020-08-25			X		X	X	X	X	X
	A/mute_swan/Poland/P071/2020_H9N2_2020-09-18			X			X	X	X	X
	A/herring_gull/Poland/P092/2020_H16N3_2020-10-28		X	X			X	X	X	X
	A/mallard/Poland/P096/2020_H9N2_2020-11-02			X			X	X	X	
	A/black-headed_gull/Poland/P075/2021_H9N7_2021-02-09			X			X	X	X	X
PASSIVE SURVEILLANCE HPAI 2020/2021 (H5N8, H5N1, H5N5)	A/tundra-bean-goose/Poland/MB132/2020_H5N8_2020-12-11		X	X			X	X	X	X
	A/swan/Poland/MB141/2020_H5N8_2020-12-17		X	X			X	X	X	X
	A/wild_goose/Poland/MB142/2020_H5N8_2020-12-17		X	X			X	X	X	X
	A/mute_swan/Poland/MB021-21_22VIR5675-11/2021_H5N8_2021-01-19		X	X			X	X	X	X
	A/tufted_duck/Poland/MB061/2021_H5N5_2021-02-01			X			X	X	X	X
	A/swan/Poland/MB122-21_22VIR5675-9/2021_H5N8_2021-02-18		X	X			X	X	X	X
	A/buzzard/Poland/MB129-21_22VIR5675-7/2021_2021-02-19		X	X			X	X	X	X
	A/mute_swan/Poland/MB131/2021_H5N8_2021-02-19		X	X			X	X	X	X
	A/swan/Poland/MB185/2021_H5N8_2021-02-26		X	X			X	X	X	X
	A/mute_swan/Poland/MB189/2021_H5N8_2021-02-26		X	X			X	X	X	X
	A/mute_swan/Poland/MB268/2021_H5N8_2021-03-09		X	X			X	X	X	X
	A/mute_swan/Poland/MB272/2021_H5N8_2021-03-09		X	X			X	X	X	X
	A/buzzard/Poland/MB277-21_22VIR5675-18/2021_H5N8_2021-03-09		X	X			X	X	X	X
	A/avian/Poland/MB292-21_22VIR5675-14/2021_H5N8_2021-03-11		X	X			X	X	X	X
	A/mute_swan/Poland/MB306-21_22VIR5675-20/2021_H5N8_2021-03-16		X	X			X	X	X	X
	A/white_stork/Poland/MB363-21_22VIR5675-15/2021_H5N8_2021-04-01		X	X			X	X	X	X
	A/mute_swan/Poland/MB372-21_22VIR5675-19/2021_H5N8_2021-04-07		X	X			X	X	X	X
	A/white_stork/Poland/MB391/2021_H5N1_2021-04-20			X			X	X	X	X
	A/mute_swan/Poland/MB396_21RS1385-19/2021_H5N8_2021-04-21		X	X			X	X	X	X
	A/white_stork/Poland/MB412_21RS1385-11/2021_H5N8_2021-05-02		X	X			X	X	X	X
PASSIVE SURVEILLANCE HPAI 2021/2022 (H5N1) + LPAIV H2N3	A/mute_swan/Poland/MB490-L1/2021_H5N1_2021-11-08			X			X	X	X	X
	A/greylag_goose/Poland/MB503_21RS3290-18/2021_H5N1_2021-11-22			X			X	X	X	X
	A/crane/Poland/MB528/2021_H5N1_2021-12-10			X			X	X	X	X
	A/wild_bird/Poland/MW542/2021_H5N1_2021-12-20	X		X			X	X		
	A/hawk/Poland/MB544/2021_H5N1_2021-12-22	X		X			X	X		
	A/mute_swan/Poland/MB550/2021_H5N1_2021-12-23			X			X	X		
	A/mute_swan/Poland/MB551/2021_H5N1_2021-12-23			X			X	X	X	X
	A/mute_swan/Poland/MB008-22_22VIR5675-16/2022_2022-01-05						X	X	X	NA
	A/mute_swan/Poland/MB020-22_22VIR5675-3/2022_H5N1_2022-01-11			X			X	X	X	X
	A/white-fronted_goose/Poland/MB028-22_22VIR5675-1/2022_2022-01-14			X			X	X	X	X
	A/mute_swan/Poland/MB034-22_22VIR5675-12/2022_H5N1_2022-01-18			X	X		X	X	X	X
	A/mute_swan/Poland/MB040-22_22VIR5675-8/2022_H5N1_2022-01-20			NA	NA			X	X	NA
	A/mute_swan/Poland/MB042-22_22VIR5675-6/2022_H5N1_2022-01-24			X			X	X		
	A/swan/Poland/MB058-22_22VIR5675-2/2022_H5N1_2022-02-03			X			X	X	X	X
	A/swan/Poland/MB078_22VIR2515-7/2022_H5N1_2022-02-15			X			X	X	X	X
	A/swan/Poland/MB083_22VIR2515-8/2022_H5N1_2022-02-18			X			X	X	X	X
	A/mute_swan/Poland/MB122/2022_H5N1_2022-04-07			X			X	X	X	
	A/herring_gull/Poland/MB138/2022_H5N1_2022-05-30			X			X	X	X	X
	A/black-headed_gull/Poland/MB139/2022_H5N1_2022-05-30			X			X	X	X	X
	A/sandwich_stern/Poland/MB142/2022_H5N1_2022-06-15			X			X	X	X	X
	A/common_tern/Poland/MB143/2022_H5N1_2022-06-15			X			X	X	X	X
	A/common_mure/Poland/MB151/2022_H5N1_2022-07-13			X			X	X	X	X
	A/swan/Poland/MB152/2022_H2N3_2022-07-15			X			X	X	X	X

NA = not applicable ("N" in the sequence)
X = present in the sequence

		PB1			PB1-F2	
		D3V	D622G	S678N	truncated to 11 aa	N66S
ACTIVE SURVEILLANCE 2018-2021 (LPAIVs)	A/mallard/Poland/P074w5/2018_H3N8_2018-09-05	X	X			X
	A/mallard/Poland/P077w1/2018_H5N2_2018-09-12	X	X			
	A/common_teal/P079w24/2018_H3-H12-N8-N5_2018_09_14	X	X			
	A/common_teal/P079w24/2018_H3-H12-N8-N5_2018_09_14	X	X			
	A/mallard/Poland/P060w2/20_H3N8_2020-08-25	X	X			X
	A/mute_swan/Poland/P071/2020_H9N2_2020-09-18	X	X			
	A/herring_gull/Poland/P092/20_H16N3_2020-10-28	X	X			
	A/mallard/Poland/P096/2020_H9N2_2020-11-02	X	X			X
	A/black-headed_gull/Poland/P075/2021_H9N7_2021-02-09	X	X			X
PASSIVE SURVEILLANCE HPAI 2020/2021 (H5N8, H5N1, H5N5)	A/tundra-bean-goose/Poland/MB132/2020_H5N8_2020-12-11	X	X		X	
	A/swan/Poland/MB141/2020_H5N8_2020-12-17	X	X		X	
	A/wild_goose/Poland/MB142/2020_H5N8_2020-12-17	X	X		X	
	A/mute_swan/Poland/MB021-21_22VIR5675-11/2021_H5N8_2021-01-19	X	X		X	
	A/tufted_duck/Poland/MB061/2021_H5N5_2021-02-01	X	X			
	A/swan/Poland/MB122-21_22VIR5675-9/2021_H5N8_2021-02-18	X	X		X	
	A/buzzard/Poland/MB129-21_22VIR5675-7/2021_2021-02-19	X	X		X	
	A/mute_swan/Poland/MB131/2021_H5N8_2021-02-19	X	X		X	
	A/swan/Poland/MB185/2021_H5N8_2021-02-26	X	X		X	
	A/mute_swan/Poland/MB189/2021_H5N8_2021-02-26	X	X		X	
	A/mute_swan/Poland/MB268/2021_H5N8_2021-03-09	X	X		X	
	A/mute_swan/Poland/MB272/2021_H5N8_2021-03-09	X	X		X	
	A/buzzard/Poland/MB277-21_22VIR5675-18/2021_H5N8_2021-03-09	X	X		X	
	A/avian/Poland/MB292-21_22VIR5675-14/2021_H5N8_2021-03-11	X	X		X	
	A/mute_swan/Poland/MB306-21_22VIR5675-20/2021_H5N8_2021-03-16	X	X		X	
	A/white_stork/Poland/MB363-21_22VIR5675-15/2021_H5N8_2021-04-01	X	X		X	
	A/mute_swan/Poland/MB372-21_22VIR5675-19/2021_H5N8_2021-04-07	X	X		X	
	A/white_stork/Poland/MB391/2021_H5N1_2021-04-20	X	X			
	A/mute_swan/Poland/MB396_21RS1385-19/2021_H5N8_2021-04-21	X	X	X	X	
	A/white_stork/Poland/MB412_21RS1385-11/2021_H5N8_2021-05-02	X	X		X	
PASSIVE SURVEILLANCE HPAI 2021/2022 (H5N1) + LPAIV H2N3	A/mute_swan/Poland/MB490-L1/2021_H5N1_2021-11-08	X	X			X
	A/greylag_goose/Poland/MB503_21RS3290-18/2021_H5N1_2021-11-22	X	X			X
	A/crane/Poland/MB528/2021_H5N1_2021-12-10	X	X			X
	A/wild_bird/Poland/MW542/2021_H5N1_2021-12-20	X	X			X
	A/hawk/Poland/MB544/2021_H5N1_2021-12-22	X	X			X
	A/mute_swan/Poland/MB550/2021_H5N1_2021-12-23	X	X			X
	A/mute_swan/Poland/MB551/2021_H5N1_2021-12-23	X	X			X
	A/mute_swan/Poland/MB008-22_22VIR5675-16/2022_2022-01-05	X	X			X
	A/mute_swan/Poland/MB020-22_22VIR5675-3/2022_H5N1_2022-01-11	X	X			X
	A/white-fronted_goose/Poland/MB028-22_22VIR5675-1/2022_2022-01-14	X	X			X
	A/mute_swan/Poland/MB034-22_22VIR5675-12/2022_H5N1_2022-01-18	X	X			X
	A/mute_swan/Poland/MB040-22_22VIR5675-8/2022_H5N1_2022-01-20	X	X			X
	A/mute_swan/Poland/MB042-22_22VIR5675-6/2022_H5N1_2022-01-24	X	X			X
	A/swan/Poland/MB058-22_22VIR5675-2/2022_H5N1_2022-02-03	X	X			X
	A/swan/Poland/MB078_22VIR2515-7/2022_H5N1_2022-02-15	X	X			X
	A/swan/Poland/MB083_22VIR2515-8/2022_H5N1_2022-02-18	X	X			X
	A/mute_swan/Poland/MB122/2022_H5N1_2022-04-07	X	X			
	A/herring_gull/Poland/MB138/2022_H5N1_2022-05-30	X	X			X
	A/black-headed_gull/Poland/MB139/2022_H5N1_2022-05-30	X	X			X
	A/sandwich_stern/Poland/MB142/2022_H5N1_2022-06-15	X	X			X
	A/common_tern/Poland/MB143/2022_H5N1_2022-06-15	X	X			X
	A/common_mure/Poland/MB151/2022_H5N1_2022-07-13	X	X			X
	A/swan/Poland/MB152/2022_H2N3_2022-07-15	X	X			X

NA = not applicable ("N" in the sequence)
X = present in the sequence

		PA					
		S37A	K158R	P190S	N383D	Q400P	N409S
ACTIVE SURVEILLANCE 2018-2021 (LPAIVs)	A/mallard/Poland/P074w5/2018_H3N8_2018-09-05	X		X	X		X
	A/mallard/Poland/P077w1/2018_H5N2_2018-09-12	X		X	X		X
	A/common_teal/P079w24/2018_H3-H12-N8-N5_2018_09_14	X		X	X		X
	A/common_teal/P079w24/2018_H3-H12-N8-N5_2018_09_14	X		X	X		X
	A/mallard/Poland/P060w2/20_H3N8_2020-08-25	X		X	X		X
	A/mute_swan/Poland/P071/2020_H9N2_2020-09-18	X		X	X		X
	A/herring_gull/Poland/P092/20_H16N3_2020-10-28	X		X	X		X
	A/mallard/Poland/P096/2020_H9N2_2020-11-02	X		X	X		X
	A/black-headed_gull/Poland/P075/2021_H9N7_2021-02-09	X		X	X		X
PASSIVE SURVEILLANCE HPAI 2020/2021 (H5N8, H5N1, H5N5)	A/tundra-bean-goose/Poland/MB132/2020_H5N8_2020-12-11	X		X	X		X
	A/swan/Poland/MB141/2020_H5N8_2020-12-17	X		X	X		X
	A/wild_goose/Poland/MB142/2020_H5N8_2020-12-17	X		X	X		X
	A/mute_swan/Poland/MB021-21_22VIR5675-11/2021_H5N8_2021-01-19	X		X	X		X
	A/tufted_duck/Poland/MB061/2021_H5N5_2021-02-01	X		X	X		X
	A/swan/Poland/MB122-21_22VIR5675-9/2021_H5N8_2021-02-18	X		X	X		X
	A/buzzard/Poland/MB129-21_22VIR5675-7/2021_2021-02-19	X		X	X		X
	A/mute_swan/Poland/MB131/2021_H5N8_2021-02-19	X		X	X		X
	A/swan/Poland/MB185/2021_H5N8_2021-02-26	X		X	X		X
	A/mute_swan/Poland/MB189/2021_H5N8_2021-02-26	X		X	X		X
	A/mute_swan/Poland/MB268/2021_H5N8_2021-03-09	X		X	X		X
	A/mute_swan/Poland/MB272/2021_H5N8_2021-03-09	X		X	X		X
	A/buzzard/Poland/MB277-21_22VIR5675-18/2021_H5N8_2021-03-09	X		X	X		X
	A/avian/Poland/MB292-21_22VIR5675-14/2021_H5N8_2021-03-11	X		X	X		X
	A/mute_swan/Poland/MB306-21_22VIR5675-20/2021_H5N8_2021-03-16	X		X	X		X
	A/white_stork/Poland/MB363-21_22VIR5675-15/2021_H5N8_2021-04-01	X		X	X		X
	A/mute_swan/Poland/MB372-21_22VIR5675-19/2021_H5N8_2021-04-07	X		X	X		X
	A/white_stork/Poland/MB391/2021_H5N1_2021-04-20	X		X	X		X
	A/mute_swan/Poland/MB396_21RS1385-19/2021_H5N8_2021-04-21	X		X	X		X
	A/white_stork/Poland/MB412_21RS1385-11/2021_H5N8_2021-05-02	X		X	X		X
PASSIVE SURVEILLANCE HPAI 2021/2022 (H5N1) + LPAIV H2N3	A/mute_swan/Poland/MB490-L1/2021_H5N1_2021-11-08	X		X	X		X
	A/greylag_goose/Poland/MB503_21RS3290-18/2021_H5N1_2021-11-22	X		X	X	X	X
	A/crane/Poland/MB528/2021_H5N1_2021-12-10	X		X	X	X	X
	A/wild_bird/Poland/MW542/2021_H5N1_2021-12-20	X		X	X	X	X
	A/hawk/Poland/MB544/2021_H5N1_2021-12-22	X		X	X	X	X
	A/mute_swan/Poland/MB550/2021_H5N1_2021-12-23	X		X	X	X	X
	A/mute_swan/Poland/MB551/2021_H5N1_2021-12-23	X		X	X	X	X
	A/mute_swan/Poland/MB008-22_22VIR5675-16/2022_2022-01-05	X		X	X	X	X
	A/mute_swan/Poland/MB020-22_22VIR5675-3/2022_H5N1_2022-01-11	X		X	X	X	X
	A/white-fronted_goose/Poland/MB028-22_22VIR5675-1/2022_2022-01-14	X	X	X	X	X	X
	A/mute_swan/Poland/MB034-22_22VIR5675-12/2022_H5N1_2022-01-18	X		X	X		X
	A/mute_swan/Poland/MB040-22_22VIR5675-8/2022_H5N1_2022-01-20	X	NA	NA	NA		X
	A/mute_swan/Poland/MB042-22_22VIR5675-6/2022_H5N1_2022-01-24	X		X	X	X	X
	A/swan/Poland/MB058-22_22VIR5675-2/2022_H5N1_2022-02-03	X	X	X	X	X	X
	A/swan/Poland/MB078_22VIR2515-7/2022_H5N1_2022-02-15	X		X	X		X
	A/swan/Poland/MB083_22VIR2515-8/2022_H5N1_2022-02-18	X		X	X	X	X
	A/mute_swan/Poland/MB122/2022_H5N1_2022-04-07	X		X	X		X
	A/herring_gull/Poland/MB138/2022_H5N1_2022-05-30	X		X	X		X
	A/black-headed_gull/Poland/MB139/2022_H5N1_2022-05-30	X		X	X		X
	A/sandwich_stern/Poland/MB142/2022_H5N1_2022-06-15	X		X	X		X
	A/common_tern/Poland/MB143/2022_H5N1_2022-06-15	X		X	X		X
	A/common_mure/Poland/MB151/2022_H5N1_2022-07-13	X		X	X		X
	A/swan/Poland/MB152/2022_H2N3_2022-07-15	X		X	X		X

NA = not applicable ("N" in the sequence)
X = present in the sequence

		H5 numbering										
		S123P	A133A	S154N	S155N	T156A	V182N	V210I	K394E	S107R + T108I	S155N + T156A	K218Q + S223R
**ACTIVE SURVEILLANCE 2018-2021 (LPAIVs)**	A/mallard/Poland/P077w1/2018_H5N2_2018-09-12	X		X	X	X	X		X		X	
**PASSIVE SURVEILLANCE HPAI 2020/2021 (H5N8)**	A/tundra-bean-goose/Poland/MB132/2020_H5N8_2020-12-11	X	X	X		X	X		X	X		X
	A/swan/Poland/MB141/2020_H5N8_2020-12-17	X	X	X		X	X		X	X		X
	A/wild_goose/Poland/MB142/2020_H5N8_2020-12-17	X	X	X		X	X		X	X		X
	A/mute_swan/Poland/MB021-21_22VIR5675-11/2021_H5N8_2021-01-19	X	X	X		X	X		X	X		X
	A/tufted_duck/Poland/MB061/2021_H5N5	X	X	X		X	X		X	X		X
	A/swan/Poland/MB122-21_22VIR5675-9/2021_H5N8_2021-02-18	X	X	X		X	X		X	X		X
	A/buzzard/Poland/MB129-21_22VIR5675-7/2021_2021-02-19	X	X	X		X	X		X	X		X
	A/mute_swan/Poland/MB131/2021_H5N8_2021-02-19	X	X	X		X	X		X	X		X
	A/swan/Poland/MB185/2021_H5N8_2021-02-26	X	X	X		X	X		X	X		X
	A/mute_swan/Poland/MB189/2021_H5N8_2021-02-26	X	X	X		X	X		X	X		X
	A/mute_swan/Poland/MB268/2021_H5N8_2021-03-09	X	X	X		X	X		X	X		X
	A/mute_swan/Poland/MB272/2021_H5N8_2021-03-09	X	X	X		X	X		X	X		X
	A/buzzard/Poland/MB277-21_22VIR5675-18/2021_H5N8_2021-03-09	X	X	X		X	X		X	X		X
	A/avian/Poland/MB292-21_22VIR5675-14/2021_H5N8_2021-03-11	X	X	X		X	X		X	X		X
	A/mute_swan/Poland/MB306-21_22VIR5675-20/2021_H5N8_2021-03-16	X	X	X		X	X		X	X		X
	A/white_stork/Poland/MB363-21_22VIR5675-15/2021_H5N8_2021-04-01	X	X	X		X	X		X	X		X
	A/mute_swan/Poland/MB372-21_22VIR5675-19/2021_H5N8_2021-04-07	X	X	X		X	X		X	X		X
	A/white_stork/Poland/MB391/2021_H5N1_2021-04-20	X	X	X		X	X		X	X		X
	A/mute_swan/Poland/MB396_21RS1385-19/2021_H5N8_2021-04-21	X	X	X		X	X		X	X		X
	A/white_stork/Poland/MB412_21RS1385-11/2021_H5N8_2021-05-02	X	X	X		X	X		X	X		X
**PASSIVE SURVEILLANCE HPAI 2021/2022 (H5N1) + LPAIV H2N3**	A/mute_swan/Poland/MB490-L1/2021_H5N1_	X	X	X		X	X		X	X		X
	A/greylag_goose/Poland/MB503_21RS3290-18/2021_H5N1_2021-11-22	X	X	X		X	X		X	X		X
	A/crane/Poland/MB528/2021_H5N1_2021-12-10	X	X	X		X	X		X	X		X
	A/wild_bird/Poland/MW542/2021_H5N1_2021-12-20	X	X	X		X	X		X	X		X
	A/hawk/Poland/MB544/2021_H5N1_2021-12-22	X	X	X		X	X		X	X		X
	A/mute_swan/Poland/MB550/2021_H5N1_2021-12-23	X	X	X		X	X		X	X		X
	A/mute_swan/Poland/MB551/2021_H5N1_2021-12-23	X	X	X		X	X		X	X		X
	A/mute_swan/Poland/MB008-22_22VIR5675-16/2022_2022-01-05	X	X	X		X	X		X	X		X
	A/mute_swan/Poland/MB020-22_22VIR5675-3/2022_H5N1_2022-01-11	X	X	X		X	X	X	X	X		X
	A/white-fronted_goose/Poland/MB028-22_22VIR5675-1/2022_2022-01-14	X	X	X		X	X		X	X		X
	A/mute_swan/Poland/MB034-22_22VIR5675-12/2022_H5N1_2022-01-18	X	X	X		X	X		X	X		X
	A/mute_swan/Poland/MB040-22_22VIR5675-8/2022_H5N1_2022-01-20	NA	NA	X		NA	NA	NA	X	NA		X
	A/mute_swan/Poland/MB042-22_22VIR5675-6/2022_H5N1_2022-01-24	X	X	X		X	X		X	X		X
	A/swan/Poland/MB058-22_22VIR5675-2/2022_H5N1_2022-02-03	X	X	X		X	X		X	X		X
	A/swan/Poland/MB078_22VIR2515-7/2022_H5N1_2022-02-15	X	X	X		X	X		X	X		X
	A/swan/Poland/MB083_22VIR2515-8/2022_H5N1_2022-02-18	X	X	X		X	X		X	X		X
	A/mute_swan/Poland/MB122/2022_H5N1_2022-04-07	X	X	X		X	X		X	X		X
	A/herring_gull/Poland/MB138/2022_H5N1_2022-05-30	X	X	X		X	X		X	X		X
	A/black-headed_gull/Poland/MB139/2022_H5N1_2022-05-30	X	X	X		X	X		X	X		X
	A/sandwich_stern/Poland/MB142/2022_H5N1_2022-06-15	X	X	X		X	X		X	X		X
	A/common_tern/Poland/MB143/2022_H5N1_2022-06-15	X	X	X		X	X		X	X		X
	A/common_mure/Poland/MB151/2022_H5N1_2022-07-13	X	X	X		X	X		X	X		X
	A/swan/Poland/MB152/2022_H2N3_2022-07-15	X	X	X		X	X		X	X		X

NA = not applicable ("N" in the sequence)
X = present in the sequence

		H3 numbering - H5 numbering [H2 numbering]					
		I155T - I151T [**I150T**]	V214I - V210I [**V209I**]	R496K - R493K [**R492K**]	V186N - V182N [**V181N**]	K393E - K394E [**K389E**]	T189A + G192R - T185A + G188R [**T184A + G187R**]
**H2**	A/swan/Poland/MB152/2022_H2N3_2022-07-15	X	X	X	X	X	X

		H5 numbering [H3 numbering]						
		Y7H [**Y17H**]	N11any [**N21any**]	I151T [**I155T**]	S155N [**S159N**]	T156A [**T160A**]	S155N [**S159N**] + T156A [**T160A**]	V214I [**V210I**]
**H3**	A/mallard/Poland/P074w5/2018_H3N8_2018-09-05	X	X [N21P]	X	X	X	X	X
	A/common_teal/P079w24/2018_**H3**-H12-N8-N5_2018_09_14	X	X [N21P]	X	X	X	X	X
	A/common_teal/P079w24/2018_**H3**-H12-N8-N5_2018_09_14	X	X [N21P]	X	X	X	X	X
	A/mallard/Poland/P060w2/2020_H3N8_2020-08-25	X	X [N21P]	X				X

		H5 numbering [H9 numbering]											
		H8Q [**H8Q**]	E75K [**E75K**]	E75K + N193K + R497K [**E75K + N187K + R487K**]	D94N [**D94N**]	S133A [**S131A**]	I151T [**I145T**]	N193K [**N187K**]	K394E [**K384E**]	R493K [**R487K**]	Q222L [**Q216L**]	N154S + Q222L [**N148S + Q216L**]	N193K + R497K [**N187K + R487K**]
**H9**	A/mute_swan/Poland/P071/2020_H9N2_2020-09-18	X	X	X	X		X	X	X	X	X	X	X
	A/mallard/Poland/P096/2020_H9N2_2020-11-02	X			X		X	X	X	X			X
	A/black-headed_gull/Poland/P075/2021_H9N7_2021-02-09	X			X	X	X		X	X			

		H5 numbering - H3 numbering [H12 numbering]			
		H8Q- H18Q [**H9Q**]	I155T - I151T [**I151T**]	T318I - T315I [**T315I**]	K393E - K394E [**K390E**]
**H12**	A/common_teal/P079w24/2018_H3-**H12**-N8-N5_2018_09_14	X	X	X	X
	A/common_teal/P079w24/2018_H3-**H12**-N8-N5_2018_09_14	X	X	X	X

		H5 numbering - H3 numbering [H16
		G228S - G224S [**G221S**]
**H16**	A/herring_gull/Poland/P092/2020_H16N3_2020-10-28	X

NA = not applicable ("N" in the sequence)
X = present in the sequence

		NP			
		I41V	M105V	A184K	N319K
ACTIVE SURVEILLANCE 2018-2021 (LPAIVs)	A/mallard/Poland/P074w5/2018_H3N8_2018-09-05		X	X	
	A/mallard/Poland/P077w1/2018_H5N2_2018-09-12			X	
	A/common_teal/P079w24/2018_H3-H12-N8-N5_2018_09_14		NA	X	
	A/common_teal/P079w24/2018_H3-H12-N8-N5_2018_09_14		NA	X	
	A/mallard/Poland/P060w2/20_H3N8_2020-08-25		X	X	
	A/mute_swan/Poland/P071/2020_H9N2_2020-09-18		X	X	
	A/herring_gull/Poland/P092/20_H16N3_2020-10-28		X	X	
	A/mallard/Poland/P096/2020_H9N2_2020-11-02			X	
	A/black-headed_gull/Poland/P075/2021_H9N7_2021-02-09		X	X	
PASSIVE SURVEILLANCE HPAI 2020/2021 (H5N8, H5N1, H5N5)	A/tundra-bean-goose/Poland/MB132/2020_H5N8_2020-12-11		X	X	
	A/swan/Poland/MB141/2020_H5N8_2020-12-17		X	X	
	A/wild_goose/Poland/MB142/2020_H5N8_2020-12-17		X	X	
	A/mute_swan/Poland/MB021-21_22VIR5675-11/2021_H5N8_2021-01-19		X	X	
	A/tufted_duck/Poland/MB061/2021_H5N5_2021-02-01			X	
	A/swan/Poland/MB122-21_22VIR5675-9/2021_H5N8_2021-02-18		X	X	
	A/buzzard/Poland/MB129-21_22VIR5675-7/2021_2021-02-19		X	X	
	A/mute_swan/Poland/MB131/2021_H5N8_2021-02-19		X	X	
	A/swan/Poland/MB185/2021_H5N8_2021-02-26		X	X	
	A/mute_swan/Poland/MB189/2021_H5N8_2021-02-26		X	X	
	A/mute_swan/Poland/MB268/2021_H5N8_2021-03-09		X	X	
	A/mute_swan/Poland/MB272/2021_H5N8_2021-03-09		X	X	
	A/buzzard/Poland/MB277-21_22VIR5675-18/2021_H5N8_2021-03-09		X	X	
	A/avian/Poland/MB292-21_22VIR5675-14/2021_H5N8_2021-03-11		X	X	
	A/mute_swan/Poland/MB306-21_22VIR5675-20/2021_H5N8_2021-03-16		X	X	
	A/white_stork/Poland/MB363-21_22VIR5675-15/2021_H5N8_2021-04-01		X	X	
	A/mute_swan/Poland/MB372-21_22VIR5675-19/2021_H5N8_2021-04-07		X	X	
	A/white_stork/Poland/MB391/2021_H5N1_2021-04-20		X	X	
	A/mute_swan/Poland/MB396_21RS1385-19/2021_H5N8_2021-04-21		X	X	
	A/white_stork/Poland/MB412_21RS1385-11/2021_H5N8_2021-05-02		X	X	
PASSIVE SURVEILLANCE HPAI 2021/2022 (H5N1) + LPAIV H2N3	A/mute_swan/Poland/MB490-L1/2021_H5N1_2021-11-08		X	X	
	A/greylag_goose/Poland/MB503_21RS3290-18/2021_H5N1_2021-11-22		X	X	
	A/crane/Poland/MB528/2021_H5N1_2021-12-10		X	X	
	A/wild_bird/Poland/MW542/2021_H5N1_2021-12-20		X	X	
	A/hawk/Poland/MB544/2021_H5N1_2021-12-22		X	X	
	A/mute_swan/Poland/MB550/2021_H5N1_2021-12-23		X	X	
	A/mute_swan/Poland/MB551/2021_H5N1_2021-12-23		X	X	
	A/mute_swan/Poland/MB008-22_22VIR5675-16/2022_2022-01-05		X	X	
	A/mute_swan/Poland/MB020-22_22VIR5675-3/2022_H5N1_2022-01-11		X	X	
	A/white-fronted_goose/Poland/MB028-22_22VIR5675-1/2022_2022-01-14			X	
	A/mute_swan/Poland/MB034-22_22VIR5675-12/2022_H5N1_2022-01-18			X	
	A/mute_swan/Poland/MB040-22_22VIR5675-8/2022_H5N1_2022-01-20		X	X	
	A/mute_swan/Poland/MB042-22_22VIR5675-6/2022_H5N1_2022-01-24			X	
	A/swan/Poland/MB058-22_22VIR5675-2/2022_H5N1_2022-02-03			X	
	A/swan/Poland/MB078_22VIR2515-7/2022_H5N1_2022-02-15			X	
	A/swan/Poland/MB083_22VIR2515-8/2022_H5N1_2022-02-18		X	X	
	A/mute_swan/Poland/MB122/2022_H5N1_2022-04-07	X		X	
	A/herring_gull/Poland/MB138/2022_H5N1_2022-05-30			X	X
	A/black-headed_gull/Poland/MB139/2022_H5N1_2022-05-30			X	X
	A/sandwich_stern/Poland/MB142/2022_H5N1_2022-06-15			X	
	A/common_tern/Poland/MB143/2022_H5N1_2022-06-15			X	
	A/common_mure/Poland/MB151/2022_H5N1_2022-07-13			X	
	A/swan/Poland/MB152/2022_H2N3_2022-07-15			X	

NA = not applicable ("N" in the sequence)
X = present in the sequence

		N2 numbering [Nx numbering]
		I117T
ACTIVE SURVEILLANCE 2018-2021 (LPAIVs)	A/mallard/Poland/P074w5/2018_H3N8_2018-09-05	
	A/mallard/Poland/P077w1/2018_H5N2_2018-09-12	X [I117T]
	A/common_teal/P079w24/2018_H3-H12-N8-N5_2018_09_14	
	A/common_teal/P079w24/2018_H3-H12-N8-N5_2018_09_14	
	A/mallard/Poland/P060w2/2020_H3N8_2020-08-25	
	A/mute_swan/Poland/P071/2020_H9N2_2020-09-18	X [I117T]
	A/herring_gull/Poland/P092/2020_H16N3_2020-10-28	X [I118T]
	A/mallard/Poland/P096/2020_H9N2_2020-11-02	X [I117T]
	A/black-headed_gull/Poland/P075/2021_H9N7_2021-02-09	X [I116T]
PASSIVE SURVEILLANCE HPAI 2020/2021 (H5N8, H5N1, H5N5)	A/tundra-bean-goose/Poland/MB132/2020_H5N8_2020-12-11	
	A/swan/Poland/MB141/2020_H5N8_2020-12-17	
	A/wild_goose/Poland/MB142/2020_H5N8_2020-12-17	
	A/mute_swan/Poland/MB021-21_22VIR5675-11/2021_H5N8_2021-01-19	
	A/tufted_duck/Poland/MB061/2021_H5N5_2021-02-01	
	A/swan/Poland/MB122-21_22VIR5675-9/2021_H5N8_2021-02-18	
	A/buzzard/Poland/MB129-21_22VIR5675-7/2021_2021-02-19	
	A/mute_swan/Poland/MB131/2021_H5N8_2021-02-19	
	A/swan/Poland/MB185/2021_H5N8_2021-02-26	
	A/mute_swan/Poland/MB189/2021_H5N8_2021-02-26	
	A/mute_swan/Poland/MB268/2021_H5N8_2021-03-09	
	A/mute_swan/Poland/MB272/2021_H5N8_2021-03-09	
	A/buzzard/Poland/MB277-21_22VIR5675-18/2021_H5N8_2021-03-09	
	A/avian/Poland/MB292-21_22VIR5675-14/2021_H5N8_2021-03-11	
	A/mute_swan/Poland/MB306-21_22VIR5675-20/2021_H5N8_2021-03-16	
	A/white_stork/Poland/MB363-21_22VIR5675-15/2021_H5N8_2021-04-01	
	A/mute_swan/Poland/MB372-21_22VIR5675-19/2021_H5N8_2021-04-07	
	A/white_stork/Poland/MB391/2021_H5N1_2021-04-20	
	A/mute_swan/Poland/MB396_21RS1385-19/2021_H5N8_2021-04-21	
	A/white_stork/Poland/MB412_21RS1385-11/2021_H5N8_2021-05-02	
PASSIVE SURVEILLANCE HPAI 2021/2022 (H5N1) + LPAIV H2N3	A/mute_swan/Poland/MB490-L1/2021_H5N1_2021-11-08	
	A/greylag_goose/Poland/MB503_21RS3290-18/2021_H5N1_2021-11-22	
	A/crane/Poland/MB528/2021_H5N1_2021-12-10	
	A/wild_bird/Poland/MW542/2021_H5N1_2021-12-20	
	A/hawk/Poland/MB544/2021_H5N1_2021-12-22	
	A/mute_swan/Poland/MB550/2021_H5N1_2021-12-23	
	A/mute_swan/Poland/MB551/2021_H5N1_2021-12-23	
	A/mute_swan/Poland/MB008-22_22VIR5675-16/2022_2022-01-05	
	A/mute_swan/Poland/MB020-22_22VIR5675-3/2022_H5N1_2022-01-11	
	A/white-fronted_goose/Poland/MB028-22_22VIR5675-1/2022_2022-01-14	
	A/mute_swan/Poland/MB034-22_22VIR5675-12/2022_H5N1_2022-01-18	
	A/mute_swan/Poland/MB040-22_22VIR5675-8/2022_H5N1_2022-01-20	
	A/mute_swan/Poland/MB042-22_22VIR5675-6/2022_H5N1_2022-01-24	
	A/swan/Poland/MB058-22_22VIR5675-2/2022_H5N1_2022-02-03	
	A/swan/Poland/MB078_22VIR2515-7/2022_H5N1_2022-02-15	
	A/swan/Poland/MB083_22VIR2515-8/2022_H5N1_2022-02-18	
	A/mute_swan/Poland/MB122/2022_H5N1_2022-04-07	
	A/herring_gull/Poland/MB138/2022_H5N1_2022-05-30	
	A/black-headed_gull/Poland/MB139/2022_H5N1_2022-05-30	
	A/sandwich_stern/Poland/MB142/2022_H5N1_2022-06-15	
	A/common_tern/Poland/MB143/2022_H5N1_2022-06-15	
	A/common_mure/Poland/MB151/2022_H5N1_2022-07-13	
	A/swan/Poland/MB152/2022_H2N3_2022-07-15	X [I118T]

NA = not applicable ("N" in the sequence)
X = present in the sequence

Nx numbering = subtype=specific numbering

		M1		
		N30D	I43M	T215A
ACTIVE SURVEILLANCE 2018-2021 (LPAIVs)	A/mallard/Poland/P074w5/2018_H3N8_2018-09-05	X	X	X
	A/mallard/Poland/P077w1/2018_H5N2_2018-09-12	X	X	X
	A/common_teal/P079w24/2018_H3-H12-N8-N5_2018_09_14	X	X	X
	A/common_teal/P079w24/2018_H3-H12-N8-N5_2018_09_14	X	X	X
	A/mallard/Poland/P060w2/20_H3N8_2020-08-25	X	X	X
	A/mute_swan/Poland/P071/2020_H9N2_2020-09-18	X	X	X
	A/herring_gull/Poland/P092/20_H16N3_2020-10-28	X	X	X
	A/mallard/Poland/P096/2020_H9N2_2020-11-02	X	X	X
	A/black-headed_gull/Poland/P075/2021_H9N7_2021-02-09	X	X	X
PASSIVE SURVEILLANCE HPAI 2020/2021 (H5N8, H5N1, H5N5)	A/tundra-bean-goose/Poland/MB132/2020_H5N8_2020-12-11	X	X	X
	A/swan/Poland/MB141/2020_H5N8_2020-12-17	X	X	X
	A/wild_goose/Poland/MB142/2020_H5N8_2020-12-17	X	X	X
	A/mute_swan/Poland/MB021-21_22VIR5675-11/2021_H5N8_2021-01-19	X	X	X
	A/tufted_duck/Poland/MB061/2021_H5N5_2021-02-01	X	X	X
	A/swan/Poland/MB122-21_22VIR5675-9/2021_H5N8_2021-02-18	X	X	X
	A/buzzard/Poland/MB129-21_22VIR5675-7/2021_2021-02-19	X	X	X
	A/mute_swan/Poland/MB131/2021_H5N8_2021-02-19	X	X	X
	A/swan/Poland/MB185/2021_H5N8_2021-02-26	X	X	X
	A/mute_swan/Poland/MB189/2021_H5N8_2021-02-26	X	X	X
	A/mute_swan/Poland/MB268/2021_H5N8_2021-03-09	X	X	X
	A/mute_swan/Poland/MB272/2021_H5N8_2021-03-09	X	X	X
	A/buzzard/Poland/MB277-21_22VIR5675-18/2021_H5N8_2021-03-09	X	X	X
	A/avian/Poland/MB292-21_22VIR5675-14/2021_H5N8_2021-03-11	X	X	X
	A/mute_swan/Poland/MB306-21_22VIR5675-20/2021_H5N8_2021-03-16	X	X	X
	A/white_stork/Poland/MB363-21_22VIR5675-15/2021_H5N8_2021-04-01	X	X	X
	A/mute_swan/Poland/MB372-21_22VIR5675-19/2021_H5N8_2021-04-07	X	X	X
	A/white_stork/Poland/MB391/2021_H5N1_2021-04-20	X	X	X
	A/mute_swan/Poland/MB396_21RS1385-19/2021_H5N8_2021-04-21	X	X	X
	A/white_stork/Poland/MB412_21RS1385-11/2021_H5N8_2021-05-02	X	X	X
PASSIVE SURVEILLANCE HPAI 2021/2022 (H5N1) + LPAIV H2N3	A/mute_swan/Poland/MB490-L1/2021_H5N1_2021-11-08	X	X	X
	A/greylag_goose/Poland/MB503_21RS3290-18/2021_H5N1_2021-11-22	X	X	X
	A/crane/Poland/MB528/2021_H5N1_2021-12-10	X	X	X
	A/wild_bird/Poland/MW542/2021_H5N1_2021-12-20	X	X	X
	A/hawk/Poland/MB544/2021_H5N1_2021-12-22	X	X	X
	A/mute_swan/Poland/MB550/2021_H5N1_2021-12-23	X	X	X
	A/mute_swan/Poland/MB551/2021_H5N1_2021-12-23	X	X	X
	A/mute_swan/Poland/MB008-22_22VIR5675-16/2022_2022-01-05	X	X	X
	A/mute_swan/Poland/MB020-22_22VIR5675-3/2022_H5N1_2022-01-11	X	X	X
	A/white-fronted_goose/Poland/MB028-22_22VIR5675-1/2022_2022-01-14	X	X	X
	A/mute_swan/Poland/MB034-22_22VIR5675-12/2022_H5N1_2022-01-18	X	X	X
	A/mute_swan/Poland/MB040-22_22VIR5675-8/2022_H5N1_2022-01-20	X	X	X
	A/mute_swan/Poland/MB042-22_22VIR5675-6/2022_H5N1_2022-01-24	X	X	X
	A/swan/Poland/MB058-22_22VIR5675-2/2022_H5N1_2022-02-03	X	X	X
	A/swan/Poland/MB078_22VIR2515-7/2022_H5N1_2022-02-15	X	X	X
	A/swan/Poland/MB083_22VIR2515-8/2022_H5N1_2022-02-18	X	X	X
	A/mute_swan/Poland/MB122/2022_H5N1_2022-04-07	X	X	X
	A/herring_gull/Poland/MB138/2022_H5N1_2022-05-30	X	X	X
	A/black-headed_gull/Poland/MB139/2022_H5N1_2022-05-30	X	X	X
	A/sandwich_stern/Poland/MB142/2022_H5N1_2022-06-15	X	X	X
	A/common_tern/Poland/MB143/2022_H5N1_2022-06-15	X	X	X
	A/common_mure/Poland/MB151/2022_H5N1_2022-07-13	X	X	X
	A/swan/Poland/MB152/2022_H2N3_2022-07-15	X	X	X

NA = not applicable ("N" in the sequence)
X = present in the sequence

no mutations in M2 segment gene detected

		NS1							
		P42S	I106M	C138F	V149A	L103F + I106M	K55E + K66E + C138F	ESEV motif (227-230aa)	truncated NS1
ACTIVE SURVEILLANCE 2018-2021 (LPAIVs)	A/mallard/Poland/P074w5/2018_H3N8_2018-09-05		X	X	X			X	
	A/mallard/Poland/P077w1/2018_H5N2_2018-09-12	X	X	X	X	X	X	X	
	A/common_teal/P079w24/2018_H3-H12-N8-N5_2018_09_14	NA	NA	NA	NA	NA	NA	NA	NA
	A/common_teal/P079w24/2018_H3-H12-N8-N5_2018_09_14	NA	NA	NA	NA	NA	NA	NA	NA
	A/mallard/Poland/P060w2/20_H3N8_2020-08-25	X	X	X	X	X	X	X	
	A/mute_swan/Poland/P071/2020_H9N2_2020-09-18	X	X	X	X	X	X	X	
	A/herring_gull/Poland/P092/20_H16N3_2020-10-28	X	X	X	X	X	X	X	
	A/mallard/Poland/P096/2020_H9N2_2020-11-02		X	X	X			X	
	A/black-headed_gull/Poland/P075/2021_H9N7_2021-02-09	X	X	X	X	X	X	X	
PASSIVE SURVEILLANCE HPAI 2020/2021 (H5N8, H5N1, H5N5)	A/tundra-bean-goose/Poland/MB132/2020_H5N8_2020-12-11	X	X	X	X	X			217 aa
	A/swan/Poland/MB141/2020_H5N8_2020-12-17	X	X	X	X	X			217 aa
	A/wild_goose/Poland/MB142/2020_H5N8_2020-12-17	X	X	X	X	X			217 aa
	A/mute_swan/Poland/MB021-21_22VIR5675-11/2021_H5N8_2021-01-19	X	X	X	X	X			217 aa
	A/tufted_duck/Poland/MB061/2021_H5N5_2021-02-01	X	X	X	X	X			217 aa
	A/swan/Poland/MB122-21_22VIR5675-9/2021_H5N8_2021-02-18	X	X	X	X	X			217 aa
	A/buzzard/Poland/MB129-21_22VIR5675-7/2021_2021-02-19	X	X	X	X	X			217 aa
	A/mute_swan/Poland/MB131/2021_H5N8_2021-02-19	X	X	X	X	X			217 aa
	A/swan/Poland/MB185/2021_H5N8_2021-02-26	X	X	X	X	X			217 aa
	A/mute_swan/Poland/MB189/2021_H5N8_2021-02-26	X	X	X	X	X			217 aa
	A/mute_swan/Poland/MB268/2021_H5N8_2021-03-09	X	X	X	X	X			217 aa
	A/mute_swan/Poland/MB272/2021_H5N8_2021-03-09	X	X	X	X	X			217 aa
	A/buzzard/Poland/MB277-21_22VIR5675-18/2021_H5N8_2021-03-09	X	X	X	X	X			217 aa
	A/avian/Poland/MB292-21_22VIR5675-14/2021_H5N8_2021-03-11	X	X	X	X	X			217 aa
	A/mute_swan/Poland/MB306-21_22VIR5675-20/2021_H5N8_2021-03-16	X	X	X	X	X			217 aa
	A/white_stork/Poland/MB363-21_22VIR5675-15/2021_H5N8_2021-04-01	X	X	X	X	X			217 aa
	A/mute_swan/Poland/MB372-21_22VIR5675-19/2021_H5N8_2021-04-07	X	X	X	X	X			217 aa
	A/white_stork/Poland/MB391/2021_H5N1_2021-04-20	X	X	X	X	X	X	X	
	A/mute_swan/Poland/MB396_21RS1385-19/2021_H5N8_2021-04-21	X	X	X	X	X			217 aa
	A/white_stork/Poland/MB412_21RS1385-11/2021_H5N8_2021-05-02	X	X	X	X	X			217 aa
PASSIVE SURVEILLANCE HPAI 2021/2022 (H5N1) + LPAIV H2N3	A/mute_swan/Poland/MB490-L1/2021_H5N1_2021-11-08	X	X	X	X	X	X	X	
	A/greylag_goose/Poland/MB503_21RS3290-18/2021_H5N1_2021-11-22	X	X	X	X	X	X	X	
	A/crane/Poland/MB528/2021_H5N1_2021-12-10	X	X	X	X	X	X	X	
	A/wild_bird/Poland/MW542/2021_H5N1_2021-12-20	X	X	X	X	X	X	X	
	A/hawk/Poland/MB544/2021_H5N1_2021-12-22	X	X	X	X	X	X	X	
	A/mute_swan/Poland/MB550/2021_H5N1_2021-12-23	X	X	X	X	X	X	X	
	A/mute_swan/Poland/MB551/2021_H5N1_2021-12-23	X	X	X	X	X	X	X	
	A/mute_swan/Poland/MB008-22_22VIR5675-16/2022_2022-01-05	X	X	X	X	X	X	X	
	A/mute_swan/Poland/MB020-22_22VIR5675-3/2022_H5N1_2022-01-11	X	X	X	X	X	X	X	
	A/white-fronted_goose/Poland/MB028-22_22VIR5675-1/2022_2022-01-14	X	X	X	X	X	X	X	
	A/mute_swan/Poland/MB034-22_22VIR5675-12/2022_H5N1_2022-01-18	X	X	X	X	X	X	X	
	A/mute_swan/Poland/MB040-22_22VIR5675-8/2022_H5N1_2022-01-20	X	X	X	X	X	X	X	
	A/mute_swan/Poland/MB042-22_22VIR5675-6/2022_H5N1_2022-01-24	X	X	X	X	X	X	X	
	A/swan/Poland/MB058-22_22VIR5675-2/2022_H5N1_2022-02-03	X	X	X	X	X	X	X	
	A/swan/Poland/MB078_22VIR2515-7/2022_H5N1_2022-02-15		X	X	X				224 aa*
	A/swan/Poland/MB083_22VIR2515-8/2022_H5N1_2022-02-18	X	X	X	X	X	X	X	
	A/mute_swan/Poland/MB122/2022_H5N1_2022-04-07	X	X	X	X	X	X	X	
	A/herring_gull/Poland/MB138/2022_H5N1_2022-05-30	X	X	X	X	X	X	X	
	A/black-headed_gull/Poland/MB139/2022_H5N1_2022-05-30	X	X	X	X	X	X	X	
	A/sandwich_stern/Poland/MB142/2022_H5N1_2022-06-15	X	X	X	X	X	X	X	
	A/common_tern/Poland/MB143/2022_H5N1_2022-06-15	X	X	X	X	X	X	X	
	A/common_mure/Poland/MB151/2022_H5N1_2022-07-13	X	X	X	X	X	X	X	
	A/swan/Poland/MB152/2022_H2N3_2022-07-15	X	X	X	X	X	X	X	

NA = not applicable ("N" in the sequence)
X = present in the sequence

no mutations in NS2 segment gene detected

*225-230 deletion
